# SIRPG promotes lung squamous cell carcinoma pathogenesis via M1 macrophages: a multi-omics study integrating data and Mendelian randomization

**DOI:** 10.3389/fonc.2024.1392417

**Published:** 2024-06-04

**Authors:** Guocai Mao, Jing Li, Nan Wang, Hongbin Yu, Shiyu Han, Mengqi Xiang, Huachuan Zhang, Daxiong Zeng, Junhong Jiang, Haitao Ma

**Affiliations:** ^1^ Department of Thoracic Surgery, Suzhou Dushu Lake Hospital, Dushu Lake Hospital Affiliated to Soochow University, Medical Centre of Soochow University, Suzhou, China; ^2^ Department of Respiratory and Critical Care Medicine, Suzhou Dushu Lake Hospital, Dushu Lake Hospital Affiliated to Soochow University, Medical Centre of Soochow University, Suzhou, China; ^3^ Department of Thoracic Surgery, The First Affiliated Hospital of Soochow University, Soochow University, Suzhou, China; ^4^ Department of Clinical Laboratory, Suzhou Dushu Lake Hospital, Dushu Lake Hospital Affiliated to Soochow University, Medical Centre of Soochow University, Suzhou, China; ^5^ Department of Oncology, Jiangsu Institute of Hematology, Suzhou, China; ^6^ Department of Medical Oncology, Sichuan Clinical Research Center for Cancer, Sichuan Cancer Hospital & Institute, Sichuan Cancer Center, Affiliated Cancer Hospital of University of Electronic Science and Technology of China, Chengdu, China; ^7^ Department of Thoracic Surgery, Sichuan Clinical Research Center for Cancer, Sichuan Cancer Hospital & Institute, Sichuan Cancer Center, Affiliated Cancer Hospital of University of Electronic Science and Technology of China, Chengdu, China; ^8^ Department of Respiratory and Critical Care Medicine, The First Affiliated Hospital of Soochow University, Soochow University, Suzhou, China

**Keywords:** squamous cell carcinoma of the lung, Mendelian randomization, RNA-seq, immune infiltration, SIRPG

## Abstract

**Background:**

Squamous cell carcinoma of the lung (LUSC) is a severe and highly lethal malignant tumor of the respiratory system, and its molecular mechanisms at the molecular level remain unc\lear.

**Methods:**

We acquired RNA-seq data from 8 surgical samples obtained from early-stage LUSC and adjacent non-cancerous tissues from 3 different centers. Utilizing Deseq2, we identified 1088 differentially expressed genes with |LogFC| > 1 and a p-value < 0.05 threshold. Furthermore, through MR analysis of Exposure Data for 26,153 Genes and 63,053 LUSC Patients, incorporating 7,838,805 SNPs as endpoints, we identified 213 genes as potential exposure factors.

**Results:**

After intersecting the results, we identified 5 differentially expressed genes, including GYPE, PODXL2, RNF182, SIRPG, and WNT7A. PODXL2 (OR 95% CI, 1.169 (1.040 to 1.313)) was identified as an exposed risk factor, with p-values less than 0.01 under the inverse variance weighted model. GO and KEGG analyses revealed enhanced ubiquitin-protein transferase activity and activation of pathways such as the mTOR signaling pathway and Wnt signaling pathway. Immune infiltration analysis showed downregulation of Plasma cells, T cells regulatory (Tregs), and Dendritic cells activated by the identified gene set, while an enhancement was observed in Macrophages M1. Furthermore, we externally validated the expression levels of these five genes using RNA-seq data from TCGA database and 11 GEO datasets of LUSC, and the results showed SIRPG could induce LUSC.

**Conclusion:**

SIRPG emerged as a noteworthy exposure risk factor for LUSC. Immune infiltration analysis highlighted Macrophages M1 and mTOR signaling pathway play an important role in LUSC.

## Introduction

Squamous cell carcinoma of the lung (LUSC) constitutes 25%-30% of all non-small cell lung cancer (NSCLC) cases, presenting predominantly as locally advanced or metastatic disease, posing significant treatment challenges (1, 2). LUSC is characterized by its genetic complexity, high mutation rates, and DNA damage, including that associated with smoking (3–5). Unlike nonsquamous NSCLC, LUSC has a low incidence of actionable driver mutations, limiting the effectiveness of targeted therapies for most patients (1). Although chemotherapy options are limited in this context (2, 6, 7), the combination of chemotherapy and immunotherapy (IO) has emerged as a new standard treatment approach (8, 9). However, durable clinical benefits from IO treatment remain elusive for many LUSC patients (8, 10, 11). The lack of novel treatments and reliable biomarkers to predict durable responses highlights the unmet clinical needs of the LUSC population (12–14).

Mendelian randomization (MR) is a methodology designed to reduce measurement errors, reverse causation, and confounding problems. It leverages genetically determined variations that are randomly allocated at birth and have established associations with modifiable risk factors to estimate the causal effects of these risk factors on disease outcomes (5, 15). Compared to observational studies, Mendelian randomization (MR) offers the advantage of mitigating confounding factors and reverse causation by utilizing genetic variations that are less influenced by external environmental changes (16). Over the past few years, several genome-wide association studies (GWAS) investigating plasma proteins have identified numerous cis-variants known as protein quantitative trait loci (pQTLs), which are linked to genes encoding plasma proteins (17–21). Consequently, these cis-pQTLs have been widely employed as genetic instruments to estimate the causal impact of plasma proteins on complex diseases. Importantly, they satisfy the three fundamental assumptions of MR: the relevance assumption, independence assumption, and exclusion restriction assumption (22). There have been numerous studies focusing on multi-omics sequencing analysis of lung adenocarcinoma (23, 24). However, it has been challenging to analyze the etiology of LUSC from an epidemiological perspective. By combining Mendelian randomization (MR) with omics analysis, we aim to improve the representativeness of the identified genes and gain insights into the causal factors of LUSC.

In this multicenter study, we collected surgical tissue samples from 8 LUSC patients, encompassing both tumor and adjacent non-cancerous tissues, gathered from three different centers. RNA was successfully extracted from these samples, and paired RNA-seq analysis was performed to identify differentially expressed genes associated with the initiation of lung cancer. Additionally, we conducted Mendelian randomization analysis using GWAS databases to investigate the exposure factors related to LUSC endpoints. The significant exposure genes were then intersected with the differentially expressed genes identified from the RNA-seq analysis, followed by mechanistic analysis. Furthermore, the findings were validated by utilizing LUSC data from the TCGA database and 11 GEO datasets. Through this research, our objective is to discover novel exposure factors and therapeutic targets for understanding the etiology of LUSC.

## Materials and methods

### Patients

In this study, we analyzed data from 8 patients who were diagnosed with early-stage LUSC and underwent surgical procedures at three different hospitals: Dushu Lake Hospital Affiliated to Soochow University, the First Affiliated Hospital of Soochow University, and Sichuan Cancer Hospital. The study duration ranged from August 3, 2021, to July 30, 2022, with LUSC diagnoses confirmed through pathological assessment. The collected data included patient parameters such as age, body mass index (BMI), gender, smoking status, staging, surgical procedure, and survival outcomes. To classify the data, the median value was utilized as the cutoff point. Patients is in the early to mid-stage who had not undergone chemotherapy. Prior to the study, informed consent was obtained from all patients or their immediate family members in adherence to ethical standards. The research protocol strictly adhered to the guidelines established by the Ethics Committee of Soochow University and followed the principles outlined in the Declaration of Helsinki.

### Sample collection and RNA-seq analysis

During the surgical procedure, LUSC patient samples were collected and preserved by immersing them in an RNA-specific storage solution.

#### Sample size and shape

Samples were carefully selected to ensure an optimal amount of RNA for subsequent RNA sequencing. Typically, tissue samples ranging from 100 mg to 500 mg were utilized. The shape of the samples was chosen to facilitate ease of handling during the sampling process, with rectangular or elliptical shapes being preferred.

#### Quality

To maintain RNA integrity, samples were handled with utmost care to keep them as fresh as possible. Immediately after sampling, samples were either refrigerated or frozen to minimize RNA degradation. Damaged or necrotic tissue was avoided to prevent any adverse effects on RNAseq results.

#### Tissue type

Our sampling focused on tumor tissue containing a sufficient number of cancer cells, in accordance with the study requirements. In lung SCC patients, tumor tissue was the primary tissue of interest. Efforts were made to minimize the inclusion of normal tissue to mitigate potential interference with subsequent RNAseq analysis.

#### Sampling procedure

Sampling procedures were carried out by experienced surgeons to ensure accuracy and efficacy. Sterile techniques were employed throughout the sampling process to prevent sample contamination. All tools and materials used were thoroughly cleaned to maintain sample integrity.

The cellular samples obtained provided around 20–30ng of mRNA, which was used to generate RNA-seq libraries utilizing the KAPA Stranded mRNA-Seq Kit compatible with the Illumina platform. Subsequently, paired-end sequencing of the libraries was performed on the Illumina HiSeq-PE150 instrument.

### Transcriptomic data, and clinical information of TCGA and GEO database

The transcriptome profiling dataset for RNA-seq analysis consisted of 501 samples obtained from patients diagnosed with LUSC. These samples were sourced from the TCGA database (https://portal.gdc.cancer.gov/) on Dec 23, 2023. We also collected 803 cases of squamous cell carcinoma of the lung from the GEO database (https://www.ncbi.nlm.nih.gov/geo/) for subsequent validation analysis using RNA-seq data (GSE11969, GSE14814, GSE157011, GSE19188, GSE29066, GSE30219, GSE3141, GSE37745, GSE41271, GSE42127, GSE50081 and GSE8894).

### Mendelian randomization analysis

In the initial stage, we extracted single nucleotide polymorphisms (SNPs) associated with LUSC as the endpoint from the GWAS data (ebi-a-GCST004750), which demonstrated a significant genome-wide impact (P < 5×10–8). To ensure the independence of instrumental variables and mitigate any bias caused by linkage disequilibrium, we conducted a linkage disequilibrium analysis using a threshold of (r2 = 0.001, kb = 5,000). The strength of each SNP as an instrumental variable was assessed through the F statistic (F > 10). Mendelian randomization pleiotropy residual sum and outlier (MR-PRESSO) were employed to identify and address outliers that may contribute to horizontal pleiotropy. Before evaluating the causal effects, we applied the Outlier-corrected method to remove outliers from the instrumental variable set and correct for potential horizontal pleiotropy.

The identified SNPs were then mapped to their respective genes and intersected with the differentially expressed genes (DEGs) to obtain intergenes. In single instrumental variable MR analysis, we utilized the Wald ratio. For scenarios involving two or more SNPs, we employed the inverse-variance-weighted (IVW) method. Egger’s regression and the weighted median methods were used as reference approaches. Sensitivity analysis was performed through leave-one-out analysis to assess the influence of individual SNPs on the association between intergenes and LUSC outcome. Horizontal pleiotropy was examined using the MR-Egger intercept, where a p-value greater than 0.05 suggests an absence of horizontal pleiotropy.

### Pathway and functional analysis

We employed the “Deseq2” package in R to identify differentially expressed genes (DEGs) between the high- and low-risk groups. Our DEG selection criteria were based on a log2 |fold change| > 1 and a false discovery rate < 0.05. To gain a deeper understanding of the biological functions and signaling pathways associated with these DEGs, we utilized various R packages including “clusterProfiler,” “org.Hs.eg.db,” and “enrichplot” to conduct an exploration of the Gene Ontology (GO) terms as well as the Kyoto Encyclopedia of Genes and Genomes (KEGG) pathways. By adopting this approach, we aimed to unravel the underlying molecular mechanisms and functional implications of the DEGs identified in our analysis.

### Estimation of tumor-infiltrating immune cells

To explore the relationship between the risk score and immune cell infiltration, we utilized the single-sample gene set enrichment analysis (ssGSEA) algorithm in R. By employing this approach, we were able to evaluate the levels of infiltration as well as the functional characteristics of immune cells within the tumor microenvironment. The outcomes of our analysis were then presented in the form of a visually informative heat map, allowing for a comprehensive visualization of the results. This methodology enabled us to gain valuable insights into the potential link between the risk score and immune cell infiltration, shedding light on the interplay between the tumor and the immune system.

### Statistical analysis

**Figure 1:**
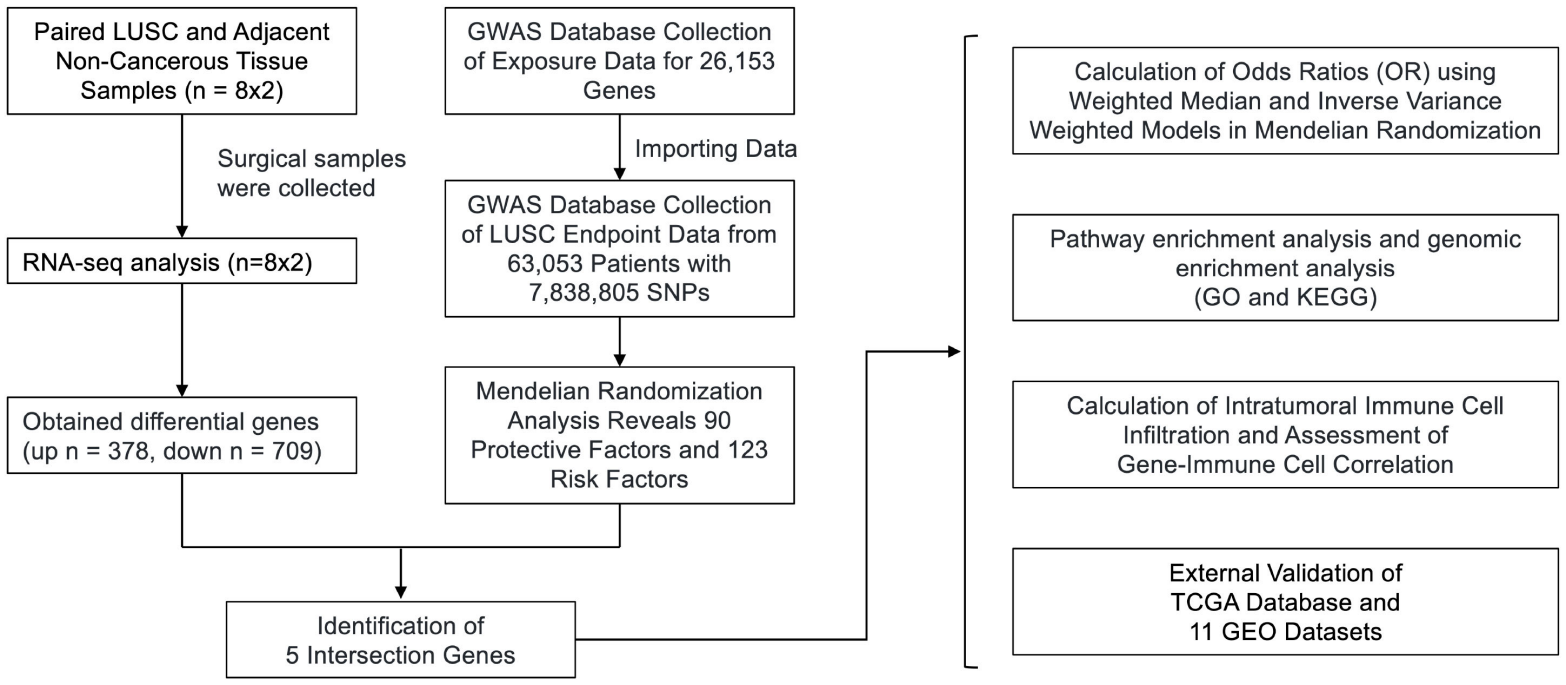
Flowchart of the study design. We obtained RNA-seq data from 8 surgical samples of LUSC and adjacent non-cancerous tissues from 3 centers. Using DESeq2, we identified 1088 differentially expressed genes with |LogFC| > 1 and p-value < 0.05. Additionally, MR analysis of Exposure Data for 26,153 Genes and 63,053 LUSC Patients with 7,838,805 SNPs as endpoints yielded 213 genes as potential exposure factors. After intersecting the results, we identified 5 differentially expressed genes, namely GYPE, PODXL2, RNF182, SIRPG, and WNT7A. PODXL2 was identified as an exposed risk factor, with p-values less than 0.01 under the inverse variance weighted model. GO and KEGG analyses revealed enhanced ubiquitin-protein transferase activity and activation of pathways such as the mTOR signaling pathway and Wnt signaling pathway. Immune infiltration analysis indicated downregulation of Plasma cells, T cells regulatory (Tregs), and Dendritic cells activated by the identified gene set, while an enhancement was observed in Macrophages M1. Furthermore, we externally validated the expression levels of these five genes using RNA-seq data from TCGA database and 11 GEO datasets of LUSC, and the results showed that SIRPG could induce LUSC.

**Figure 2:**
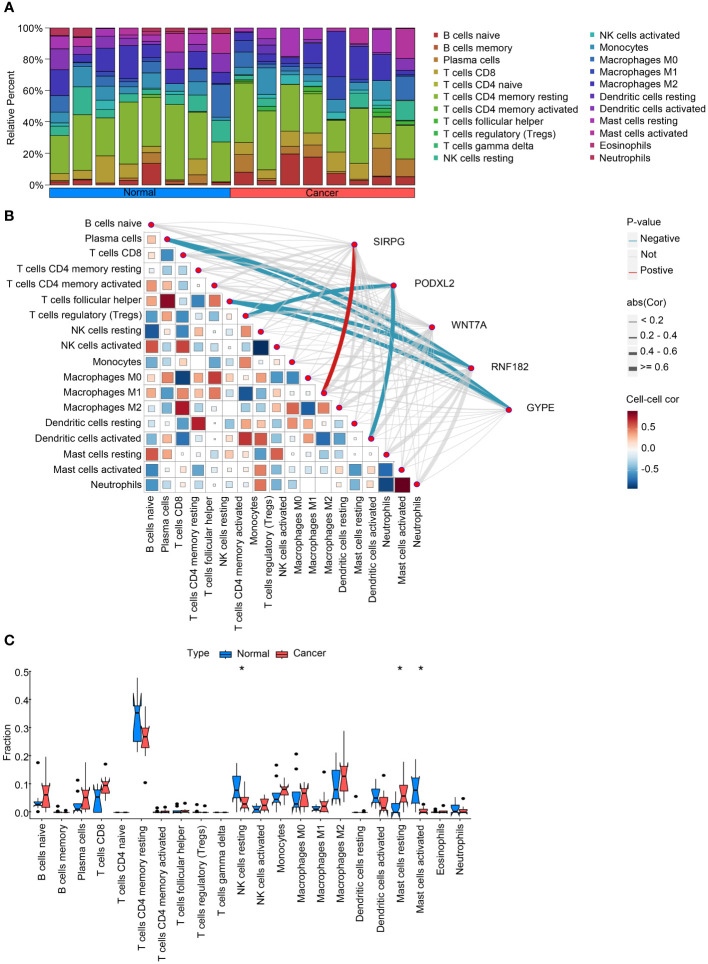
Analysis of intratumoral immune cell infiltration and correlation with intersecting genes. **(A)** provides a bar chart depicting the proportion of immune cell infiltration in both cancerous and adjacent non-cancerous samples, offering insights into the differences in immune cell composition between the two tissue types. **(B)** presents a heatmap illustrating the correlation between immune cell infiltration scores and the expression levels of intersecting genes, providing information on potential associations between gene expression and immune cell infiltration. **(C)** compares the expression levels of immune cell infiltration scores in cancerous and adjacent non-cancerous samples, highlighting any differences in immune cell infiltration patterns between the two tissue types. These analyses contribute to our understanding of the interplay between intratumoral immune cell infiltration and intersecting genes in the context of lung squamous cell carcinoma. *P<0.05.

**Figure 3:**
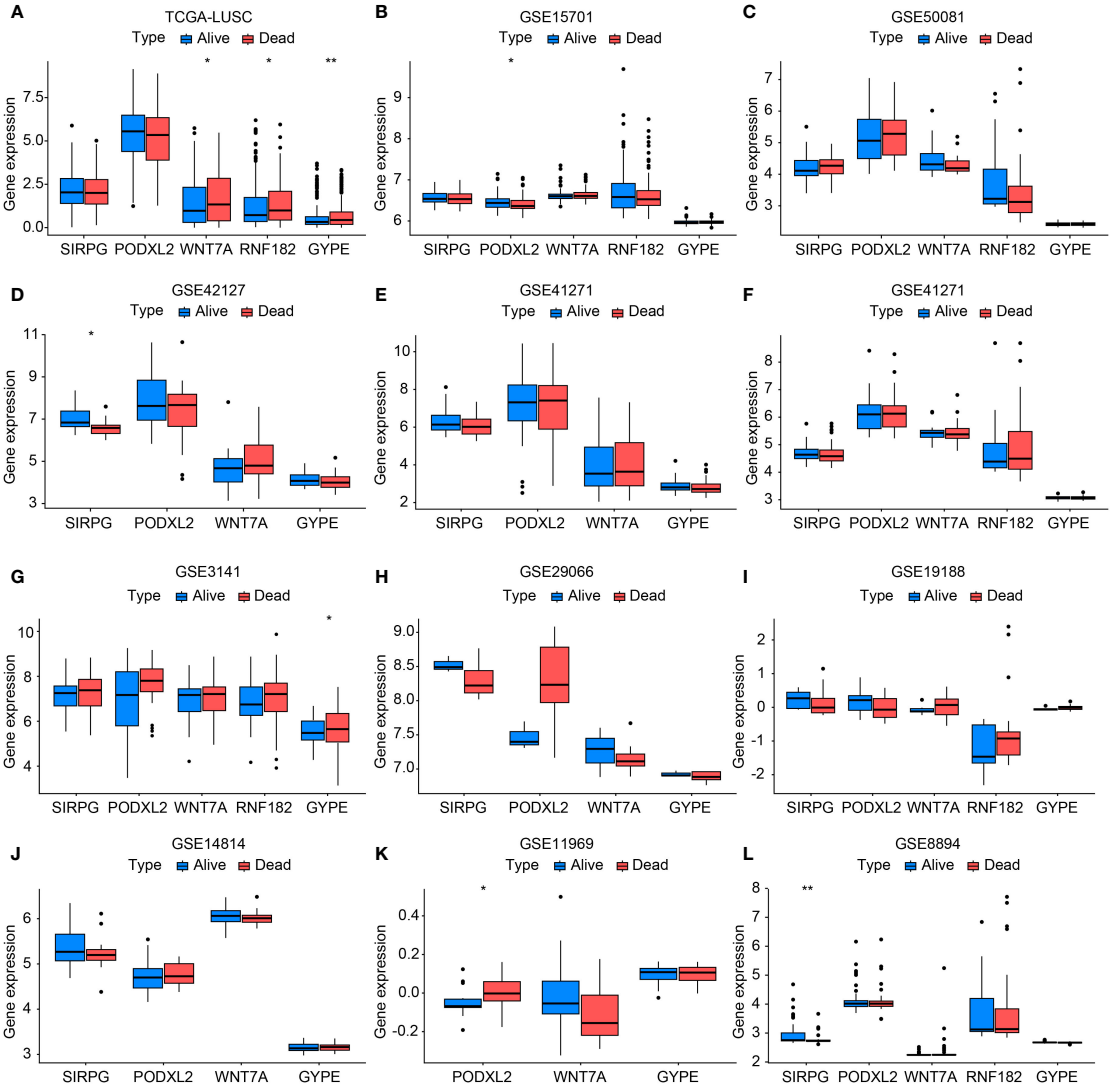
Bar chart validating the expression levels between TCGA-LUSC and RNA-seq data (GSE11969, GSE14814, GSE157011, GSE19188, GSE29066, GSE30219, GSE3141, GSE37745, GSE41271, GSE42127, GSE50081, and GSE8894) in terms of progression/death and survival groups **(A-L)**. In this bar chart, we compare the expression levels of the genes of interest between The Cancer Genome Atlas-Lung Squamous Cell Carcinoma (TCGA-LUSC) dataset and various RNA-seq datasets representing different clinical outcomes, including progression/death and survival groups. The comparison aims to validate the expression patterns observed in TCGA-LUSC across multiple independent datasets, providing robustness to our findings and enhancing the reliability of the identified gene signatures associated with disease progression and patient survival. Each bar represents the mean expression level of the genes in the respective dataset and clinical outcome group, with error bars indicating standard deviation or standard error where applicable. *P<0.05, **P<0.01.

Statistical power is set at 0.90. Forest plots were employed to present the significance of prognostic covariates. To perform functional enrichment analysis of Gene Ontology (GO) terms and Kyoto Encyclopedia of Genes and Genomes (KEGG) pathways, we utilized the “clusterProfiler” package. The generation of heat maps for cluster analysis was accomplished using the “Pheatmap” package. For the analysis of differences between two groups of quantitative data, we applied the Wilcoxon rank-sum test. In order to establish a prognostic risk model, statistical analyses were conducted using RStudio (R version 4.3.2) and a variety of R packages such as “rms,” “ggplot2,” “risk regression,” “PredictABLE,” and “survminer.” The logical sequence of the statistical analysis performed in the article is illustrated in **Figure 1**, as depicted in the flowchart. Through this comprehensive approach, we aimed to ensure robust statistical analysis while minimizing redundancy in our methodologies. We utilized the CIBERSORT package in R to calculate immune infiltration scores and estimate the abundance of different immune cell types in tumor tissues, such as infiltrating T cells, B cells, macrophages, etc. For the statistical analysis in **Figures 2C**, **3**, when comparing immune infiltration scores between different groups, we employed either the t-test or Wilcoxon rank-sum test, depending on the distribution of the data and assumptions of normality. These tests were chosen to assess the significance of differences in immune infiltration levels between experimental conditions.

## Results

### Clinical analysis

A total of 8 patients diagnosed with early-stage LUSC were included in this study, covering a period from August 3, 2021, to July 30, 2022. At the time of analysis, none of the patients had succumbed to mortality. However, disease progression was observed in 1 patient, accounting for 12.0% of the cohort. Among the participants, there were 5 male individuals, representing 62.0% of the sample. Additionally, 5 patients, or 62.0% of the group, were over the age of 65. It is worth noting that a history of smoking was reported by 6 individuals, constituting 75.0% of the cohort. Moreover, 5 patients (62.0%) exhibited an ECOG performance status score of 1. Surgical intervention was performed on all patients, with 6 individuals (75.0%) classified as stage I-II and the remaining 2 patients (25.0%) classified as stage III-IV, as stated in **Supplementary Table S1**.

### Integration analysis of DEGs from RNA-seq and Mendelian randomization for cross-gene identification

**Figure 4:**
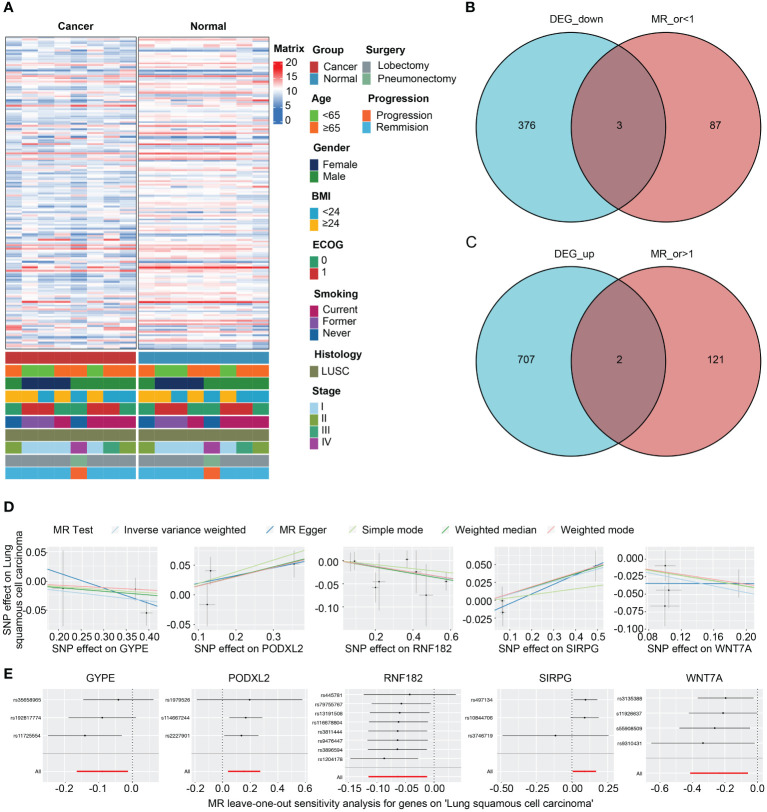
Intersection of DEGs from RNA-seq analysis and effective exposure genes from MR. **(A)** Integrated heatmap of 8 LUSC patients with cancer and adjacent non-cancerous tissues. **(B)** Venn diagrams of the intersection between DEGs upregulated and downregulated, as well as MR analysis. **(C)** Scatter plot showing the intersection of 5 genes, which were analyzed for single nucleotide polymorphism (SNP) Mendelian randomization in relation to the risk of LUSC (lung squamous cell carcinoma). **(D)** Leave one out plot demonstrates the influential outlier after excluding a specific SNP. Consistent evidence for a causal effect of the exposure gene on the risk of LUSC was found even when this variant was excluded from the analysis. Effect estimates are reported per standard deviation increase in the exposure variable, and error bars represent 95% confidence intervals.

**Figure 4A** presents the analysis of RNA-seq data from the patients, and a heatmap was created to visualize the expression levels of genes that exhibit differential expression. The findings indicate notable variances in gene expression levels between tumor tissue and adjacent non-tumor tissue in cases of squamous cell carcinoma of the lung. We initially utilized the Deseq2 package in R software, based on our previously established methods, to identify differentially expressed genes (DEGs) with |LogFc| > 1 and a p-value less than 0.05. We detected 709 up-regulated DEGs and 379 down-regulated DEGs. Furthermore, we screened 213 genes associated with the LUSC endpoint in the GWAS database. By conducting a cross-tabulation analysis between the two databases, we identified 3 down-regulated genes (WNT7A, RNF182, GYPE) in **Figure 4B** and 2 up-regulated genes (SIRPG, PODXL2) in **Figure 4C** that were common to both databases. The scatter plot displays the analysis of SIRPG and PODXL2 genes as risk factors, and WNT7A, RNF182, and GYPE genes as protective factors, in a Mendelian randomization analysis for the endpoint of LUSC patients (n = 63,053) (**Figure 4D**). Even after excluding the SNPs located within these genes from the analysis, consistent evidence of a causal effect between these genes and the risk of developing LUSC was observed (**Figure 4E**). In addition, we conducted sensitivity analysis on all included genes. Please refer to the **Supplementary Materials** for detailed information. The reported effect estimate represents the effect of an increase in exposure level by one standard deviation (SD), and the error bars represent the 95% confidence interval. We also plotted the MR (Mendelian Randomization) effect size of the five genes under the IVW (Inverse Variance Weighted) model, and generated a funnel plot for the IVW analysis, demonstrating a balanced distribution of variables (**Supplementary Figures S1A, B**).

**Figure 5:**
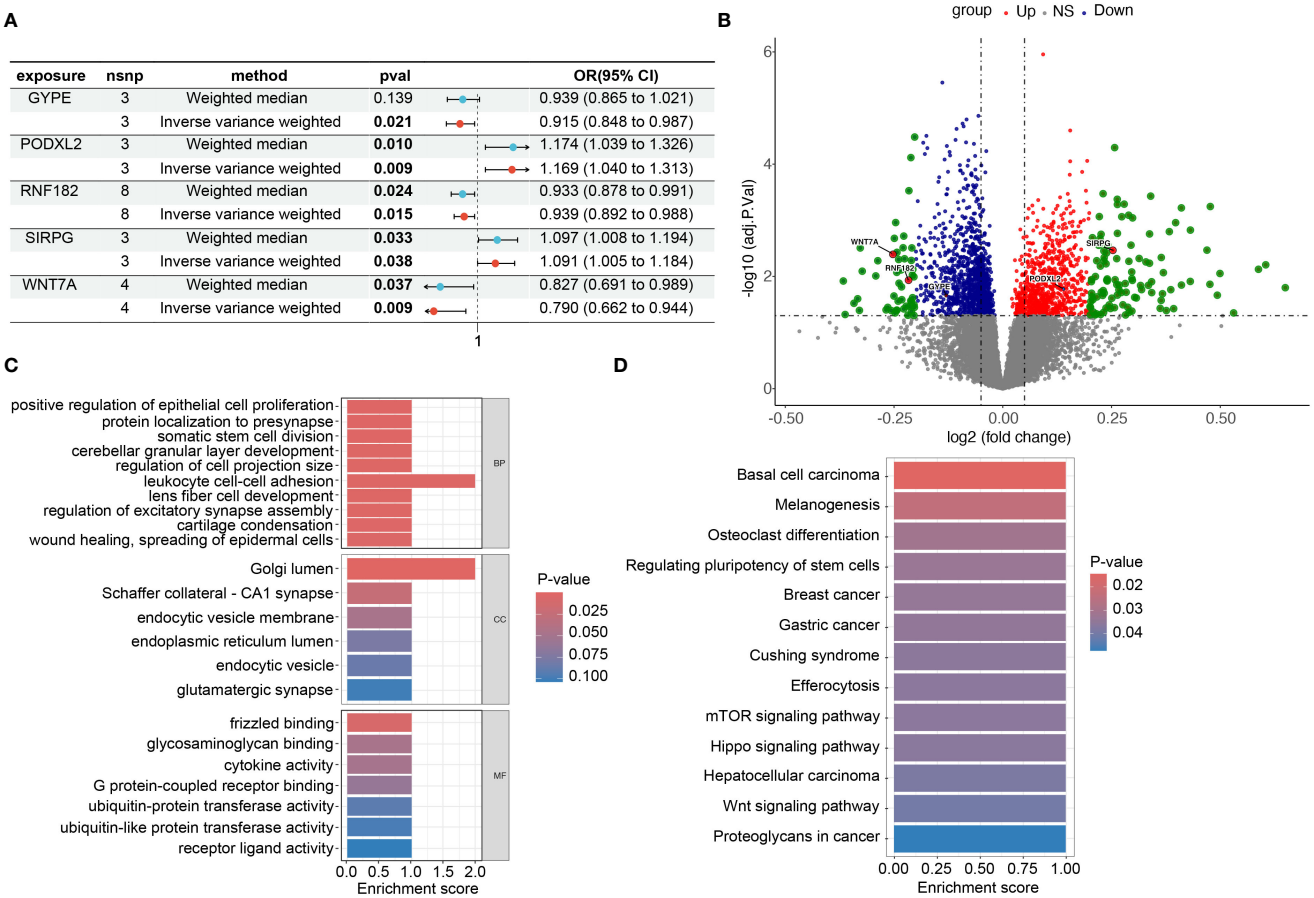
Risk and omics analysis of intersecting genes. **(A)** It shows forest plots of the risk estimates for the five intersecting genes obtained using both the Mendelian Randomization-Egger (ME) and Inverse Variance-Weighted (IVW) models. **(B)** volcano plot to illustrate the Differentially Expressed Genes (DEG), specifically highlighting the positions of the chosen genes. This plot, labeled as **Figures 3B** in our manuscript. Each point on the plot represents a gene, with the chosen genes ‘GYPE’, ‘PODXL2’, ‘RNF182’, ‘SIRPG’, and ‘WNT7A’ indicated and labeled accordingly. **(C, D)** display barplots representing the enriched Gene Ontology (GO) terms and Kyoto Encyclopedia of Genes and Genomes (KEGG) terms, respectively. These analyses provide insights into the potential biological functions and pathways associated with the intersecting genes identified in our study.

We constructed forest plots for the OR values calculated by two Mendelian randomization models, Weighted Median (WM) and Inverse Variance Weighted (IVW), for these five genes. The results showed that SIRPG (WM: OR(95%CI)=1.097 (1.008 to 1.194), p=0.033; IVW: OR(95%CI)=1.091 (1.005 to 1.184), p=0.038) and PODXL2 (WM: OR(95%CI)=1.174 (1.039 to 1.326), p=0.010; IVW: OR(95%CI)=1.169 (1.040 to 1.313), p=0.009), WNT7A (WM: OR(95%CI)=0.827 (0.691 to 0.989), p=0.037; IVW: OR(95%CI)=0.790 (0.662 to 0.944), p=0.009), RNF182 (WM: OR(95%CI)=0.933 (0.878 to 0.991), p=0.024; IVW: OR(95%CI)=0.939 (0.892 to 0.988), p=0.015), and GYPE (WM: OR(95%CI)=0.939 (0.865 to 1.021), p=0.139; IVW: OR(95%CI)=0.915 (0.848 to 0.987), p=0.021) were associated with tumor risk based on their differential expression patterns. (**Figure 5A**). SIRPG was detected by qPCR and the level was increased in the LUSC group (**Supplementary Figure S1E**).

### Pathway enrichment analysis and genomic enrichment analysis

To investigate the biological functions and pathways related to these 5 differentially expressed genes (DEGs) (**Figure 5B**), we initially presented their chromosomal locations (**Supplementary Figure S4**) and conducted enrichment analyses using Gene Ontology (GO) and Kyoto Encyclopedia of Genes and Genomes (KEGG). In terms of biological processes, the DEGs were significantly enriched in various categories, including cerebellar granular layer development, lens fiber cell development, and protein localization to the presynapse. Within the cellular component category, enrichment was observed in specific areas such as Schaffer collateral - CA1 synapse, endocytic vesicle membrane, and glutamatergic synapse. In relation to molecular function, the DEGs exhibited significant enrichment in diverse functions like glycosaminoglycan binding, G protein-coupled receptor binding, and ubiquitin-protein transferase activity (**Figure 5C**). Furthermore, the KEGG analysis revealed associations with multiple signaling pathways, including the mTOR signaling pathway, Hippo signaling pathway, and Wnt signaling pathway (**Figure 5D**). Through this comprehensive analysis, we aimed to gain insights into the potential biological roles and underlying pathways associated with these DEGs while minimizing repetition in the description of our findings. In addition, we grouped these five genes based on their expression levels and utilized the GSEA (Gene Set Enrichment Analysis) method to analyze the pathways affected by upregulation and downregulation of gene expression. We found that GYPE, PODXL2, and WNT7A all have regulatory effects on the JAK-STAT pathway (**Supplementary Figure S1C**). Through our analysis in Reactome, we have identified potential interactions between SIRPG and CD47.

### Prediction of intratumoral immune cell infiltration and immunotherapy response

To investigate the disparities among these gene modifications, we conducted GSVA analysis and immune infiltration analysis for each patient. The overall findings demonstrated a decrease in T cell activity and an increase in B cell activity within cancerous tissues (**Figure 2A**). Moreover, we performed correlation analysis between these five genes and pertinent immune cells. The outcomes revealed that RNF182 and GYPE suppressed the function of T cells follicular helper and Plasma cells, as well as T cells regulatory (Tregs) and Dendritic cells. Furthermore, SIRPG stimulated the activity of Macrophages M1 (**Figure 2B**). Furthermore, bar graphs were utilized to compare the immune cell scores between tumor and adjacent tissues. The results indicated an augmented activity of NK cells and Mast cells within the LUSC group (**Figure 2C**).

### External validation of TCGA and GEO databases

For external validation, we obtained a total of 501 samples from patients diagnosed with LUSC from the TCGA database on Dec 23, 2023. Additionally, we collected an additional 803 cases of squamous cell carcinoma of the lung from the GEO database. **Supplementary Table S2** displayed specific details of the data information. The results showed that the expression levels of these five genes in the progression/death group and survival group of lung cancer patients were consistent with the overall trend observed in our center’s RNA-seq sequencing. Among them, TCGA-LUSC, GSE15701, GSE42127, GSE3141, GSE11969, and GSE8894 databases exhibited statistically significant gene expressions (**Figures 3A–L**).

## Discussion

In this study conducted across multiple centers, we collected RNA-seq data from 8 surgical samples of LUSC and adjacent non-cancerous tissues. Differential gene expression analysis using Deseq2 revealed 1088 genes that exhibited significant differences (|LogFC| > 1, p-value < 0.05). Additionally, MR analysis using a large dataset of exposure data and LUSC patient information identified 213 potential exposure factors.

Further analysis by intersecting the results highlighted five differentially expressed genes: GYPE, PODXL2, RNF182, SIRPG, and WNT7A. Notably, PODXL2 was identified as an exposed risk factor associated with LUSC progression [OR 95% CI, 1.169 (1.040 to 1.313)], demonstrating statistical significance under the inverse variance weighted model (p < 0.01). Functional analyses, including GO and KEGG, uncovered pathways such as the mTOR signaling pathway and Wnt signaling pathway, along with enhanced ubiquitin-protein transferase activity.

Based on the above results, SIRPG emerged as a significant risk factor for LUSC. Immune infiltration analysis highlighted the role of Macrophages M1 and the mTOR signaling pathway in LUSC. These findings suggest that immune cell infiltration patterns may have prognostic significance in LUSC patients. Furthermore, the study results may have implications for predicting the response of LUSC patients to immunotherapy or other targeted treatments.

Moreover, immune infiltration analysis revealed downregulation of Plasma cells, T cells regulatory (Tregs), and Dendritic cells associated with the identified gene set, while Macrophages M1 exhibited enhanced infiltration.

To validate our findings, we performed external validation using RNA-seq data from the TCGA database and 11 GEO datasets of LUSC, which consistently supported our initial results.

Mendelian Randomization (MR) is a powerful analytical method used in genetic epidemiology. It leverages genetic variants, which are determined randomly and fixed at conception, as instrumental variables to study the impact of modifiable factors on health and disease outcomes (15, 25). The increasing popularity of genome-wide association studies (GWAS) has facilitated the broader adoption of MR, offering several advantages that help overcome limitations associated with observational studies in nutritional epidemiology. By utilizing genetic markers that are strongly associated with exposures, MR allows for the examination of causal relationships between these exposures and health or disease outcomes (26, 27). One key strength of MR is its ability to mitigate confounding biases caused by behavioral or environmental exposures and reverse causation (28). By using genetic variants as proxies for exposures, MR provides a more reliable estimate of the true effects of these exposures on health outcomes. This approach offers valuable insights into the potential impact of modifiable factors on disease risk and can inform public health strategies and interventions.

Our study utilized Mendelian Randomization analysis to identify 213 genes and intersect them with the differentially expressed genes in our center’s tissue samples, resulting in the identification of effective differential genes associated with lung squamous cell carcinoma (LUSC). Subsequent GSEA analysis based on these findings revealed multiple genes that are involved in regulating the JAK-STAT pathway. Numerous studies have demonstrated the crucial involvement of JAK/STAT1, JAK/STAT3, and JAK/STAT5 signaling pathways in tumor progression. Aberrant expression and genetic mutations of JAK family members have been linked to the development and occurrence of lung cancer. Notably, Xu et al. (29) observed upregulation of JAK2 gene expression in tumor tissues, which significantly correlated with lymph node metastasis. Elevated JAK2 expression was found to enhance tumor cell proliferation, metastasis, and invasion, while its downregulation yielded opposing effects. Additionally, JAK2 gene mutations have been identified in LUSC, suggesting an association between JAK2 mutations and lung cancer progression, as well as poor prognosis and drug resistance. Another investigation (30) highlighted the relationship between JAK2/JAK3 mutations in lung cancer and the expression of programmed cell death ligand-1 (PD-L1), implying potential benefits of immunotherapy for patients harboring JAK3 gene mutations. Furthermore, NSCLC patients exhibited significantly increased phosphorylation levels of JAK1, and its high expression correlated with unfavorable prognosis, emphasizing the utility of phosphorylated JAK1 as a predictive marker for NSCLC treatment (31). Our discovery of intersecting genes that modulate the JAK-STAT pathway suggests their potential as therapeutic targets.

Our research also revealed that the intersecting genes obtained showed a strong correlation with immune infiltration scores in the samples. Specifically, these genes were found to have a significant regulatory effect on Regulatory T cells (Tregs), Macrophages M1 and plasma cells. Tregs are a type of immune inhibitory cell population present in the tumor microenvironment (TME). They express CD4 and the transcription factor forkhead box protein P3 (FoxP3) and play a crucial role in maintaining immune homeostasis by regulating peripheral tolerance and suppressing autoimmune diseases through diverse immunosuppressive mechanisms (32, 33). Studies using mouse models have shown that Tregs can effectively inhibit the response of antitumor effector T cells and impede the elimination of tumors by endogenous tumor-specific effector T cells (34, 35). In various murine and human cancers, the proportion of Tregs among tumor-infiltrating lymphocytes (TILs) is generally higher compared to normal tissues and blood. Additionally, recent research has linked increased infiltration of Tregs expressing specific genes to a poor prognosis in non-small cell lung cancer (NSCLC) (36, 37). Despite multiple attempts, clinical approaches aimed at selectively depleting or inhibiting Tregs in tumors have not been successful, potentially due to the lack of Treg specificity or the inability to target functionally relevant subsets of Tregs. Therefore, gaining a comprehensive understanding of the phenotype and regulatory gene expression of Tregs within tumor-infiltrating lymphocytes (TIL-Tregs) is critical for developing effective immunotherapy strategies.

Macrophages can exhibit different phenotypes, including classically activated type 1 (M1) macrophages and alternatively activated type 2 (M2) macrophages. M1 macrophages are polarized by lipopolysaccharide (LPS), interferon-gamma (IFN-γ), or tumor necrosis factor (TNF), leading to the secretion of pro-inflammatory cytokines and the ability to eliminate microorganisms and tumor cells. Notabl (38, 39)y, Dong et al. demonstrated that gamma-aminobutyric acid (GABA), a neurotransmitter commonly found in tumors, promotes the polarization of M2 macrophages through JAK1/STAT6 activation while inhibiting M1 polarization via JAK2/STAT3 inhibition, thus promoting tumor progression. Furthermore, GABA enhances tumor neovascularization by upregulating the expression of FGF2 in macrophages (40). Our findings also suggest that both M1 macrophages and the JAK-STAT pathway play regulatory roles in lung squamous cell carcinoma.

SIRPG plays a crucial role in regulating the progression of lung cancer. It has been shown that SIRPG can modulate the activation and function of macrophages by binding to its ligand. Specifically, the binding of SIRPG to its ligand promotes the differentiation of macrophages into the M1 phenotype. M1 macrophages exhibit pro-inflammatory and anti-tumor effects, playing a significant role in inhibiting tumor growth and metastasis in lung cancer. These macrophages release various cytokines and effector molecules such as IL-12, TNF-α, and NO, which suppress tumor cell proliferation, induce apoptosis, and activate other anti-tumor immune cells. Furthermore, SIRPG can also influence the development of lung cancer by regulating inflammation and immune cell infiltration. It may regulate the production of inflammatory factors and chemokines in the tumor microenvironment, as well as the migration and infiltration of immune cells, thereby impacting tumor growth and metastasis. It is important to note that although there have been studies on the role of SIRPG in regulating M1 macrophages in lung cancer, this field is still evolving, and further research is needed to fully understand the specific mechanisms involved (41). Moreover, this study also demonstrated that the expression of this gene is upregulated in lung cancer. Therefore, inhibitors targeting this gene could potentially serve as targeted therapeutic agents for lung cancer (41).

**Figure 6:**
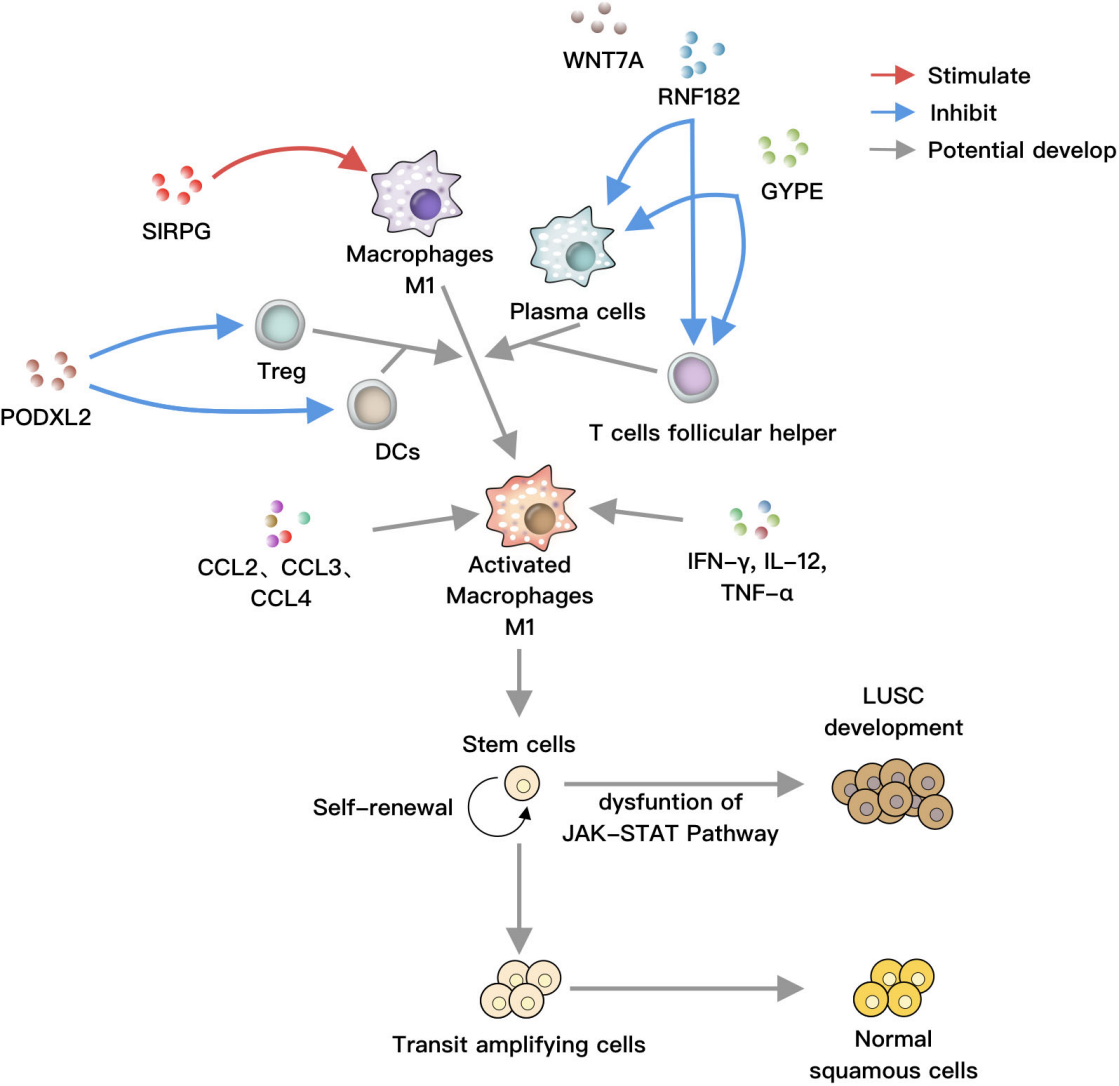
SIRPG positively regulates M1 macrophages, while RNF182 and GYPE negatively regulate plasma cells and T helper cells, respectively. PODXL2 negatively modulates T regulatory cells and dendritic cells (DCs). These cells can collectively act on M1 macrophages, which are activated by soluble factors such as IFN-γ, IL-12, TNF-α, as well as chemokines like CCL2, CCL3, and CCL4. Under normal circumstances, stem cells undergo self-renewal, proliferation, and differentiation into bronchial epithelial cells. However, under the influence of activated M1 macrophages, the JAK-STAT pathway is disrupted, leading to the differentiation of stem cells towards LUSC, ultimately resulting in lung cancer.

Based on our study results and previous literature, we hypothesize the following: SIRPG activates M1 macrophages, while RNF182 and GYPE inhibit plasma cells and T helper cells. PODXL2 suppresses Treg and DCs. These cells, along with soluble factors and chemokines, disrupt the JAK-STAT pathway, promoting LUSC formation (**Figure 6**).

The methods of MR also have their limitations. Hardware constraints: MR devices require powerful processors and batteries, which limits their portability and introduces concerns about weight and comfort. Dependency on external sensors: Many MR systems rely on external cameras or sensors to track movement and position, adding complexity and potentially limiting the user’s freedom of movement. Interaction challenges: Interacting with virtual objects in MR environments can be challenging, as current input methods are not as intuitive or precise as physical interactions. Limited content availability: Despite advancements, there is still a limited library of MR content available, which restricts the variety and range of experiences users can have. Therefore, we need more clinical real-world data for validation.

## Conclusion

In conclusion, our study on early-stage LUSC identified differentially expressed genes and potential exposure factors associated with tumor progression. Among the five highlighted genes, SIRPG was found to be an exposed risk factor for LUSC. Functional analysis revealed relevant signaling pathways and enriched activities. Immune infiltration analysis indicated alterations in various immune cell populations. External validation using multiple datasets consistently supported our findings. These results provide valuable insights into the molecular mechanisms and potential therapeutic targets in LUSC.

## Data availability statement

The datasets generated and/or analyzed during the current study are publicly available in the Gene Expression Omnibus (GEO) repository. The data can be accessed at the following link: https://www.ncbi.nlm.nih.gov/geo/query/acc.cgi?acc=GSE268175, with the accession number GSE268175.

## Ethics statement

The studies involving humans were approved by Ethics Committee of Soochow University. The studies were conducted in accordance with the local legislation and institutional requirements. Written informed consent for participation in this study was provided by the participants’ legal guardians/next of kin. Written informed consent was obtained from the individual(s), and minor(s)’ legal guardian/next of kin, for the publication of any potentially identifiable images or data included in this article.

## Author contributions

GM: Data curation, Formal analysis, Methodology, Writing – original draft. JL: Data curation, Formal analysis, Methodology, Project administration, Resources, Visualization, Writing – original draft. NW: Formal analysis, Methodology, Project administration, Validation, Writing – original draft. HY: Data curation, Methodology, Resources, Visualization, Writing – original draft. SH: Formal analysis, Project administration, Writing – original draft. MX: Project administration, Resources, Writing – original draft. HZ: Methodology, Project administration, Validation, Writing – original draft. DZ: Methodology, Project administration, Validation, Visualization, Writing – review & editing. JJ: Conceptualization, Formal analysis, Funding acquisition, Investigation, Resources, Supervision, Visualization, Writing – original draft, Writing – review & editing. HM: Formal analysis, Project administration, Resources, Supervision, Writing – review & editing.
