# Supplementary figures and images for: SIRPG promotes lung squamous cell carcinoma pathogenesis via M1 macrophages: a multi-omics study integrating data and Mendelian randomization
Source: Front Oncol. 2024 Jun 4;14:1392417. doi: 10.3389/fonc.2024.1392417 (PMC11183323; doi:10.3389/fonc.2024.1392417)

Figure S1  
A

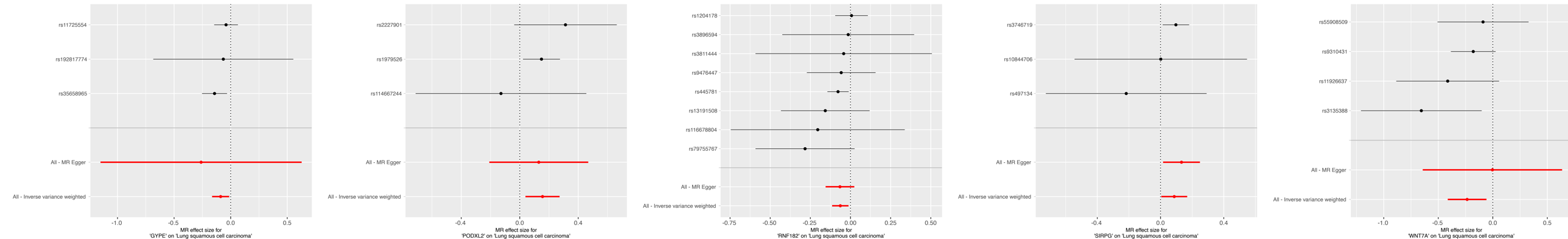

B

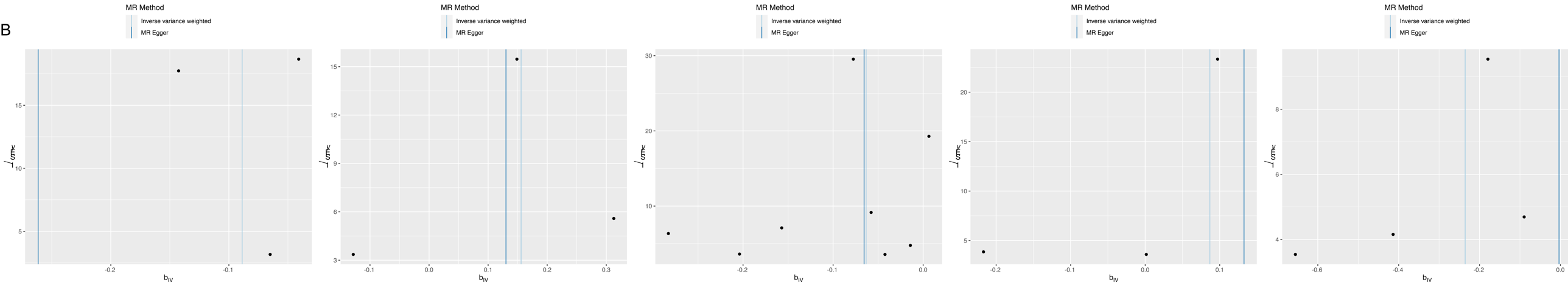

C

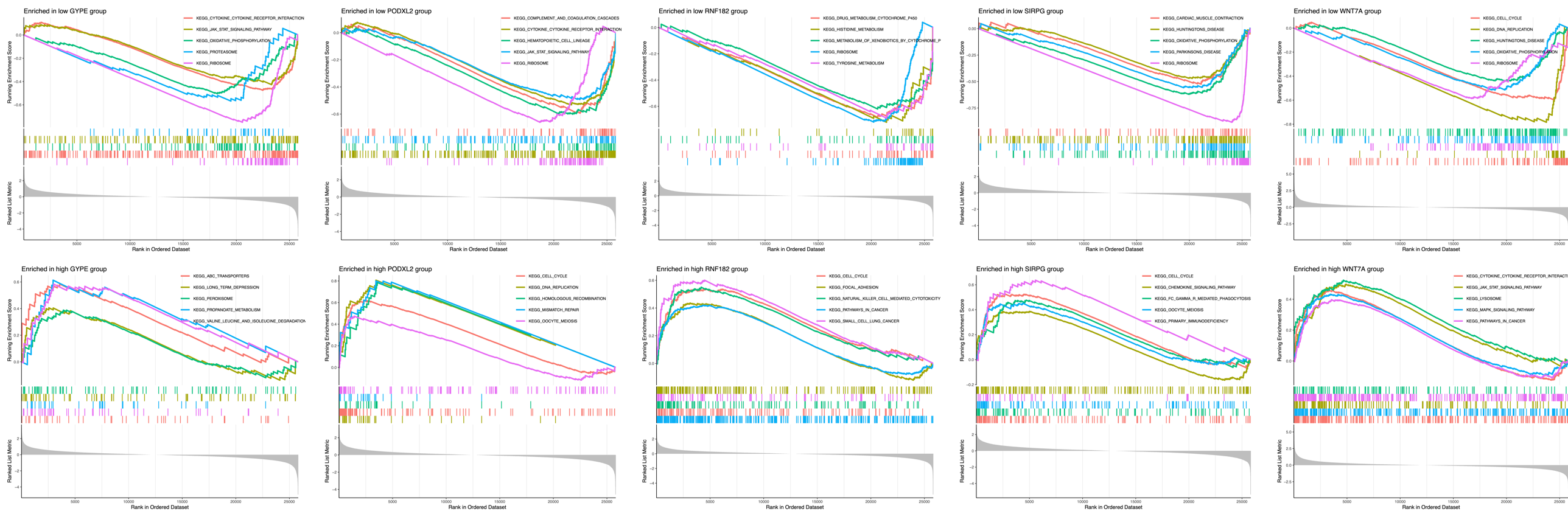

D

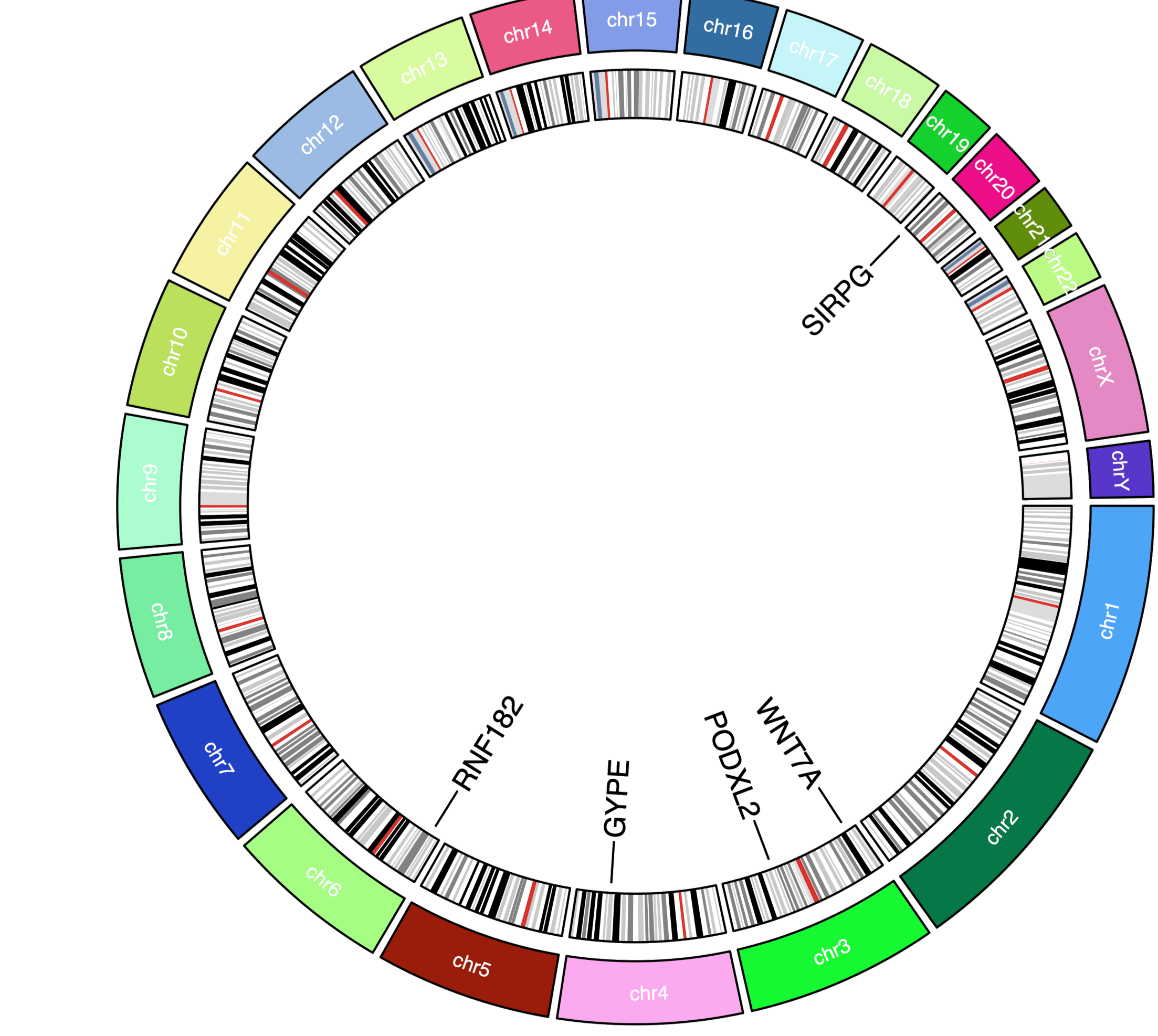

E

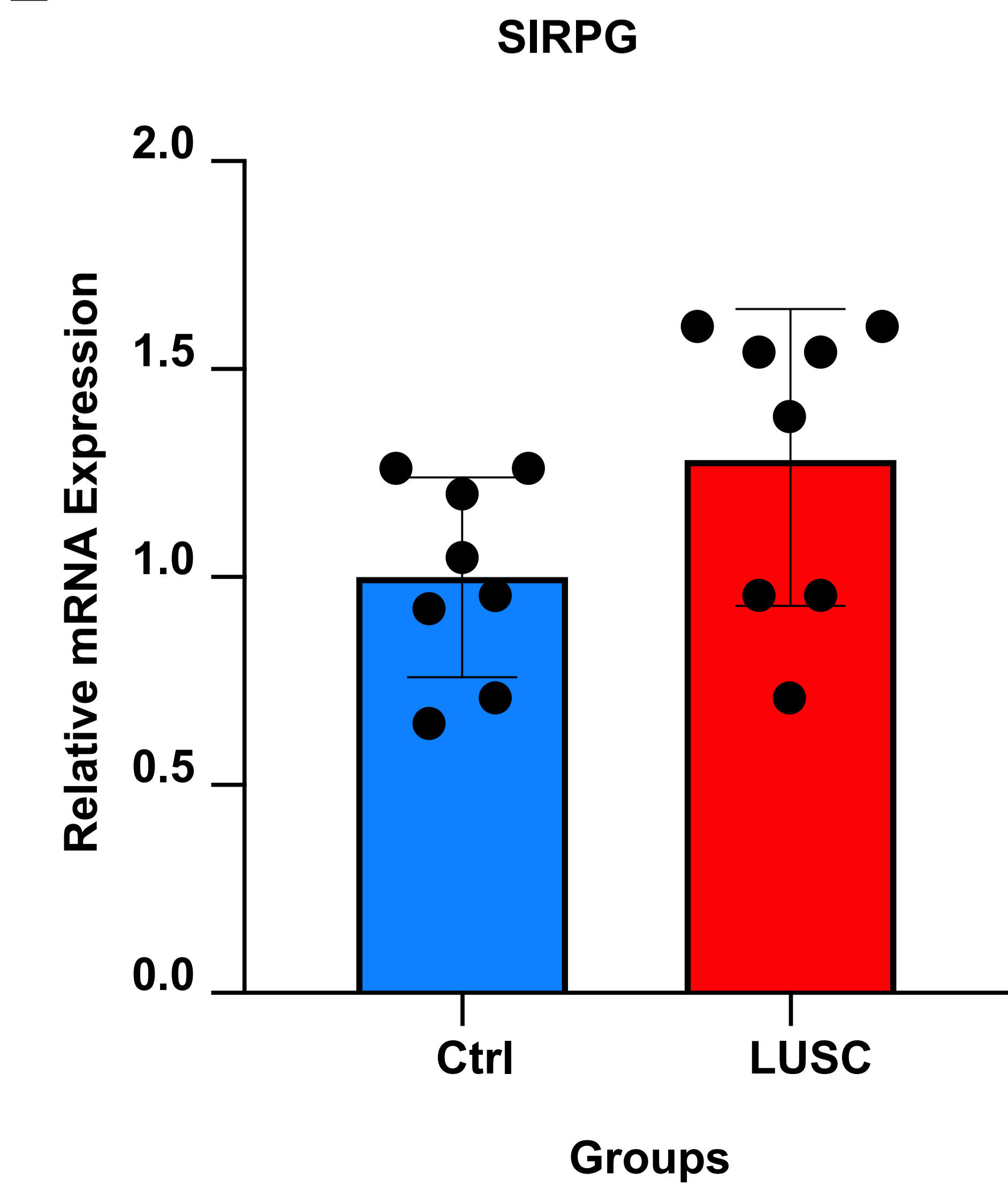

Supplement: Supplementary file 1 [file DataSheet_1.pdf]
