# Supplementary material for: SIRPG promotes lung squamous cell carcinoma pathogenesis via M1 macrophages: a multi-omics study integrating data and Mendelian randomization
Source: Front Oncol. 2024 Jun 4;14:1392417. doi: 10.3389/fonc.2024.1392417 (PMC11183323; doi:10.3389/fonc.2024.1392417)
Supplement: Supplementary file 2 [file DataSheet_2.pdf]

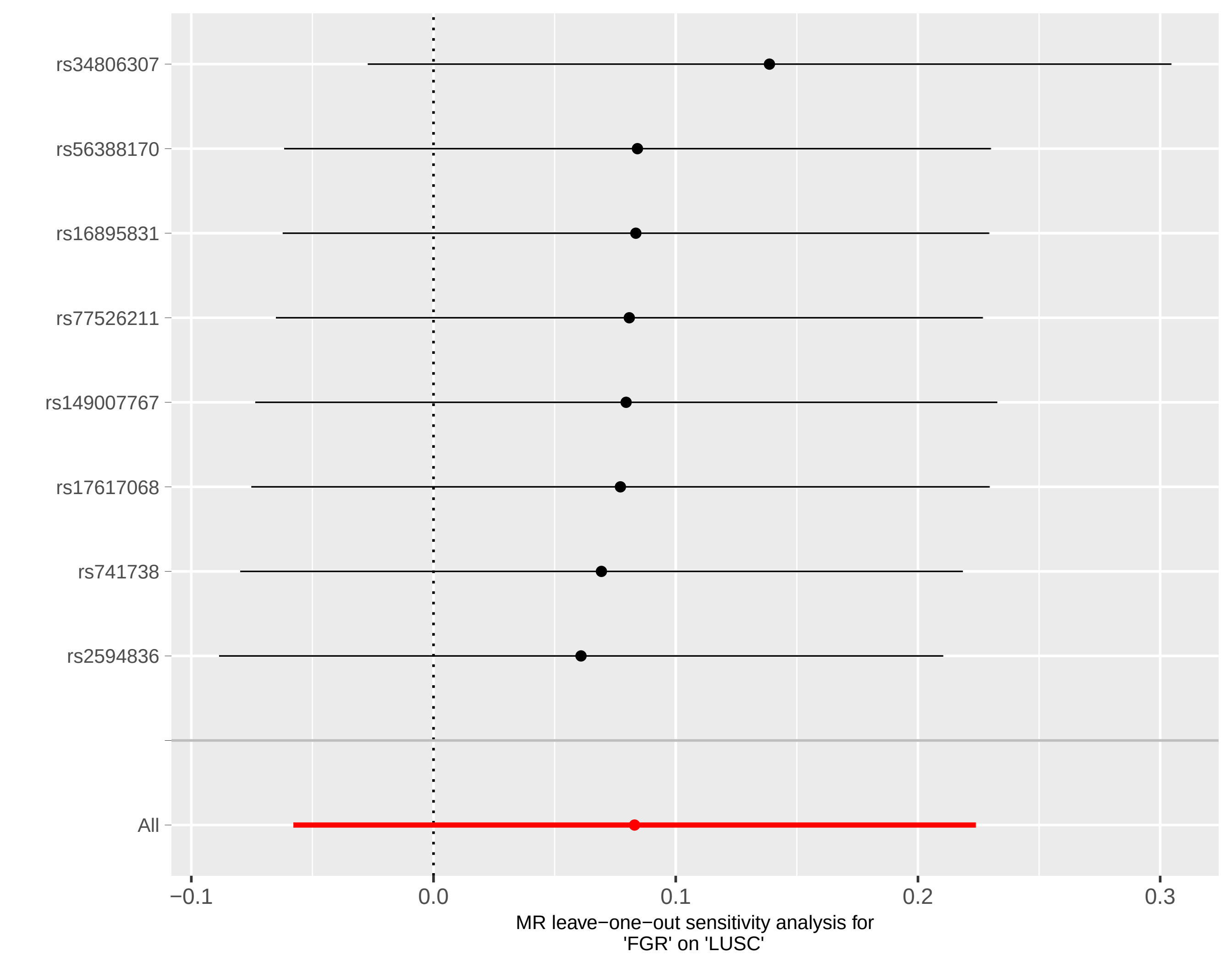
rs34806307
rs56388170
rs16895831
rs77526211
rs149007767
rs17617068
rs741738
rs2594836
All
−0.1
0.0
0.1
0.2
0.3
MR leave−one−out sensitivity analysis for
'FGR' on 'LUSC'

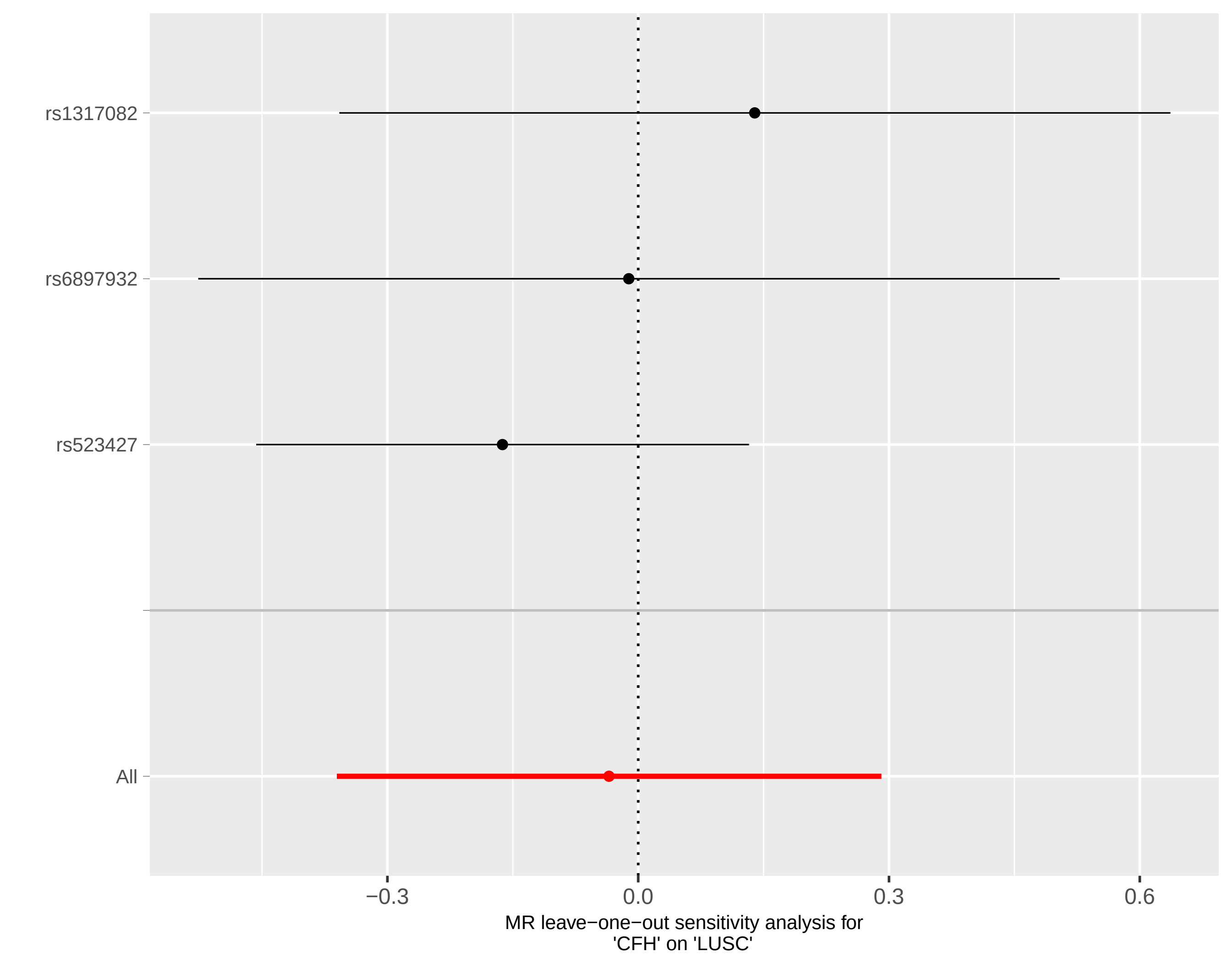
rs1317082
rs6897932
rs523427
All
−0.3
0.0
0.3
0.6
MR leave−one−out sensitivity analysis for
'CFH' on 'LUSC'

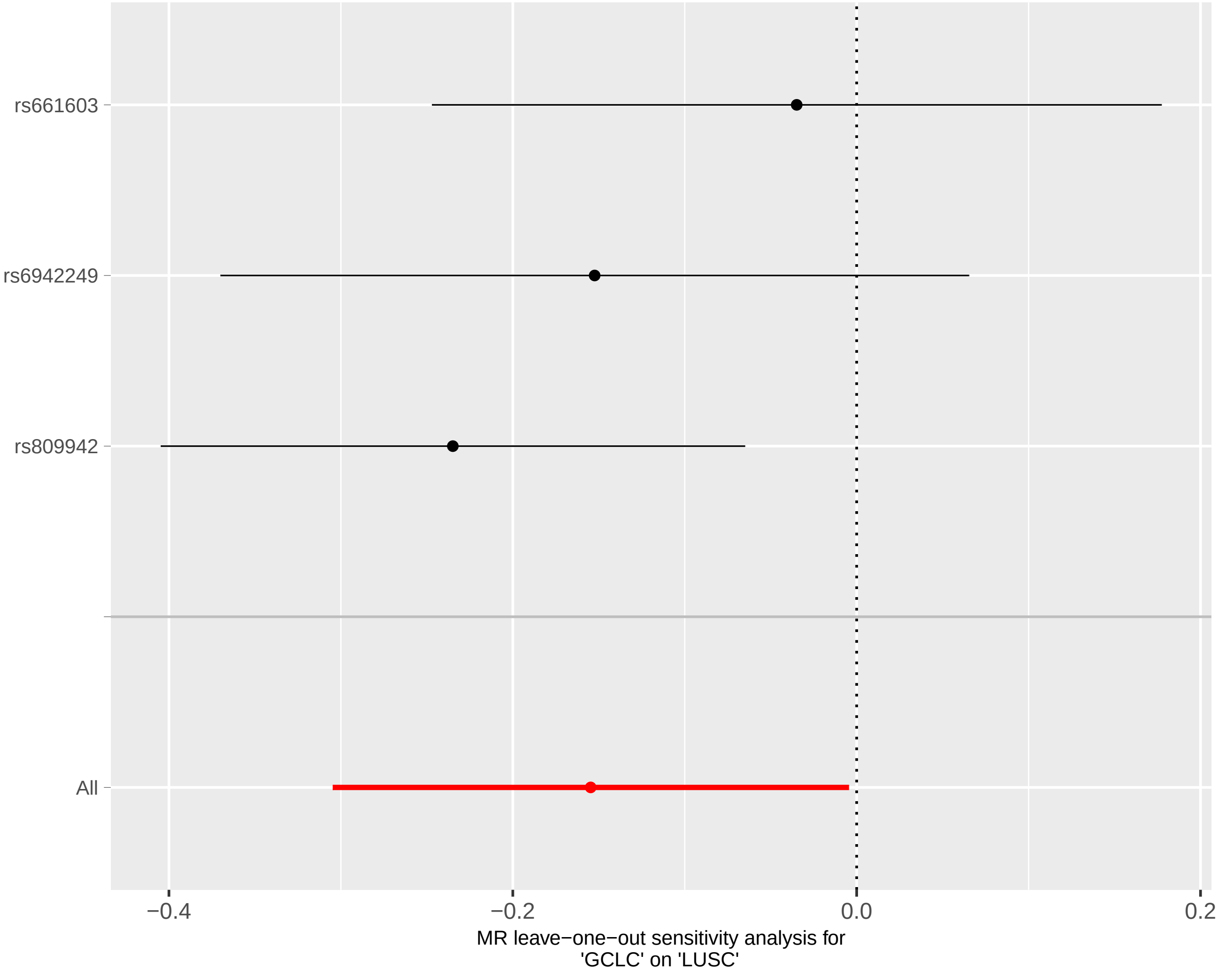
rs661603
rs6942249
rs809942
All
−0.4
−0.2
0.0
0.2
MR leave−one−out sensitivity analysis for
'GCLC' on 'LUSC'

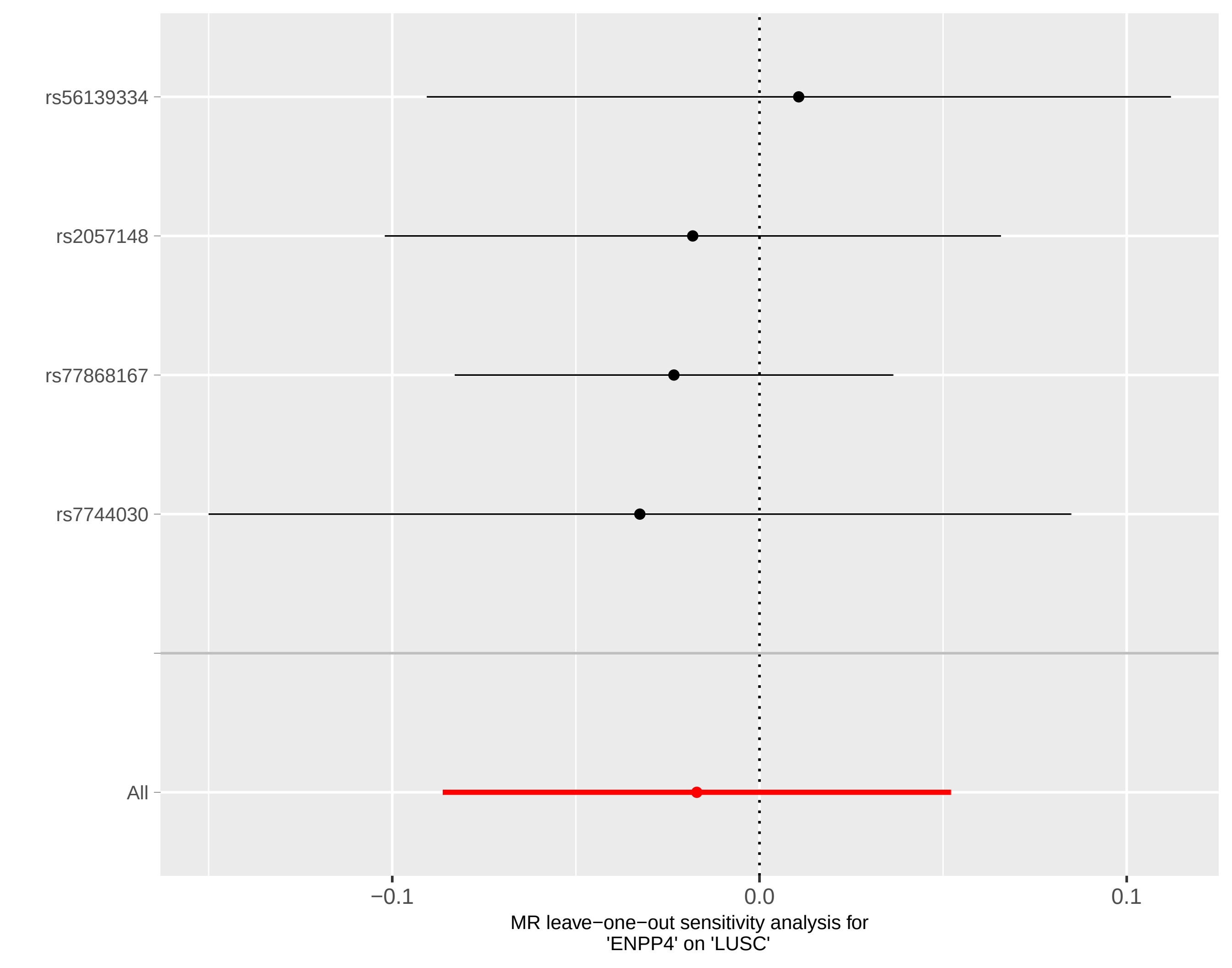

rs56139334
rs2057148
rs77868167
rs7744030
All
−0.1
0.0
0.1
MR leave−one−out sensitivity analysis for
'ENPP4' on 'LUSC'

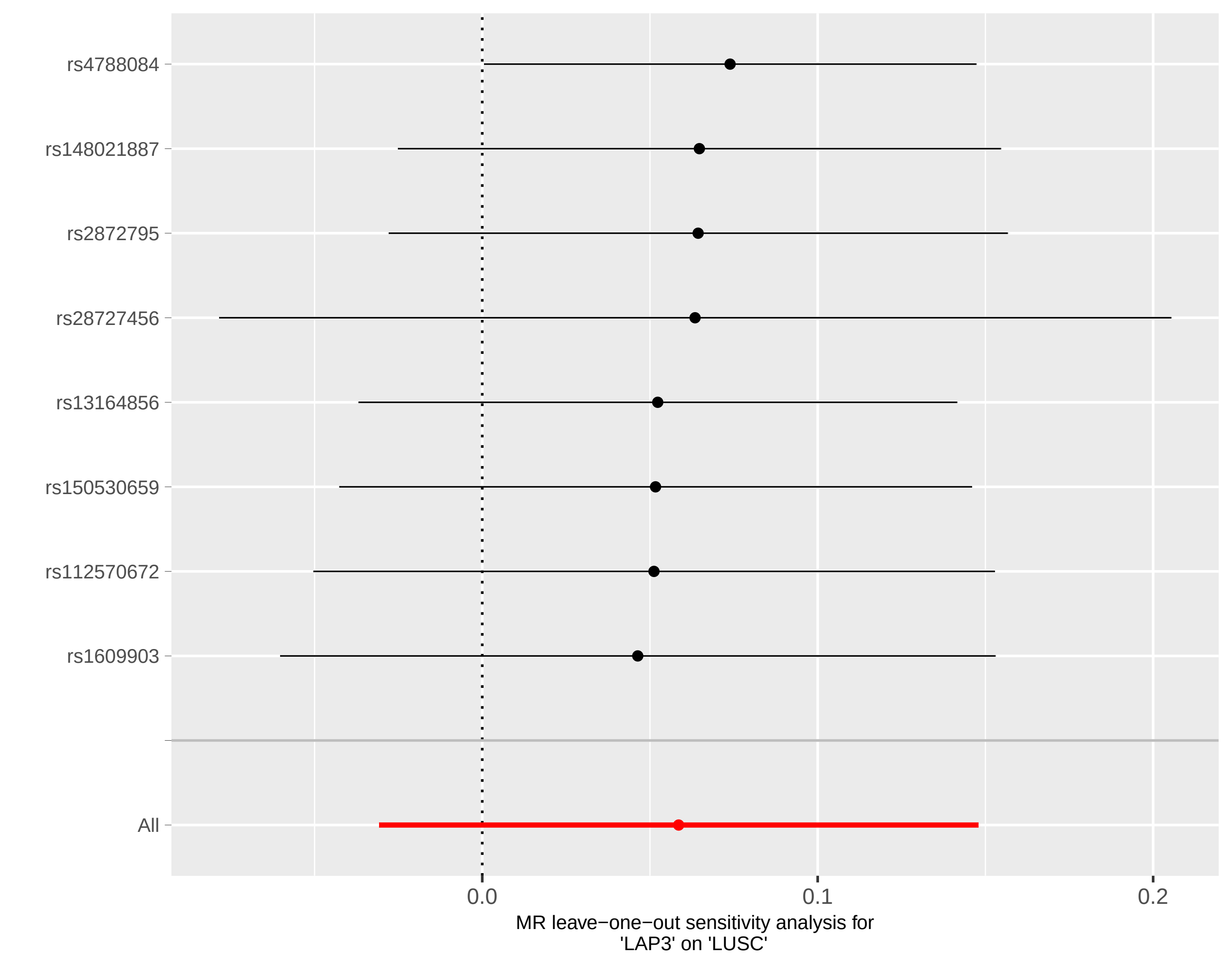
rs4788084
rs148021887
rs2872795
rs28727456
rs13164856
rs150530659
rs112570672
rs1609903
All
0.0
0.1
0.2
MR leave−one−out sensitivity analysis for
'LAP3' on 'LUSC'

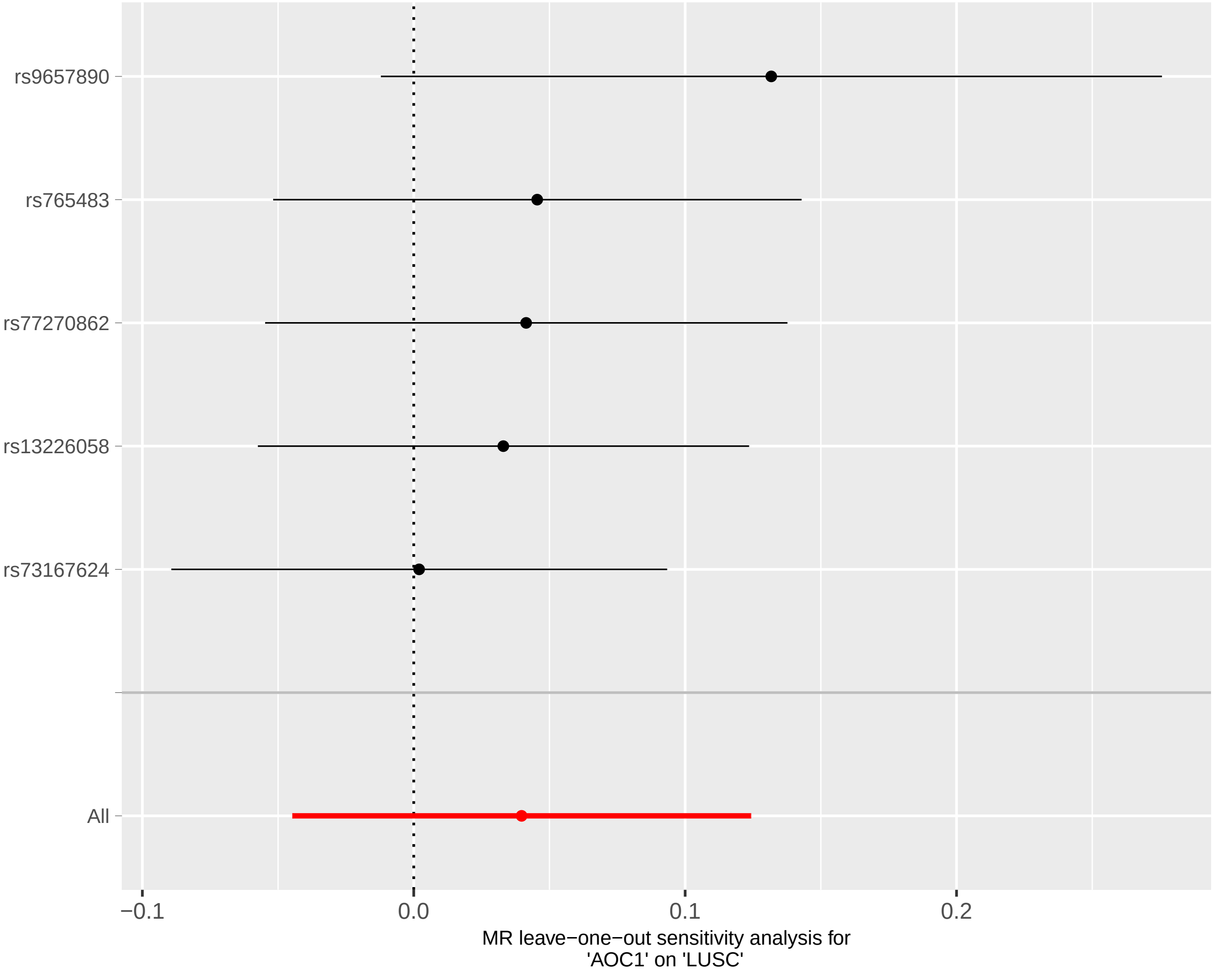

rs9657890
rs765483
rs77270862
rs13226058
rs73167624
All
−0.1
0.0
0.1
0.2
MR leave−one−out sensitivity analysis for
'AOC1' on 'LUSC'

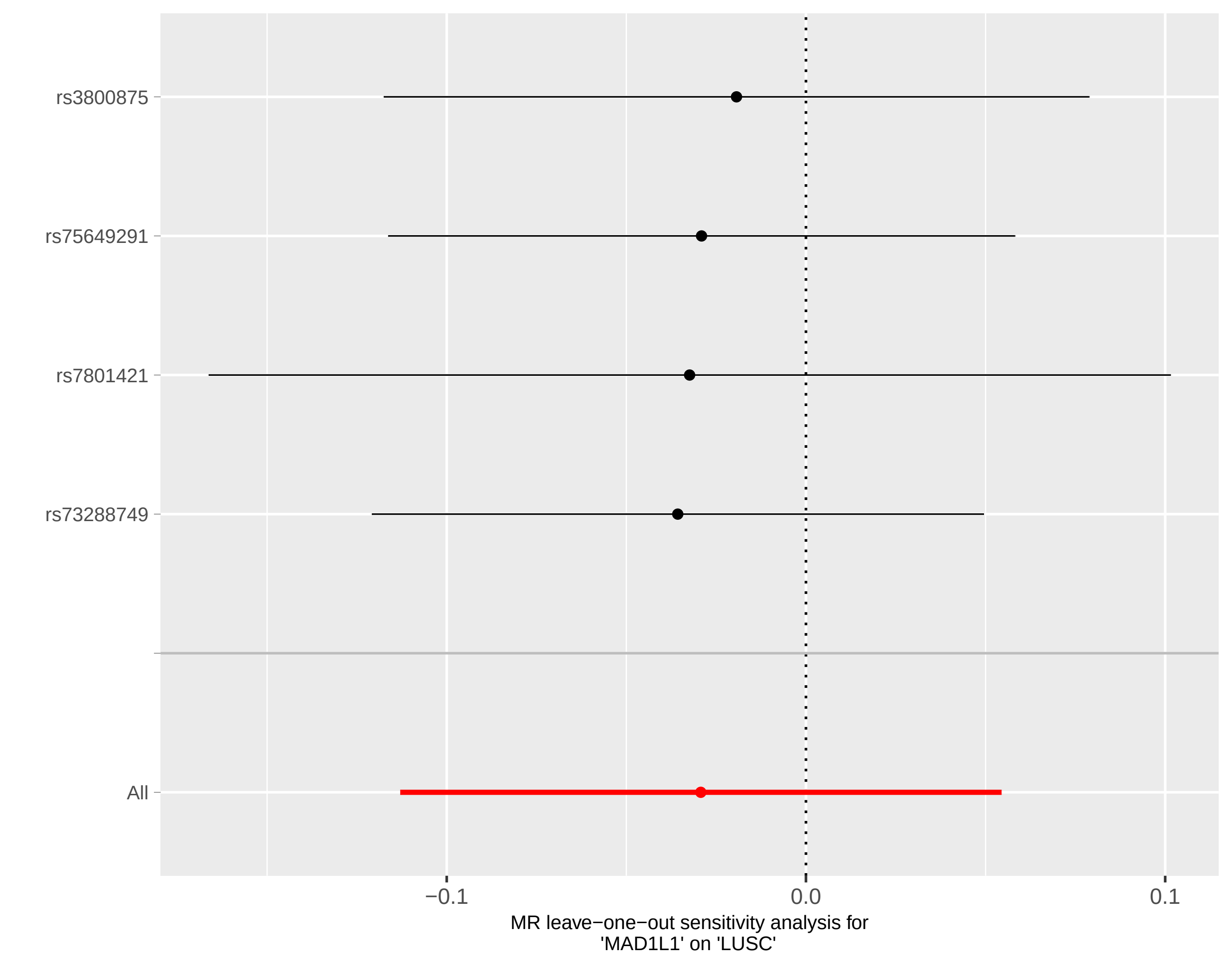
rs3800875
rs75649291
rs7801421
rs73288749
All
−0.1
0.0
0.1
MR leave−one−out sensitivity analysis for
'MAD1L1' on 'LUSC'

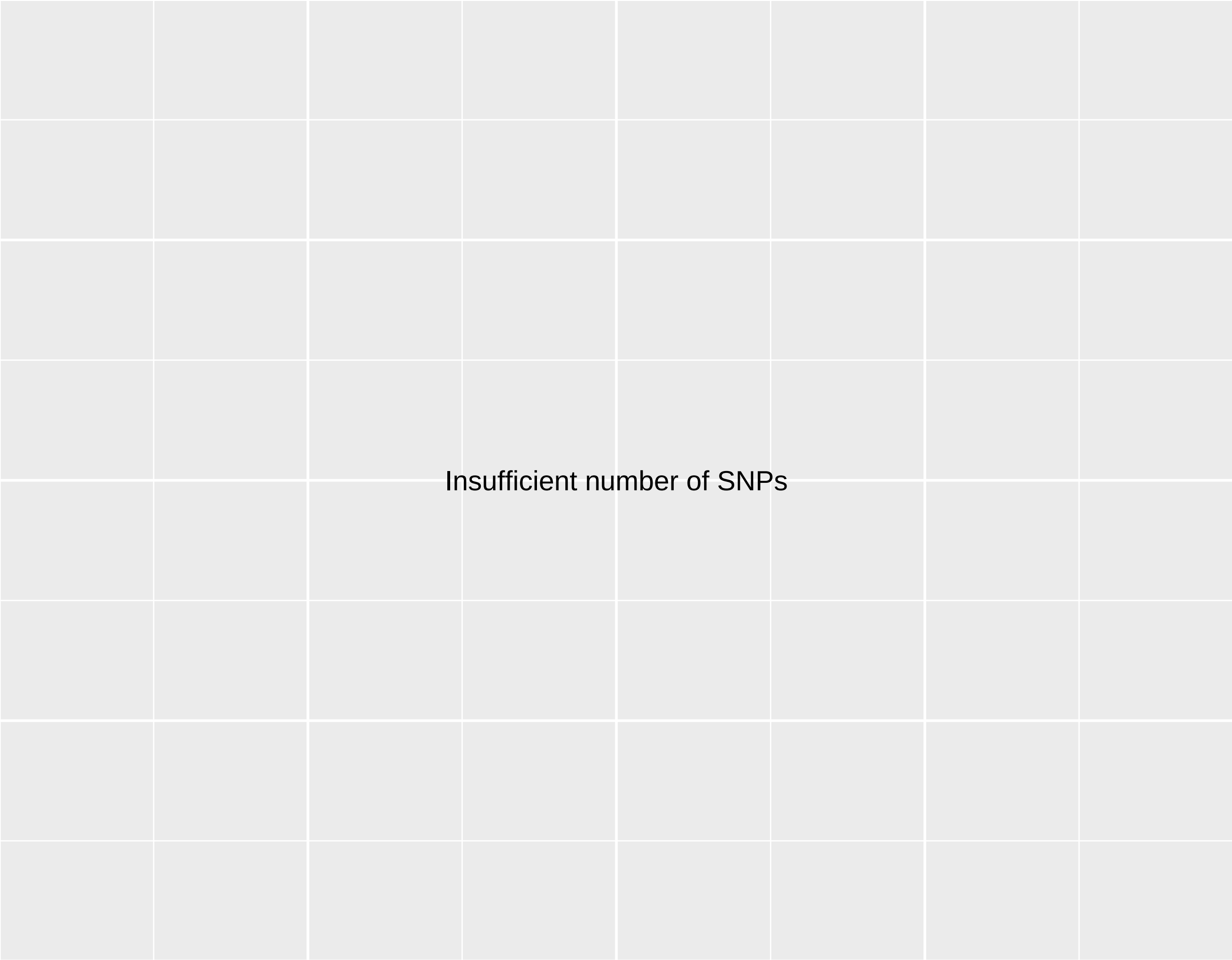
Insufficient number of SNPs

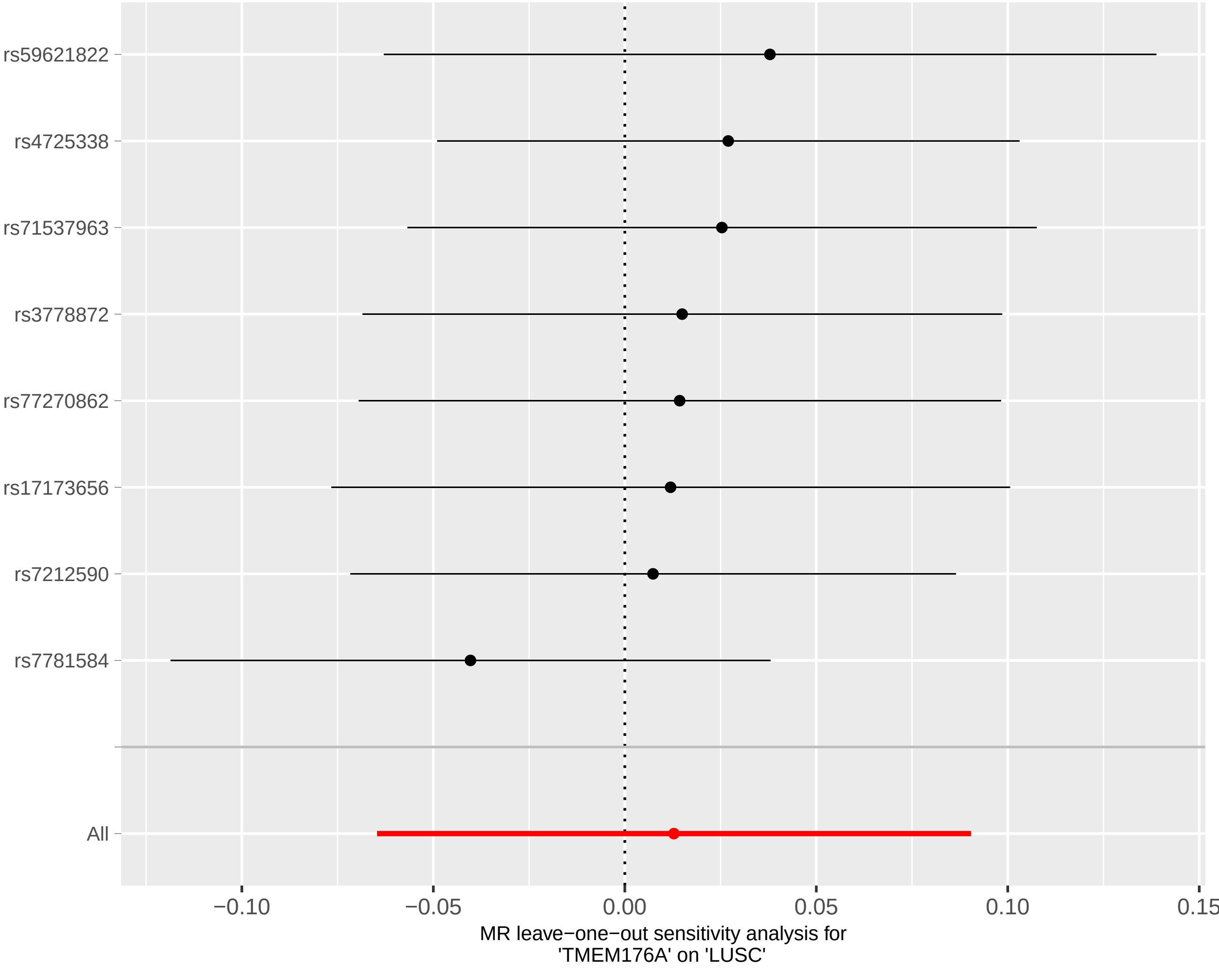

rs59621822
rs4725338
rs71537963
rs3778872
rs77270862
rs17173656
rs7212590
rs7781584
All
−0.10
−0.05
0.00
0.05
0.10
0.15
MR leave−one−out sensitivity analysis for
'TMEM176A' on 'LUSC'

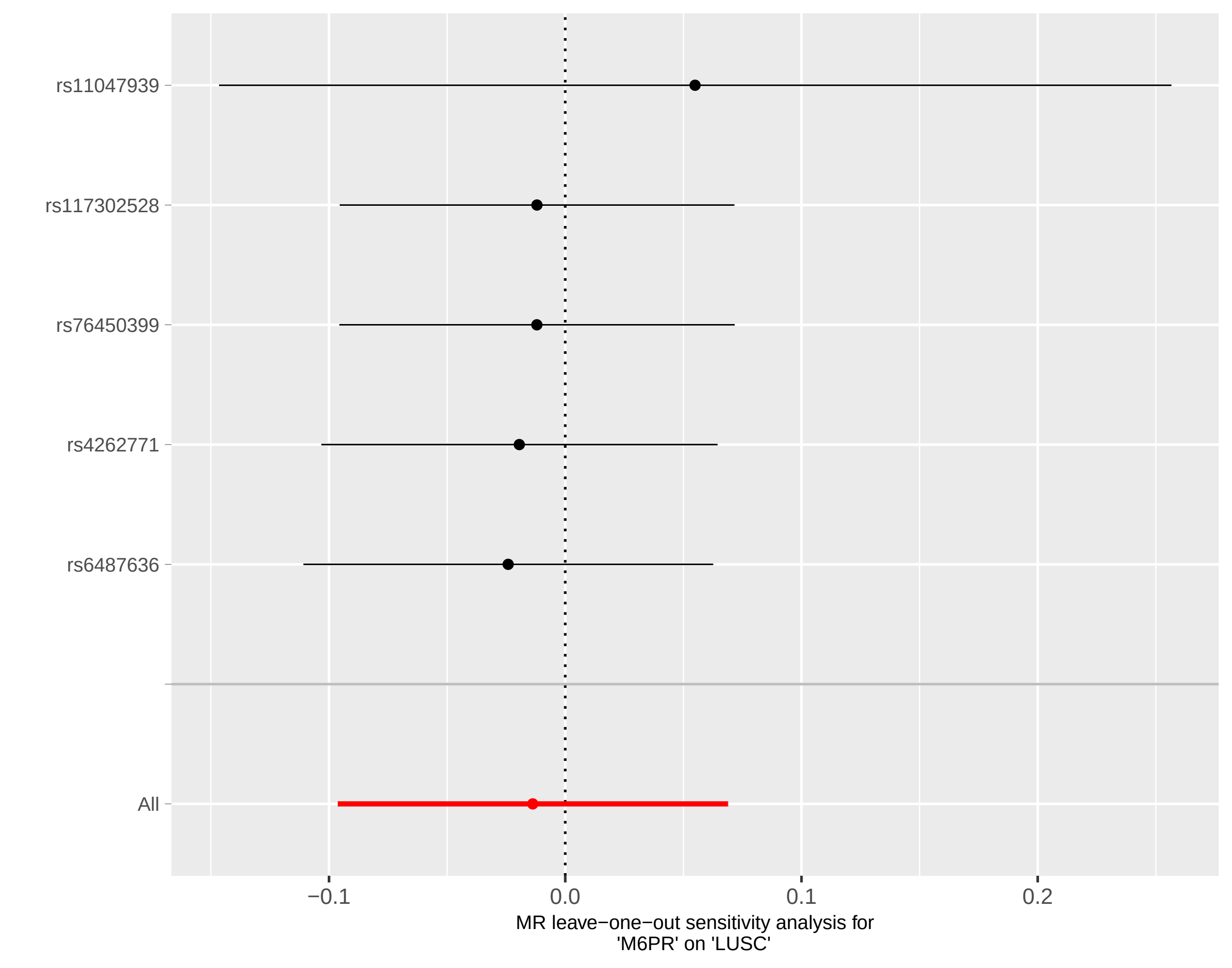
rs11047939
rs117302528
rs76450399
rs4262771
rs6487636
All
−0.1
0.0
0.1
0.2
MR leave−one−out sensitivity analysis for
'M6PR' on 'LUSC'

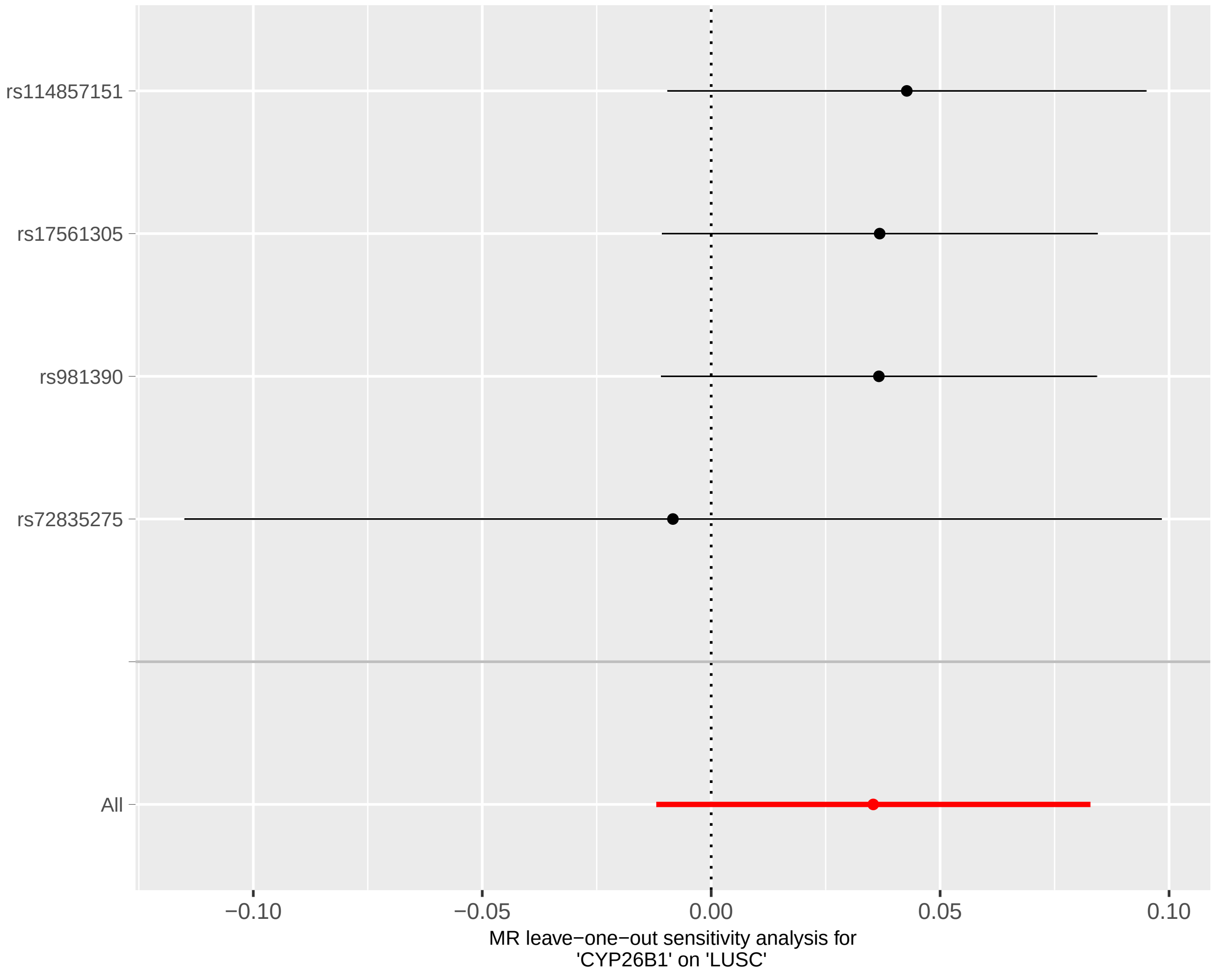
rs114857151
rs17561305
rs981390
rs72835275
All
−0.10
−0.05
0.00
0.05
0.10
MR leave−one−out sensitivity analysis for
'CYP26B1' on 'LUSC'

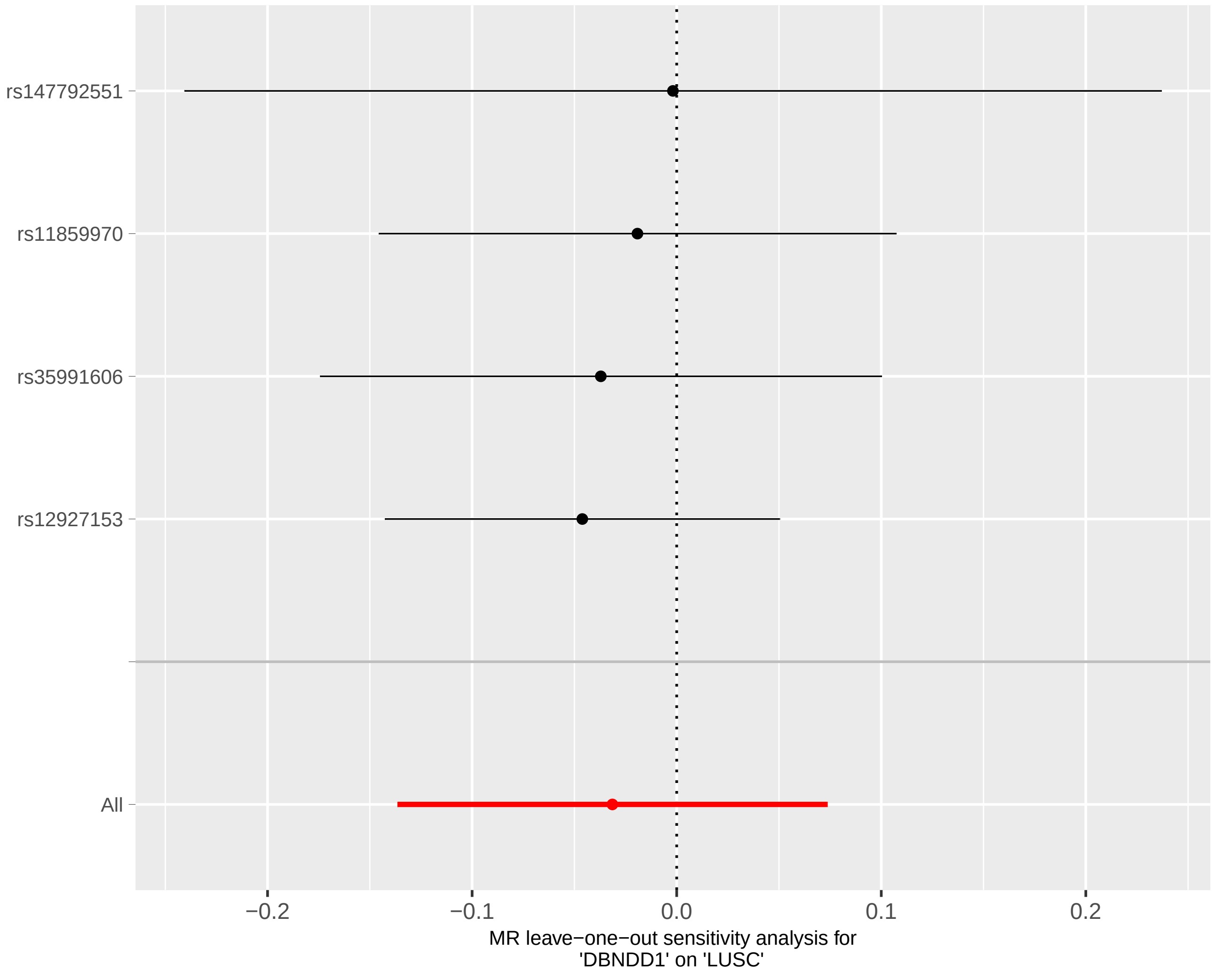

rs147792551
rs11859970
rs35991606
rs12927153
All
−0.2
−0.1
0.0
0.1
0.2
MR leave−one−out sensitivity analysis for
'DBNDD1' on 'LUSC'

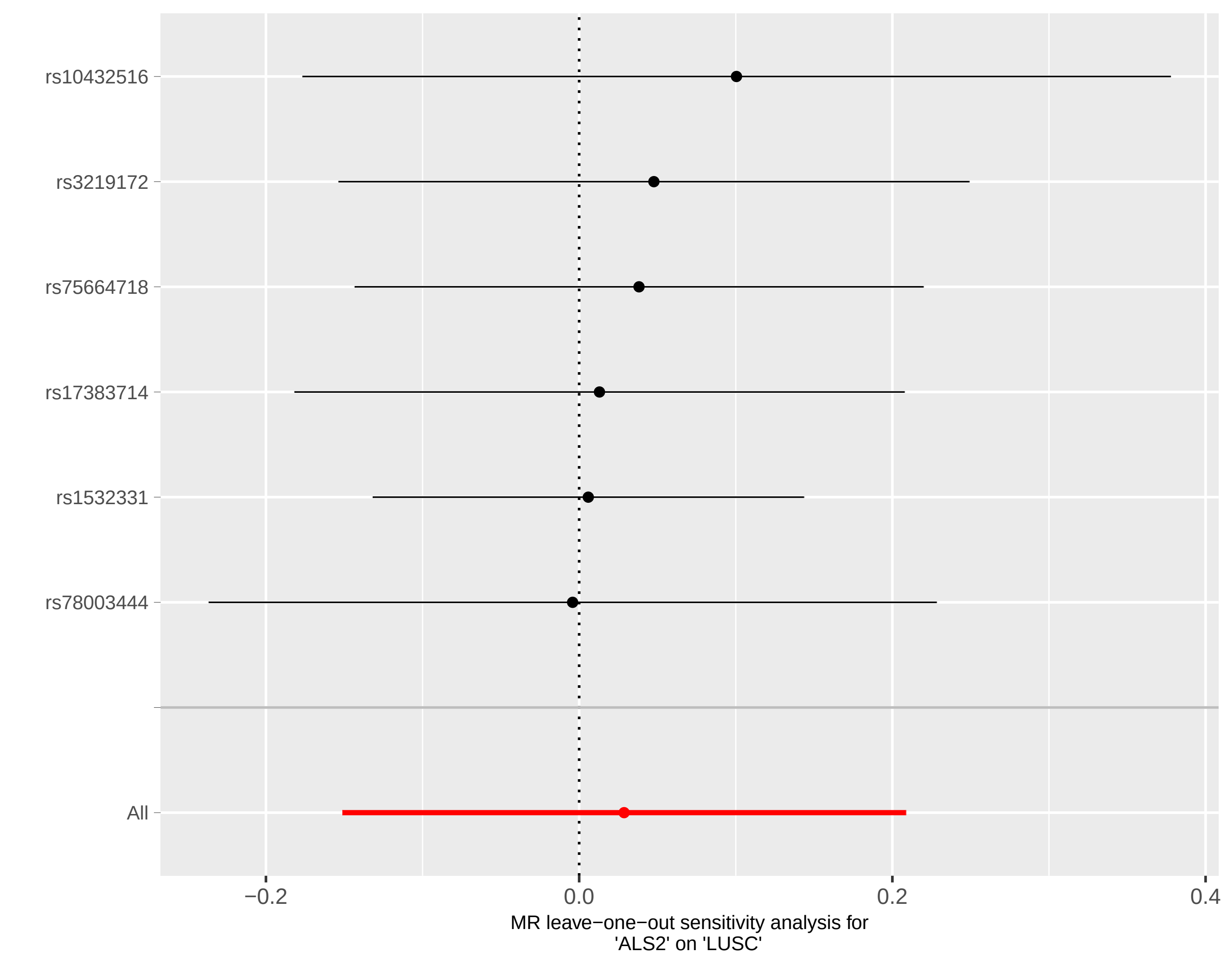
rs10432516
rs3219172
rs75664718
rs17383714
rs1532331
rs78003444
All
−0.2
0.0
0.2
0.4
MR leave−one−out sensitivity analysis for
'ALS2' on 'LUSC'

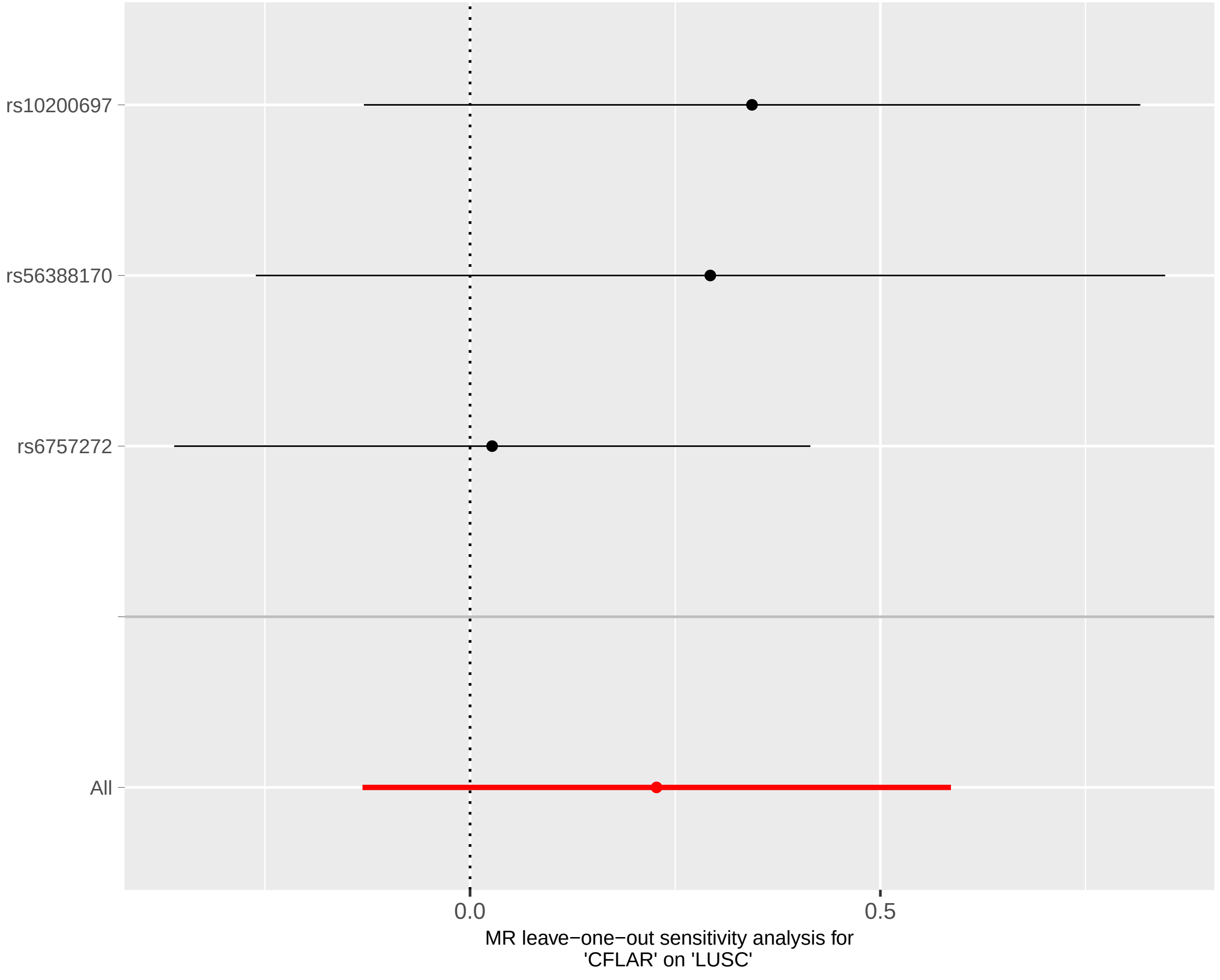

rs10200697
rs56388170
rs6757272
All
0.0
0.5
MR leave−one−out sensitivity analysis for
'CFLAR' on 'LUSC'

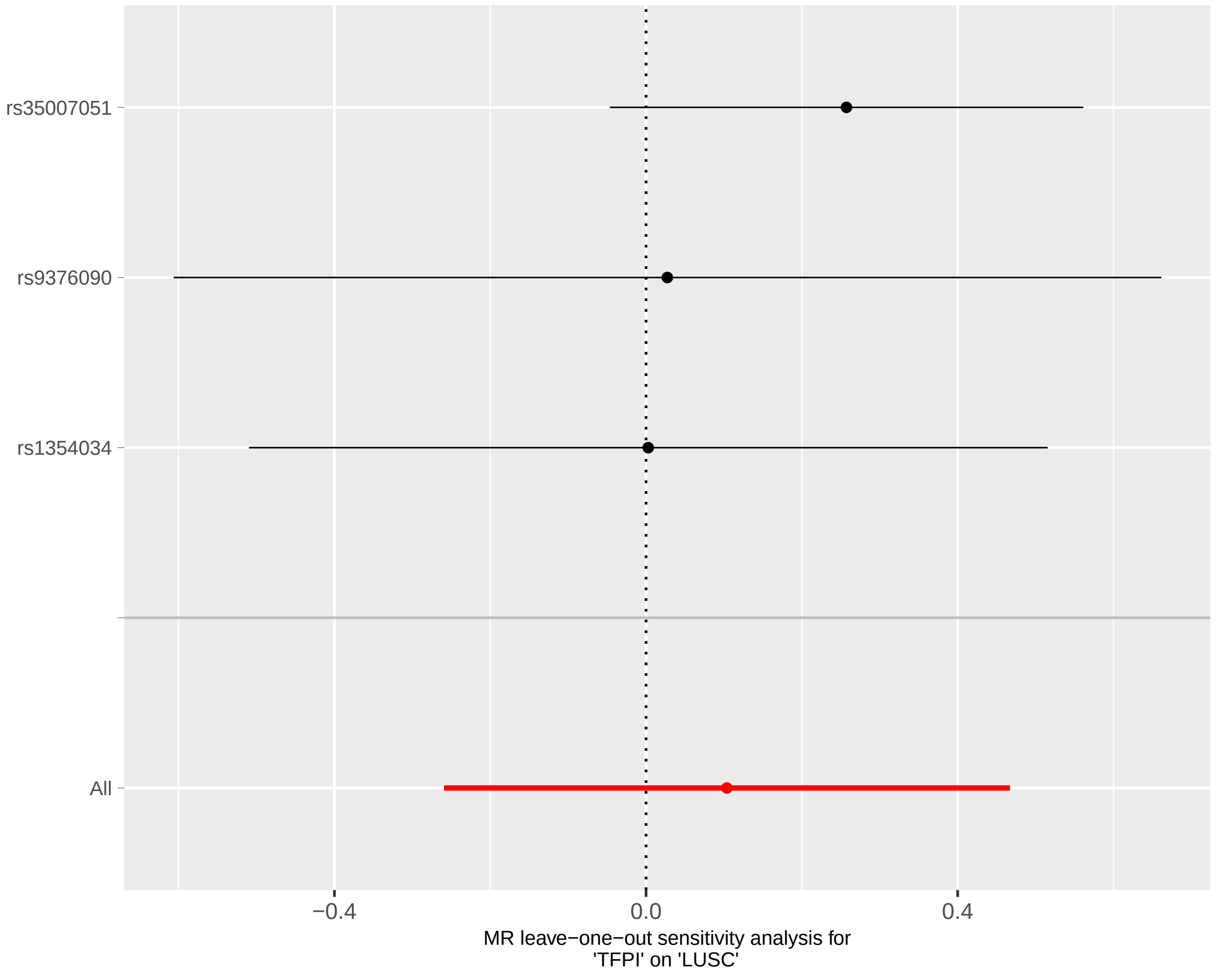

rs35007051
rs9376090
rs1354034
All
−0.4
0.0
0.4
MR leave−one−out sensitivity analysis for
'TFPI' on 'LUSC'

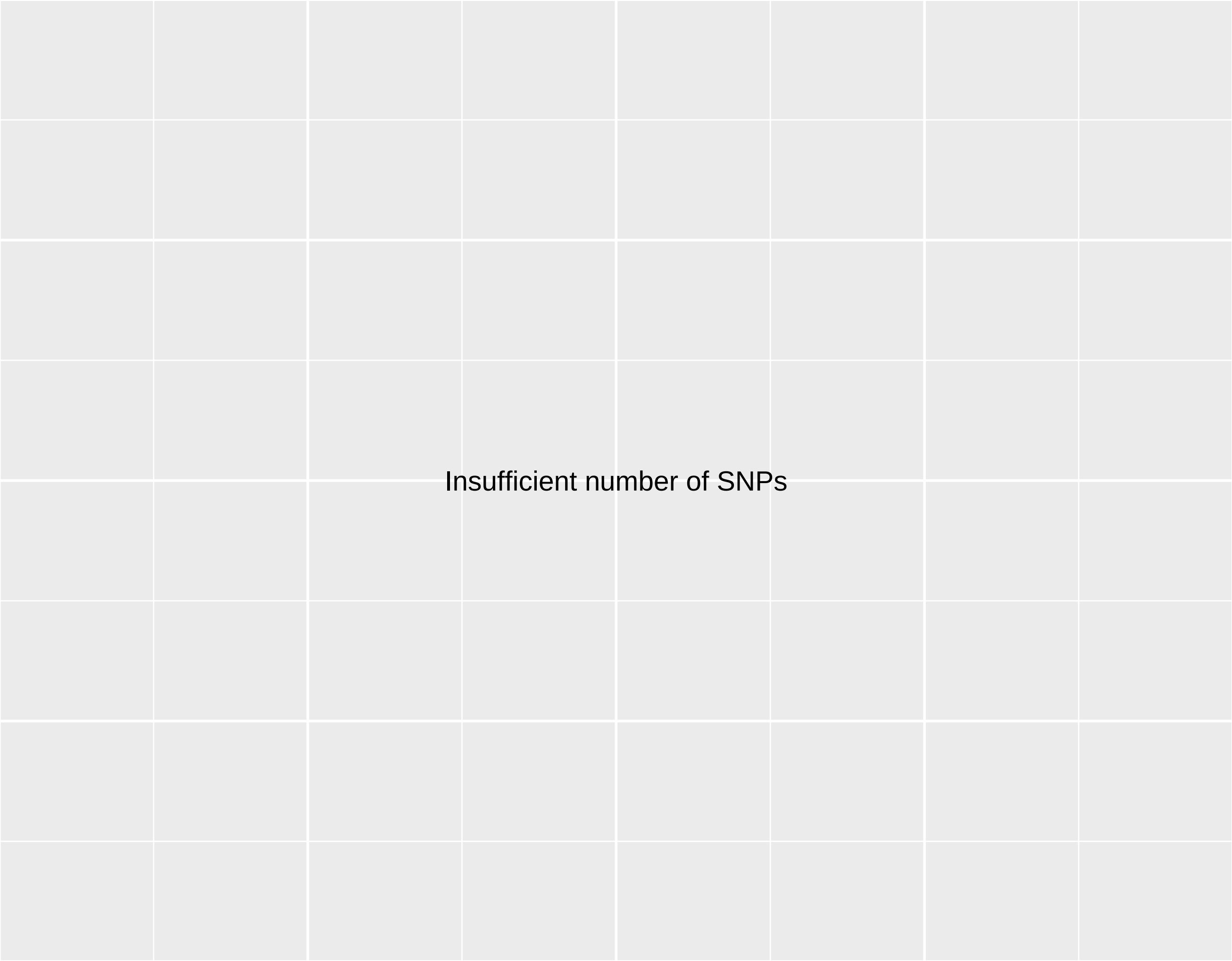
Insufficient number of SNPs

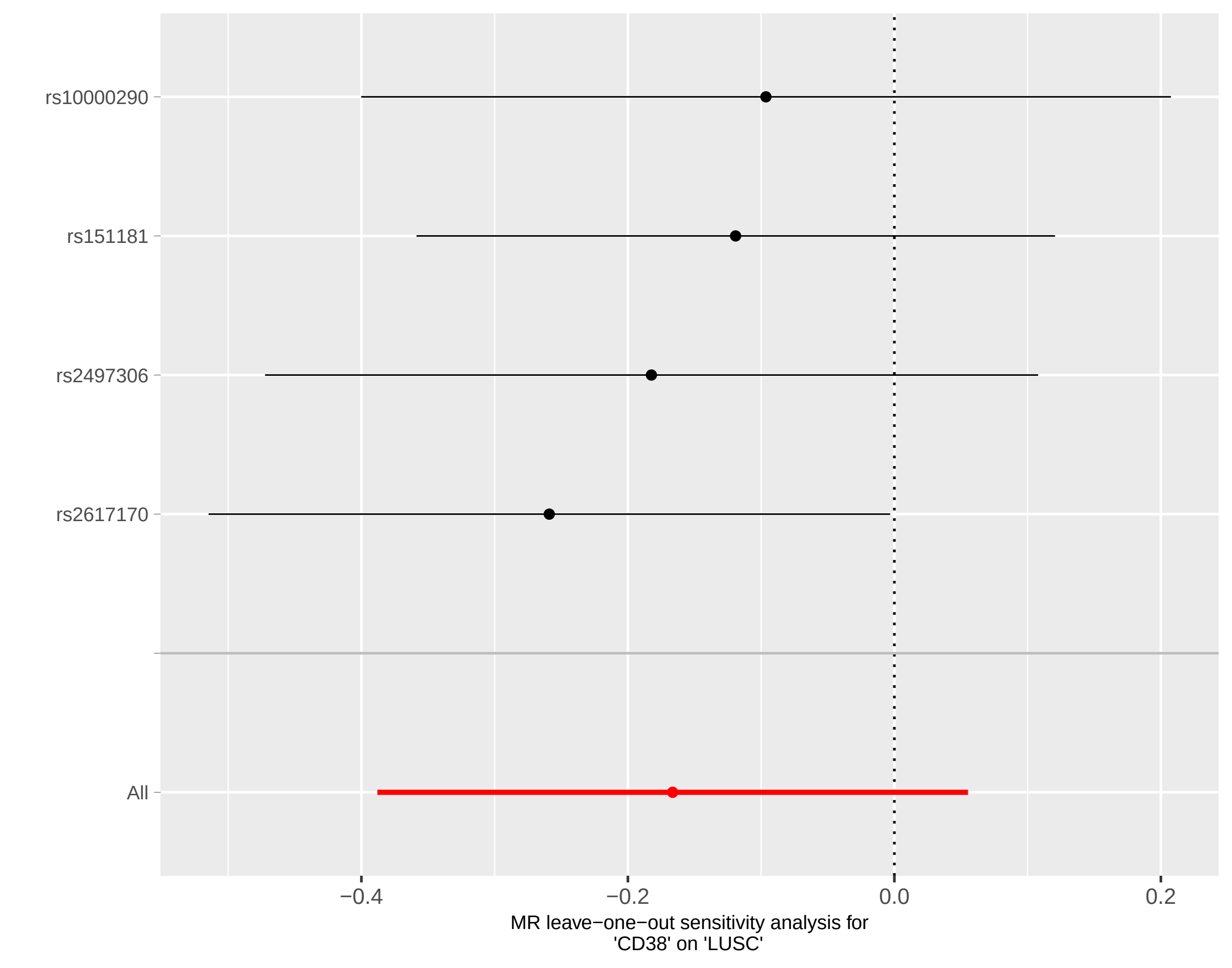

rs10000290
rs151181
rs2497306
rs2617170
All
−0.4
−0.2
0.0
0.2
MR leave−one−out sensitivity analysis for
'CD38' on 'LUSC'

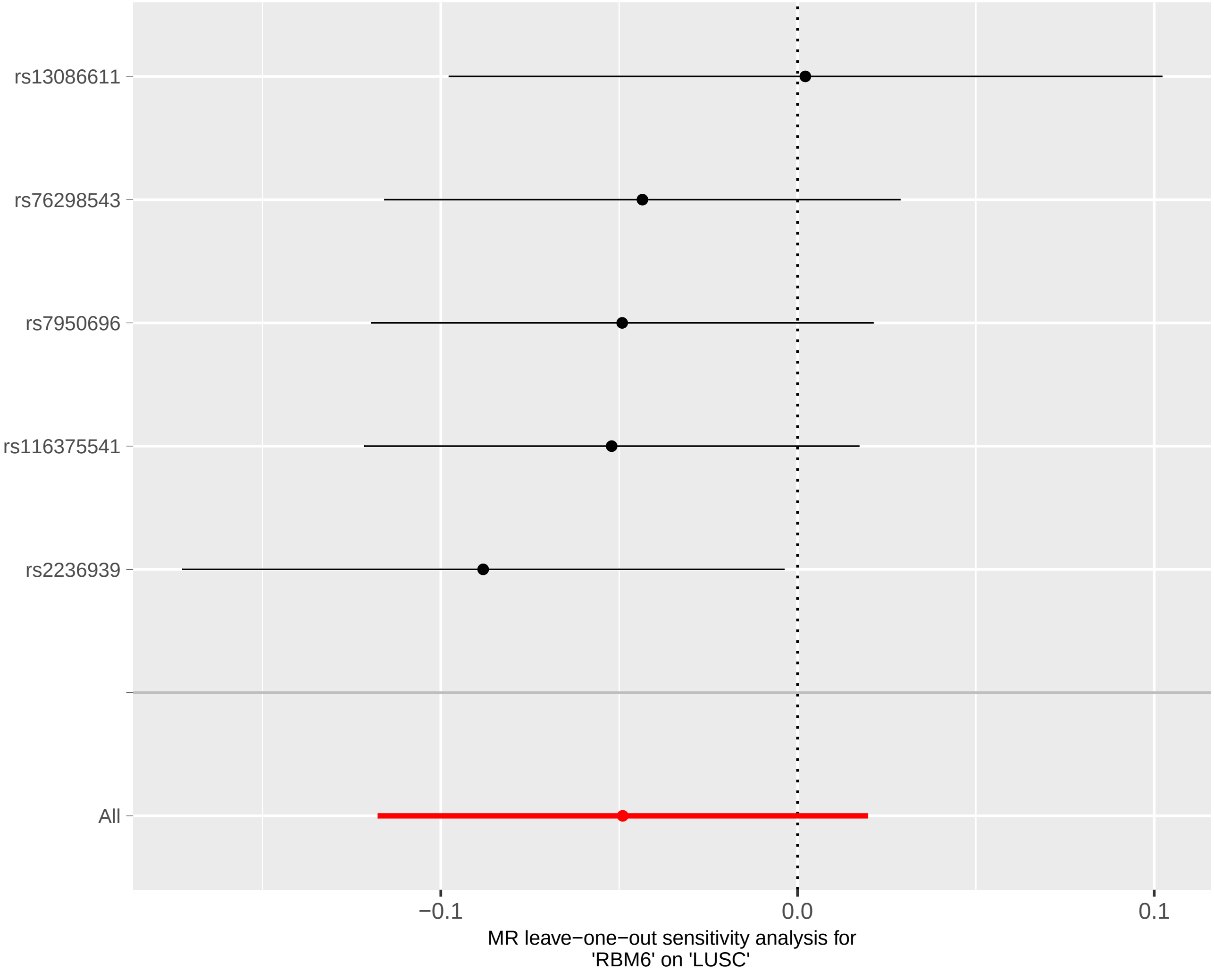
rs13086611
rs76298543
rs7950696
rs116375541
rs2236939
All
−0.1
0.0
0.1
MR leave−one−out sensitivity analysis for
'RBM6' on 'LUSC'

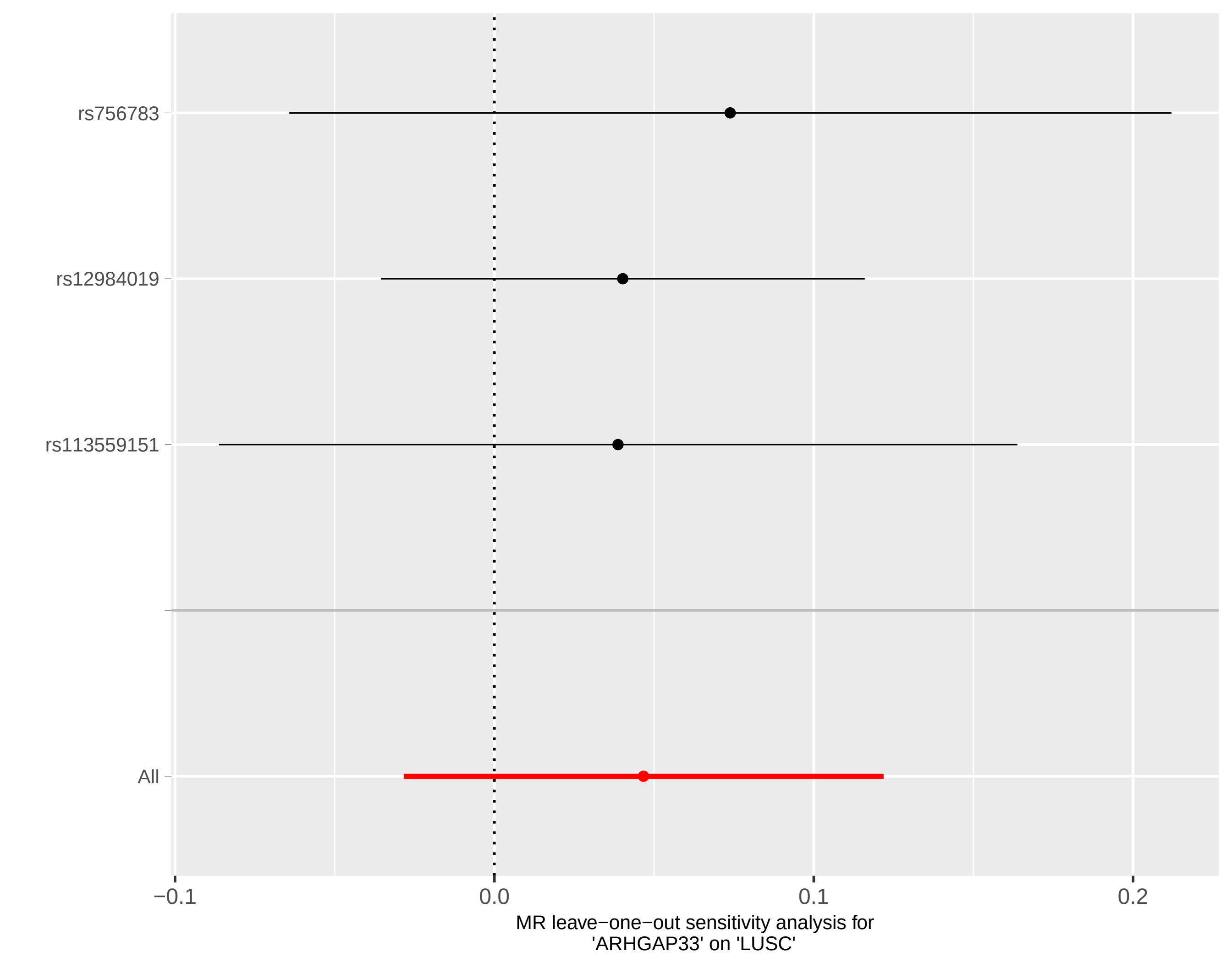
rs756783
rs12984019
rs113559151
All
−0.1
0.0
0.1
0.2
MR leave−one−out sensitivity analysis for
'ARHGAP33' on 'LUSC'

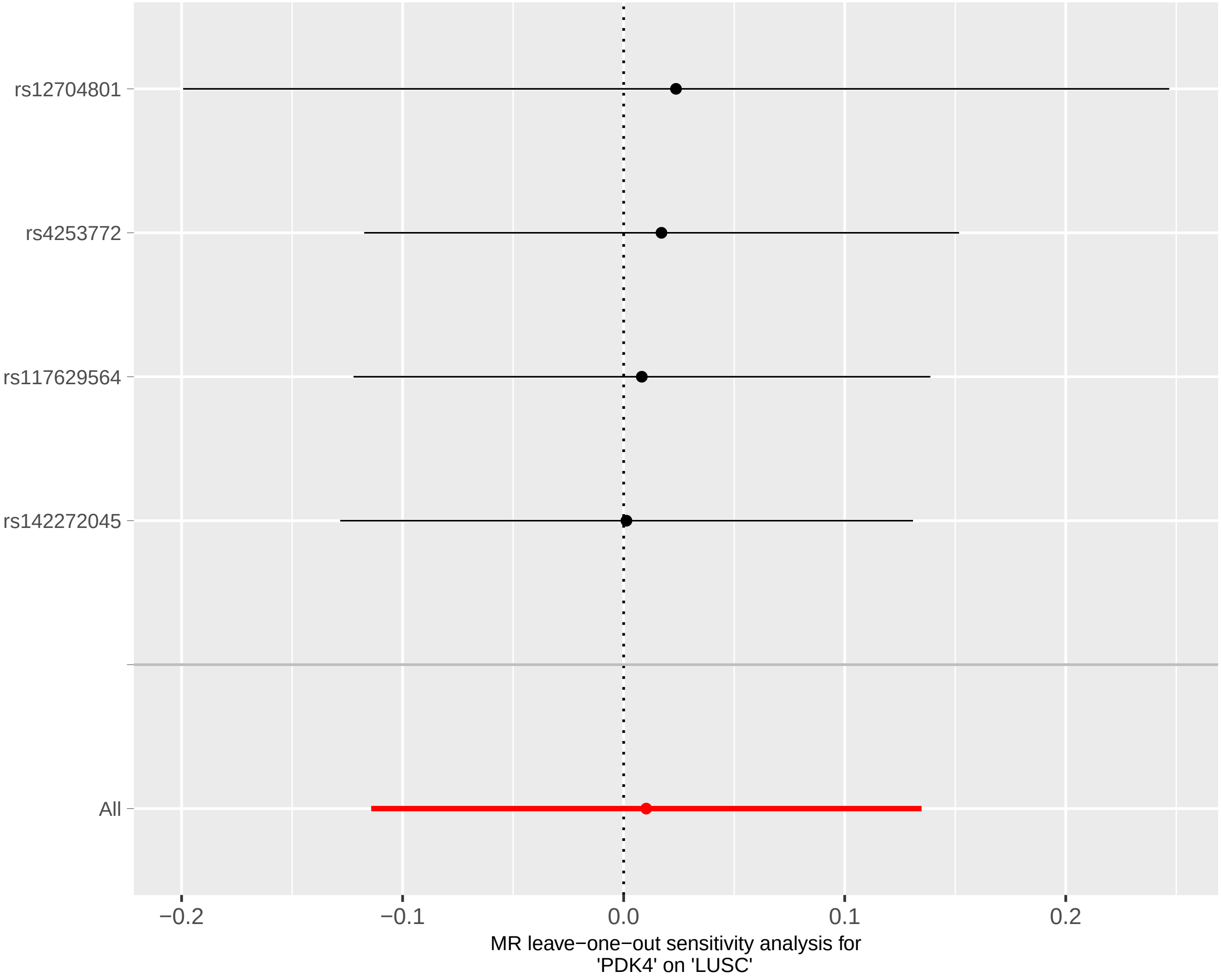

rs12704801
rs4253772
rs117629564
rs142272045
All
−0.2
−0.1
0.0
0.1
0.2
MR leave−one−out sensitivity analysis for
'PDK4' on 'LUSC'

Insufficient number of SNPs

Insufficient number of SNPs

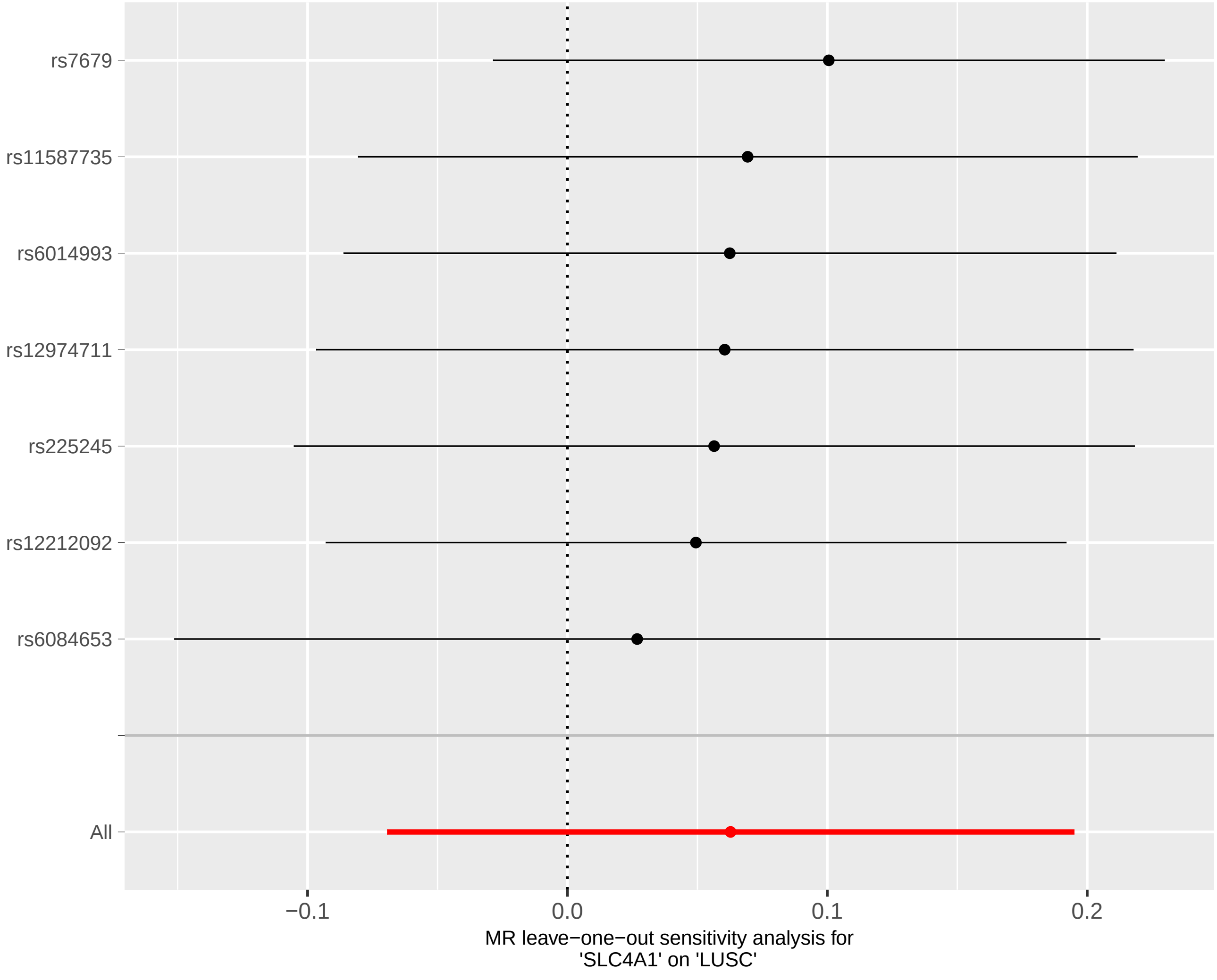
rs7679
rs11587735
rs6014993
rs12974711
rs225245
rs12212092
rs6084653
All
−0.1
0.0
0.1
0.2
MR leave−one−out sensitivity analysis for
'SLC4A1' on 'LUSC'

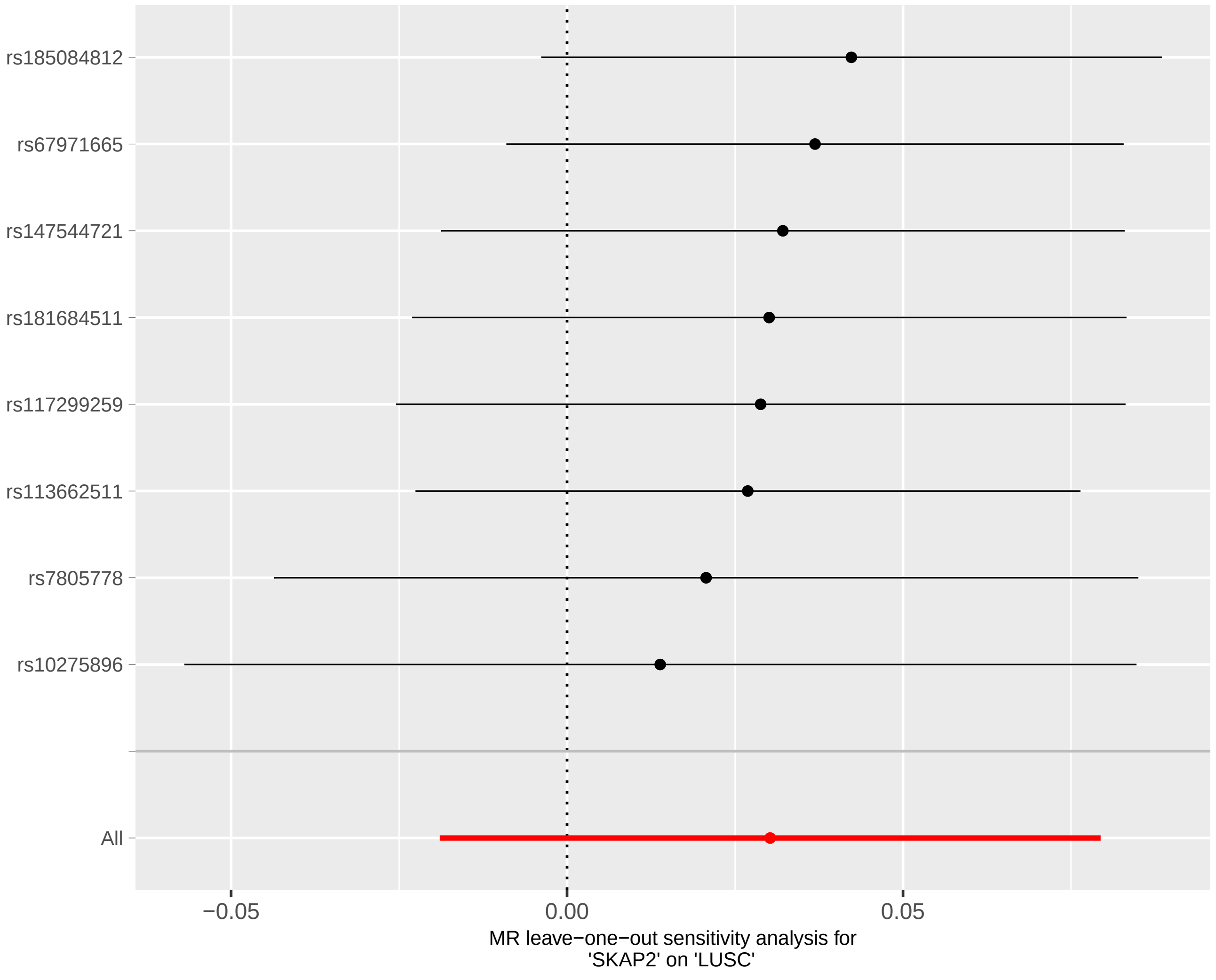
rs185084812
rs67971665
rs147544721
rs181684511
rs117299259
rs113662511
rs7805778
rs10275896
All
−0.05
0.00
0.05
MR leave−one−out sensitivity analysis for
'SKAP2' on 'LUSC'

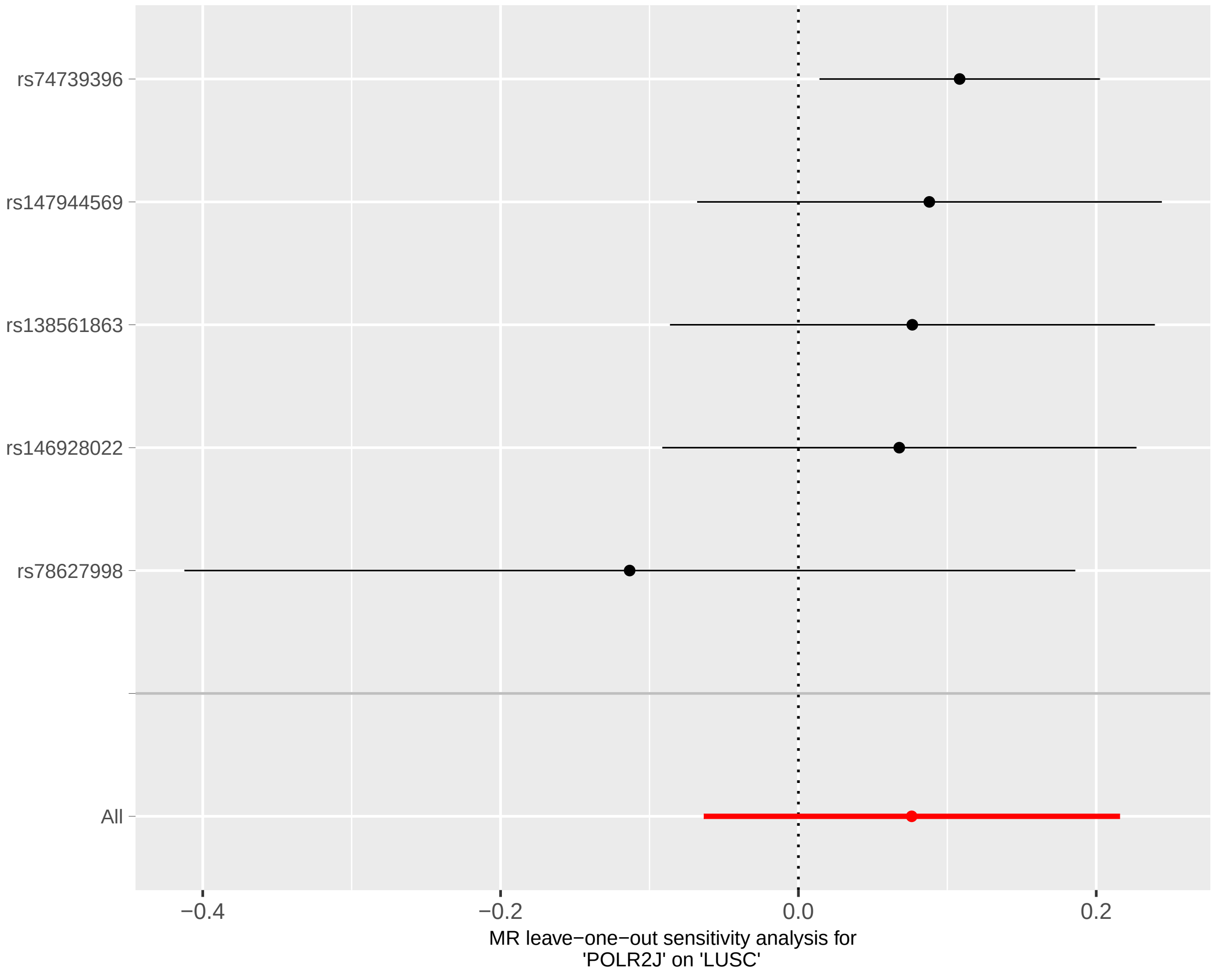
rs74739396
rs147944569
rs138561863
rs146928022
rs78627998
All
−0.4
−0.2
0.0
0.2
MR leave−one−out sensitivity analysis for
'POLR2J' on 'LUSC'

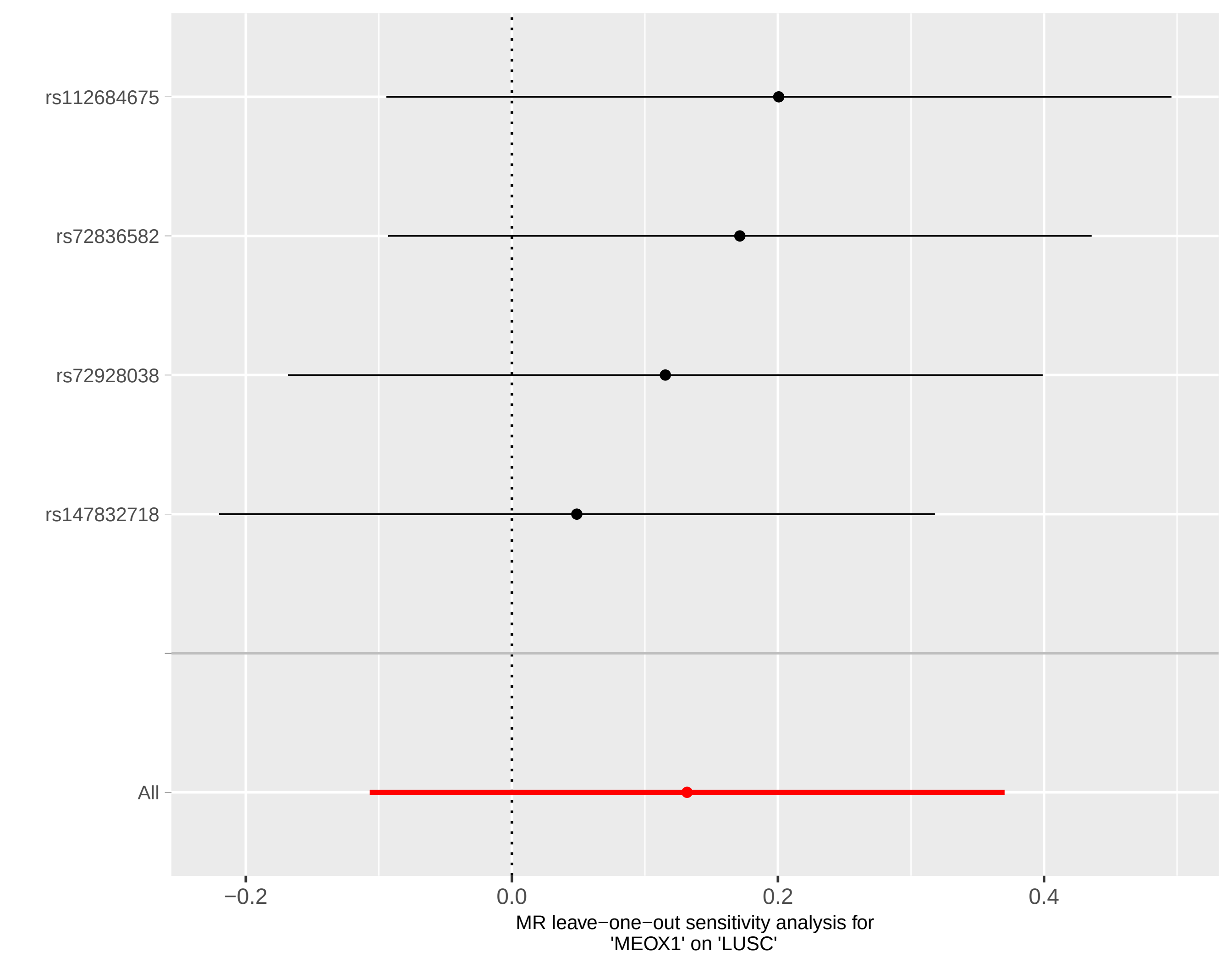
rs112684675
rs72836582
rs72928038
rs147832718
All
−0.2
0.0
0.2
0.4
MR leave−one−out sensitivity analysis for
'MEOX1' on 'LUSC'

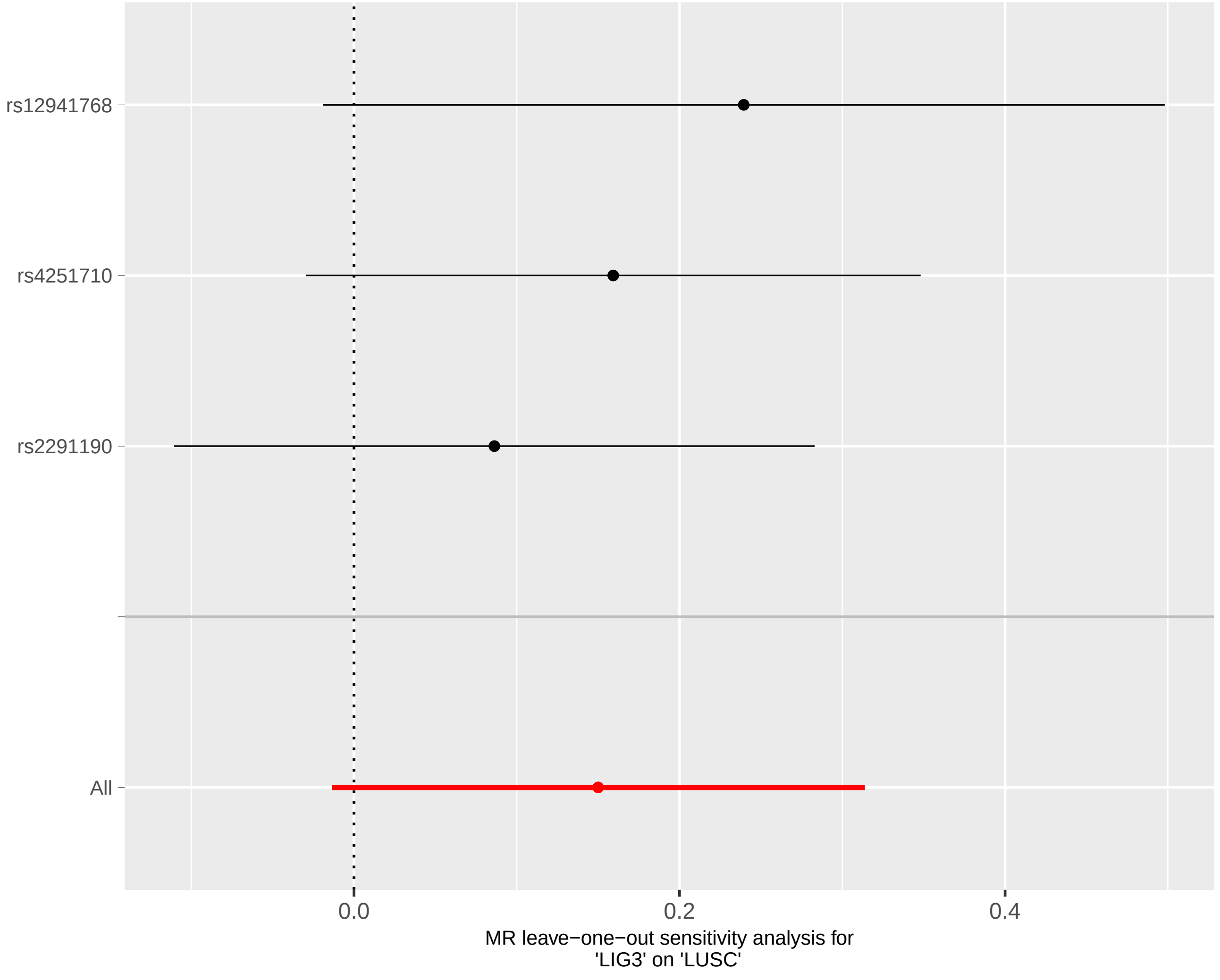
rs12941768
rs4251710
rs2291190
All
0.0
0.2
0.4
MR leave−one−out sensitivity analysis for
'LIG3' on 'LUSC'

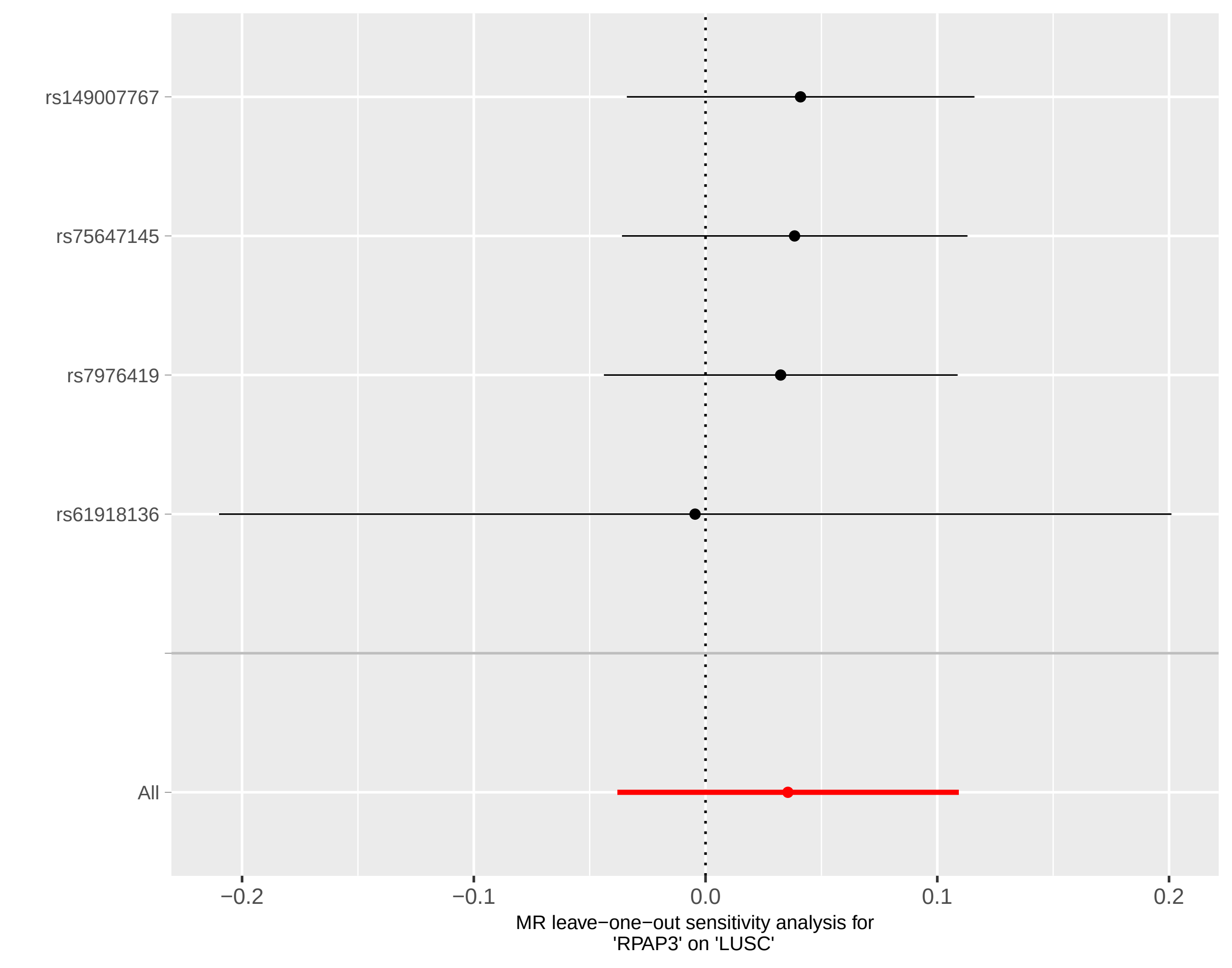
rs149007767
rs75647145
rs7976419
rs61918136
All
−0.2
−0.1
0.0
0.1
0.2
MR leave−one−out sensitivity analysis for
'RPAP3' on 'LUSC'

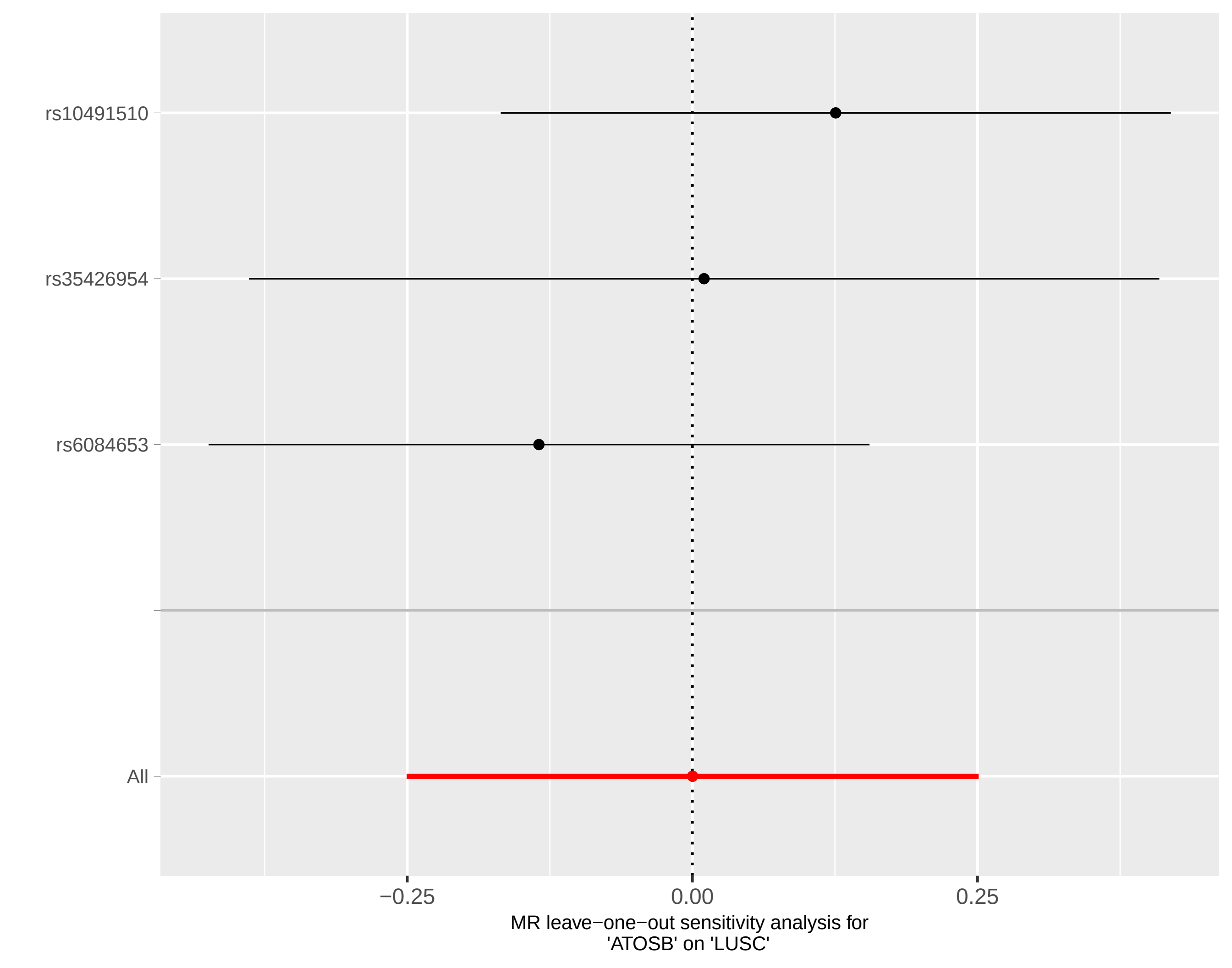
rs10491510
rs35426954
rs6084653
All
−0.25
0.00
0.25
MR leave−one−out sensitivity analysis for
'ATOSB' on 'LUSC'

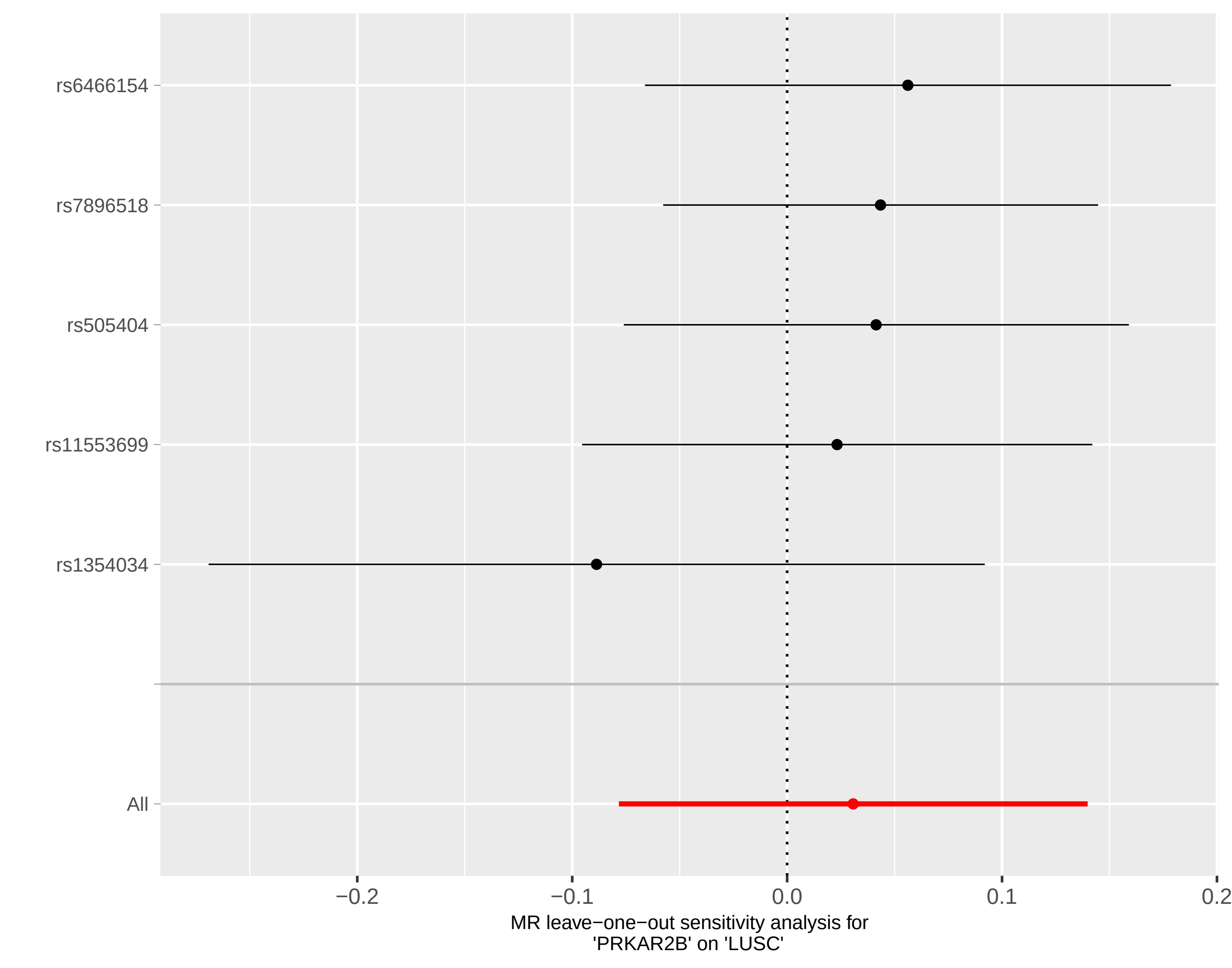

rs6466154
rs7896518
rs505404
rs11553699
rs1354034
All
−0.2
−0.1
0.0
0.1
0.2
MR leave−one−out sensitivity analysis for
'PRKAR2B' on 'LUSC'

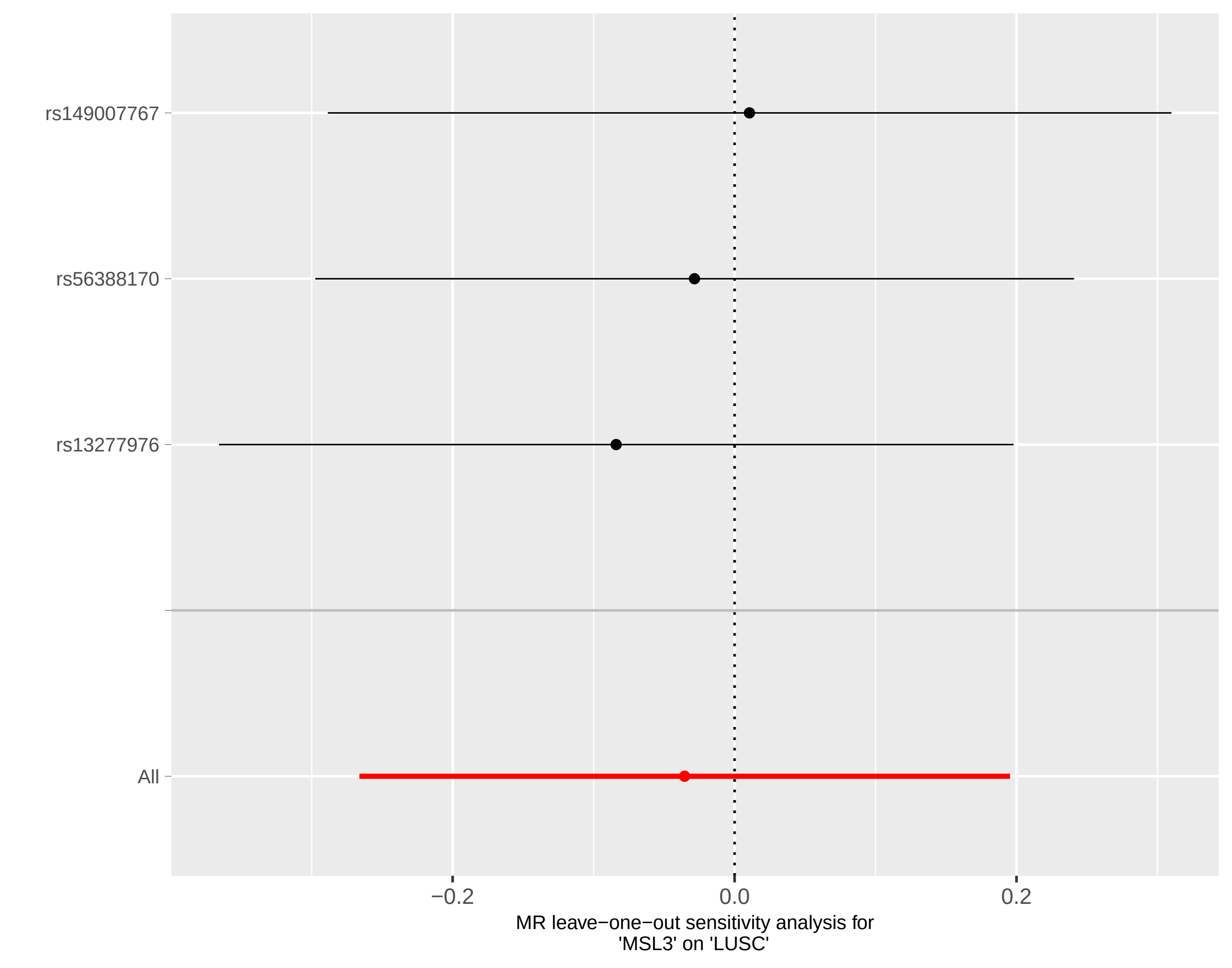

rs149007767
rs56388170
rs13277976
All
−0.2
0.0
0.2
MR leave−one−out sensitivity analysis for
'MSL3' on 'LUSC'

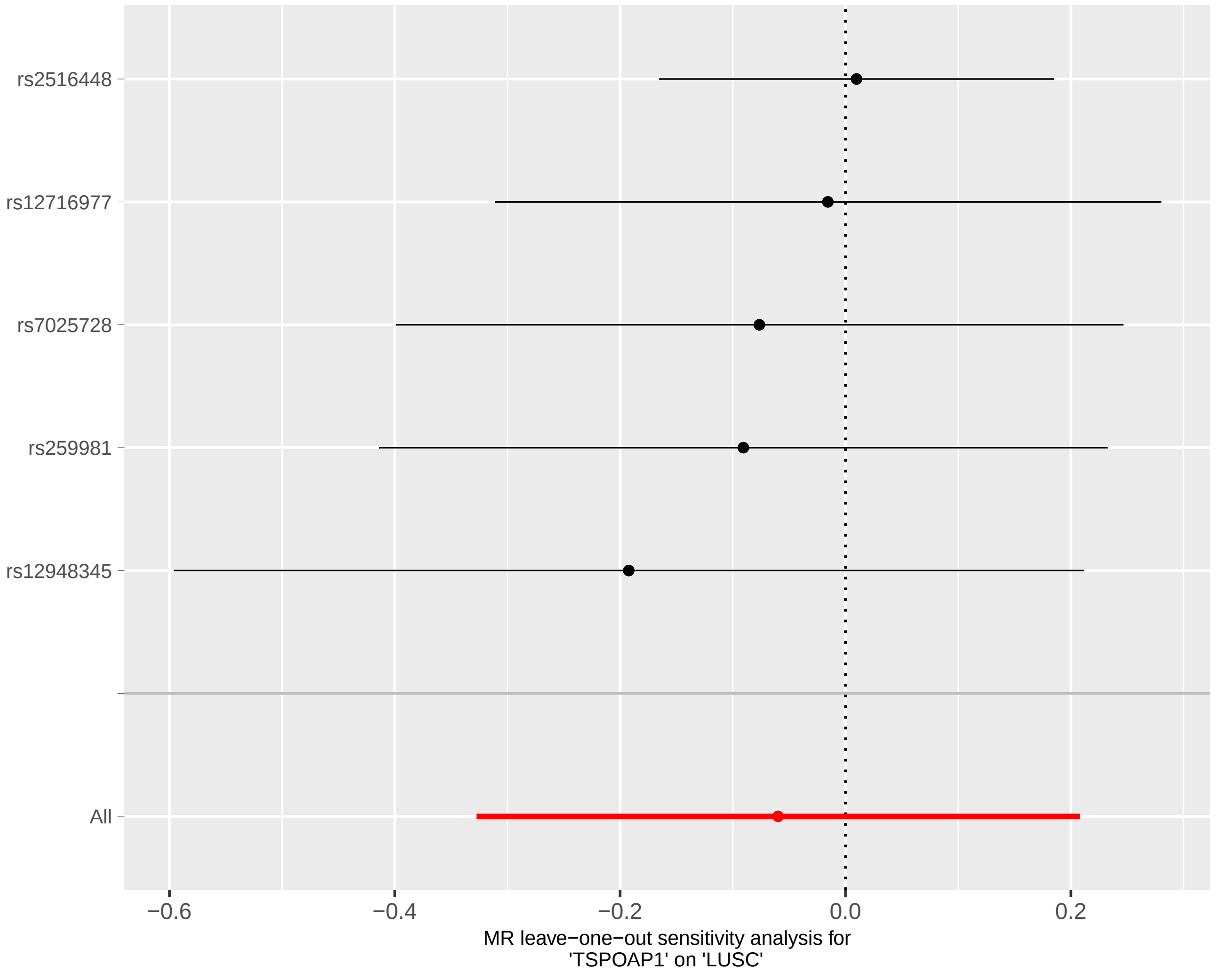

rs2516448
rs12716977
rs7025728
rs259981
rs12948345
All
−0.6
−0.4
−0.2
0.0
0.2
MR leave−one−out sensitivity analysis for
'TSPOAP1' on 'LUSC'

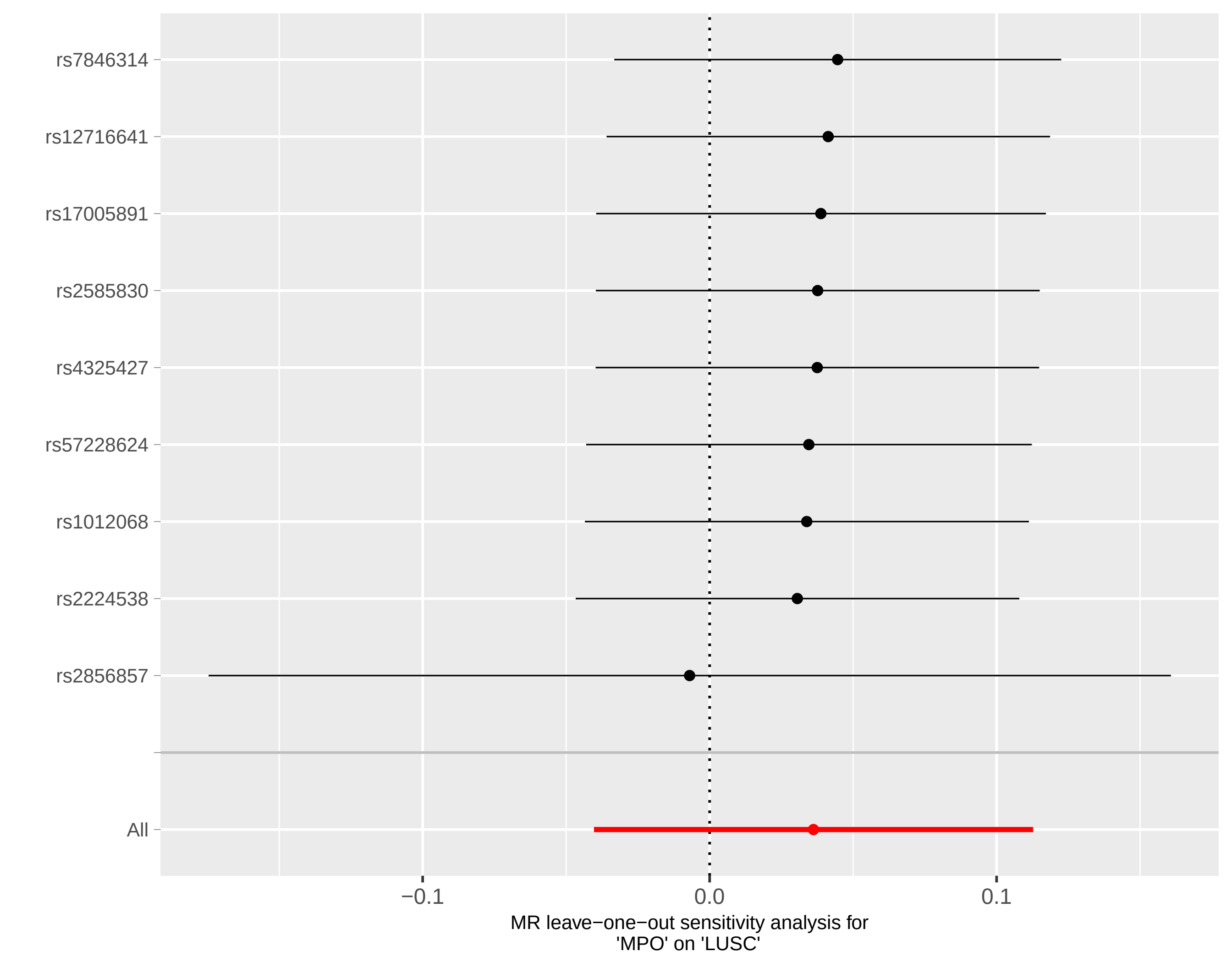
rs7846314
rs12716641
rs17005891
rs2585830
rs4325427
rs57228624
rs1012068
rs2224538
rs2856857
All
−0.1
0.0
0.1
MR leave−one−out sensitivity analysis for
'MPO' on 'LUSC'

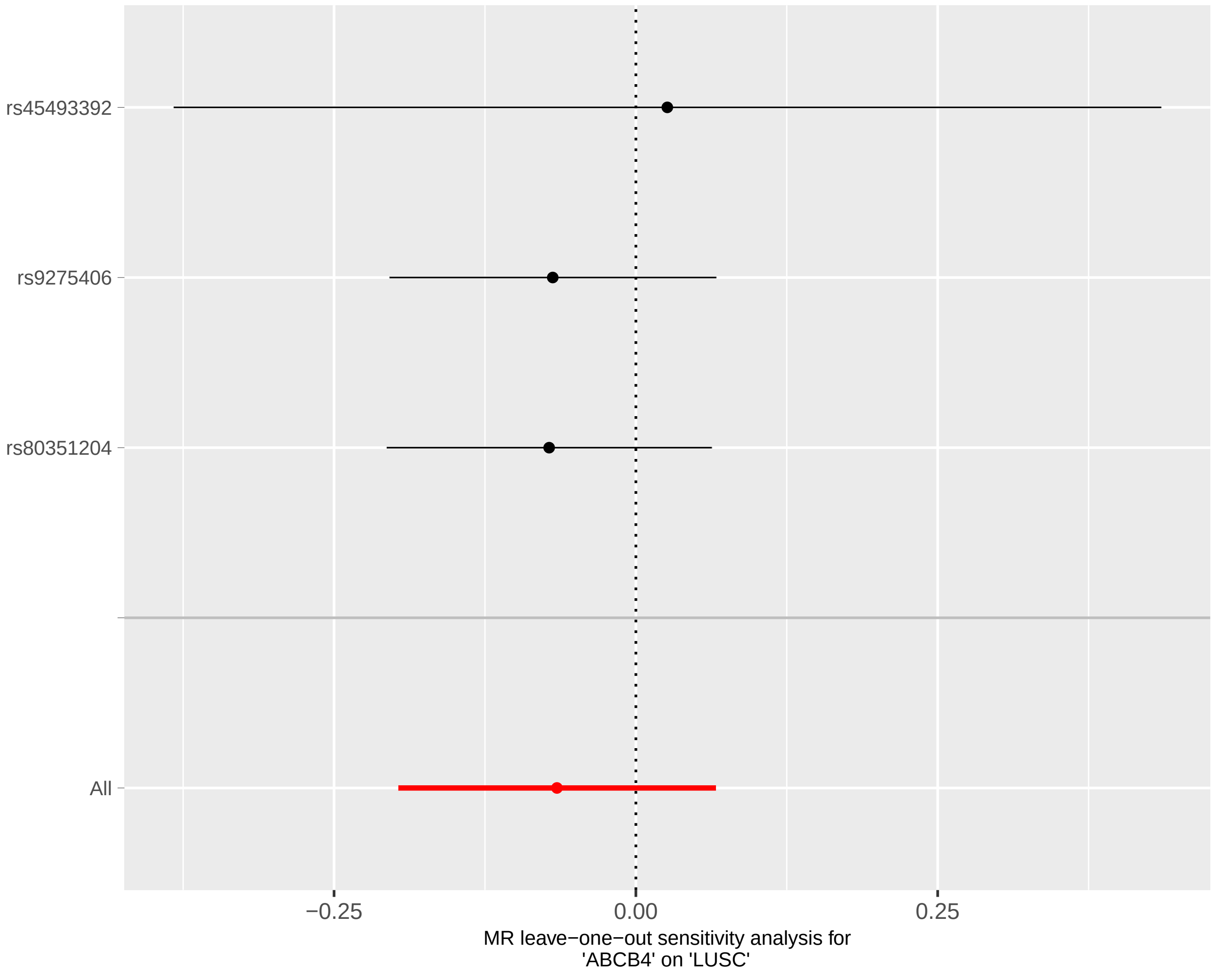
rs45493392
rs9275406
rs80351204
All
−0.25
0.00
0.25
MR leave−one−out sensitivity analysis for
'ABCB4' on 'LUSC'

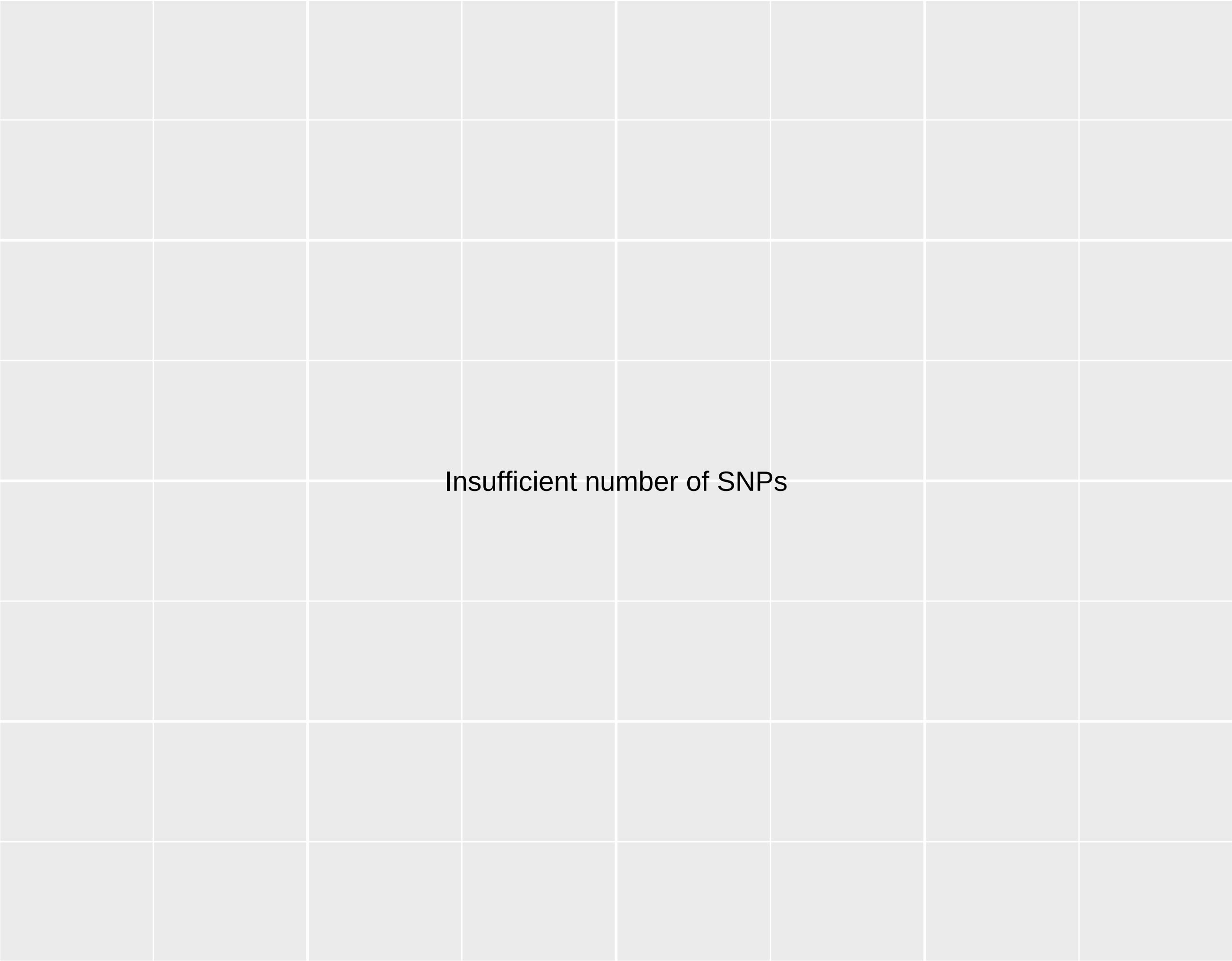
Insufficient number of SNPs

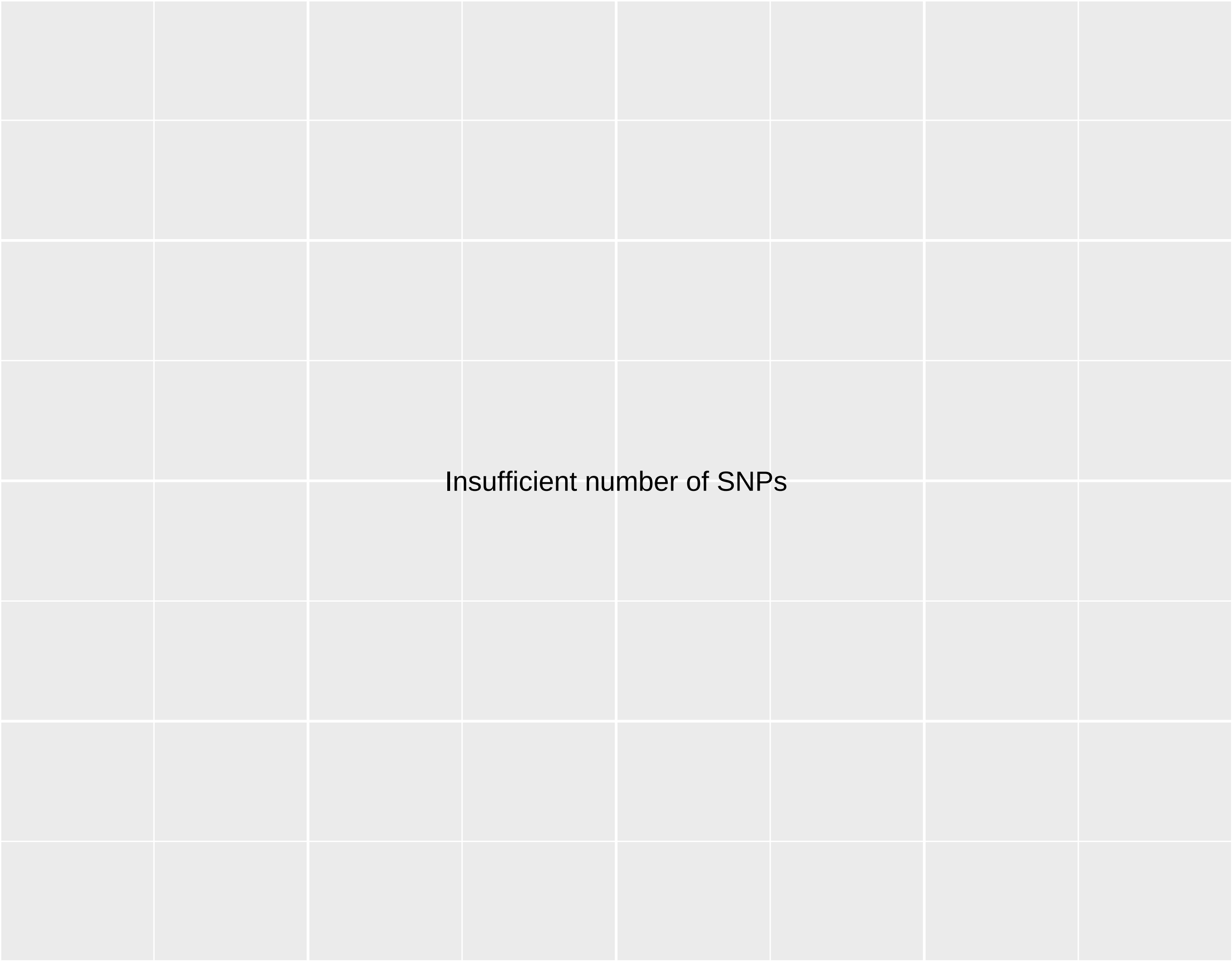
Insufficient number of SNPs

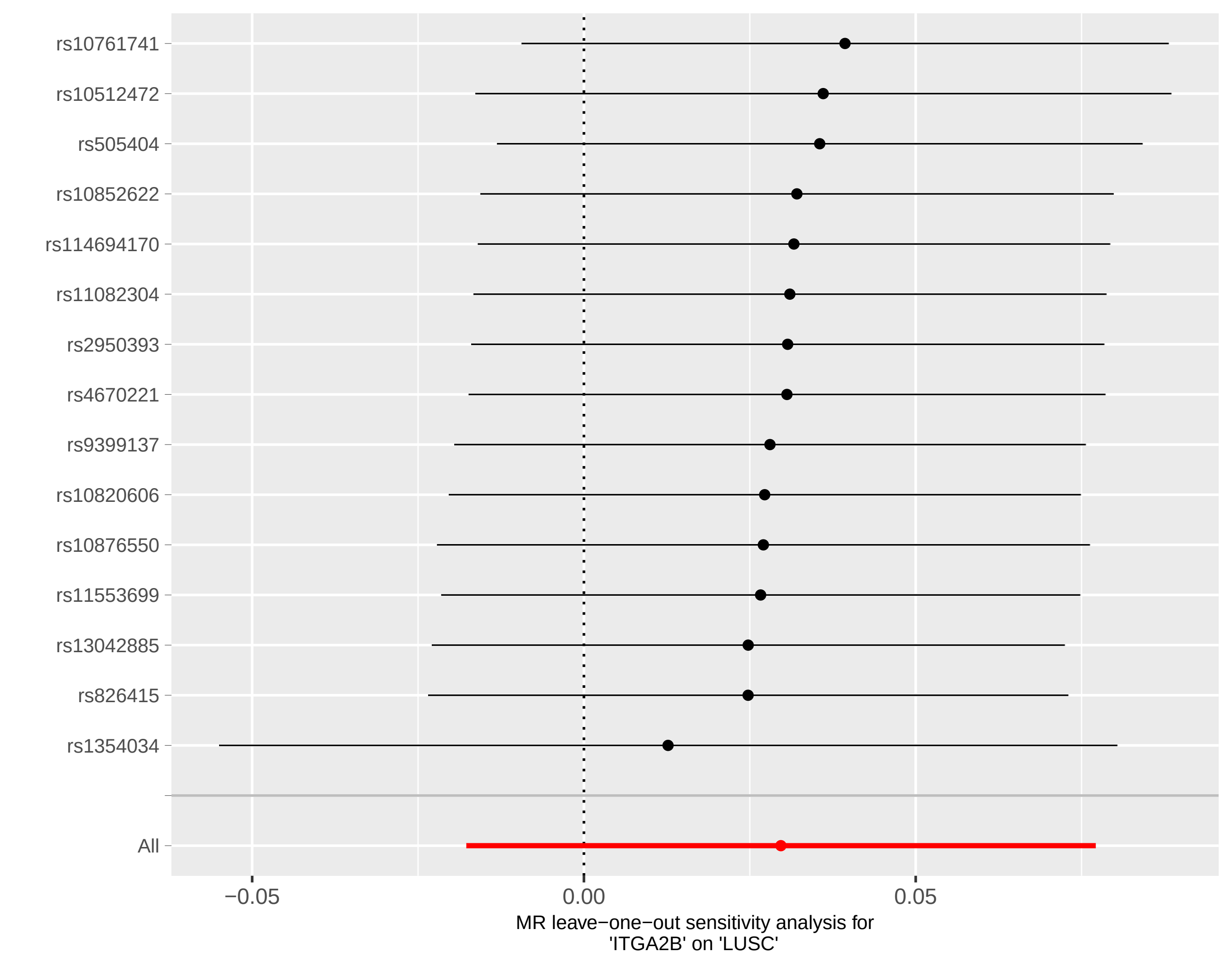

rs10761741
rs10512472
rs505404
rs10852622
rs114694170
rs11082304
rs2950393
rs4670221
rs9399137
rs10820606
rs10876550
rs11553699
rs13042885
rs826415
rs1354034
All
−0.05
0.00
0.05
MR leave−one−out sensitivity analysis for
'ITGA2B' on 'LUSC'

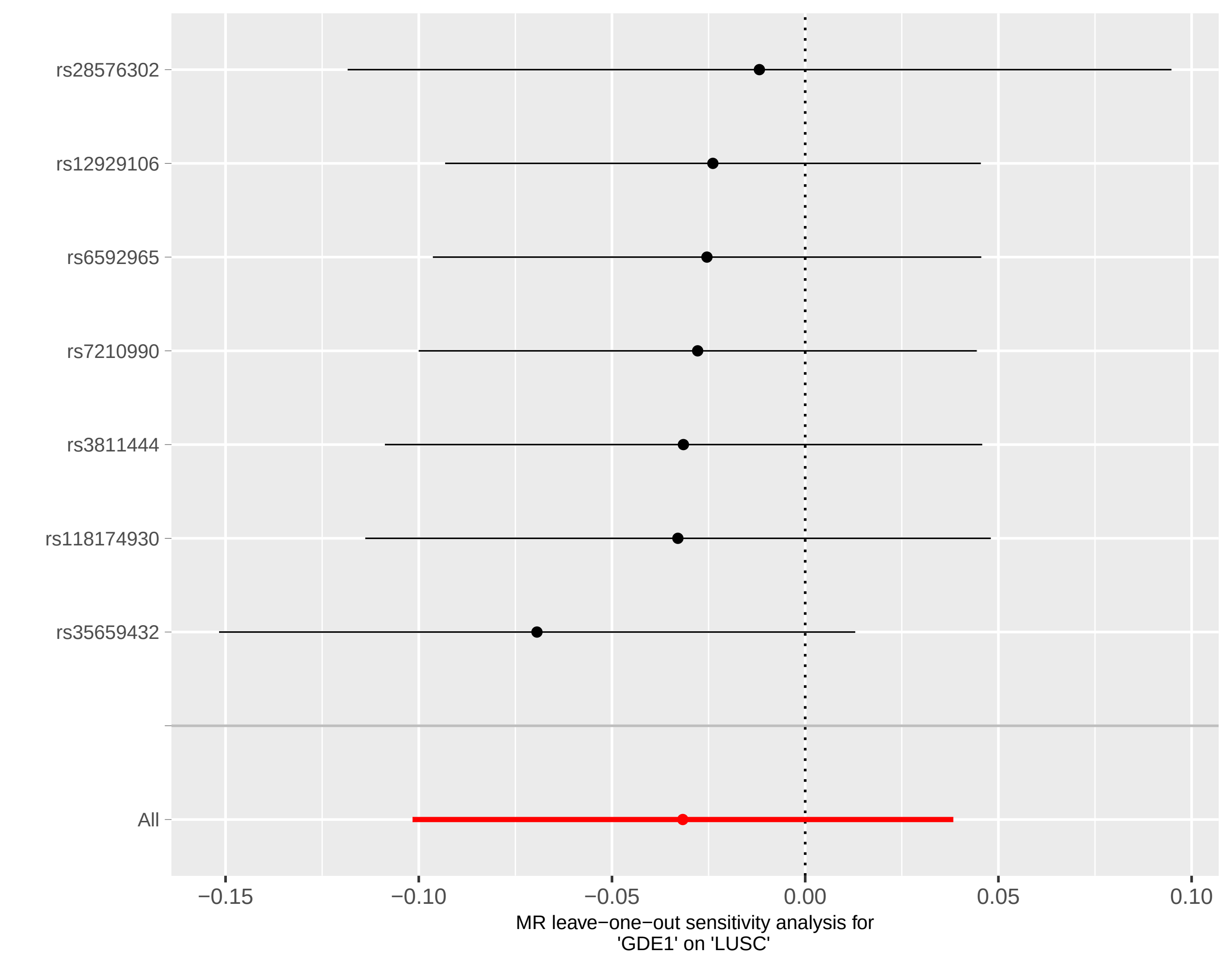

rs28576302
rs12929106
rs6592965
rs7210990
rs3811444
rs118174930
rs35659432
All
−0.15
−0.10
−0.05
0.00
0.05
0.10
MR leave−one−out sensitivity analysis for
'GDE1' on 'LUSC'

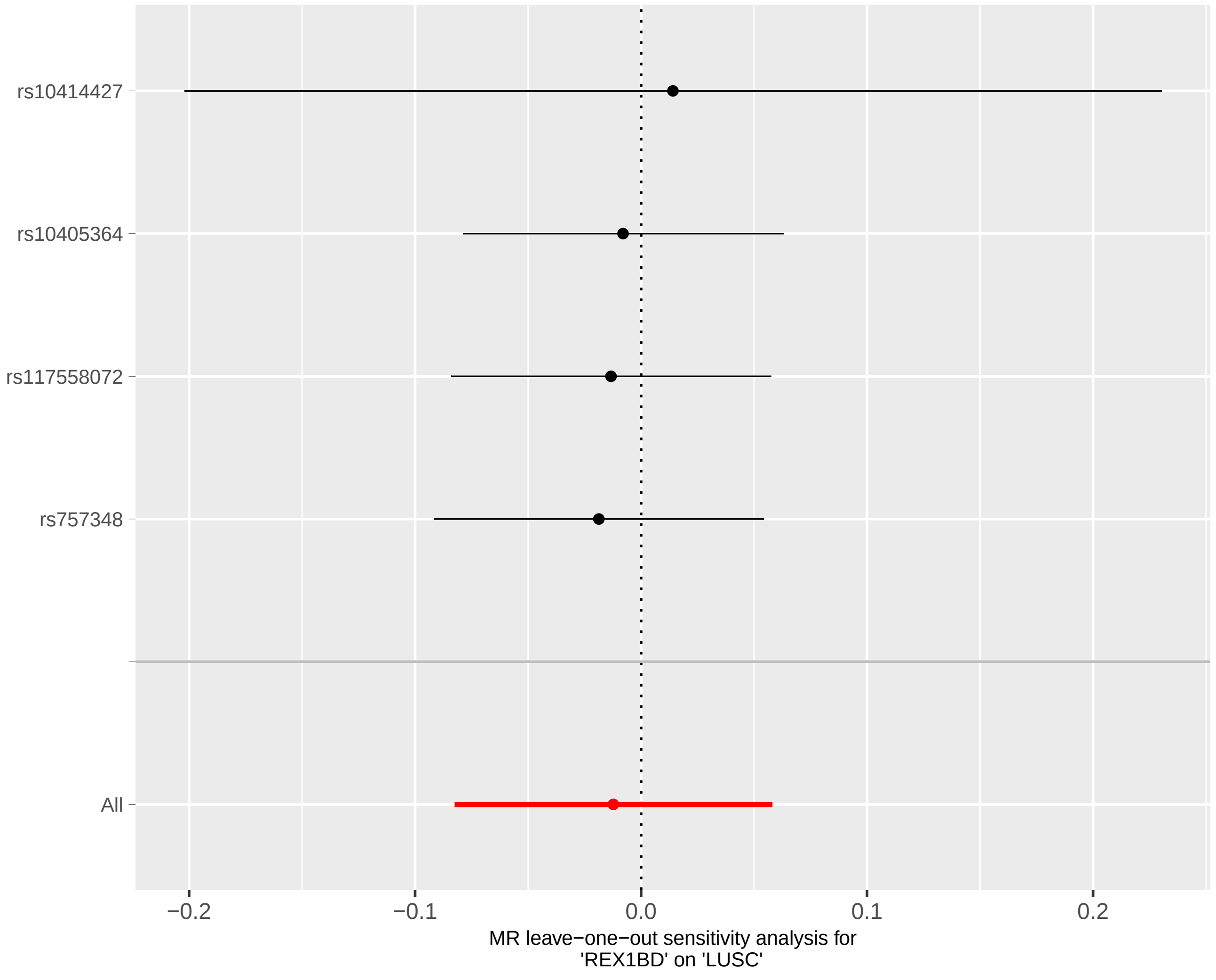
rs10414427
rs10405364
rs117558072
rs757348
All
−0.2
−0.1
0.0
0.1
0.2
MR leave−one−out sensitivity analysis for
'REX1BD' on 'LUSC'

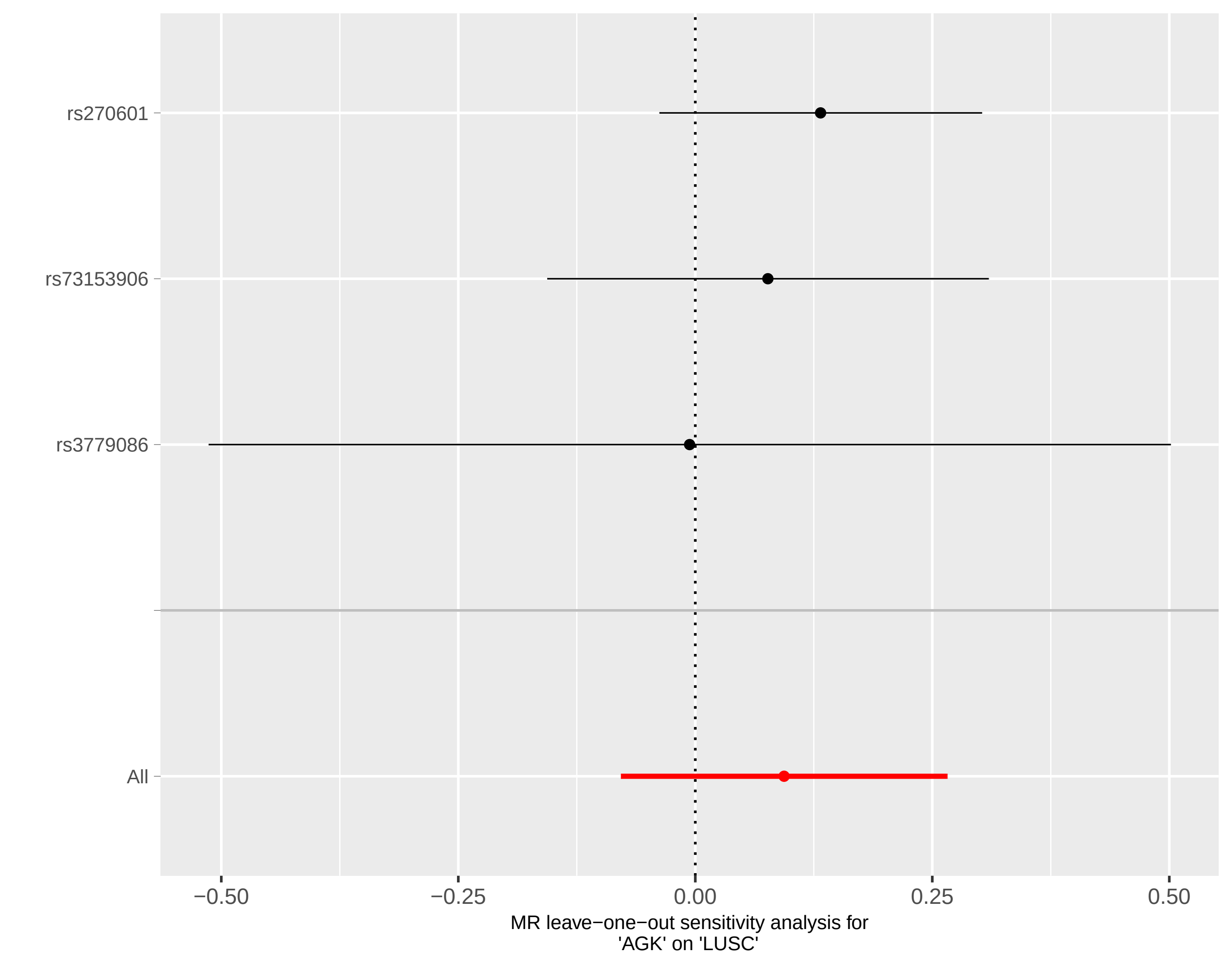
rs270601
rs73153906
rs3779086
All
−0.50
−0.25
0.00
0.25
0.50
MR leave−one−out sensitivity analysis for
'AGK' on 'LUSC'

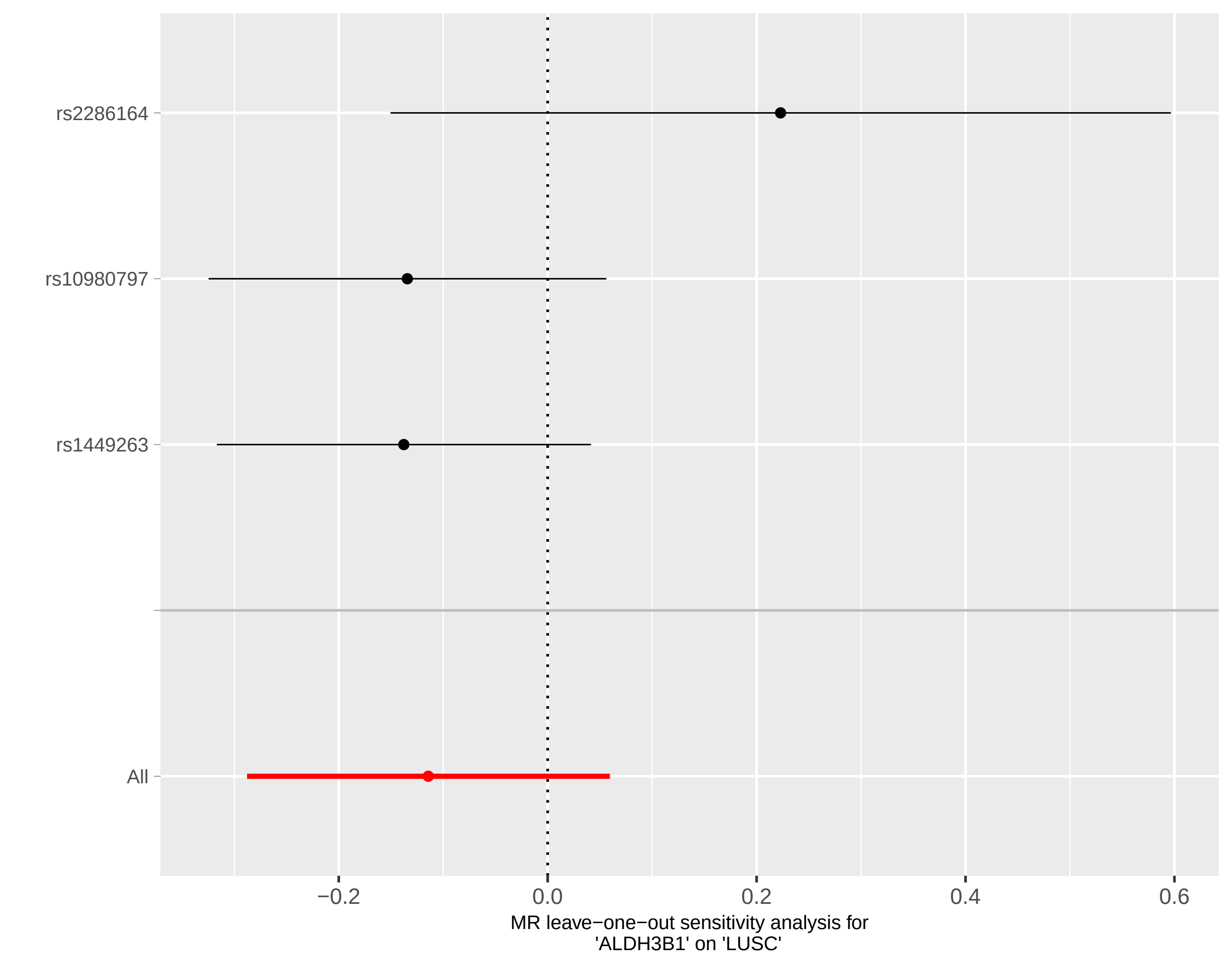
rs2286164
rs10980797
rs1449263
All
−0.2
0.0
0.2
0.4
0.6
MR leave−one−out sensitivity analysis for
'ALDH3B1' on 'LUSC'

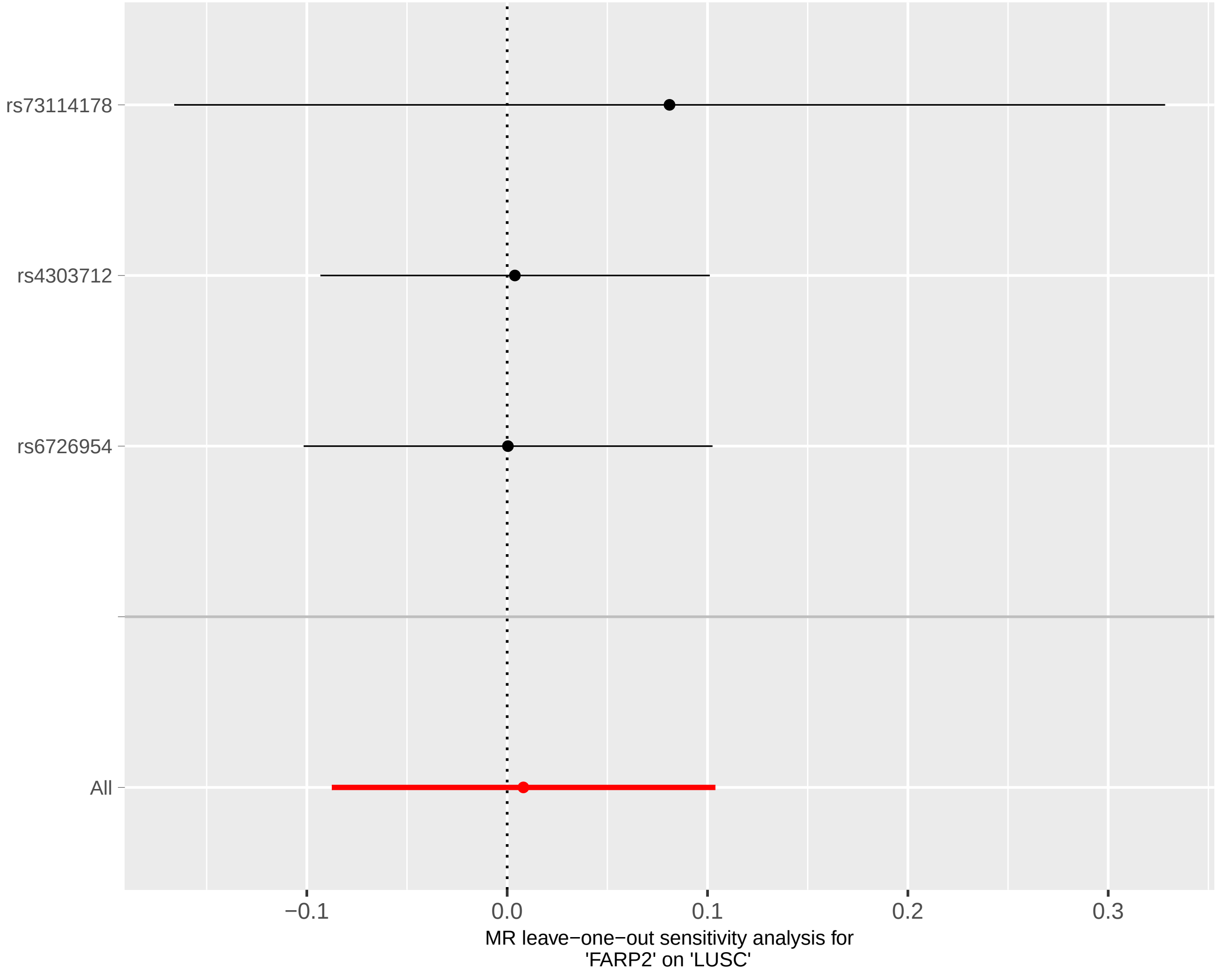

rs73114178
rs4303712
rs6726954
All
−0.1
0.0
0.1
0.2
0.3
MR leave−one−out sensitivity analysis for
'FARP2' on 'LUSC'

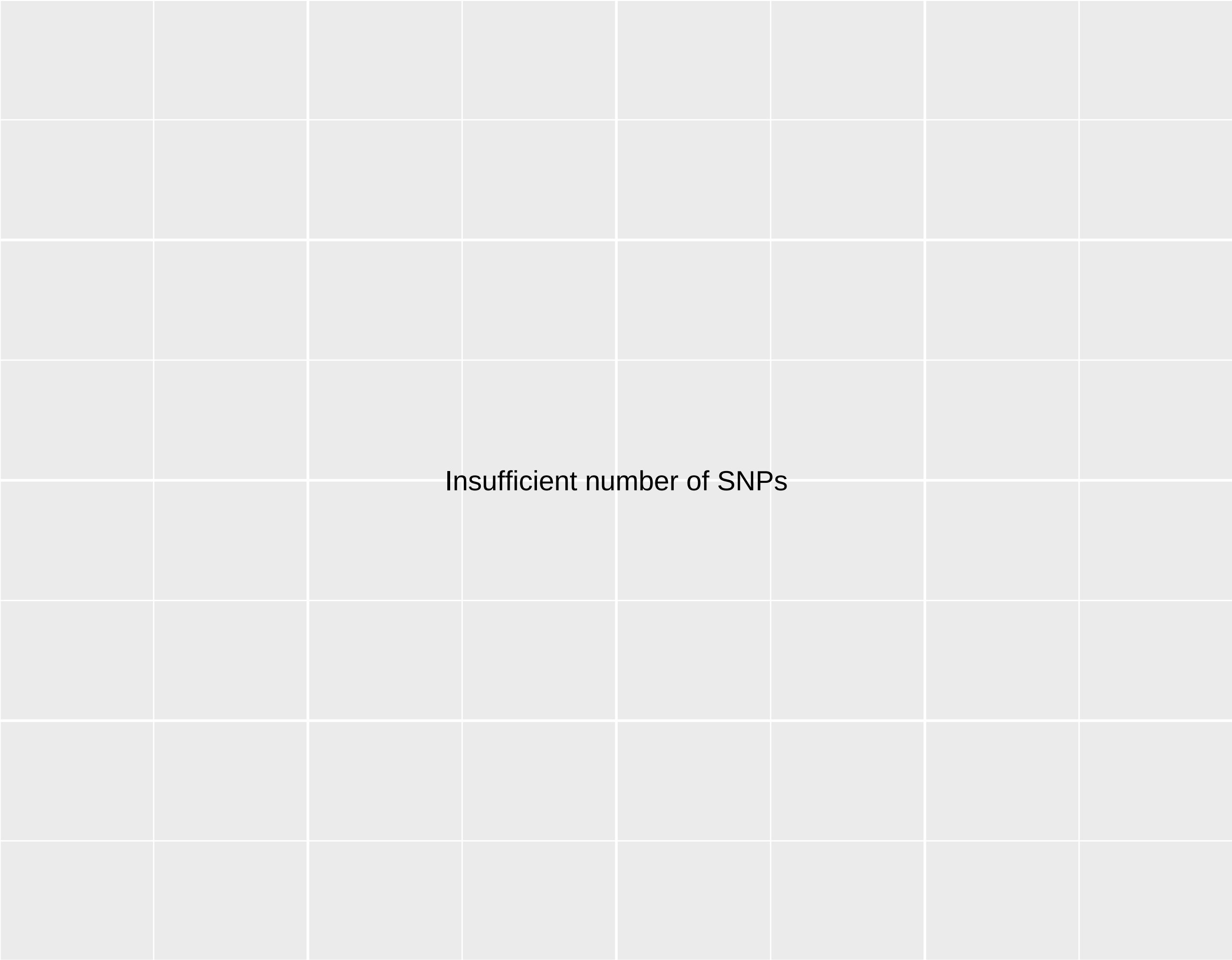
Insufficient number of SNPs

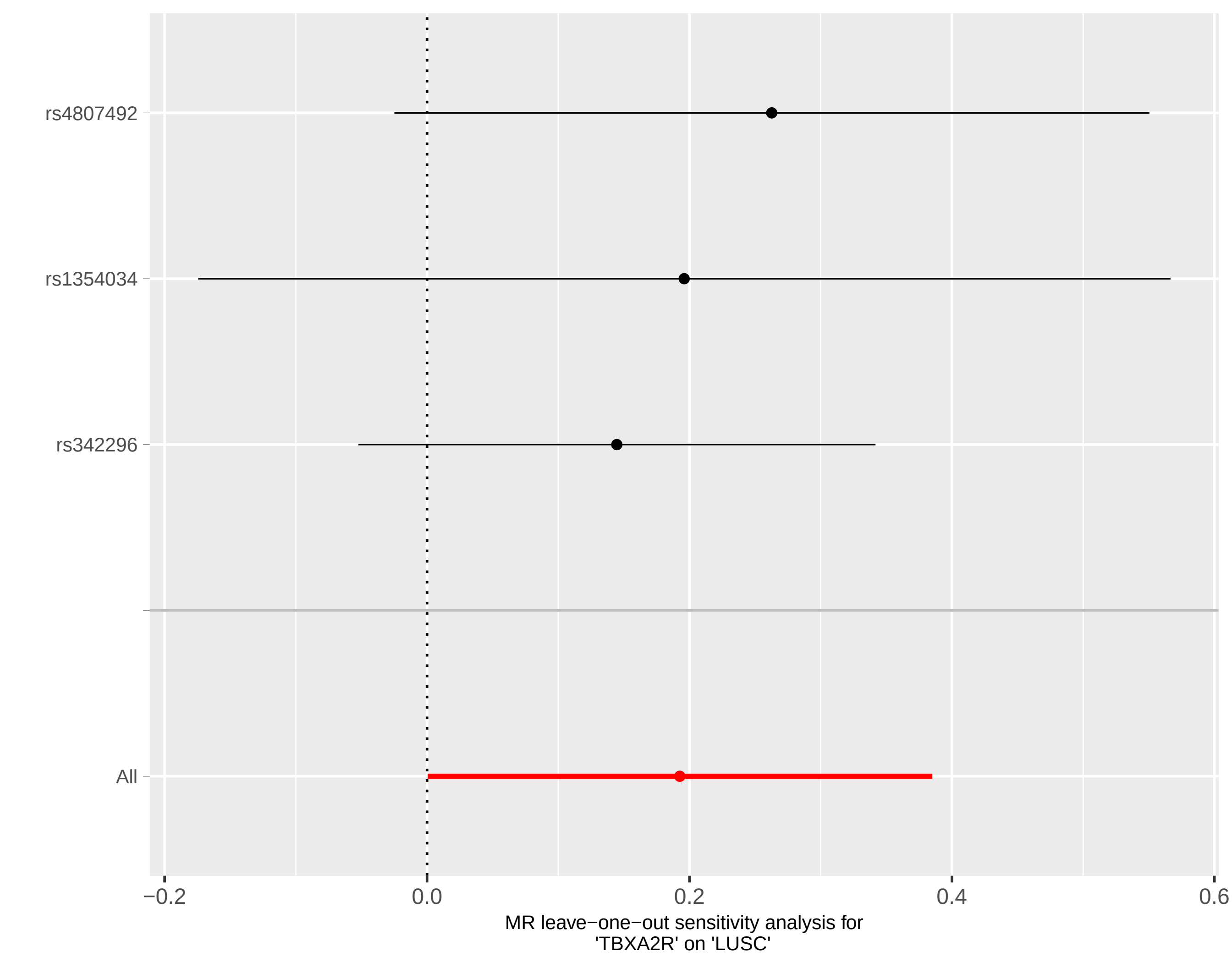
rs4807492
rs1354034
rs342296
All
−0.2
0.0
0.2
0.4
0.6
MR leave−one−out sensitivity analysis for
'TBXA2R' on 'LUSC'

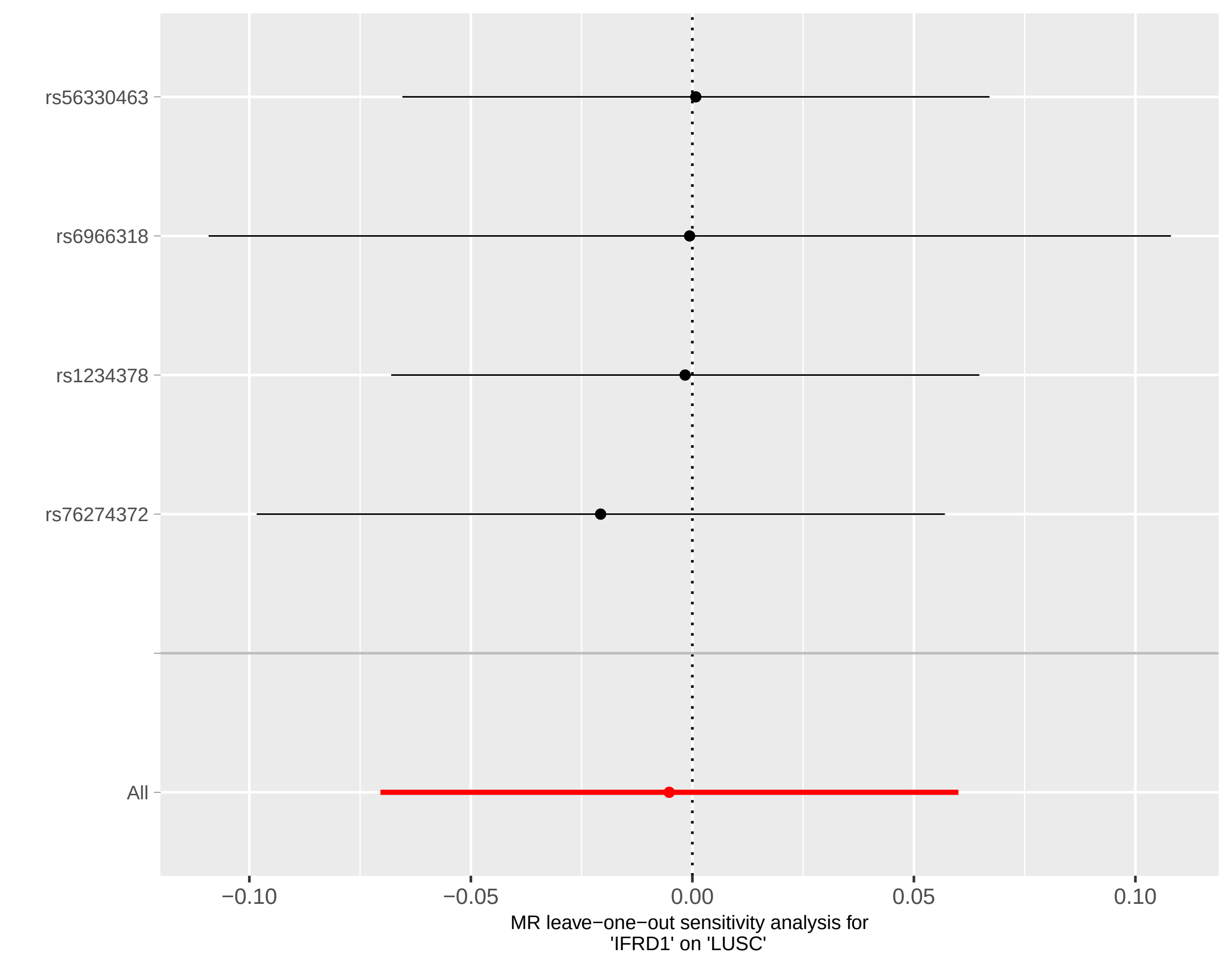
rs56330463
rs6966318
rs1234378
rs76274372
All
−0.10
−0.05
0.00
0.05
0.10
MR leave−one−out sensitivity analysis for
'IFRD1' on 'LUSC'

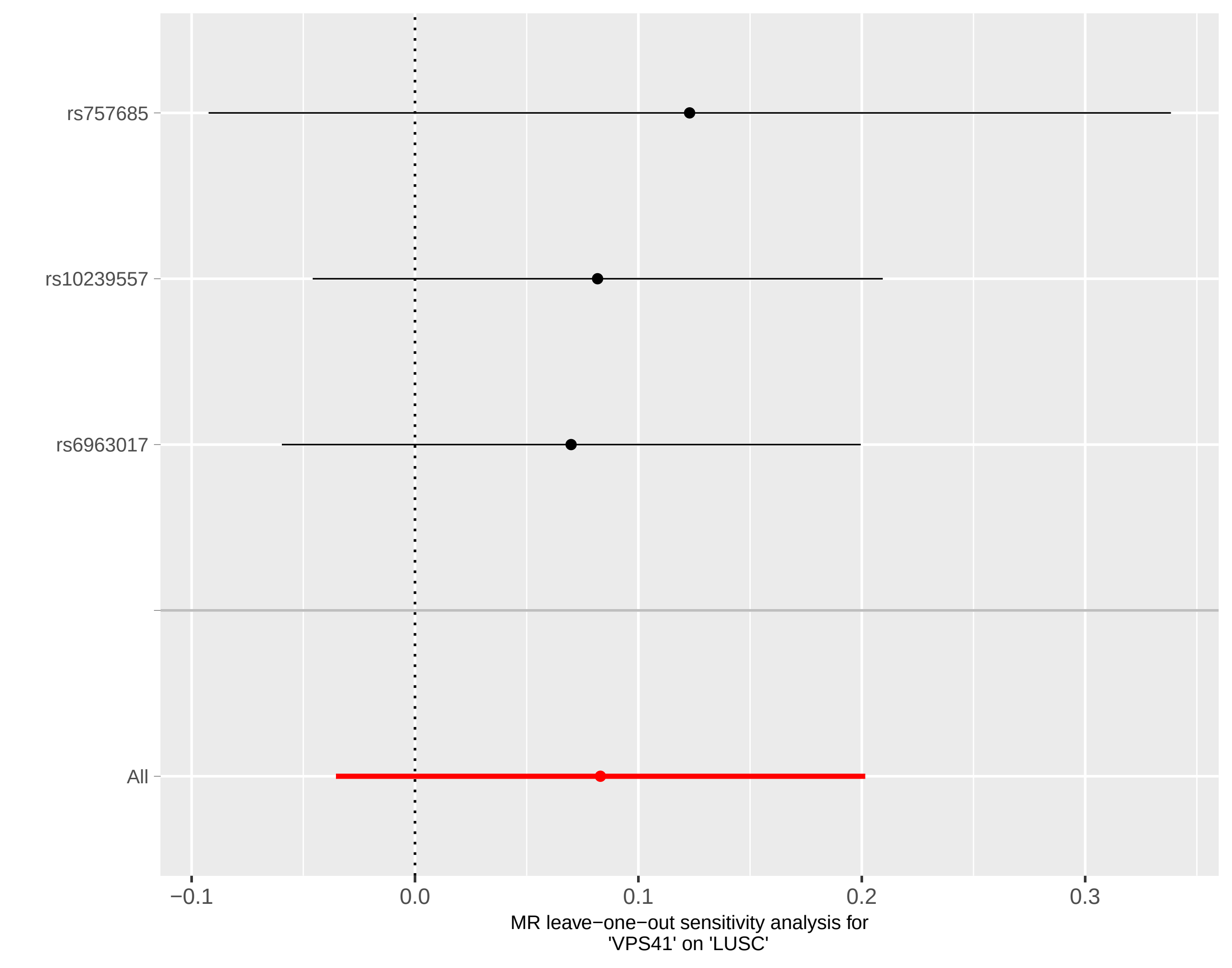

rs757685
rs10239557
rs6963017
All
−0.1
0.0
0.1
0.2
0.3
MR leave−one−out sensitivity analysis for
'VPS41' on 'LUSC'

Insufficient number of SNPs

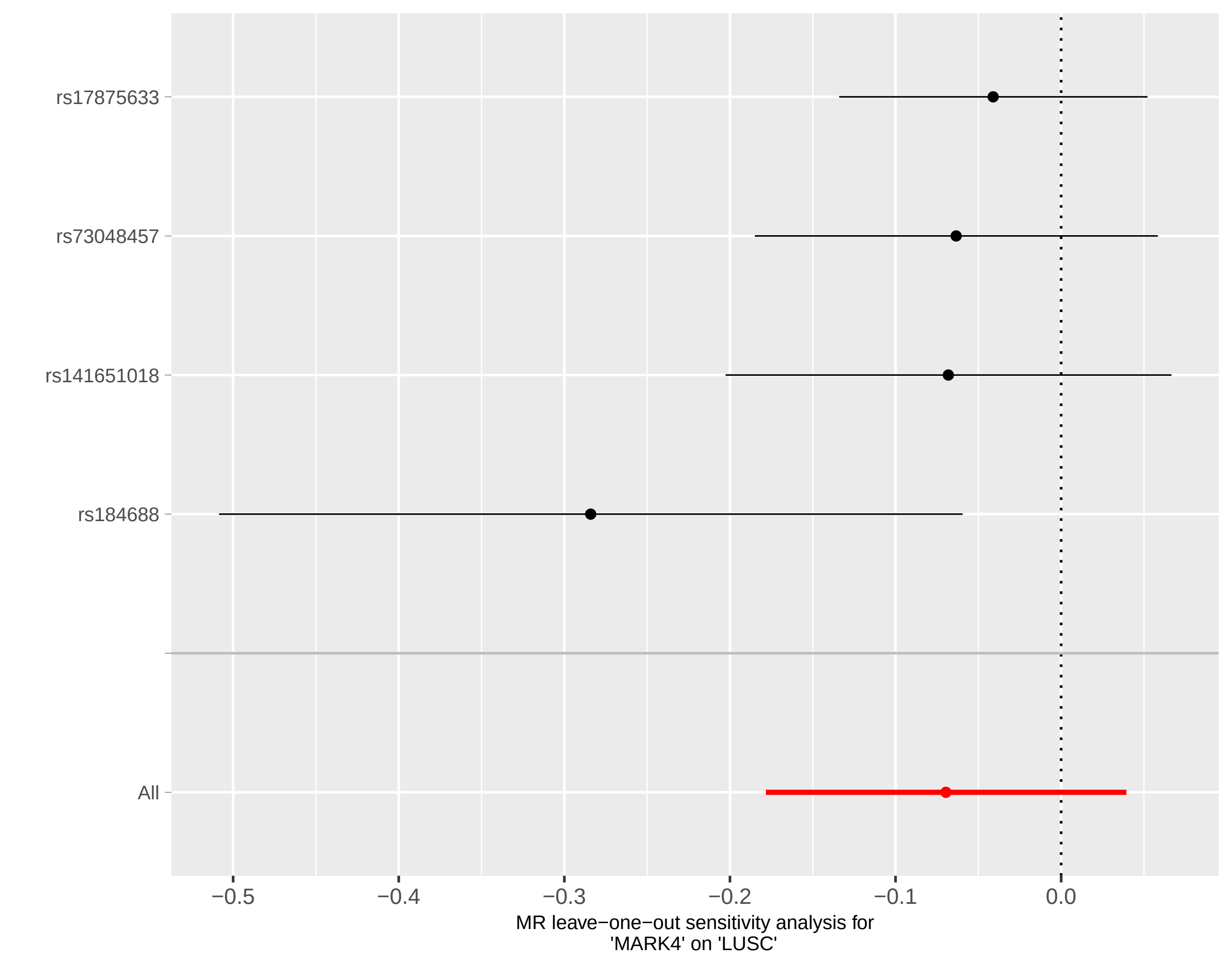

rs17875633
rs73048457
rs141651018
rs184688
All
−0.5
−0.4
−0.3
−0.2
−0.1
0.0
MR leave−one−out sensitivity analysis for
'MARK4' on 'LUSC'

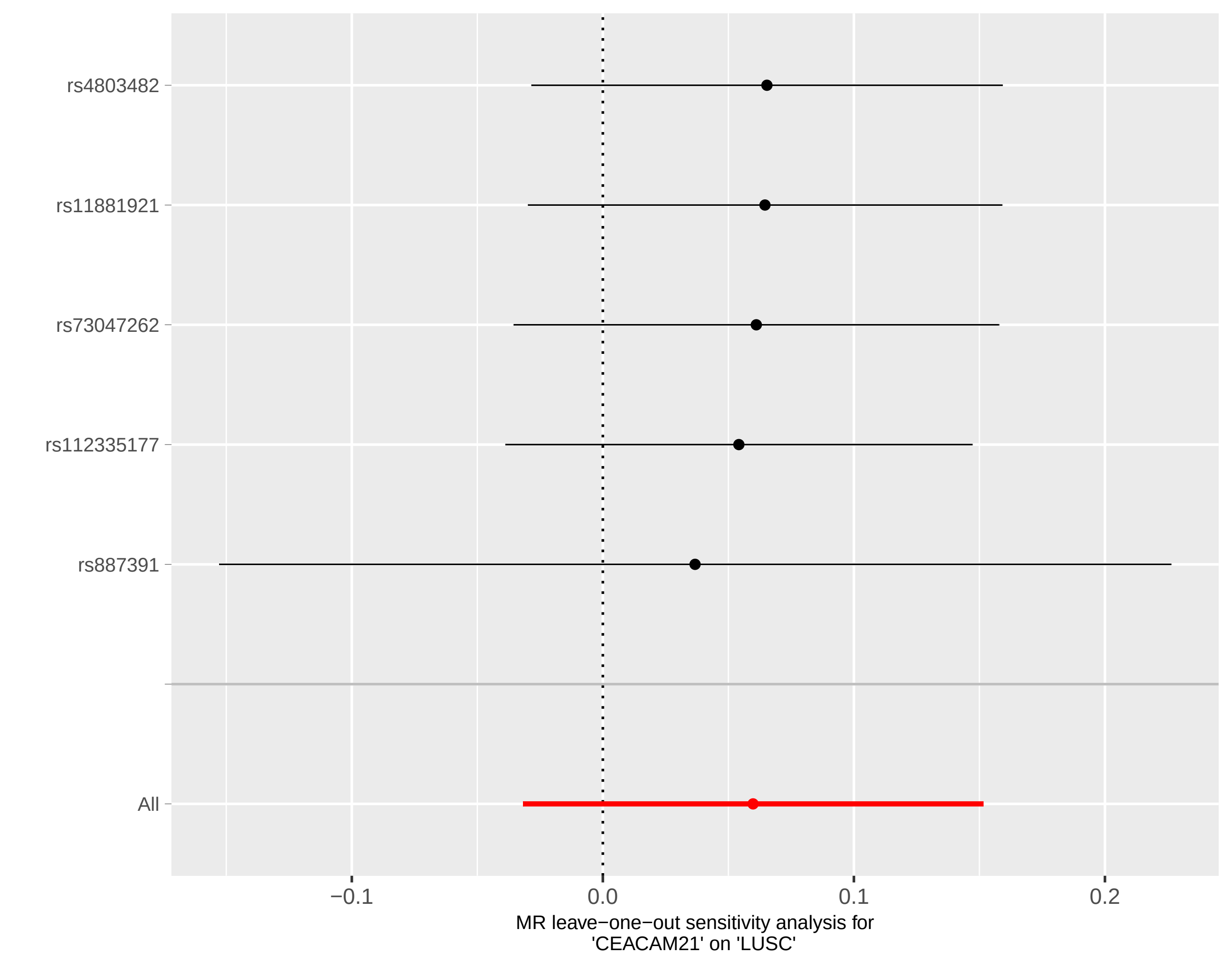
rs4803482
rs11881921
rs73047262
rs112335177
rs887391
All
−0.1
0.0
0.1
0.2
MR leave−one−out sensitivity analysis for
'CEACAM21' on 'LUSC'

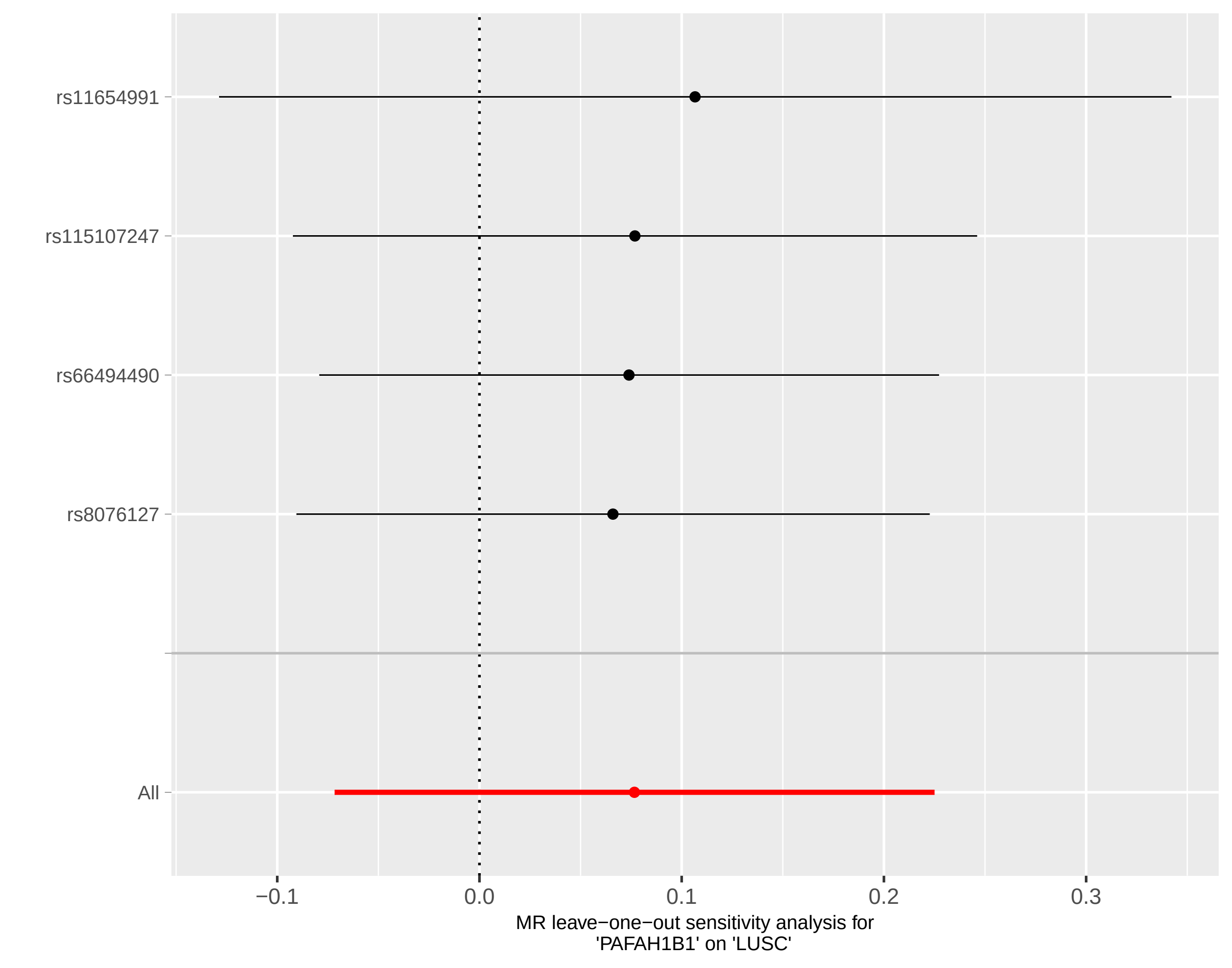
rs11654991
rs115107247
rs66494490
rs8076127
All
−0.1
0.0
0.1
0.2
0.3
MR leave−one−out sensitivity analysis for
'PAFAH1B1' on 'LUSC'

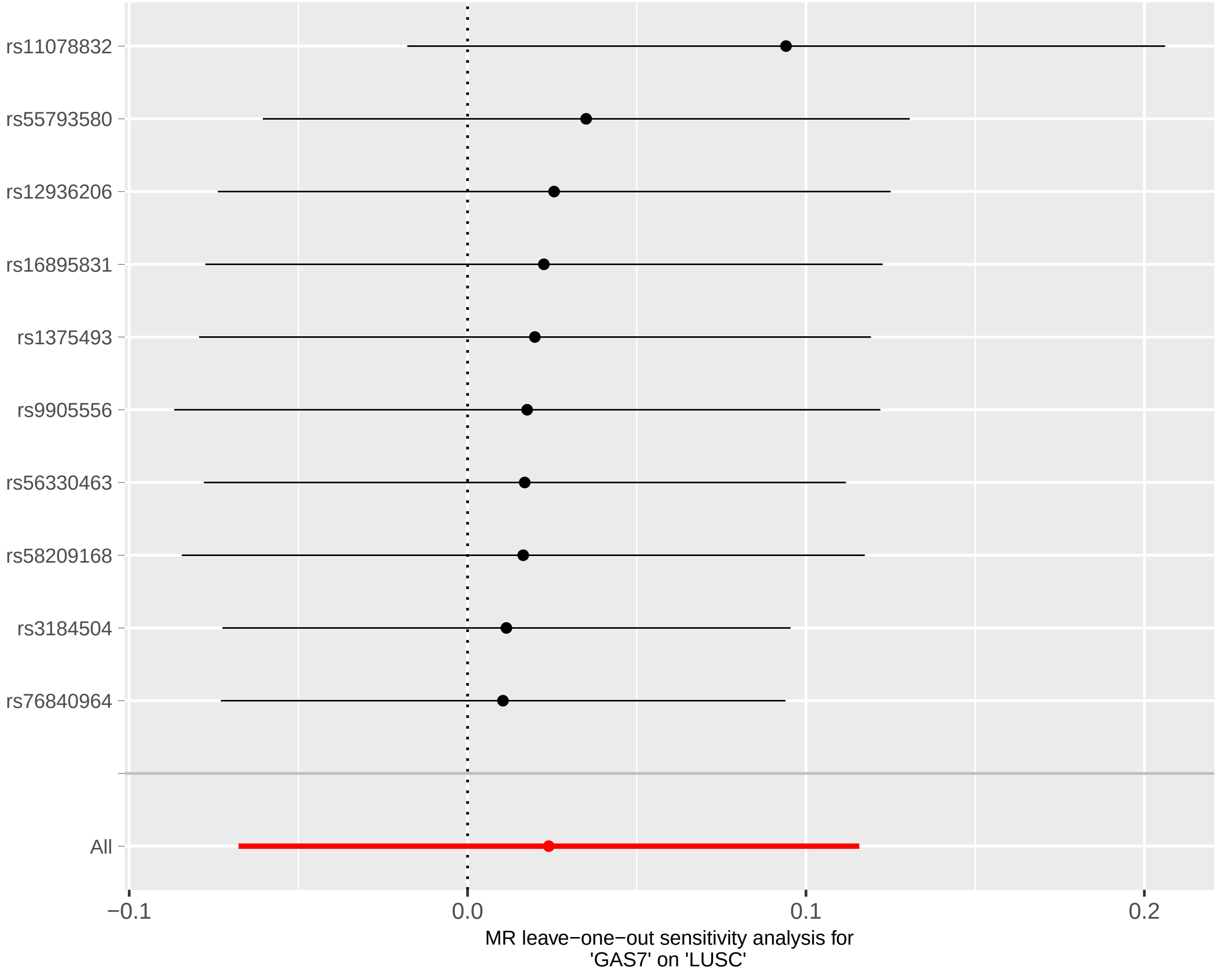
rs11078832
rs55793580
rs12936206
rs16895831
rs1375493
rs9905556
rs56330463
rs58209168
rs3184504
rs76840964
All
−0.1
0.0
0.1
0.2
MR leave−one−out sensitivity analysis for
'GAS7' on 'LUSC'

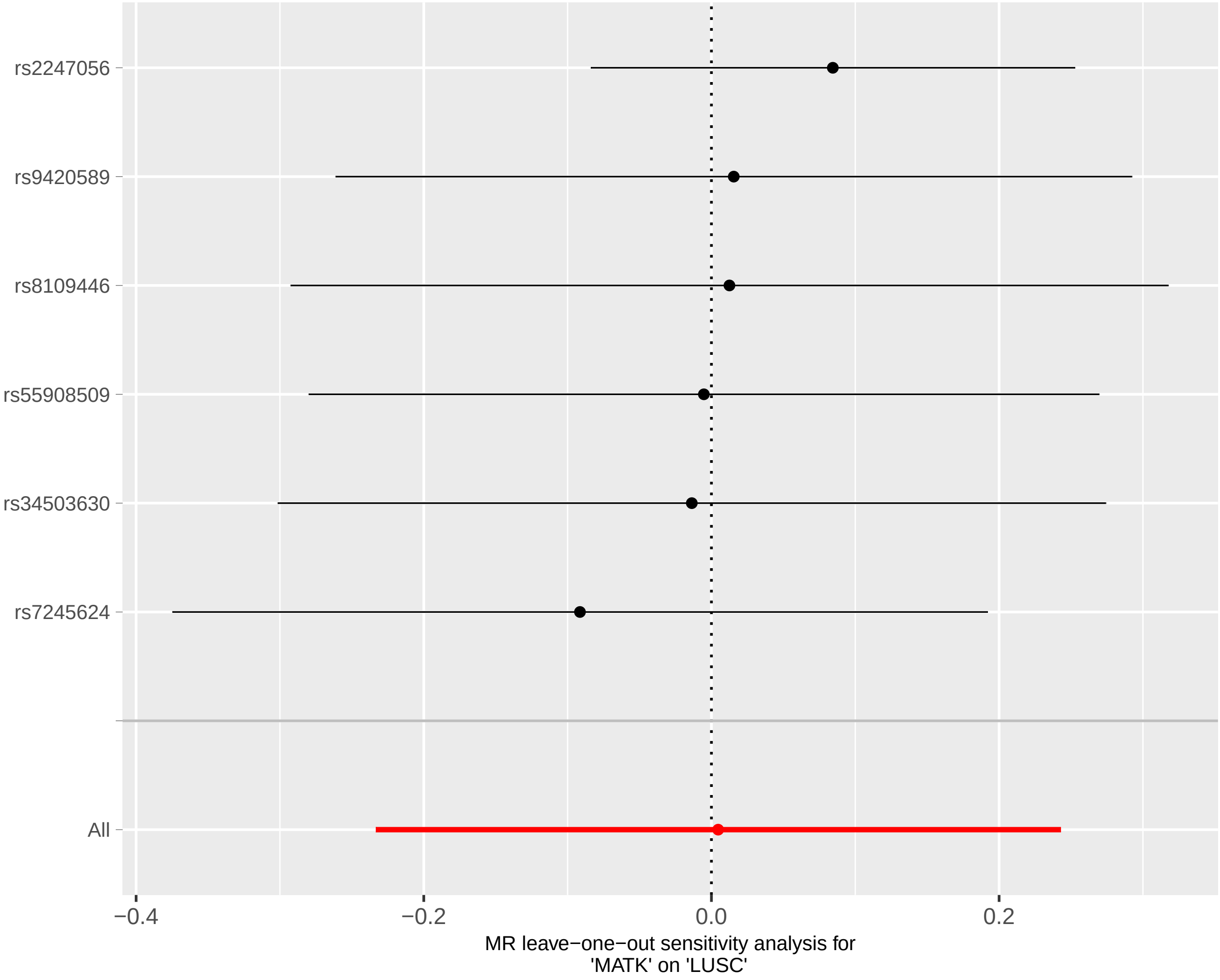
rs2247056
rs9420589
rs8109446
rs55908509
rs34503630
rs7245624
All
−0.4
−0.2
0.0
0.2
MR leave−one−out sensitivity analysis for
'MATK' on 'LUSC'

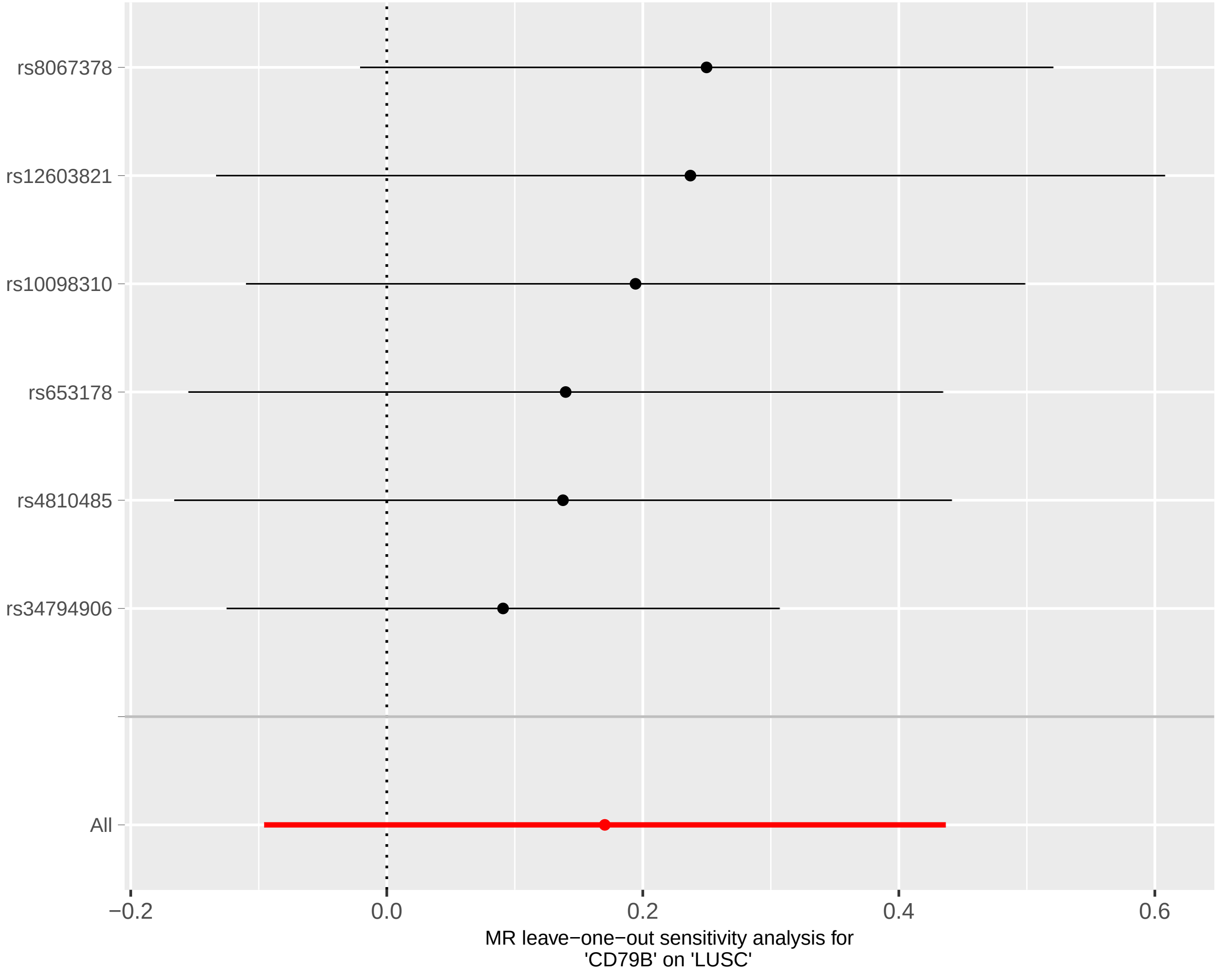
rs8067378
rs12603821
rs10098310
rs653178
rs4810485
rs34794906
All
−0.2
0.0
0.2
0.4
0.6
MR leave−one−out sensitivity analysis for
'CD79B' on 'LUSC'

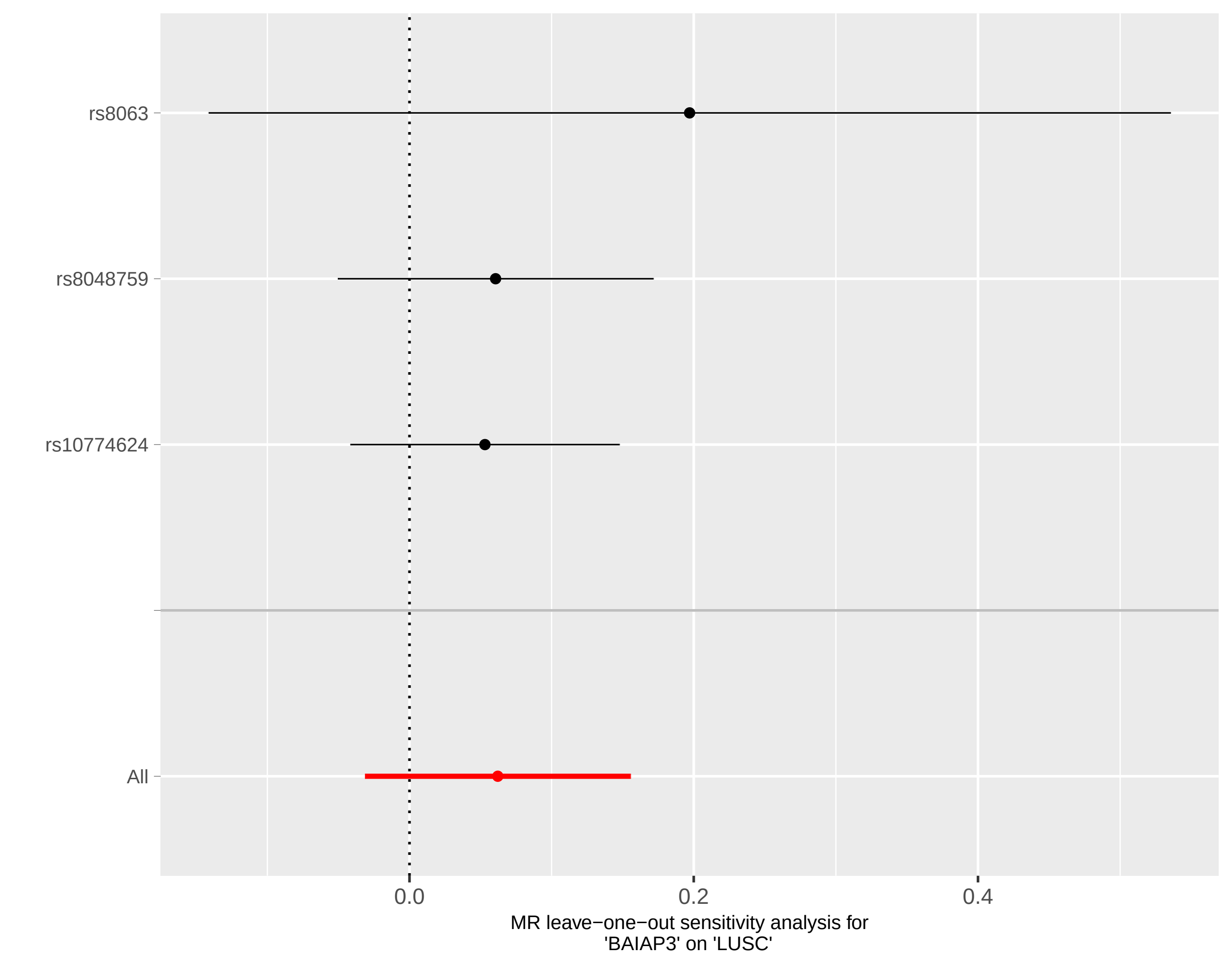

rs8063
rs8048759
rs10774624
All
0.0
0.2
0.4
MR leave−one−out sensitivity analysis for
'BAIAP3' on 'LUSC'

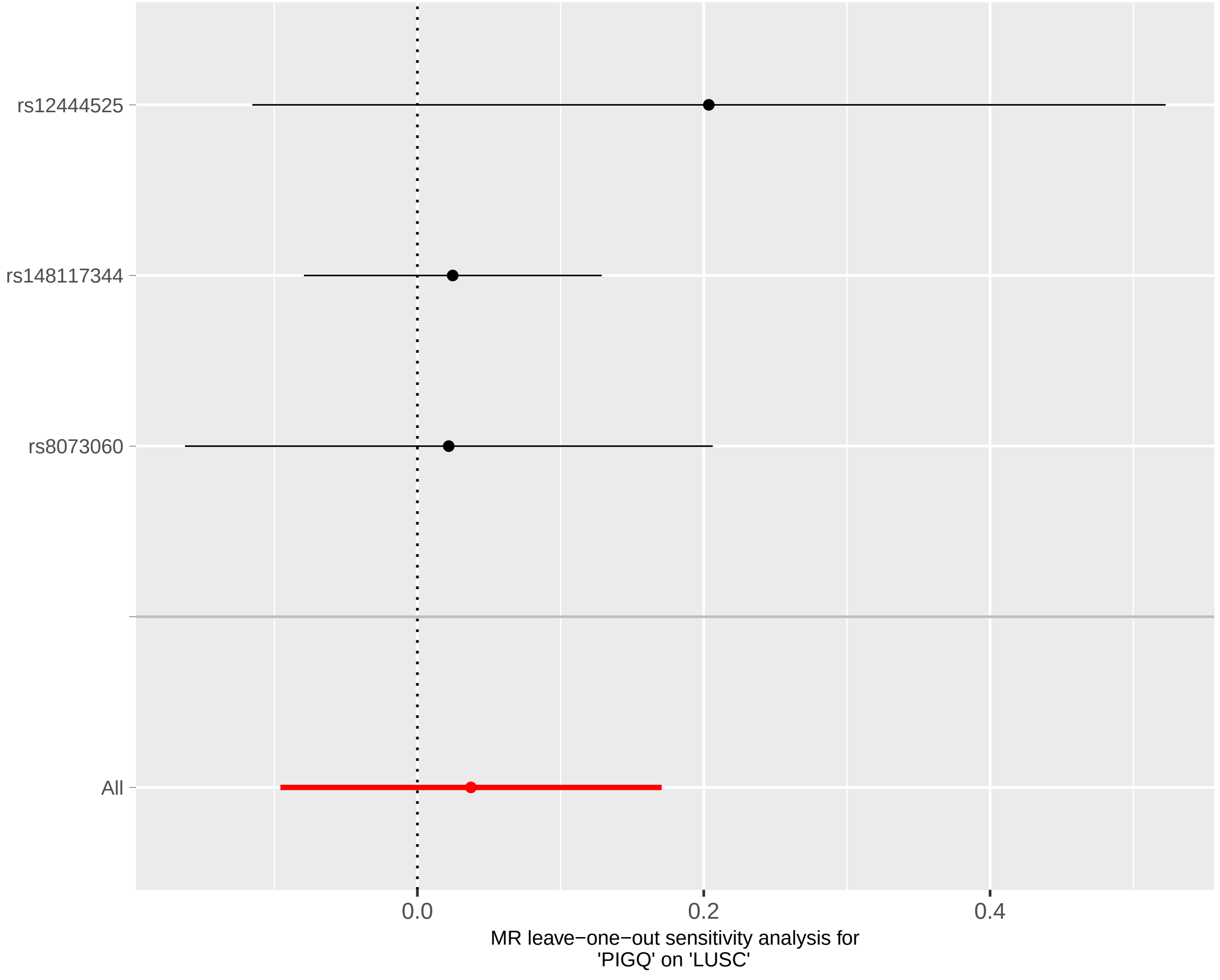

rs12444525
rs148117344
rs8073060
All
0.0
0.2
0.4
MR leave−one−out sensitivity analysis for
'PIGQ' on 'LUSC'

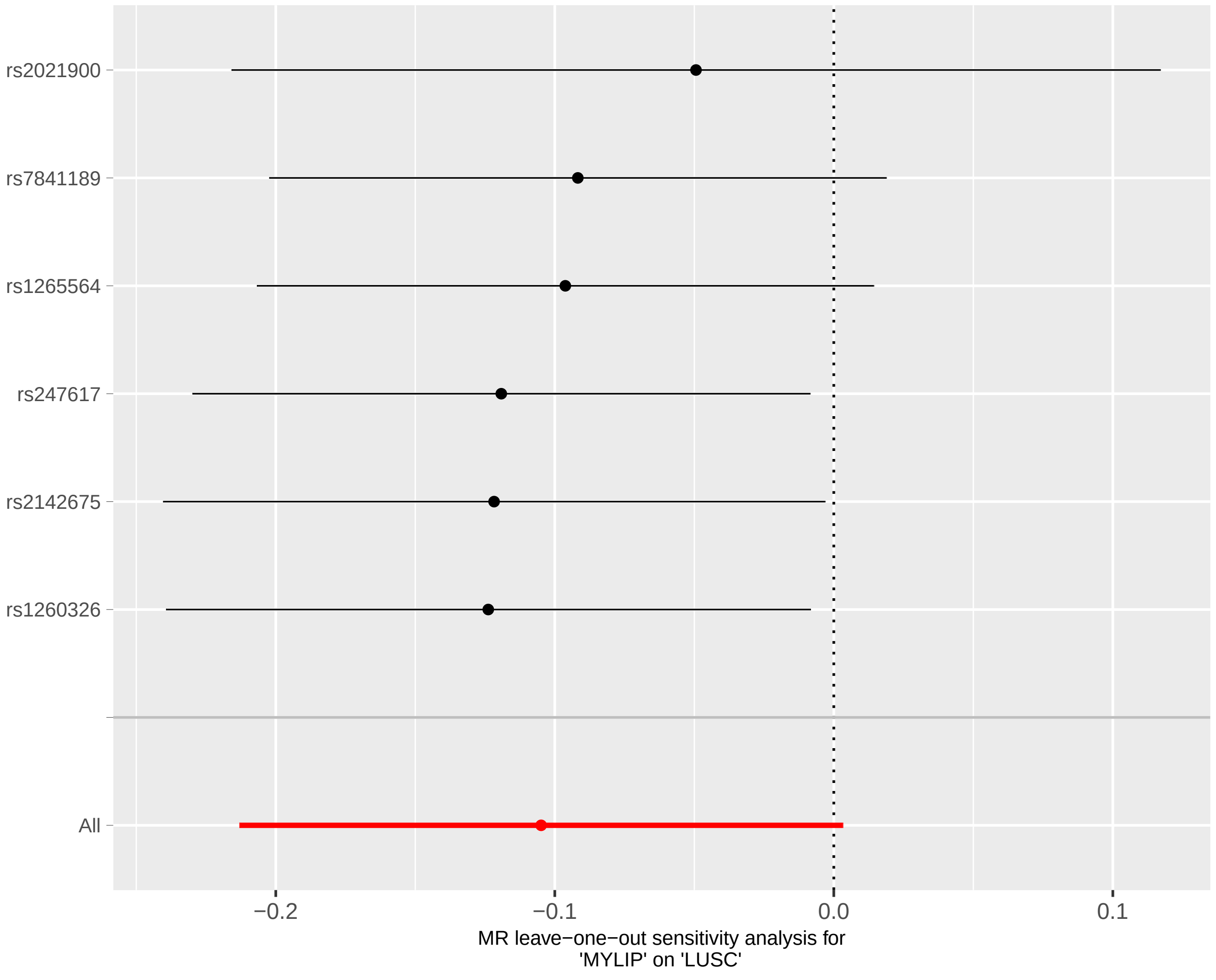
rs2021900
rs7841189
rs1265564
rs247617
rs2142675
rs1260326
All
−0.2
−0.1
0.0
0.1
MR leave−one−out sensitivity analysis for
'MYLIP' on 'LUSC'

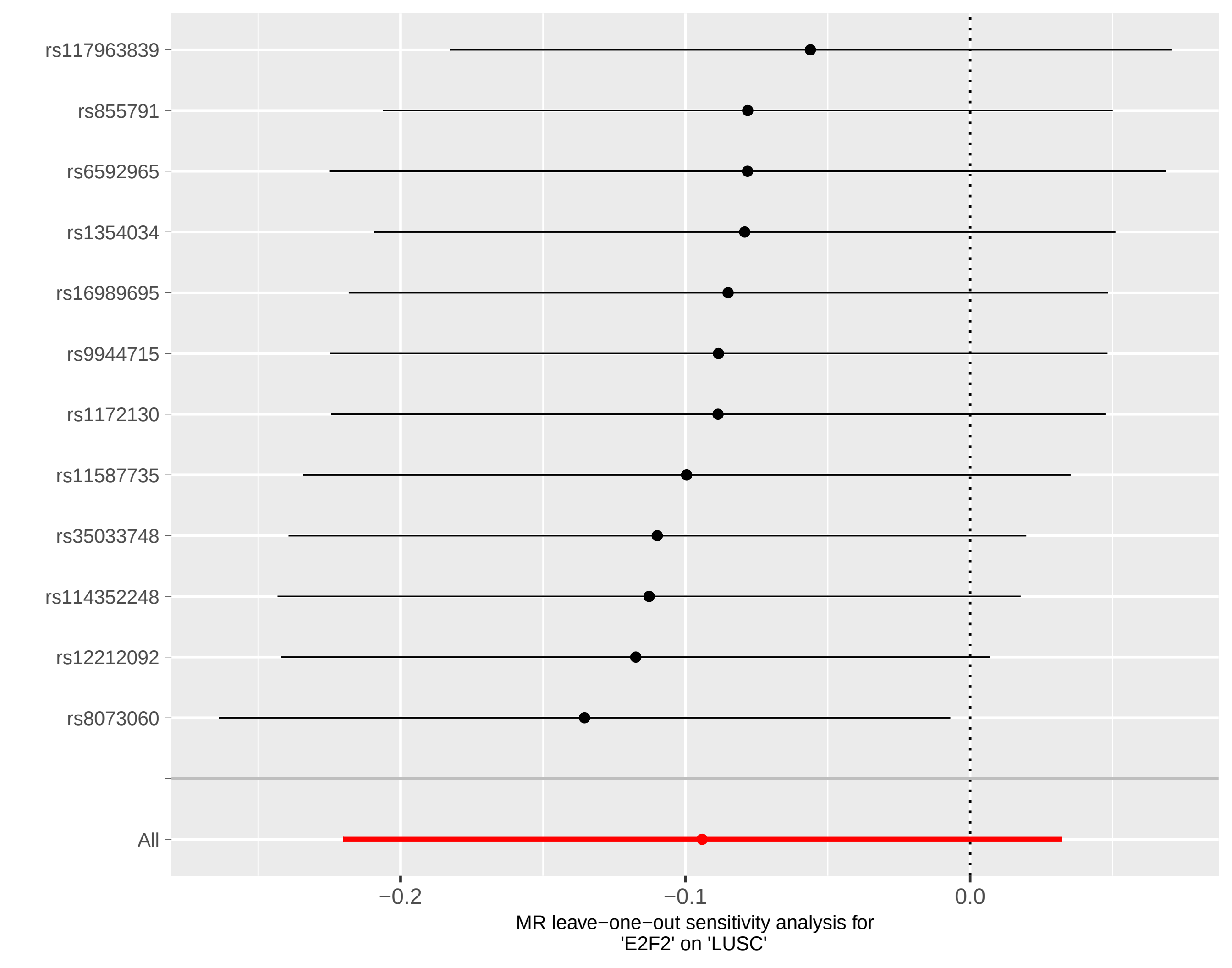
rs117963839
rs855791
rs6592965
rs1354034
rs16989695
rs9944715
rs1172130
rs11587735
rs35033748
rs114352248
rs12212092
rs8073060
All
−0.2
−0.1
0.0
MR leave−one−out sensitivity analysis for
'E2F2' on 'LUSC'

Insufficient number of SNPs

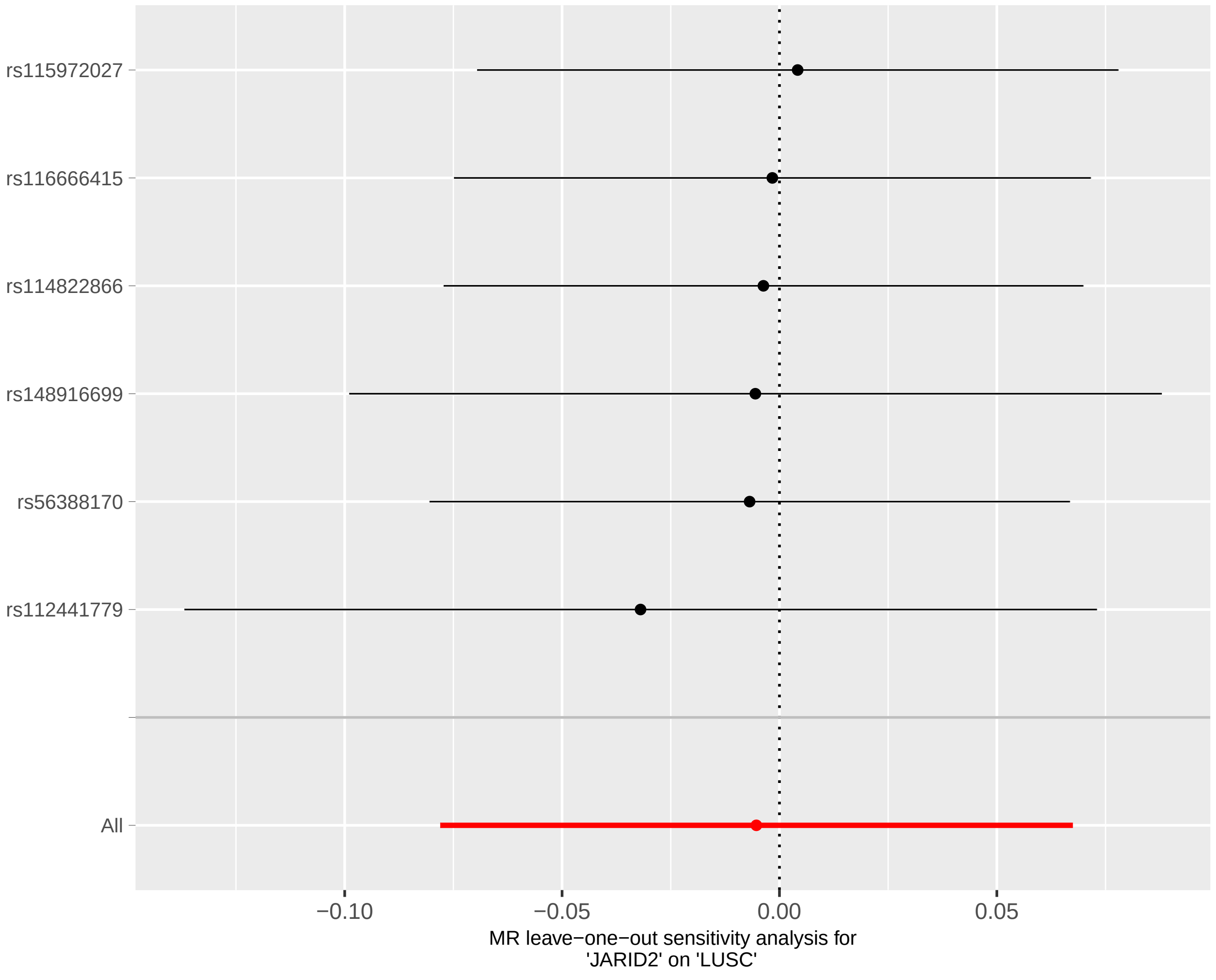
rs115972027
rs116666415
rs114822866
rs148916699
rs56388170
rs112441779
All
−0.10
−0.05
0.00
0.05
MR leave−one−out sensitivity analysis for
'JARID2' on 'LUSC'

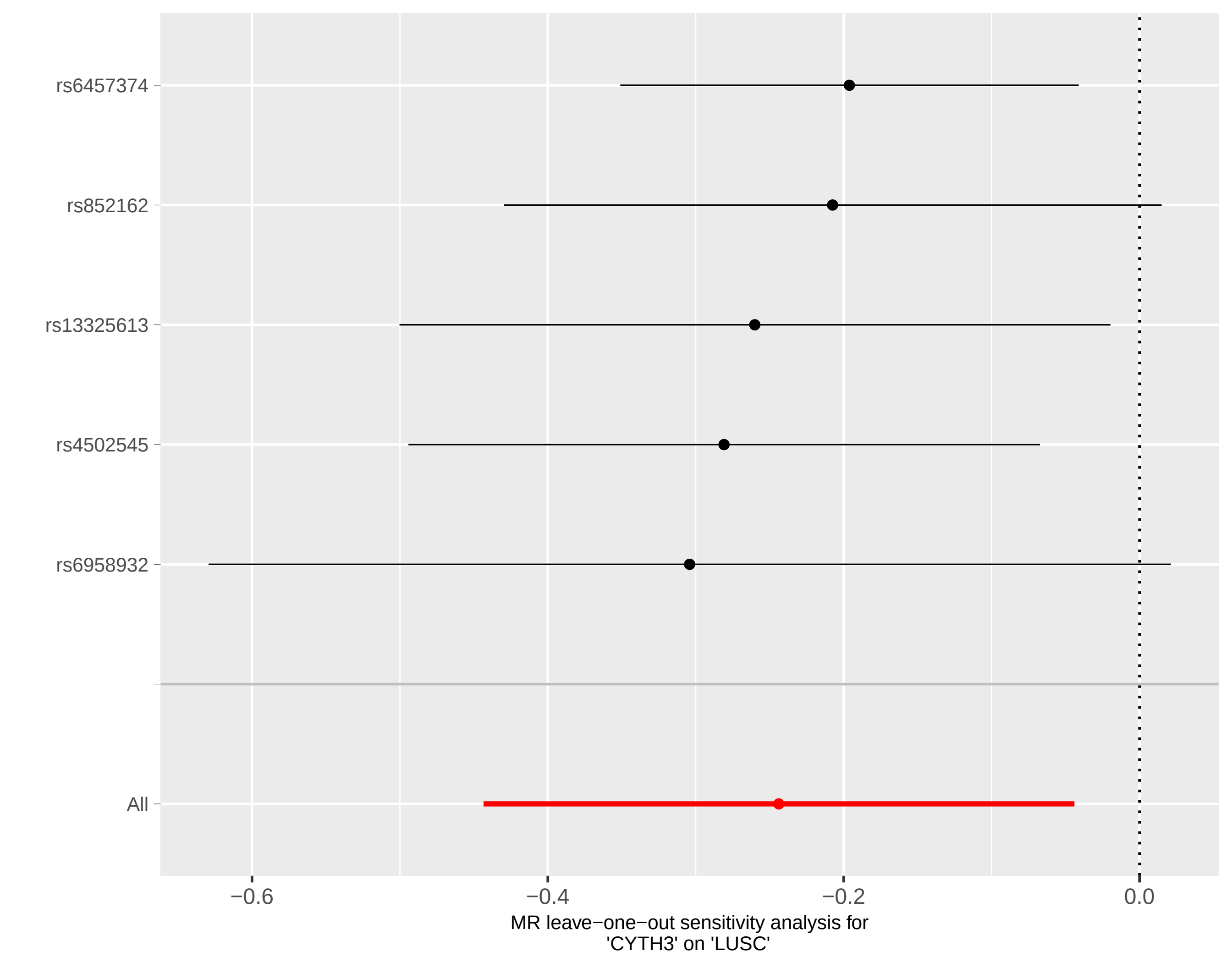

rs6457374
rs852162
rs13325613
rs4502545
rs6958932
All
−0.6
−0.4
−0.2
0.0
MR leave−one−out sensitivity analysis for
'CYTH3' on 'LUSC'

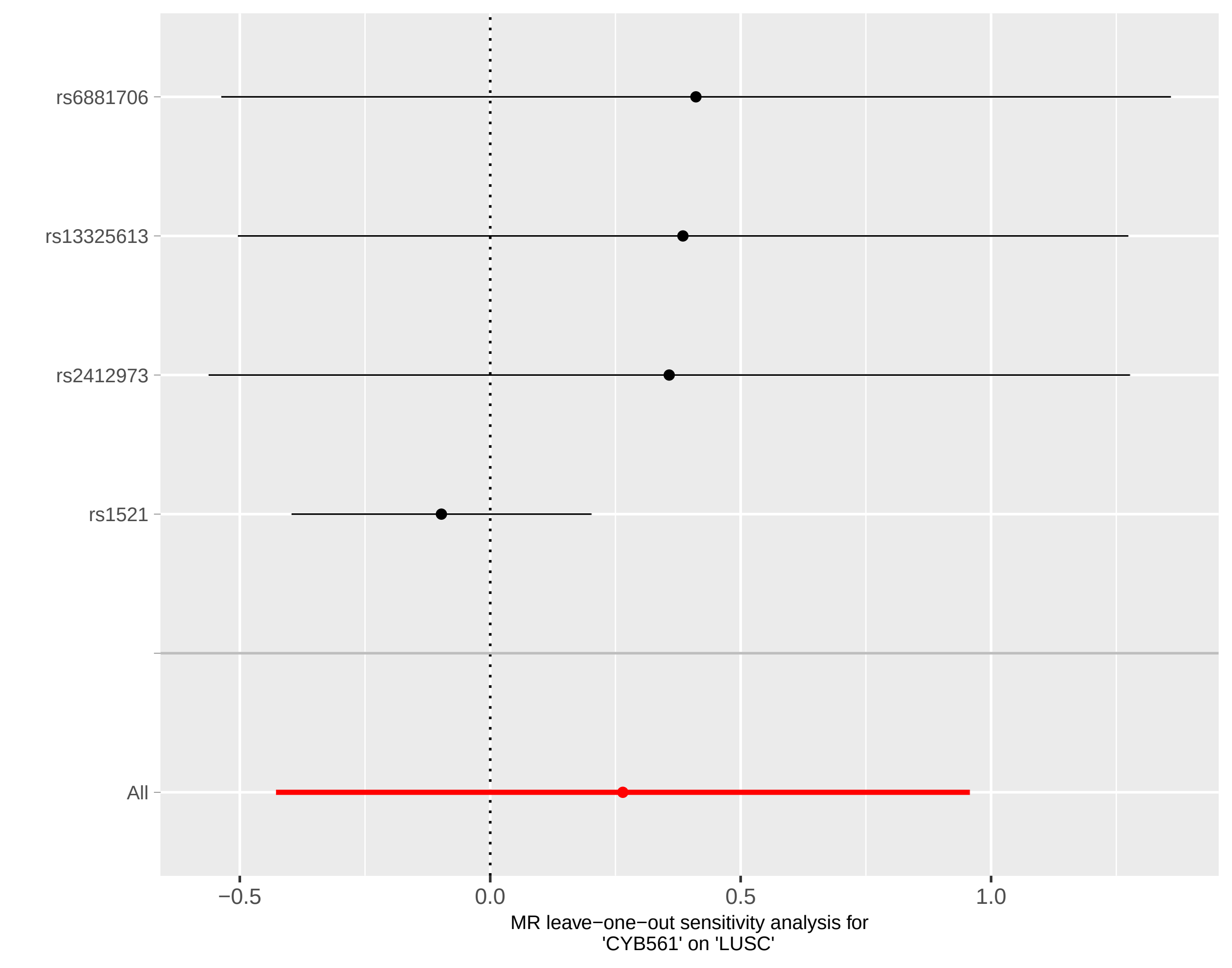
rs6881706
rs13325613
rs2412973
rs1521
All
−0.5
0.0
0.5
1.0
MR leave−one−out sensitivity analysis for
'CYB561' on 'LUSC'

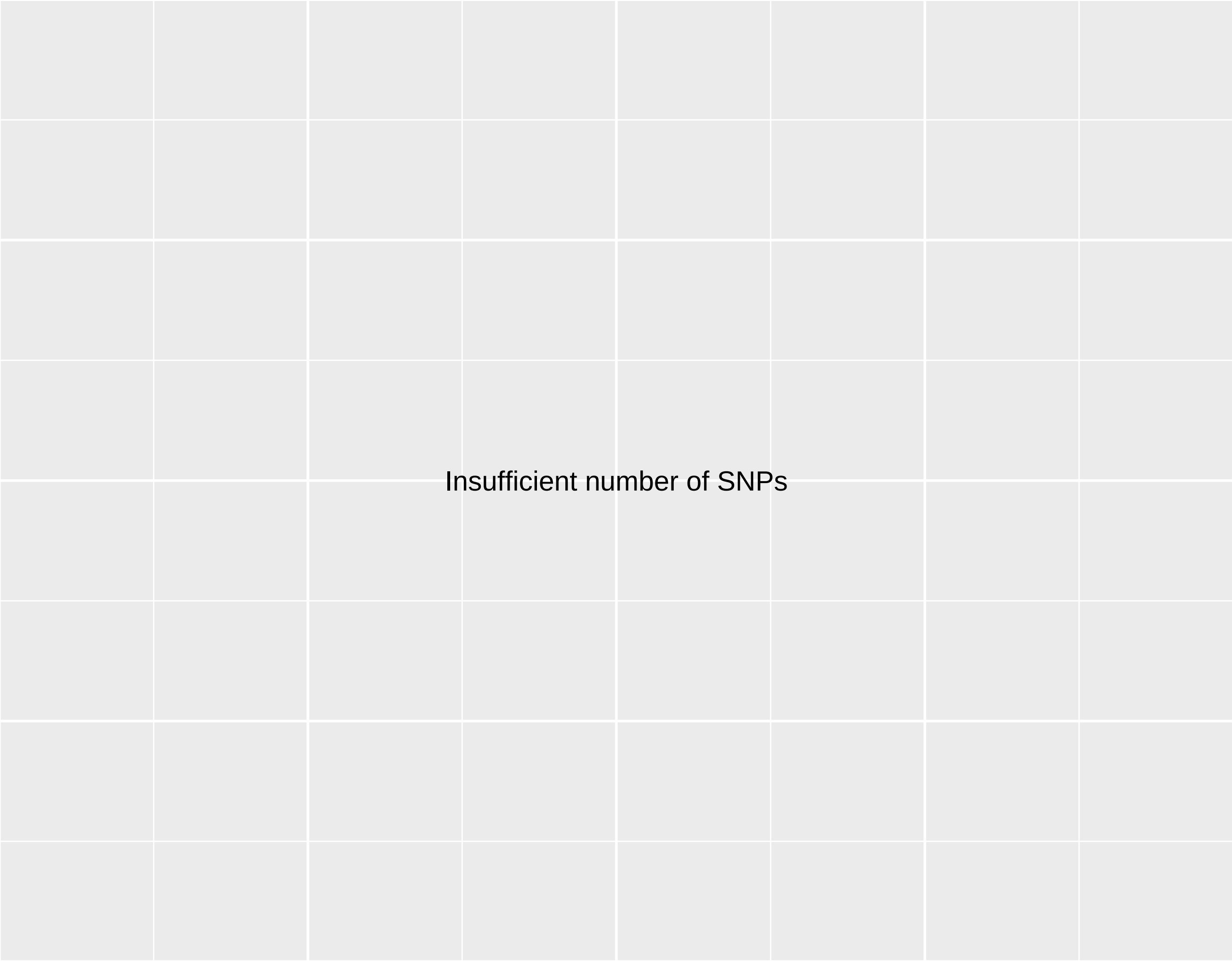
Insufficient number of SNPs

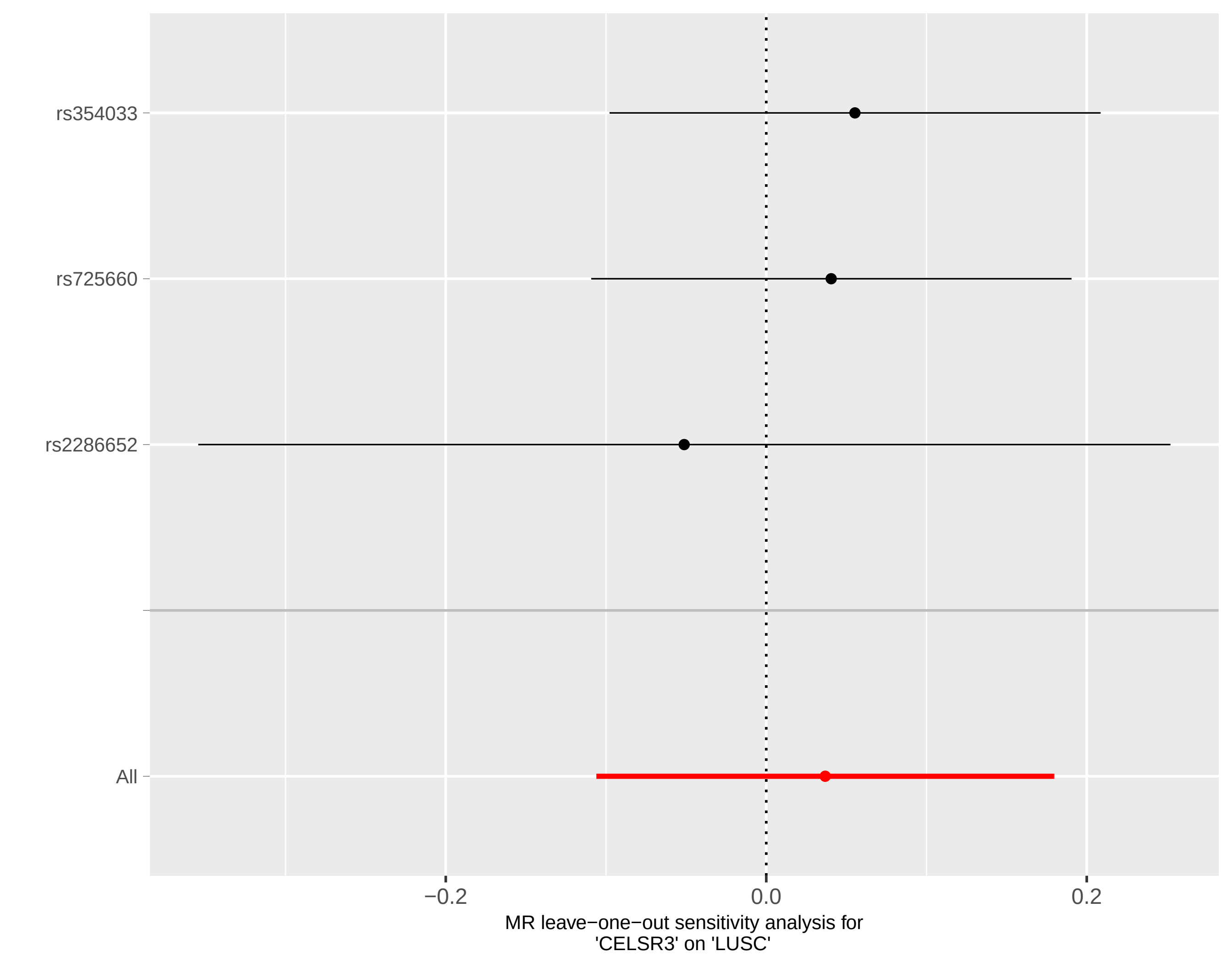
rs354033
rs725660
rs2286652
All
−0.2
0.0
0.2
MR leave−one−out sensitivity analysis for
'CELSR3' on 'LUSC'

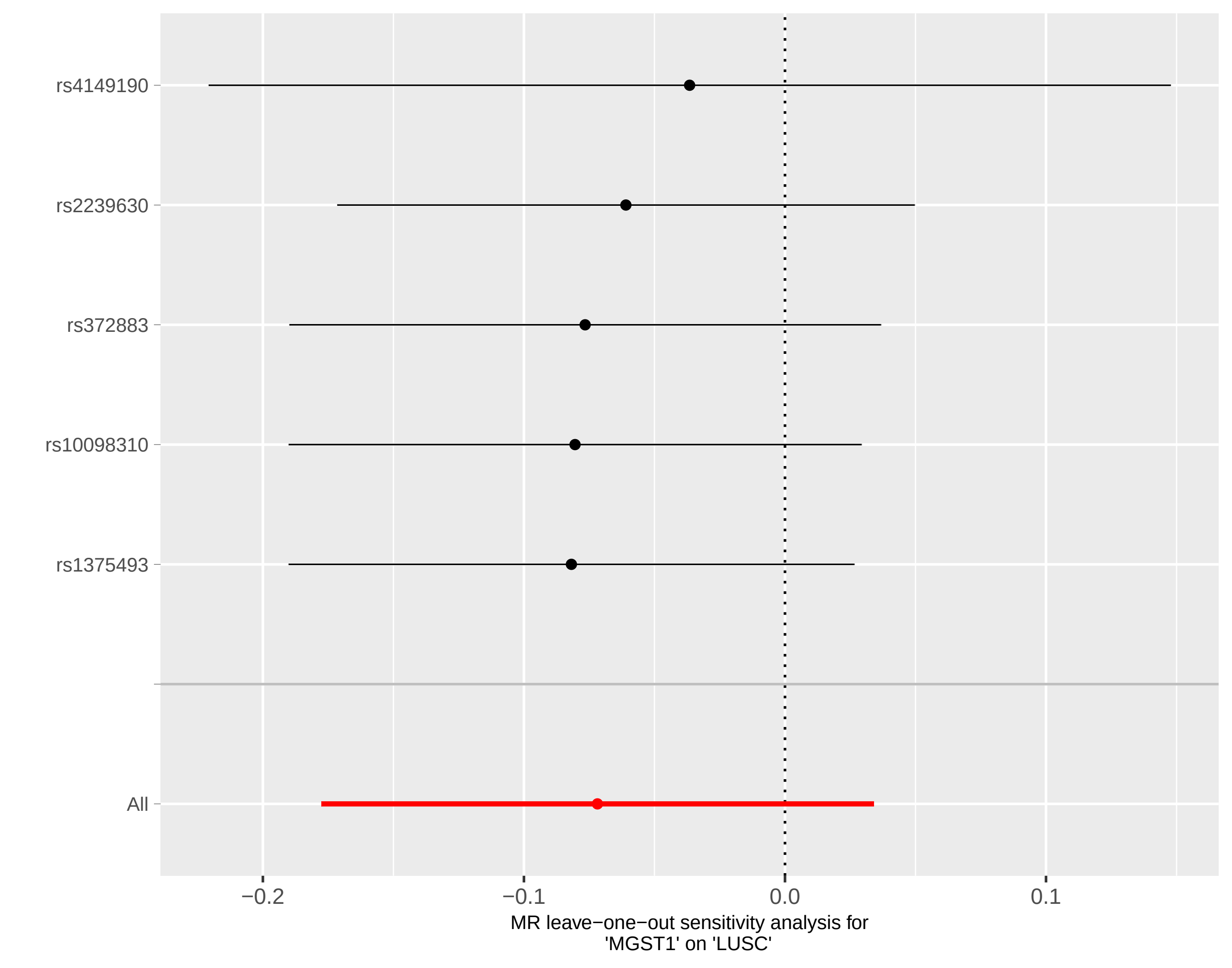

rs4149190
rs2239630
rs372883
rs10098310
rs1375493
All
−0.2
−0.1
0.0
0.1
MR leave−one−out sensitivity analysis for
'MGST1' on 'LUSC'

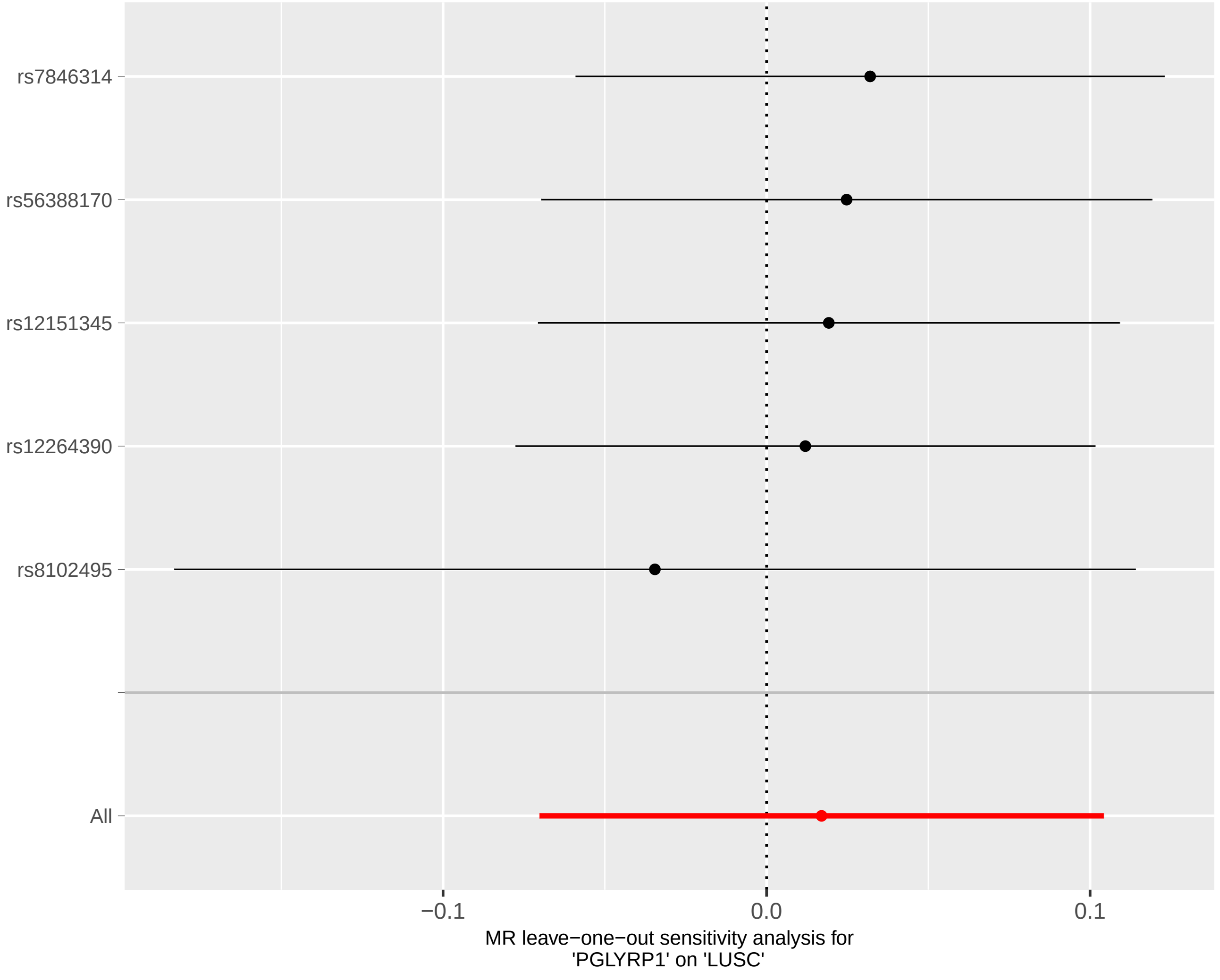
rs7846314
rs56388170
rs12151345
rs12264390
rs8102495
All
−0.1
0.0
0.1
MR leave−one−out sensitivity analysis for
'PGLYRP1' on 'LUSC'

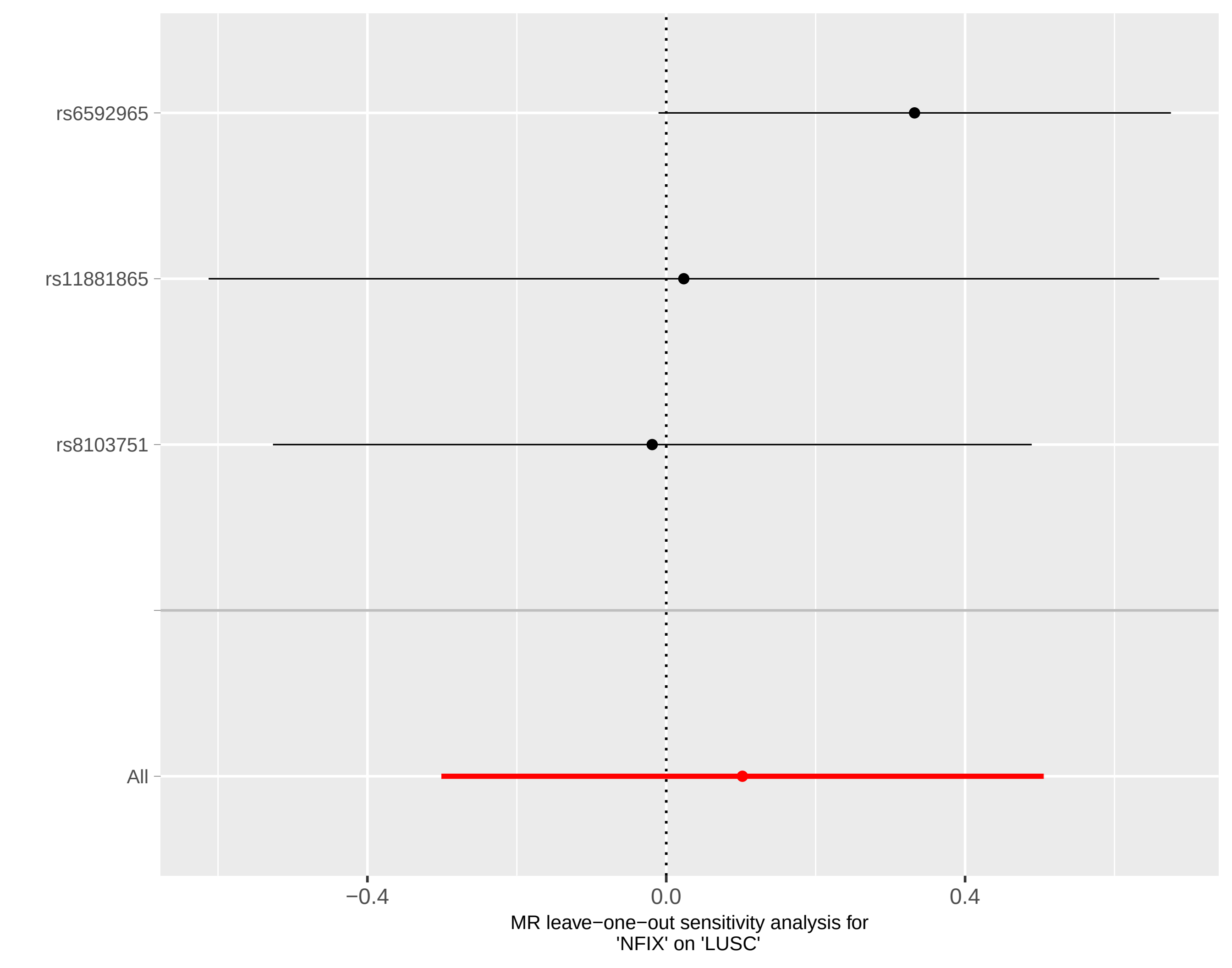
rs6592965
rs11881865
rs8103751
All
−0.4
0.0
0.4
MR leave−one−out sensitivity analysis for
'NFIX' on 'LUSC'

Insufficient number of SNPs

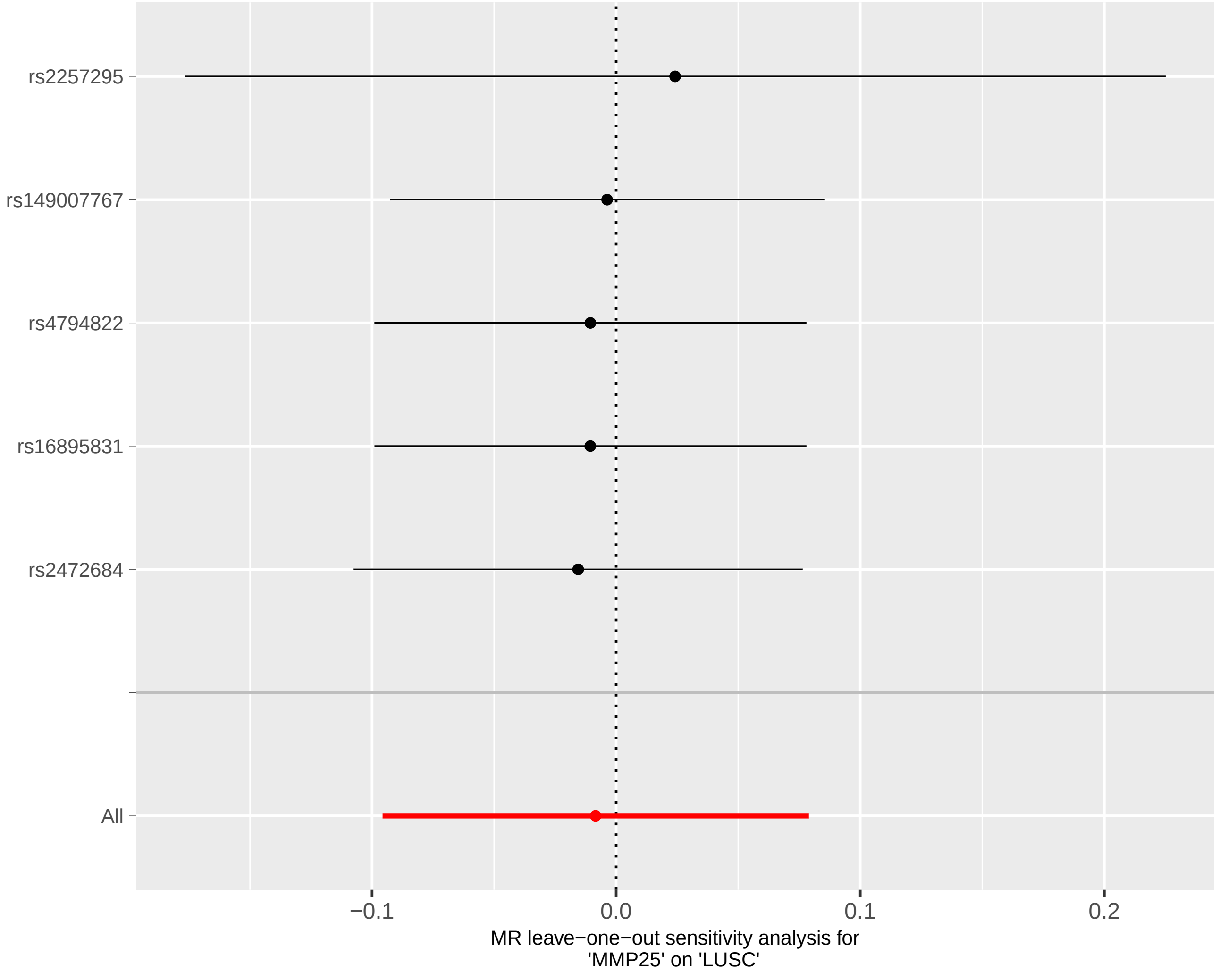

rs2257295
rs149007767
rs4794822
rs16895831
rs2472684
All
−0.1
0.0
0.1
0.2
MR leave−one−out sensitivity analysis for
'MMP25' on 'LUSC'

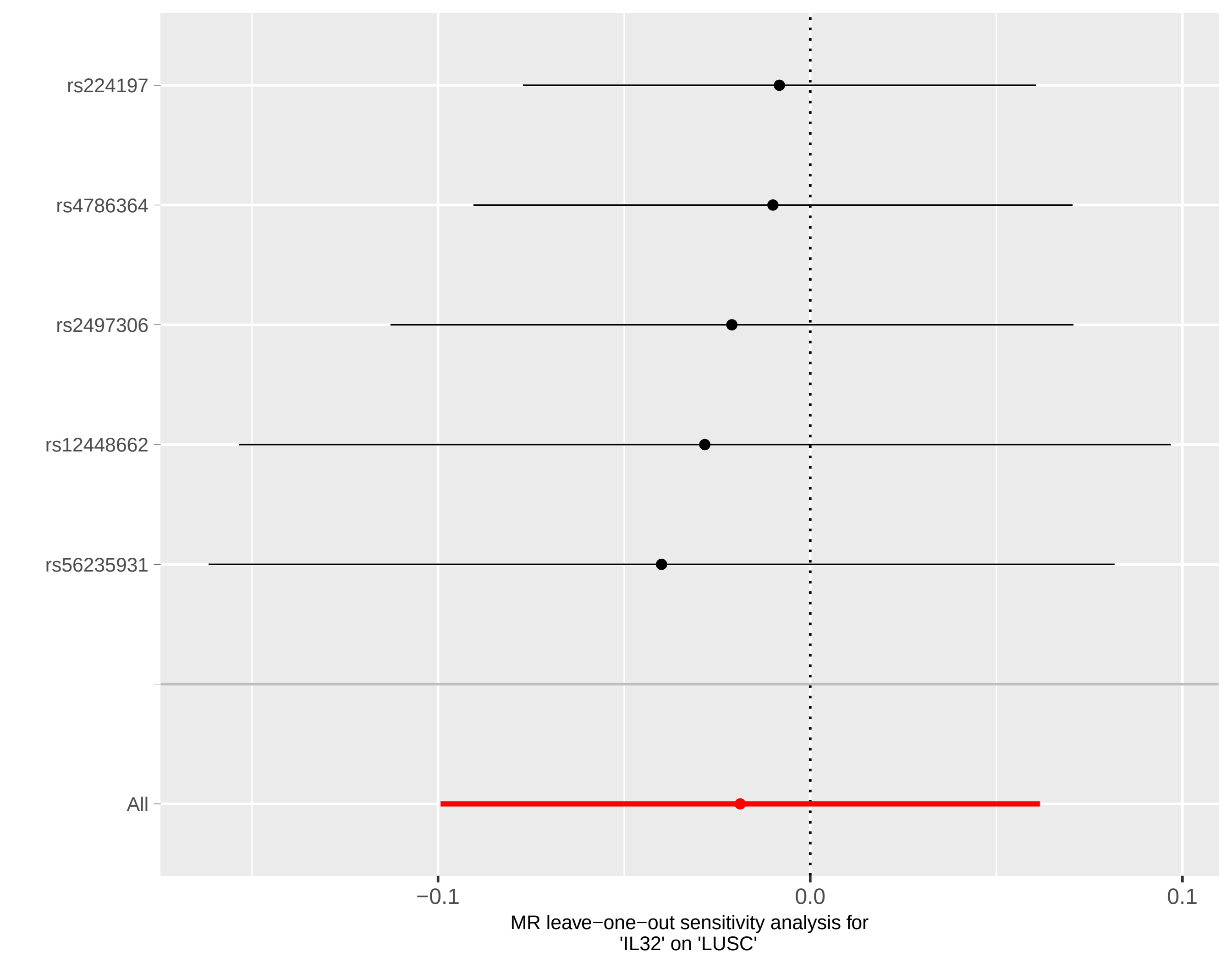
rs224197
rs4786364
rs2497306
rs12448662
rs56235931
All
−0.1
0.0
0.1
MR leave−one−out sensitivity analysis for
'IL32' on 'LUSC'

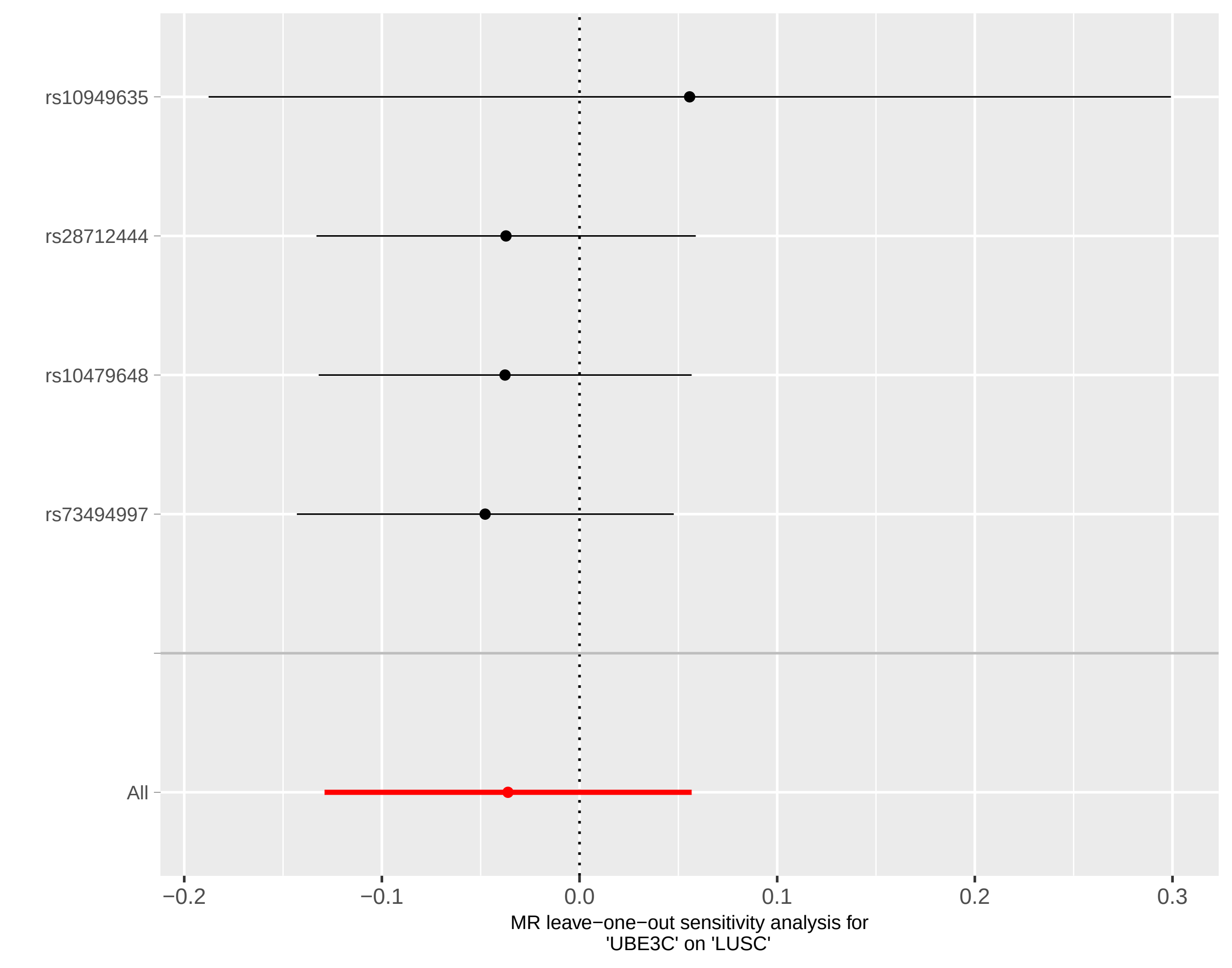

rs10949635
rs28712444
rs10479648
rs73494997
All
−0.2
−0.1
0.0
0.1
0.2
0.3
MR leave−one−out sensitivity analysis for
'UBE3C' on 'LUSC'

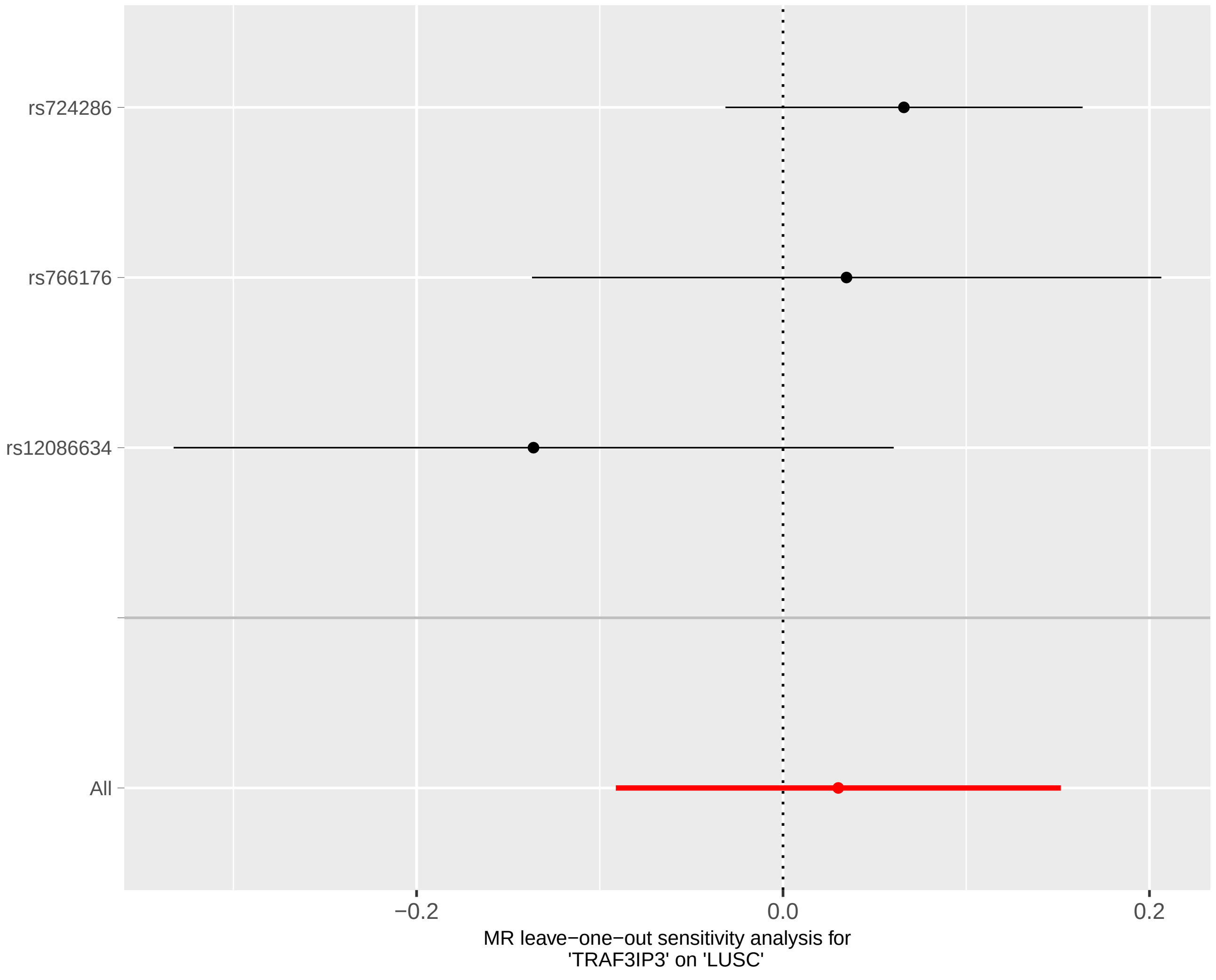
rs724286
rs766176
rs12086634
All
−0.2
0.0
0.2
MR leave−one−out sensitivity analysis for
'TRAF3IP3' on 'LUSC'

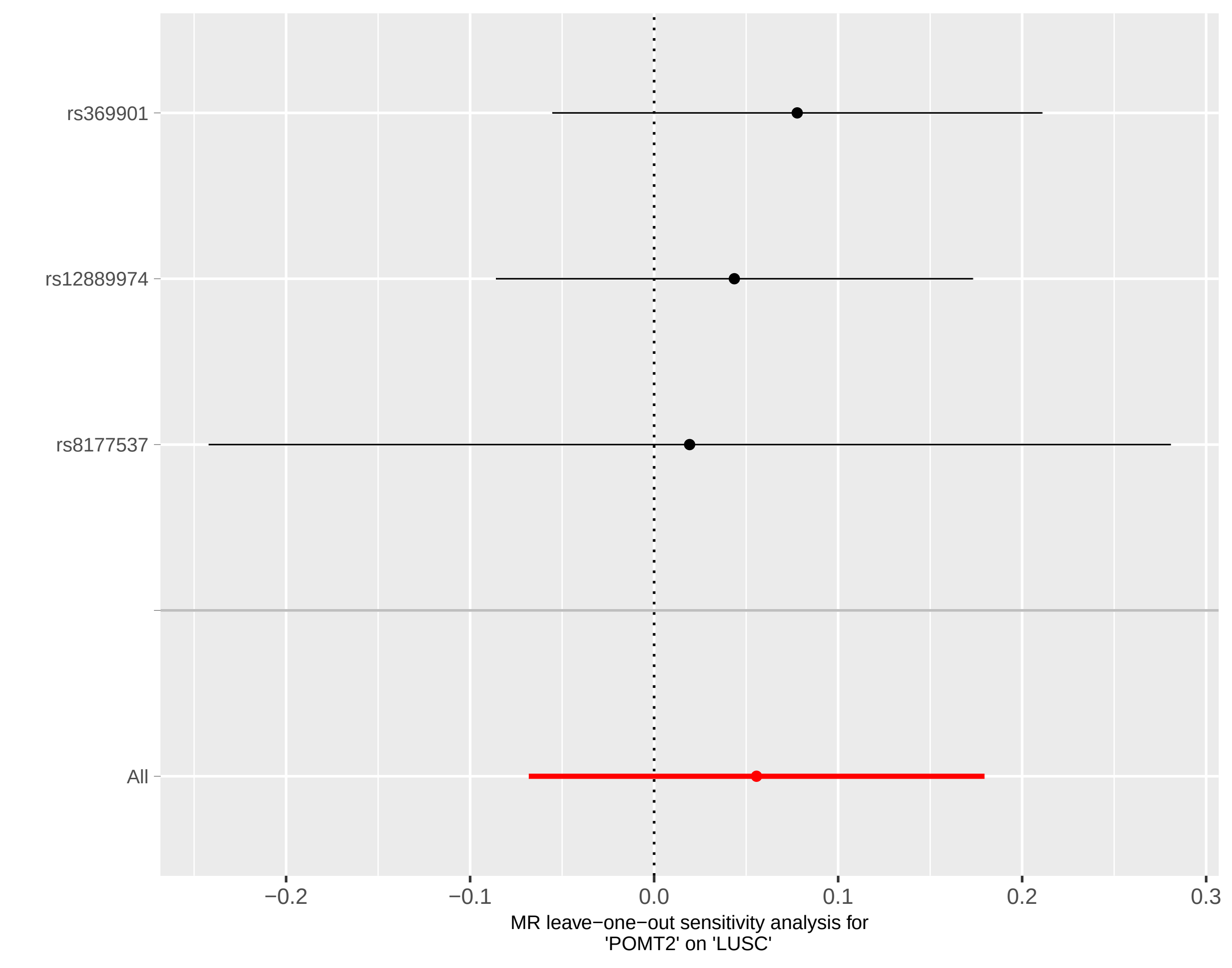
rs369901
rs12889974
rs8177537
All
−0.2
−0.1
0.0
0.1
0.2
0.3
MR leave−one−out sensitivity analysis for
'POMT2' on 'LUSC'

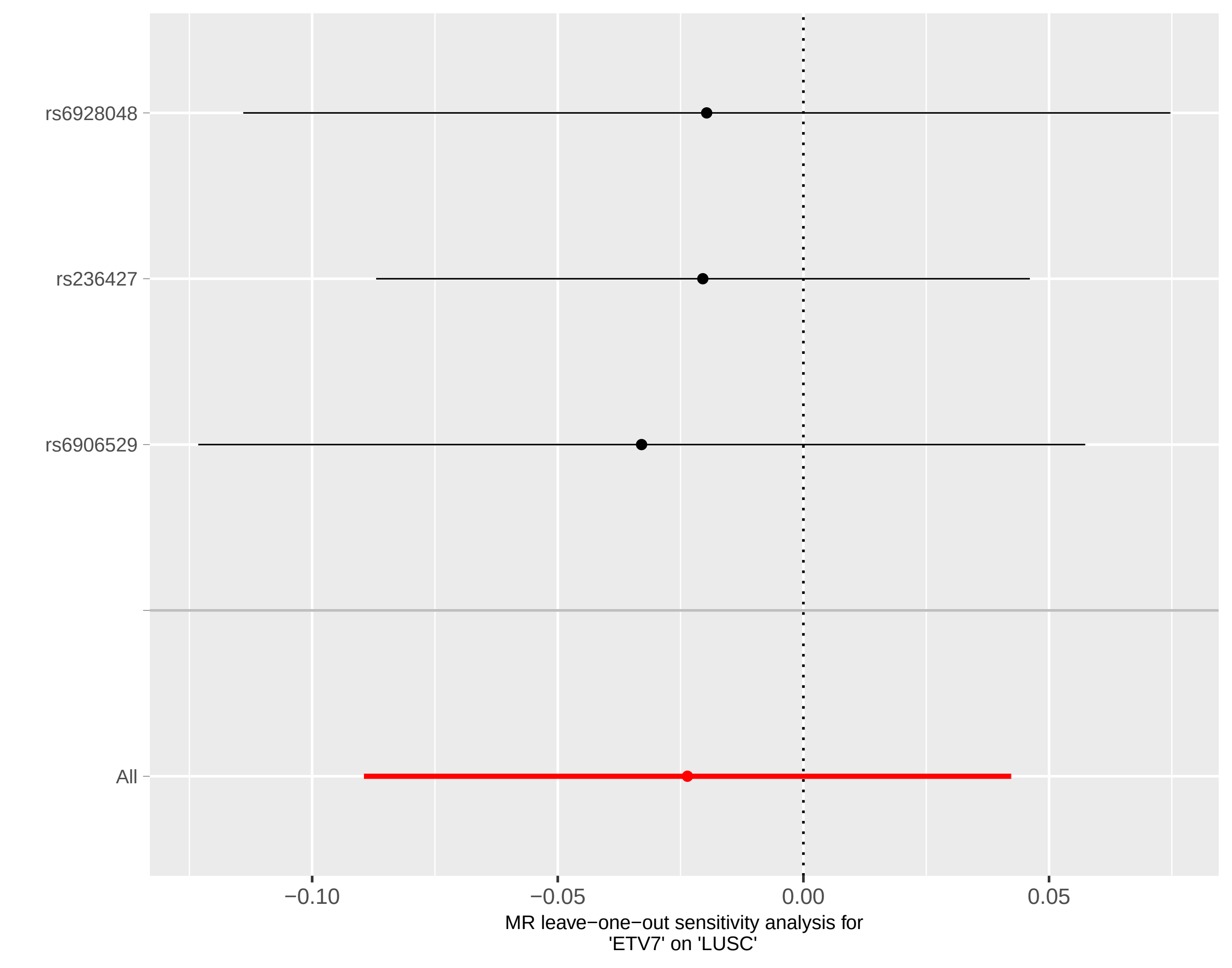

rs6928048
rs236427
rs6906529
All
−0.10
−0.05
0.00
0.05
MR leave−one−out sensitivity analysis for
'ETV7' on 'LUSC'

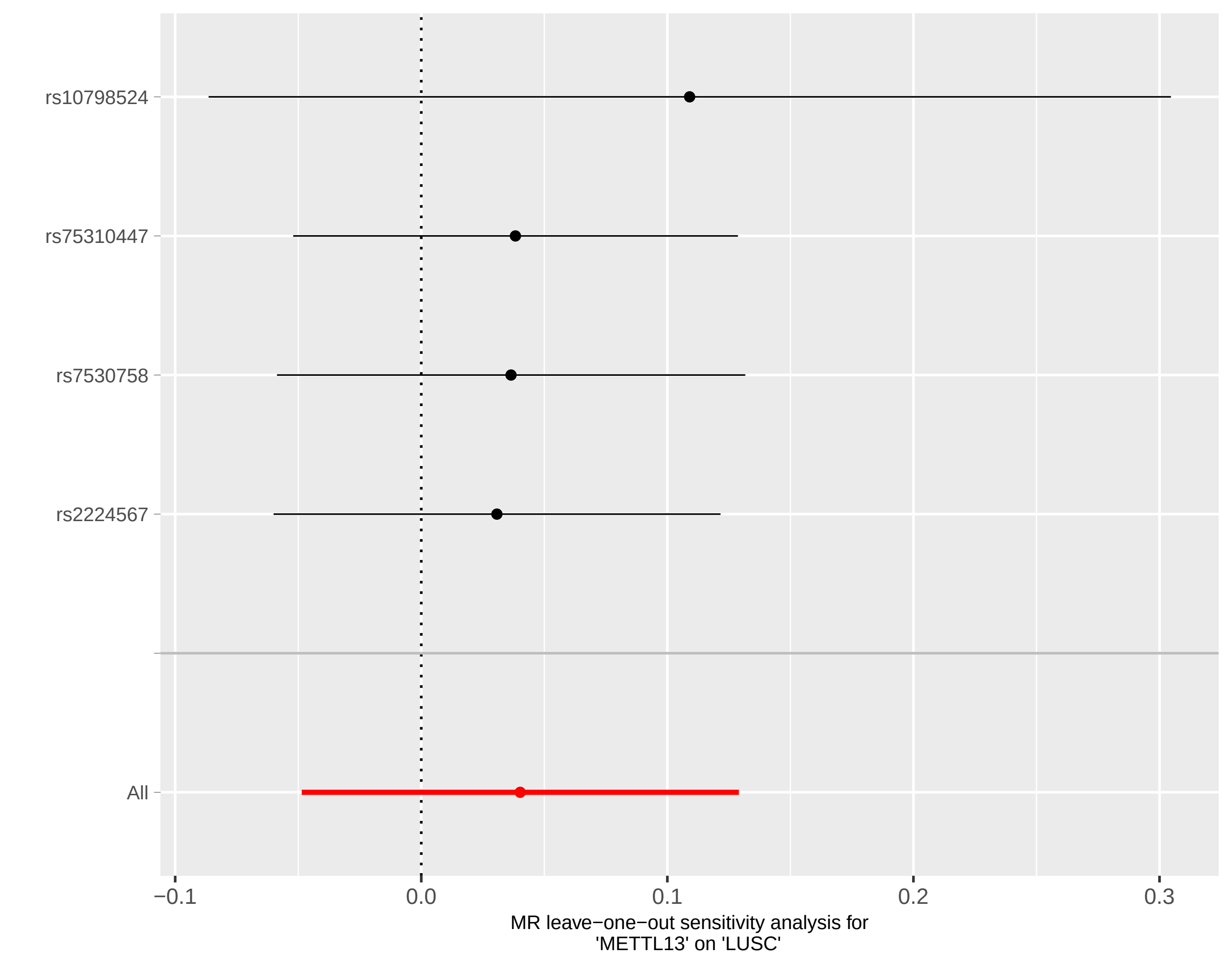
rs10798524
rs75310447
rs7530758
rs2224567
All
−0.1
0.0
0.1
0.2
0.3
MR leave−one−out sensitivity analysis for
'METTL13' on 'LUSC'

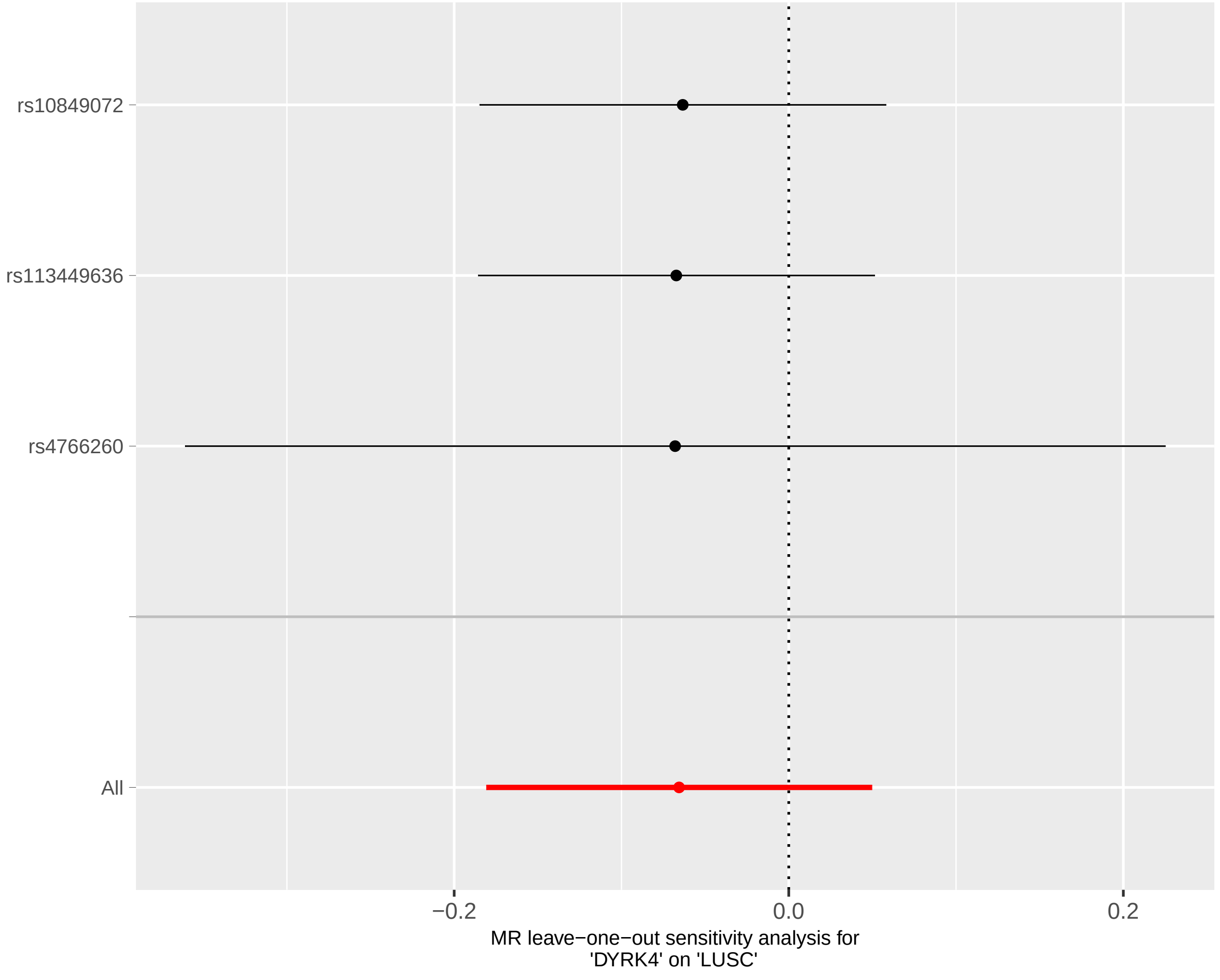
rs10849072
rs113449636
rs4766260
All
−0.2
0.0
0.2
MR leave−one−out sensitivity analysis for
'DYRK4' on 'LUSC'

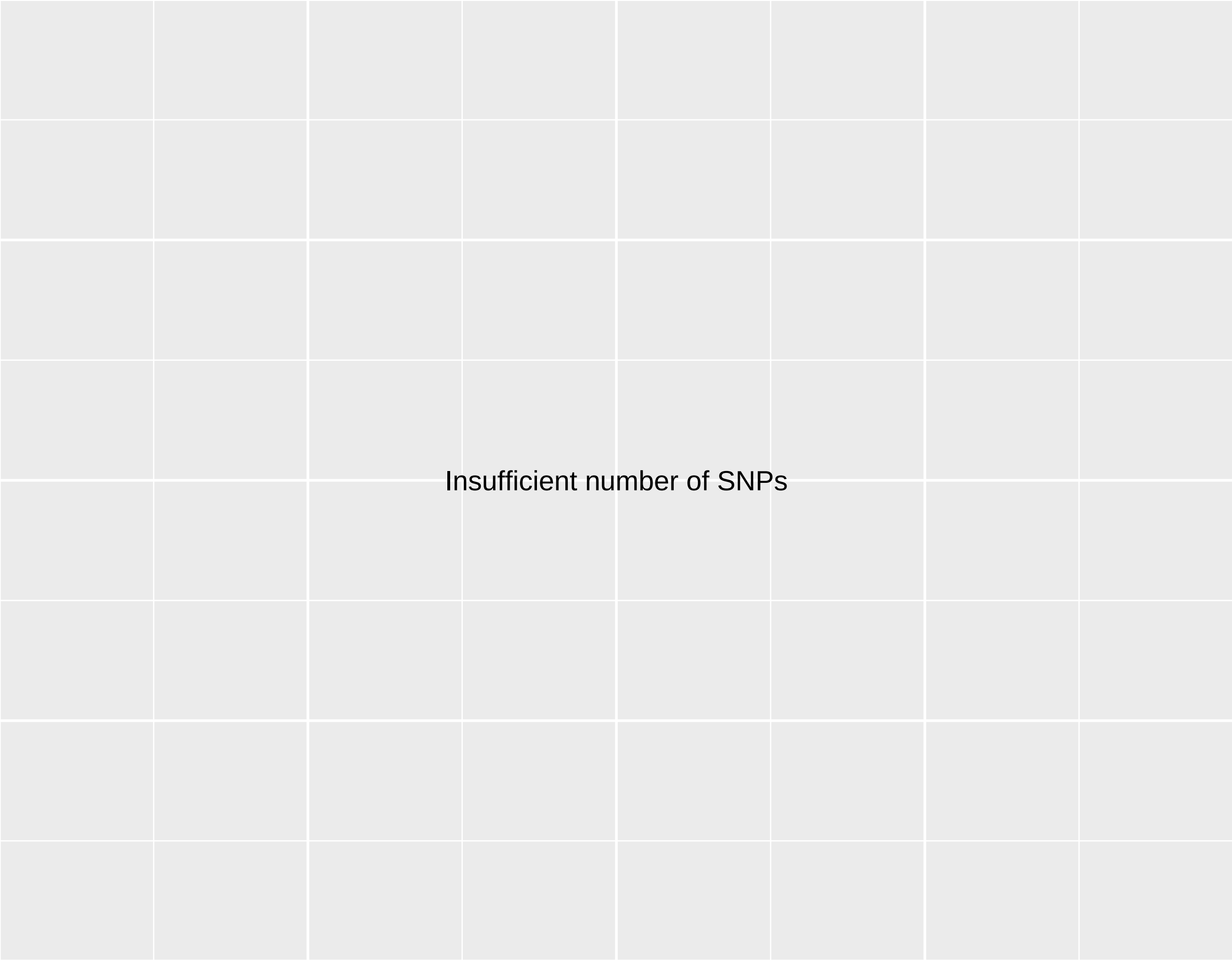
Insufficient number of SNPs

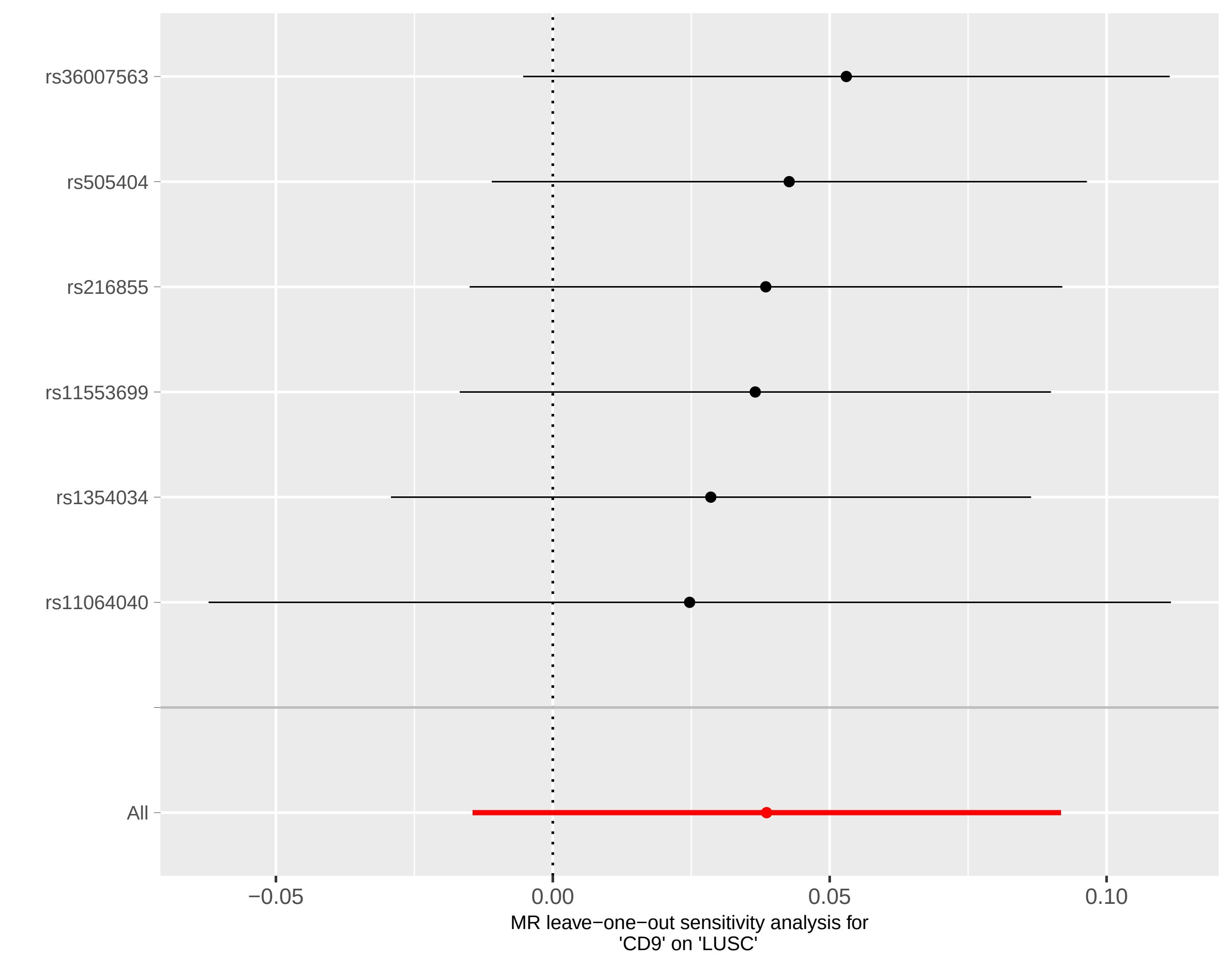

rs36007563
rs505404
rs216855
rs11553699
rs1354034
rs11064040
All
−0.05
0.00
0.05
0.10
MR leave−one−out sensitivity analysis for
'CD9' on 'LUSC'

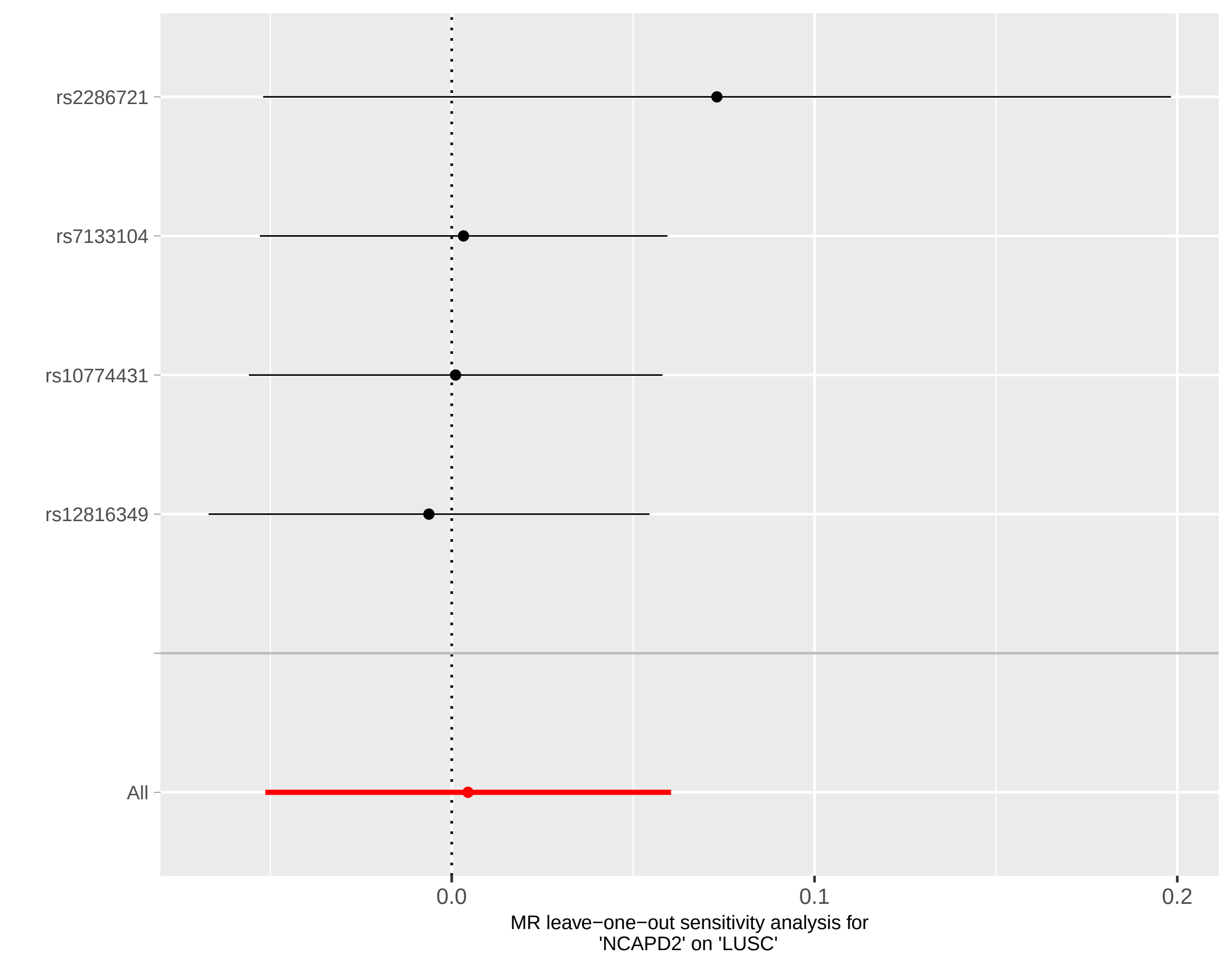
rs2286721
rs7133104
rs10774431
rs12816349
All
0.0
0.1
0.2
MR leave−one−out sensitivity analysis for
'NCAPD2' on 'LUSC'

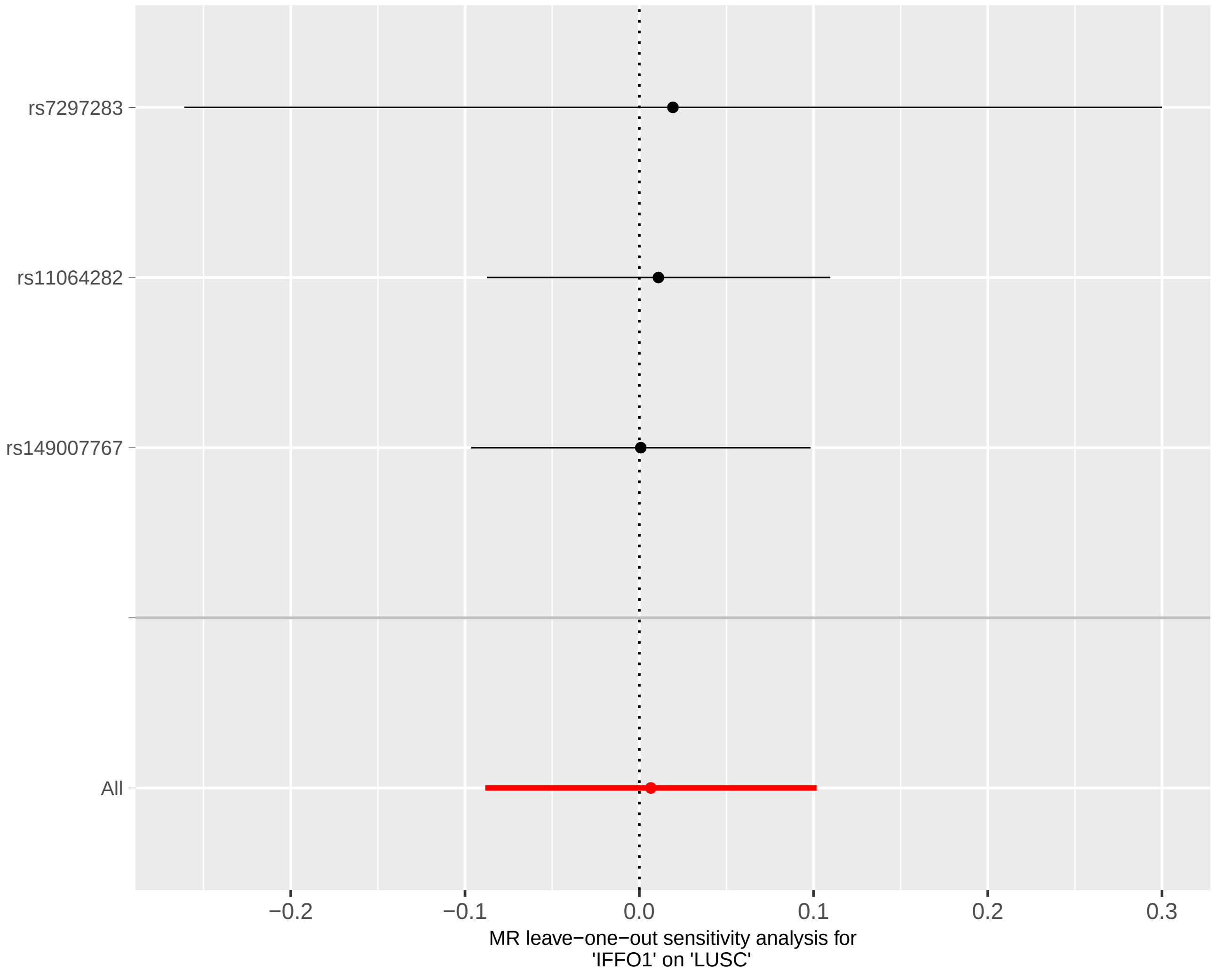

rs7297283
rs11064282
rs149007767
All
−0.2
−0.1
0.0
0.1
0.2
0.3
MR leave−one−out sensitivity analysis for
'IFFO1' on 'LUSC'

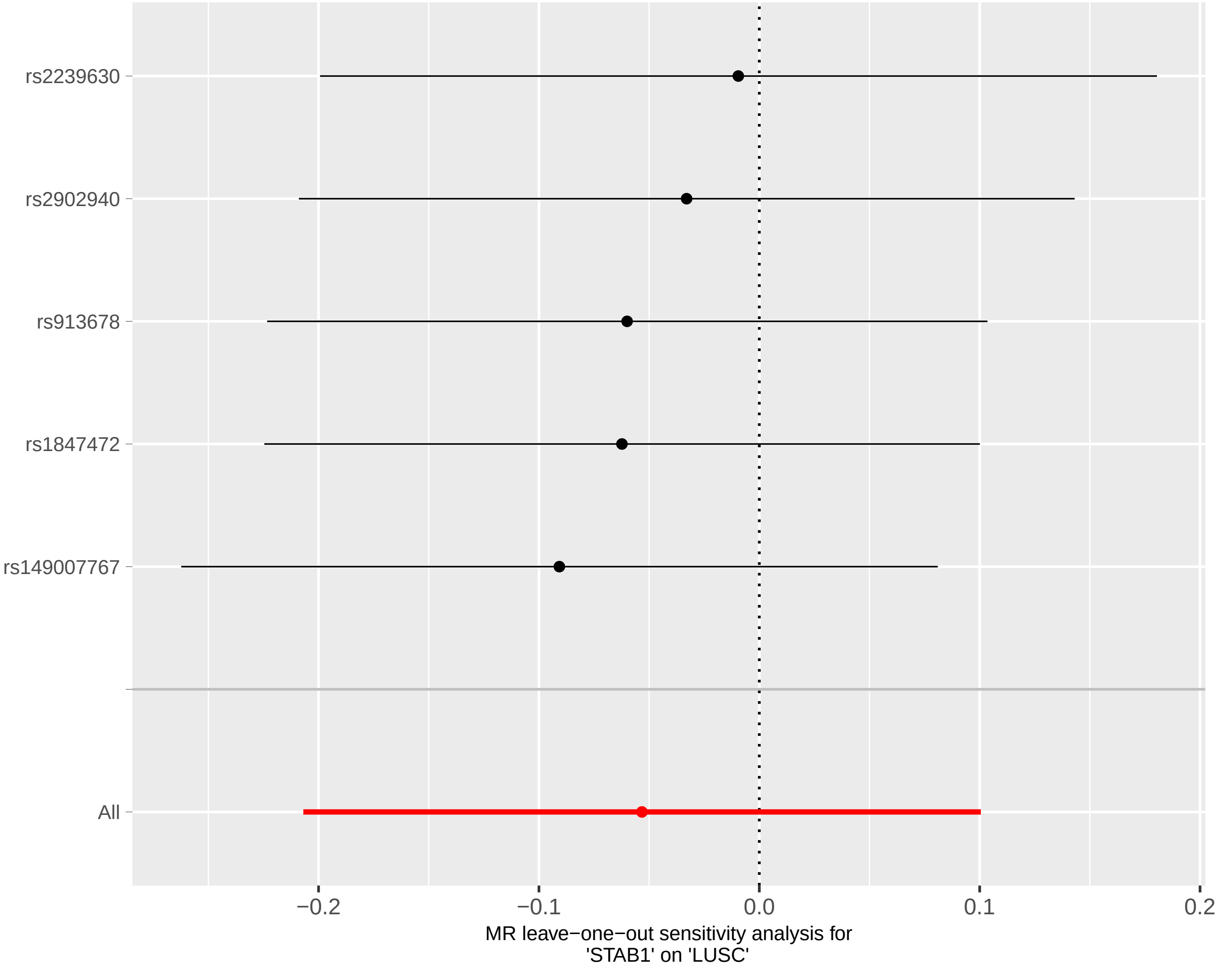
rs2239630
rs2902940
rs913678
rs1847472
rs149007767
All
−0.2
−0.1
0.0
0.1
0.2
MR leave−one−out sensitivity analysis for
'STAB1' on 'LUSC'

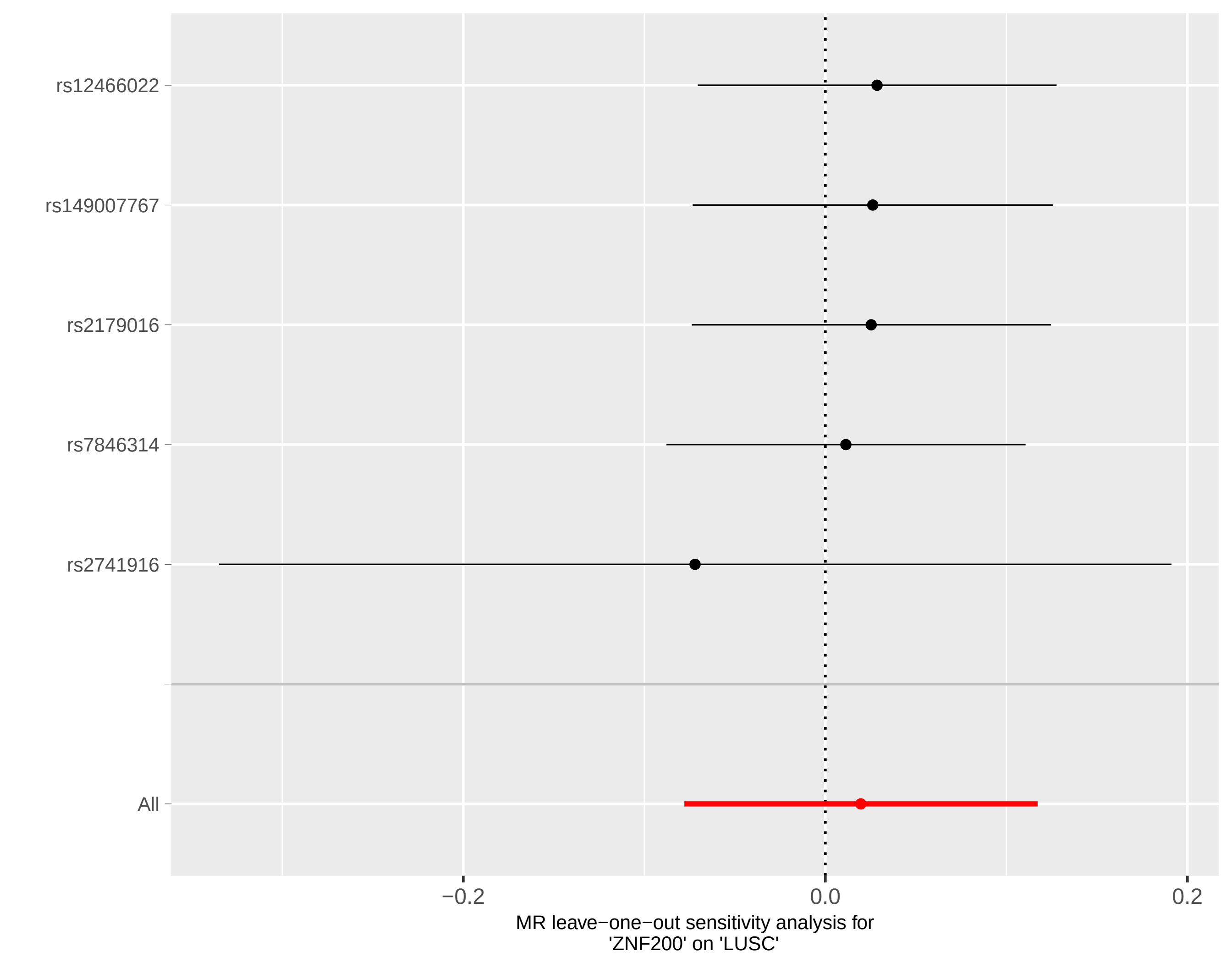
rs12466022
rs149007767
rs2179016
rs7846314
rs2741916
All
−0.2
0.0
0.2
MR leave−one−out sensitivity analysis for
'ZNF200' on 'LUSC'

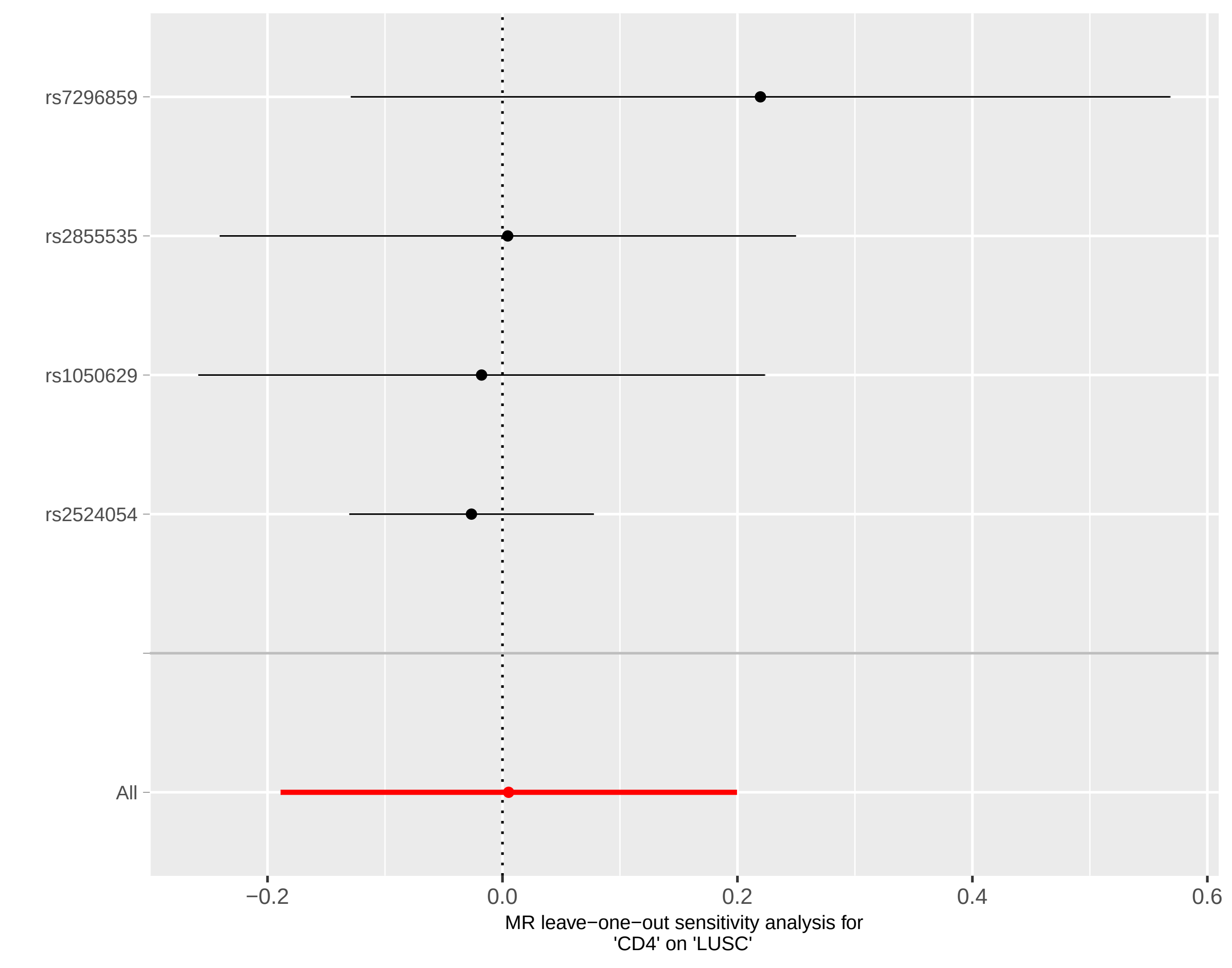

rs7296859
rs2855535
rs1050629
rs2524054
All
−0.2
0.0
0.2
0.4
0.6
MR leave−one−out sensitivity analysis for
'CD4' on 'LUSC'

Insufficient number of SNPs

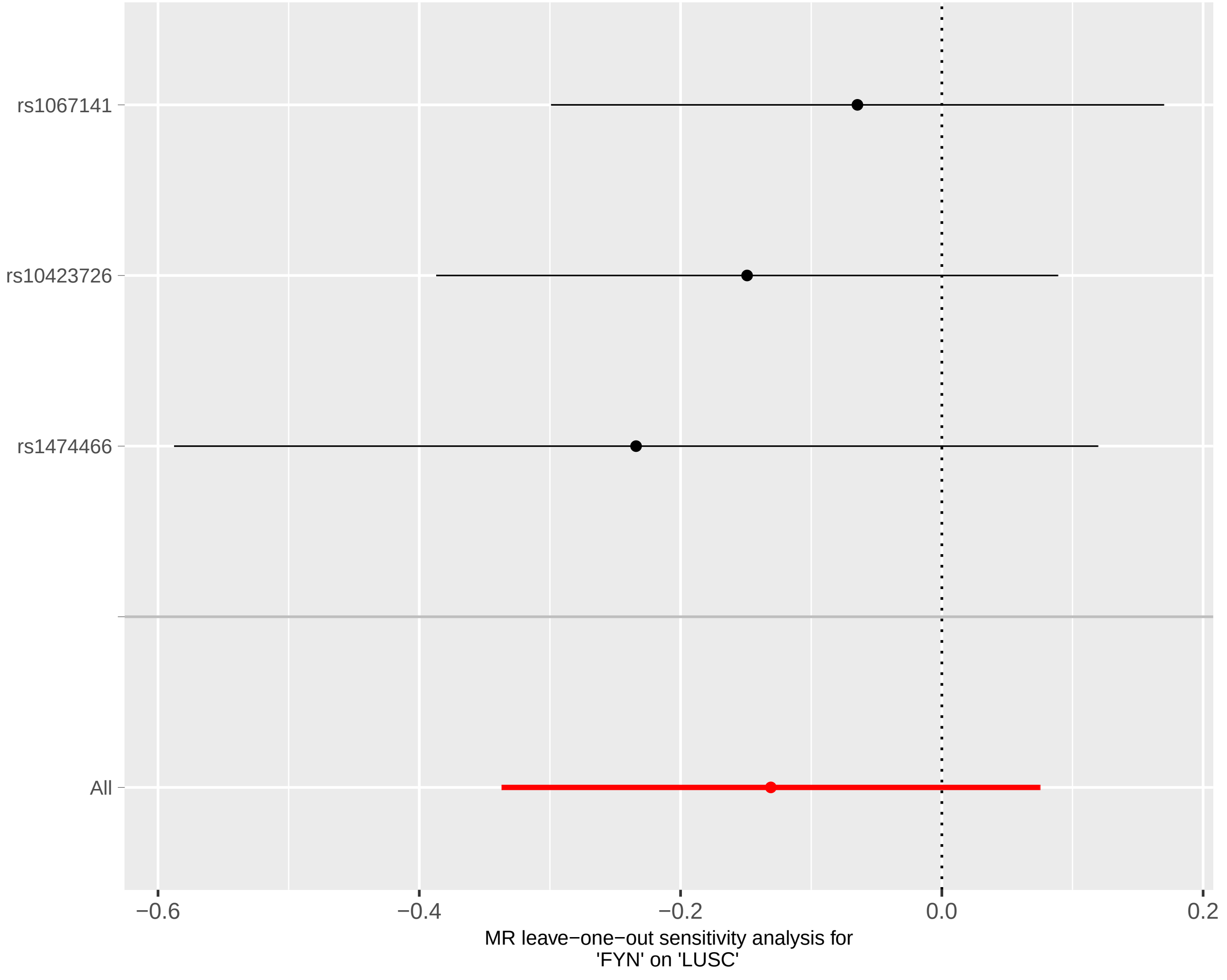
rs1067141
rs10423726
rs1474466
All
−0.6
−0.4
−0.2
0.0
0.2
MR leave−one−out sensitivity analysis for
'FYN' on 'LUSC'

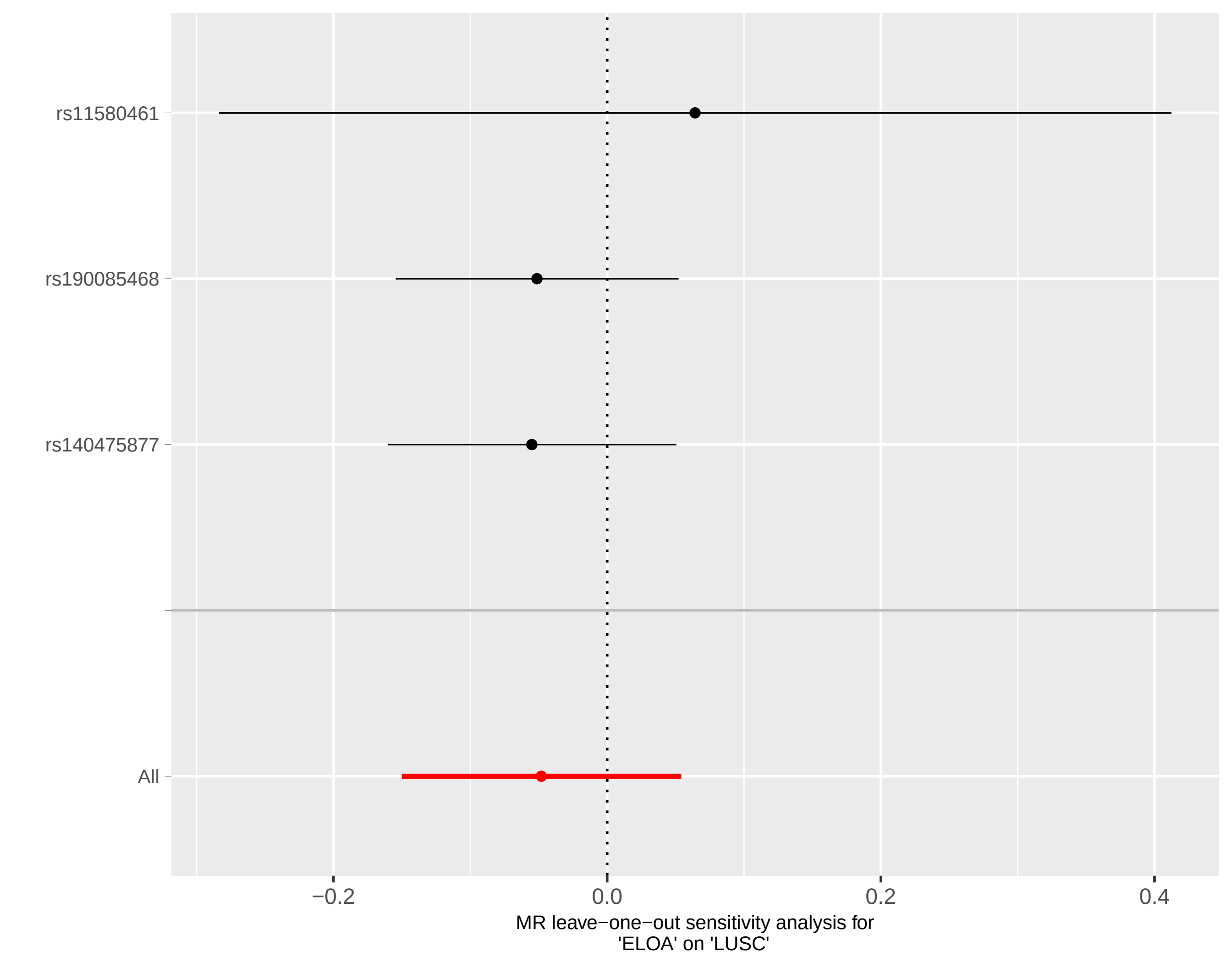

rs11580461
rs190085468
rs140475877
All
−0.2
0.0
0.2
0.4
MR leave−one−out sensitivity analysis for
'ELOA' on 'LUSC'

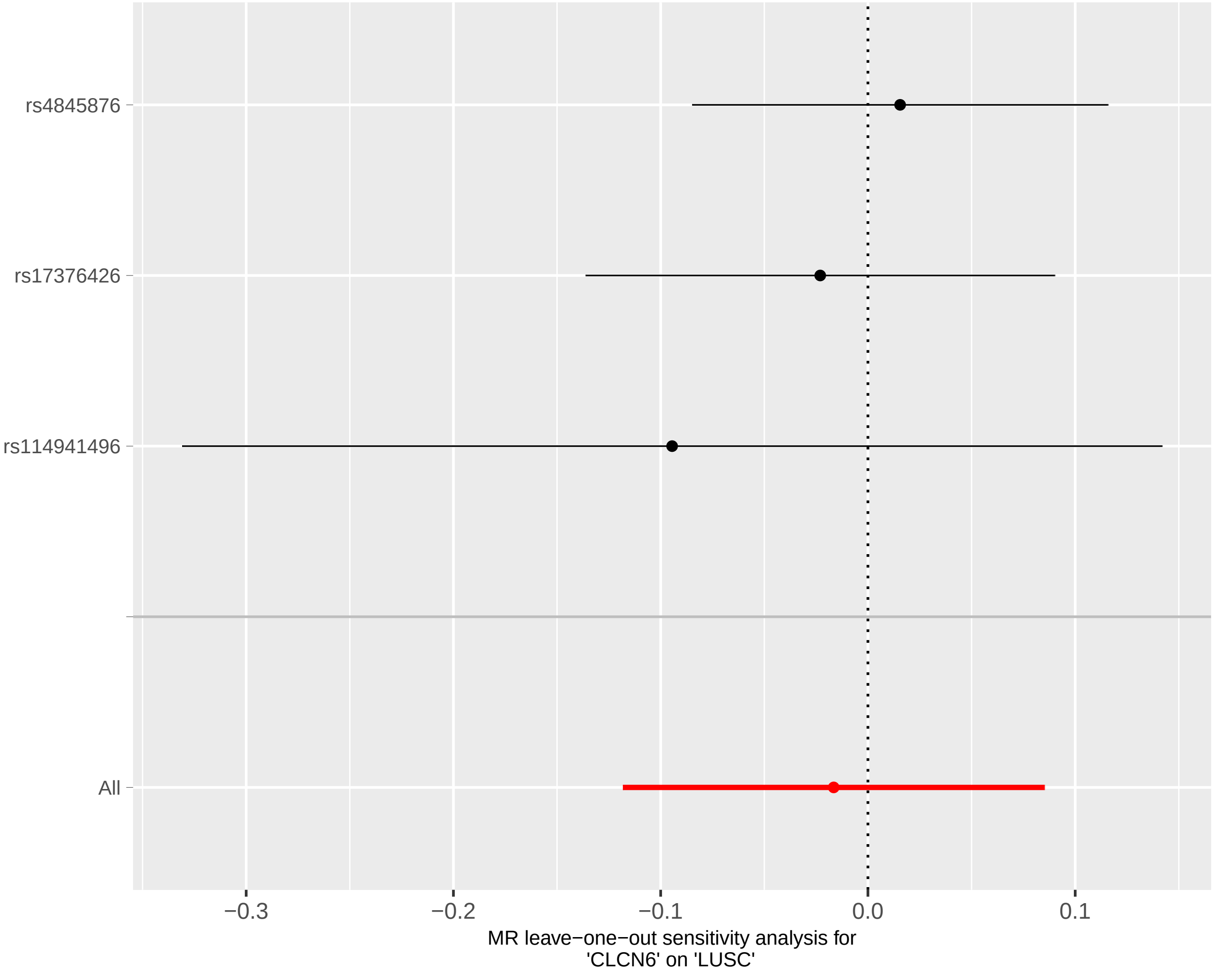

rs4845876
rs17376426
rs114941496
All
−0.3
−0.2
−0.1
0.0
0.1
MR leave−one−out sensitivity analysis for
'CLCN6' on 'LUSC'

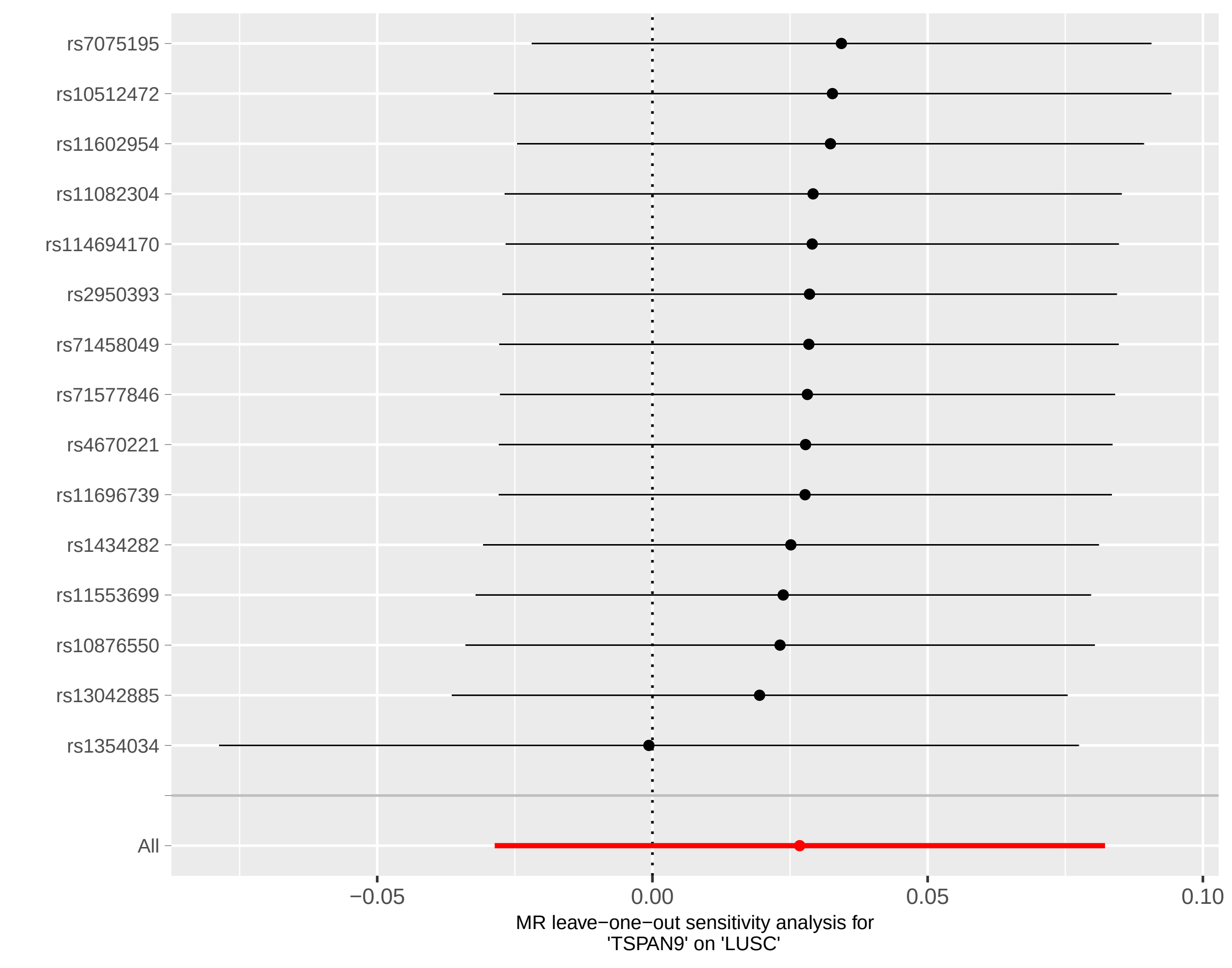

rs7075195
rs10512472
rs11602954
rs11082304
rs114694170
rs2950393
rs71458049
rs71577846
rs4670221
rs11696739
rs1434282
rs11553699
rs10876550
rs13042885
rs1354034
All
−0.05
0.00
0.05
0.10
MR leave−one−out sensitivity analysis for
'TSPAN9' on 'LUSC'

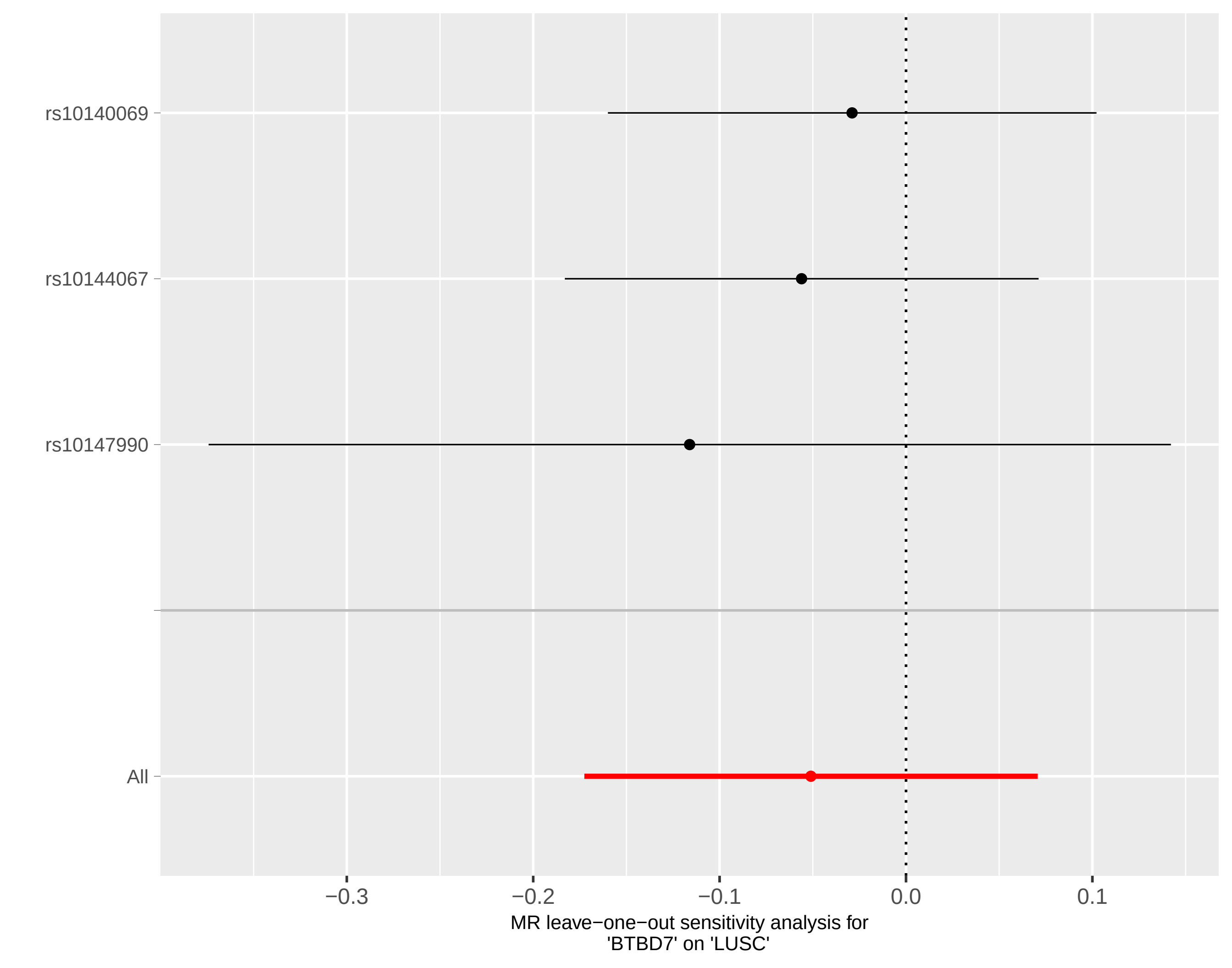

rs10140069
rs10144067
rs10147990
All
−0.3
−0.2
−0.1
0.0
0.1
MR leave−one−out sensitivity analysis for
'BTBD7' on 'LUSC'

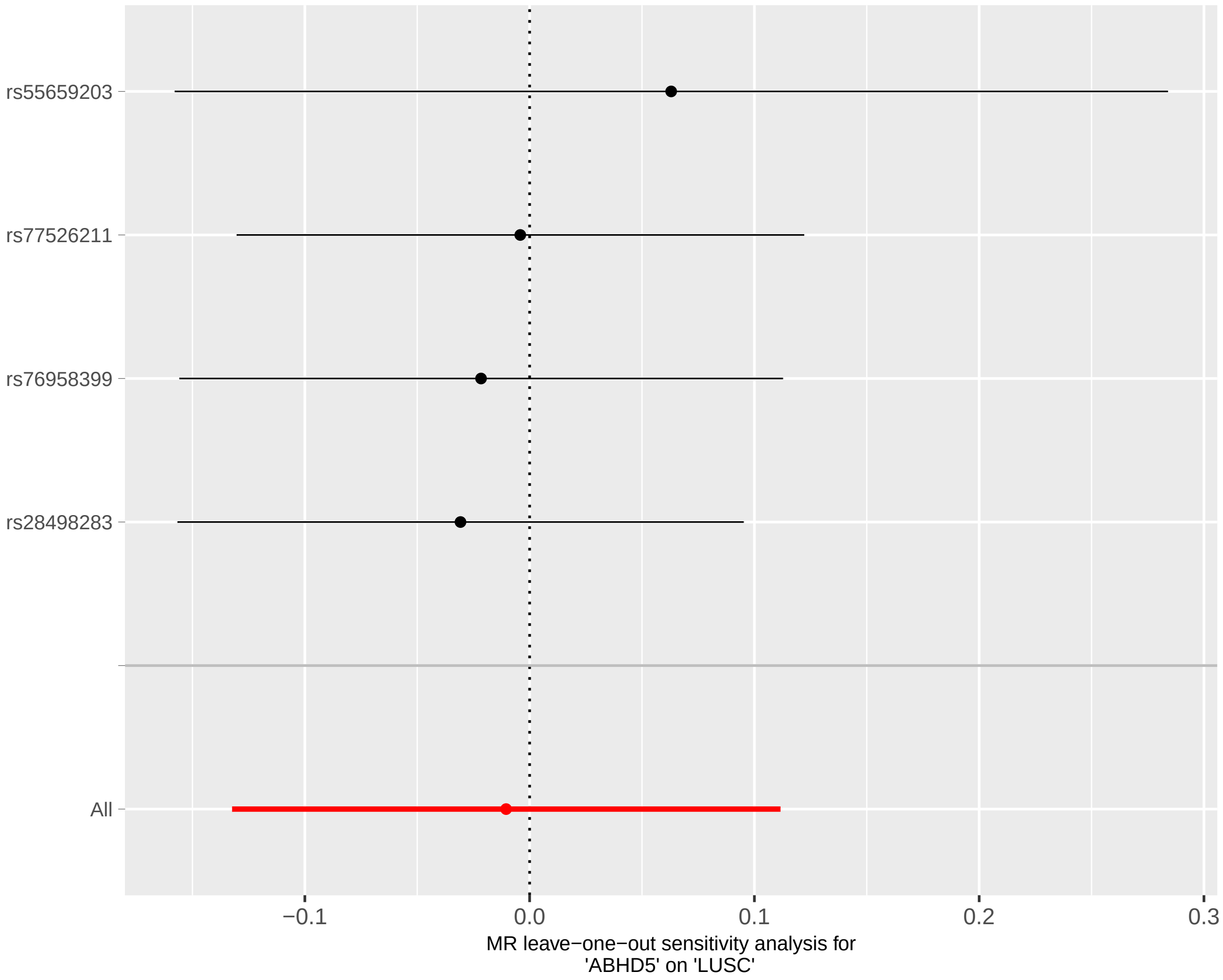

rs55659203
rs77526211
rs76958399
rs28498283
All
−0.1
0.0
0.1
0.2
0.3
MR leave−one−out sensitivity analysis for
'ABHD5' on 'LUSC'

Insufficient number of SNPs

Insufficient number of SNPs

Insufficient number of SNPs

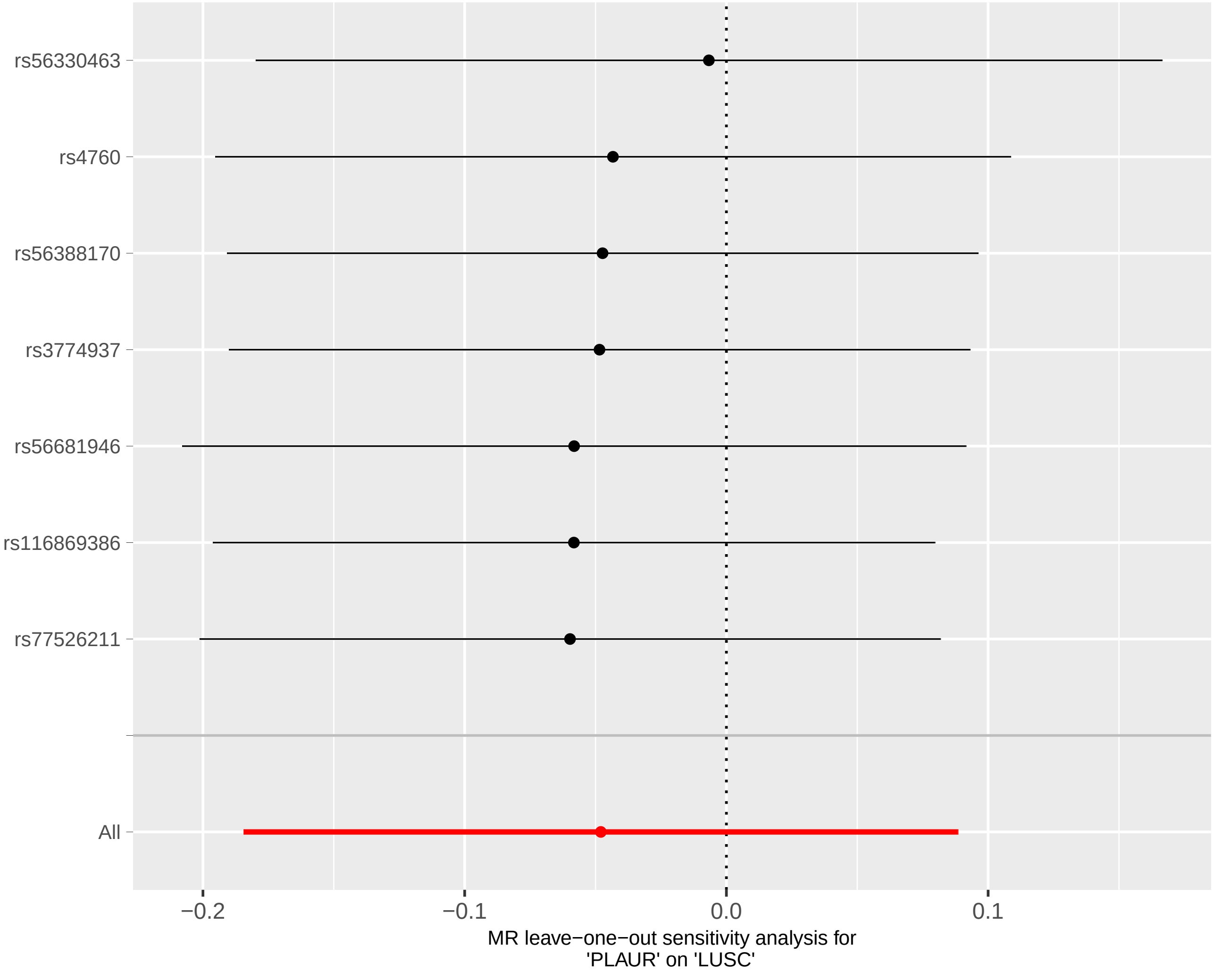

rs56330463
rs4760
rs56388170
rs3774937
rs56681946
rs116869386
rs77526211
All
−0.2
−0.1
0.0
0.1
MR leave−one−out sensitivity analysis for
'PLAUR' on 'LUSC'

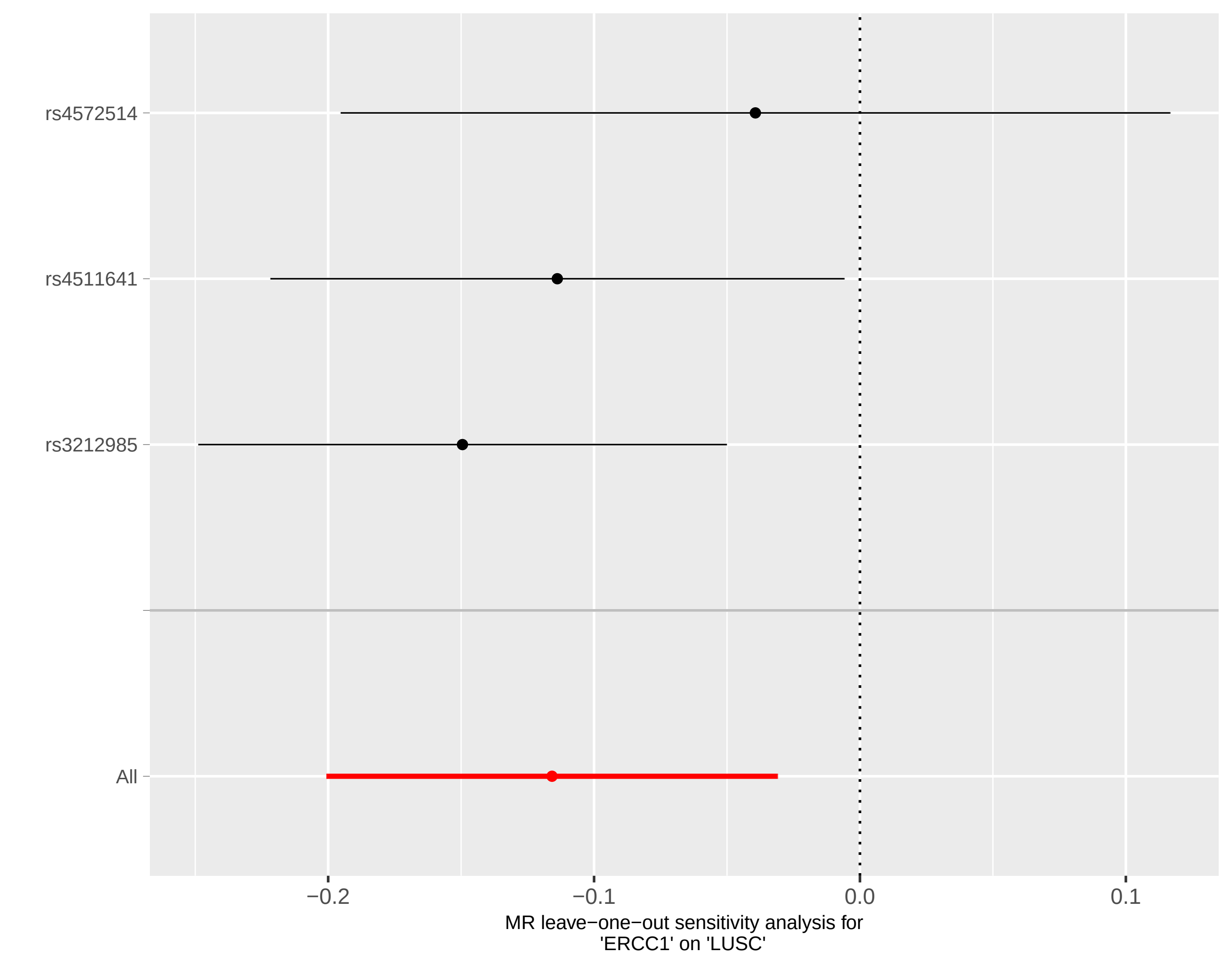
rs4572514
rs4511641
rs3212985
All
−0.2
−0.1
0.0
0.1
MR leave−one−out sensitivity analysis for
'ERCC1' on 'LUSC'

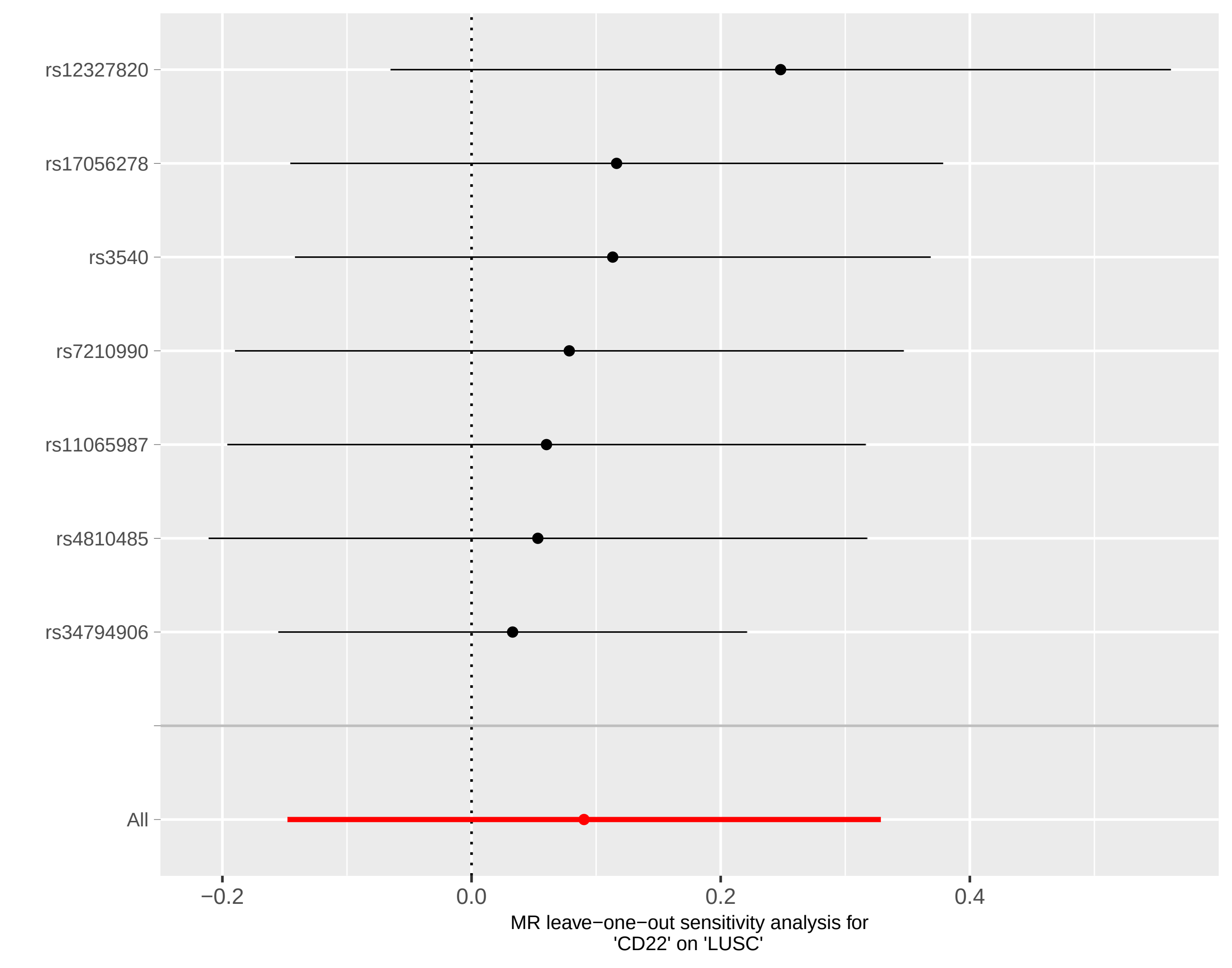
rs12327820
rs17056278
rs3540
rs7210990
rs11065987
rs4810485
rs34794906
All
−0.2
0.0
0.2
0.4
MR leave−one−out sensitivity analysis for
'CD22' on 'LUSC'

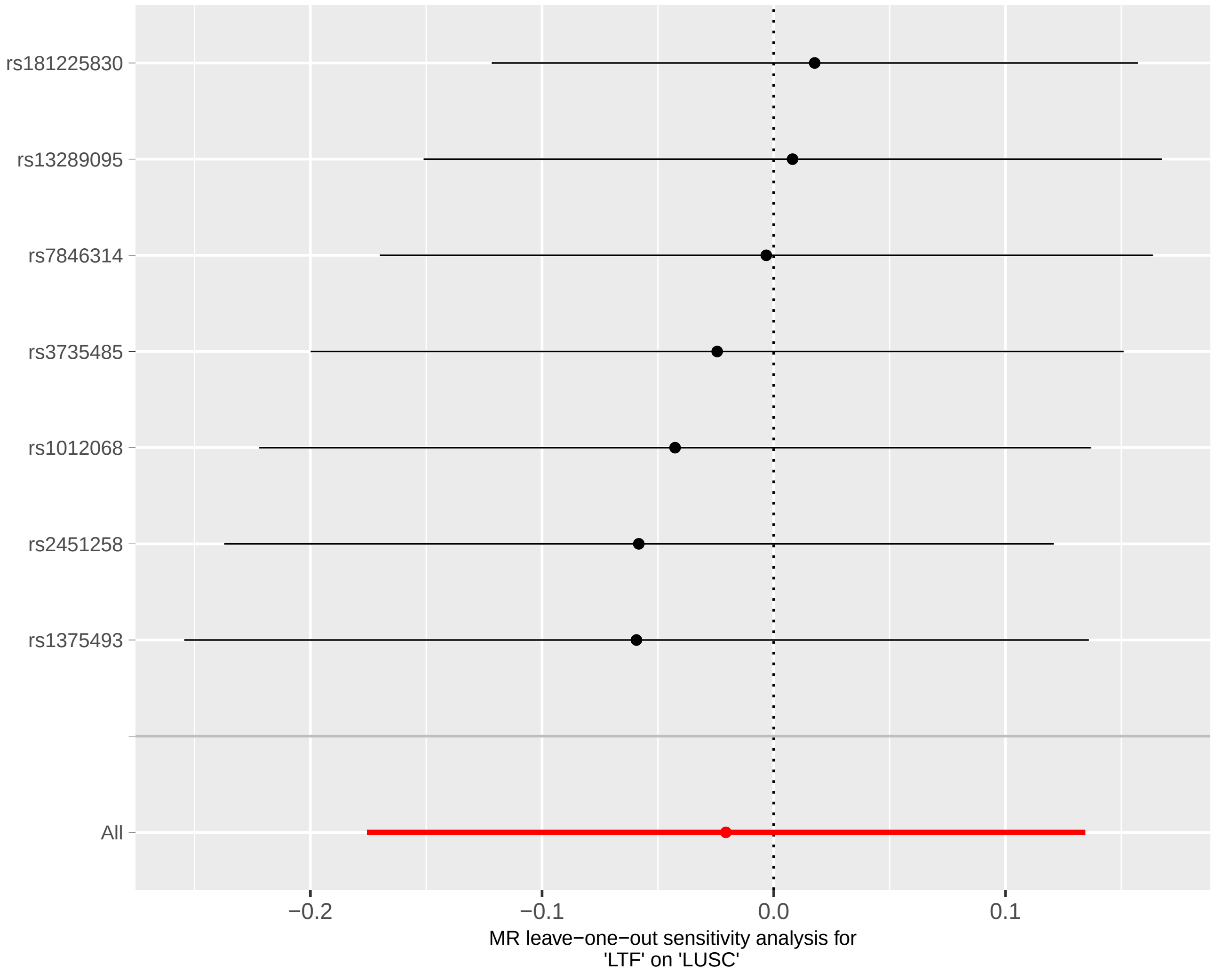
rs181225830
rs13289095
rs7846314
rs3735485
rs1012068
rs2451258
rs1375493
All
−0.2
−0.1
0.0
0.1
MR leave−one−out sensitivity analysis for
'LTF' on 'LUSC'

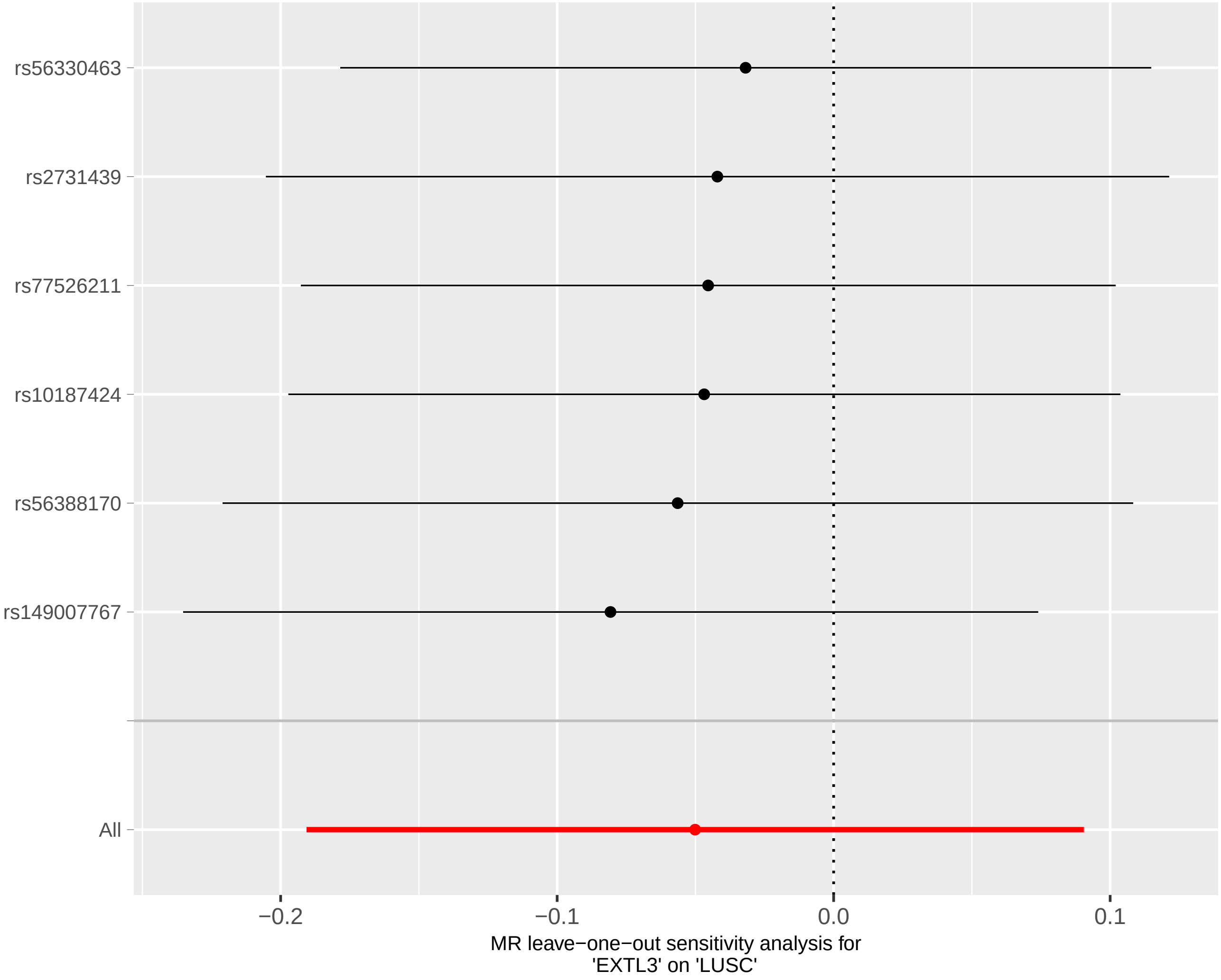

rs56330463
rs2731439
rs77526211
rs10187424
rs56388170
rs149007767
All
−0.2
−0.1
0.0
0.1
MR leave−one−out sensitivity analysis for
'EXTL3' on 'LUSC'

Insufficient number of SNPs

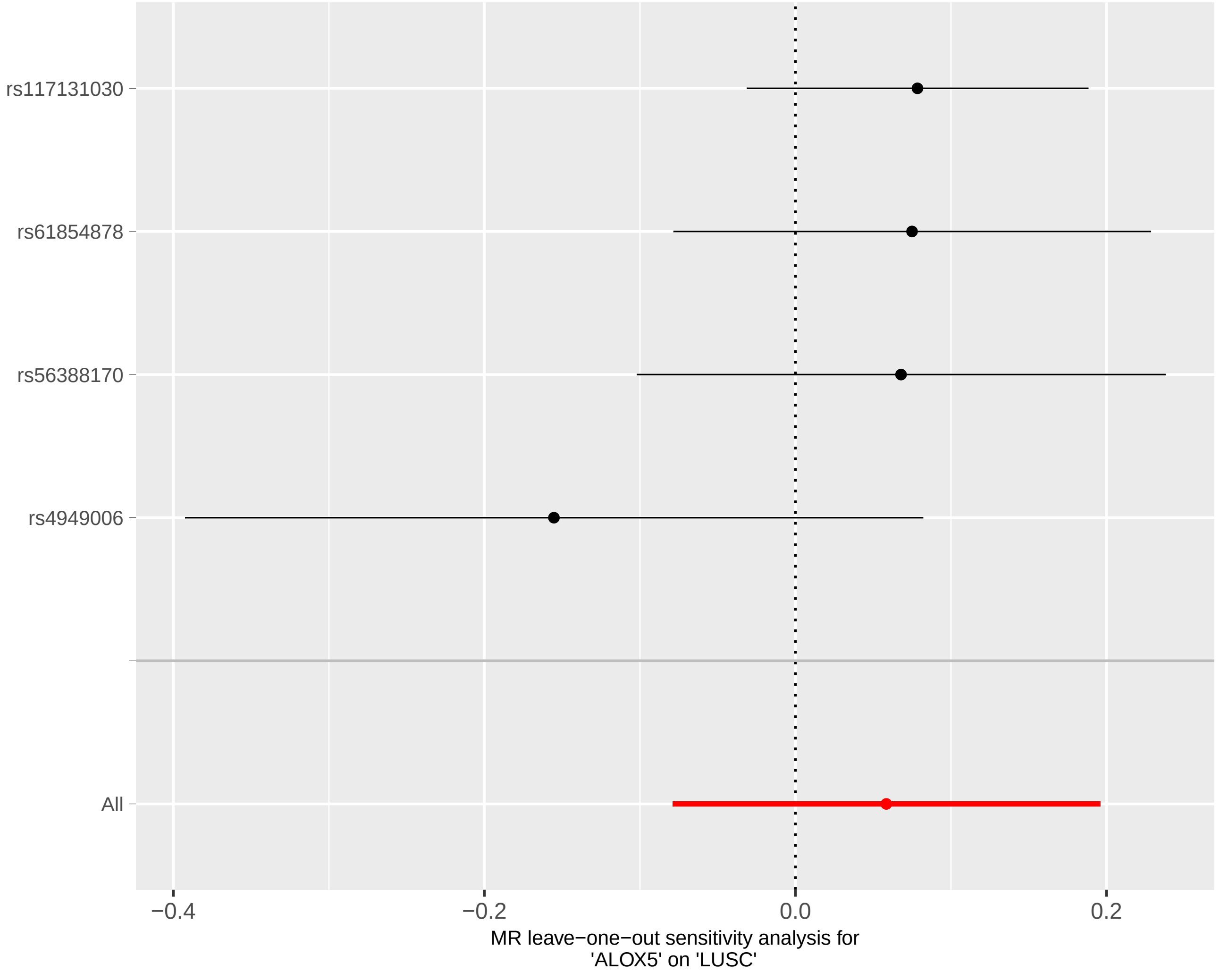
rs117131030
rs61854878
rs56388170
rs4949006
All
−0.4
−0.2
0.0
0.2
MR leave−one−out sensitivity analysis for
'ALOX5' on 'LUSC'

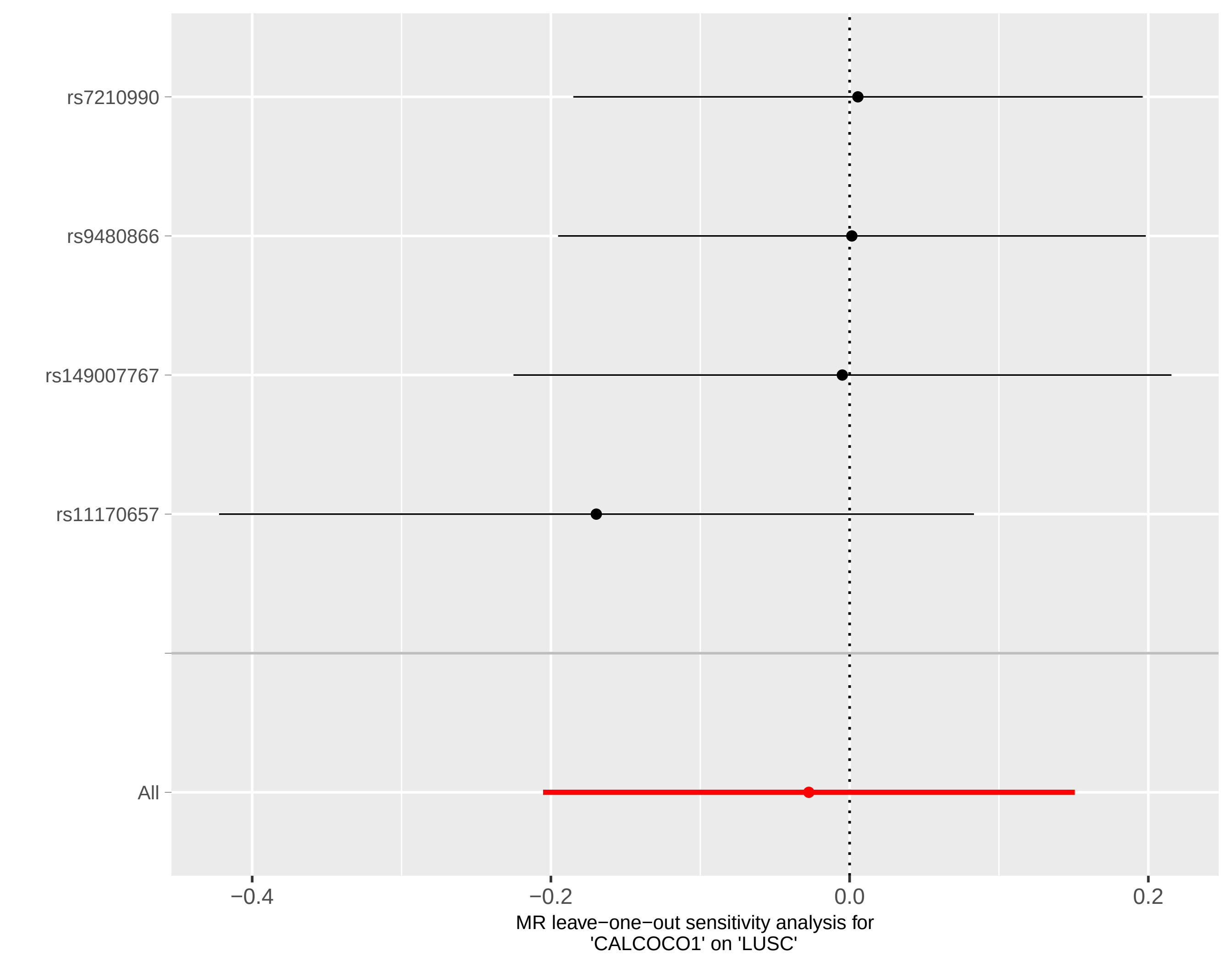

rs7210990
rs9480866
rs149007767
rs11170657
All
−0.4
−0.2
0.0
0.2
MR leave−one−out sensitivity analysis for
'CALCOCO1' on 'LUSC'

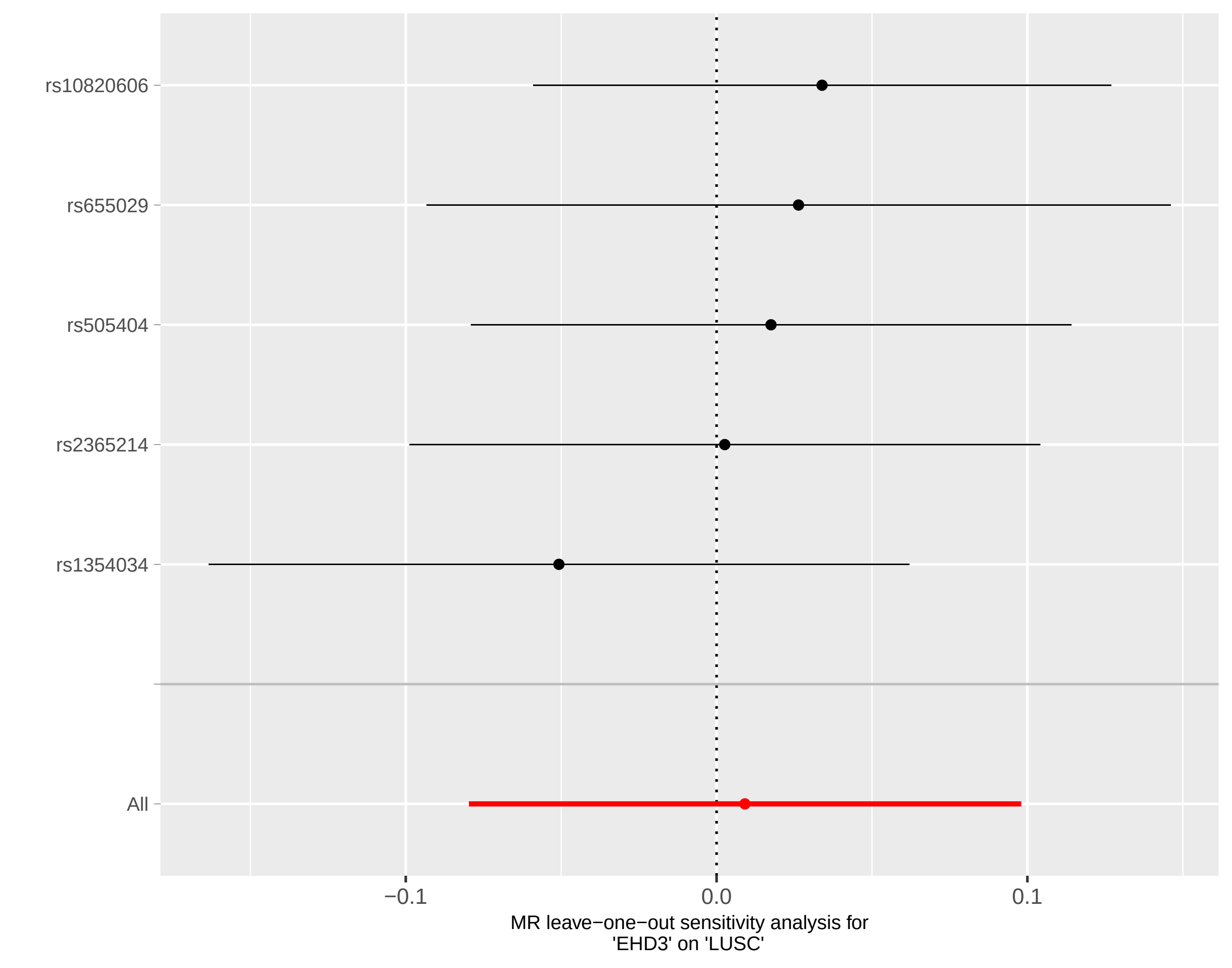
rs10820606
rs655029
rs505404
rs2365214
rs1354034
All
−0.1
0.0
0.1
MR leave−one−out sensitivity analysis for
'EHD3' on 'LUSC'

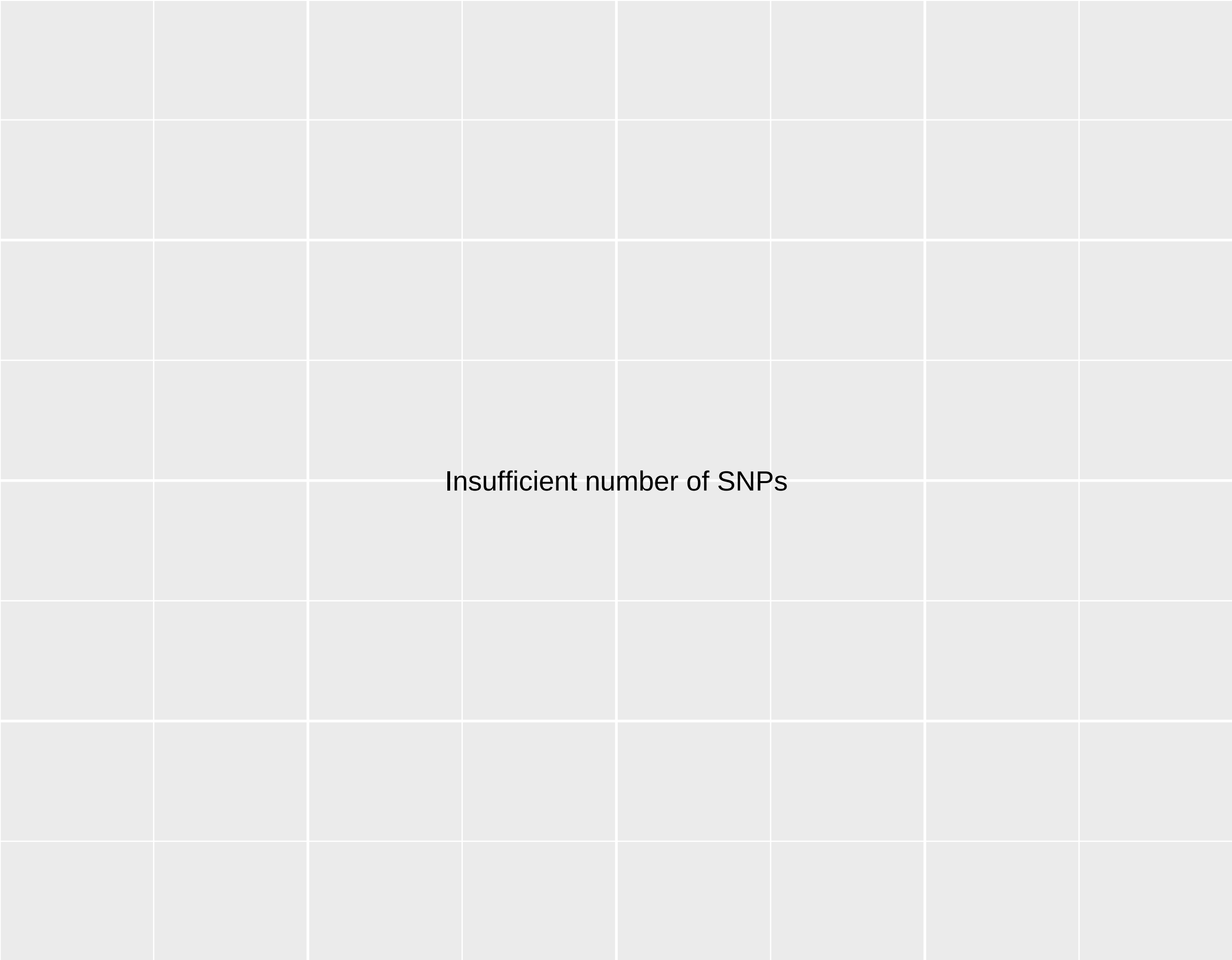
Insufficient number of SNPs

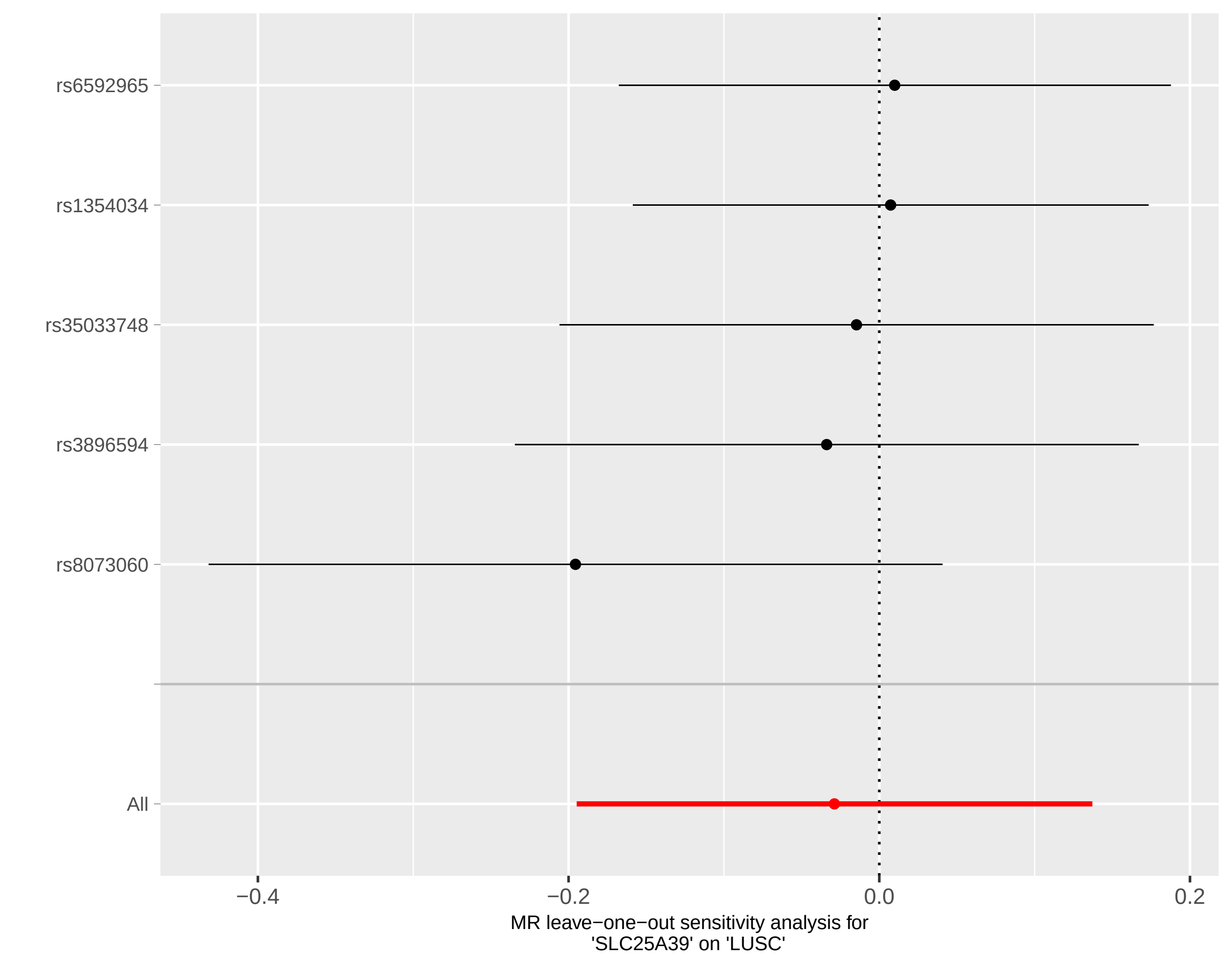

rs6592965
rs1354034
rs35033748
rs3896594
rs8073060
All
−0.4
−0.2
0.0
0.2
MR leave−one−out sensitivity analysis for
'SLC25A39' on 'LUSC'

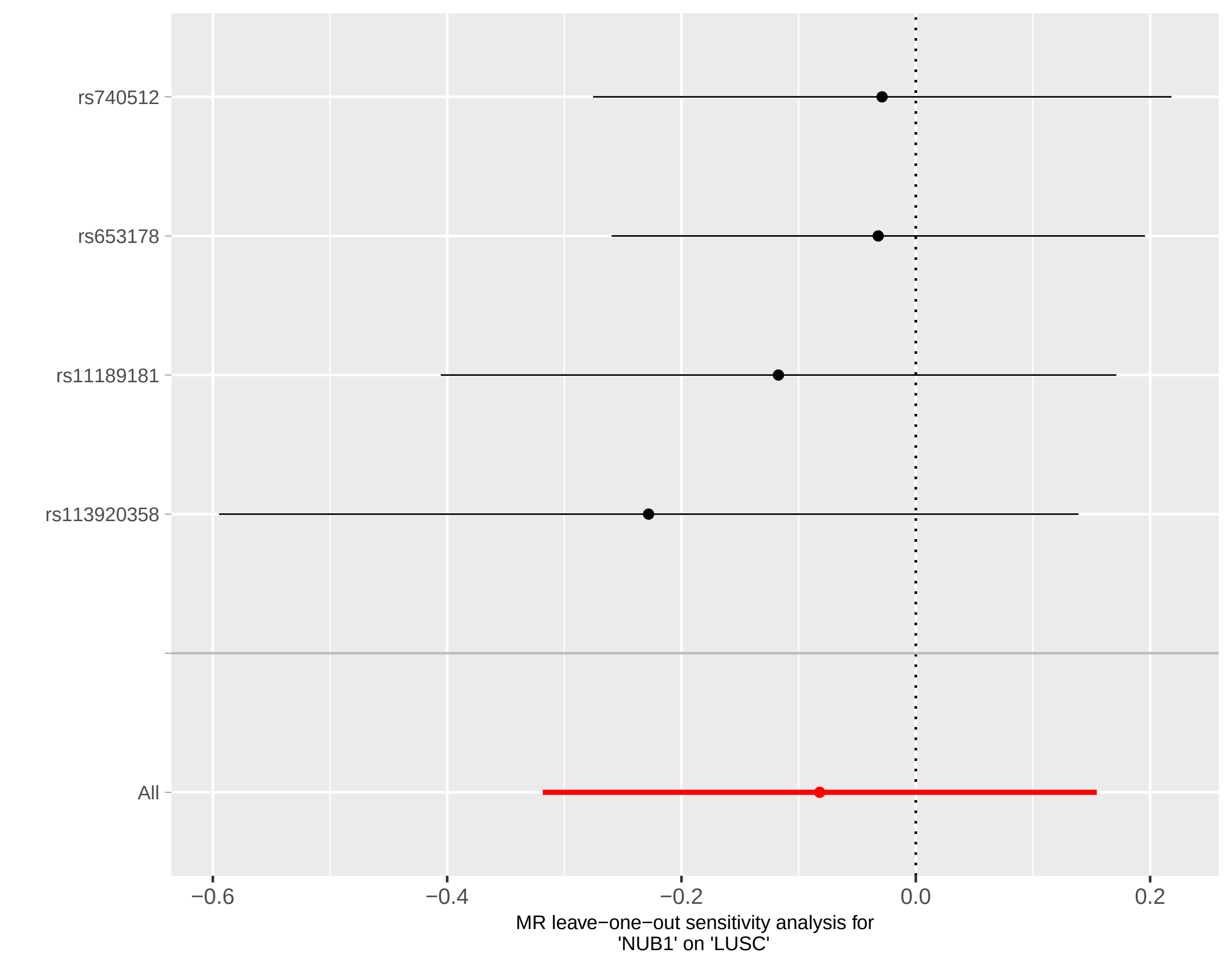

rs740512
rs653178
rs11189181
rs113920358
All
−0.6
−0.4
−0.2
0.0
0.2
MR leave−one−out sensitivity analysis for
'NUB1' on 'LUSC'

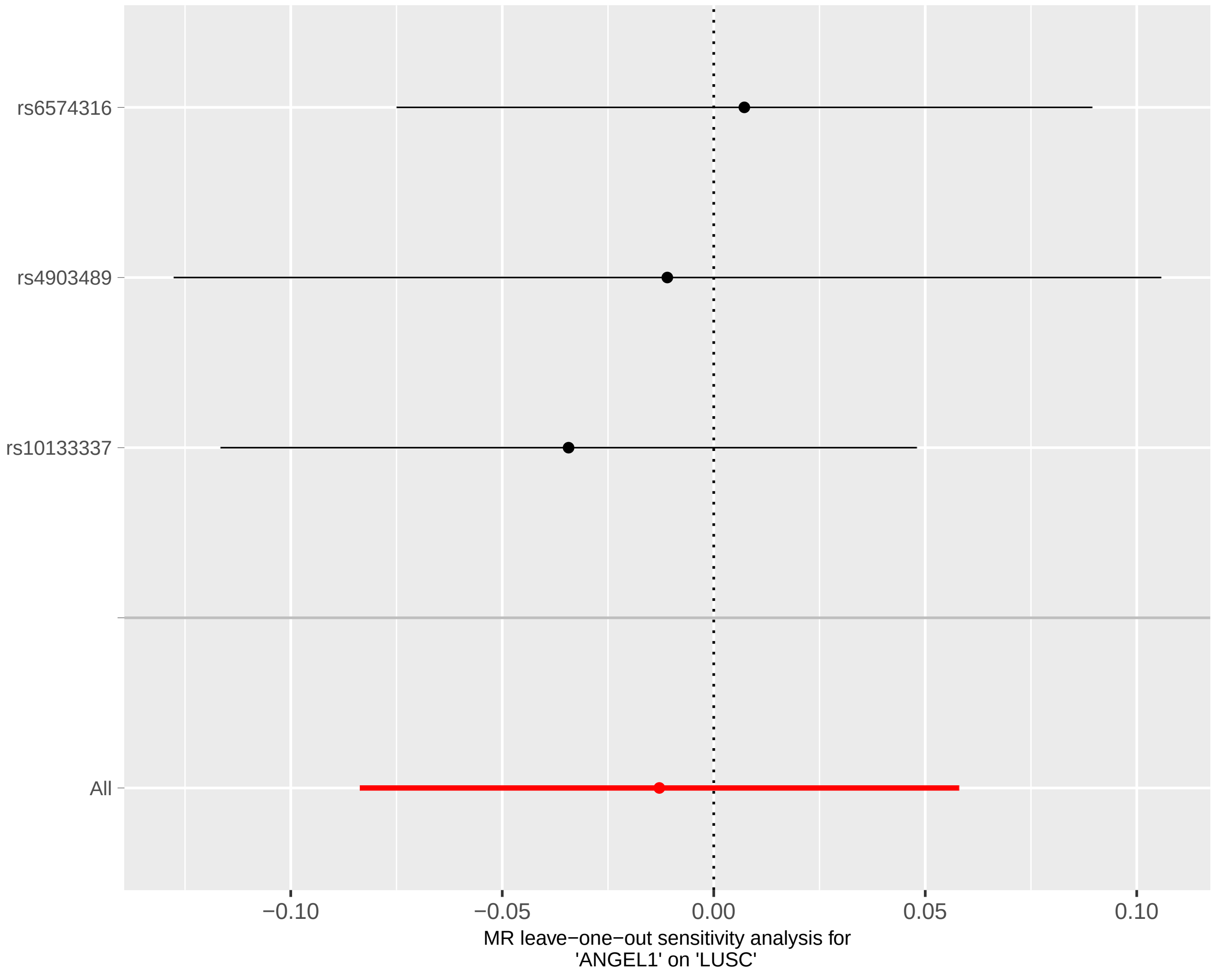

rs6574316
rs4903489
rs10133337
All
−0.10
−0.05
0.00
0.05
0.10
MR leave−one−out sensitivity analysis for
'ANGEL1' on 'LUSC'

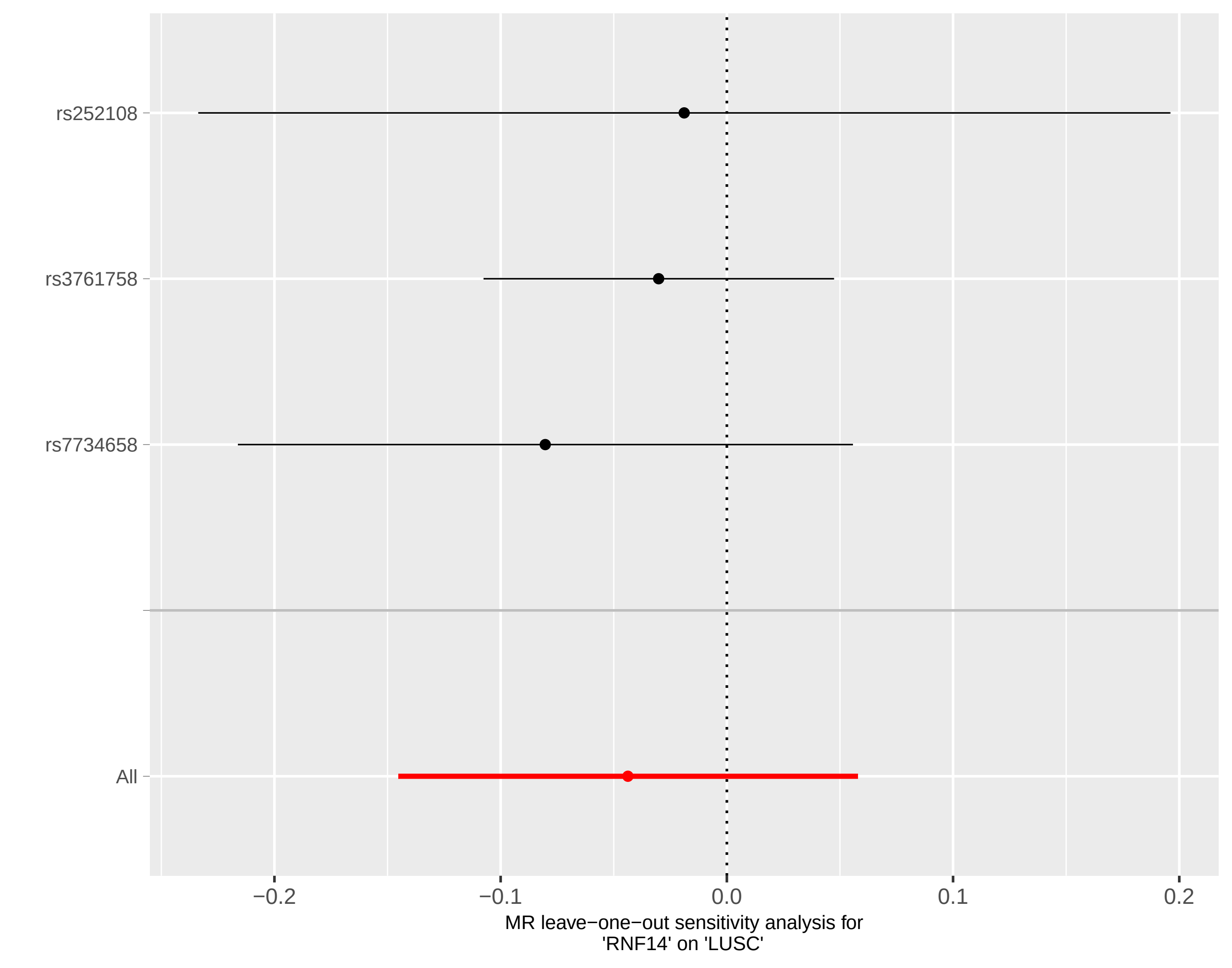
rs252108
rs3761758
rs7734658
All
−0.2
−0.1
0.0
0.1
0.2
MR leave−one−out sensitivity analysis for
'RNF14' on 'LUSC'

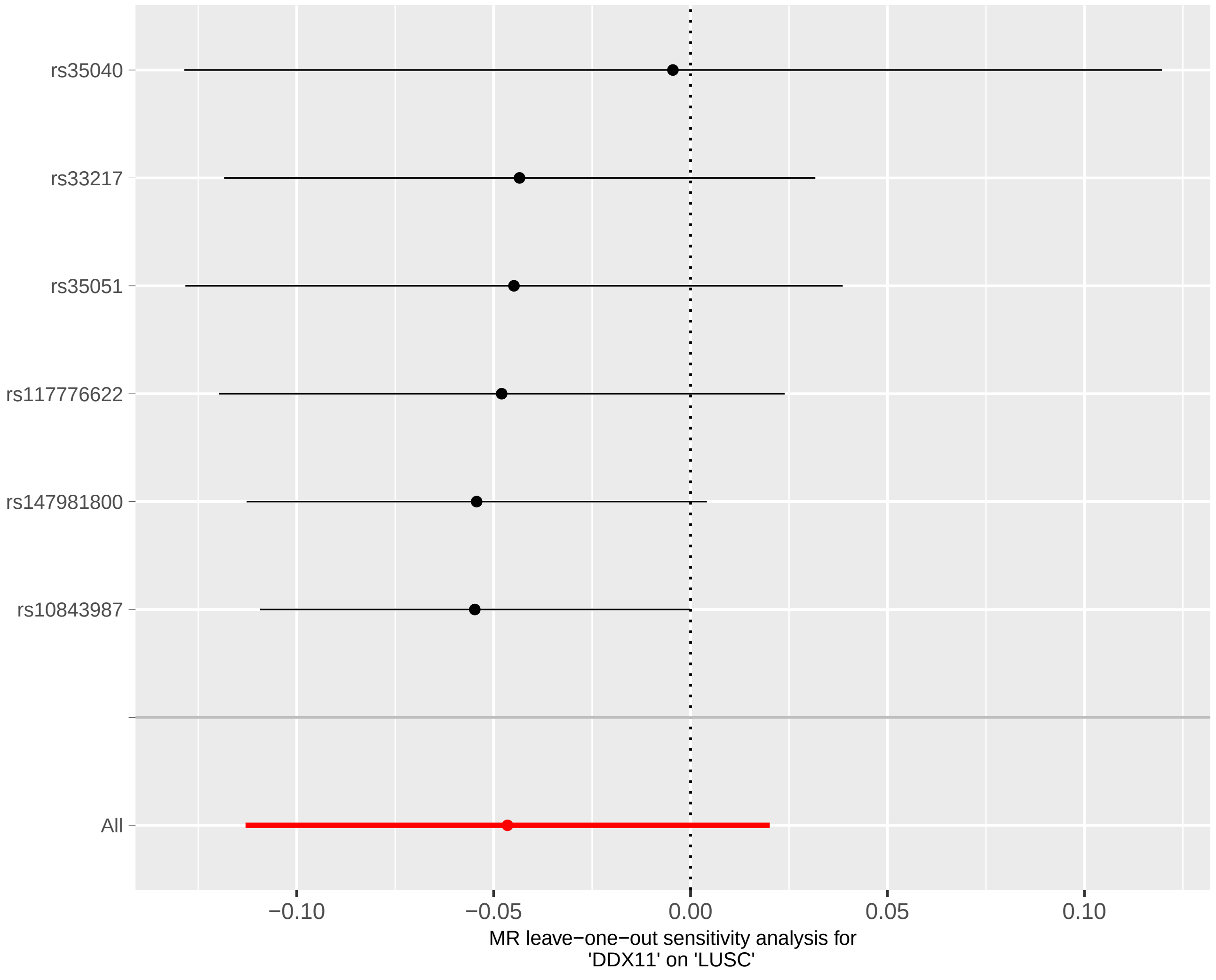
rs35040
rs33217
rs35051
rs117776622
rs147981800
rs10843987
All
−0.10
−0.05
0.00
0.05
0.10
MR leave−one−out sensitivity analysis for
'DDX11' on 'LUSC'

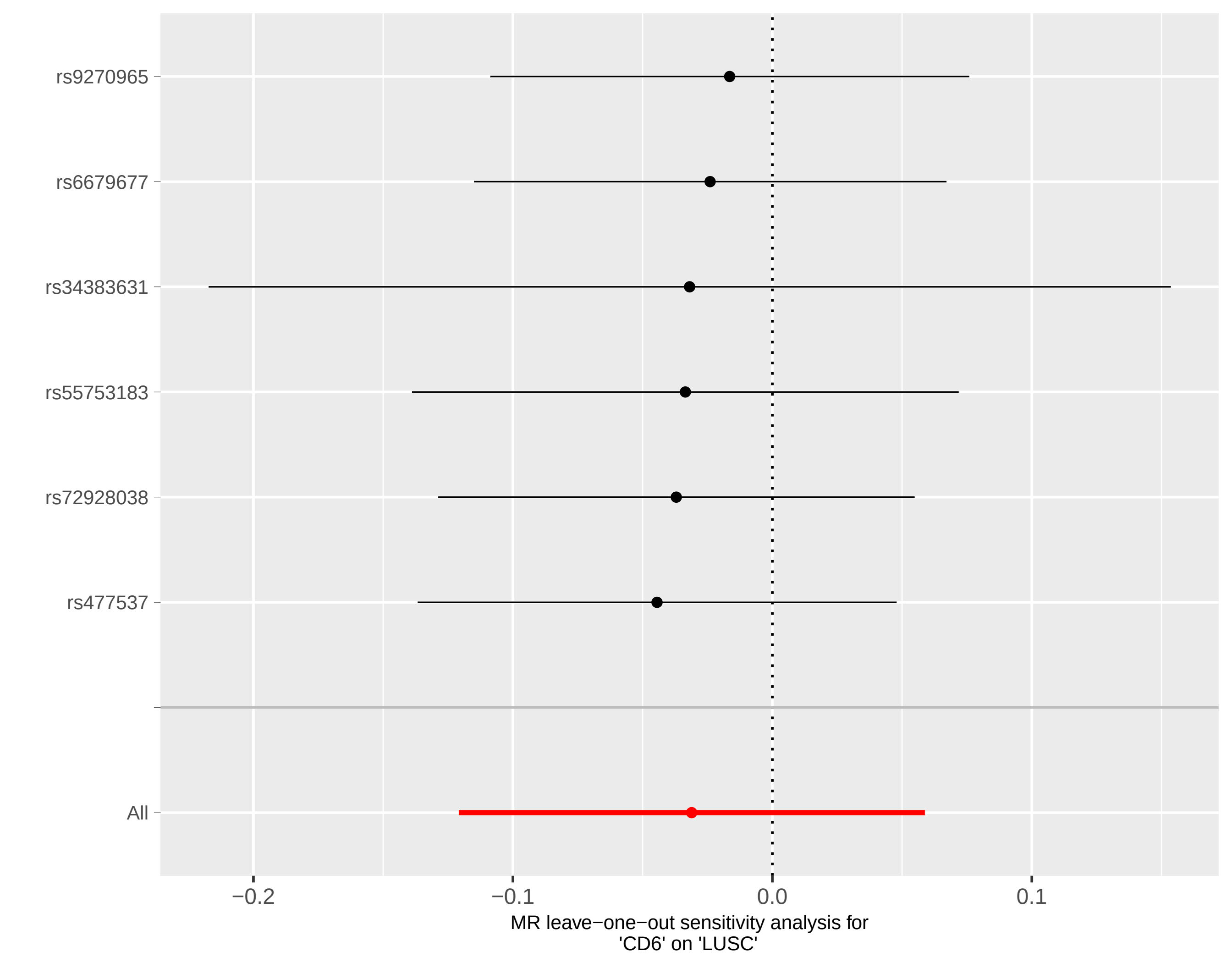

rs9270965
rs6679677
rs34383631
rs55753183
rs72928038
rs477537
All
−0.2
−0.1
0.0
0.1
MR leave−one−out sensitivity analysis for
'CD6' on 'LUSC'

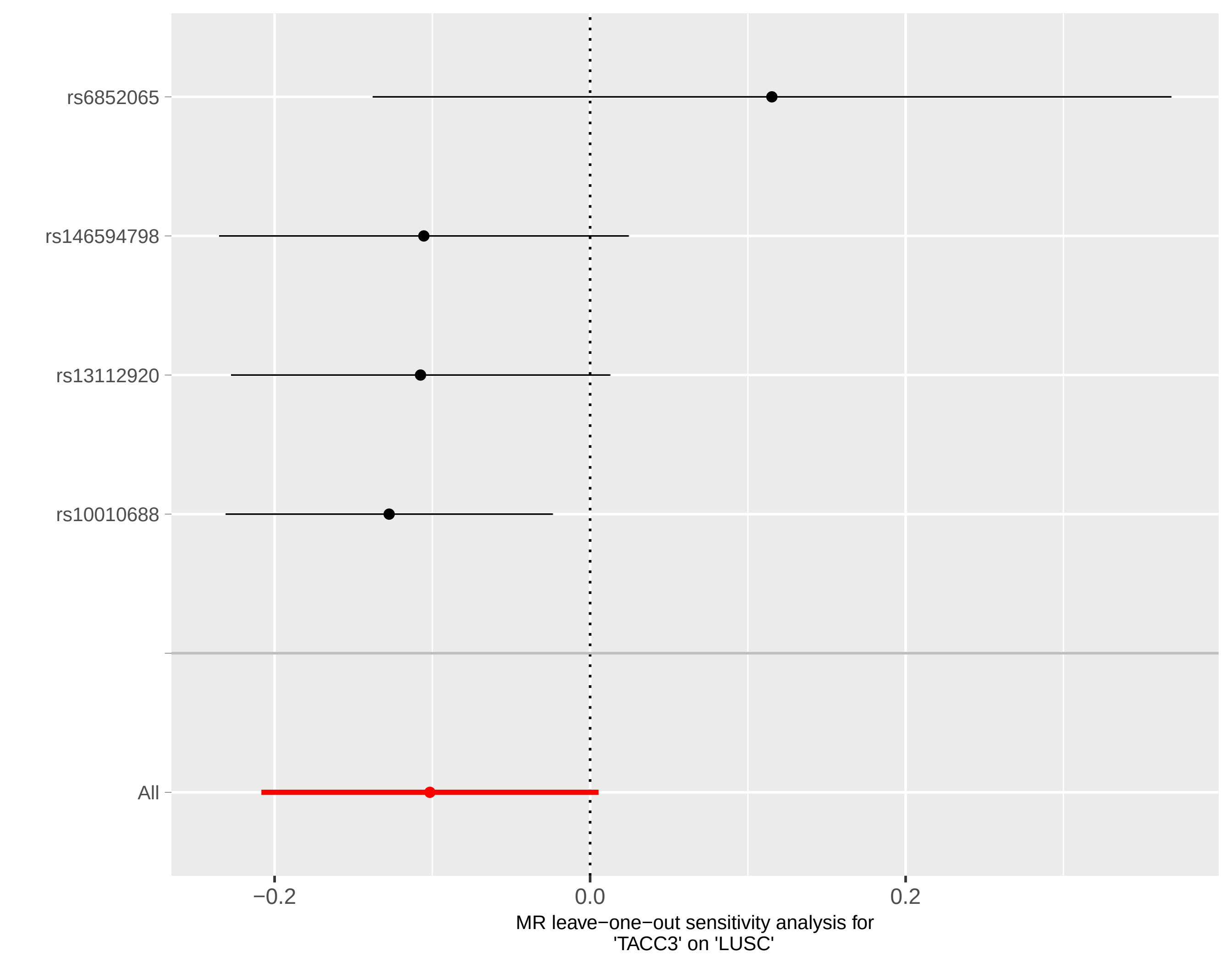

rs6852065
rs146594798
rs13112920
rs10010688
All
−0.2
0.0
0.2
MR leave−one−out sensitivity analysis for
'TACC3' on 'LUSC'

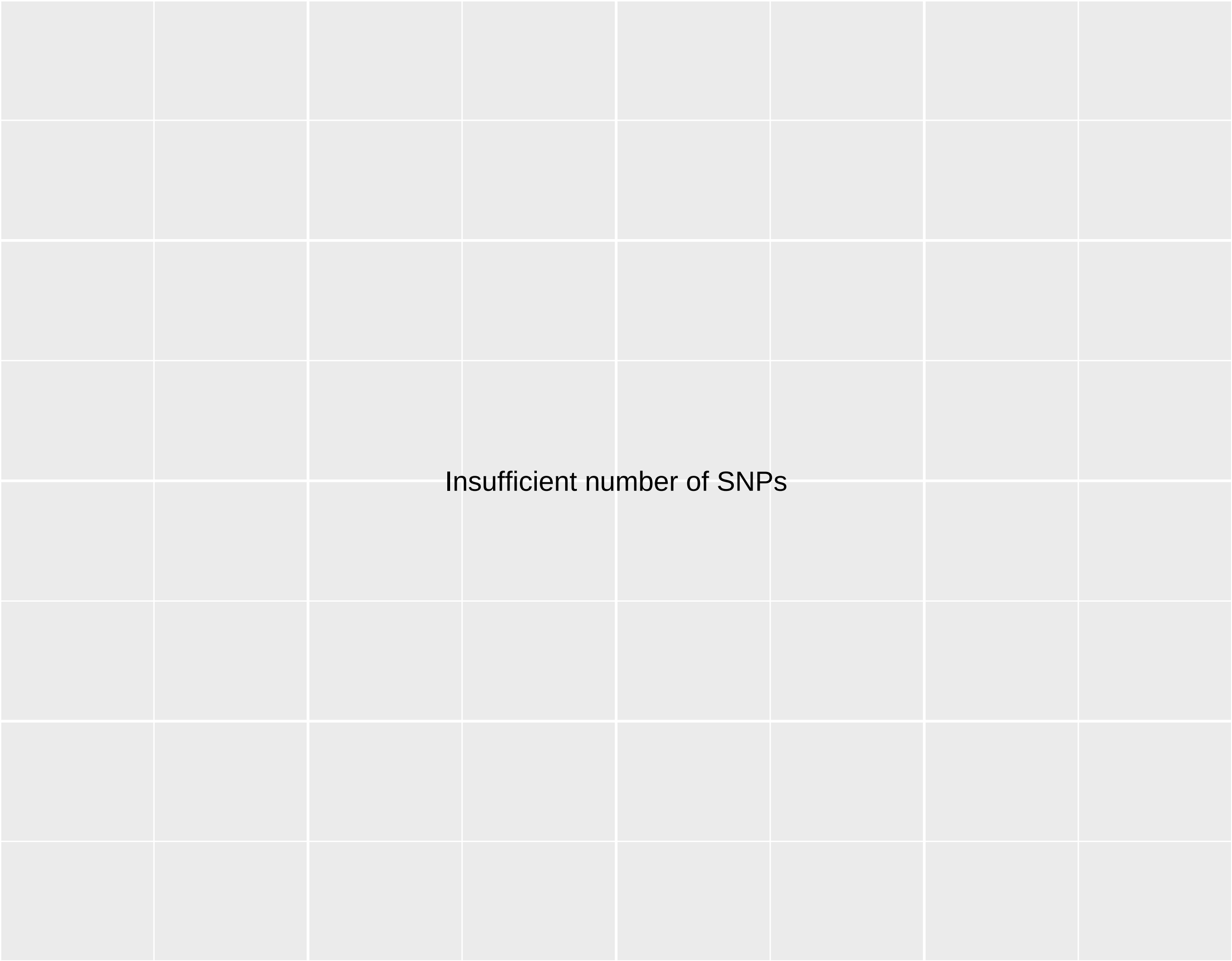
Insufficient number of SNPs

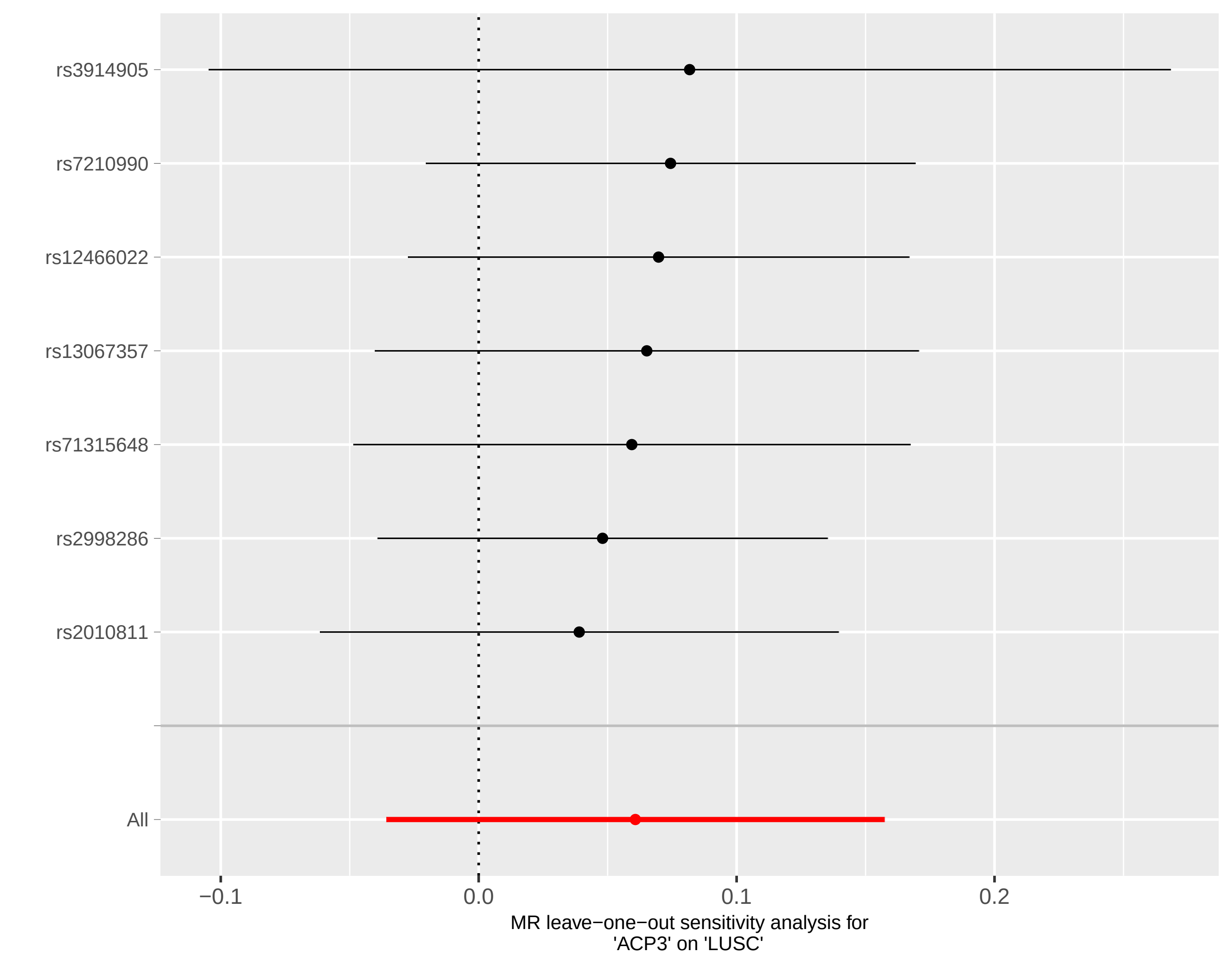
rs3914905
rs7210990
rs12466022
rs13067357
rs71315648
rs2998286
rs2010811
All
−0.1
0.0
0.1
0.2
MR leave−one−out sensitivity analysis for
'ACP3' on 'LUSC'

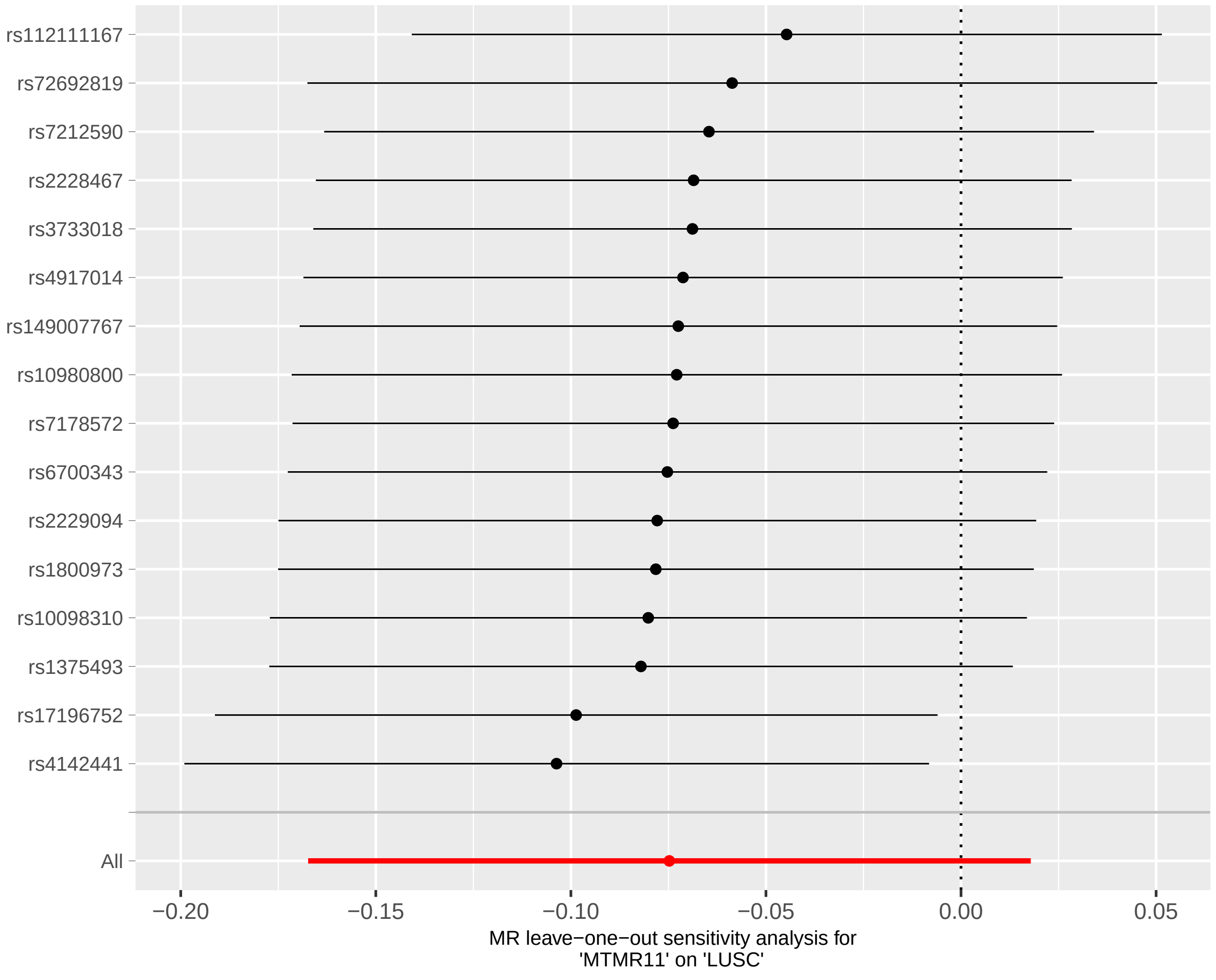
rs112111167
rs72692819
rs7212590
rs2228467
rs3733018
rs4917014
rs149007767
rs10980800
rs7178572
rs6700343
rs2229094
rs1800973
rs10098310
rs1375493
rs17196752
rs4142441
All
−0.20
−0.15
−0.10
−0.05
0.00
0.05
MR leave−one−out sensitivity analysis for
'MTMR11' on 'LUSC'

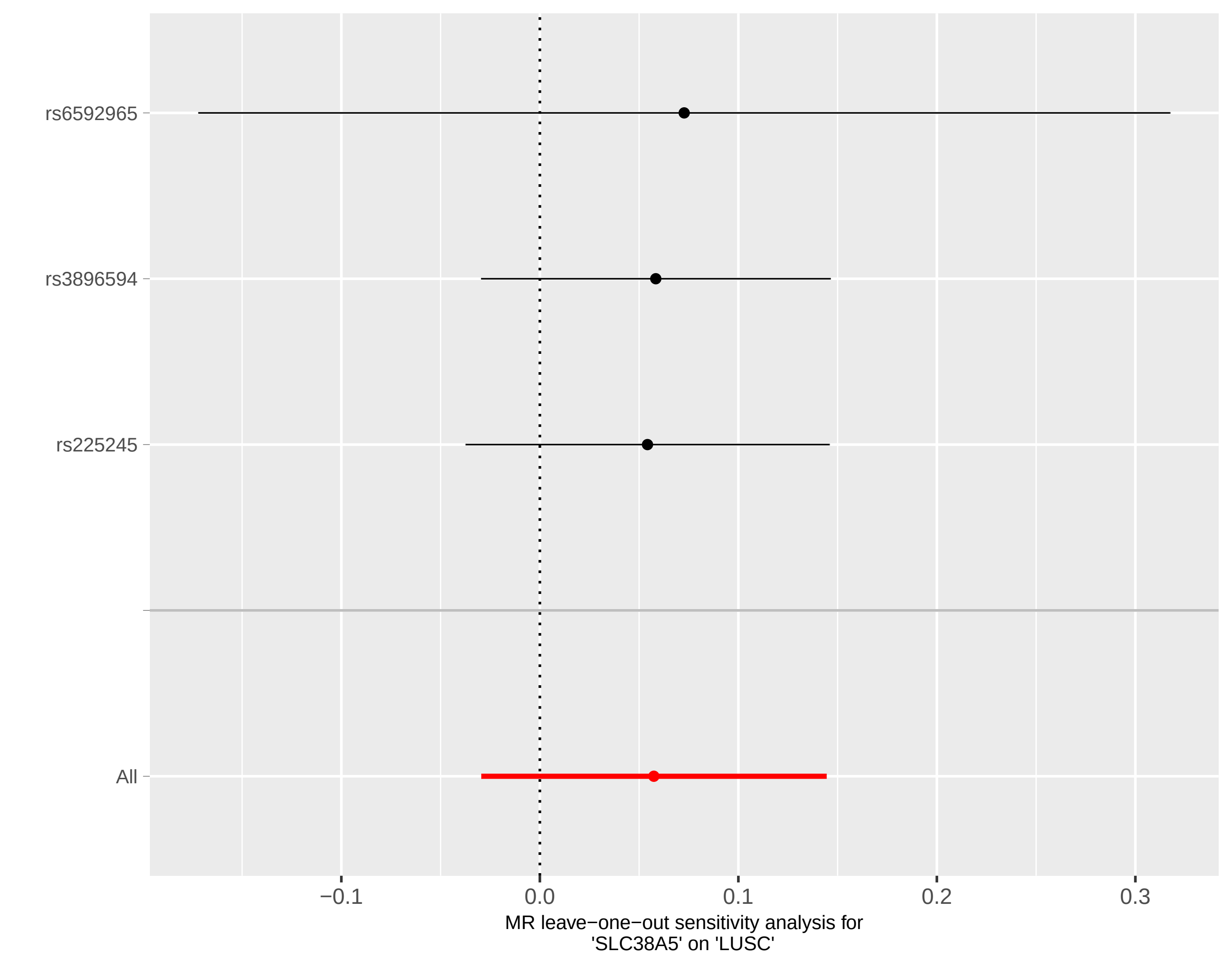

rs6592965
rs3896594
rs225245
All
−0.1
0.0
0.1
0.2
0.3
MR leave−one−out sensitivity analysis for
'SLC38A5' on 'LUSC'

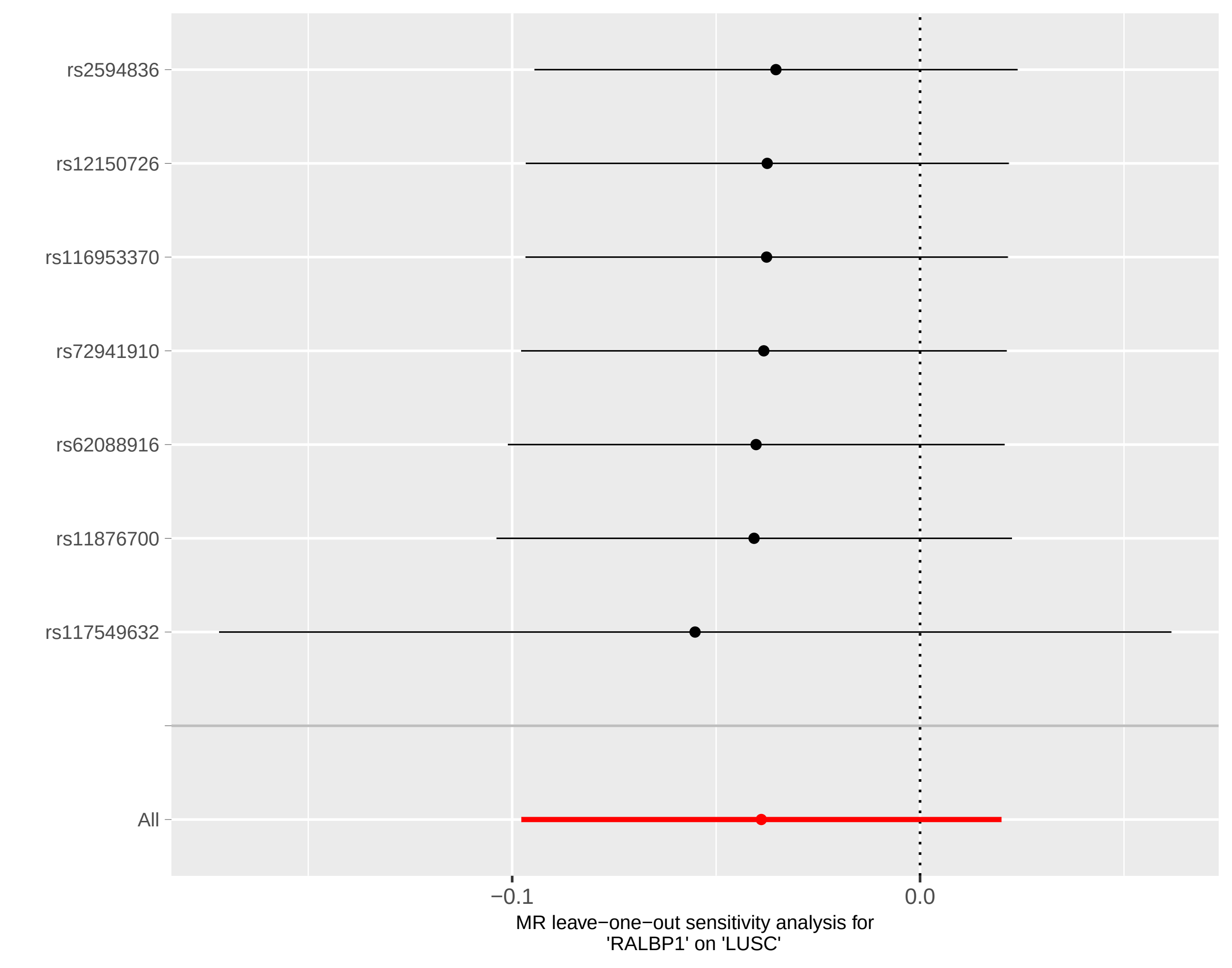
rs2594836
rs12150726
rs116953370
rs72941910
rs62088916
rs11876700
rs117549632
All
−0.1
0.0
MR leave−one−out sensitivity analysis for
'RALBP1' on 'LUSC'

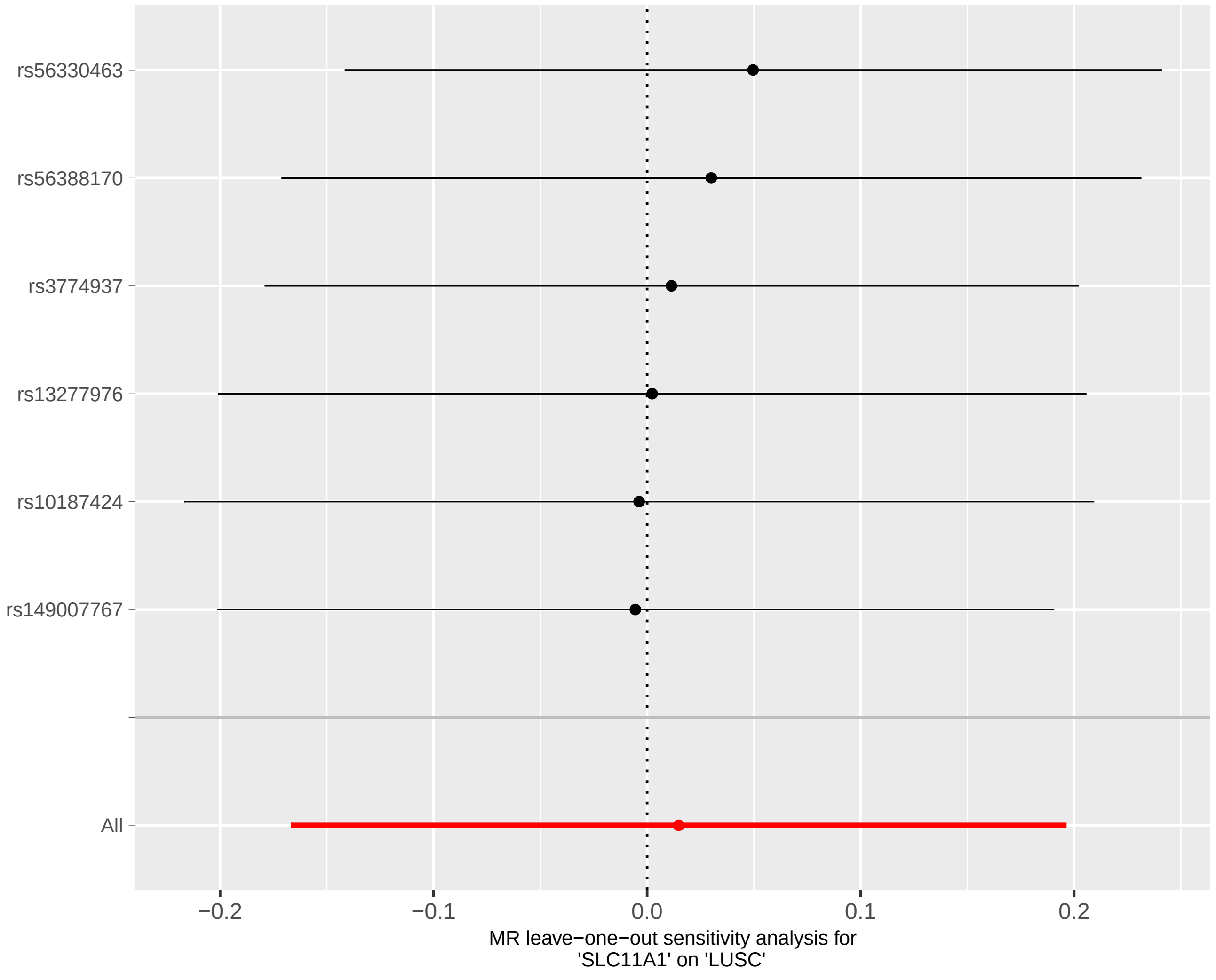
rs56330463
rs56388170
rs3774937
rs13277976
rs10187424
rs149007767
All
−0.2
−0.1
0.0
0.1
0.2
MR leave−one−out sensitivity analysis for
'SLC11A1' on 'LUSC'

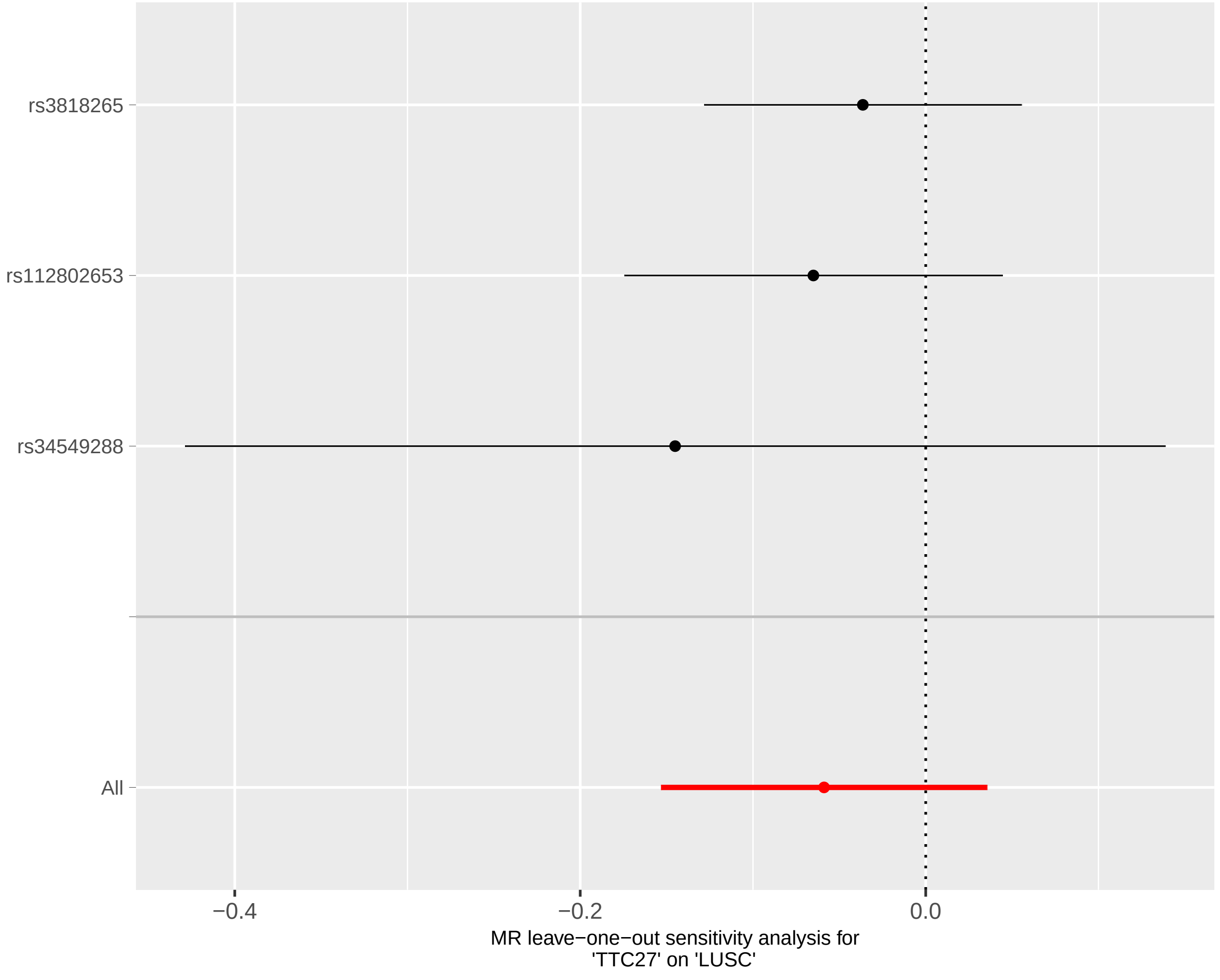
rs3818265
rs112802653
rs34549288
All
−0.4
−0.2
0.0
MR leave−one−out sensitivity analysis for
'TTC27' on 'LUSC'

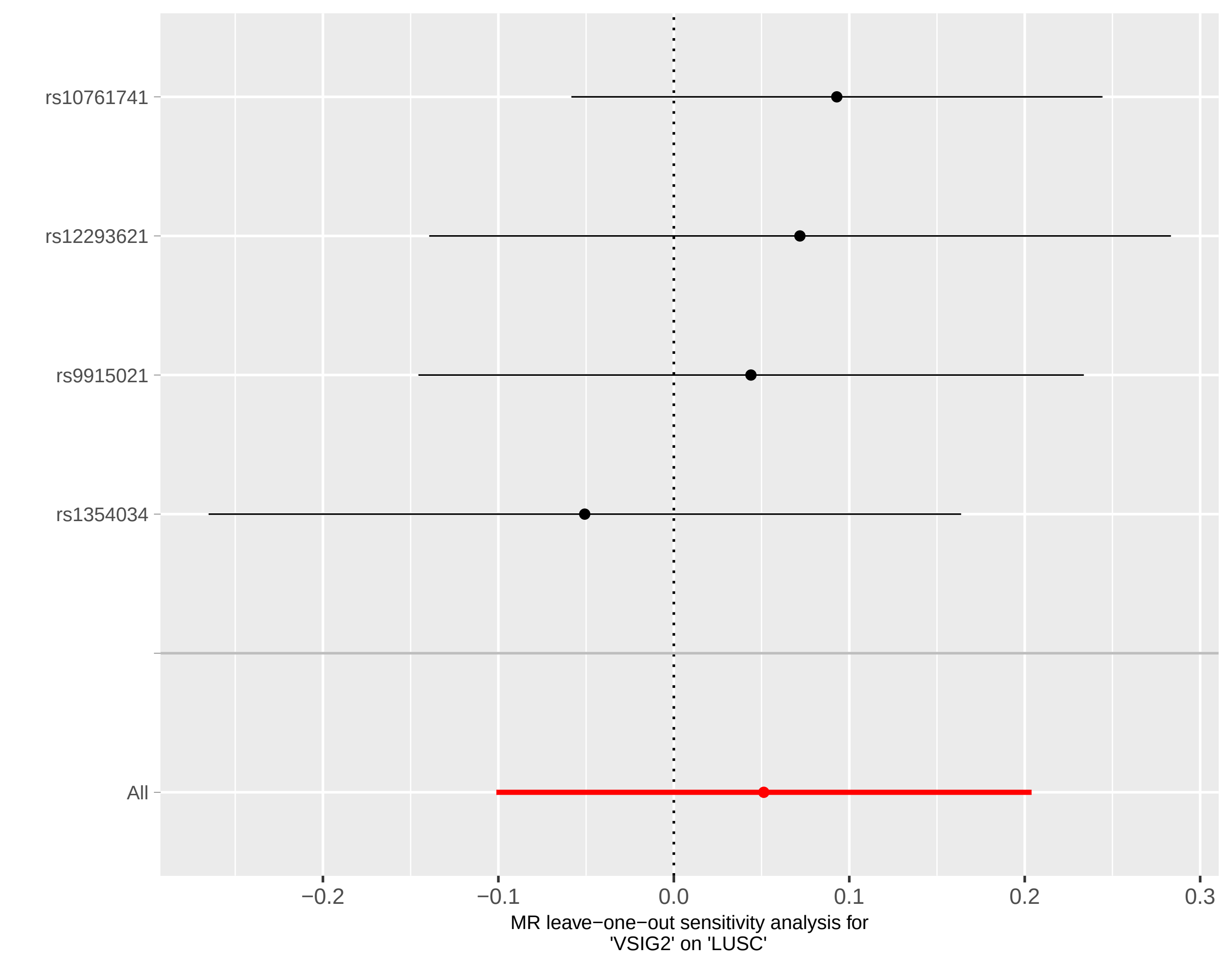

rs10761741
rs12293621
rs9915021
rs1354034
All
−0.2
−0.1
0.0
0.1
0.2
0.3
MR leave−one−out sensitivity analysis for
'VSIG2' on 'LUSC'

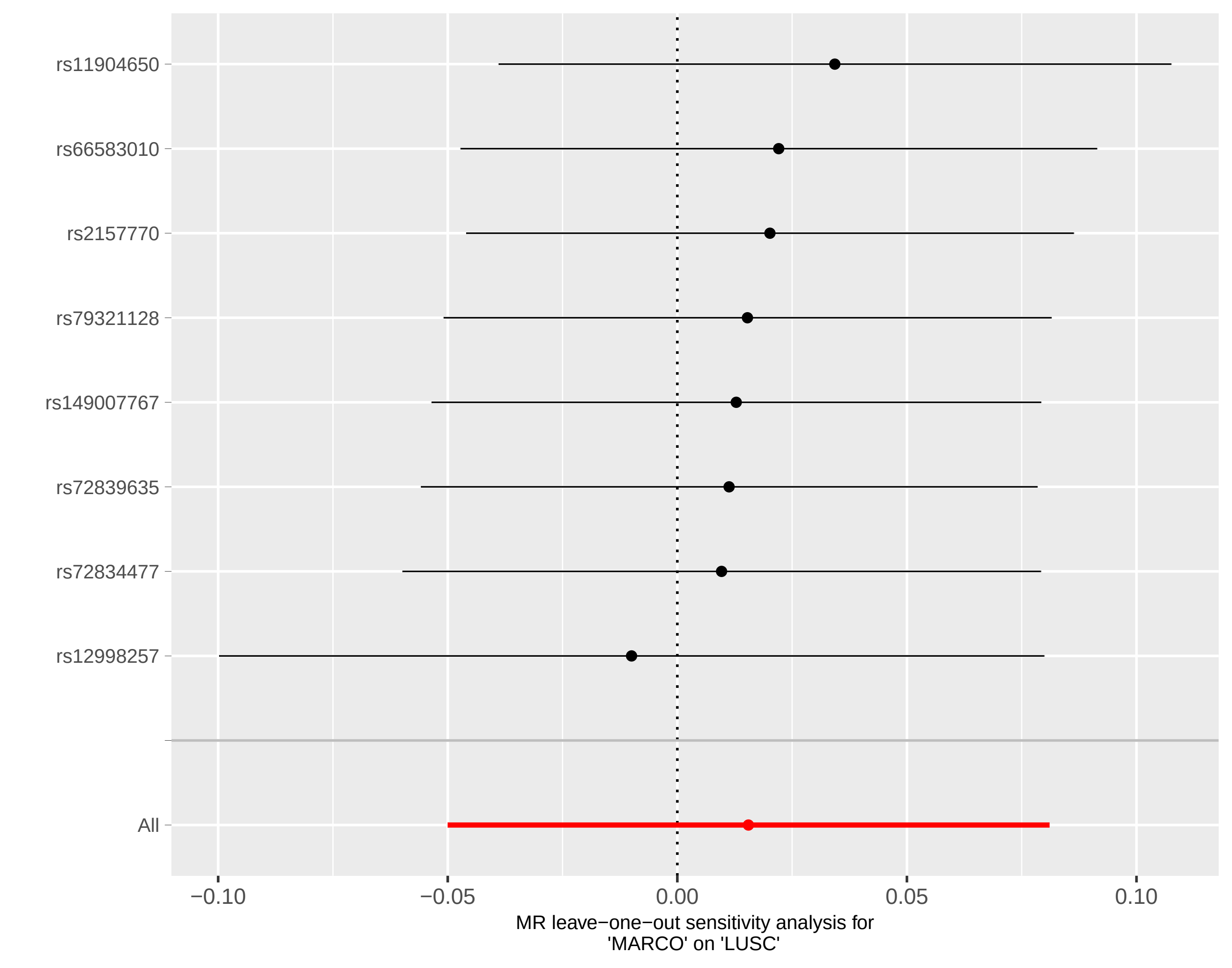
rs11904650
rs66583010
rs2157770
rs79321128
rs149007767
rs72839635
rs72834477
rs12998257
All
−0.10
−0.05
0.00
0.05
0.10
MR leave−one−out sensitivity analysis for
'MARCO' on 'LUSC'

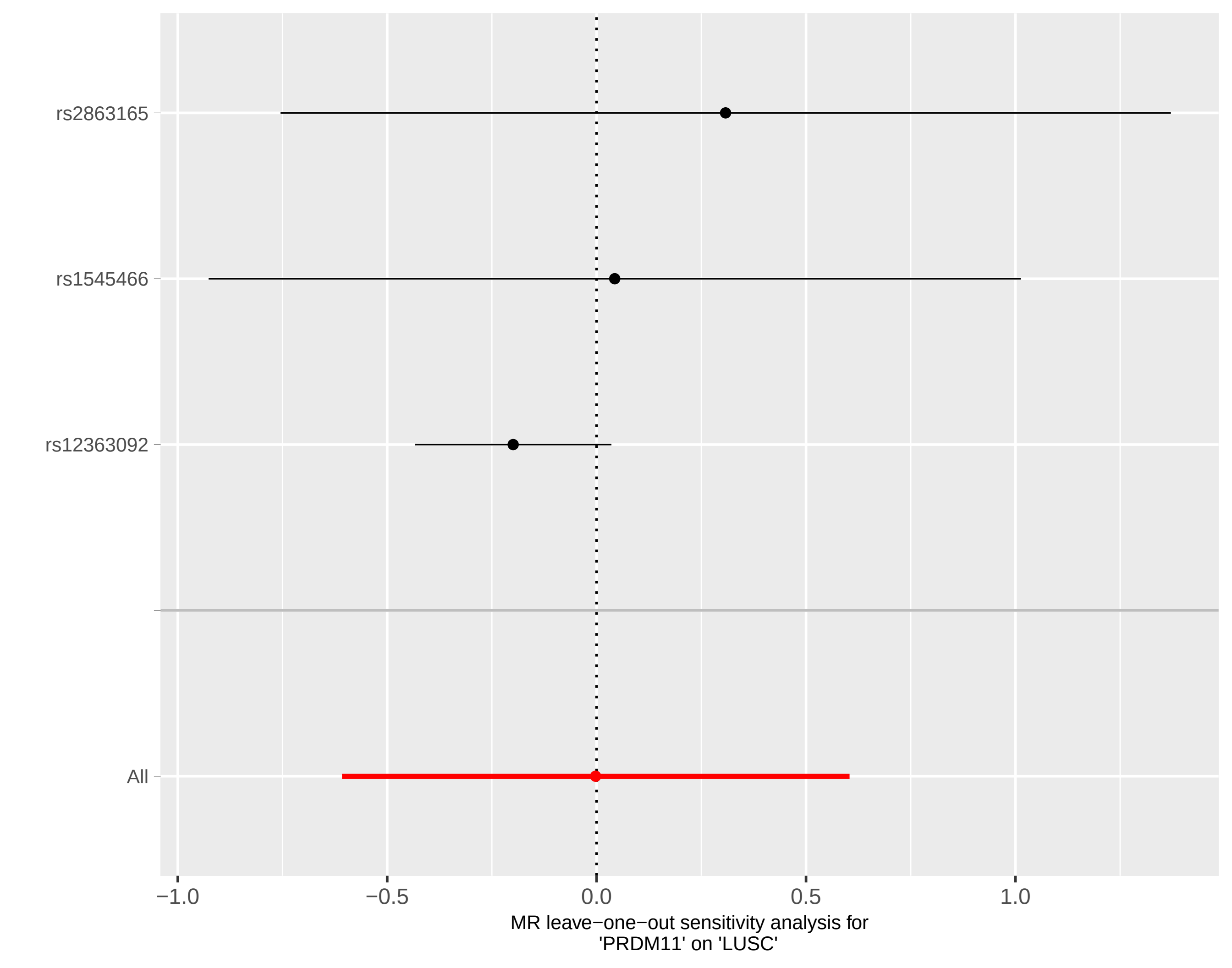
rs2863165
rs1545466
rs12363092
All
−1.0
−0.5
0.0
0.5
1.0
MR leave−one−out sensitivity analysis for
'PRDM11' on 'LUSC'

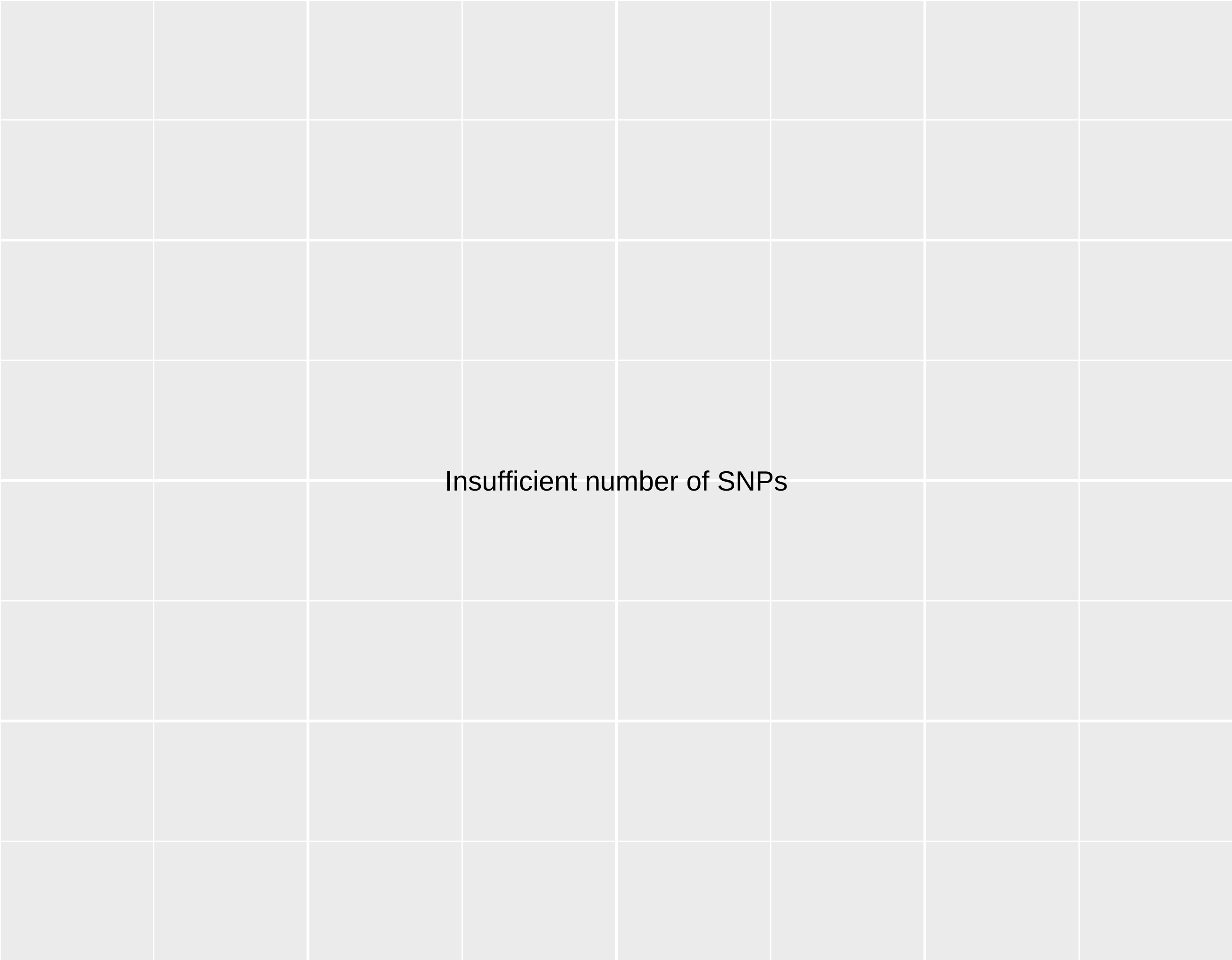
Insufficient number of SNPs

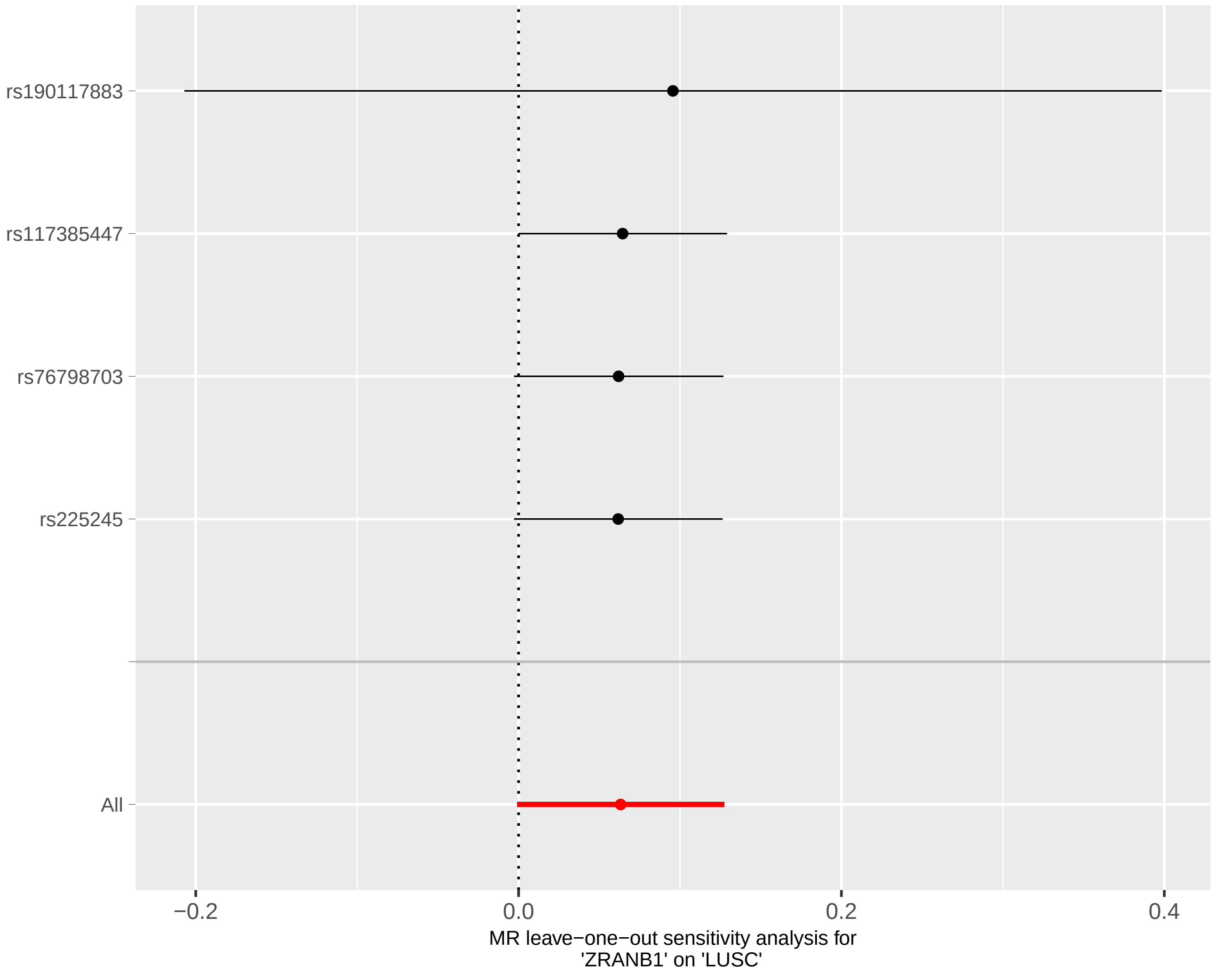
rs190117883
rs117385447
rs76798703
rs225245
All
−0.2
0.0
0.2
0.4
MR leave−one−out sensitivity analysis for
'ZRANB1' on 'LUSC'

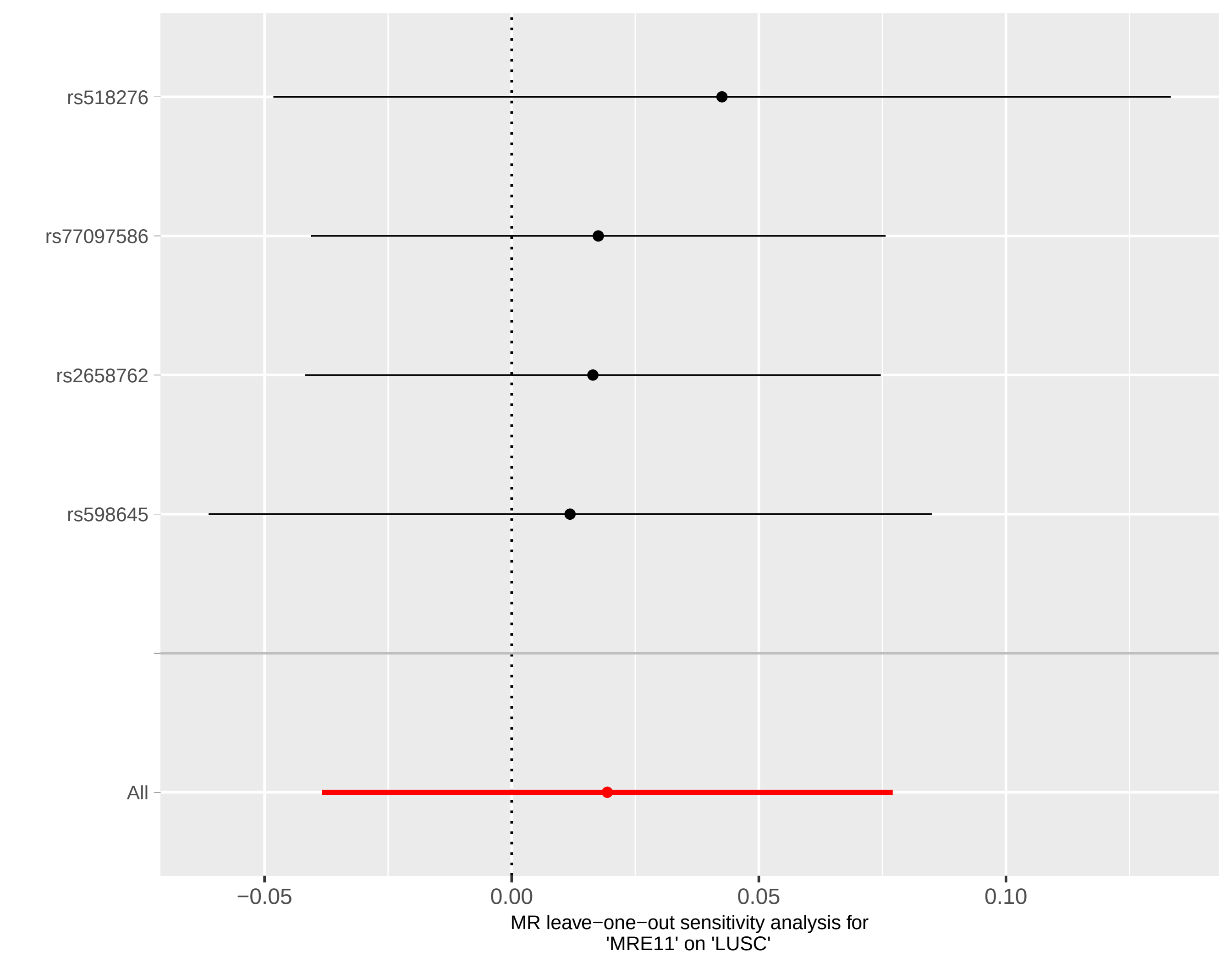
rs518276
rs77097586
rs2658762
rs598645
All
−0.05
0.00
0.05
0.10
MR leave−one−out sensitivity analysis for
'MRE11' on 'LUSC'

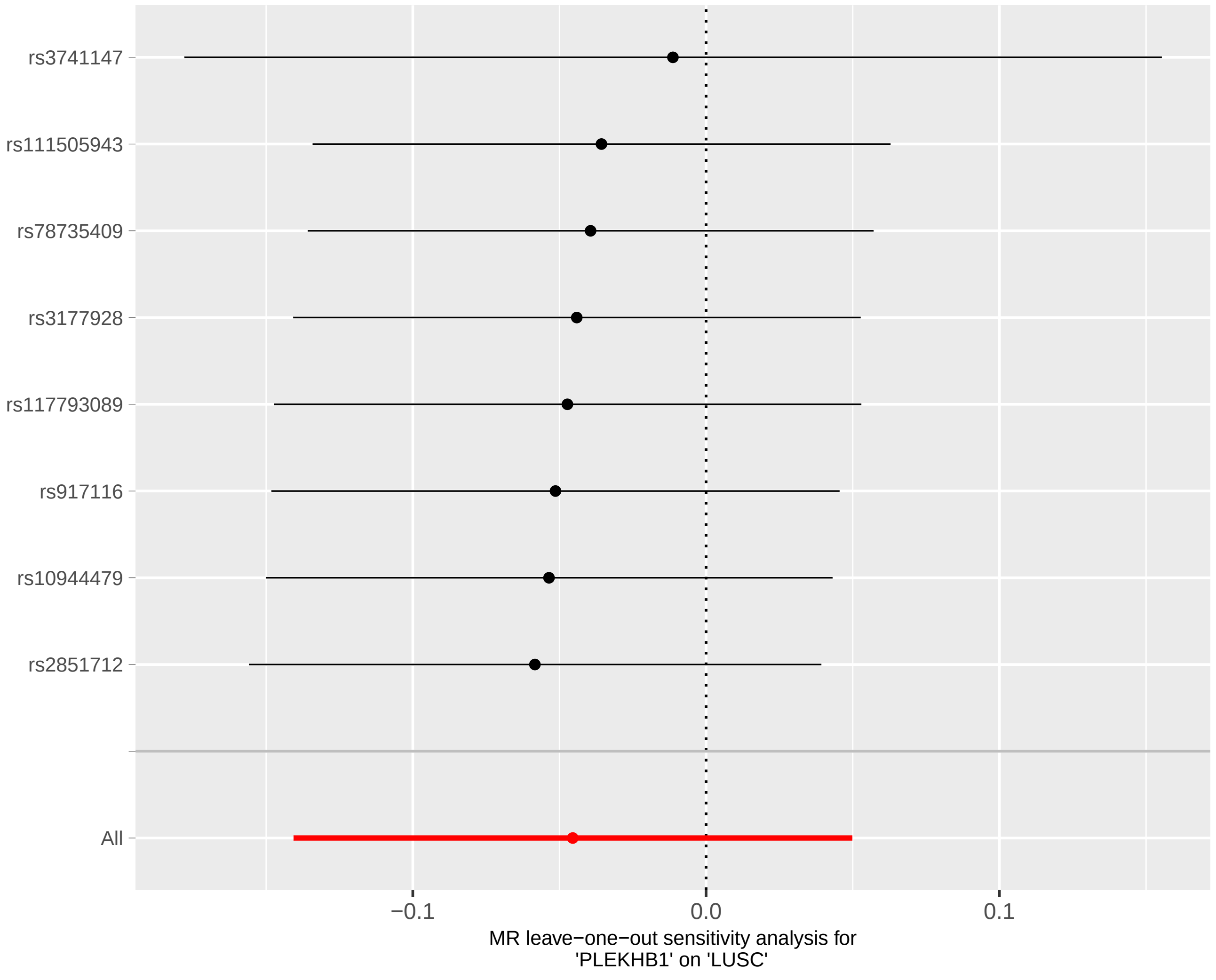
rs3741147
rs111505943
rs78735409
rs3177928
rs117793089
rs917116
rs10944479
rs2851712
All
−0.1
0.0
0.1
MR leave−one−out sensitivity analysis for
'PLEKHB1' on 'LUSC'

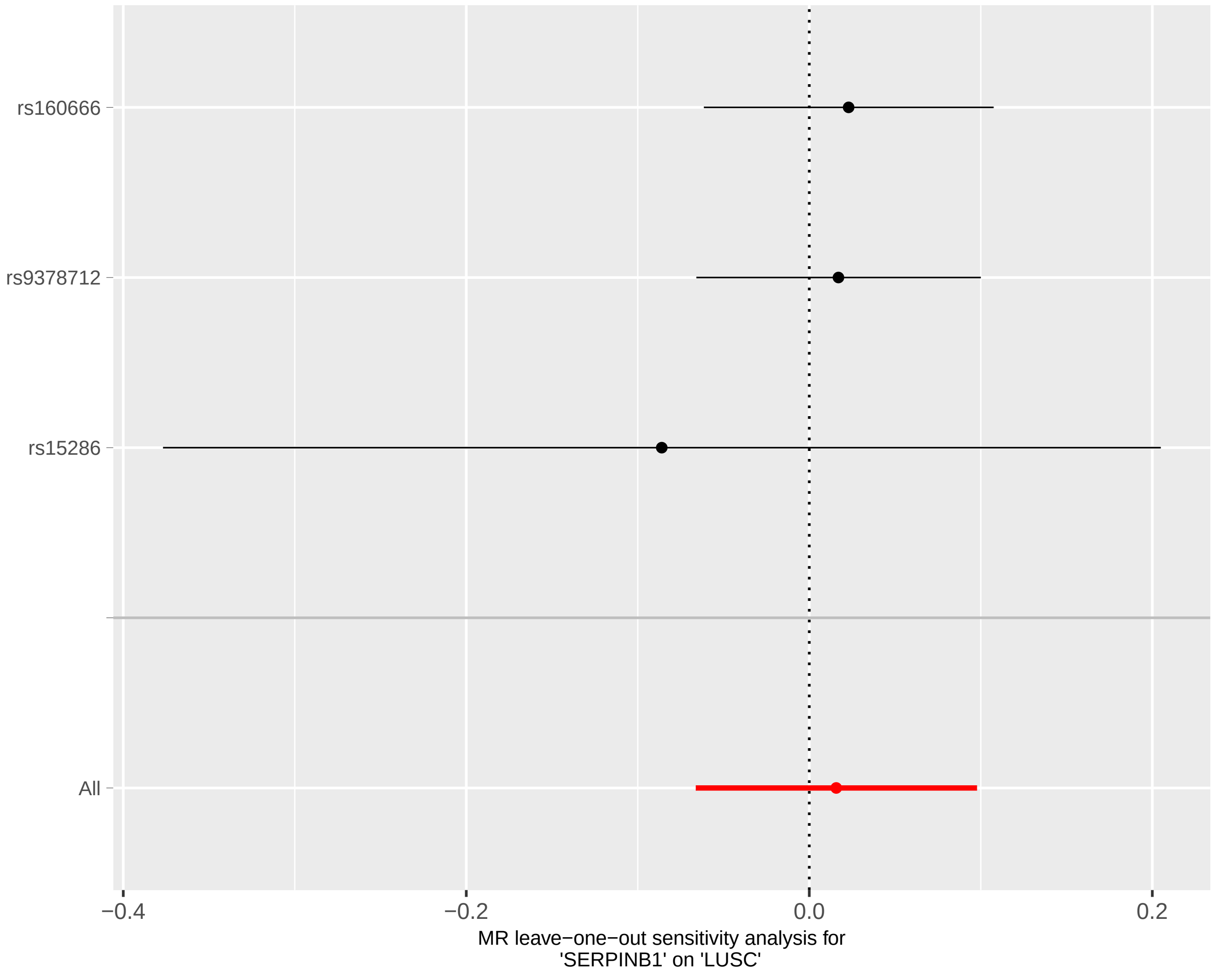
rs160666
rs9378712
rs15286
All
−0.4
−0.2
0.0
0.2
MR leave−one−out sensitivity analysis for
'SERPINB1' on 'LUSC'

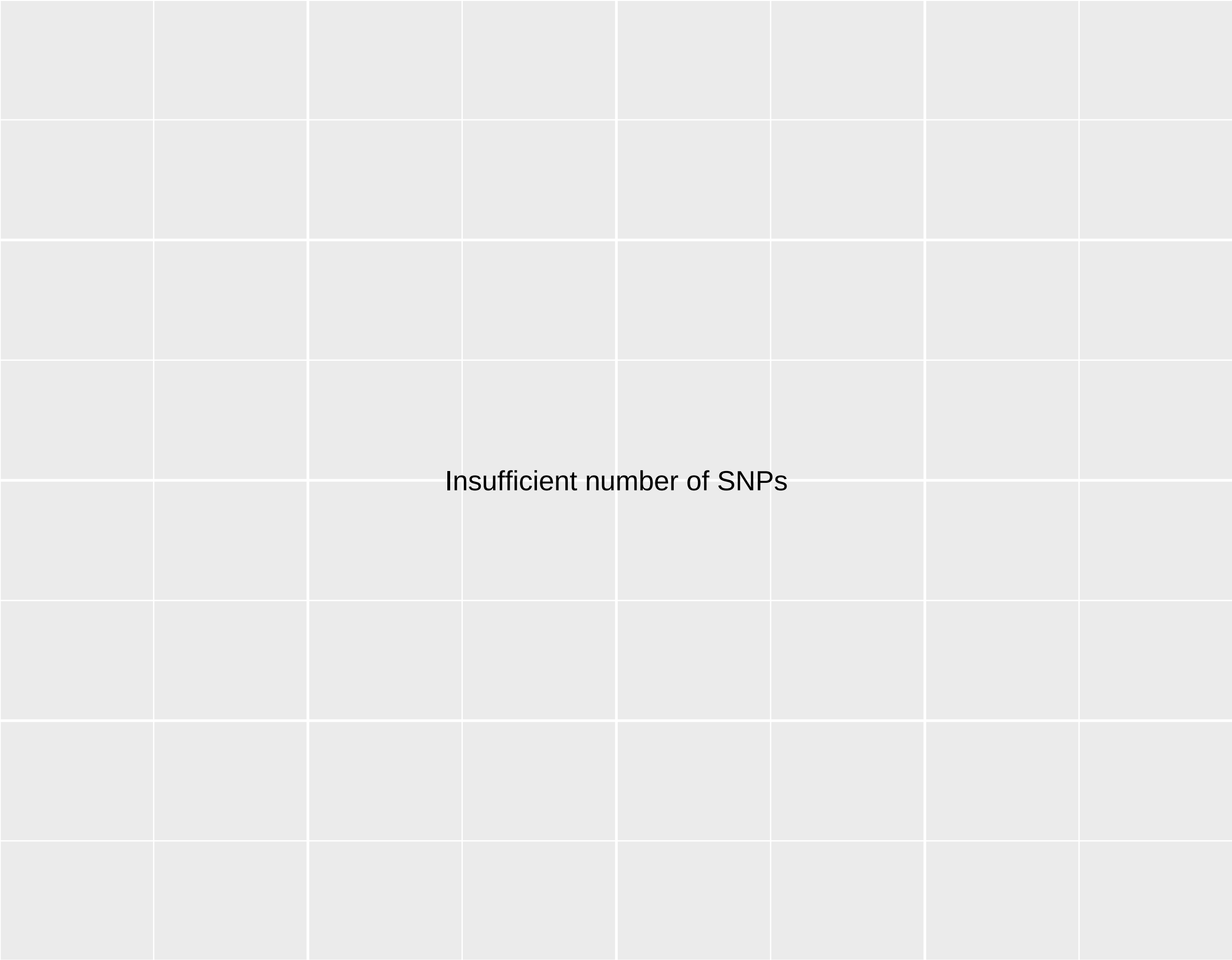
Insufficient number of SNPs

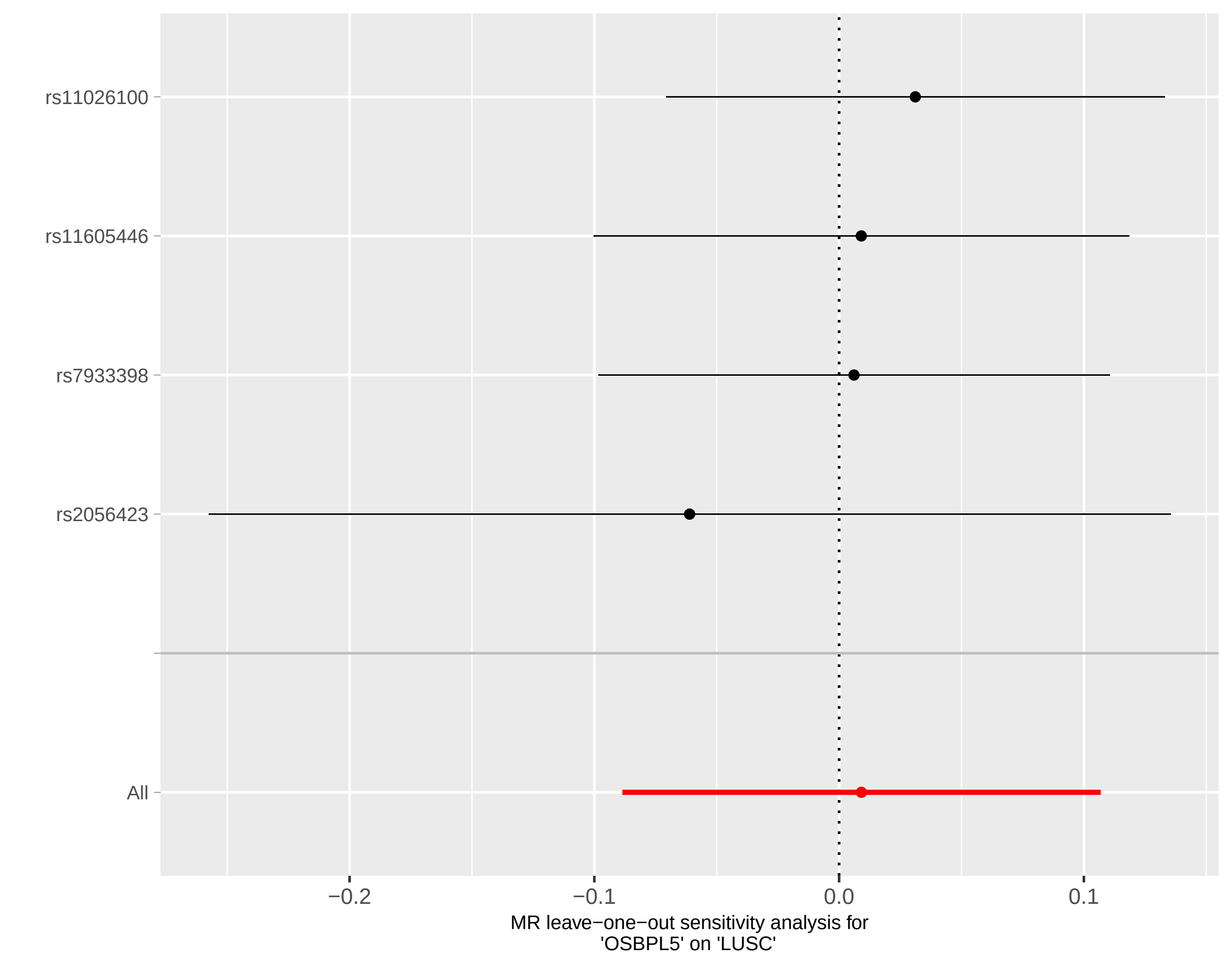
rs11026100
rs11605446
rs7933398
rs2056423
All
−0.2
−0.1
0.0
0.1
MR leave−one−out sensitivity analysis for
'OSBPL5' on 'LUSC'

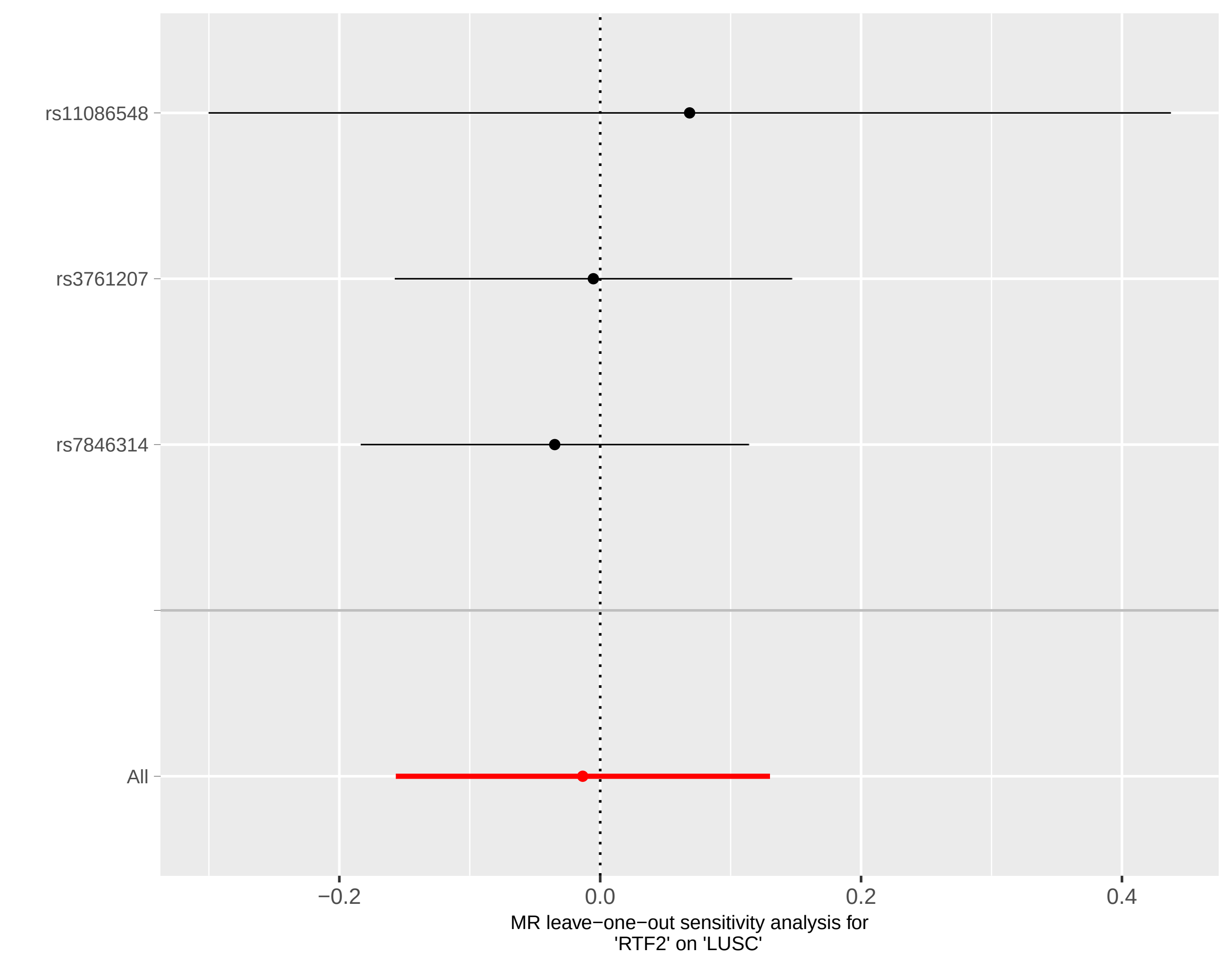
rs11086548
rs3761207
rs7846314
All
−0.2
0.0
0.2
0.4
MR leave−one−out sensitivity analysis for
'RTF2' on 'LUSC'

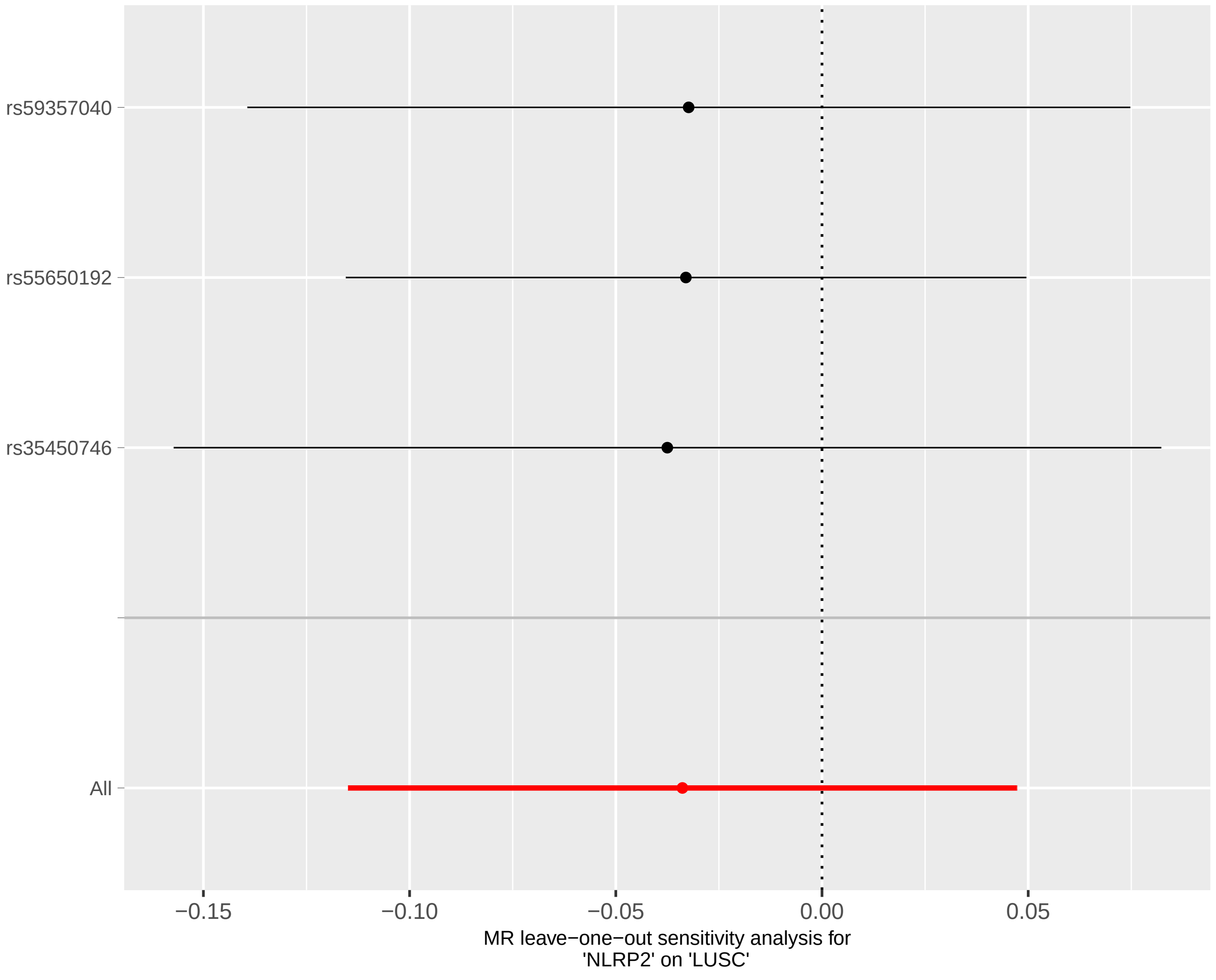
rs59357040
rs55650192
rs35450746
All
−0.15
−0.10
−0.05
0.00
0.05
MR leave−one−out sensitivity analysis for
'NLRP2' on 'LUSC'

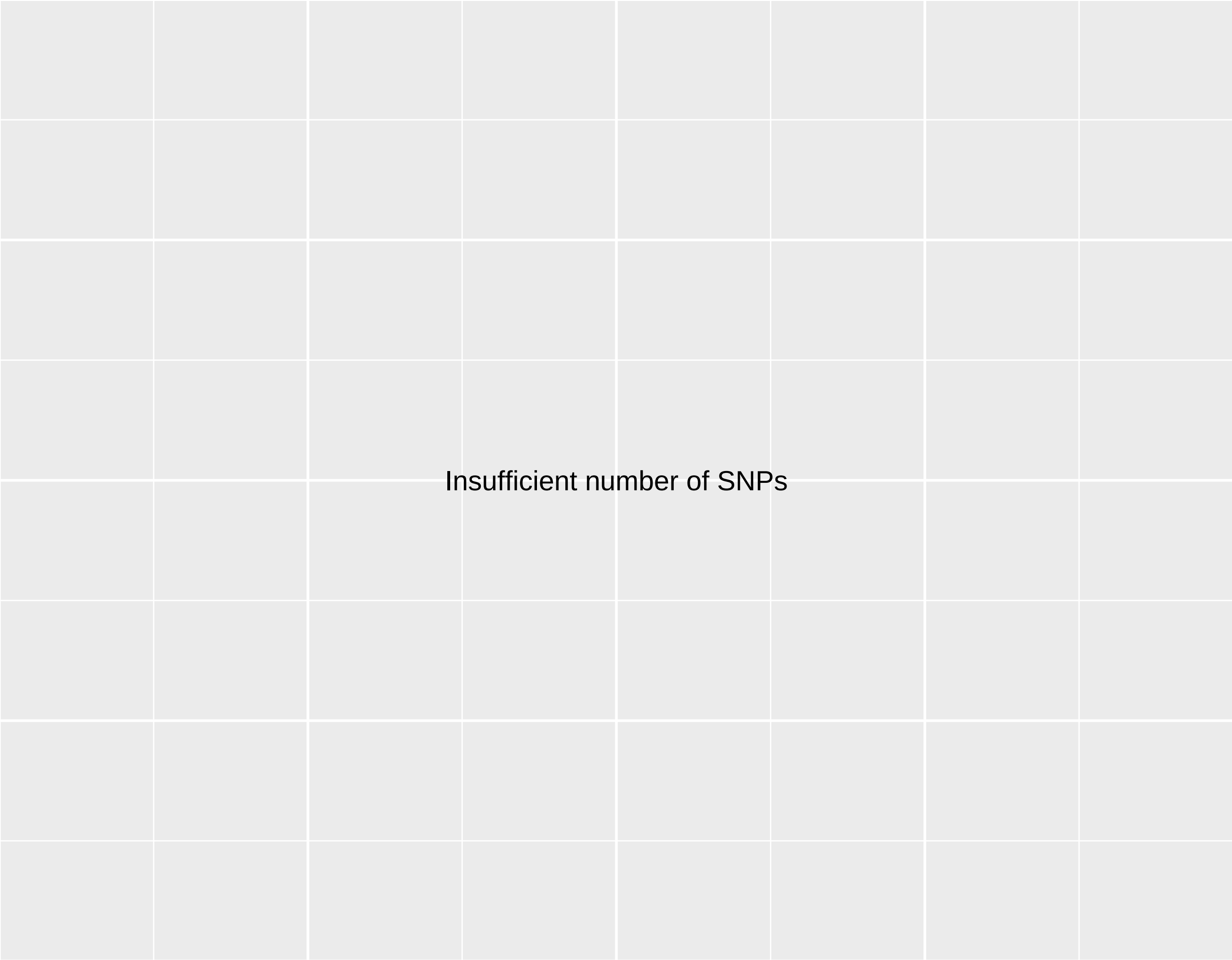
Insufficient number of SNPs

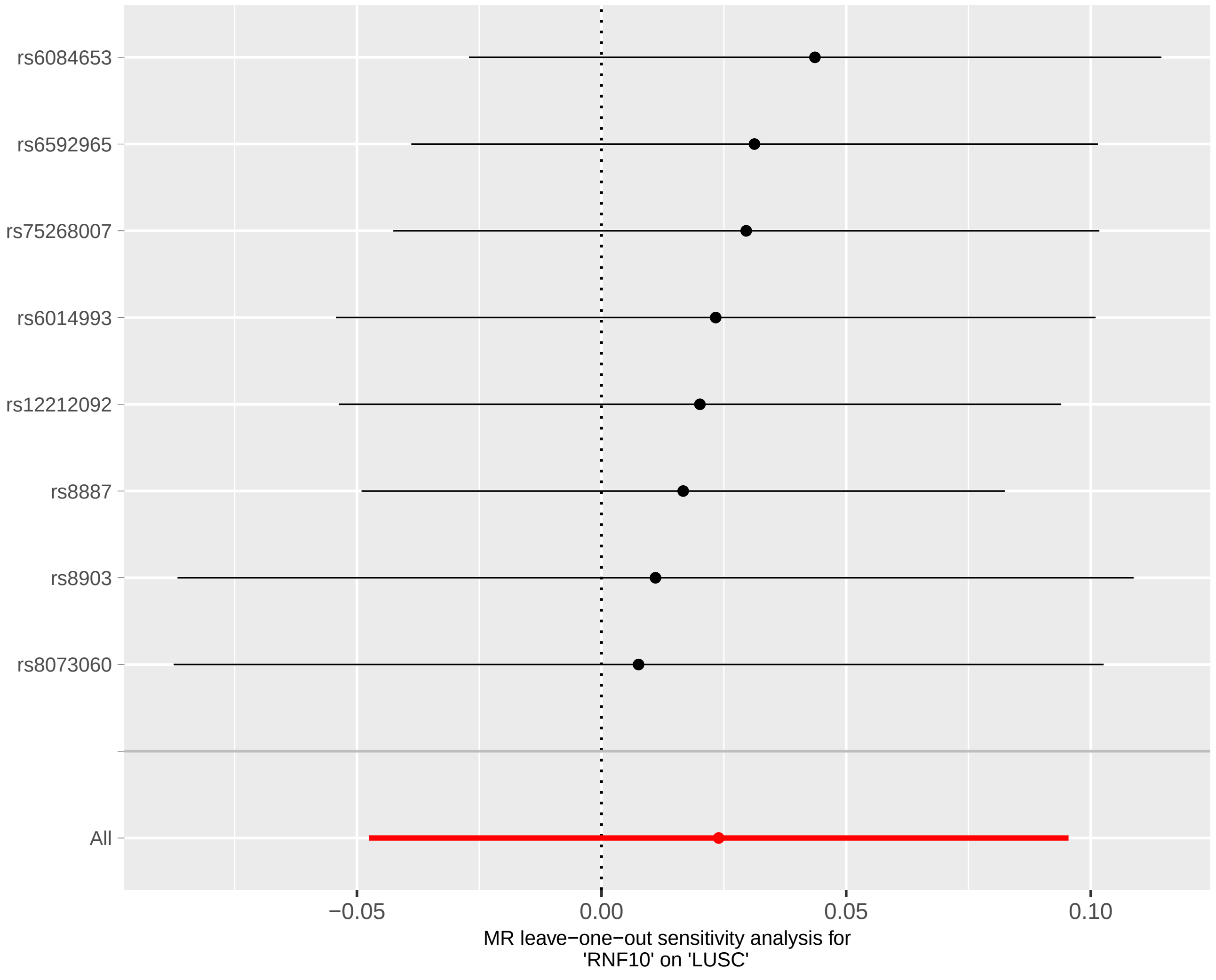
rs6084653
rs6592965
rs75268007
rs6014993
rs12212092
rs8887
rs8903
rs8073060
All
−0.05
0.00
0.05
0.10
MR leave−one−out sensitivity analysis for
'RNF10' on 'LUSC'

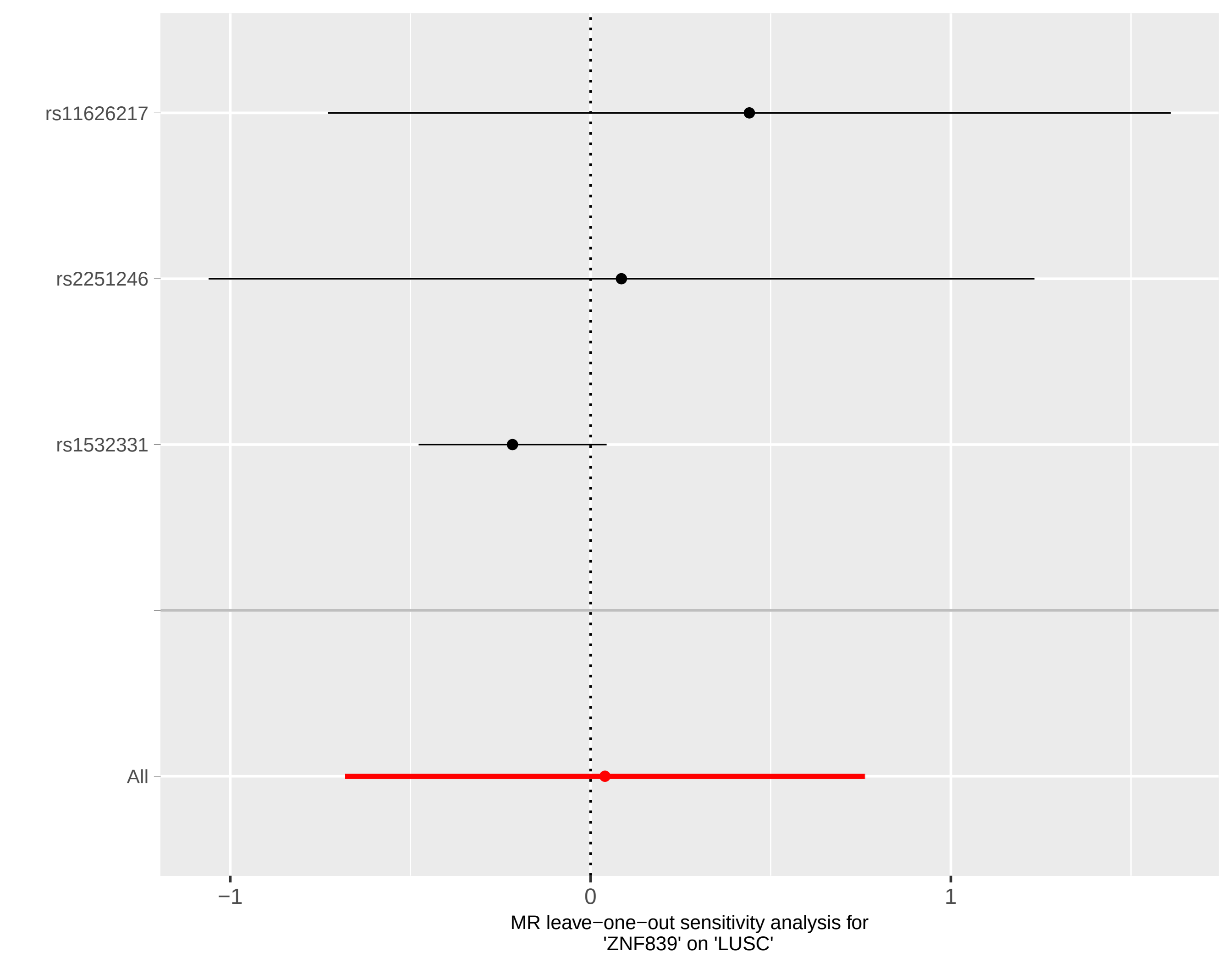
rs11626217
rs2251246
rs1532331
All
−1
0
1
MR leave−one−out sensitivity analysis for
'ZNF839' on 'LUSC'

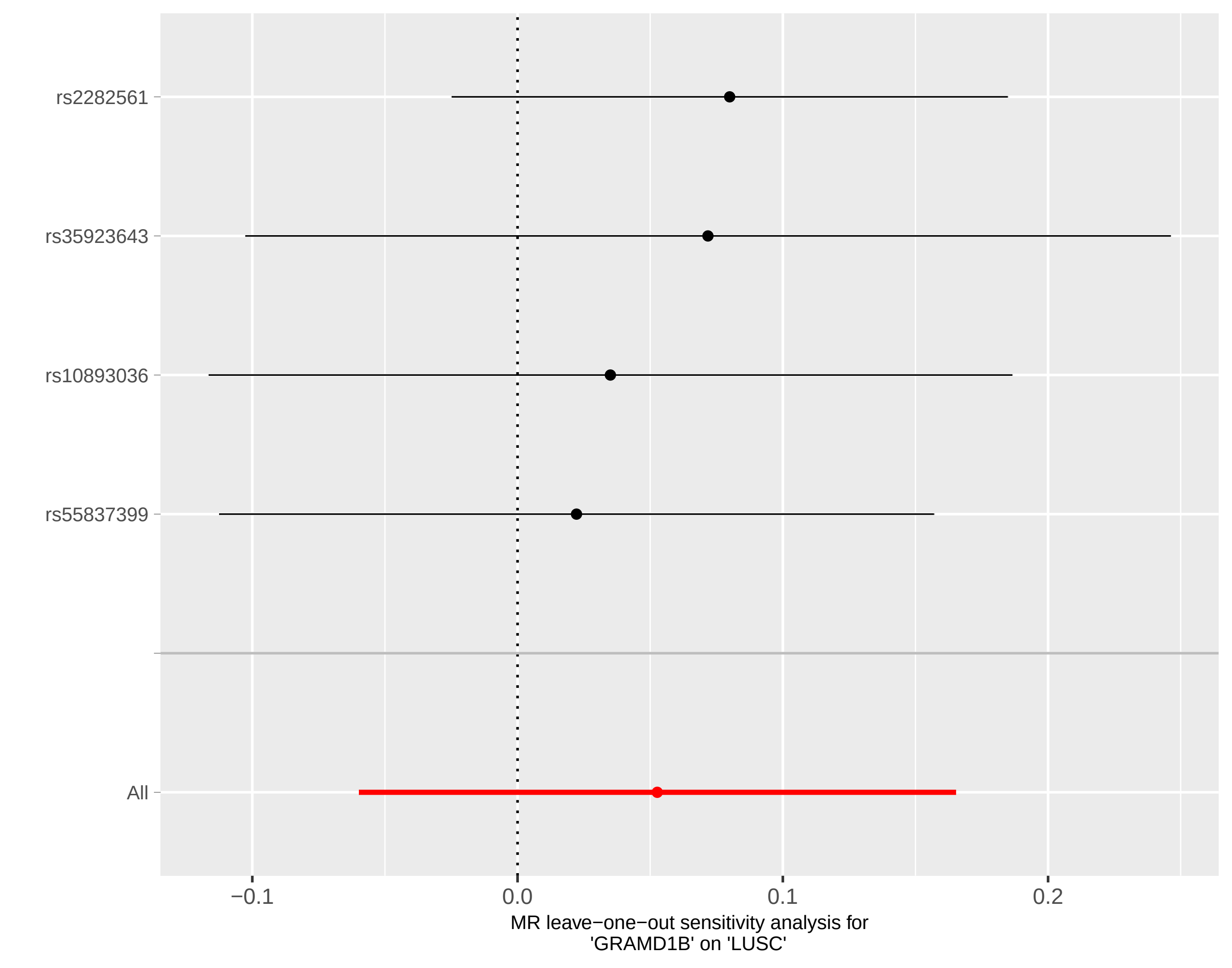
rs2282561
rs35923643
rs10893036
rs55837399
All
−0.1
0.0
0.1
0.2
MR leave−one−out sensitivity analysis for
'GRAMD1B' on 'LUSC'

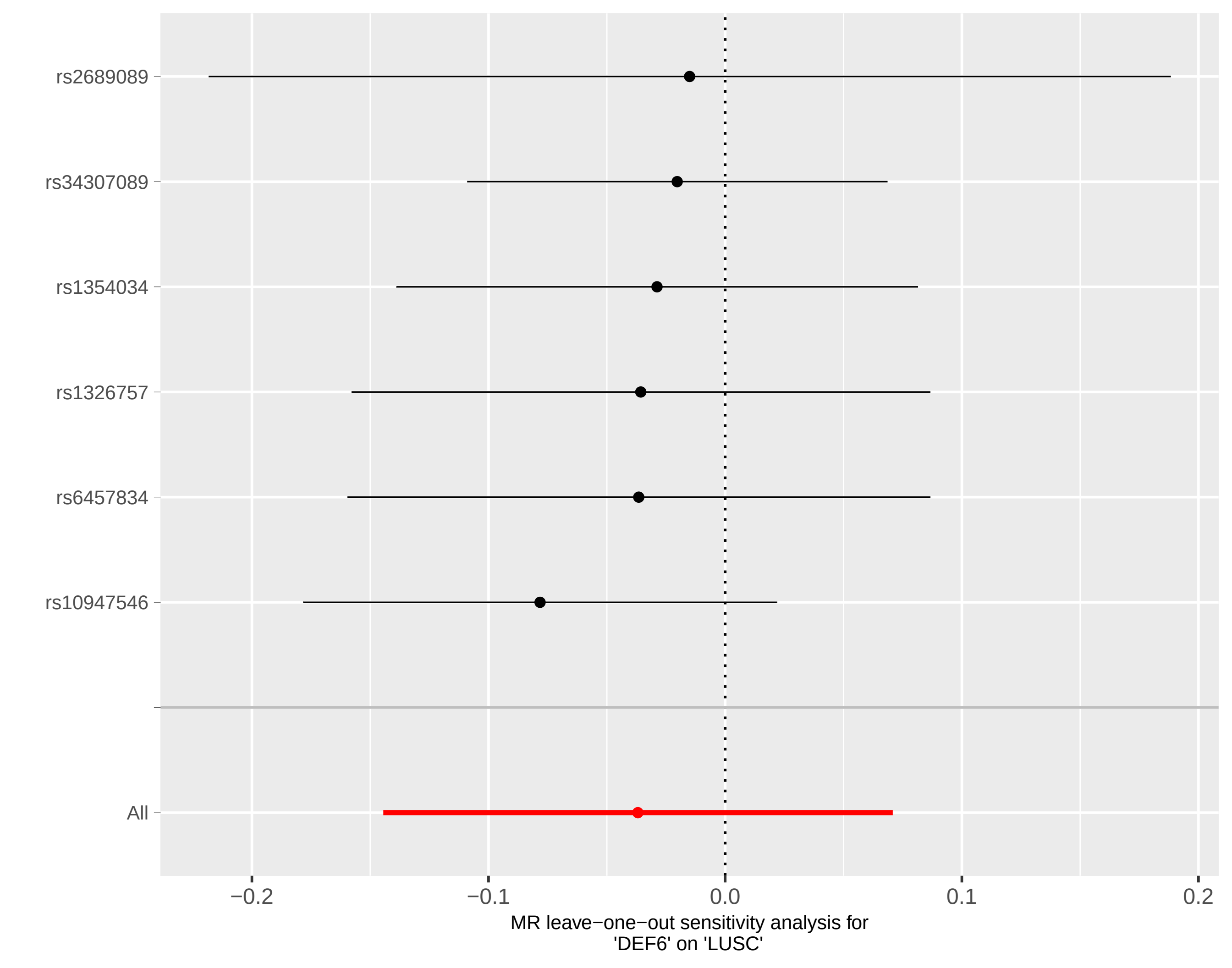
rs2689089
rs34307089
rs1354034
rs1326757
rs6457834
rs10947546
All
−0.2
−0.1
0.0
0.1
0.2
MR leave−one−out sensitivity analysis for
'DEF6' on 'LUSC'

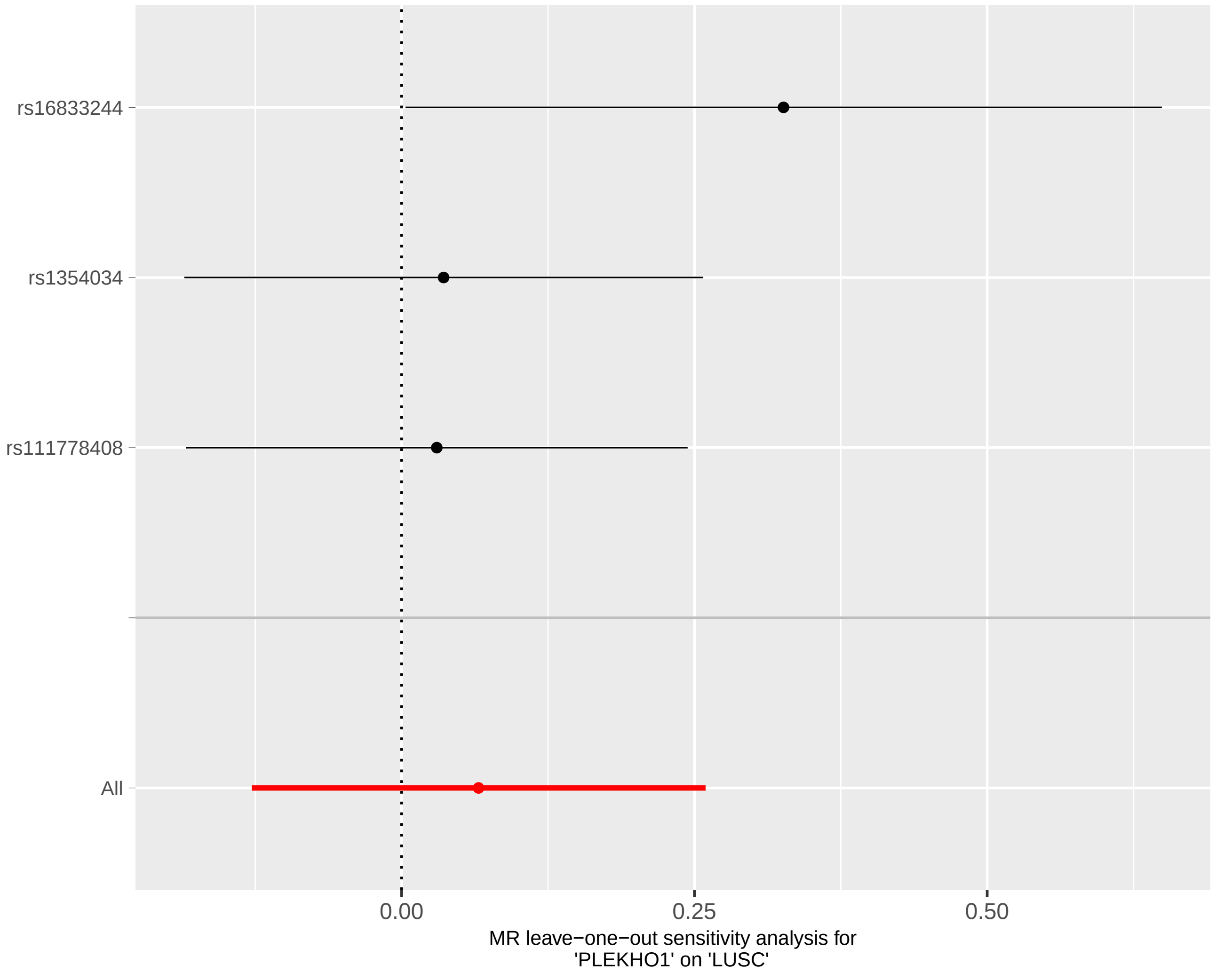

rs16833244
rs1354034
rs111778408
All
0.00
0.25
0.50
MR leave−one−out sensitivity analysis for
'PLEKHO1' on 'LUSC'

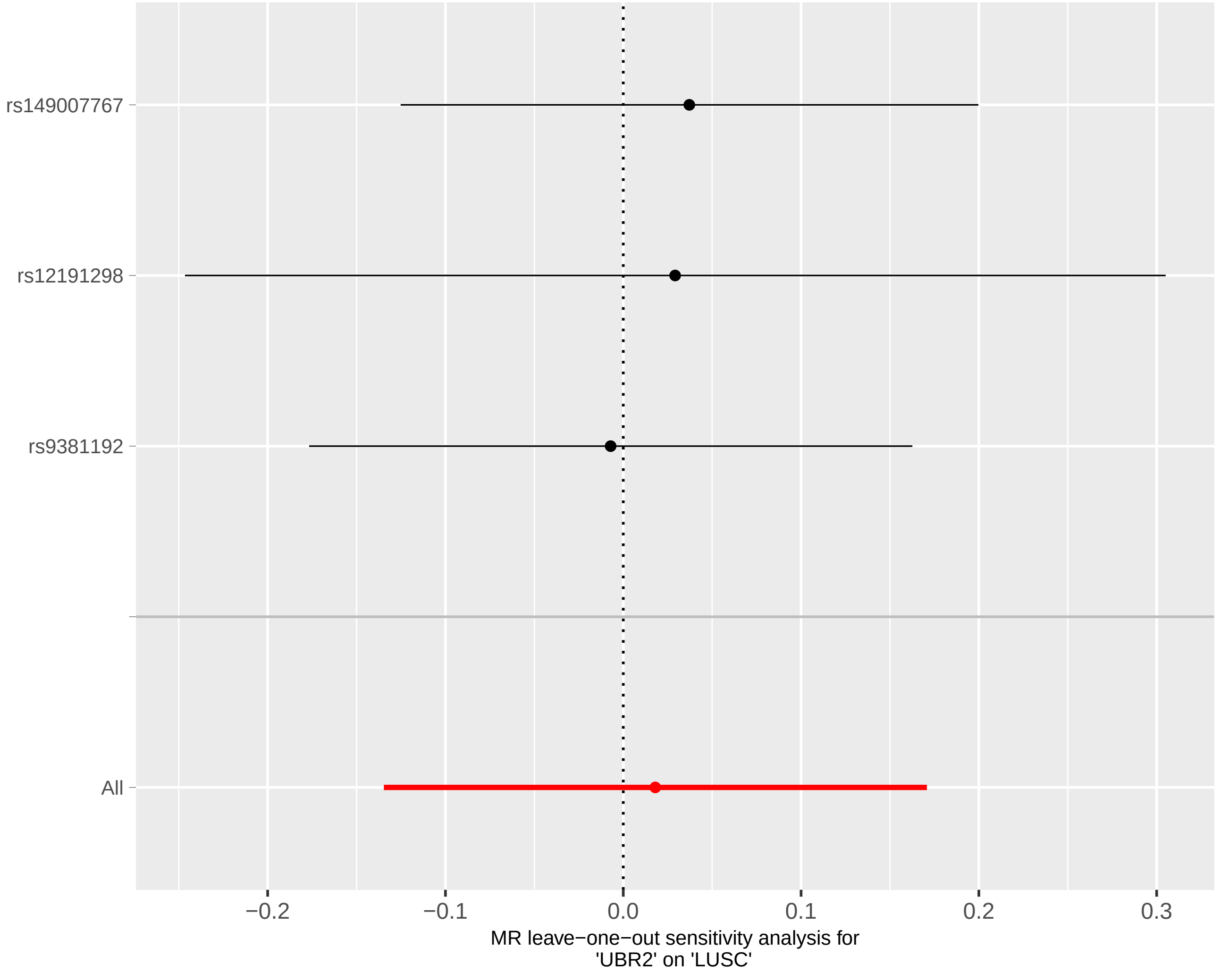
rs149007767
rs12191298
rs9381192
All
−0.2
−0.1
0.0
0.1
0.2
0.3
MR leave−one−out sensitivity analysis for
'UBR2' on 'LUSC'

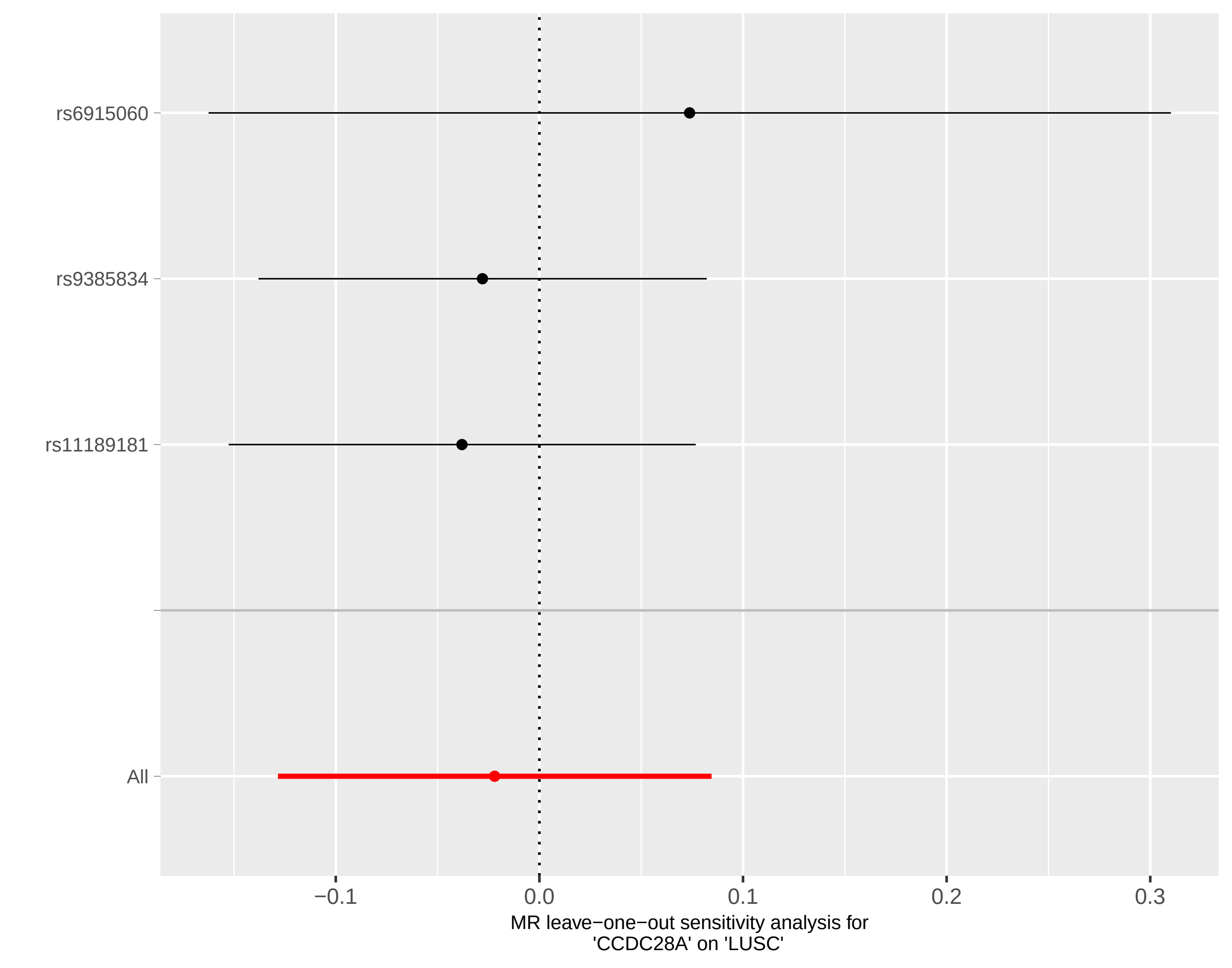

rs6915060
rs9385834
rs11189181
All
−0.1
0.0
0.1
0.2
0.3
MR leave−one−out sensitivity analysis for
'CCDC28A' on 'LUSC'

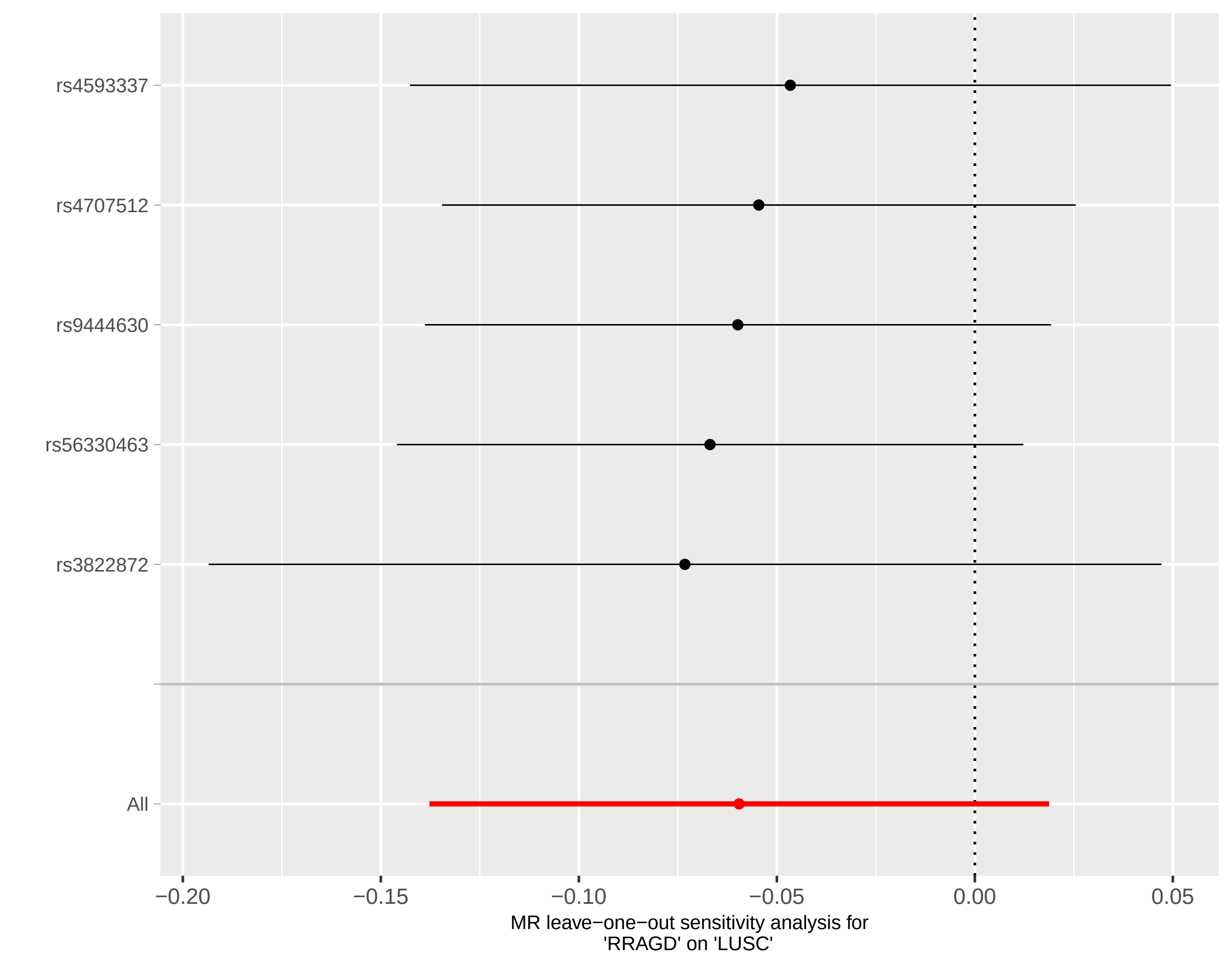
rs4593337
rs4707512
rs9444630
rs56330463
rs3822872
All
−0.20
−0.15
−0.10
−0.05
0.00
0.05
MR leave−one−out sensitivity analysis for
'RRAGD' on 'LUSC'

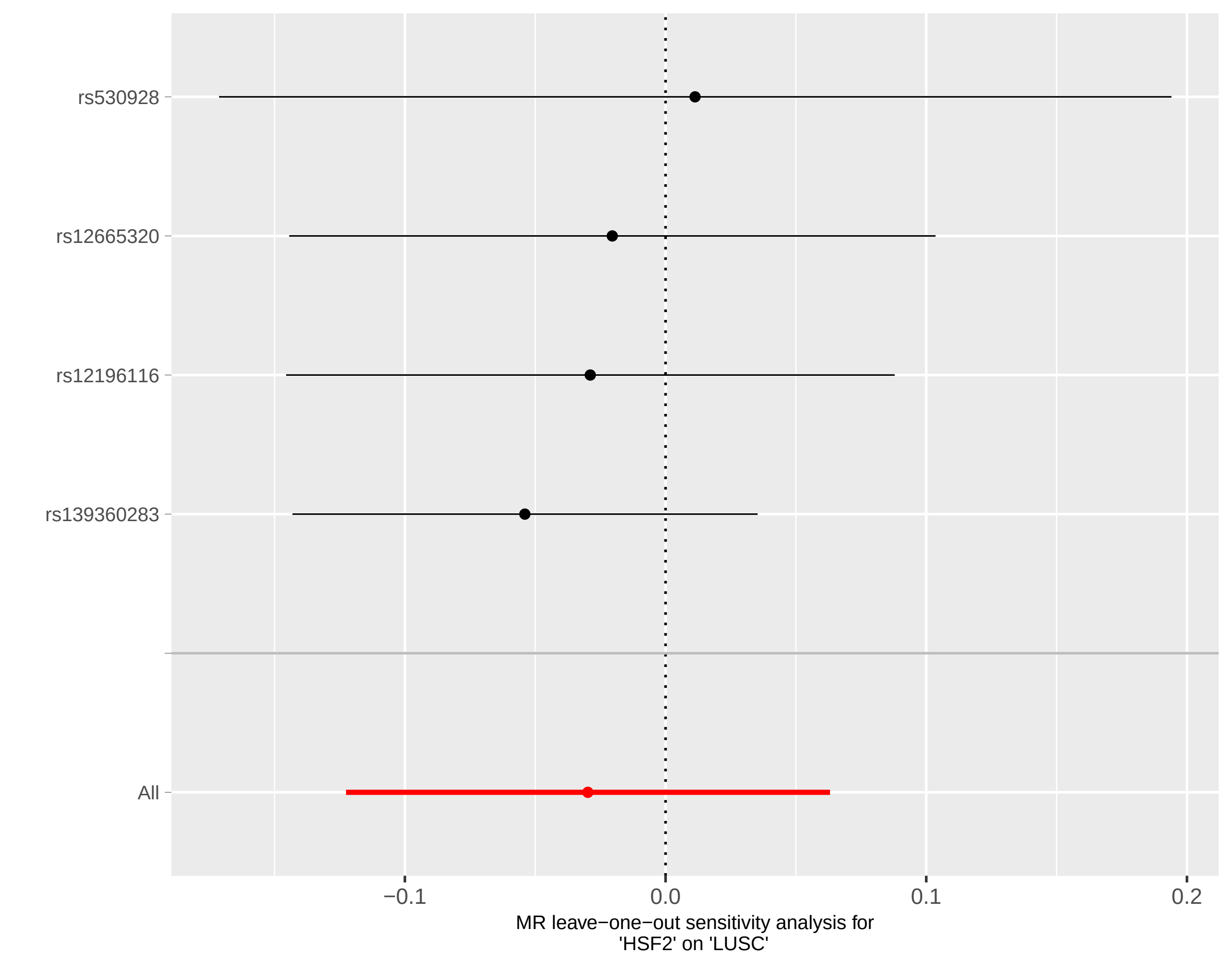

rs530928
rs12665320
rs12196116
rs139360283
All
−0.1
0.0
0.1
0.2
MR leave−one−out sensitivity analysis for
'HSF2' on 'LUSC'

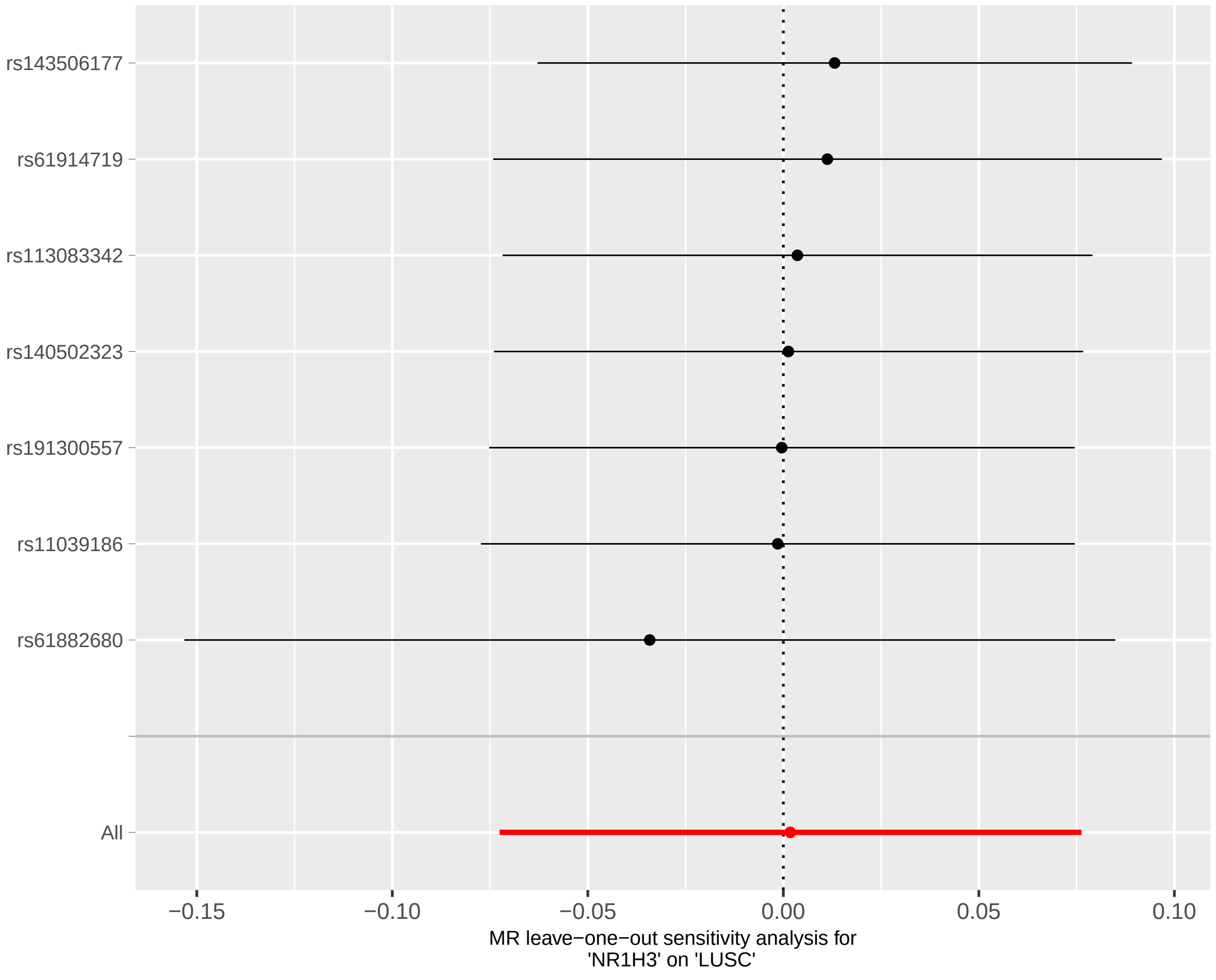
rs143506177
rs61914719
rs113083342
rs140502323
rs191300557
rs11039186
rs61882680
All
−0.15
−0.10
−0.05
0.00
0.05
0.10
MR leave−one−out sensitivity analysis for
'NR1H3' on 'LUSC'

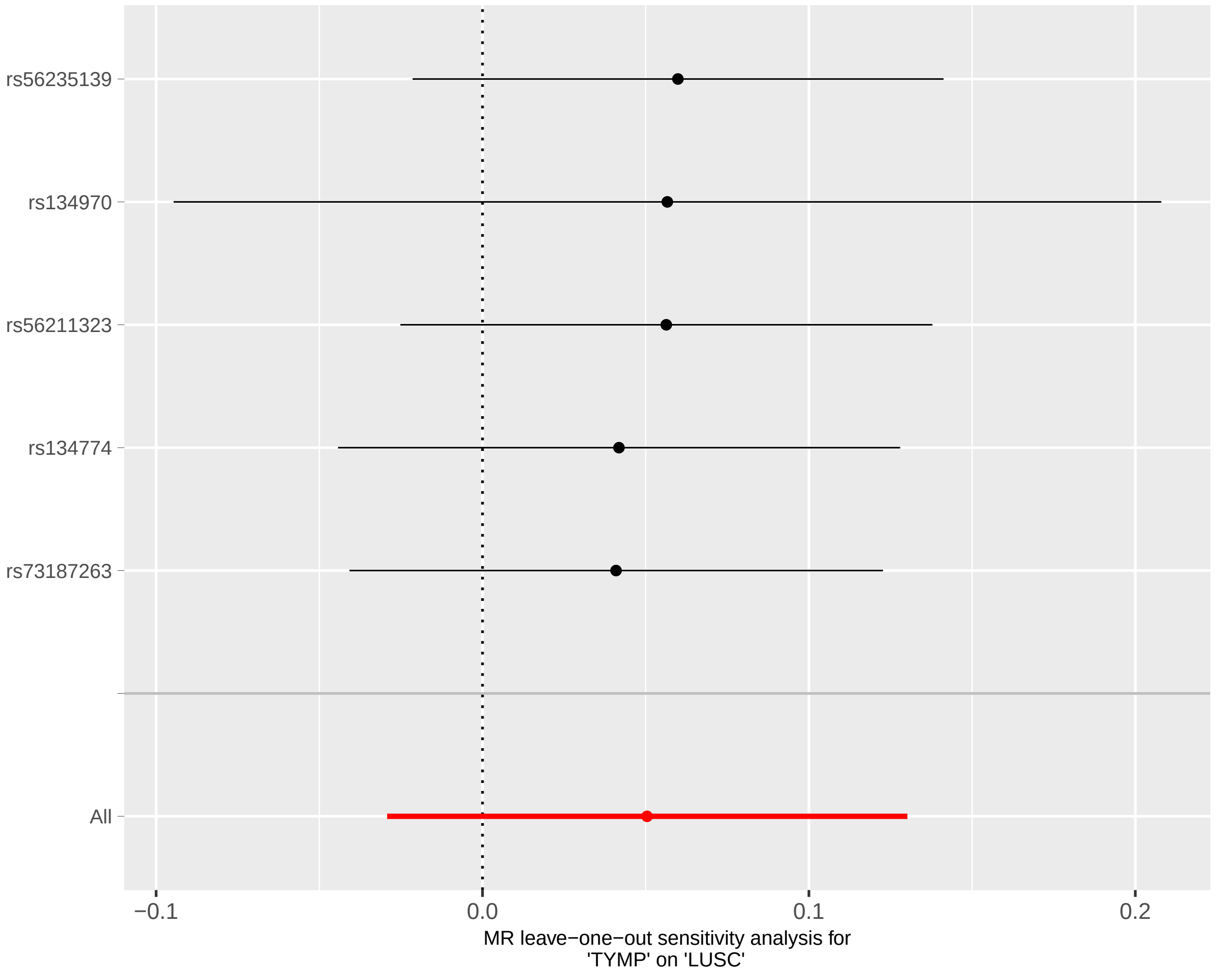
rs56235139
rs134970
rs56211323
rs134774
rs73187263
All
−0.1
0.0
0.1
0.2
MR leave−one−out sensitivity analysis for
'TYMP' on 'LUSC'

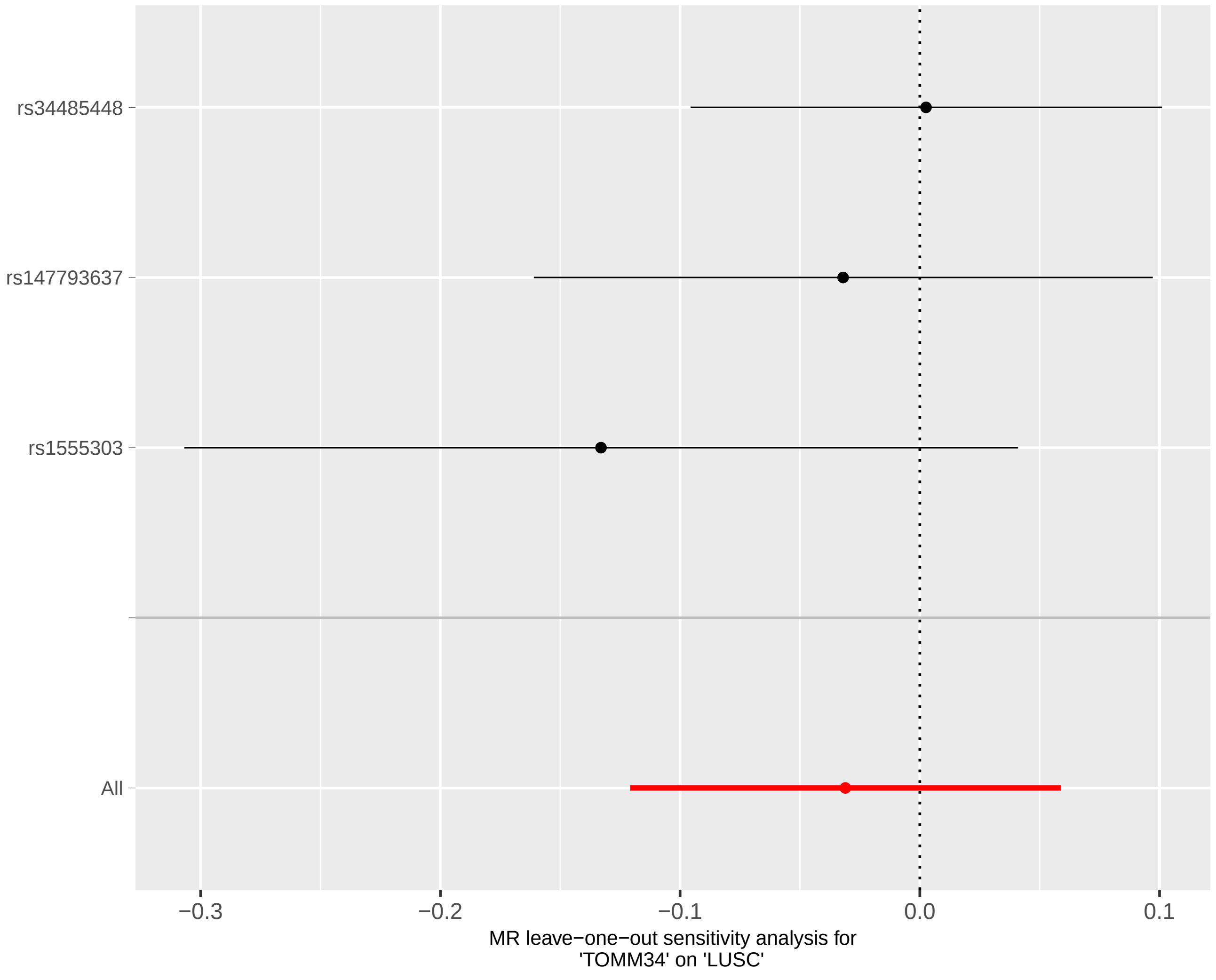

rs34485448
rs147793637
rs1555303
All
−0.3
−0.2
−0.1
0.0
0.1
MR leave−one−out sensitivity analysis for
'TOMM34' on 'LUSC'

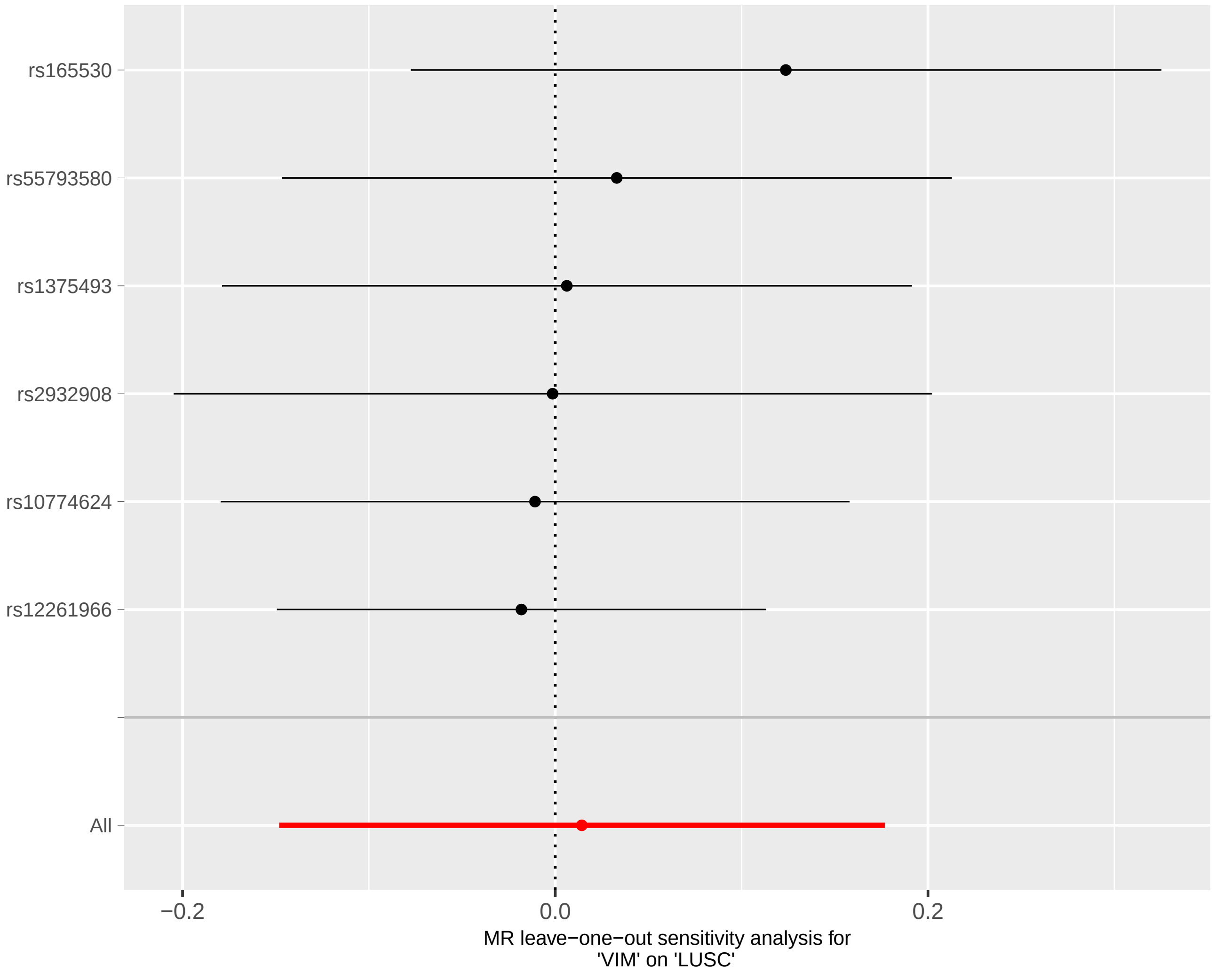
rs165530
rs55793580
rs1375493
rs2932908
rs10774624
rs12261966
All
−0.2
0.0
0.2
MR leave−one−out sensitivity analysis for
'VIM' on 'LUSC'

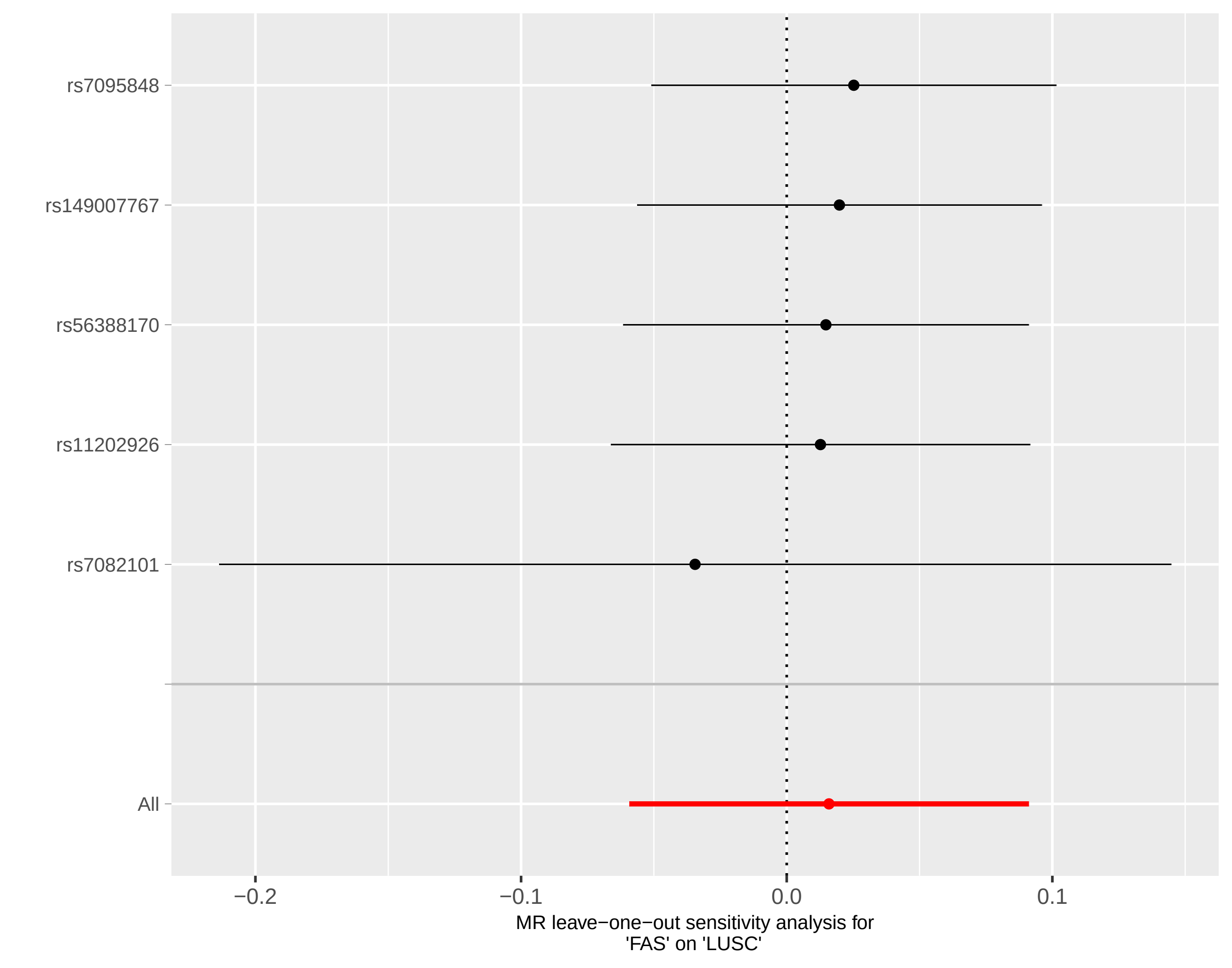

rs7095848
rs149007767
rs56388170
rs11202926
rs7082101
All
−0.2
−0.1
0.0
0.1
MR leave−one−out sensitivity analysis for
'FAS' on 'LUSC'

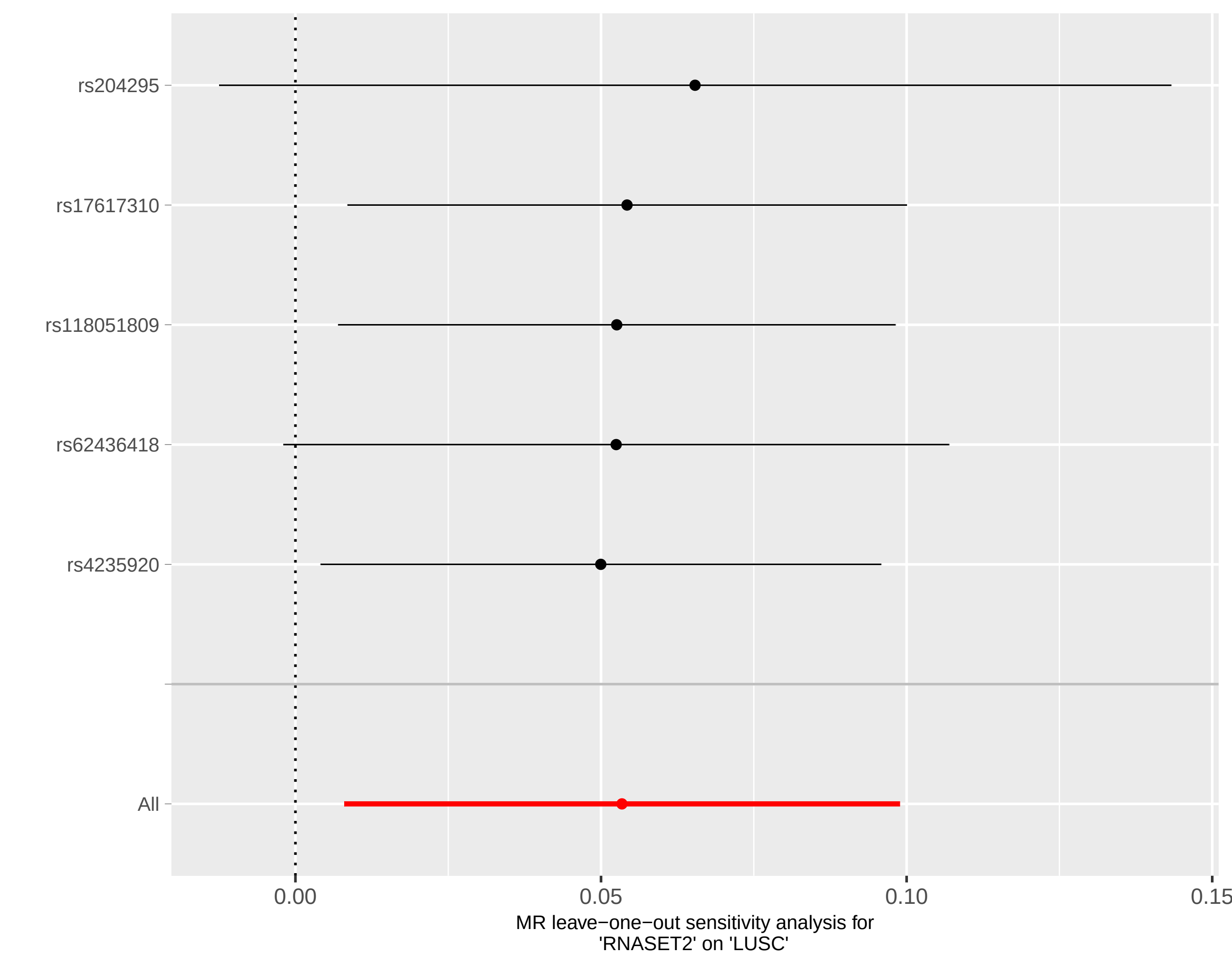

rs204295
rs17617310
rs118051809
rs62436418
rs4235920
All
0.00
0.05
0.10
0.15
MR leave−one−out sensitivity analysis for
'RNASET2' on 'LUSC'

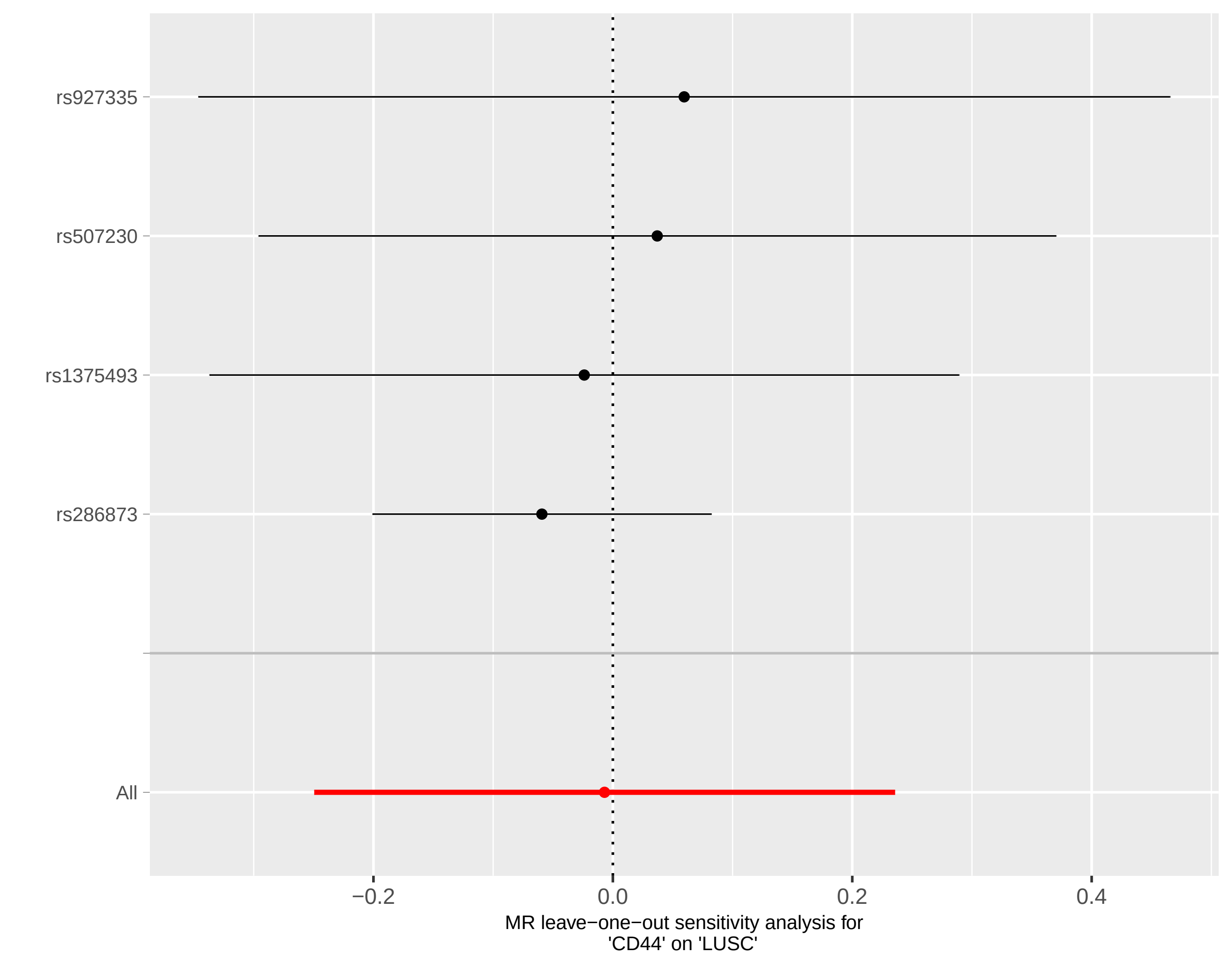

rs927335
rs507230
rs1375493
rs286873
All
−0.2
0.0
0.2
0.4
MR leave−one−out sensitivity analysis for
'CD44' on 'LUSC'

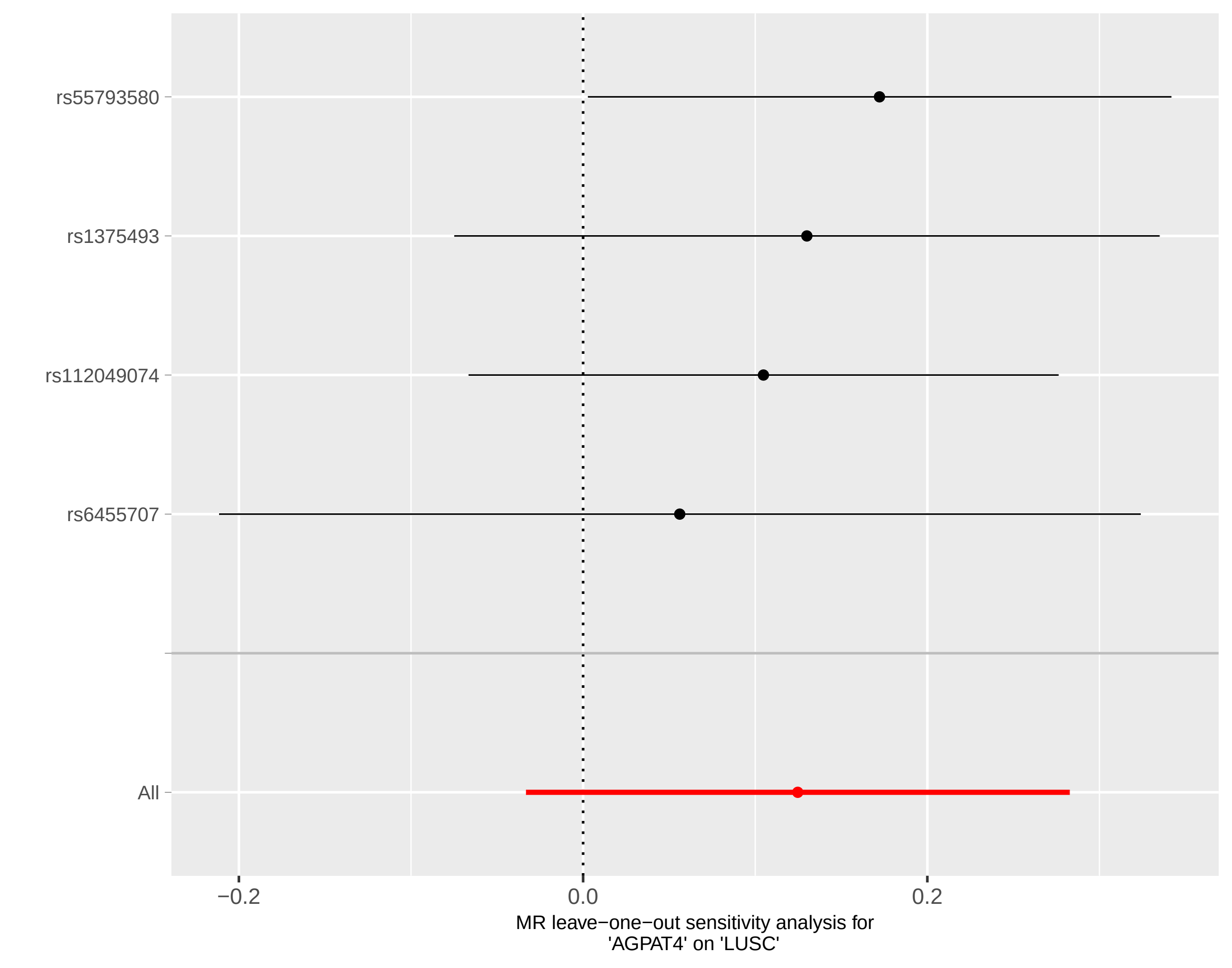

rs55793580
rs1375493
rs112049074
rs6455707
All
−0.2
0.0
0.2
MR leave−one−out sensitivity analysis for
'AGPAT4' on 'LUSC'

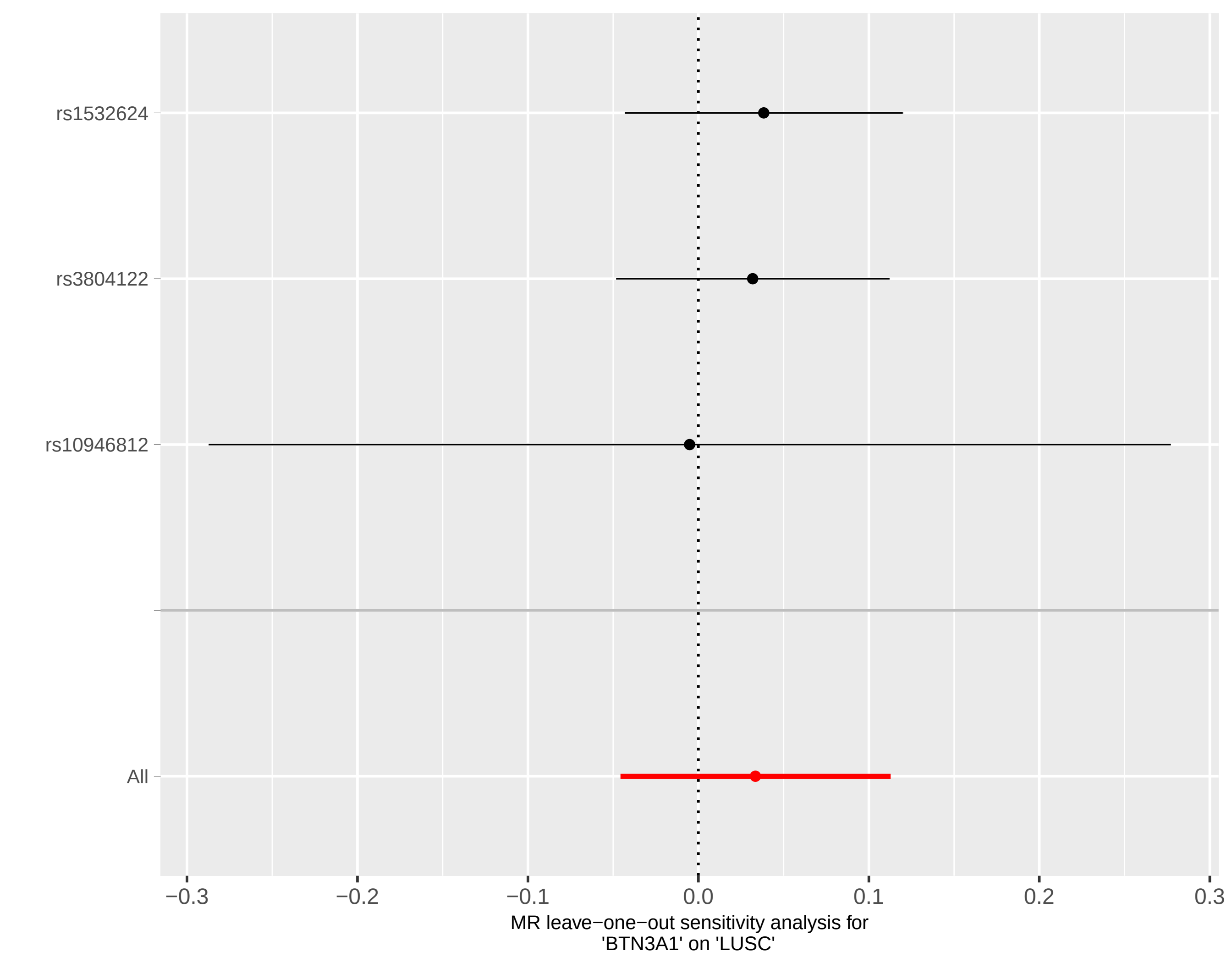
rs1532624
rs3804122
rs10946812
All
−0.3
−0.2
−0.1
0.0
0.1
0.2
0.3
MR leave−one−out sensitivity analysis for
'BTN3A1' on 'LUSC'

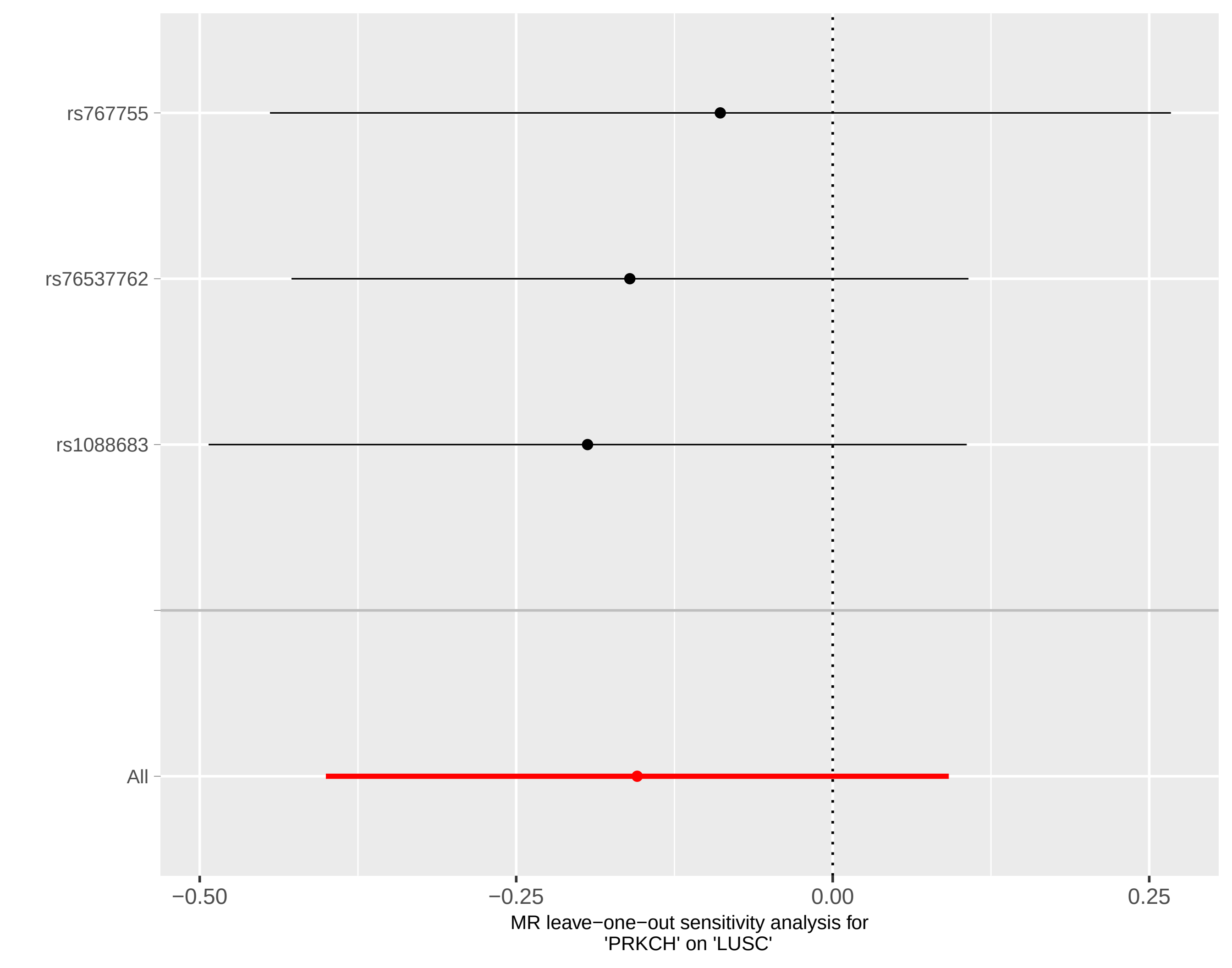
rs767755
rs76537762
rs1088683
All
−0.50
−0.25
0.00
0.25
MR leave−one−out sensitivity analysis for
'PRKCH' on 'LUSC'

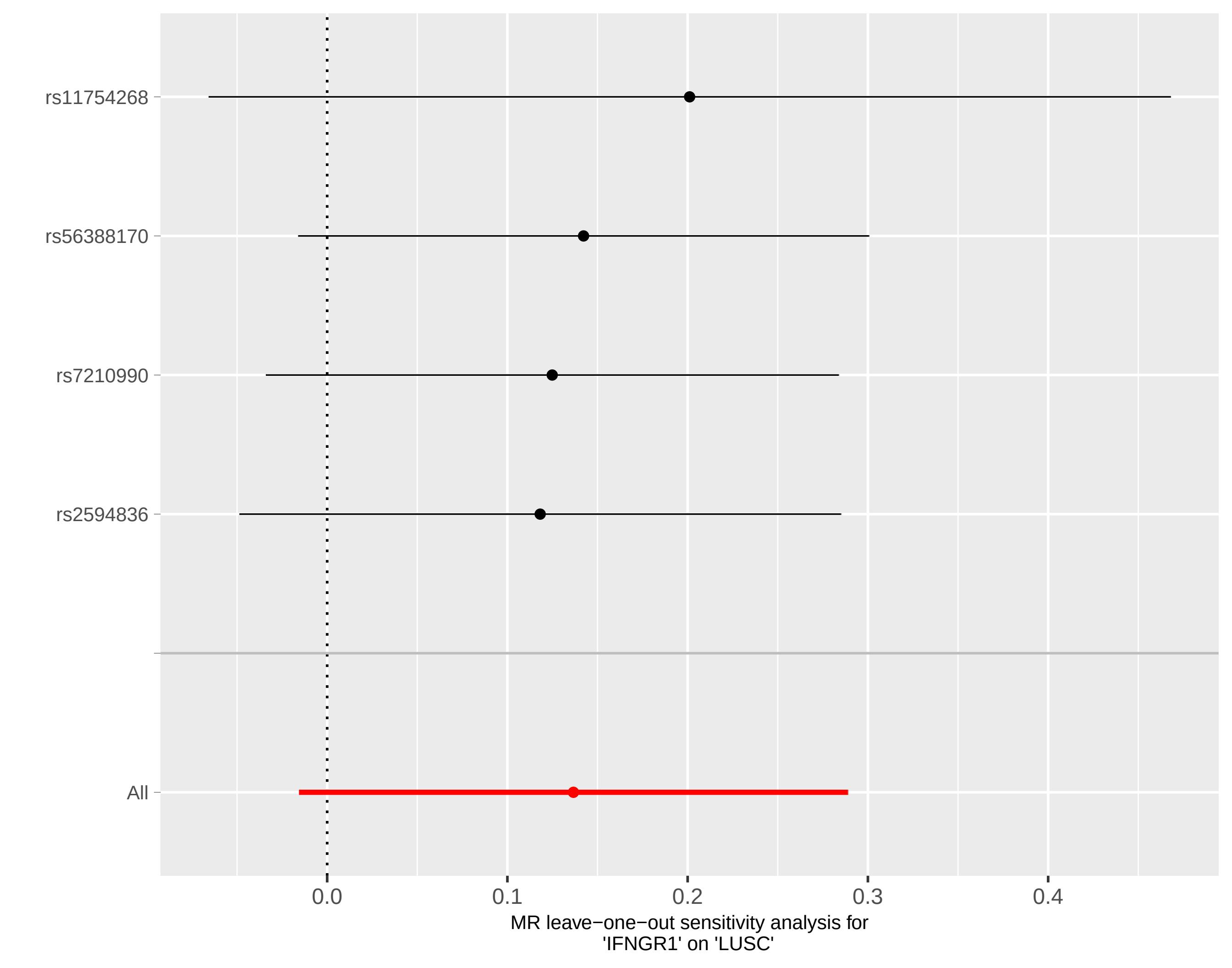
rs11754268
rs56388170
rs7210990
rs2594836
All
0.0
0.1
0.2
0.3
0.4
MR leave−one−out sensitivity analysis for
'IFNGR1' on 'LUSC'

Insufficient number of SNPs

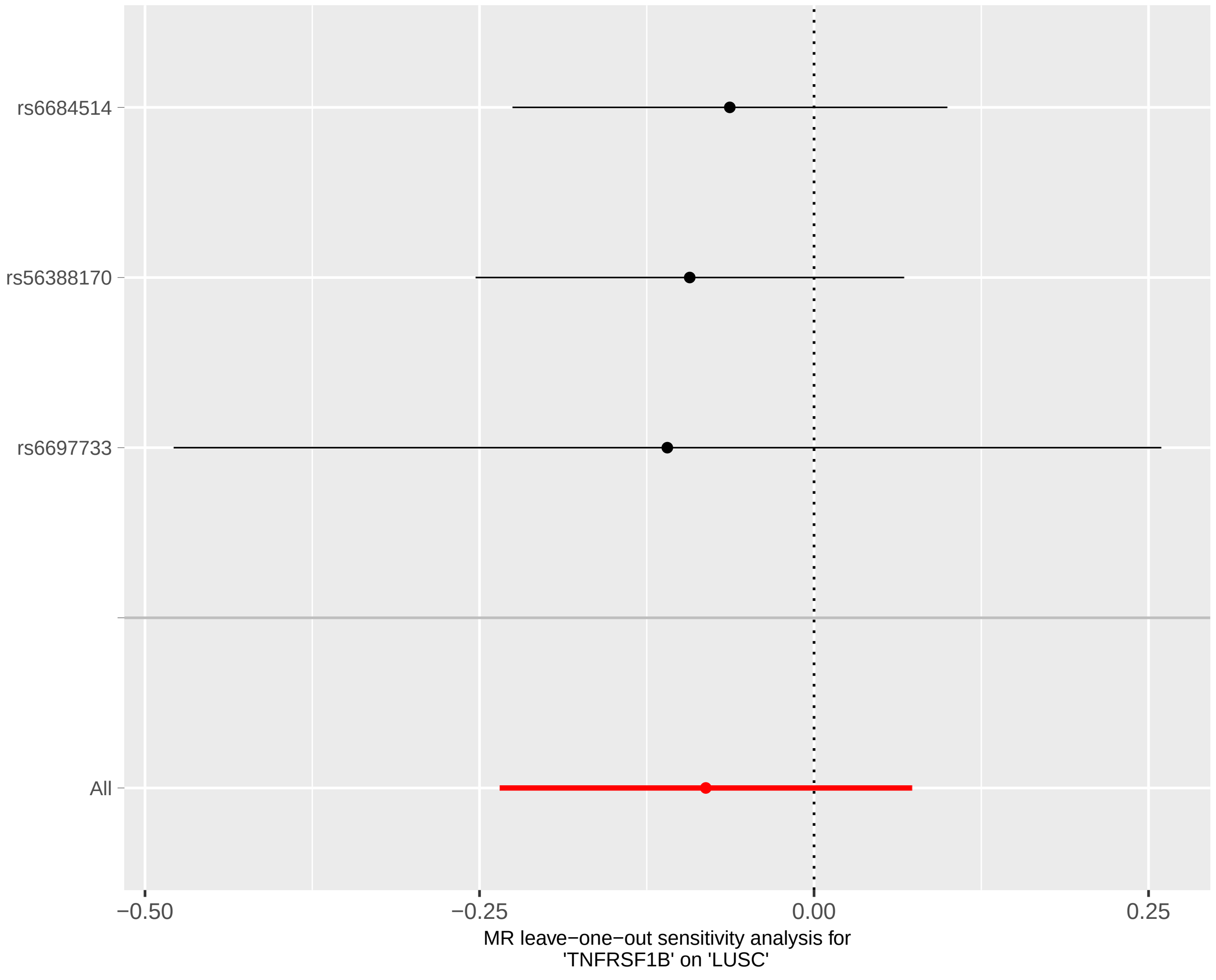
rs6684514
rs56388170
rs6697733
All
−0.50
−0.25
0.00
0.25
MR leave−one−out sensitivity analysis for
'TNFRSF1B' on 'LUSC'

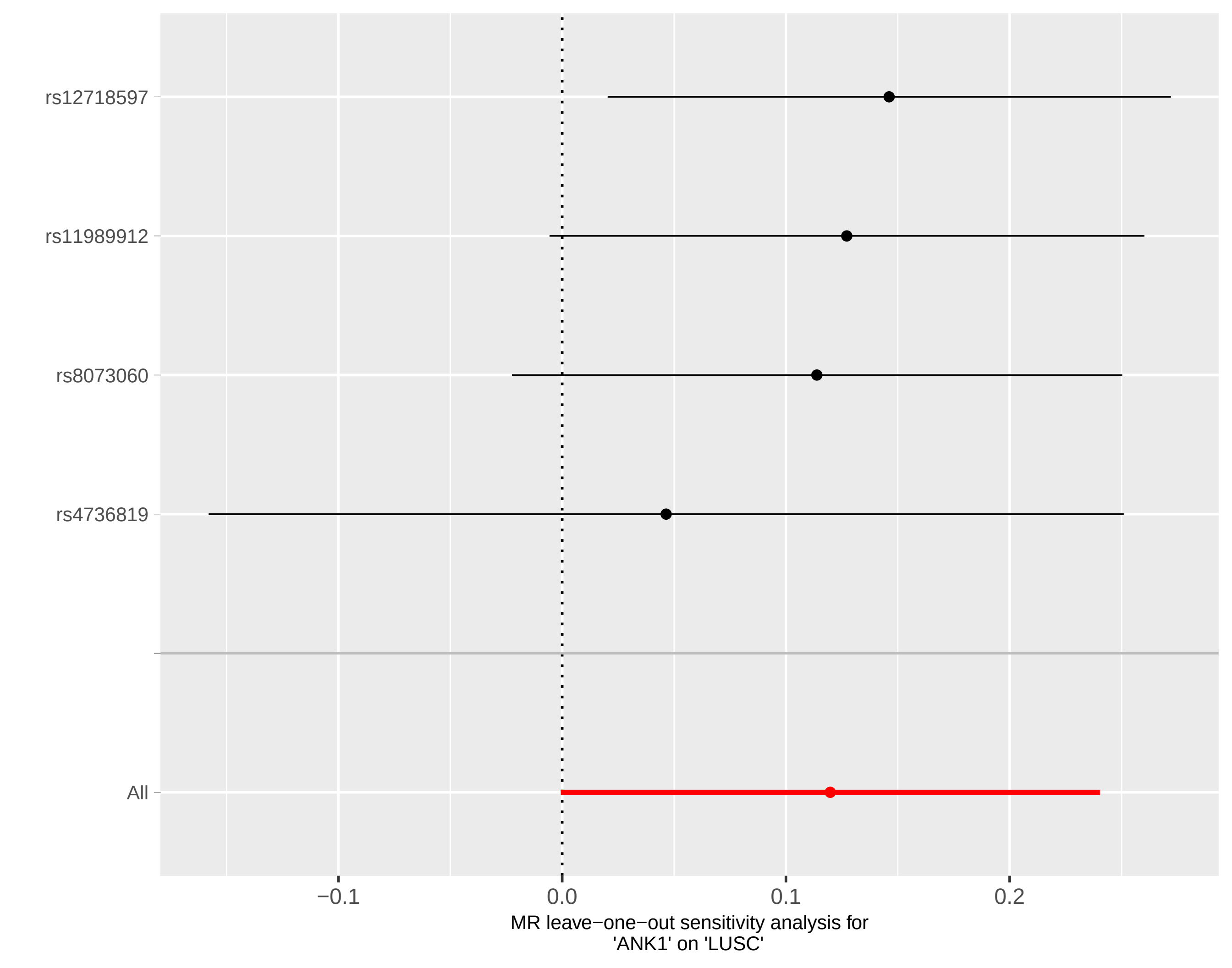

rs12718597
rs11989912
rs8073060
rs4736819
All
−0.1
0.0
0.1
0.2
MR leave−one−out sensitivity analysis for
'ANK1' on 'LUSC'

Insufficient number of SNPs

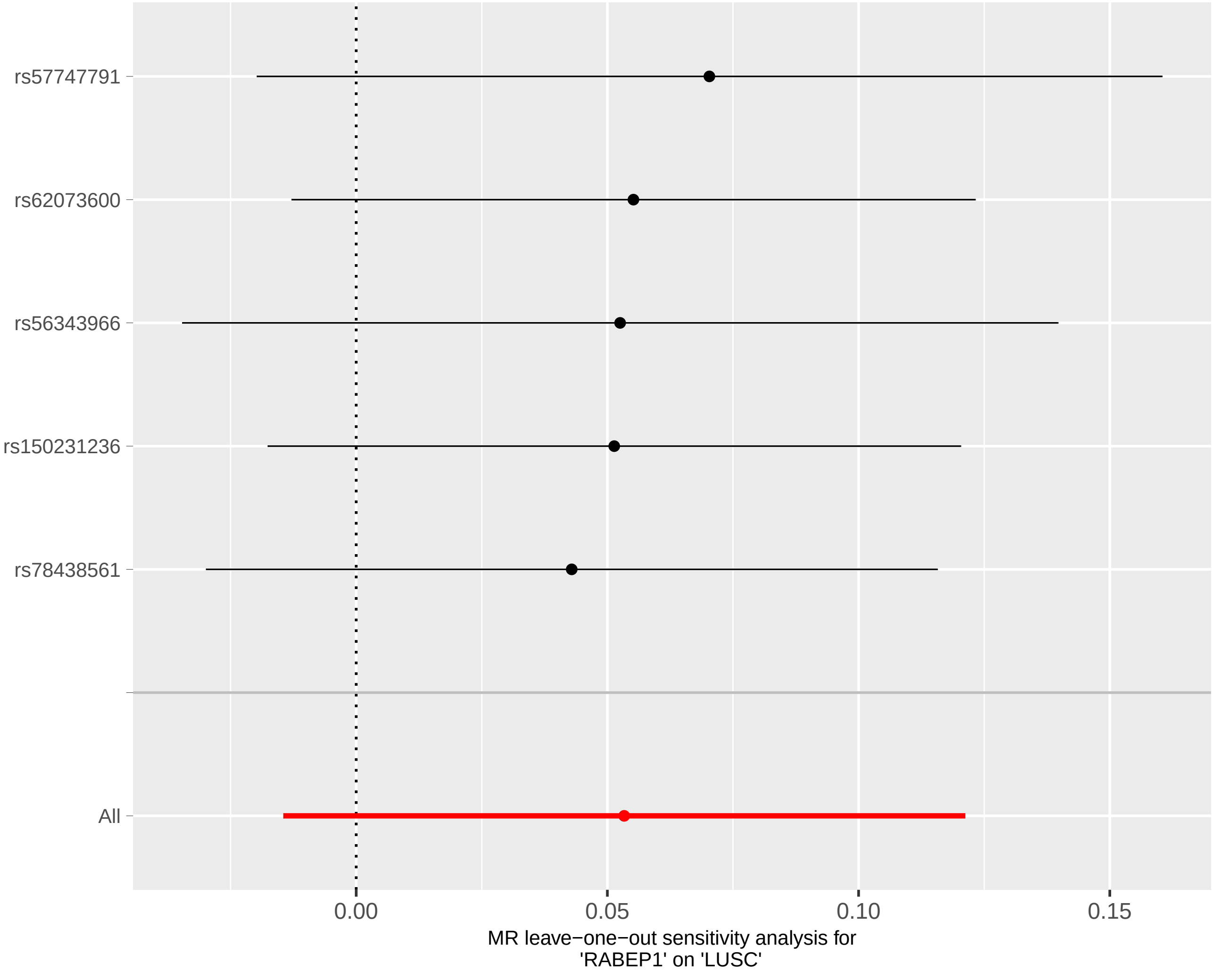

rs57747791
rs62073600
rs56343966
rs150231236
rs78438561
All
0.00
0.05
0.10
0.15
MR leave−one−out sensitivity analysis for
'RABEP1' on 'LUSC'

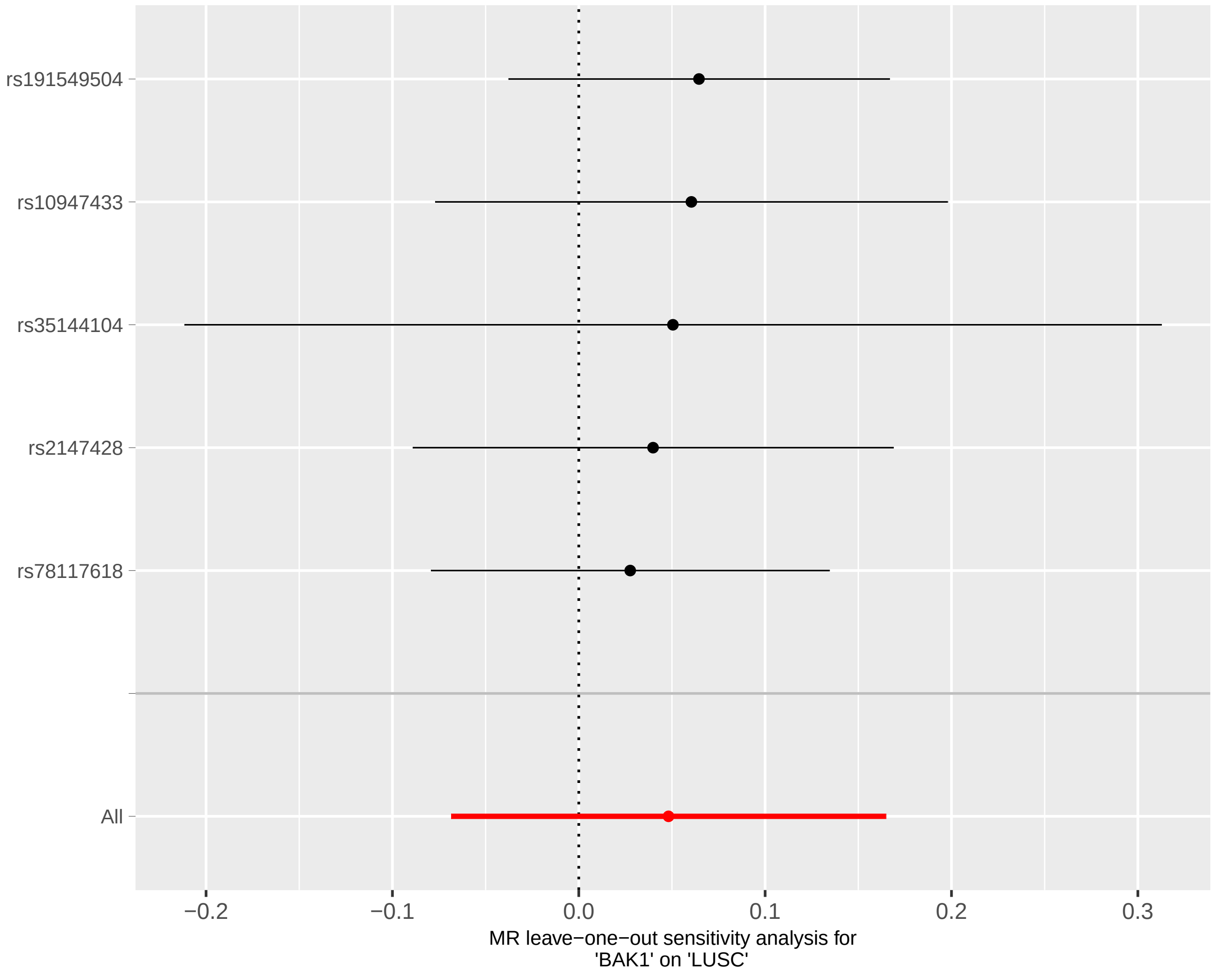
rs191549504
rs10947433
rs35144104
rs2147428
rs78117618
All
−0.2
−0.1
0.0
0.1
0.2
0.3
MR leave−one−out sensitivity analysis for
'BAK1' on 'LUSC'

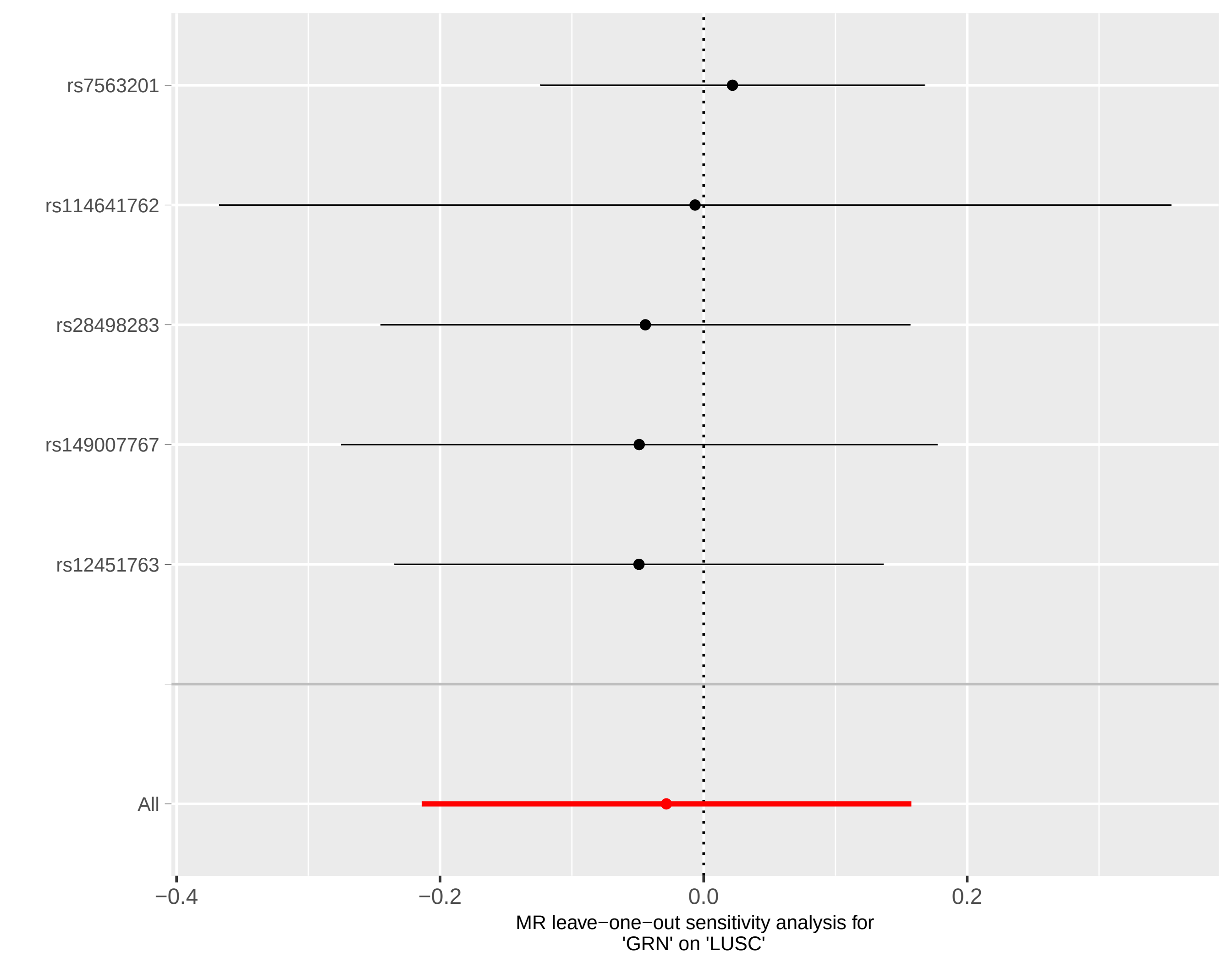
rs7563201
rs114641762
rs28498283
rs149007767
rs12451763
All
−0.4
−0.2
0.0
0.2
MR leave−one−out sensitivity analysis for
'GRN' on 'LUSC'

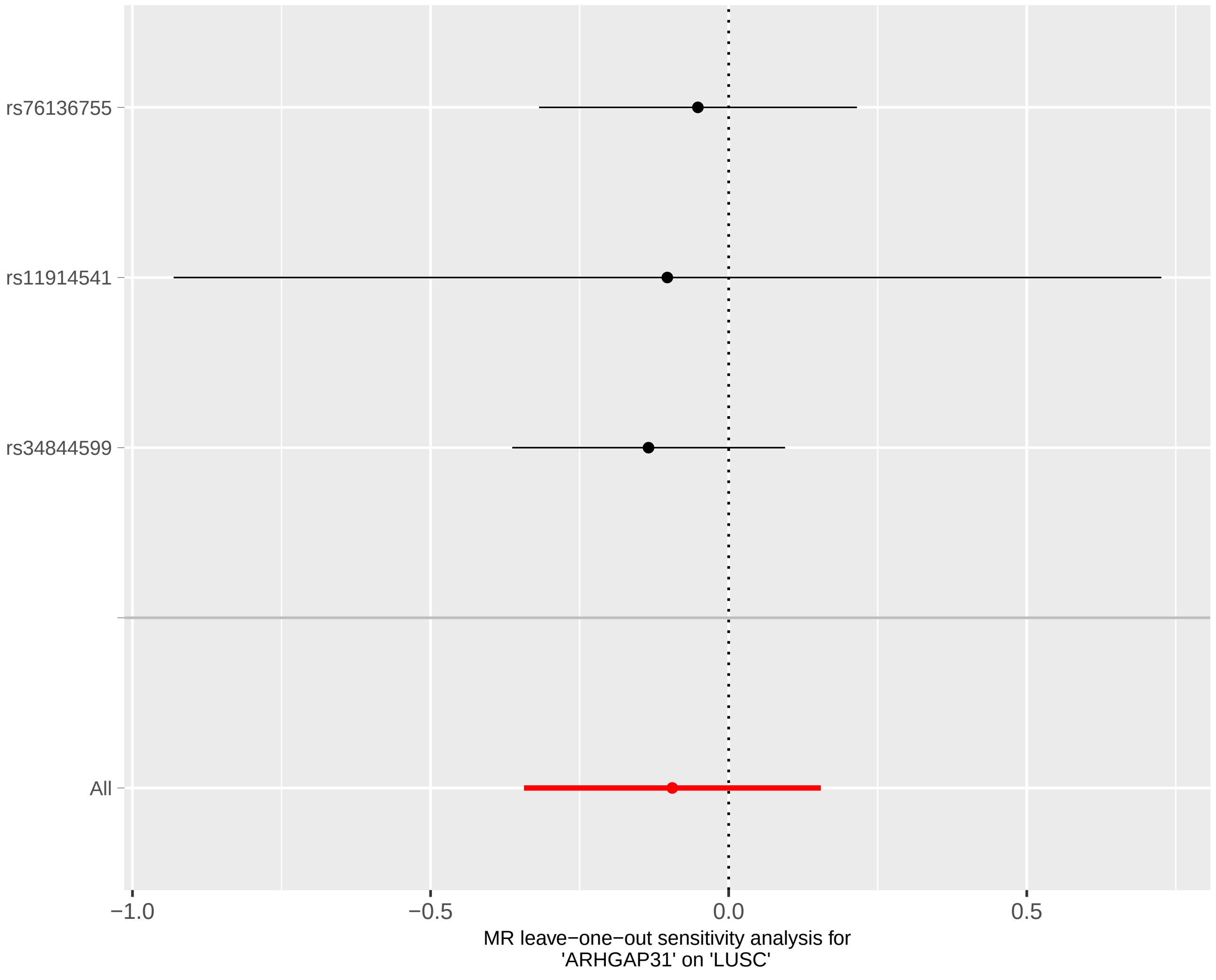
rs76136755
rs11914541
rs34844599
All
−1.0
−0.5
0.0
0.5
MR leave−one−out sensitivity analysis for
'ARHGAP31' on 'LUSC'

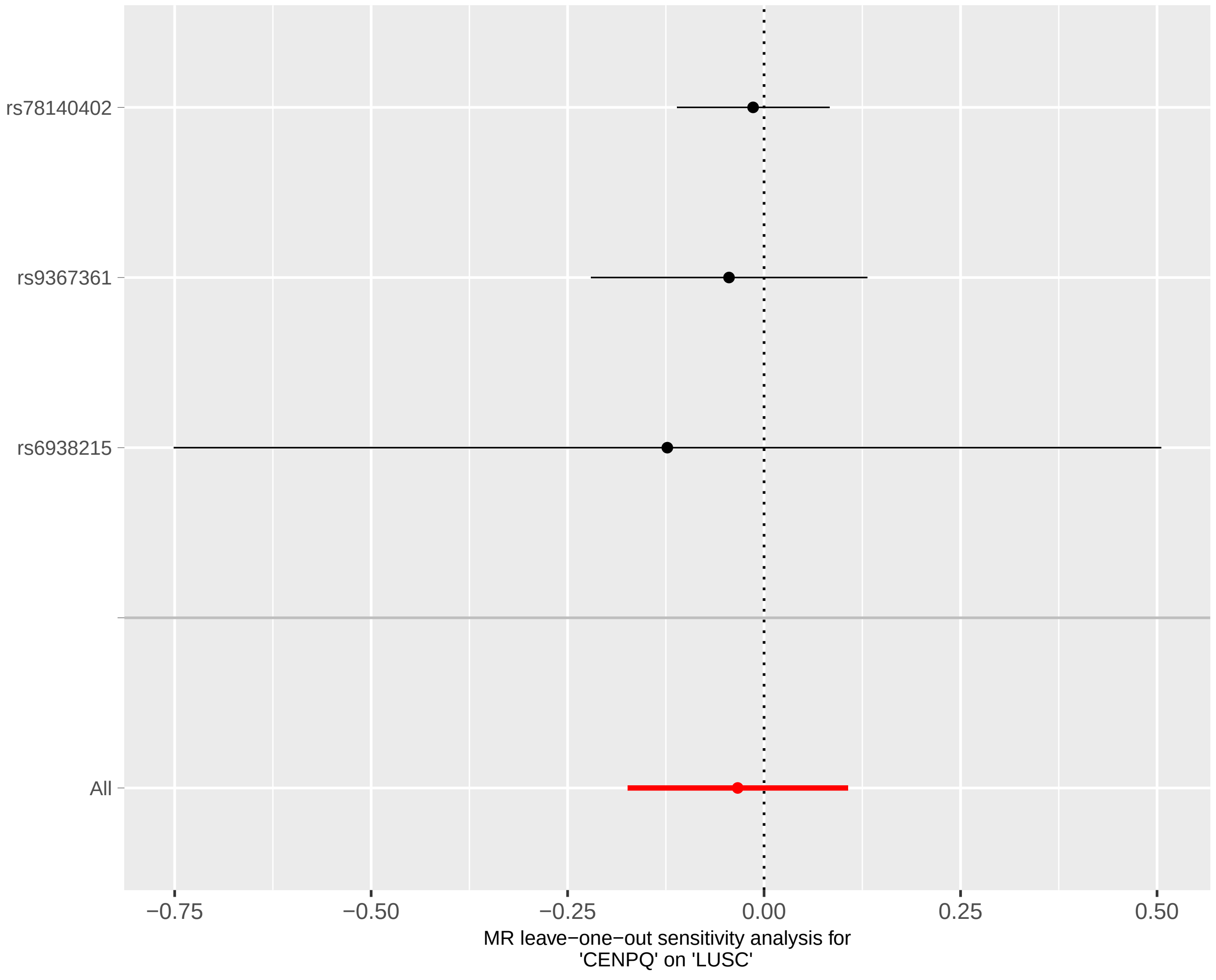
rs78140402
rs9367361
rs6938215
All
−0.75
−0.50
−0.25
0.00
0.25
0.50
MR leave−one−out sensitivity analysis for
'CENPQ' on 'LUSC'

Insufficient number of SNPs

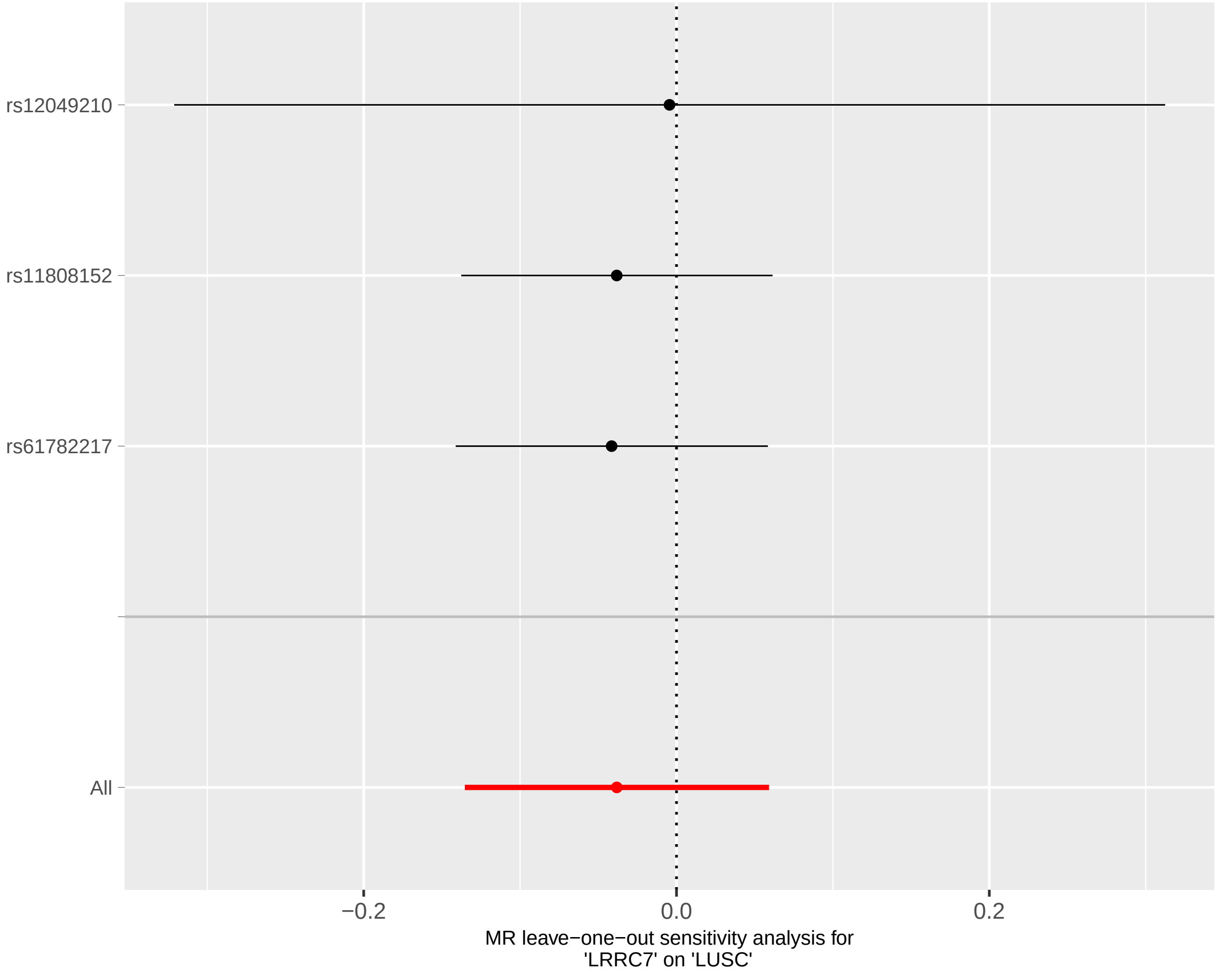
rs12049210
rs11808152
rs61782217
All
−0.2
0.0
0.2
MR leave−one−out sensitivity analysis for
'LRRC7' on 'LUSC'

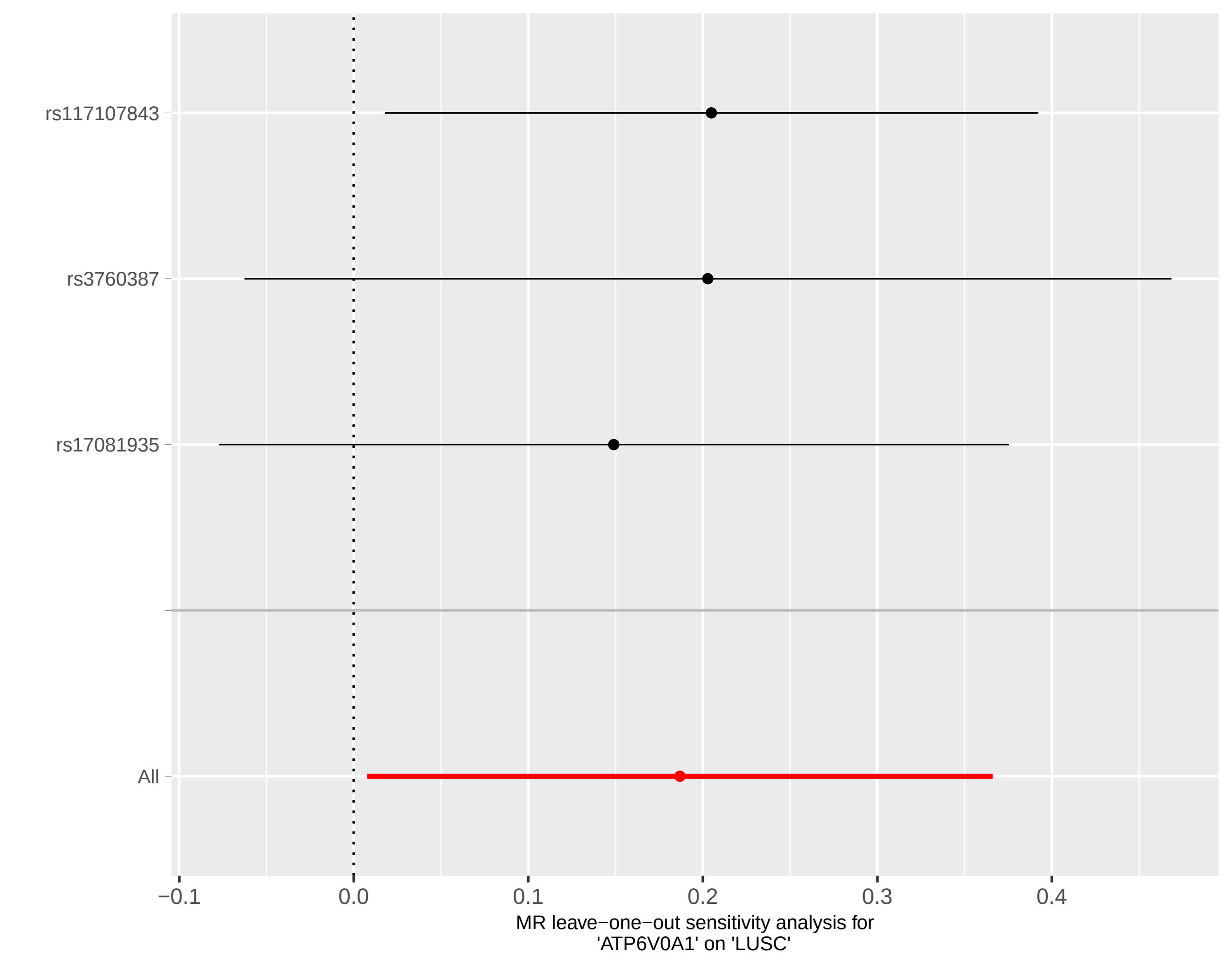
rs117107843
rs3760387
rs17081935
All
−0.1
0.0
0.1
0.2
0.3
0.4
MR leave−one−out sensitivity analysis for
'ATP6V0A1' on 'LUSC'

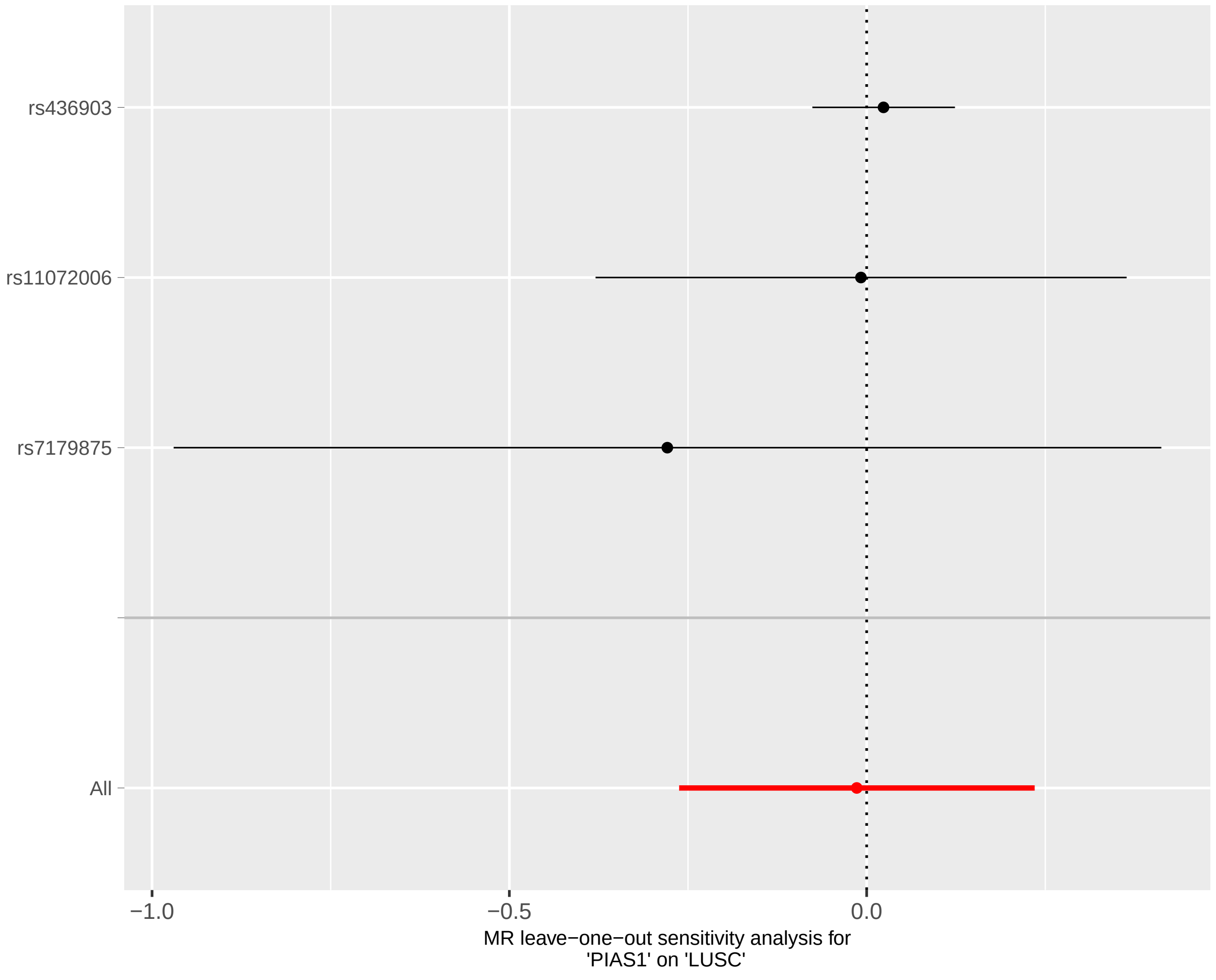

rs436903
rs11072006
rs7179875
All
−1.0
−0.5
0.0
MR leave−one−out sensitivity analysis for
'PIAS1' on 'LUSC'

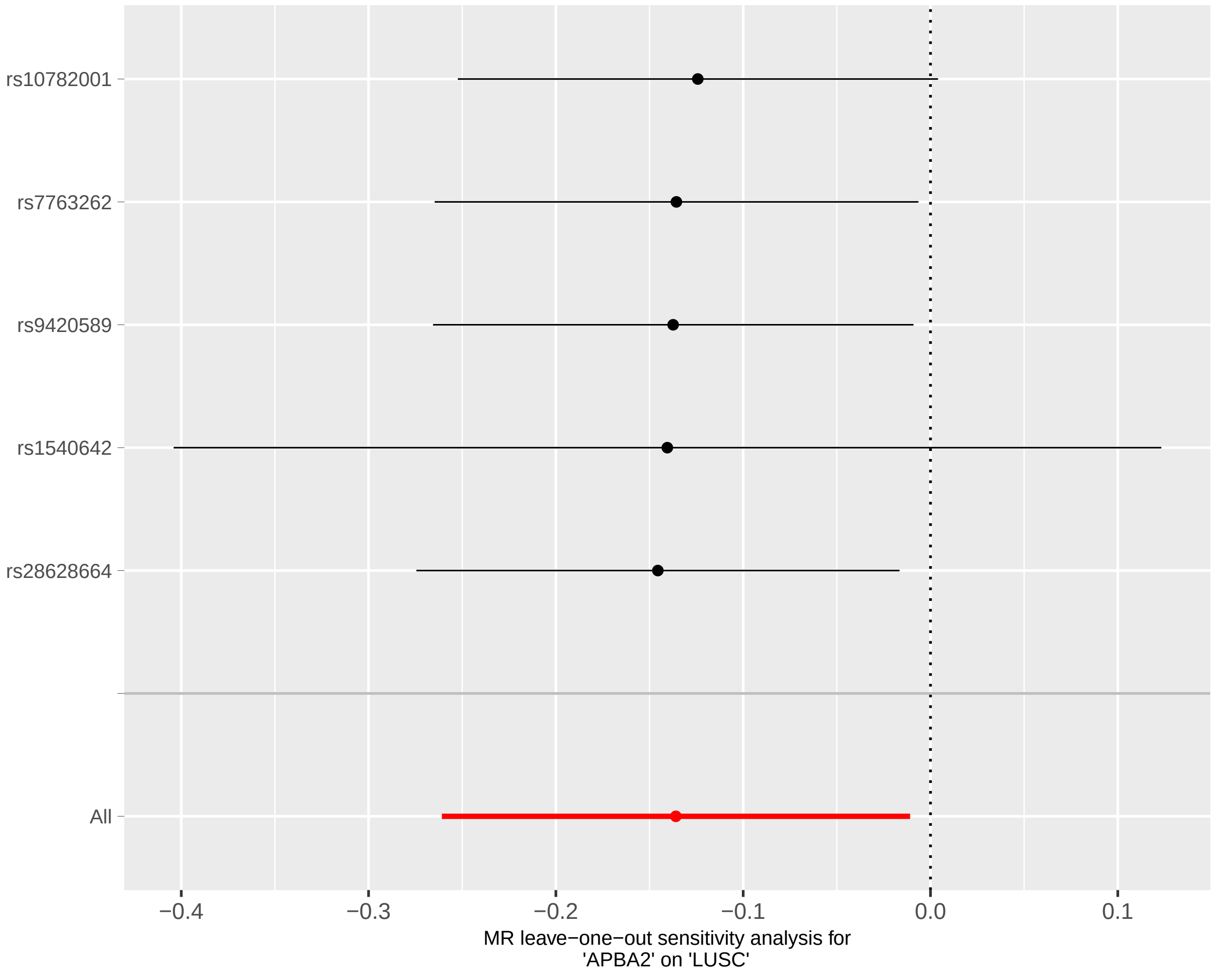
rs10782001
rs7763262
rs9420589
rs1540642
rs28628664
All
−0.4
−0.3
−0.2
−0.1
0.0
0.1
MR leave−one−out sensitivity analysis for
'APBA2' on 'LUSC'

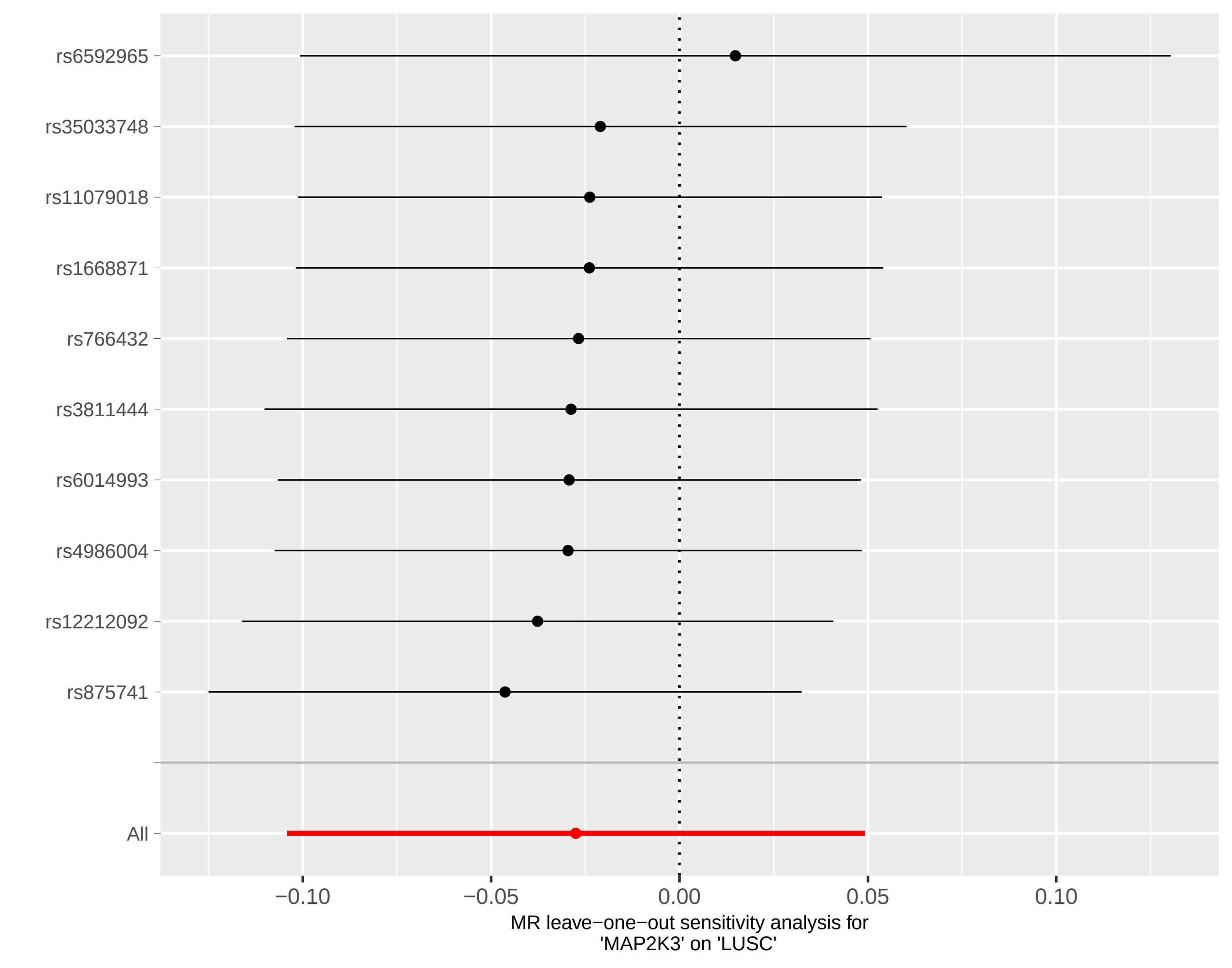

rs6592965
rs35033748
rs11079018
rs1668871
rs766432
rs3811444
rs6014993
rs4986004
rs12212092
rs875741
All
−0.10
−0.05
0.00
0.05
0.10
MR leave−one−out sensitivity analysis for
'MAP2K3' on 'LUSC'

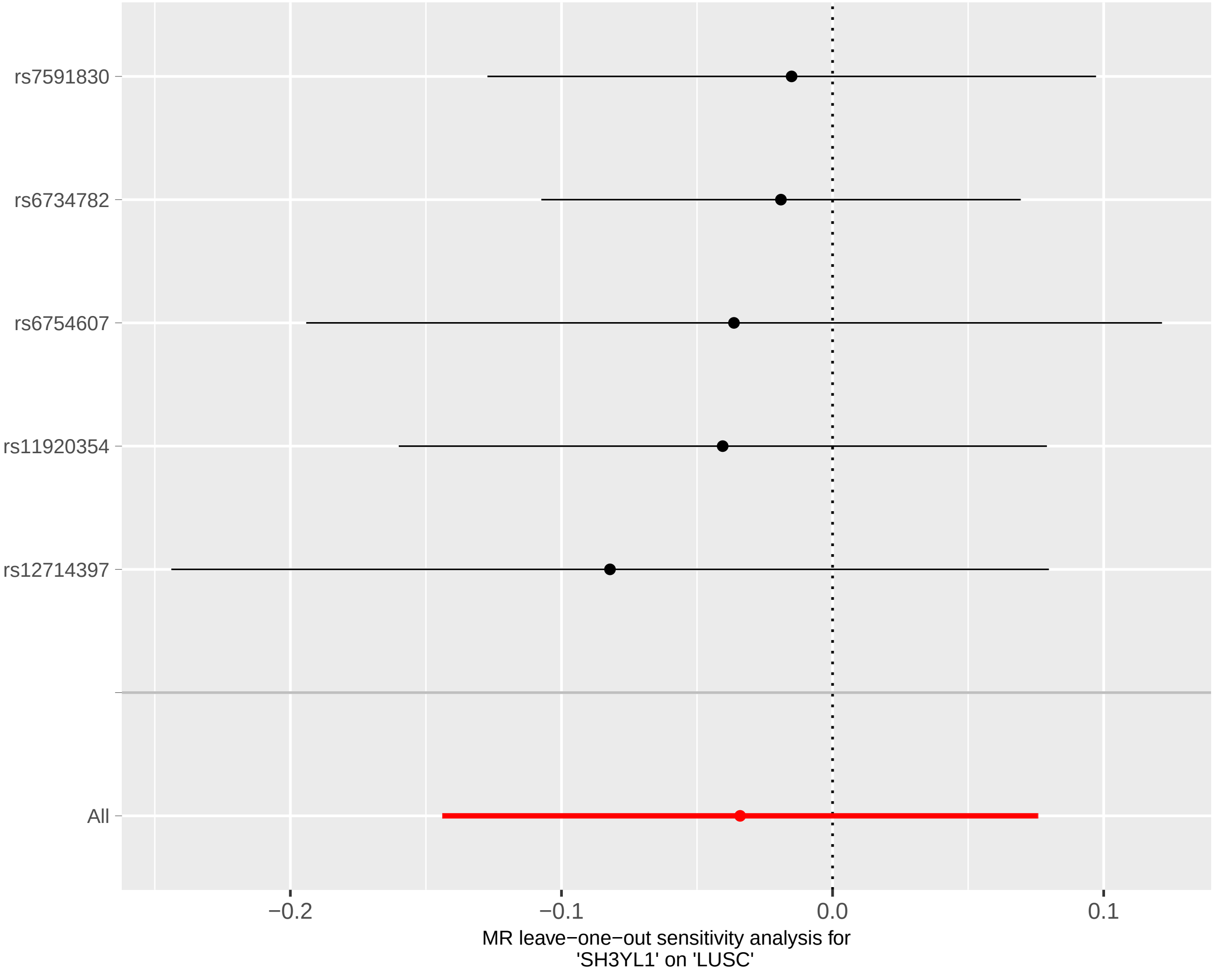

rs7591830
rs6734782
rs6754607
rs11920354
rs12714397
All
−0.2
−0.1
0.0
0.1
MR leave−one−out sensitivity analysis for
'SH3YL1' on 'LUSC'

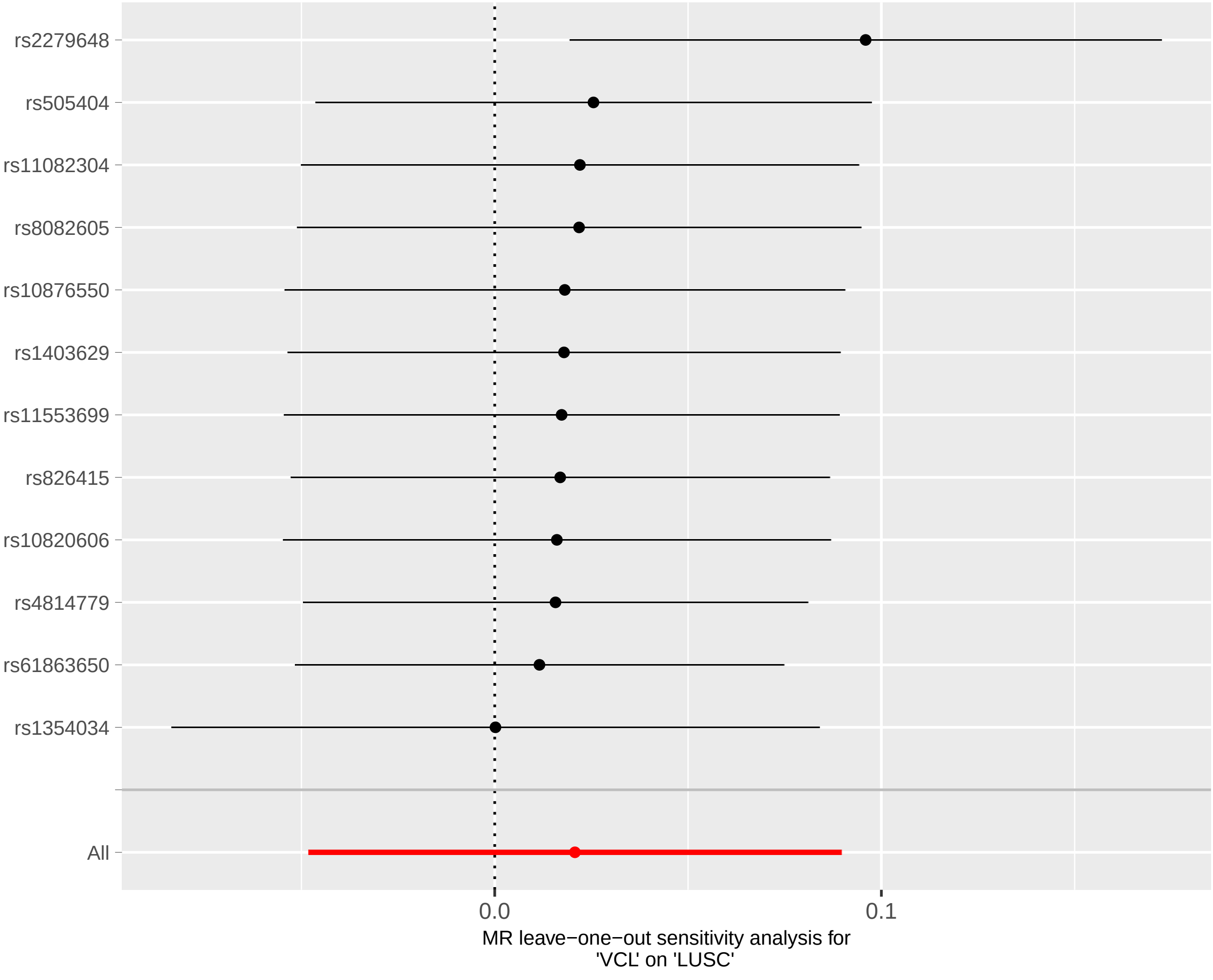
rs2279648
rs505404
rs11082304
rs8082605
rs10876550
rs1403629
rs11553699
rs826415
rs10820606
rs4814779
rs61863650
rs1354034
All
0.0
0.1
MR leave−one−out sensitivity analysis for
'VCL' on 'LUSC'

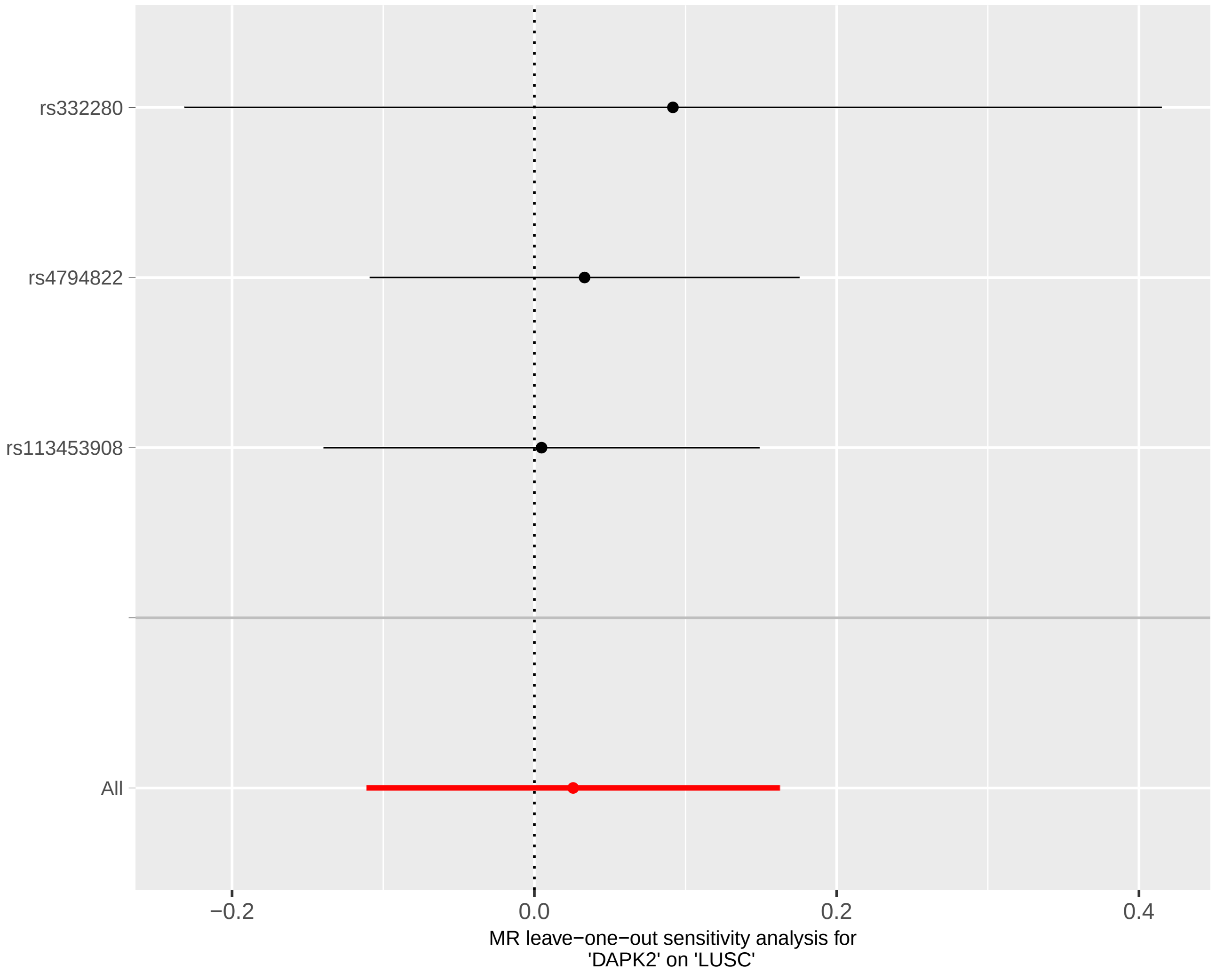

rs332280
rs4794822
rs113453908
All
−0.2
0.0
0.2
0.4
MR leave−one−out sensitivity analysis for
'DAPK2' on 'LUSC'

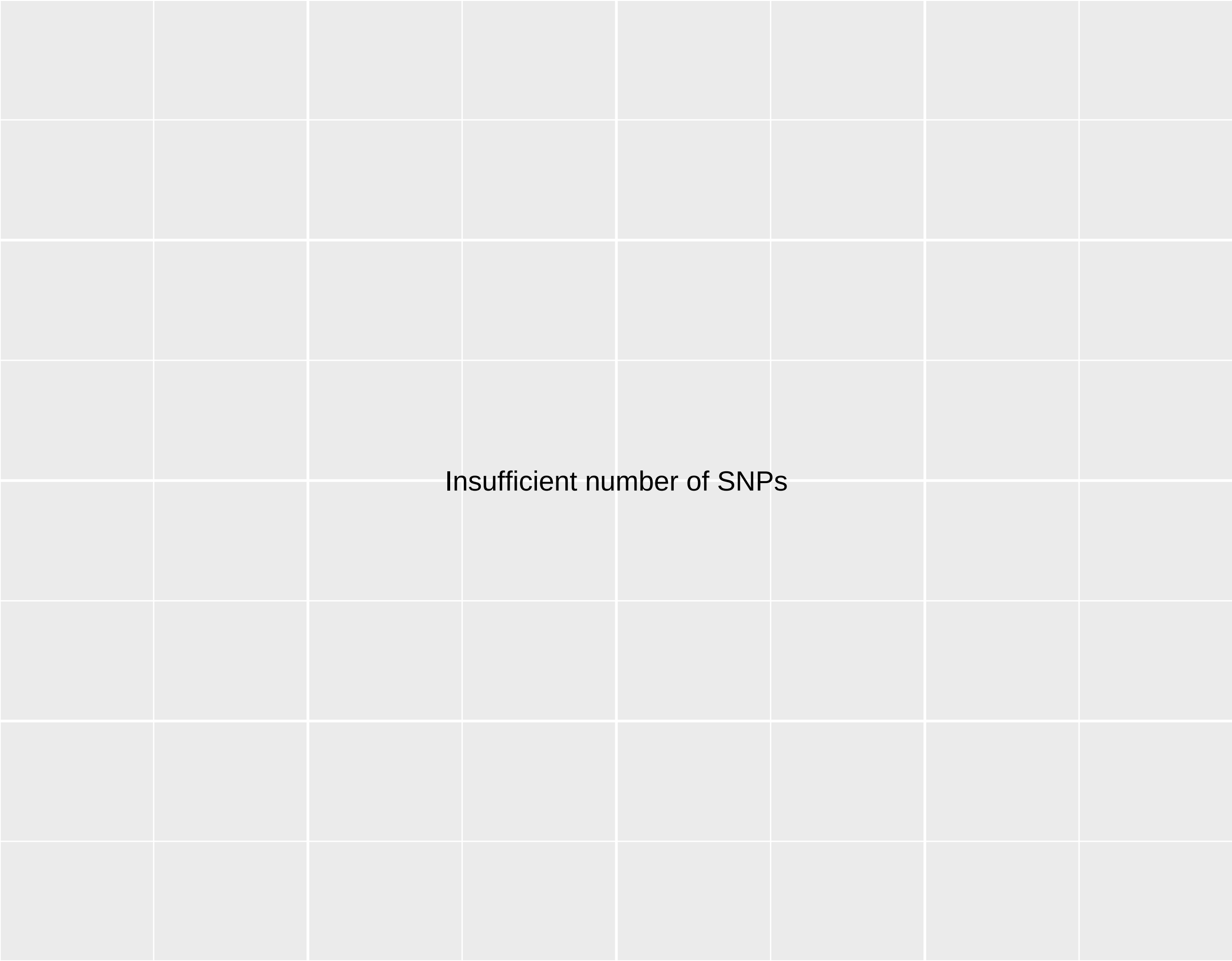
Insufficient number of SNPs

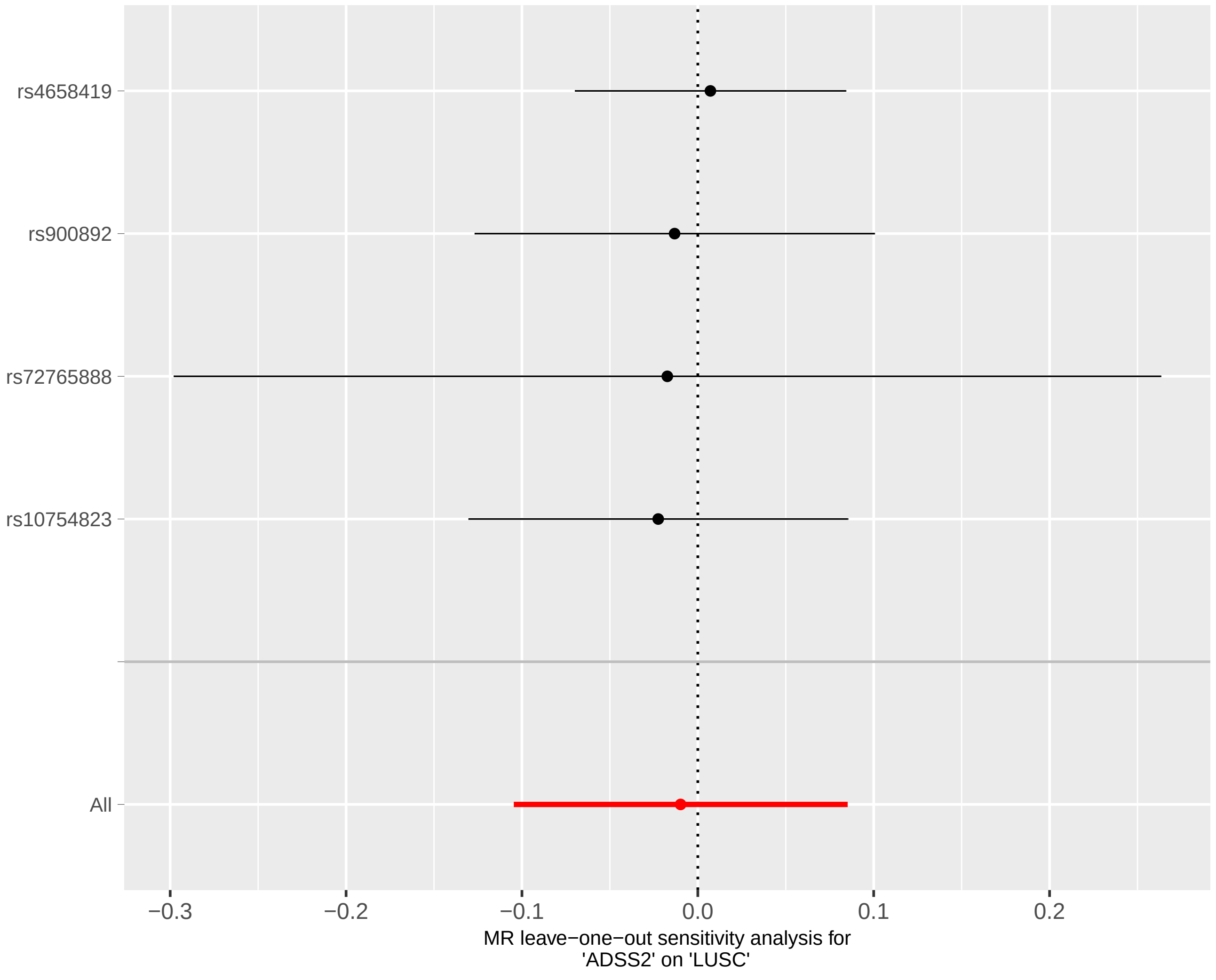
rs4658419
rs900892
rs72765888
rs10754823
All
−0.3
−0.2
−0.1
0.0
0.1
0.2
MR leave−one−out sensitivity analysis for
'ADSS2' on 'LUSC'

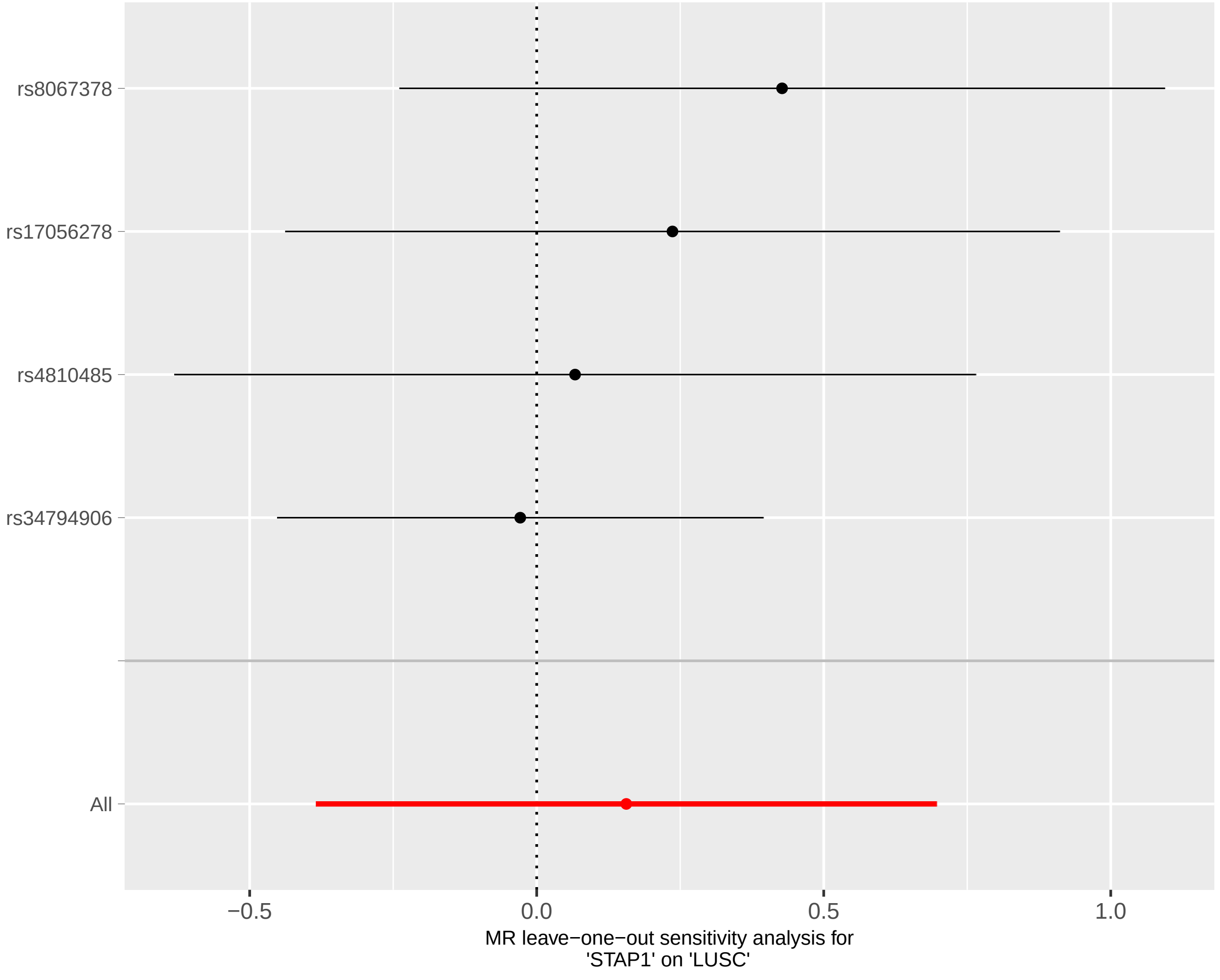
rs8067378
rs17056278
rs4810485
rs34794906
All
−0.5
0.0
0.5
1.0
MR leave−one−out sensitivity analysis for
'STAP1' on 'LUSC'

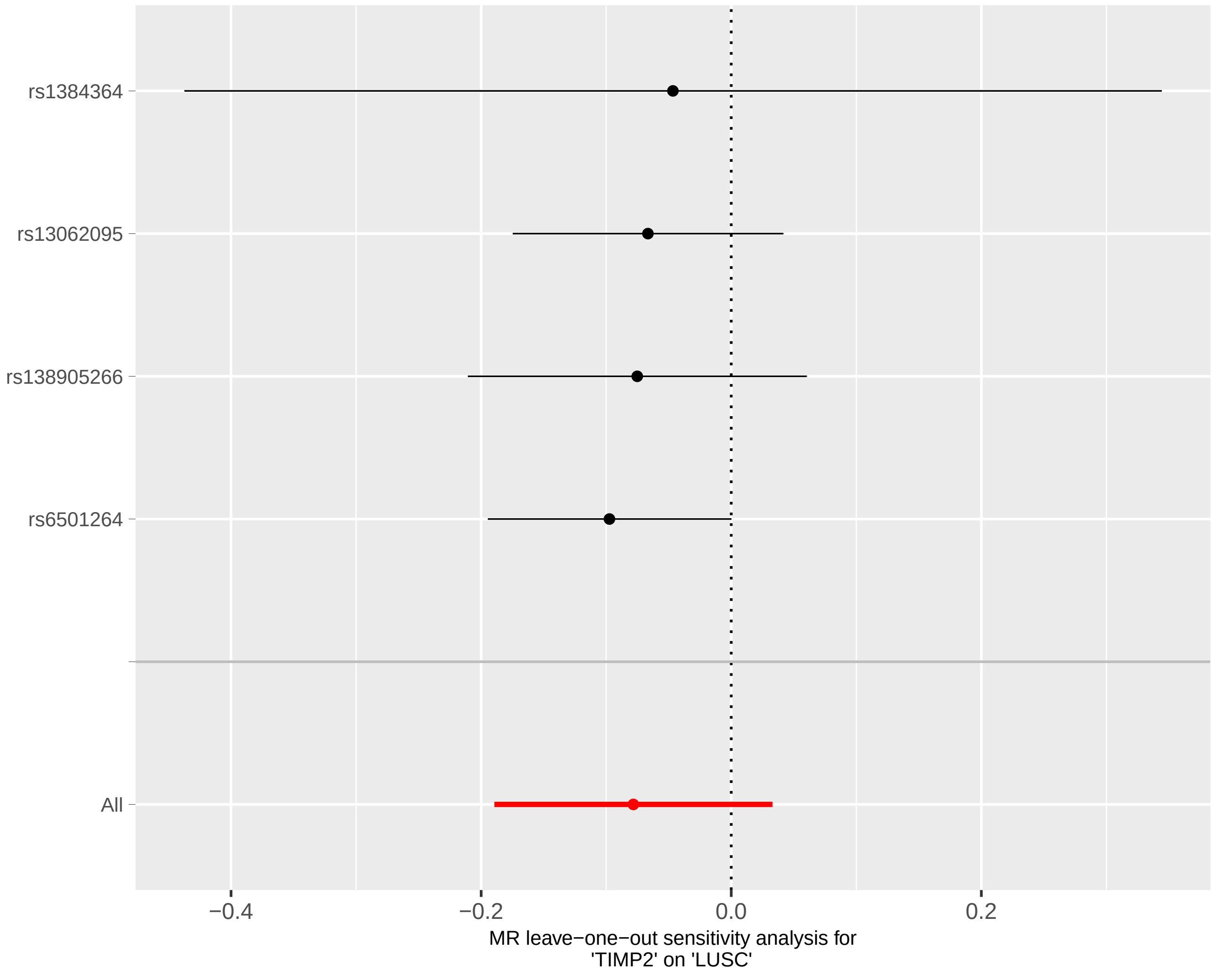

rs1384364
rs13062095
rs138905266
rs6501264
All
−0.4
−0.2
0.0
0.2
MR leave−one−out sensitivity analysis for
'TIMP2' on 'LUSC'

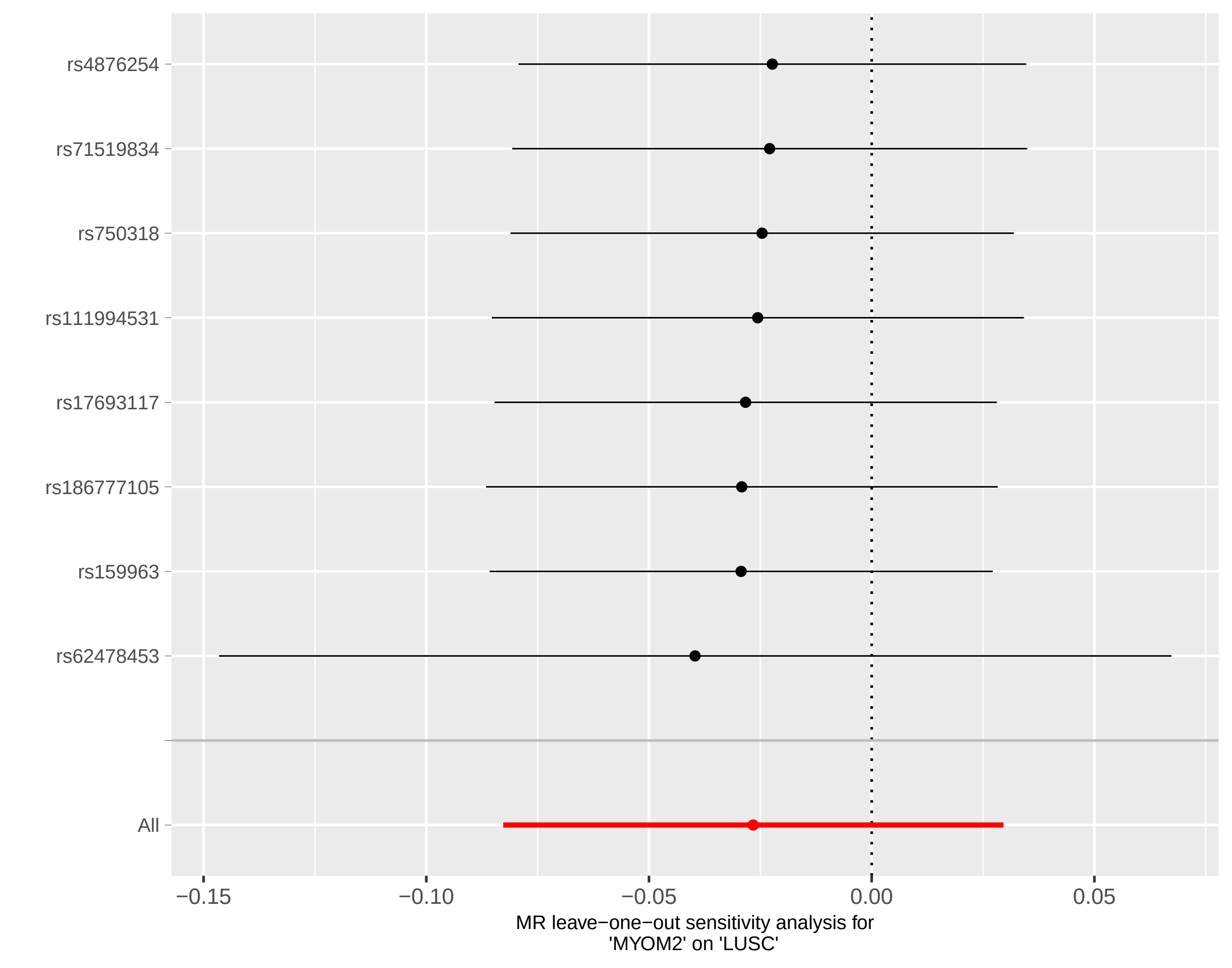
rs4876254
rs71519834
rs750318
rs111994531
rs17693117
rs186777105
rs159963
rs62478453
All
−0.15
−0.10
−0.05
0.00
0.05
MR leave−one−out sensitivity analysis for
'MYOM2' on 'LUSC'

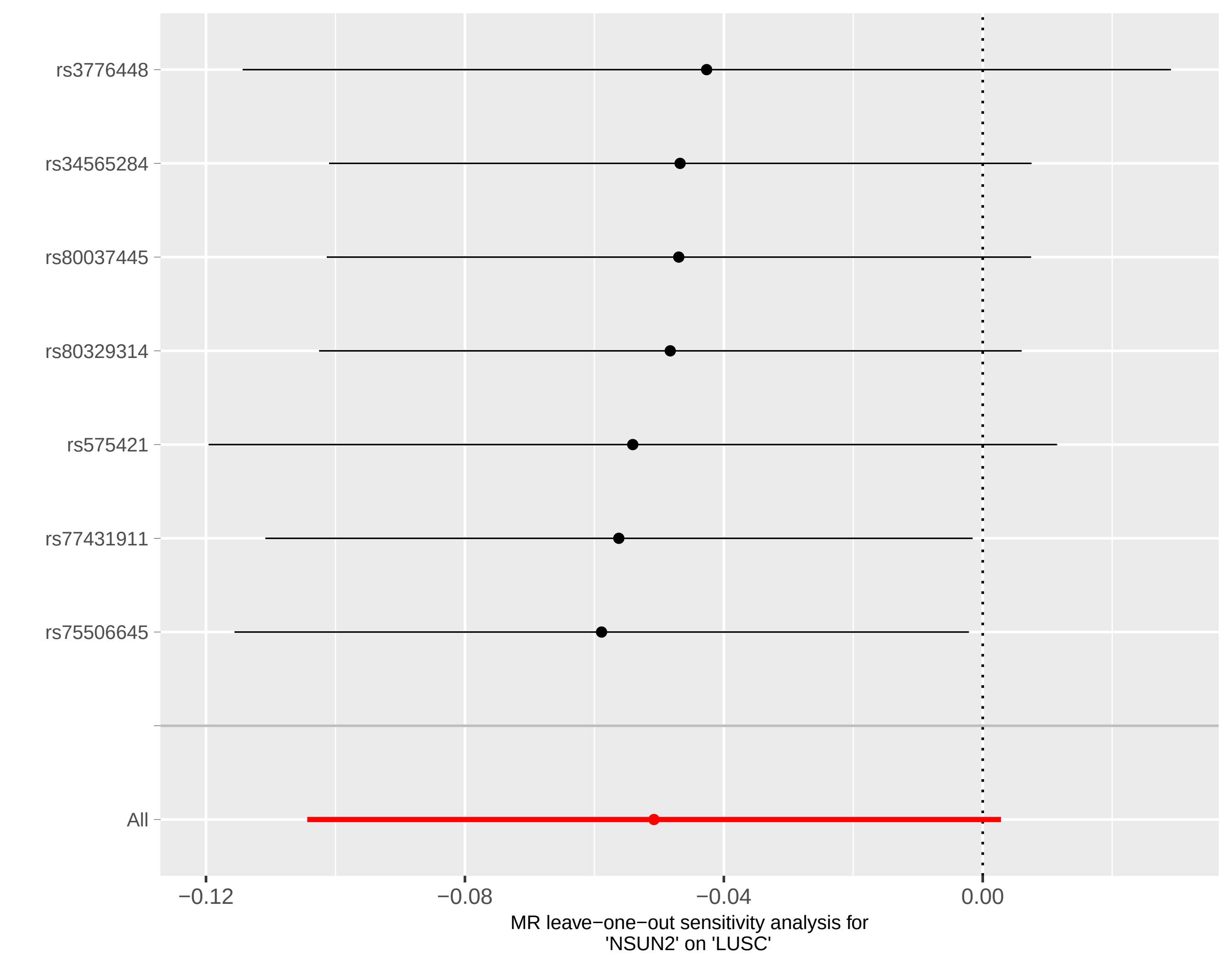

rs3776448
rs34565284
rs80037445
rs80329314
rs575421
rs77431911
rs75506645
All
−0.12
−0.08
−0.04
0.00
MR leave−one−out sensitivity analysis for
'NSUN2' on 'LUSC'

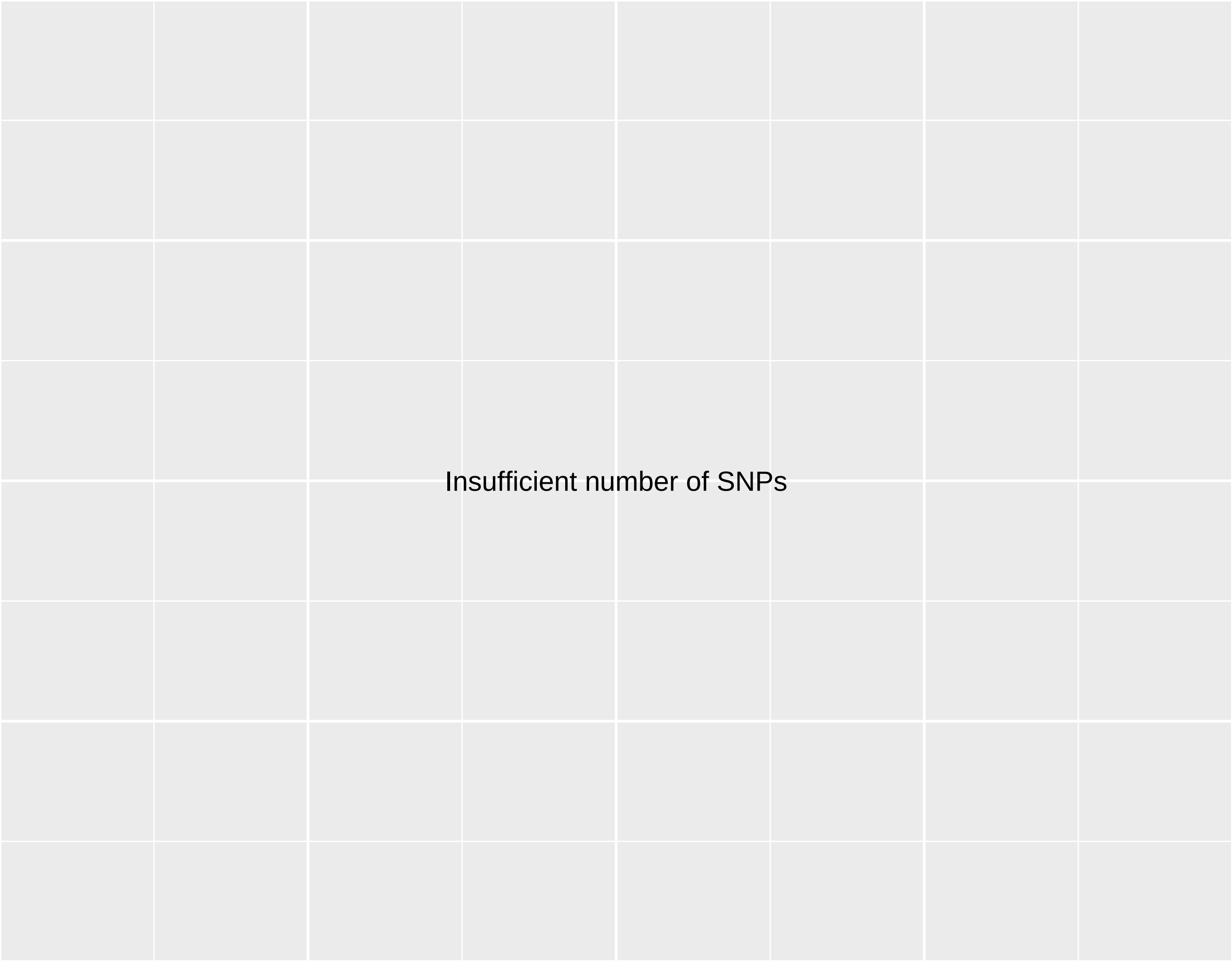
Insufficient number of SNPs

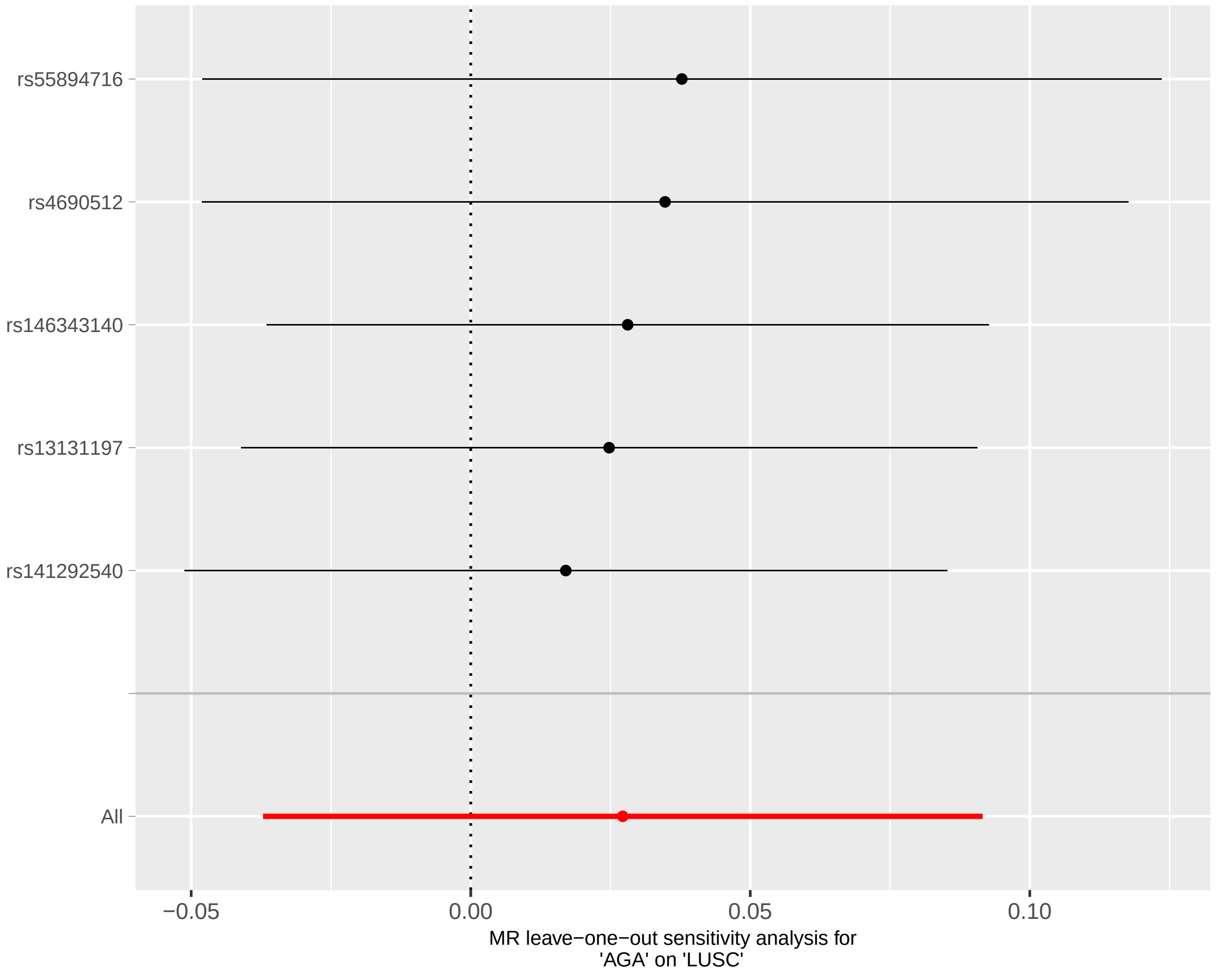

rs55894716
rs4690512
rs146343140
rs13131197
rs141292540
All
−0.05
0.00
0.05
0.10
MR leave−one−out sensitivity analysis for
'AGA' on 'LUSC'

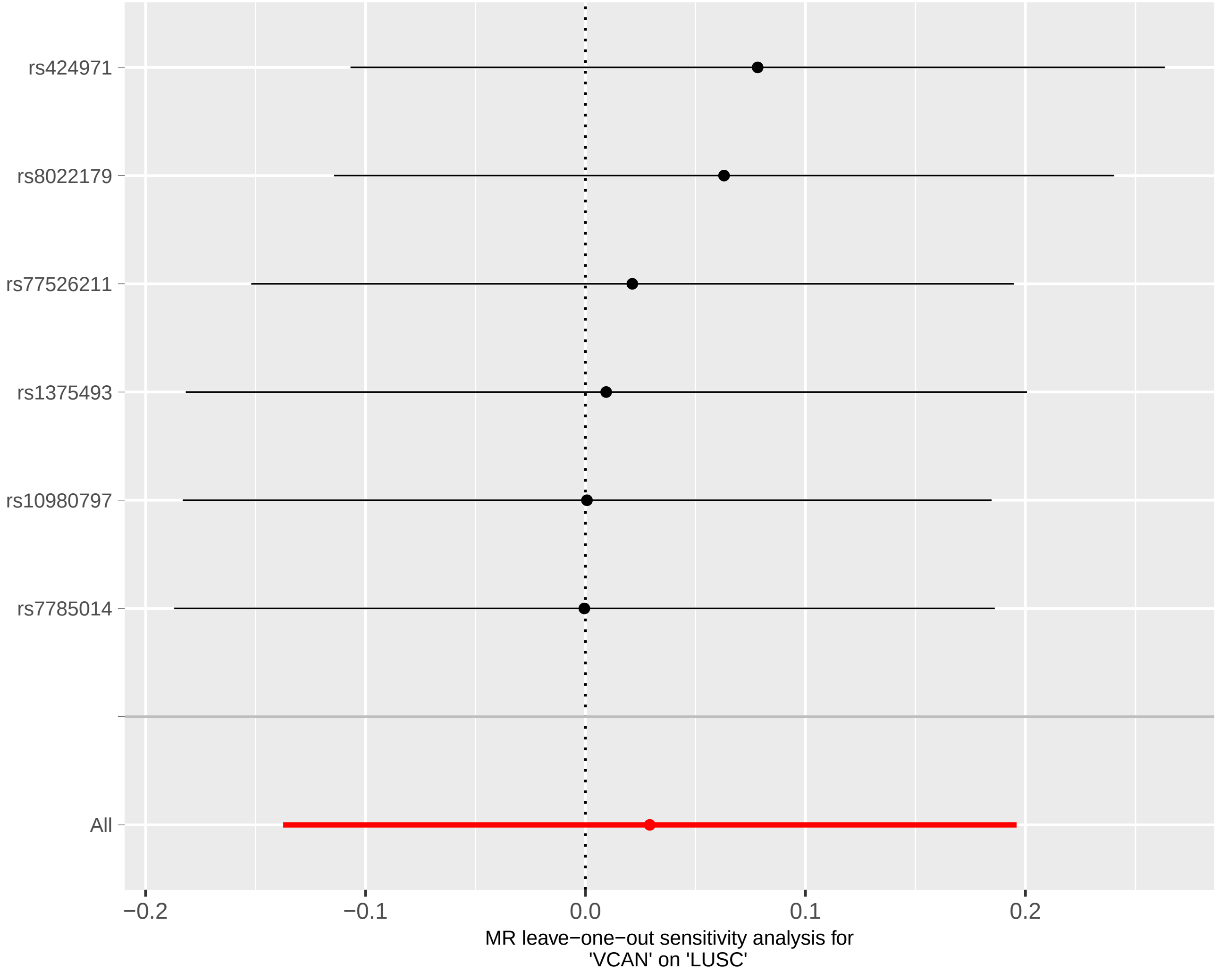

rs424971
rs8022179
rs77526211
rs1375493
rs10980797
rs7785014
All
−0.2
−0.1
0.0
0.1
0.2
MR leave−one−out sensitivity analysis for
'VCAN' on 'LUSC'

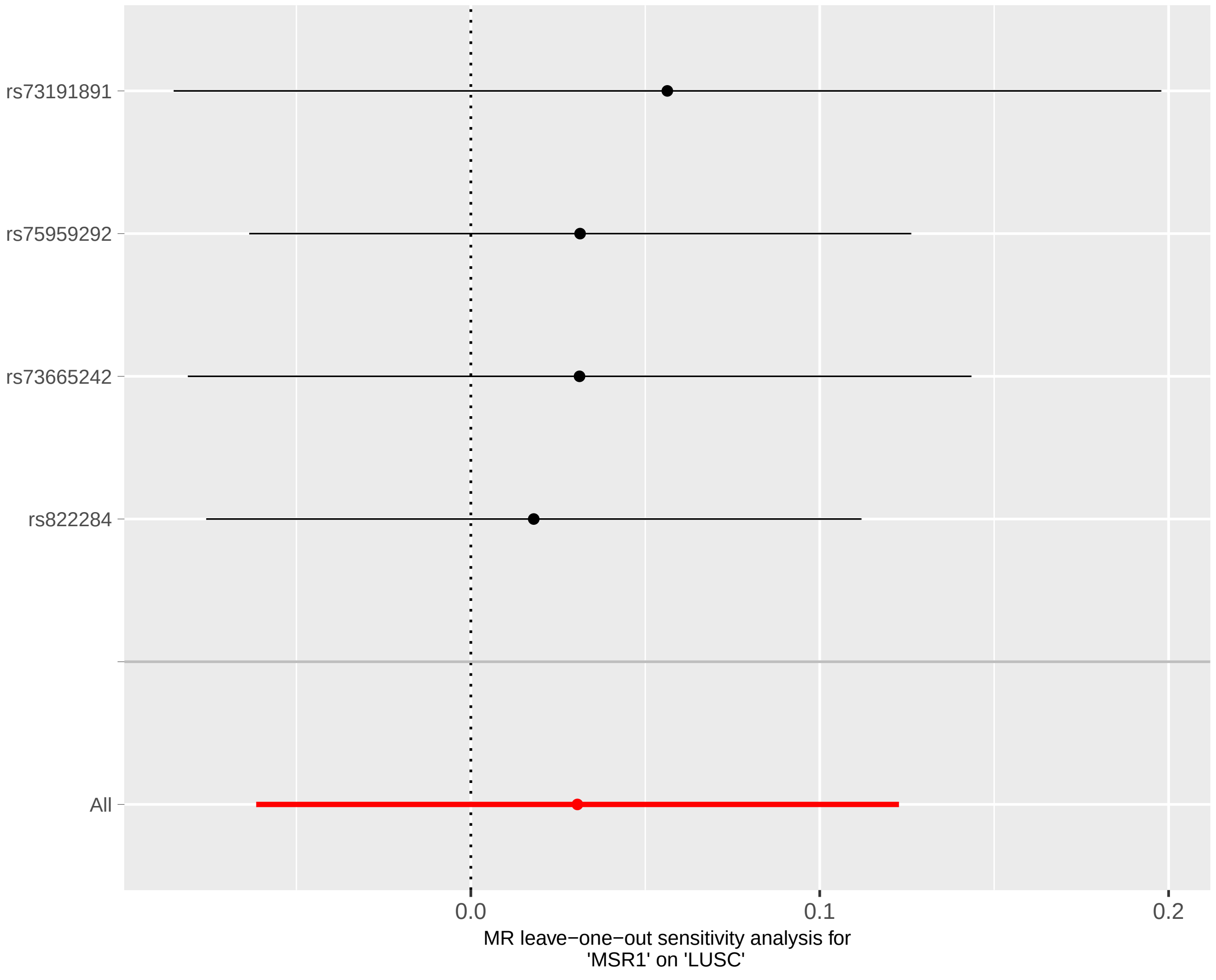
rs73191891
rs75959292
rs73665242
rs822284
All
0.0
0.1
0.2
MR leave−one−out sensitivity analysis for
'MSR1' on 'LUSC'

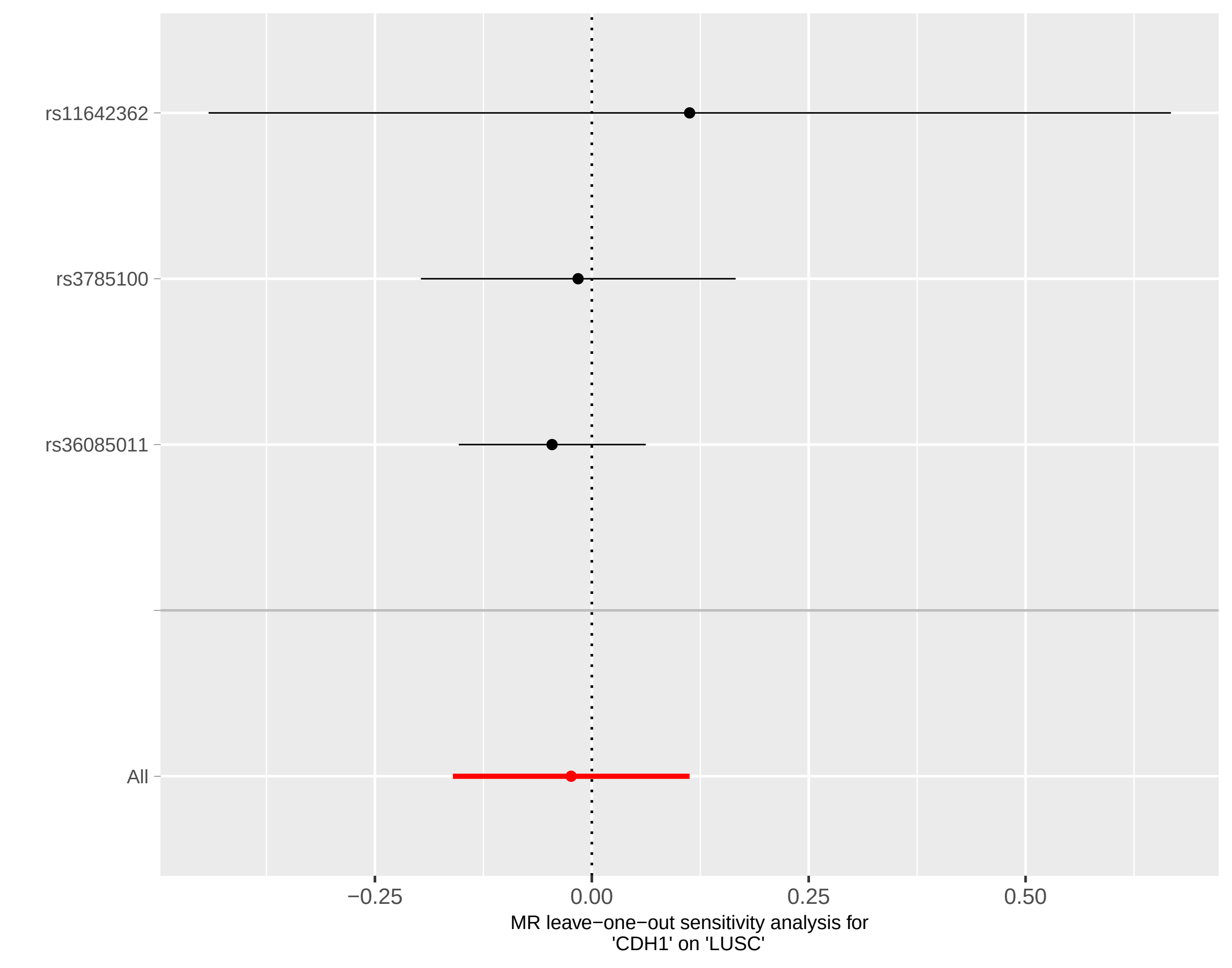
rs11642362
rs3785100
rs36085011
All
−0.25
0.00
0.25
0.50
MR leave−one−out sensitivity analysis for
'CDH1' on 'LUSC'

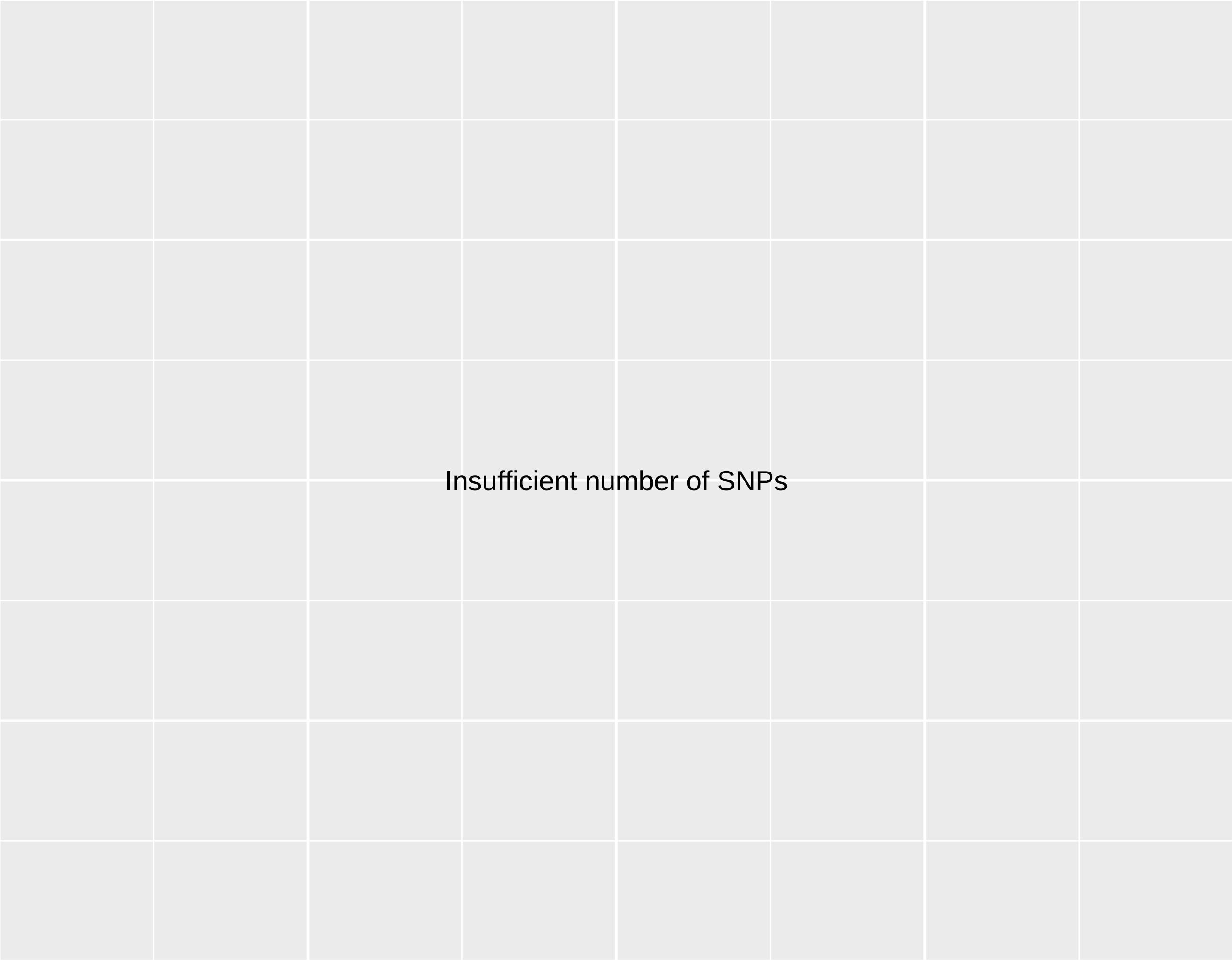
Insufficient number of SNPs

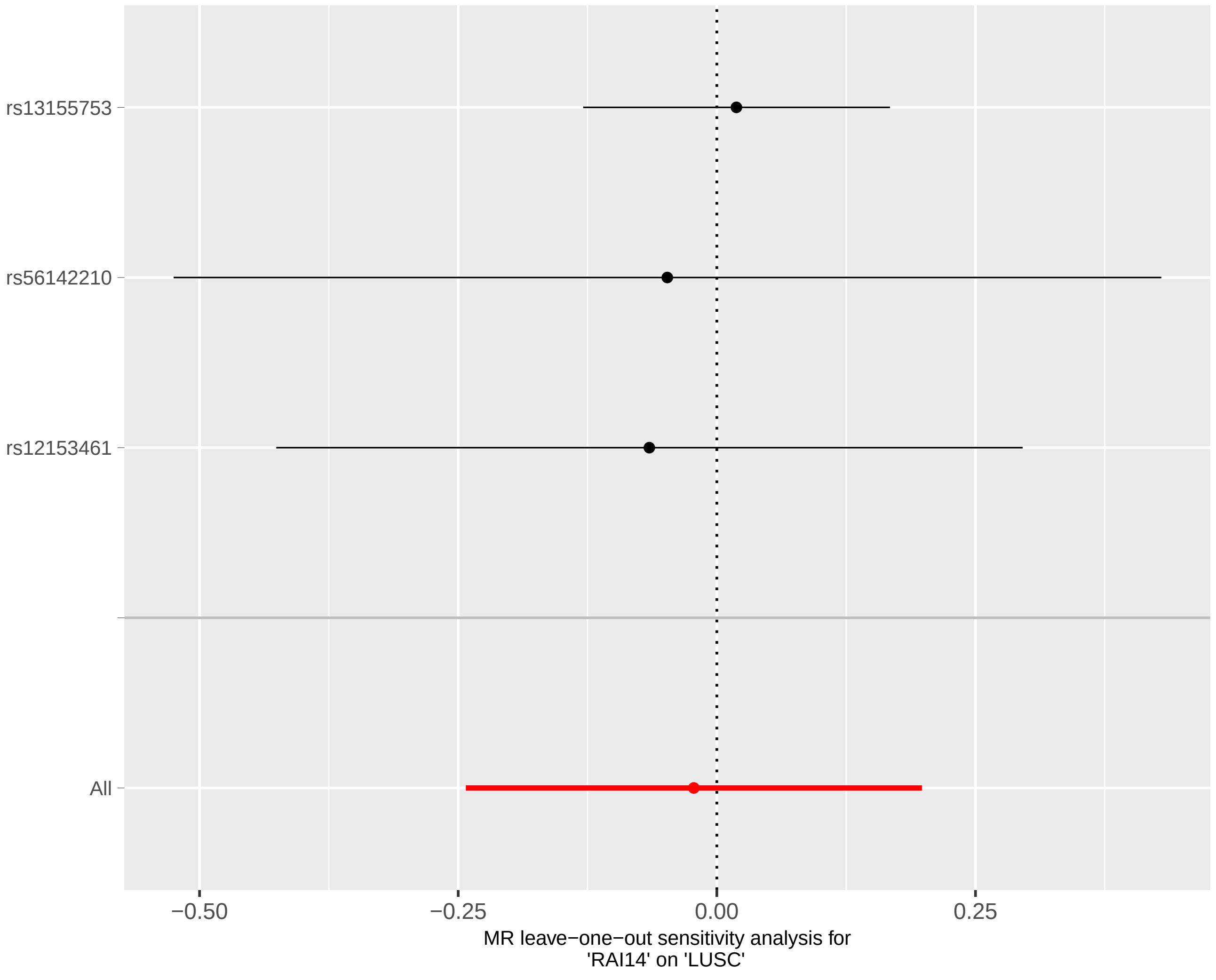

rs13155753
rs56142210
rs12153461
All
−0.50
−0.25
0.00
0.25
MR leave−one−out sensitivity analysis for
'RAI14' on 'LUSC'

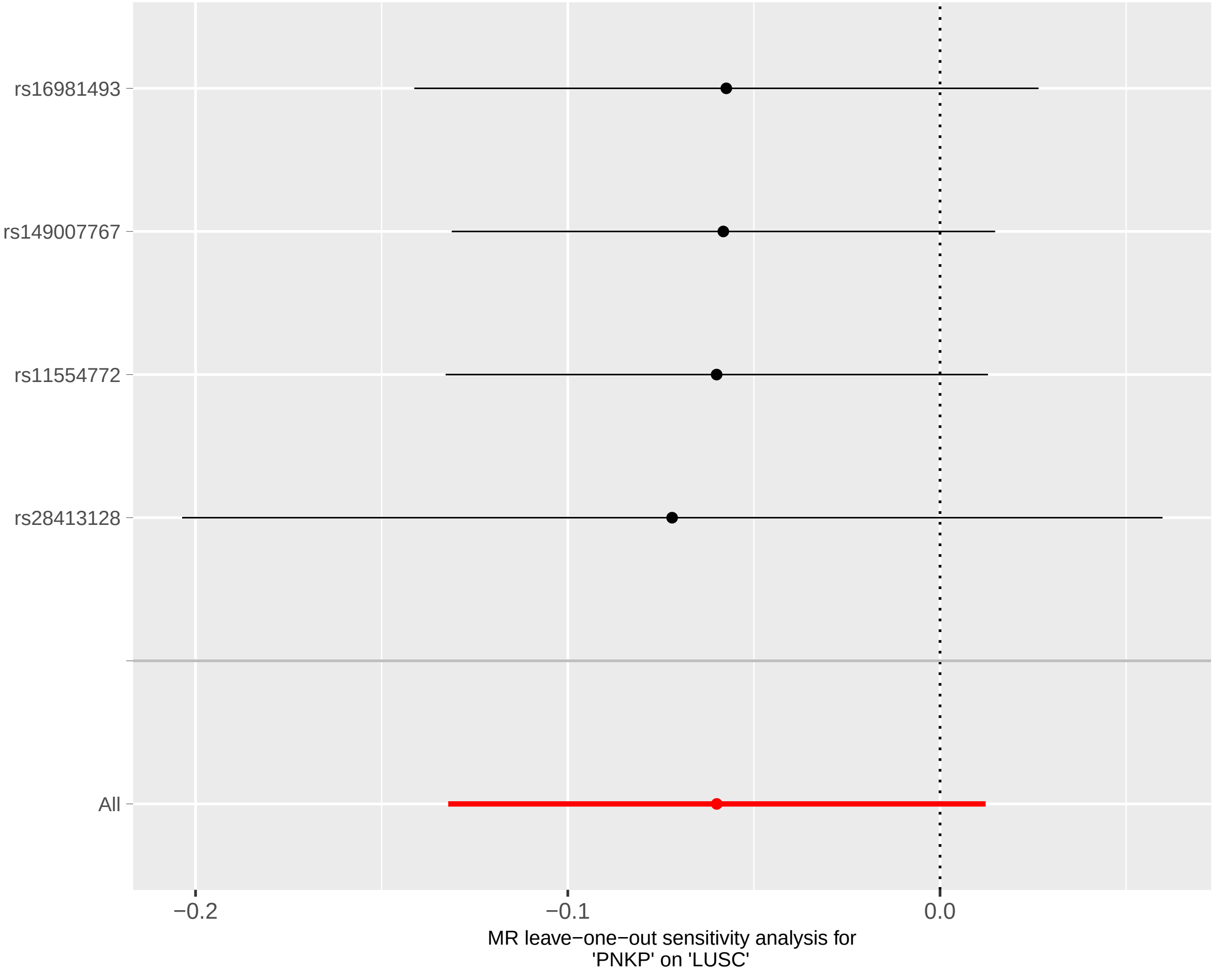
rs16981493
rs149007767
rs11554772
rs28413128
All
−0.2
−0.1
0.0
MR leave−one−out sensitivity analysis for
'PNKP' on 'LUSC'

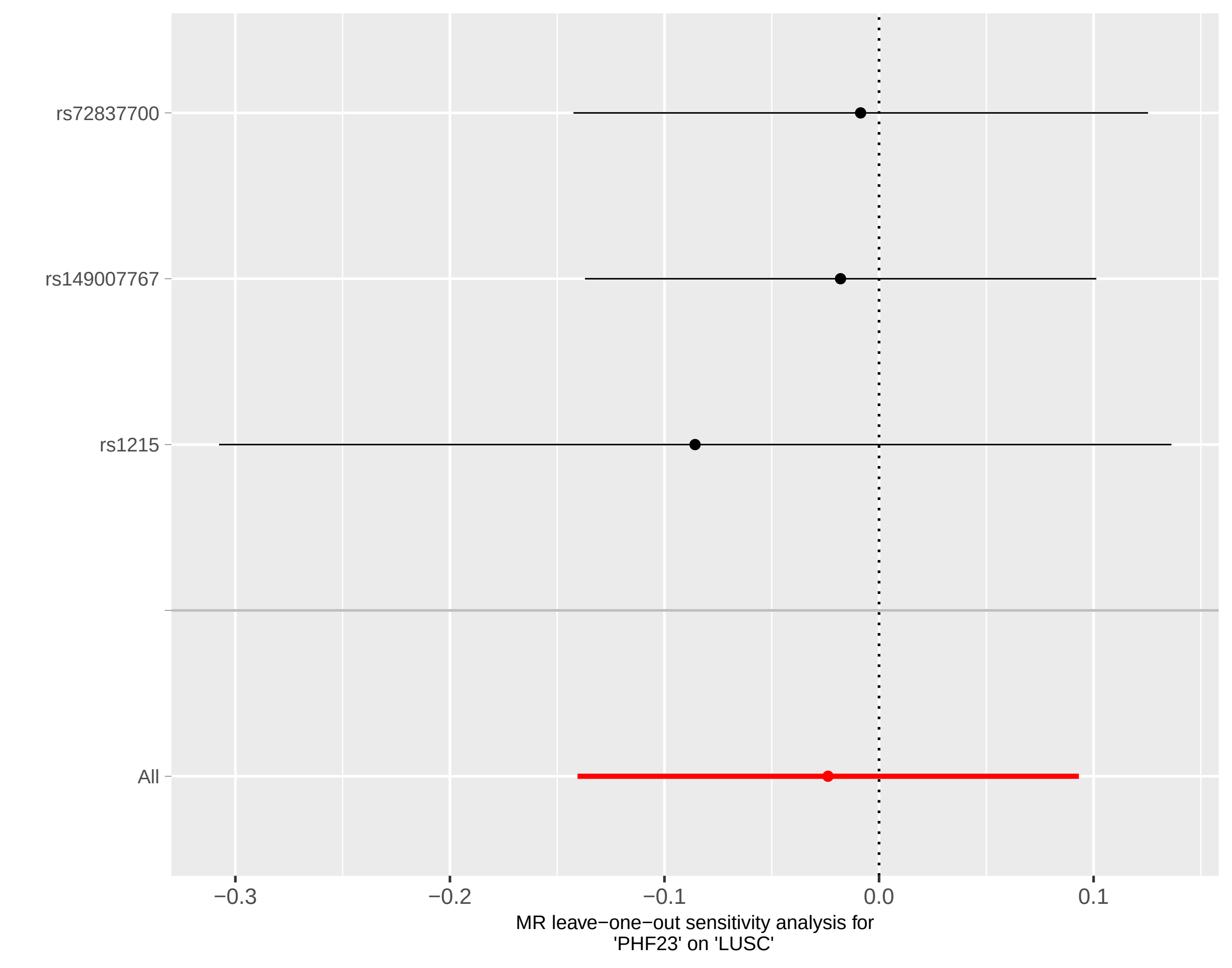
rs72837700
rs149007767
rs1215
All
−0.3
−0.2
−0.1
0.0
0.1
MR leave−one−out sensitivity analysis for
'PHF23' on 'LUSC'

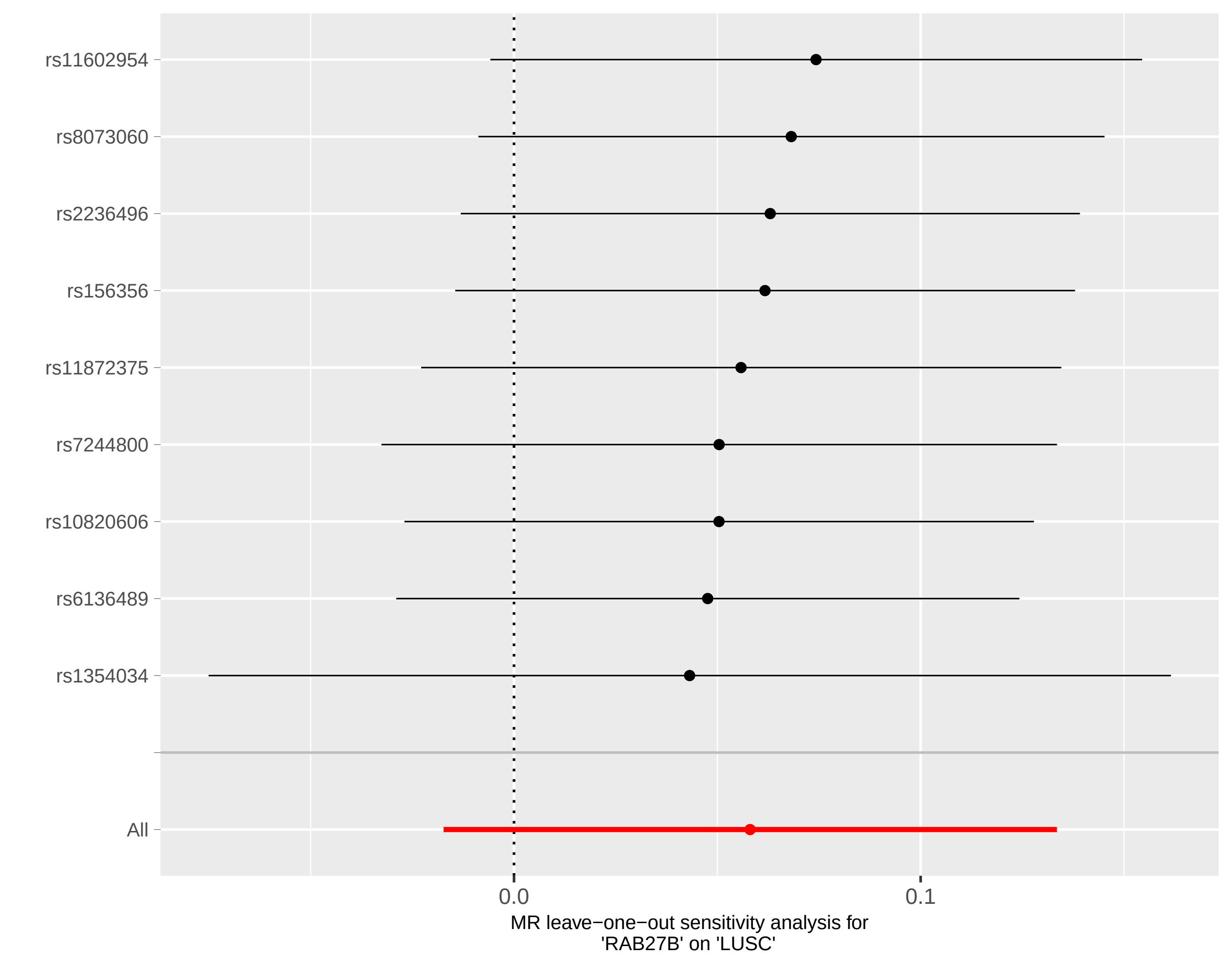
rs11602954
rs8073060
rs2236496
rs156356
rs11872375
rs7244800
rs10820606
rs6136489
rs1354034
All
0.0
0.1
MR leave−one−out sensitivity analysis for
'RAB27B' on 'LUSC'

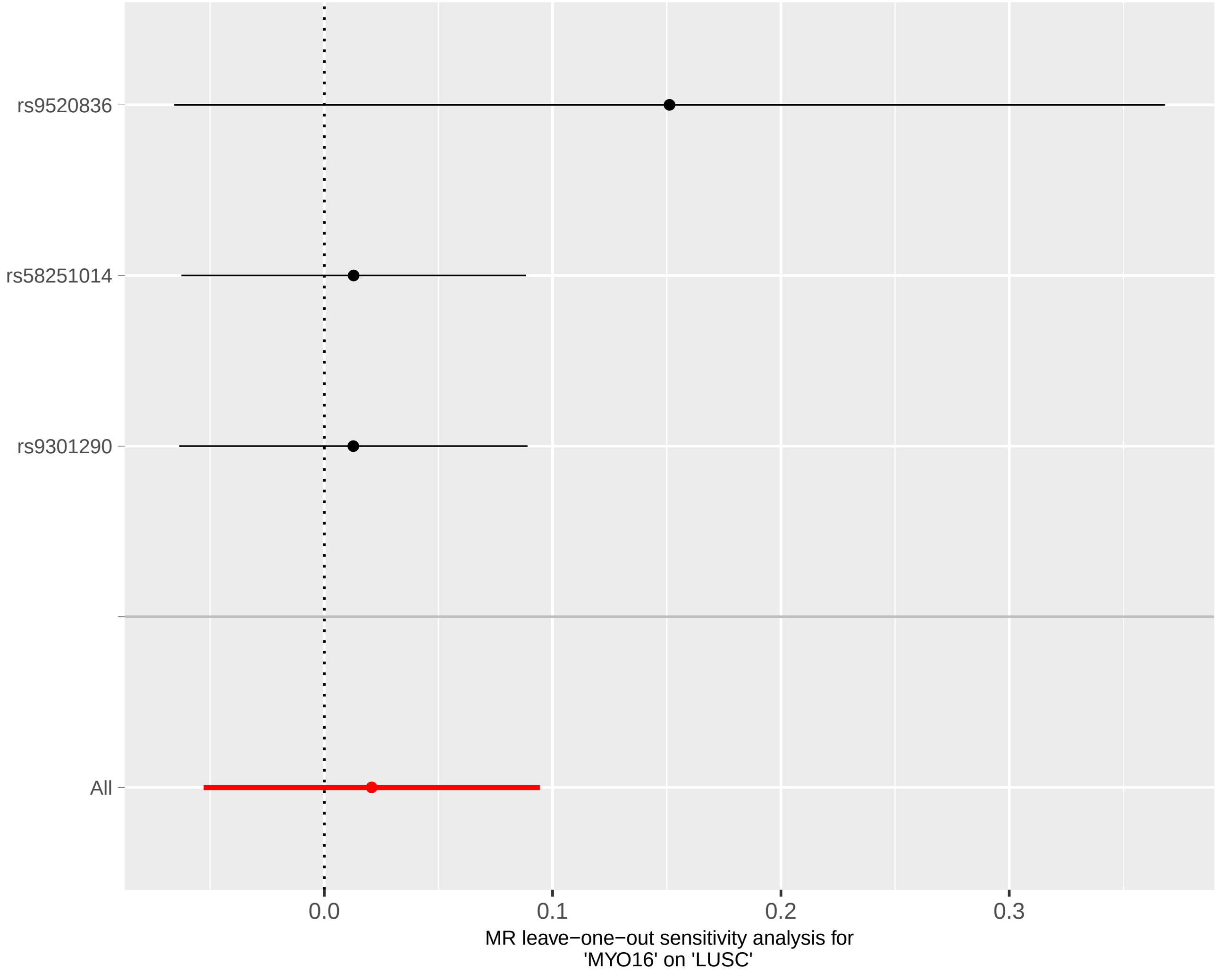

rs9520836
rs58251014
rs9301290
All
0.0
0.1
0.2
0.3
MR leave−one−out sensitivity analysis for
'MYO16' on 'LUSC'

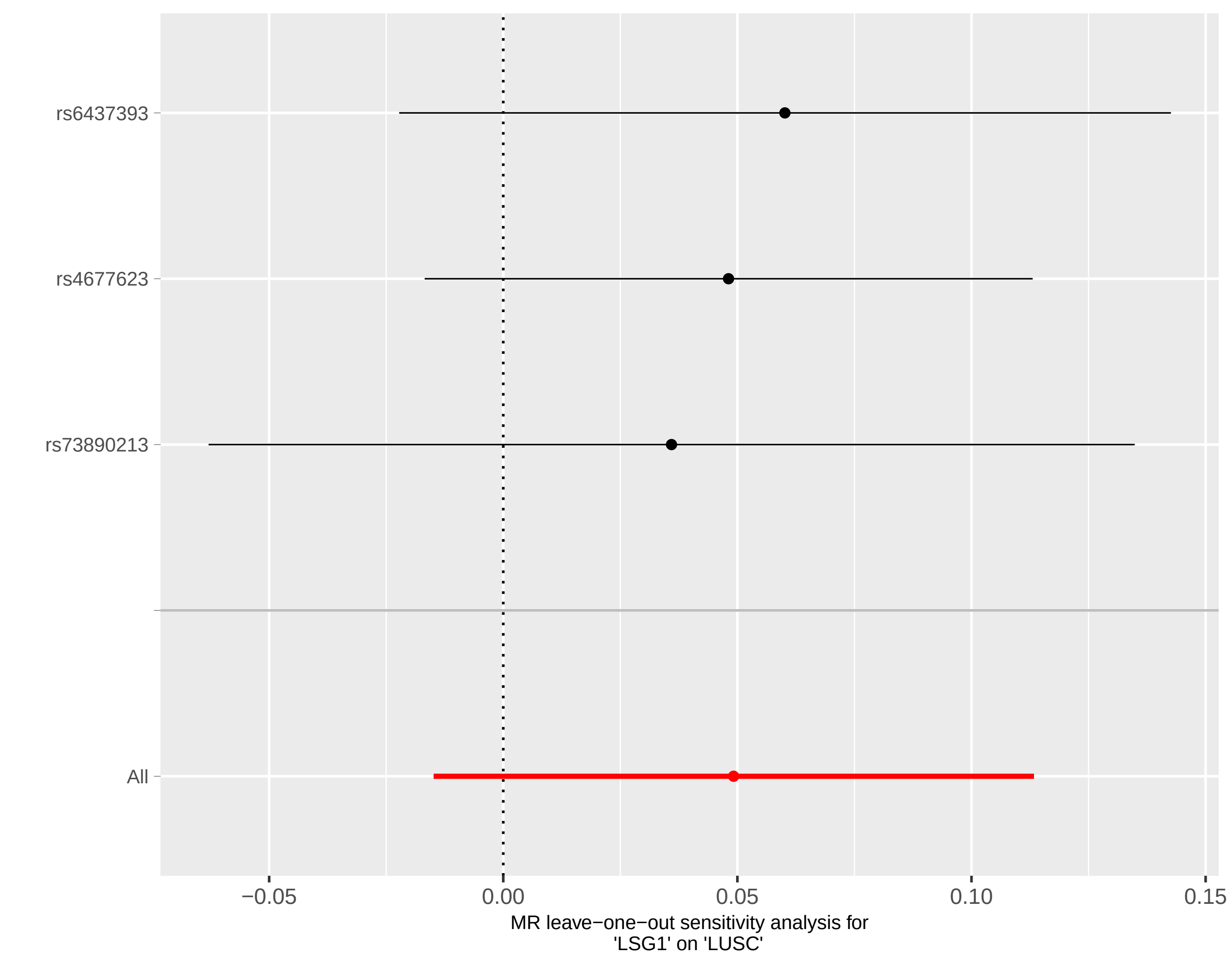
rs6437393
rs4677623
rs73890213
All
−0.05
0.00
0.05
0.10
0.15
MR leave−one−out sensitivity analysis for
'LSG1' on 'LUSC'

Insufficient number of SNPs

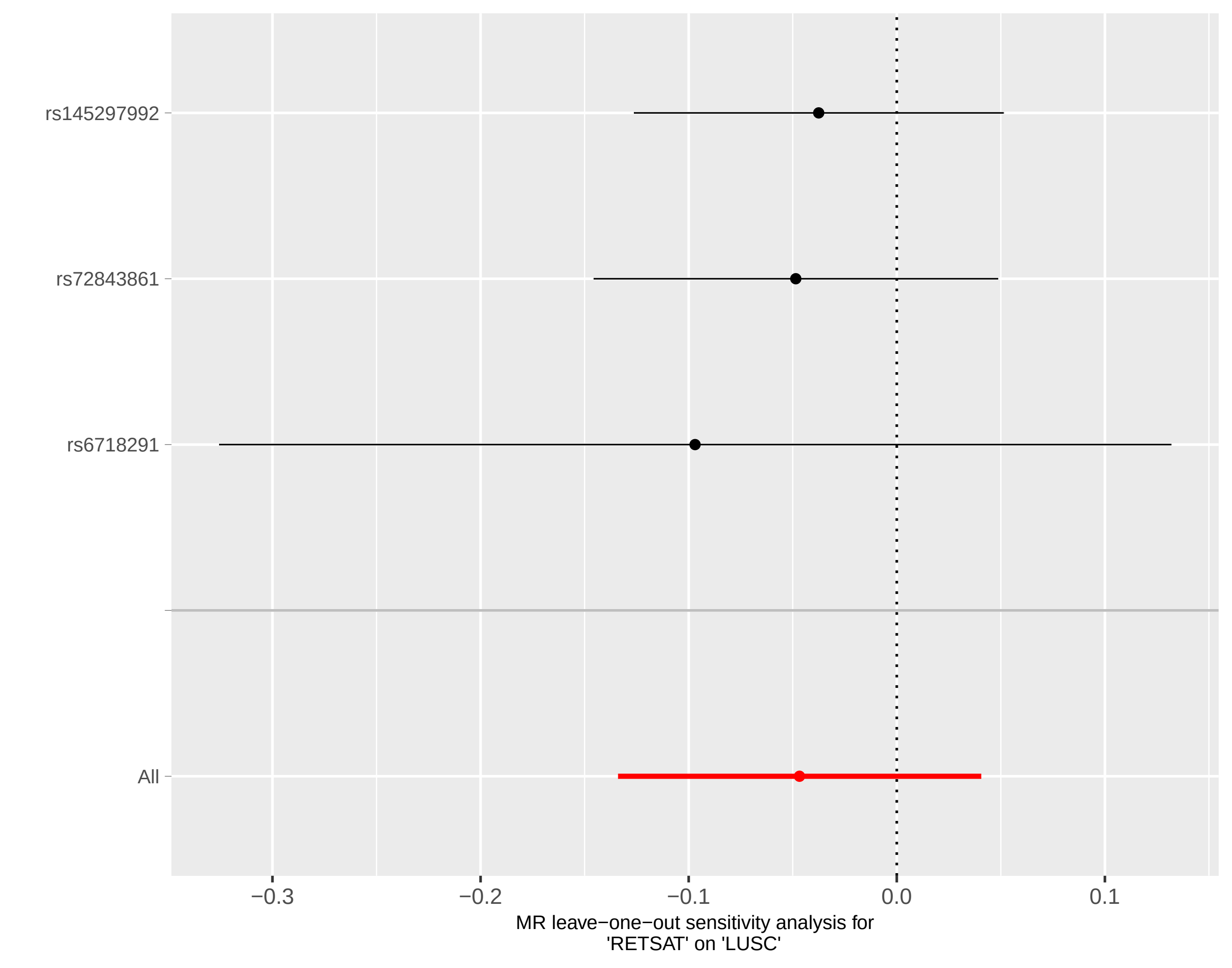

rs145297992
rs72843861
rs6718291
All
−0.3
−0.2
−0.1
0.0
0.1
MR leave−one−out sensitivity analysis for
'RETSAT' on 'LUSC'

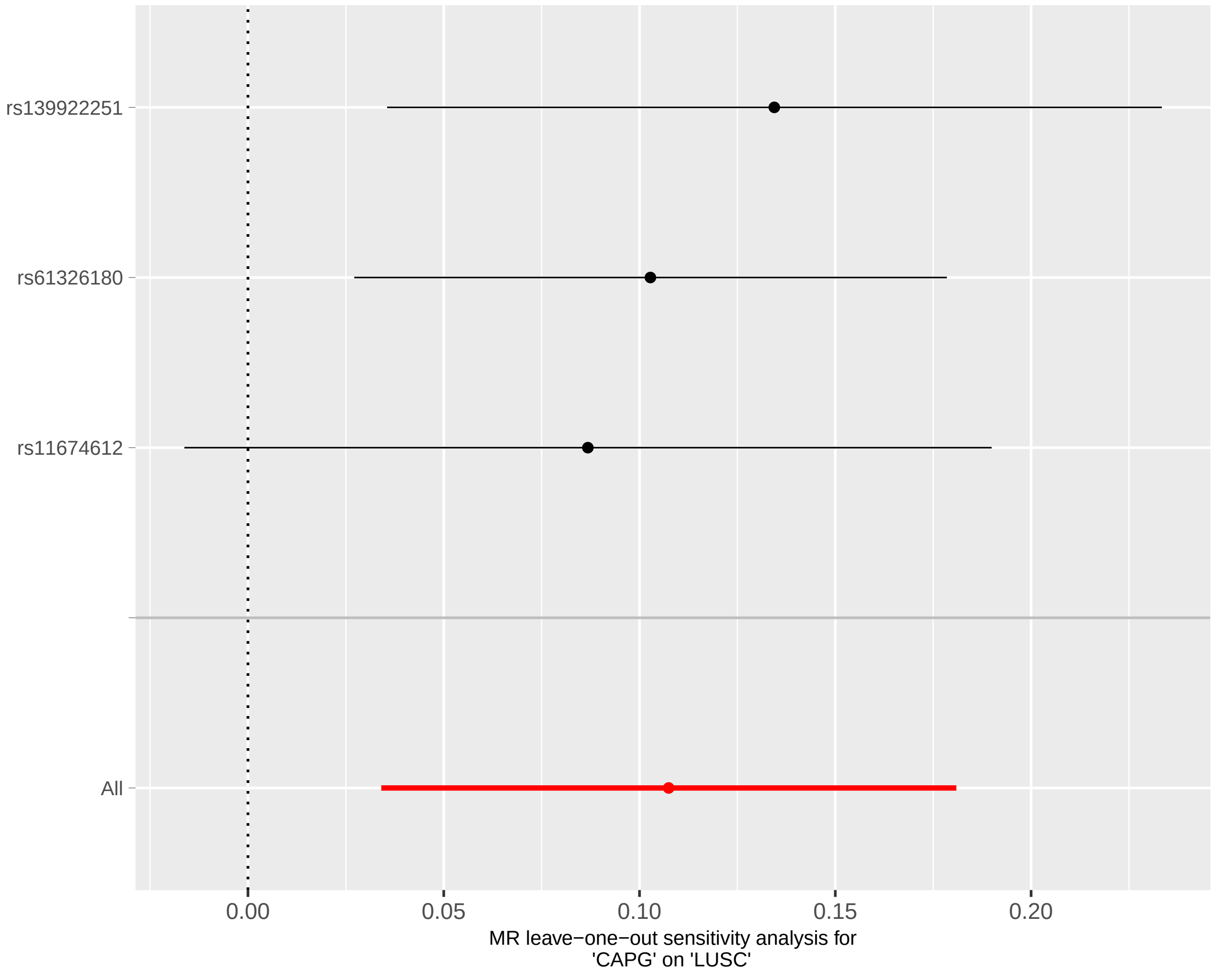
rs139922251
rs61326180
rs11674612
All
0.00
0.05
0.10
0.15
0.20
MR leave−one−out sensitivity analysis for
'CAPG' on 'LUSC'

Insufficient number of SNPs

Insufficient number of SNPs

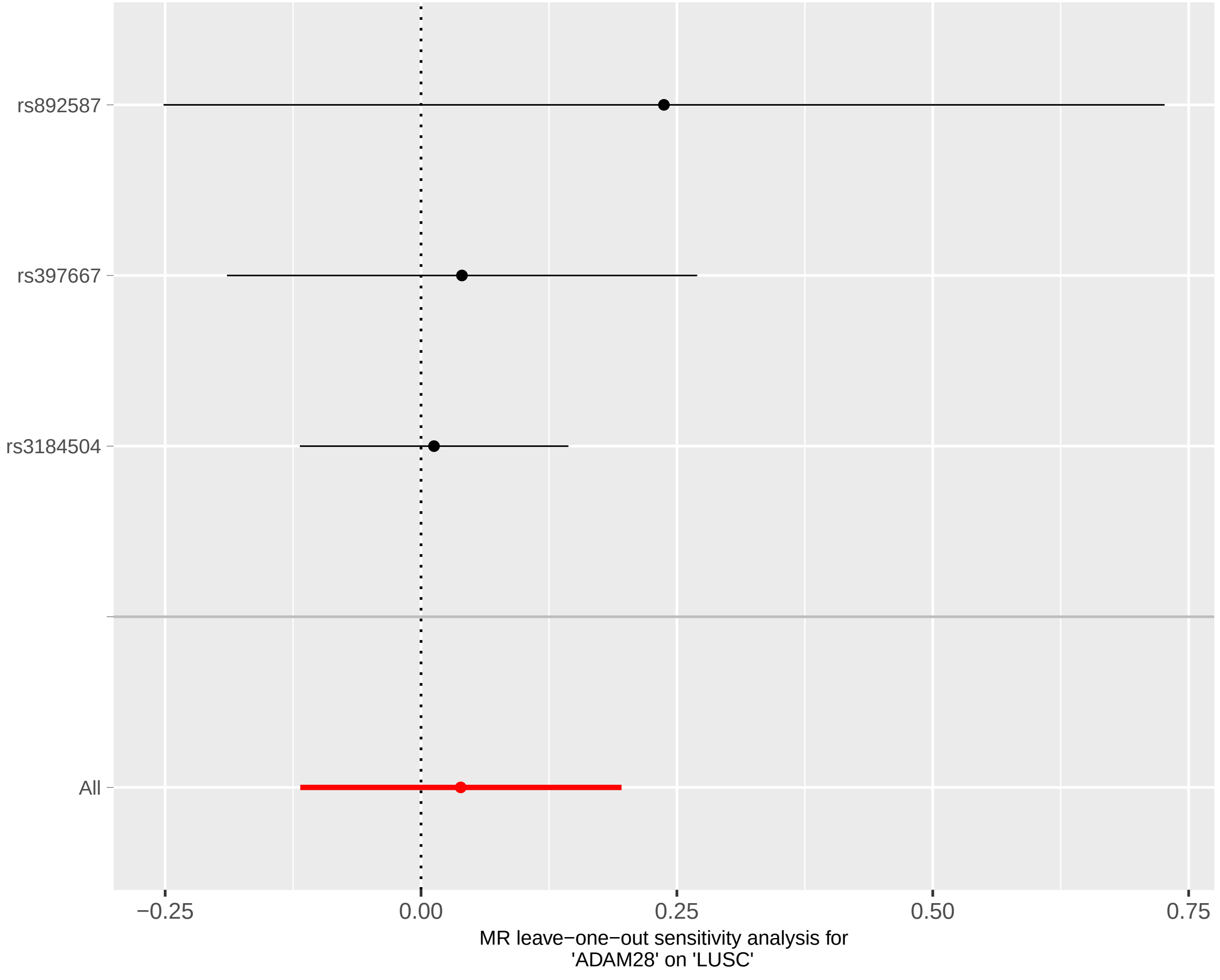
rs892587
rs397667
rs3184504
All
−0.25
0.00
0.25
0.50
0.75
MR leave−one−out sensitivity analysis for
'ADAM28' on 'LUSC'

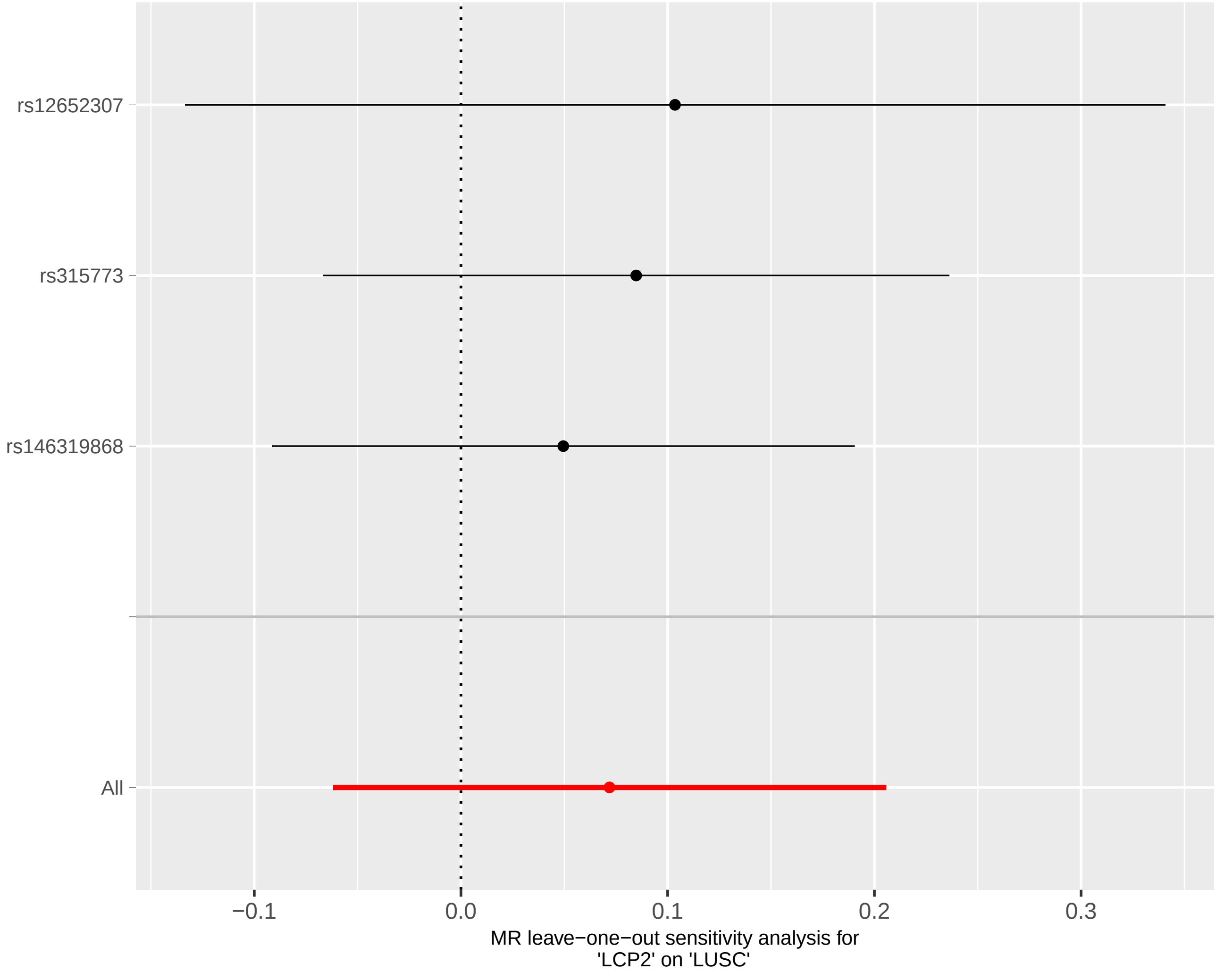
rs12652307
rs315773
rs146319868
All
−0.1
0.0
0.1
0.2
0.3
MR leave−one−out sensitivity analysis for
'LCP2' on 'LUSC'

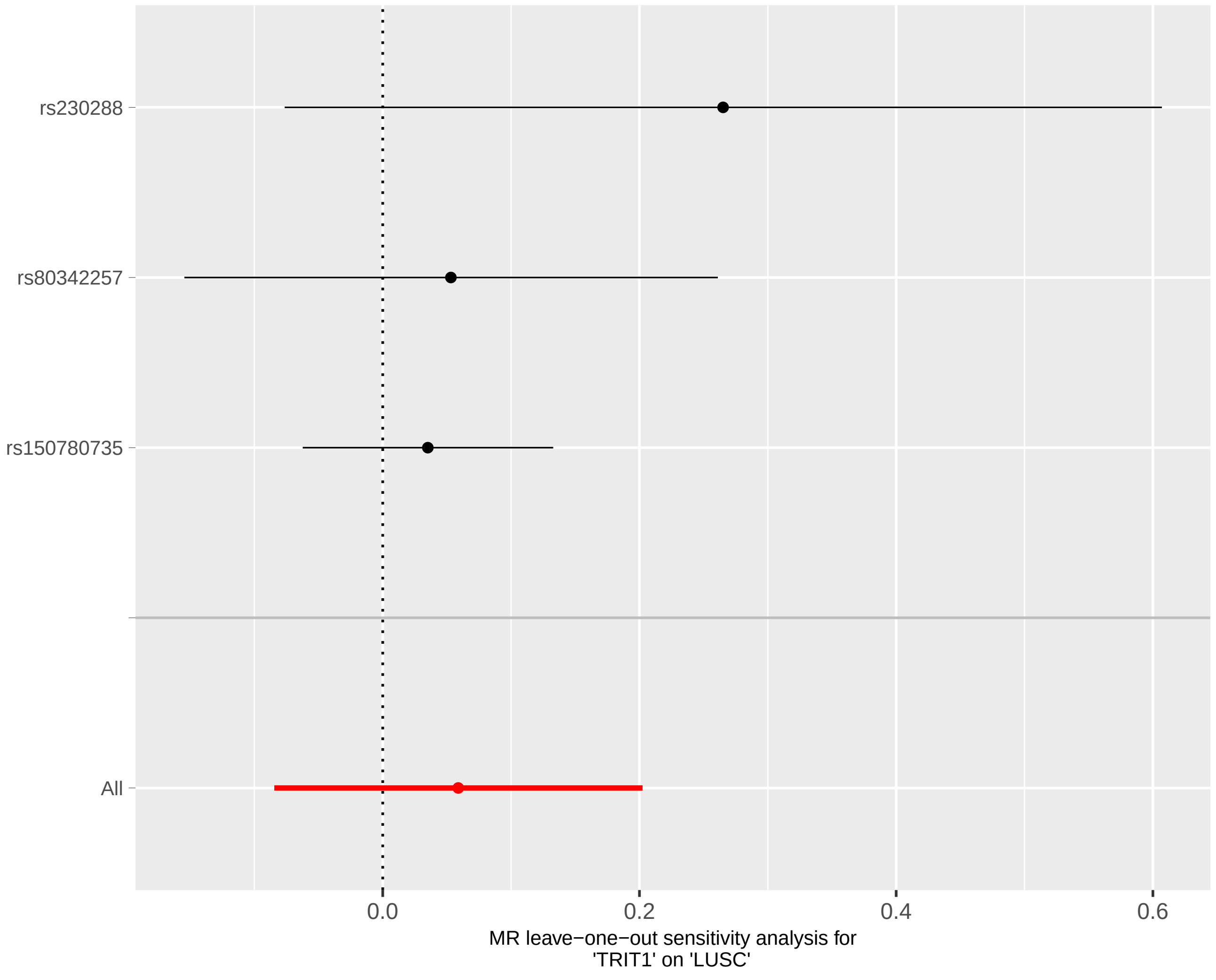
rs230288
rs80342257
rs150780735
All
0.0
0.2
0.4
0.6
MR leave−one−out sensitivity analysis for
'TRIT1' on 'LUSC'

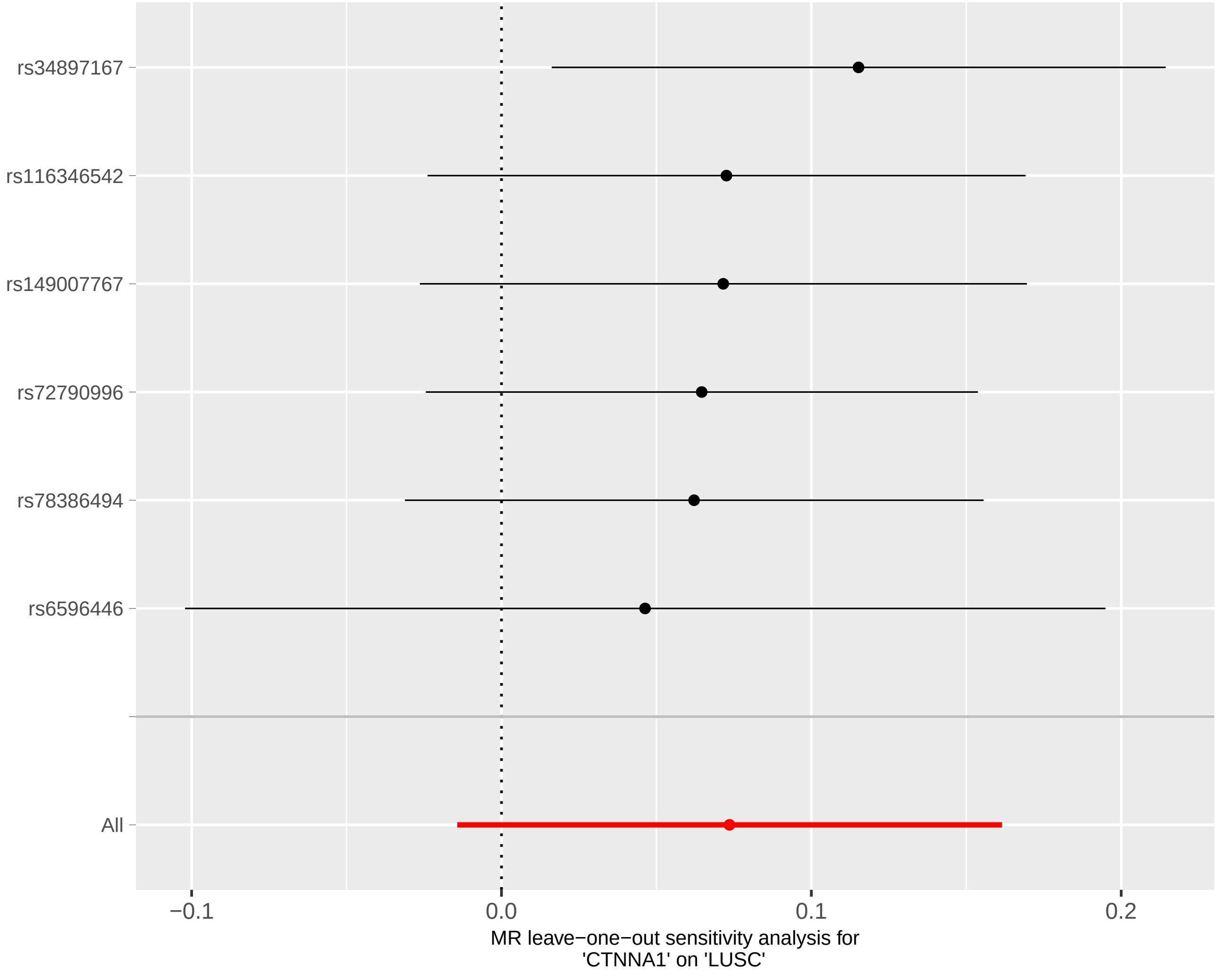
rs34897167
rs116346542
rs149007767
rs72790996
rs78386494
rs6596446
All
−0.1
0.0
0.1
0.2
MR leave−one−out sensitivity analysis for
'CTNNA1' on 'LUSC'

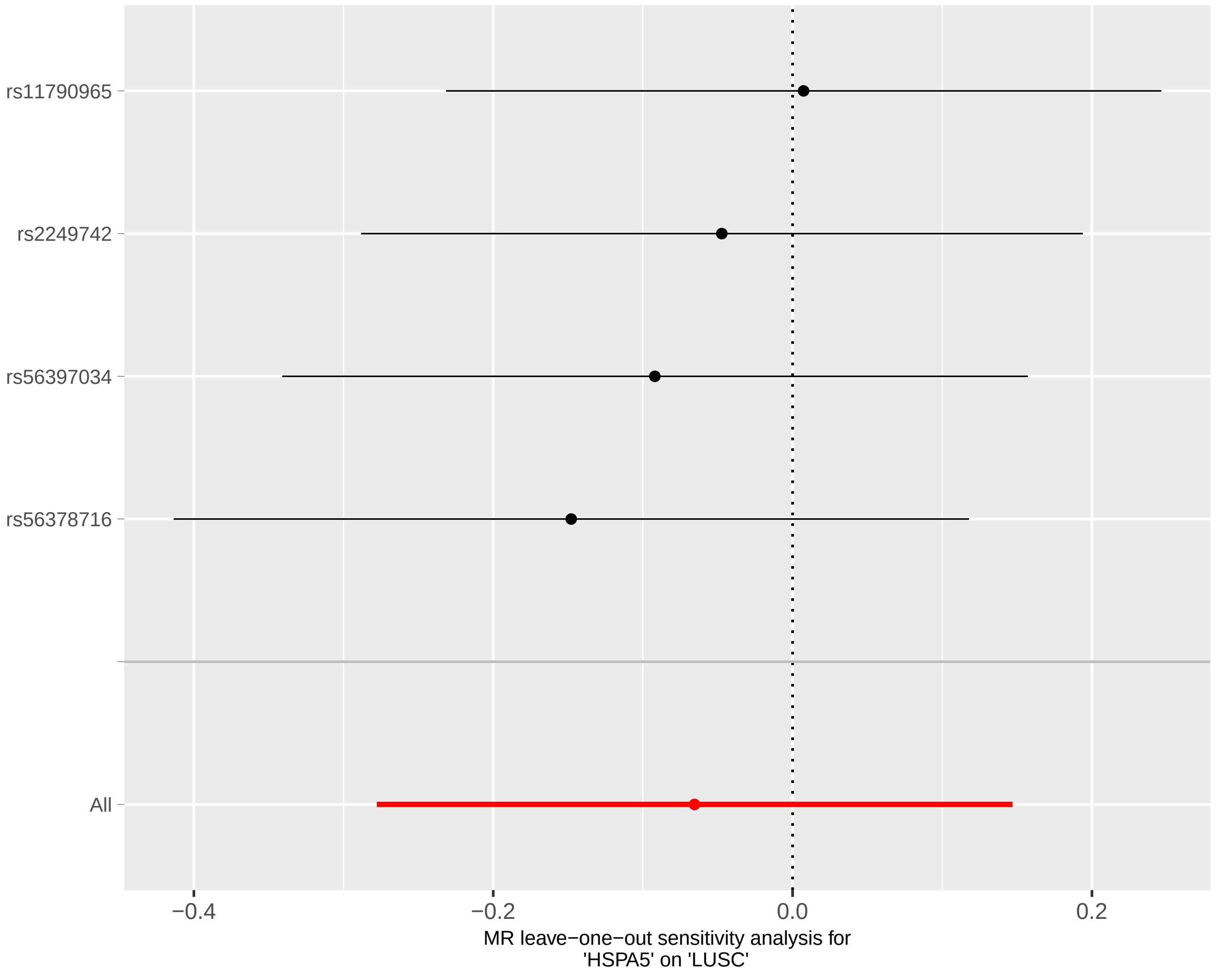
rs11790965
rs2249742
rs56397034
rs56378716
All
−0.4
−0.2
0.0
0.2
MR leave−one−out sensitivity analysis for
'HSPA5' on 'LUSC'

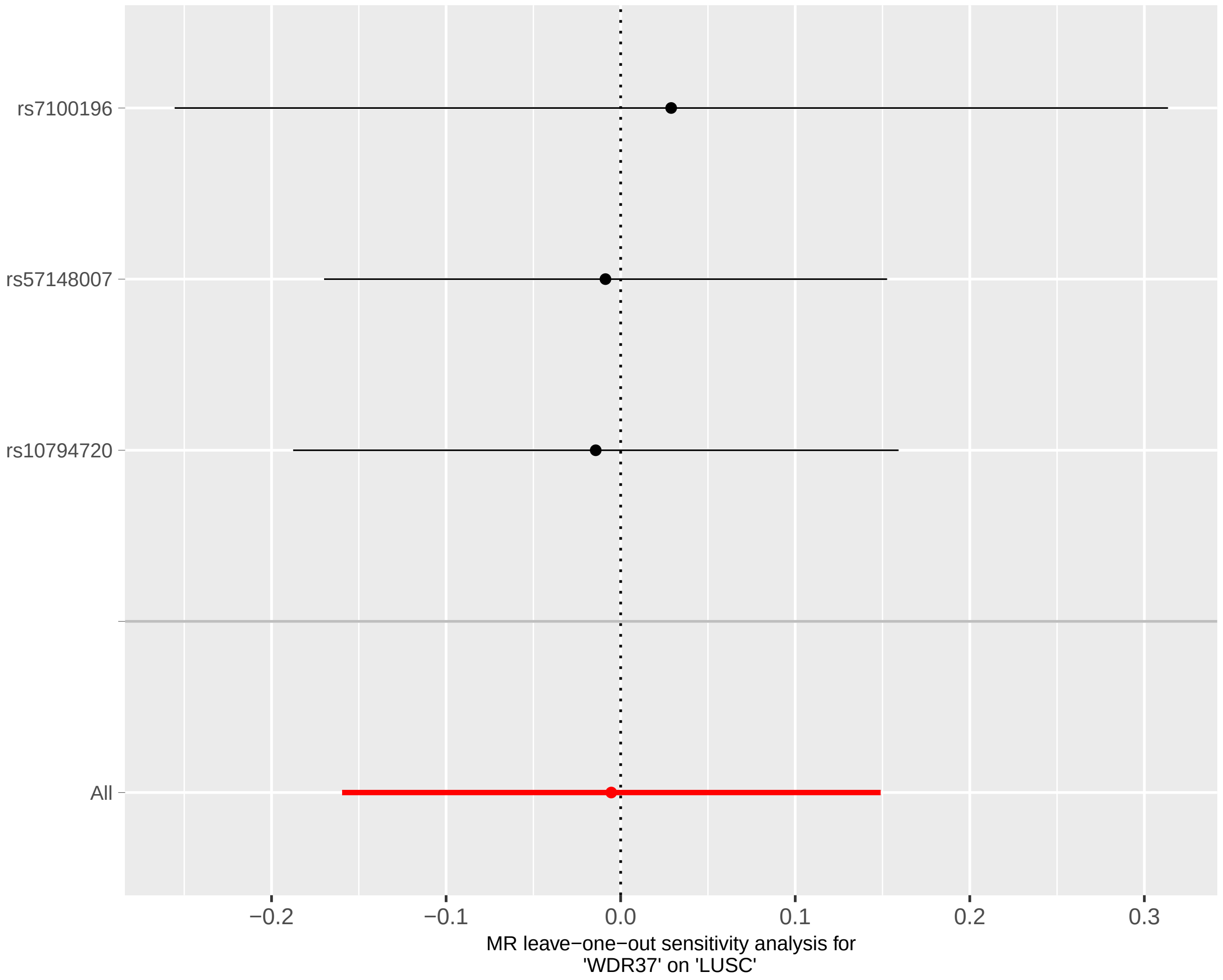
rs7100196
rs57148007
rs10794720
All
−0.2
−0.1
0.0
0.1
0.2
0.3
MR leave−one−out sensitivity analysis for
'WDR37' on 'LUSC'

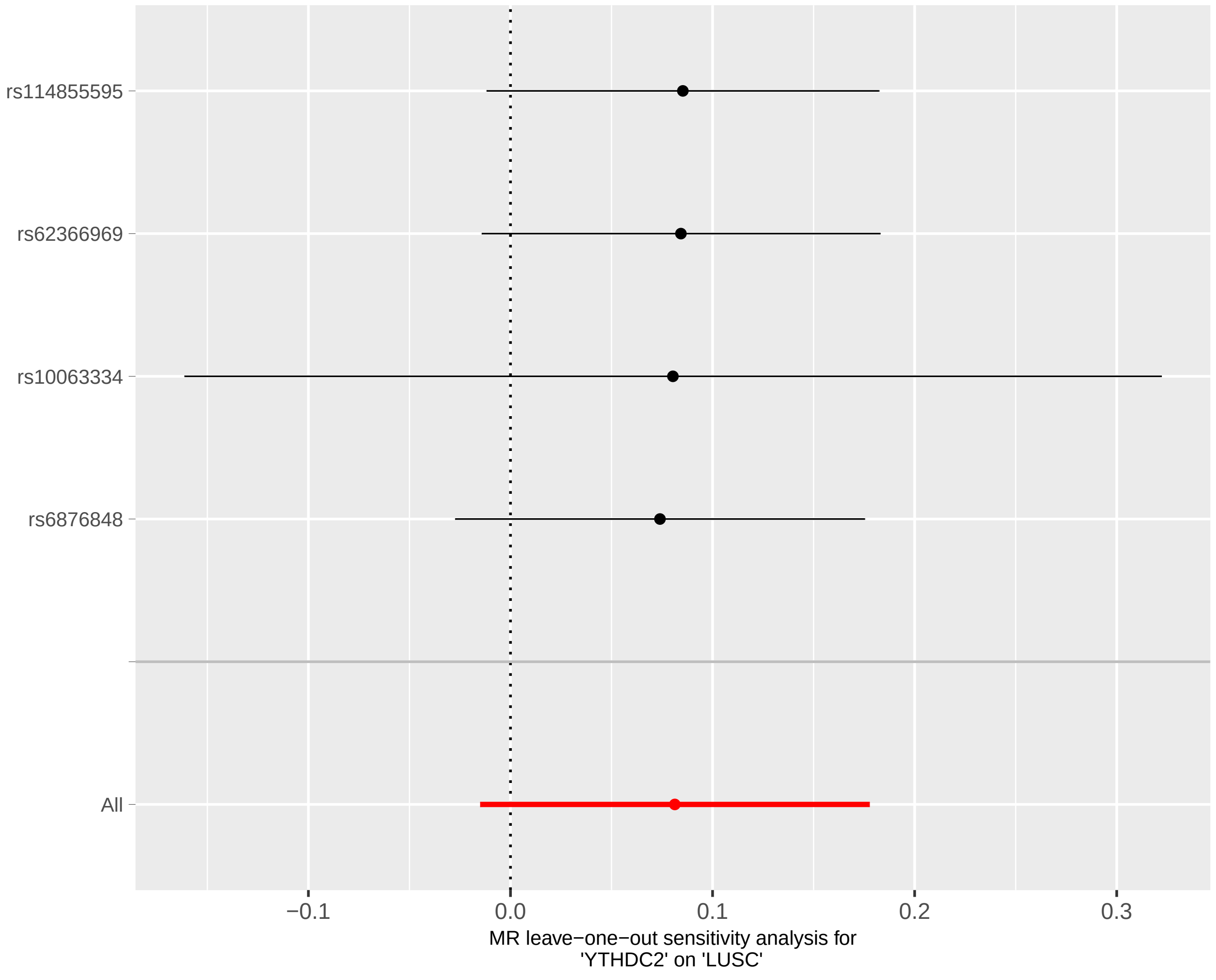
rs114855595
rs62366969
rs10063334
rs6876848
All
−0.1
0.0
0.1
0.2
0.3
MR leave−one−out sensitivity analysis for
'YTHDC2' on 'LUSC'

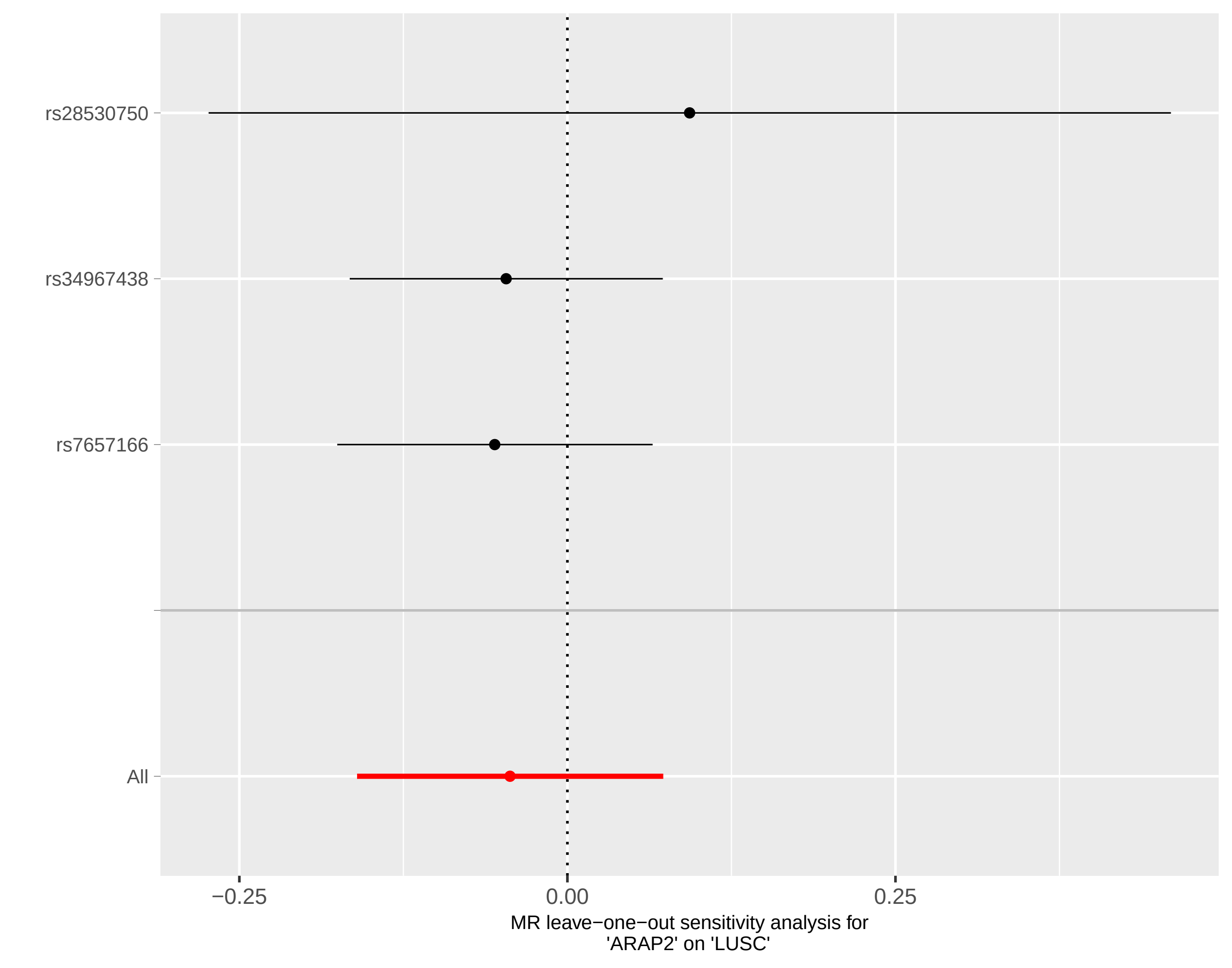

rs28530750
rs34967438
rs7657166
All
−0.25
0.00
0.25
MR leave−one−out sensitivity analysis for
'ARAP2' on 'LUSC'

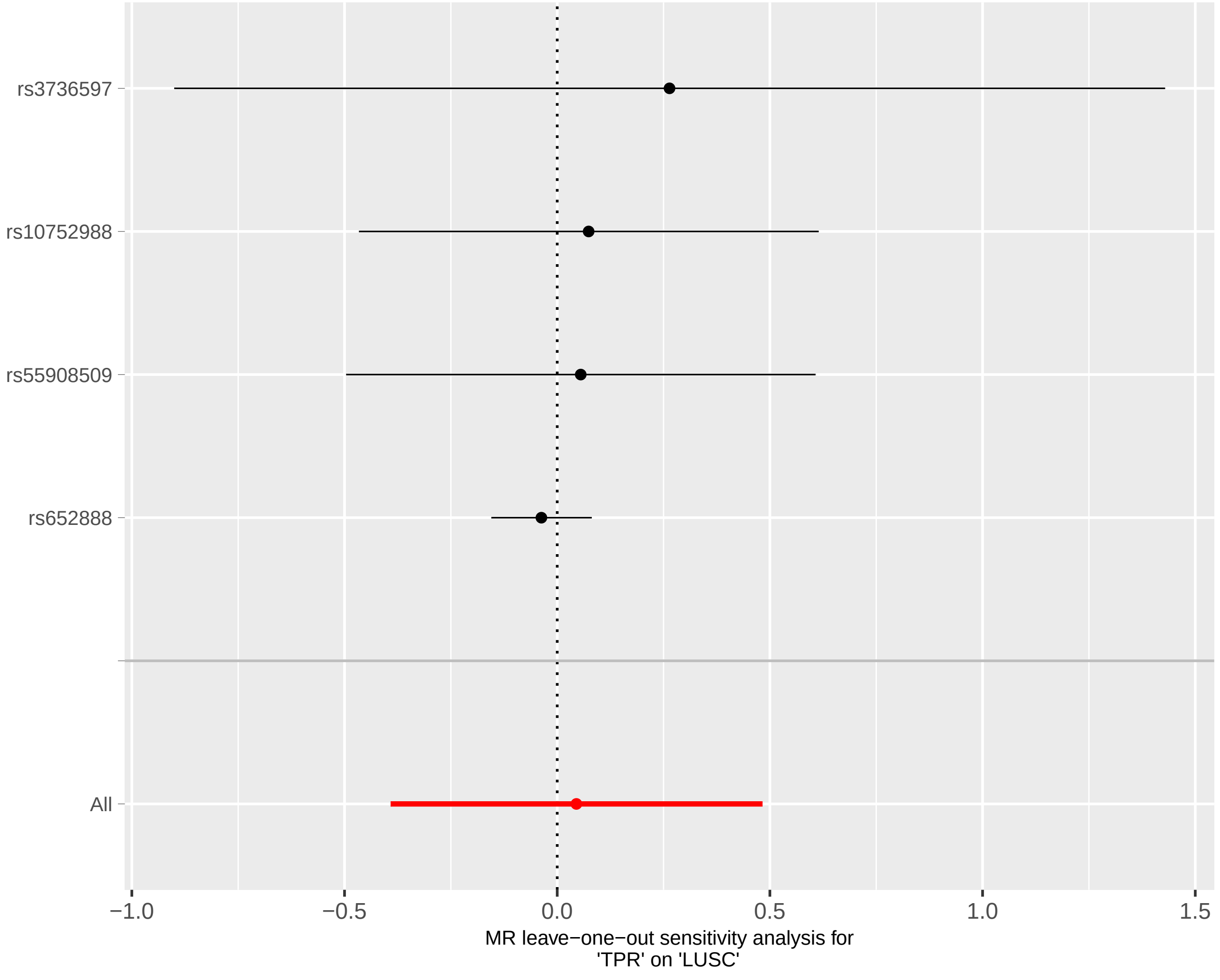
rs3736597
rs10752988
rs55908509
rs652888
All
−1.0
−0.5
0.0
0.5
1.0
1.5
MR leave−one−out sensitivity analysis for
'TPR' on 'LUSC'

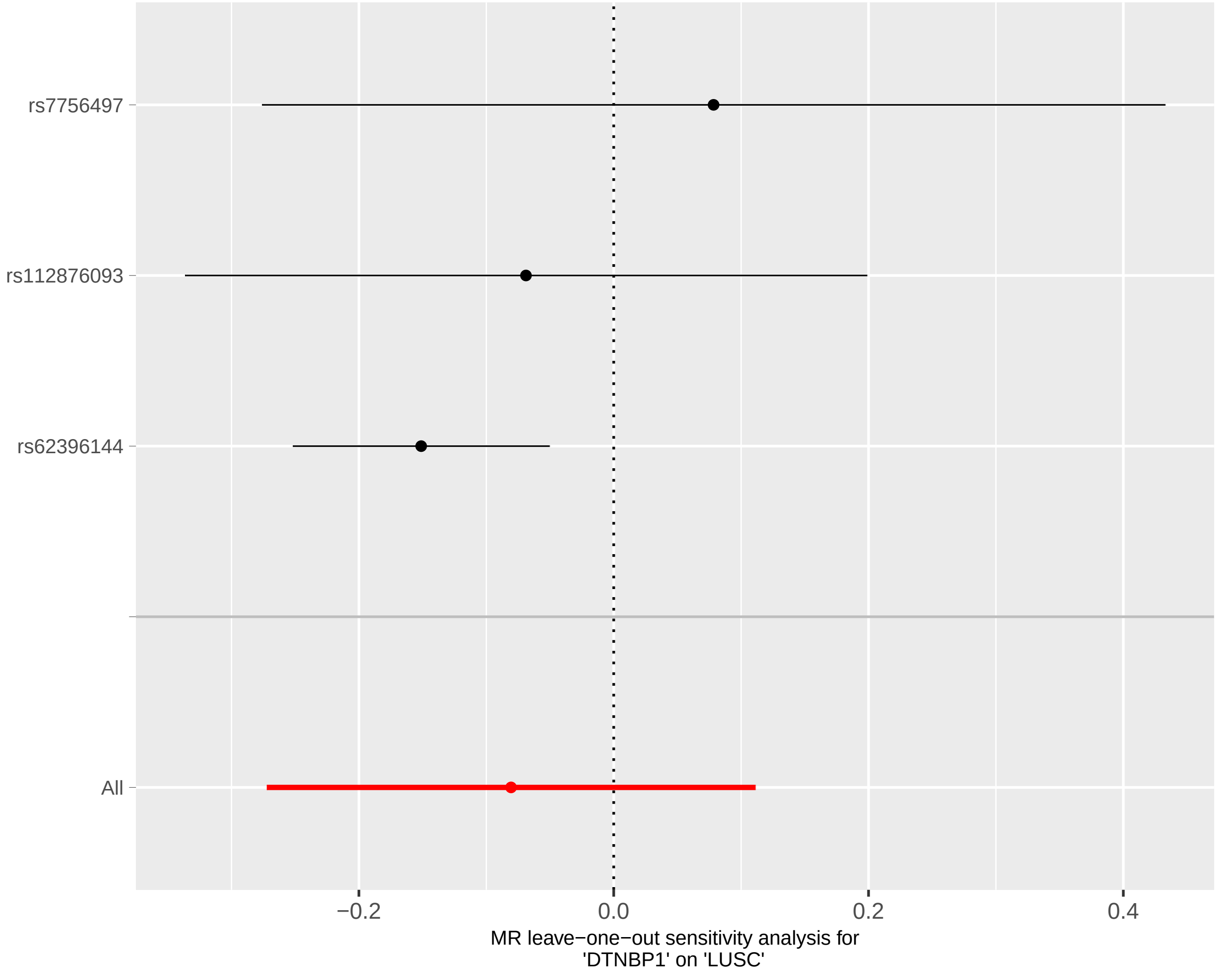

rs7756497
rs112876093
rs62396144
All
−0.2
0.0
0.2
0.4
MR leave−one−out sensitivity analysis for
'DTNBP1' on 'LUSC'

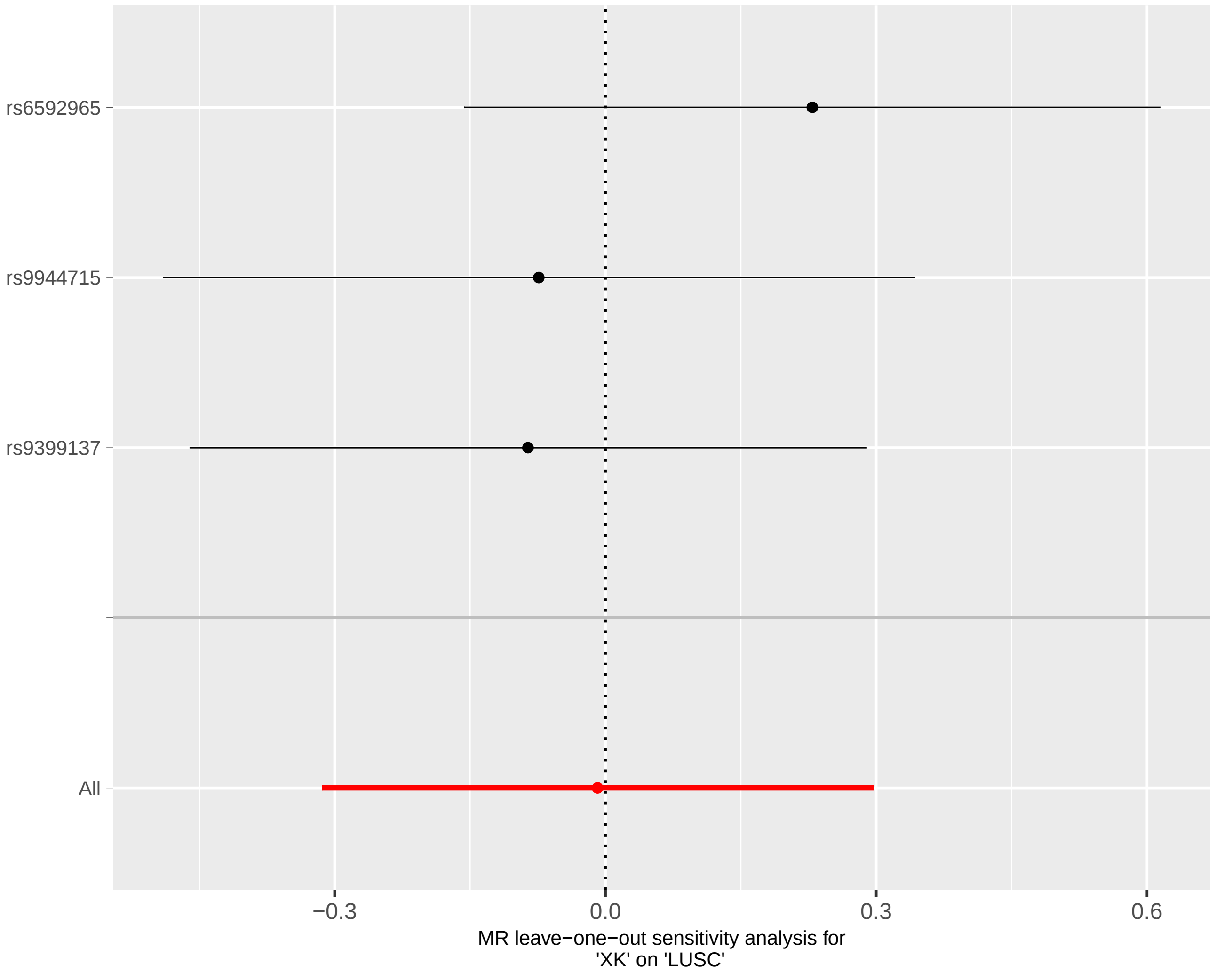
rs6592965
rs9944715
rs9399137
All
−0.3
0.0
0.3
0.6
MR leave−one−out sensitivity analysis for
'XK' on 'LUSC'

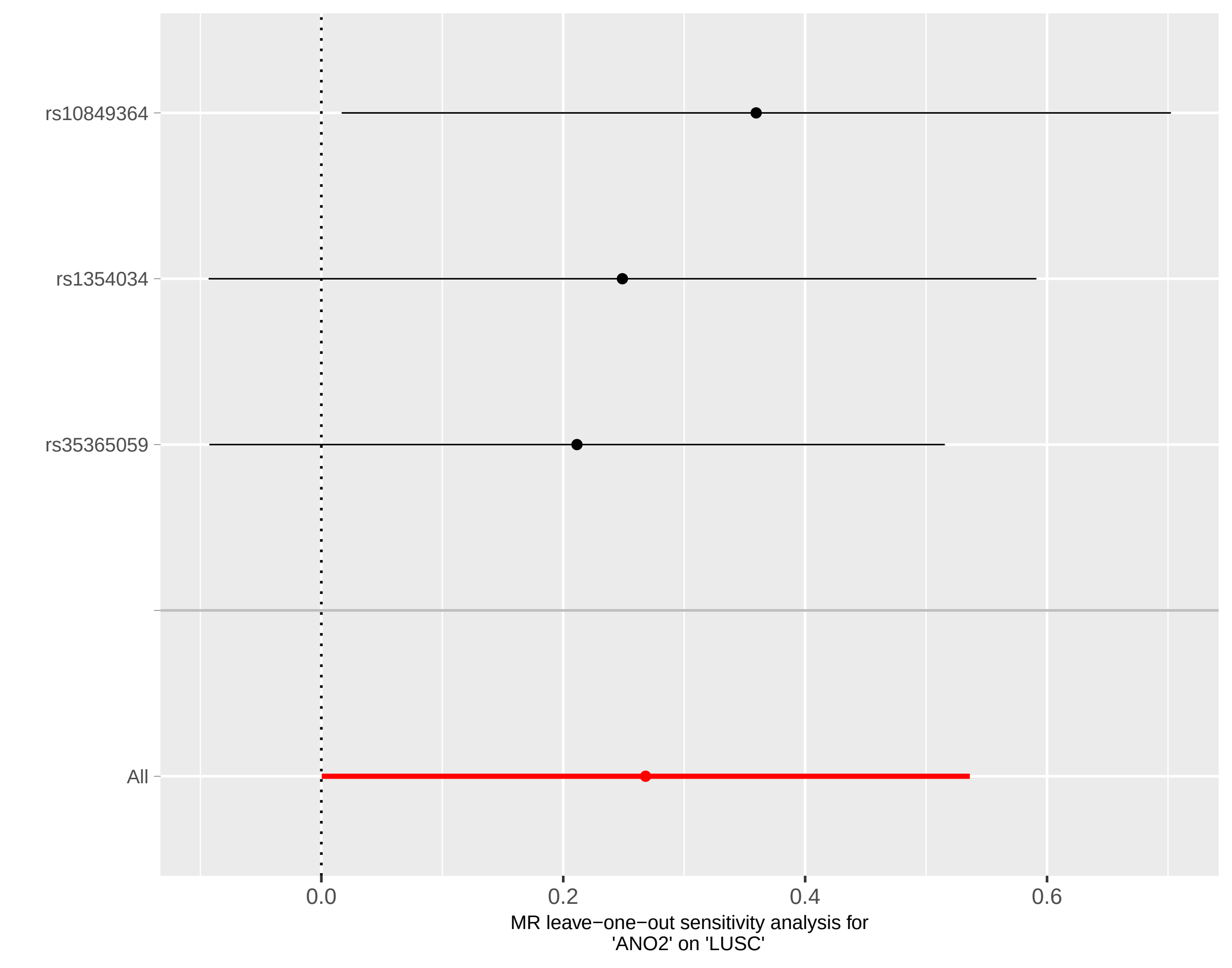
rs10849364
rs1354034
rs35365059
All
0.0
0.2
0.4
0.6
MR leave−one−out sensitivity analysis for
'ANO2' on 'LUSC'

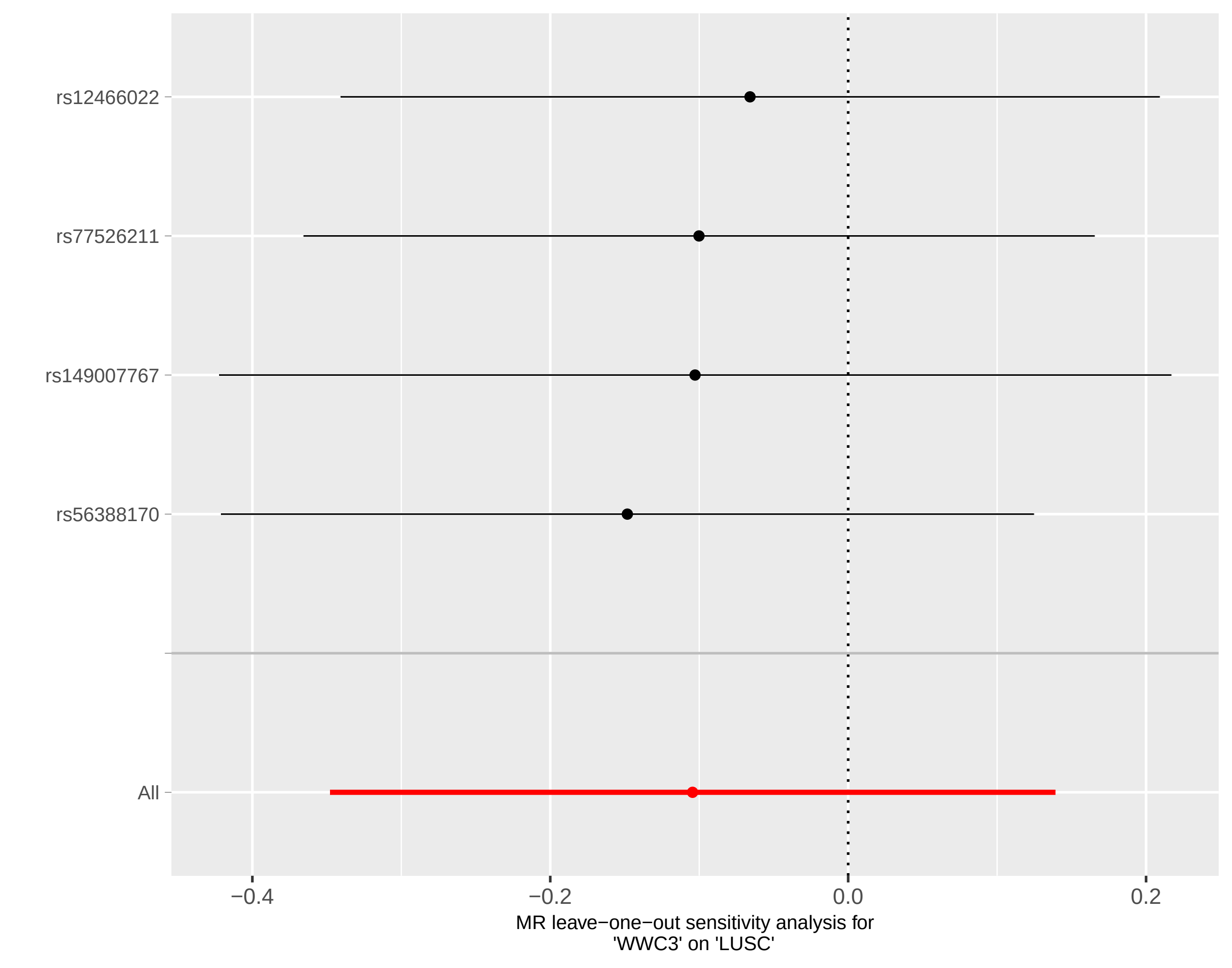
rs12466022
rs77526211
rs149007767
rs56388170
All
−0.4
−0.2
0.0
0.2
MR leave−one−out sensitivity analysis for
'WWC3' on 'LUSC'

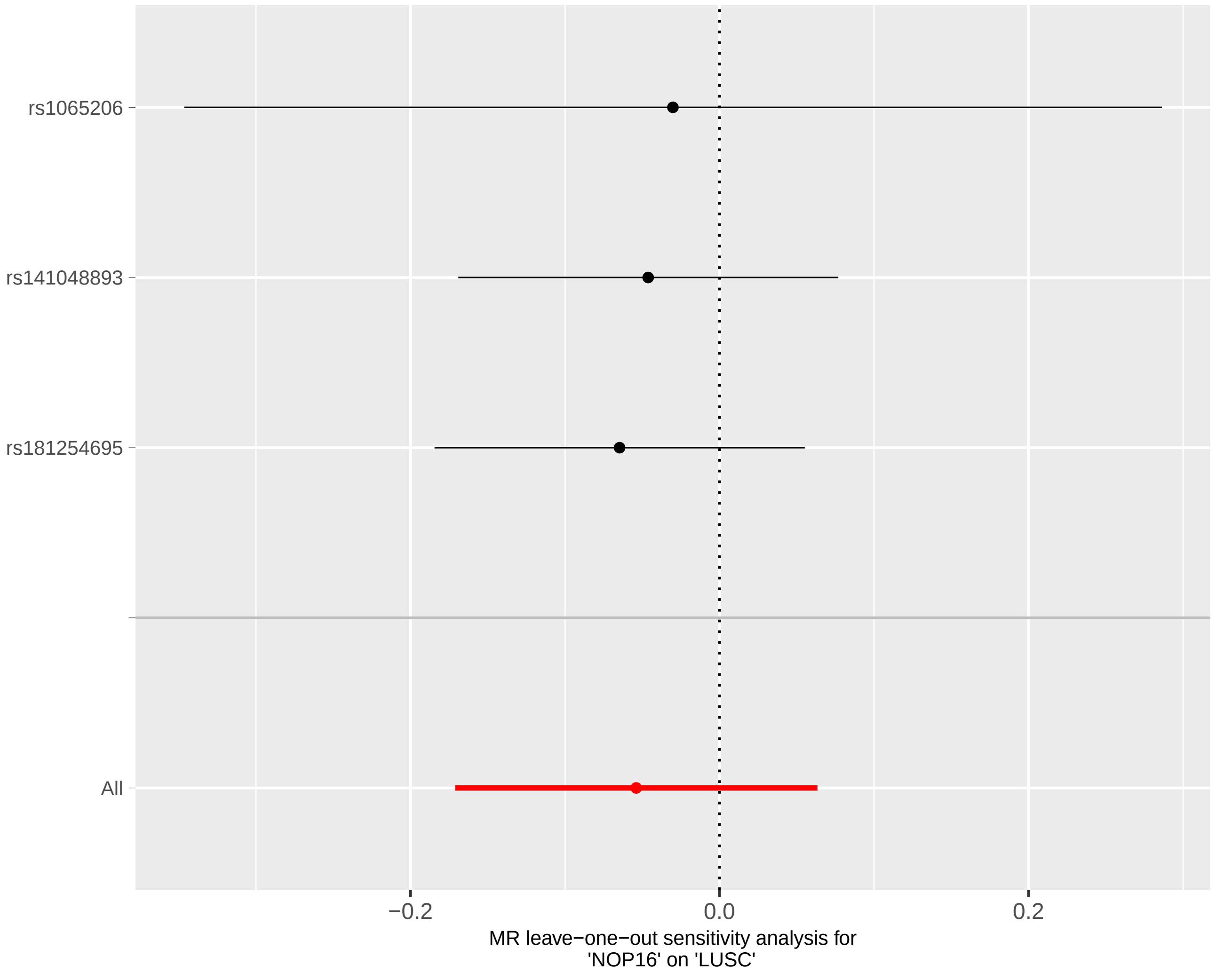
rs1065206
rs141048893
rs181254695
All
−0.2
0.0
0.2
MR leave−one−out sensitivity analysis for
'NOP16' on 'LUSC'

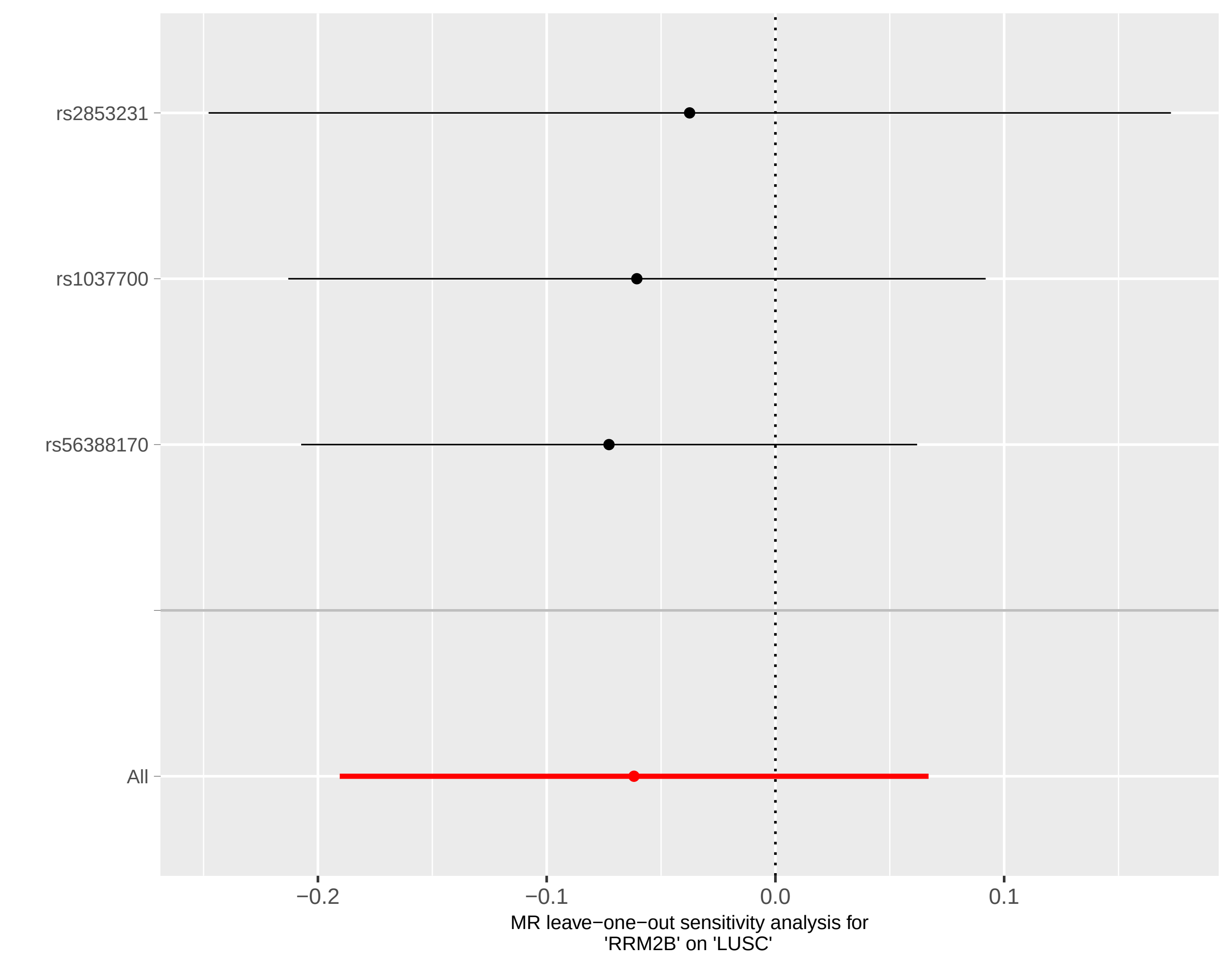
rs2853231
rs1037700
rs56388170
All
−0.2
−0.1
0.0
0.1
MR leave−one−out sensitivity analysis for
'RRM2B' on 'LUSC'

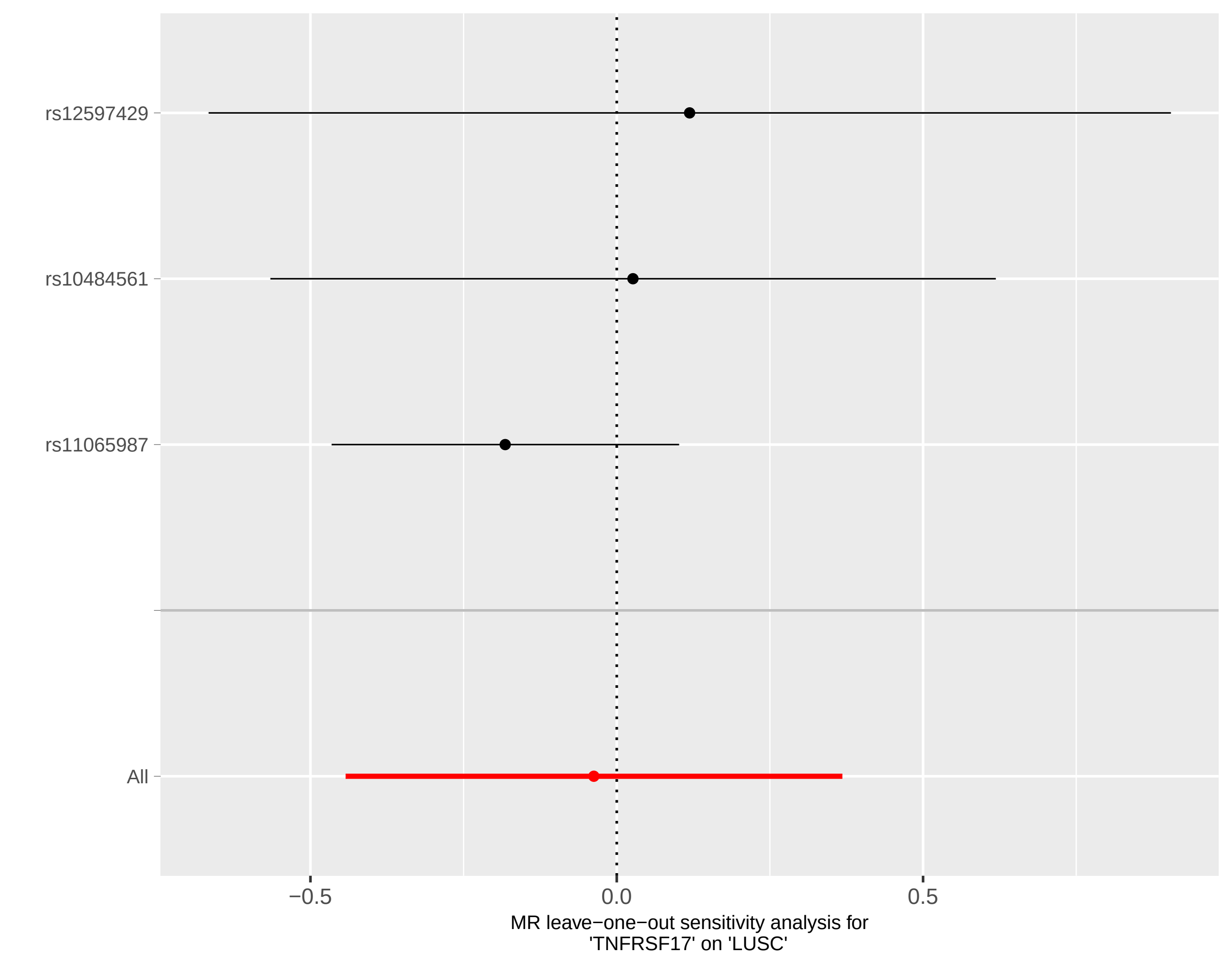
rs12597429
rs10484561
rs11065987
All
−0.5
0.0
0.5
MR leave−one−out sensitivity analysis for
'TNFRSF17' on 'LUSC'

Insufficient number of SNPs

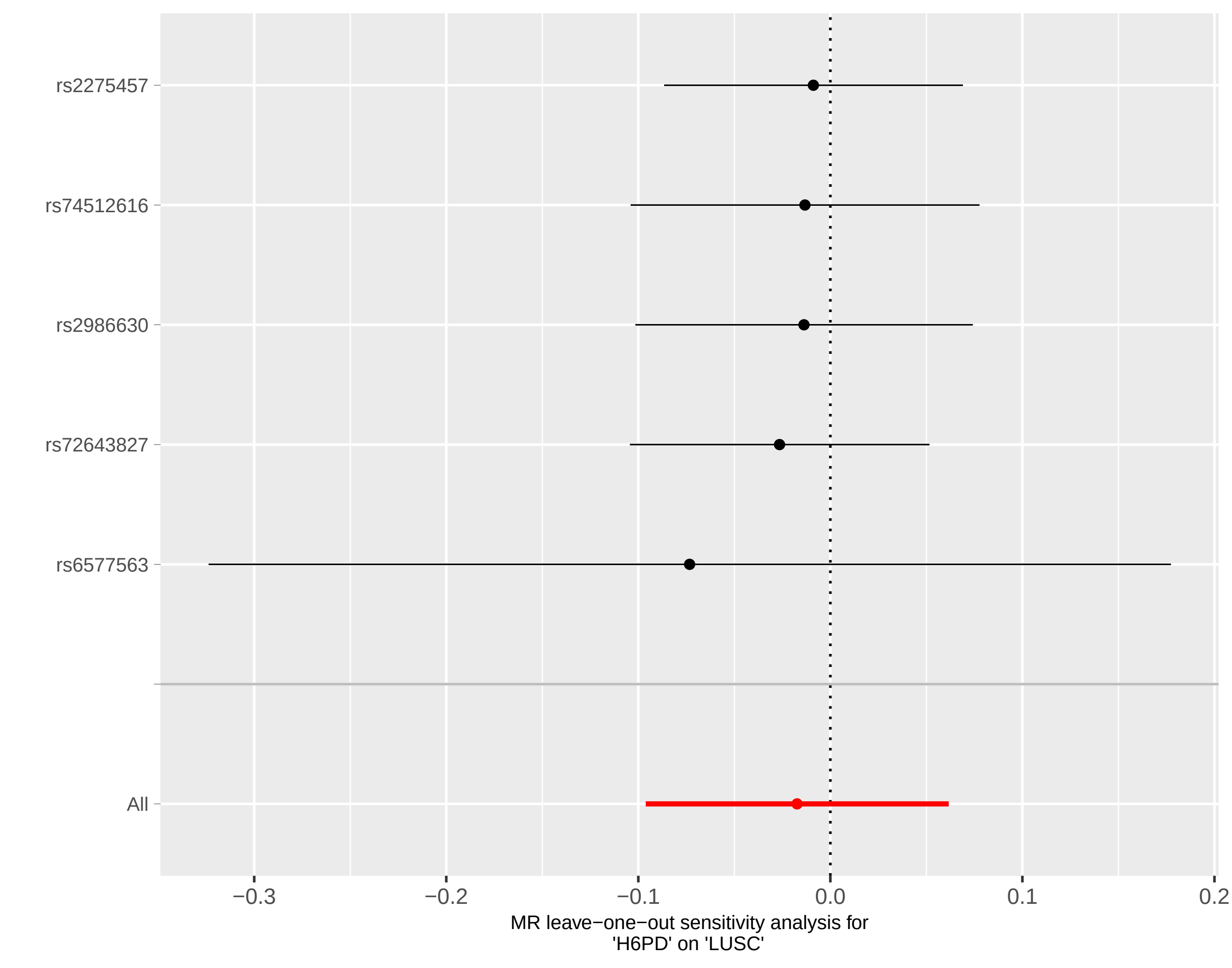
rs2275457
rs74512616
rs2986630
rs72643827
rs6577563
All
−0.3
−0.2
−0.1
0.0
0.1
0.2
MR leave−one−out sensitivity analysis for
'H6PD' on 'LUSC'

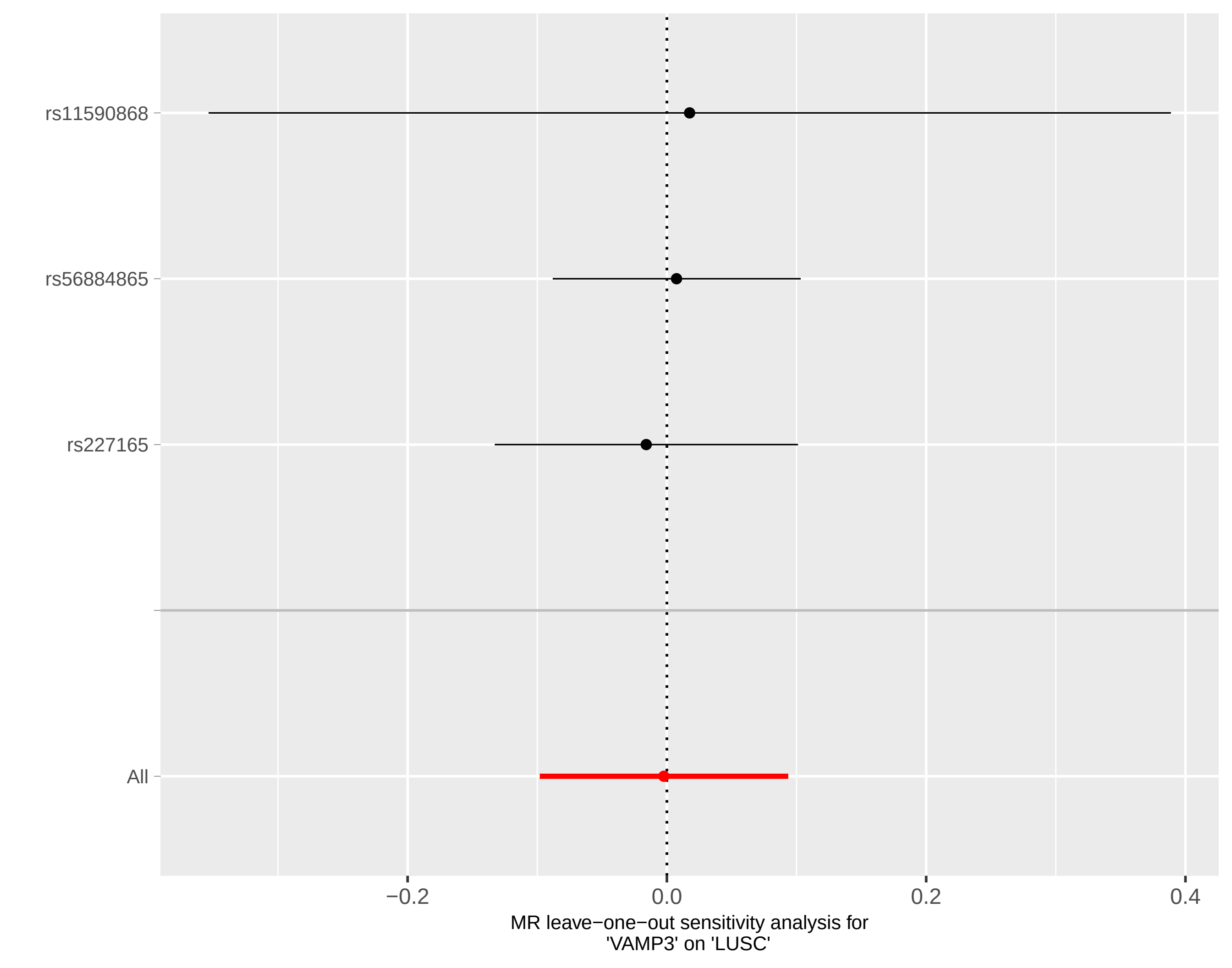
rs11590868
rs56884865
rs227165
All
−0.2
0.0
0.2
0.4
MR leave−one−out sensitivity analysis for
'VAMP3' on 'LUSC'

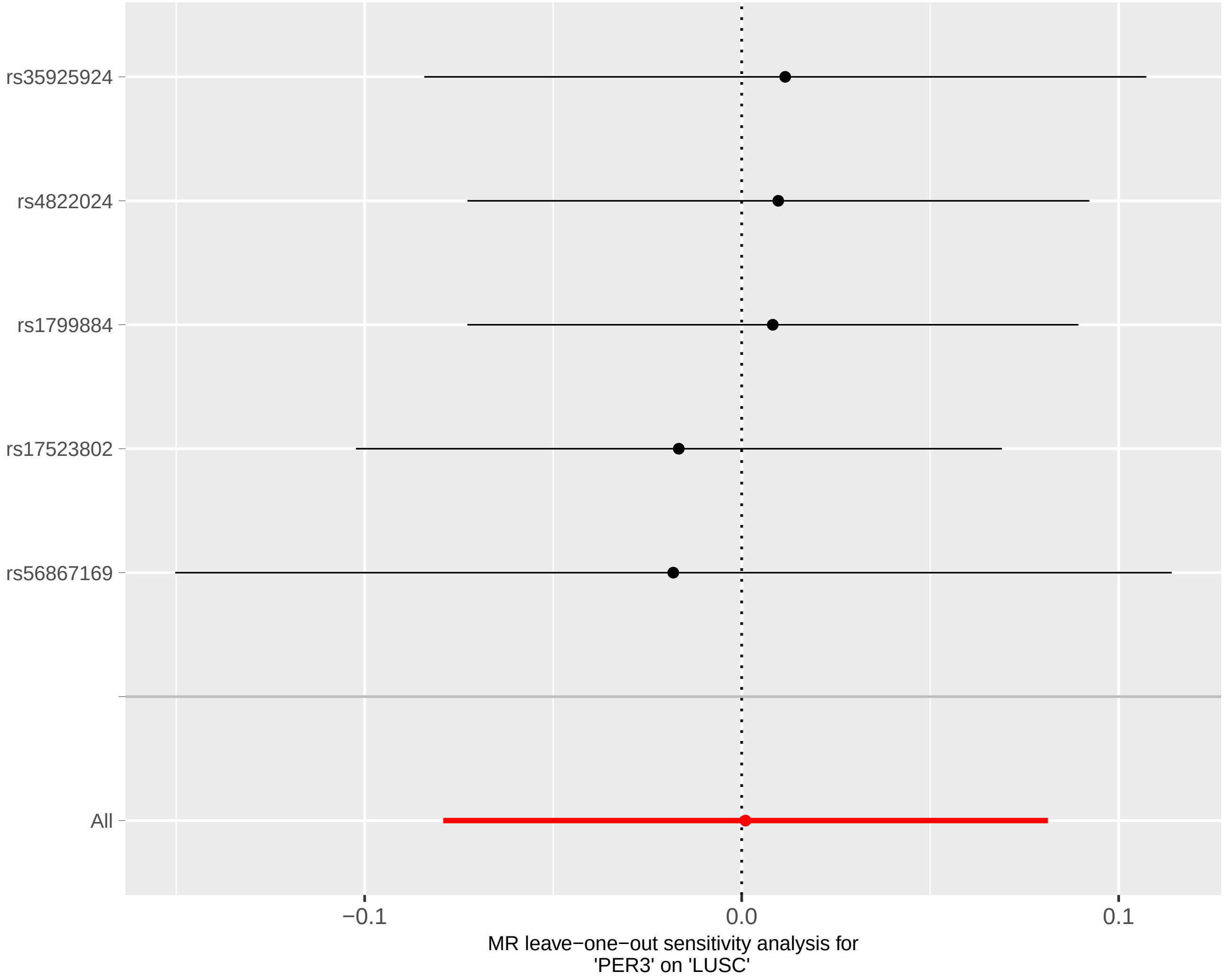
rs35925924
rs4822024
rs1799884
rs17523802
rs56867169
All
−0.1
0.0
0.1
MR leave−one−out sensitivity analysis for
'PER3' on 'LUSC'

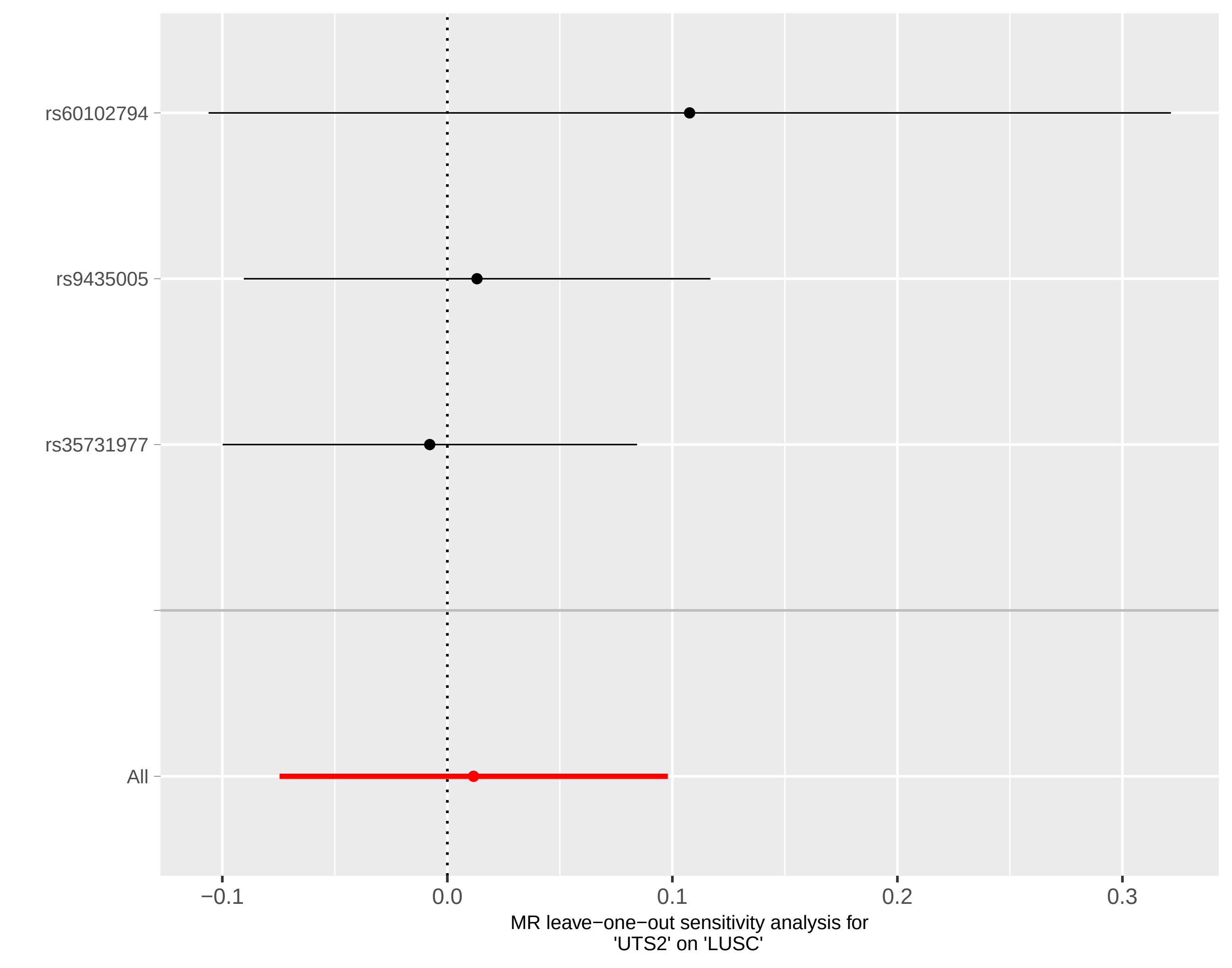
rs60102794
rs9435005
rs35731977
All
−0.1
0.0
0.1
0.2
0.3
MR leave−one−out sensitivity analysis for
'UTS2' on 'LUSC'

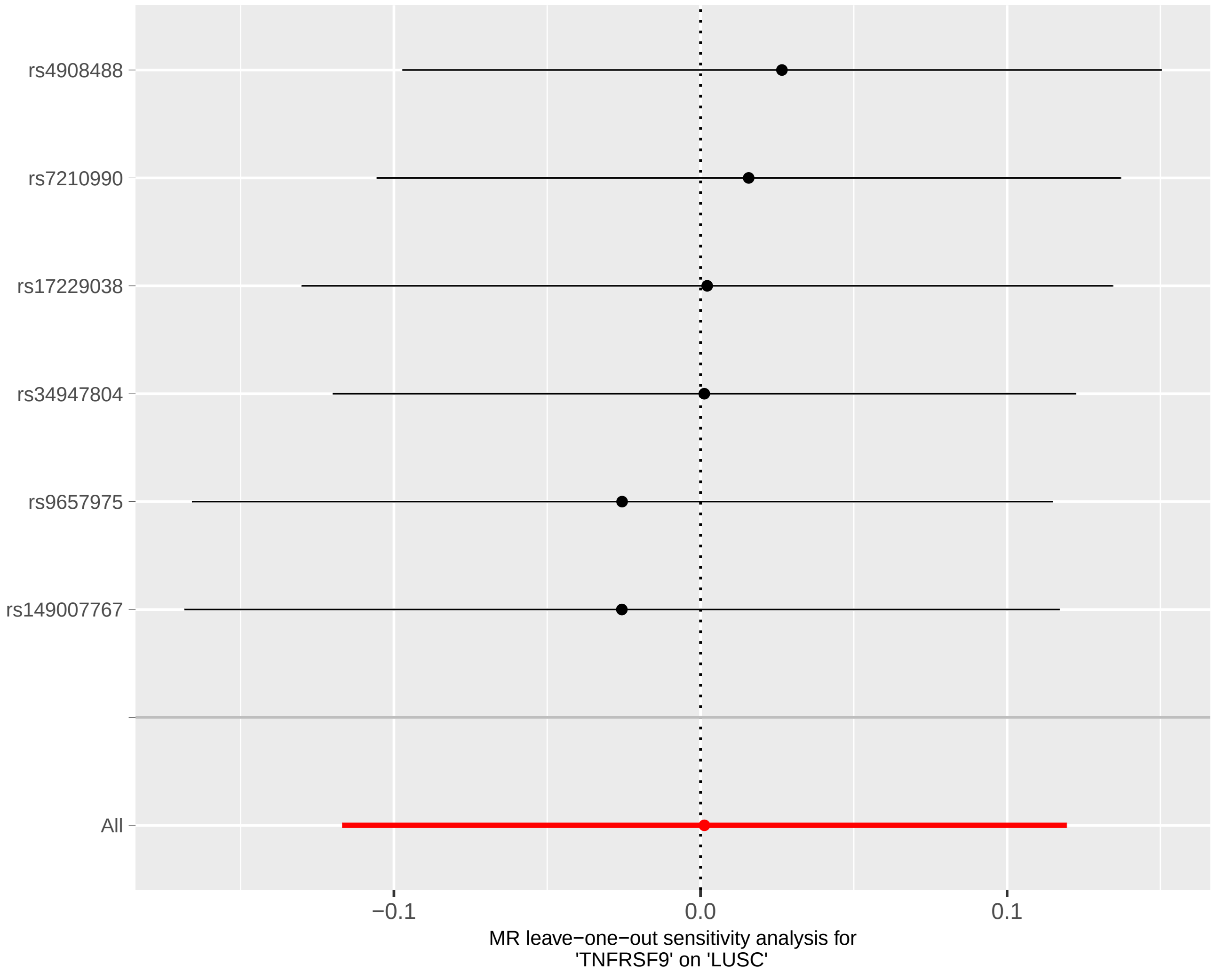
rs4908488
rs7210990
rs17229038
rs34947804
rs9657975
rs149007767
All
−0.1
0.0
0.1
MR leave−one−out sensitivity analysis for
'TNFRSF9' on 'LUSC'

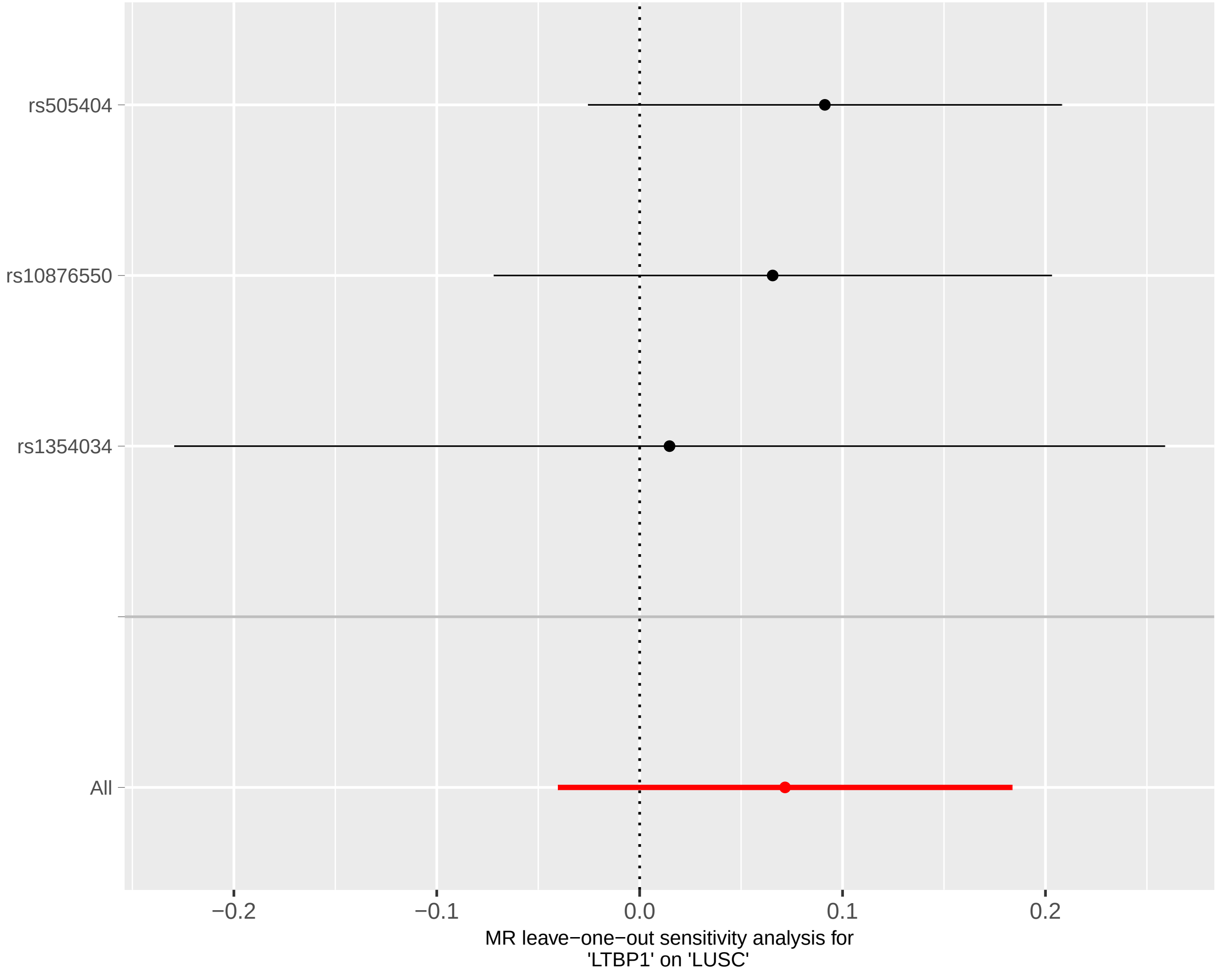
rs505404
rs10876550
rs1354034
All
−0.2
−0.1
0.0
0.1
0.2
MR leave−one−out sensitivity analysis for
'LTBP1' on 'LUSC'

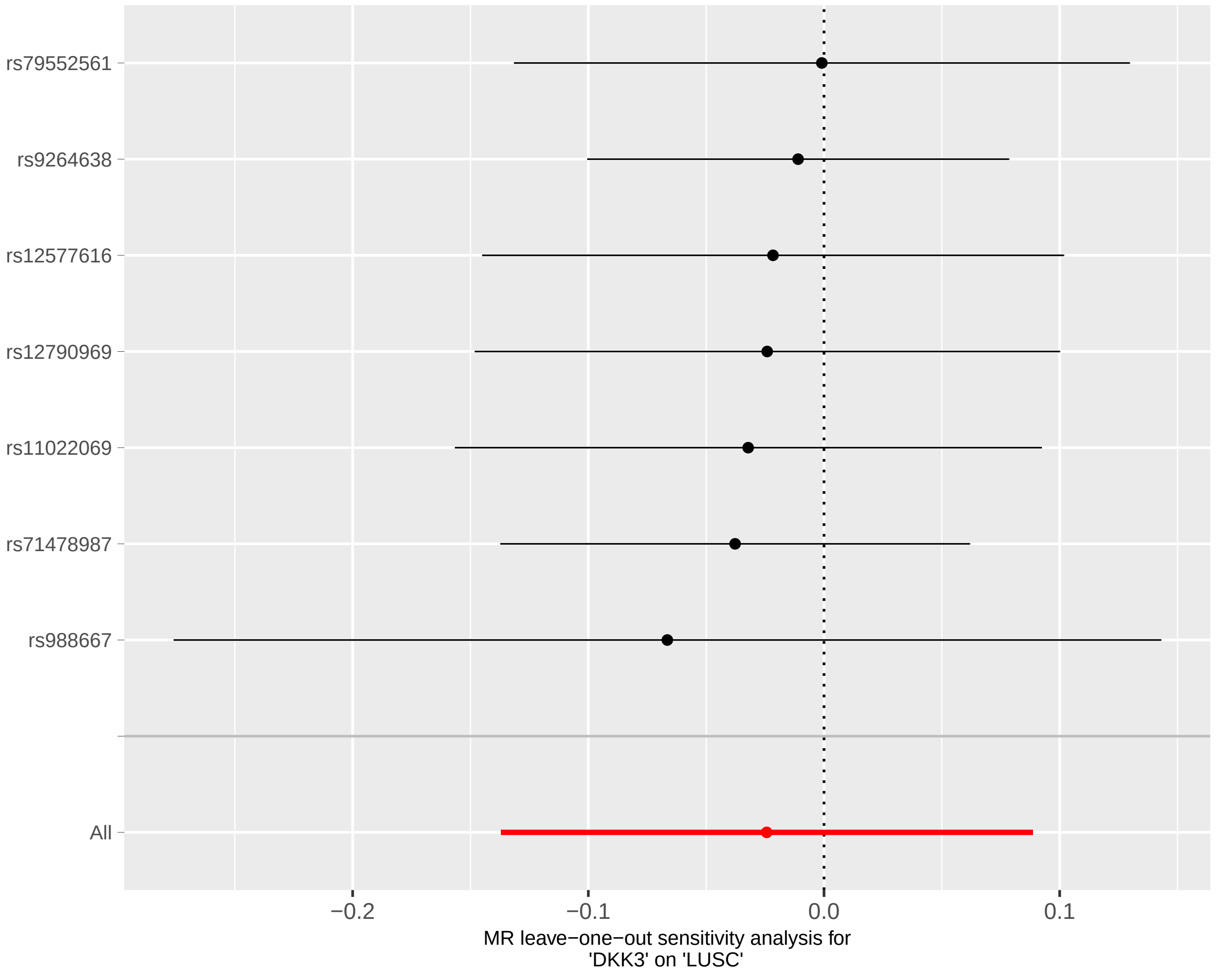
rs79552561
rs9264638
rs12577616
rs12790969
rs11022069
rs71478987
rs988667
All
−0.2
−0.1
0.0
0.1
MR leave−one−out sensitivity analysis for
'DKK3' on 'LUSC'

Insufficient number of SNPs

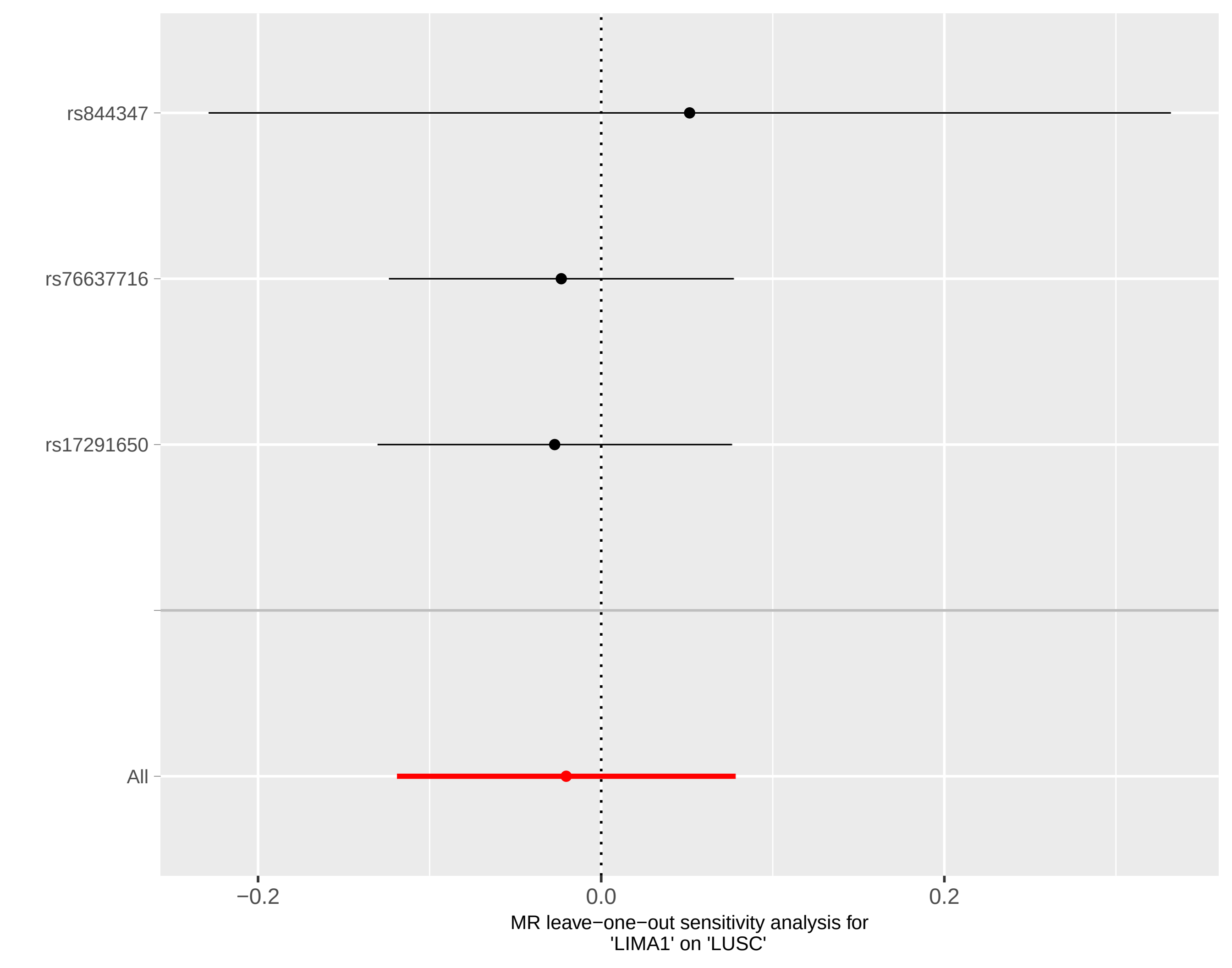
rs844347
rs76637716
rs17291650
All
−0.2
0.0
0.2
MR leave−one−out sensitivity analysis for
'LIMA1' on 'LUSC'

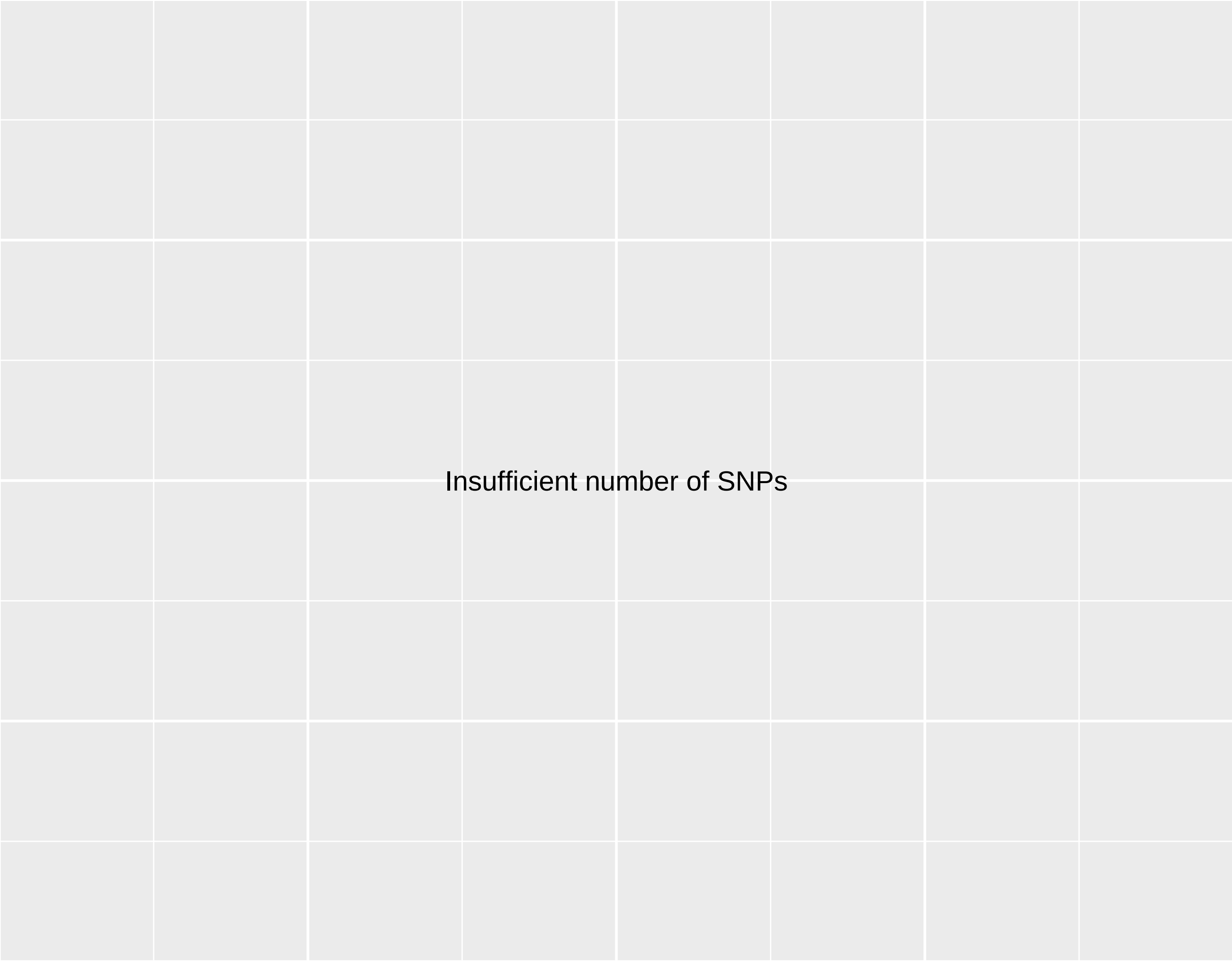
Insufficient number of SNPs

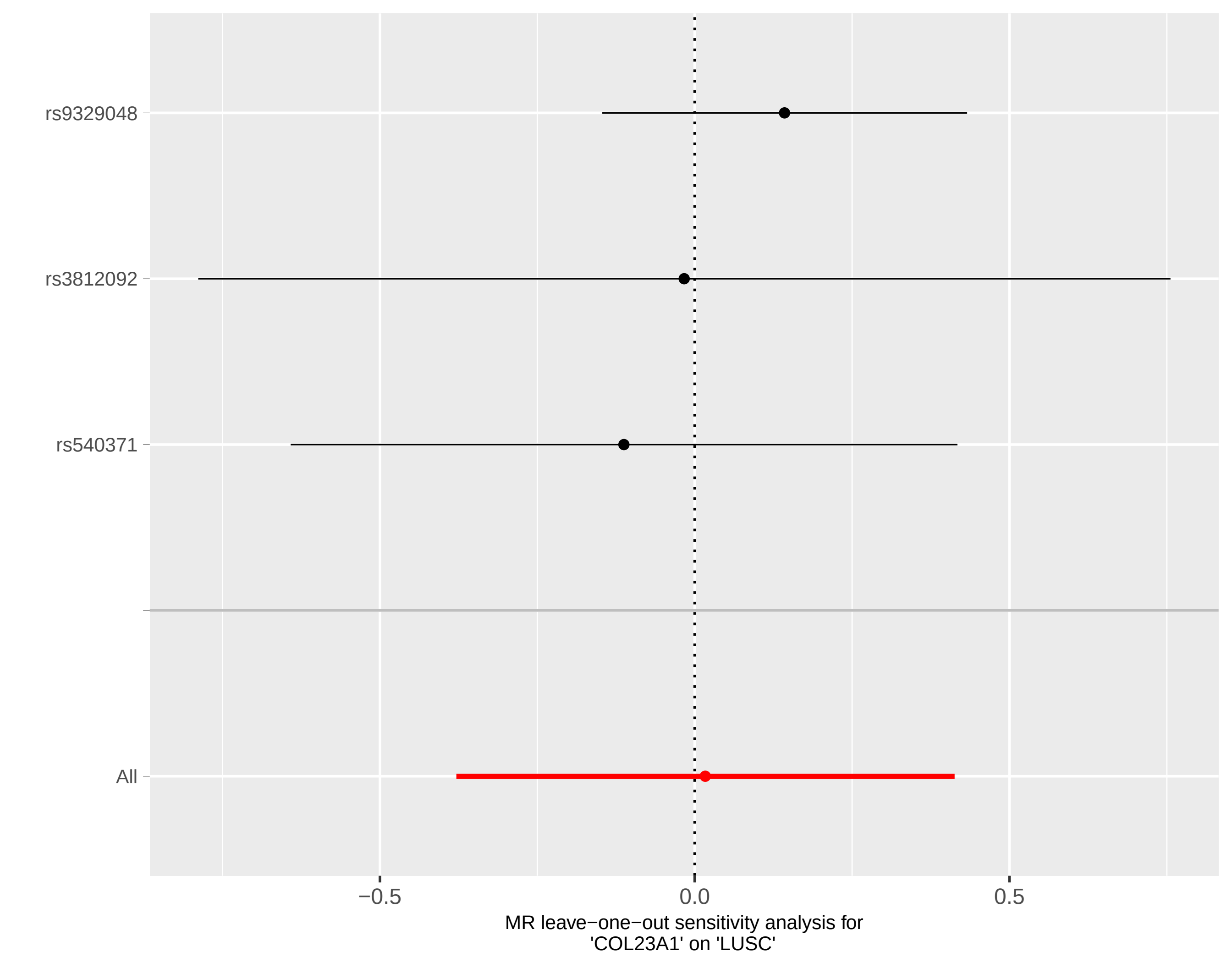
rs9329048
rs3812092
rs540371
All
−0.5
0.0
0.5
MR leave−one−out sensitivity analysis for
'COL23A1' on 'LUSC'

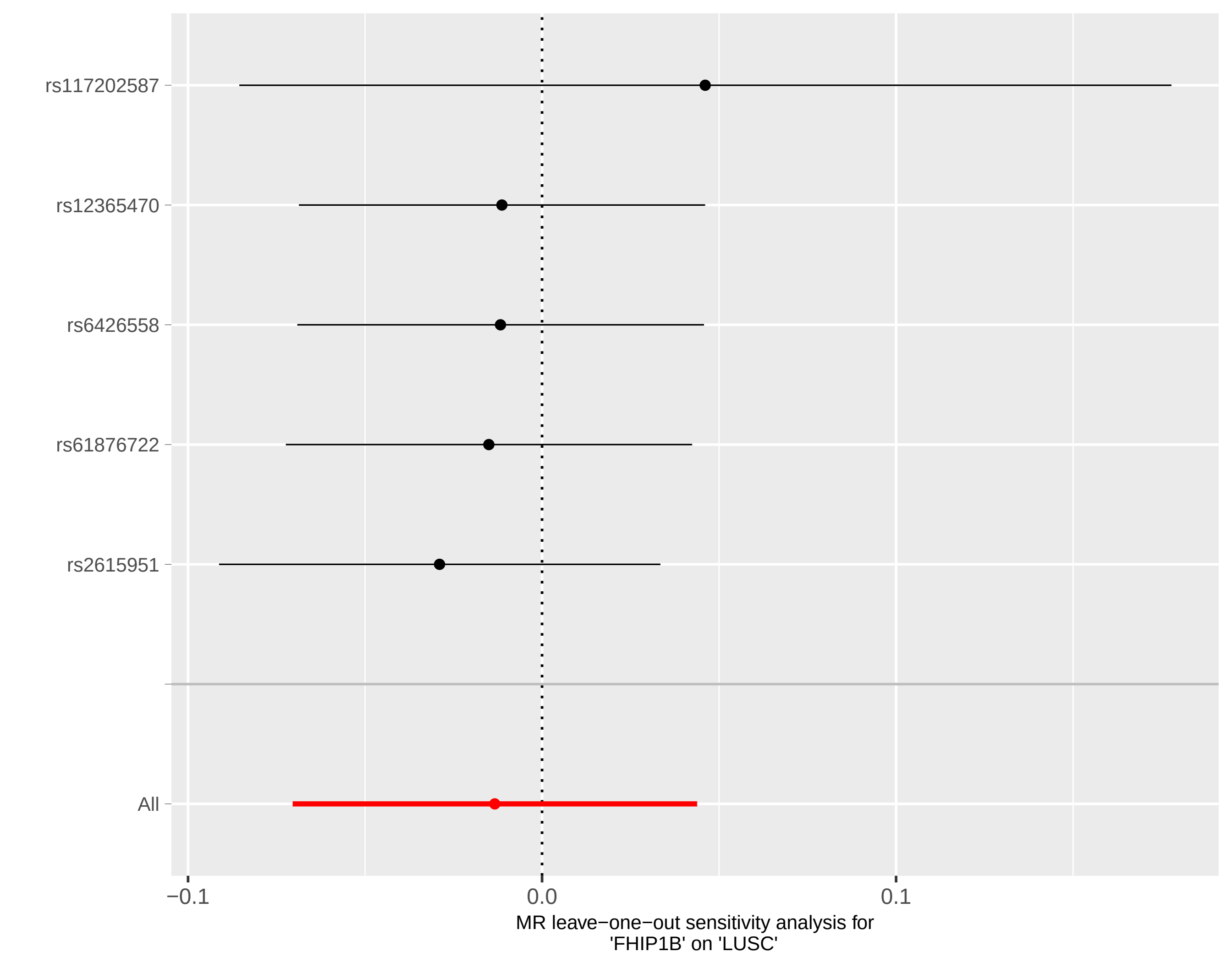
rs117202587
rs12365470
rs6426558
rs61876722
rs2615951
All
−0.1
0.0
0.1
MR leave−one−out sensitivity analysis for
'FHIP1B' on 'LUSC'

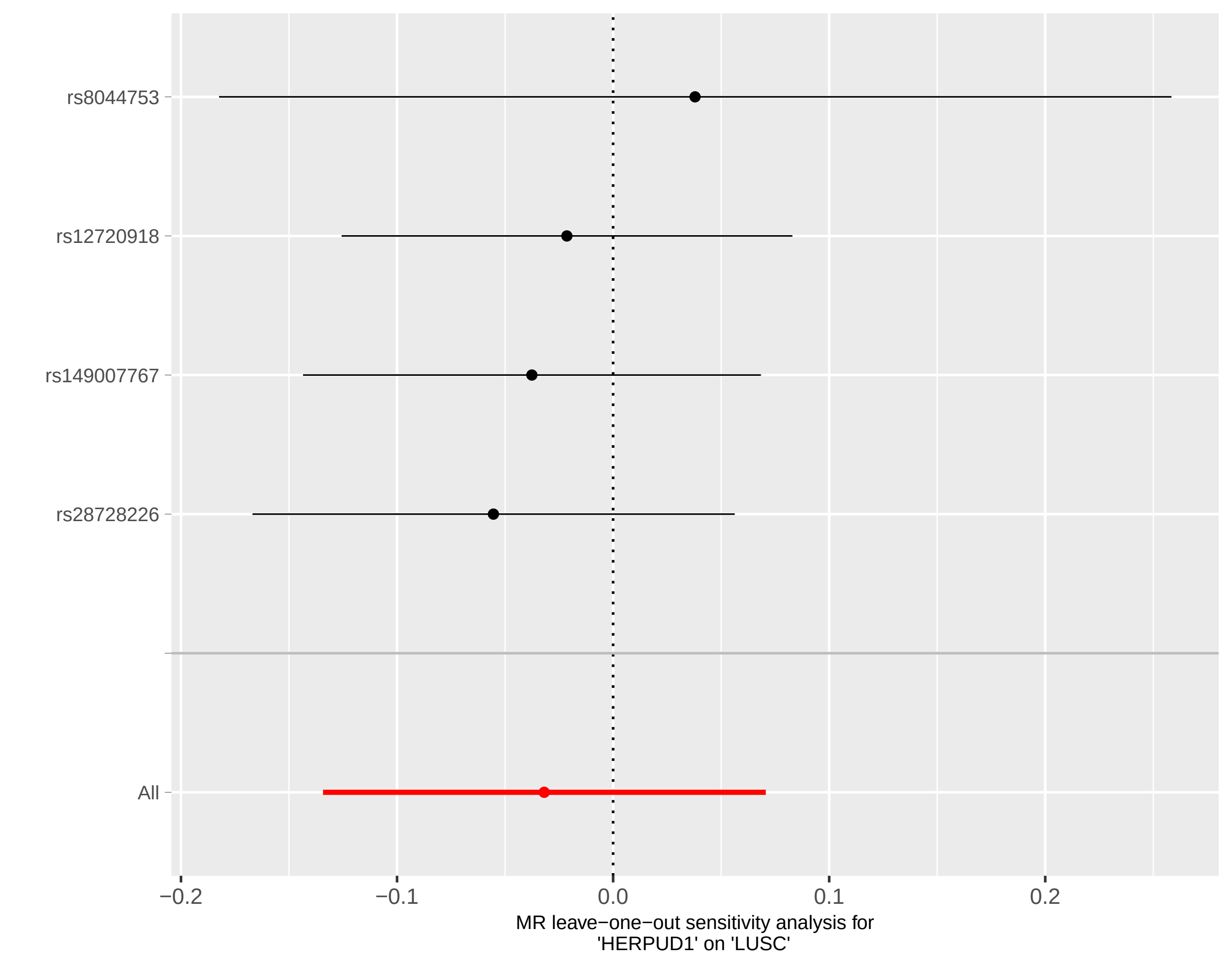

rs8044753
rs12720918
rs149007767
rs28728226
All
−0.2
−0.1
0.0
0.1
0.2
MR leave−one−out sensitivity analysis for
'HERPUD1' on 'LUSC'

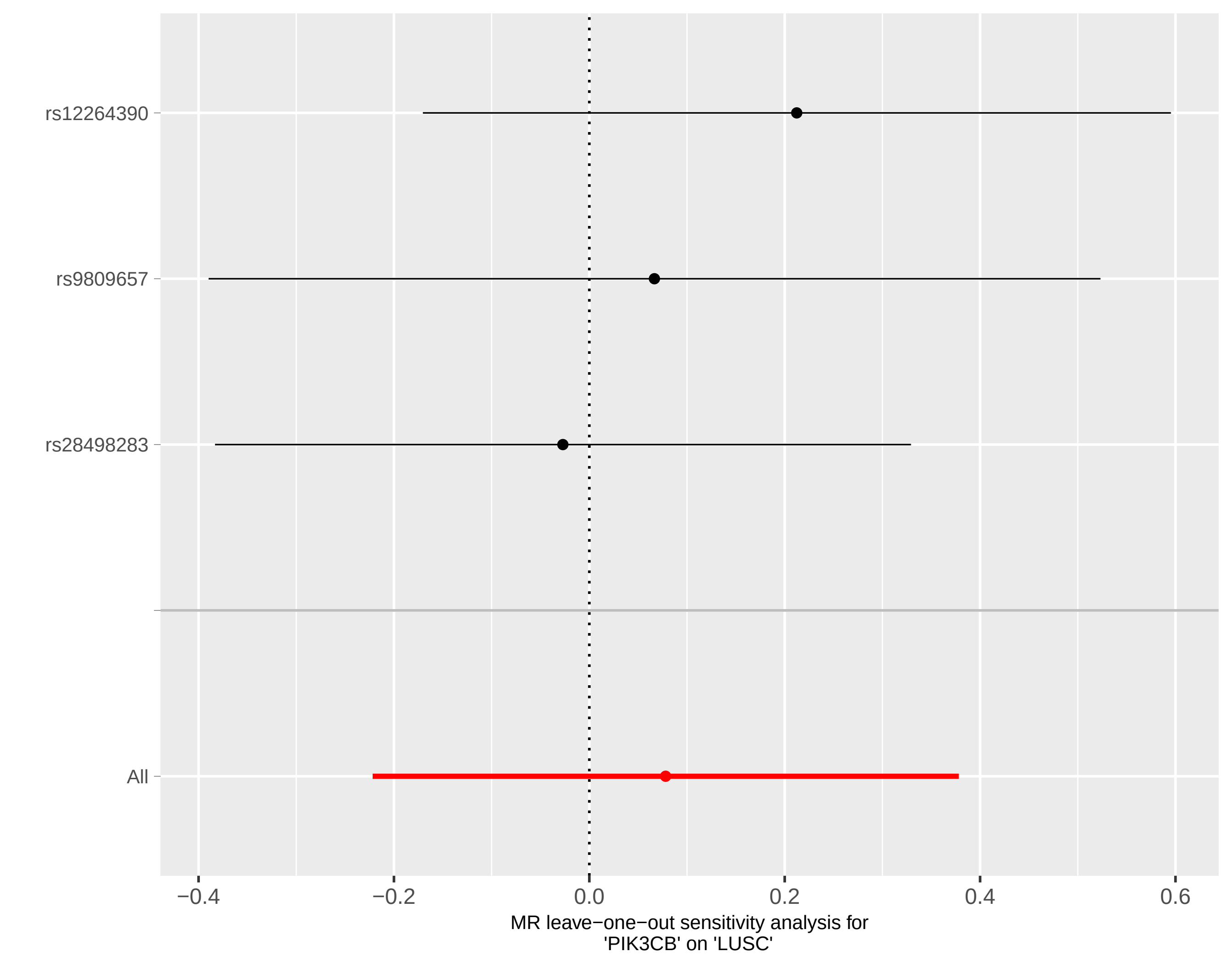
rs12264390
rs9809657
rs28498283
All
−0.4
−0.2
0.0
0.2
0.4
0.6
MR leave−one−out sensitivity analysis for
'PIK3CB' on 'LUSC'

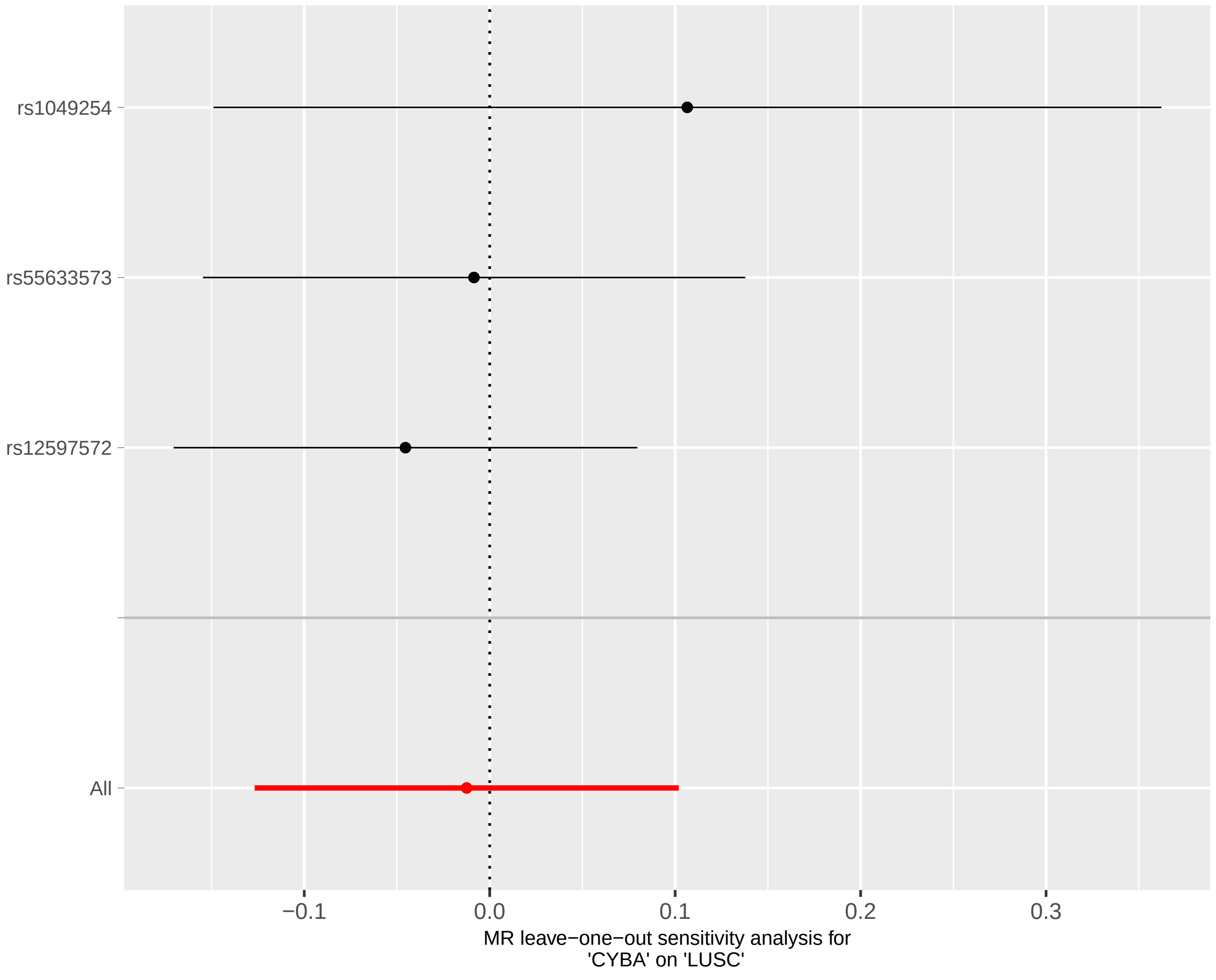
rs1049254
rs55633573
rs12597572
All
−0.1
0.0
0.1
0.2
0.3
MR leave−one−out sensitivity analysis for
'CYBA' on 'LUSC'

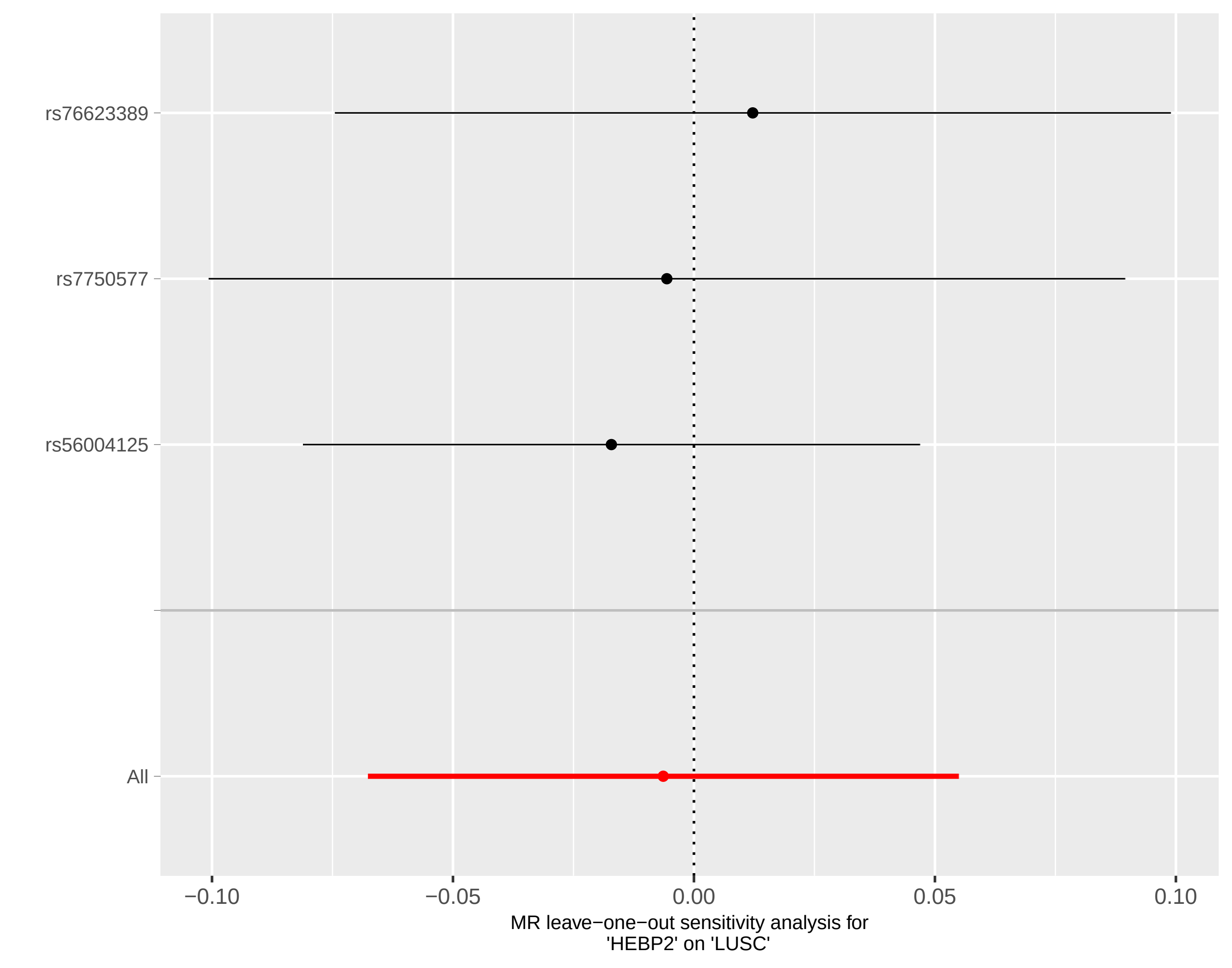

rs76623389
rs7750577
rs56004125
All
−0.10
−0.05
0.00
0.05
0.10
MR leave−one−out sensitivity analysis for
'HEBP2' on 'LUSC'

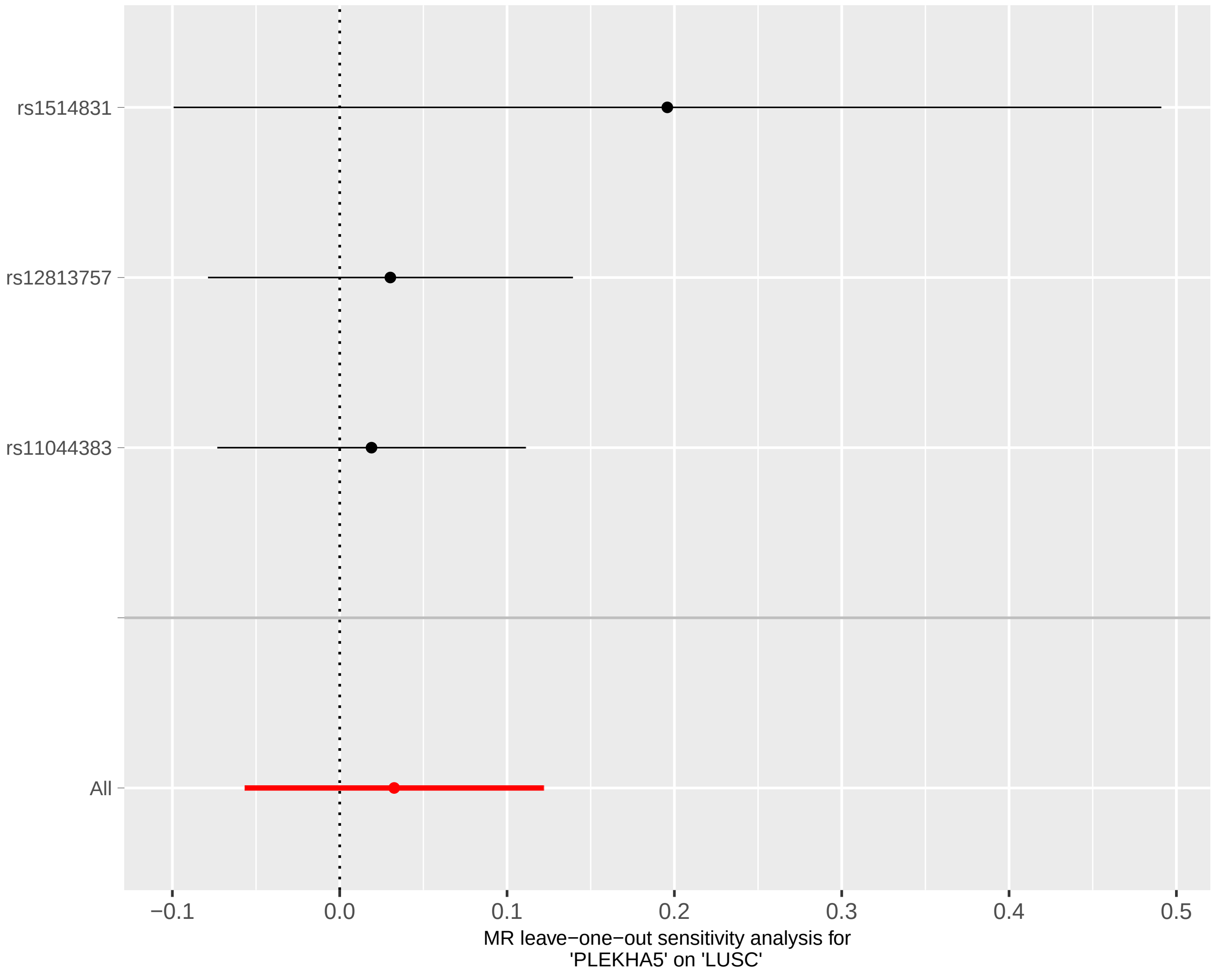
rs1514831
rs12813757
rs11044383
All
−0.1
0.0
0.1
0.2
0.3
0.4
0.5
MR leave−one−out sensitivity analysis for
'PLEKHA5' on 'LUSC'

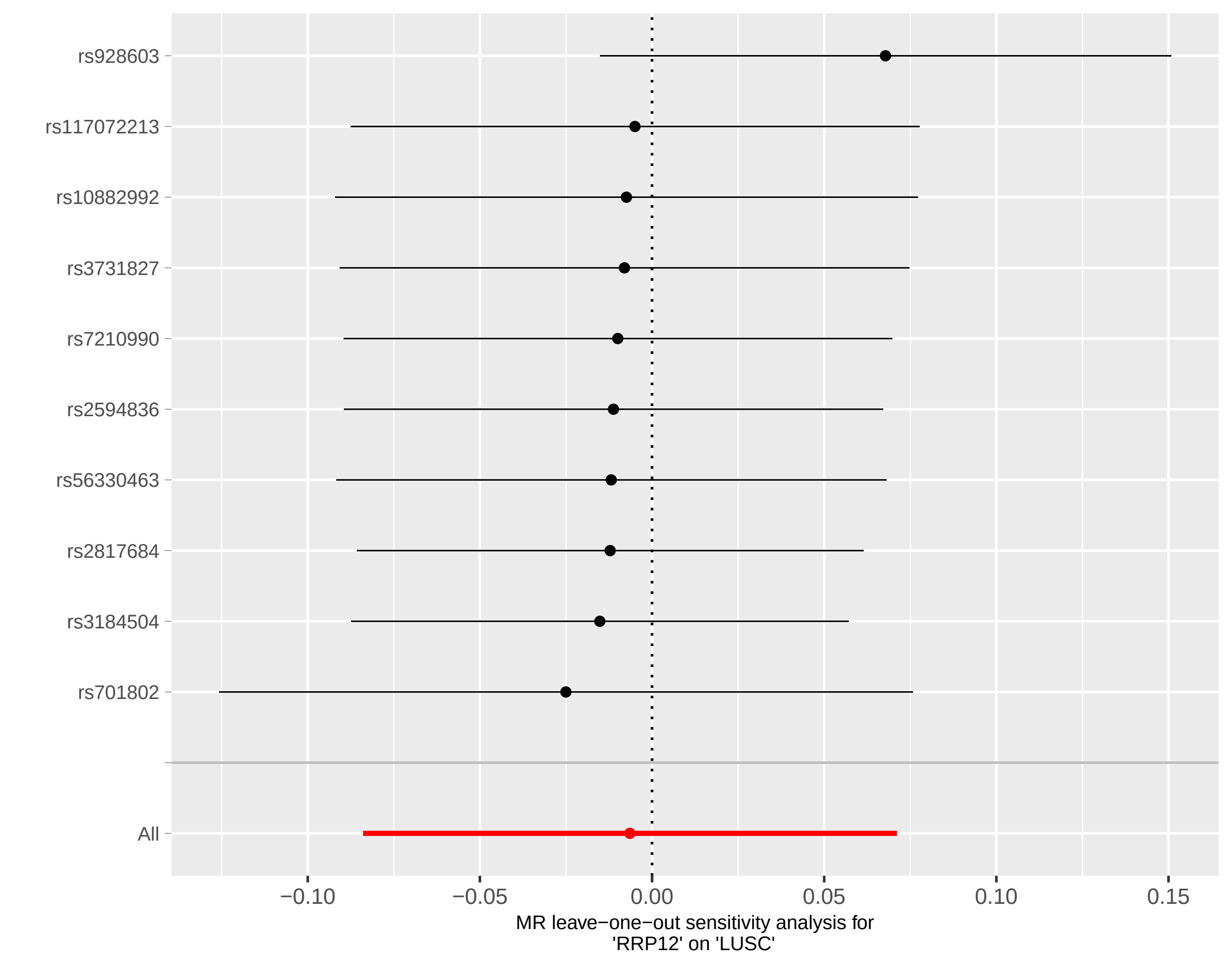
rs928603
rs117072213
rs10882992
rs3731827
rs7210990
rs2594836
rs56330463
rs2817684
rs3184504
rs701802
All
−0.10
−0.05
0.00
0.05
0.10
0.15
MR leave−one−out sensitivity analysis for
'RRP12' on 'LUSC'

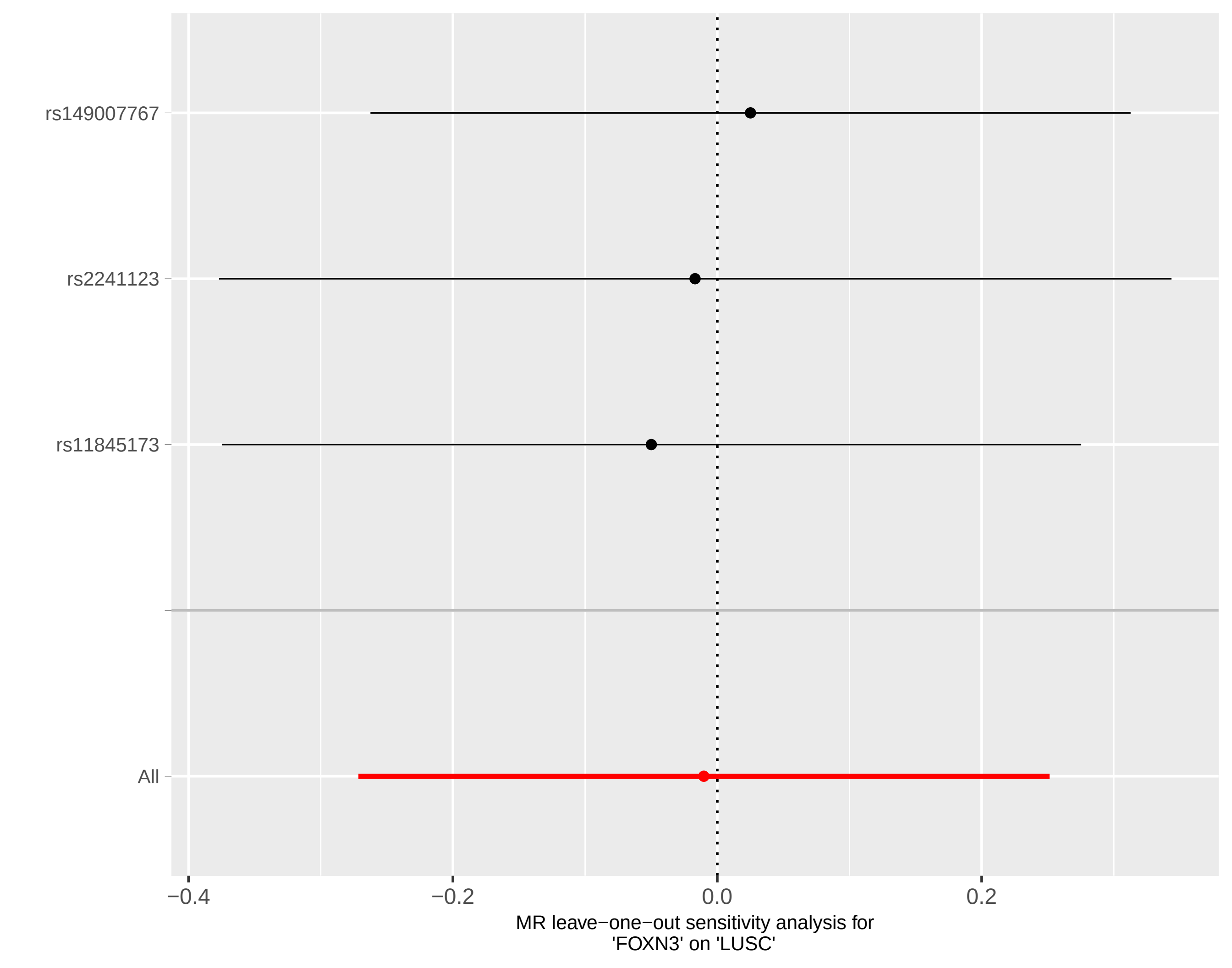
rs149007767
rs2241123
rs11845173
All
−0.4
−0.2
0.0
0.2
MR leave−one−out sensitivity analysis for
'FOXN3' on 'LUSC'

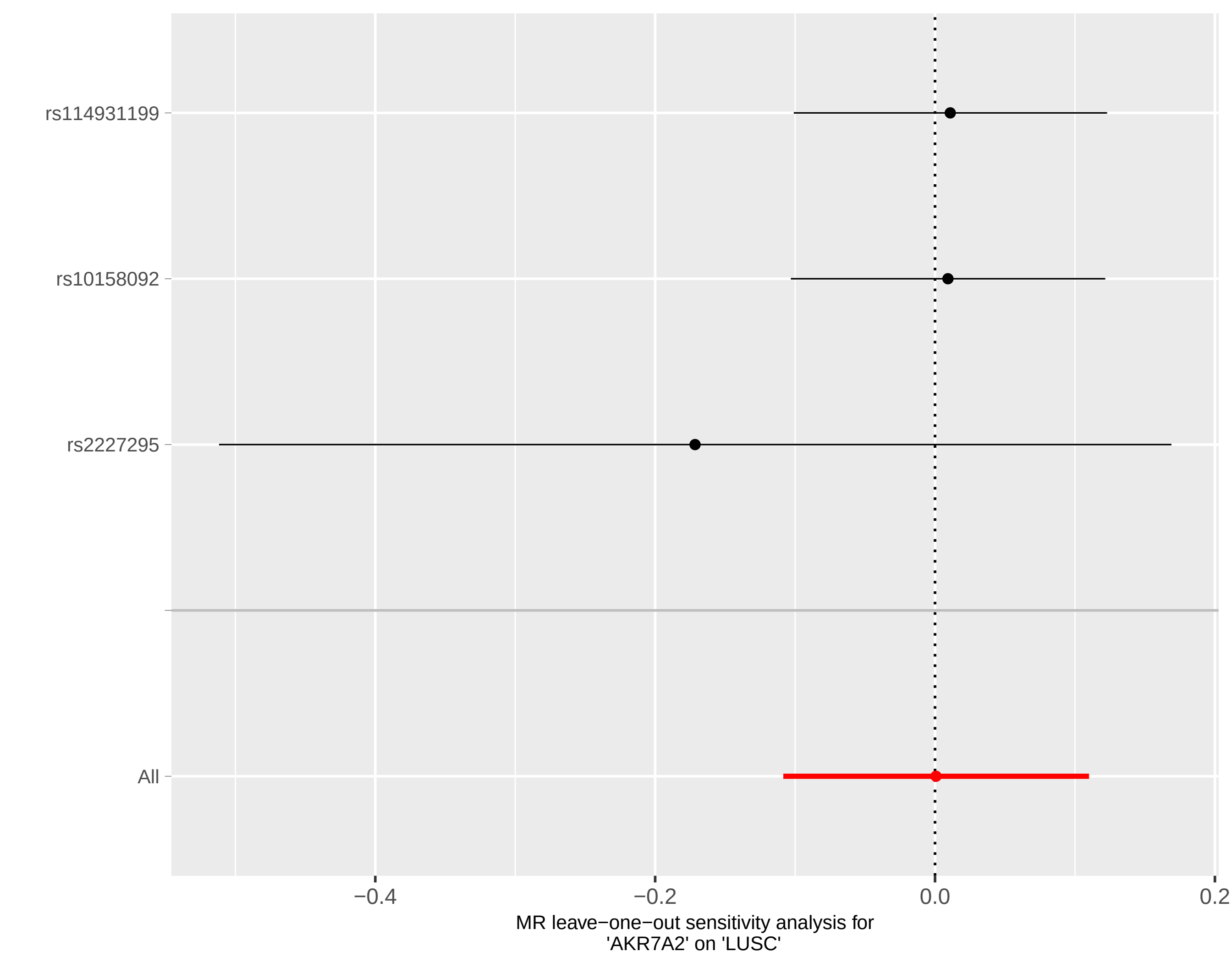
rs114931199
rs10158092
rs2227295
All
−0.4
−0.2
0.0
0.2
MR leave−one−out sensitivity analysis for
'AKR7A2' on 'LUSC'

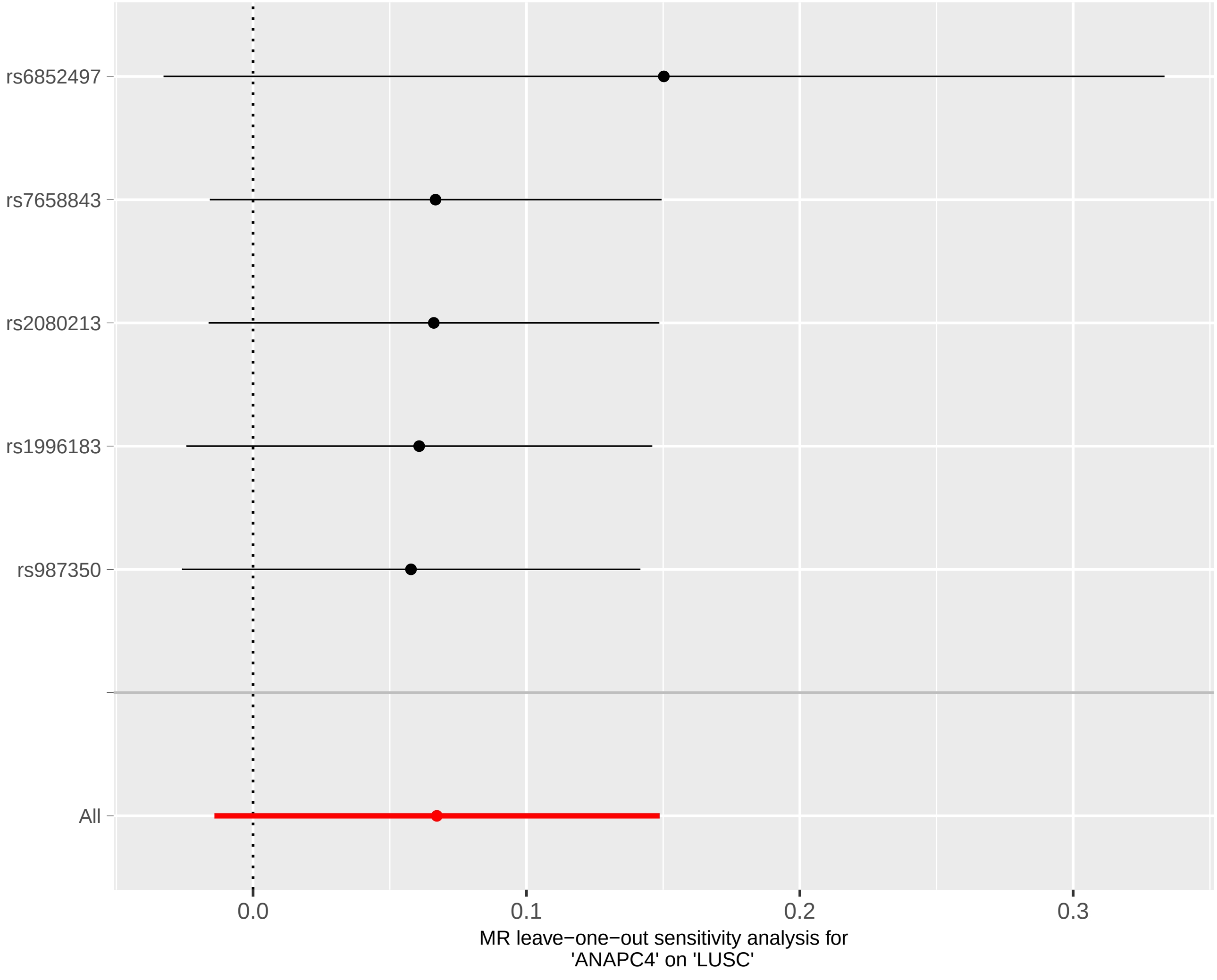
rs6852497
rs7658843
rs2080213
rs1996183
rs987350
All
0.0
0.1
0.2
0.3
MR leave−one−out sensitivity analysis for
'ANAPC4' on 'LUSC'

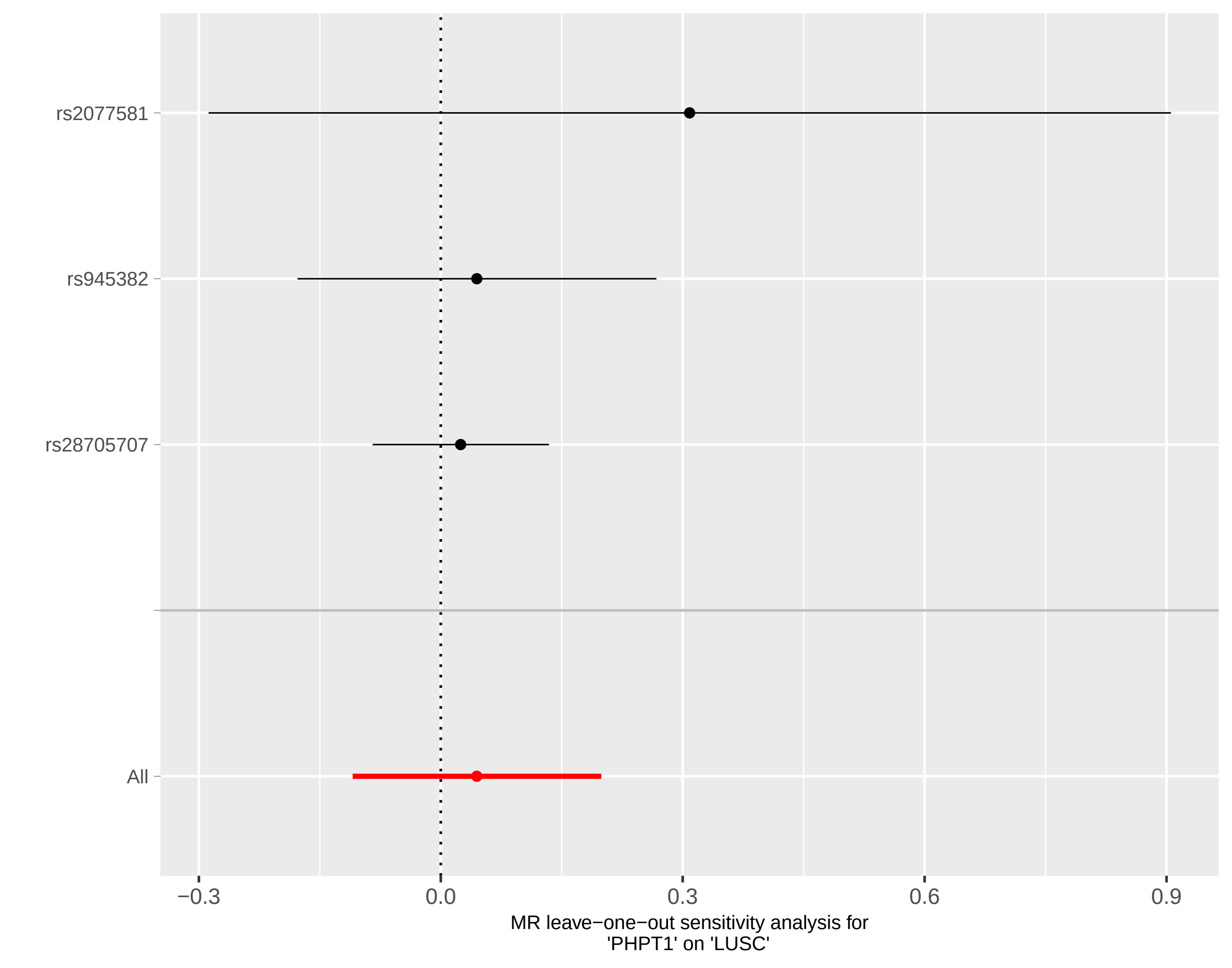
rs2077581
rs945382
rs28705707
All
−0.3
0.0
0.3
0.6
0.9
MR leave−one−out sensitivity analysis for
'PHPT1' on 'LUSC'

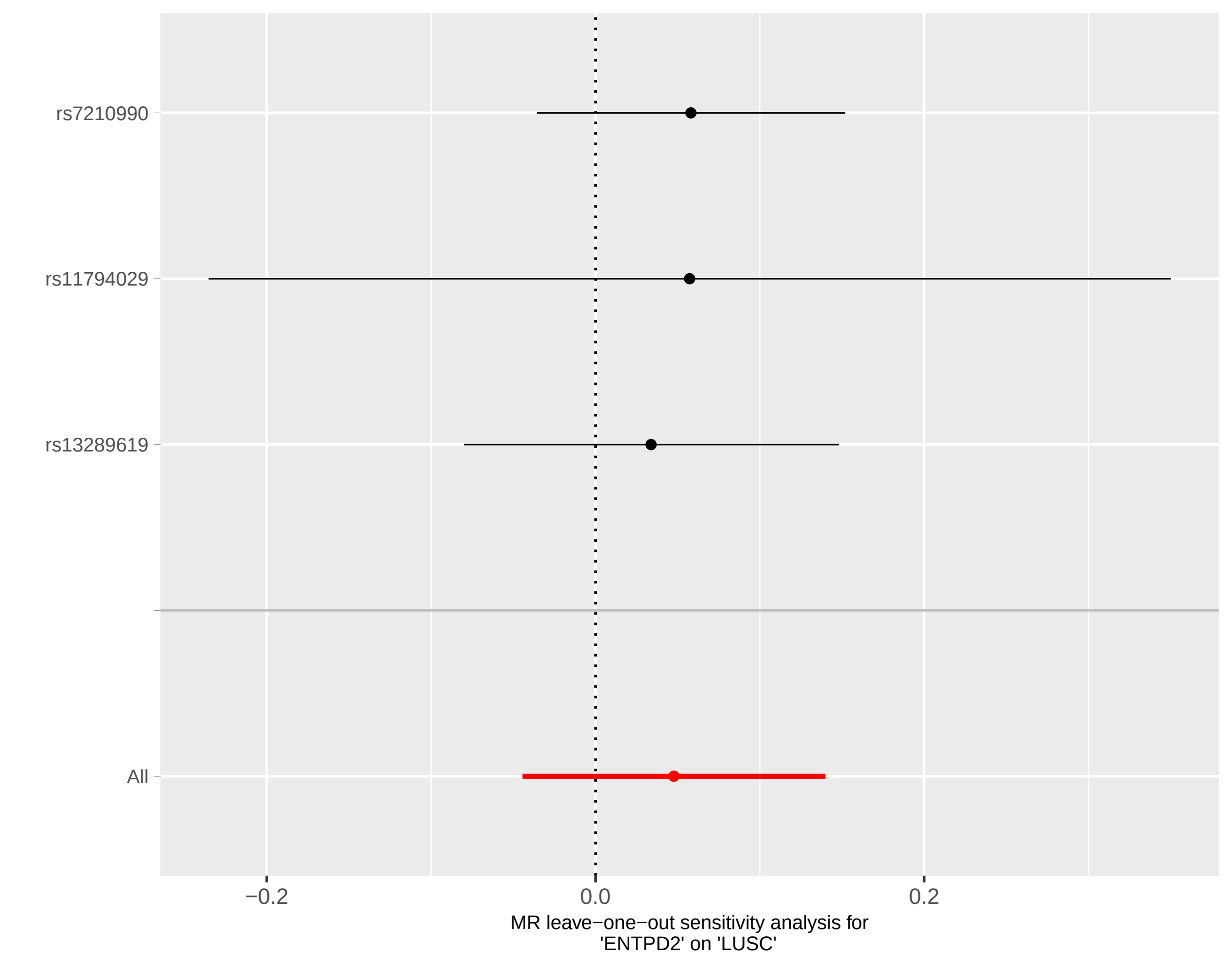

rs7210990
rs11794029
rs13289619
All
−0.2
0.0
0.2
MR leave−one−out sensitivity analysis for
'ENTPD2' on 'LUSC'

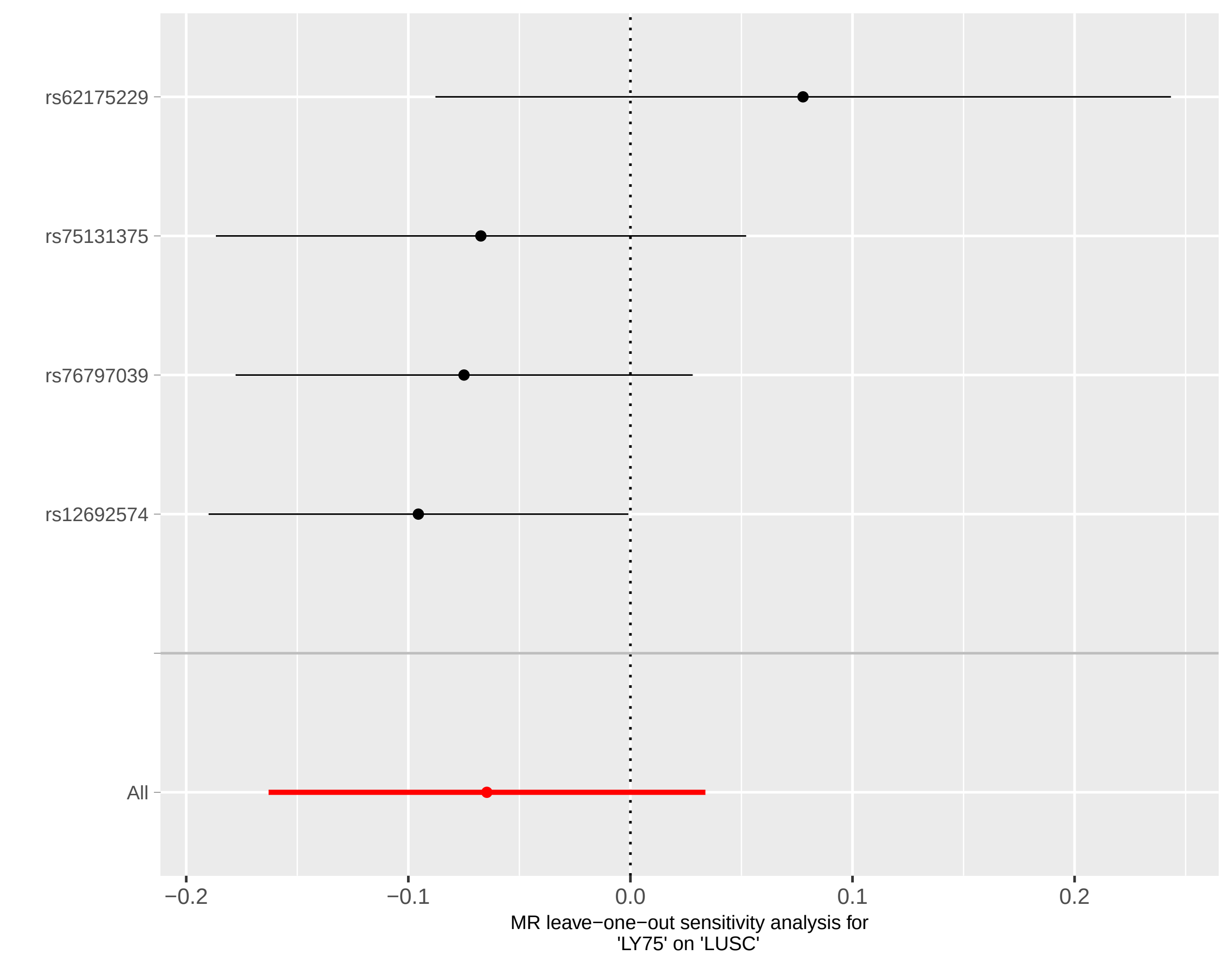
rs62175229
rs75131375
rs76797039
rs12692574
All
−0.2
−0.1
0.0
0.1
0.2
MR leave−one−out sensitivity analysis for
'LY75' on 'LUSC'

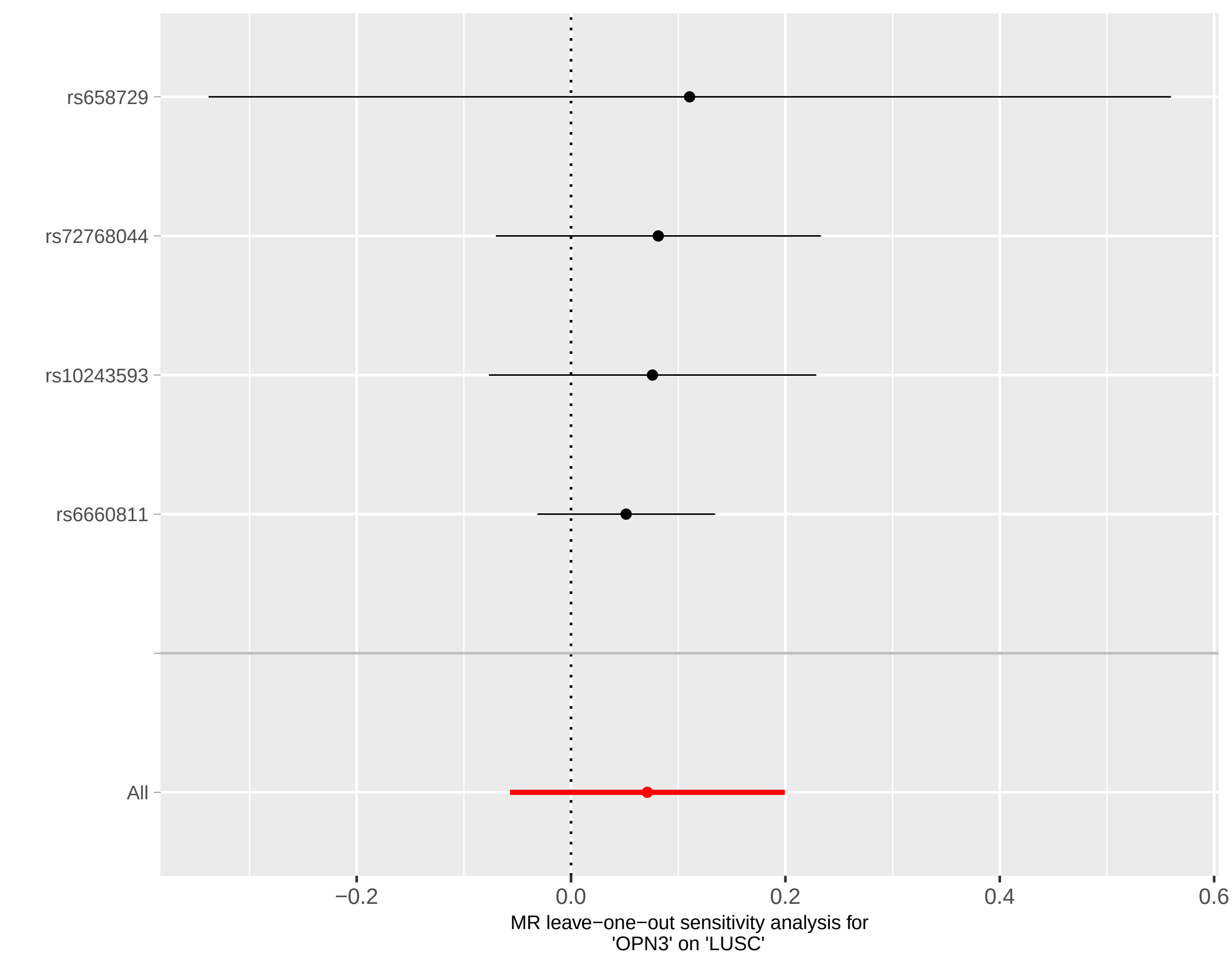

rs658729
rs72768044
rs10243593
rs6660811
All
−0.2
0.0
0.2
0.4
0.6
MR leave−one−out sensitivity analysis for
'OPN3' on 'LUSC'

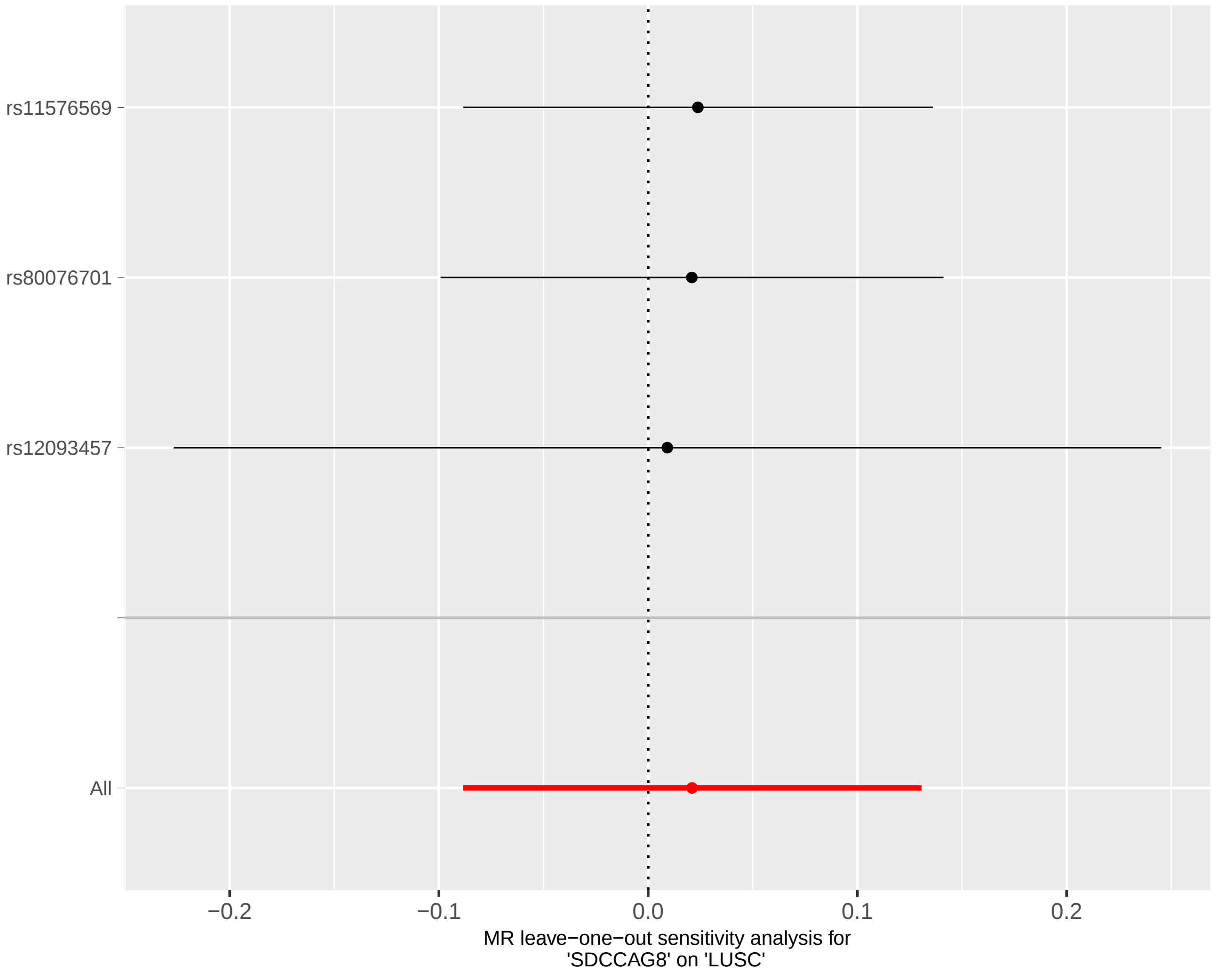

rs11576569
rs80076701
rs12093457
All
−0.2
−0.1
0.0
0.1
0.2
MR leave−one−out sensitivity analysis for
'SDCCAG8' on 'LUSC'

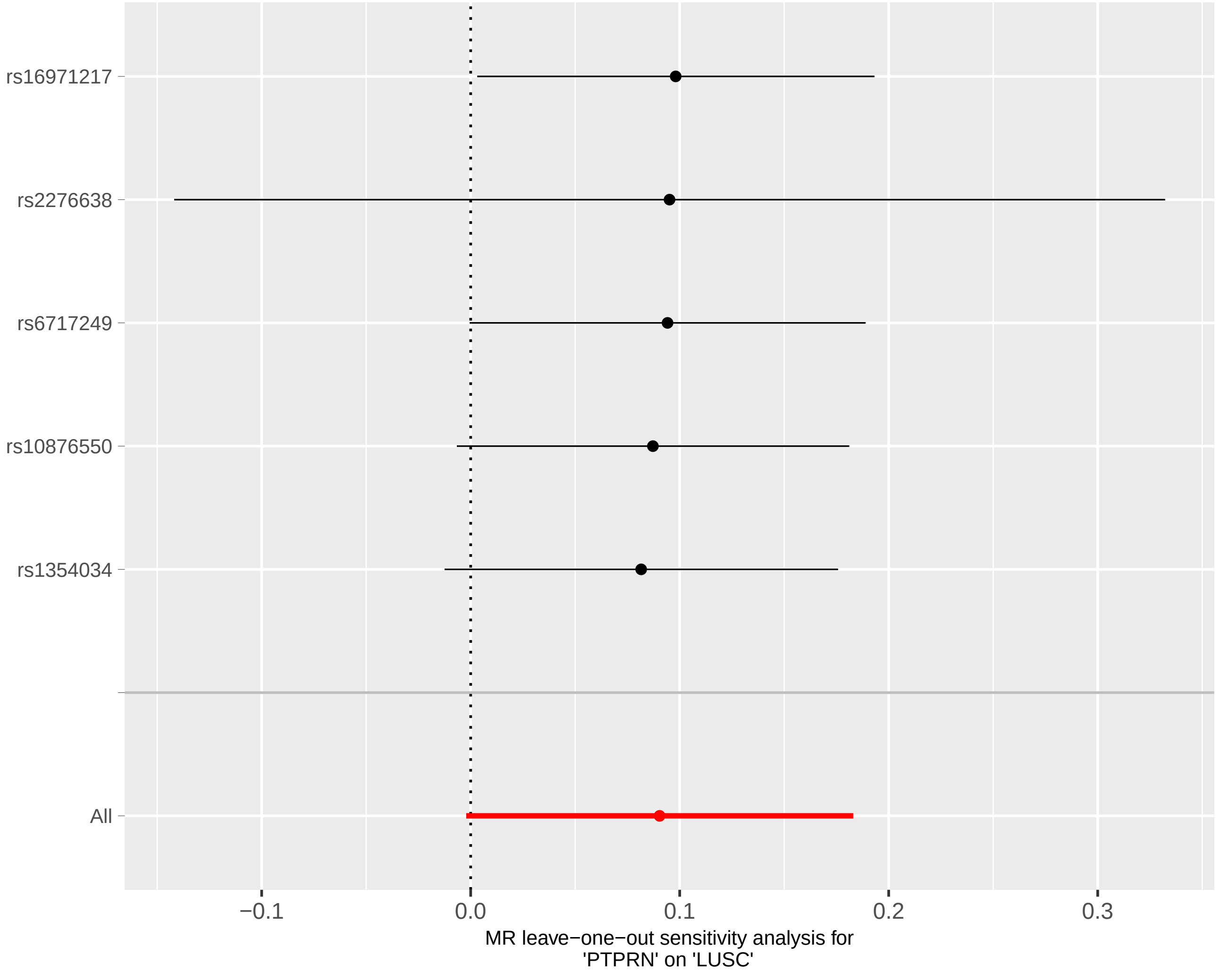

rs16971217
rs2276638
rs6717249
rs10876550
rs1354034
All
−0.1
0.0
0.1
0.2
0.3
MR leave−one−out sensitivity analysis for
'PTPRN' on 'LUSC'

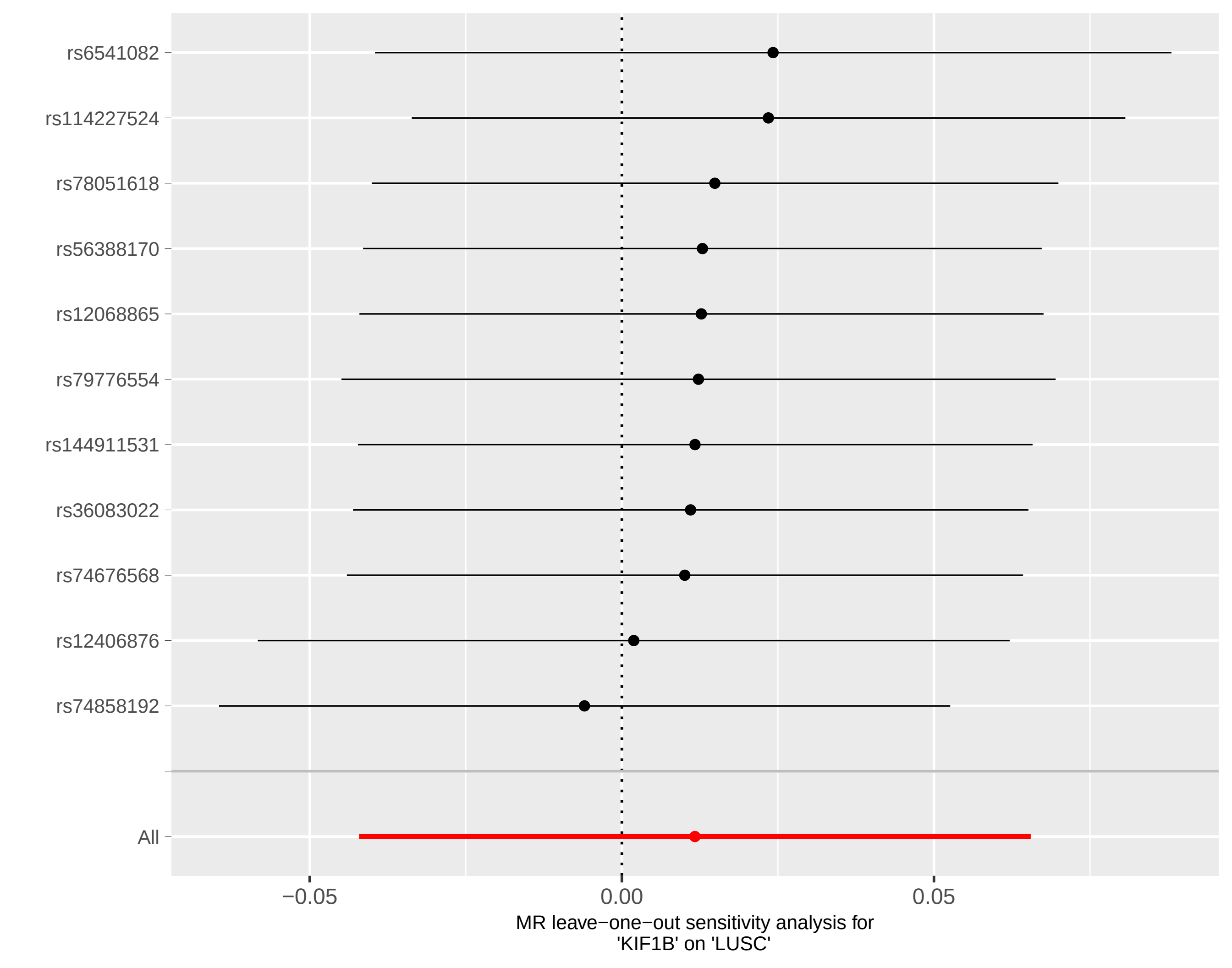
rs6541082
rs114227524
rs78051618
rs56388170
rs12068865
rs79776554
rs144911531
rs36083022
rs74676568
rs12406876
rs74858192
All
−0.05
0.00
0.05
MR leave−one−out sensitivity analysis for
'KIF1B' on 'LUSC'

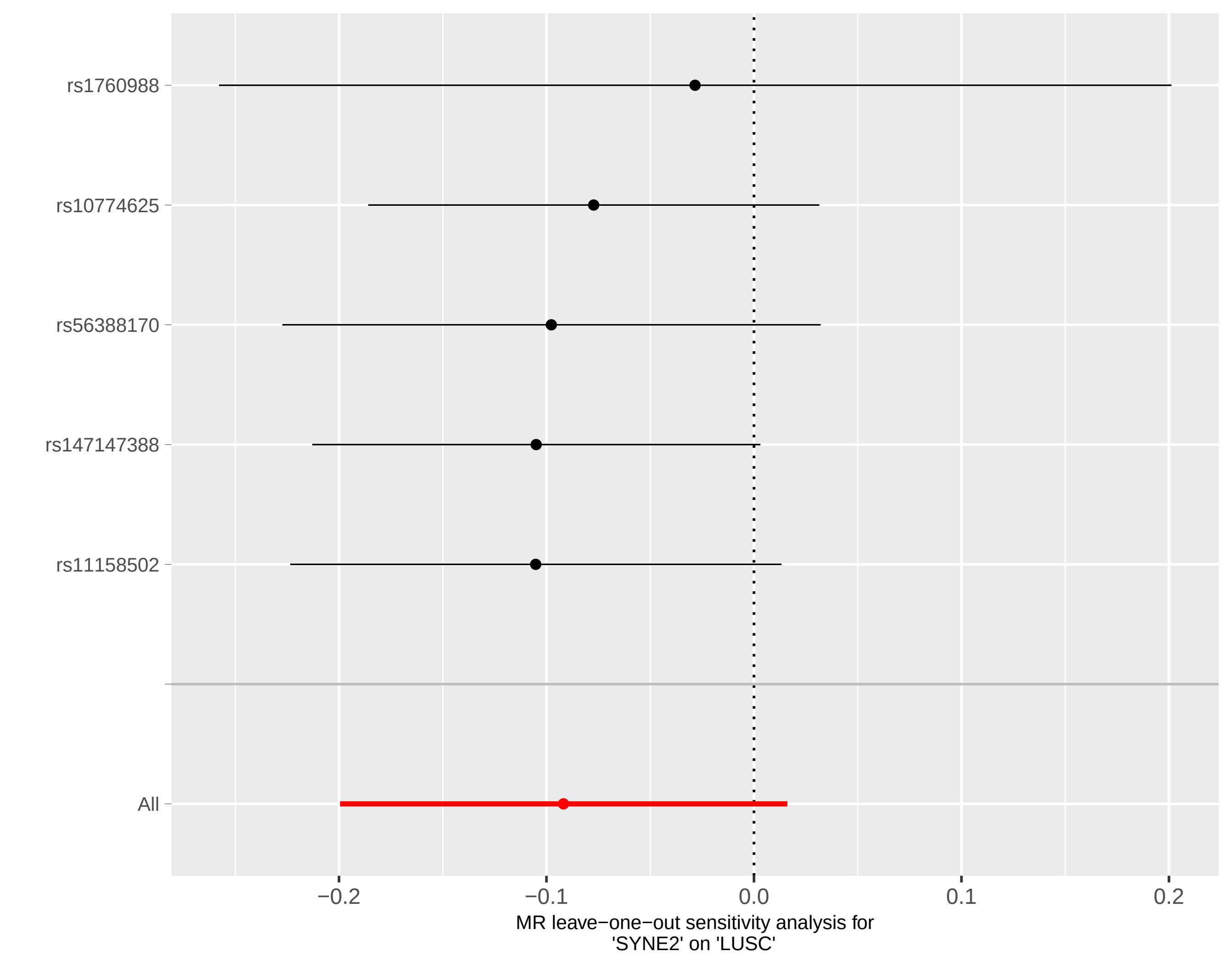

rs1760988
rs10774625
rs56388170
rs147147388
rs11158502
All
−0.2
−0.1
0.0
0.1
0.2
MR leave−one−out sensitivity analysis for
'SYNE2' on 'LUSC'

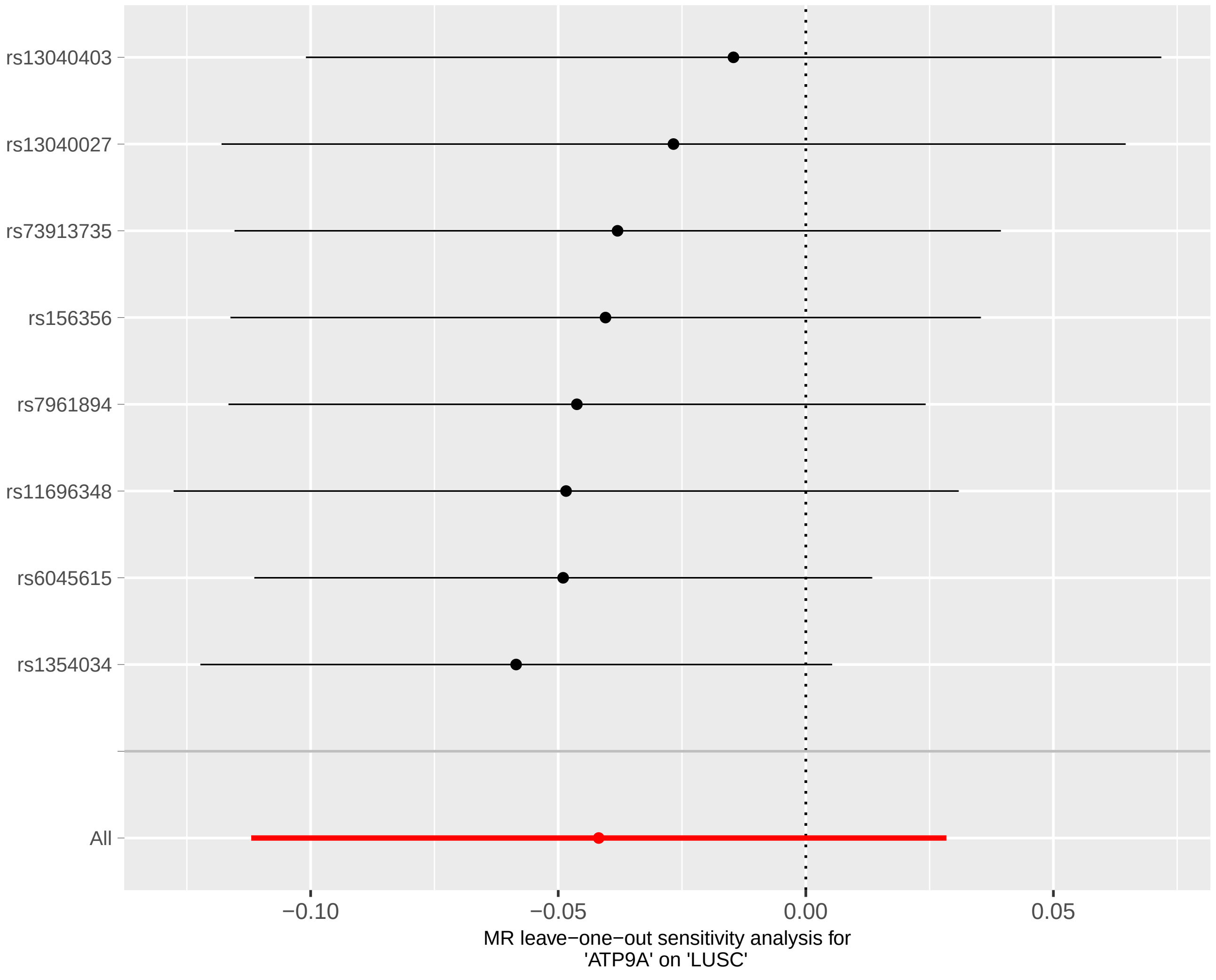

rs13040403
rs13040027
rs73913735
rs156356
rs7961894
rs11696348
rs6045615
rs1354034
All
−0.10
−0.05
0.00
0.05
MR leave−one−out sensitivity analysis for
'ATP9A' on 'LUSC'

Insufficient number of SNPs

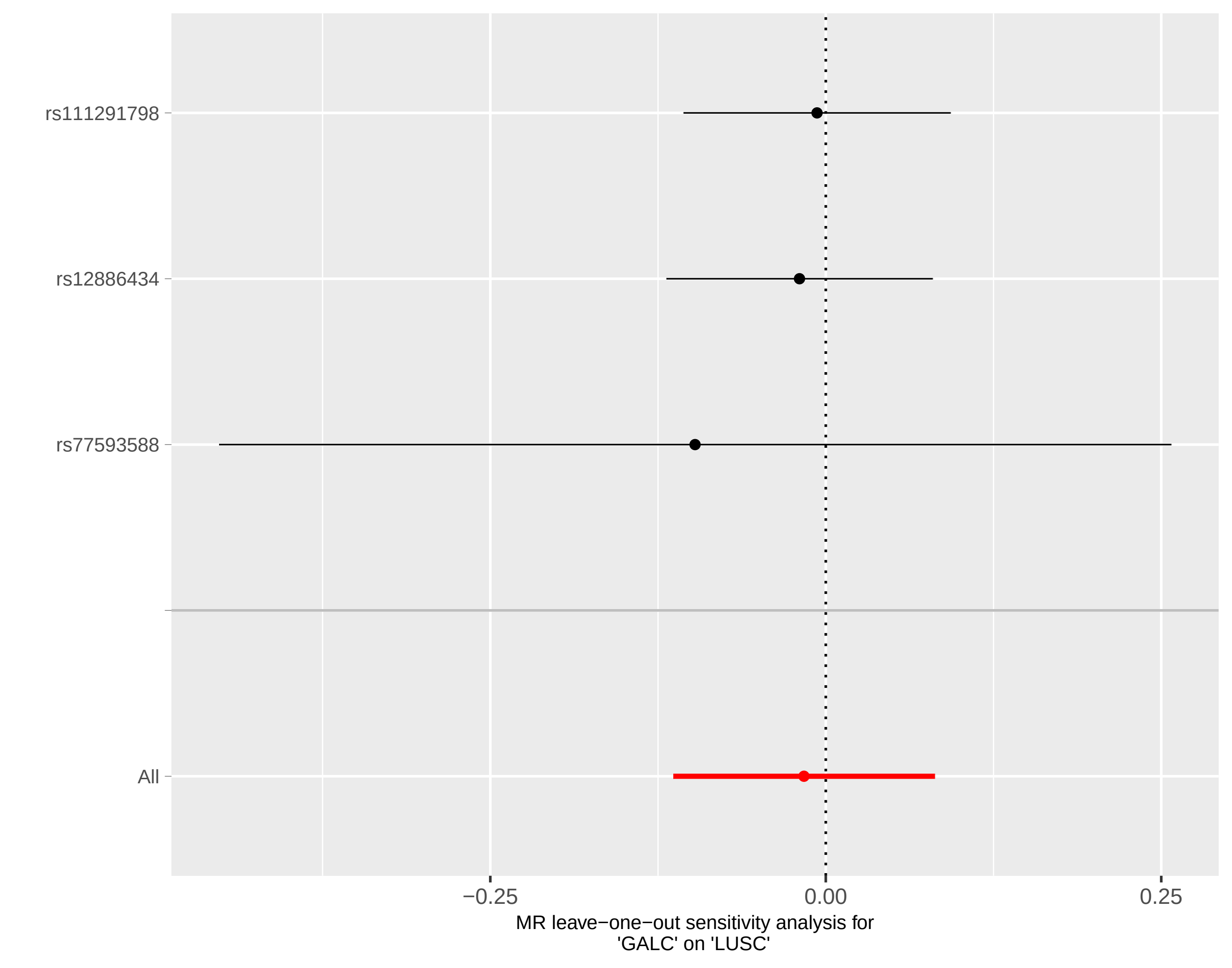

rs111291798
rs12886434
rs77593588
All
−0.25
0.00
0.25
MR leave−one−out sensitivity analysis for
'GALC' on 'LUSC'

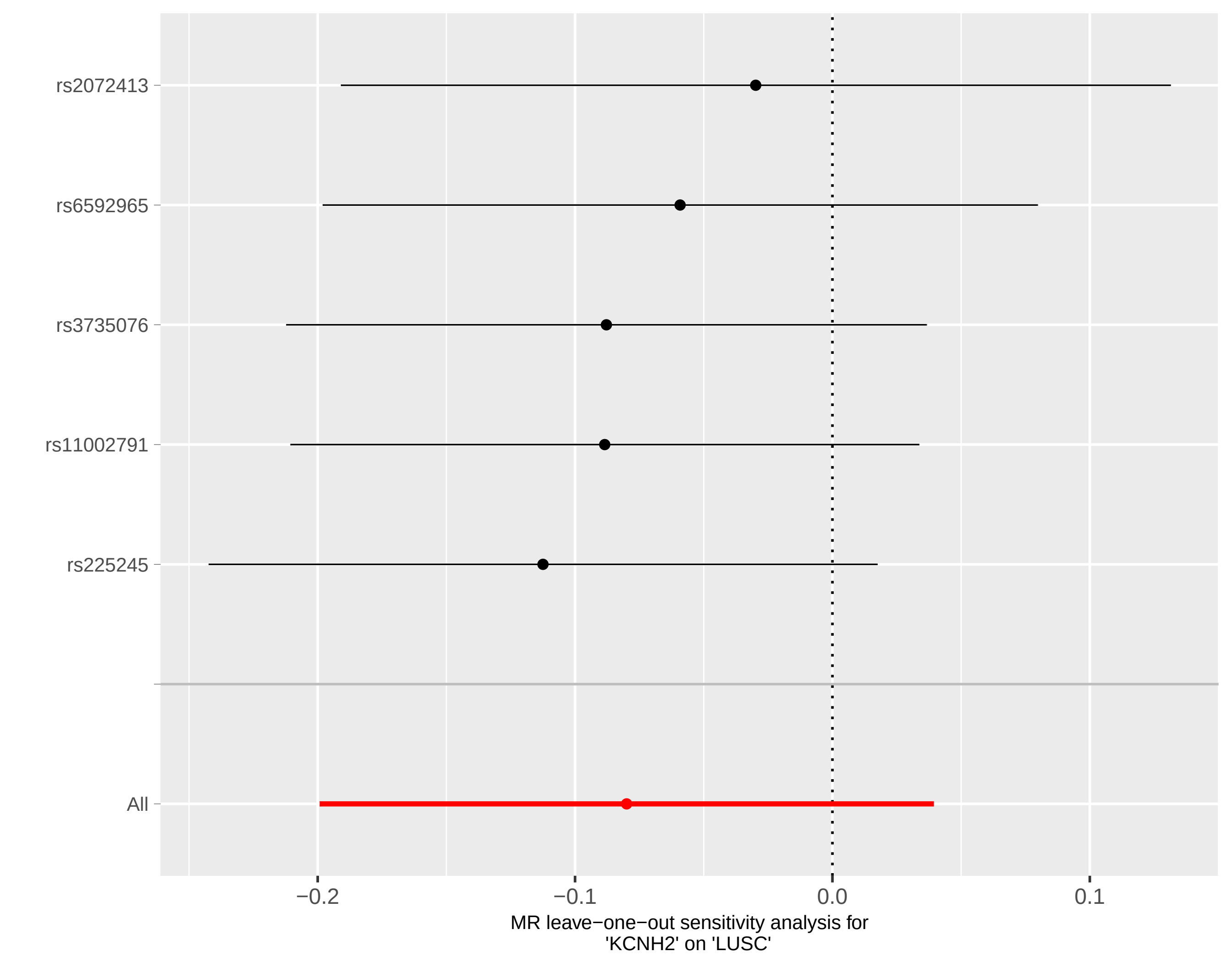
rs2072413
rs6592965
rs3735076
rs11002791
rs225245
All
−0.2
−0.1
0.0
0.1
MR leave−one−out sensitivity analysis for
'KCNH2' on 'LUSC'

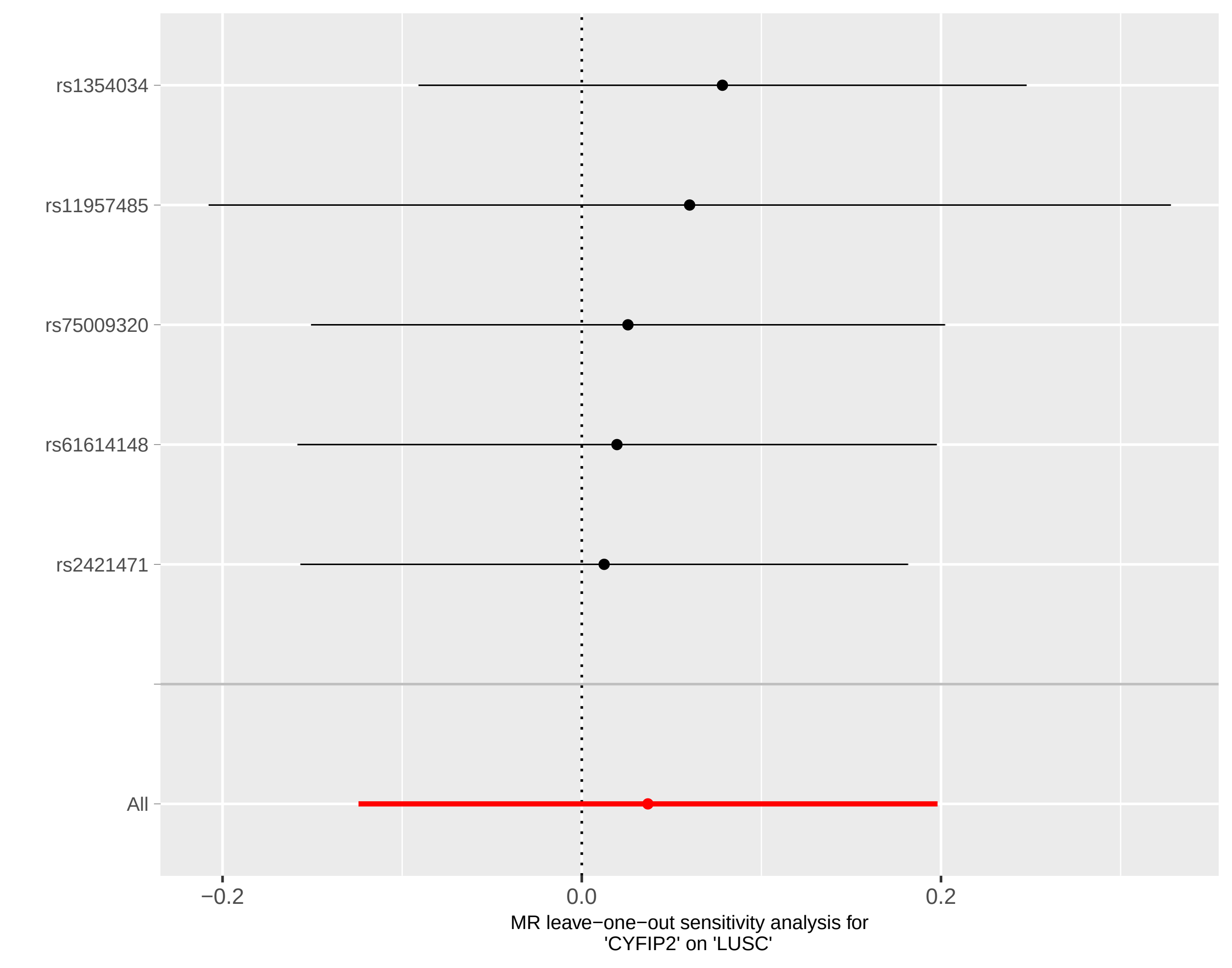
rs1354034
rs11957485
rs75009320
rs61614148
rs2421471
All
−0.2
0.0
0.2
MR leave−one−out sensitivity analysis for
'CYFIP2' on 'LUSC'

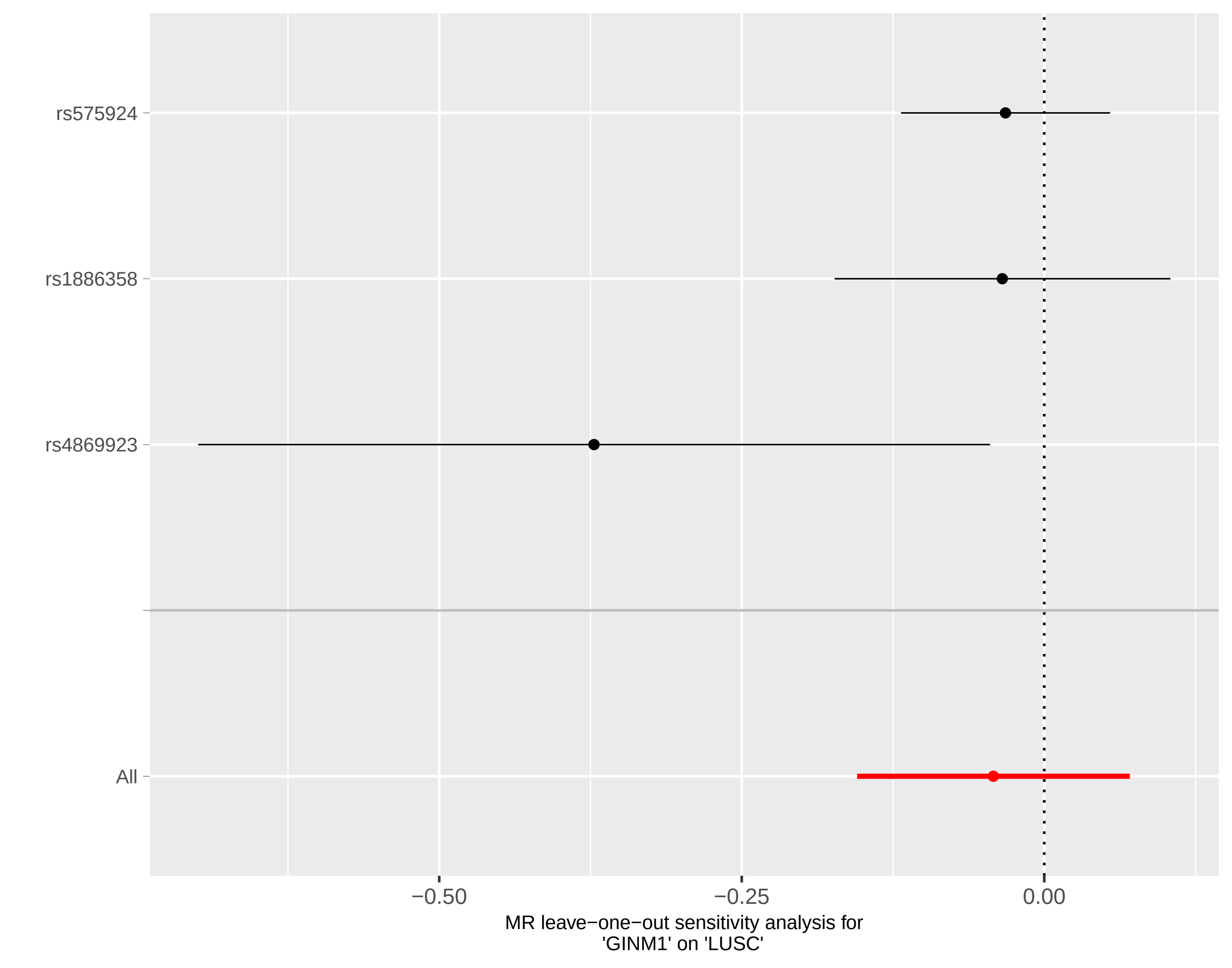

rs575924
rs1886358
rs4869923
All
−0.50
−0.25
0.00
MR leave−one−out sensitivity analysis for
'GINM1' on 'LUSC'

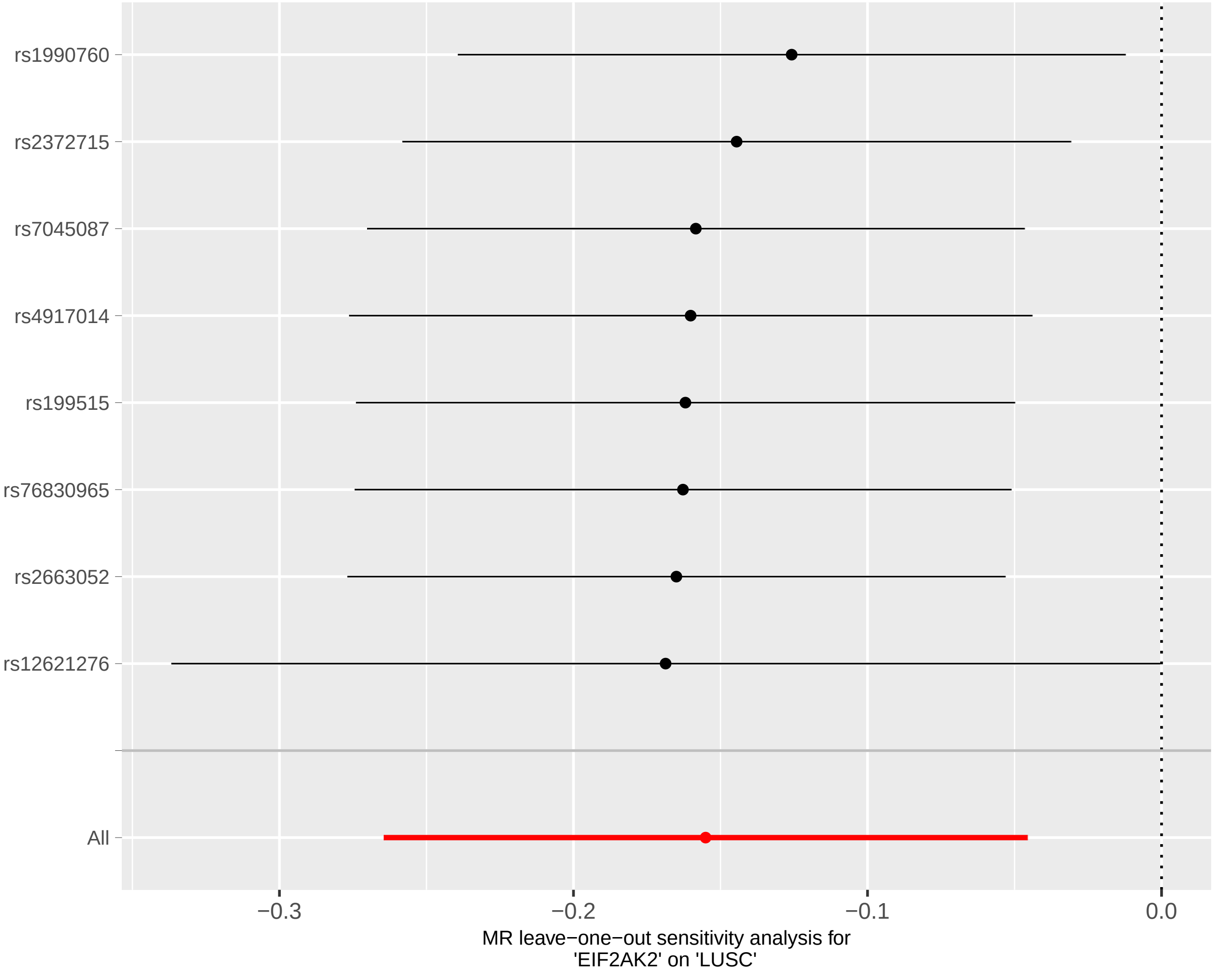

rs1990760
rs2372715
rs7045087
rs4917014
rs199515
rs76830965
rs2663052
rs12621276
All
−0.3
−0.2
−0.1
0.0
MR leave−one−out sensitivity analysis for
'EIF2AK2' on 'LUSC'

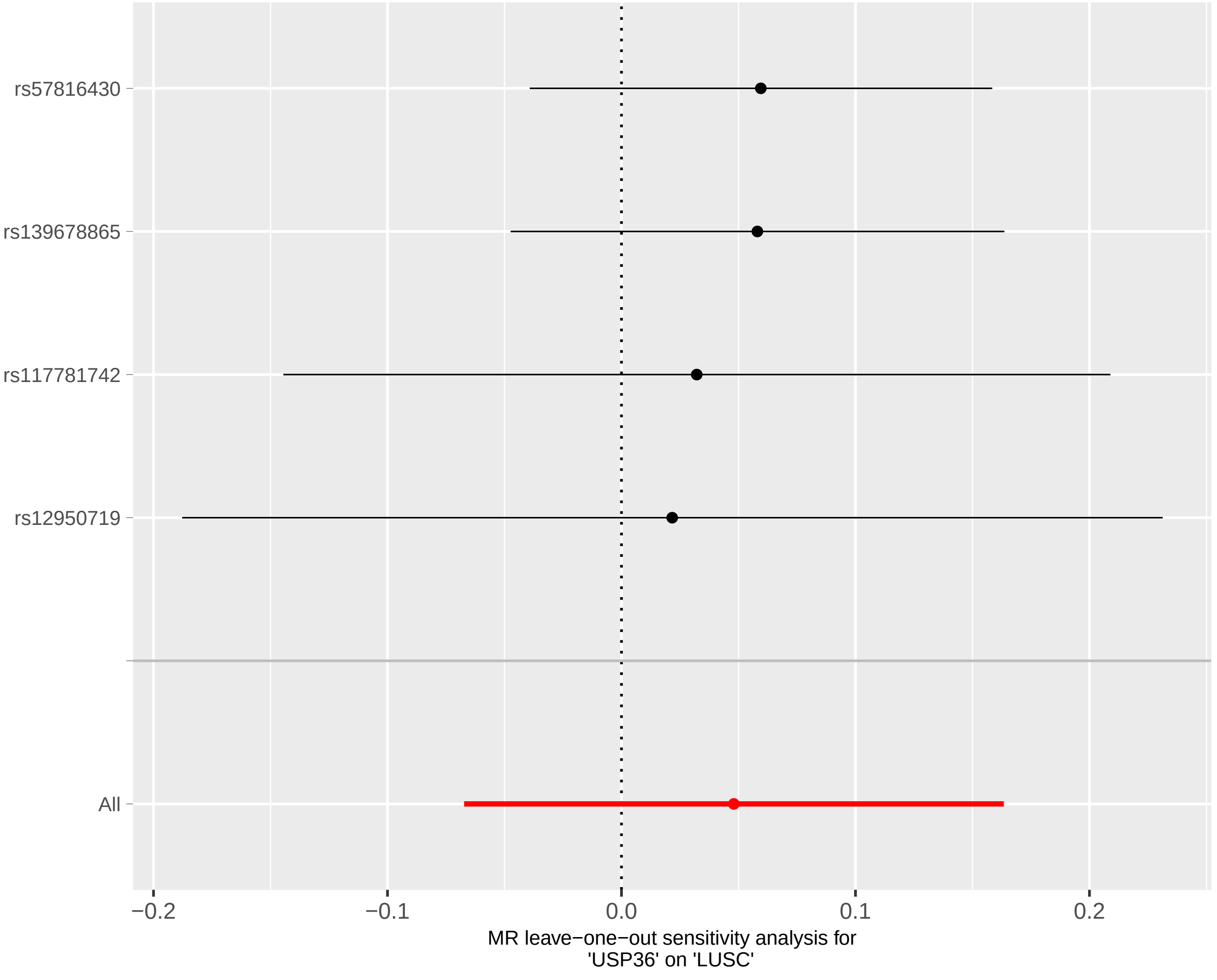

rs57816430
rs139678865
rs117781742
rs12950719
All
−0.2
−0.1
0.0
0.1
0.2
MR leave−one−out sensitivity analysis for
'USP36' on 'LUSC'

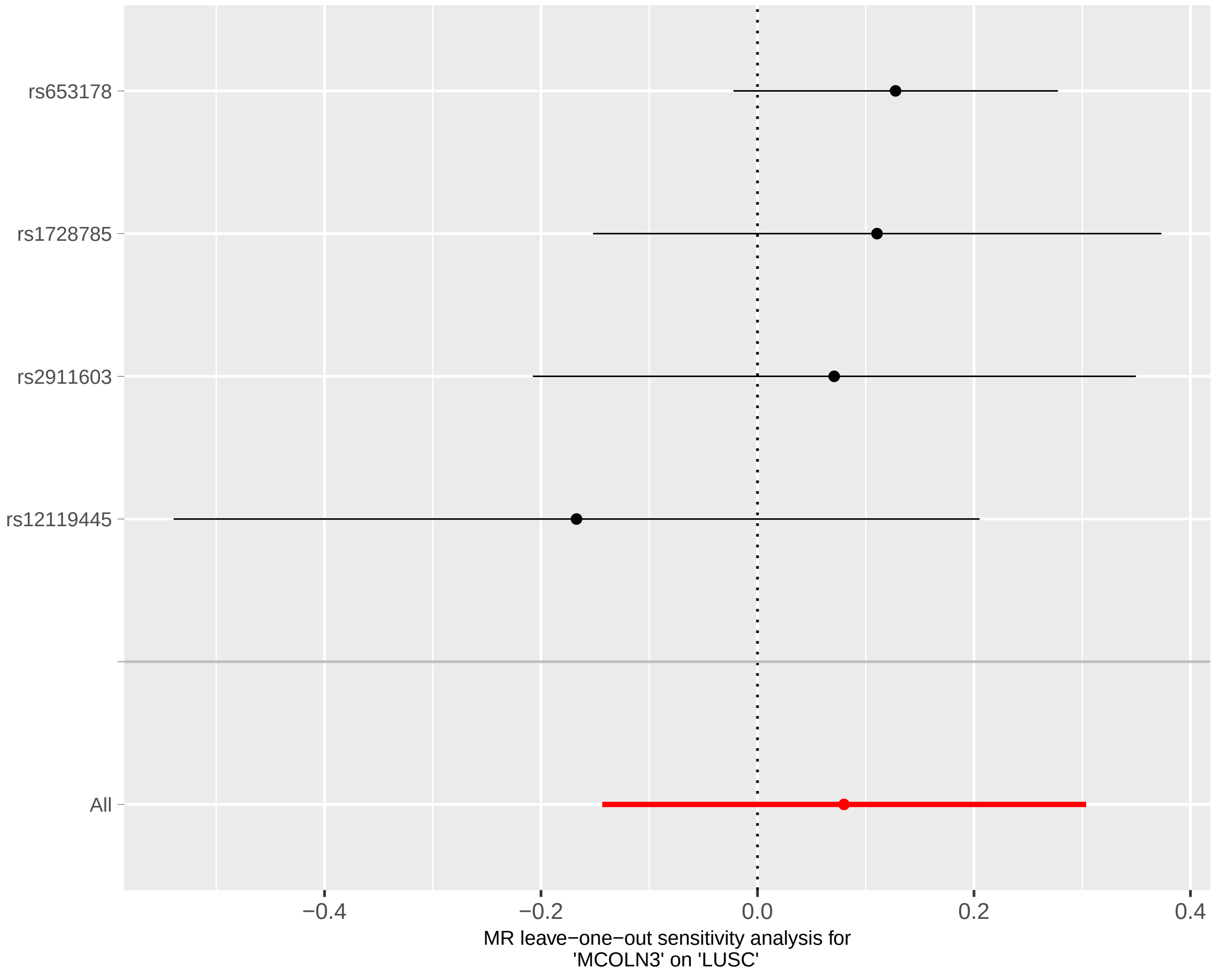
rs653178
rs1728785
rs2911603
rs12119445
All
−0.4
−0.2
0.0
0.2
0.4
MR leave−one−out sensitivity analysis for
'MCOLN3' on 'LUSC'

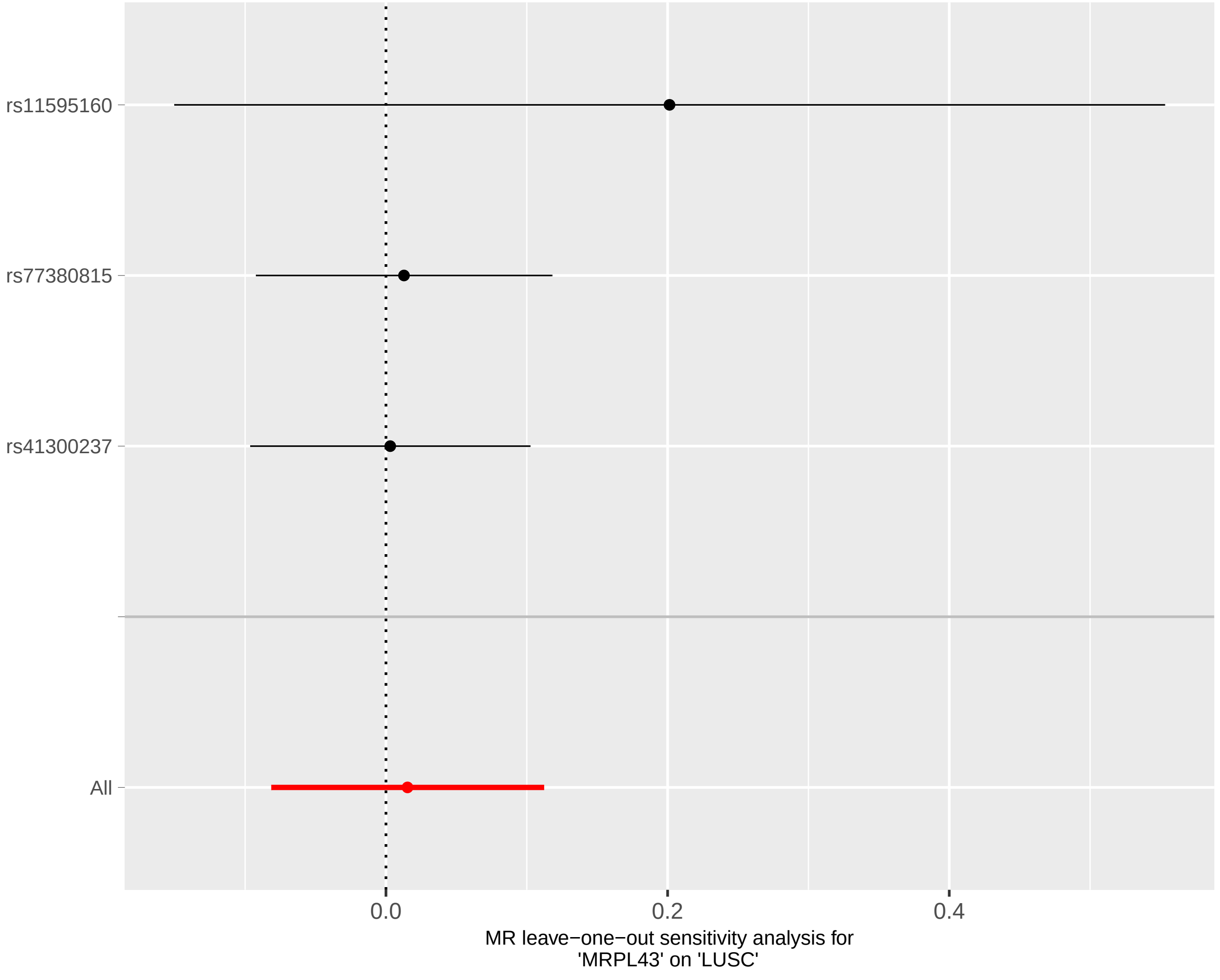

rs11595160
rs77380815
rs41300237
All
0.0
0.2
0.4
MR leave−one−out sensitivity analysis for
'MRPL43' on 'LUSC'

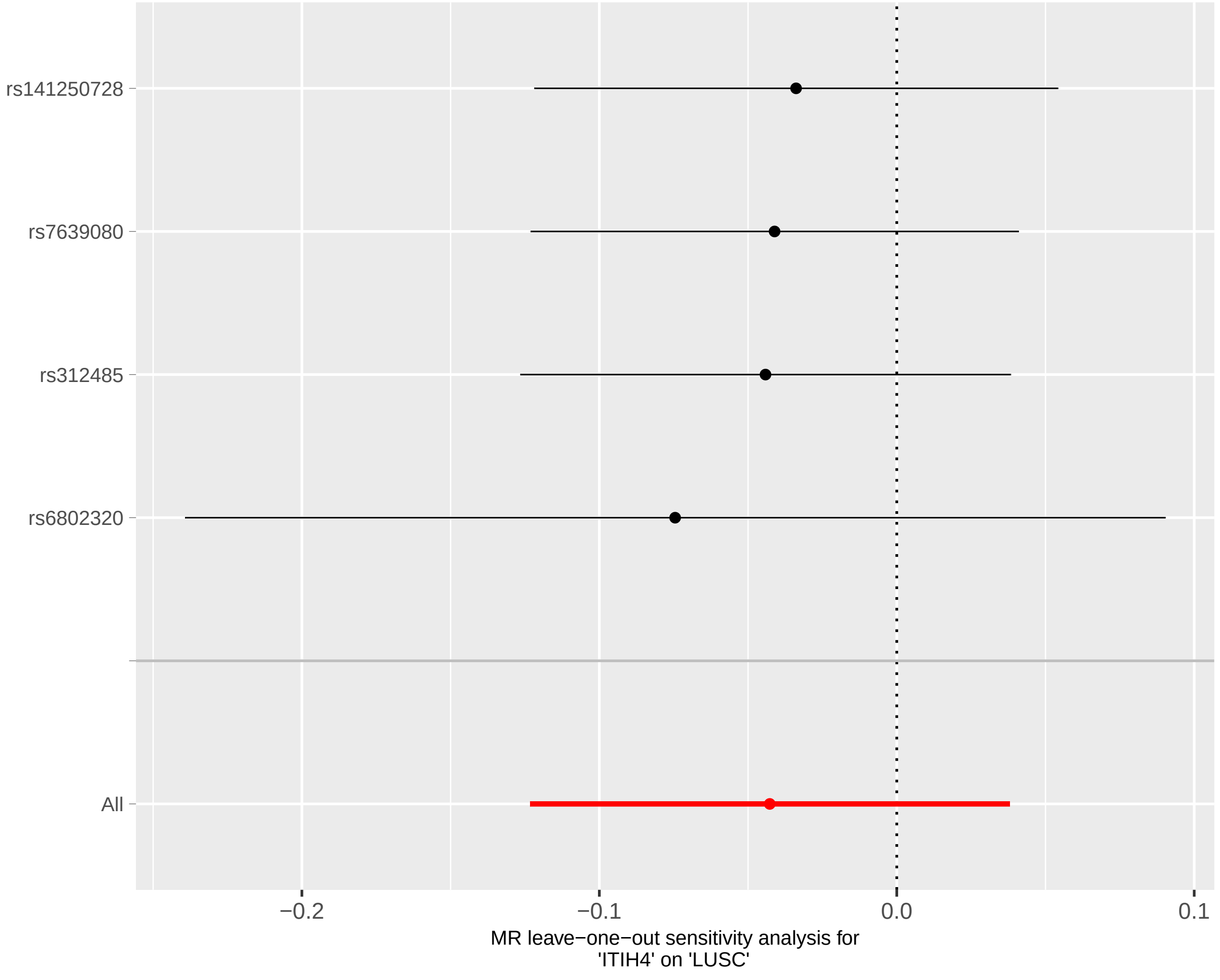

rs141250728
rs7639080
rs312485
rs6802320
All
−0.2
−0.1
0.0
0.1
MR leave−one−out sensitivity analysis for
'ITIH4' on 'LUSC'

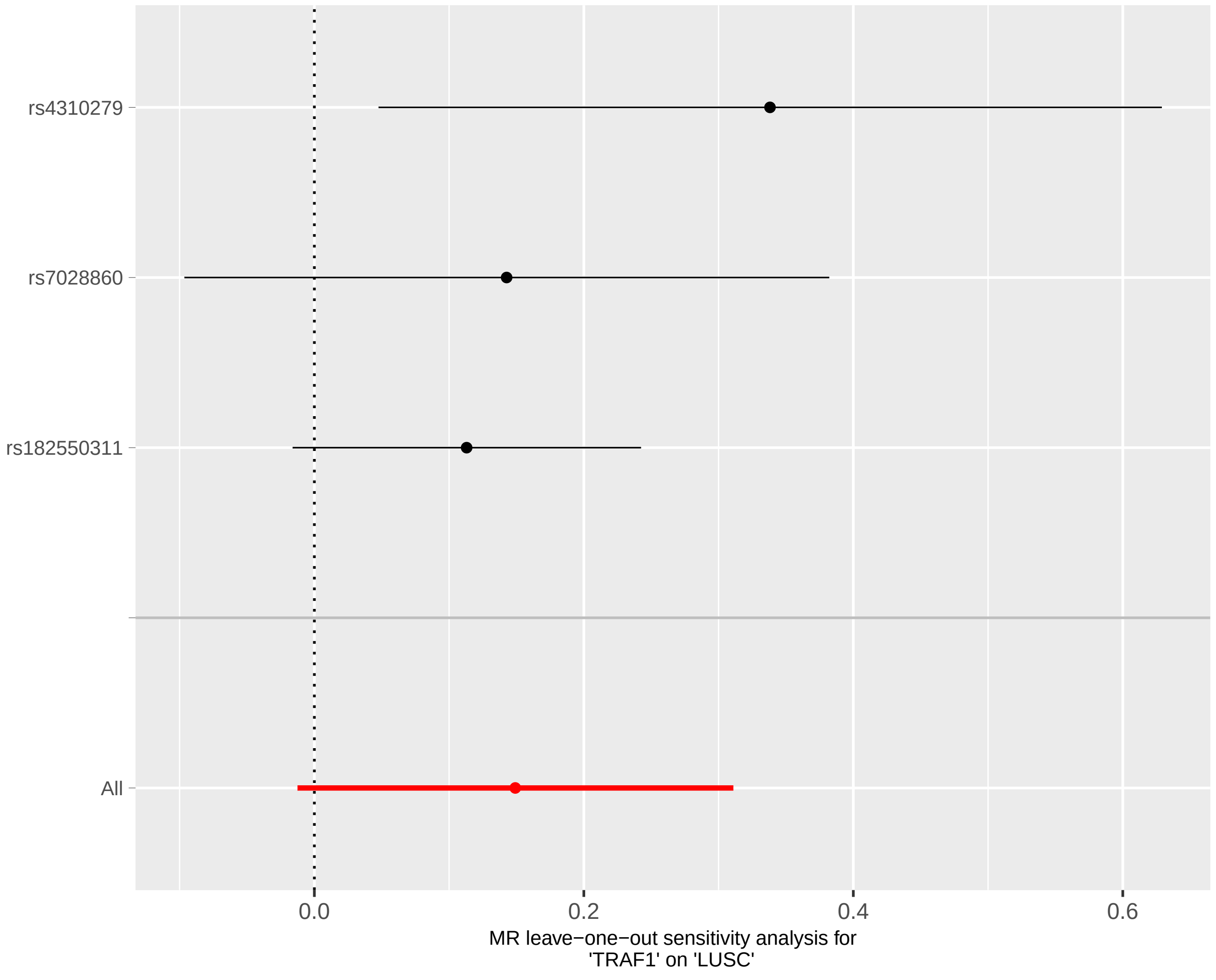
rs4310279
rs7028860
rs182550311
All
0.0
0.2
0.4
0.6
MR leave−one−out sensitivity analysis for
'TRAF1' on 'LUSC'

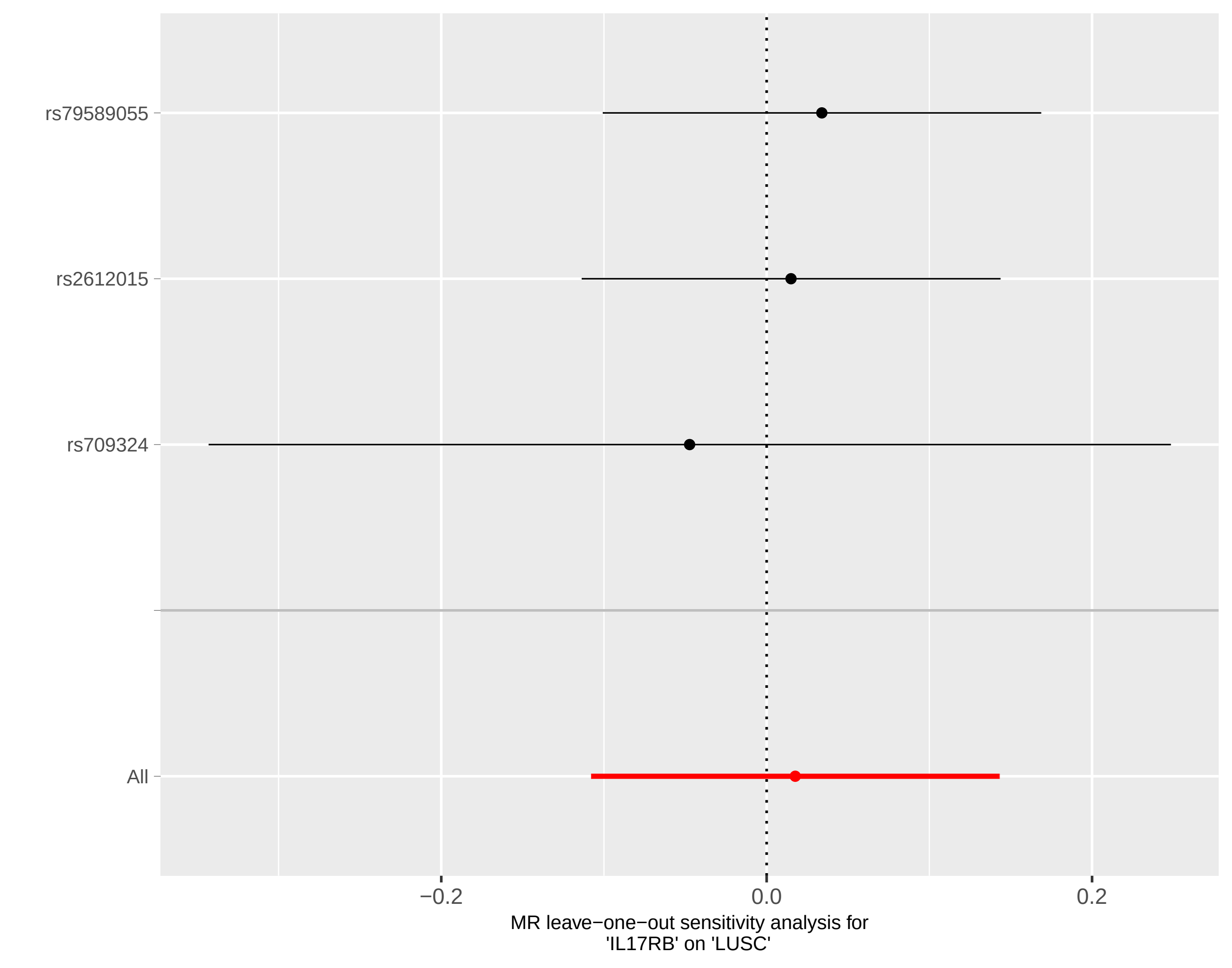

rs79589055
rs2612015
rs709324
All
−0.2
0.0
0.2
MR leave−one−out sensitivity analysis for
'IL17RB' on 'LUSC'

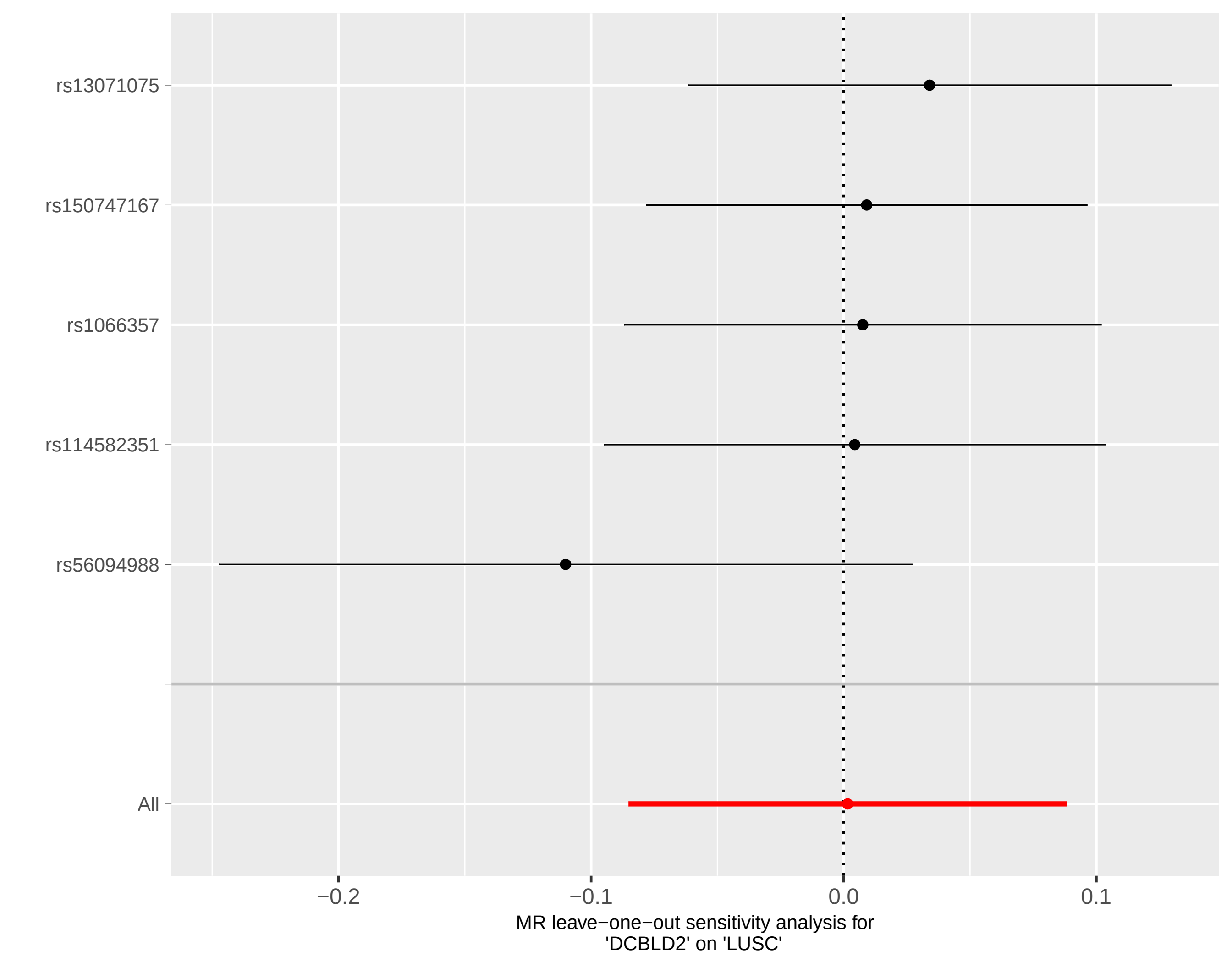
rs13071075
rs150747167
rs1066357
rs114582351
rs56094988
All
−0.2
−0.1
0.0
0.1
MR leave−one−out sensitivity analysis for
'DCBLD2' on 'LUSC'

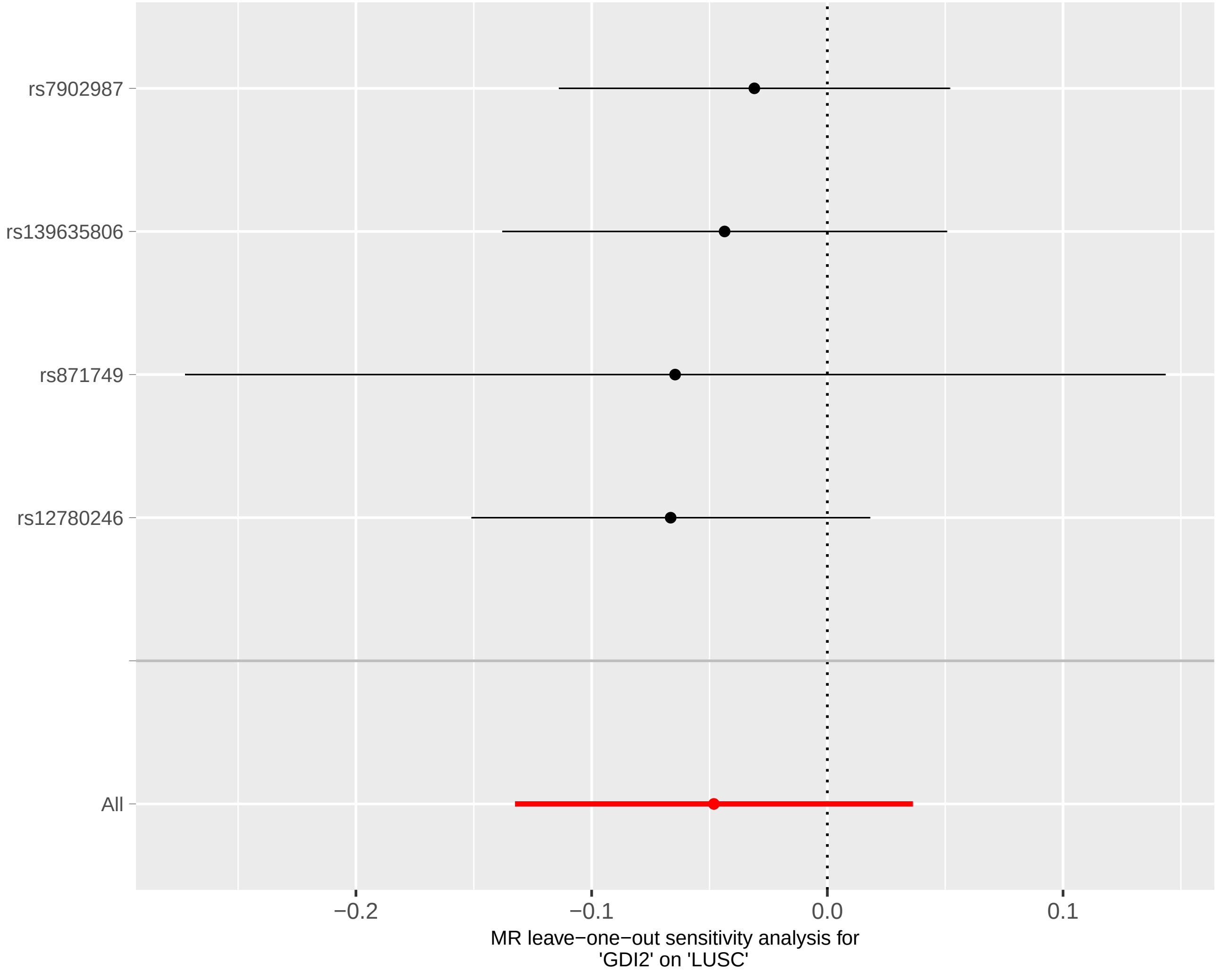
rs7902987
rs139635806
rs871749
rs12780246
All
−0.2
−0.1
0.0
0.1
MR leave−one−out sensitivity analysis for
'GDI2' on 'LUSC'

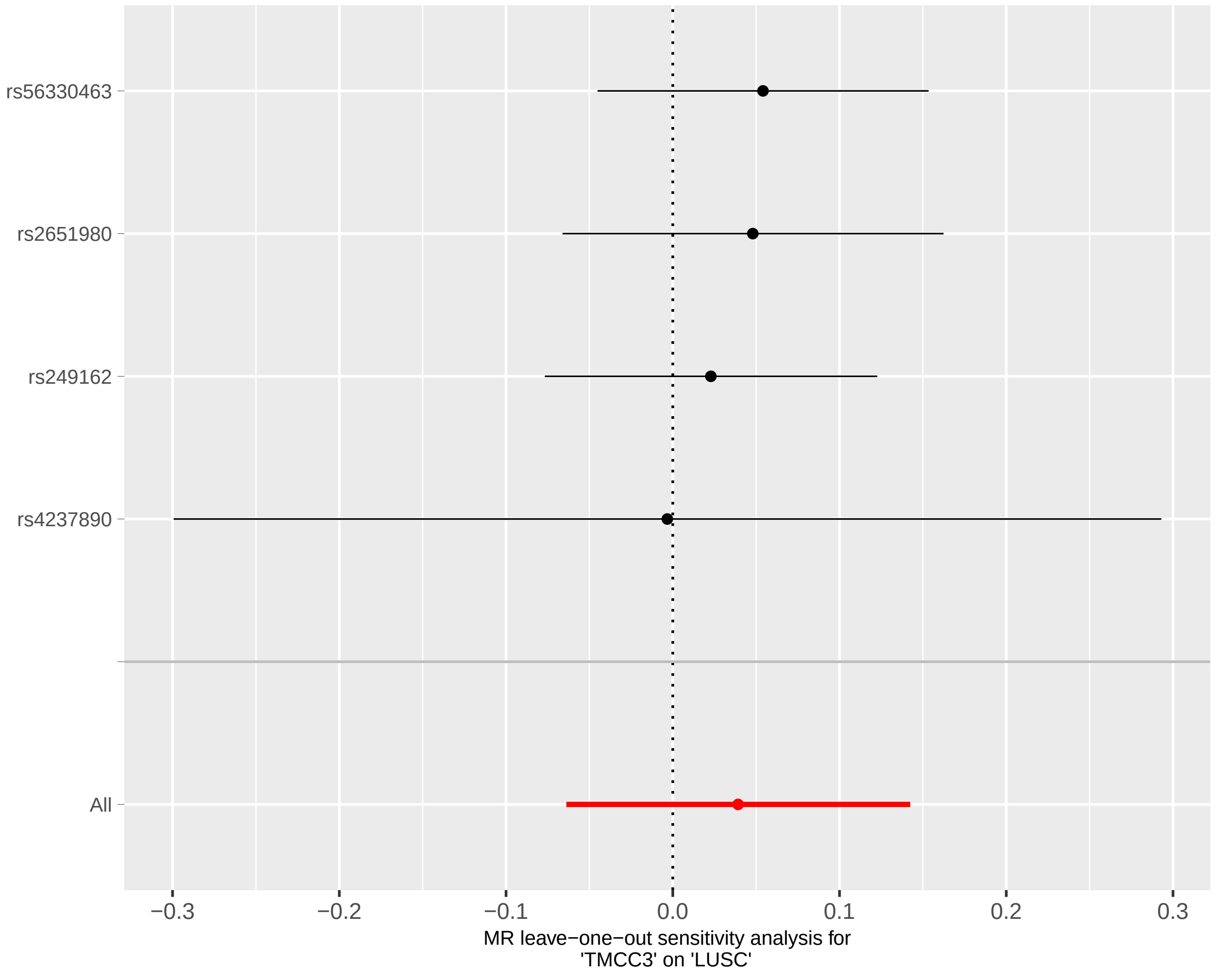
rs56330463
rs2651980
rs249162
rs4237890
All
−0.3
−0.2
−0.1
0.0
0.1
0.2
0.3
MR leave−one−out sensitivity analysis for
'TMCC3' on 'LUSC'

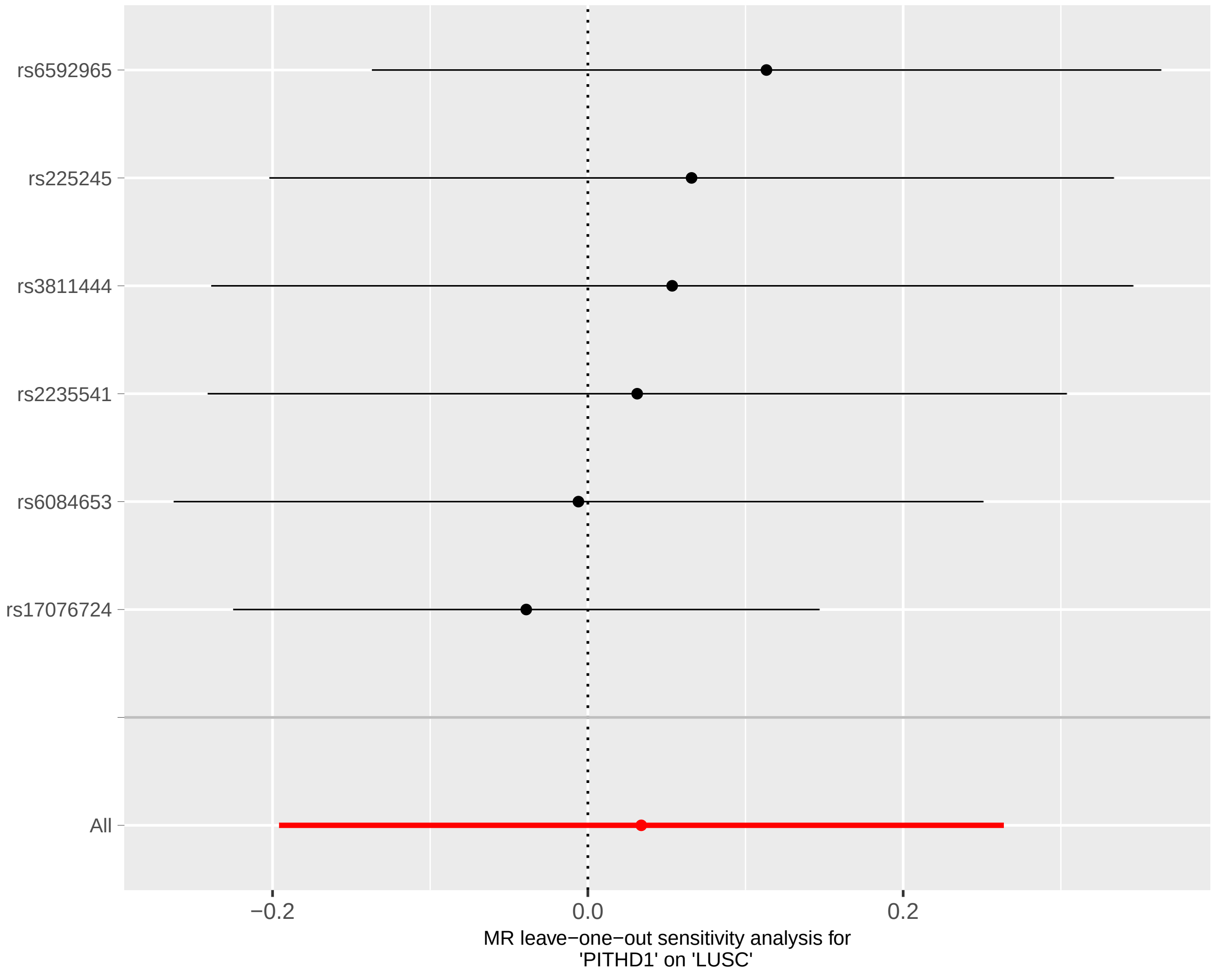
rs6592965
rs225245
rs3811444
rs2235541
rs6084653
rs17076724
All
−0.2
0.0
0.2
MR leave−one−out sensitivity analysis for
'PITHD1' on 'LUSC'

Insufficient number of SNPs

Insufficient number of SNPs

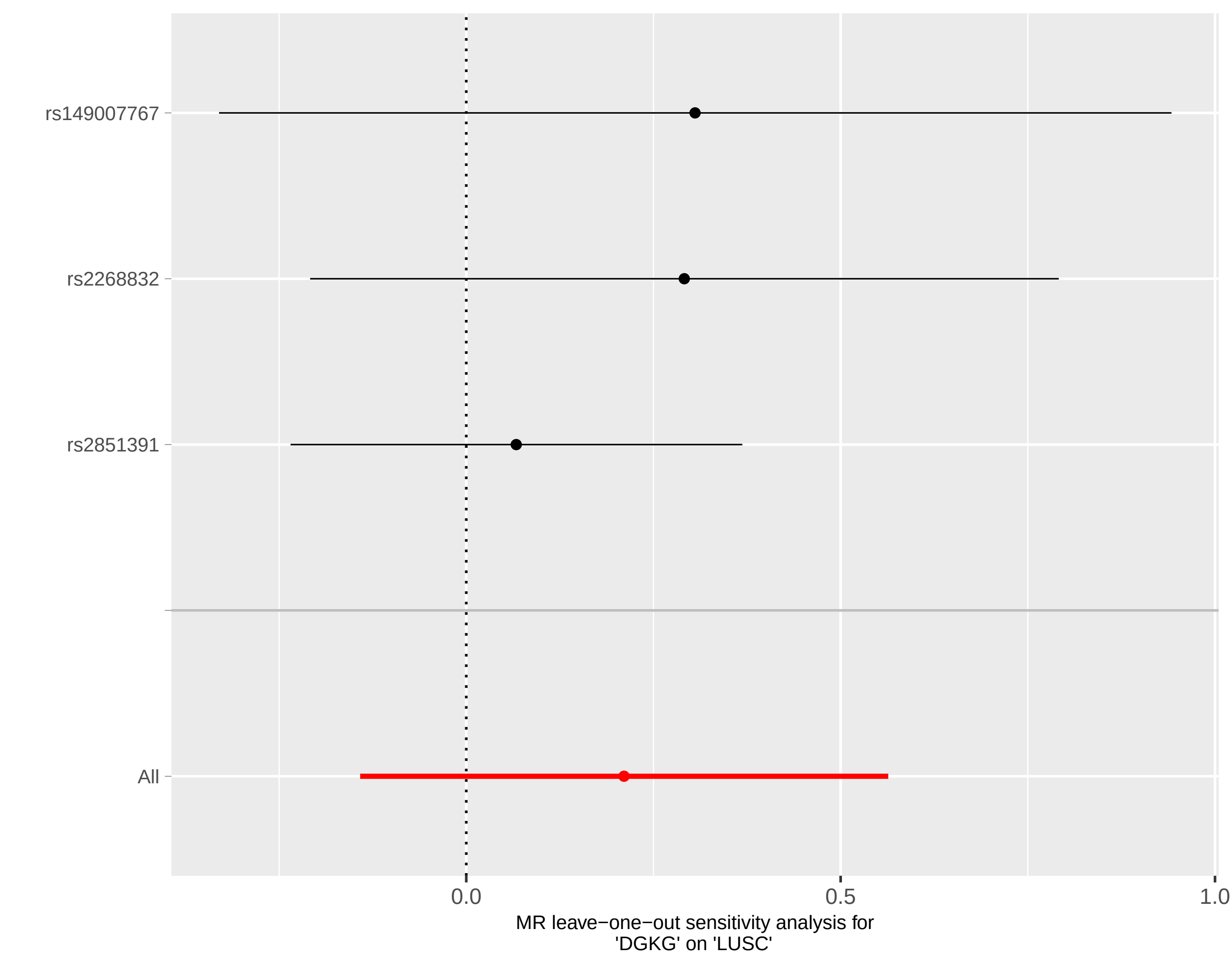
rs149007767
rs2268832
rs2851391
All
0.0
0.5
1.0
MR leave−one−out sensitivity analysis for
'DGKG' on 'LUSC'

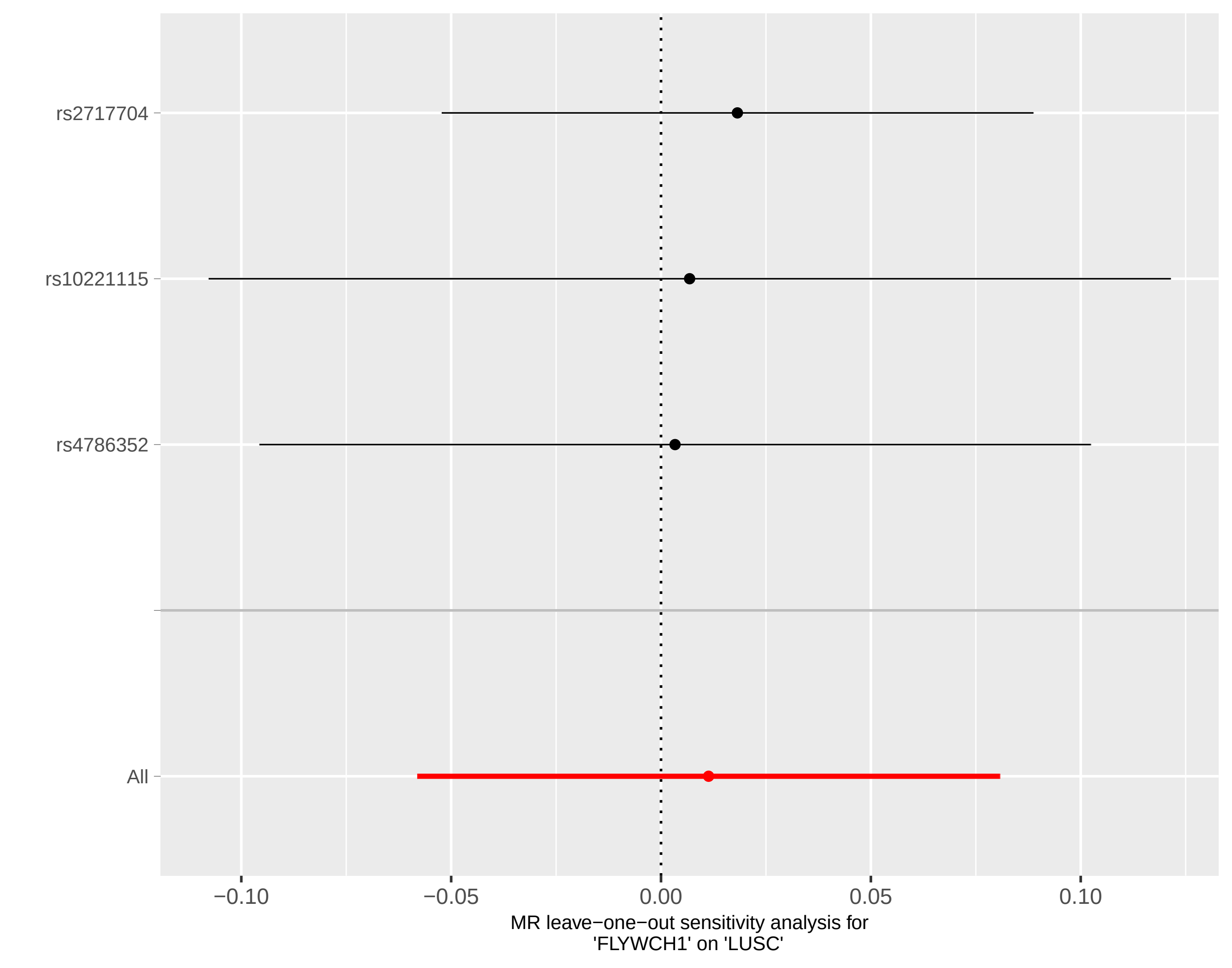

rs2717704
rs10221115
rs4786352
All
−0.10
−0.05
0.00
0.05
0.10
MR leave−one−out sensitivity analysis for
'FLYWCH1' on 'LUSC'

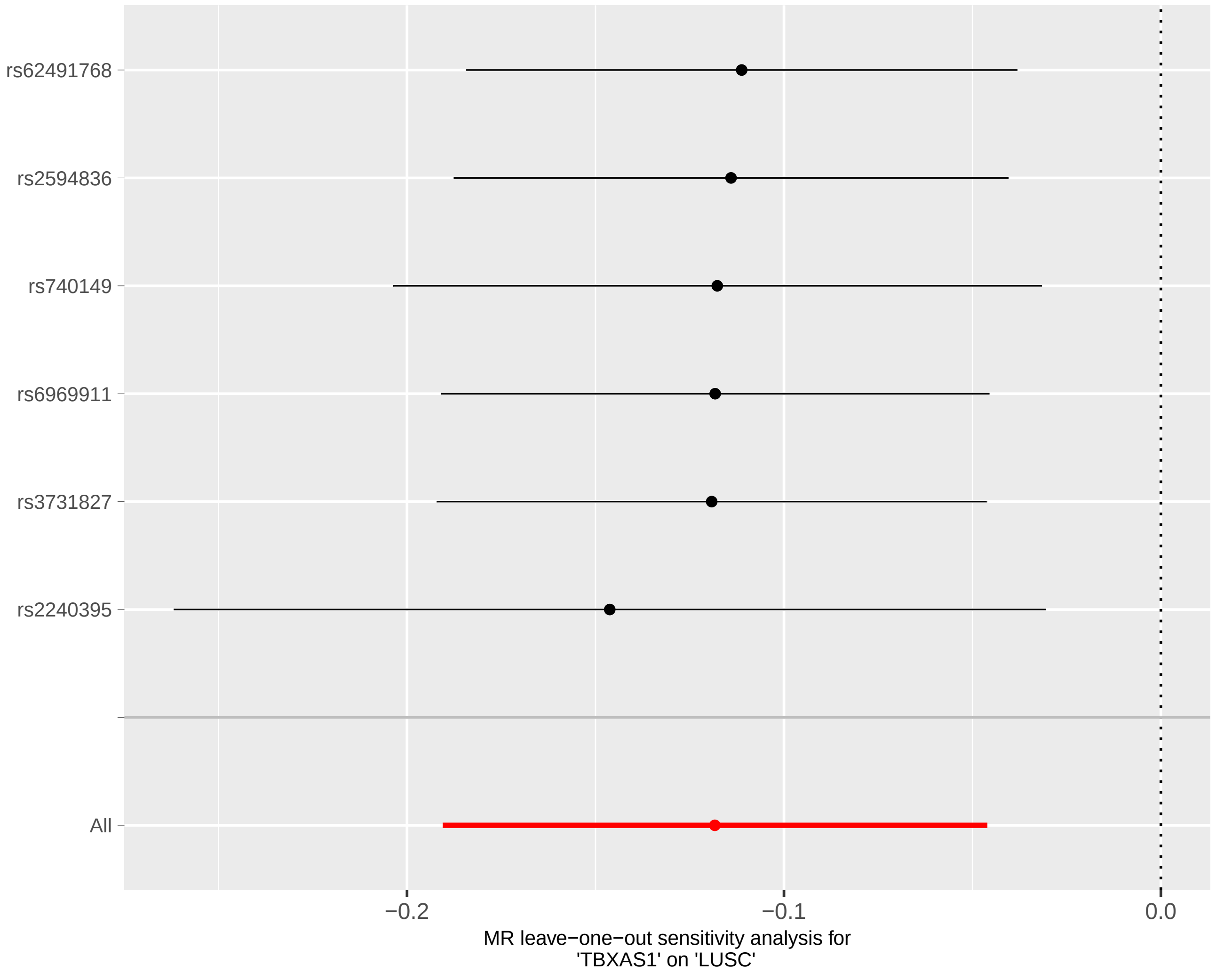

rs62491768
rs2594836
rs740149
rs6969911
rs3731827
rs2240395
All
−0.2
−0.1
0.0
MR leave−one−out sensitivity analysis for
'TBXAS1' on 'LUSC'

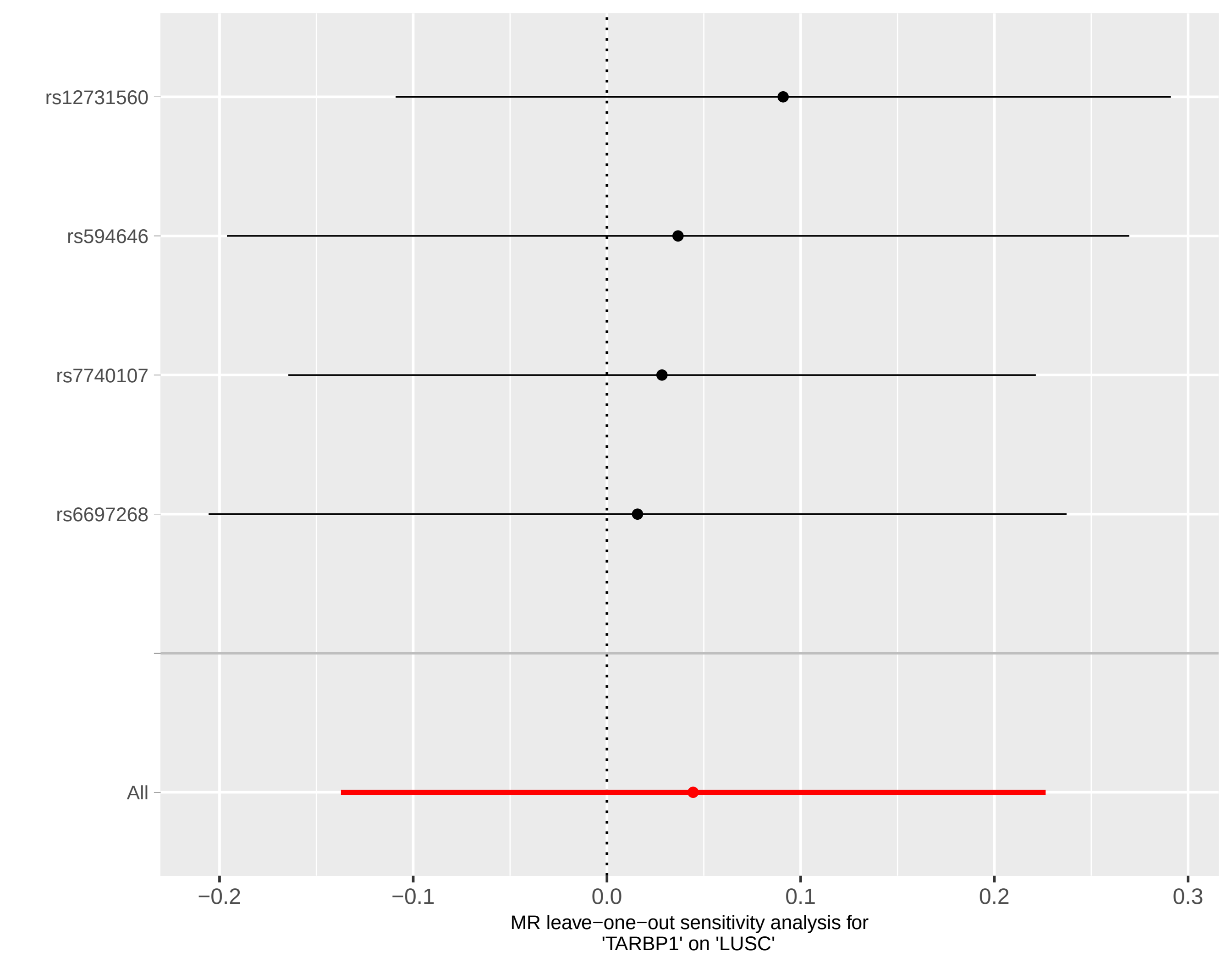
rs12731560
rs594646
rs7740107
rs6697268
All
−0.2
−0.1
0.0
0.1
0.2
0.3
MR leave−one−out sensitivity analysis for
'TARBP1' on 'LUSC'

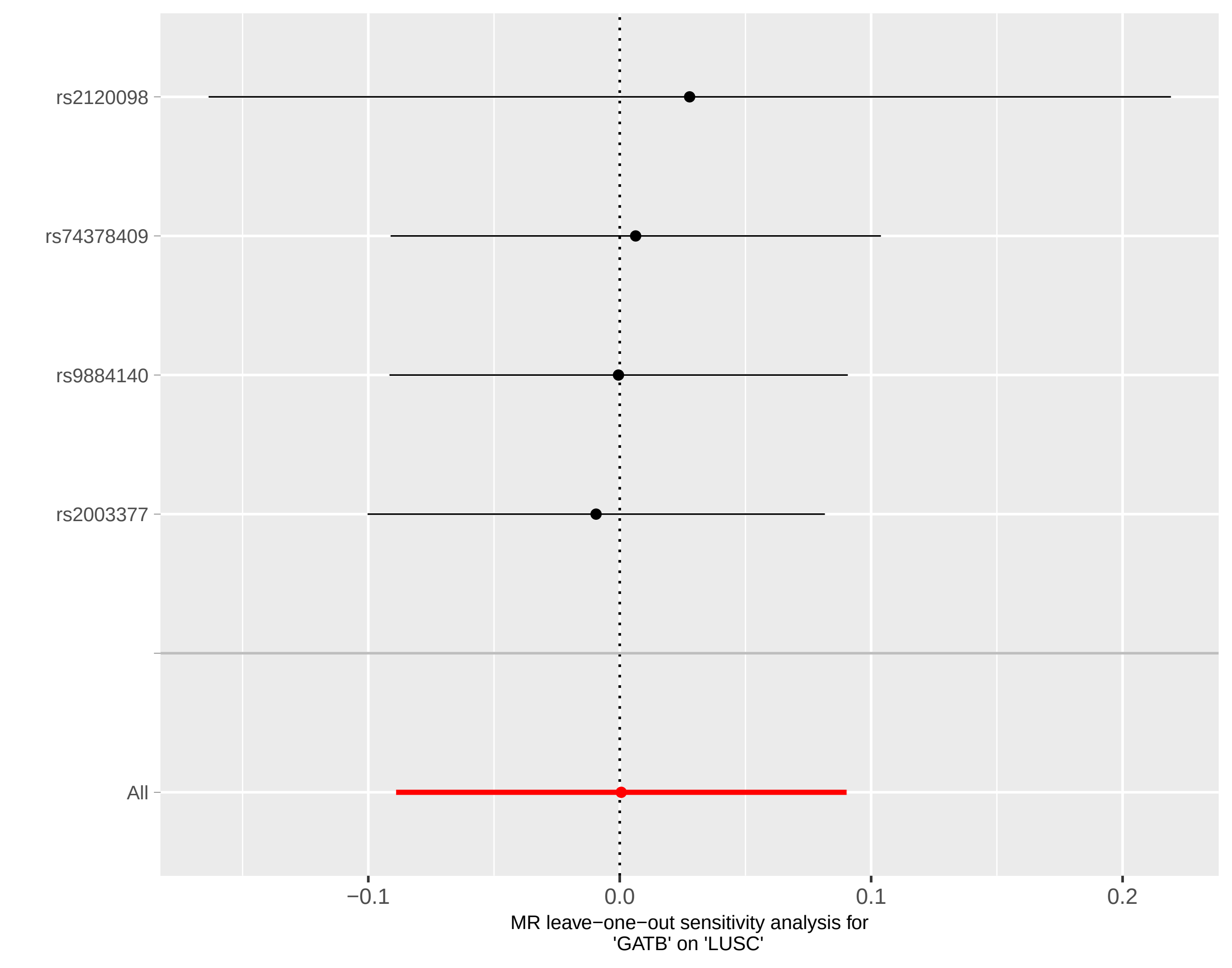
rs2120098
rs74378409
rs9884140
rs2003377
All
−0.1
0.0
0.1
0.2
MR leave−one−out sensitivity analysis for
'GATB' on 'LUSC'

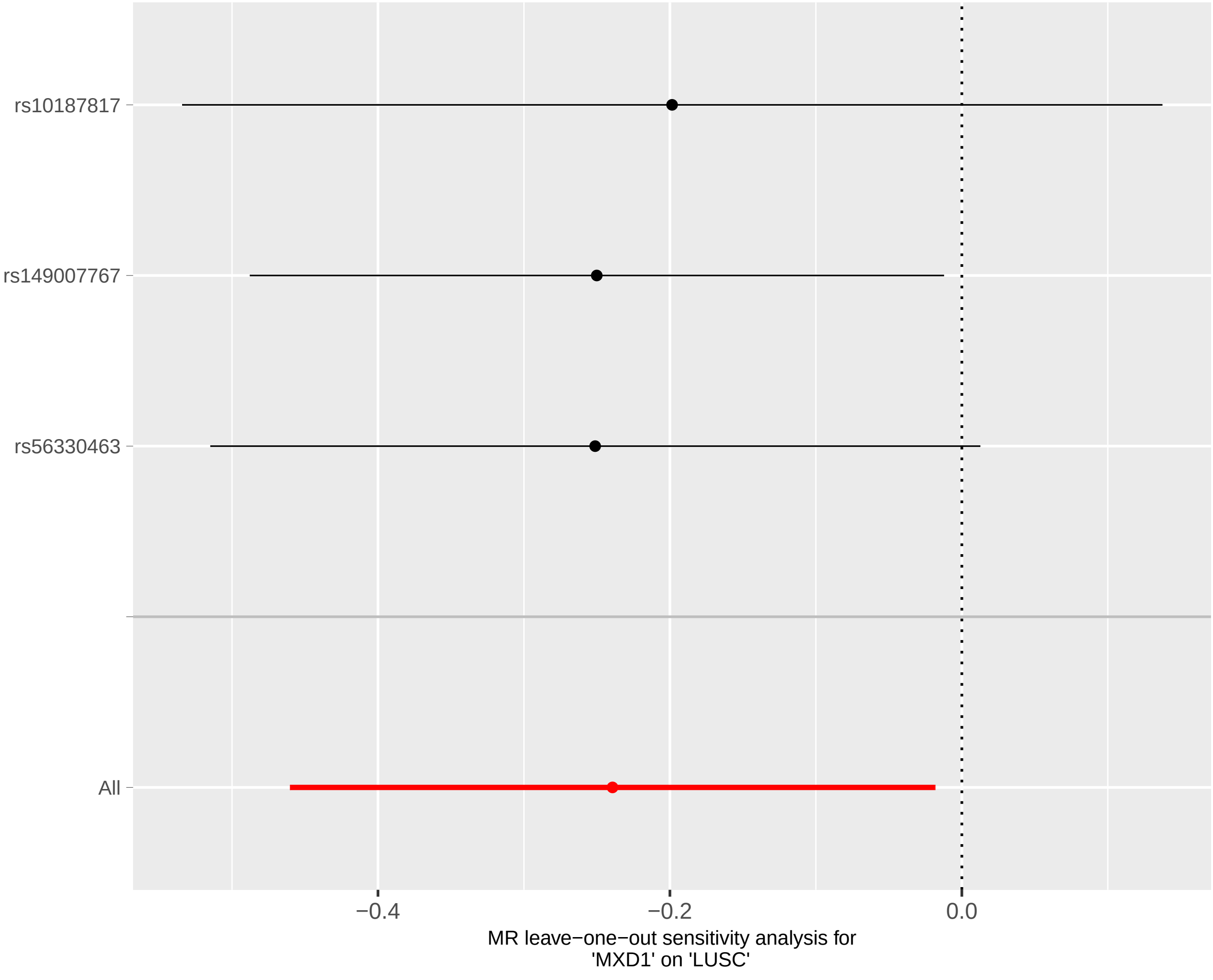
rs10187817
rs149007767
rs56330463
All
−0.4
−0.2
0.0
MR leave−one−out sensitivity analysis for
'MXD1' on 'LUSC'

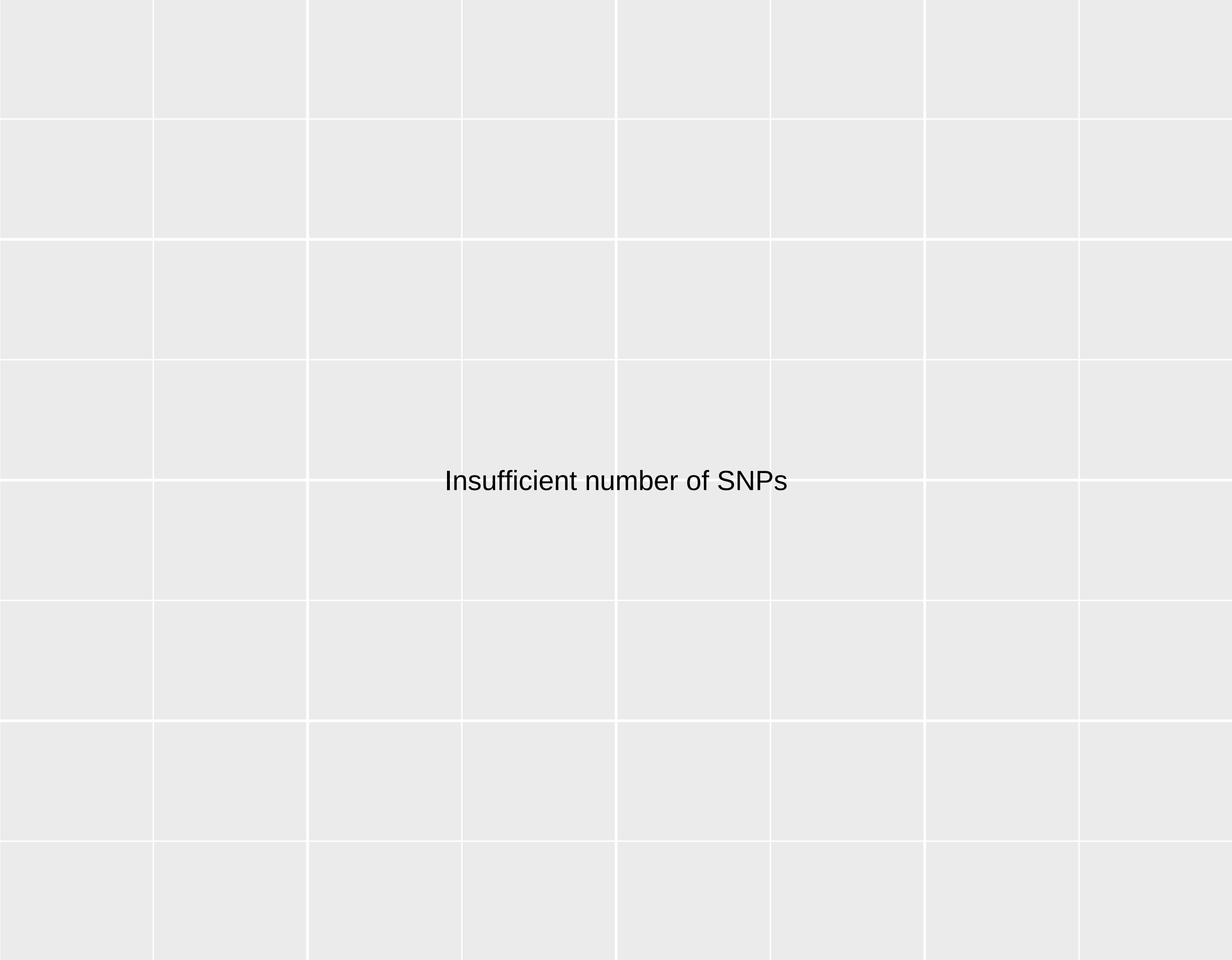
Insufficient number of SNPs

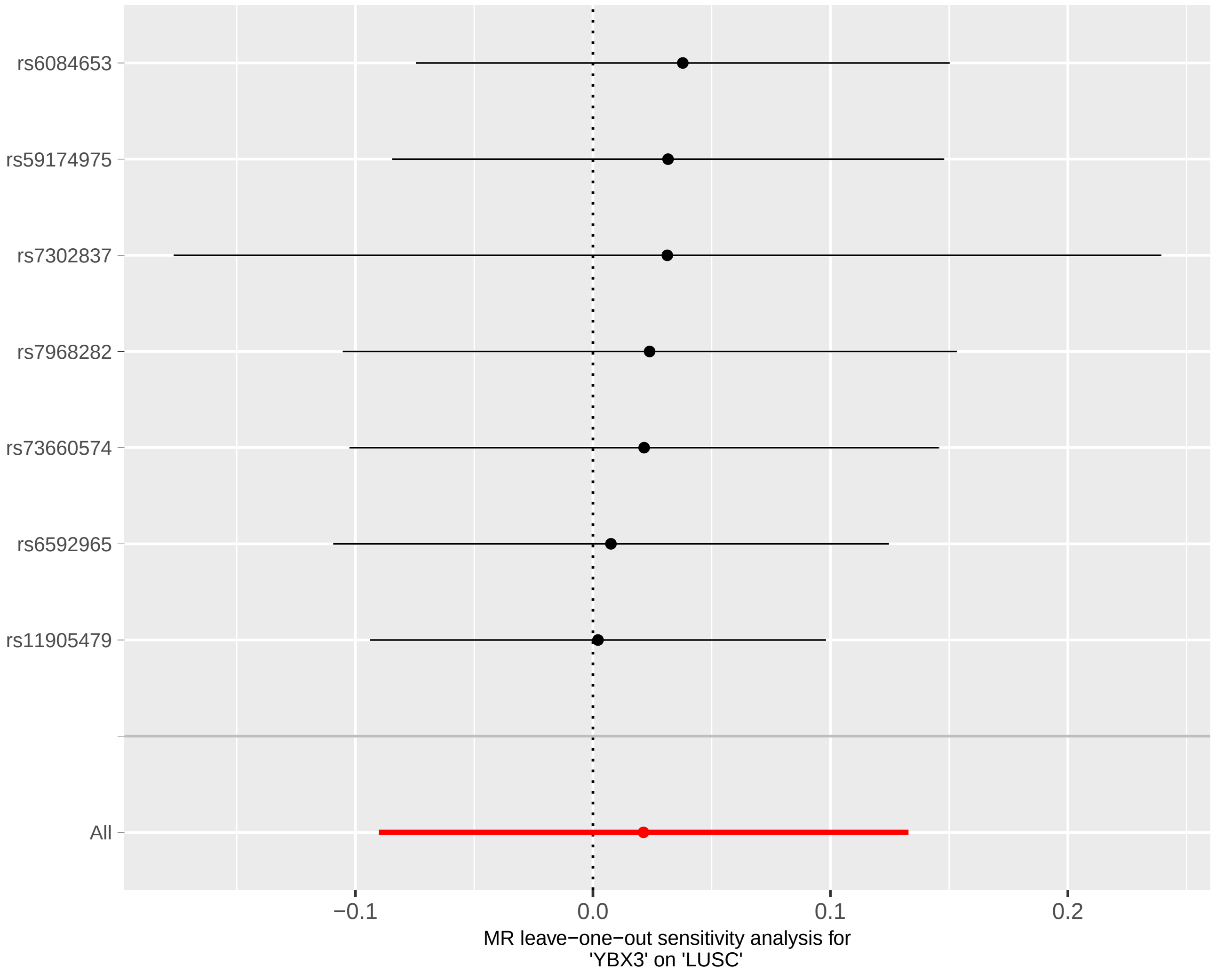

rs6084653
rs59174975
rs7302837
rs7968282
rs73660574
rs6592965
rs11905479
All
−0.1
0.0
0.1
0.2
MR leave−one−out sensitivity analysis for
'YBX3' on 'LUSC'

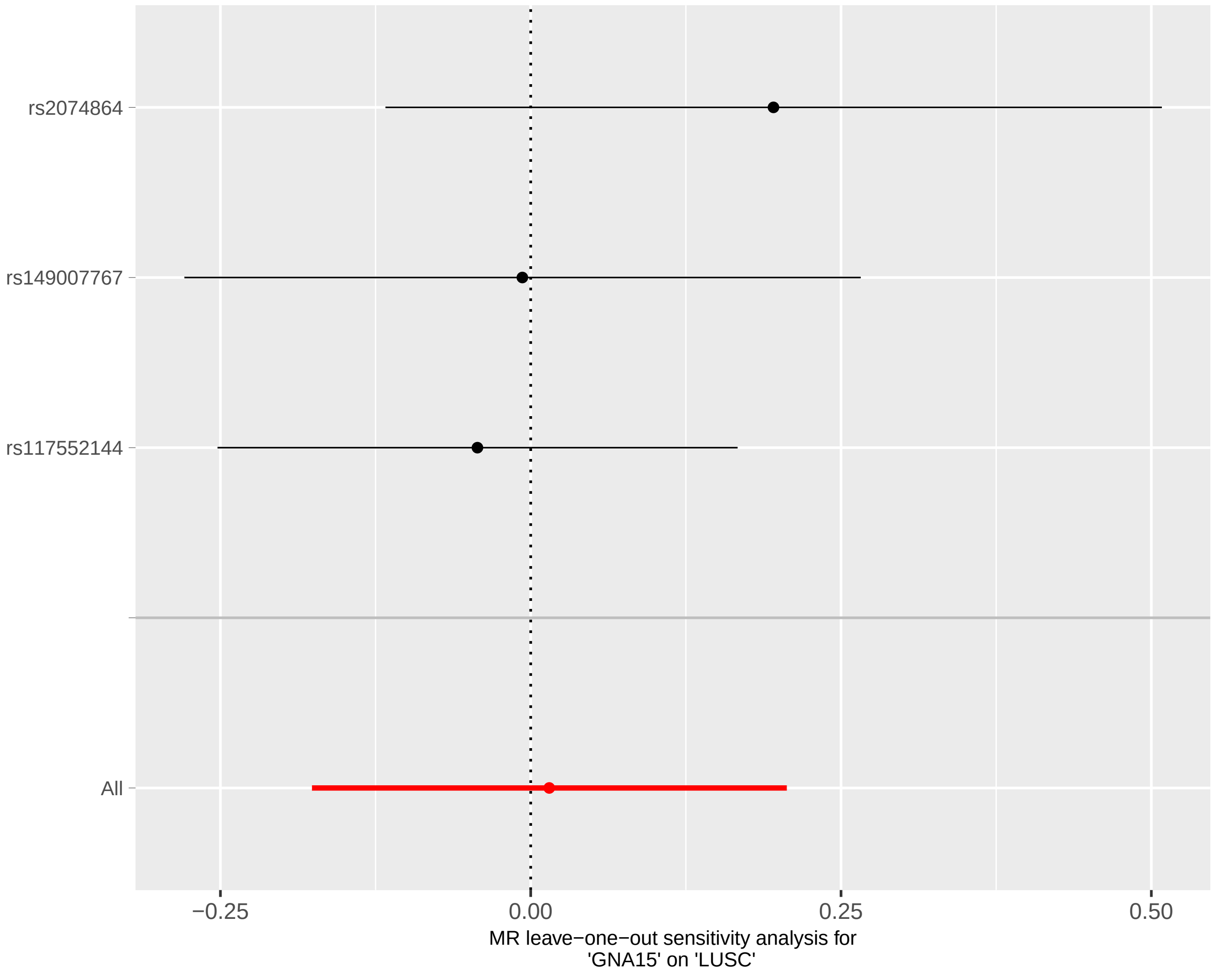

rs2074864
rs149007767
rs117552144
All
−0.25
0.00
0.25
0.50
MR leave−one−out sensitivity analysis for
'GNA15' on 'LUSC'

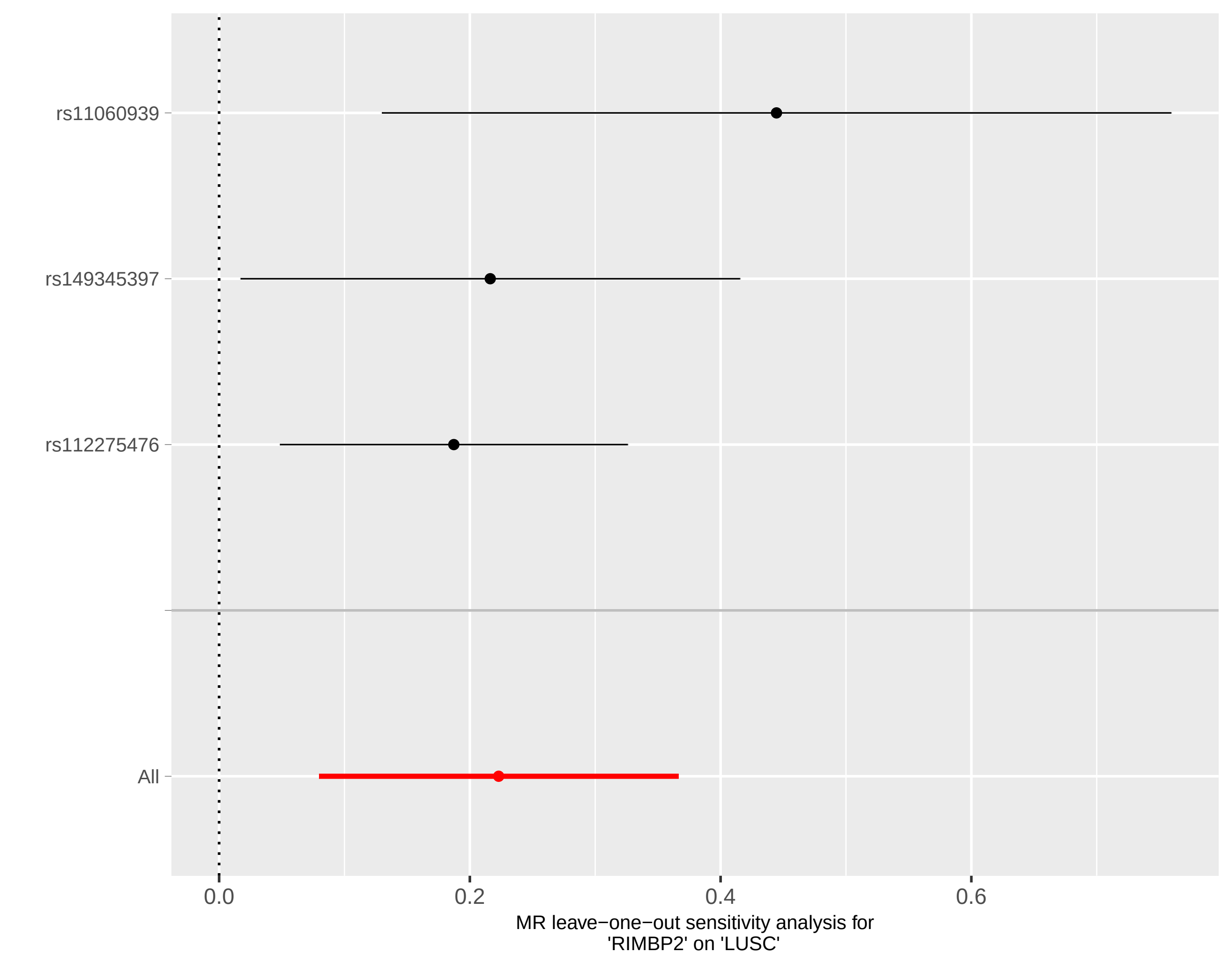

rs11060939
rs149345397
rs112275476
All
0.0
0.2
0.4
0.6
MR leave−one−out sensitivity analysis for
'RIMBP2' on 'LUSC'

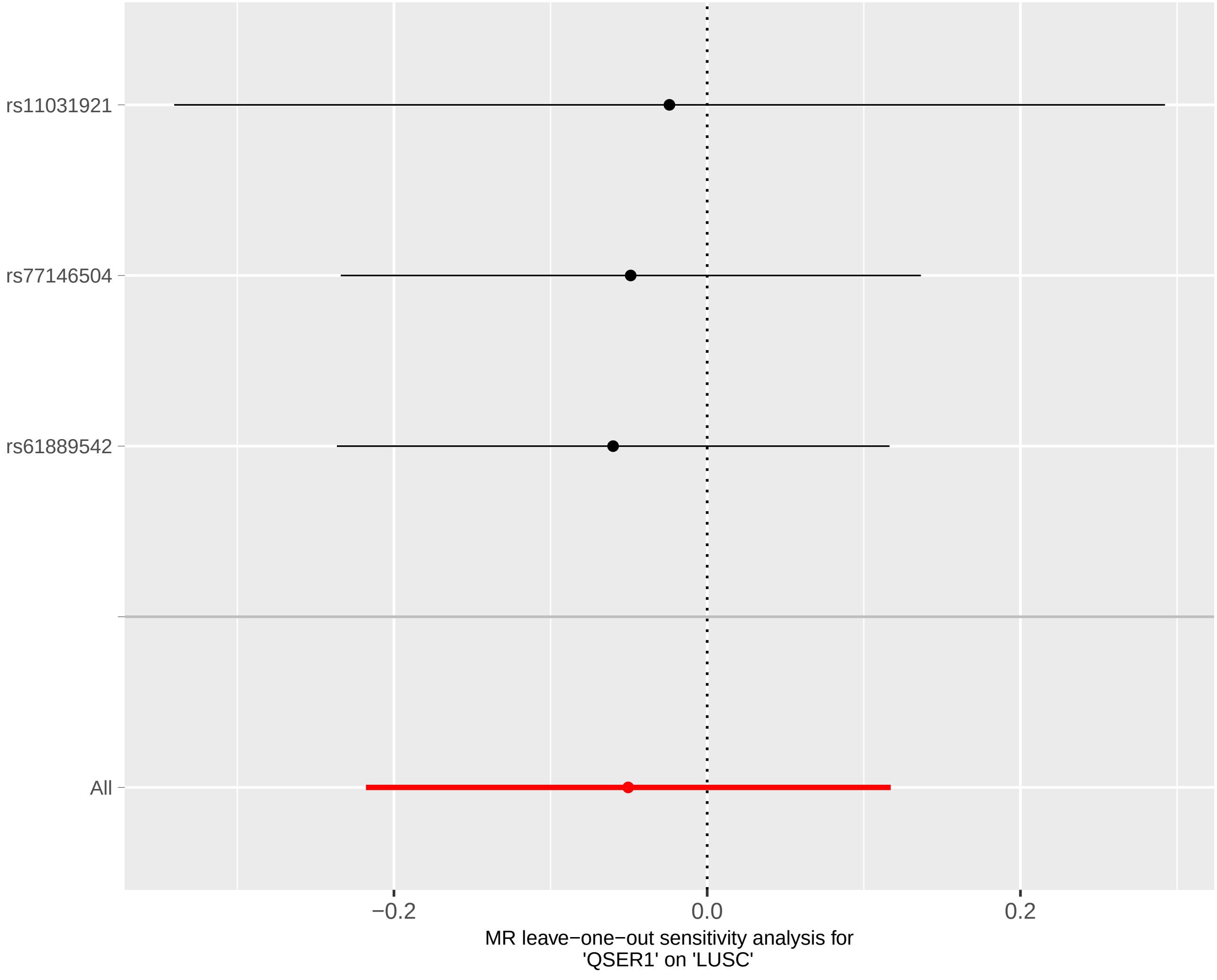

rs11031921
rs77146504
rs61889542
All
−0.2
0.0
0.2
MR leave−one−out sensitivity analysis for
'QSER1' on 'LUSC'

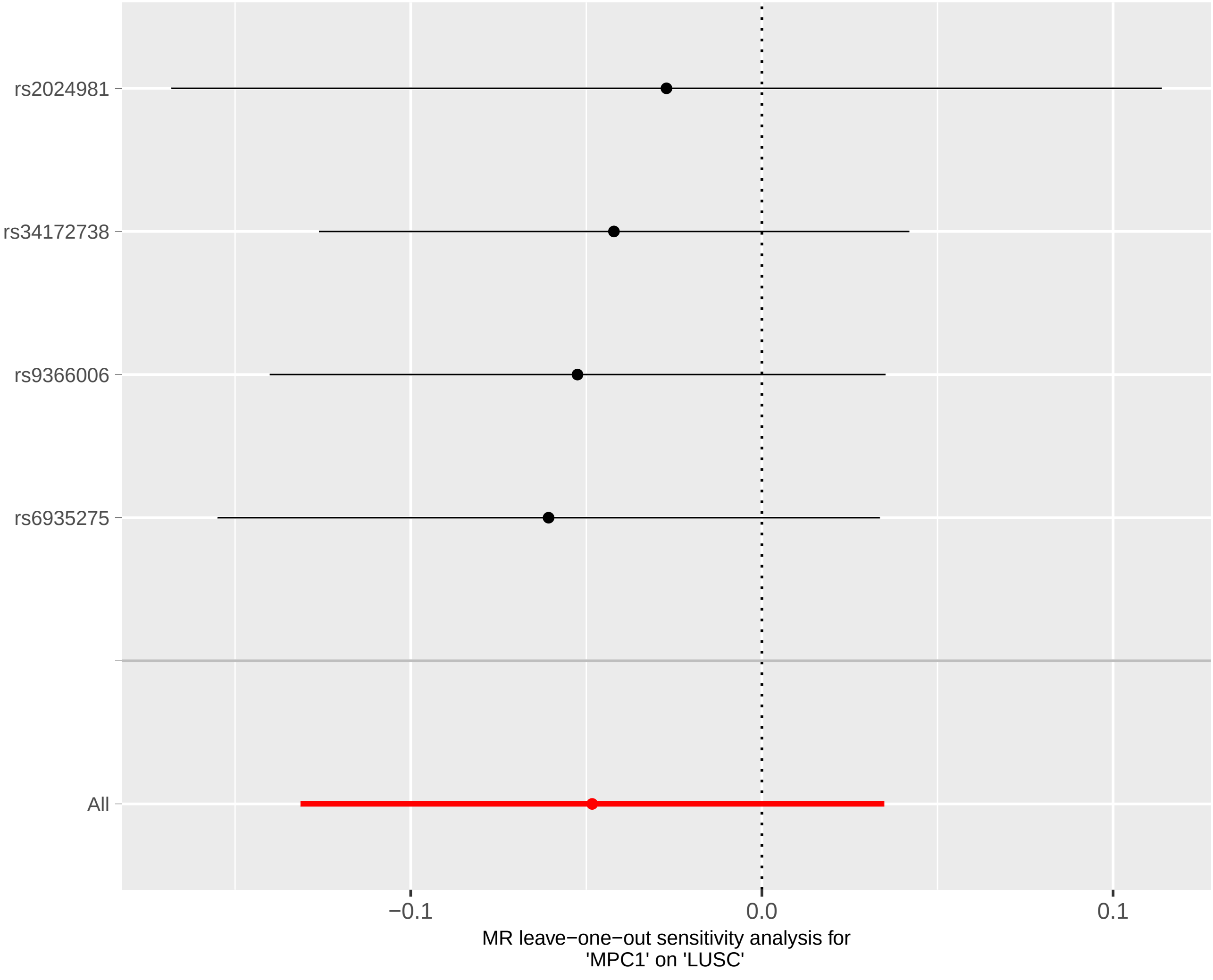

rs2024981
rs34172738
rs9366006
rs6935275
All
−0.1
0.0
0.1
MR leave−one−out sensitivity analysis for
'MPC1' on 'LUSC'

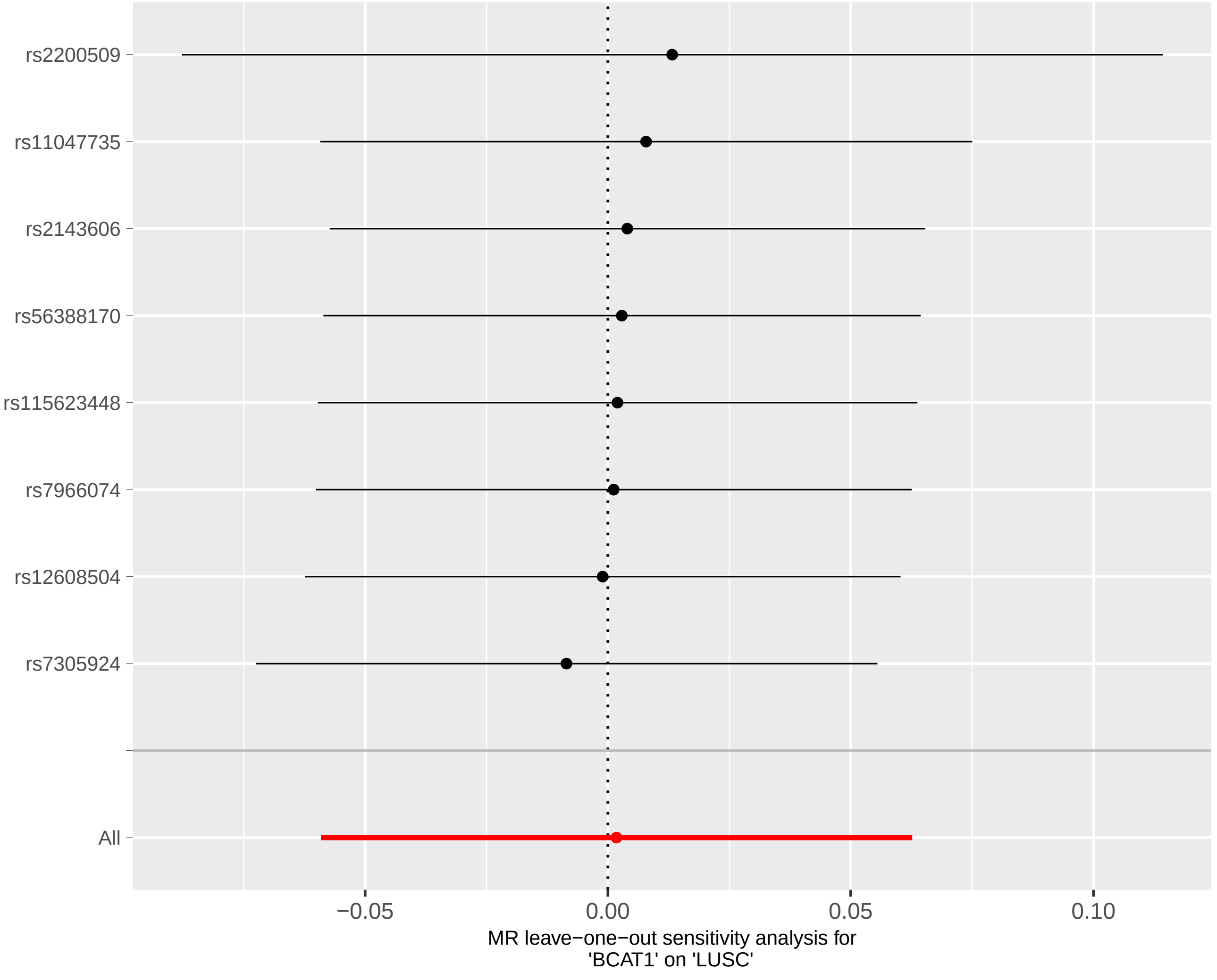
rs2200509
rs11047735
rs2143606
rs56388170
rs115623448
rs7966074
rs12608504
rs7305924
All
−0.05
0.00
0.05
0.10
MR leave−one−out sensitivity analysis for
'BCAT1' on 'LUSC'

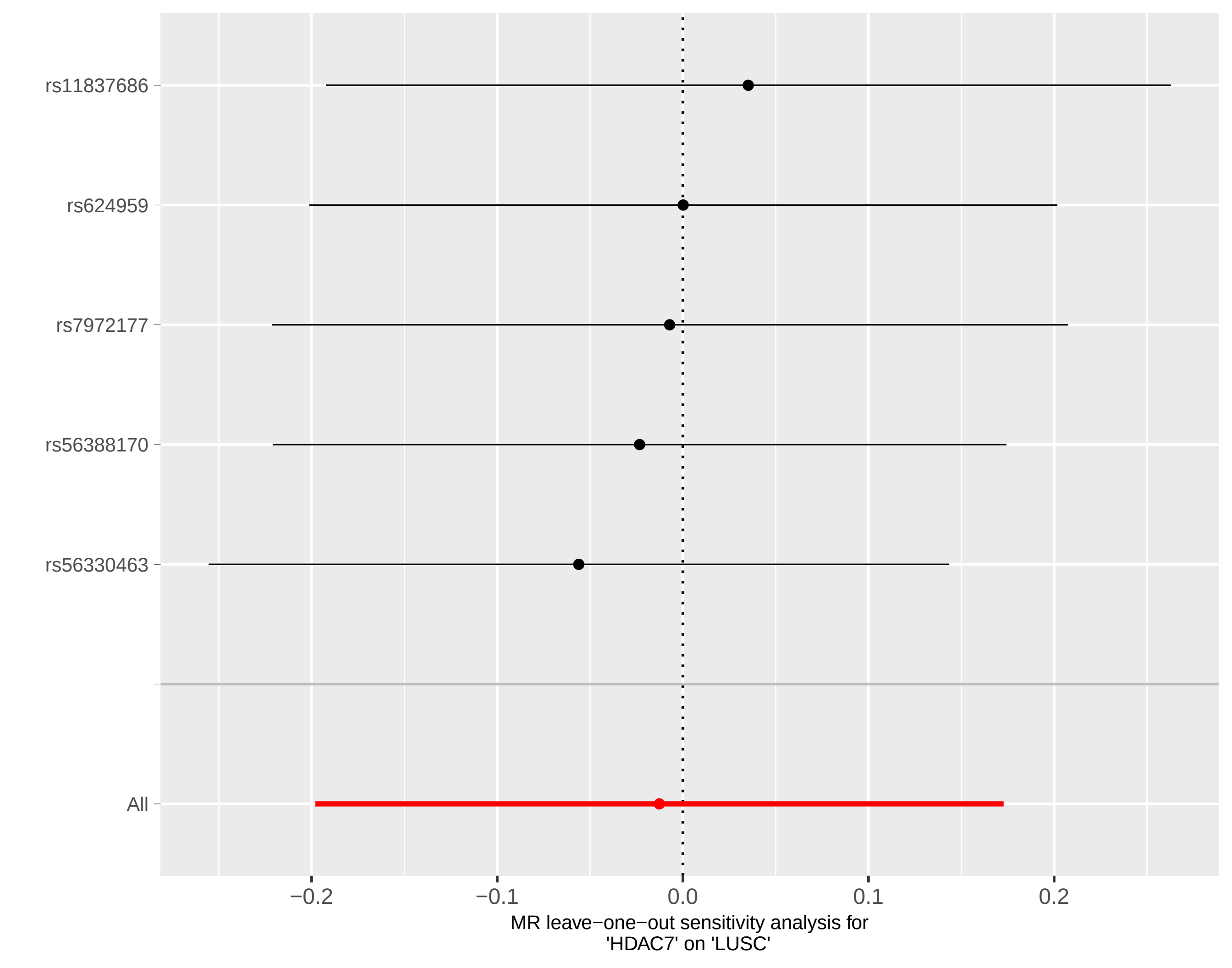
rs11837686
rs624959
rs7972177
rs56388170
rs56330463
All
−0.2
−0.1
0.0
0.1
0.2
MR leave−one−out sensitivity analysis for
'HDAC7' on 'LUSC'

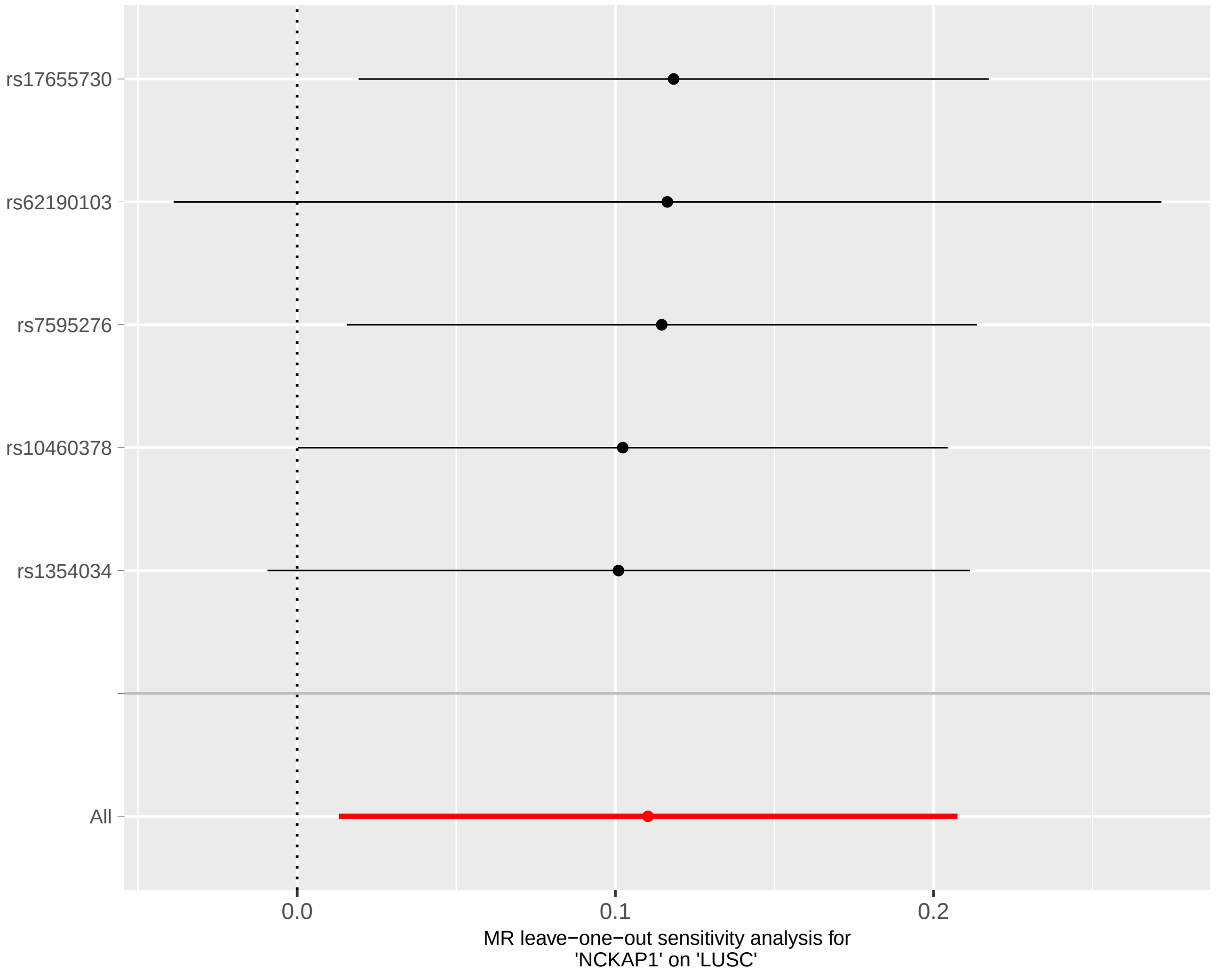

rs17655730
rs62190103
rs7595276
rs10460378
rs1354034
All
0.0
0.1
0.2
MR leave−one−out sensitivity analysis for
'NCKAP1' on 'LUSC'

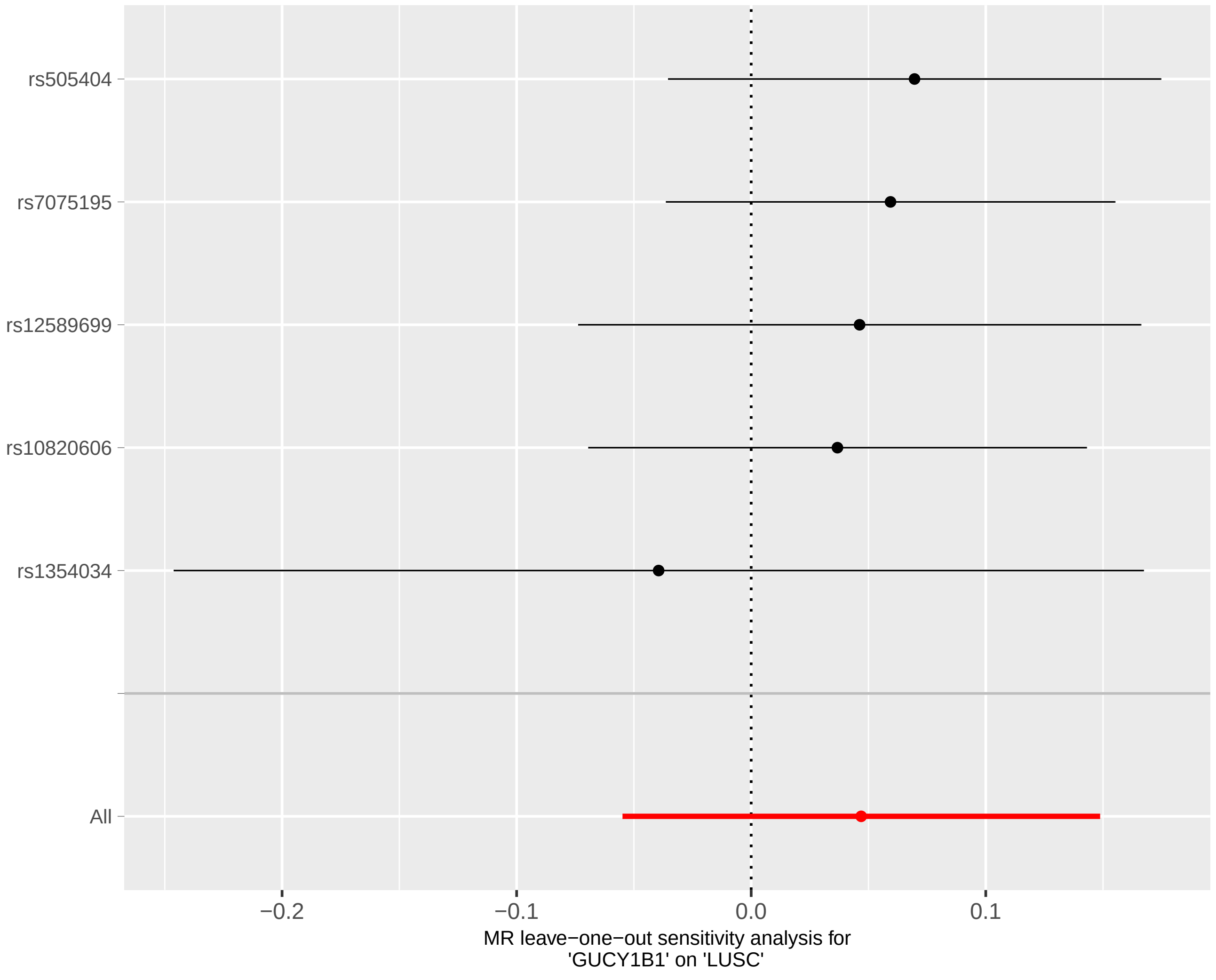
rs505404
rs7075195
rs12589699
rs10820606
rs1354034
All
−0.2
−0.1
0.0
0.1
MR leave−one−out sensitivity analysis for
'GUCY1B1' on 'LUSC'

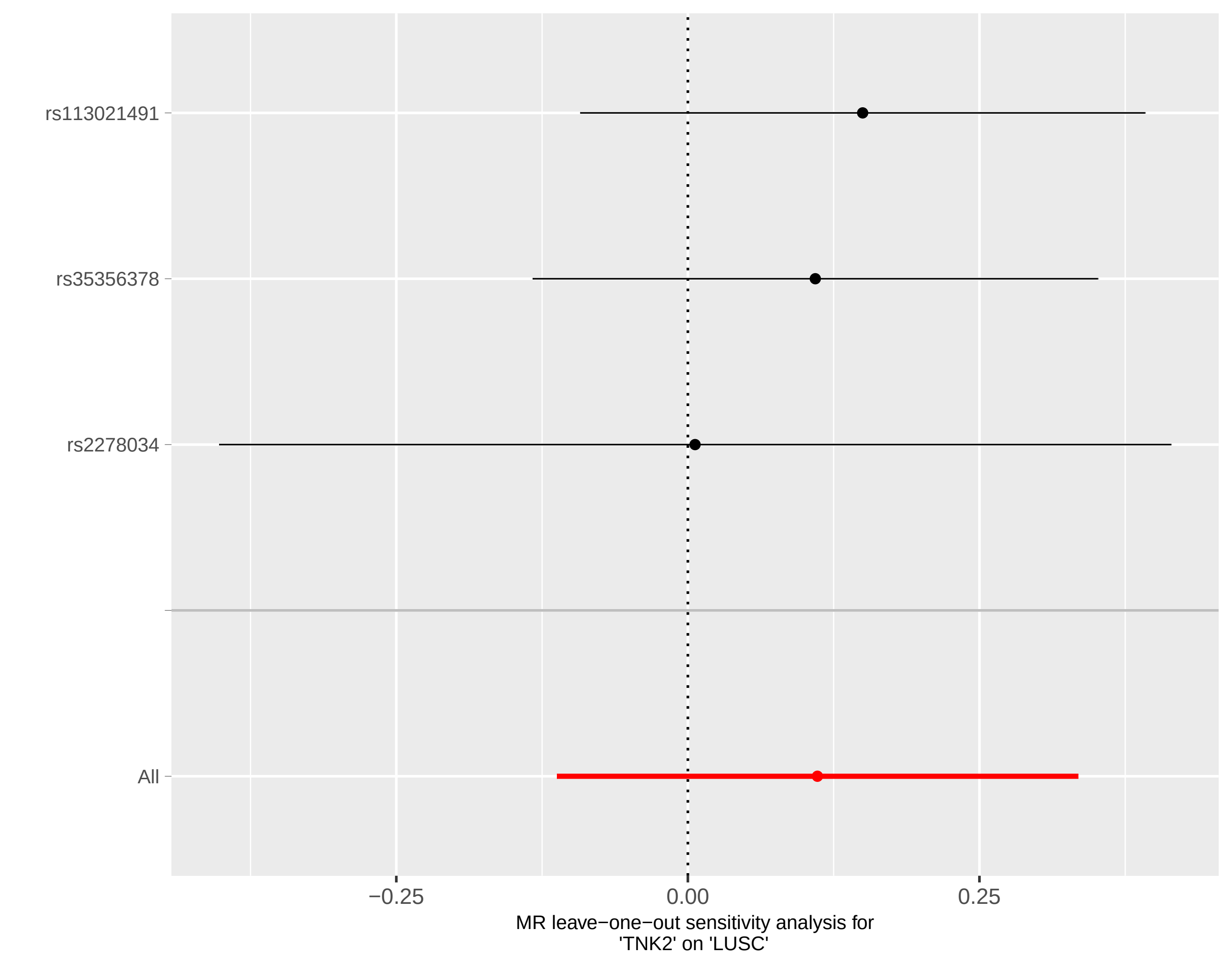

rs113021491
rs35356378
rs2278034
All
−0.25
0.00
0.25
MR leave−one−out sensitivity analysis for
'TNK2' on 'LUSC'

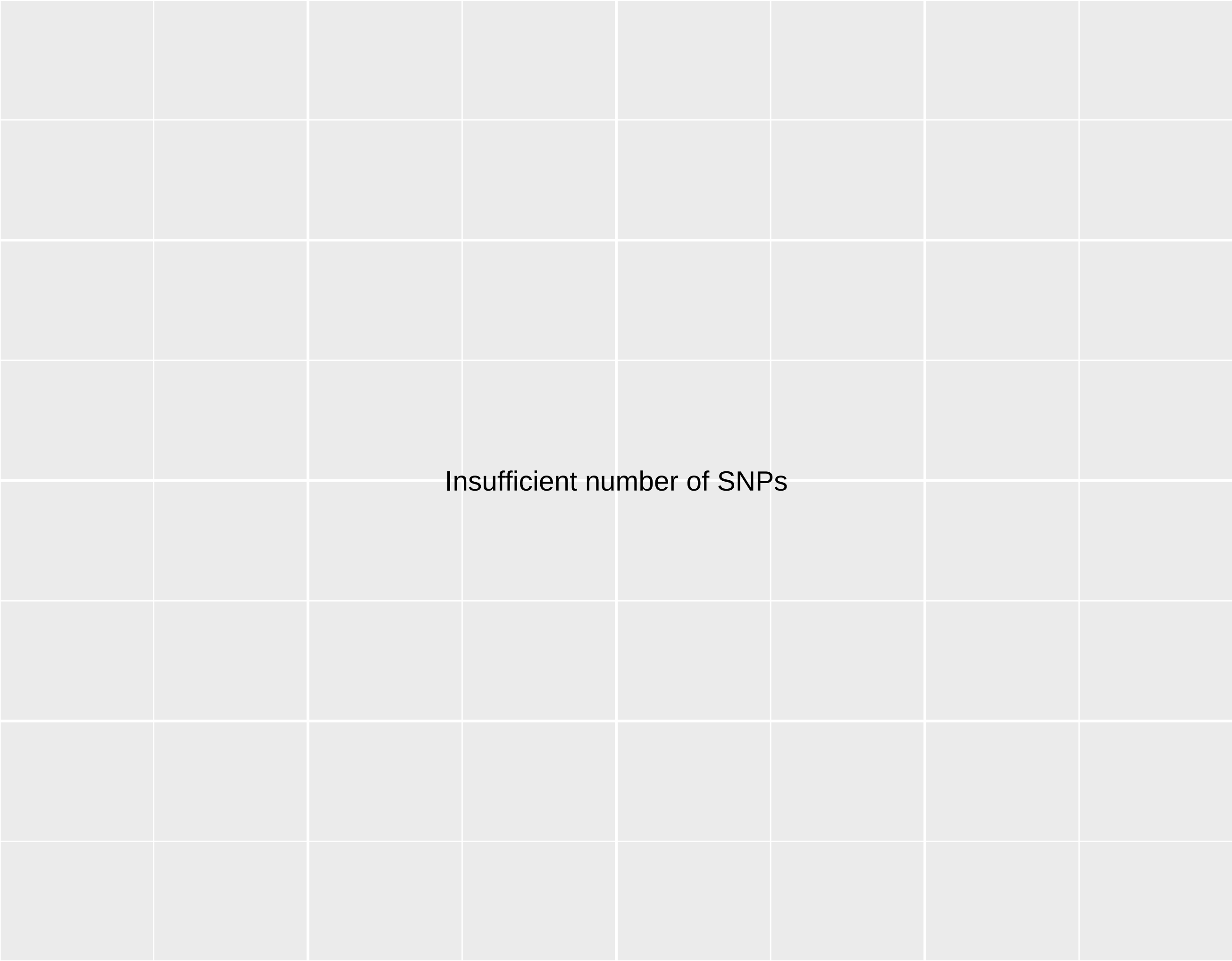
Insufficient number of SNPs

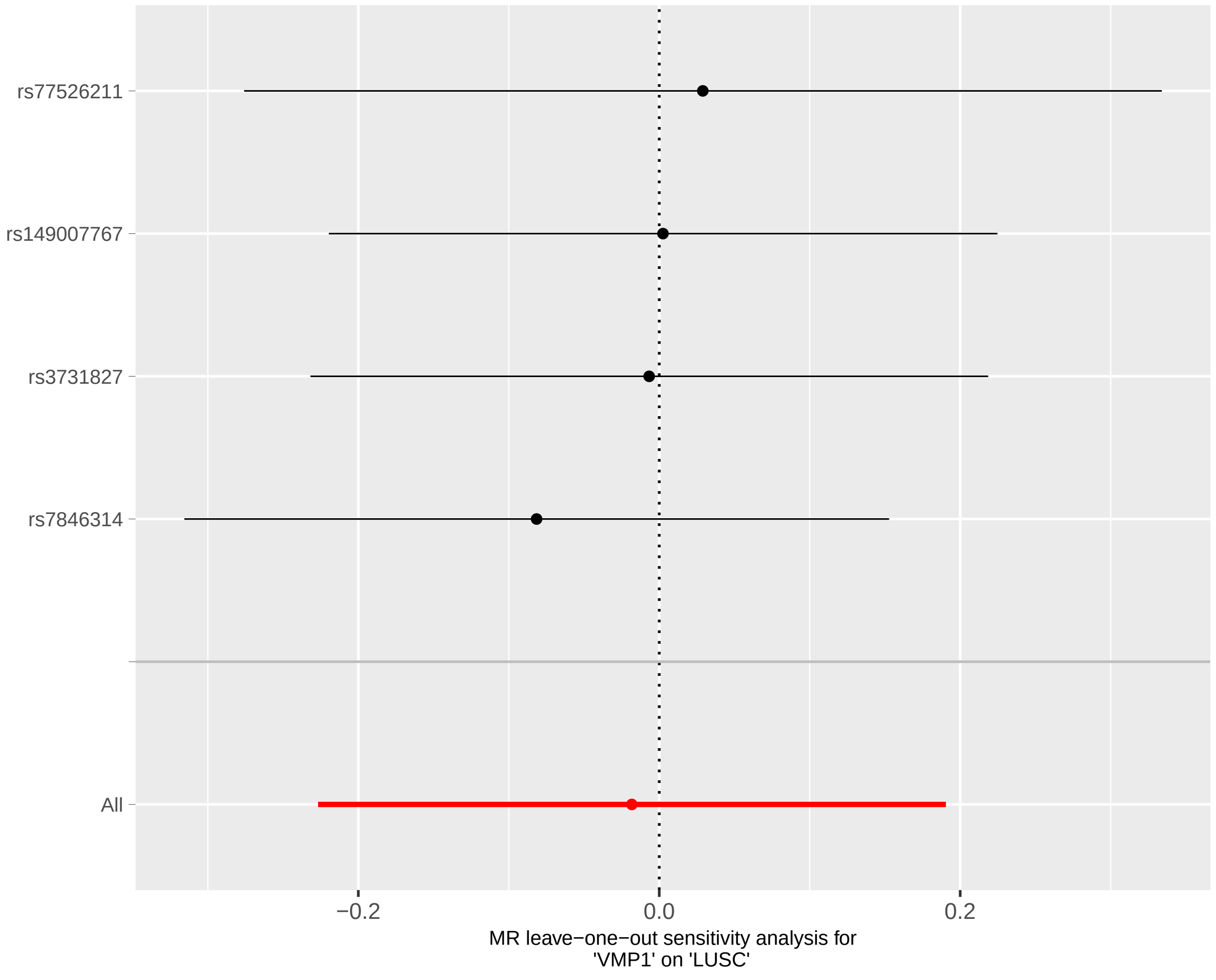
rs77526211
rs149007767
rs3731827
rs7846314
All
−0.2
0.0
0.2
MR leave−one−out sensitivity analysis for
'VMP1' on 'LUSC'

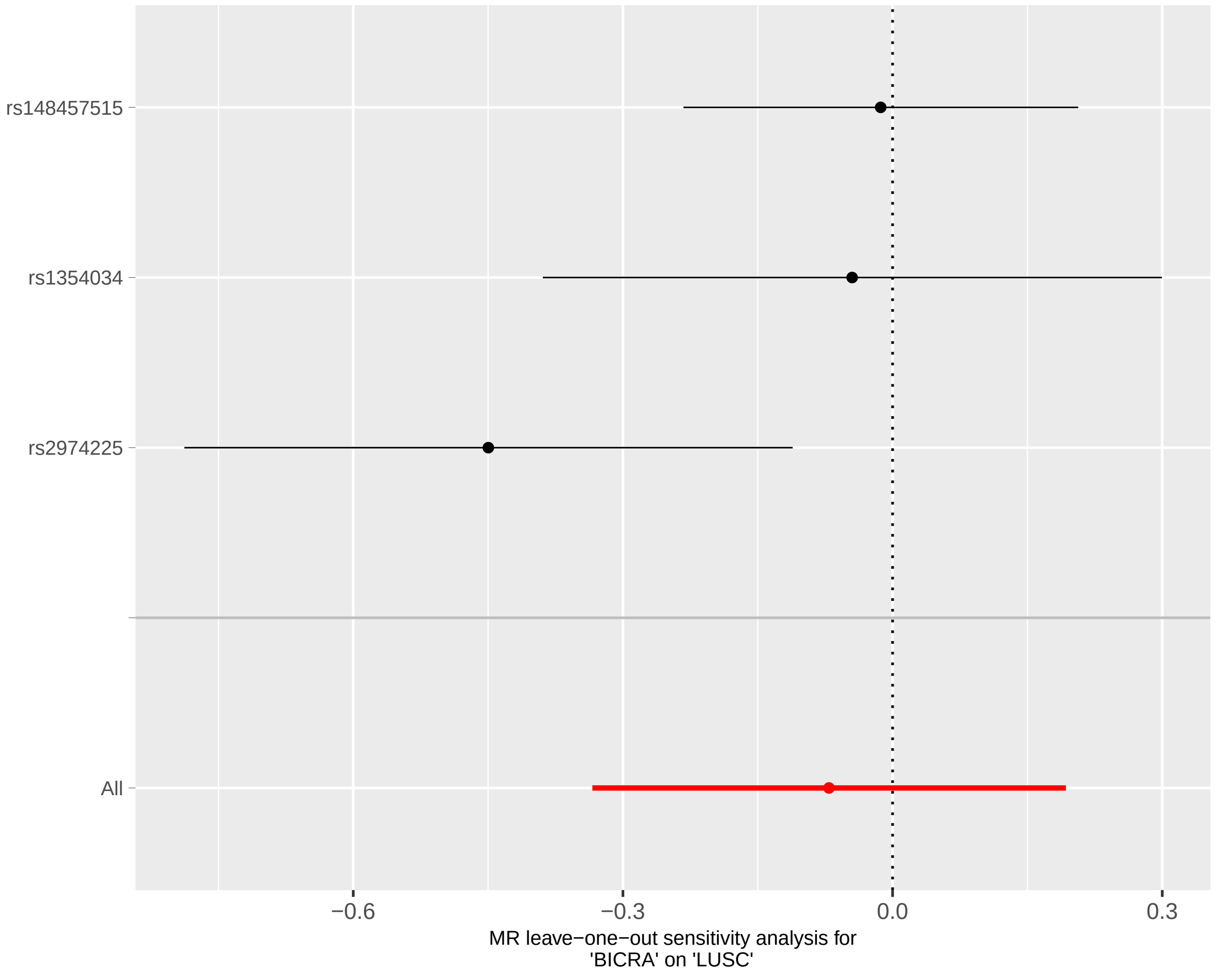
rs148457515
rs1354034
rs2974225
All
−0.6
−0.3
0.0
0.3
MR leave−one−out sensitivity analysis for
'BICRA' on 'LUSC'

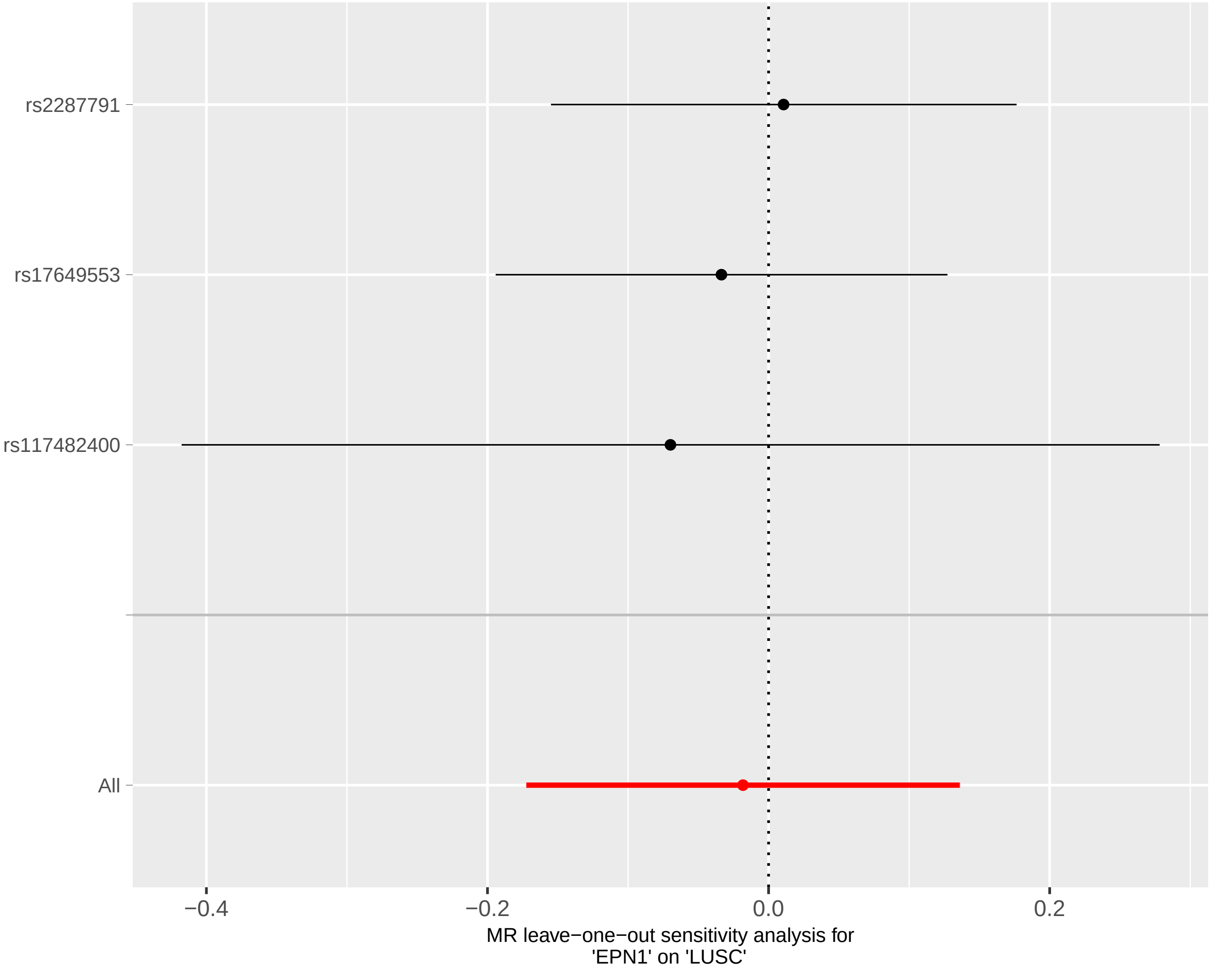

rs2287791
rs17649553
rs117482400
All
−0.4
−0.2
0.0
0.2
MR leave−one−out sensitivity analysis for
'EPN1' on 'LUSC'

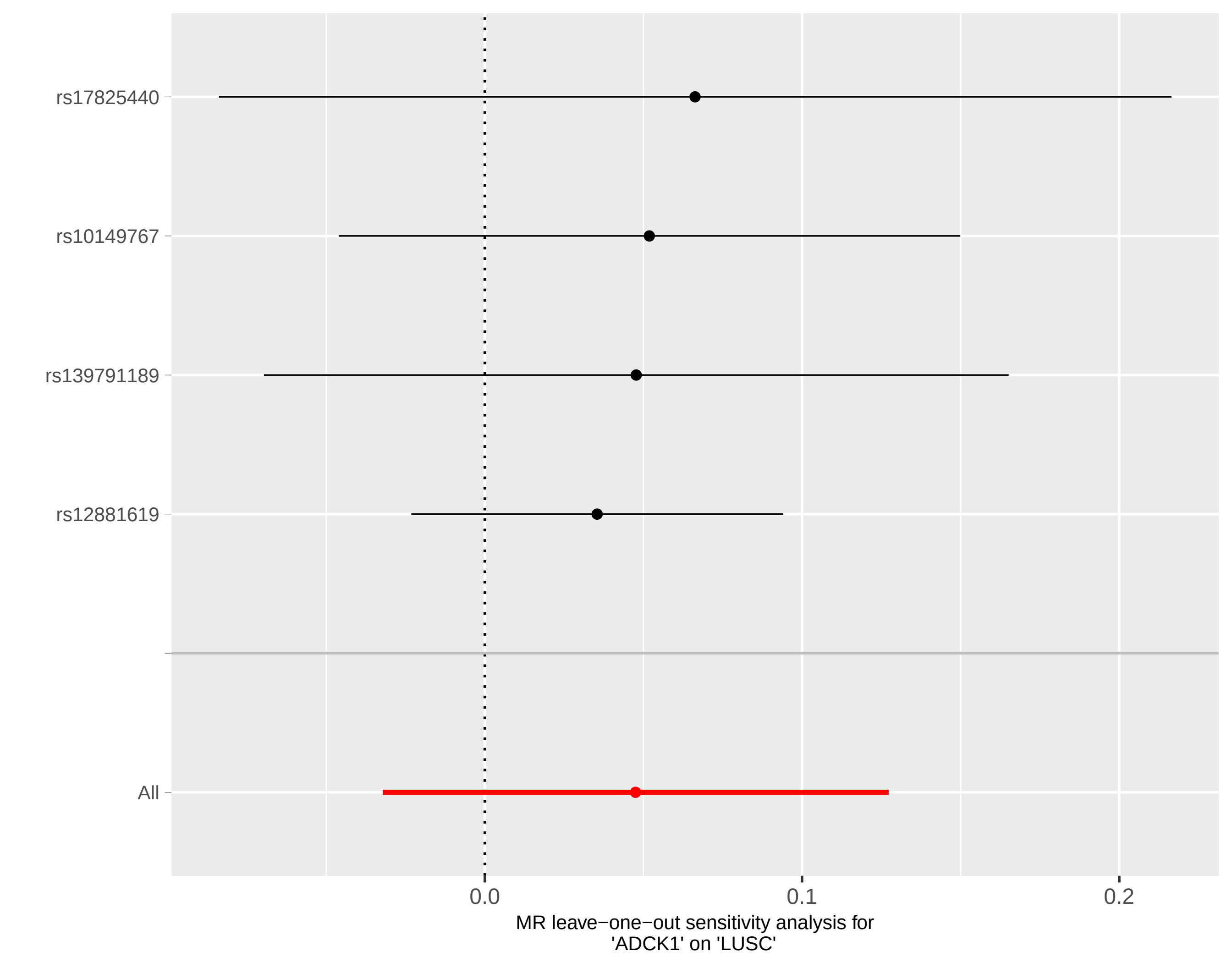

rs17825440
rs10149767
rs139791189
rs12881619
All
0.0
0.1
0.2
MR leave−one−out sensitivity analysis for
'ADCK1' on 'LUSC'

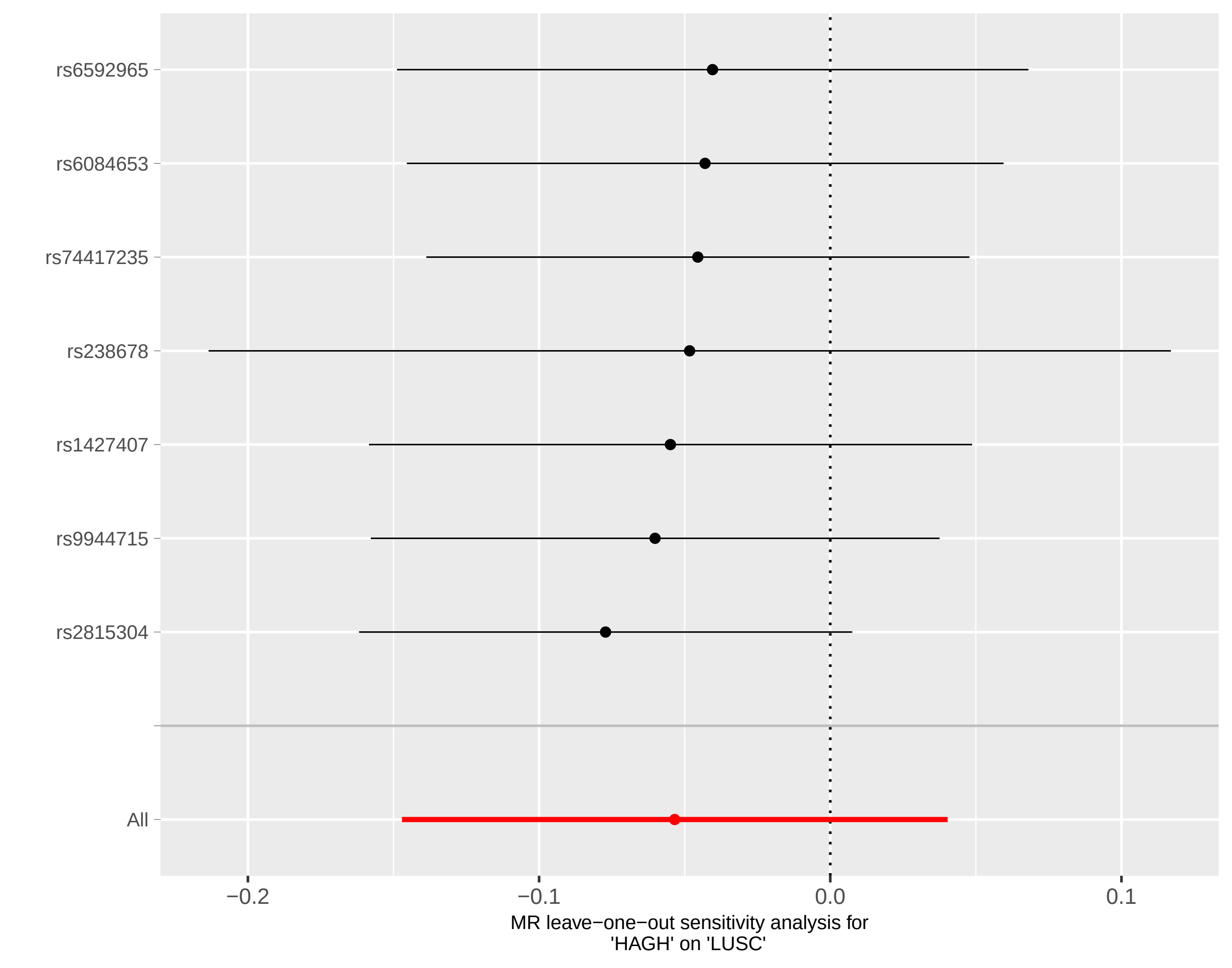
rs6592965
rs6084653
rs74417235
rs238678
rs1427407
rs9944715
rs2815304
All
−0.2
−0.1
0.0
0.1
MR leave−one−out sensitivity analysis for
'HAGH' on 'LUSC'

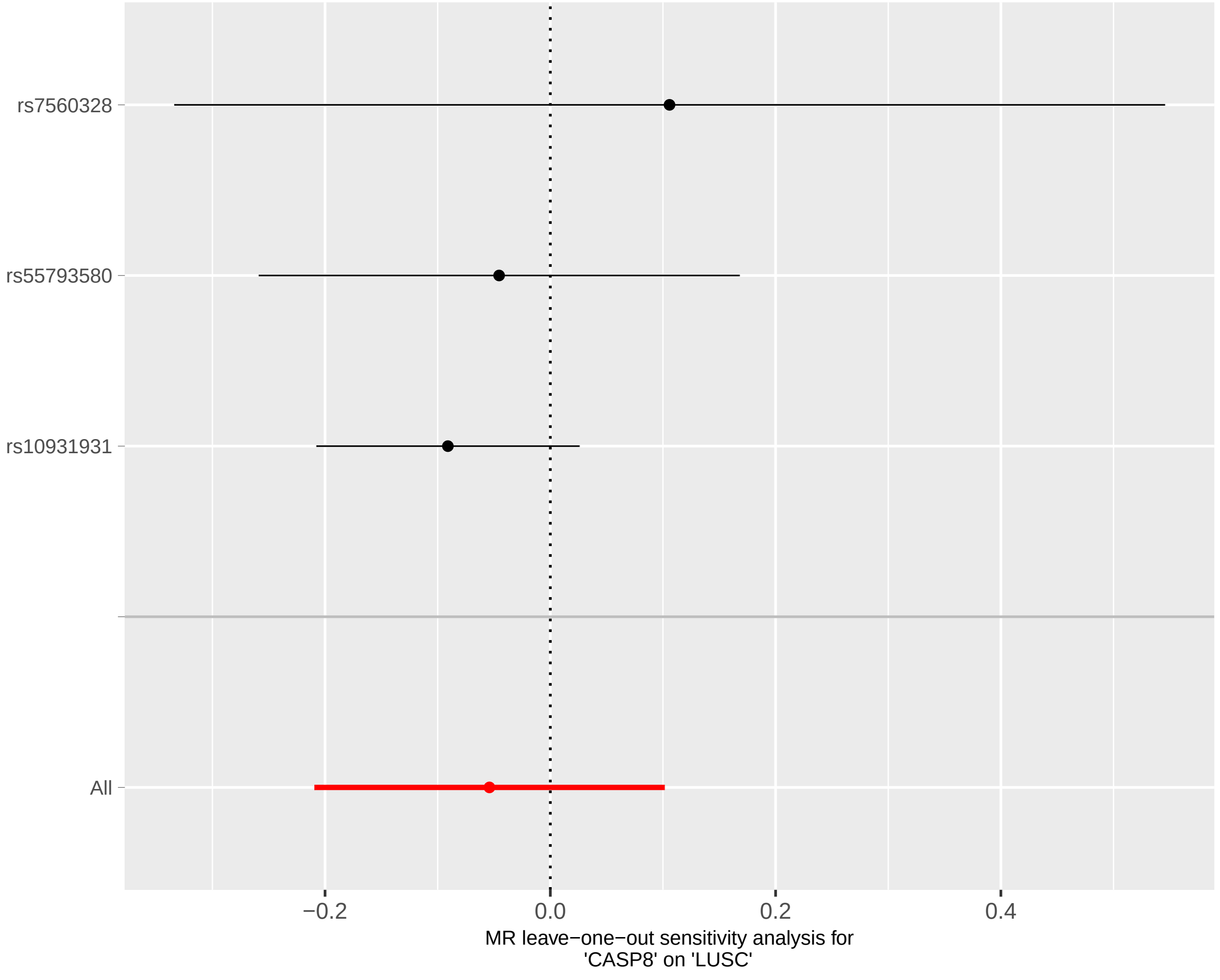
rs7560328
rs55793580
rs10931931
All
−0.2
0.0
0.2
0.4
MR leave−one−out sensitivity analysis for
'CASP8' on 'LUSC'

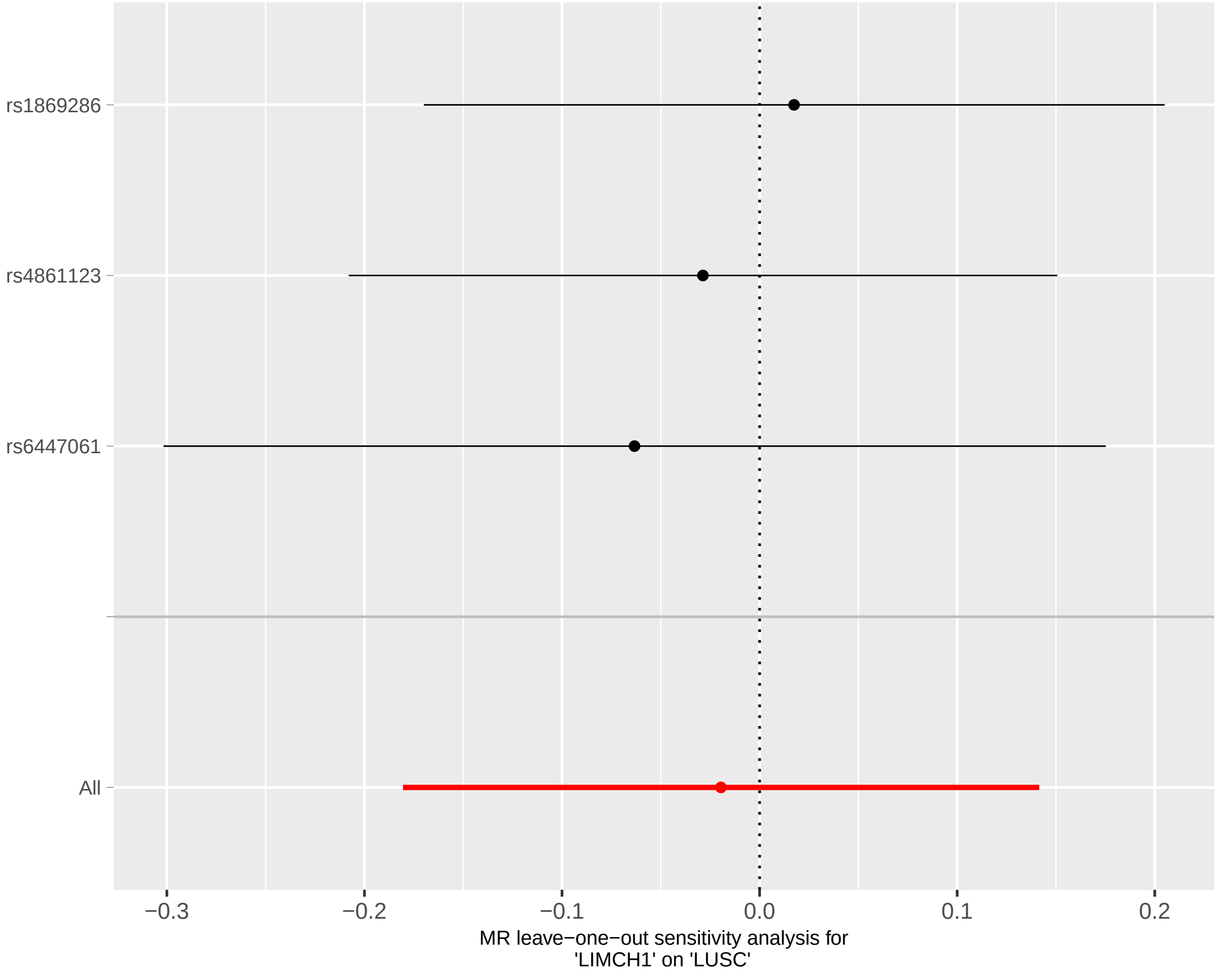
rs1869286
rs4861123
rs6447061
All
−0.3
−0.2
−0.1
0.0
0.1
0.2
MR leave−one−out sensitivity analysis for
'LIMCH1' on 'LUSC'

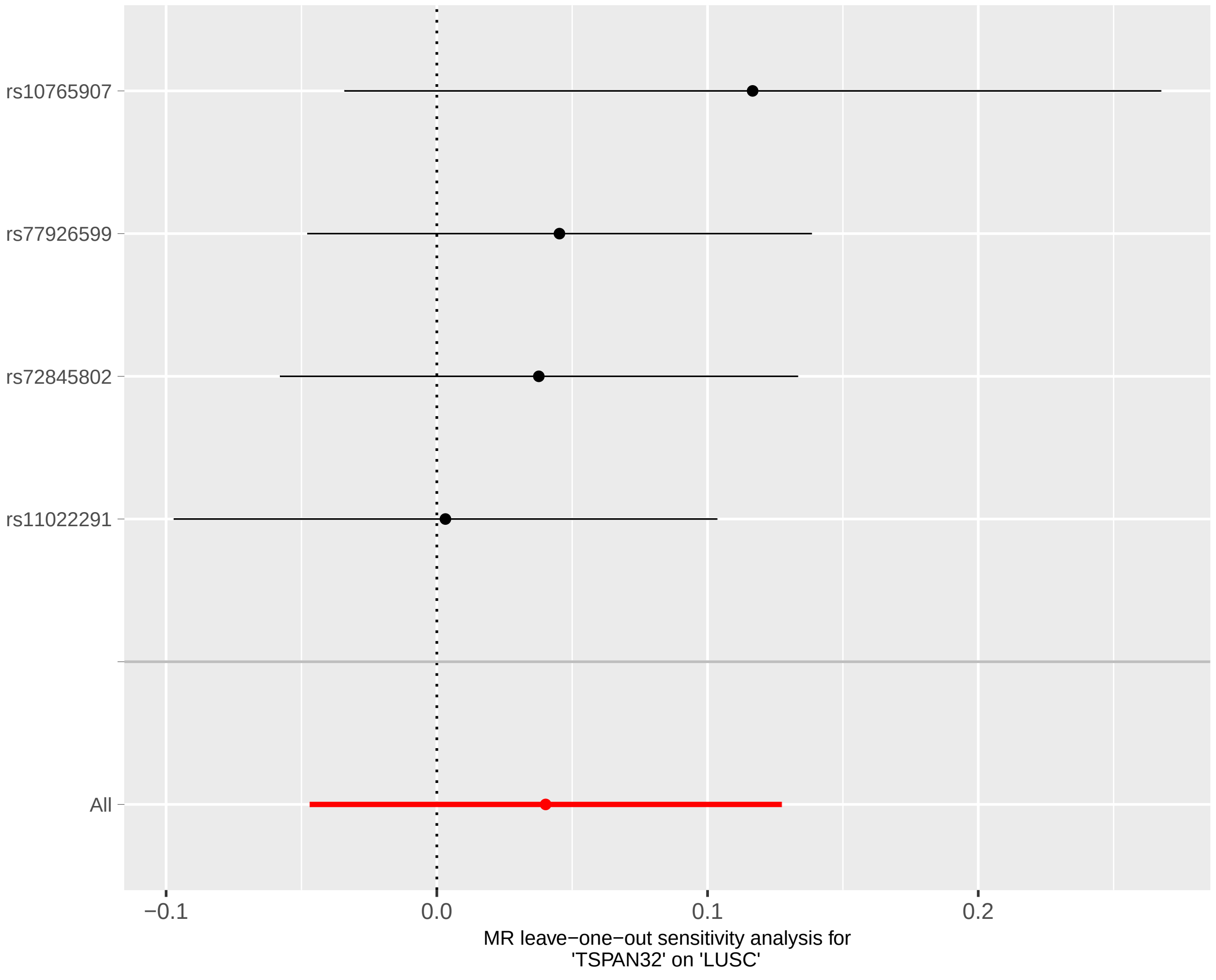
rs10765907
rs77926599
rs72845802
rs11022291
All
−0.1
0.0
0.1
0.2
MR leave−one−out sensitivity analysis for
'TSPAN32' on 'LUSC'

Insufficient number of SNPs

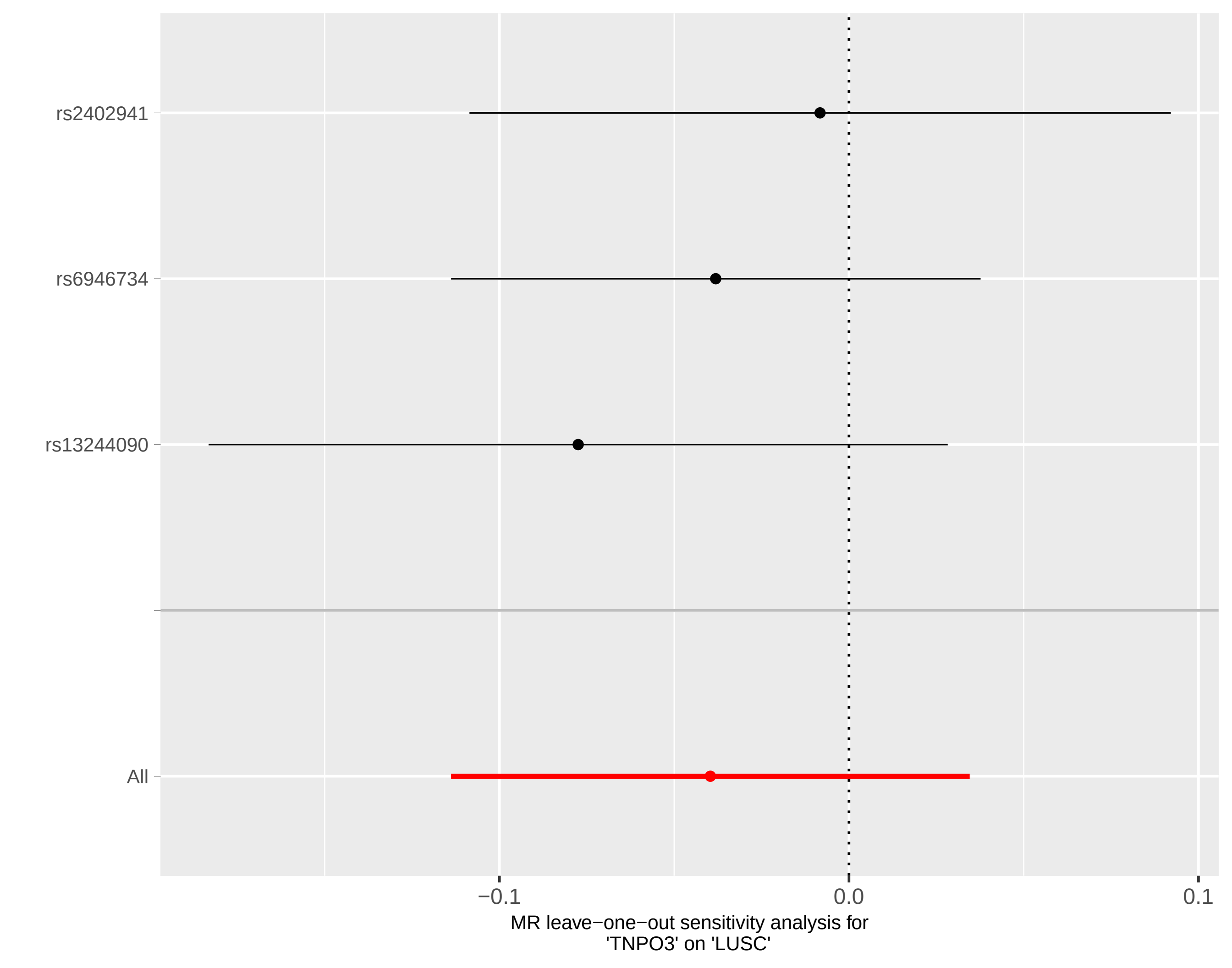
rs2402941
rs6946734
rs13244090
All
−0.1
0.0
0.1
MR leave−one−out sensitivity analysis for
'TNPO3' on 'LUSC'

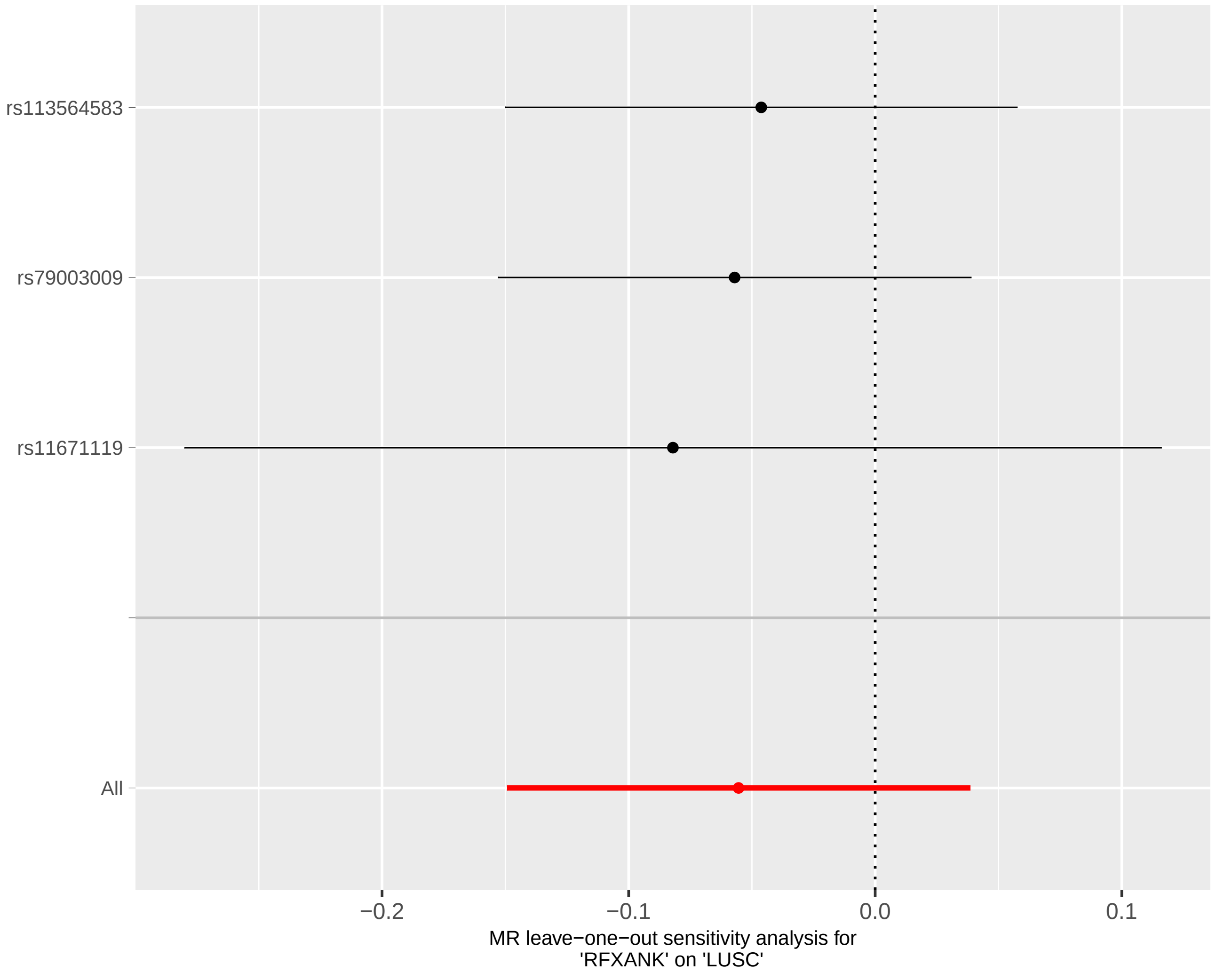

rs113564583
rs79003009
rs11671119
All
−0.2
−0.1
0.0
0.1
MR leave−one−out sensitivity analysis for
'RFXANK' on 'LUSC'

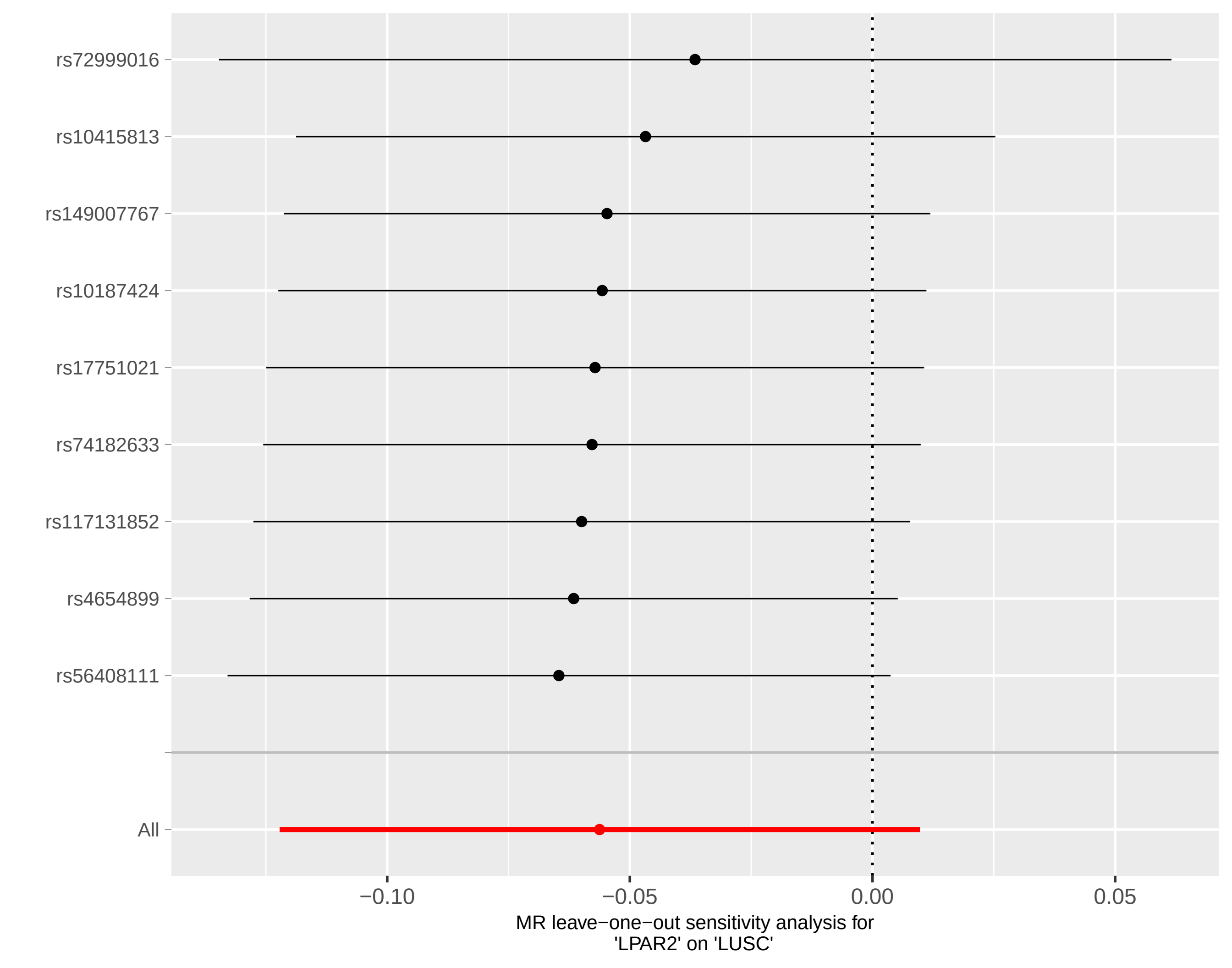
rs72999016
rs10415813
rs149007767
rs10187424
rs17751021
rs74182633
rs117131852
rs4654899
rs56408111
All
−0.10
−0.05
0.00
0.05
MR leave−one−out sensitivity analysis for
'LPAR2' on 'LUSC'

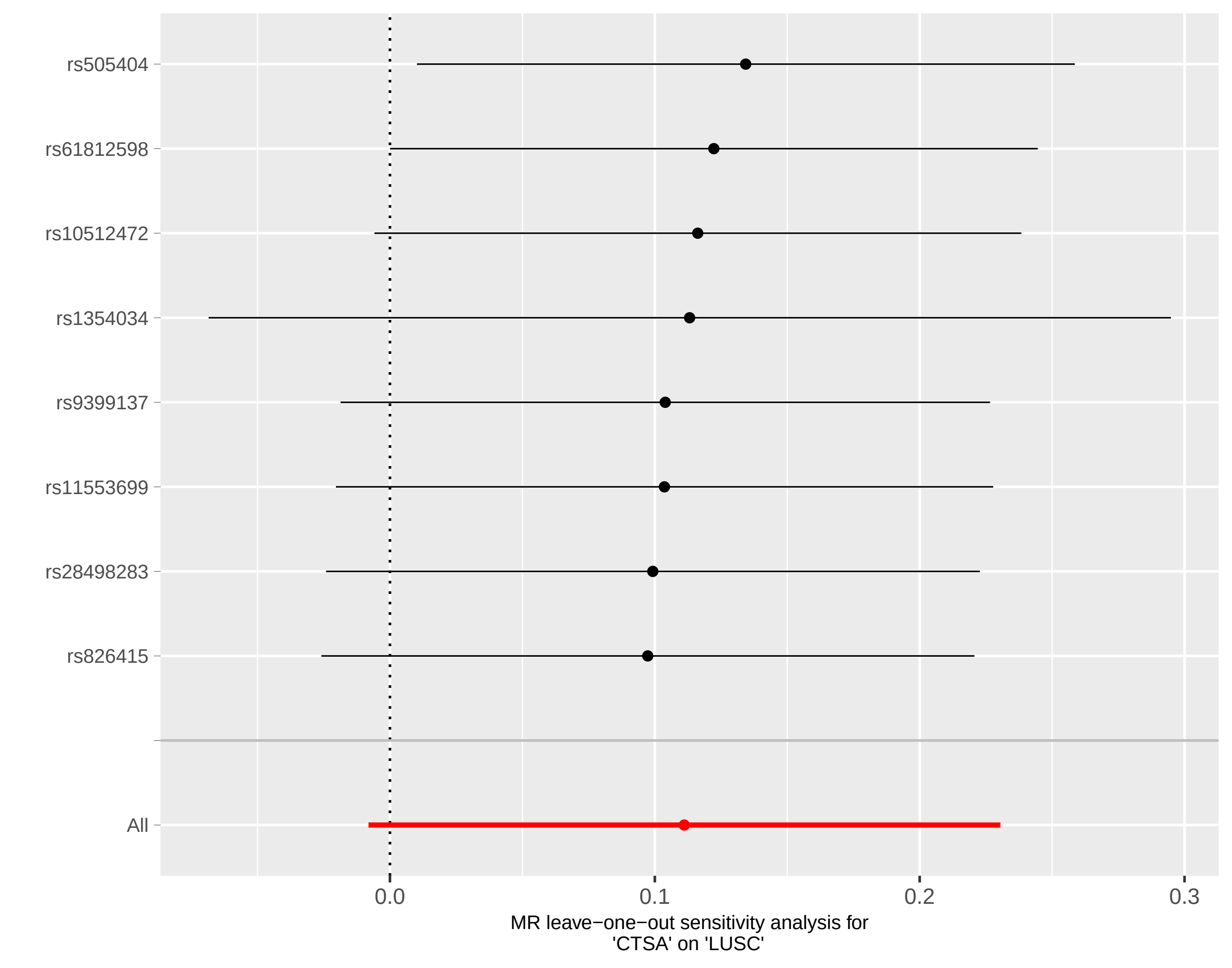
rs505404
rs61812598
rs10512472
rs1354034
rs9399137
rs11553699
rs28498283
rs826415
All
0.0
0.1
0.2
0.3
MR leave−one−out sensitivity analysis for
'CTSA' on 'LUSC'

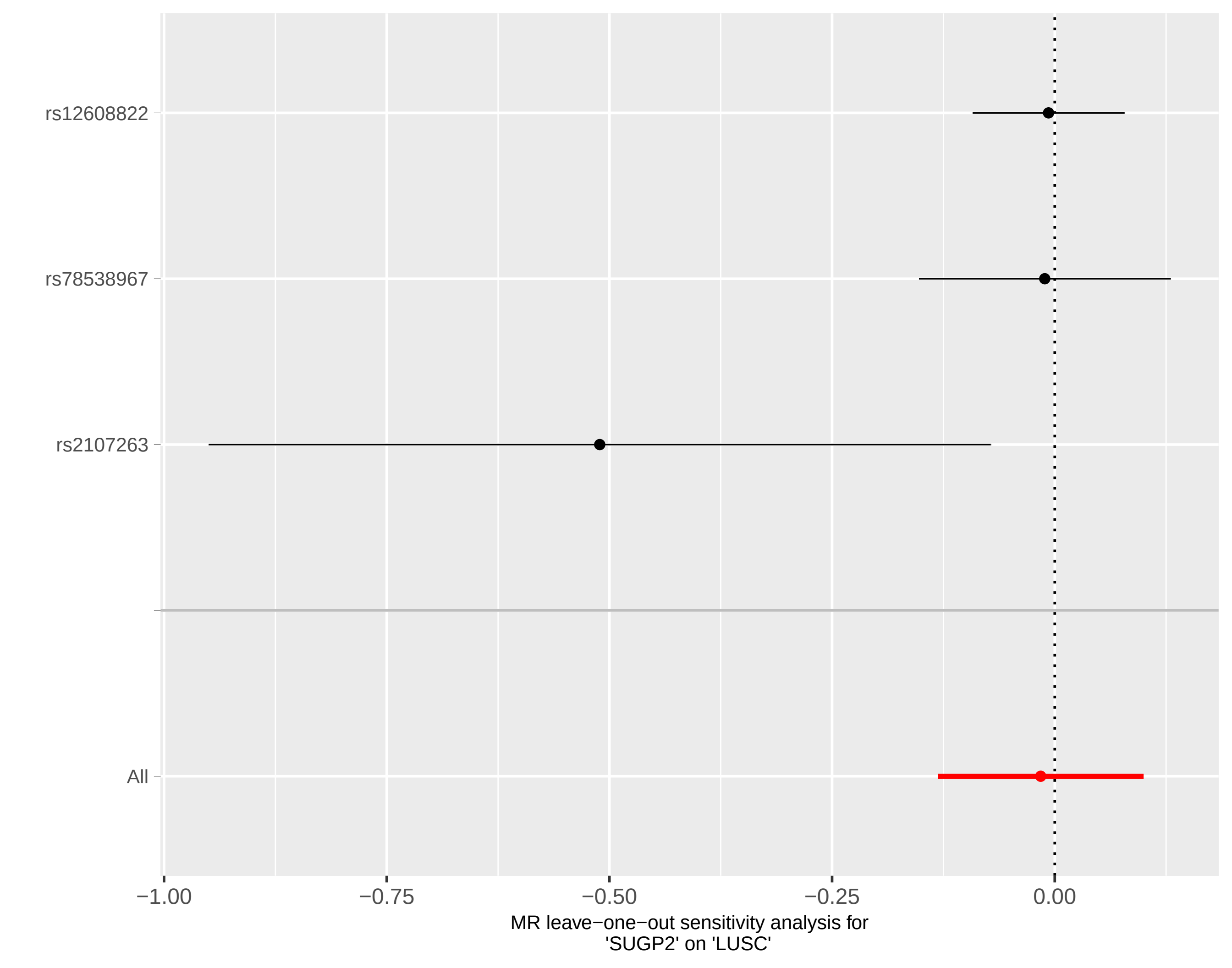
rs12608822
rs78538967
rs2107263
All
−1.00
−0.75
−0.50
−0.25
0.00
MR leave−one−out sensitivity analysis for
'SUGP2' on 'LUSC'

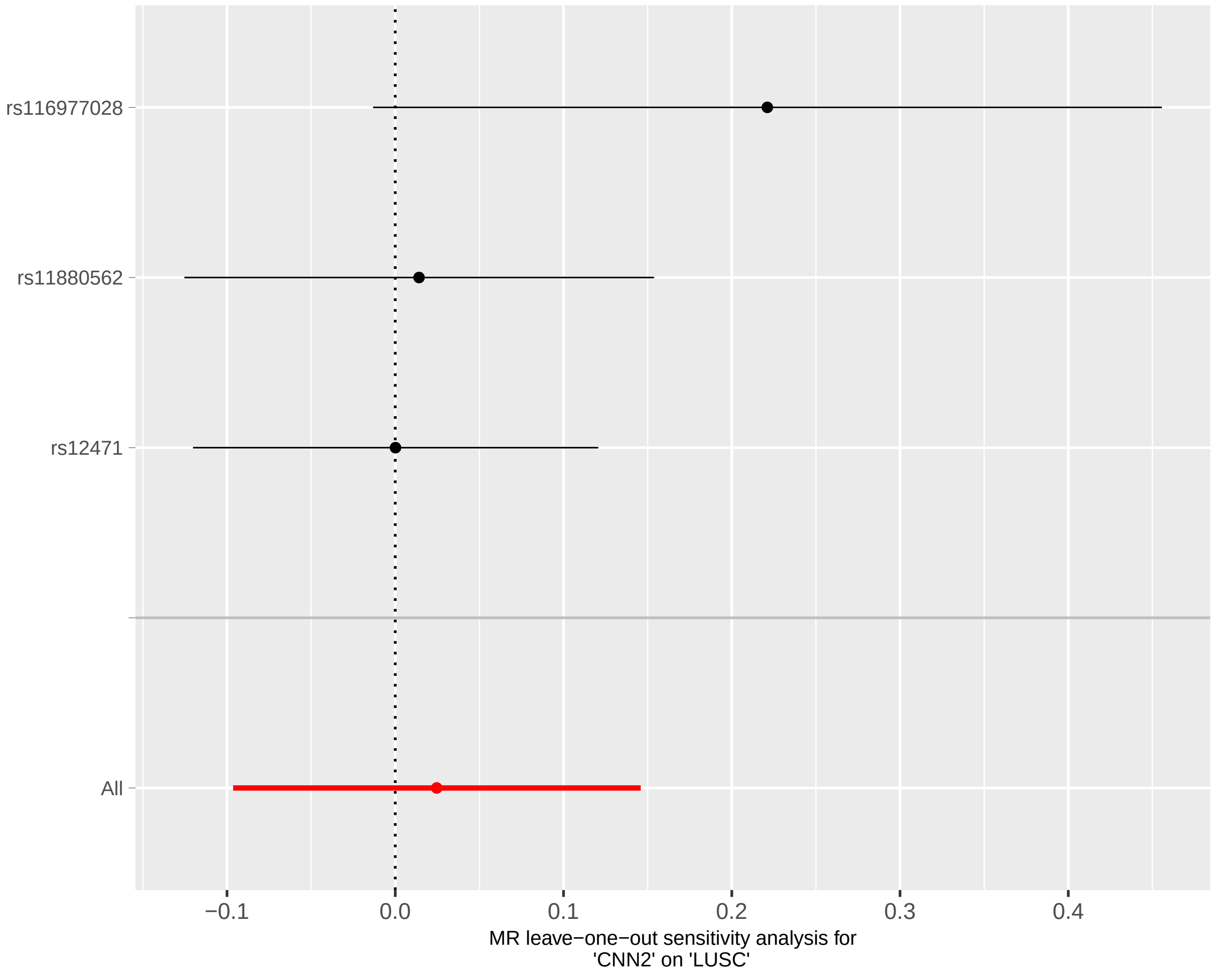

rs116977028
rs11880562
rs12471
All
−0.1
0.0
0.1
0.2
0.3
0.4
MR leave−one−out sensitivity analysis for
'CNN2' on 'LUSC'

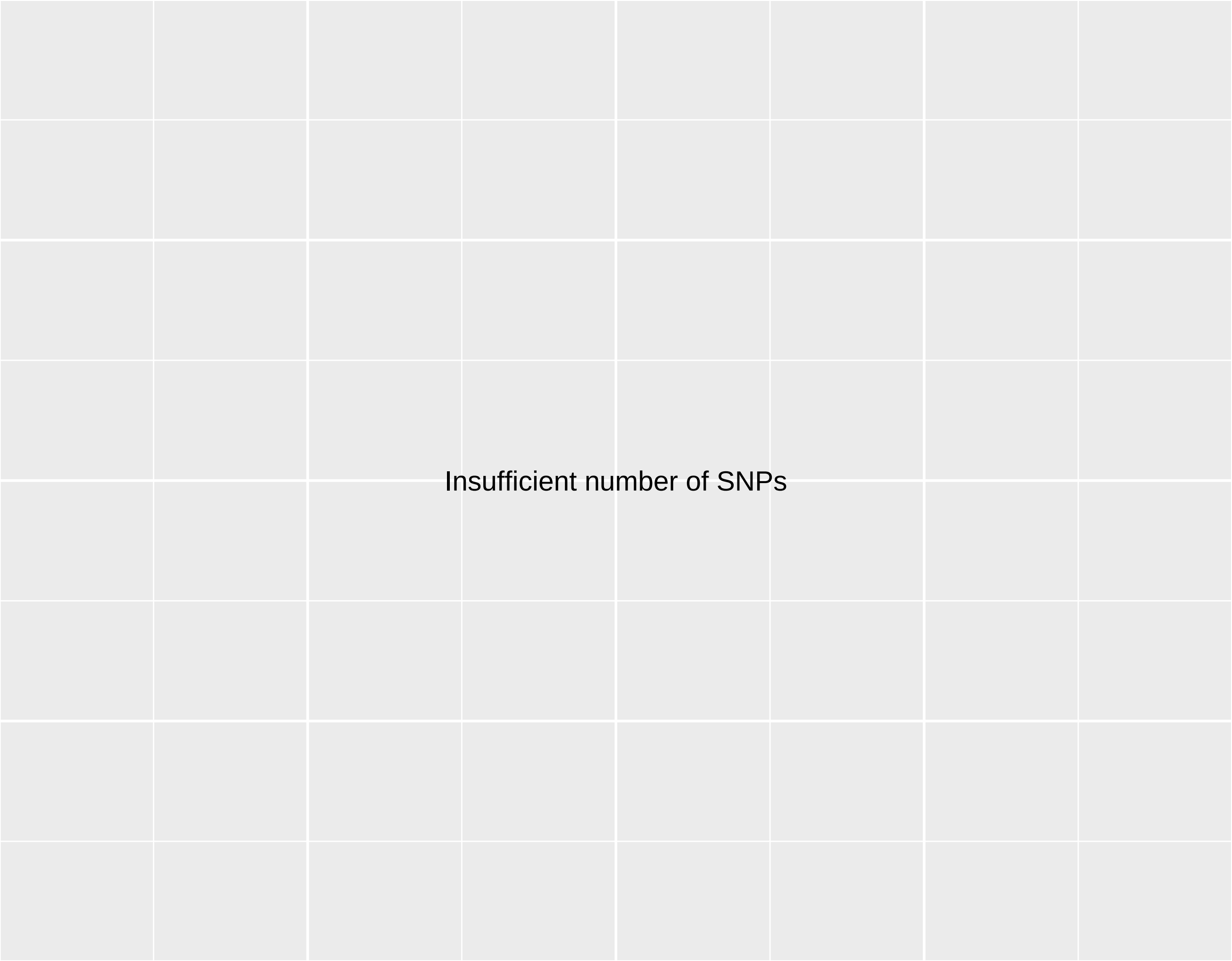
Insufficient number of SNPs

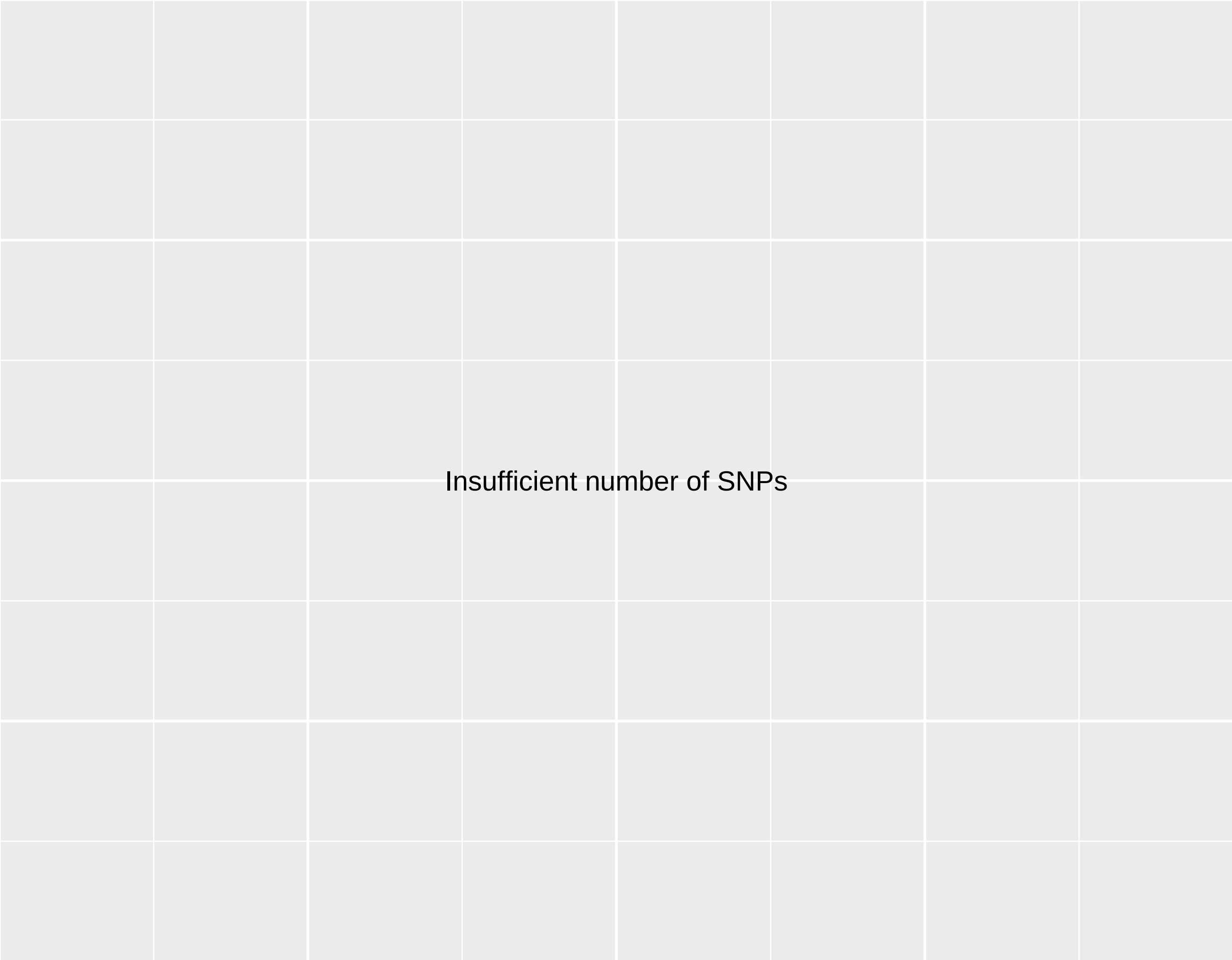
Insufficient number of SNPs

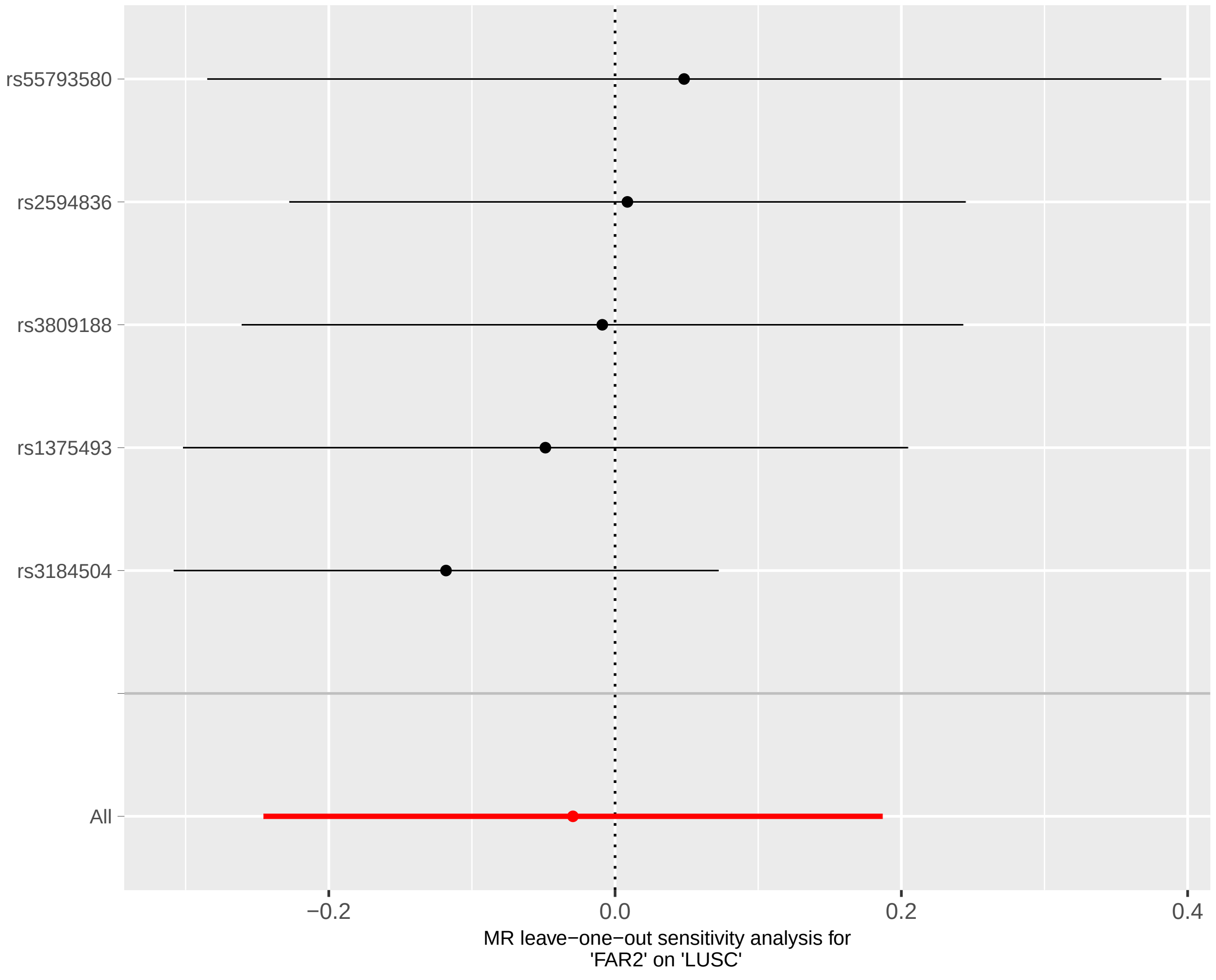
rs55793580
rs2594836
rs3809188
rs1375493
rs3184504
All
−0.2
0.0
0.2
0.4
MR leave−one−out sensitivity analysis for
'FAR2' on 'LUSC'

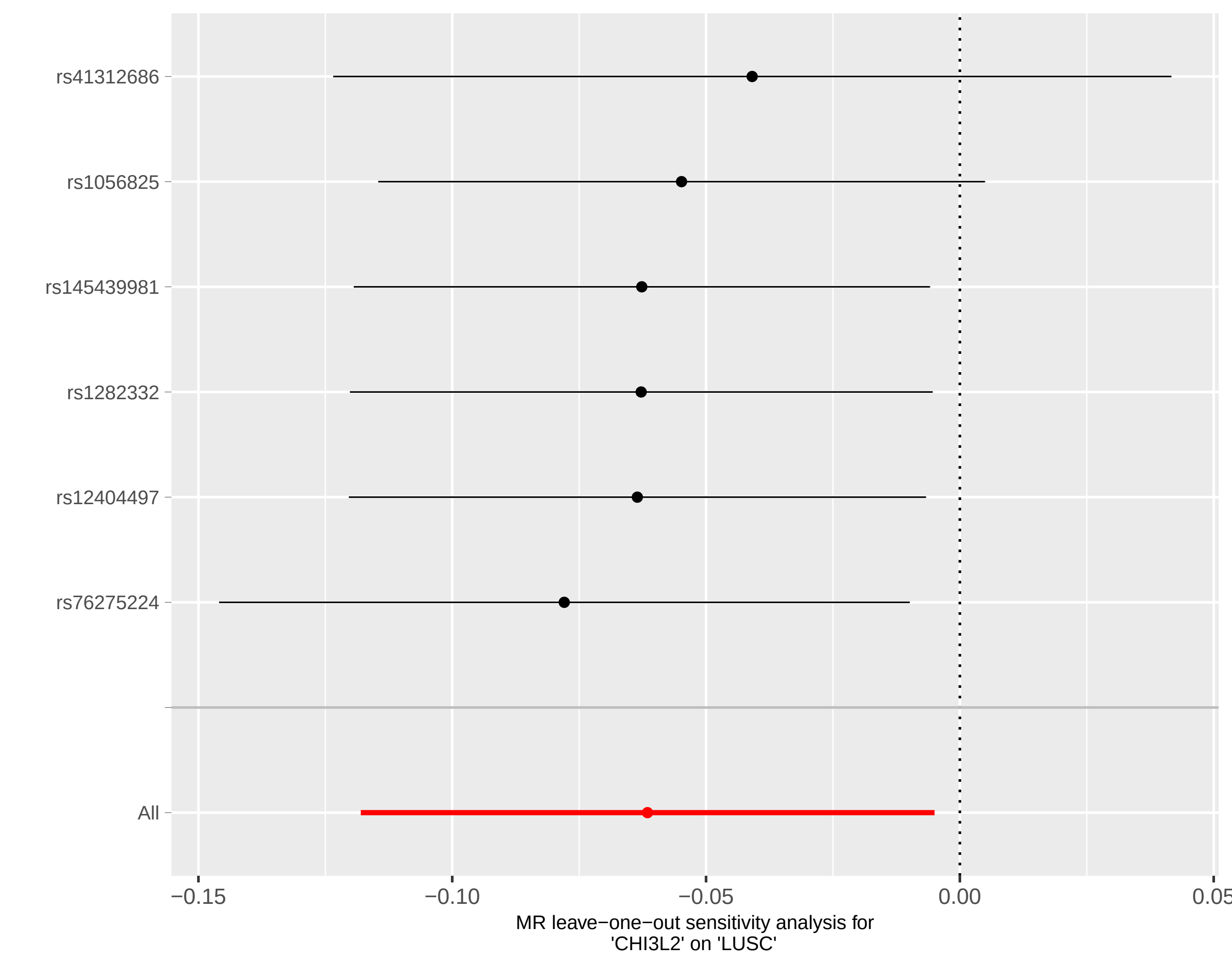
rs41312686
rs1056825
rs145439981
rs1282332
rs12404497
rs76275224
All
−0.15
−0.10
−0.05
0.00
0.05
MR leave−one−out sensitivity analysis for
'CHI3L2' on 'LUSC'

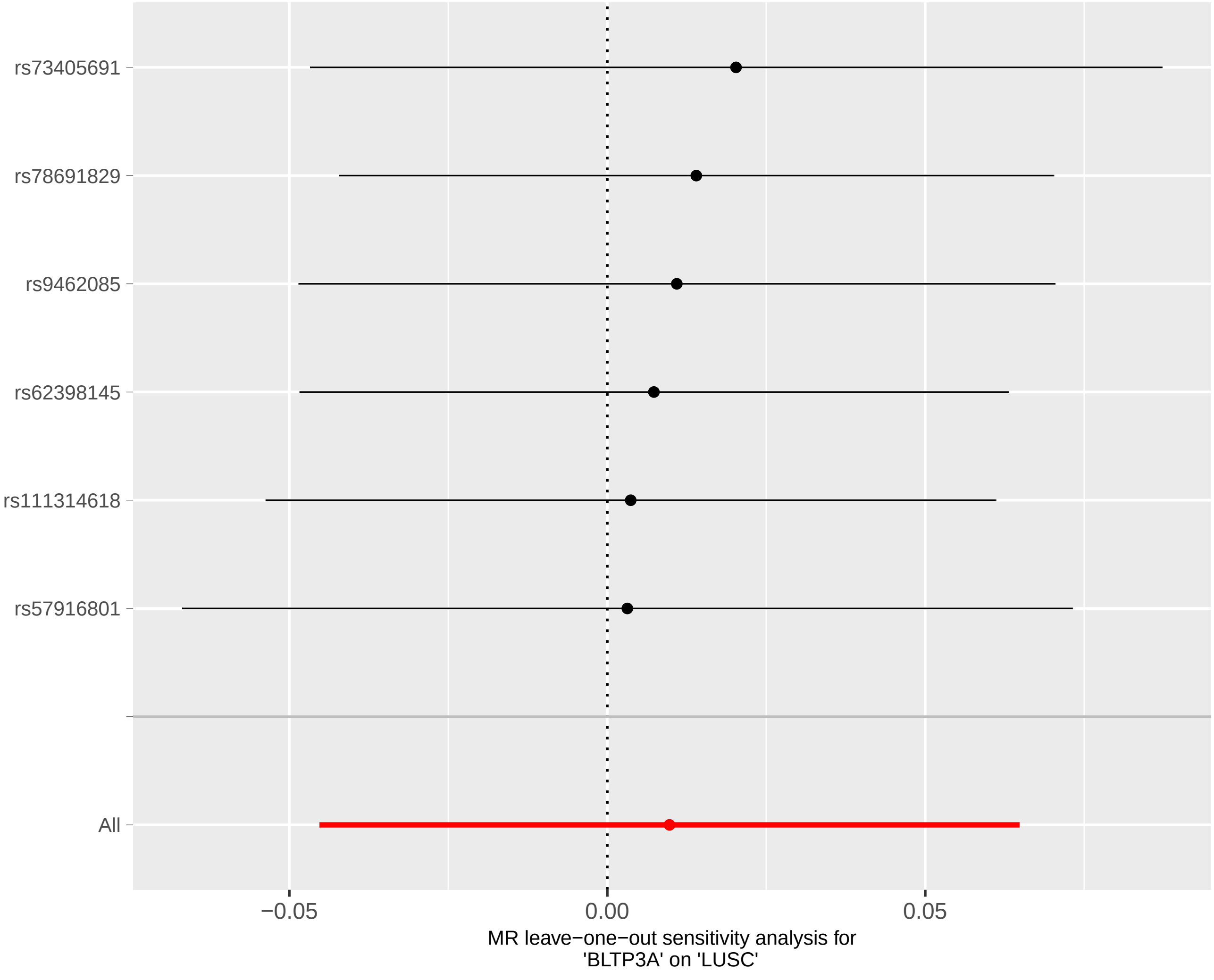
rs73405691
rs78691829
rs9462085
rs62398145
rs111314618
rs57916801
All
−0.05
0.00
0.05
MR leave−one−out sensitivity analysis for
'BLTP3A' on 'LUSC'

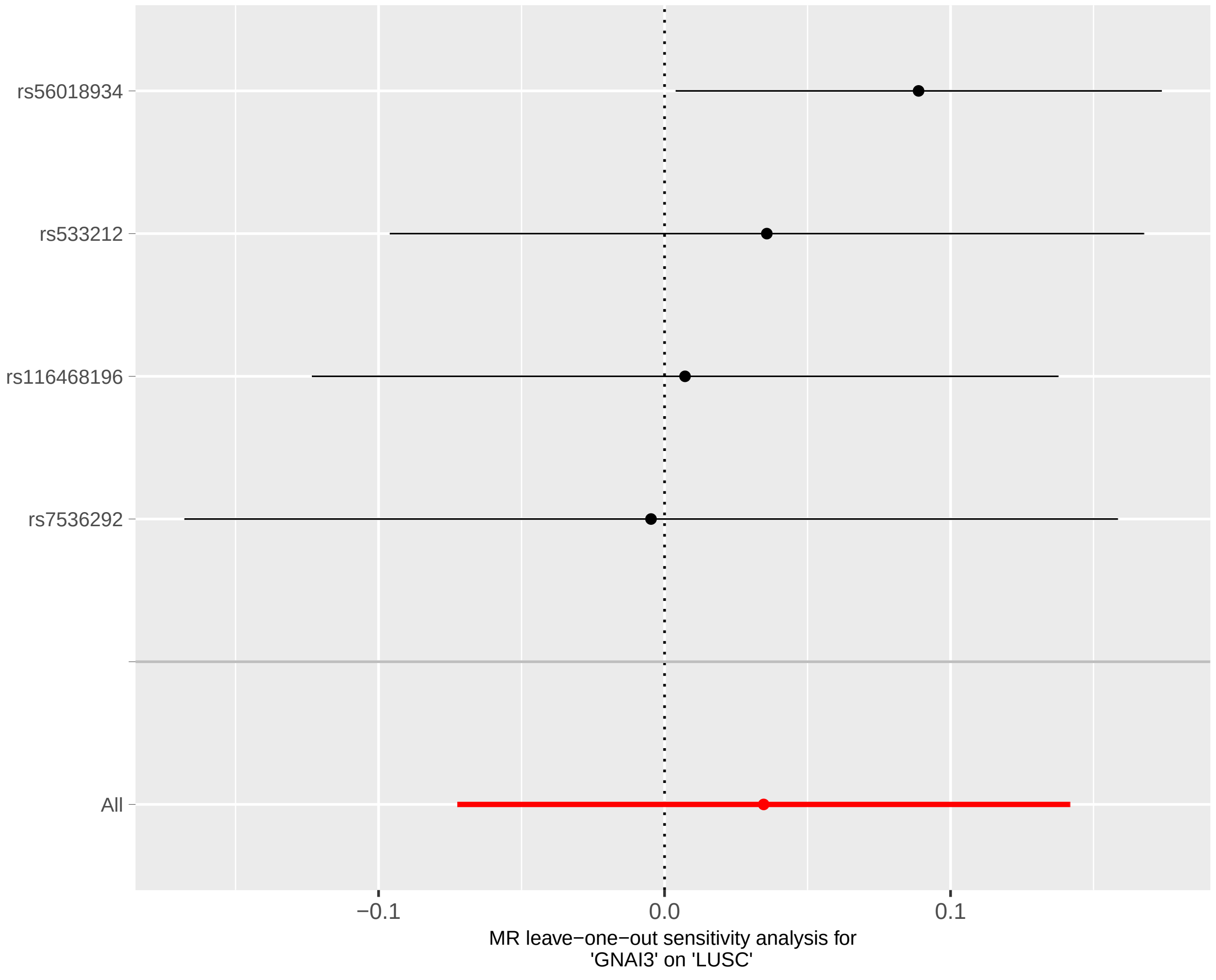

rs56018934
rs533212
rs116468196
rs7536292
All
−0.1
0.0
0.1
MR leave−one−out sensitivity analysis for
'GNAI3' on 'LUSC'

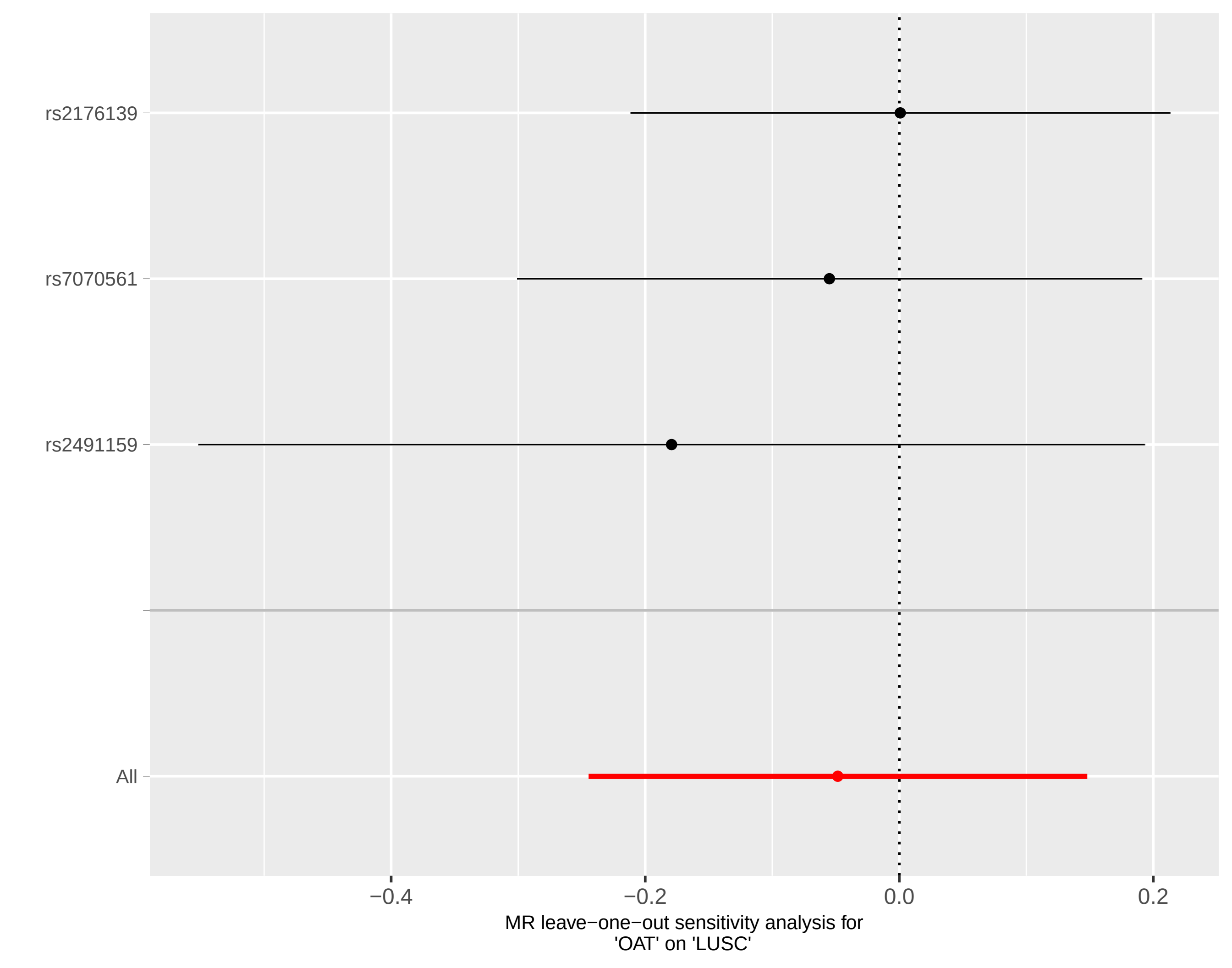

rs2176139
rs7070561
rs2491159
All
−0.4
−0.2
0.0
0.2
MR leave−one−out sensitivity analysis for
'OAT' on 'LUSC'

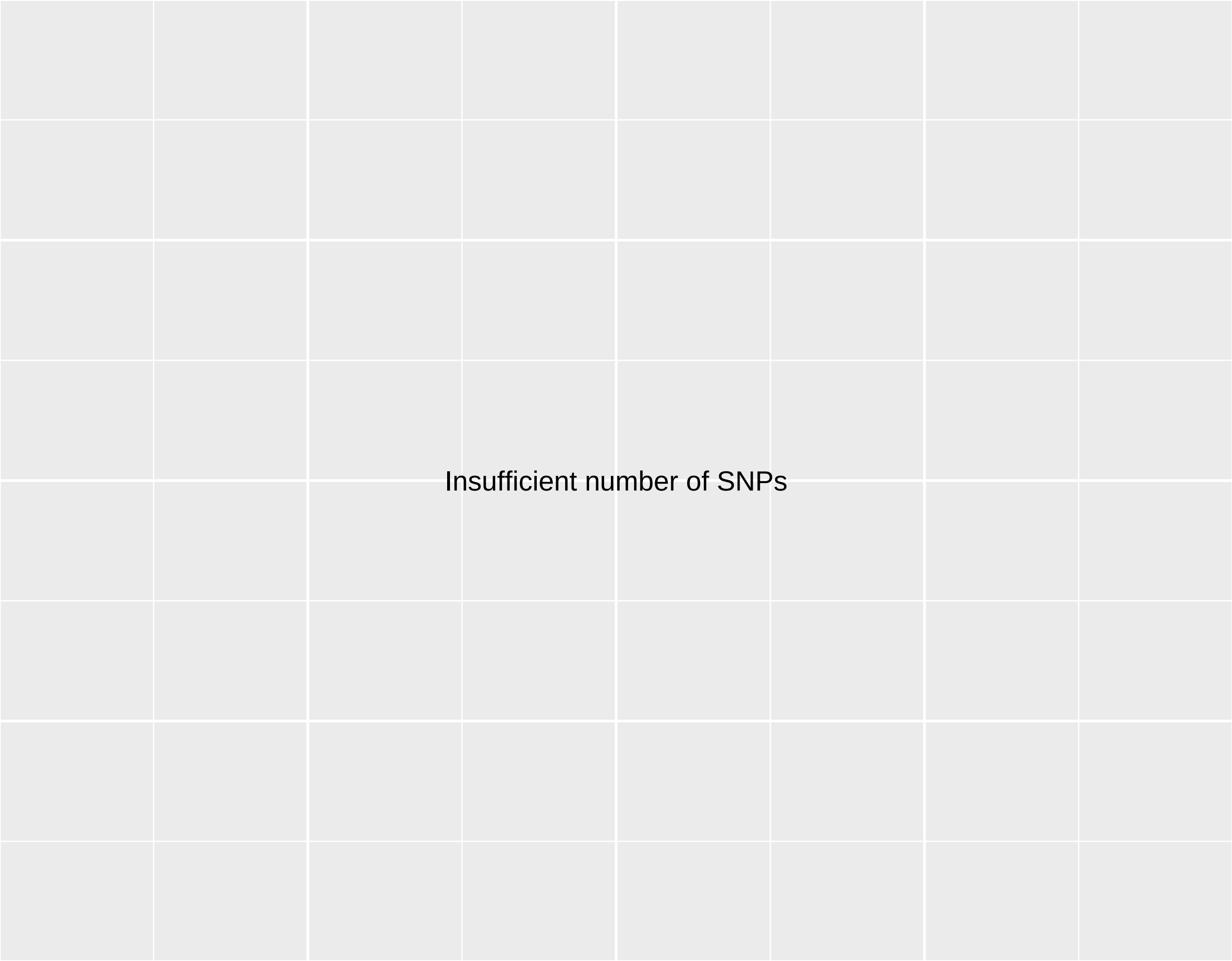
Insufficient number of SNPs

Insufficient number of SNPs

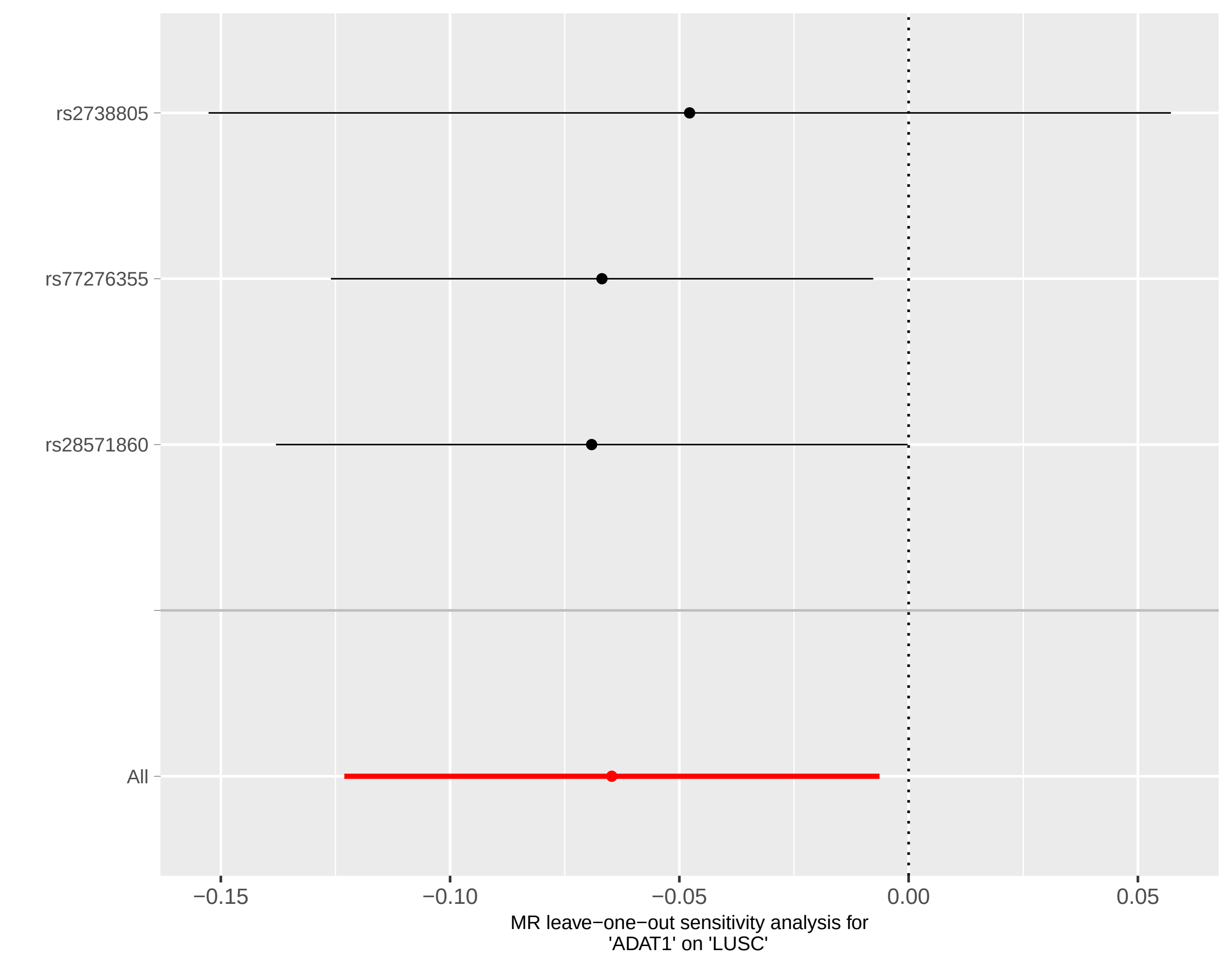
rs2738805
rs77276355
rs28571860
All
−0.15
−0.10
−0.05
0.00
0.05
MR leave−one−out sensitivity analysis for
'ADAT1' on 'LUSC'

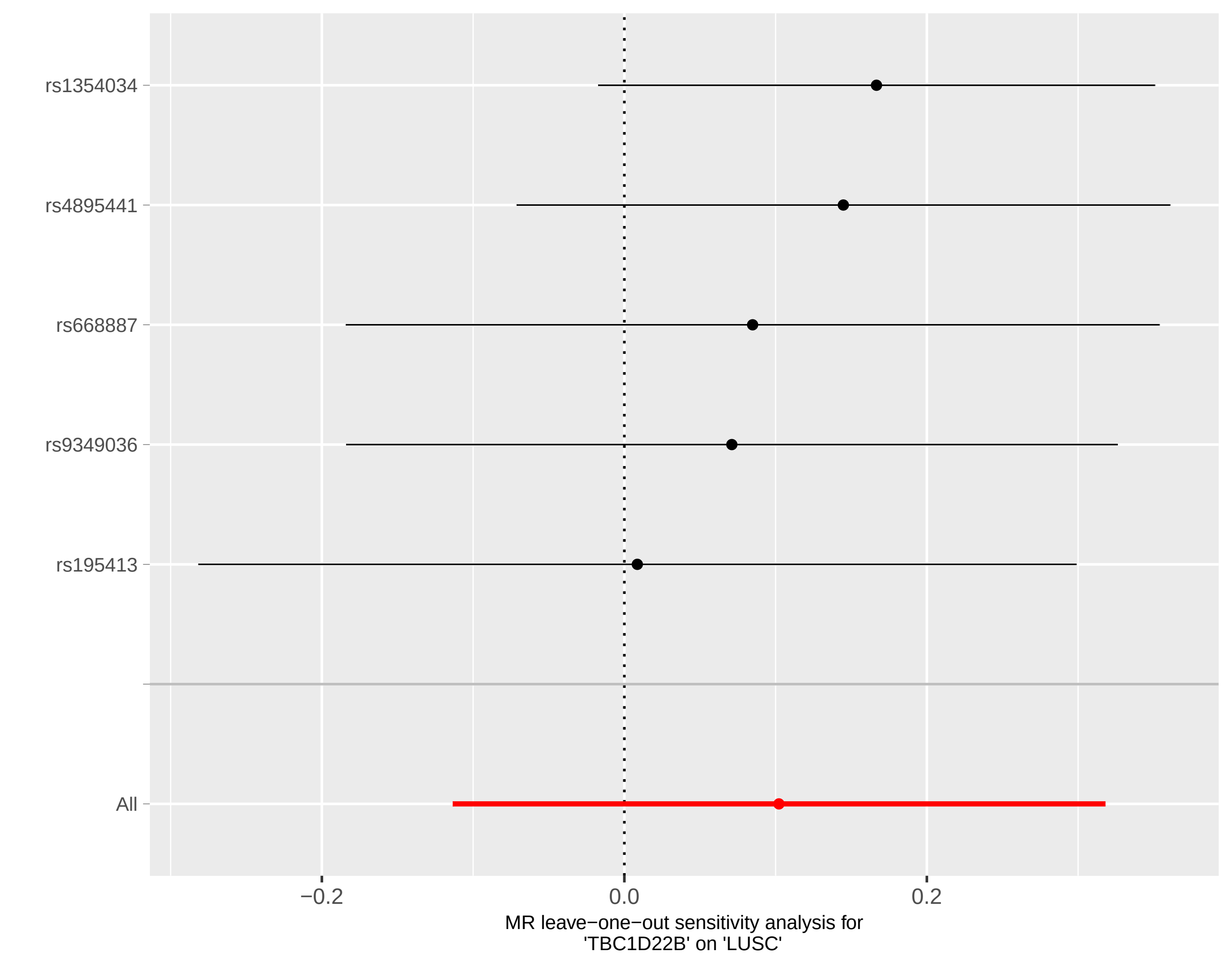
rs1354034
rs4895441
rs668887
rs9349036
rs195413
All
−0.2
0.0
0.2
MR leave−one−out sensitivity analysis for
'TBC1D22B' on 'LUSC'

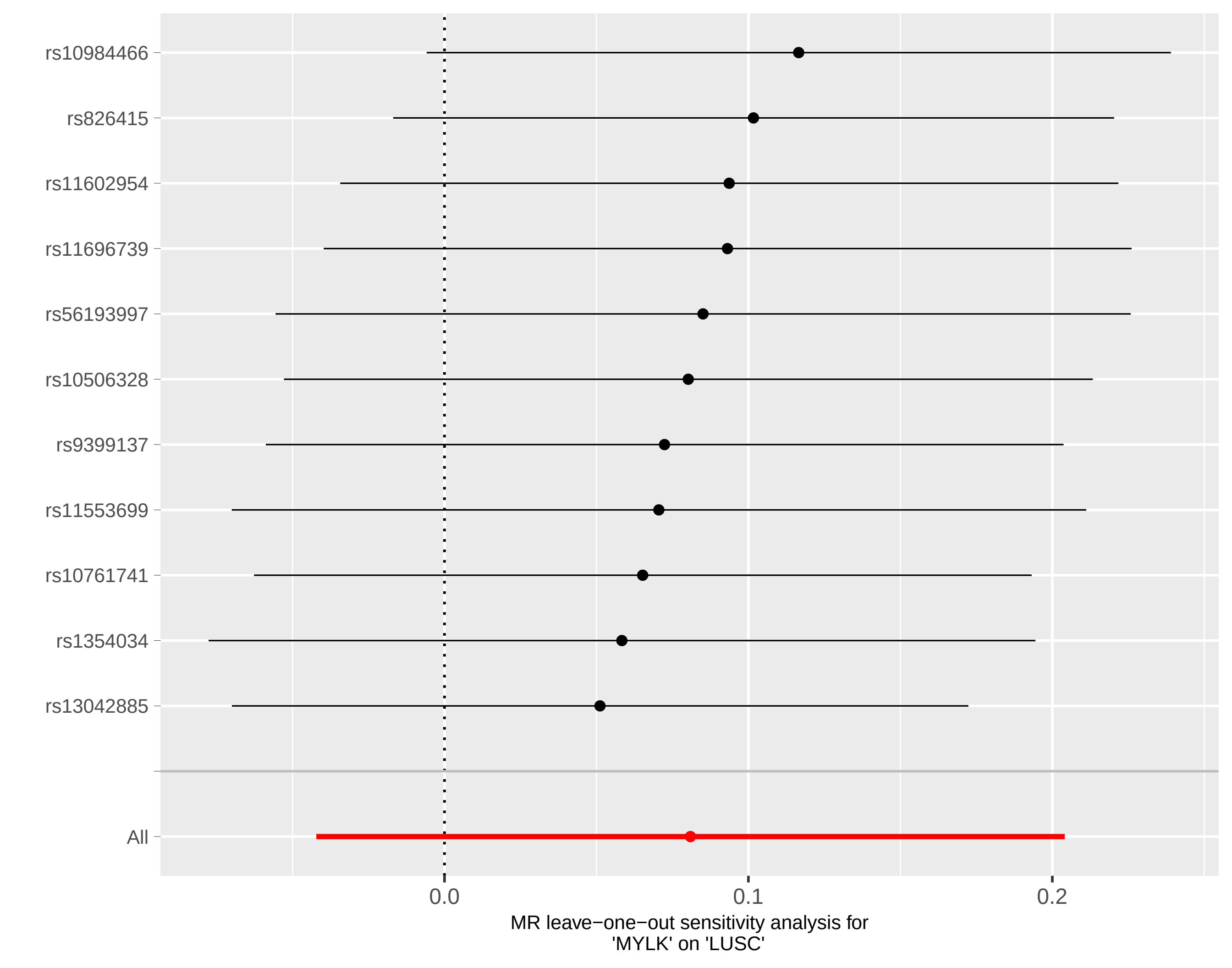
rs10984466
rs826415
rs11602954
rs11696739
rs56193997
rs10506328
rs9399137
rs11553699
rs10761741
rs1354034
rs13042885
All
0.0
0.1
0.2
MR leave−one−out sensitivity analysis for
'MYLK' on 'LUSC'

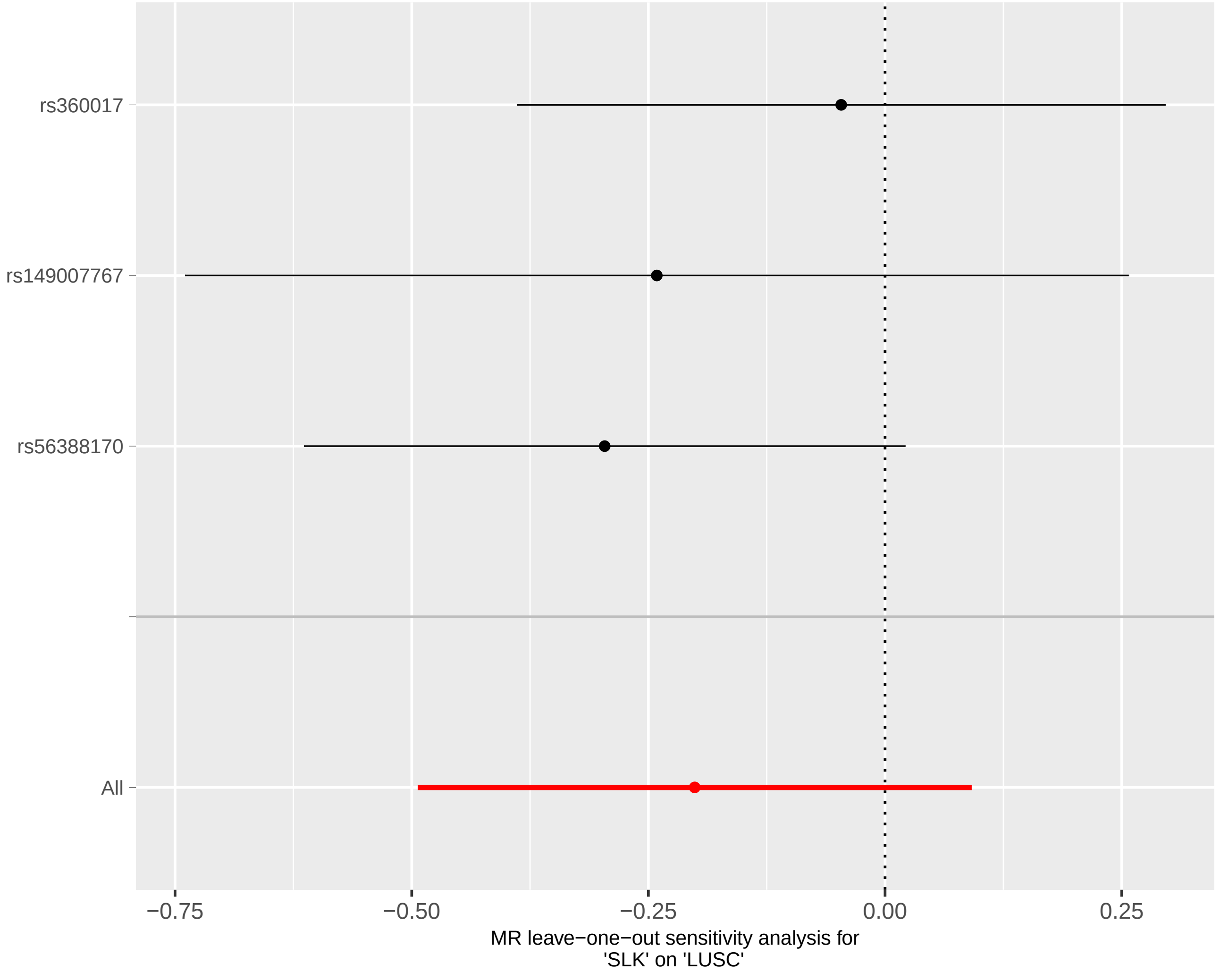
rs360017
rs149007767
rs56388170
All
−0.75
−0.50
−0.25
0.00
0.25
MR leave−one−out sensitivity analysis for
'SLK' on 'LUSC'

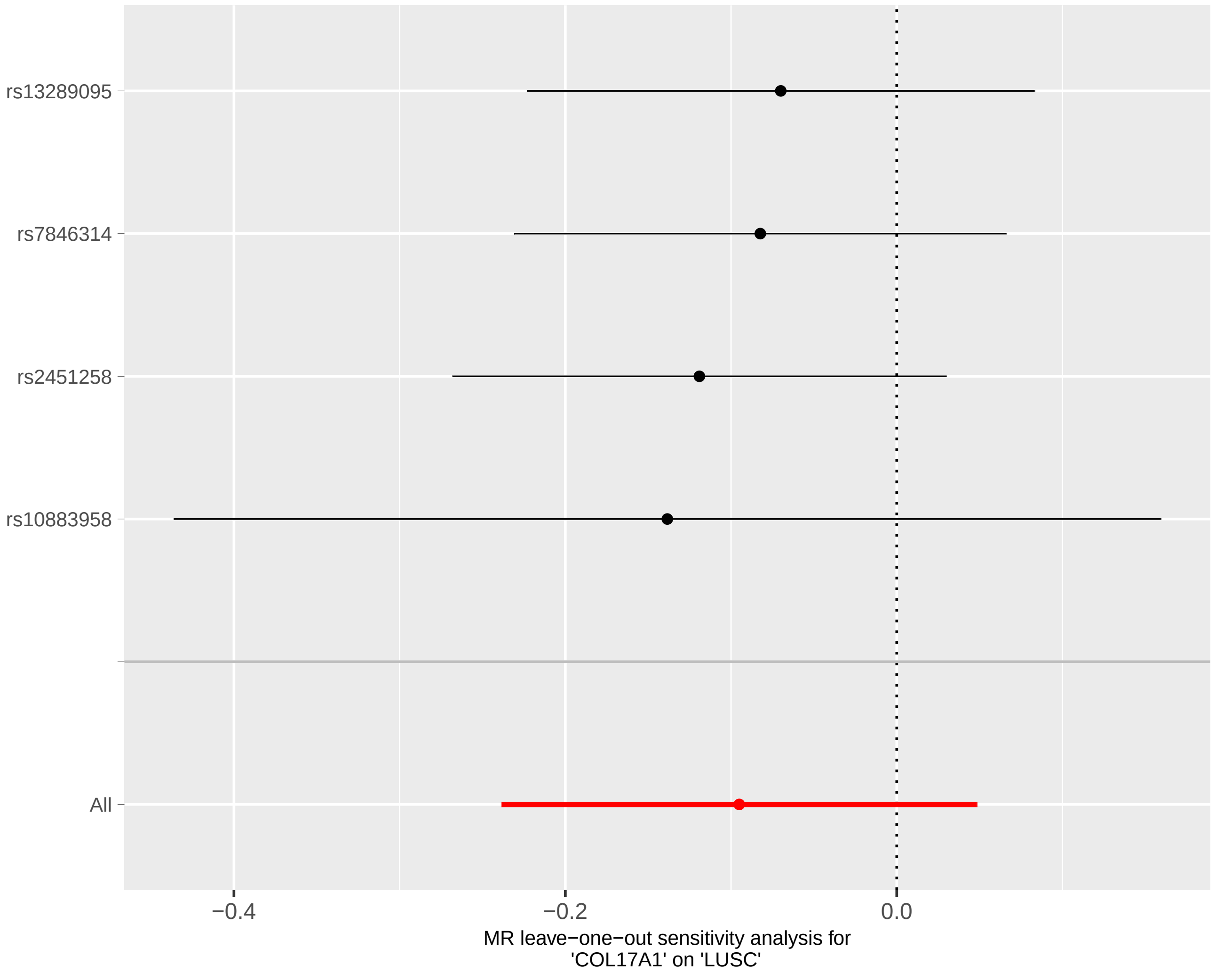
rs13289095
rs7846314
rs2451258
rs10883958
All
−0.4
−0.2
0.0
MR leave−one−out sensitivity analysis for
'COL17A1' on 'LUSC'

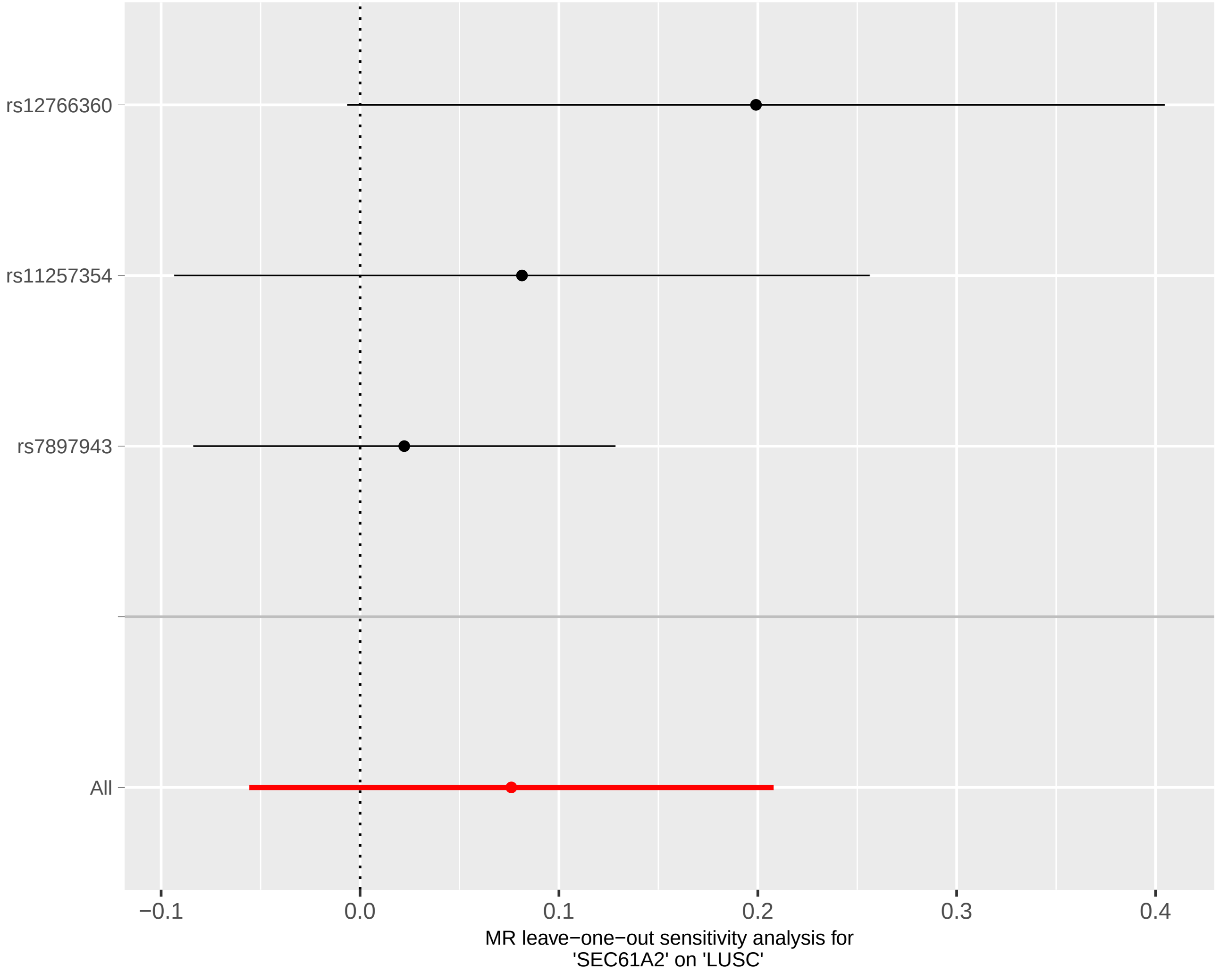
rs12766360
rs11257354
rs7897943
All
−0.1
0.0
0.1
0.2
0.3
0.4
MR leave−one−out sensitivity analysis for
'SEC61A2' on 'LUSC'

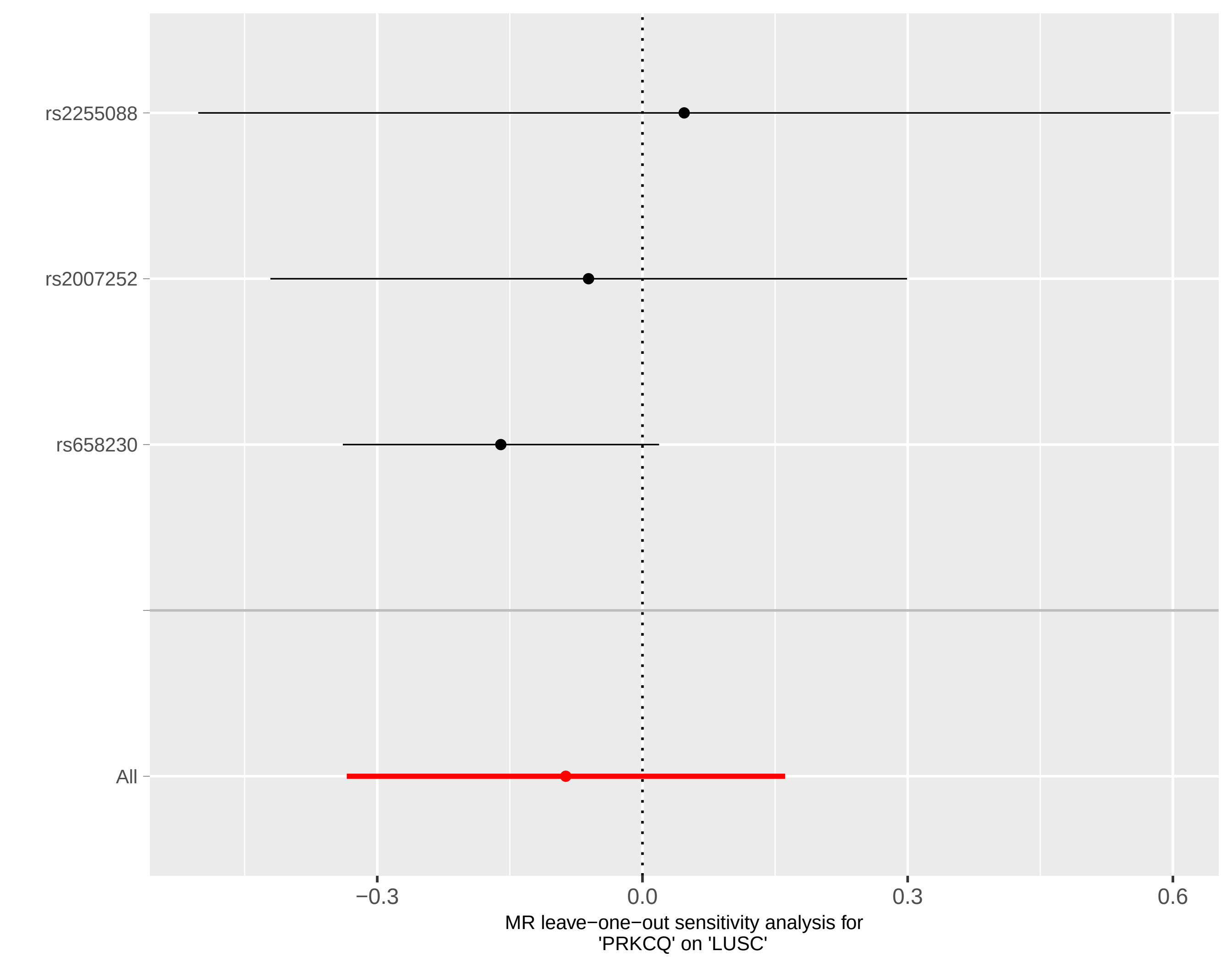

rs2255088
rs2007252
rs658230
All
−0.3
0.0
0.3
0.6
MR leave−one−out sensitivity analysis for
'PRKCQ' on 'LUSC'

Insufficient number of SNPs

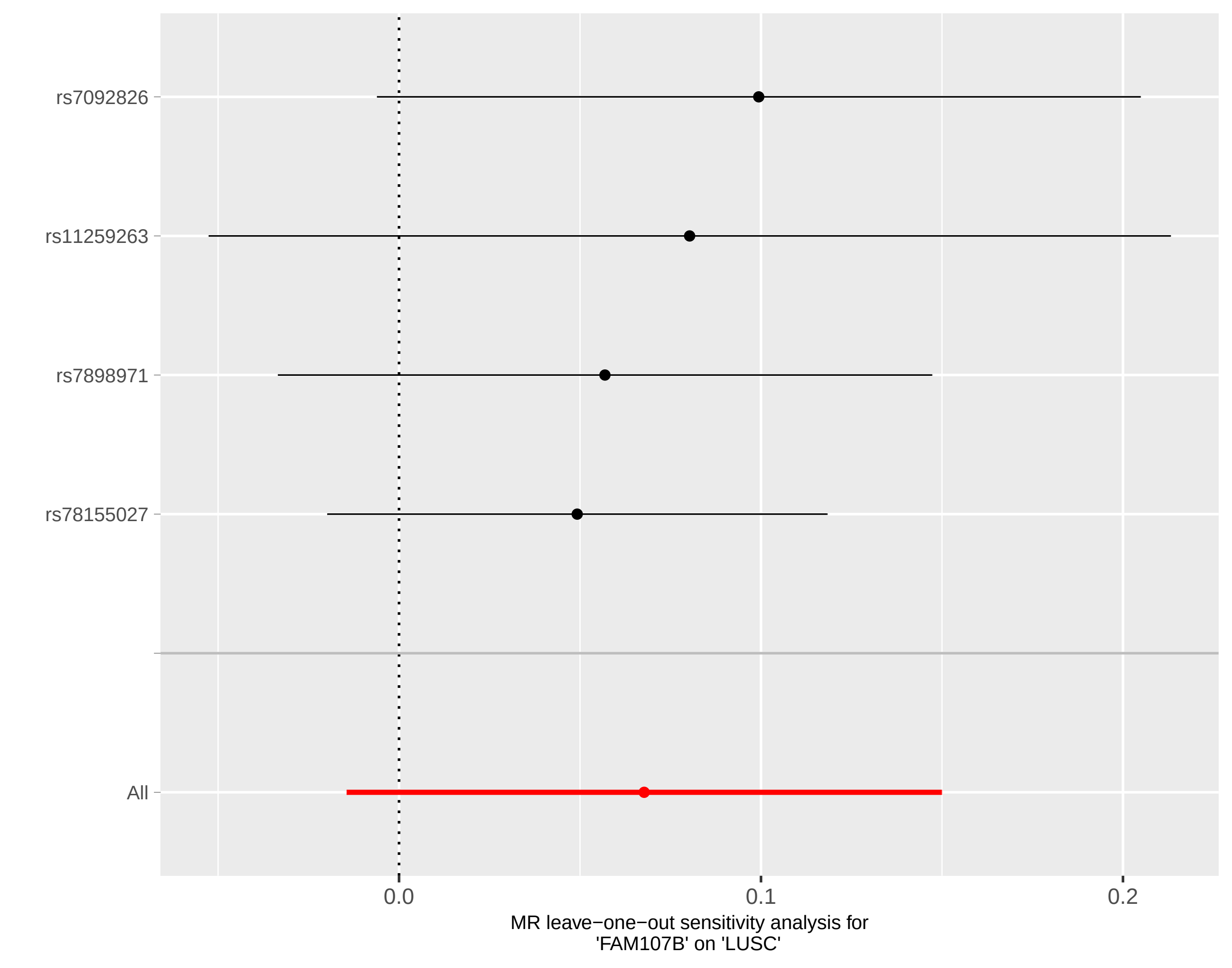
rs7092826
rs11259263
rs7898971
rs78155027
All
0.0
0.1
0.2
MR leave−one−out sensitivity analysis for
'FAM107B' on 'LUSC'

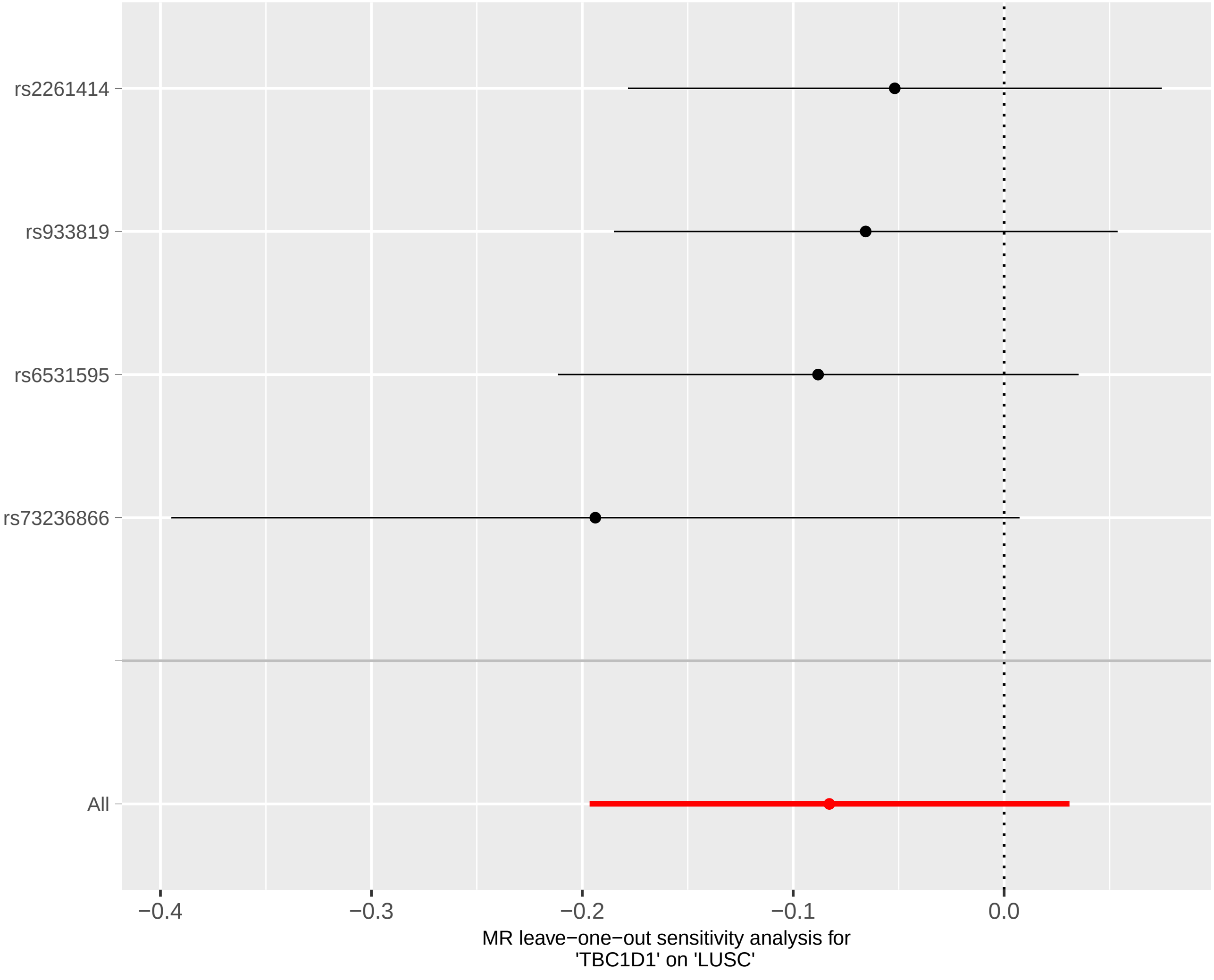
rs2261414
rs933819
rs6531595
rs73236866
All
−0.4
−0.3
−0.2
−0.1
0.0
MR leave−one−out sensitivity analysis for
'TBC1D1' on 'LUSC'

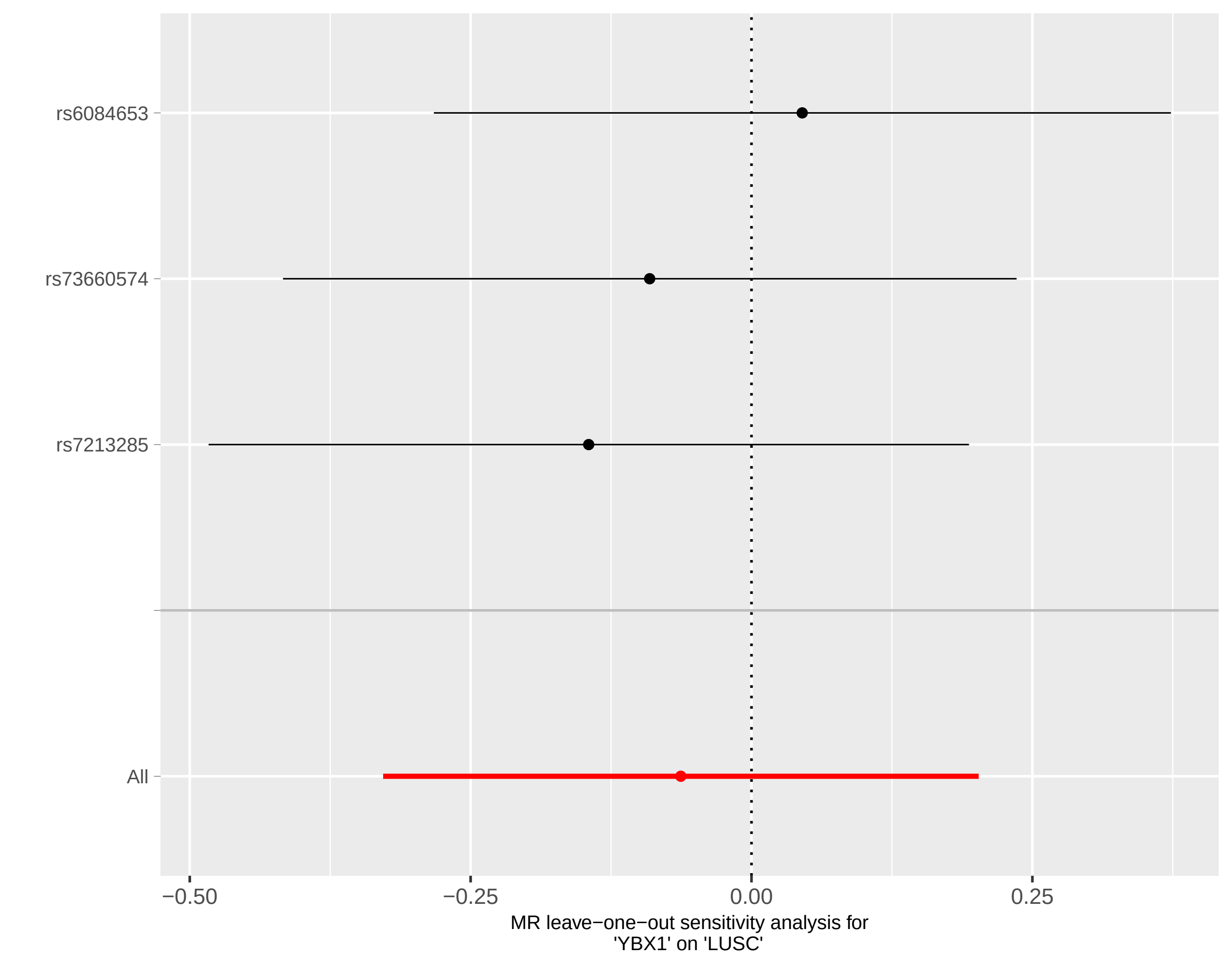

rs6084653
rs73660574
rs7213285
All
−0.50
−0.25
0.00
0.25
MR leave−one−out sensitivity analysis for
'YBX1' on 'LUSC'

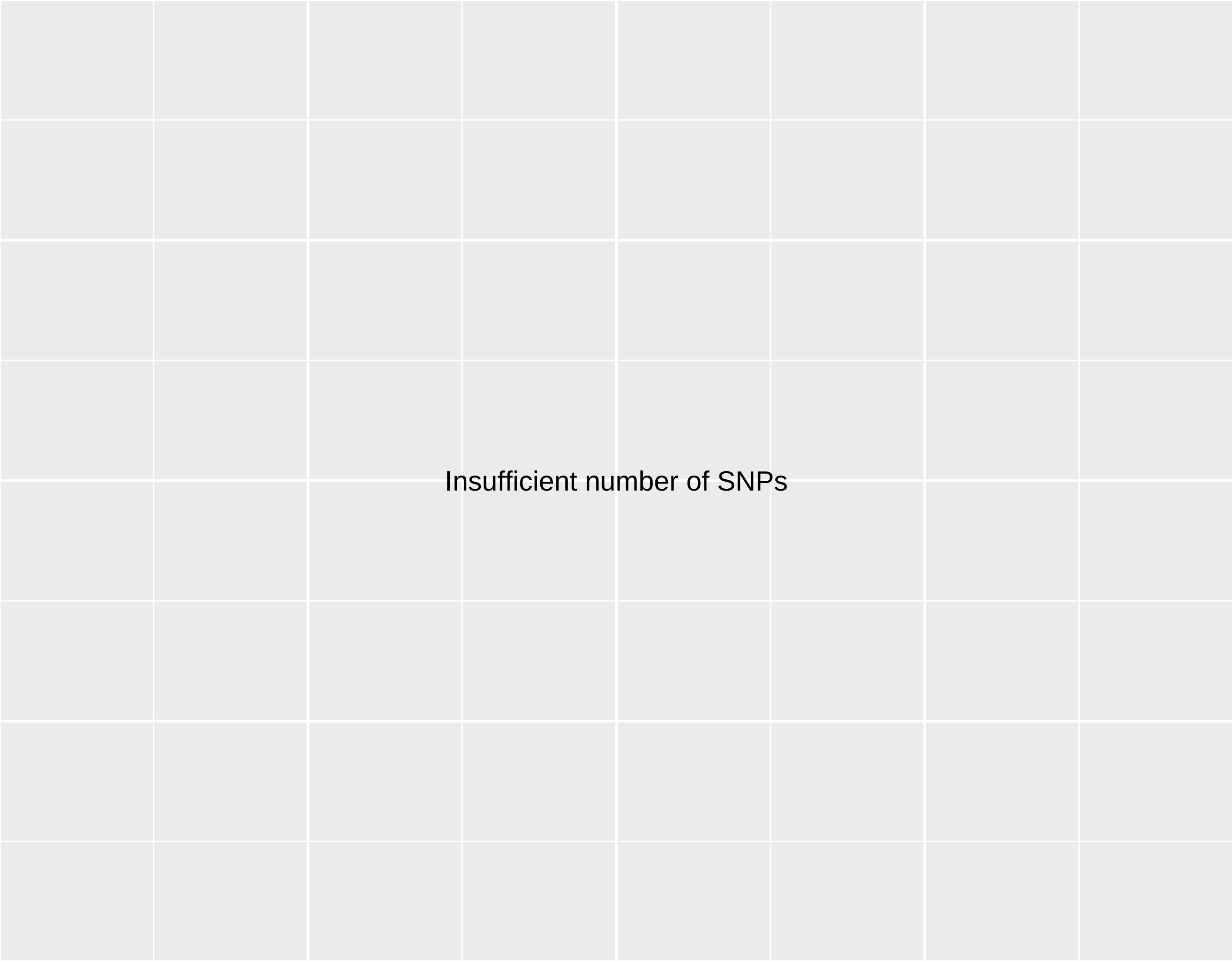
Insufficient number of SNPs

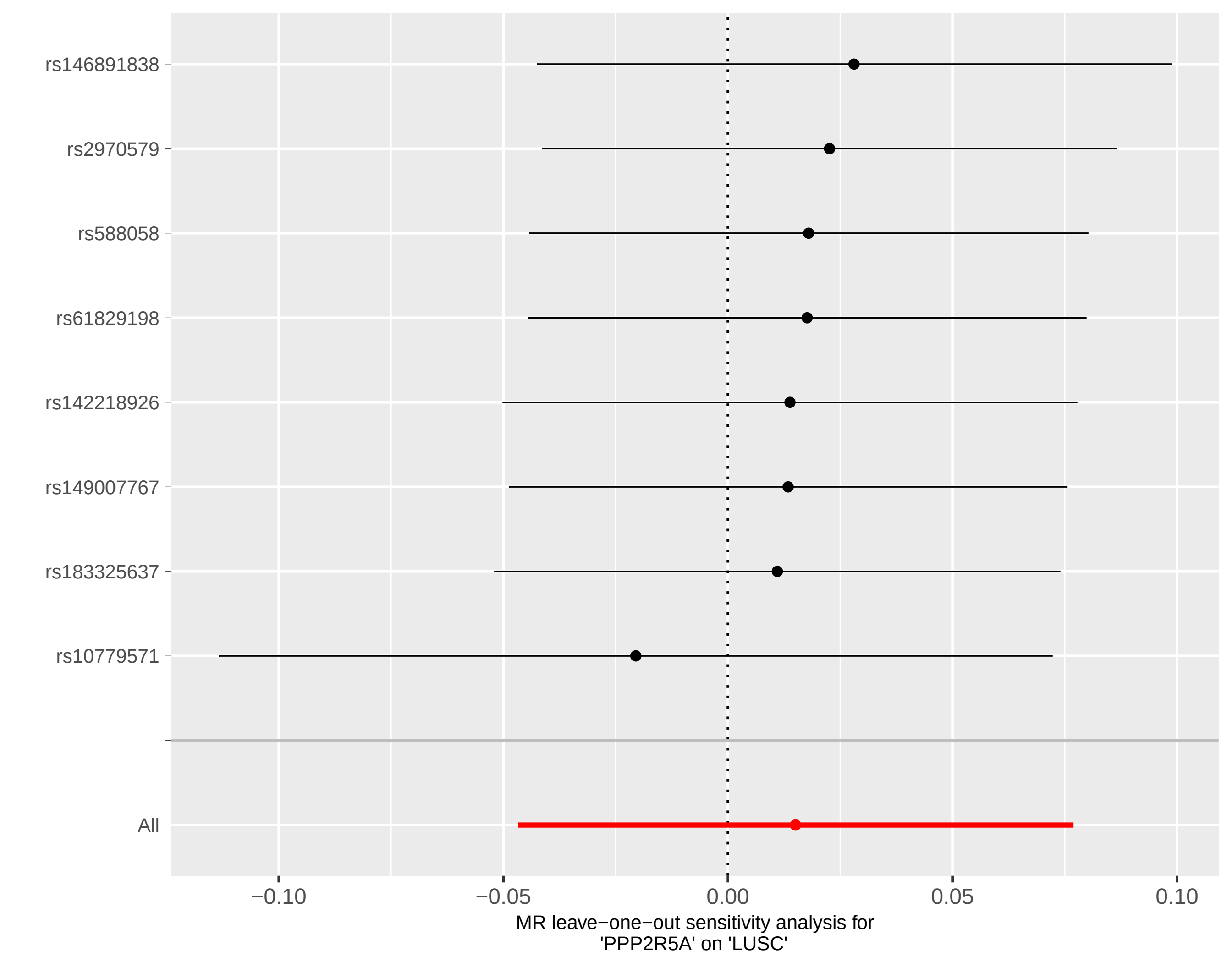
rs146891838
rs2970579
rs588058
rs61829198
rs142218926
rs149007767
rs183325637
rs10779571
All
−0.10
−0.05
0.00
0.05
0.10
MR leave−one−out sensitivity analysis for
'PPP2R5A' on 'LUSC'

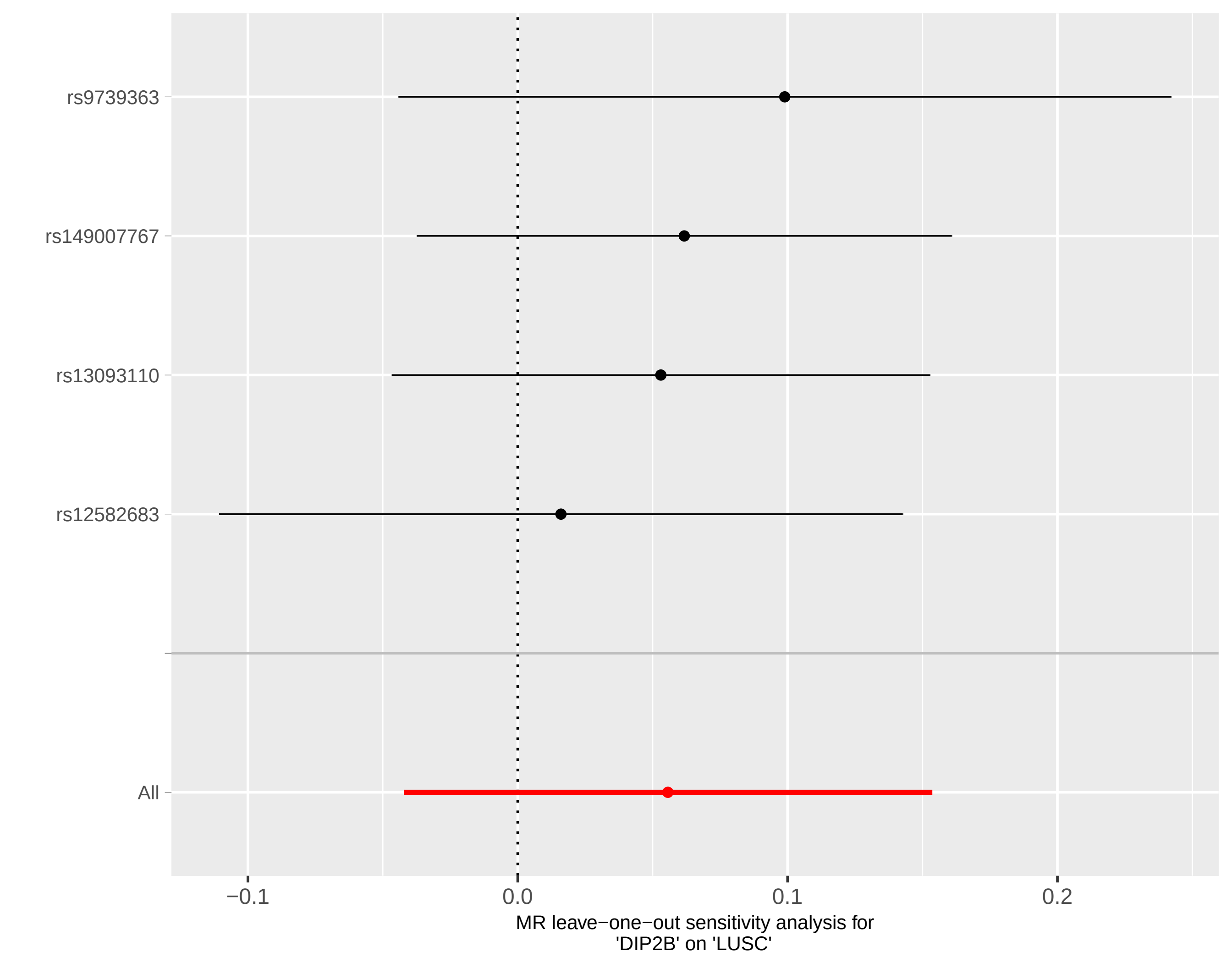
rs9739363
rs149007767
rs13093110
rs12582683
All
−0.1
0.0
0.1
0.2
MR leave−one−out sensitivity analysis for
'DIP2B' on 'LUSC'

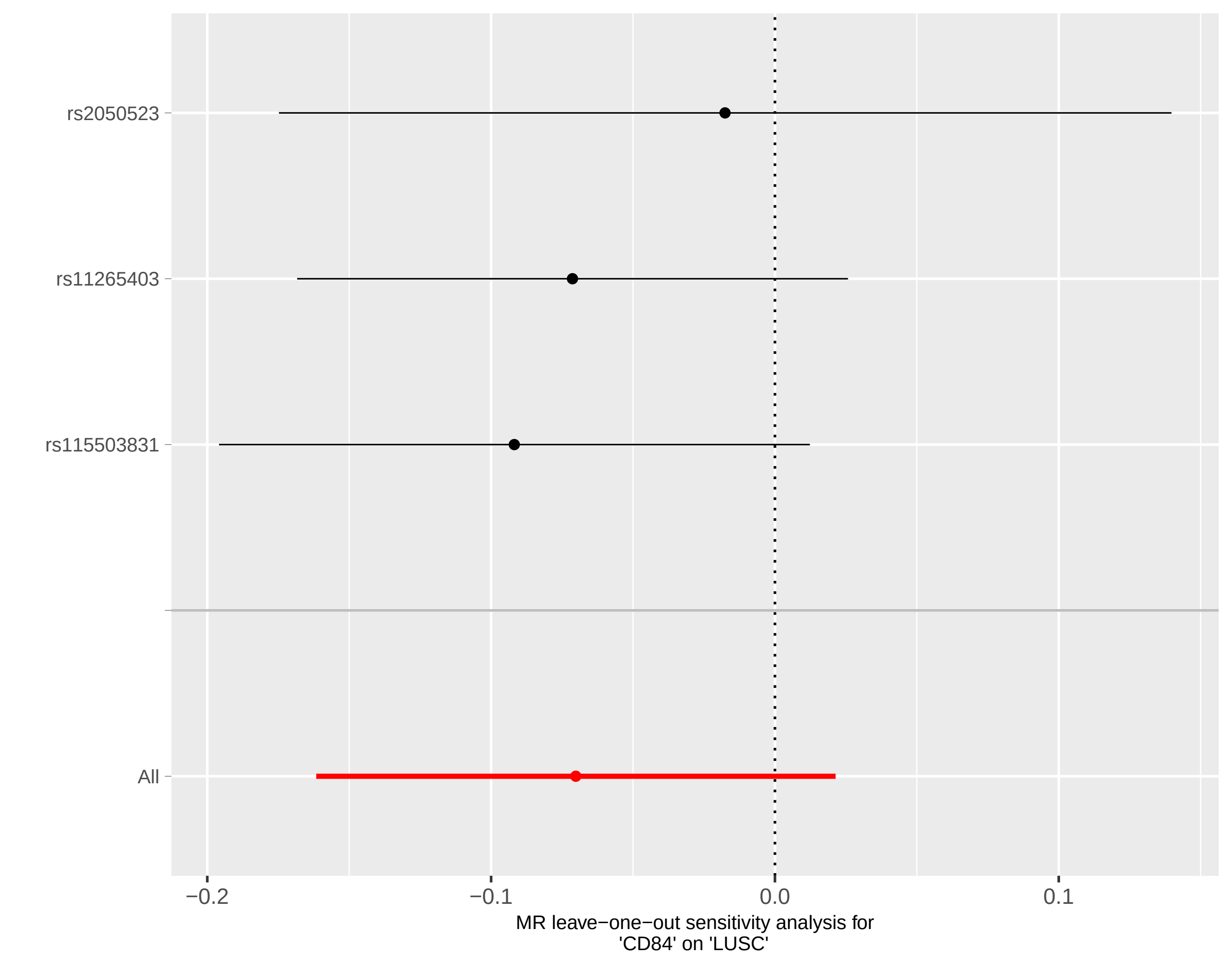

rs2050523
rs11265403
rs115503831
All
−0.2
−0.1
0.0
0.1
MR leave−one−out sensitivity analysis for
'CD84' on 'LUSC'

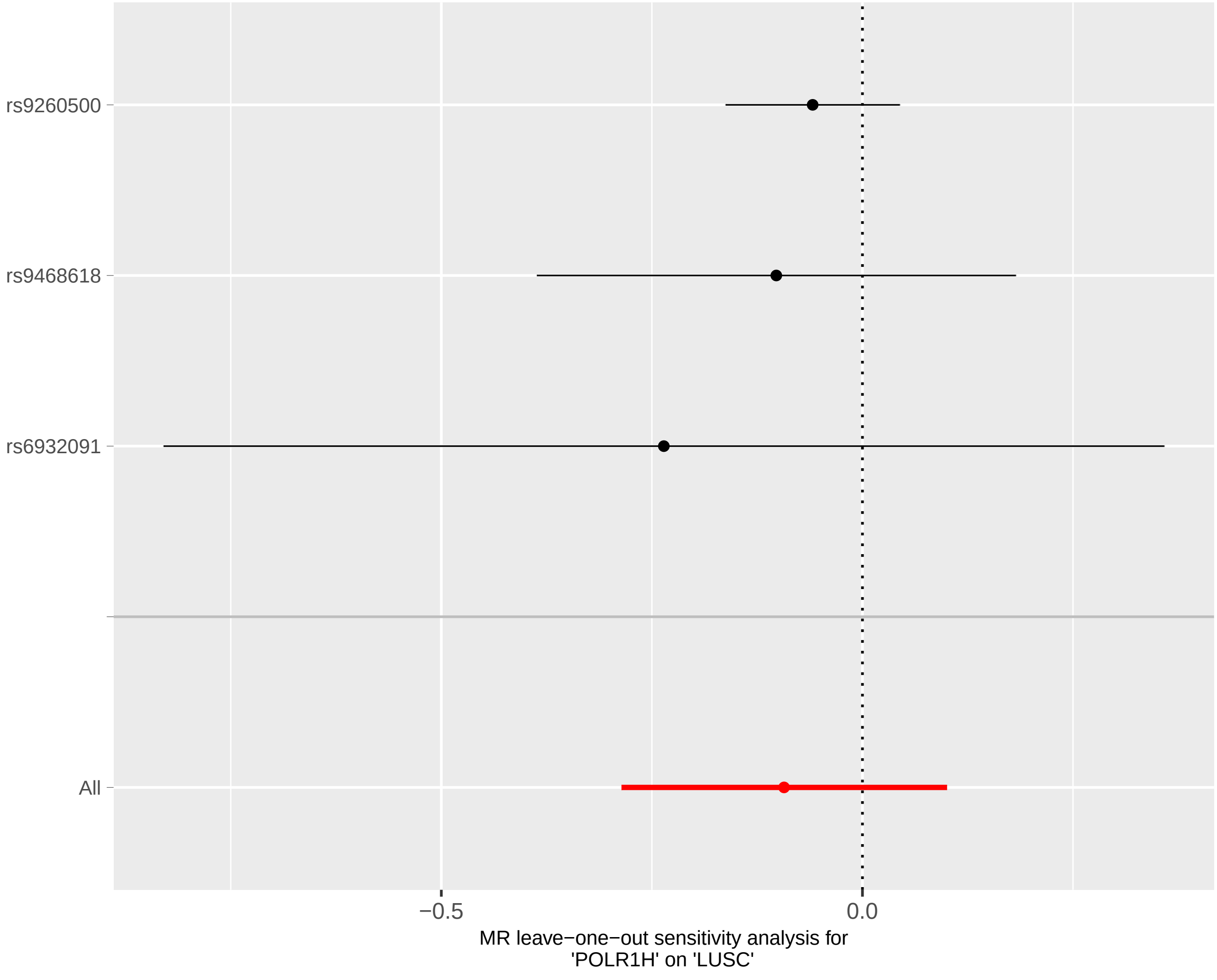

rs9260500
rs9468618
rs6932091
All
−0.5
0.0
MR leave−one−out sensitivity analysis for
'POLR1H' on 'LUSC'

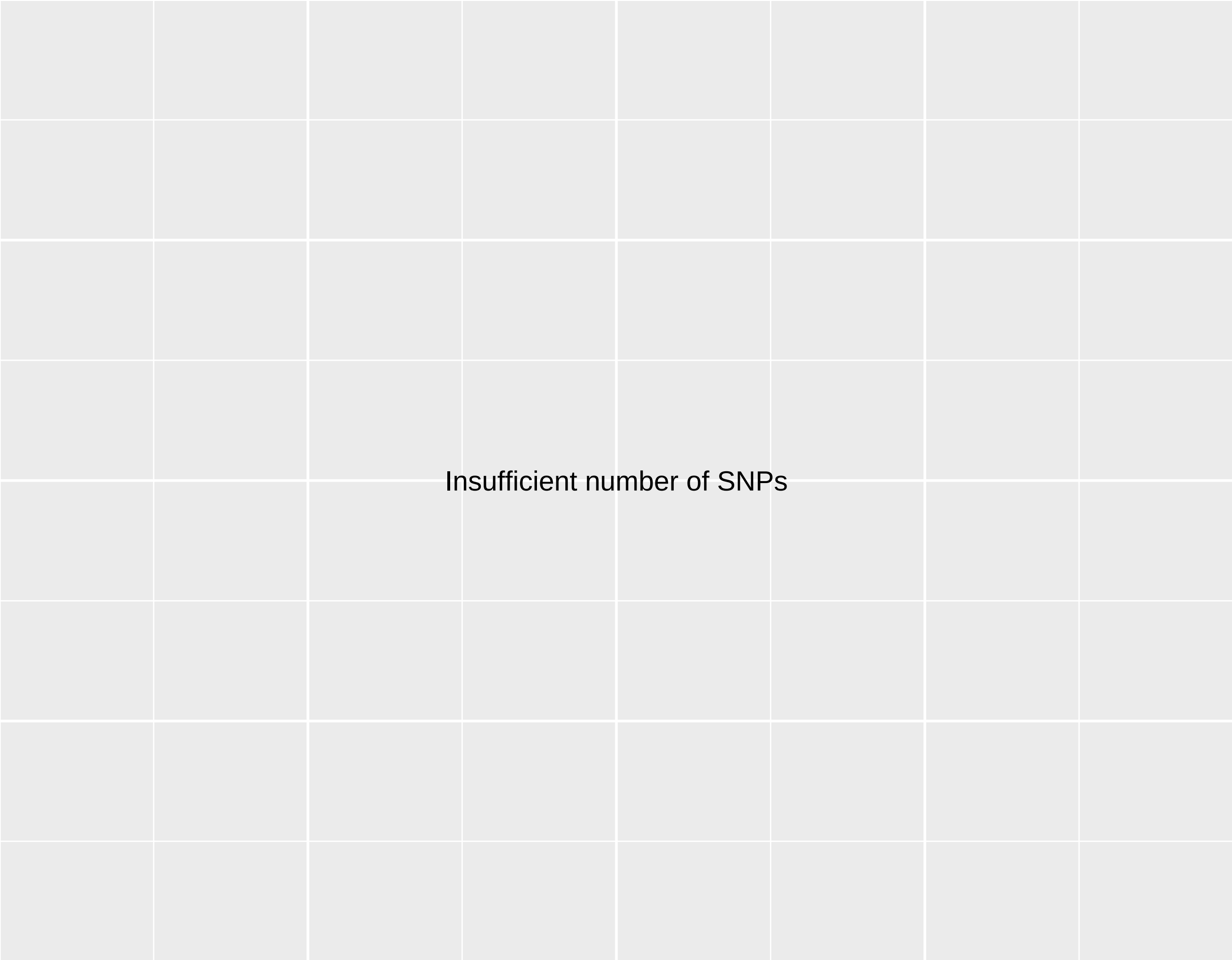
Insufficient number of SNPs

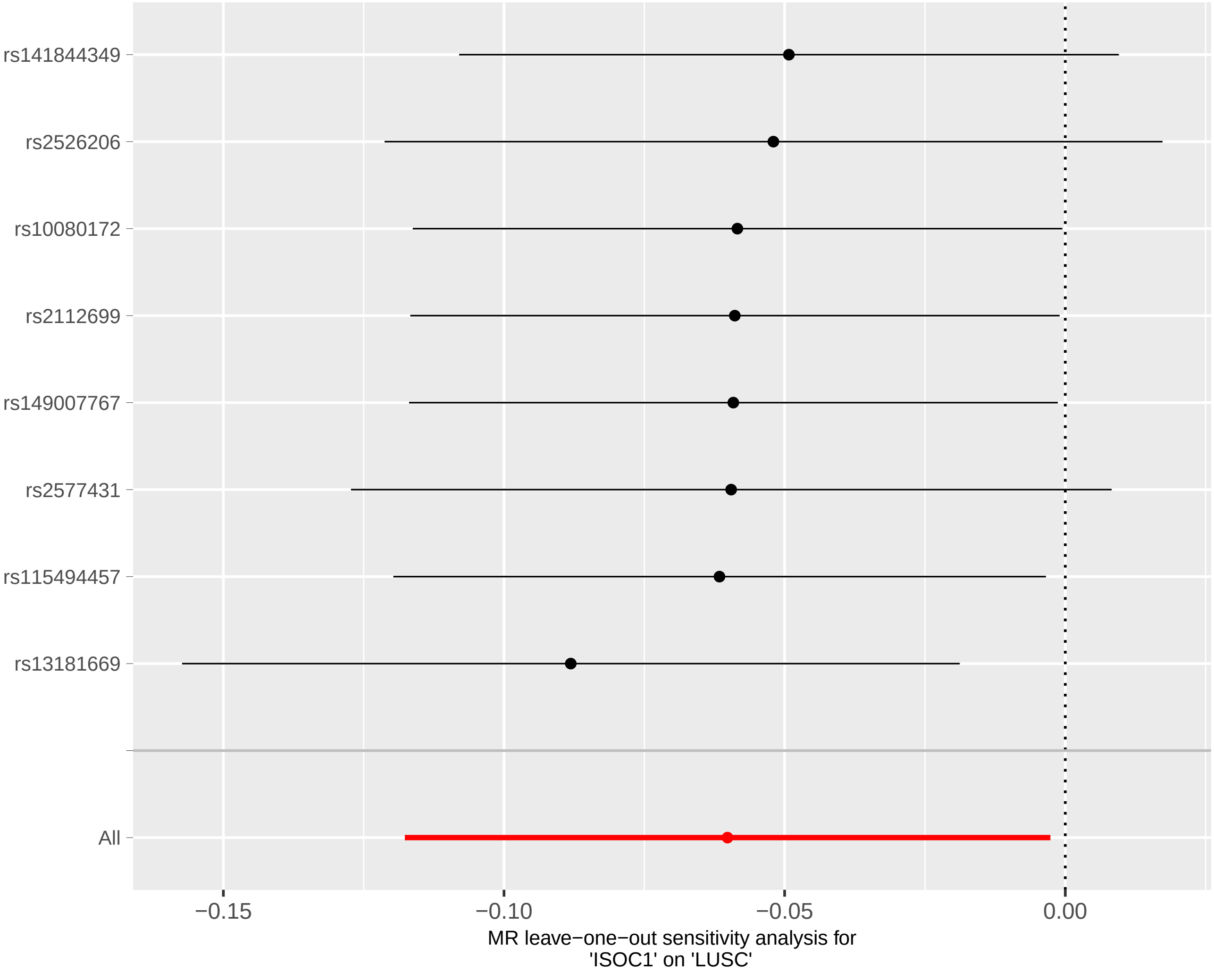
rs141844349
rs2526206
rs10080172
rs2112699
rs149007767
rs2577431
rs115494457
rs13181669
All
−0.15
−0.10
−0.05
0.00
MR leave−one−out sensitivity analysis for
'ISOC1' on 'LUSC'

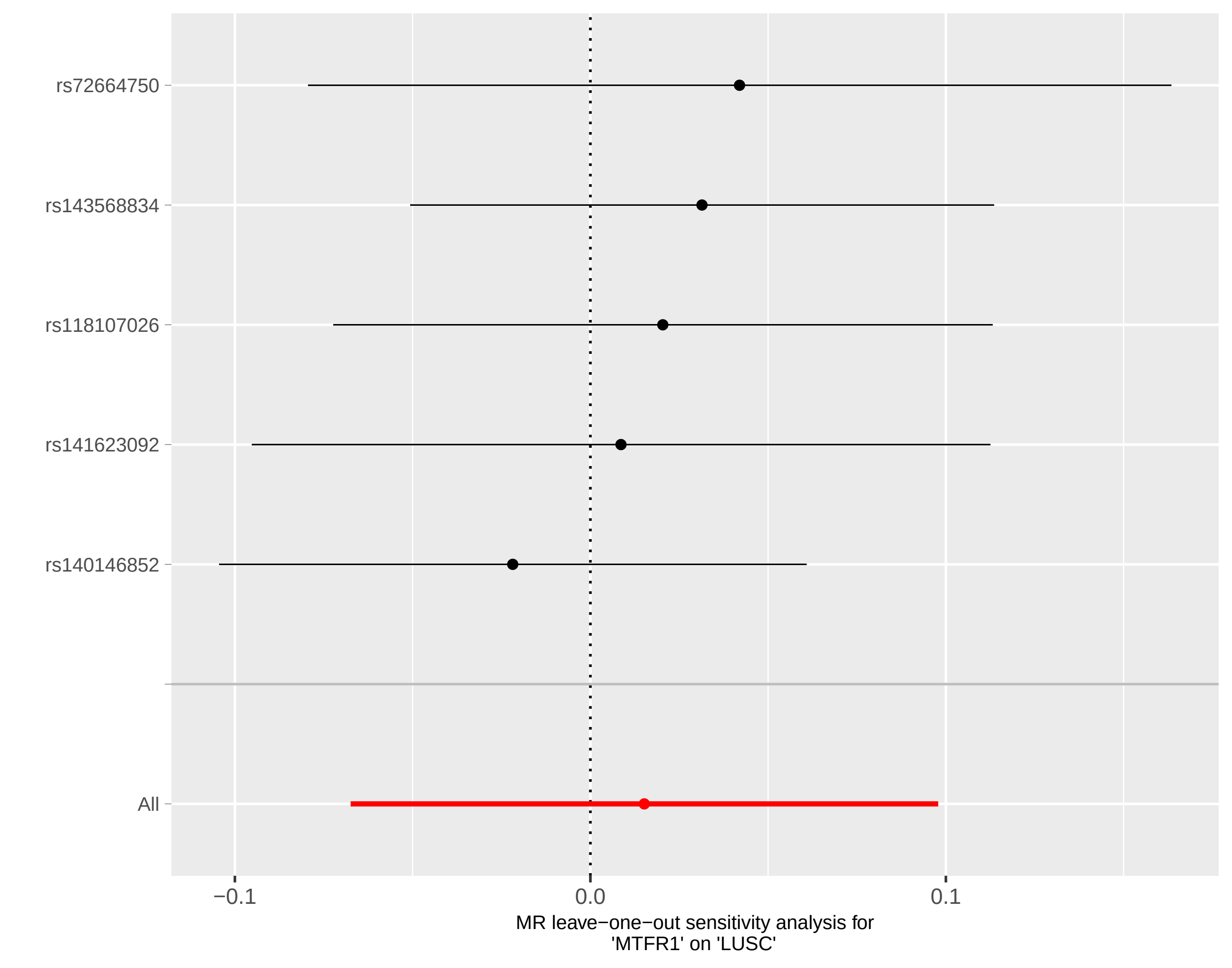
rs72664750
rs143568834
rs118107026
rs141623092
rs140146852
All
−0.1
0.0
0.1
MR leave−one−out sensitivity analysis for
'MTFR1' on 'LUSC'

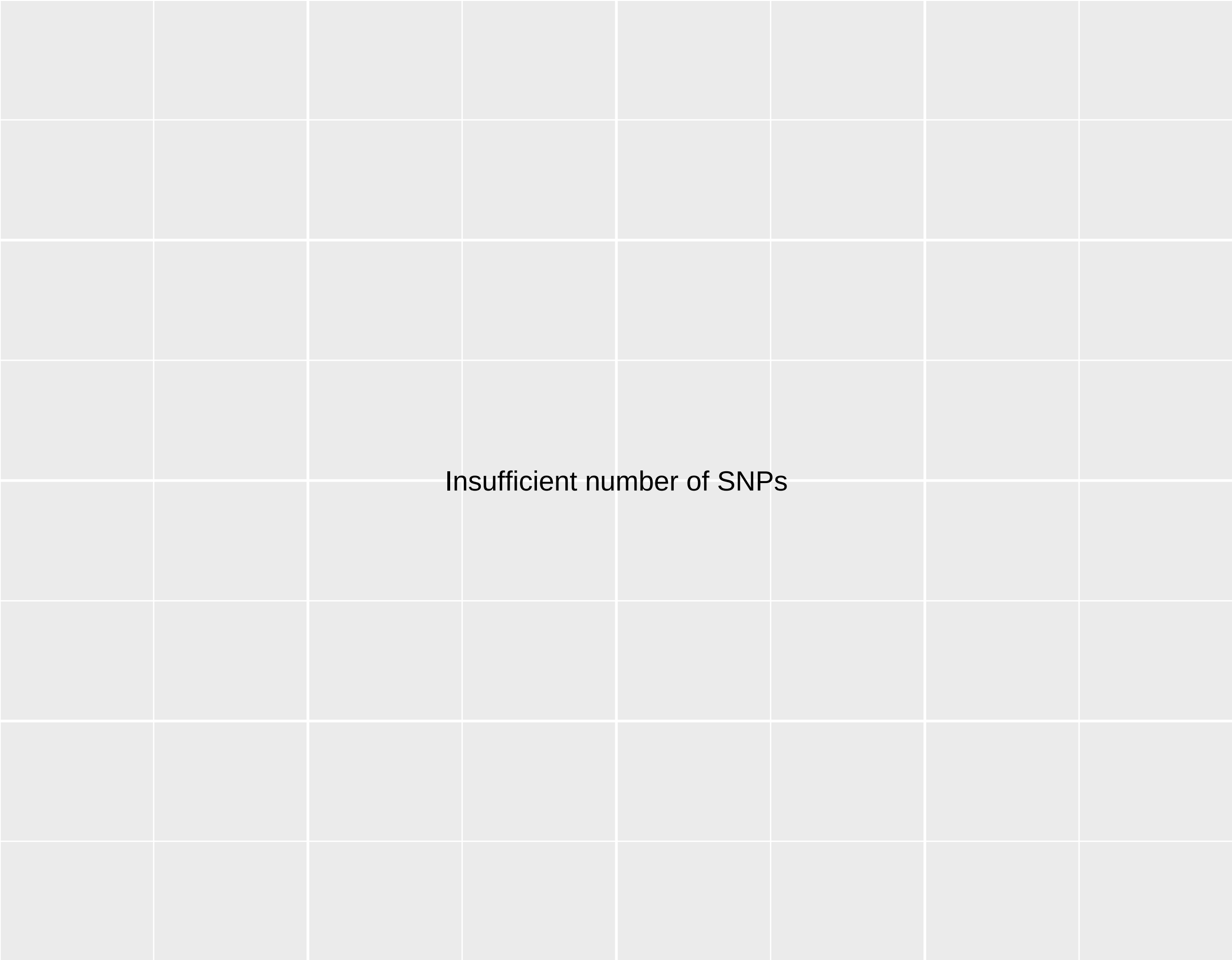
Insufficient number of SNPs

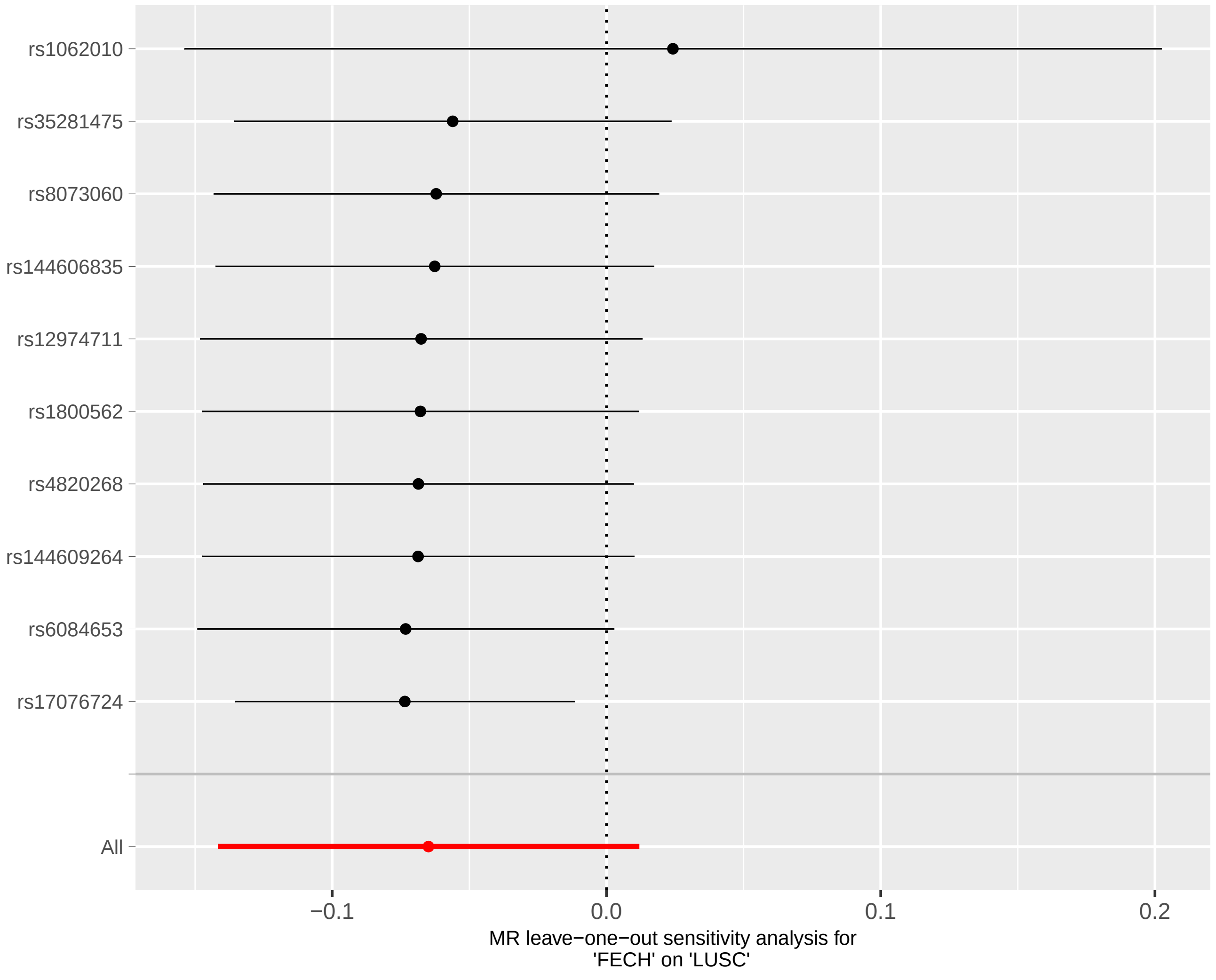

rs1062010
rs35281475
rs8073060
rs144606835
rs12974711
rs1800562
rs4820268
rs144609264
rs6084653
rs17076724
All
−0.1
0.0
0.1
0.2
MR leave−one−out sensitivity analysis for
'FECH' on 'LUSC'

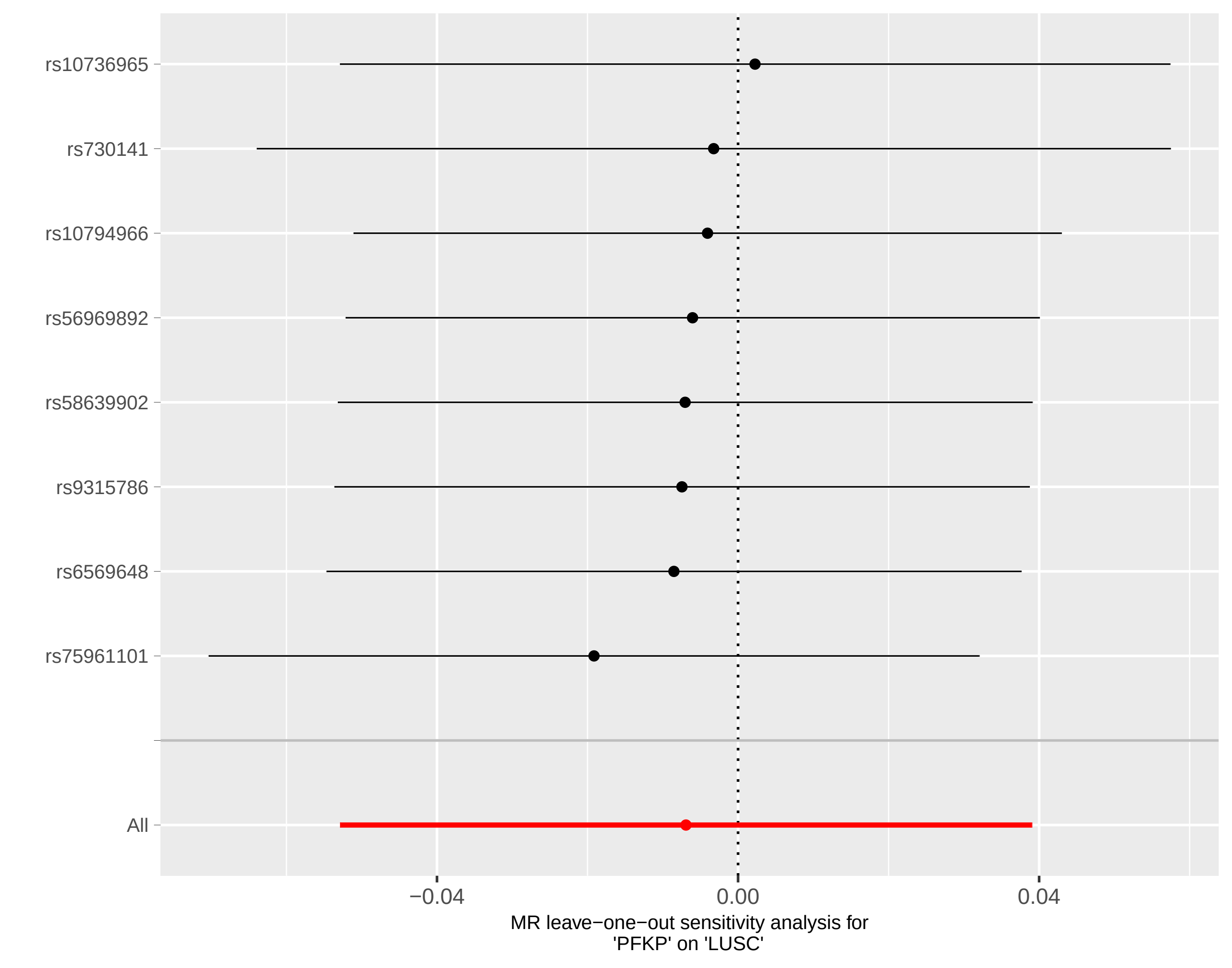
rs10736965
rs730141
rs10794966
rs56969892
rs58639902
rs9315786
rs6569648
rs75961101
All
−0.04
0.00
0.04
MR leave−one−out sensitivity analysis for
'PFKP' on 'LUSC'

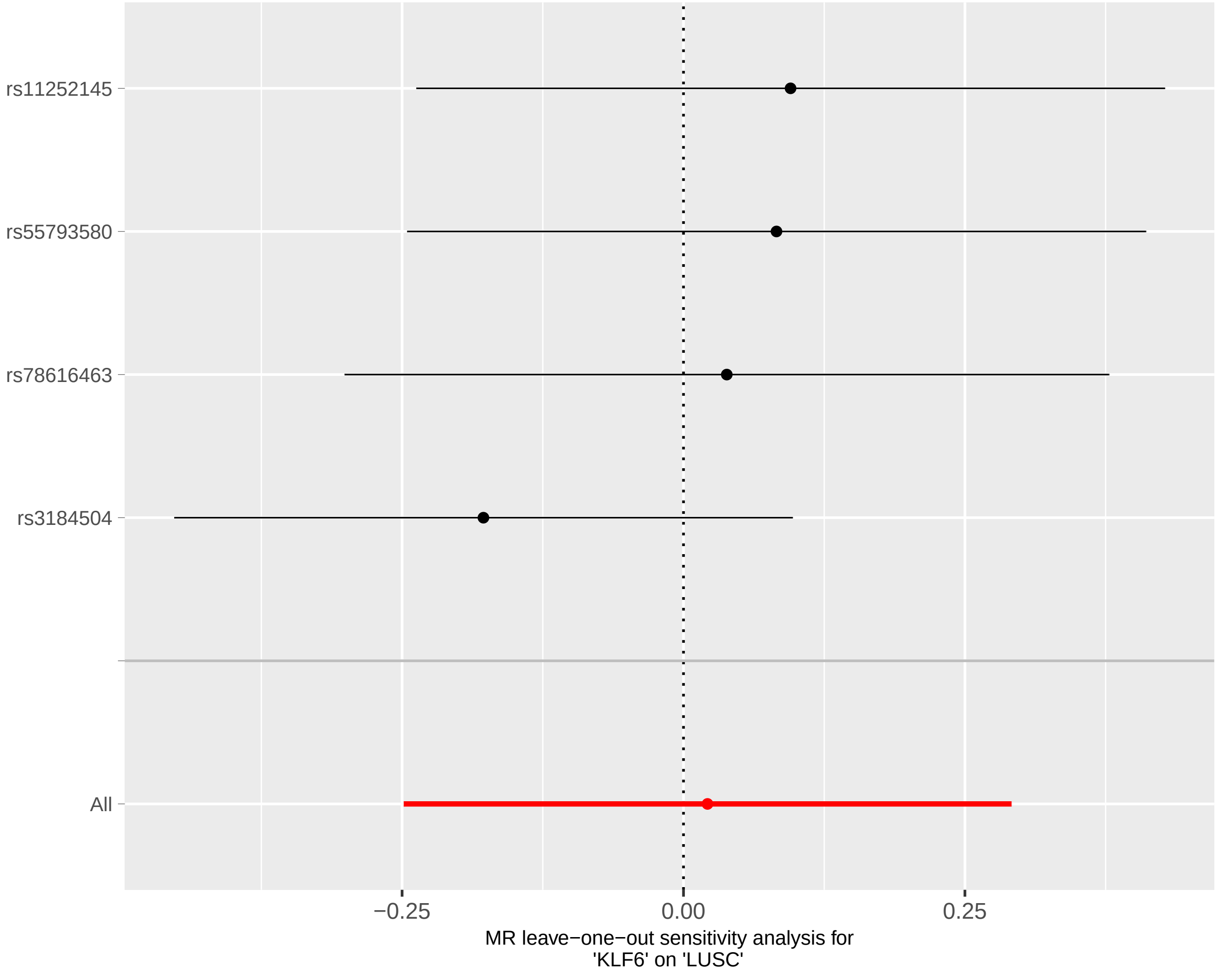
rs11252145
rs55793580
rs78616463
rs3184504
All
−0.25
0.00
0.25
MR leave−one−out sensitivity analysis for
'KLF6' on 'LUSC'

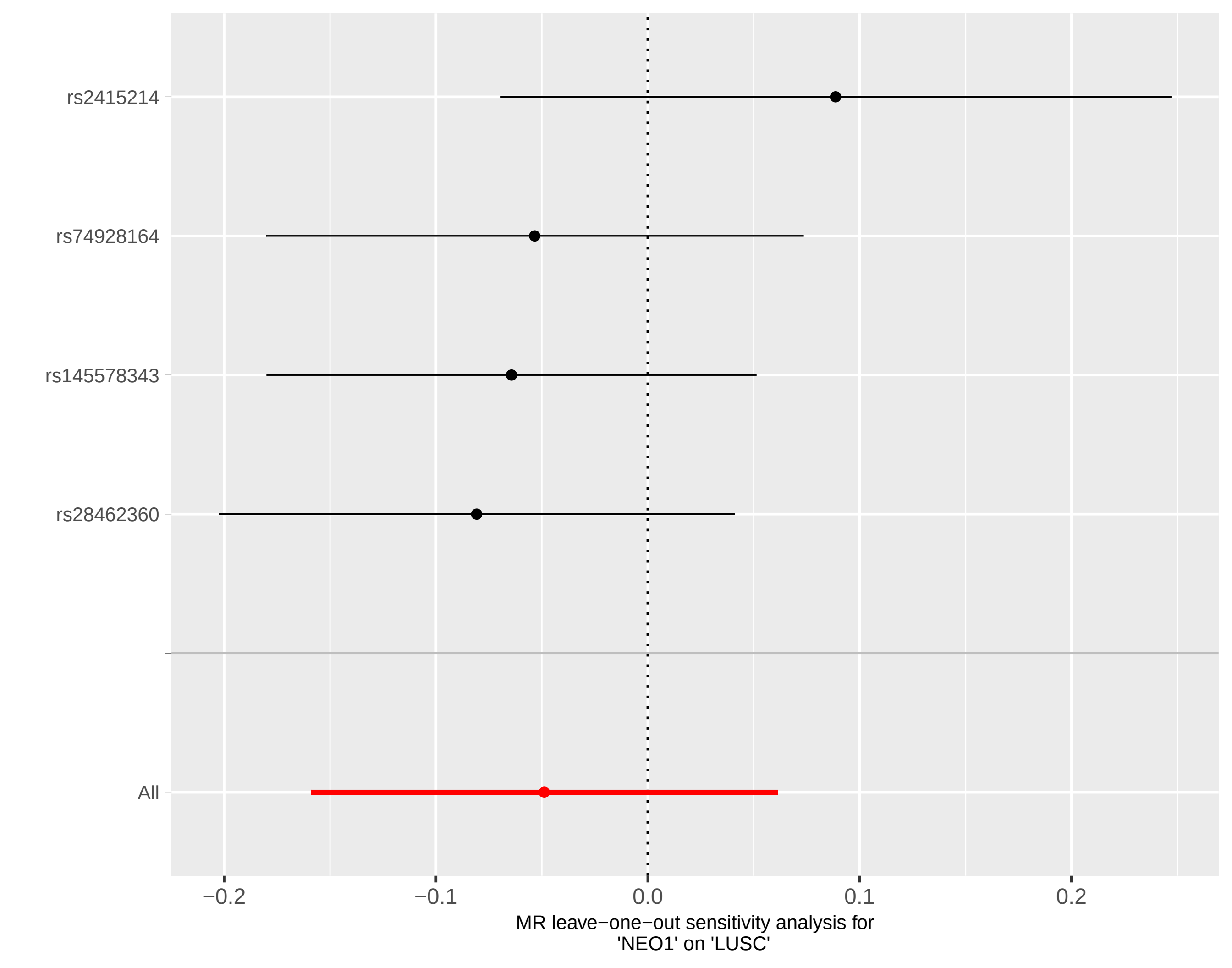
rs2415214
rs74928164
rs145578343
rs28462360
All
−0.2
−0.1
0.0
0.1
0.2
MR leave−one−out sensitivity analysis for
'NEO1' on 'LUSC'

Insufficient number of SNPs

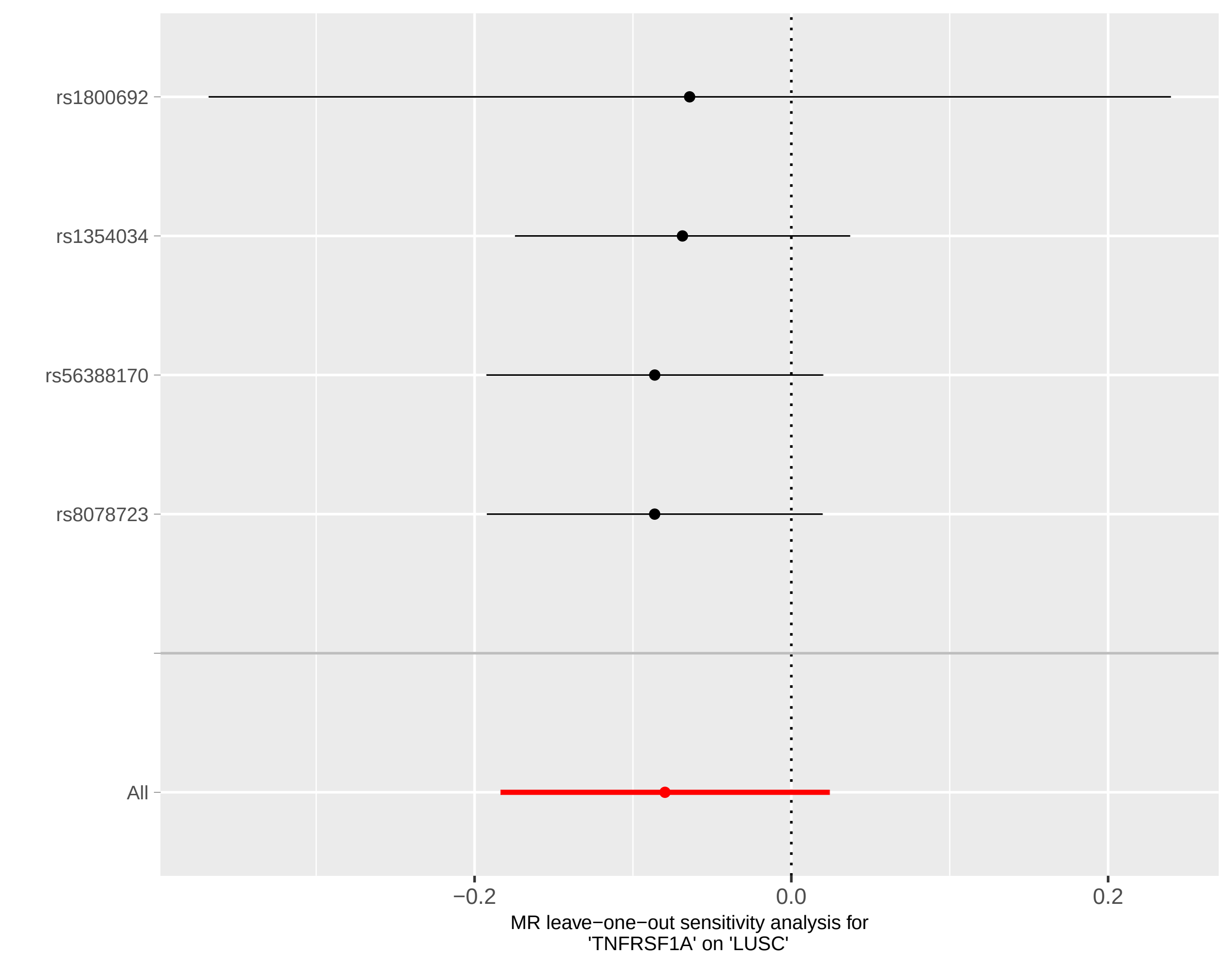

rs1800692
rs1354034
rs56388170
rs8078723
All
−0.2
0.0
0.2
MR leave−one−out sensitivity analysis for
'TNFRSF1A' on 'LUSC'

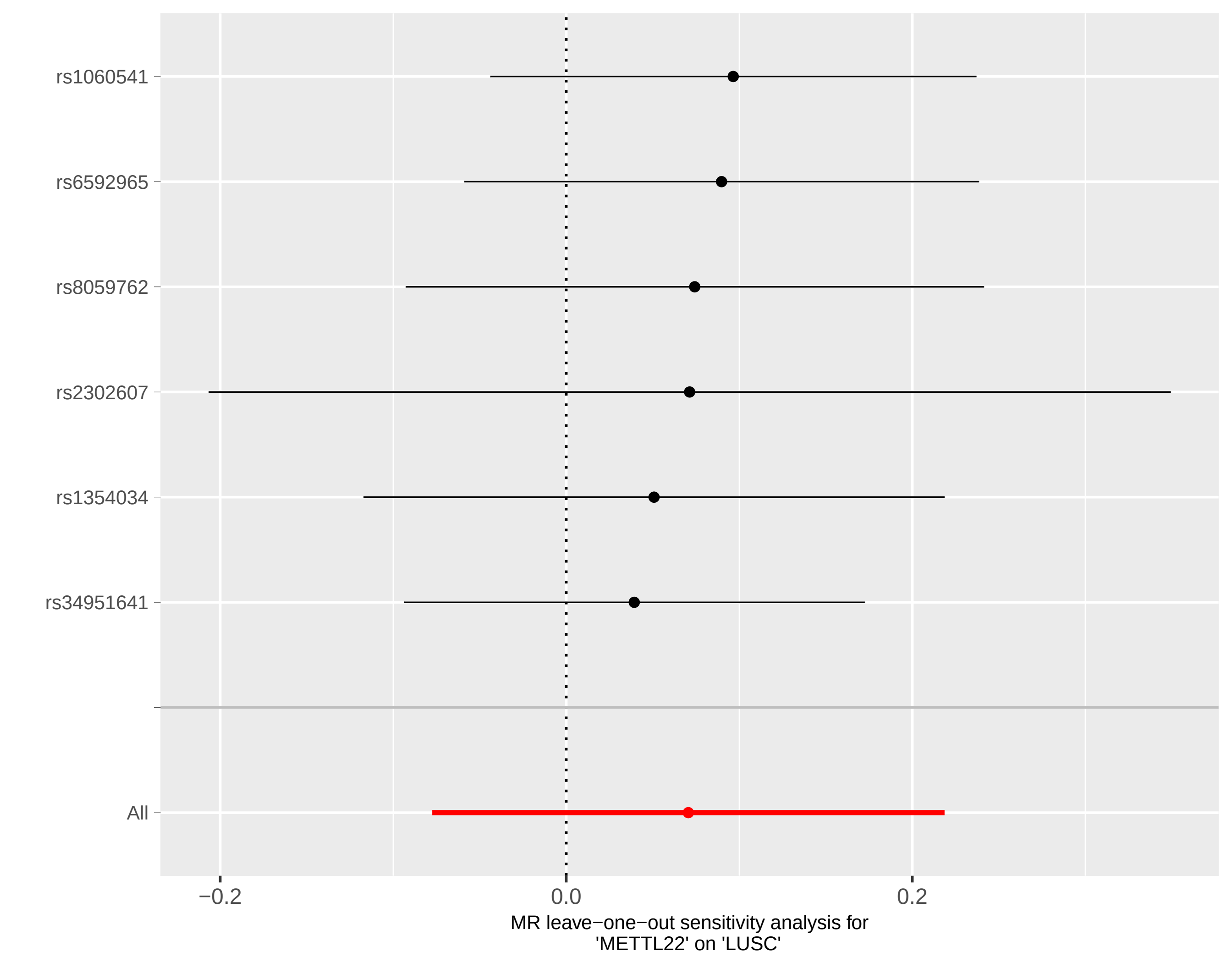

rs1060541
rs6592965
rs8059762
rs2302607
rs1354034
rs34951641
All
−0.2
0.0
0.2
MR leave−one−out sensitivity analysis for
'METTL22' on 'LUSC'

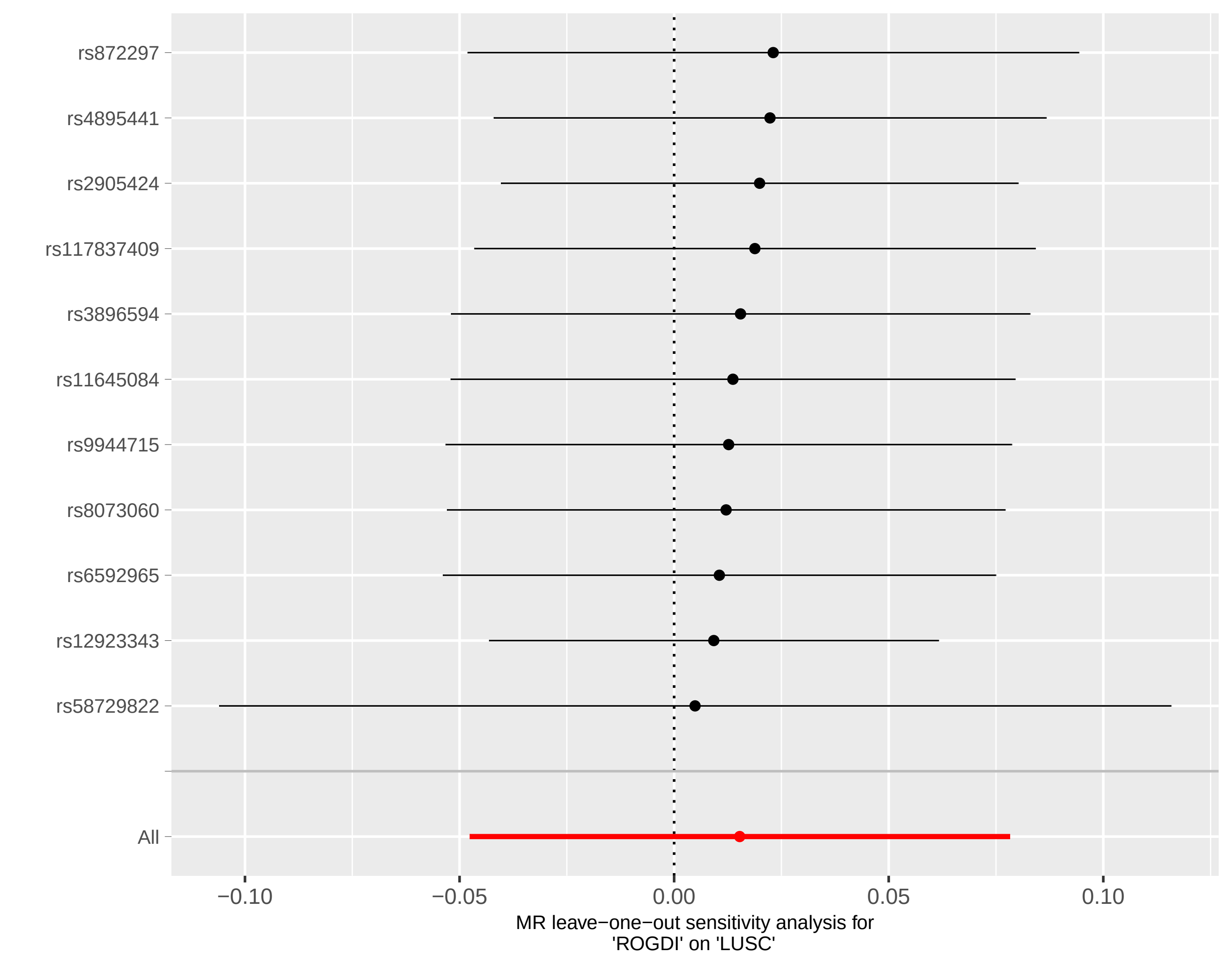
rs872297
rs4895441
rs2905424
rs117837409
rs3896594
rs11645084
rs9944715
rs8073060
rs6592965
rs12923343
rs58729822
All
−0.10
−0.05
0.00
0.05
0.10
MR leave−one−out sensitivity analysis for
'ROGDI' on 'LUSC'

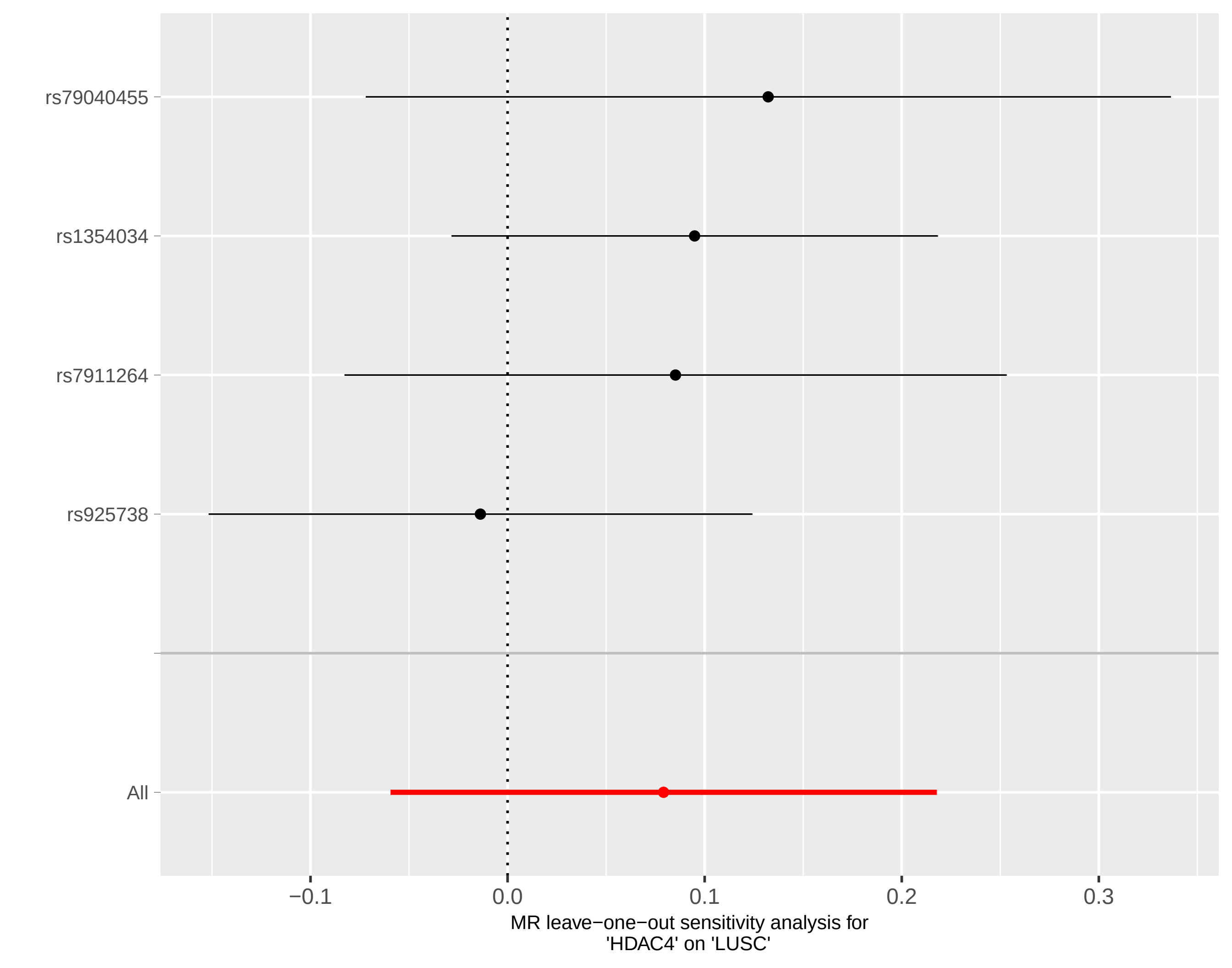

rs79040455
rs1354034
rs7911264
rs925738
All
−0.1
0.0
0.1
0.2
0.3
MR leave−one−out sensitivity analysis for
'HDAC4' on 'LUSC'

Insufficient number of SNPs

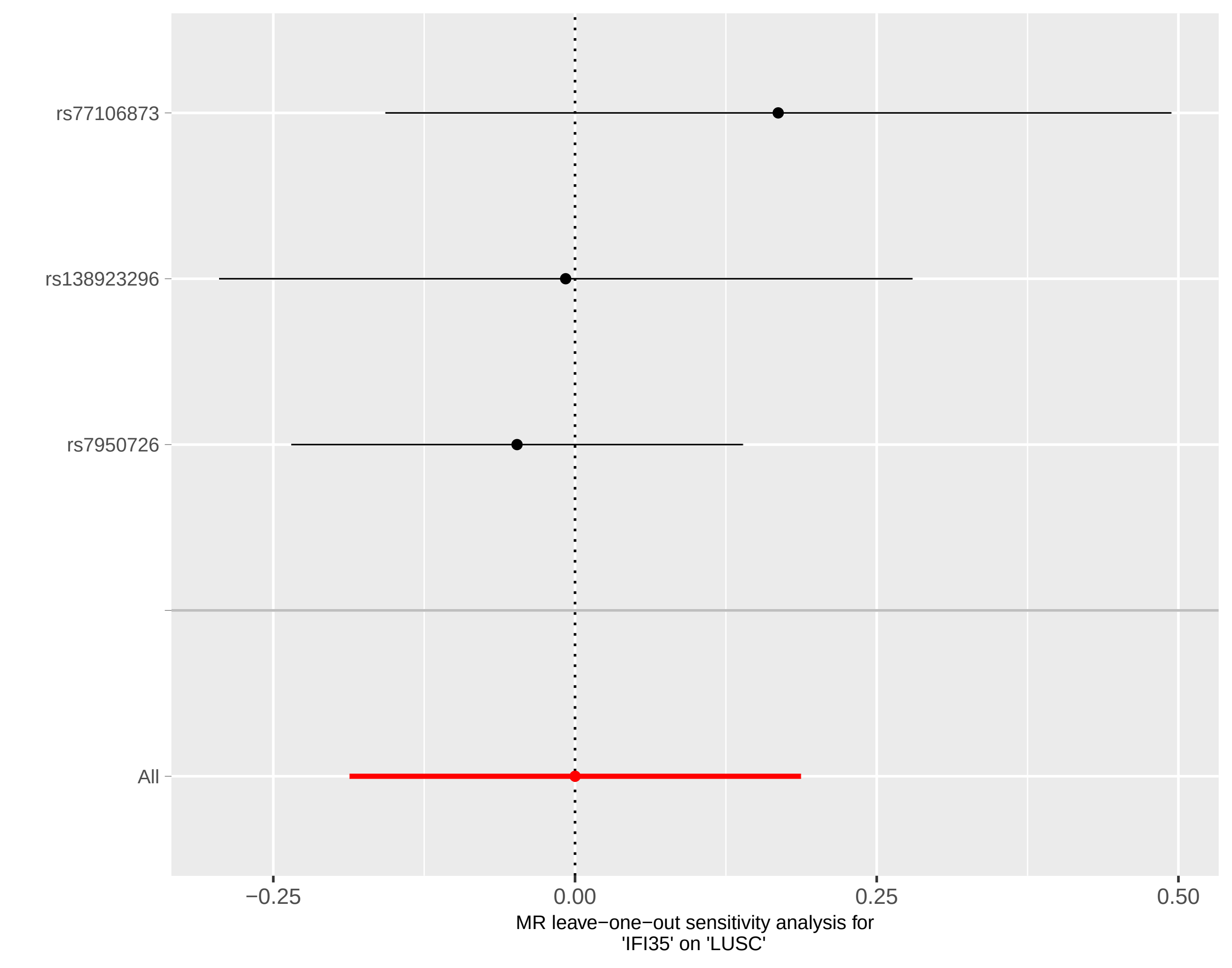
rs77106873
rs138923296
rs7950726
All
−0.25
0.00
0.25
0.50
MR leave−one−out sensitivity analysis for
'IFI35' on 'LUSC'

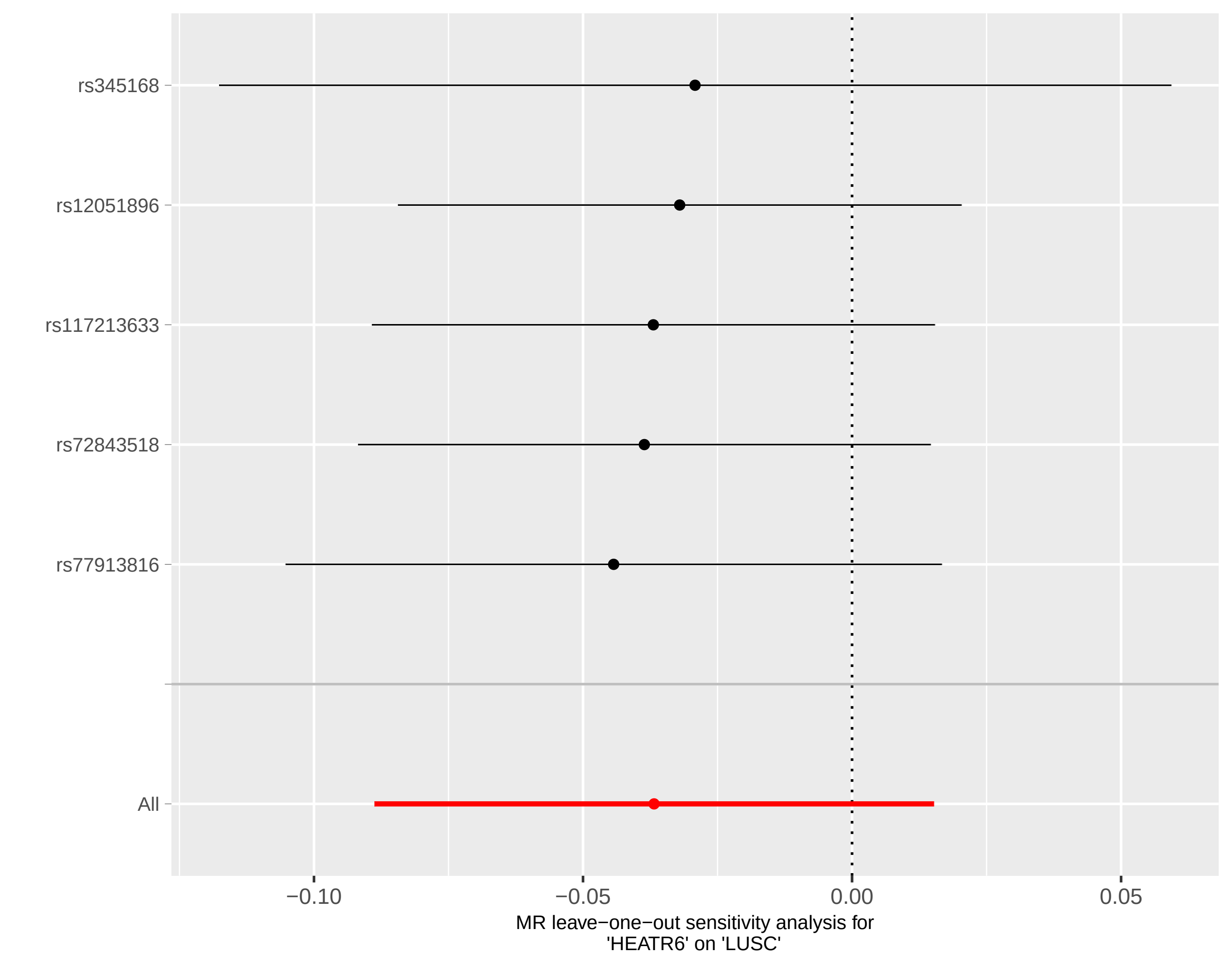
rs345168
rs12051896
rs117213633
rs72843518
rs77913816
All
−0.10
−0.05
0.00
0.05
MR leave−one−out sensitivity analysis for
'HEATR6' on 'LUSC'

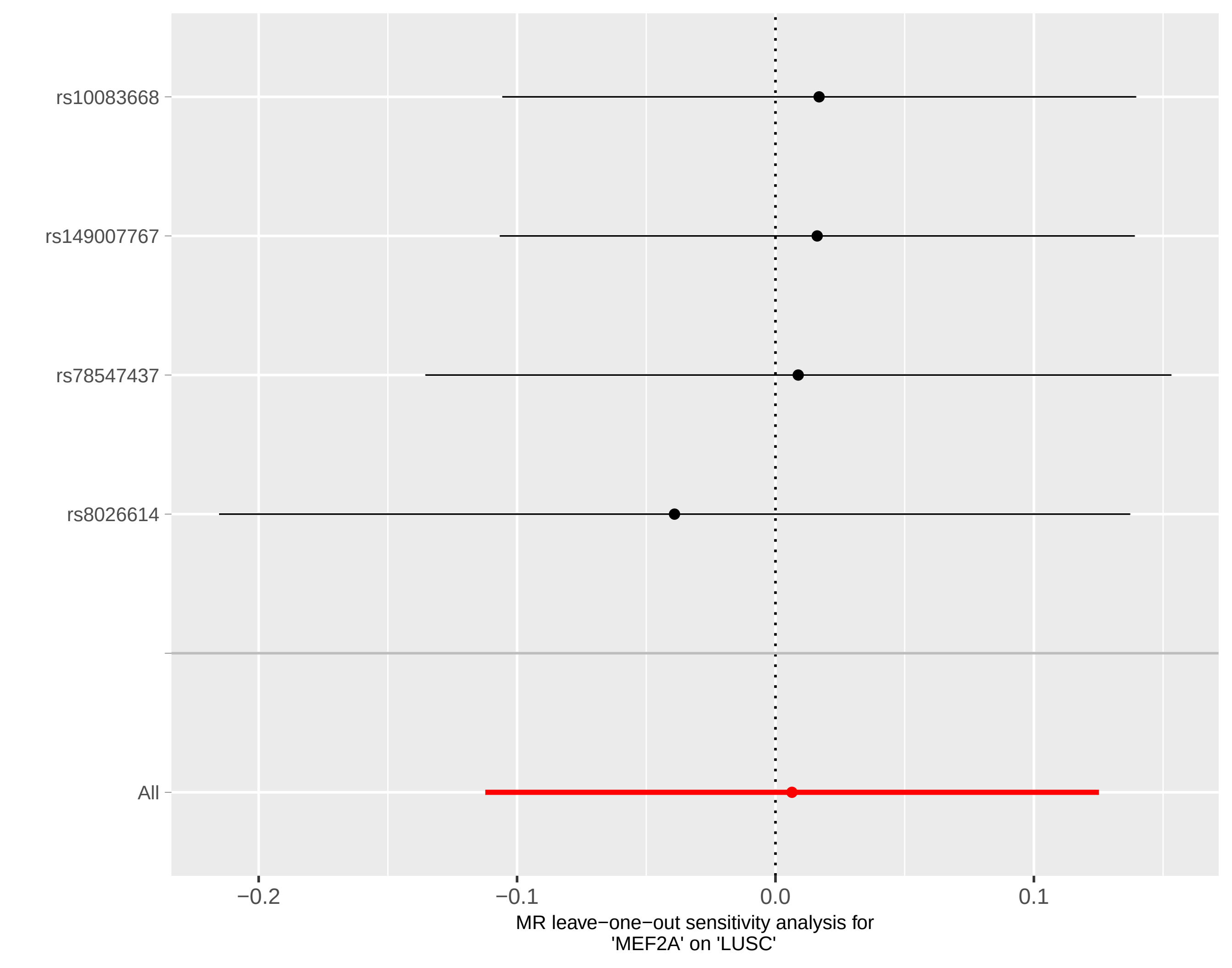
rs10083668
rs149007767
rs78547437
rs8026614
All
−0.2
−0.1
0.0
0.1
MR leave−one−out sensitivity analysis for
'MEF2A' on 'LUSC'

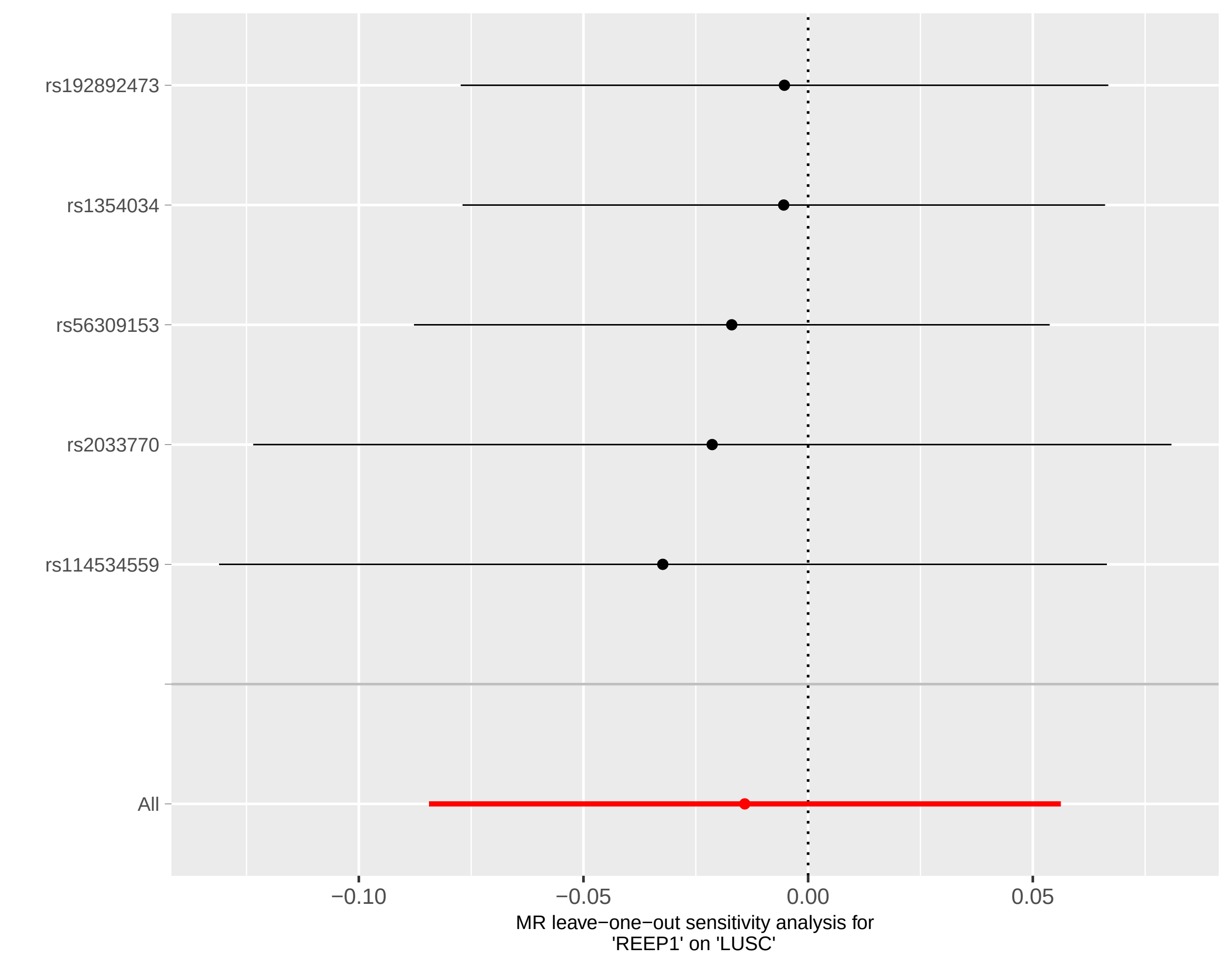

rs192892473
rs1354034
rs56309153
rs2033770
rs114534559
All
−0.10
−0.05
0.00
0.05
MR leave−one−out sensitivity analysis for
'REEP1' on 'LUSC'

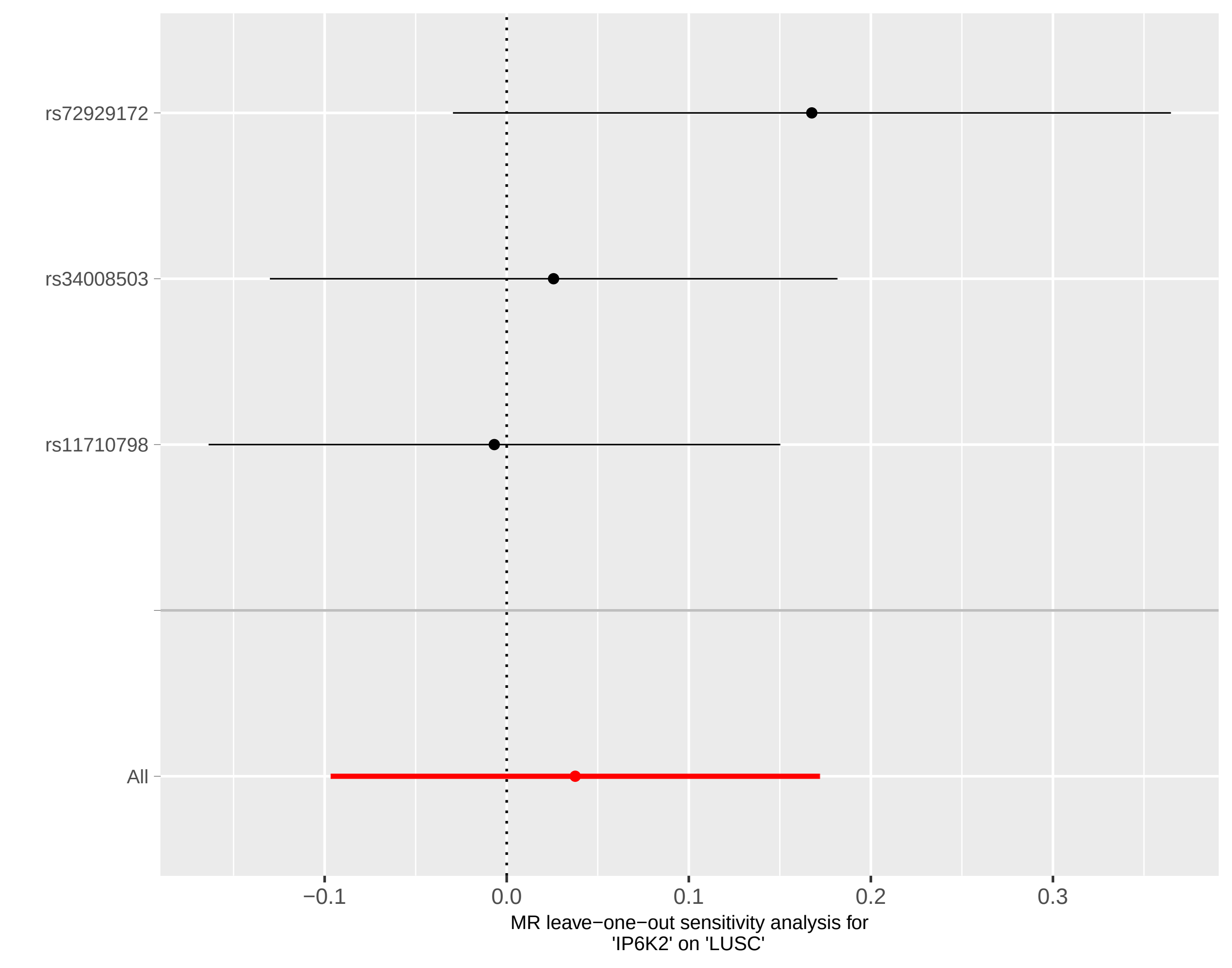
rs72929172
rs34008503
rs11710798
All
−0.1
0.0
0.1
0.2
0.3
MR leave−one−out sensitivity analysis for
'IP6K2' on 'LUSC'

Insufficient number of SNPs

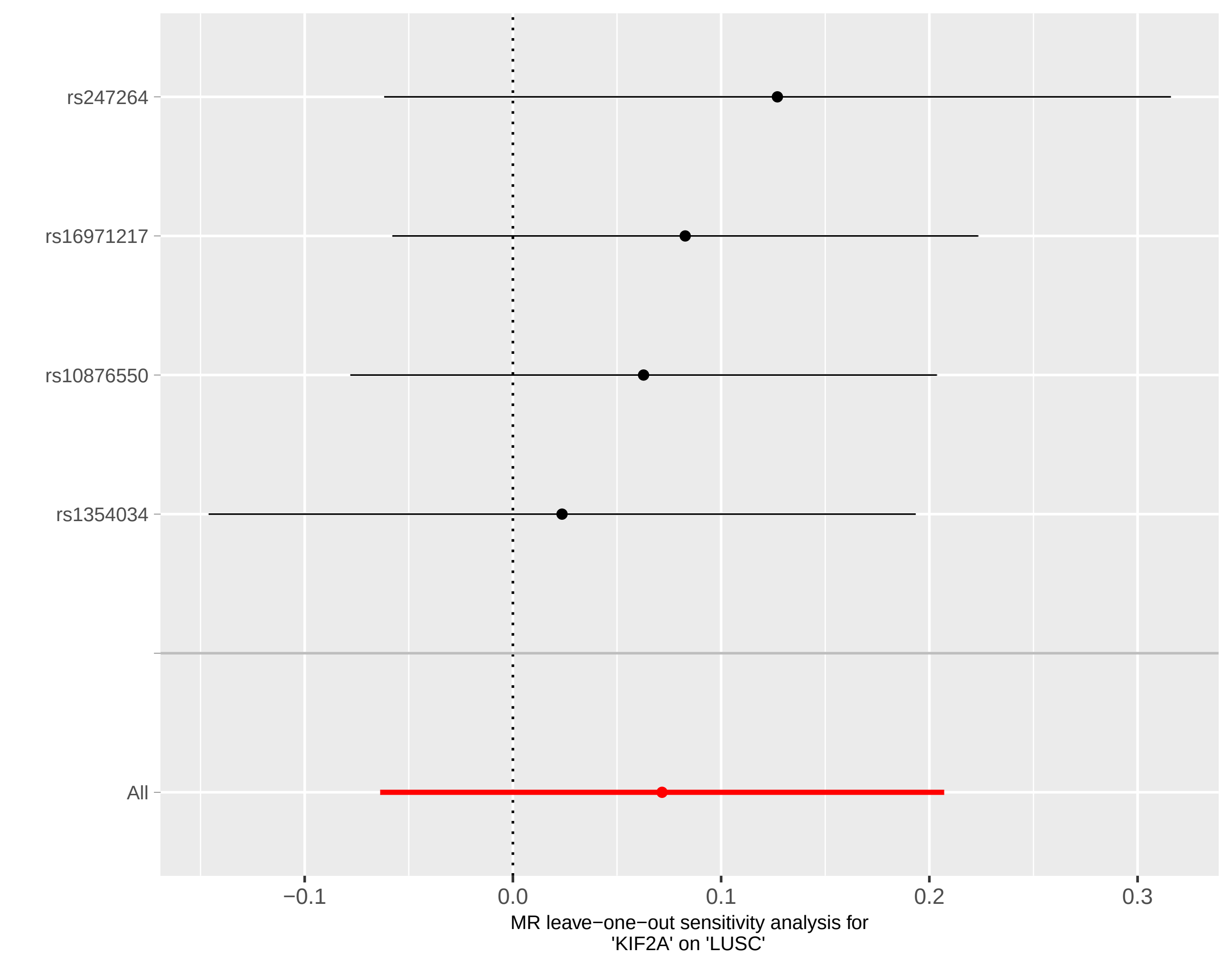
rs247264
rs16971217
rs10876550
rs1354034
All
−0.1
0.0
0.1
0.2
0.3
MR leave−one−out sensitivity analysis for
'KIF2A' on 'LUSC'

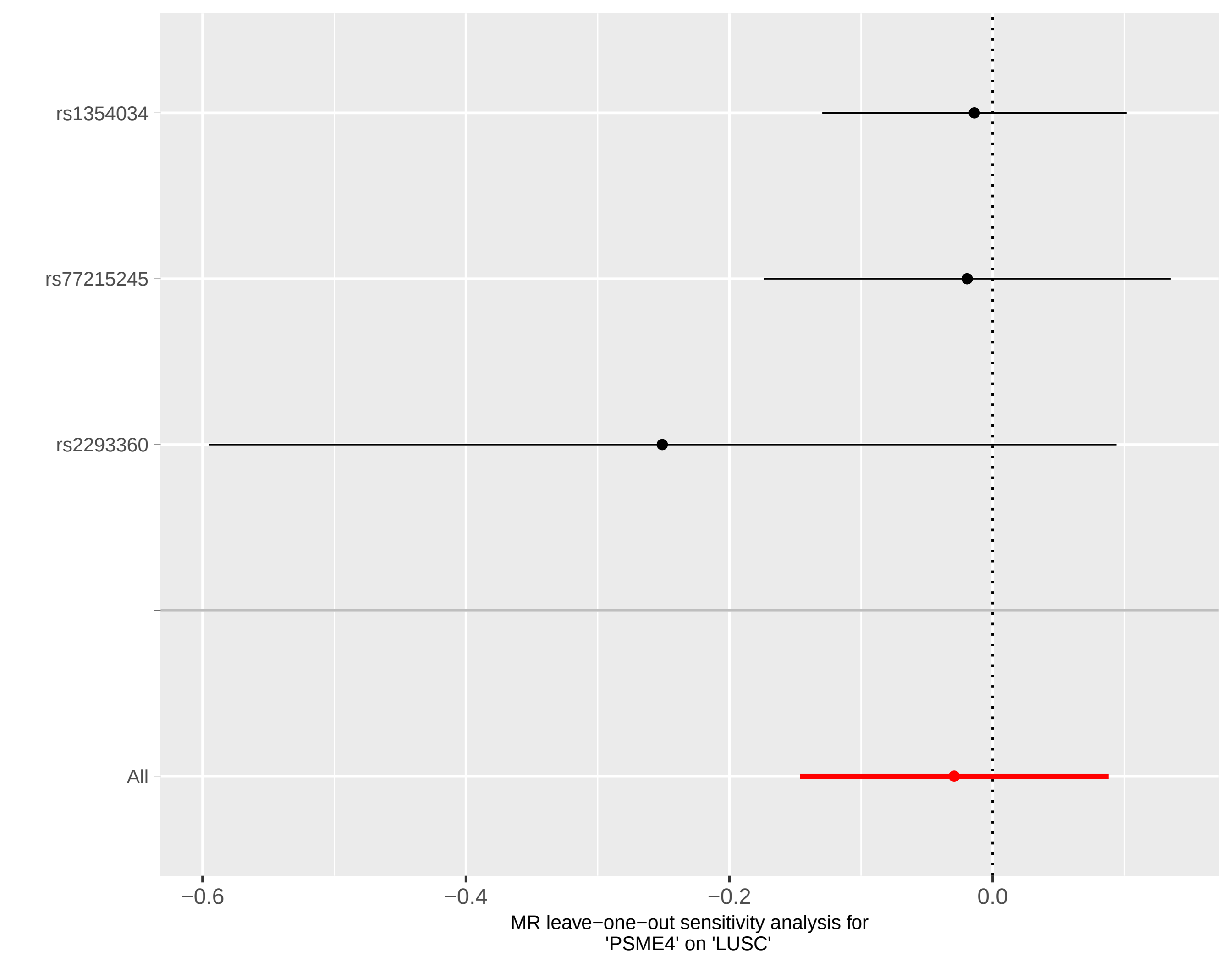
rs1354034
rs77215245
rs2293360
All
−0.6
−0.4
−0.2
0.0
MR leave−one−out sensitivity analysis for
'PSME4' on 'LUSC'

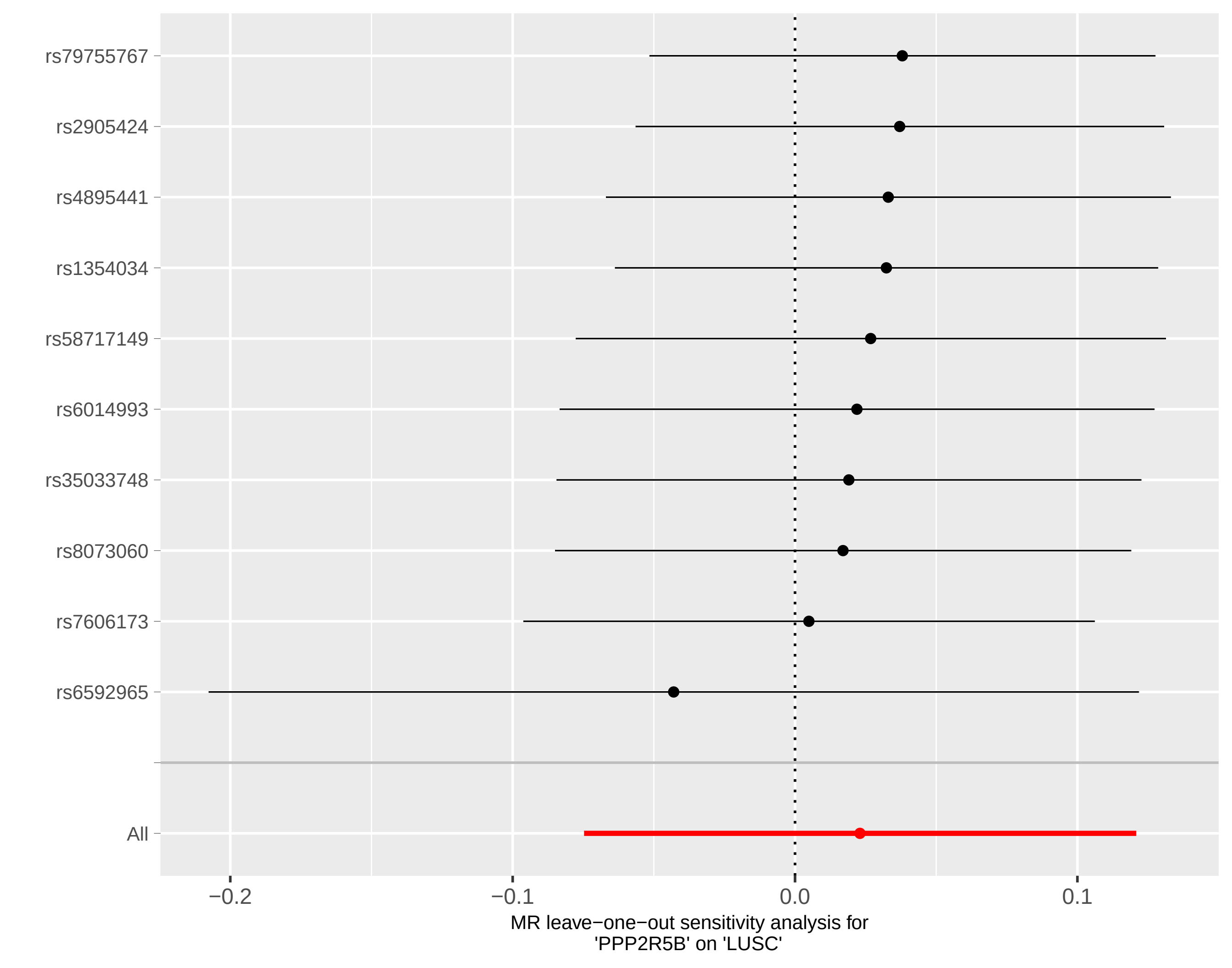
rs79755767
rs2905424
rs4895441
rs1354034
rs58717149
rs6014993
rs35033748
rs8073060
rs7606173
rs6592965
All
−0.2
−0.1
0.0
0.1
MR leave−one−out sensitivity analysis for
'PPP2R5B' on 'LUSC'

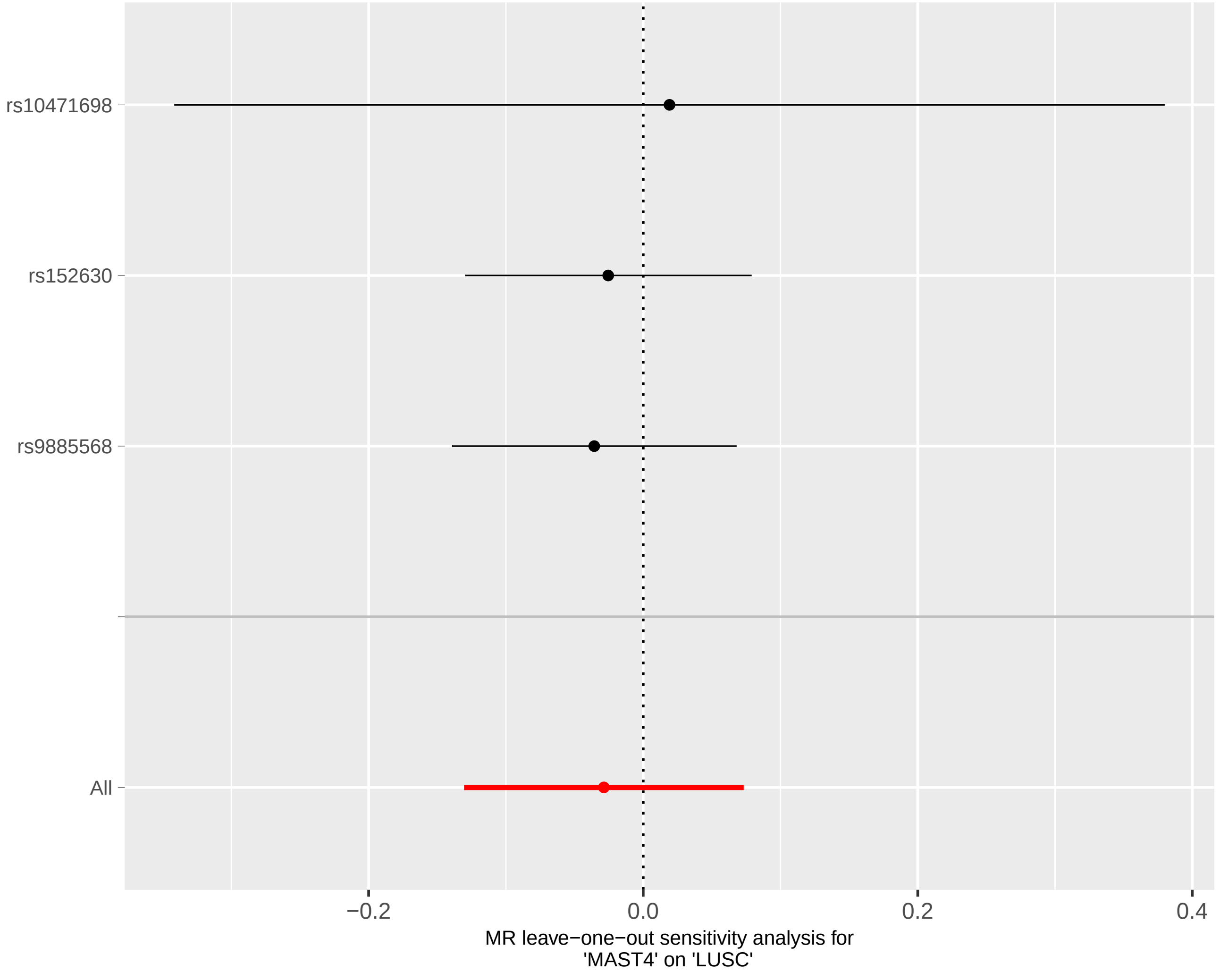
rs10471698
rs152630
rs9885568
All
−0.2
0.0
0.2
0.4
MR leave−one−out sensitivity analysis for
'MAST4' on 'LUSC'

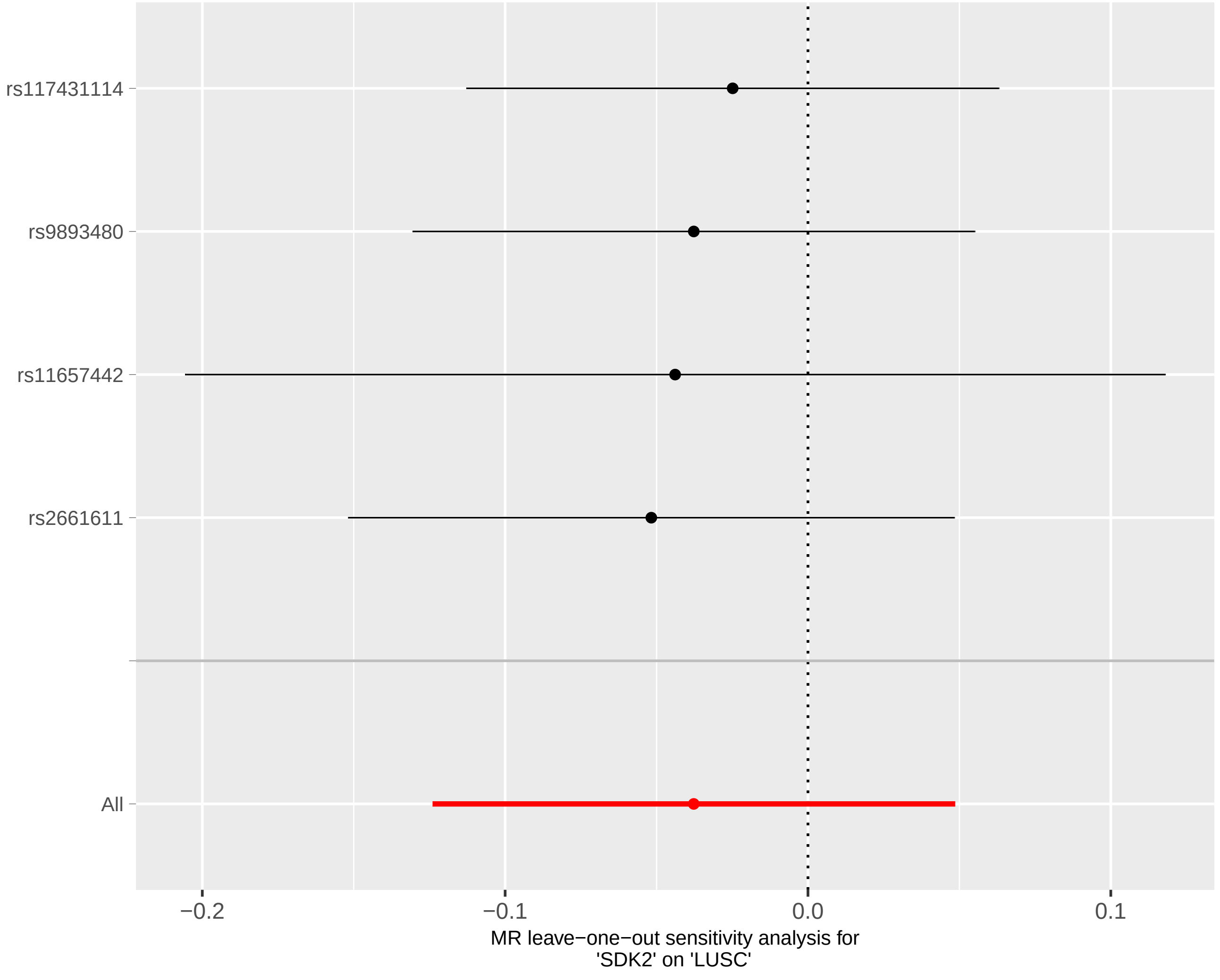

rs117431114
rs9893480
rs11657442
rs2661611
All
−0.2
−0.1
0.0
0.1
MR leave−one−out sensitivity analysis for
'SDK2' on 'LUSC'

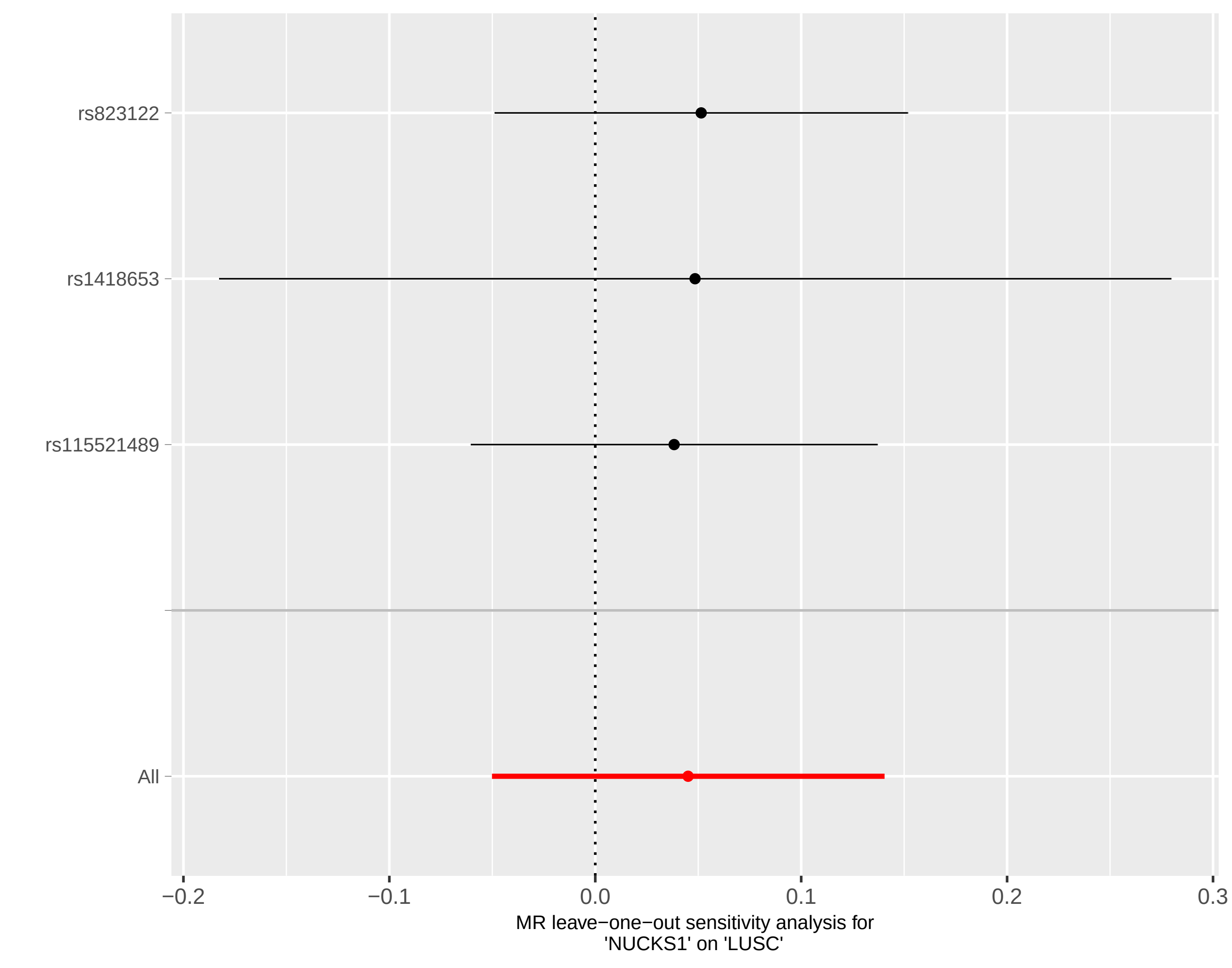

rs823122
rs1418653
rs115521489
All
−0.2
−0.1
0.0
0.1
0.2
0.3
MR leave−one−out sensitivity analysis for
'NUCKS1' on 'LUSC'

Insufficient number of SNPs

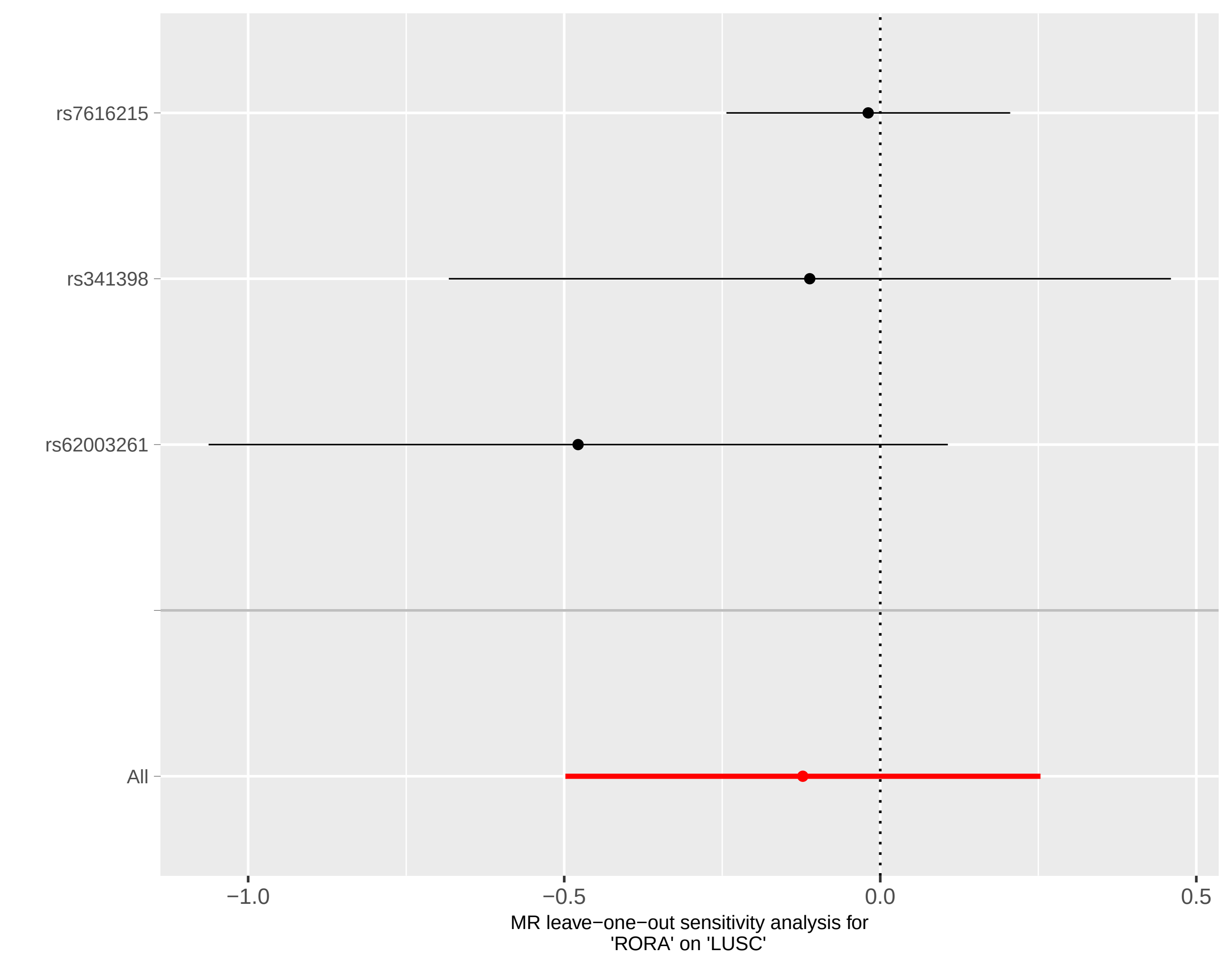

rs7616215
rs341398
rs62003261
All
−1.0
−0.5
0.0
0.5
MR leave−one−out sensitivity analysis for
'RORA' on 'LUSC'

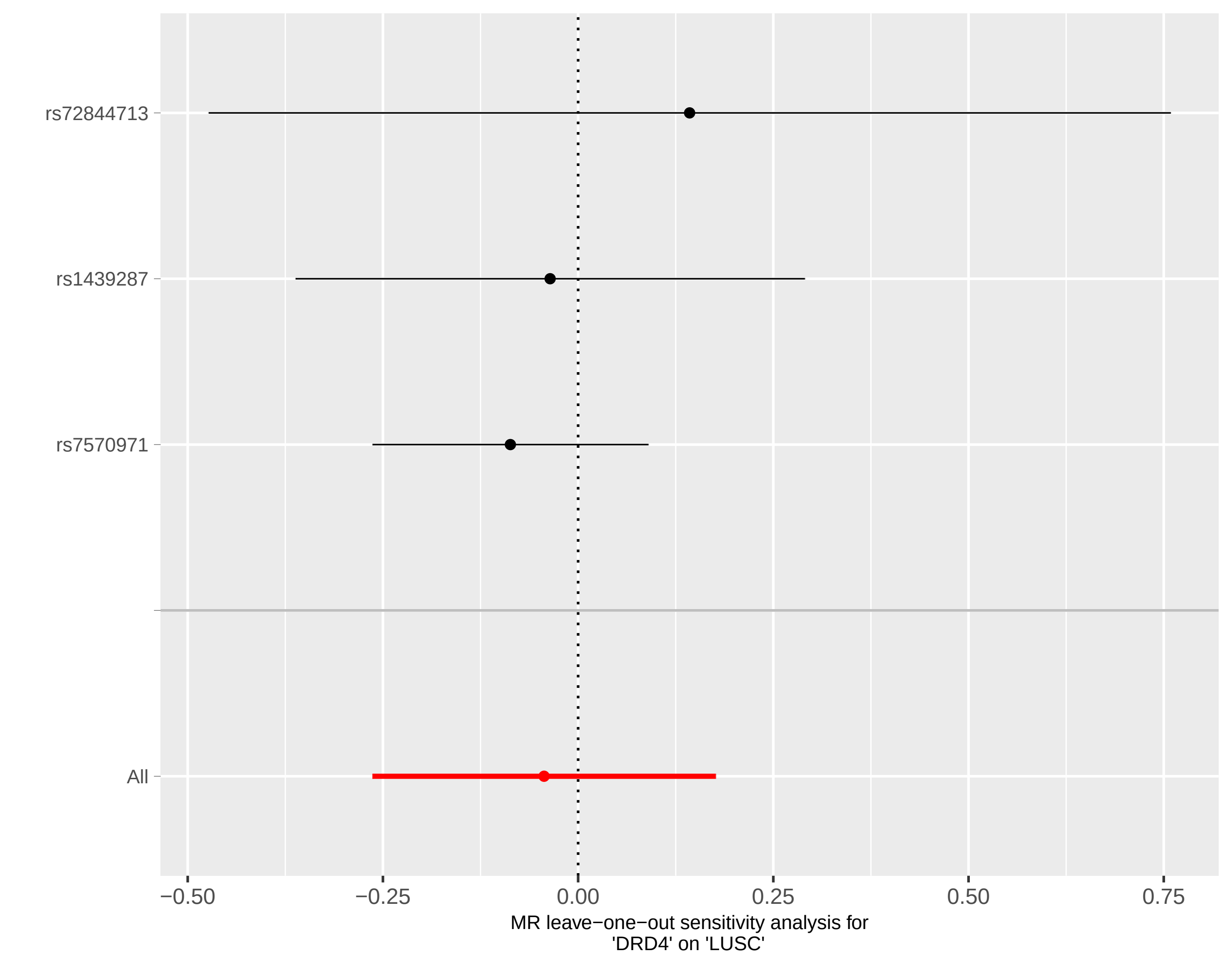
rs72844713
rs1439287
rs7570971
All
−0.50
−0.25
0.00
0.25
0.50
0.75
MR leave−one−out sensitivity analysis for
'DRD4' on 'LUSC'

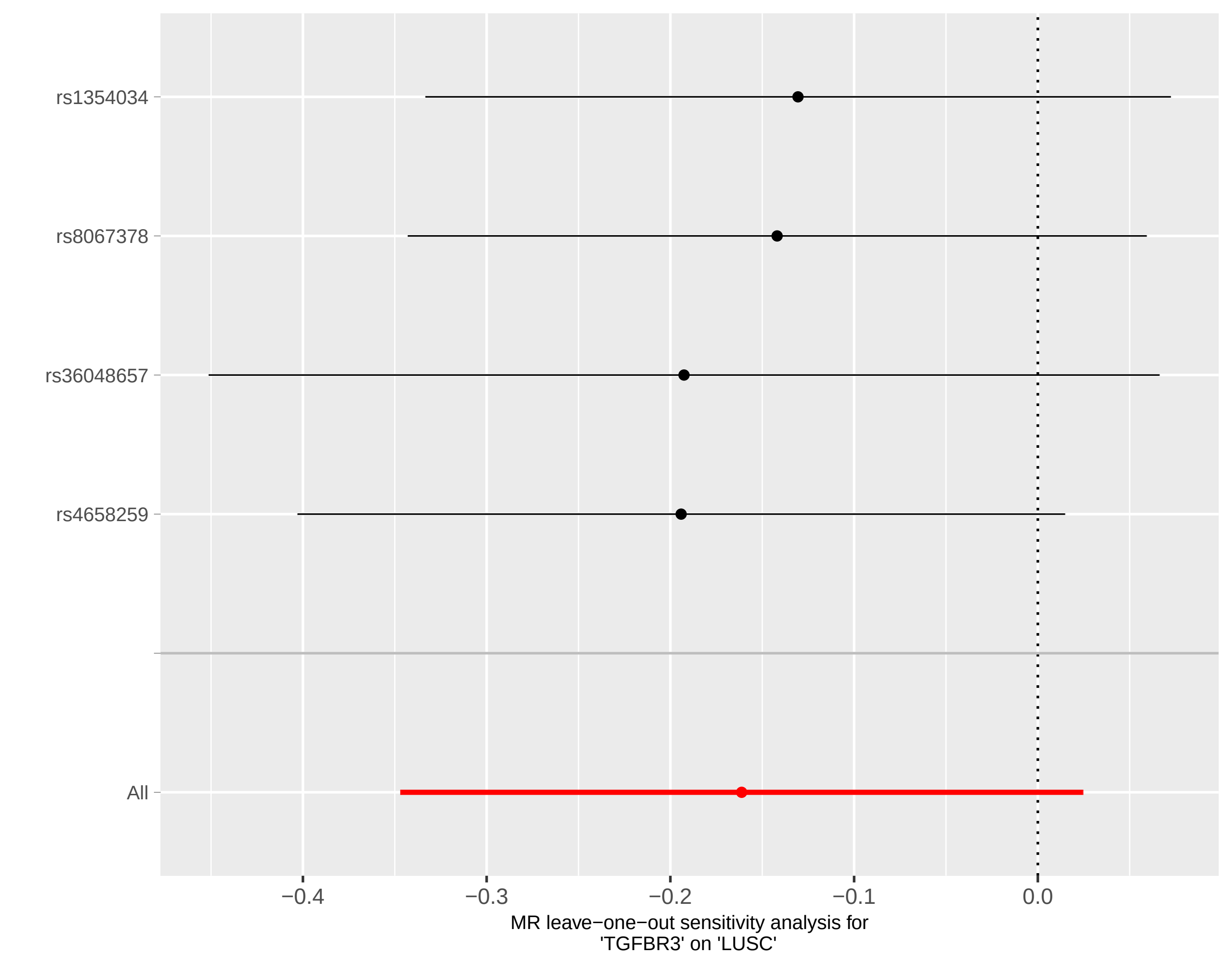
rs1354034
rs8067378
rs36048657
rs4658259
All
−0.4
−0.3
−0.2
−0.1
0.0
MR leave−one−out sensitivity analysis for
'TGFBR3' on 'LUSC'

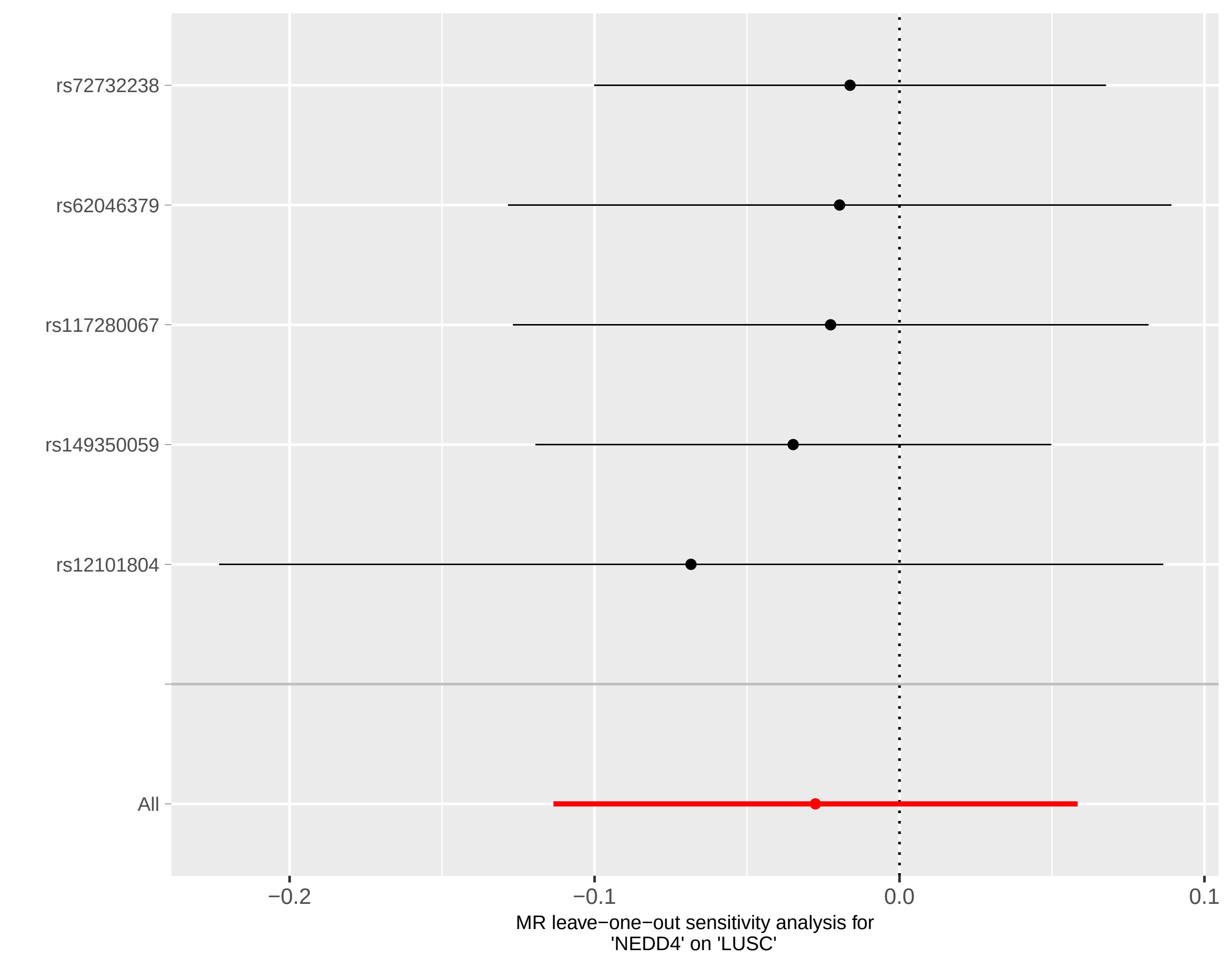
rs72732238
rs62046379
rs117280067
rs149350059
rs12101804
All
−0.2
−0.1
0.0
0.1
MR leave−one−out sensitivity analysis for
'NEDD4' on 'LUSC'

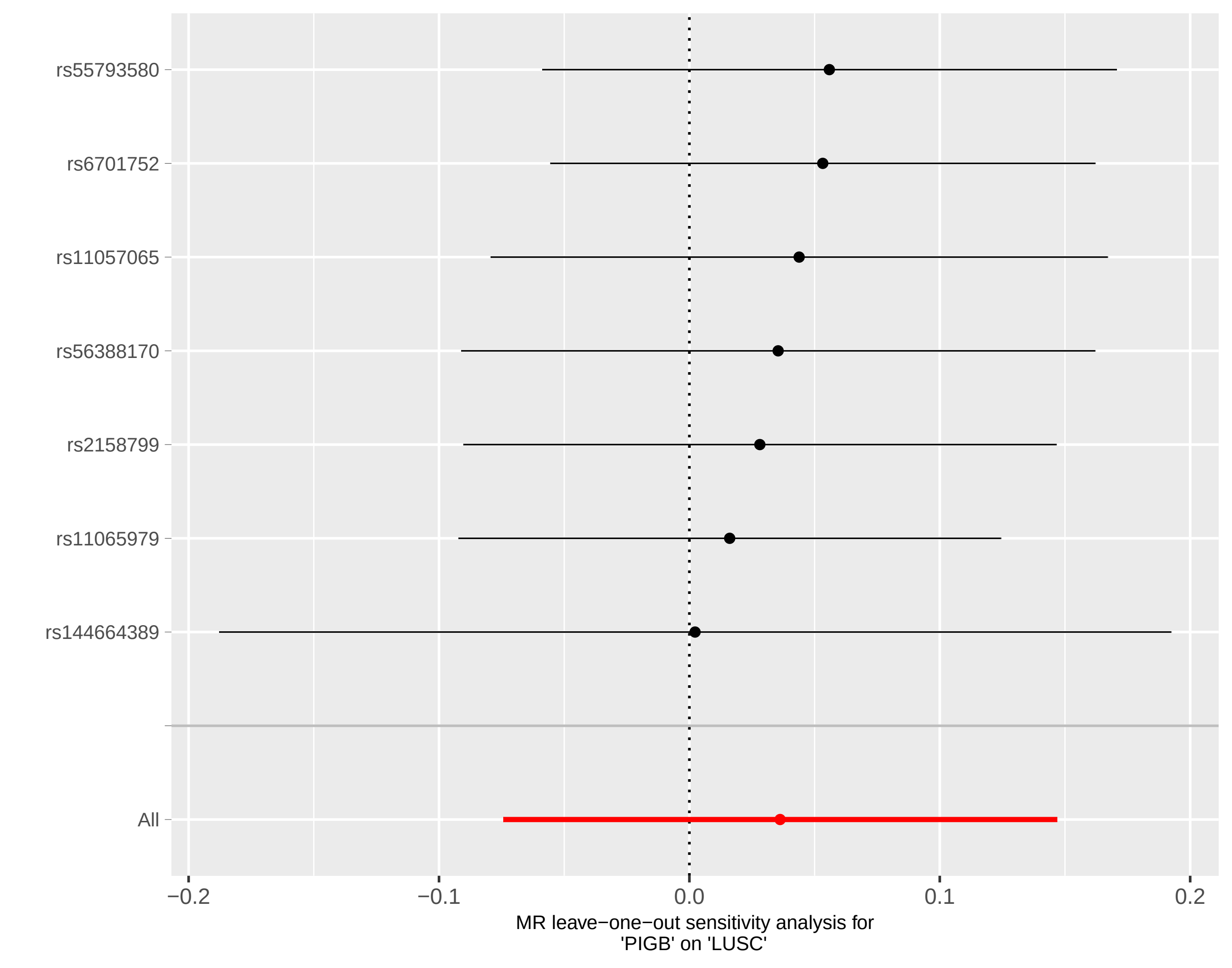
rs55793580
rs6701752
rs11057065
rs56388170
rs2158799
rs11065979
rs144664389
All
−0.2
−0.1
0.0
0.1
0.2
MR leave−one−out sensitivity analysis for
'PIGB' on 'LUSC'

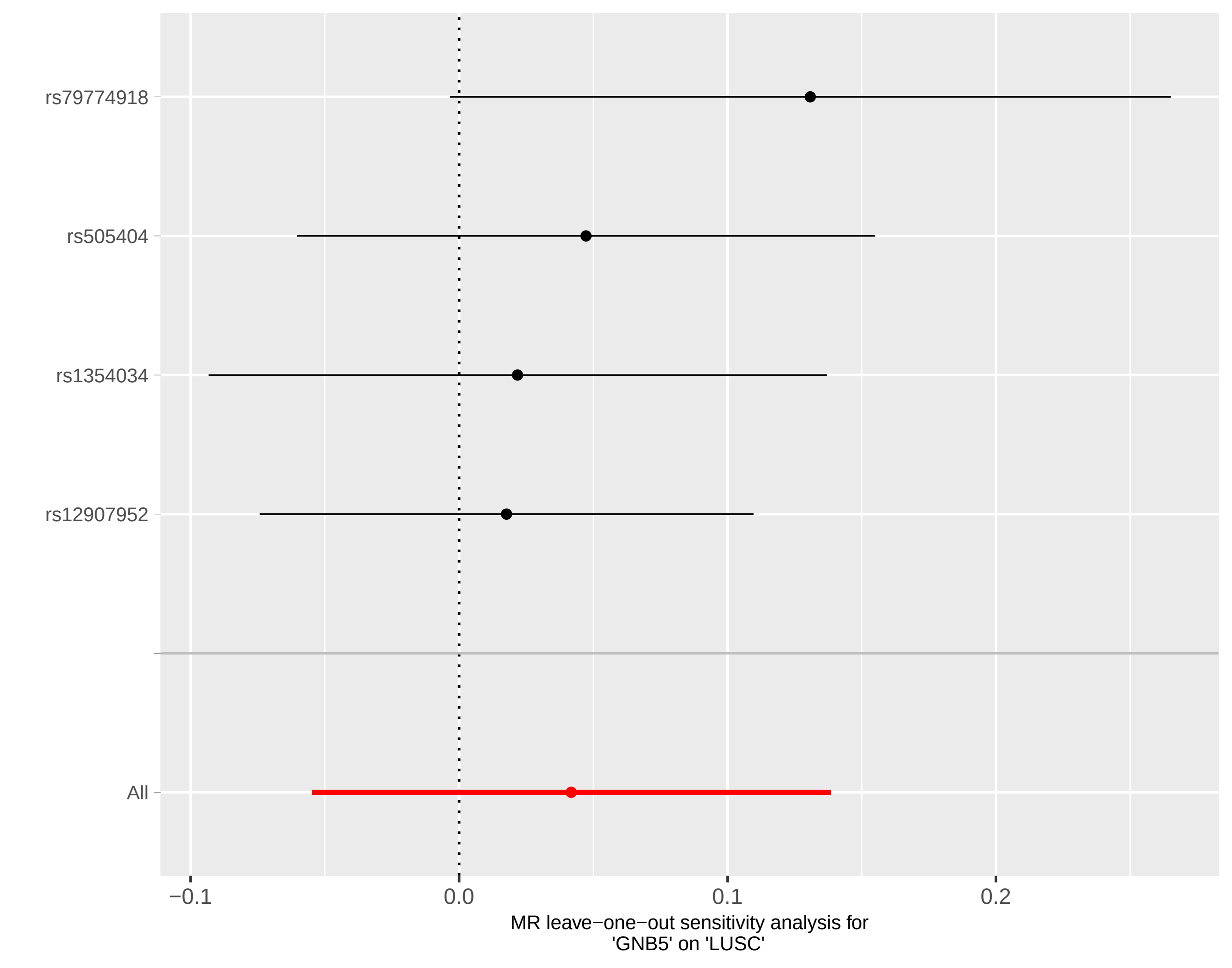
rs79774918
rs505404
rs1354034
rs12907952
All
−0.1
0.0
0.1
0.2
MR leave−one−out sensitivity analysis for
'GNB5' on 'LUSC'

Insufficient number of SNPs

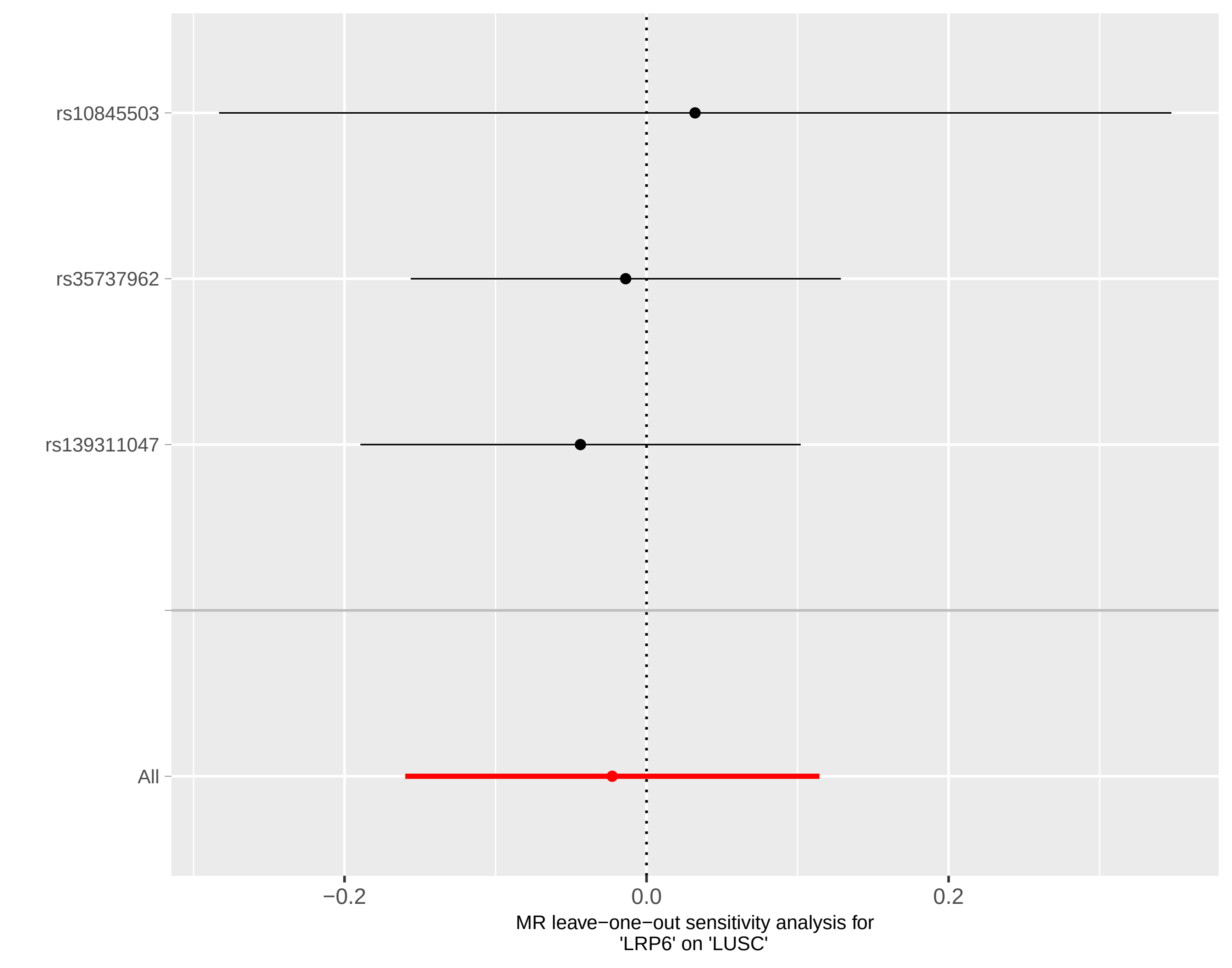

rs10845503
rs35737962
rs139311047
All
−0.2
0.0
0.2
MR leave−one−out sensitivity analysis for
'LRP6' on 'LUSC'

Insufficient number of SNPs

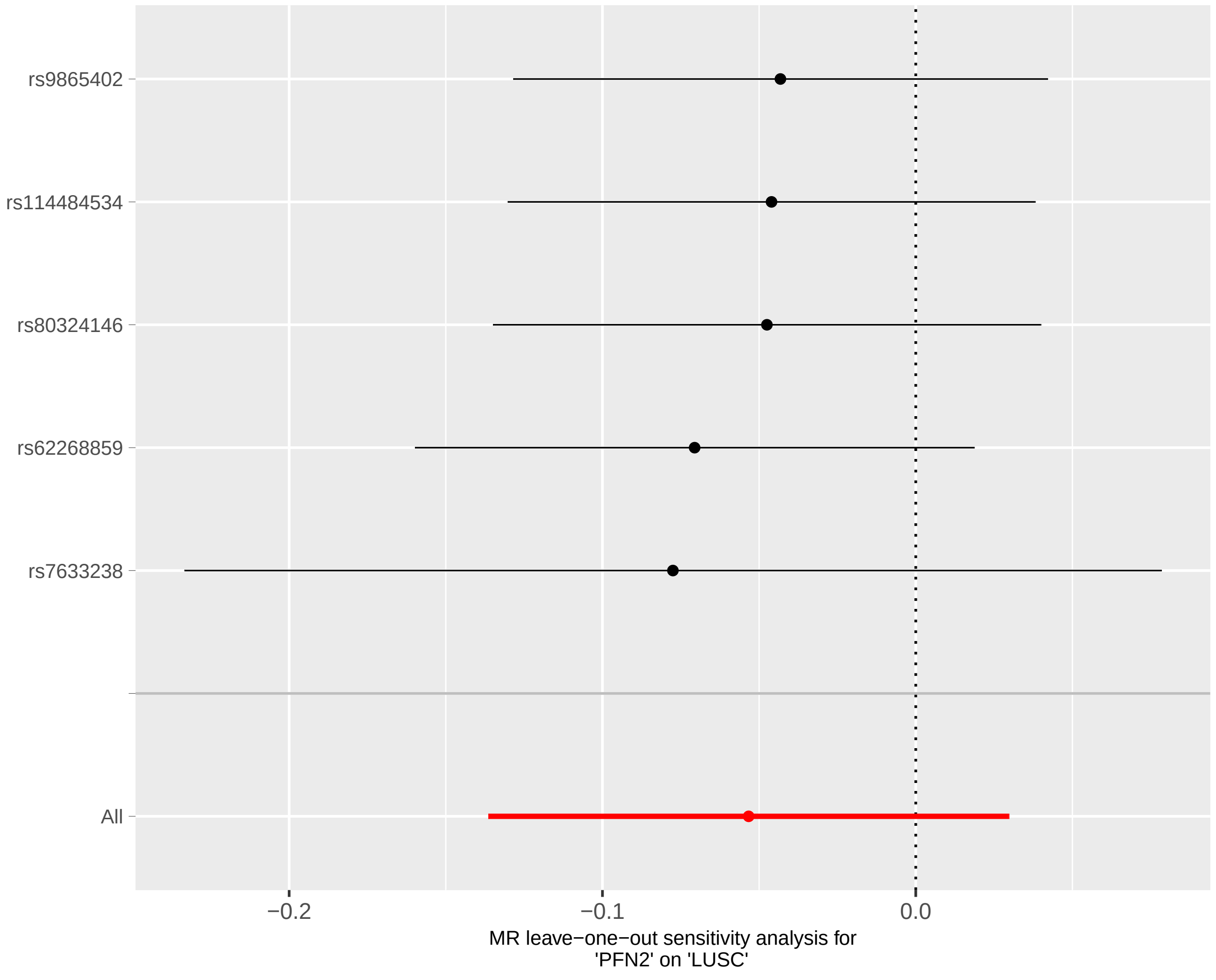

rs9865402
rs114484534
rs80324146
rs62268859
rs7633238
All
−0.2
−0.1
0.0
MR leave−one−out sensitivity analysis for
'PFN2' on 'LUSC'

Insufficient number of SNPs

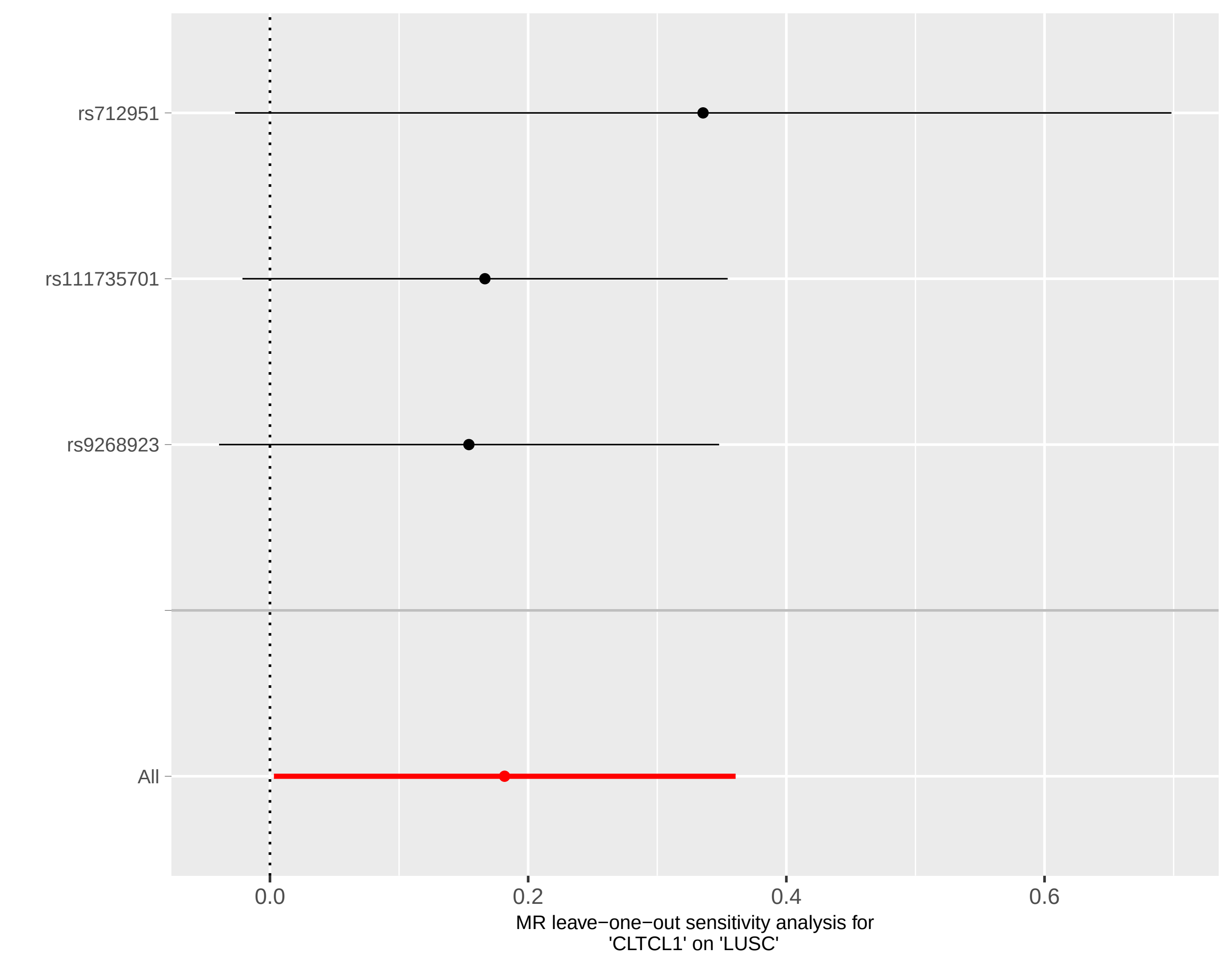

rs712951
rs111735701
rs9268923
All
0.0
0.2
0.4
0.6
MR leave−one−out sensitivity analysis for
'CLTCL1' on 'LUSC'

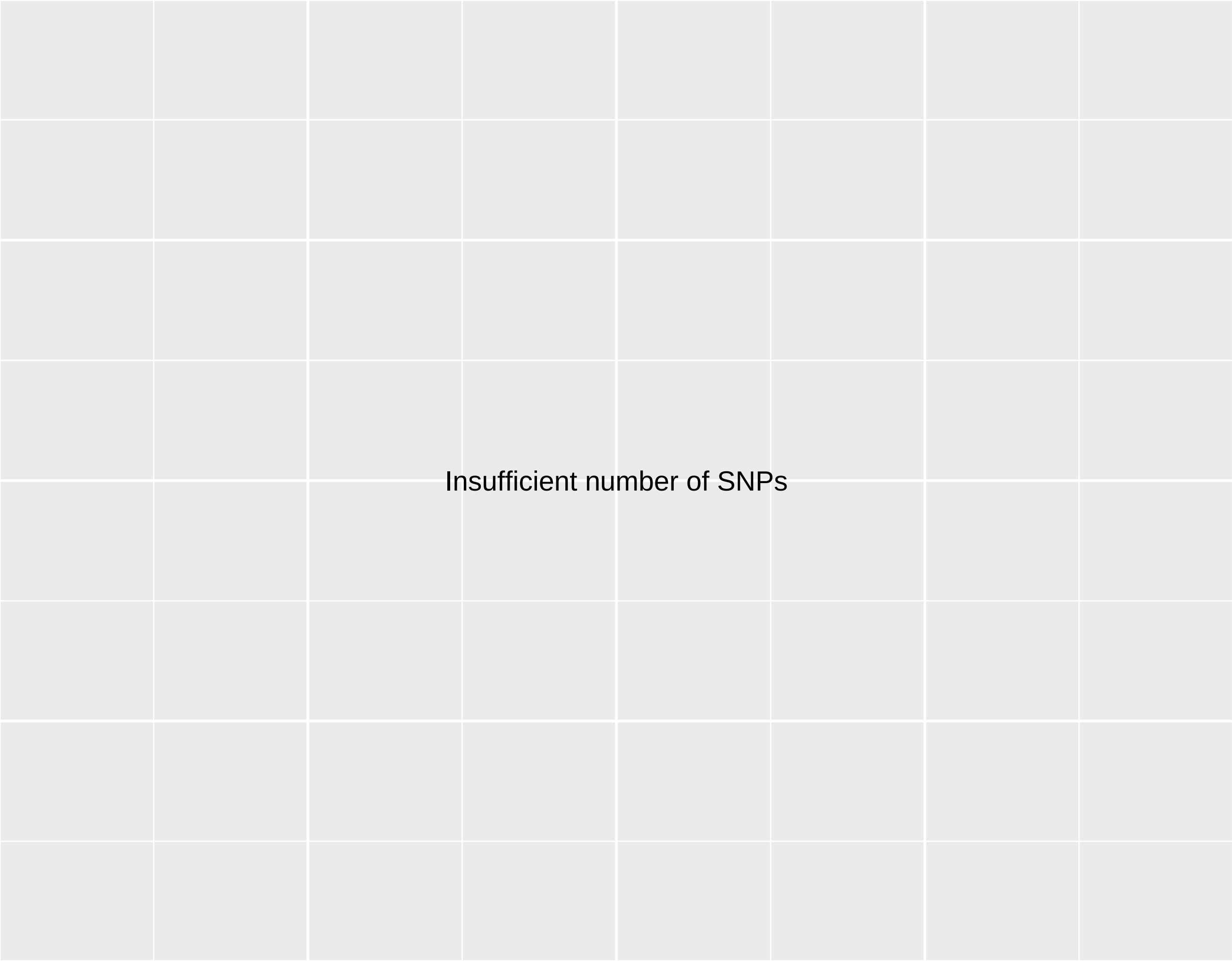
Insufficient number of SNPs

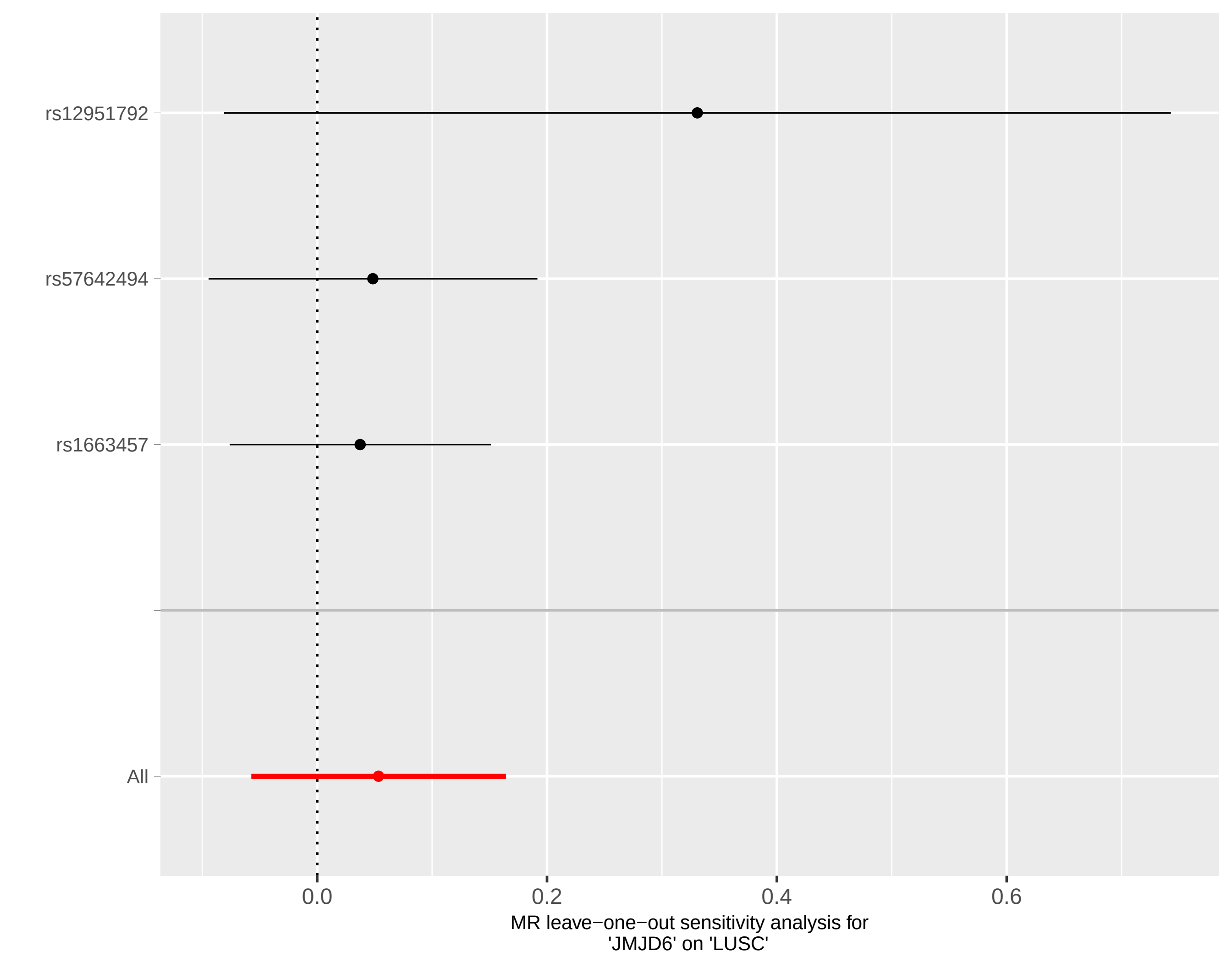
rs12951792
rs57642494
rs1663457
All
0.0
0.2
0.4
0.6
MR leave−one−out sensitivity analysis for
'JMJD6' on 'LUSC'

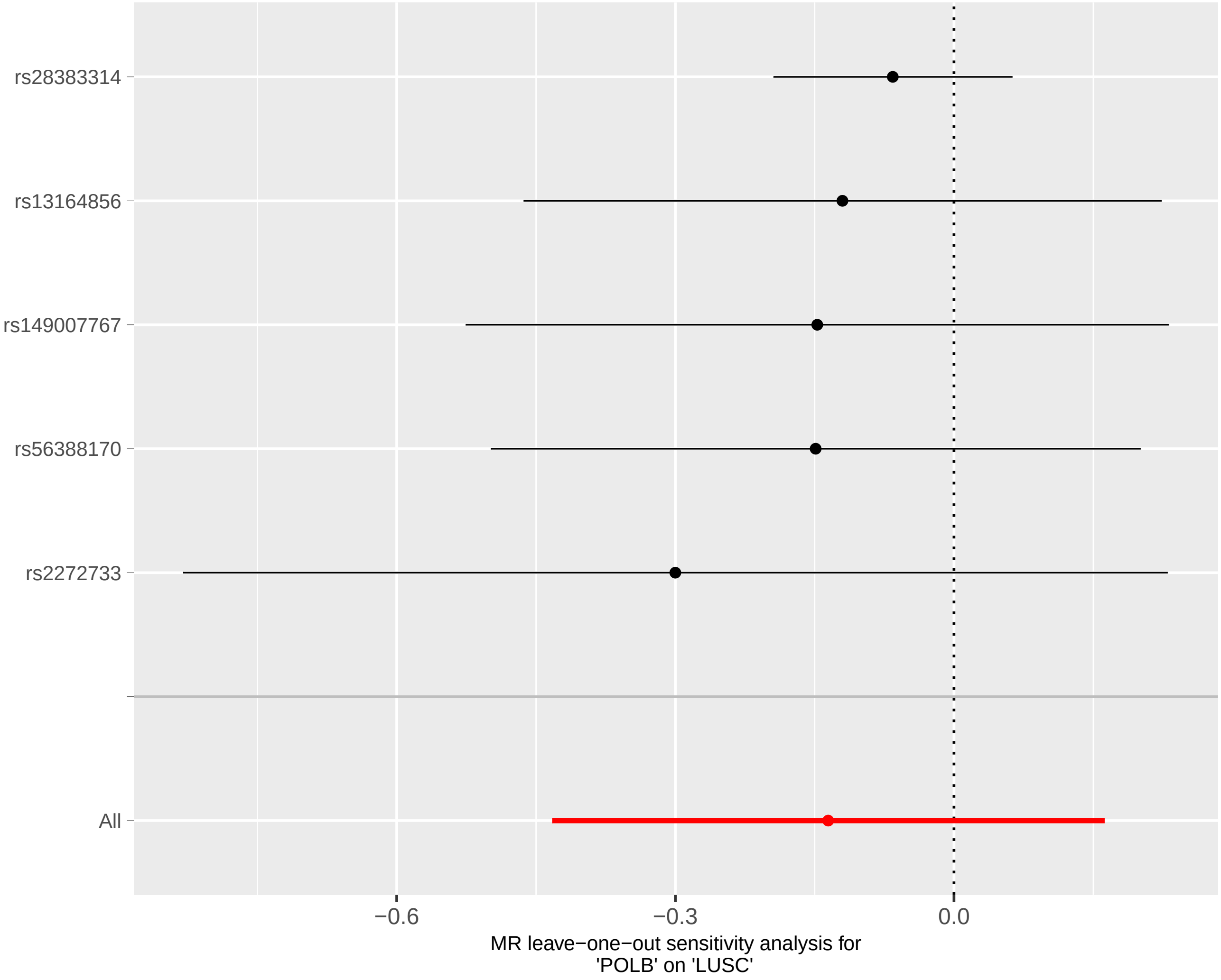
rs28383314
rs13164856
rs149007767
rs56388170
rs2272733
All
−0.6
−0.3
0.0
MR leave−one−out sensitivity analysis for
'POLB' on 'LUSC'

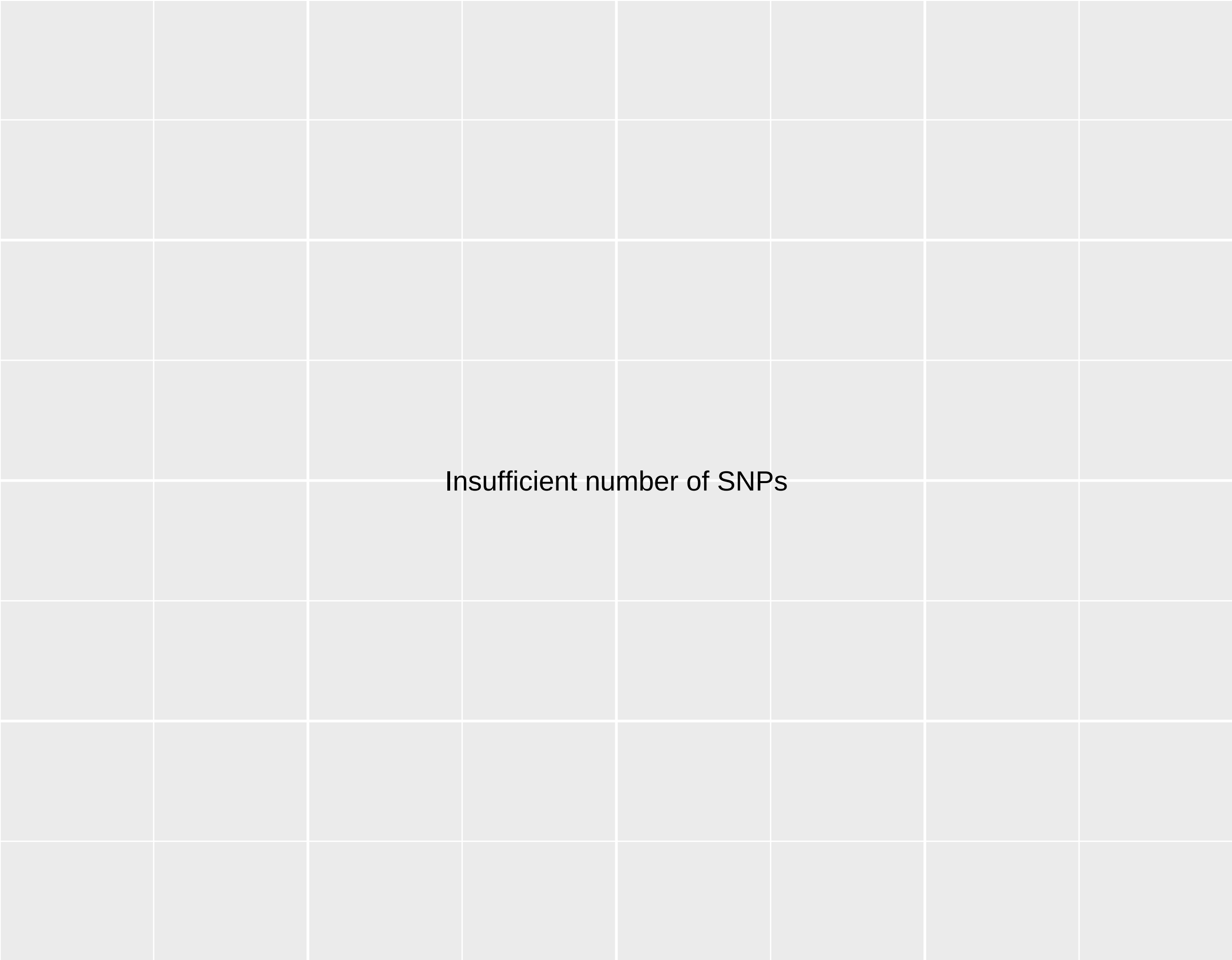
Insufficient number of SNPs

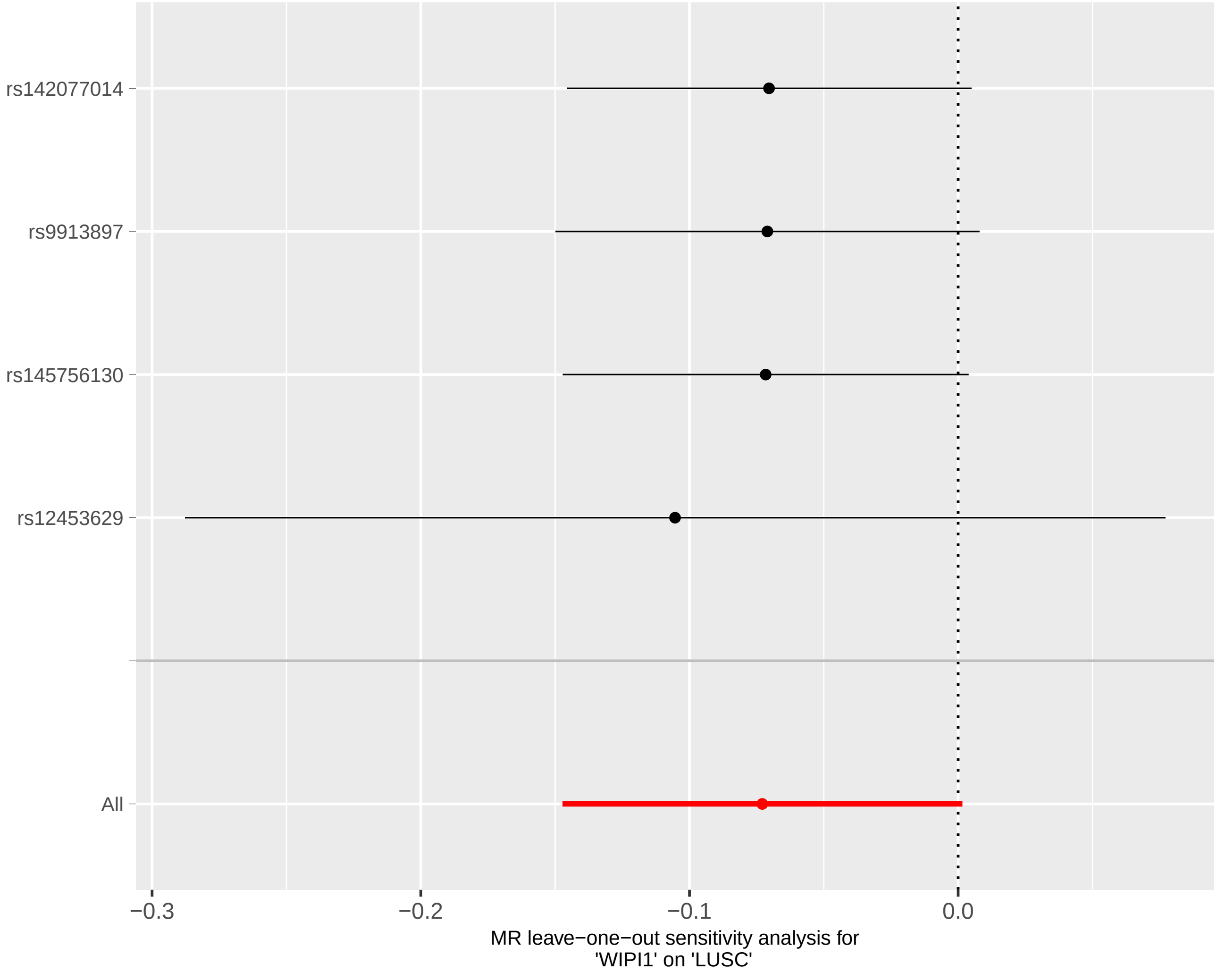

rs142077014
rs9913897
rs145756130
rs12453629
All
−0.3
−0.2
−0.1
0.0
MR leave−one−out sensitivity analysis for
'WIPI1' on 'LUSC'

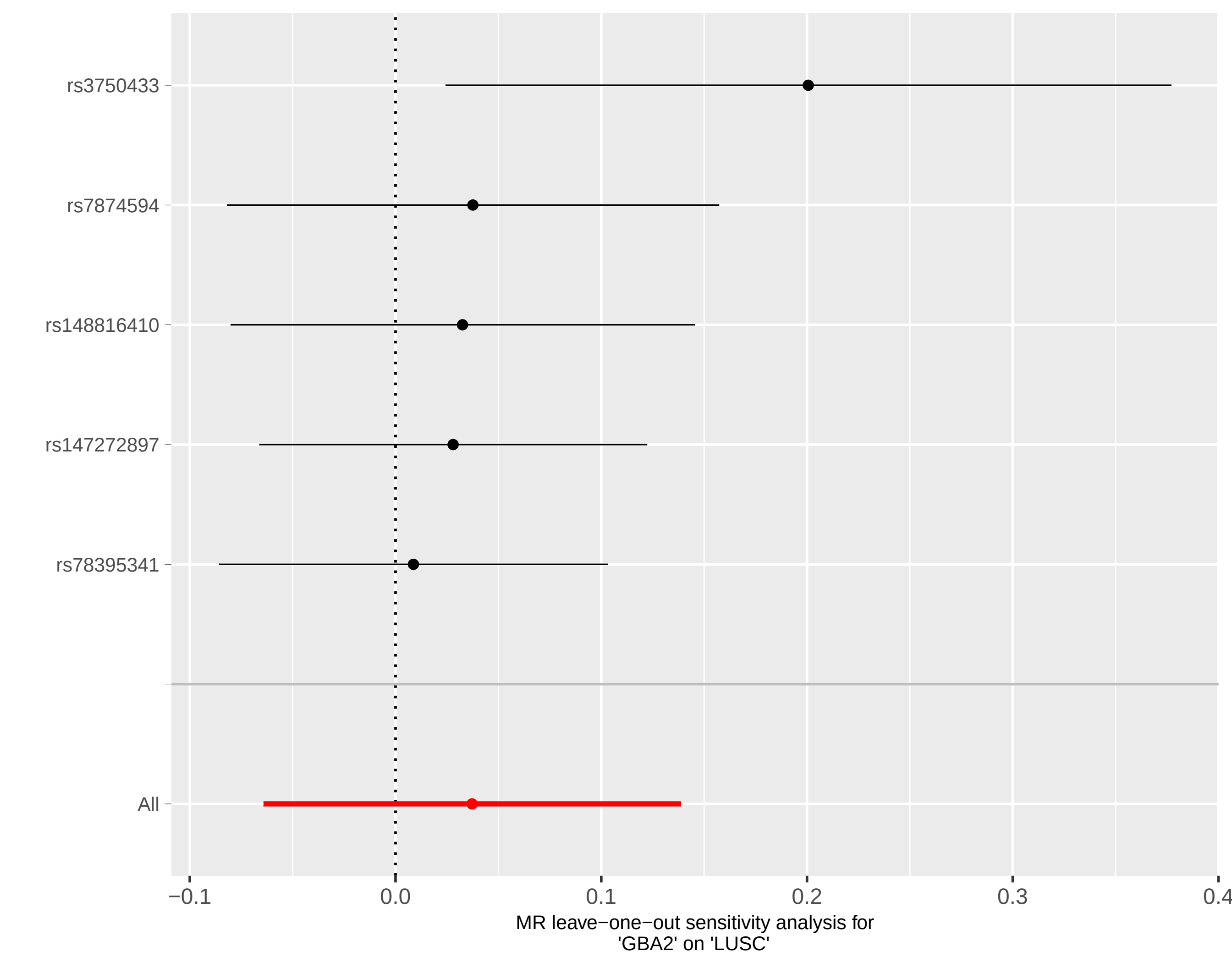
rs3750433
rs7874594
rs148816410
rs147272897
rs78395341
All
−0.1
0.0
0.1
0.2
0.3
0.4
MR leave−one−out sensitivity analysis for
'GBA2' on 'LUSC'

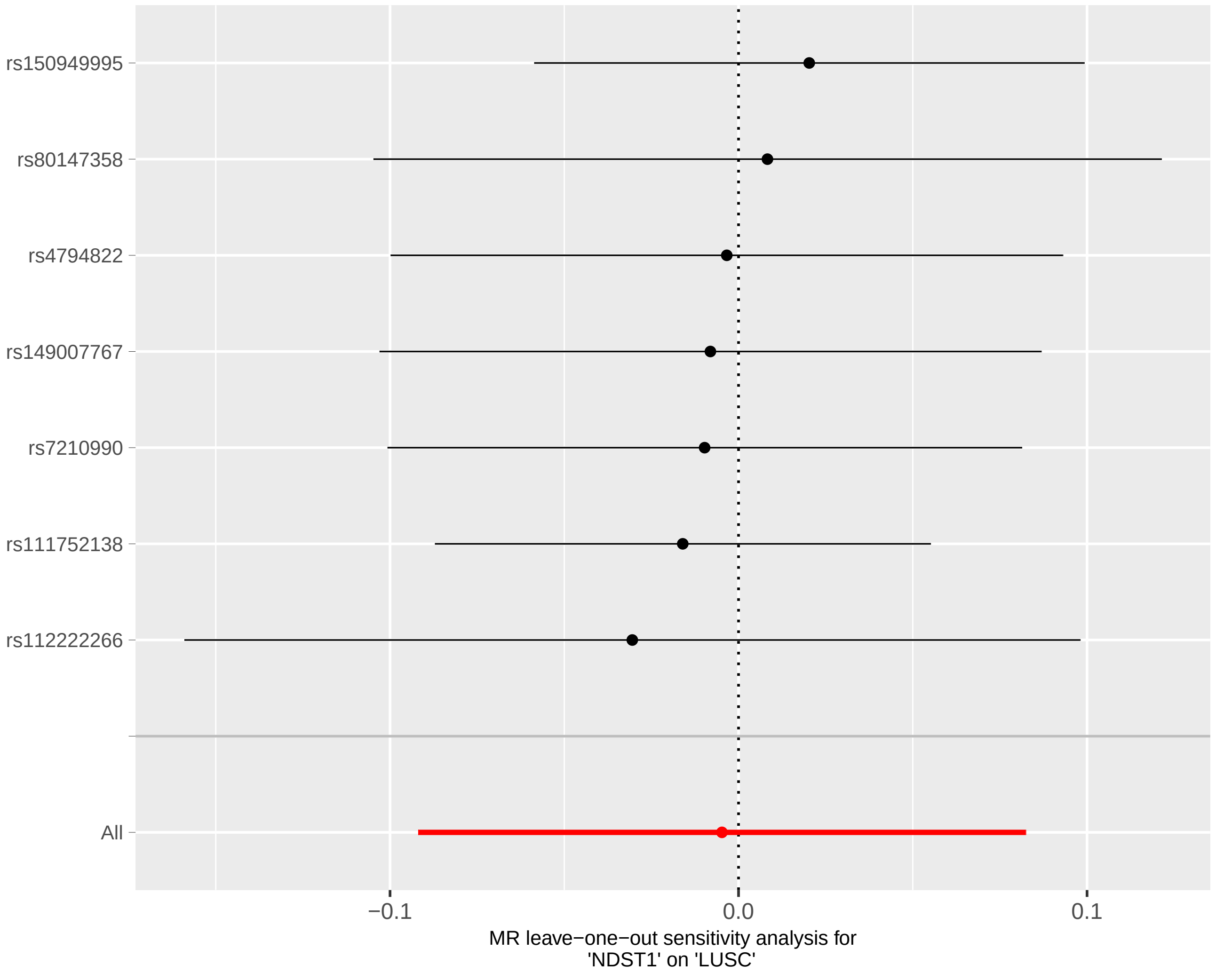
rs150949995
rs80147358
rs4794822
rs149007767
rs7210990
rs111752138
rs112222266
All
−0.1
0.0
0.1
MR leave−one−out sensitivity analysis for
'NDST1' on 'LUSC'

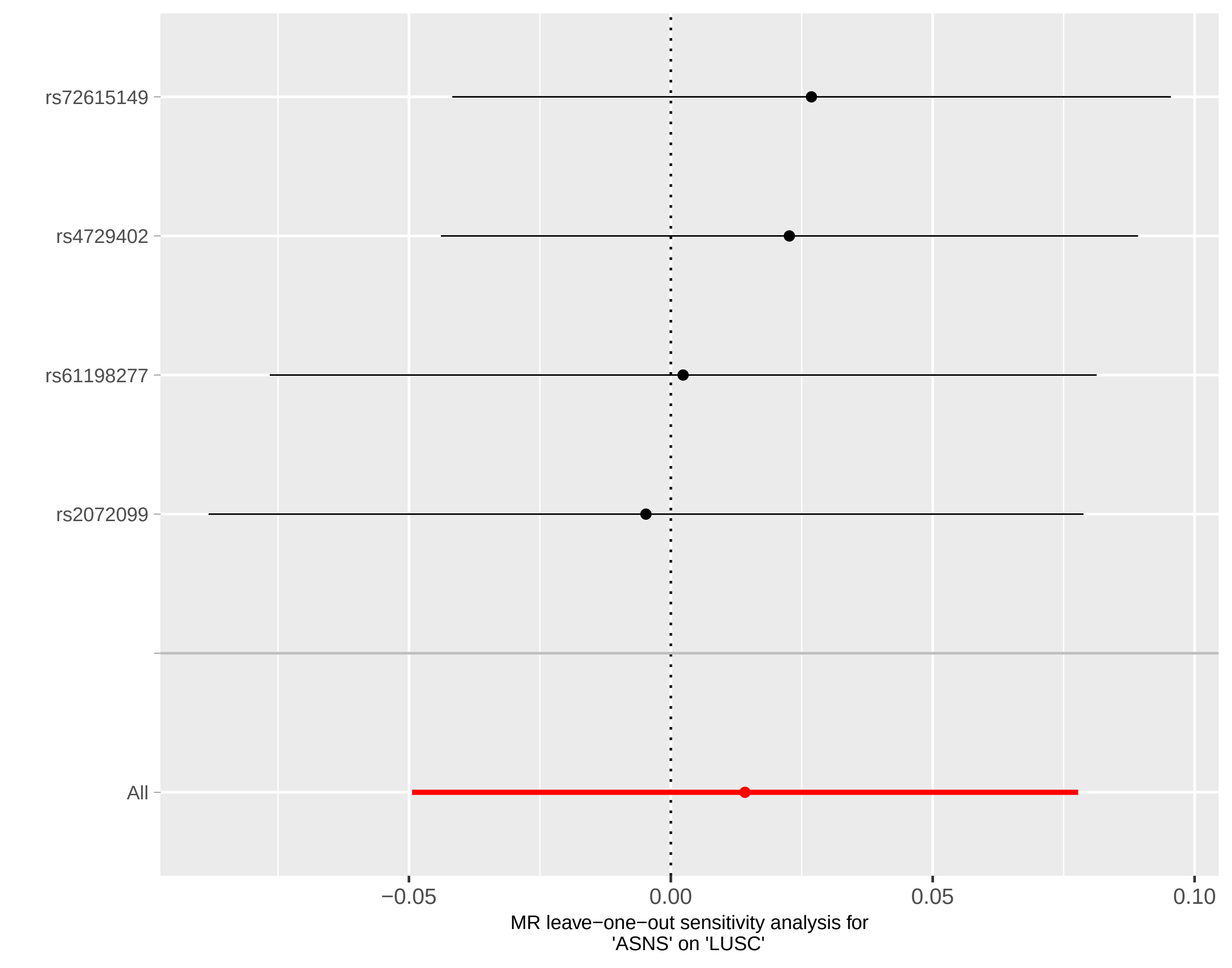
rs72615149
rs4729402
rs61198277
rs2072099
All
−0.05
0.00
0.05
0.10
MR leave−one−out sensitivity analysis for
'ASNS' on 'LUSC'

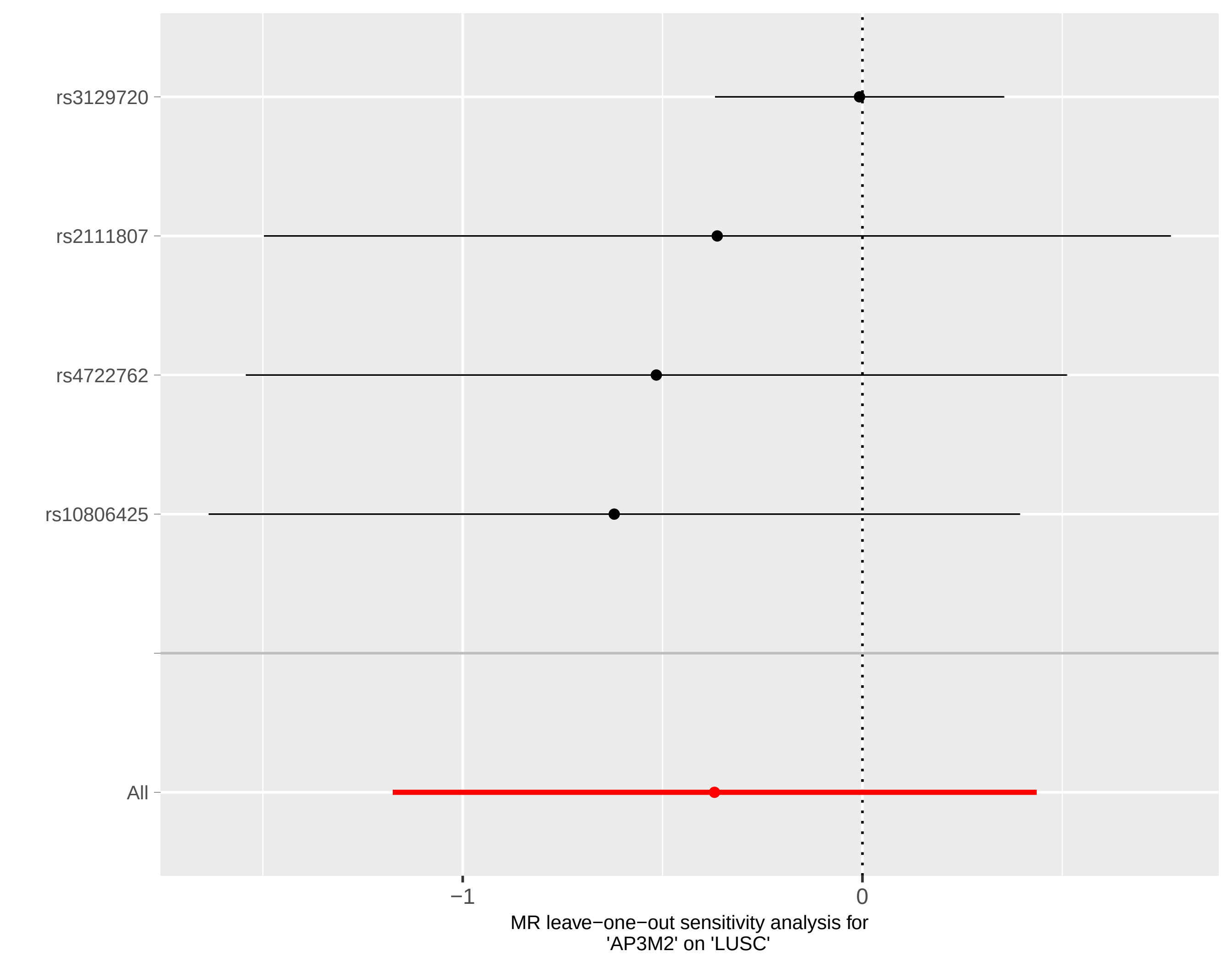
rs3129720
rs2111807
rs4722762
rs10806425
All
−1
0
MR leave−one−out sensitivity analysis for
'AP3M2' on 'LUSC'

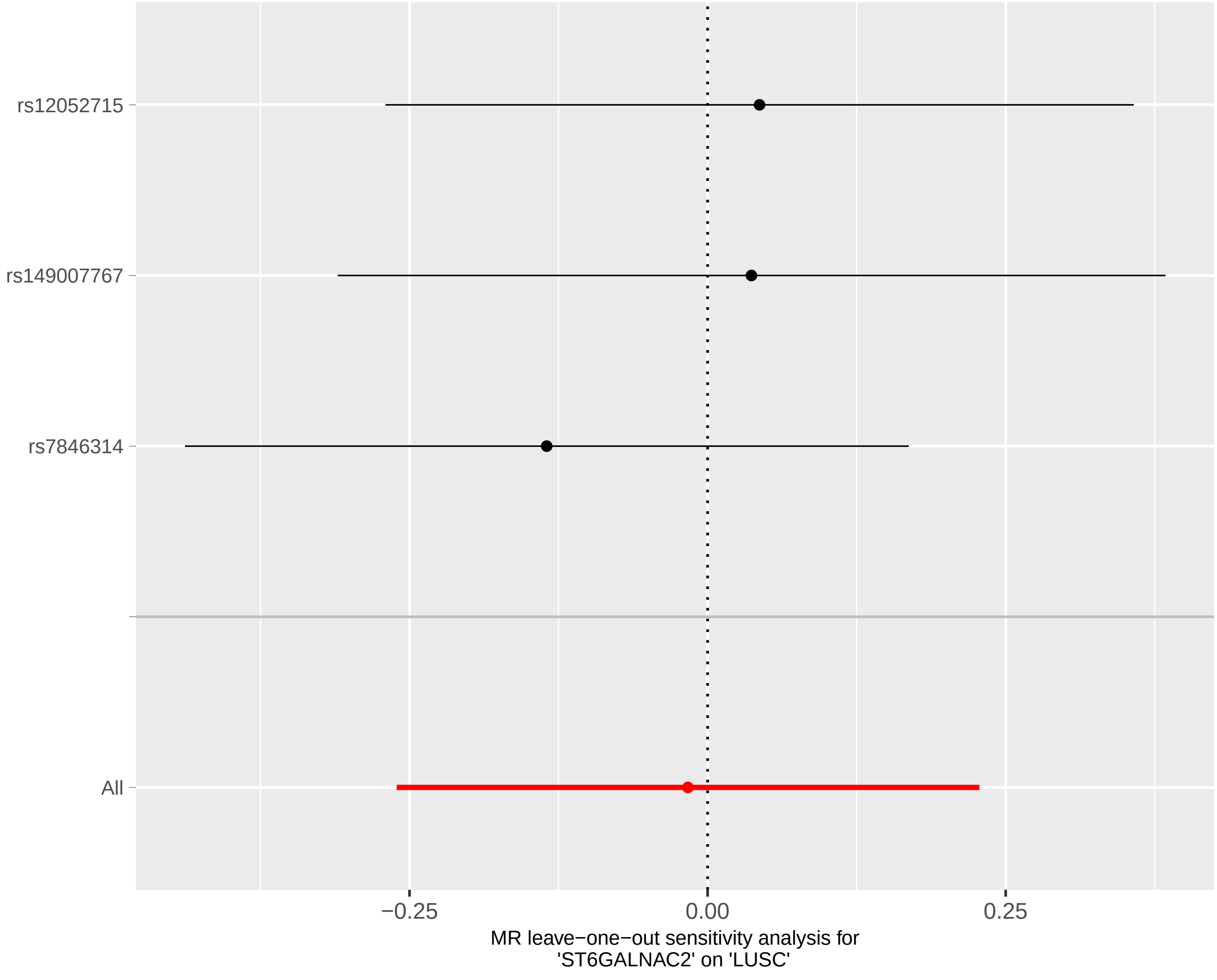
rs12052715
rs149007767
rs7846314
All
−0.25
0.00
0.25
MR leave−one−out sensitivity analysis for
'ST6GALNAC2' on 'LUSC'

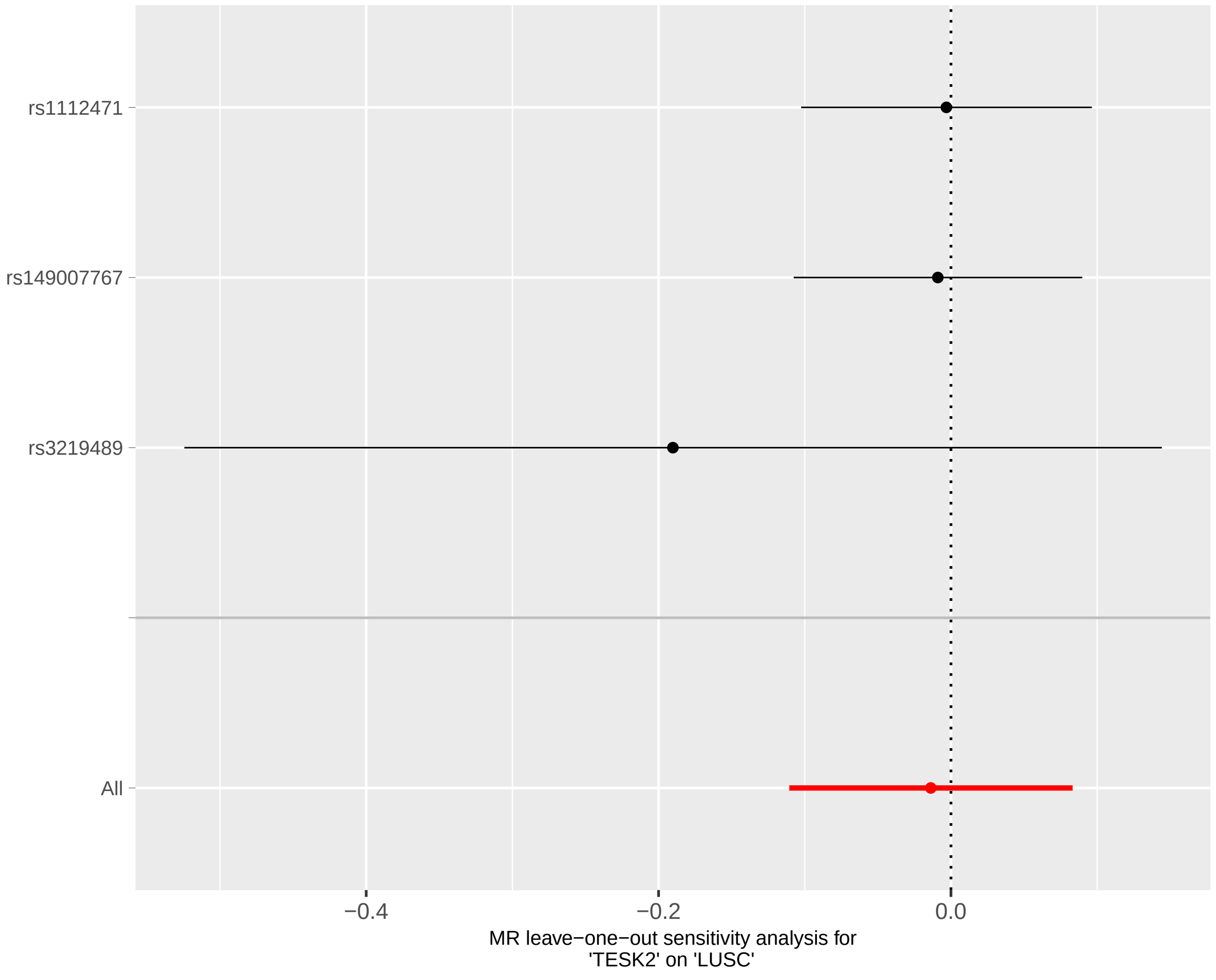
rs1112471
rs149007767
rs3219489
All
−0.4
−0.2
0.0
MR leave−one−out sensitivity analysis for
'TESK2' on 'LUSC'

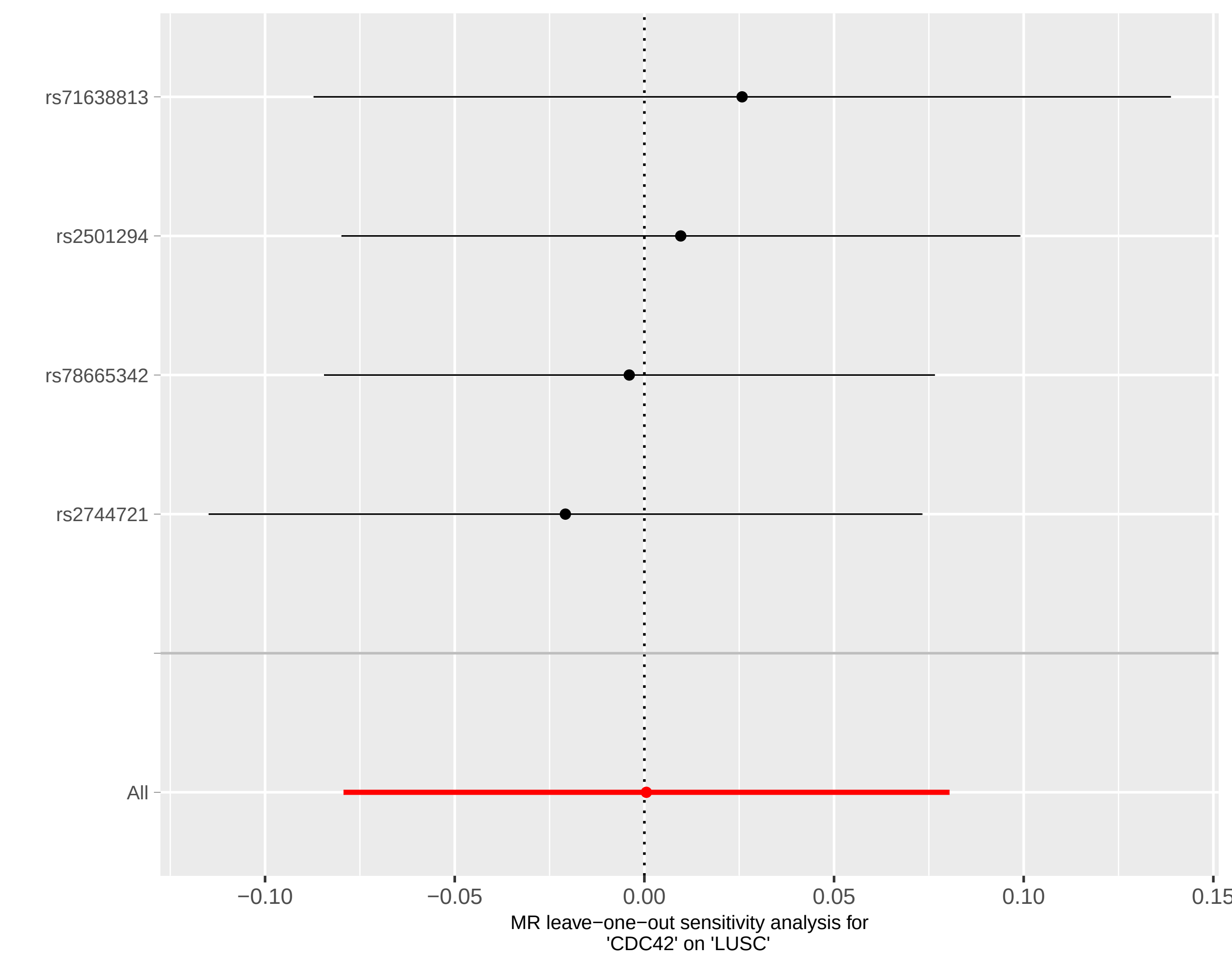

rs71638813
rs2501294
rs78665342
rs2744721
All
−0.10
−0.05
0.00
0.05
0.10
0.15
MR leave−one−out sensitivity analysis for
'CDC42' on 'LUSC'

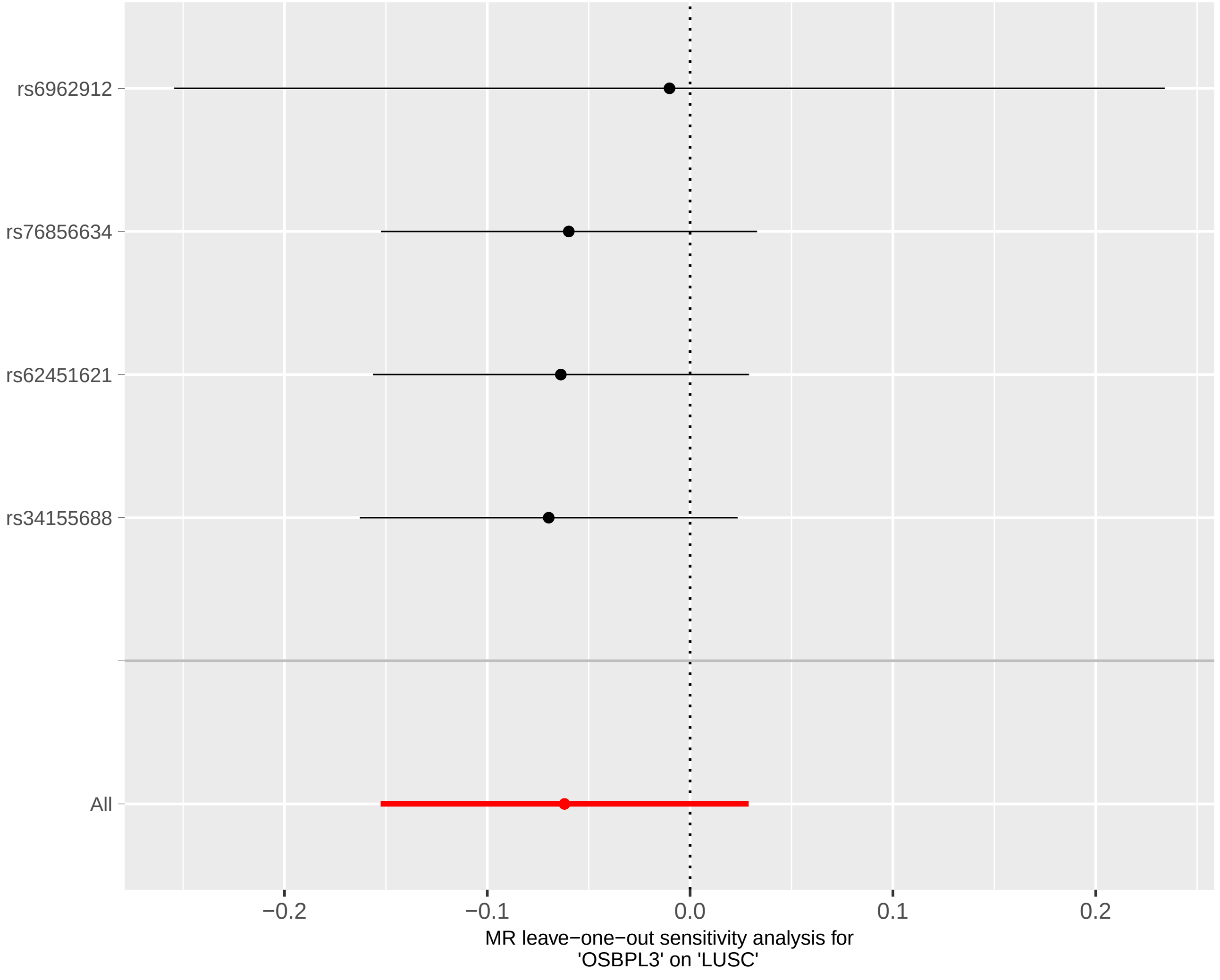
rs6962912
rs76856634
rs62451621
rs34155688
All
−0.2
−0.1
0.0
0.1
0.2
MR leave−one−out sensitivity analysis for
'OSBPL3' on 'LUSC'

Insufficient number of SNPs

Insufficient number of SNPs

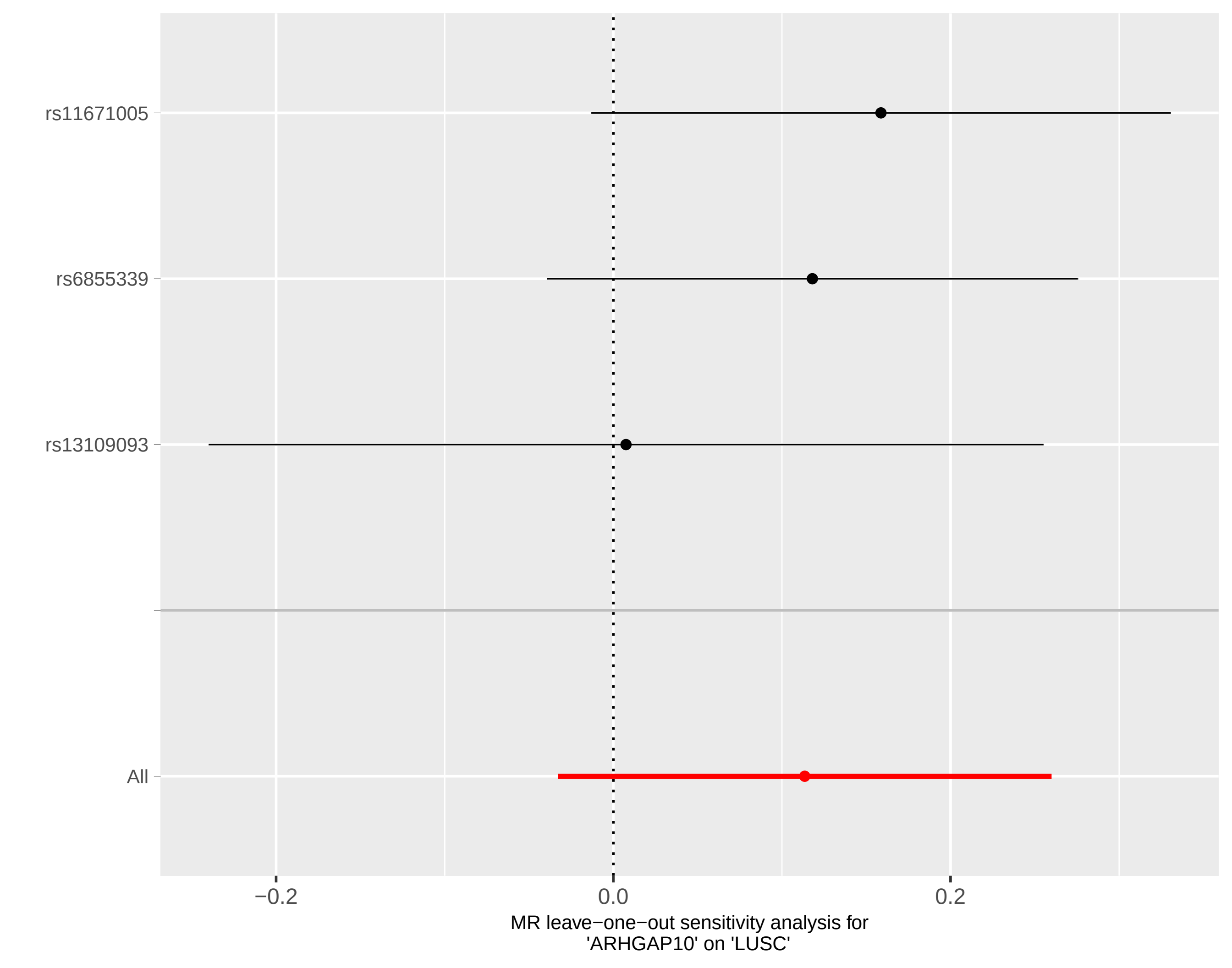
rs11671005
rs6855339
rs13109093
All
−0.2
0.0
0.2
MR leave−one−out sensitivity analysis for
'ARHGAP10' on 'LUSC'

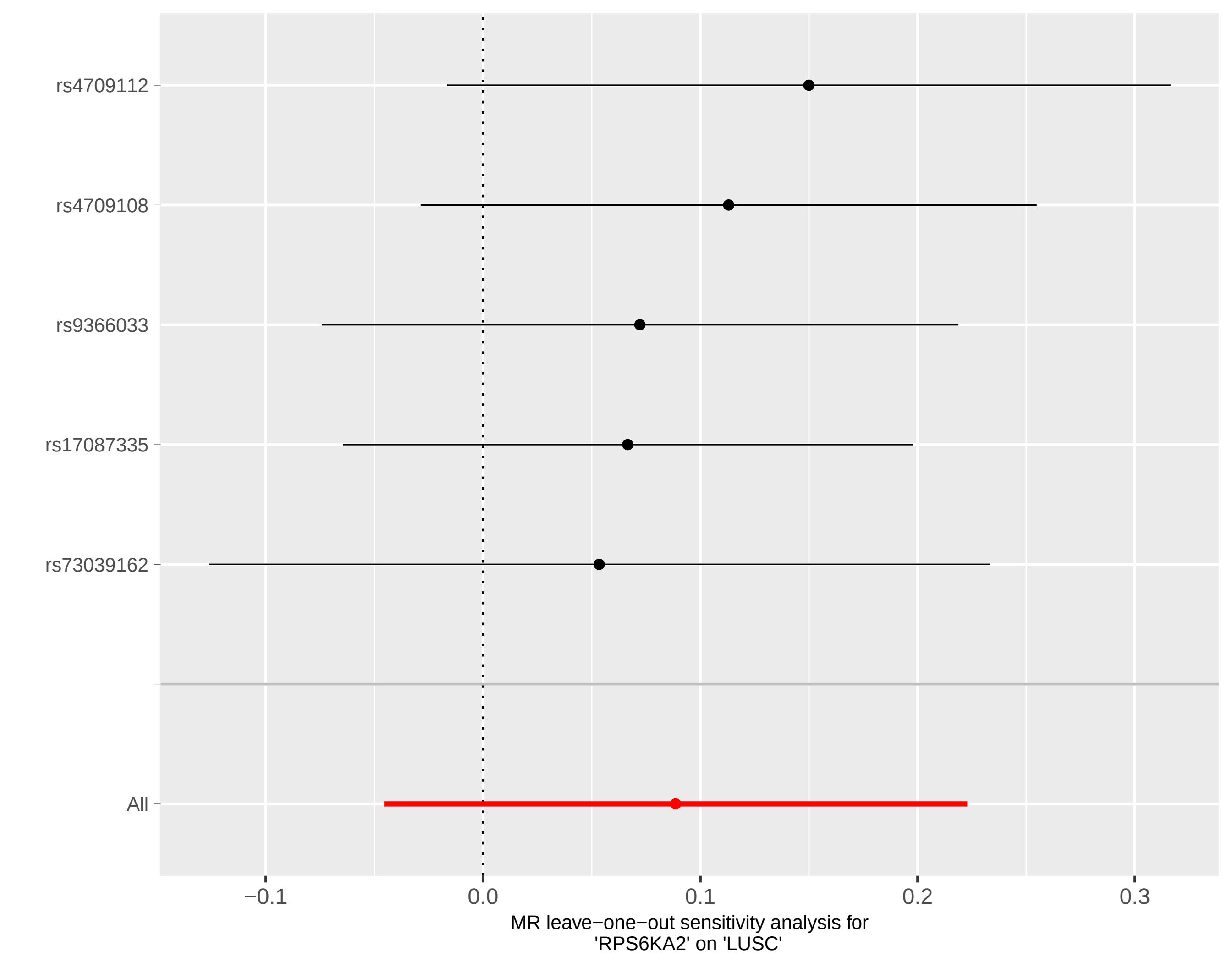

rs4709112
rs4709108
rs9366033
rs17087335
rs73039162
All
−0.1
0.0
0.1
0.2
0.3
MR leave−one−out sensitivity analysis for
'RPS6KA2' on 'LUSC'

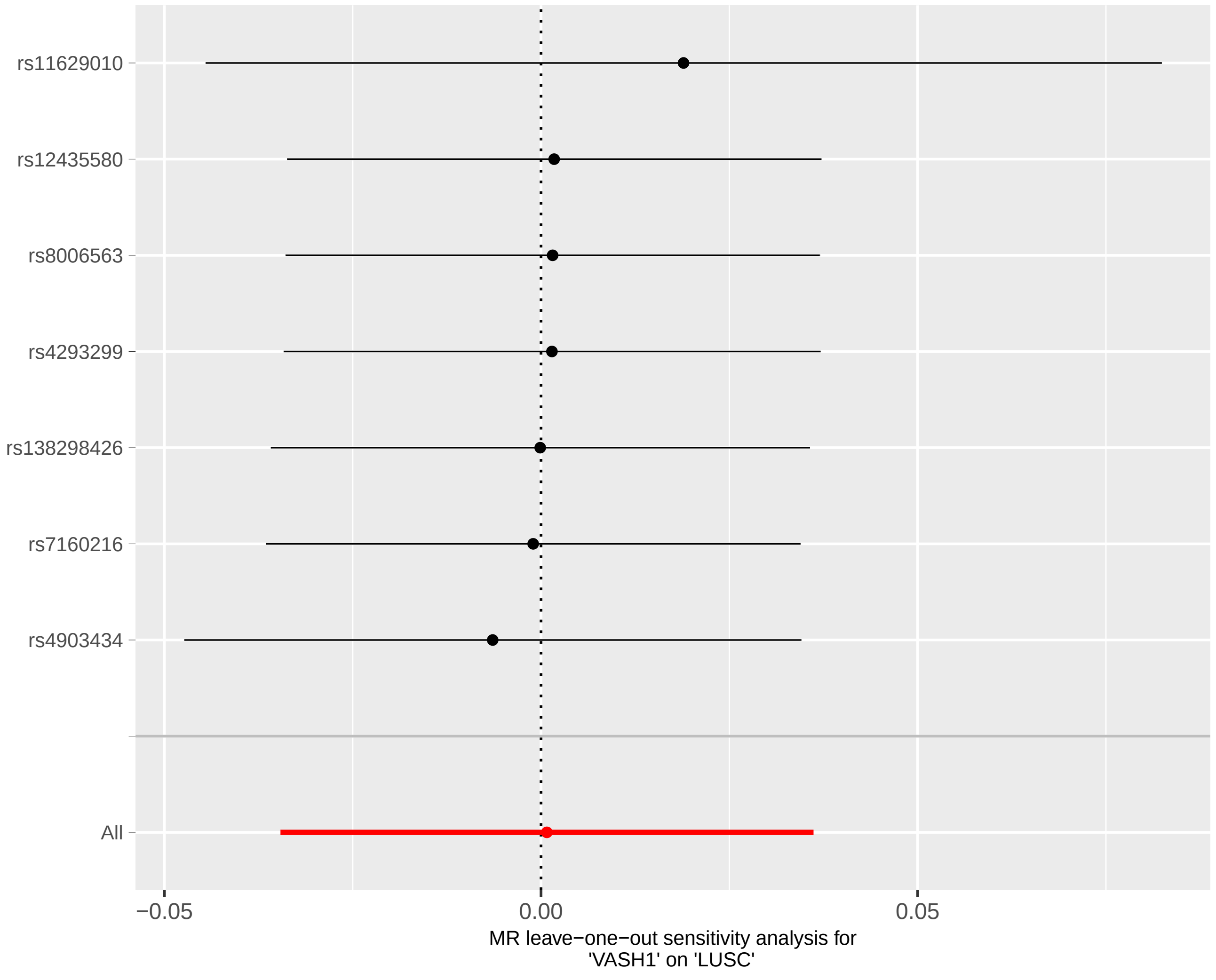
rs11629010
rs12435580
rs8006563
rs4293299
rs138298426
rs7160216
rs4903434
All
−0.05
0.00
0.05
MR leave−one−out sensitivity analysis for
'VASH1' on 'LUSC'

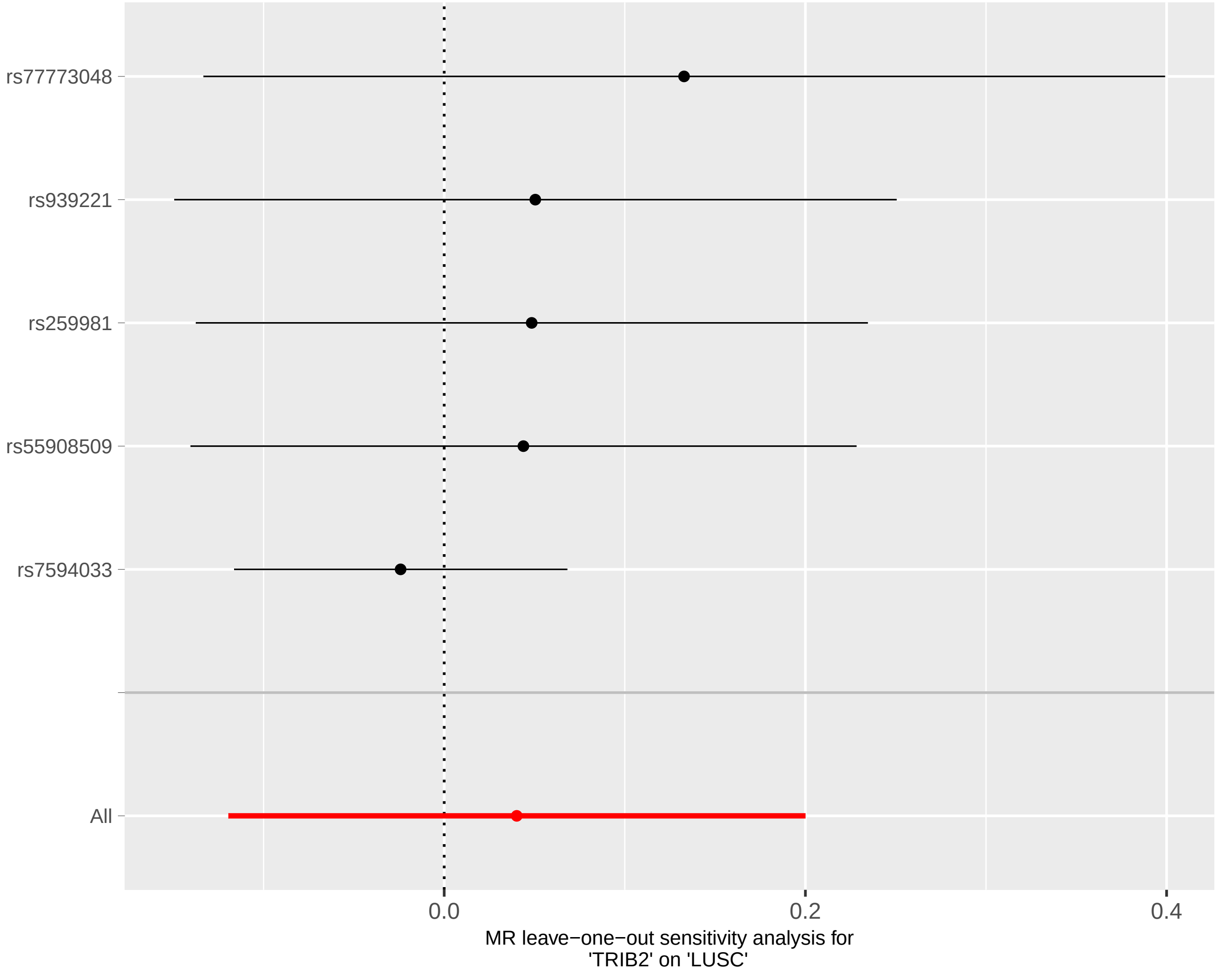

rs77773048
rs939221
rs259981
rs55908509
rs7594033
All
0.0
0.2
0.4
MR leave−one−out sensitivity analysis for
'TRIB2' on 'LUSC'

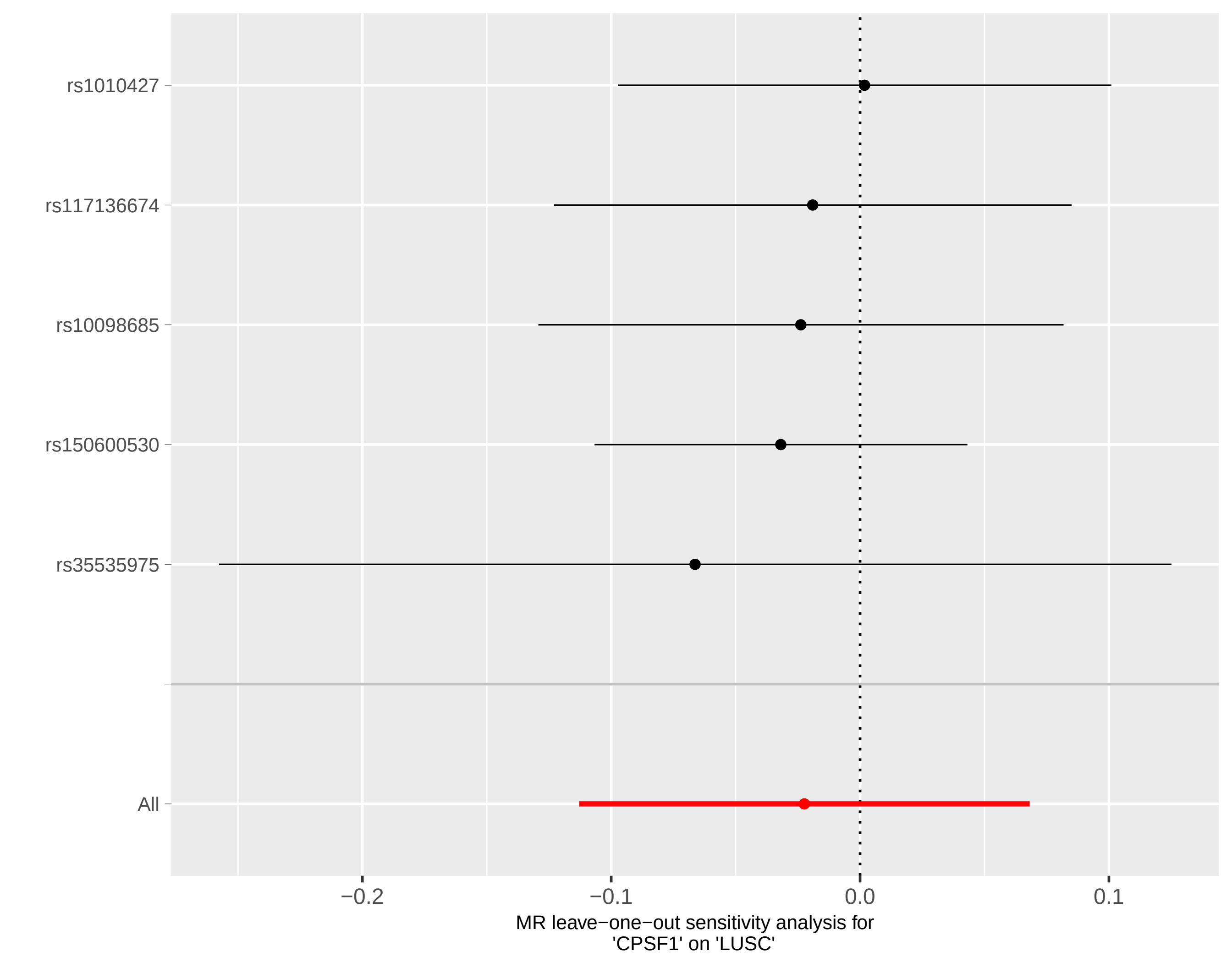
rs1010427
rs117136674
rs10098685
rs150600530
rs35535975
All
−0.2
−0.1
0.0
0.1
MR leave−one−out sensitivity analysis for
'CPSF1' on 'LUSC'

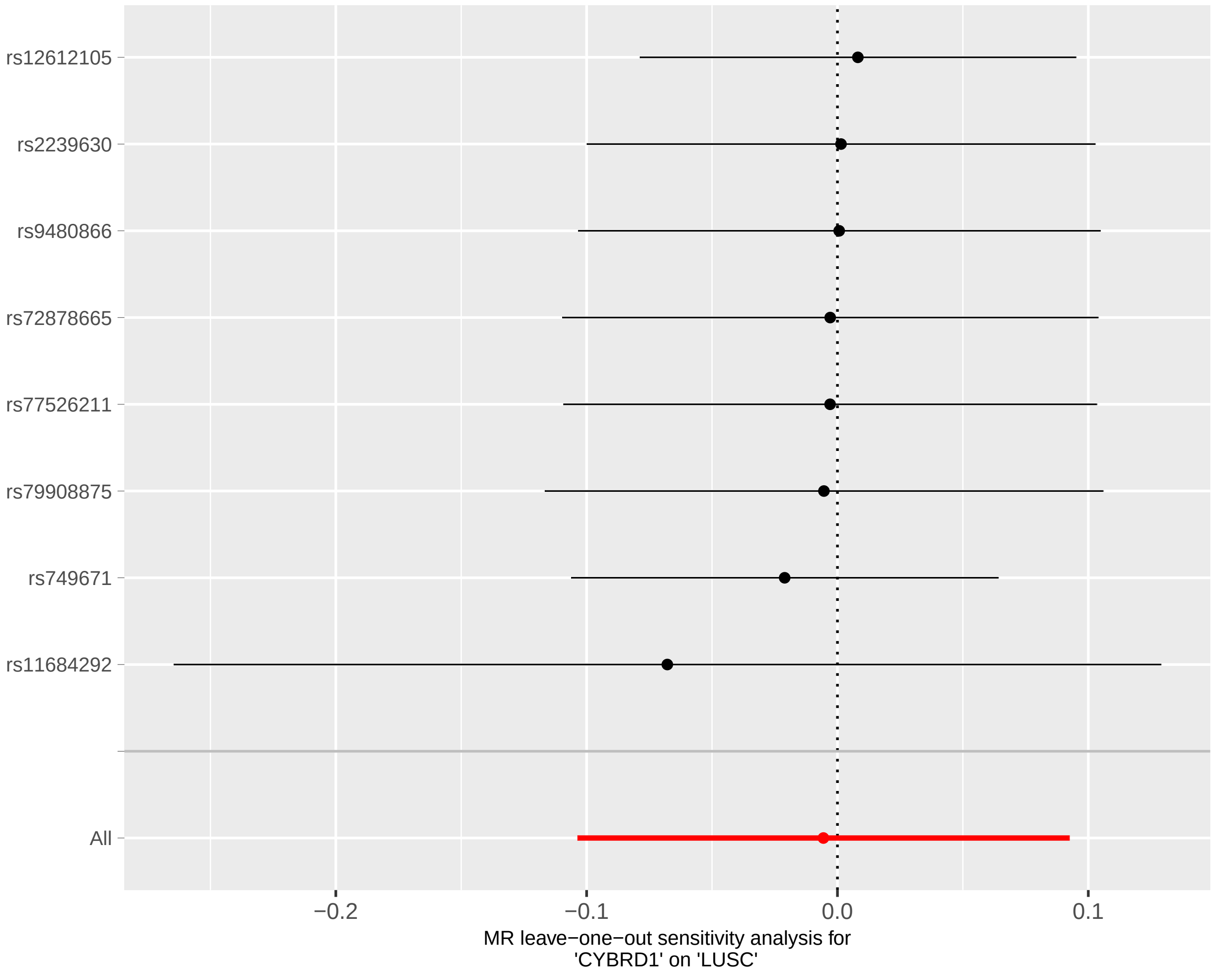

rs12612105
rs2239630
rs9480866
rs72878665
rs77526211
rs79908875
rs749671
rs11684292
All
−0.2
−0.1
0.0
0.1
MR leave−one−out sensitivity analysis for
'CYBRD1' on 'LUSC'

Insufficient number of SNPs

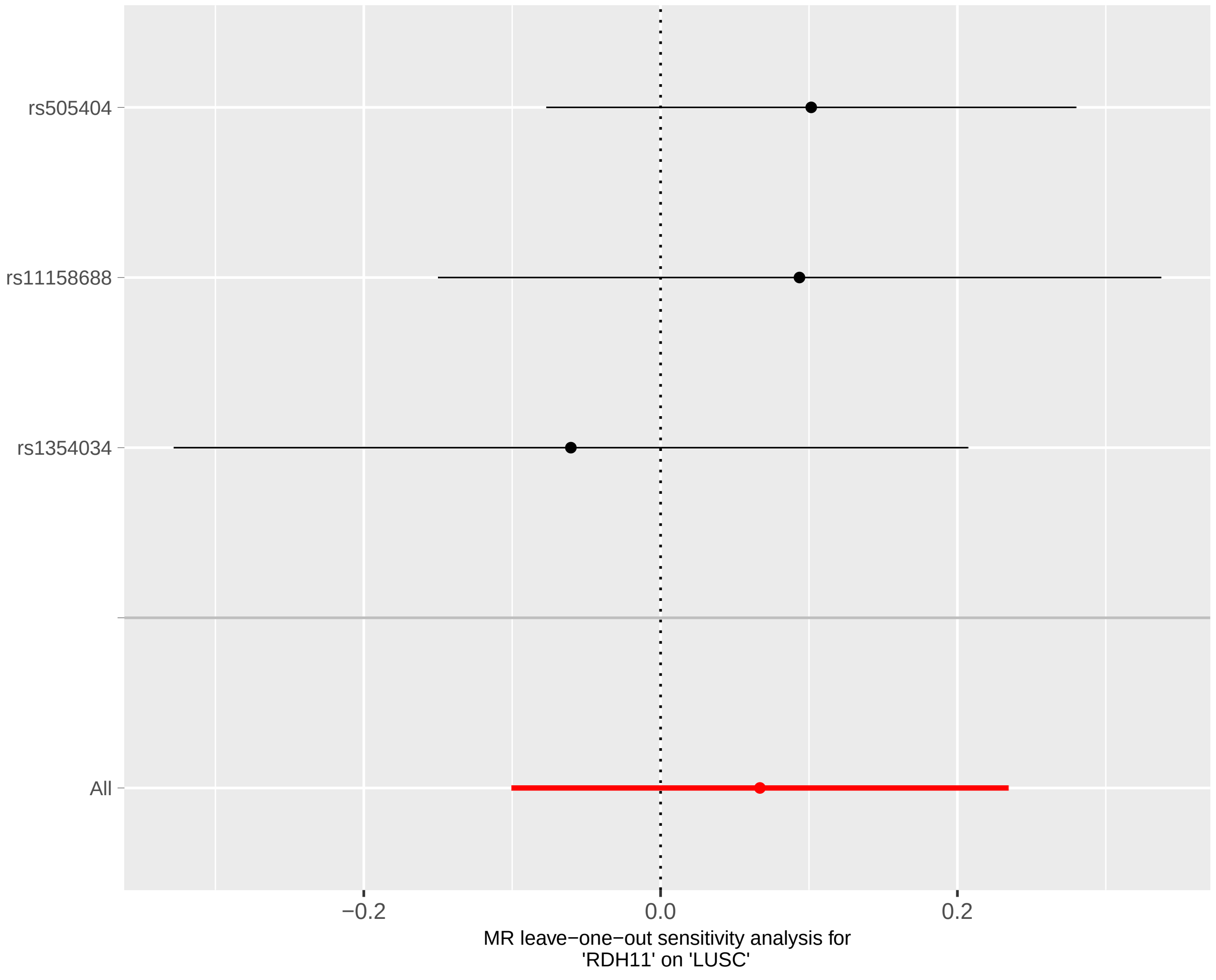
rs505404
rs11158688
rs1354034
All
−0.2
0.0
0.2
MR leave−one−out sensitivity analysis for
'RDH11' on 'LUSC'

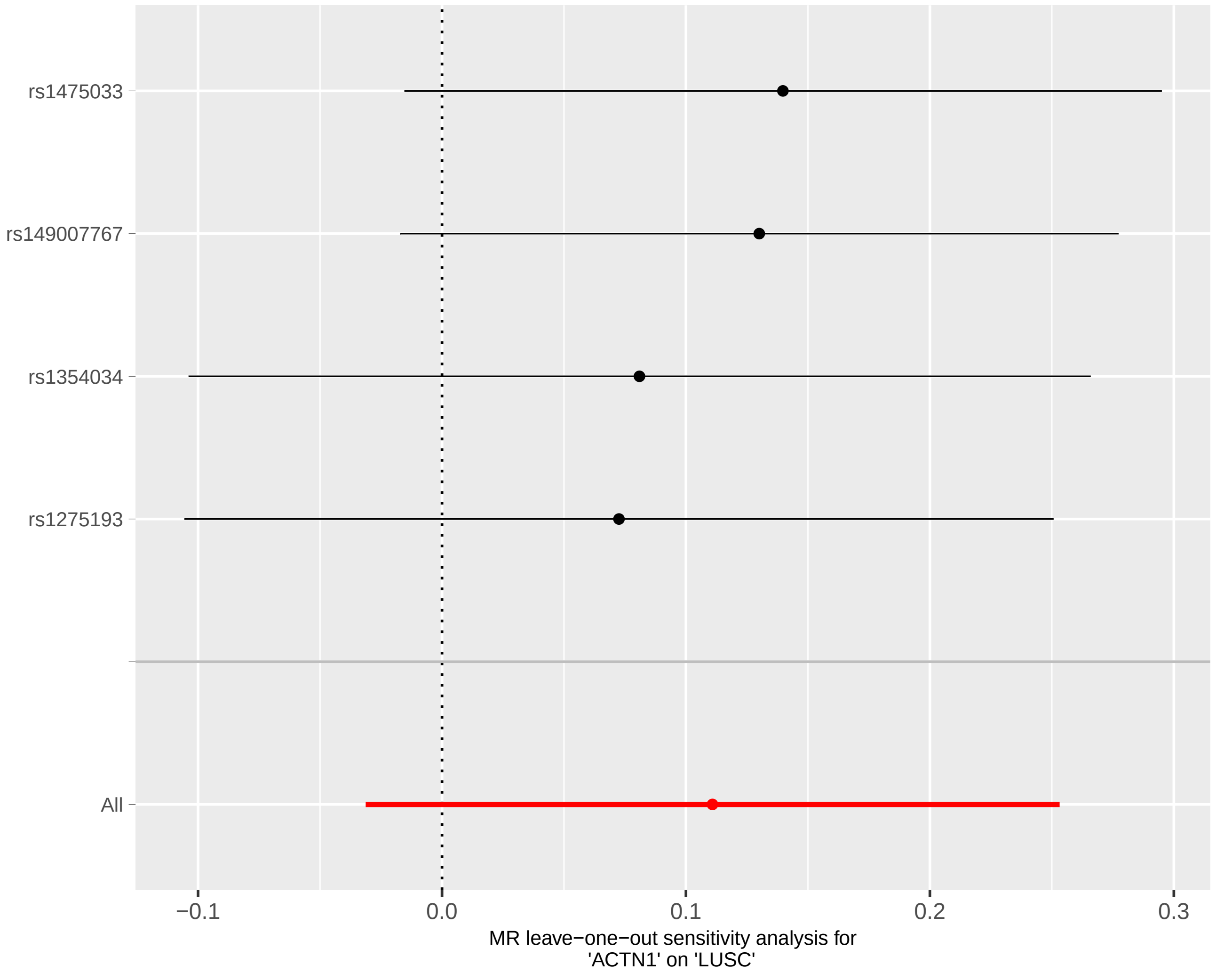
rs1475033
rs149007767
rs1354034
rs1275193
All
−0.1
0.0
0.1
0.2
0.3
MR leave−one−out sensitivity analysis for
'ACTN1' on 'LUSC'

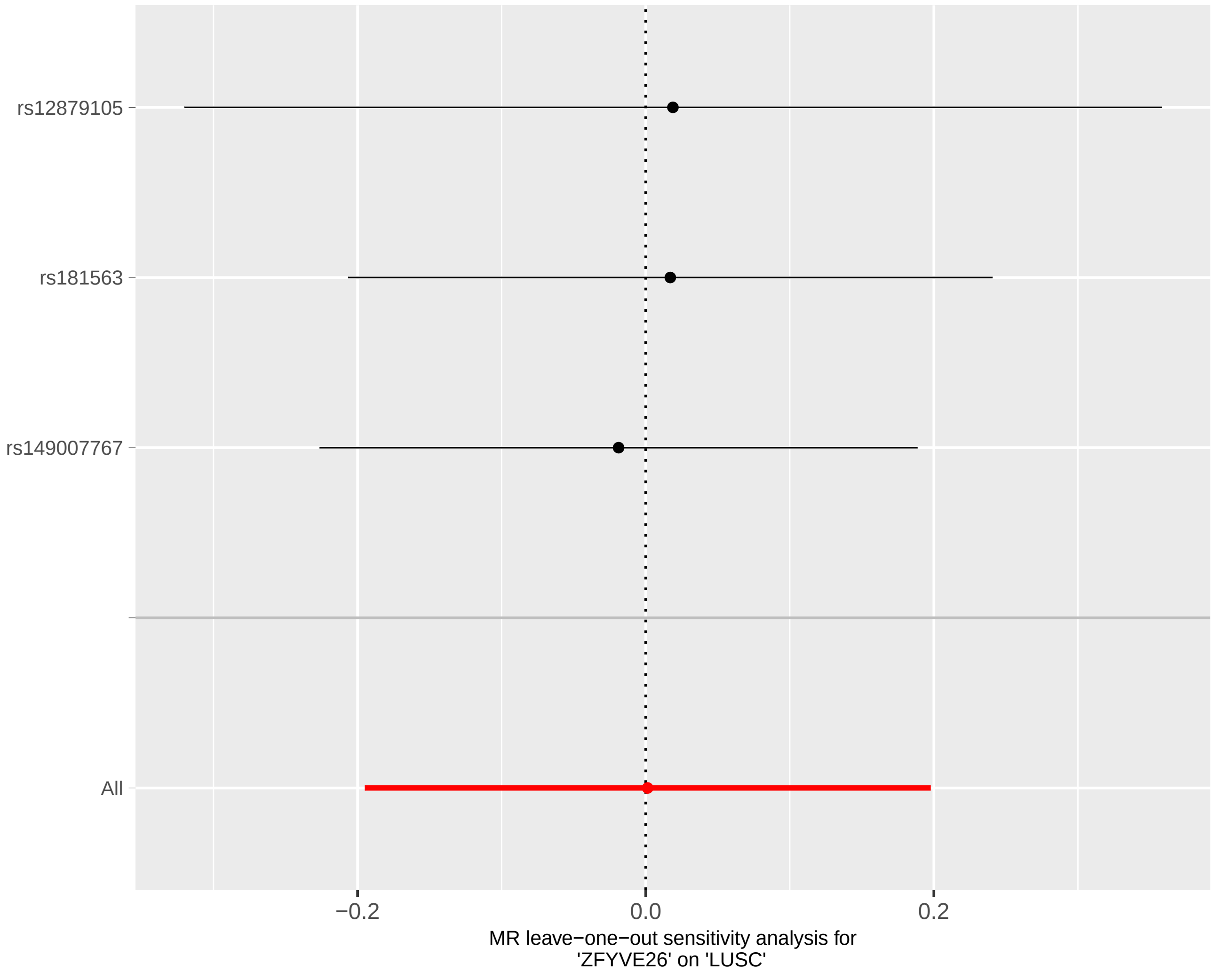

rs12879105
rs181563
rs149007767
All
−0.2
0.0
0.2
MR leave−one−out sensitivity analysis for
'ZFYVE26' on 'LUSC'

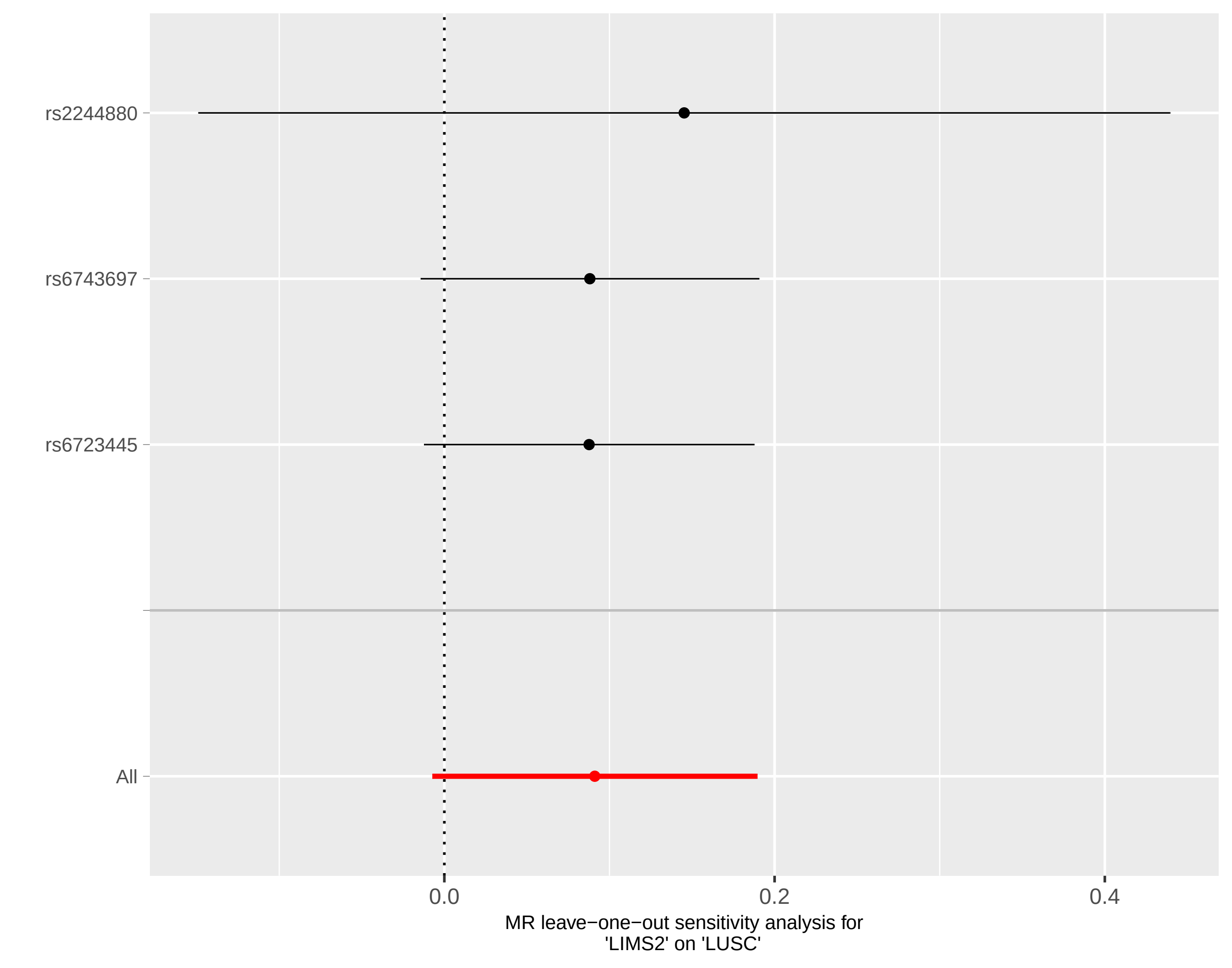
rs2244880
rs6743697
rs6723445
All
0.0
0.2
0.4
MR leave−one−out sensitivity analysis for
'LIMS2' on 'LUSC'

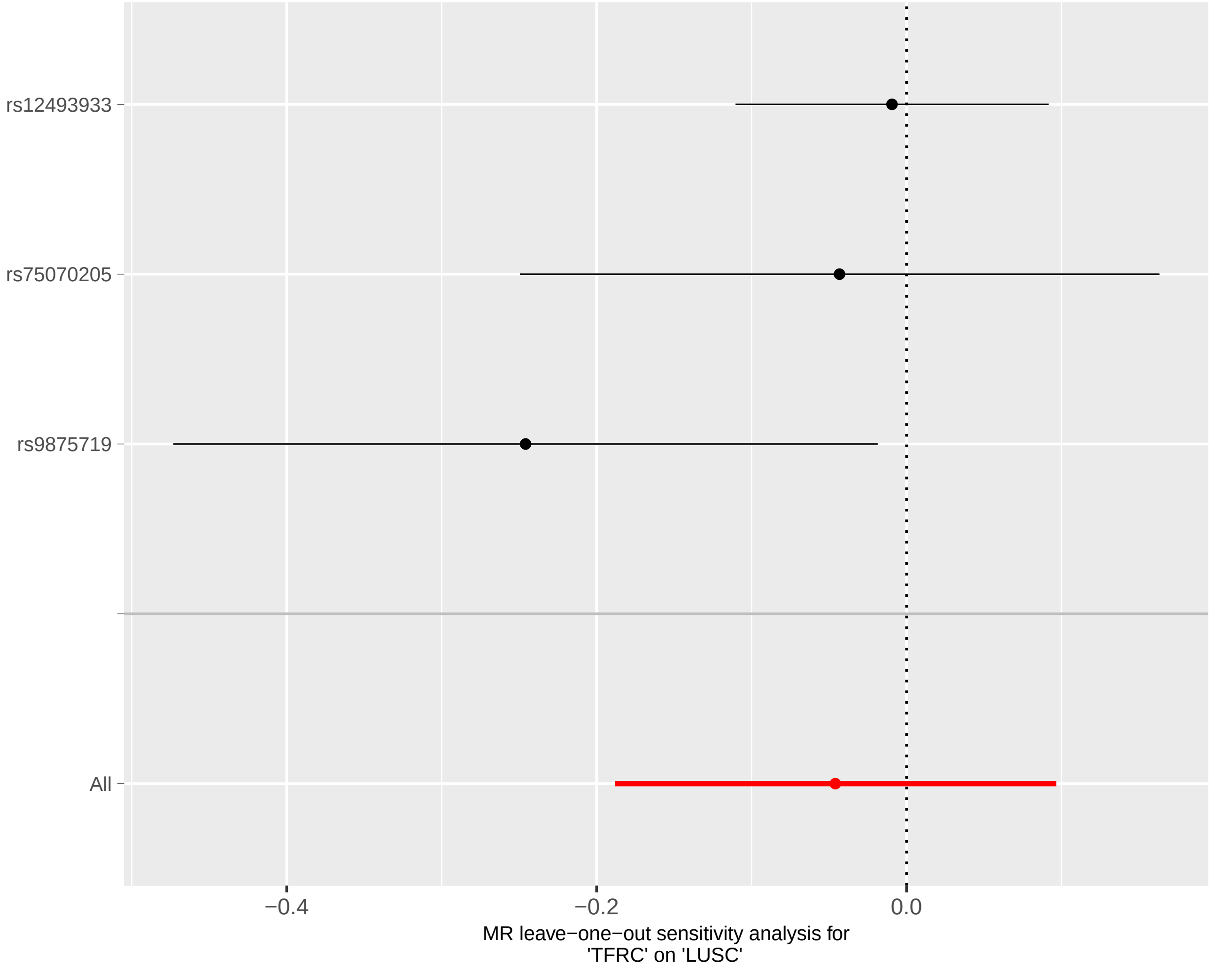
rs12493933
rs75070205
rs9875719
All
−0.4
−0.2
0.0
MR leave−one−out sensitivity analysis for
'TFRC' on 'LUSC'

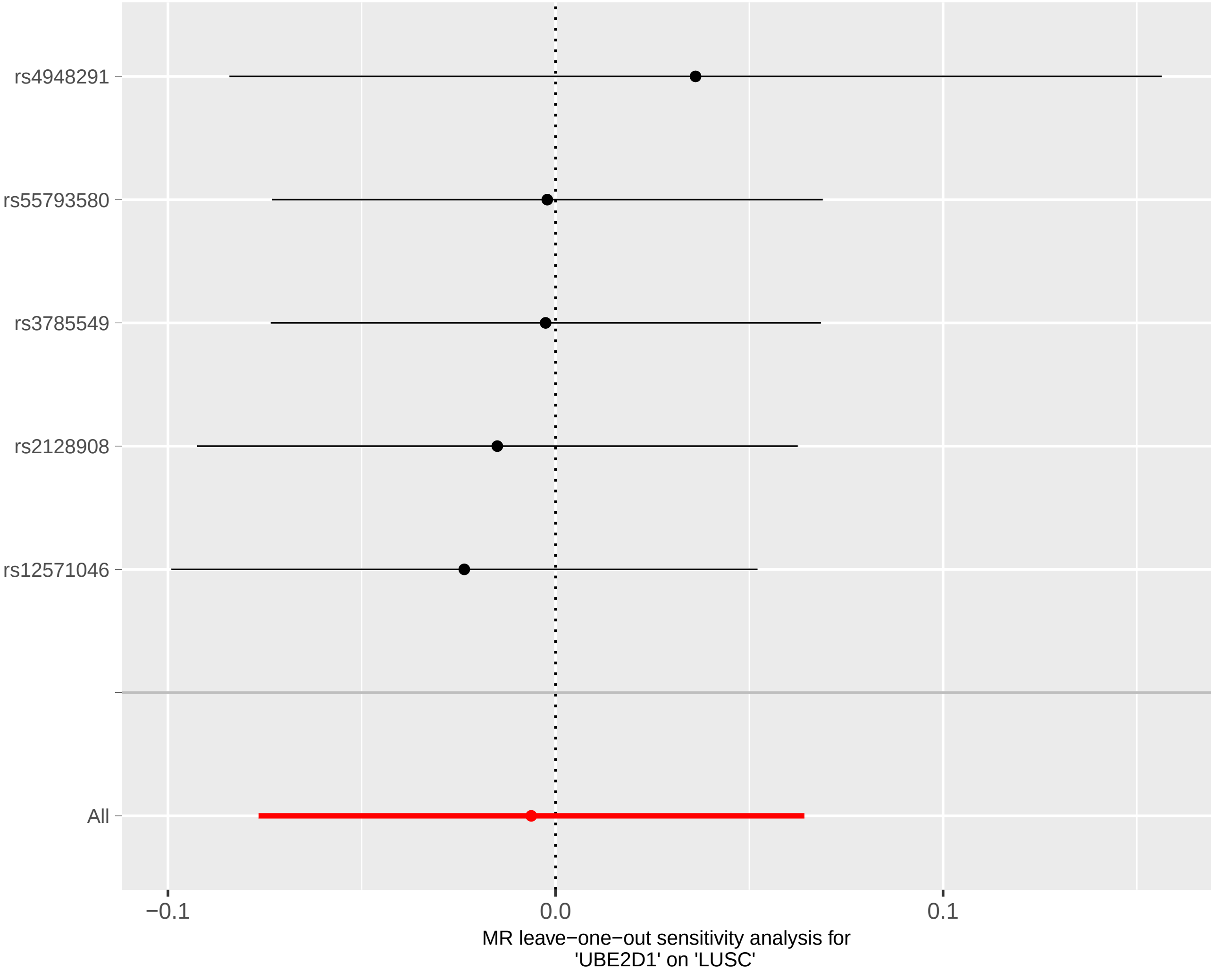
rs4948291
rs55793580
rs3785549
rs2128908
rs12571046
All
−0.1
0.0
0.1
MR leave−one−out sensitivity analysis for
'UBE2D1' on 'LUSC'

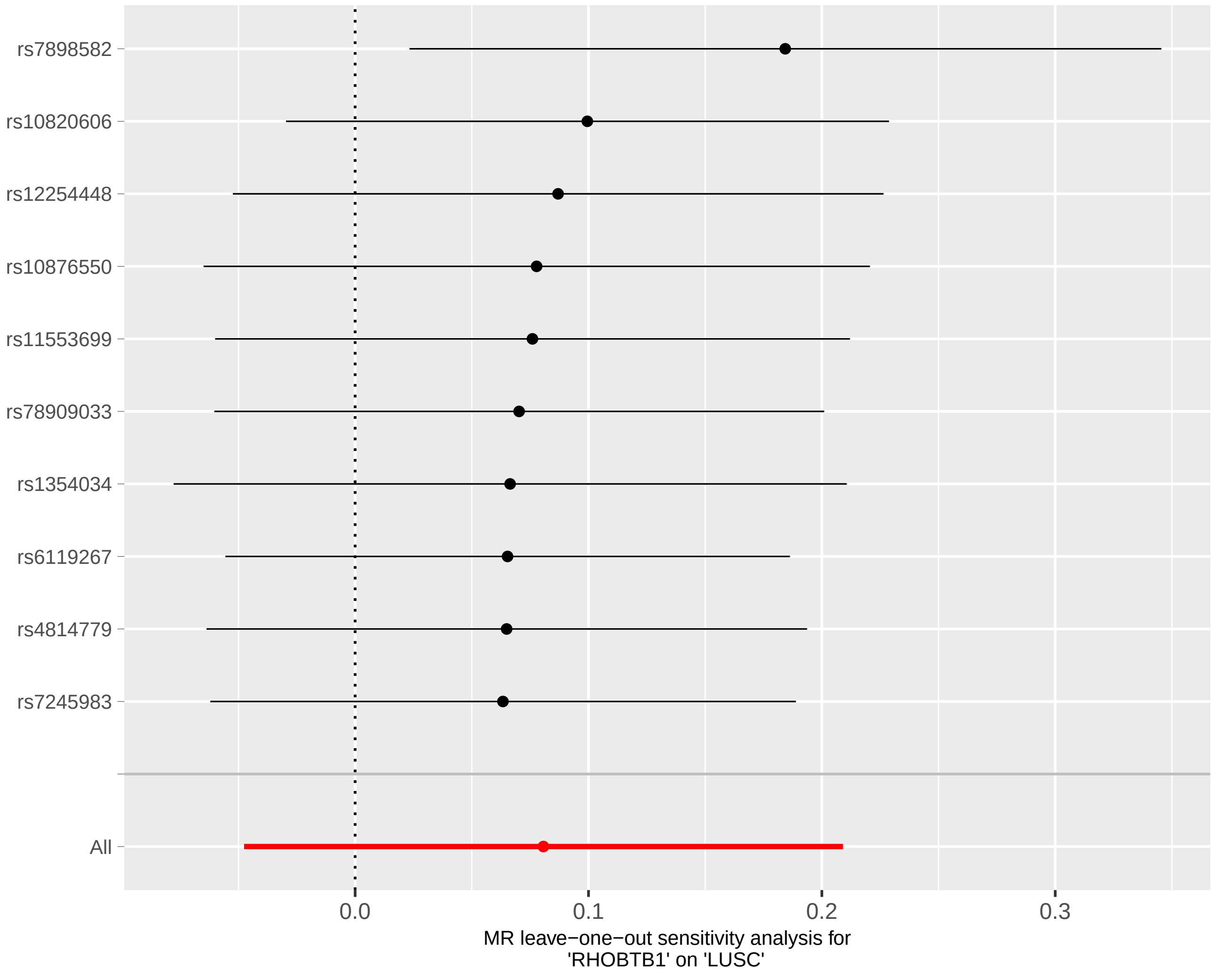
rs7898582
rs10820606
rs12254448
rs10876550
rs11553699
rs78909033
rs1354034
rs6119267
rs4814779
rs7245983
All
0.0
0.1
0.2
0.3
MR leave−one−out sensitivity analysis for
'RHOBTB1' on 'LUSC'

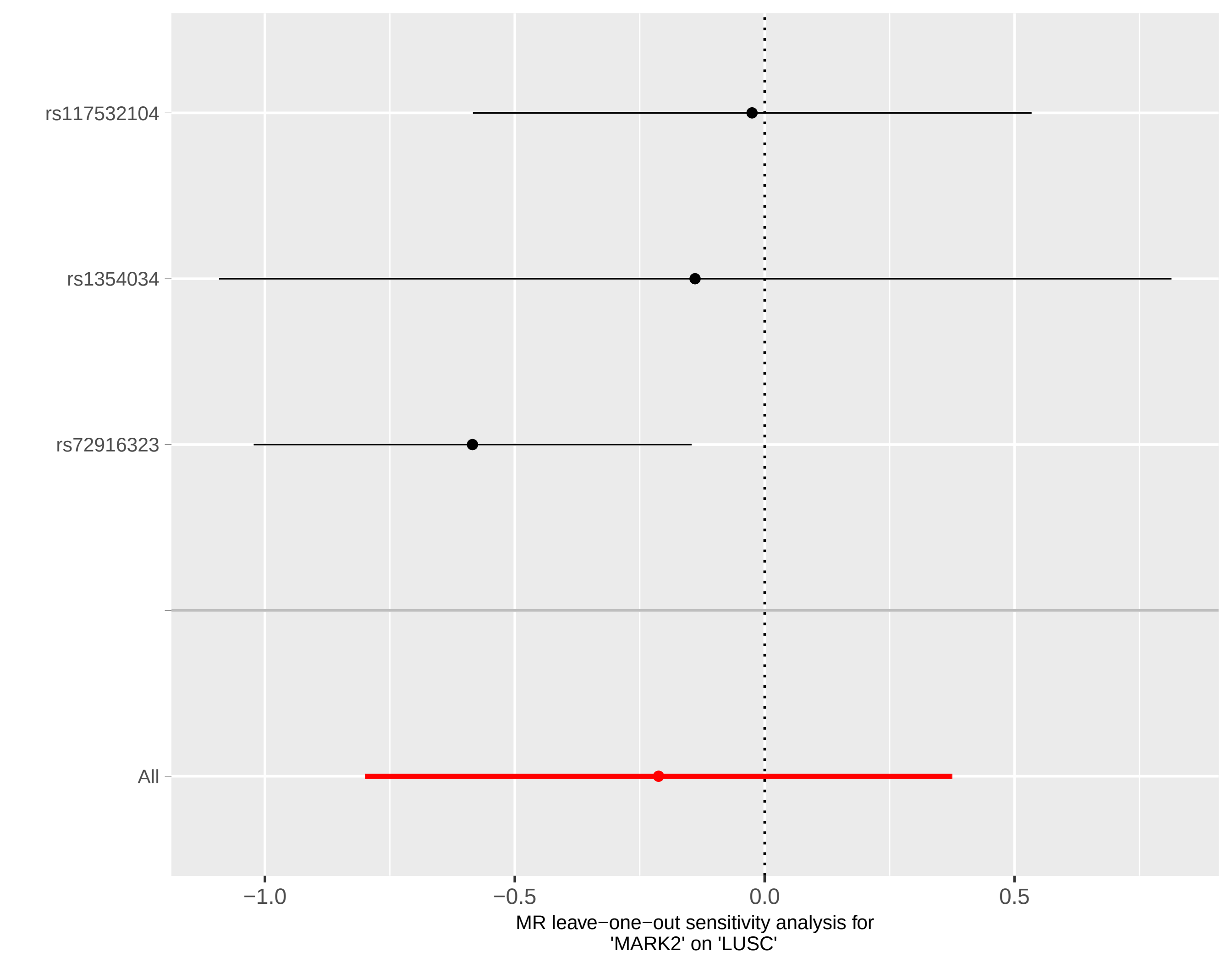

rs117532104
rs1354034
rs72916323
All
−1.0
−0.5
0.0
0.5
MR leave−one−out sensitivity analysis for
'MARK2' on 'LUSC'

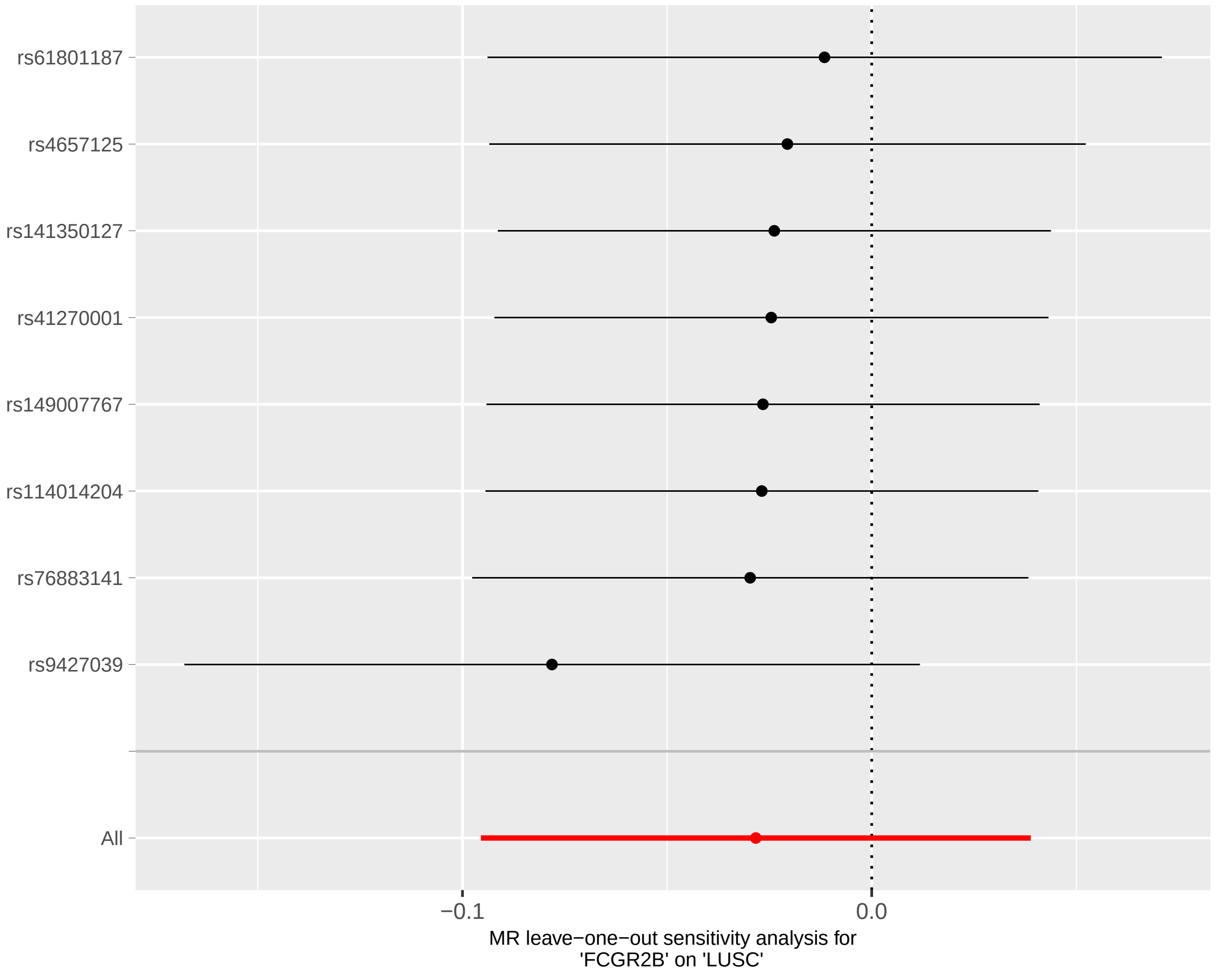
rs61801187
rs4657125
rs141350127
rs41270001
rs149007767
rs114014204
rs76883141
rs9427039
All
−0.1
0.0
MR leave−one−out sensitivity analysis for
'FCGR2B' on 'LUSC'

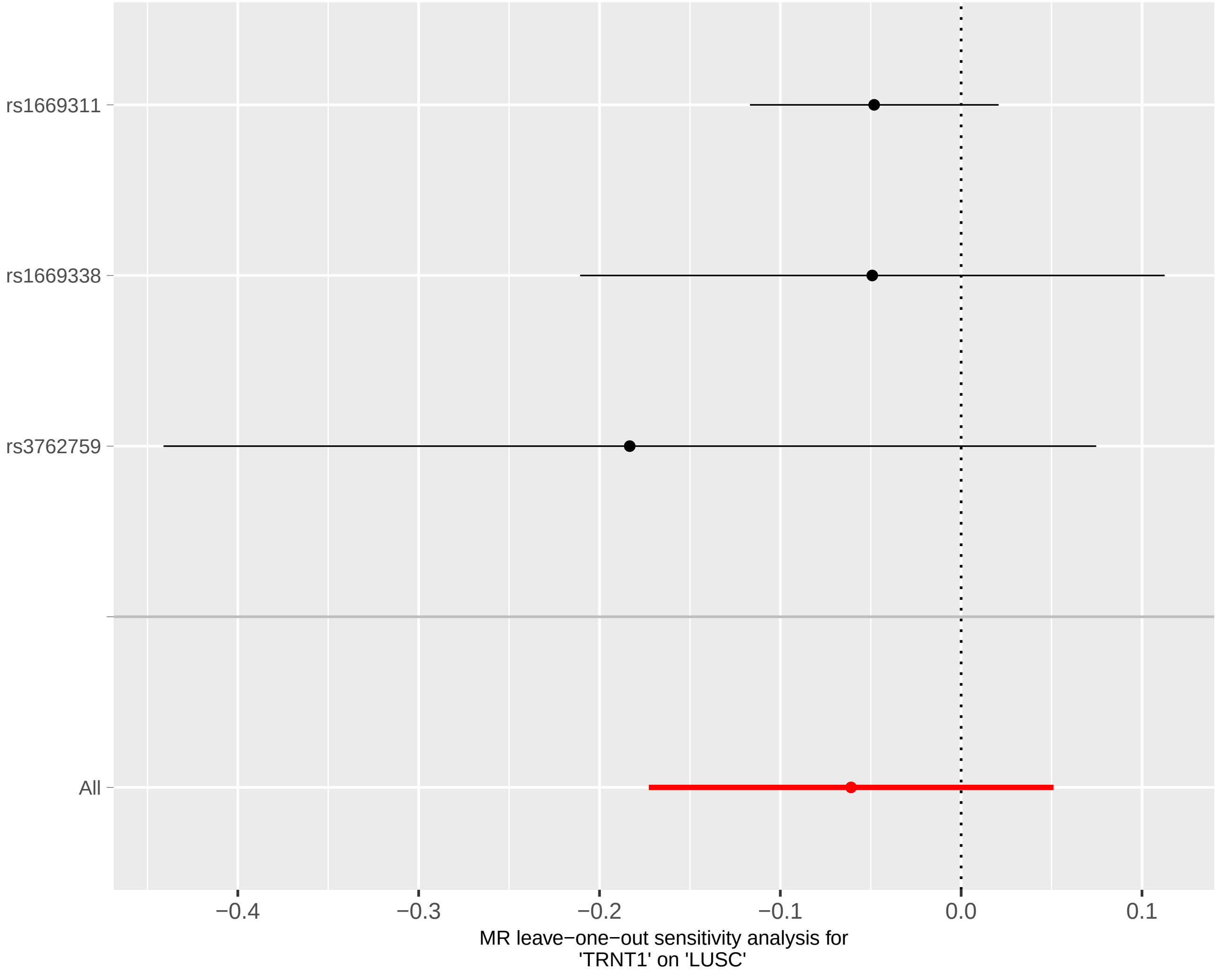
rs1669311
rs1669338
rs3762759
All
−0.4
−0.3
−0.2
−0.1
0.0
0.1
MR leave−one−out sensitivity analysis for
'TRNT1' on 'LUSC'

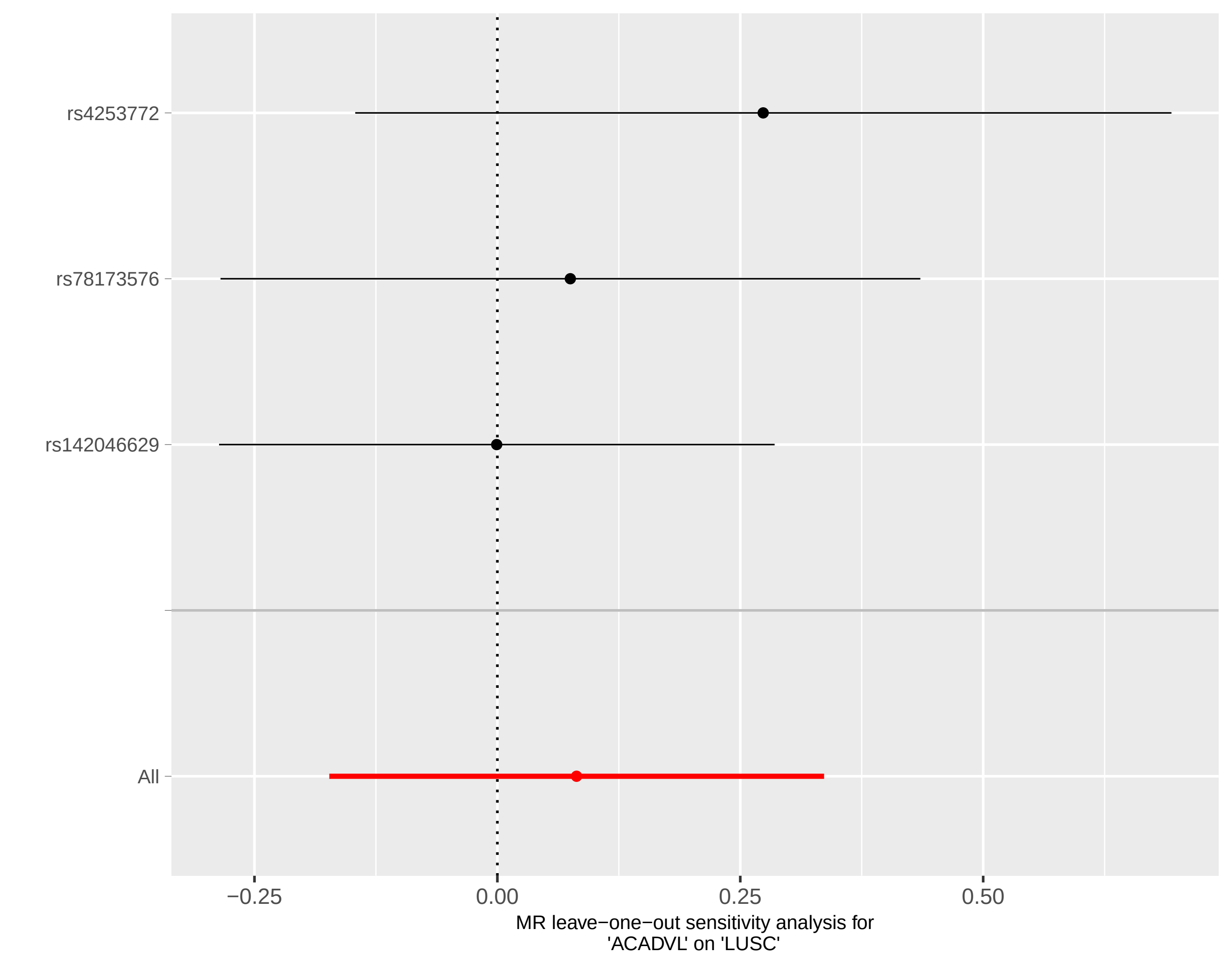
rs4253772
rs78173576
rs142046629
All
−0.25
0.00
0.25
0.50
MR leave−one−out sensitivity analysis for
'ACADVL' on 'LUSC'

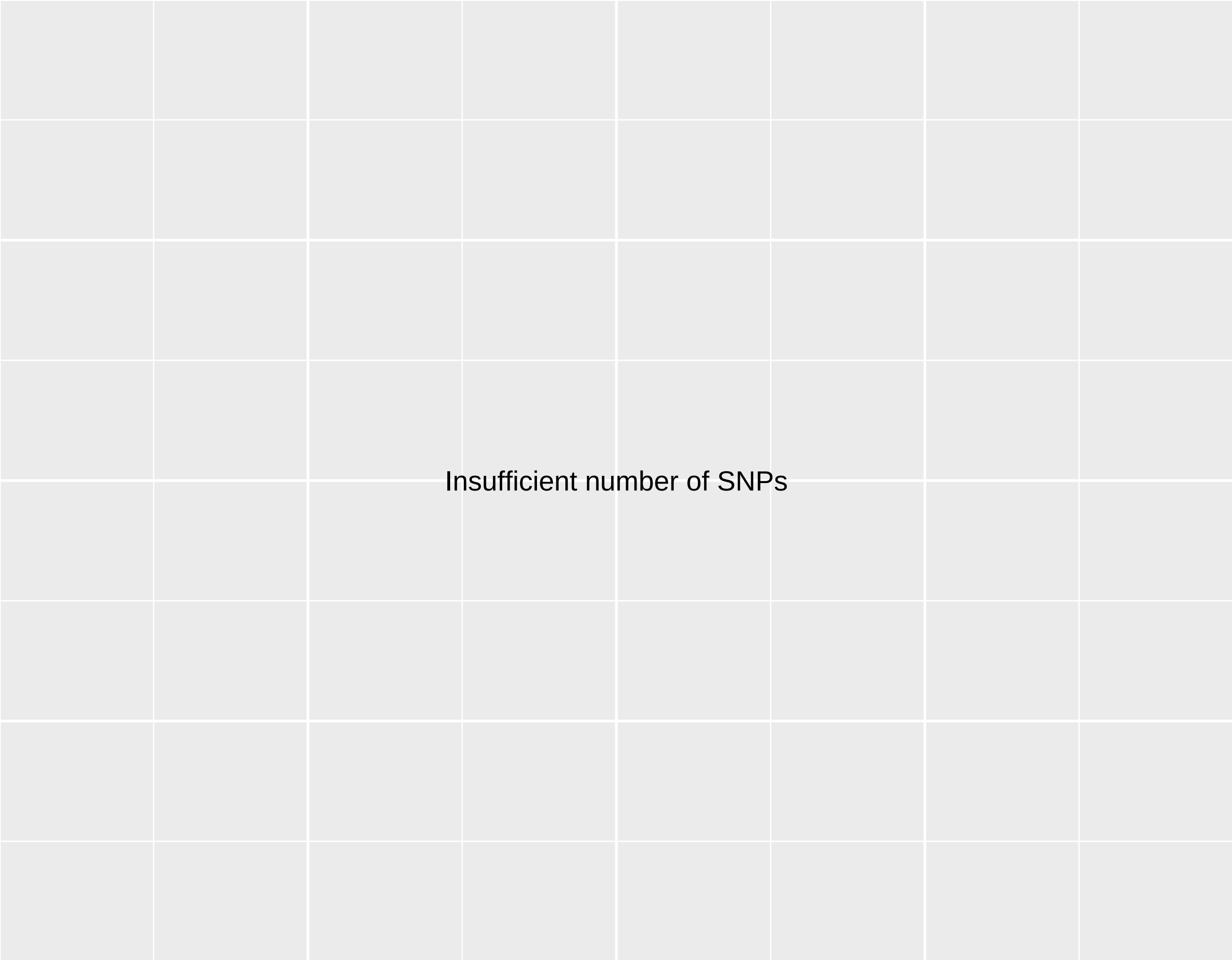
Insufficient number of SNPs

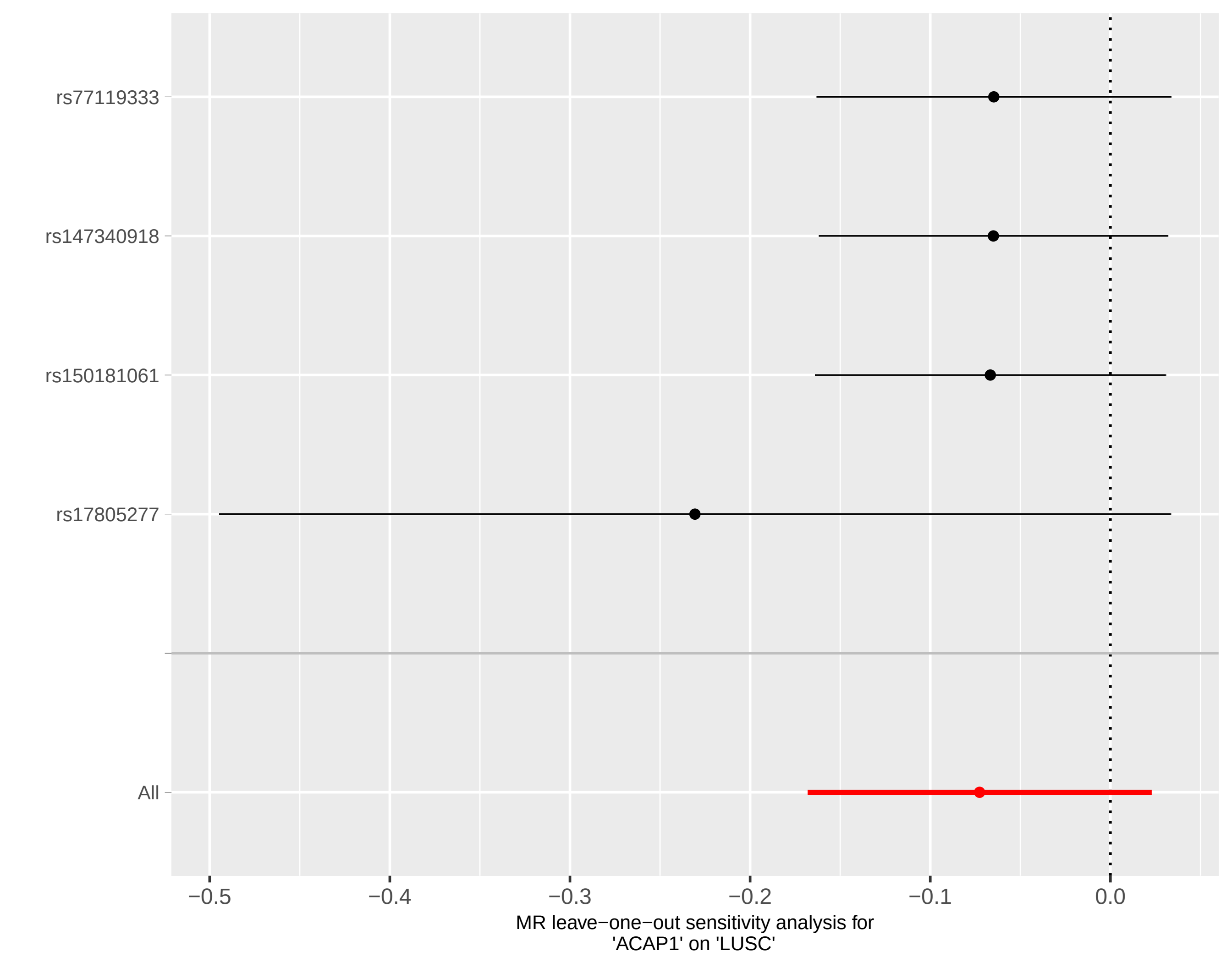

rs77119333
rs147340918
rs150181061
rs17805277
All
−0.5
−0.4
−0.3
−0.2
−0.1
0.0
MR leave−one−out sensitivity analysis for
'ACAP1' on 'LUSC'

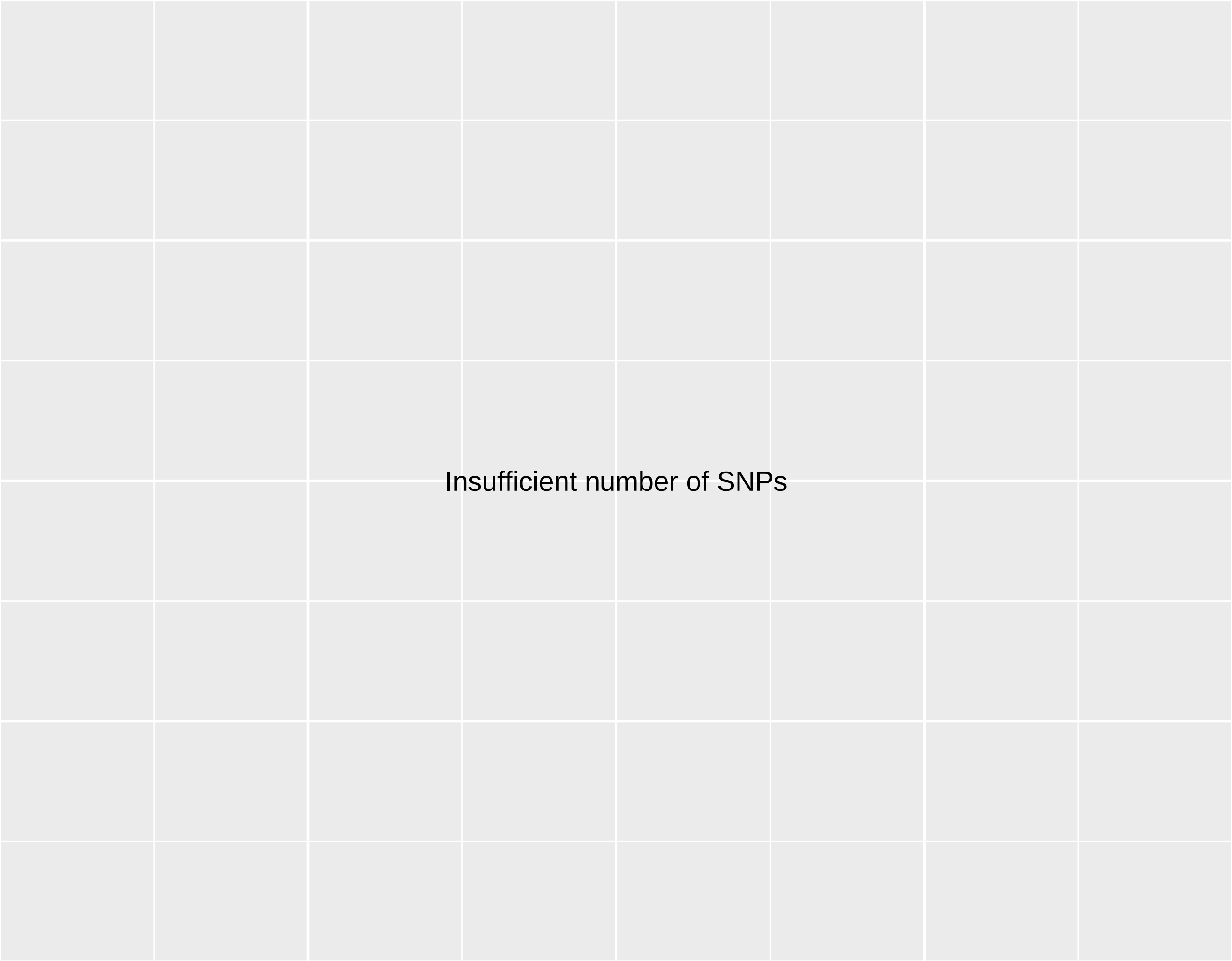
Insufficient number of SNPs

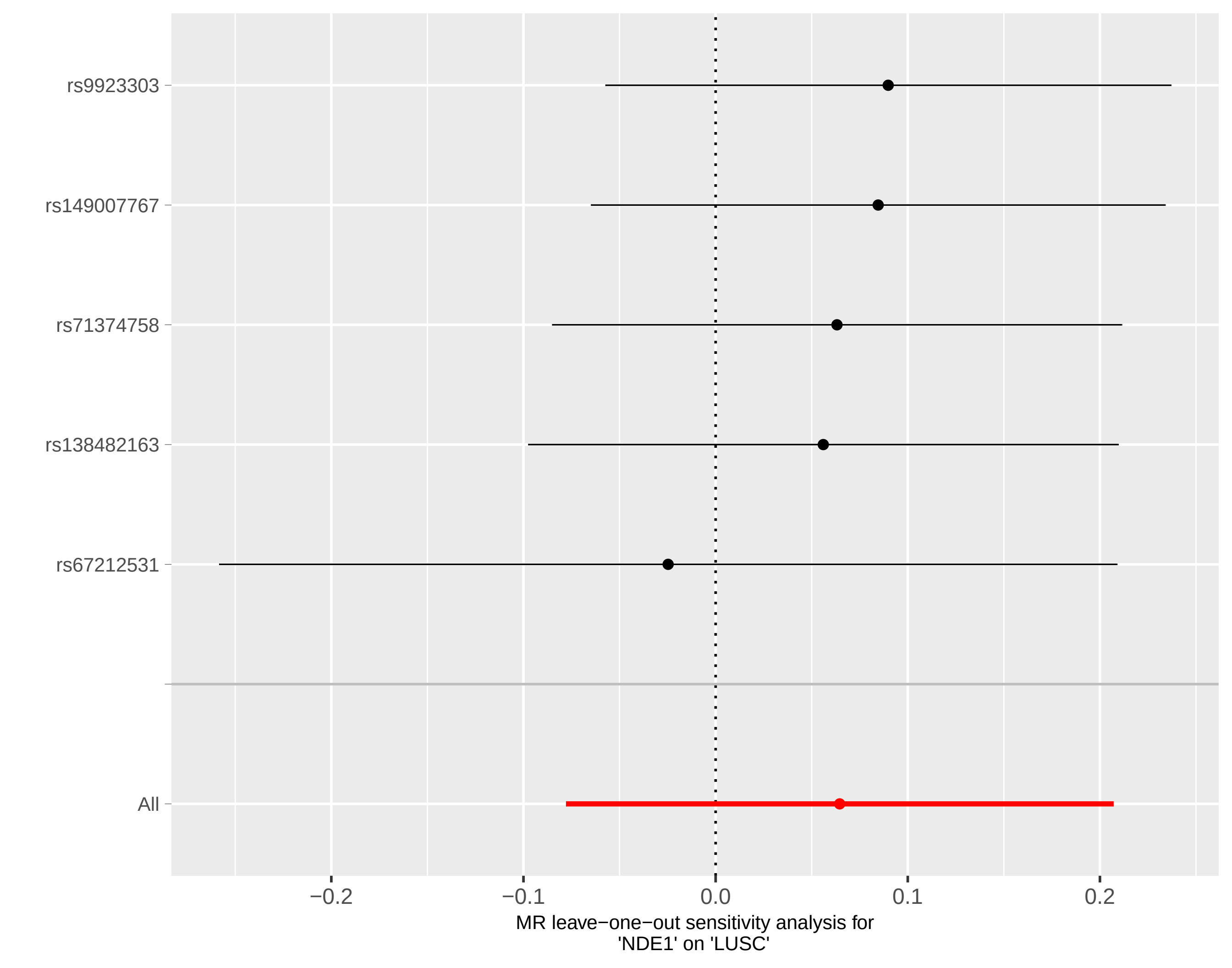
rs9923303
rs149007767
rs71374758
rs138482163
rs67212531
All
−0.2
−0.1
0.0
0.1
0.2
MR leave−one−out sensitivity analysis for
'NDE1' on 'LUSC'

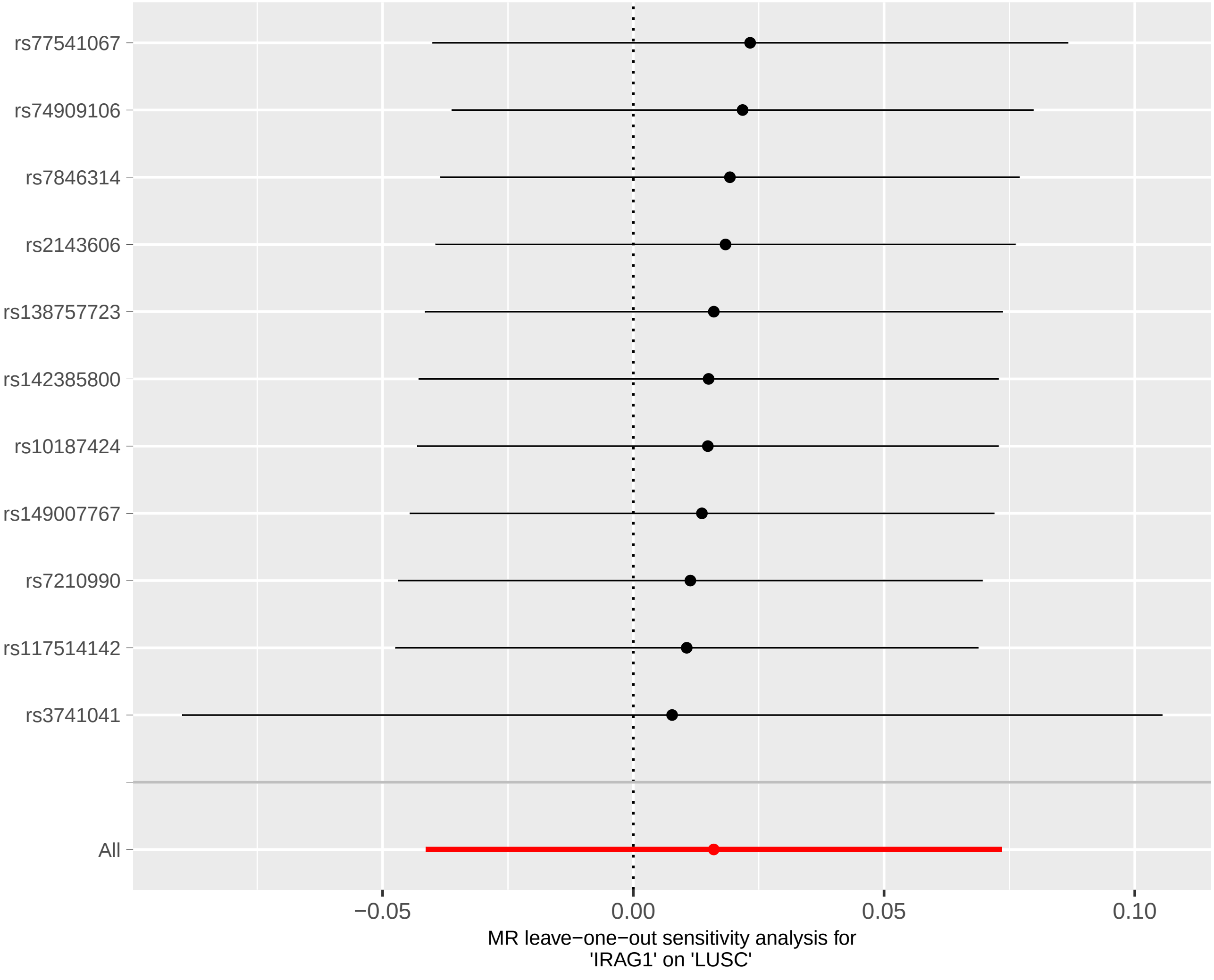
rs77541067
rs74909106
rs7846314
rs2143606
rs138757723
rs142385800
rs10187424
rs149007767
rs7210990
rs117514142
rs3741041
All
−0.05
0.00
0.05
0.10
MR leave−one−out sensitivity analysis for
'IRAG1' on 'LUSC'

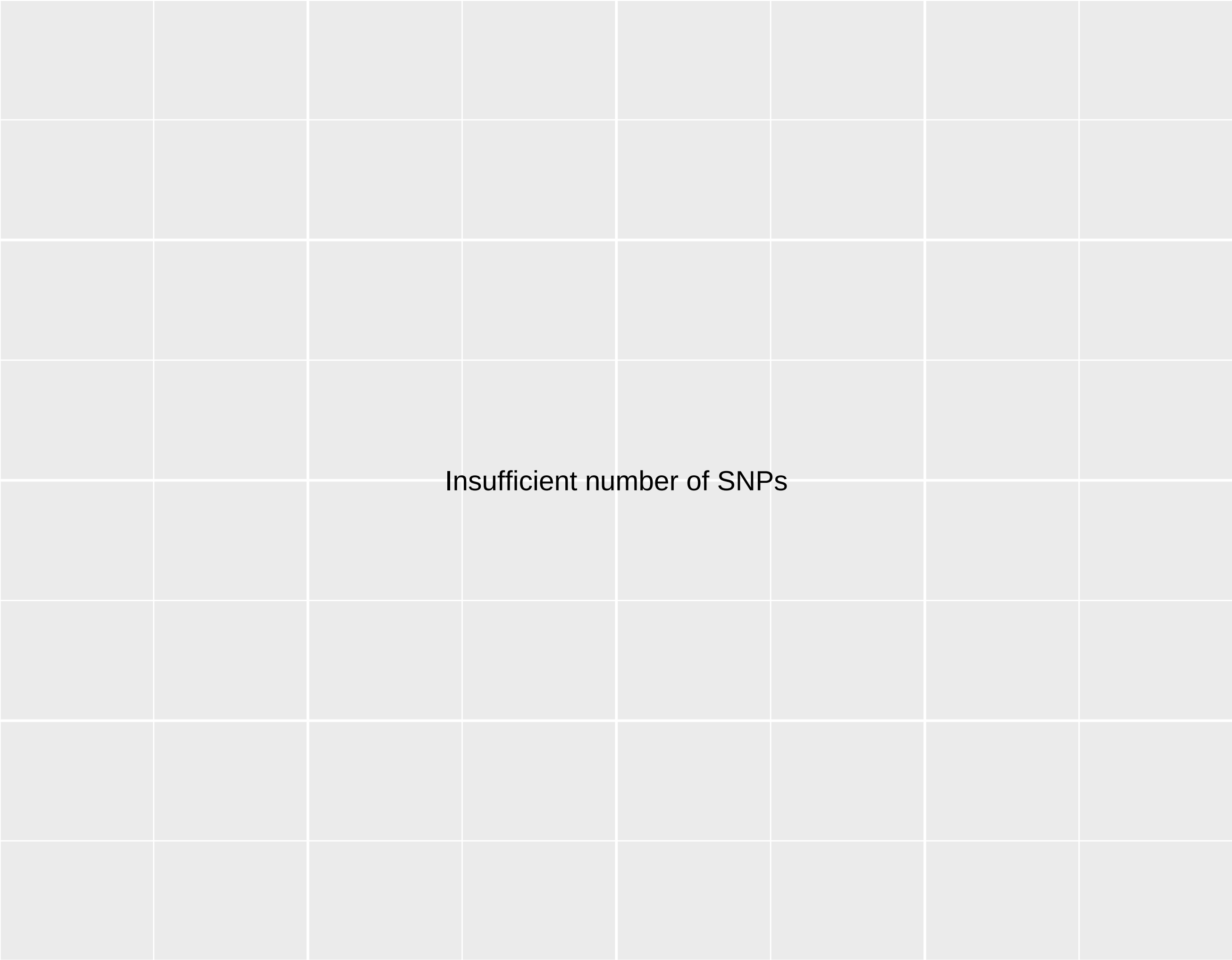
Insufficient number of SNPs

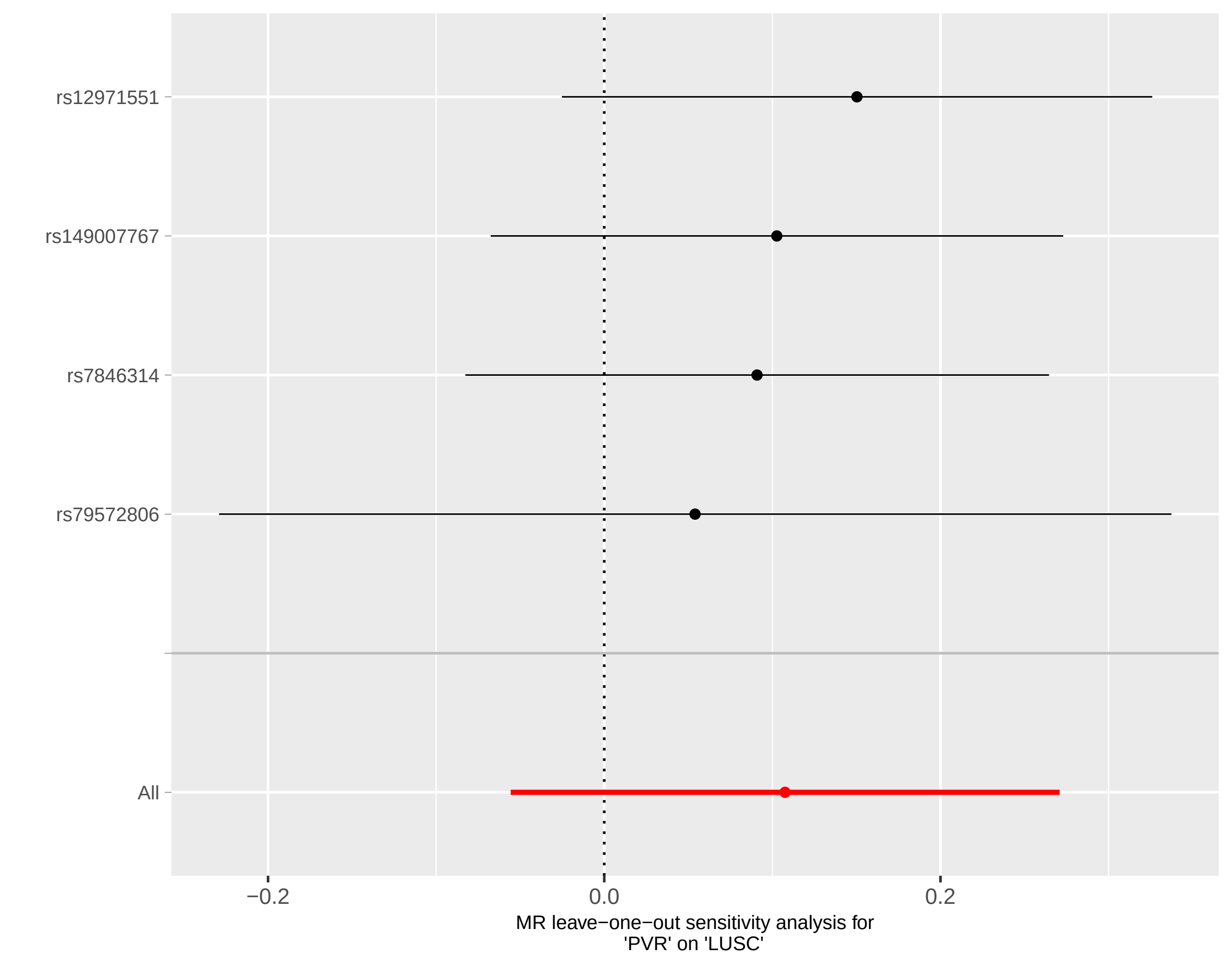
rs12971551
rs149007767
rs7846314
rs79572806
All
−0.2
0.0
0.2
MR leave−one−out sensitivity analysis for
'PVR' on 'LUSC'

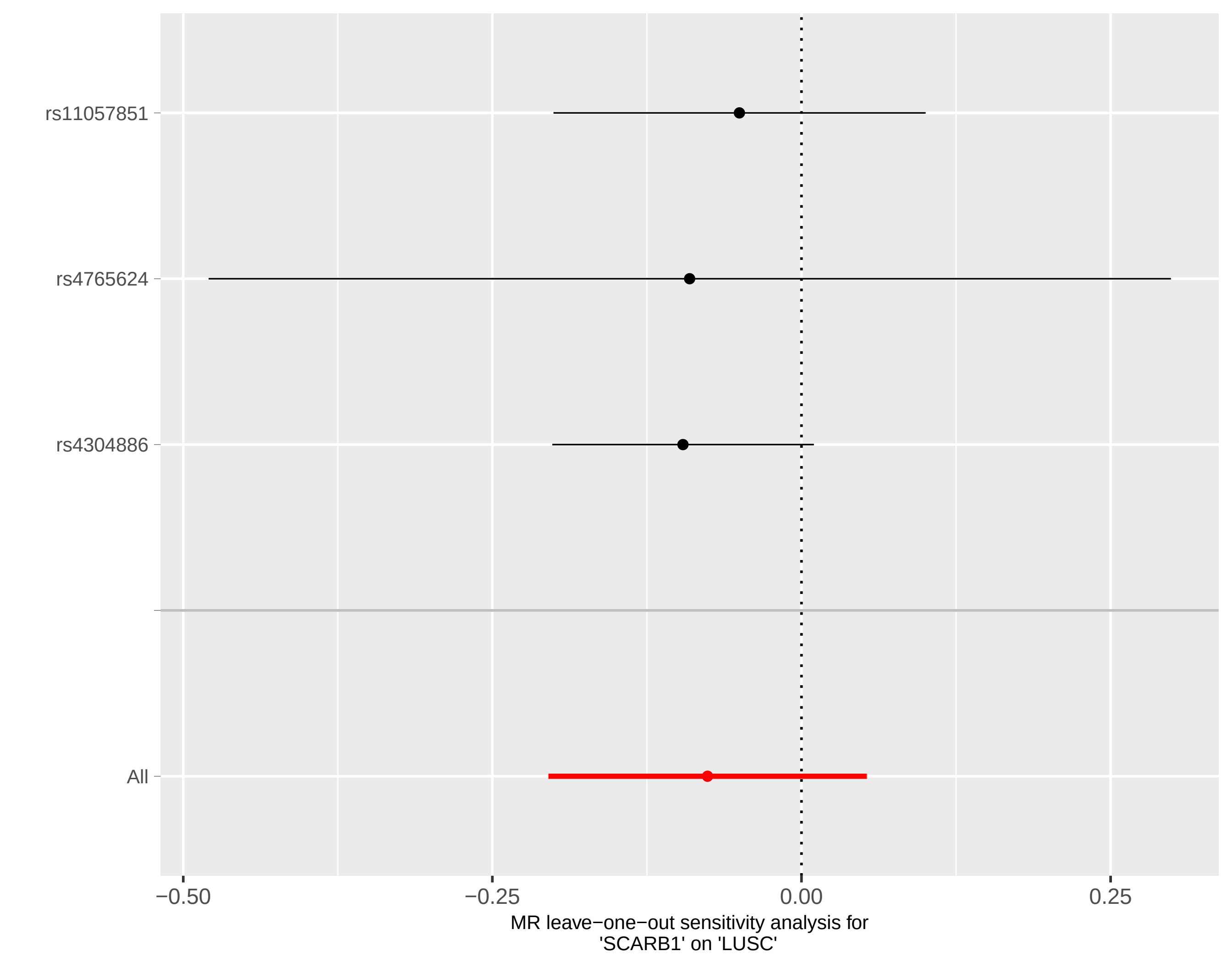
rs11057851
rs4765624
rs4304886
All
−0.50
−0.25
0.00
0.25
MR leave−one−out sensitivity analysis for
'SCARB1' on 'LUSC'

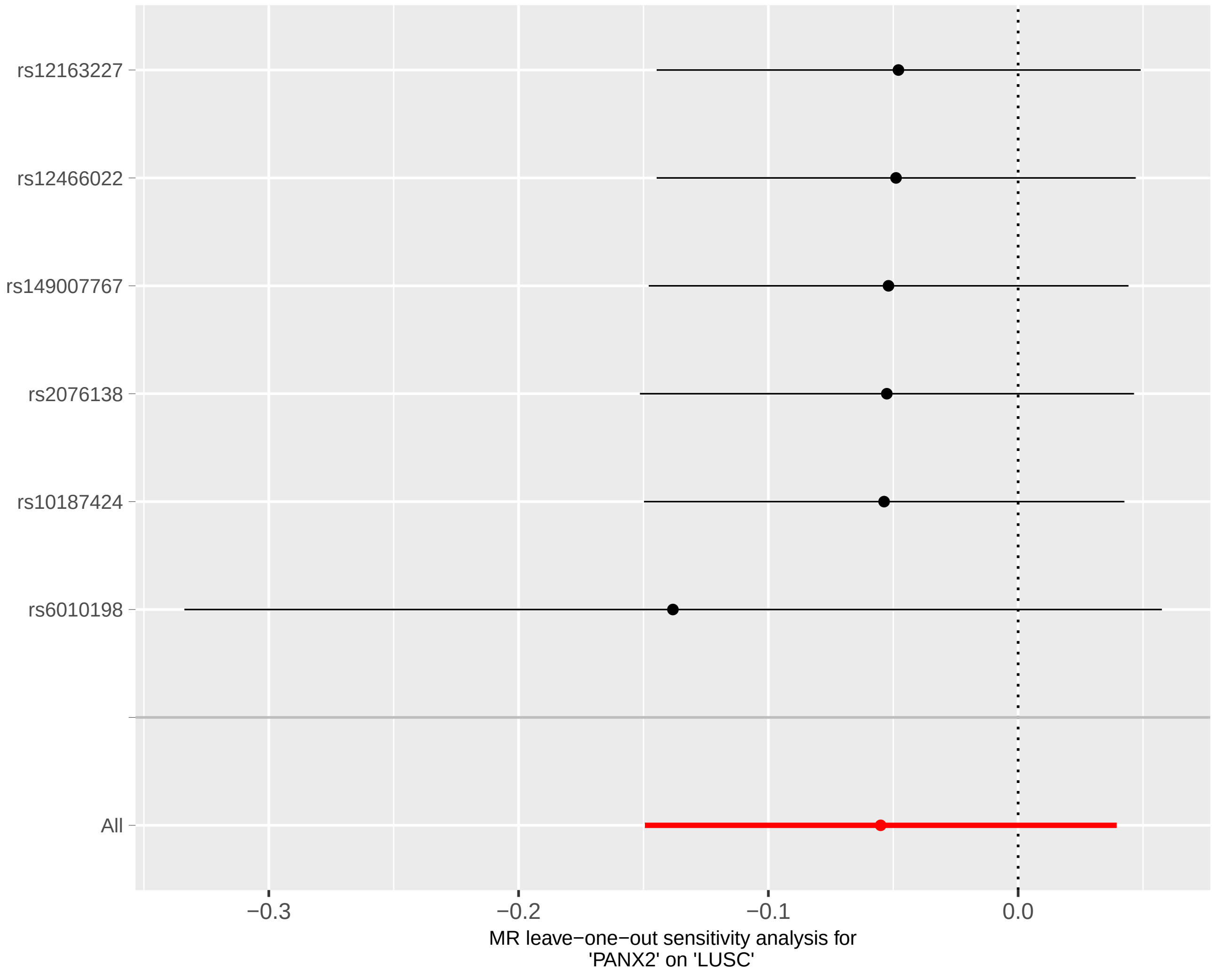
rs12163227
rs12466022
rs149007767
rs2076138
rs10187424
rs6010198
All
−0.3
−0.2
−0.1
0.0
MR leave−one−out sensitivity analysis for
'PANX2' on 'LUSC'

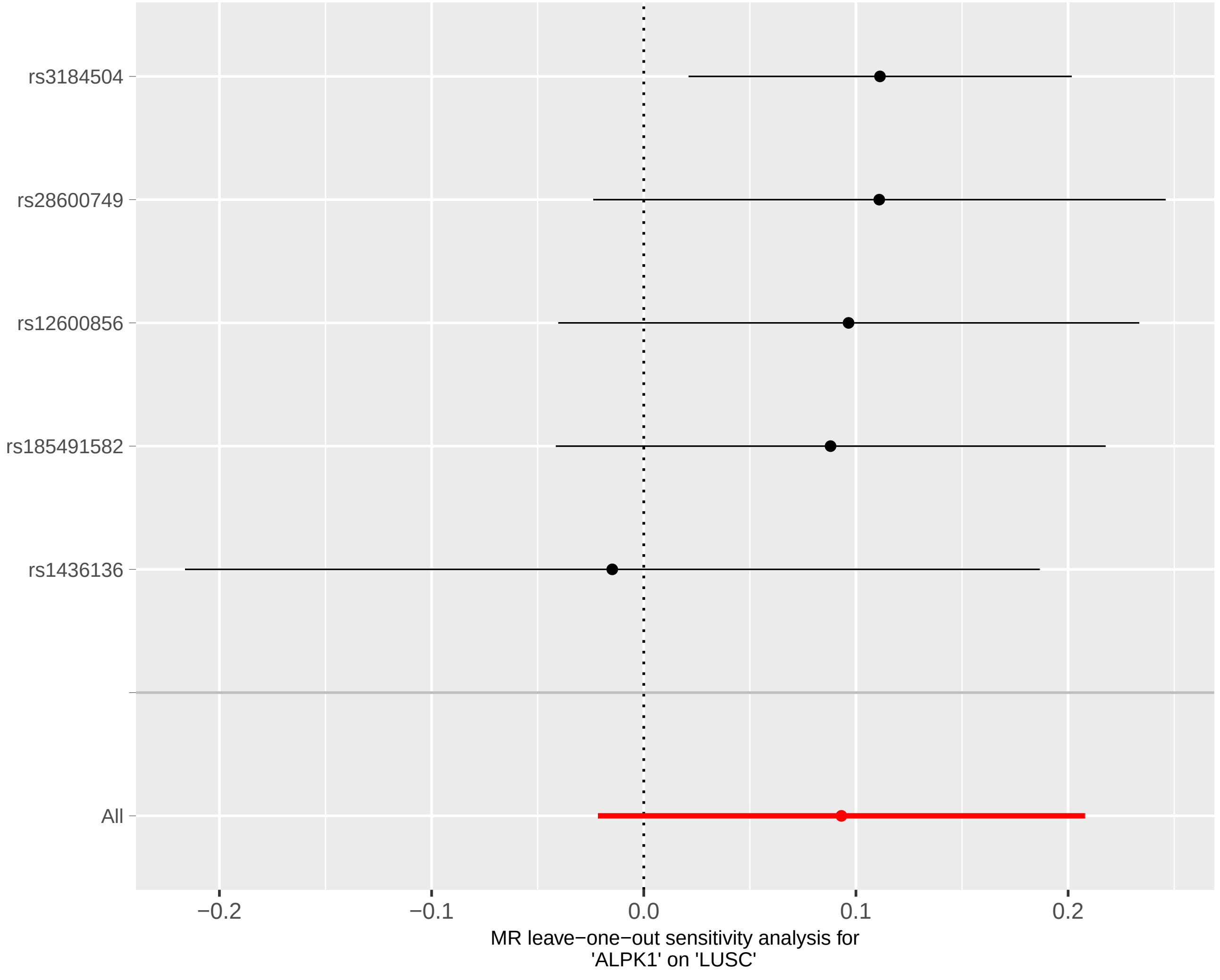

rs3184504
rs28600749
rs12600856
rs185491582
rs1436136
All
−0.2
−0.1
0.0
0.1
0.2
MR leave−one−out sensitivity analysis for
'ALPK1' on 'LUSC'

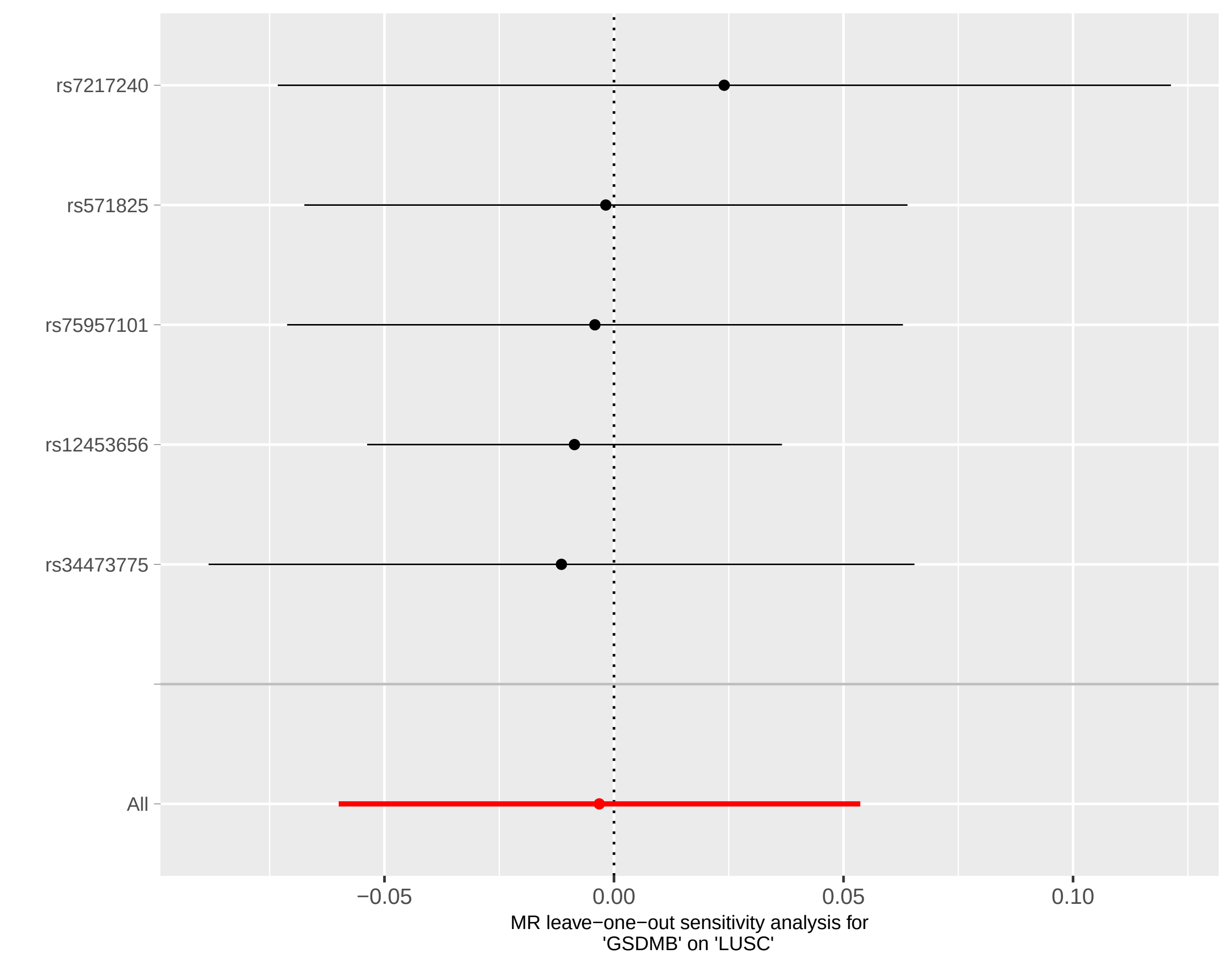

rs7217240
rs571825
rs75957101
rs12453656
rs34473775
All
−0.05
0.00
0.05
0.10
MR leave−one−out sensitivity analysis for
'GSDMB' on 'LUSC'

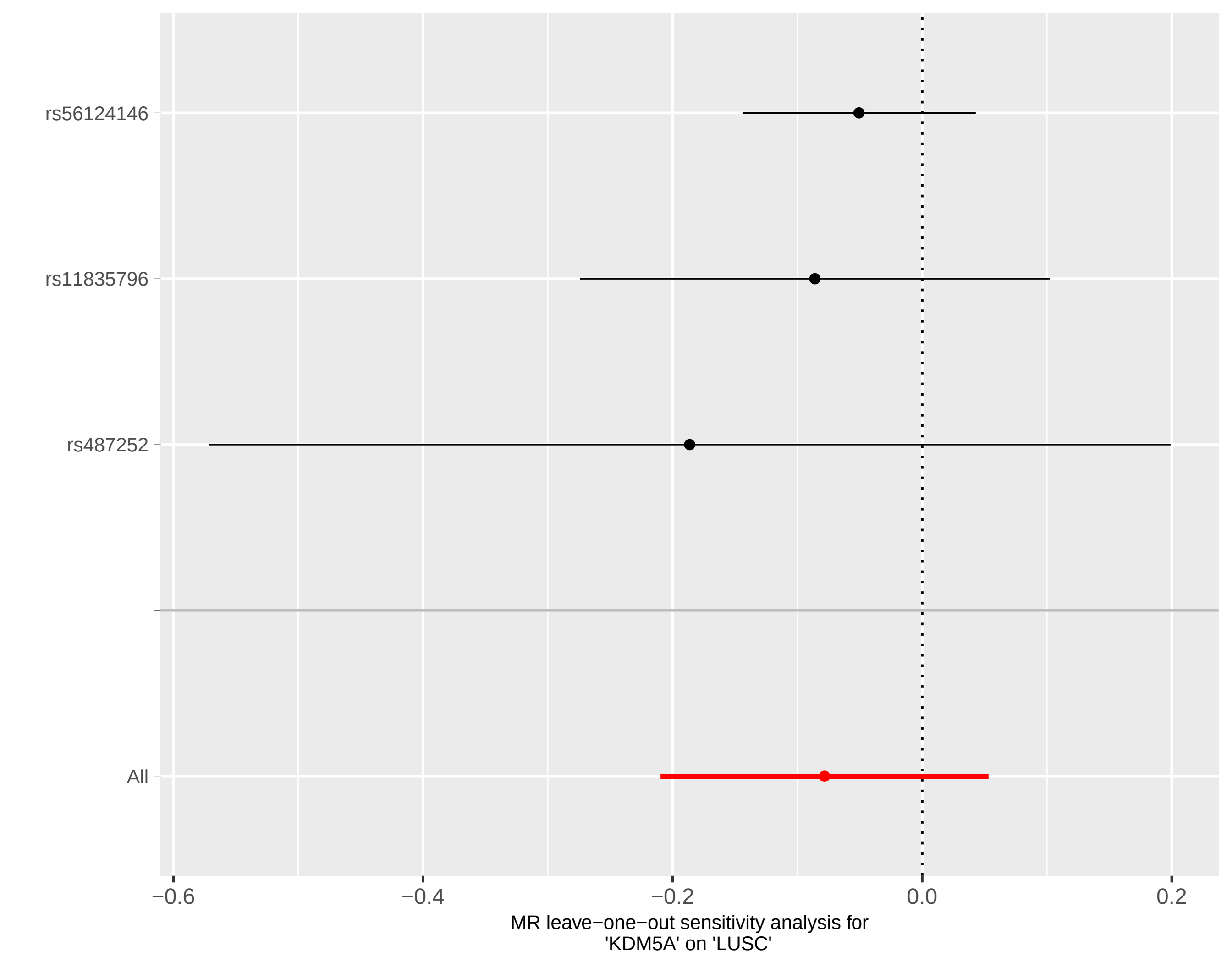

rs56124146
rs11835796
rs487252
All
−0.6
−0.4
−0.2
0.0
0.2
MR leave−one−out sensitivity analysis for
'KDM5A' on 'LUSC'

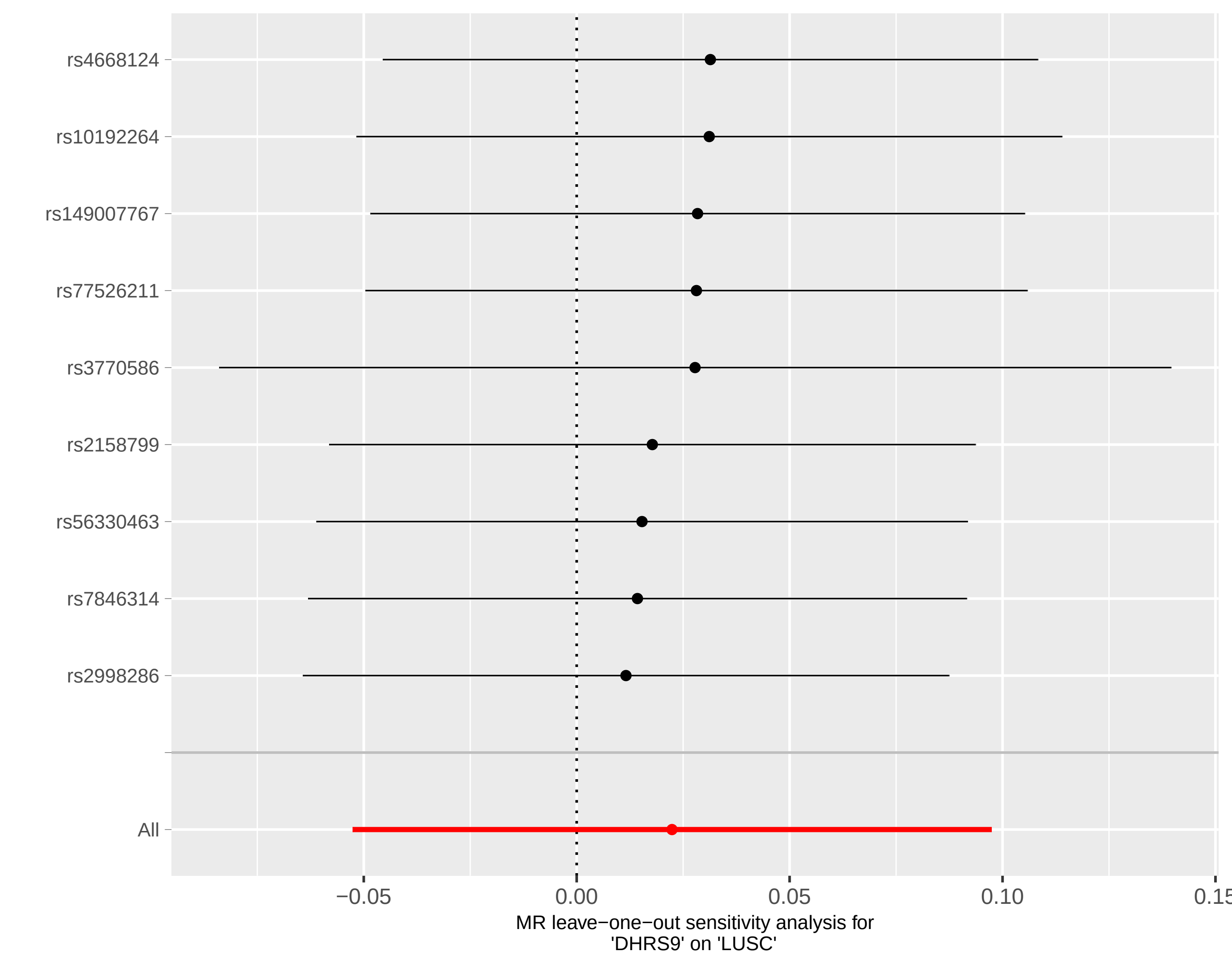

rs4668124
rs10192264
rs149007767
rs77526211
rs3770586
rs2158799
rs56330463
rs7846314
rs2998286
All
−0.05
0.00
0.05
0.10
0.15
MR leave−one−out sensitivity analysis for
'DHRS9' on 'LUSC'

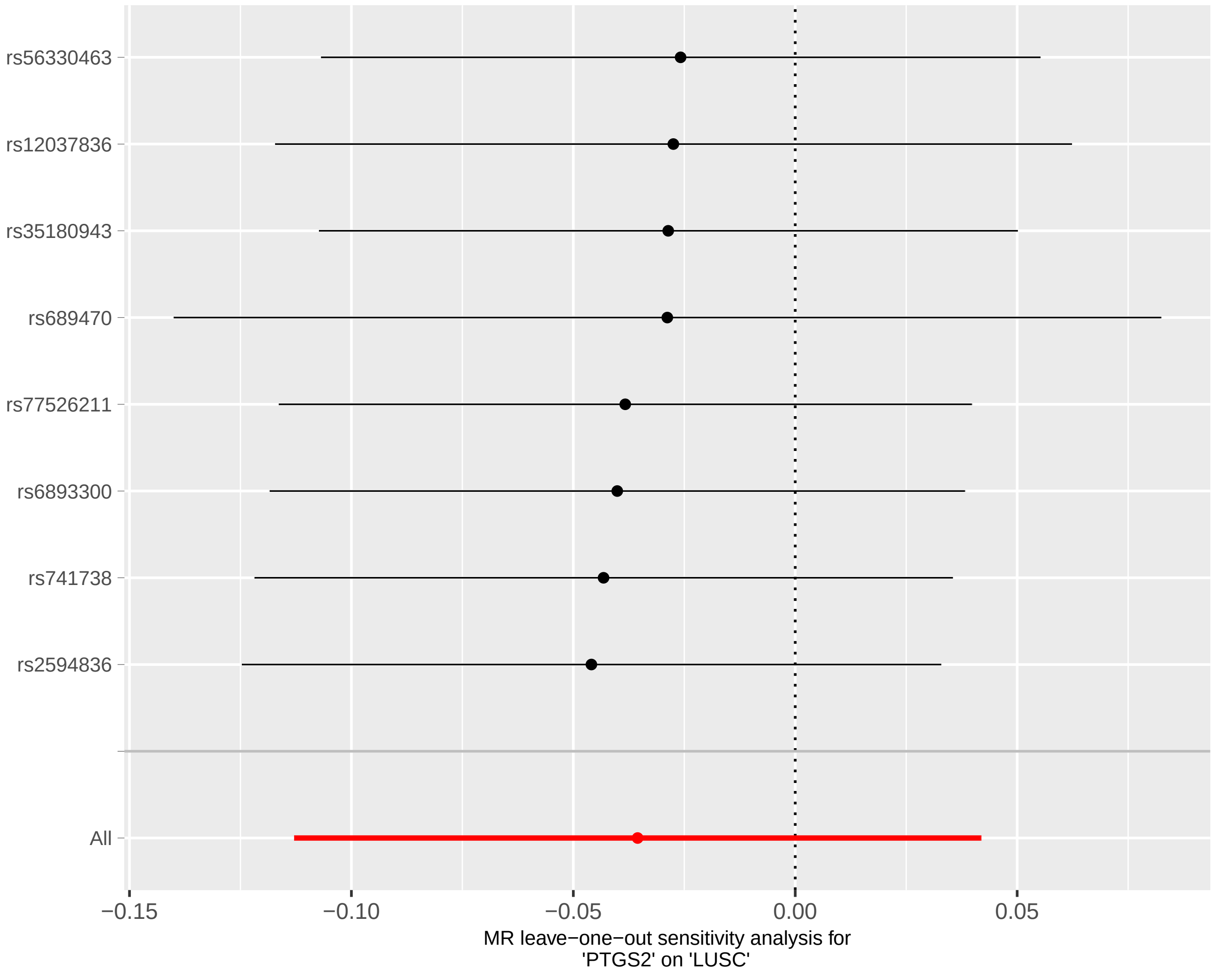

rs56330463
rs12037836
rs35180943
rs689470
rs77526211
rs6893300
rs741738
rs2594836
All
−0.15
−0.10
−0.05
0.00
0.05
MR leave−one−out sensitivity analysis for
'PTGS2' on 'LUSC'

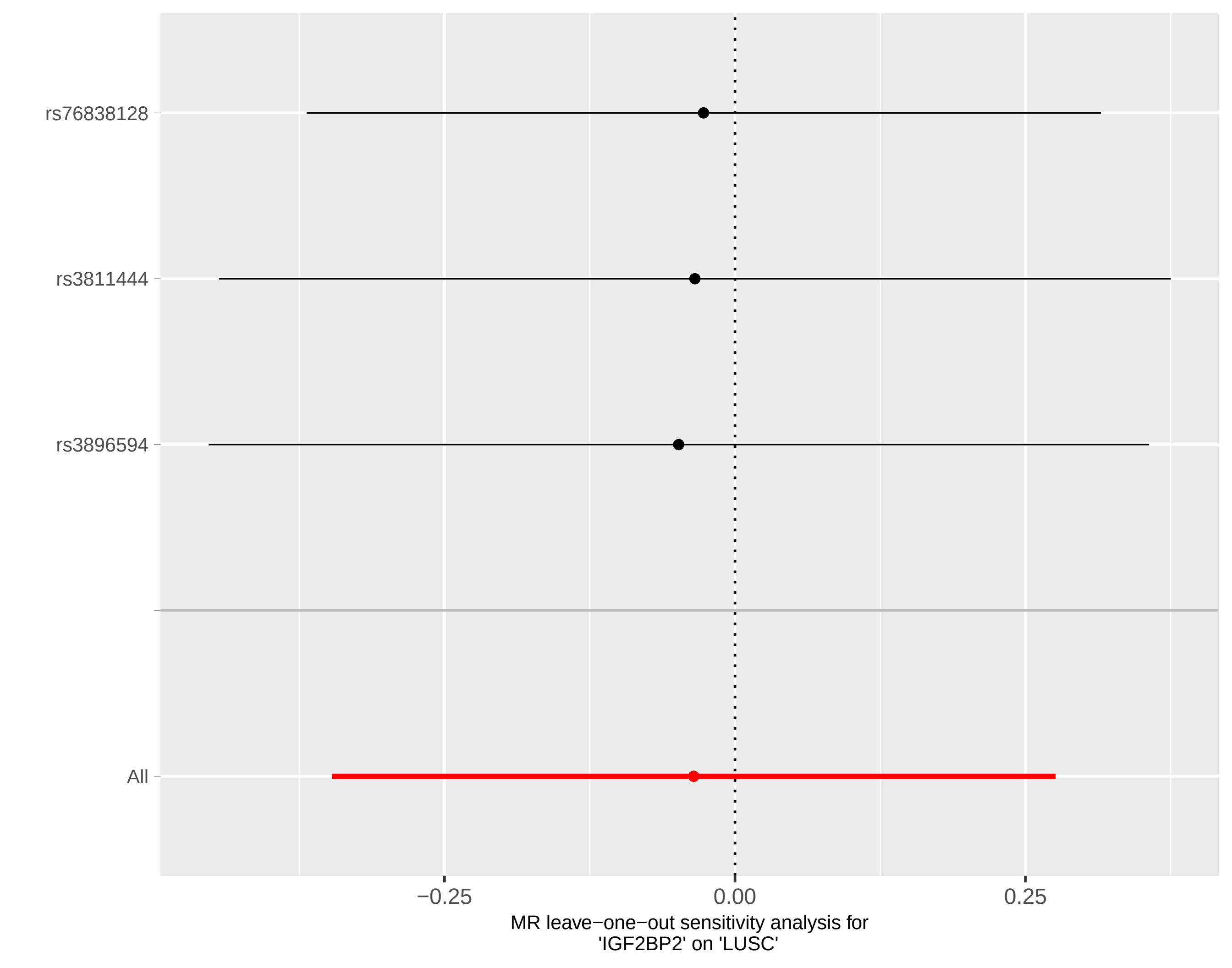

rs76838128
rs3811444
rs3896594
All
−0.25
0.00
0.25
MR leave−one−out sensitivity analysis for
'IGF2BP2' on 'LUSC'

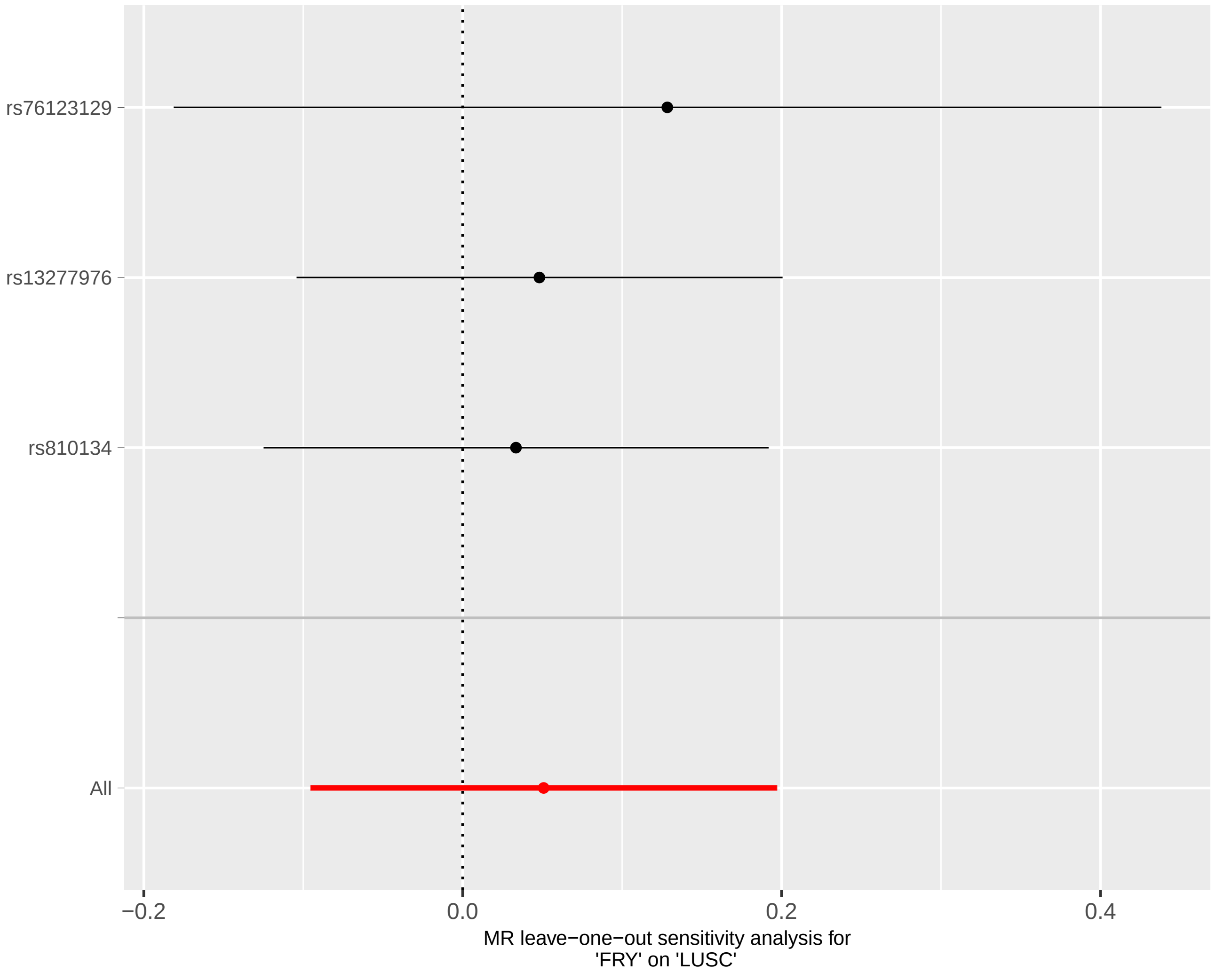
rs76123129
rs13277976
rs810134
All
−0.2
0.0
0.2
0.4
MR leave−one−out sensitivity analysis for
'FRY' on 'LUSC'

Insufficient number of SNPs

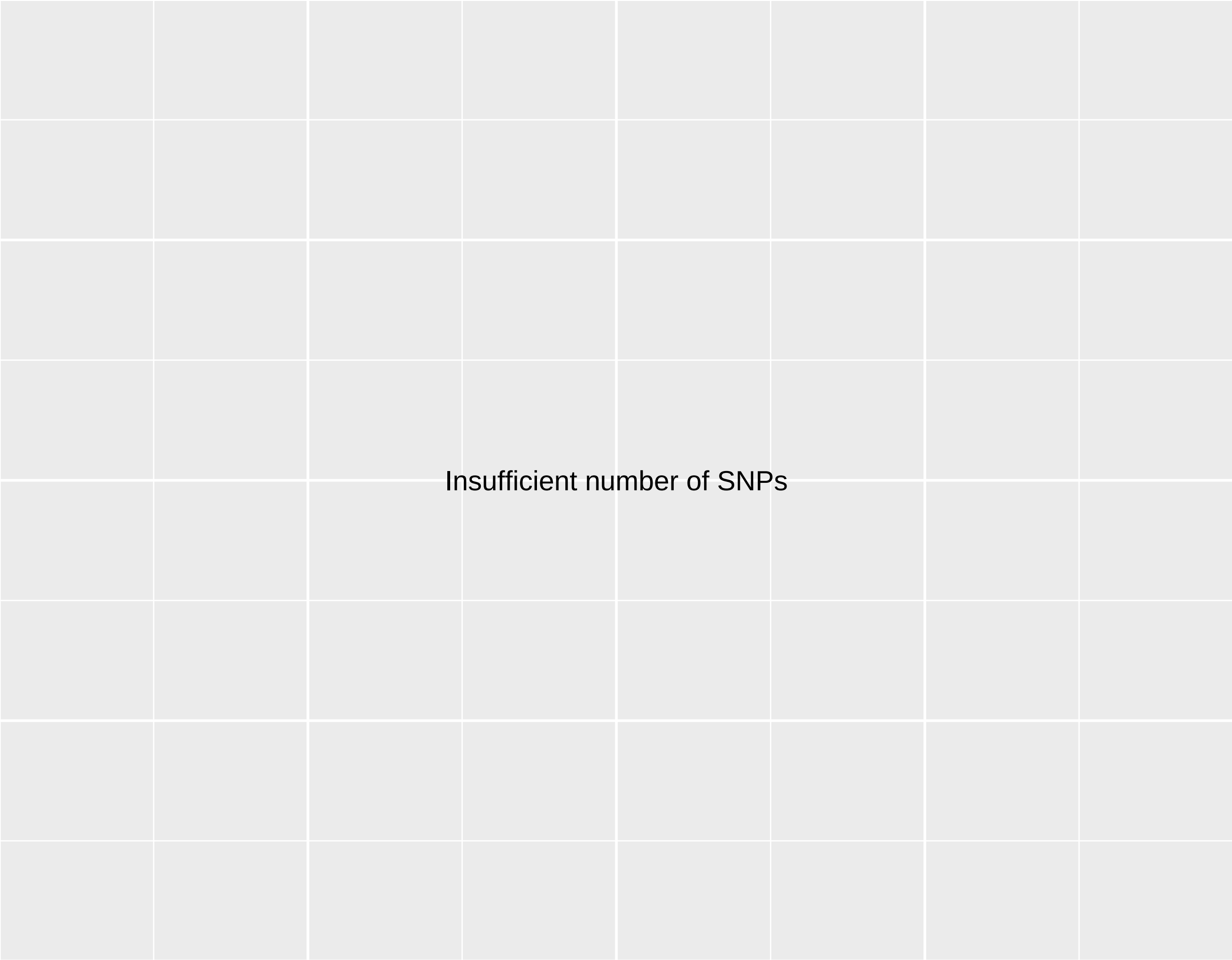
Insufficient number of SNPs

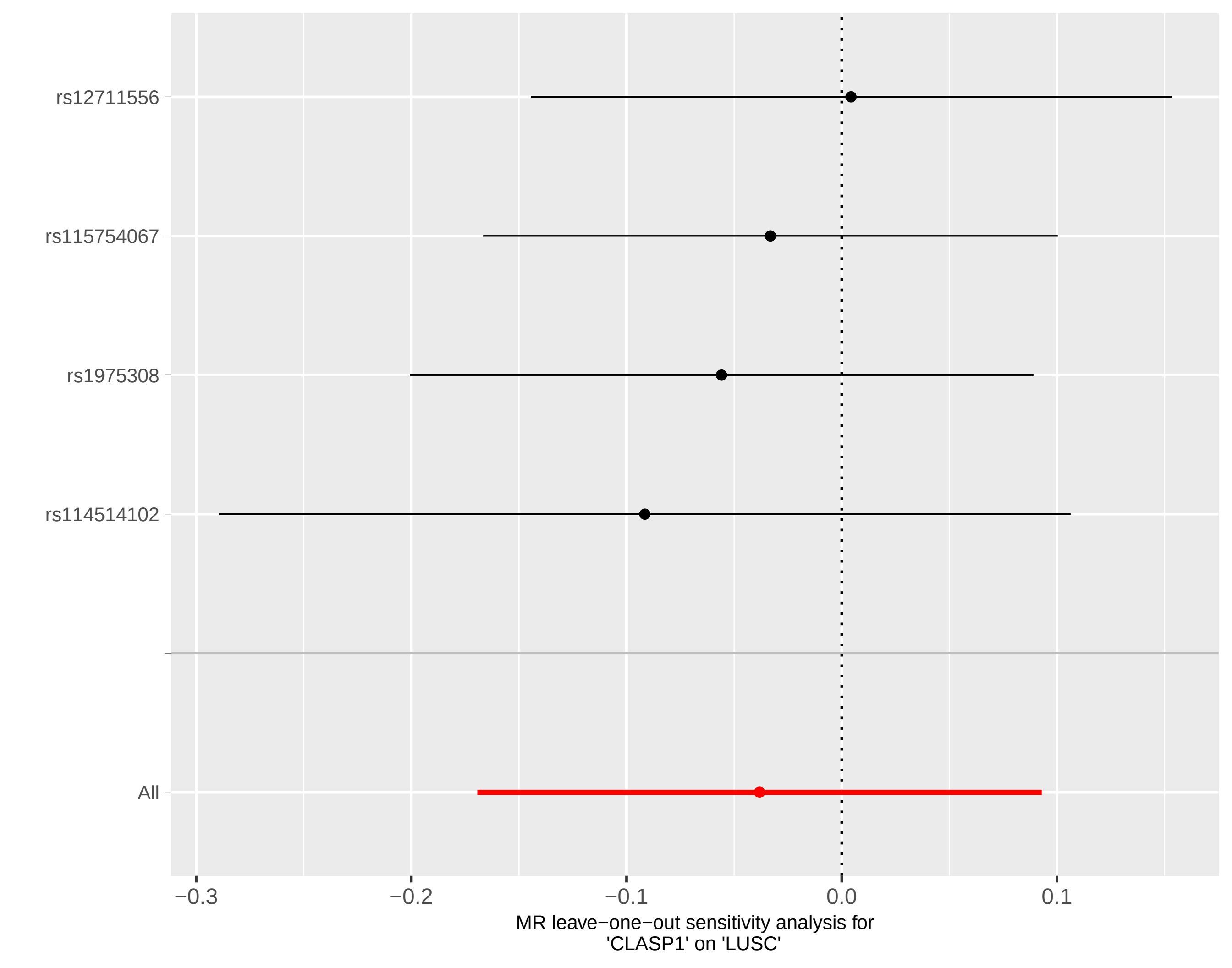
rs12711556
rs115754067
rs1975308
rs114514102
All
−0.3
−0.2
−0.1
0.0
0.1
MR leave−one−out sensitivity analysis for
'CLASP1' on 'LUSC'

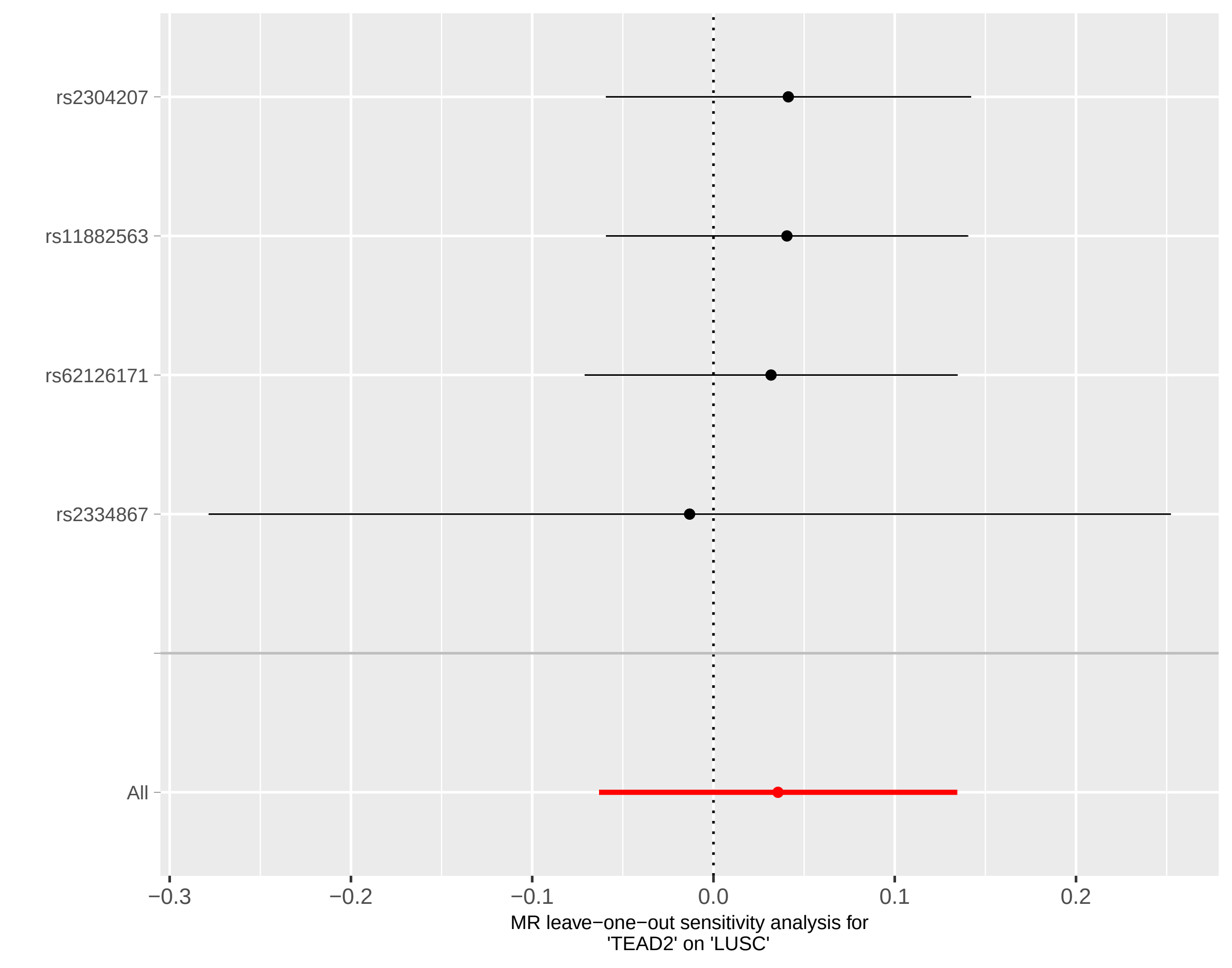

rs2304207
rs11882563
rs62126171
rs2334867
All
−0.3
−0.2
−0.1
0.0
0.1
0.2
MR leave−one−out sensitivity analysis for
'TEAD2' on 'LUSC'

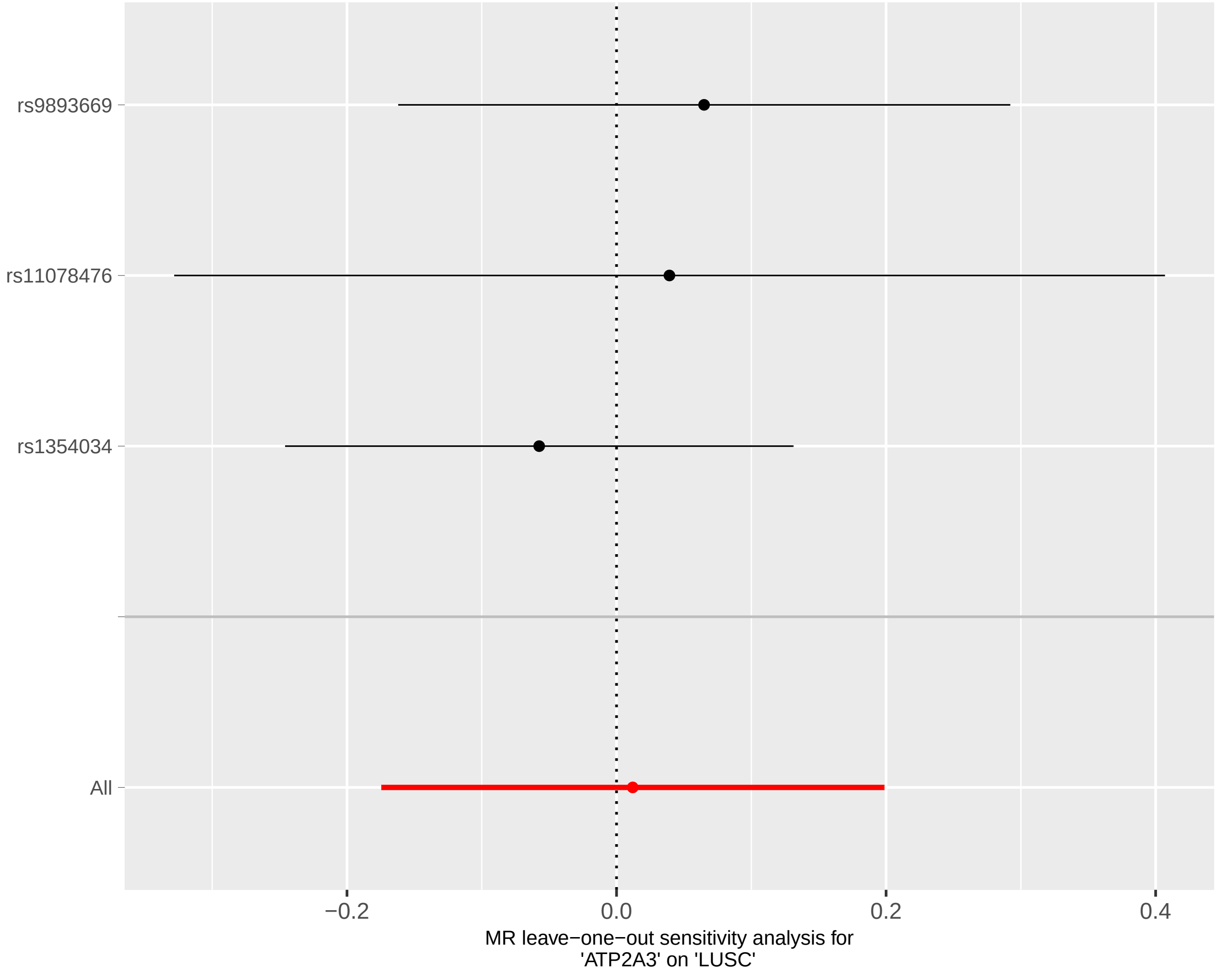

rs9893669
rs11078476
rs1354034
All
−0.2
0.0
0.2
0.4
MR leave−one−out sensitivity analysis for
'ATP2A3' on 'LUSC'

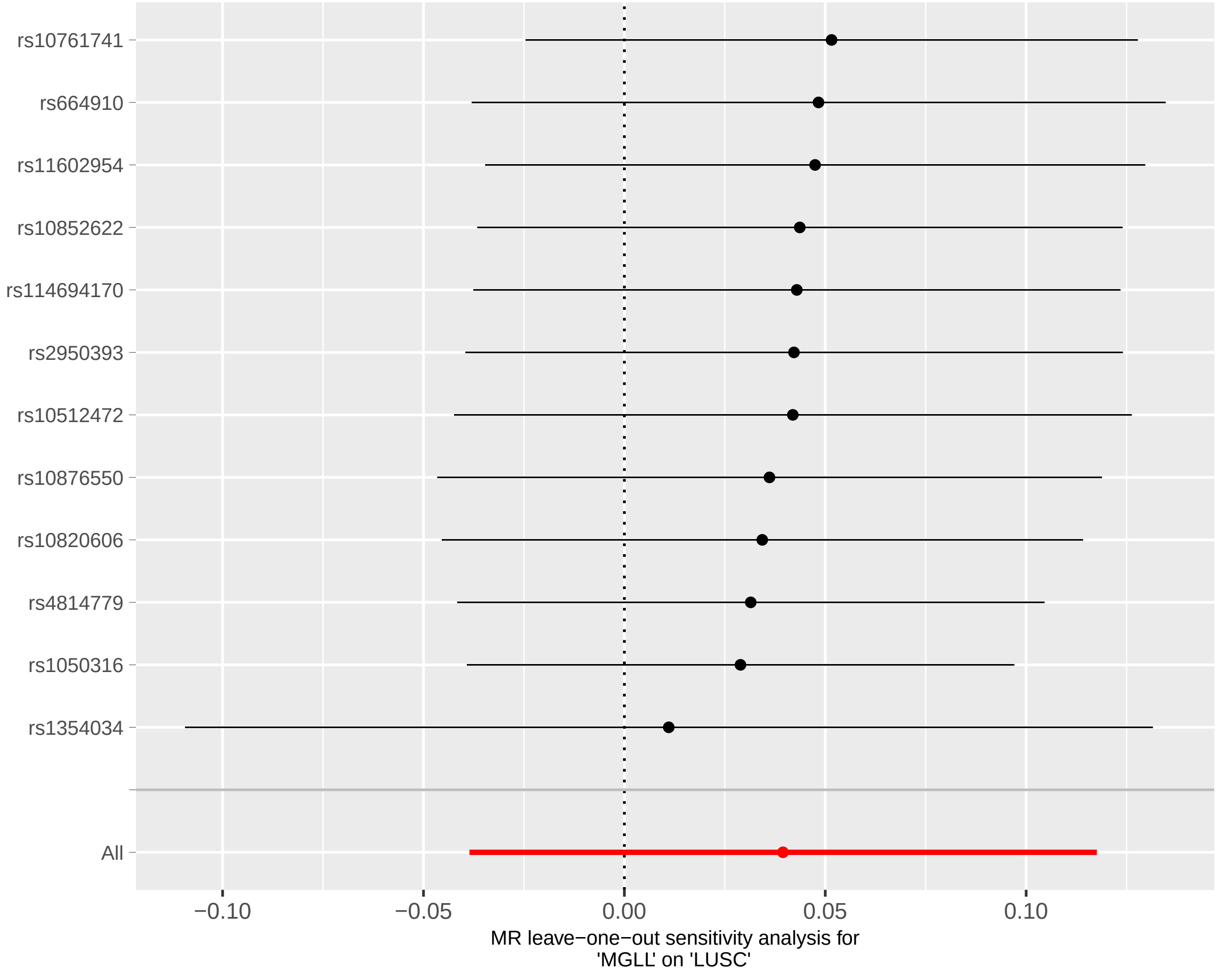
rs10761741
rs664910
rs11602954
rs10852622
rs114694170
rs2950393
rs10512472
rs10876550
rs10820606
rs4814779
rs1050316
rs1354034
All
−0.10
−0.05
0.00
0.05
0.10
MR leave−one−out sensitivity analysis for
'MGLL' on 'LUSC'

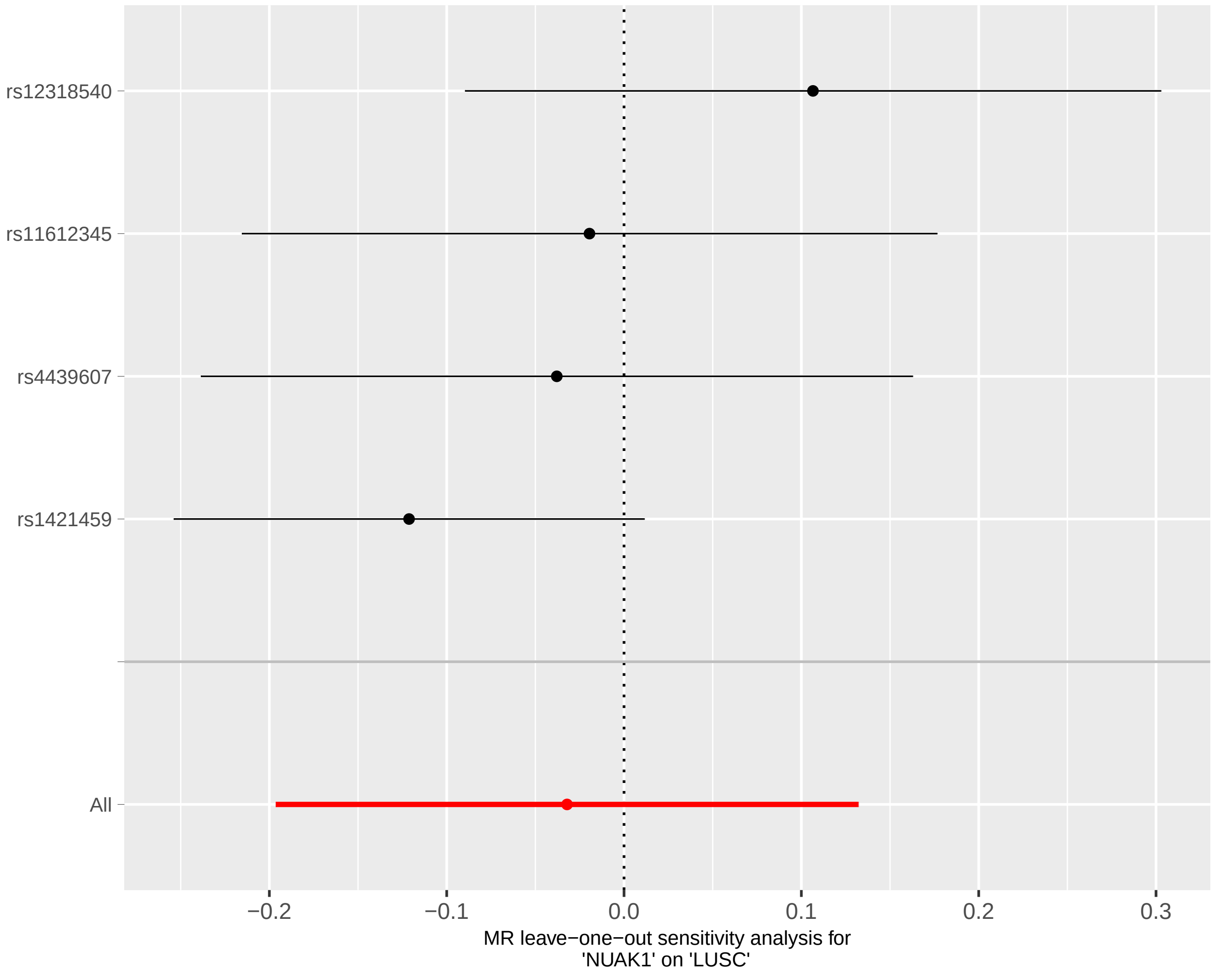

rs12318540
rs11612345
rs4439607
rs1421459
All
−0.2
−0.1
0.0
0.1
0.2
0.3
MR leave−one−out sensitivity analysis for
'NUAK1' on 'LUSC'

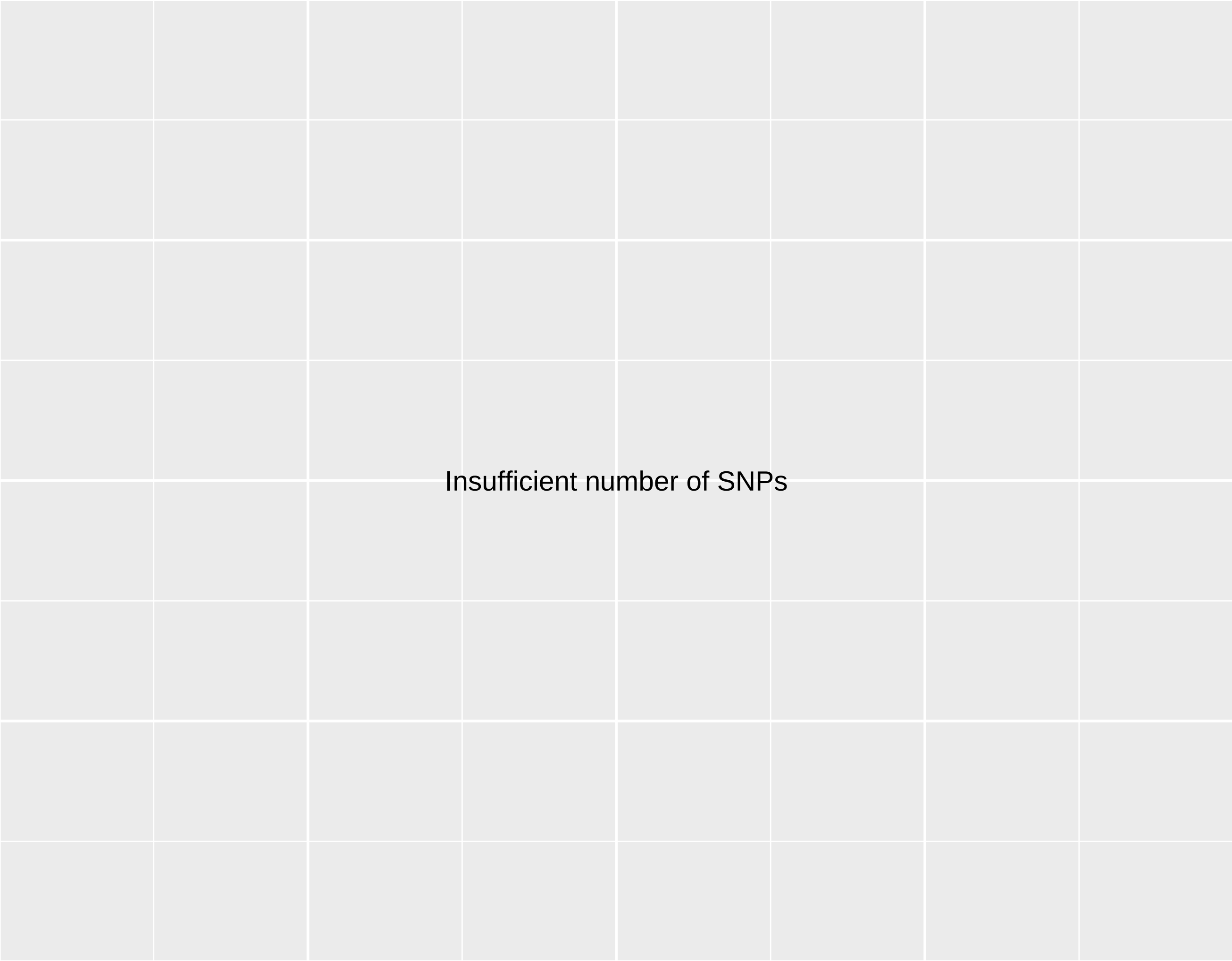
Insufficient number of SNPs

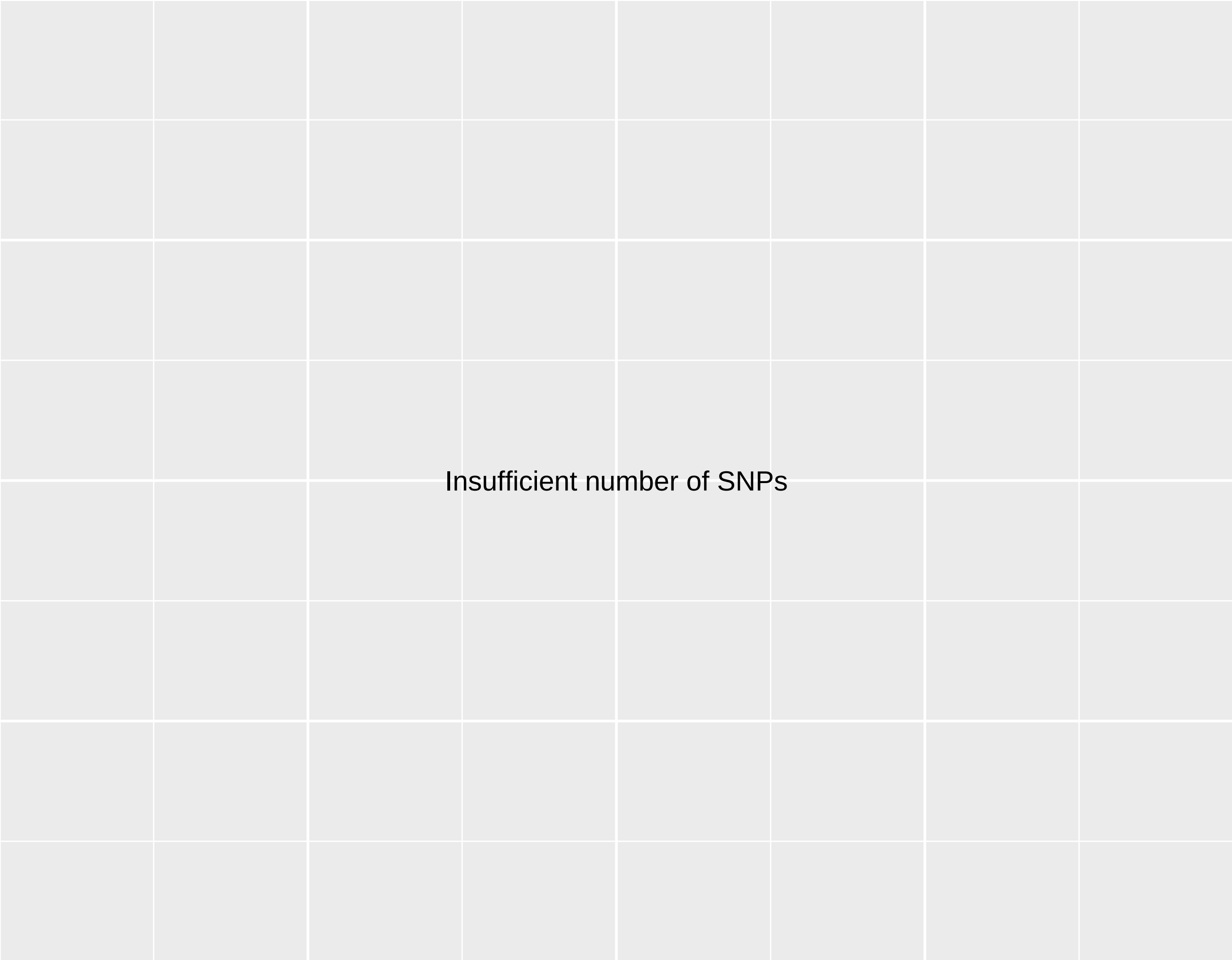
Insufficient number of SNPs

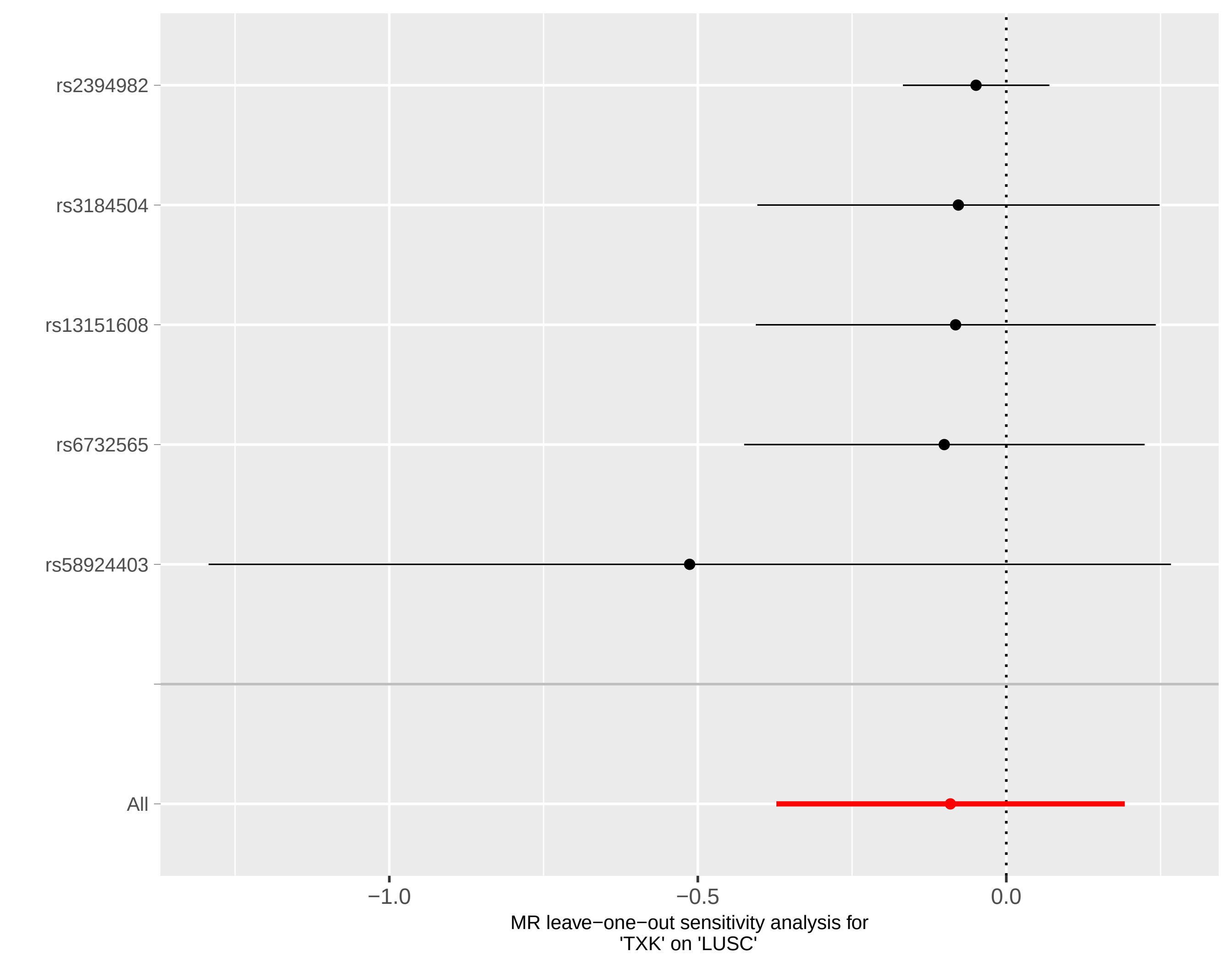

rs2394982
rs3184504
rs13151608
rs6732565
rs58924403
All
−1.0
−0.5
0.0
MR leave−one−out sensitivity analysis for
'TXK' on 'LUSC'

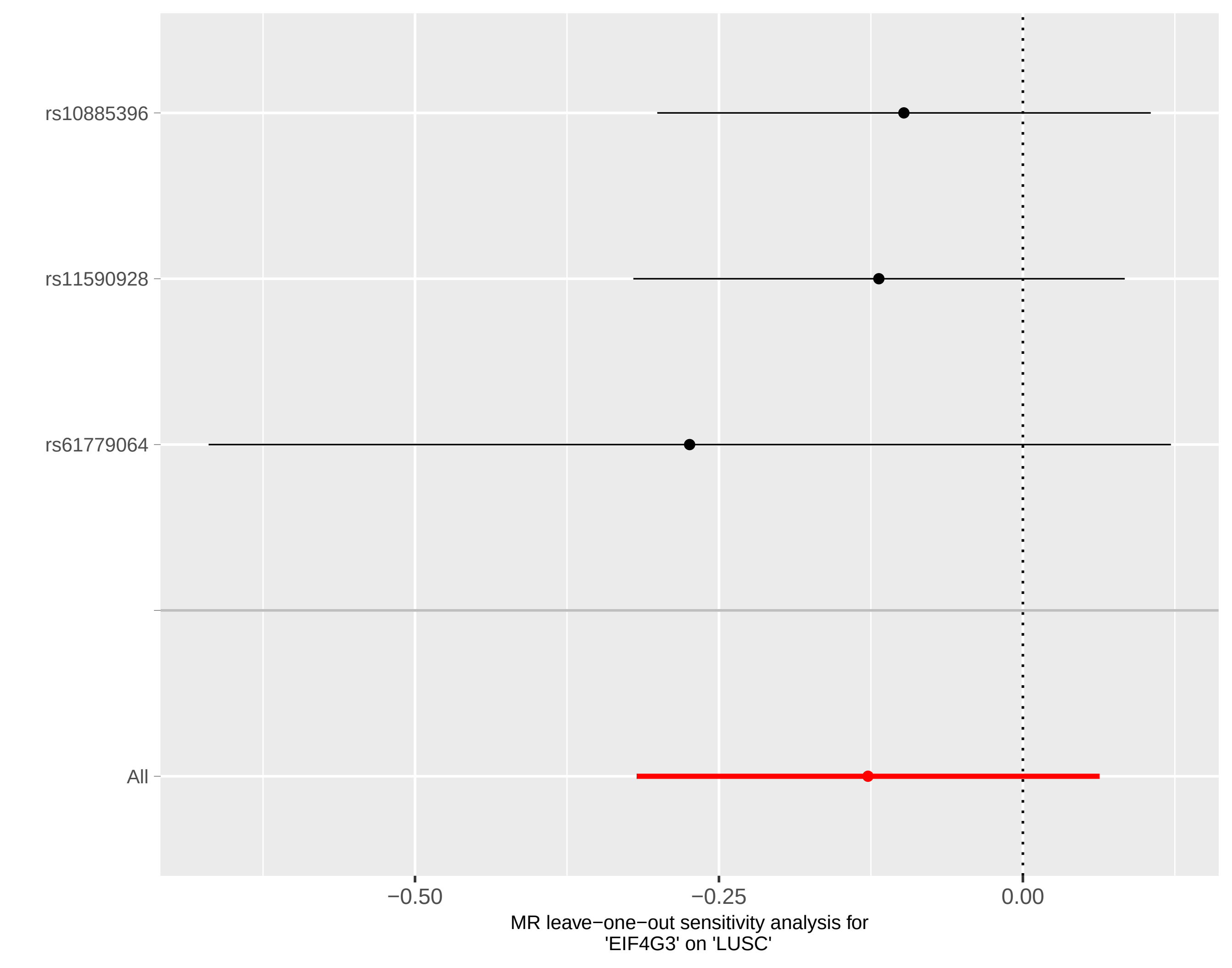
rs10885396
rs11590928
rs61779064
All
−0.50
−0.25
0.00
MR leave−one−out sensitivity analysis for
'EIF4G3' on 'LUSC'

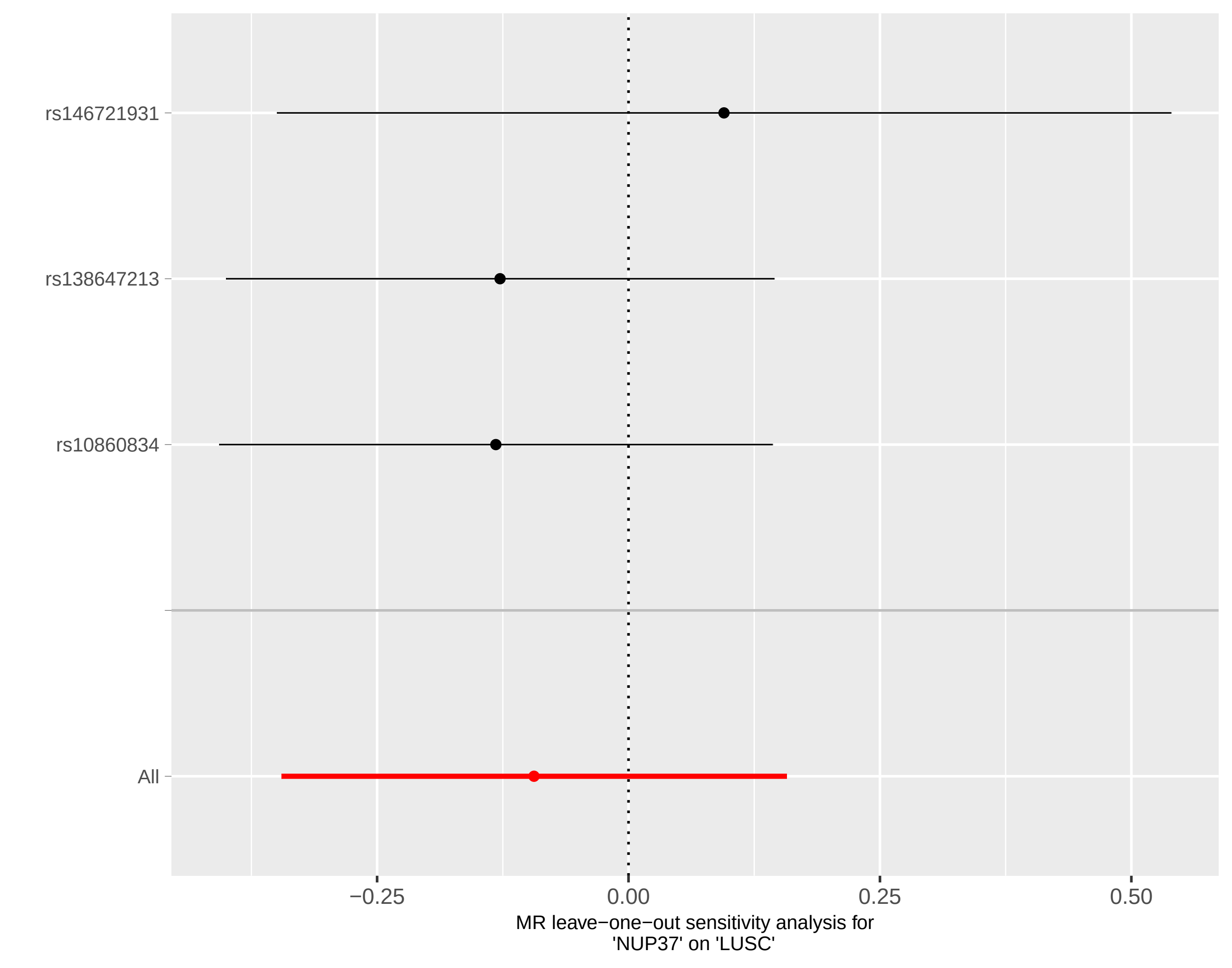
rs146721931
rs138647213
rs10860834
All
−0.25
0.00
0.25
0.50
MR leave−one−out sensitivity analysis for
'NUP37' on 'LUSC'

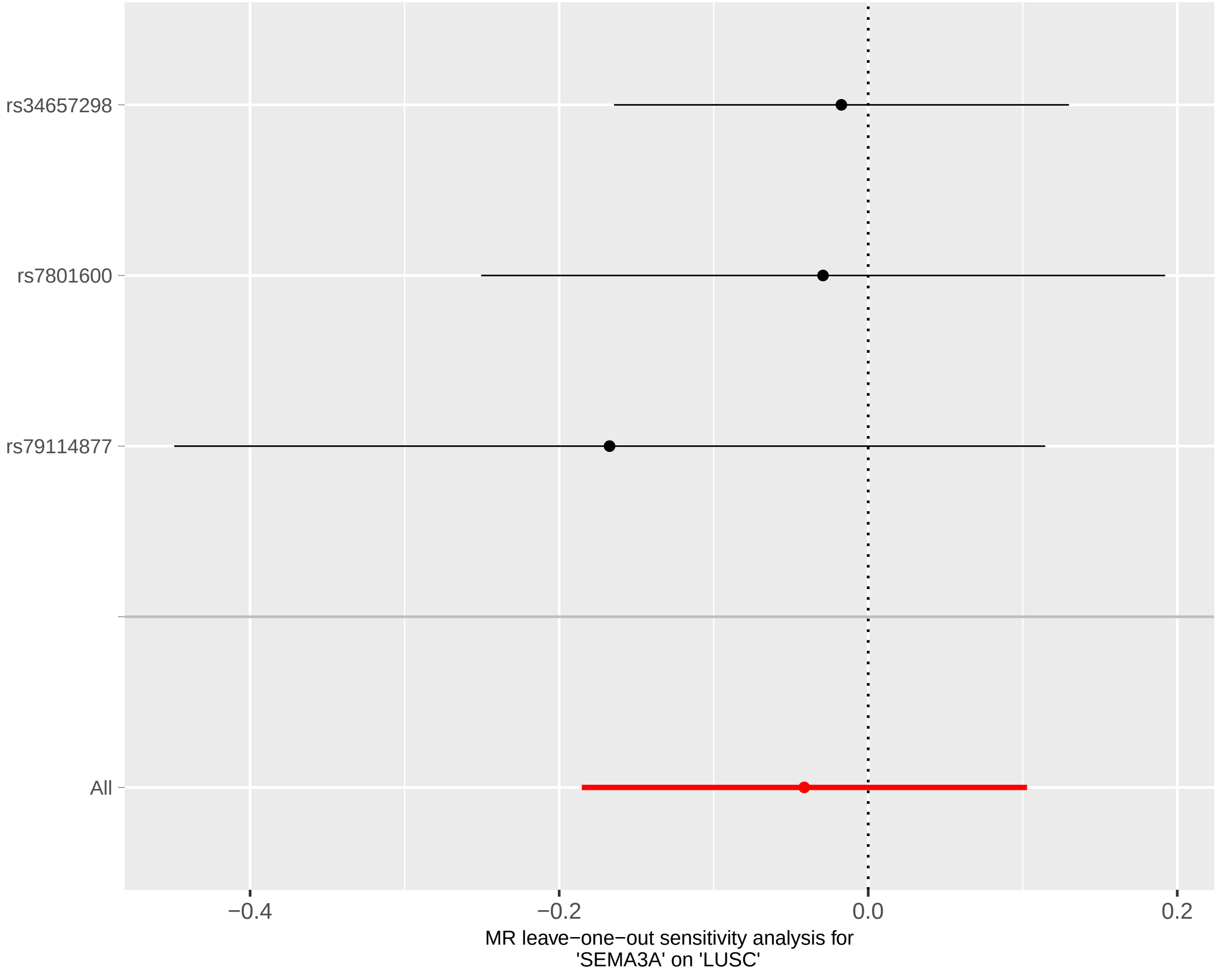
rs34657298
rs7801600
rs79114877
All
−0.4
−0.2
0.0
0.2
MR leave−one−out sensitivity analysis for
'SEMA3A' on 'LUSC'

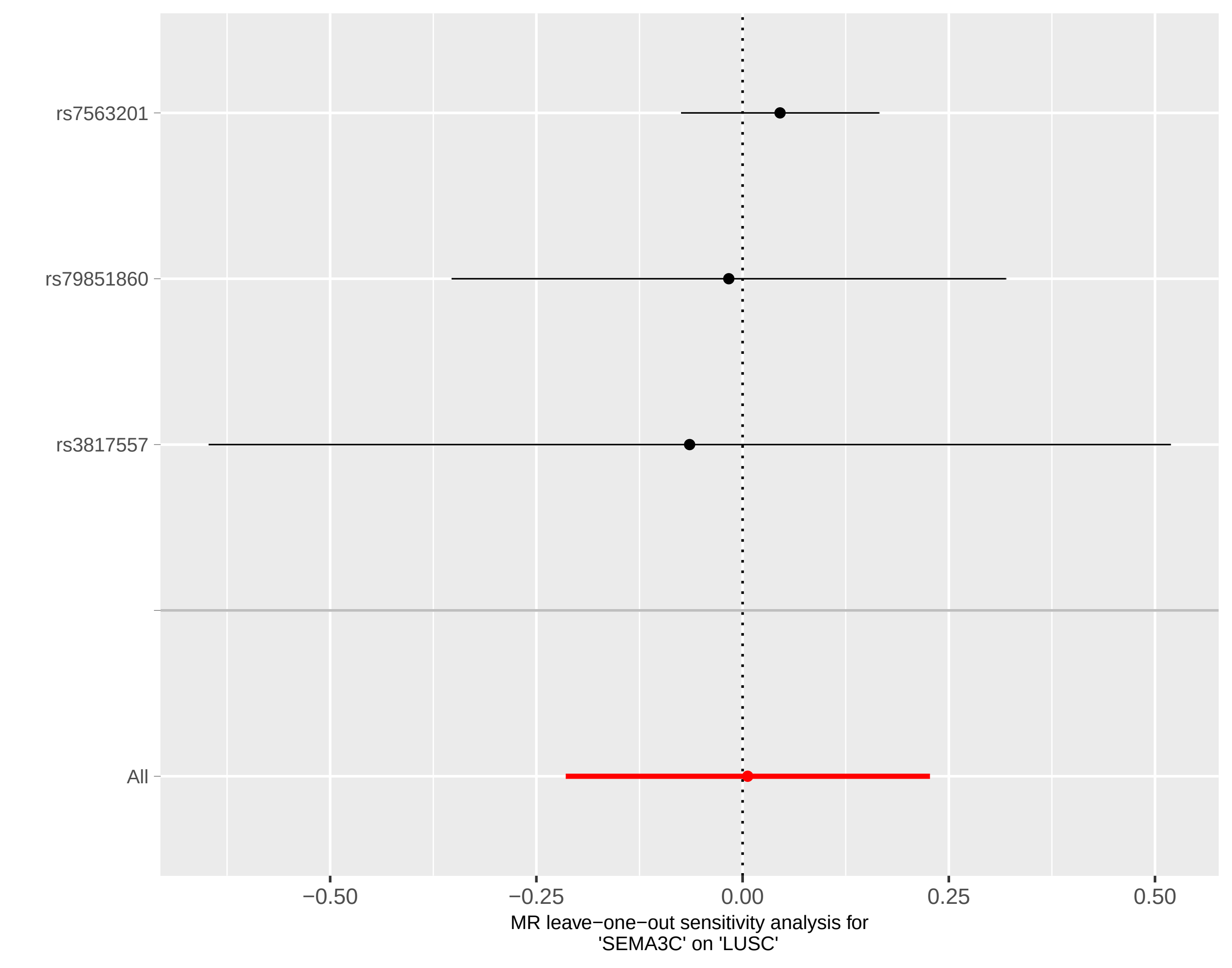
rs7563201
rs79851860
rs3817557
All
−0.50
−0.25
0.00
0.25
0.50
MR leave−one−out sensitivity analysis for
'SEMA3C' on 'LUSC'

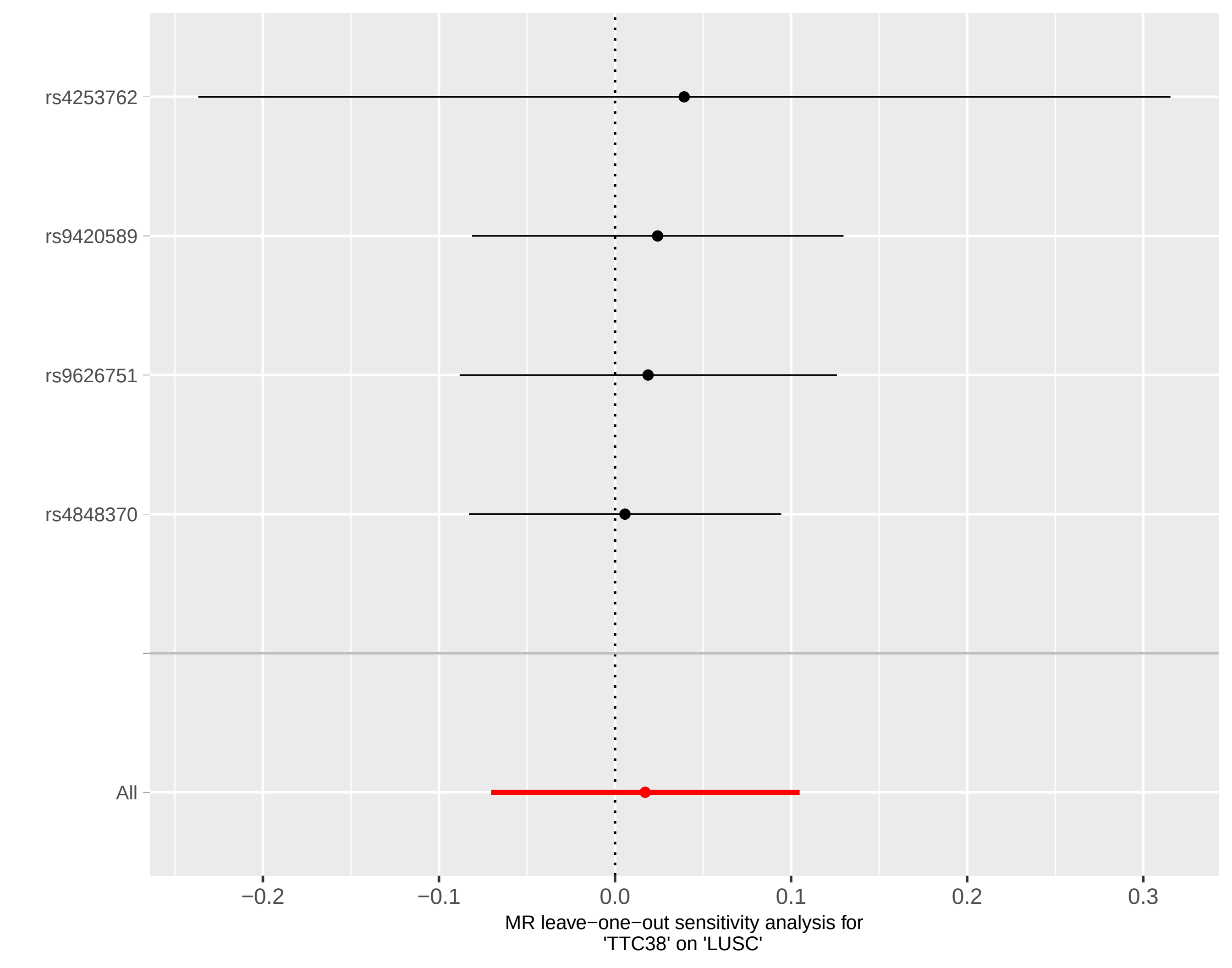
rs4253762
rs9420589
rs9626751
rs4848370
All
−0.2
−0.1
0.0
0.1
0.2
0.3
MR leave−one−out sensitivity analysis for
'TTC38' on 'LUSC'

Insufficient number of SNPs

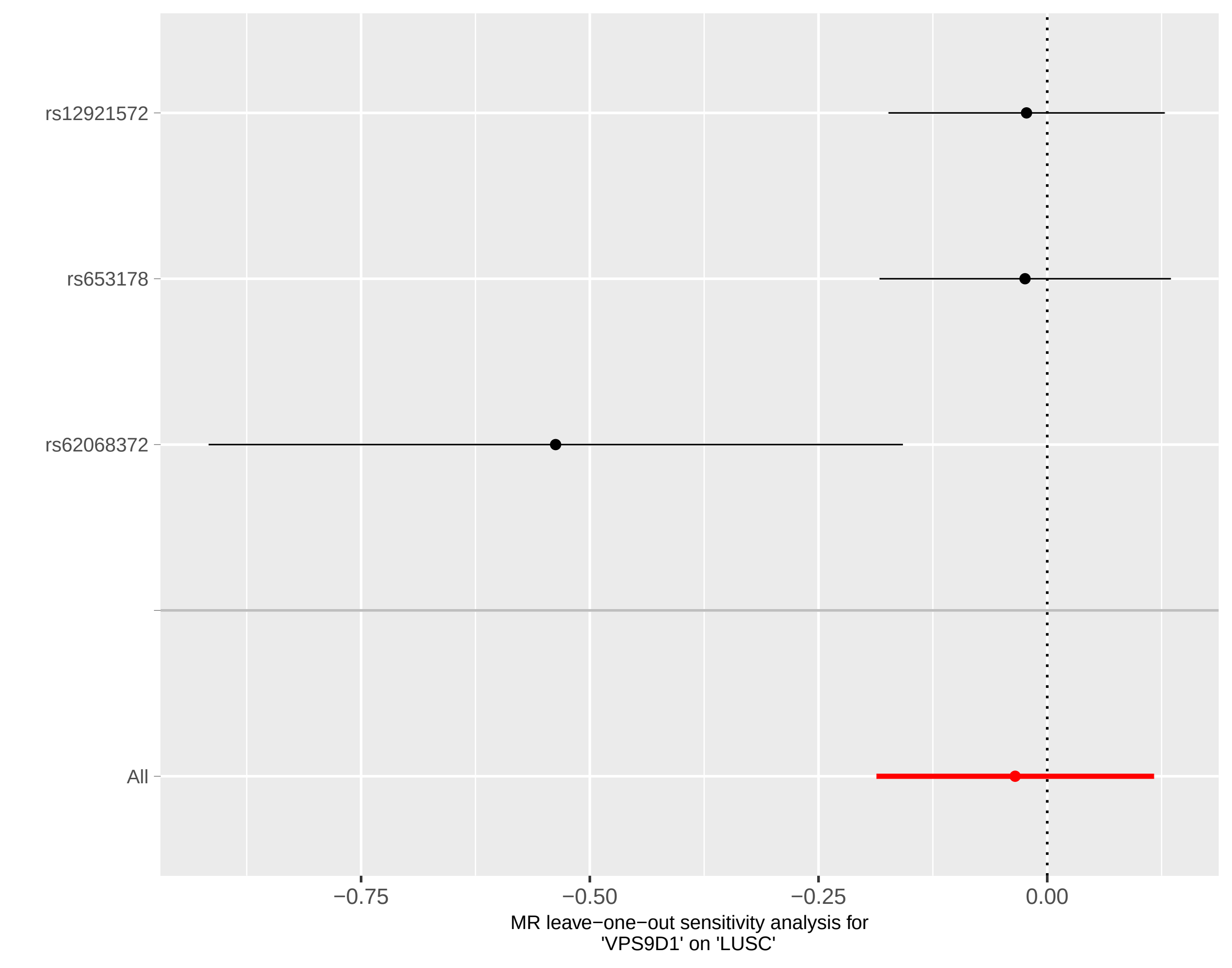
rs12921572
rs653178
rs62068372
All
−0.75
−0.50
−0.25
0.00
MR leave−one−out sensitivity analysis for
'VPS9D1' on 'LUSC'

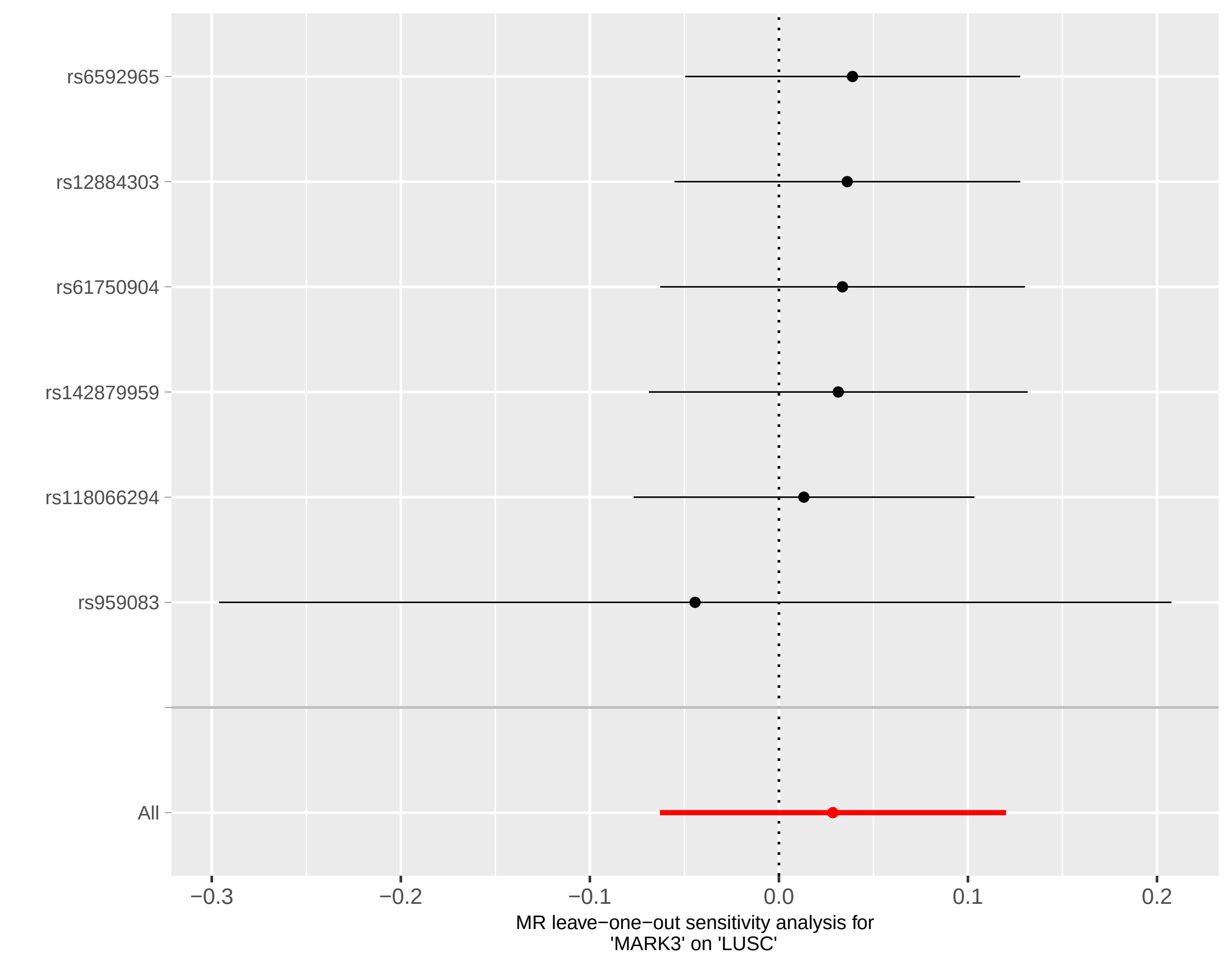
rs6592965
rs12884303
rs61750904
rs142879959
rs118066294
rs959083
All
−0.3
−0.2
−0.1
0.0
0.1
0.2
MR leave−one−out sensitivity analysis for
'MARK3' on 'LUSC'

Insufficient number of SNPs

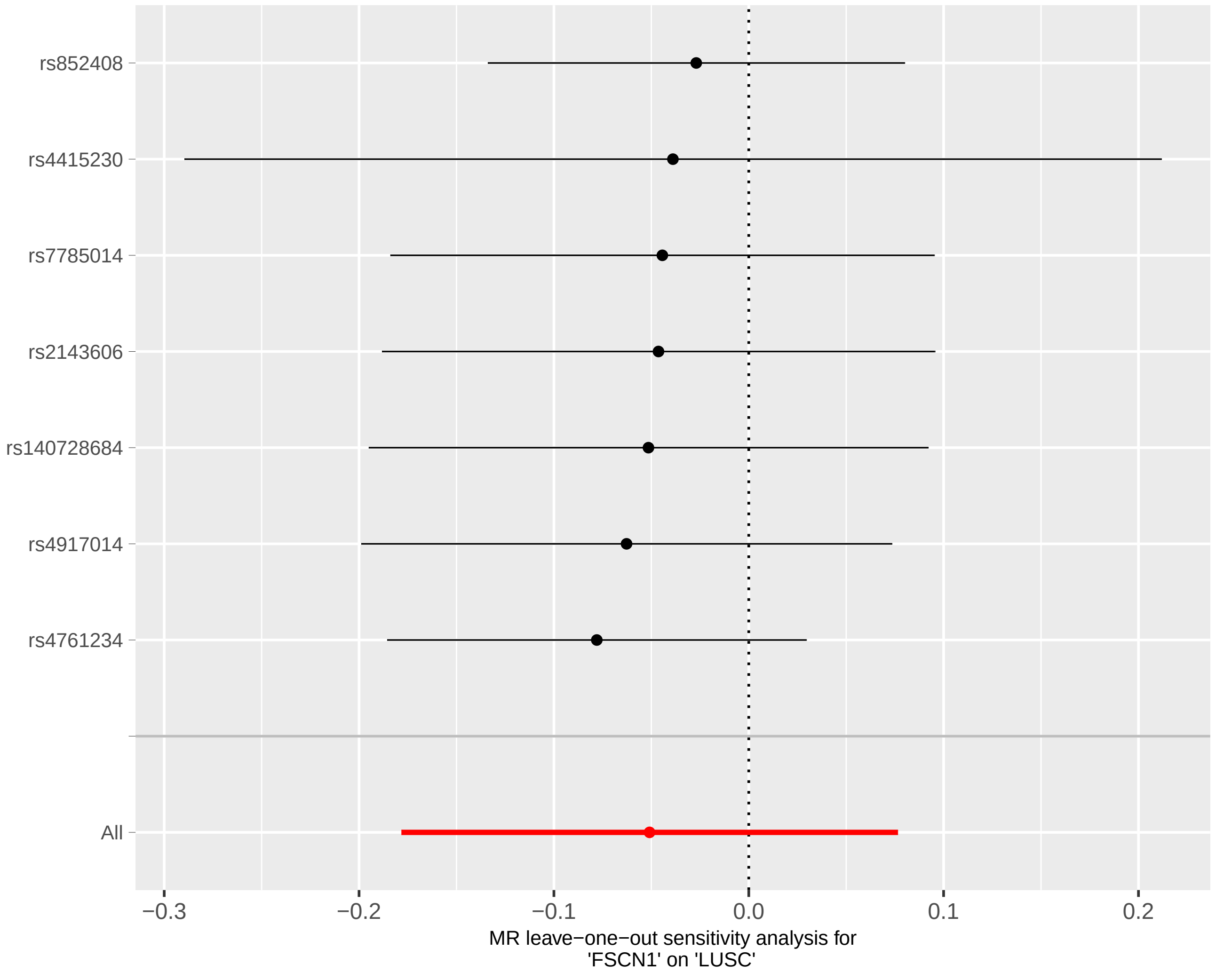

rs852408
rs4415230
rs7785014
rs2143606
rs140728684
rs4917014
rs4761234
All
−0.3
−0.2
−0.1
0.0
0.1
0.2
MR leave−one−out sensitivity analysis for
'FSCN1' on 'LUSC'

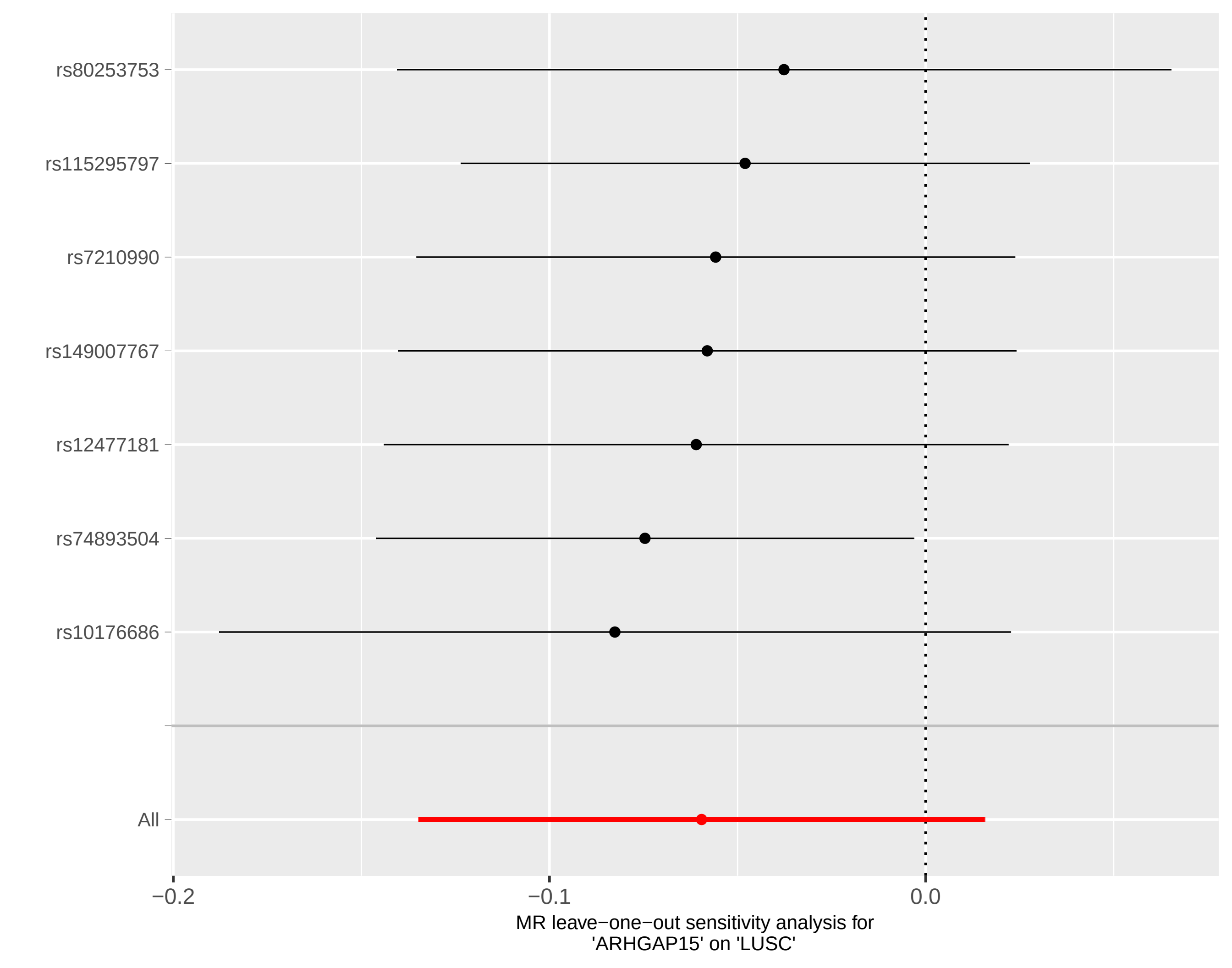
rs80253753
rs115295797
rs7210990
rs149007767
rs12477181
rs74893504
rs10176686
All
−0.2
−0.1
0.0
MR leave−one−out sensitivity analysis for
'ARHGAP15' on 'LUSC'

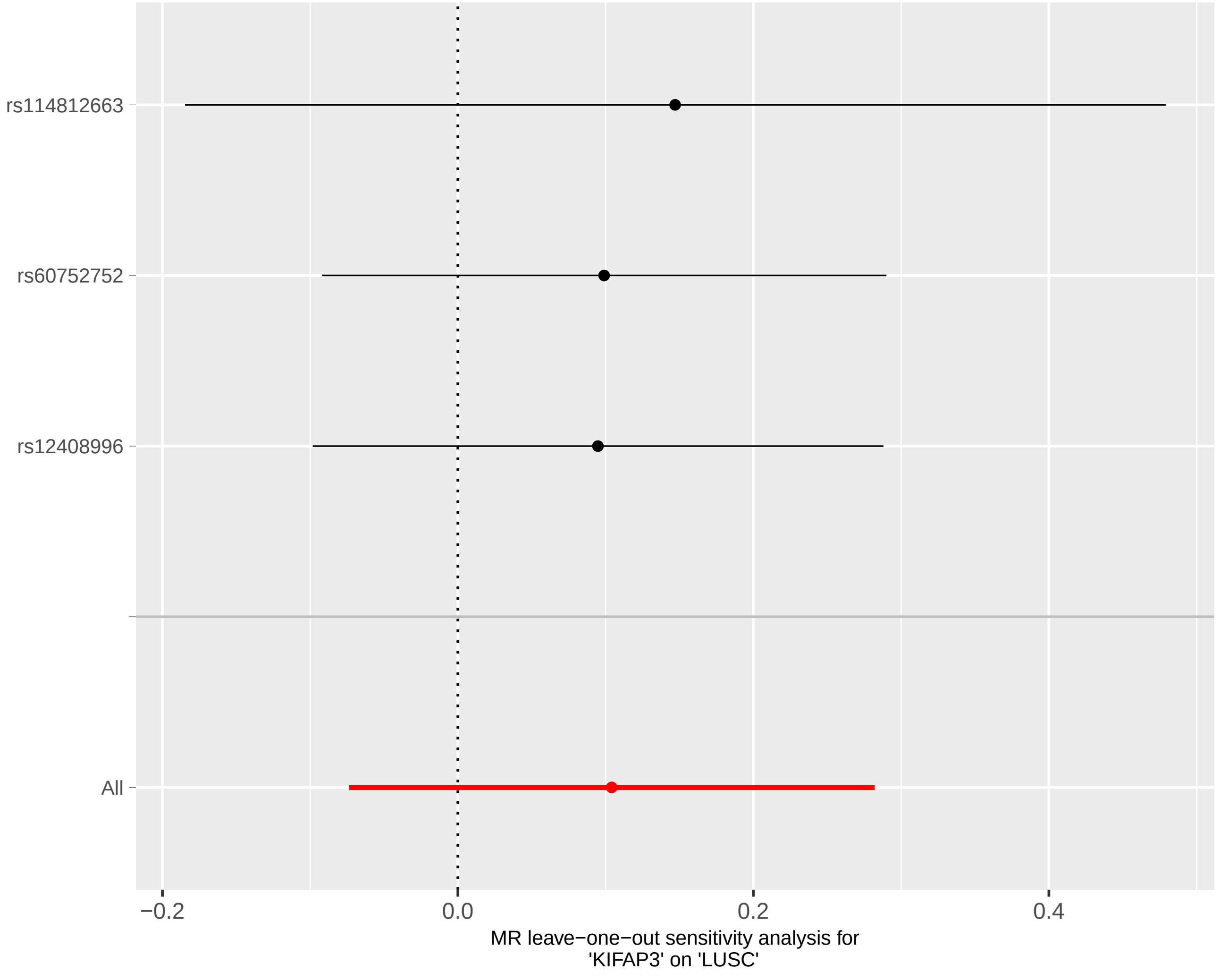
rs114812663
rs60752752
rs12408996
All
−0.2
0.0
0.2
0.4
MR leave−one−out sensitivity analysis for
'KIFAP3' on 'LUSC'

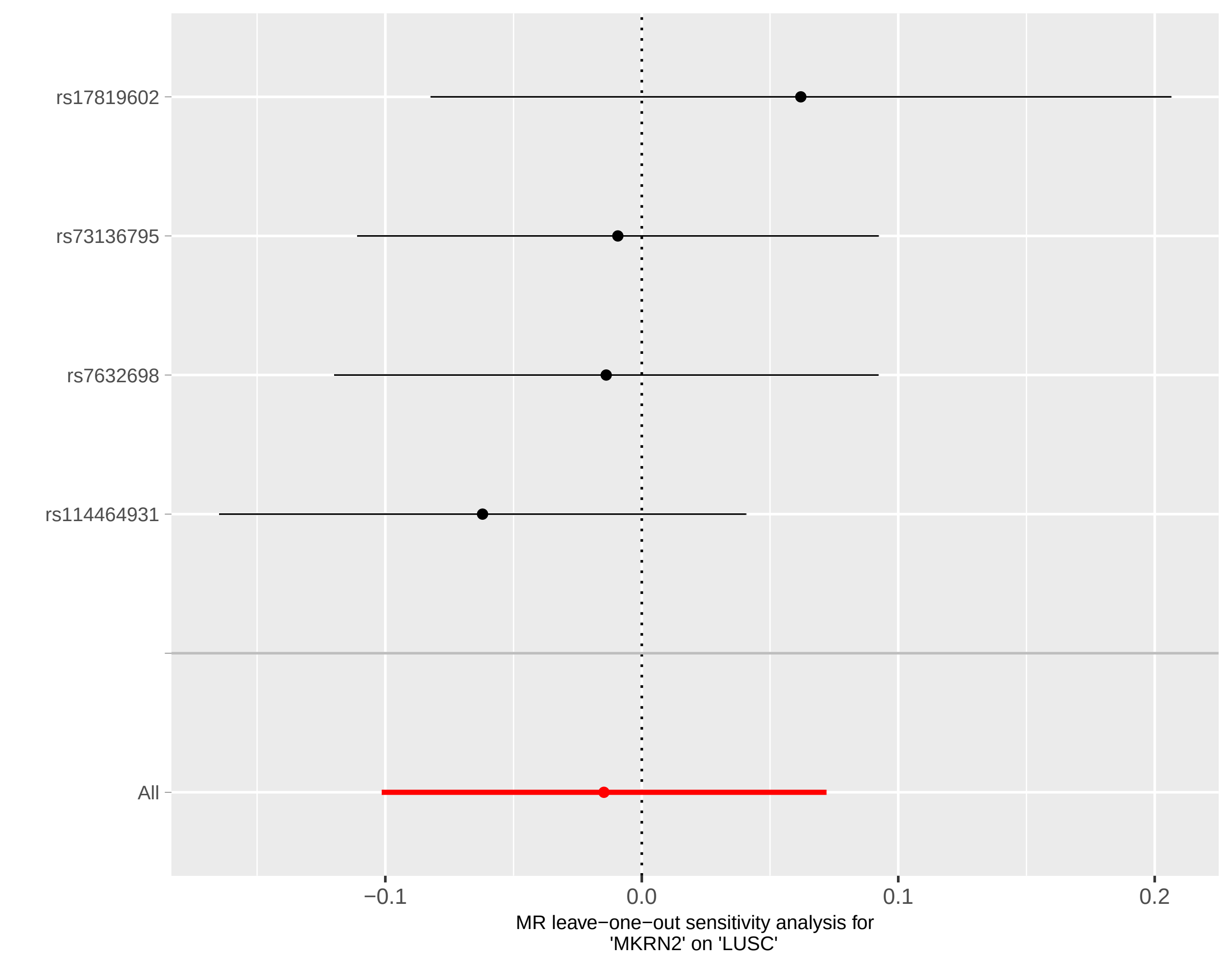
rs17819602
rs73136795
rs7632698
rs114464931
All
−0.1
0.0
0.1
0.2
MR leave−one−out sensitivity analysis for
'MKRN2' on 'LUSC'

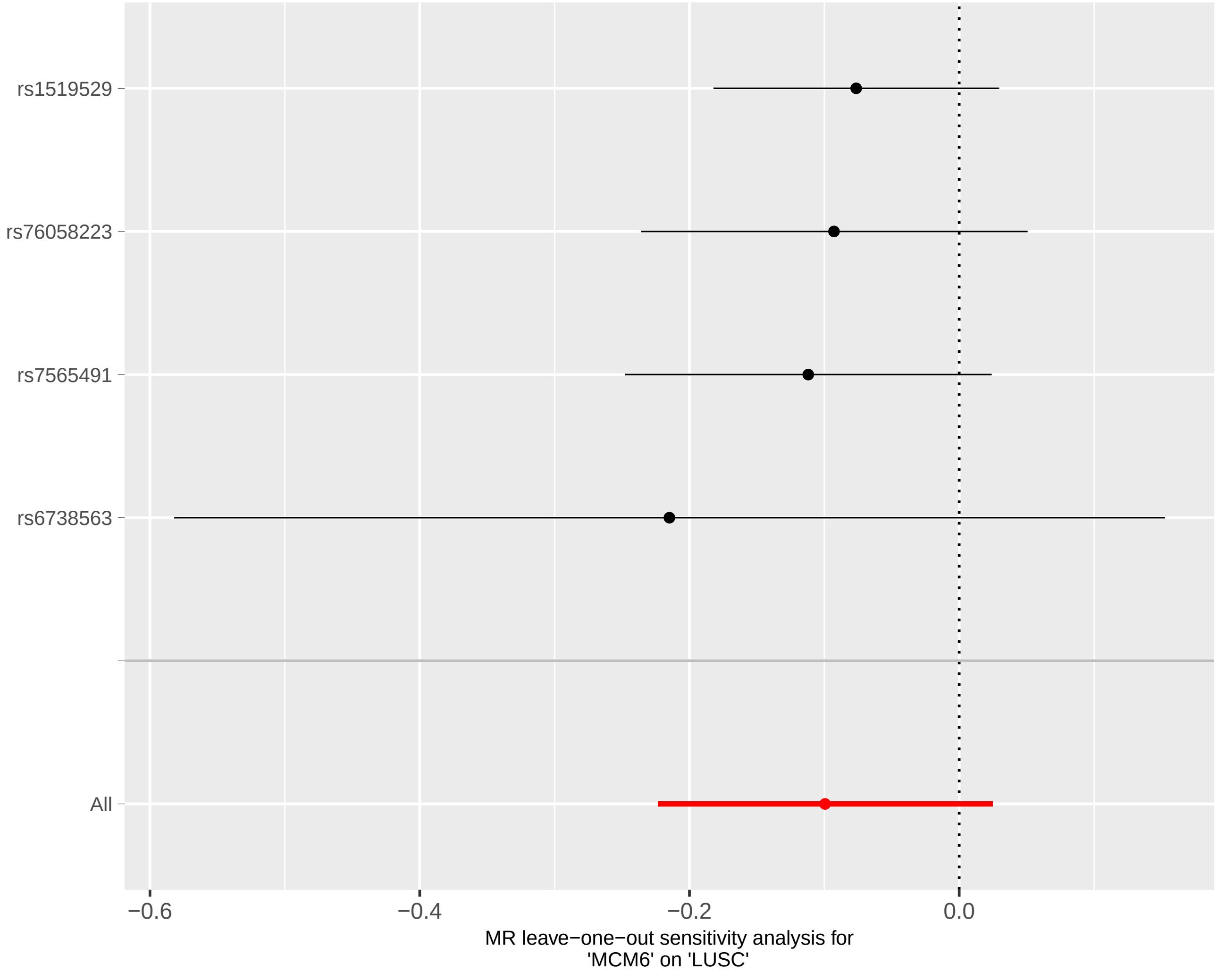

rs1519529
rs76058223
rs7565491
rs6738563
All
−0.6
−0.4
−0.2
0.0
MR leave−one−out sensitivity analysis for
'MCM6' on 'LUSC'

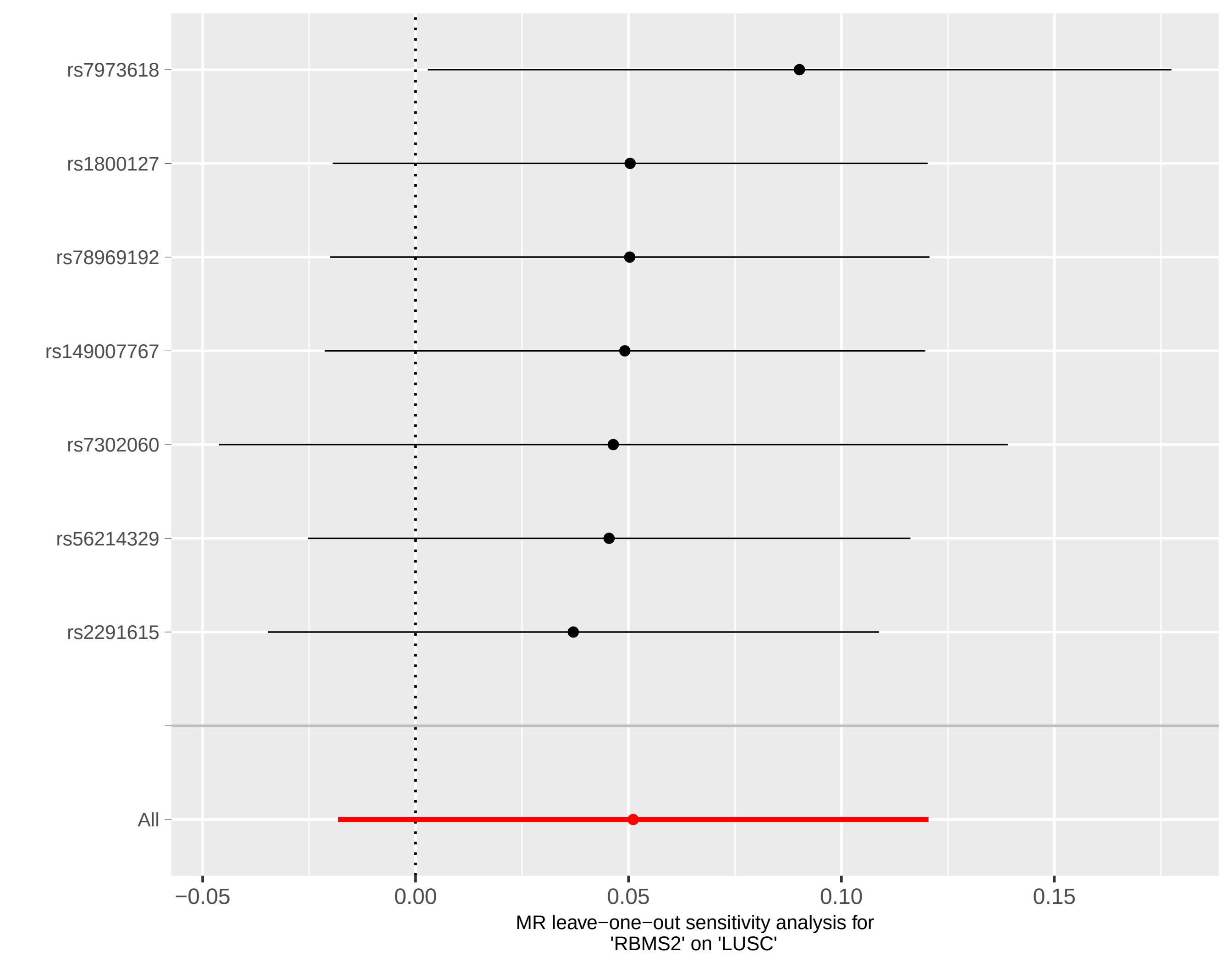

rs7973618
rs1800127
rs78969192
rs149007767
rs7302060
rs56214329
rs2291615
All
−0.05
0.00
0.05
0.10
0.15
MR leave−one−out sensitivity analysis for
'RBMS2' on 'LUSC'

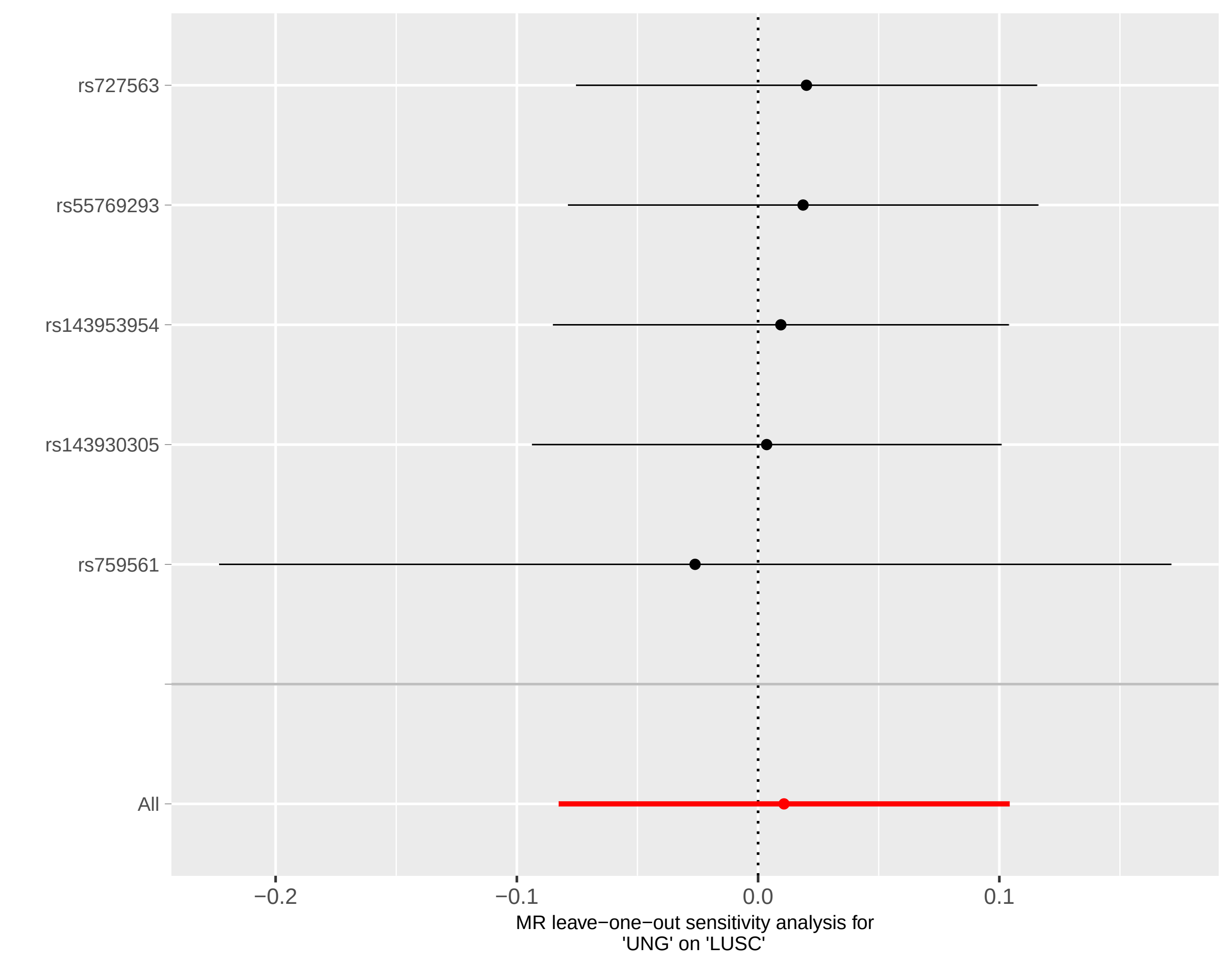

rs727563
rs55769293
rs143953954
rs143930305
rs759561
All
−0.2
−0.1
0.0
0.1
MR leave−one−out sensitivity analysis for
'UNG' on 'LUSC'

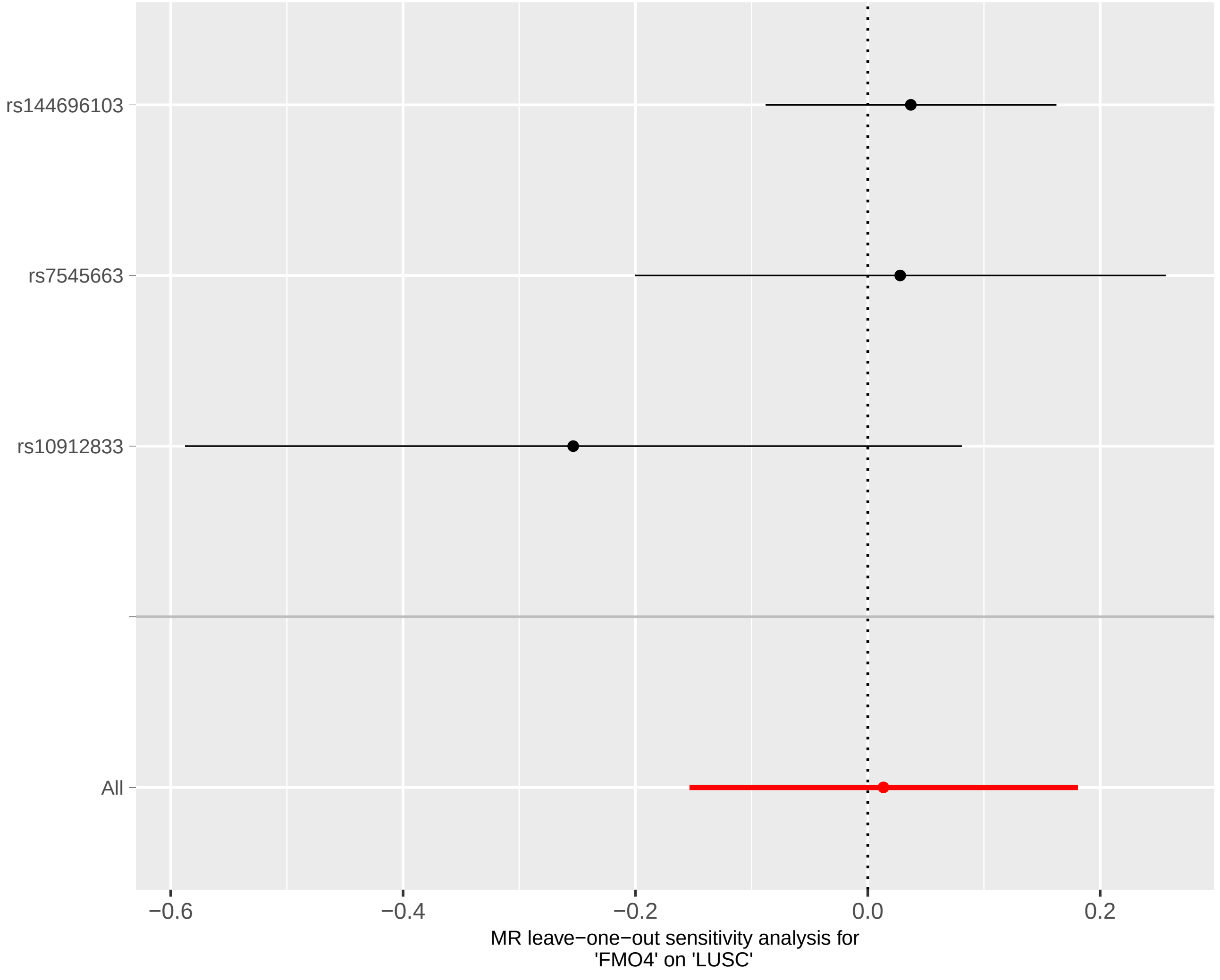

rs144696103
rs7545663
rs10912833
All
−0.6
−0.4
−0.2
0.0
0.2
MR leave−one−out sensitivity analysis for
'FMO4' on 'LUSC'

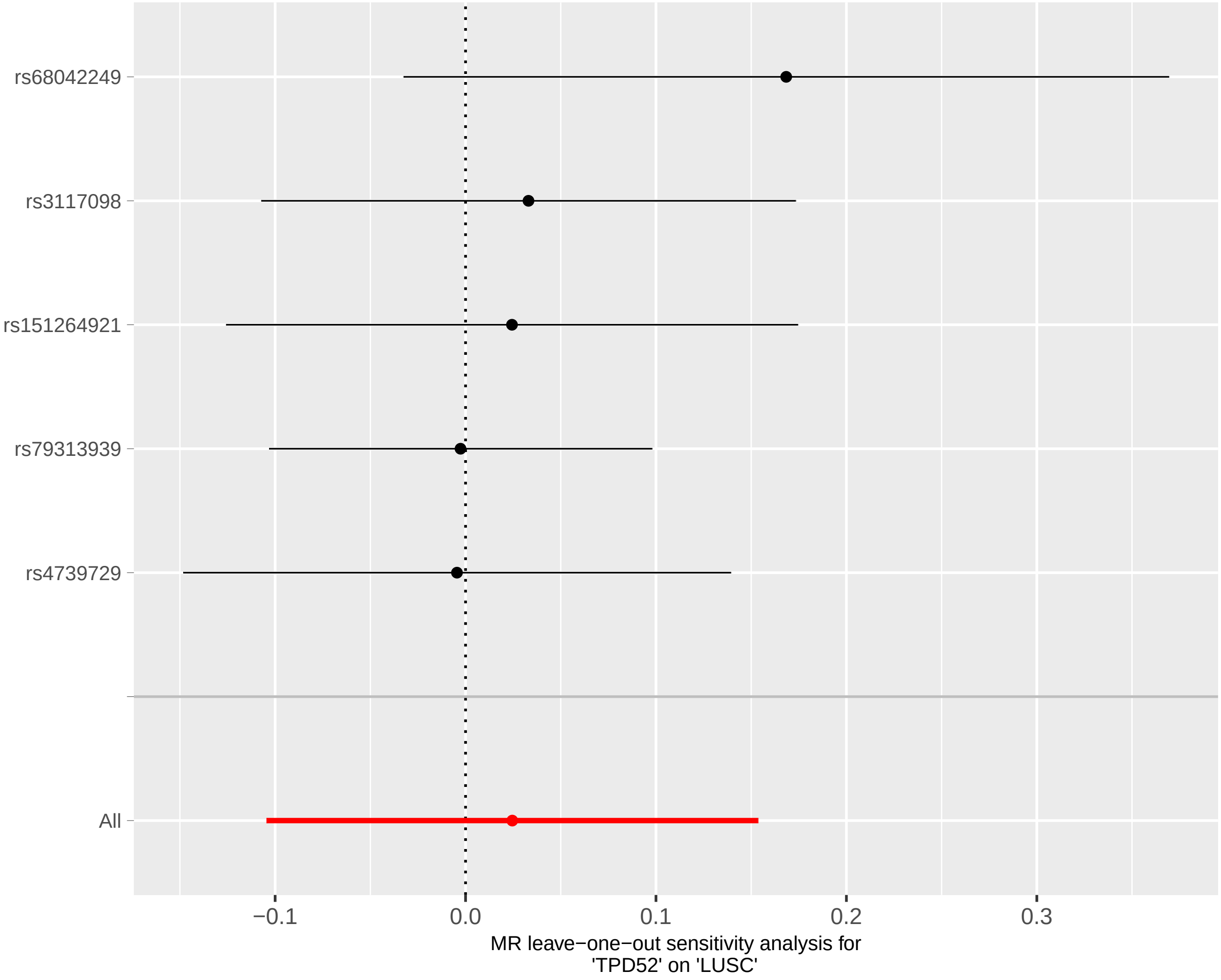

rs68042249
rs3117098
rs151264921
rs79313939
rs4739729
All
−0.1
0.0
0.1
0.2
0.3
MR leave−one−out sensitivity analysis for
'TPD52' on 'LUSC'

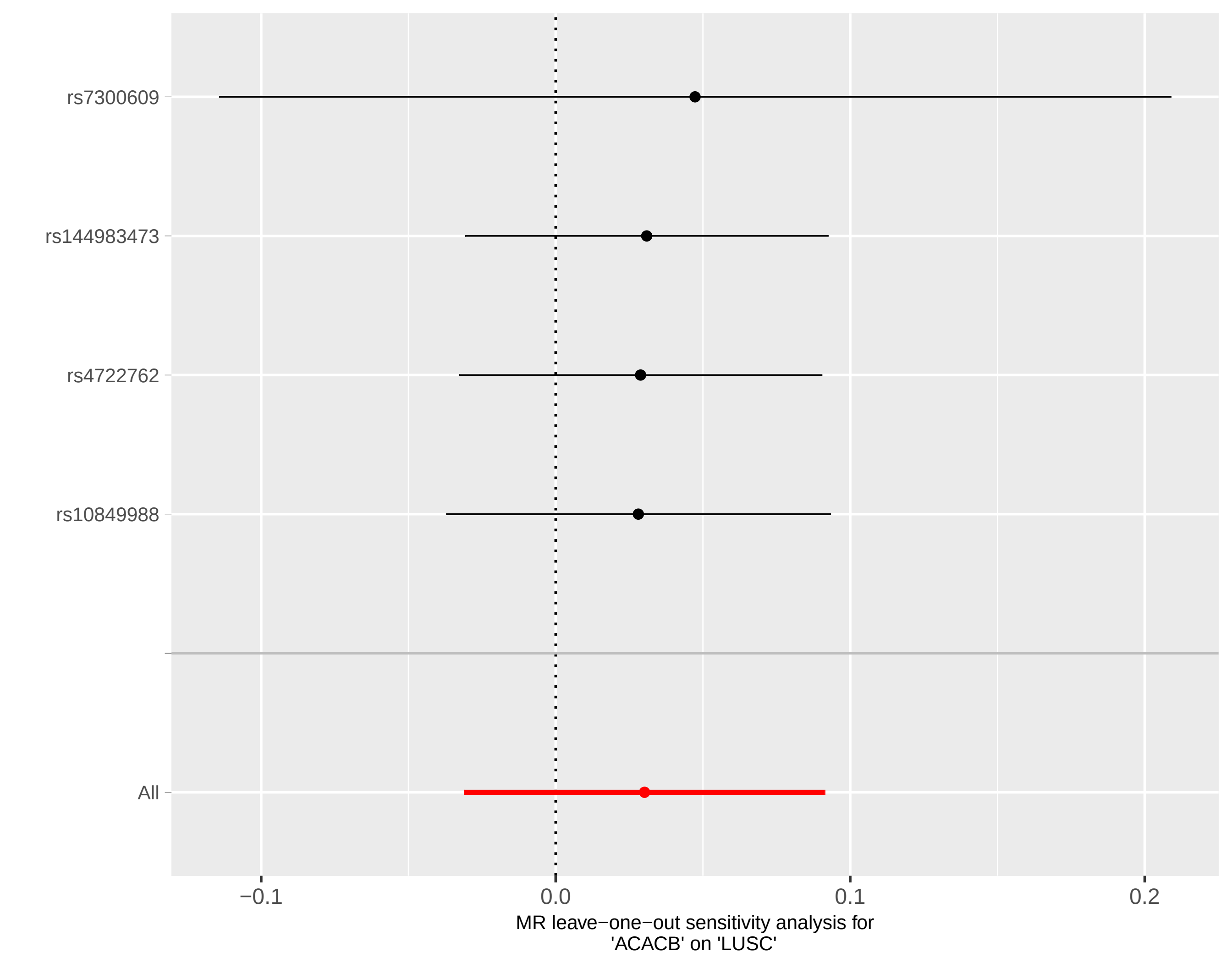
rs7300609
rs144983473
rs4722762
rs10849988
All
−0.1
0.0
0.1
0.2
MR leave−one−out sensitivity analysis for
'ACACB' on 'LUSC'

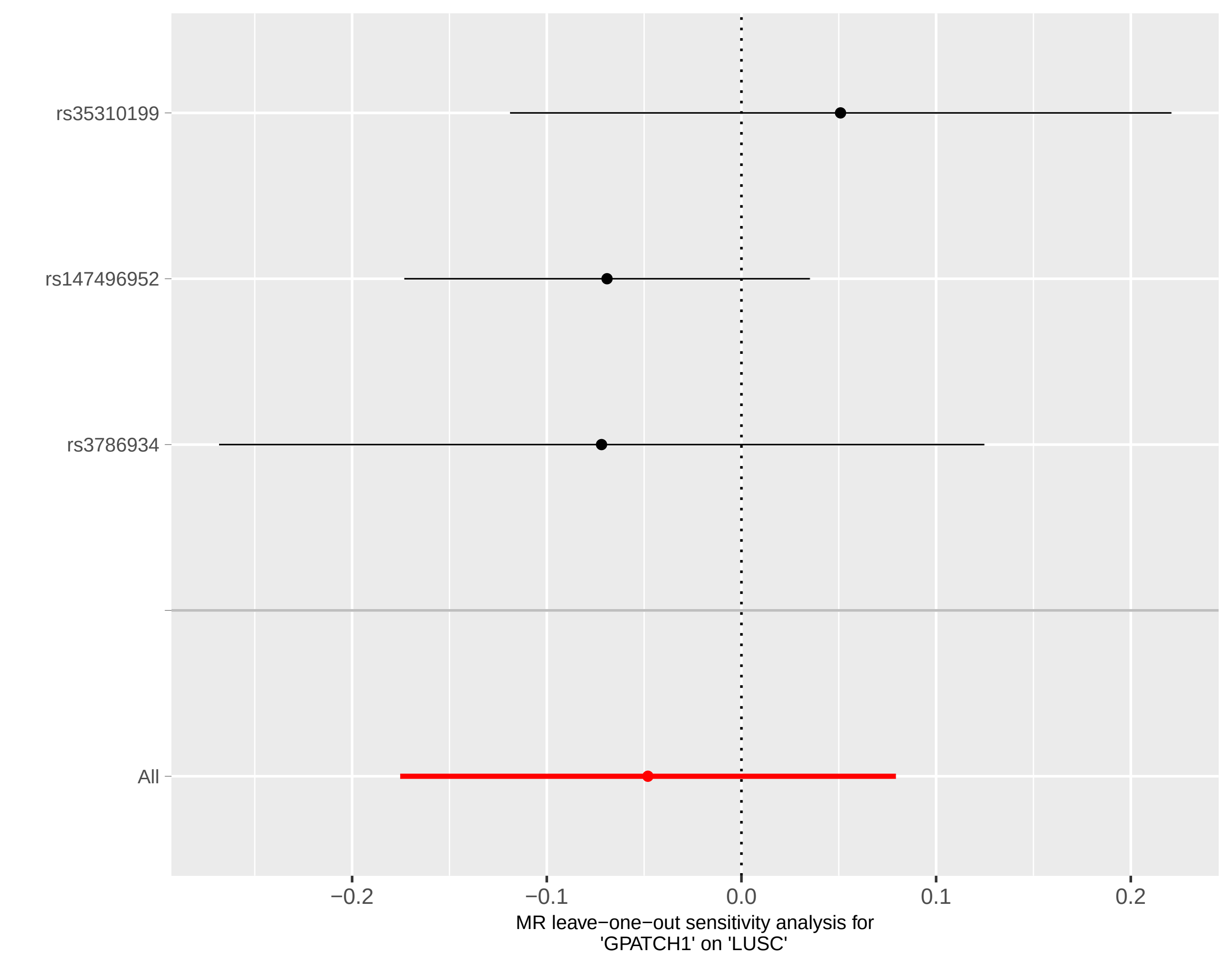
rs35310199
rs147496952
rs3786934
All
−0.2
−0.1
0.0
0.1
0.2
MR leave−one−out sensitivity analysis for
'GPATCH1' on 'LUSC'

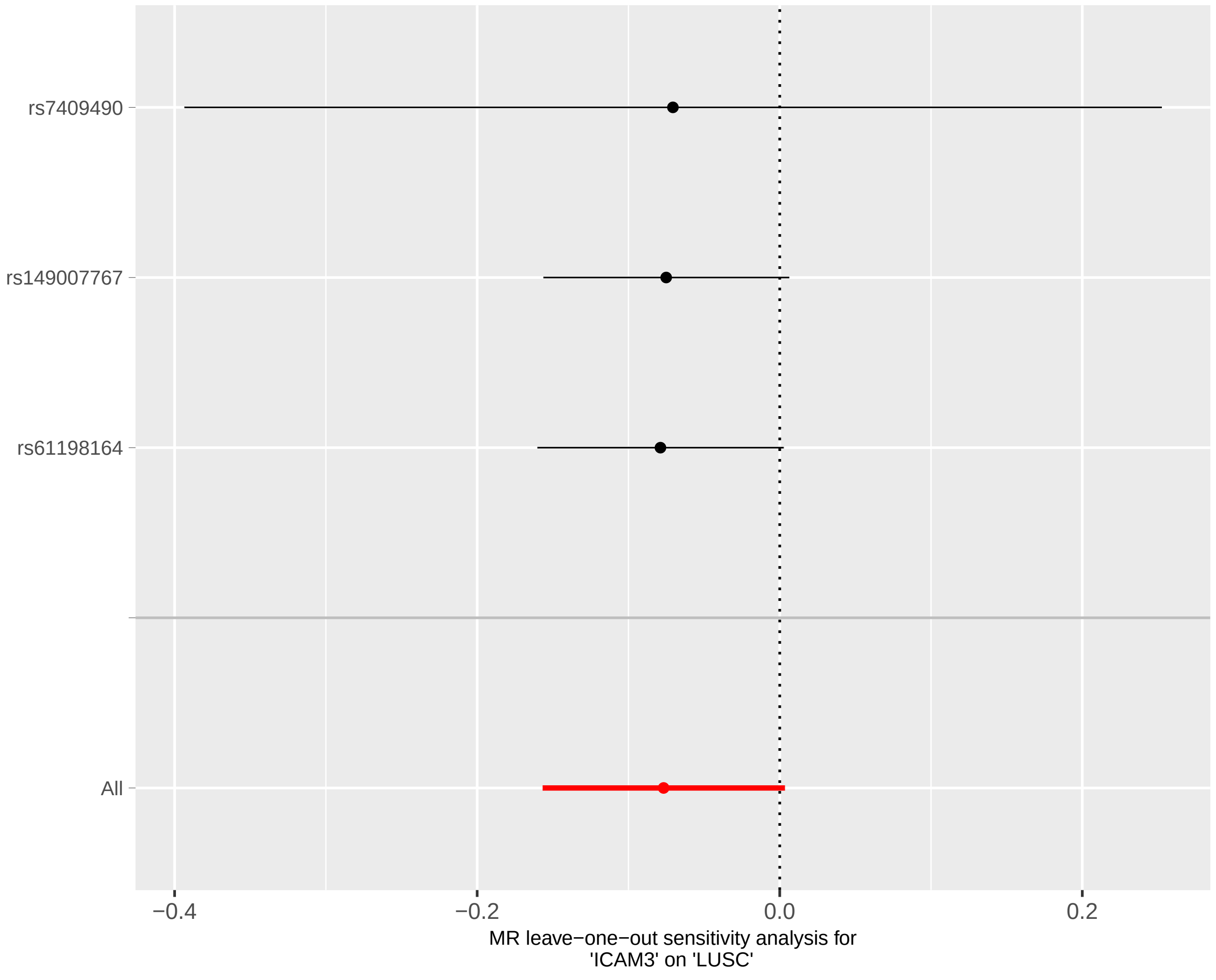
rs7409490
rs149007767
rs61198164
All
−0.4
−0.2
0.0
0.2
MR leave−one−out sensitivity analysis for
'ICAM3' on 'LUSC'

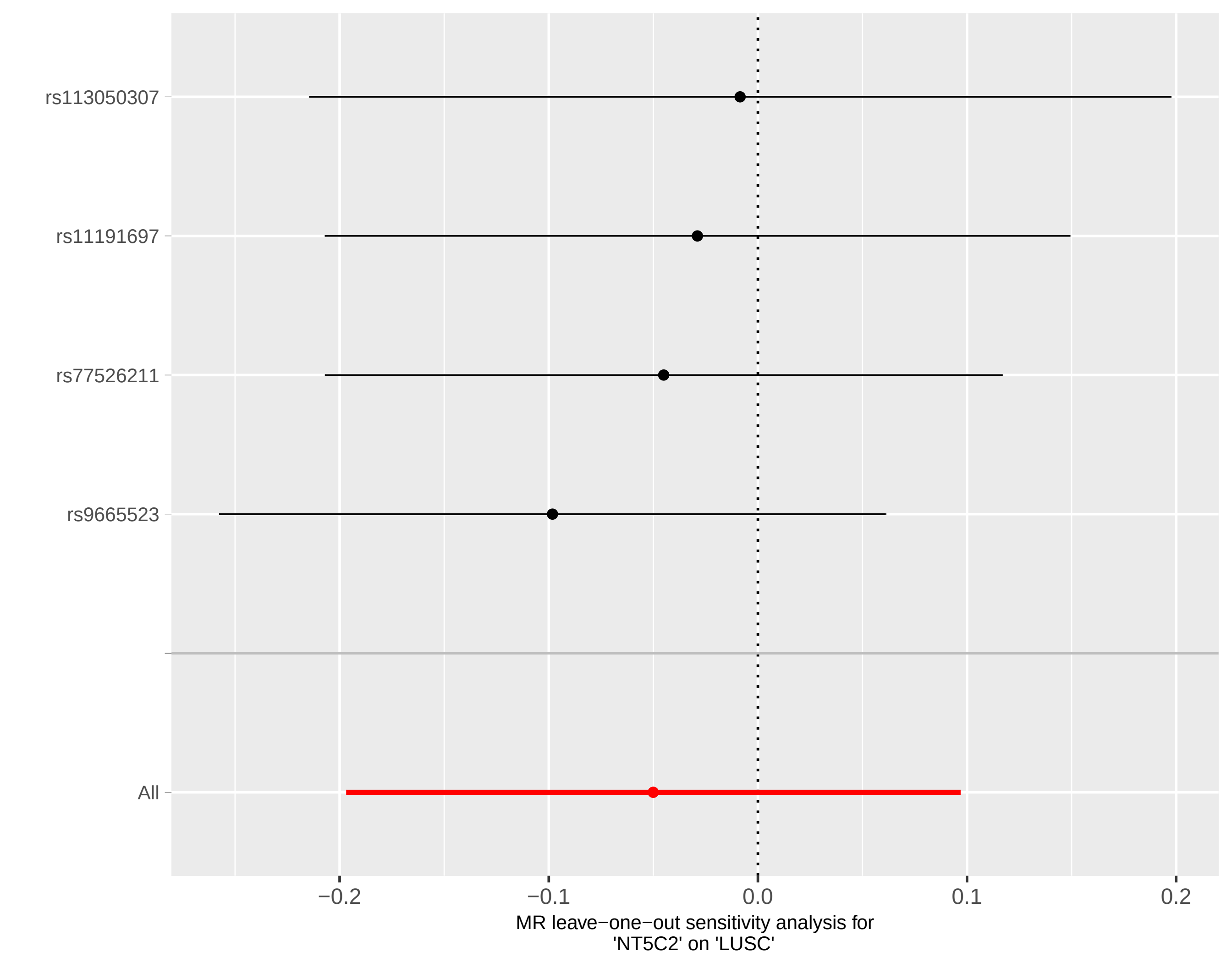

rs113050307
rs11191697
rs77526211
rs9665523
All
−0.2
−0.1
0.0
0.1
0.2
MR leave−one−out sensitivity analysis for
'NT5C2' on 'LUSC'

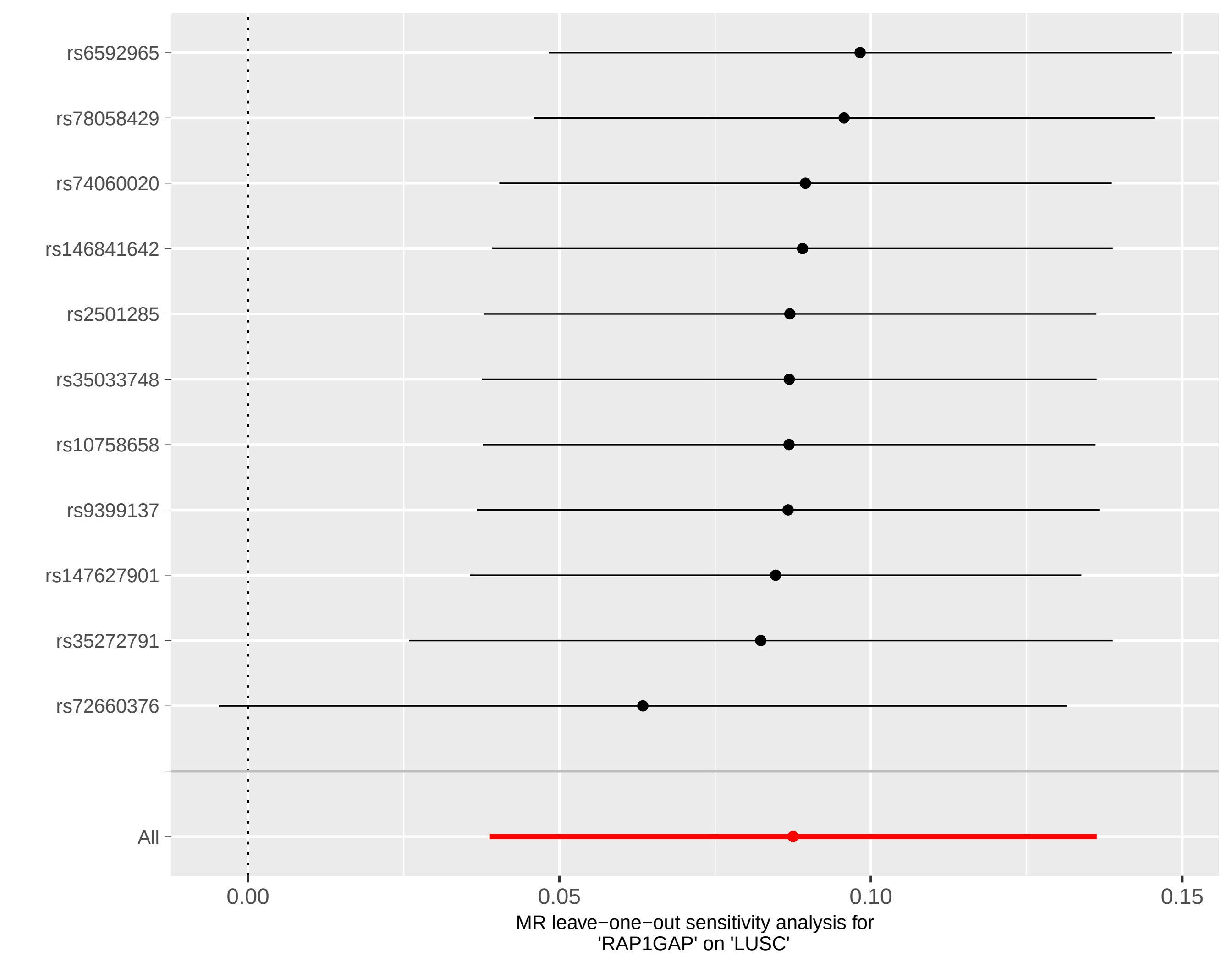
rs6592965
rs78058429
rs74060020
rs146841642
rs2501285
rs35033748
rs10758658
rs9399137
rs147627901
rs35272791
rs72660376
All
0.00
0.05
0.10
0.15
MR leave−one−out sensitivity analysis for
'RAP1GAP' on 'LUSC'

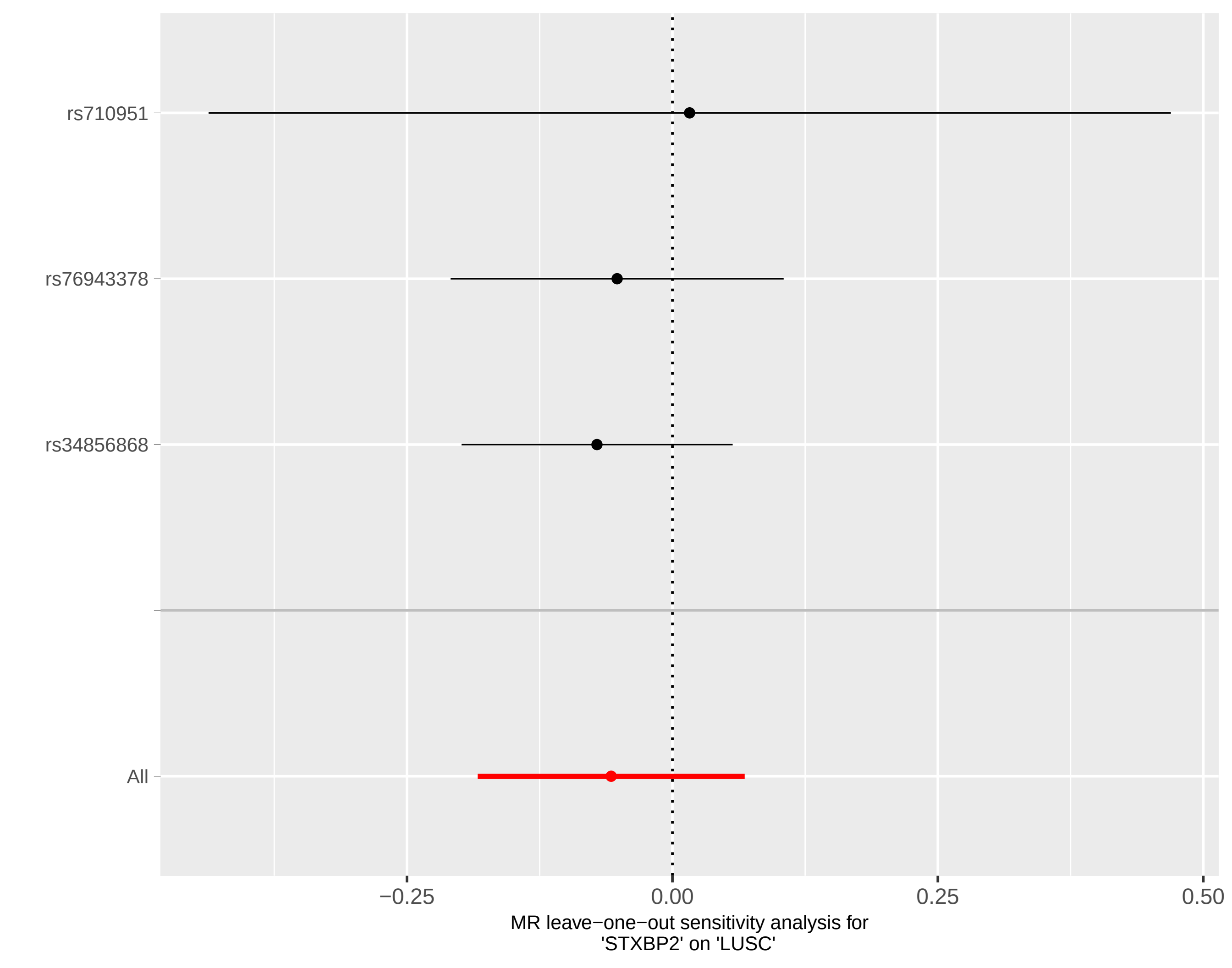

rs710951
rs76943378
rs34856868
All
−0.25
0.00
0.25
0.50
MR leave−one−out sensitivity analysis for
'STXBP2' on 'LUSC'

Insufficient number of SNPs

Insufficient number of SNPs

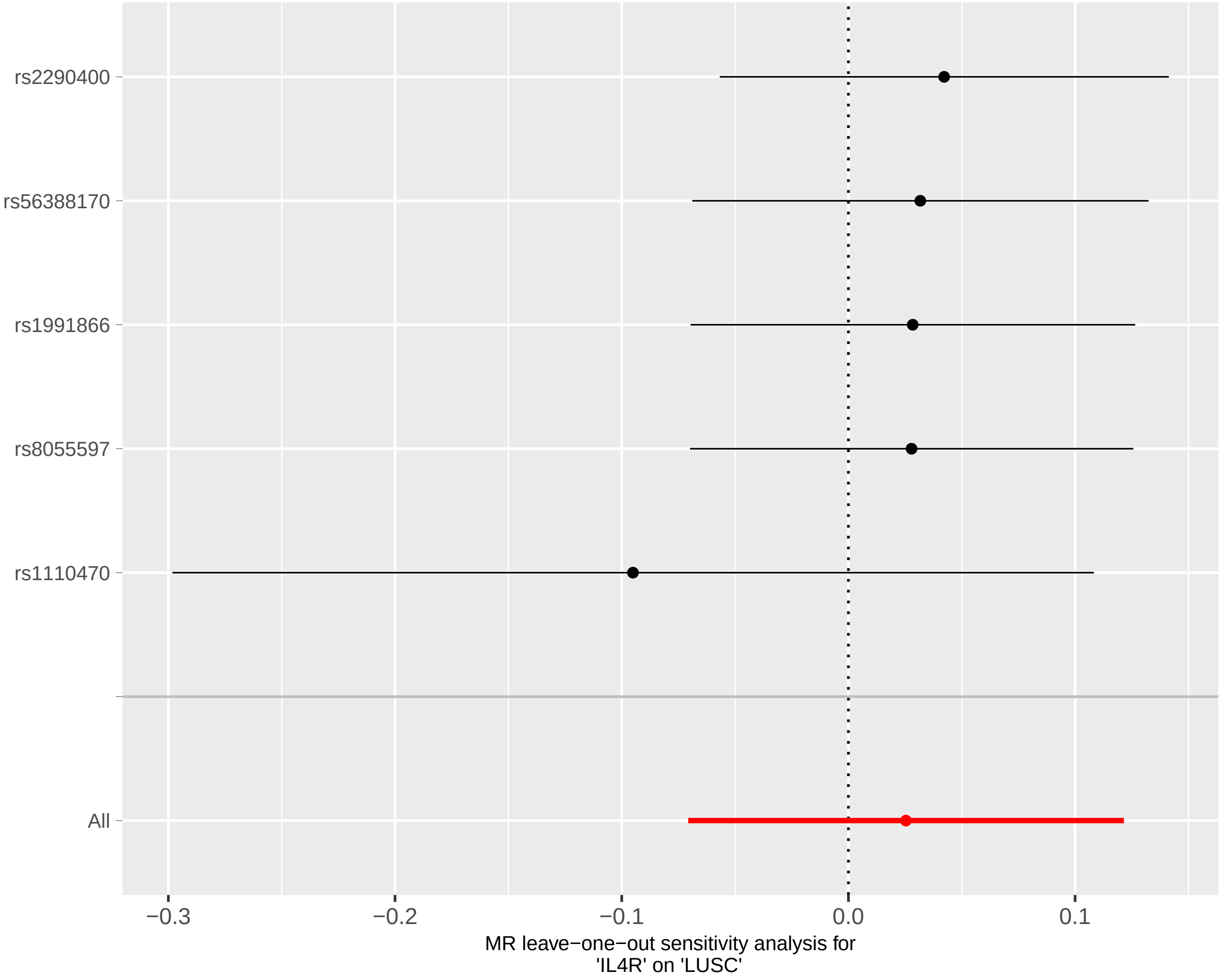
rs2290400
rs56388170
rs1991866
rs8055597
rs1110470
All
−0.3
−0.2
−0.1
0.0
0.1
MR leave−one−out sensitivity analysis for
'IL4R' on 'LUSC'

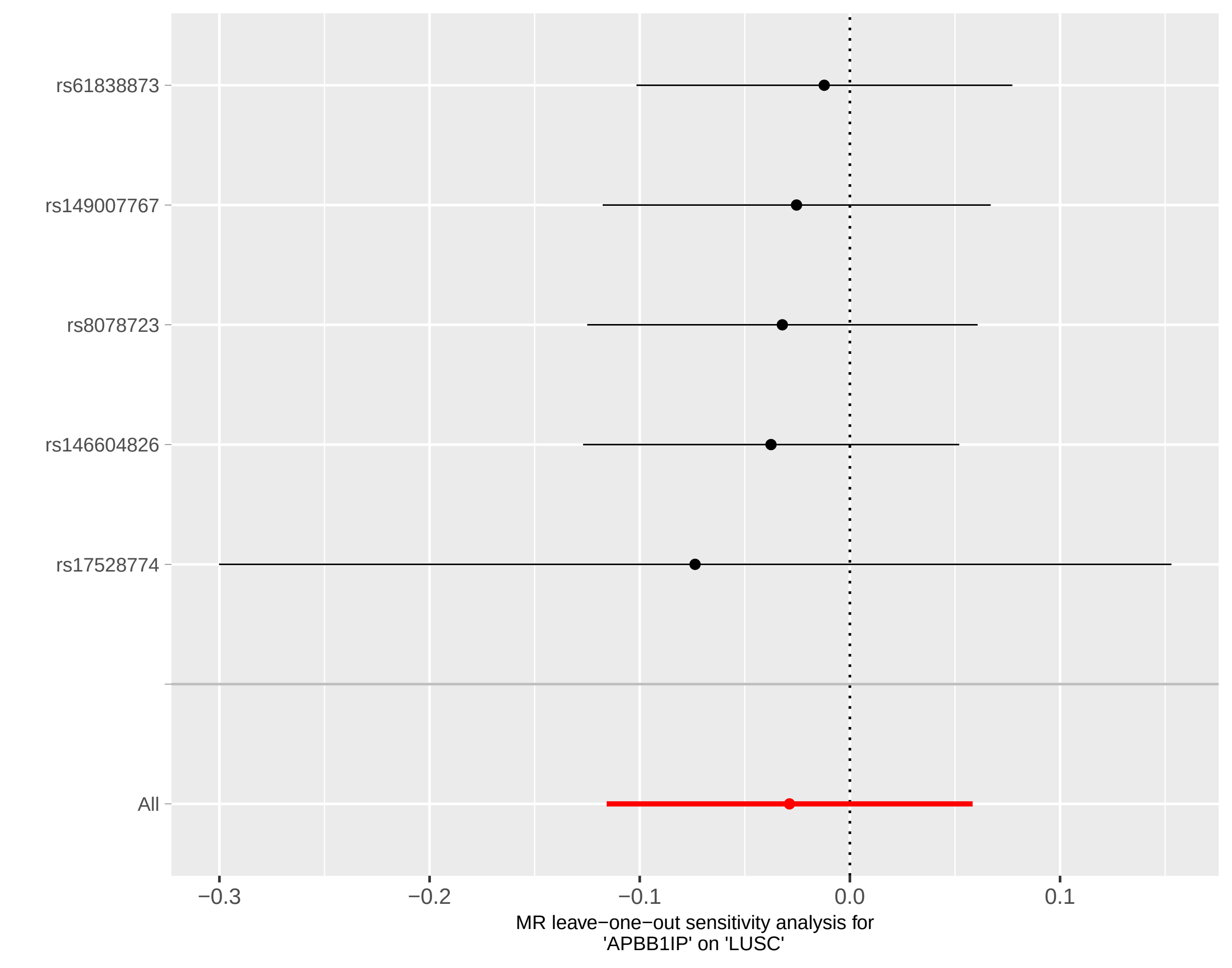

rs61838873
rs149007767
rs8078723
rs146604826
rs17528774
All
−0.3
−0.2
−0.1
0.0
0.1
MR leave−one−out sensitivity analysis for
'APBB1IP' on 'LUSC'

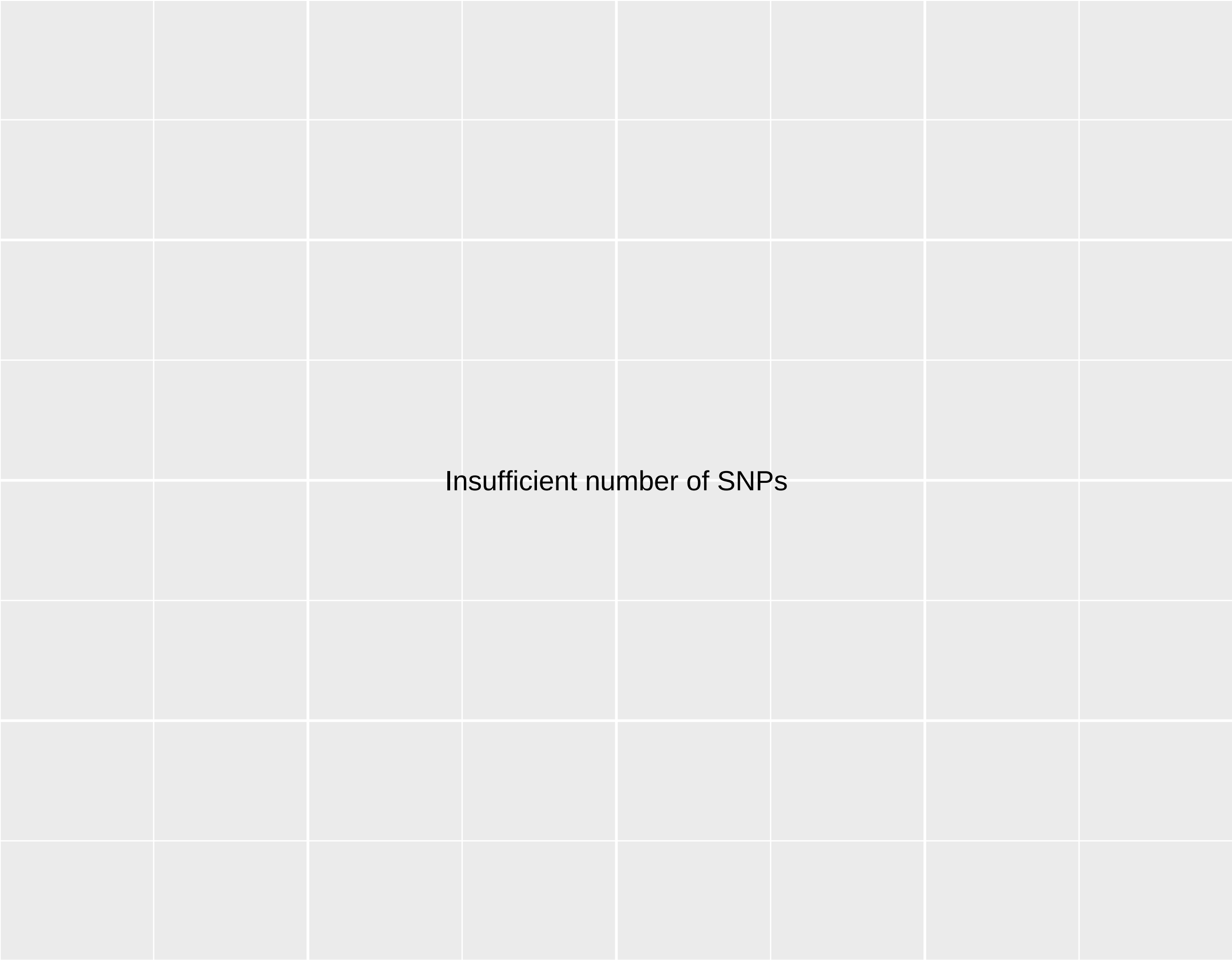
Insufficient number of SNPs

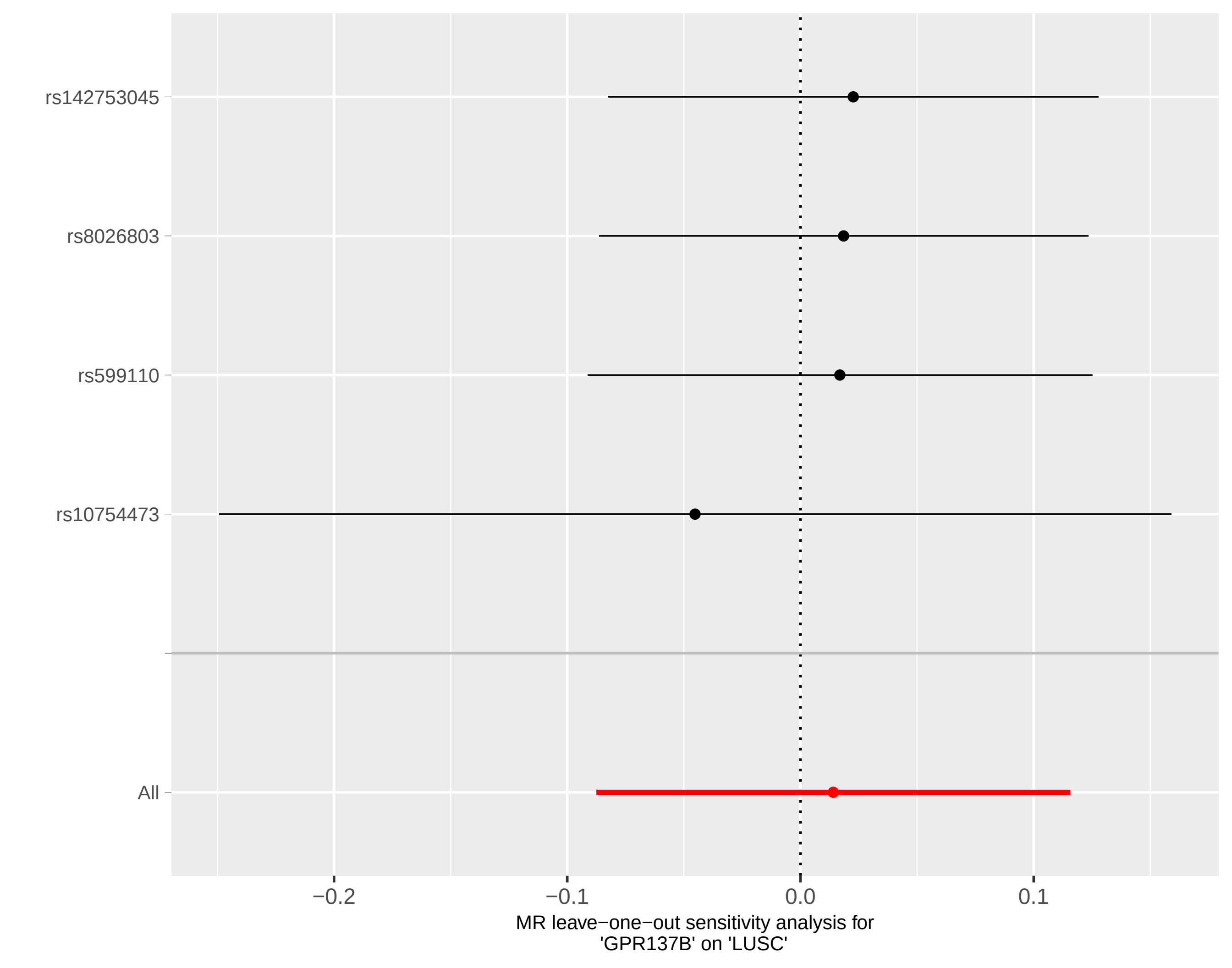

rs142753045
rs8026803
rs599110
rs10754473
All
−0.2
−0.1
0.0
0.1
MR leave−one−out sensitivity analysis for
'GPR137B' on 'LUSC'

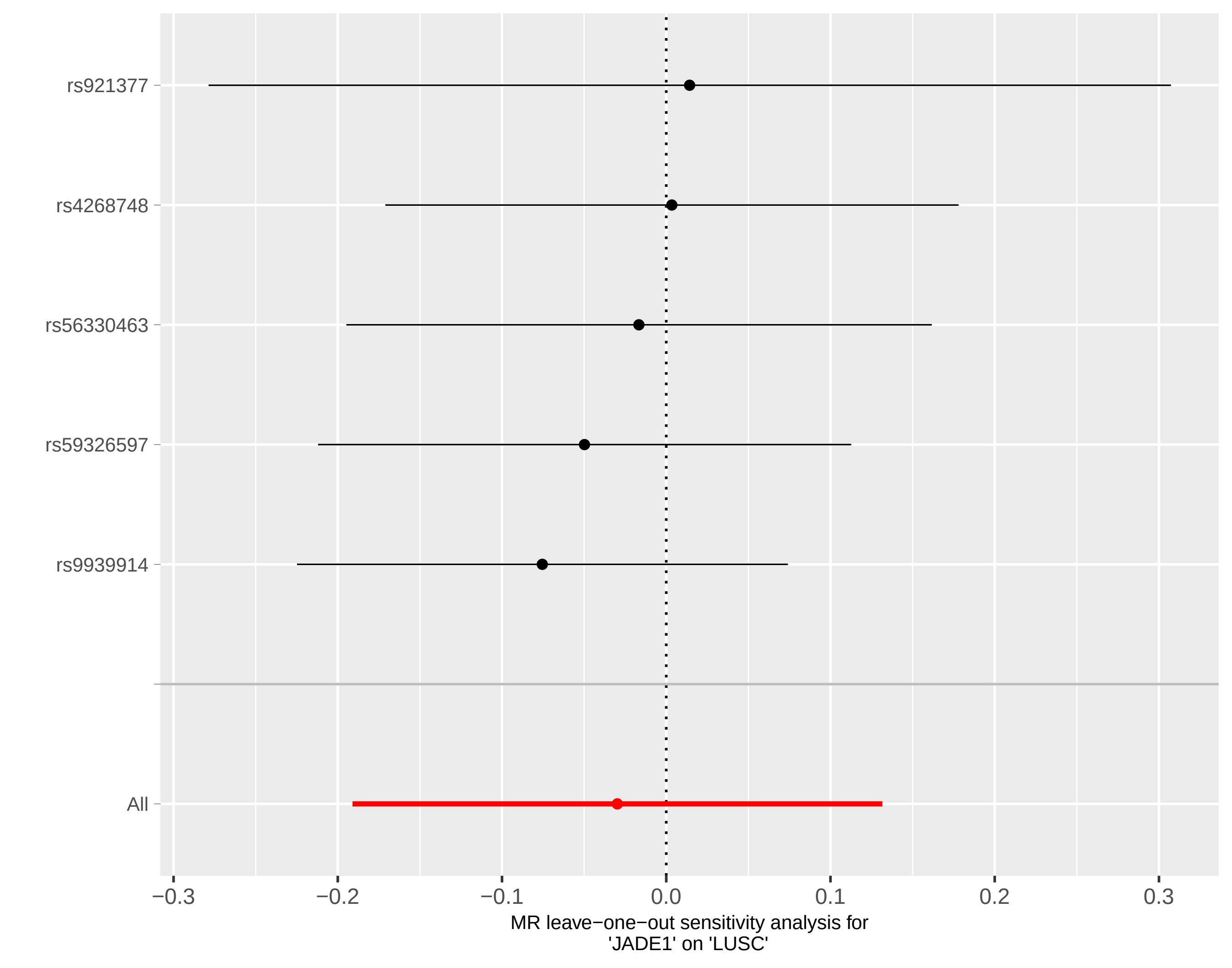
rs921377
rs4268748
rs56330463
rs59326597
rs9939914
All
−0.3
−0.2
−0.1
0.0
0.1
0.2
0.3
MR leave−one−out sensitivity analysis for
'JADE1' on 'LUSC'

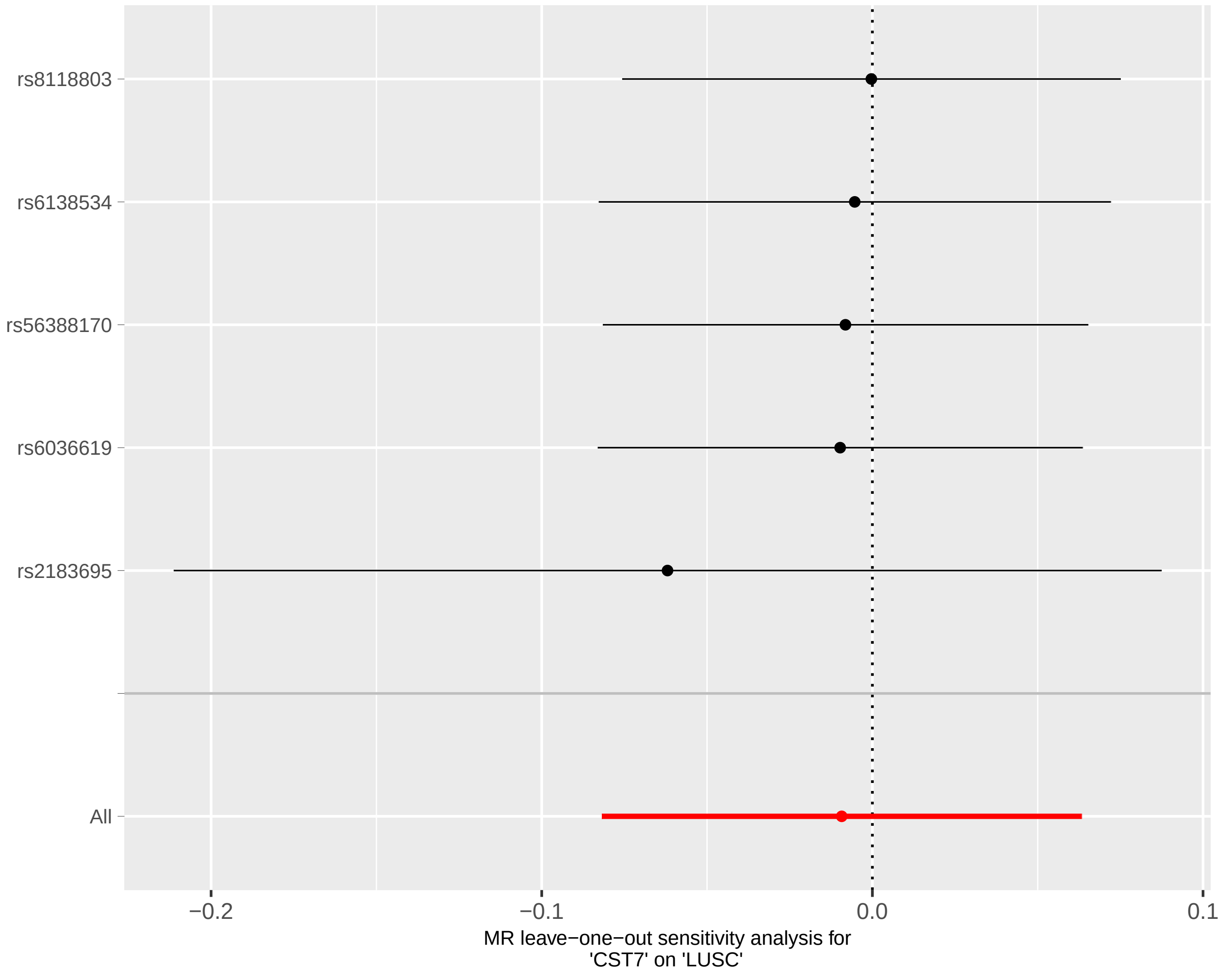

rs8118803
rs6138534
rs56388170
rs6036619
rs2183695
All
−0.2
−0.1
0.0
0.1
MR leave−one−out sensitivity analysis for
'CST7' on 'LUSC'

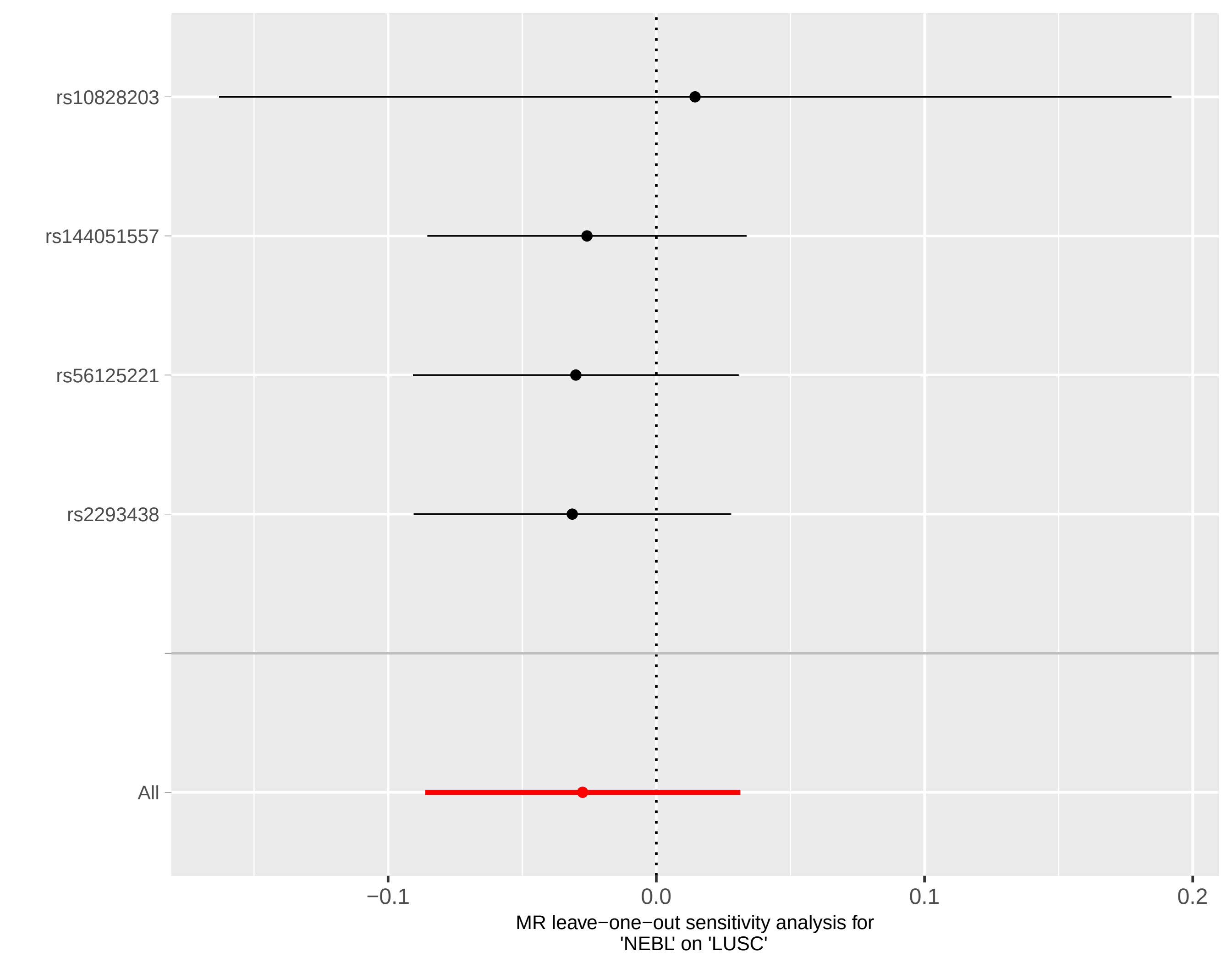
rs10828203
rs144051557
rs56125221
rs2293438
All
−0.1
0.0
0.1
0.2
MR leave−one−out sensitivity analysis for
'NEBL' on 'LUSC'

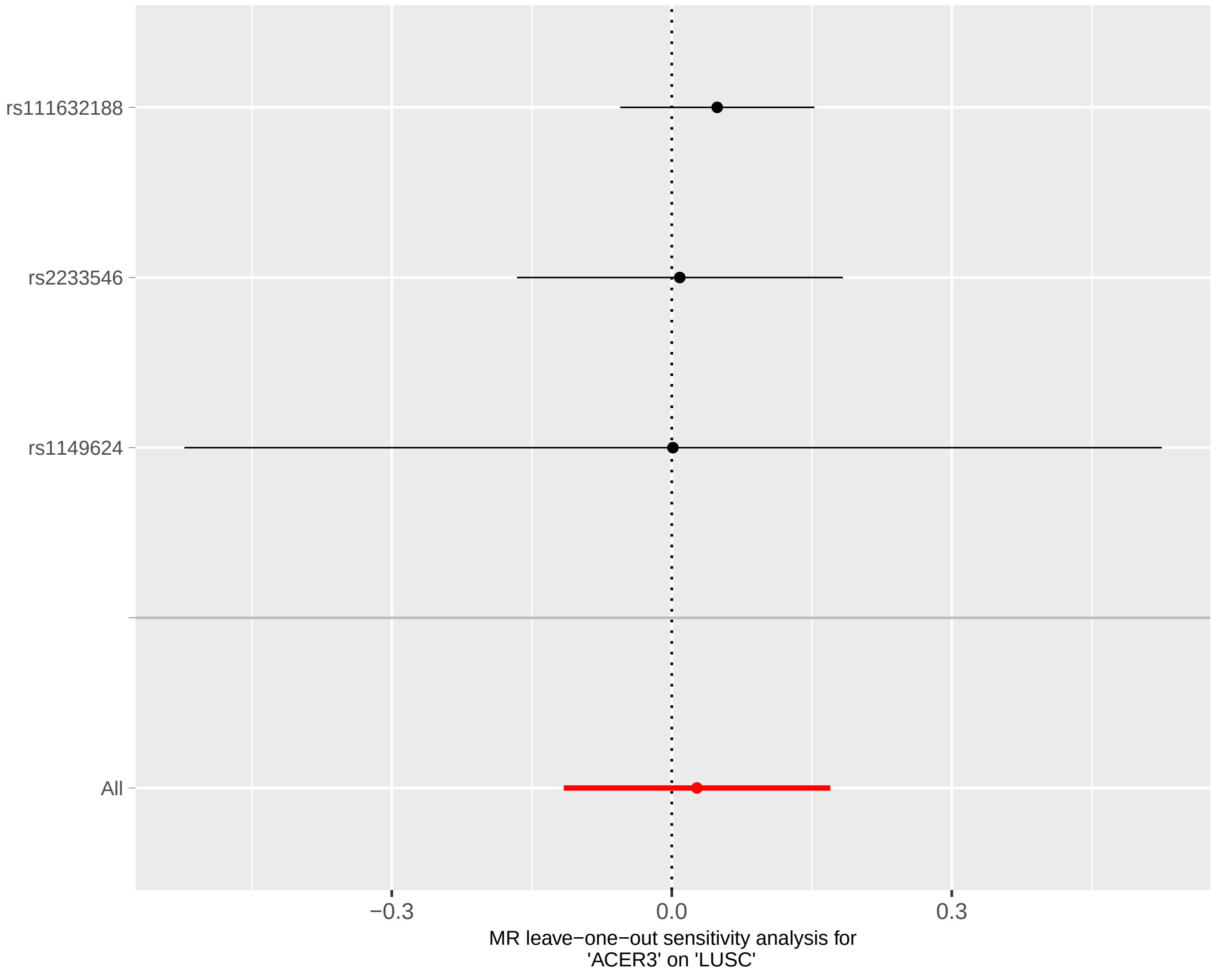

rs111632188
rs2233546
rs1149624
All
−0.3
0.0
0.3
MR leave−one−out sensitivity analysis for
'ACER3' on 'LUSC'

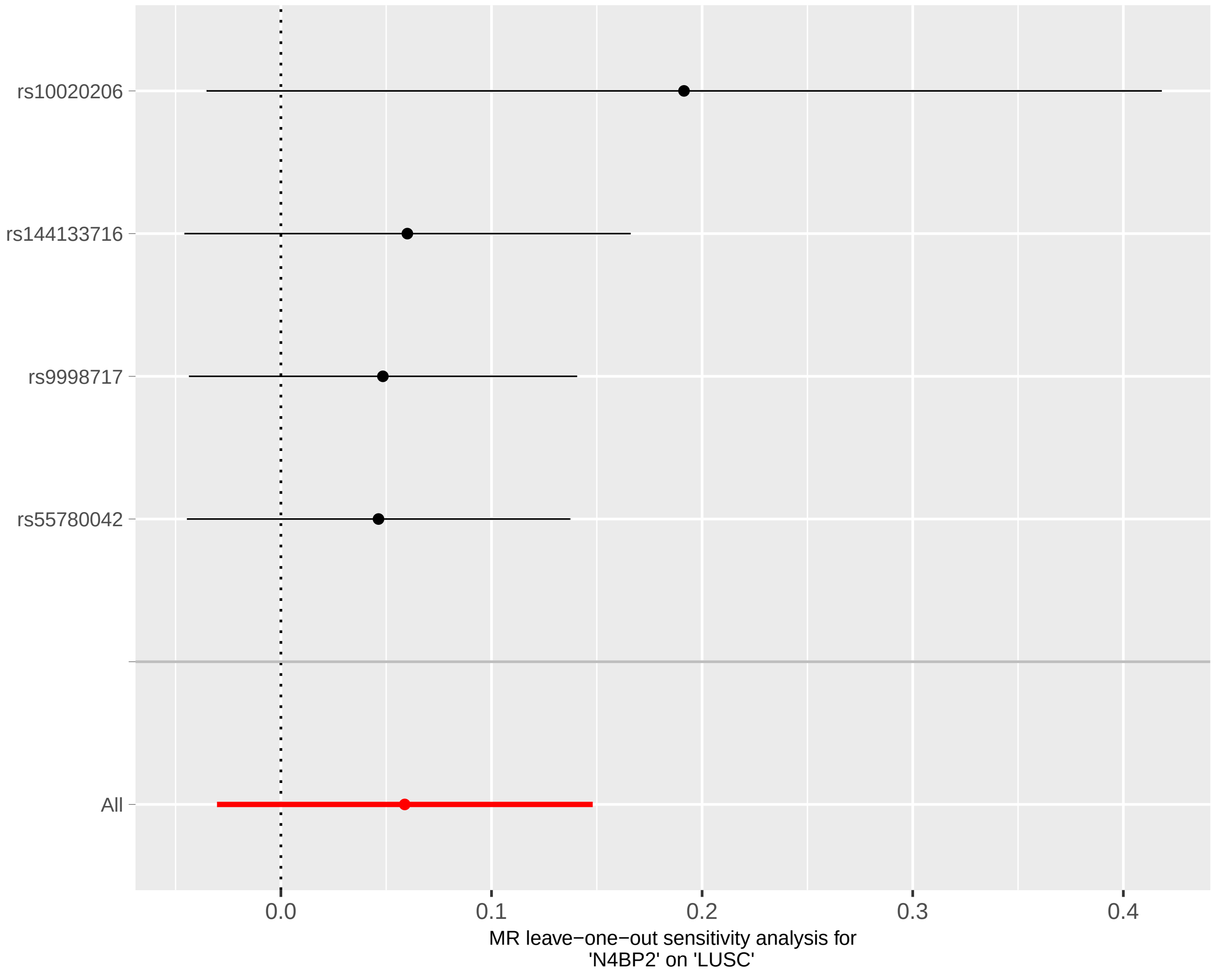
rs10020206
rs144133716
rs9998717
rs55780042
All
0.0
0.1
0.2
0.3
0.4
MR leave−one−out sensitivity analysis for
'N4BP2' on 'LUSC'

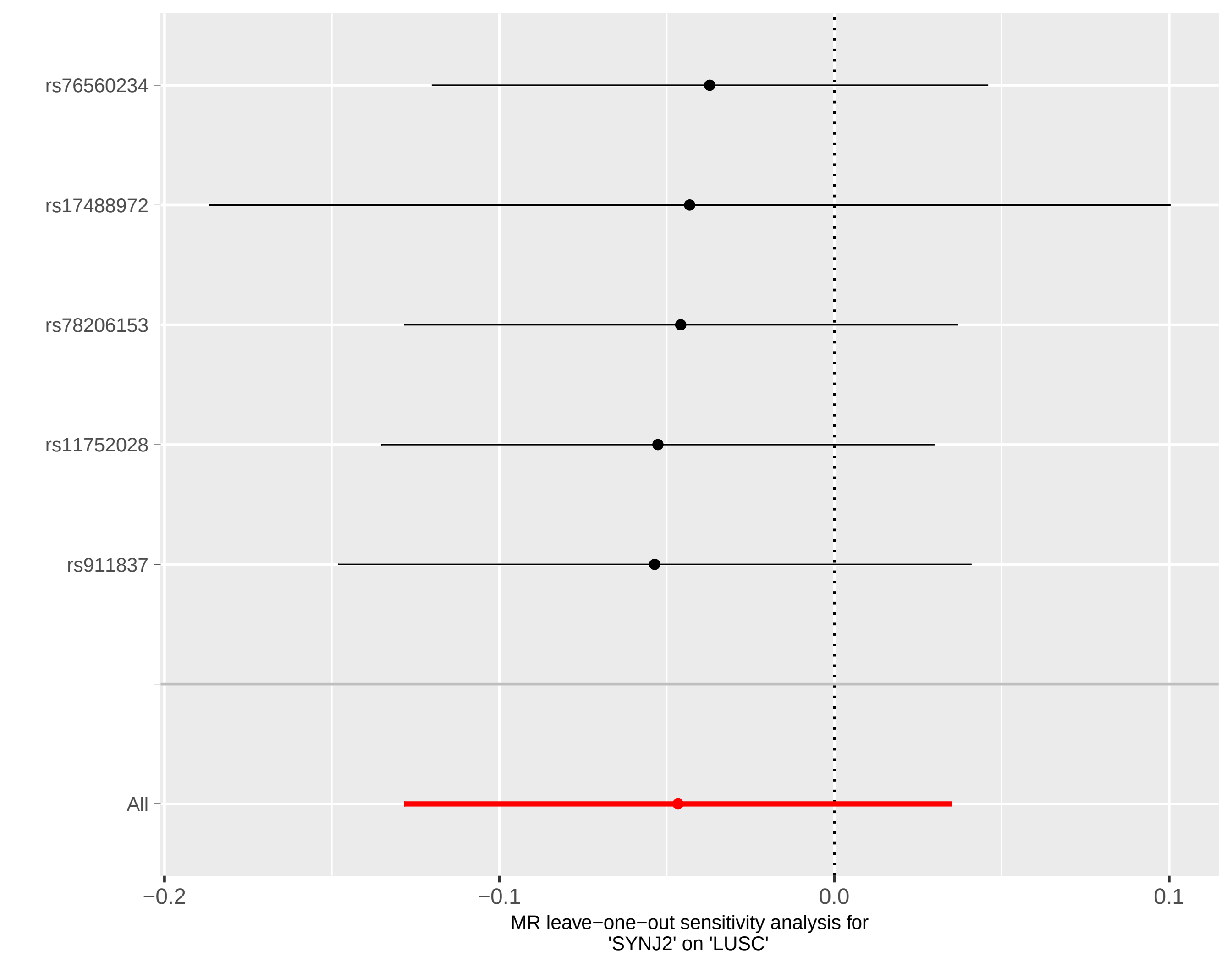

rs76560234
rs17488972
rs78206153
rs11752028
rs911837
All
−0.2
−0.1
0.0
0.1
MR leave−one−out sensitivity analysis for
'SYNJ2' on 'LUSC'

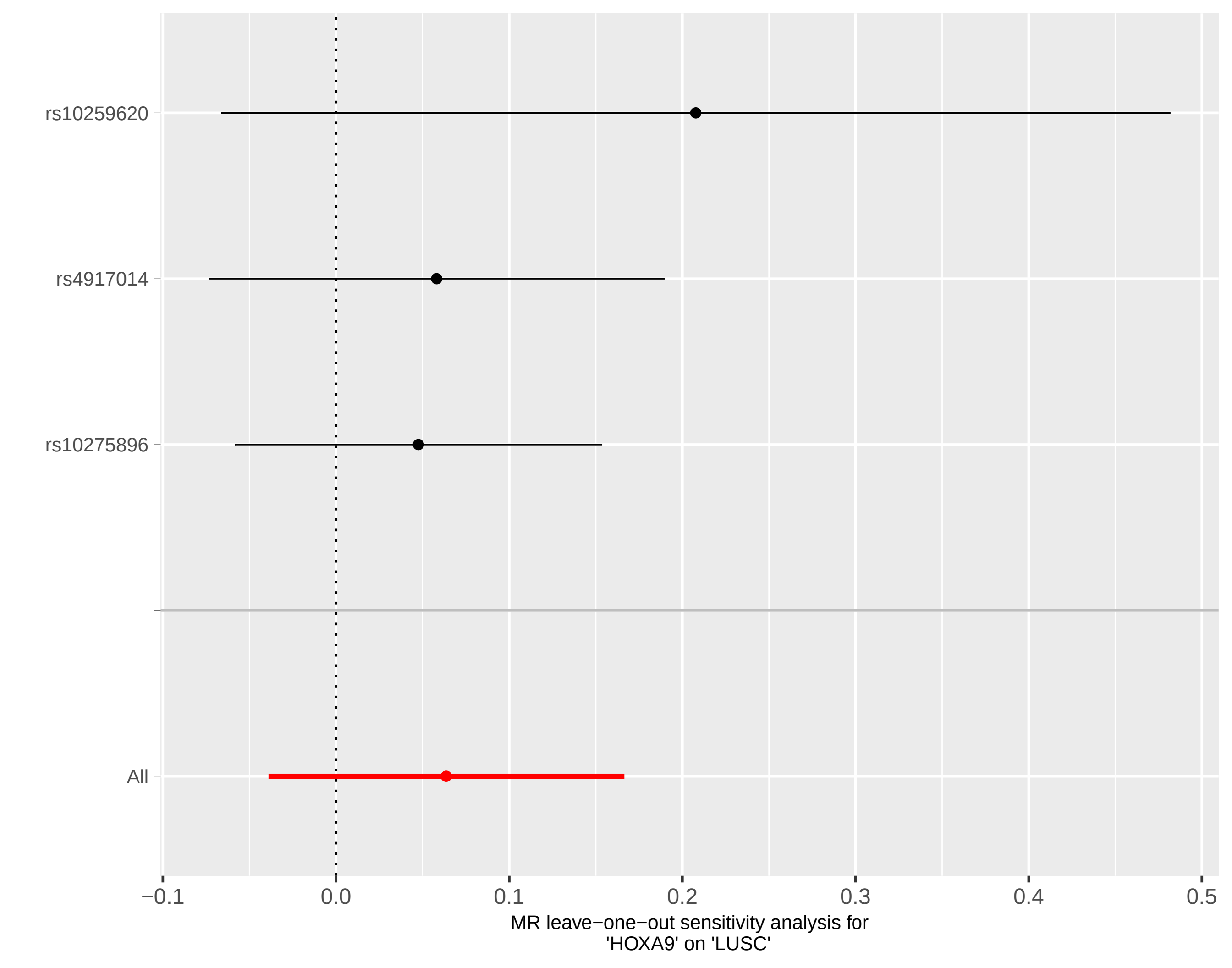

rs10259620
rs4917014
rs10275896
All
−0.1
0.0
0.1
0.2
0.3
0.4
0.5
MR leave−one−out sensitivity analysis for
'HOXA9' on 'LUSC'

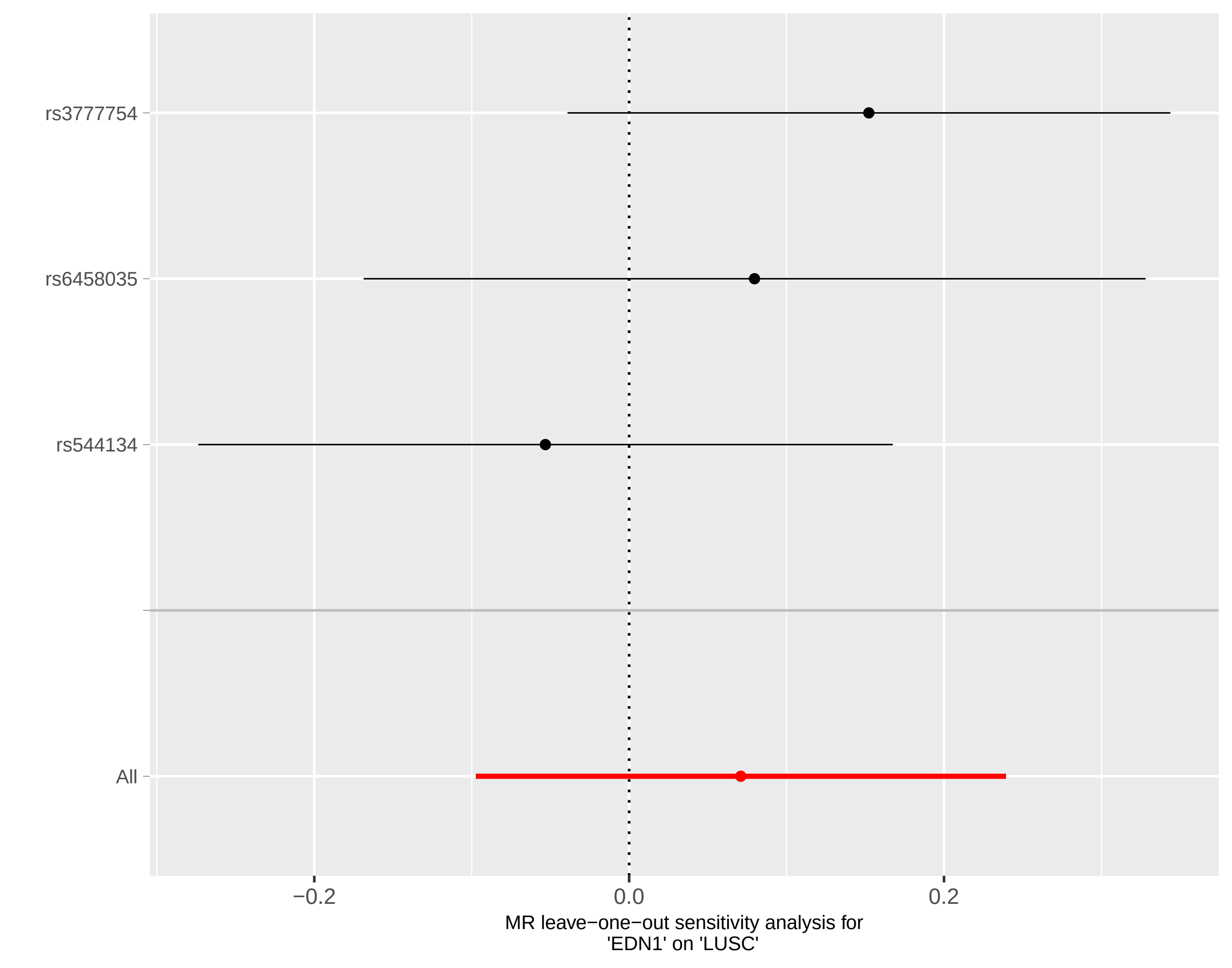
rs3777754
rs6458035
rs544134
All
−0.2
0.0
0.2
MR leave−one−out sensitivity analysis for
'EDN1' on 'LUSC'

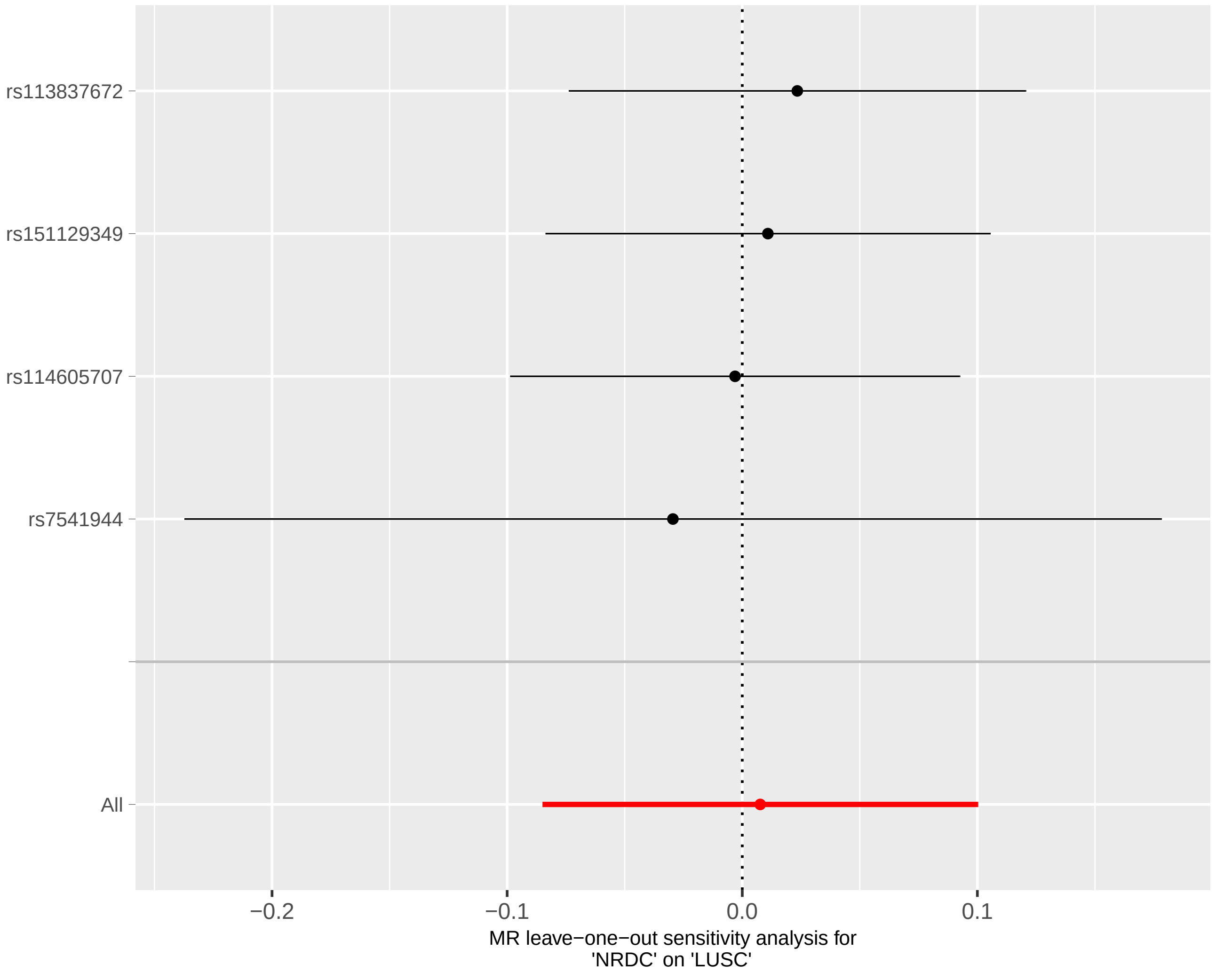
rs113837672
rs151129349
rs114605707
rs7541944
All
−0.2
−0.1
0.0
0.1
MR leave−one−out sensitivity analysis for
'NRDC' on 'LUSC'

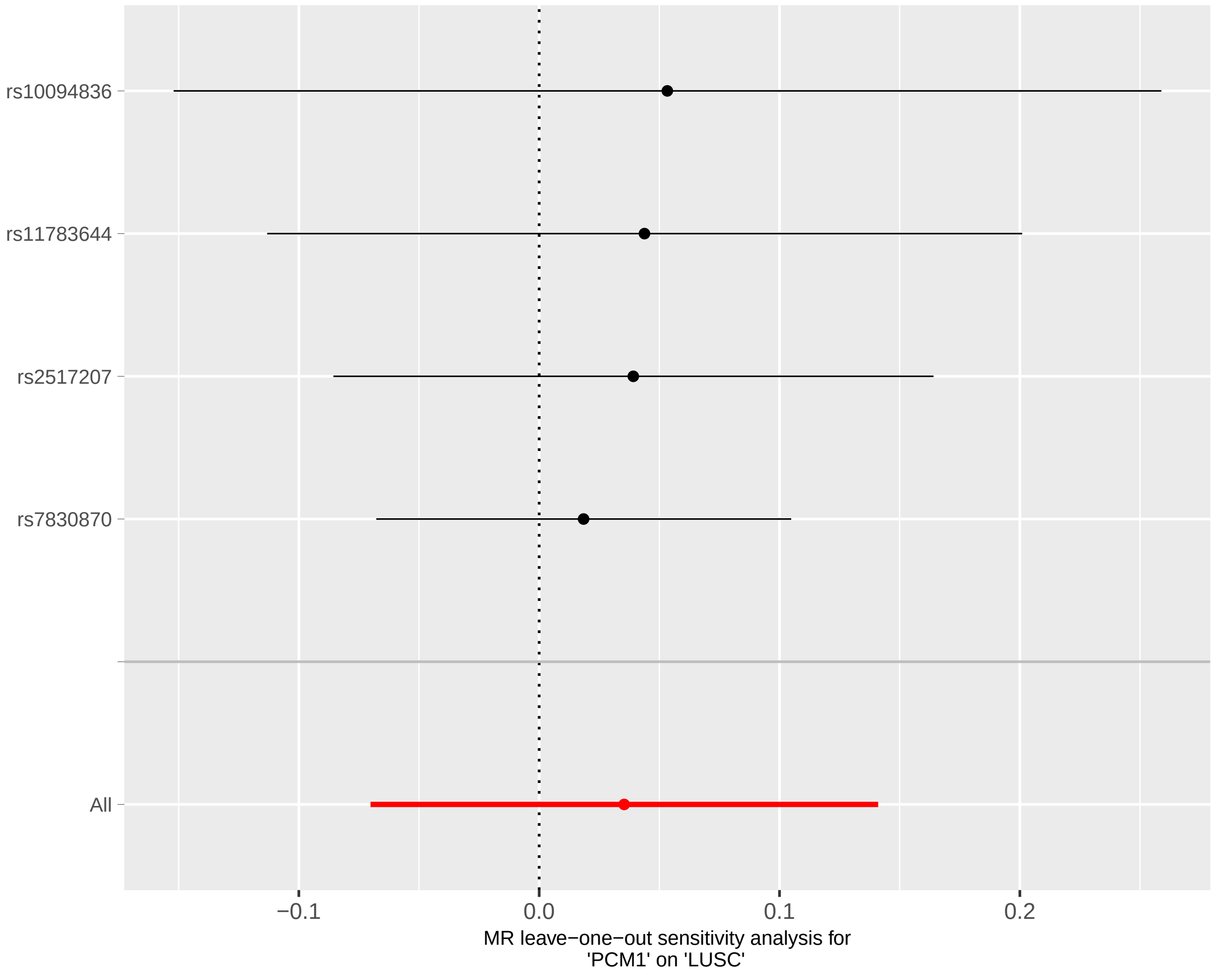
rs10094836
rs11783644
rs2517207
rs7830870
All
−0.1
0.0
0.1
0.2
MR leave−one−out sensitivity analysis for
'PCM1' on 'LUSC'

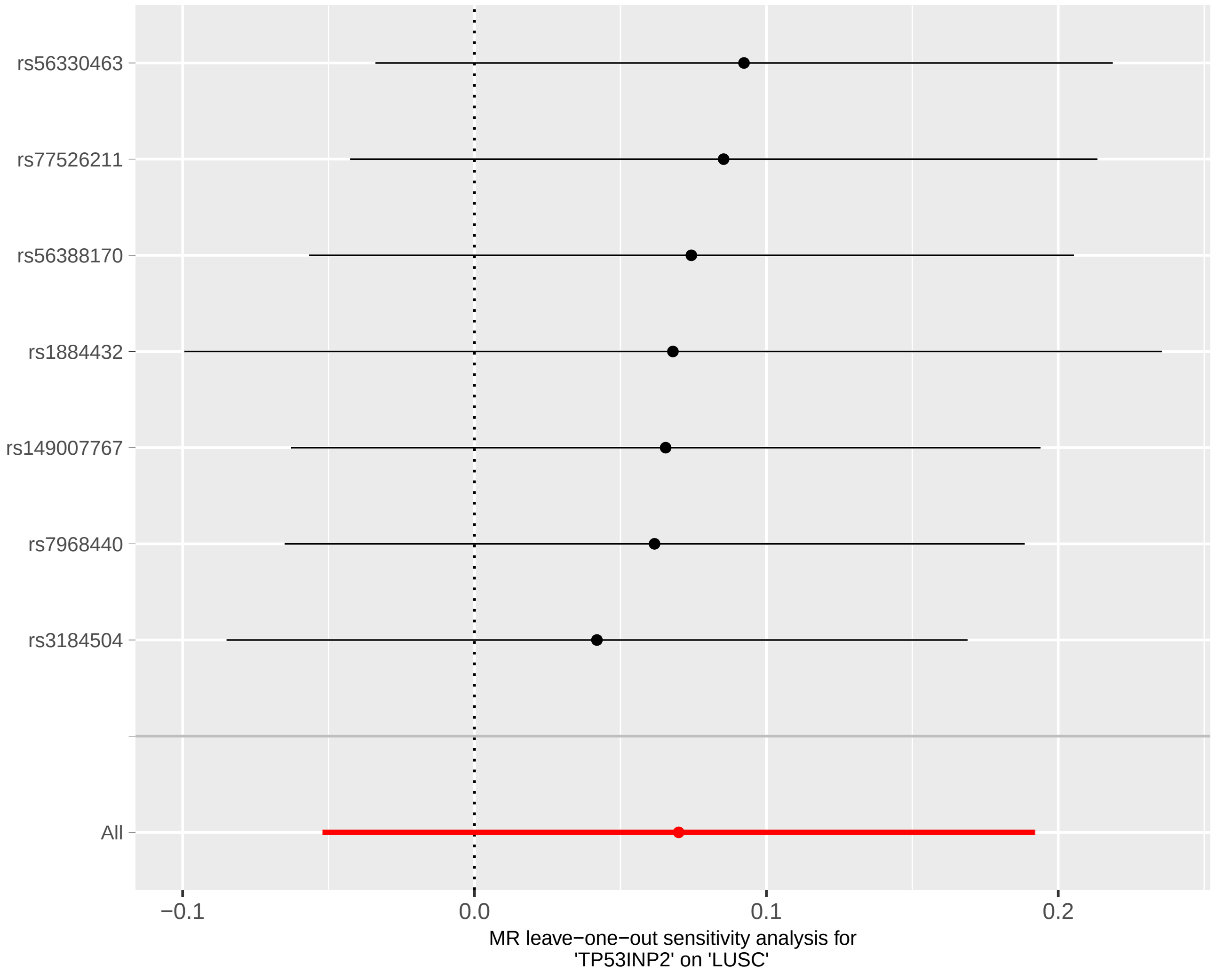

rs56330463
rs77526211
rs56388170
rs1884432
rs149007767
rs7968440
rs3184504
All
−0.1
0.0
0.1
0.2
MR leave−one−out sensitivity analysis for
'TP53INP2' on 'LUSC'

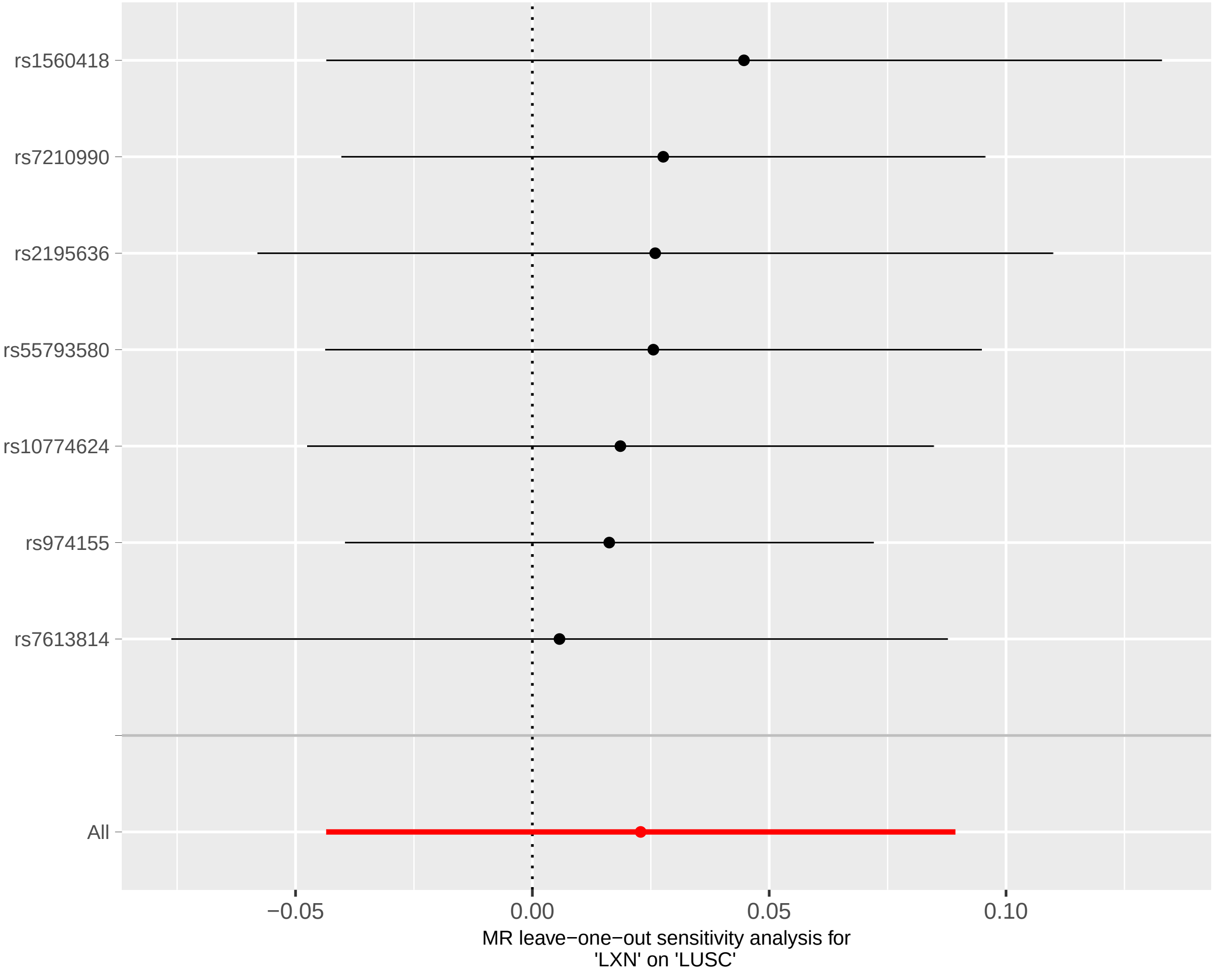

rs1560418
rs7210990
rs2195636
rs55793580
rs10774624
rs974155
rs7613814
All
−0.05
0.00
0.05
0.10
MR leave−one−out sensitivity analysis for
'LXN' on 'LUSC'

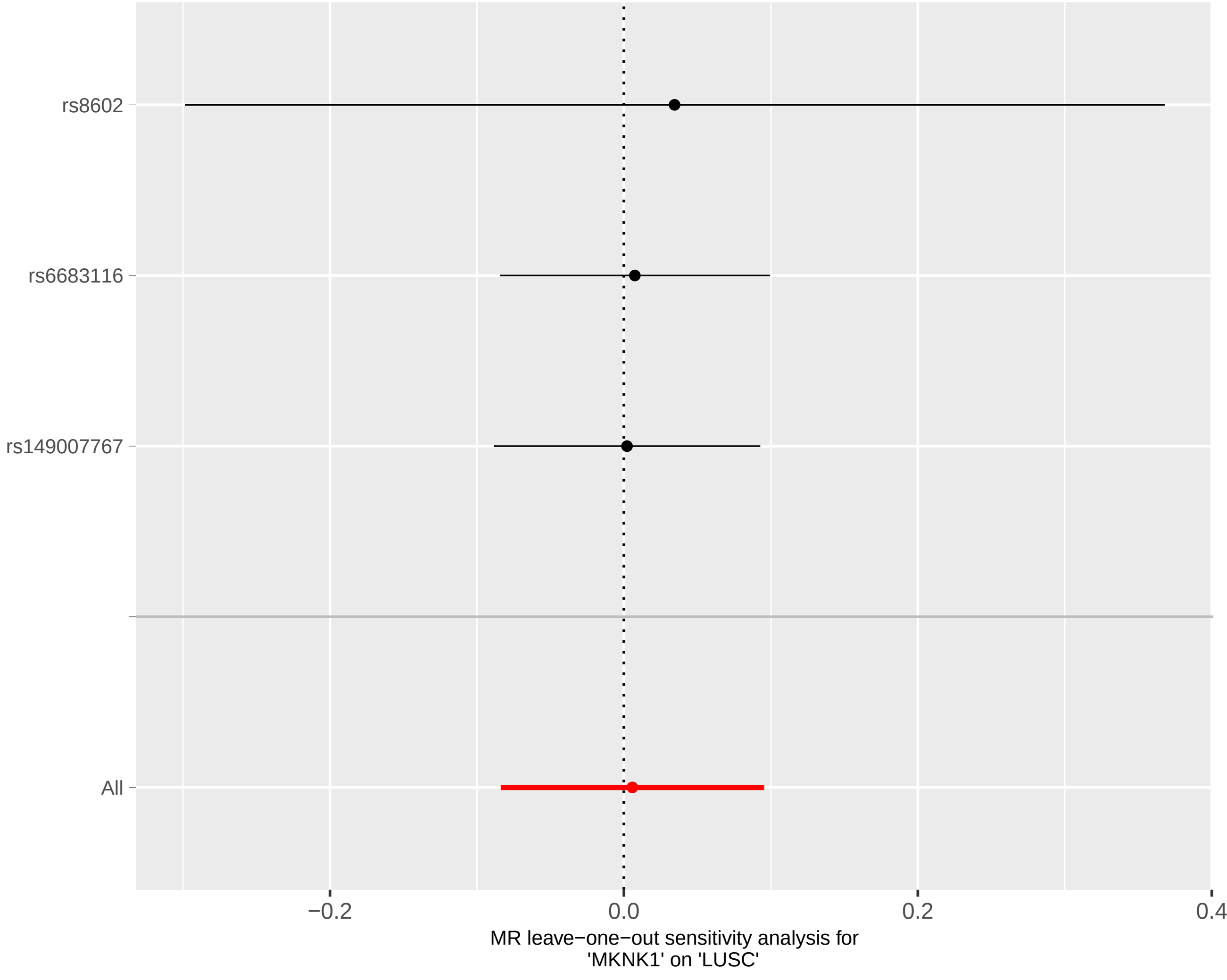
rs8602
rs6683116
rs149007767
All
−0.2
0.0
0.2
0.4
MR leave−one−out sensitivity analysis for
'MKNK1' on 'LUSC'

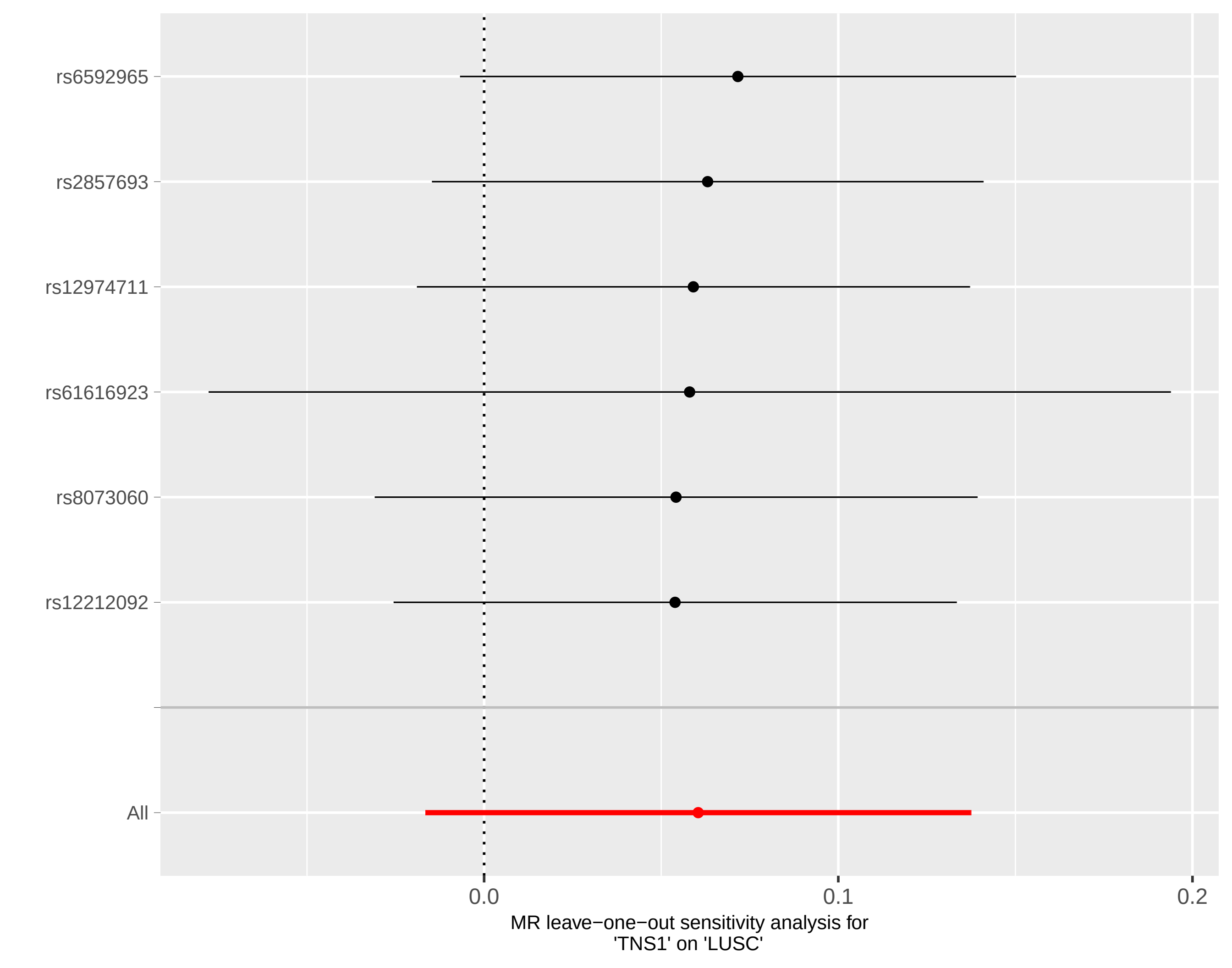

rs6592965
rs2857693
rs12974711
rs61616923
rs8073060
rs12212092
All
0.0
0.1
0.2
MR leave−one−out sensitivity analysis for
'TNS1' on 'LUSC'

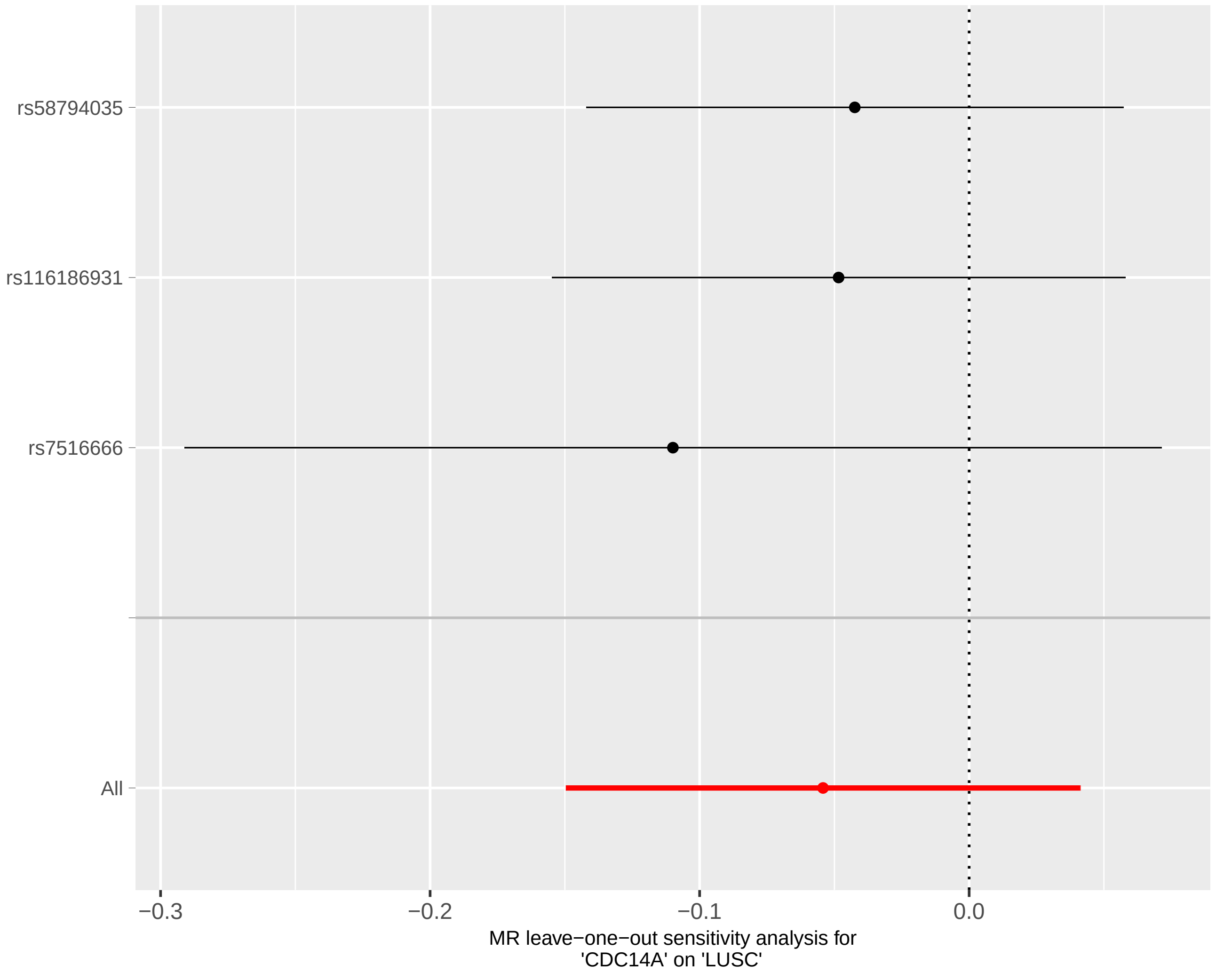
rs58794035
rs116186931
rs7516666
All
−0.3
−0.2
−0.1
0.0
MR leave−one−out sensitivity analysis for
'CDC14A' on 'LUSC'

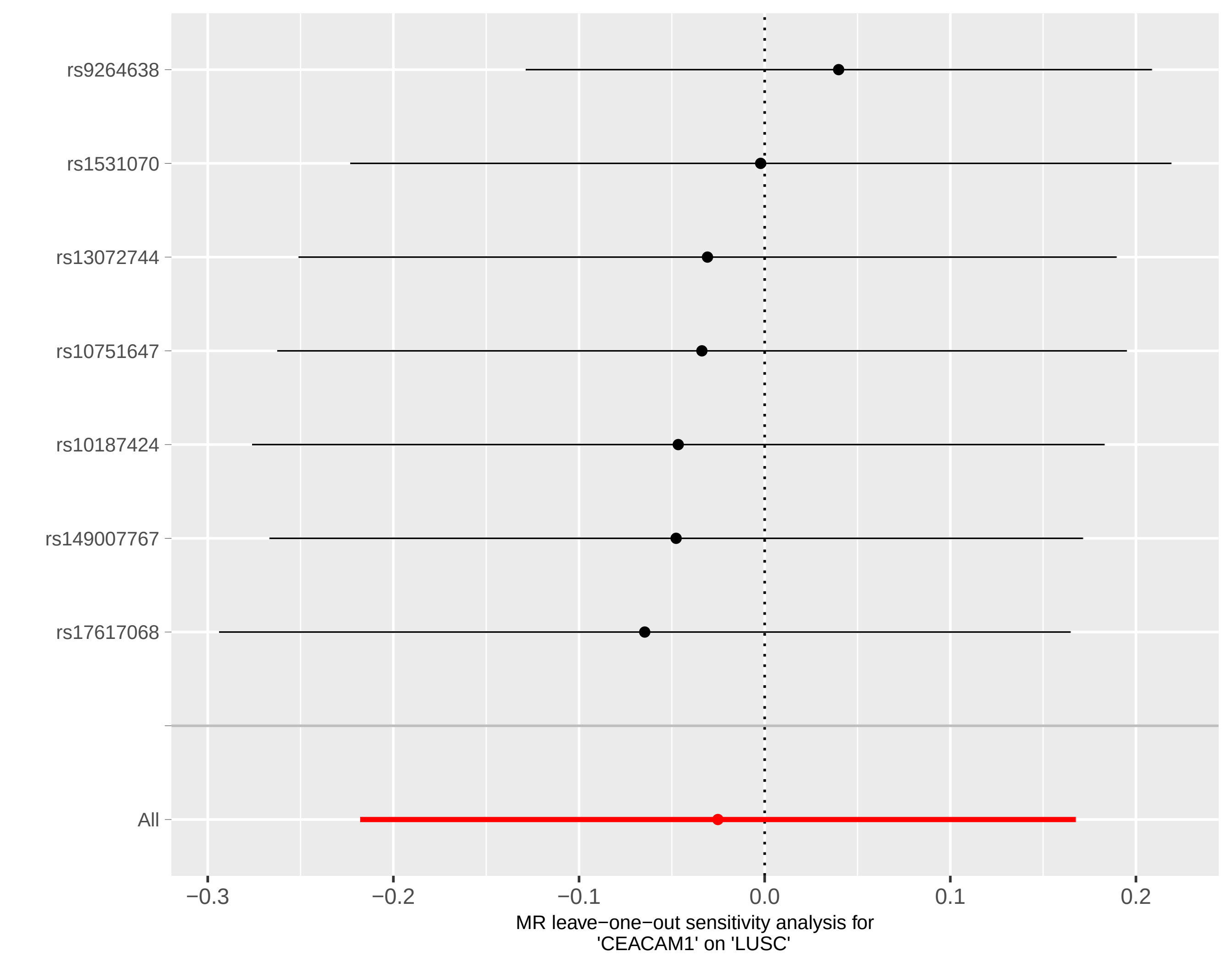
rs9264638
rs1531070
rs13072744
rs10751647
rs10187424
rs149007767
rs17617068
All
−0.3
−0.2
−0.1
0.0
0.1
0.2
MR leave−one−out sensitivity analysis for
'CEACAM1' on 'LUSC'

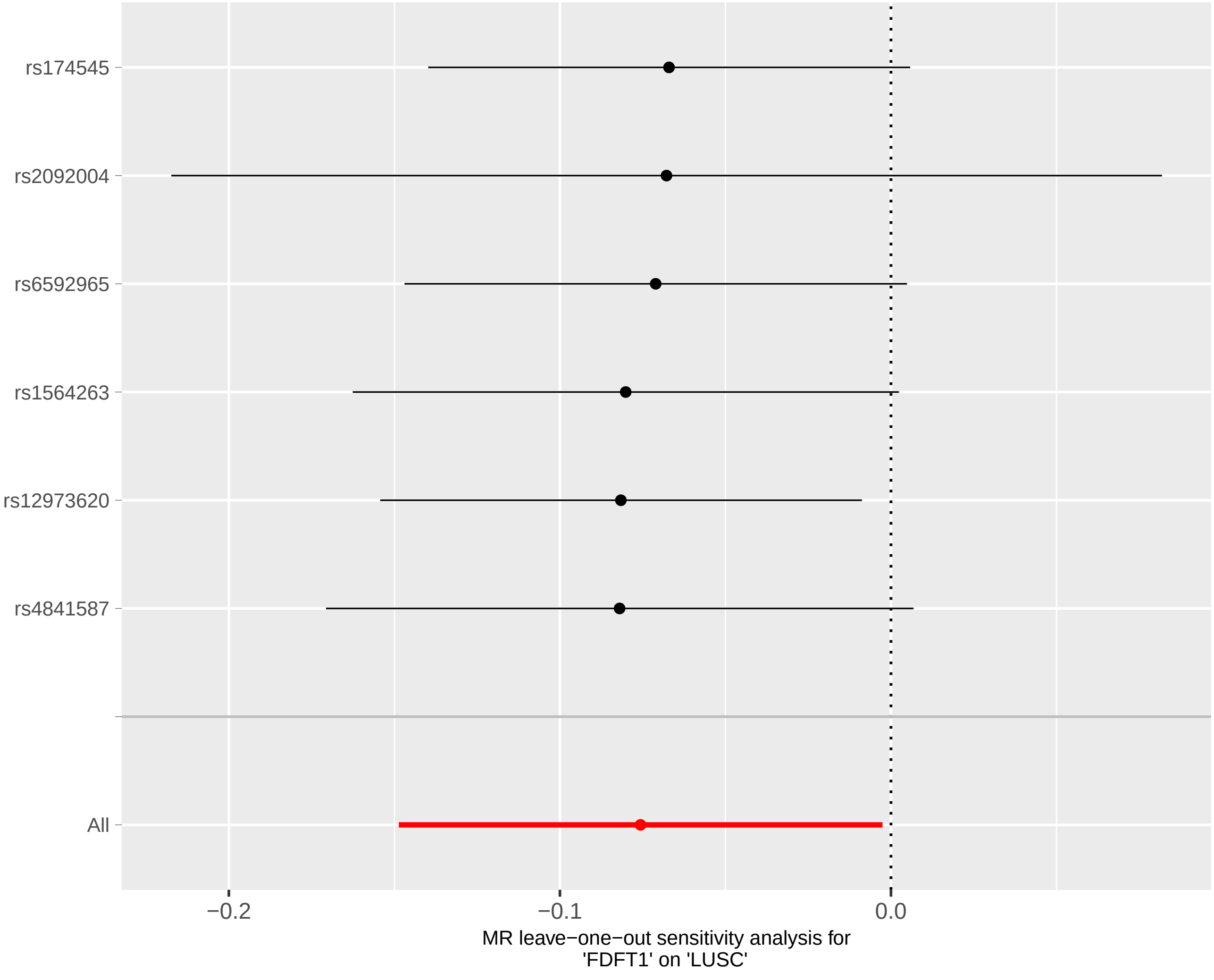

rs174545
rs2092004
rs6592965
rs1564263
rs12973620
rs4841587
All
−0.2
−0.1
0.0
MR leave−one−out sensitivity analysis for
'FDFT1' on 'LUSC'

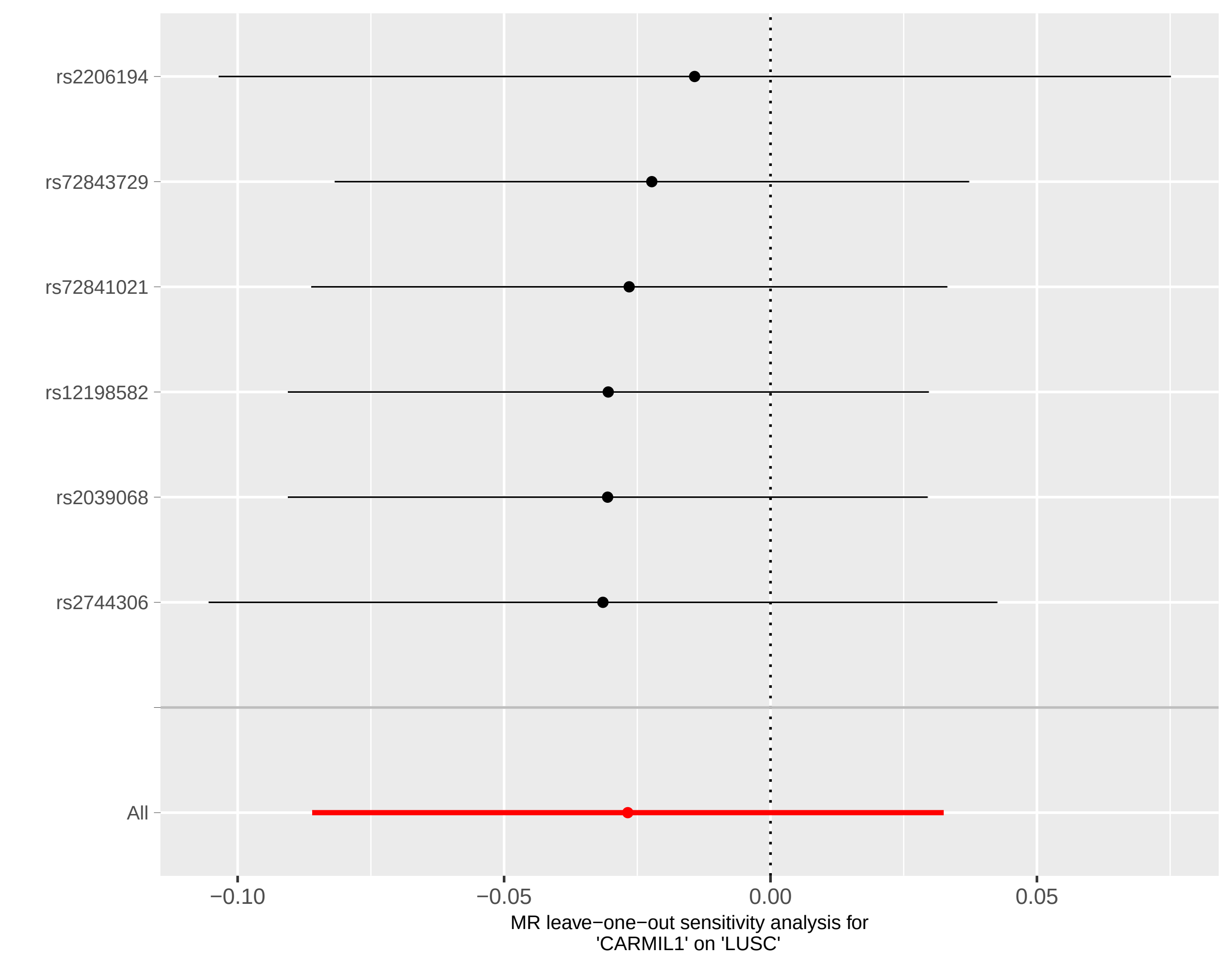
rs2206194
rs72843729
rs72841021
rs12198582
rs2039068
rs2744306
All
−0.10
−0.05
0.00
0.05
MR leave−one−out sensitivity analysis for
'CARMIL1' on 'LUSC'

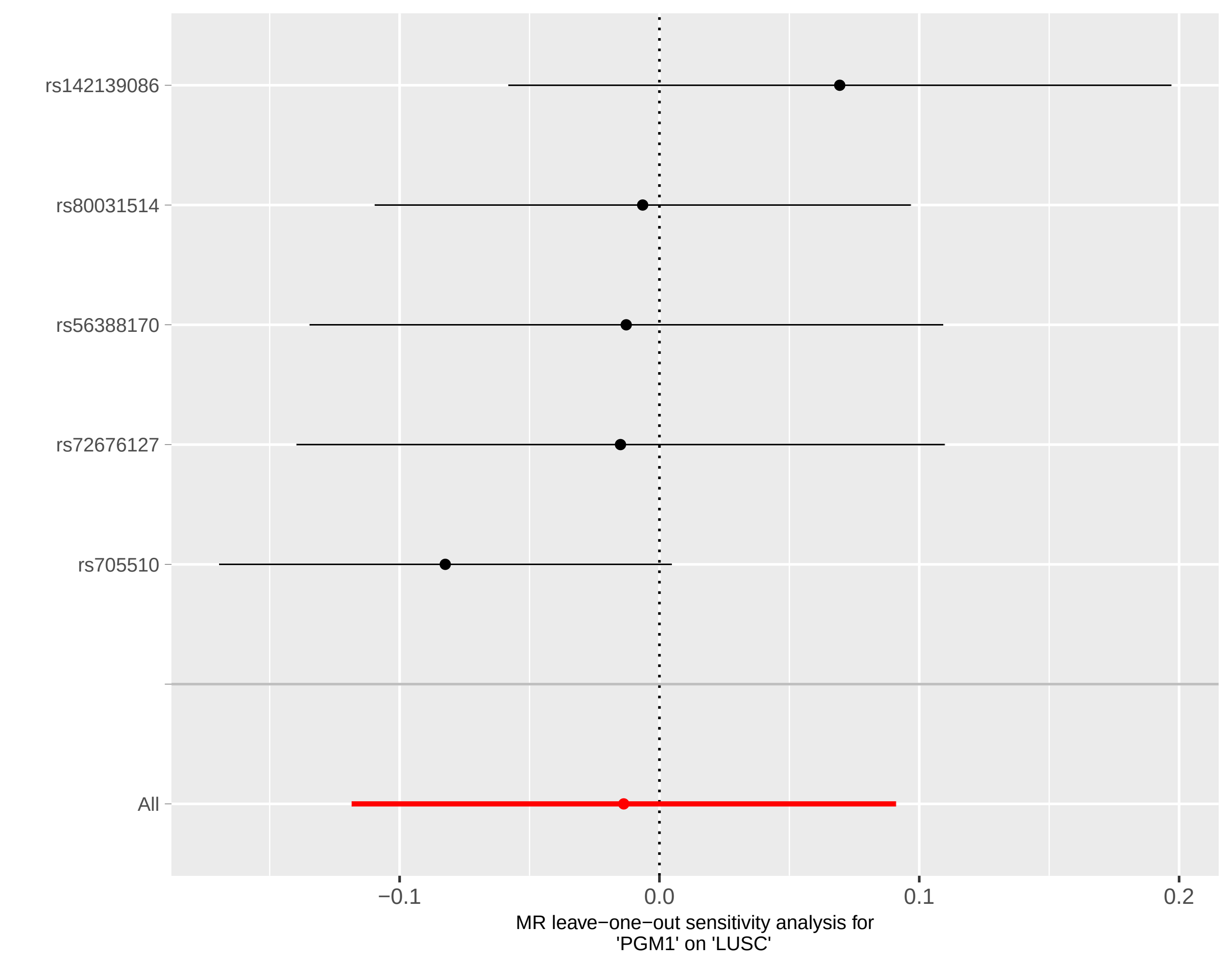

rs142139086
rs80031514
rs56388170
rs72676127
rs705510
All
−0.1
0.0
0.1
0.2
MR leave−one−out sensitivity analysis for
'PGM1' on 'LUSC'

Insufficient number of SNPs

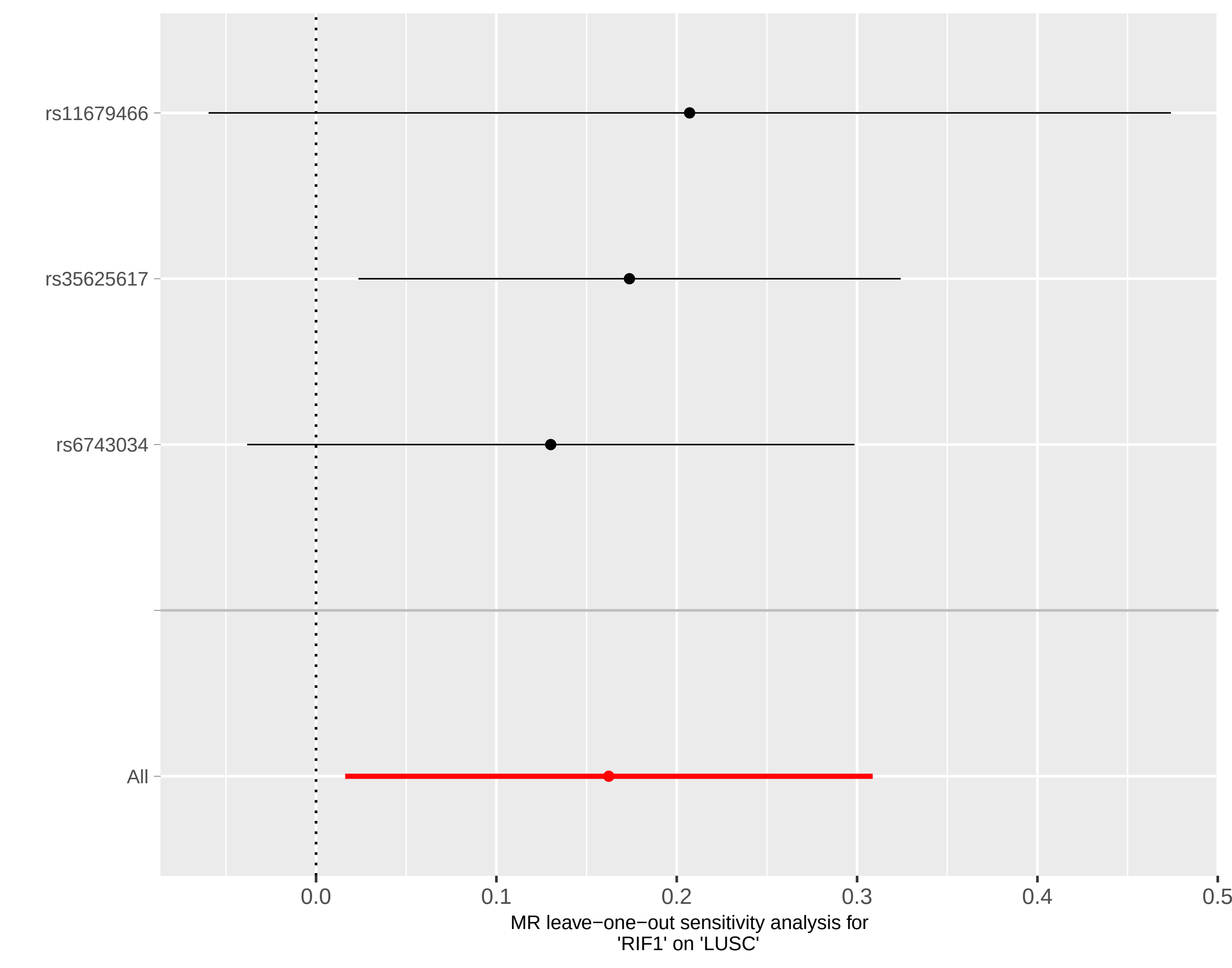

rs11679466
rs35625617
rs6743034
All
0.0
0.1
0.2
0.3
0.4
0.5
MR leave−one−out sensitivity analysis for
'RIF1' on 'LUSC'

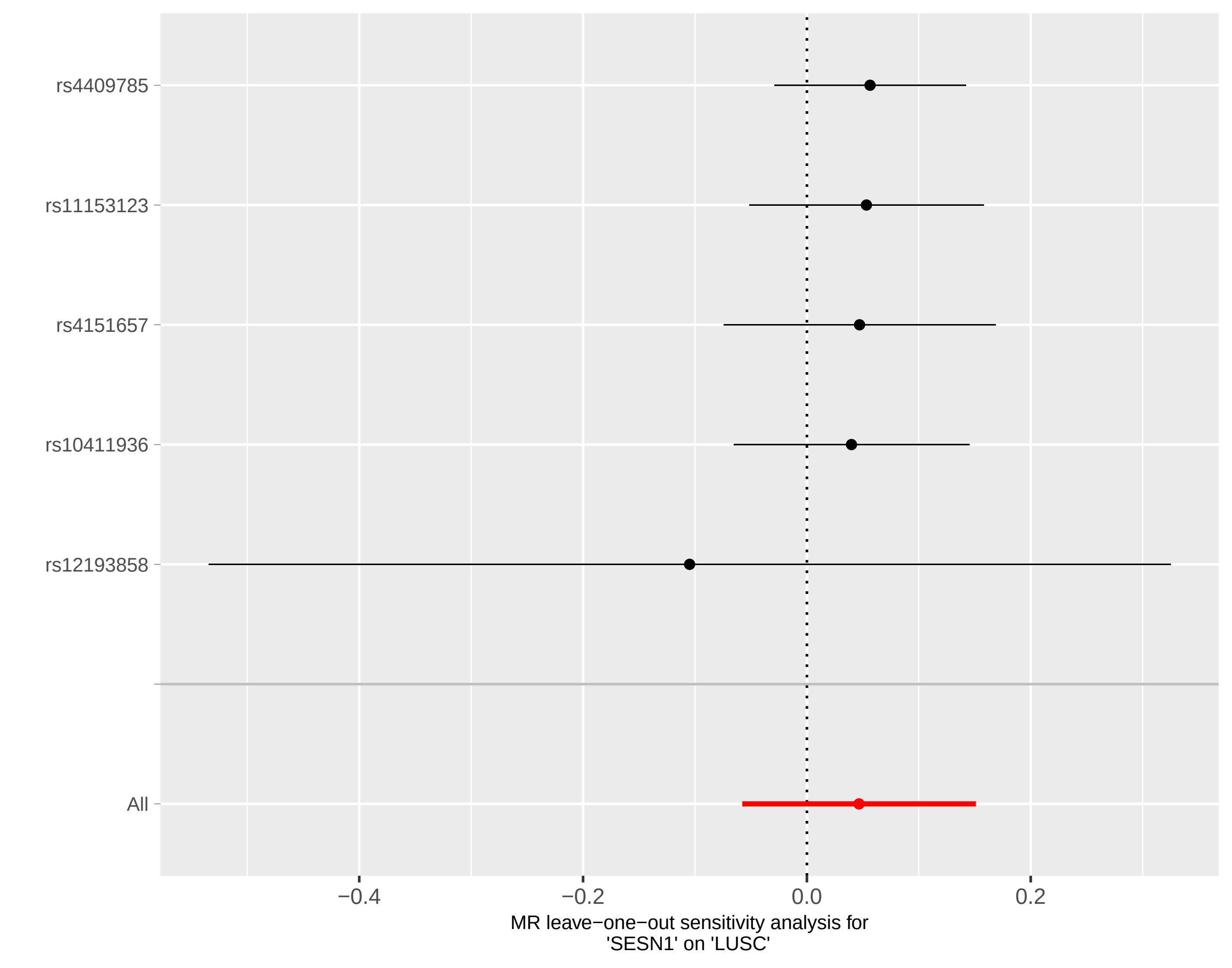
rs4409785
rs11153123
rs4151657
rs10411936
rs12193858
All
−0.4
−0.2
0.0
0.2
MR leave−one−out sensitivity analysis for
'SESN1' on 'LUSC'

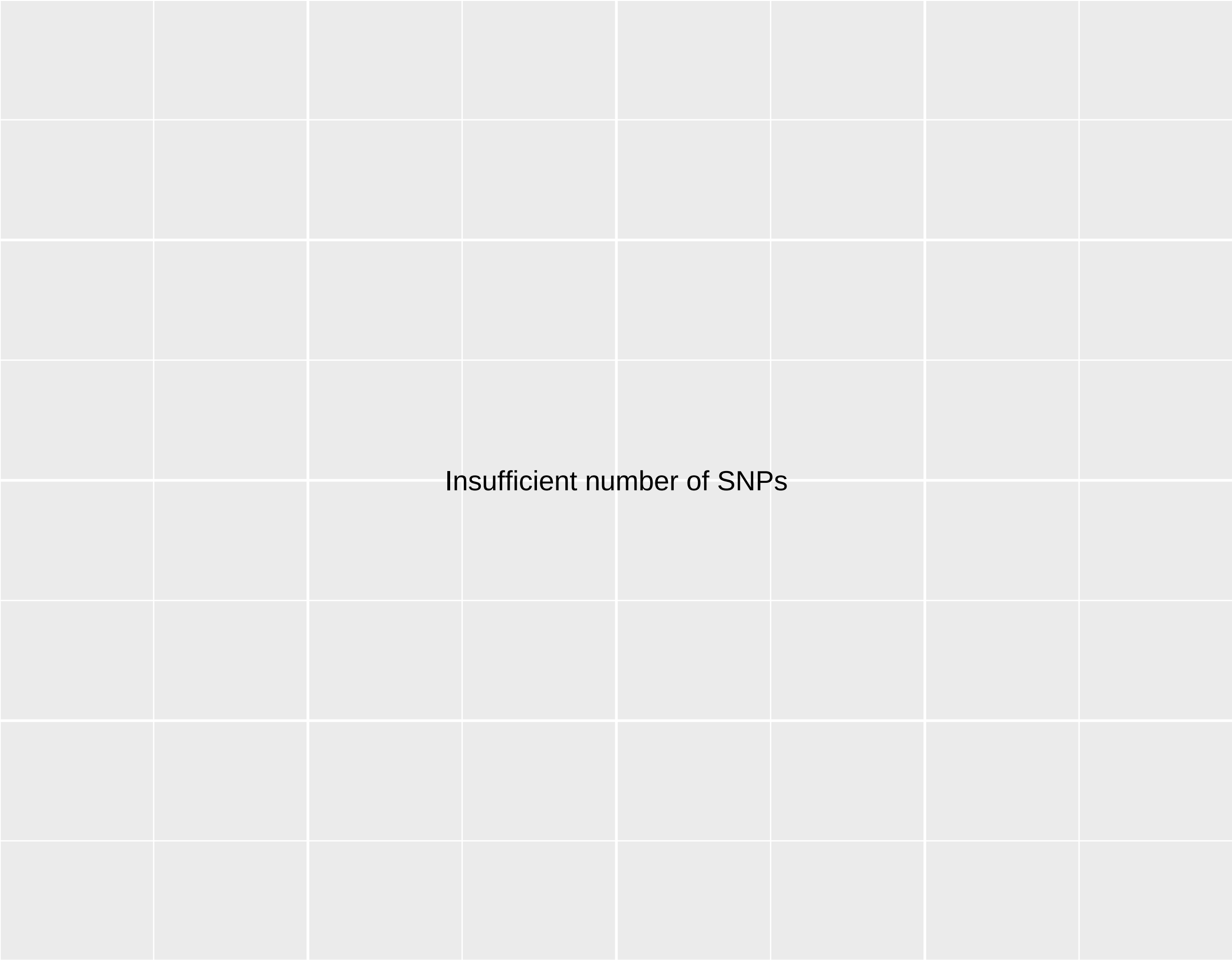
Insufficient number of SNPs

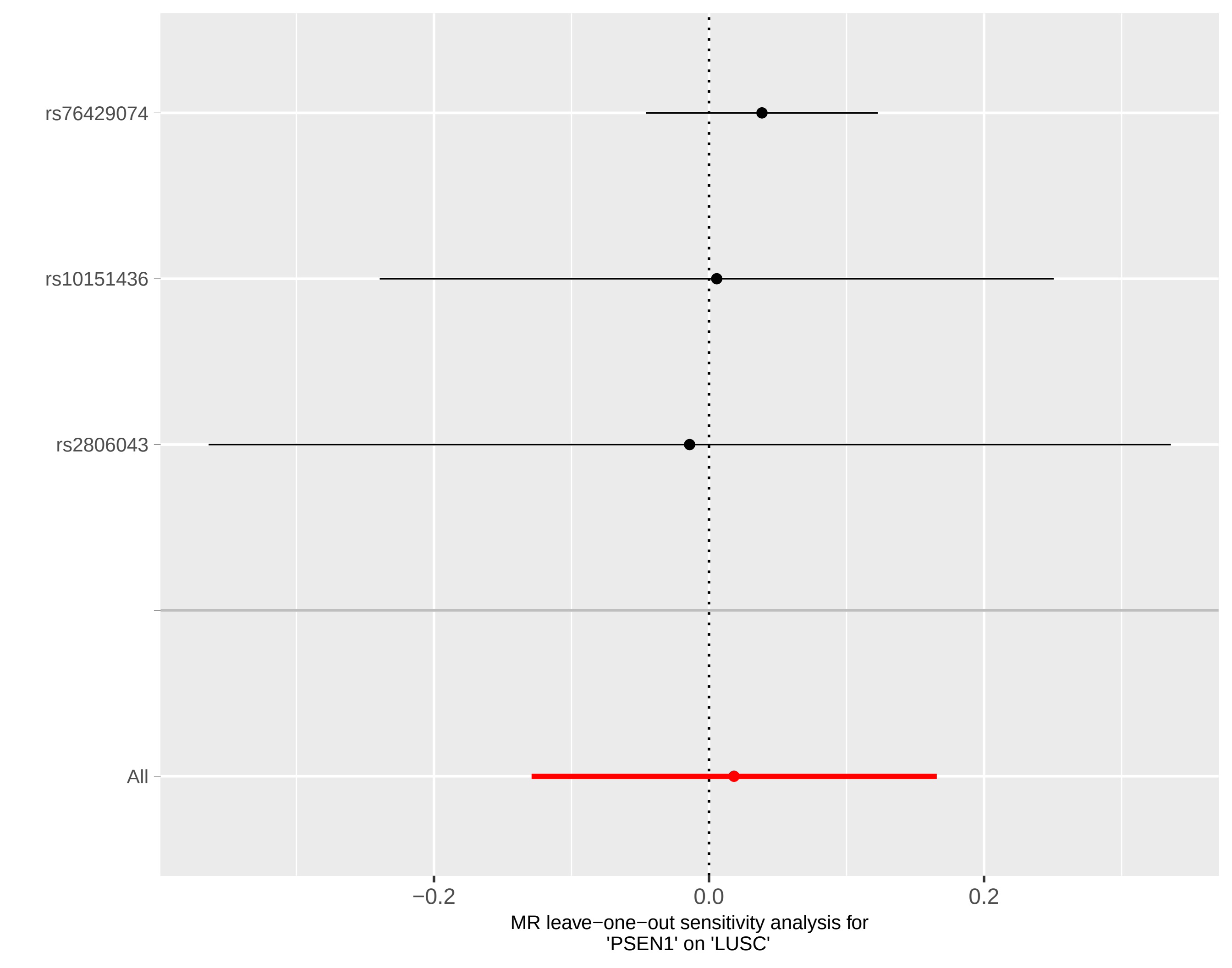

rs76429074
rs10151436
rs2806043
All
−0.2
0.0
0.2
MR leave−one−out sensitivity analysis for
'PSEN1' on 'LUSC'

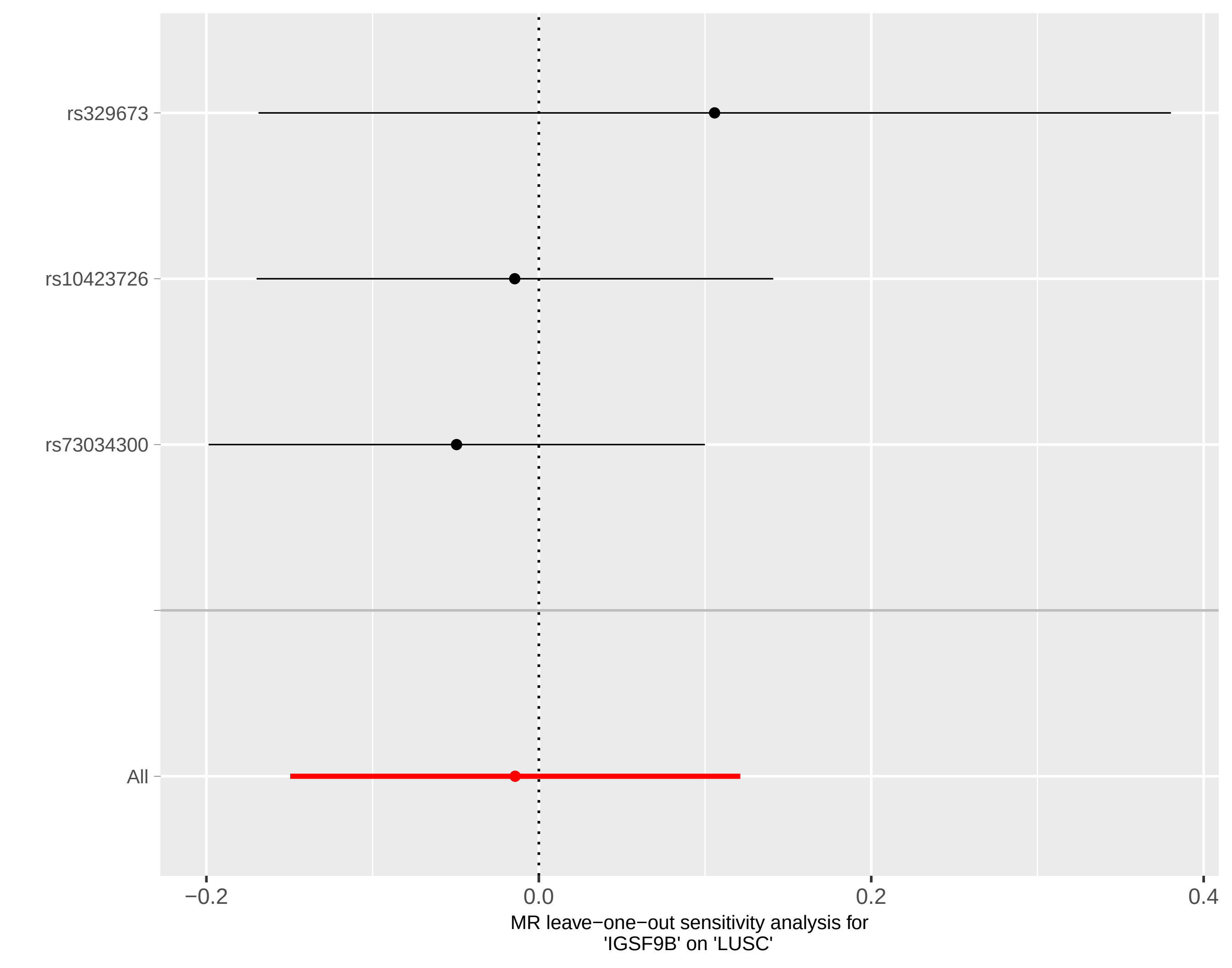
rs329673
rs10423726
rs73034300
All
−0.2
0.0
0.2
0.4
MR leave−one−out sensitivity analysis for
'IGSF9B' on 'LUSC'

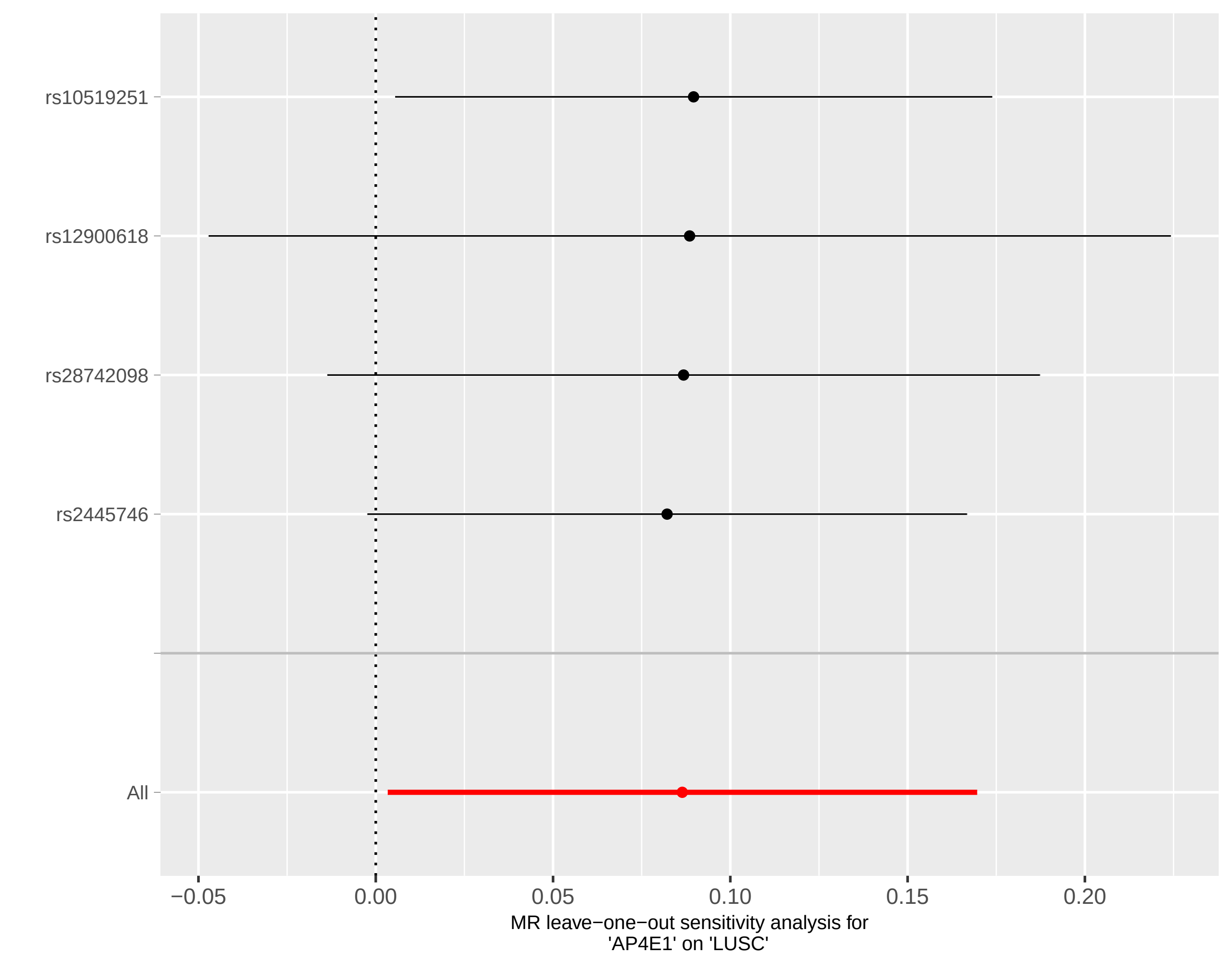
rs10519251
rs12900618
rs28742098
rs2445746
All
−0.05
0.00
0.05
0.10
0.15
0.20
MR leave−one−out sensitivity analysis for
'AP4E1' on 'LUSC'

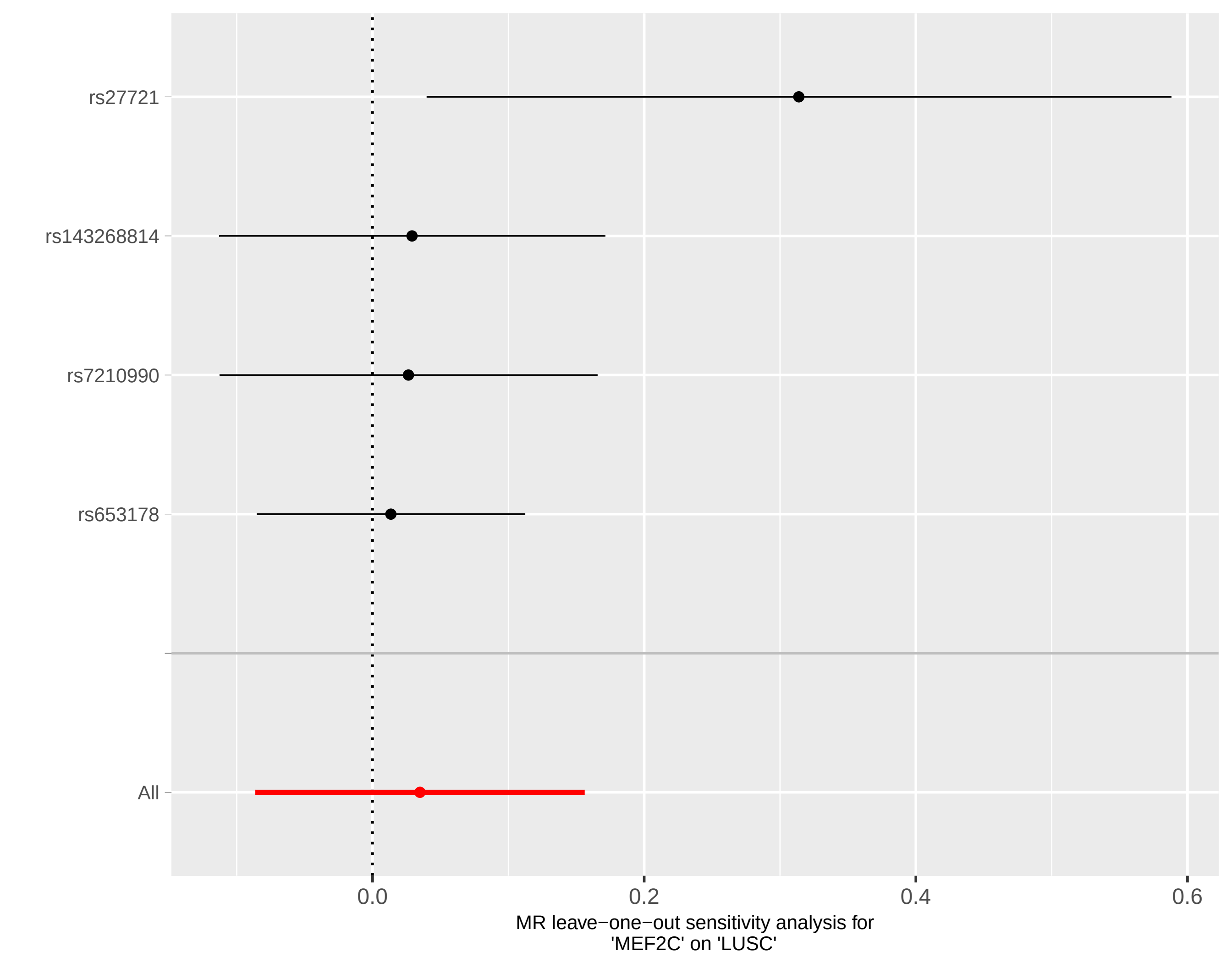

rs27721
rs143268814
rs7210990
rs653178
All
0.0
0.2
0.4
0.6
MR leave−one−out sensitivity analysis for
'MEF2C' on 'LUSC'

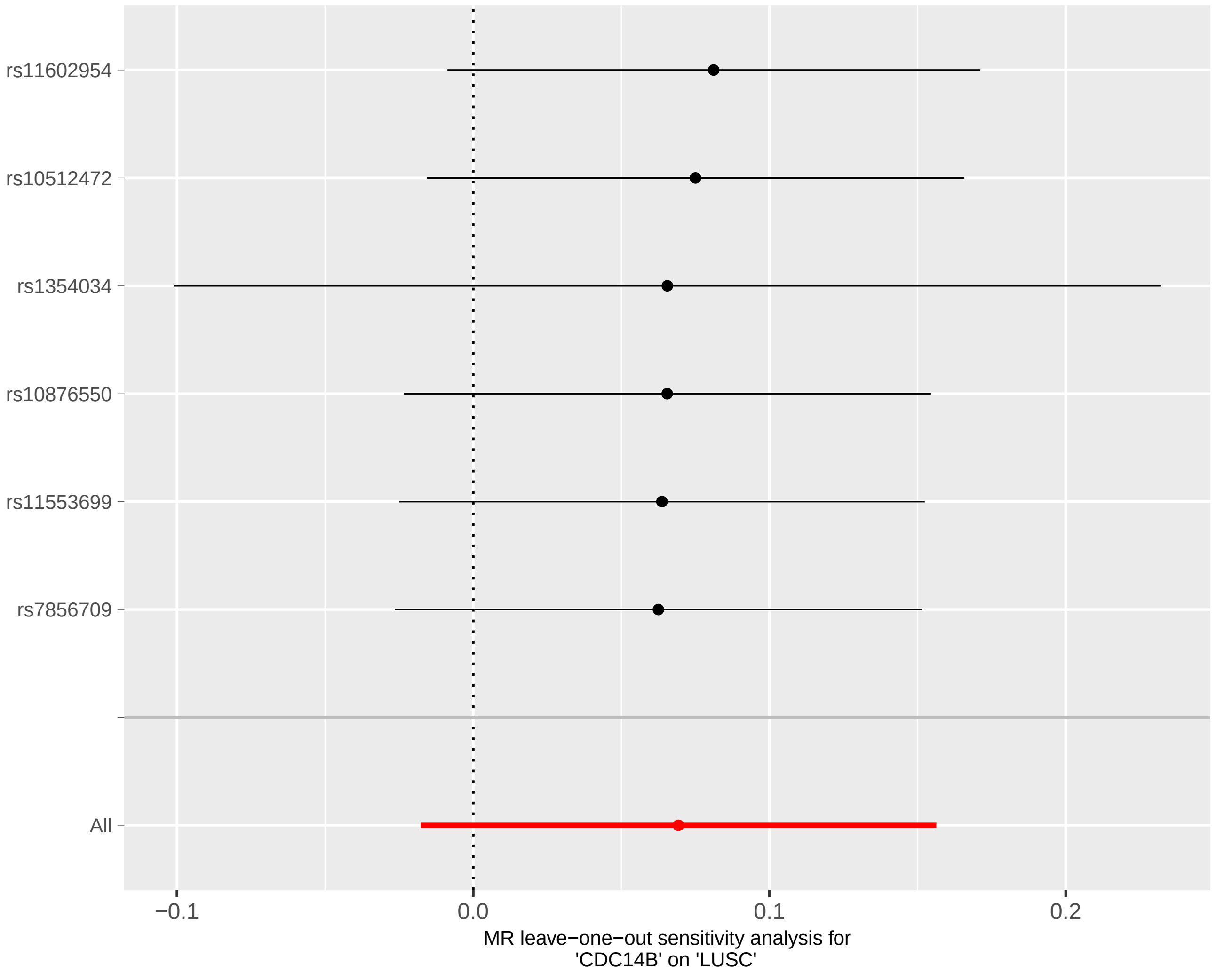
rs11602954
rs10512472
rs1354034
rs10876550
rs11553699
rs7856709
All
−0.1
0.0
0.1
0.2
MR leave−one−out sensitivity analysis for
'CDC14B' on 'LUSC'

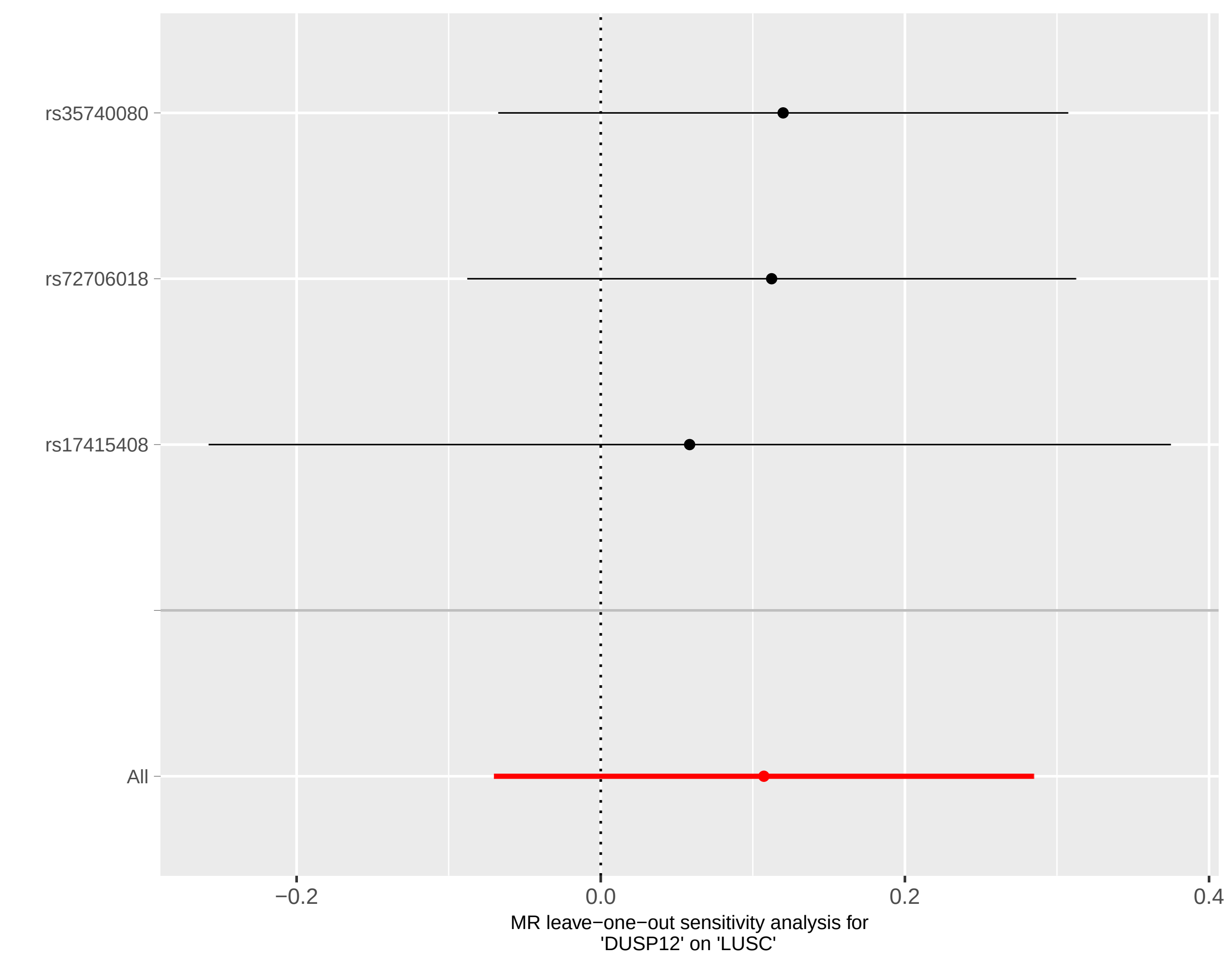

rs35740080
rs72706018
rs17415408
All
−0.2
0.0
0.2
0.4
MR leave−one−out sensitivity analysis for
'DUSP12' on 'LUSC'

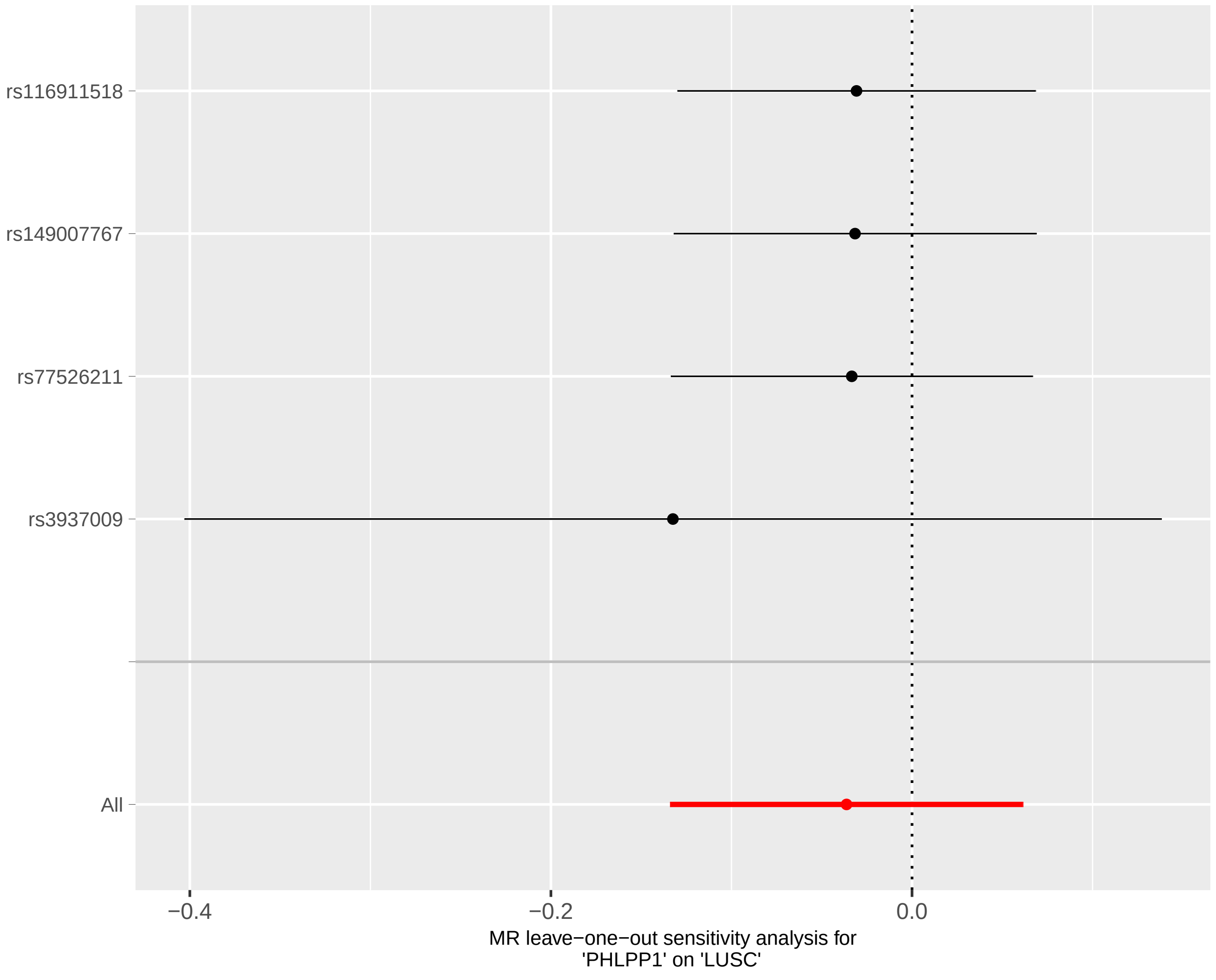

rs116911518
rs149007767
rs77526211
rs3937009
All
−0.4
−0.2
0.0
MR leave−one−out sensitivity analysis for
'PHLPP1' on 'LUSC'

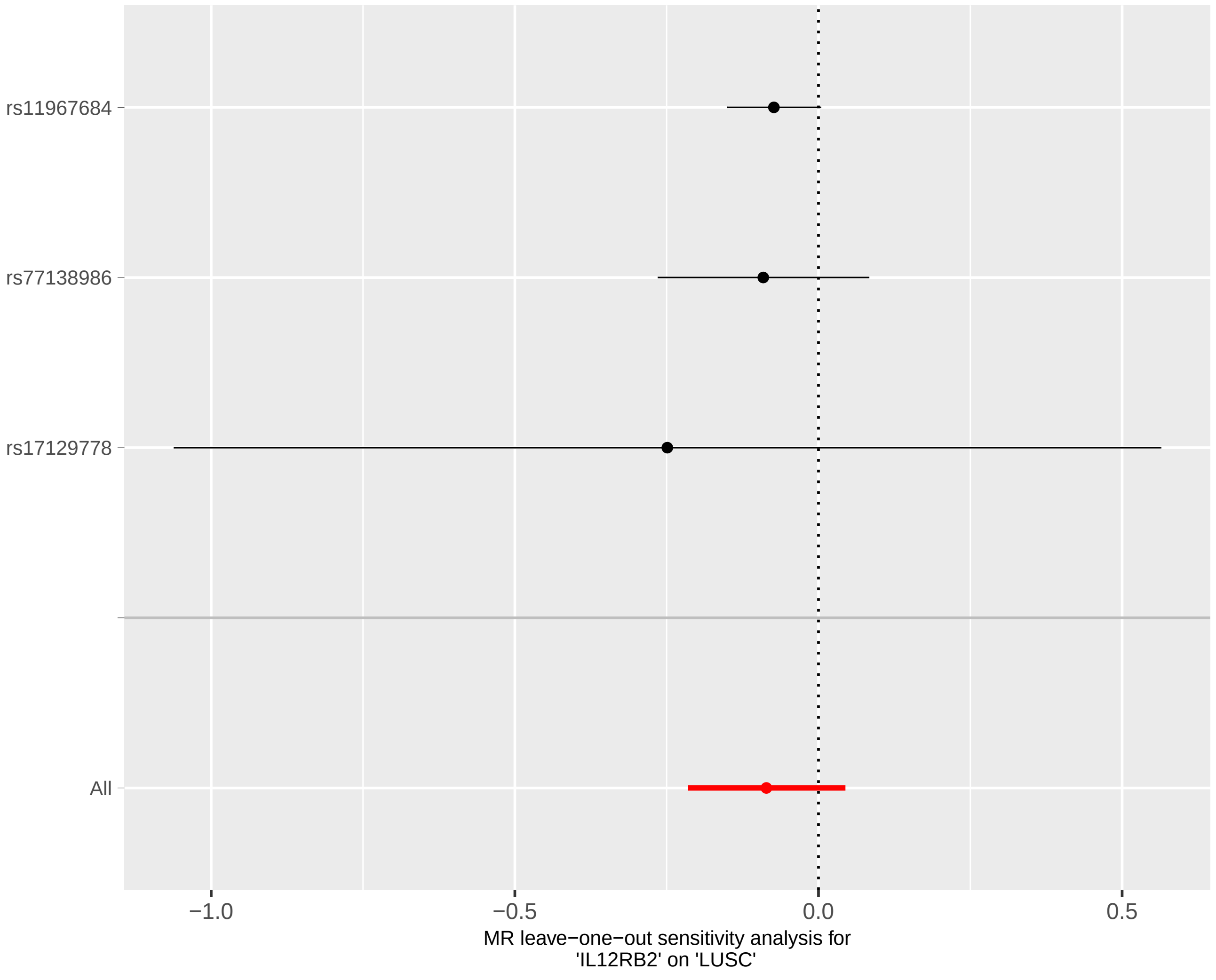

rs11967684
rs77138986
rs17129778
All
−1.0
−0.5
0.0
0.5
MR leave−one−out sensitivity analysis for
'IL12RB2' on 'LUSC'

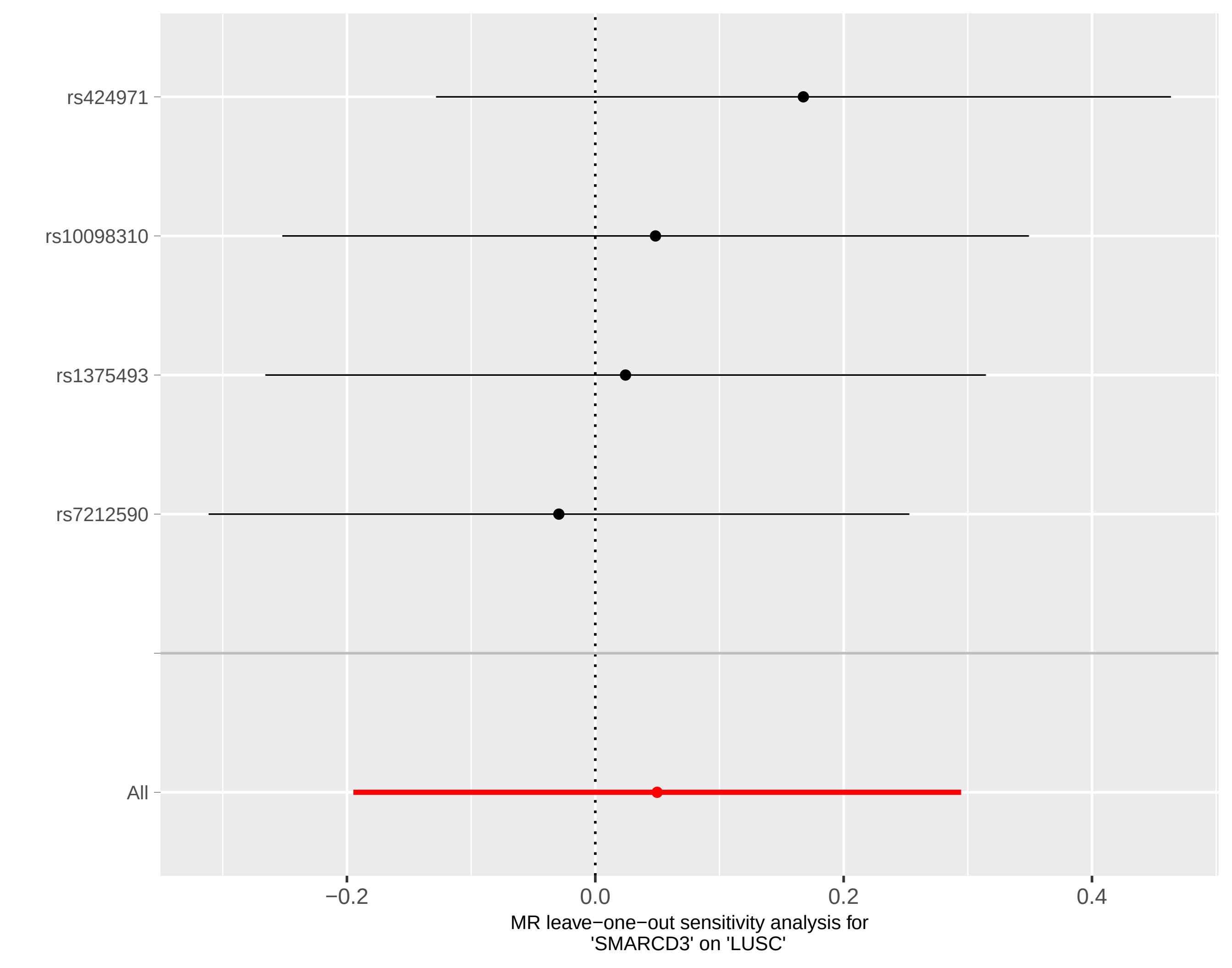
rs424971
rs10098310
rs1375493
rs7212590
All
−0.2
0.0
0.2
0.4
MR leave−one−out sensitivity analysis for
'SMARCD3' on 'LUSC'

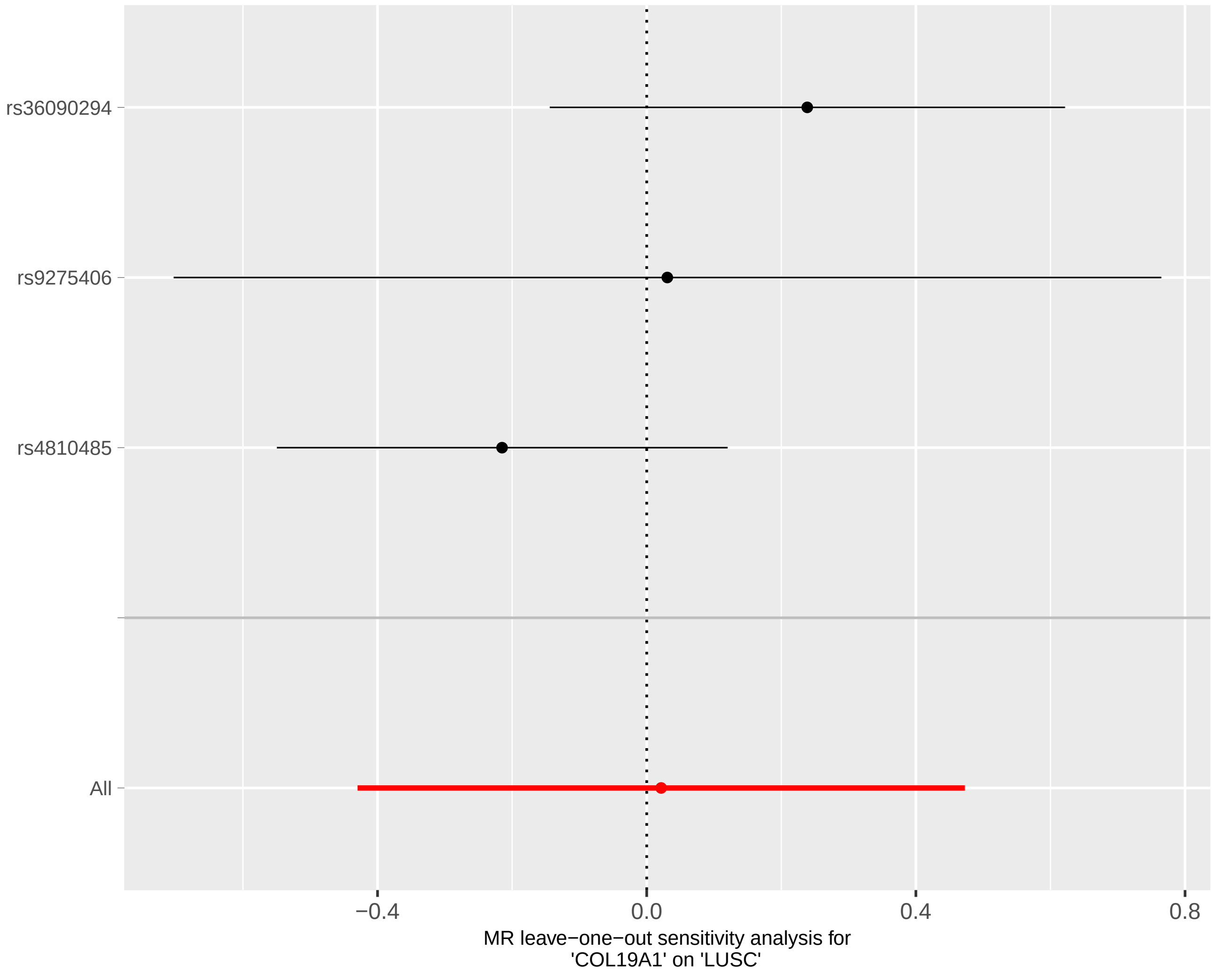

rs36090294
rs9275406
rs4810485
All
−0.4
0.0
0.4
0.8
MR leave−one−out sensitivity analysis for
'COL19A1' on 'LUSC'

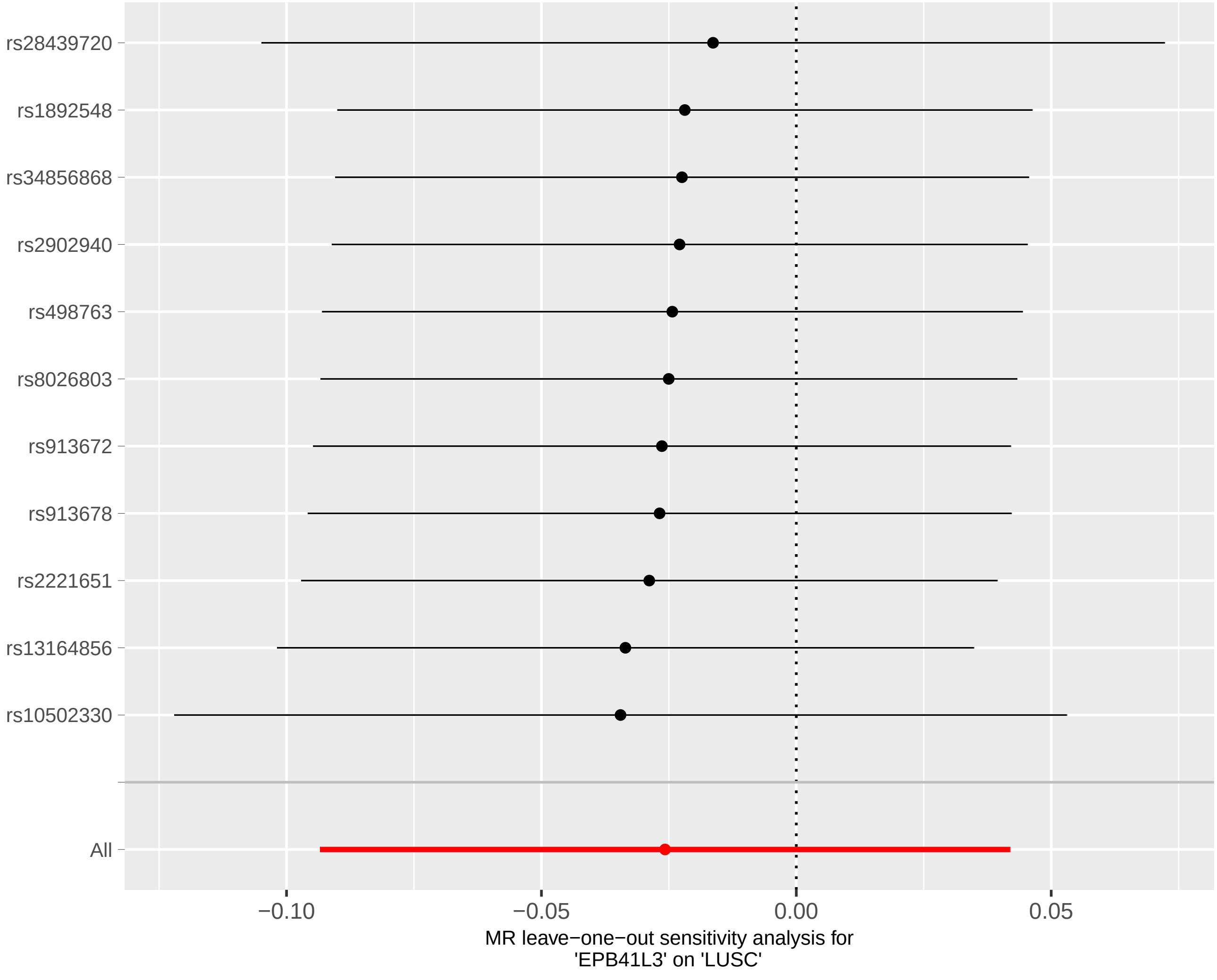

rs28439720
rs1892548
rs34856868
rs2902940
rs498763
rs8026803
rs913672
rs913678
rs2221651
rs13164856
rs10502330
All
−0.10
−0.05
0.00
0.05
MR leave−one−out sensitivity analysis for
'EPB41L3' on 'LUSC'

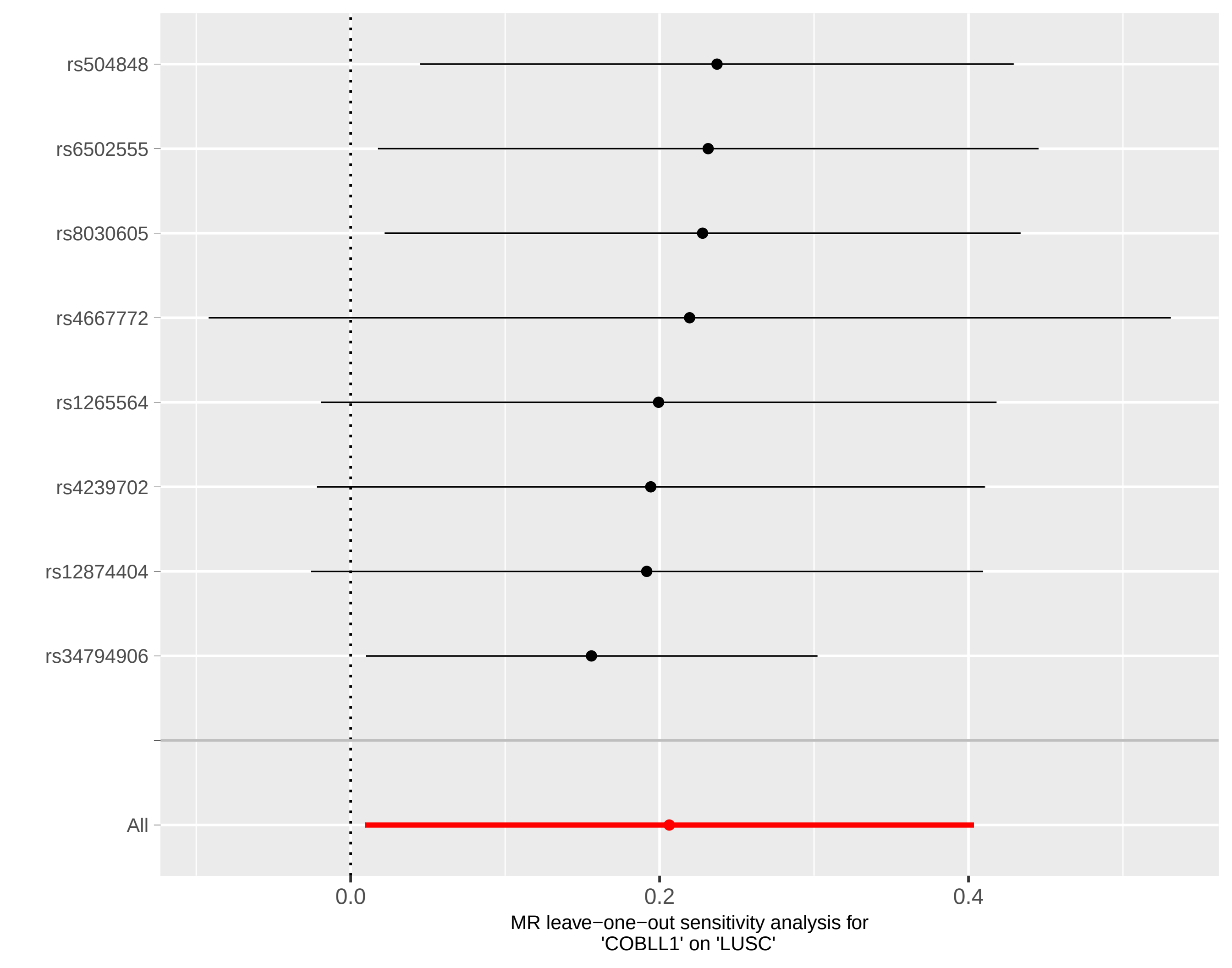
rs504848
rs6502555
rs8030605
rs4667772
rs1265564
rs4239702
rs12874404
rs34794906
All
0.0
0.2
0.4
MR leave−one−out sensitivity analysis for
'COBLL1' on 'LUSC'

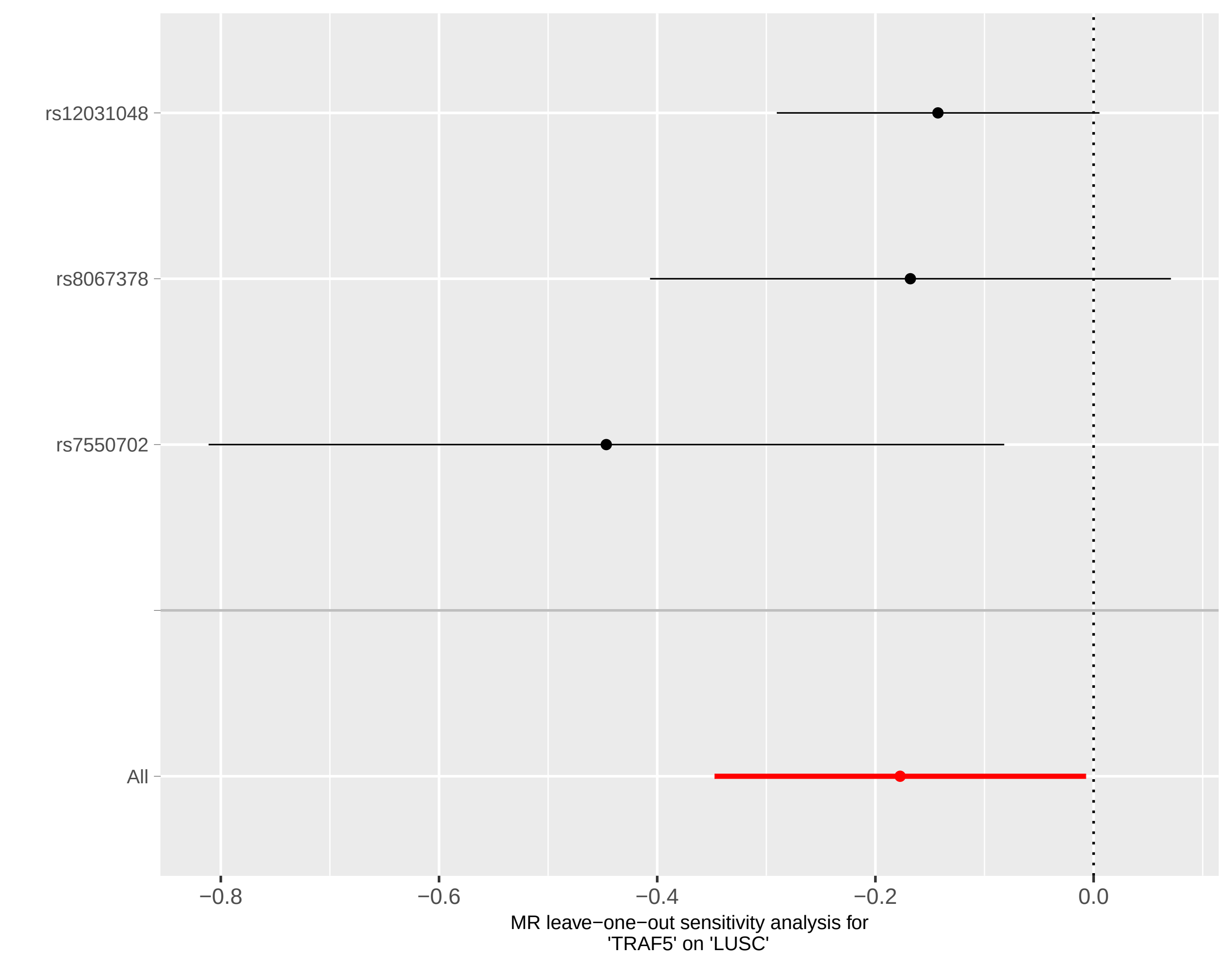

rs12031048
rs8067378
rs7550702
All
−0.8
−0.6
−0.4
−0.2
0.0
MR leave−one−out sensitivity analysis for
'TRAF5' on 'LUSC'

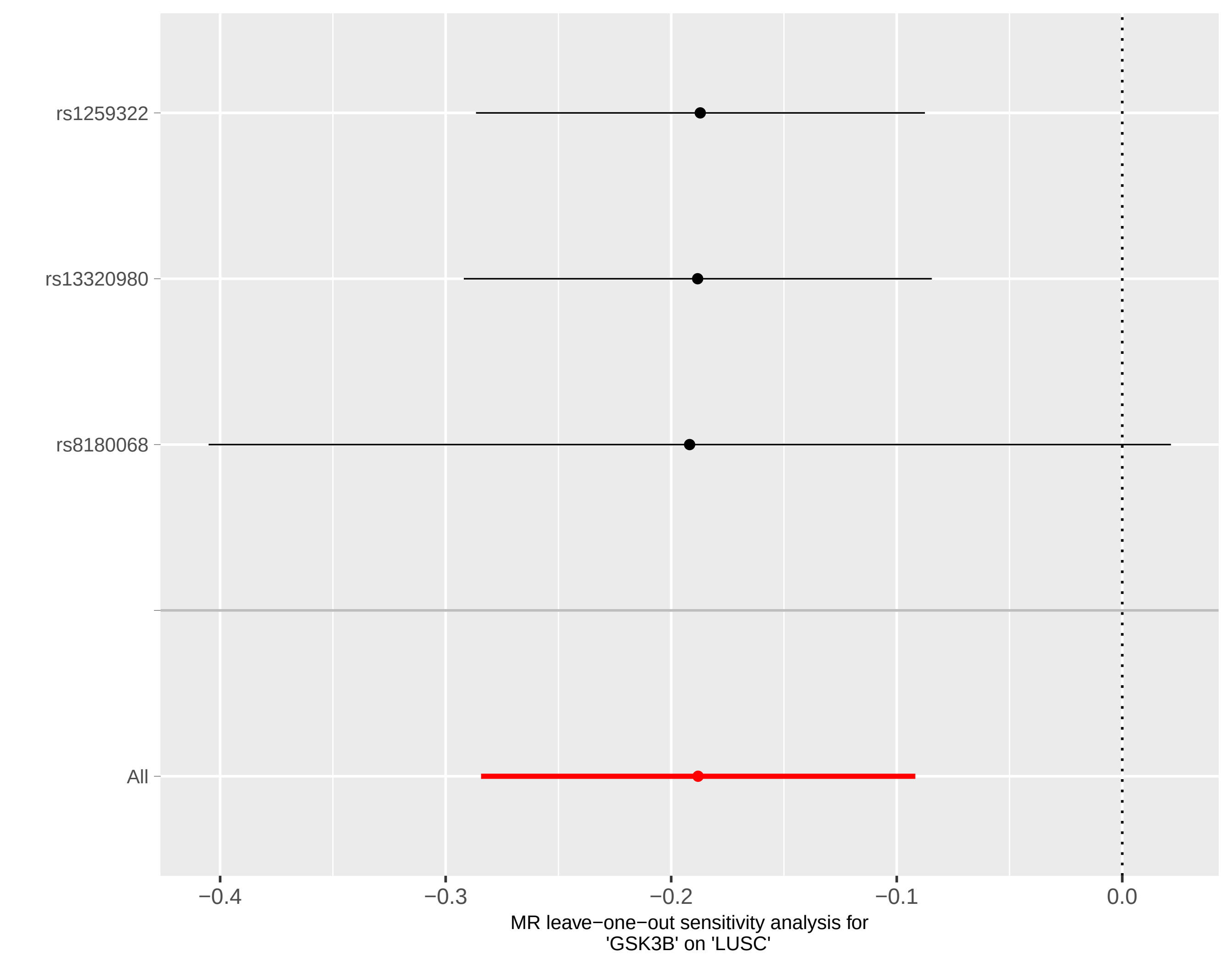
rs1259322
rs13320980
rs8180068
All
−0.4
−0.3
−0.2
−0.1
0.0
MR leave−one−out sensitivity analysis for
'GSK3B' on 'LUSC'

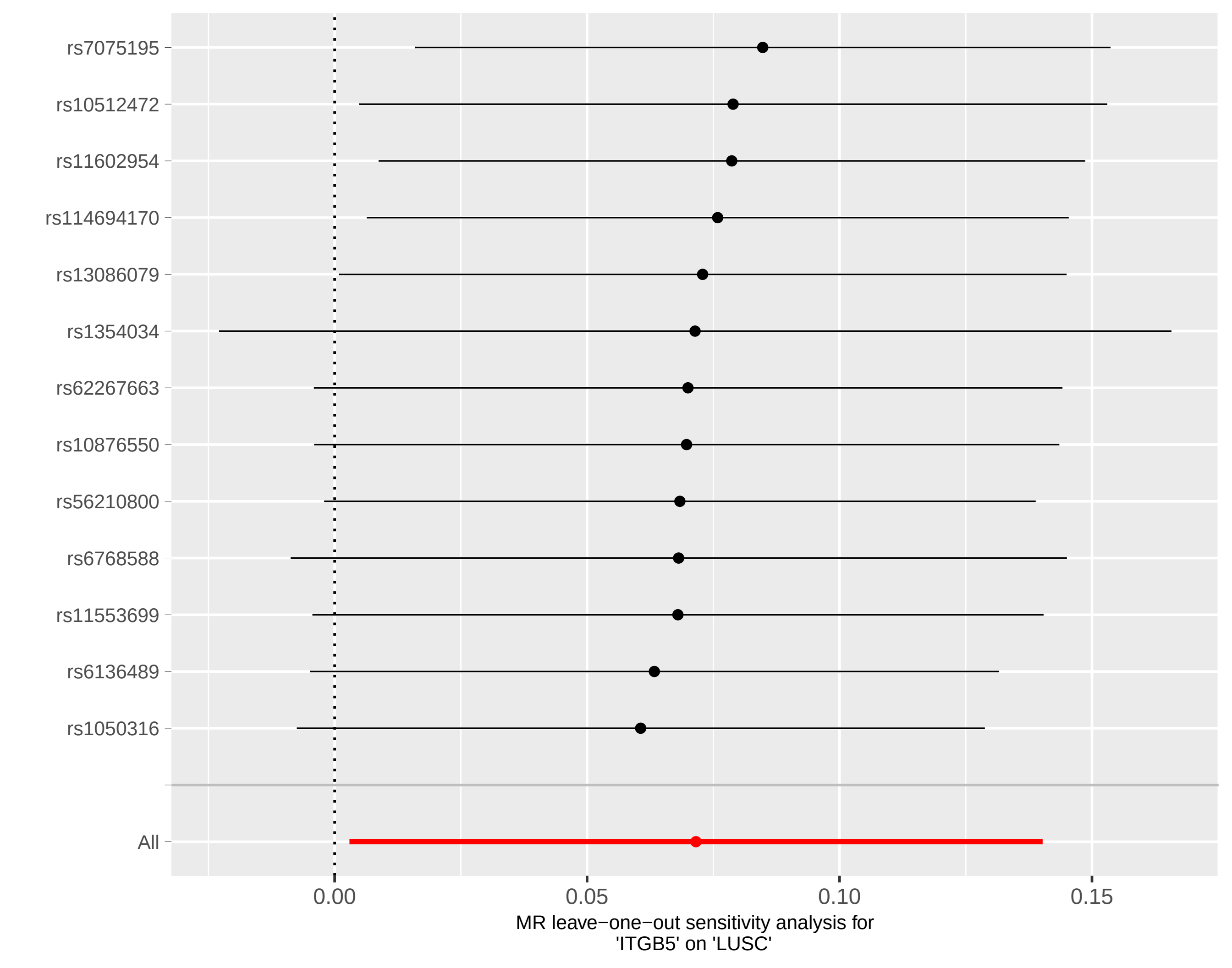

rs7075195
rs10512472
rs11602954
rs114694170
rs13086079
rs1354034
rs62267663
rs10876550
rs56210800
rs6768588
rs11553699
rs6136489
rs1050316
All
0.00
0.05
0.10
0.15
MR leave−one−out sensitivity analysis for
'ITGB5' on 'LUSC'

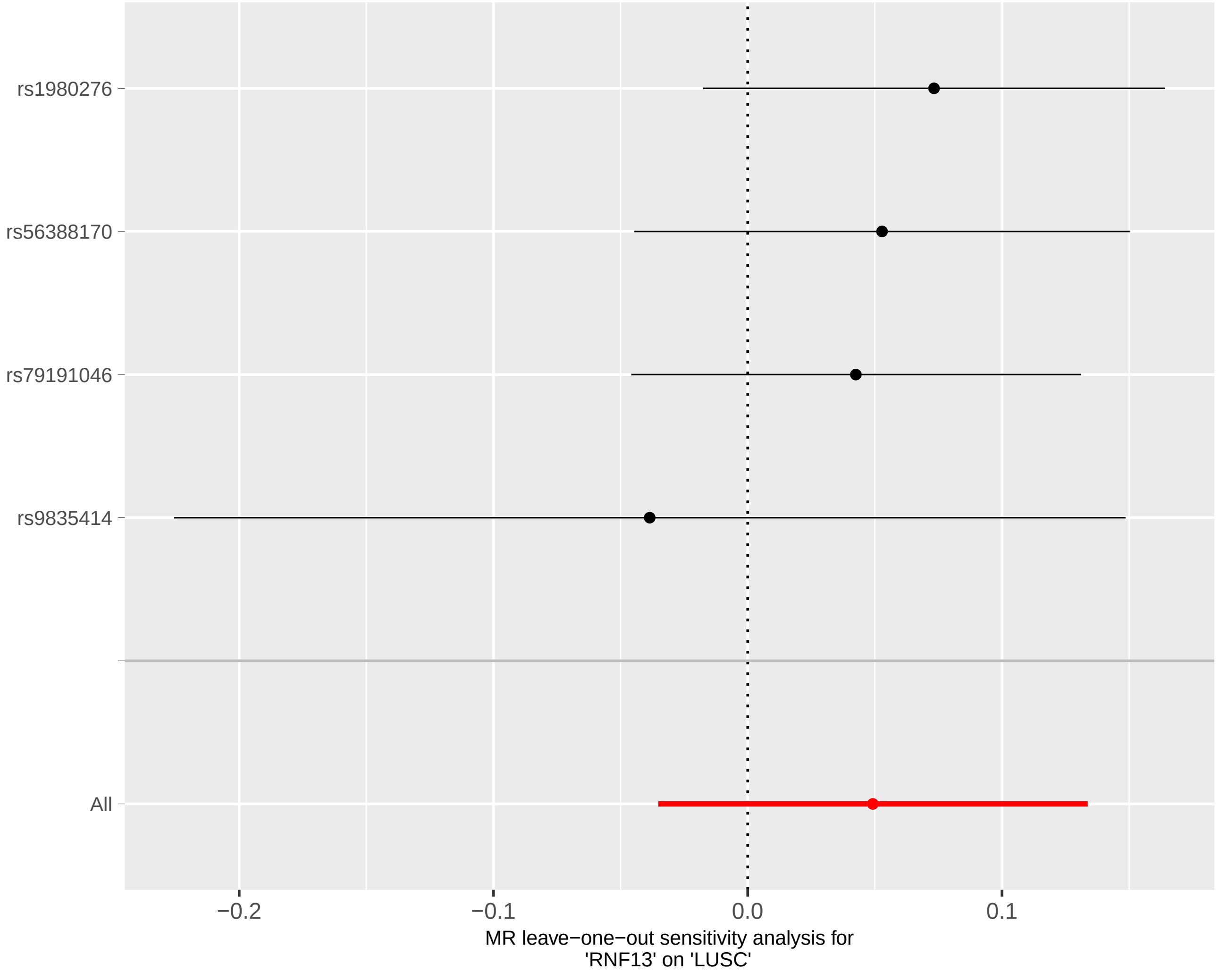

rs1980276
rs56388170
rs79191046
rs9835414
All
−0.2
−0.1
0.0
0.1
MR leave−one−out sensitivity analysis for
'RNF13' on 'LUSC'

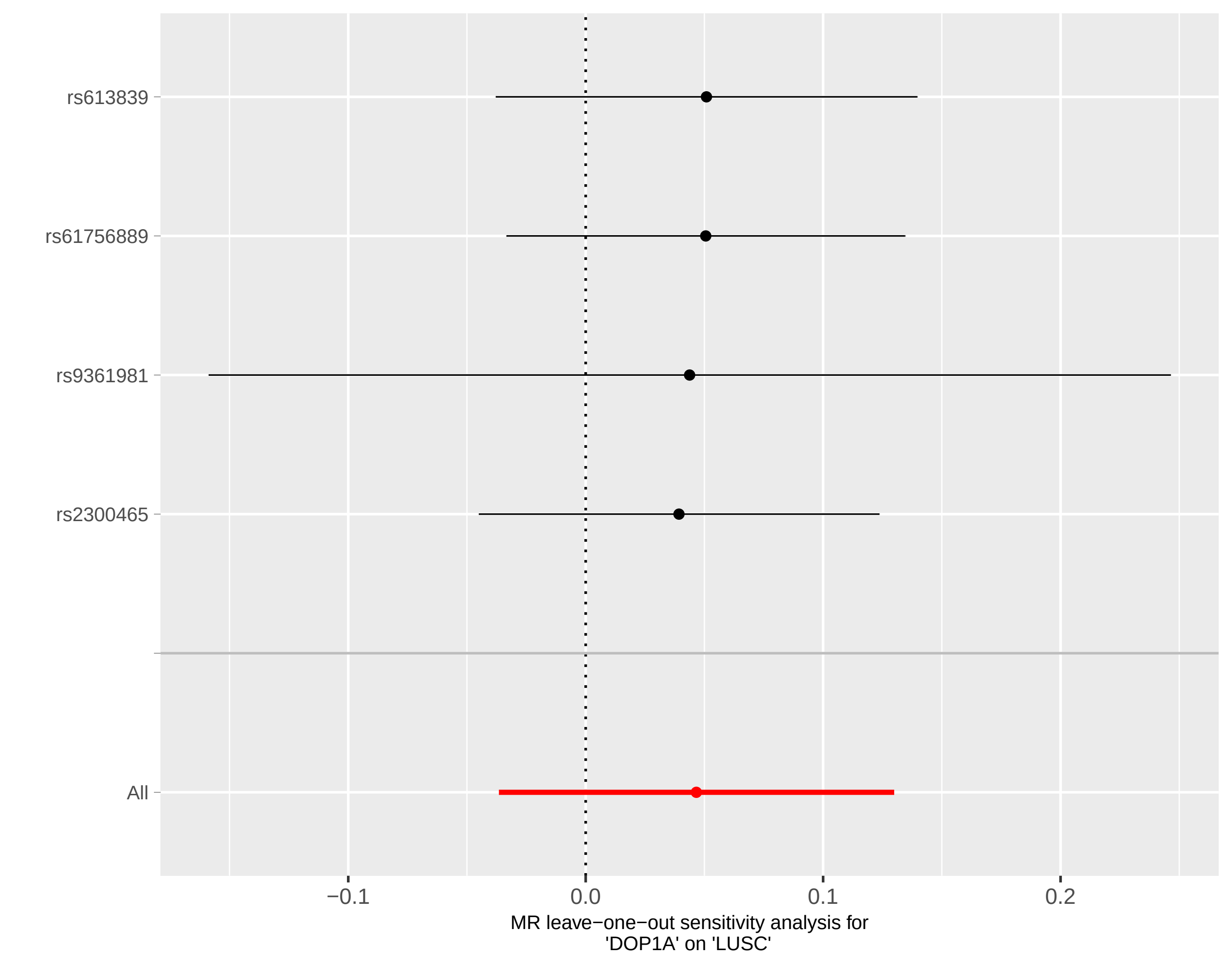
rs613839
rs61756889
rs9361981
rs2300465
All
−0.1
0.0
0.1
0.2
MR leave−one−out sensitivity analysis for
'DOP1A' on 'LUSC'

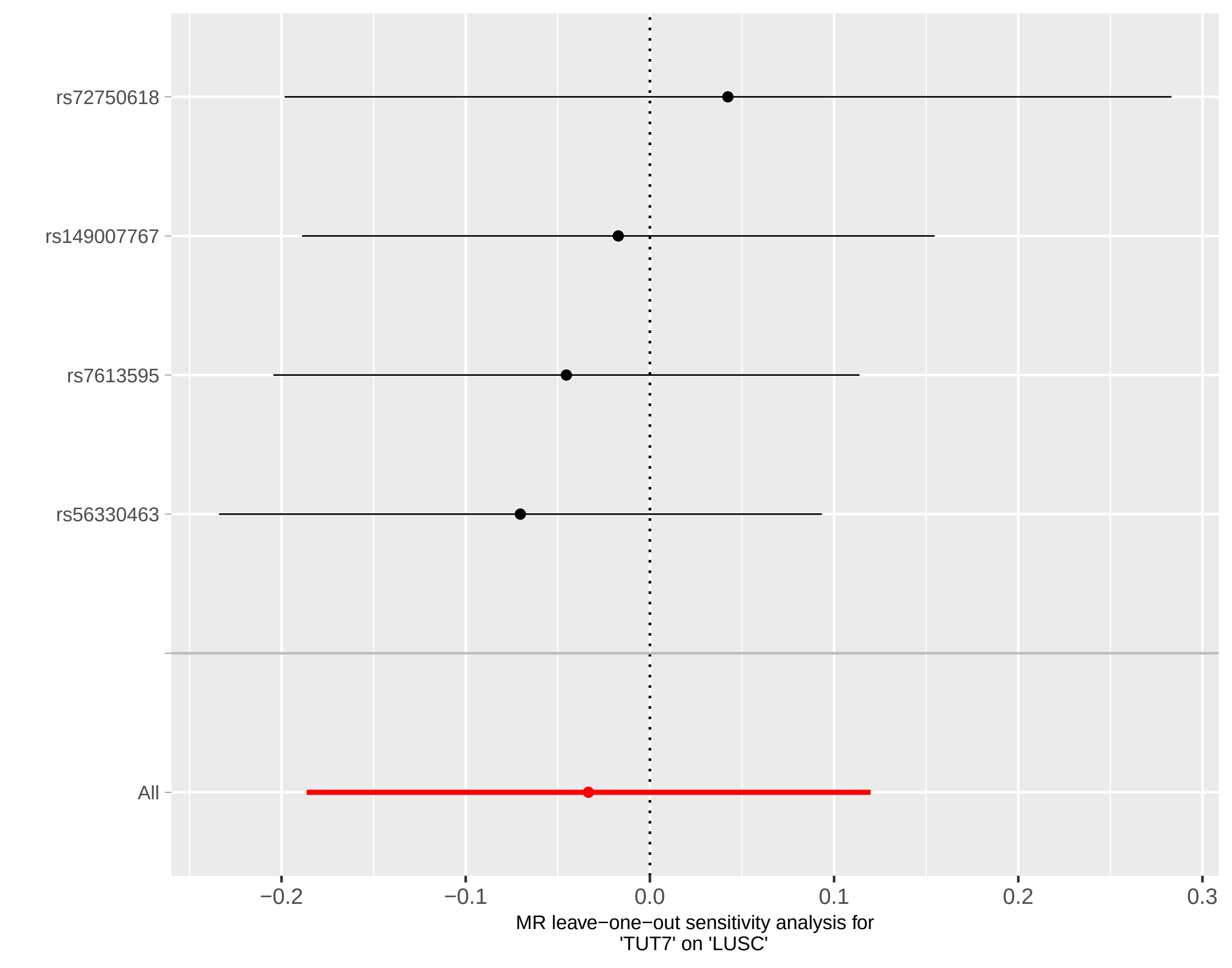
rs72750618
rs149007767
rs7613595
rs56330463
All
−0.2
−0.1
0.0
0.1
0.2
0.3
MR leave−one−out sensitivity analysis for
'TUT7' on 'LUSC'

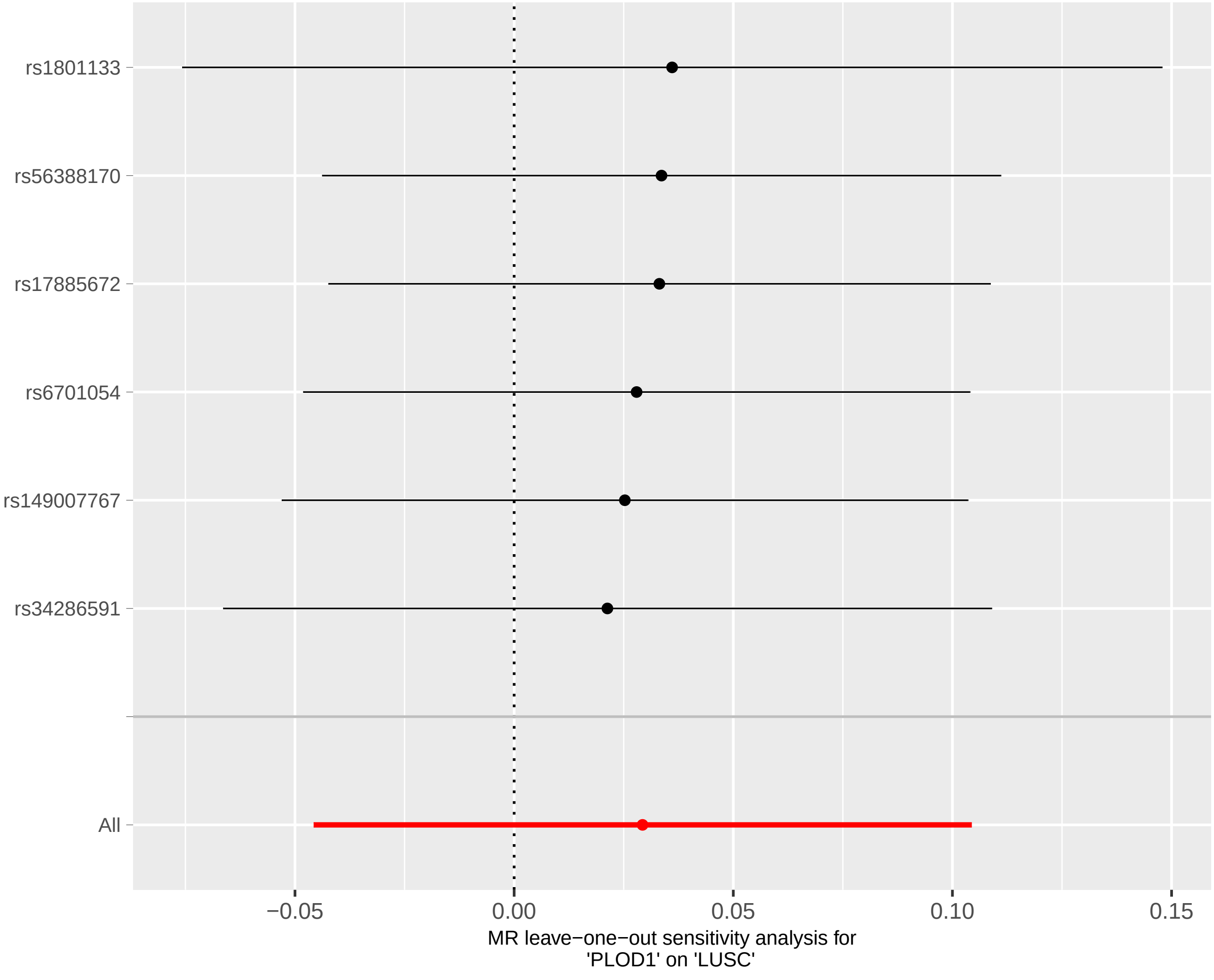
rs1801133
rs56388170
rs17885672
rs6701054
rs149007767
rs34286591
All
−0.05
0.00
0.05
0.10
0.15
MR leave−one−out sensitivity analysis for
'PLOD1' on 'LUSC'

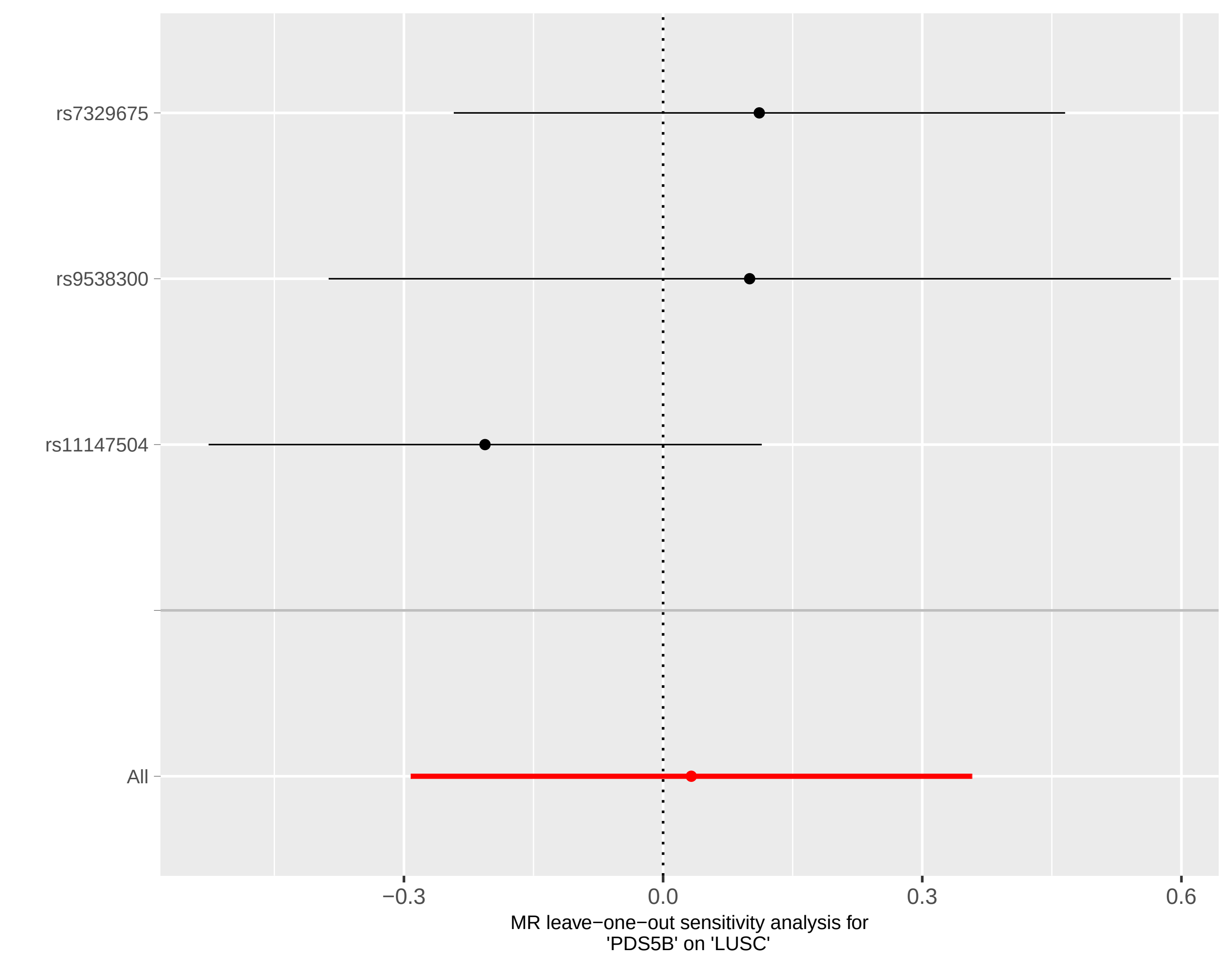
rs7329675
rs9538300
rs11147504
All
−0.3
0.0
0.3
0.6
MR leave−one−out sensitivity analysis for
'PDS5B' on 'LUSC'

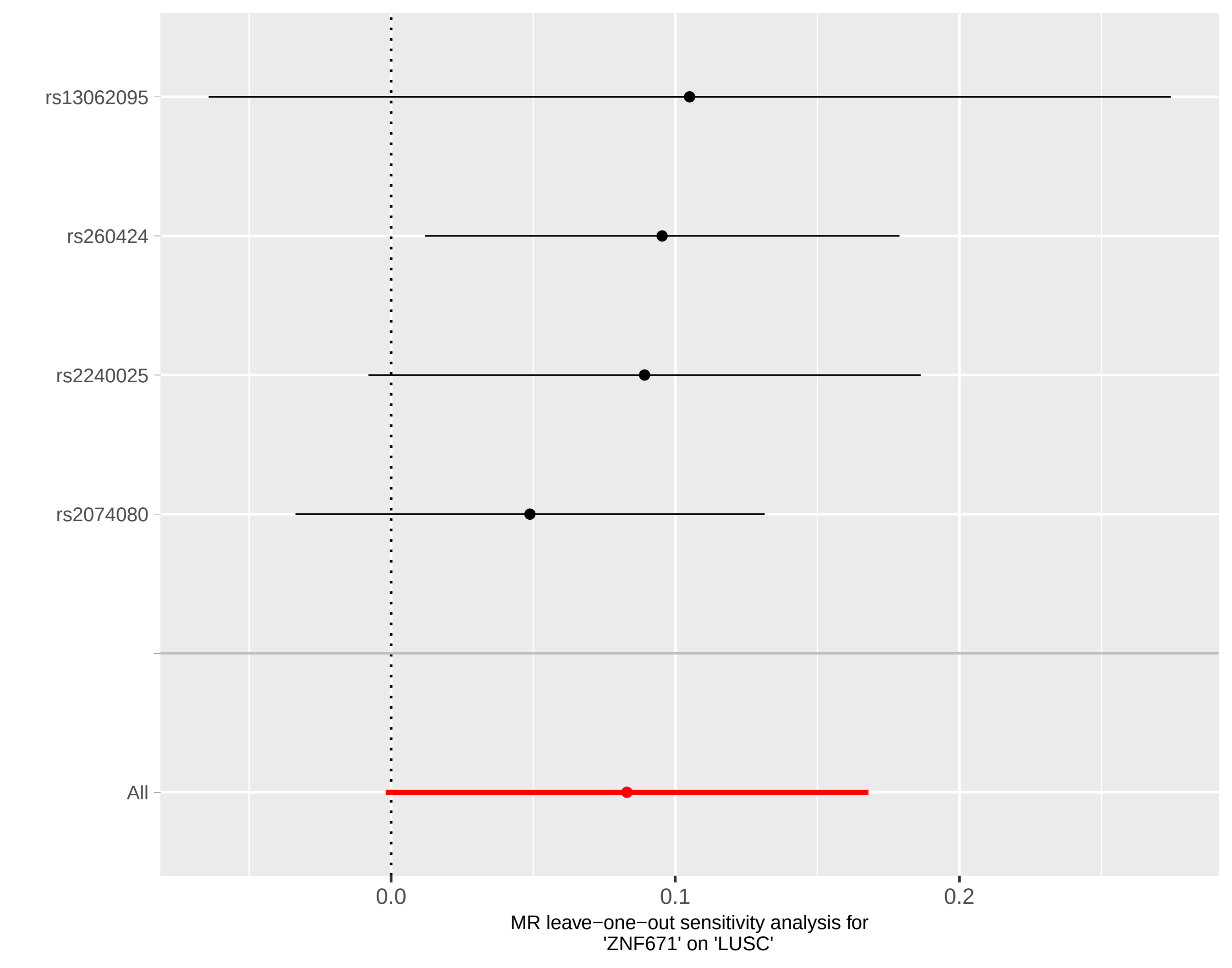

rs13062095
rs260424
rs2240025
rs2074080
All
0.0
0.1
0.2
MR leave−one−out sensitivity analysis for
'ZNF671' on 'LUSC'

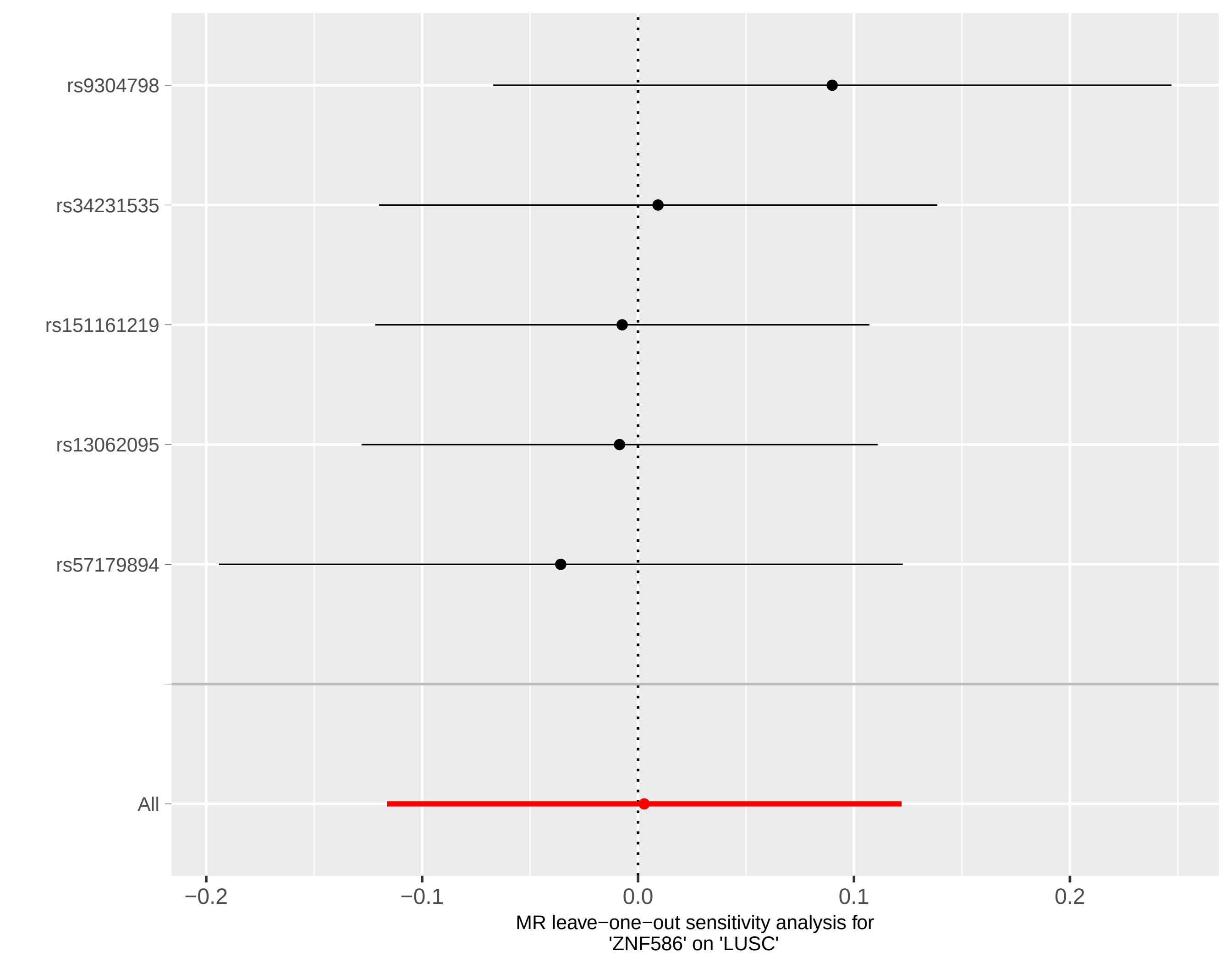
rs9304798
rs34231535
rs151161219
rs13062095
rs57179894
All
−0.2
−0.1
0.0
0.1
0.2
MR leave−one−out sensitivity analysis for
'ZNF586' on 'LUSC'

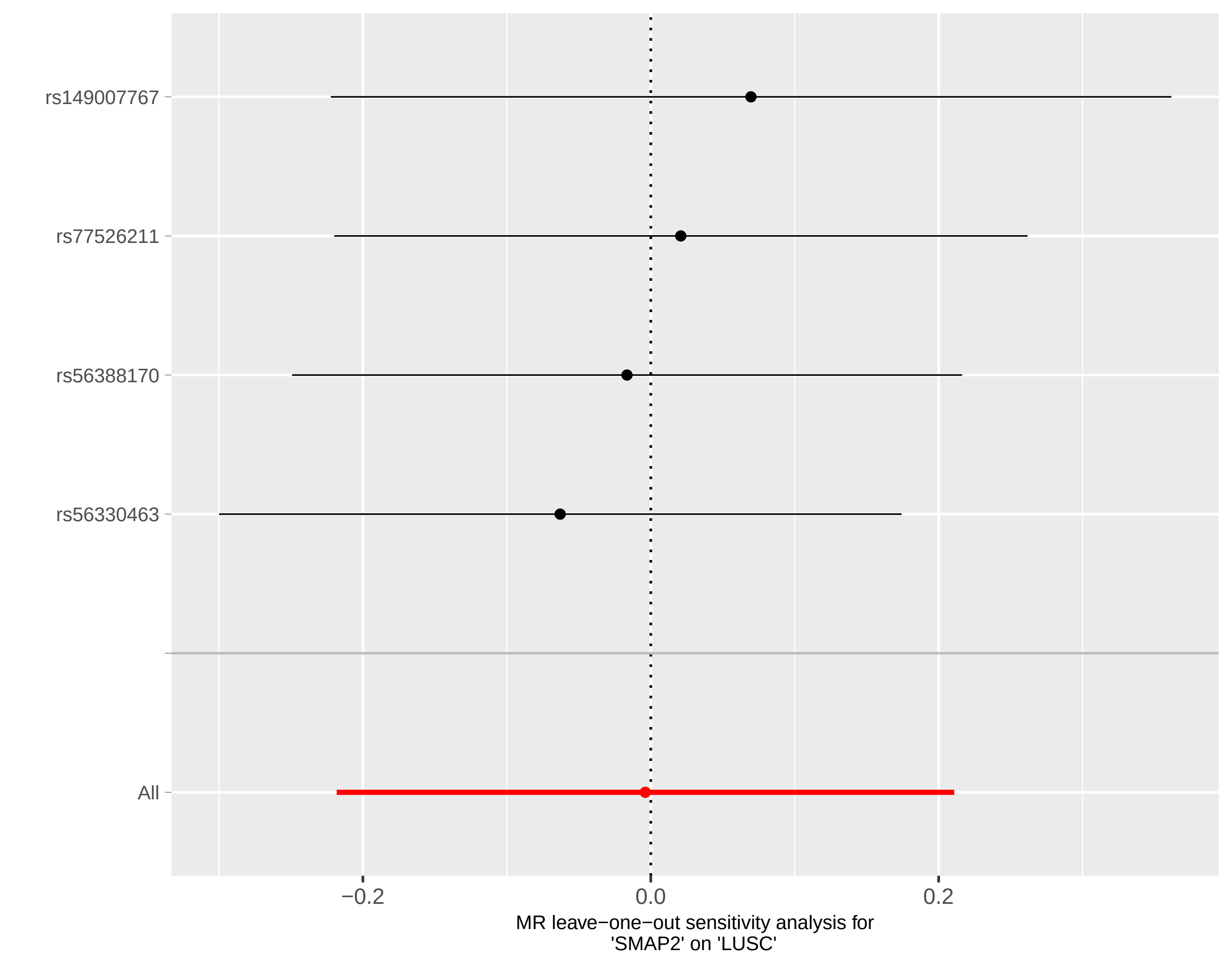

rs149007767
rs77526211
rs56388170
rs56330463
All
−0.2
0.0
0.2
MR leave−one−out sensitivity analysis for
'SMAP2' on 'LUSC'

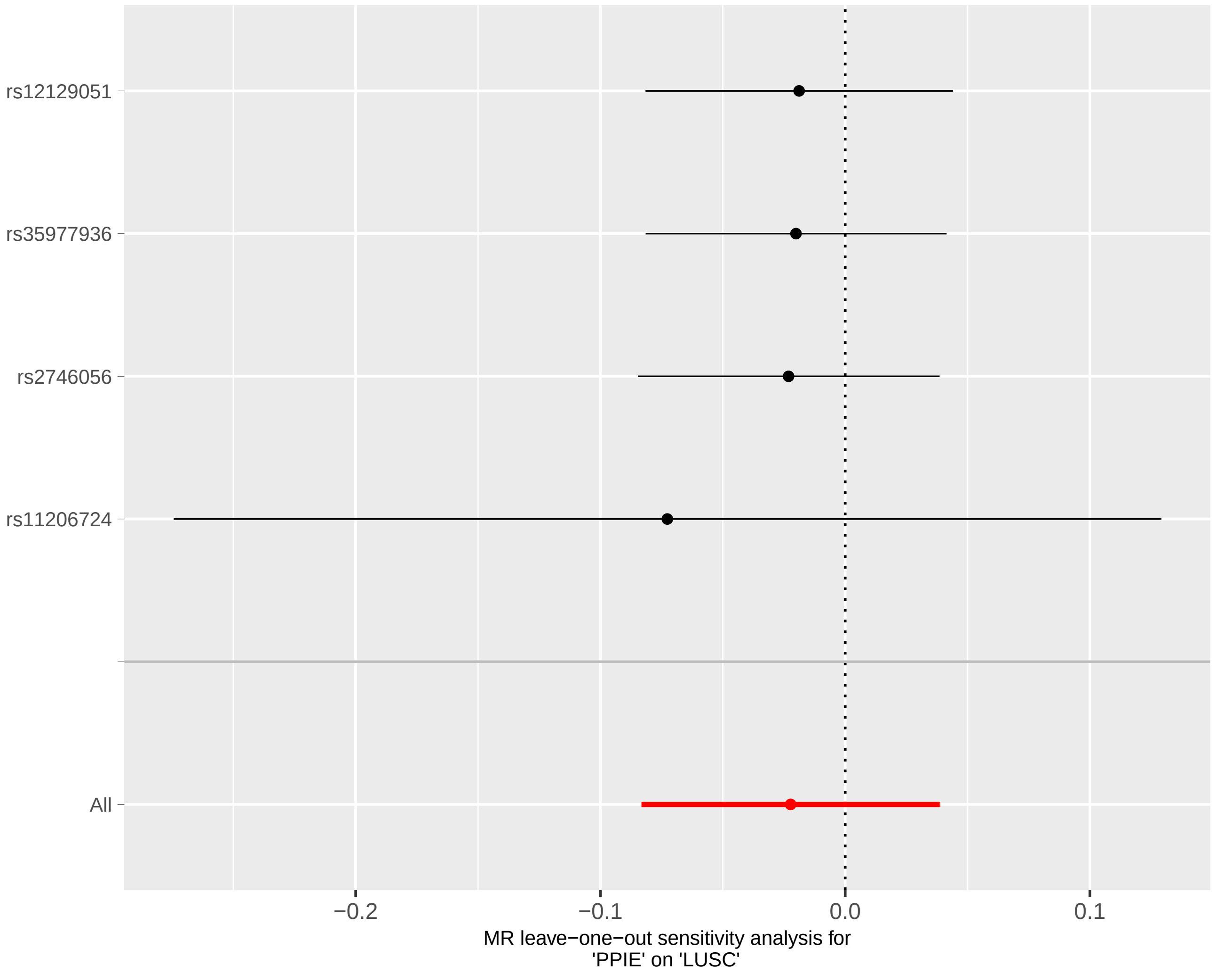
rs12129051
rs35977936
rs2746056
rs11206724
All
−0.2
−0.1
0.0
0.1
MR leave−one−out sensitivity analysis for
'PPIE' on 'LUSC'

Insufficient number of SNPs

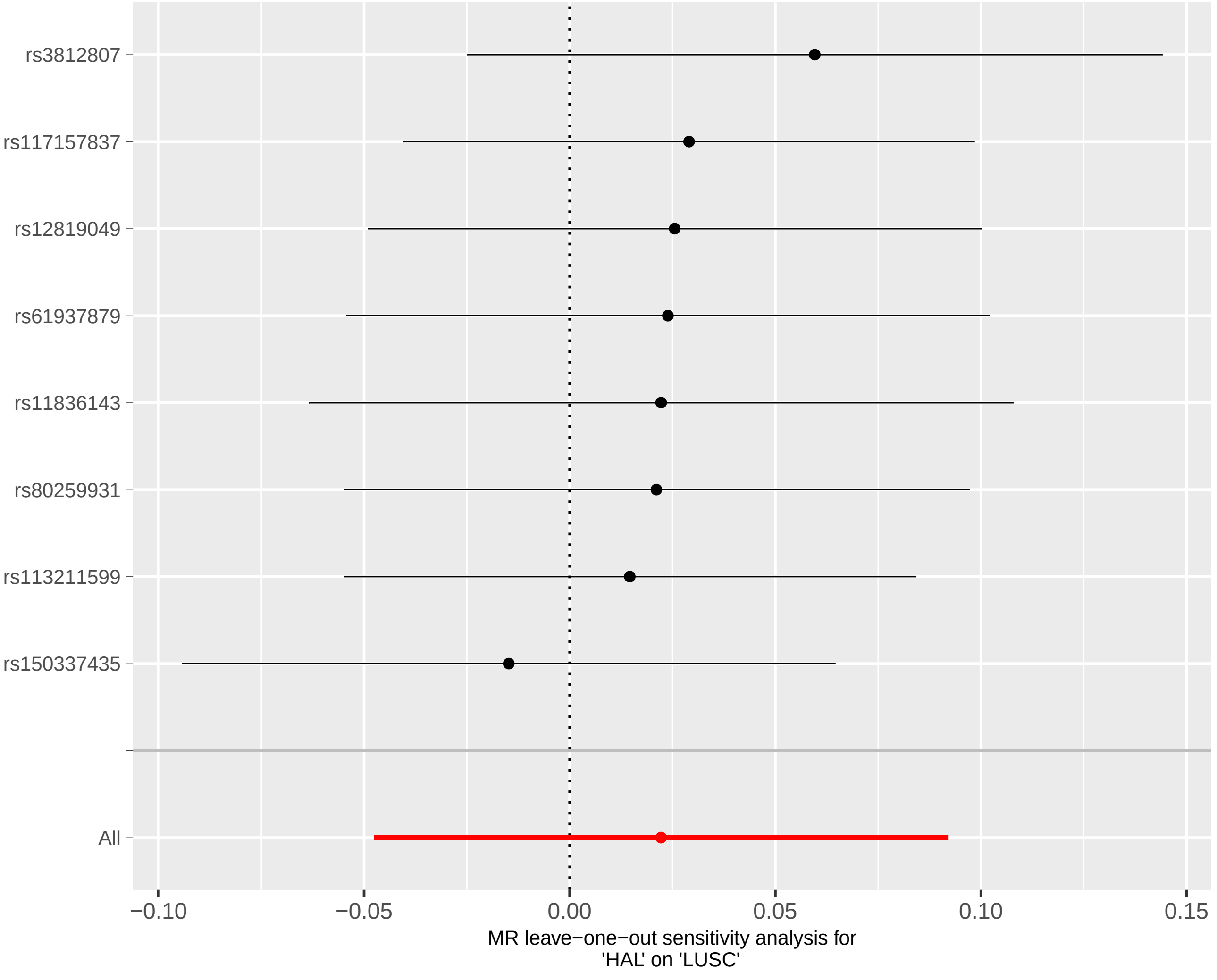
rs3812807
rs117157837
rs12819049
rs61937879
rs11836143
rs80259931
rs113211599
rs150337435
All
−0.10
−0.05
0.00
0.05
0.10
0.15
MR leave−one−out sensitivity analysis for
'HAL' on 'LUSC'

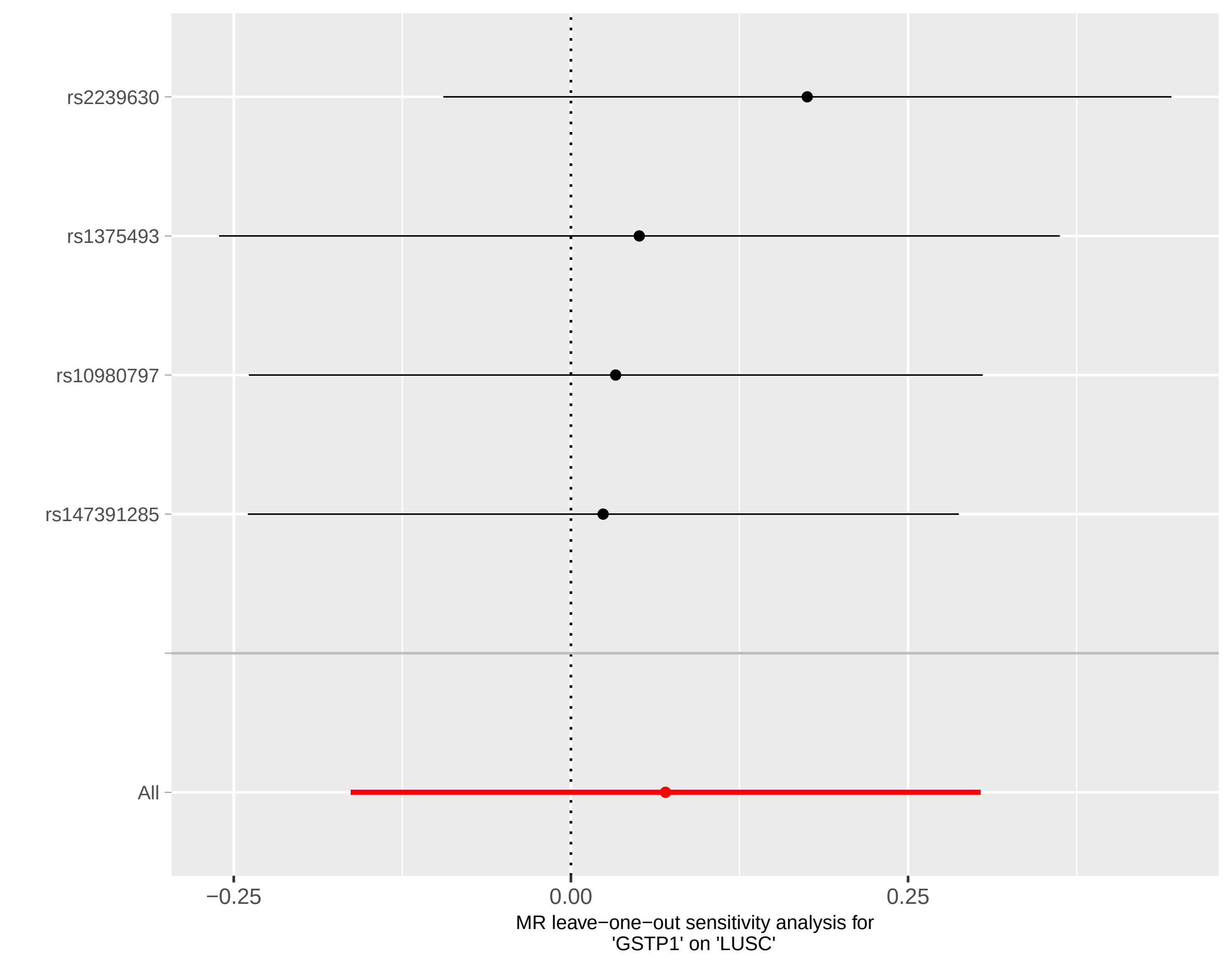
rs2239630
rs1375493
rs10980797
rs147391285
All
−0.25
0.00
0.25
MR leave−one−out sensitivity analysis for
'GSTP1' on 'LUSC'

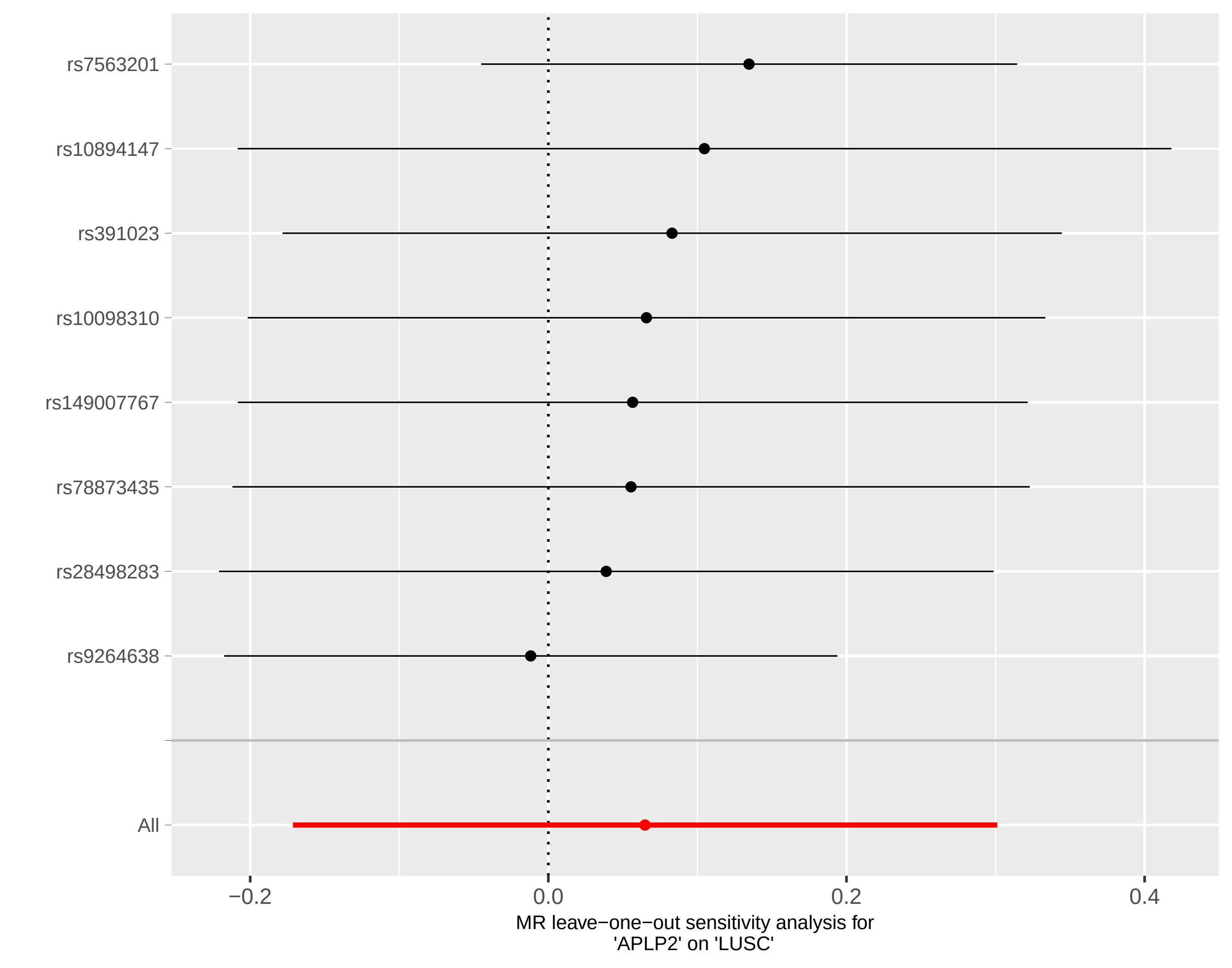
rs7563201
rs10894147
rs391023
rs10098310
rs149007767
rs78873435
rs28498283
rs9264638
All
−0.2
0.0
0.2
0.4
MR leave−one−out sensitivity analysis for
'APLP2' on 'LUSC'

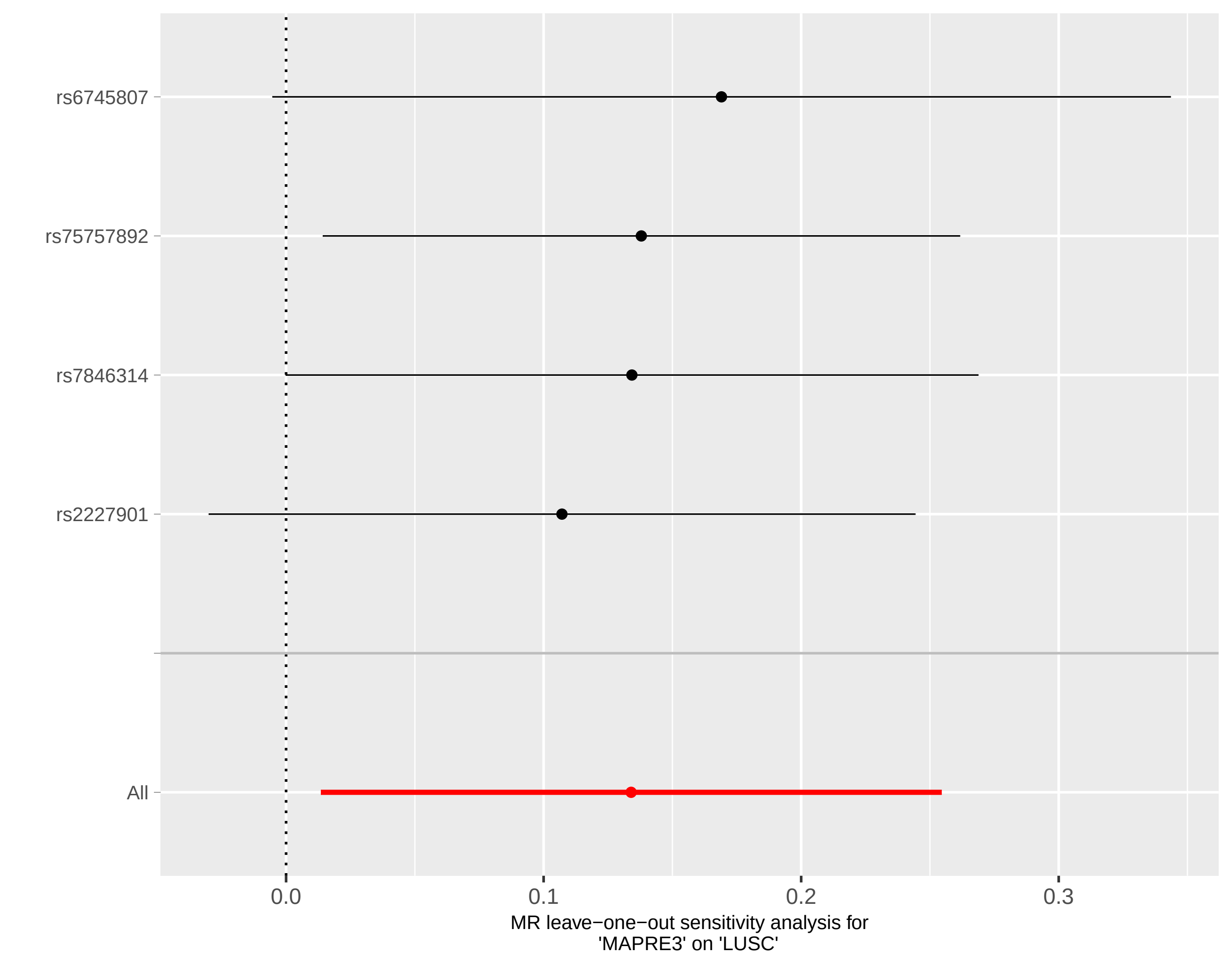
rs6745807
rs75757892
rs7846314
rs2227901
All
0.0
0.1
0.2
0.3
MR leave−one−out sensitivity analysis for
'MAPRE3' on 'LUSC'

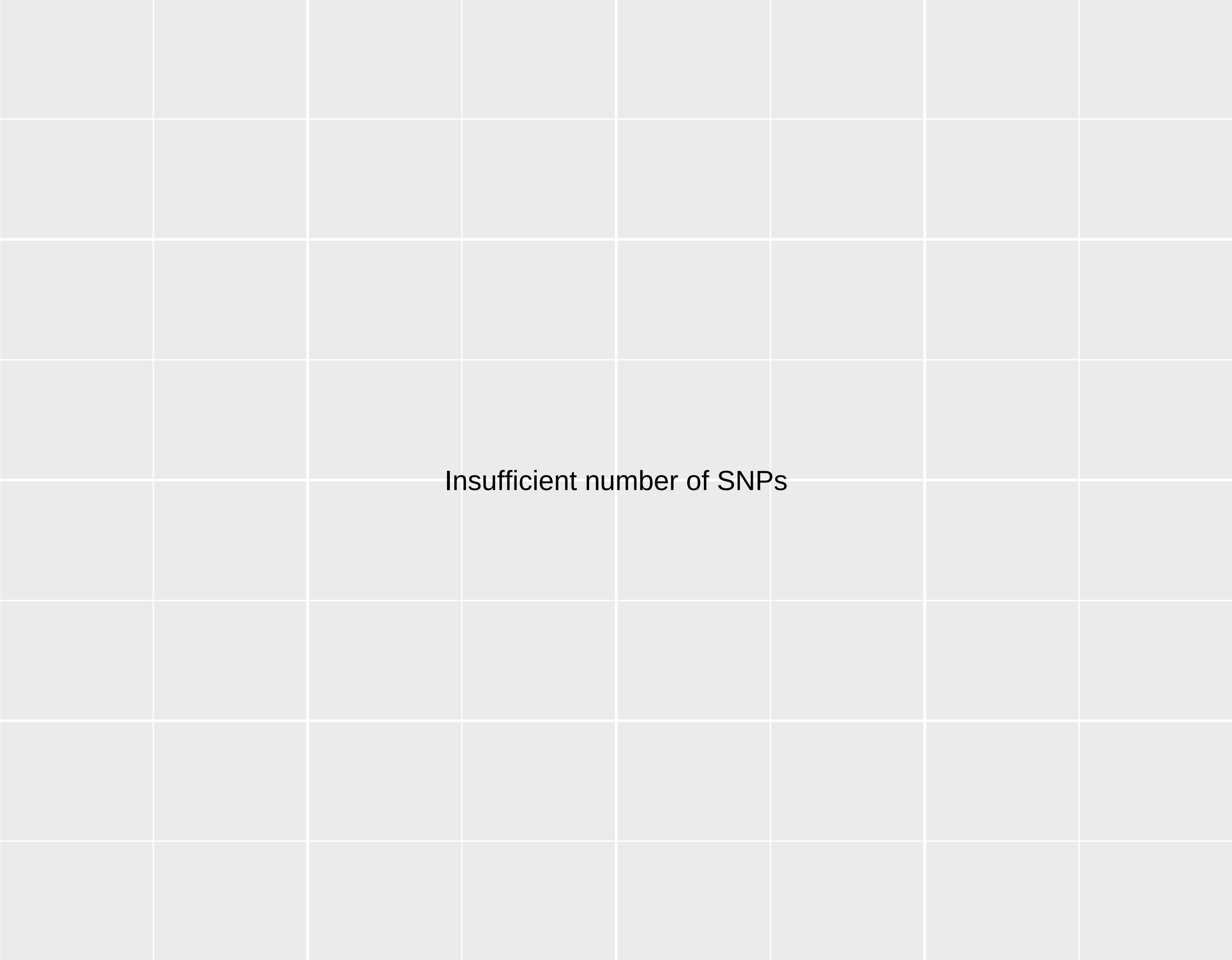
Insufficient number of SNPs

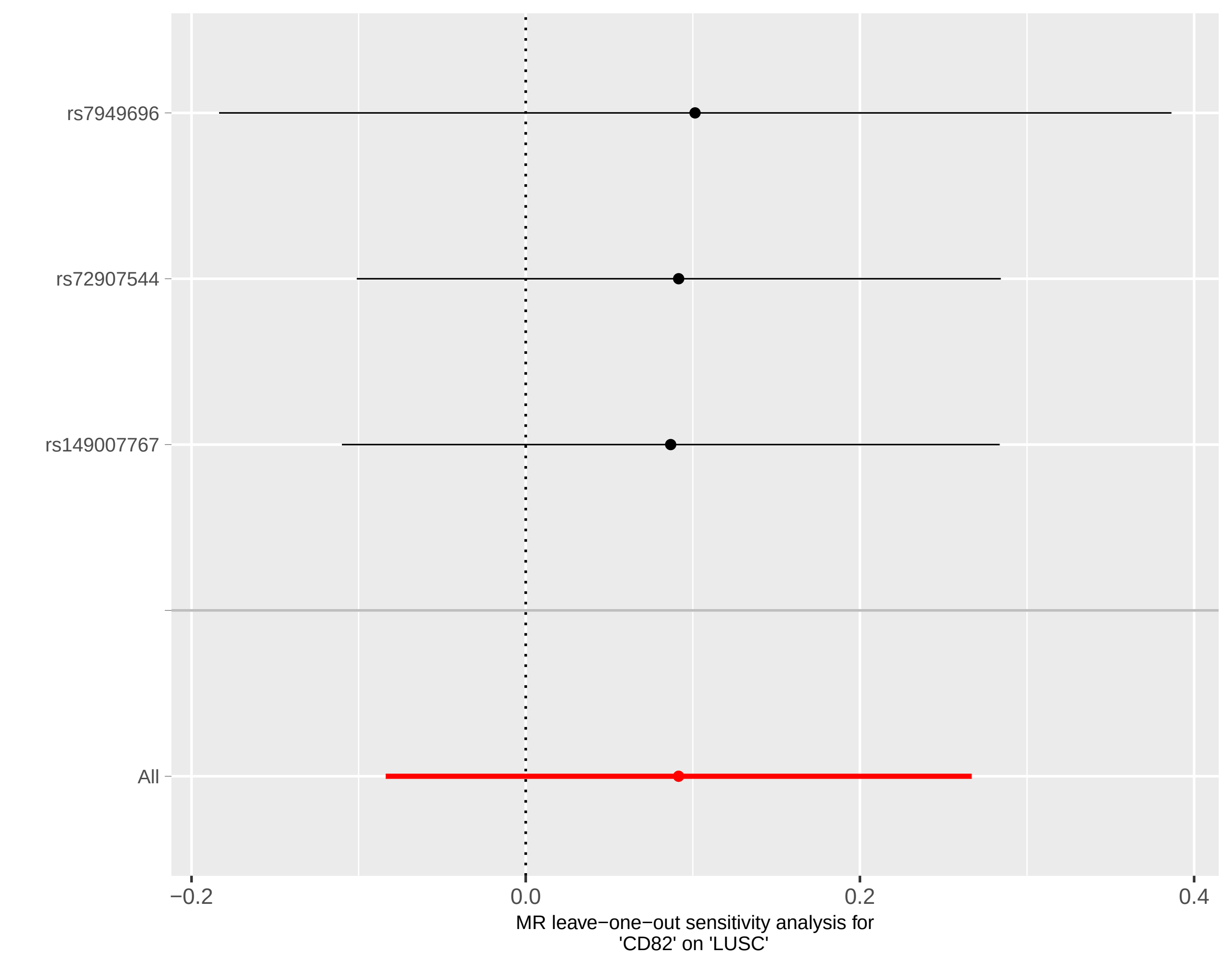
rs7949696
rs72907544
rs149007767
All
−0.2
0.0
0.2
0.4
MR leave−one−out sensitivity analysis for
'CD82' on 'LUSC'

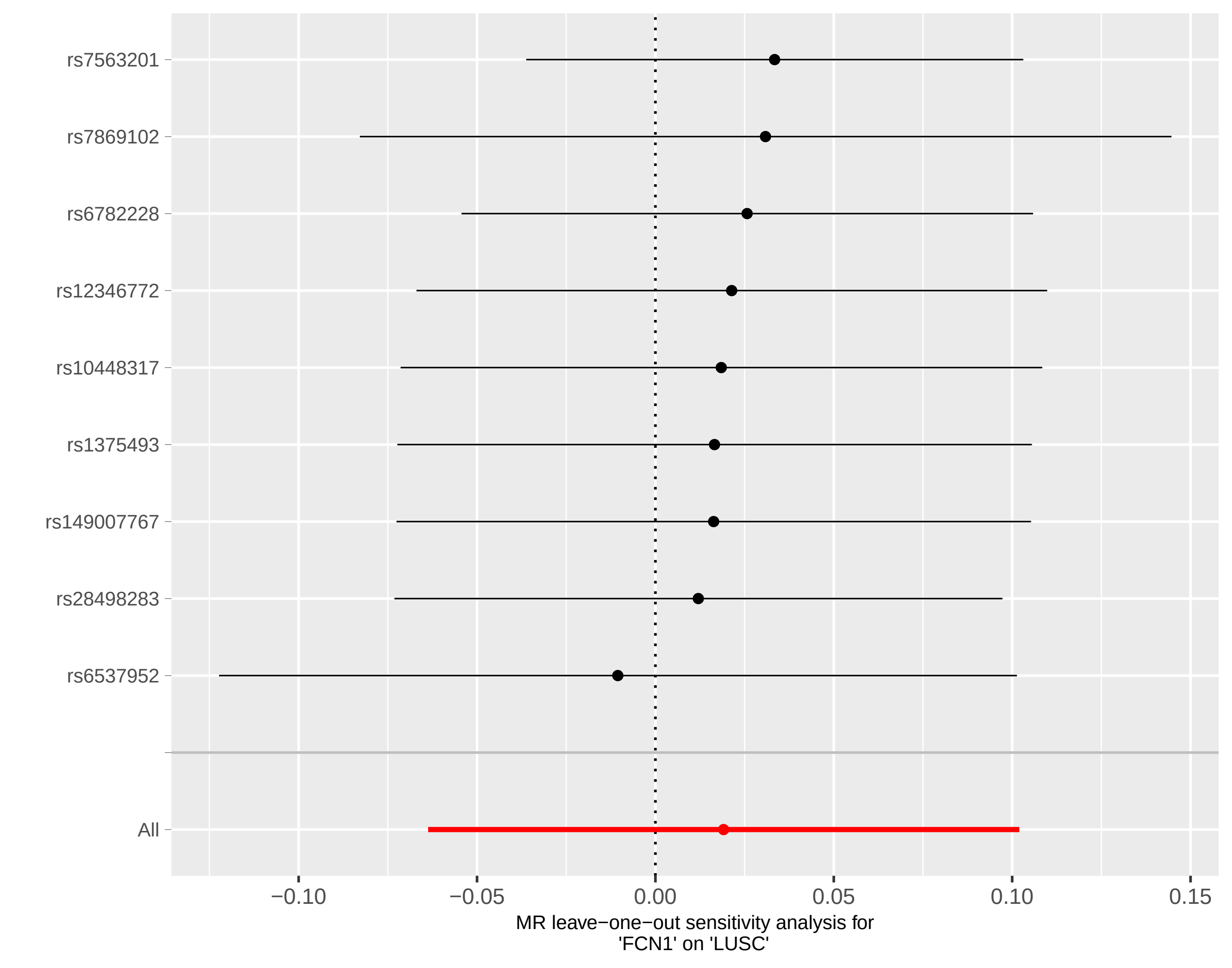
rs7563201
rs7869102
rs6782228
rs12346772
rs10448317
rs1375493
rs149007767
rs28498283
rs6537952
All
−0.10
−0.05
0.00
0.05
0.10
0.15
MR leave−one−out sensitivity analysis for
'FCN1' on 'LUSC'

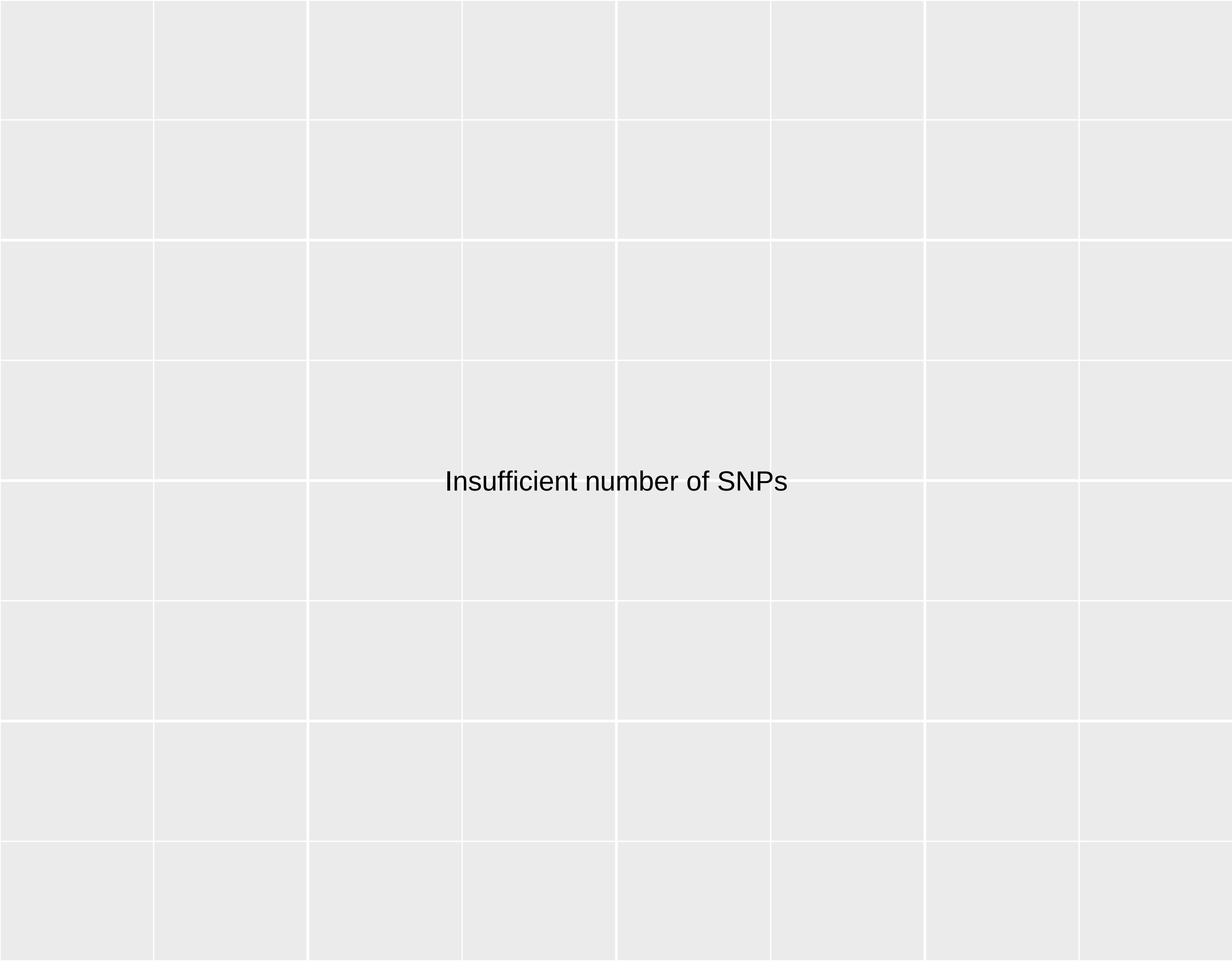
Insufficient number of SNPs

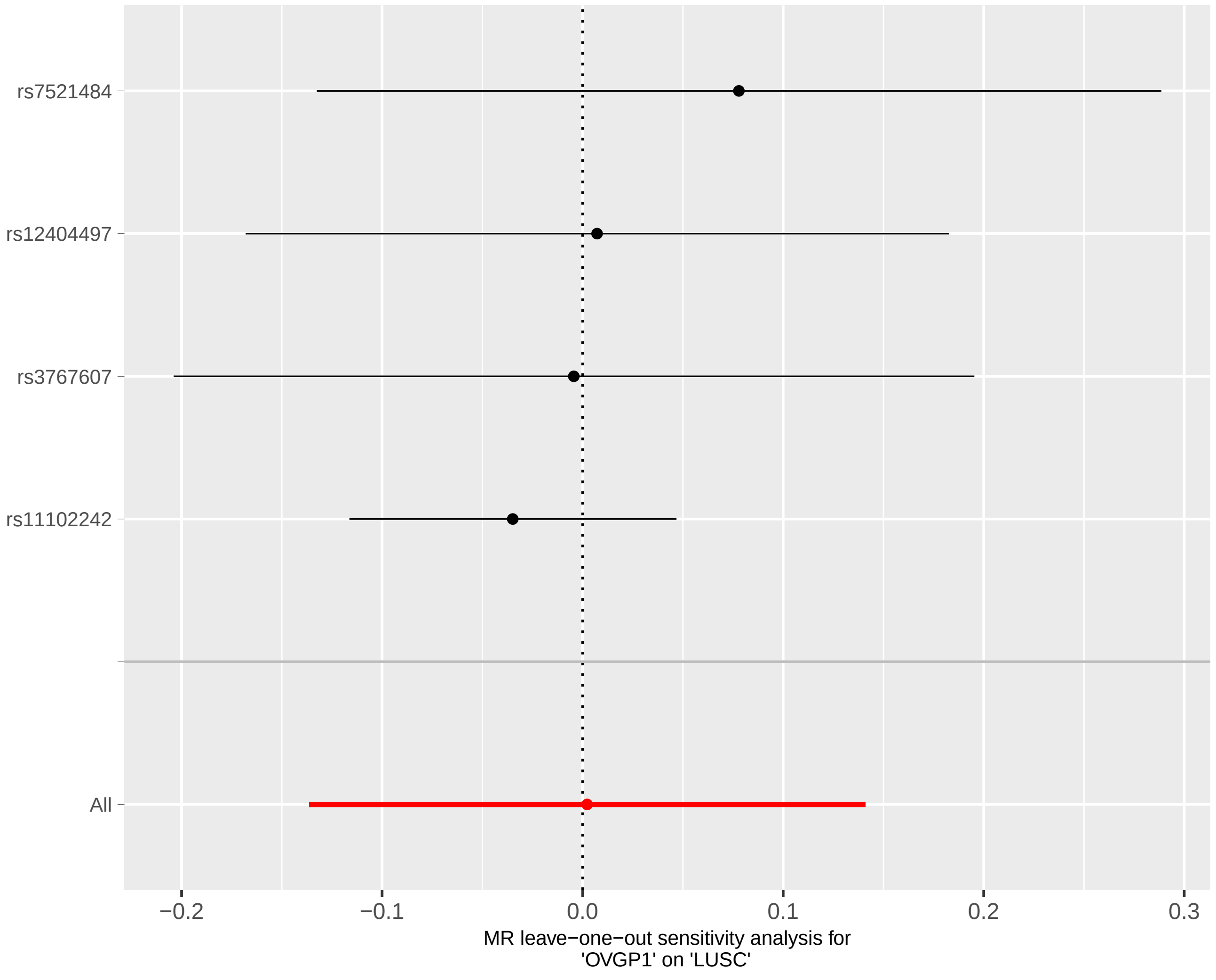
rs7521484
rs12404497
rs3767607
rs11102242
All
−0.2
−0.1
0.0
0.1
0.2
0.3
MR leave−one−out sensitivity analysis for
'OVGP1' on 'LUSC'

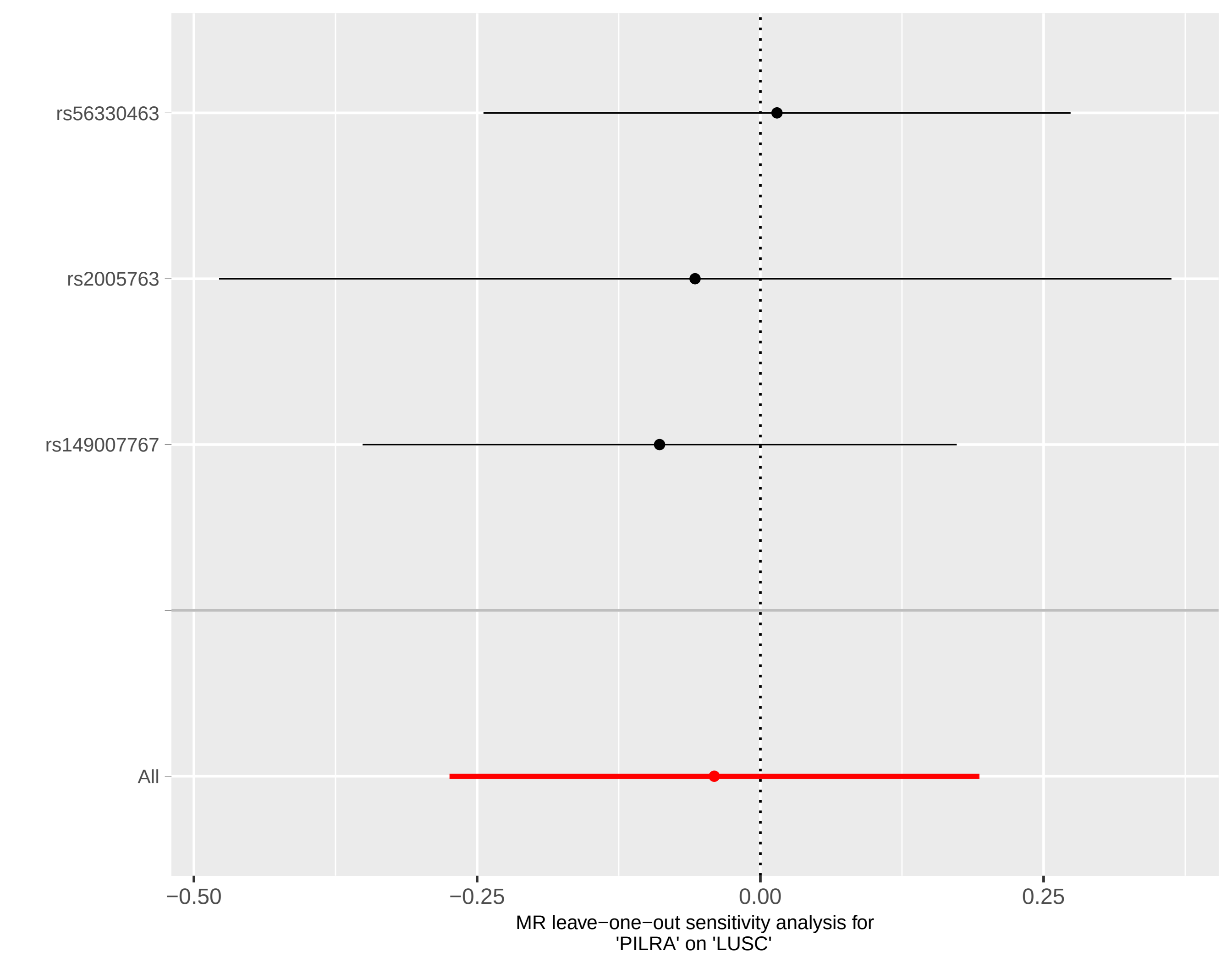
rs56330463
rs2005763
rs149007767
All
−0.50
−0.25
0.00
0.25
MR leave−one−out sensitivity analysis for
'PILRA' on 'LUSC'

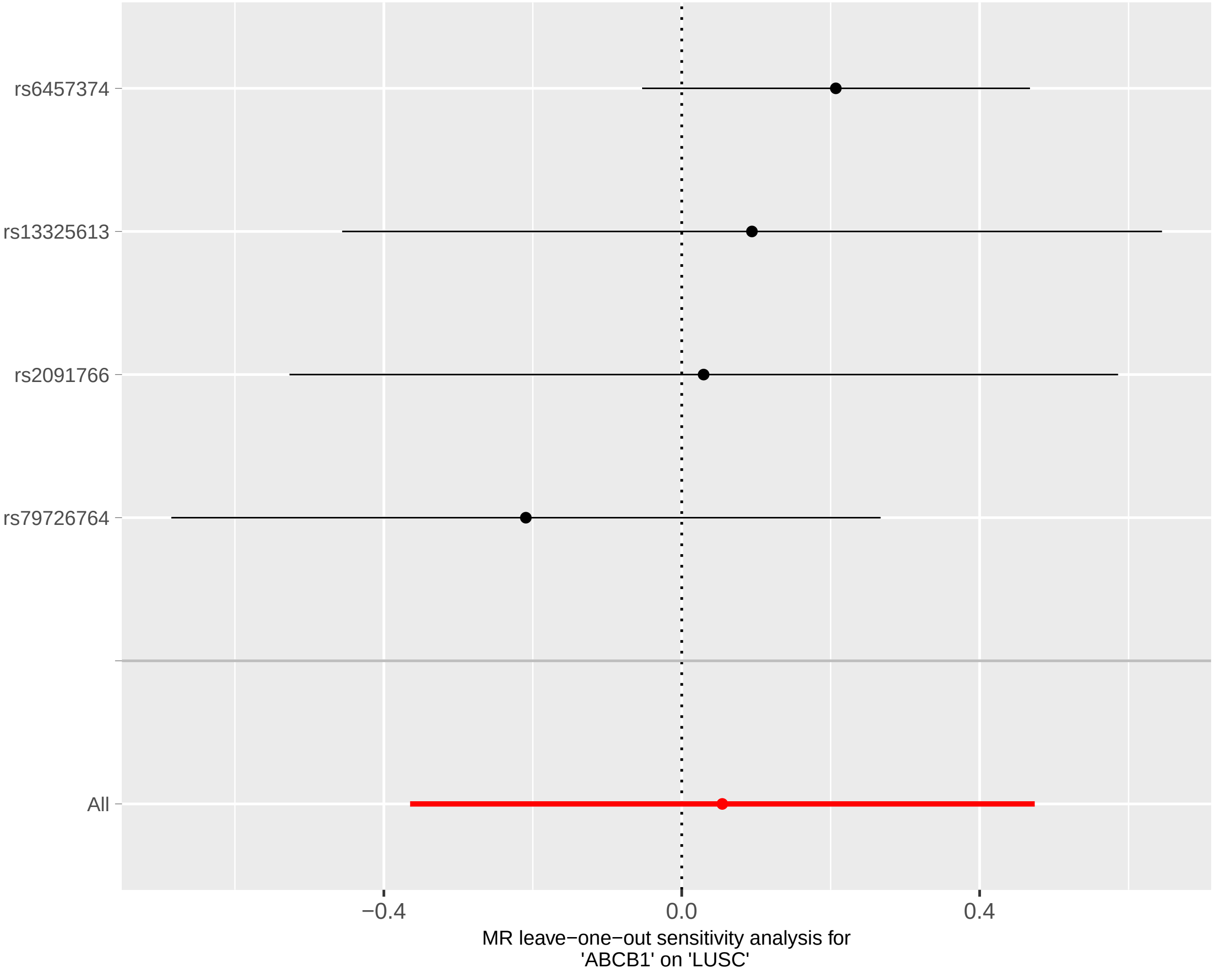
rs6457374
rs13325613
rs2091766
rs79726764
All
−0.4
0.0
0.4
MR leave−one−out sensitivity analysis for
'ABCB1' on 'LUSC'

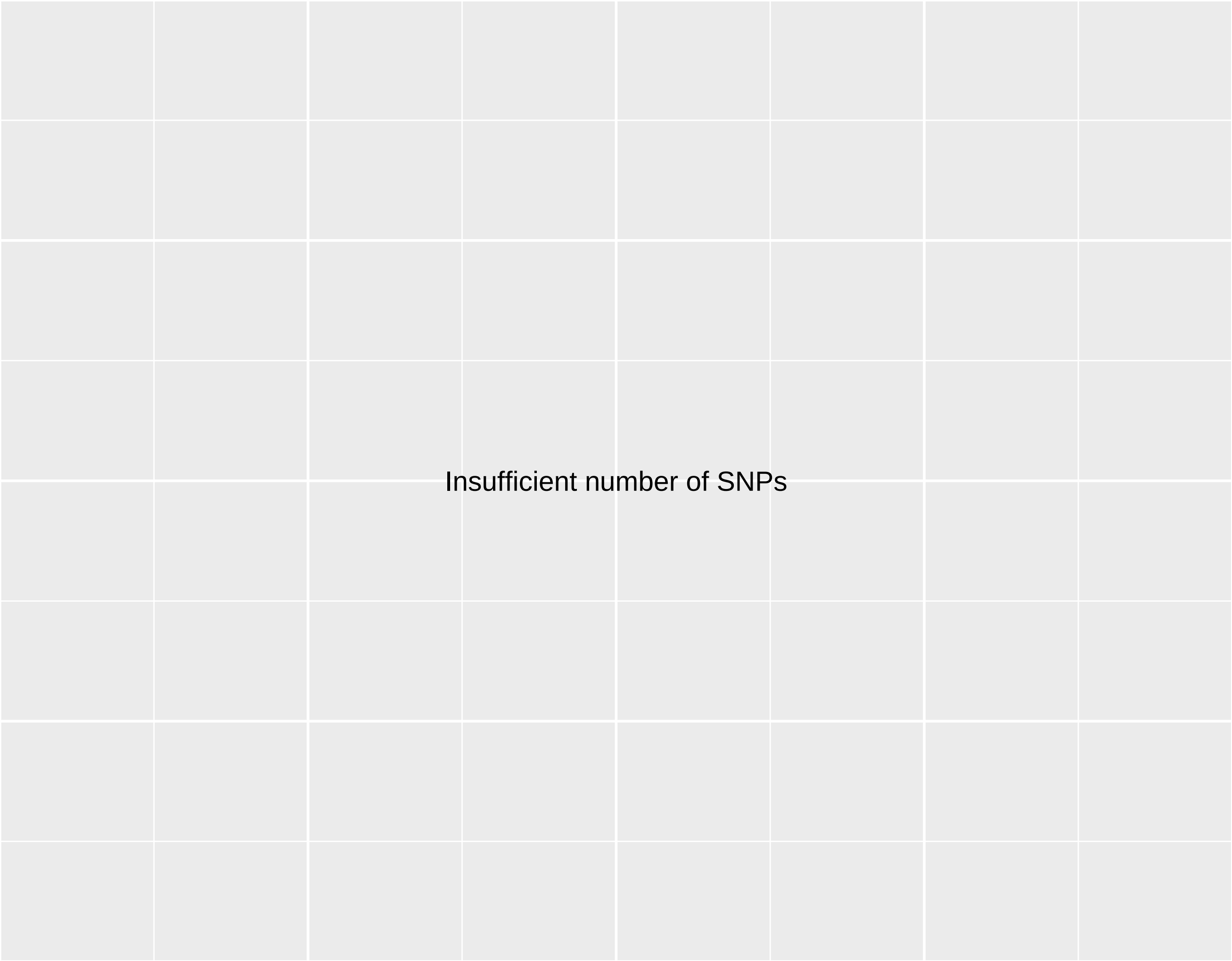
Insufficient number of SNPs

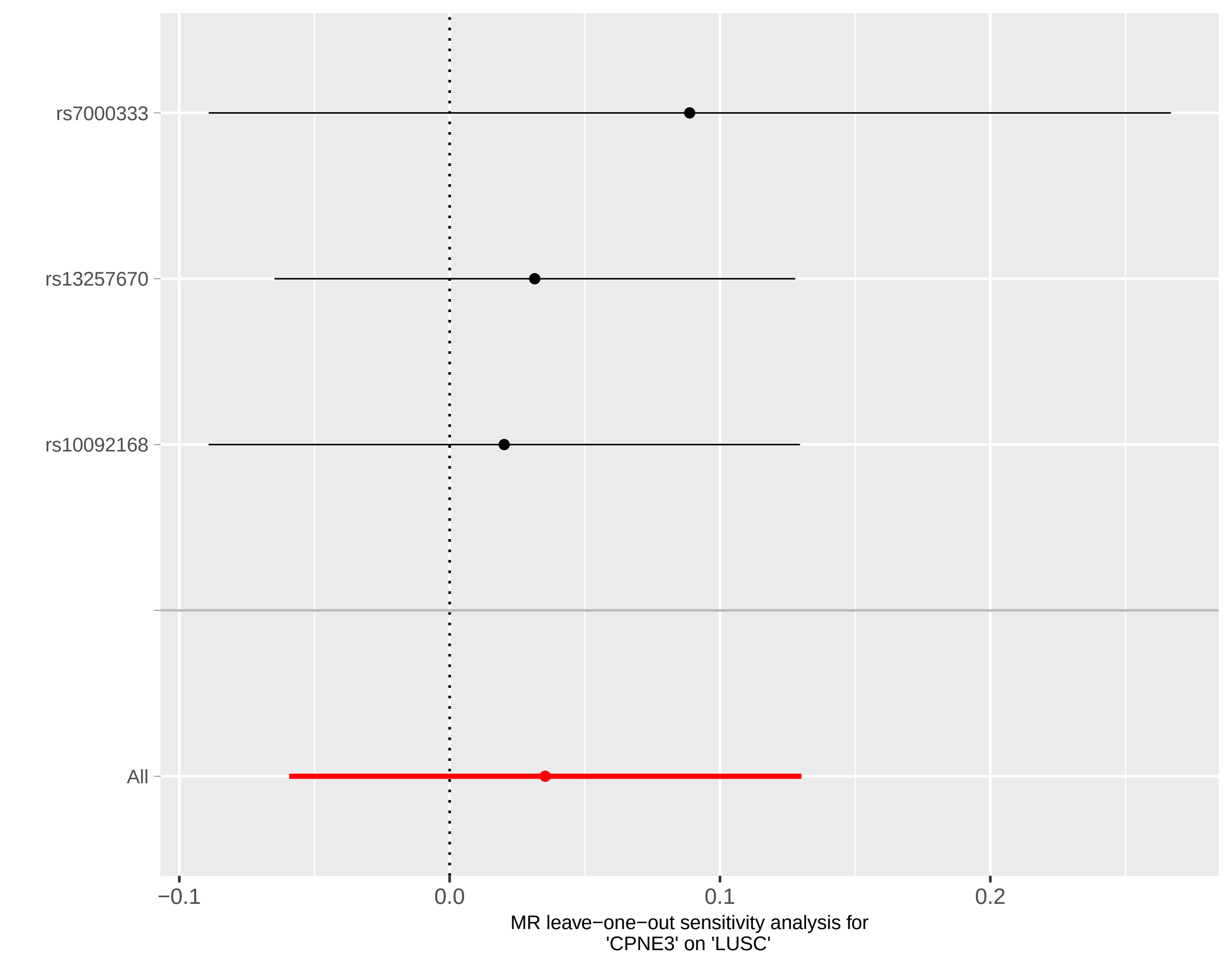

rs7000333
rs13257670
rs10092168
All
−0.1
0.0
0.1
0.2
MR leave−one−out sensitivity analysis for
'CPNE3' on 'LUSC'

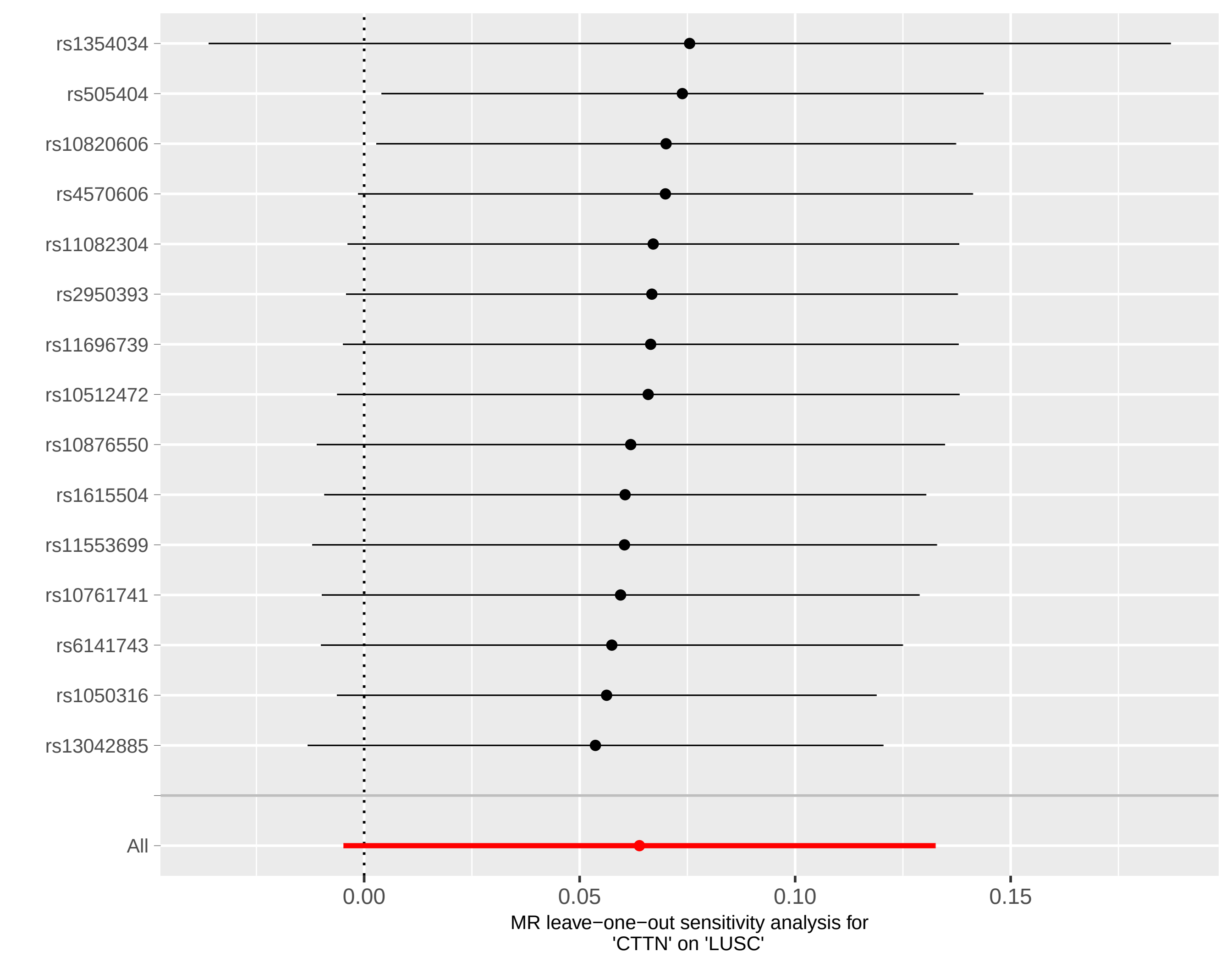

rs1354034
rs505404
rs10820606
rs4570606
rs11082304
rs2950393
rs11696739
rs10512472
rs10876550
rs1615504
rs11553699
rs10761741
rs6141743
rs1050316
rs13042885
All
0.00
0.05
0.10
0.15
MR leave−one−out sensitivity analysis for
'CTTN' on 'LUSC'

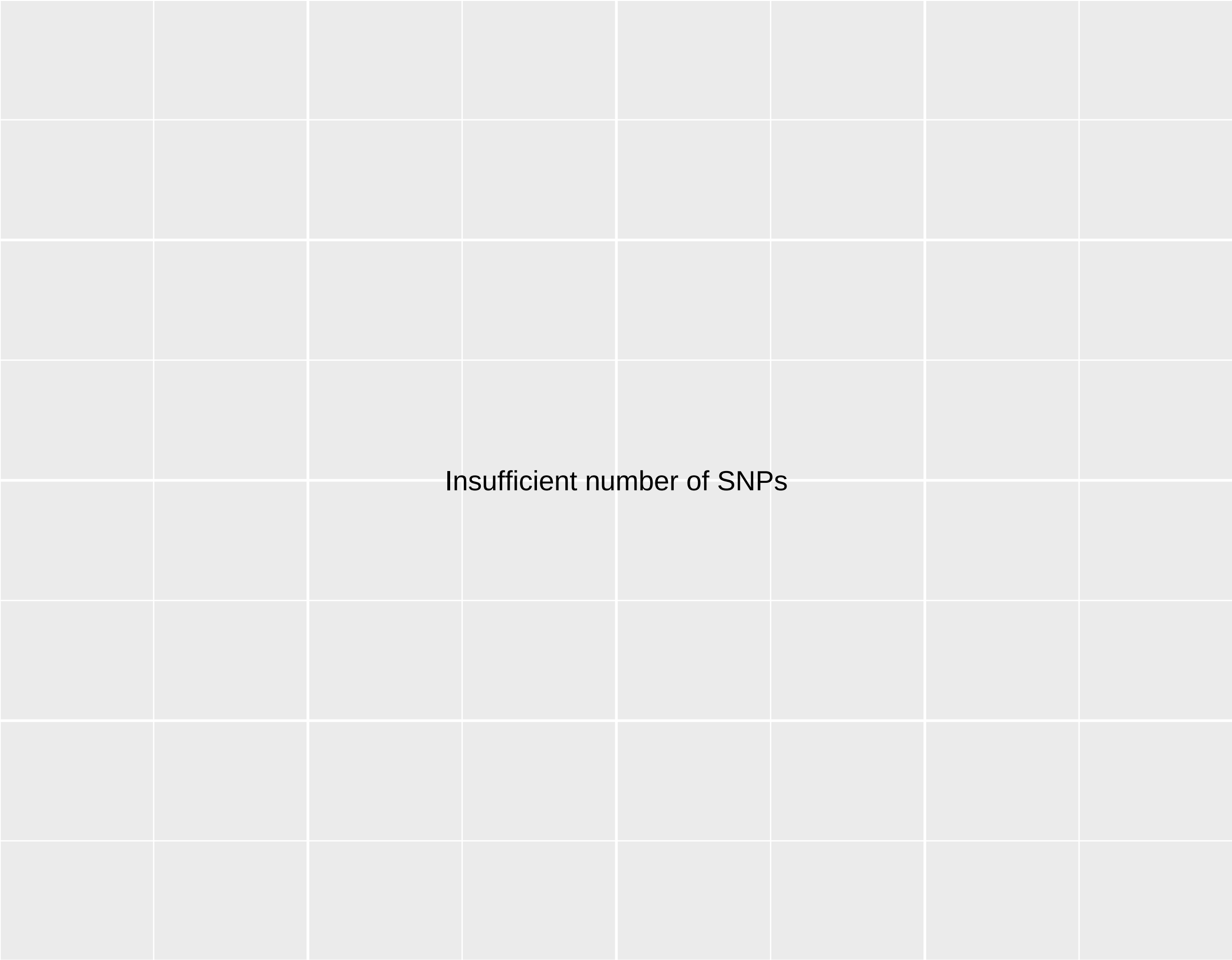
Insufficient number of SNPs

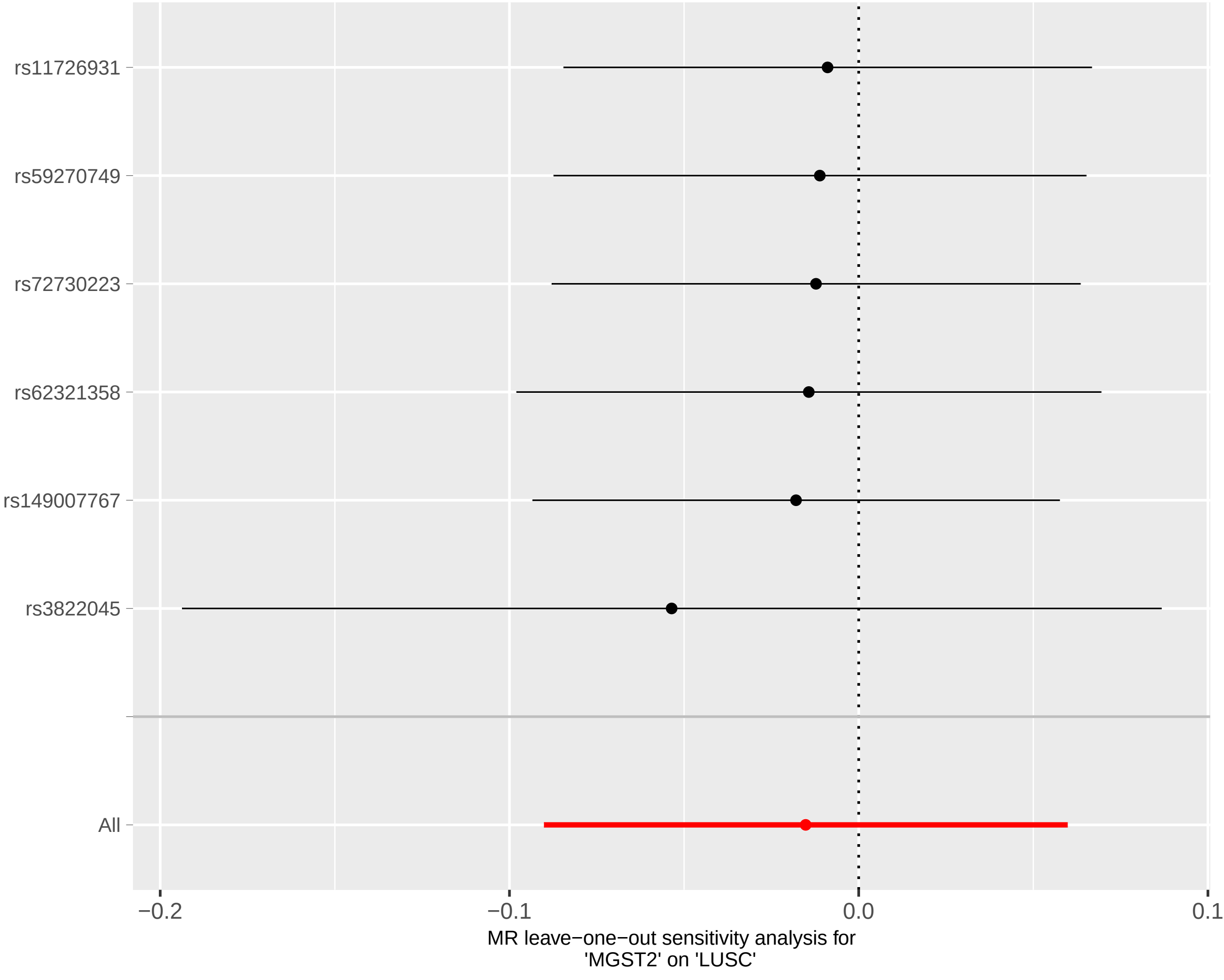

rs11726931
rs59270749
rs72730223
rs62321358
rs149007767
rs3822045
All
−0.2
−0.1
0.0
0.1
MR leave−one−out sensitivity analysis for
'MGST2' on 'LUSC'

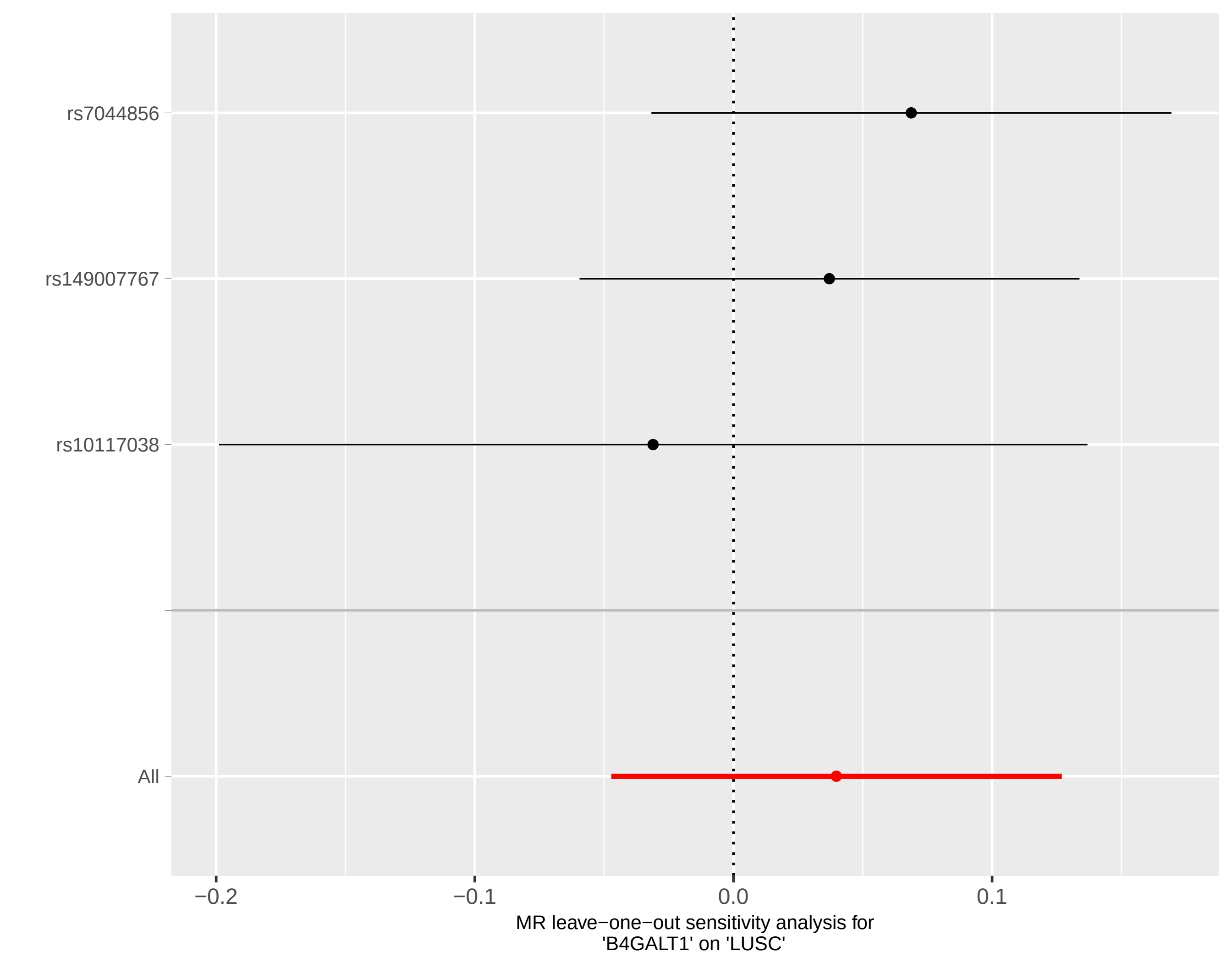

rs7044856
rs149007767
rs10117038
All
−0.2
−0.1
0.0
0.1
MR leave−one−out sensitivity analysis for
'B4GALT1' on 'LUSC'

Insufficient number of SNPs

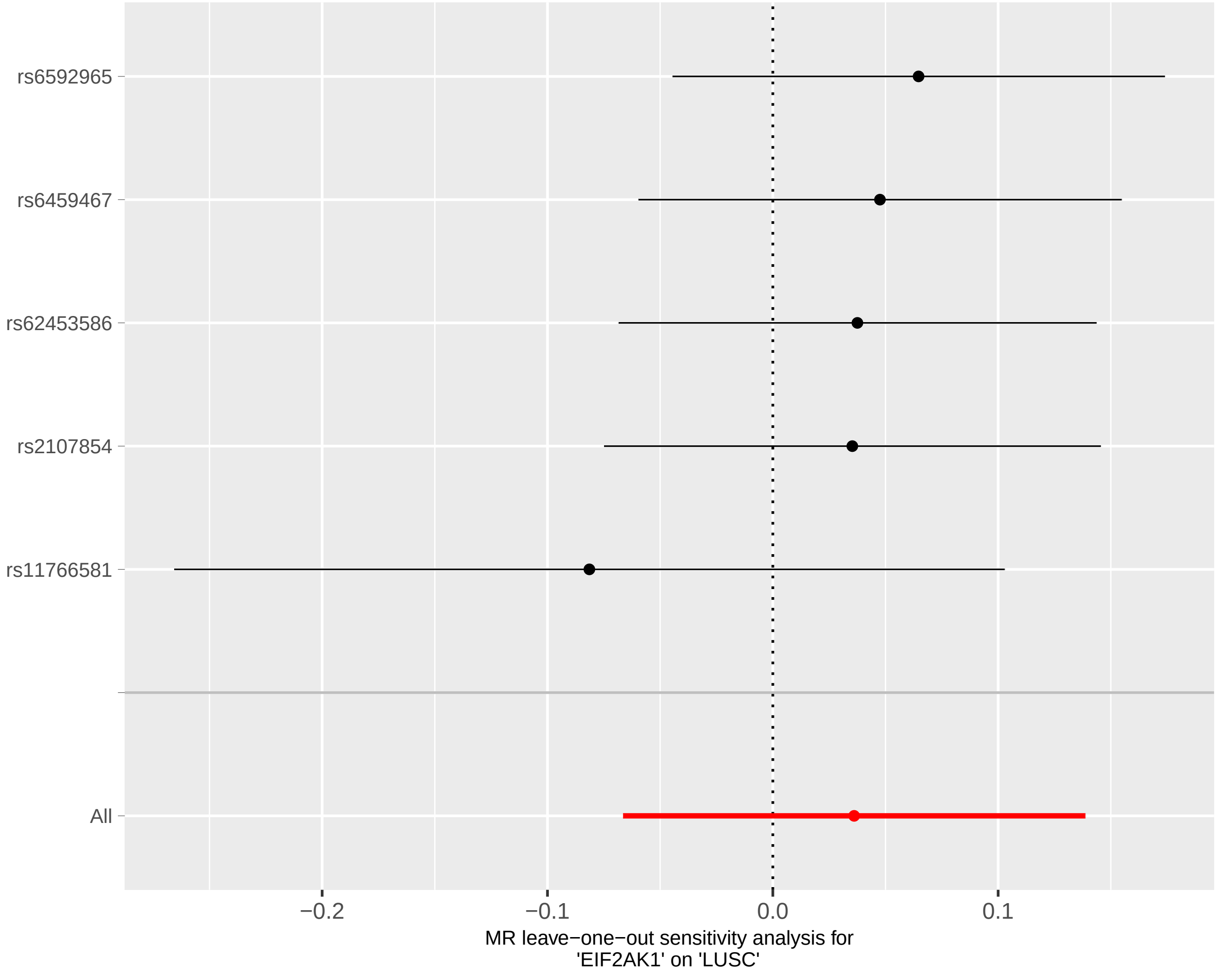
rs6592965
rs6459467
rs62453586
rs2107854
rs11766581
All
−0.2
−0.1
0.0
0.1
MR leave−one−out sensitivity analysis for
'EIF2AK1' on 'LUSC'

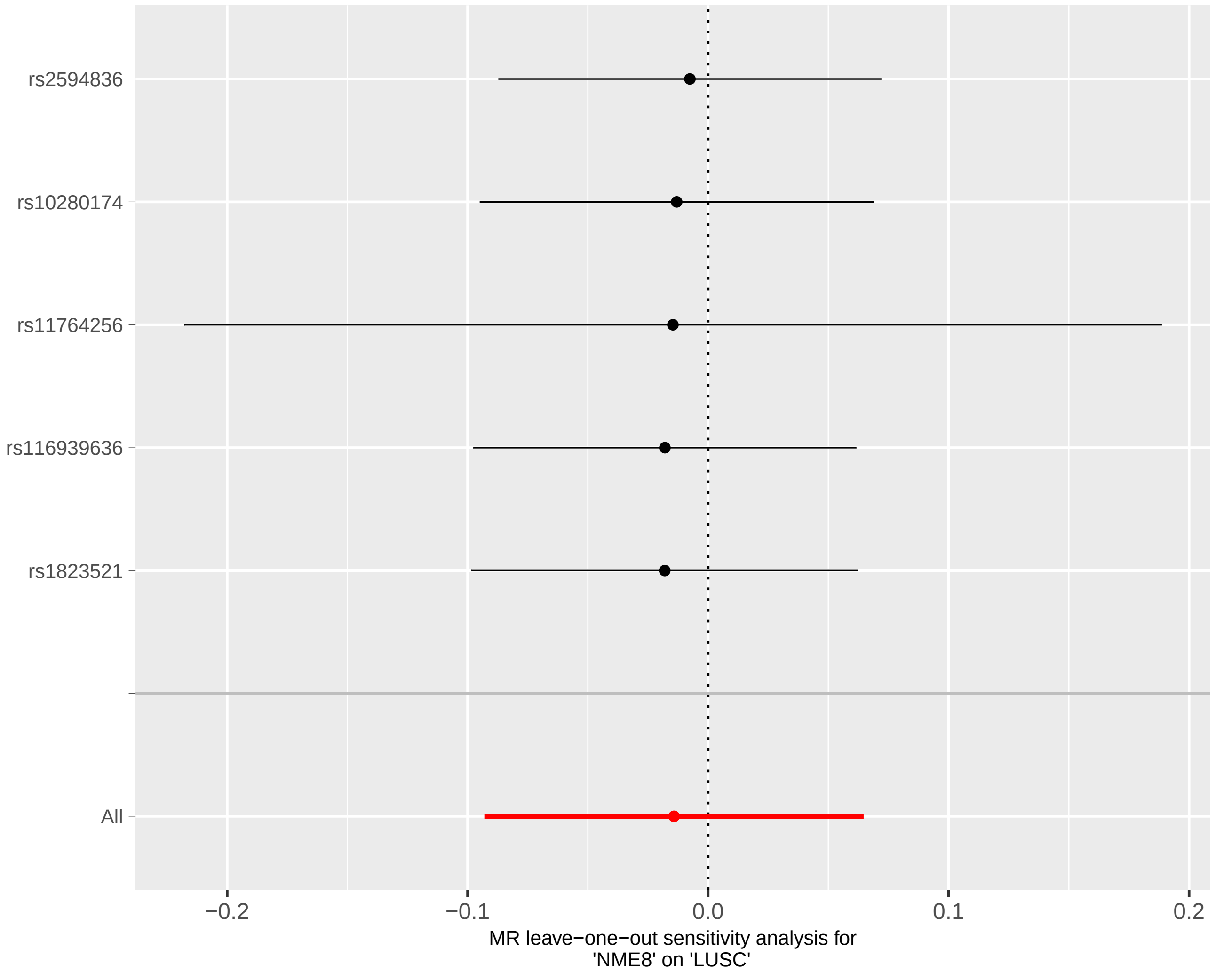

rs2594836
rs10280174
rs11764256
rs116939636
rs1823521
All
−0.2
−0.1
0.0
0.1
0.2
MR leave−one−out sensitivity analysis for
'NME8' on 'LUSC'

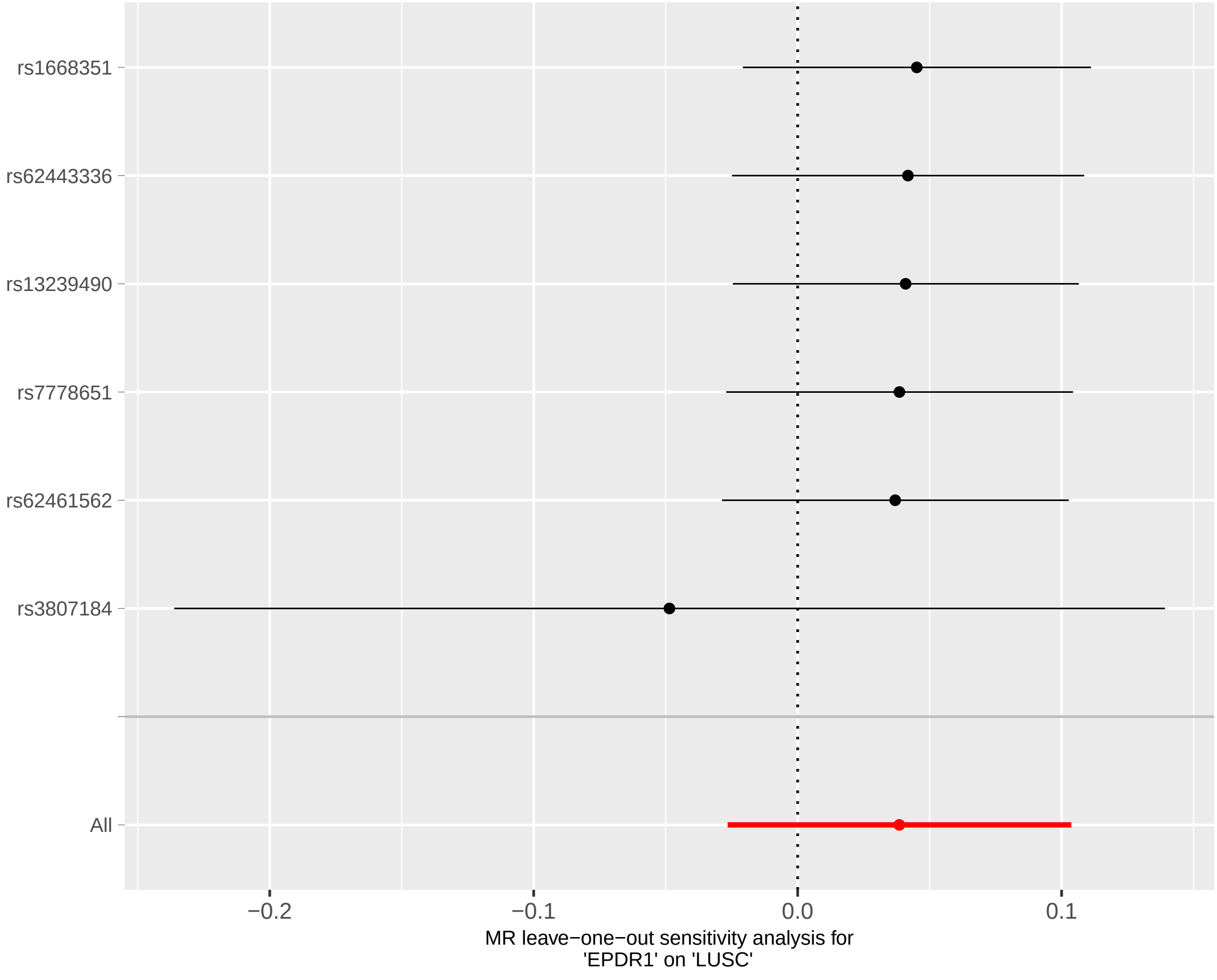
rs1668351
rs62443336
rs13239490
rs7778651
rs62461562
rs3807184
All
−0.2
−0.1
0.0
0.1
MR leave−one−out sensitivity analysis for
'EPDR1' on 'LUSC'

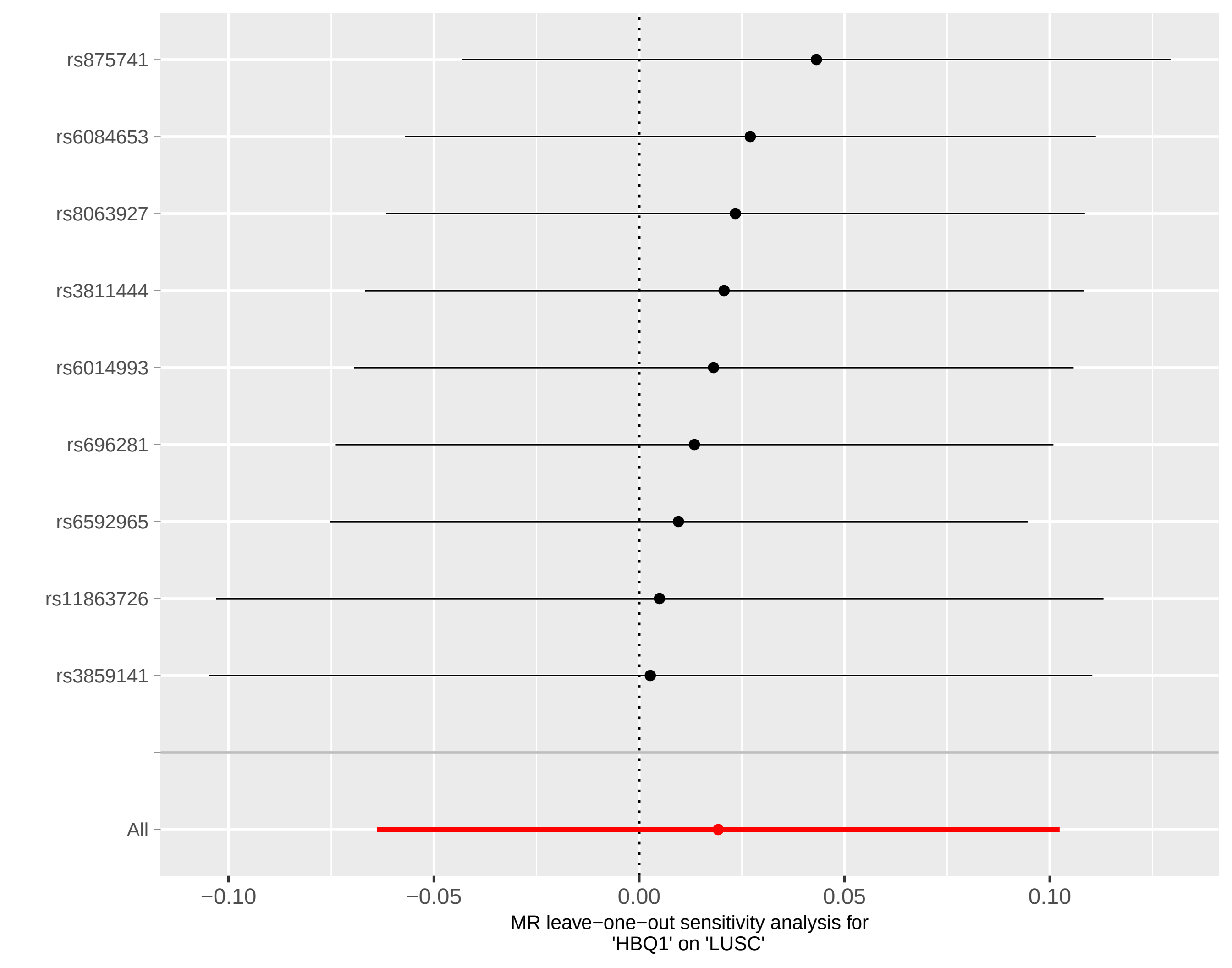
rs875741
rs6084653
rs8063927
rs3811444
rs6014993
rs696281
rs6592965
rs11863726
rs3859141
All
−0.10
−0.05
0.00
0.05
0.10
MR leave−one−out sensitivity analysis for
'HBQ1' on 'LUSC'

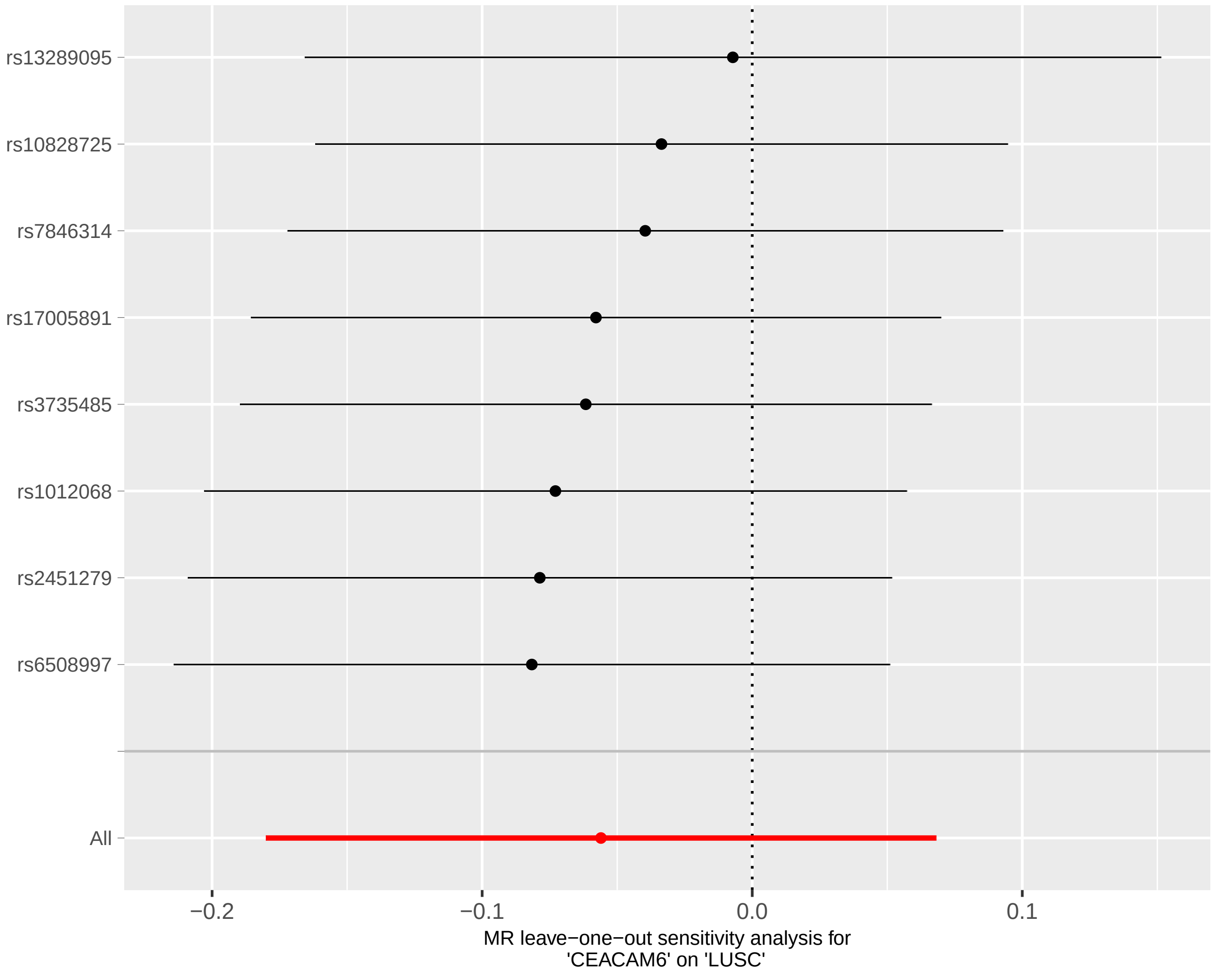

rs13289095
rs10828725
rs7846314
rs17005891
rs3735485
rs1012068
rs2451279
rs6508997
All
−0.2
−0.1
0.0
0.1
MR leave−one−out sensitivity analysis for
'CEACAM6' on 'LUSC'

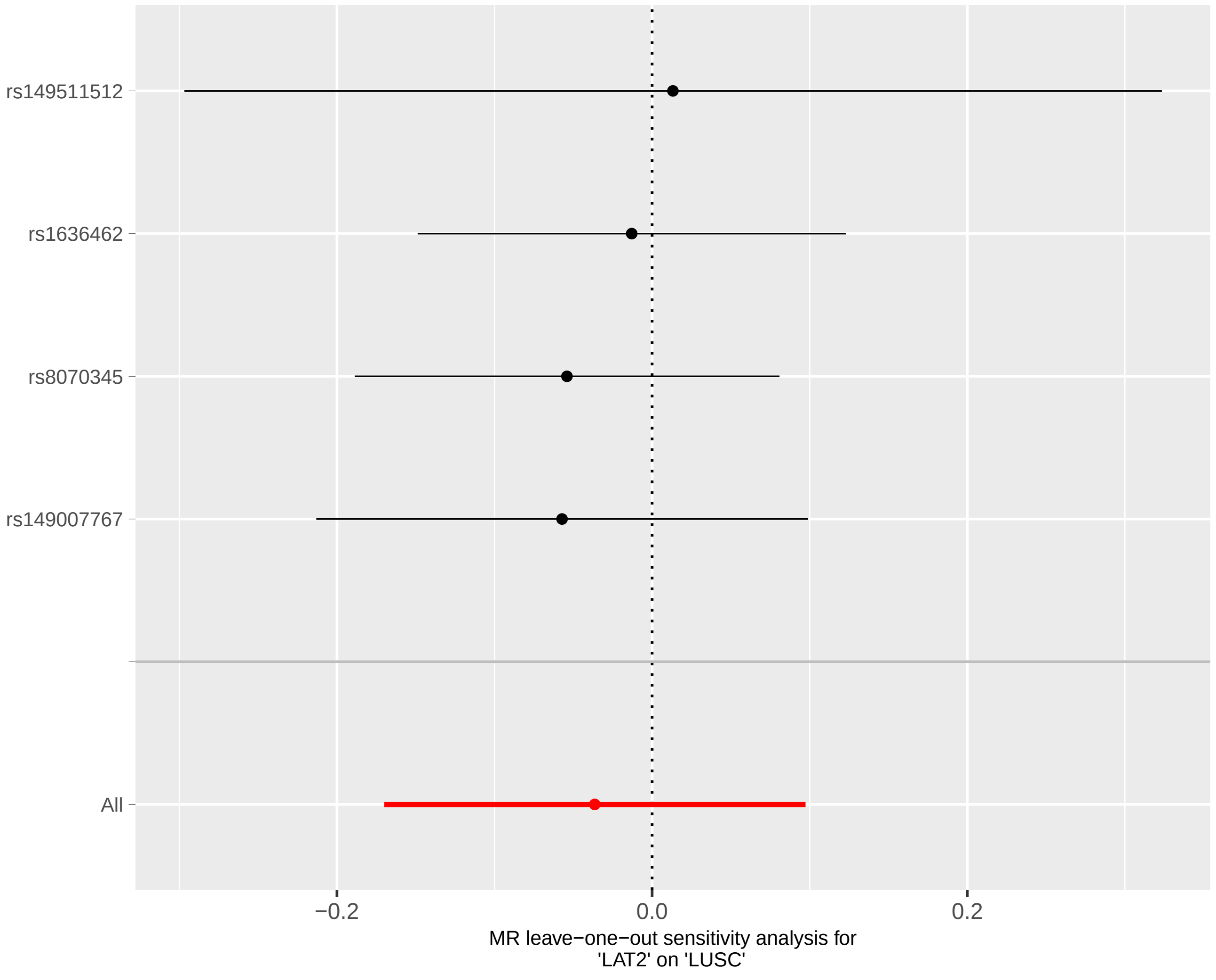

rs149511512
rs1636462
rs8070345
rs149007767
All
−0.2
0.0
0.2
MR leave−one−out sensitivity analysis for
'LAT2' on 'LUSC'

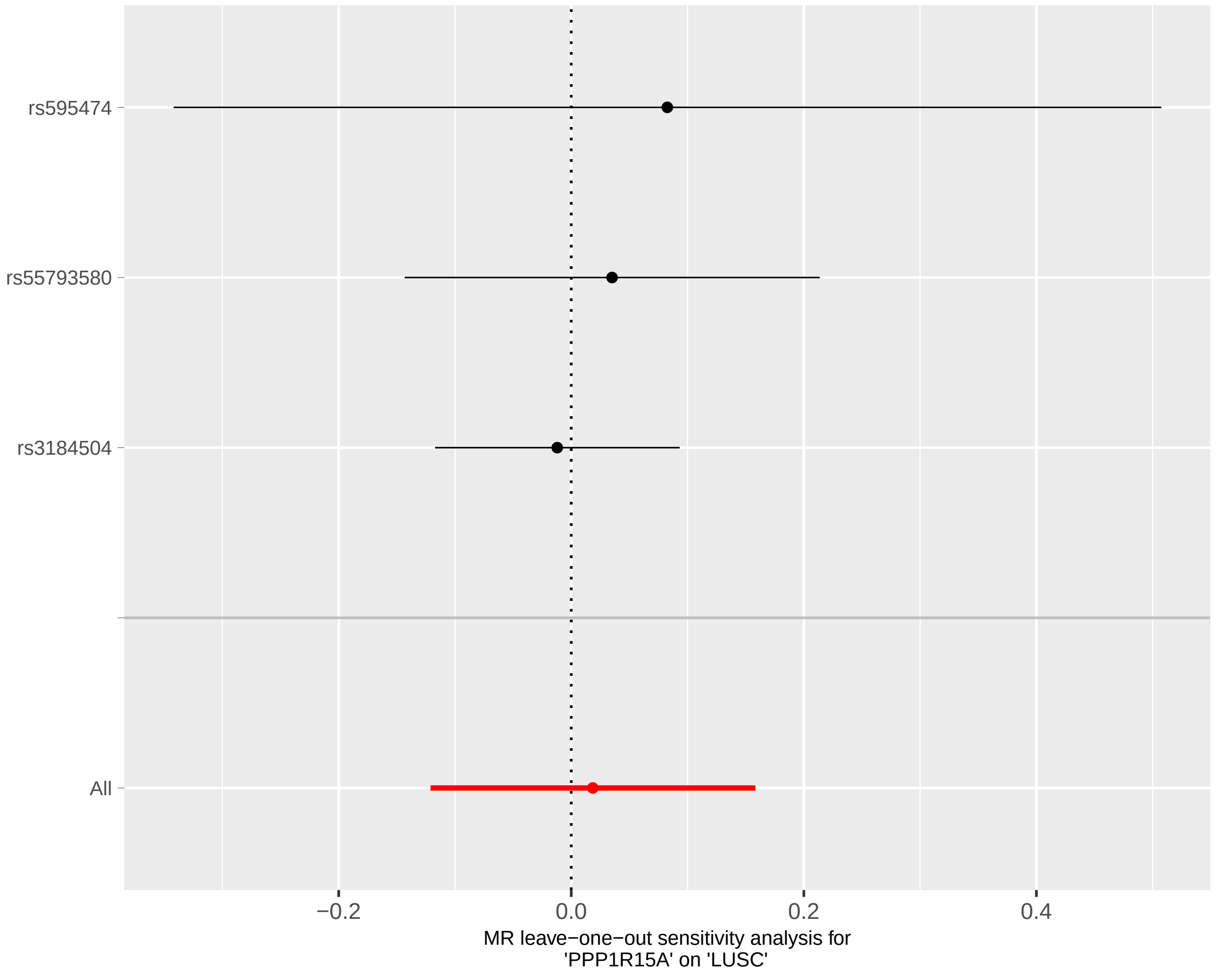

rs595474
rs55793580
rs3184504
All
−0.2
0.0
0.2
0.4
MR leave−one−out sensitivity analysis for
'PPP1R15A' on 'LUSC'

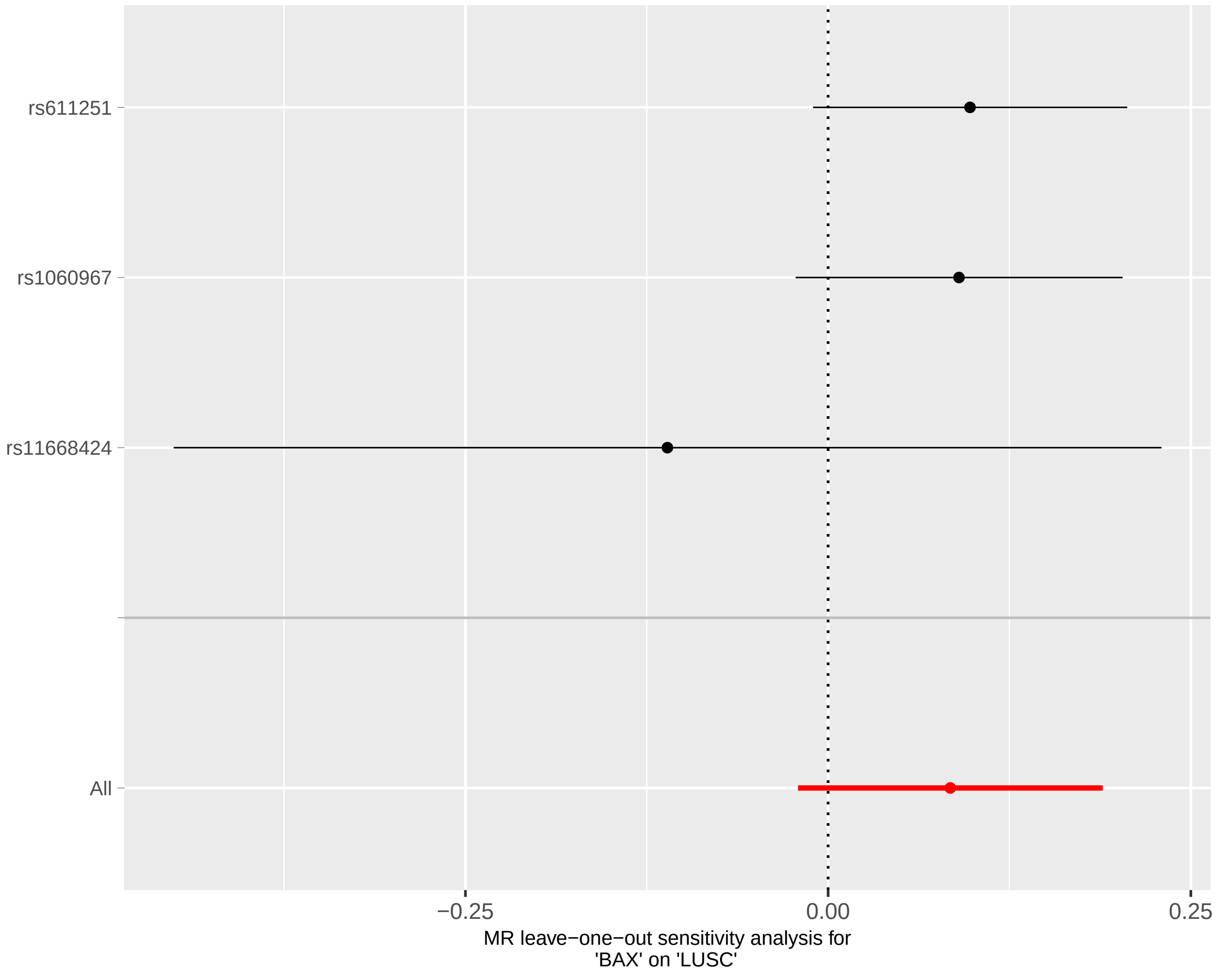
rs611251
rs1060967
rs11668424
All
−0.25
0.00
0.25
MR leave−one−out sensitivity analysis for
'BAX' on 'LUSC'

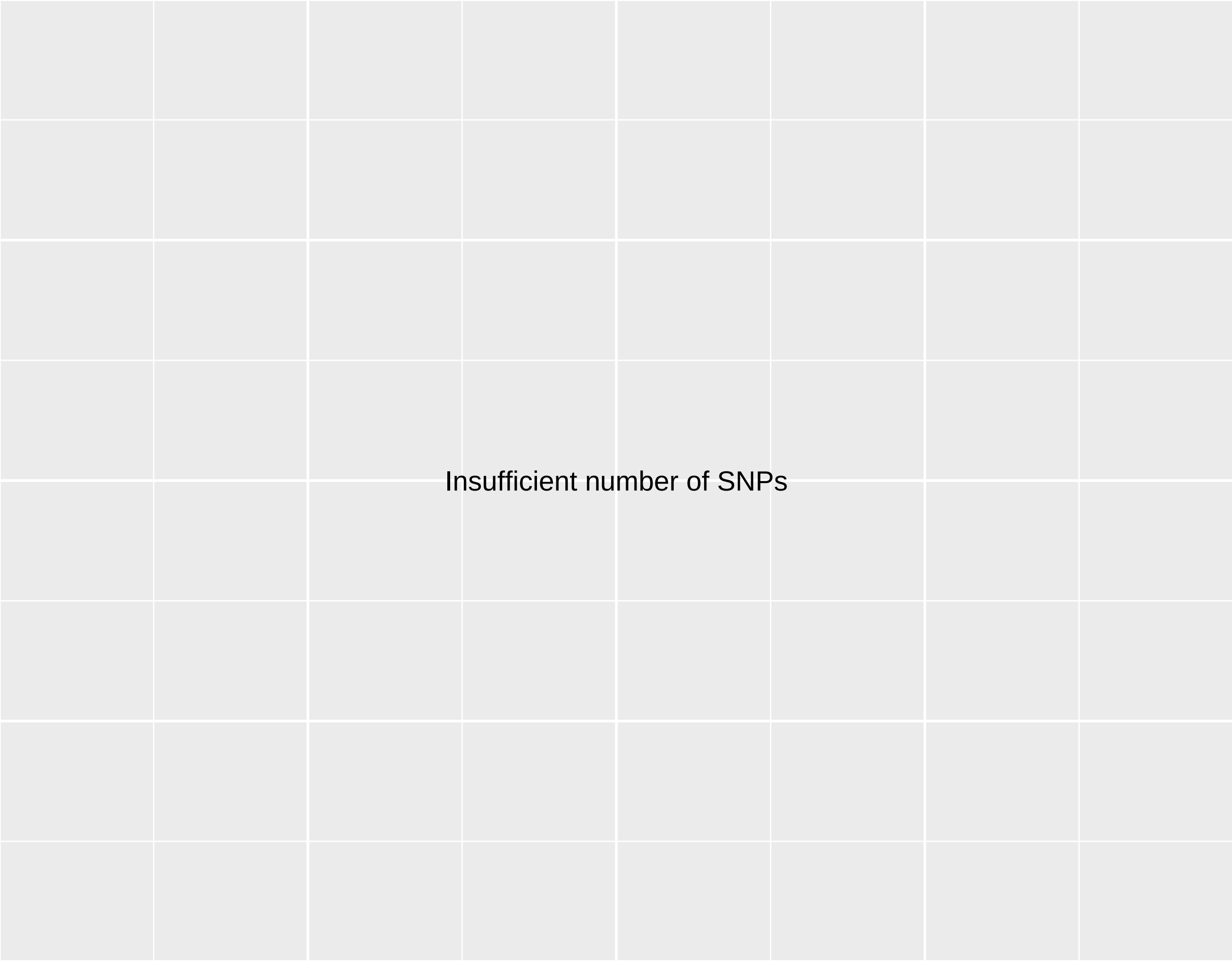
Insufficient number of SNPs

Insufficient number of SNPs

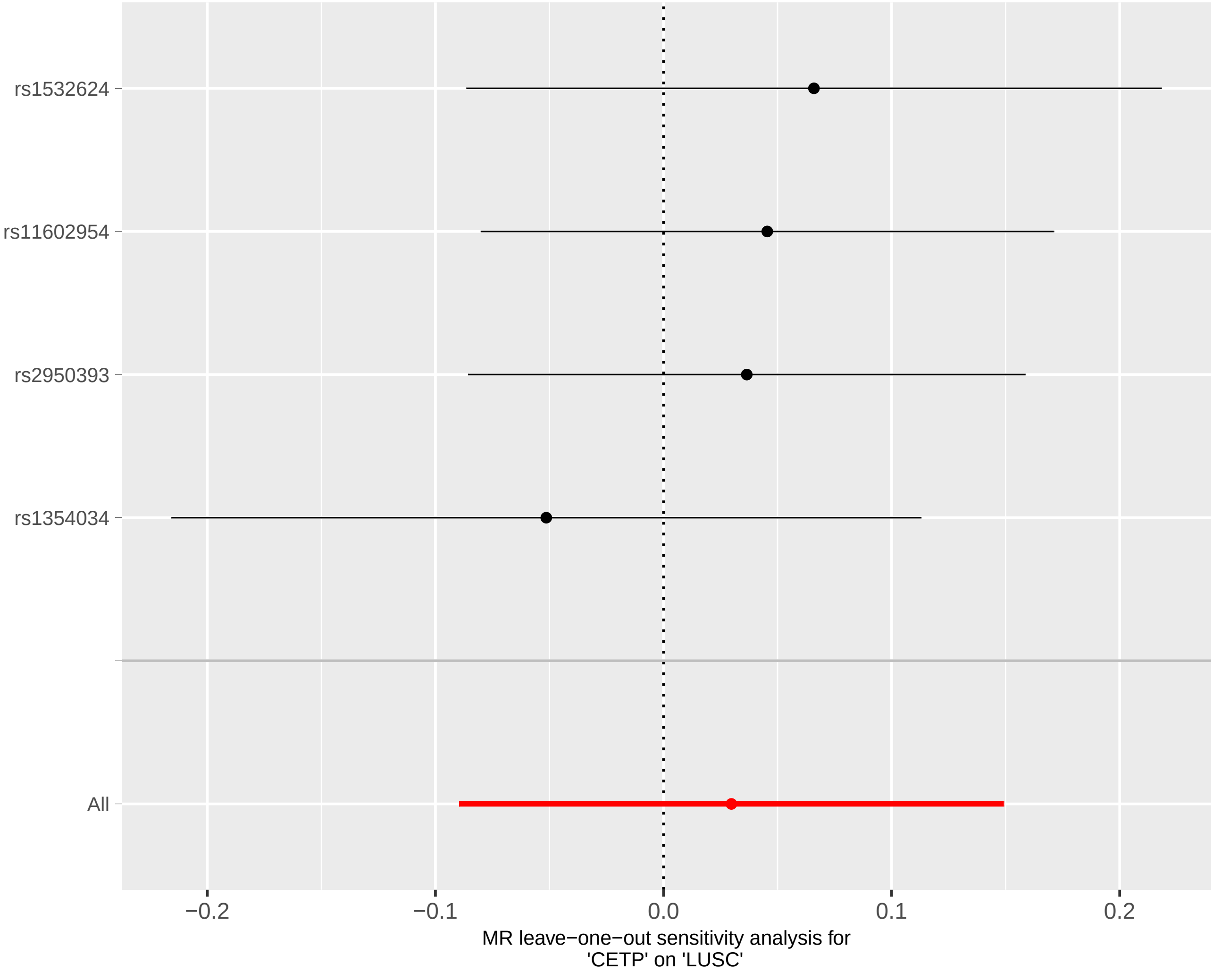

rs1532624
rs11602954
rs2950393
rs1354034
All
−0.2
−0.1
0.0
0.1
0.2
MR leave−one−out sensitivity analysis for
'CETP' on 'LUSC'

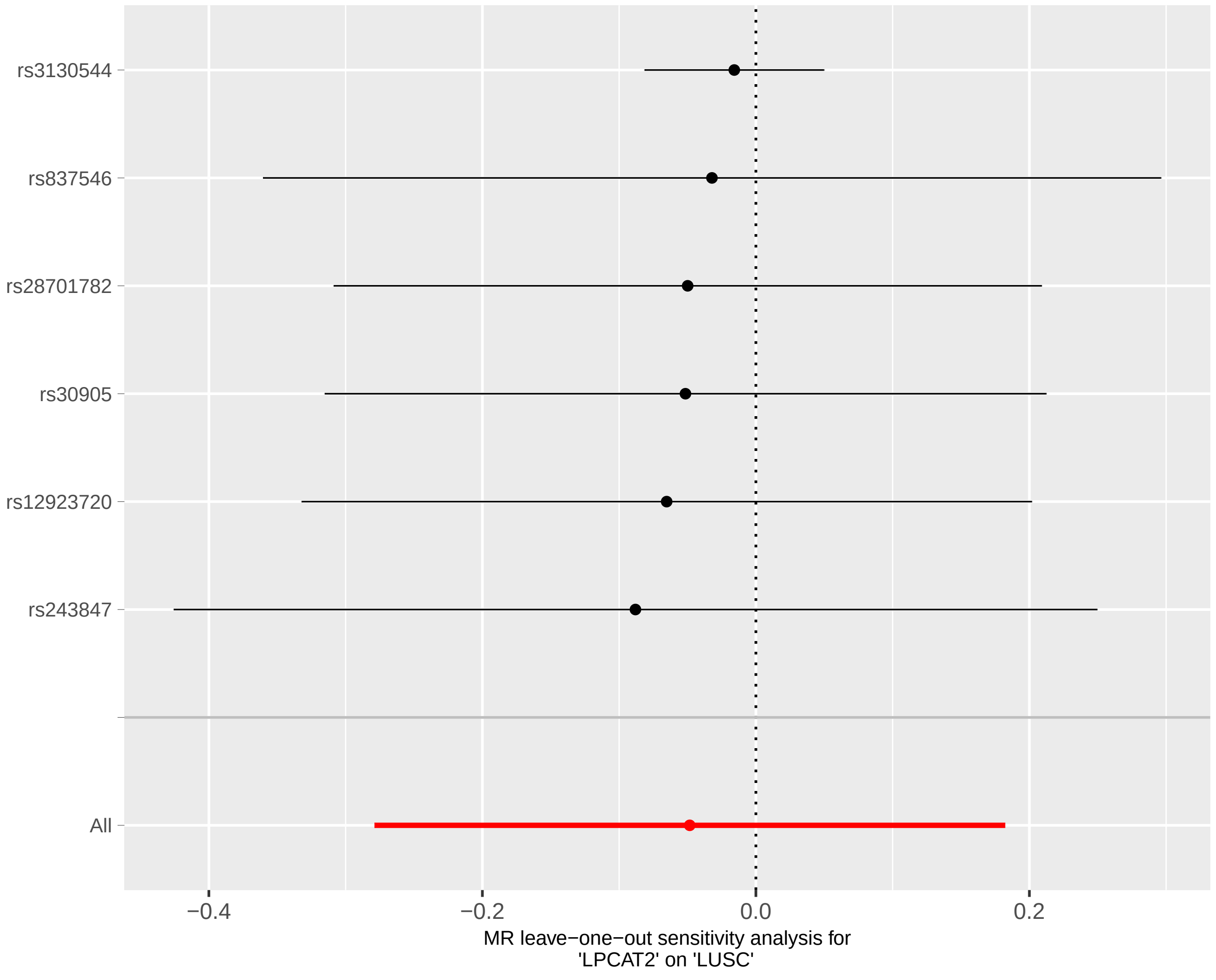
rs3130544
rs837546
rs28701782
rs30905
rs12923720
rs243847
All
−0.4
−0.2
0.0
0.2
MR leave−one−out sensitivity analysis for
'LPCAT2' on 'LUSC'

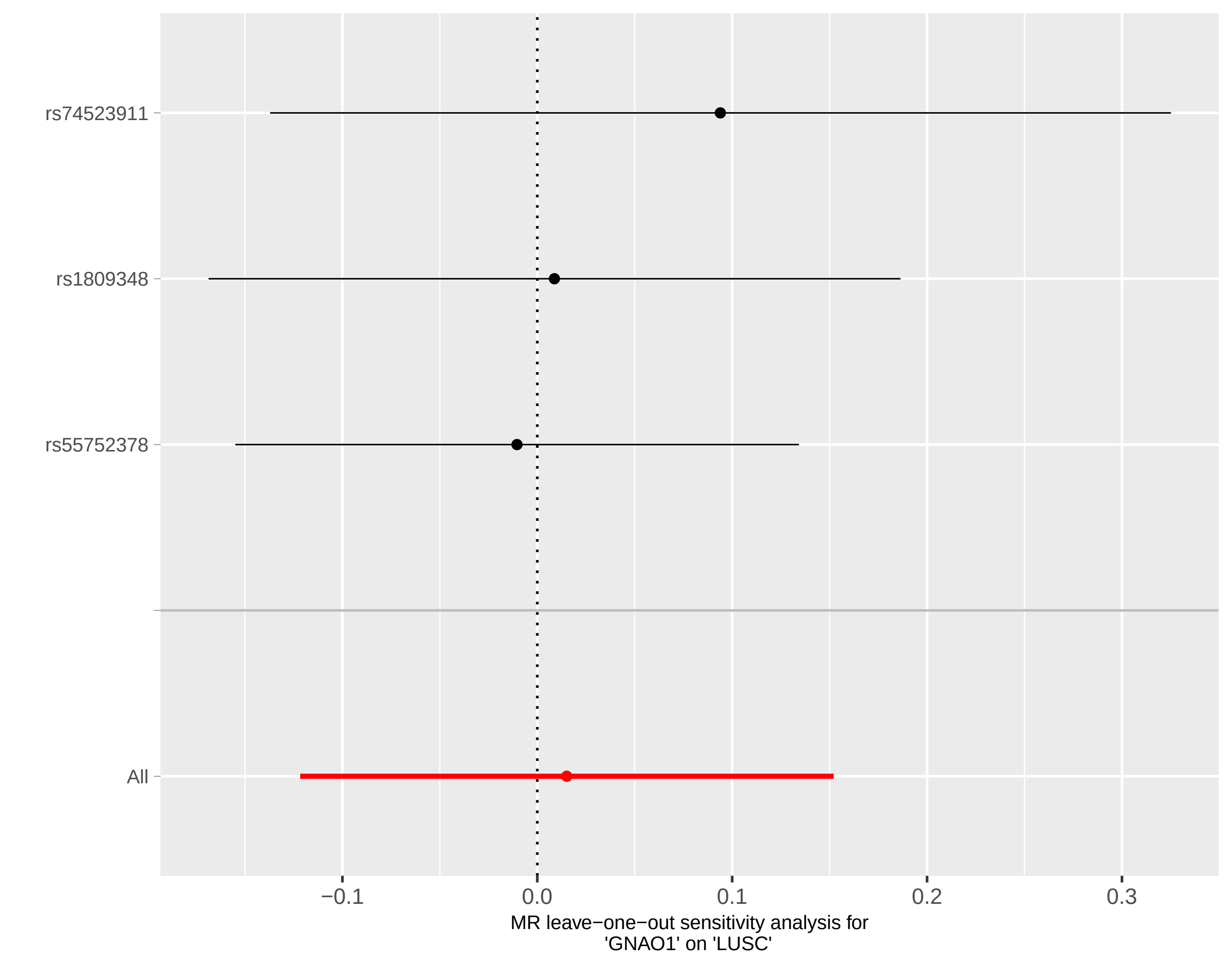

rs74523911
rs1809348
rs55752378
All
−0.1
0.0
0.1
0.2
0.3
MR leave−one−out sensitivity analysis for
'GNAO1' on 'LUSC'

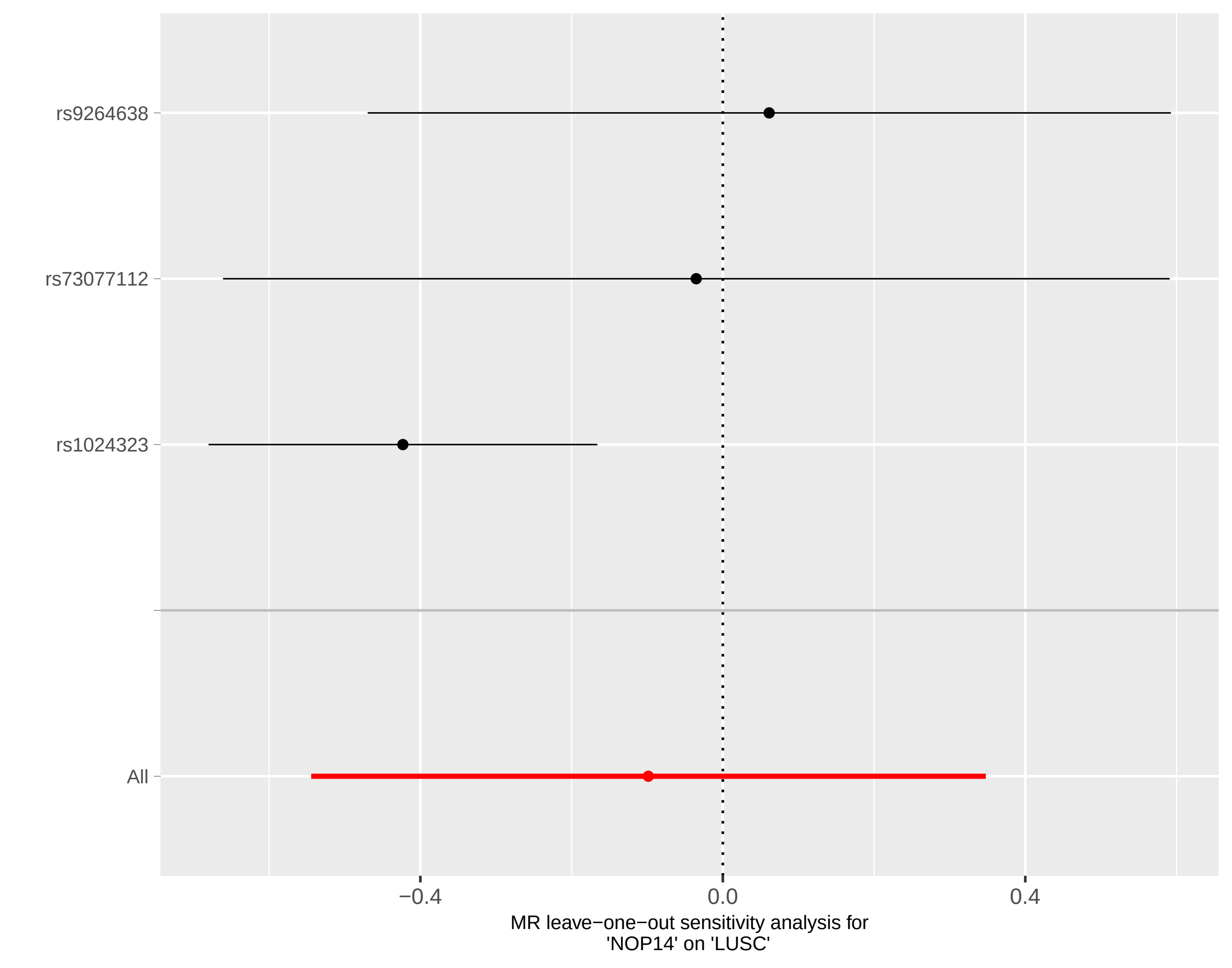

rs9264638
rs73077112
rs1024323
All
−0.4
0.0
0.4
MR leave−one−out sensitivity analysis for
'NOP14' on 'LUSC'

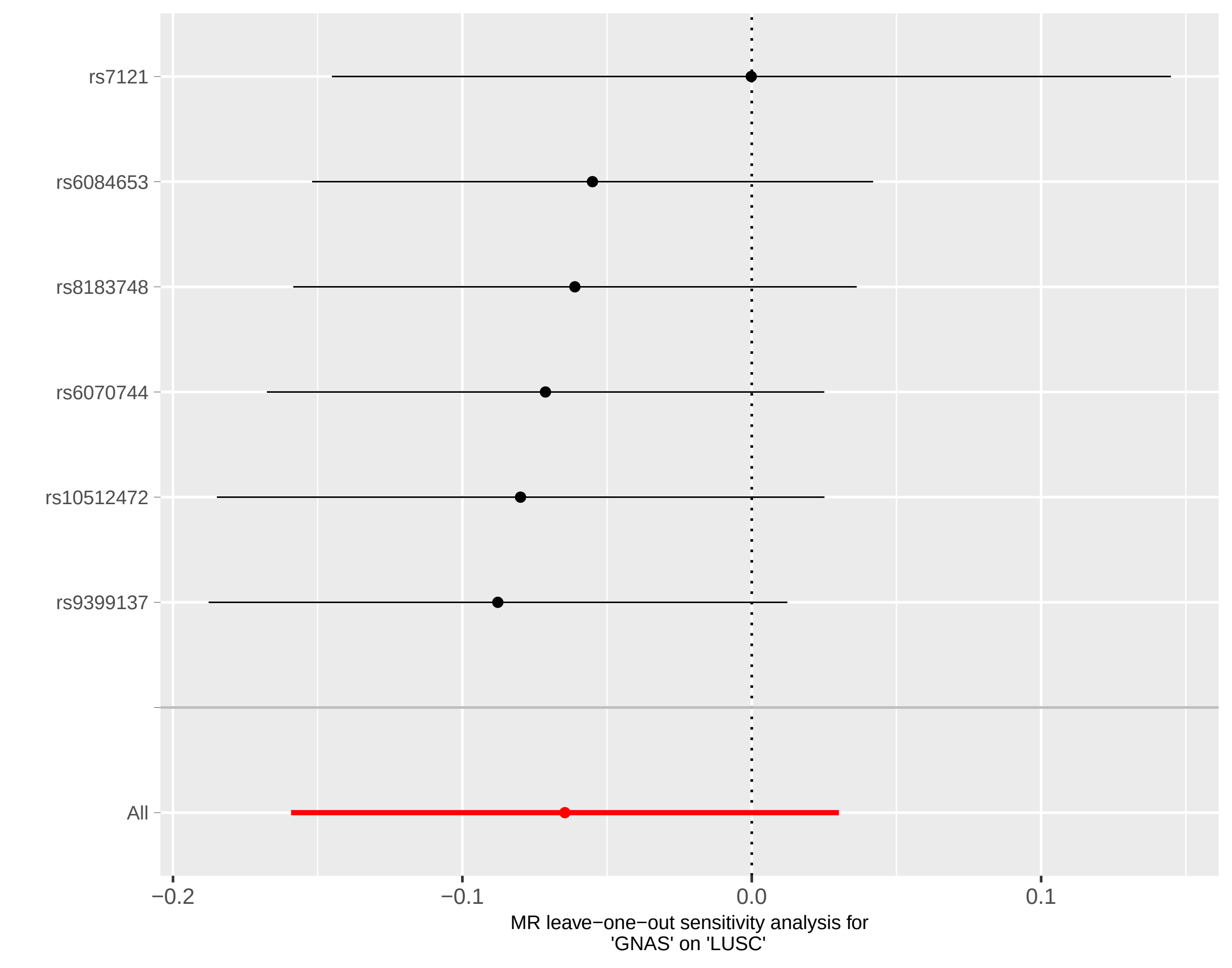

rs7121
rs6084653
rs8183748
rs6070744
rs10512472
rs9399137
All
−0.2
−0.1
0.0
0.1
MR leave−one−out sensitivity analysis for
'GNAS' on 'LUSC'

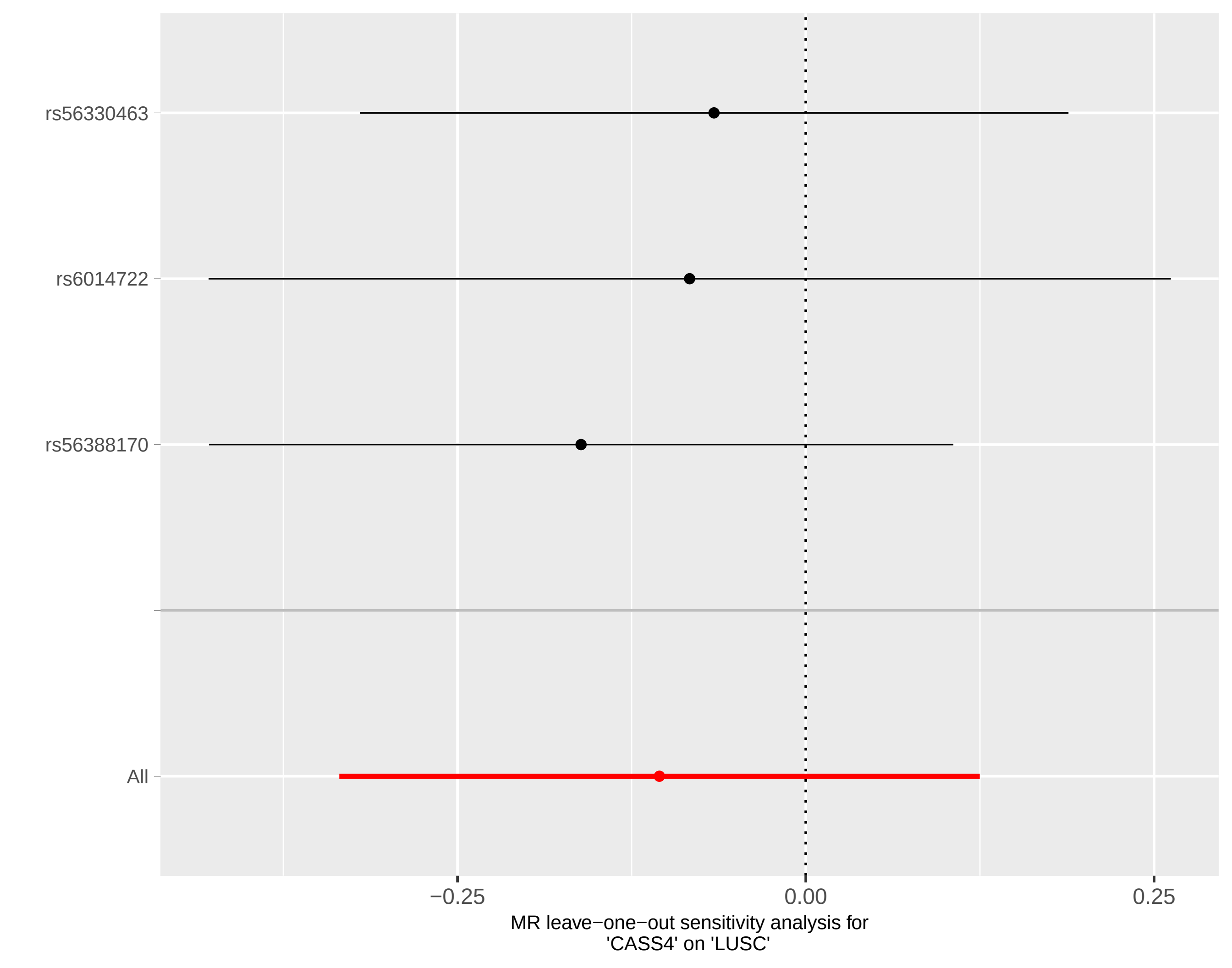
rs56330463
rs6014722
rs56388170
All
−0.25
0.00
0.25
MR leave−one−out sensitivity analysis for
'CASS4' on 'LUSC'

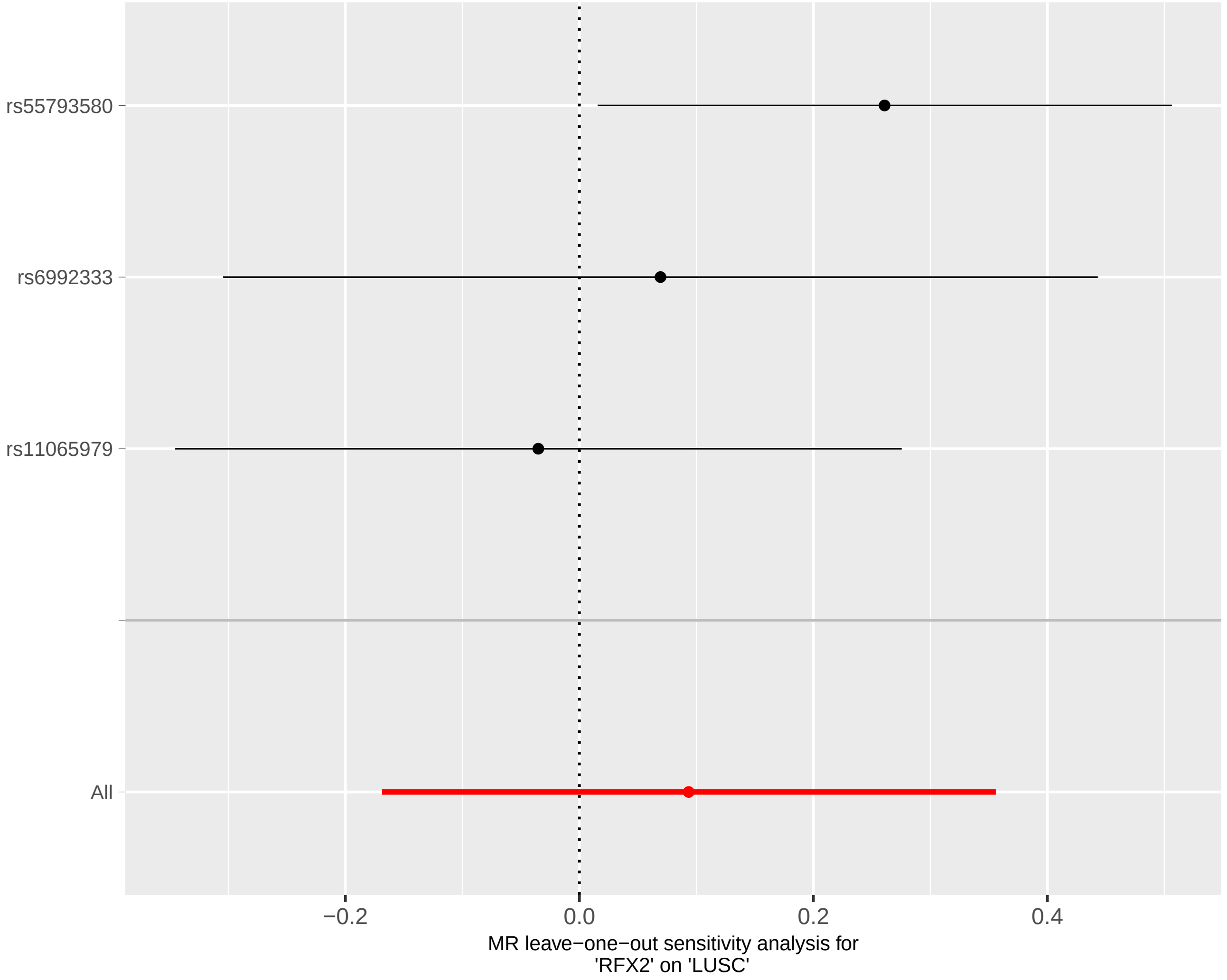
rs55793580
rs6992333
rs11065979
All
−0.2
0.0
0.2
0.4
MR leave−one−out sensitivity analysis for
'RFX2' on 'LUSC'

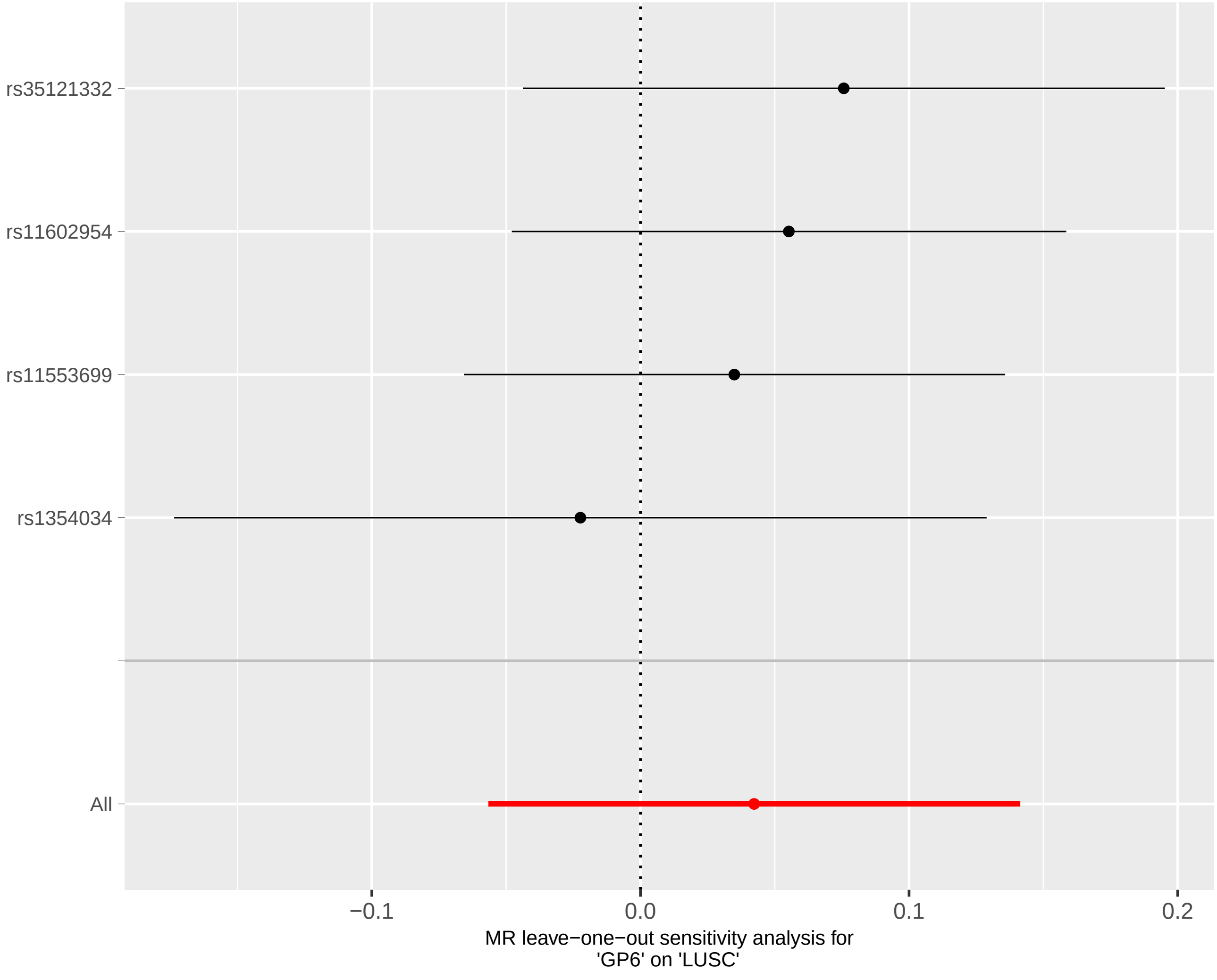
rs35121332
rs11602954
rs11553699
rs1354034
All
−0.1
0.0
0.1
0.2
MR leave−one−out sensitivity analysis for
'GP6' on 'LUSC'

Insufficient number of SNPs

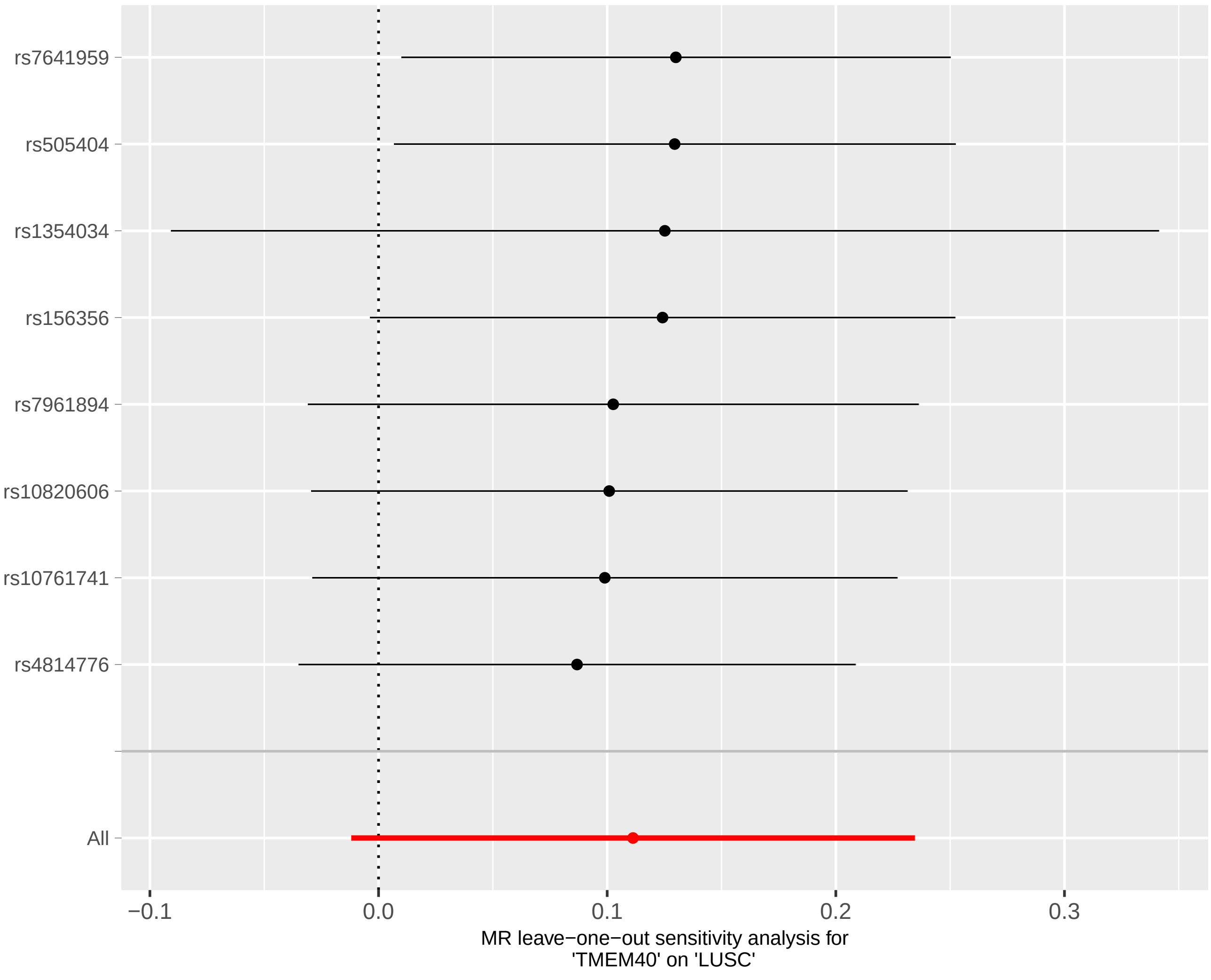
rs7641959
rs505404
rs1354034
rs156356
rs7961894
rs10820606
rs10761741
rs4814776
All
−0.1
0.0
0.1
0.2
0.3
MR leave−one−out sensitivity analysis for
'TMEM40' on 'LUSC'

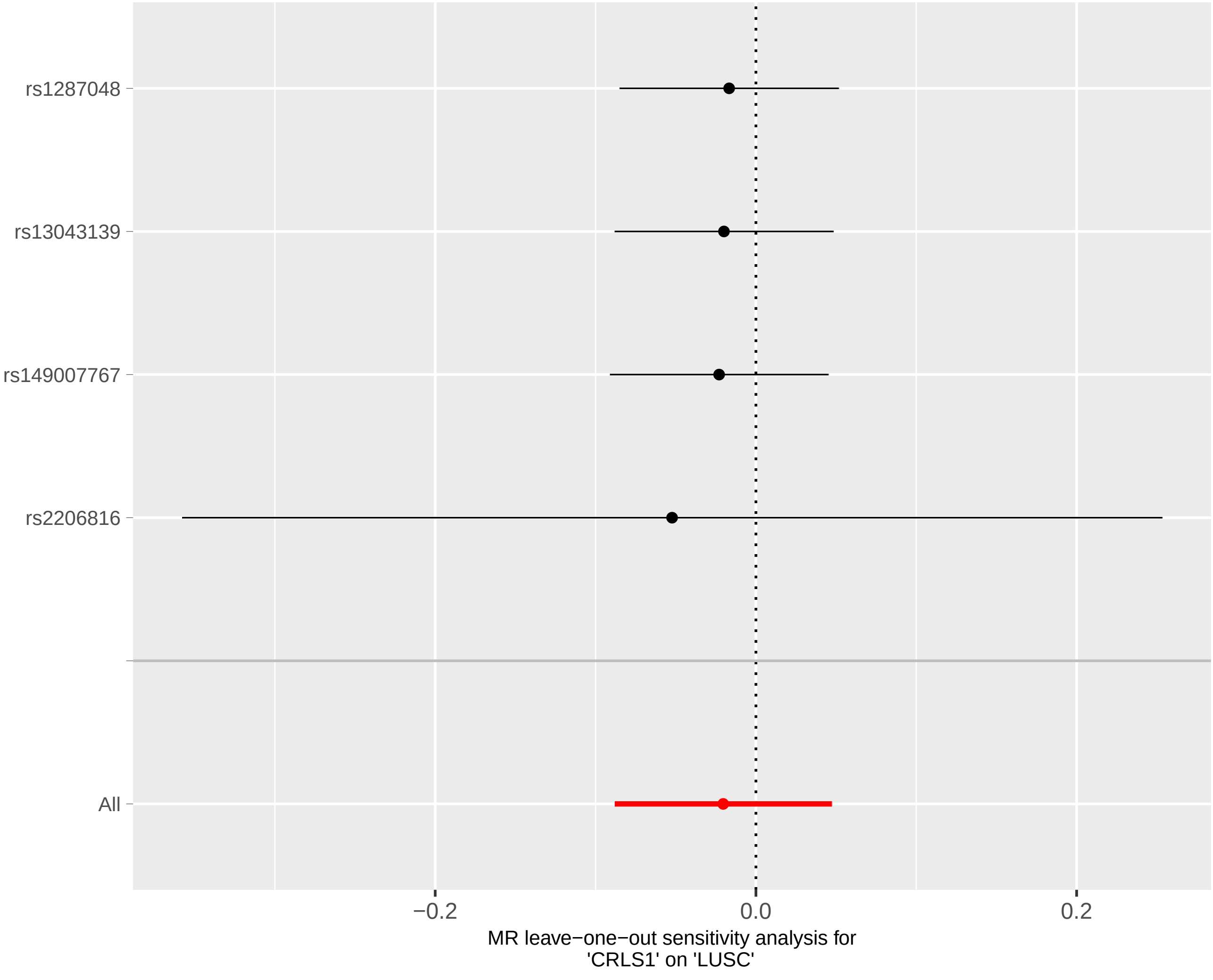
rs1287048
rs13043139
rs149007767
rs2206816
All
−0.2
0.0
0.2
MR leave−one−out sensitivity analysis for
'CRLS1' on 'LUSC'

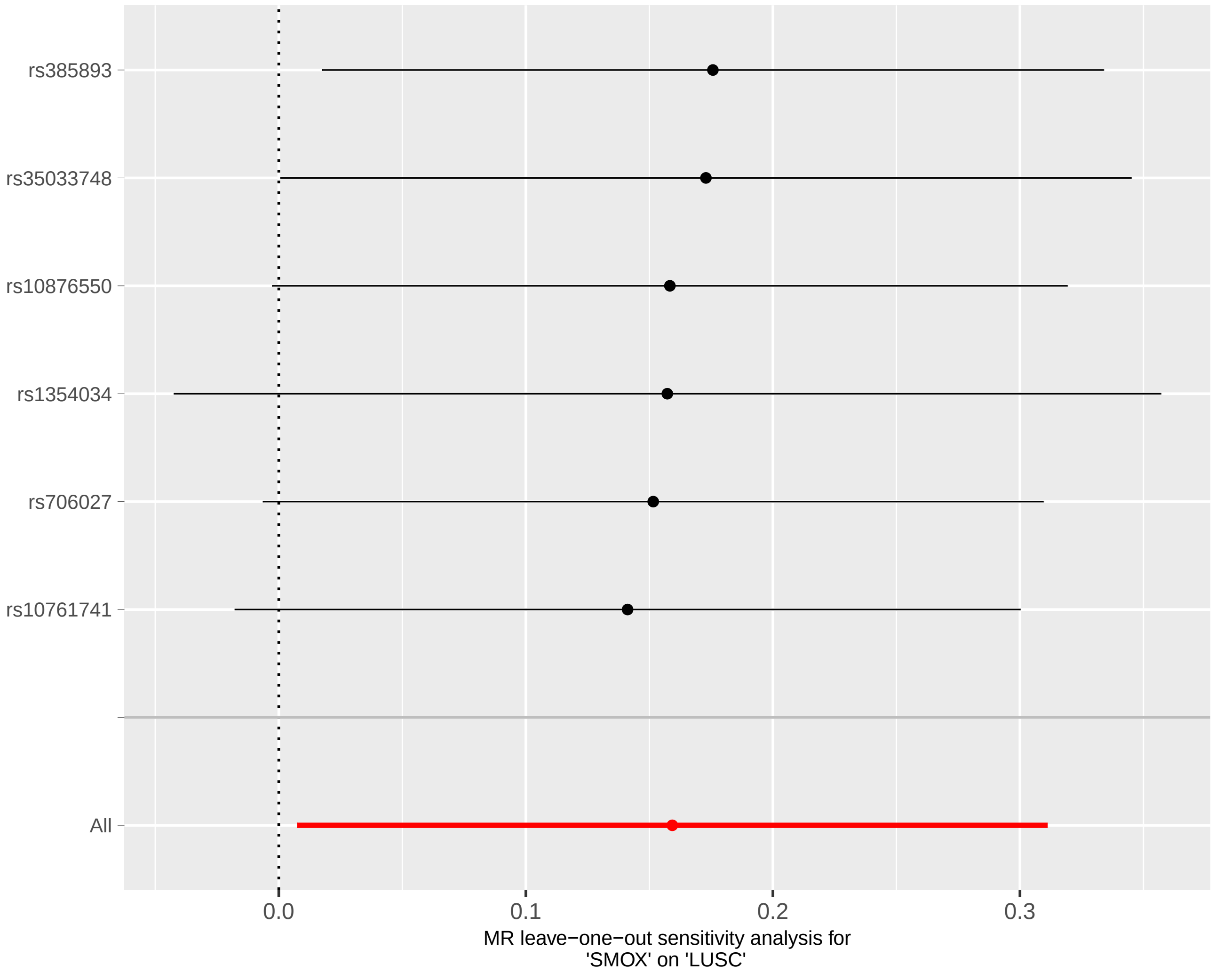
rs385893
rs35033748
rs10876550
rs1354034
rs706027
rs10761741
All
0.0
0.1
0.2
0.3
MR leave−one−out sensitivity analysis for
'SMOX' on 'LUSC'

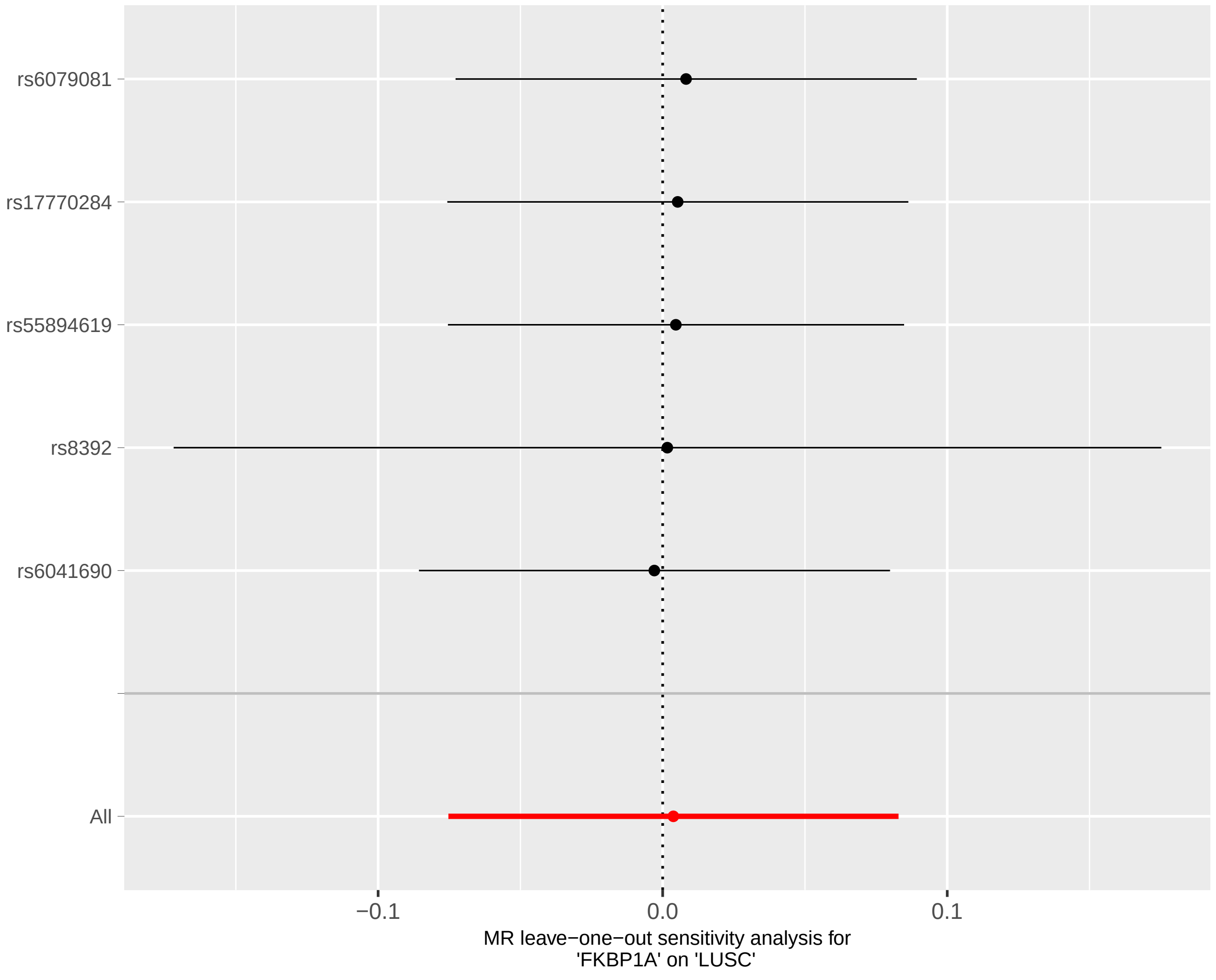
rs6079081
rs17770284
rs55894619
rs8392
rs6041690
All
−0.1
0.0
0.1
MR leave−one−out sensitivity analysis for
'FKBP1A' on 'LUSC'

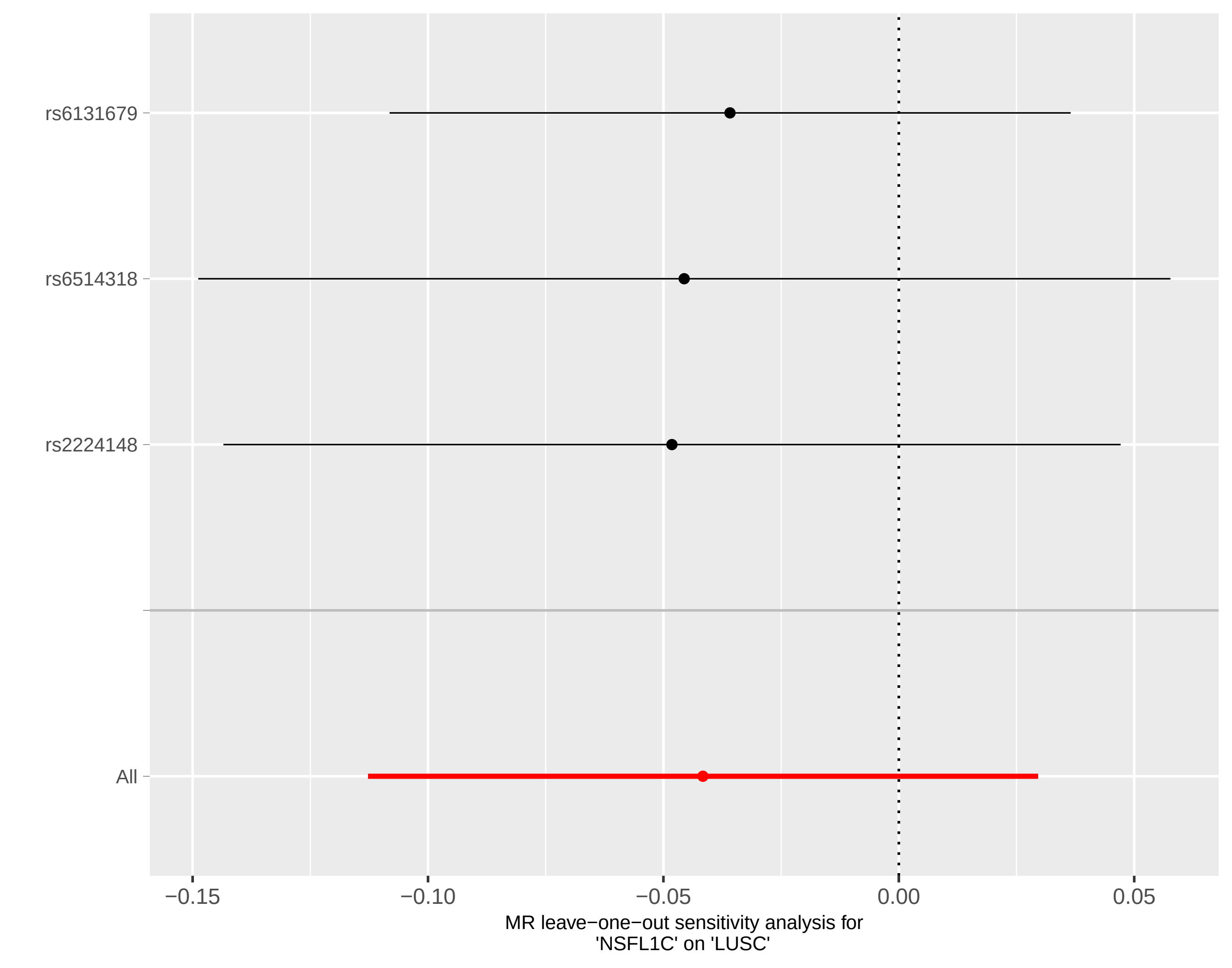

rs6131679
rs6514318
rs2224148
All
−0.15
−0.10
−0.05
0.00
0.05
MR leave−one−out sensitivity analysis for
'NSFL1C' on 'LUSC'

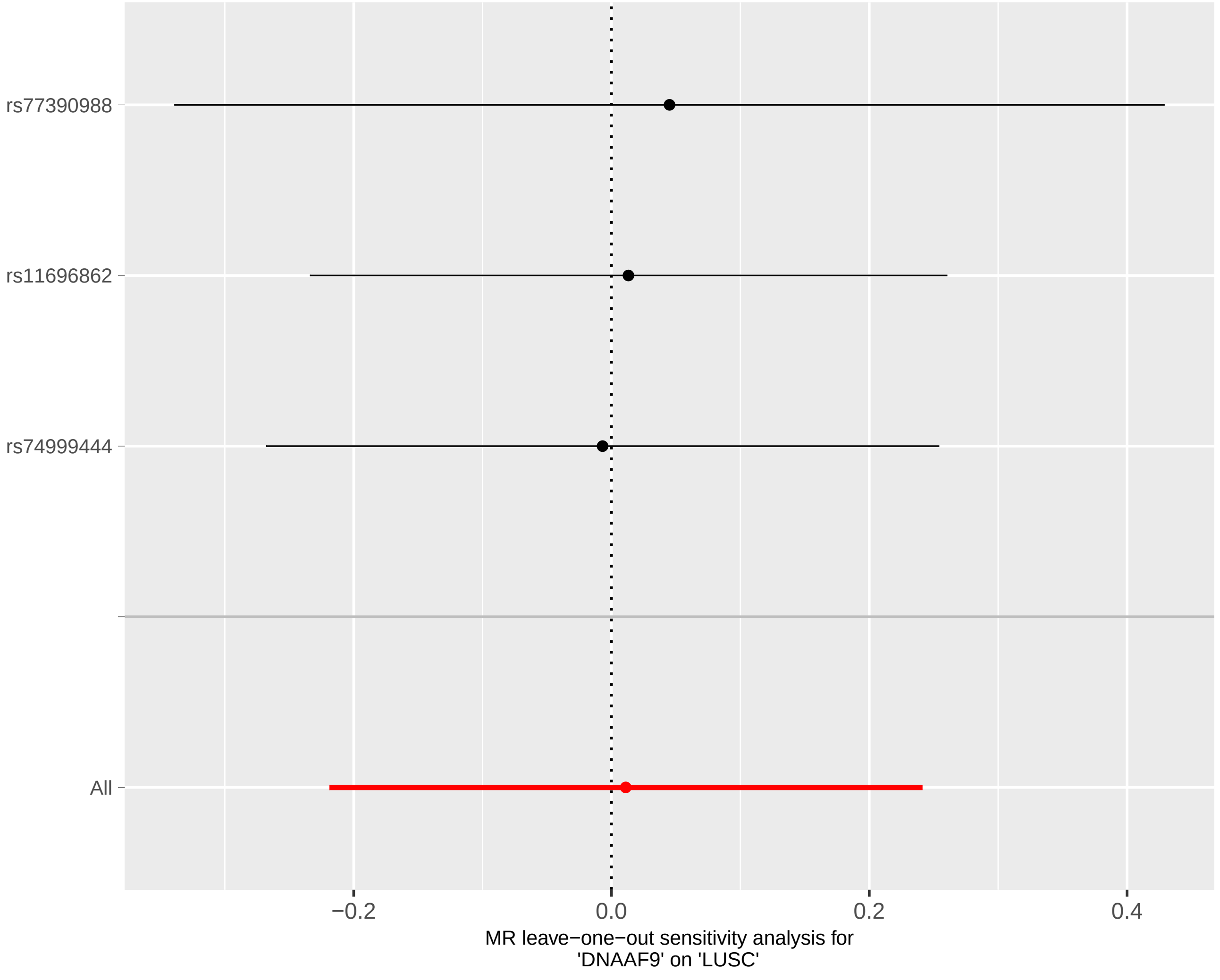

rs77390988
rs11696862
rs74999444
All
−0.2
0.0
0.2
0.4
MR leave−one−out sensitivity analysis for
'DNAAF9' on 'LUSC'

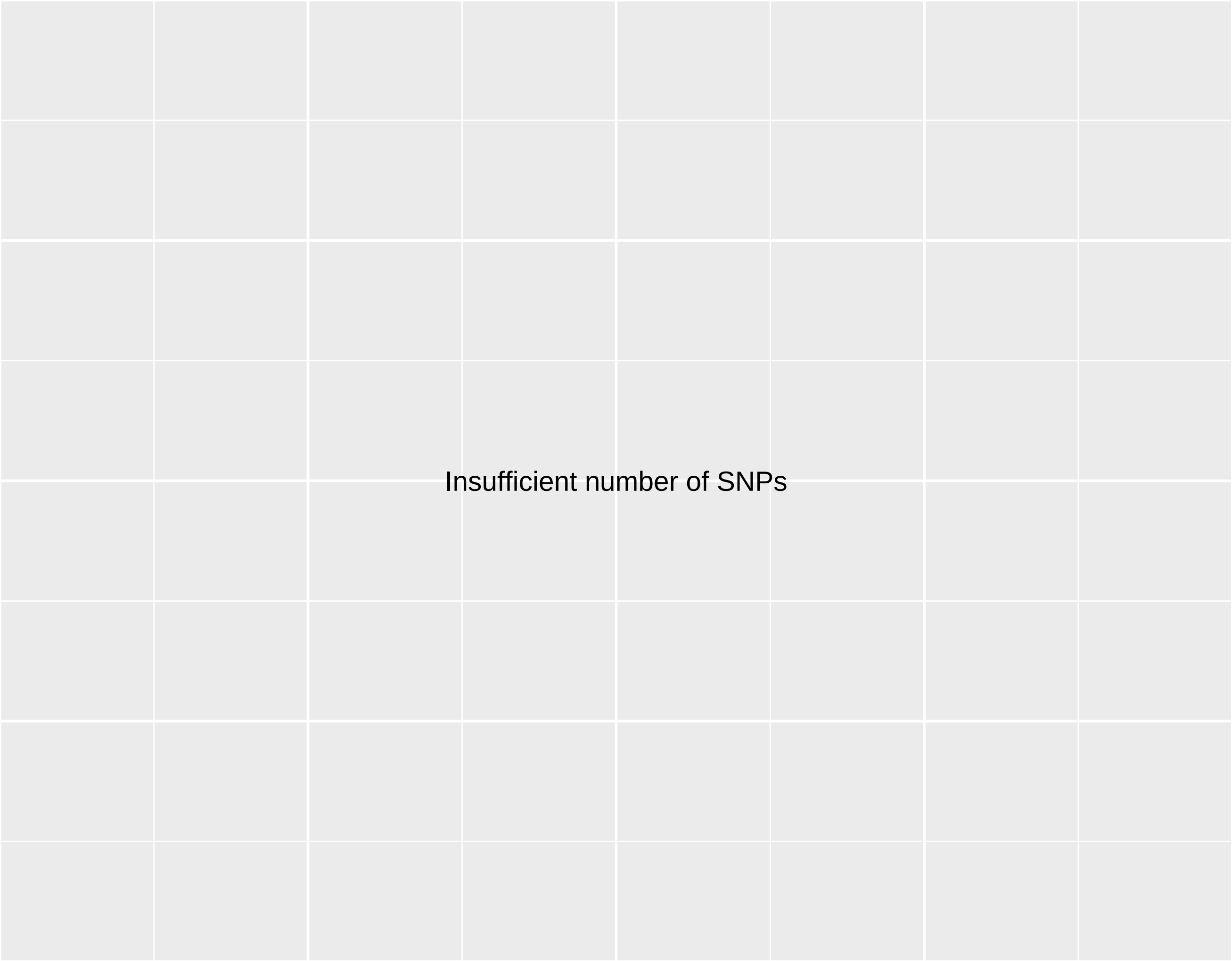
Insufficient number of SNPs

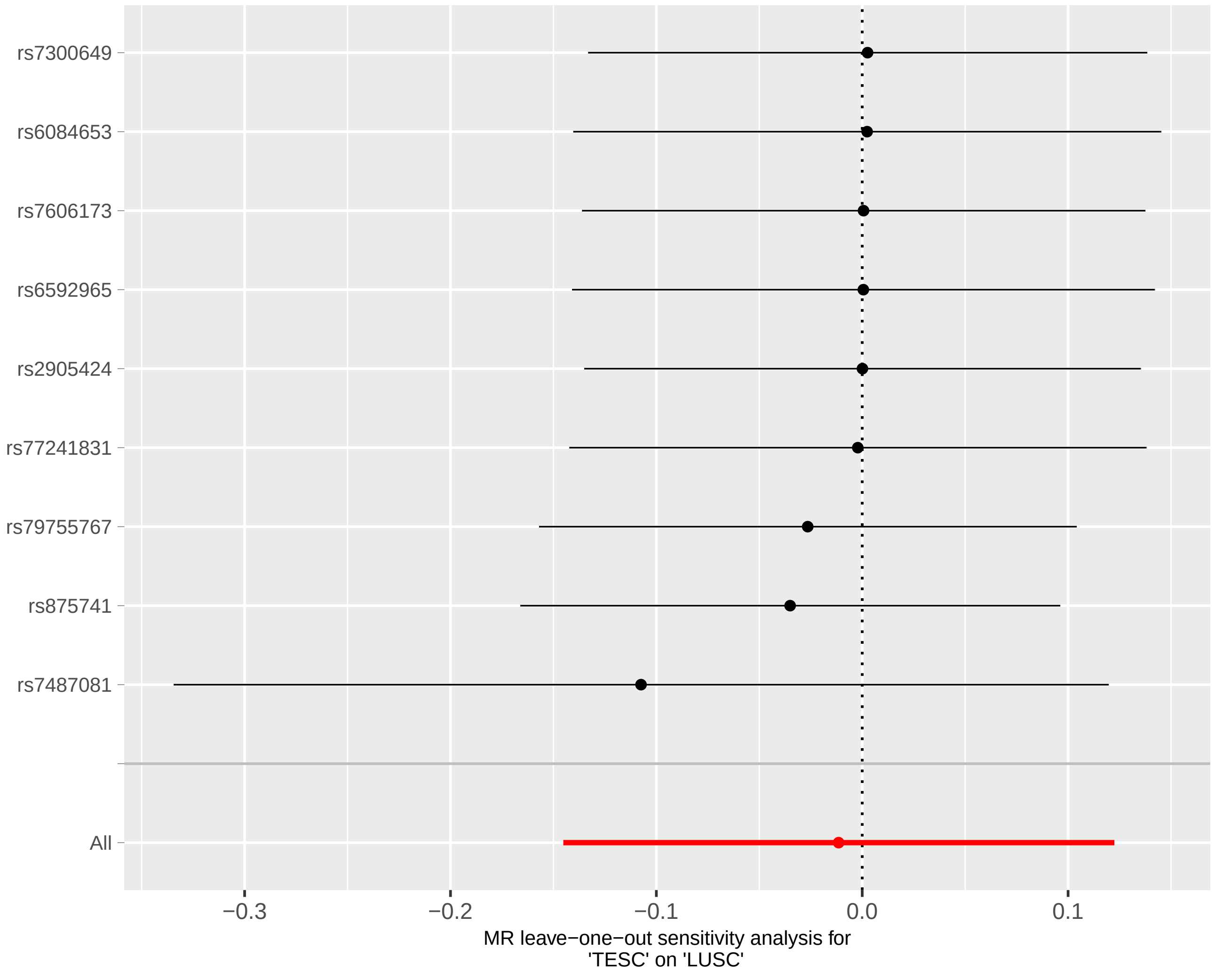

rs7300649
rs6084653
rs7606173
rs6592965
rs2905424
rs77241831
rs79755767
rs875741
rs7487081
All
−0.3
−0.2
−0.1
0.0
0.1
MR leave−one−out sensitivity analysis for
'TESC' on 'LUSC'

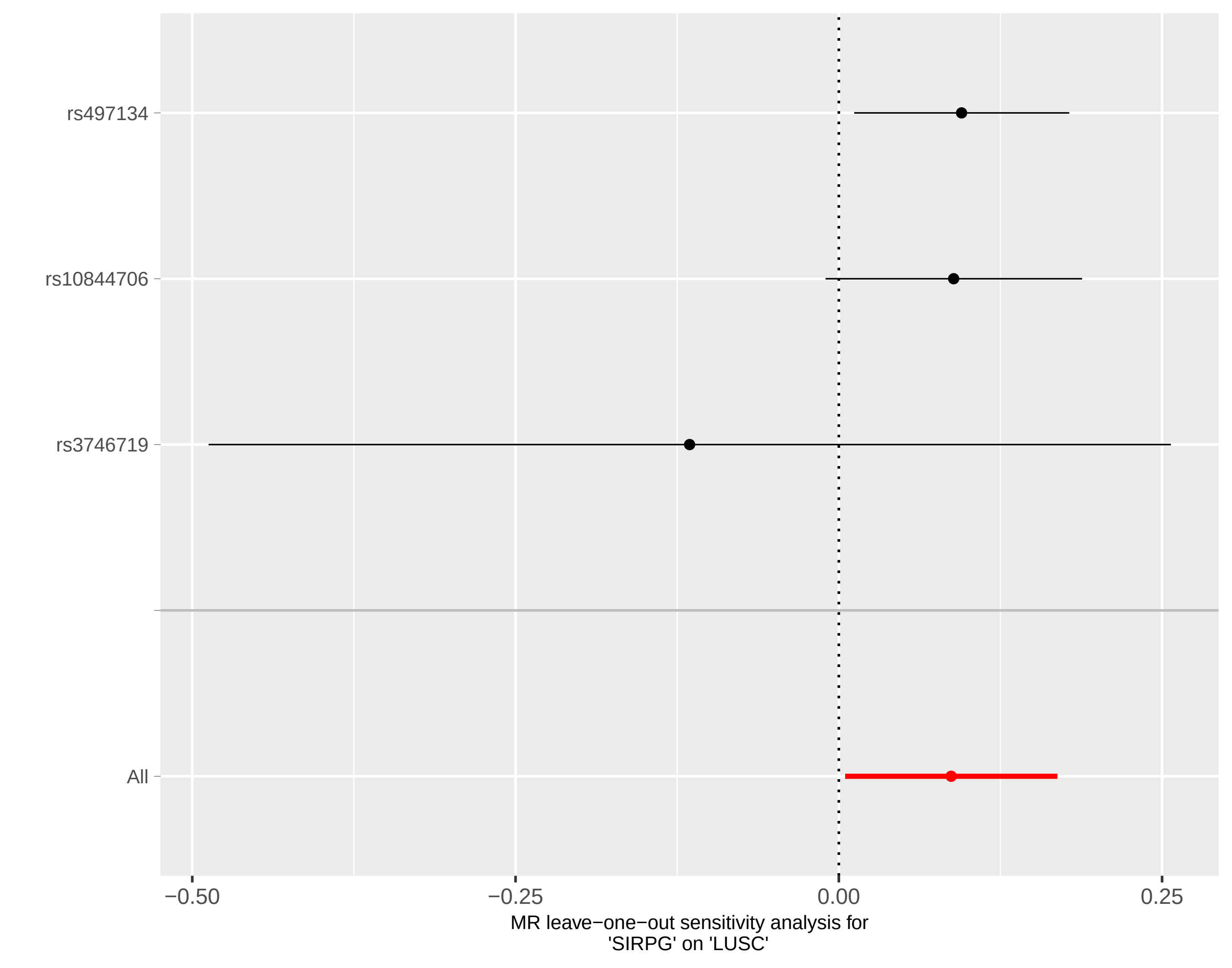

rs497134
rs10844706
rs3746719
All
−0.50
−0.25
0.00
0.25
MR leave−one−out sensitivity analysis for
'SIRPG' on 'LUSC'

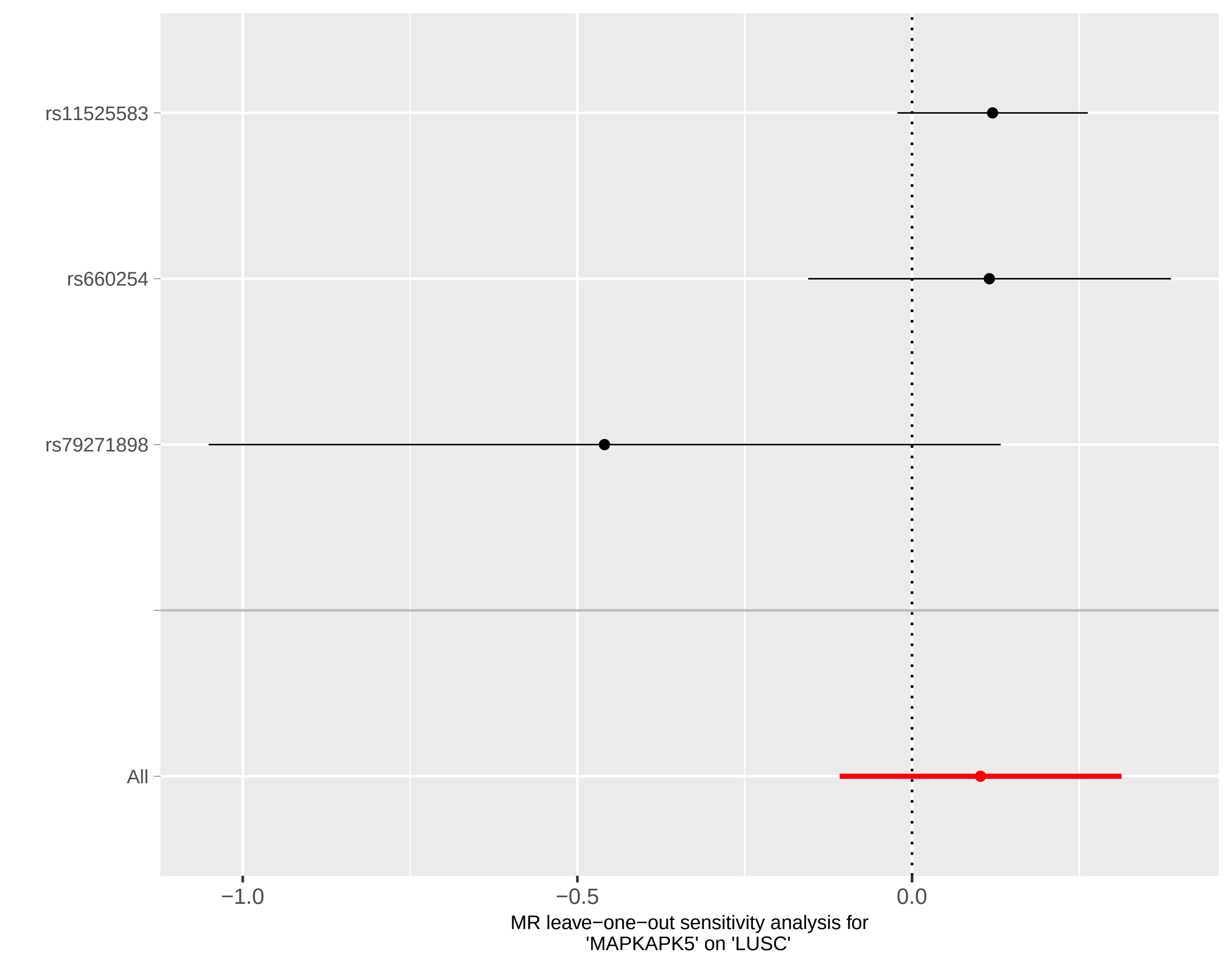
rs11525583
rs660254
rs79271898
All
−1.0
−0.5
0.0
MR leave−one−out sensitivity analysis for
'MAPKAPK5' on 'LUSC'

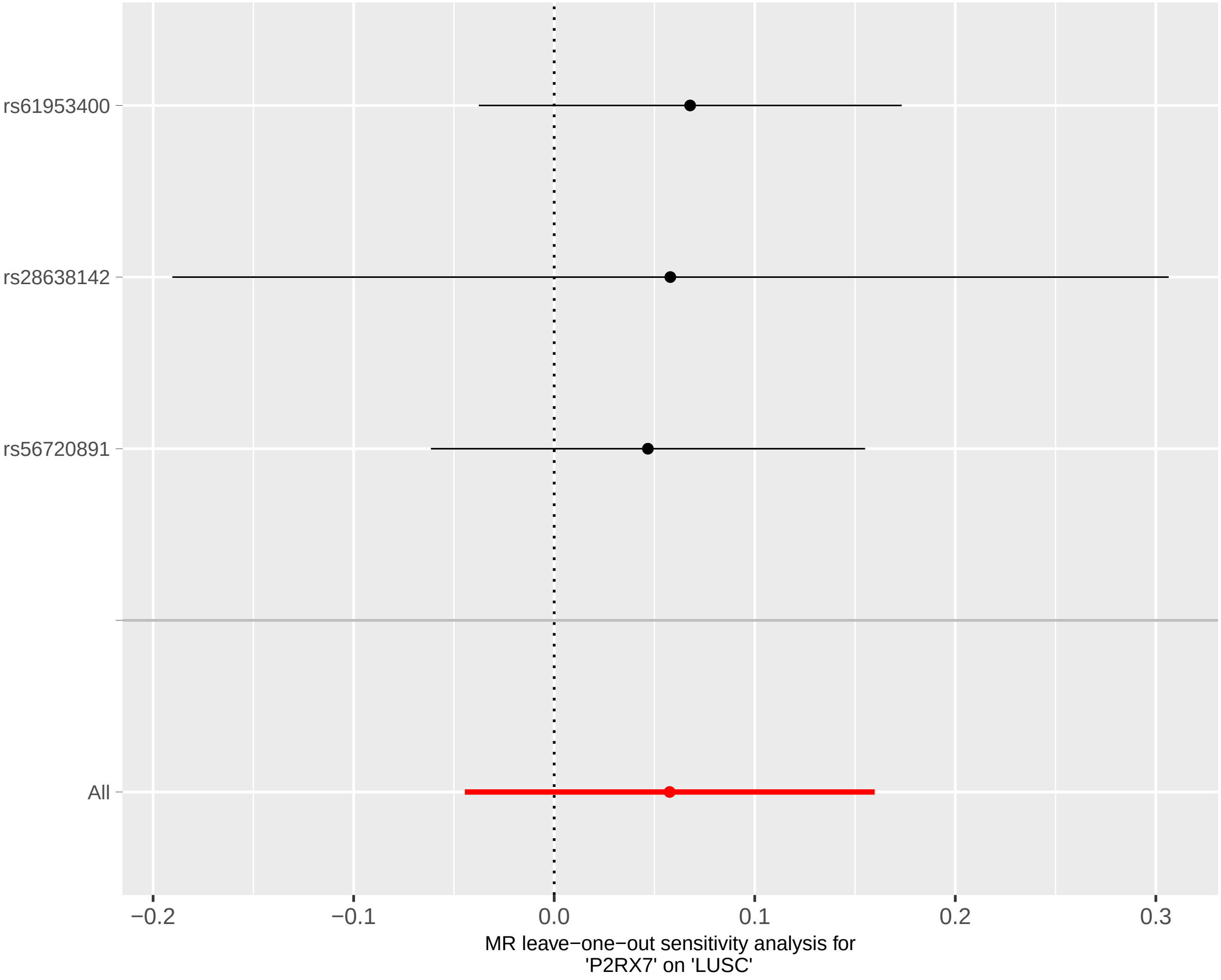
rs61953400
rs28638142
rs56720891
All
−0.2
−0.1
0.0
0.1
0.2
0.3
MR leave−one−out sensitivity analysis for
'P2RX7' on 'LUSC'

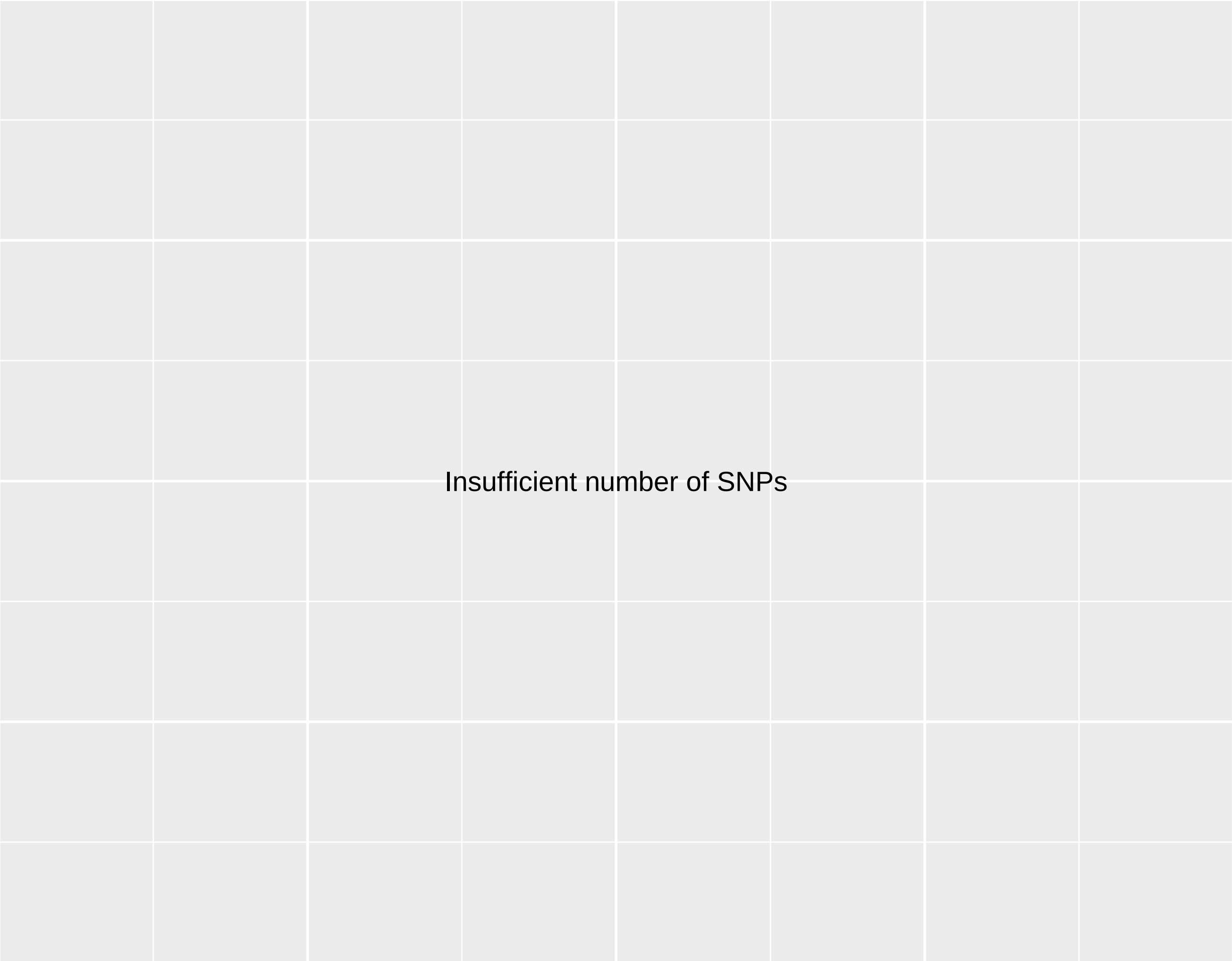
Insufficient number of SNPs

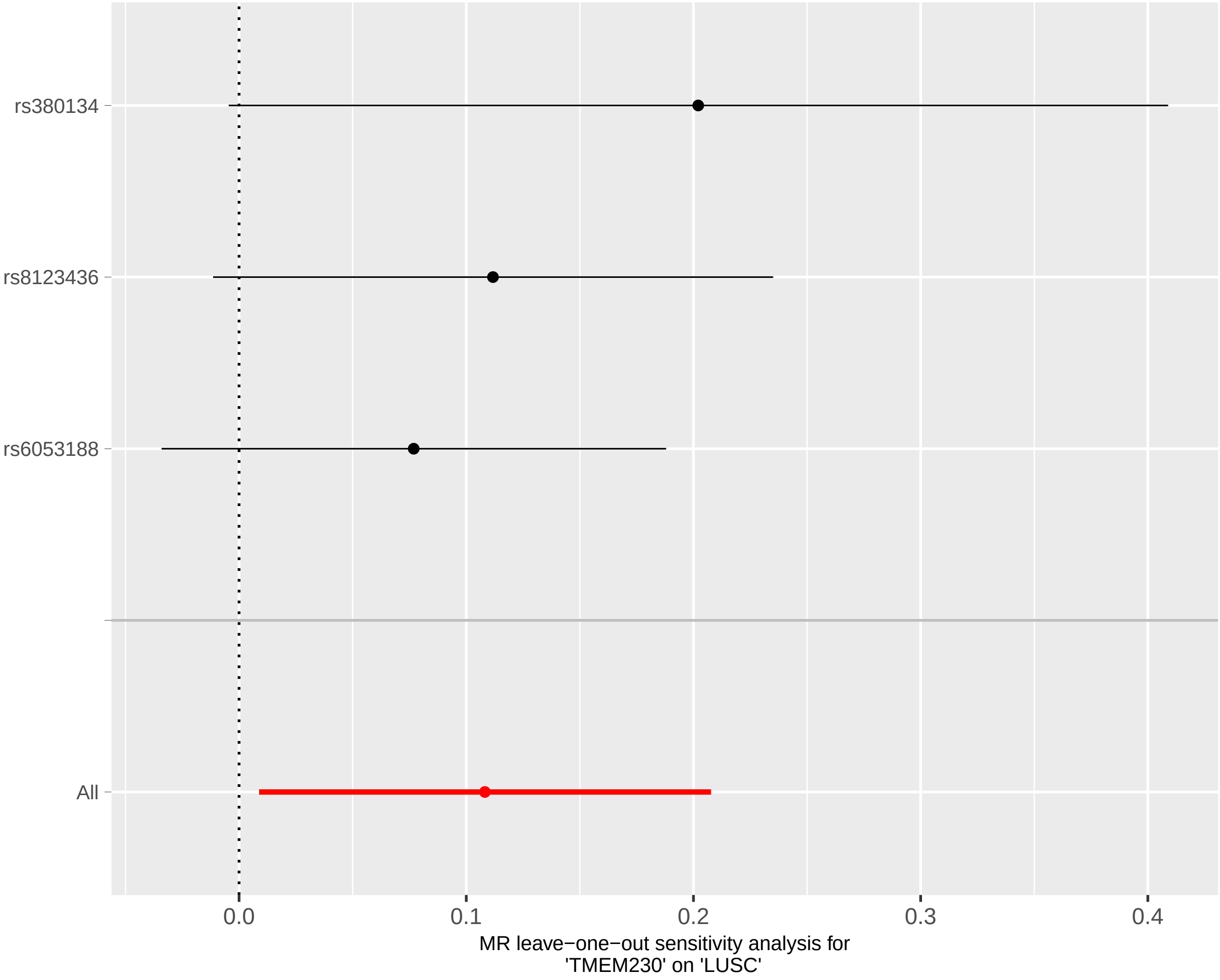

rs380134
rs8123436
rs6053188
All
0.0
0.1
0.2
0.3
0.4
MR leave−one−out sensitivity analysis for
'TMEM230' on 'LUSC'

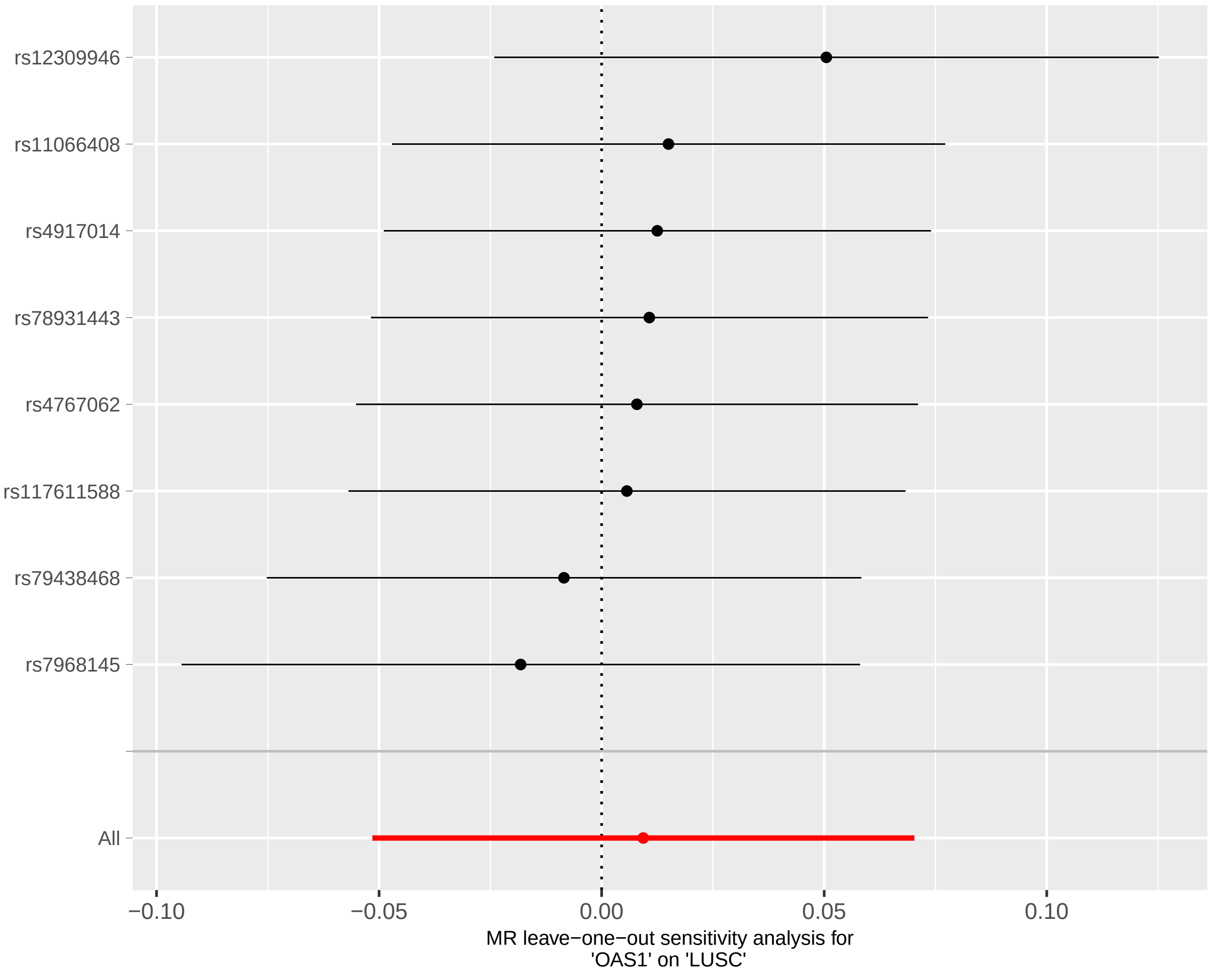
rs12309946
rs11066408
rs4917014
rs78931443
rs4767062
rs117611588
rs79438468
rs7968145
All
−0.10
−0.05
0.00
0.05
0.10
MR leave−one−out sensitivity analysis for
'OAS1' on 'LUSC'

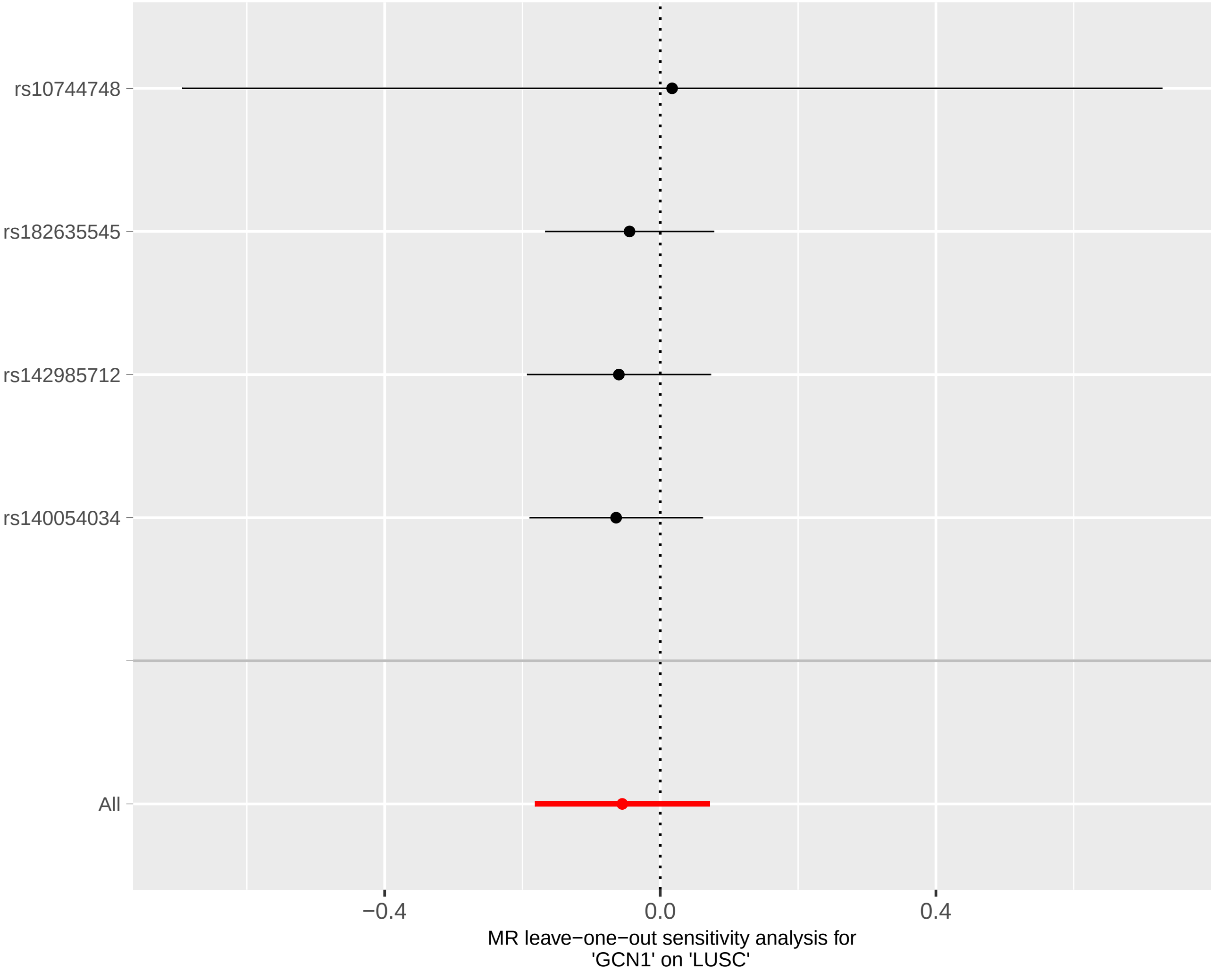
rs10744748
rs182635545
rs142985712
rs140054034
All
−0.4
0.0
0.4
MR leave−one−out sensitivity analysis for
'GCN1' on 'LUSC'

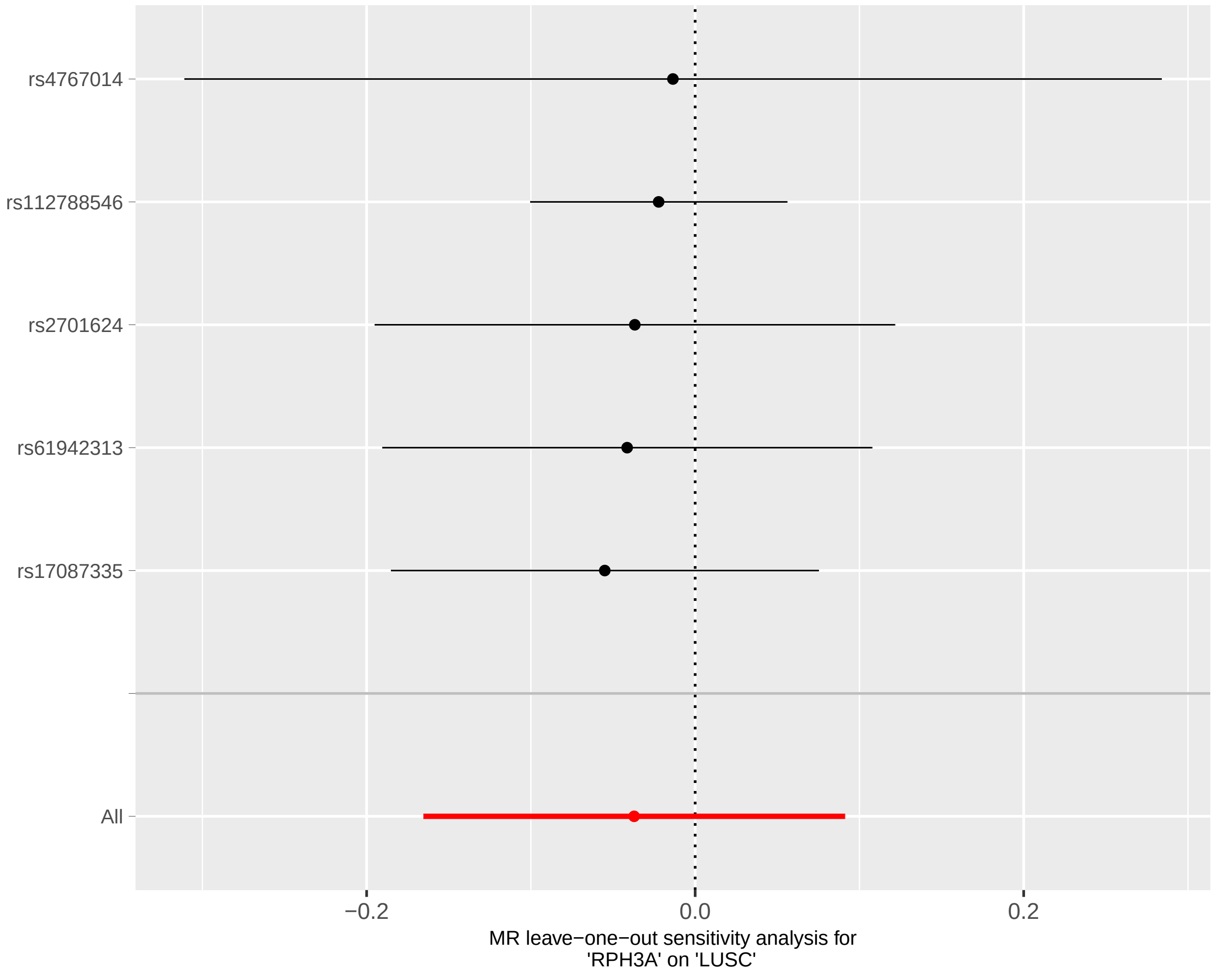

rs4767014
rs112788546
rs2701624
rs61942313
rs17087335
All
−0.2
0.0
0.2
MR leave−one−out sensitivity analysis for
'RPH3A' on 'LUSC'

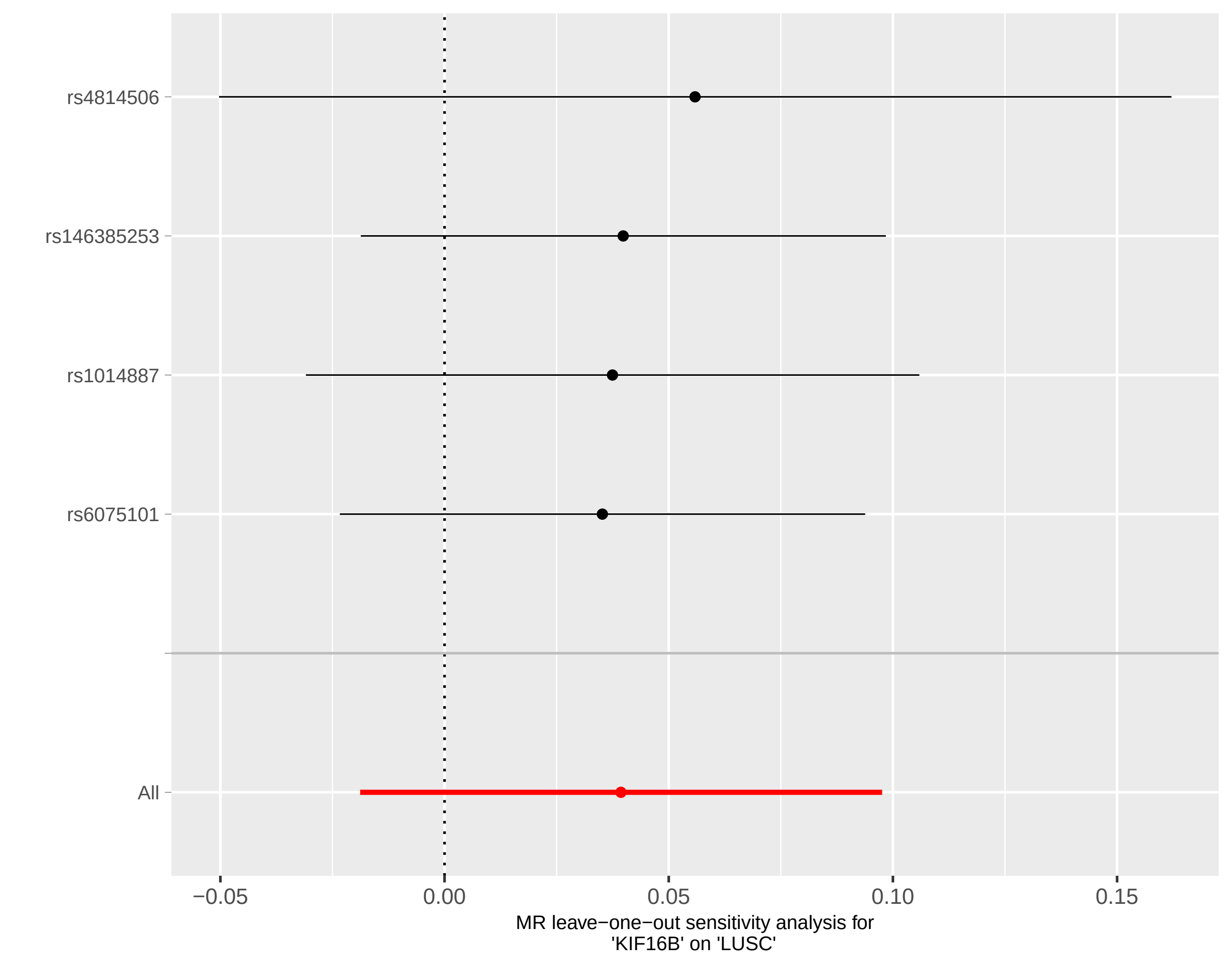
rs4814506
rs146385253
rs1014887
rs6075101
All
−0.05
0.00
0.05
0.10
0.15
MR leave−one−out sensitivity analysis for
'KIF16B' on 'LUSC'

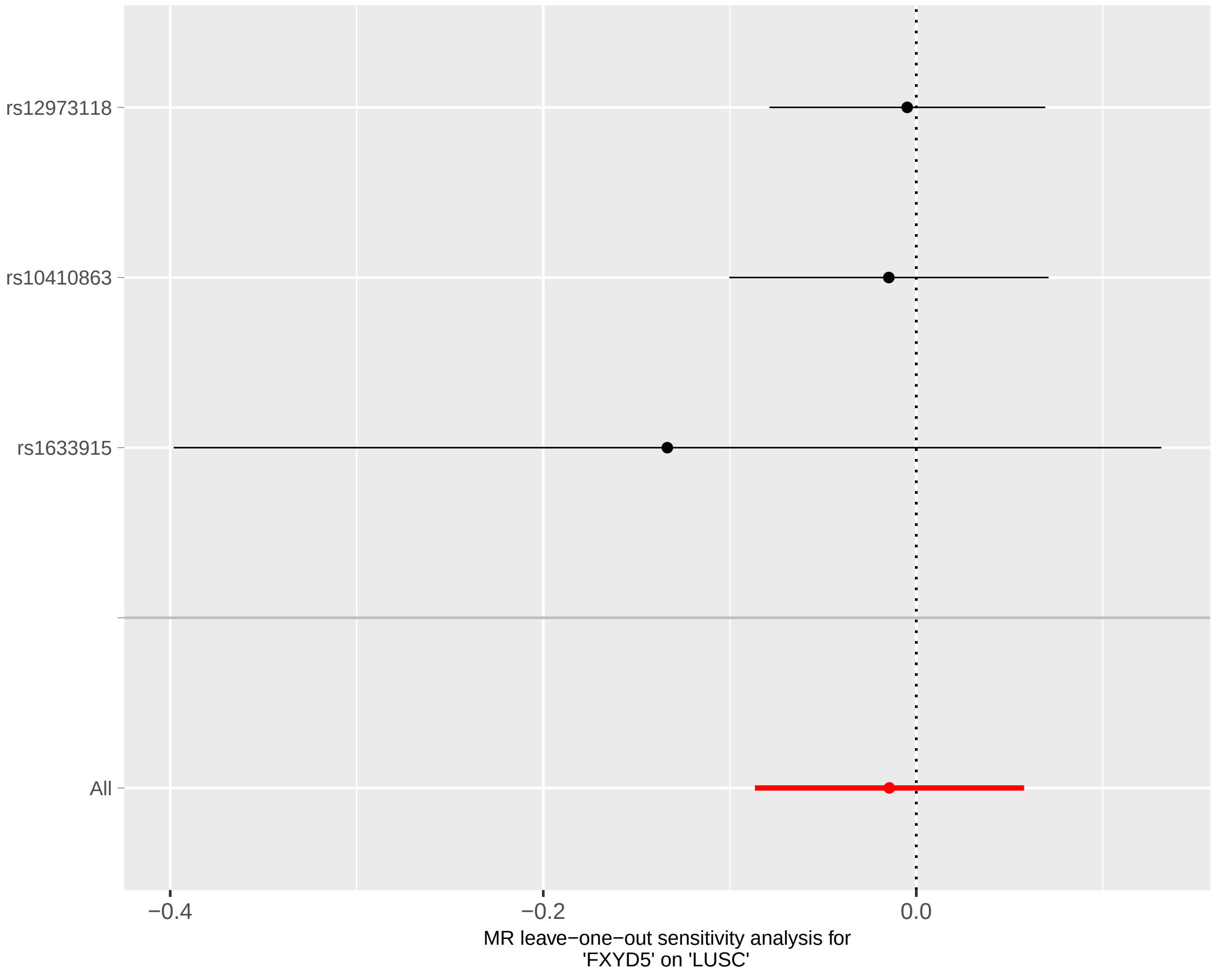
rs12973118
rs10410863
rs1633915
All
−0.4
−0.2
0.0
MR leave−one−out sensitivity analysis for
'FXYD5' on 'LUSC'

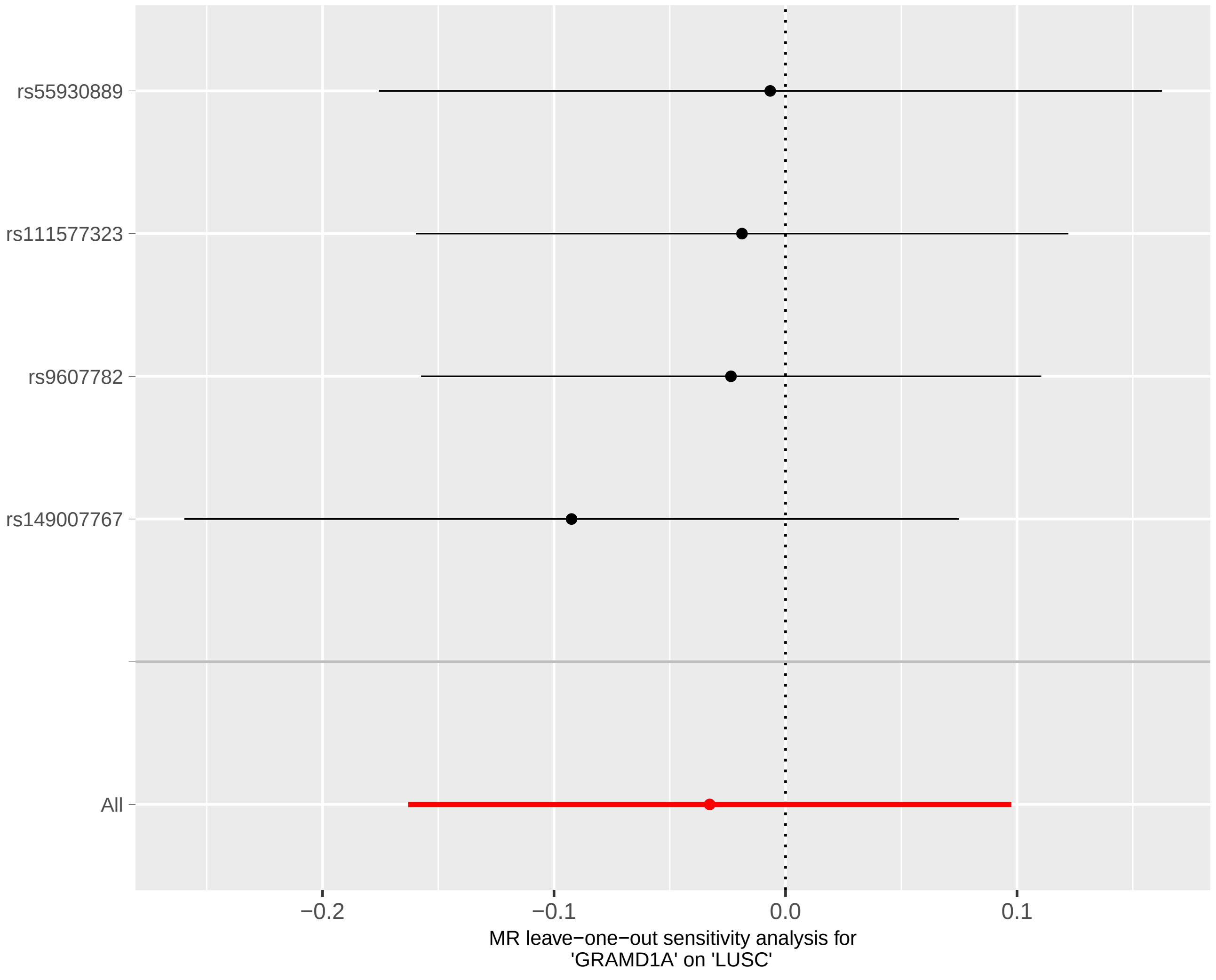

rs55930889
rs111577323
rs9607782
rs149007767
All
−0.2
−0.1
0.0
0.1
MR leave−one−out sensitivity analysis for
'GRAMD1A' on 'LUSC'

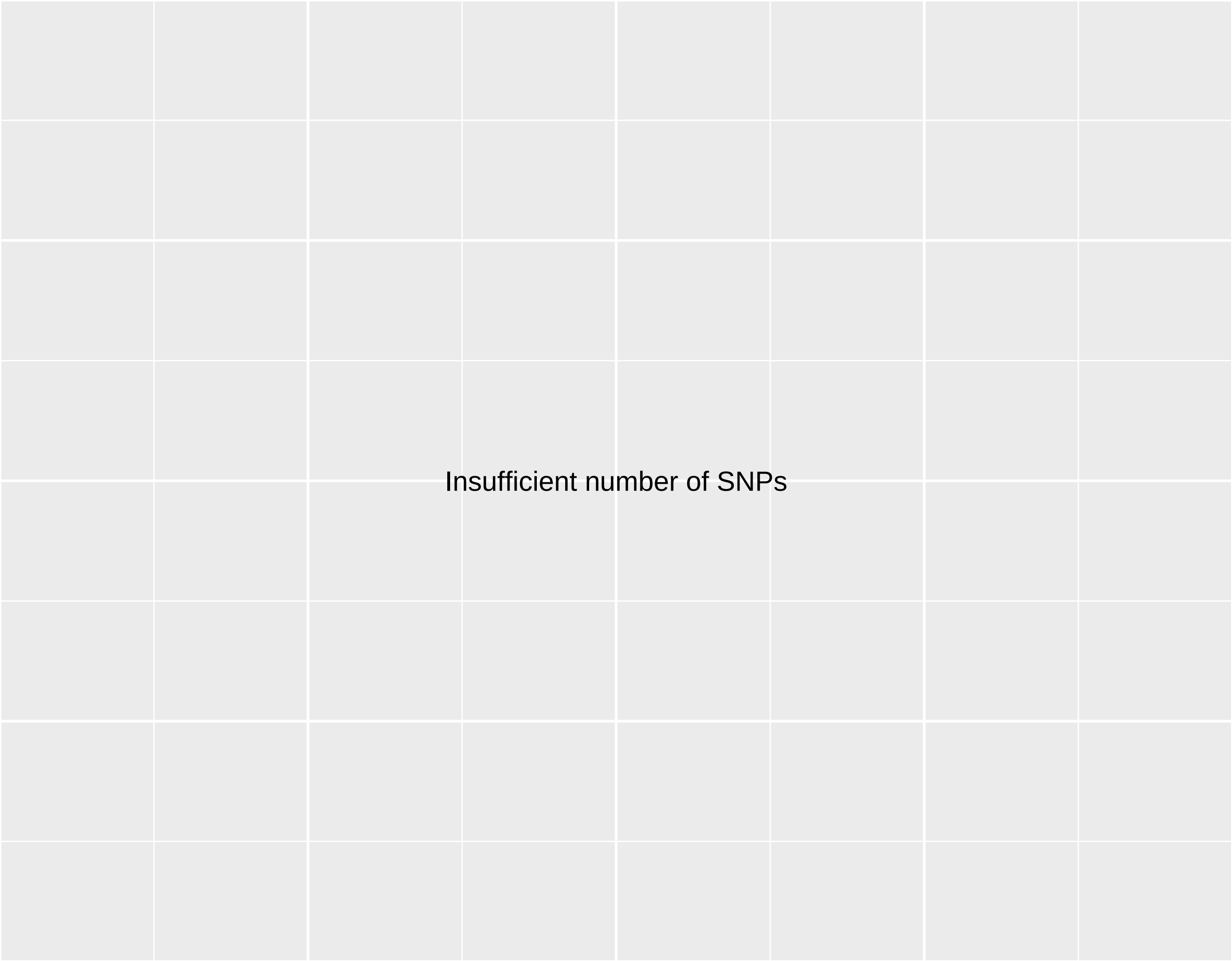
Insufficient number of SNPs

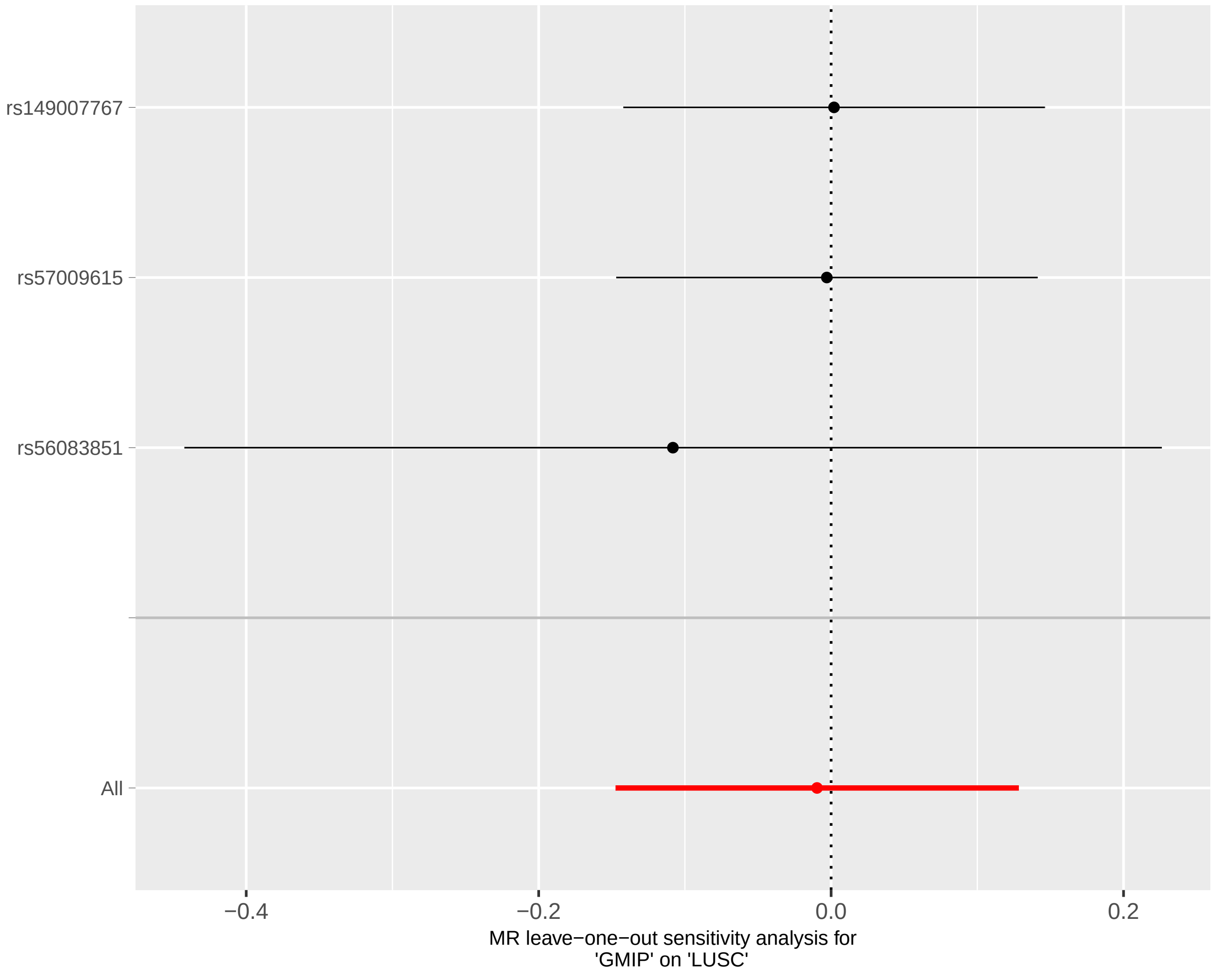
rs149007767
rs57009615
rs56083851
All
−0.4
−0.2
0.0
0.2
MR leave−one−out sensitivity analysis for
'GMIP' on 'LUSC'

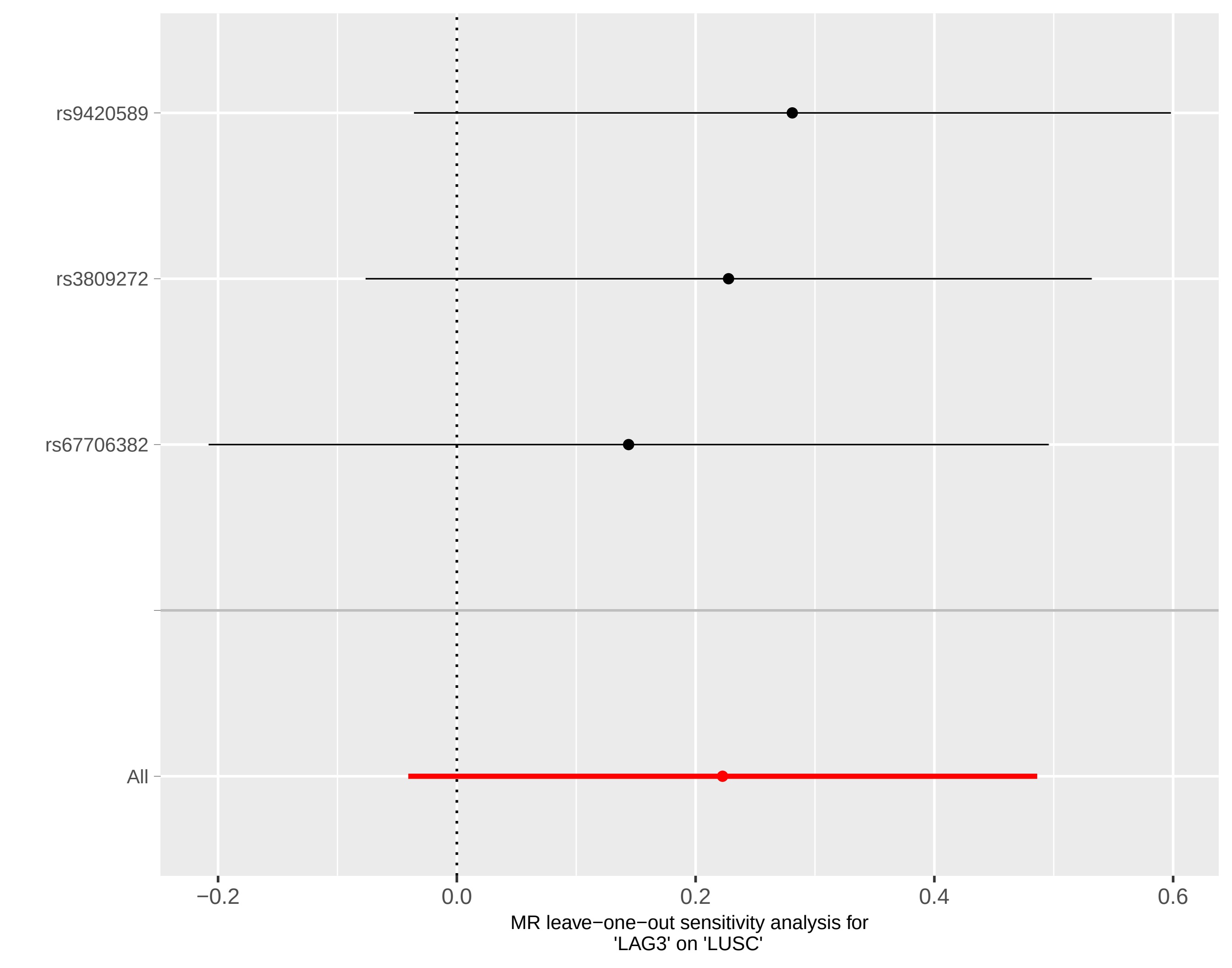
rs9420589
rs3809272
rs67706382
All
−0.2
0.0
0.2
0.4
0.6
MR leave−one−out sensitivity analysis for
'LAG3' on 'LUSC'

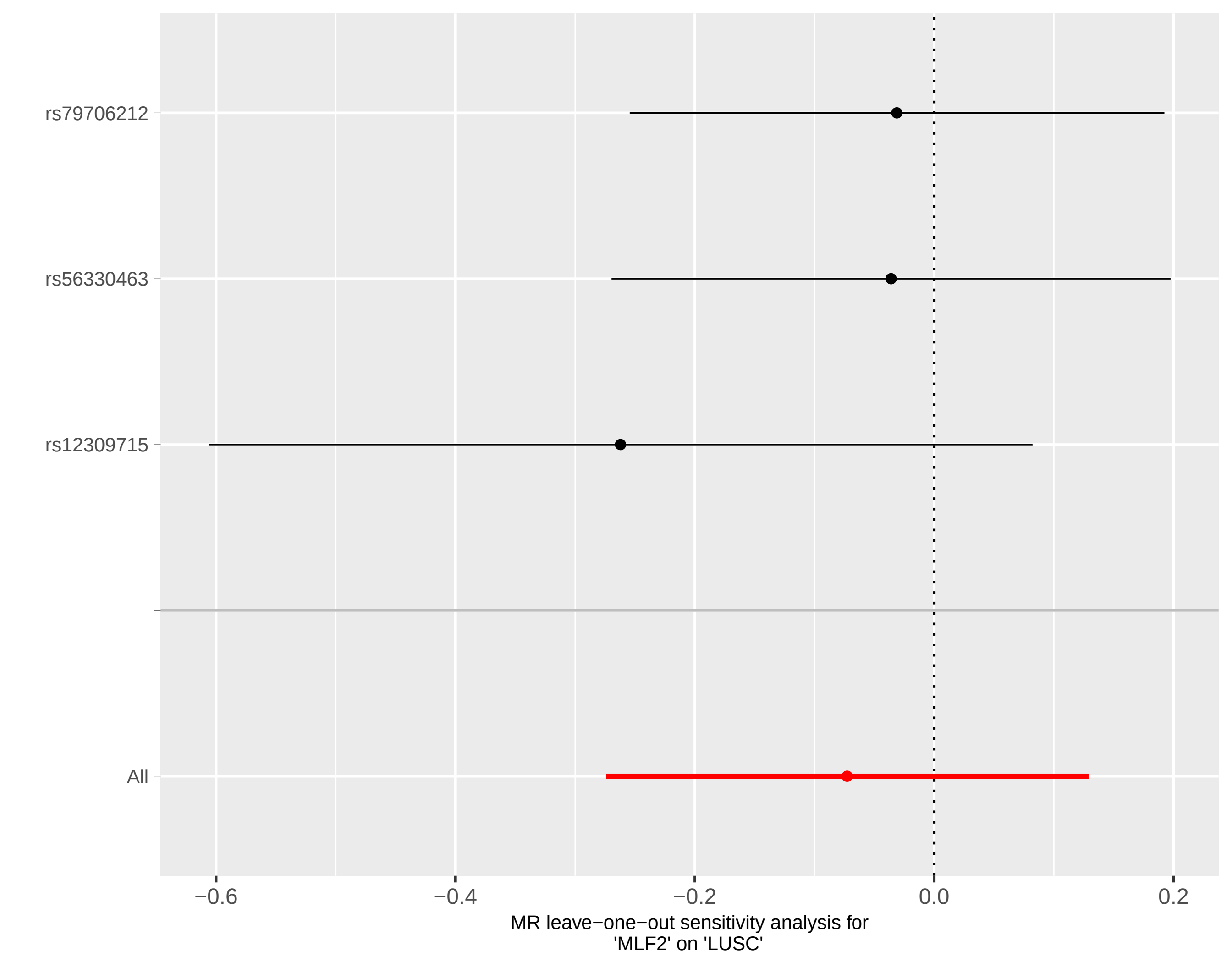

rs79706212
rs56330463
rs12309715
All
−0.6
−0.4
−0.2
0.0
0.2
MR leave−one−out sensitivity analysis for
'MLF2' on 'LUSC'

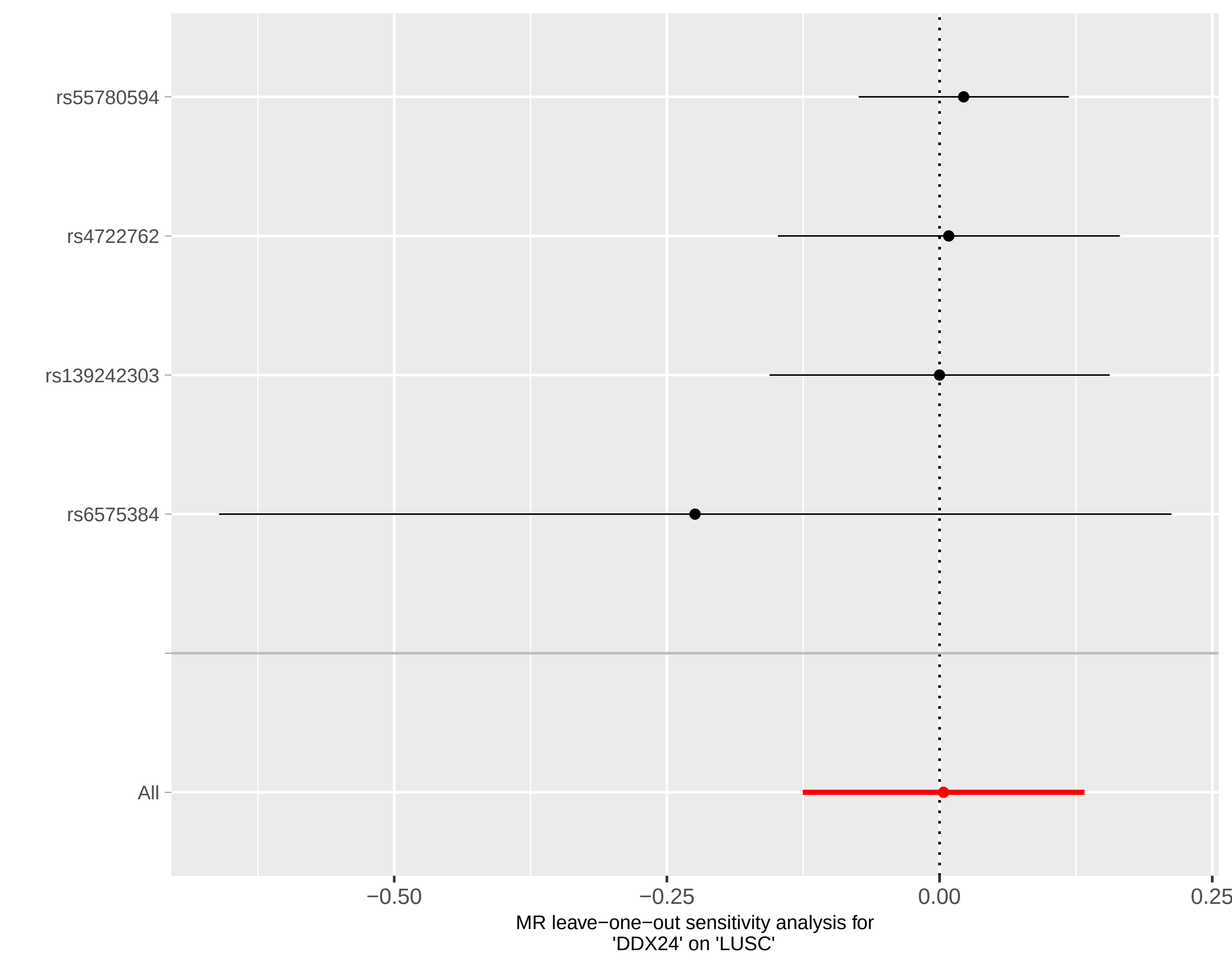
rs55780594
rs4722762
rs139242303
rs6575384
All
−0.50
−0.25
0.00
0.25
MR leave−one−out sensitivity analysis for
'DDX24' on 'LUSC'

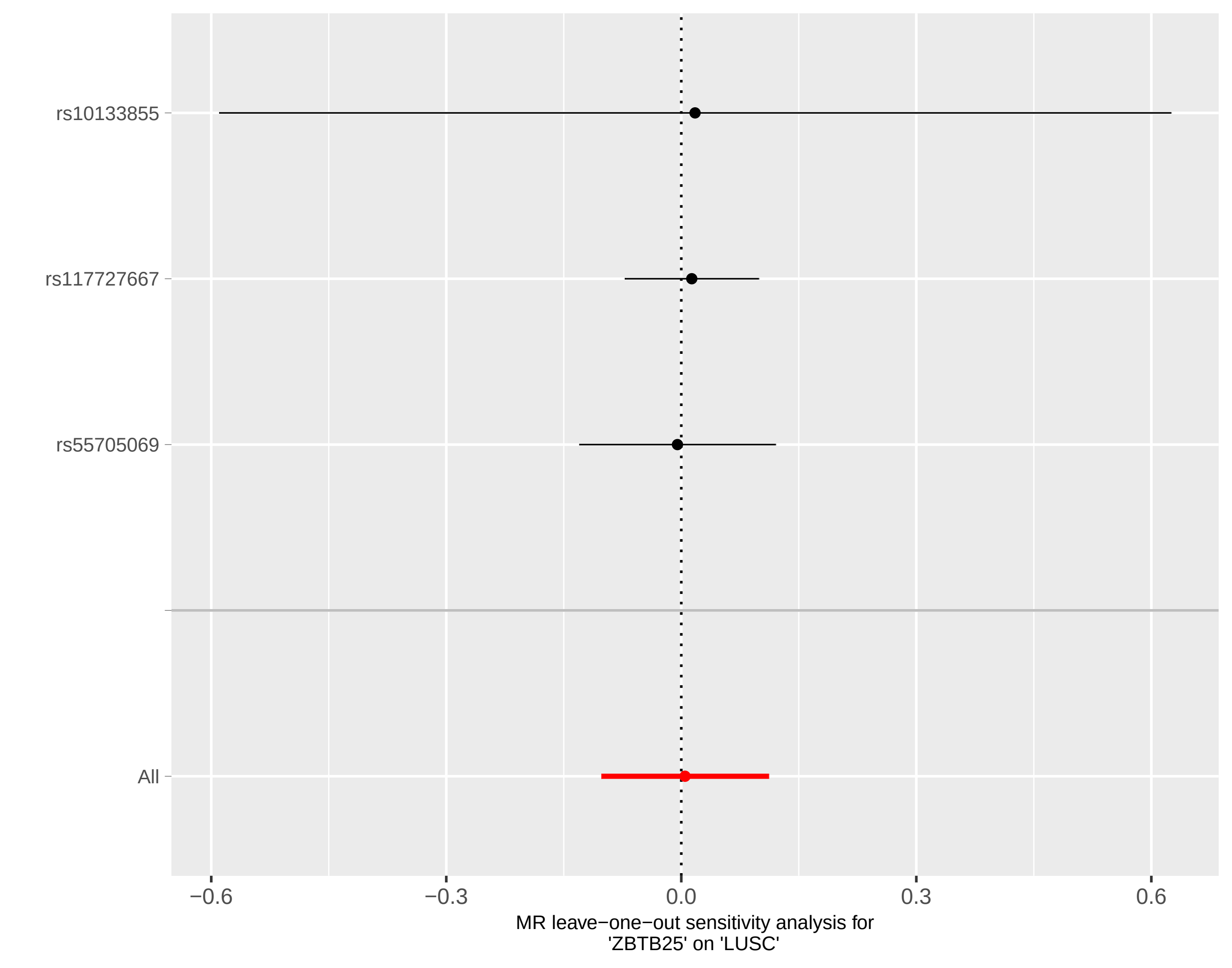
rs10133855
rs117727667
rs55705069
All
−0.6
−0.3
0.0
0.3
0.6
MR leave−one−out sensitivity analysis for
'ZBTB25' on 'LUSC'

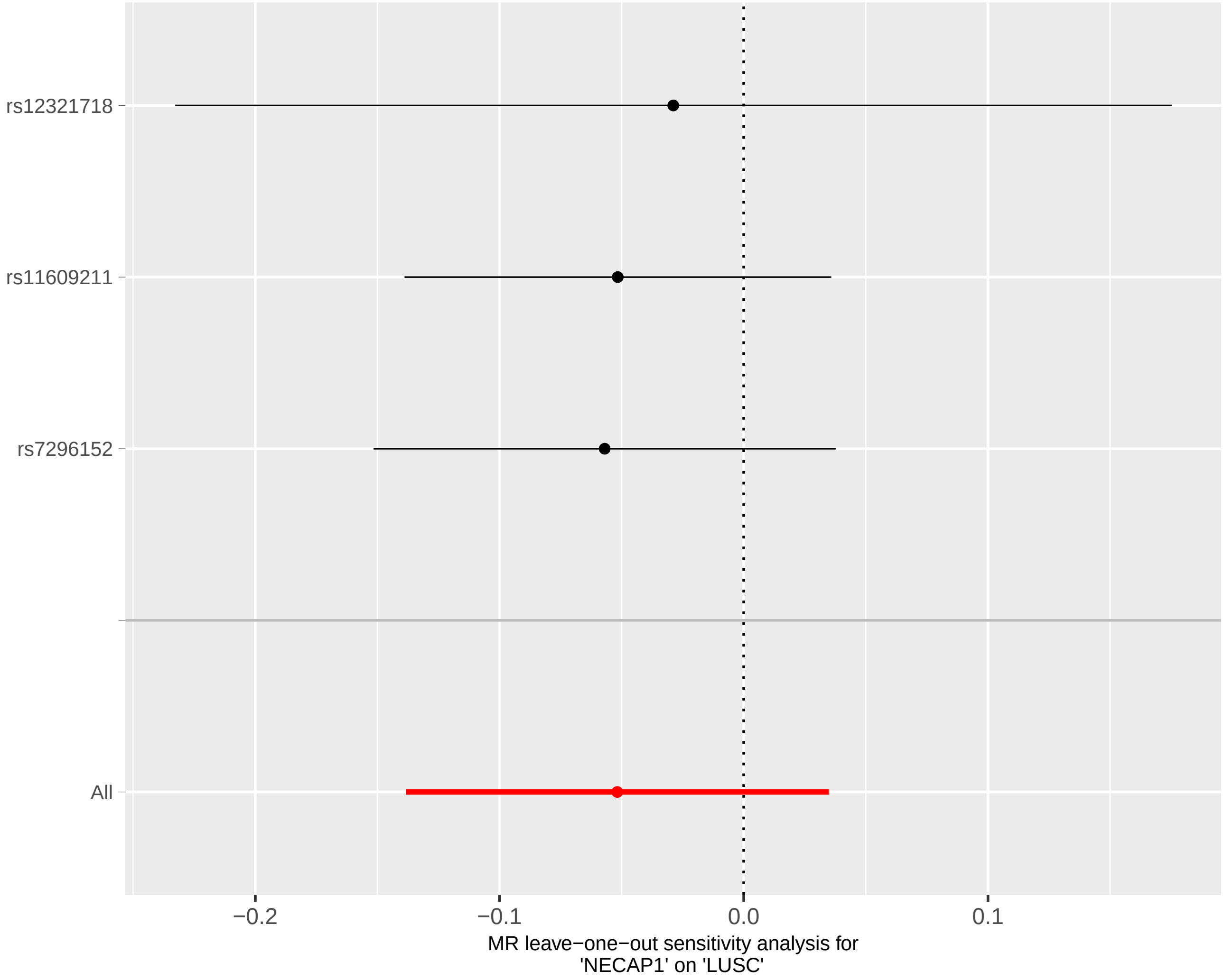

rs12321718
rs11609211
rs7296152
All
−0.2
−0.1
0.0
0.1
MR leave−one−out sensitivity analysis for
'NECAP1' on 'LUSC'

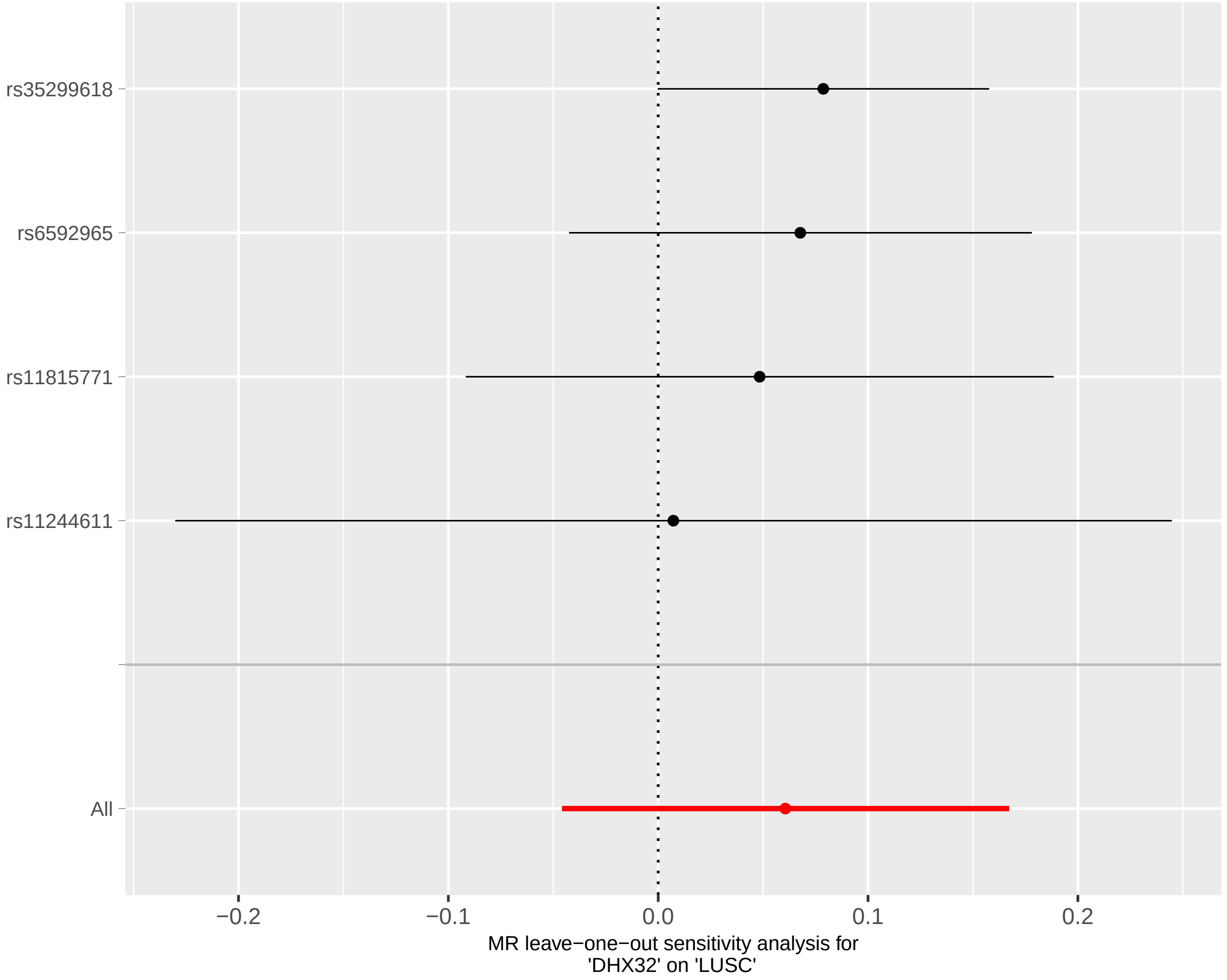
rs35299618
rs6592965
rs11815771
rs11244611
All
−0.2
−0.1
0.0
0.1
0.2
MR leave−one−out sensitivity analysis for
'DHX32' on 'LUSC'

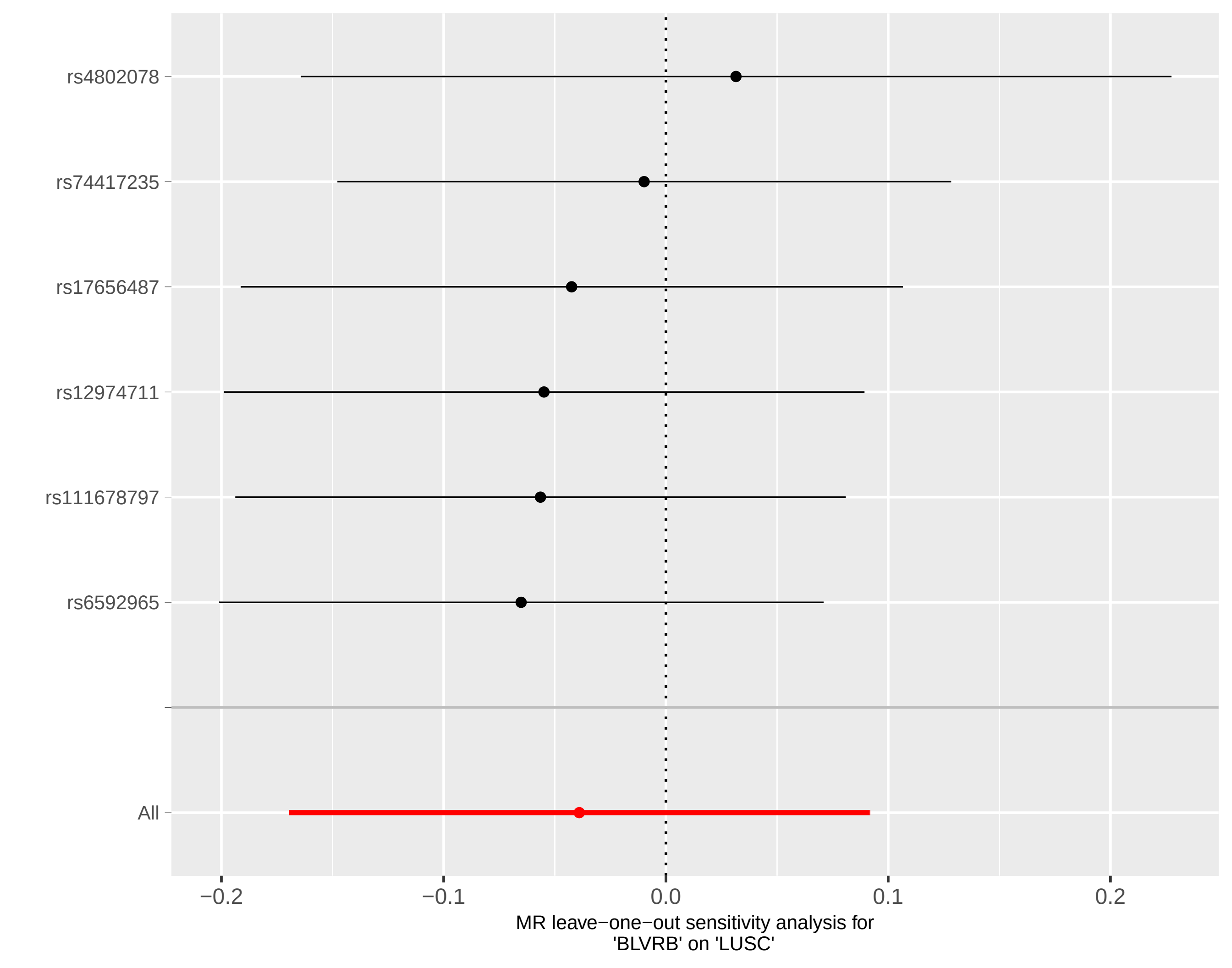
rs4802078
rs74417235
rs17656487
rs12974711
rs111678797
rs6592965
All
−0.2
−0.1
0.0
0.1
0.2
MR leave−one−out sensitivity analysis for
'BLVRB' on 'LUSC'

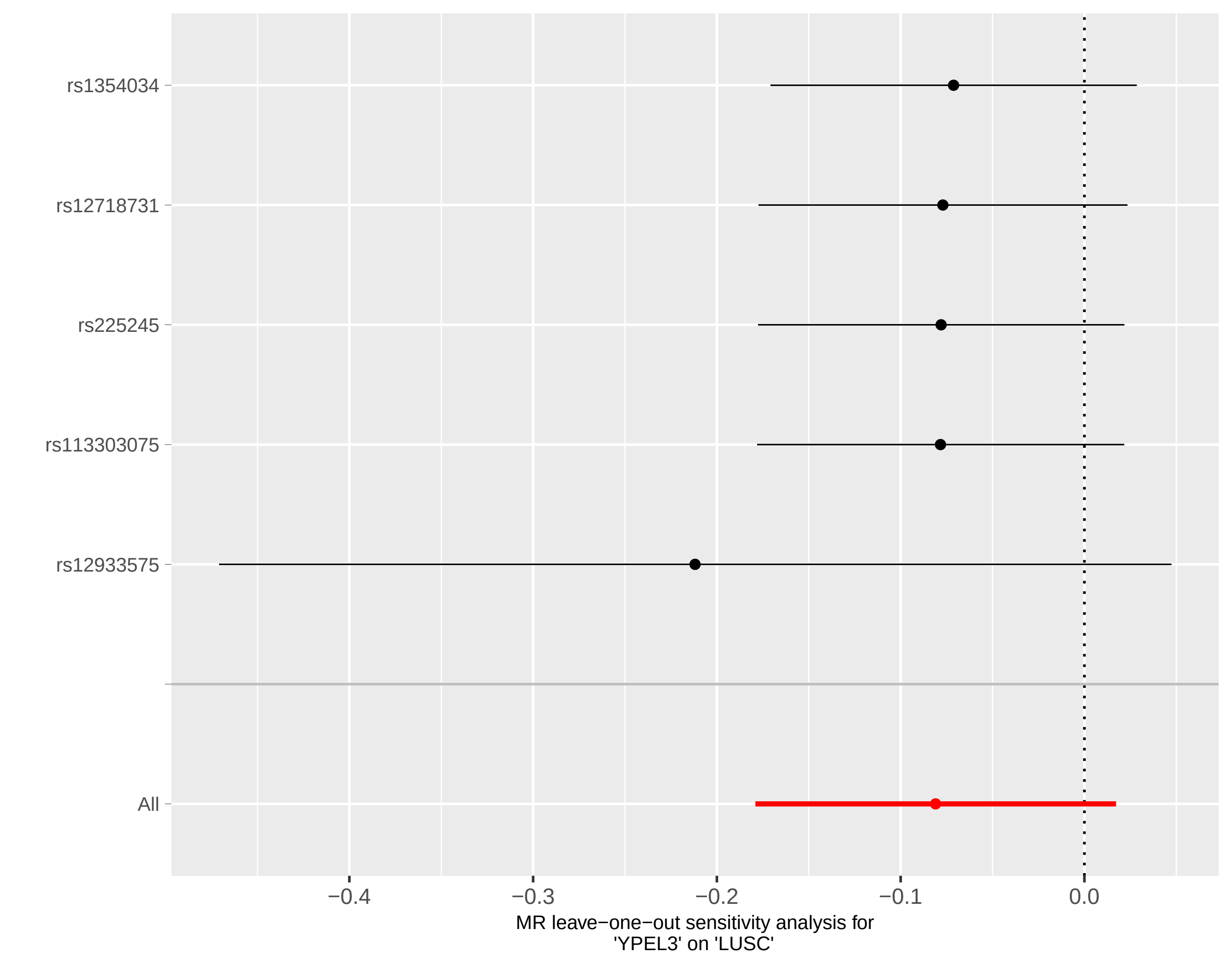
rs1354034
rs12718731
rs225245
rs113303075
rs12933575
All
−0.4
−0.3
−0.2
−0.1
0.0
MR leave−one−out sensitivity analysis for
'YPEL3' on 'LUSC'

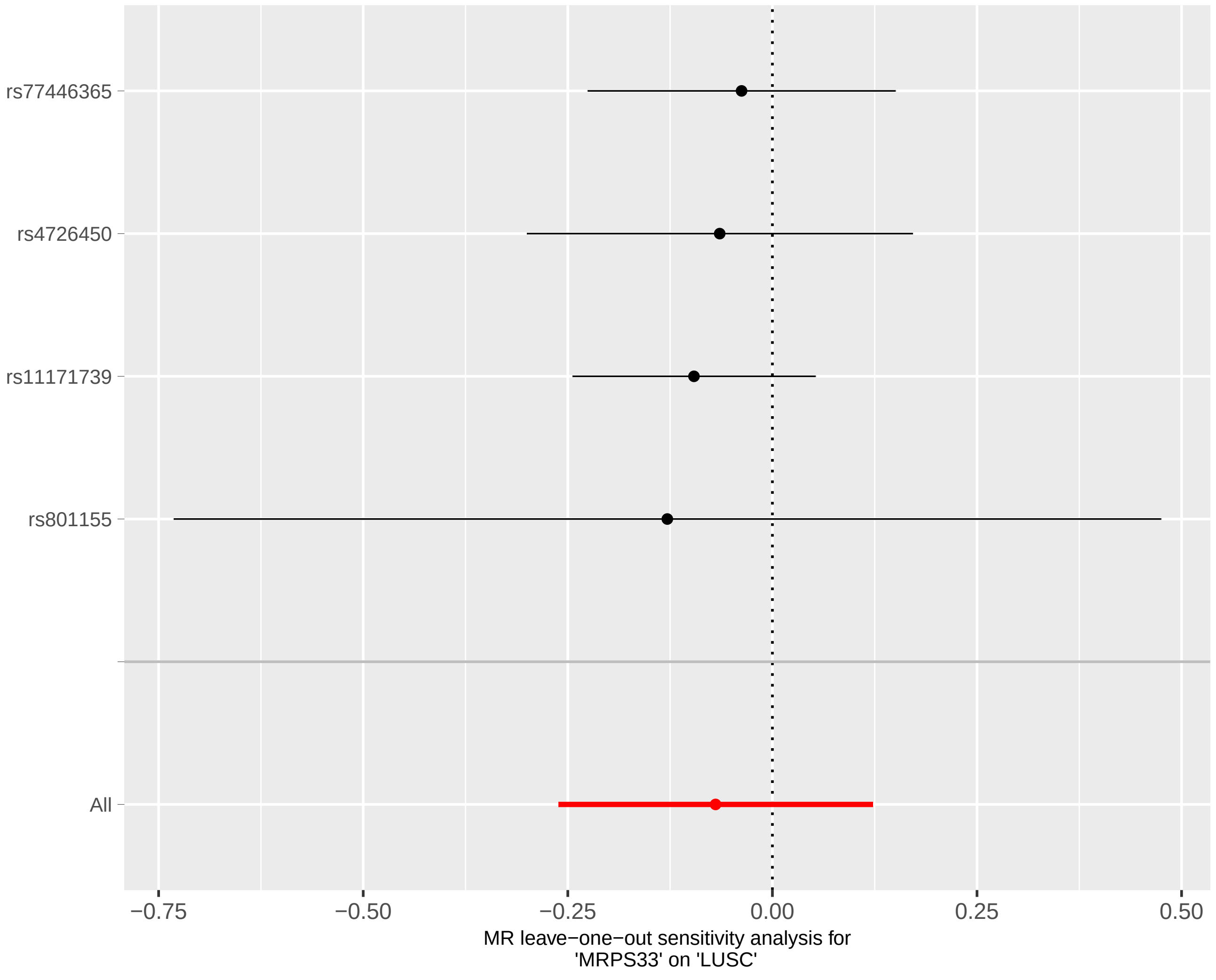

rs77446365
rs4726450
rs11171739
rs801155
All
−0.75
−0.50
−0.25
0.00
0.25
0.50
MR leave−one−out sensitivity analysis for
'MRPS33' on 'LUSC'

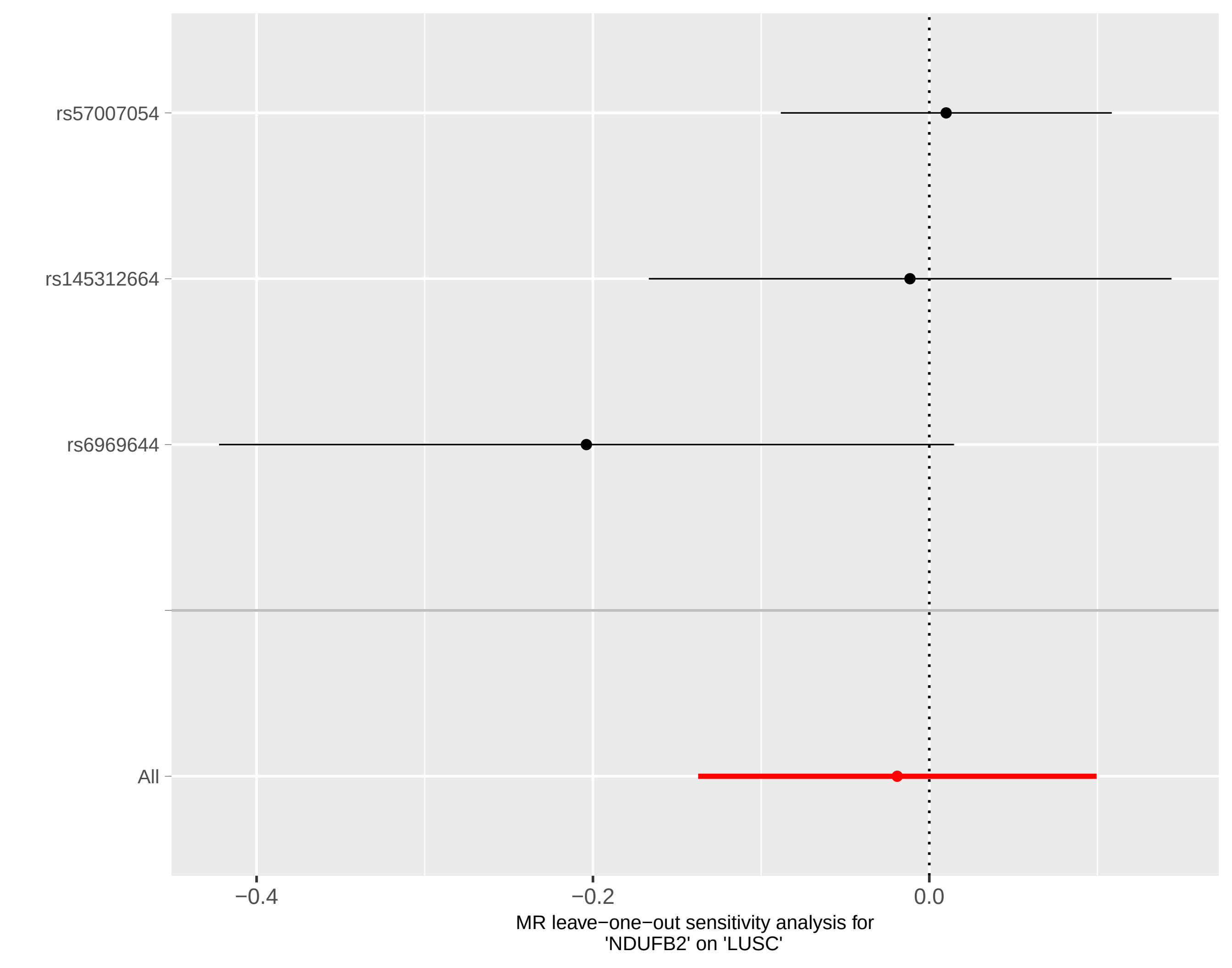
rs57007054
rs145312664
rs6969644
All
−0.4
−0.2
0.0
MR leave−one−out sensitivity analysis for
'NDUFB2' on 'LUSC'

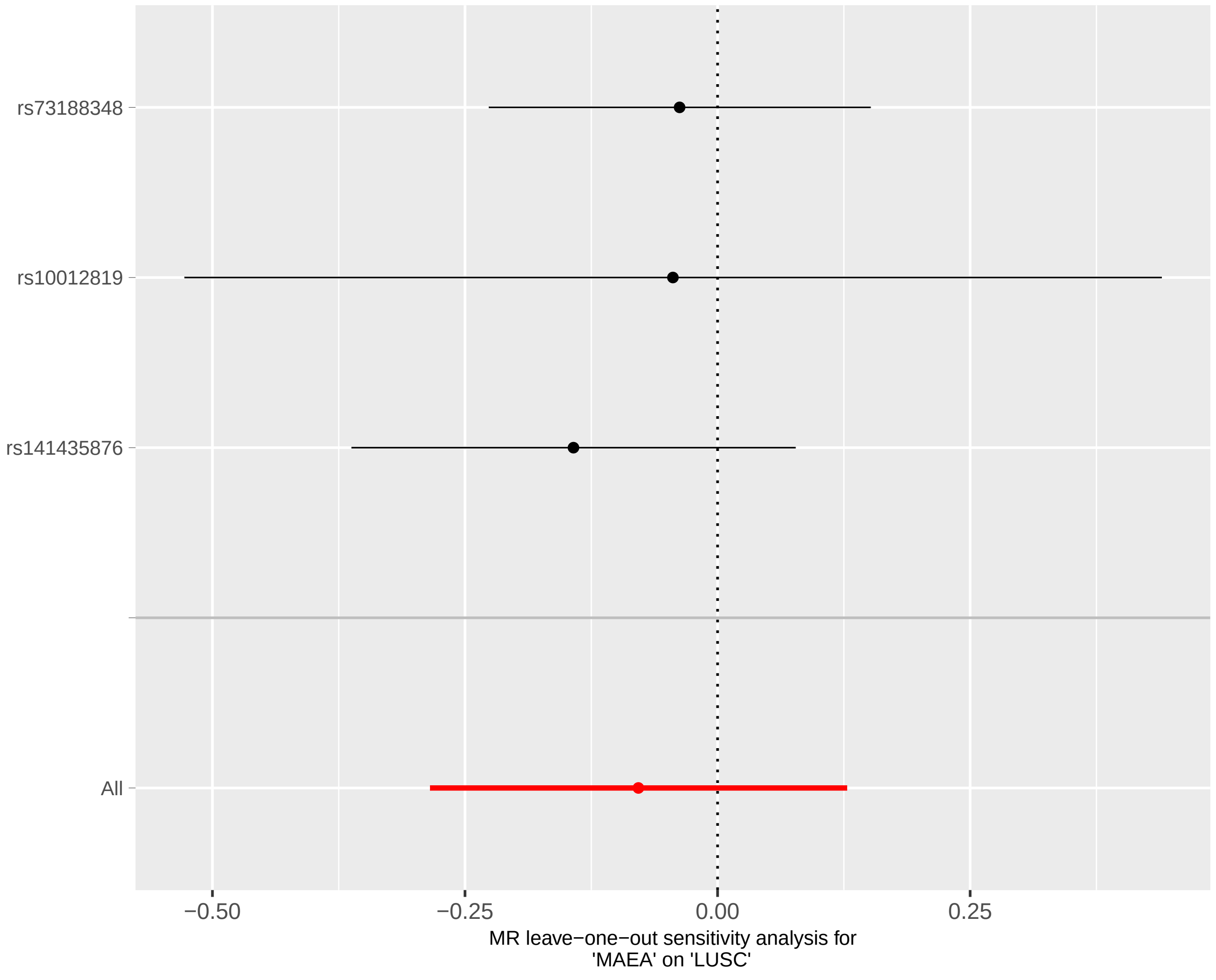
rs73188348
rs10012819
rs141435876
All
−0.50
−0.25
0.00
0.25
MR leave−one−out sensitivity analysis for
'MAEA' on 'LUSC'

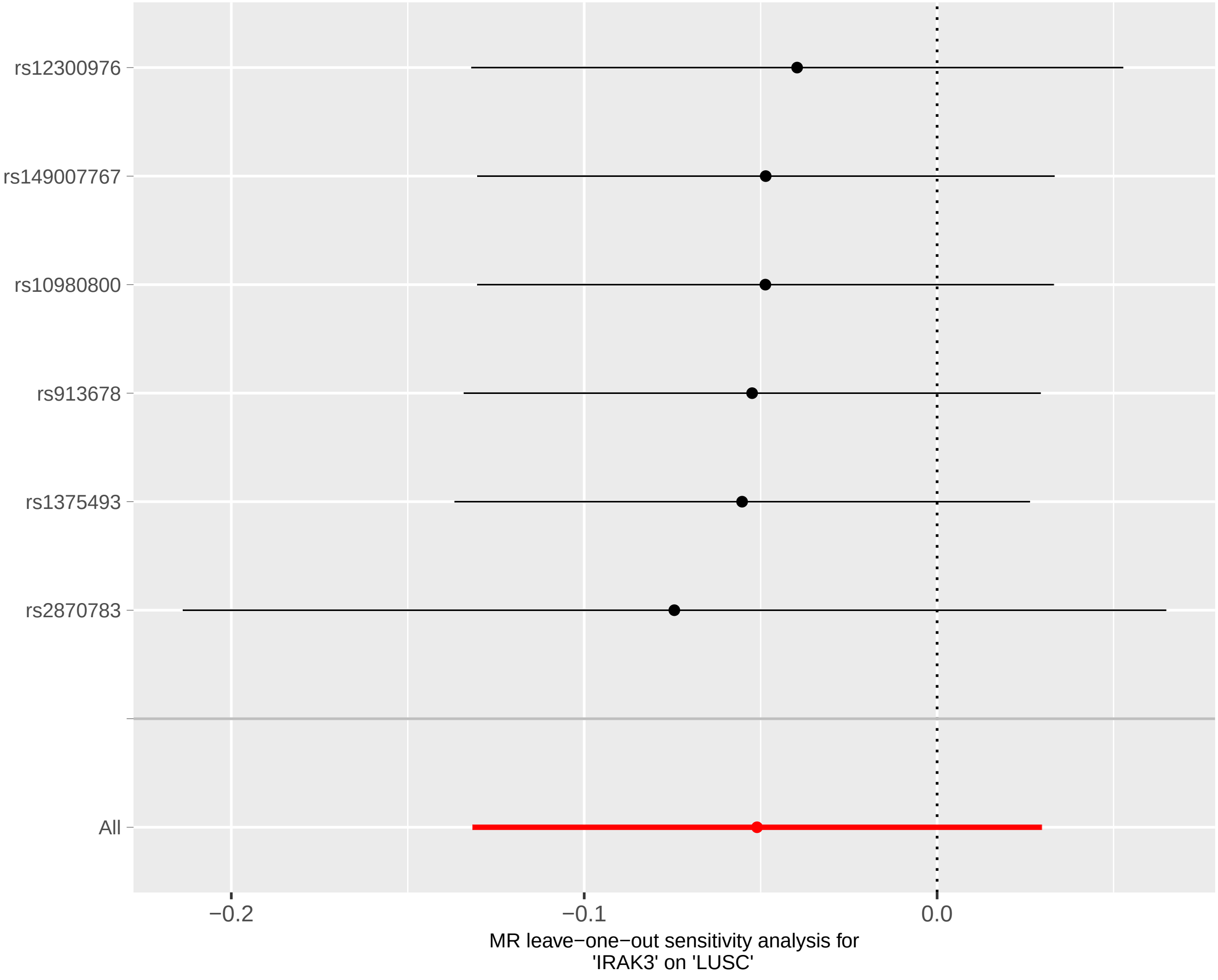

rs12300976
rs149007767
rs10980800
rs913678
rs1375493
rs2870783
All
−0.2
−0.1
0.0
MR leave−one−out sensitivity analysis for
'IRAK3' on 'LUSC'

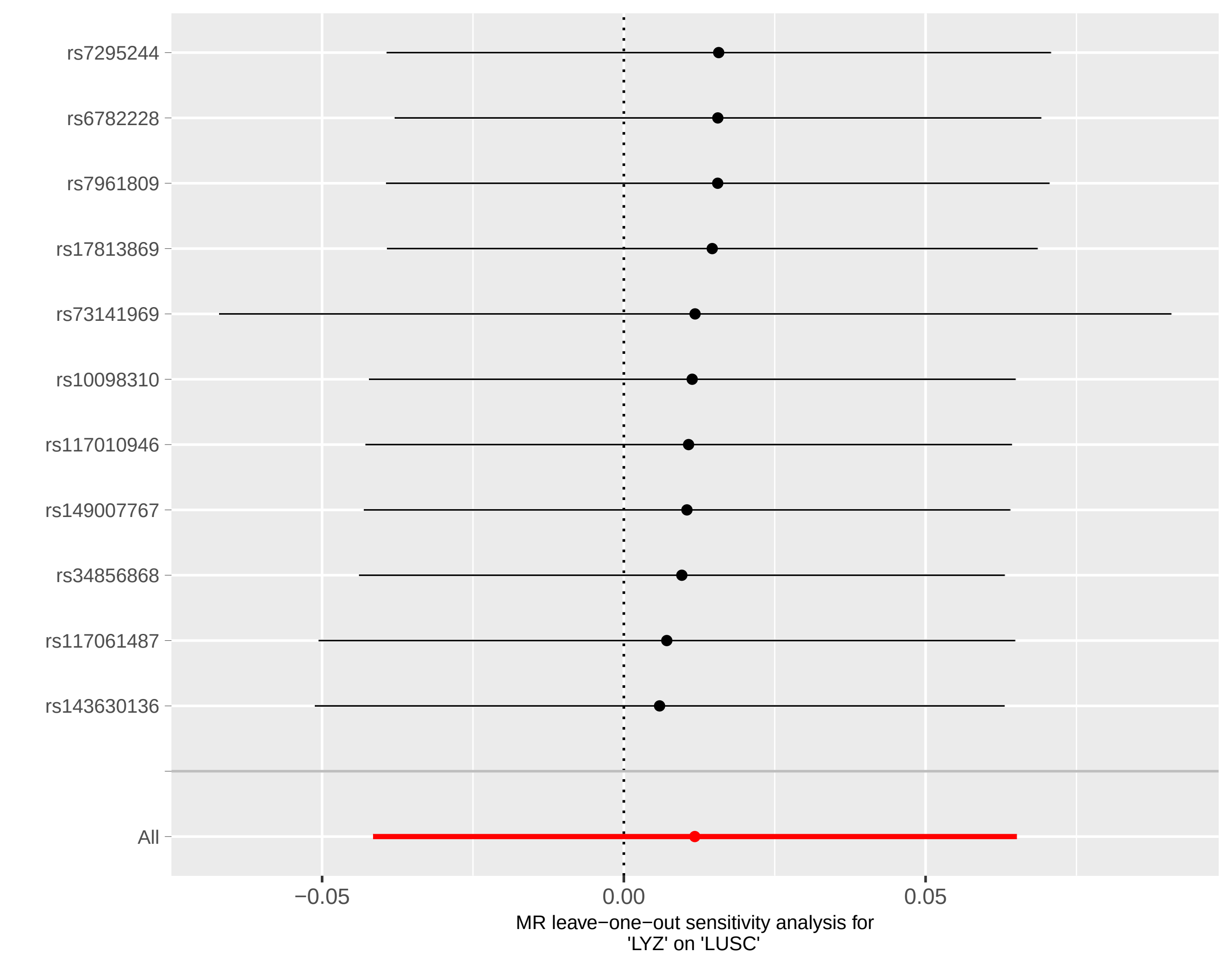
rs7295244
rs6782228
rs7961809
rs17813869
rs73141969
rs10098310
rs117010946
rs149007767
rs34856868
rs117061487
rs143630136
All
−0.05
0.00
0.05
MR leave−one−out sensitivity analysis for
'LYZ' on 'LUSC'

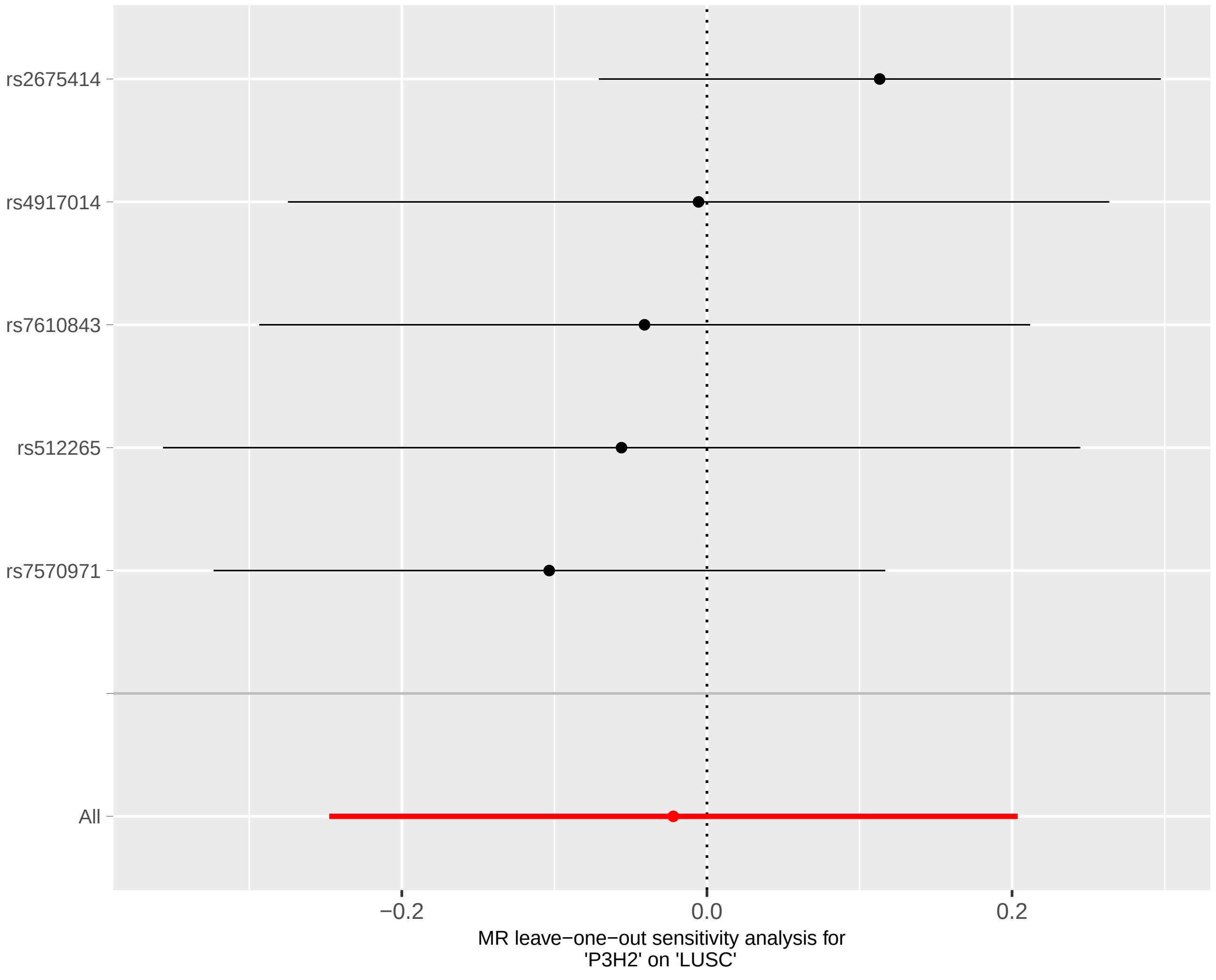

rs2675414
rs4917014
rs7610843
rs512265
rs7570971
All
−0.2
0.0
0.2
MR leave−one−out sensitivity analysis for
'P3H2' on 'LUSC'

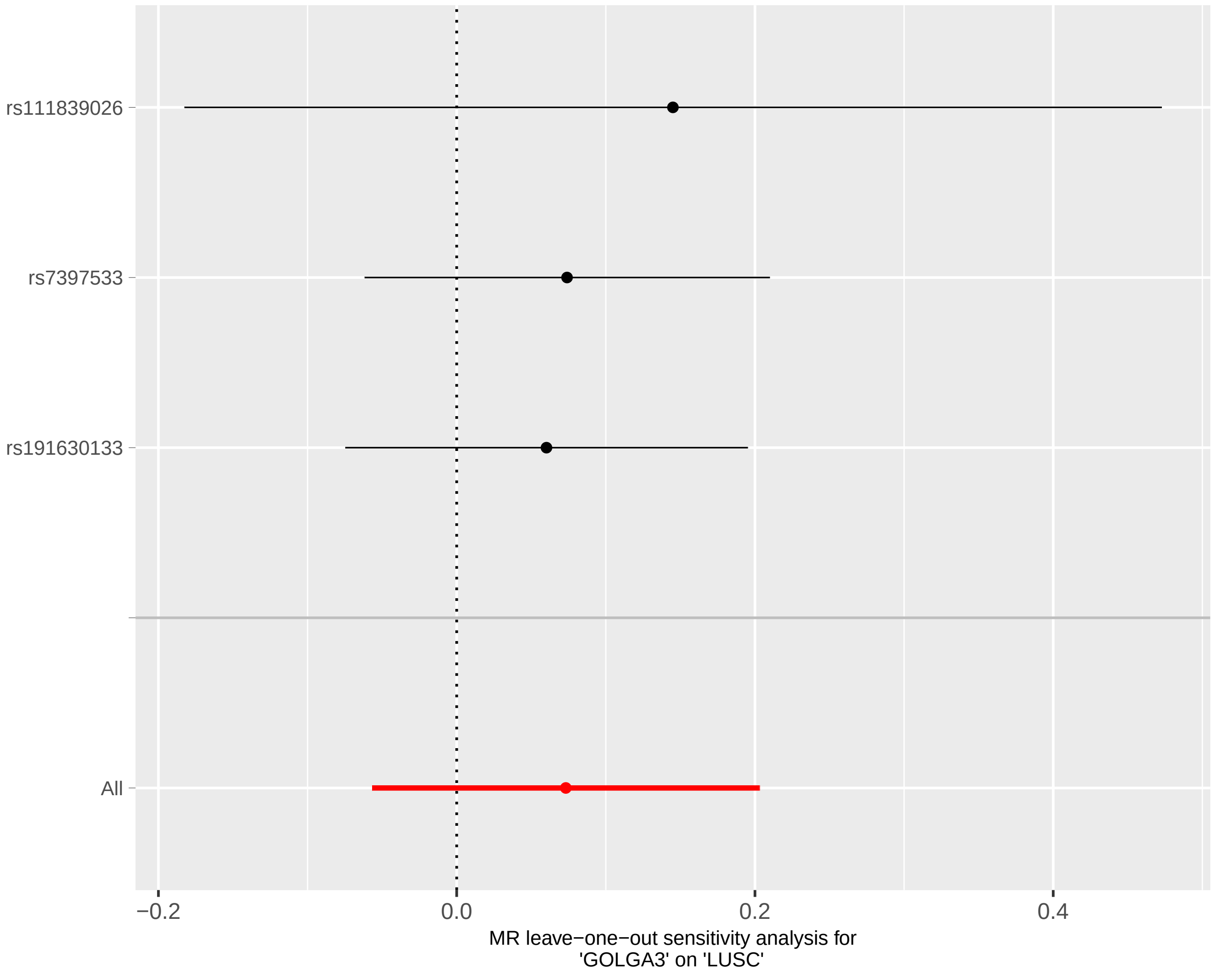
rs111839026
rs7397533
rs191630133
All
−0.2
0.0
0.2
0.4
MR leave−one−out sensitivity analysis for
'GOLGA3' on 'LUSC'

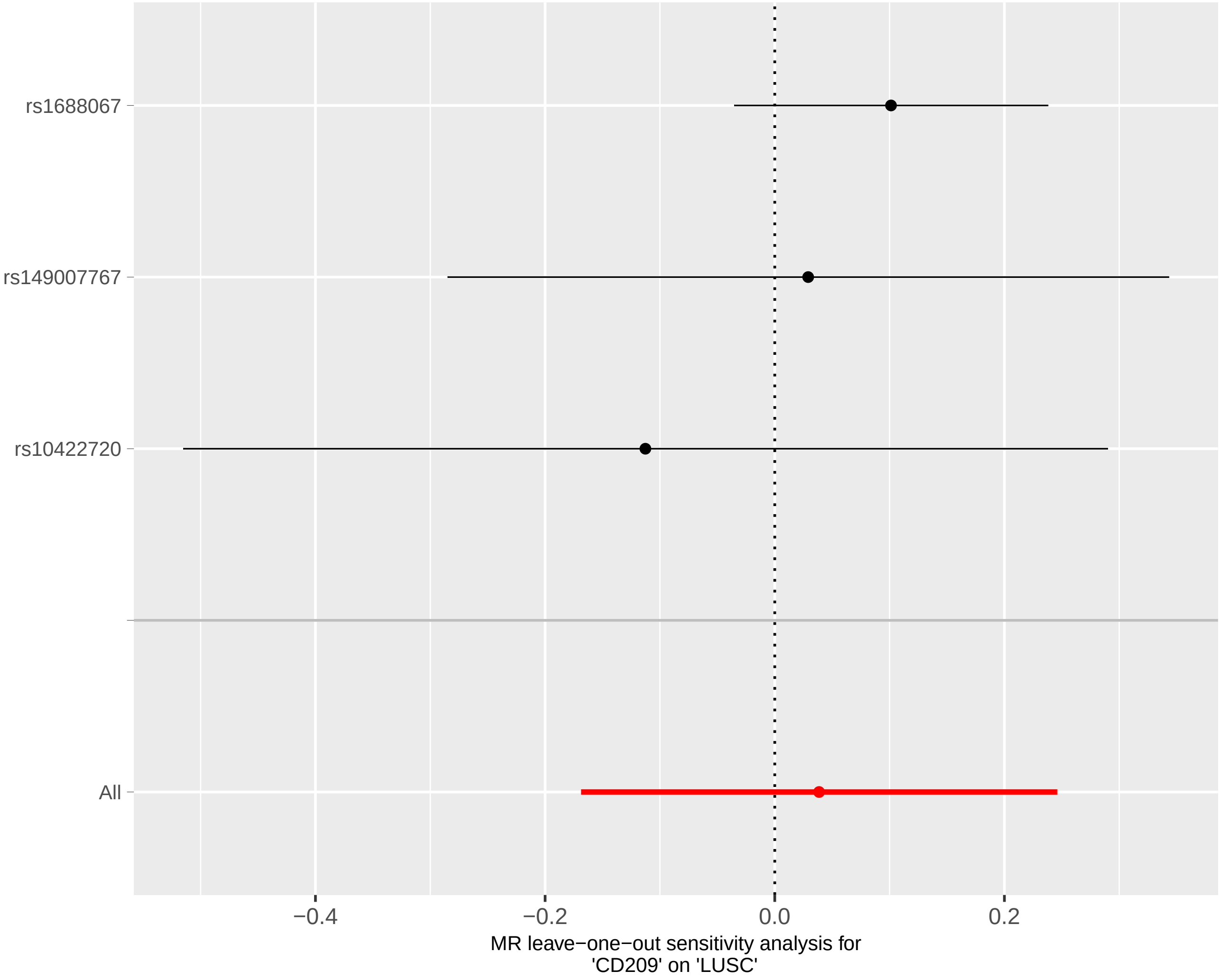
rs1688067
rs149007767
rs10422720
All
−0.4
−0.2
0.0
0.2
MR leave−one−out sensitivity analysis for
'CD209' on 'LUSC'

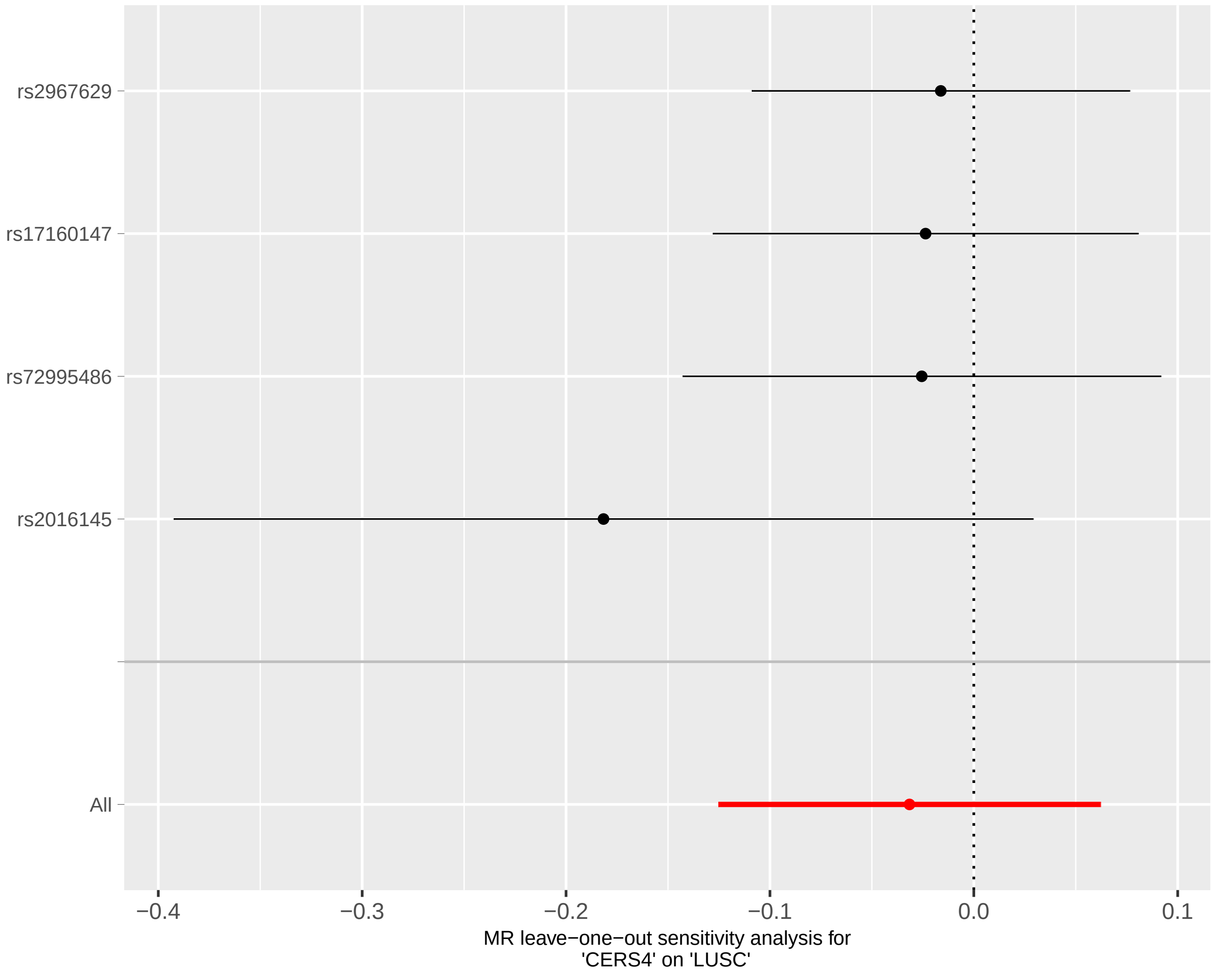
rs2967629
rs17160147
rs72995486
rs2016145
All
−0.4
−0.3
−0.2
−0.1
0.0
0.1
MR leave−one−out sensitivity analysis for
'CERS4' on 'LUSC'

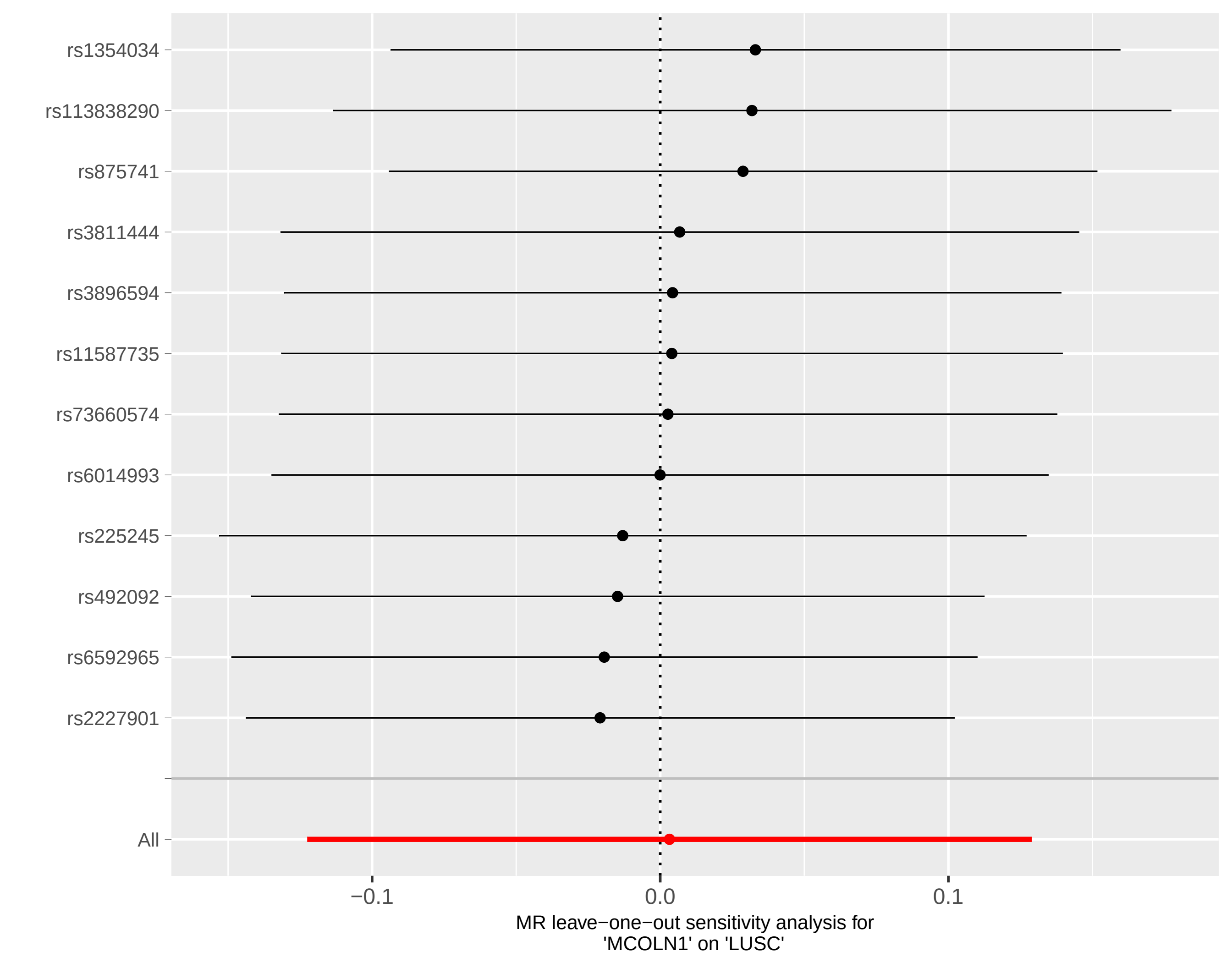
rs1354034
rs113838290
rs875741
rs3811444
rs3896594
rs11587735
rs73660574
rs6014993
rs225245
rs492092
rs6592965
rs2227901
All
−0.1
0.0
0.1
MR leave−one−out sensitivity analysis for
'MCOLN1' on 'LUSC'

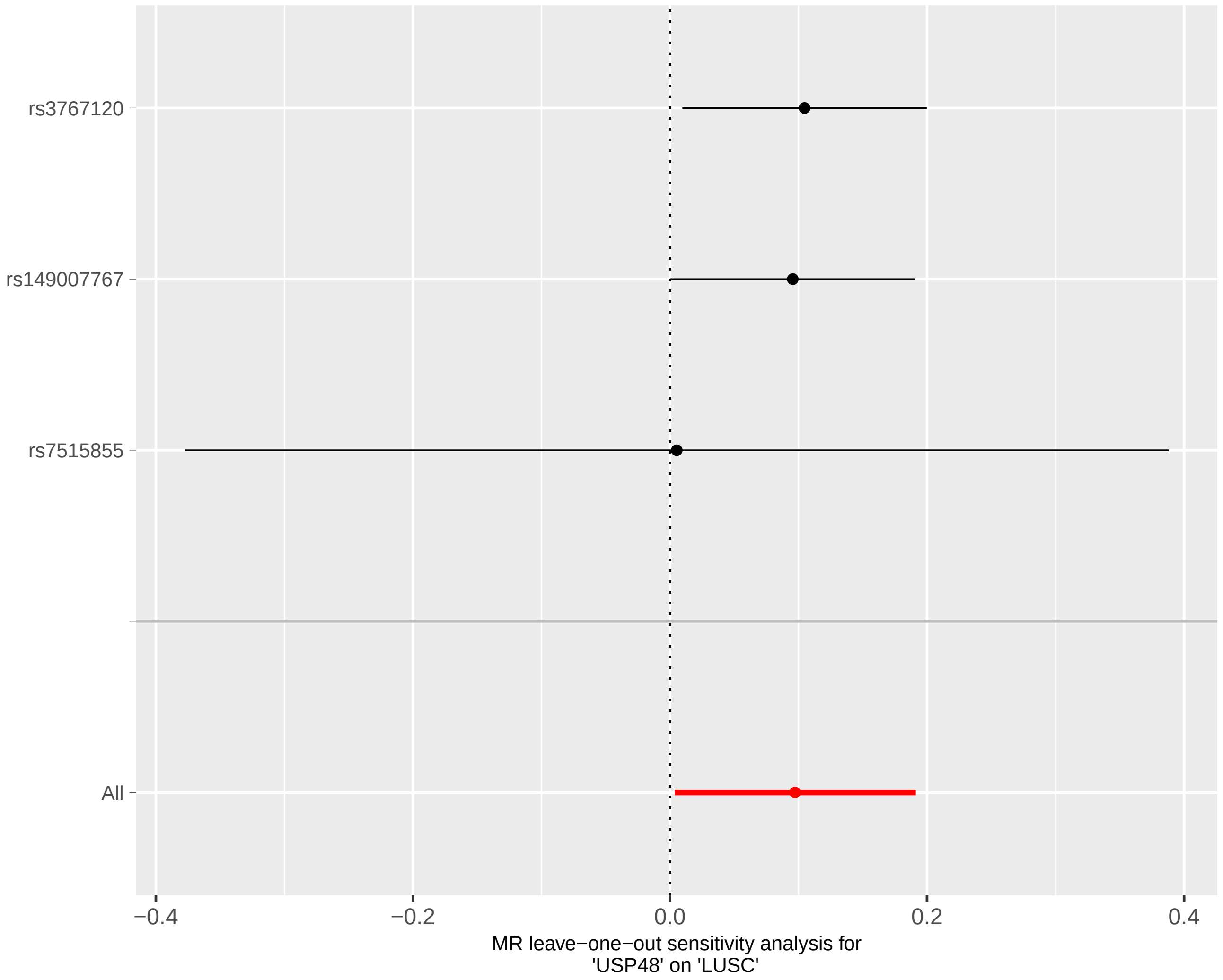
rs3767120
rs149007767
rs7515855
All
−0.4
−0.2
0.0
0.2
0.4
MR leave−one−out sensitivity analysis for
'USP48' on 'LUSC'

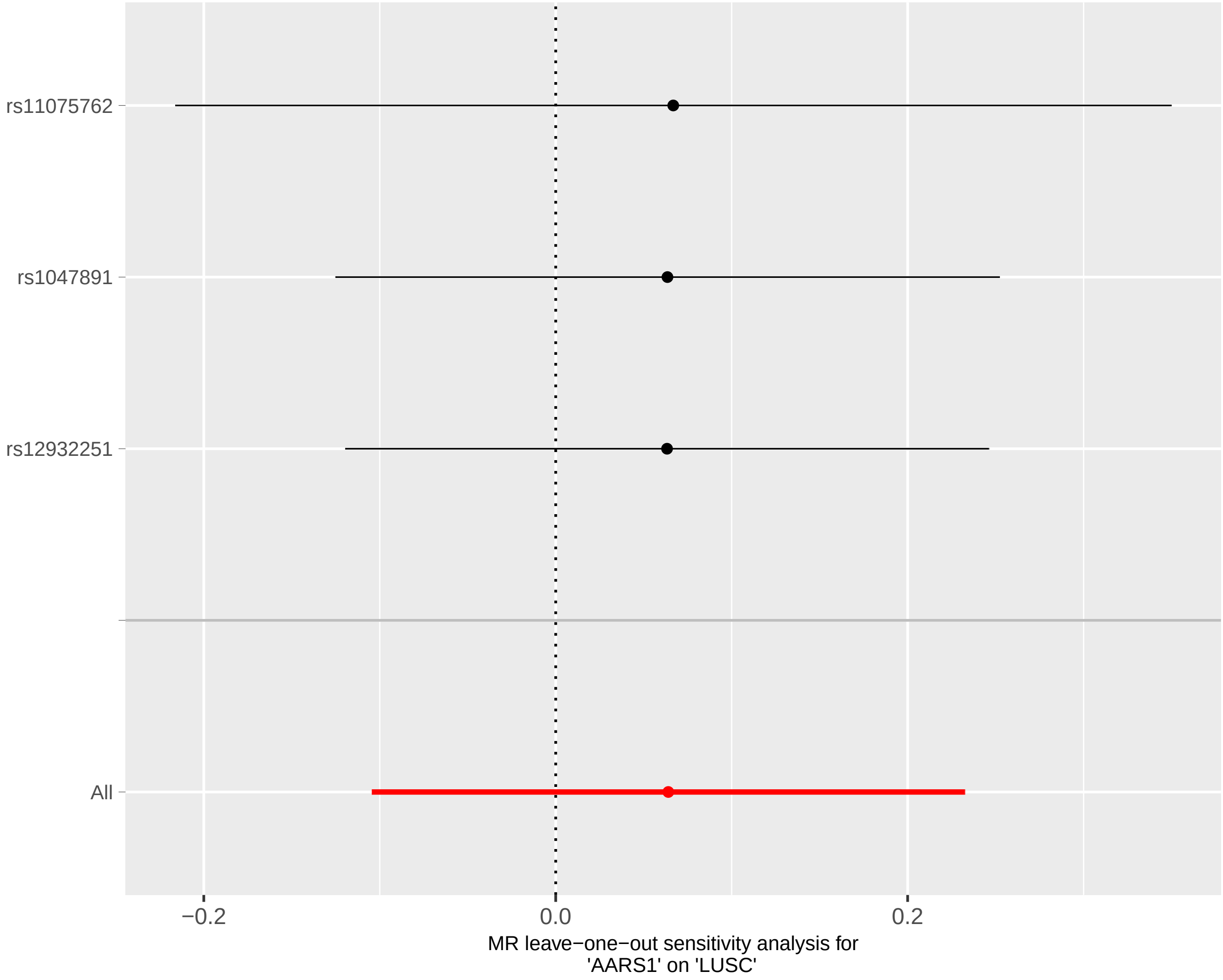
rs11075762
rs1047891
rs12932251
All
−0.2
0.0
0.2
MR leave−one−out sensitivity analysis for
'AARS1' on 'LUSC'

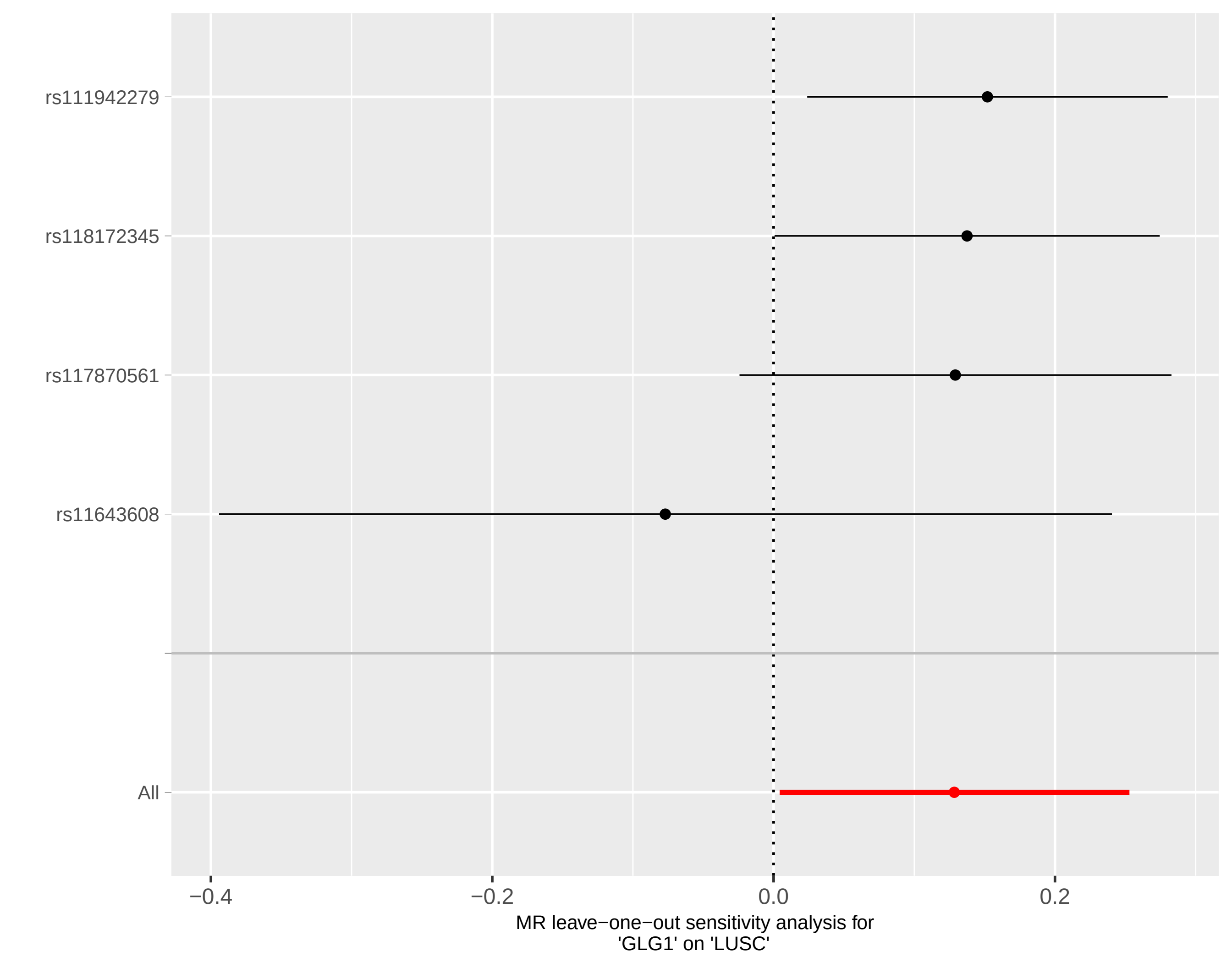
rs111942279
rs118172345
rs117870561
rs11643608
All
−0.4
−0.2
0.0
0.2
MR leave−one−out sensitivity analysis for
'GLG1' on 'LUSC'

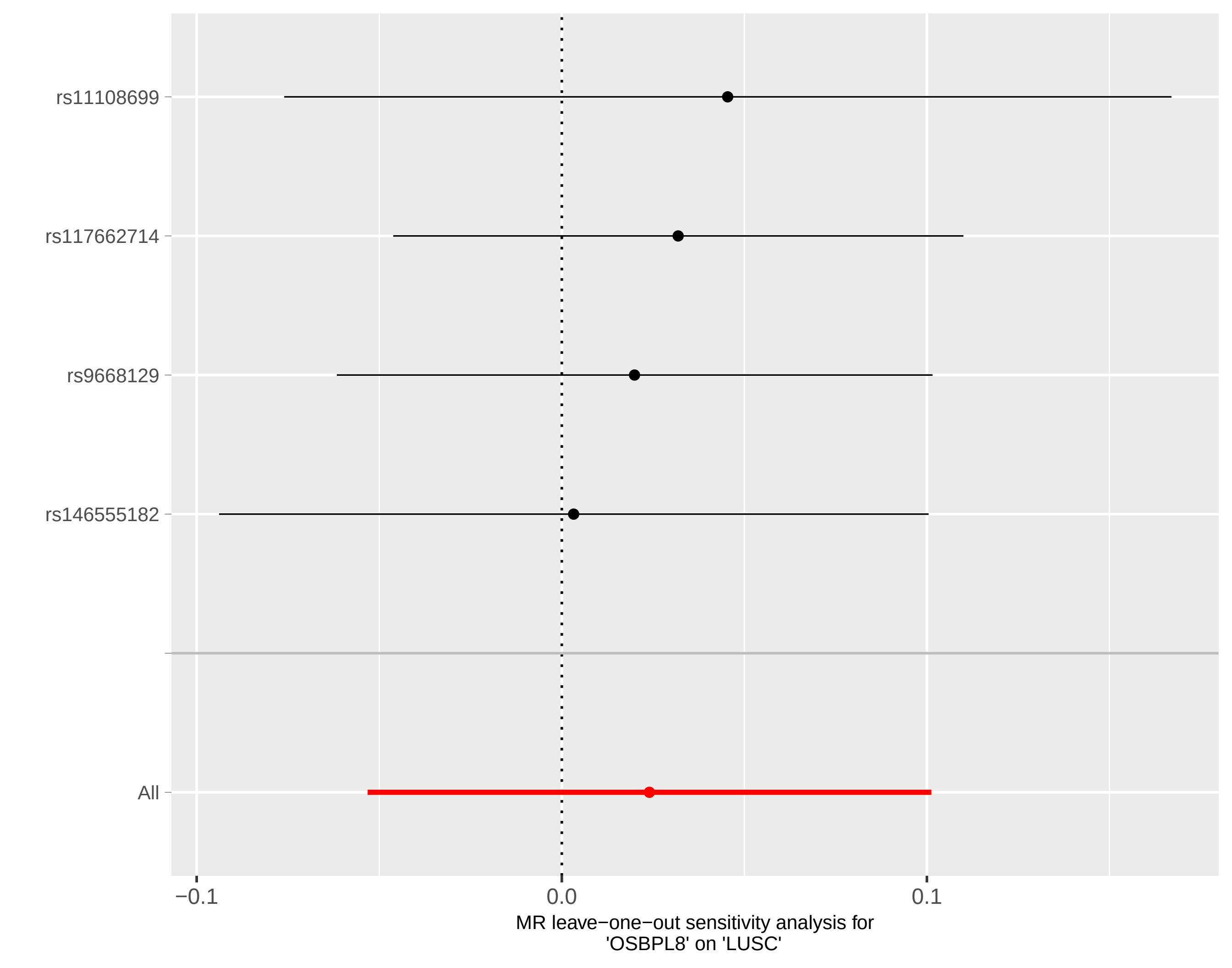

rs11108699
rs117662714
rs9668129
rs146555182
All
−0.1
0.0
0.1
MR leave−one−out sensitivity analysis for
'OSBPL8' on 'LUSC'

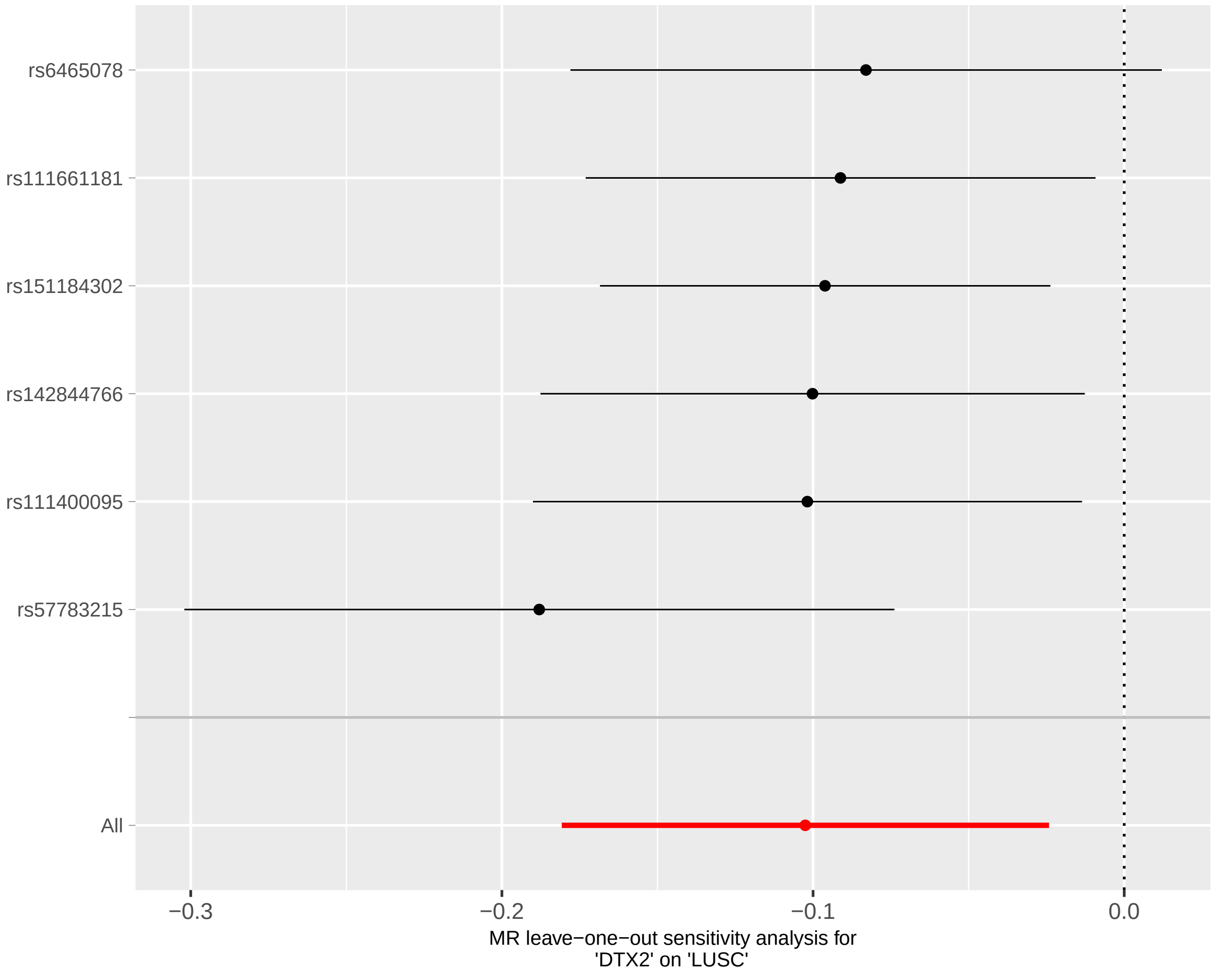
rs6465078
rs111661181
rs151184302
rs142844766
rs111400095
rs57783215
All
−0.3
−0.2
−0.1
0.0
MR leave−one−out sensitivity analysis for
'DTX2' on 'LUSC'

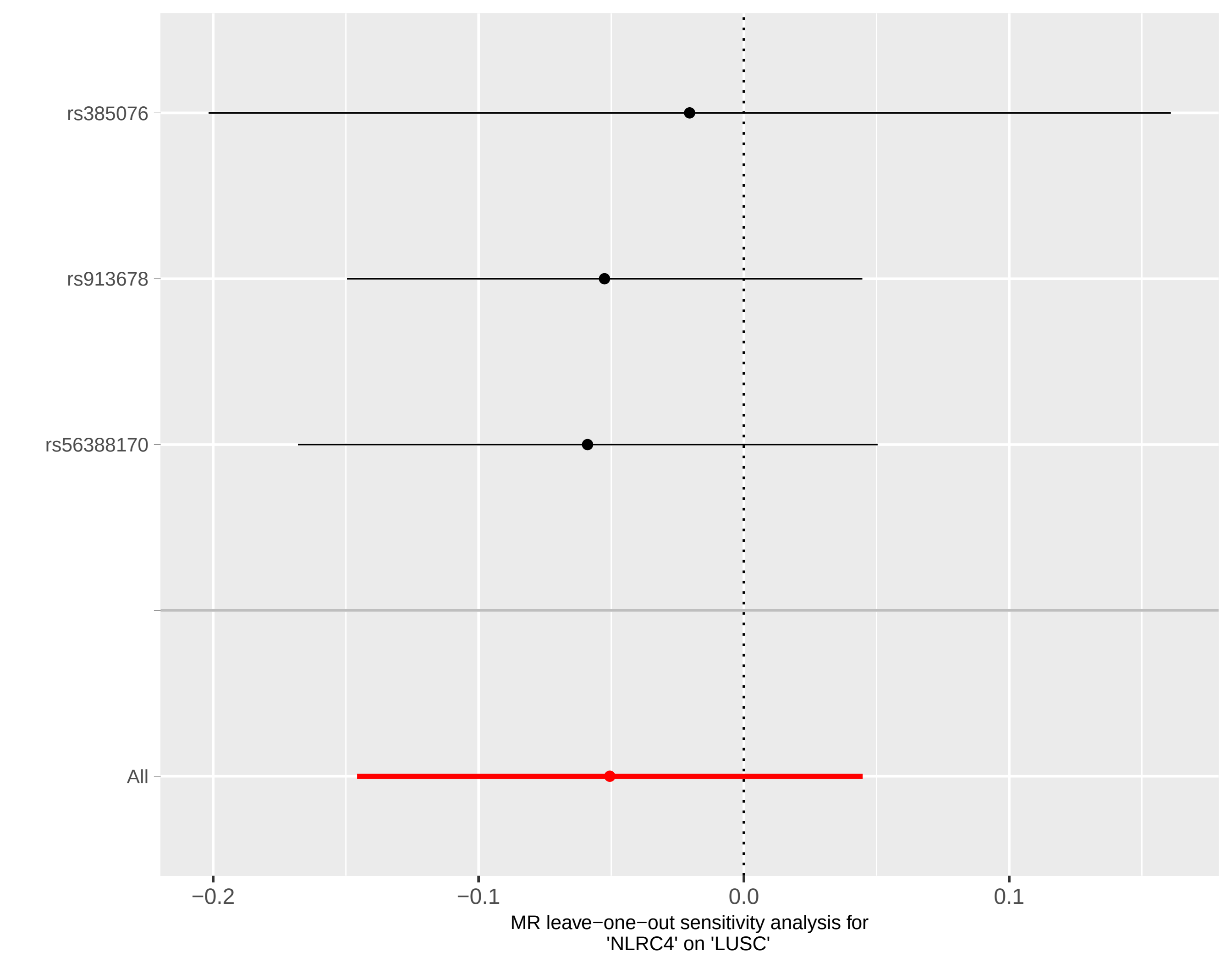
rs385076
rs913678
rs56388170
All
−0.2
−0.1
0.0
0.1
MR leave−one−out sensitivity analysis for
'NLRC4' on 'LUSC'

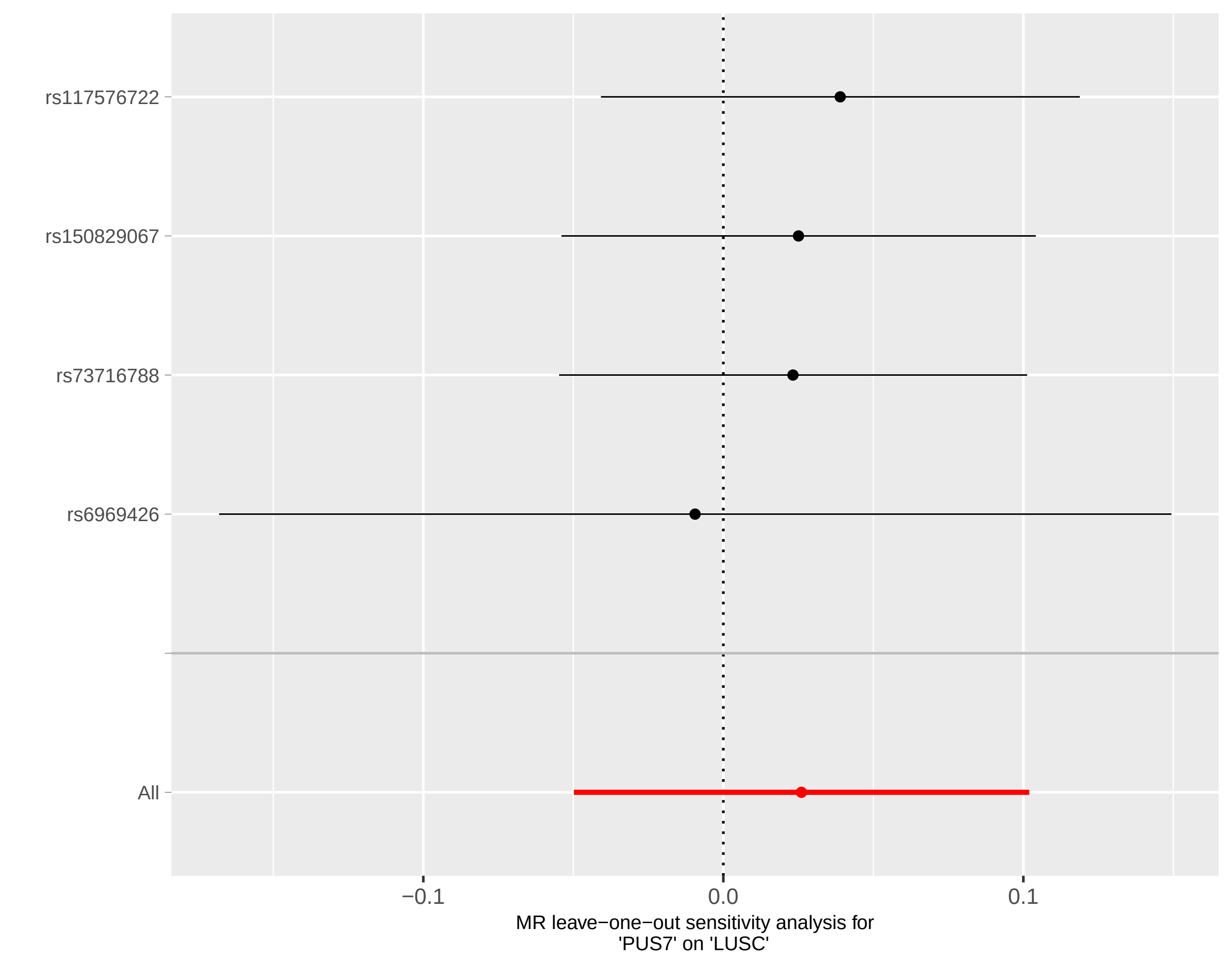

rs117576722
rs150829067
rs73716788
rs6969426
All
−0.1
0.0
0.1
MR leave−one−out sensitivity analysis for
'PUS7' on 'LUSC'

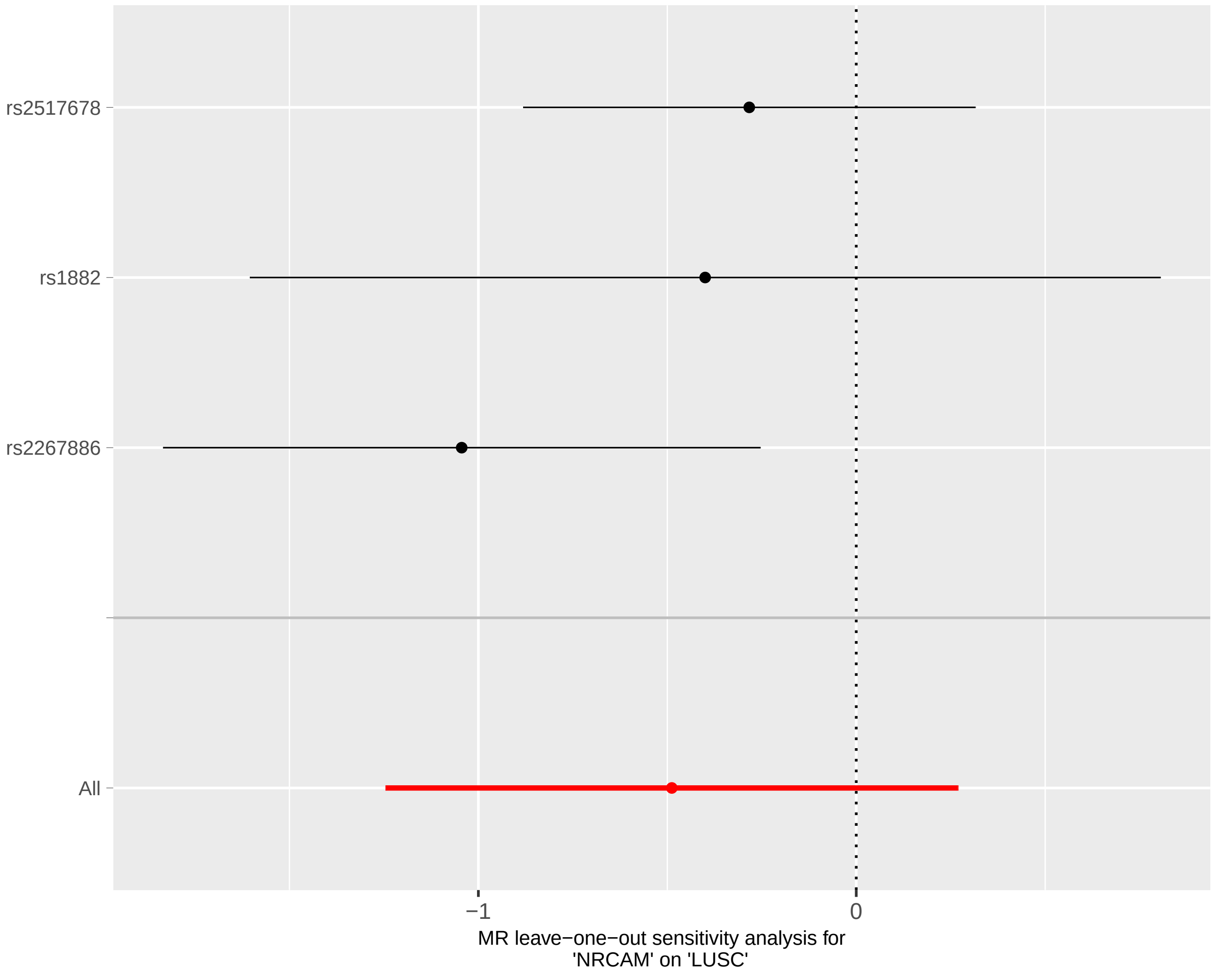
rs2517678
rs1882
rs2267886
All
−1
0
MR leave−one−out sensitivity analysis for
'NRCAM' on 'LUSC'

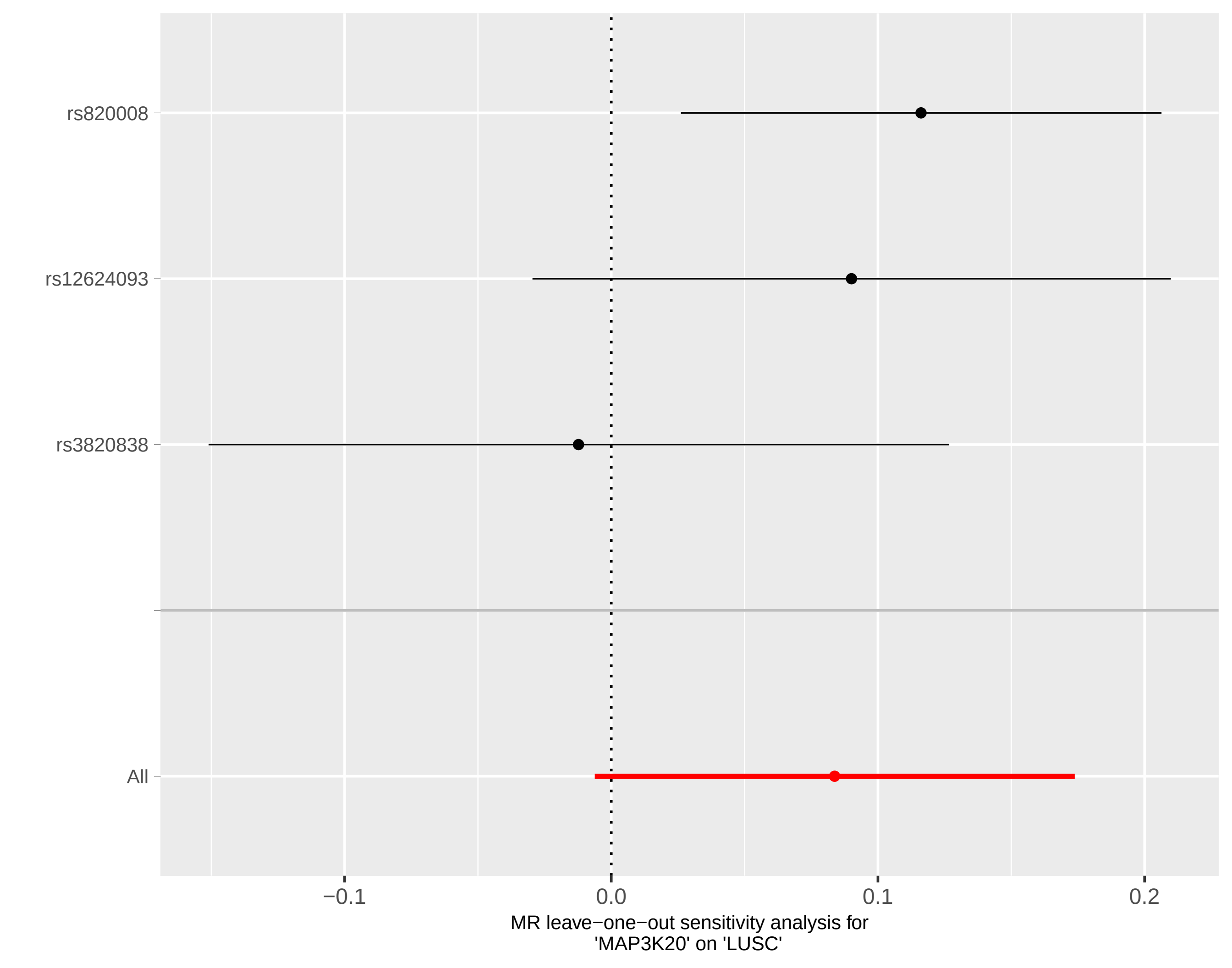

rs820008
rs12624093
rs3820838
All
−0.1
0.0
0.1
0.2
MR leave−one−out sensitivity analysis for
'MAP3K20' on 'LUSC'

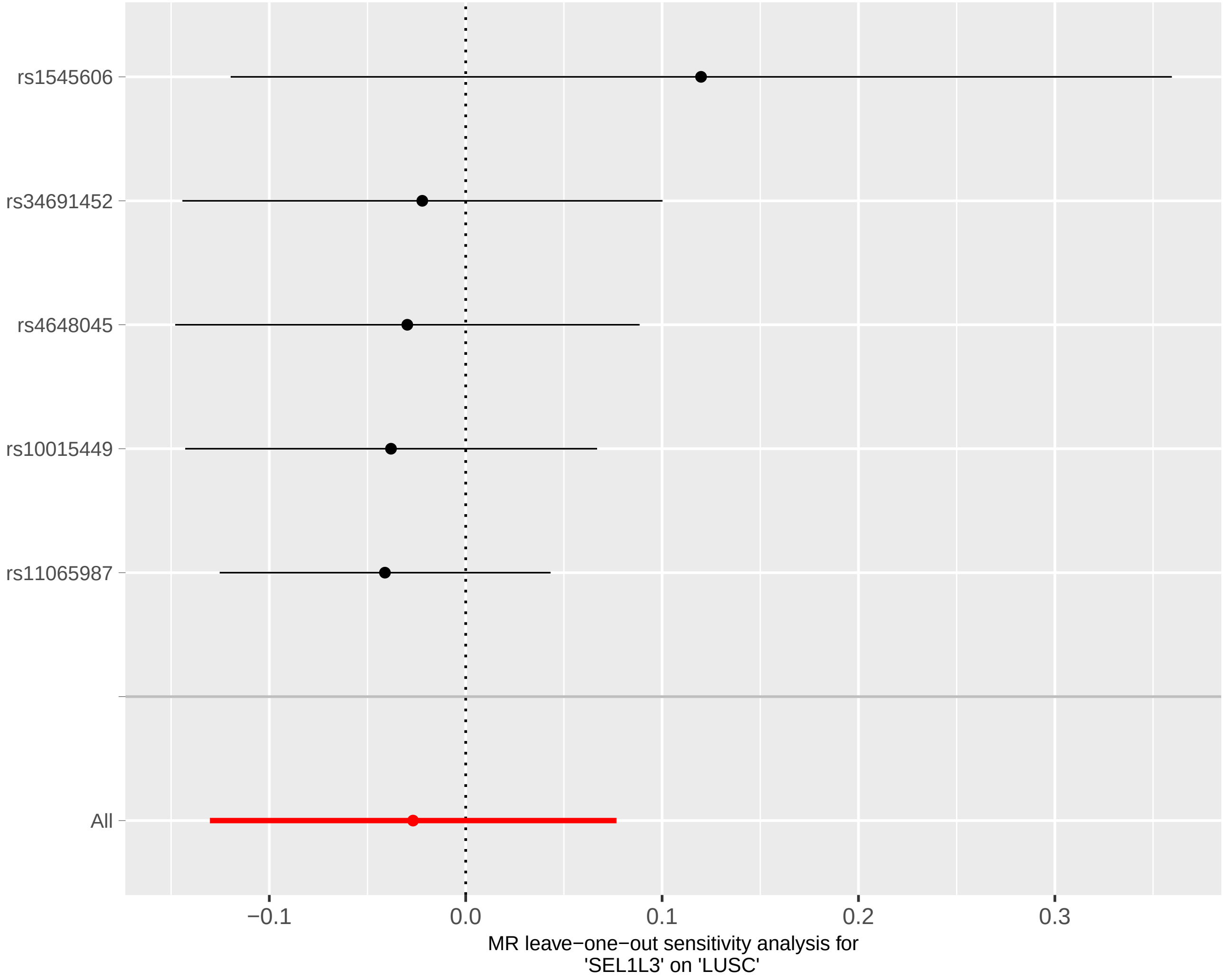

rs1545606
rs34691452
rs4648045
rs10015449
rs11065987
All
−0.1
0.0
0.1
0.2
0.3
MR leave−one−out sensitivity analysis for
'SEL1L3' on 'LUSC'

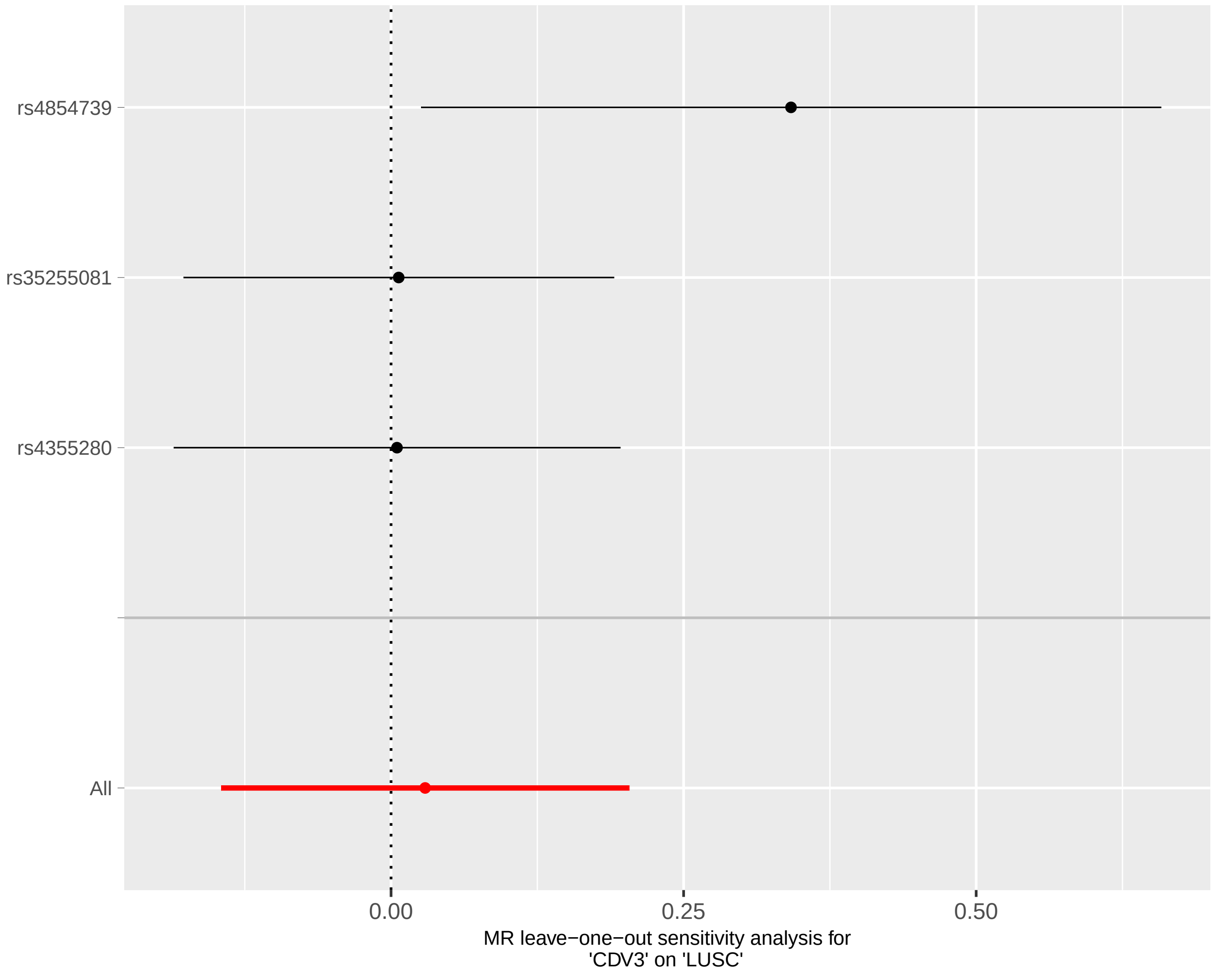

rs4854739
rs35255081
rs4355280
All
0.00
0.25
0.50
MR leave−one−out sensitivity analysis for
'CDV3' on 'LUSC'

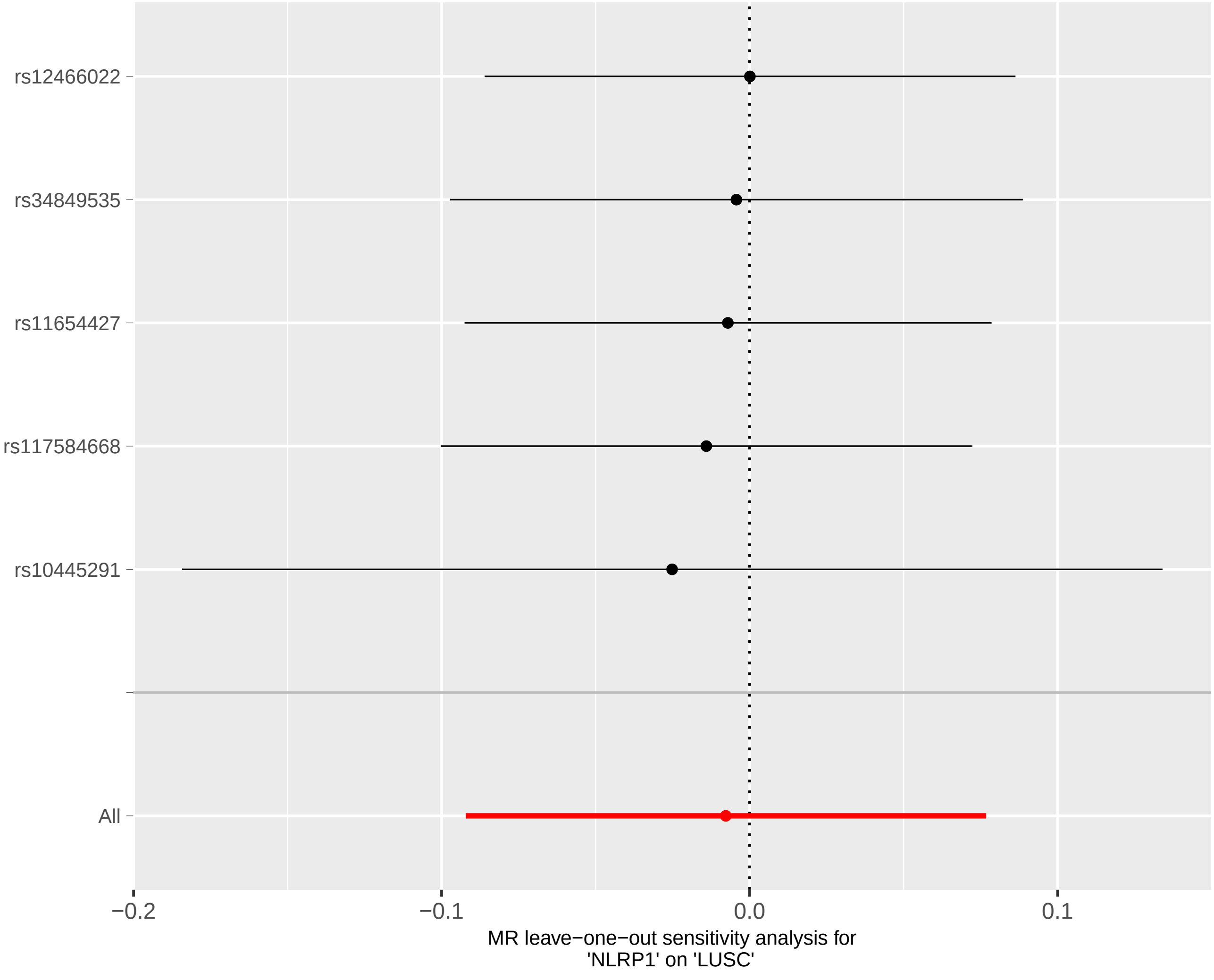
rs12466022
rs34849535
rs11654427
rs117584668
rs10445291
All
−0.2
−0.1
0.0
0.1
MR leave−one−out sensitivity analysis for
'NLRP1' on 'LUSC'

Insufficient number of SNPs

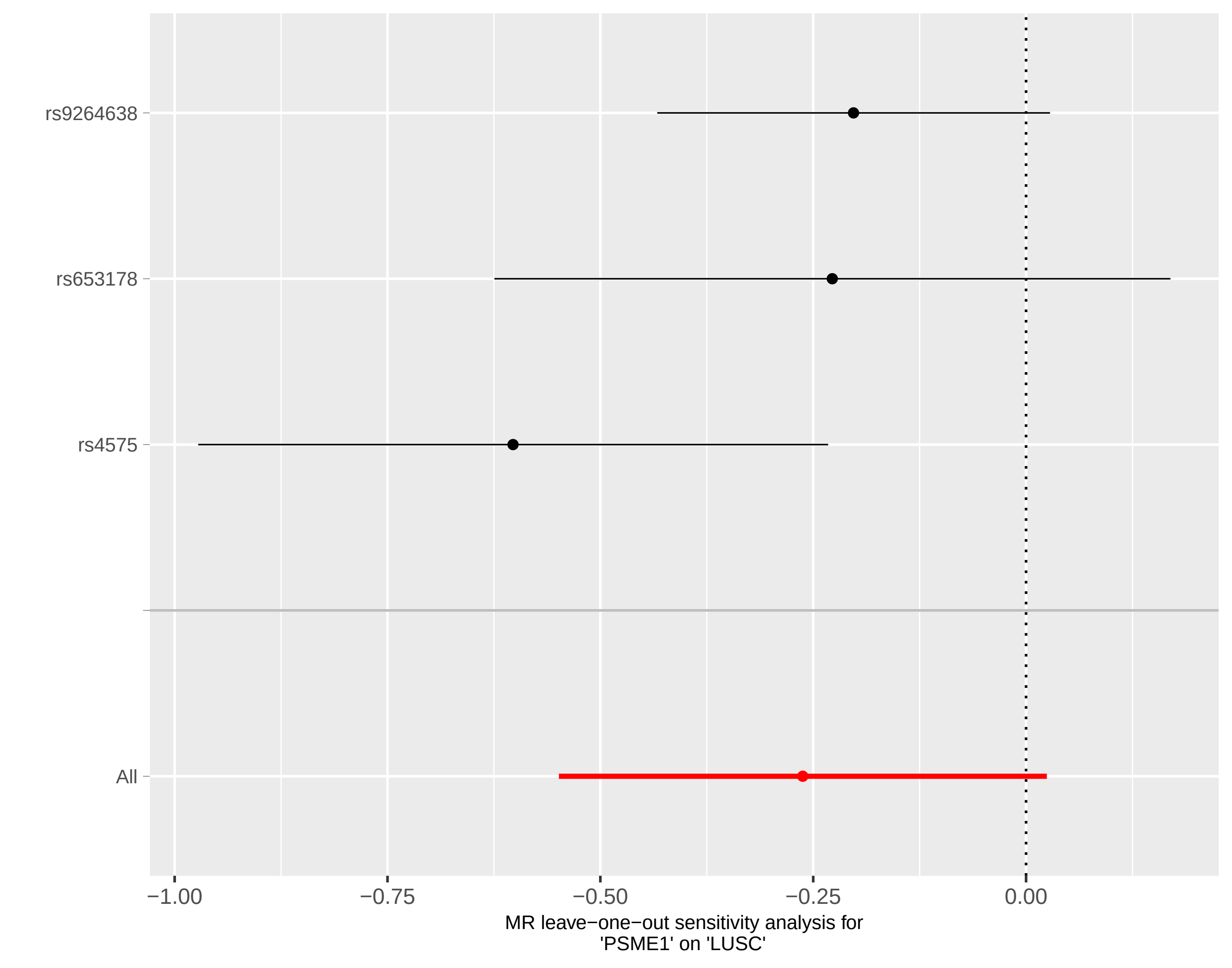
rs9264638
rs653178
rs4575
All
−1.00
−0.75
−0.50
−0.25
0.00
MR leave−one−out sensitivity analysis for
'PSME1' on 'LUSC'

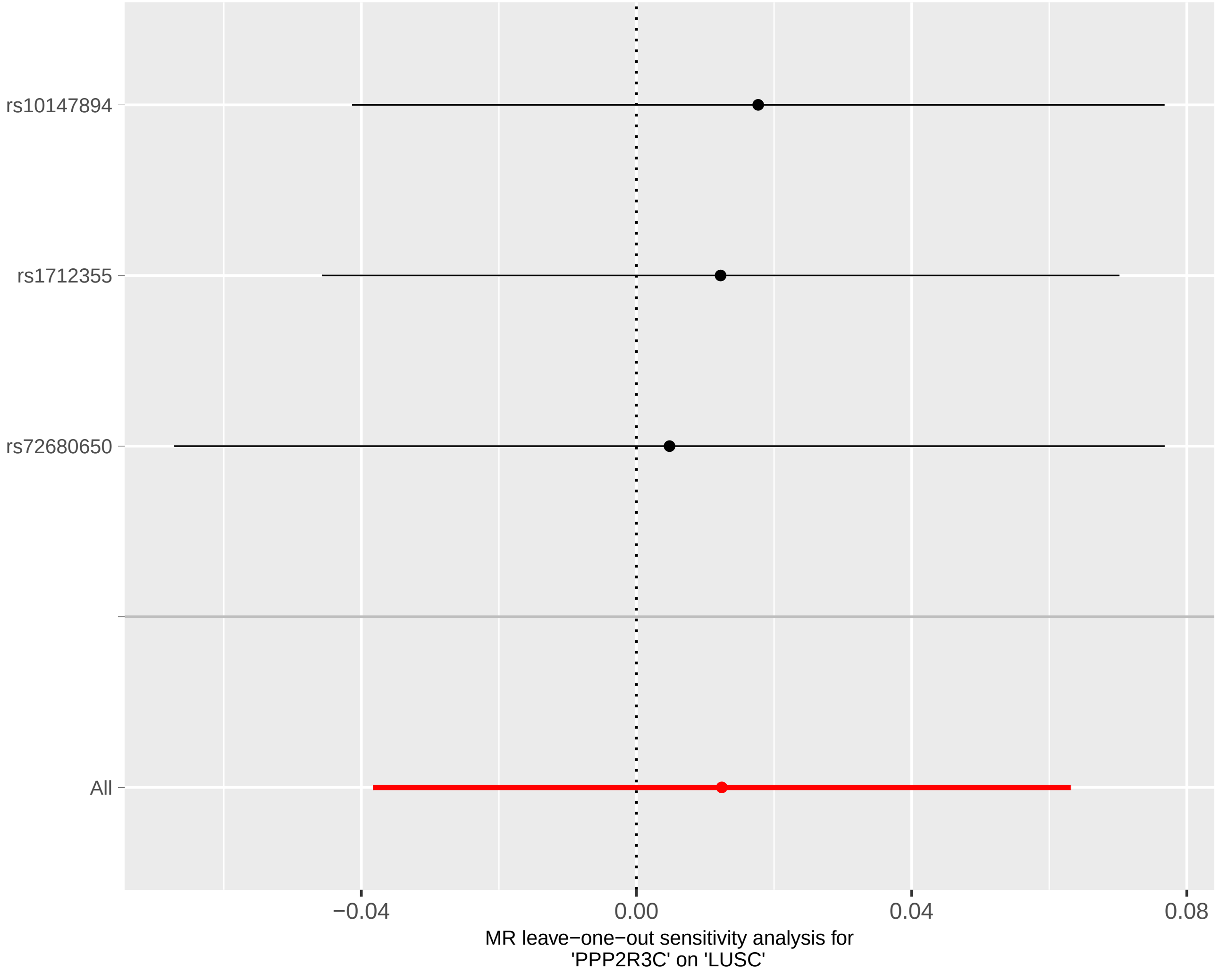

rs10147894
rs1712355
rs72680650
All
−0.04
0.00
0.04
0.08
MR leave−one−out sensitivity analysis for
'PPP2R3C' on 'LUSC'

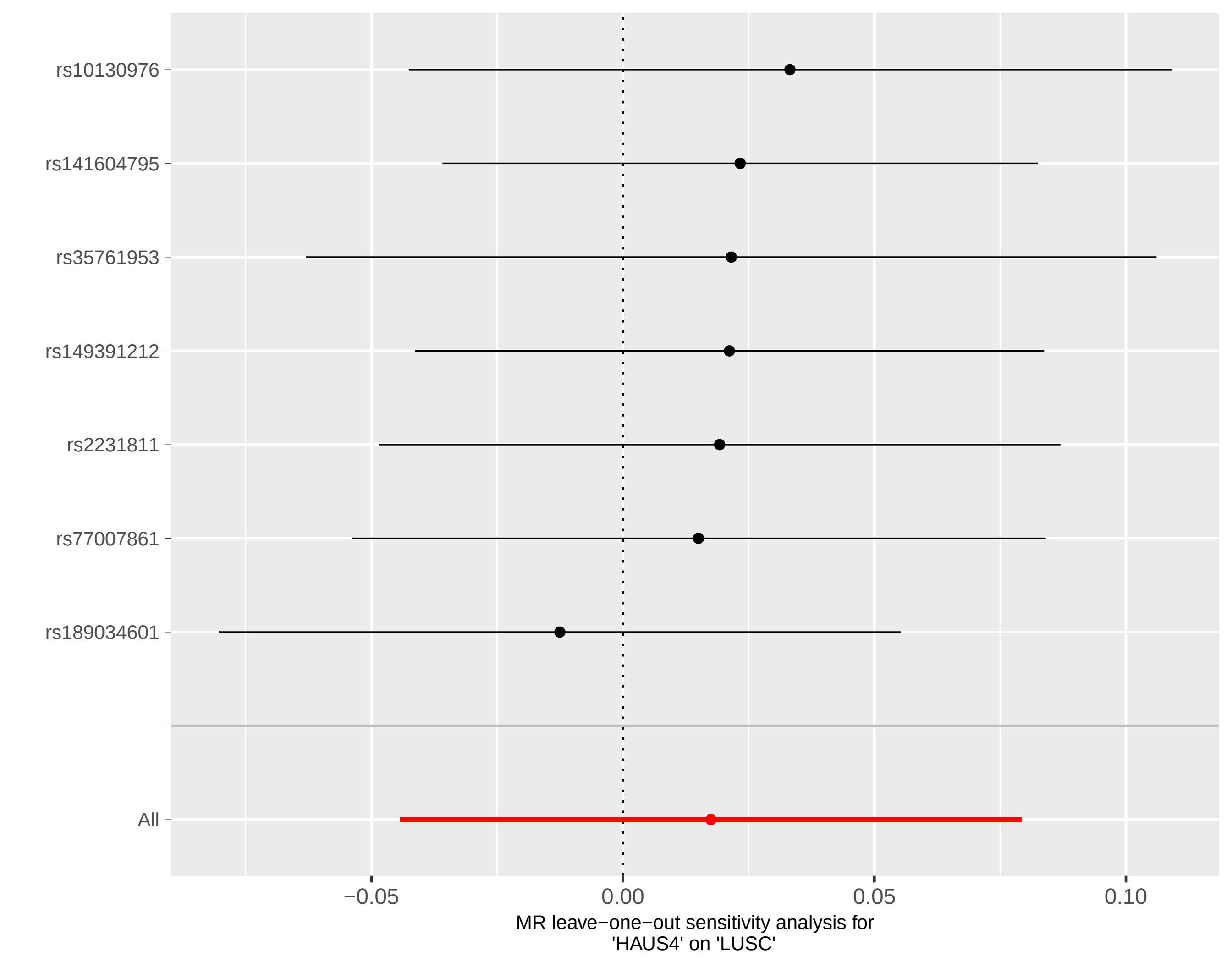

rs10130976
rs141604795
rs35761953
rs149391212
rs2231811
rs77007861
rs189034601
All
−0.05
0.00
0.05
0.10
MR leave−one−out sensitivity analysis for
'HAUS4' on 'LUSC'

Insufficient number of SNPs

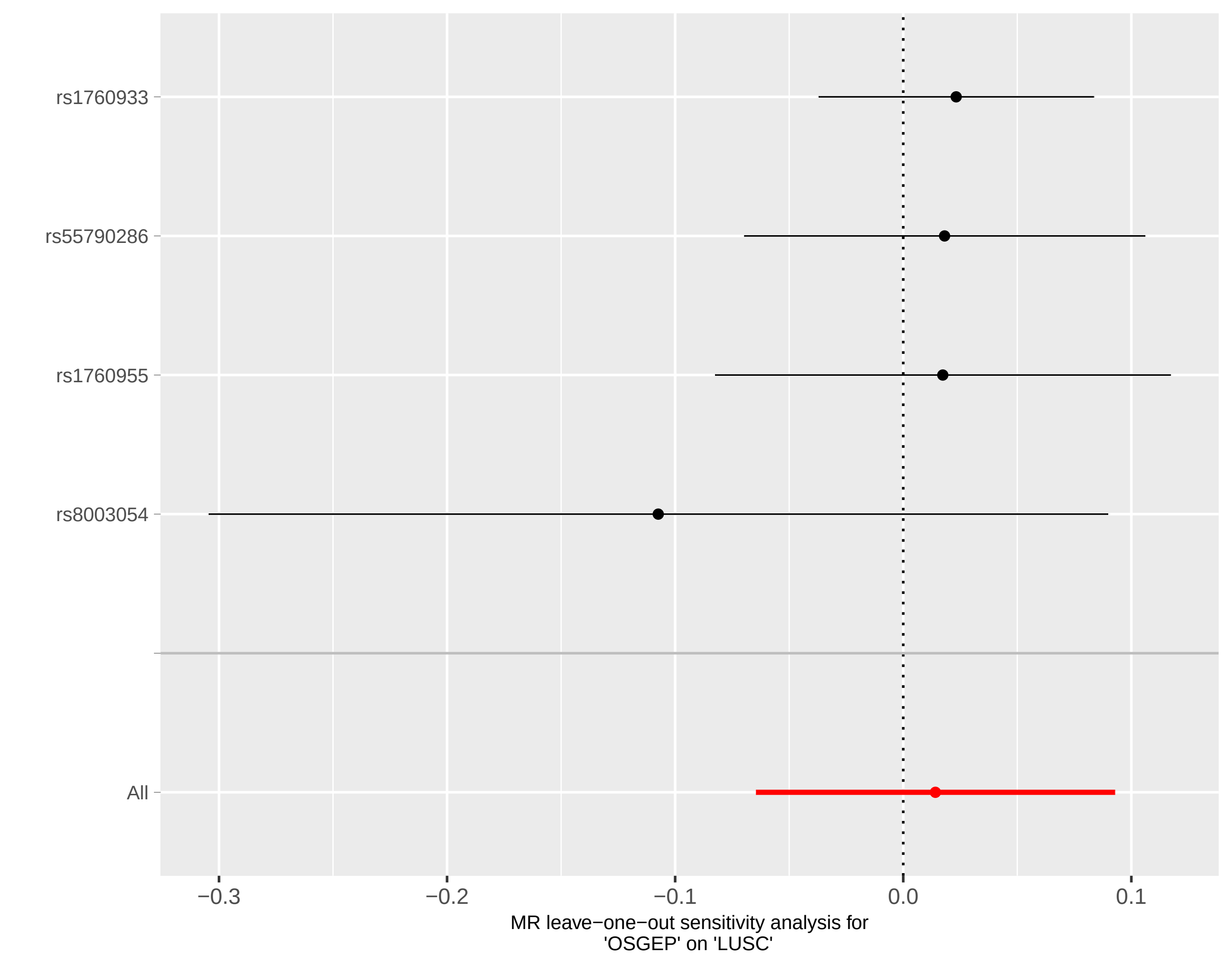

rs1760933
rs55790286
rs1760955
rs8003054
All
−0.3
−0.2
−0.1
0.0
0.1
MR leave−one−out sensitivity analysis for
'OSGEP' on 'LUSC'

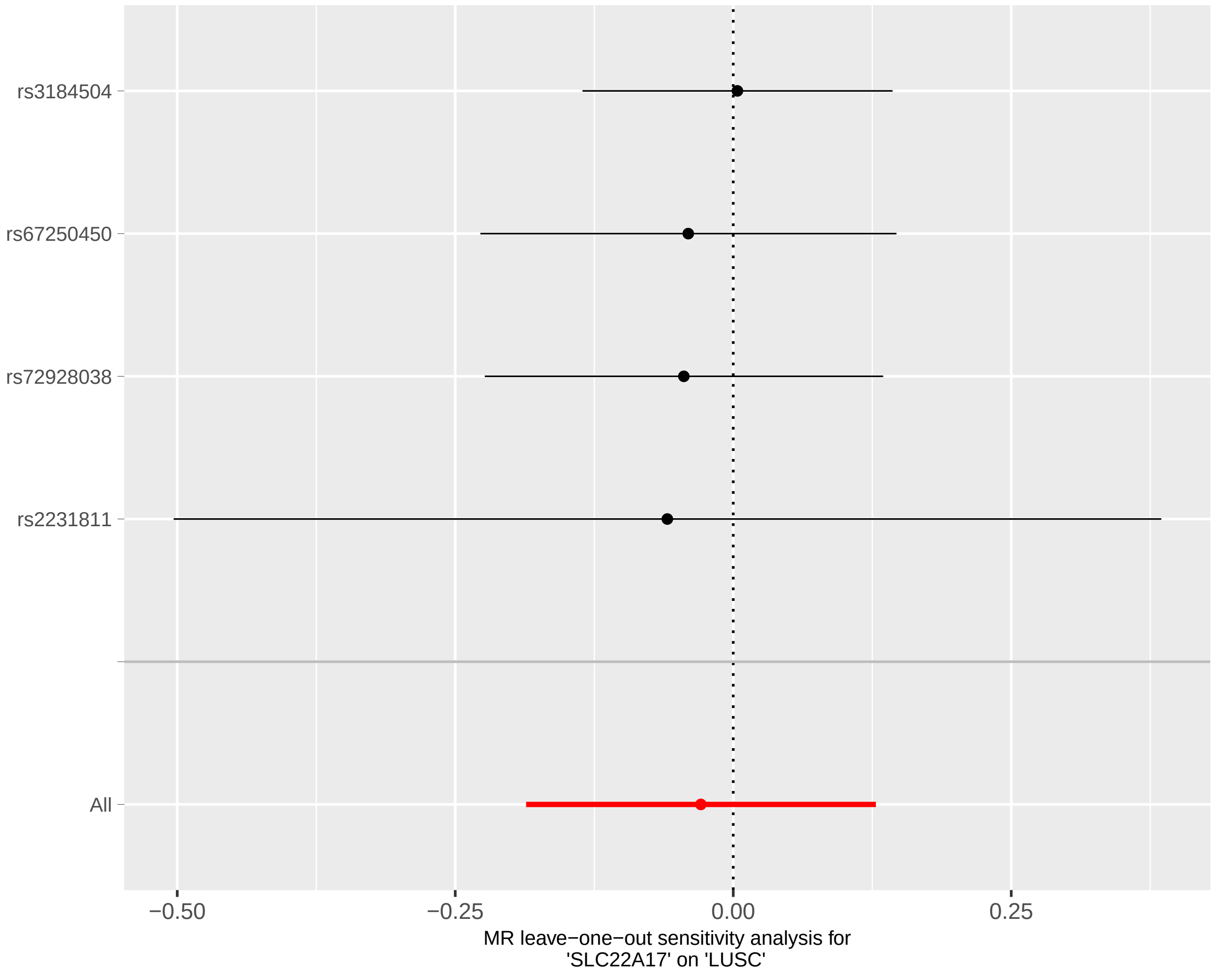
rs3184504
rs67250450
rs72928038
rs2231811
All
−0.50
−0.25
0.00
0.25
MR leave−one−out sensitivity analysis for
'SLC22A17' on 'LUSC'

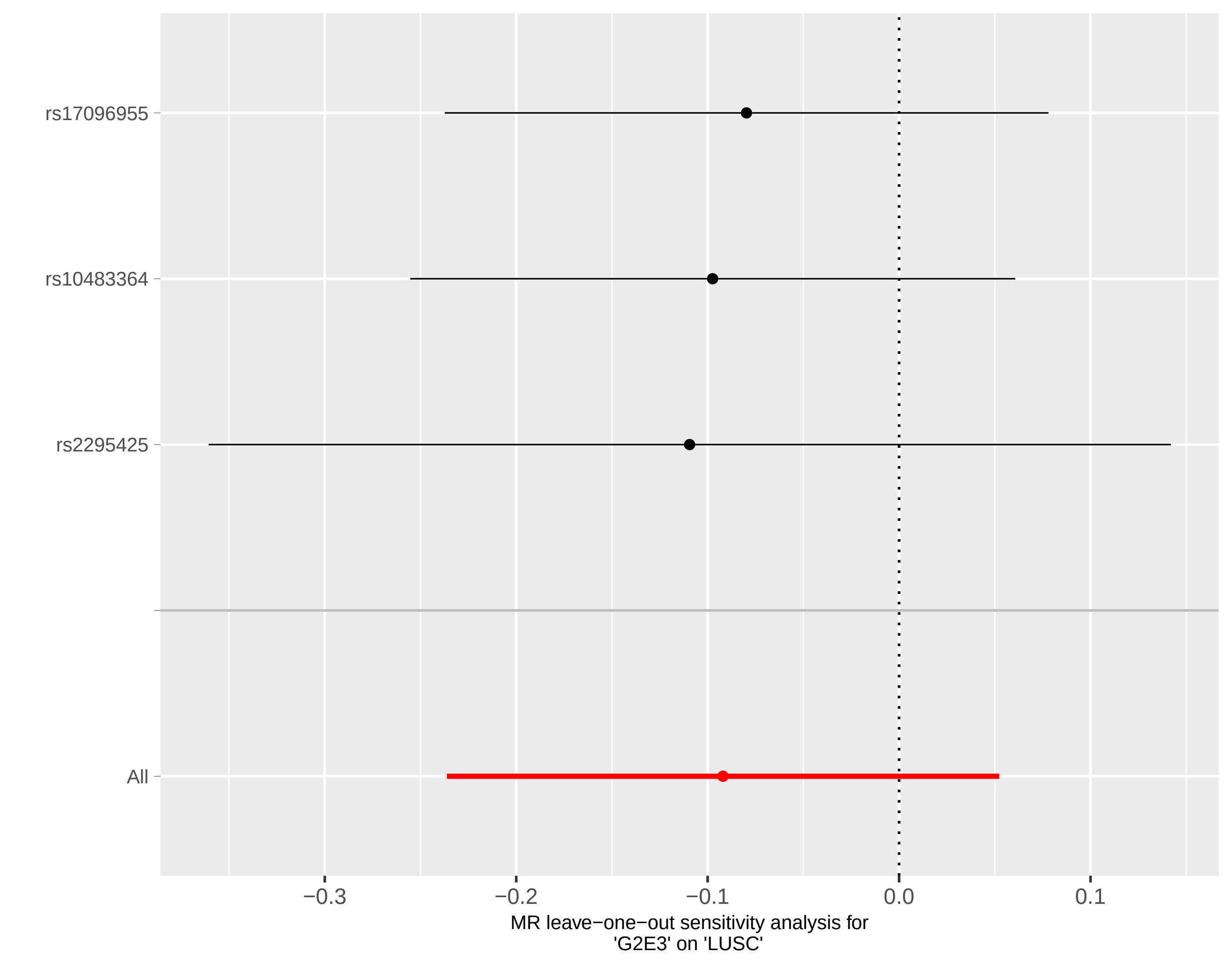
rs17096955
rs10483364
rs2295425
All
−0.3
−0.2
−0.1
0.0
0.1
MR leave−one−out sensitivity analysis for
'G2E3' on 'LUSC'

Insufficient number of SNPs

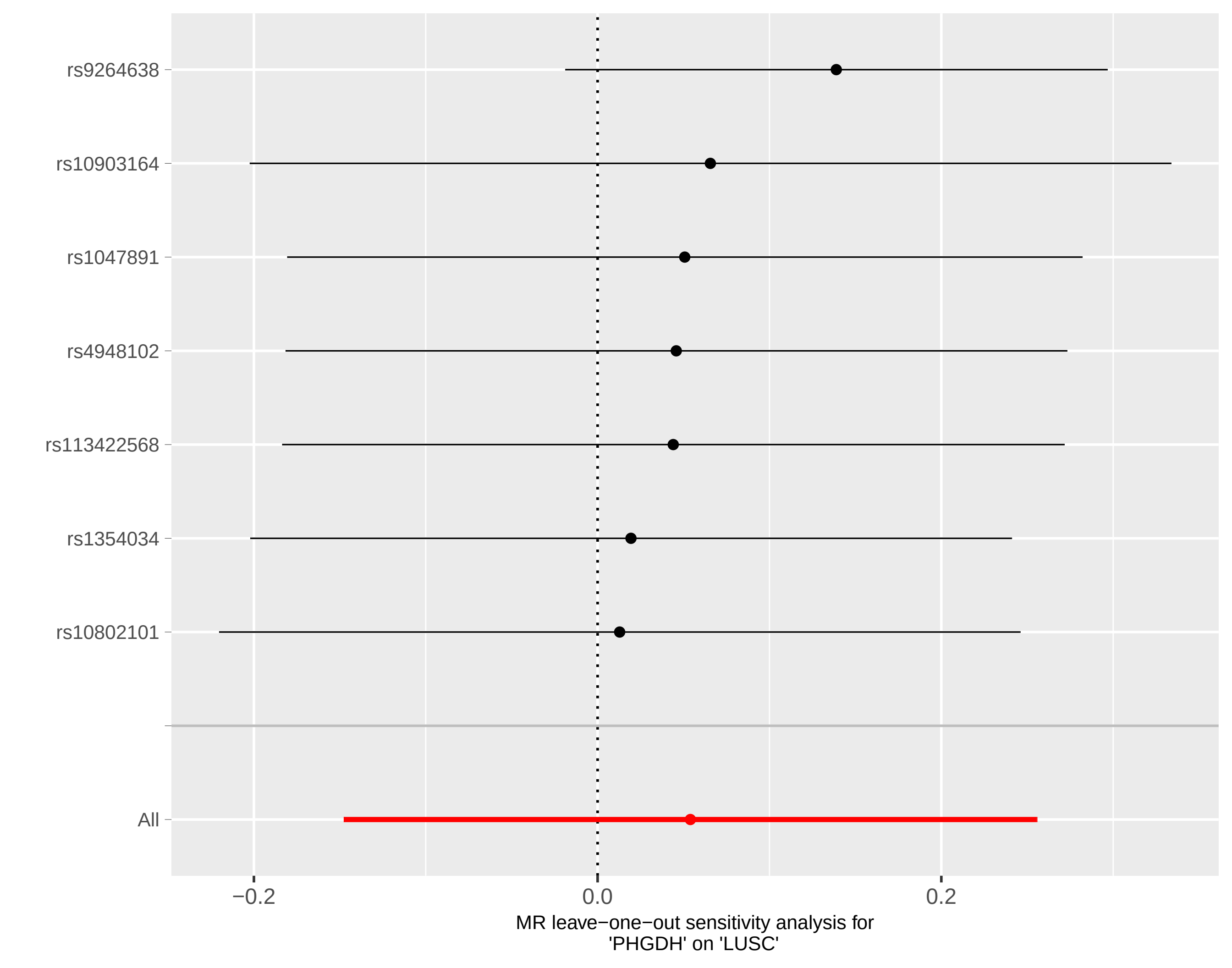
rs9264638
rs10903164
rs1047891
rs4948102
rs113422568
rs1354034
rs10802101
All
−0.2
0.0
0.2
MR leave−one−out sensitivity analysis for
'PHGDH' on 'LUSC'

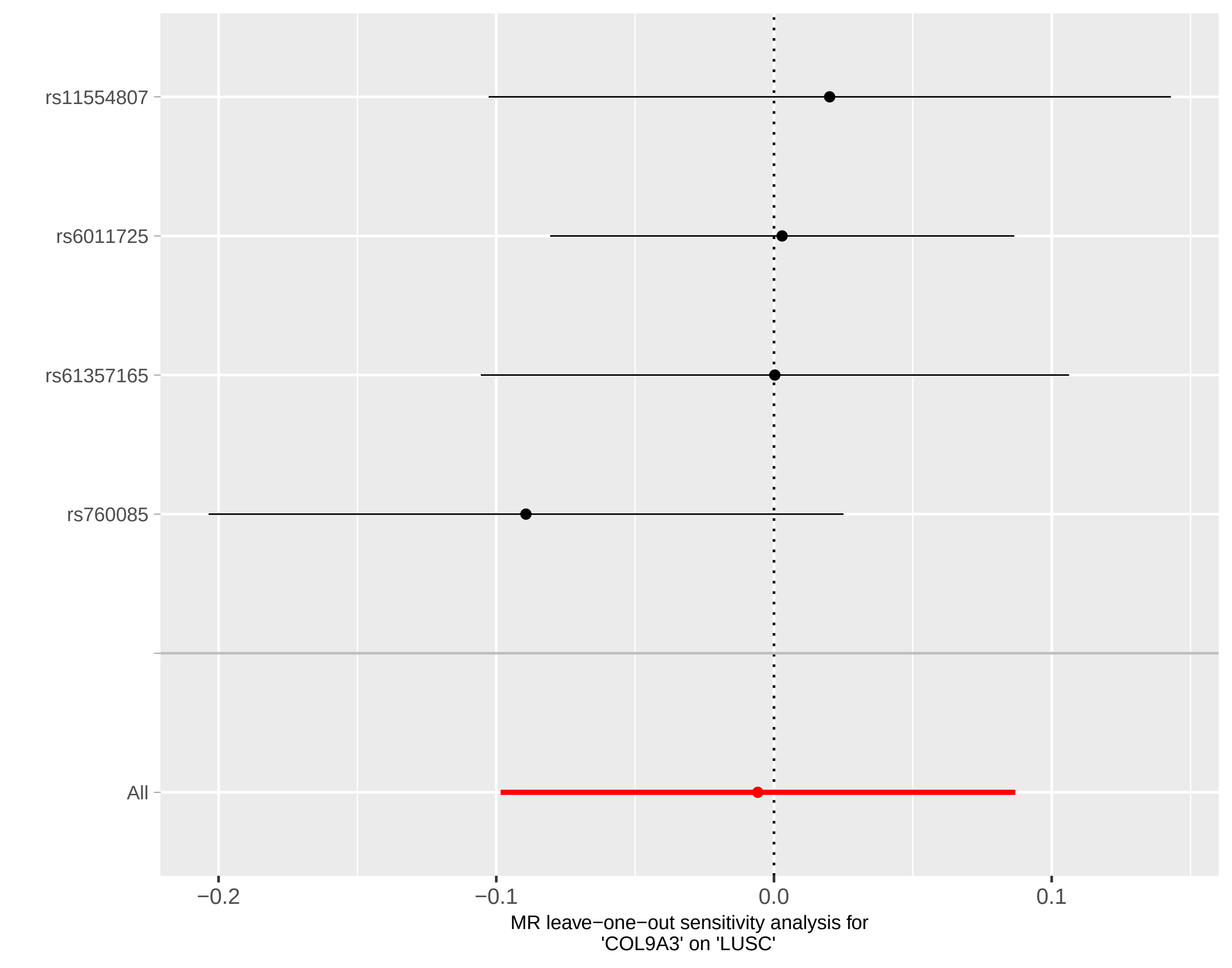
rs11554807
rs6011725
rs61357165
rs760085
All
−0.2
−0.1
0.0
0.1
MR leave−one−out sensitivity analysis for
'COL9A3' on 'LUSC'

Insufficient number of SNPs

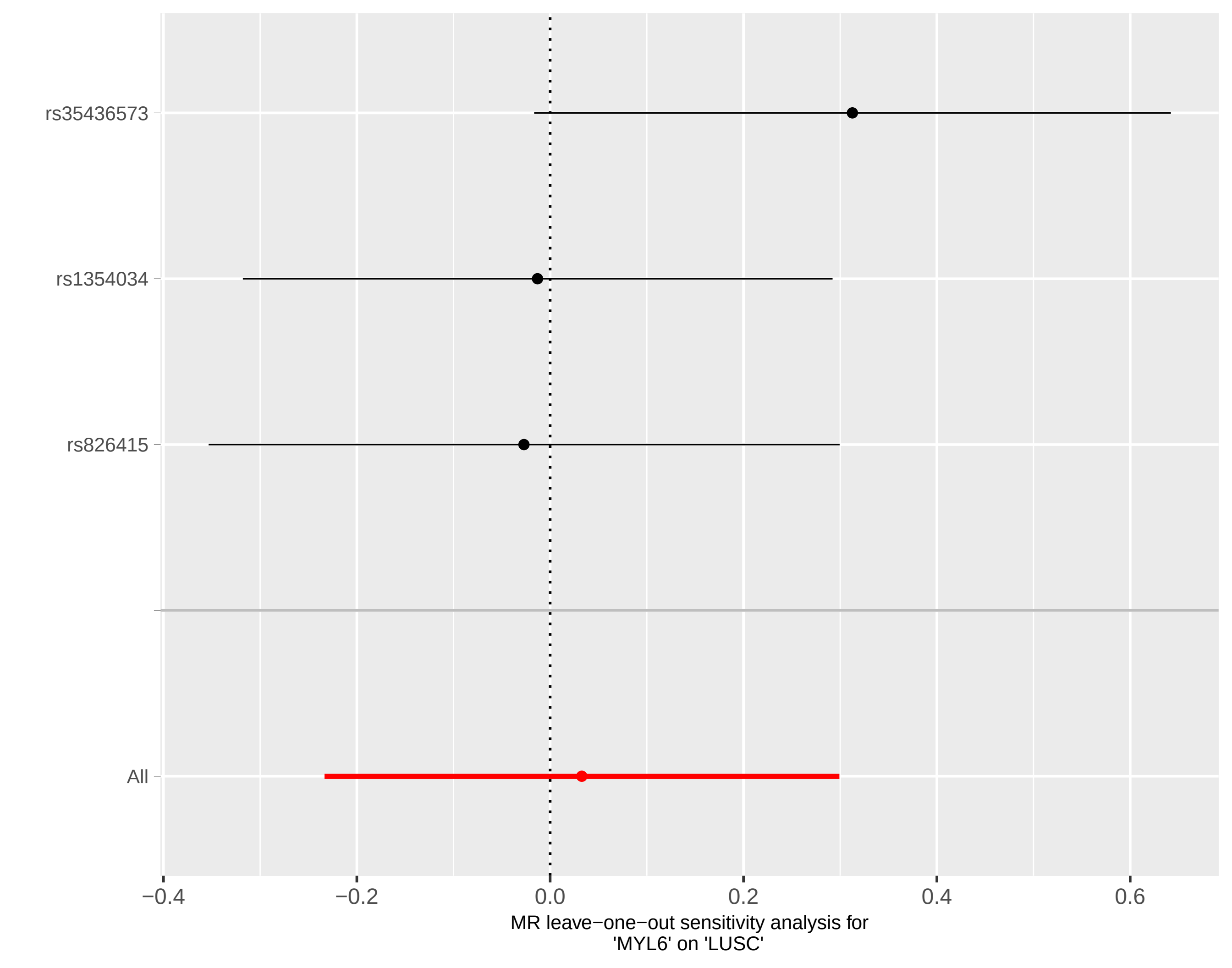
rs35436573
rs1354034
rs826415
All
−0.4
−0.2
0.0
0.2
0.4
0.6
MR leave−one−out sensitivity analysis for
'MYL6' on 'LUSC'

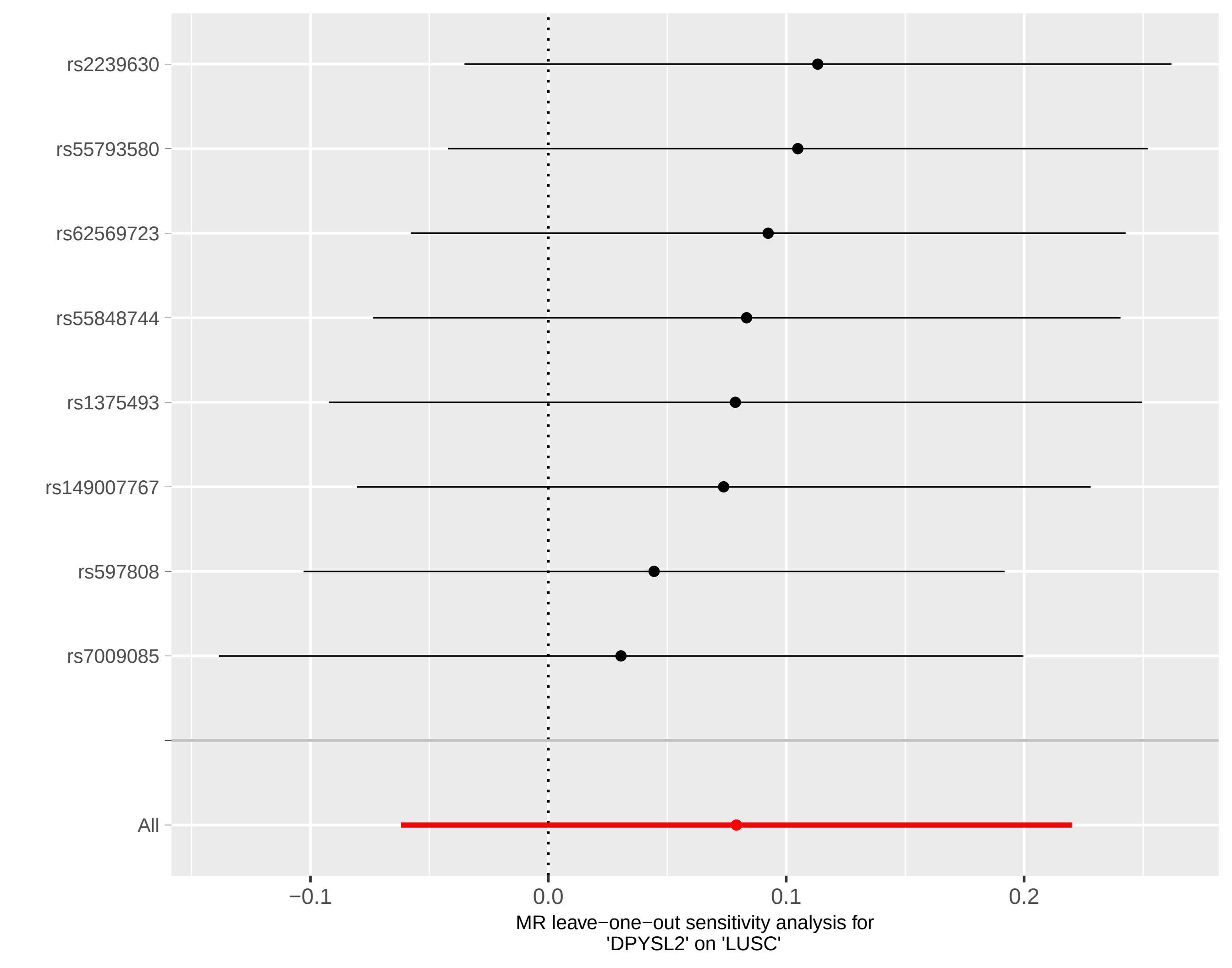
rs2239630
rs55793580
rs62569723
rs55848744
rs1375493
rs149007767
rs597808
rs7009085
All
−0.1
0.0
0.1
0.2
MR leave−one−out sensitivity analysis for
'DPYSL2' on 'LUSC'

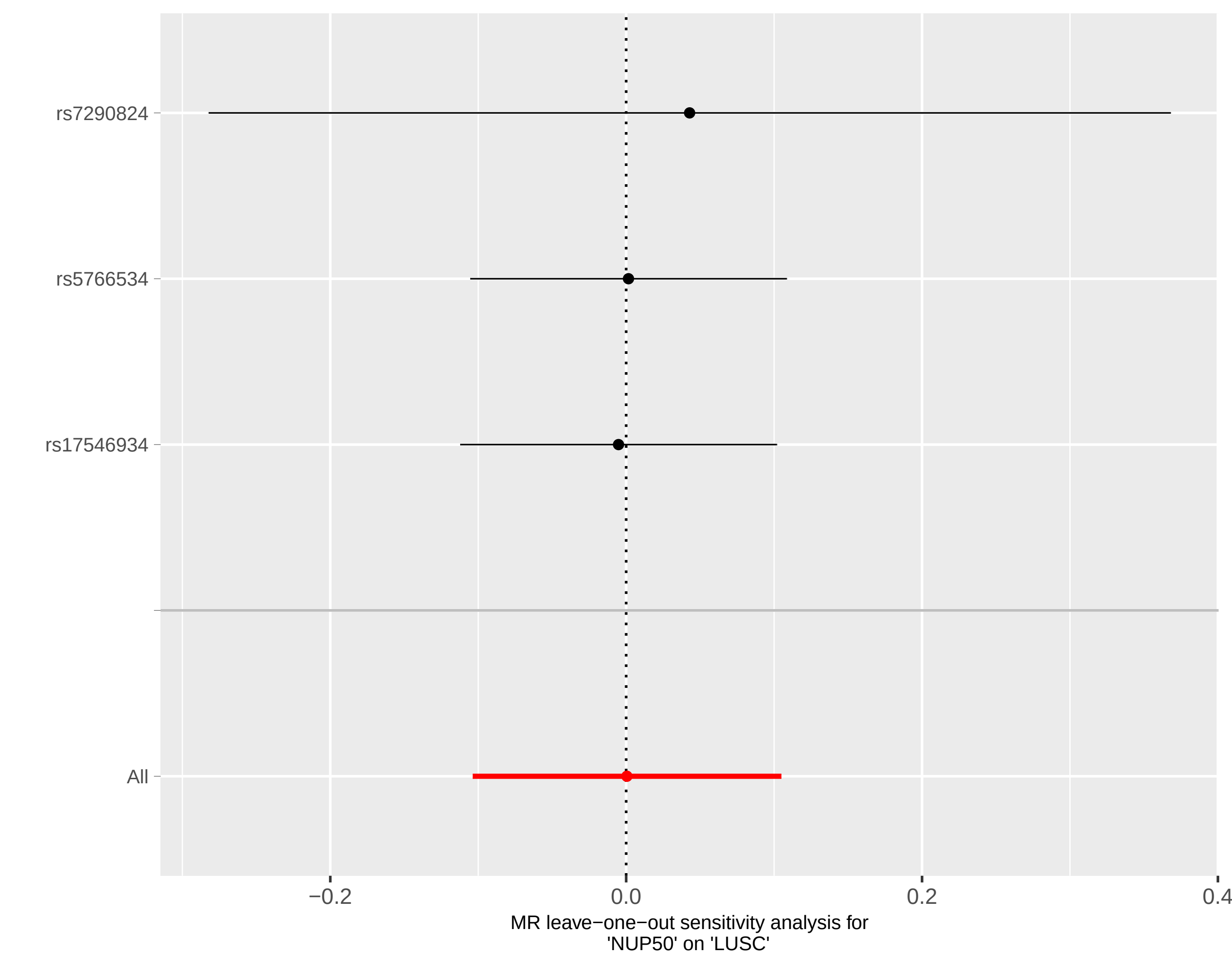

rs7290824
rs5766534
rs17546934
All
−0.2
0.0
0.2
0.4
MR leave−one−out sensitivity analysis for
'NUP50' on 'LUSC'

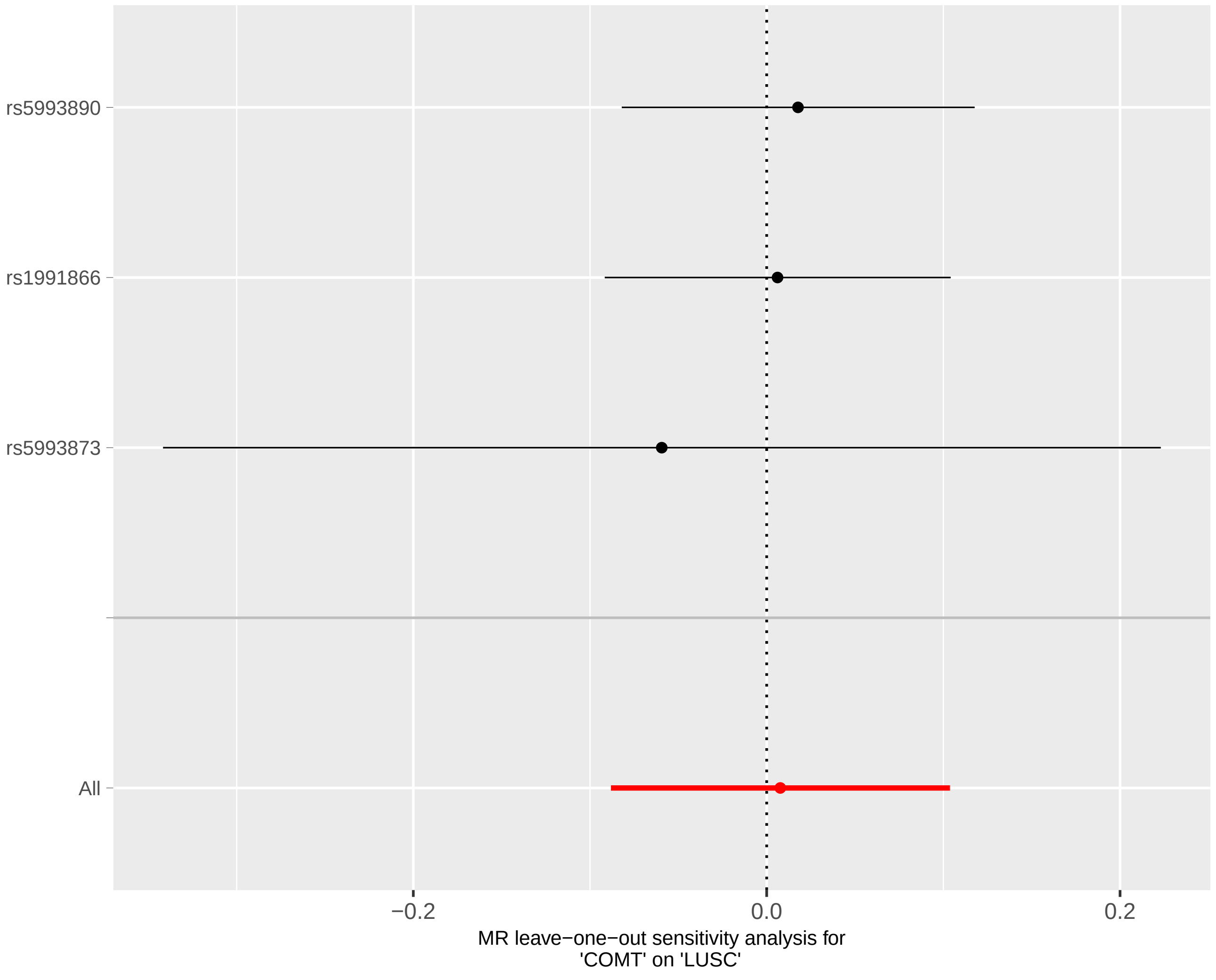
rs5993890
rs1991866
rs5993873
All
−0.2
0.0
0.2
MR leave−one−out sensitivity analysis for
'COMT' on 'LUSC'

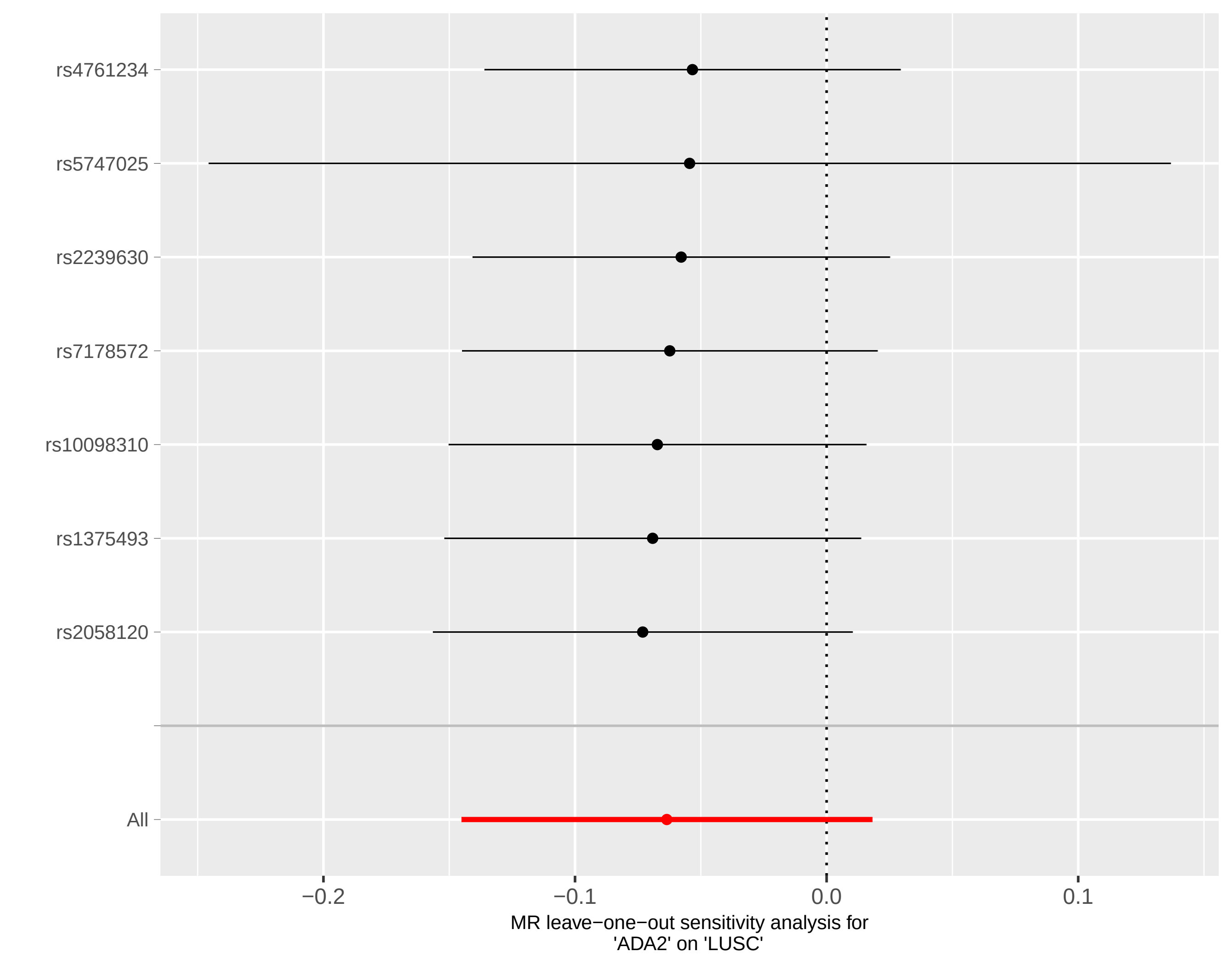

rs4761234
rs5747025
rs2239630
rs7178572
rs10098310
rs1375493
rs2058120
All
−0.2
−0.1
0.0
0.1
MR leave−one−out sensitivity analysis for
'ADA2' on 'LUSC'

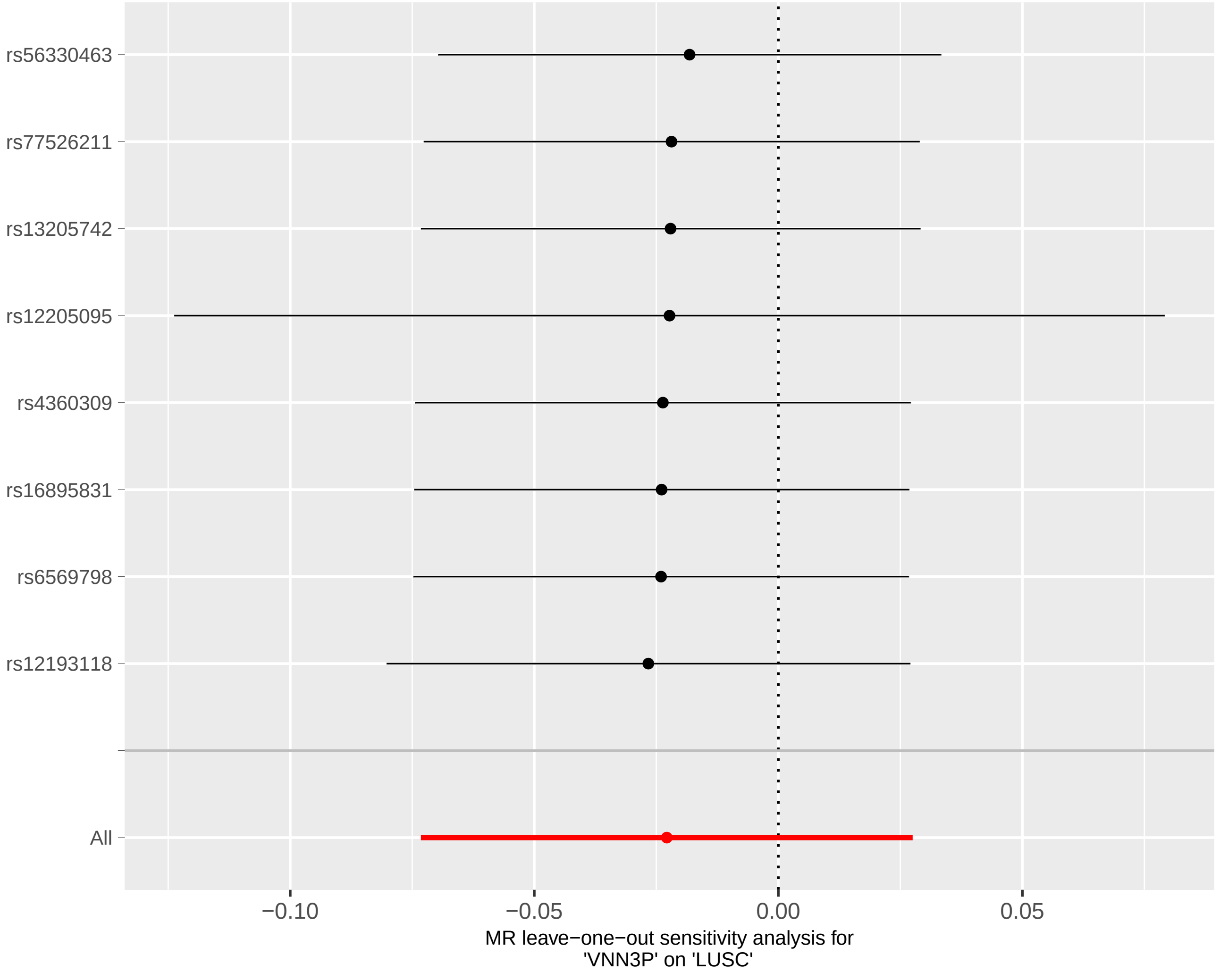

rs56330463
rs77526211
rs13205742
rs12205095
rs4360309
rs16895831
rs6569798
rs12193118
All
−0.10
−0.05
0.00
0.05
MR leave−one−out sensitivity analysis for
'VNN3P' on 'LUSC'

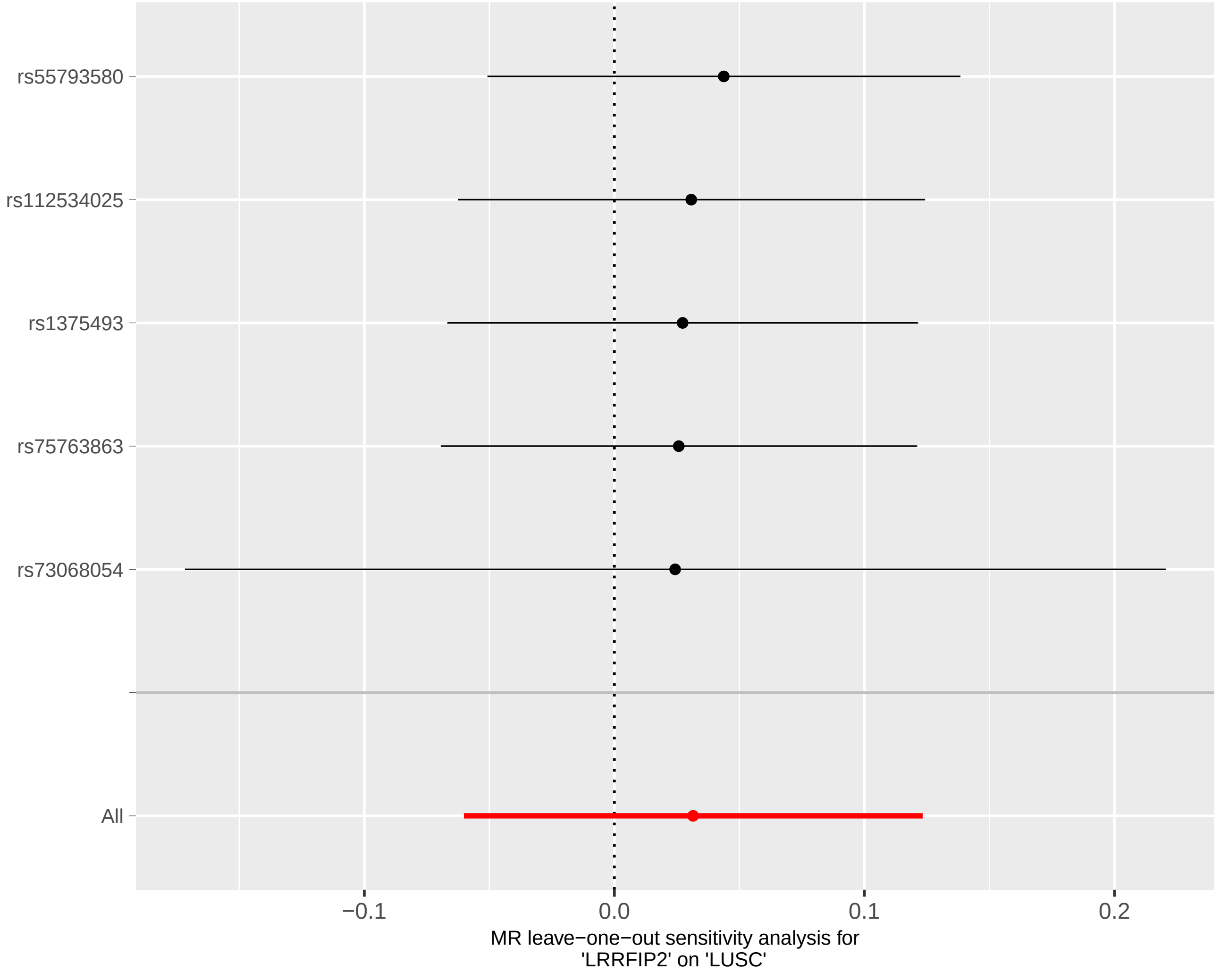
rs55793580
rs112534025
rs1375493
rs75763863
rs73068054
All
−0.1
0.0
0.1
0.2
MR leave−one−out sensitivity analysis for
'LRRFIP2' on 'LUSC'

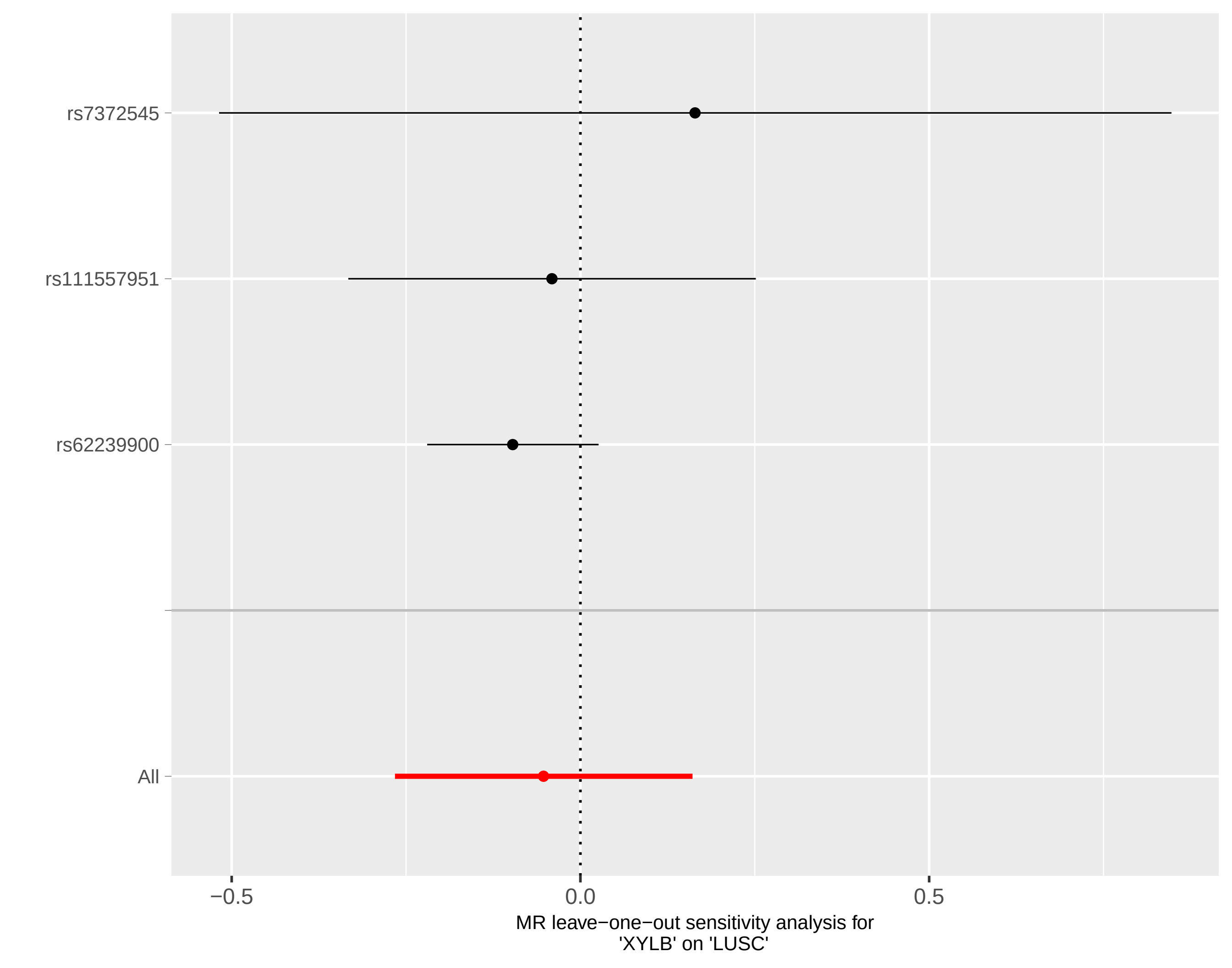
rs7372545
rs111557951
rs62239900
All
−0.5
0.0
0.5
MR leave−one−out sensitivity analysis for
'XYLB' on 'LUSC'

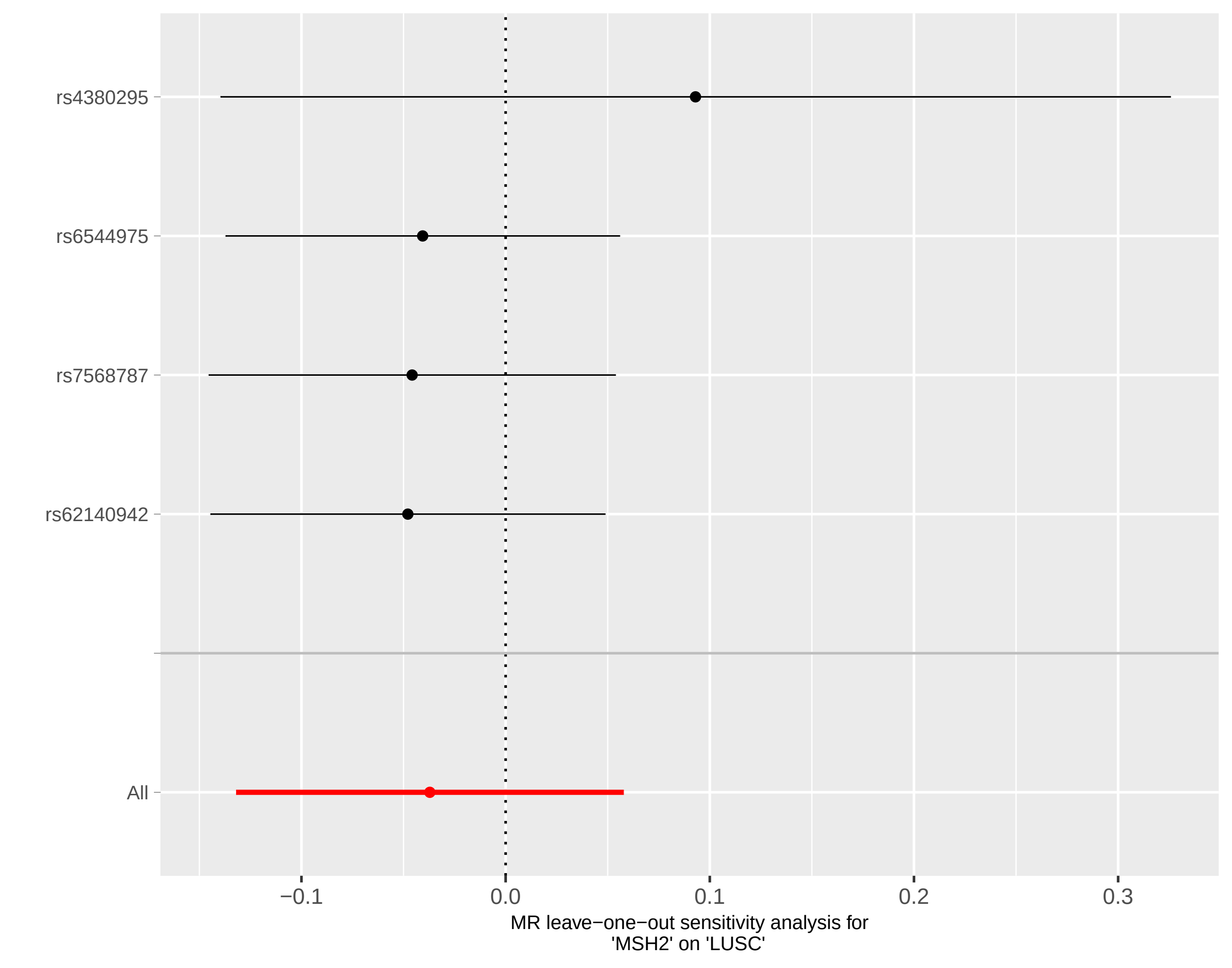
rs4380295
rs6544975
rs7568787
rs62140942
All
−0.1
0.0
0.1
0.2
0.3
MR leave−one−out sensitivity analysis for
'MSH2' on 'LUSC'

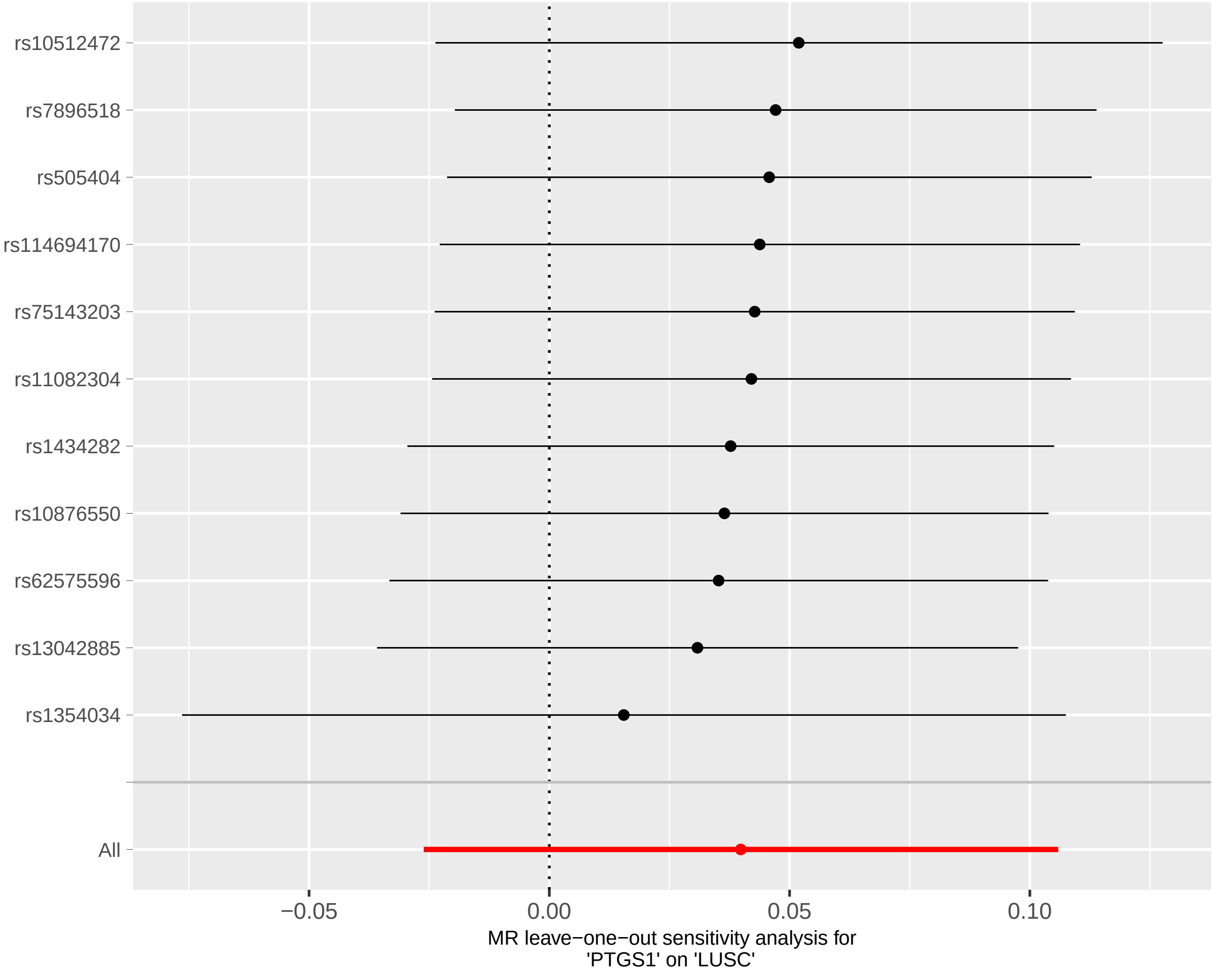

rs10512472
rs7896518
rs505404
rs114694170
rs75143203
rs11082304
rs1434282
rs10876550
rs62575596
rs13042885
rs1354034
All
−0.05
0.00
0.05
0.10
MR leave−one−out sensitivity analysis for
'PTGS1' on 'LUSC'

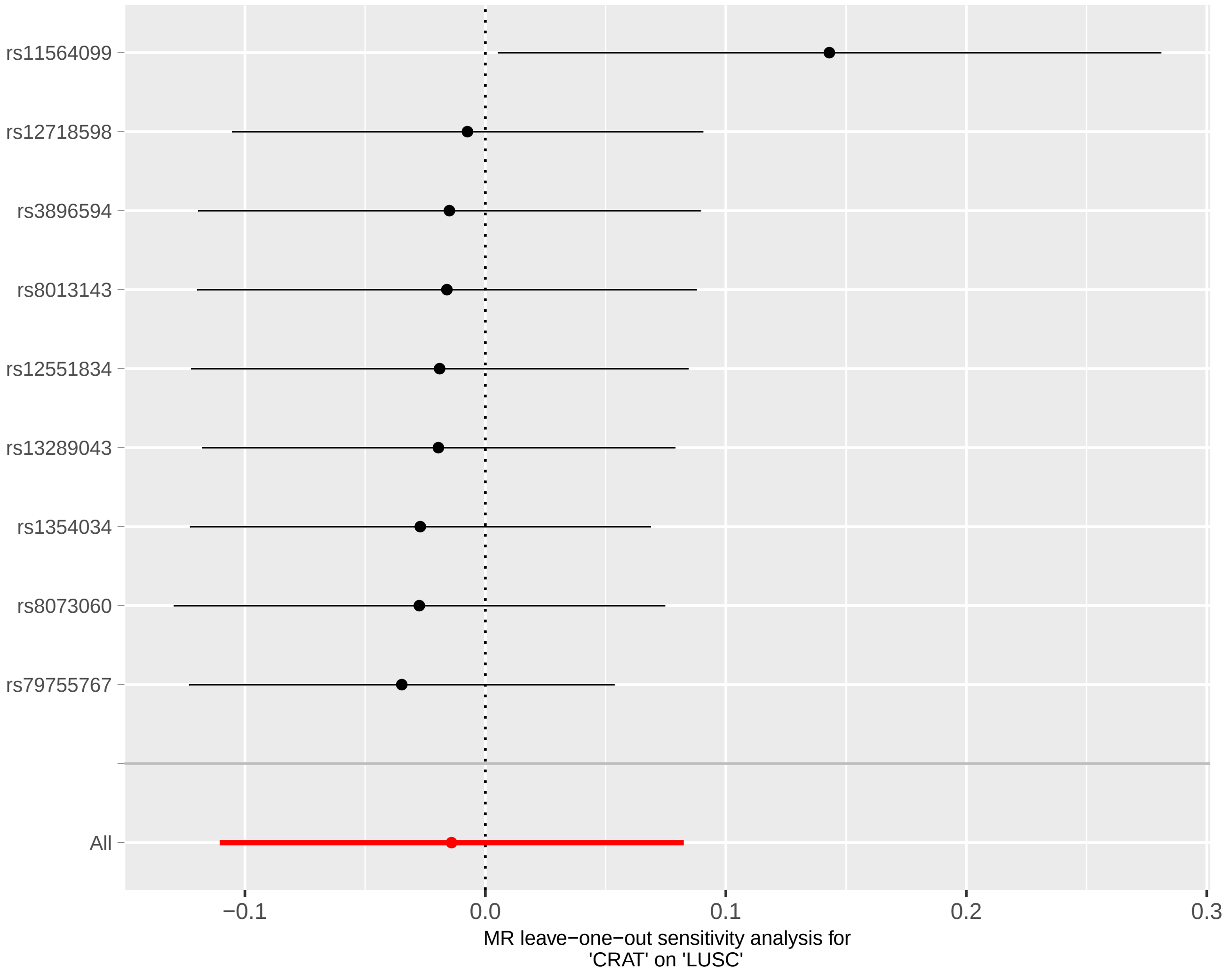
rs11564099
rs12718598
rs3896594
rs8013143
rs12551834
rs13289043
rs1354034
rs8073060
rs79755767
All
−0.1
0.0
0.1
0.2
0.3
MR leave−one−out sensitivity analysis for
'CRAT' on 'LUSC'

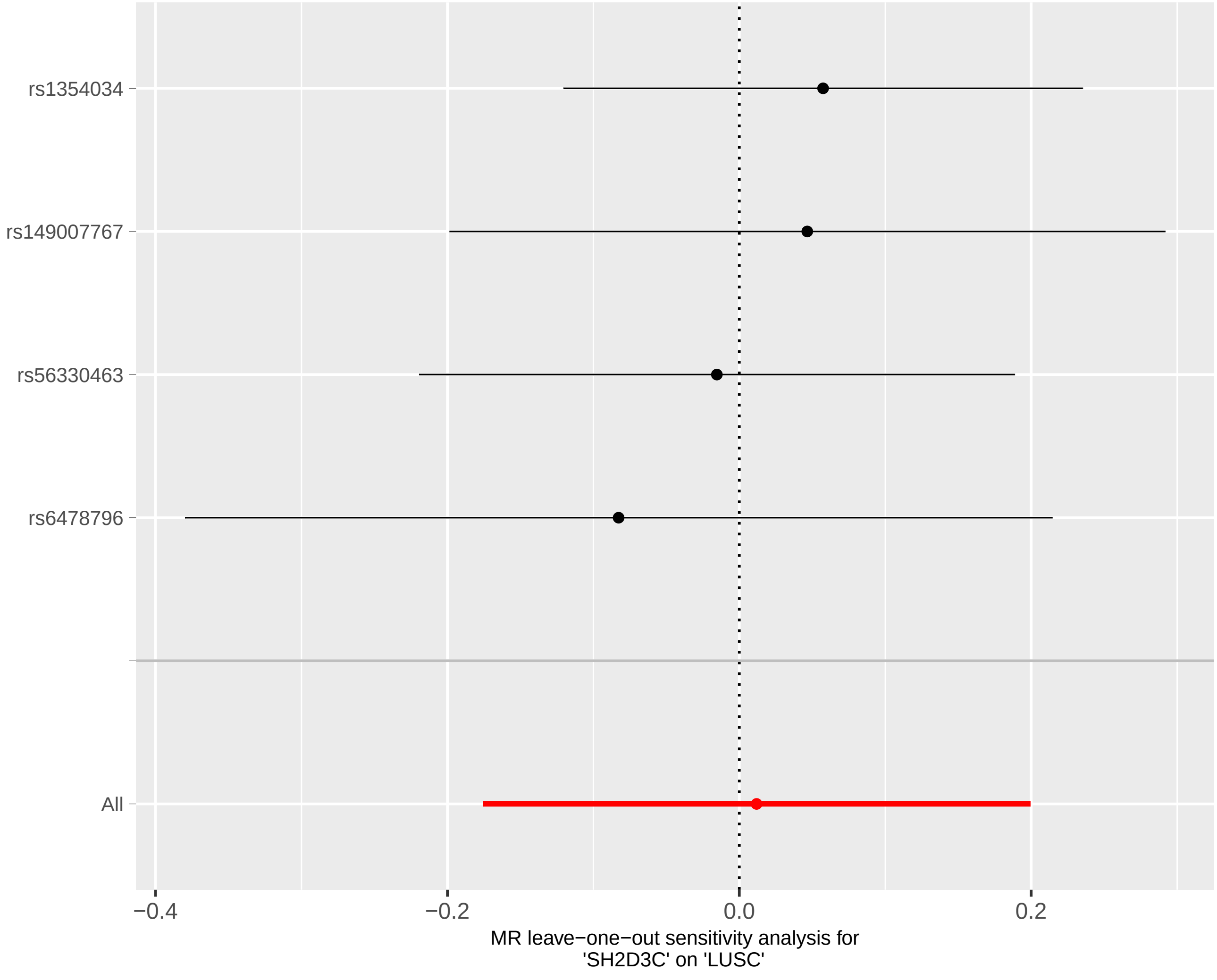

rs1354034
rs149007767
rs56330463
rs6478796
All
−0.4
−0.2
0.0
0.2
MR leave−one−out sensitivity analysis for
'SH2D3C' on 'LUSC'

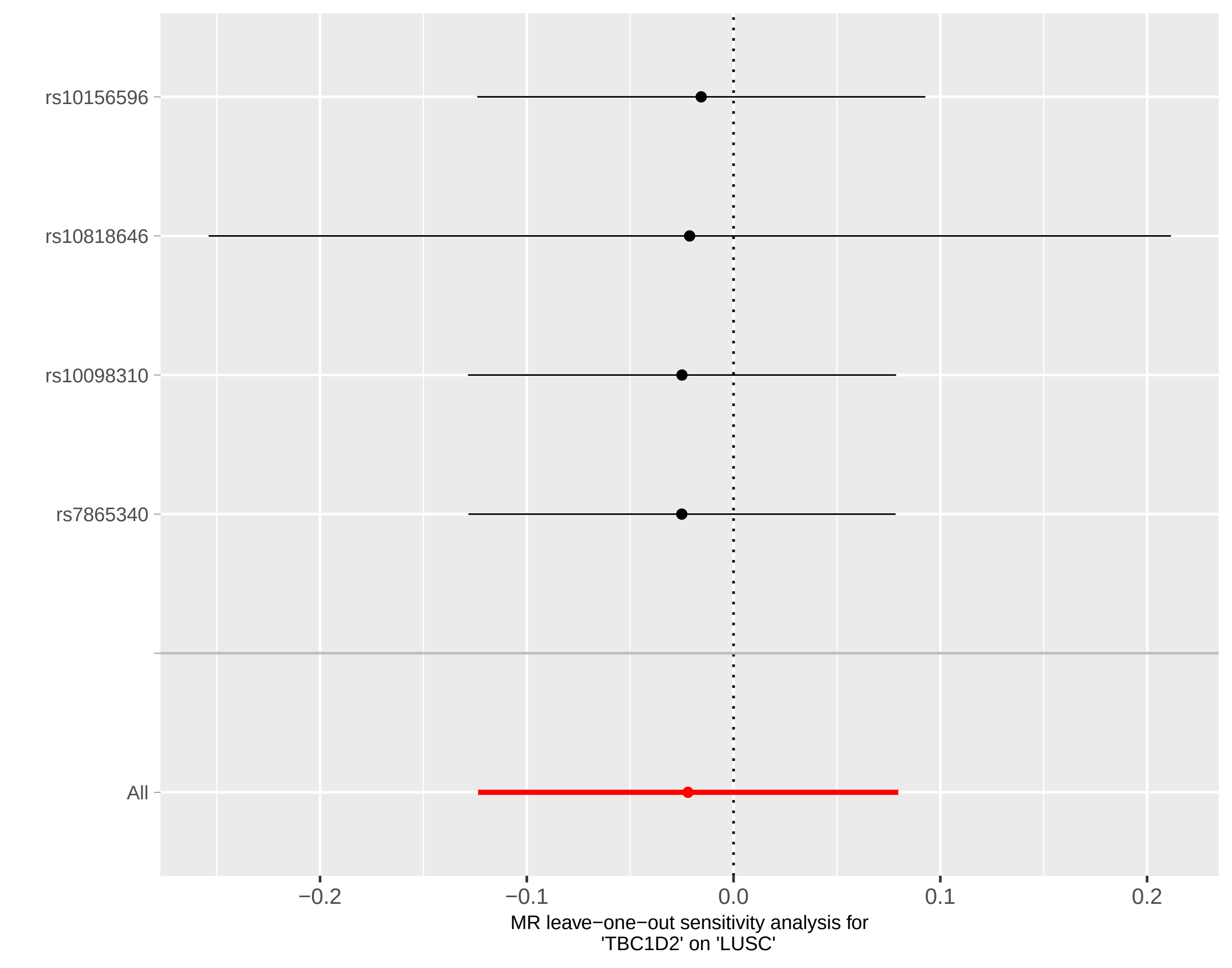
rs10156596
rs10818646
rs10098310
rs7865340
All
−0.2
−0.1
0.0
0.1
0.2
MR leave−one−out sensitivity analysis for
'TBC1D2' on 'LUSC'

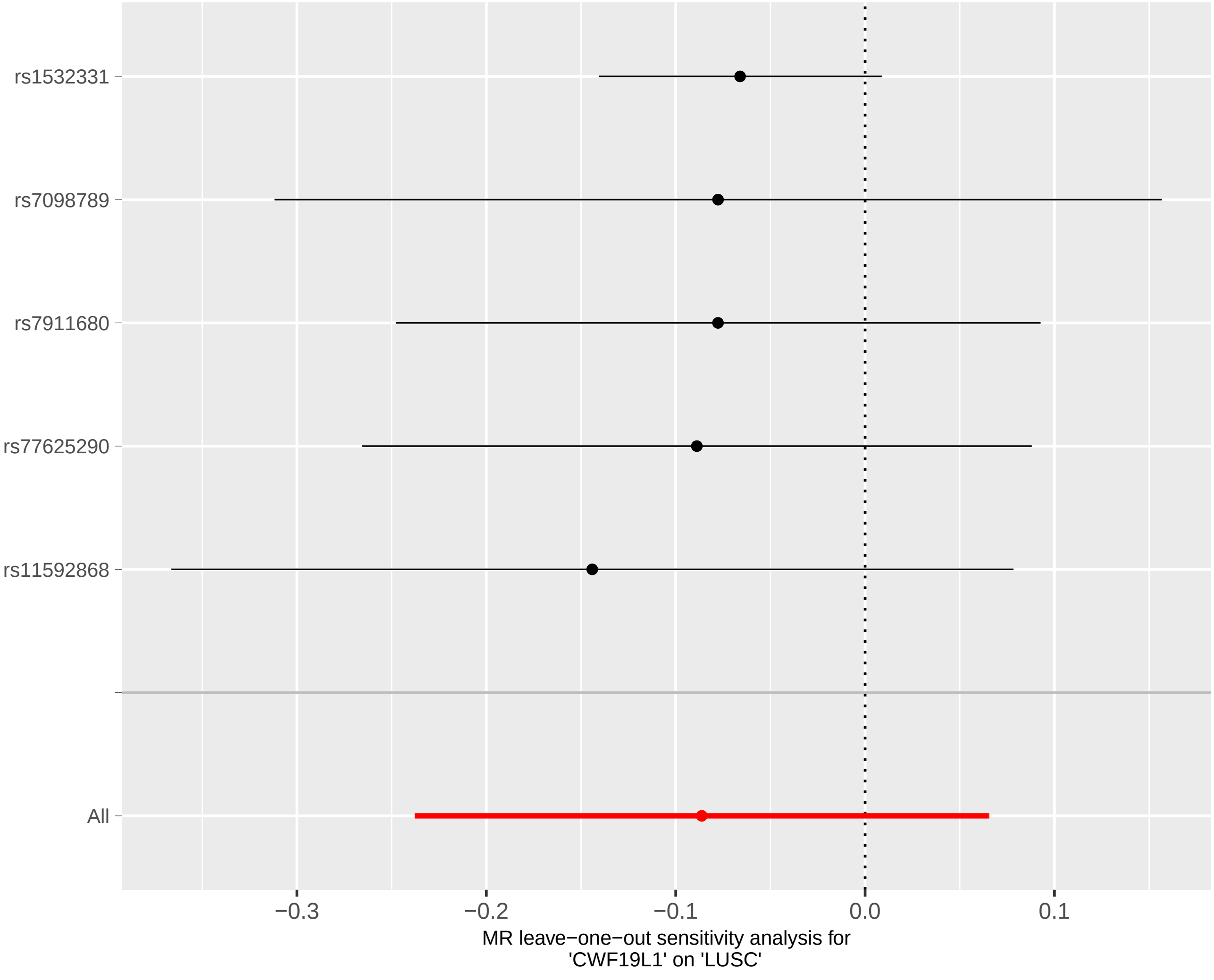
rs1532331
rs7098789
rs7911680
rs77625290
rs11592868
All
−0.3
−0.2
−0.1
0.0
0.1
MR leave−one−out sensitivity analysis for
'CWF19L1' on 'LUSC'

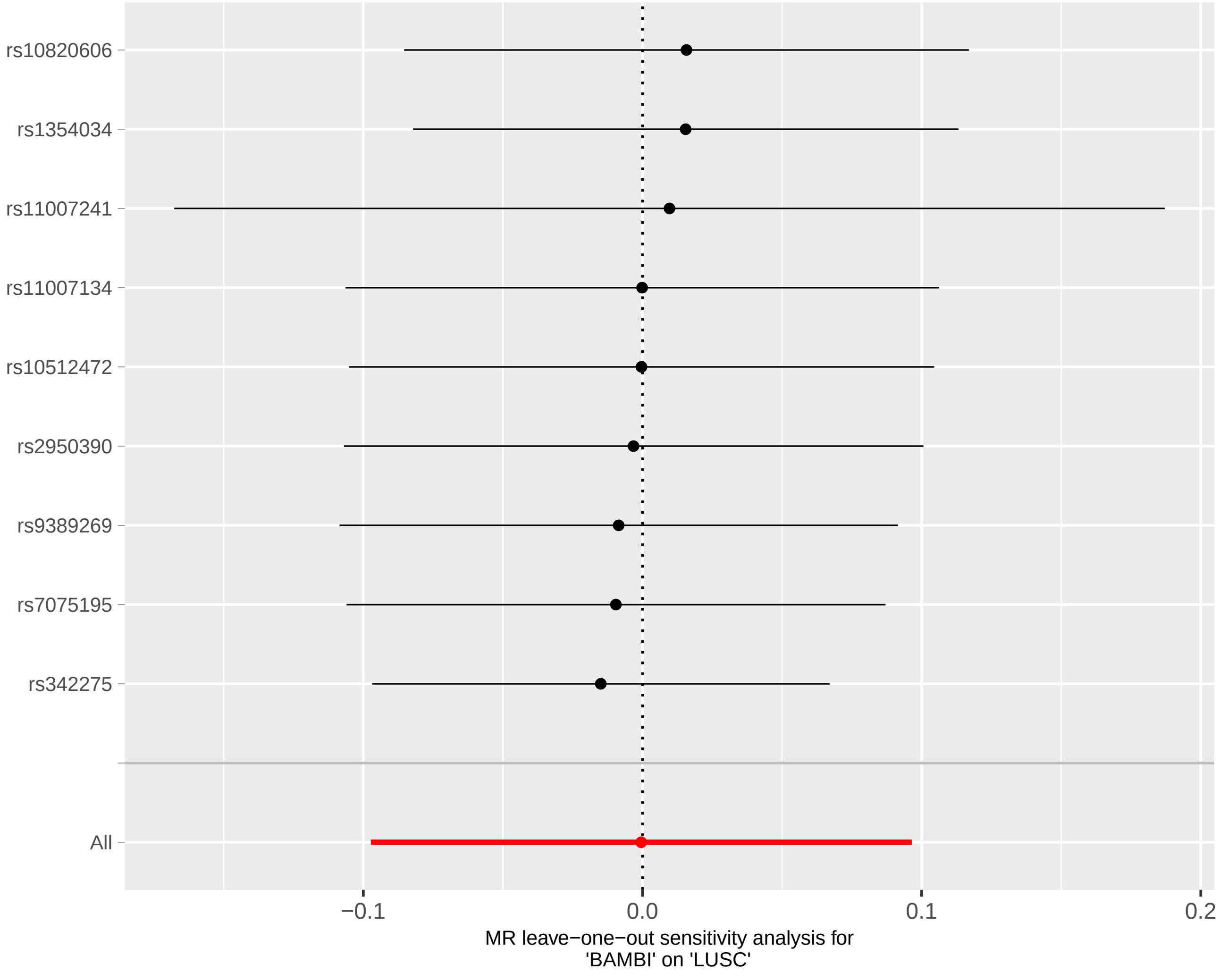
rs10820606
rs1354034
rs11007241
rs11007134
rs10512472
rs2950390
rs9389269
rs7075195
rs342275
All
−0.1
0.0
0.1
0.2
MR leave−one−out sensitivity analysis for
'BAMBI' on 'LUSC'

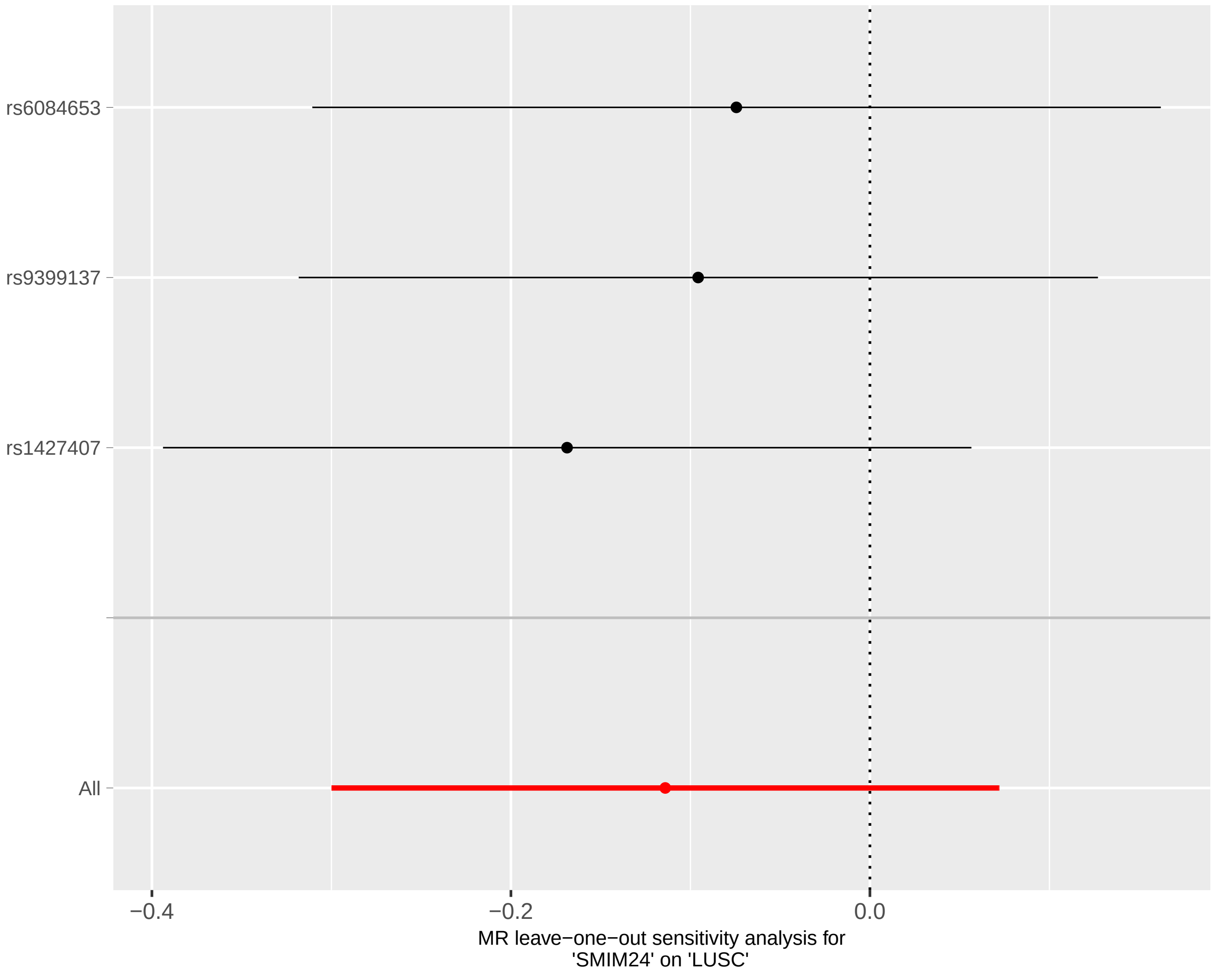

rs6084653
rs9399137
rs1427407
All
−0.4
−0.2
0.0
MR leave−one−out sensitivity analysis for
'SMIM24' on 'LUSC'

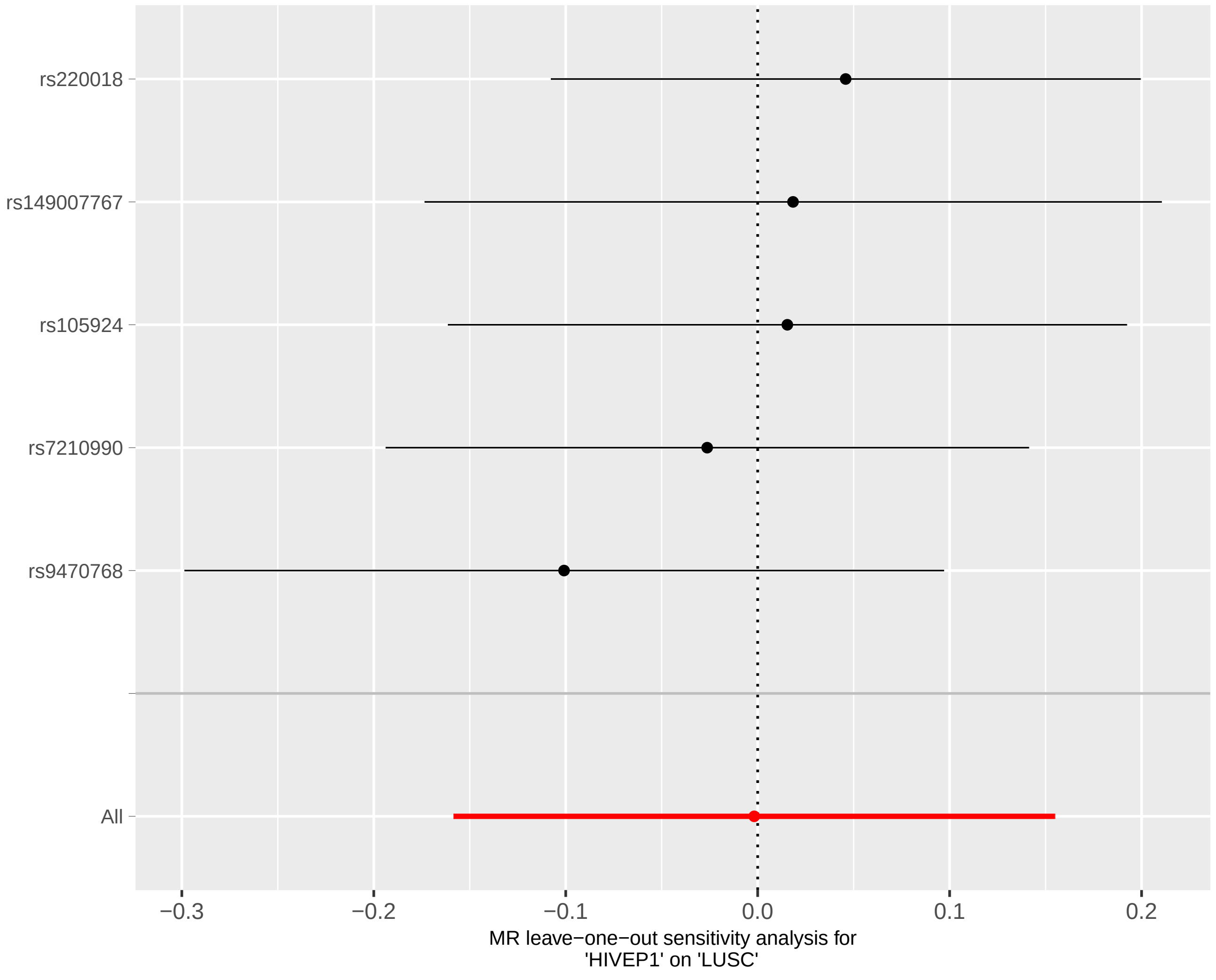
rs220018
rs149007767
rs105924
rs7210990
rs9470768
All
−0.3
−0.2
−0.1
0.0
0.1
0.2
MR leave−one−out sensitivity analysis for
'HIVEP1' on 'LUSC'

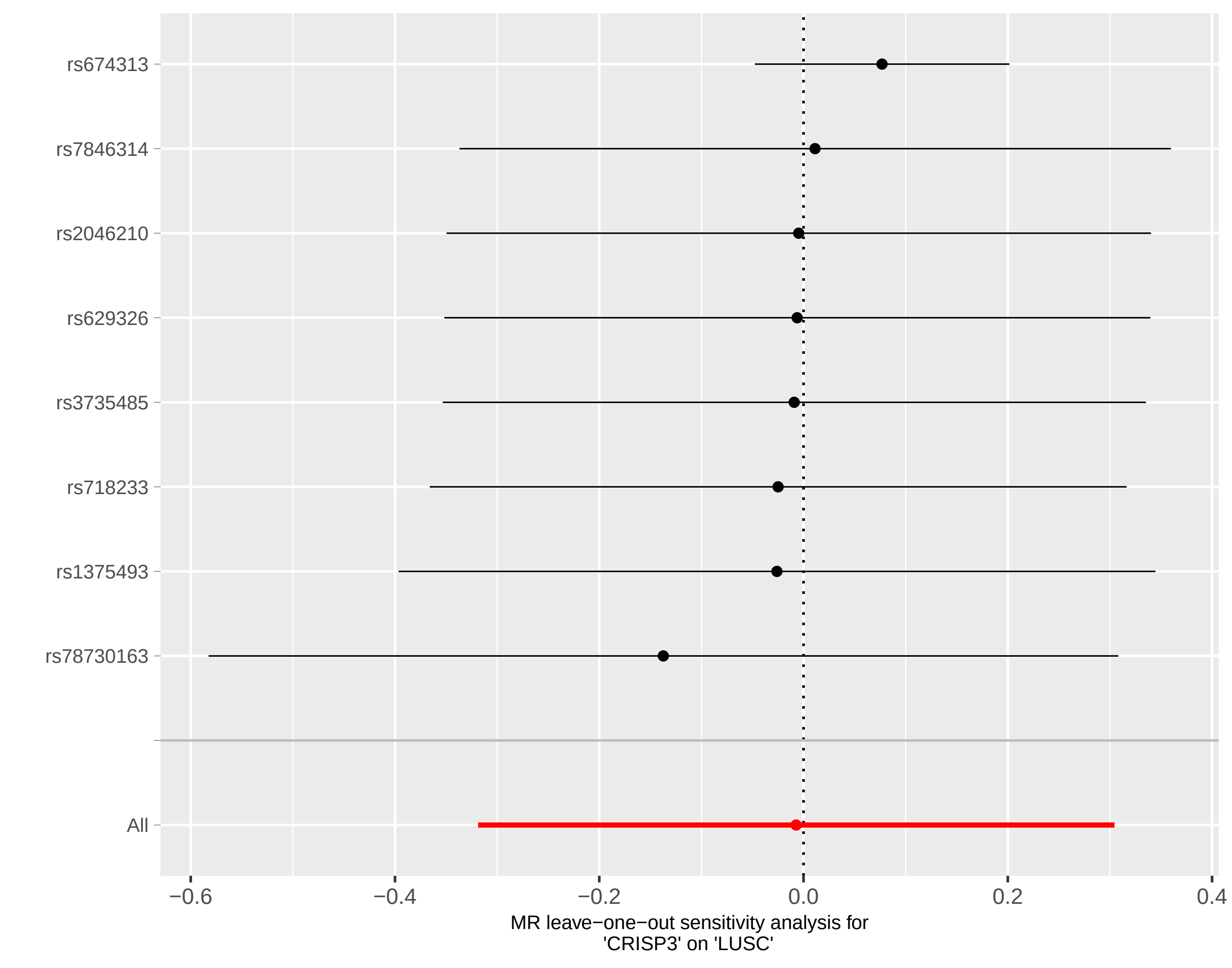
rs674313
rs7846314
rs2046210
rs629326
rs3735485
rs718233
rs1375493
rs78730163
All
−0.6
−0.4
−0.2
0.0
0.2
0.4
MR leave−one−out sensitivity analysis for
'CRISP3' on 'LUSC'

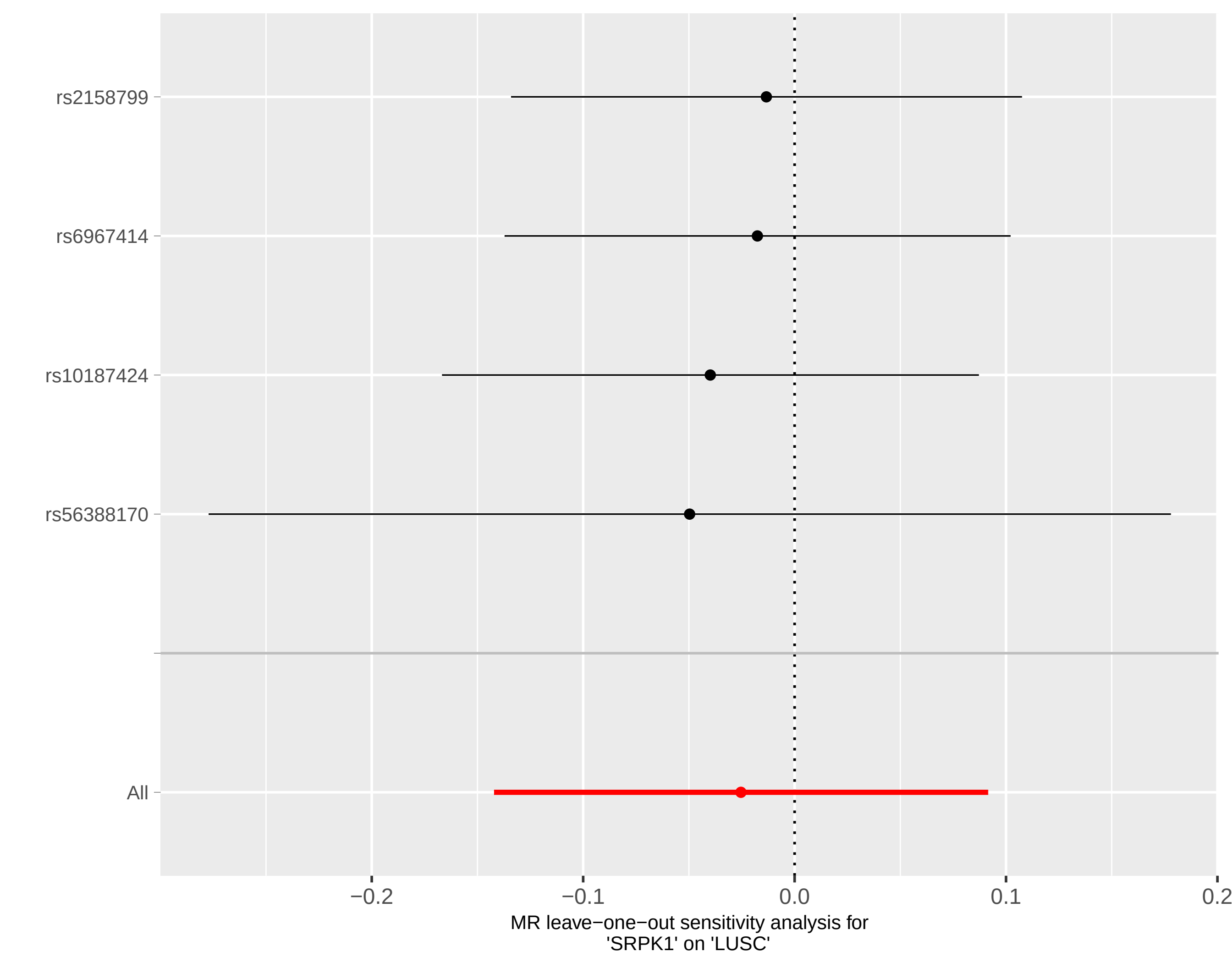
rs2158799
rs6967414
rs10187424
rs56388170
All
−0.2
−0.1
0.0
0.1
0.2
MR leave−one−out sensitivity analysis for
'SRPK1' on 'LUSC'

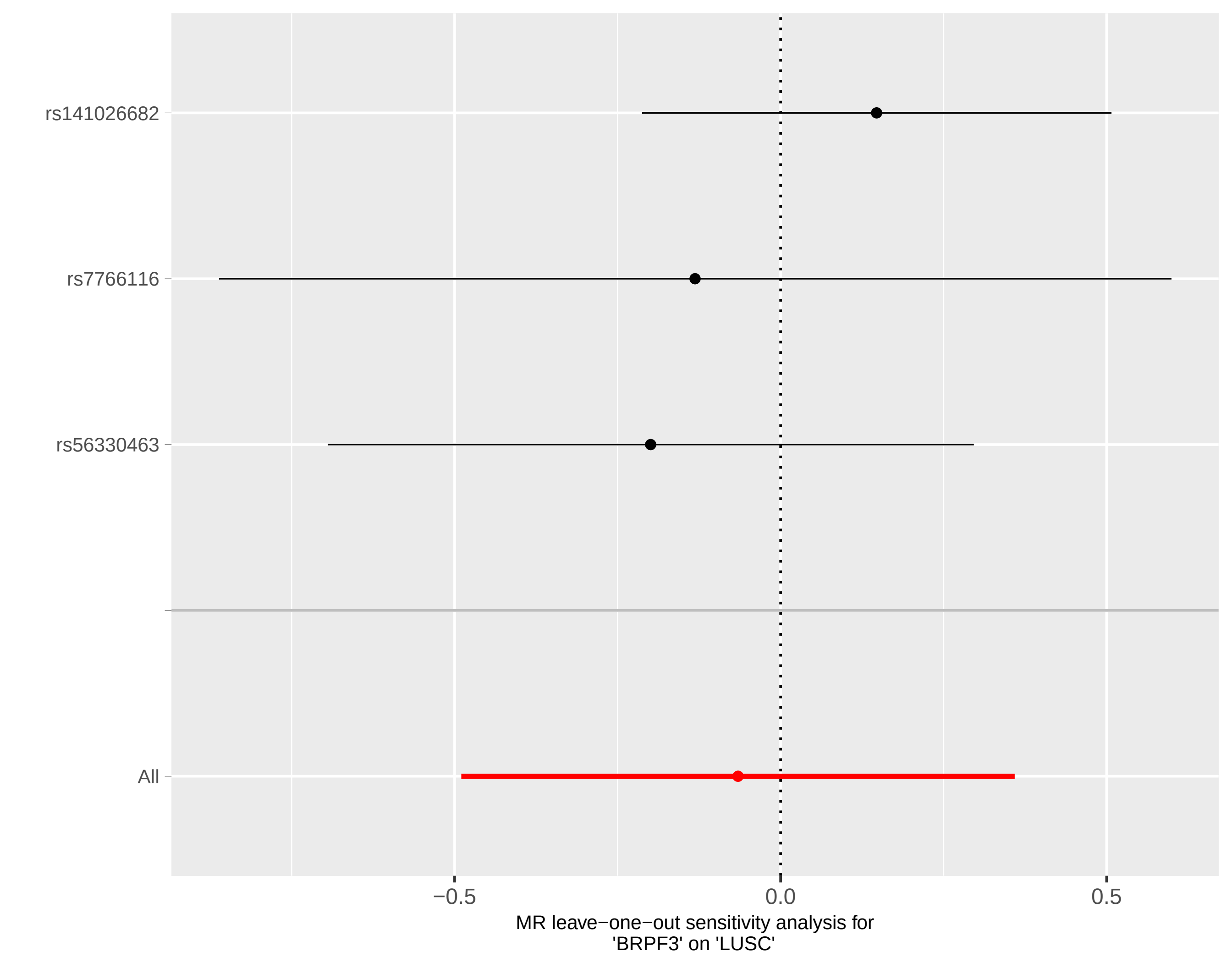
rs141026682
rs7766116
rs56330463
All
−0.5
0.0
0.5
MR leave−one−out sensitivity analysis for
'BRPF3' on 'LUSC'

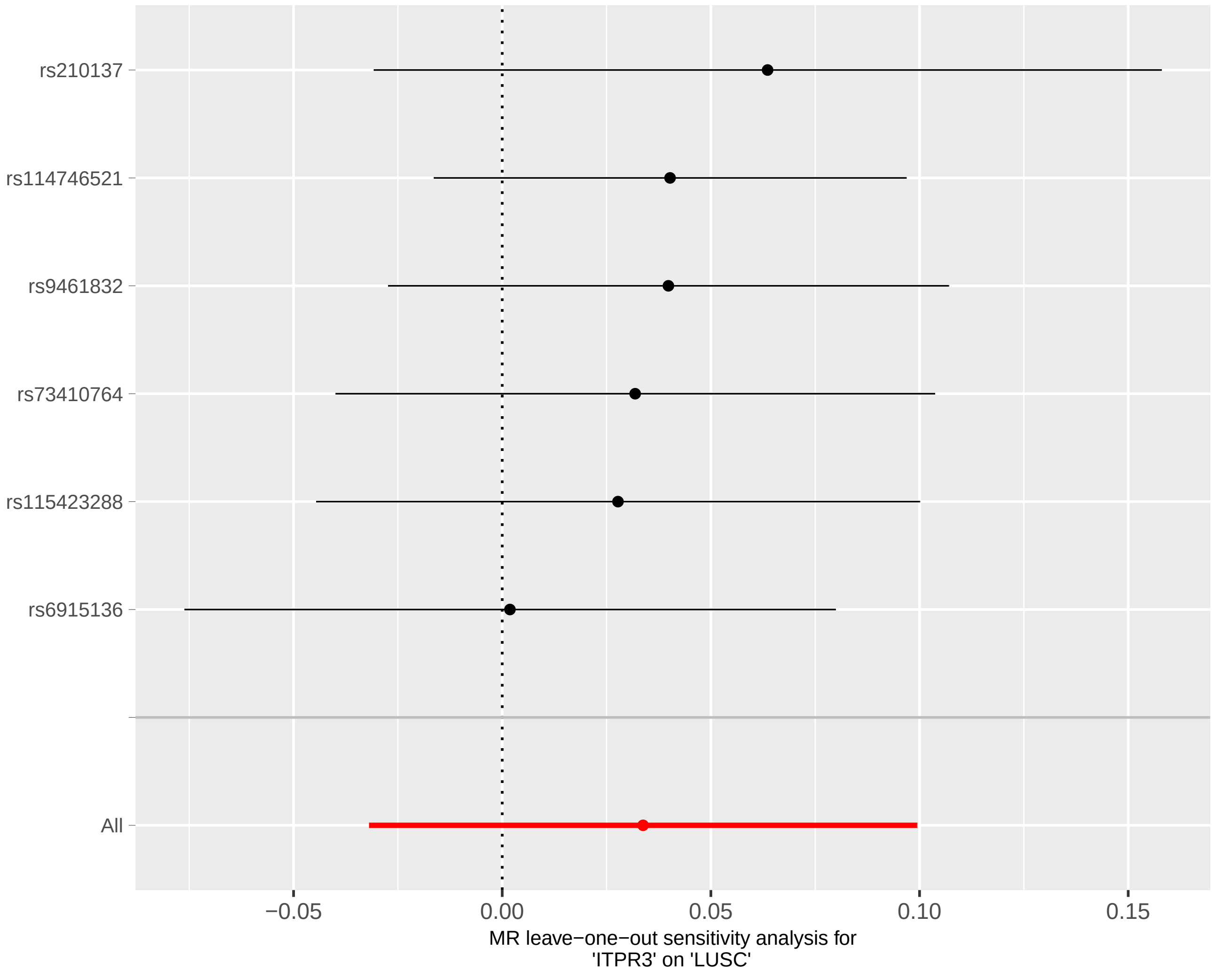
rs210137
rs114746521
rs9461832
rs73410764
rs115423288
rs6915136
All
−0.05
0.00
0.05
0.10
0.15
MR leave−one−out sensitivity analysis for
'ITPR3' on 'LUSC'

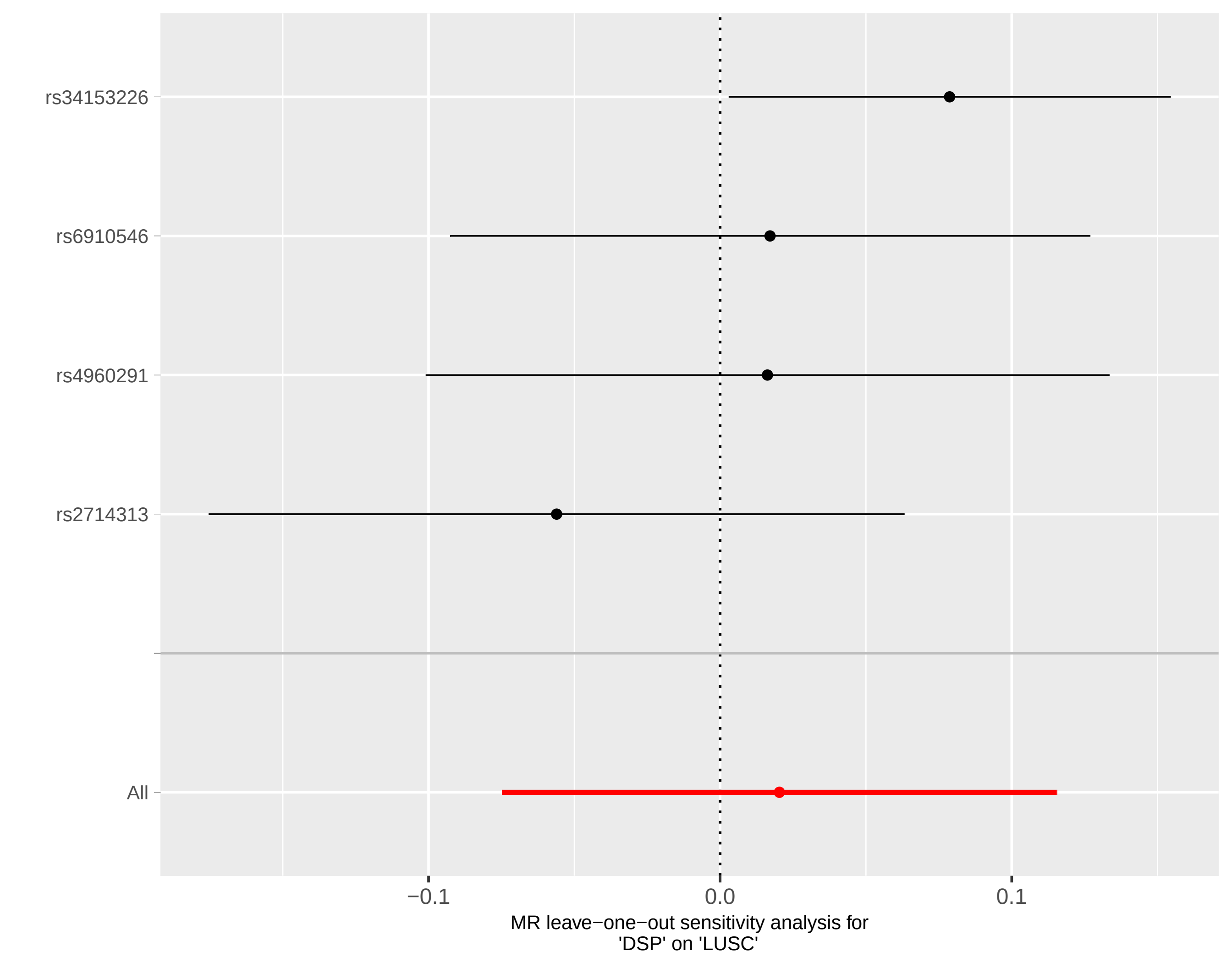

rs34153226
rs6910546
rs4960291
rs2714313
All
−0.1
0.0
0.1
MR leave−one−out sensitivity analysis for
'DSP' on 'LUSC'

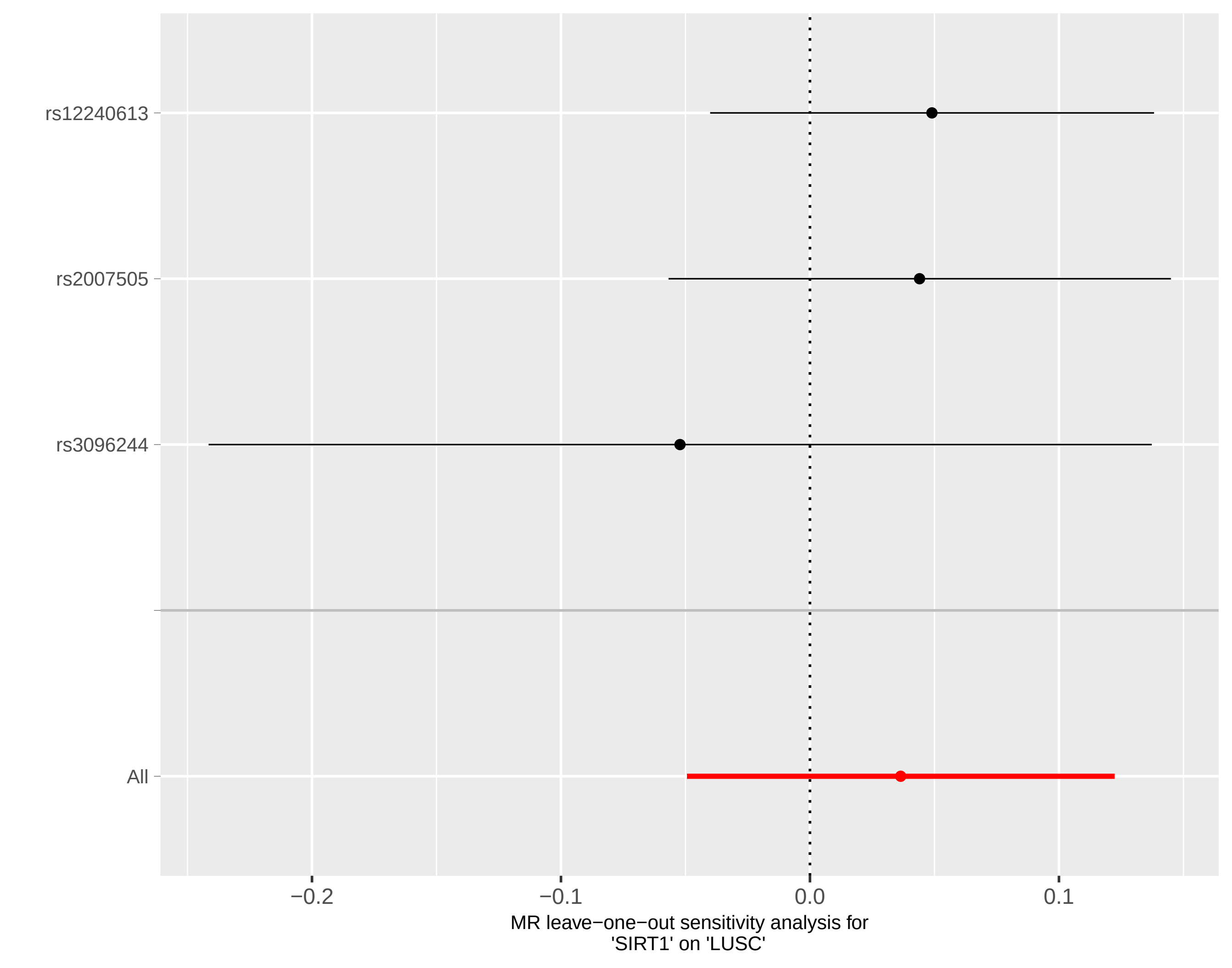

rs12240613
rs2007505
rs3096244
All
−0.2
−0.1
0.0
0.1
MR leave−one−out sensitivity analysis for
'SIRT1' on 'LUSC'

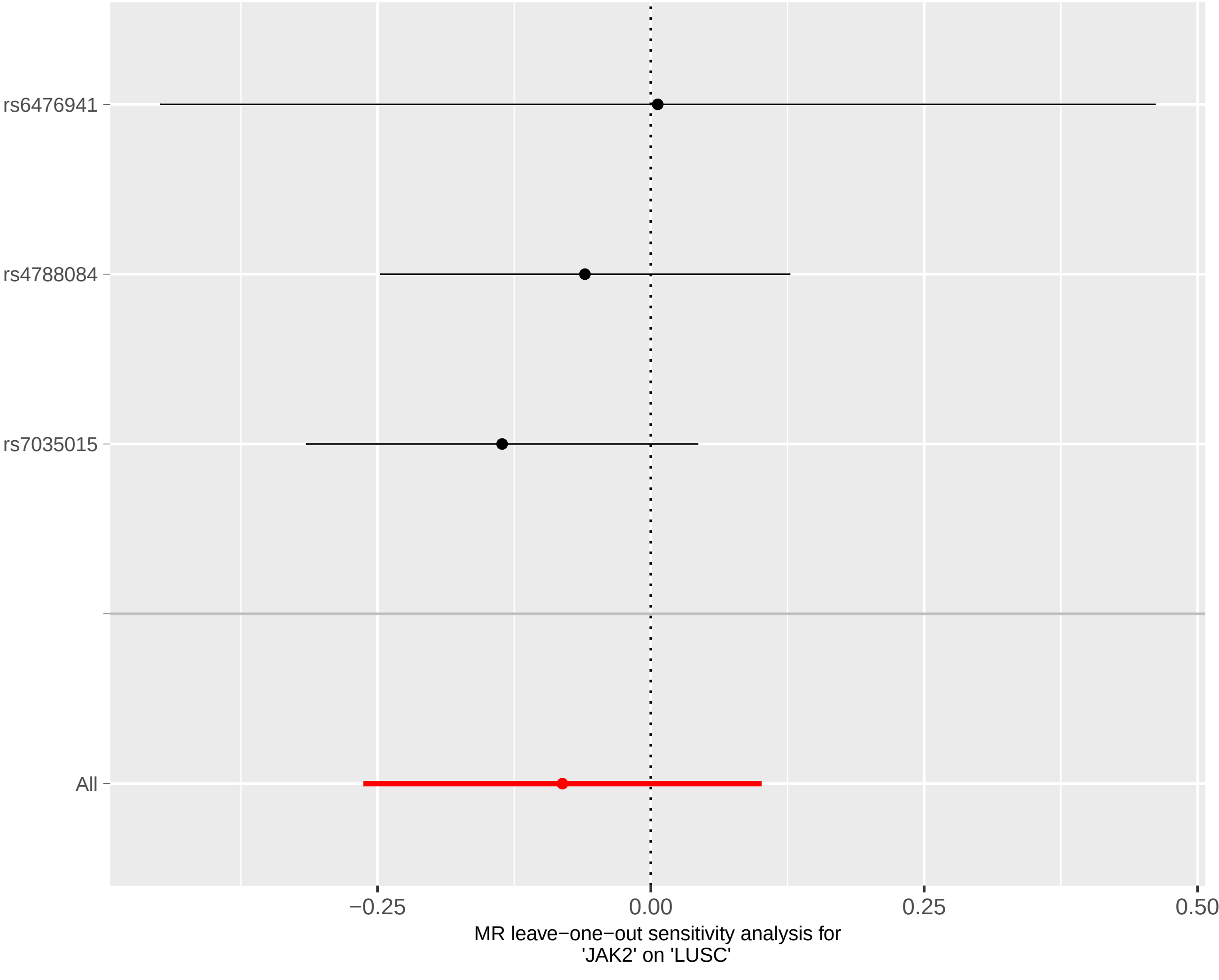
rs6476941
rs4788084
rs7035015
All
−0.25
0.00
0.25
0.50
MR leave−one−out sensitivity analysis for
'JAK2' on 'LUSC'

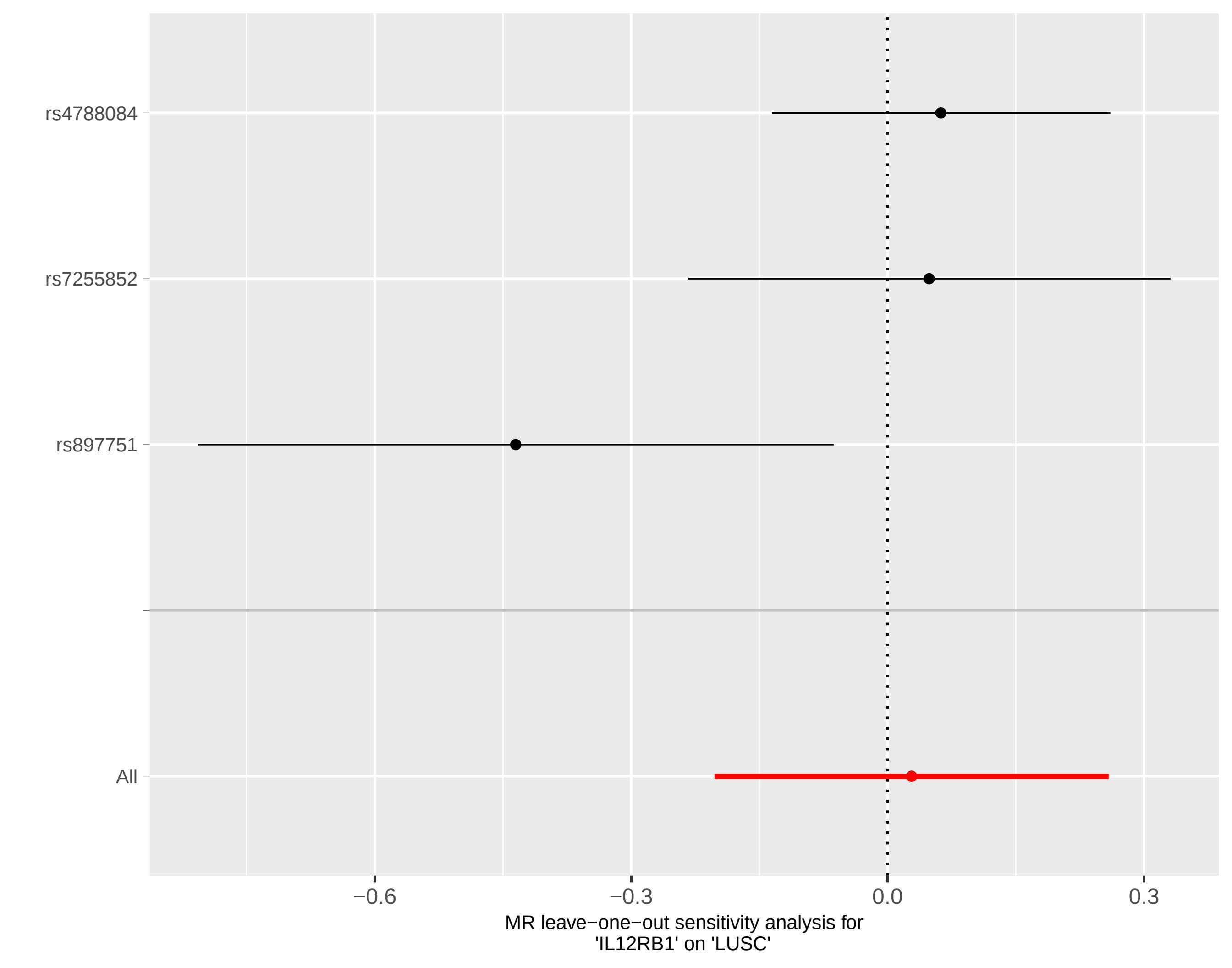
rs4788084
rs7255852
rs897751
All
−0.6
−0.3
0.0
0.3
MR leave−one−out sensitivity analysis for
'IL12RB1' on 'LUSC'

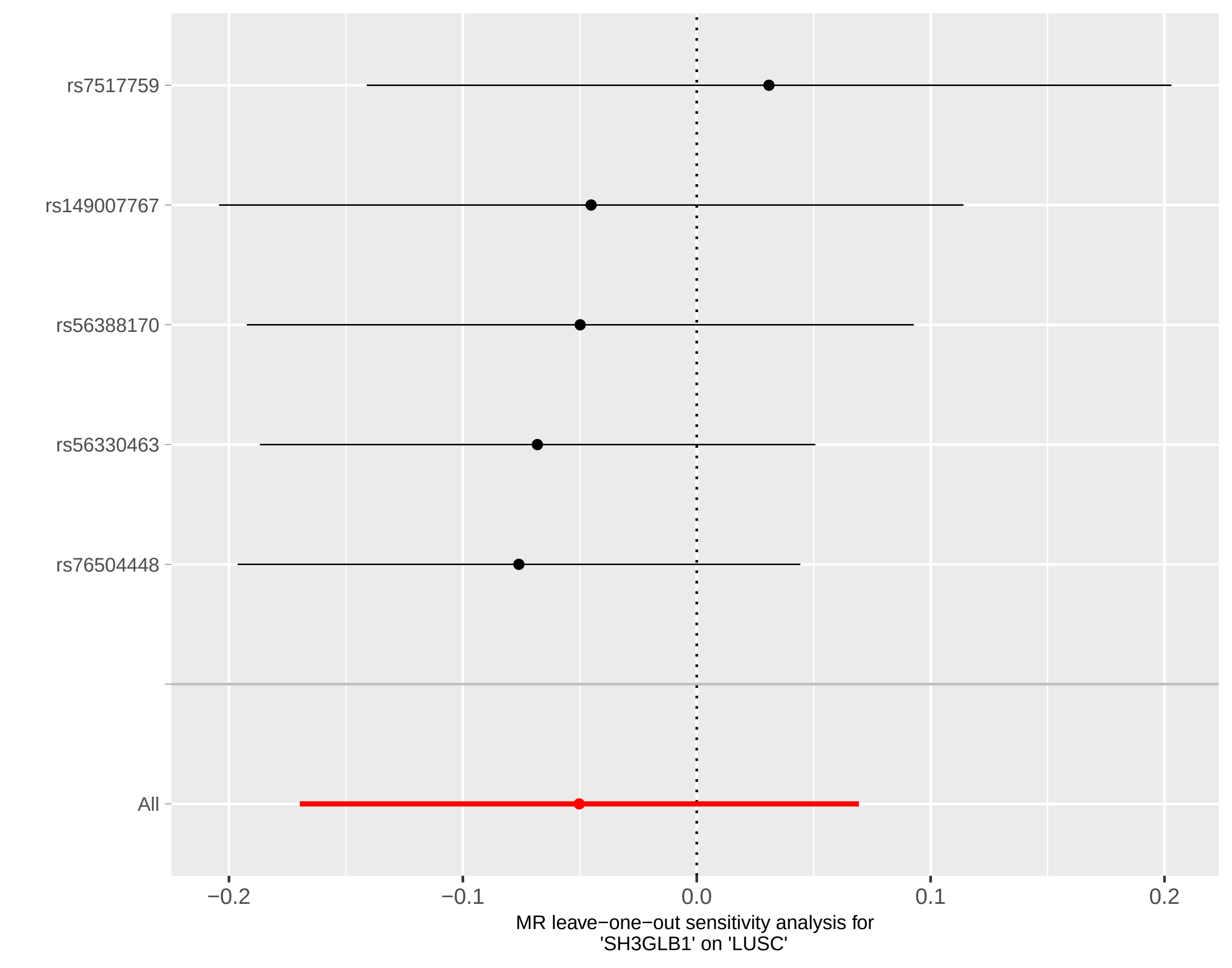
rs7517759
rs149007767
rs56388170
rs56330463
rs76504448
All
−0.2
−0.1
0.0
0.1
0.2
MR leave−one−out sensitivity analysis for
'SH3GLB1' on 'LUSC'

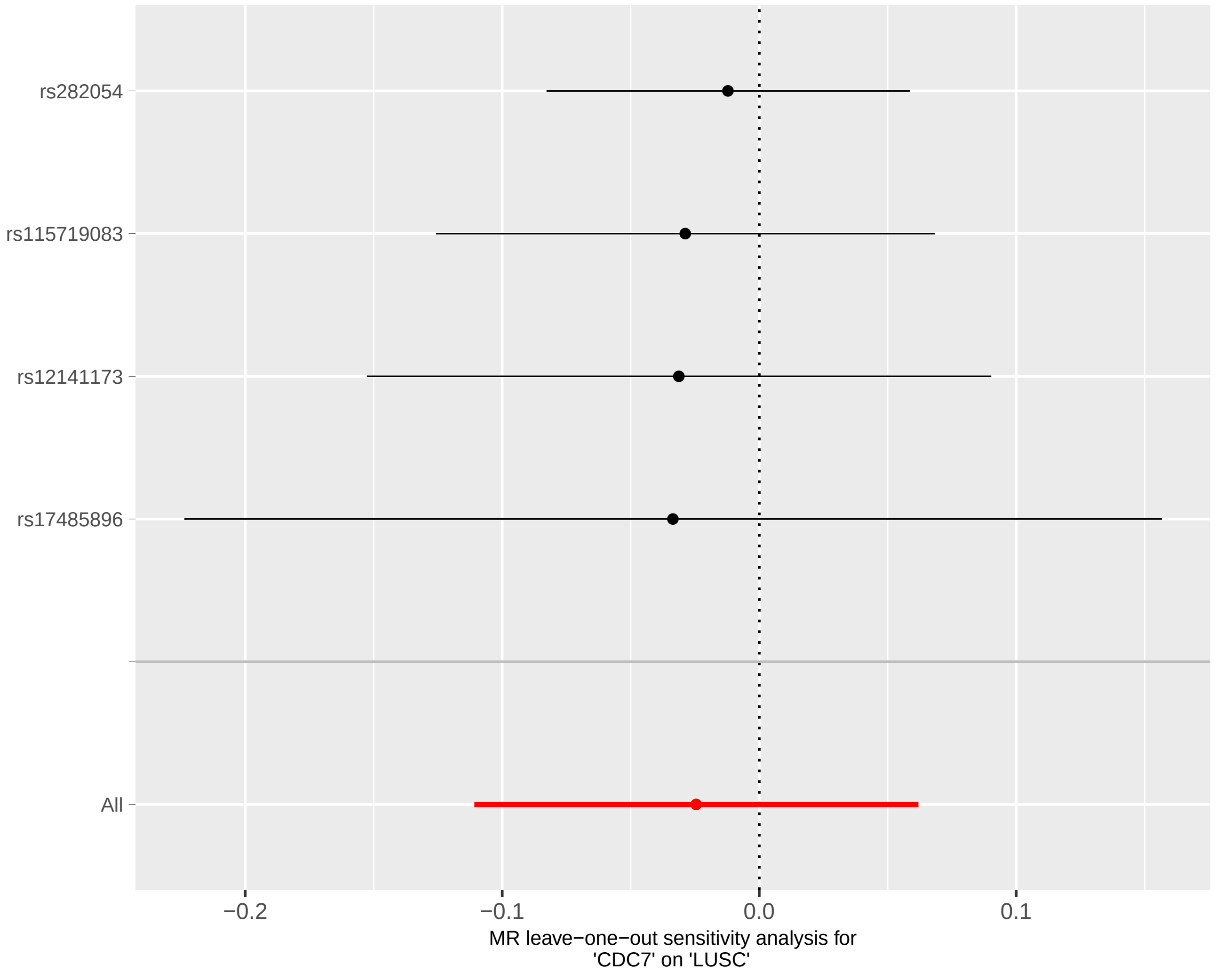

rs282054
rs115719083
rs12141173
rs17485896
All
−0.2
−0.1
0.0
0.1
MR leave−one−out sensitivity analysis for
'CDC7' on 'LUSC'

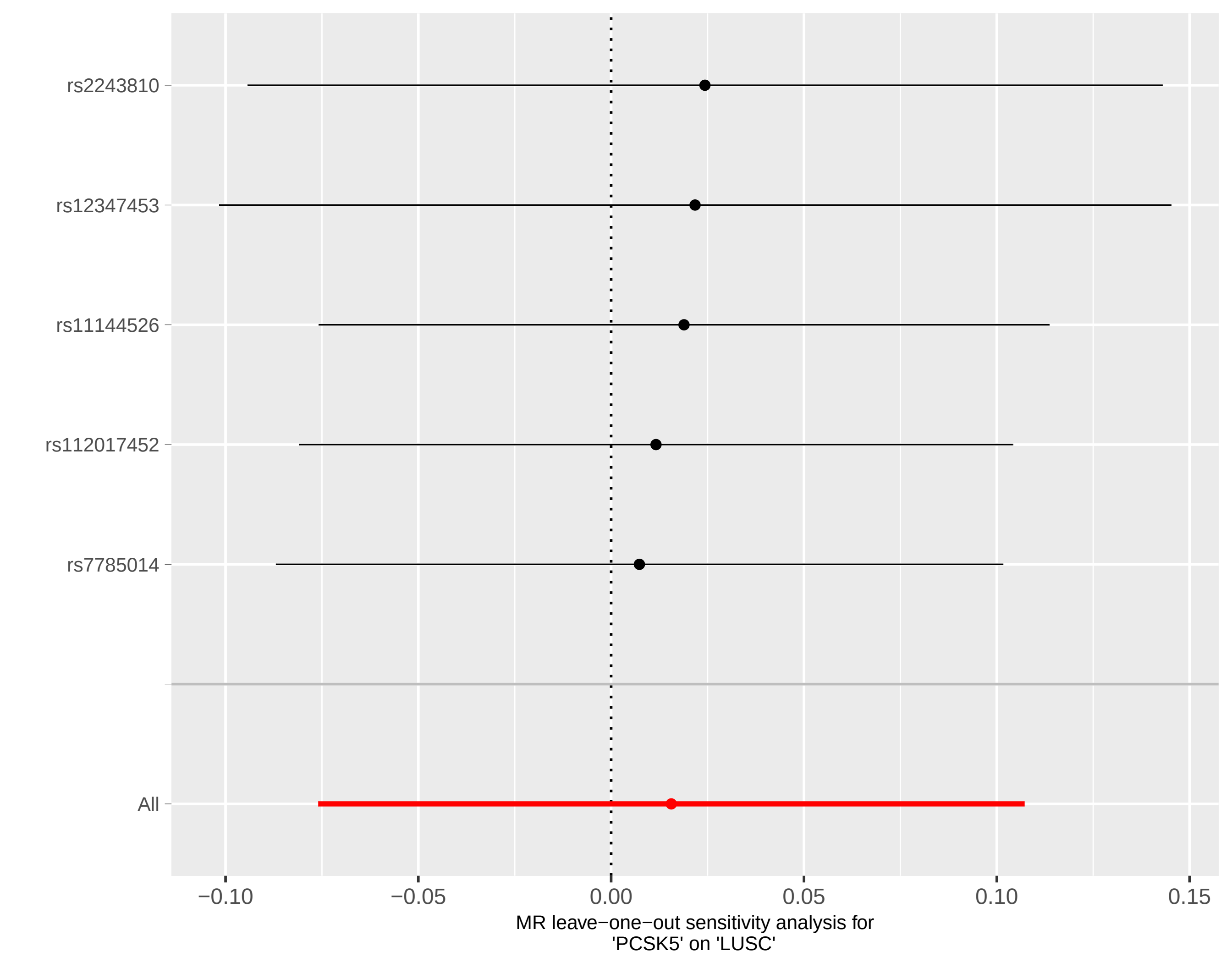
rs2243810
rs12347453
rs11144526
rs112017452
rs7785014
All
−0.10
−0.05
0.00
0.05
0.10
0.15
MR leave−one−out sensitivity analysis for
'PCSK5' on 'LUSC'

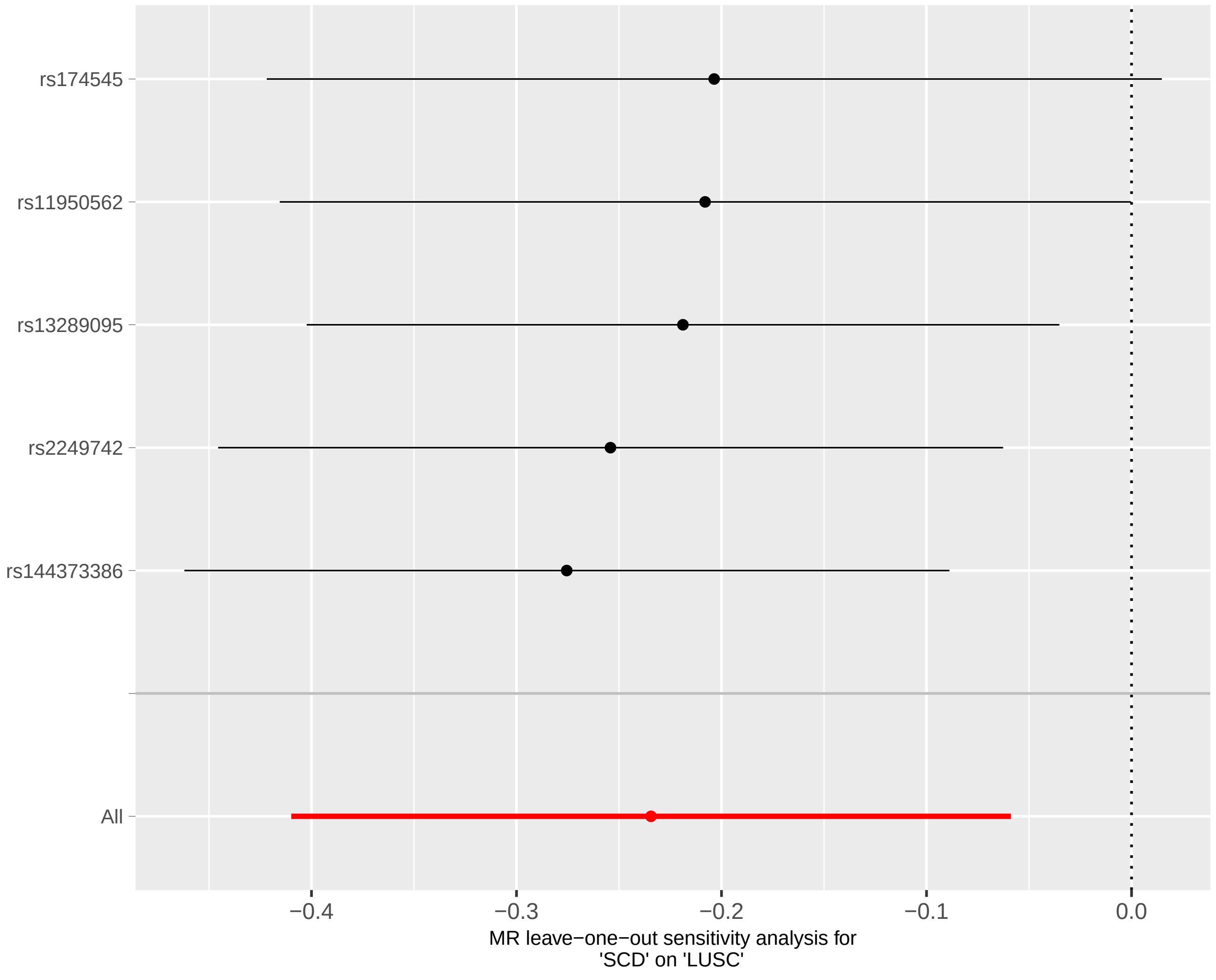

rs174545
rs11950562
rs13289095
rs2249742
rs144373386
All
−0.4
−0.3
−0.2
−0.1
0.0
MR leave−one−out sensitivity analysis for
'SCD' on 'LUSC'

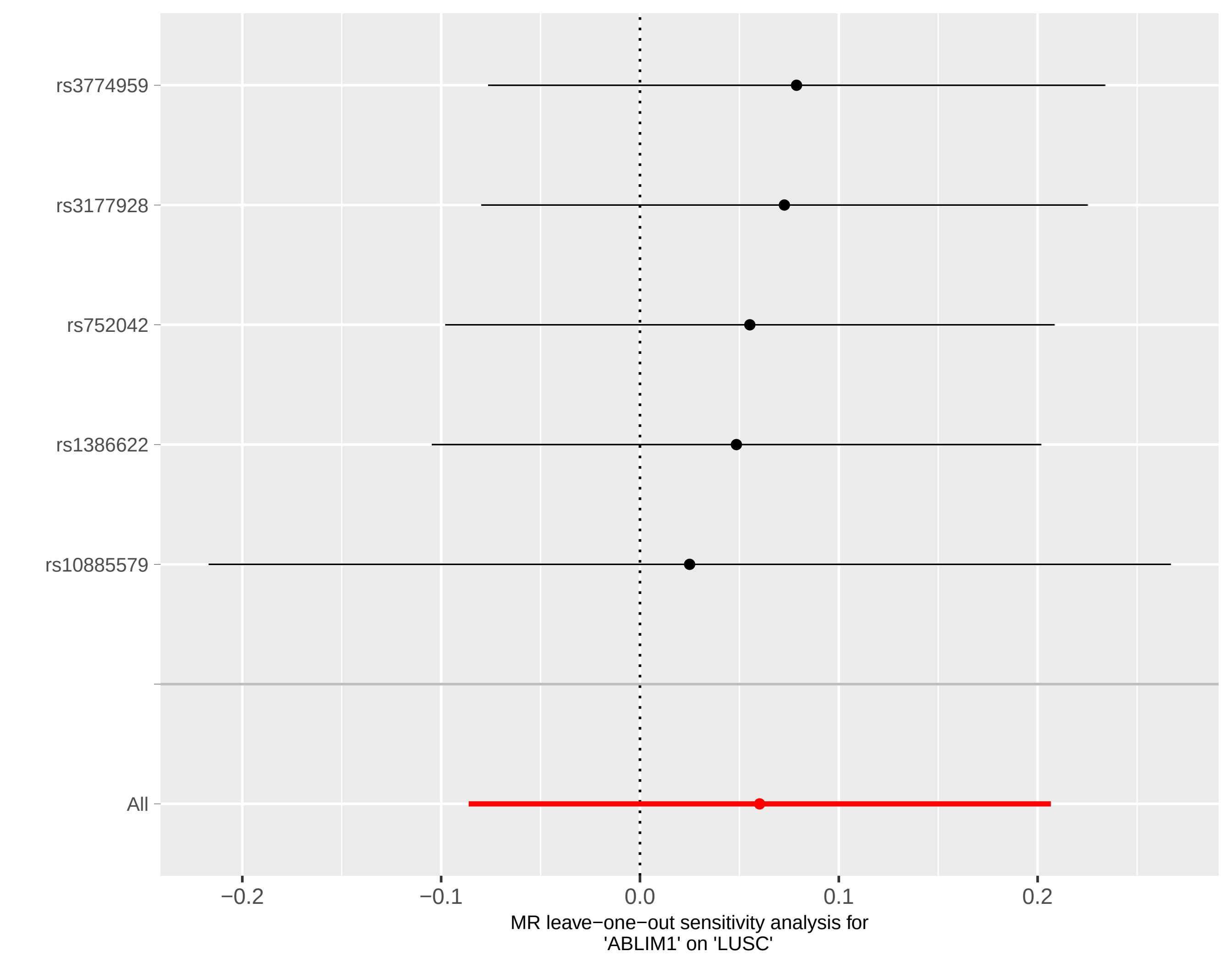
rs3774959
rs3177928
rs752042
rs1386622
rs10885579
All
−0.2
−0.1
0.0
0.1
0.2
MR leave−one−out sensitivity analysis for
'ABLIM1' on 'LUSC'

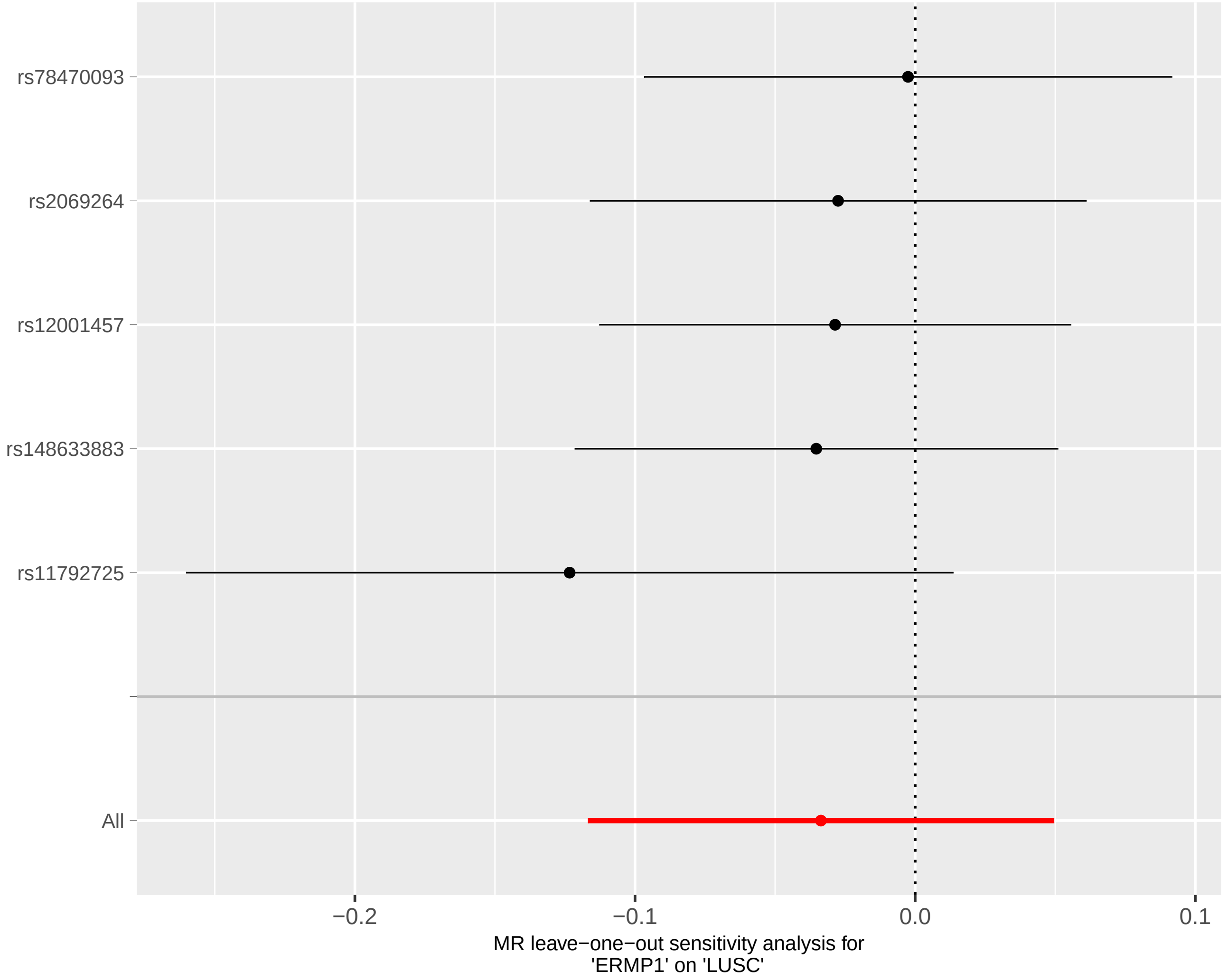
rs78470093
rs2069264
rs12001457
rs148633883
rs11792725
All
−0.2
−0.1
0.0
0.1
MR leave−one−out sensitivity analysis for
'ERMP1' on 'LUSC'

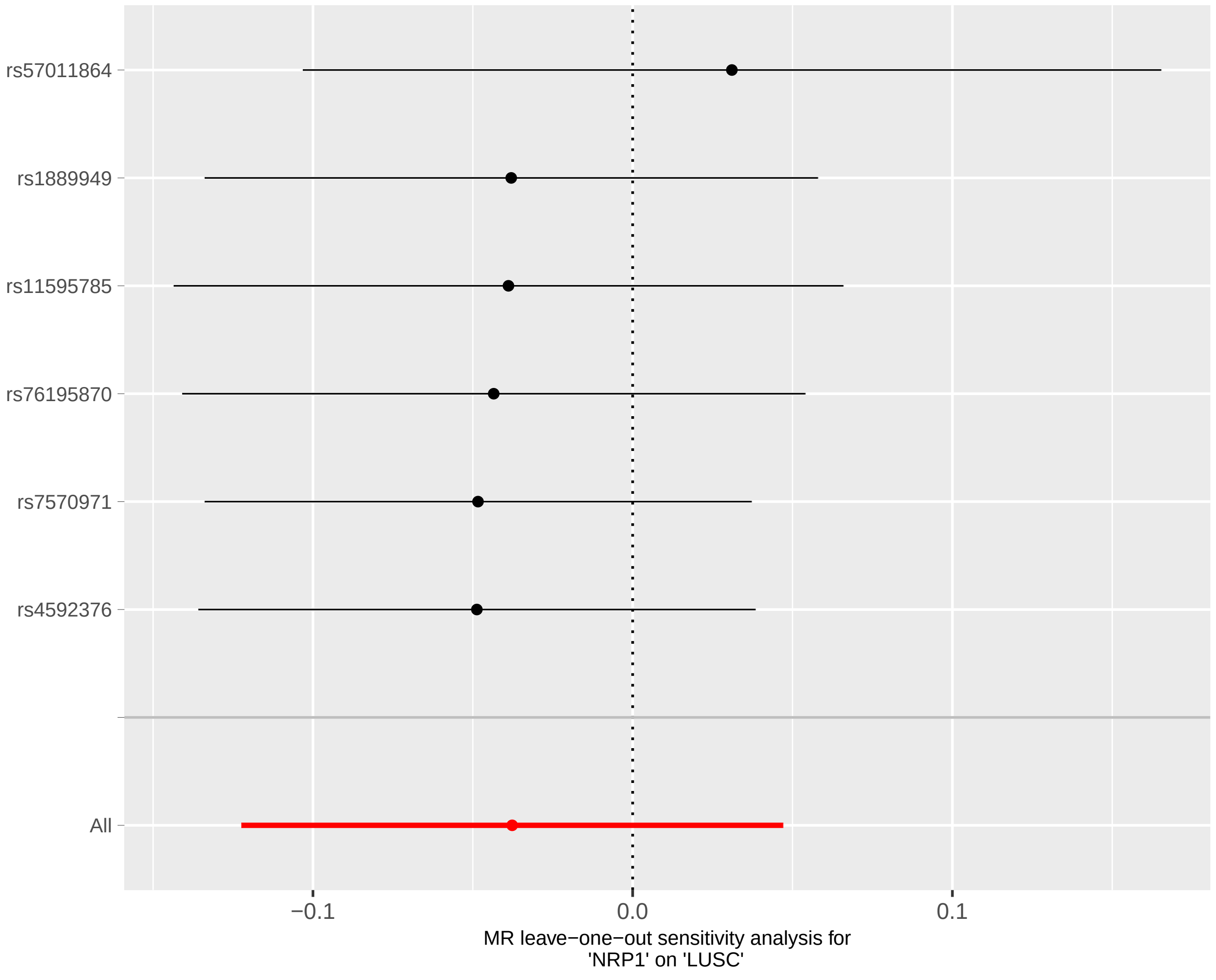
rs57011864
rs1889949
rs11595785
rs76195870
rs7570971
rs4592376
All
−0.1
0.0
0.1
MR leave−one−out sensitivity analysis for
'NRP1' on 'LUSC'

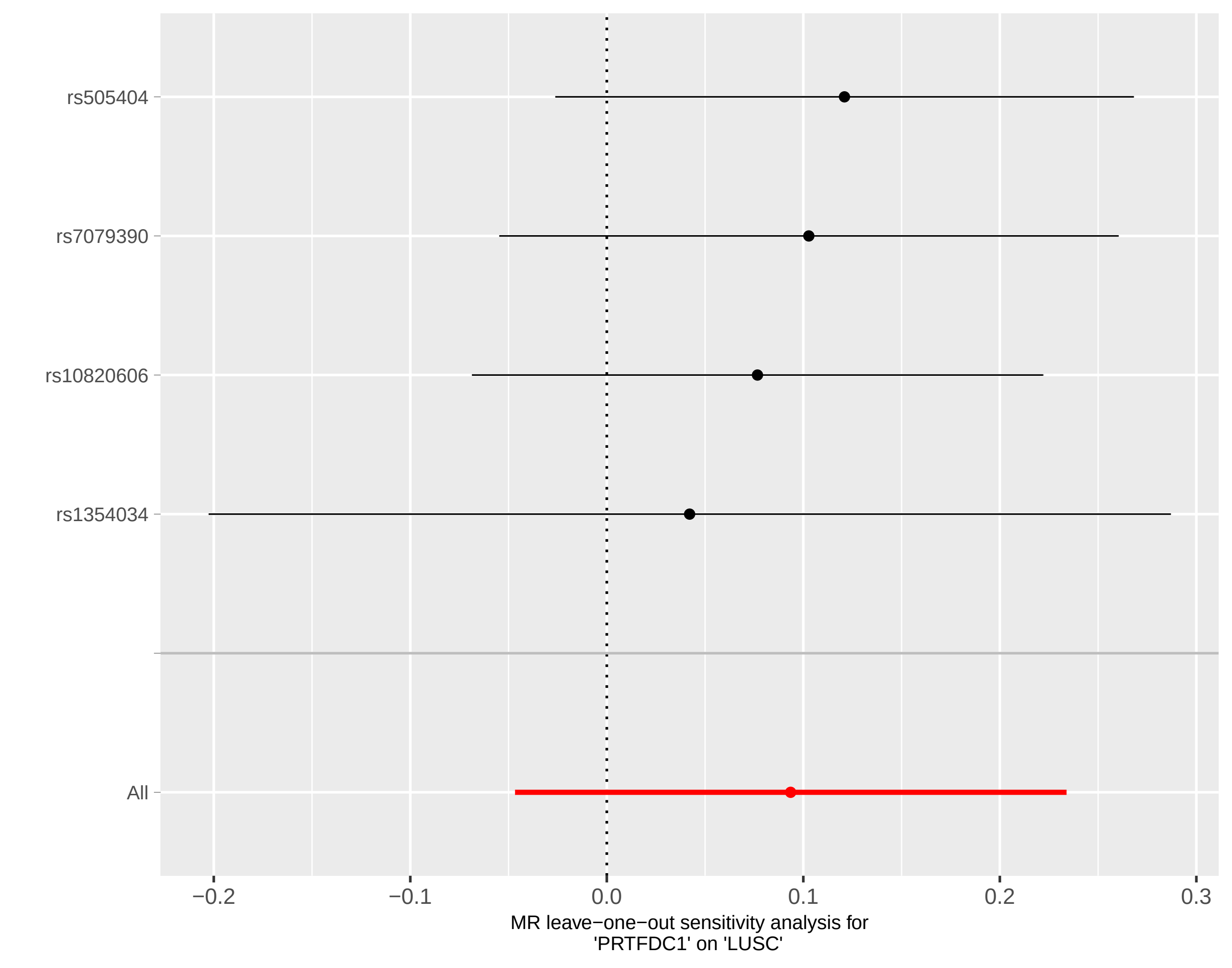

rs505404
rs7079390
rs10820606
rs1354034
All
−0.2
−0.1
0.0
0.1
0.2
0.3
MR leave−one−out sensitivity analysis for
'PRTFDC1' on 'LUSC'

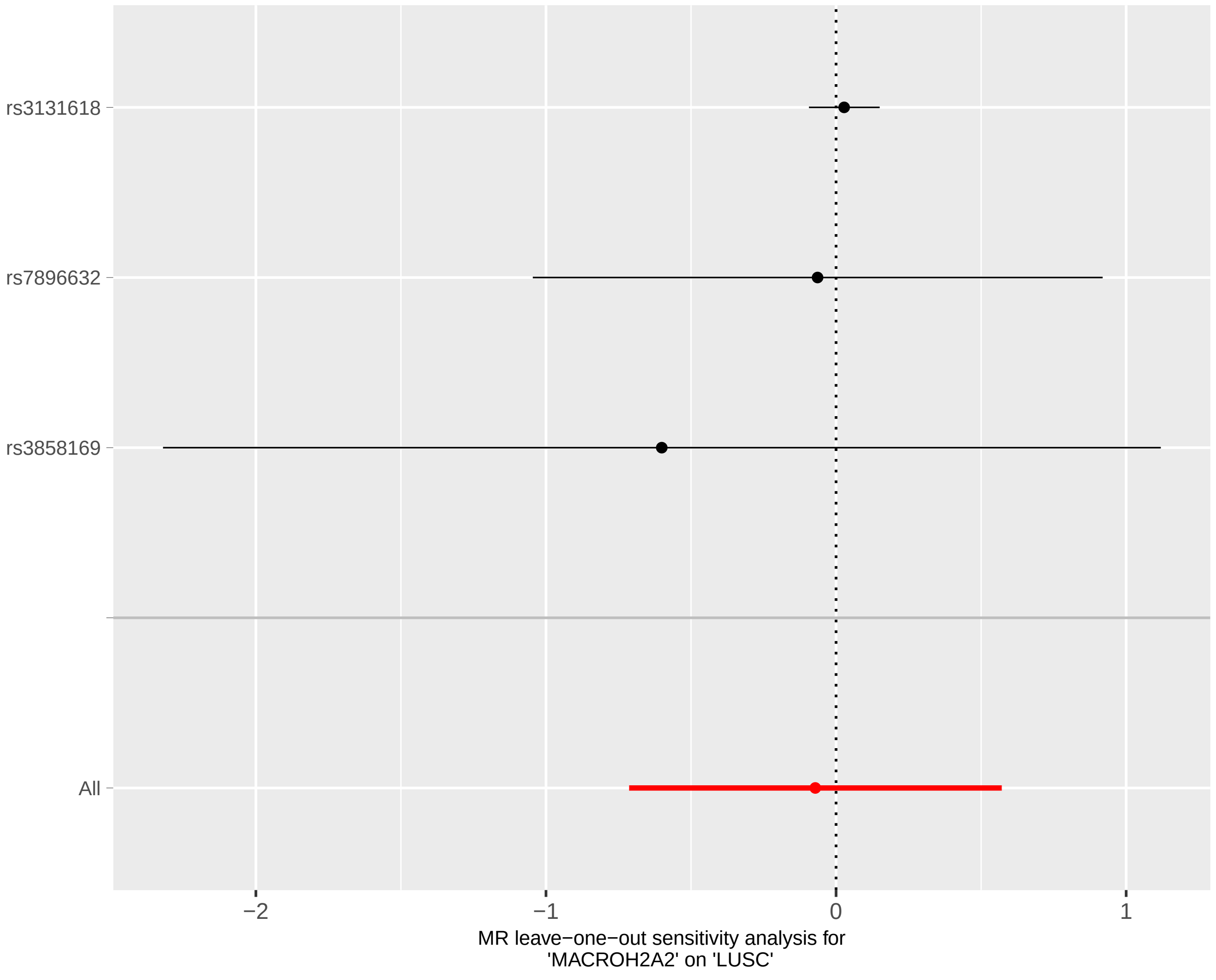

rs3131618
rs7896632
rs3858169
All
−2
−1
0
1
MR leave−one−out sensitivity analysis for
'MACROH2A2' on 'LUSC'

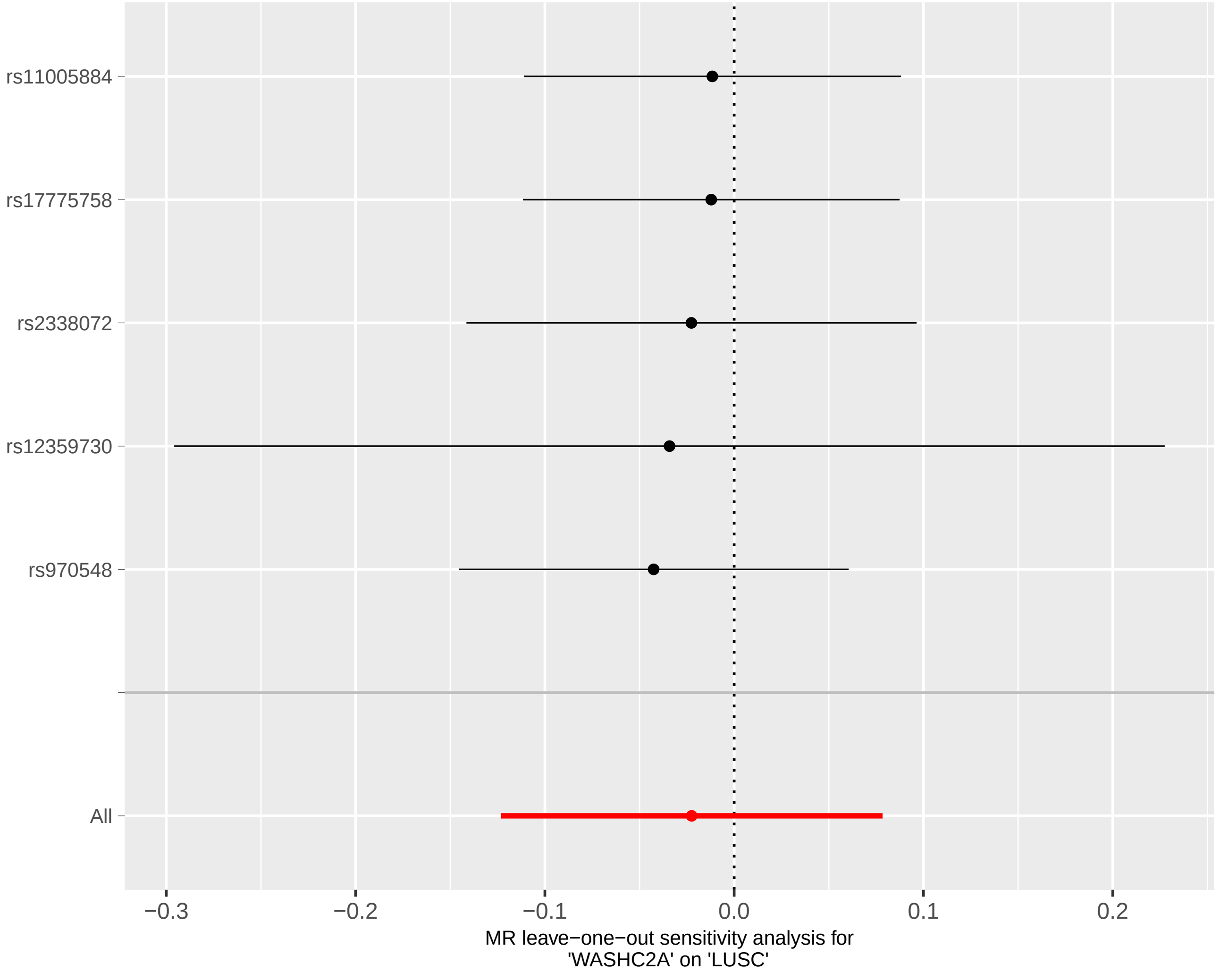
rs11005884
rs17775758
rs2338072
rs12359730
rs970548
All
−0.3
−0.2
−0.1
0.0
0.1
0.2
MR leave−one−out sensitivity analysis for
'WASHC2A' on 'LUSC'

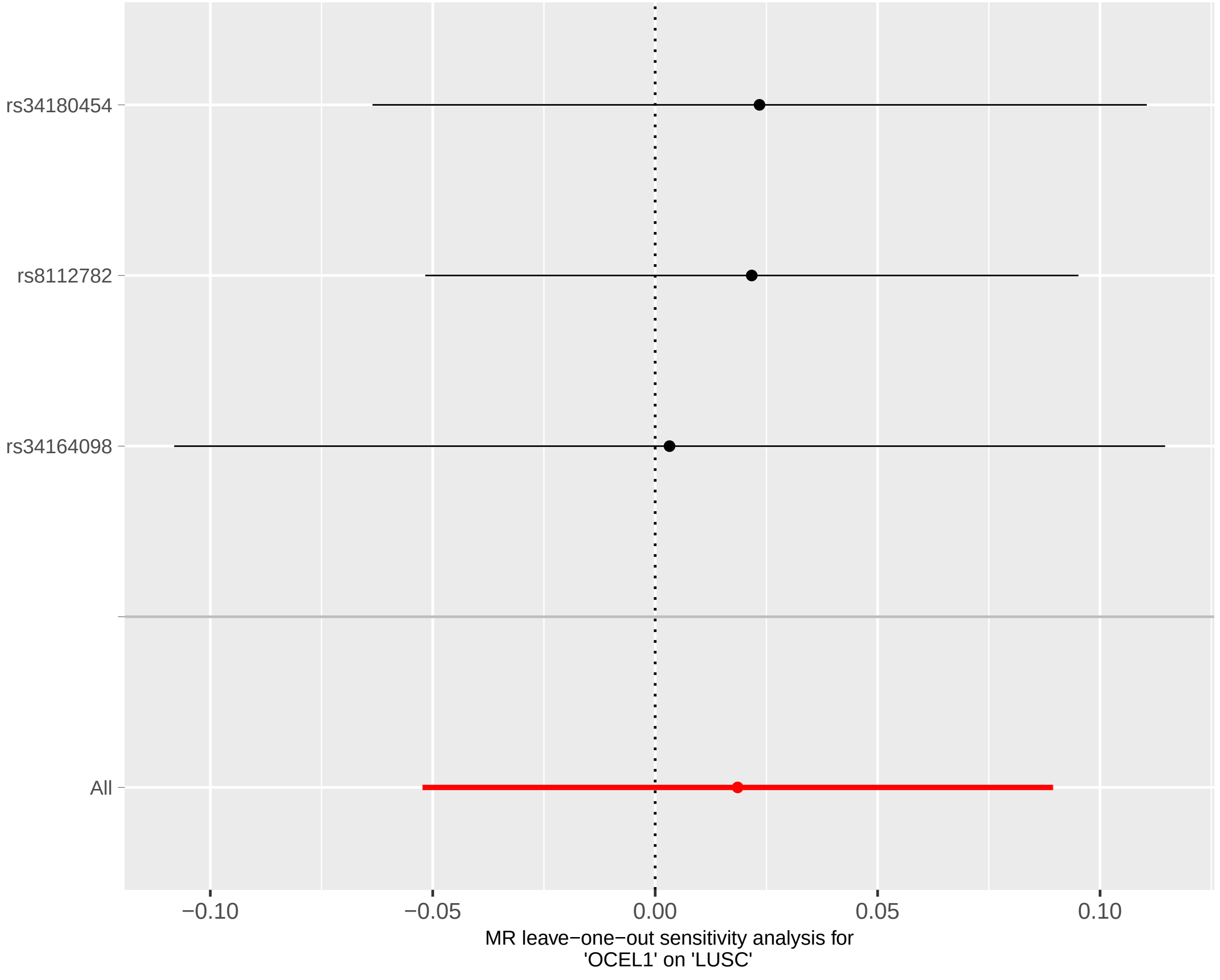

rs34180454
rs8112782
rs34164098
All
−0.10
−0.05
0.00
0.05
0.10
MR leave−one−out sensitivity analysis for
'OCEL1' on 'LUSC'

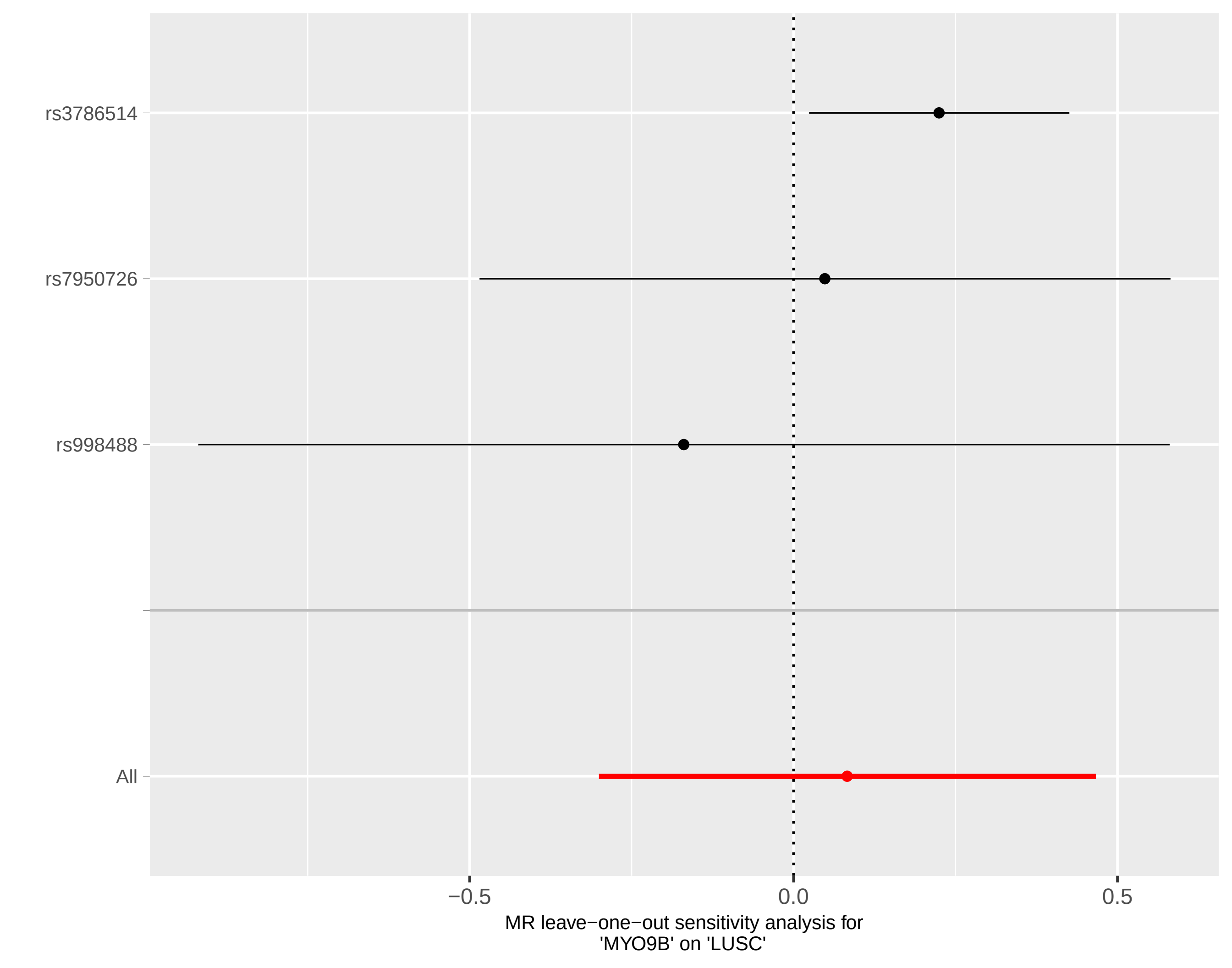

rs3786514
rs7950726
rs998488
All
−0.5
0.0
0.5
MR leave−one−out sensitivity analysis for
'MYO9B' on 'LUSC'

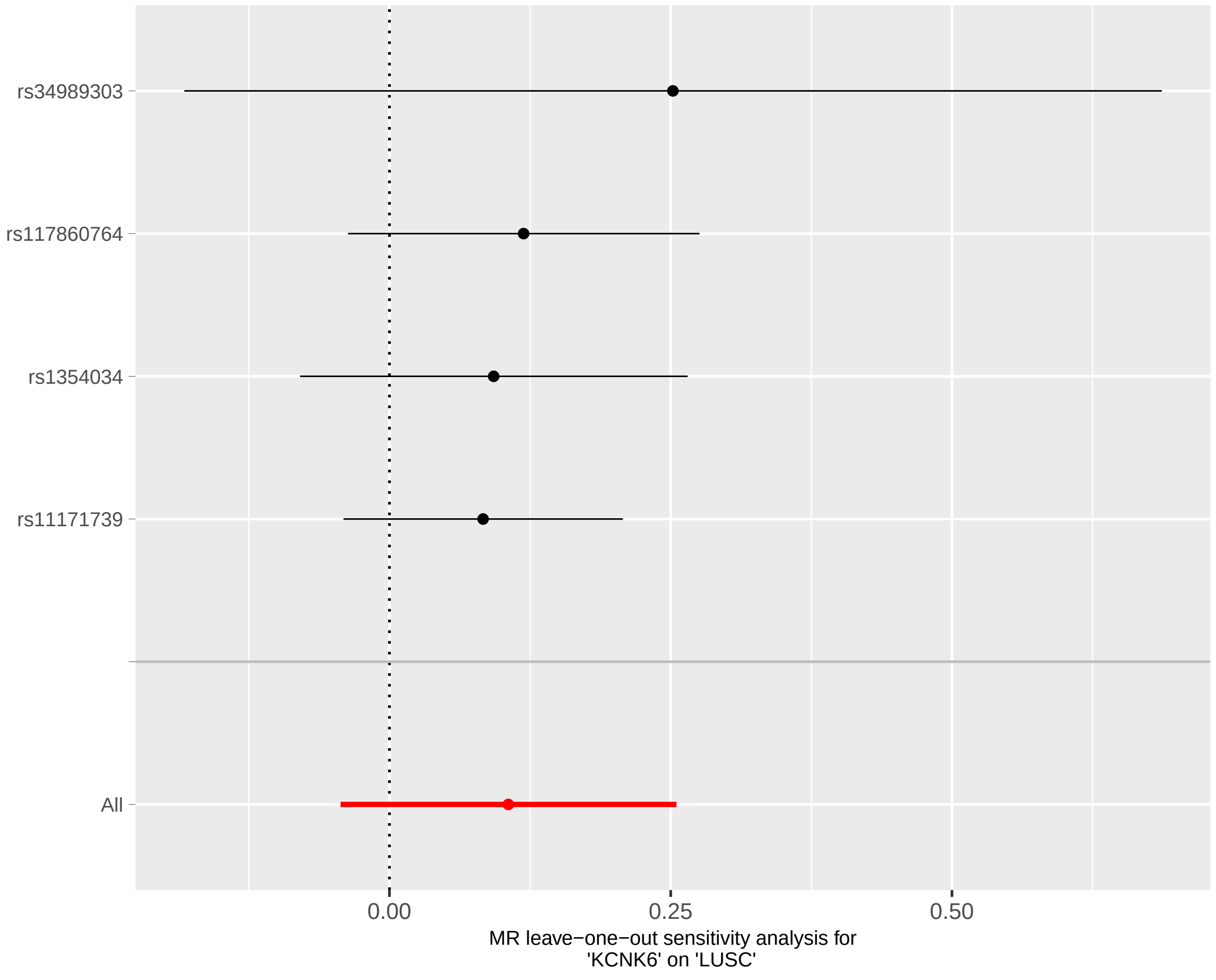
rs34989303
rs117860764
rs1354034
rs11171739
All
0.00
0.25
0.50
MR leave−one−out sensitivity analysis for
'KCNK6' on 'LUSC'

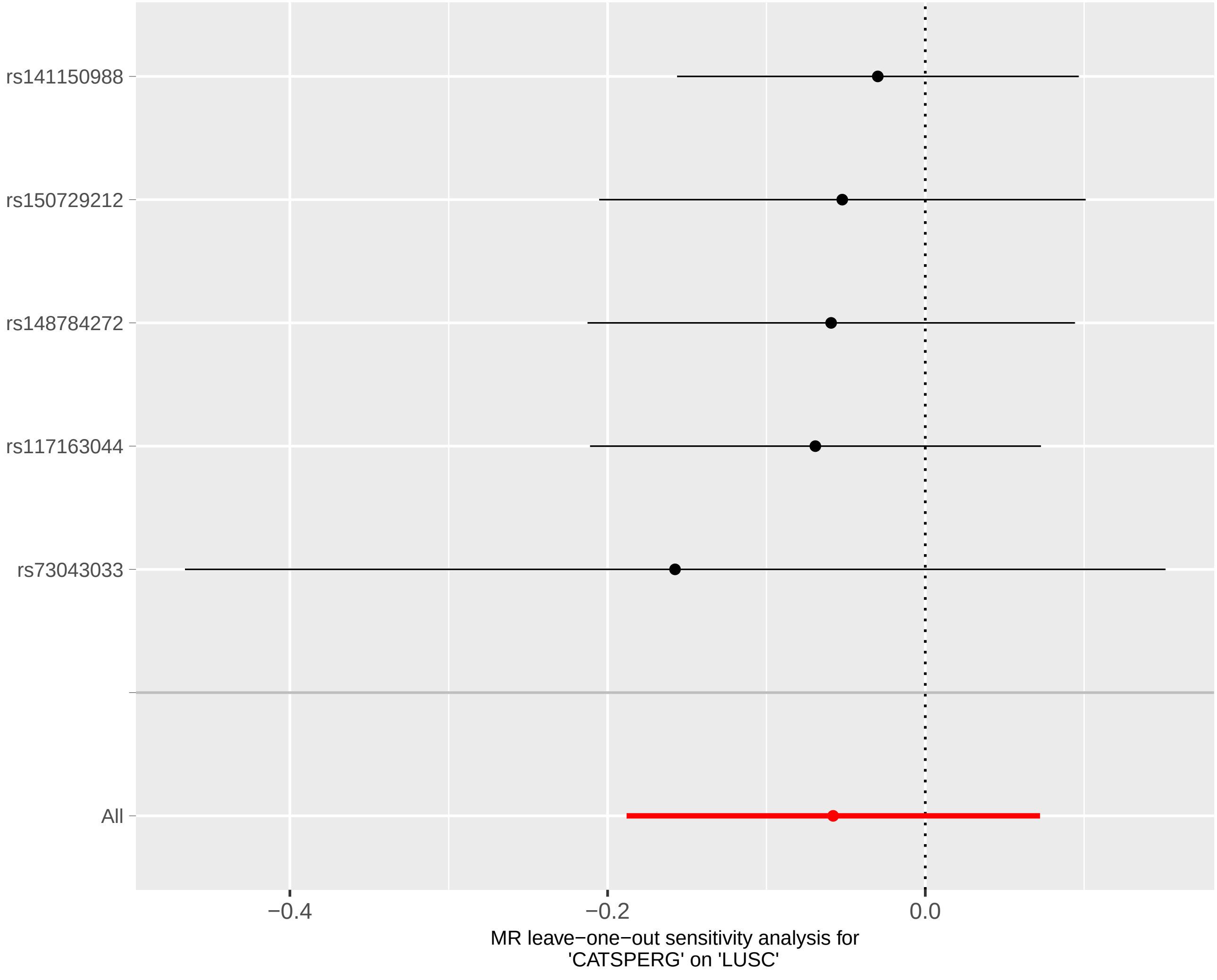
rs141150988
rs150729212
rs148784272
rs117163044
rs73043033
All
−0.4
−0.2
0.0
MR leave−one−out sensitivity analysis for
'CATSPERG' on 'LUSC'

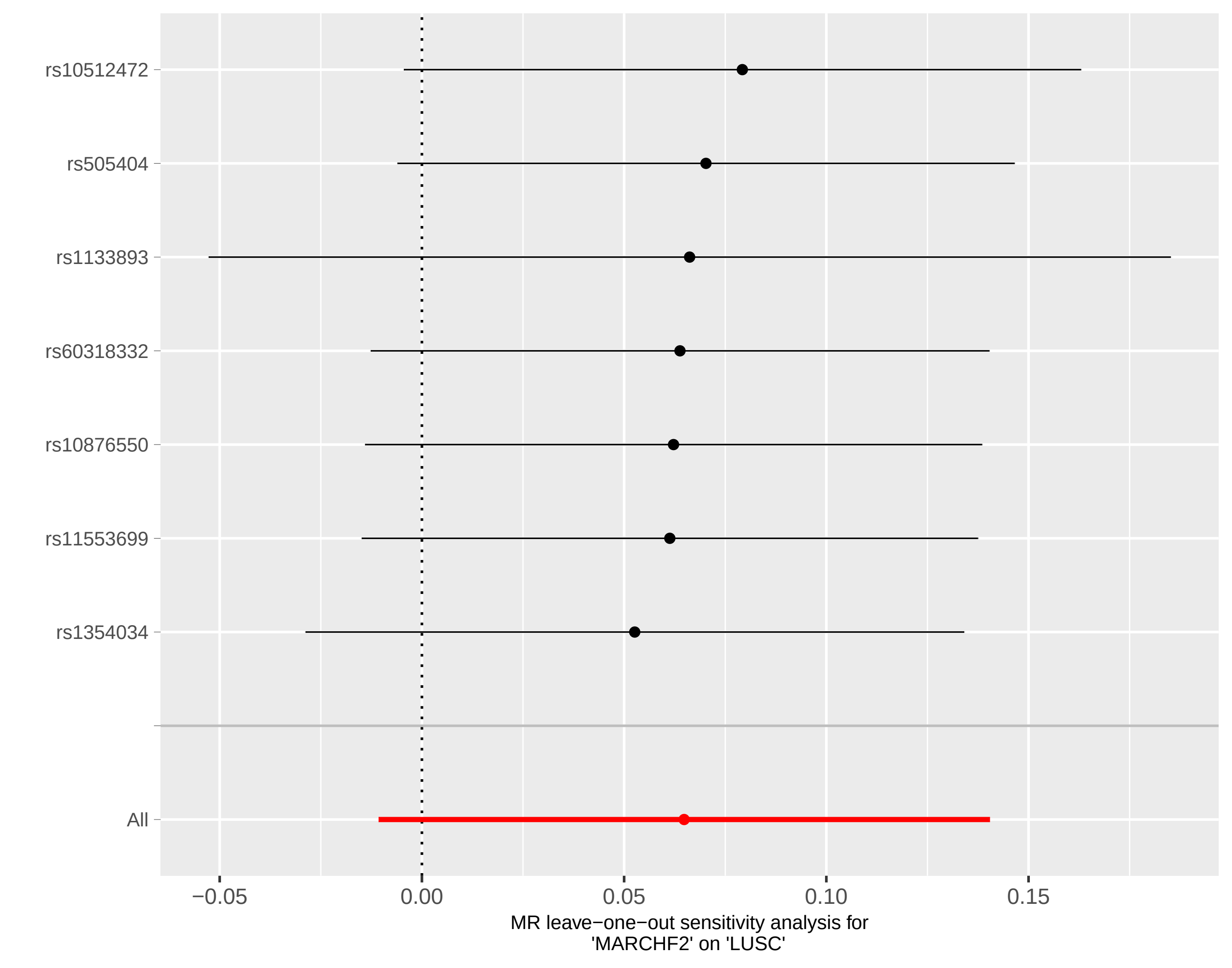

rs10512472
rs505404
rs1133893
rs60318332
rs10876550
rs11553699
rs1354034
All
−0.05
0.00
0.05
0.10
0.15
MR leave−one−out sensitivity analysis for
'MARCHF2' on 'LUSC'

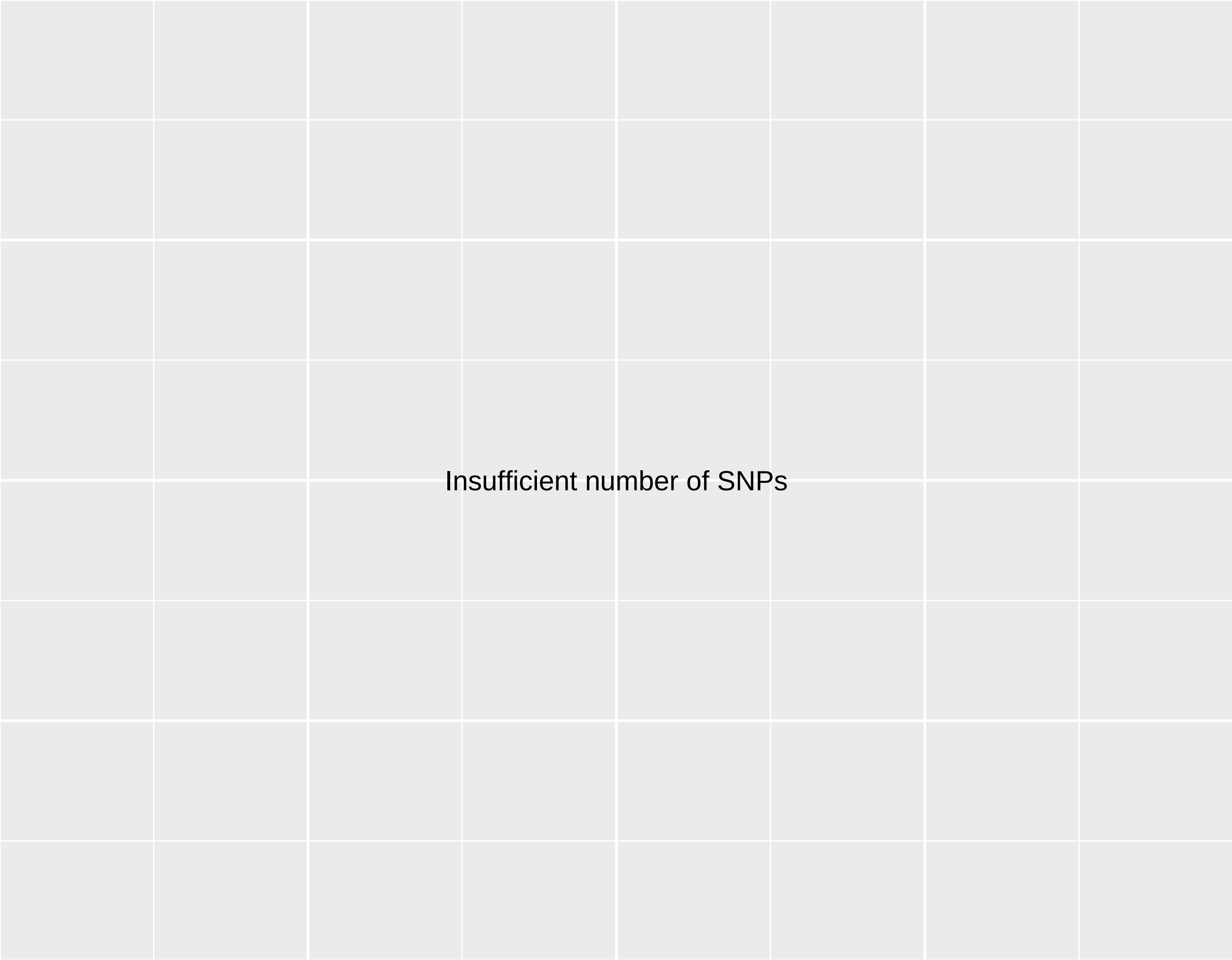
Insufficient number of SNPs

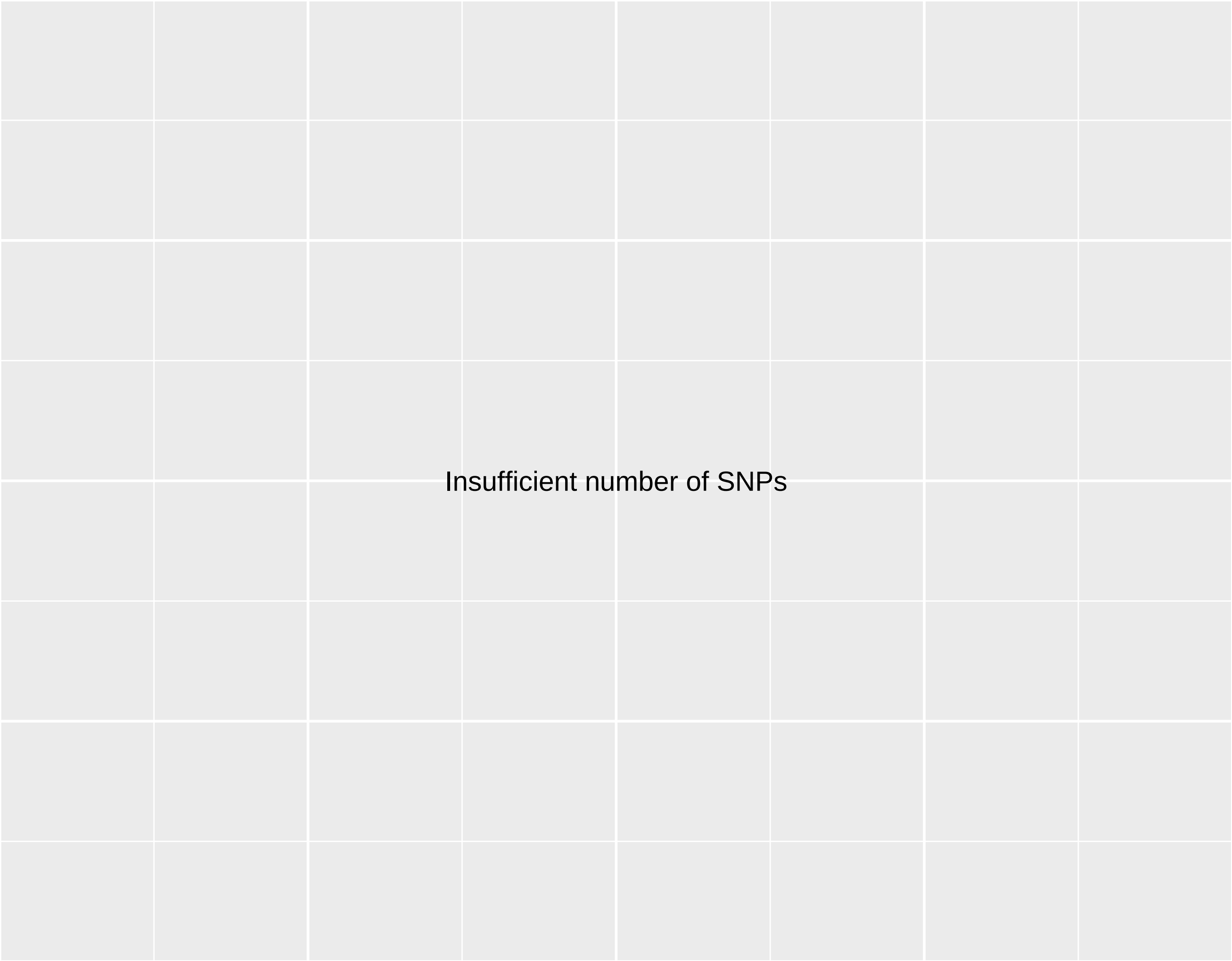
Insufficient number of SNPs

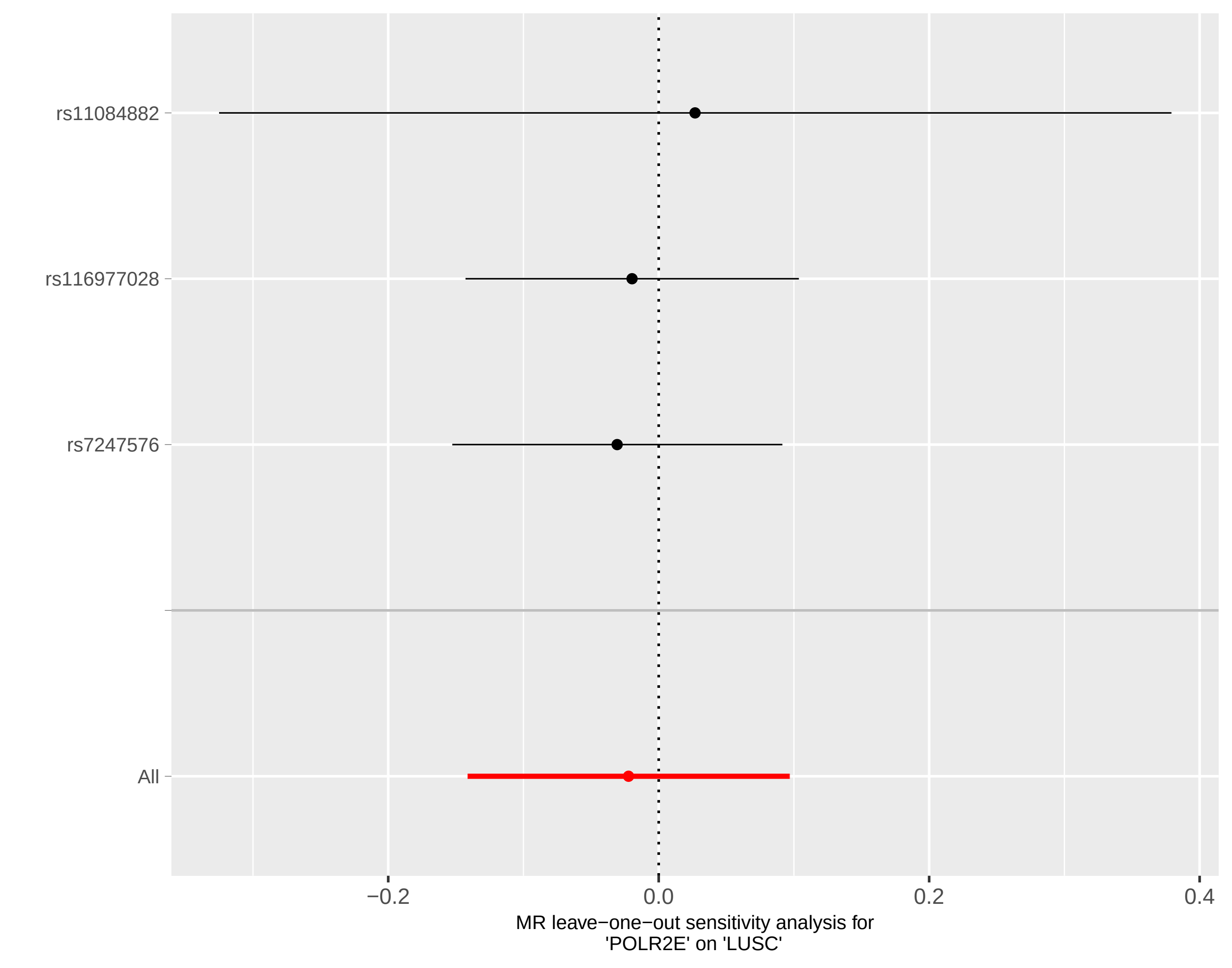

rs11084882
rs116977028
rs7247576
All
−0.2
0.0
0.2
0.4
MR leave−one−out sensitivity analysis for
'POLR2E' on 'LUSC'

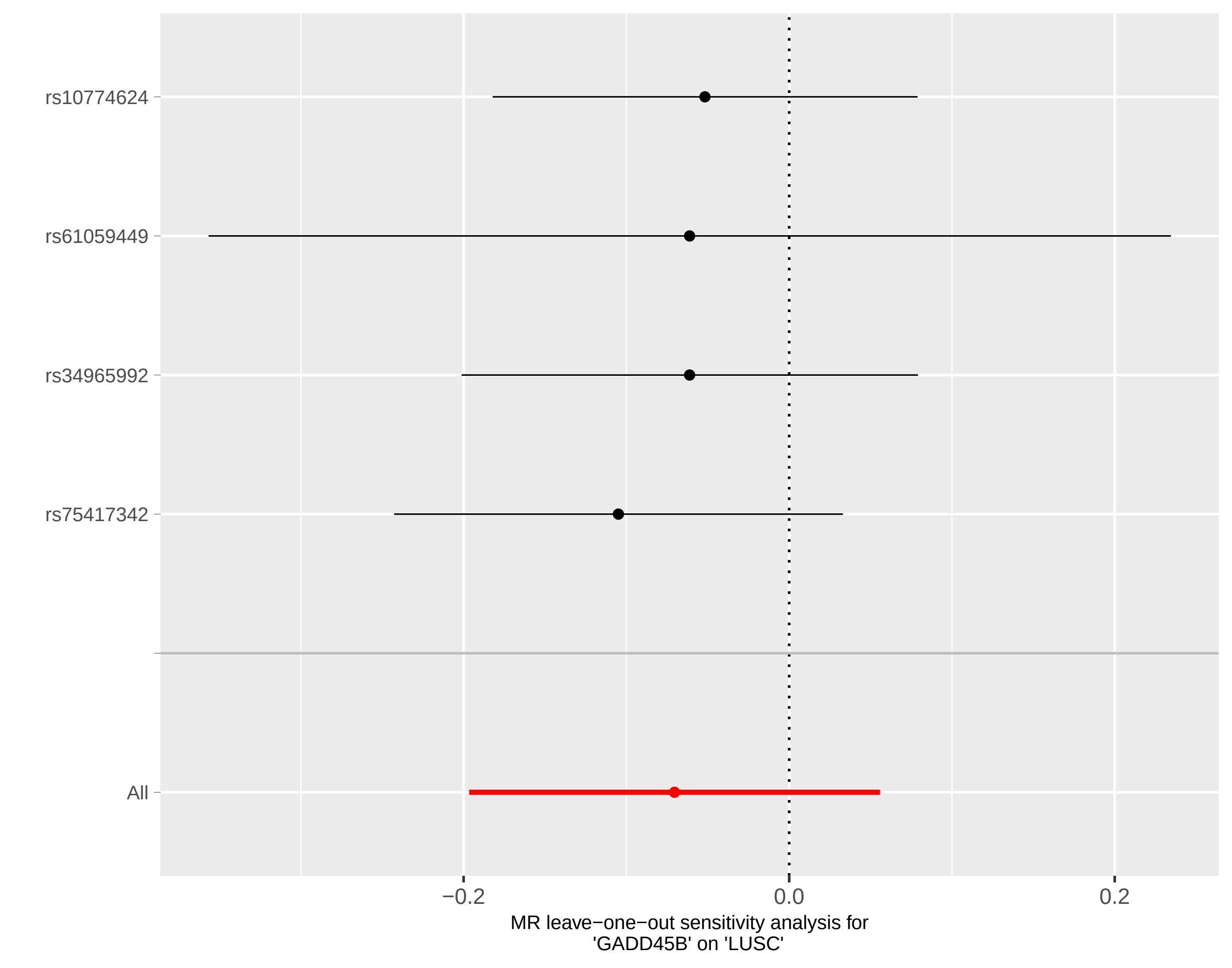

rs10774624
rs61059449
rs34965992
rs75417342
All
−0.2
0.0
0.2
MR leave−one−out sensitivity analysis for
'GADD45B' on 'LUSC'

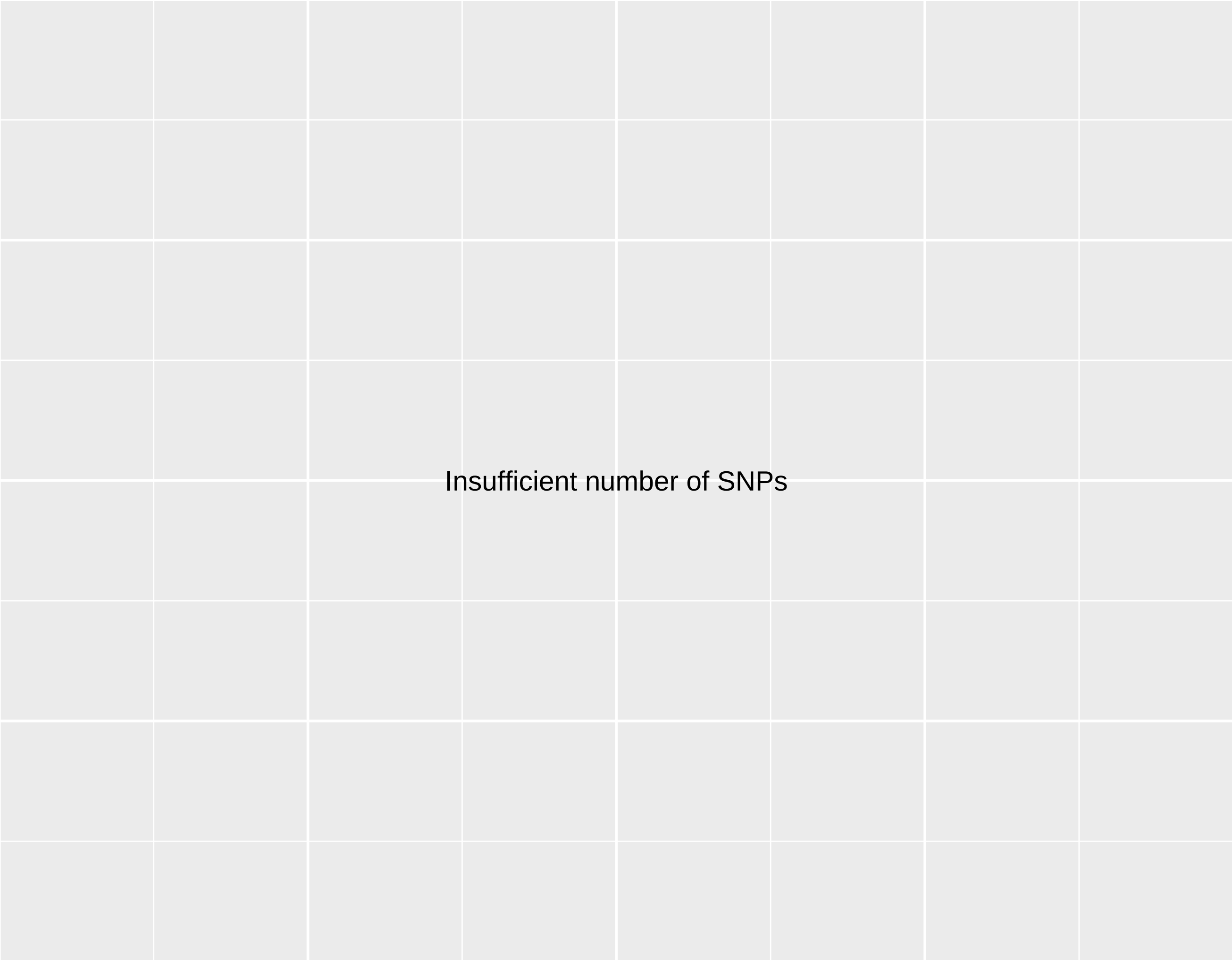
Insufficient number of SNPs

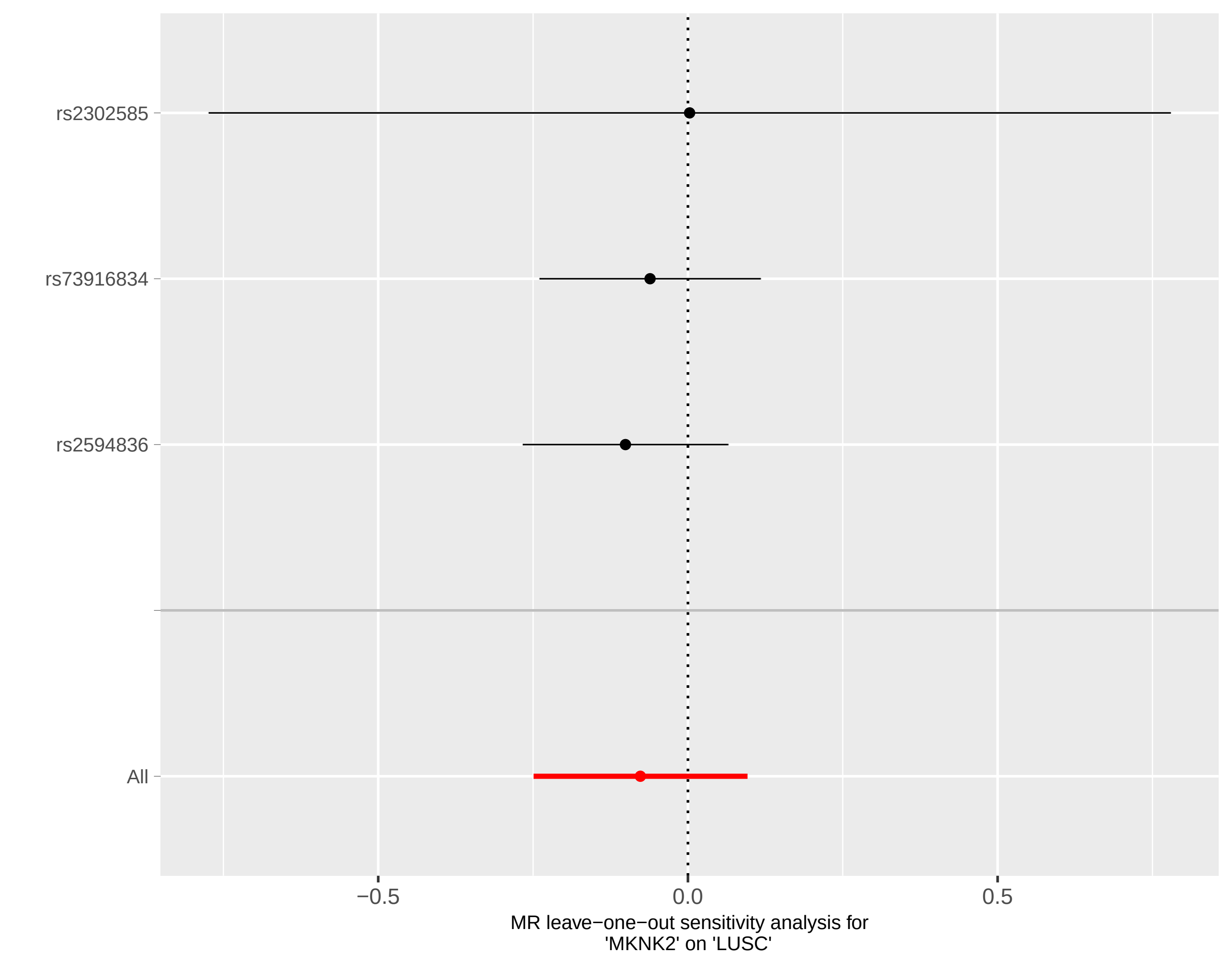
rs2302585
rs73916834
rs2594836
All
−0.5
0.0
0.5
MR leave−one−out sensitivity analysis for
'MKNK2' on 'LUSC'

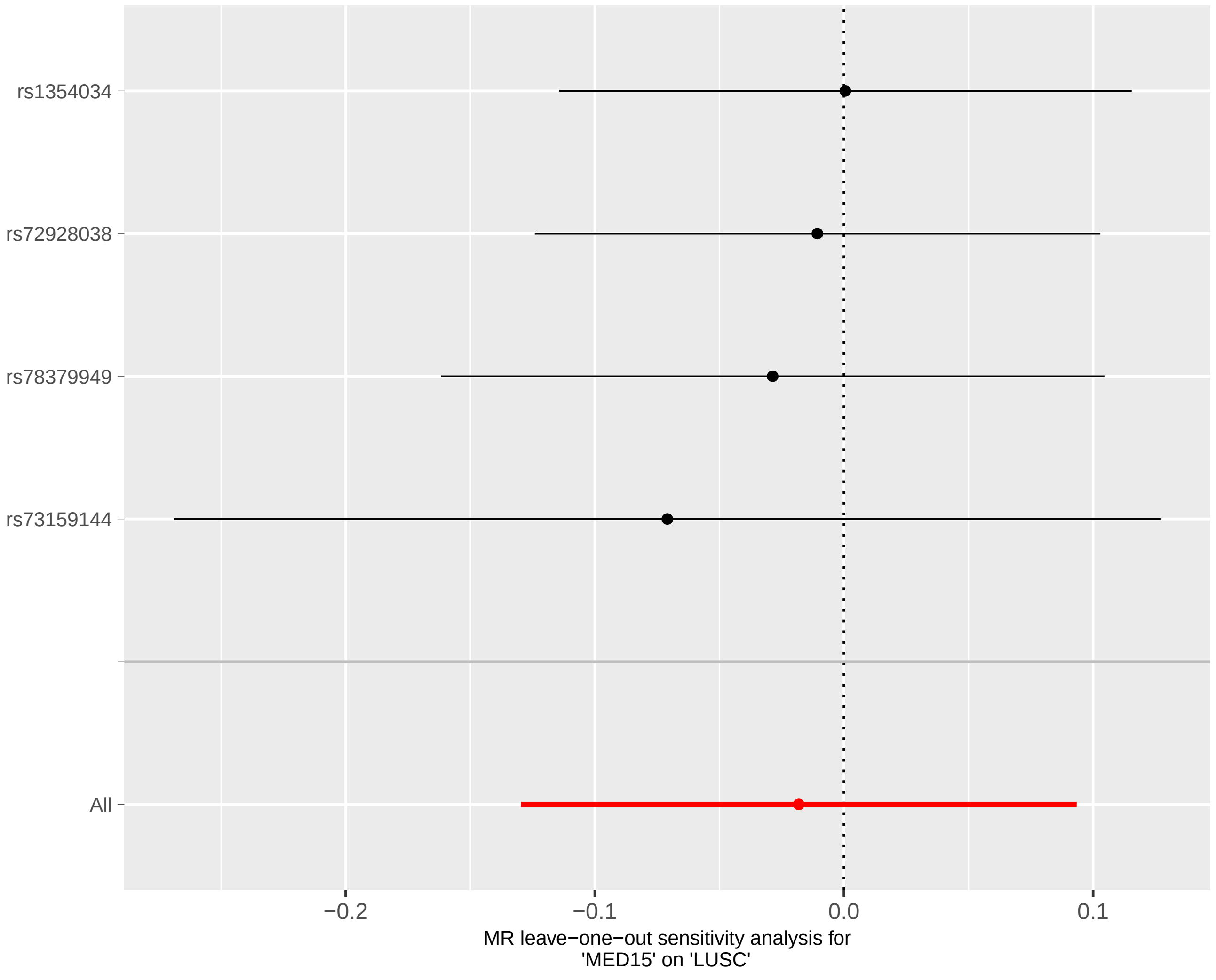

rs1354034
rs72928038
rs78379949
rs73159144
All
−0.2
−0.1
0.0
0.1
MR leave−one−out sensitivity analysis for
'MED15' on 'LUSC'

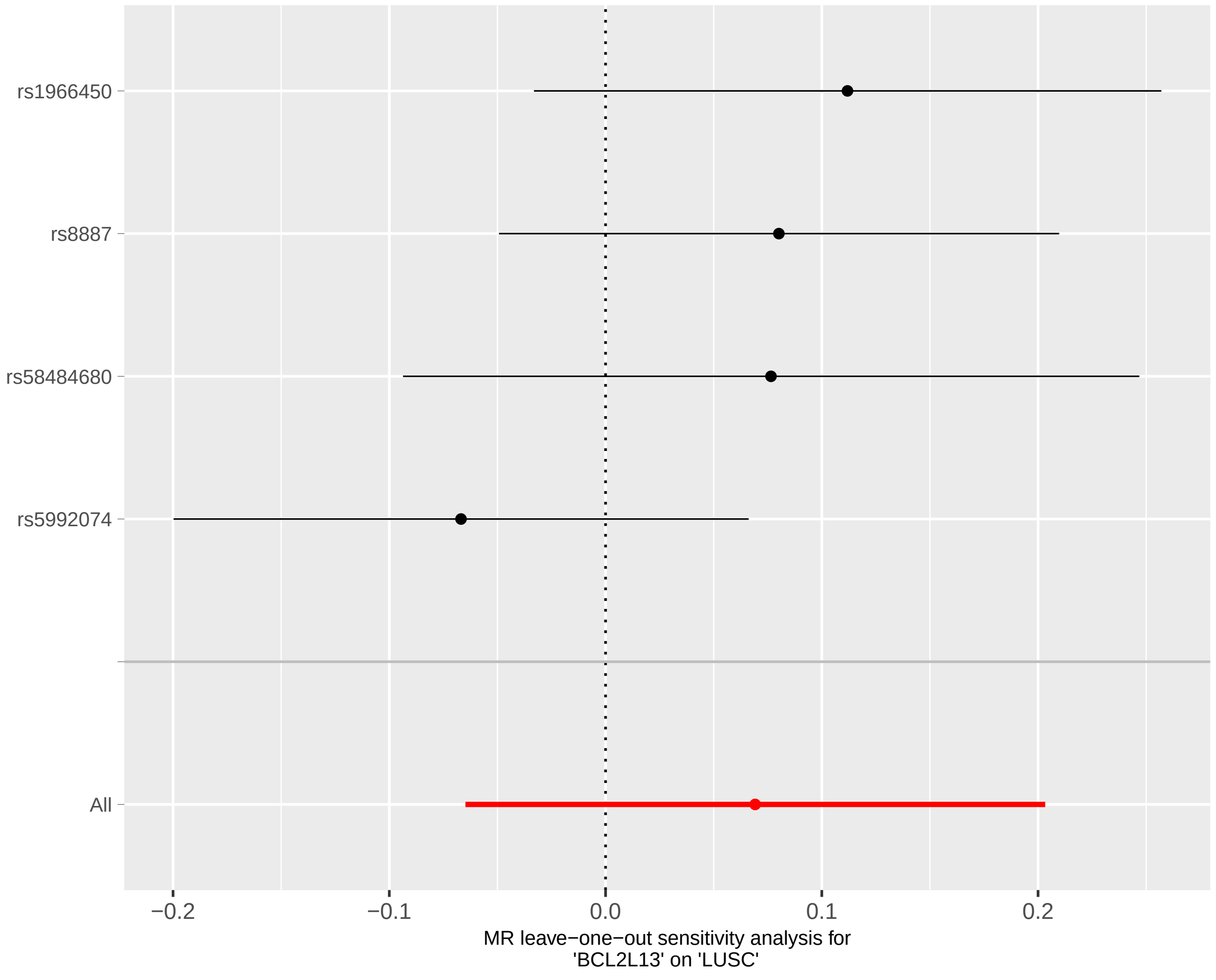
rs1966450
rs8887
rs58484680
rs5992074
All
−0.2
−0.1
0.0
0.1
0.2
MR leave−one−out sensitivity analysis for
'BCL2L13' on 'LUSC'

Insufficient number of SNPs

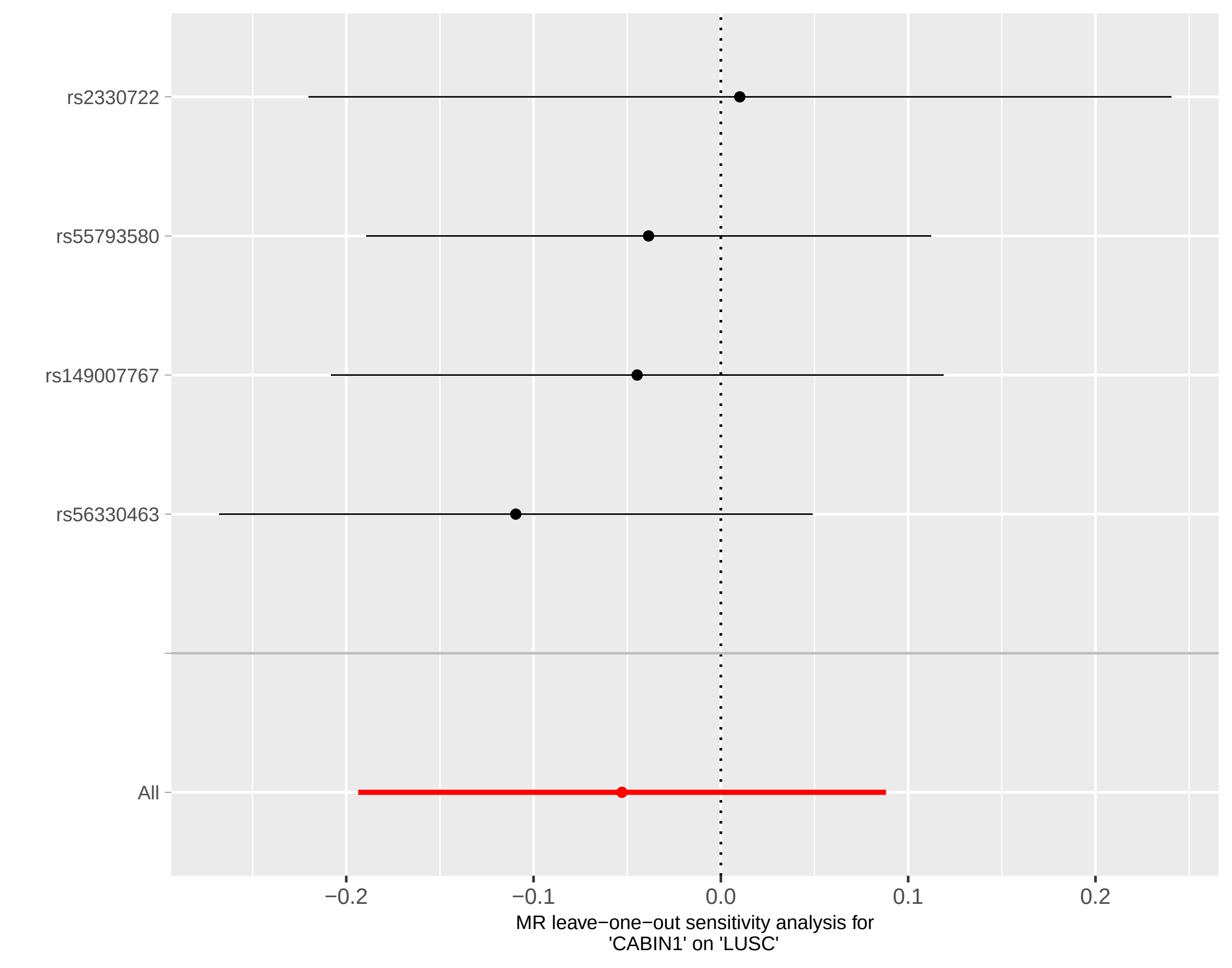
rs2330722
rs55793580
rs149007767
rs56330463
All
−0.2
−0.1
0.0
0.1
0.2
MR leave−one−out sensitivity analysis for
'CABIN1' on 'LUSC'

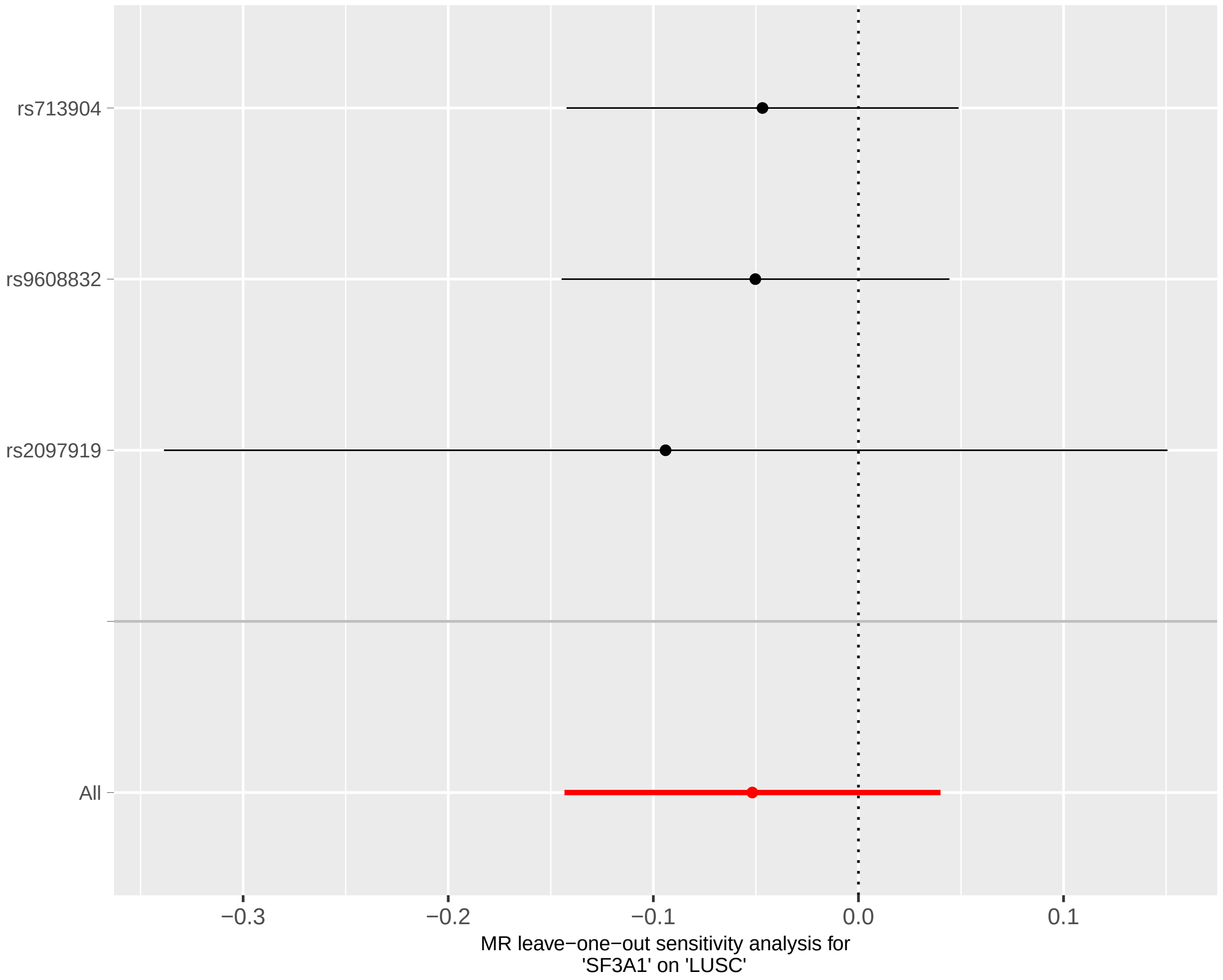
rs713904
rs9608832
rs2097919
All
−0.3
−0.2
−0.1
0.0
0.1
MR leave−one−out sensitivity analysis for
'SF3A1' on 'LUSC'

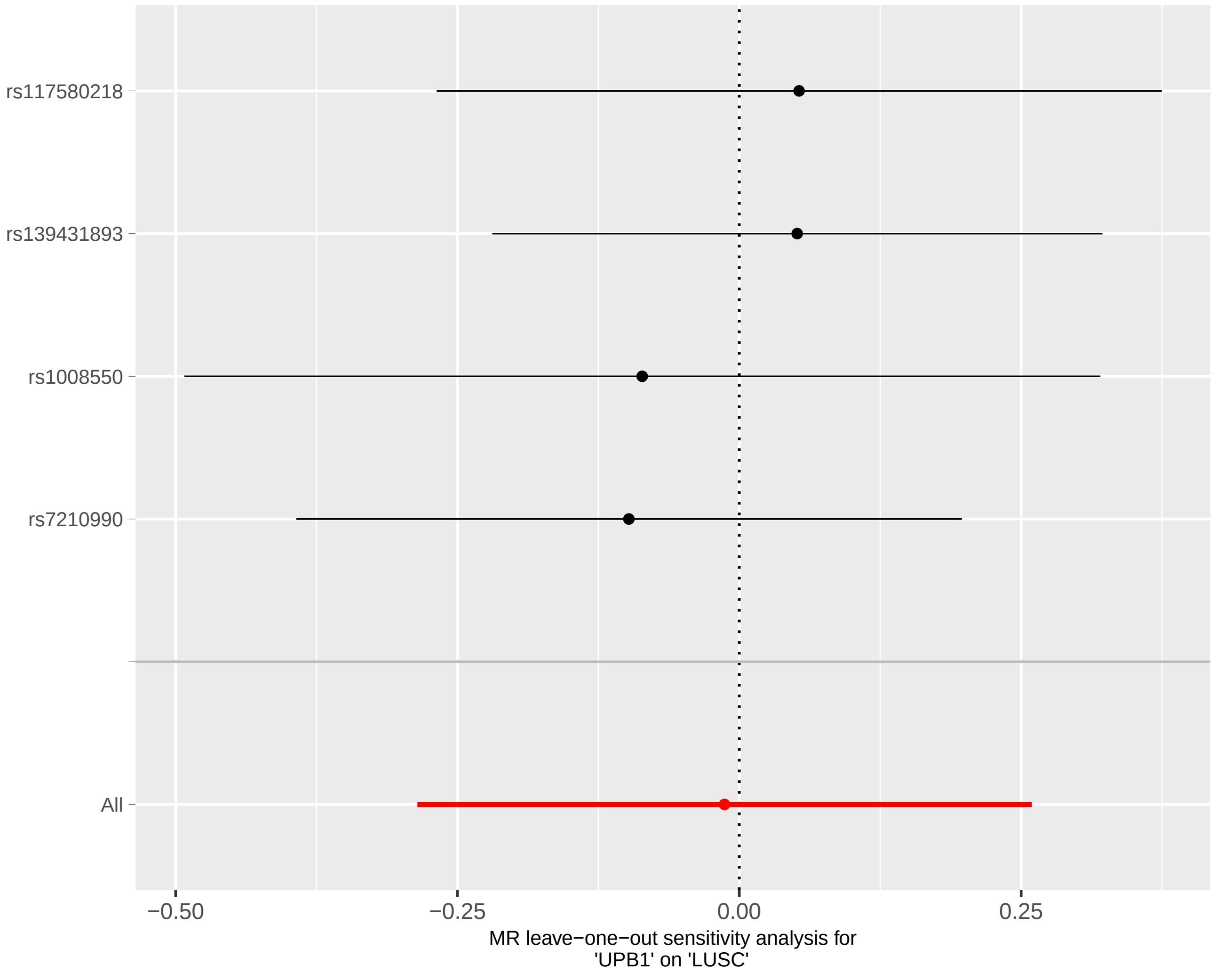
rs117580218
rs139431893
rs1008550
rs7210990
All
−0.50
−0.25
0.00
0.25
MR leave−one−out sensitivity analysis for
'UPB1' on 'LUSC'

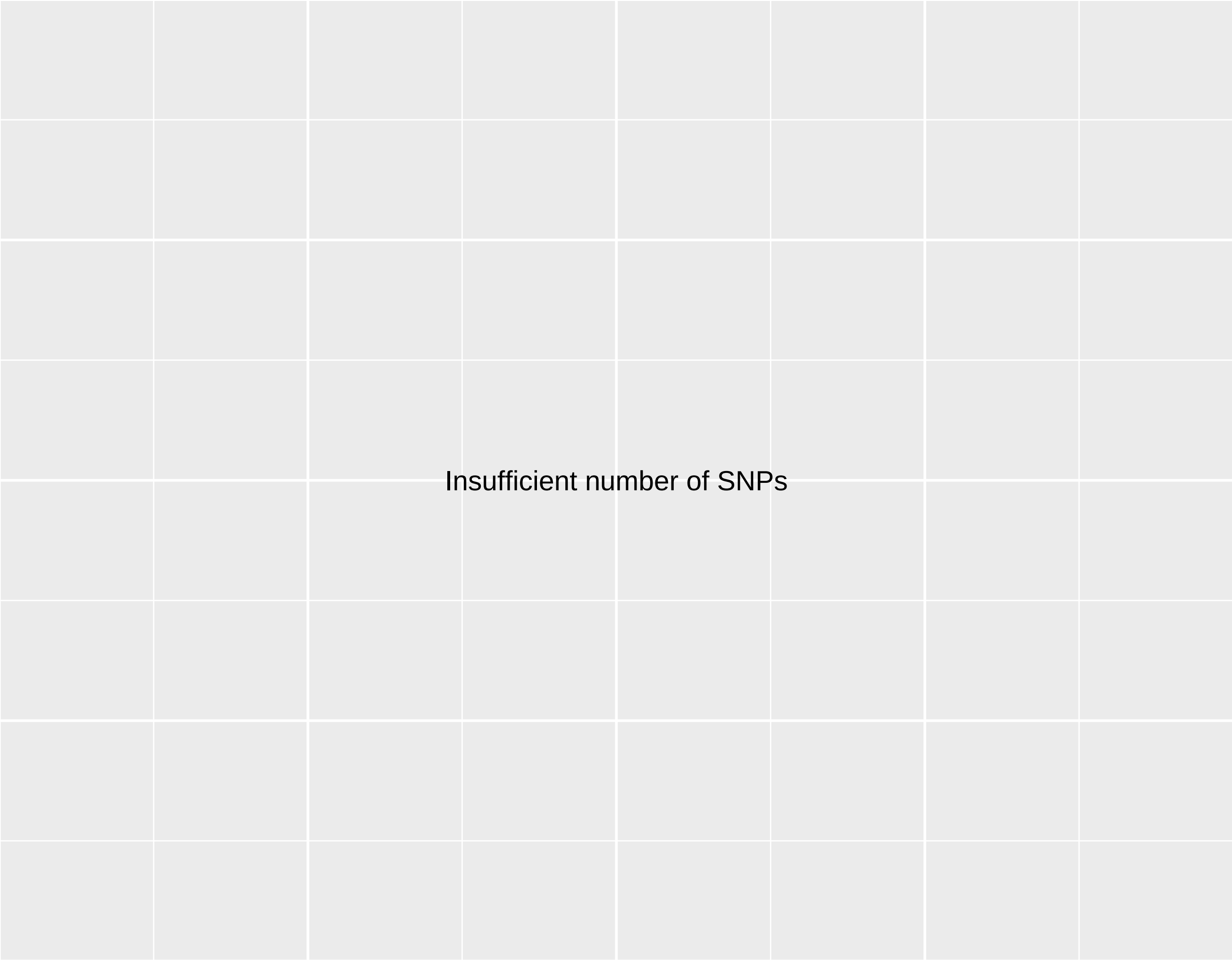
Insufficient number of SNPs

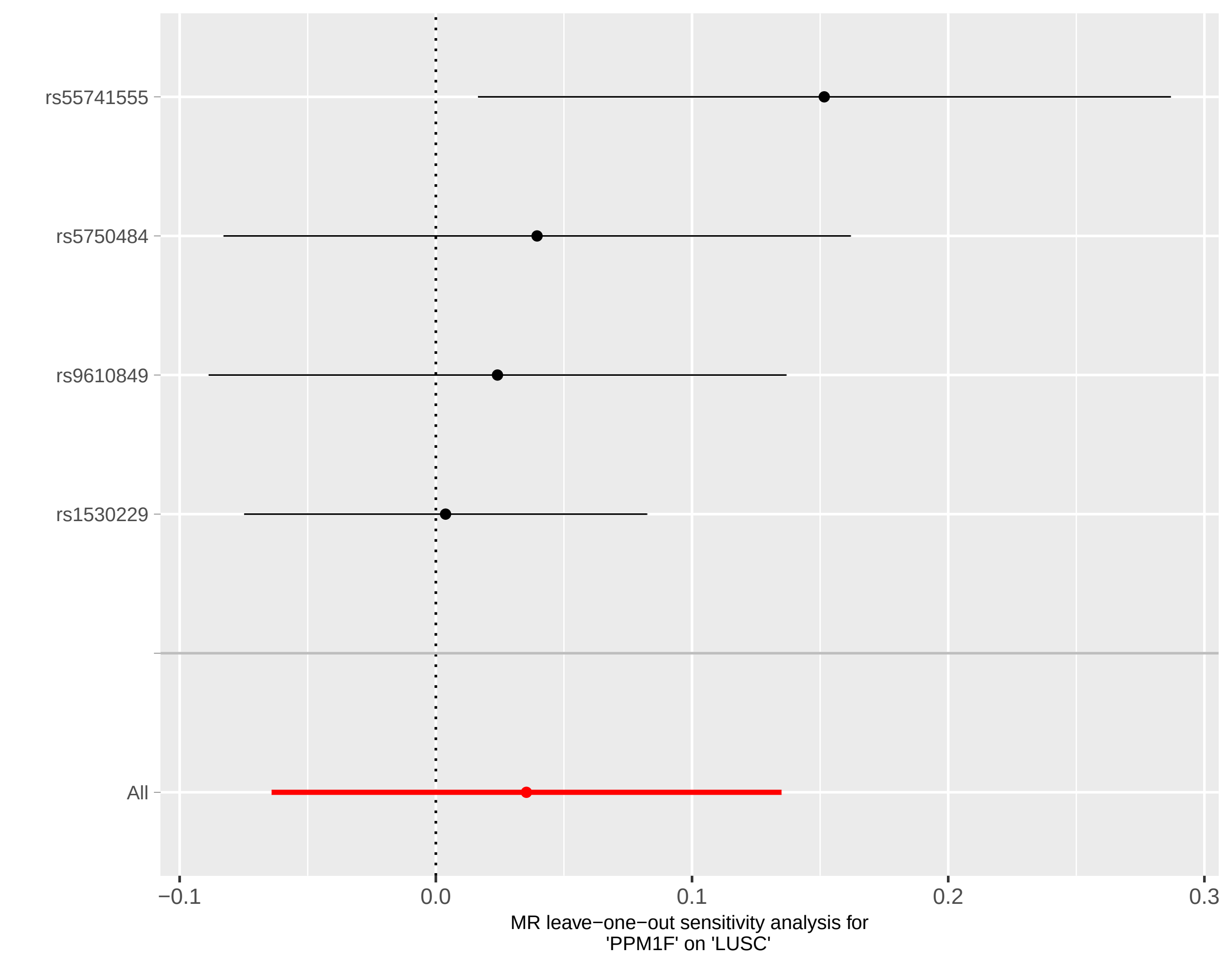
rs55741555
rs5750484
rs9610849
rs1530229
All
−0.1
0.0
0.1
0.2
0.3
MR leave−one−out sensitivity analysis for
'PPM1F' on 'LUSC'

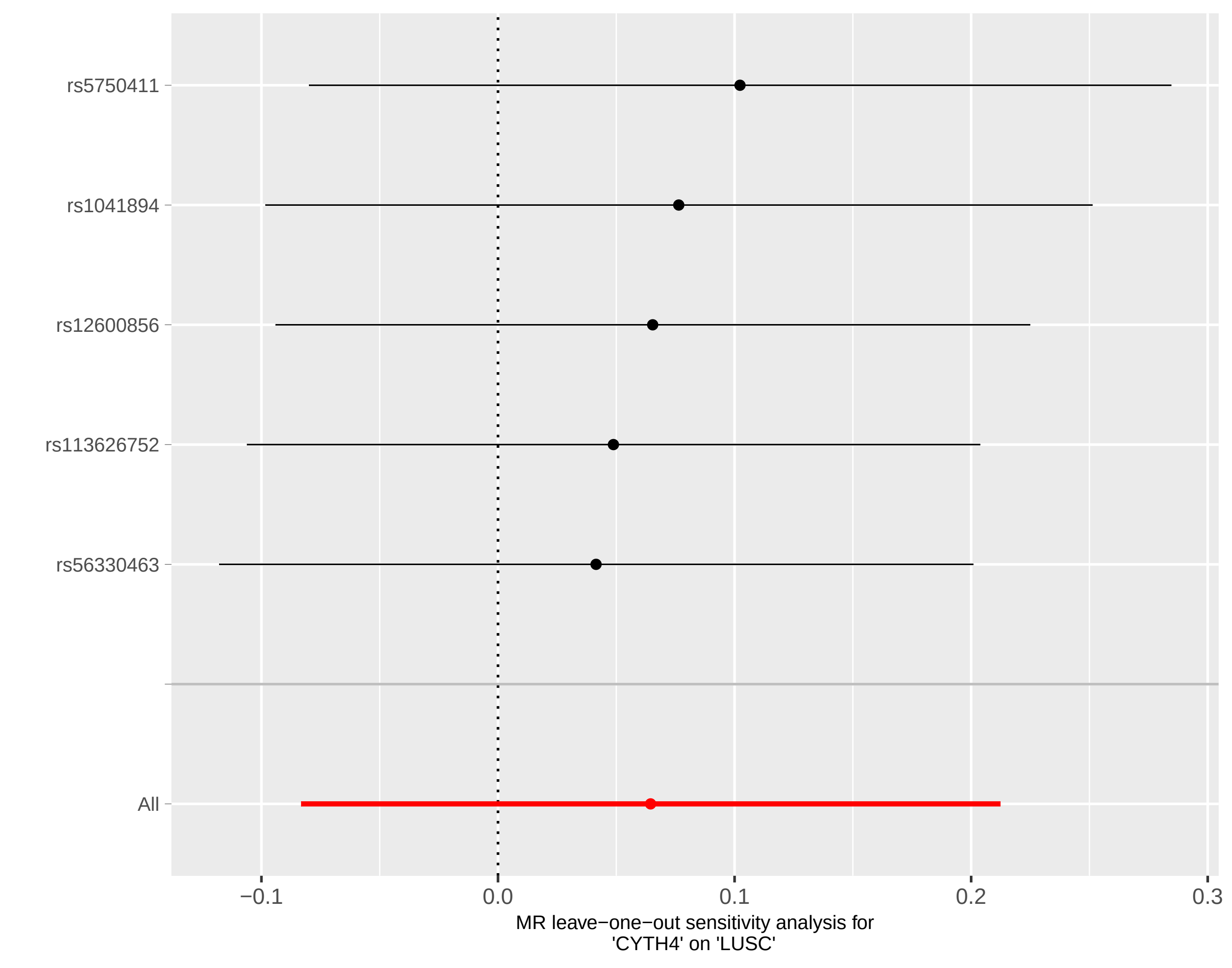
rs5750411
rs1041894
rs12600856
rs113626752
rs56330463
All
−0.1
0.0
0.1
0.2
0.3
MR leave−one−out sensitivity analysis for
'CYTH4' on 'LUSC'

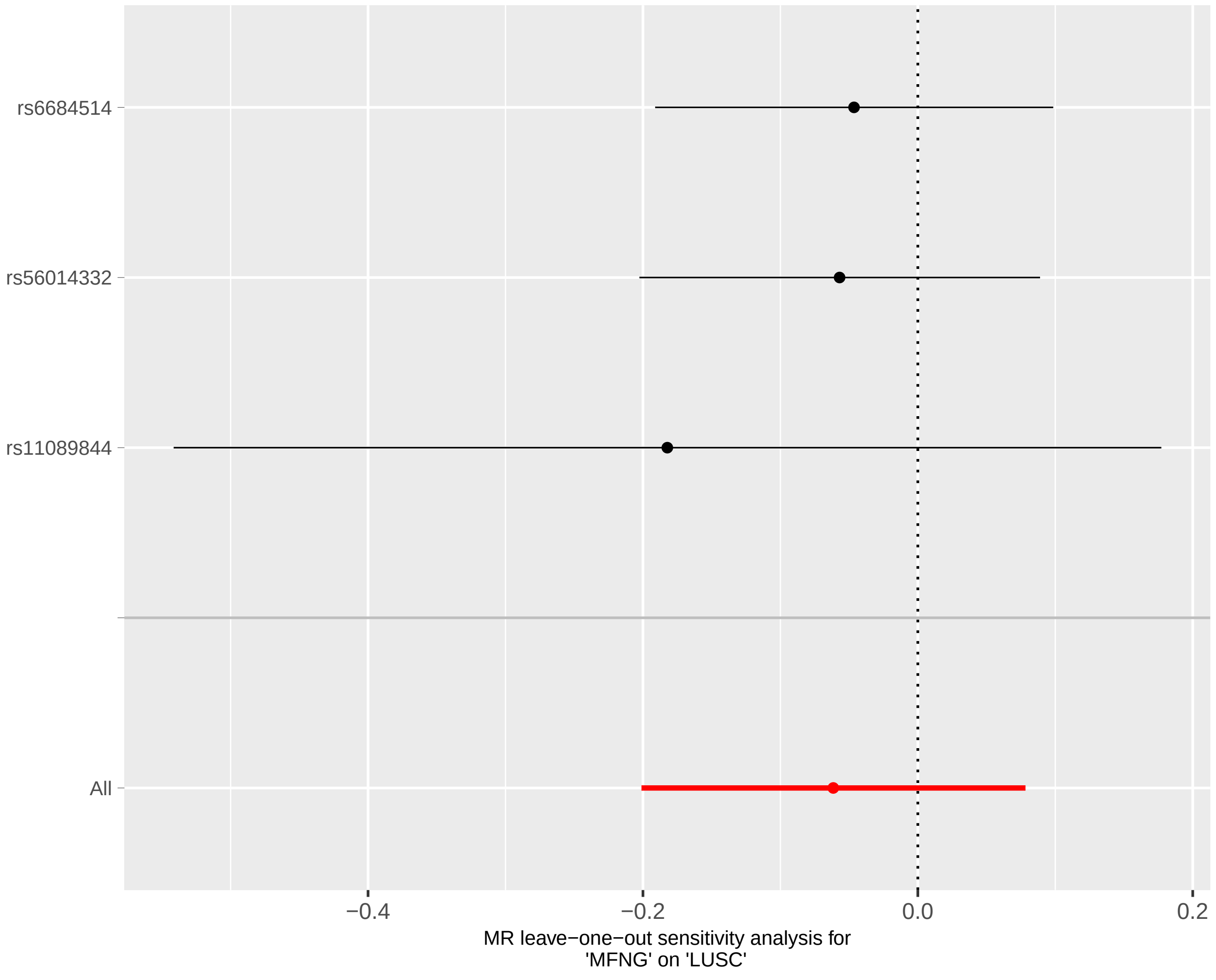

rs6684514
rs56014332
rs11089844
All
−0.4
−0.2
0.0
0.2
MR leave−one−out sensitivity analysis for
'MFNG' on 'LUSC'

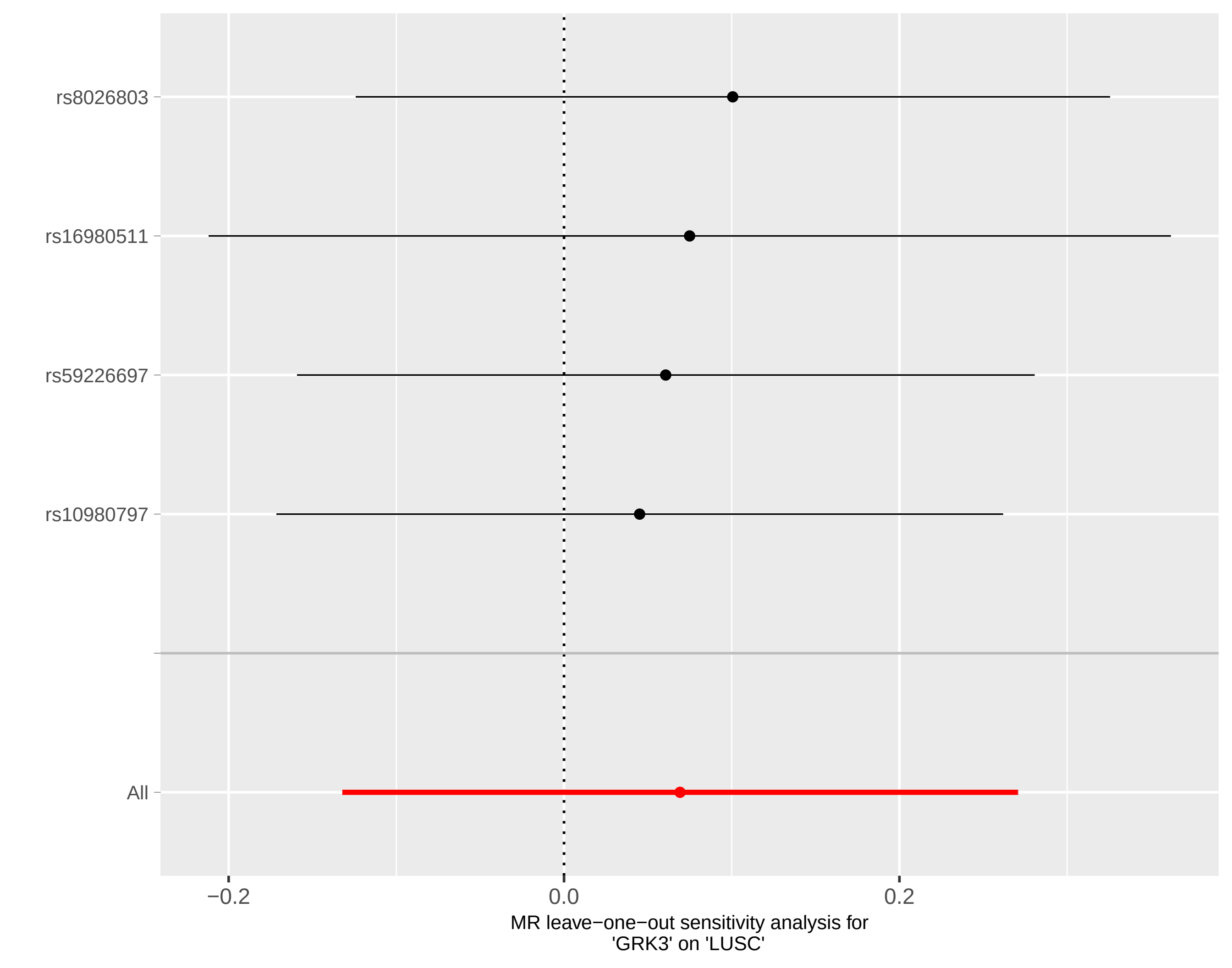
rs8026803
rs16980511
rs59226697
rs10980797
All
−0.2
0.0
0.2
MR leave−one−out sensitivity analysis for
'GRK3' on 'LUSC'

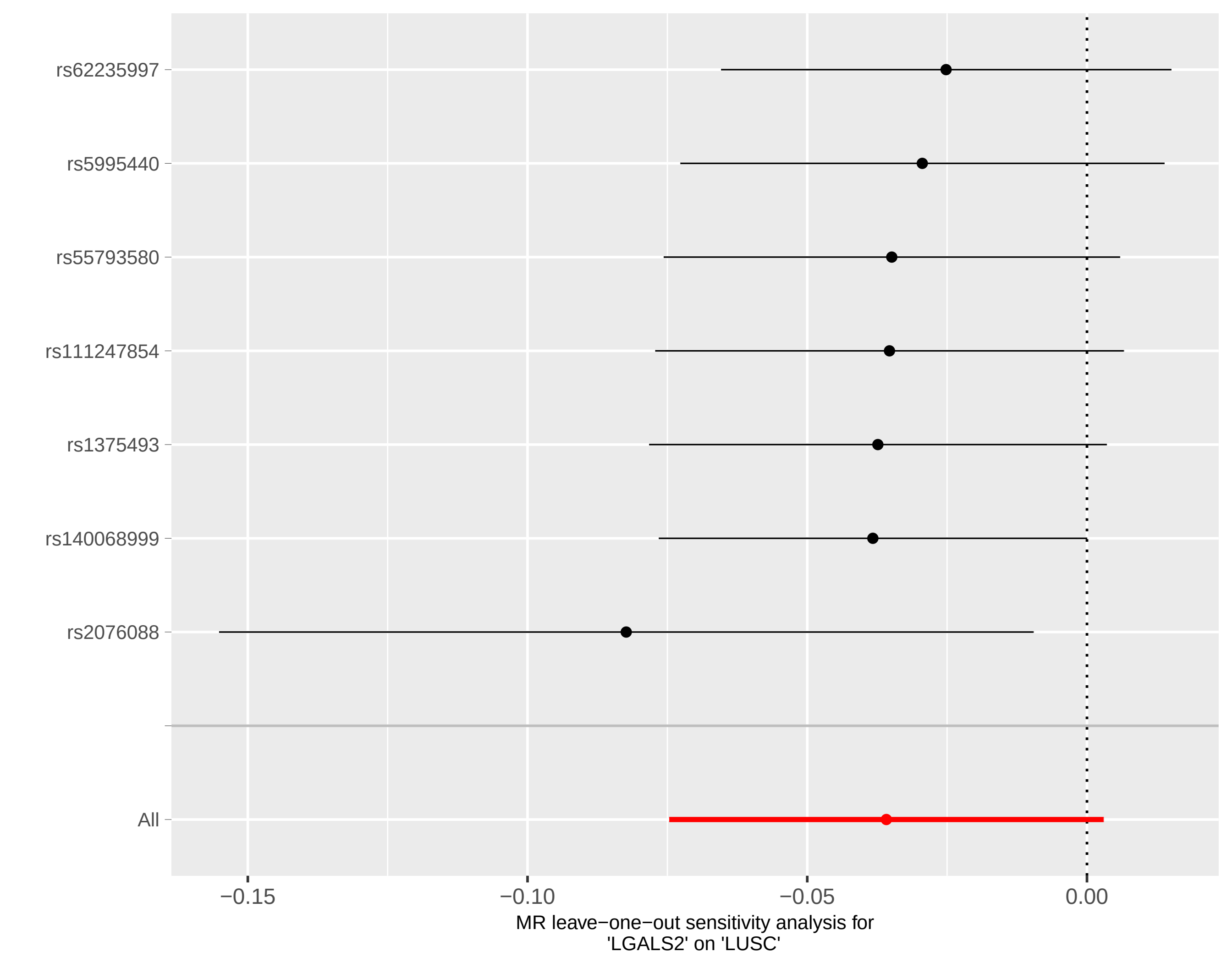
rs62235997
rs5995440
rs55793580
rs111247854
rs1375493
rs140068999
rs2076088
All
−0.15
−0.10
−0.05
0.00
MR leave−one−out sensitivity analysis for
'LGALS2' on 'LUSC'

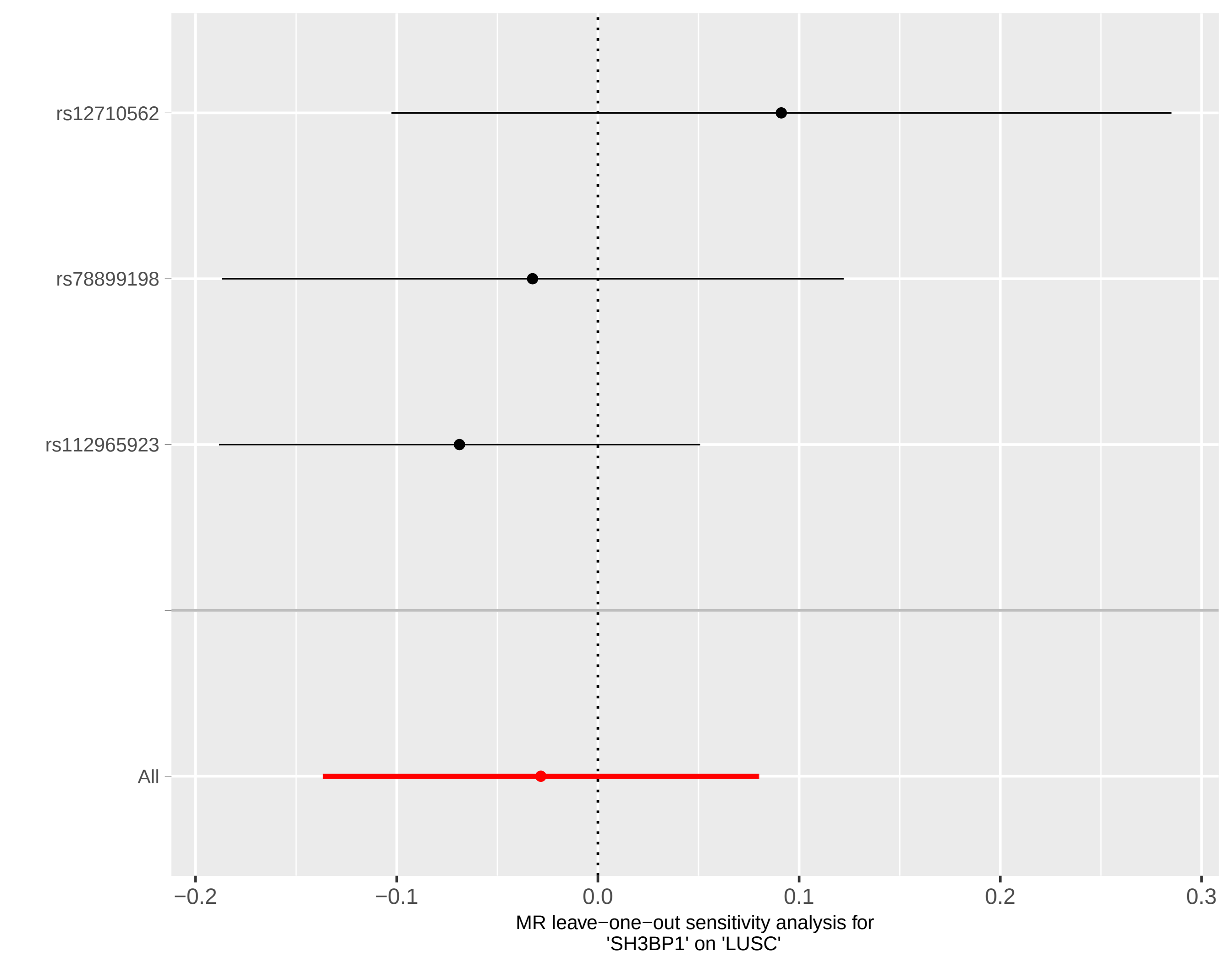
rs12710562
rs78899198
rs112965923
All
−0.2
−0.1
0.0
0.1
0.2
0.3
MR leave−one−out sensitivity analysis for
'SH3BP1' on 'LUSC'

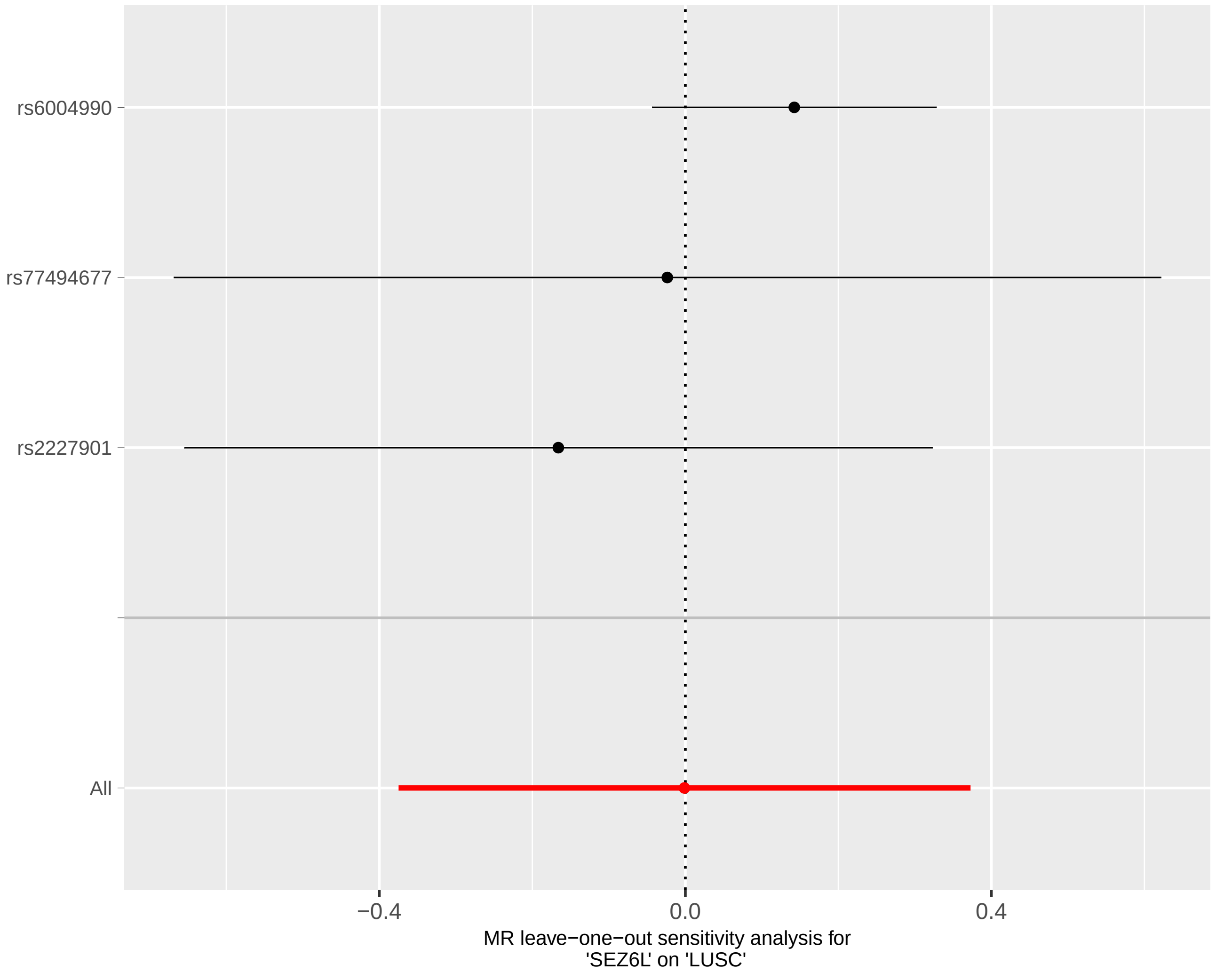
rs6004990
rs77494677
rs2227901
All
−0.4
0.0
0.4
MR leave−one−out sensitivity analysis for
'SEZ6L' on 'LUSC'

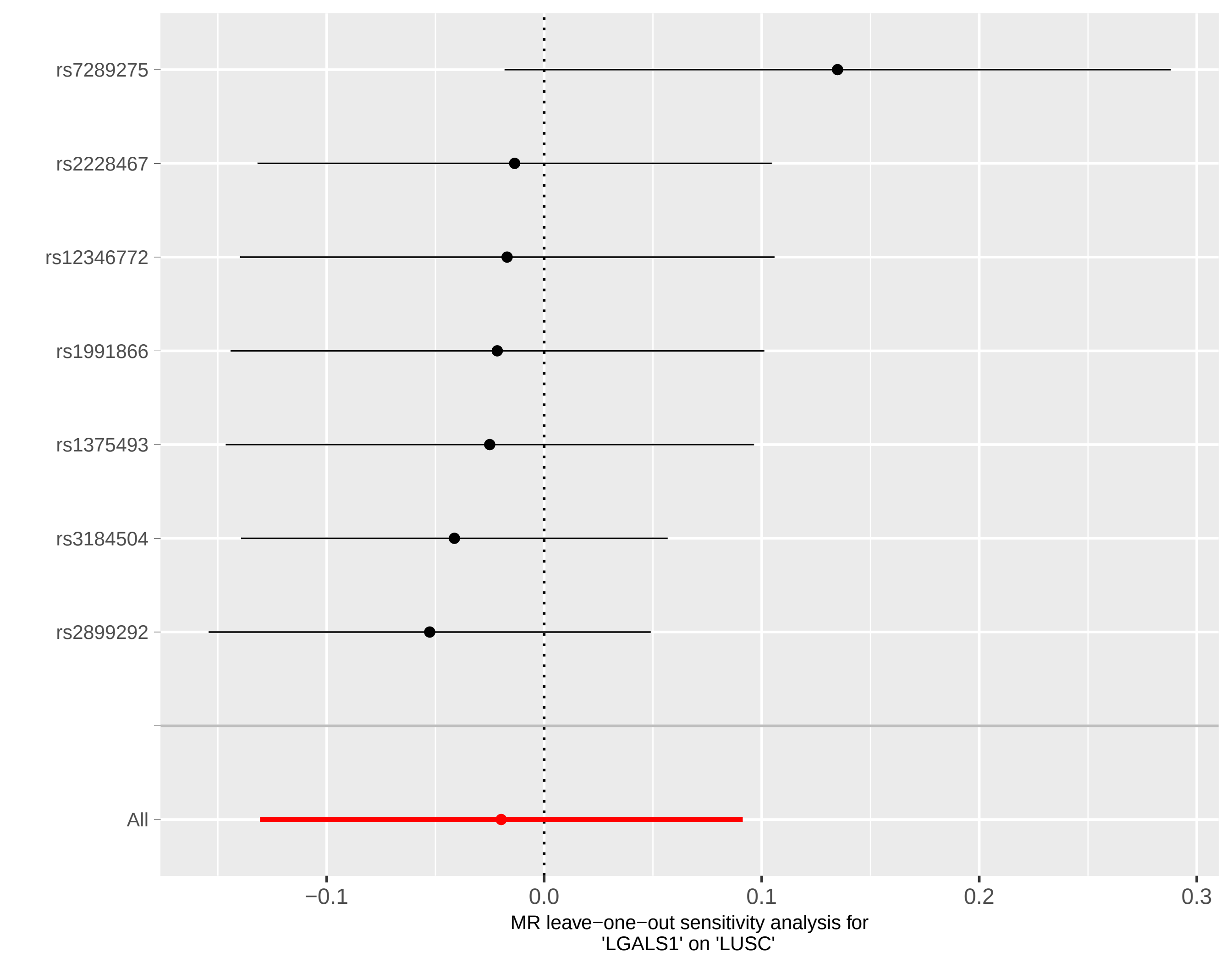
rs7289275
rs2228467
rs12346772
rs1991866
rs1375493
rs3184504
rs2899292
All
−0.1
0.0
0.1
0.2
0.3
MR leave−one−out sensitivity analysis for
'LGALS1' on 'LUSC'

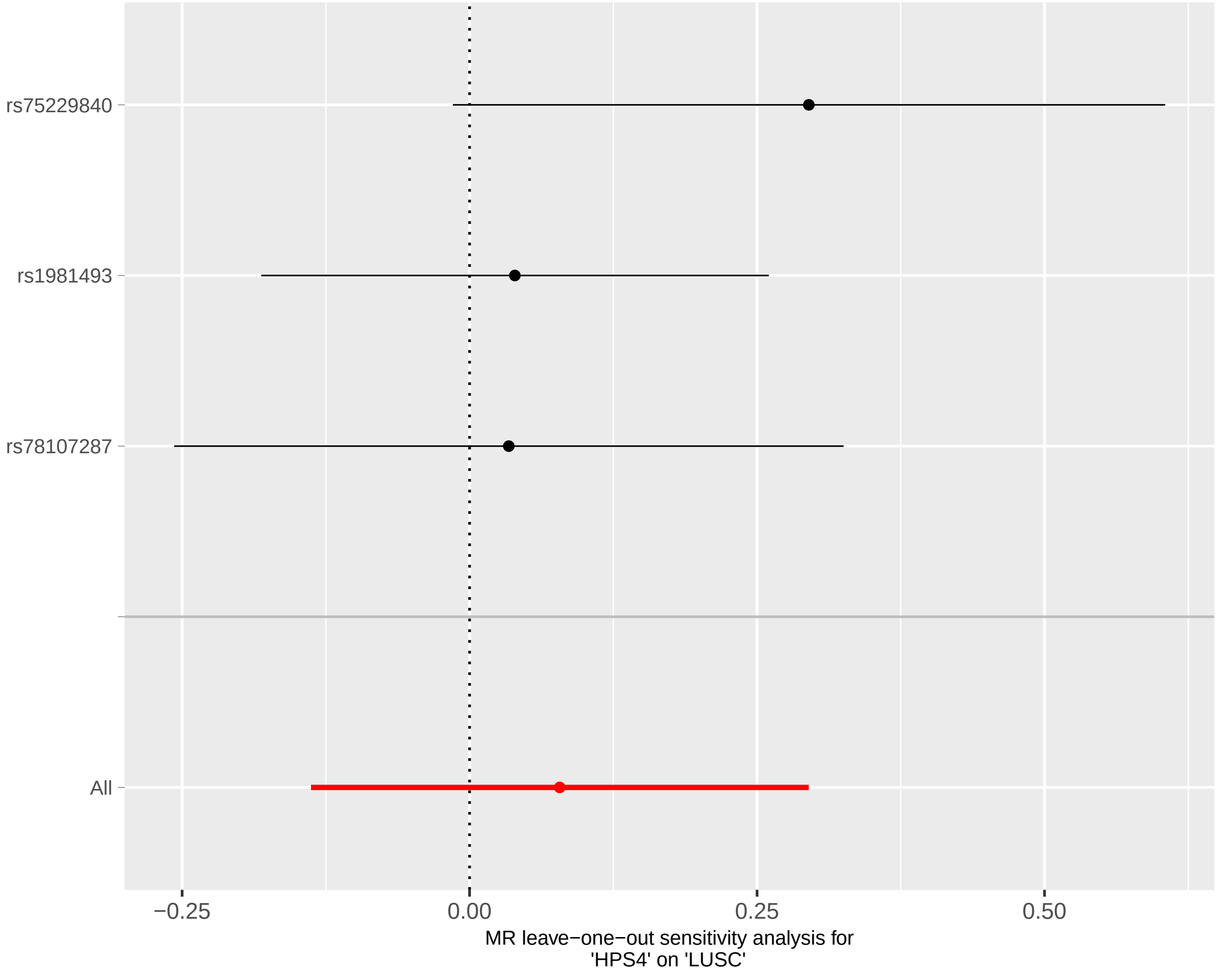
rs75229840
rs1981493
rs78107287
All
−0.25
0.00
0.25
0.50
MR leave−one−out sensitivity analysis for
'HPS4' on 'LUSC'

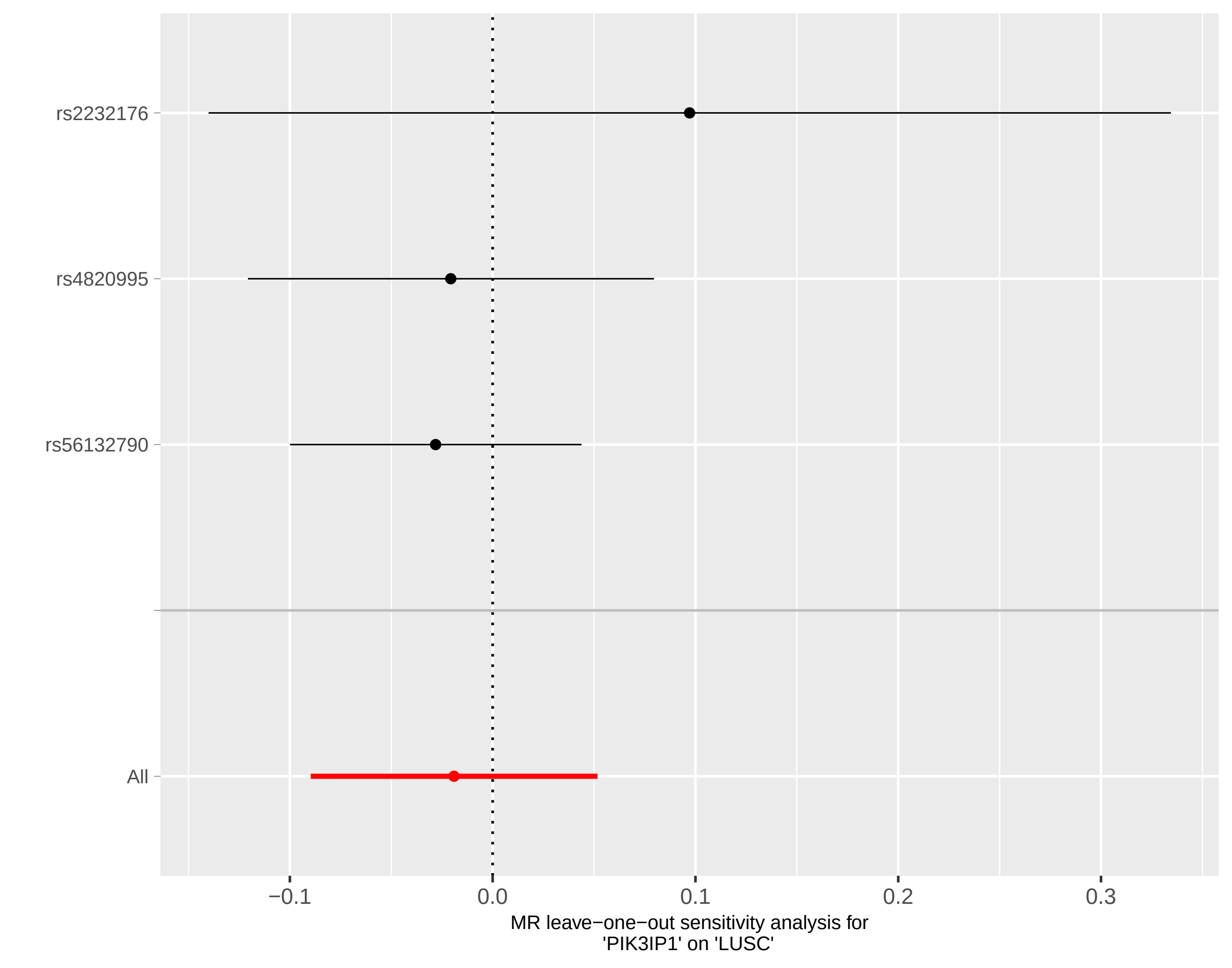
rs2232176
rs4820995
rs56132790
All
−0.1
0.0
0.1
0.2
0.3
MR leave−one−out sensitivity analysis for
'PIK3IP1' on 'LUSC'

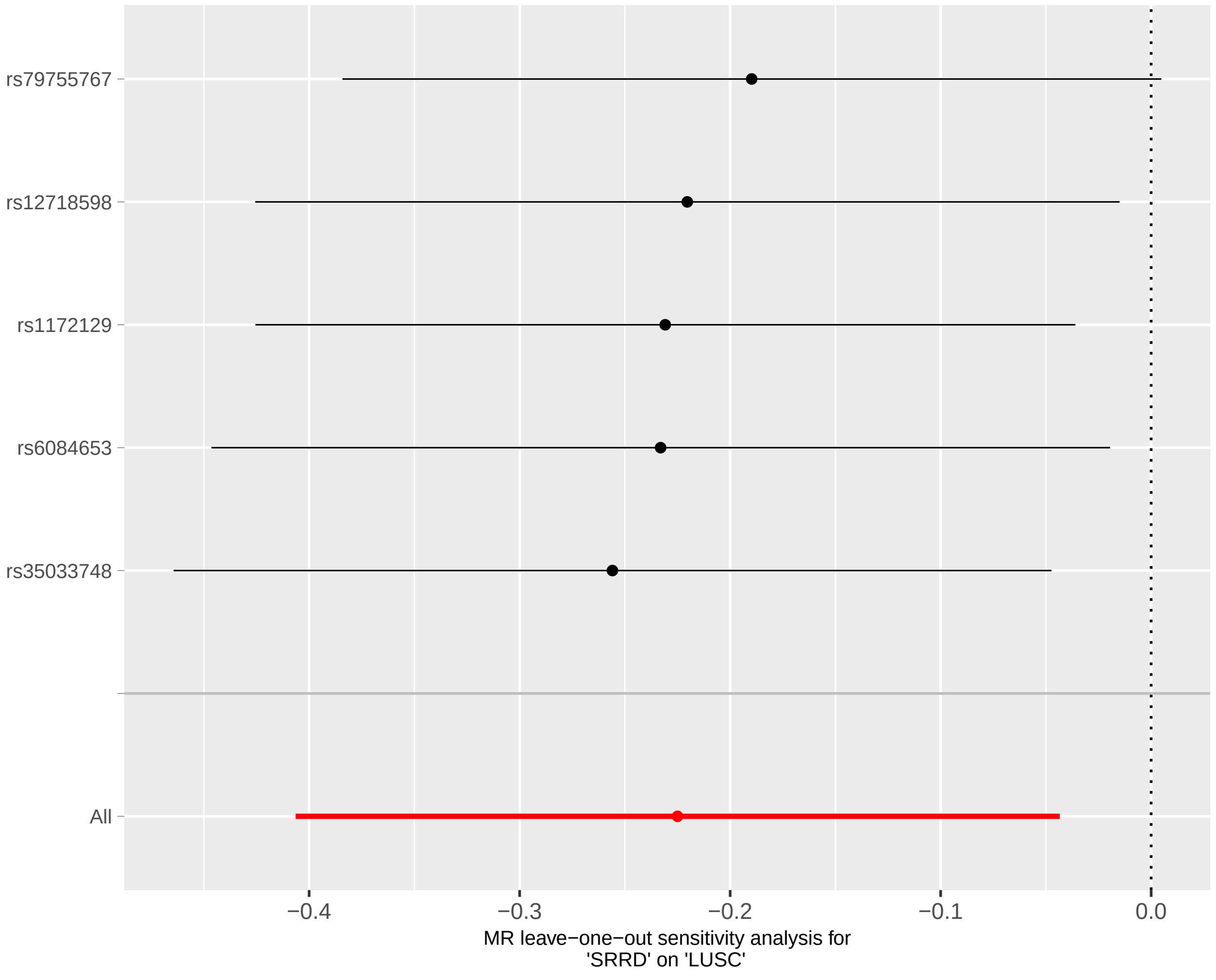
rs79755767
rs12718598
rs1172129
rs6084653
rs35033748
All
−0.4
−0.3
−0.2
−0.1
0.0
MR leave−one−out sensitivity analysis for
'SRRD' on 'LUSC'

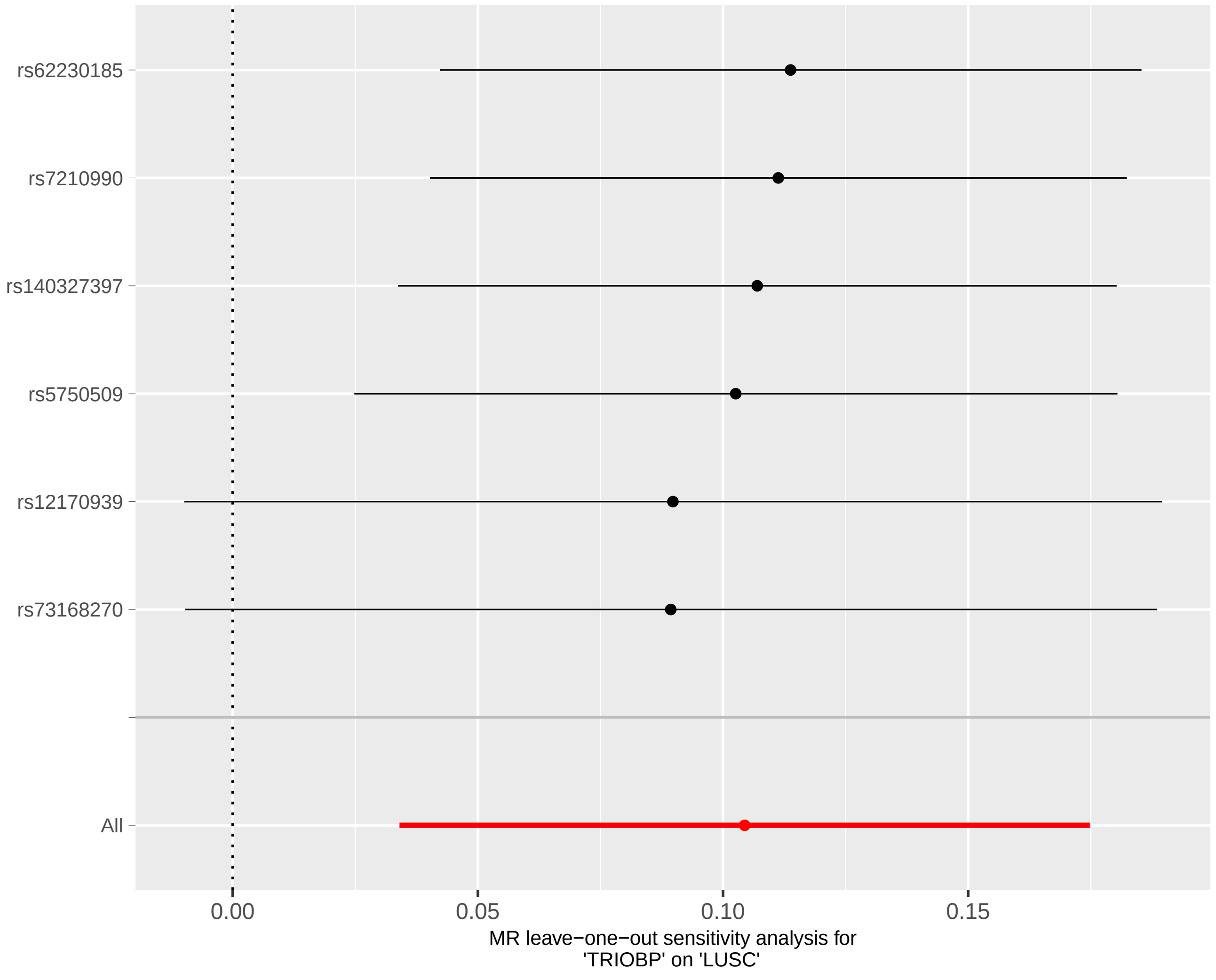
rs62230185
rs7210990
rs140327397
rs5750509
rs12170939
rs73168270
All
0.00
0.05
0.10
0.15
MR leave−one−out sensitivity analysis for
'TRIOBP' on 'LUSC'

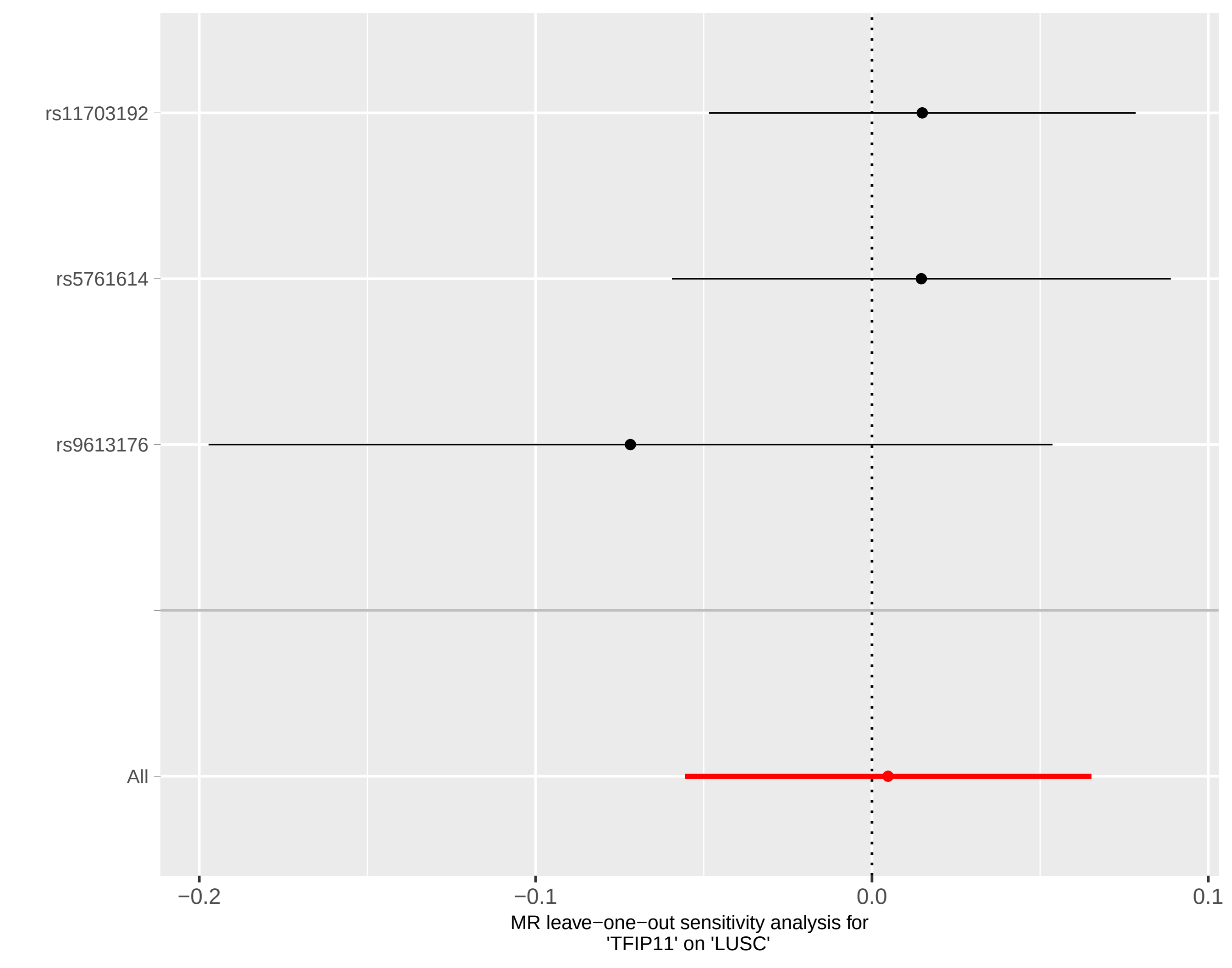
rs11703192
rs5761614
rs9613176
All
−0.2
−0.1
0.0
0.1
MR leave−one−out sensitivity analysis for
'TFIP11' on 'LUSC'

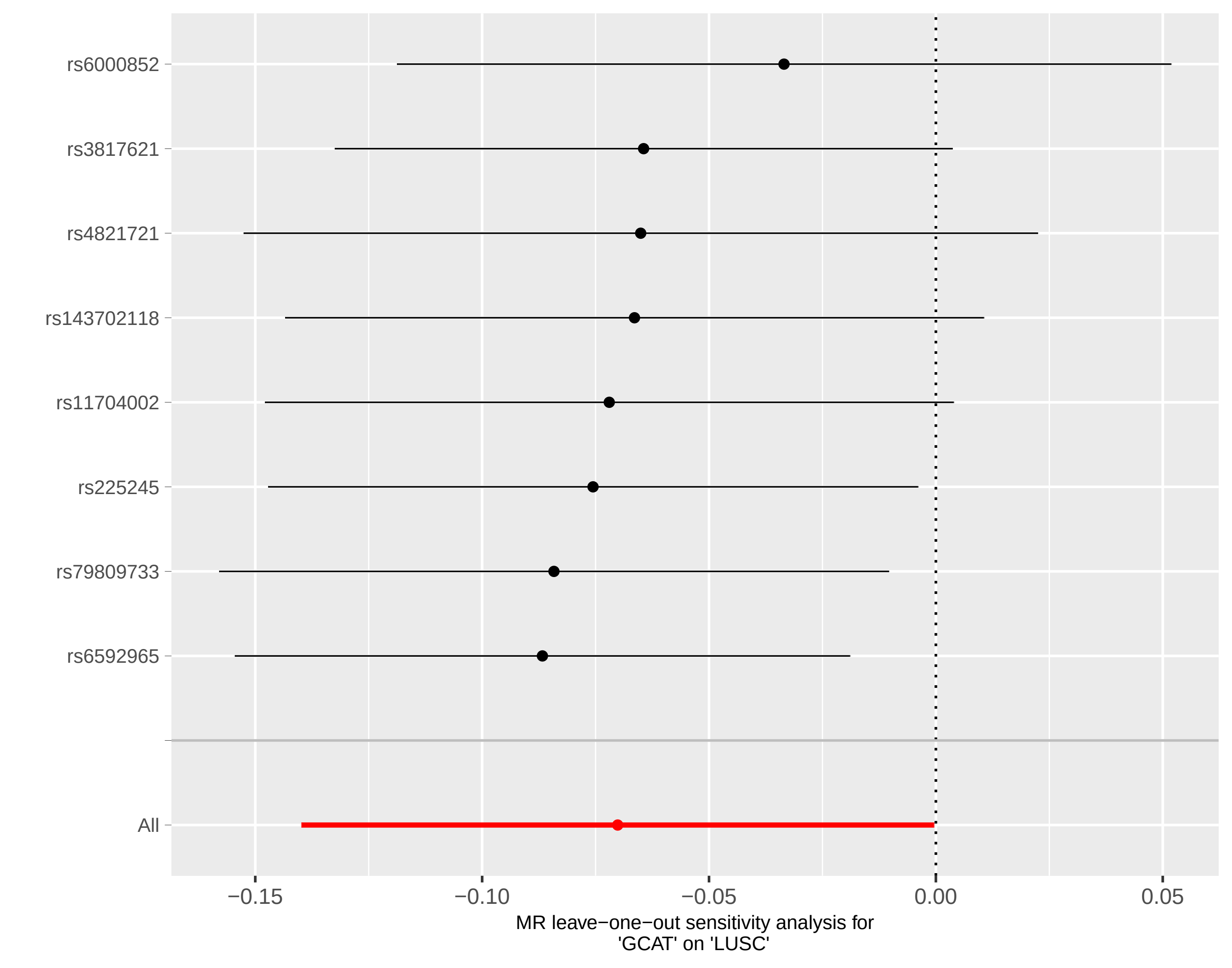

rs6000852
rs3817621
rs4821721
rs143702118
rs11704002
rs225245
rs79809733
rs6592965
All
−0.15
−0.10
−0.05
0.00
0.05
MR leave−one−out sensitivity analysis for
'GCAT' on 'LUSC'

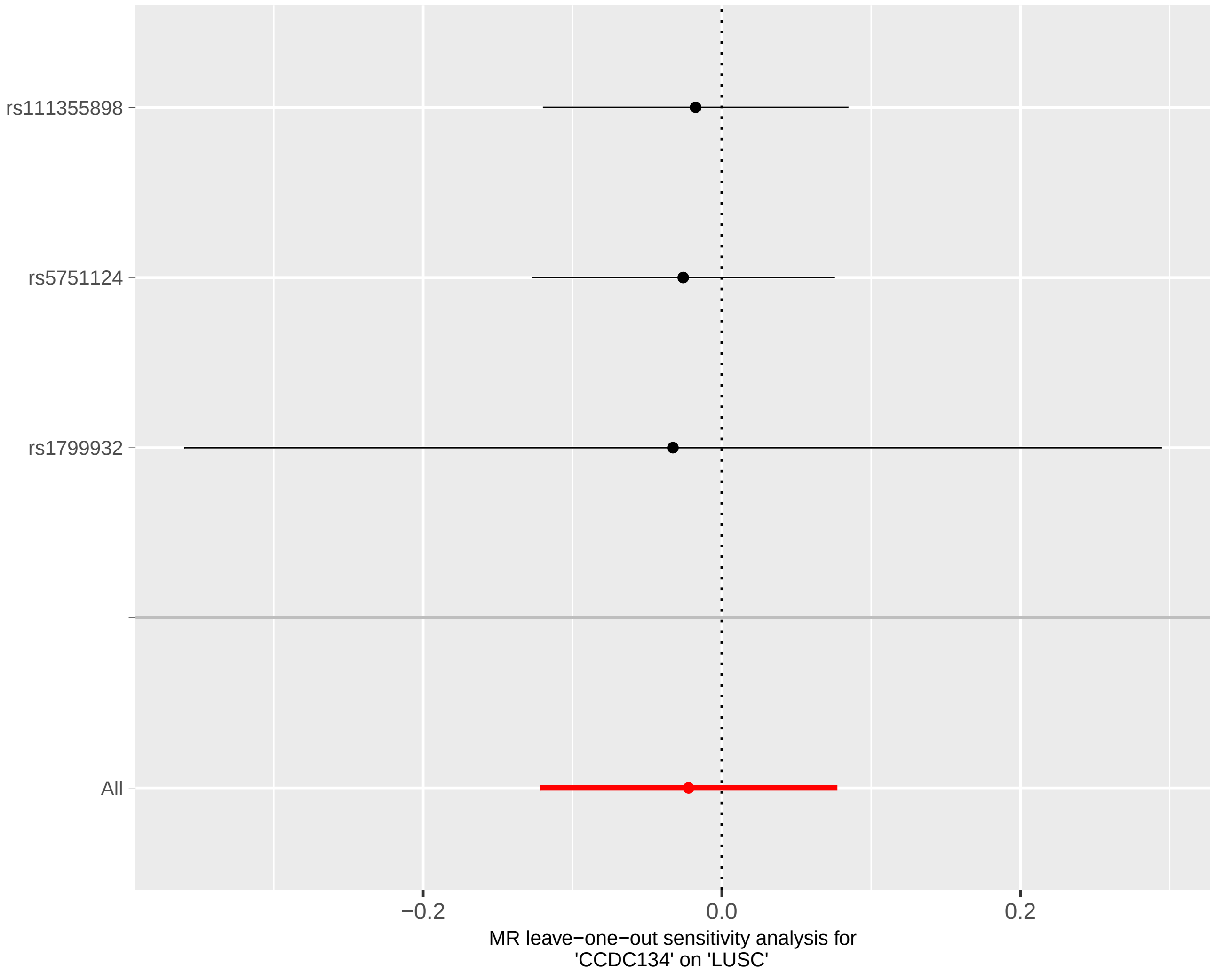

rs111355898
rs5751124
rs1799932
All
−0.2
0.0
0.2
MR leave−one−out sensitivity analysis for
'CCDC134' on 'LUSC'

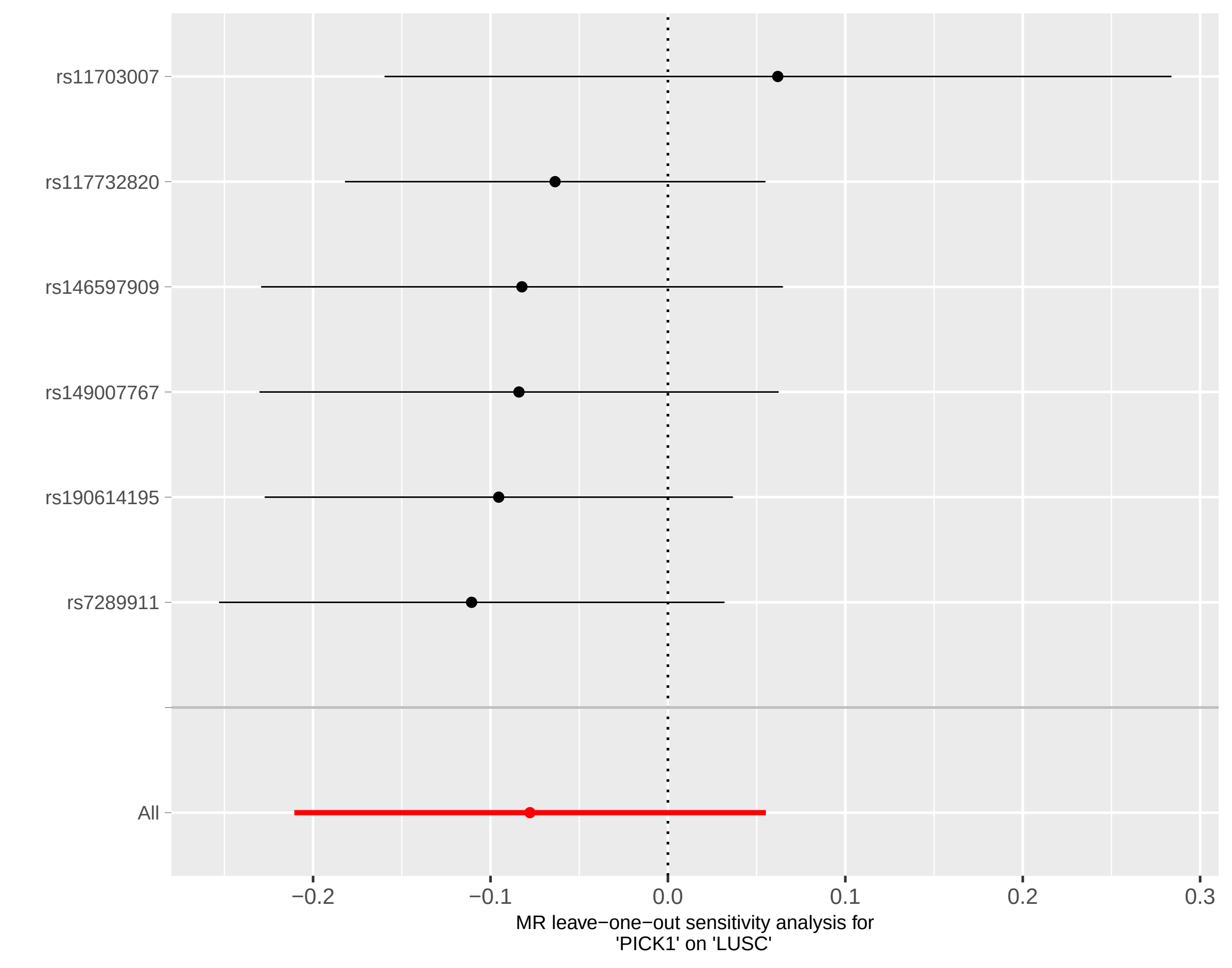
rs11703007
rs117732820
rs146597909
rs149007767
rs190614195
rs7289911
All
−0.2
−0.1
0.0
0.1
0.2
0.3
MR leave−one−out sensitivity analysis for
'PICK1' on 'LUSC'

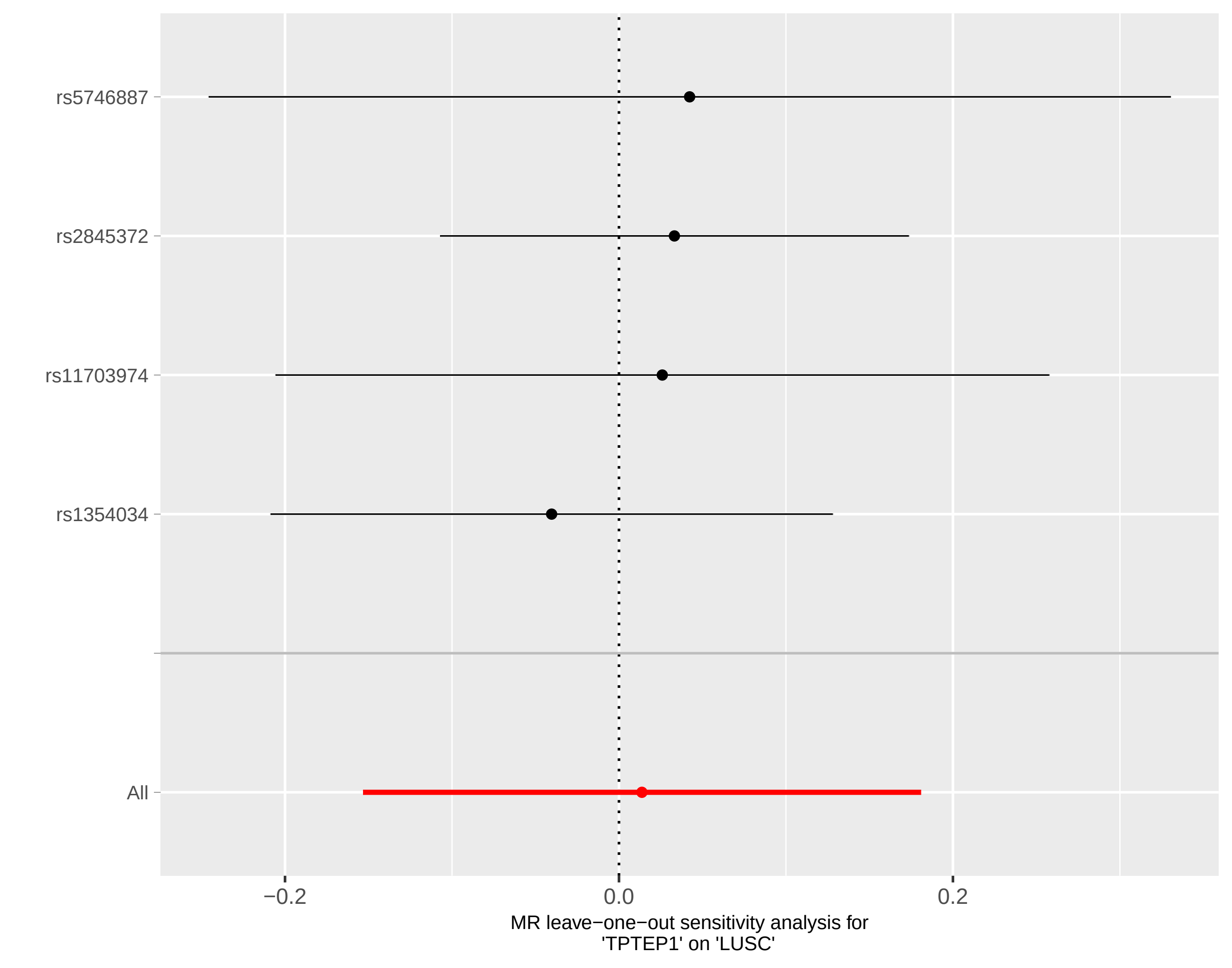
rs5746887
rs2845372
rs11703974
rs1354034
All
−0.2
0.0
0.2
MR leave−one−out sensitivity analysis for
'TPTEP1' on 'LUSC'

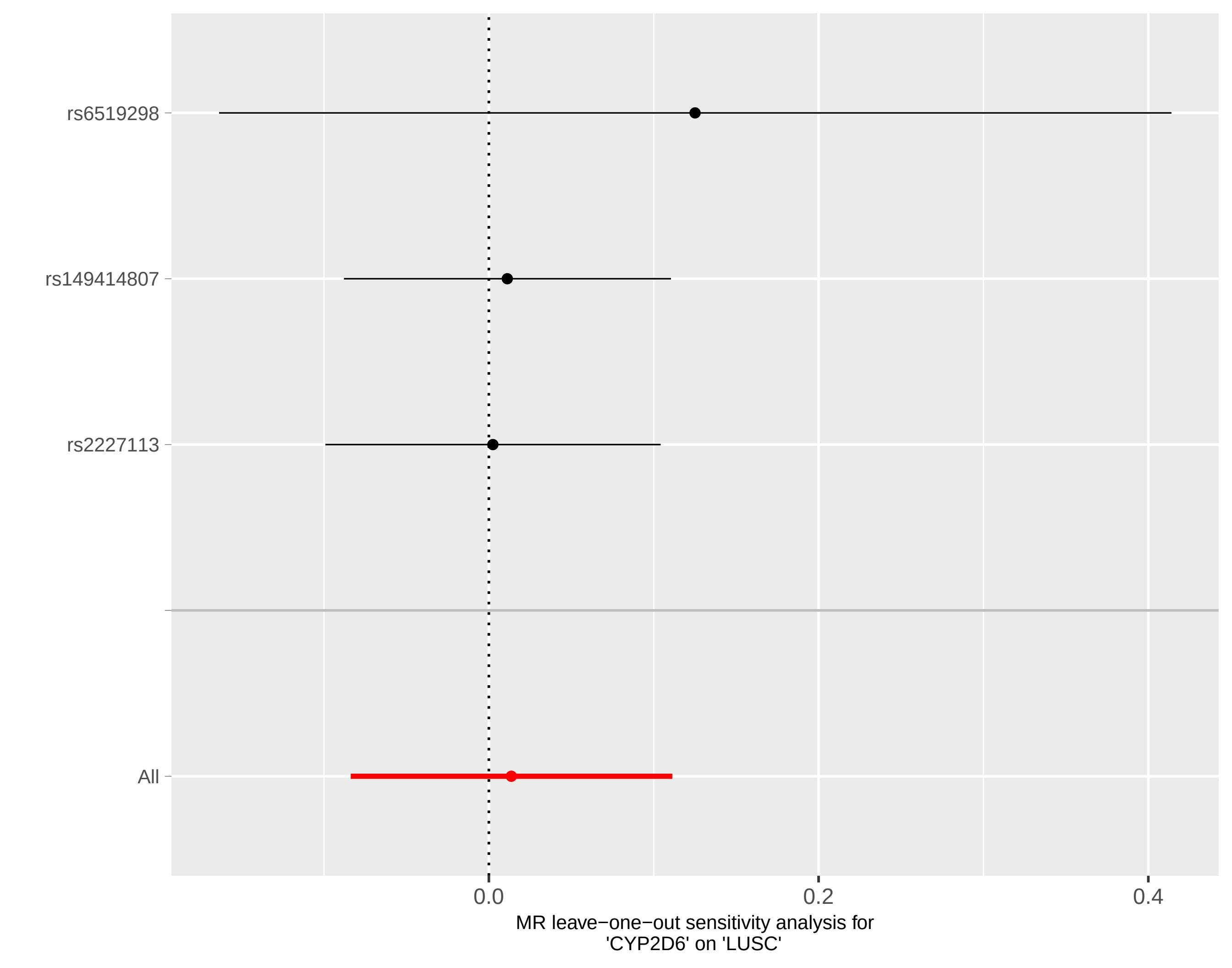
rs6519298
rs149414807
rs2227113
All
0.0
0.2
0.4
MR leave−one−out sensitivity analysis for
'CYP2D6' on 'LUSC'

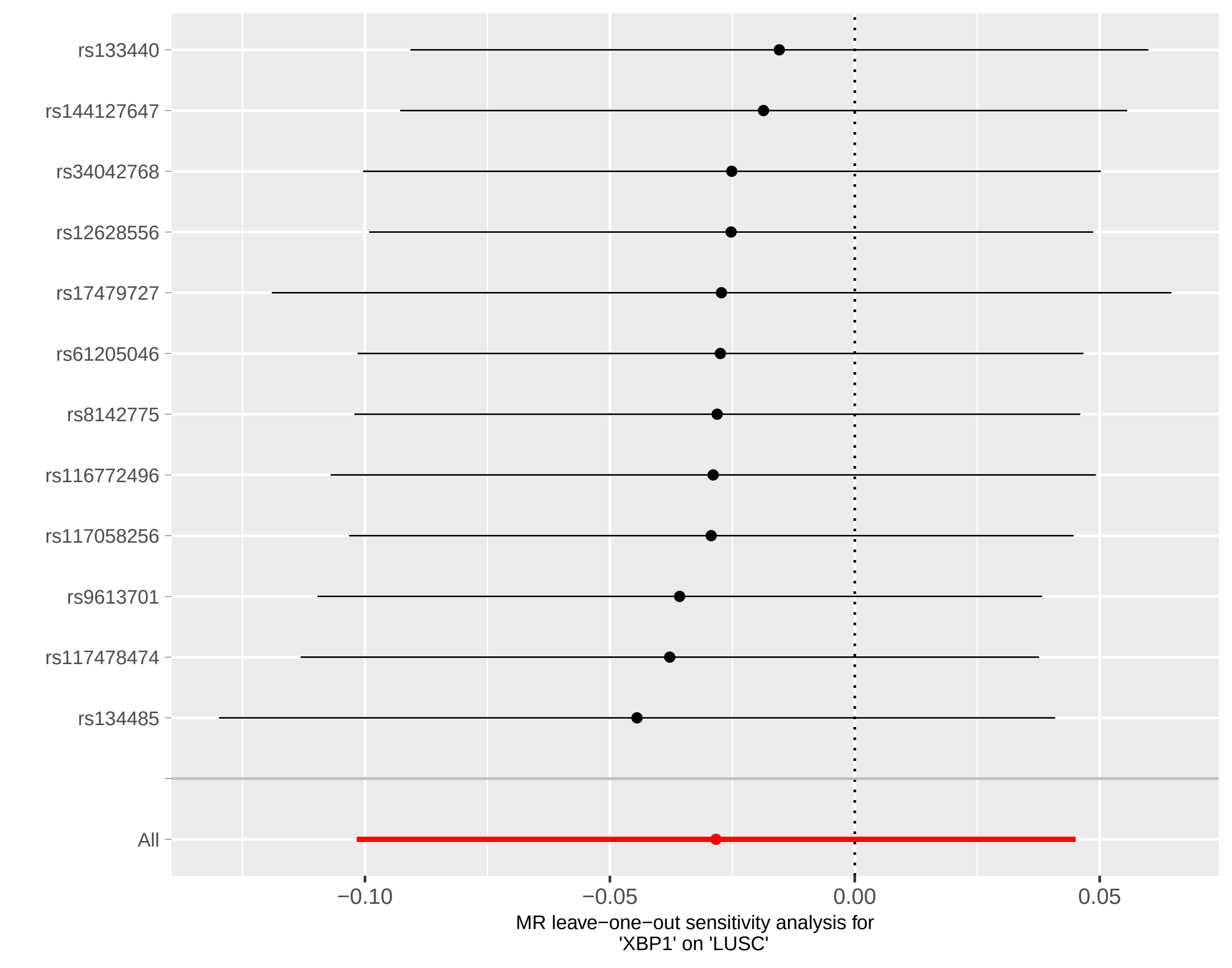
rs133440
rs144127647
rs34042768
rs12628556
rs17479727
rs61205046
rs8142775
rs116772496
rs117058256
rs9613701
rs117478474
rs134485
All
−0.10
−0.05
0.00
0.05
MR leave−one−out sensitivity analysis for
'XBP1' on 'LUSC'

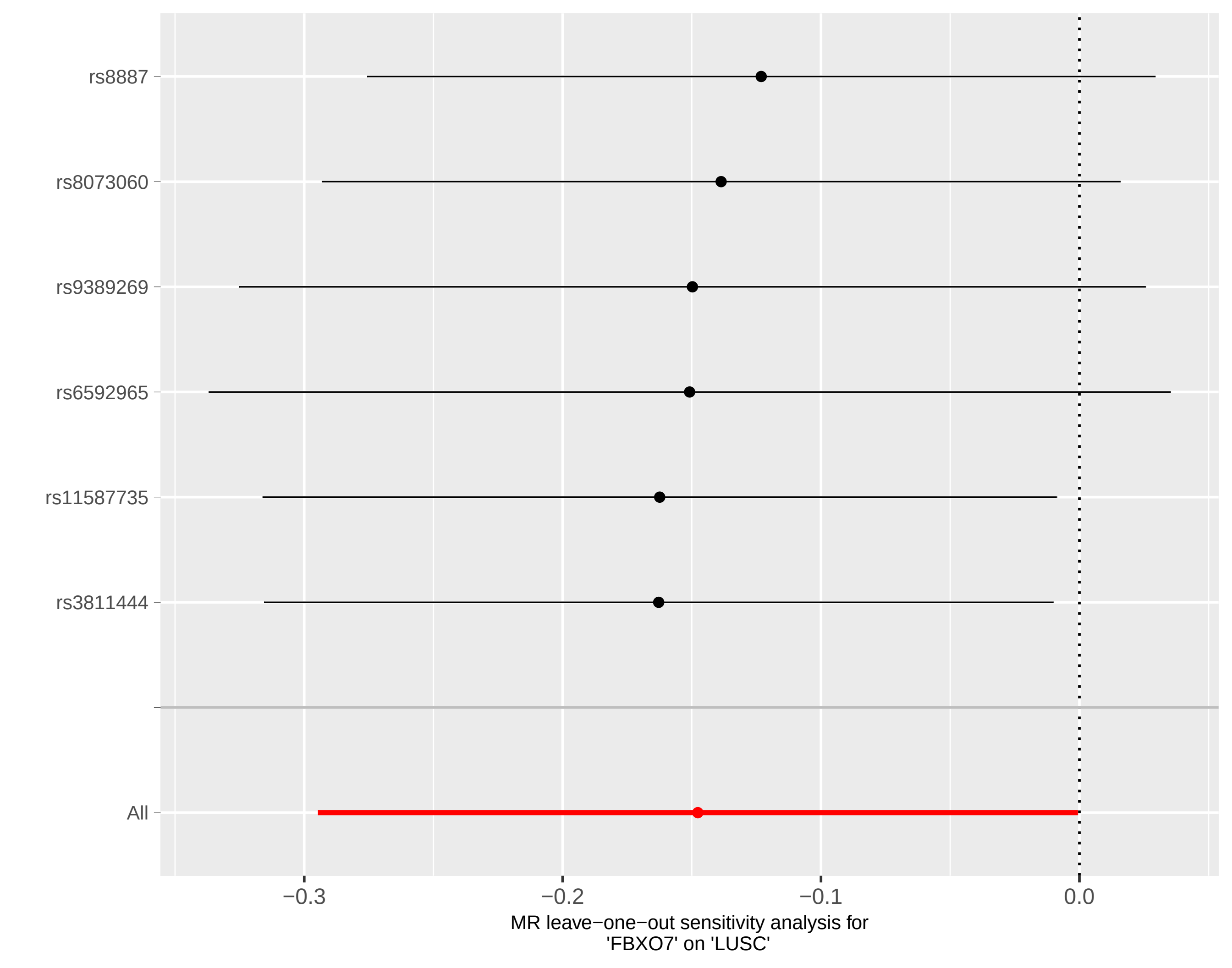
rs8887
rs8073060
rs9389269
rs6592965
rs11587735
rs3811444
All
−0.3
−0.2
−0.1
0.0
MR leave−one−out sensitivity analysis for
'FBXO7' on 'LUSC'

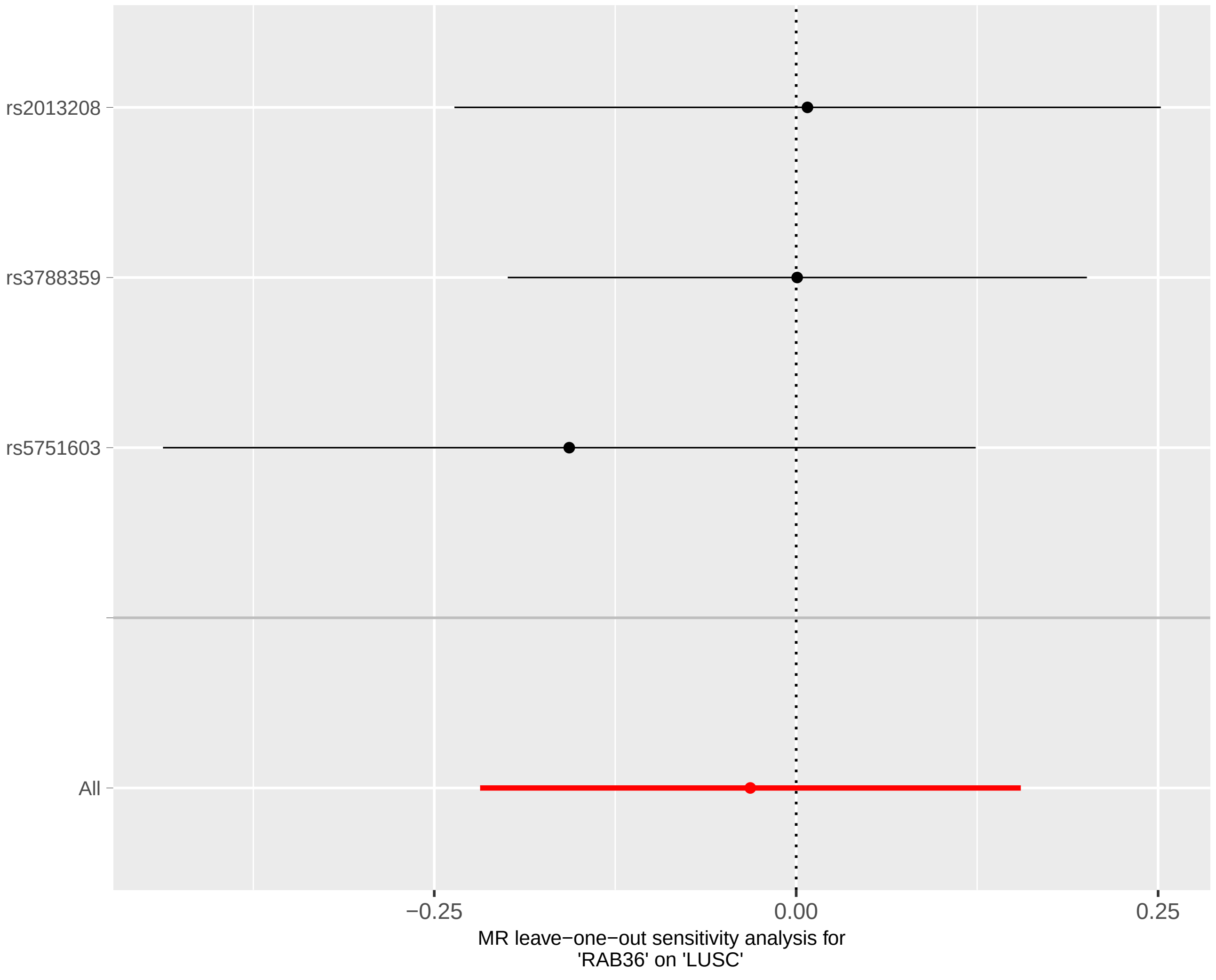
rs2013208
rs3788359
rs5751603
All
−0.25
0.00
0.25
MR leave−one−out sensitivity analysis for
'RAB36' on 'LUSC'

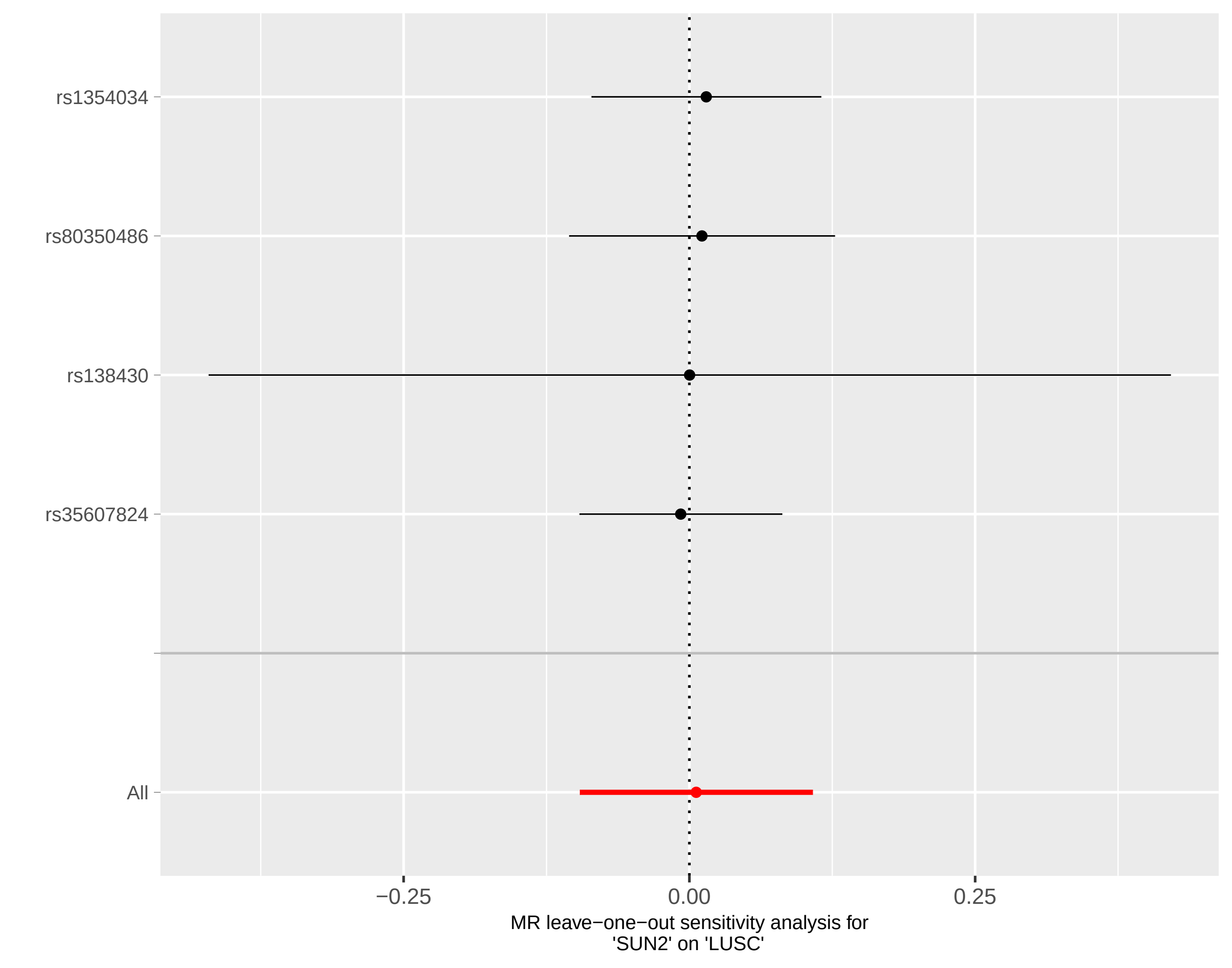
rs1354034
rs80350486
rs138430
rs35607824
All
−0.25
0.00
0.25
MR leave−one−out sensitivity analysis for
'SUN2' on 'LUSC'

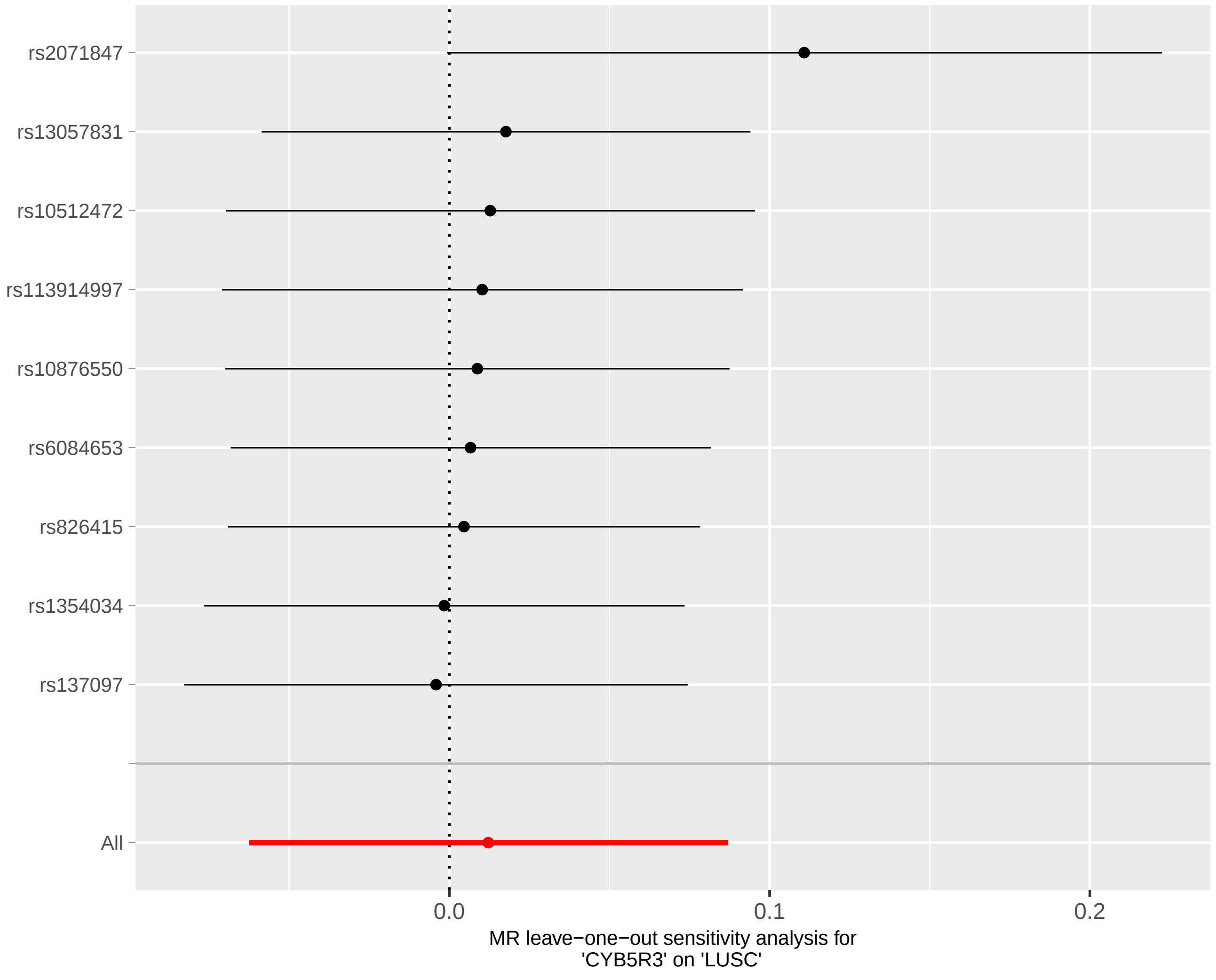
rs2071847
rs13057831
rs10512472
rs113914997
rs10876550
rs6084653
rs826415
rs1354034
rs137097
All
0.0
0.1
0.2
MR leave−one−out sensitivity analysis for
'CYB5R3' on 'LUSC'

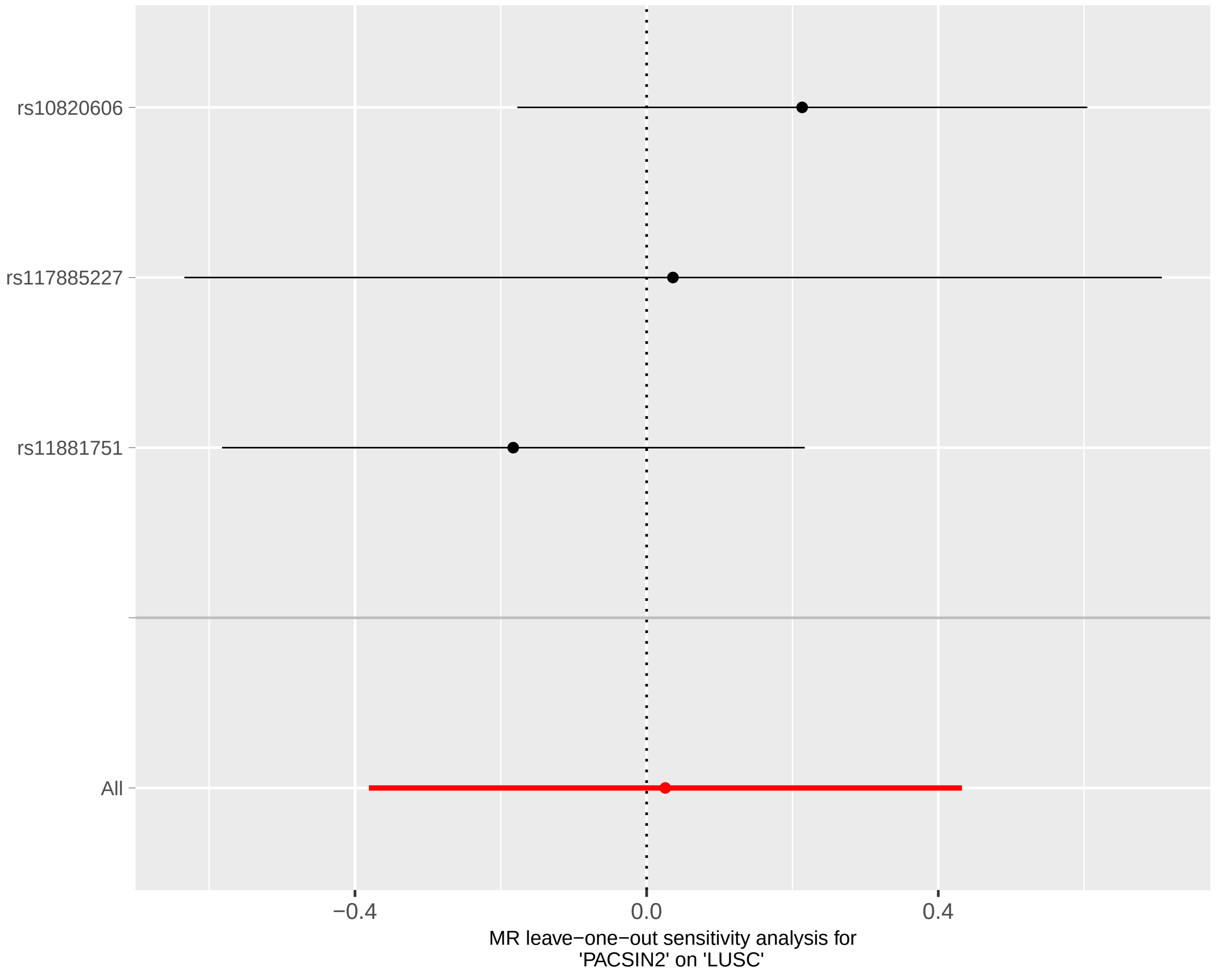

rs10820606
rs117885227
rs11881751
All
−0.4
0.0
0.4
MR leave−one−out sensitivity analysis for
'PACSIN2' on 'LUSC'

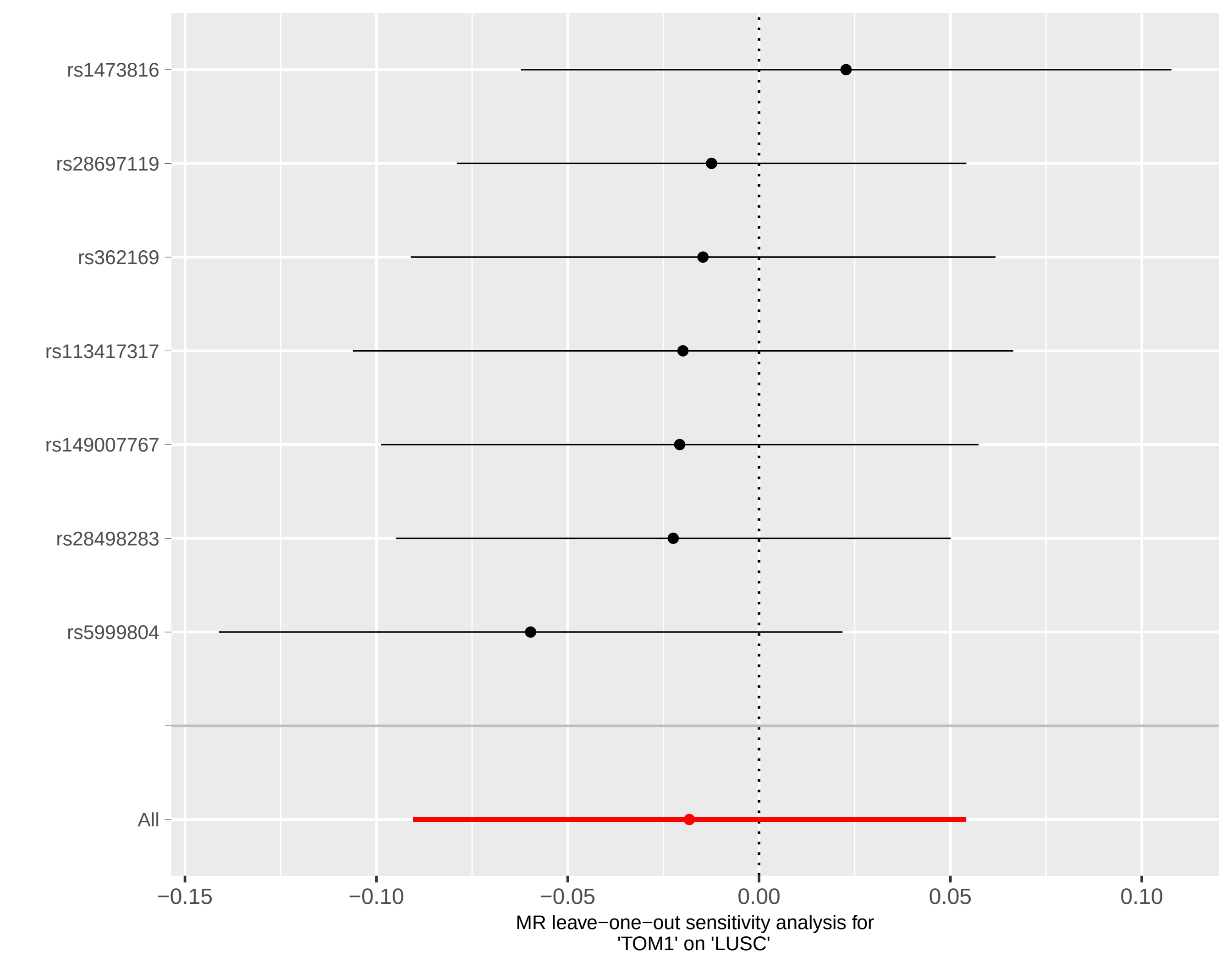
rs1473816
rs28697119
rs362169
rs113417317
rs149007767
rs28498283
rs5999804
All
−0.15
−0.10
−0.05
0.00
0.05
0.10
MR leave−one−out sensitivity analysis for
'TOM1' on 'LUSC'

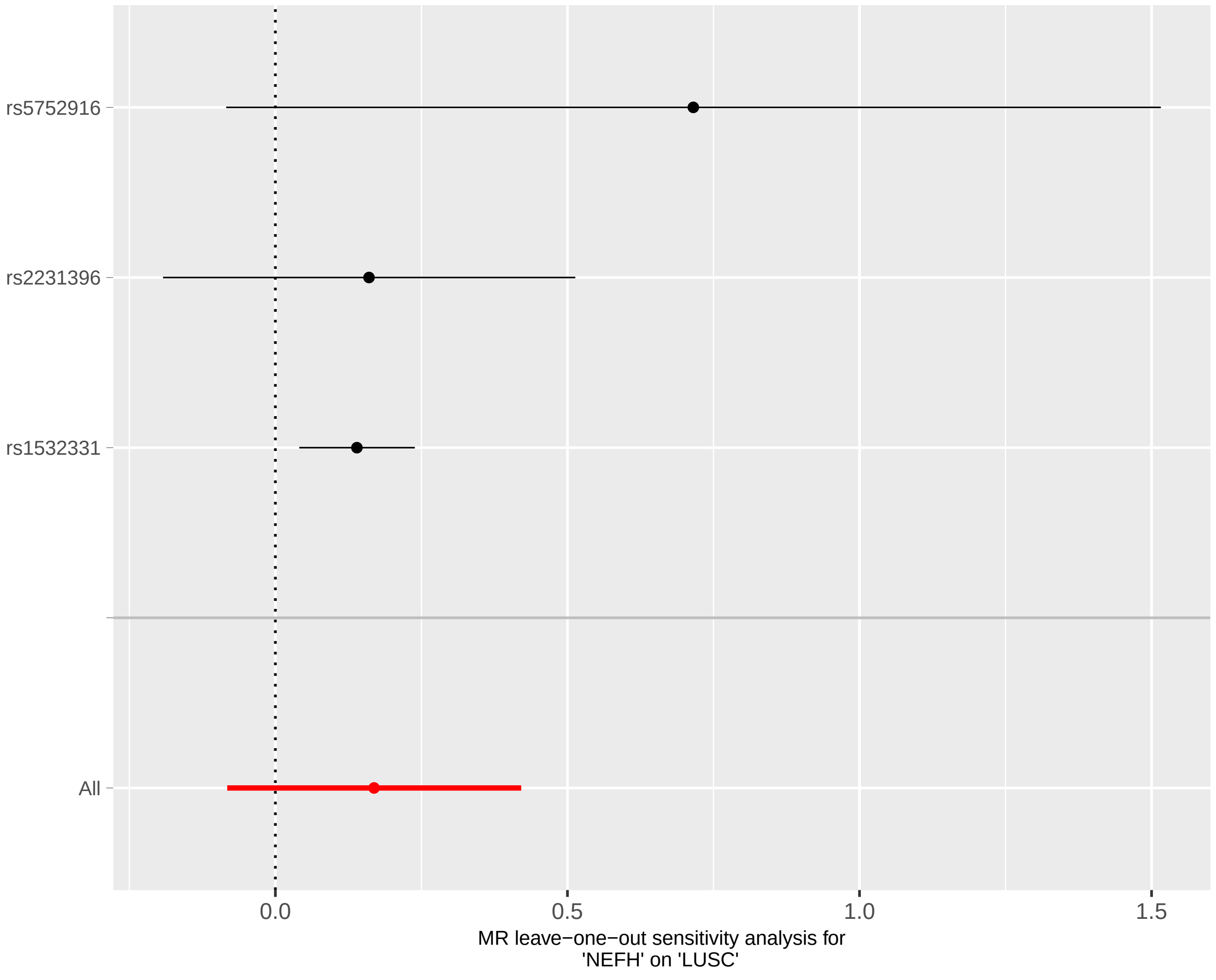
rs5752916
rs2231396
rs1532331
All
0.0
0.5
1.0
1.5
MR leave−one−out sensitivity analysis for
'NEFH' on 'LUSC'

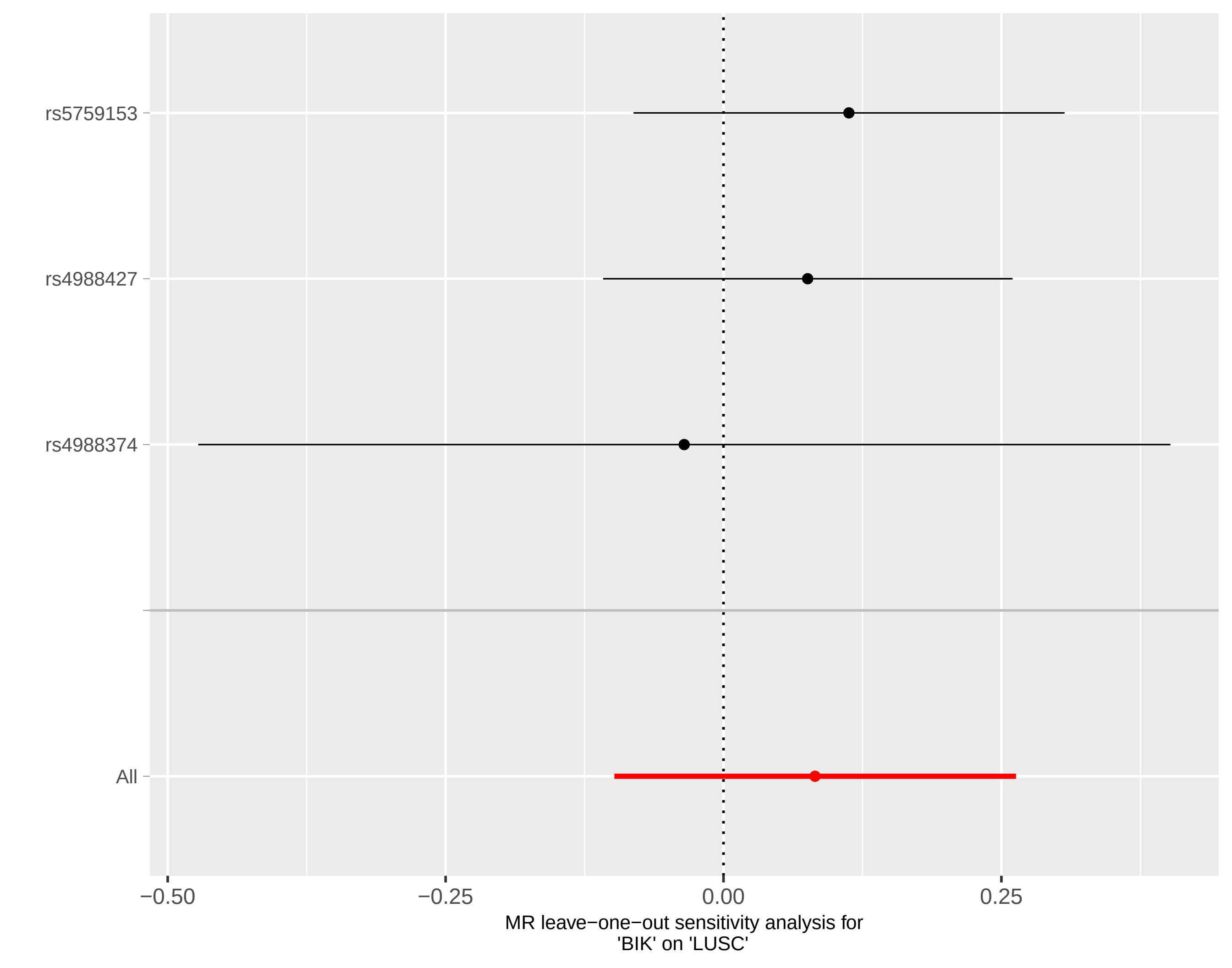
rs5759153
rs4988427
rs4988374
All
−0.50
−0.25
0.00
0.25
MR leave−one−out sensitivity analysis for
'BIK' on 'LUSC'

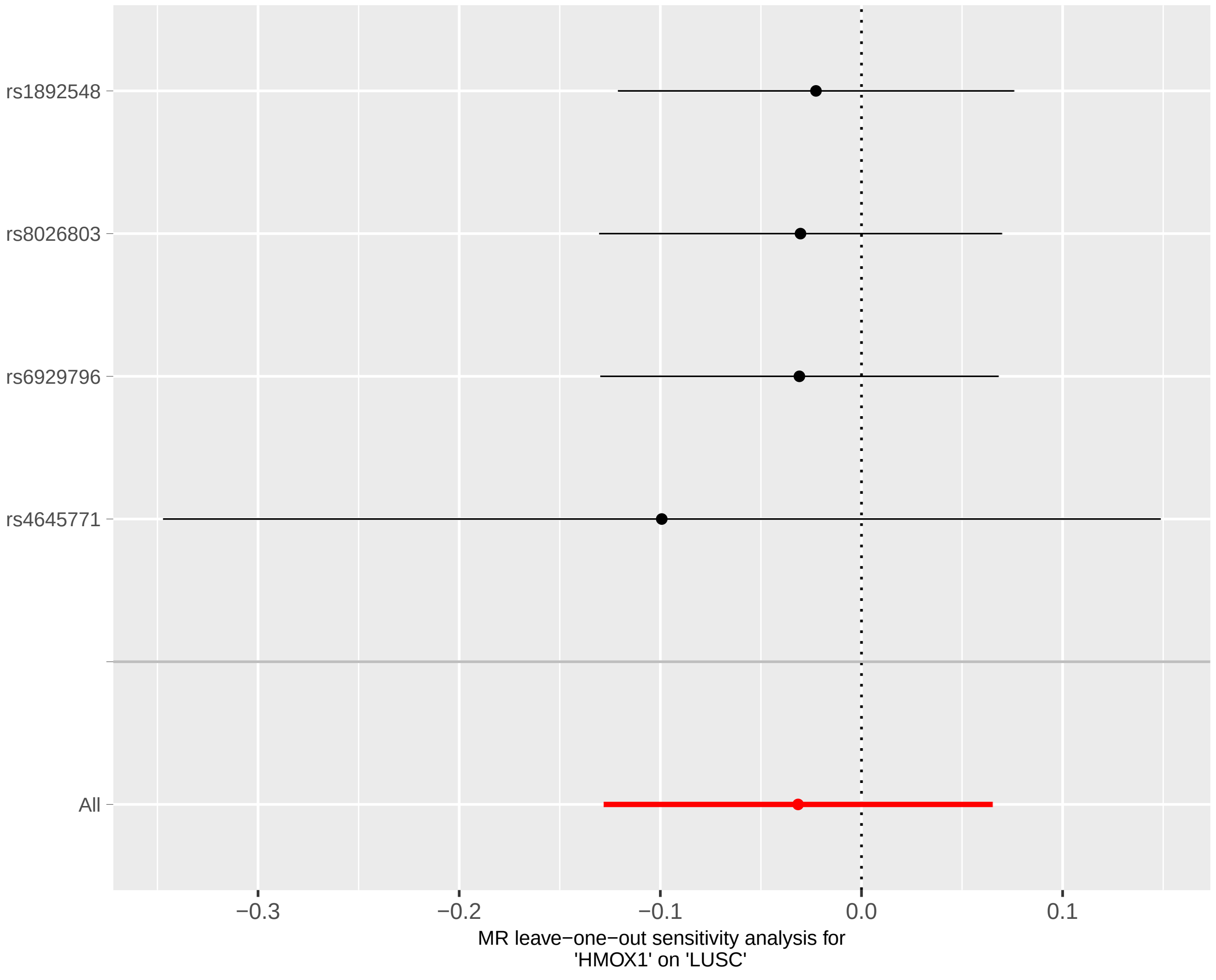
rs1892548
rs8026803
rs6929796
rs4645771
All
−0.3
−0.2
−0.1
0.0
0.1
MR leave−one−out sensitivity analysis for
'HMOX1' on 'LUSC'

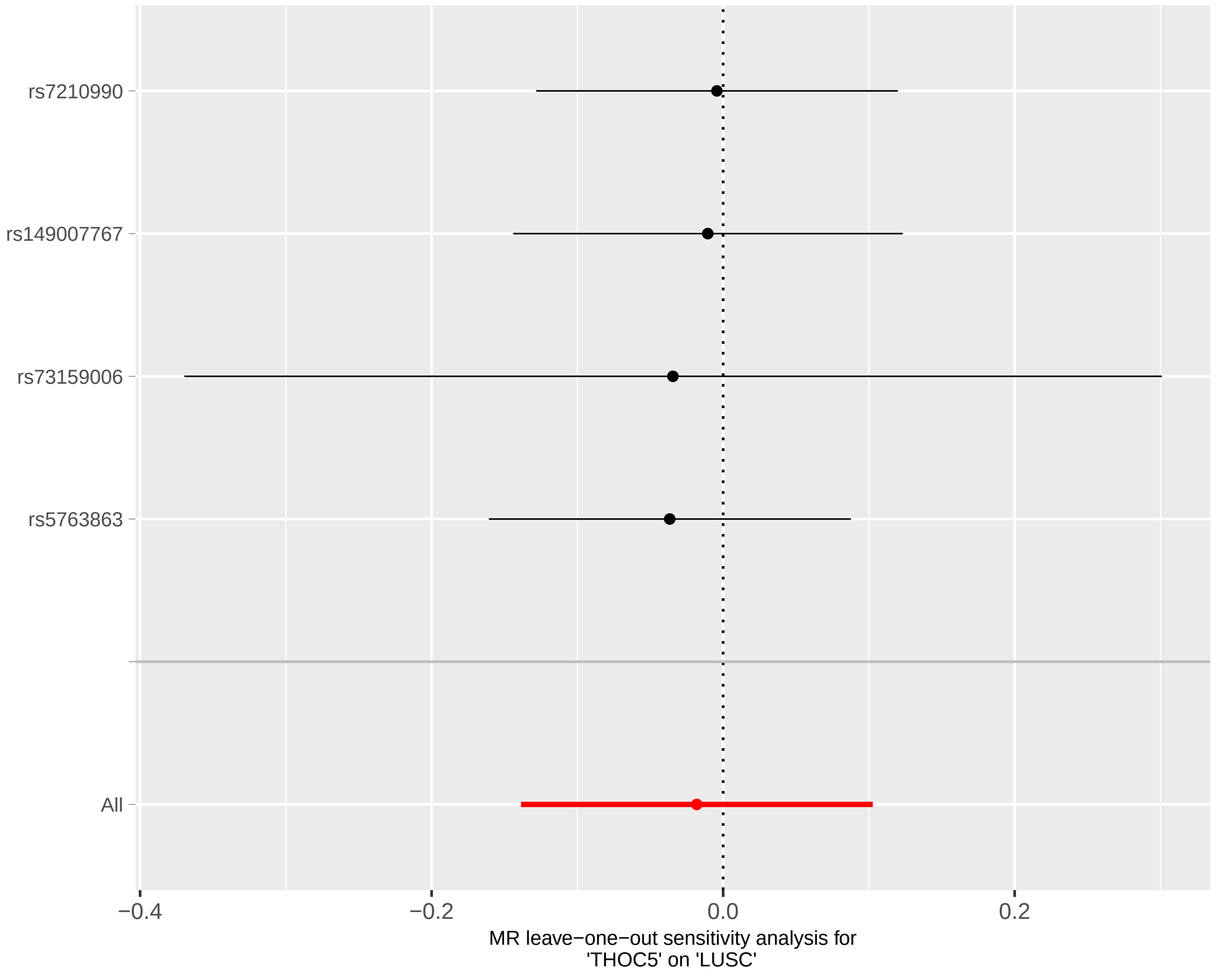
rs7210990
rs149007767
rs73159006
rs5763863
All
−0.4
−0.2
0.0
0.2
MR leave−one−out sensitivity analysis for
'THOC5' on 'LUSC'

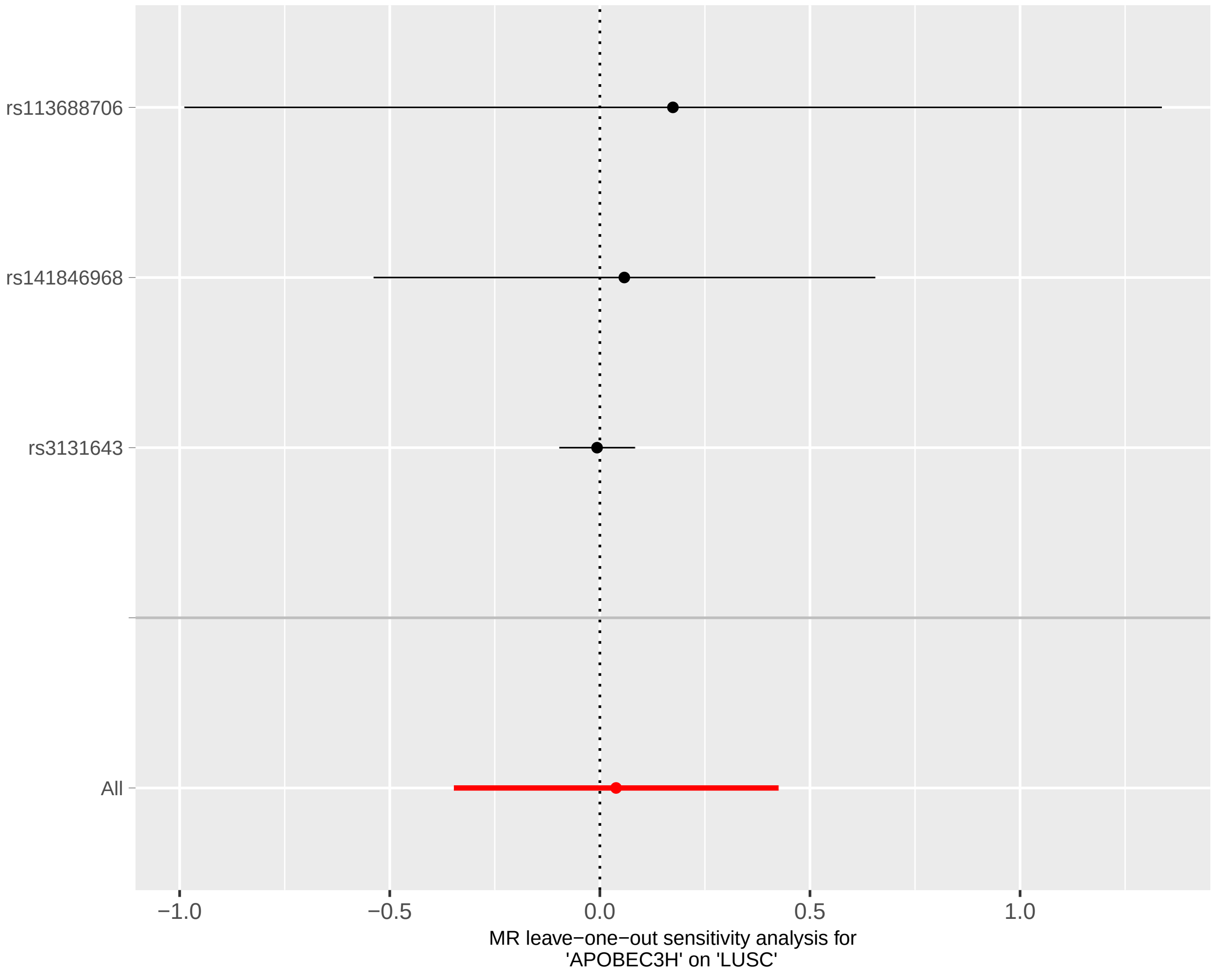
rs113688706
rs141846968
rs3131643
All
−1.0
−0.5
0.0
0.5
1.0
MR leave−one−out sensitivity analysis for
'APOBEC3H' on 'LUSC'

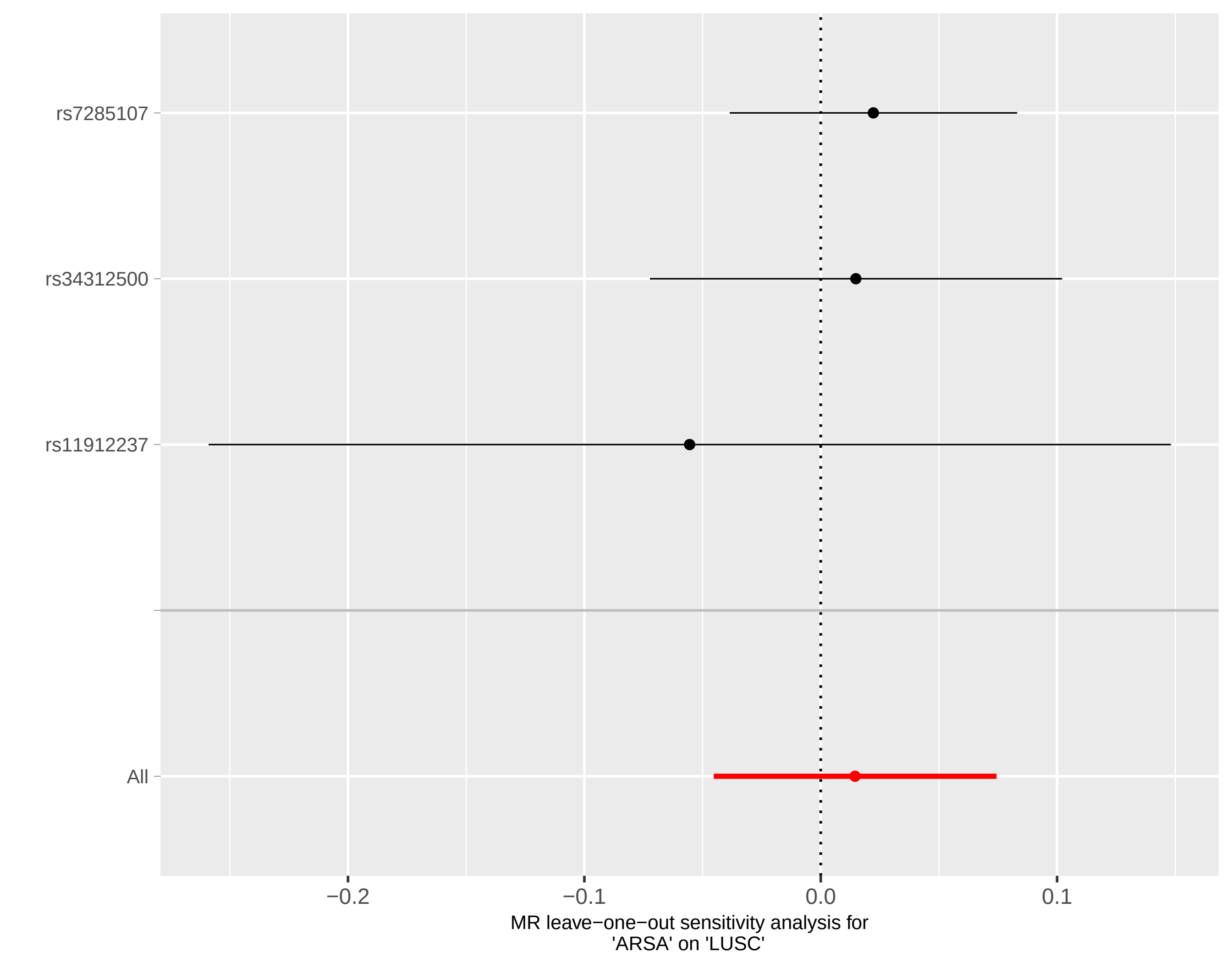

rs7285107
rs34312500
rs11912237
All
−0.2
−0.1
0.0
0.1
MR leave−one−out sensitivity analysis for
'ARSA' on 'LUSC'

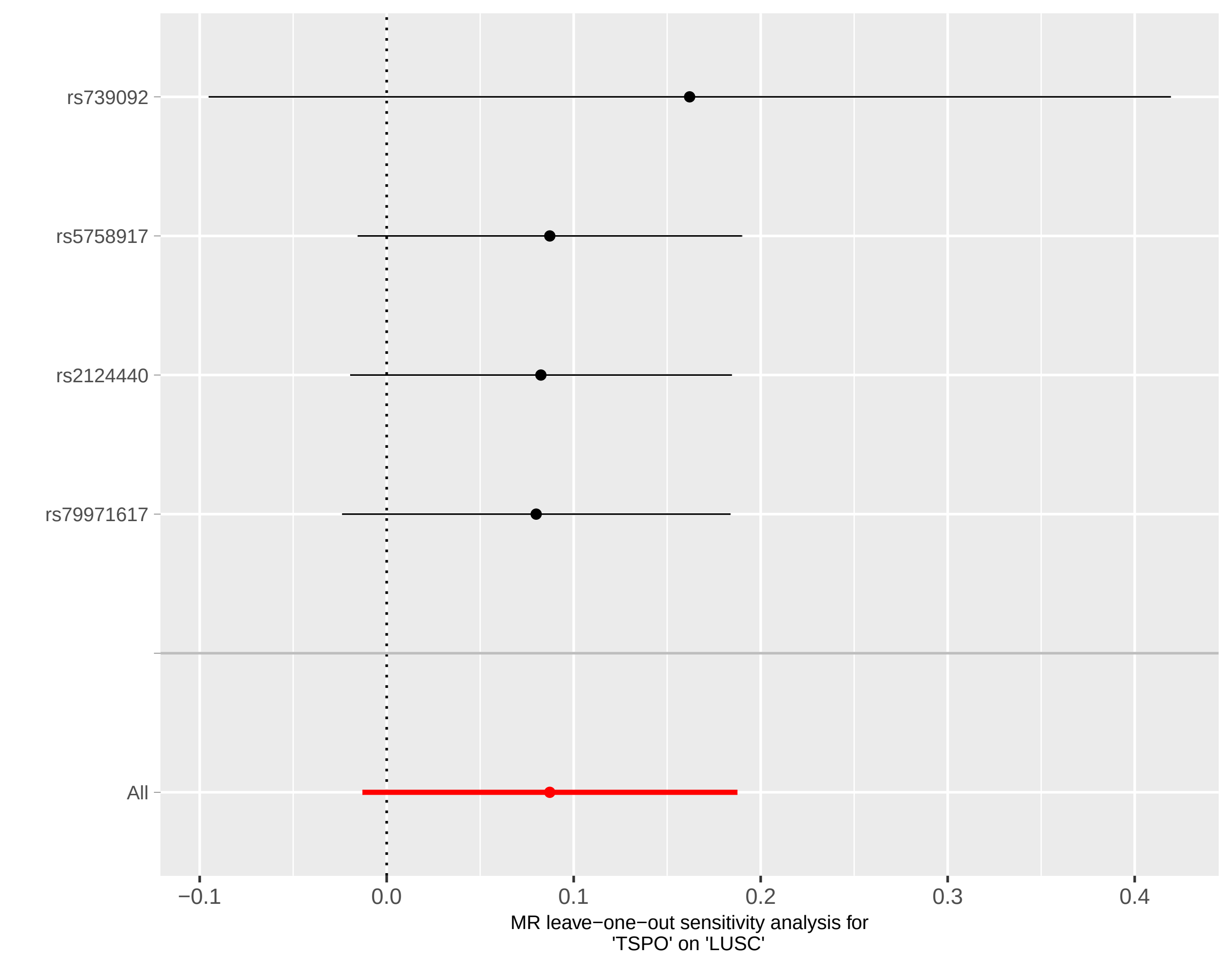

rs739092
rs5758917
rs2124440
rs79971617
All
−0.1
0.0
0.1
0.2
0.3
0.4
MR leave−one−out sensitivity analysis for
'TSPO' on 'LUSC'

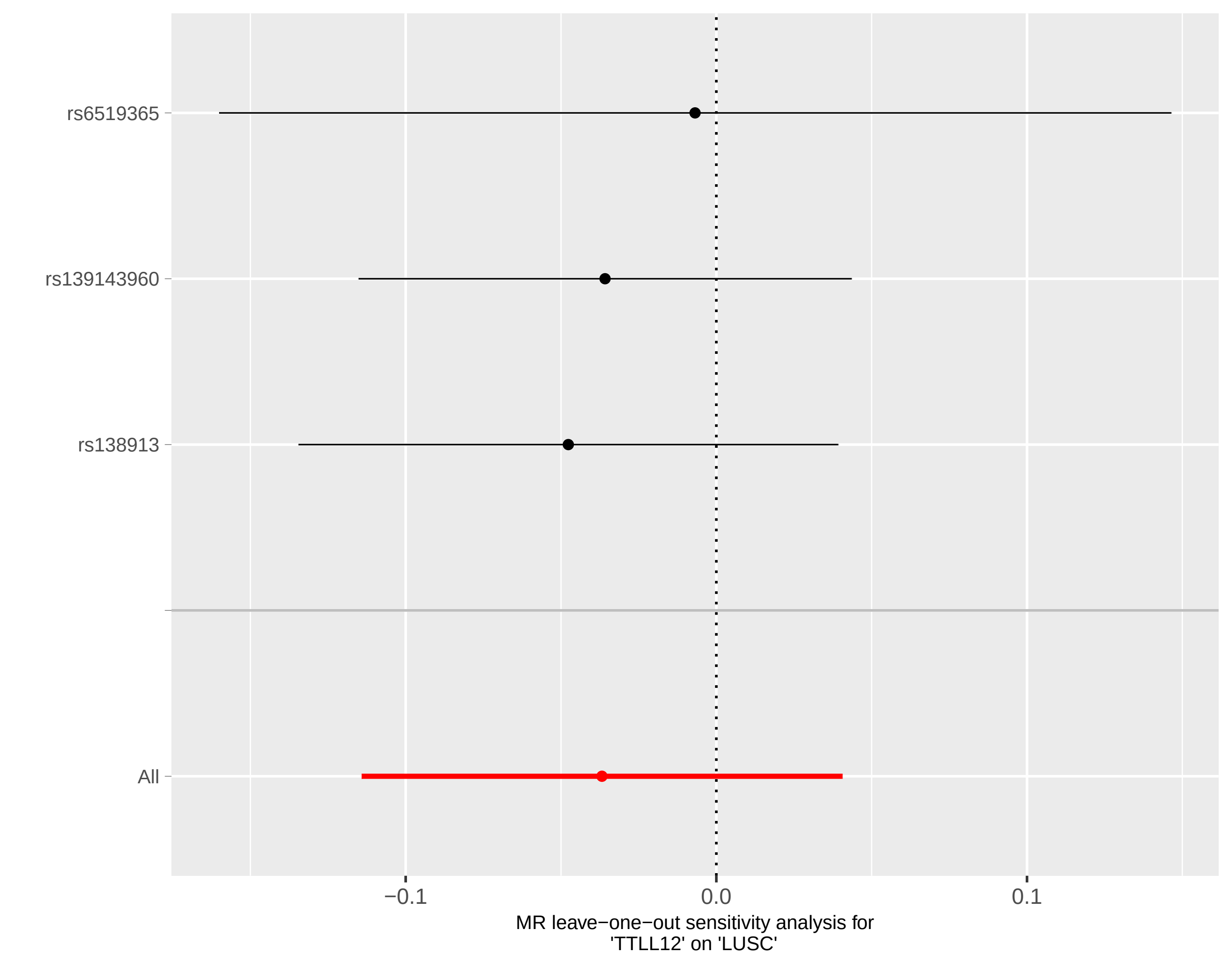

rs6519365
rs139143960
rs138913
All
−0.1
0.0
0.1
MR leave−one−out sensitivity analysis for
'TTLL12' on 'LUSC'

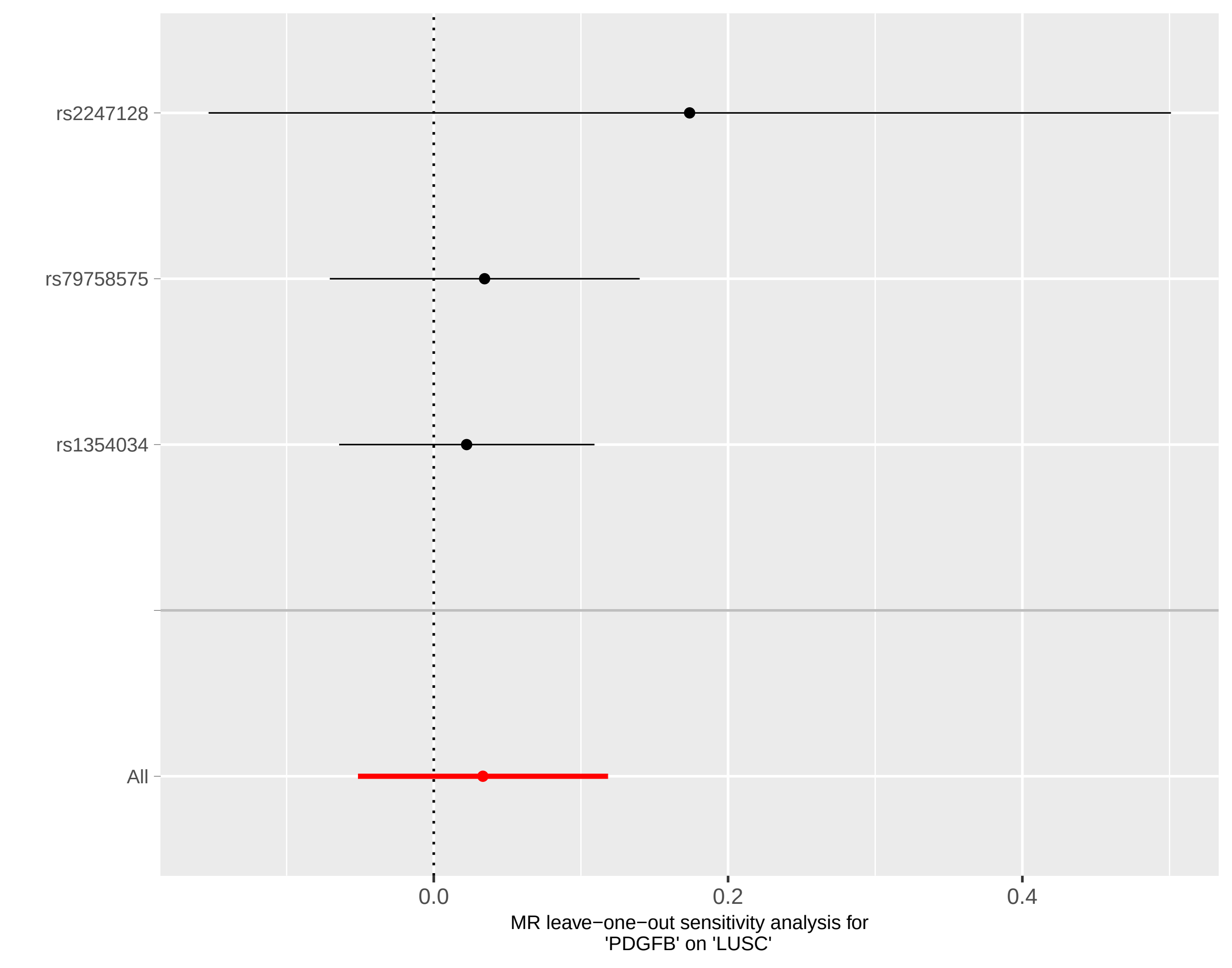
rs2247128
rs79758575
rs1354034
All
0.0
0.2
0.4
MR leave−one−out sensitivity analysis for
'PDGFB' on 'LUSC'

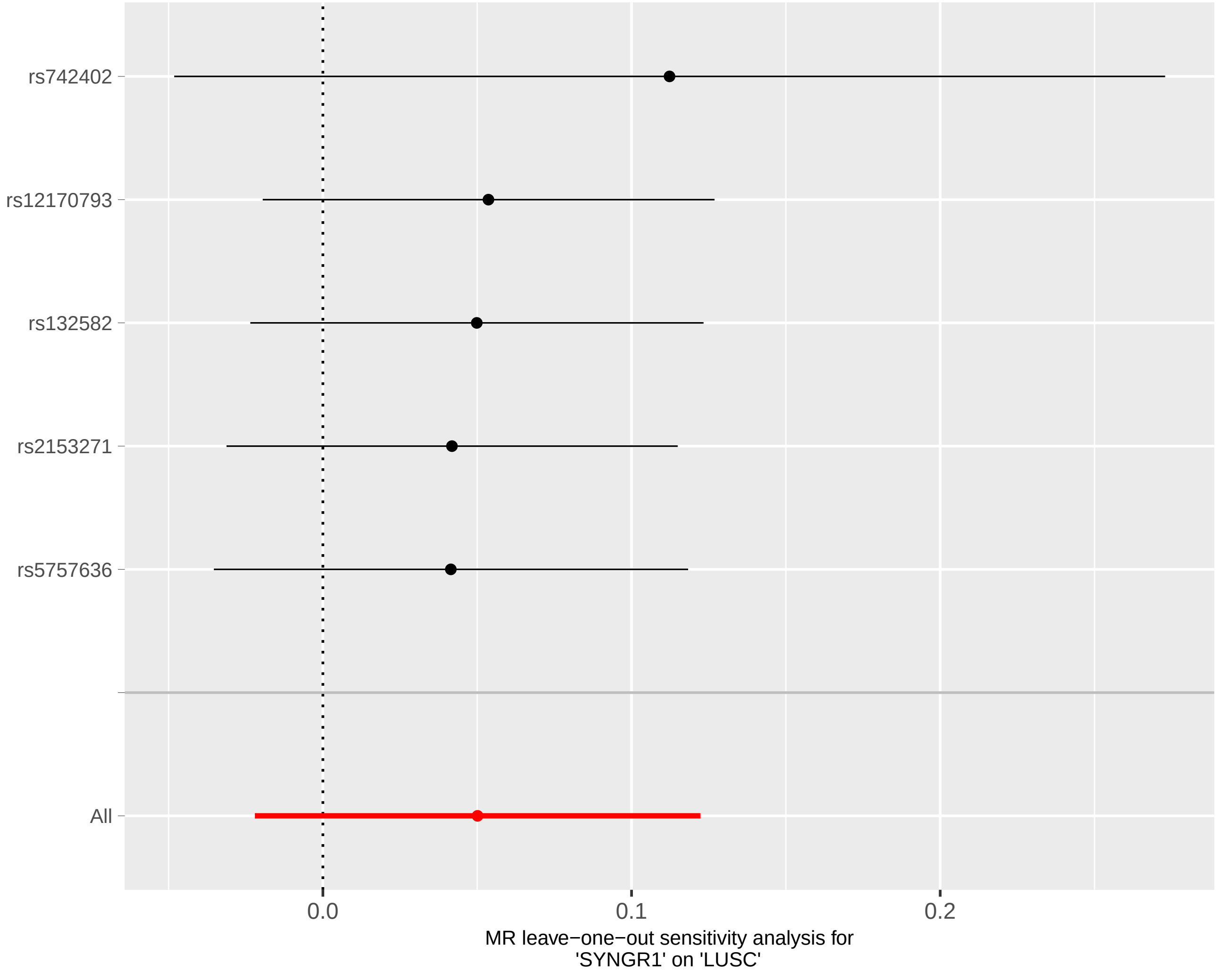
rs742402
rs12170793
rs132582
rs2153271
rs5757636
All
0.0
0.1
0.2
MR leave−one−out sensitivity analysis for
'SYNGR1' on 'LUSC'

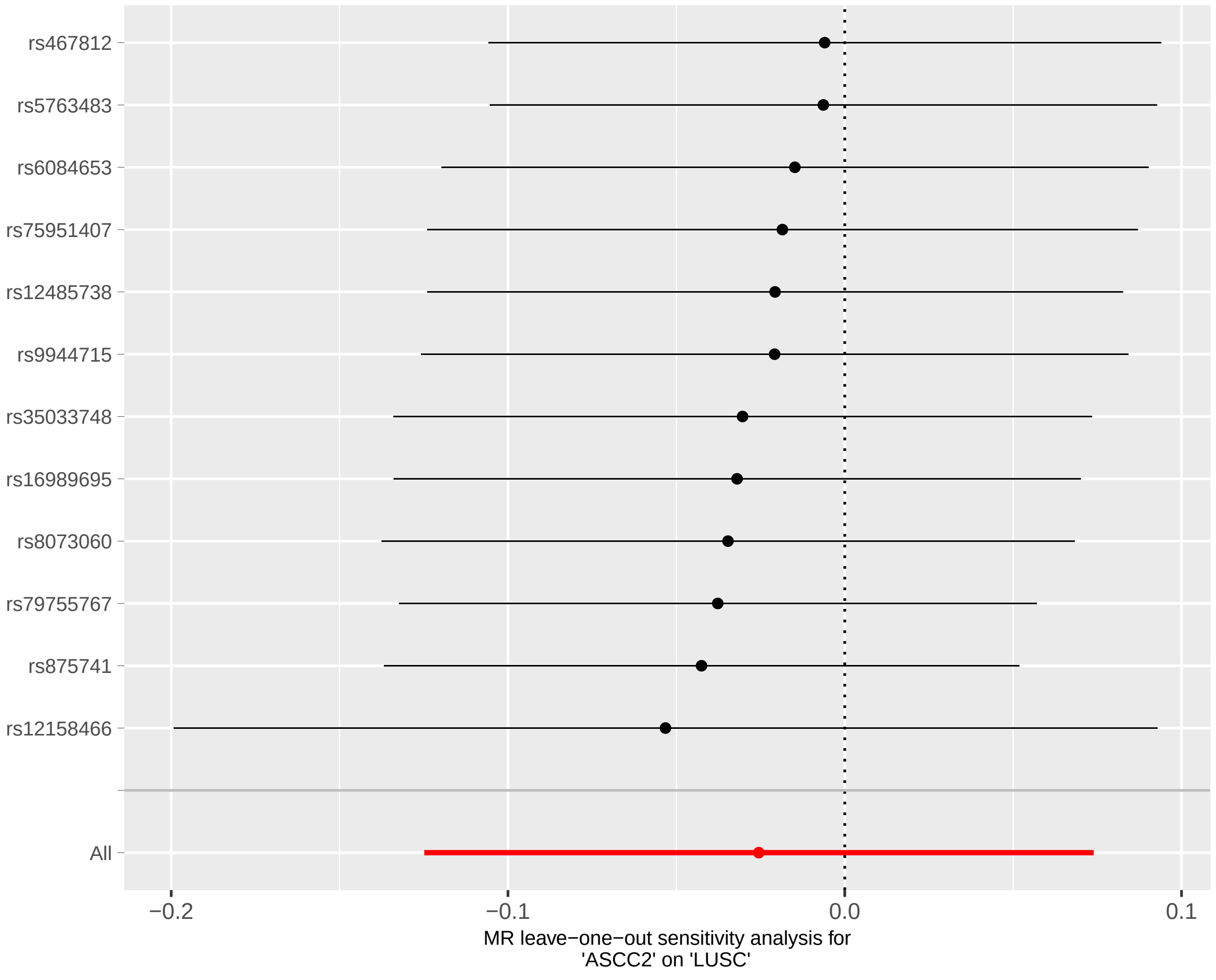
rs467812
rs5763483
rs6084653
rs75951407
rs12485738
rs9944715
rs35033748
rs16989695
rs8073060
rs79755767
rs875741
rs12158466
All
−0.2
−0.1
0.0
0.1
MR leave−one−out sensitivity analysis for
'ASCC2' on 'LUSC'

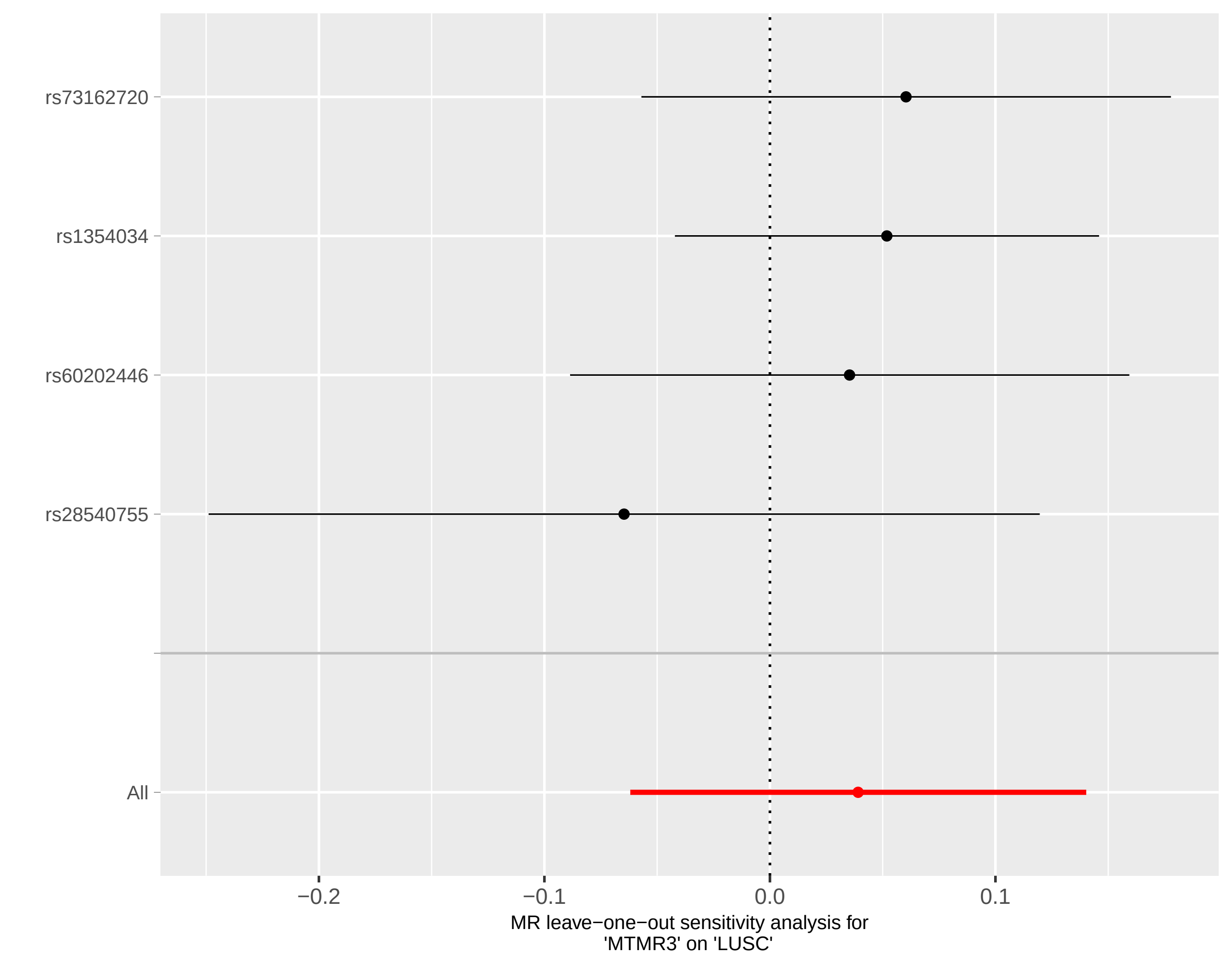
rs73162720
rs1354034
rs60202446
rs28540755
All
−0.2
−0.1
0.0
0.1
MR leave−one−out sensitivity analysis for
'MTMR3' on 'LUSC'

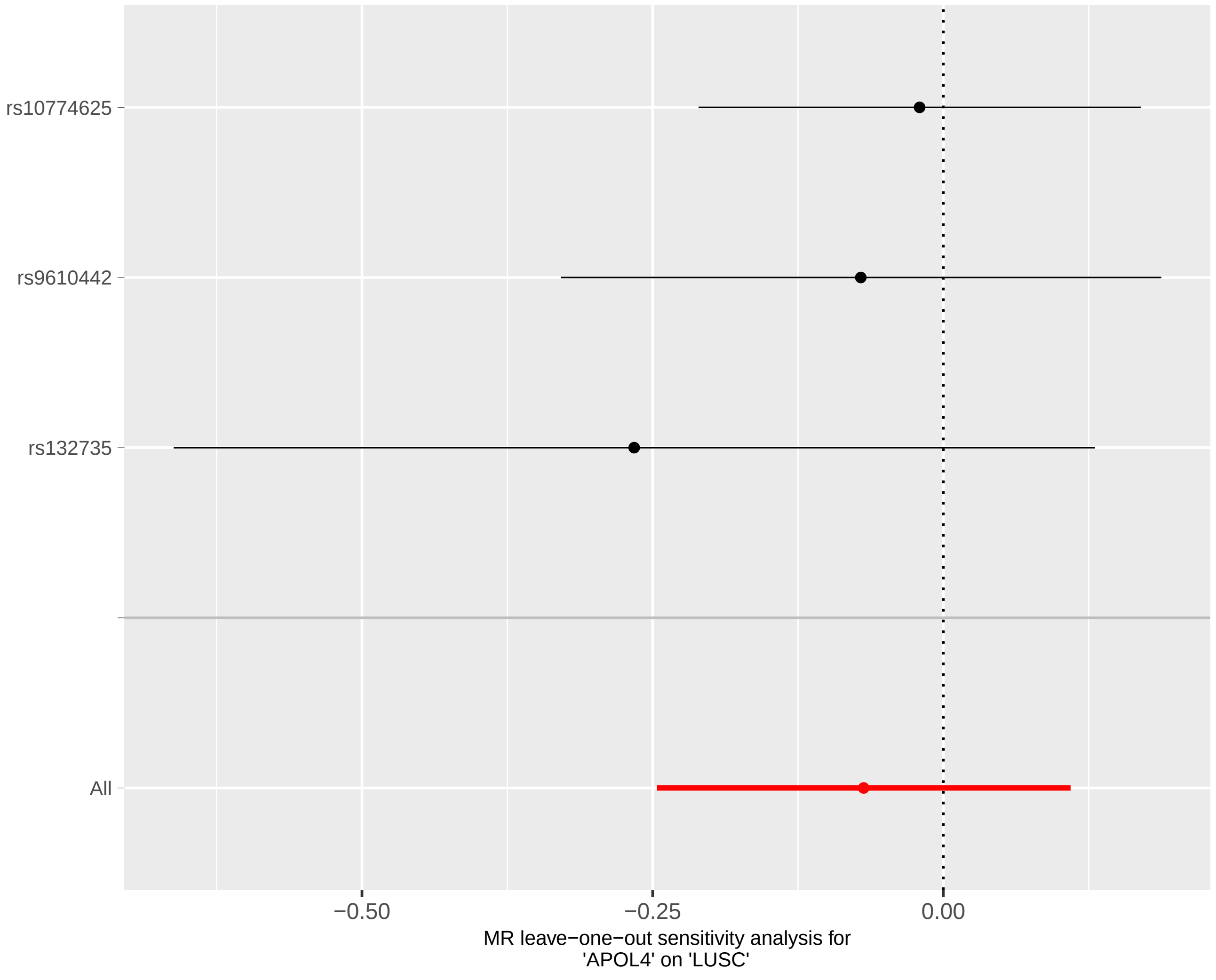
rs10774625
rs9610442
rs132735
All
−0.50
−0.25
0.00
MR leave−one−out sensitivity analysis for
'APOL4' on 'LUSC'

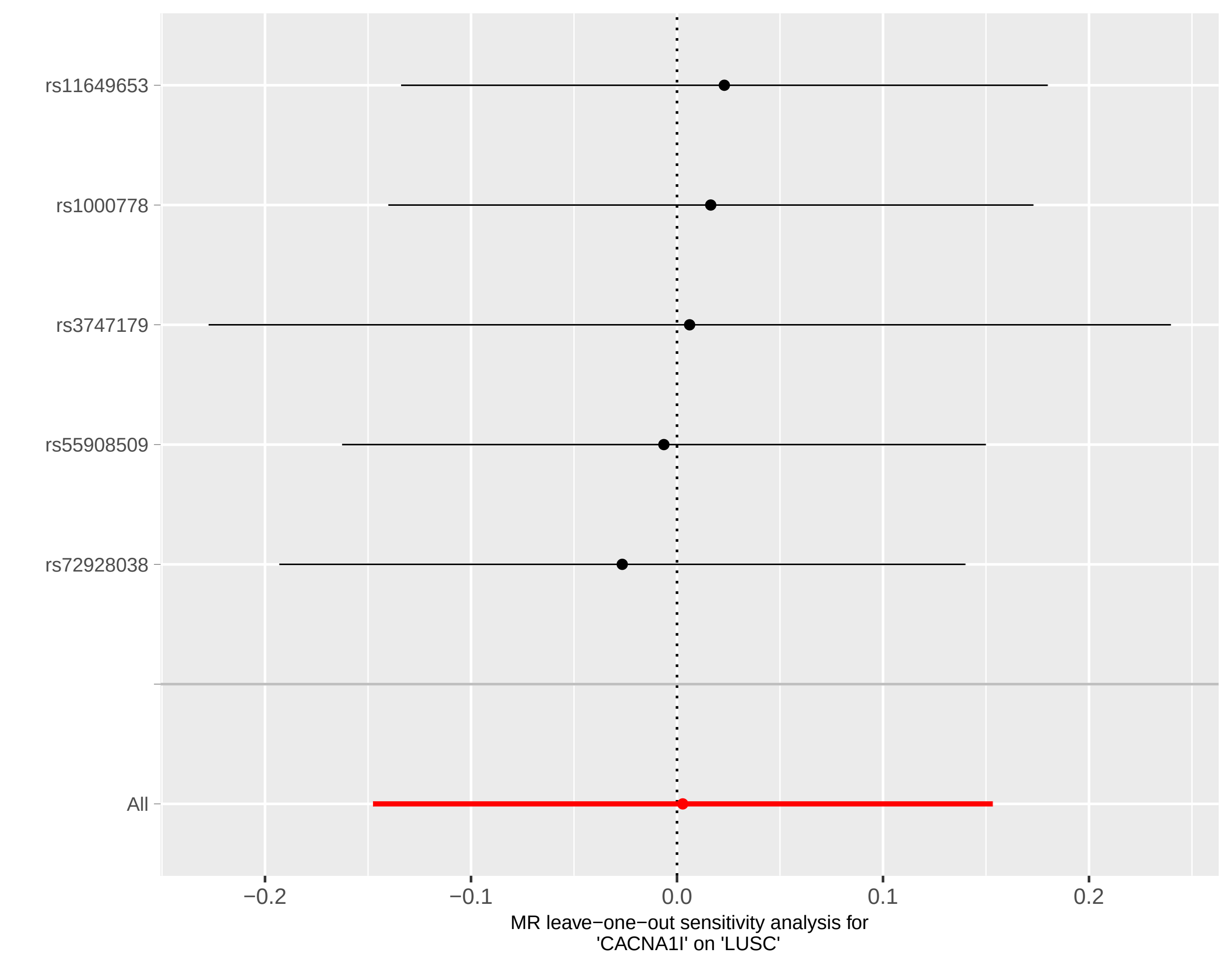

rs11649653
rs1000778
rs3747179
rs55908509
rs72928038
All
−0.2
−0.1
0.0
0.1
0.2
MR leave−one−out sensitivity analysis for
'CACNA1I' on 'LUSC'

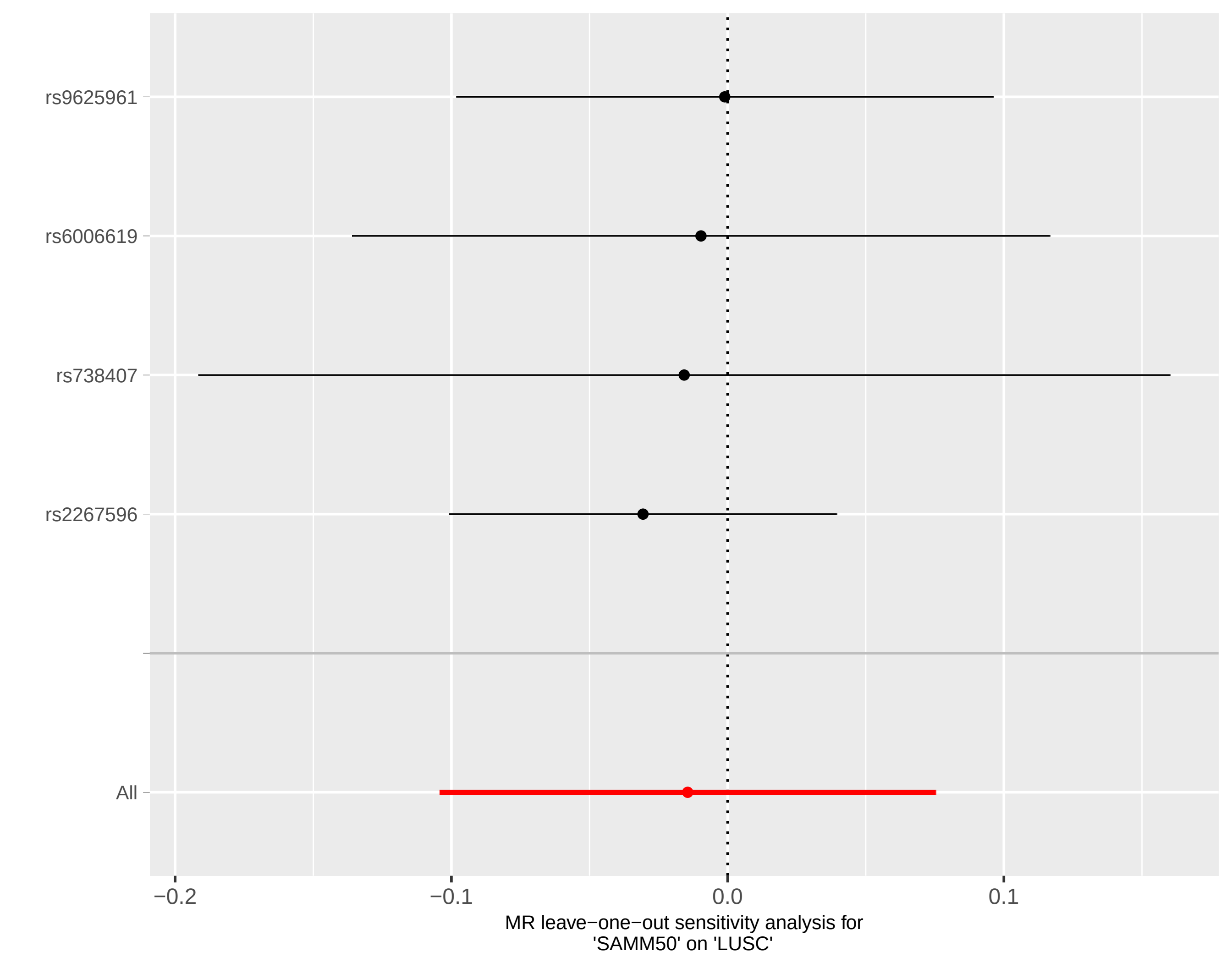

rs9625961
rs6006619
rs738407
rs2267596
All
−0.2
−0.1
0.0
0.1
MR leave−one−out sensitivity analysis for
'SAMM50' on 'LUSC'

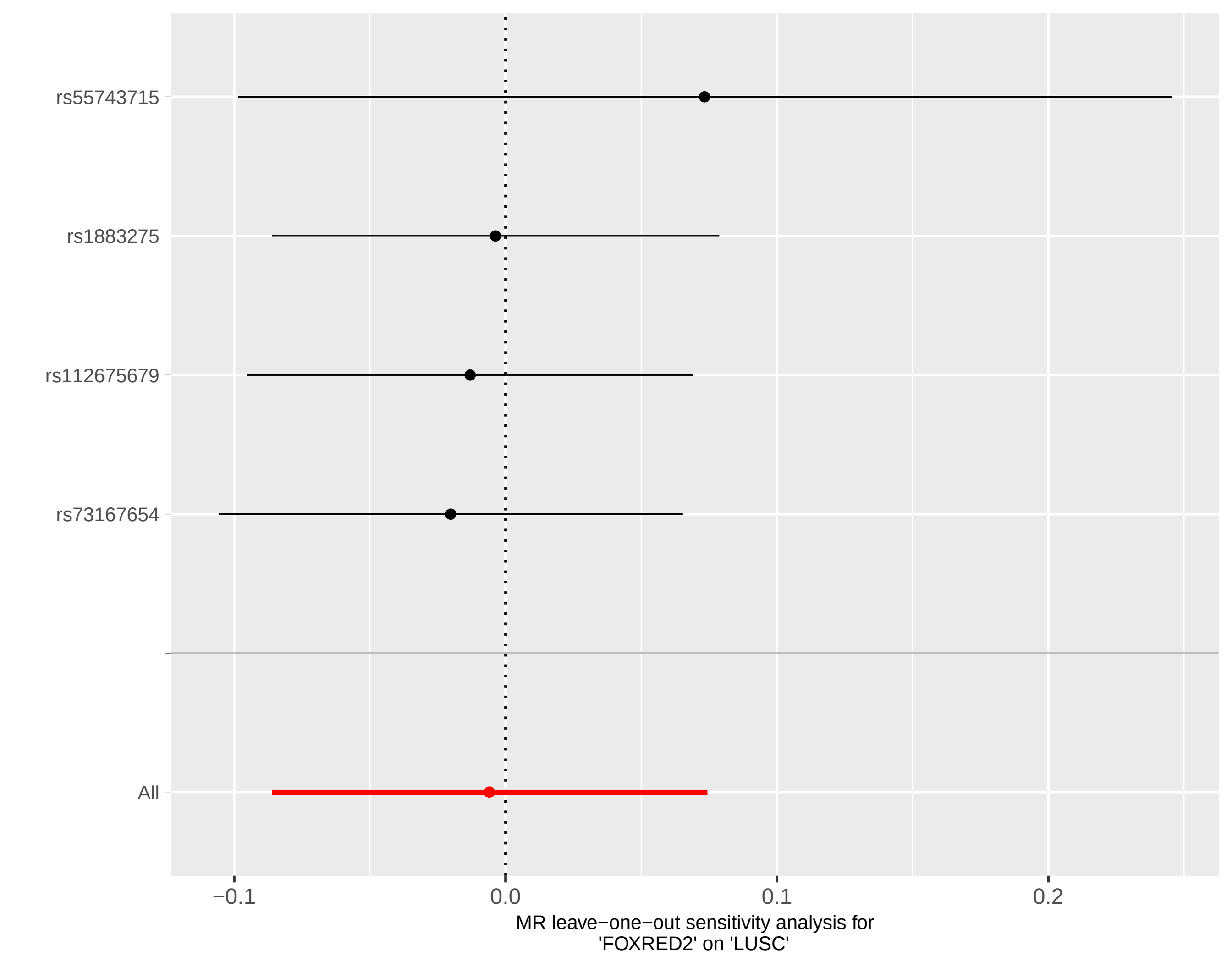
rs55743715
rs1883275
rs112675679
rs73167654
All
−0.1
0.0
0.1
0.2
MR leave−one−out sensitivity analysis for
'FOXRED2' on 'LUSC'

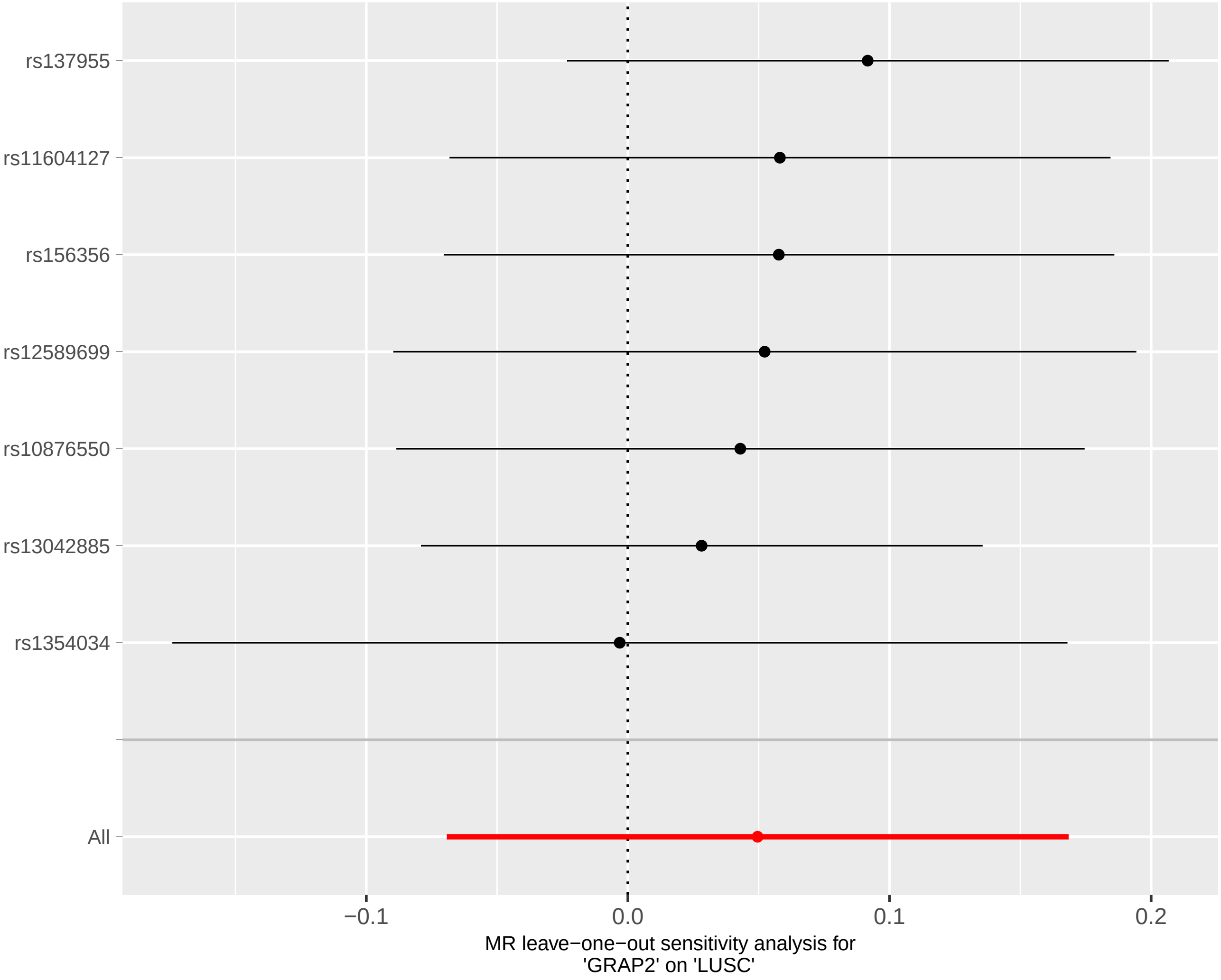
rs137955
rs11604127
rs156356
rs12589699
rs10876550
rs13042885
rs1354034
All
−0.1
0.0
0.1
0.2
MR leave−one−out sensitivity analysis for
'GRAP2' on 'LUSC'

Insufficient number of SNPs

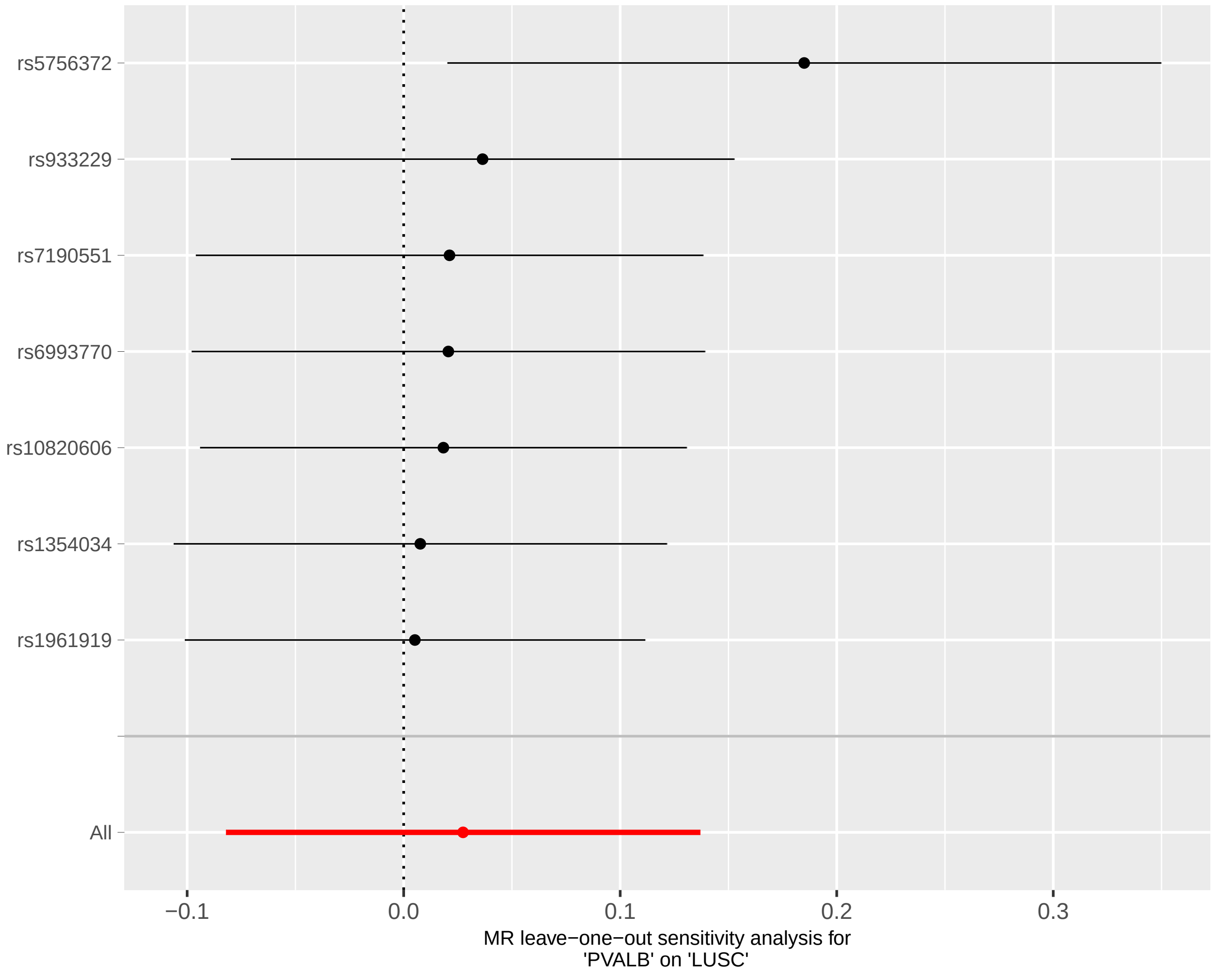
rs5756372
rs933229
rs7190551
rs6993770
rs10820606
rs1354034
rs1961919
All
−0.1
0.0
0.1
0.2
0.3
MR leave−one−out sensitivity analysis for
'PVALB' on 'LUSC'

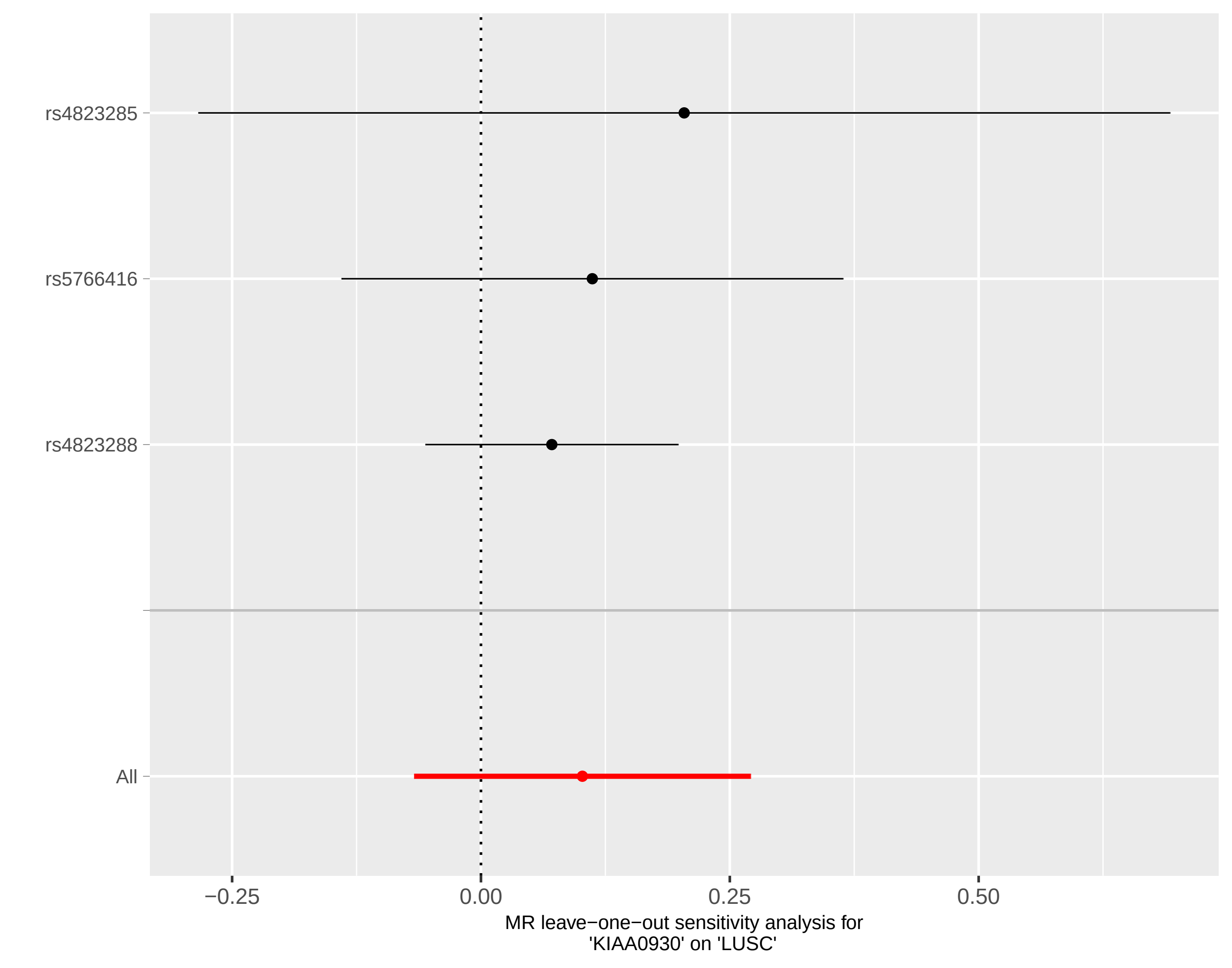
rs4823285
rs5766416
rs4823288
All
−0.25
0.00
0.25
0.50
MR leave−one−out sensitivity analysis for
'KIAA0930' on 'LUSC'

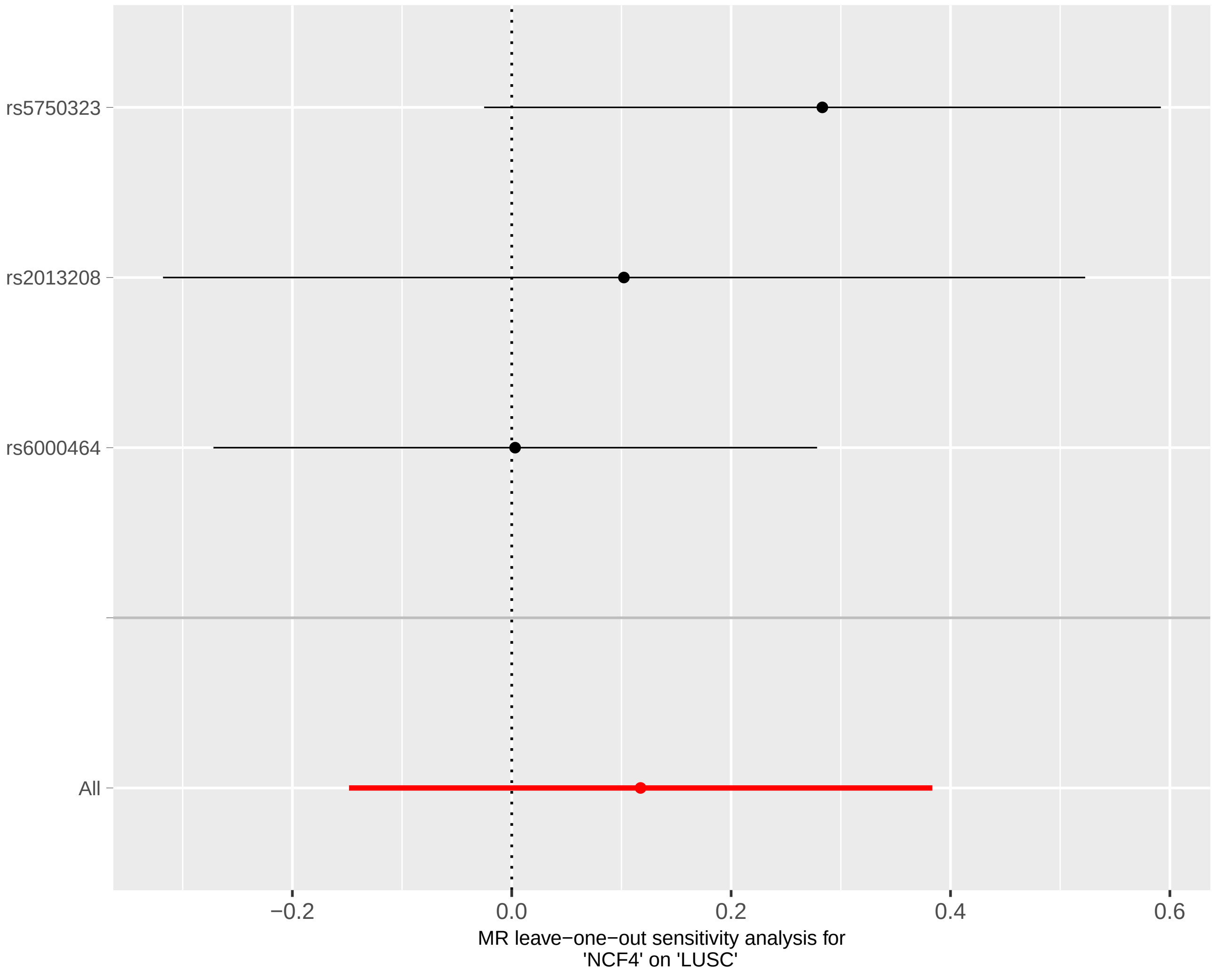
rs5750323
rs2013208
rs6000464
All
−0.2
0.0
0.2
0.4
0.6
MR leave−one−out sensitivity analysis for
'NCF4' on 'LUSC'

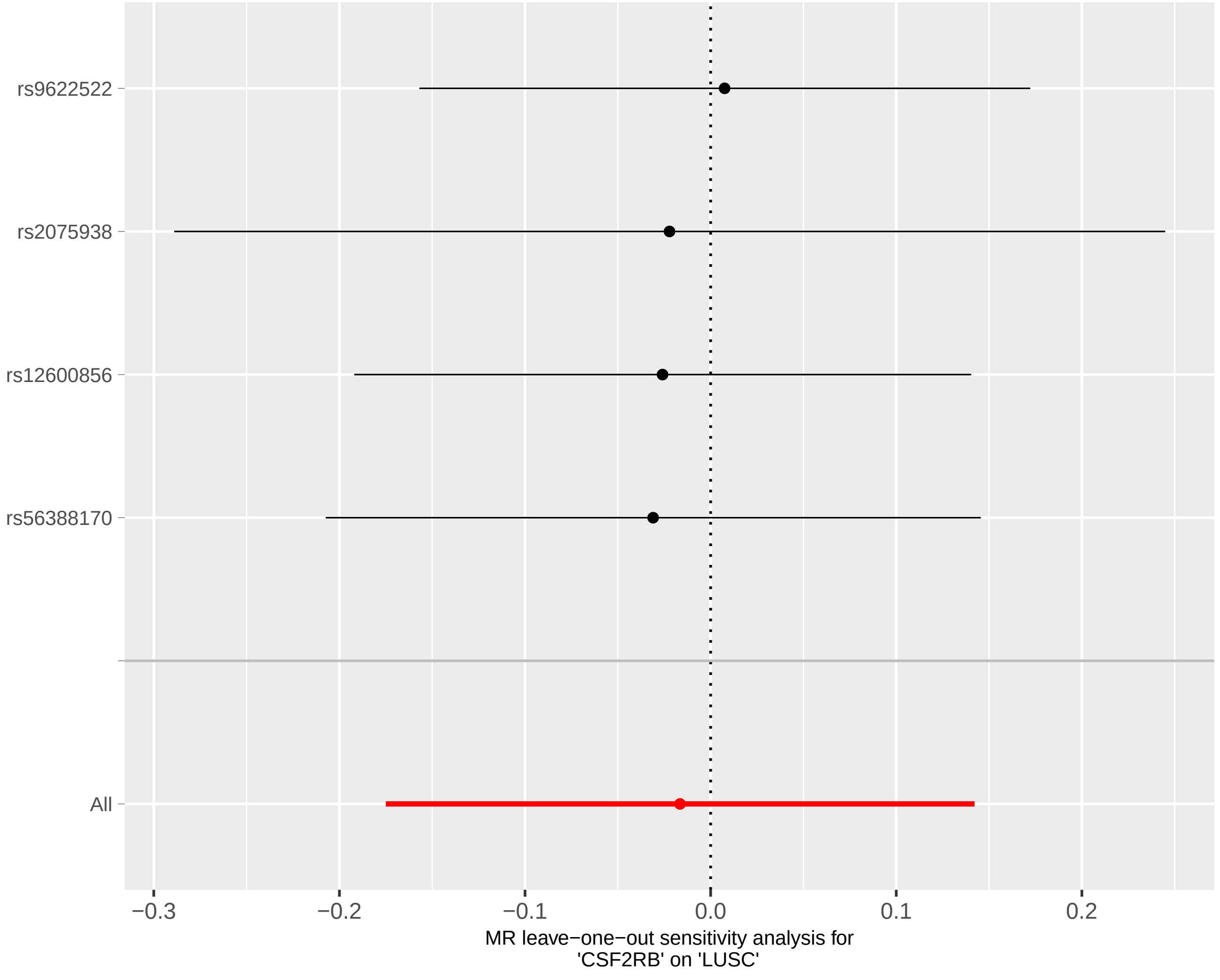
rs9622522
rs2075938
rs12600856
rs56388170
All
−0.3
−0.2
−0.1
0.0
0.1
0.2
MR leave−one−out sensitivity analysis for
'CSF2RB' on 'LUSC'

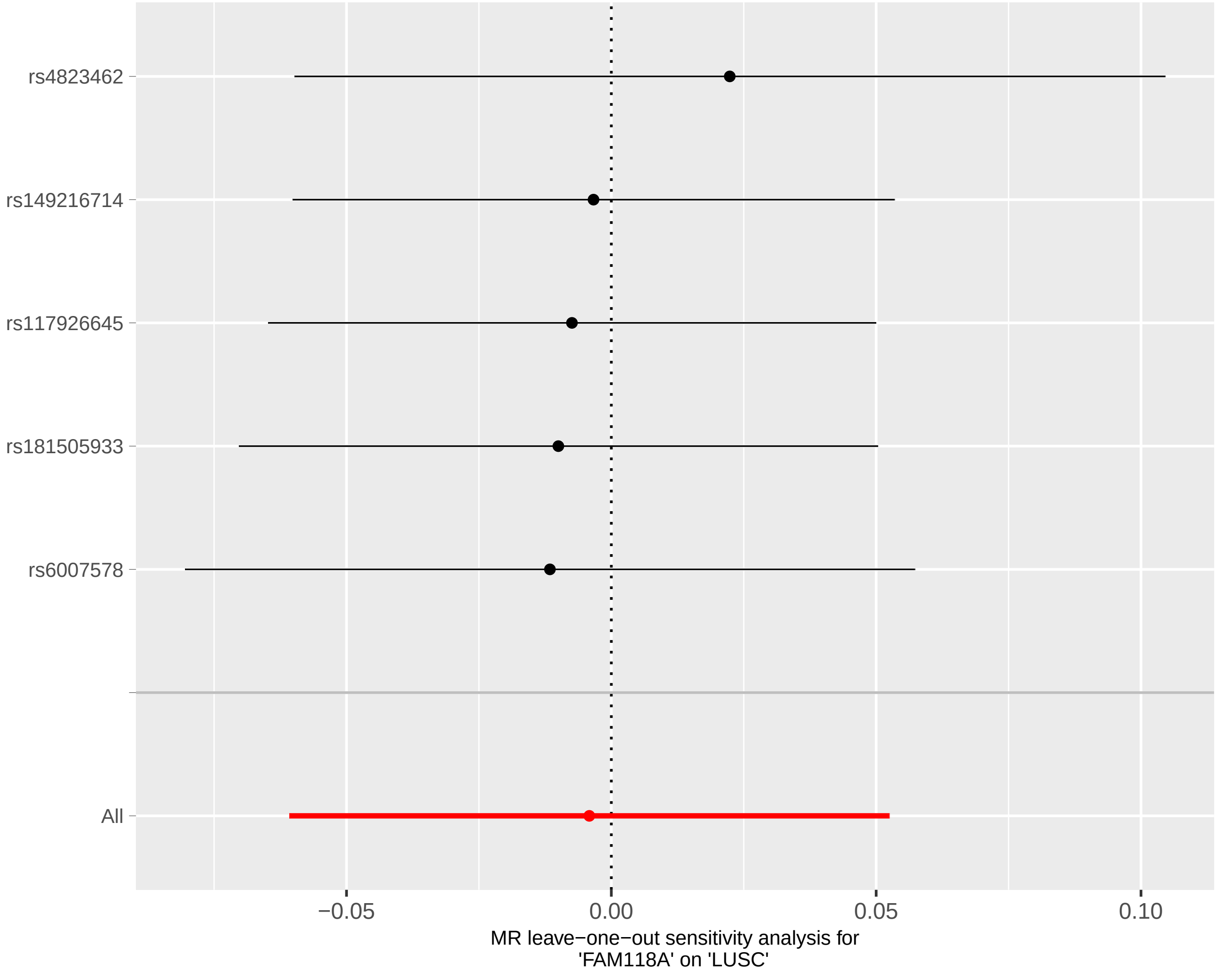
rs4823462
rs149216714
rs117926645
rs181505933
rs6007578
All
−0.05
0.00
0.05
0.10
MR leave−one−out sensitivity analysis for
'FAM118A' on 'LUSC'

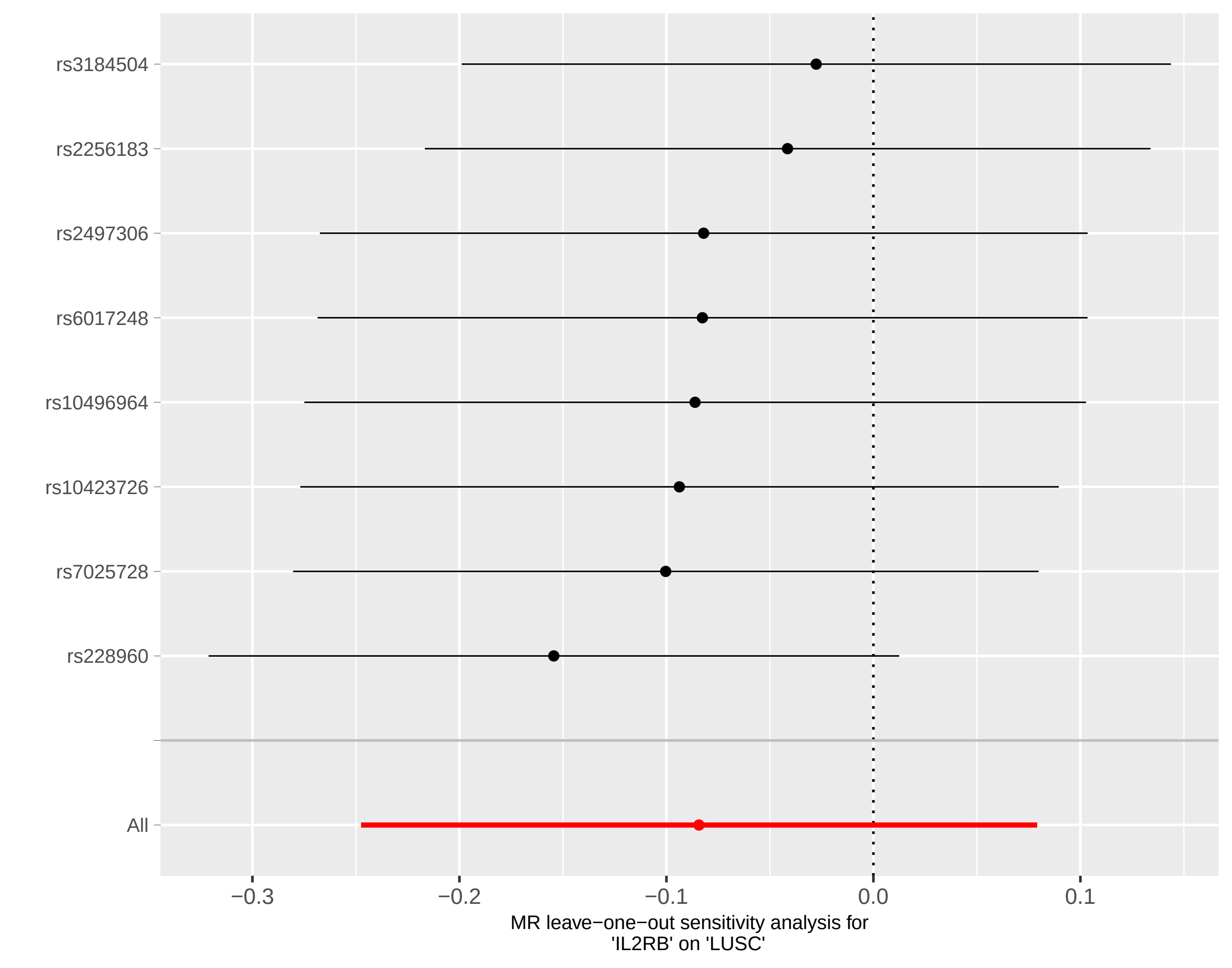

rs3184504
rs2256183
rs2497306
rs6017248
rs10496964
rs10423726
rs7025728
rs228960
All
−0.3
−0.2
−0.1
0.0
0.1
MR leave−one−out sensitivity analysis for
'IL2RB' on 'LUSC'

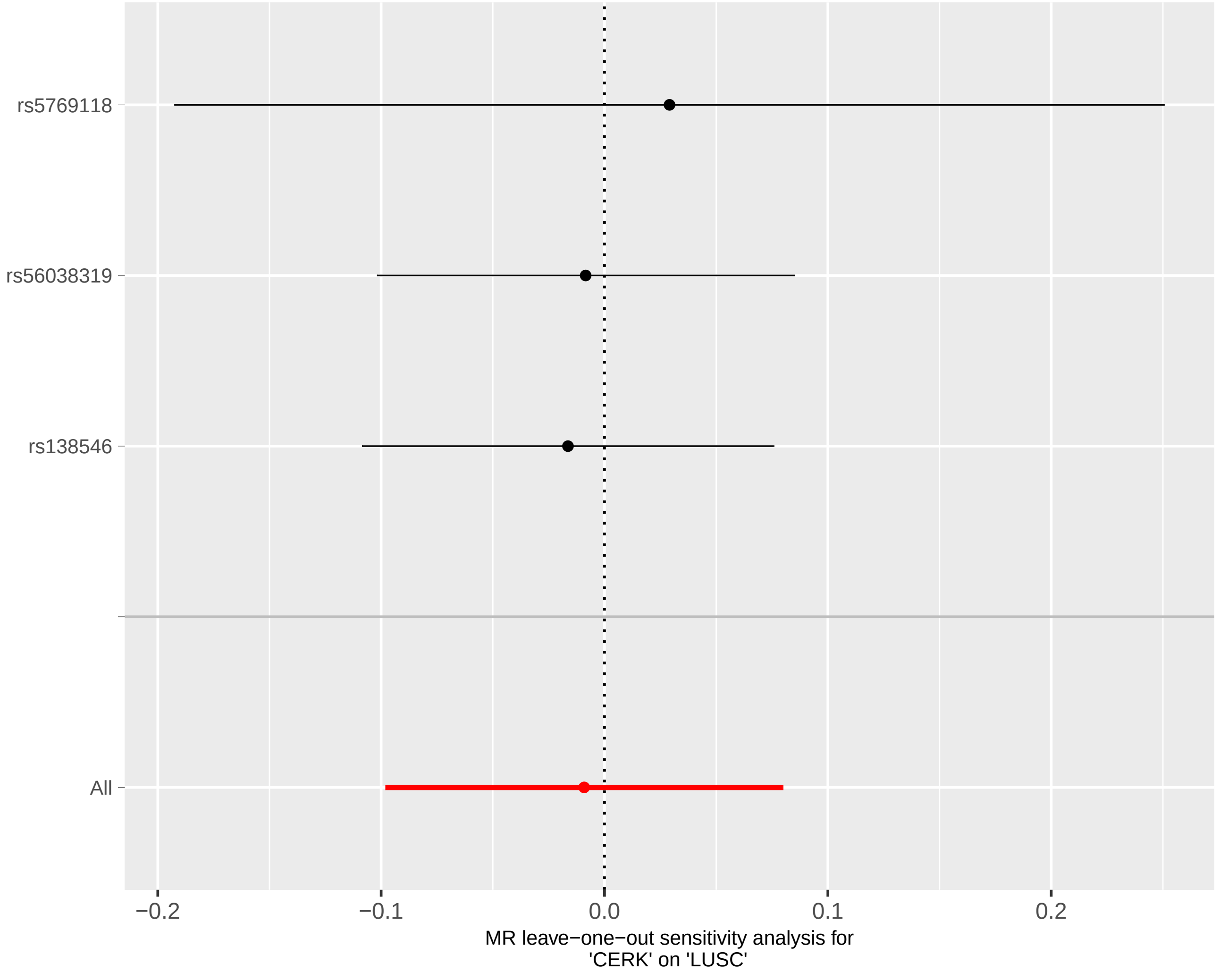
rs5769118
rs56038319
rs138546
All
−0.2
−0.1
0.0
0.1
0.2
MR leave−one−out sensitivity analysis for
'CERK' on 'LUSC'

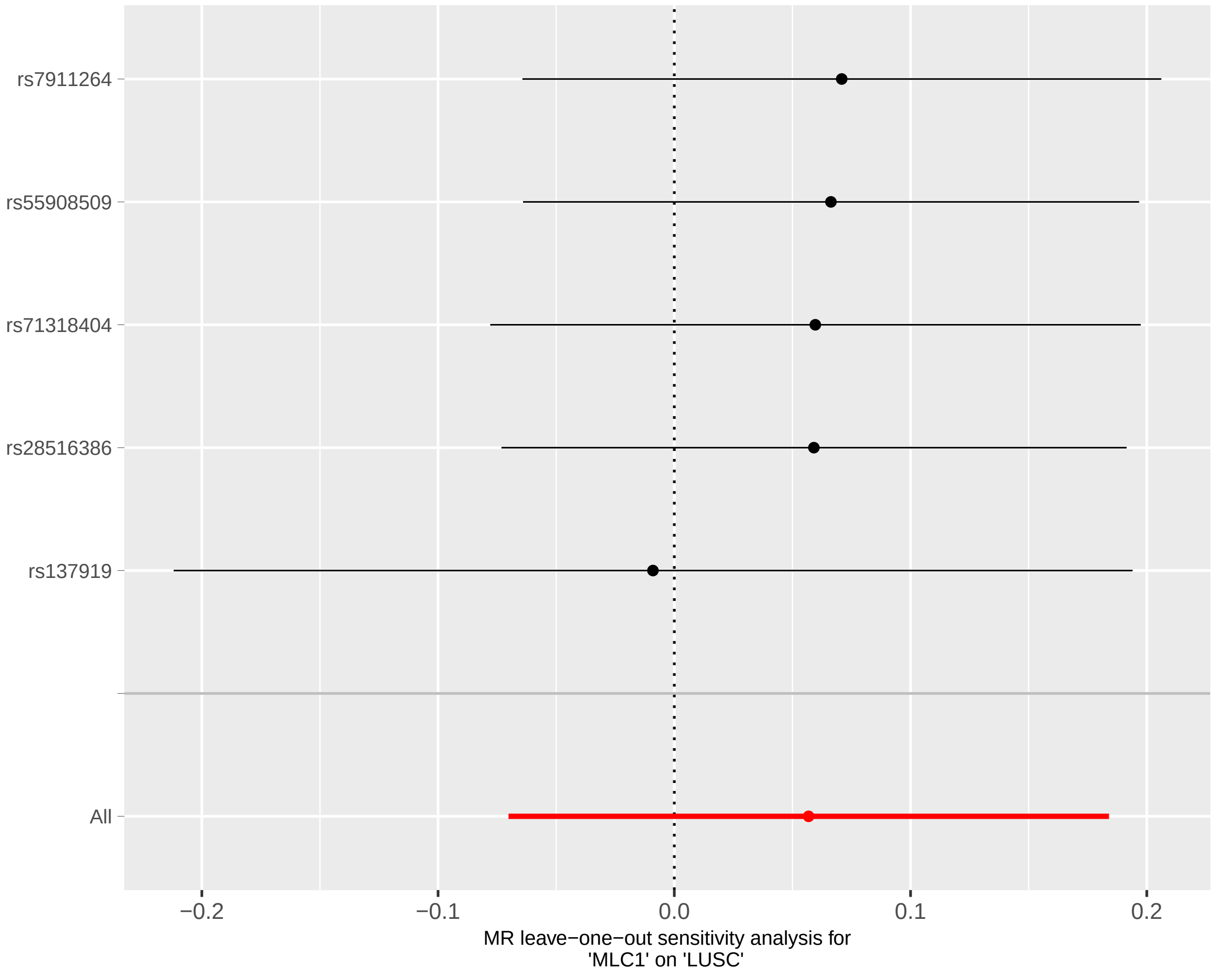

rs7911264
rs55908509
rs71318404
rs28516386
rs137919
All
−0.2
−0.1
0.0
0.1
0.2
MR leave−one−out sensitivity analysis for
'MLC1' on 'LUSC'

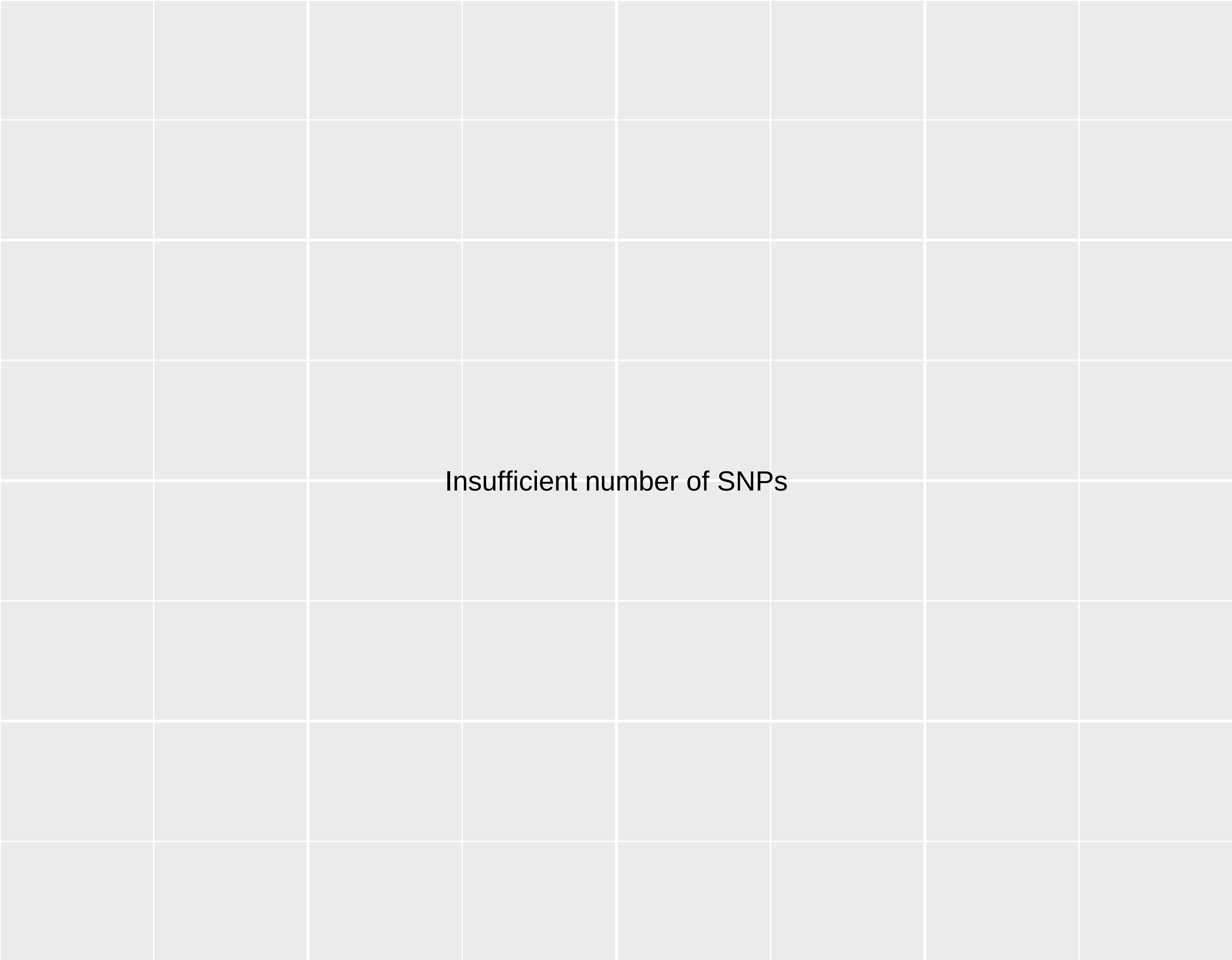
Insufficient number of SNPs

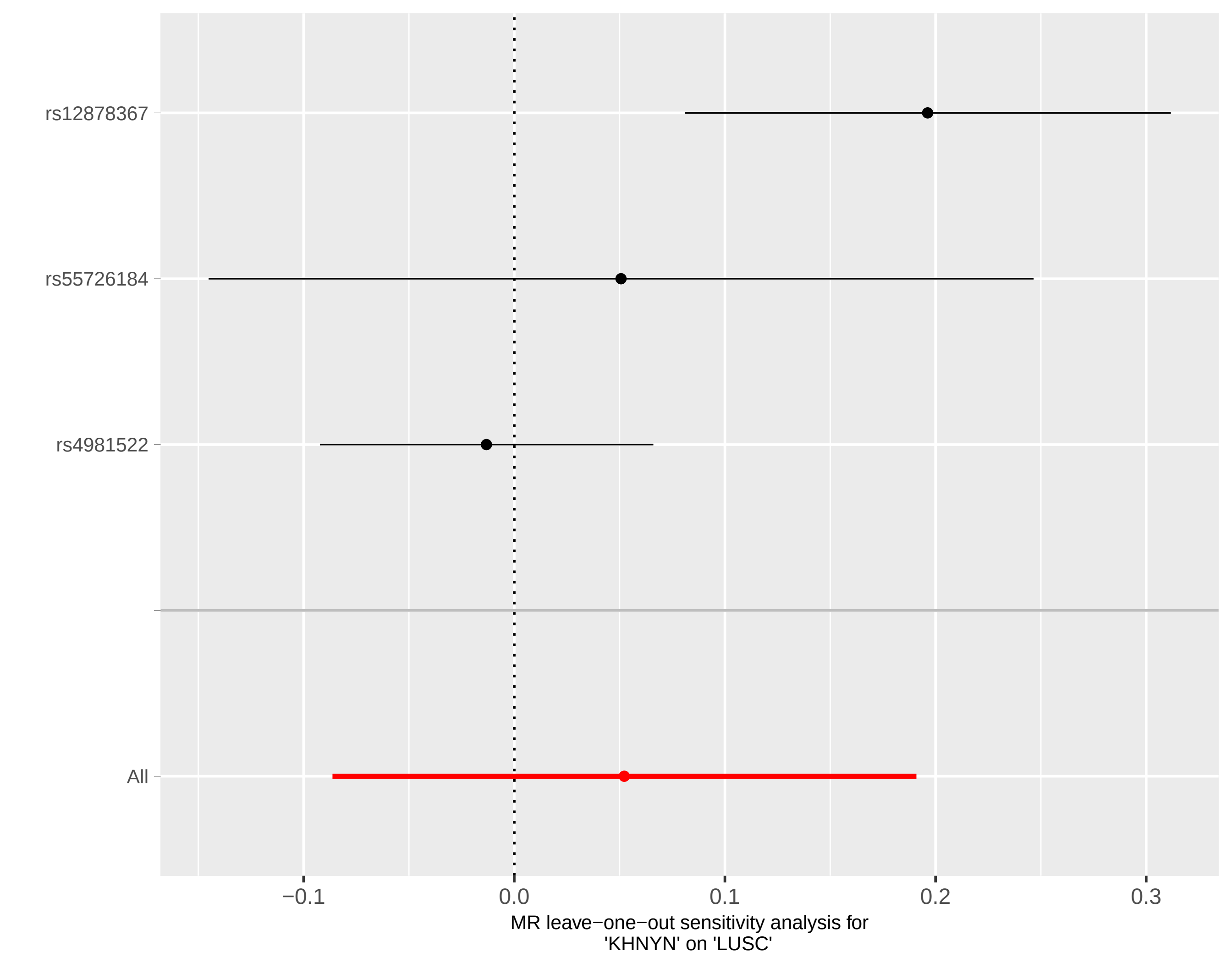
rs12878367
rs55726184
rs4981522
All
−0.1
0.0
0.1
0.2
0.3
MR leave−one−out sensitivity analysis for
'KHNYN' on 'LUSC'

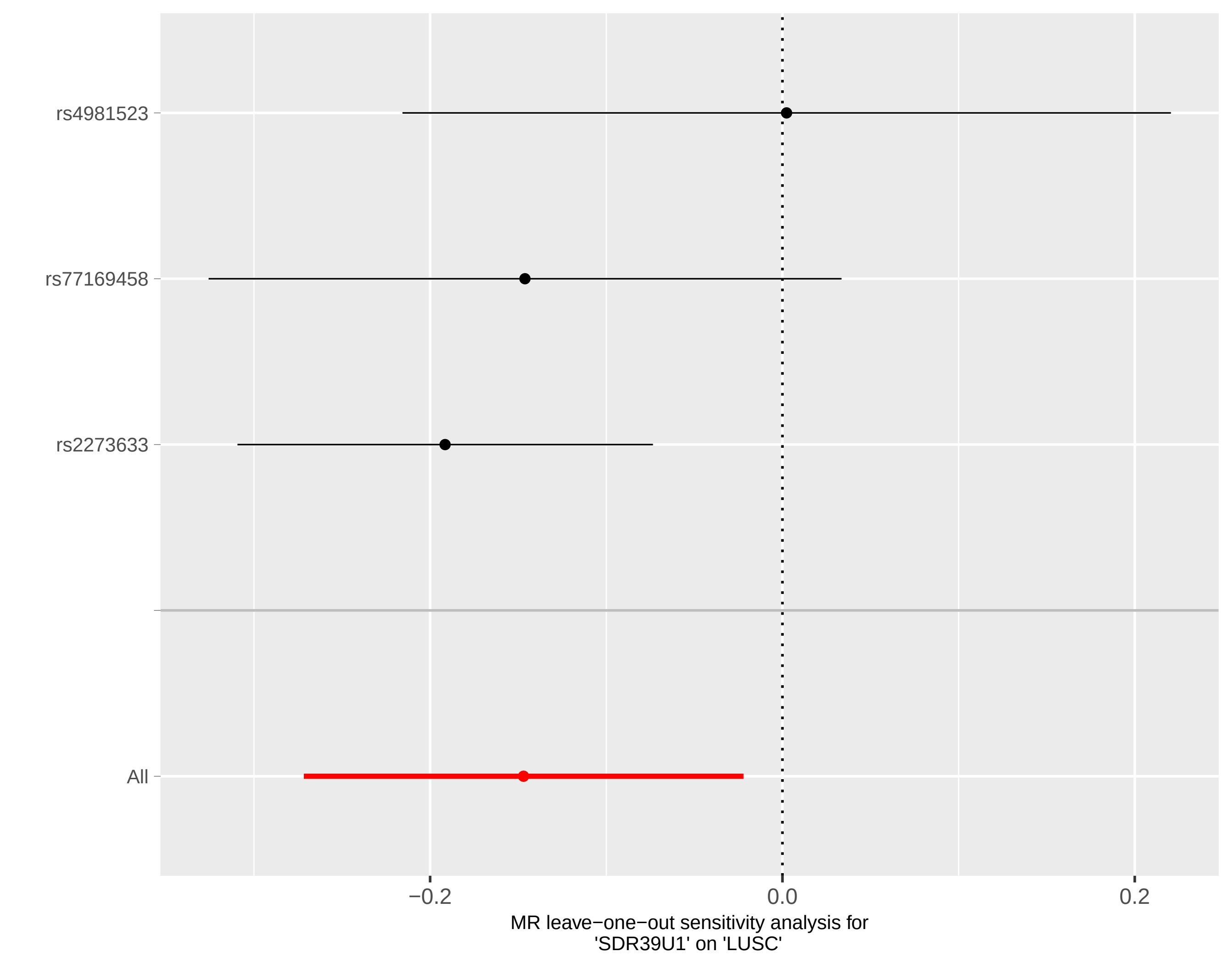
rs4981523
rs77169458
rs2273633
All
−0.2
0.0
0.2
MR leave−one−out sensitivity analysis for
'SDR39U1' on 'LUSC'

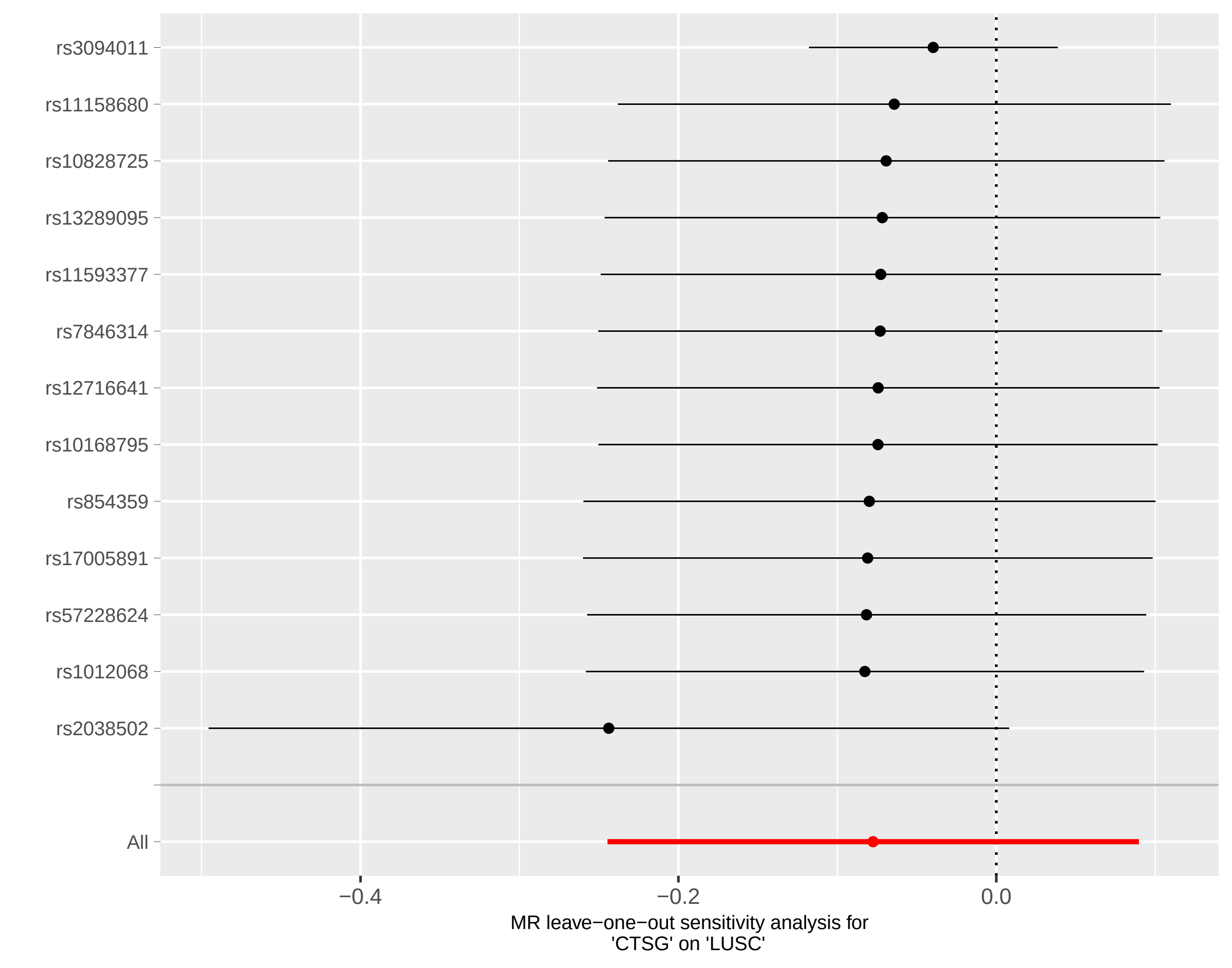

rs3094011
rs11158680
rs10828725
rs13289095
rs11593377
rs7846314
rs12716641
rs10168795
rs854359
rs17005891
rs57228624
rs1012068
rs2038502
All
−0.4
−0.2
0.0
MR leave−one−out sensitivity analysis for
'CTSG' on 'LUSC'

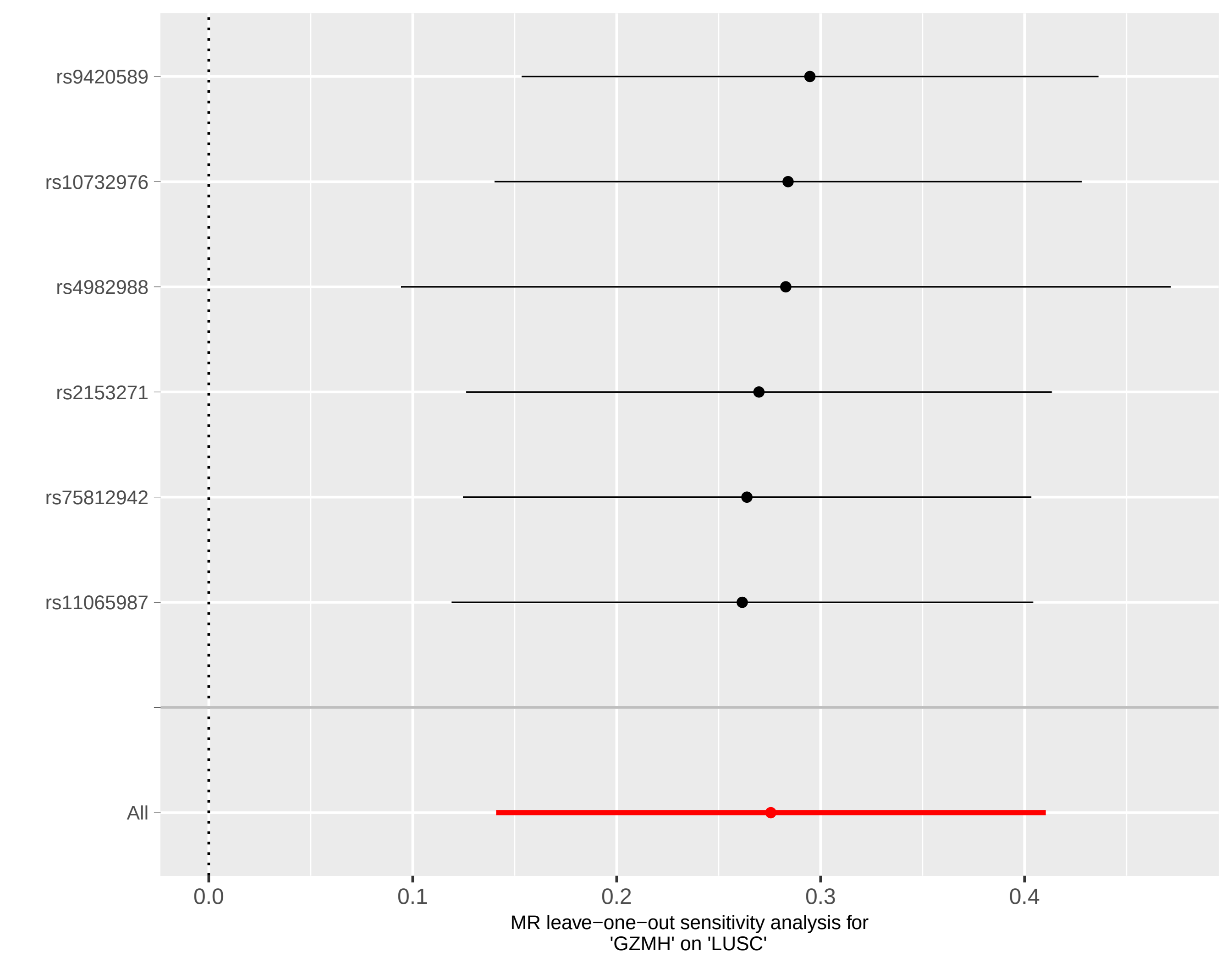
rs9420589
rs10732976
rs4982988
rs2153271
rs75812942
rs11065987
All
0.0
0.1
0.2
0.3
0.4
MR leave−one−out sensitivity analysis for
'GZMH' on 'LUSC'

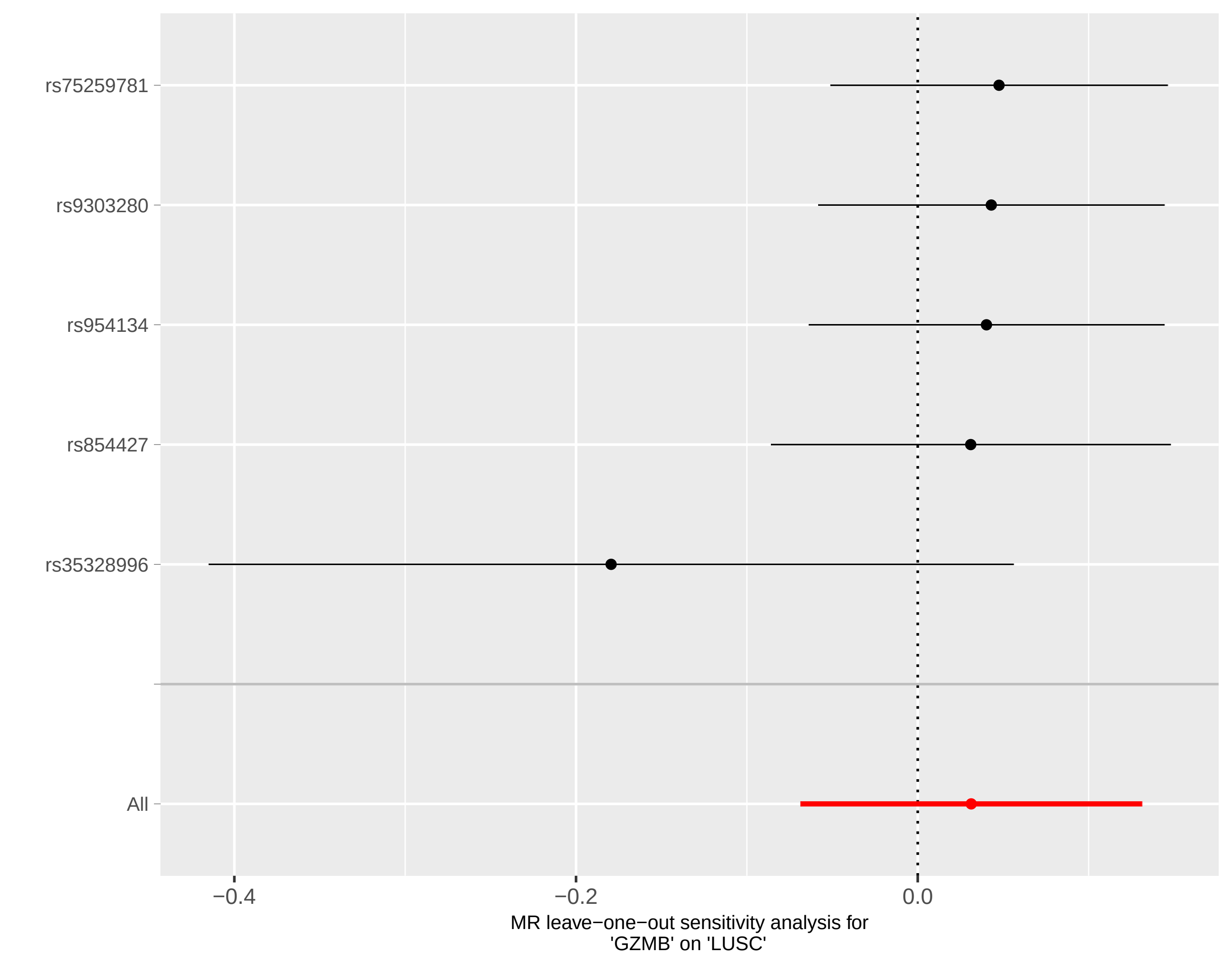

rs75259781
rs9303280
rs954134
rs854427
rs35328996
All
−0.4
−0.2
0.0
MR leave−one−out sensitivity analysis for
'GZMB' on 'LUSC'

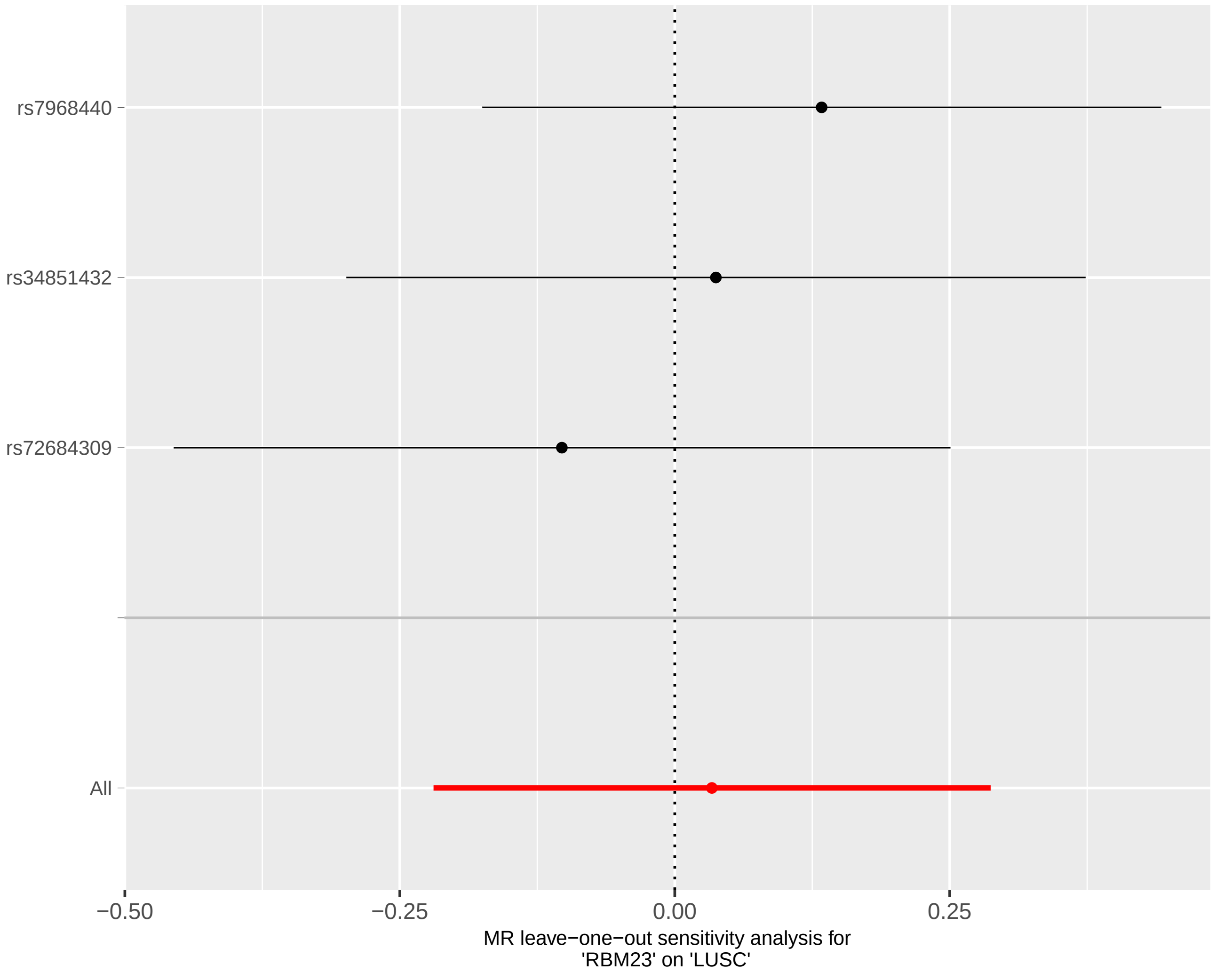

rs7968440
rs34851432
rs72684309
All
−0.50
−0.25
0.00
0.25
MR leave−one−out sensitivity analysis for
'RBM23' on 'LUSC'

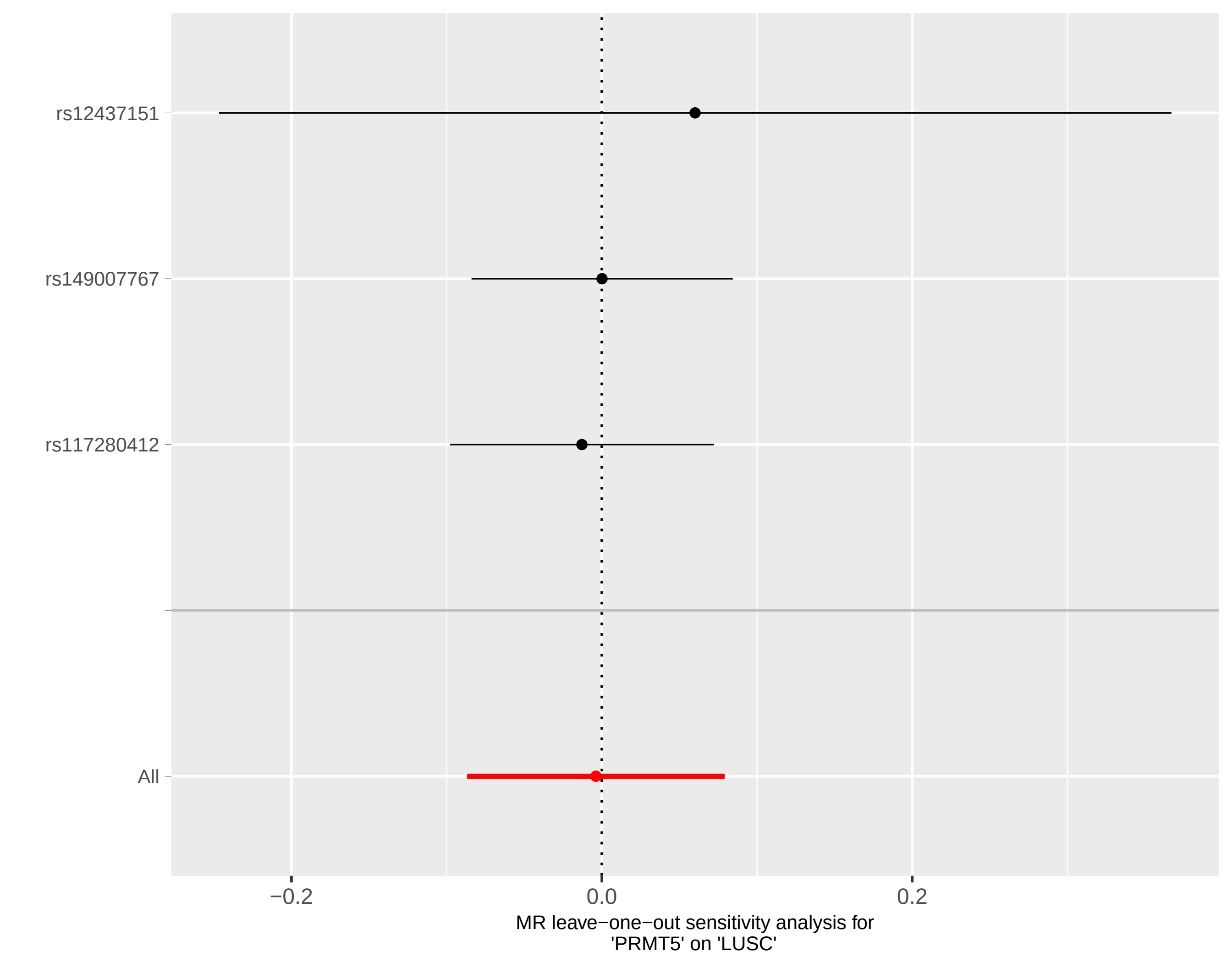
rs12437151
rs149007767
rs117280412
All
−0.2
0.0
0.2
MR leave−one−out sensitivity analysis for
'PRMT5' on 'LUSC'

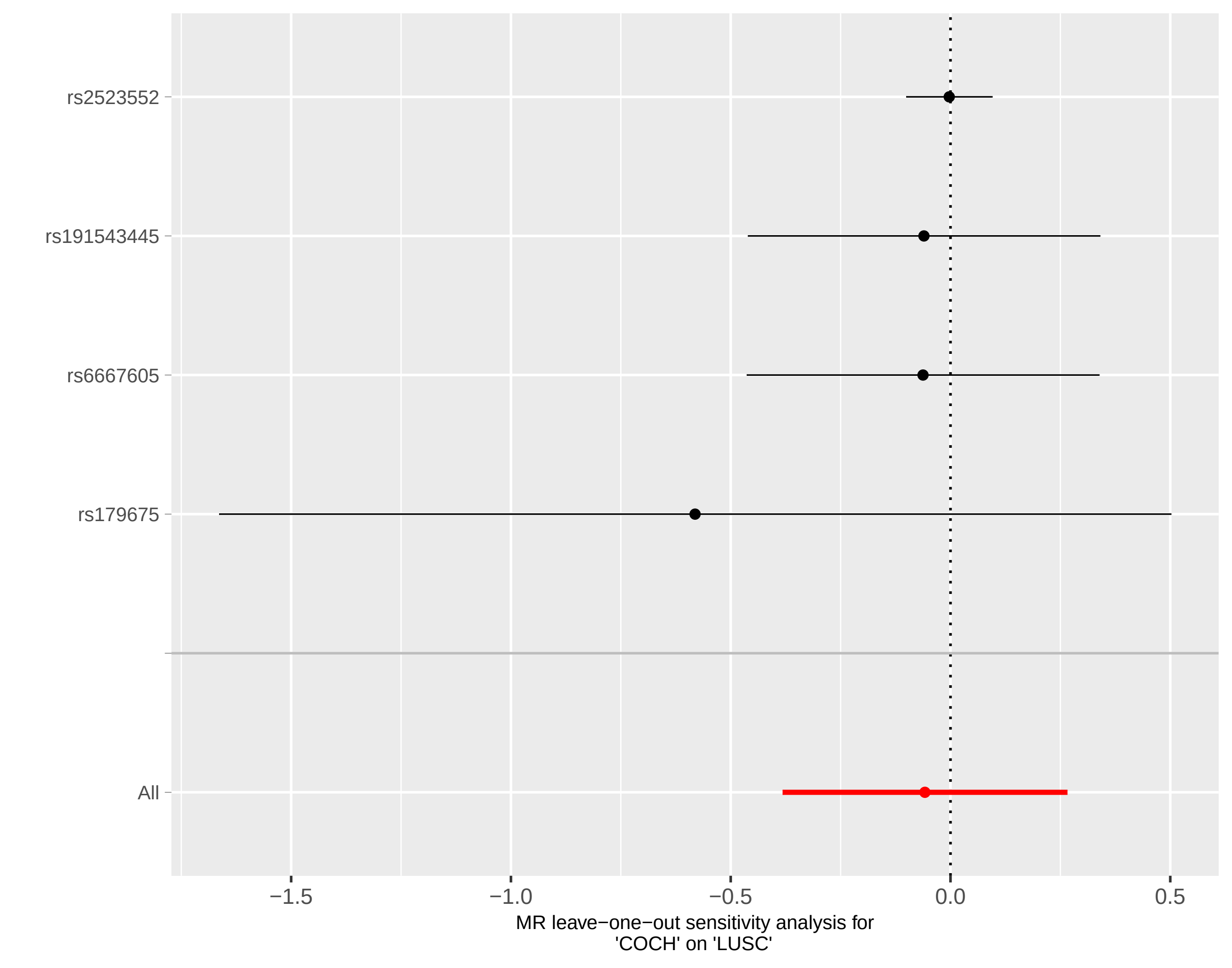

rs2523552
rs191543445
rs6667605
rs179675
All
−1.5
−1.0
−0.5
0.0
0.5
MR leave−one−out sensitivity analysis for
'COCH' on 'LUSC'

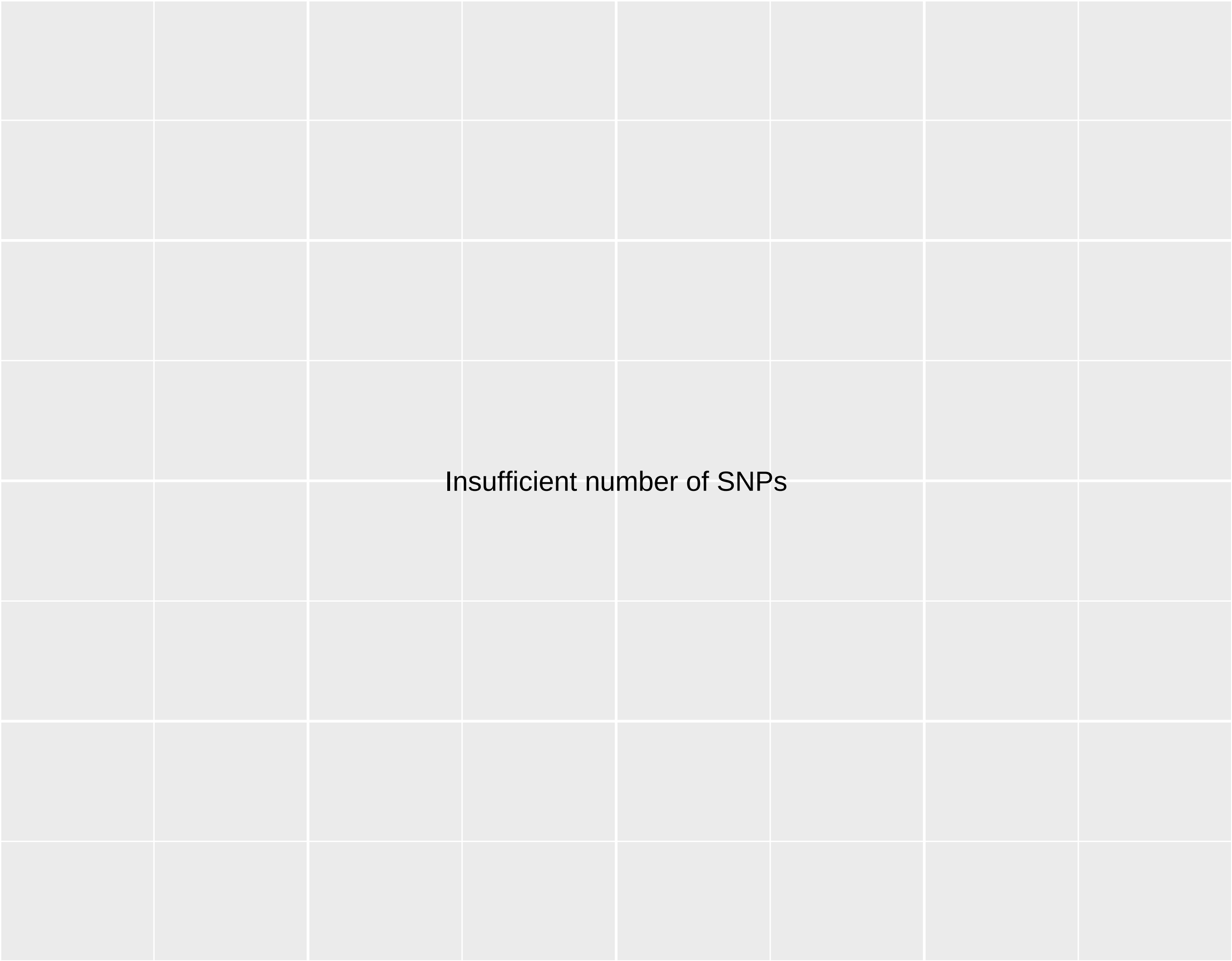
Insufficient number of SNPs

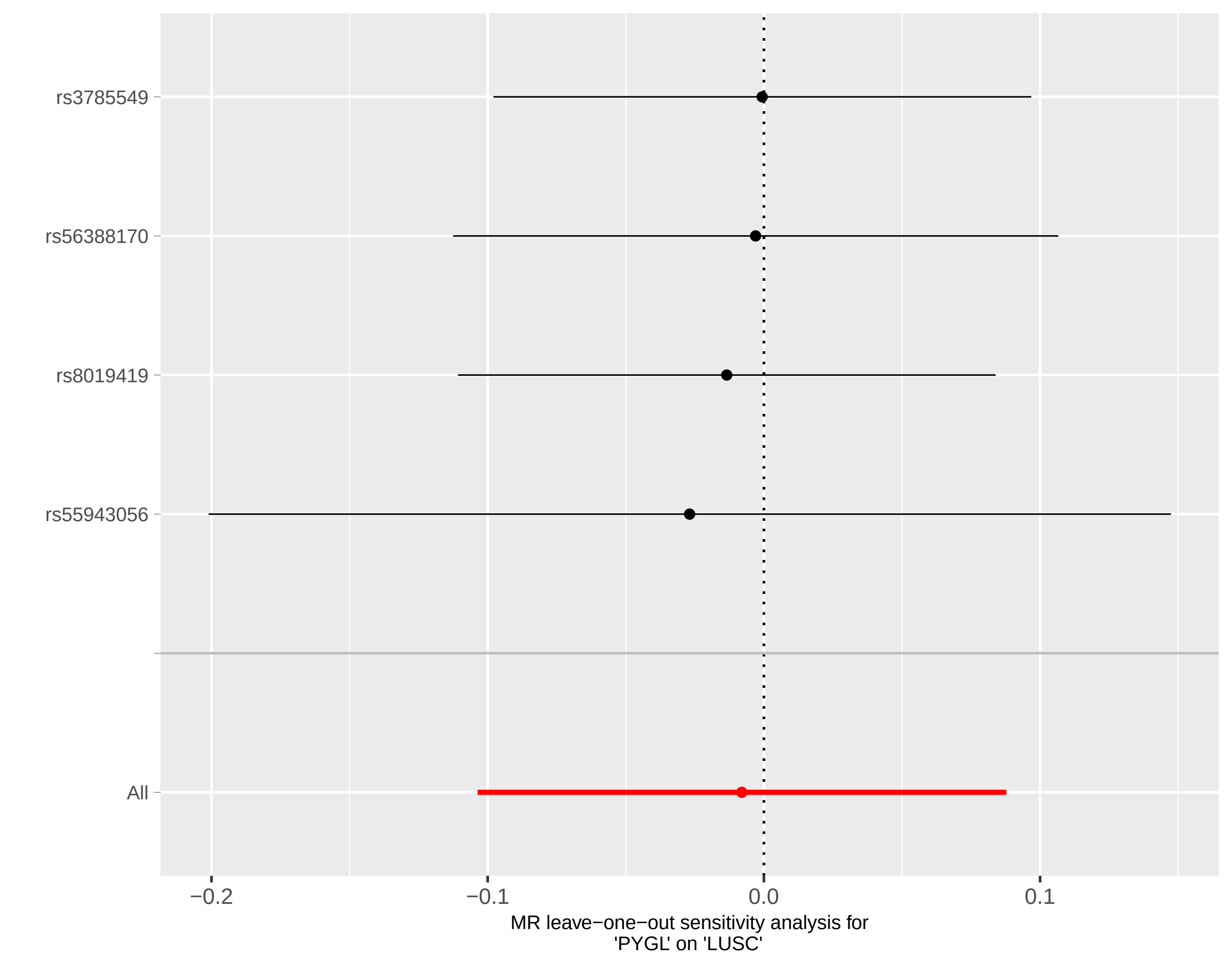
rs3785549
rs56388170
rs8019419
rs55943056
All
−0.2
−0.1
0.0
0.1
MR leave−one−out sensitivity analysis for
'PYGL' on 'LUSC'

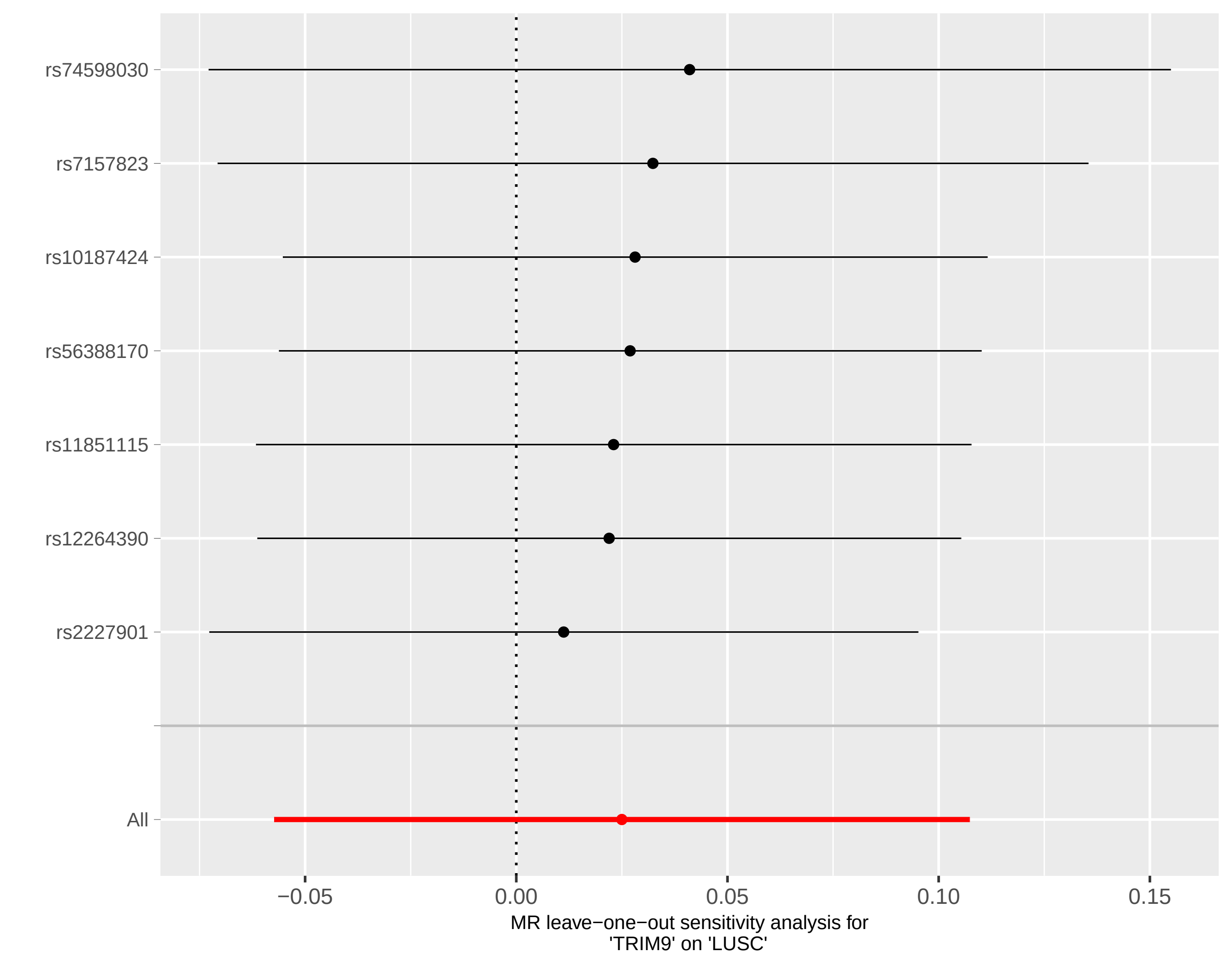
rs74598030
rs7157823
rs10187424
rs56388170
rs11851115
rs12264390
rs2227901
All
−0.05
0.00
0.05
0.10
0.15
MR leave−one−out sensitivity analysis for
'TRIM9' on 'LUSC'

Insufficient number of SNPs

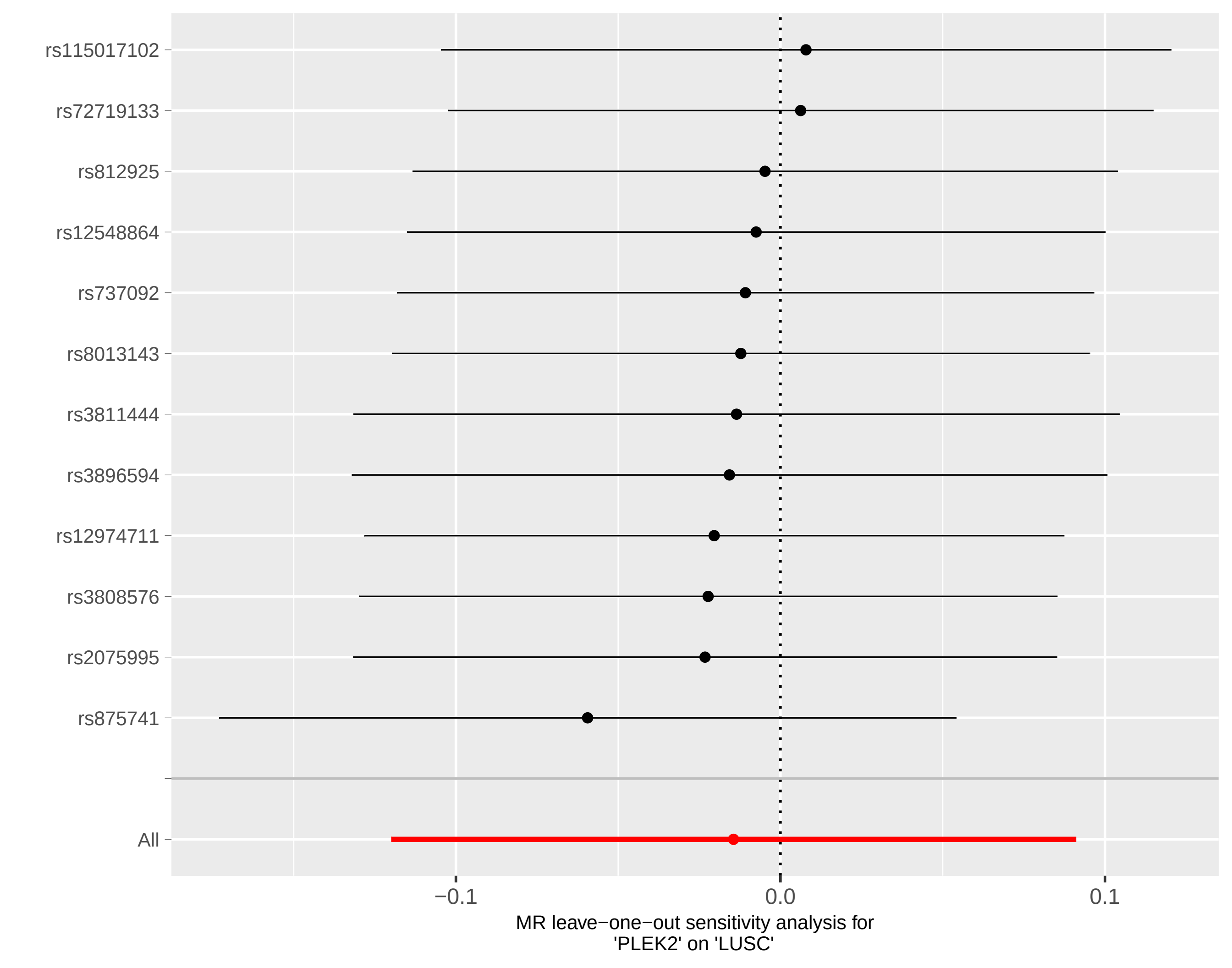
rs115017102
rs72719133
rs812925
rs12548864
rs737092
rs8013143
rs3811444
rs3896594
rs12974711
rs3808576
rs2075995
rs875741
All
−0.1
0.0
0.1
MR leave−one−out sensitivity analysis for
'PLEK2' on 'LUSC'

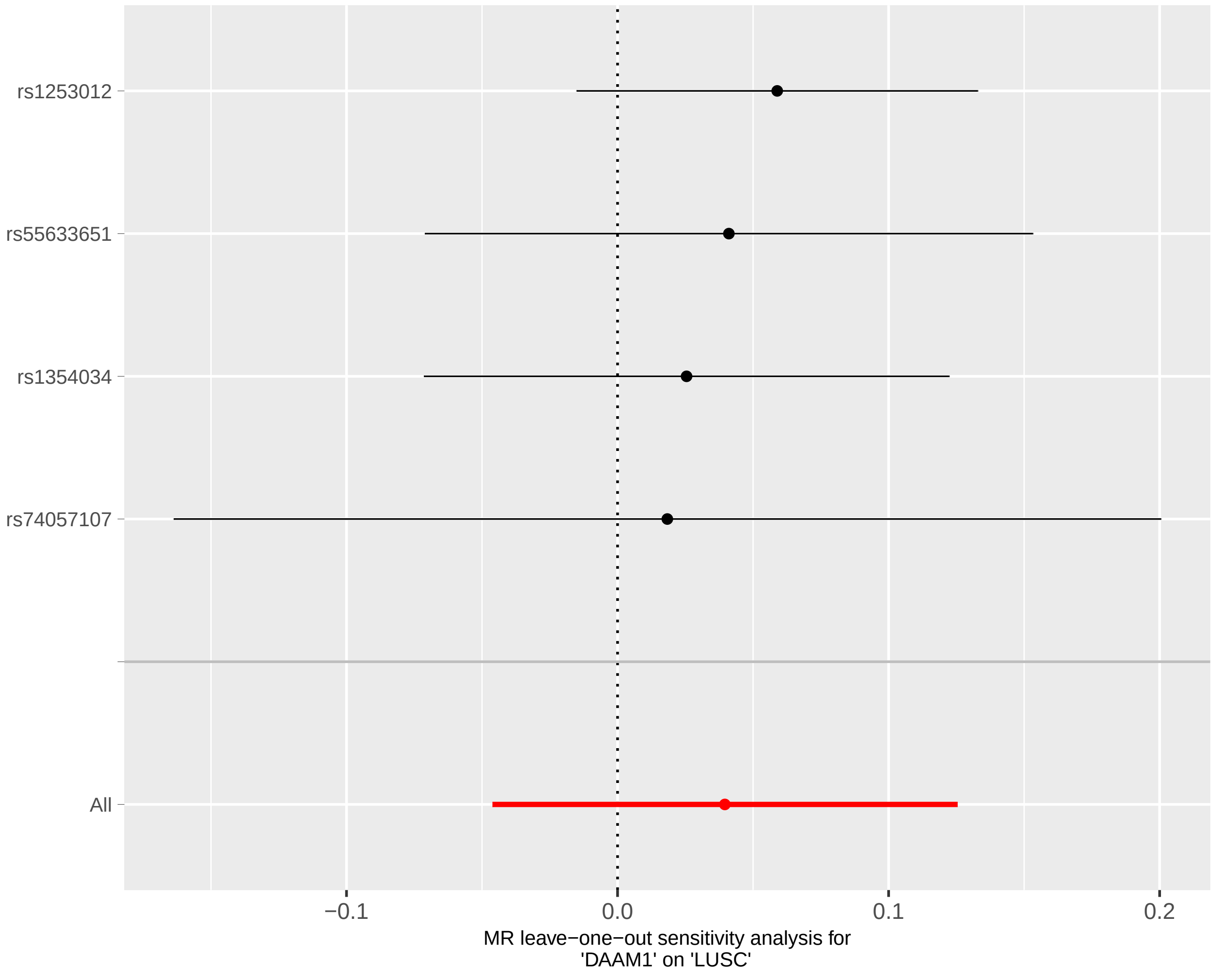

rs1253012
rs55633651
rs1354034
rs74057107
All
−0.1
0.0
0.1
0.2
MR leave−one−out sensitivity analysis for
'DAAM1' on 'LUSC'

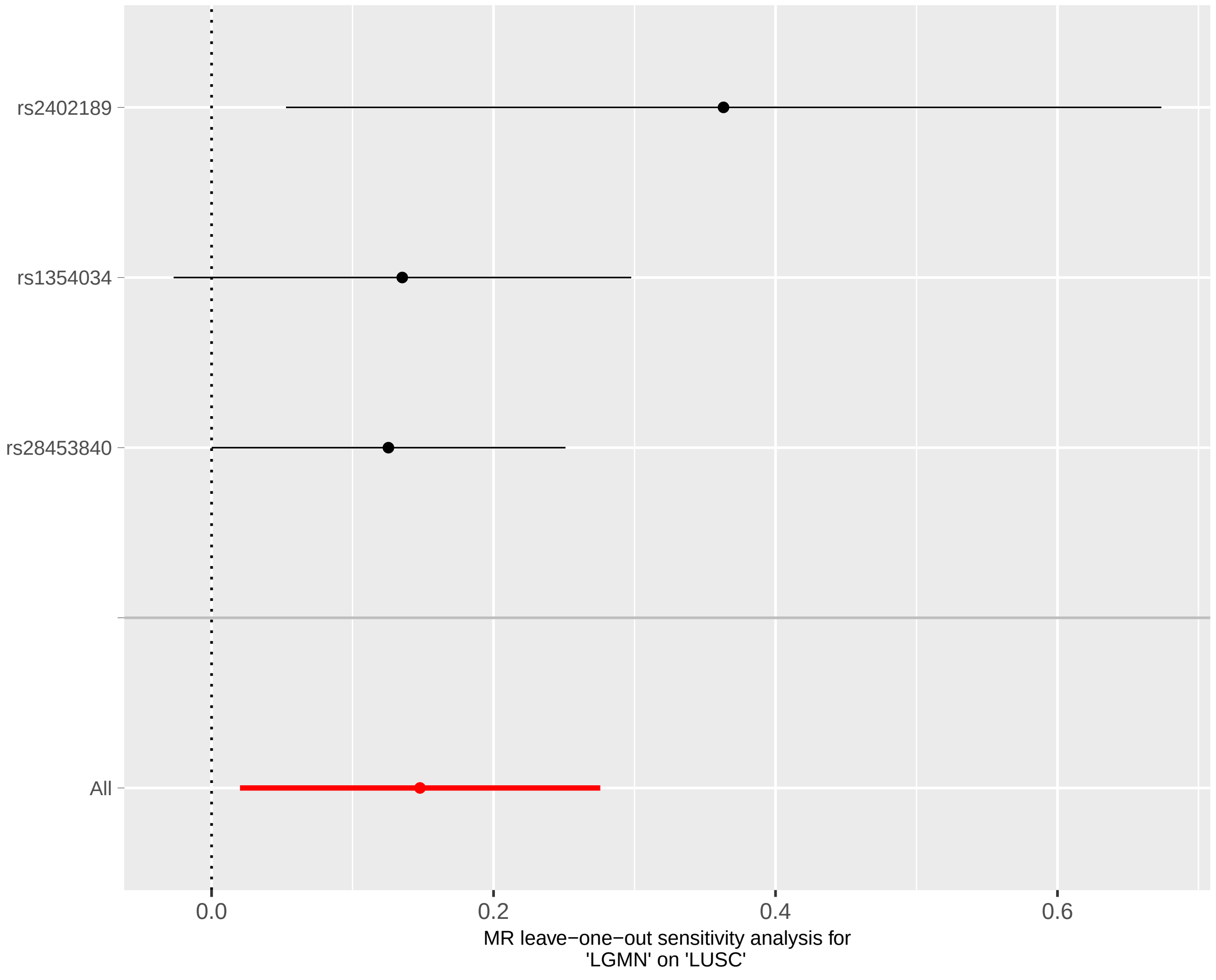
rs2402189
rs1354034
rs28453840
All
0.0
0.2
0.4
0.6
MR leave−one−out sensitivity analysis for
'LGMN' on 'LUSC'

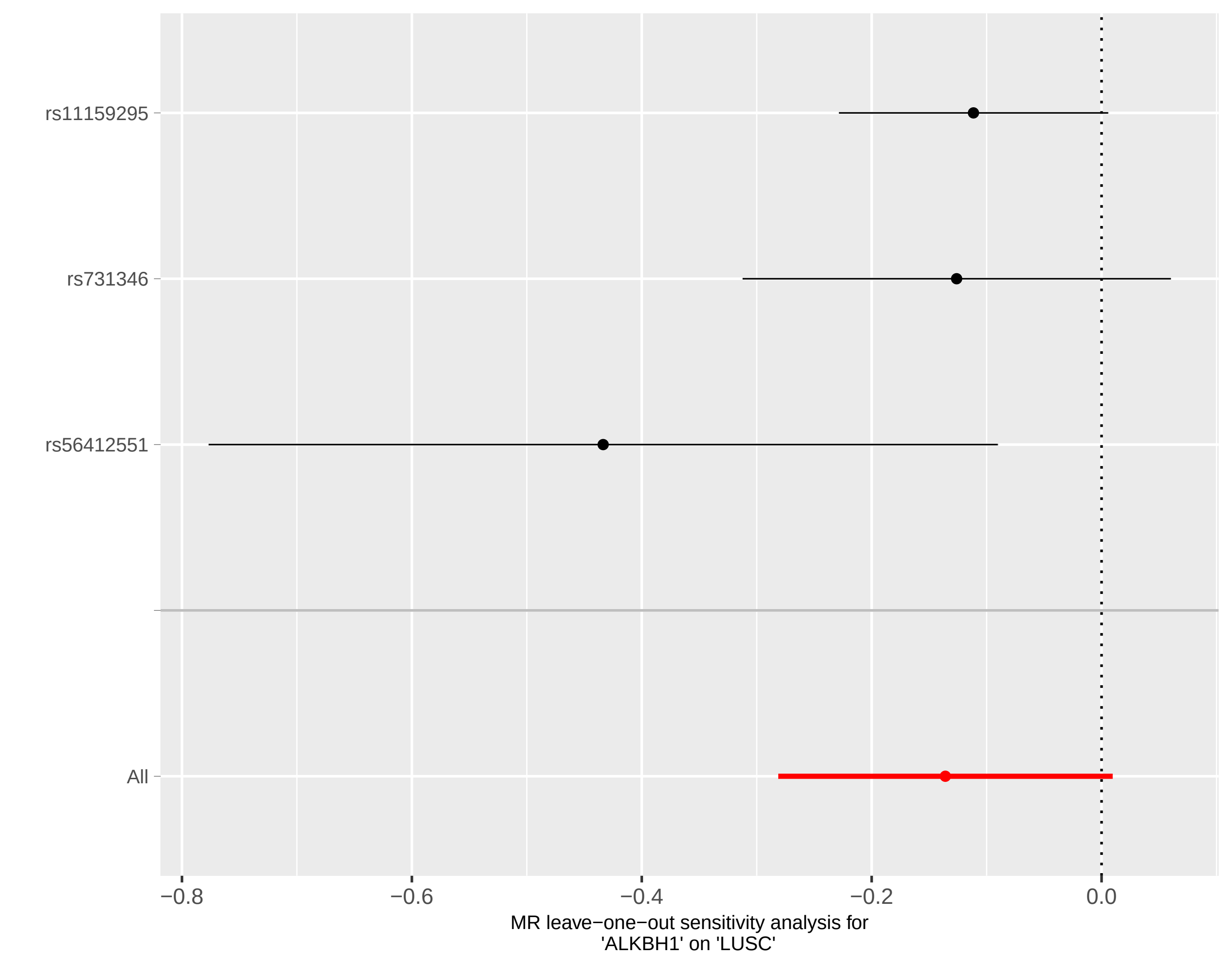

rs11159295
rs731346
rs56412551
All
−0.8
−0.6
−0.4
−0.2
0.0
MR leave−one−out sensitivity analysis for
'ALKBH1' on 'LUSC'

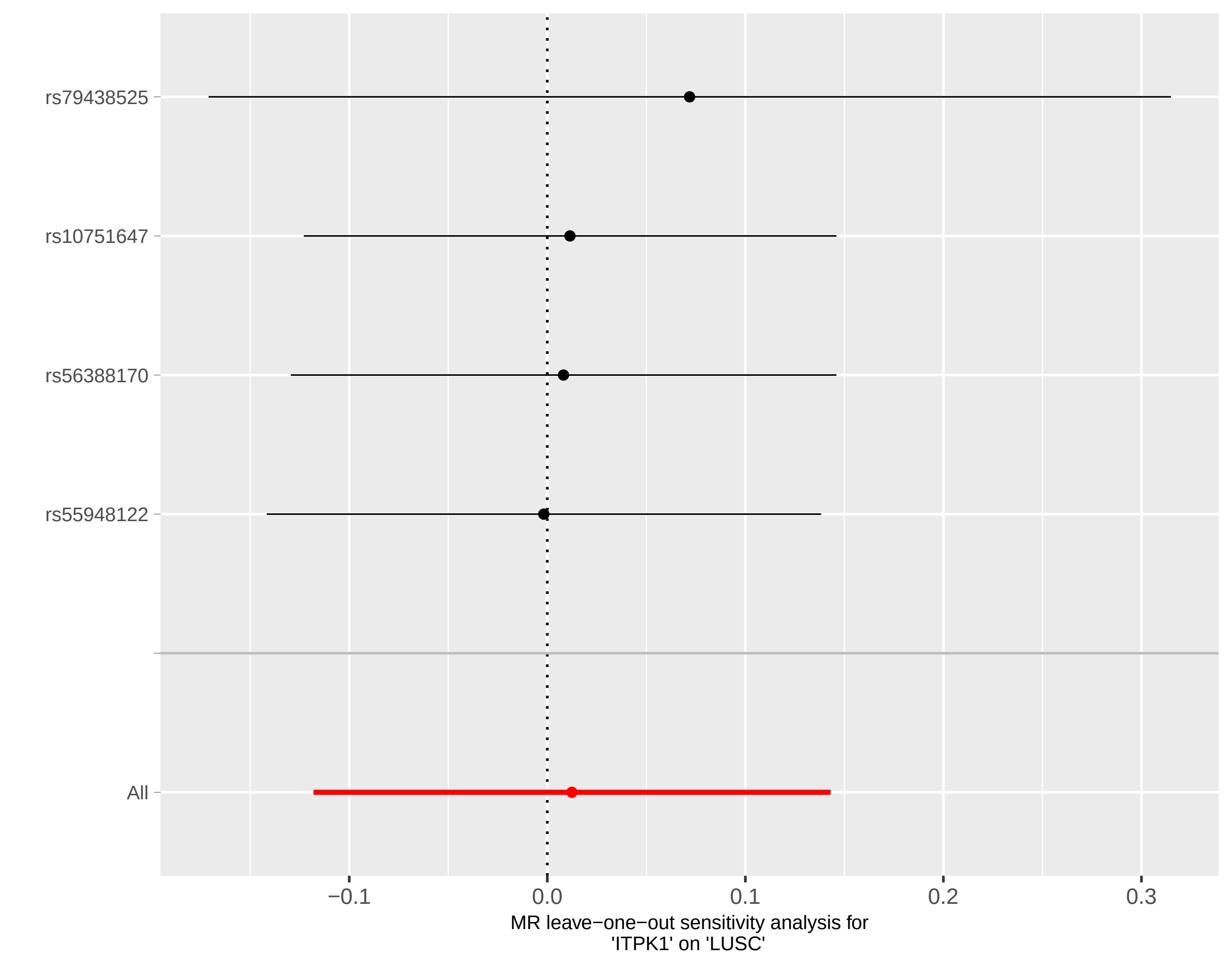

rs79438525
rs10751647
rs56388170
rs55948122
All
−0.1
0.0
0.1
0.2
0.3
MR leave−one−out sensitivity analysis for
'ITPK1' on 'LUSC'

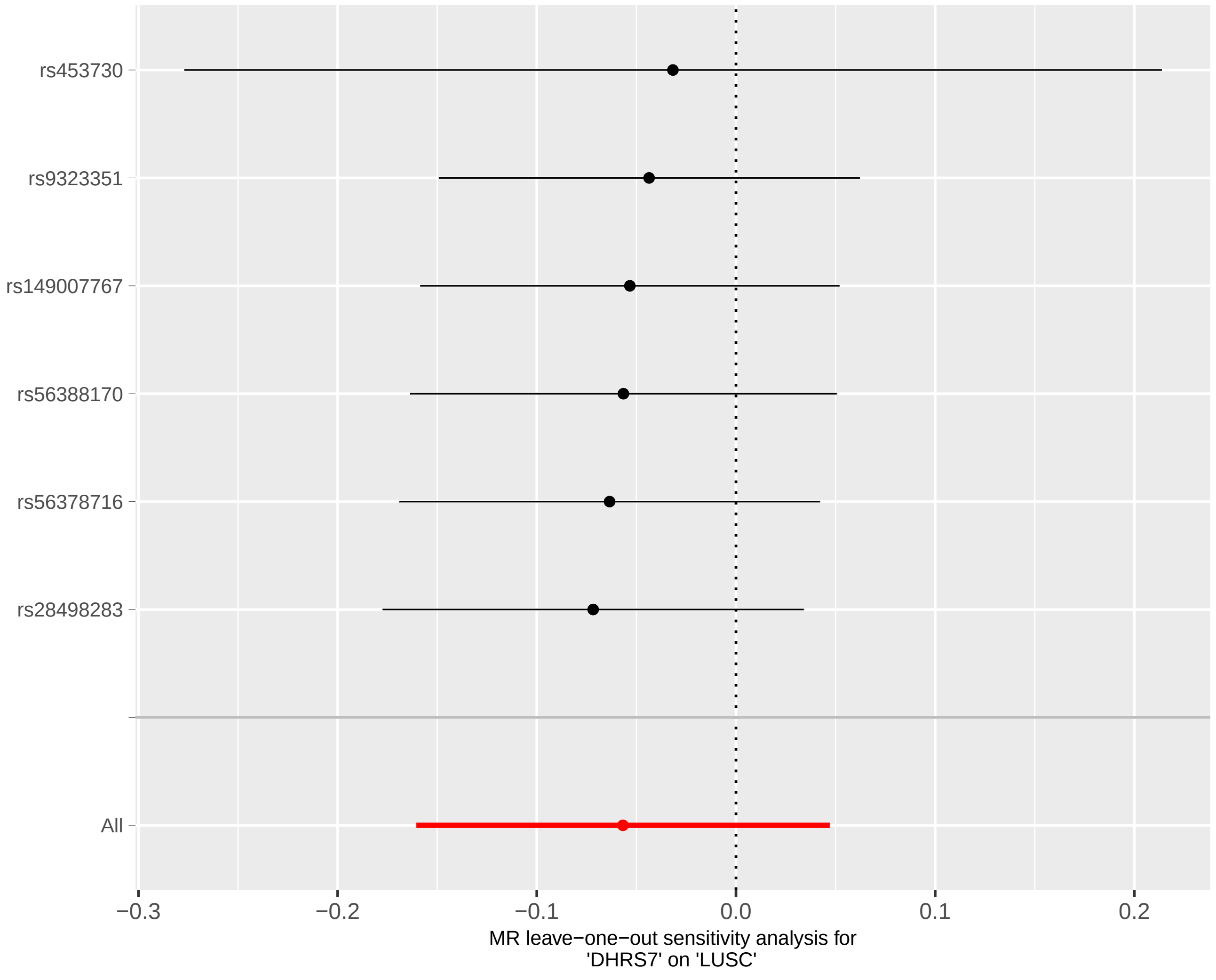
rs453730
rs9323351
rs149007767
rs56388170
rs56378716
rs28498283
All
−0.3
−0.2
−0.1
0.0
0.1
0.2
MR leave−one−out sensitivity analysis for
'DHRS7' on 'LUSC'

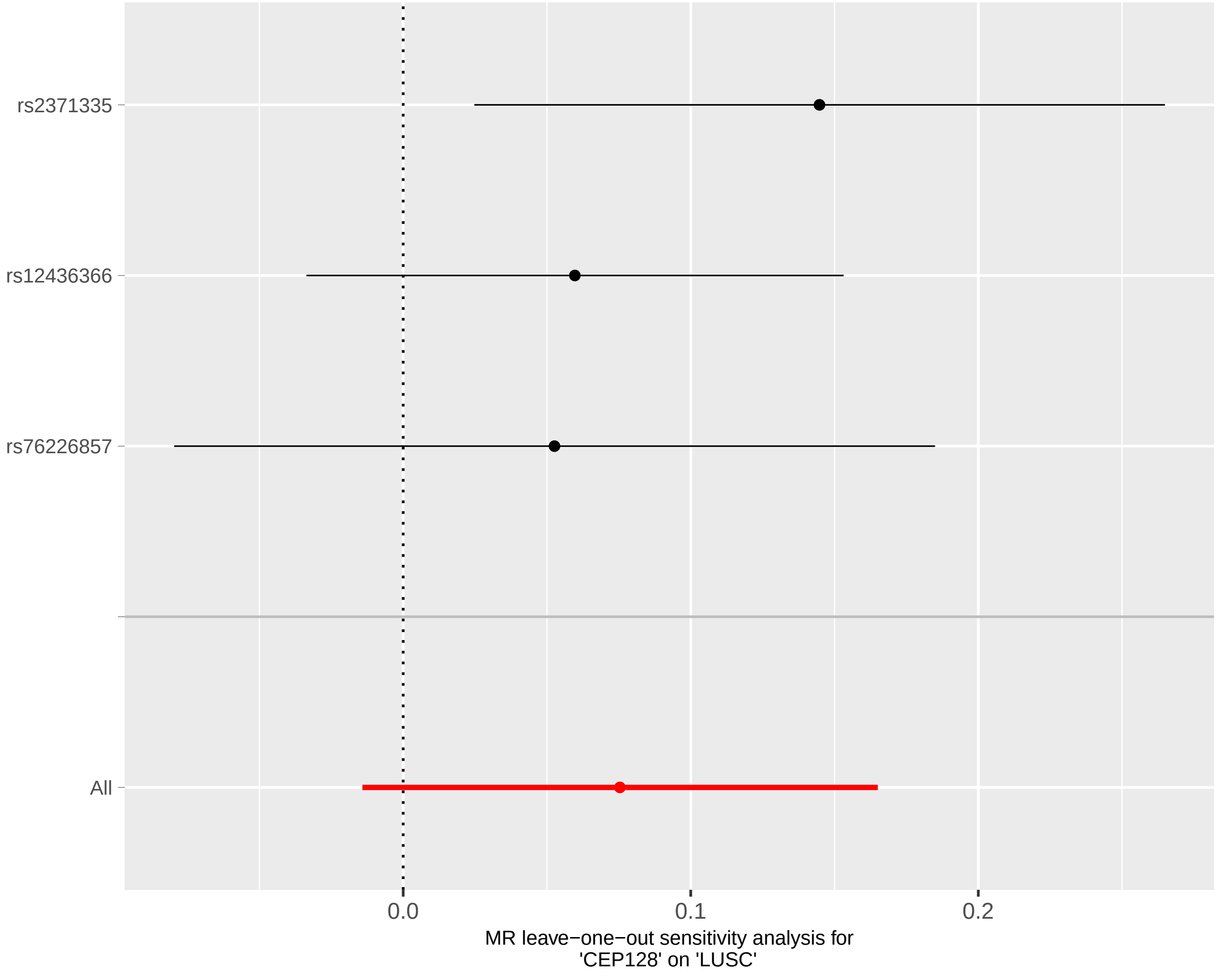
rs2371335
rs12436366
rs76226857
All
0.0
0.1
0.2
MR leave−one−out sensitivity analysis for
'CEP128' on 'LUSC'

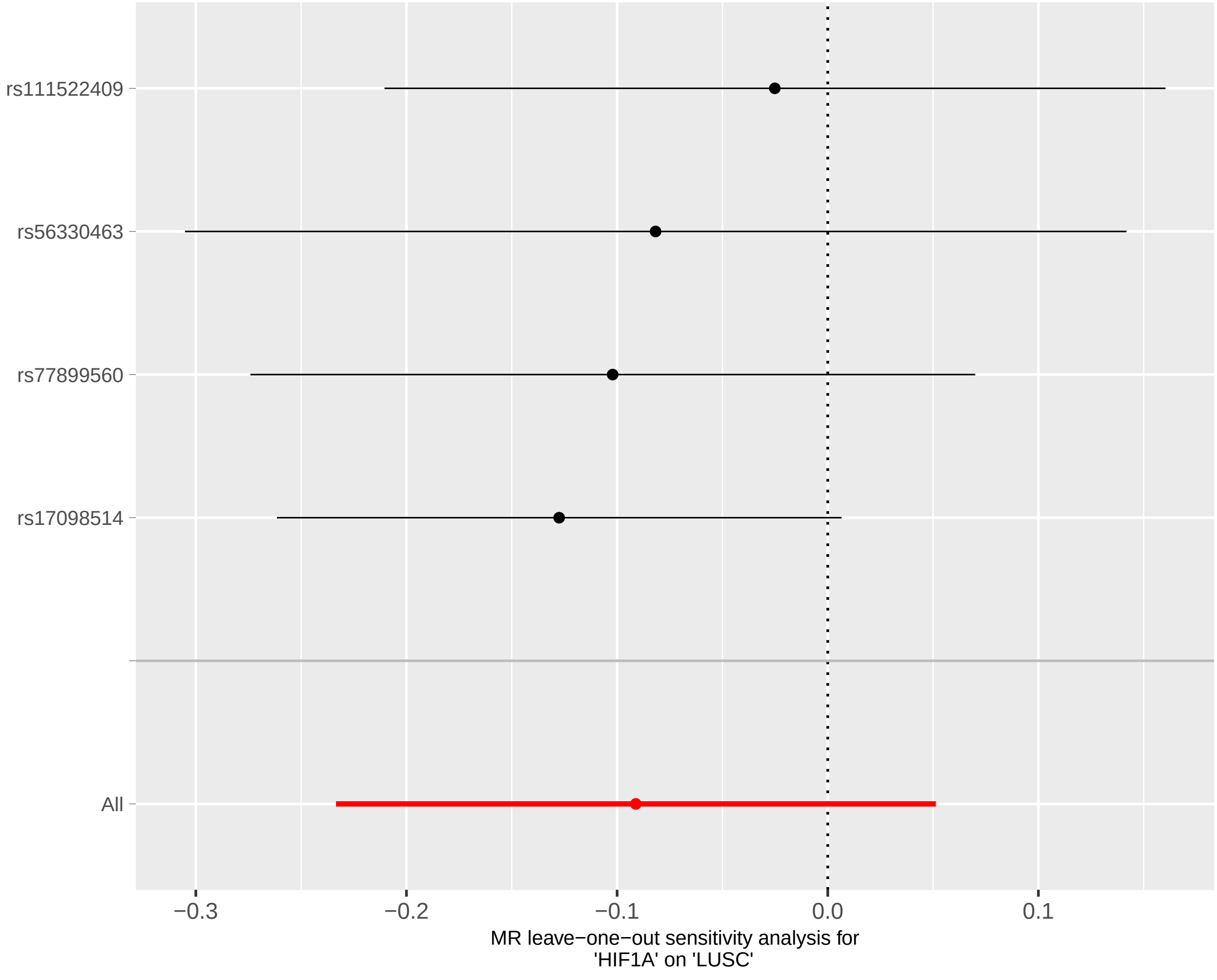
rs111522409
rs56330463
rs77899560
rs17098514
All
−0.3
−0.2
−0.1
0.0
0.1
MR leave−one−out sensitivity analysis for
'HIF1A' on 'LUSC'

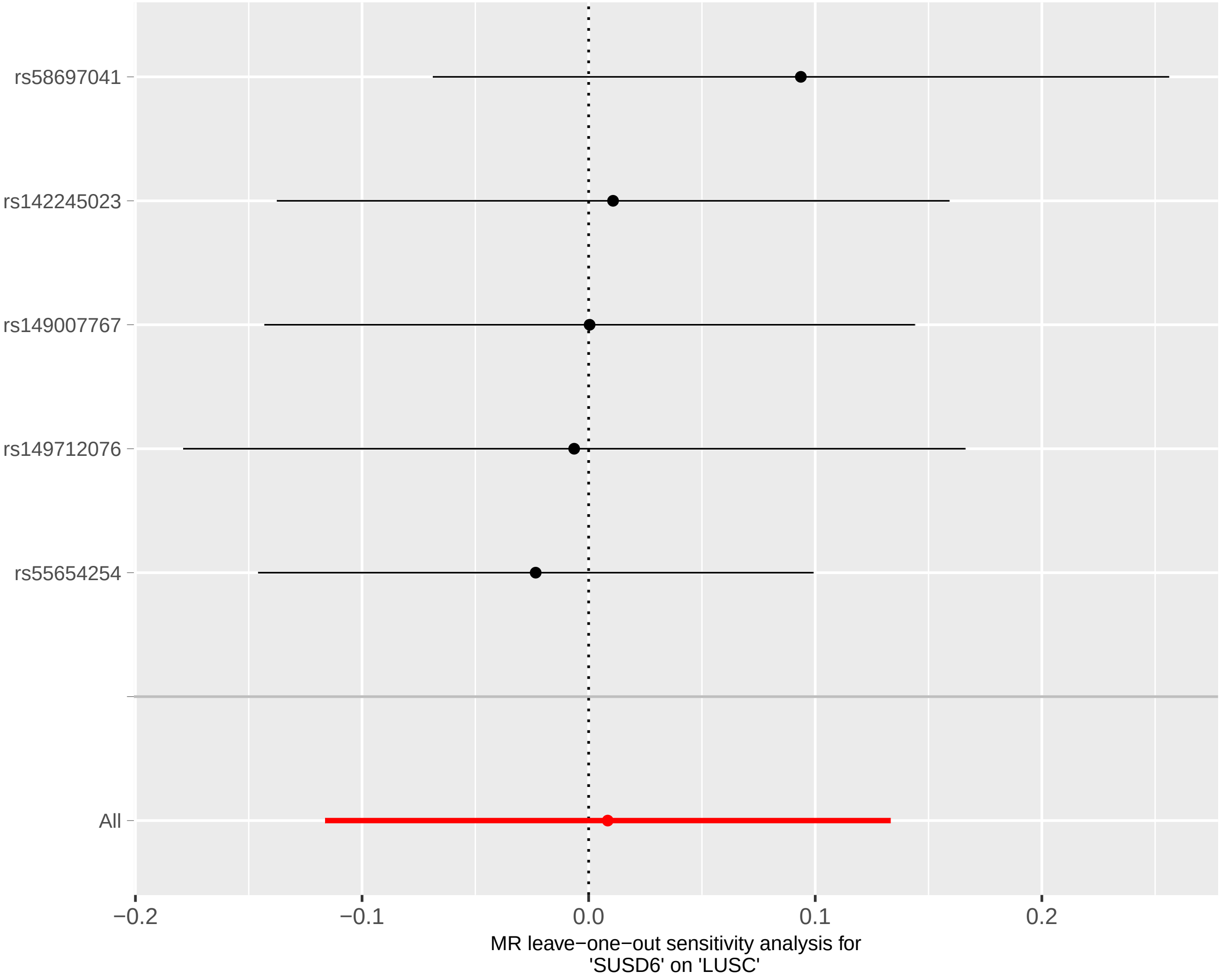

rs58697041
rs142245023
rs149007767
rs149712076
rs55654254
All
−0.2
−0.1
0.0
0.1
0.2
MR leave−one−out sensitivity analysis for
'SUSD6' on 'LUSC'

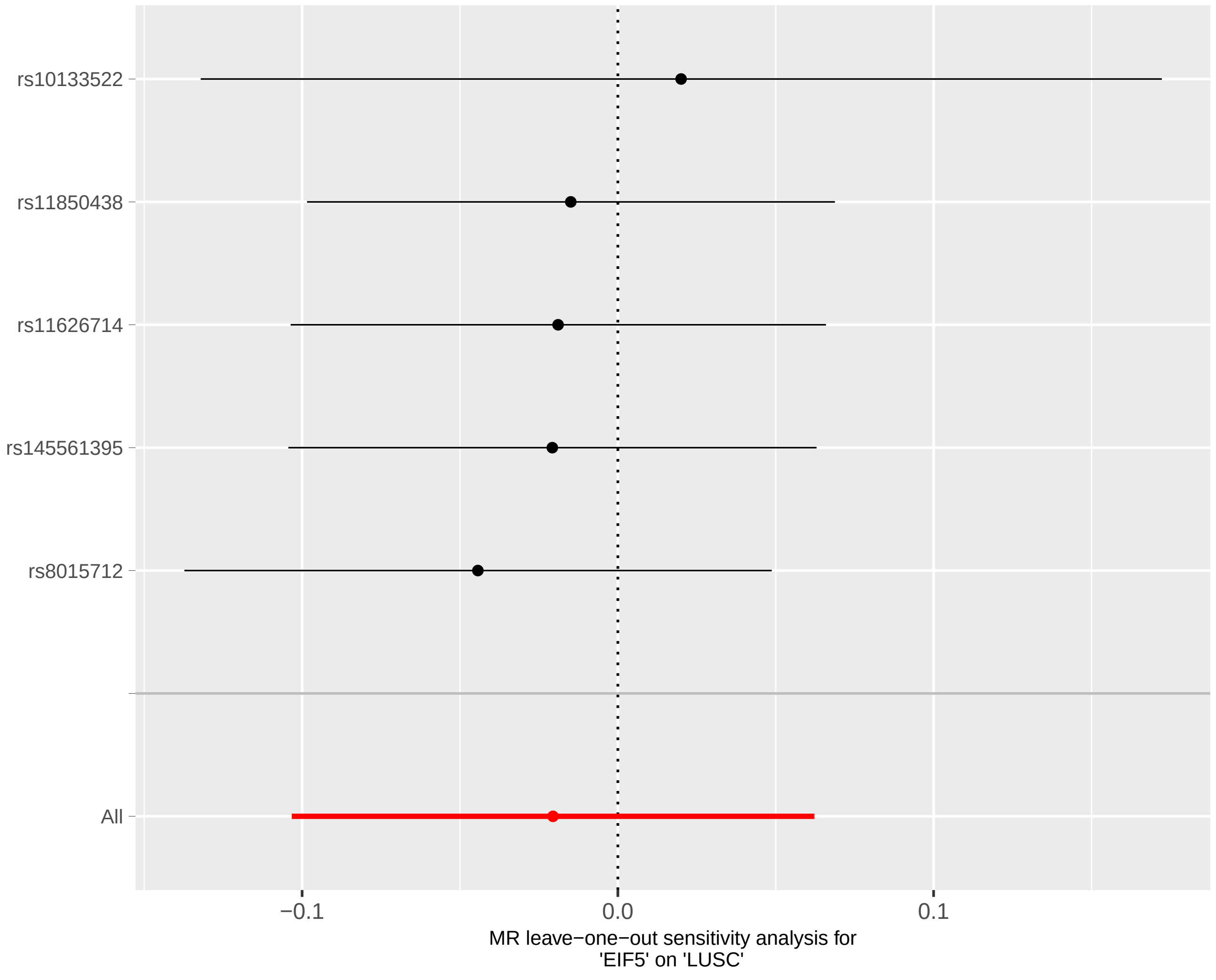

rs10133522
rs11850438
rs11626714
rs145561395
rs8015712
All
−0.1
0.0
0.1
MR leave−one−out sensitivity analysis for
'EIF5' on 'LUSC'

Insufficient number of SNPs

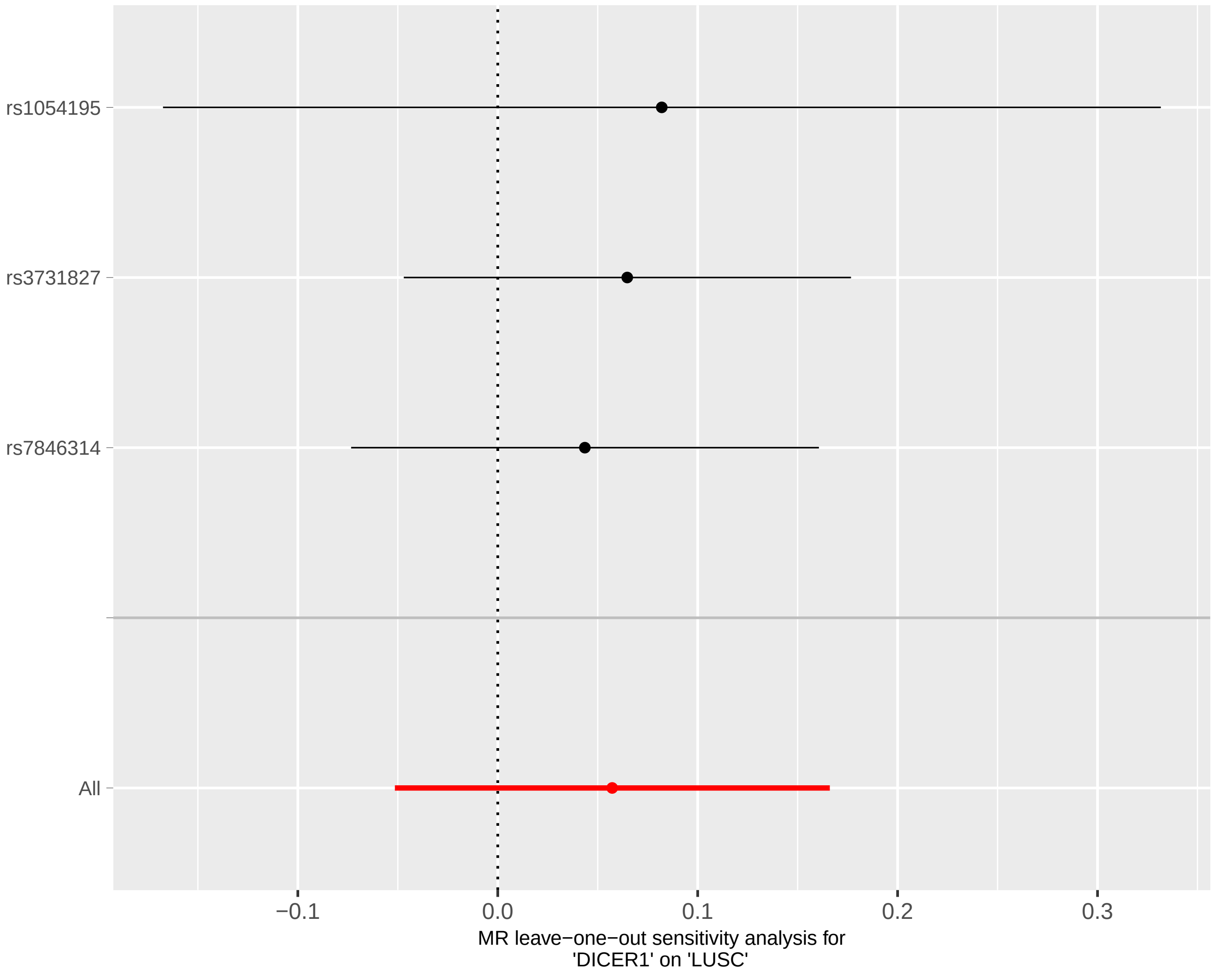

rs1054195
rs3731827
rs7846314
All
−0.1
0.0
0.1
0.2
0.3
MR leave−one−out sensitivity analysis for
'DICER1' on 'LUSC'

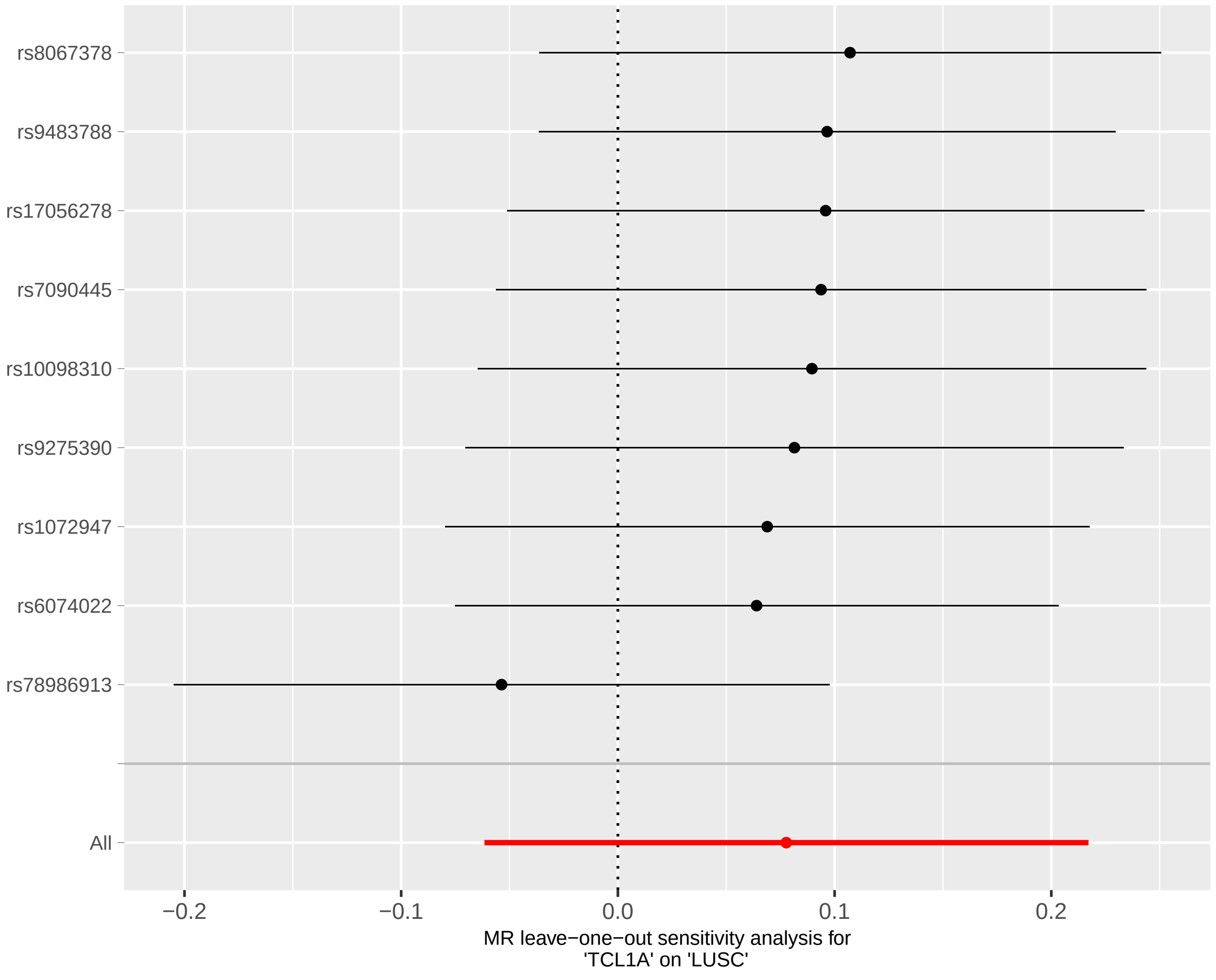

rs8067378
rs9483788
rs17056278
rs7090445
rs10098310
rs9275390
rs1072947
rs6074022
rs78986913
All
−0.2
−0.1
0.0
0.1
0.2
MR leave−one−out sensitivity analysis for
'TCL1A' on 'LUSC'

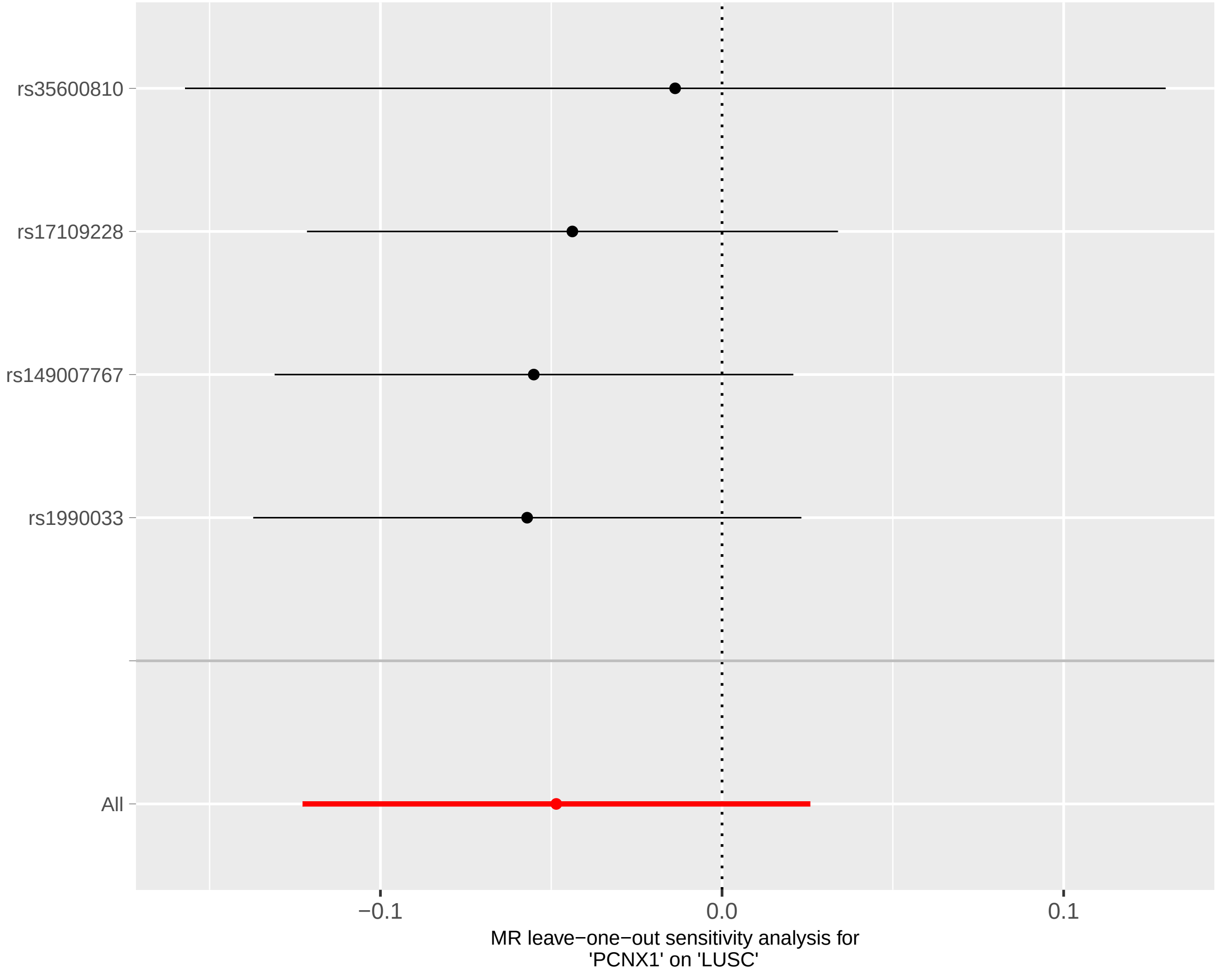
rs35600810
rs17109228
rs149007767
rs1990033
All
−0.1
0.0
0.1
MR leave−one−out sensitivity analysis for
'PCNX1' on 'LUSC'

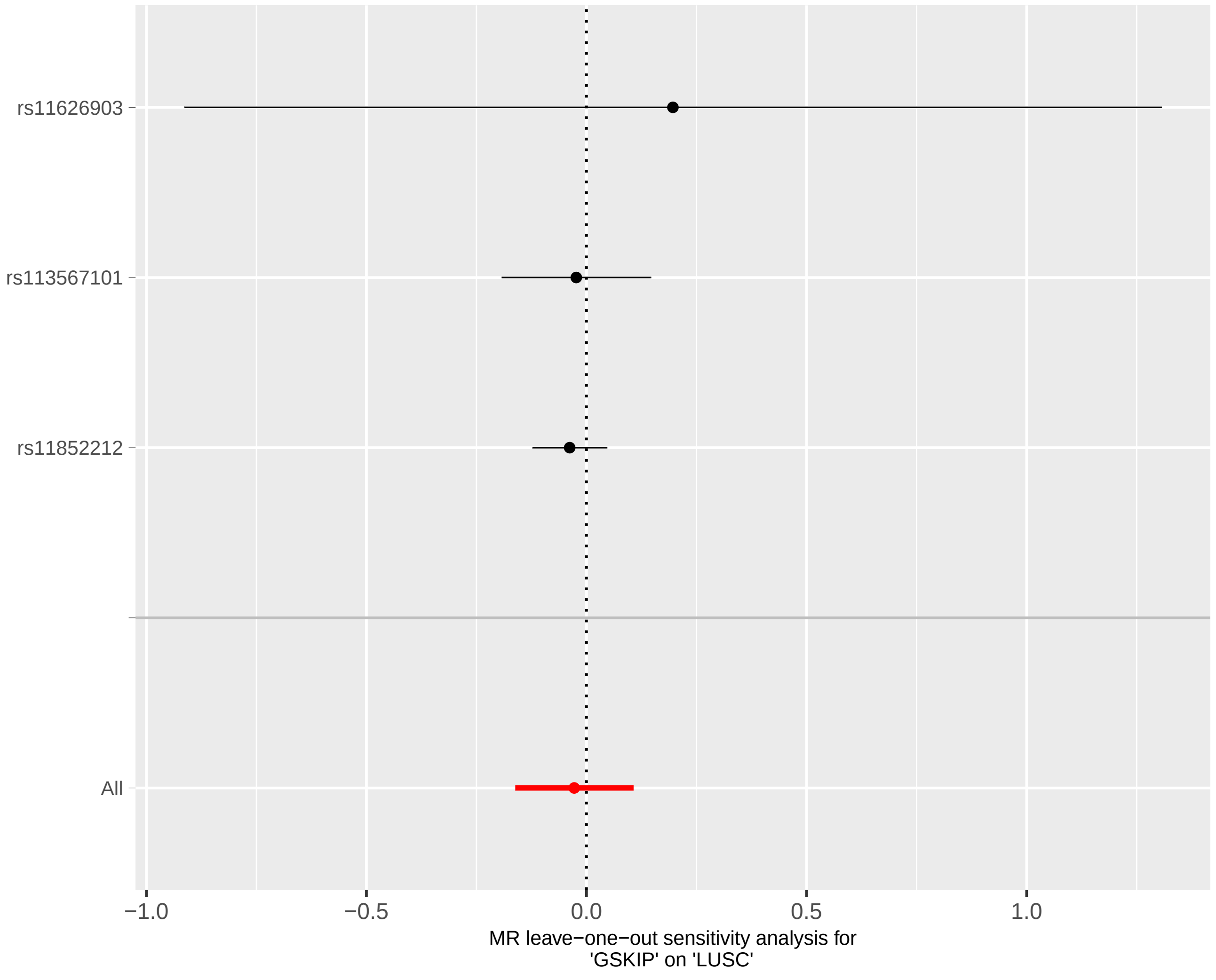

rs11626903
rs113567101
rs11852212
All
−1.0
−0.5
0.0
0.5
1.0
MR leave−one−out sensitivity analysis for
'GSKIP' on 'LUSC'

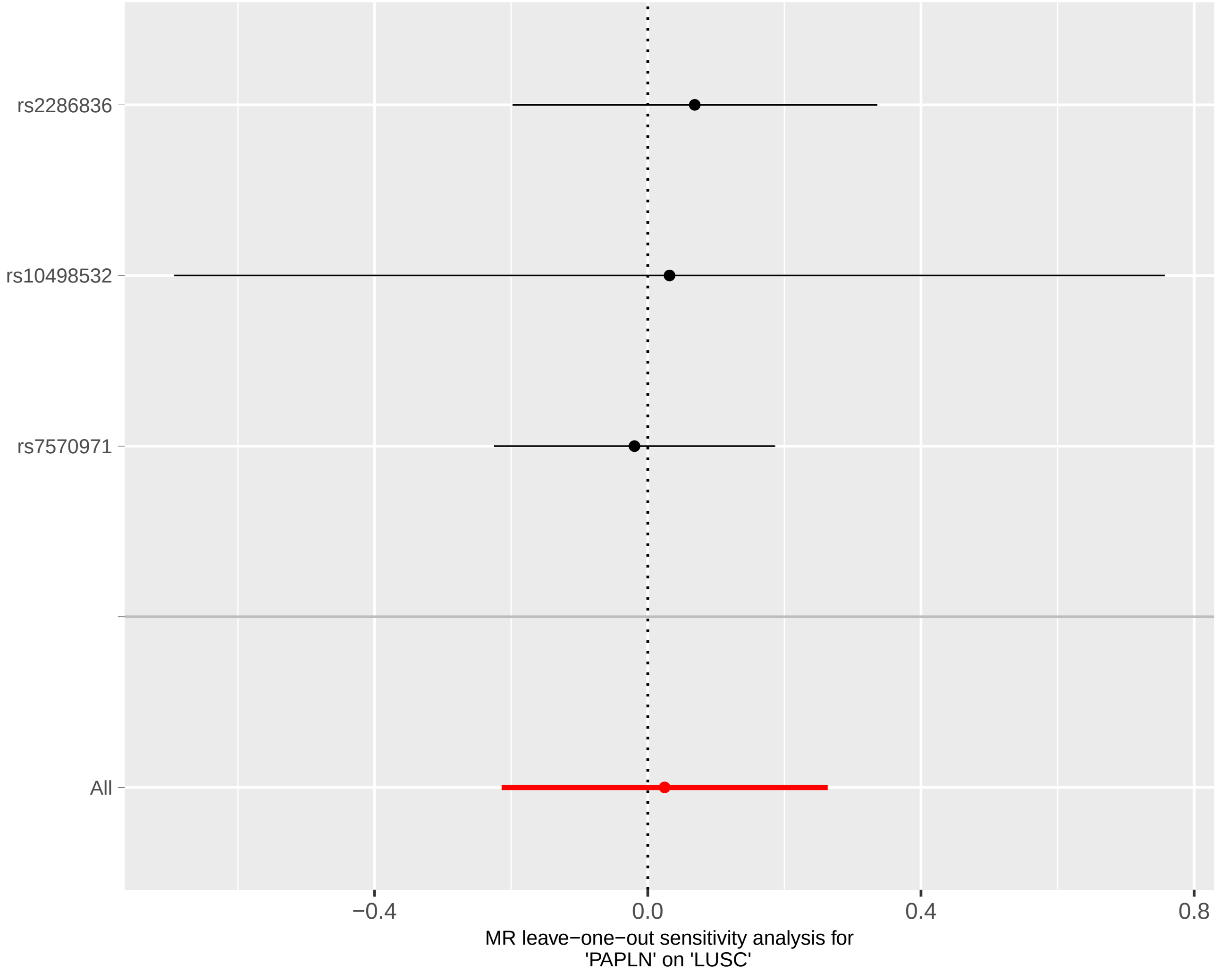
rs2286836
rs10498532
rs7570971
All
−0.4
0.0
0.4
0.8
MR leave−one−out sensitivity analysis for
'PAPLN' on 'LUSC'

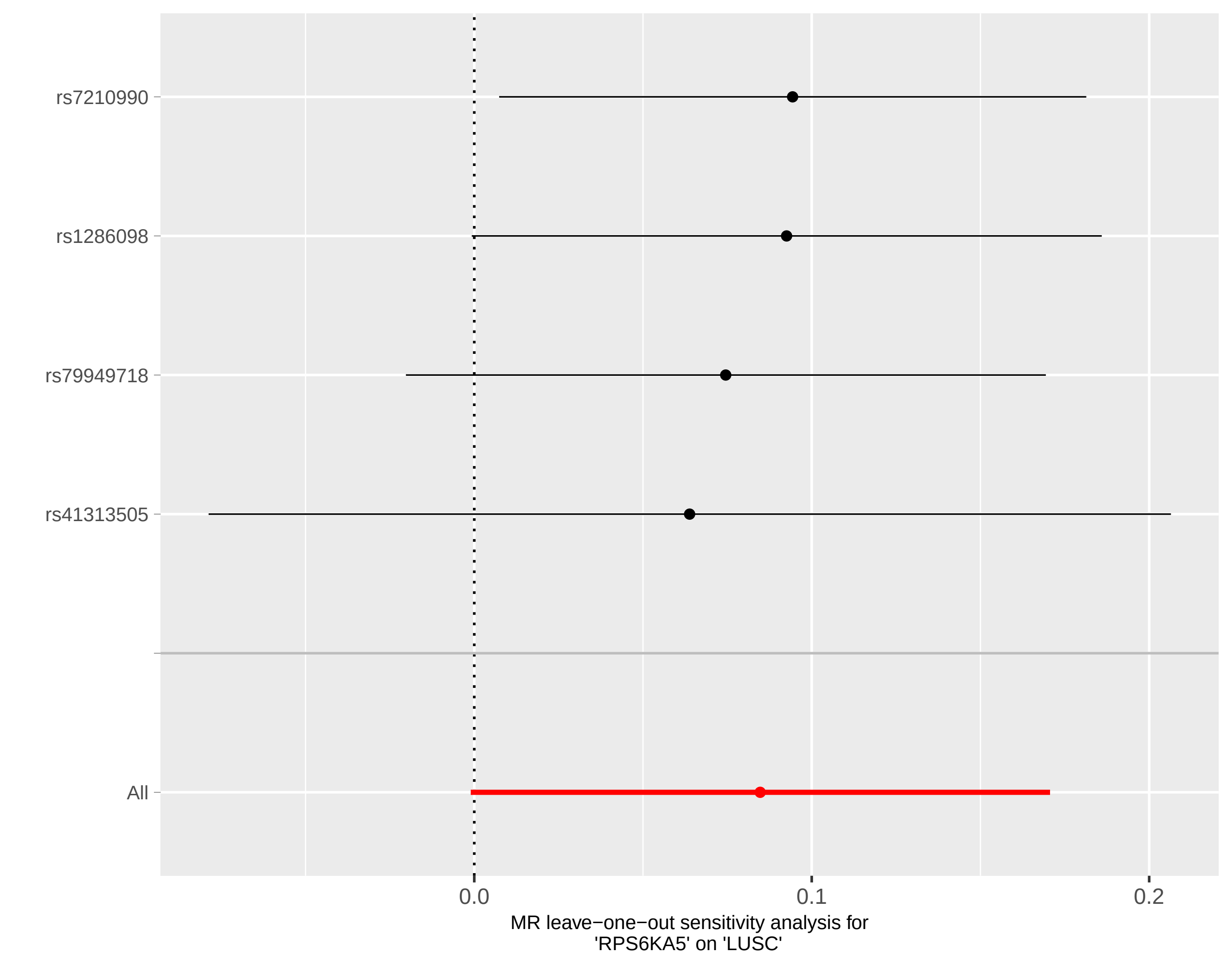

rs7210990
rs1286098
rs79949718
rs41313505
All
0.0
0.1
0.2
MR leave−one−out sensitivity analysis for
'RPS6KA5' on 'LUSC'

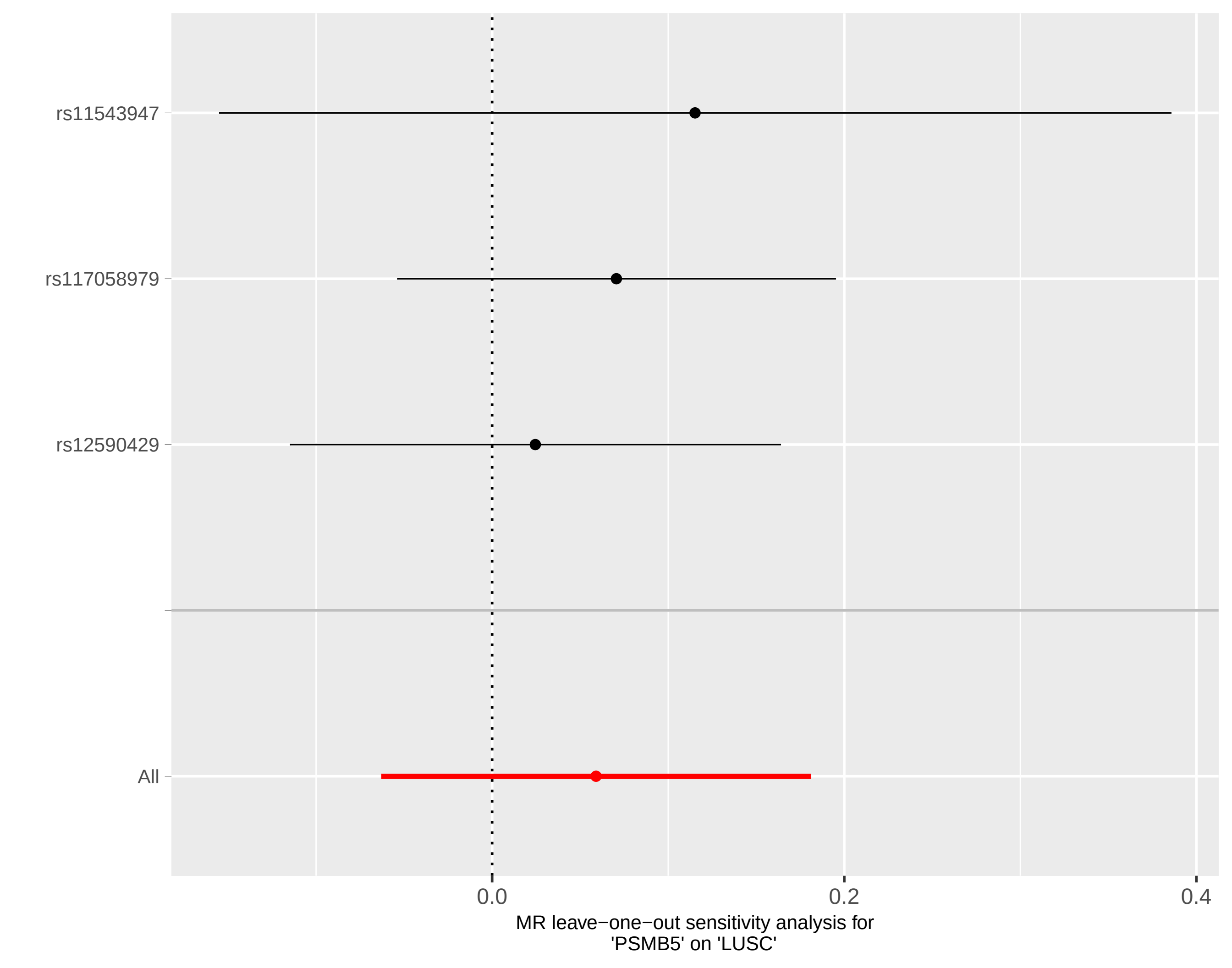

rs11543947
rs117058979
rs12590429
All
0.0
0.2
0.4
MR leave−one−out sensitivity analysis for
'PSMB5' on 'LUSC'

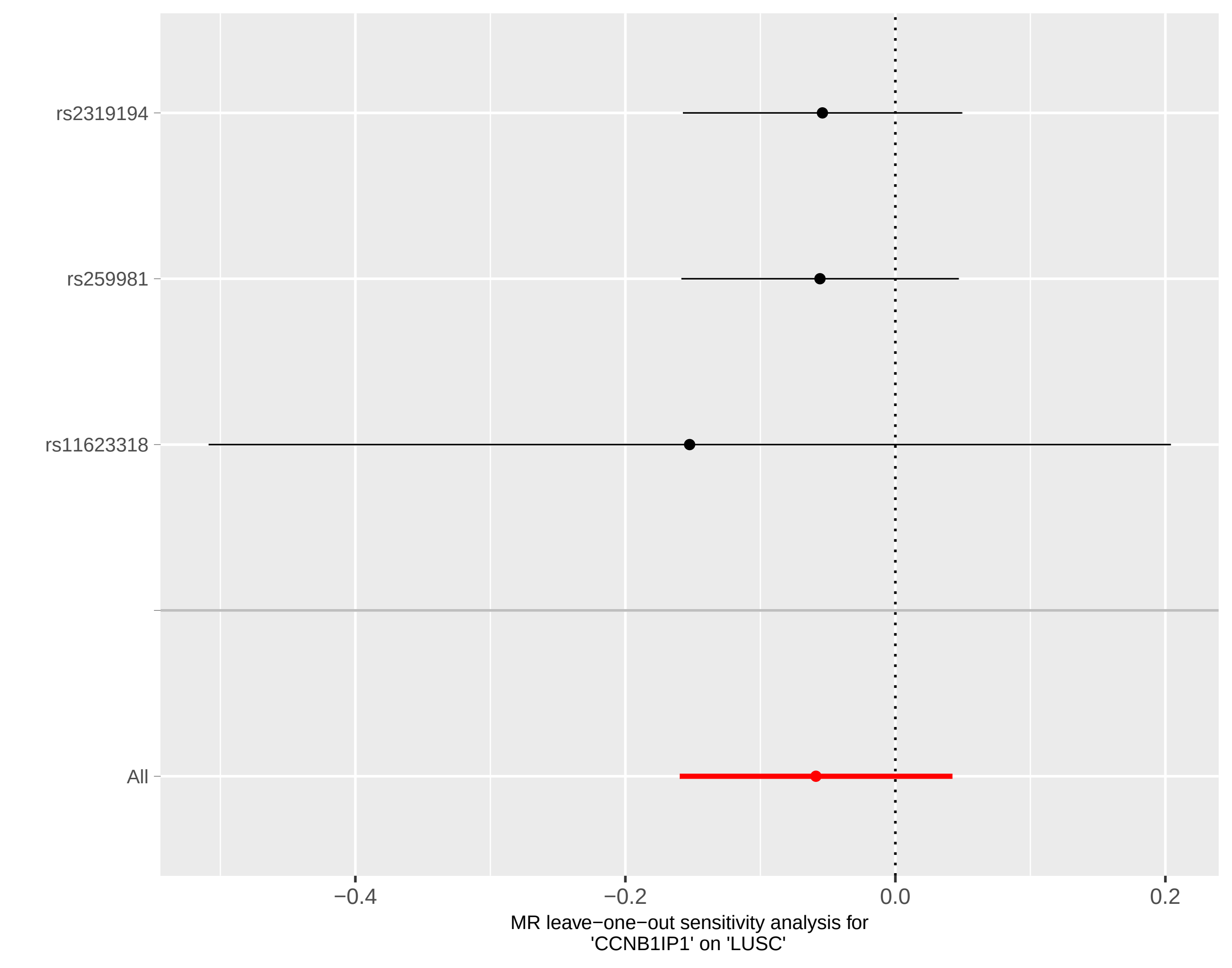

rs2319194
rs259981
rs11623318
All
−0.4
−0.2
0.0
0.2
MR leave−one−out sensitivity analysis for
'CCNB1IP1' on 'LUSC'

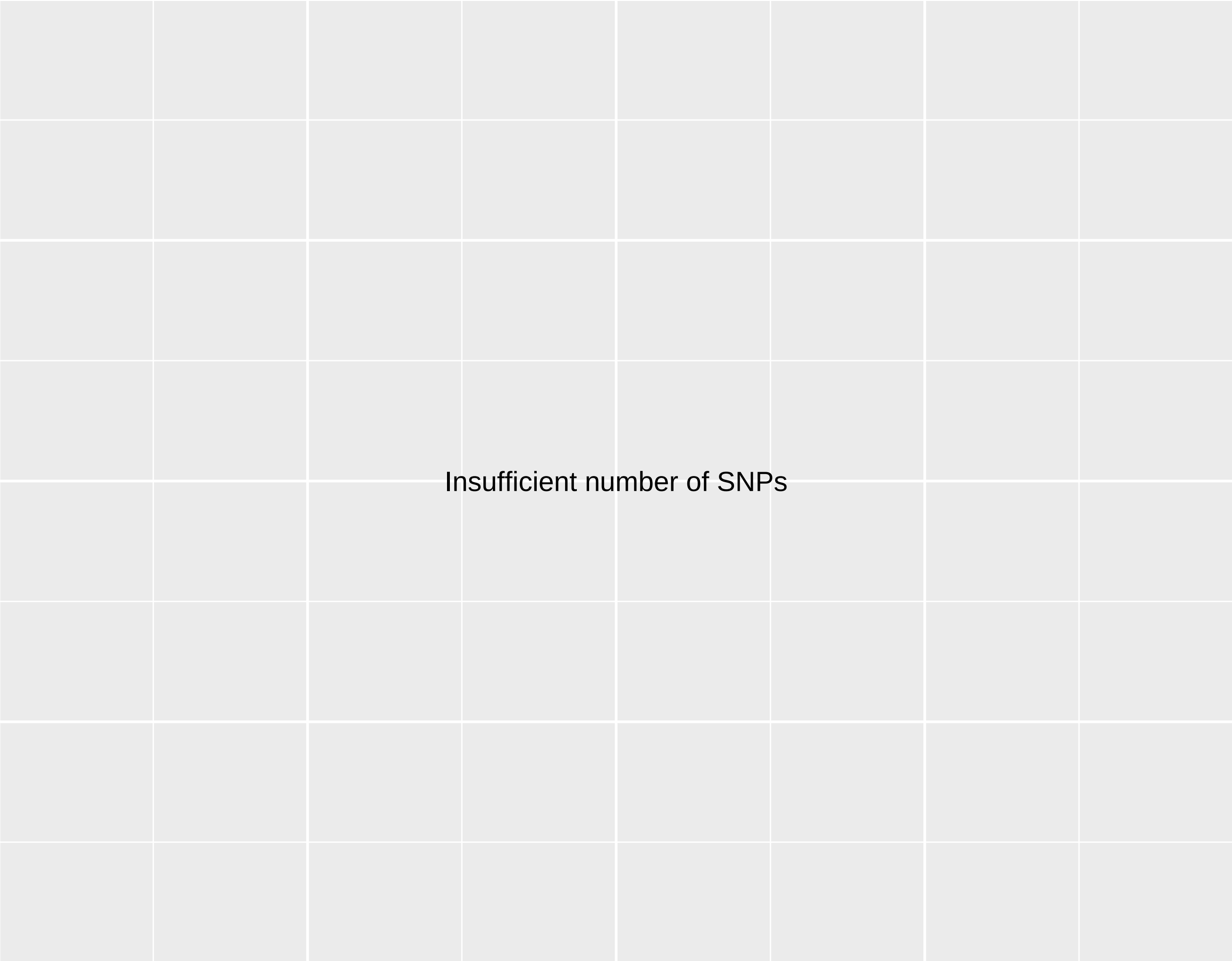
Insufficient number of SNPs

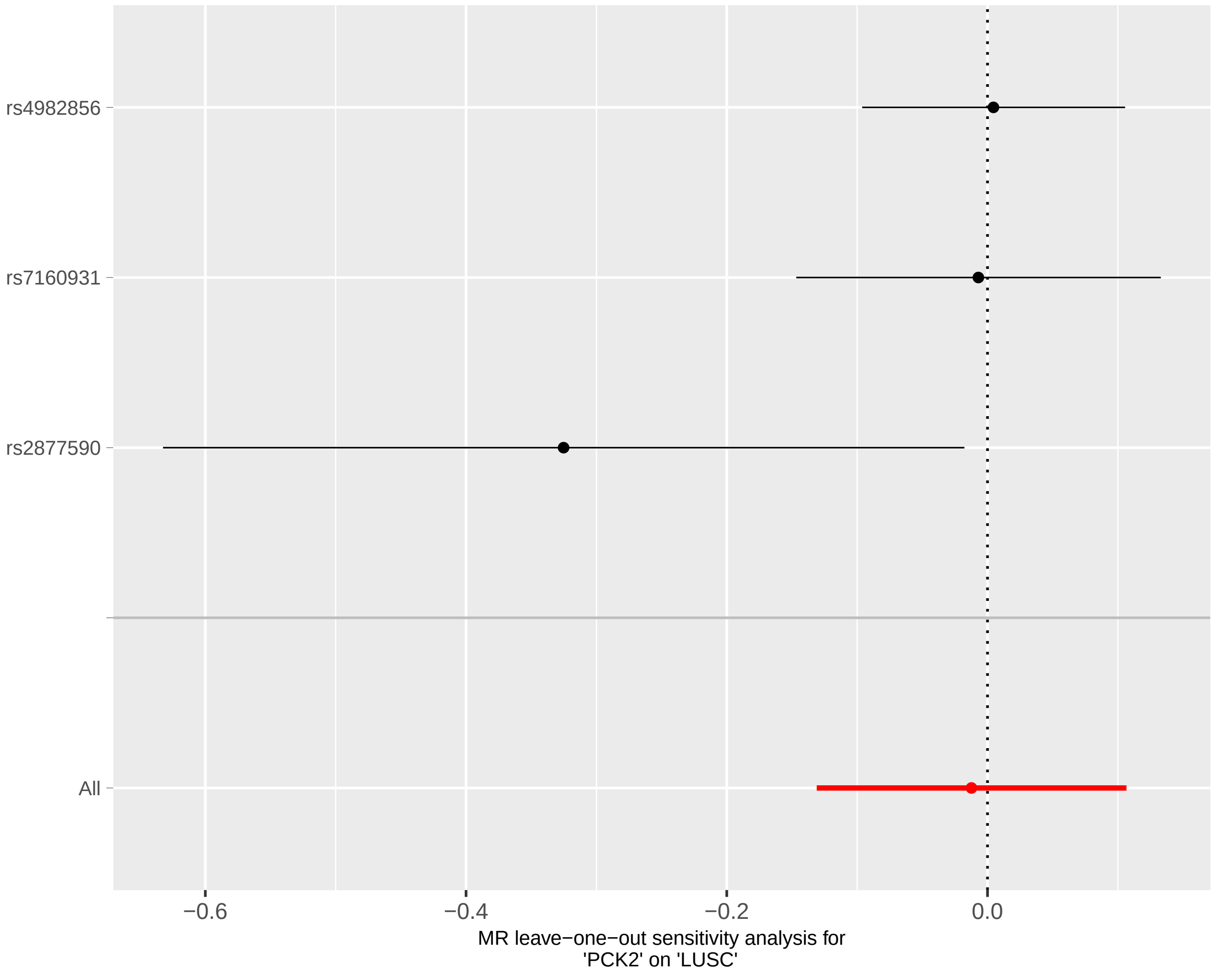
rs4982856
rs7160931
rs2877590
All
−0.6
−0.4
−0.2
0.0
MR leave−one−out sensitivity analysis for
'PCK2' on 'LUSC'

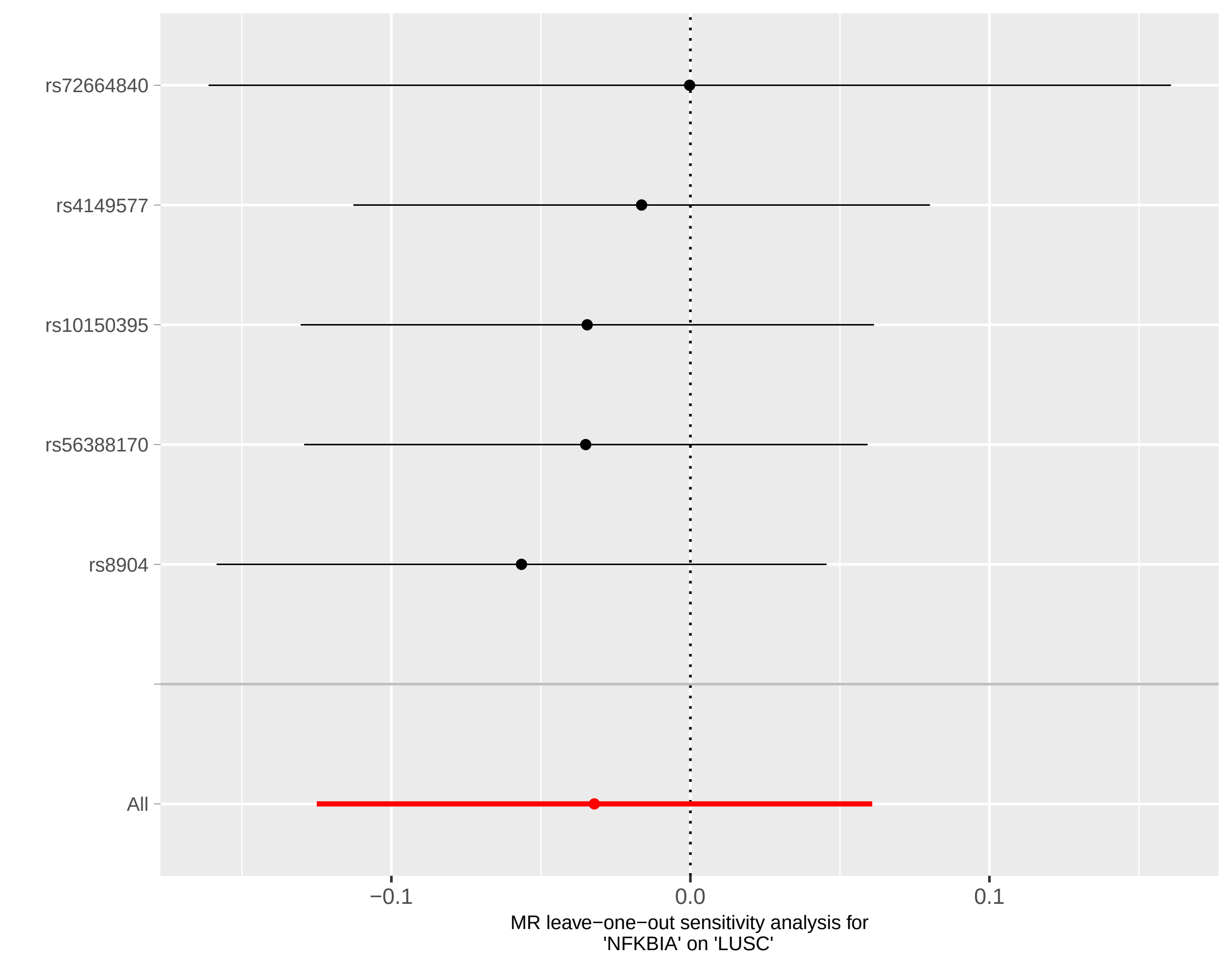
rs72664840
rs4149577
rs10150395
rs56388170
rs8904
All
−0.1
0.0
0.1
MR leave−one−out sensitivity analysis for
'NFKBIA' on 'LUSC'

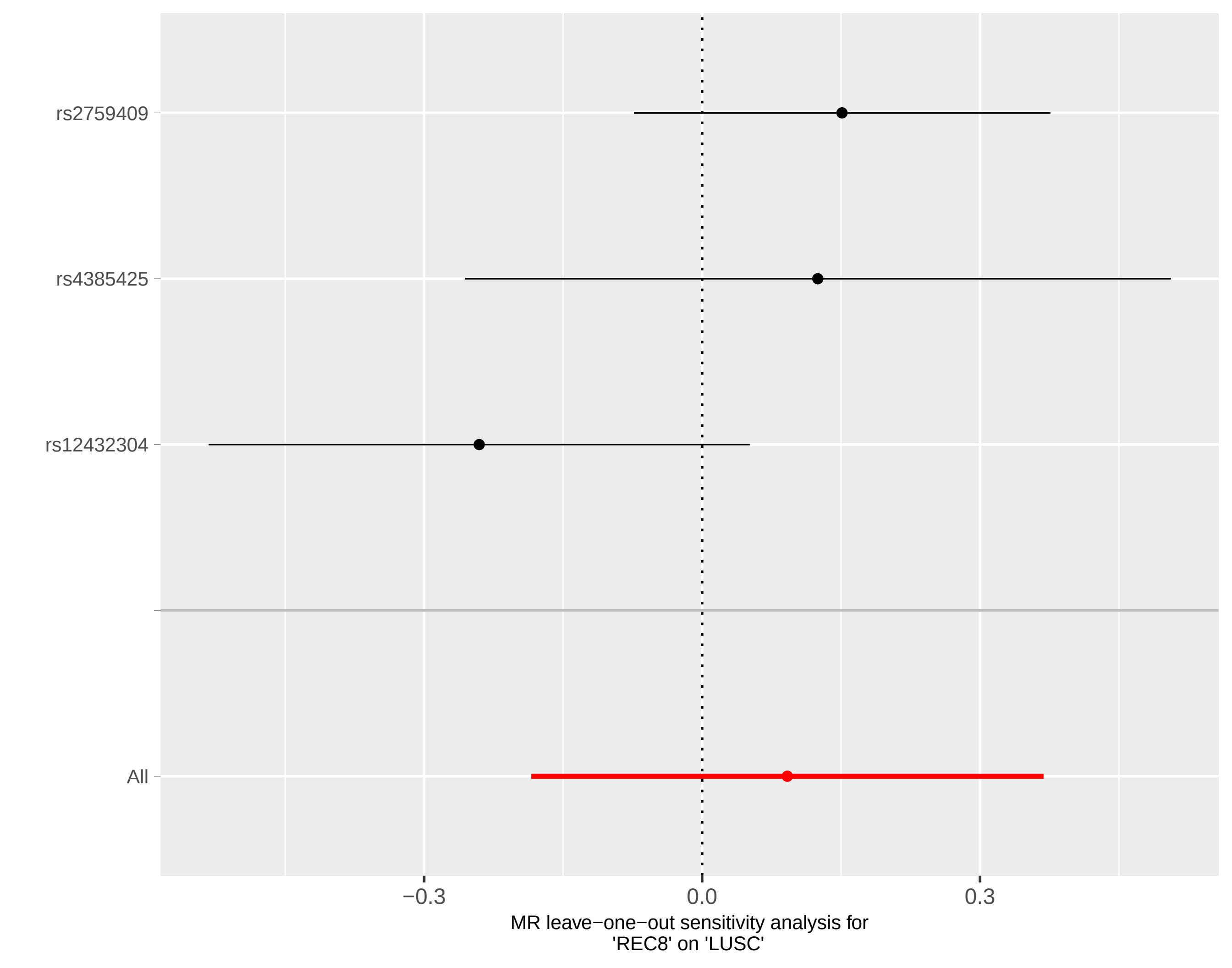

rs2759409
rs4385425
rs12432304
All
−0.3
0.0
0.3
MR leave−one−out sensitivity analysis for
'REC8' on 'LUSC'

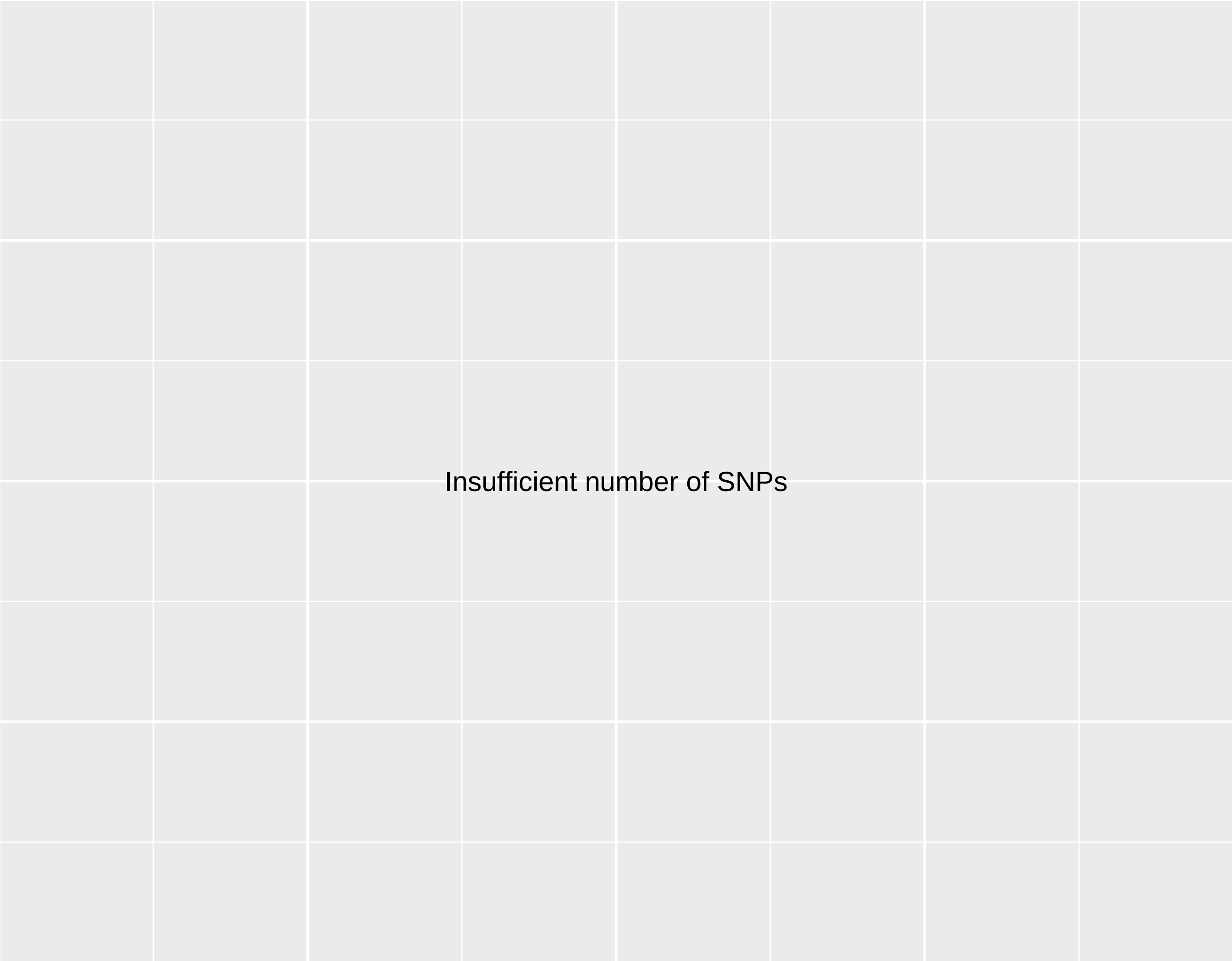
Insufficient number of SNPs

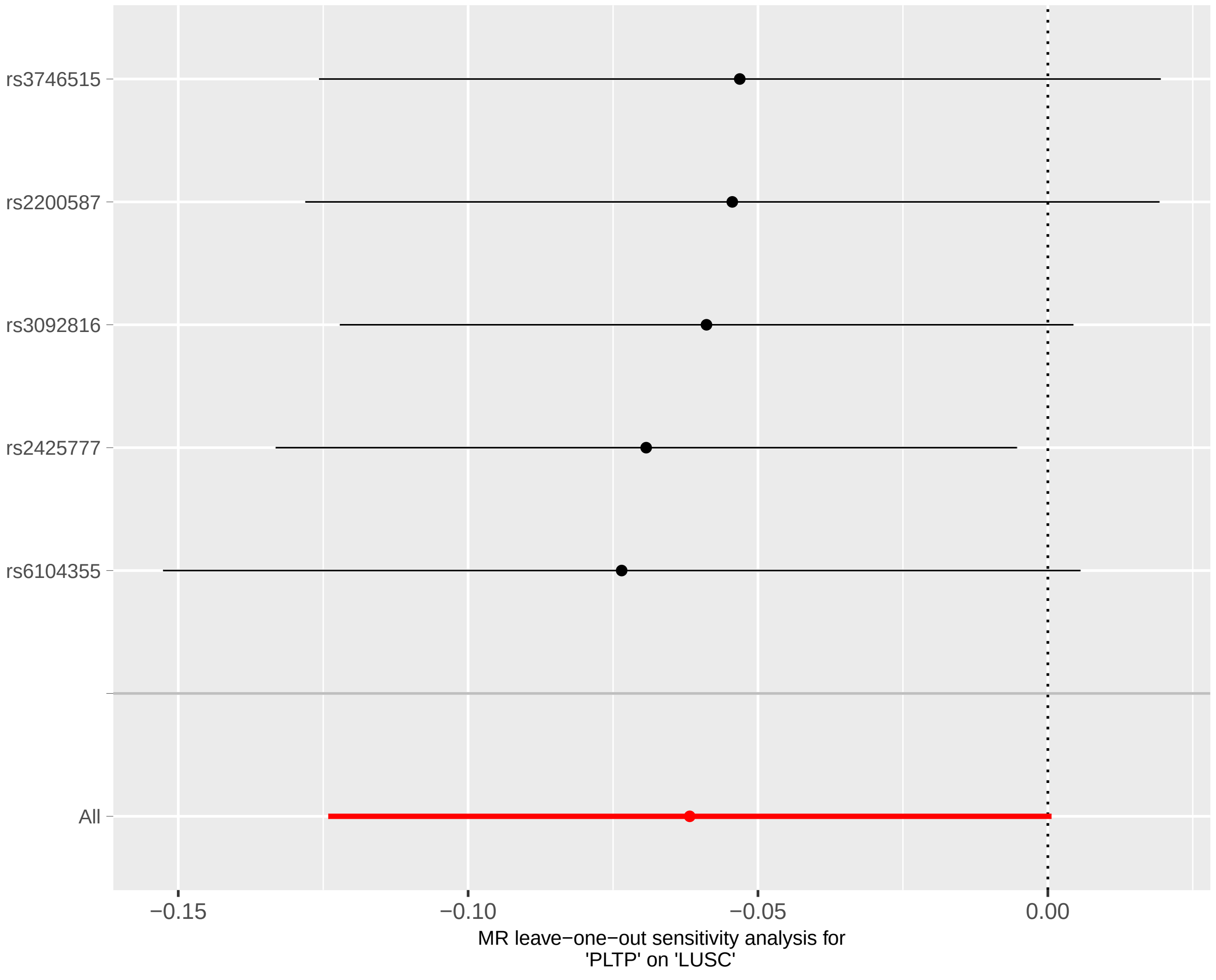

rs3746515
rs2200587
rs3092816
rs2425777
rs6104355
All
−0.15
−0.10
−0.05
0.00
MR leave−one−out sensitivity analysis for
'PLTP' on 'LUSC'

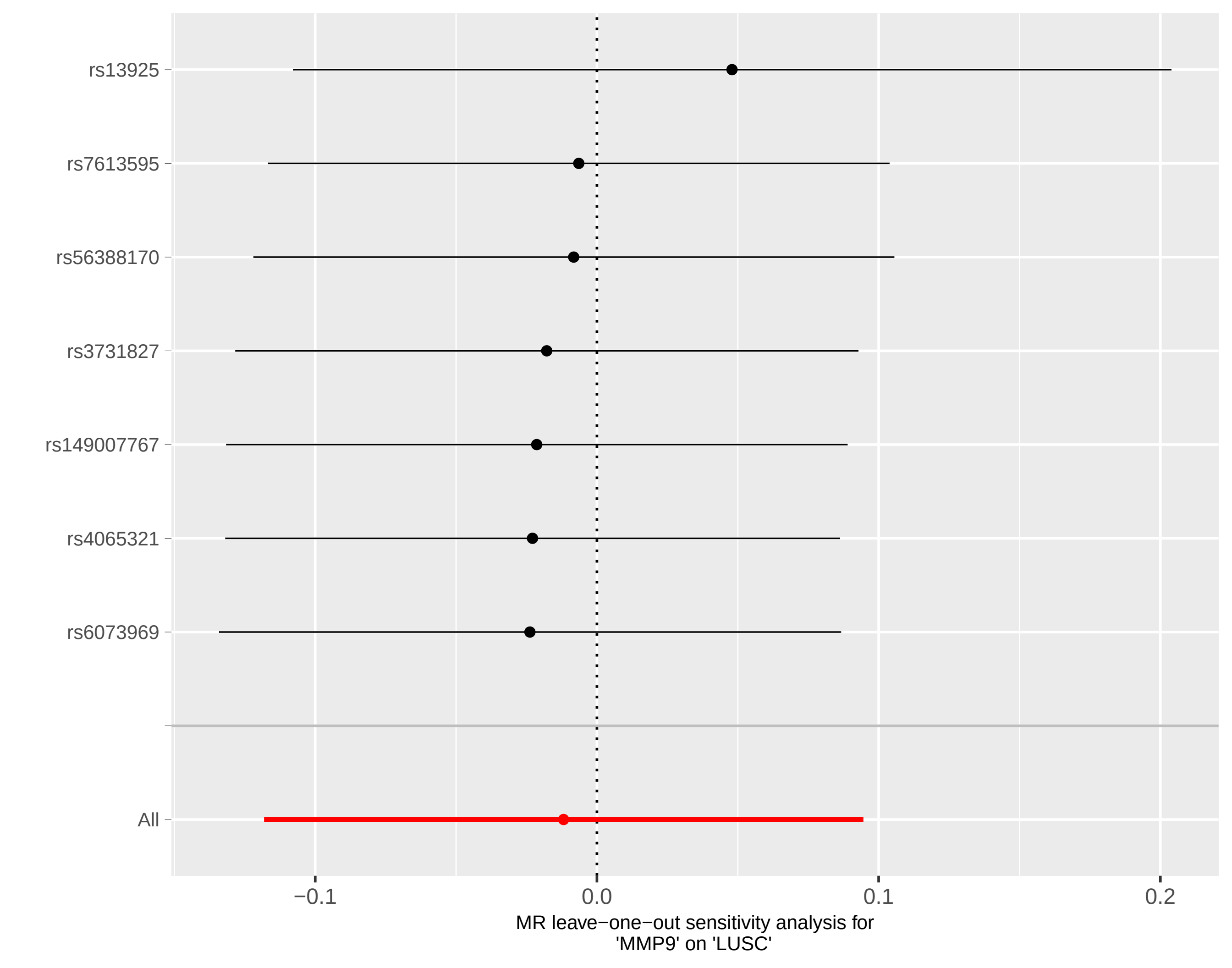
rs13925
rs7613595
rs56388170
rs3731827
rs149007767
rs4065321
rs6073969
All
−0.1
0.0
0.1
0.2
MR leave−one−out sensitivity analysis for
'MMP9' on 'LUSC'

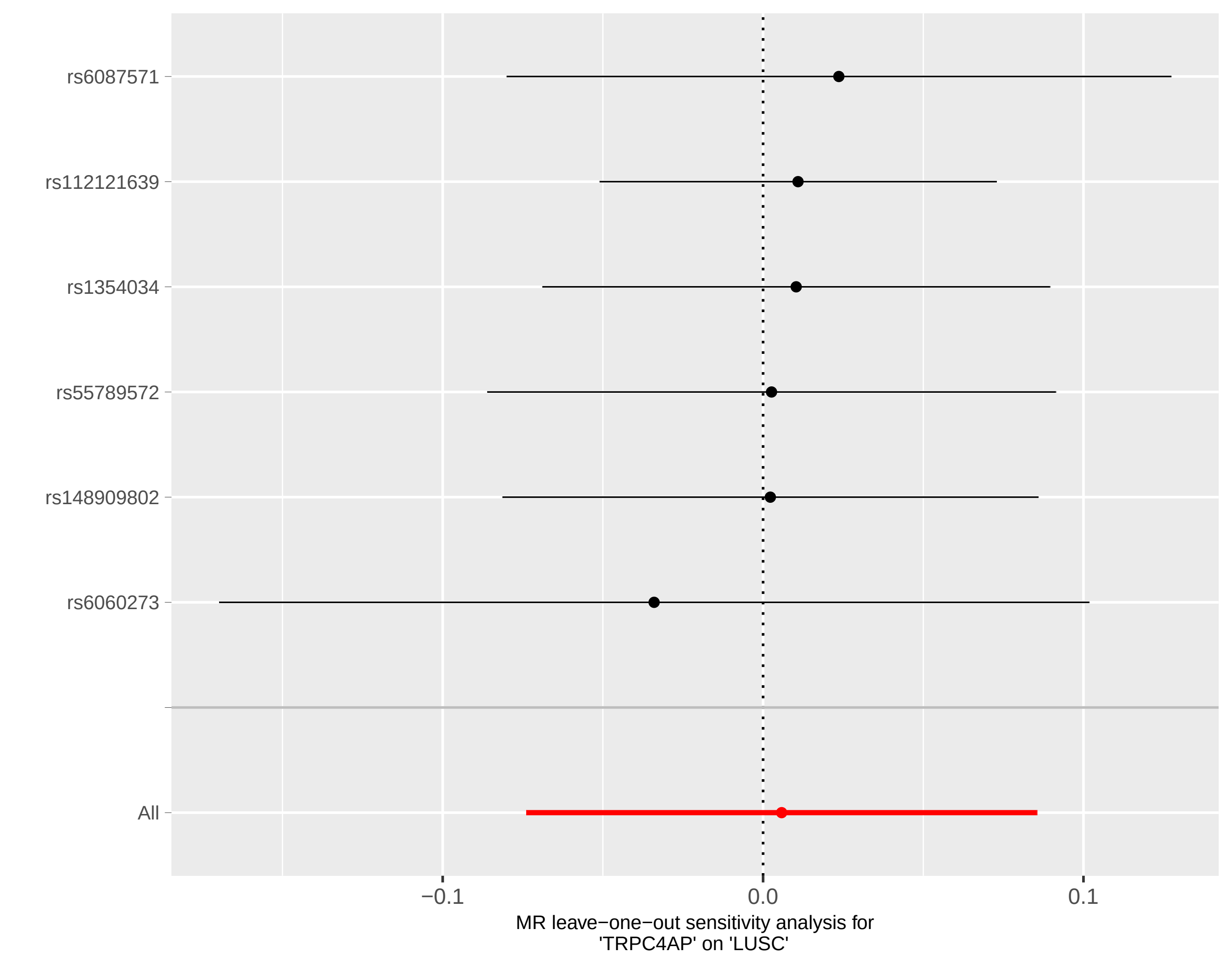
rs6087571
rs112121639
rs1354034
rs55789572
rs148909802
rs6060273
All
−0.1
0.0
0.1
MR leave−one−out sensitivity analysis for
'TRPC4AP' on 'LUSC'

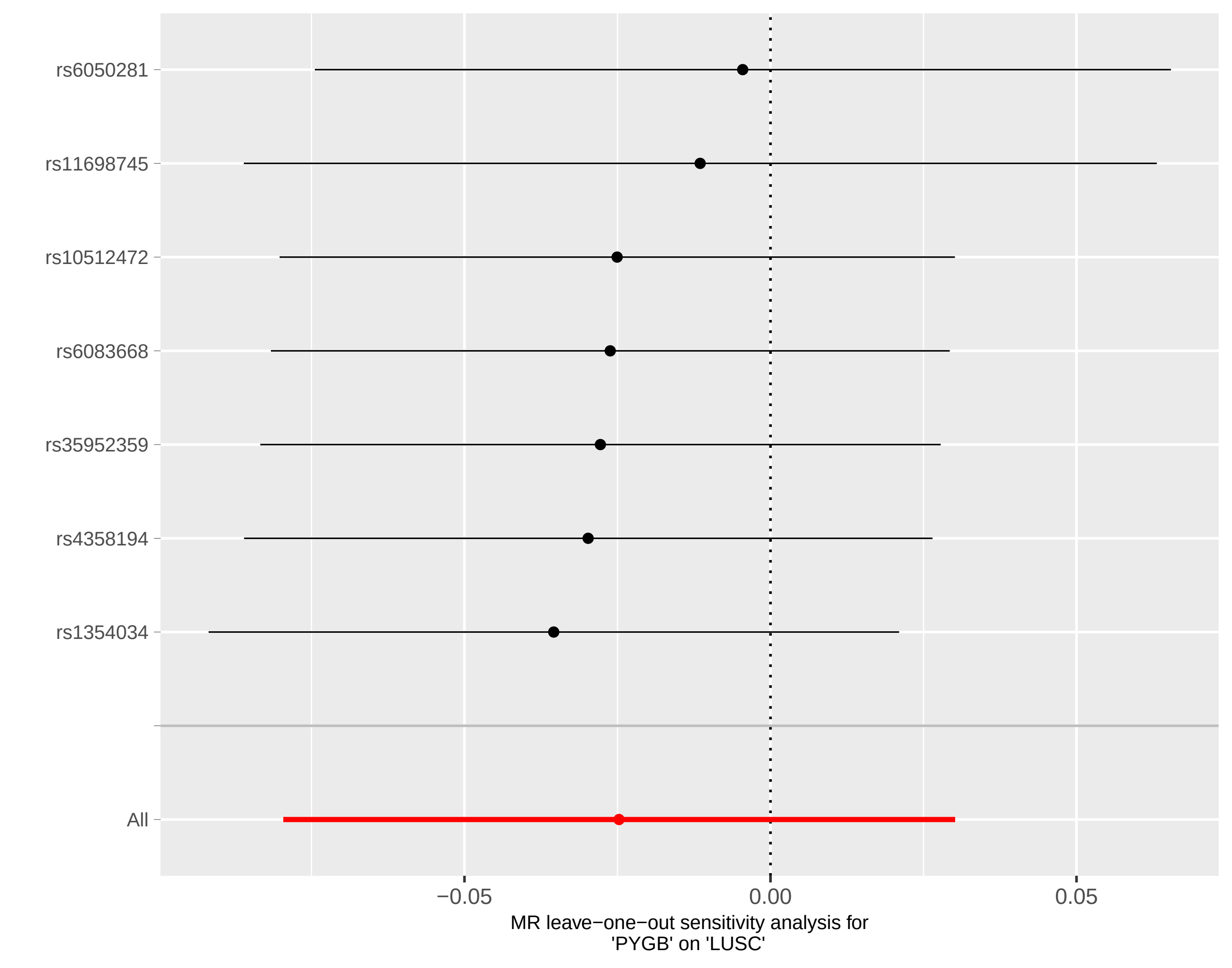
rs6050281
rs11698745
rs10512472
rs6083668
rs35952359
rs4358194
rs1354034
All
−0.05
0.00
0.05
MR leave−one−out sensitivity analysis for
'PYGB' on 'LUSC'

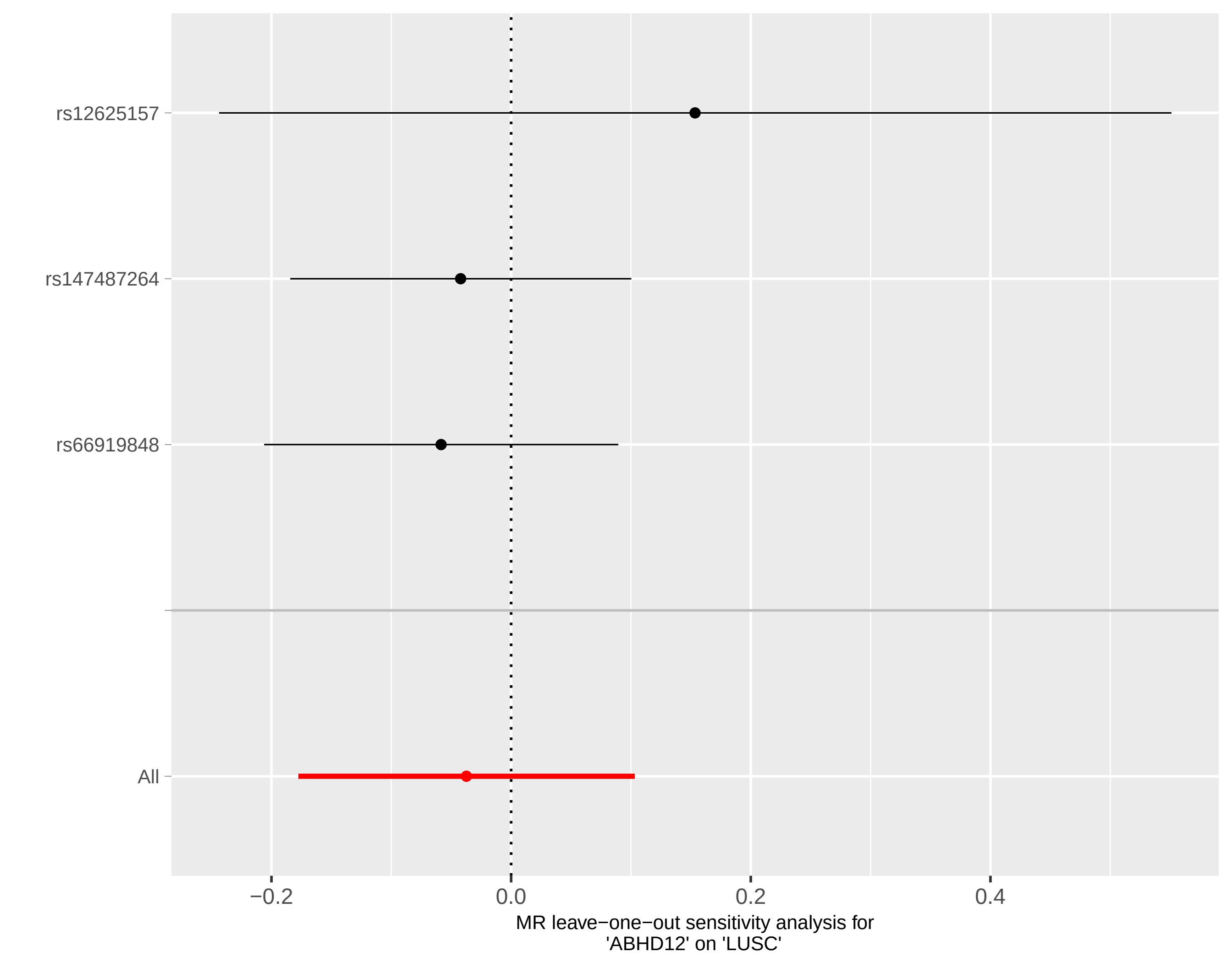
rs12625157
rs147487264
rs66919848
All
−0.2
0.0
0.2
0.4
MR leave−one−out sensitivity analysis for
'ABHD12' on 'LUSC'

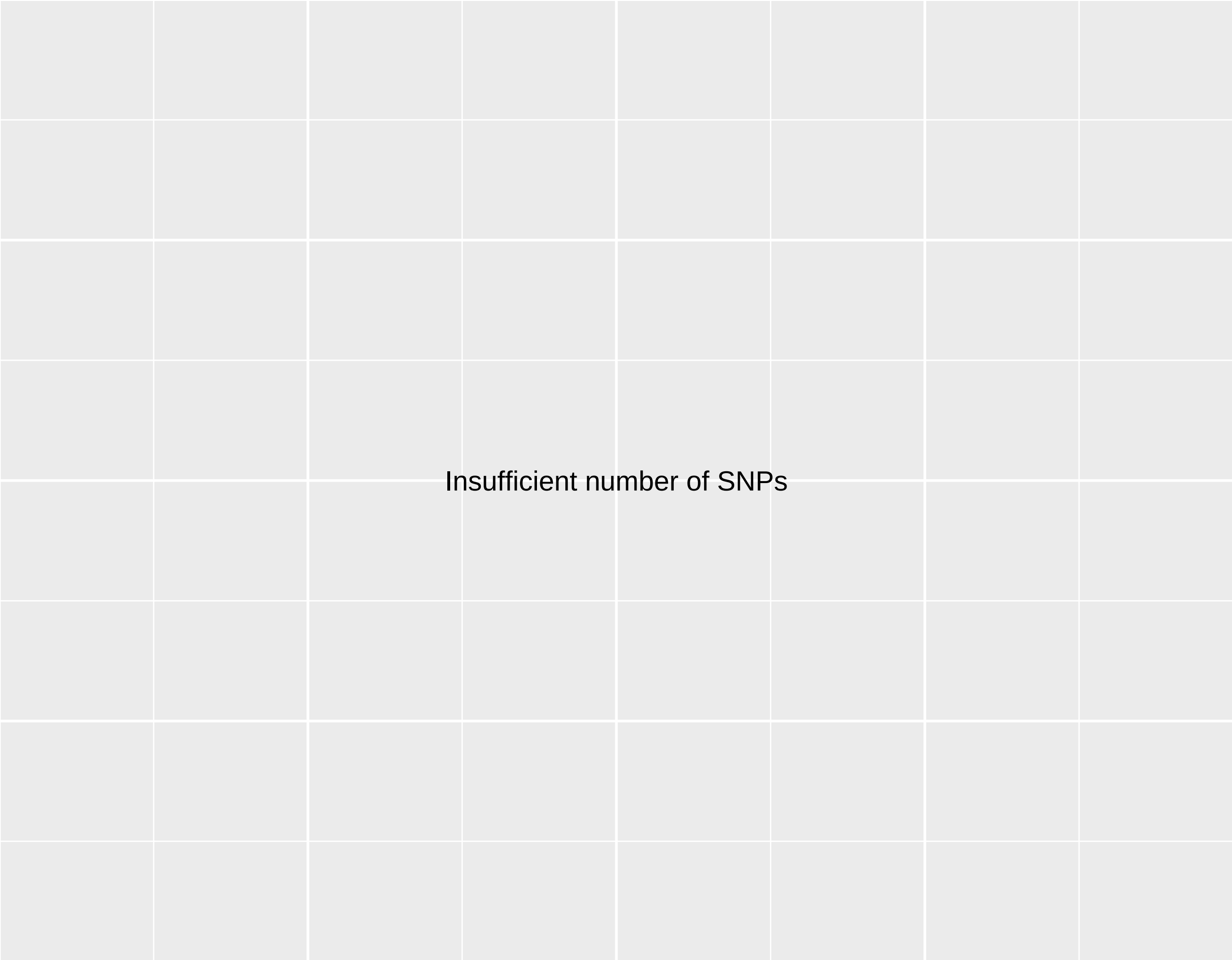
Insufficient number of SNPs

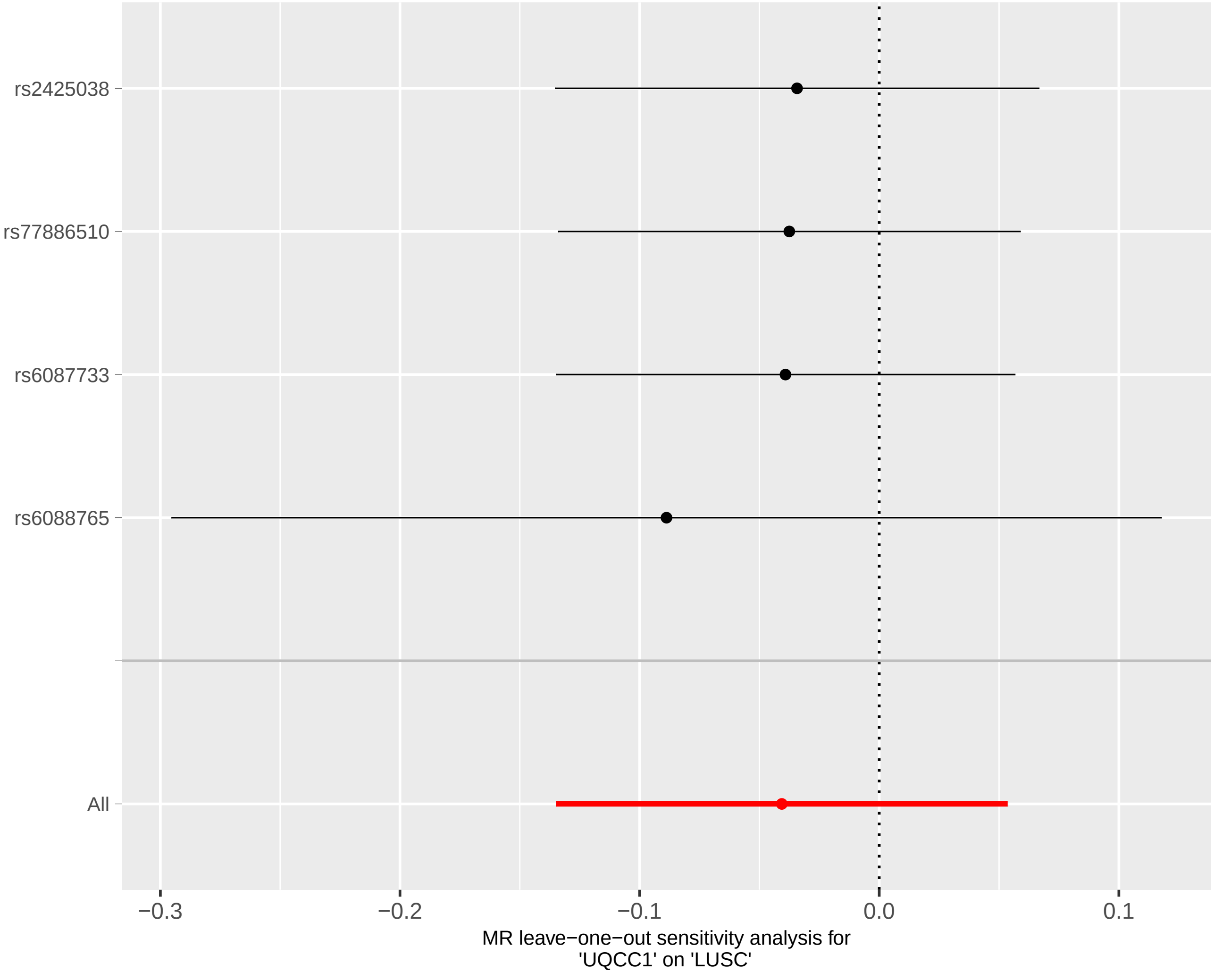
rs2425038
rs77886510
rs6087733
rs6088765
All
−0.3
−0.2
−0.1
0.0
0.1
MR leave−one−out sensitivity analysis for
'UQCC1' on 'LUSC'

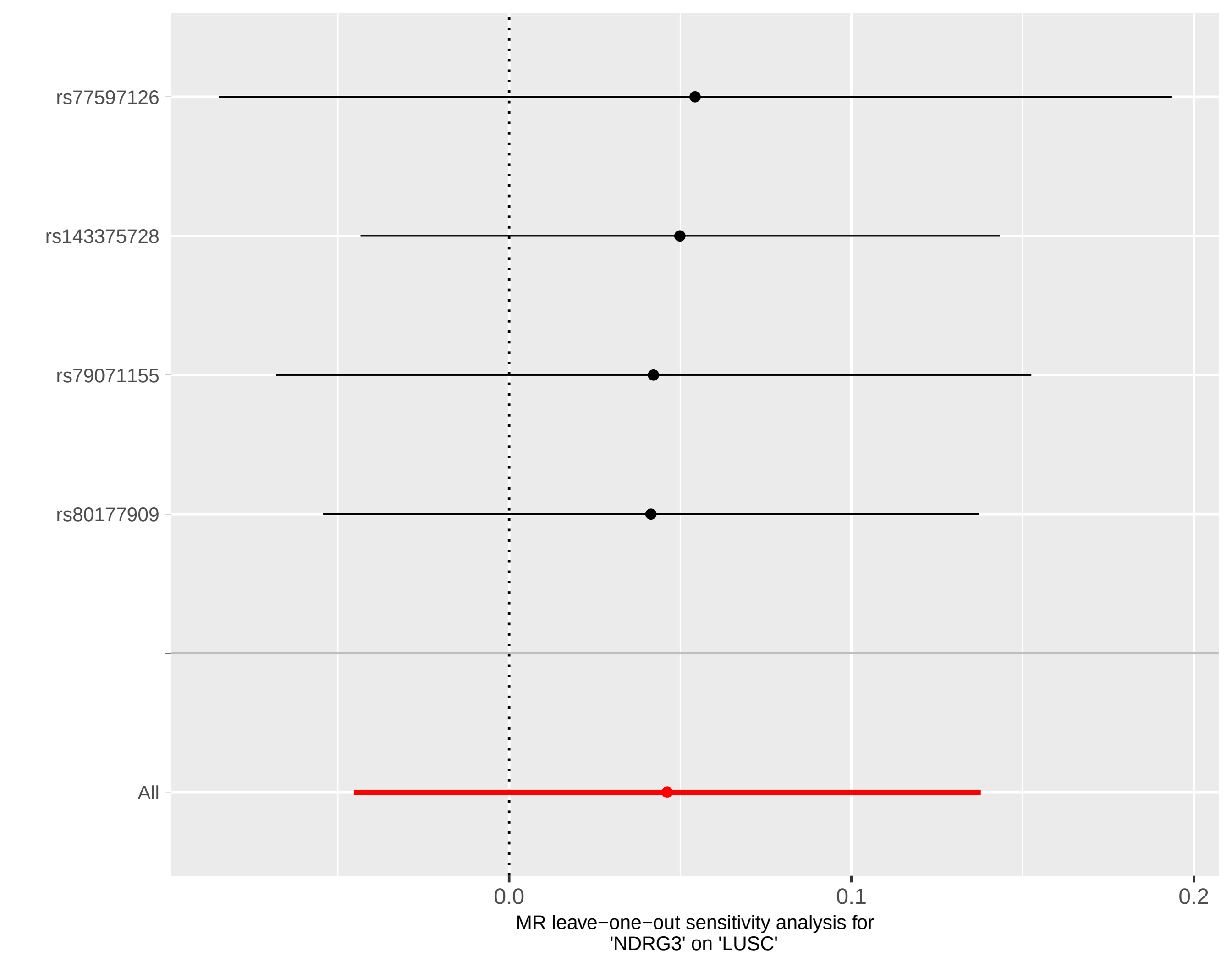
rs77597126
rs143375728
rs79071155
rs80177909
All
0.0
0.1
0.2
MR leave−one−out sensitivity analysis for
'NDRG3' on 'LUSC'

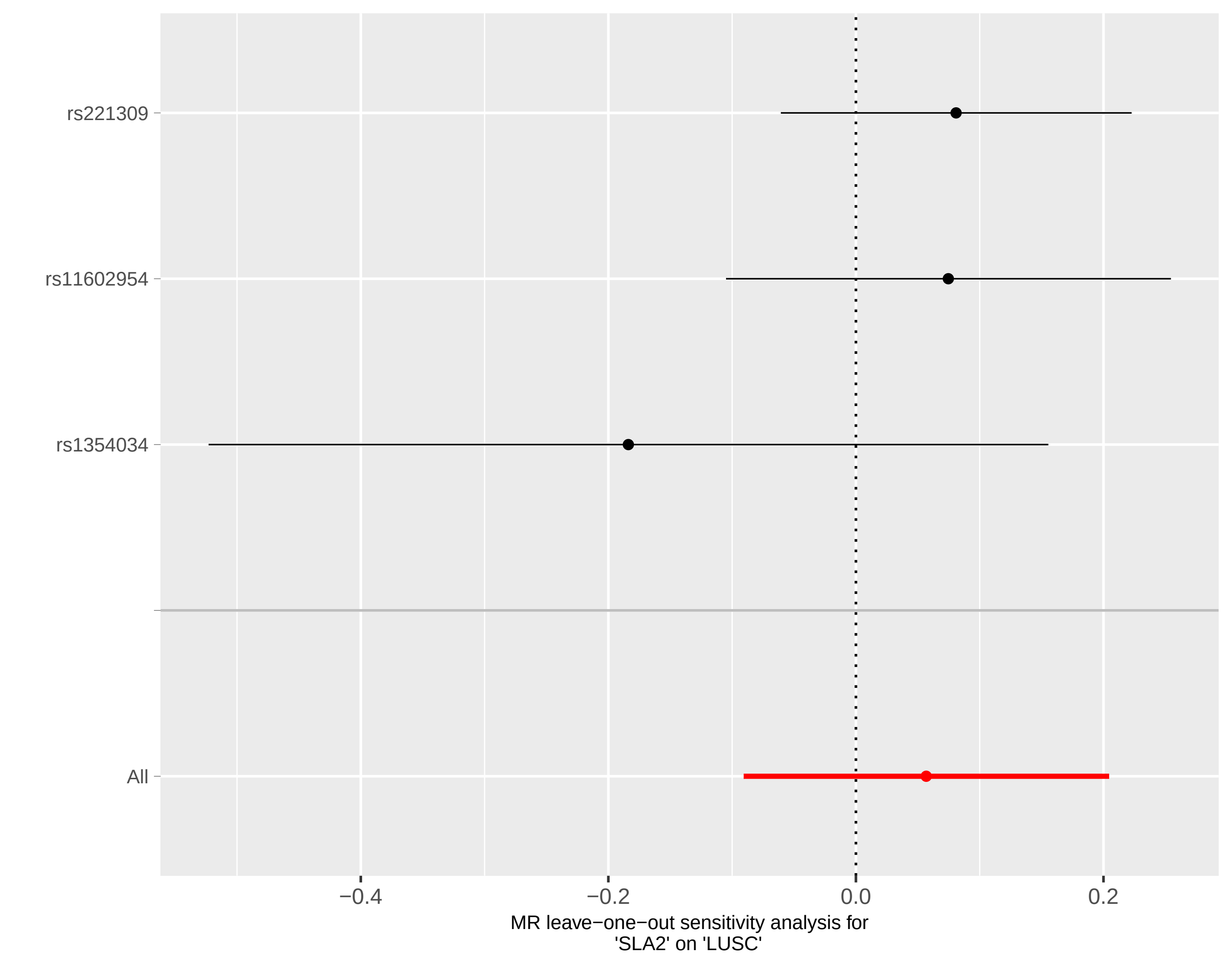
rs221309
rs11602954
rs1354034
All
−0.4
−0.2
0.0
0.2
MR leave−one−out sensitivity analysis for
'SLA2' on 'LUSC'

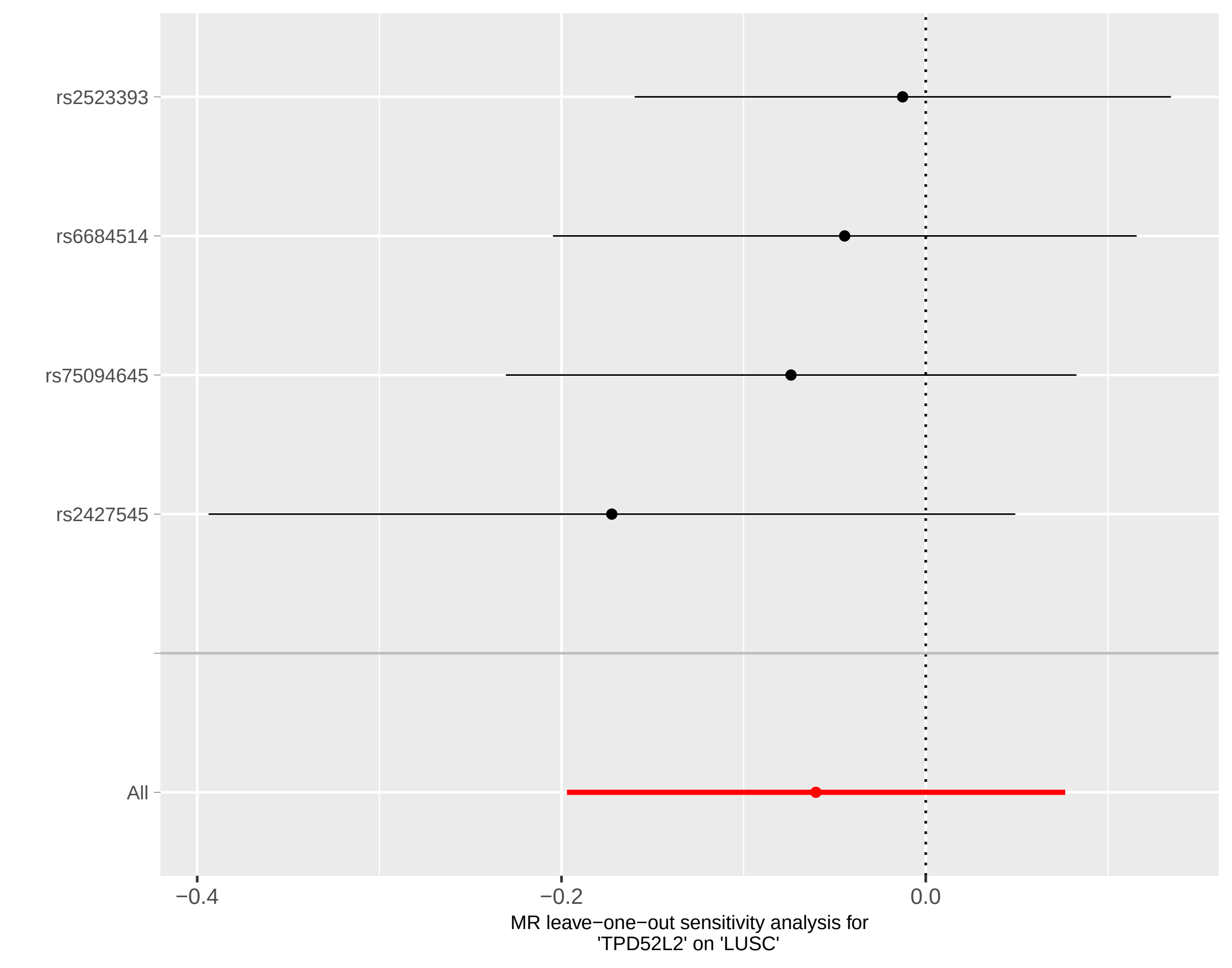
rs2523393
rs6684514
rs75094645
rs2427545
All
−0.4
−0.2
0.0
MR leave−one−out sensitivity analysis for
'TPD52L2' on 'LUSC'

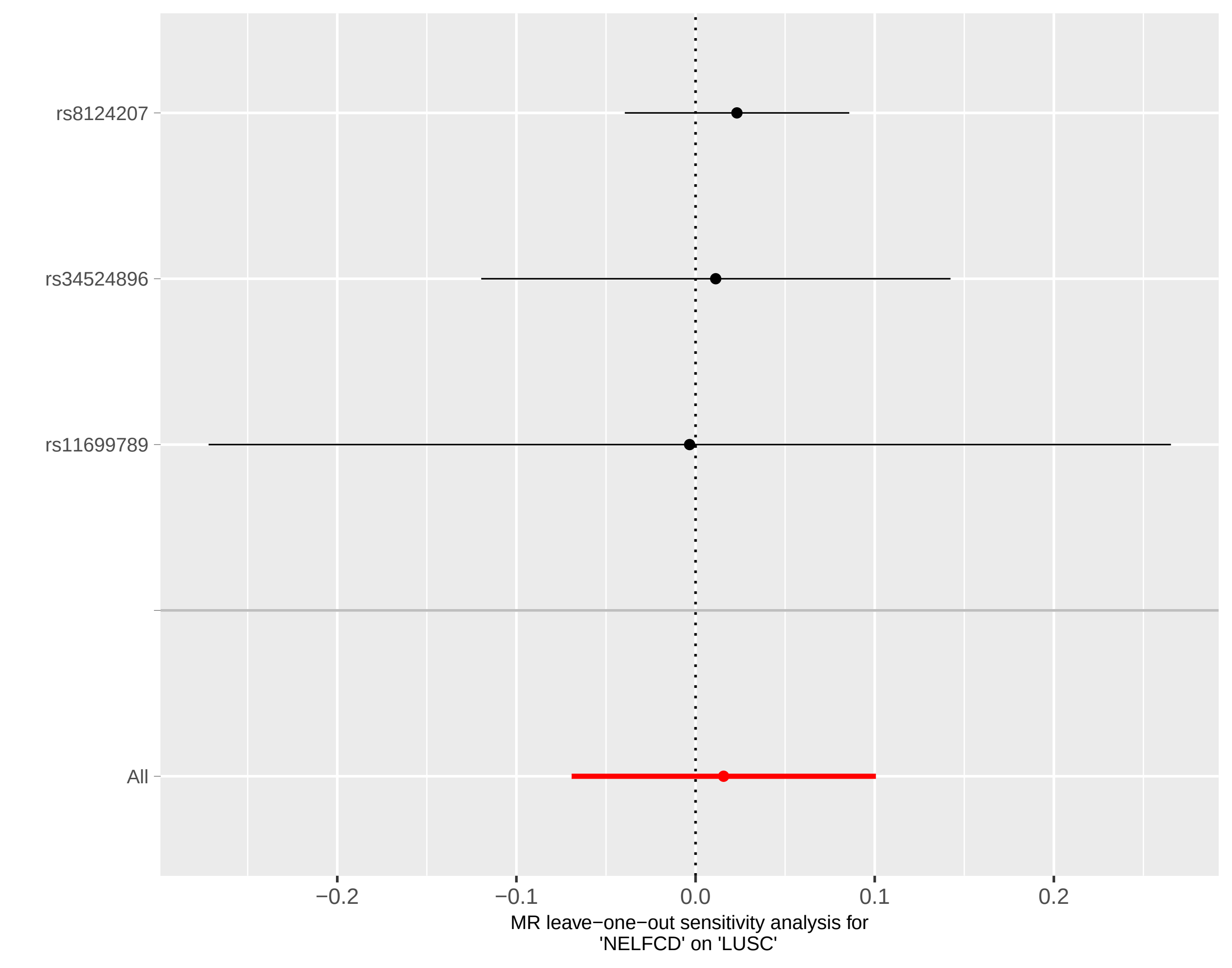

rs8124207
rs34524896
rs11699789
All
−0.2
−0.1
0.0
0.1
0.2
MR leave−one−out sensitivity analysis for
'NELFCD' on 'LUSC'

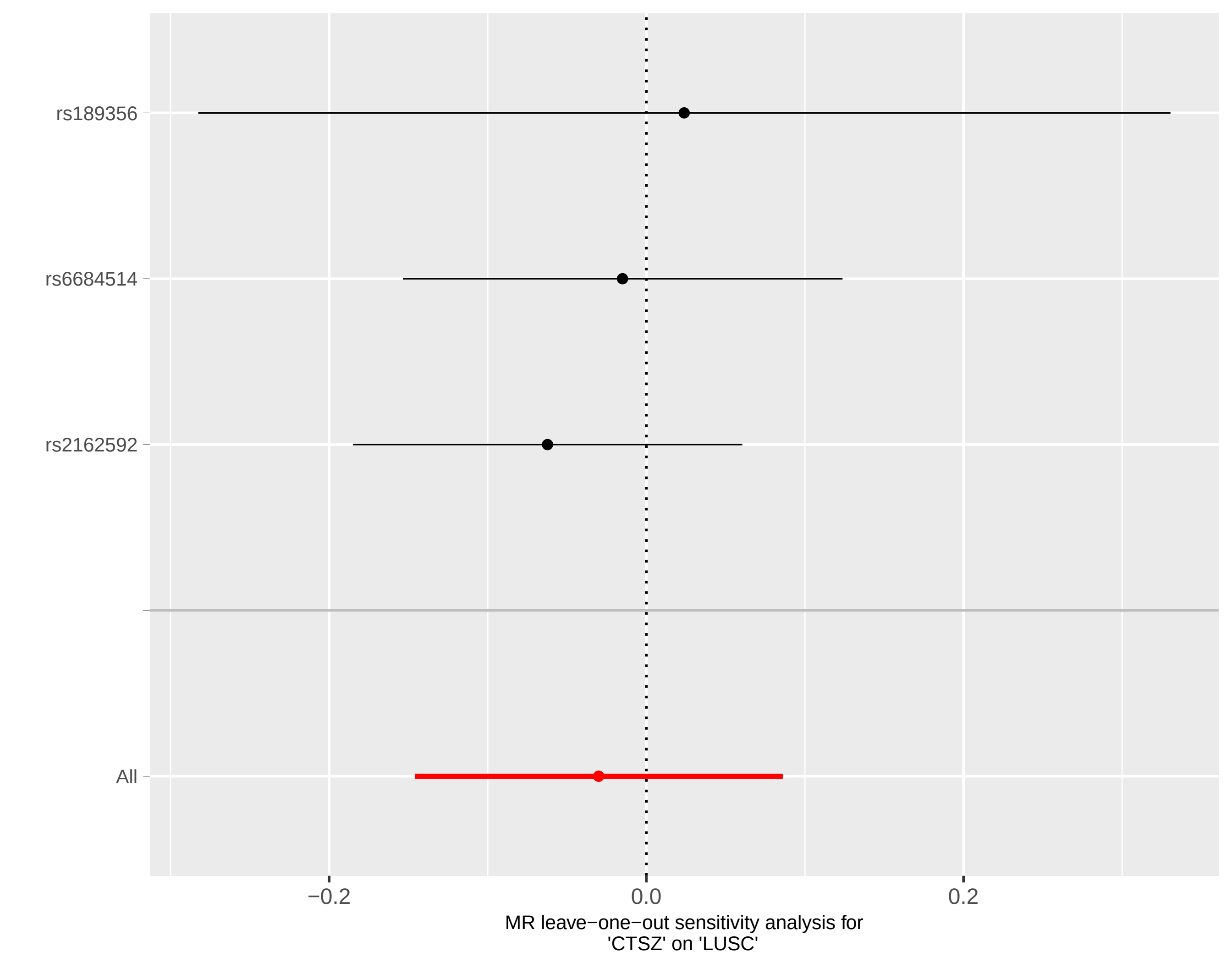
rs189356
rs6684514
rs2162592
All
−0.2
0.0
0.2
MR leave−one−out sensitivity analysis for
'CTSZ' on 'LUSC'

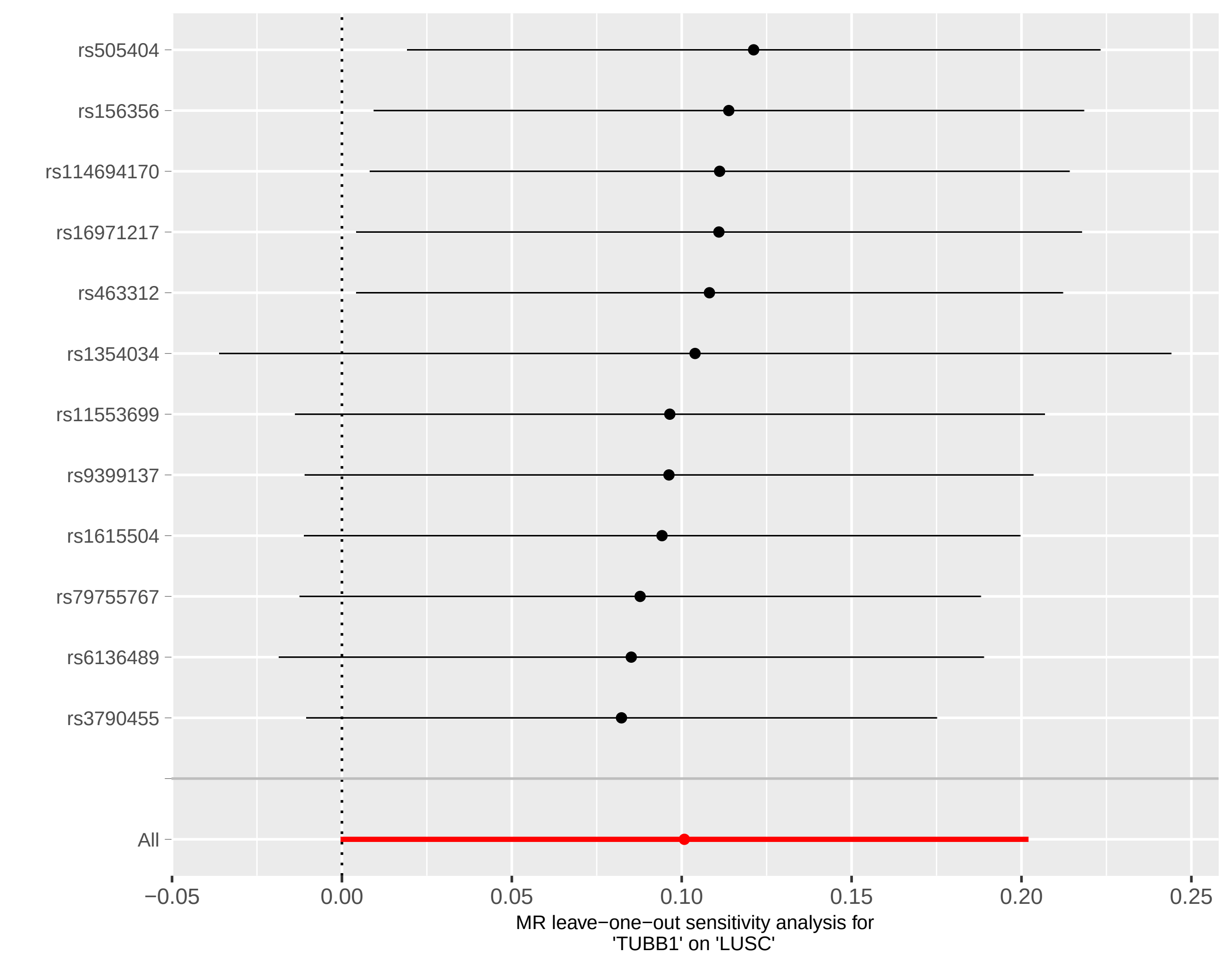
rs505404
rs156356
rs114694170
rs16971217
rs463312
rs1354034
rs11553699
rs9399137
rs1615504
rs79755767
rs6136489
rs3790455
All
−0.05
0.00
0.05
0.10
0.15
0.20
0.25
MR leave−one−out sensitivity analysis for
'TUBB1' on 'LUSC'

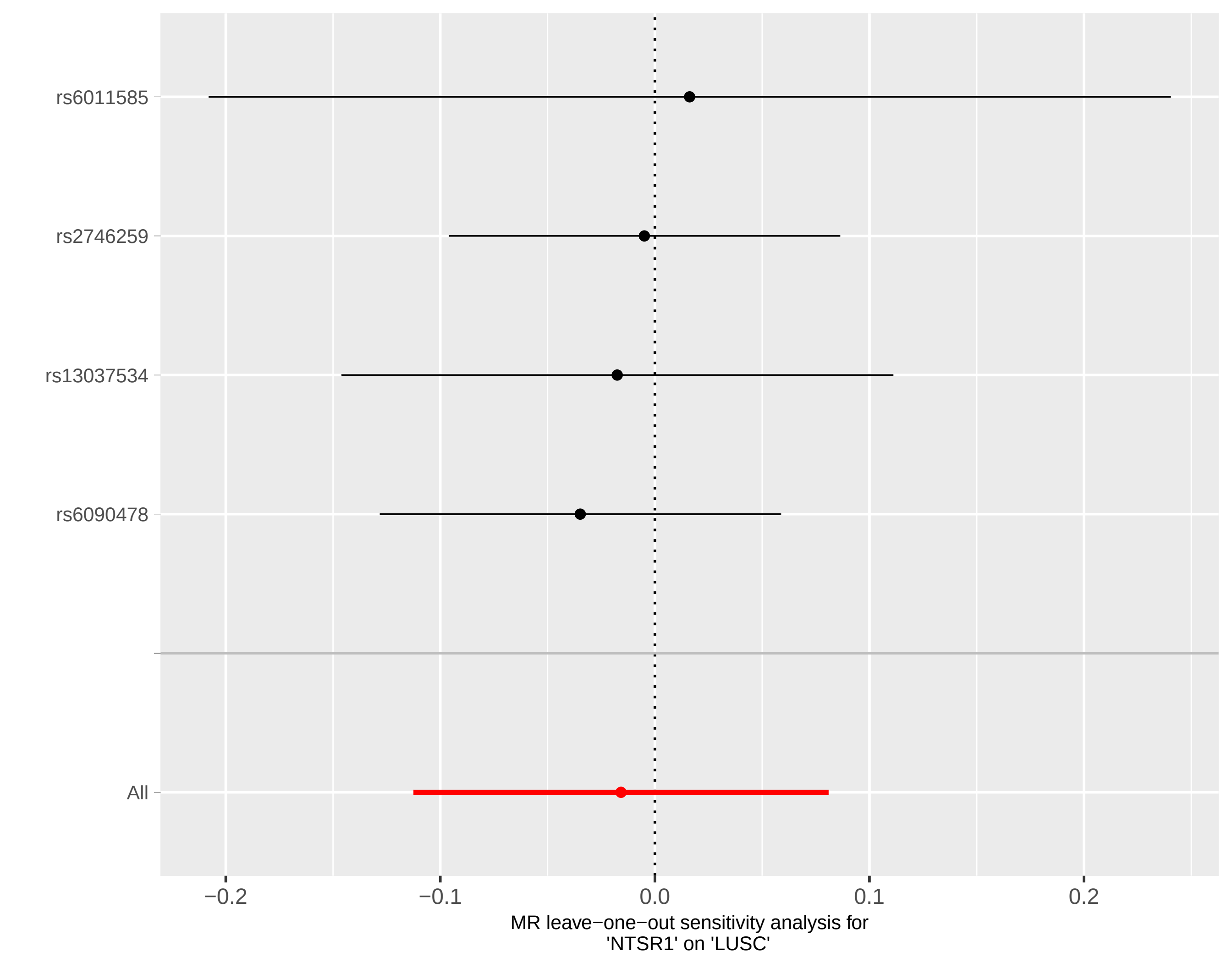

rs6011585
rs2746259
rs13037534
rs6090478
All
−0.2
−0.1
0.0
0.1
0.2
MR leave−one−out sensitivity analysis for
'NTSR1' on 'LUSC'

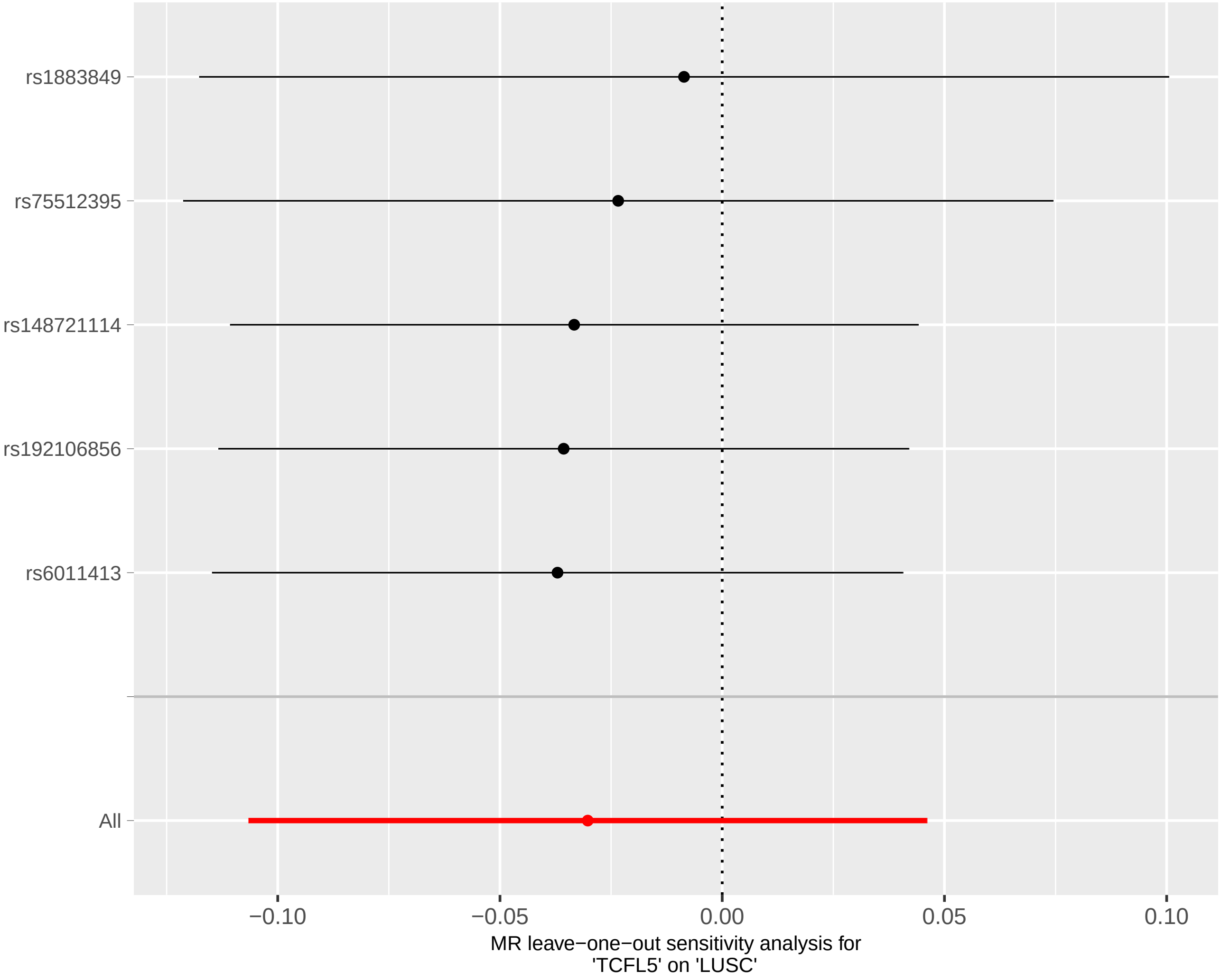
rs1883849
rs75512395
rs148721114
rs192106856
rs6011413
All
−0.10
−0.05
0.00
0.05
0.10
MR leave−one−out sensitivity analysis for
'TCFL5' on 'LUSC'

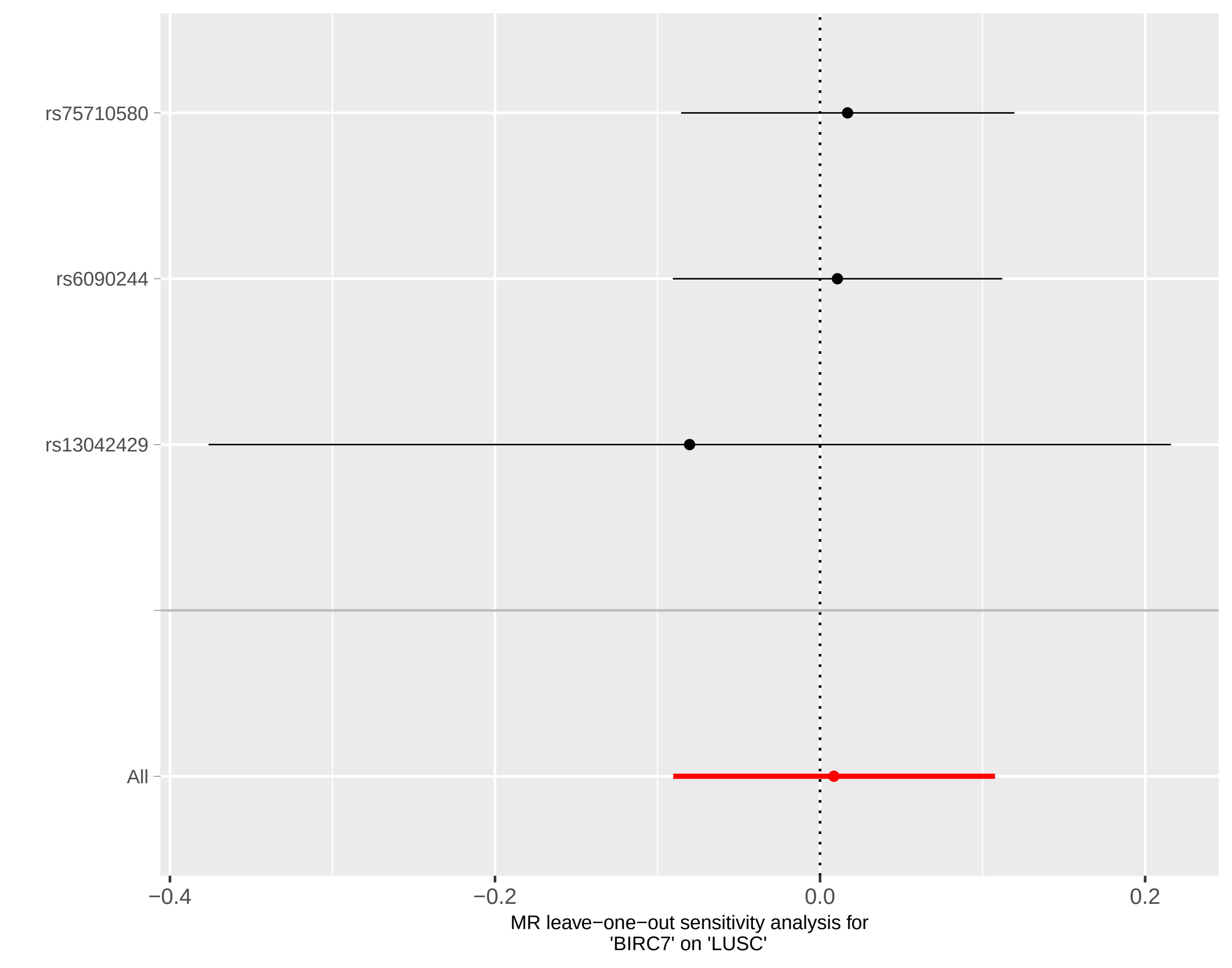

rs75710580
rs6090244
rs13042429
All
−0.4
−0.2
0.0
0.2
MR leave−one−out sensitivity analysis for
'BIRC7' on 'LUSC'

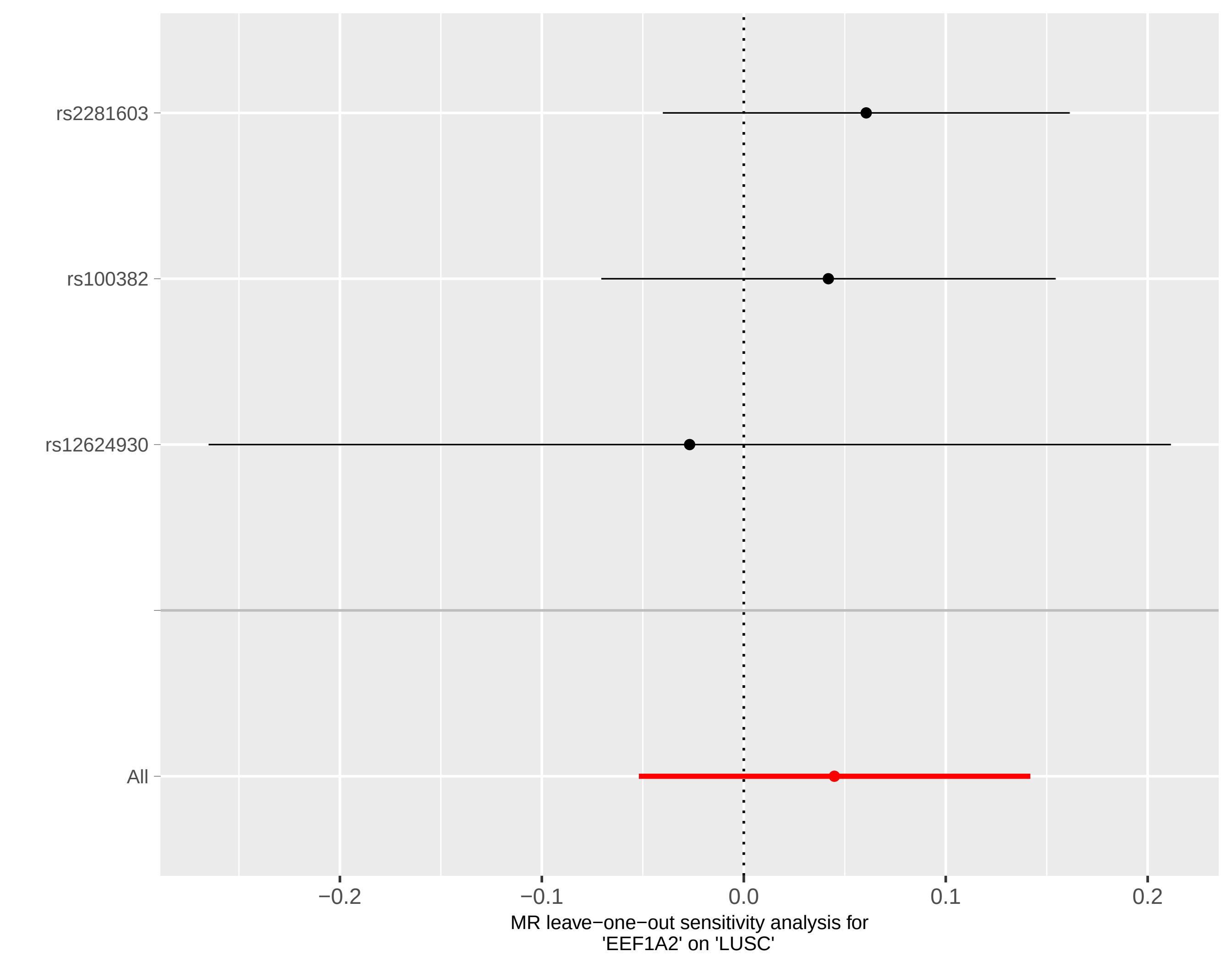
rs2281603
rs100382
rs12624930
All
−0.2
−0.1
0.0
0.1
0.2
MR leave−one−out sensitivity analysis for
'EEF1A2' on 'LUSC'

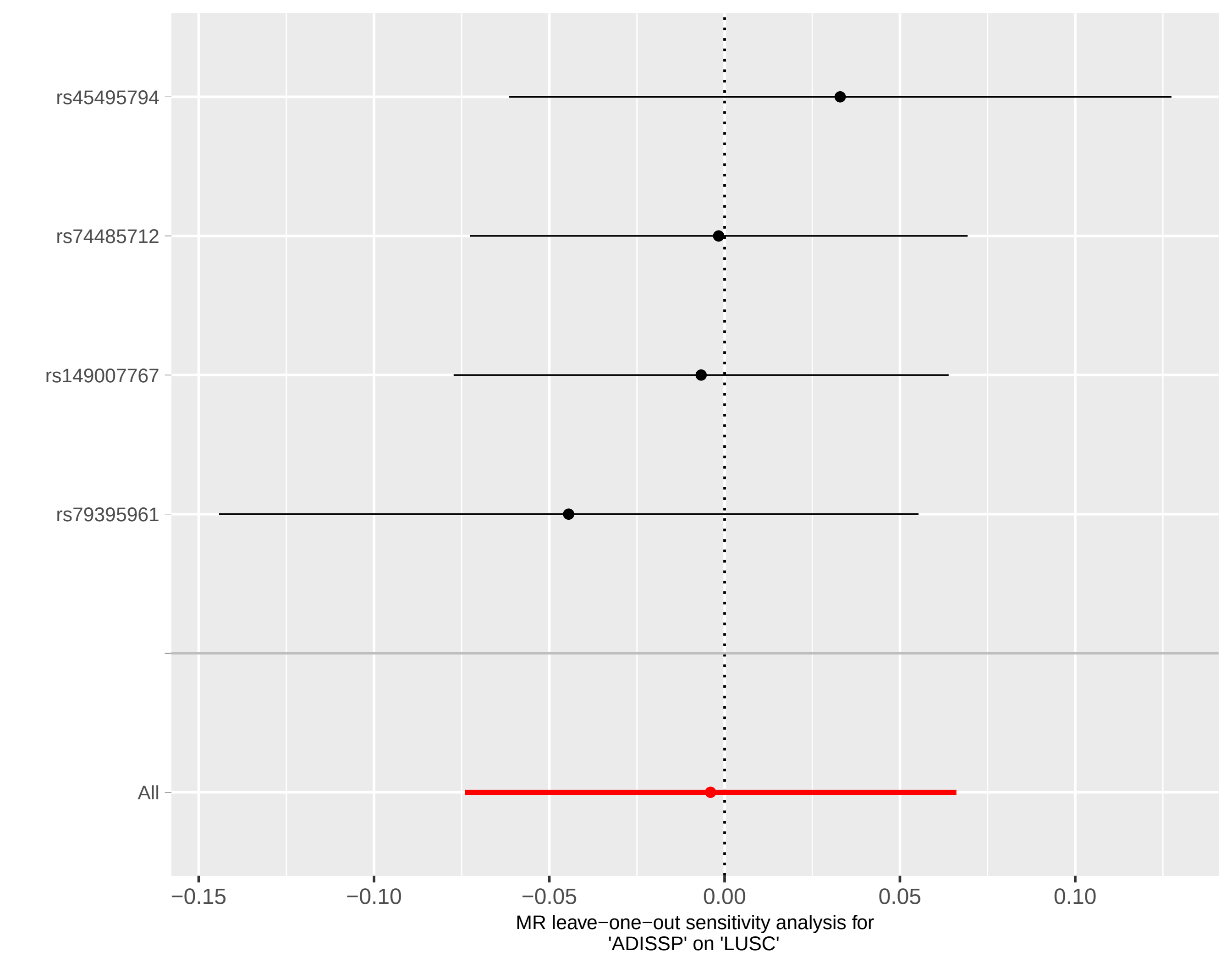
rs45495794
rs74485712
rs149007767
rs79395961
All
−0.15
−0.10
−0.05
0.00
0.05
0.10
MR leave−one−out sensitivity analysis for
'ADISSP' on 'LUSC'

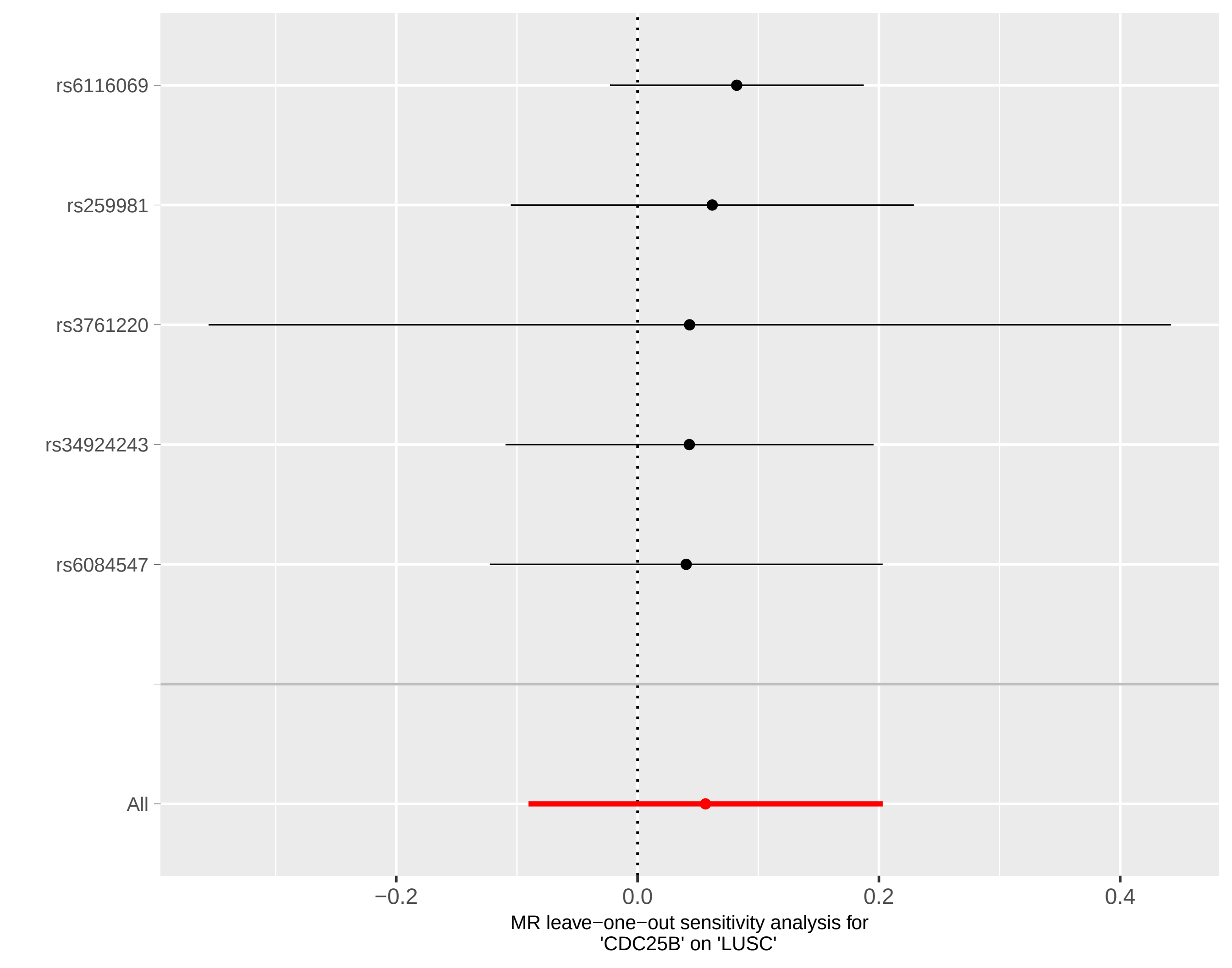
rs6116069
rs259981
rs3761220
rs34924243
rs6084547
All
−0.2
0.0
0.2
0.4
MR leave−one−out sensitivity analysis for
'CDC25B' on 'LUSC'

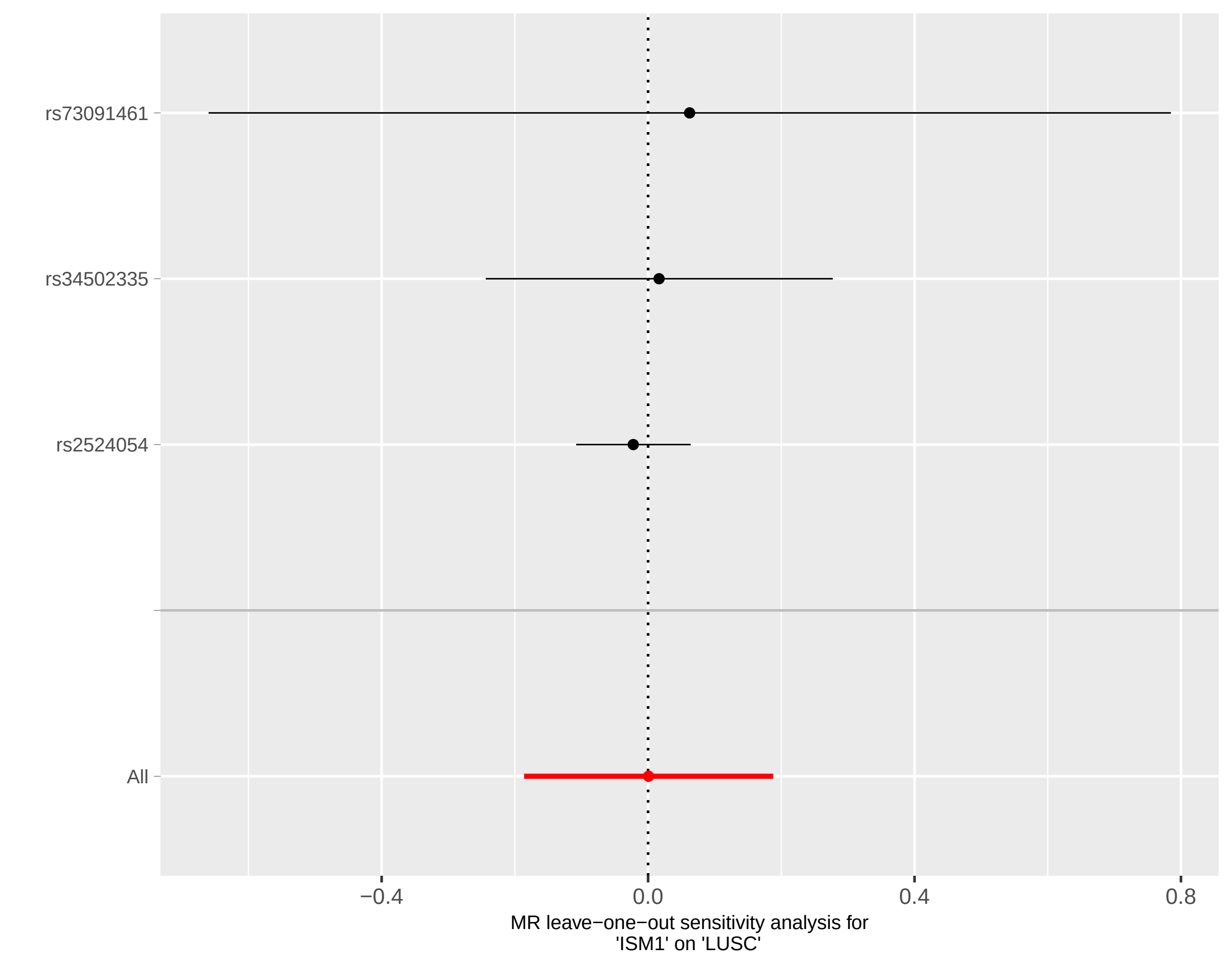

rs73091461
rs34502335
rs2524054
All
−0.4
0.0
0.4
0.8
MR leave−one−out sensitivity analysis for
'ISM1' on 'LUSC'

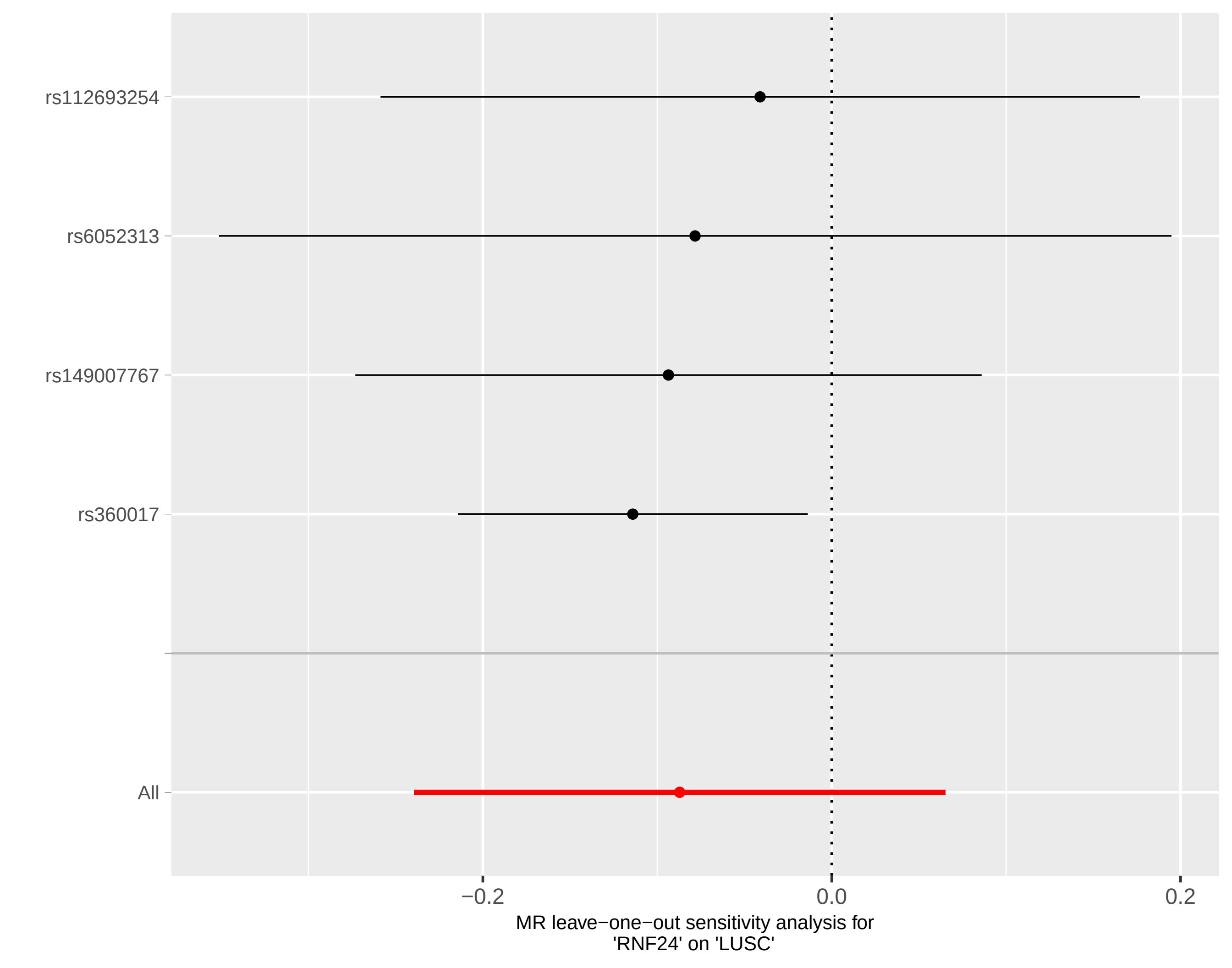
rs112693254
rs6052313
rs149007767
rs360017
All
−0.2
0.0
0.2
MR leave−one−out sensitivity analysis for
'RNF24' on 'LUSC'

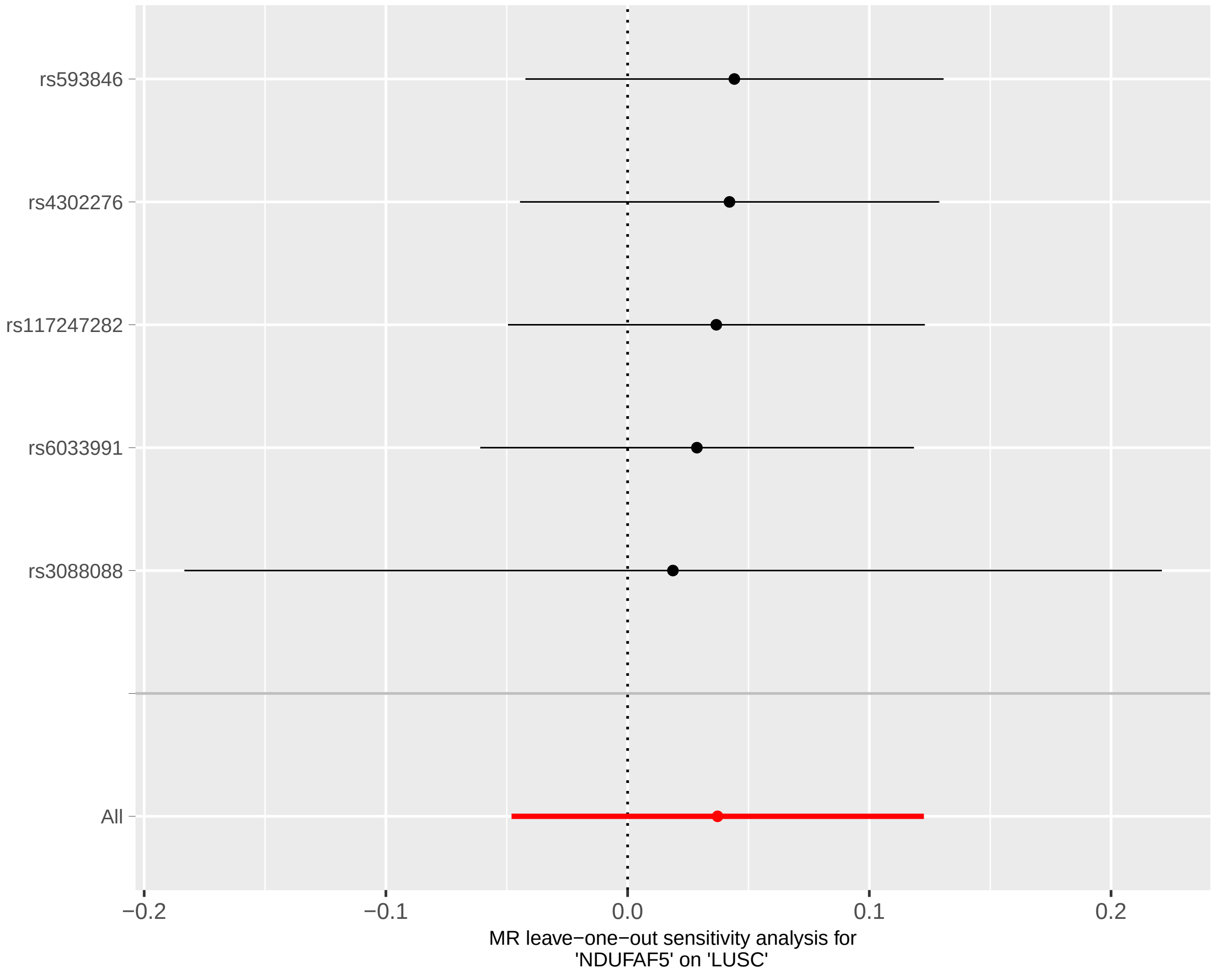

rs593846
rs4302276
rs117247282
rs6033991
rs3088088
All
−0.2
−0.1
0.0
0.1
0.2
MR leave−one−out sensitivity analysis for
'NDUFAF5' on 'LUSC'

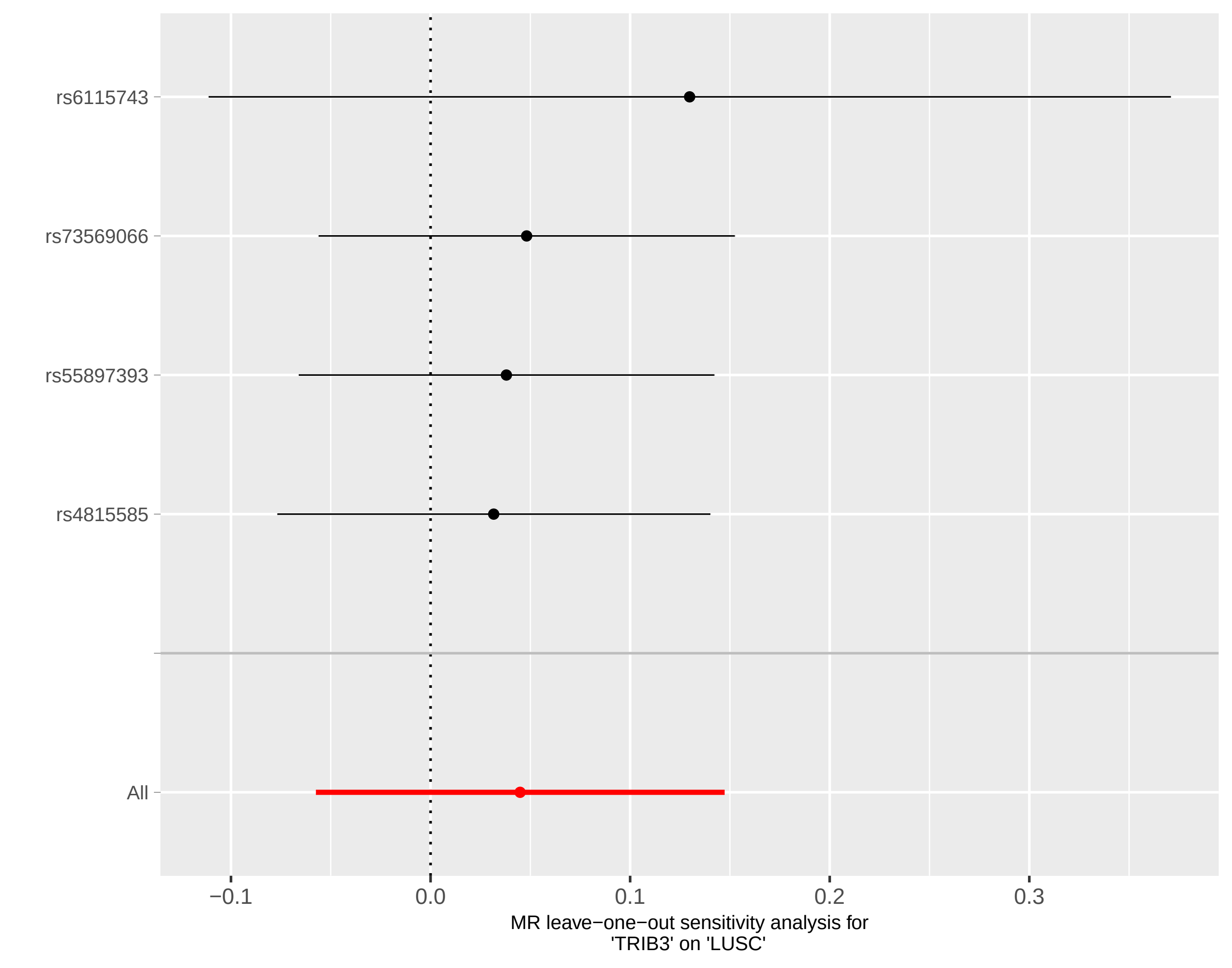

rs6115743
rs73569066
rs55897393
rs4815585
All
−0.1
0.0
0.1
0.2
0.3
MR leave−one−out sensitivity analysis for
'TRIB3' on 'LUSC'

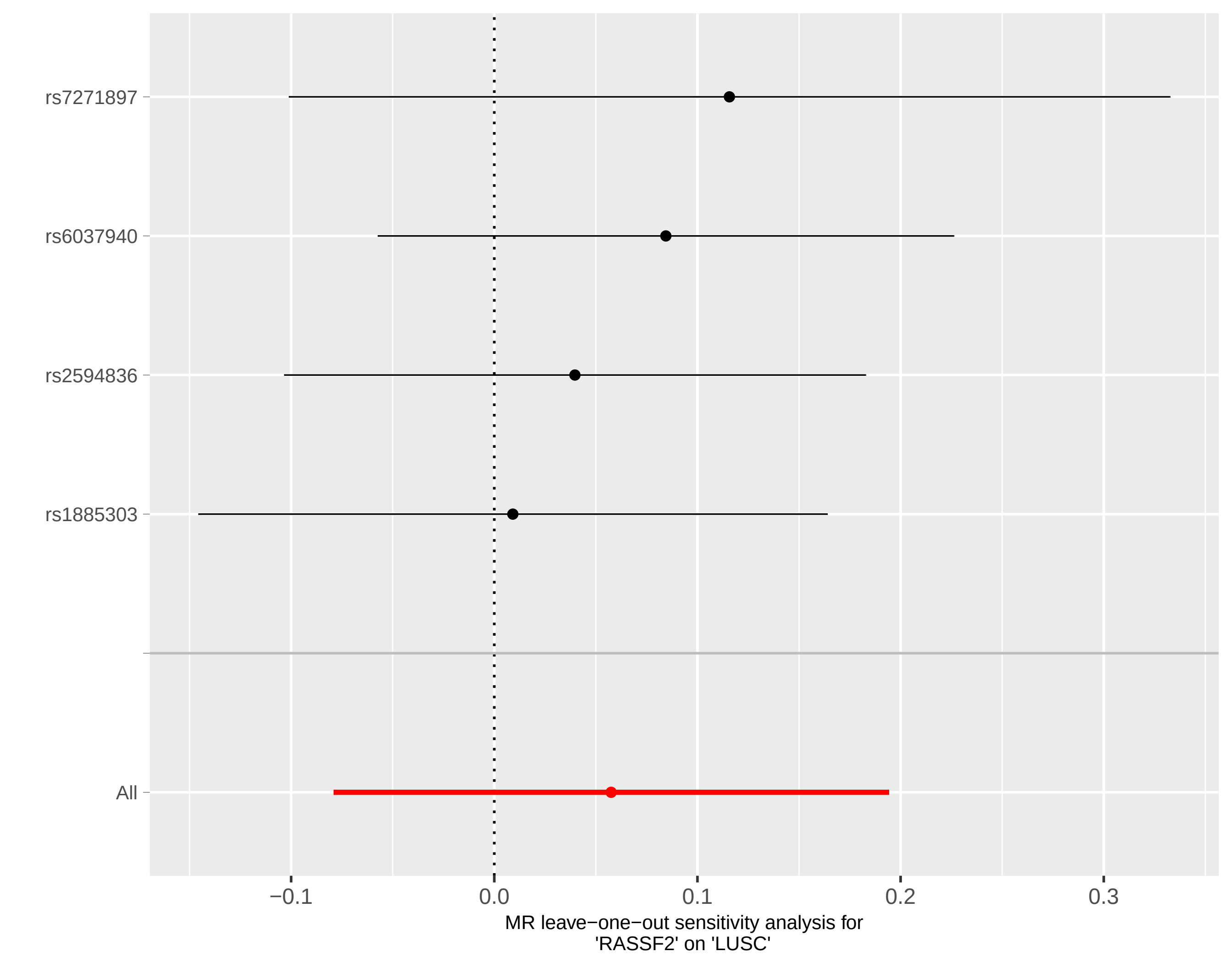
rs7271897
rs6037940
rs2594836
rs1885303
All
−0.1
0.0
0.1
0.2
0.3
MR leave−one−out sensitivity analysis for
'RASSF2' on 'LUSC'

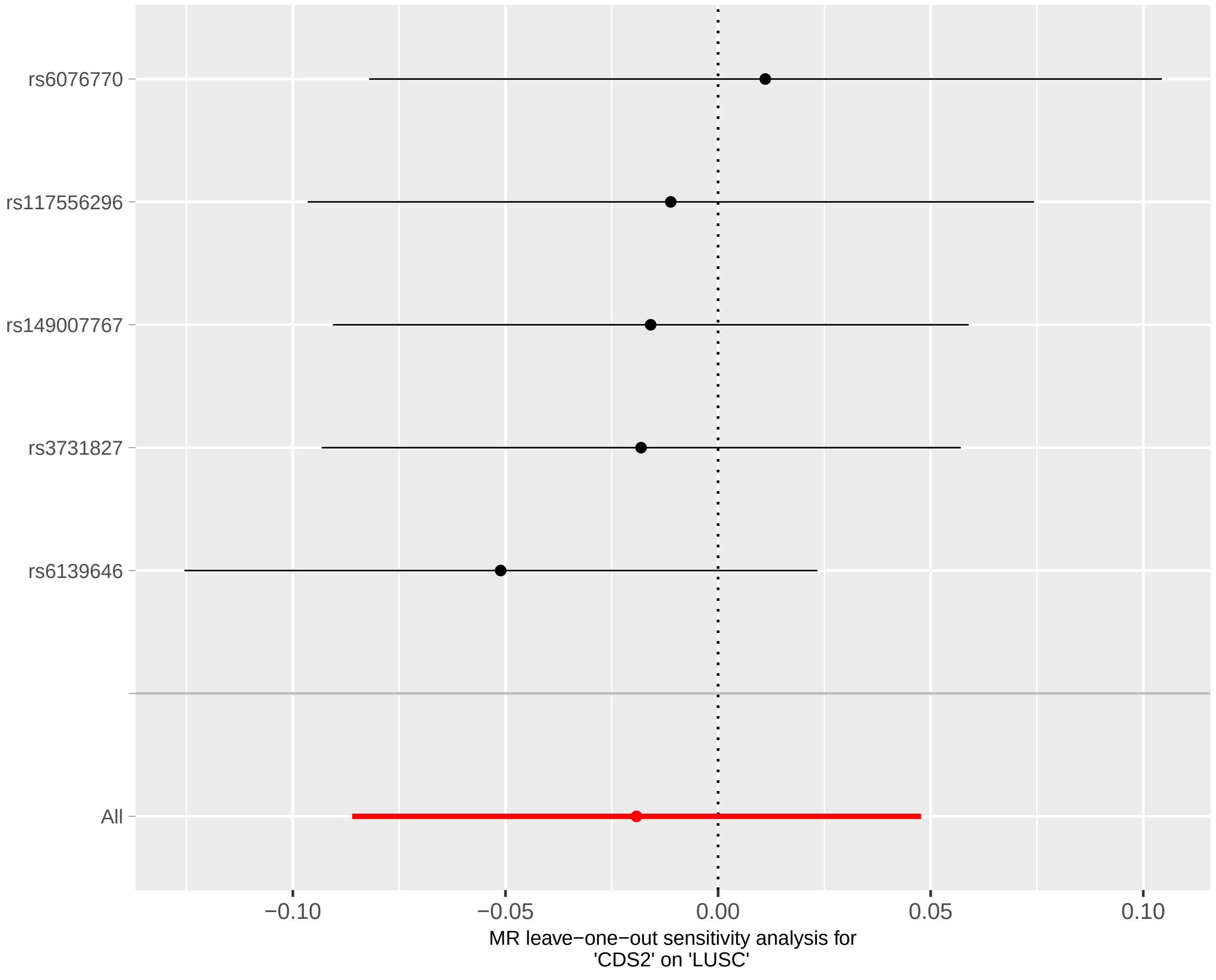

rs6076770
rs117556296
rs149007767
rs3731827
rs6139646
All
−0.10
−0.05
0.00
0.05
0.10
MR leave−one−out sensitivity analysis for
'CDS2' on 'LUSC'

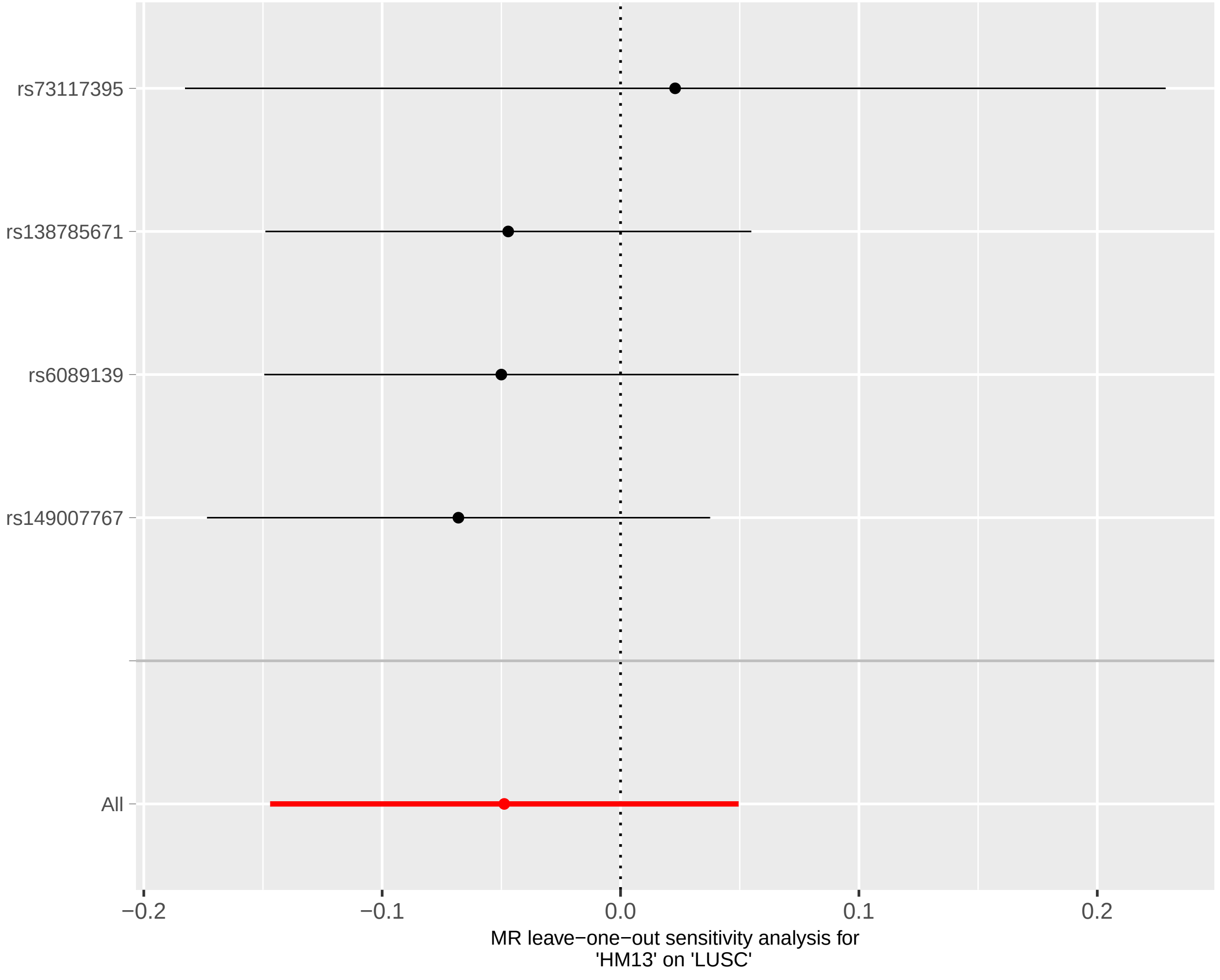
rs73117395
rs138785671
rs6089139
rs149007767
All
−0.2
−0.1
0.0
0.1
0.2
MR leave−one−out sensitivity analysis for
'HM13' on 'LUSC'

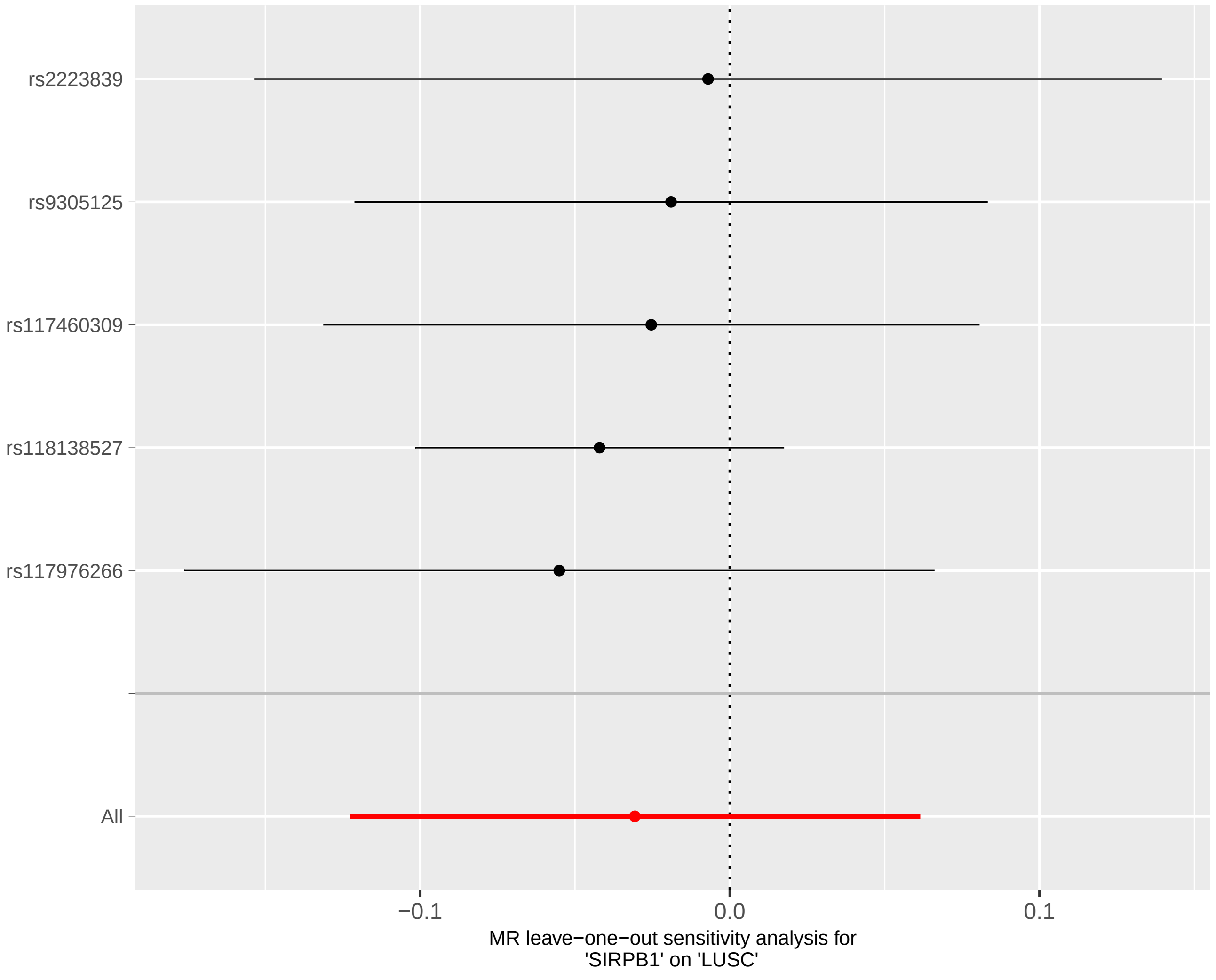

rs2223839
rs9305125
rs117460309
rs118138527
rs117976266
All
−0.1
0.0
0.1
MR leave−one−out sensitivity analysis for
'SIRPB1' on 'LUSC'

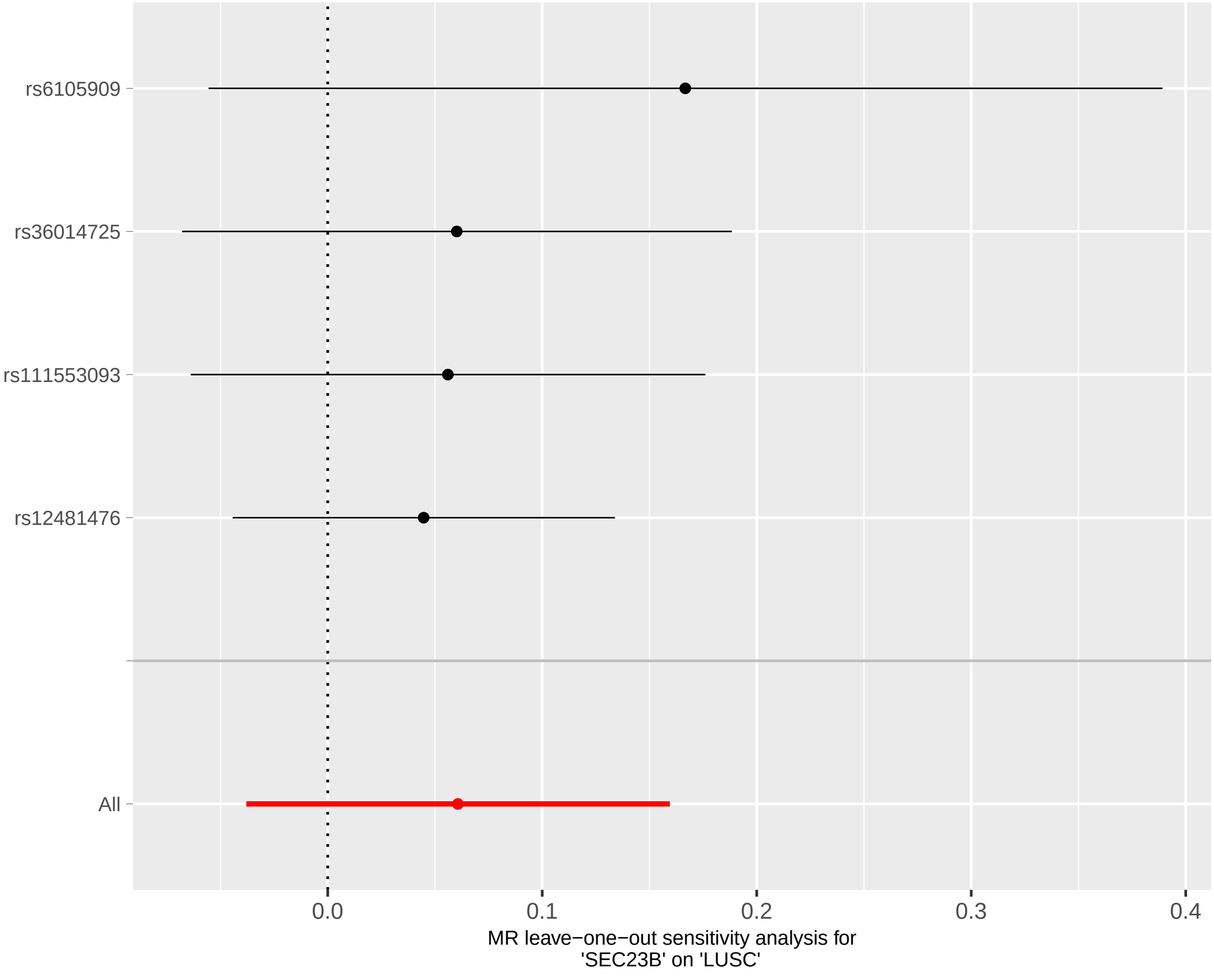

rs6105909
rs36014725
rs111553093
rs12481476
All
0.0
0.1
0.2
0.3
0.4
MR leave−one−out sensitivity analysis for
'SEC23B' on 'LUSC'

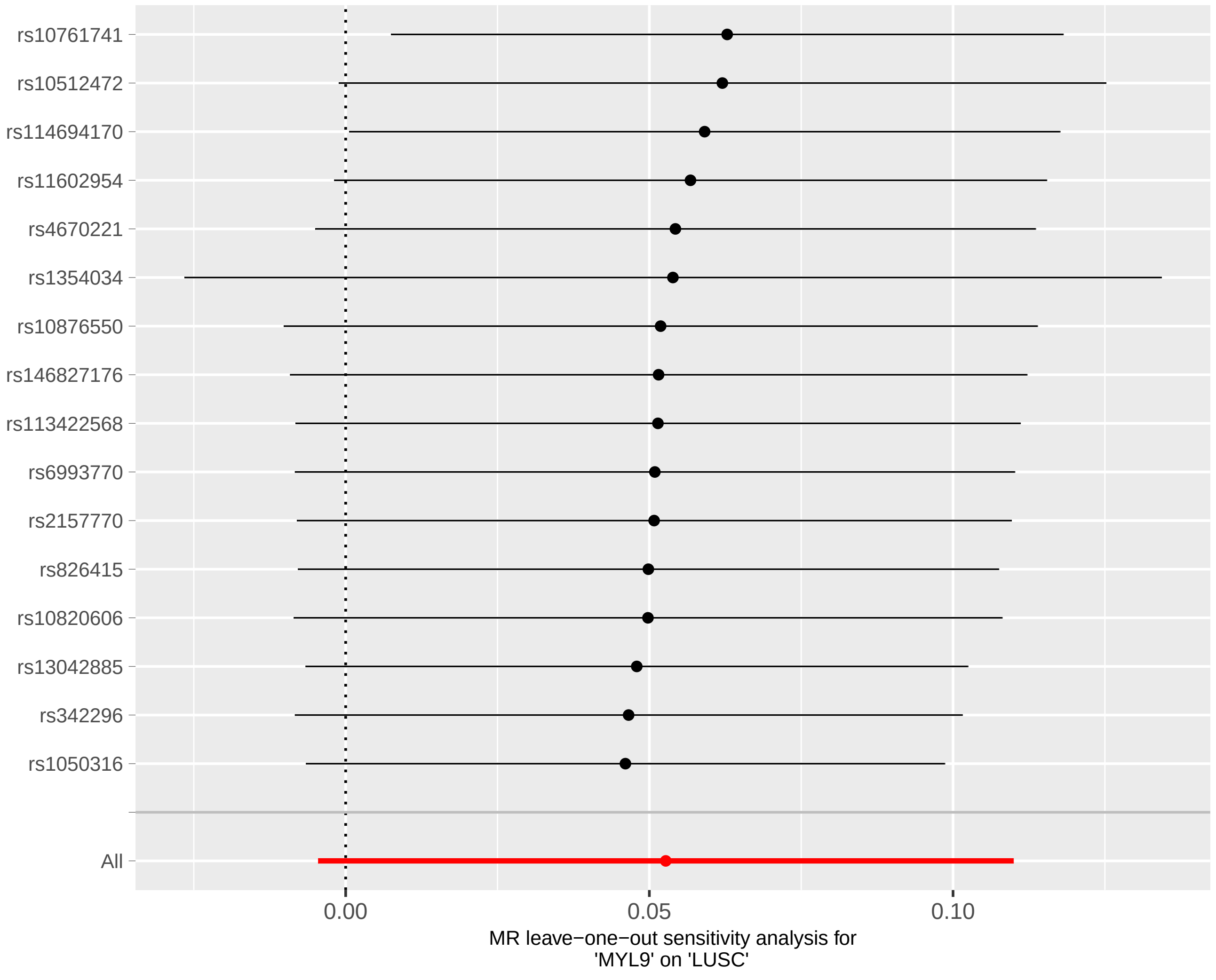

rs10761741
rs10512472
rs114694170
rs11602954
rs4670221
rs1354034
rs10876550
rs146827176
rs113422568
rs6993770
rs2157770
rs826415
rs10820606
rs13042885
rs342296
rs1050316
All
0.00
0.05
0.10
MR leave−one−out sensitivity analysis for
'MYL9' on 'LUSC'

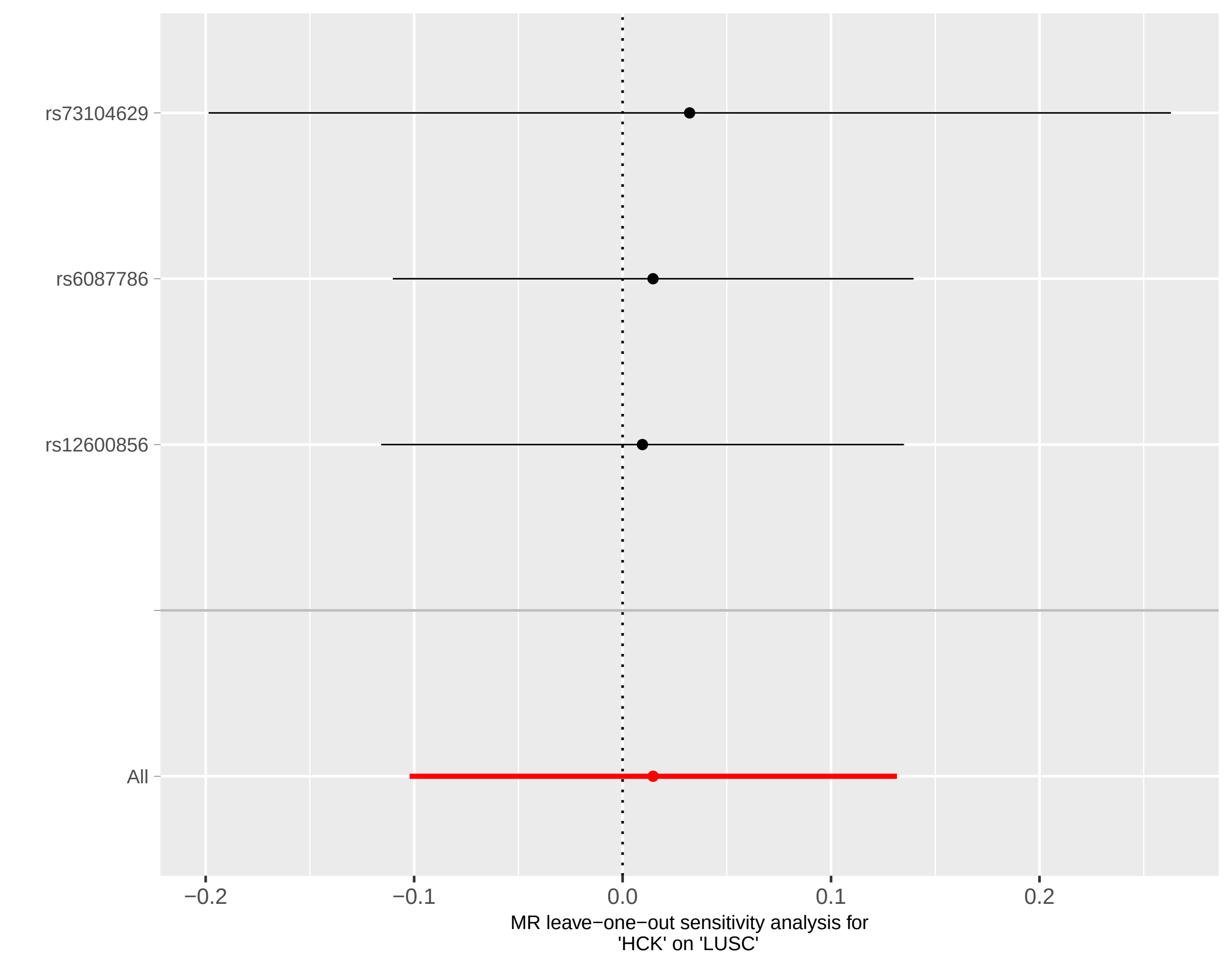
rs73104629
rs6087786
rs12600856
All
−0.2
−0.1
0.0
0.1
0.2
MR leave−one−out sensitivity analysis for
'HCK' on 'LUSC'

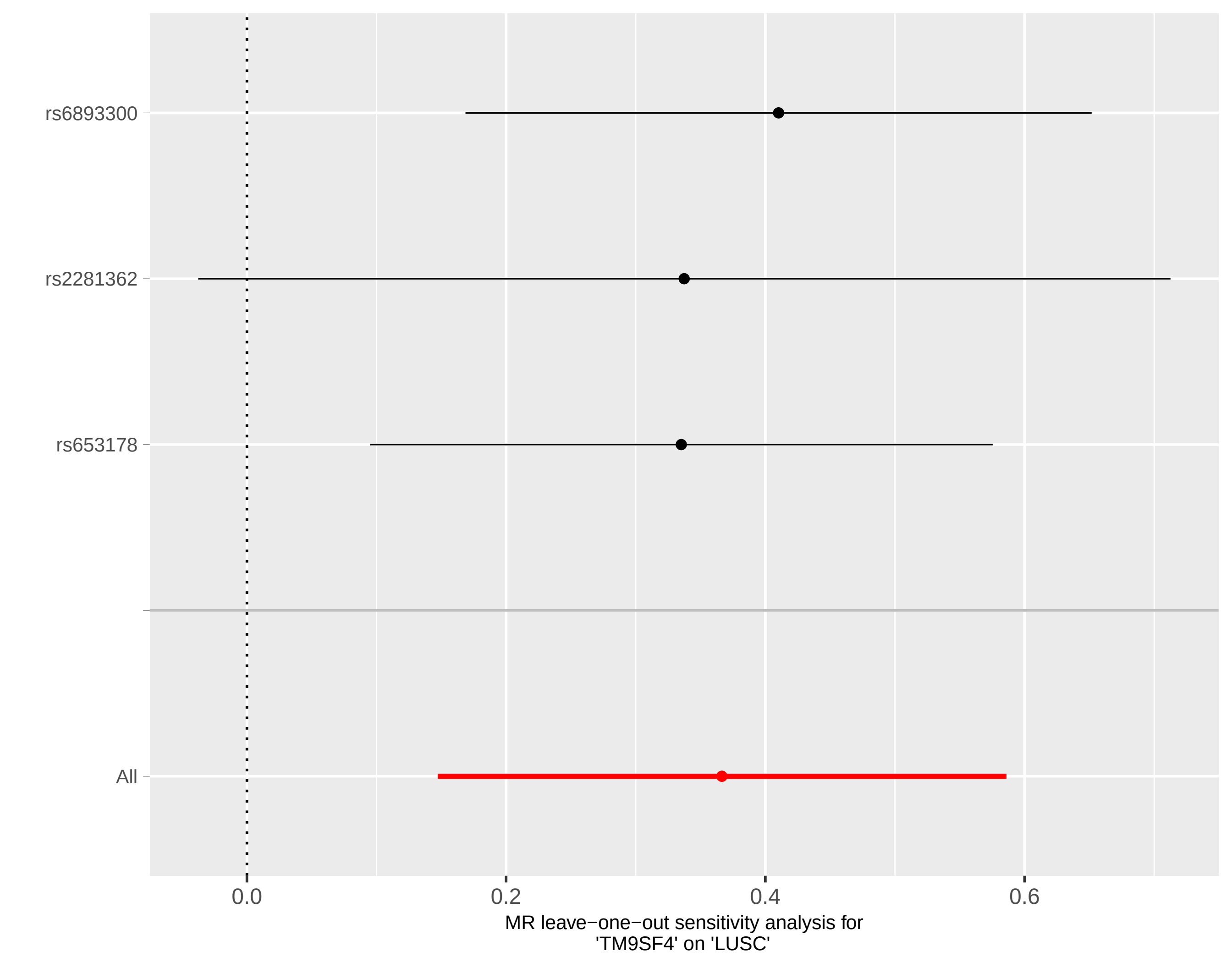

rs6893300
rs2281362
rs653178
All
0.0
0.2
0.4
0.6
MR leave−one−out sensitivity analysis for
'TM9SF4' on 'LUSC'

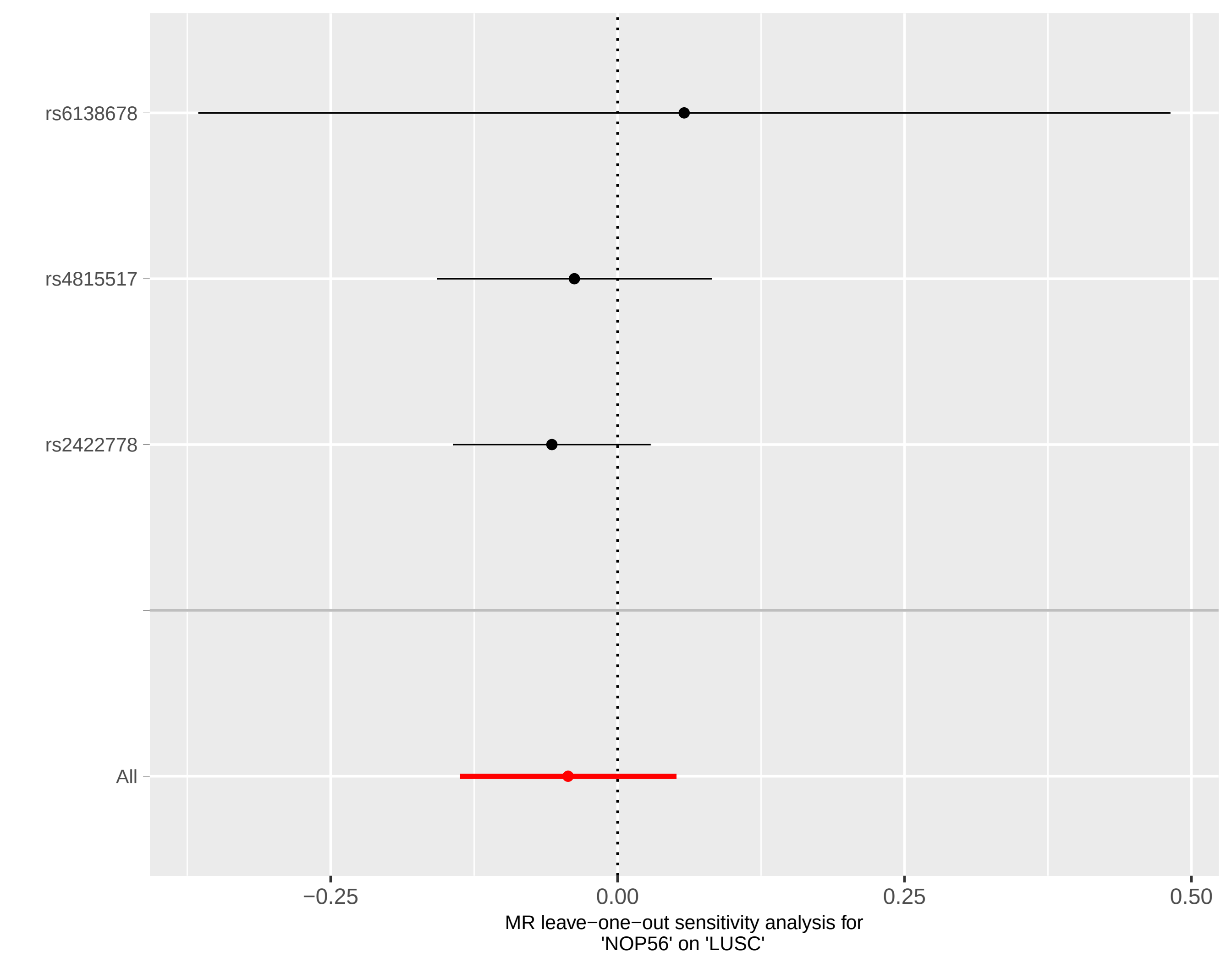
rs6138678
rs4815517
rs2422778
All
−0.25
0.00
0.25
0.50
MR leave−one−out sensitivity analysis for
'NOP56' on 'LUSC'

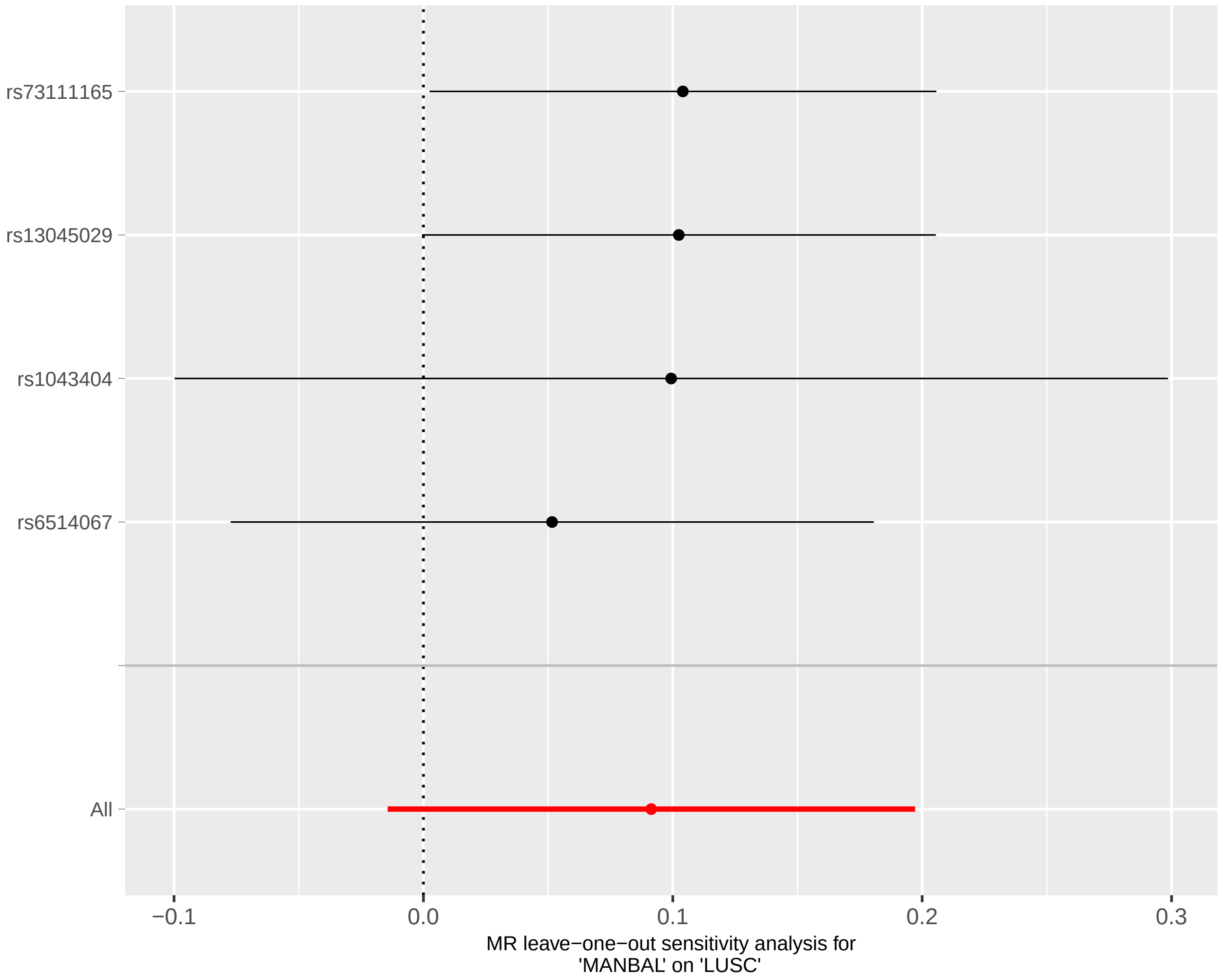

rs73111165
rs13045029
rs1043404
rs6514067
All
−0.1
0.0
0.1
0.2
0.3
MR leave−one−out sensitivity analysis for
'MANBAL' on 'LUSC'

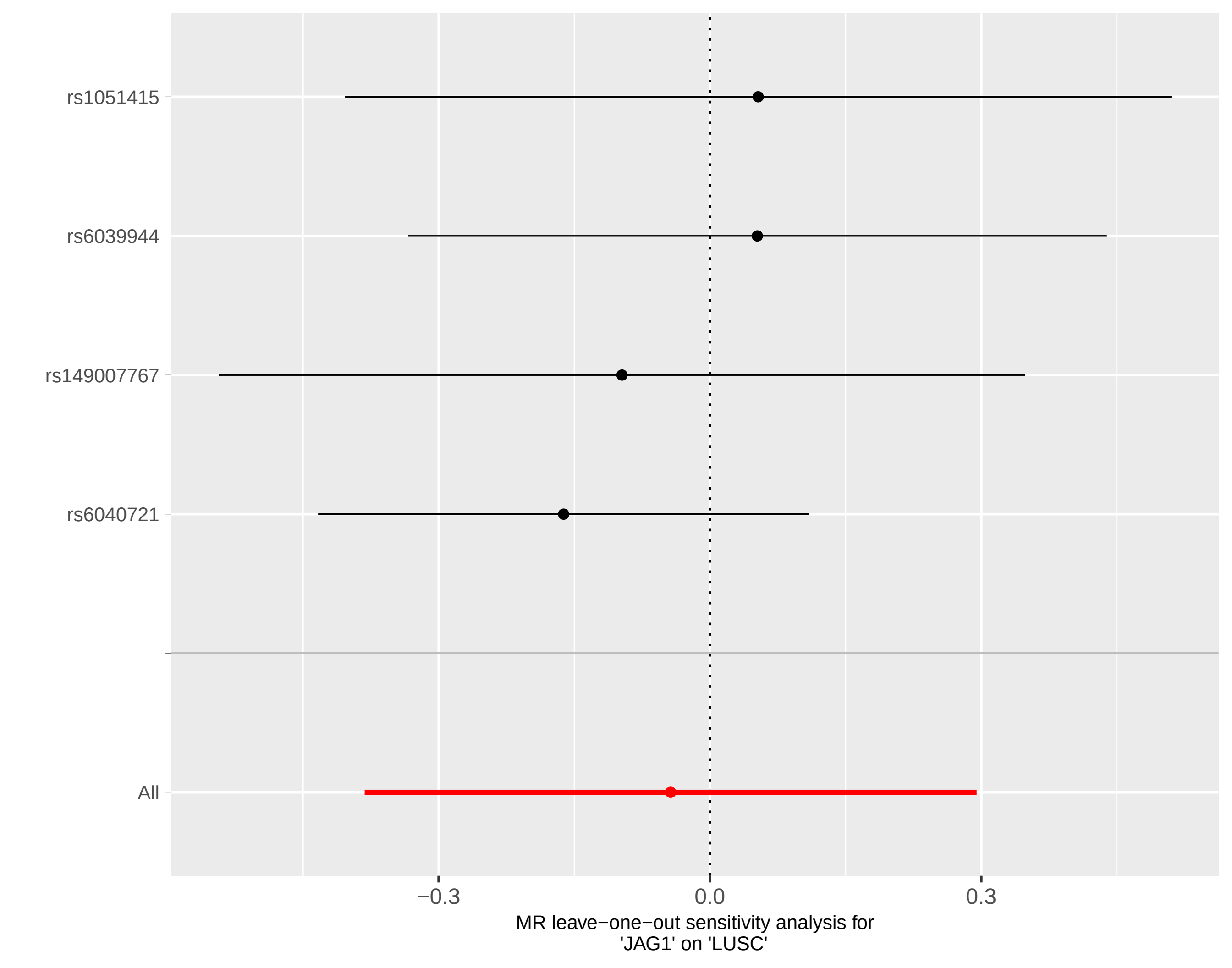

rs1051415
rs6039944
rs149007767
rs6040721
All
−0.3
0.0
0.3
MR leave−one−out sensitivity analysis for
'JAG1' on 'LUSC'

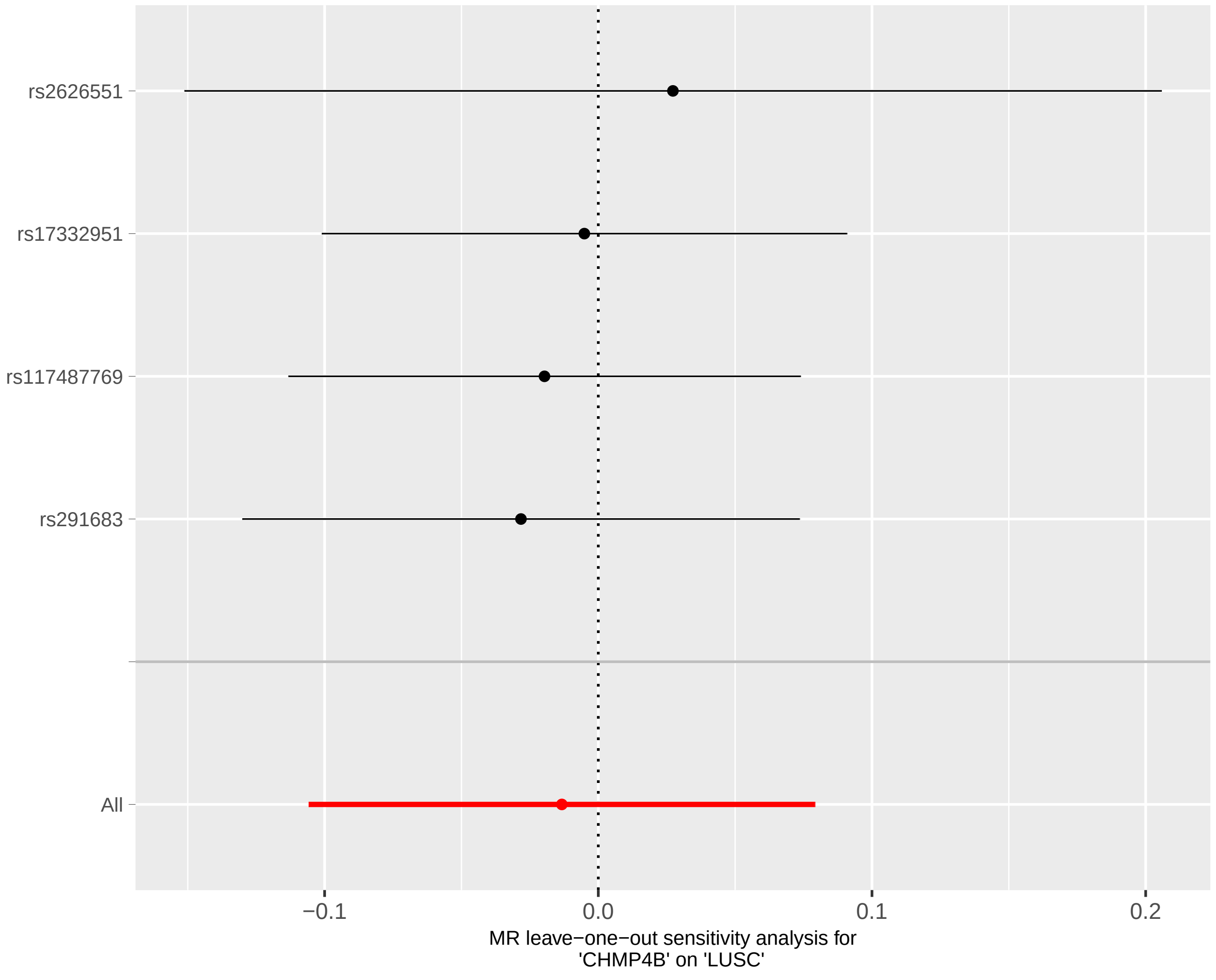
rs2626551
rs17332951
rs117487769
rs291683
All
−0.1
0.0
0.1
0.2
MR leave−one−out sensitivity analysis for
'CHMP4B' on 'LUSC'

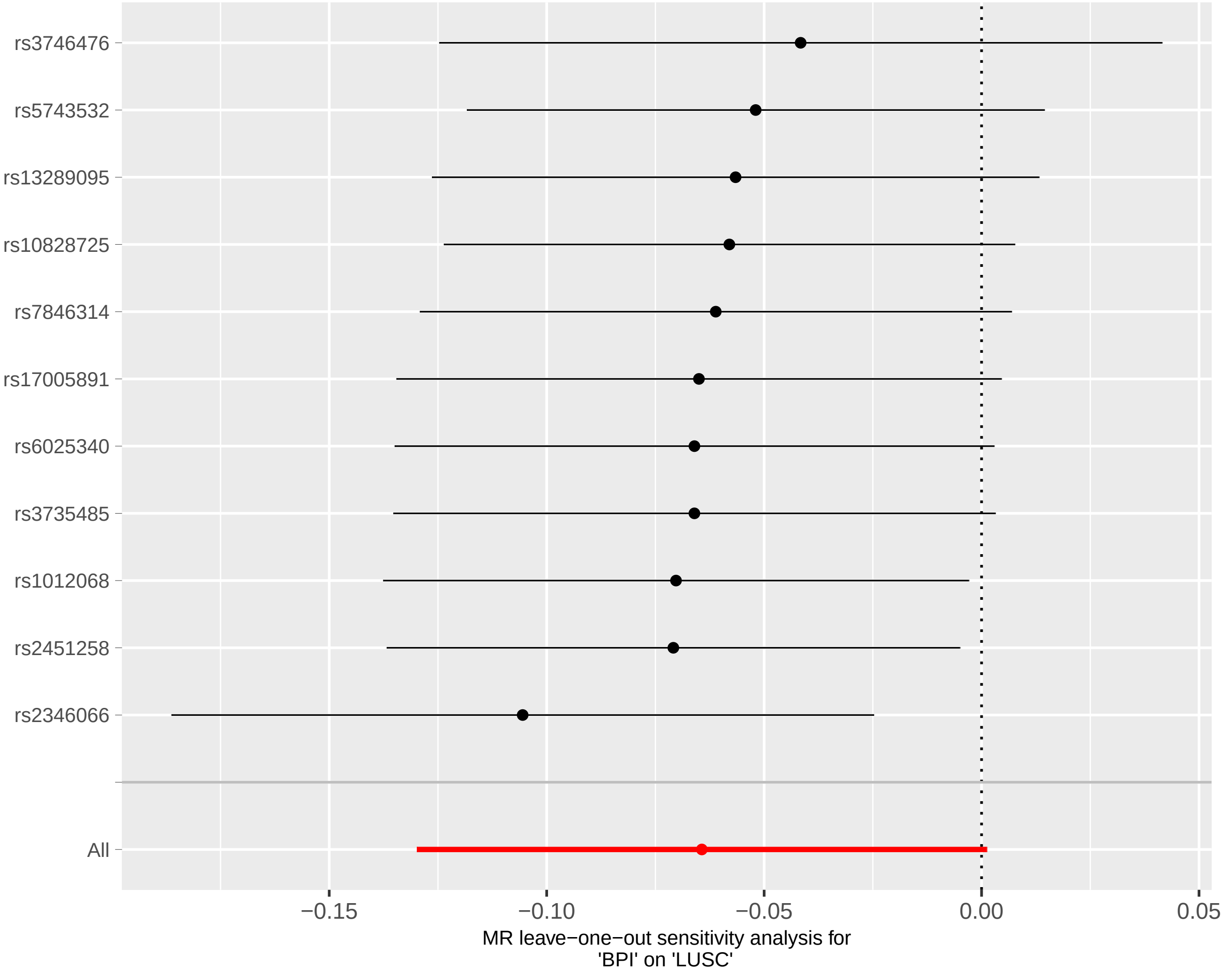

rs3746476
rs5743532
rs13289095
rs10828725
rs7846314
rs17005891
rs6025340
rs3735485
rs1012068
rs2451258
rs2346066
All
−0.15
−0.10
−0.05
0.00
0.05
MR leave−one−out sensitivity analysis for
'BPI' on 'LUSC'

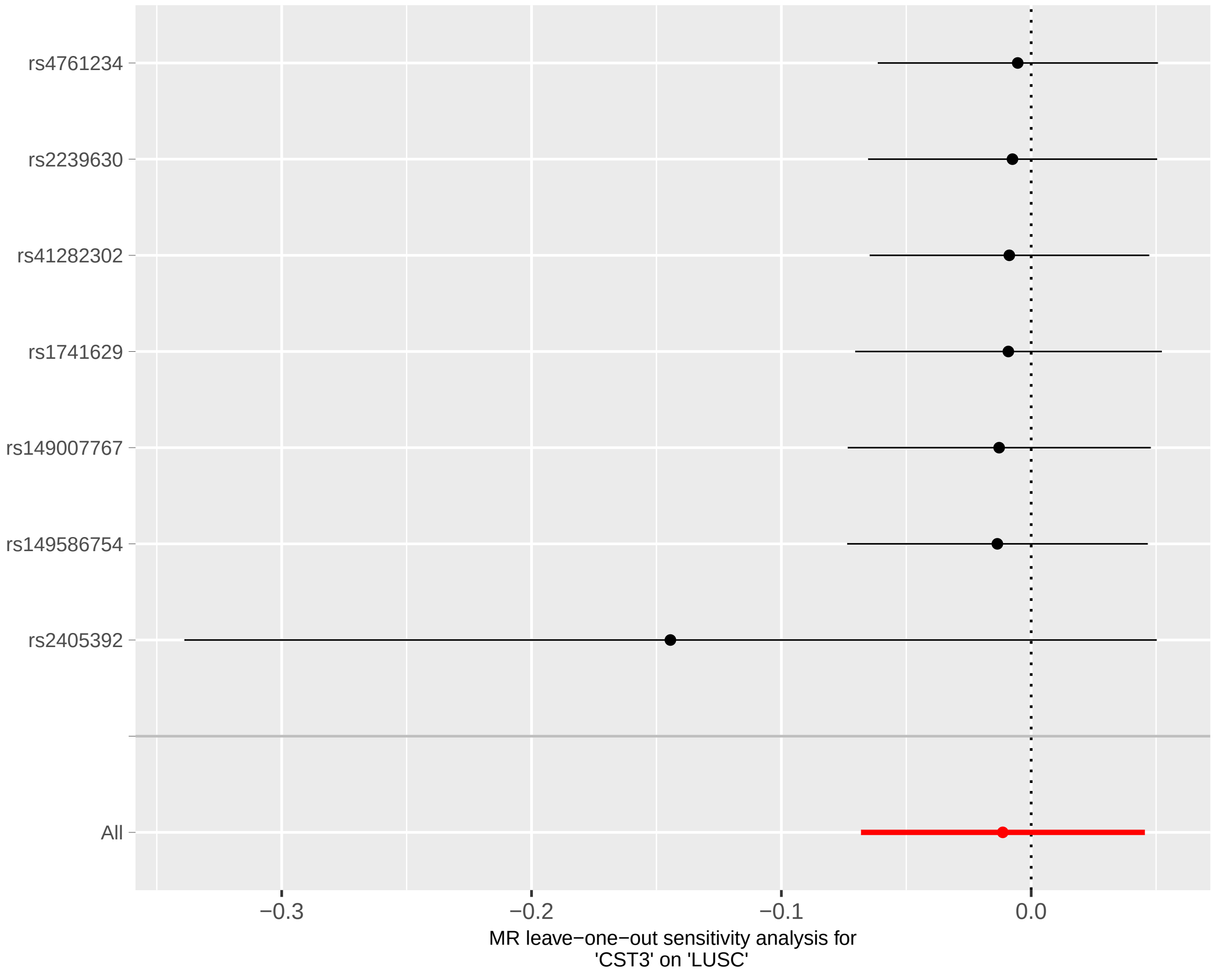
rs4761234
rs2239630
rs41282302
rs1741629
rs149007767
rs149586754
rs2405392
All
−0.3
−0.2
−0.1
0.0
MR leave−one−out sensitivity analysis for
'CST3' on 'LUSC'

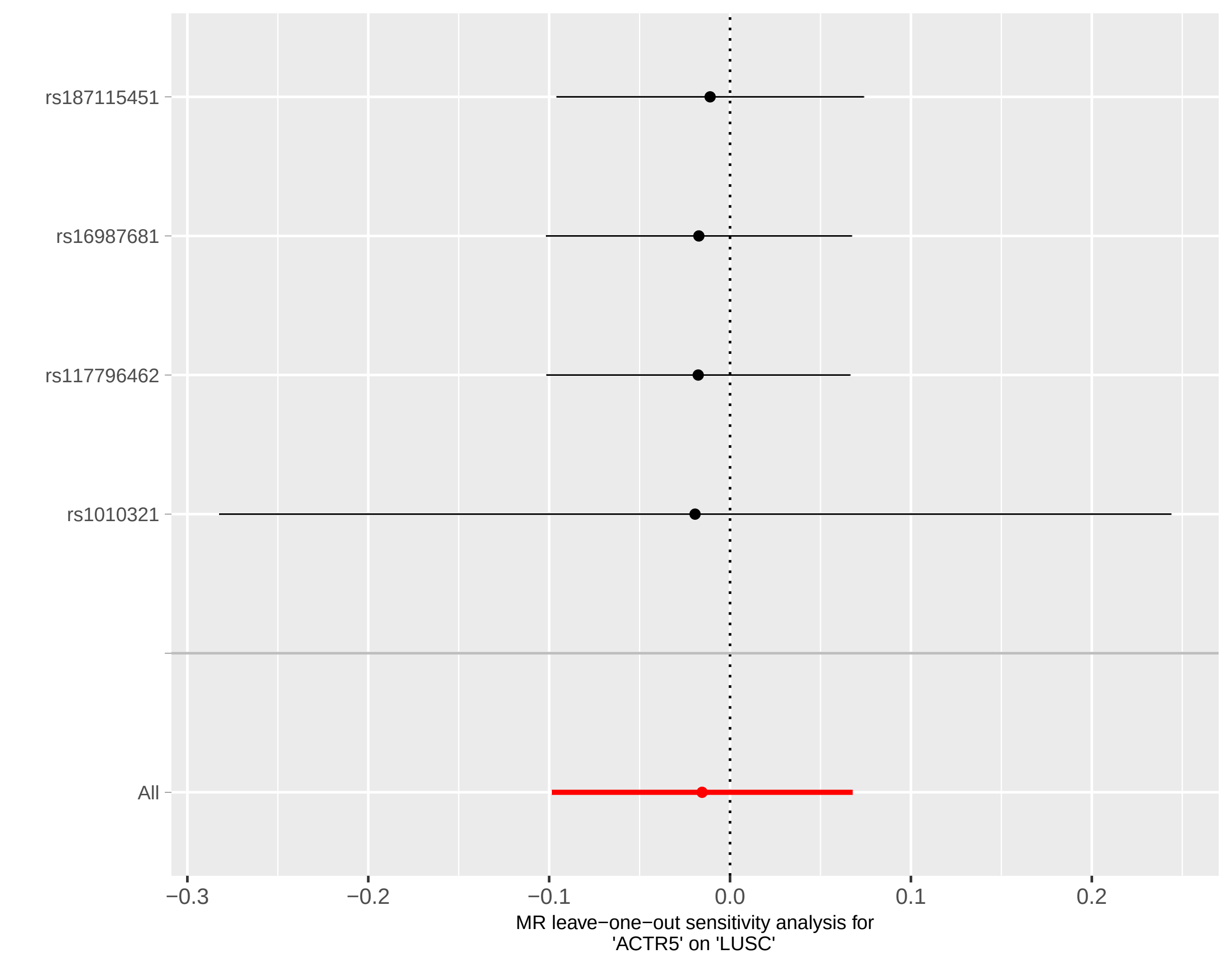

rs187115451
rs16987681
rs117796462
rs1010321
All
−0.3
−0.2
−0.1
0.0
0.1
0.2
MR leave−one−out sensitivity analysis for
'ACTR5' on 'LUSC'

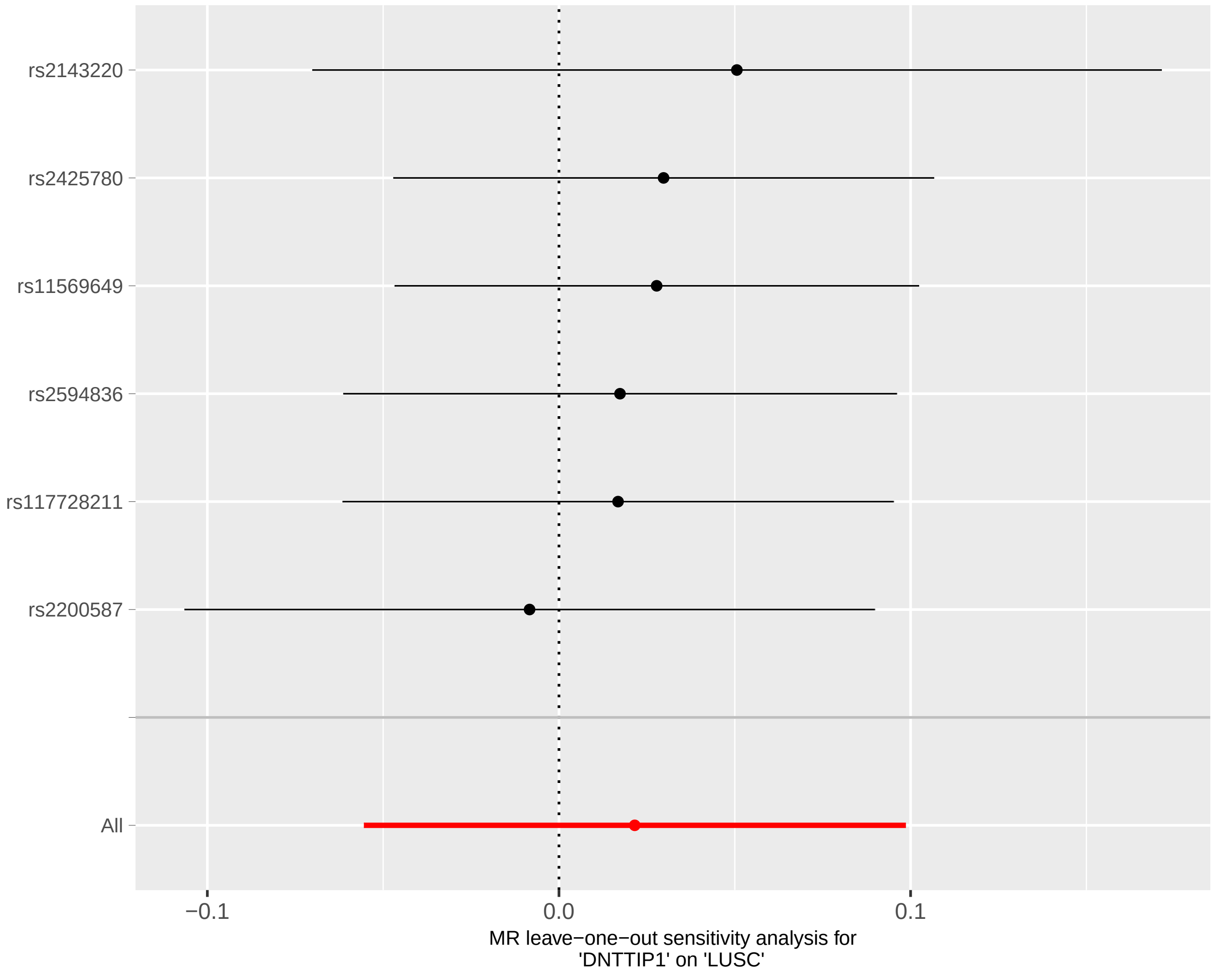

rs2143220
rs2425780
rs11569649
rs2594836
rs117728211
rs2200587
All
−0.1
0.0
0.1
MR leave−one−out sensitivity analysis for
'DNTTIP1' on 'LUSC'

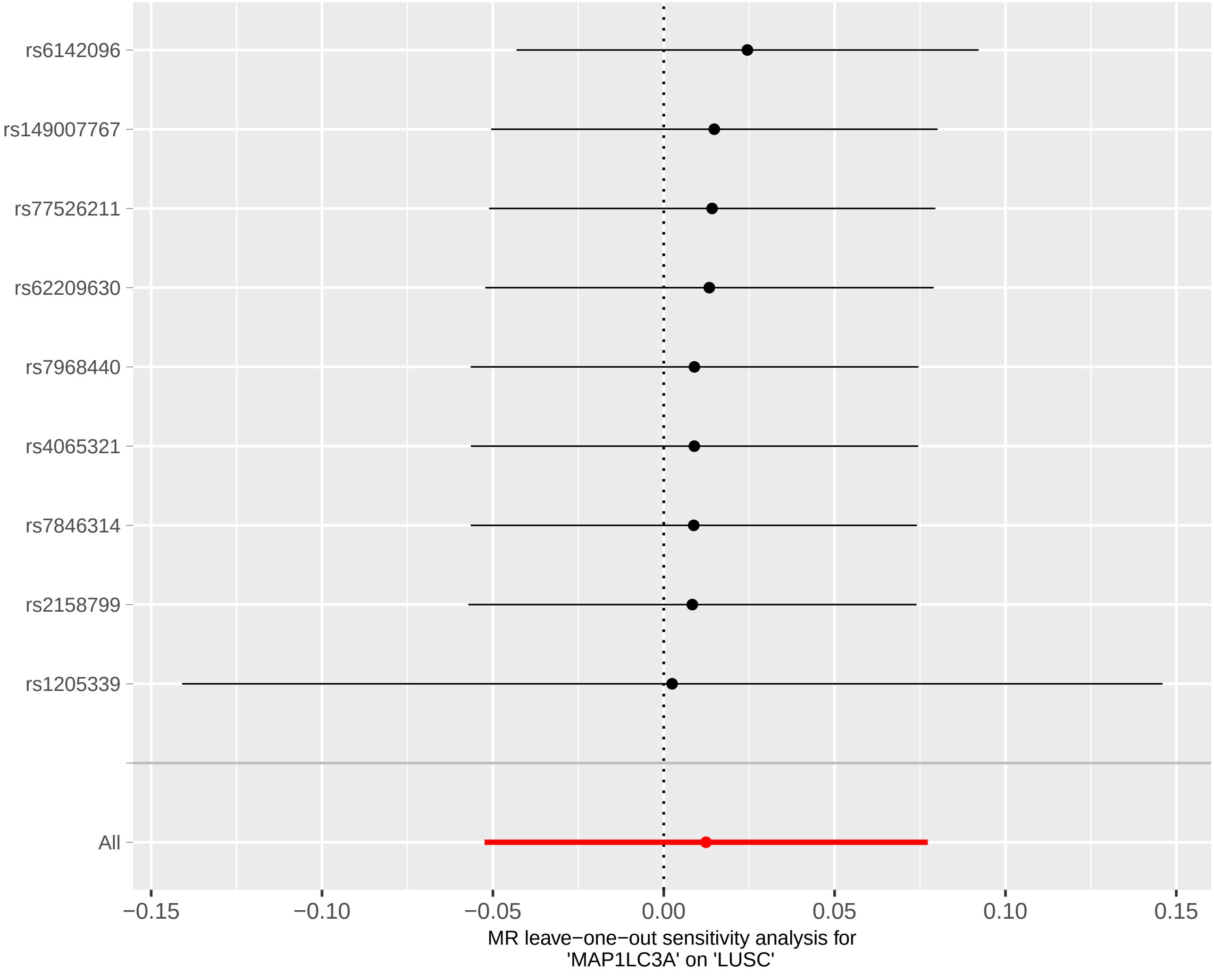

rs6142096
rs149007767
rs77526211
rs62209630
rs7968440
rs4065321
rs7846314
rs2158799
rs1205339
All
−0.15
−0.10
−0.05
0.00
0.05
0.10
0.15
MR leave−one−out sensitivity analysis for
'MAP1LC3A' on 'LUSC'

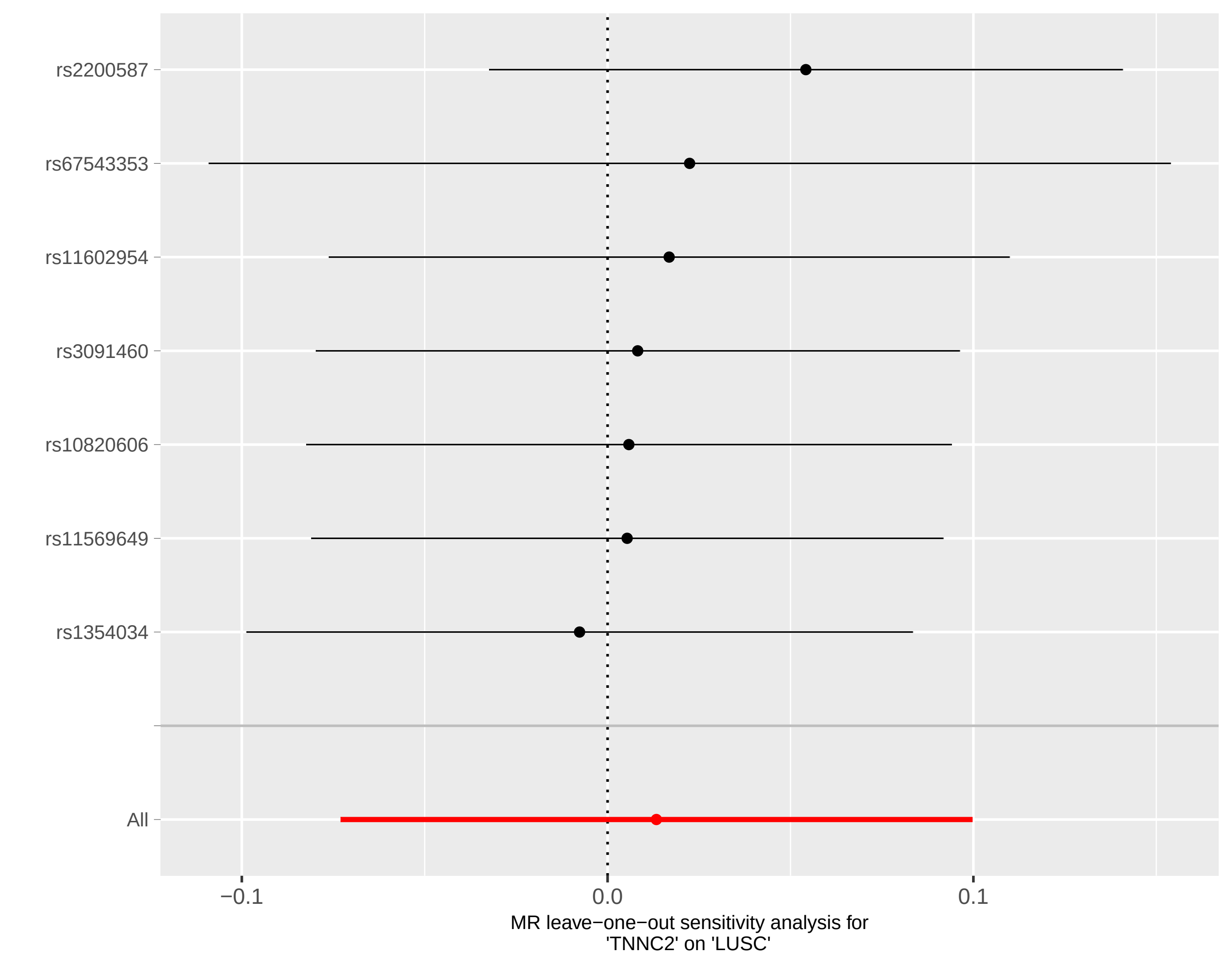
rs2200587
rs67543353
rs11602954
rs3091460
rs10820606
rs11569649
rs1354034
All
−0.1
0.0
0.1
MR leave−one−out sensitivity analysis for
'TNNC2' on 'LUSC'

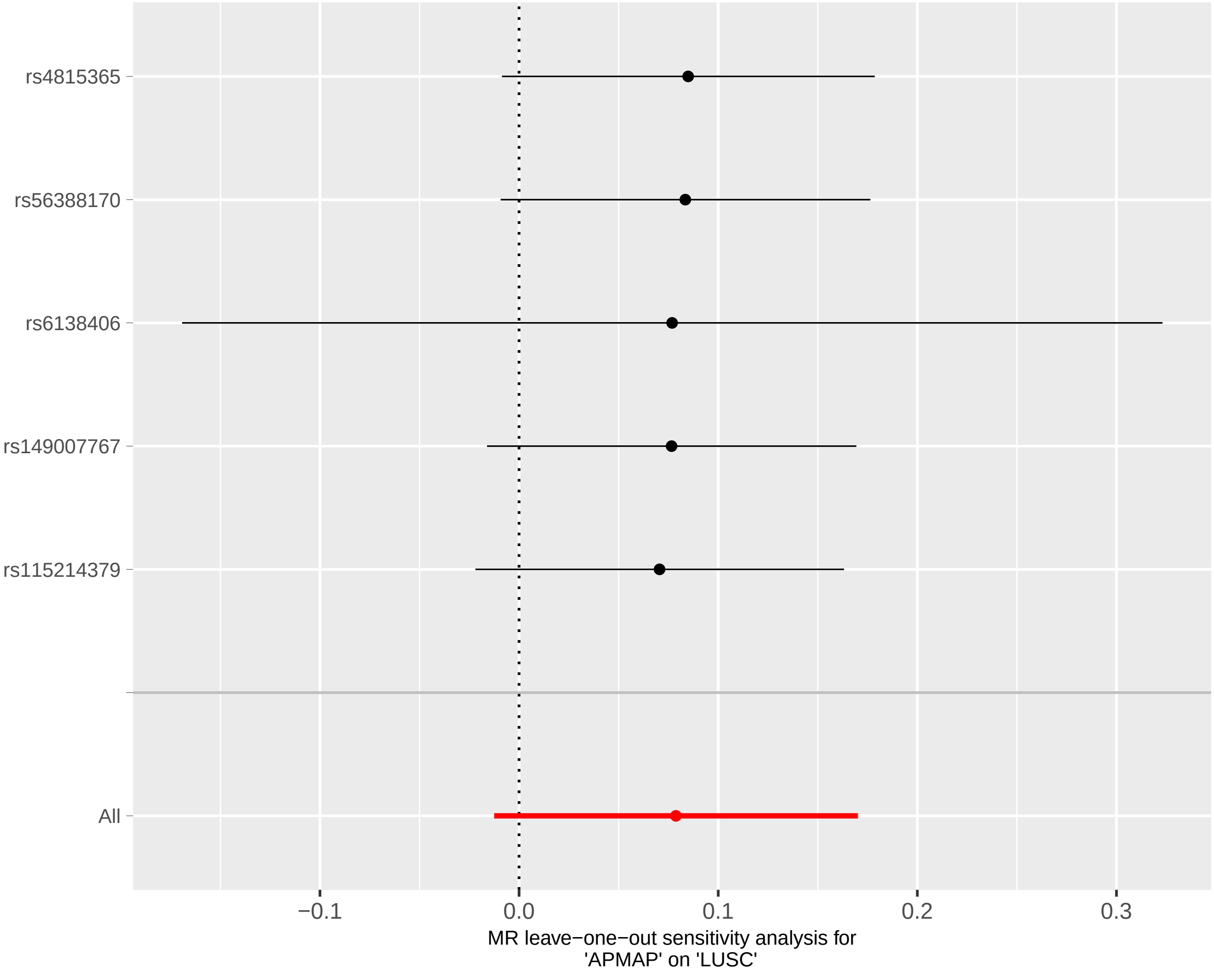
rs4815365
rs56388170
rs6138406
rs149007767
rs115214379
All
−0.1
0.0
0.1
0.2
0.3
MR leave−one−out sensitivity analysis for
'APMAP' on 'LUSC'

Insufficient number of SNPs

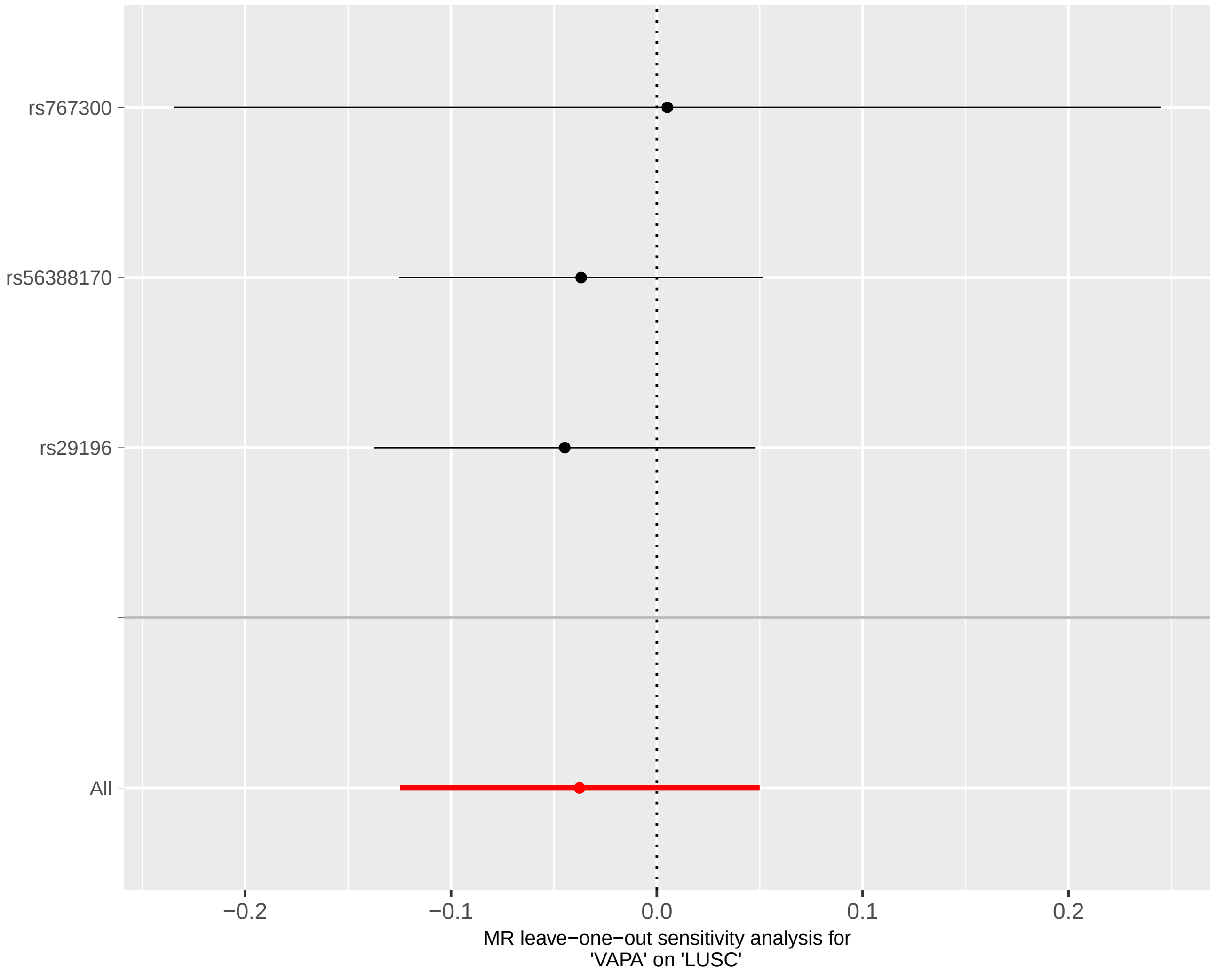
rs767300
rs56388170
rs29196
All
−0.2
−0.1
0.0
0.1
0.2
MR leave−one−out sensitivity analysis for
'VAPA' on 'LUSC'

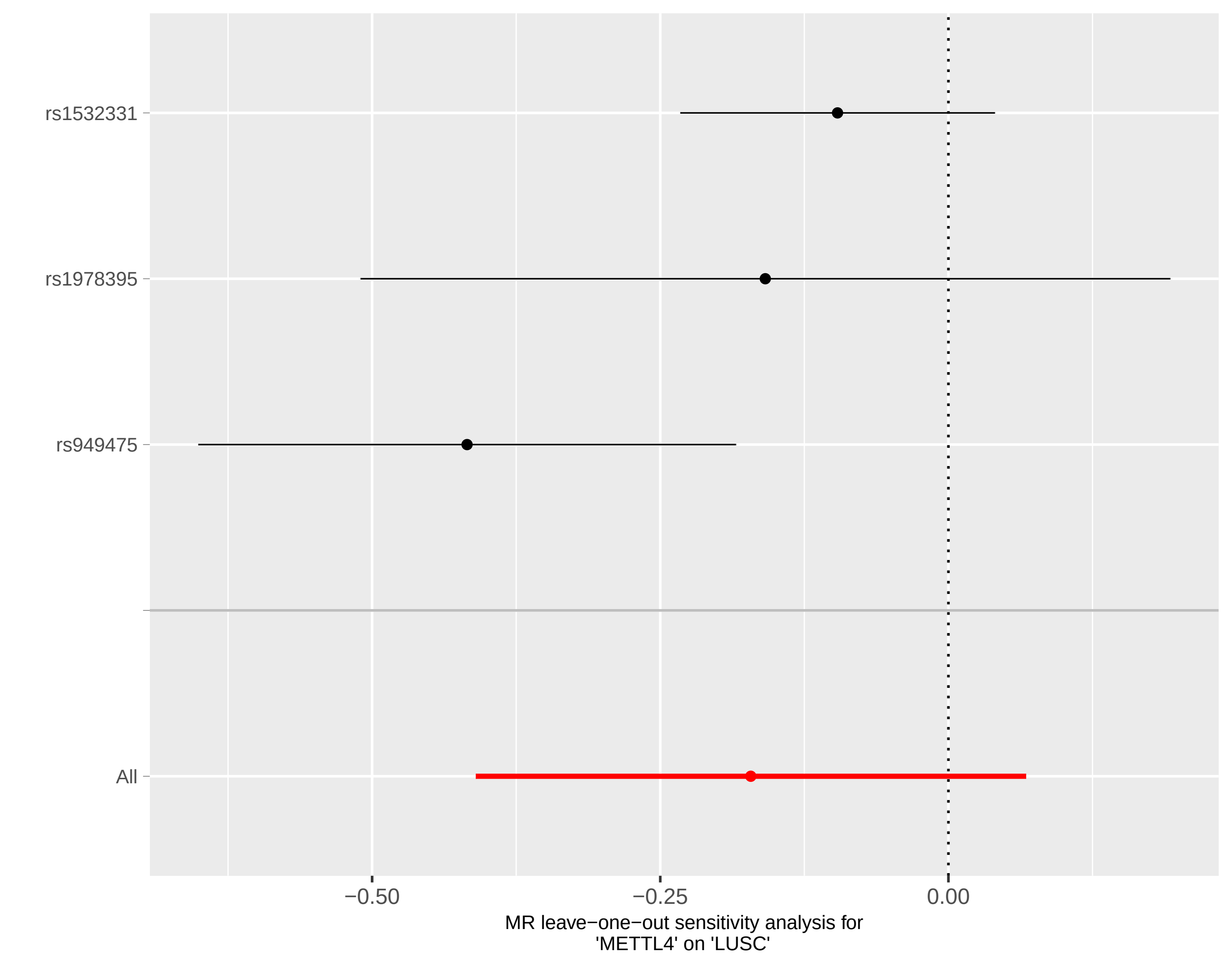

rs1532331
rs1978395
rs949475
All
−0.50
−0.25
0.00
MR leave−one−out sensitivity analysis for
'METTL4' on 'LUSC'

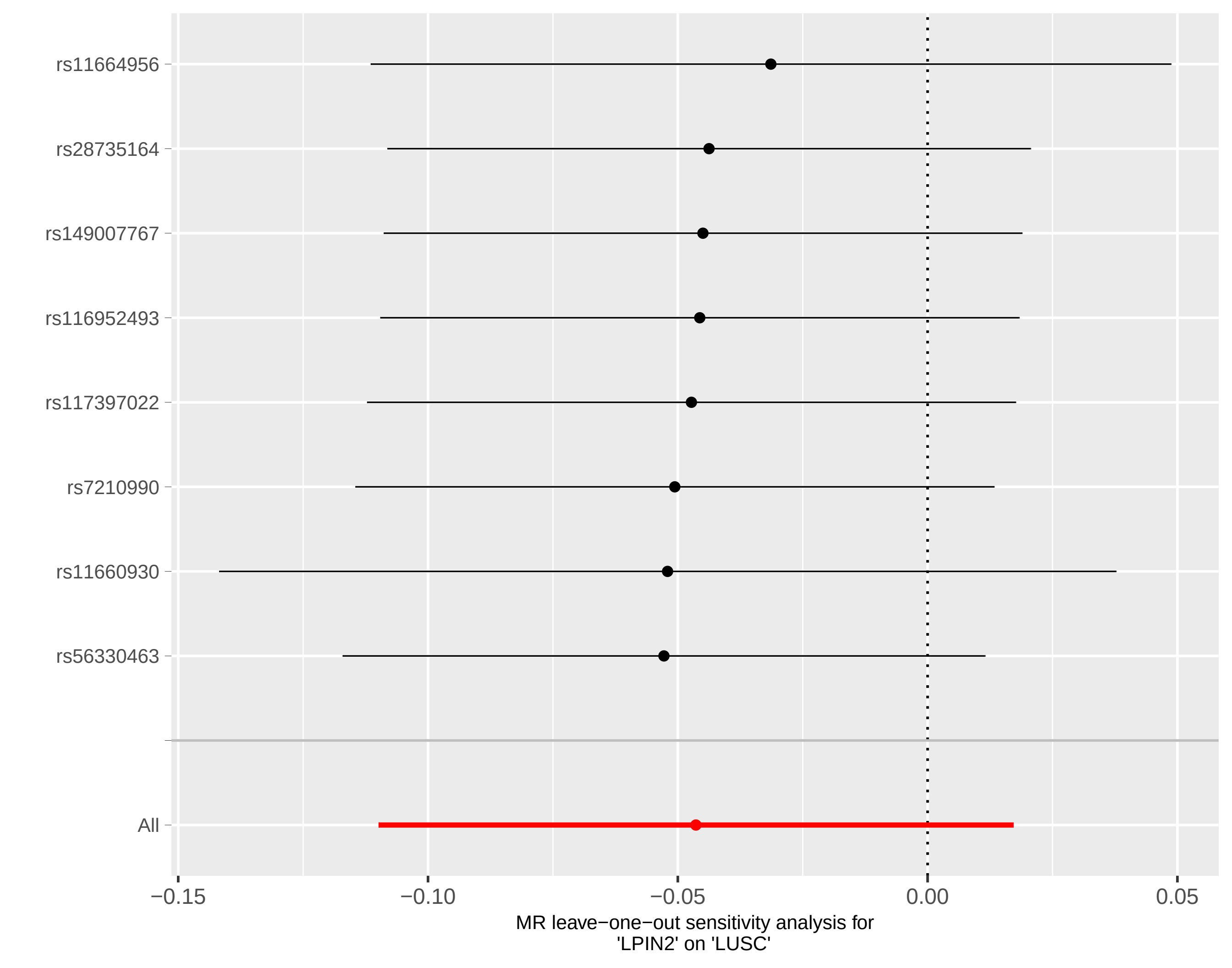
rs11664956
rs28735164
rs149007767
rs116952493
rs117397022
rs7210990
rs11660930
rs56330463
All
−0.15
−0.10
−0.05
0.00
0.05
MR leave−one−out sensitivity analysis for
'LPIN2' on 'LUSC'

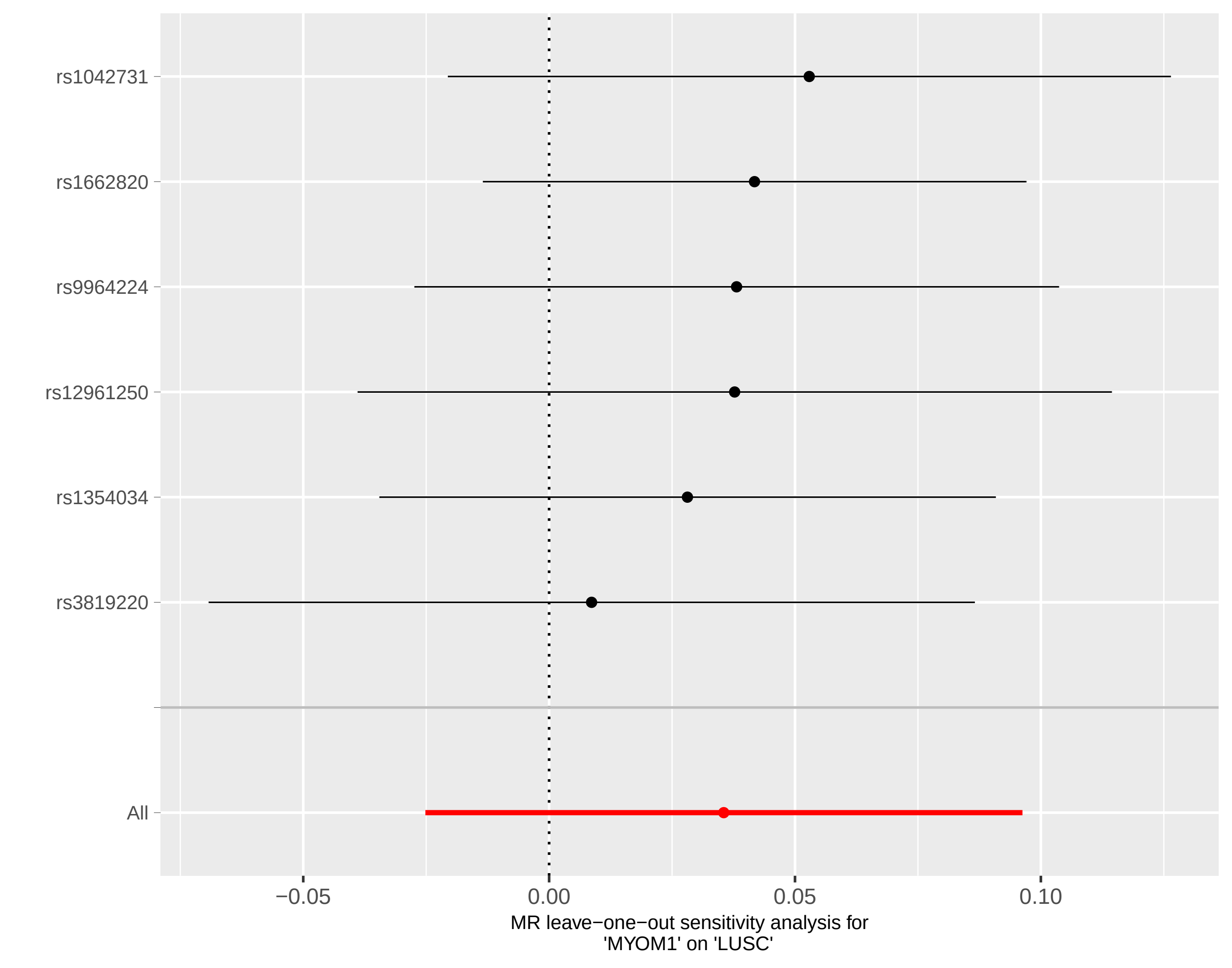

rs1042731
rs1662820
rs9964224
rs12961250
rs1354034
rs3819220
All
−0.05
0.00
0.05
0.10
MR leave−one−out sensitivity analysis for
'MYOM1' on 'LUSC'

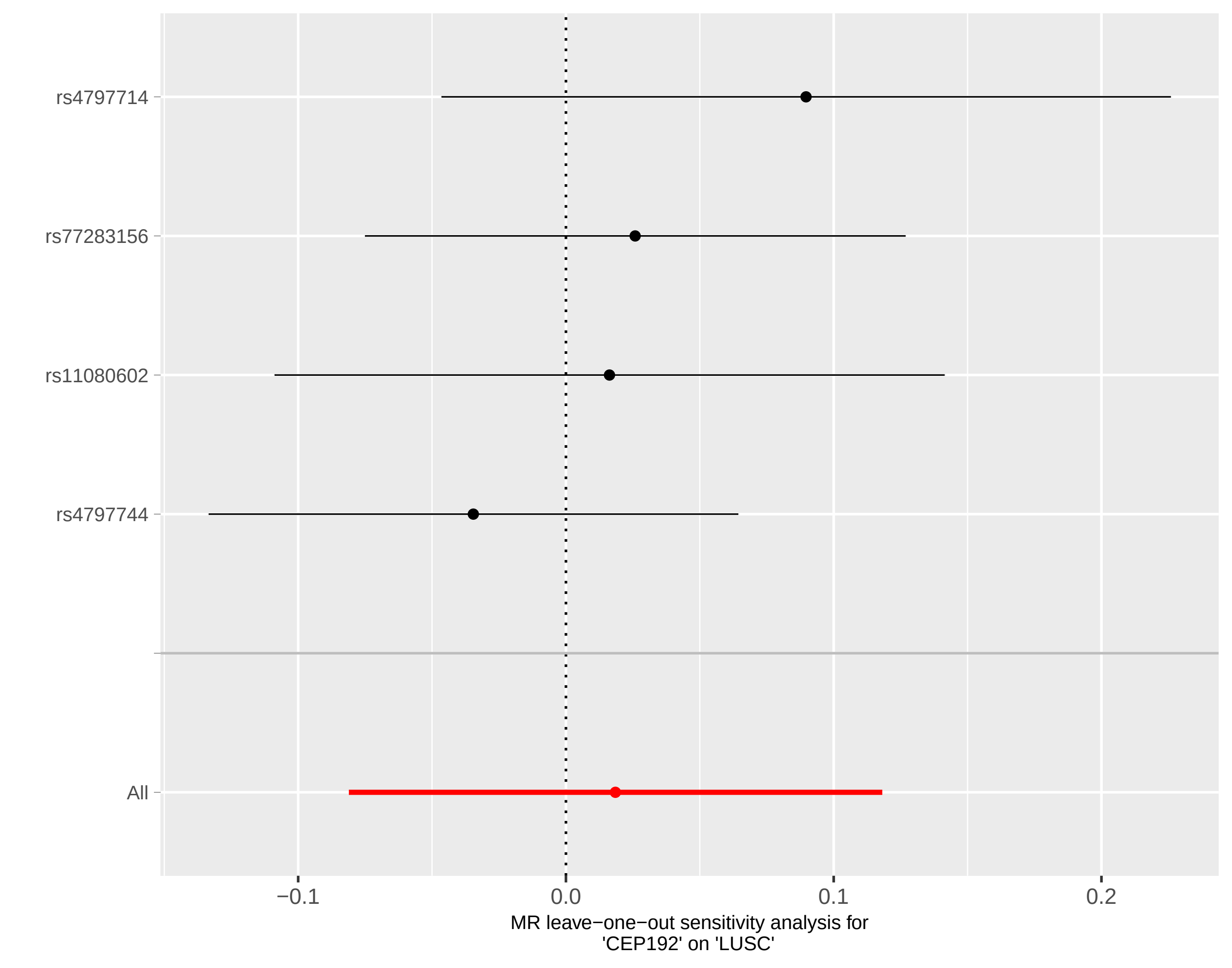

rs4797714
rs77283156
rs11080602
rs4797744
All
−0.1
0.0
0.1
0.2
MR leave−one−out sensitivity analysis for
'CEP192' on 'LUSC'

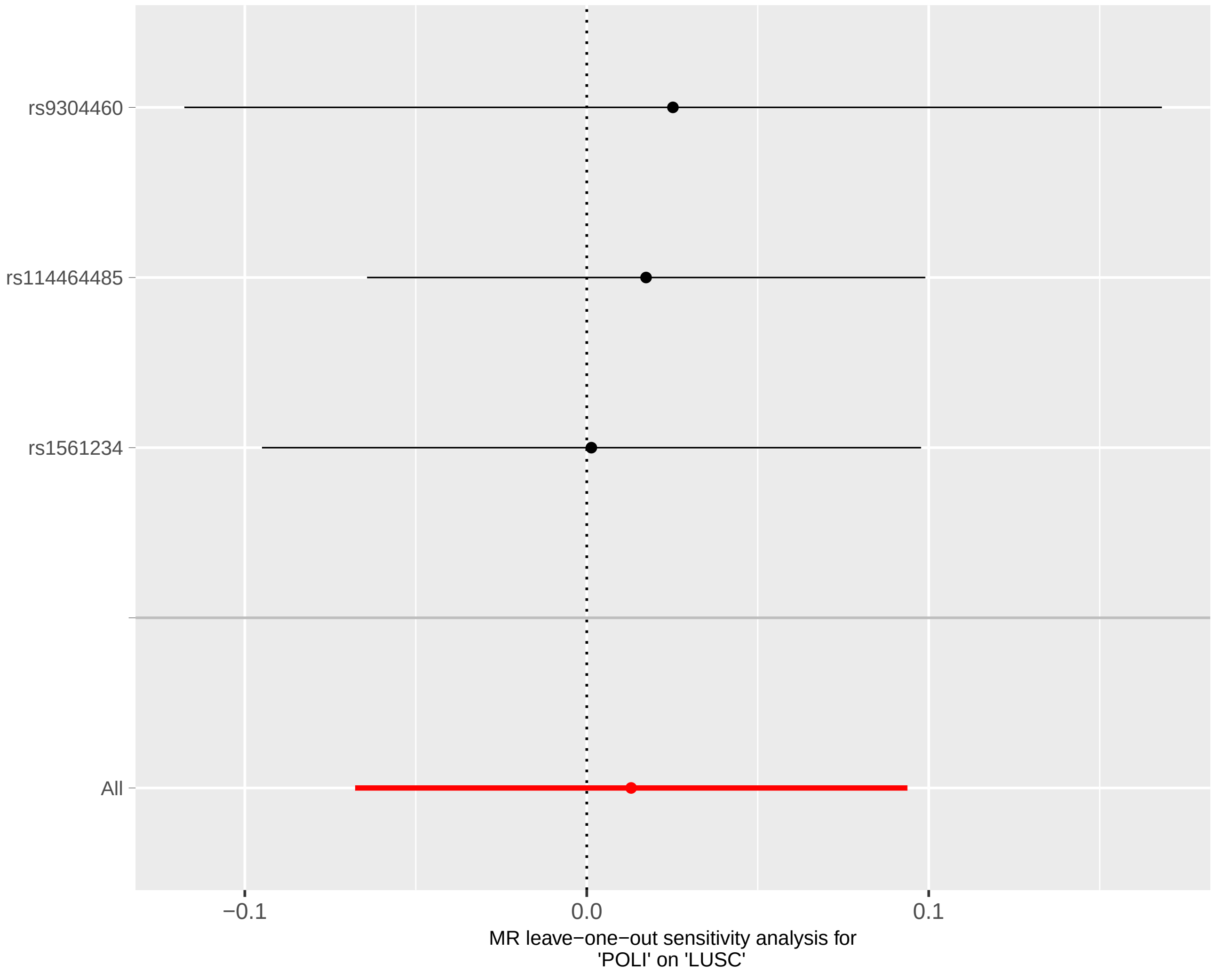

rs9304460
rs114464485
rs1561234
All
−0.1
0.0
0.1
MR leave−one−out sensitivity analysis for
'POLI' on 'LUSC'

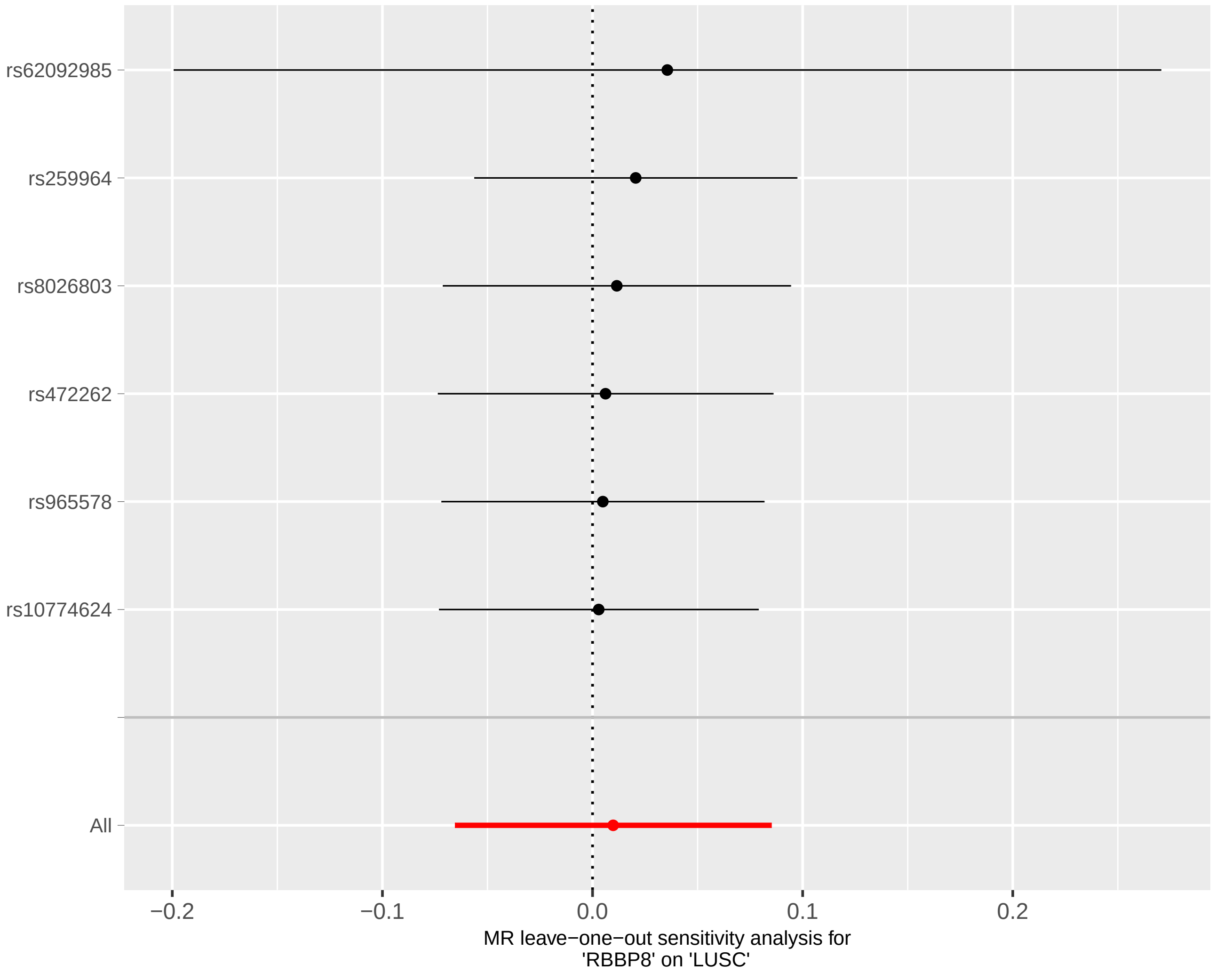

rs62092985
rs259964
rs8026803
rs472262
rs965578
rs10774624
All
−0.2
−0.1
0.0
0.1
0.2
MR leave−one−out sensitivity analysis for
'RBBP8' on 'LUSC'

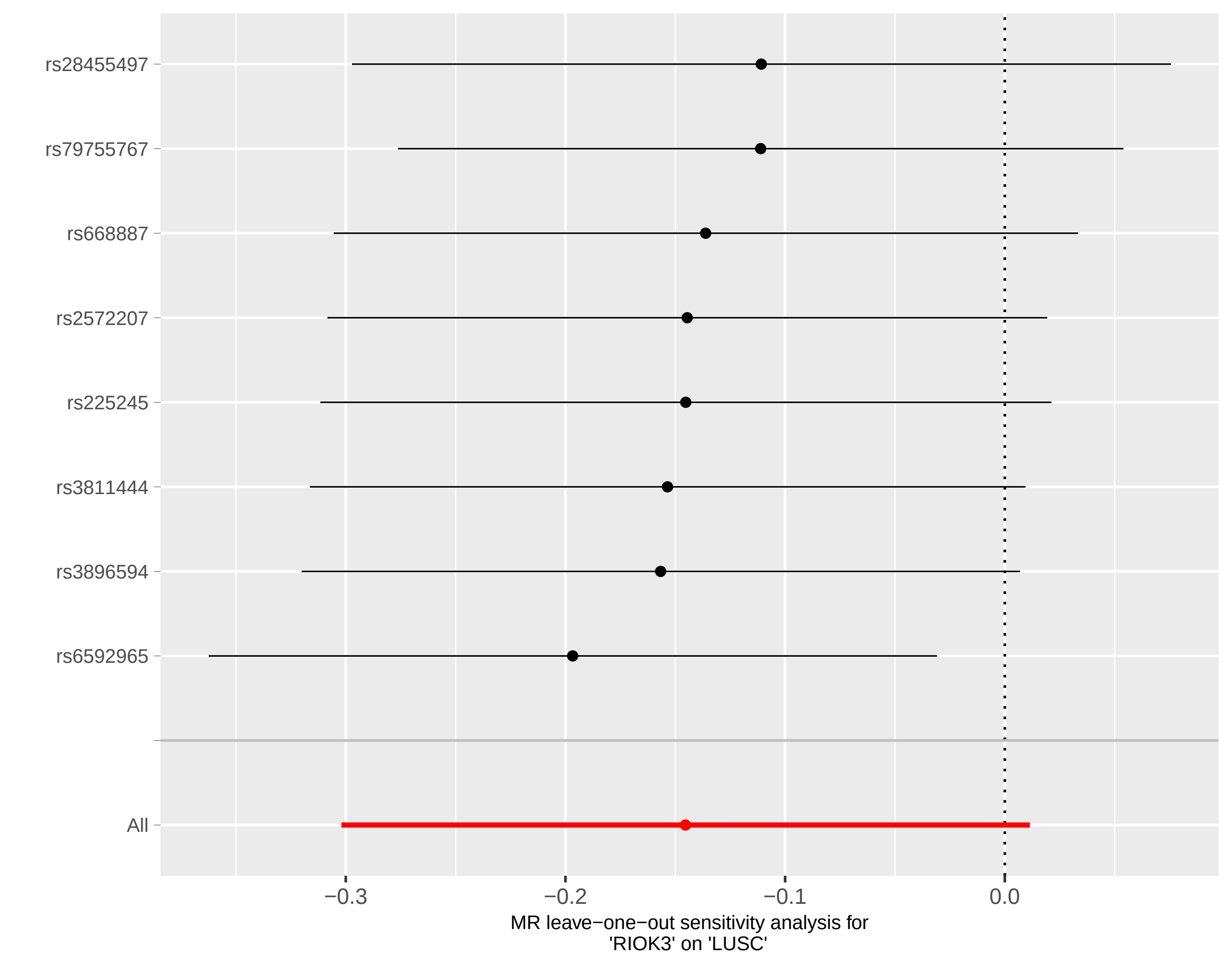

rs28455497
rs79755767
rs668887
rs2572207
rs225245
rs3811444
rs3896594
rs6592965
All
−0.3
−0.2
−0.1
0.0
MR leave−one−out sensitivity analysis for
'RIOK3' on 'LUSC'

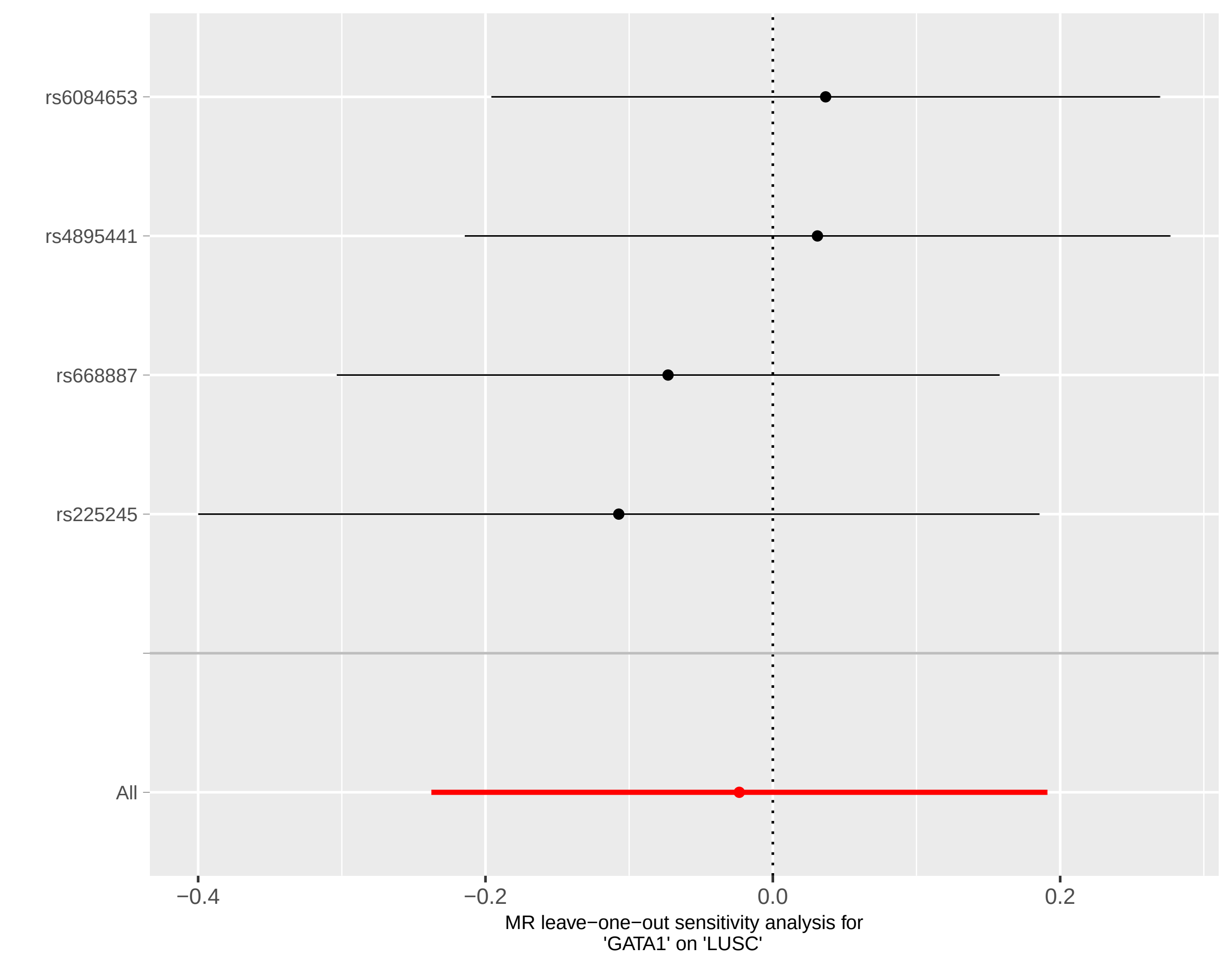
rs6084653
rs4895441
rs668887
rs225245
All
−0.4
−0.2
0.0
0.2
MR leave−one−out sensitivity analysis for
'GATA1' on 'LUSC'

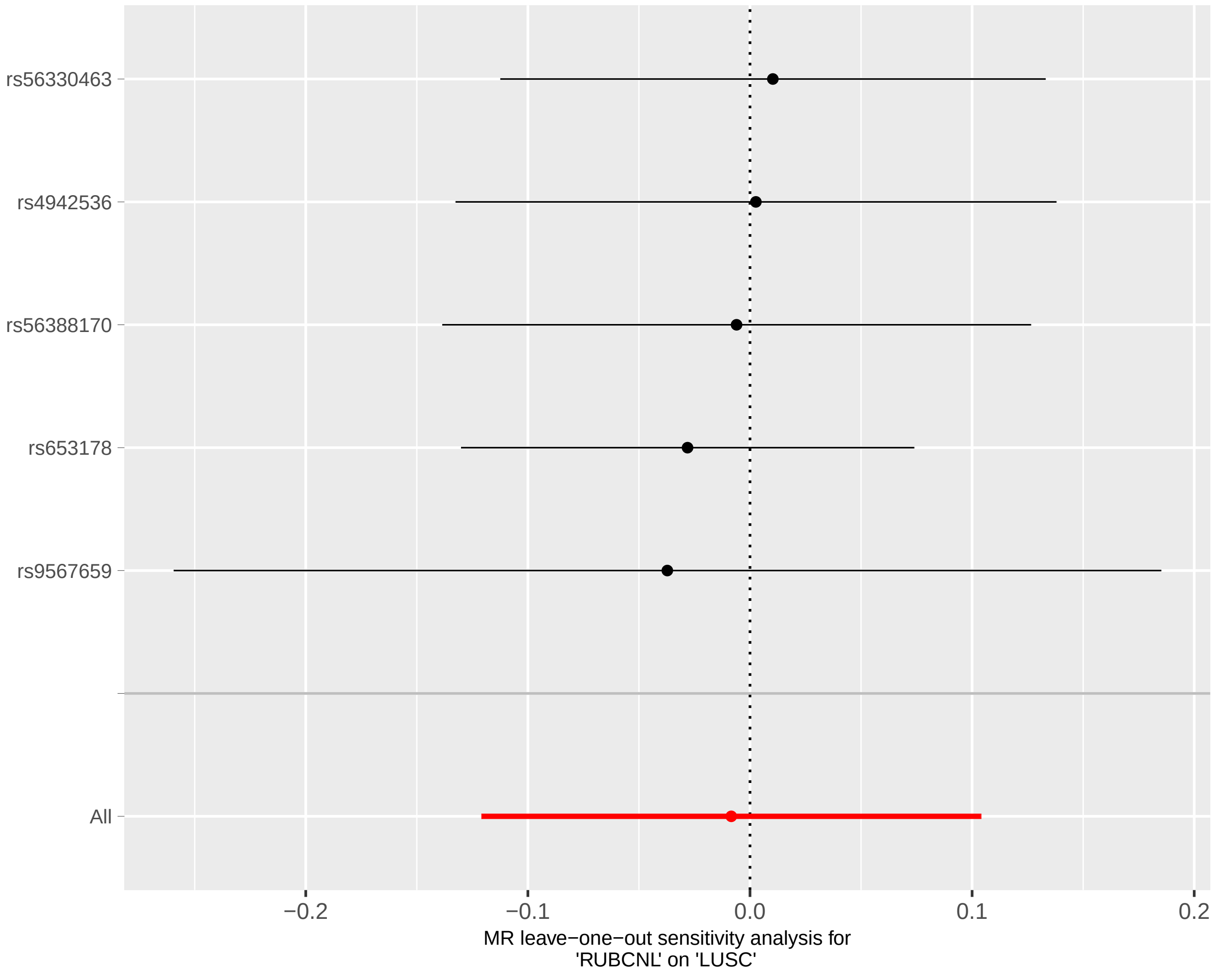

rs56330463
rs4942536
rs56388170
rs653178
rs9567659
All
−0.2
−0.1
0.0
0.1
0.2
MR leave−one−out sensitivity analysis for
'RUBCNL' on 'LUSC'

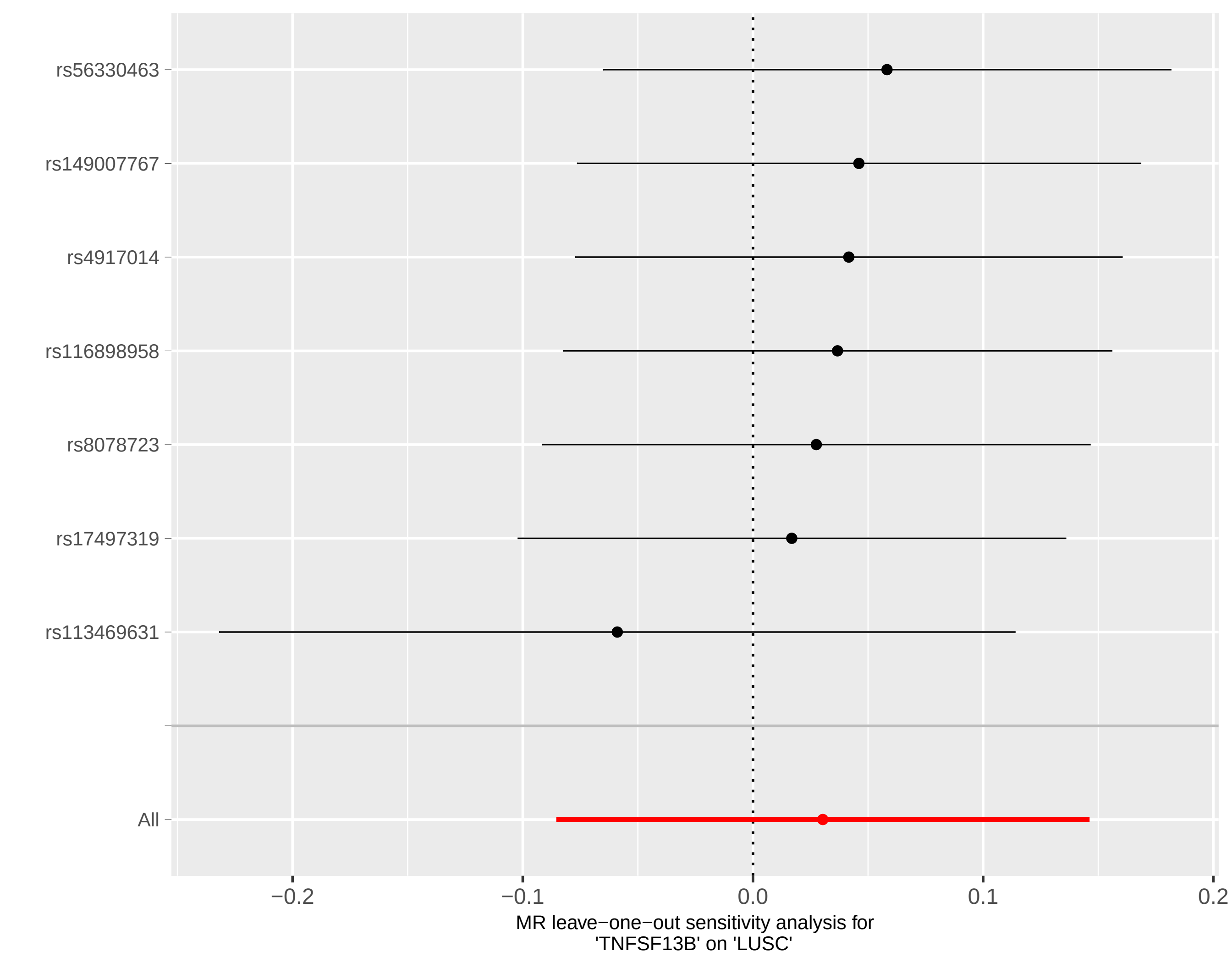

rs56330463
rs149007767
rs4917014
rs116898958
rs8078723
rs17497319
rs113469631
All
−0.2
−0.1
0.0
0.1
0.2
MR leave−one−out sensitivity analysis for
'TNFSF13B' on 'LUSC'

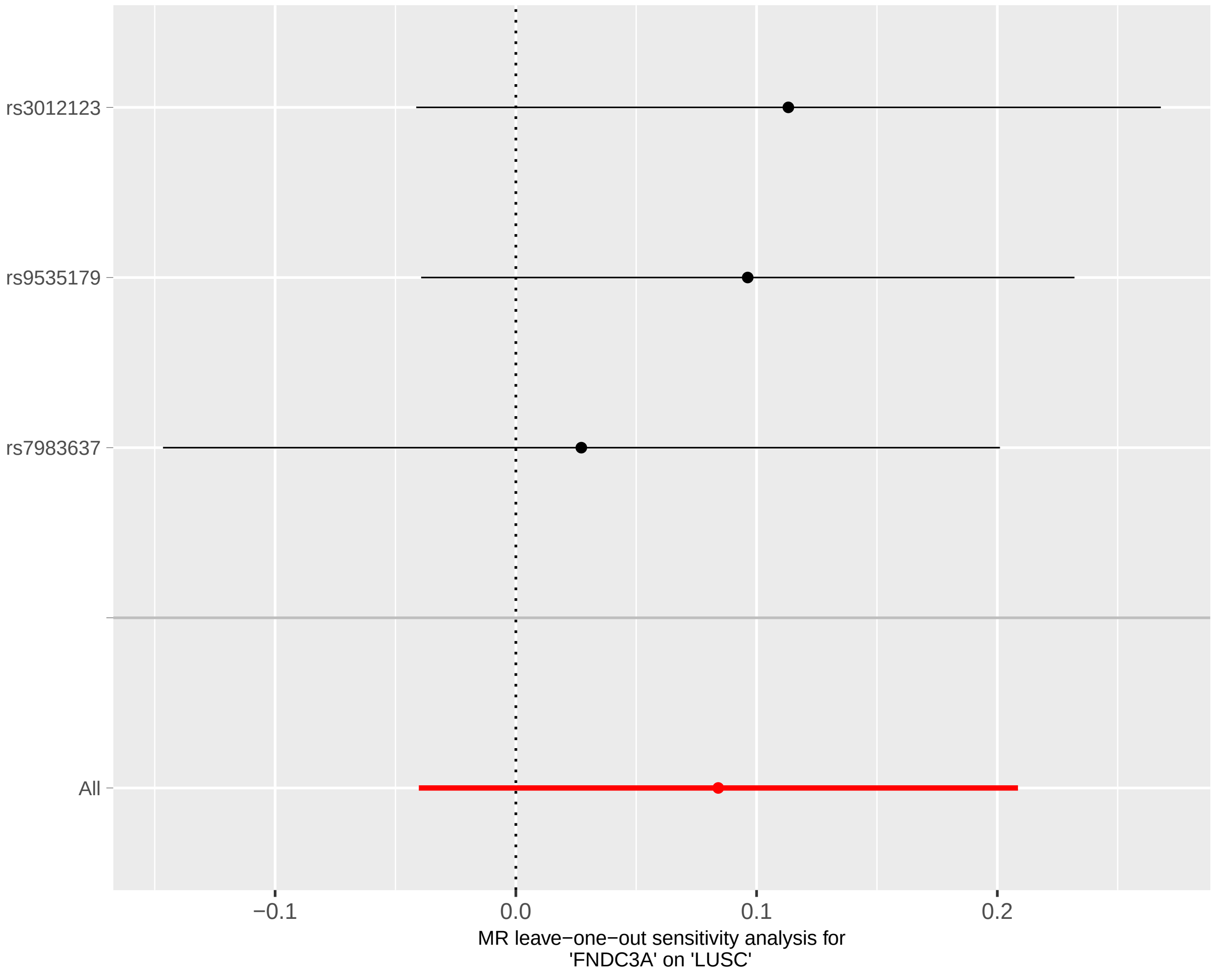
rs3012123
rs9535179
rs7983637
All
−0.1
0.0
0.1
0.2
MR leave−one−out sensitivity analysis for
'FNDC3A' on 'LUSC'

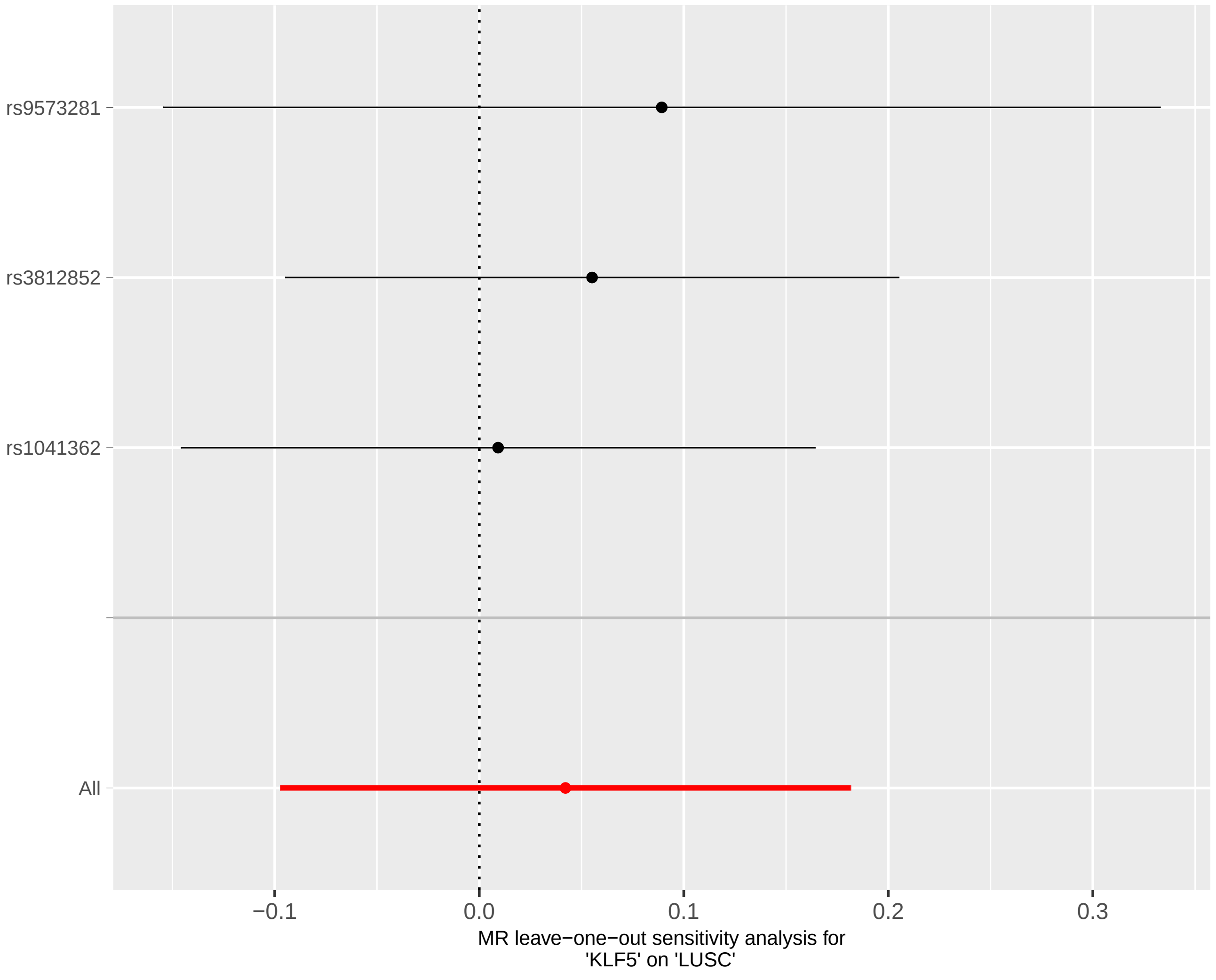
rs9573281
rs3812852
rs1041362
All
−0.1
0.0
0.1
0.2
0.3
MR leave−one−out sensitivity analysis for
'KLF5' on 'LUSC'

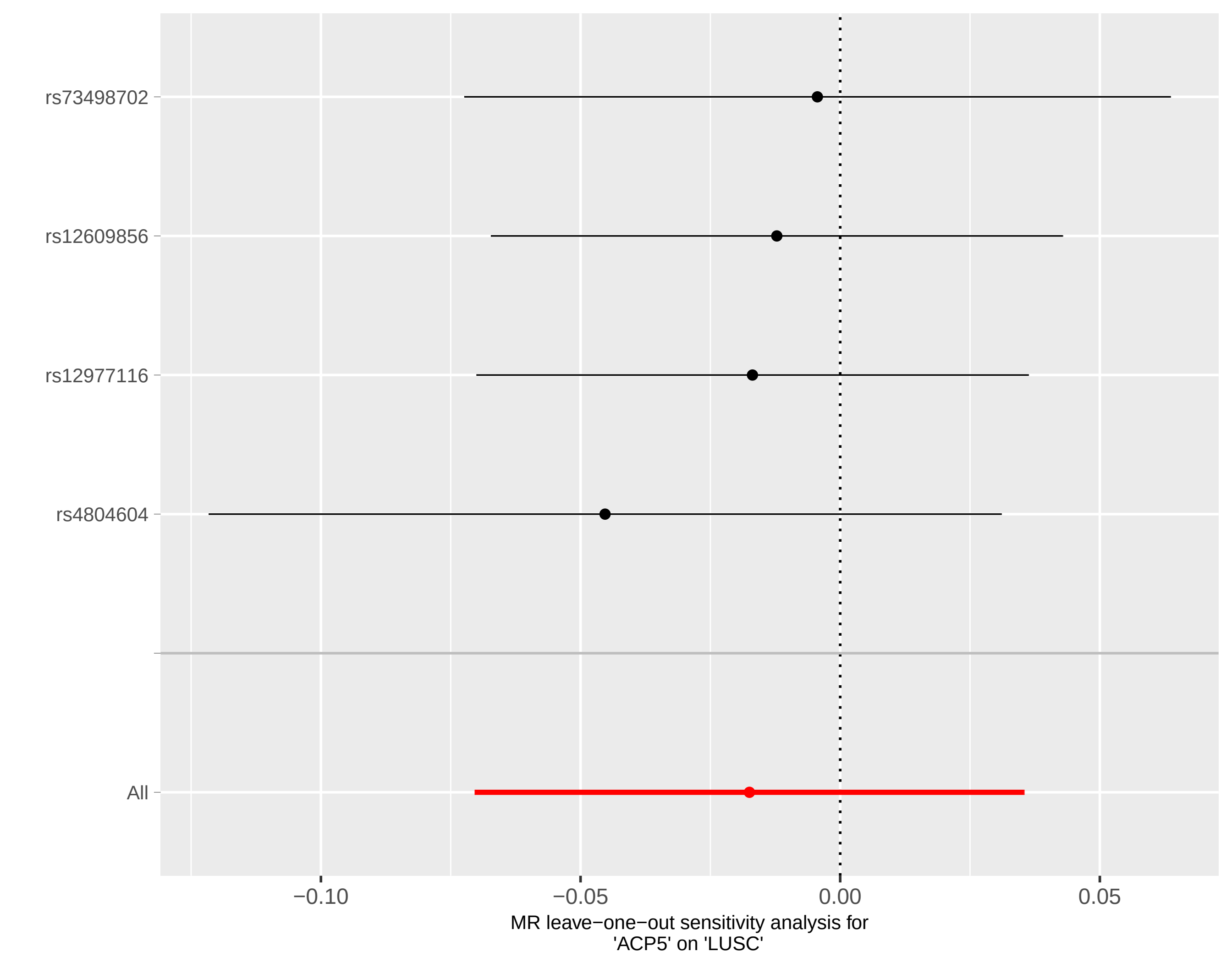

rs73498702
rs12609856
rs12977116
rs4804604
All
−0.10
−0.05
0.00
0.05
MR leave−one−out sensitivity analysis for
'ACP5' on 'LUSC'

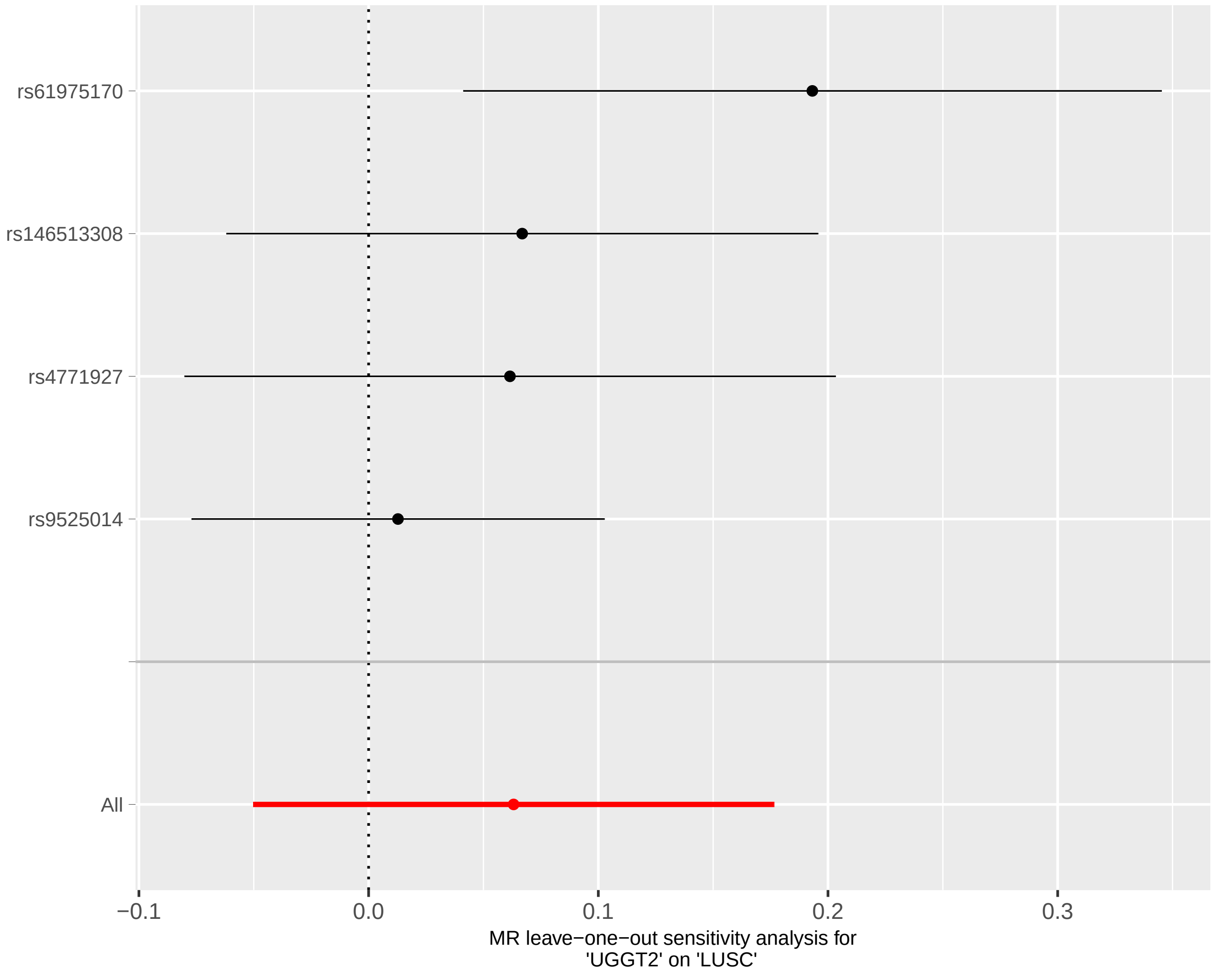

rs61975170
rs146513308
rs4771927
rs9525014
All
−0.1
0.0
0.1
0.2
0.3
MR leave−one−out sensitivity analysis for
'UGGT2' on 'LUSC'

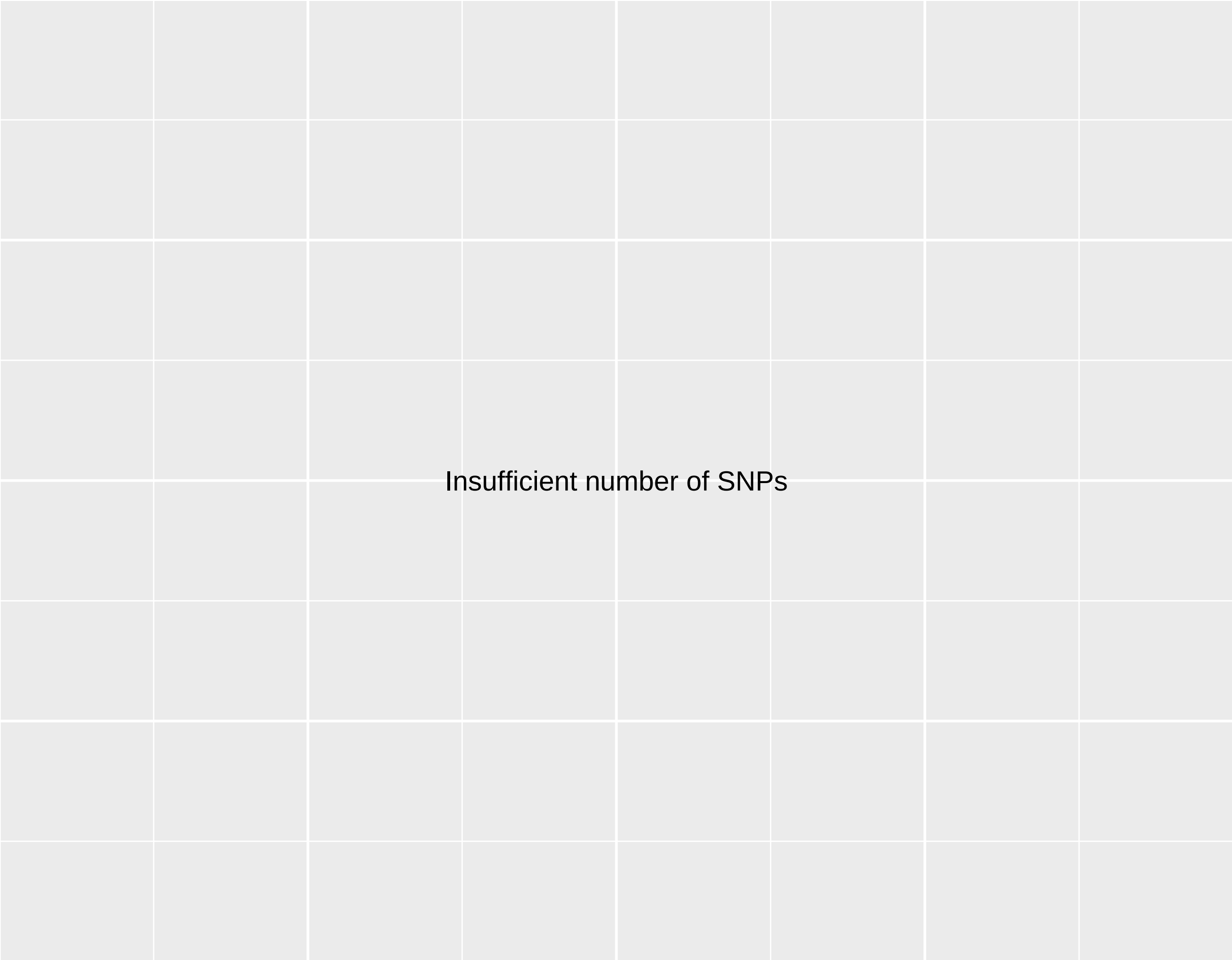
Insufficient number of SNPs

Insufficient number of SNPs

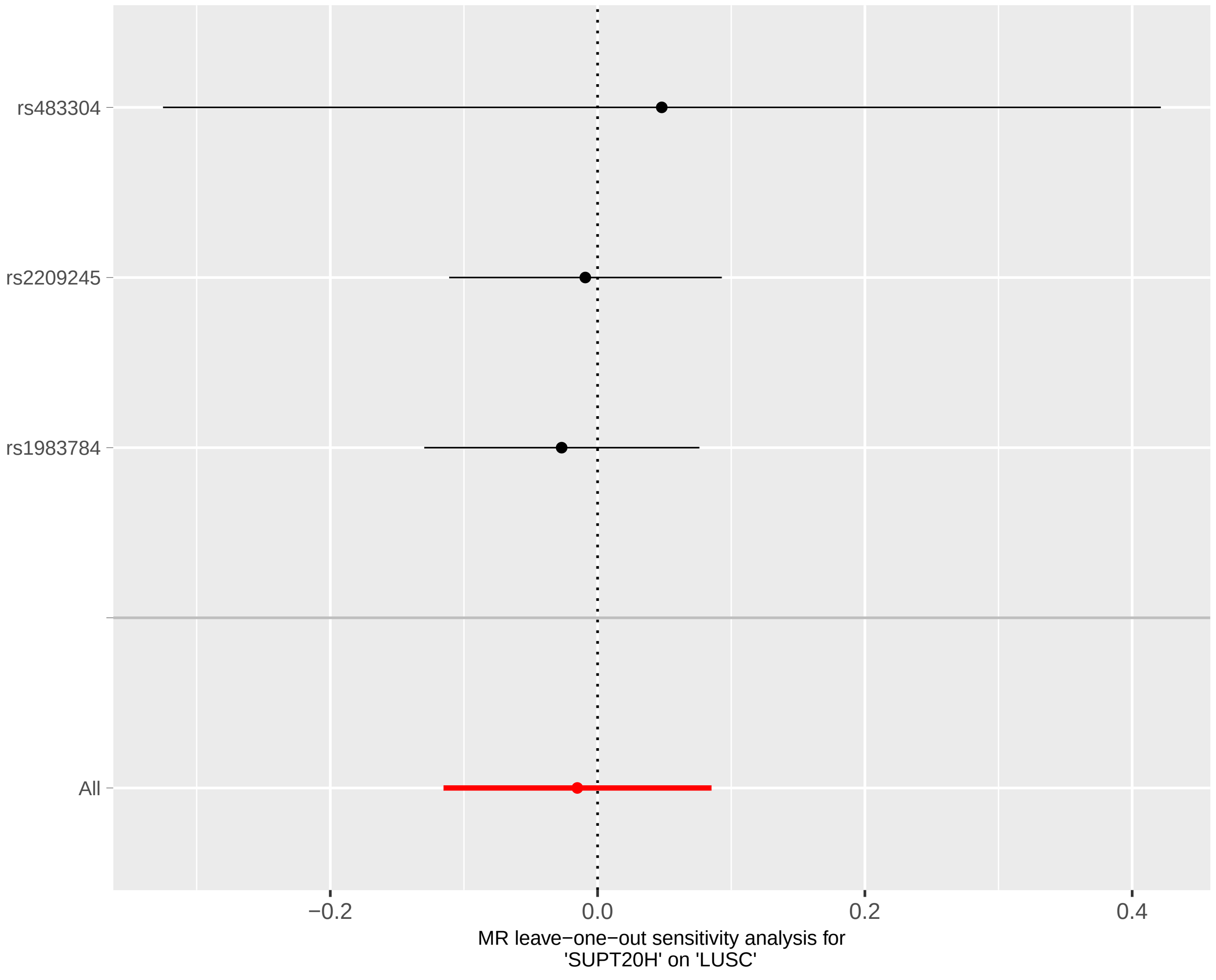
rs483304
rs2209245
rs1983784
All
−0.2
0.0
0.2
0.4
MR leave−one−out sensitivity analysis for
'SUPT20H' on 'LUSC'

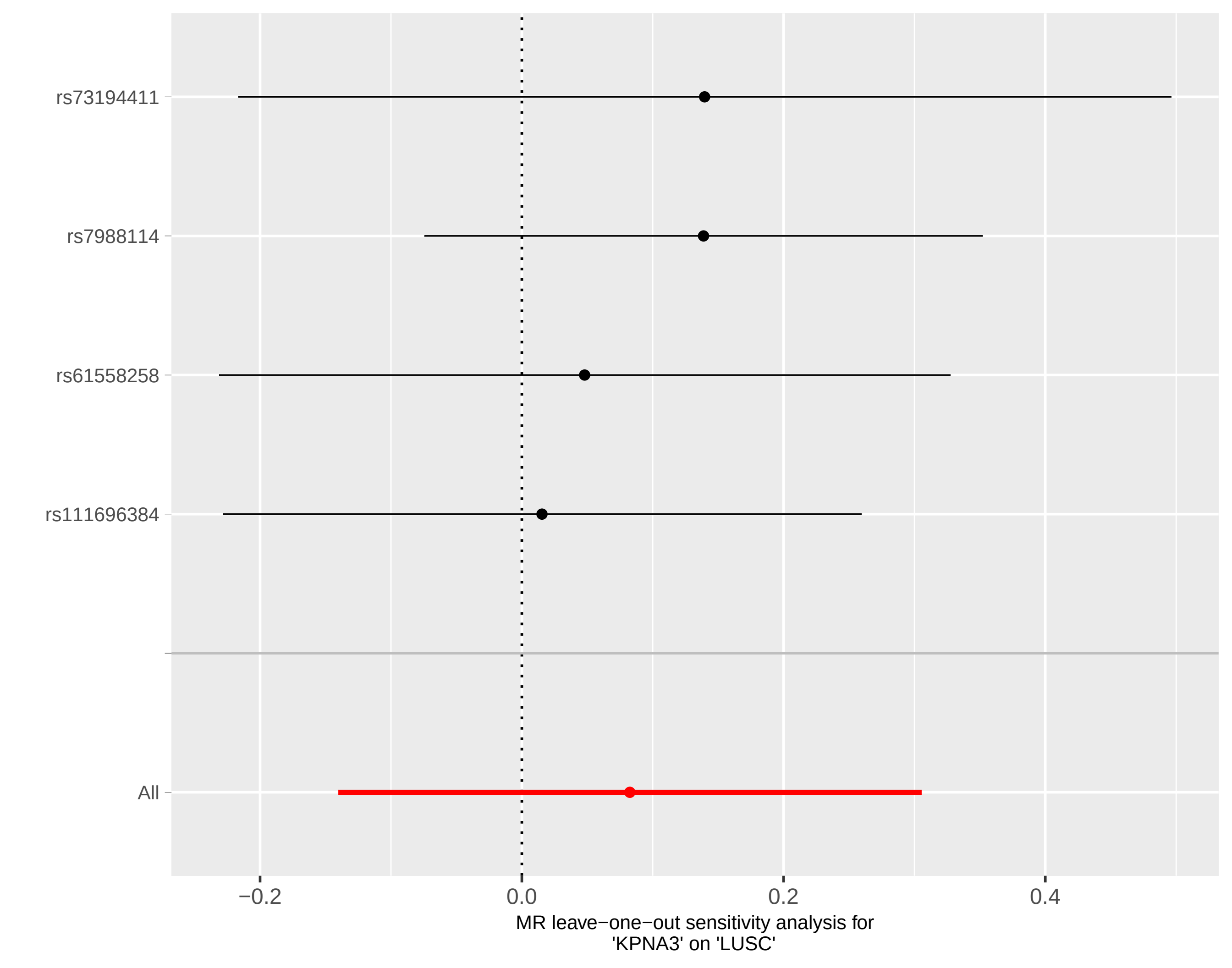
rs73194411
rs7988114
rs61558258
rs111696384
All
−0.2
0.0
0.2
0.4
MR leave−one−out sensitivity analysis for
'KPNA3' on 'LUSC'

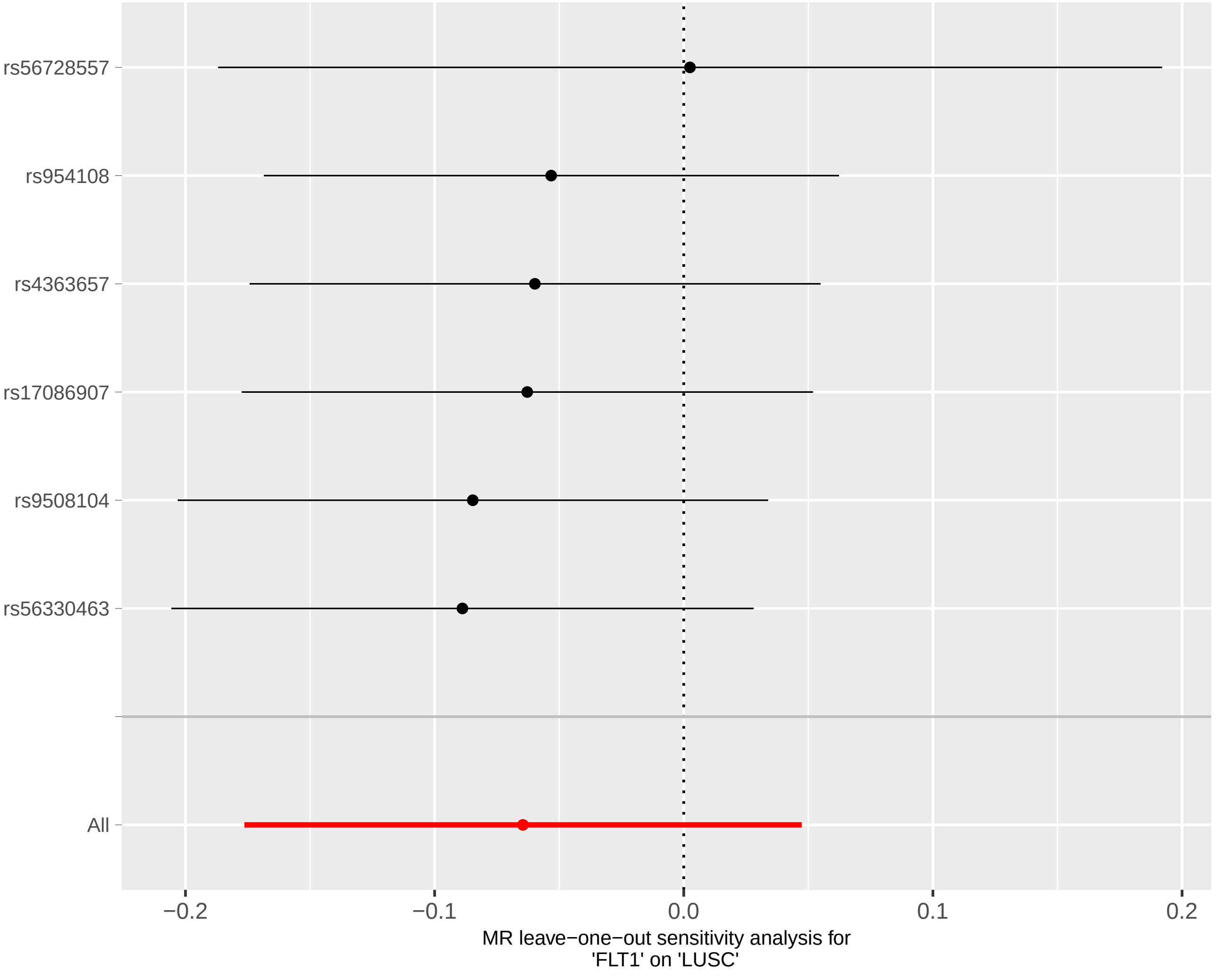
rs56728557
rs954108
rs4363657
rs17086907
rs9508104
rs56330463
All
−0.2
−0.1
0.0
0.1
0.2
MR leave−one−out sensitivity analysis for
'FLT1' on 'LUSC'

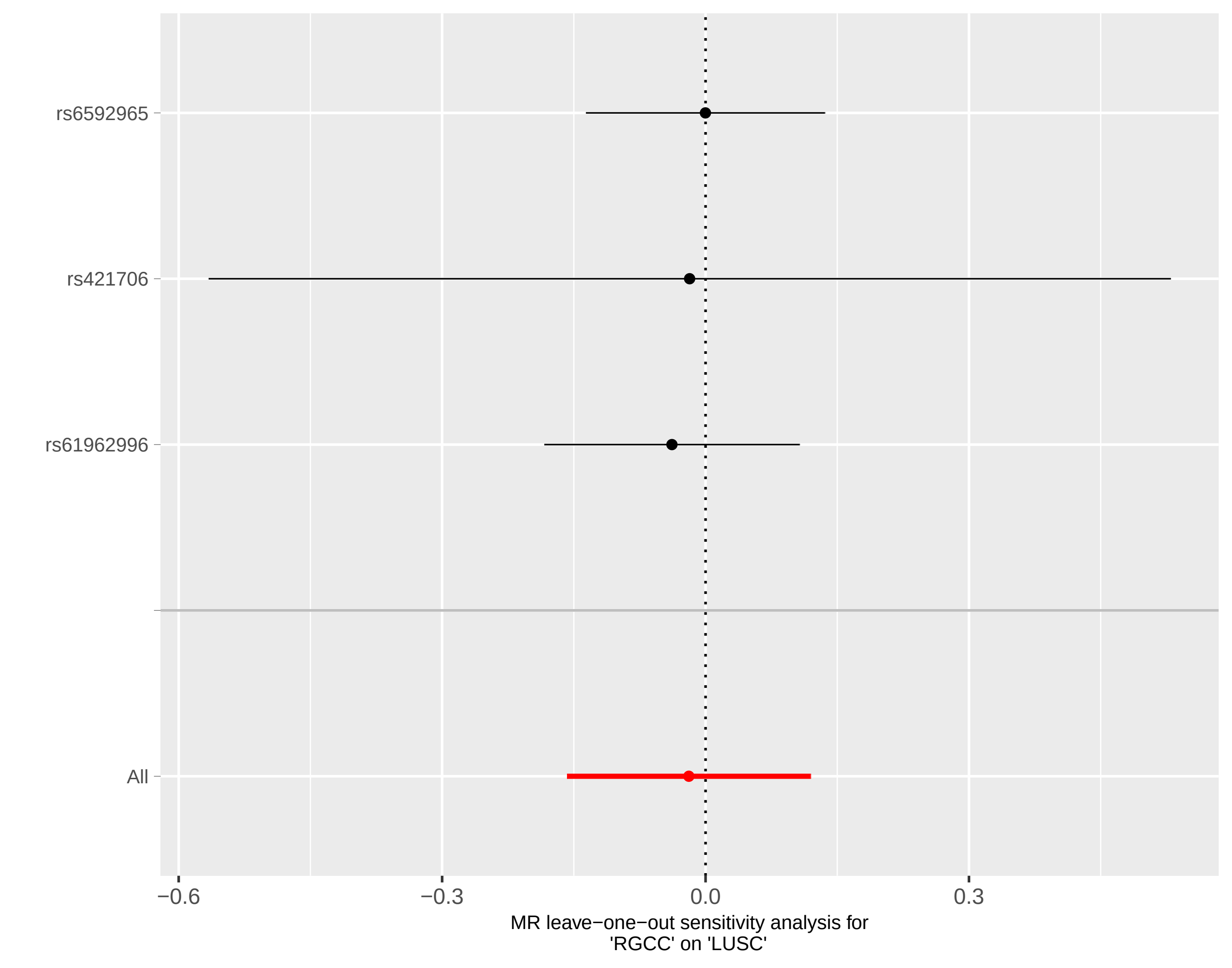
rs6592965
rs421706
rs61962996
All
−0.6
−0.3
0.0
0.3
MR leave−one−out sensitivity analysis for
'RGCC' on 'LUSC'

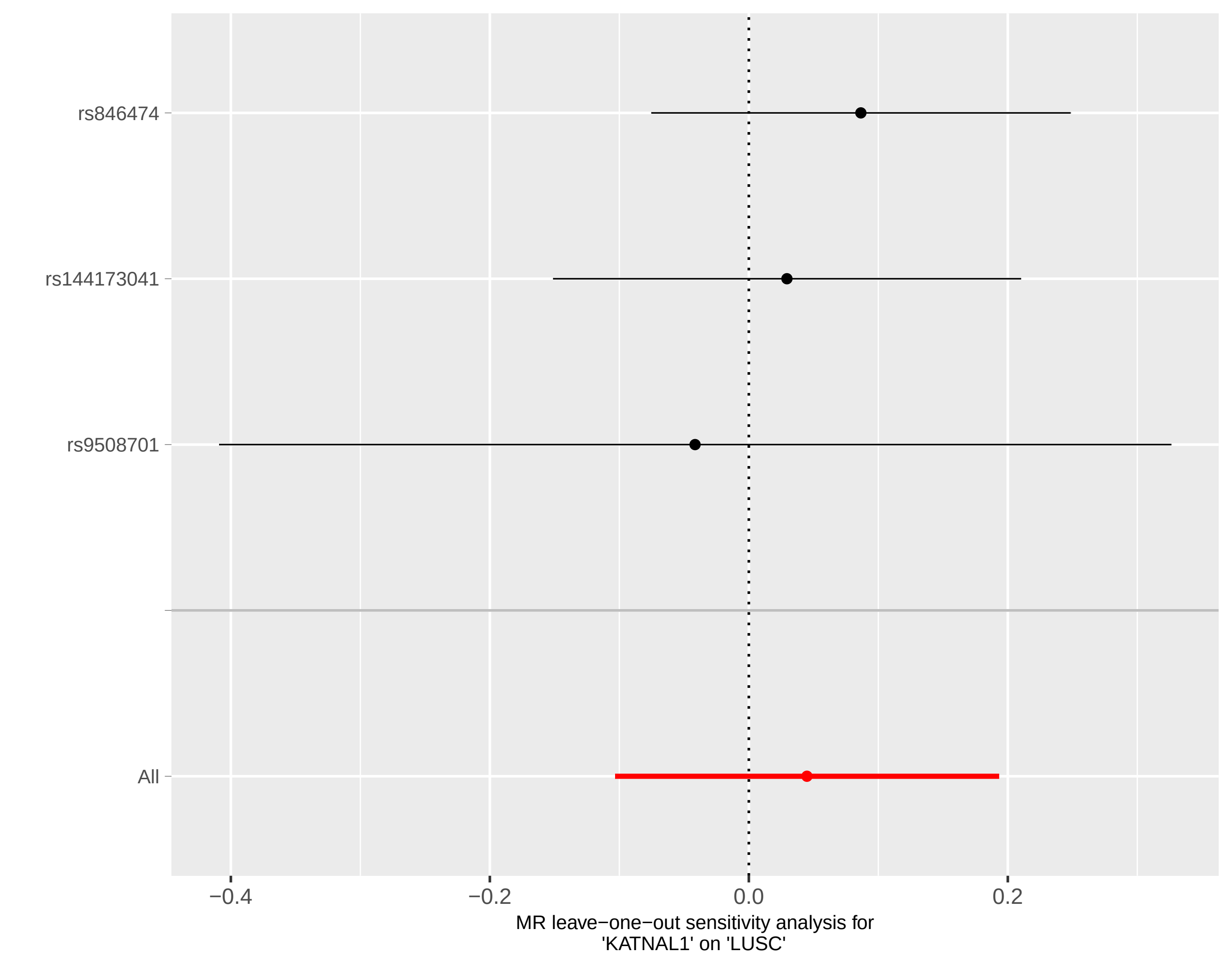
rs846474
rs144173041
rs9508701
All
−0.4
−0.2
0.0
0.2
MR leave−one−out sensitivity analysis for
'KATNAL1' on 'LUSC'

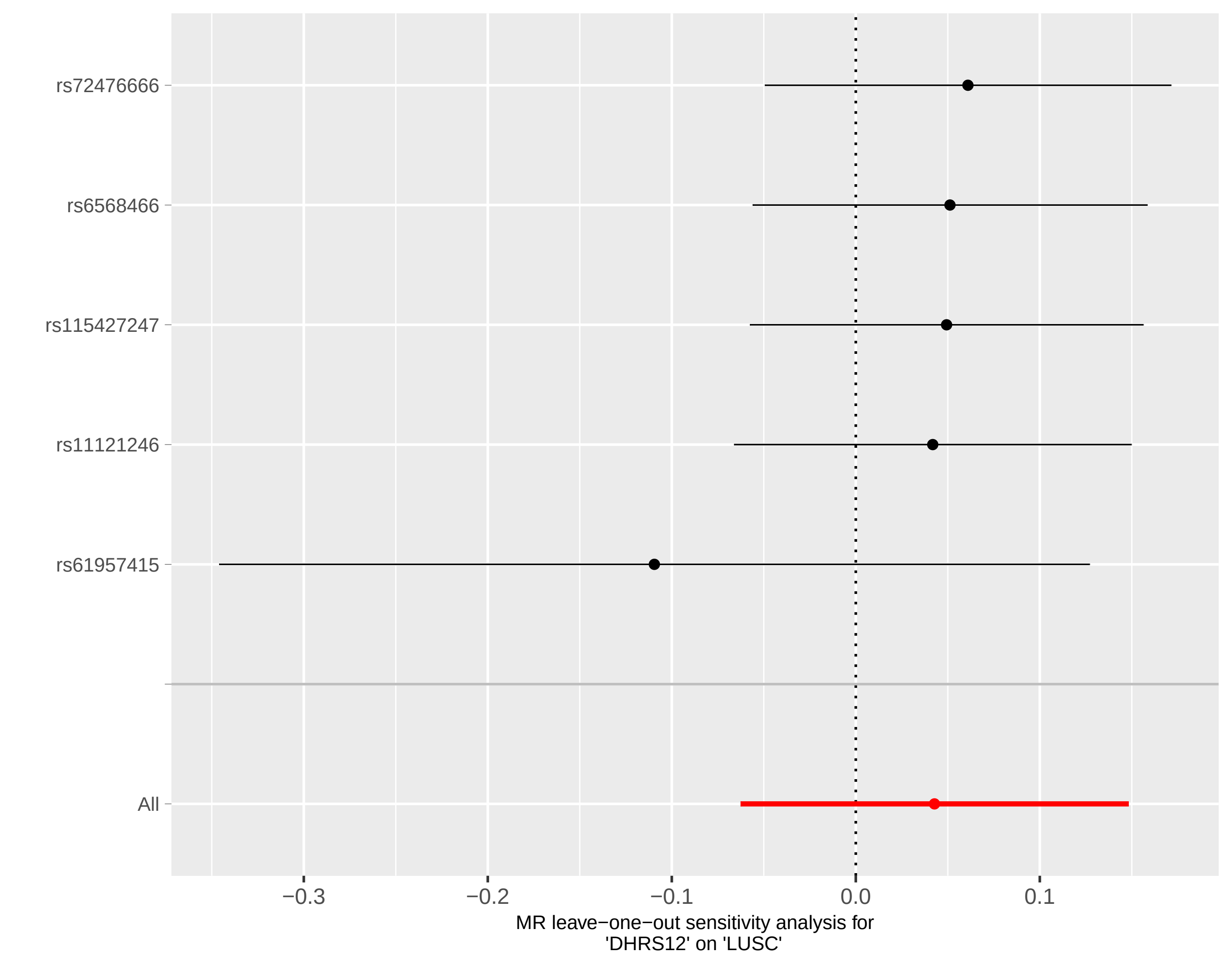
rs72476666
rs6568466
rs115427247
rs11121246
rs61957415
All
−0.3
−0.2
−0.1
0.0
0.1
MR leave−one−out sensitivity analysis for
'DHRS12' on 'LUSC'

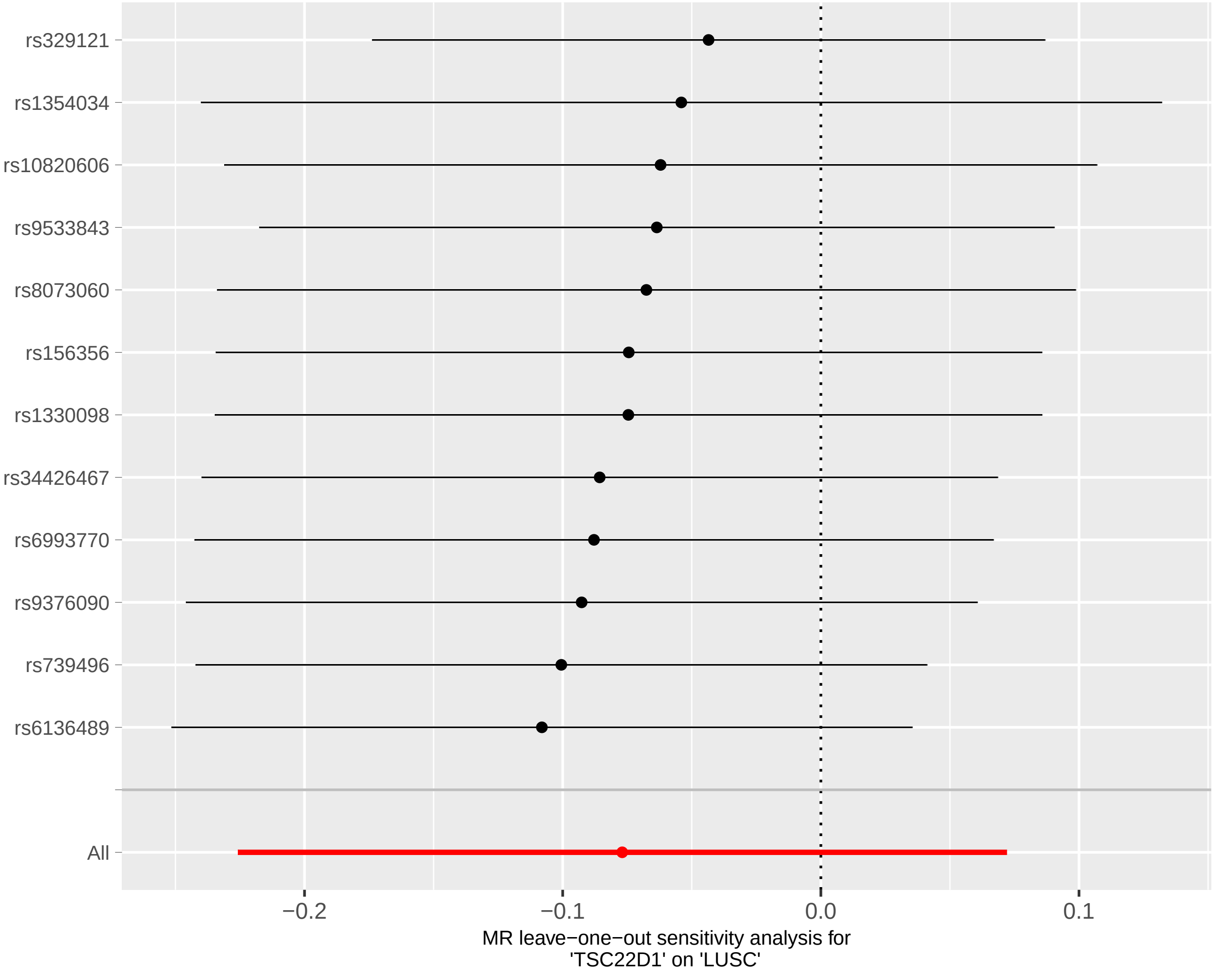
rs329121
rs1354034
rs10820606
rs9533843
rs8073060
rs156356
rs1330098
rs34426467
rs6993770
rs9376090
rs739496
rs6136489
All
−0.2
−0.1
0.0
0.1
MR leave−one−out sensitivity analysis for
'TSC22D1' on 'LUSC'

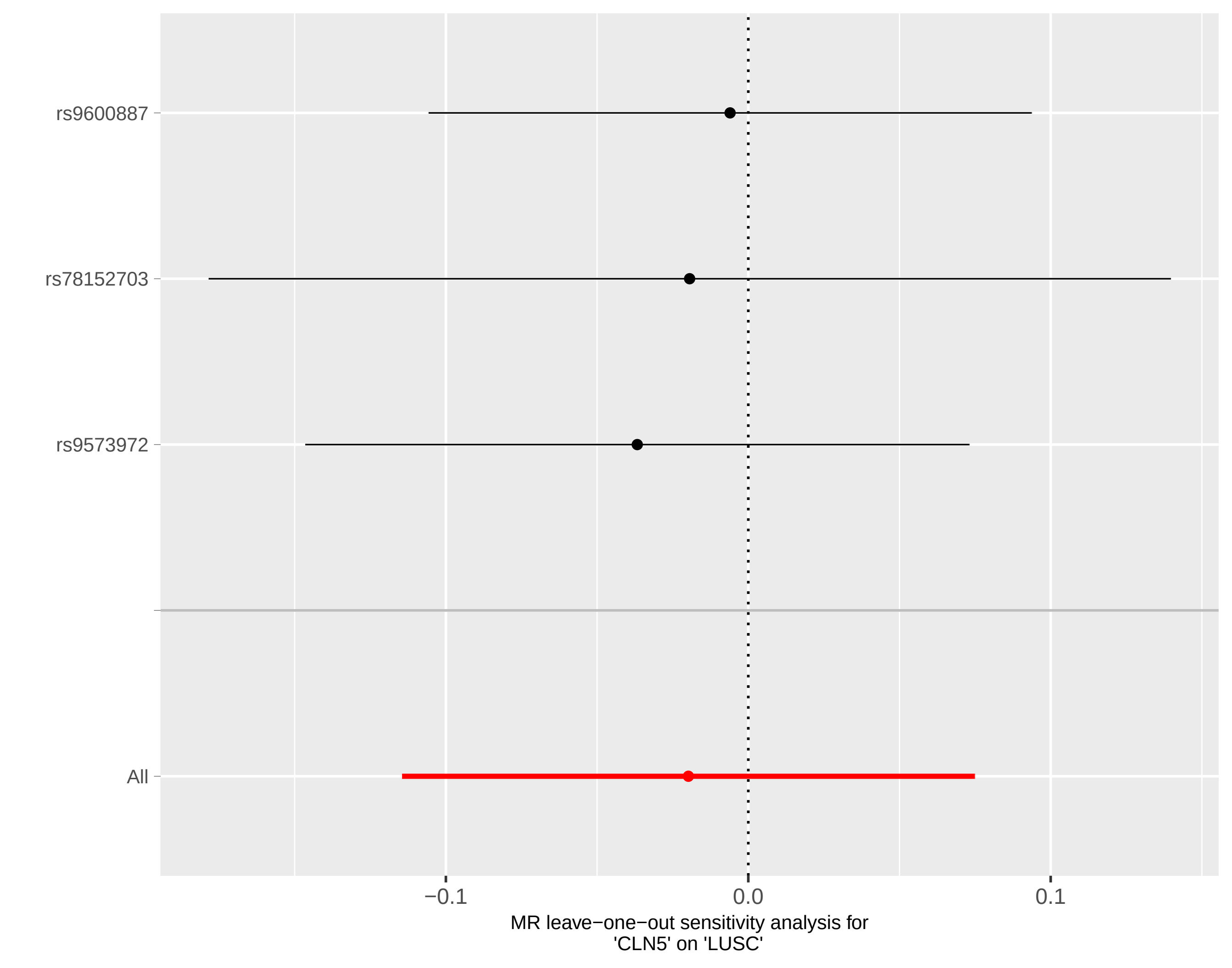
rs9600887
rs78152703
rs9573972
All
−0.1
0.0
0.1
MR leave−one−out sensitivity analysis for
'CLN5' on 'LUSC'

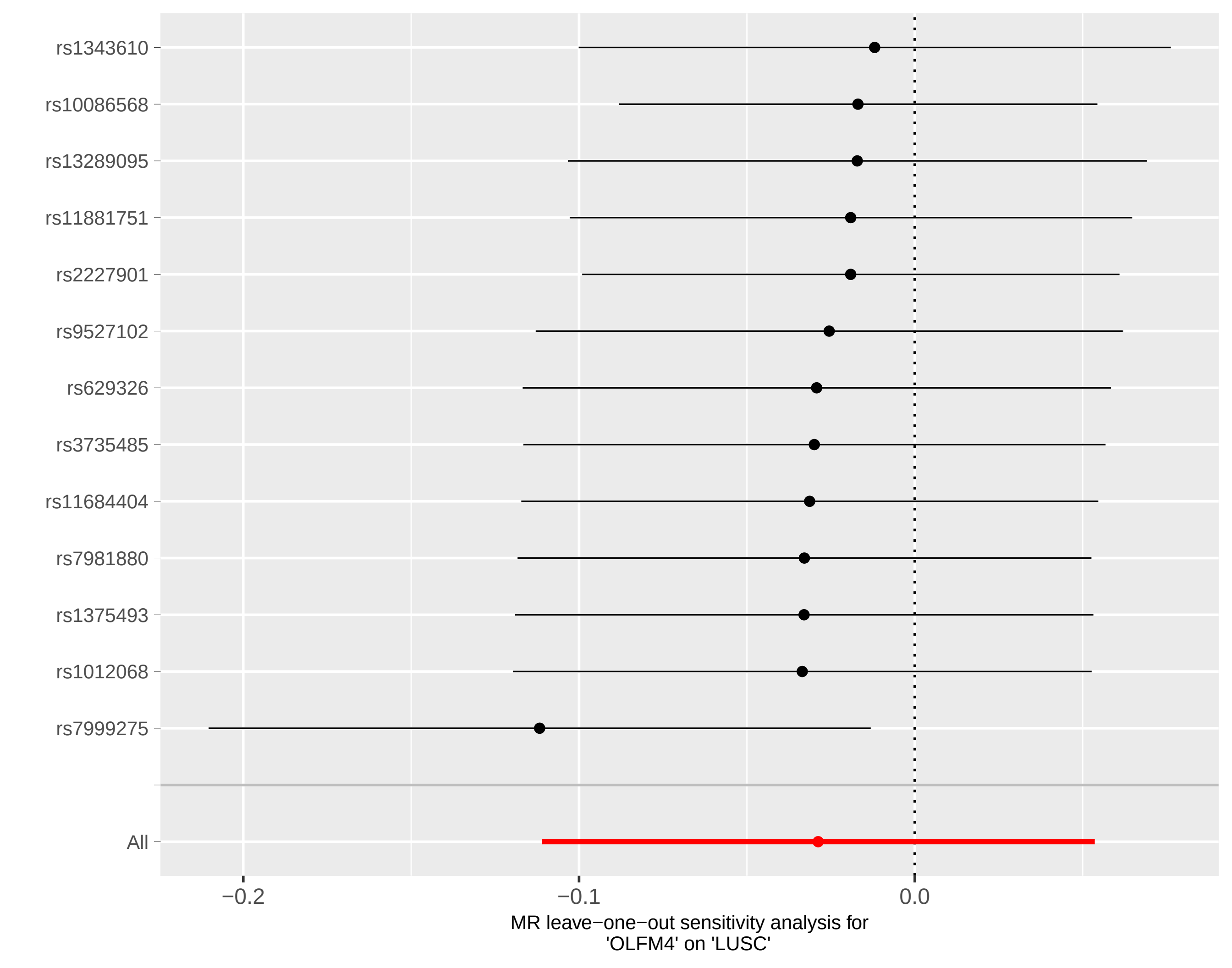

rs1343610
rs10086568
rs13289095
rs11881751
rs2227901
rs9527102
rs629326
rs3735485
rs11684404
rs7981880
rs1375493
rs1012068
rs7999275
All
−0.2
−0.1
0.0
MR leave−one−out sensitivity analysis for
'OLFM4' on 'LUSC'

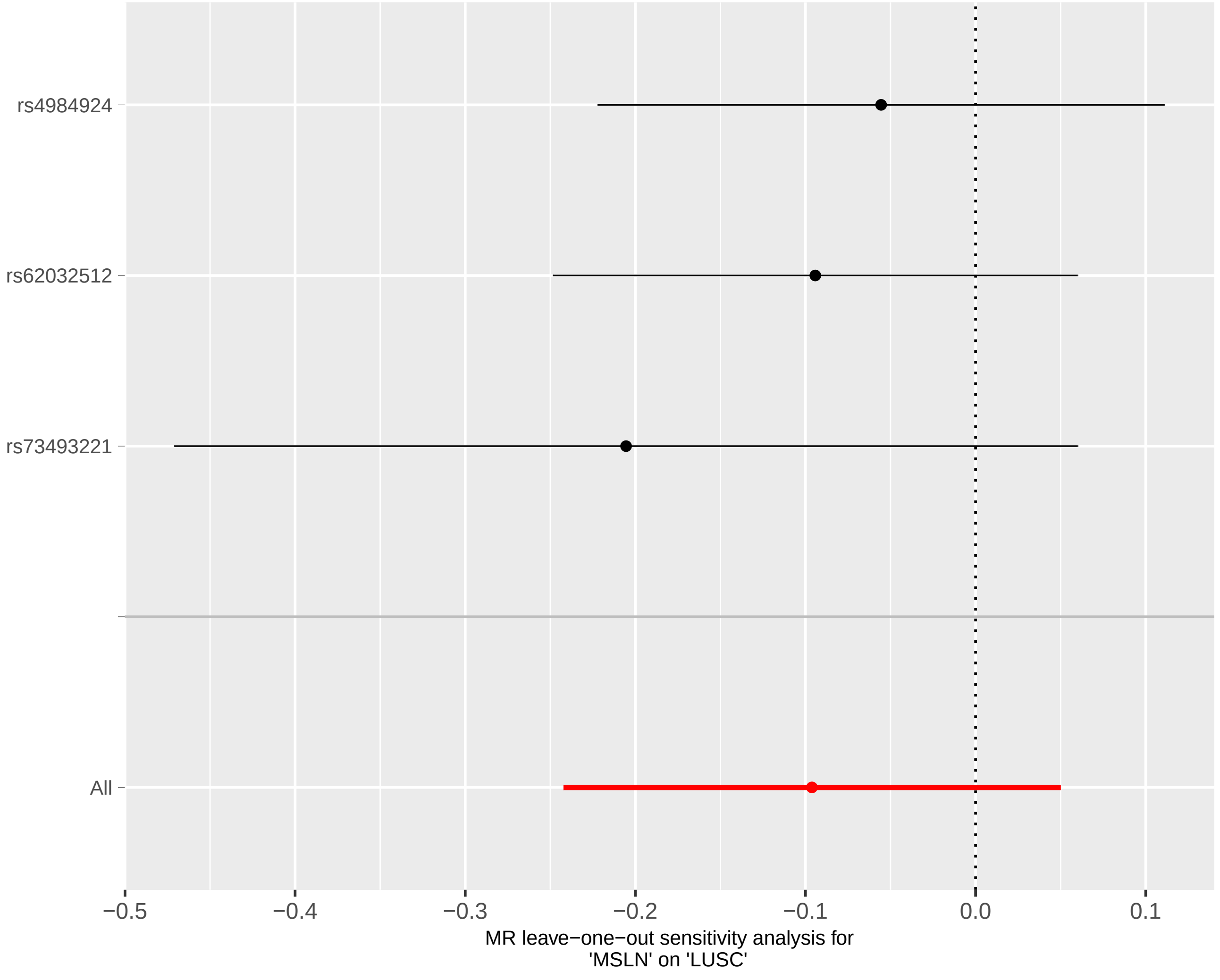
rs4984924
rs62032512
rs73493221
All
−0.5
−0.4
−0.3
−0.2
−0.1
0.0
0.1
MR leave−one−out sensitivity analysis for
'MSLN' on 'LUSC'

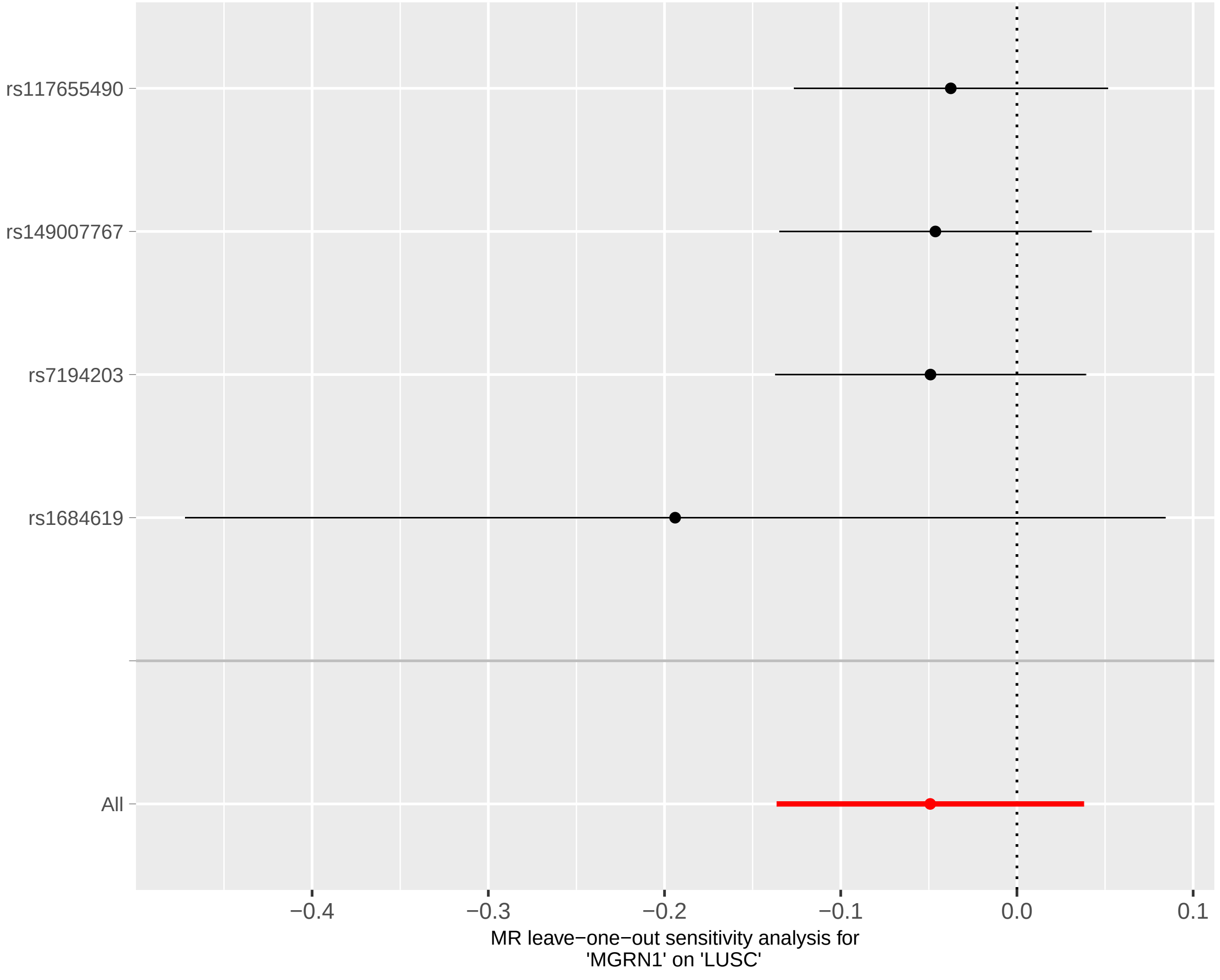
rs117655490
rs149007767
rs7194203
rs1684619
All
−0.4
−0.3
−0.2
−0.1
0.0
0.1
MR leave−one−out sensitivity analysis for
'MGRN1' on 'LUSC'

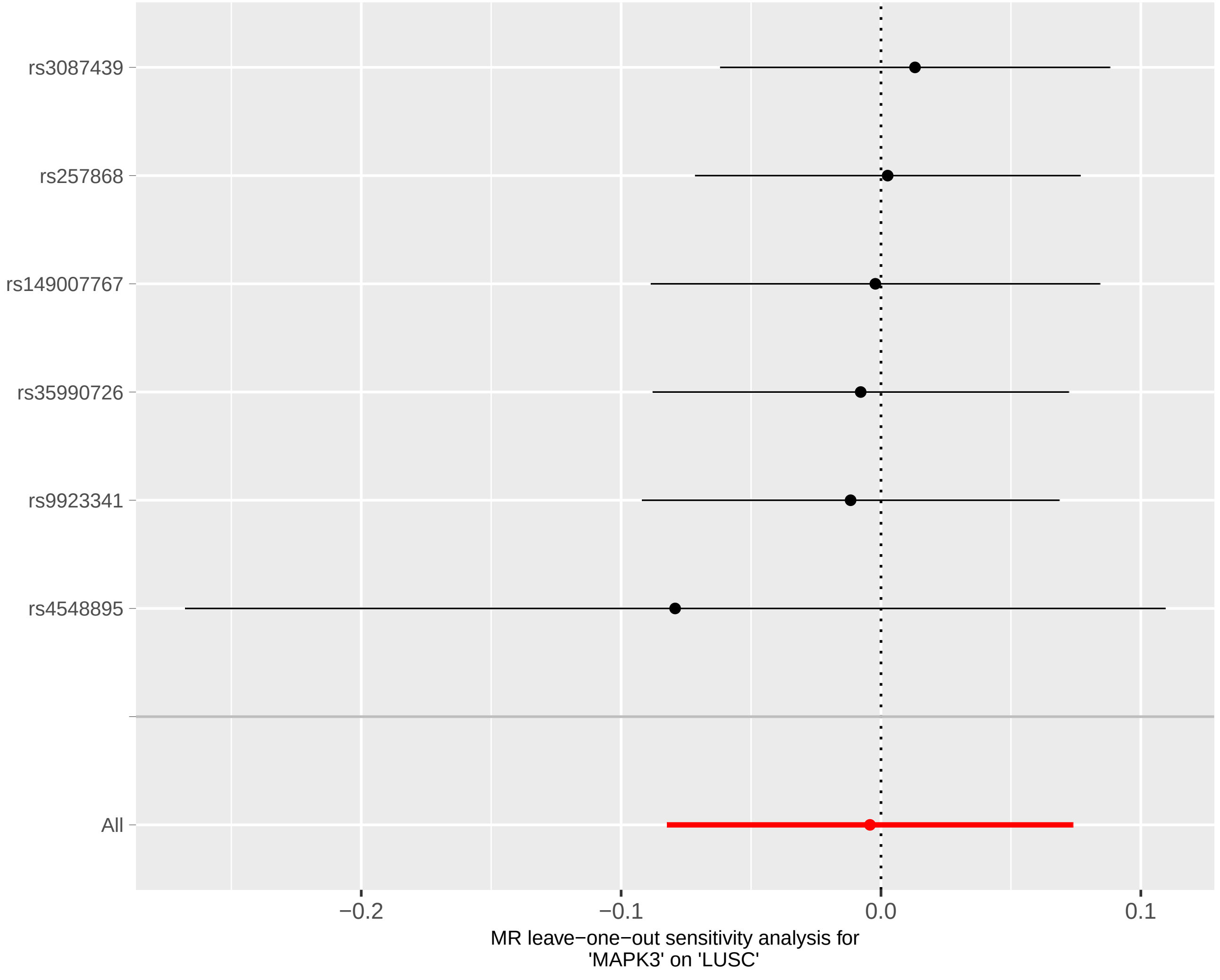

rs3087439
rs257868
rs149007767
rs35990726
rs9923341
rs4548895
All
−0.2
−0.1
0.0
0.1
MR leave−one−out sensitivity analysis for
'MAPK3' on 'LUSC'

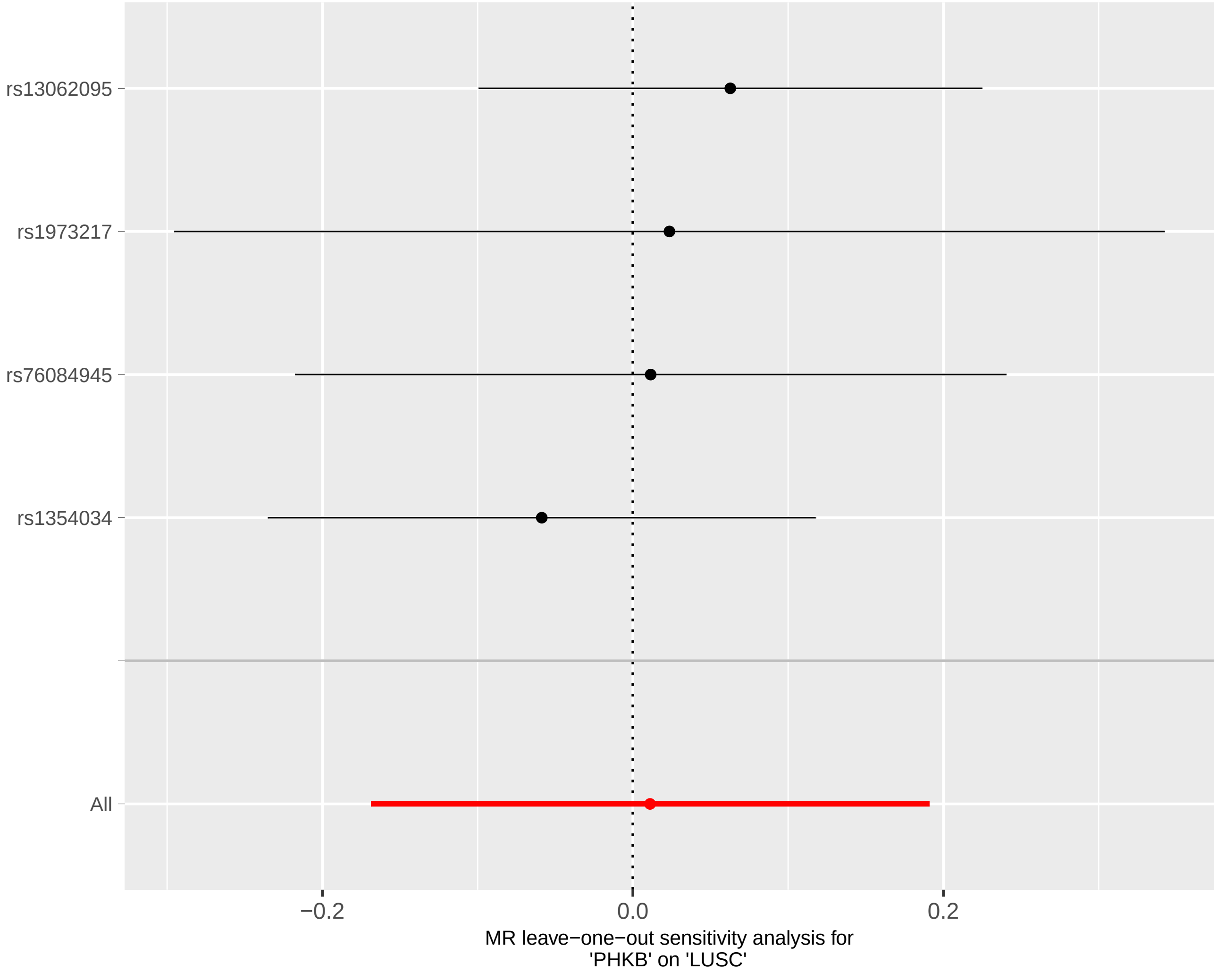
rs13062095
rs1973217
rs76084945
rs1354034
All
−0.2
0.0
0.2
MR leave−one−out sensitivity analysis for
'PHKB' on 'LUSC'

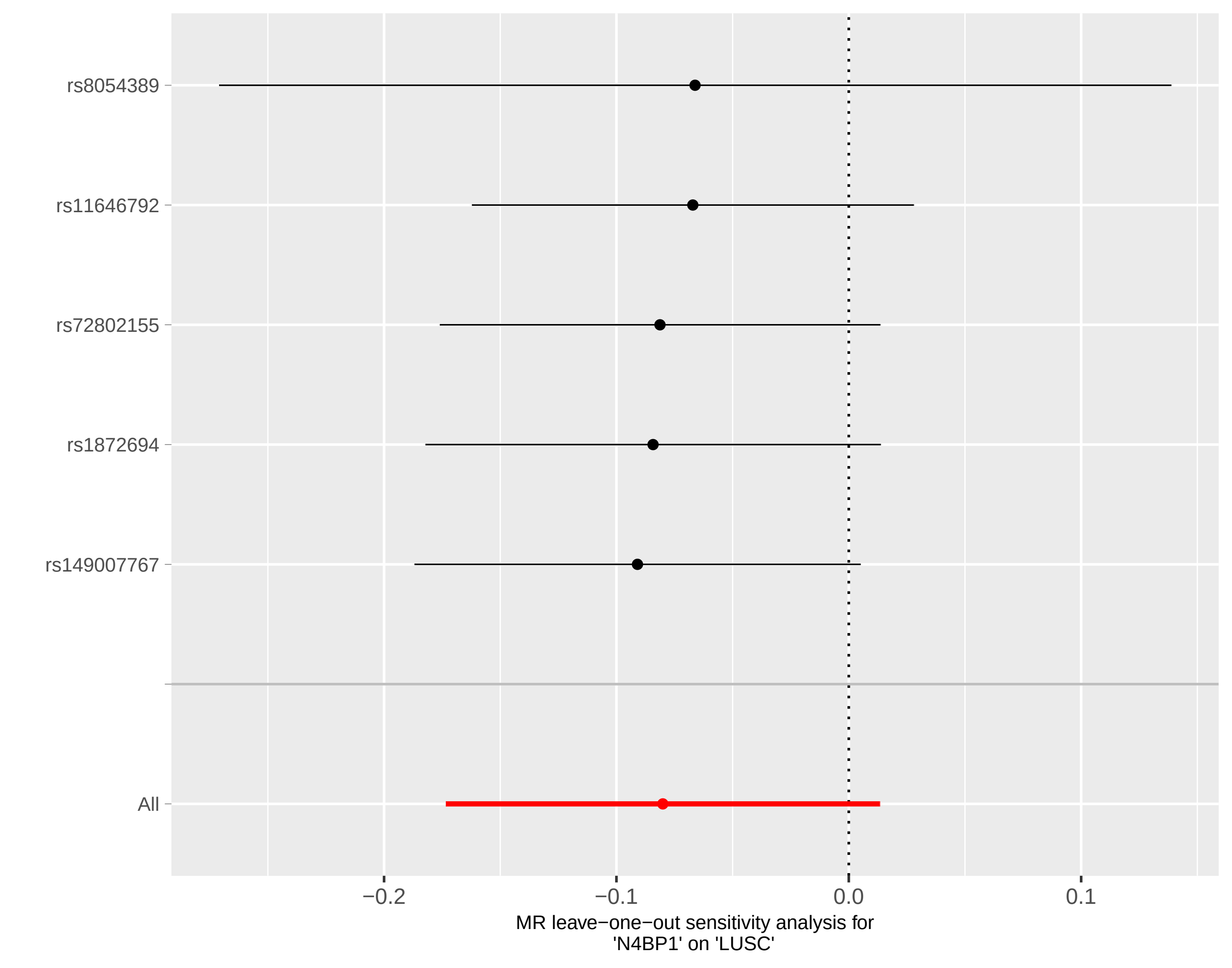

rs8054389
rs11646792
rs72802155
rs1872694
rs149007767
All
−0.2
−0.1
0.0
0.1
MR leave−one−out sensitivity analysis for
'N4BP1' on 'LUSC'

Insufficient number of SNPs

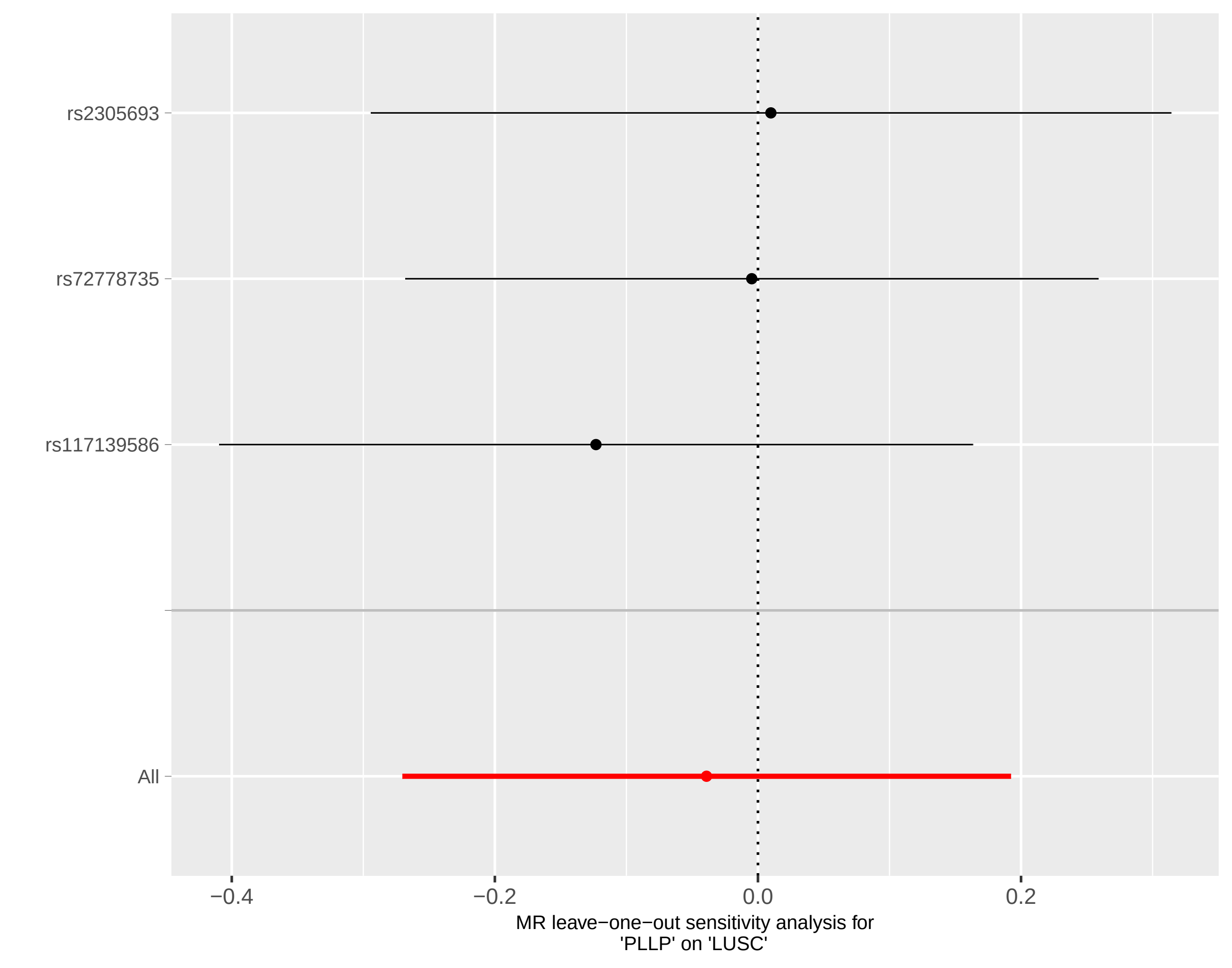
rs2305693
rs72778735
rs117139586
All
−0.4
−0.2
0.0
0.2
MR leave−one−out sensitivity analysis for
'PLLP' on 'LUSC'

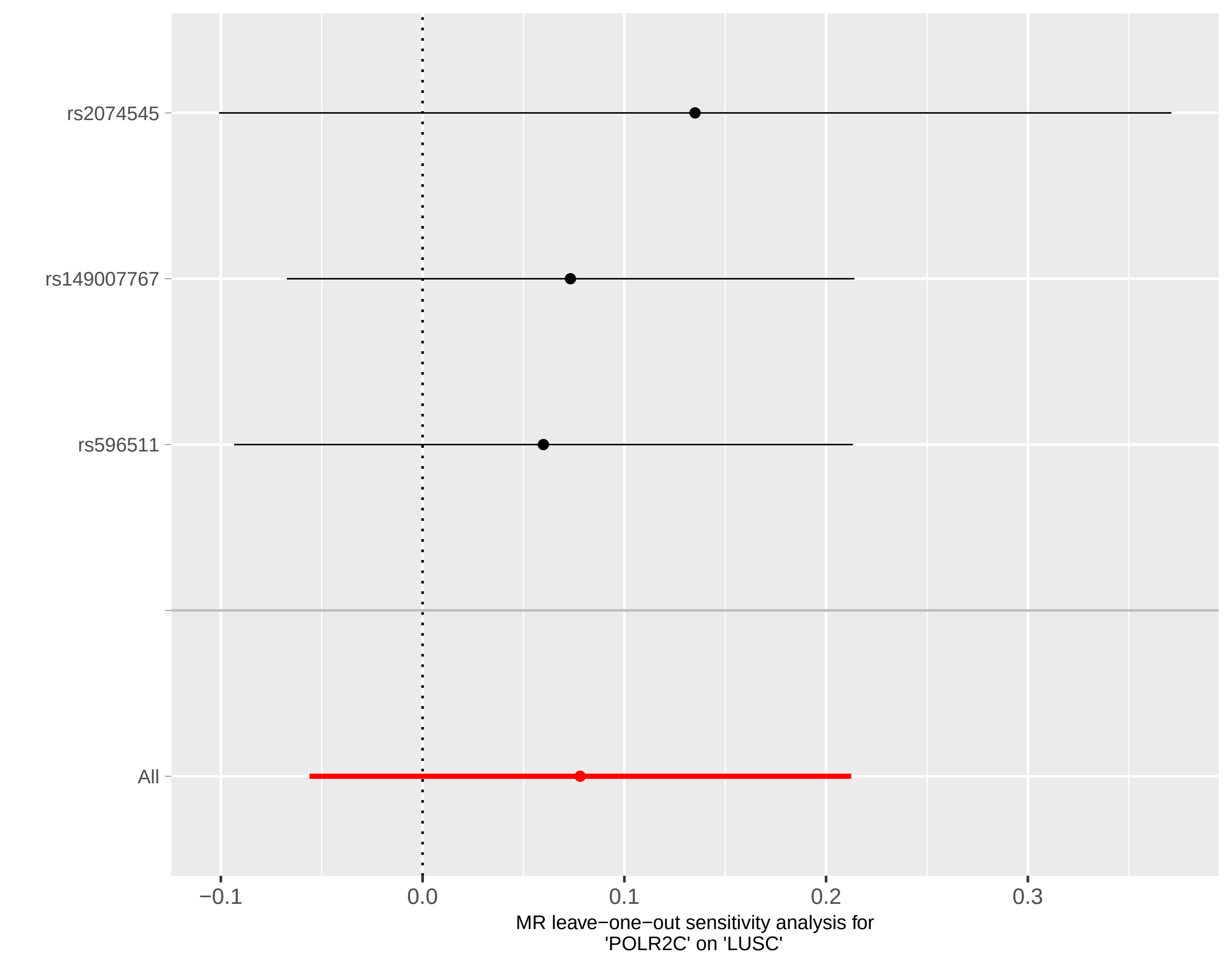
rs2074545
rs149007767
rs596511
All
−0.1
0.0
0.1
0.2
0.3
MR leave−one−out sensitivity analysis for
'POLR2C' on 'LUSC'

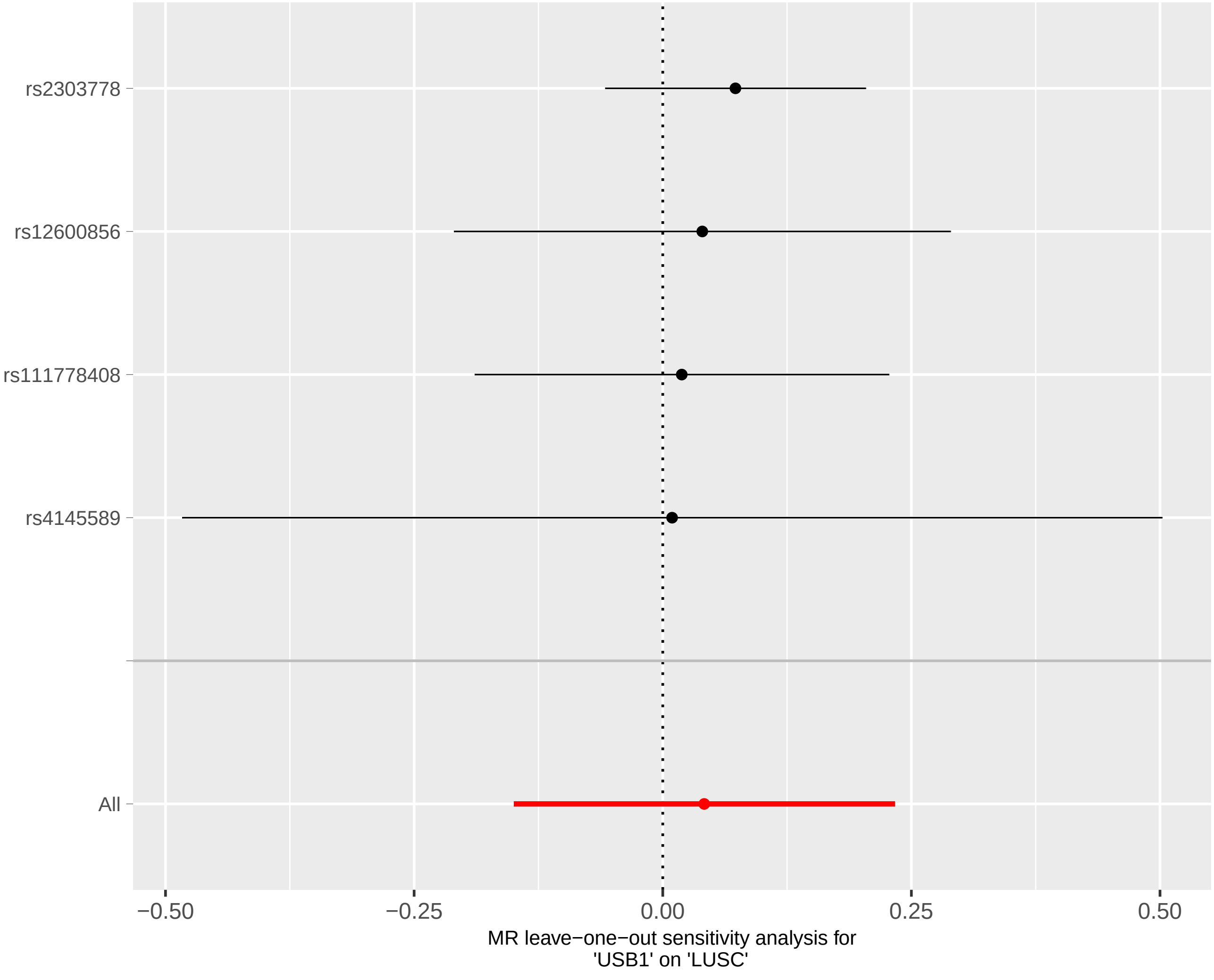
rs2303778
rs12600856
rs111778408
rs4145589
All
−0.50
−0.25
0.00
0.25
0.50
MR leave−one−out sensitivity analysis for
'USB1' on 'LUSC'

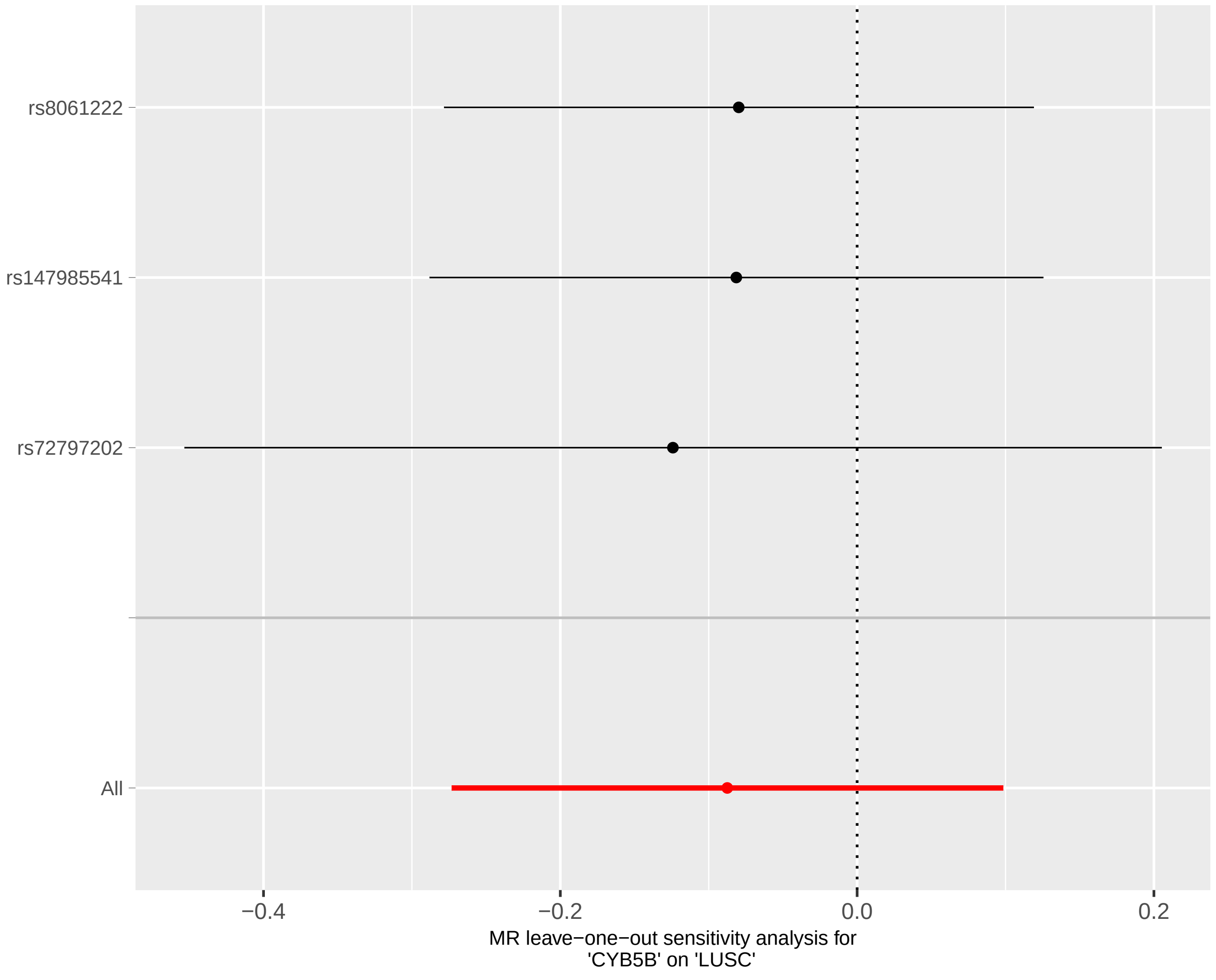
rs8061222
rs147985541
rs72797202
All
−0.4
−0.2
0.0
0.2
MR leave−one−out sensitivity analysis for
'CYB5B' on 'LUSC'

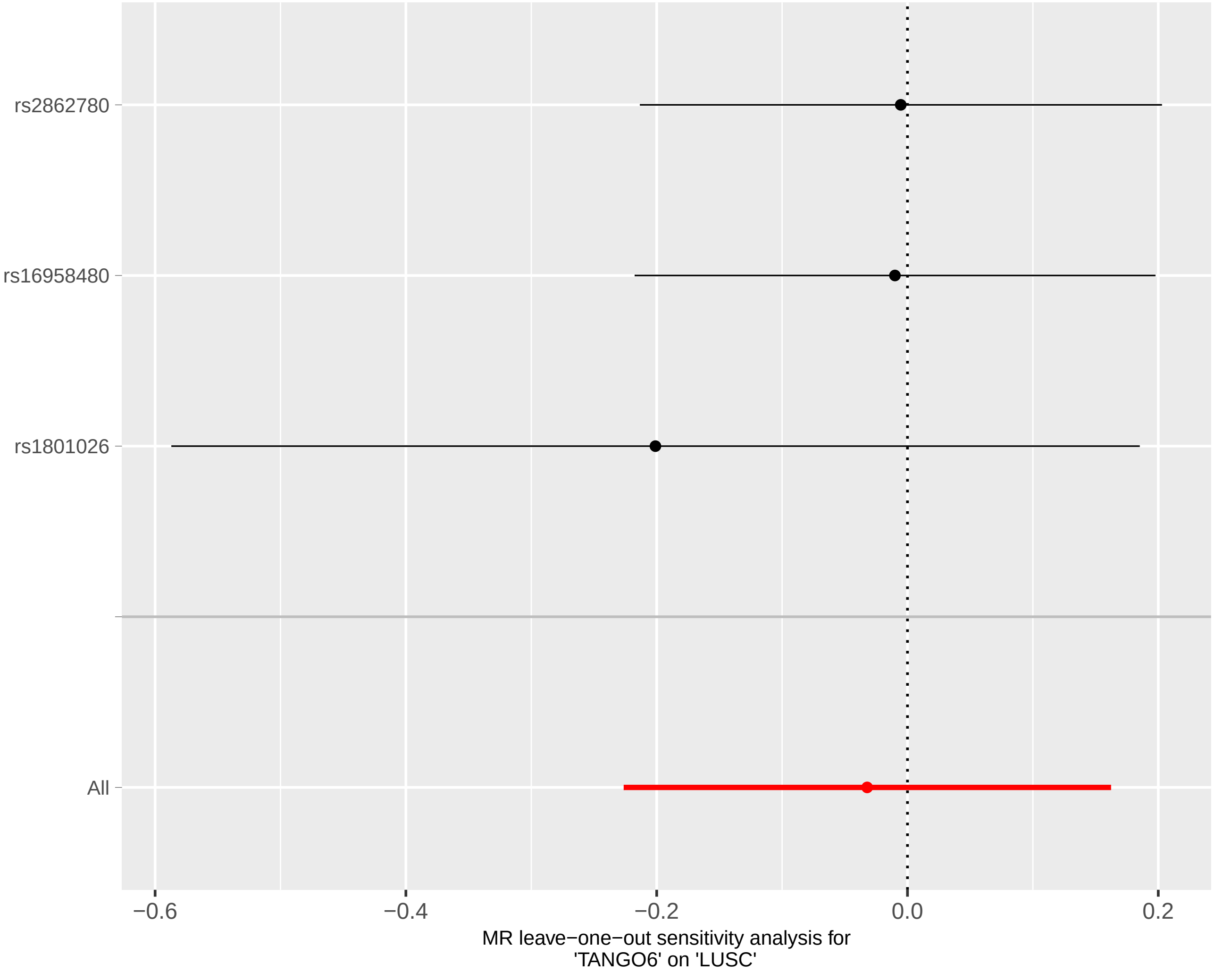

rs2862780
rs16958480
rs1801026
All
−0.6
−0.4
−0.2
0.0
0.2
MR leave−one−out sensitivity analysis for
'TANGO6' on 'LUSC'

Insufficient number of SNPs

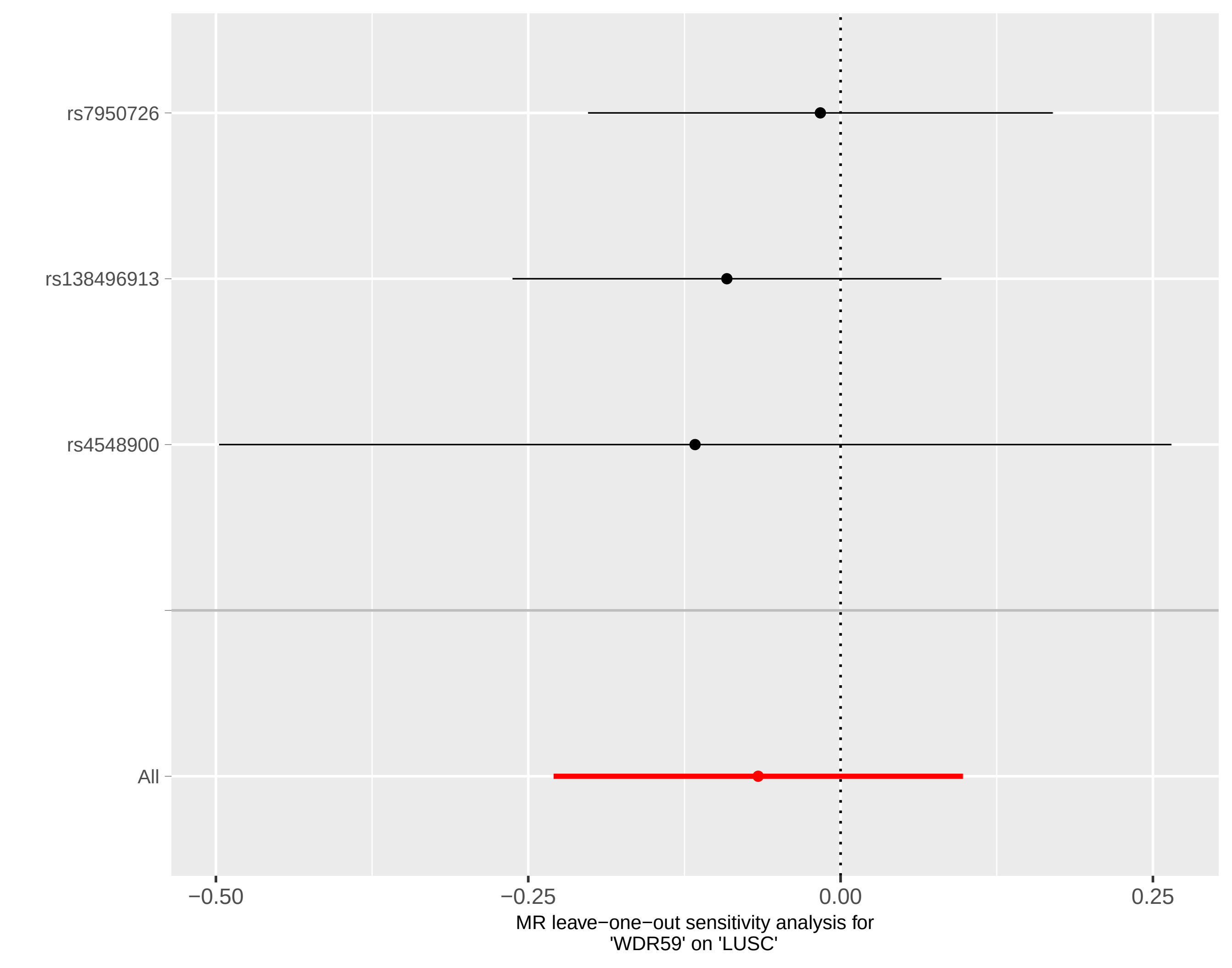
rs7950726
rs138496913
rs4548900
All
−0.50
−0.25
0.00
0.25
MR leave−one−out sensitivity analysis for
'WDR59' on 'LUSC'

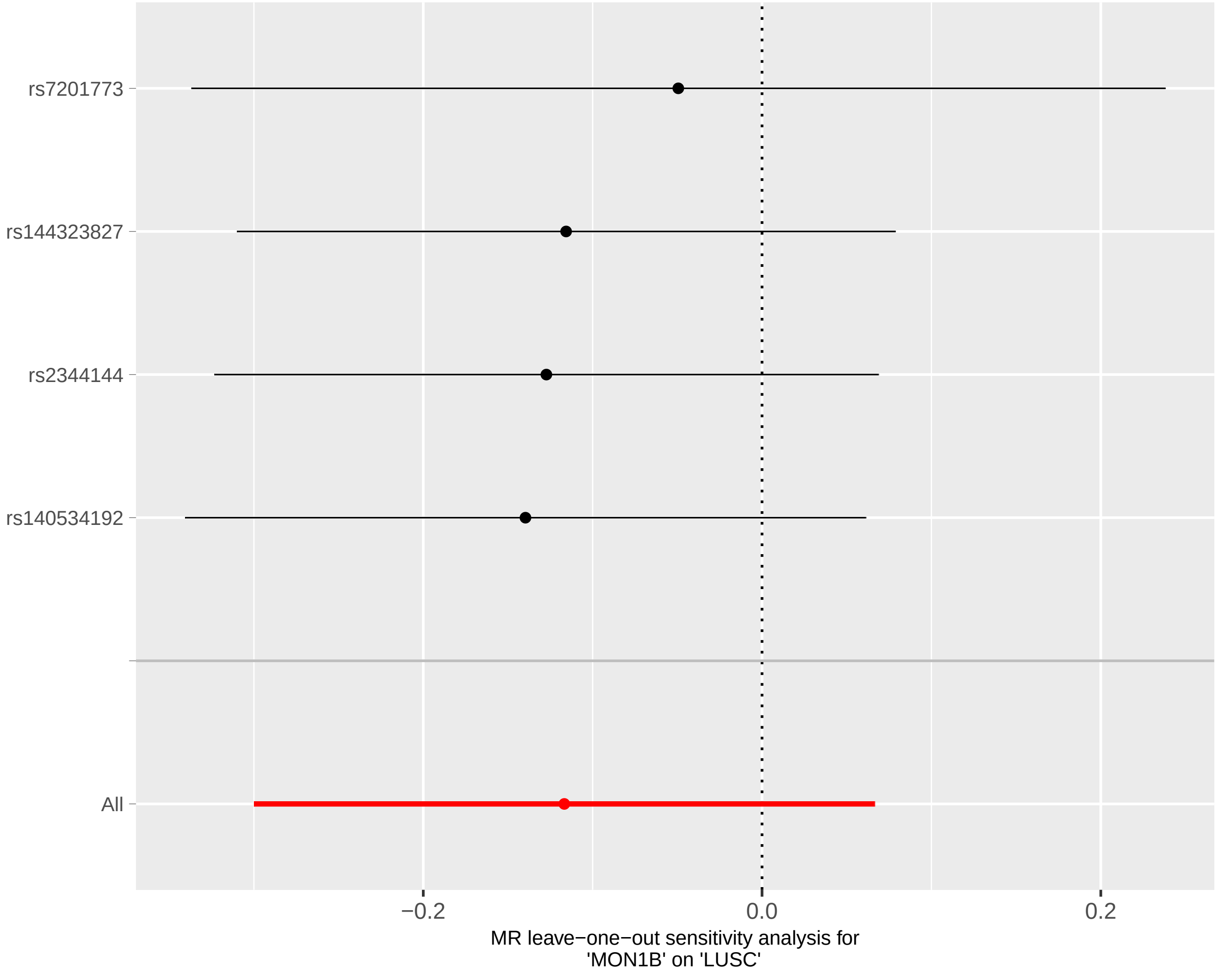
rs7201773
rs144323827
rs2344144
rs140534192
All
−0.2
0.0
0.2
MR leave−one−out sensitivity analysis for
'MON1B' on 'LUSC'

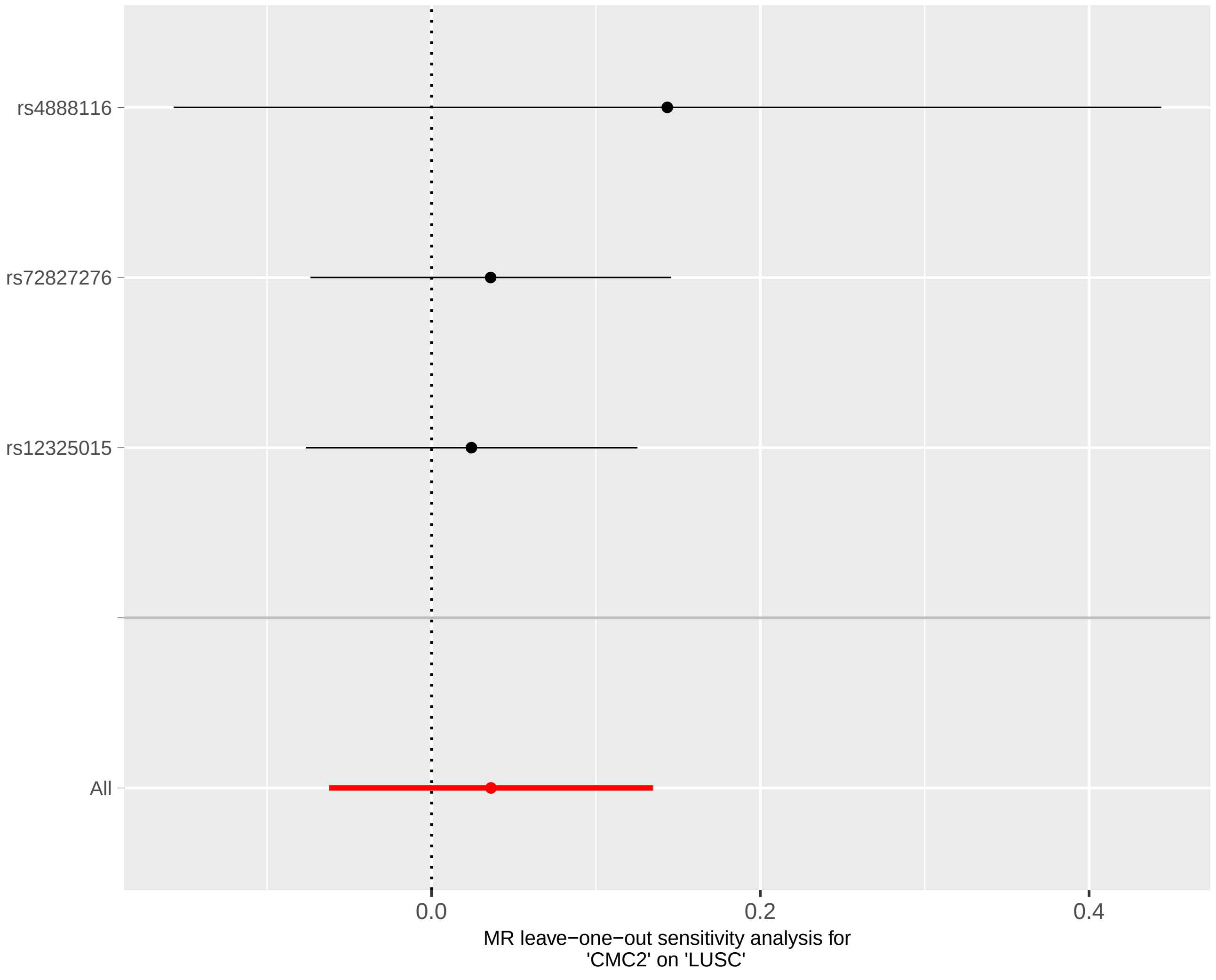
rs4888116
rs72827276
rs12325015
All
0.0
0.2
0.4
MR leave−one−out sensitivity analysis for
'CMC2' on 'LUSC'

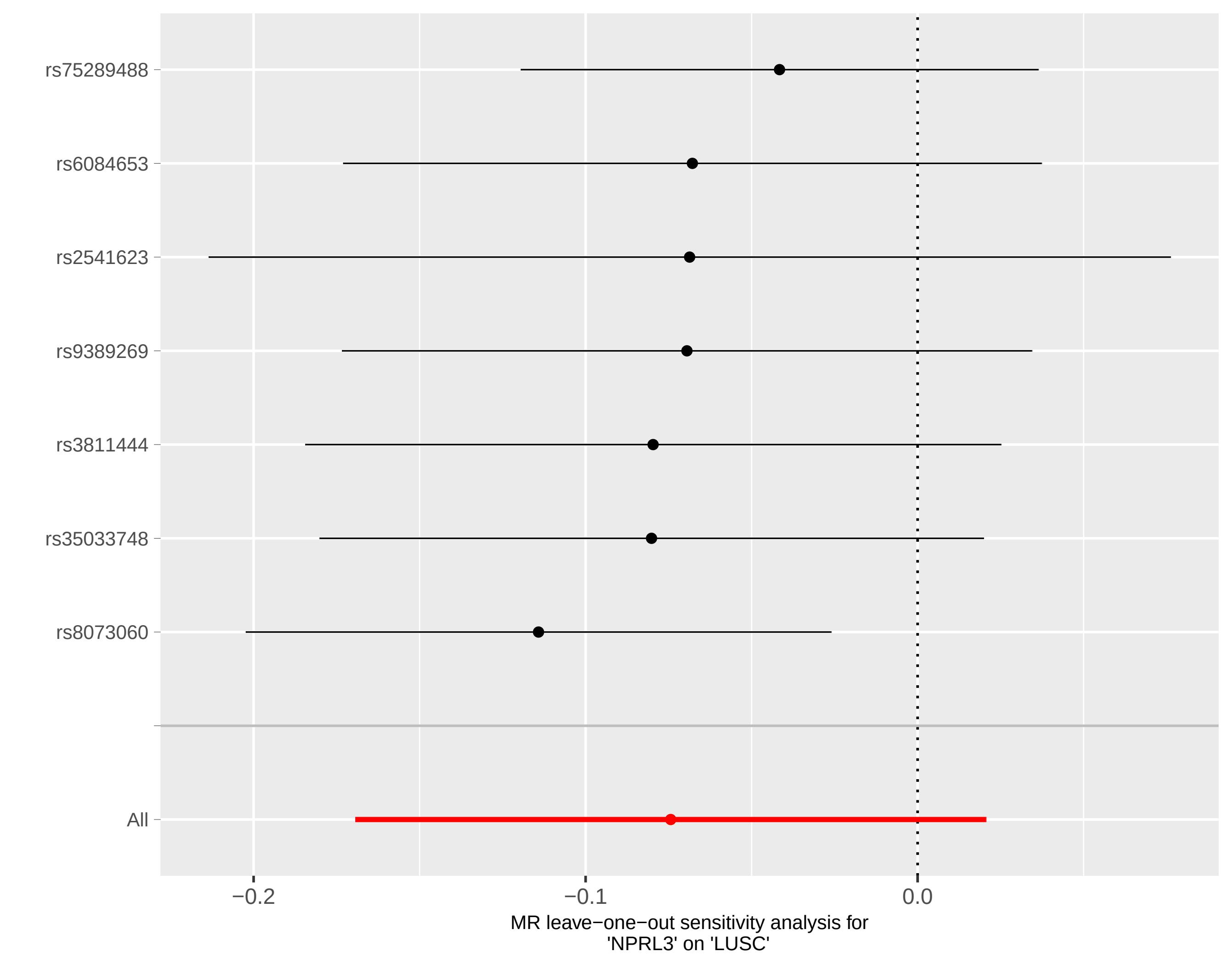
rs75289488
rs6084653
rs2541623
rs9389269
rs3811444
rs35033748
rs8073060
All
−0.2
−0.1
0.0
MR leave−one−out sensitivity analysis for
'NPRL3' on 'LUSC'

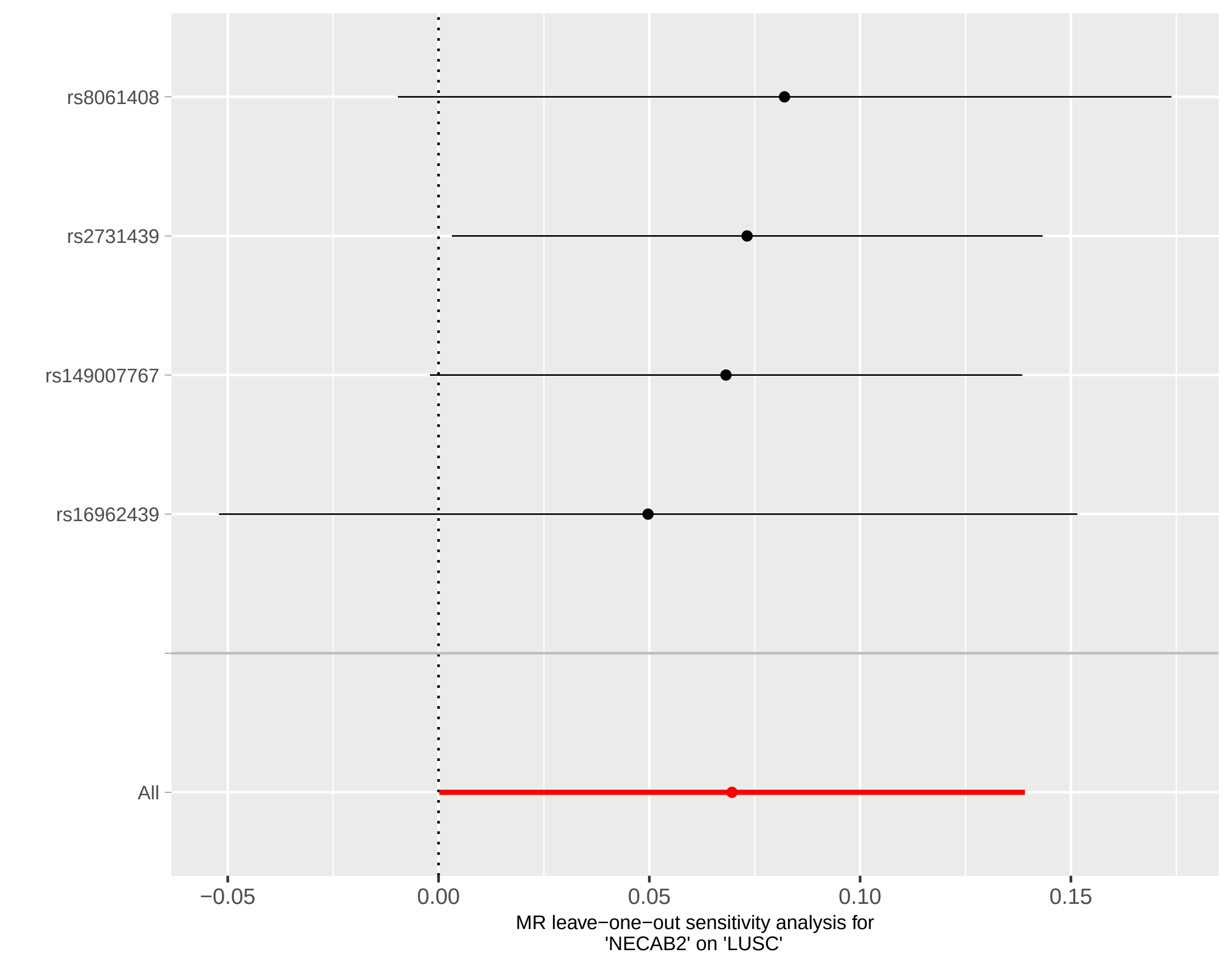
rs8061408
rs2731439
rs149007767
rs16962439
All
−0.05
0.00
0.05
0.10
0.15
MR leave−one−out sensitivity analysis for
'NECAB2' on 'LUSC'

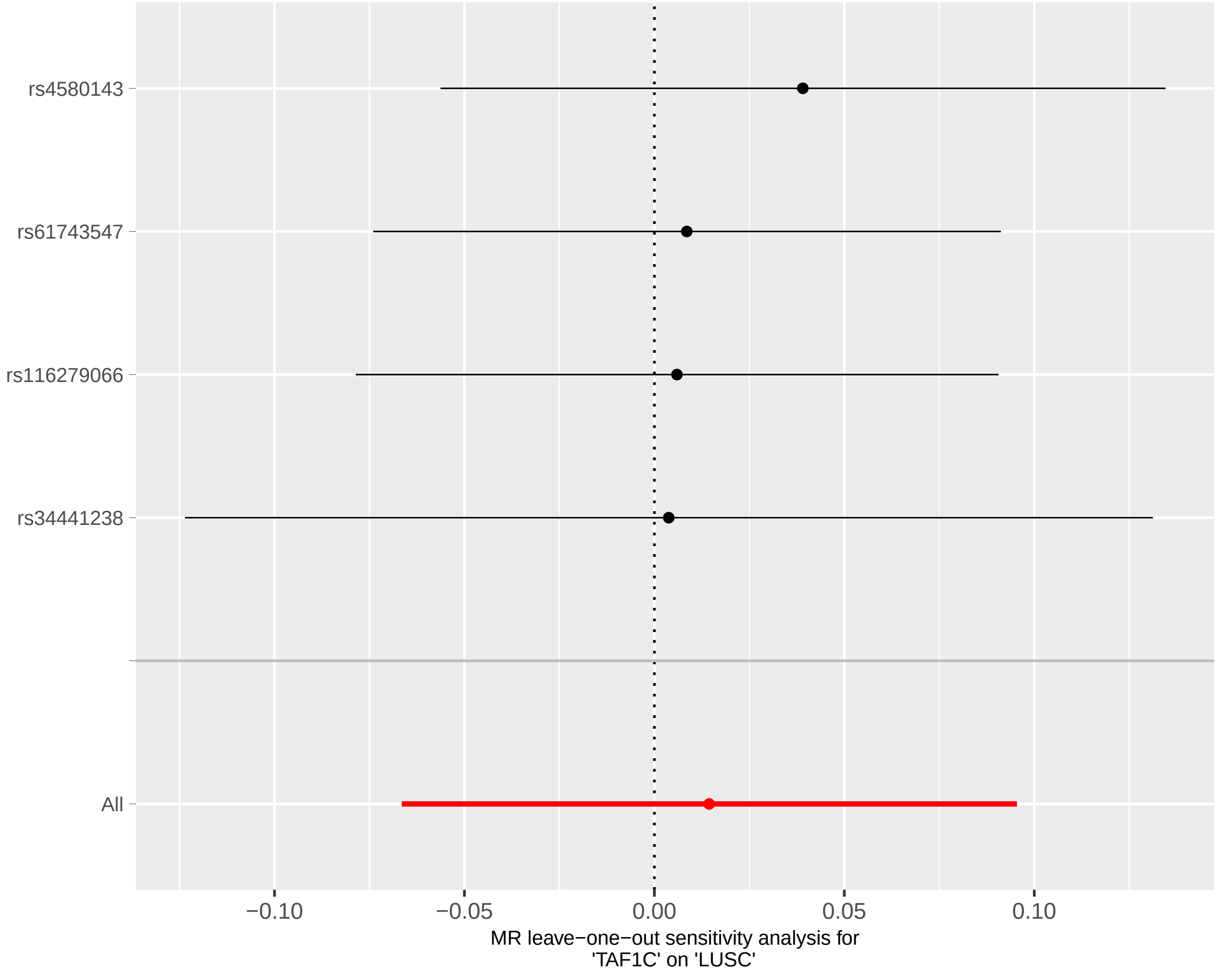

rs4580143
rs61743547
rs116279066
rs34441238
All
−0.10
−0.05
0.00
0.05
0.10
MR leave−one−out sensitivity analysis for
'TAF1C' on 'LUSC'

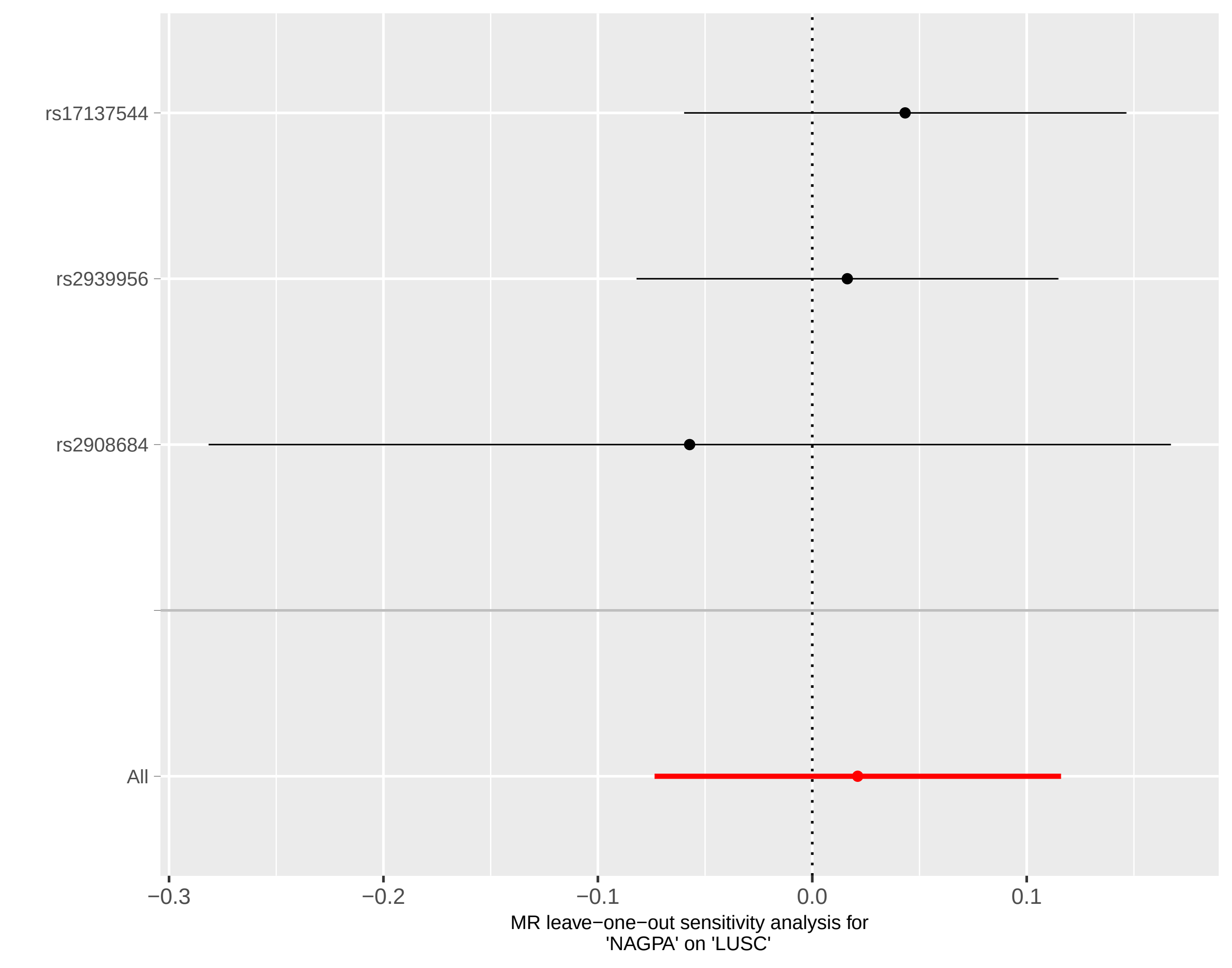
rs17137544
rs2939956
rs2908684
All
−0.3
−0.2
−0.1
0.0
0.1
MR leave−one−out sensitivity analysis for
'NAGPA' on 'LUSC'

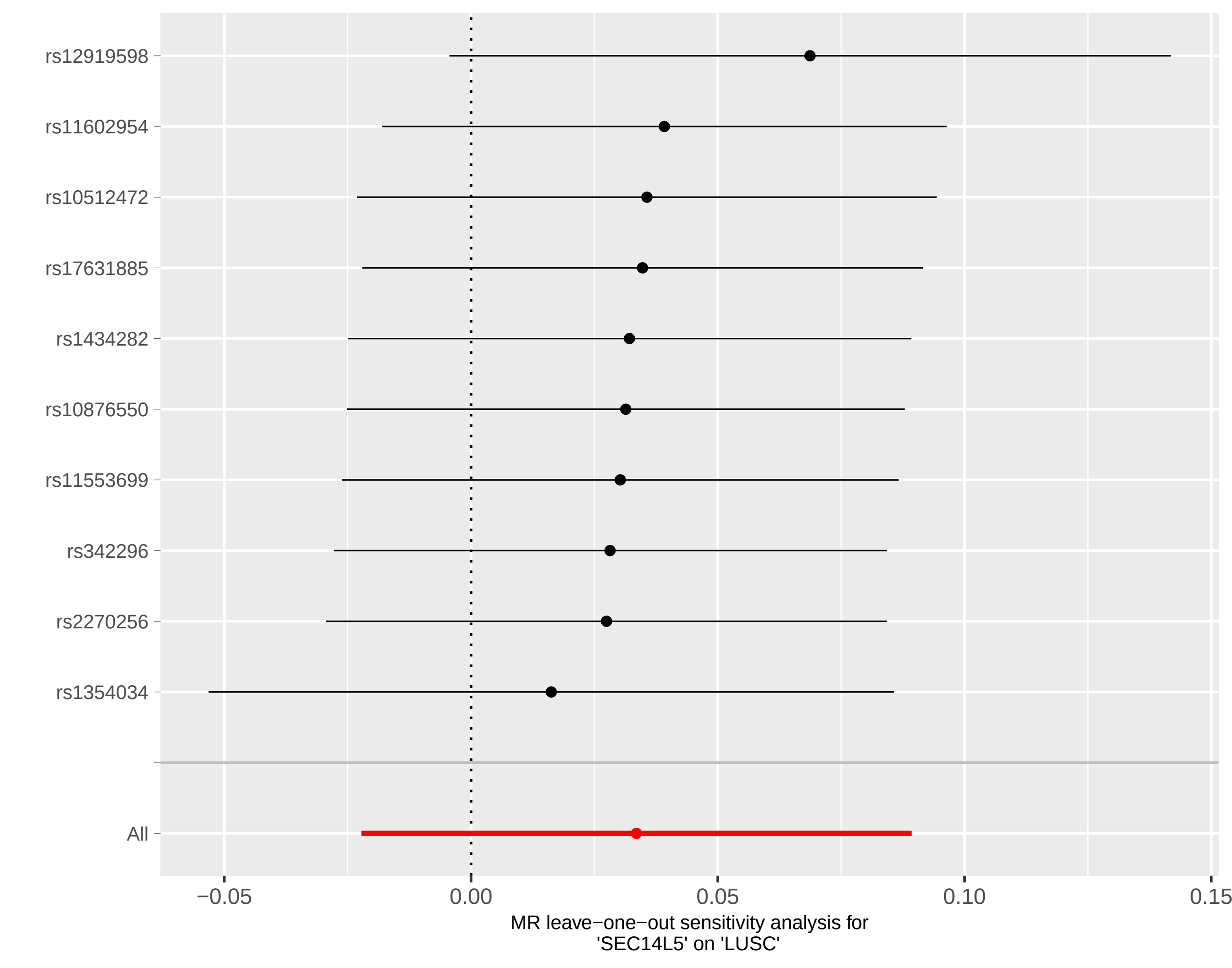
rs12919598
rs11602954
rs10512472
rs17631885
rs1434282
rs10876550
rs11553699
rs342296
rs2270256
rs1354034
All
−0.05
0.00
0.05
0.10
0.15
MR leave−one−out sensitivity analysis for
'SEC14L5' on 'LUSC'

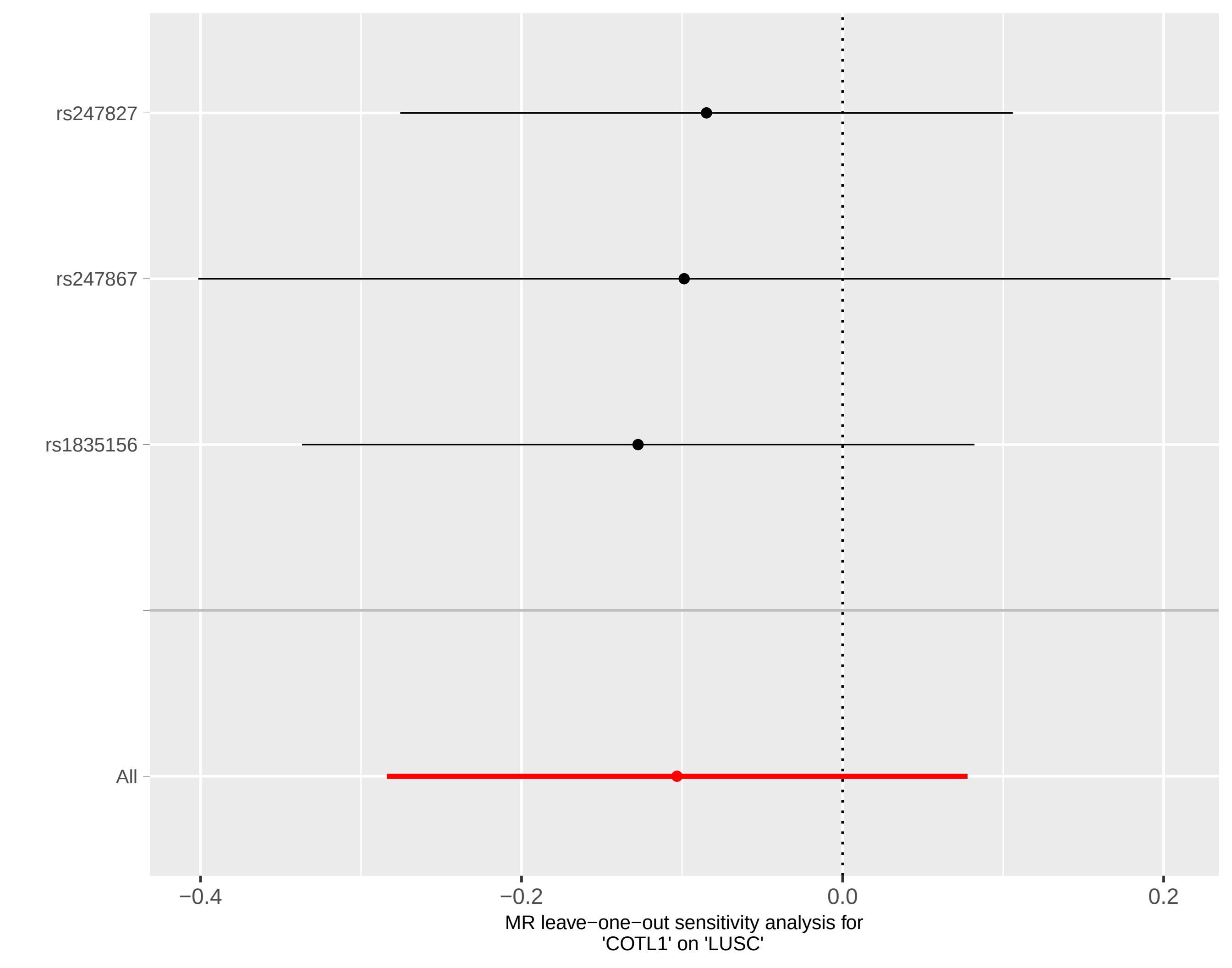
rs247827
rs247867
rs1835156
All
−0.4
−0.2
0.0
0.2
MR leave−one−out sensitivity analysis for
'COTL1' on 'LUSC'

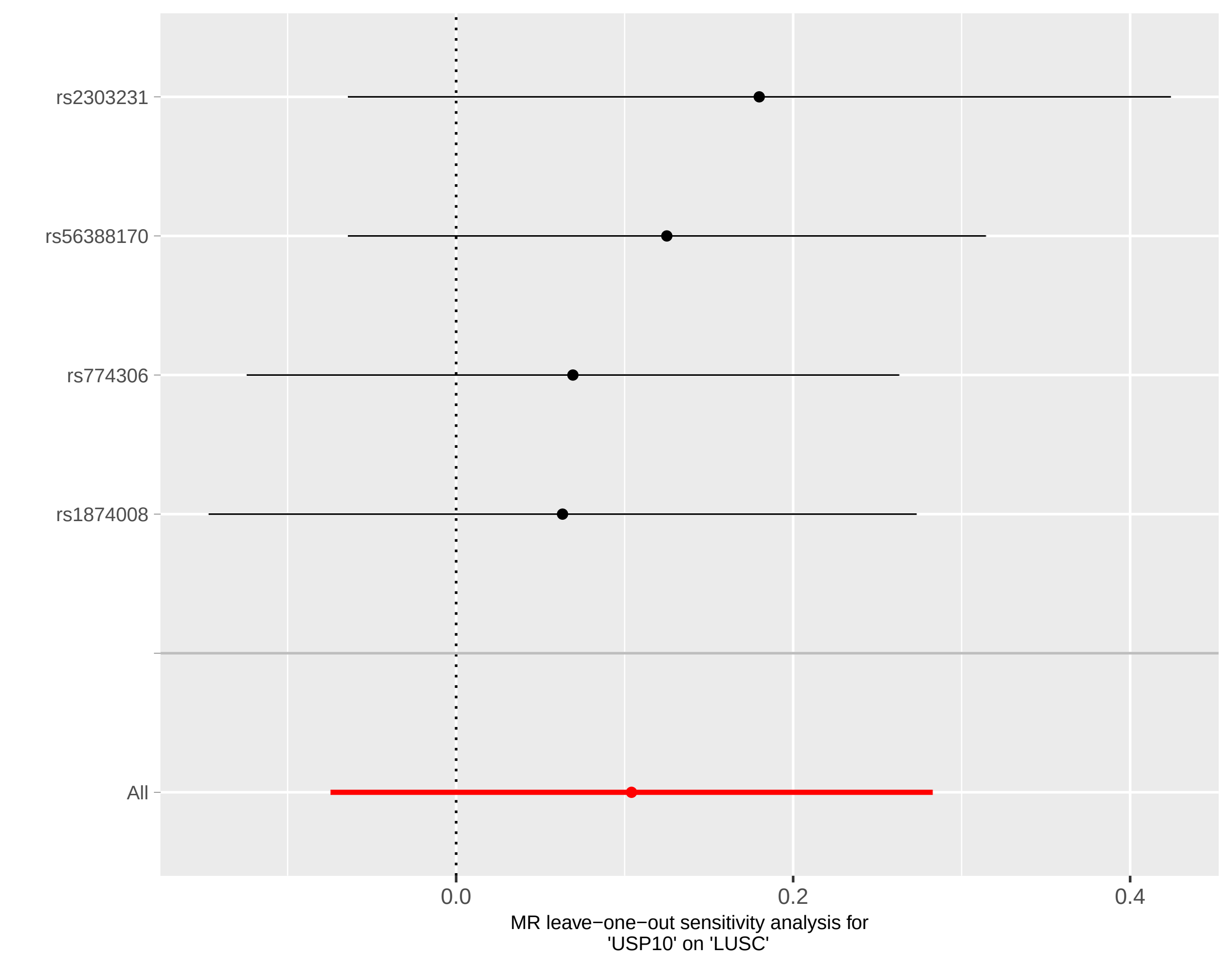

rs2303231
rs56388170
rs774306
rs1874008
All
0.0
0.2
0.4
MR leave−one−out sensitivity analysis for
'USP10' on 'LUSC'

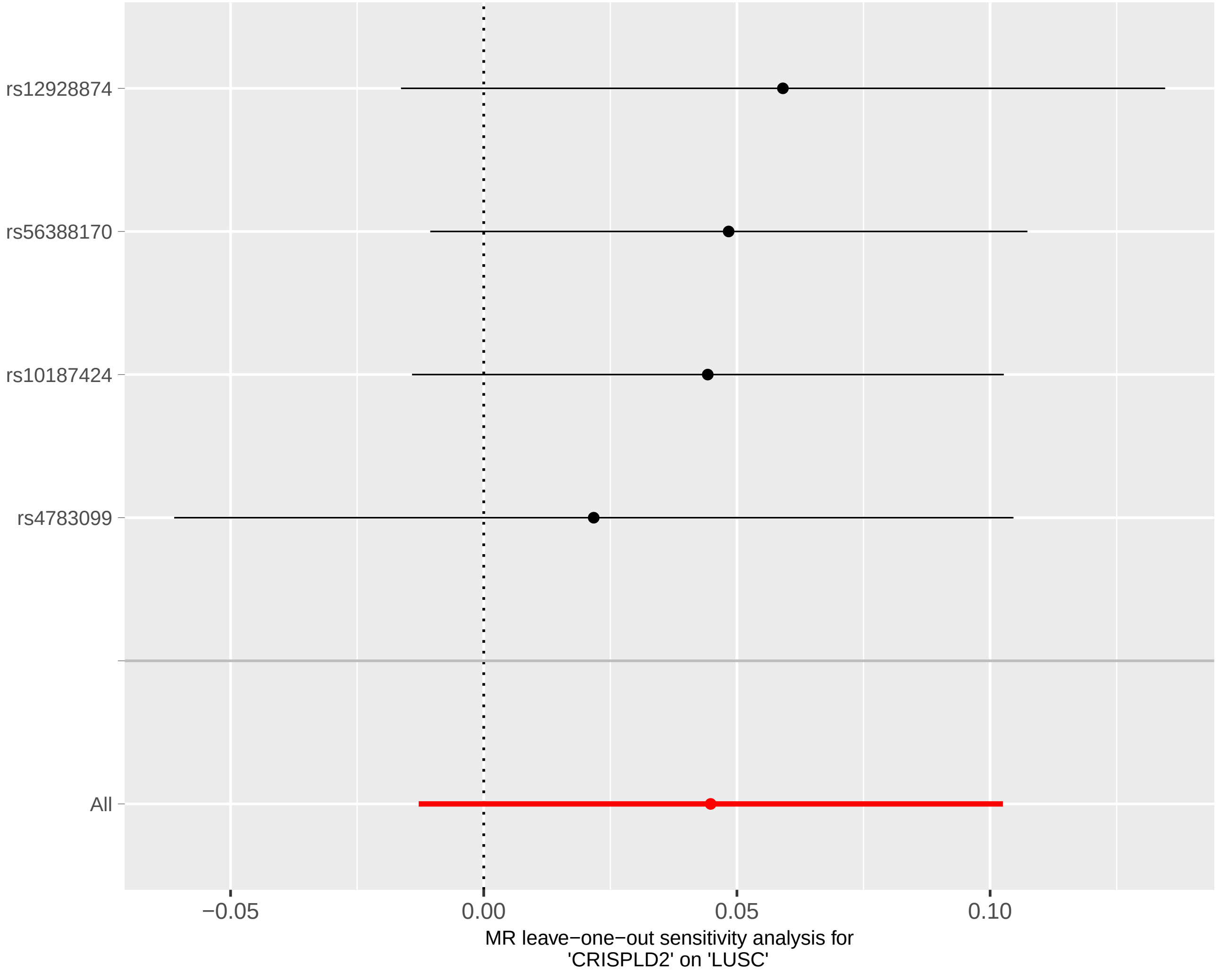
rs12928874
rs56388170
rs10187424
rs4783099
All
−0.05
0.00
0.05
0.10
MR leave−one−out sensitivity analysis for
'CRISPLD2' on 'LUSC'

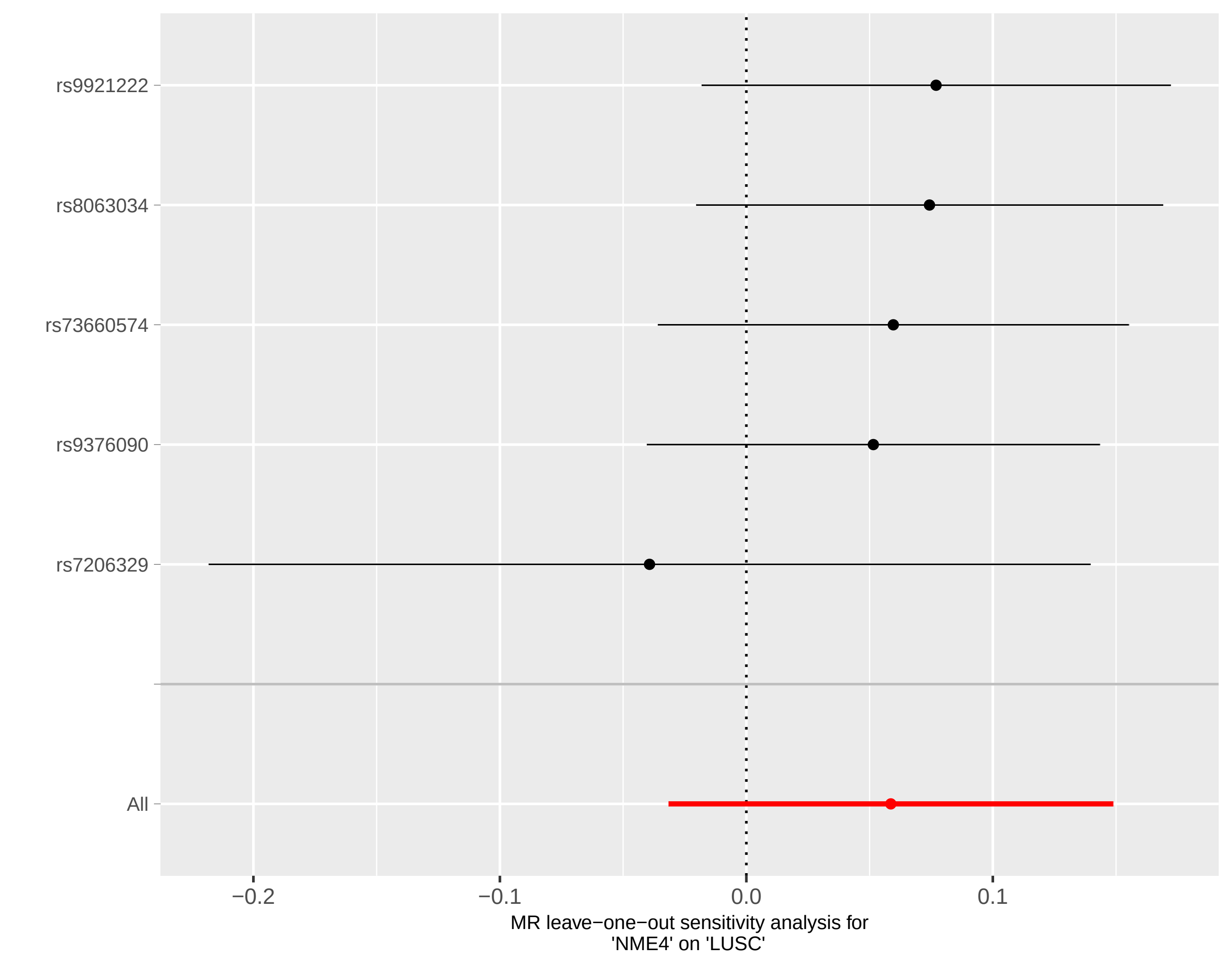
rs9921222
rs8063034
rs73660574
rs9376090
rs7206329
All
−0.2
−0.1
0.0
0.1
MR leave−one−out sensitivity analysis for
'NME4' on 'LUSC'

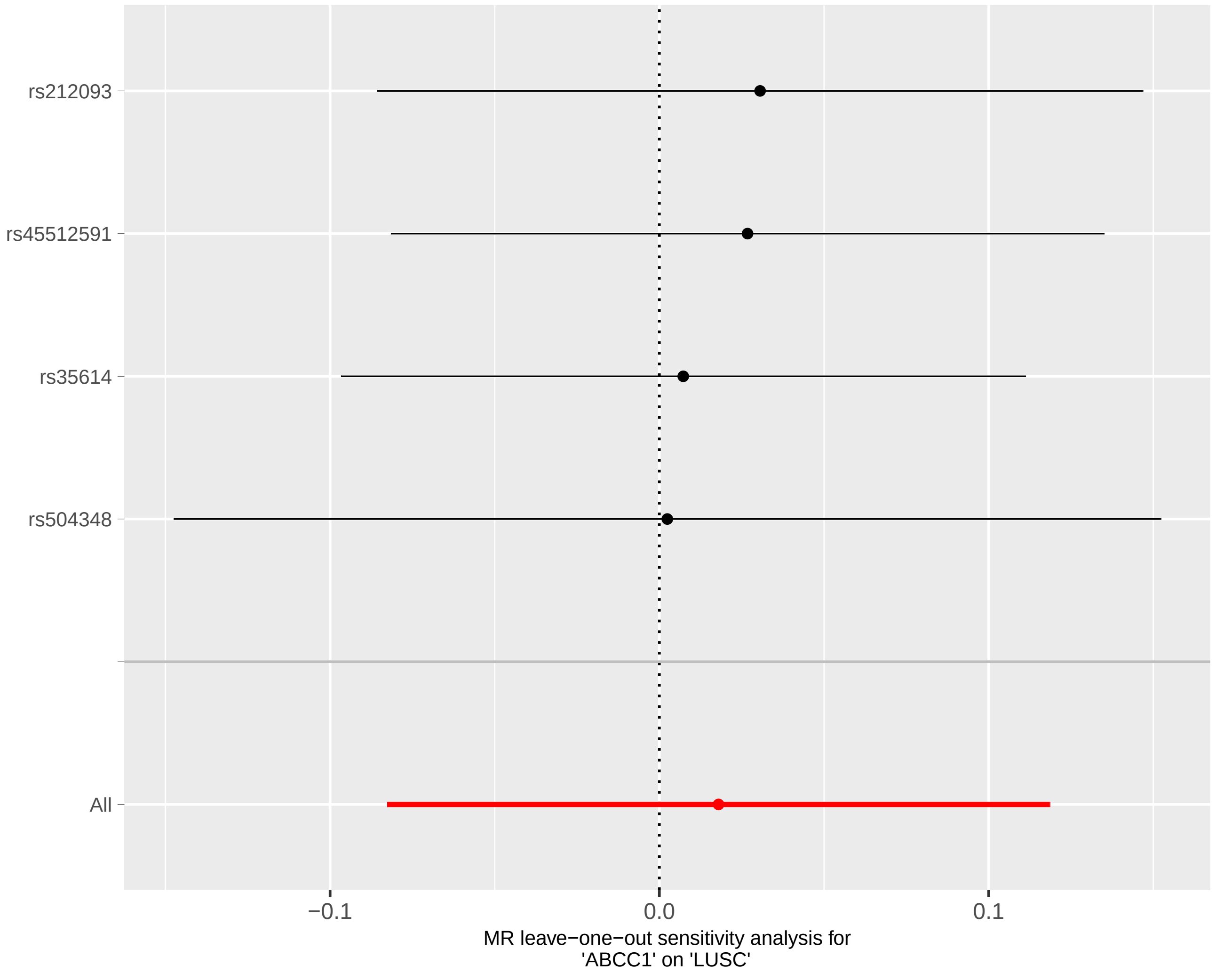

rs212093
rs45512591
rs35614
rs504348
All
−0.1
0.0
0.1
MR leave−one−out sensitivity analysis for
'ABCC1' on 'LUSC'

Insufficient number of SNPs

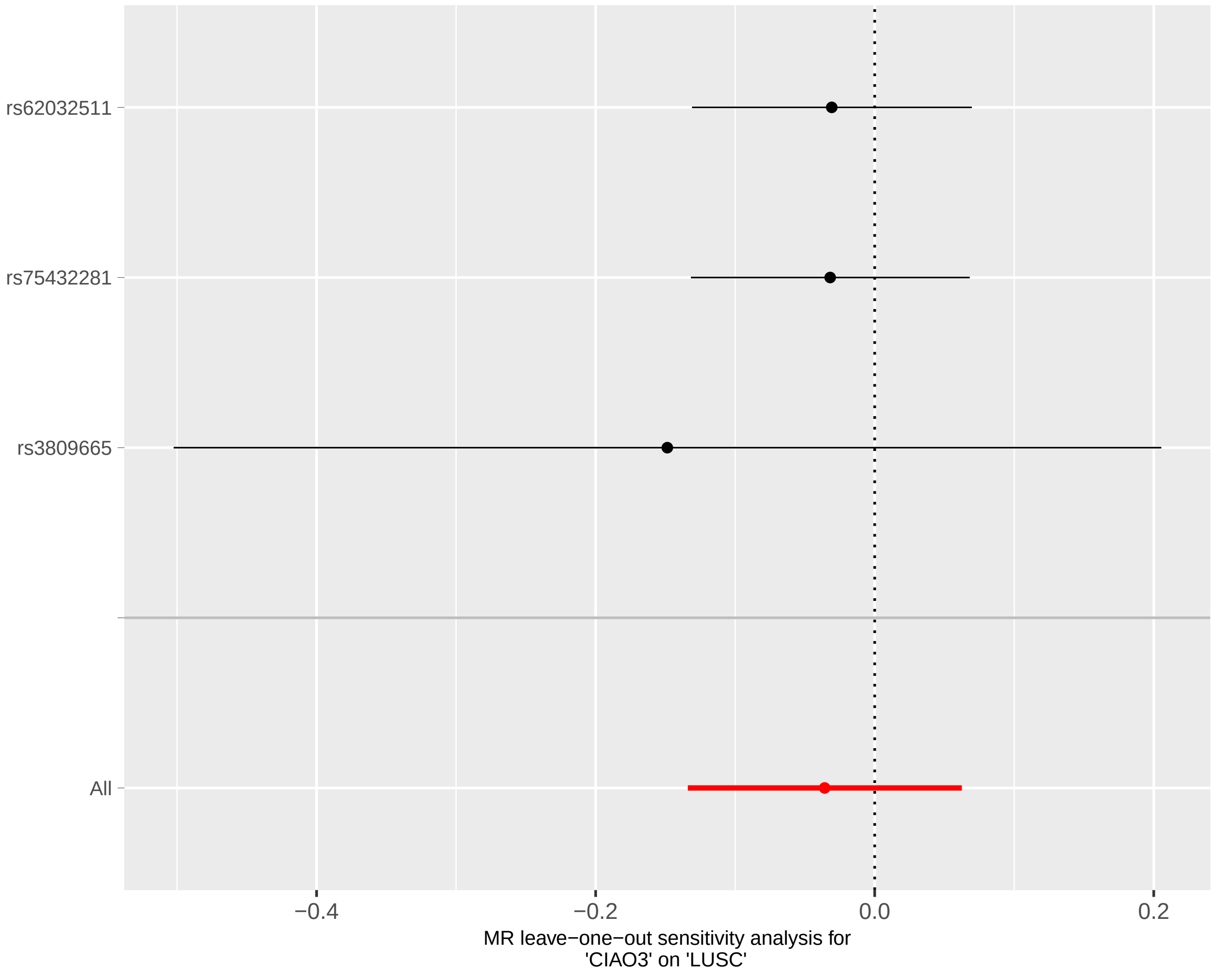
rs62032511
rs75432281
rs3809665
All
−0.4
−0.2
0.0
0.2
MR leave−one−out sensitivity analysis for
'CIAO3' on 'LUSC'

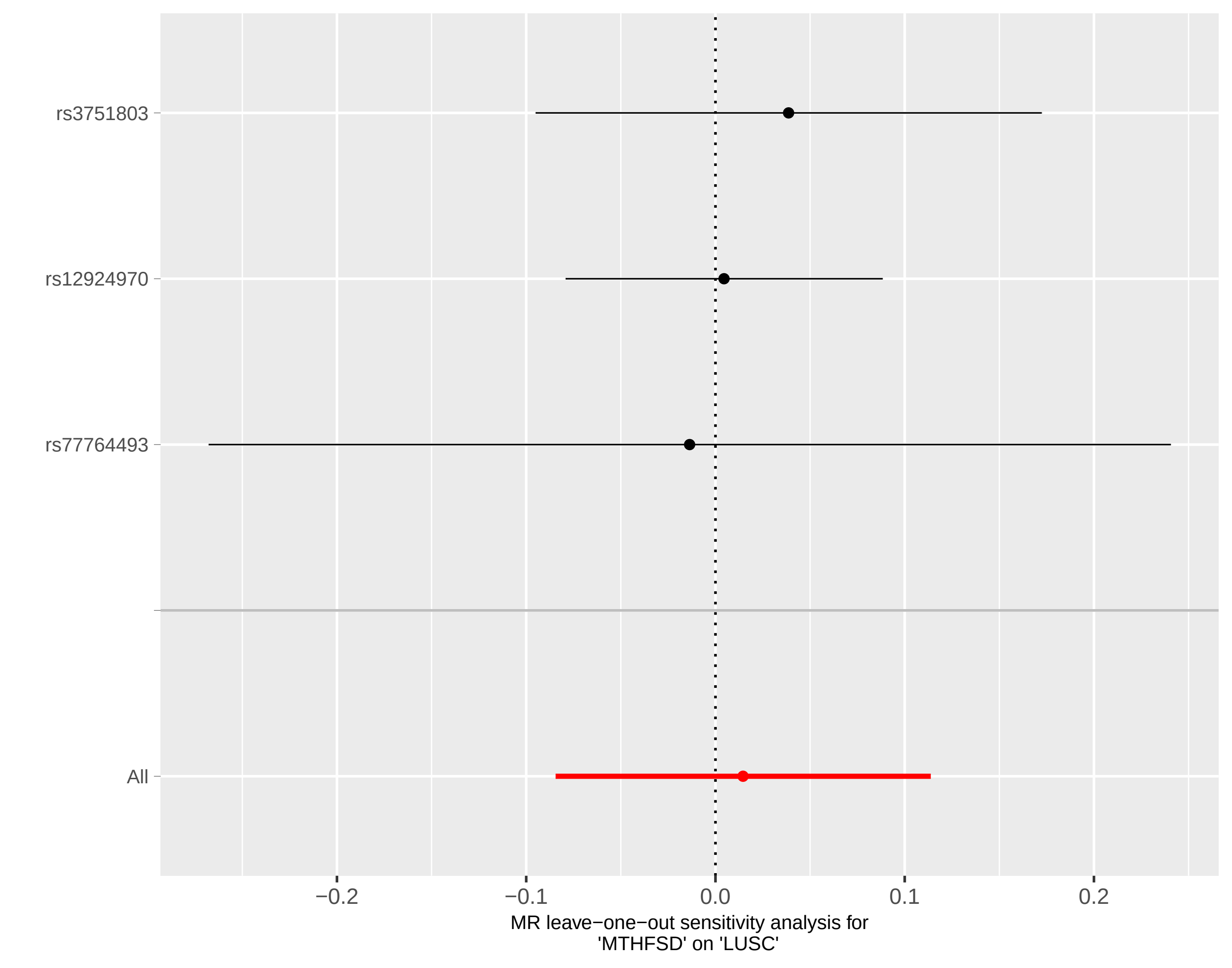

rs3751803
rs12924970
rs77764493
All
−0.2
−0.1
0.0
0.1
0.2
MR leave−one−out sensitivity analysis for
'MTHFSD' on 'LUSC'

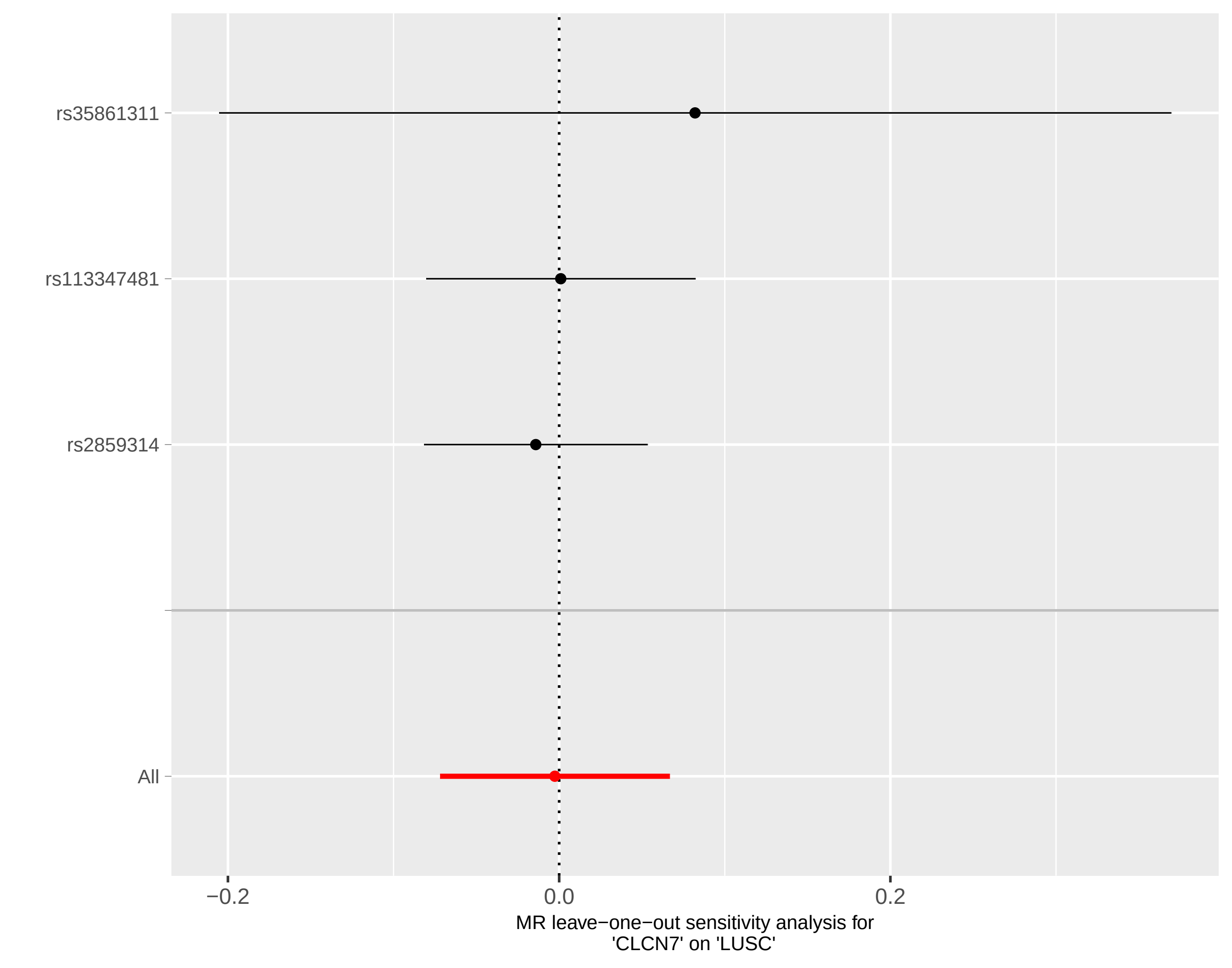
rs35861311
rs113347481
rs2859314
All
−0.2
0.0
0.2
MR leave−one−out sensitivity analysis for
'CLCN7' on 'LUSC'

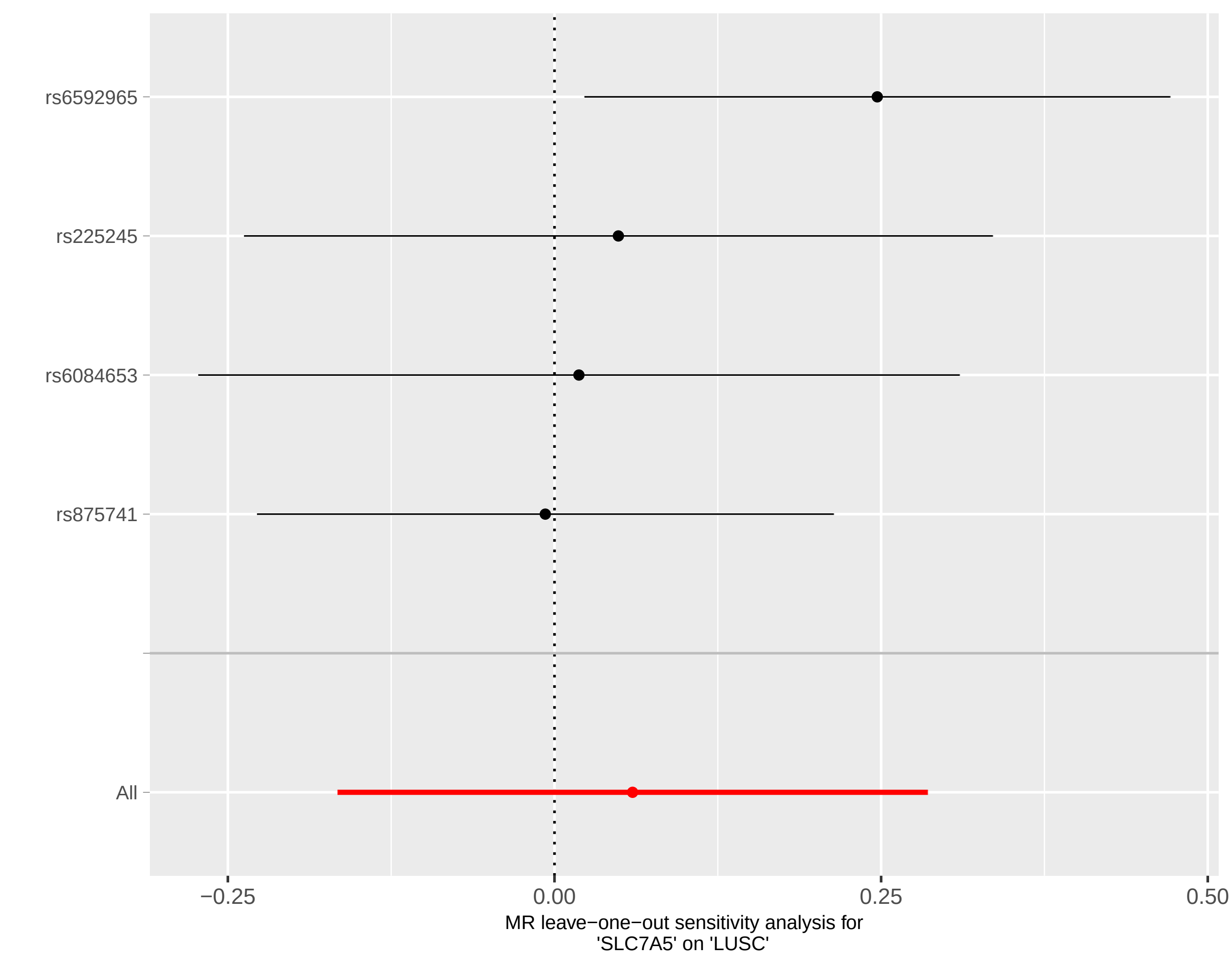
rs6592965
rs225245
rs6084653
rs875741
All
−0.25
0.00
0.25
0.50
MR leave−one−out sensitivity analysis for
'SLC7A5' on 'LUSC'

Insufficient number of SNPs

Insufficient number of SNPs

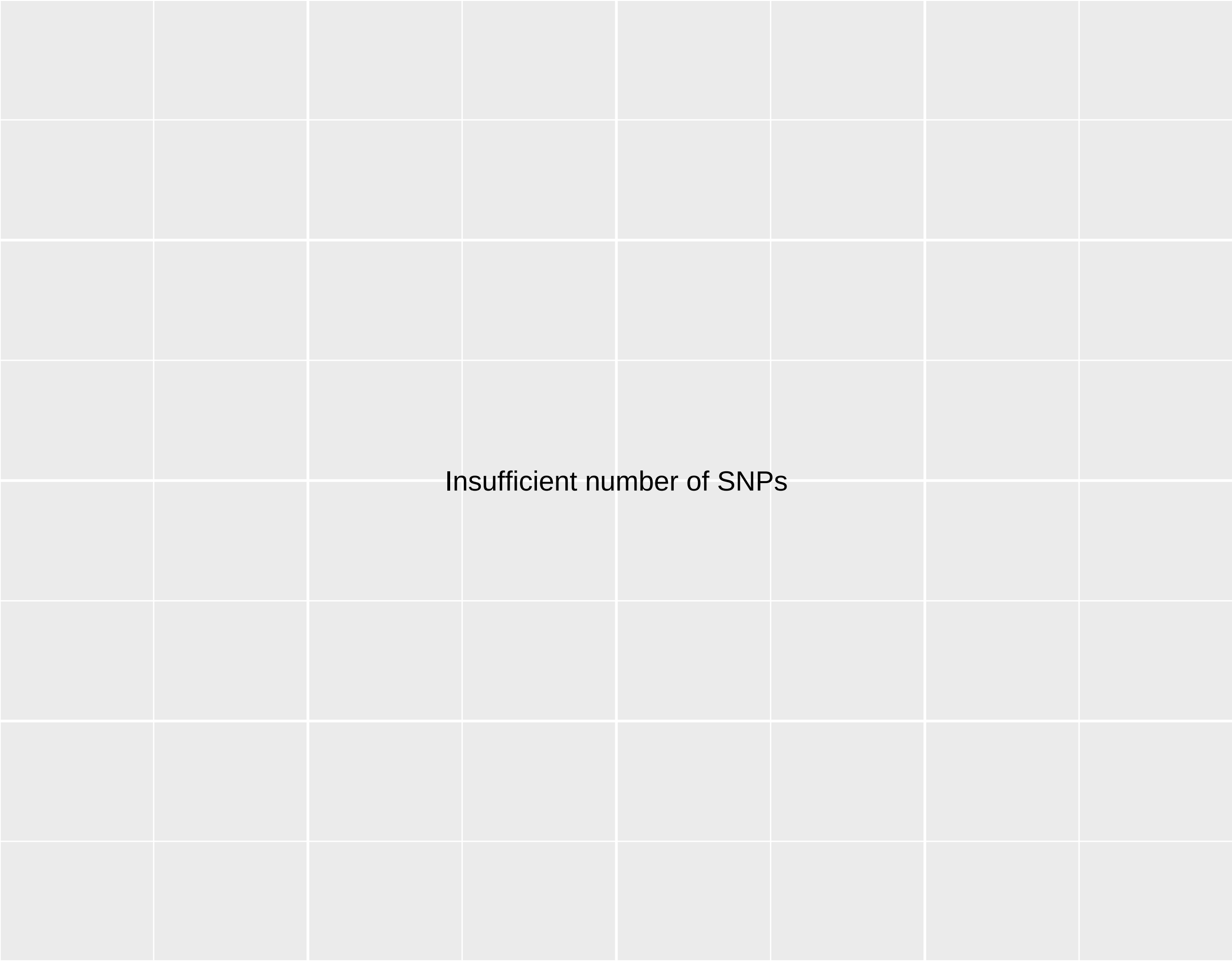
Insufficient number of SNPs

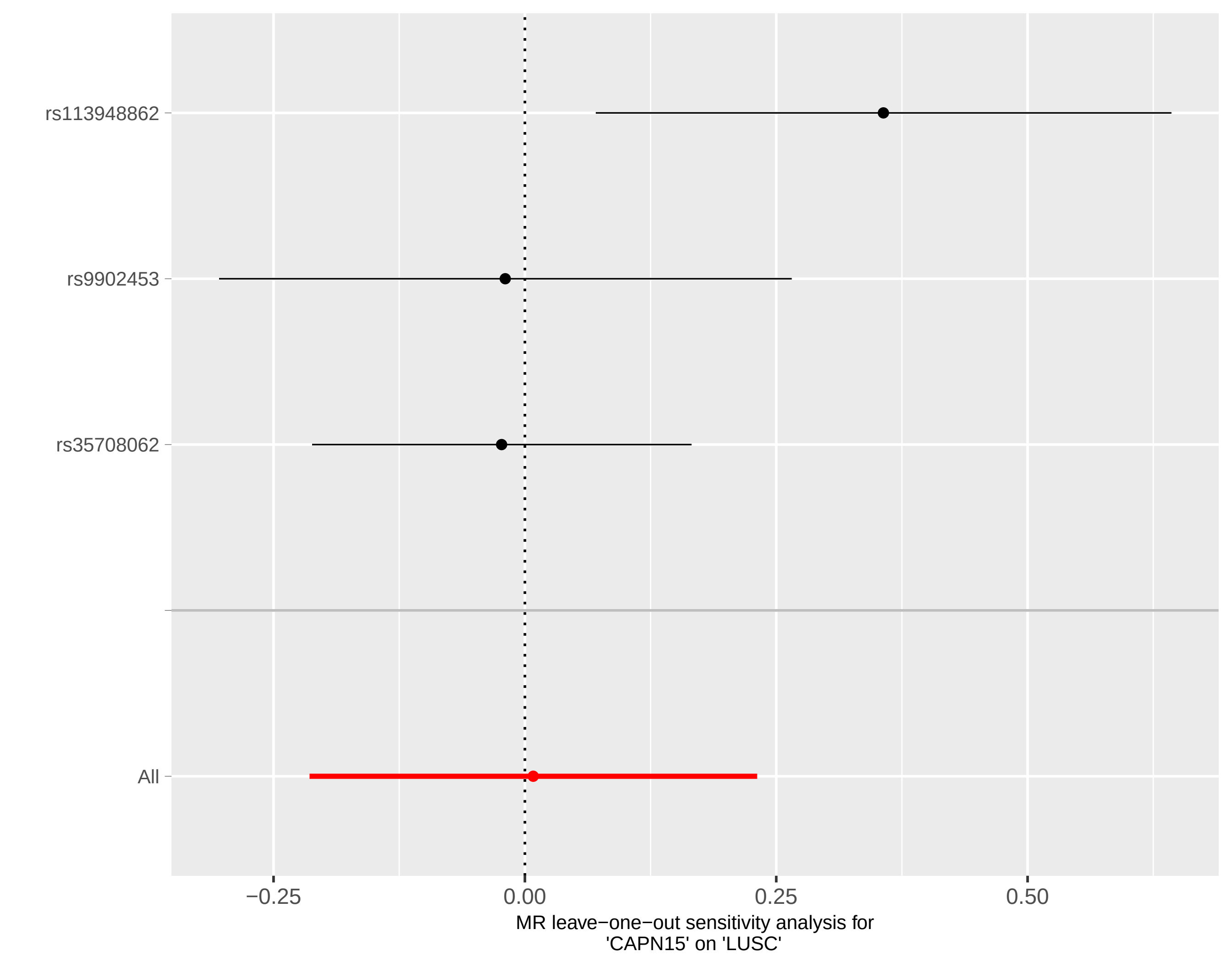
rs113948862
rs9902453
rs35708062
All
−0.25
0.00
0.25
0.50
MR leave−one−out sensitivity analysis for
'CAPN15' on 'LUSC'

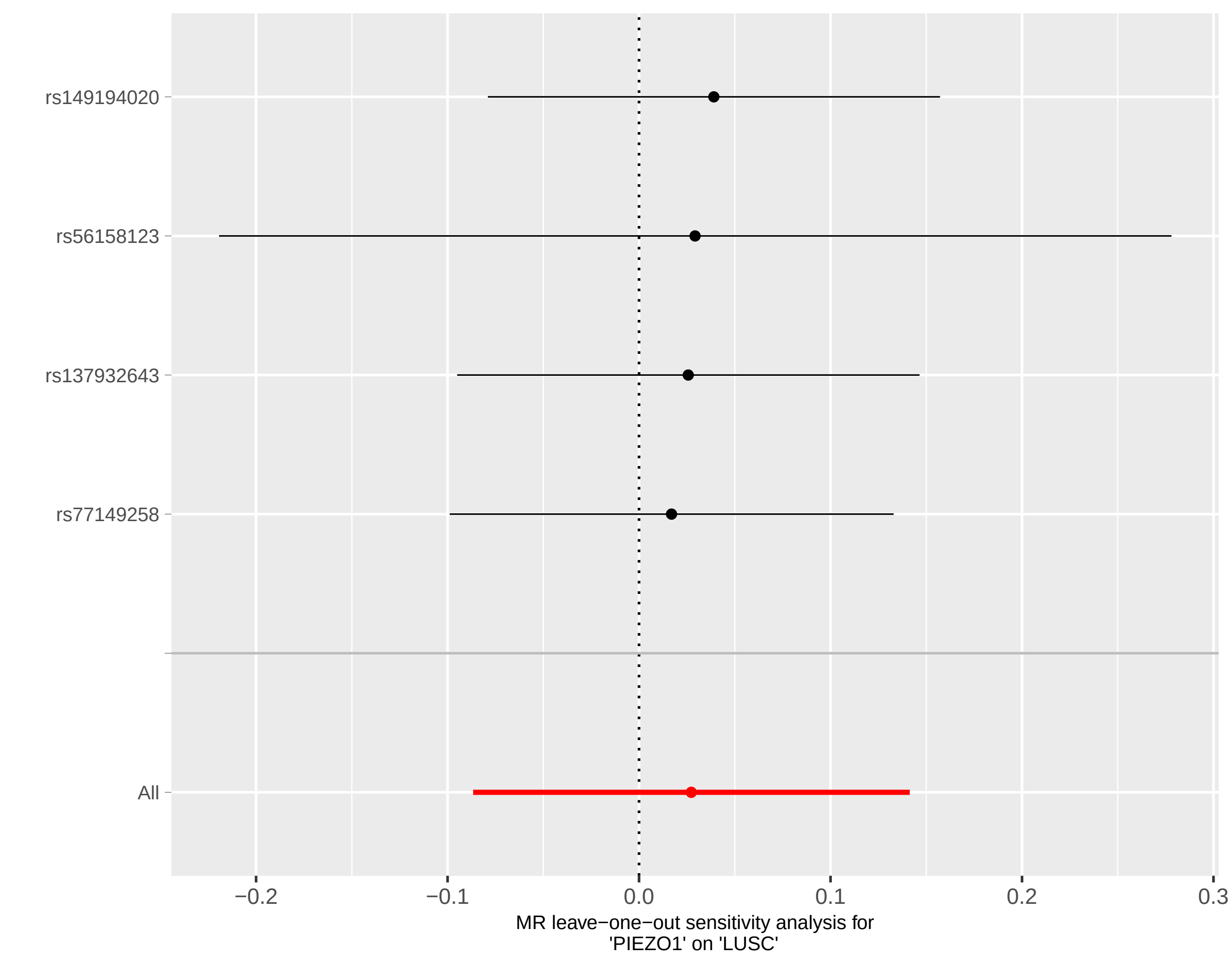

rs149194020
rs56158123
rs137932643
rs77149258
All
−0.2
−0.1
0.0
0.1
0.2
0.3
MR leave−one−out sensitivity analysis for
'PIEZO1' on 'LUSC'

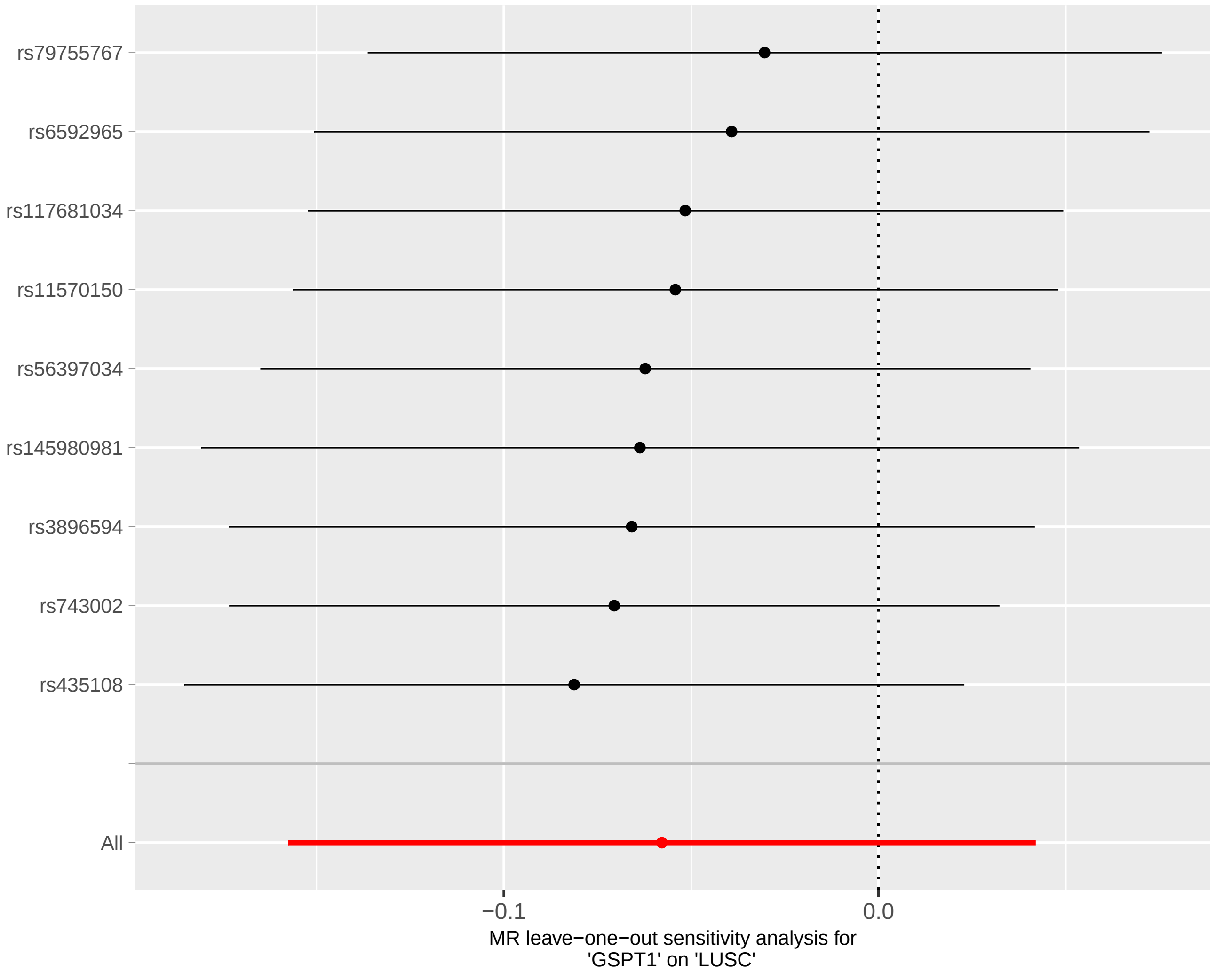
rs79755767
rs6592965
rs117681034
rs11570150
rs56397034
rs145980981
rs3896594
rs743002
rs435108
All
−0.1
0.0
MR leave−one−out sensitivity analysis for
'GSPT1' on 'LUSC'

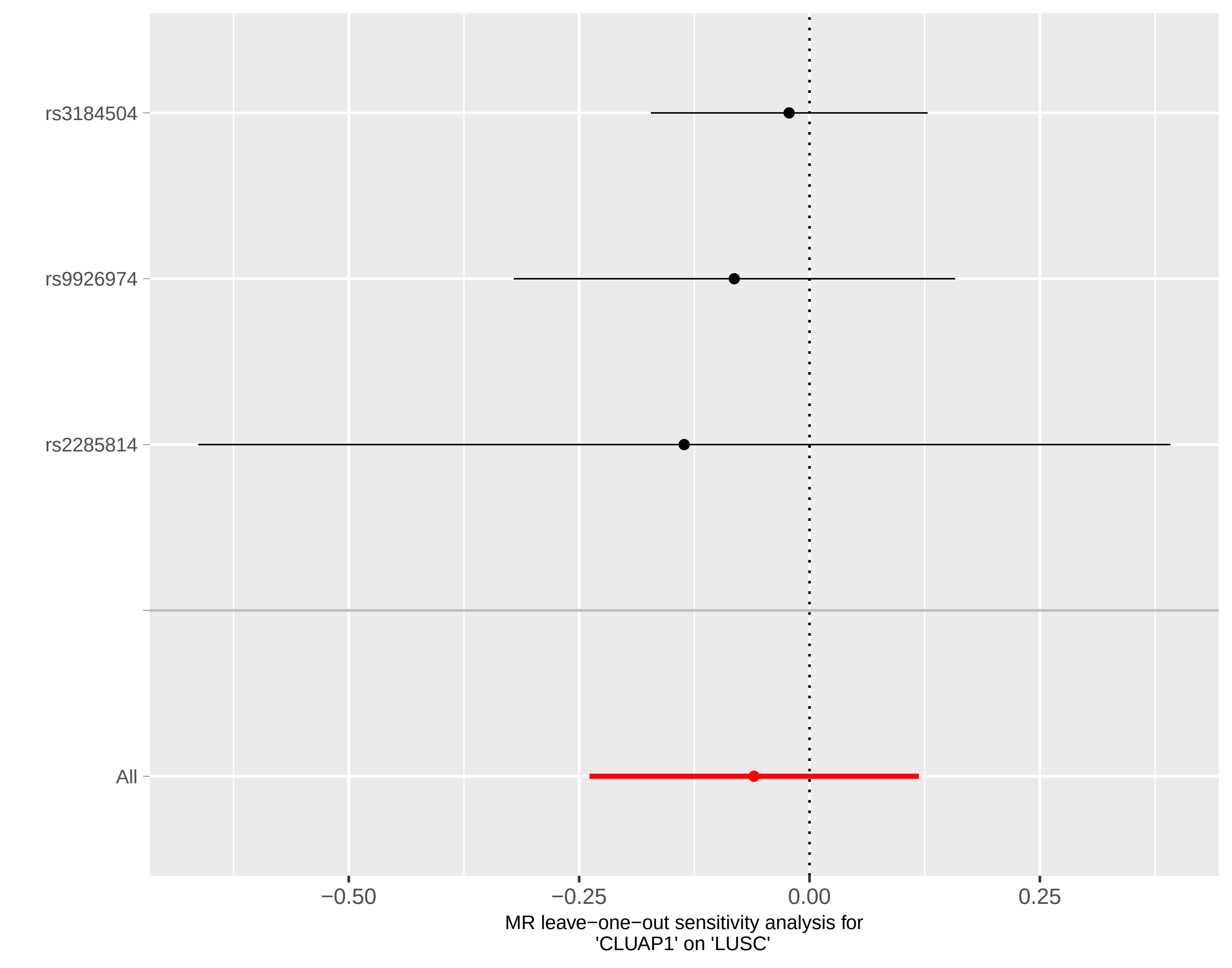

rs3184504
rs9926974
rs2285814
All
−0.50
−0.25
0.00
0.25
MR leave−one−out sensitivity analysis for
'CLUAP1' on 'LUSC'

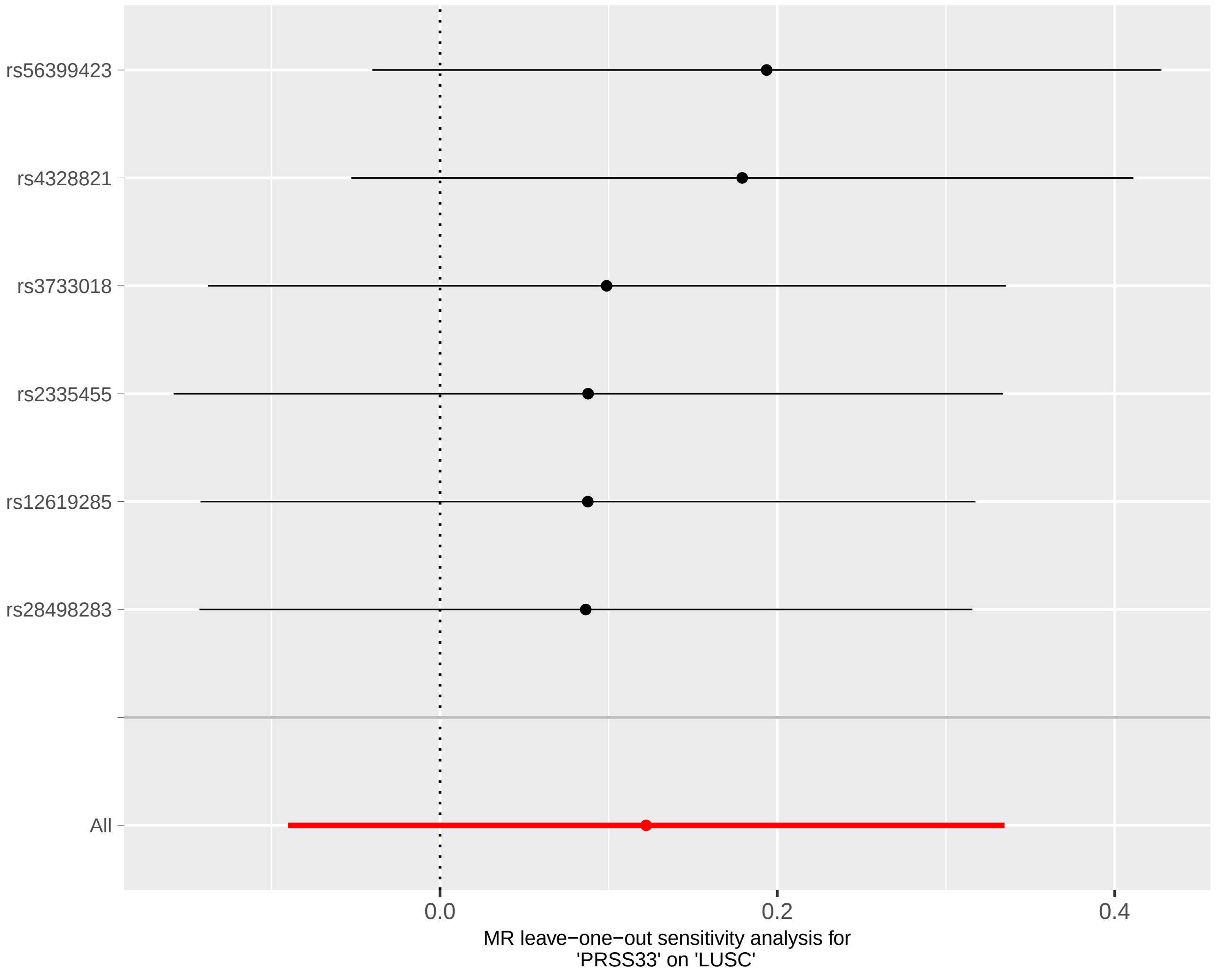
rs56399423
rs4328821
rs3733018
rs2335455
rs12619285
rs28498283
All
0.0
0.2
0.4
MR leave−one−out sensitivity analysis for
'PRSS33' on 'LUSC'

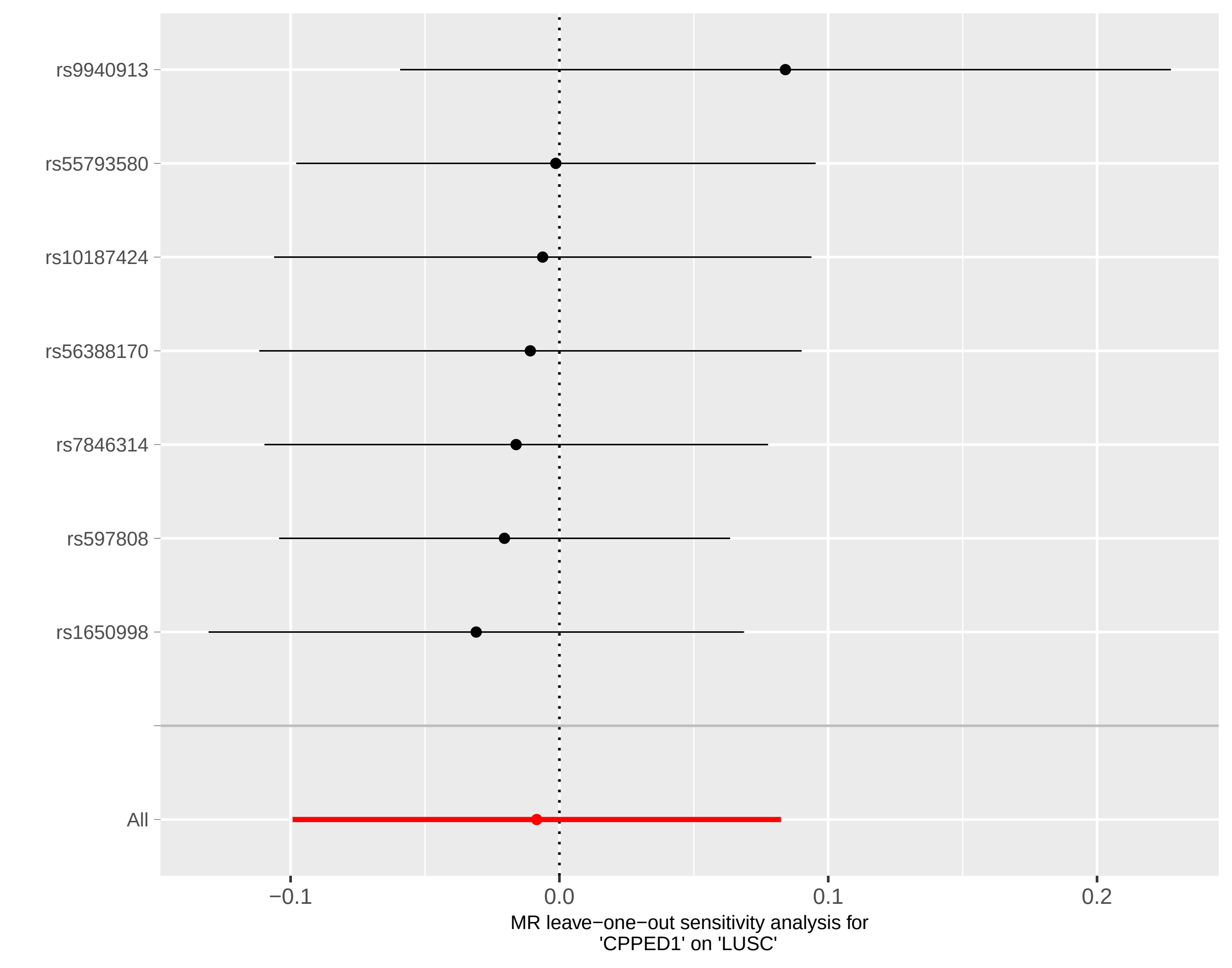
rs9940913
rs55793580
rs10187424
rs56388170
rs7846314
rs597808
rs1650998
All
−0.1
0.0
0.1
0.2
MR leave−one−out sensitivity analysis for
'CPPED1' on 'LUSC'

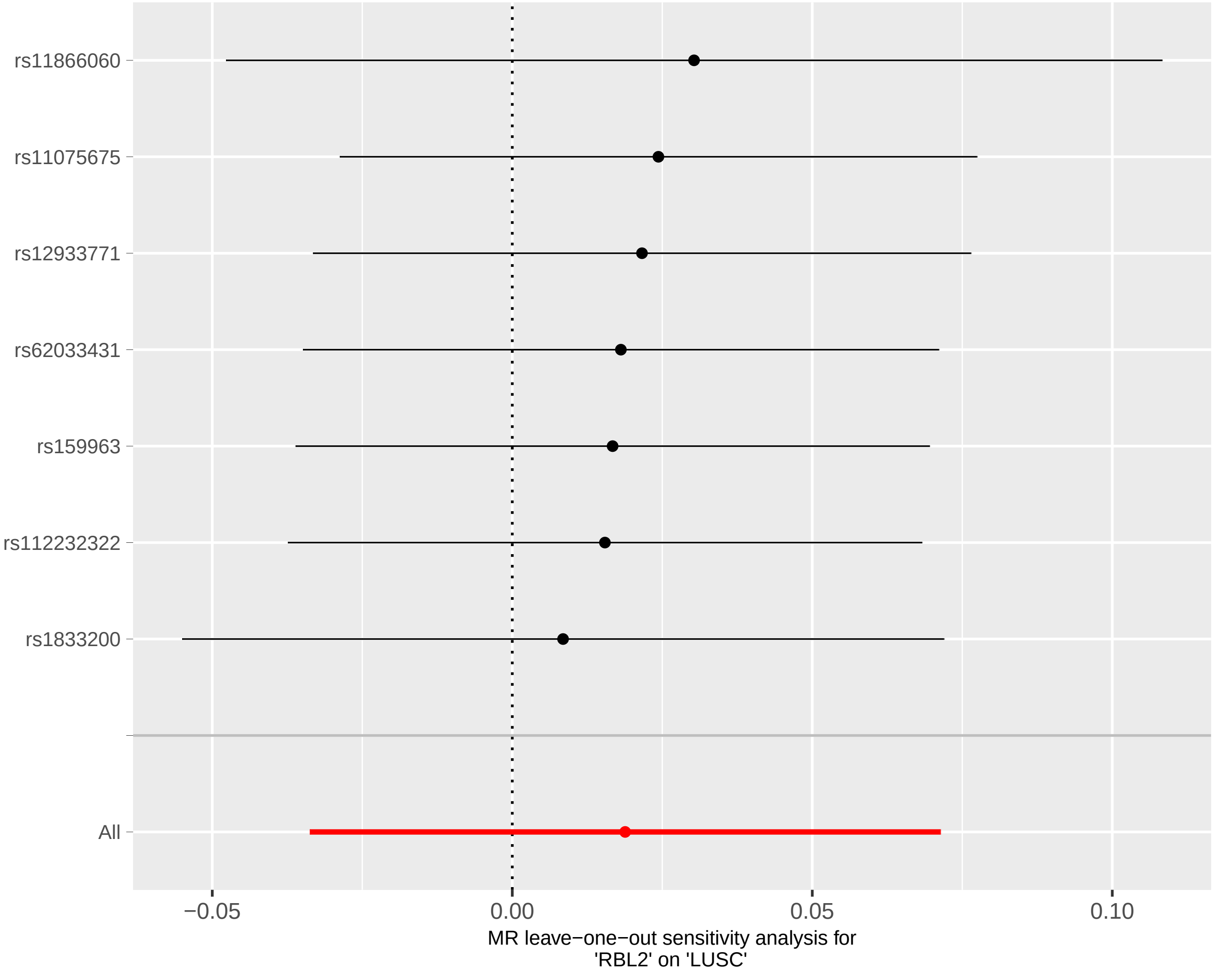

rs11866060
rs11075675
rs12933771
rs62033431
rs159963
rs112232322
rs1833200
All
−0.05
0.00
0.05
0.10
MR leave−one−out sensitivity analysis for
'RBL2' on 'LUSC'

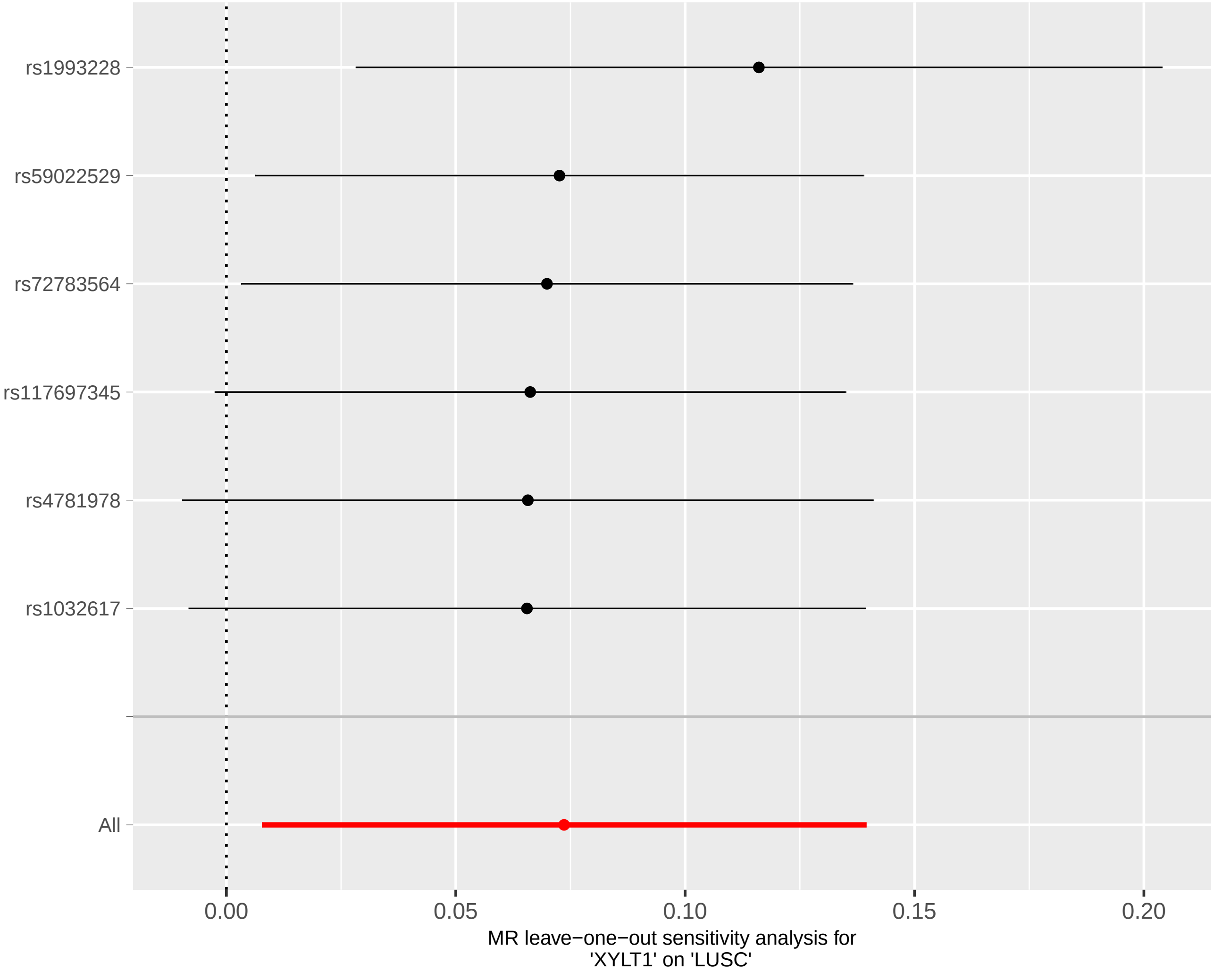

rs1993228
rs59022529
rs72783564
rs117697345
rs4781978
rs1032617
All
0.00
0.05
0.10
0.15
0.20
MR leave−one−out sensitivity analysis for
'XYLT1' on 'LUSC'

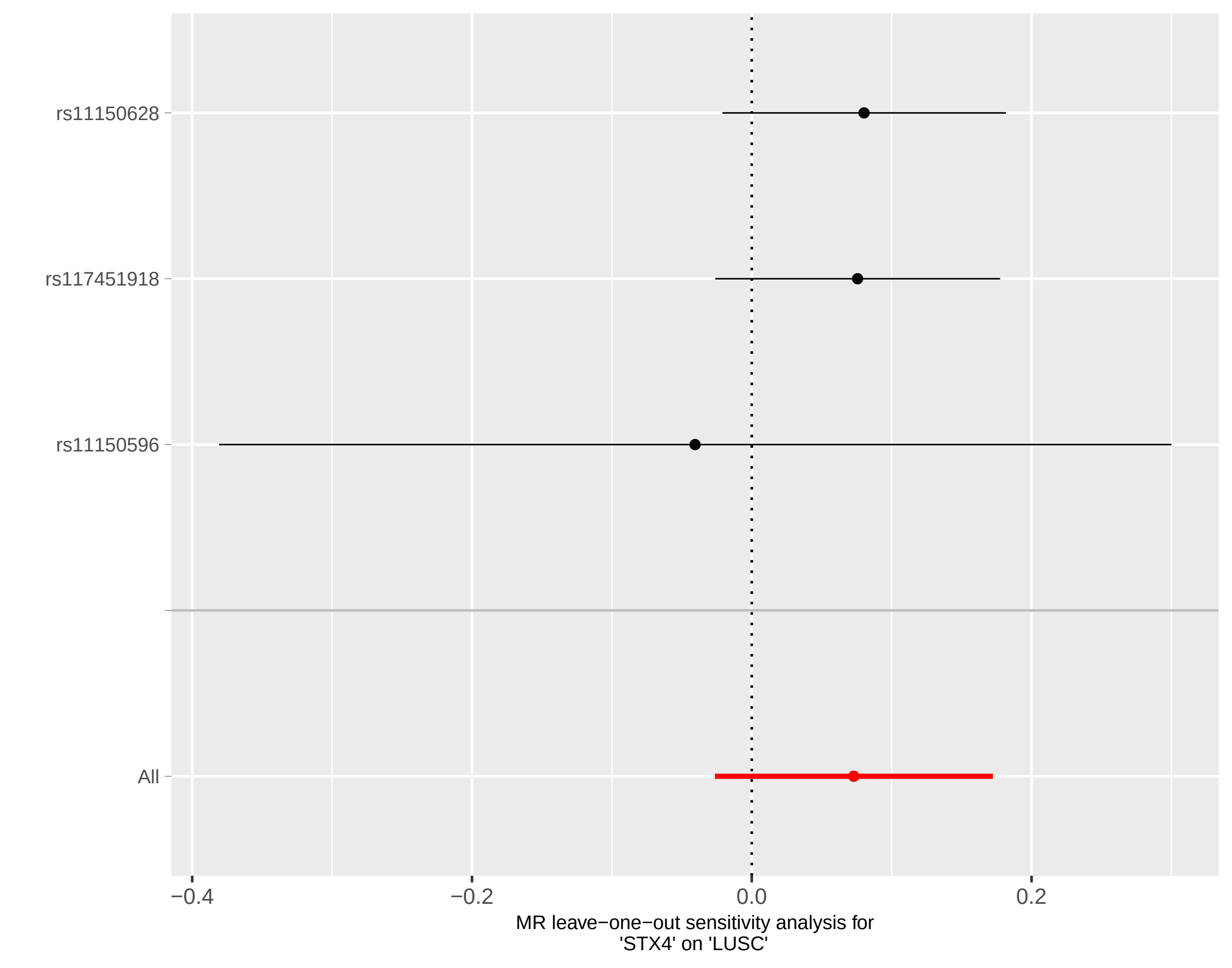
rs11150628
rs117451918
rs11150596
All
−0.4
−0.2
0.0
0.2
MR leave−one−out sensitivity analysis for
'STX4' on 'LUSC'

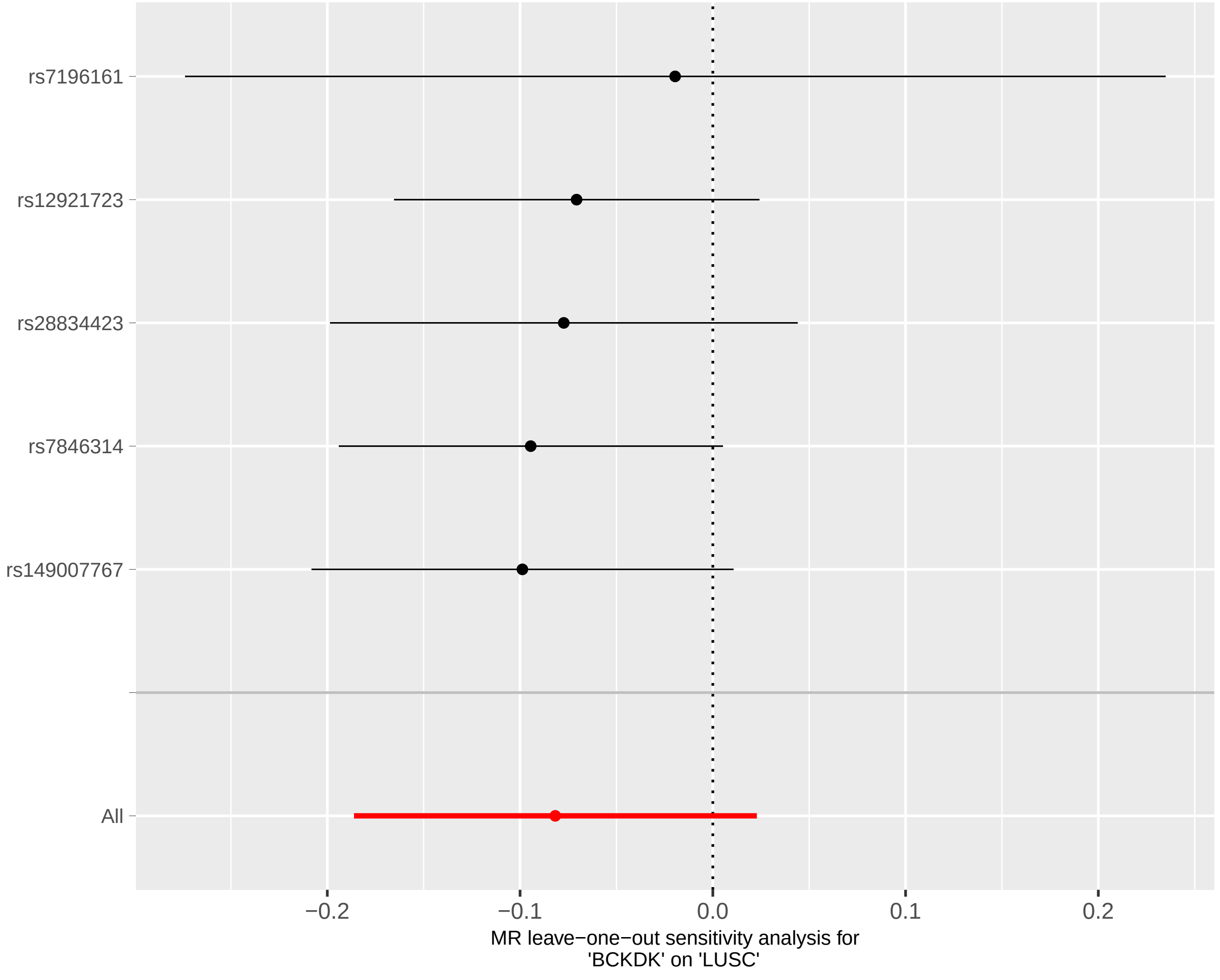
rs7196161
rs12921723
rs28834423
rs7846314
rs149007767
All
−0.2
−0.1
0.0
0.1
0.2
MR leave−one−out sensitivity analysis for
'BCKDK' on 'LUSC'

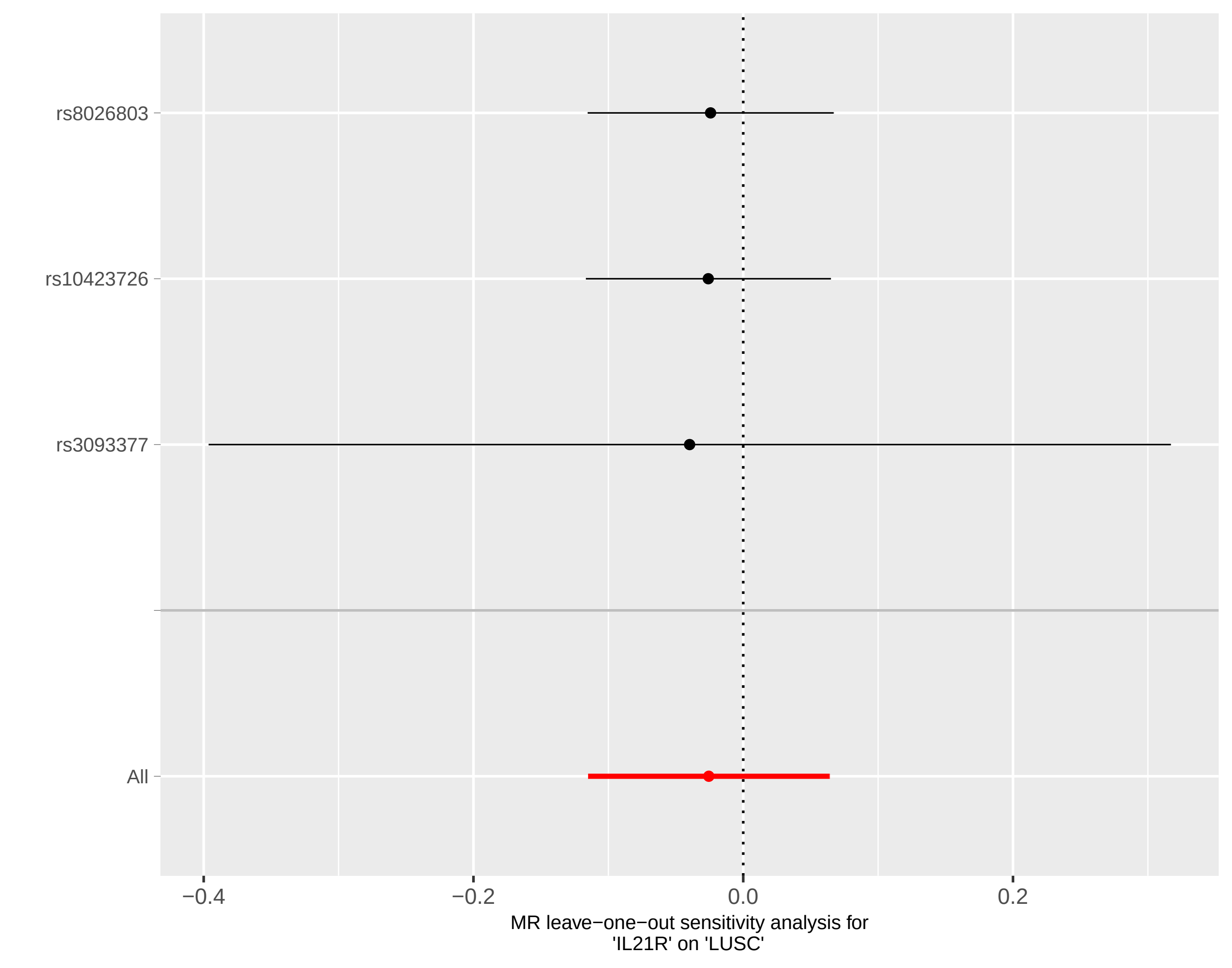
rs8026803
rs10423726
rs3093377
All
−0.4
−0.2
0.0
0.2
MR leave−one−out sensitivity analysis for
'IL21R' on 'LUSC'

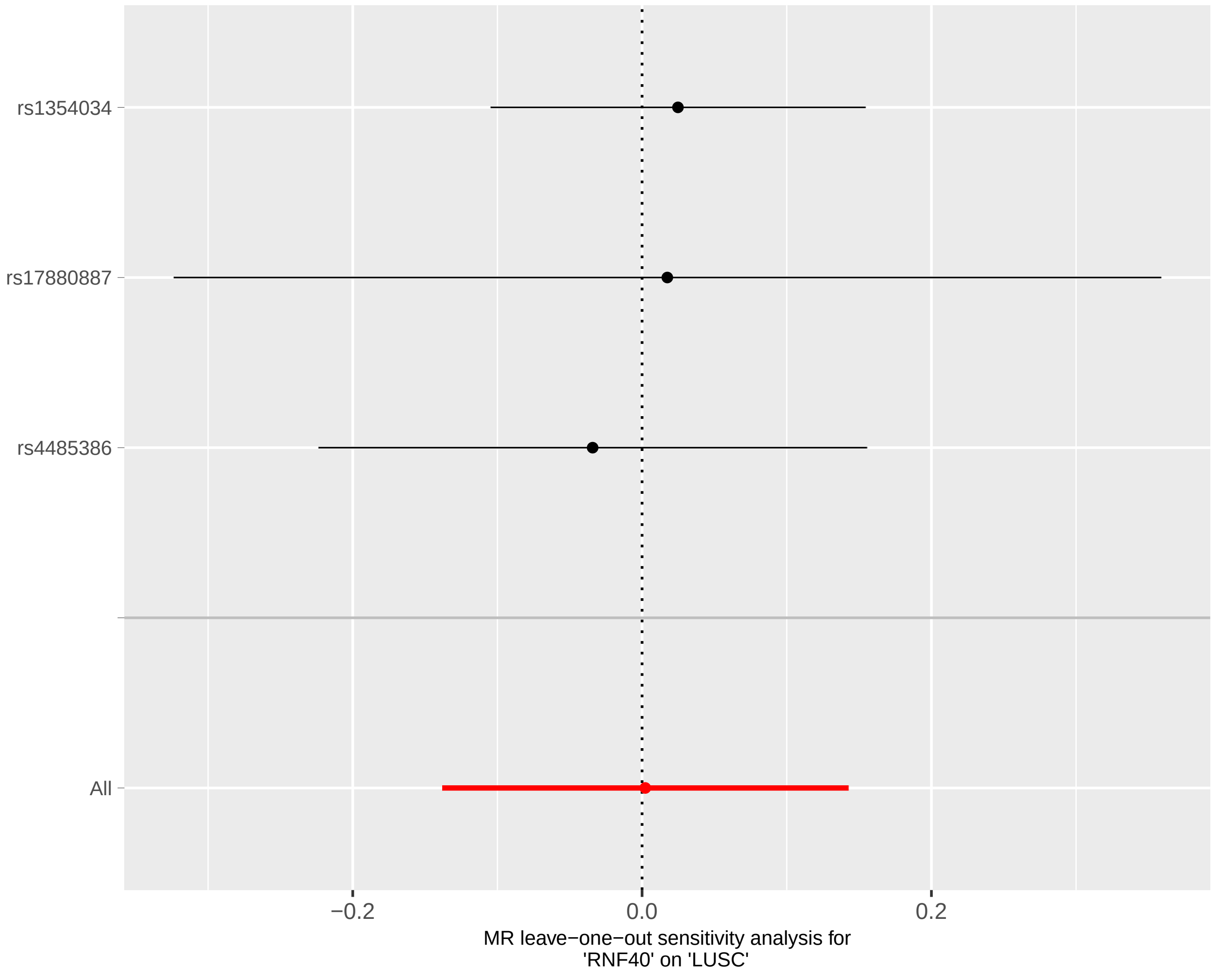

rs1354034
rs17880887
rs4485386
All
−0.2
0.0
0.2
MR leave−one−out sensitivity analysis for
'RNF40' on 'LUSC'

Insufficient number of SNPs

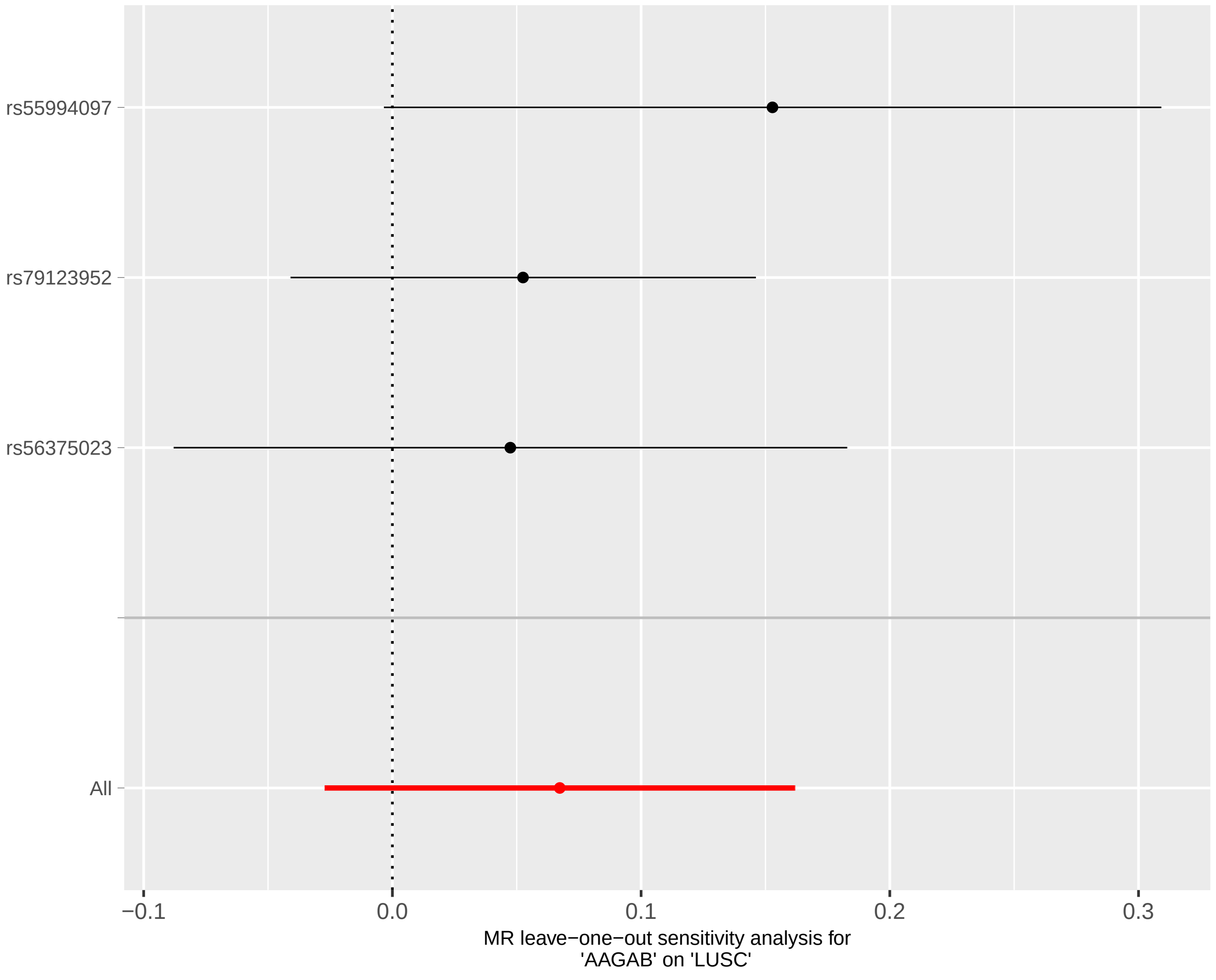
rs55994097
rs79123952
rs56375023
All
−0.1
0.0
0.1
0.2
0.3
MR leave−one−out sensitivity analysis for
'AAGAB' on 'LUSC'

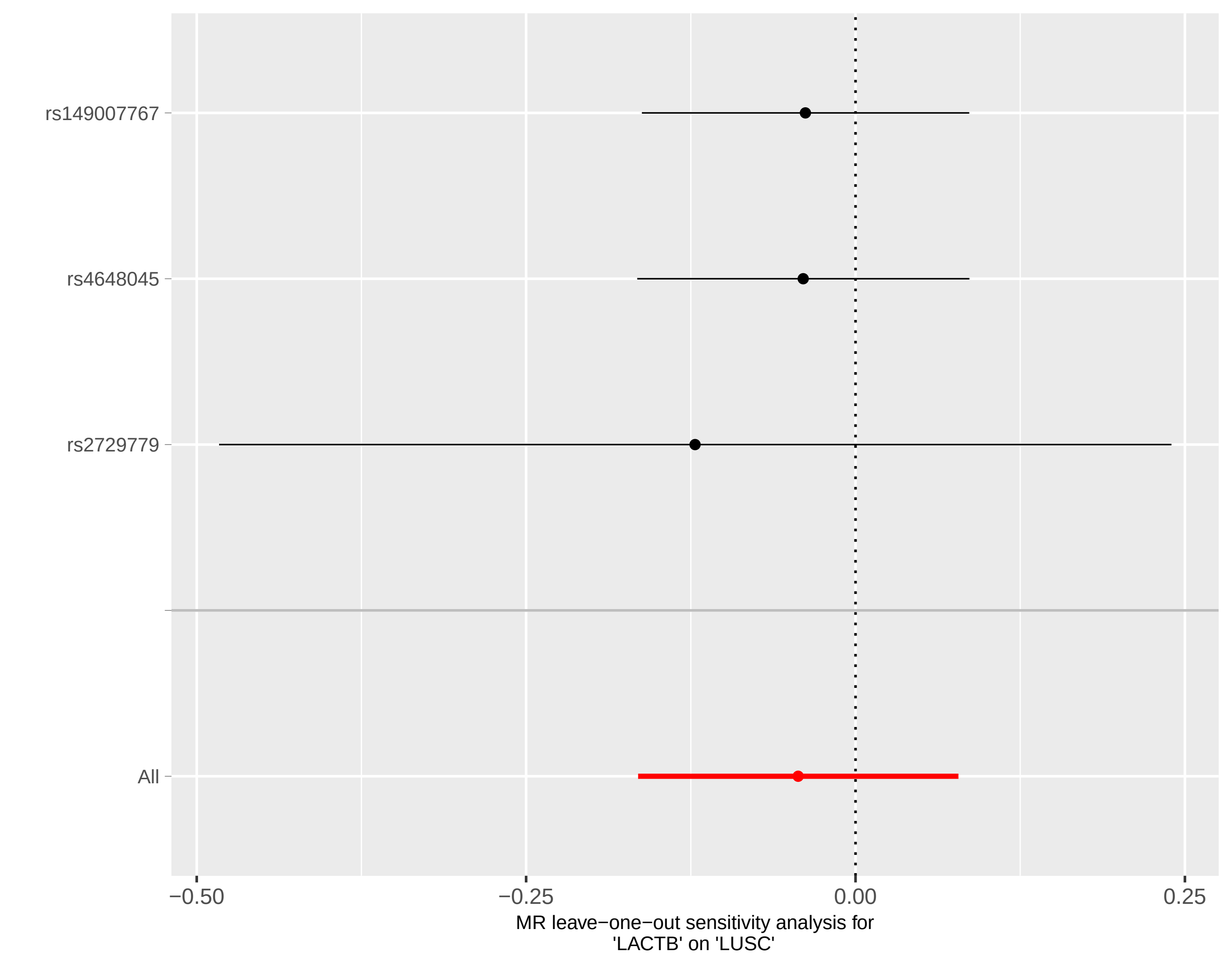
rs149007767
rs4648045
rs2729779
All
−0.50
−0.25
0.00
0.25
MR leave−one−out sensitivity analysis for
'LACTB' on 'LUSC'

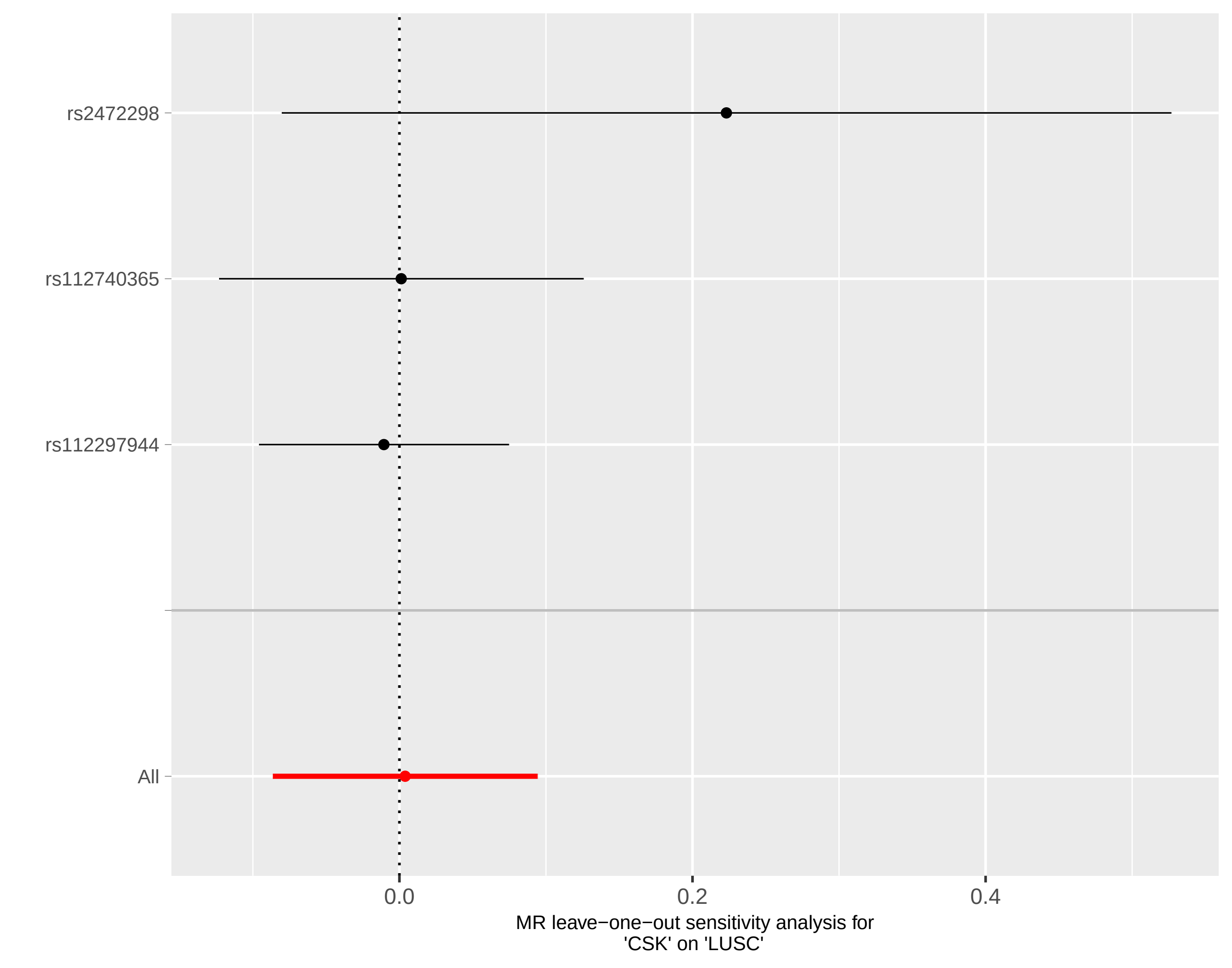

rs2472298
rs112740365
rs112297944
All
0.0
0.2
0.4
MR leave−one−out sensitivity analysis for
'CSK' on 'LUSC'

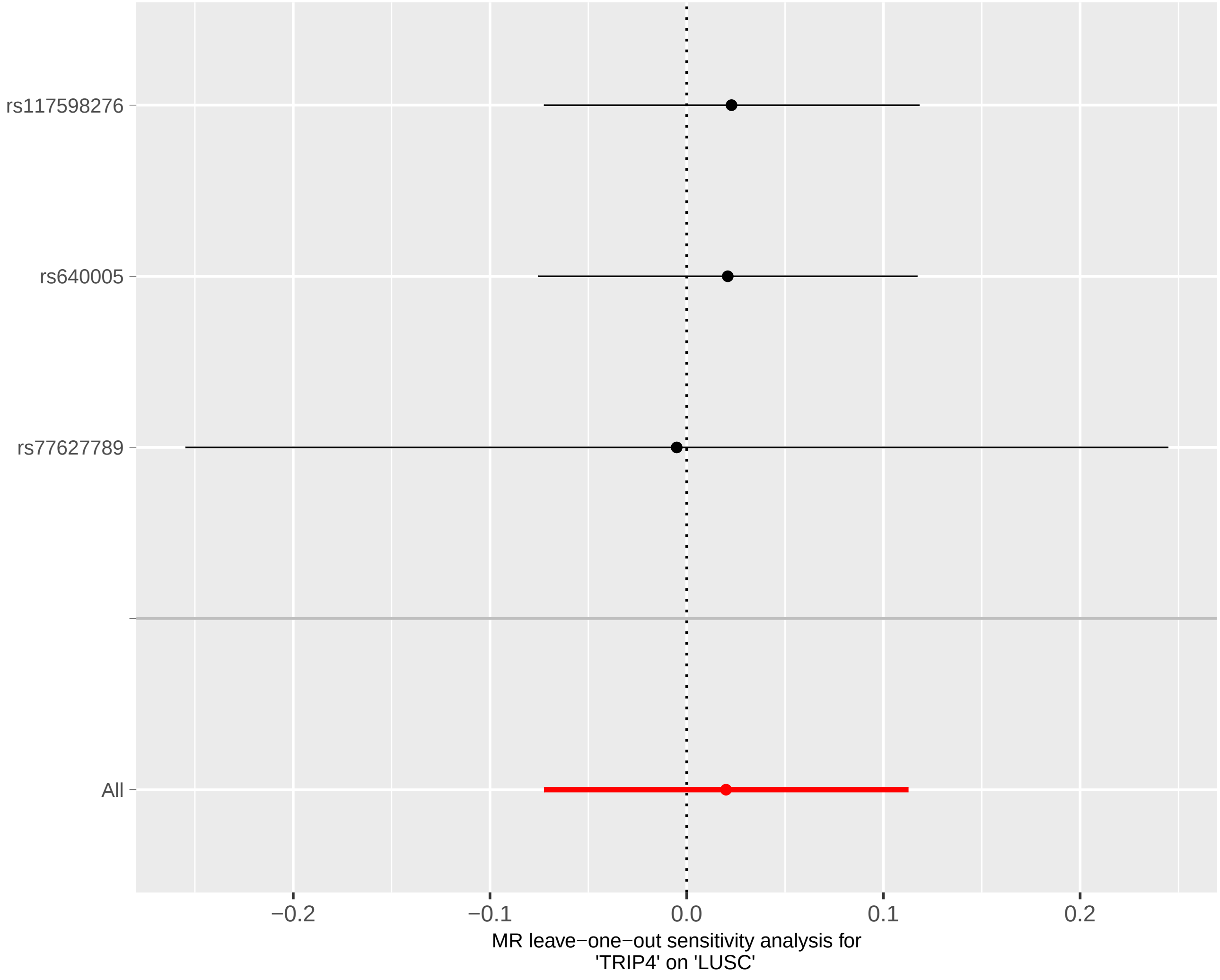
rs117598276
rs640005
rs77627789
All
−0.2
−0.1
0.0
0.1
0.2
MR leave−one−out sensitivity analysis for
'TRIP4' on 'LUSC'

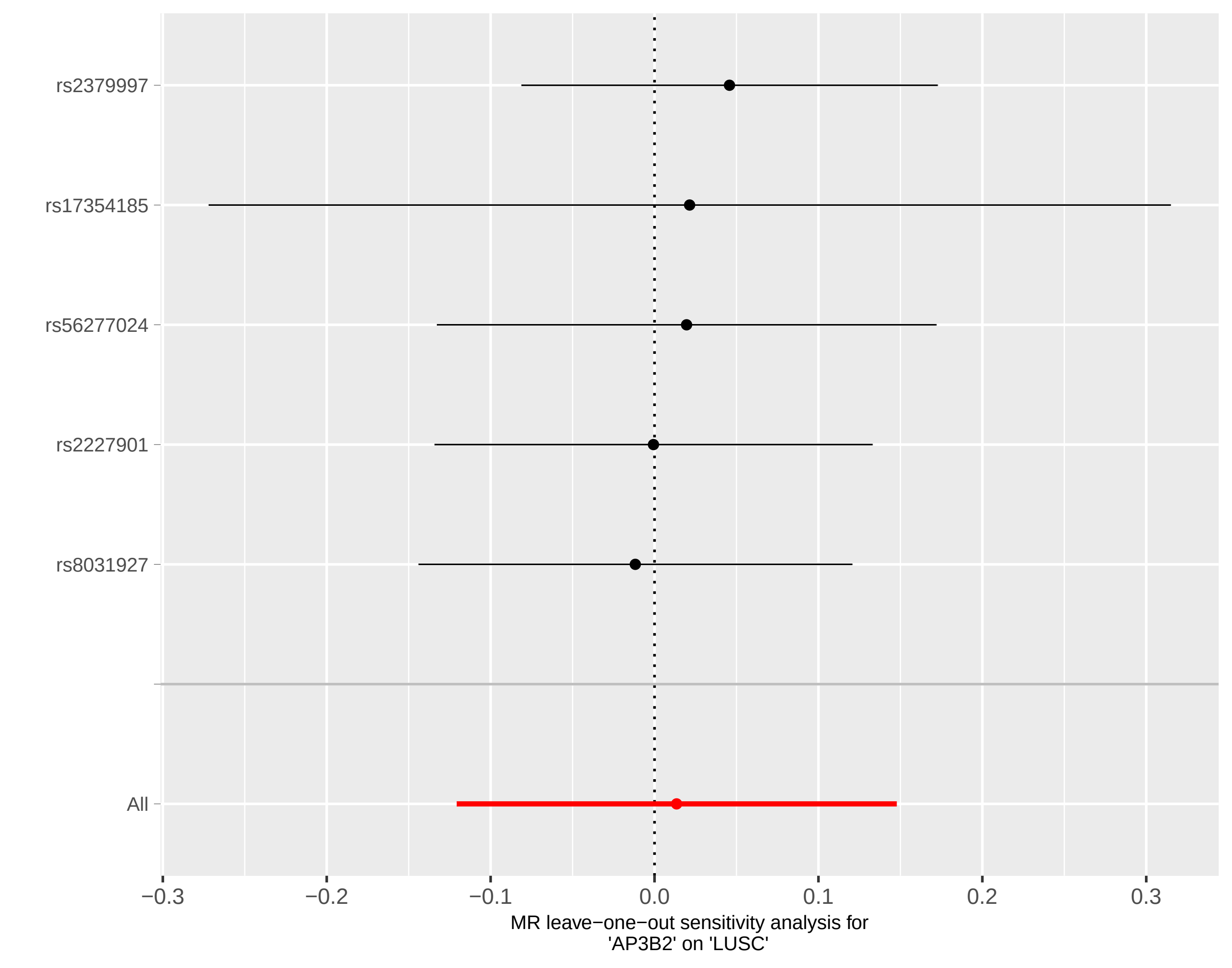

rs2379997
rs17354185
rs56277024
rs2227901
rs8031927
All
−0.3
−0.2
−0.1
0.0
0.1
0.2
0.3
MR leave−one−out sensitivity analysis for
'AP3B2' on 'LUSC'

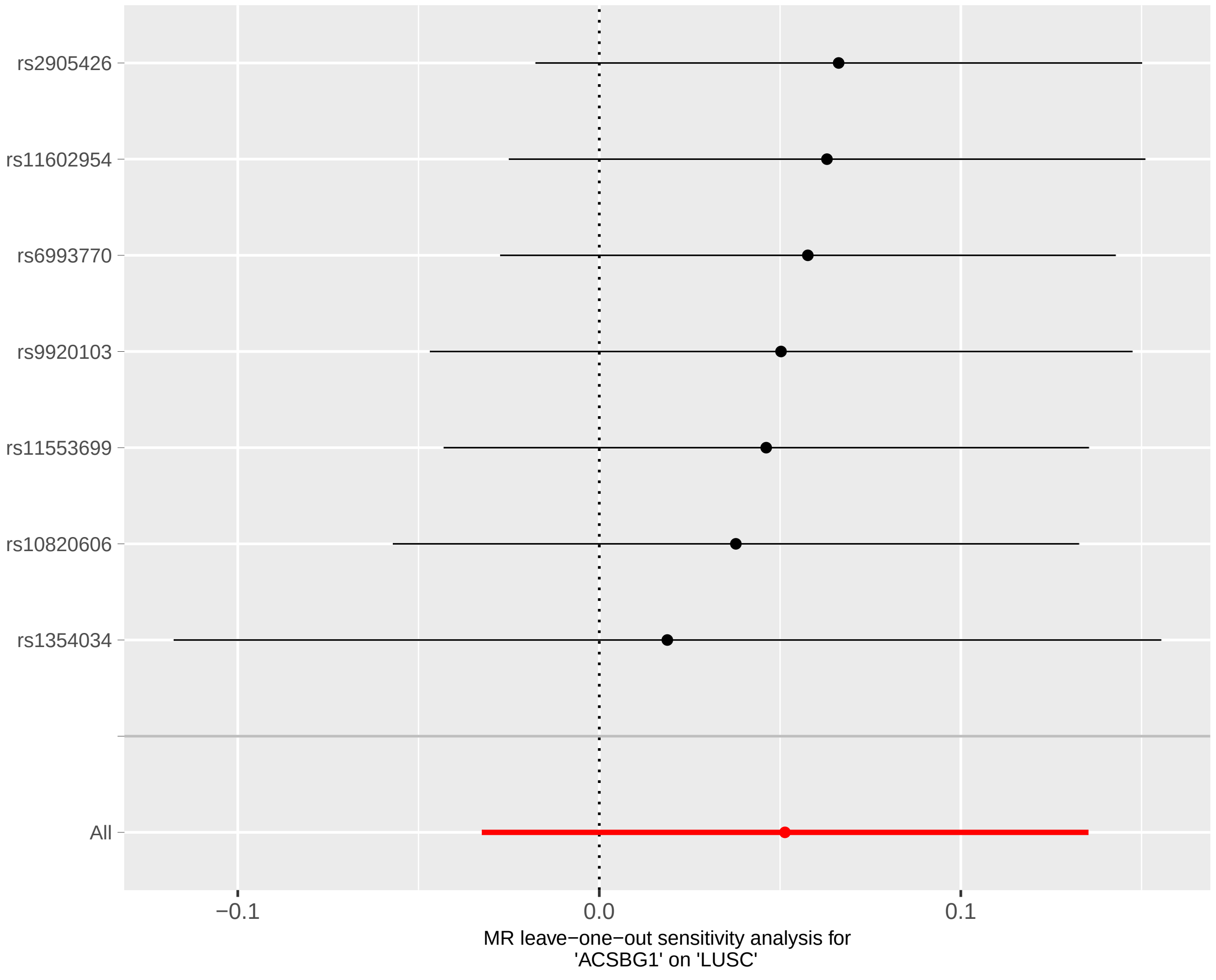
rs2905426
rs11602954
rs6993770
rs9920103
rs11553699
rs10820606
rs1354034
All
−0.1
0.0
0.1
MR leave−one−out sensitivity analysis for
'ACSBG1' on 'LUSC'

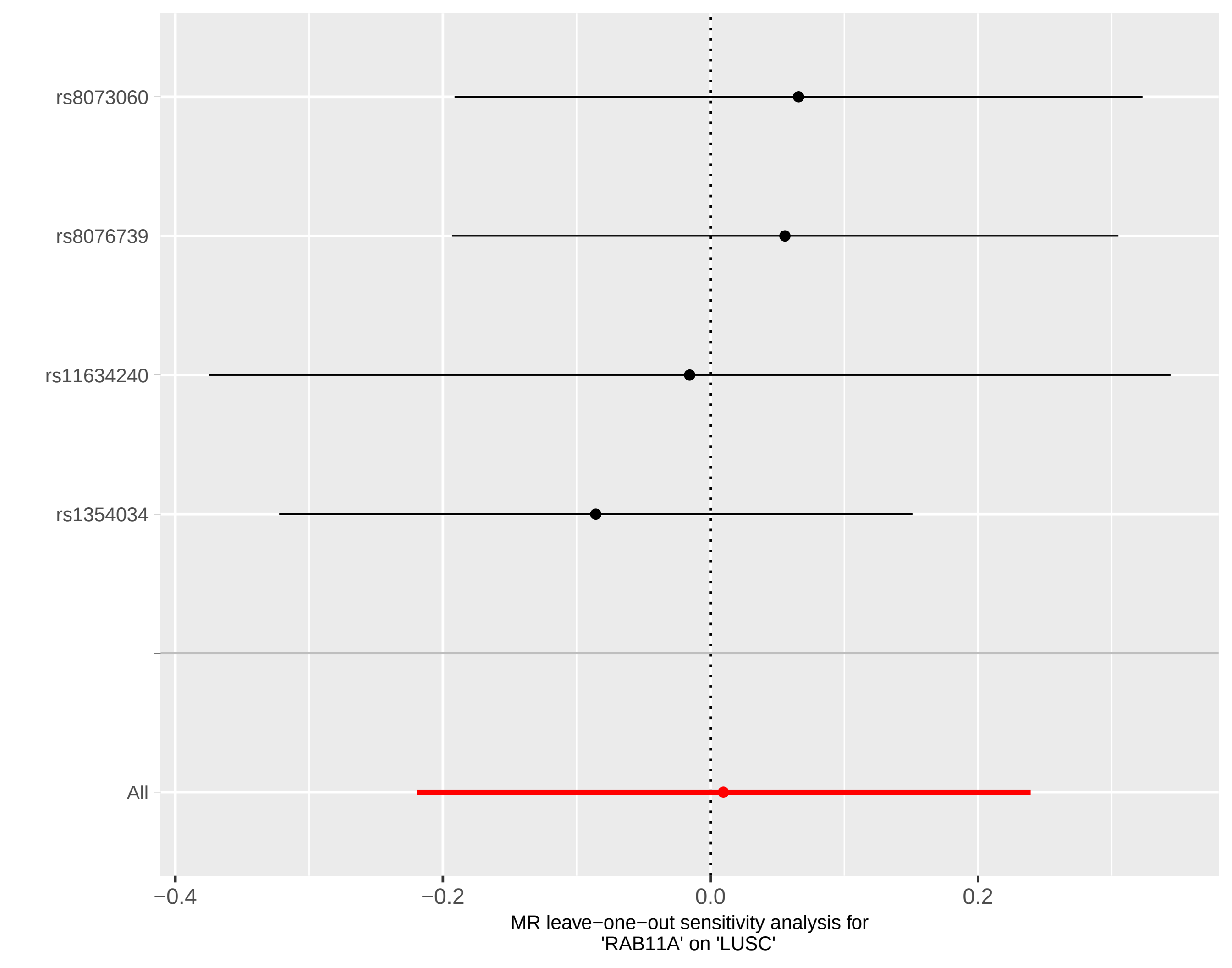

rs8073060
rs8076739
rs11634240
rs1354034
All
−0.4
−0.2
0.0
0.2
MR leave−one−out sensitivity analysis for
'RAB11A' on 'LUSC'

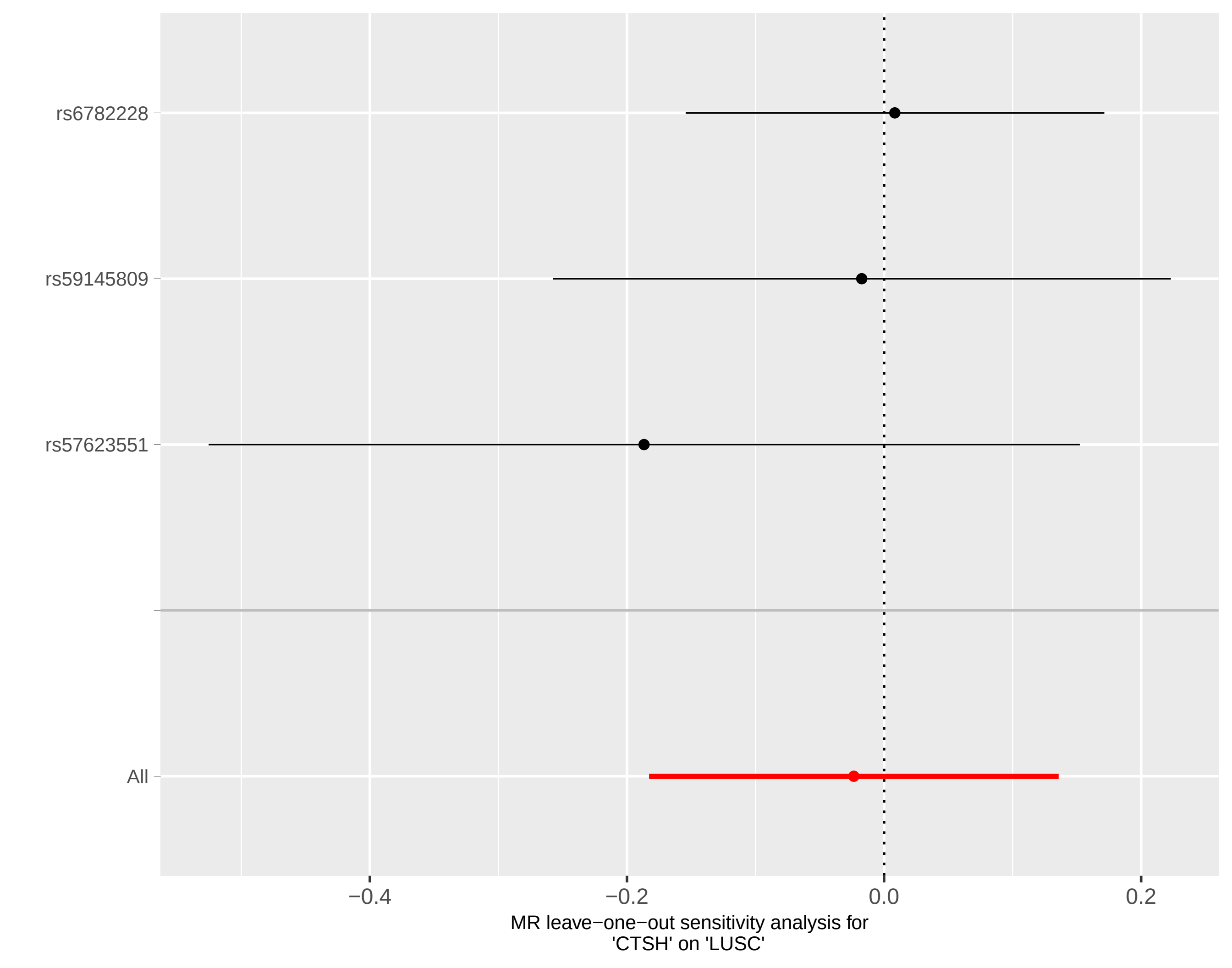

rs6782228
rs59145809
rs57623551
All
−0.4
−0.2
0.0
0.2
MR leave−one−out sensitivity analysis for
'CTSH' on 'LUSC'

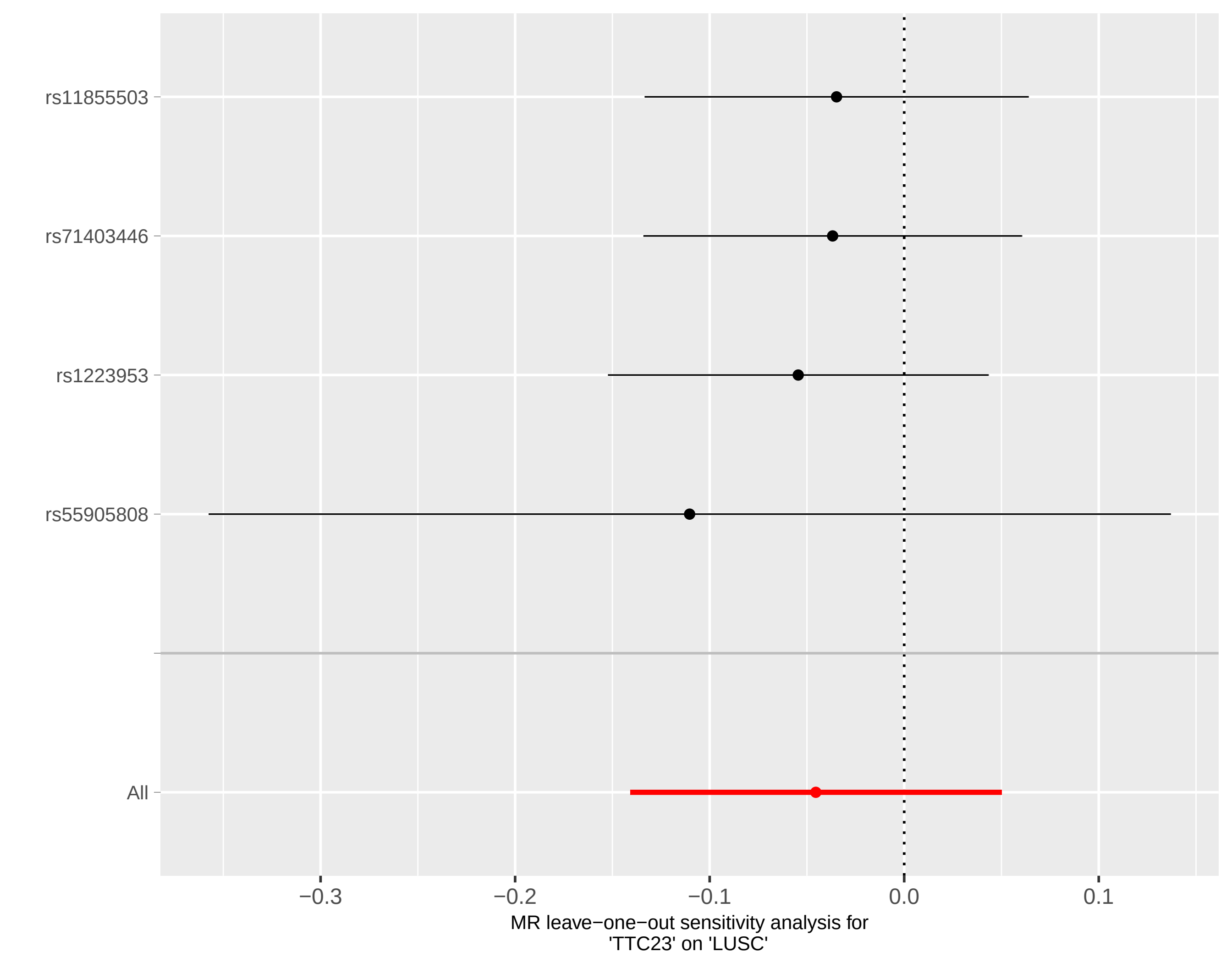
rs11855503
rs71403446
rs1223953
rs55905808
All
−0.3
−0.2
−0.1
0.0
0.1
MR leave−one−out sensitivity analysis for
'TTC23' on 'LUSC'

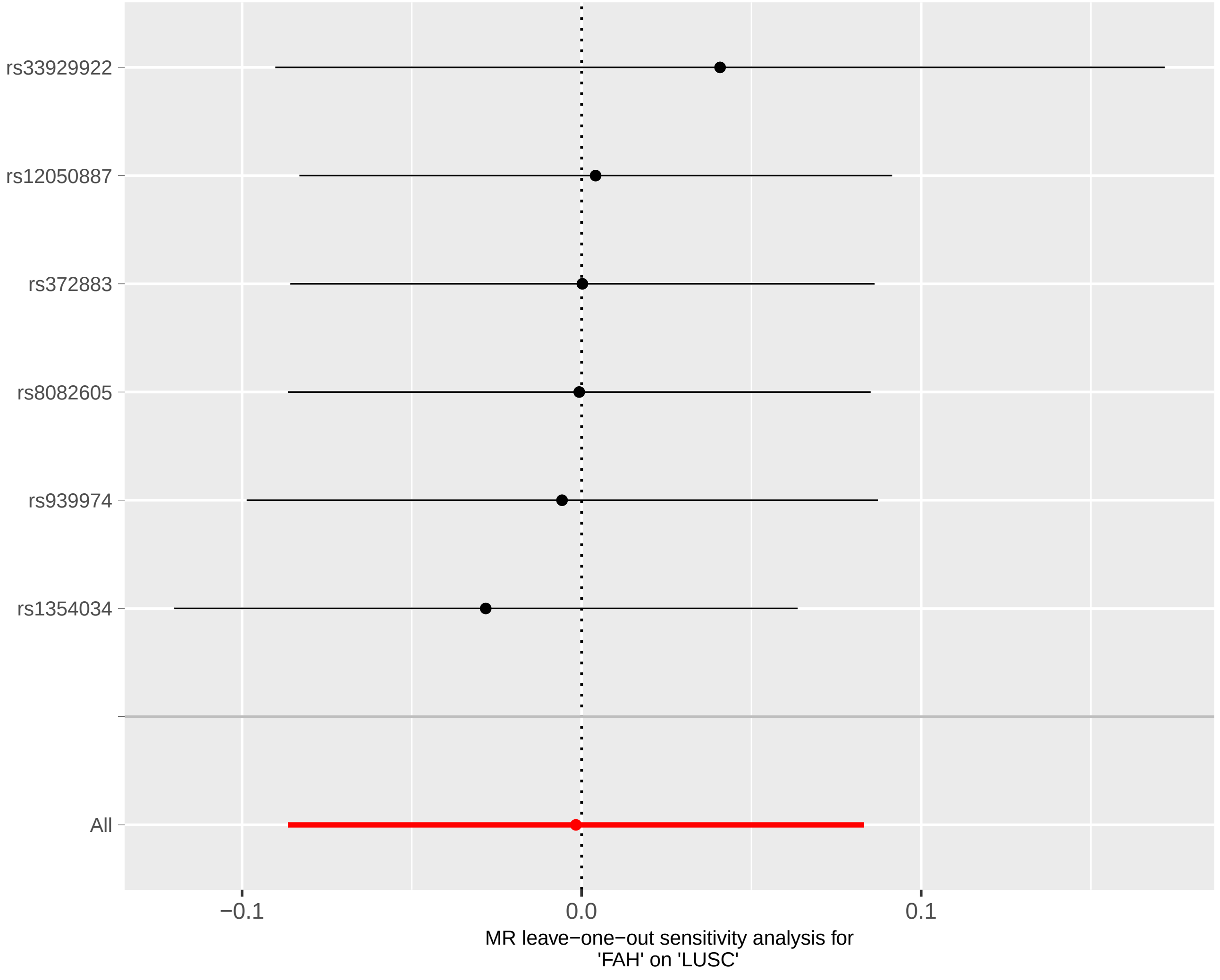
rs33929922
rs12050887
rs372883
rs8082605
rs939974
rs1354034
All
−0.1
0.0
0.1
MR leave−one−out sensitivity analysis for
'FAH' on 'LUSC'

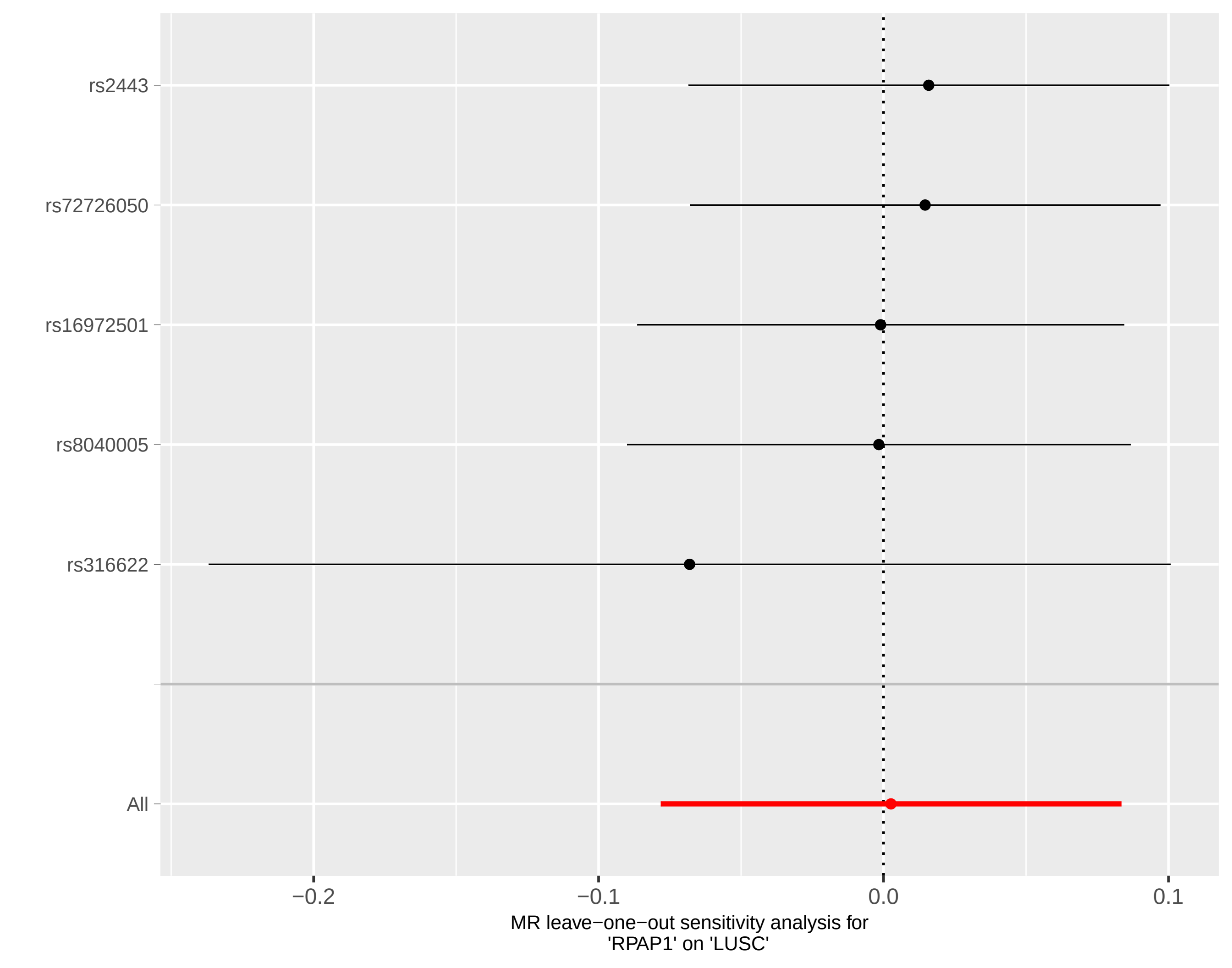

rs2443
rs72726050
rs16972501
rs8040005
rs316622
All
−0.2
−0.1
0.0
0.1
MR leave−one−out sensitivity analysis for
'RPAP1' on 'LUSC'

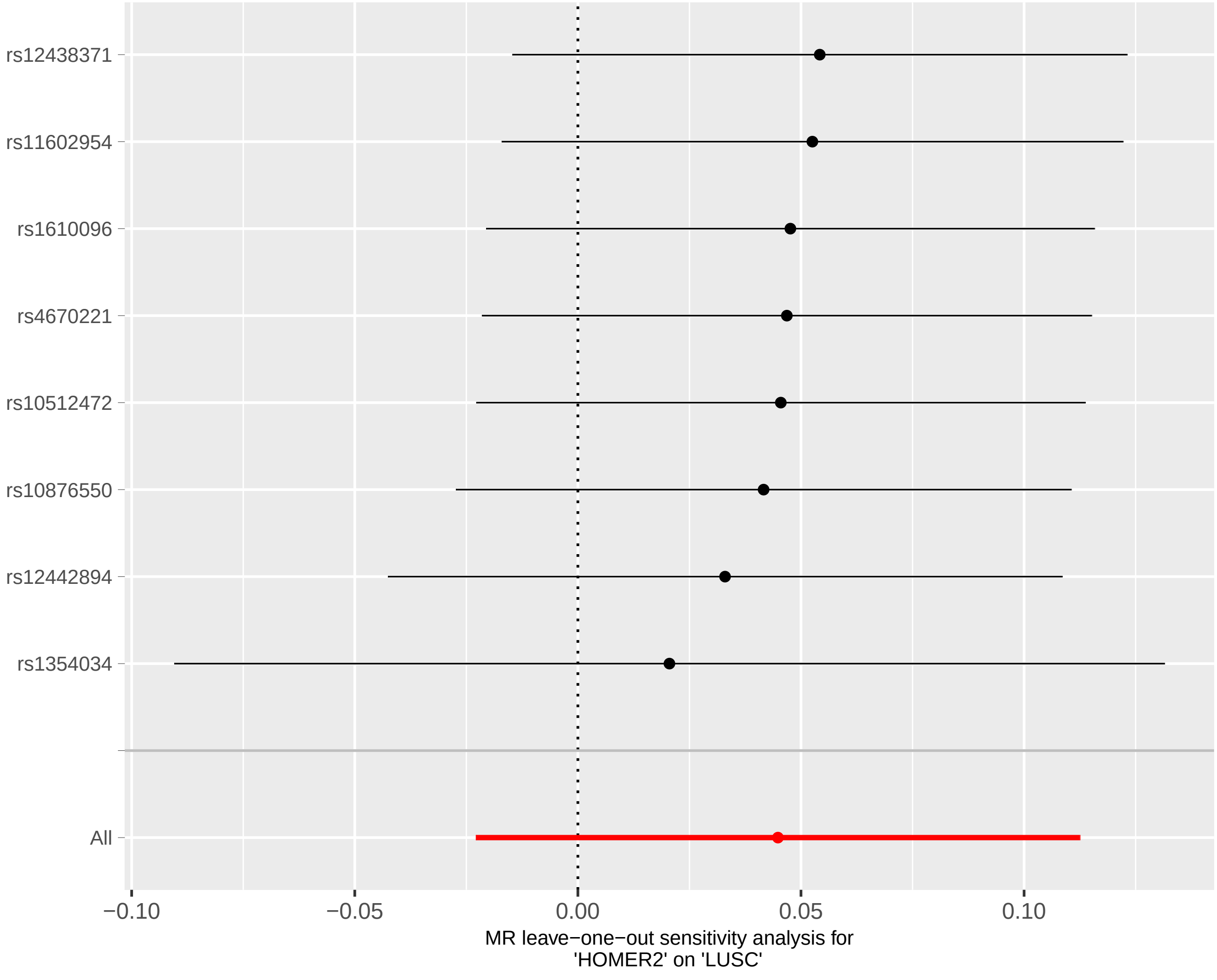

rs12438371
rs11602954
rs1610096
rs4670221
rs10512472
rs10876550
rs12442894
rs1354034
All
−0.10
−0.05
0.00
0.05
0.10
MR leave−one−out sensitivity analysis for
'HOMER2' on 'LUSC'

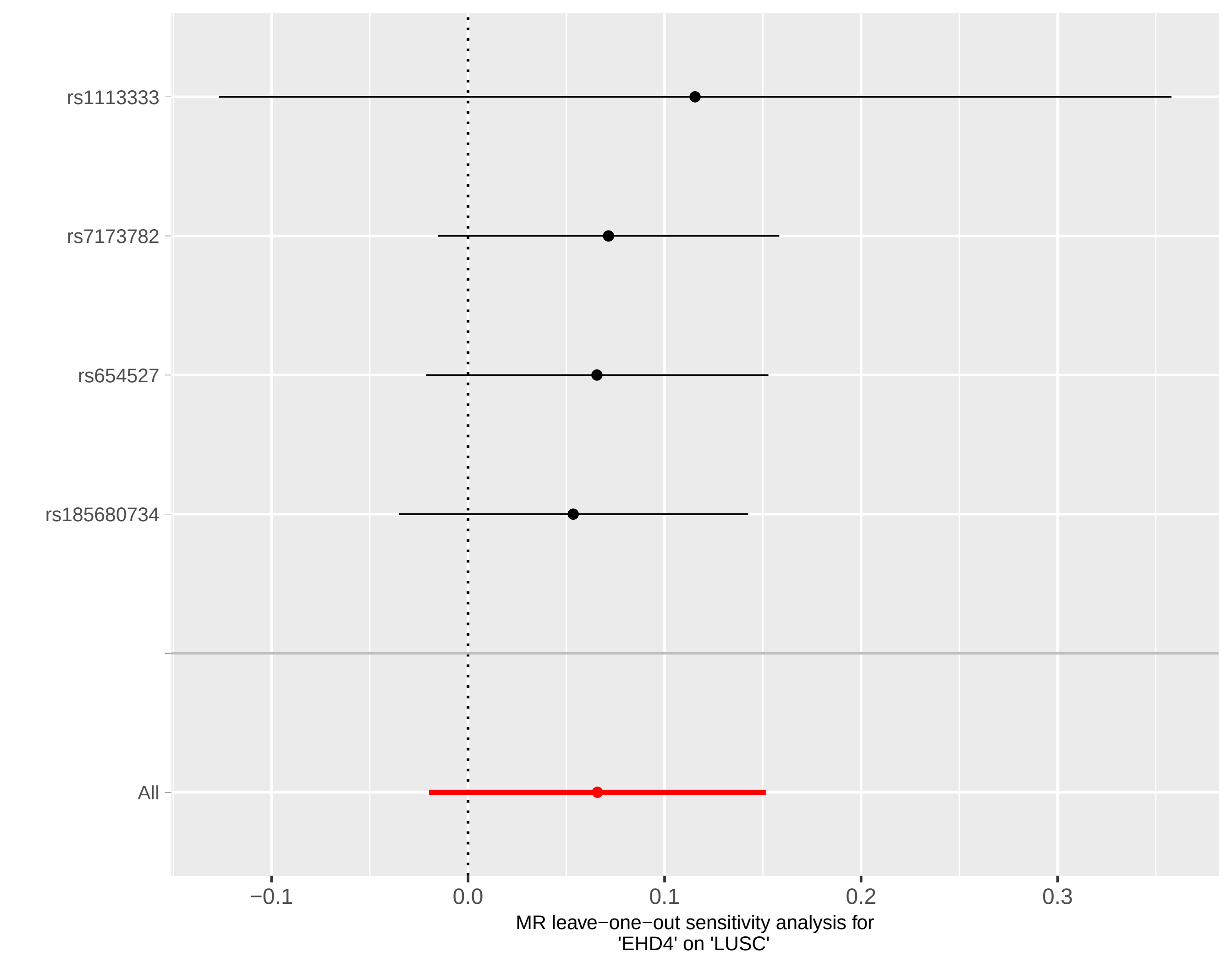
rs1113333
rs7173782
rs654527
rs185680734
All
−0.1
0.0
0.1
0.2
0.3
MR leave−one−out sensitivity analysis for
'EHD4' on 'LUSC'

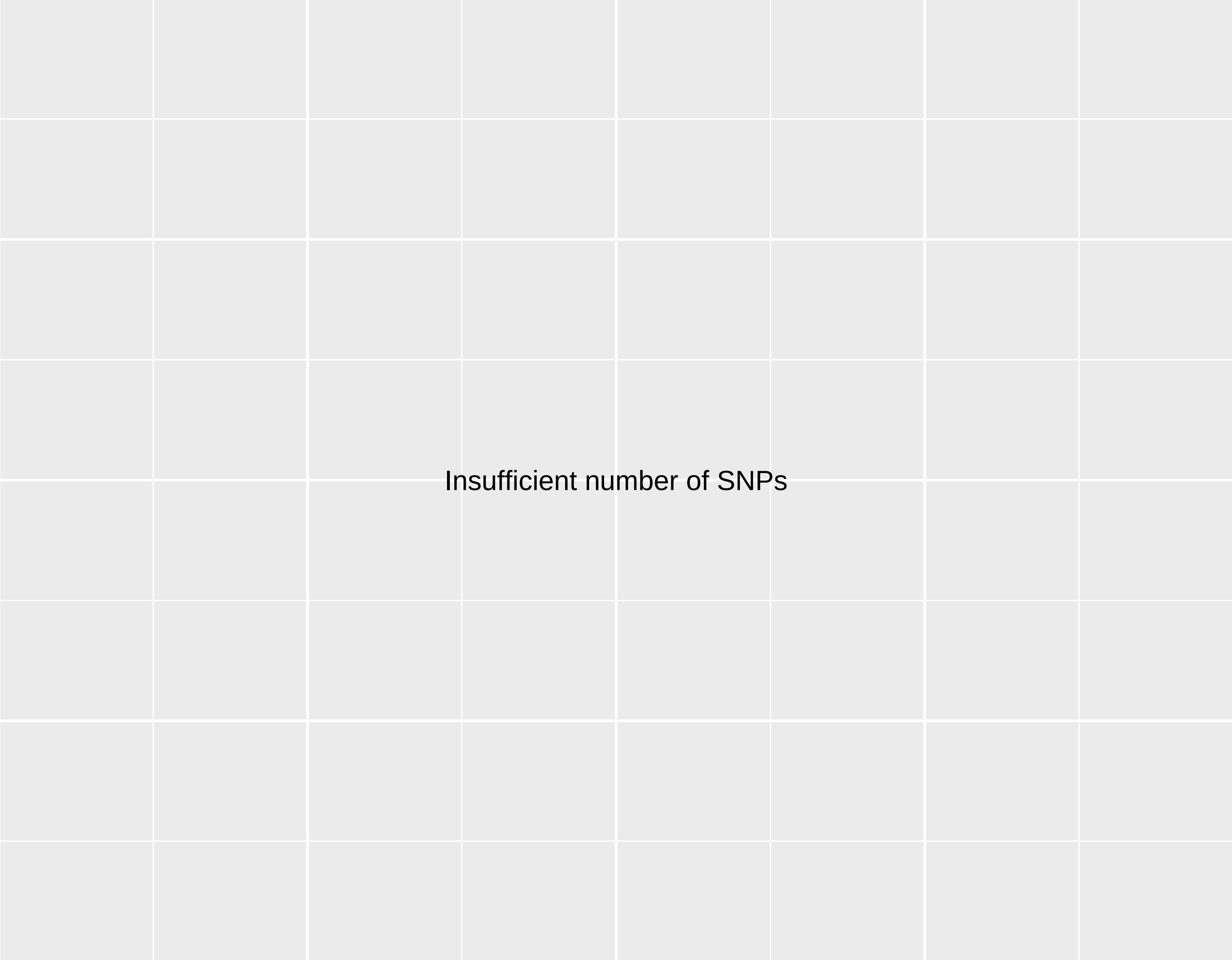
Insufficient number of SNPs

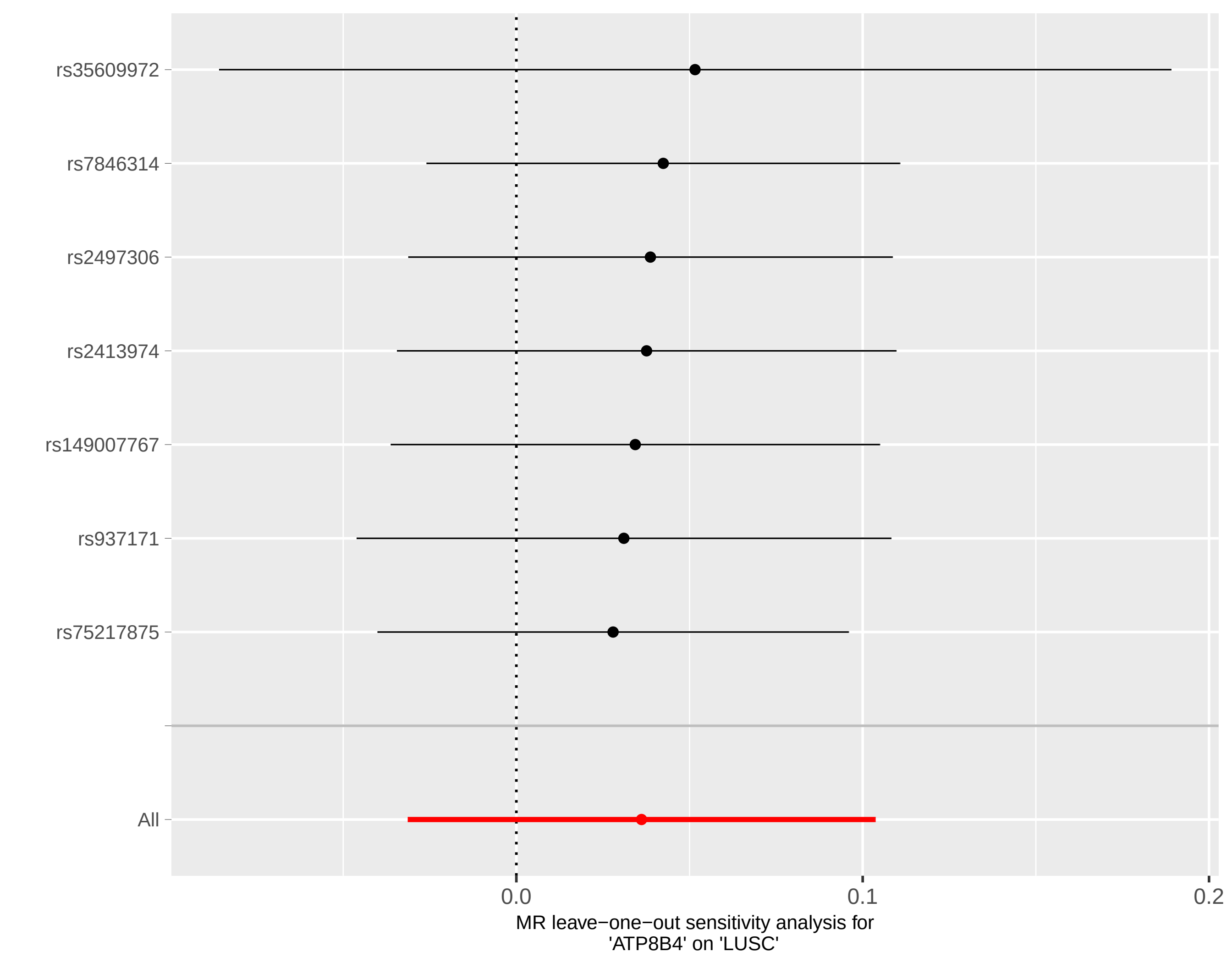

rs35609972
rs7846314
rs2497306
rs2413974
rs149007767
rs937171
rs75217875
All
0.0
0.1
0.2
MR leave−one−out sensitivity analysis for
'ATP8B4' on 'LUSC'

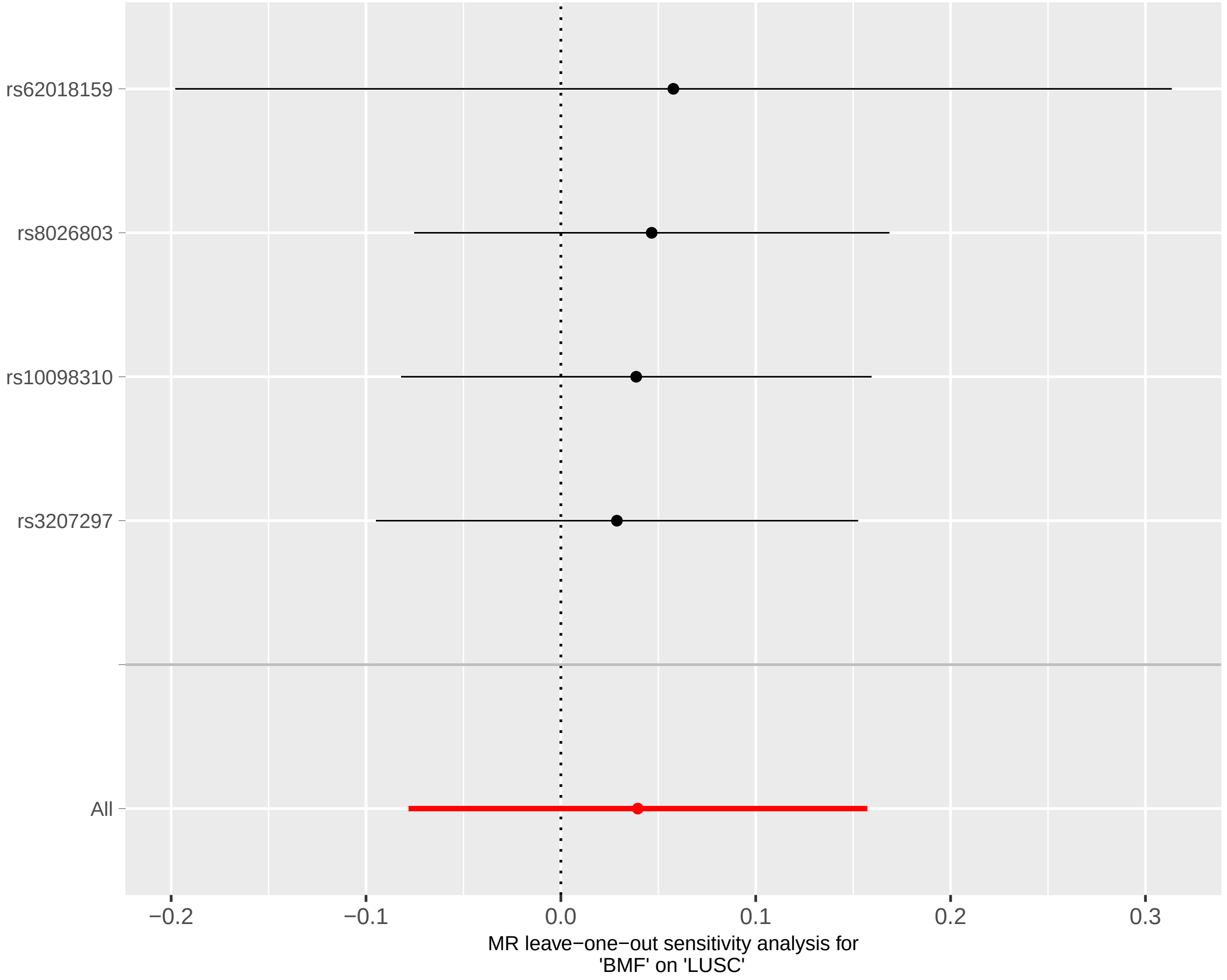

rs62018159
rs8026803
rs10098310
rs3207297
All
−0.2
−0.1
0.0
0.1
0.2
0.3
MR leave−one−out sensitivity analysis for
'BMF' on 'LUSC'

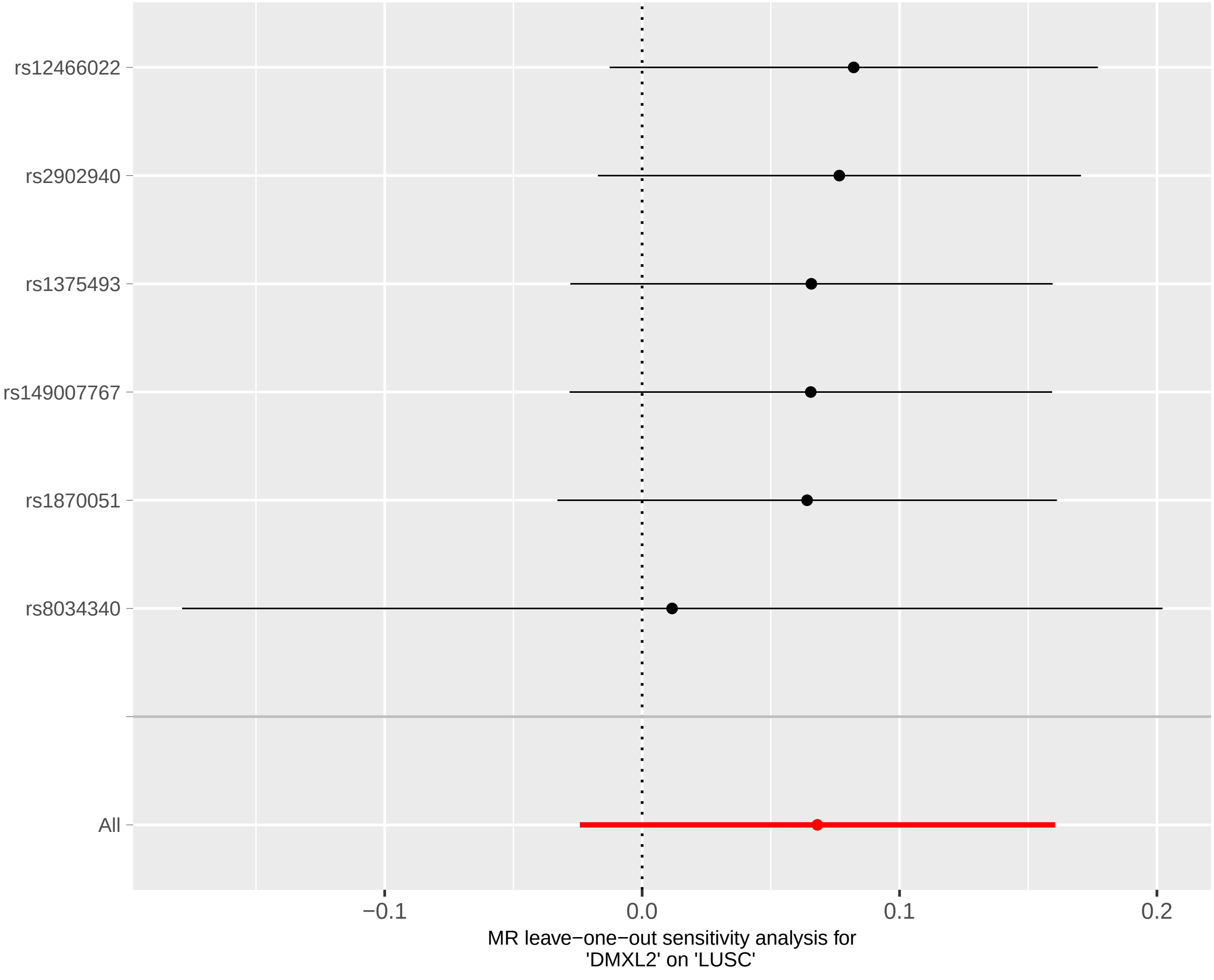

rs12466022
rs2902940
rs1375493
rs149007767
rs1870051
rs8034340
All
−0.1
0.0
0.1
0.2
MR leave−one−out sensitivity analysis for
'DMXL2' on 'LUSC'

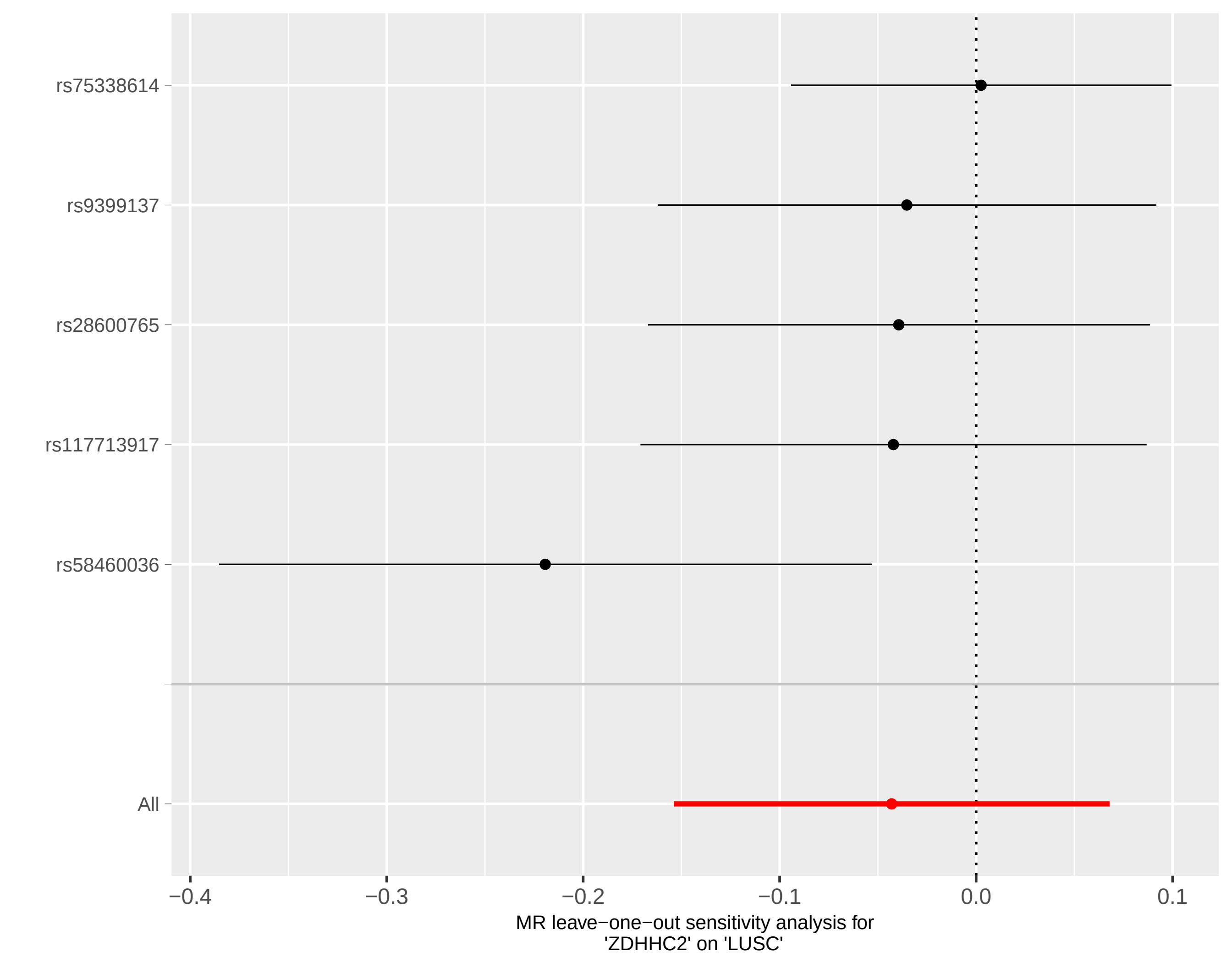

rs75338614
rs9399137
rs28600765
rs117713917
rs58460036
All
−0.4
−0.3
−0.2
−0.1
0.0
0.1
MR leave−one−out sensitivity analysis for
'ZDHHC2' on 'LUSC'

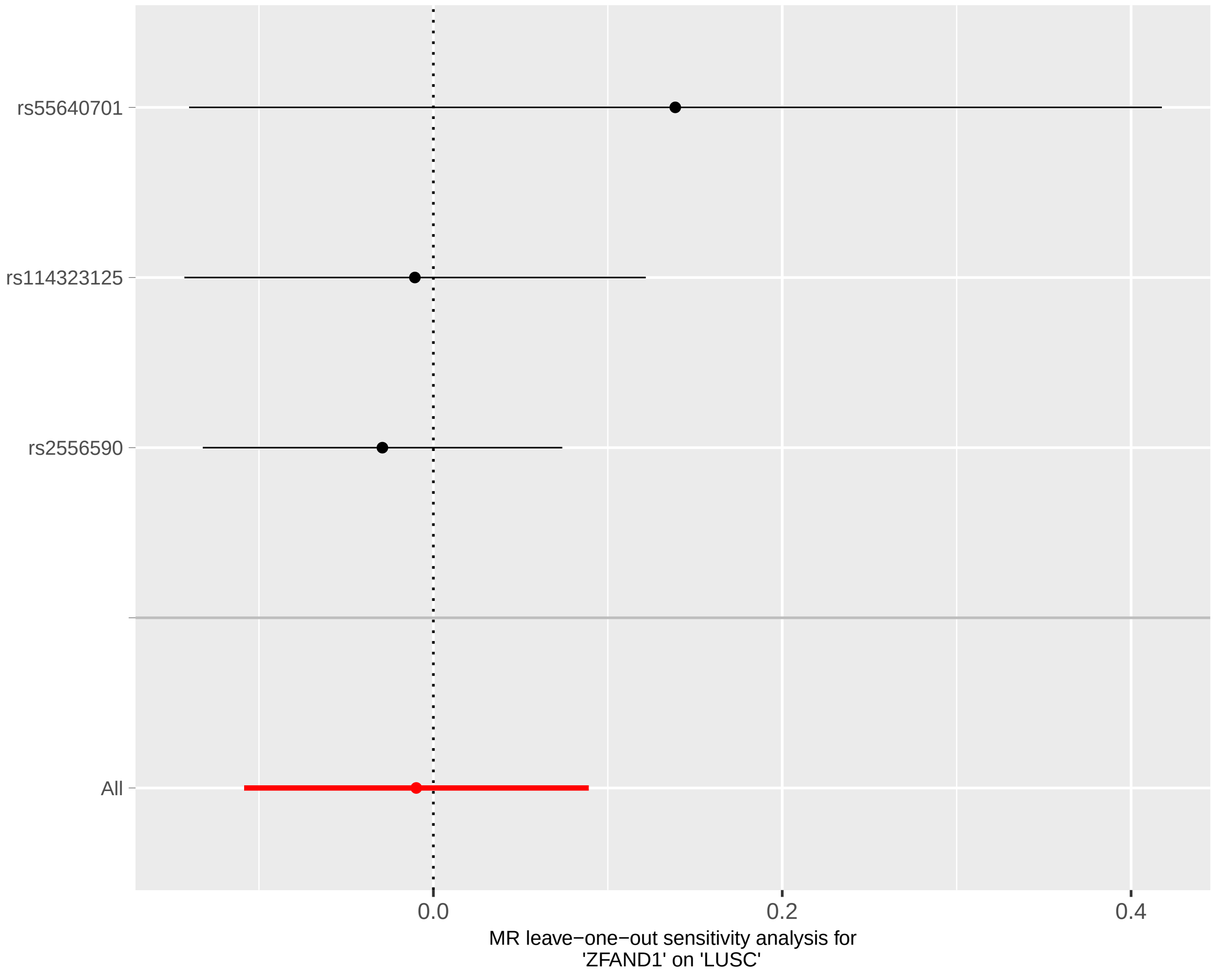
rs55640701
rs114323125
rs2556590
All
0.0
0.2
0.4
MR leave−one−out sensitivity analysis for
'ZFAND1' on 'LUSC'

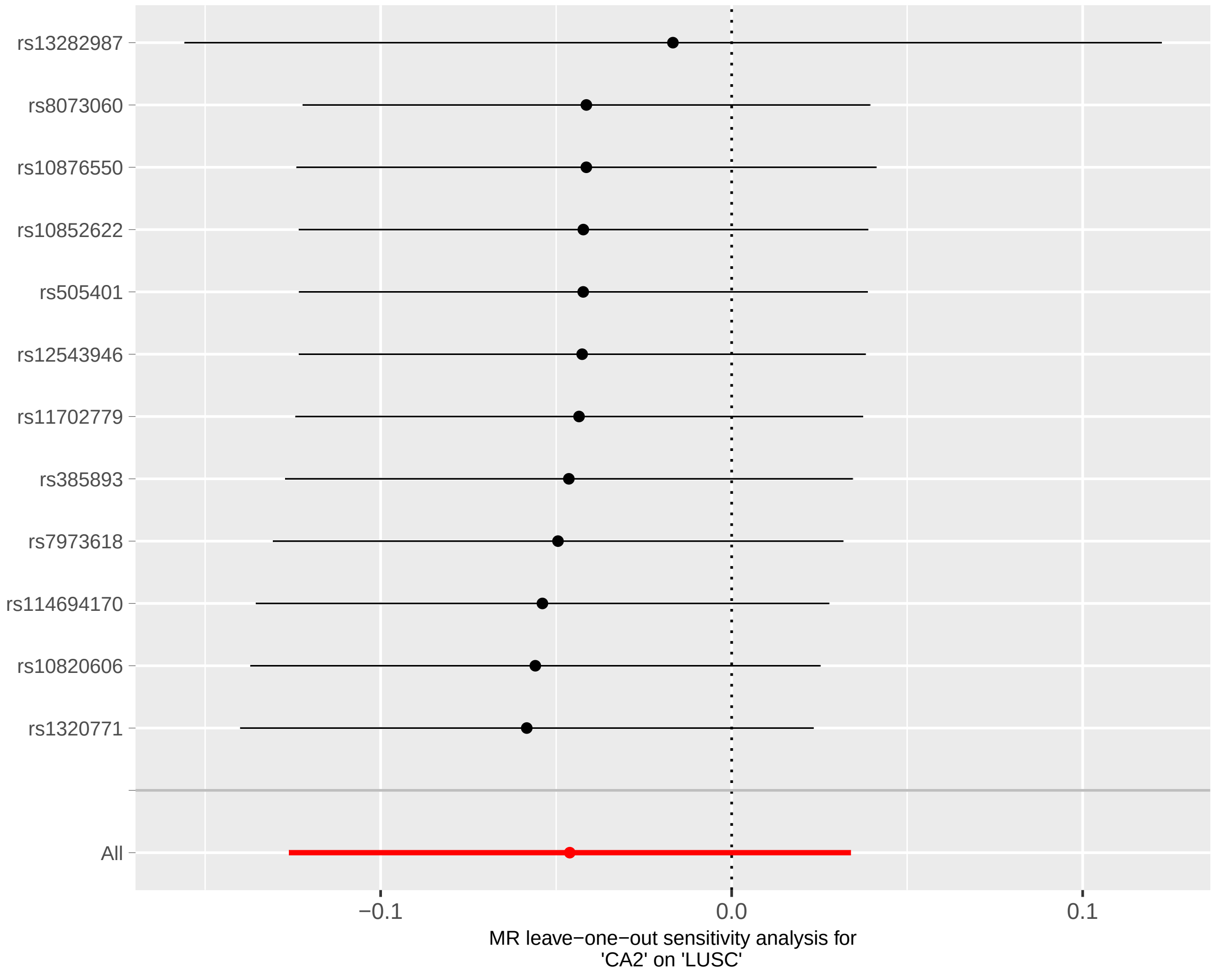

rs13282987
rs8073060
rs10876550
rs10852622
rs505401
rs12543946
rs11702779
rs385893
rs7973618
rs114694170
rs10820606
rs1320771
All
−0.1
0.0
0.1
MR leave−one−out sensitivity analysis for
'CA2' on 'LUSC'

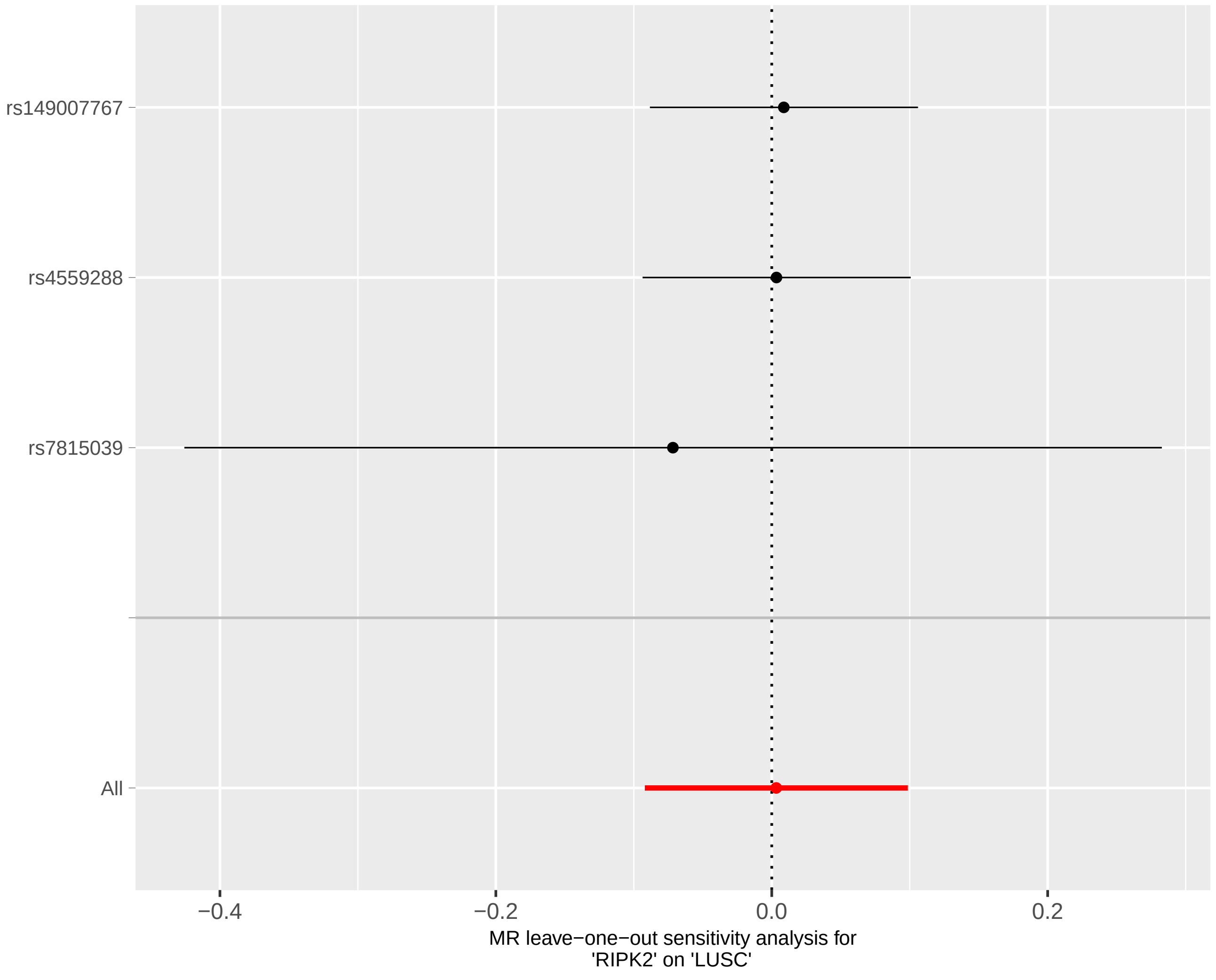
rs149007767
rs4559288
rs7815039
All
−0.4
−0.2
0.0
0.2
MR leave−one−out sensitivity analysis for
'RIPK2' on 'LUSC'

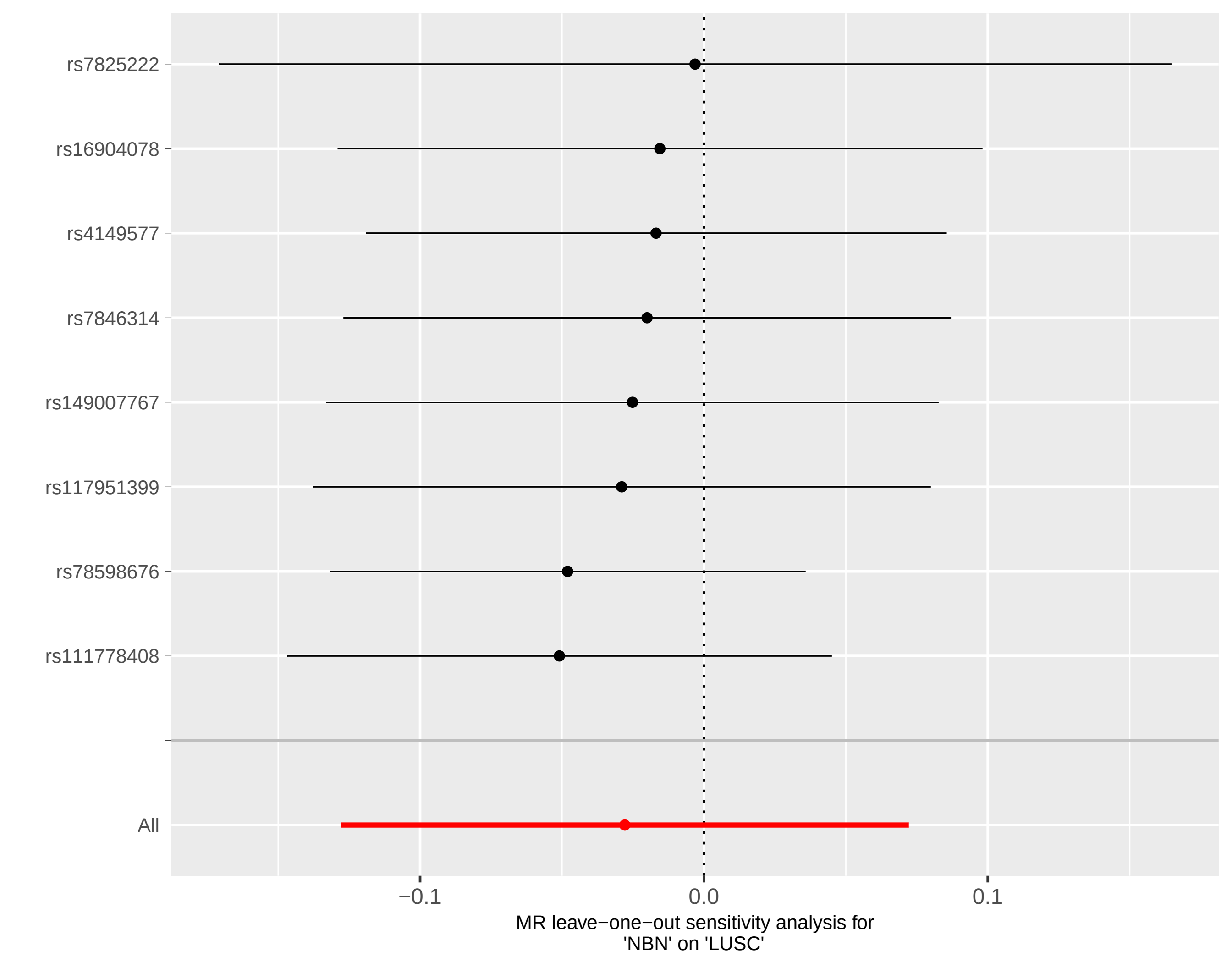

rs7825222
rs16904078
rs4149577
rs7846314
rs149007767
rs117951399
rs78598676
rs111778408
All
−0.1
0.0
0.1
MR leave−one−out sensitivity analysis for
'NBN' on 'LUSC'

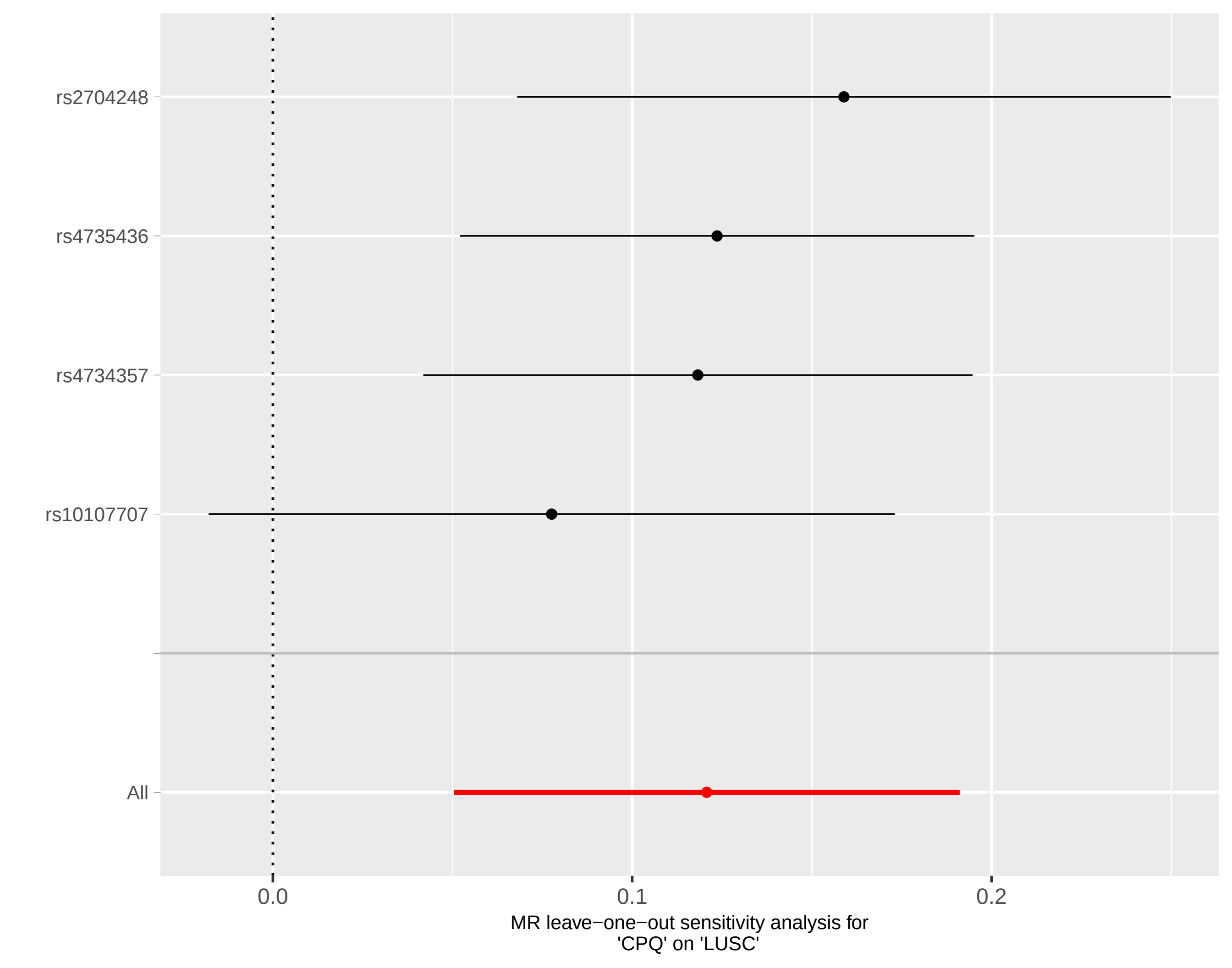
rs2704248
rs4735436
rs4734357
rs10107707
All
0.0
0.1
0.2
MR leave−one−out sensitivity analysis for
'CPQ' on 'LUSC'

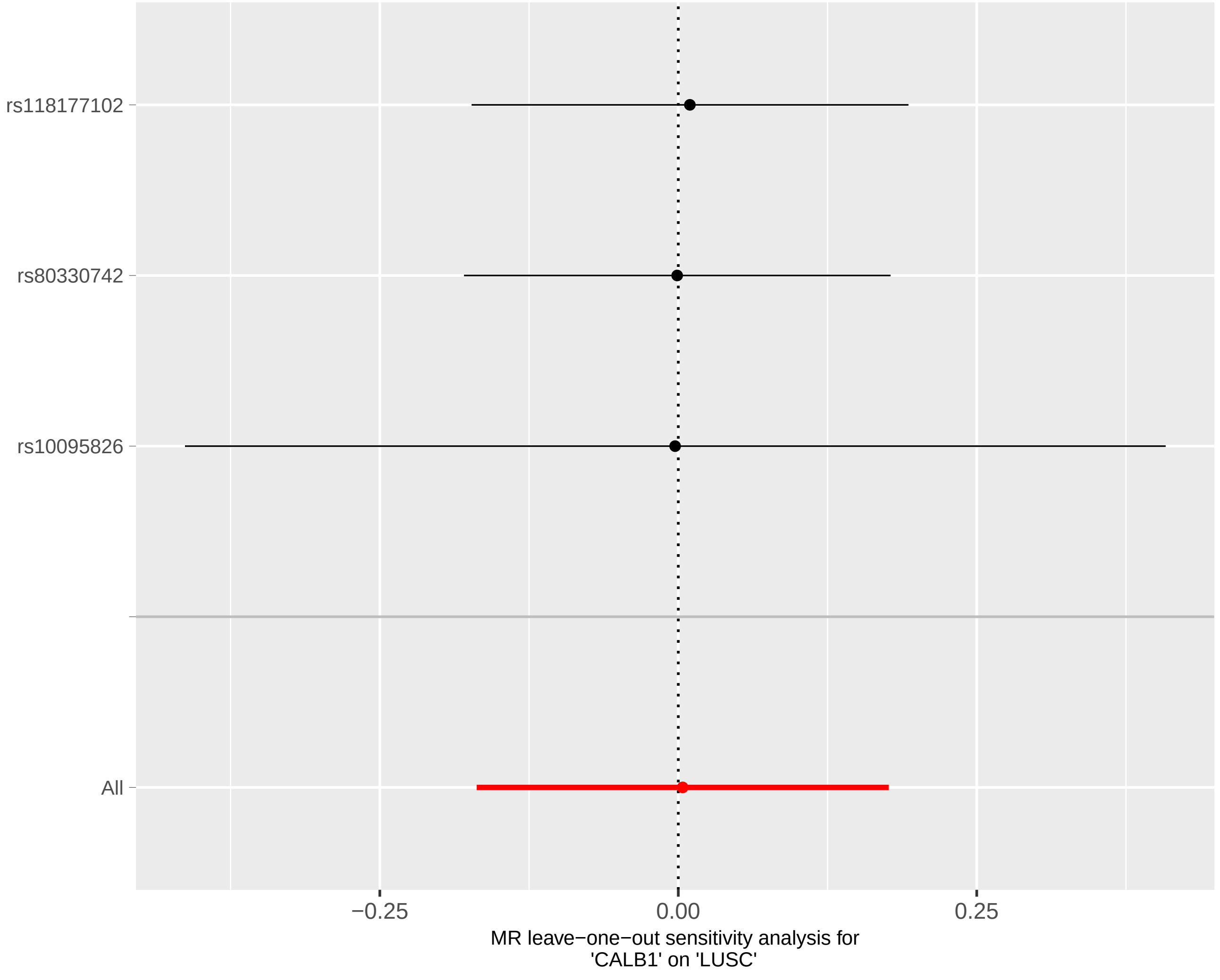
rs118177102
rs80330742
rs10095826
All
−0.25
0.00
0.25
MR leave−one−out sensitivity analysis for
'CALB1' on 'LUSC'

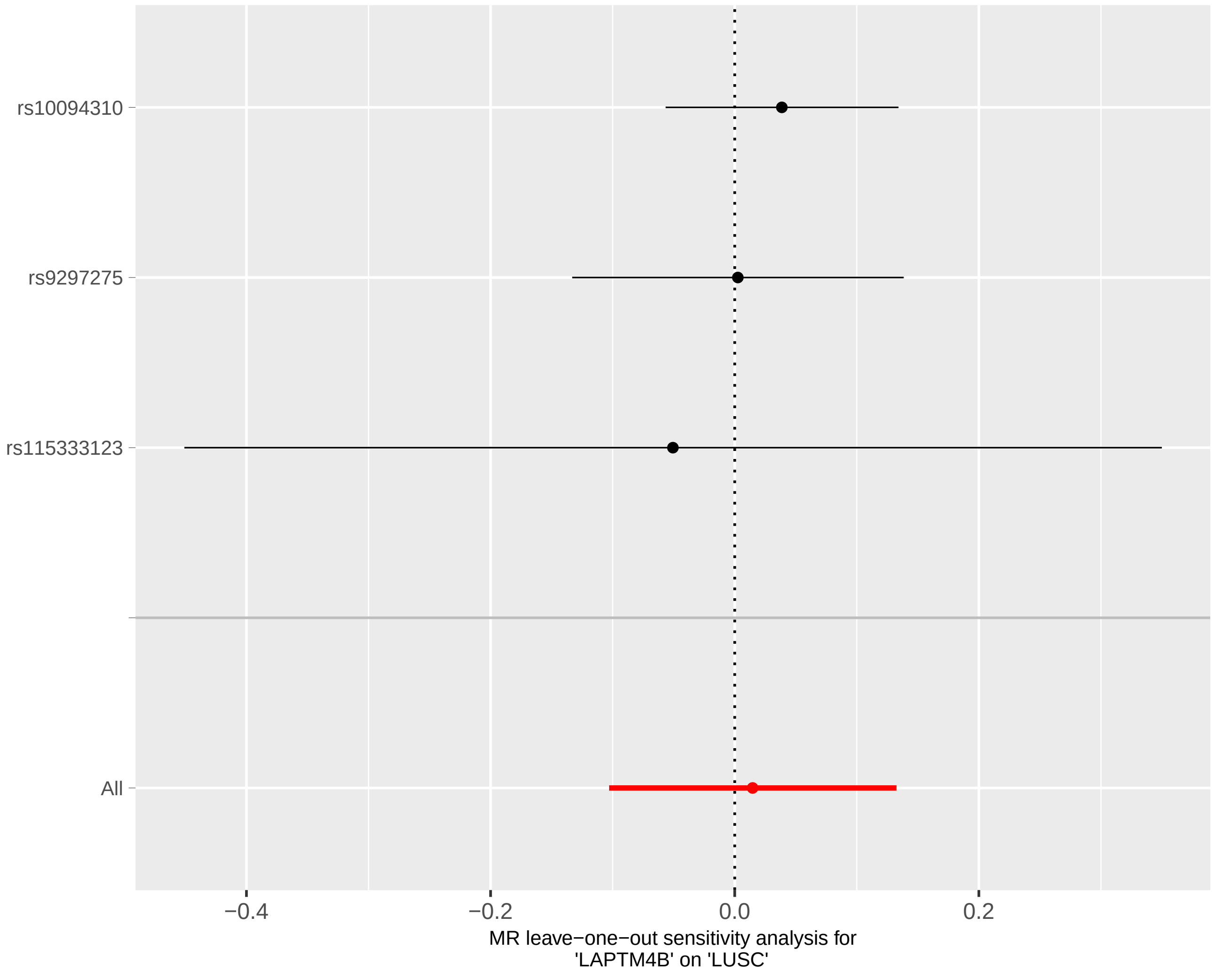
rs10094310
rs9297275
rs115333123
All
−0.4
−0.2
0.0
0.2
MR leave−one−out sensitivity analysis for
'LAPTM4B' on 'LUSC'

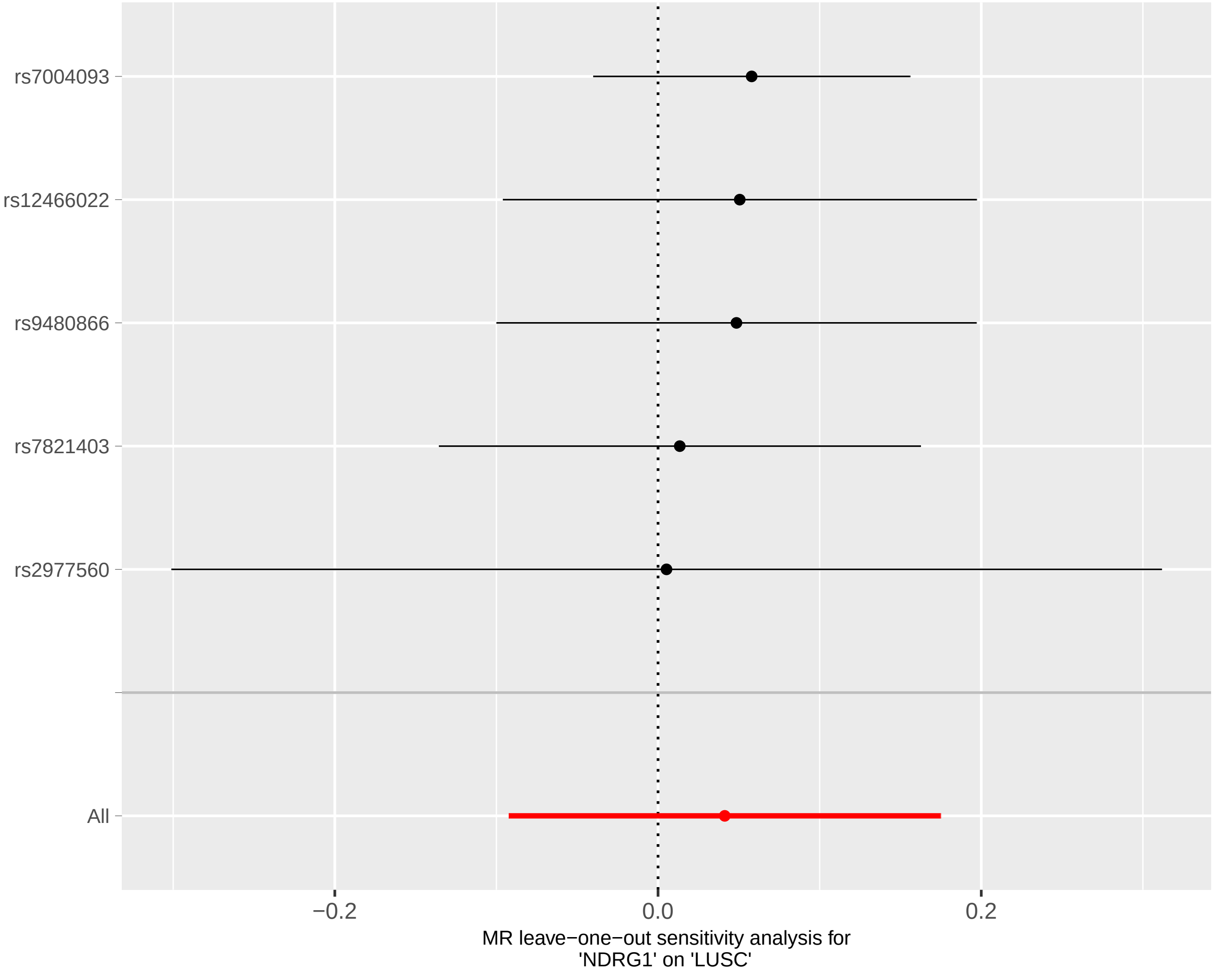
rs7004093
rs12466022
rs9480866
rs7821403
rs2977560
All
−0.2
0.0
0.2
MR leave−one−out sensitivity analysis for
'NDRG1' on 'LUSC'

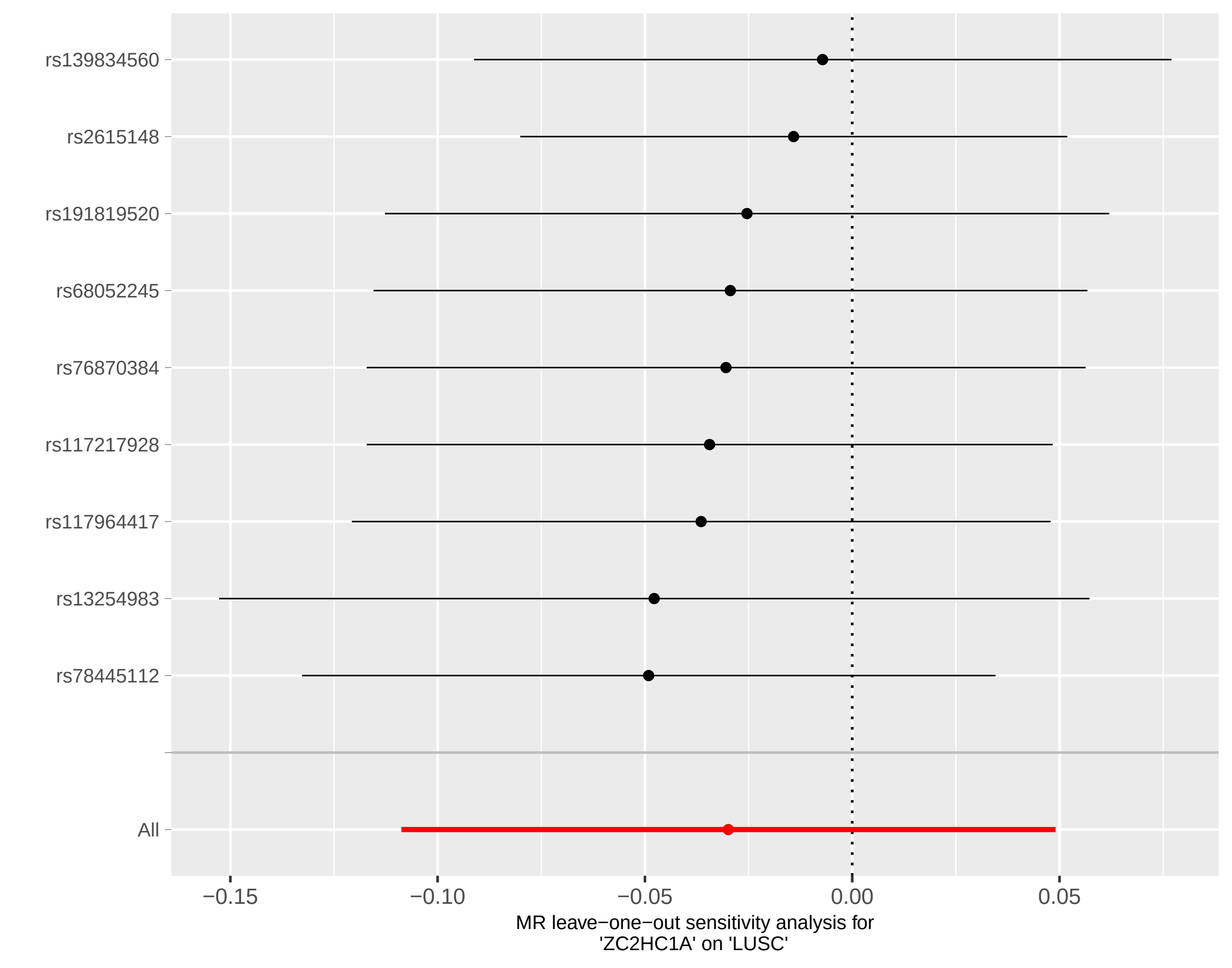
rs139834560
rs2615148
rs191819520
rs68052245
rs76870384
rs117217928
rs117964417
rs13254983
rs78445112
All
−0.15
−0.10
−0.05
0.00
0.05
MR leave−one−out sensitivity analysis for
'ZC2HC1A' on 'LUSC'

Insufficient number of SNPs

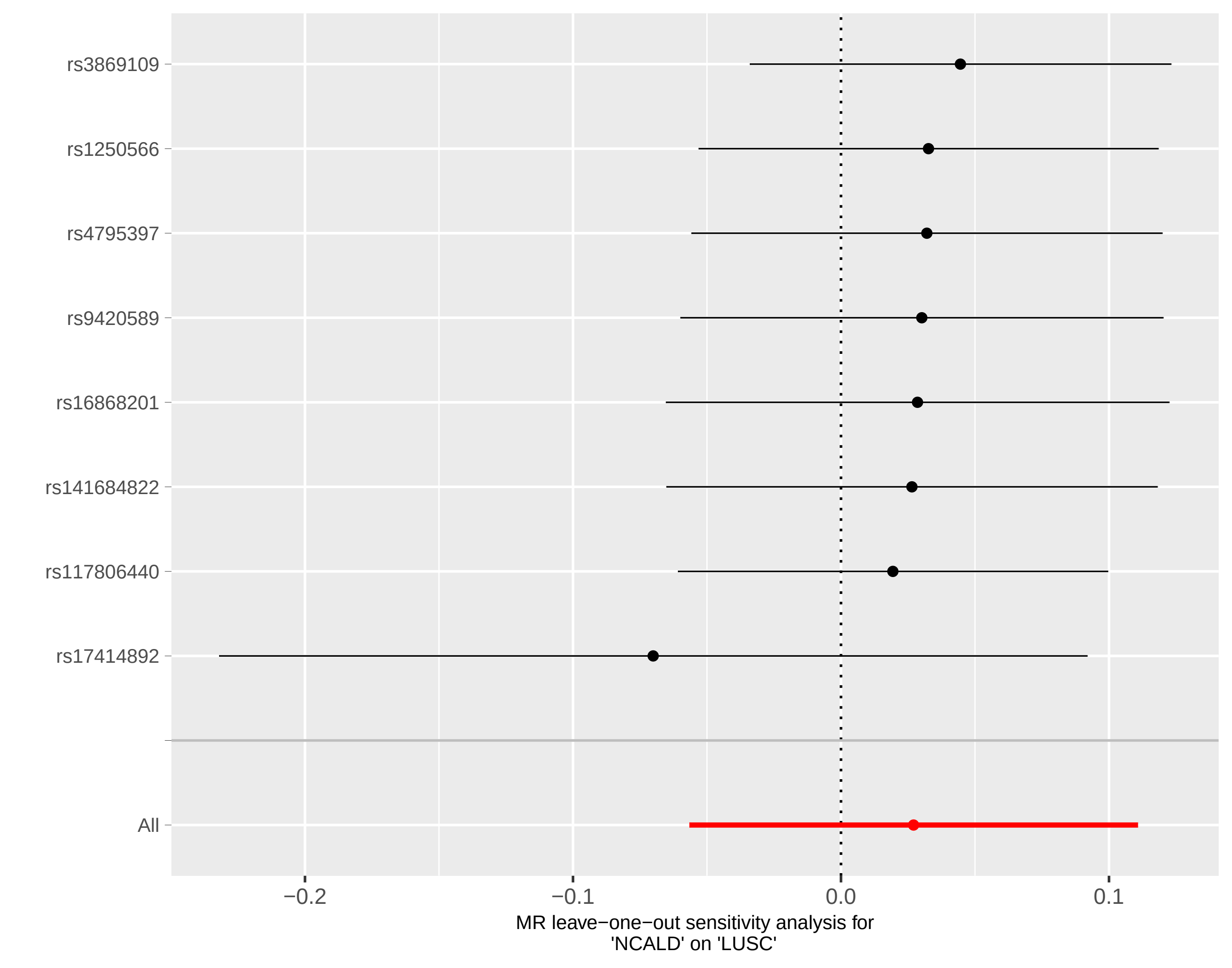

rs3869109
rs1250566
rs4795397
rs9420589
rs16868201
rs141684822
rs117806440
rs17414892
All
−0.2
−0.1
0.0
0.1
MR leave−one−out sensitivity analysis for
'NCALD' on 'LUSC'

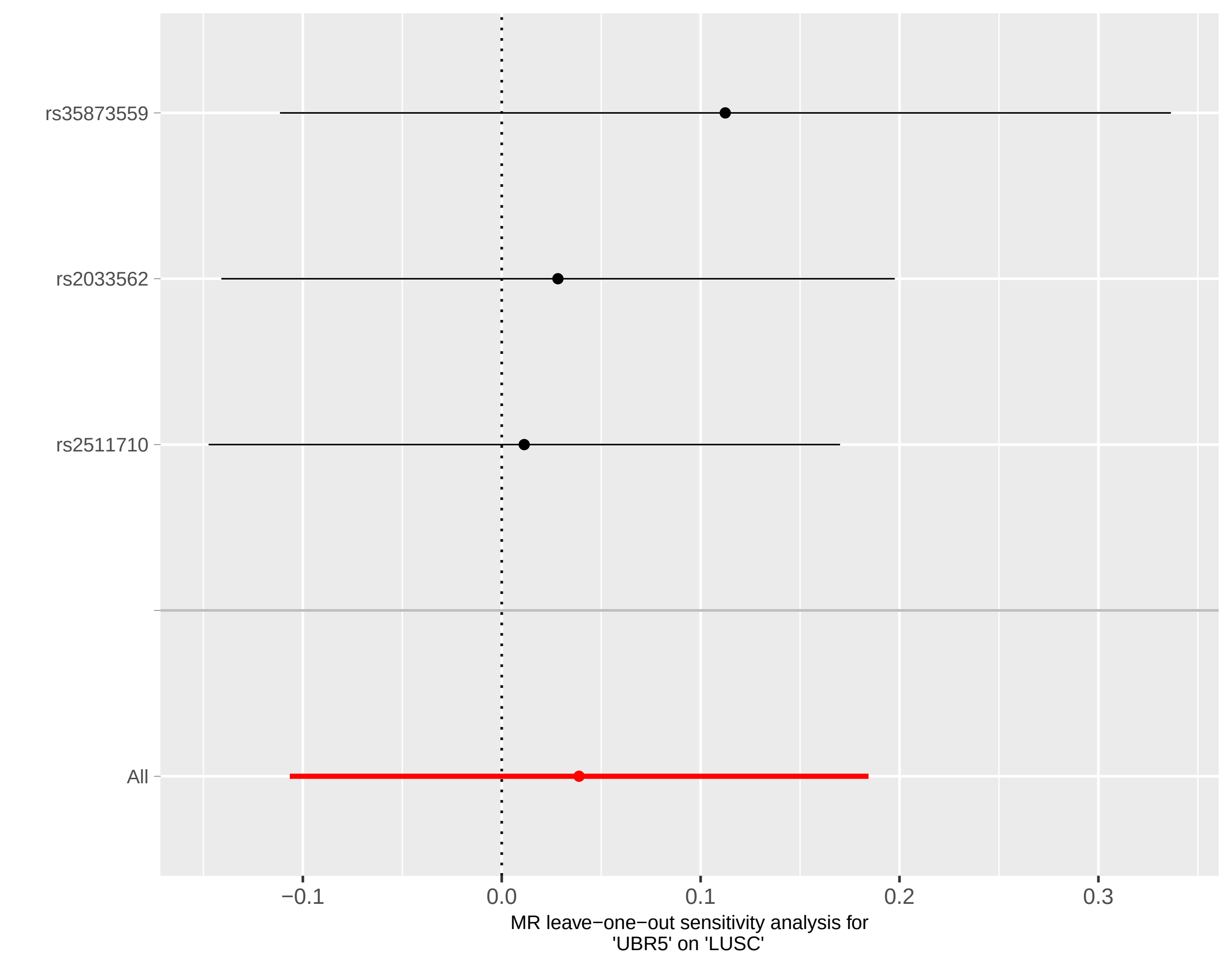

rs35873559
rs2033562
rs2511710
All
−0.1
0.0
0.1
0.2
0.3
MR leave−one−out sensitivity analysis for
'UBR5' on 'LUSC'

Insufficient number of SNPs

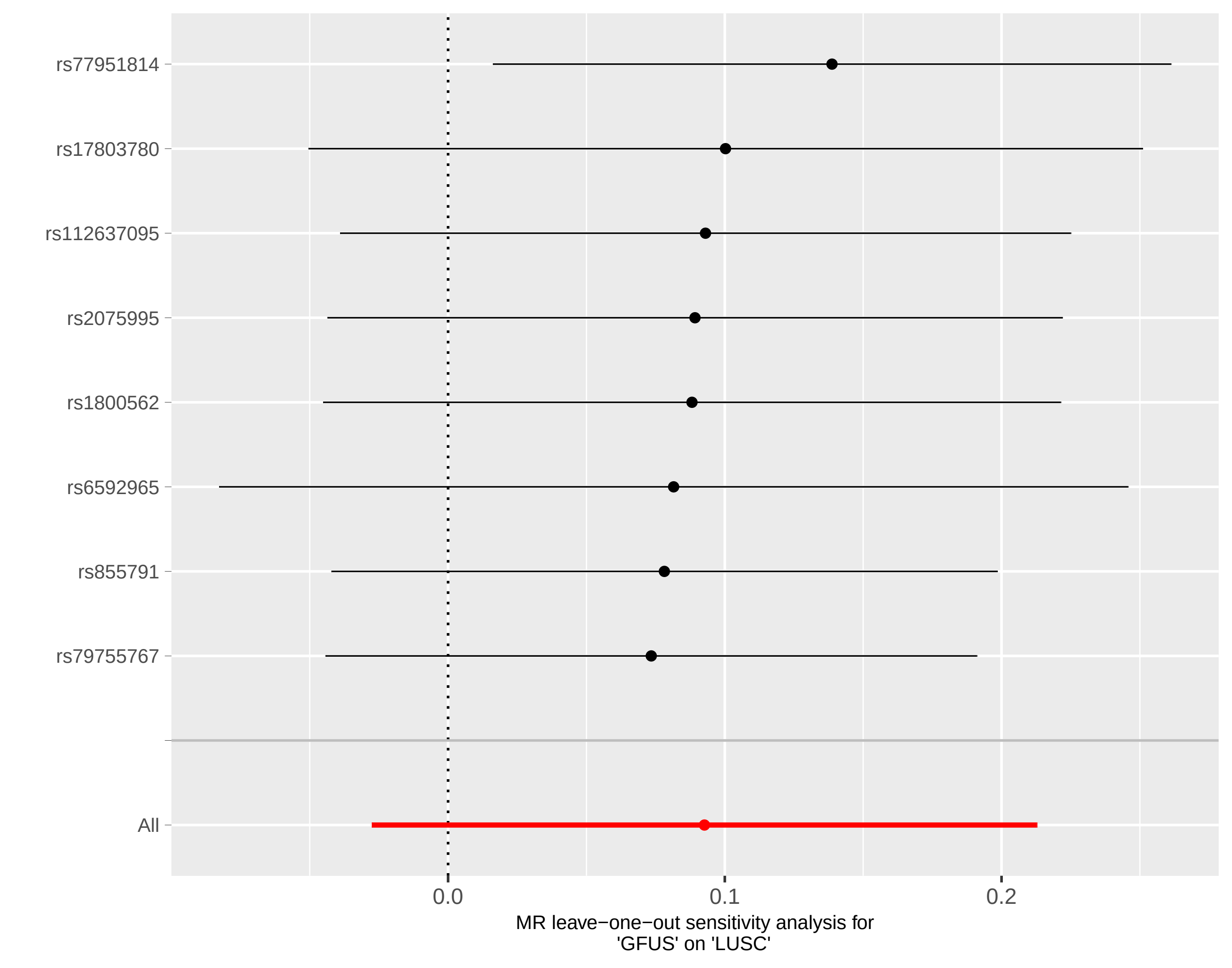
rs77951814
rs17803780
rs112637095
rs2075995
rs1800562
rs6592965
rs855791
rs79755767
All
0.0
0.1
0.2
MR leave−one−out sensitivity analysis for
'GFUS' on 'LUSC'

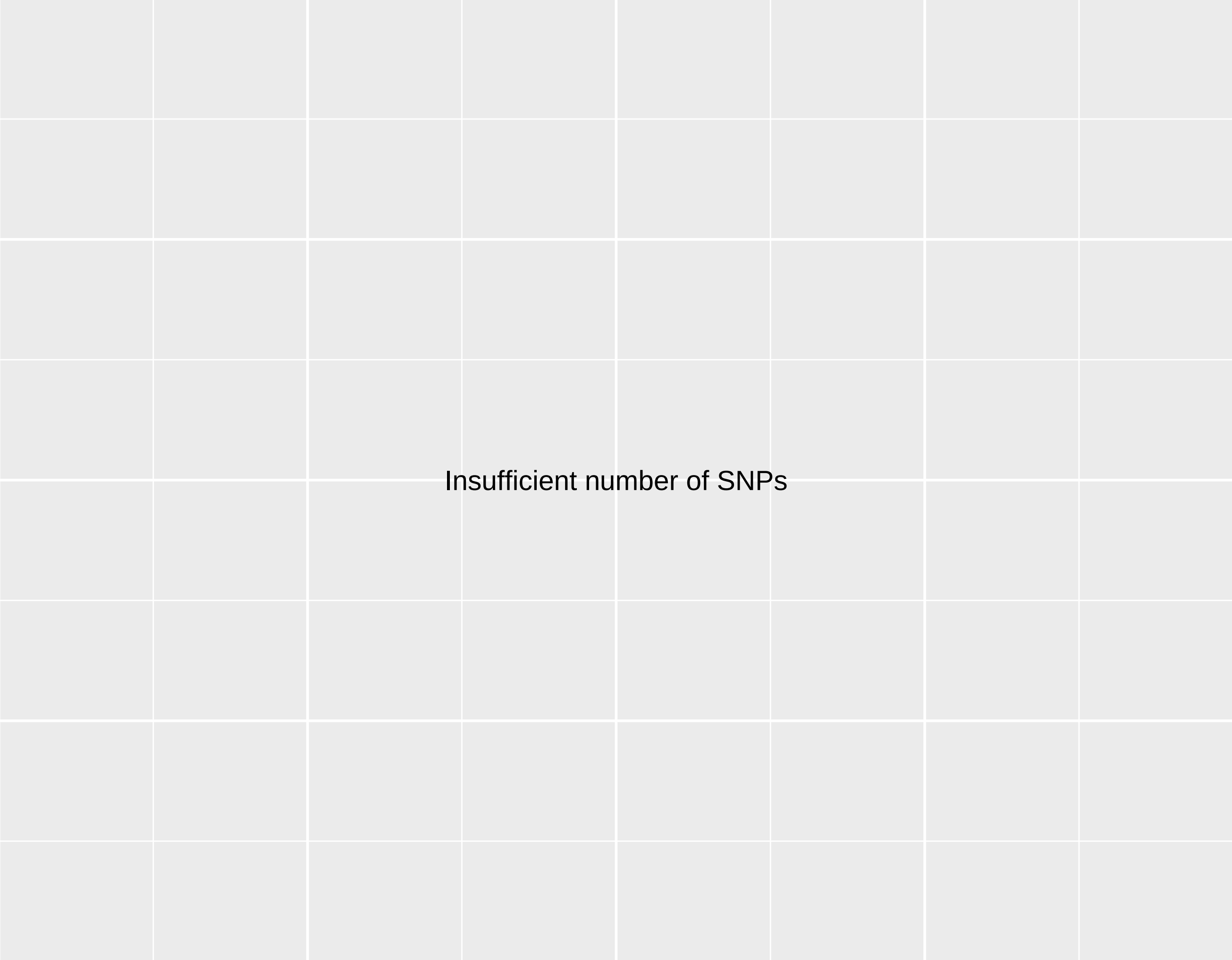
Insufficient number of SNPs

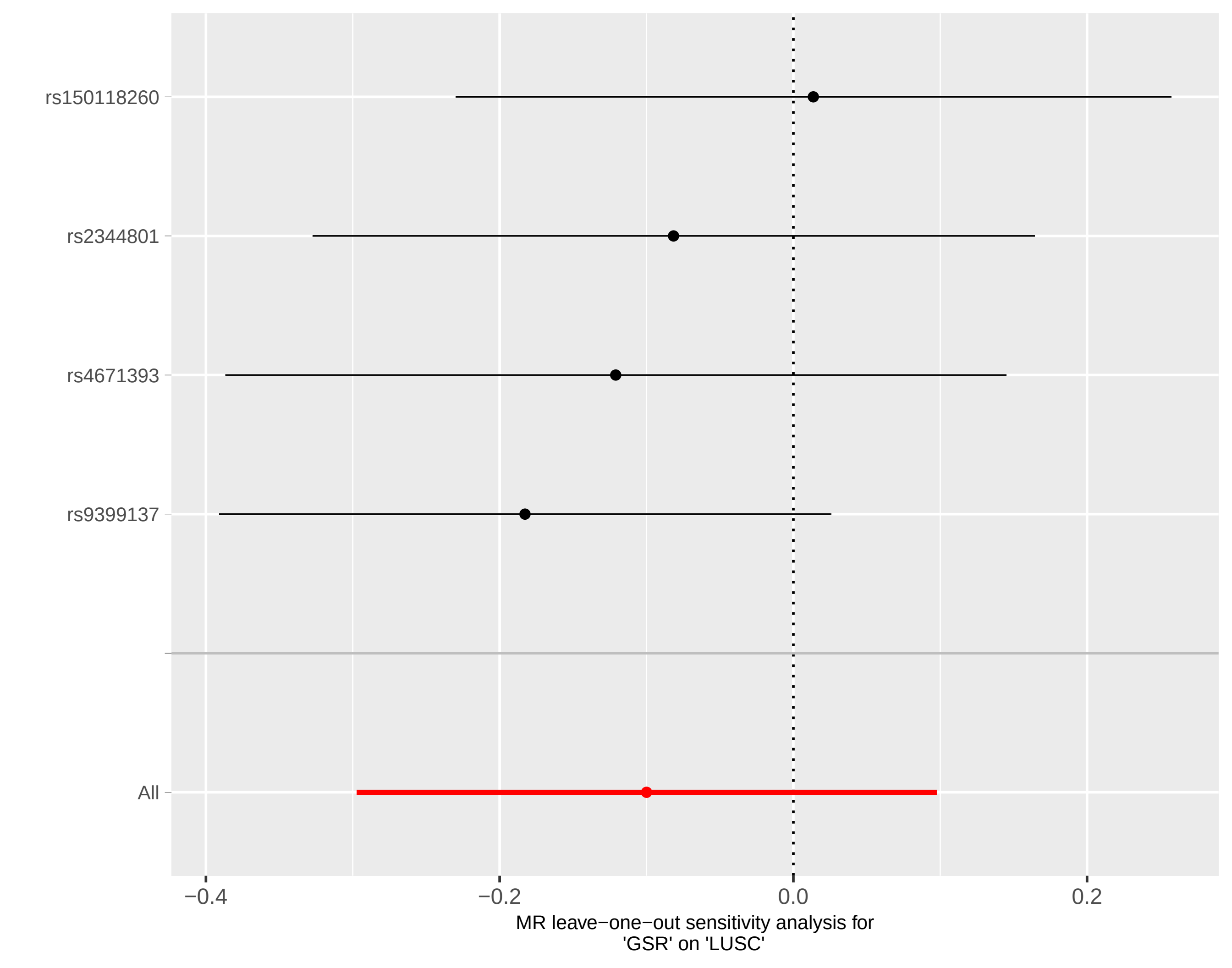
rs150118260
rs2344801
rs4671393
rs9399137
All
−0.4
−0.2
0.0
0.2
MR leave−one−out sensitivity analysis for
'GSR' on 'LUSC'

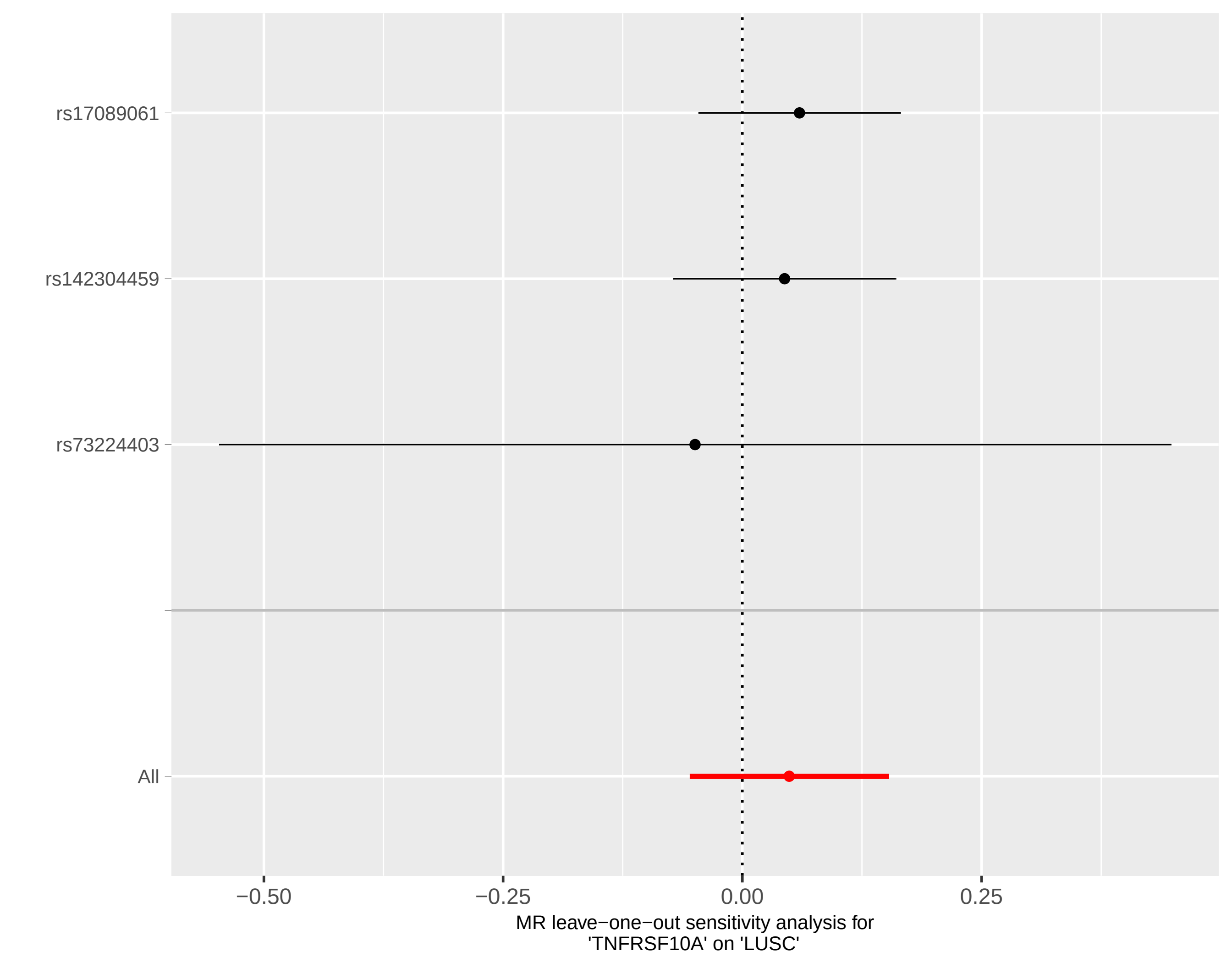

rs17089061
rs142304459
rs73224403
All
−0.50
−0.25
0.00
0.25
MR leave−one−out sensitivity analysis for
'TNFRSF10A' on 'LUSC'

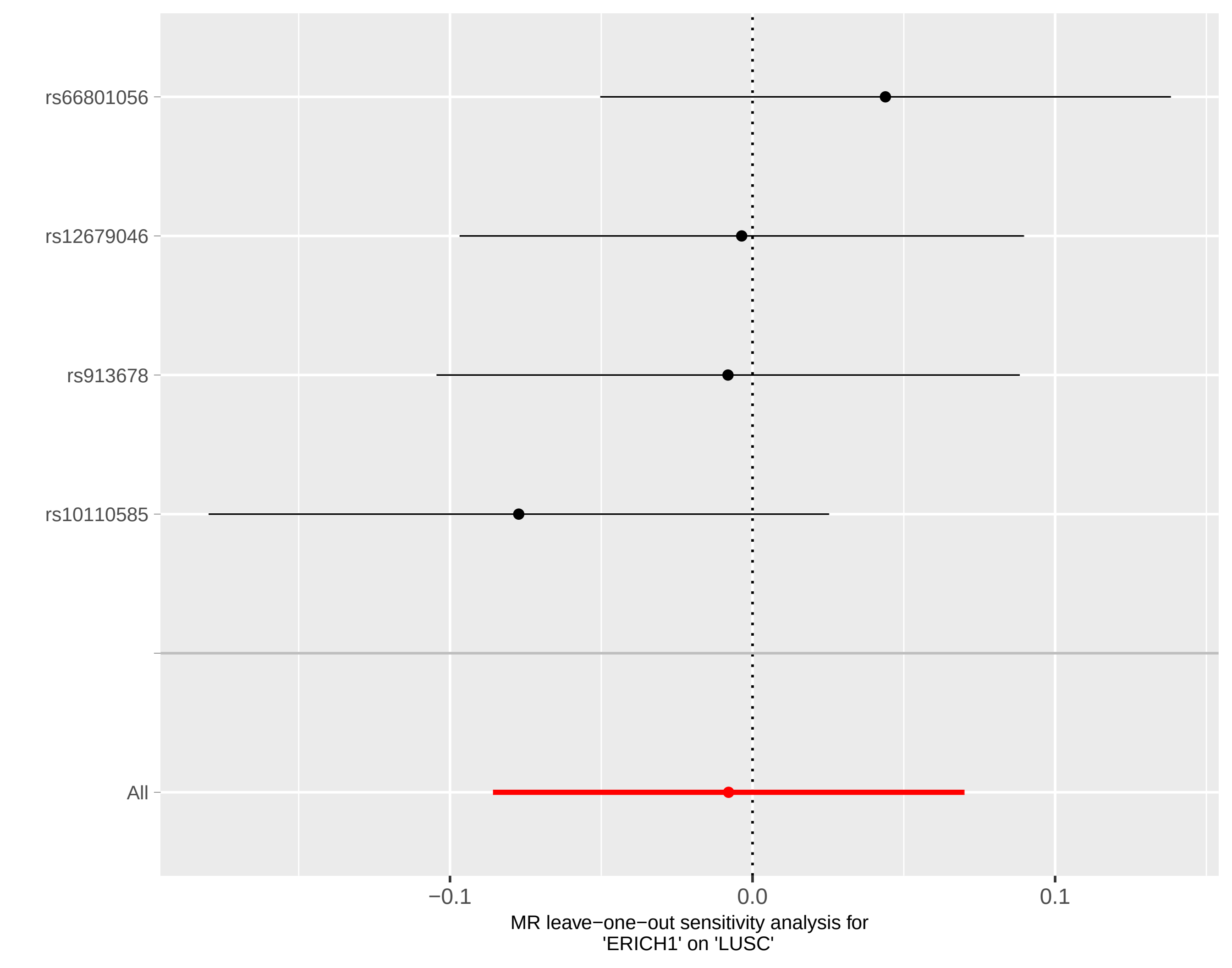
rs66801056
rs12679046
rs913678
rs10110585
All
−0.1
0.0
0.1
MR leave−one−out sensitivity analysis for
'ERICH1' on 'LUSC'

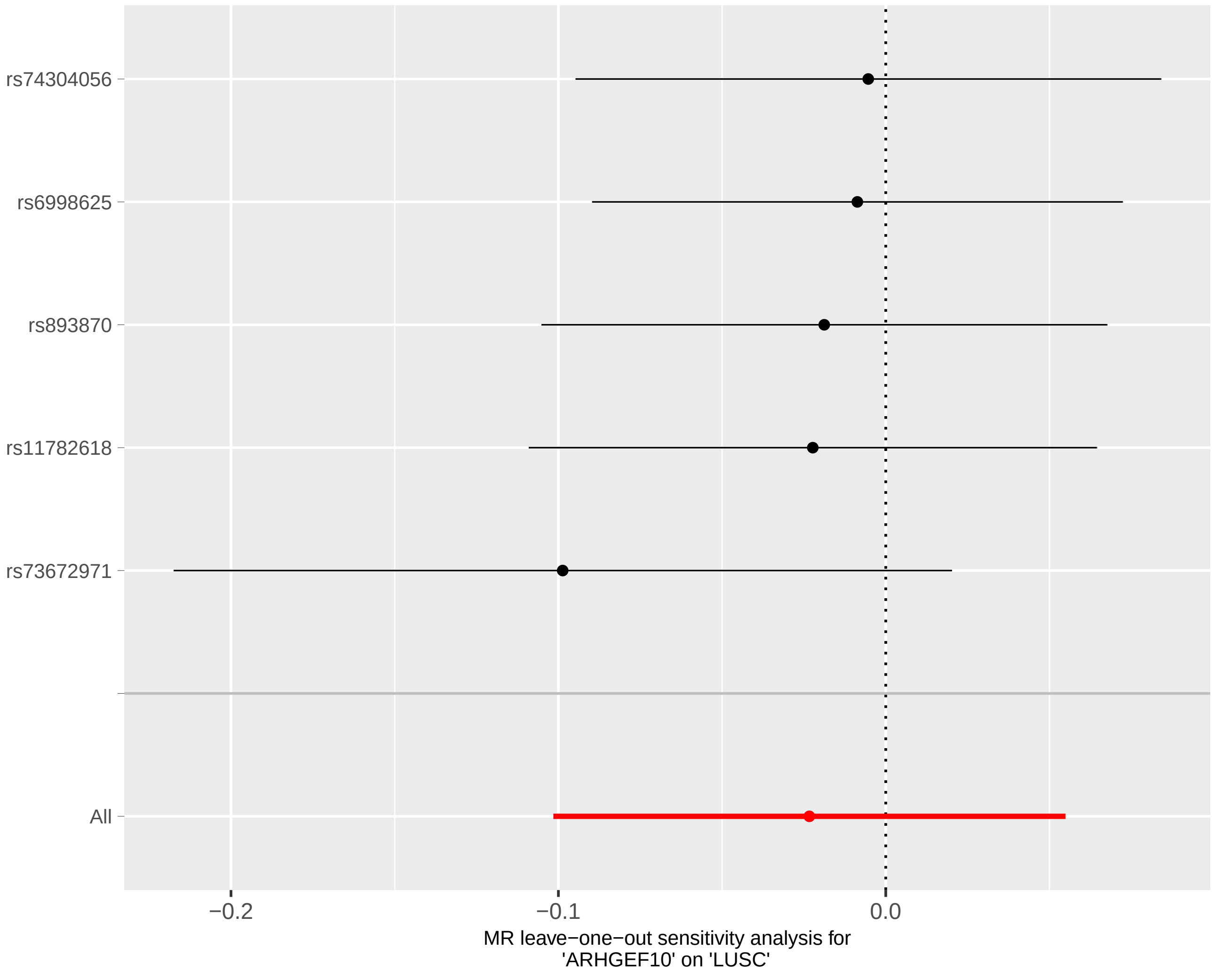
rs74304056
rs6998625
rs893870
rs11782618
rs73672971
All
−0.2
−0.1
0.0
MR leave−one−out sensitivity analysis for
'ARHGEF10' on 'LUSC'

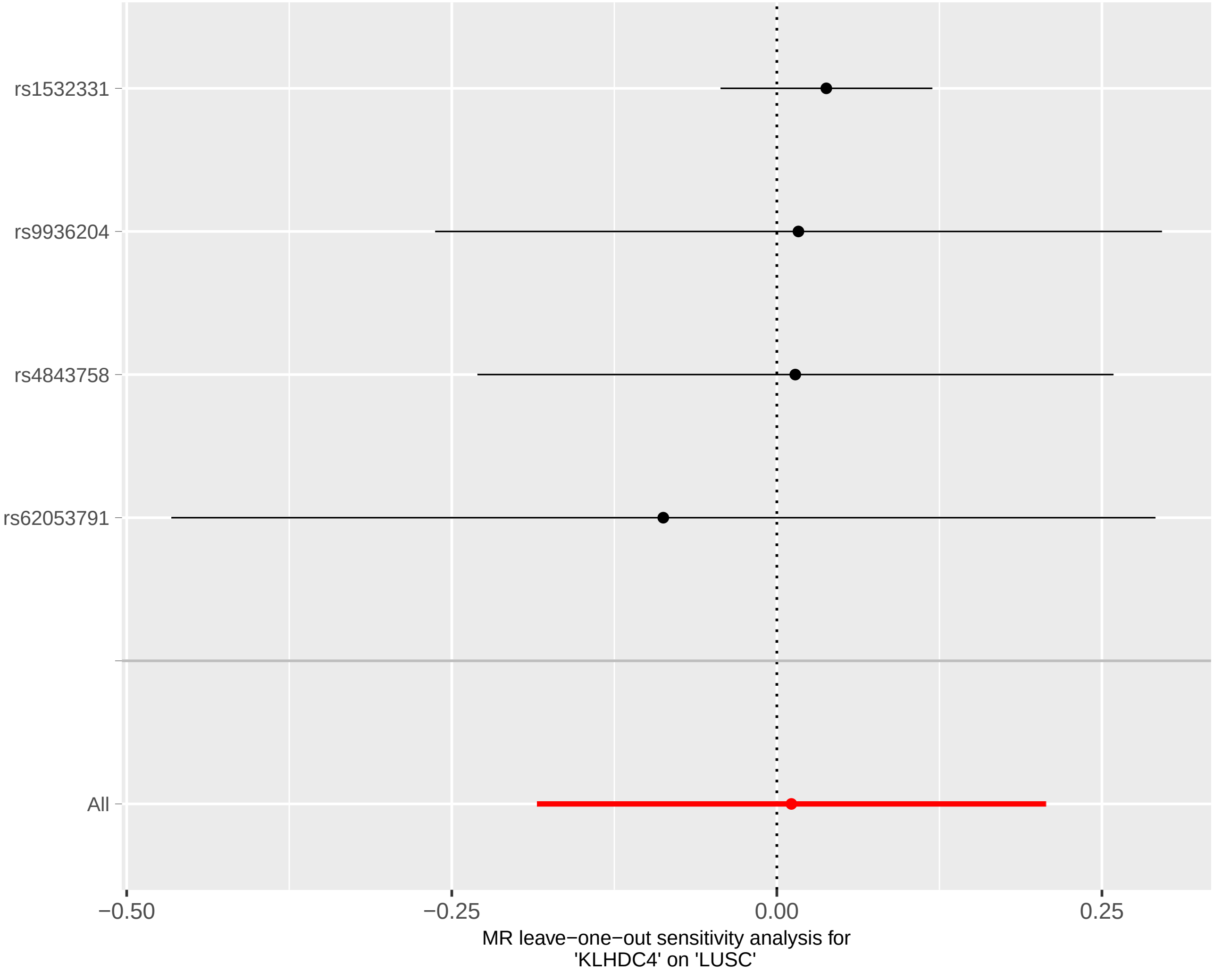

rs1532331
rs9936204
rs4843758
rs62053791
All
−0.50
−0.25
0.00
0.25
MR leave−one−out sensitivity analysis for
'KLHDC4' on 'LUSC'

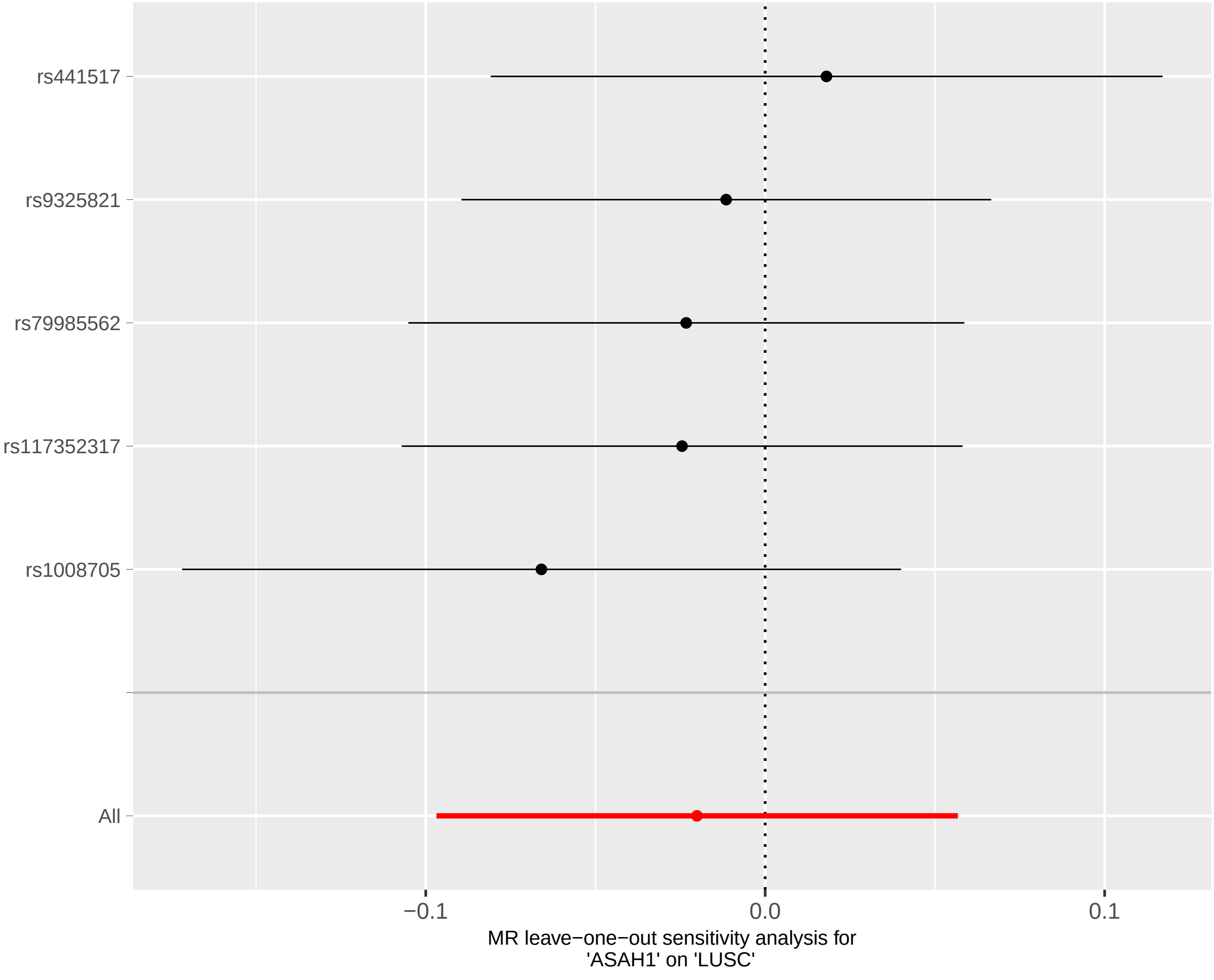
rs441517
rs9325821
rs79985562
rs117352317
rs1008705
All
−0.1
0.0
0.1
MR leave−one−out sensitivity analysis for
'ASAH1' on 'LUSC'

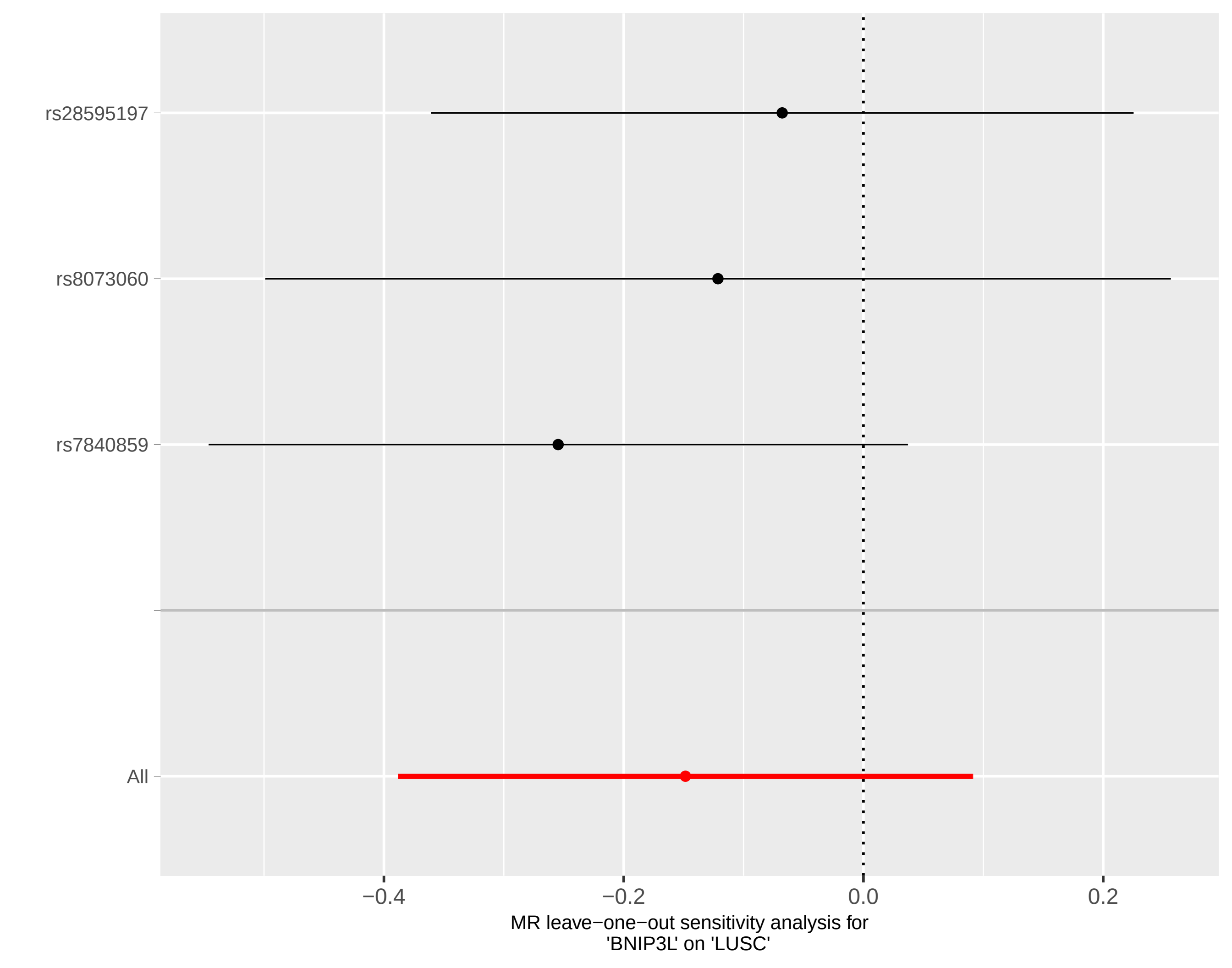

rs28595197
rs8073060
rs7840859
All
−0.4
−0.2
0.0
0.2
MR leave−one−out sensitivity analysis for
'BNIP3L' on 'LUSC'

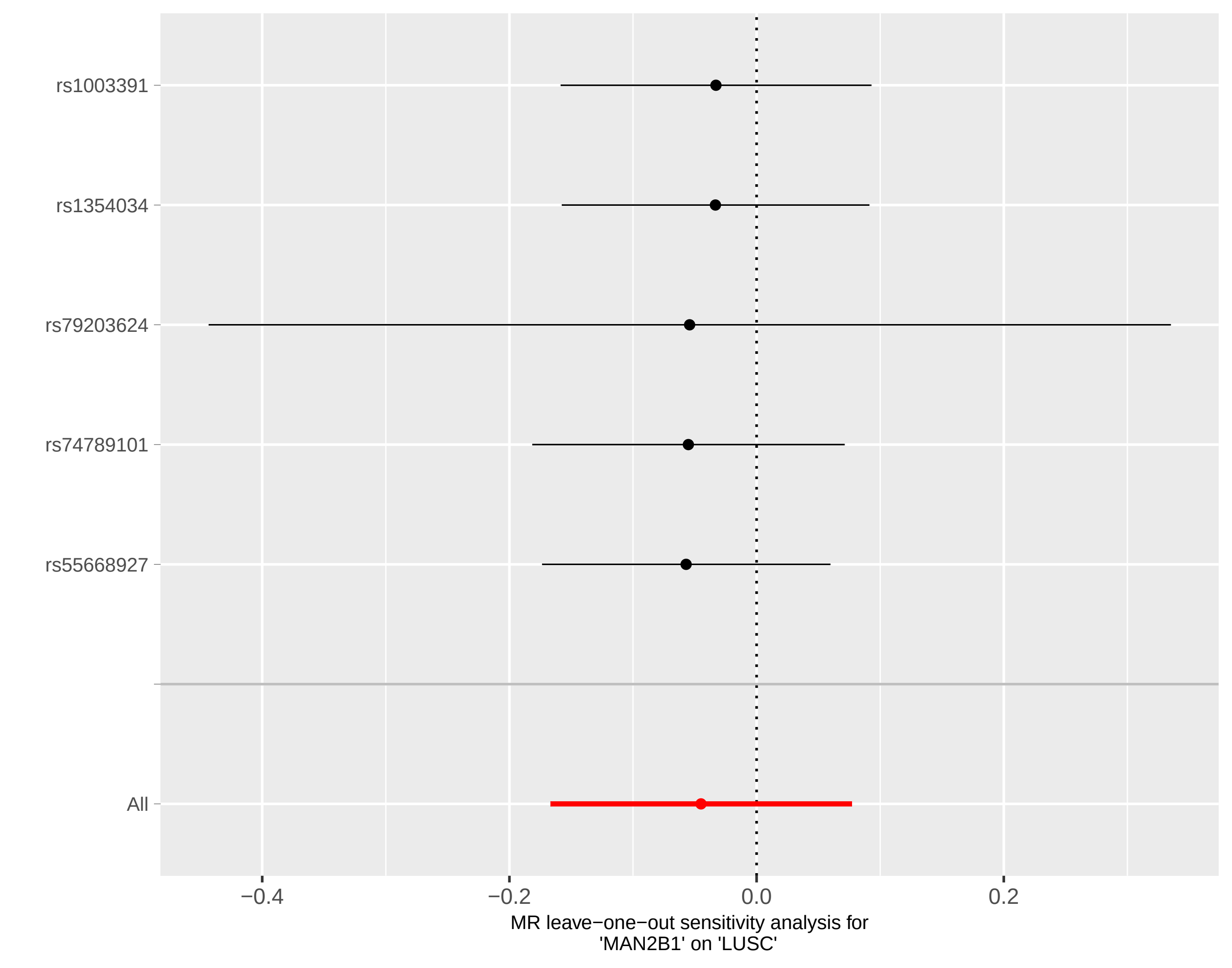
rs1003391
rs1354034
rs79203624
rs74789101
rs55668927
All
−0.4
−0.2
0.0
0.2
MR leave−one−out sensitivity analysis for
'MAN2B1' on 'LUSC'

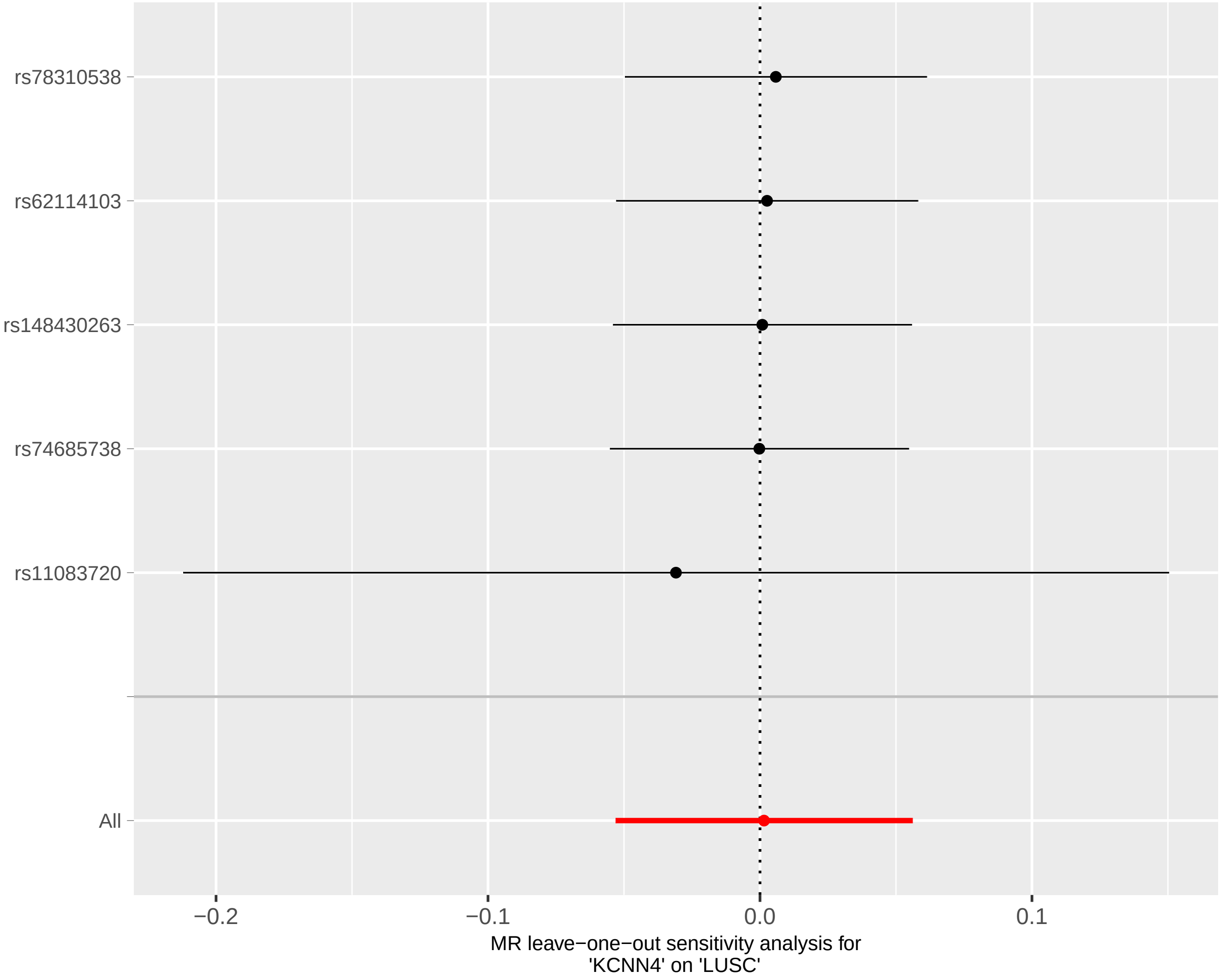
rs78310538
rs62114103
rs148430263
rs74685738
rs11083720
All
−0.2
−0.1
0.0
0.1
MR leave−one−out sensitivity analysis for
'KCNN4' on 'LUSC'

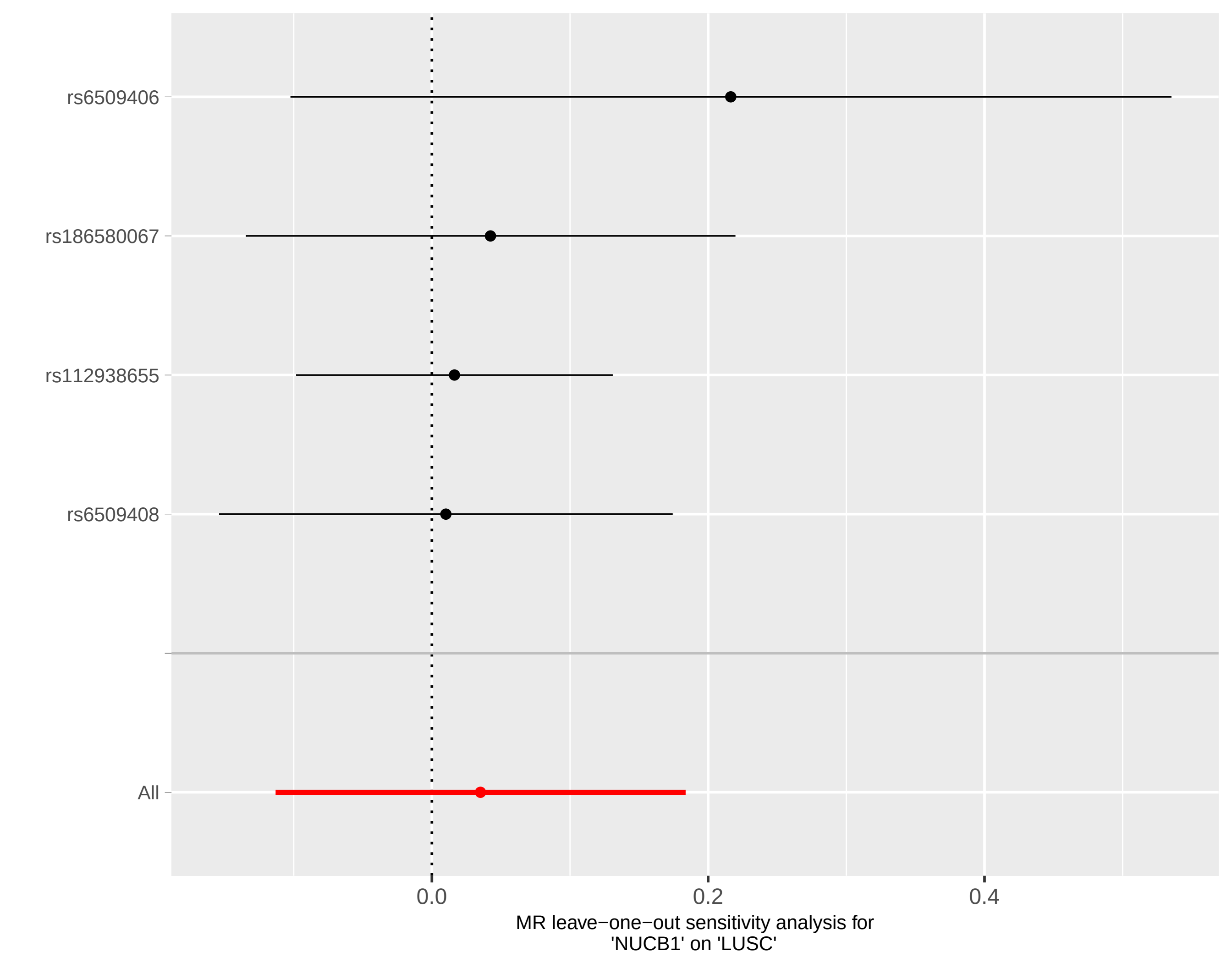
rs6509406
rs186580067
rs112938655
rs6509408
All
0.0
0.2
0.4
MR leave−one−out sensitivity analysis for
'NUCB1' on 'LUSC'

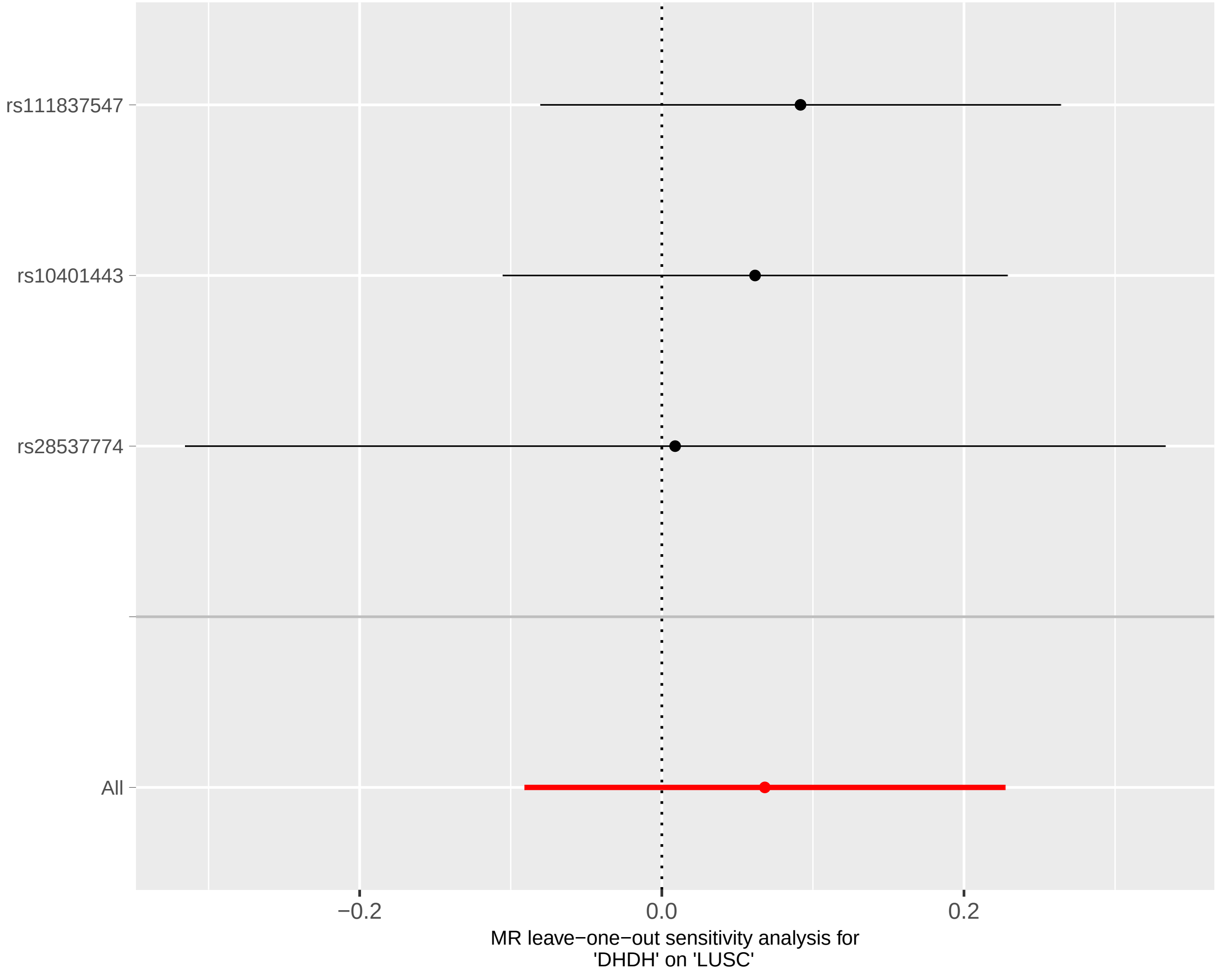
rs111837547
rs10401443
rs28537774
All
−0.2
0.0
0.2
MR leave−one−out sensitivity analysis for
'DHDH' on 'LUSC'

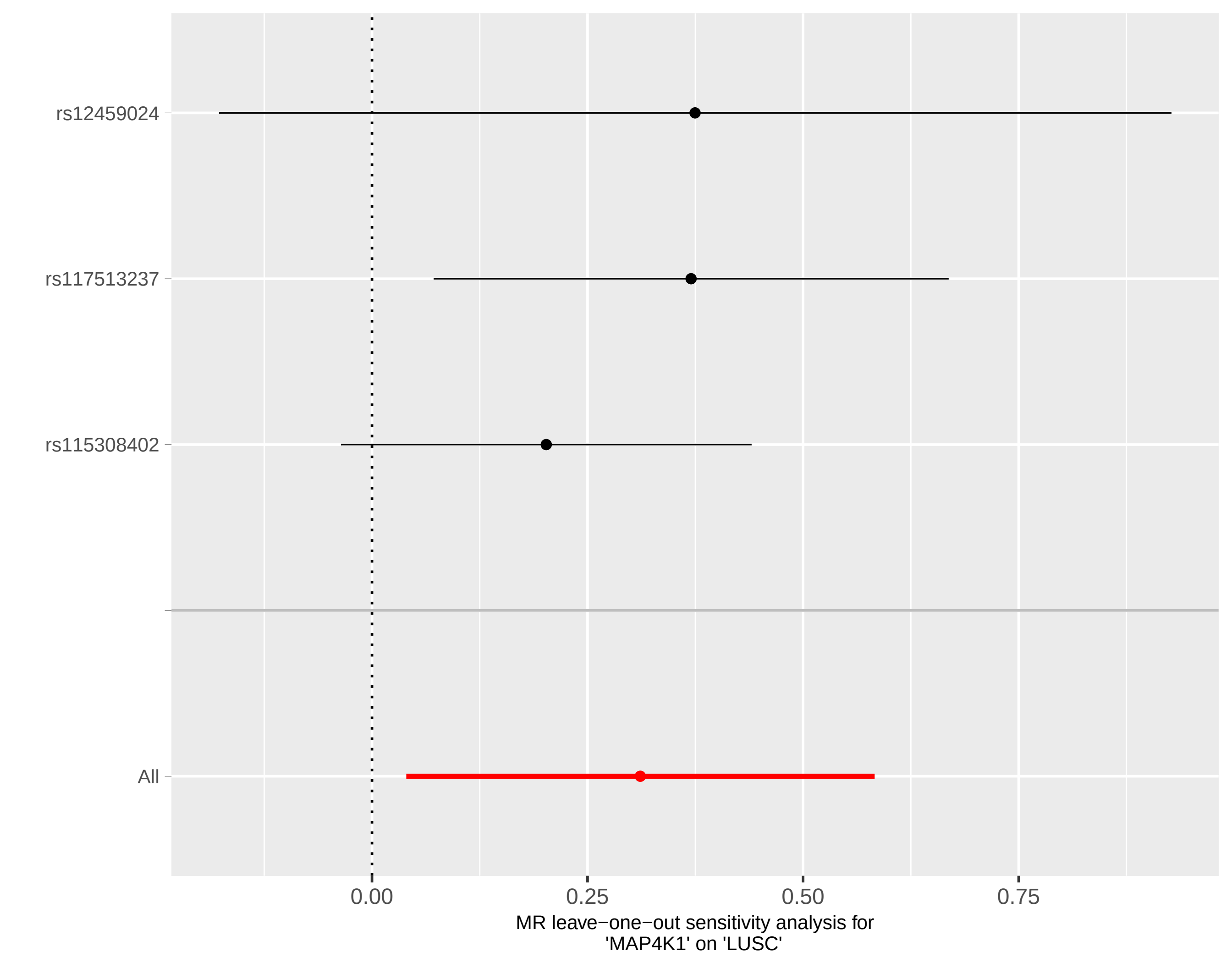
rs12459024
rs117513237
rs115308402
All
0.00
0.25
0.50
0.75
MR leave−one−out sensitivity analysis for
'MAP4K1' on 'LUSC'

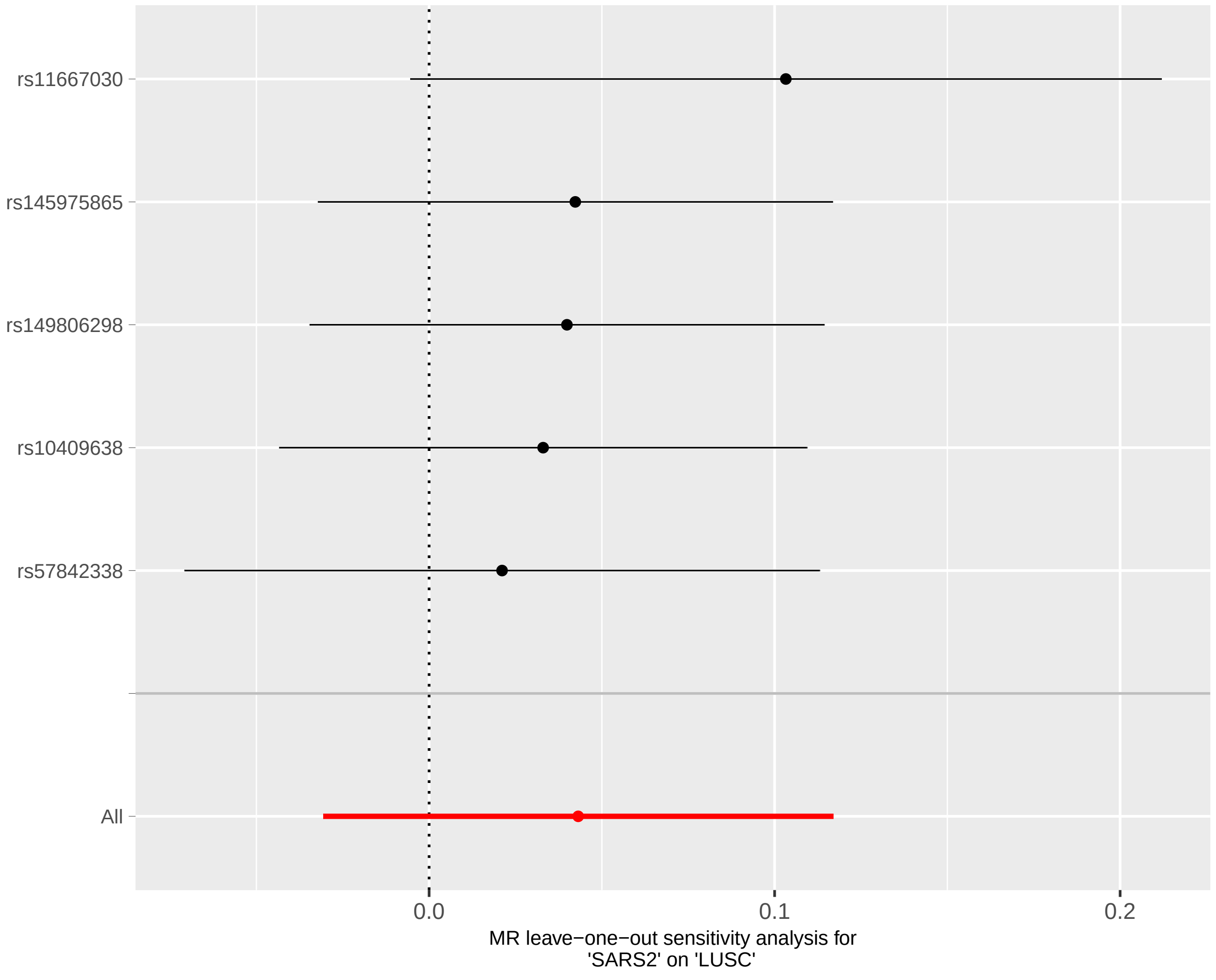
rs11667030
rs145975865
rs149806298
rs10409638
rs57842338
All
0.0
0.1
0.2
MR leave−one−out sensitivity analysis for
'SARS2' on 'LUSC'

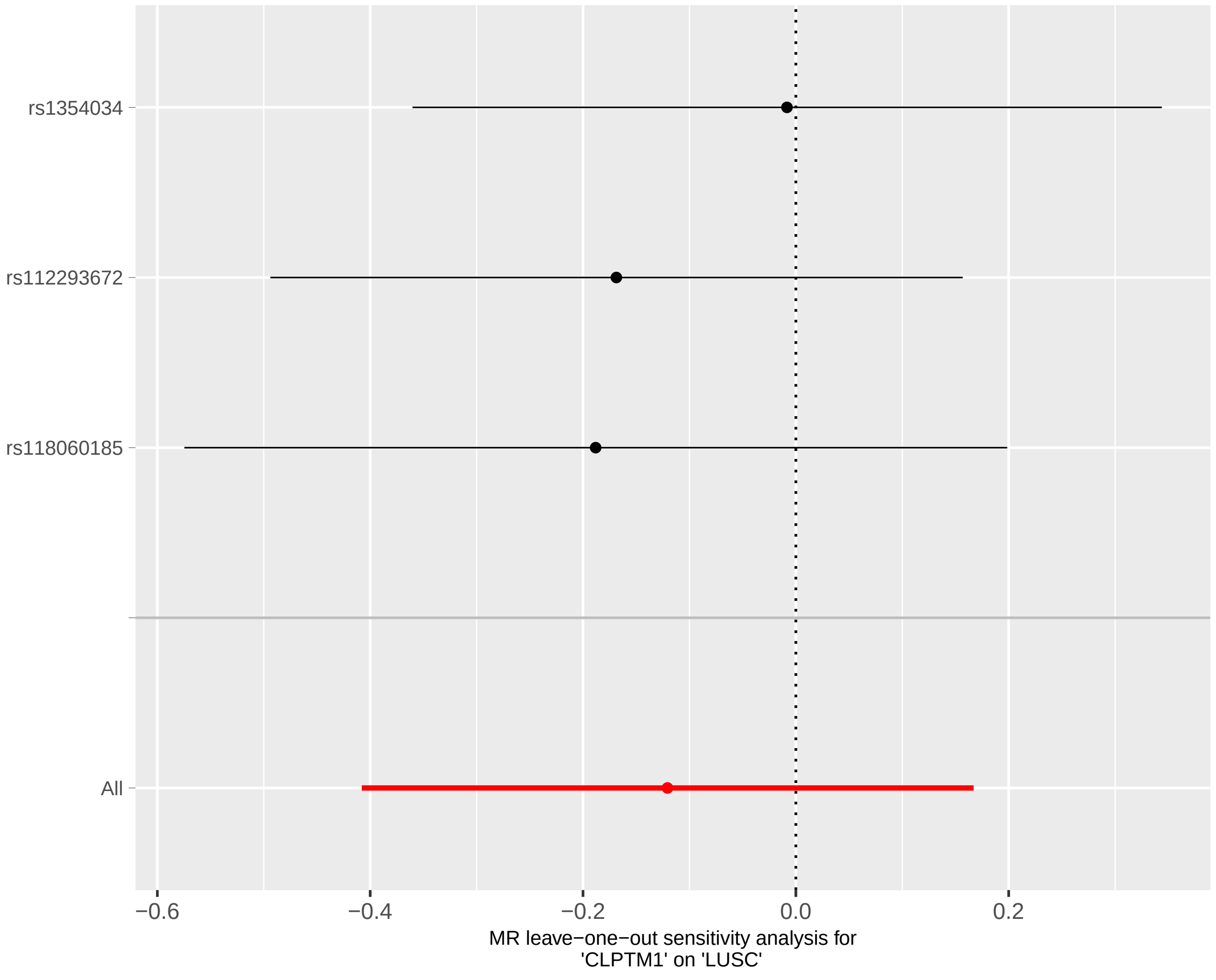
rs1354034
rs112293672
rs118060185
All
−0.6
−0.4
−0.2
0.0
0.2
MR leave−one−out sensitivity analysis for
'CLPTM1' on 'LUSC'

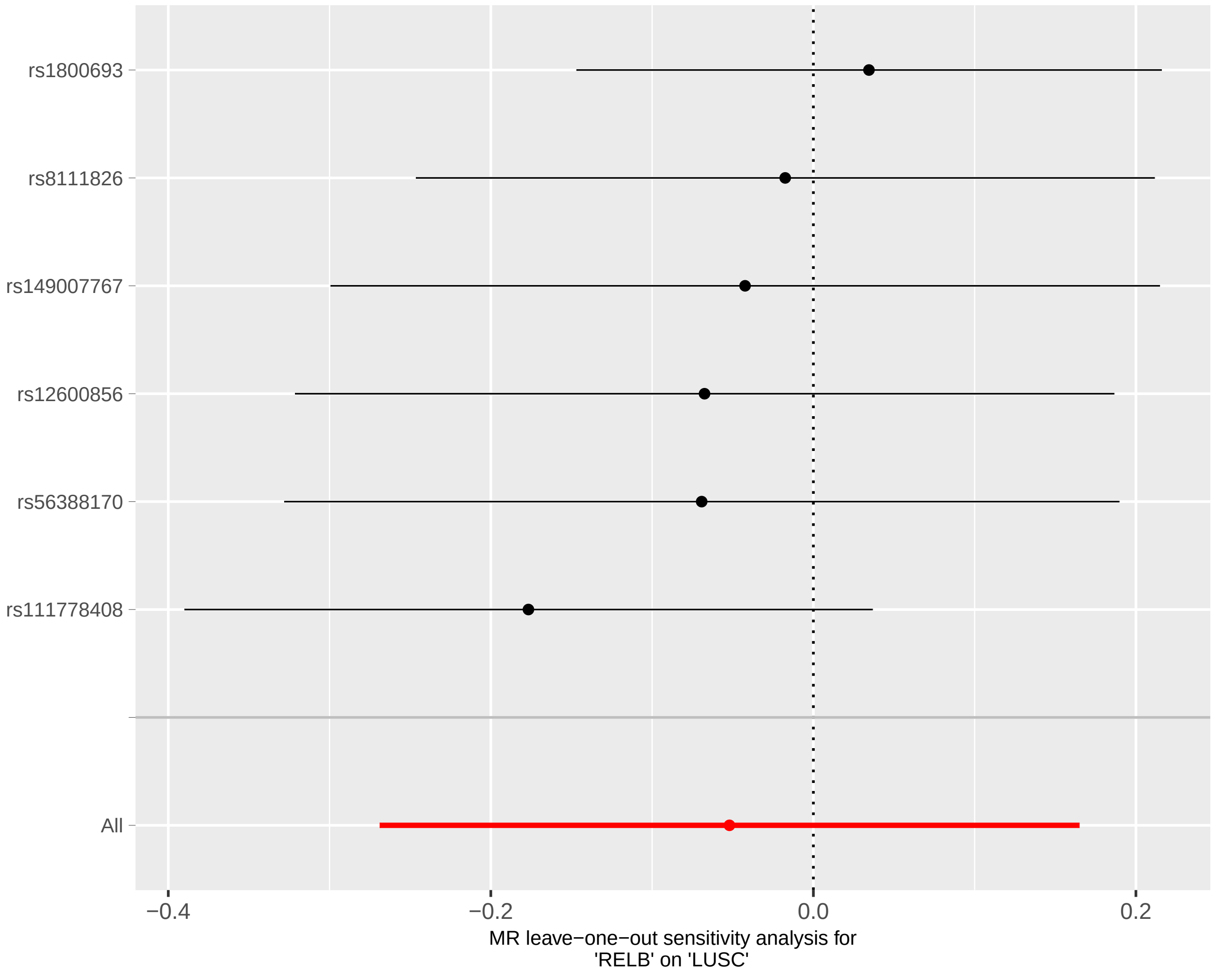
rs1800693
rs8111826
rs149007767
rs12600856
rs56388170
rs111778408
All
−0.4
−0.2
0.0
0.2
MR leave−one−out sensitivity analysis for
'RELB' on 'LUSC'

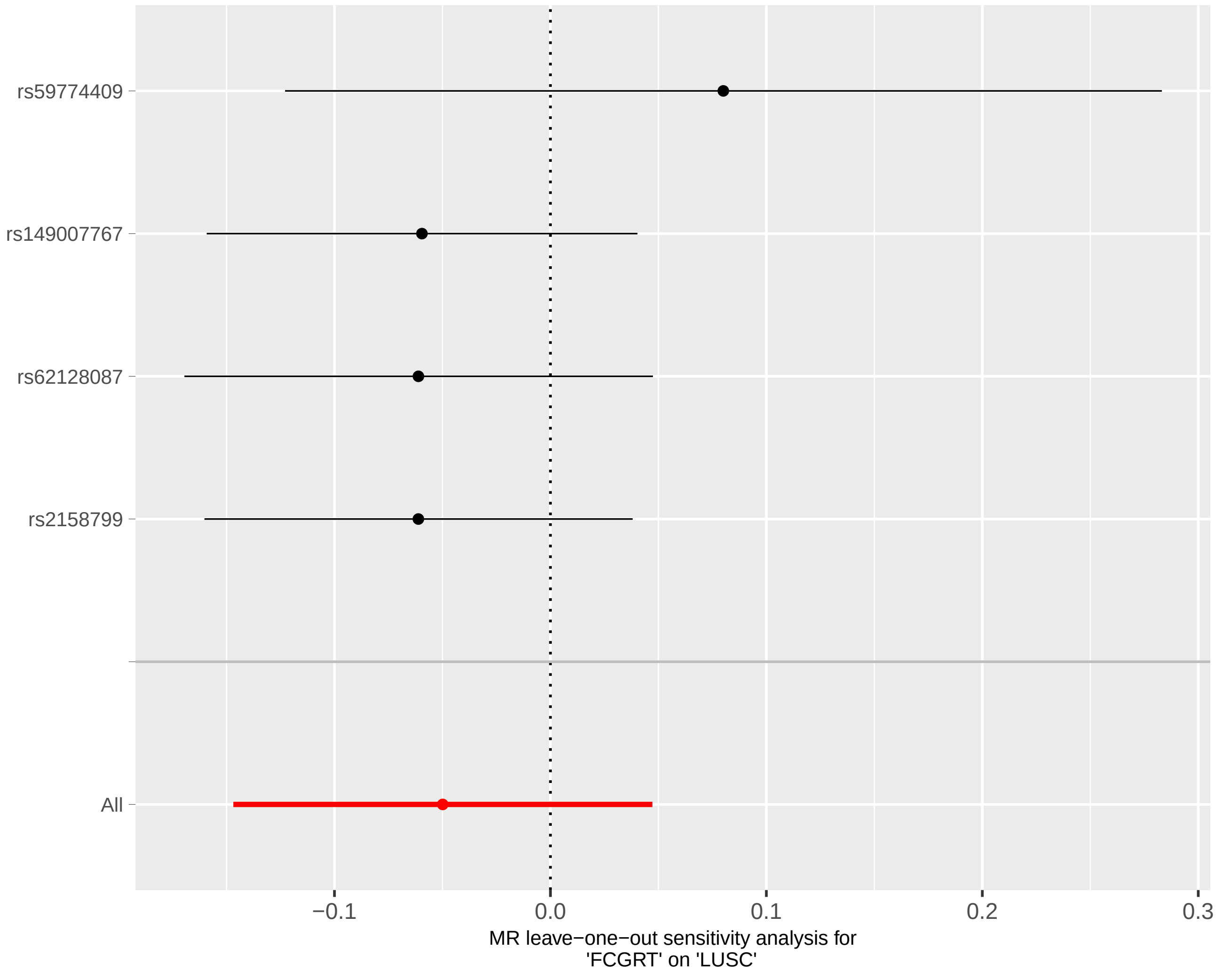

rs59774409
rs149007767
rs62128087
rs2158799
All
−0.1
0.0
0.1
0.2
0.3
MR leave−one−out sensitivity analysis for
'FCGRT' on 'LUSC'

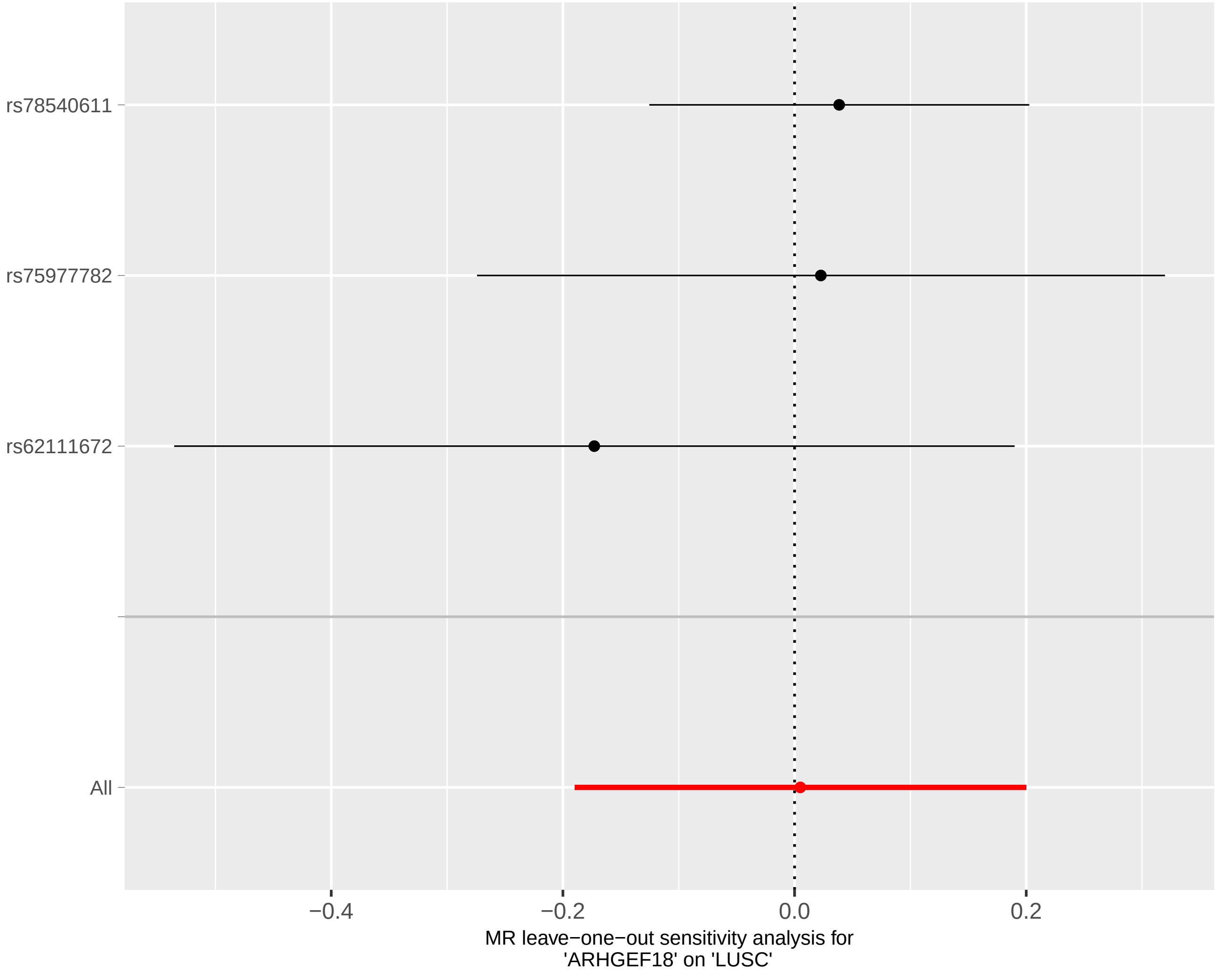
rs78540611
rs75977782
rs62111672
All
−0.4
−0.2
0.0
0.2
MR leave−one−out sensitivity analysis for
'ARHGEF18' on 'LUSC'

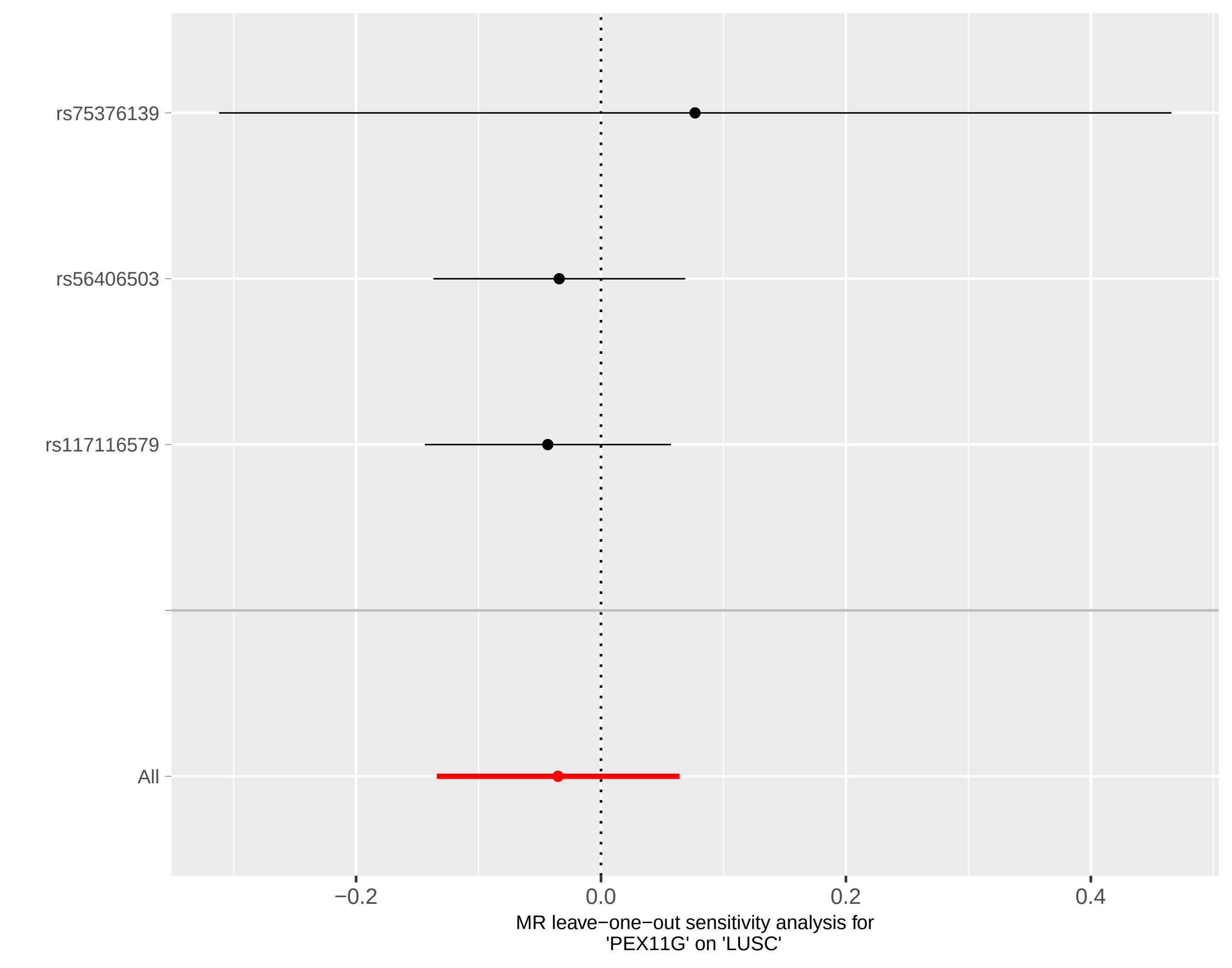

rs75376139
rs56406503
rs117116579
All
−0.2
0.0
0.2
0.4
MR leave−one−out sensitivity analysis for
'PEX11G' on 'LUSC'

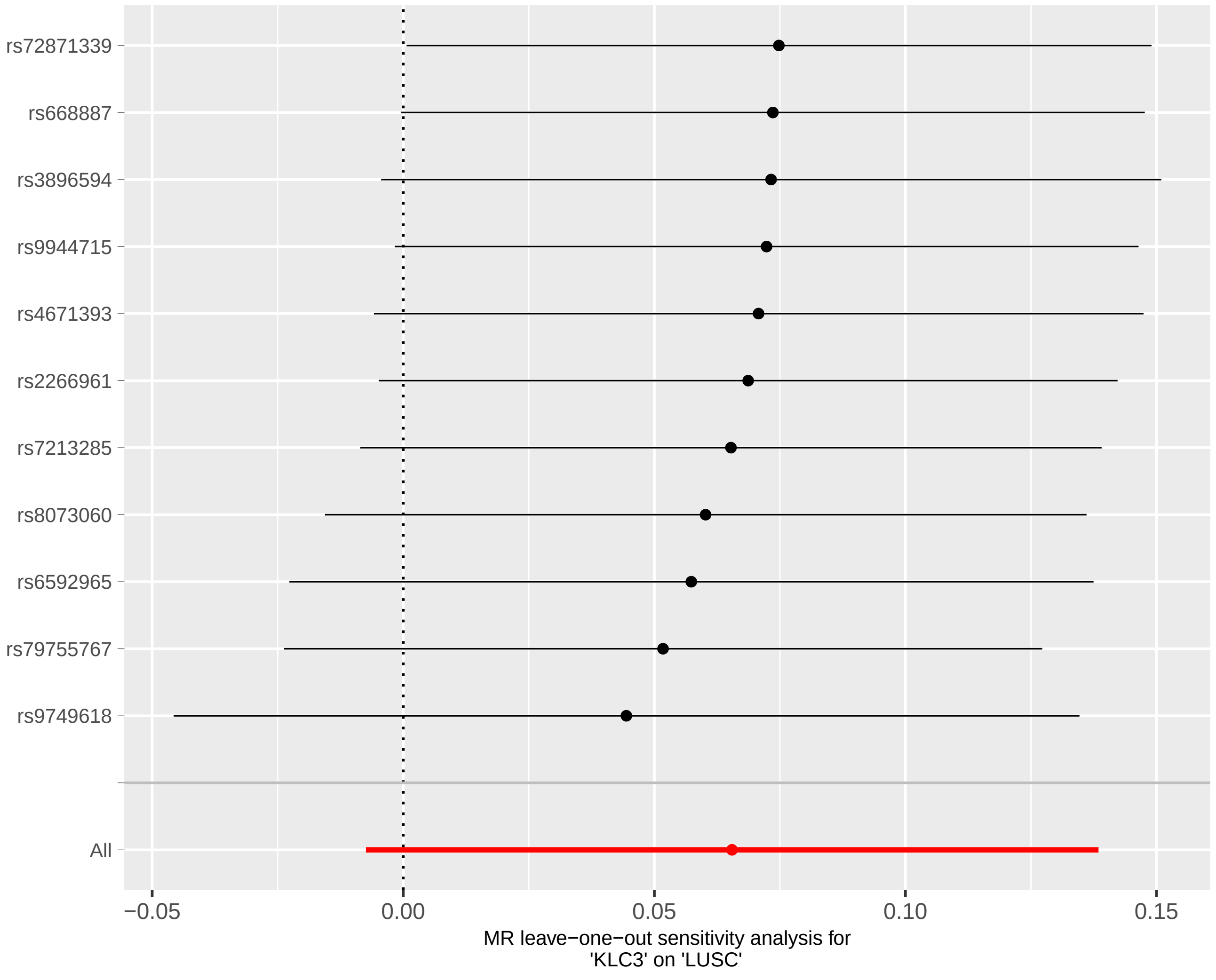
rs72871339
rs668887
rs3896594
rs9944715
rs4671393
rs2266961
rs7213285
rs8073060
rs6592965
rs79755767
rs9749618
All
−0.05
0.00
0.05
0.10
0.15
MR leave−one−out sensitivity analysis for
'KLC3' on 'LUSC'

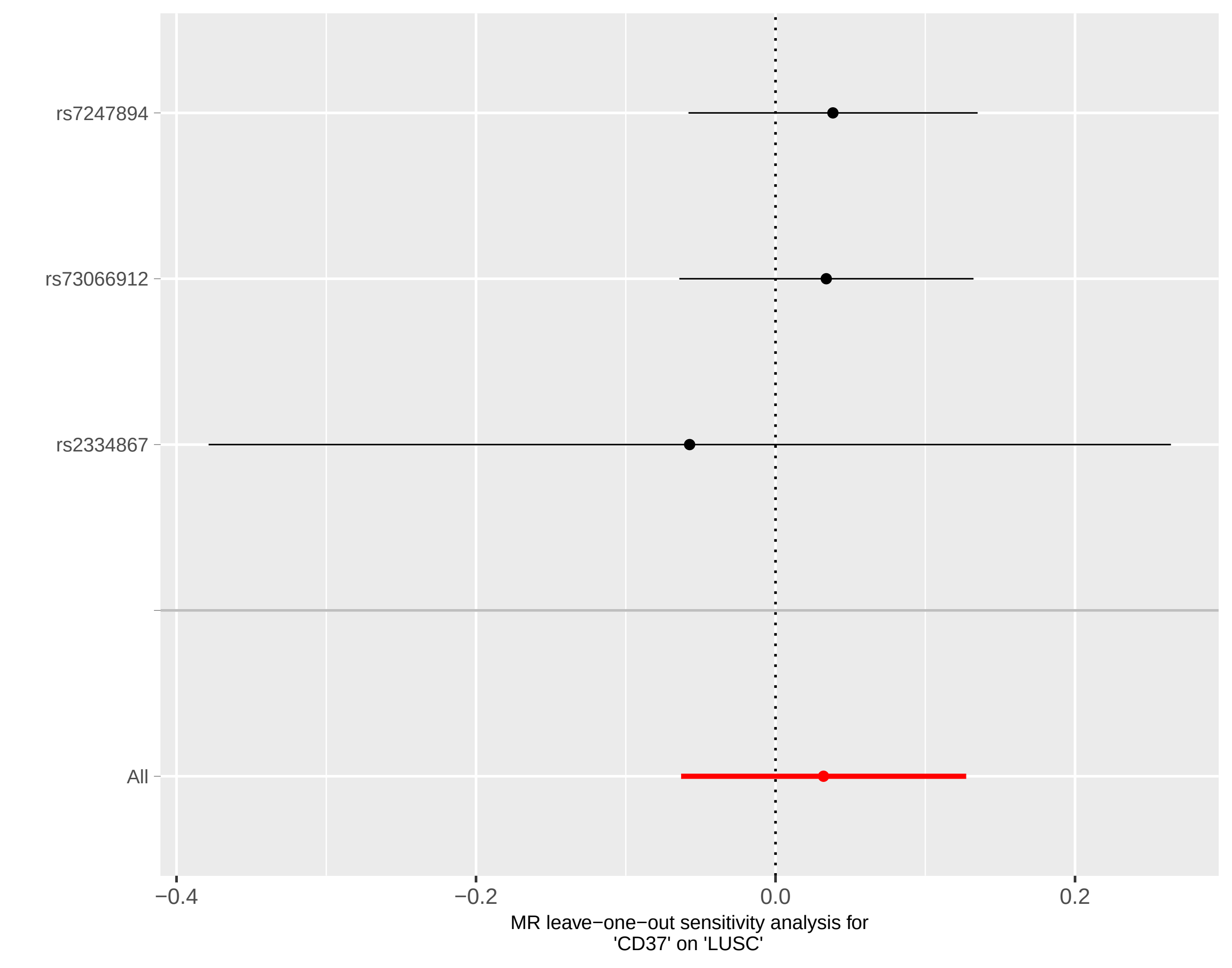
rs7247894
rs73066912
rs2334867
All
−0.4
−0.2
0.0
0.2
MR leave−one−out sensitivity analysis for
'CD37' on 'LUSC'

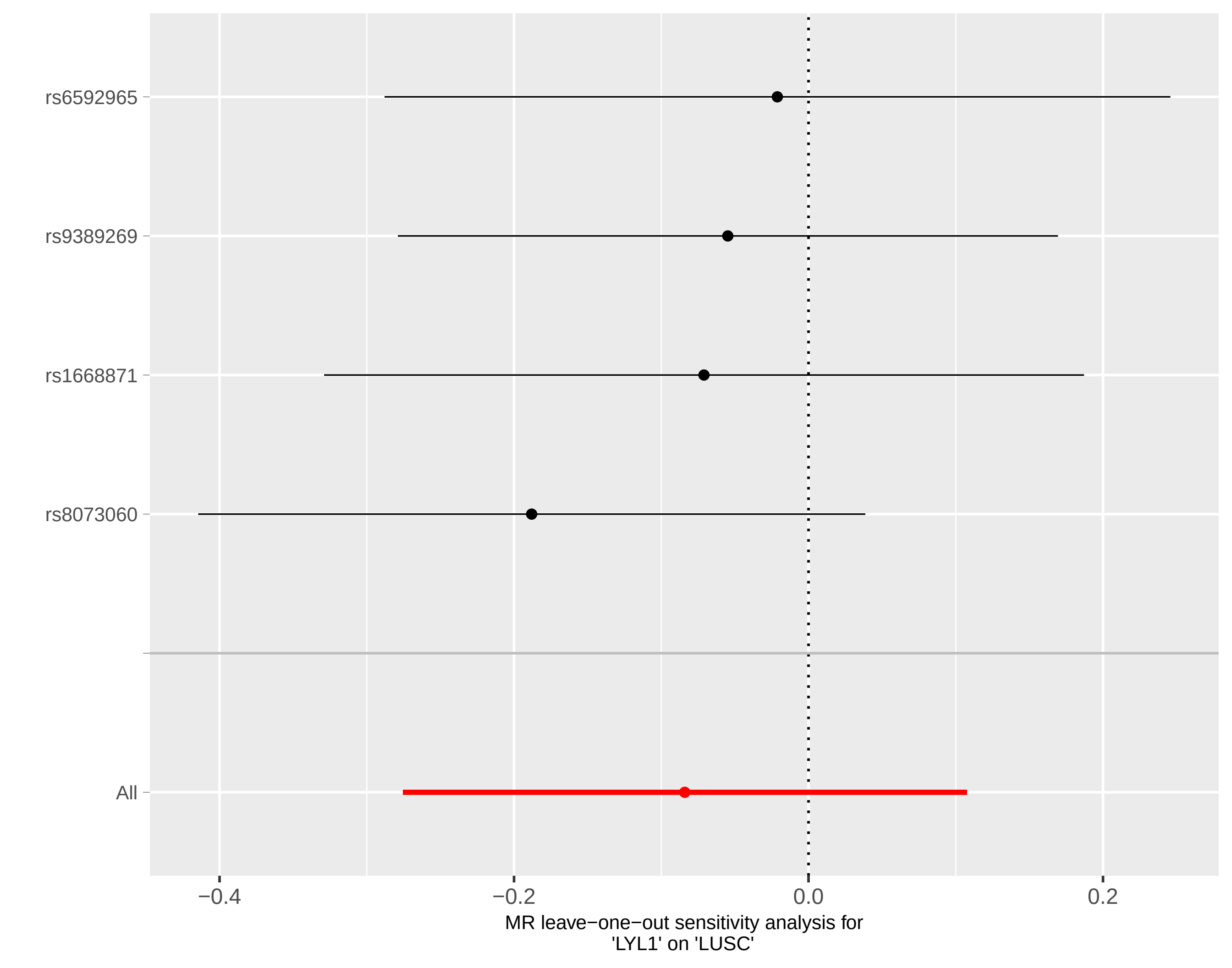

rs6592965
rs9389269
rs1668871
rs8073060
All
−0.4
−0.2
0.0
0.2
MR leave−one−out sensitivity analysis for
'LYL1' on 'LUSC'

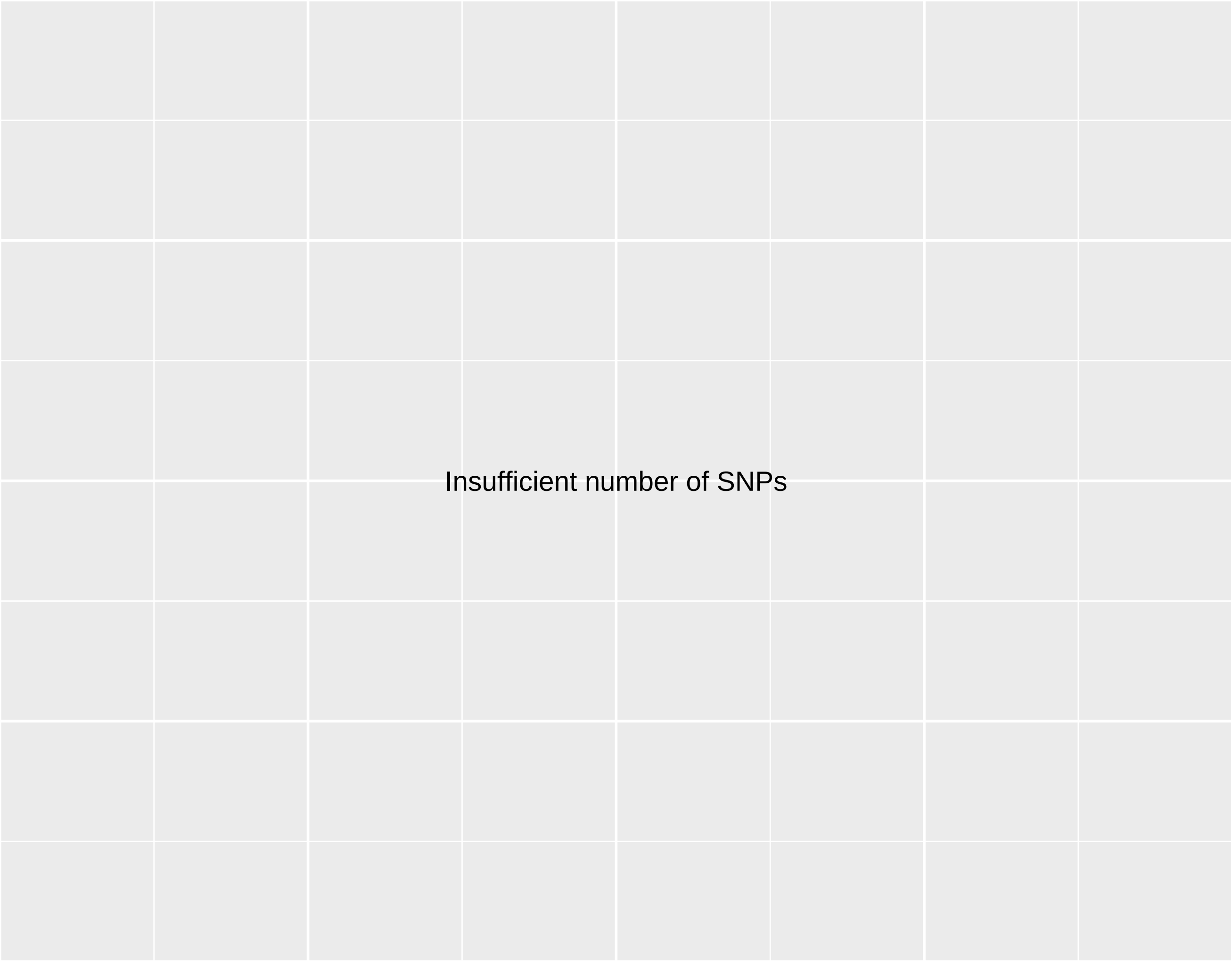
Insufficient number of SNPs

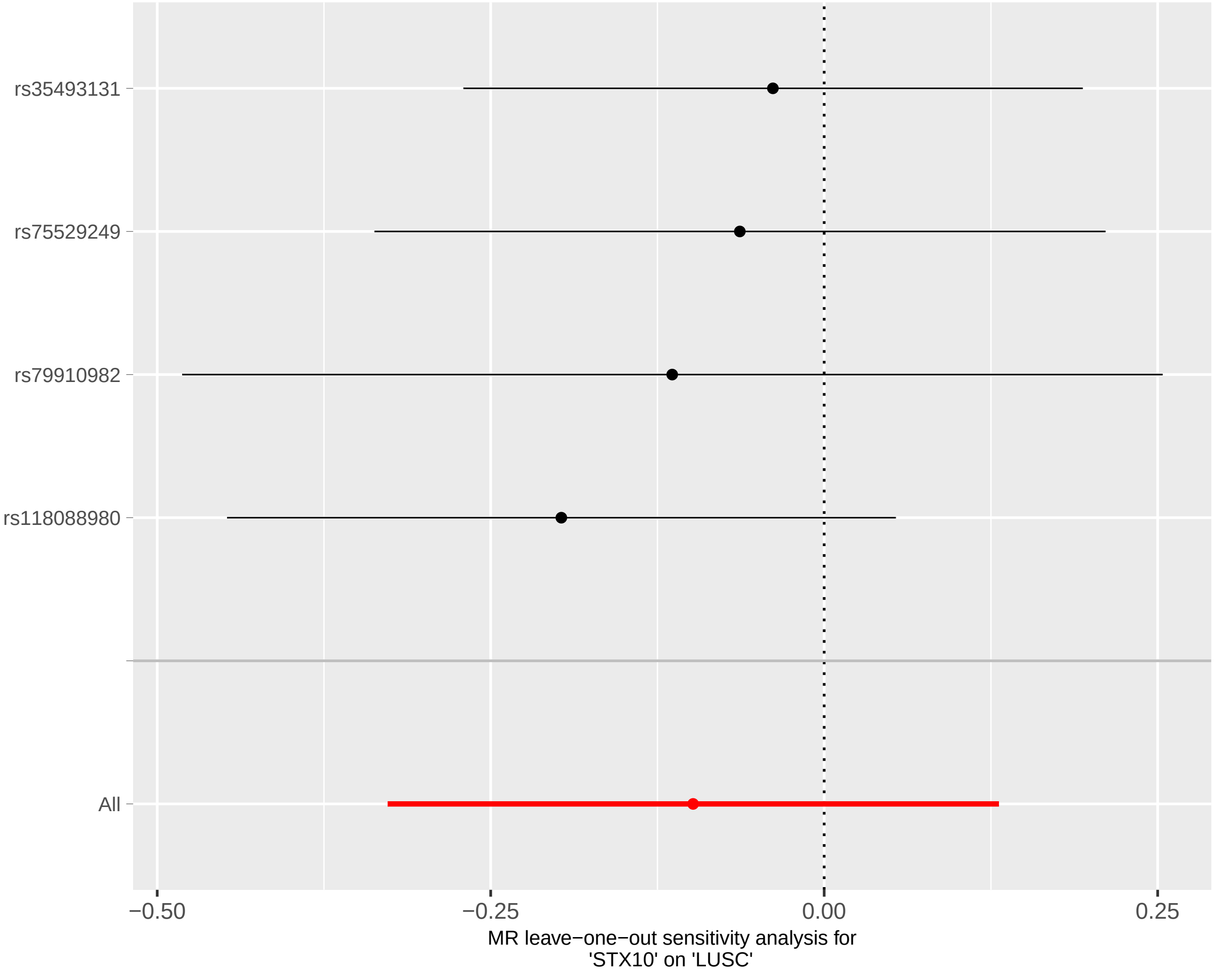

rs35493131
rs75529249
rs79910982
rs118088980
All
−0.50
−0.25
0.00
0.25
MR leave−one−out sensitivity analysis for
'STX10' on 'LUSC'

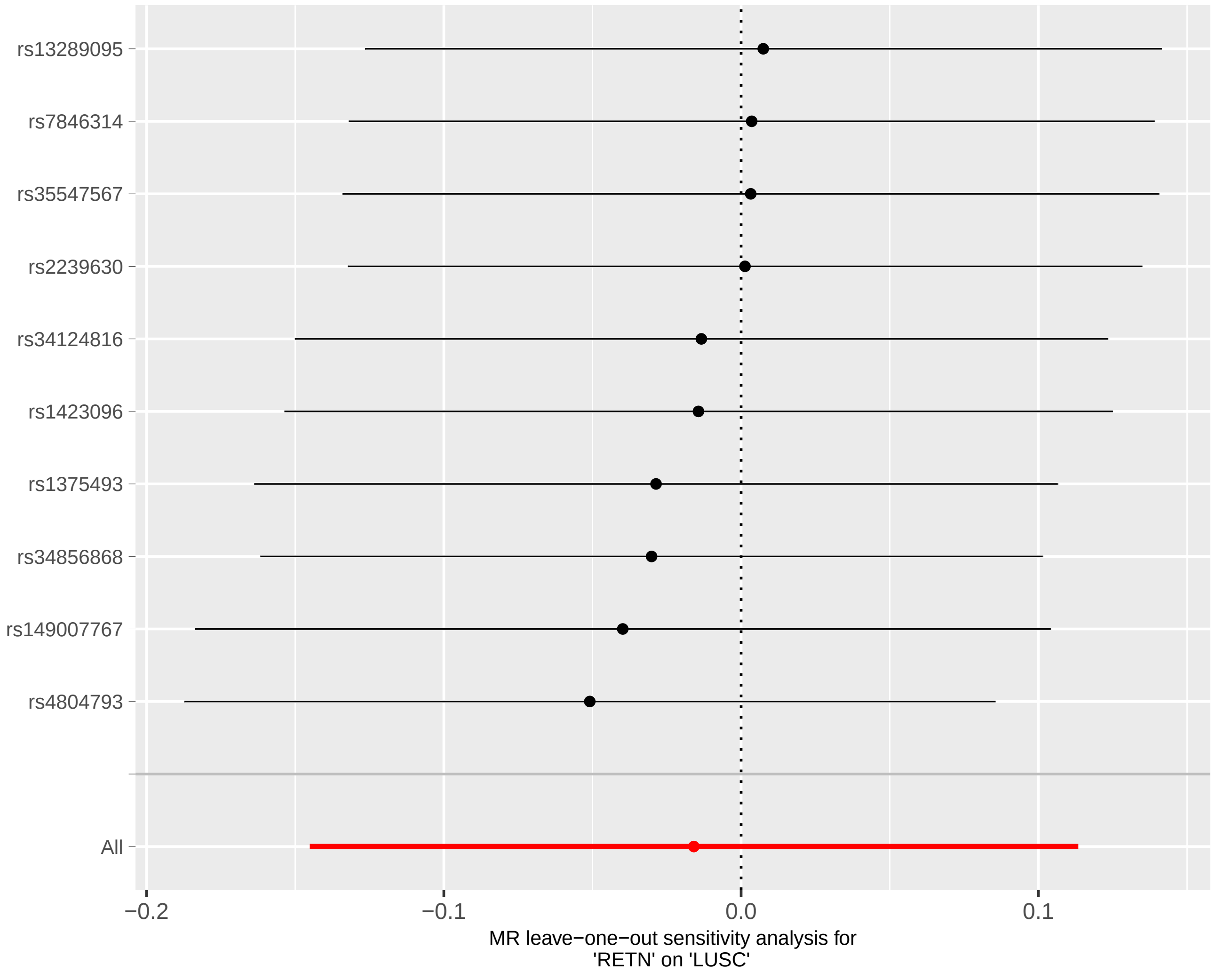
rs13289095
rs7846314
rs35547567
rs2239630
rs34124816
rs1423096
rs1375493
rs34856868
rs149007767
rs4804793
All
−0.2
−0.1
0.0
0.1
MR leave−one−out sensitivity analysis for
'RETN' on 'LUSC'

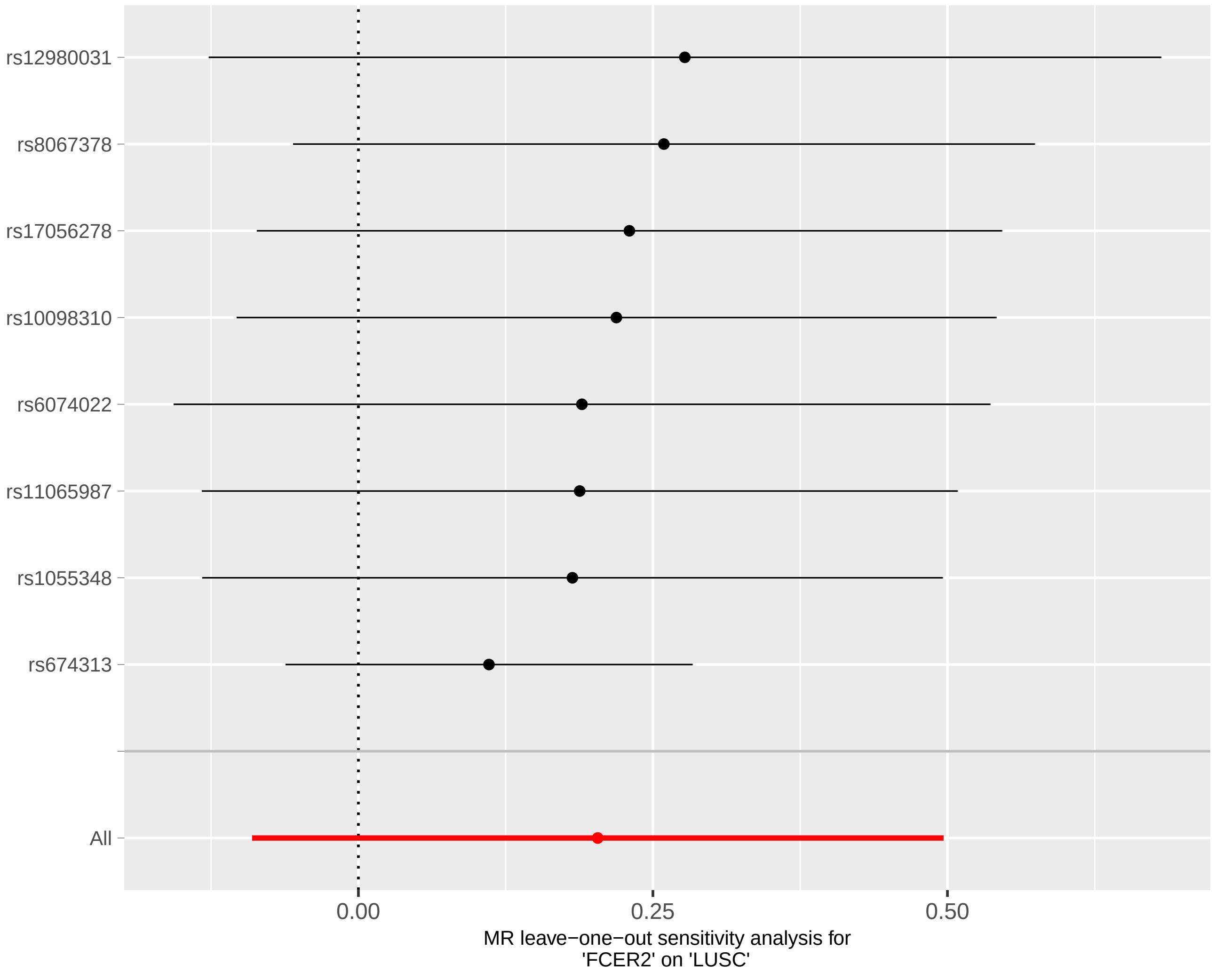

rs12980031
rs8067378
rs17056278
rs10098310
rs6074022
rs11065987
rs1055348
rs674313
All
0.00
0.25
0.50
MR leave−one−out sensitivity analysis for
'FCER2' on 'LUSC'

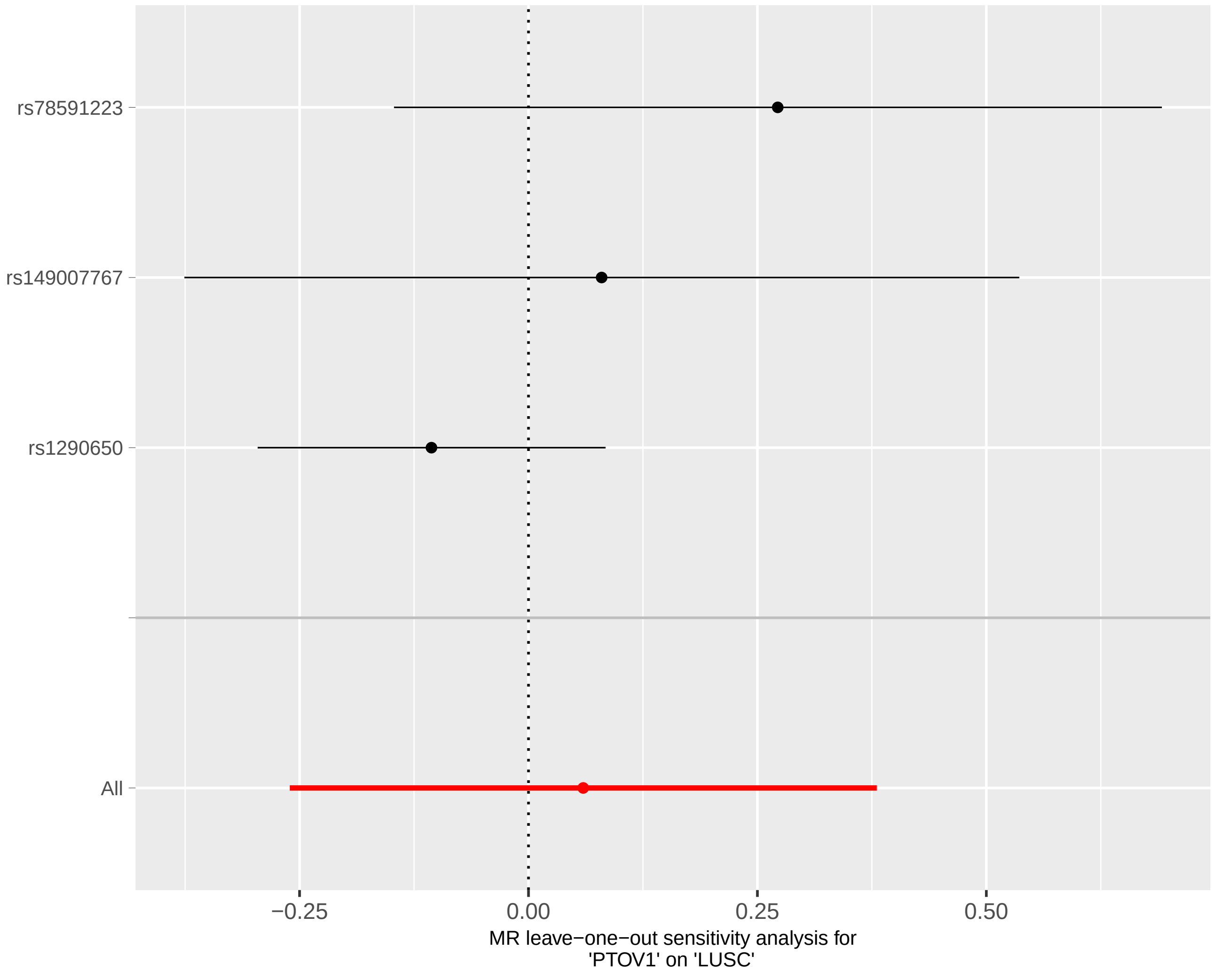

rs78591223
rs149007767
rs1290650
All
−0.25
0.00
0.25
0.50
MR leave−one−out sensitivity analysis for
'PTOV1' on 'LUSC'

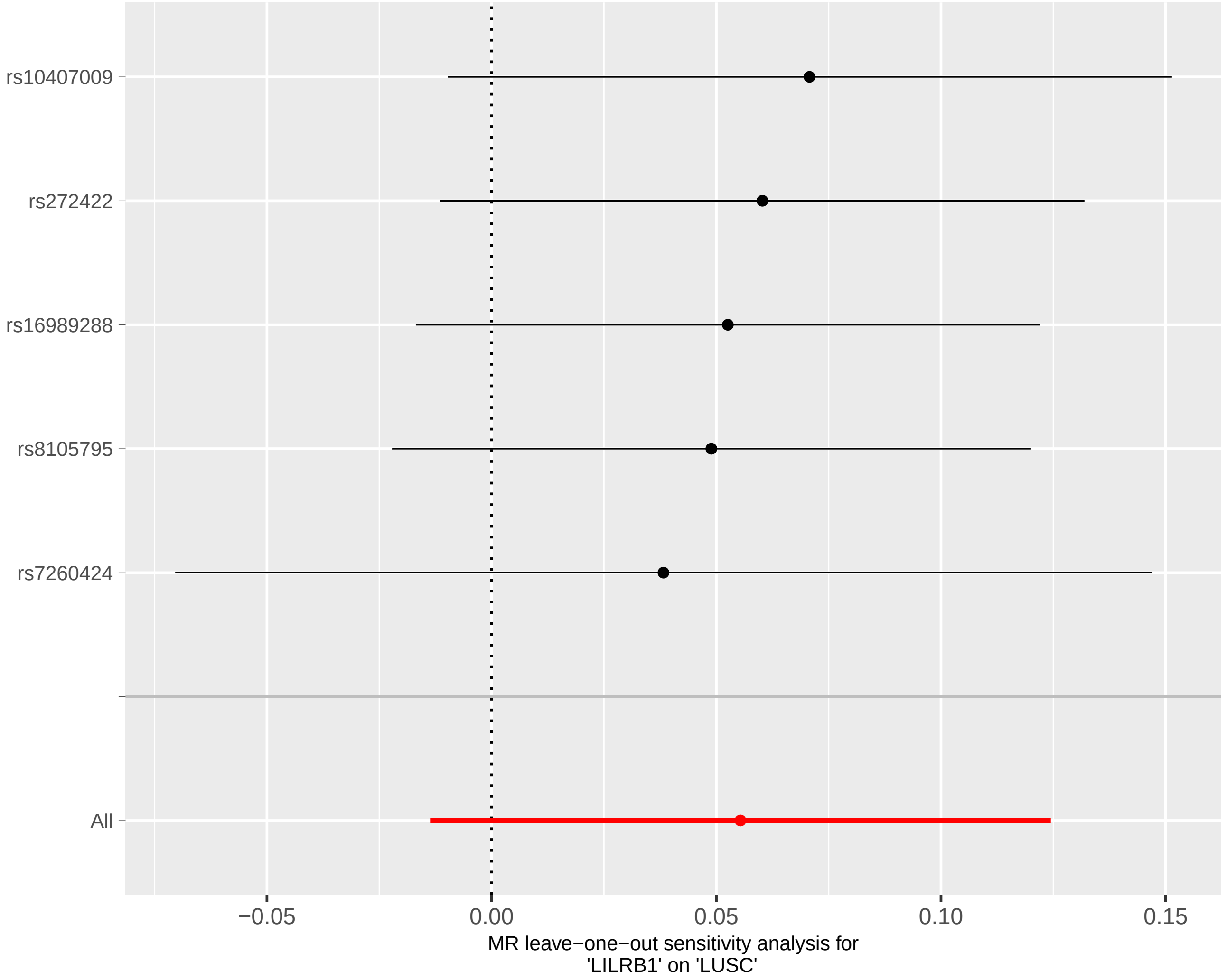
rs10407009
rs272422
rs16989288
rs8105795
rs7260424
All
−0.05
0.00
0.05
0.10
0.15
MR leave−one−out sensitivity analysis for
'LILRB1' on 'LUSC'

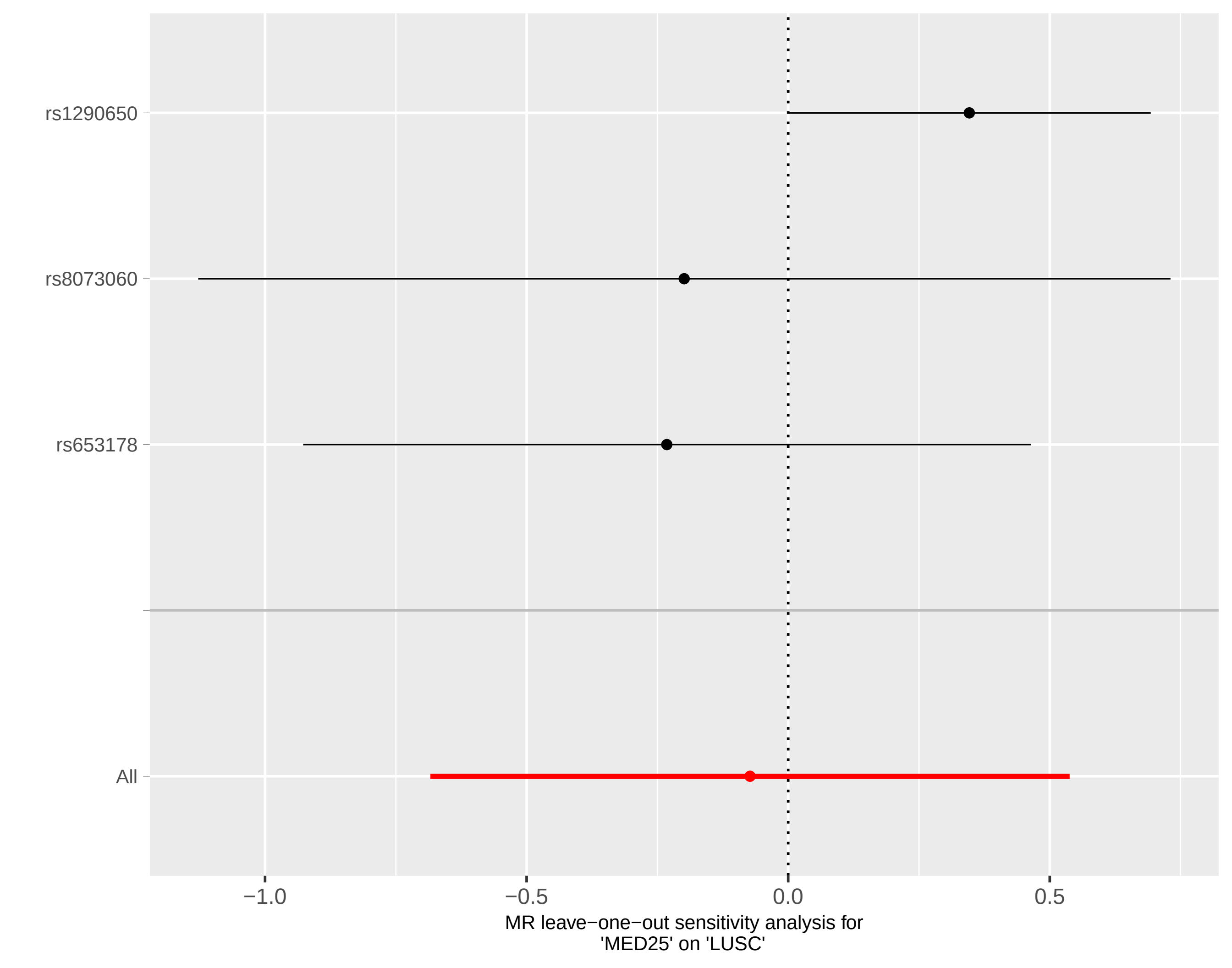

rs1290650
rs8073060
rs653178
All
−1.0
−0.5
0.0
0.5
MR leave−one−out sensitivity analysis for
'MED25' on 'LUSC'

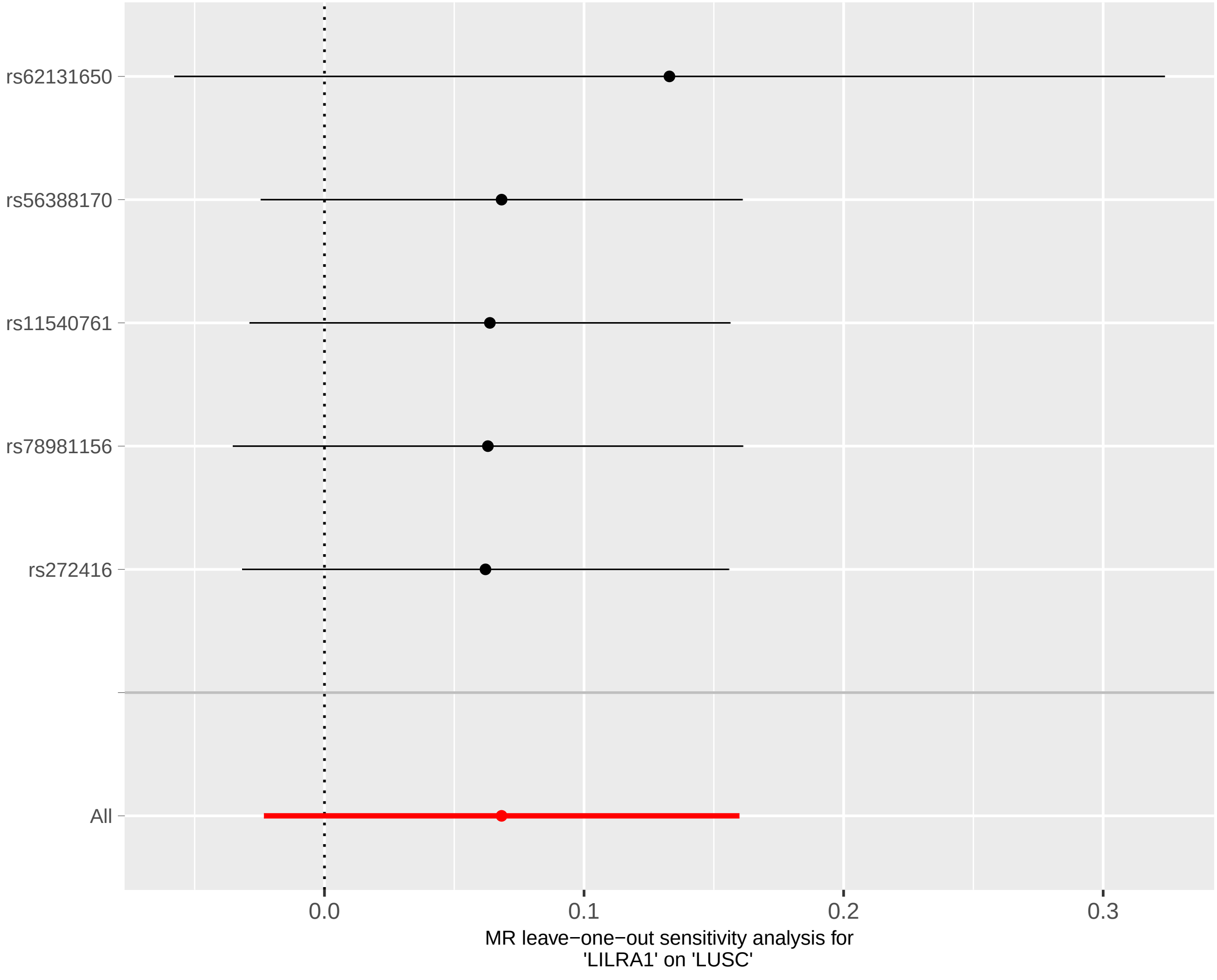
rs62131650
rs56388170
rs11540761
rs78981156
rs272416
All
0.0
0.1
0.2
0.3
MR leave−one−out sensitivity analysis for
'LILRA1' on 'LUSC'

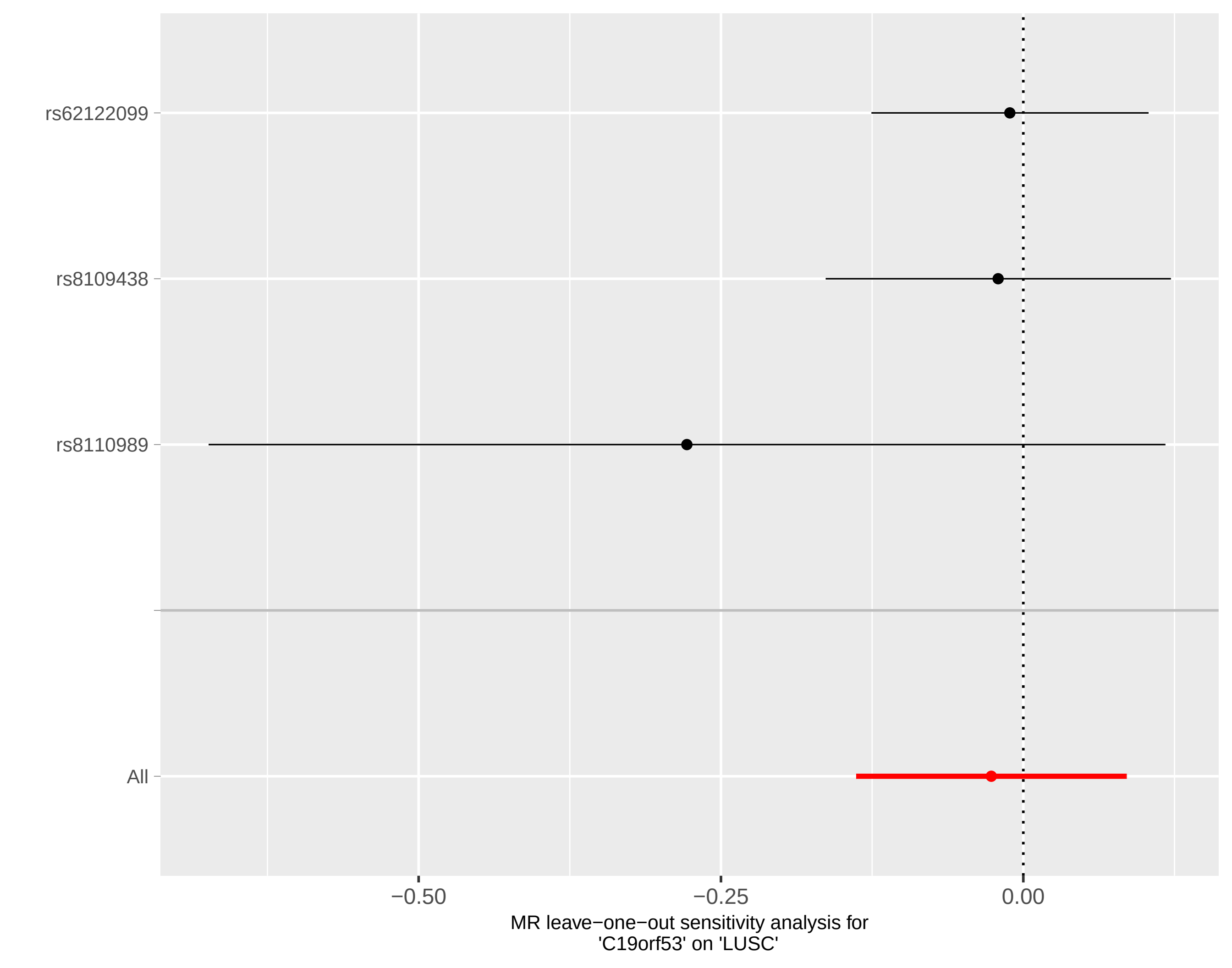

rs62122099
rs8109438
rs8110989
All
−0.50
−0.25
0.00
MR leave−one−out sensitivity analysis for
'C19orf53' on 'LUSC'

Insufficient number of SNPs

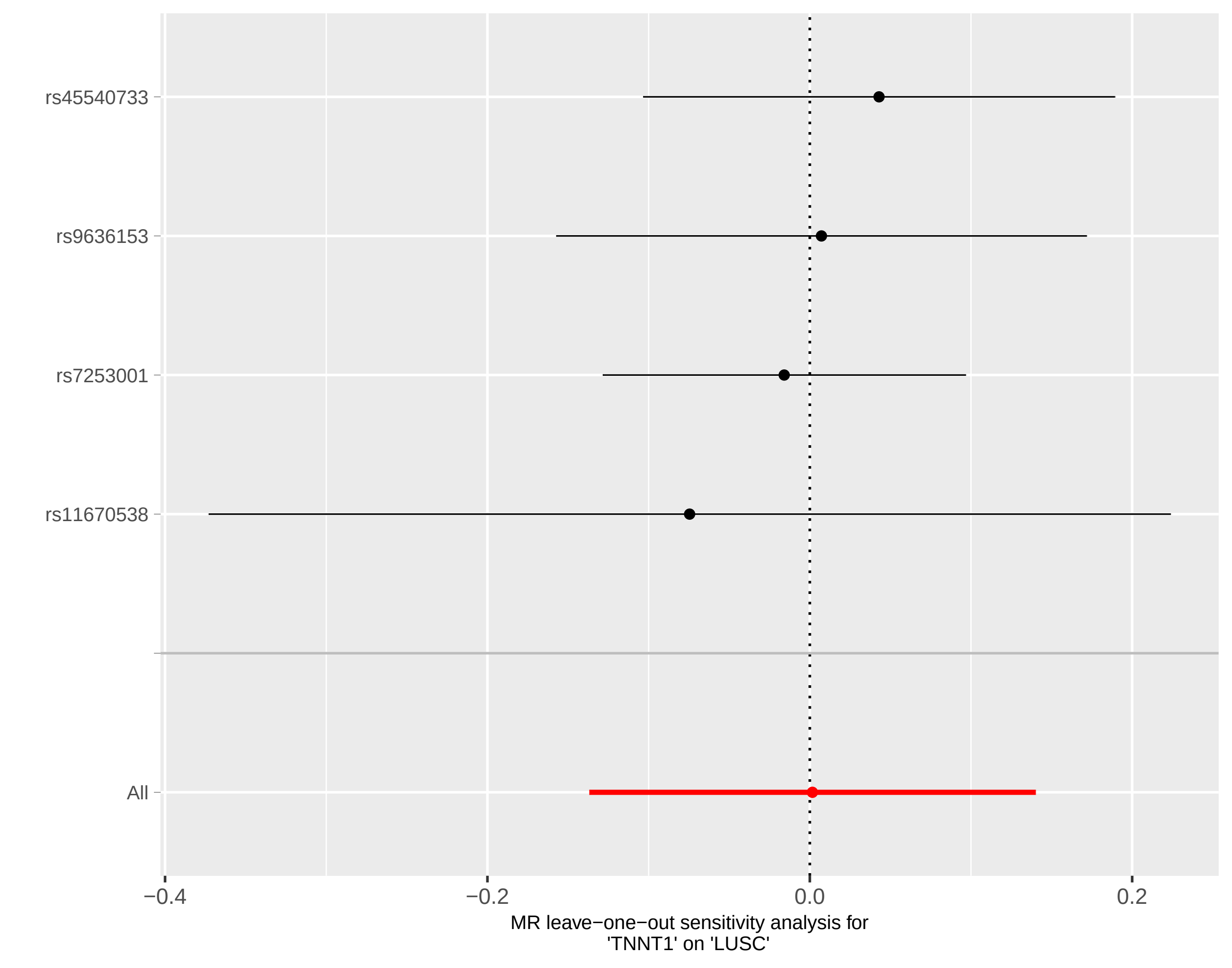
rs45540733
rs9636153
rs7253001
rs11670538
All
−0.4
−0.2
0.0
0.2
MR leave−one−out sensitivity analysis for
'TNNT1' on 'LUSC'

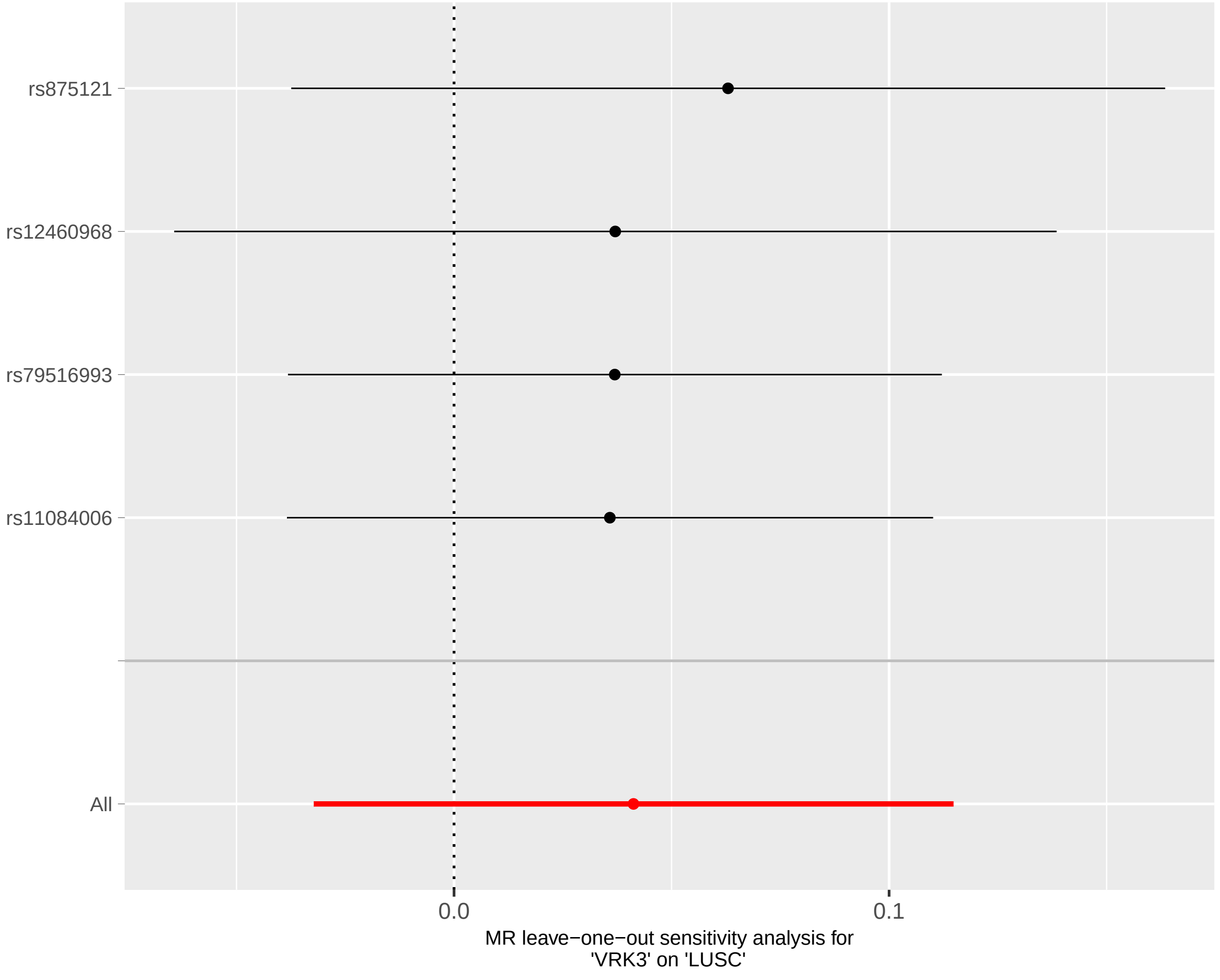
rs875121
rs12460968
rs79516993
rs11084006
All
0.0
0.1
MR leave−one−out sensitivity analysis for
'VRK3' on 'LUSC'

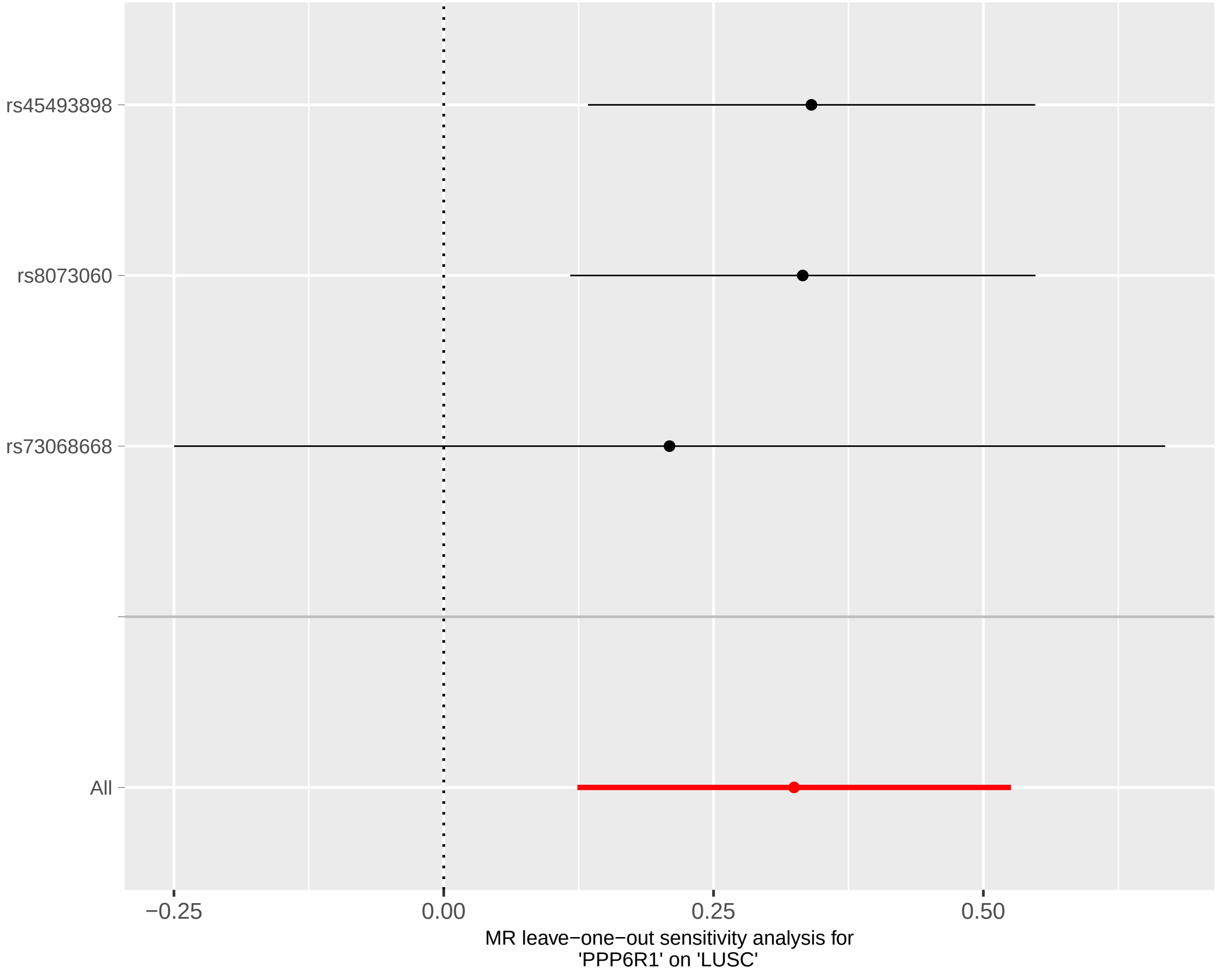

rs45493898
rs8073060
rs73068668
All
−0.25
0.00
0.25
0.50
MR leave−one−out sensitivity analysis for
'PPP6R1' on 'LUSC'

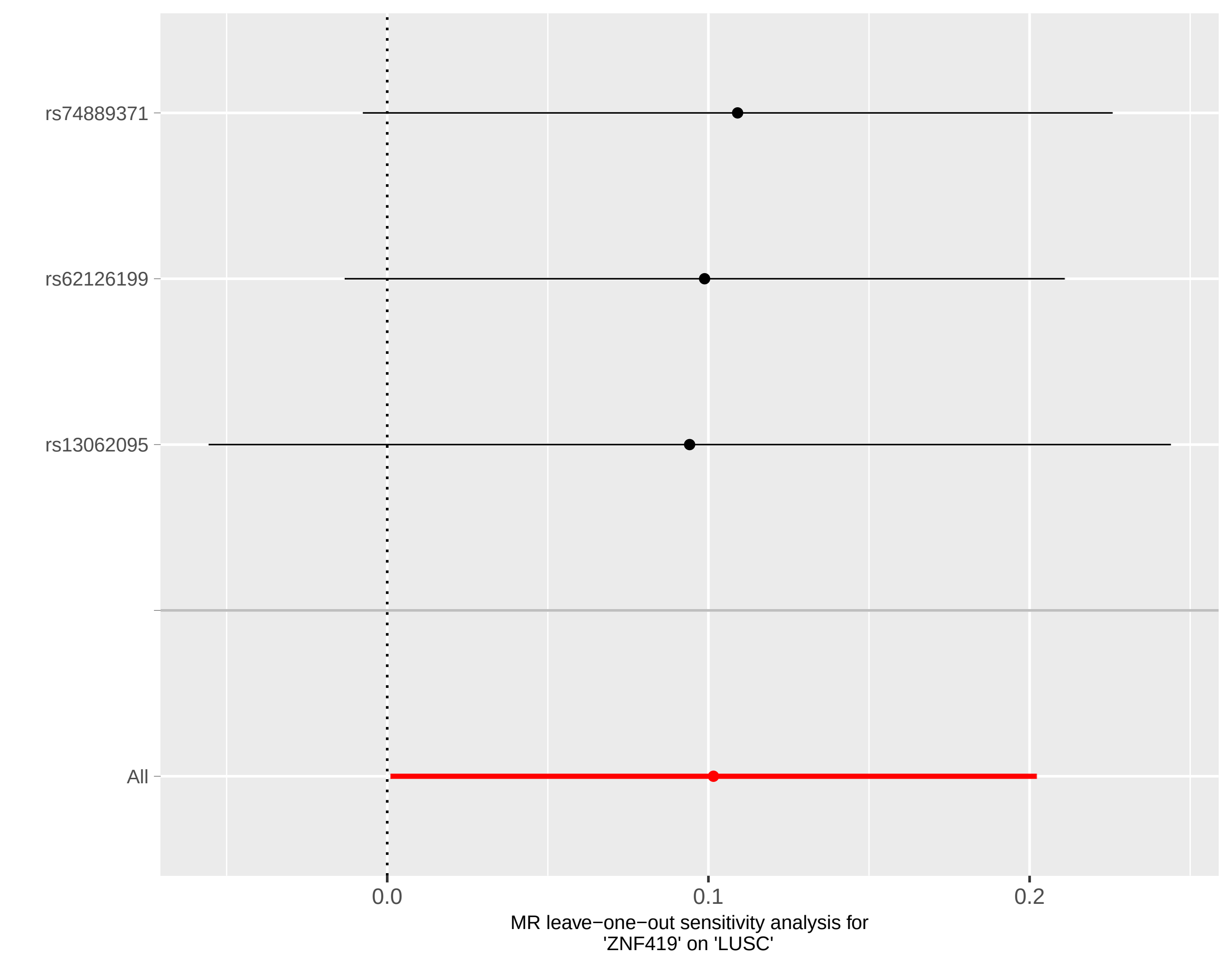

rs74889371
rs62126199
rs13062095
All
0.0
0.1
0.2
MR leave−one−out sensitivity analysis for
'ZNF419' on 'LUSC'

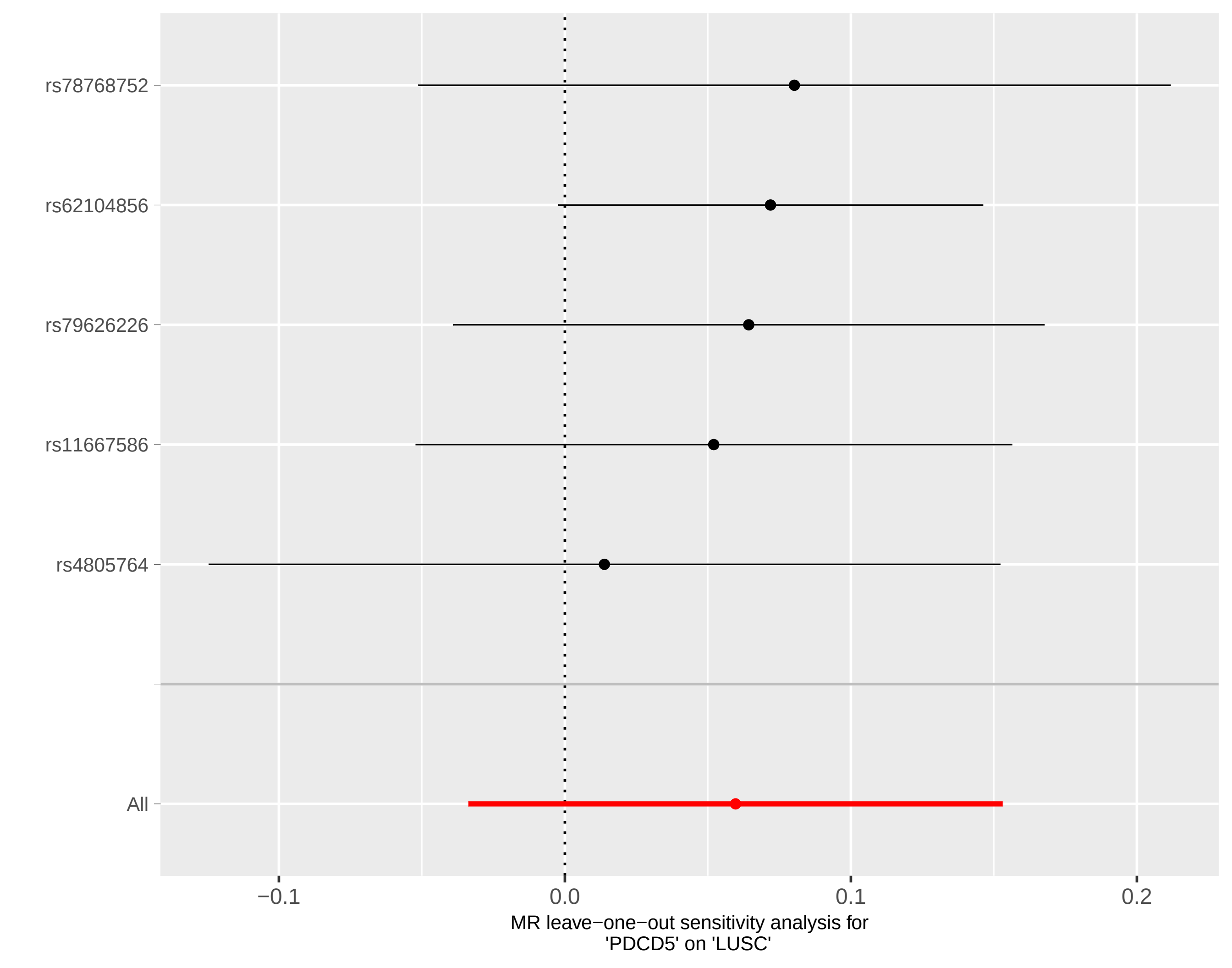
rs78768752
rs62104856
rs79626226
rs11667586
rs4805764
All
−0.1
0.0
0.1
0.2
MR leave−one−out sensitivity analysis for
'PDCD5' on 'LUSC'

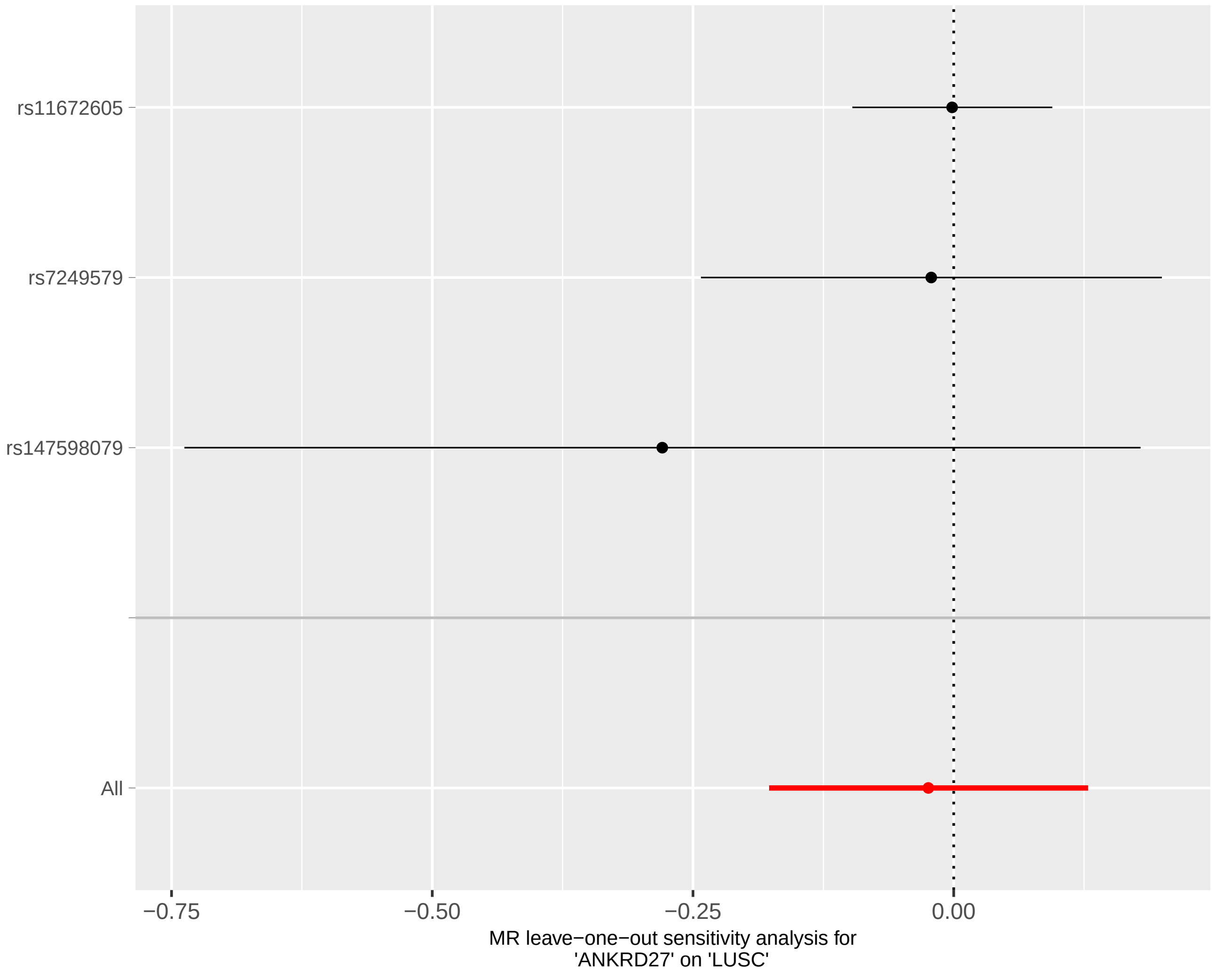
rs11672605
rs7249579
rs147598079
All
−0.75
−0.50
−0.25
0.00
MR leave−one−out sensitivity analysis for
'ANKRD27' on 'LUSC'

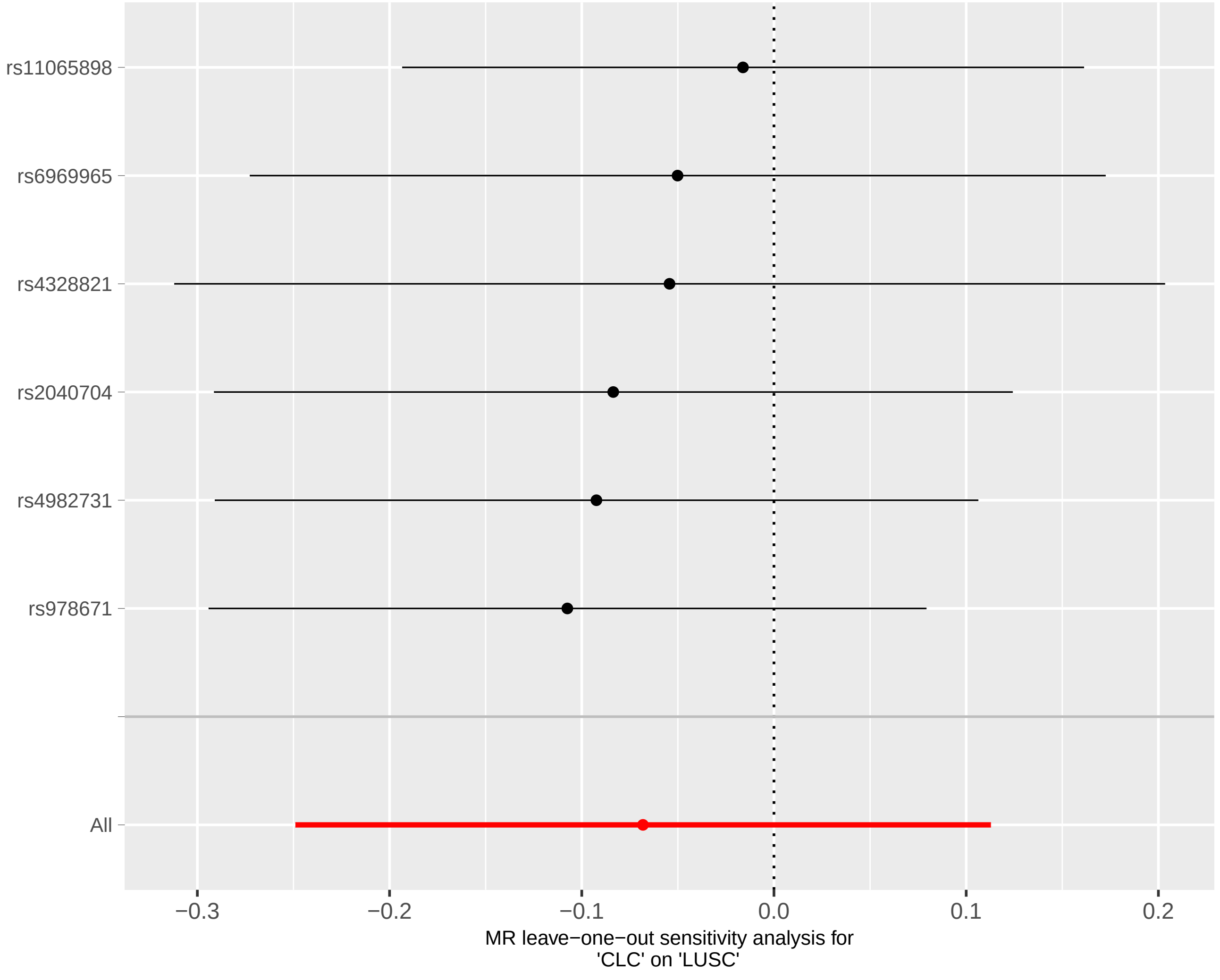
rs11065898
rs6969965
rs4328821
rs2040704
rs4982731
rs978671
All
−0.3
−0.2
−0.1
0.0
0.1
0.2
MR leave−one−out sensitivity analysis for
'CLC' on 'LUSC'

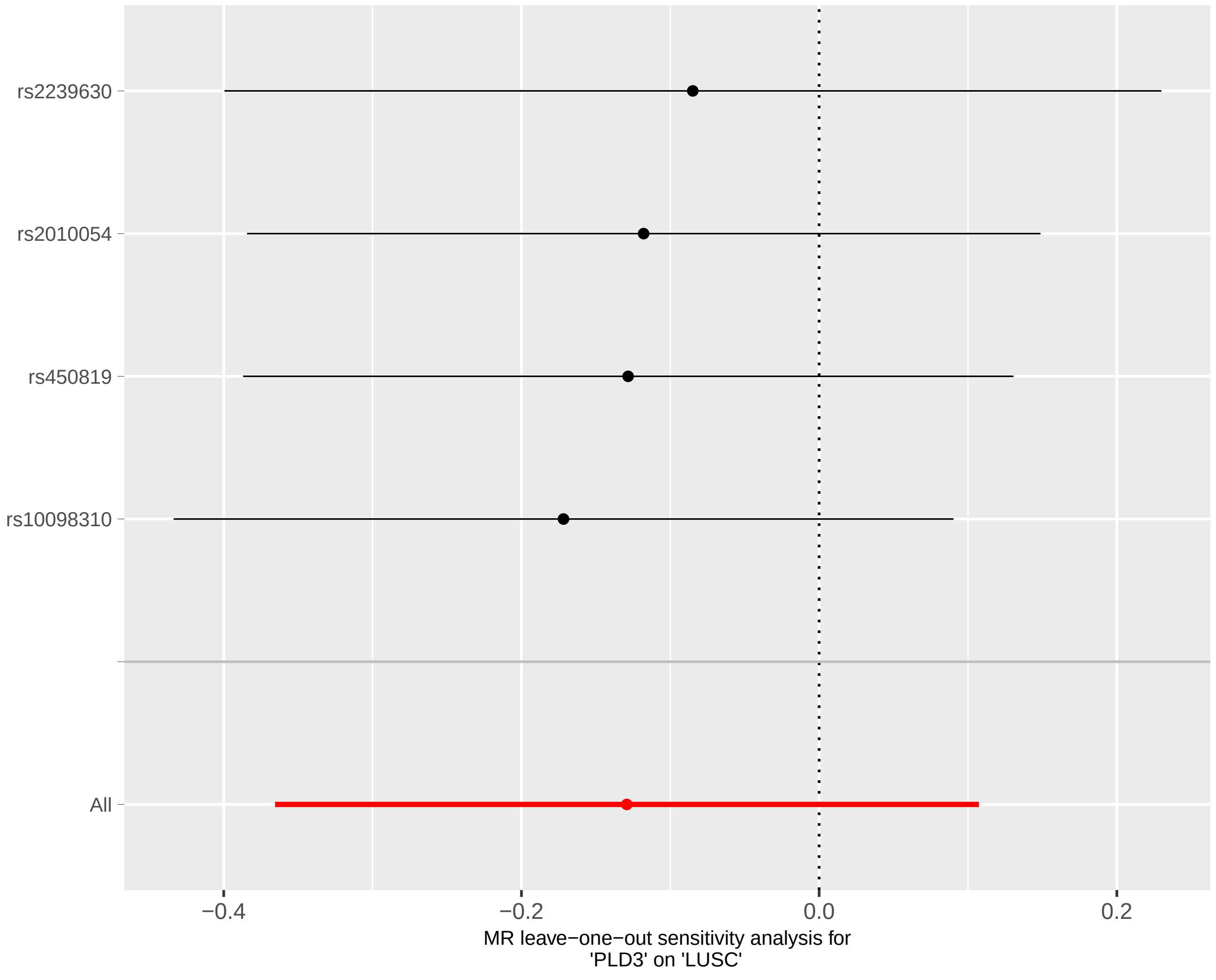

rs2239630
rs2010054
rs450819
rs10098310
All
−0.4
−0.2
0.0
0.2
MR leave−one−out sensitivity analysis for
'PLD3' on 'LUSC'

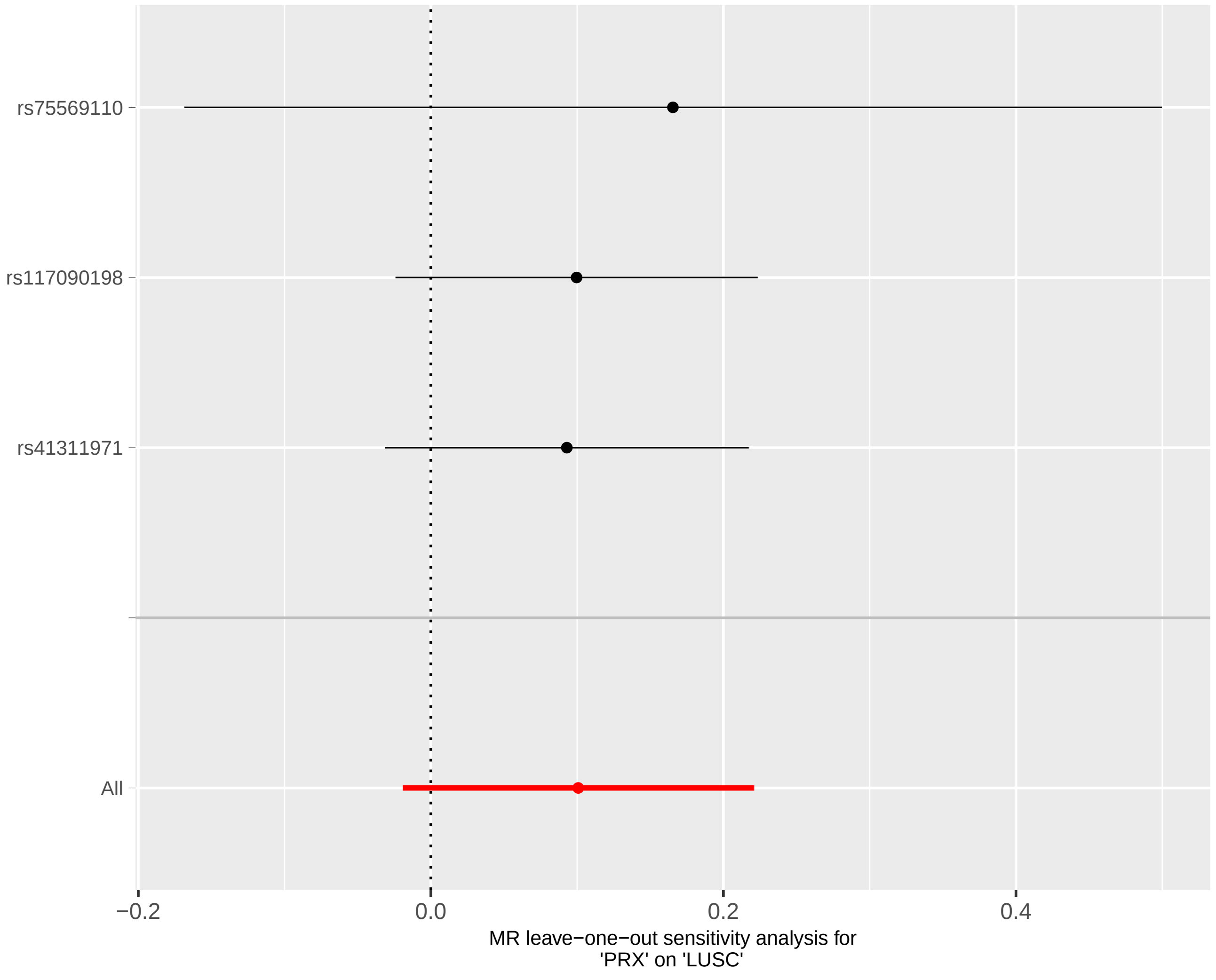
rs75569110
rs117090198
rs41311971
All
−0.2
0.0
0.2
0.4
MR leave−one−out sensitivity analysis for
'PRX' on 'LUSC'

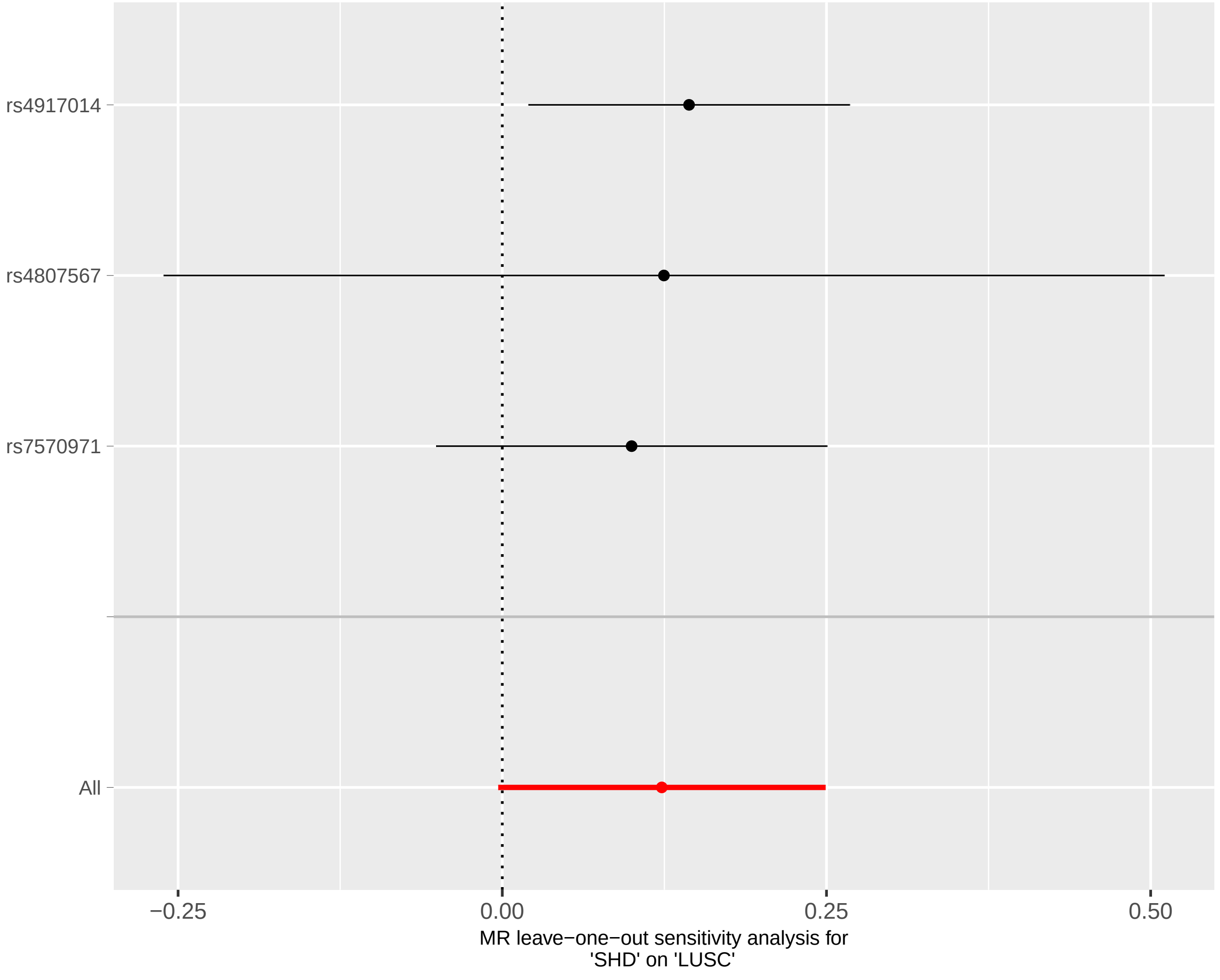
rs4917014
rs4807567
rs7570971
All
−0.25
0.00
0.25
0.50
MR leave−one−out sensitivity analysis for
'SHD' on 'LUSC'

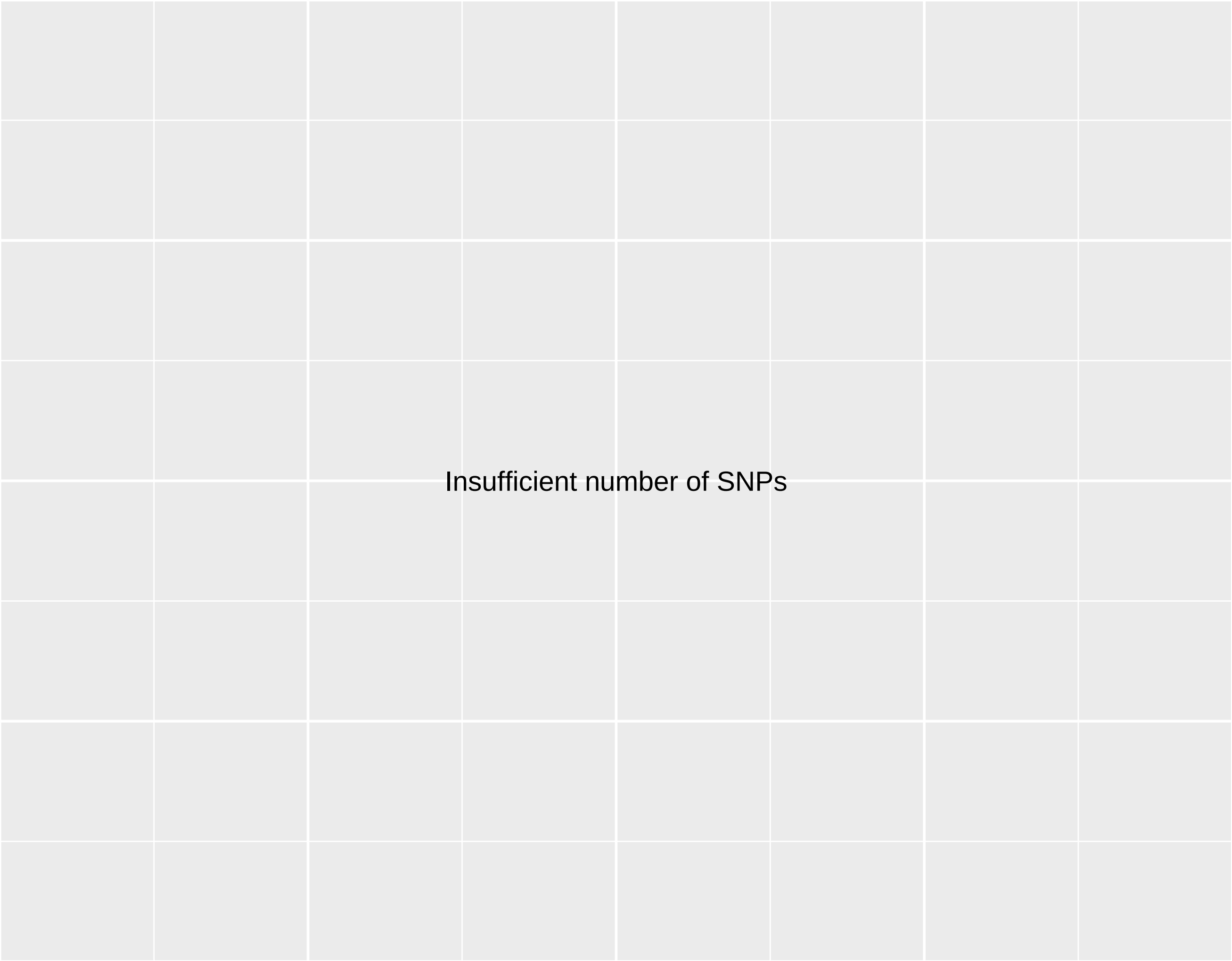
Insufficient number of SNPs

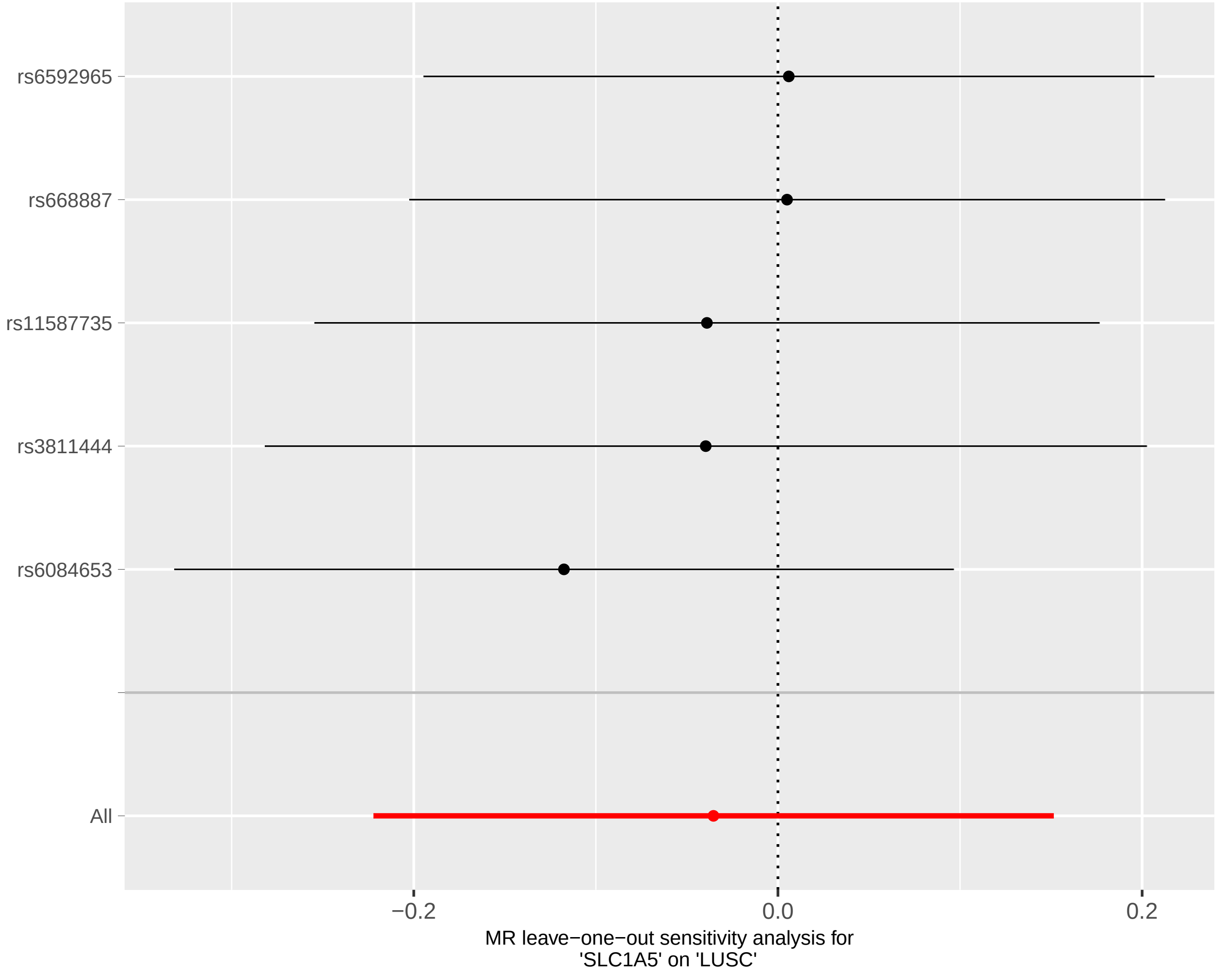
rs6592965
rs668887
rs11587735
rs3811444
rs6084653
All
−0.2
0.0
0.2
MR leave−one−out sensitivity analysis for
'SLC1A5' on 'LUSC'

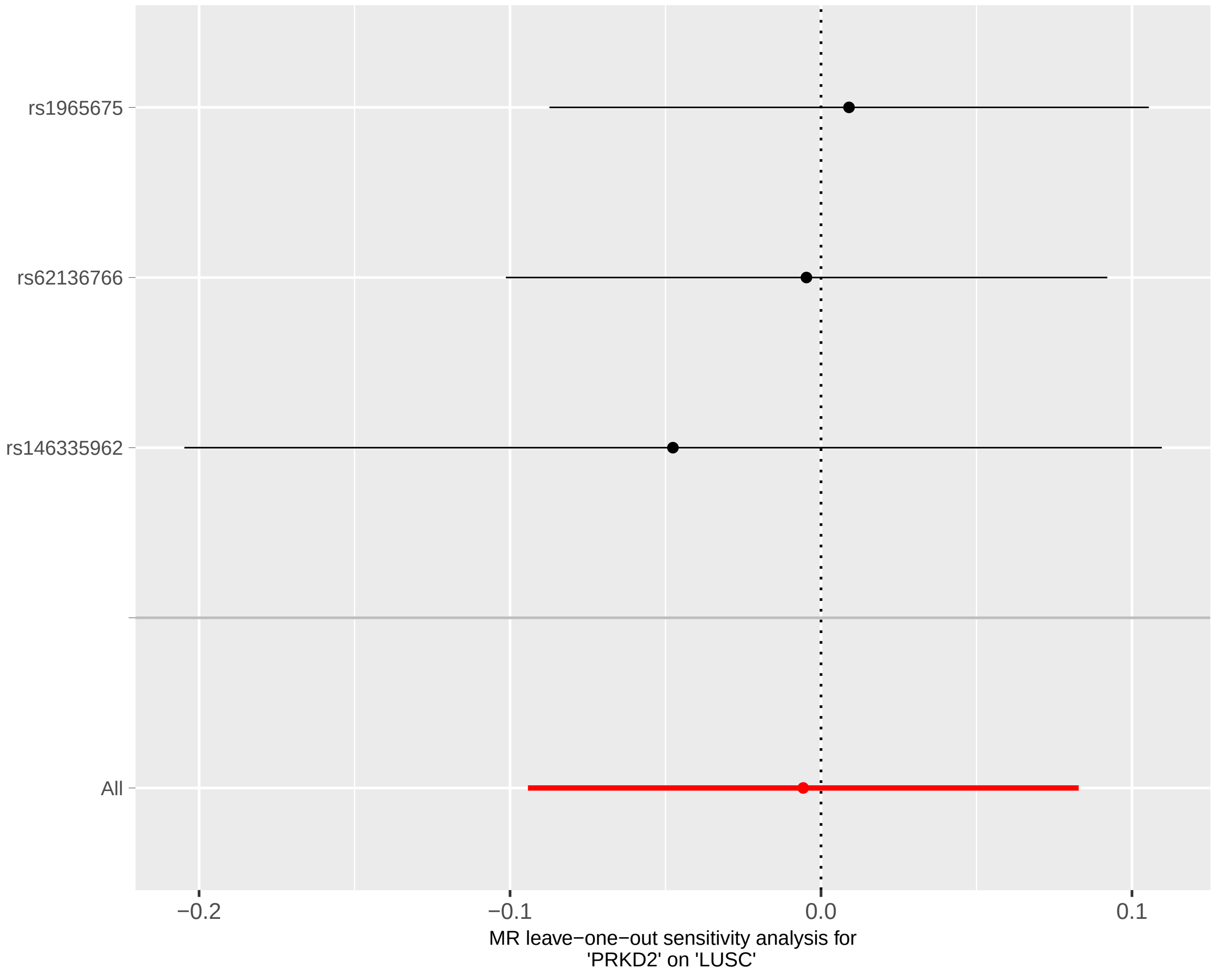
rs1965675
rs62136766
rs146335962
All
−0.2
−0.1
0.0
0.1
MR leave−one−out sensitivity analysis for
'PRKD2' on 'LUSC'

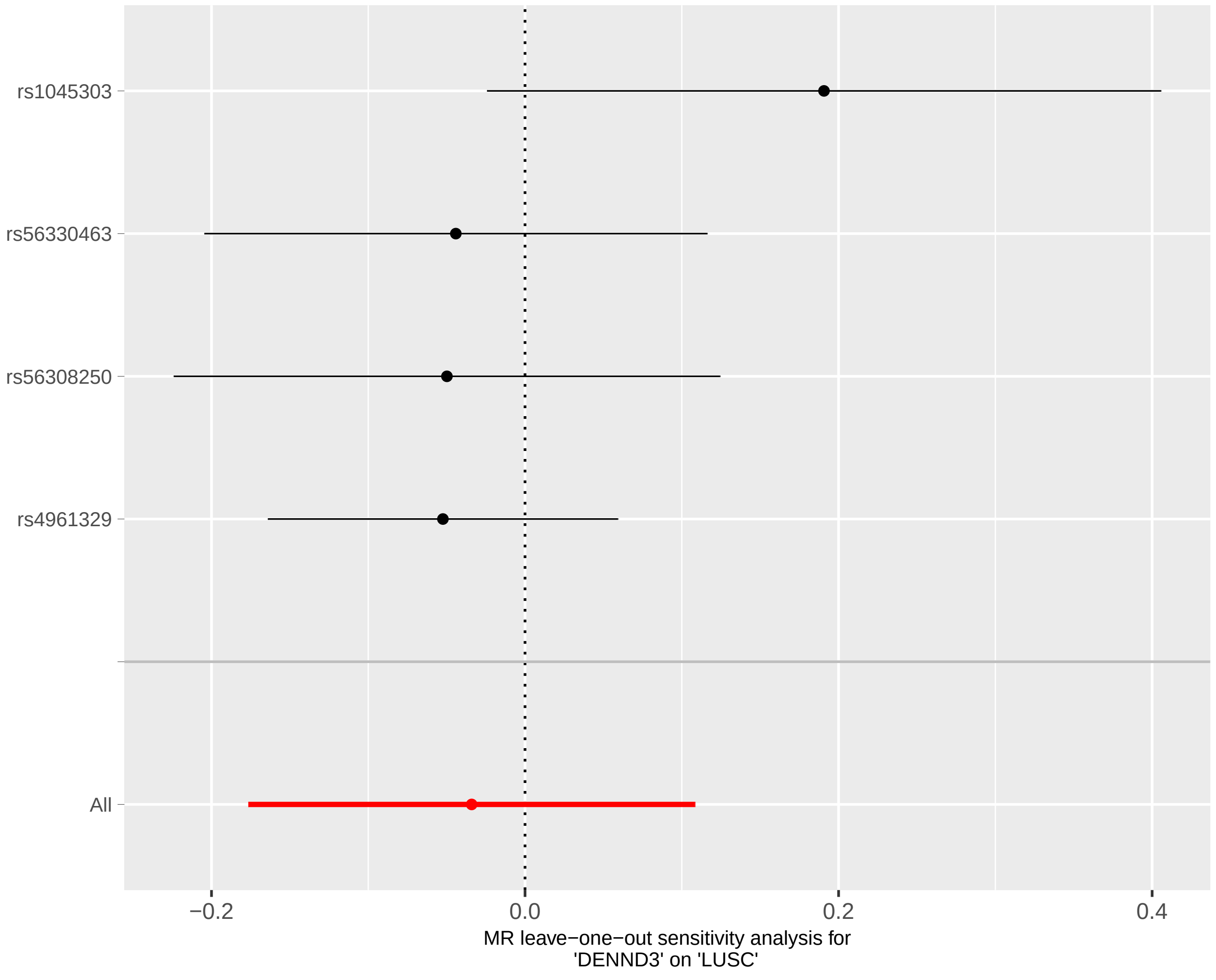

rs1045303
rs56330463
rs56308250
rs4961329
All
−0.2
0.0
0.2
0.4
MR leave−one−out sensitivity analysis for
'DENND3' on 'LUSC'

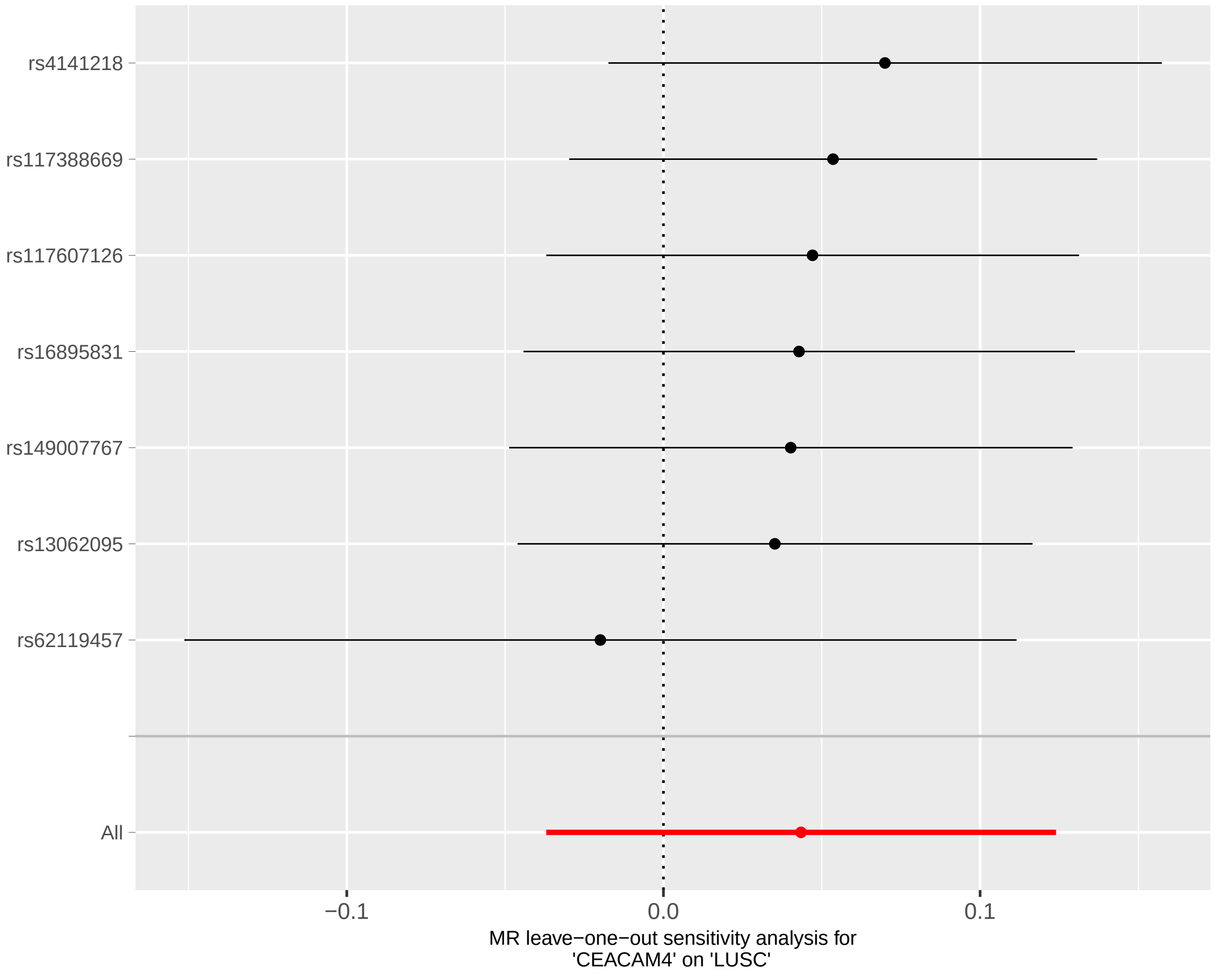
rs4141218
rs117388669
rs117607126
rs16895831
rs149007767
rs13062095
rs62119457
All
−0.1
0.0
0.1
MR leave−one−out sensitivity analysis for
'CEACAM4' on 'LUSC'

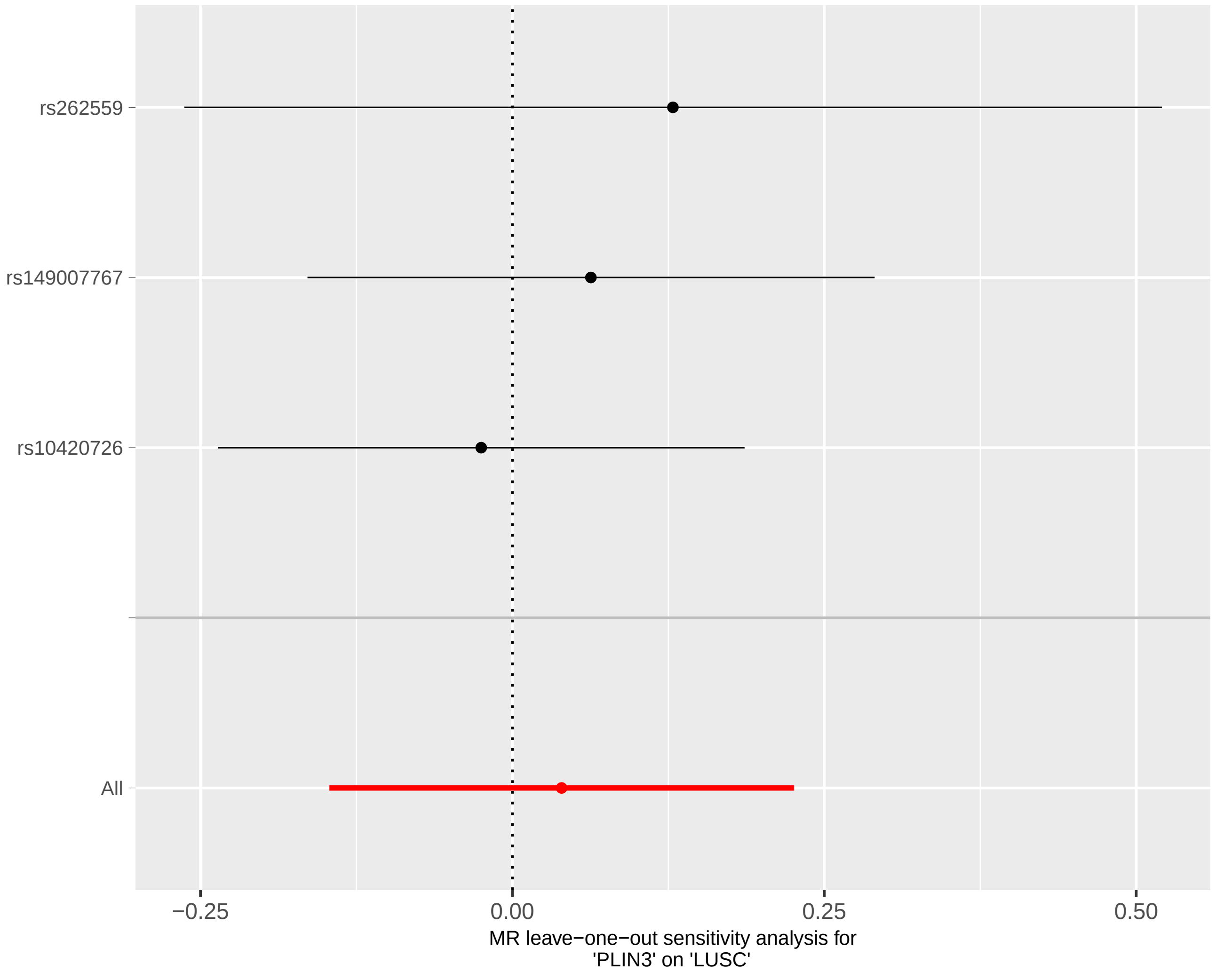
rs262559
rs149007767
rs10420726
All
−0.25
0.00
0.25
0.50
MR leave−one−out sensitivity analysis for
'PLIN3' on 'LUSC'

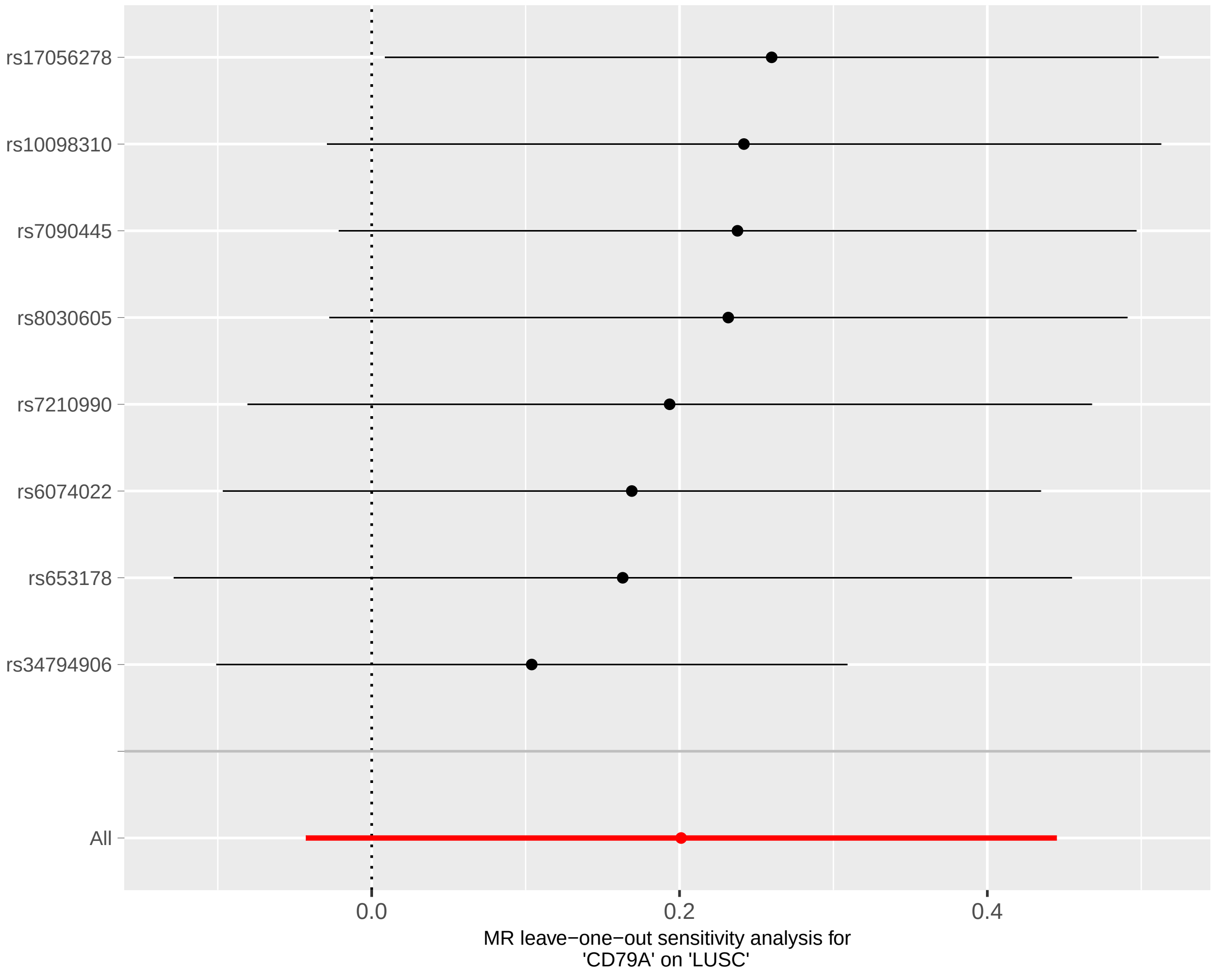
rs17056278
rs10098310
rs7090445
rs8030605
rs7210990
rs6074022
rs653178
rs34794906
All
0.0
0.2
0.4
MR leave−one−out sensitivity analysis for
'CD79A' on 'LUSC'

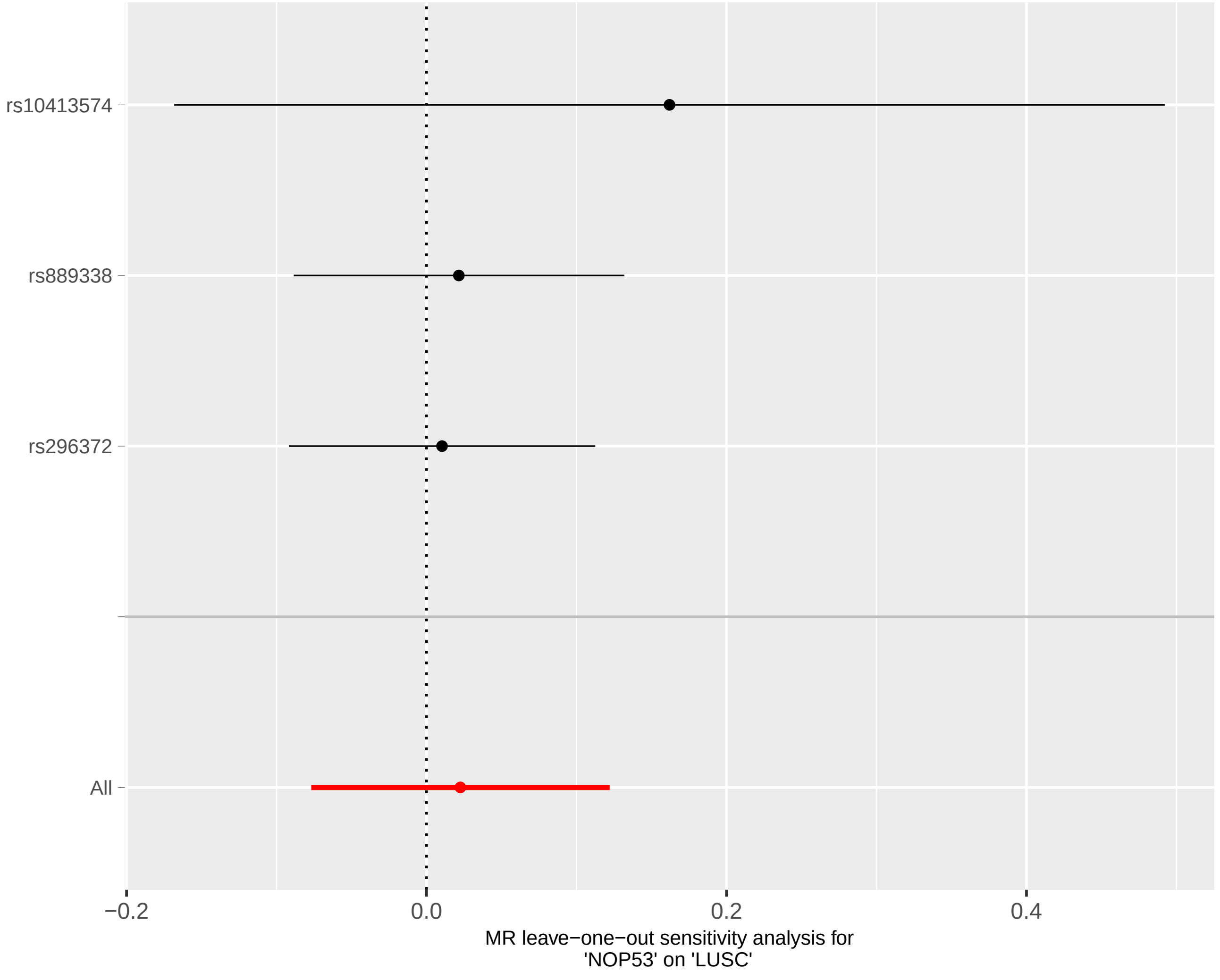

rs10413574
rs889338
rs296372
All
−0.2
0.0
0.2
0.4
MR leave−one−out sensitivity analysis for
'NOP53' on 'LUSC'

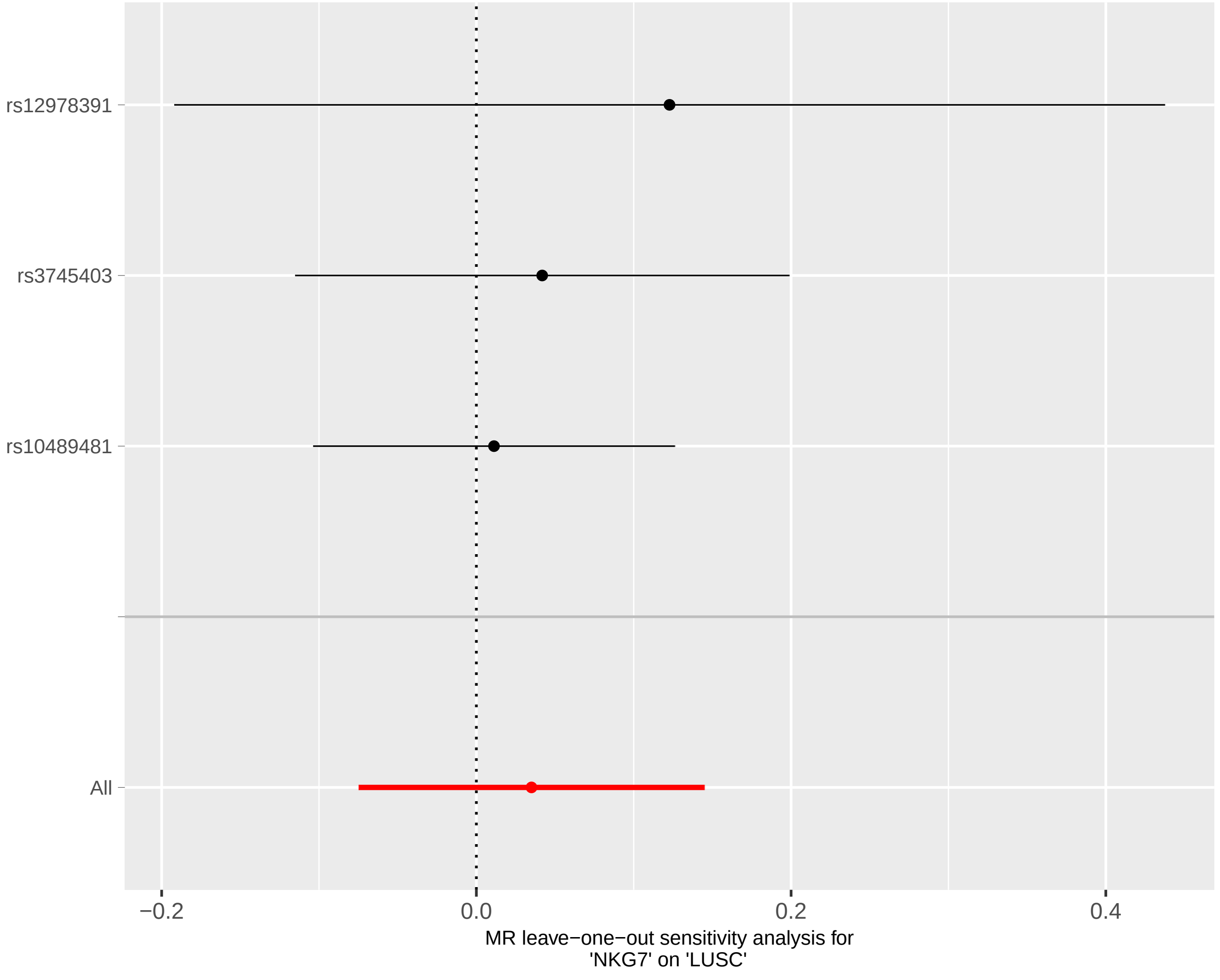
rs12978391
rs3745403
rs10489481
All
−0.2
0.0
0.2
0.4
MR leave−one−out sensitivity analysis for
'NKG7' on 'LUSC'

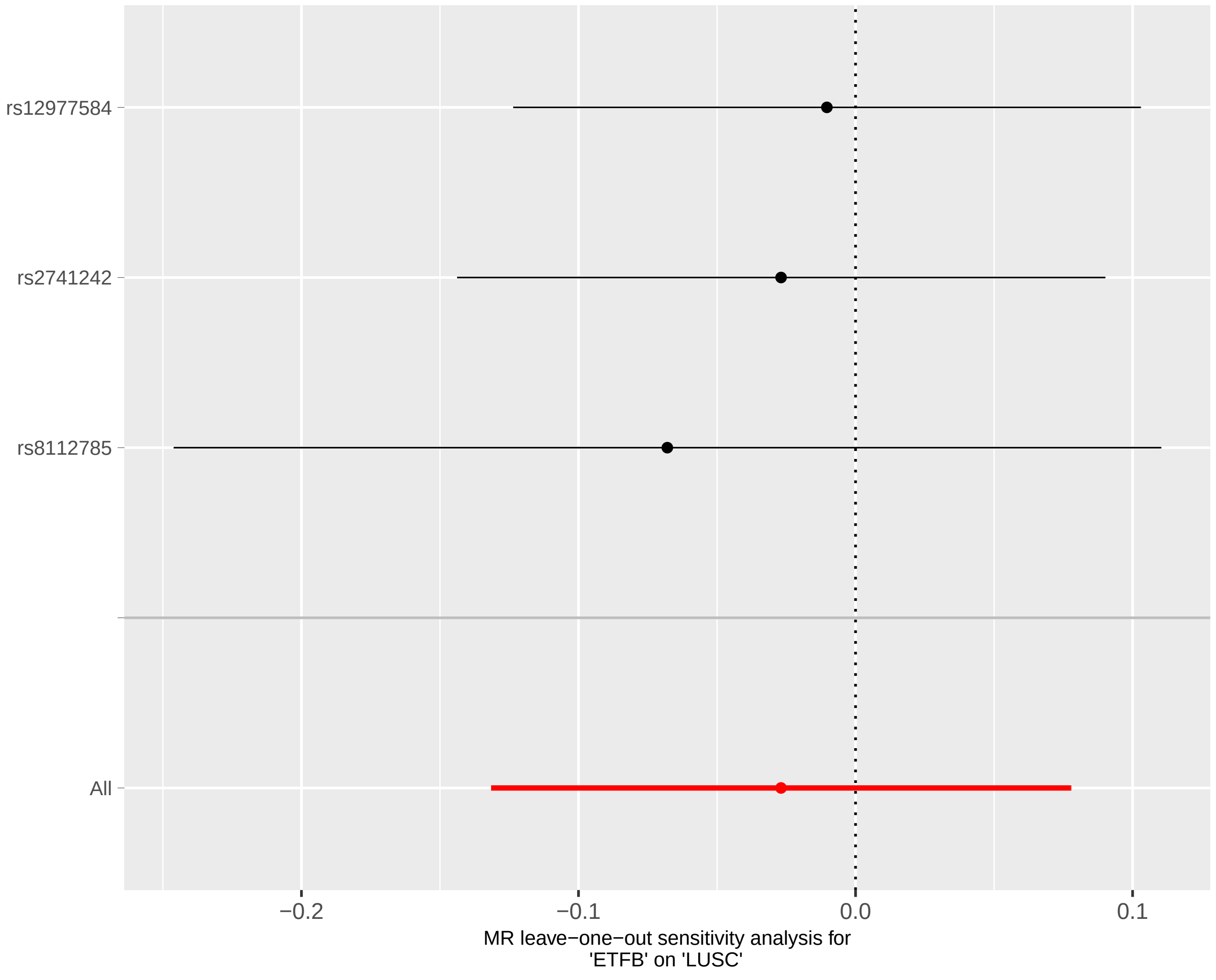
rs12977584
rs2741242
rs8112785
All
−0.2
−0.1
0.0
0.1
MR leave−one−out sensitivity analysis for
'ETFB' on 'LUSC'

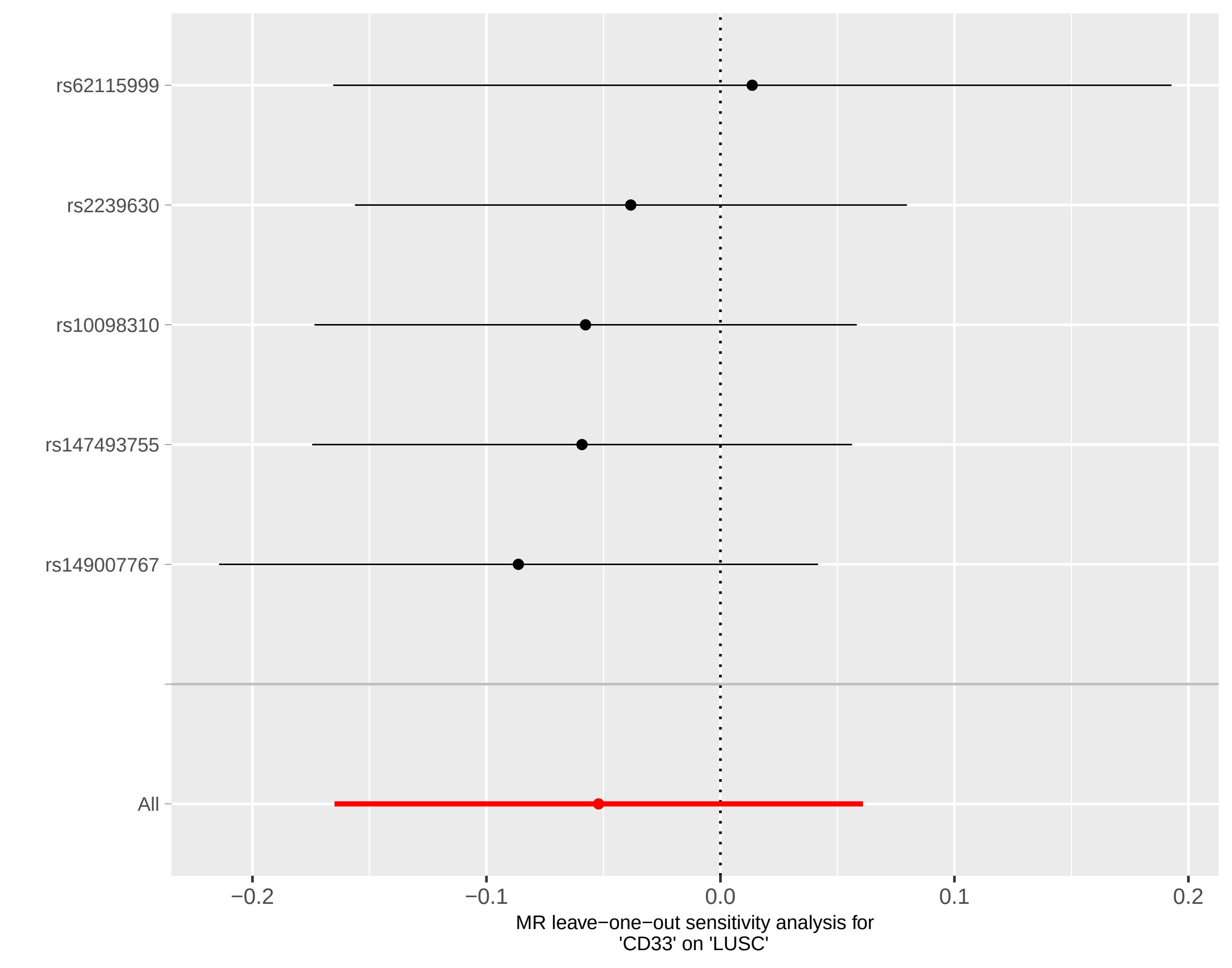
rs62115999
rs2239630
rs10098310
rs147493755
rs149007767
All
−0.2
−0.1
0.0
0.1
0.2
MR leave−one−out sensitivity analysis for
'CD33' on 'LUSC'

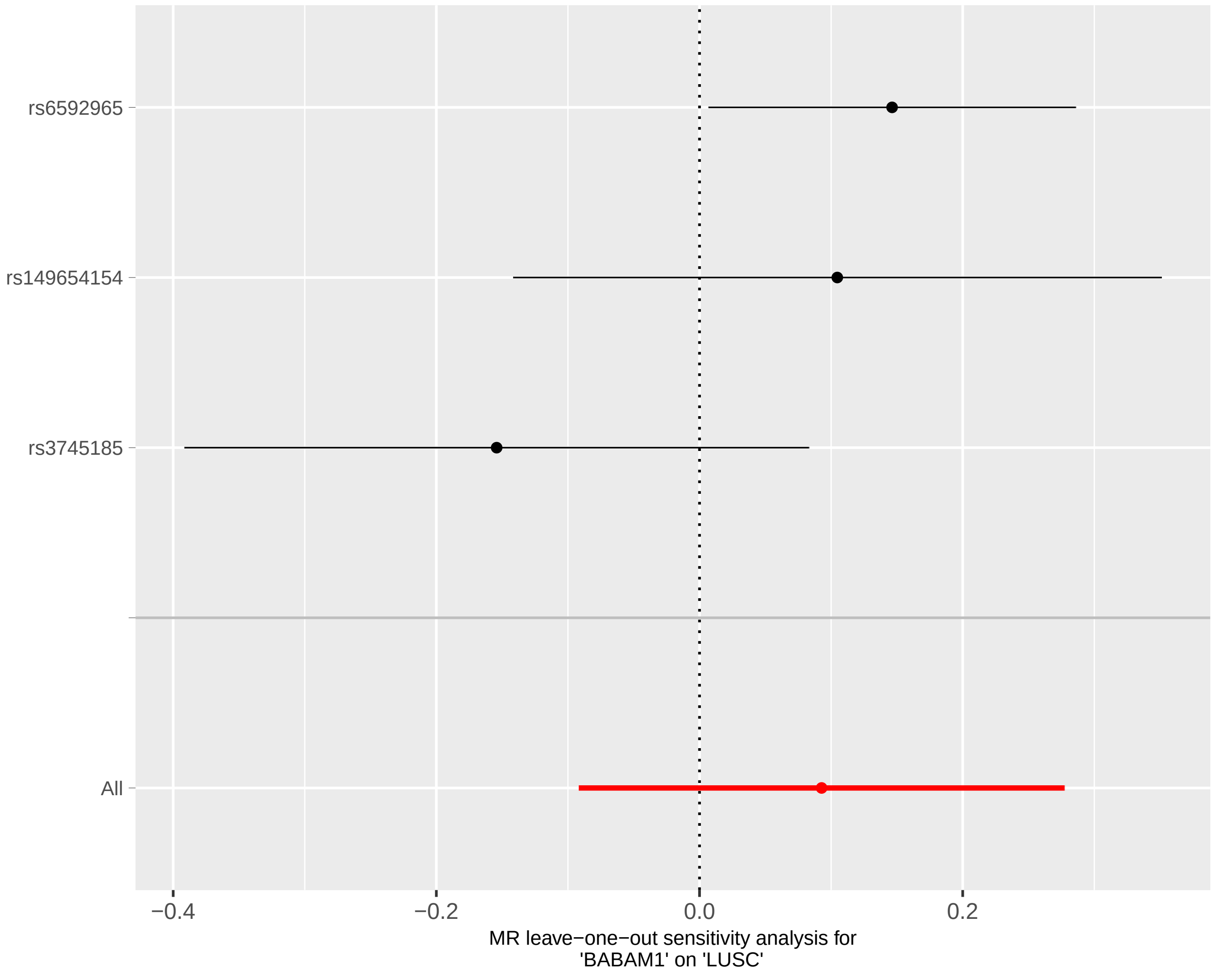
rs6592965
rs149654154
rs3745185
All
−0.4
−0.2
0.0
0.2
MR leave−one−out sensitivity analysis for
'BABAM1' on 'LUSC'

Insufficient number of SNPs

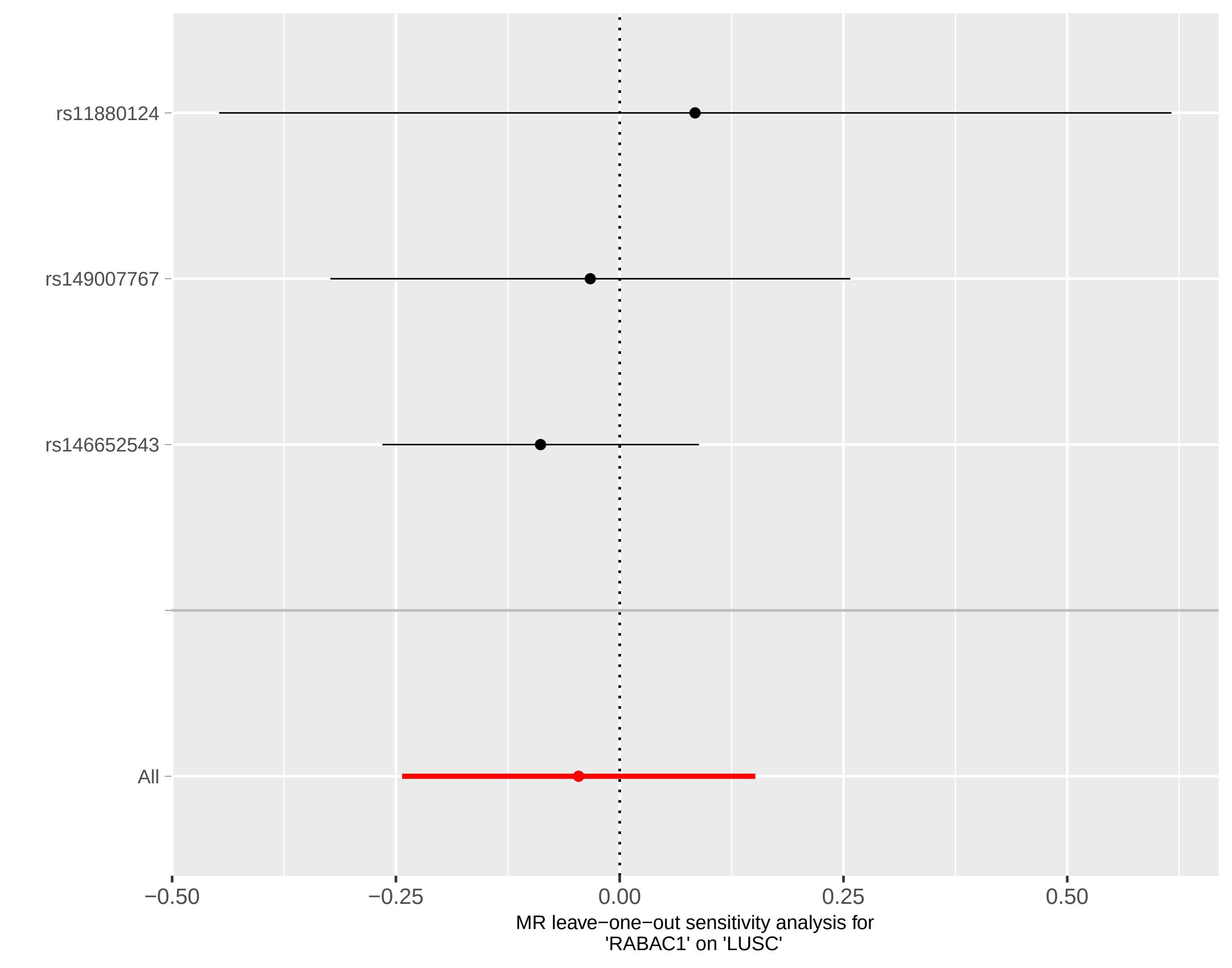
rs11880124
rs149007767
rs146652543
All
−0.50
−0.25
0.00
0.25
0.50
MR leave−one−out sensitivity analysis for
'RABAC1' on 'LUSC'

Insufficient number of SNPs

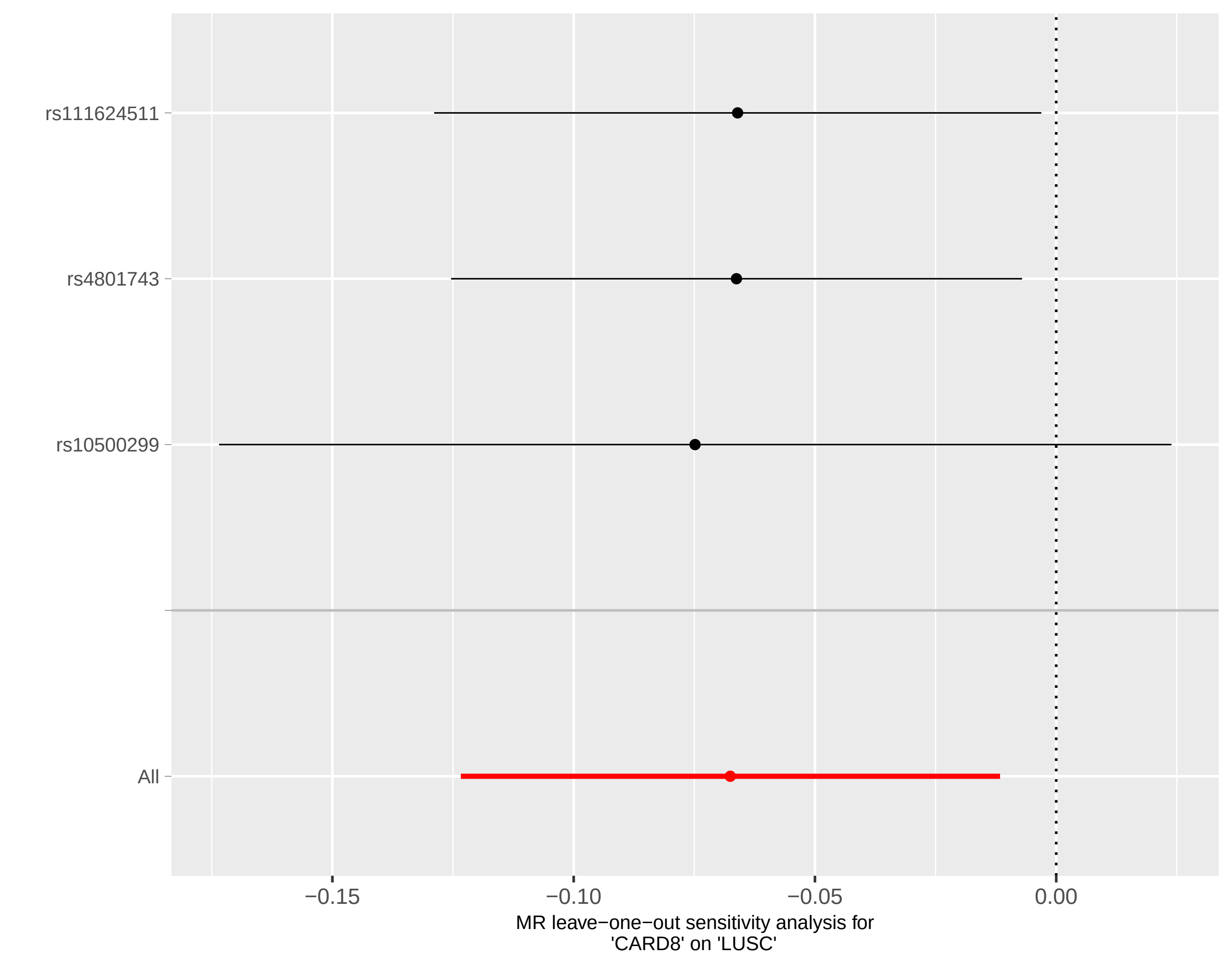
rs111624511
rs4801743
rs10500299
All
−0.15
−0.10
−0.05
0.00
MR leave−one−out sensitivity analysis for
'CARD8' on 'LUSC'

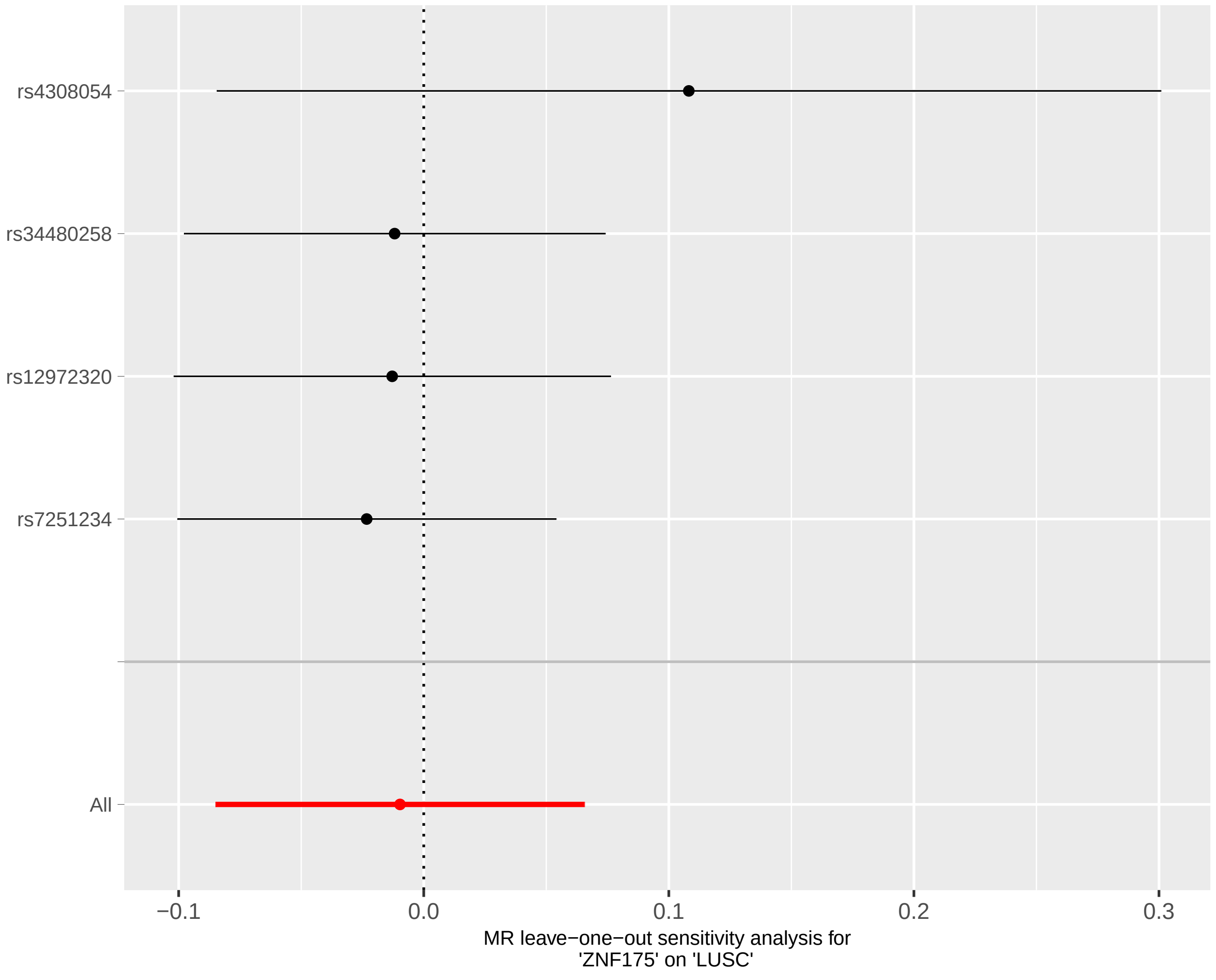
rs4308054
rs34480258
rs12972320
rs7251234
All
−0.1
0.0
0.1
0.2
0.3
MR leave−one−out sensitivity analysis for
'ZNF175' on 'LUSC'

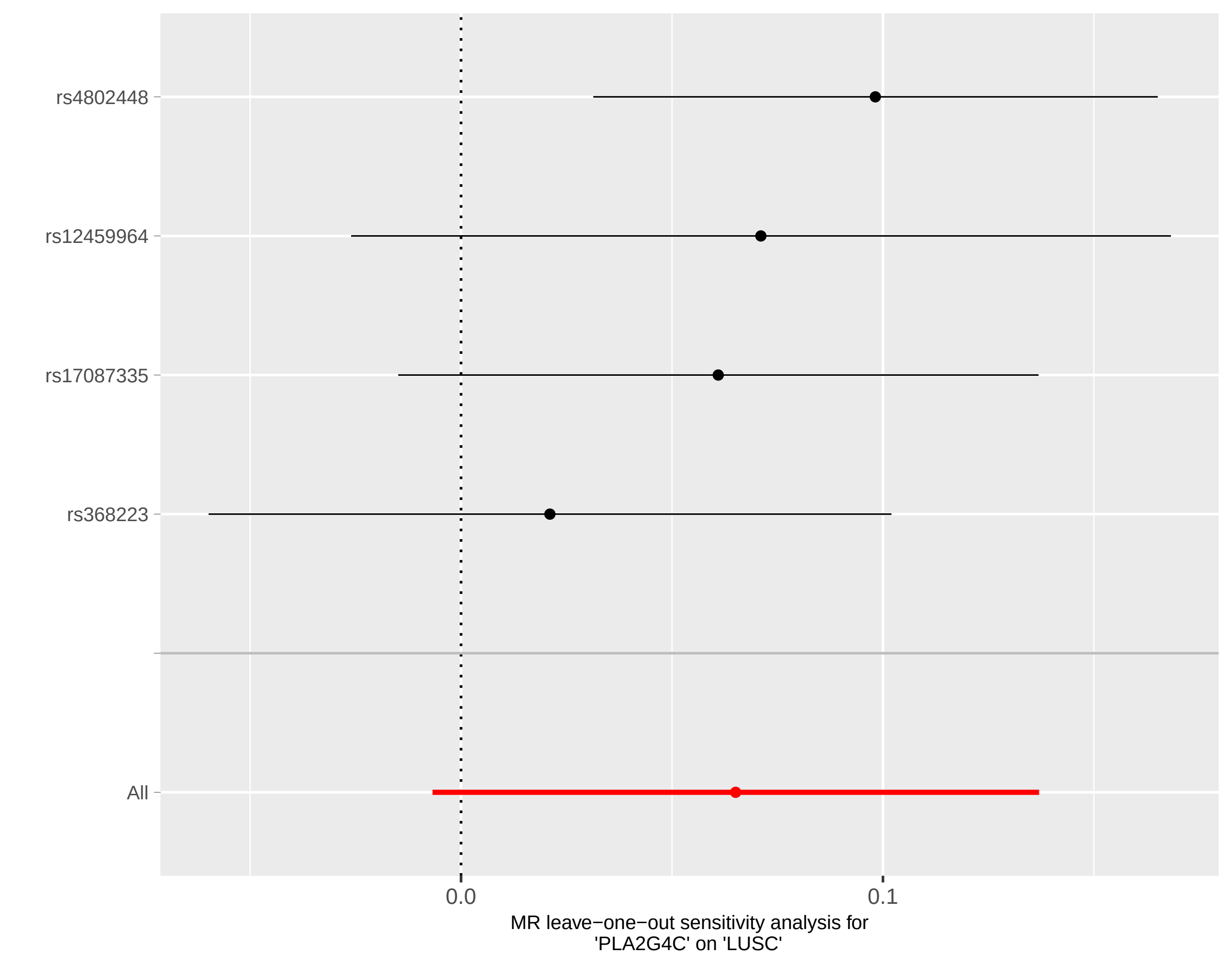
rs4802448
rs12459964
rs17087335
rs368223
All
0.0
0.1
MR leave−one−out sensitivity analysis for
'PLA2G4C' on 'LUSC'

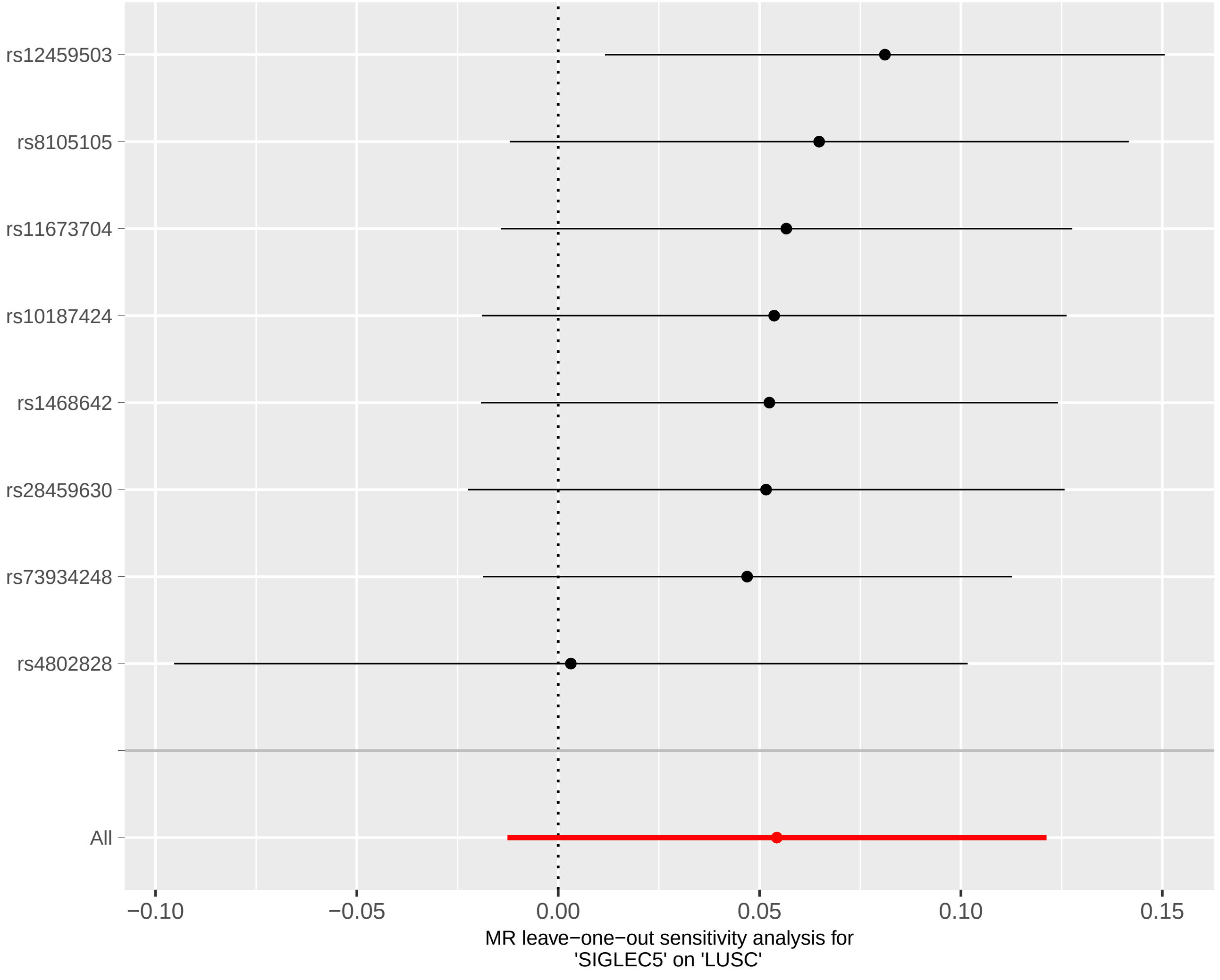
rs12459503
rs8105105
rs11673704
rs10187424
rs1468642
rs28459630
rs73934248
rs4802828
All
−0.10
−0.05
0.00
0.05
0.10
0.15
MR leave−one−out sensitivity analysis for
'SIGLEC5' on 'LUSC'

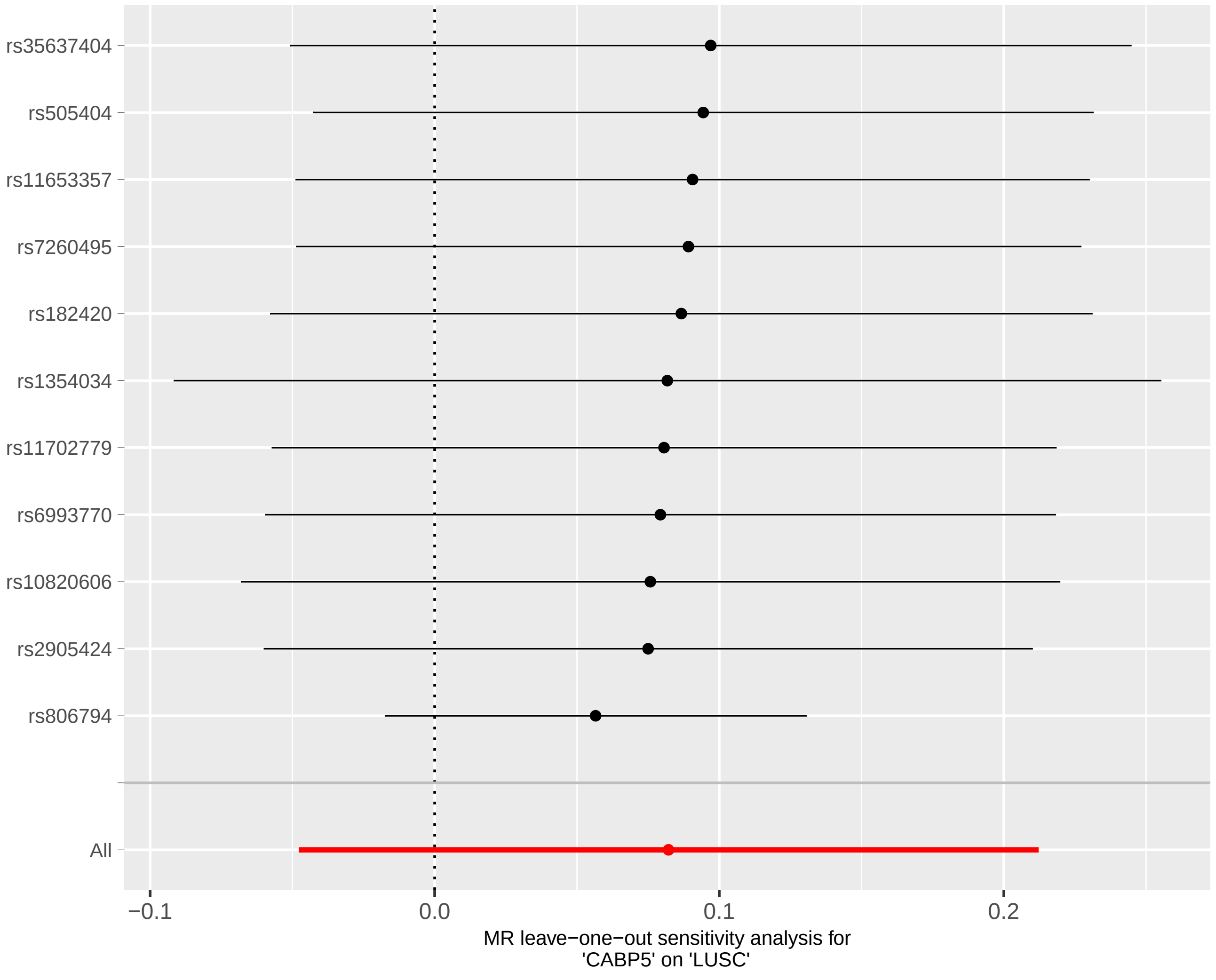
rs35637404
rs505404
rs11653357
rs7260495
rs182420
rs1354034
rs11702779
rs6993770
rs10820606
rs2905424
rs806794
All
−0.1
0.0
0.1
0.2
MR leave−one−out sensitivity analysis for
'CABP5' on 'LUSC'

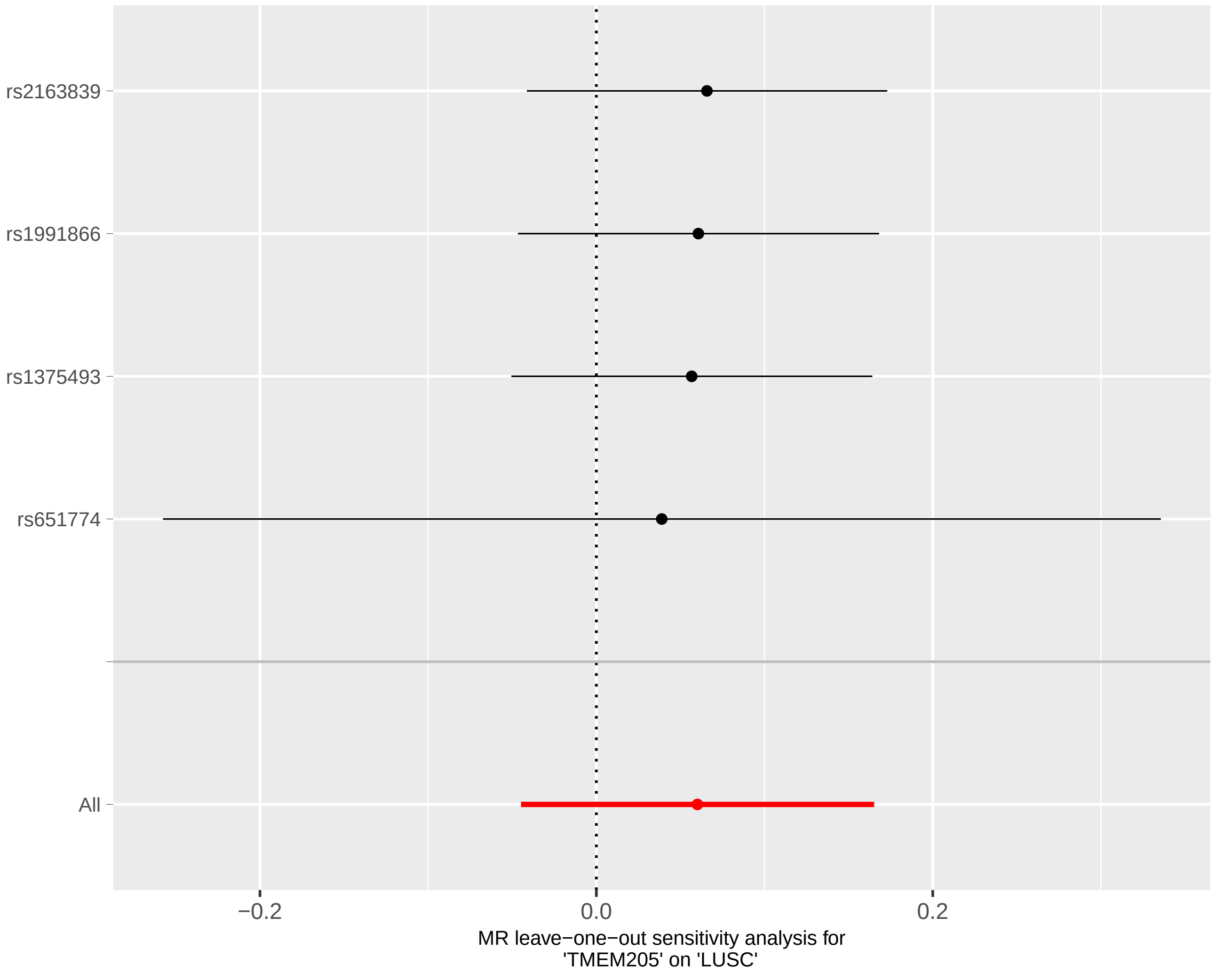
rs2163839
rs1991866
rs1375493
rs651774
All
−0.2
0.0
0.2
MR leave−one−out sensitivity analysis for
'TMEM205' on 'LUSC'

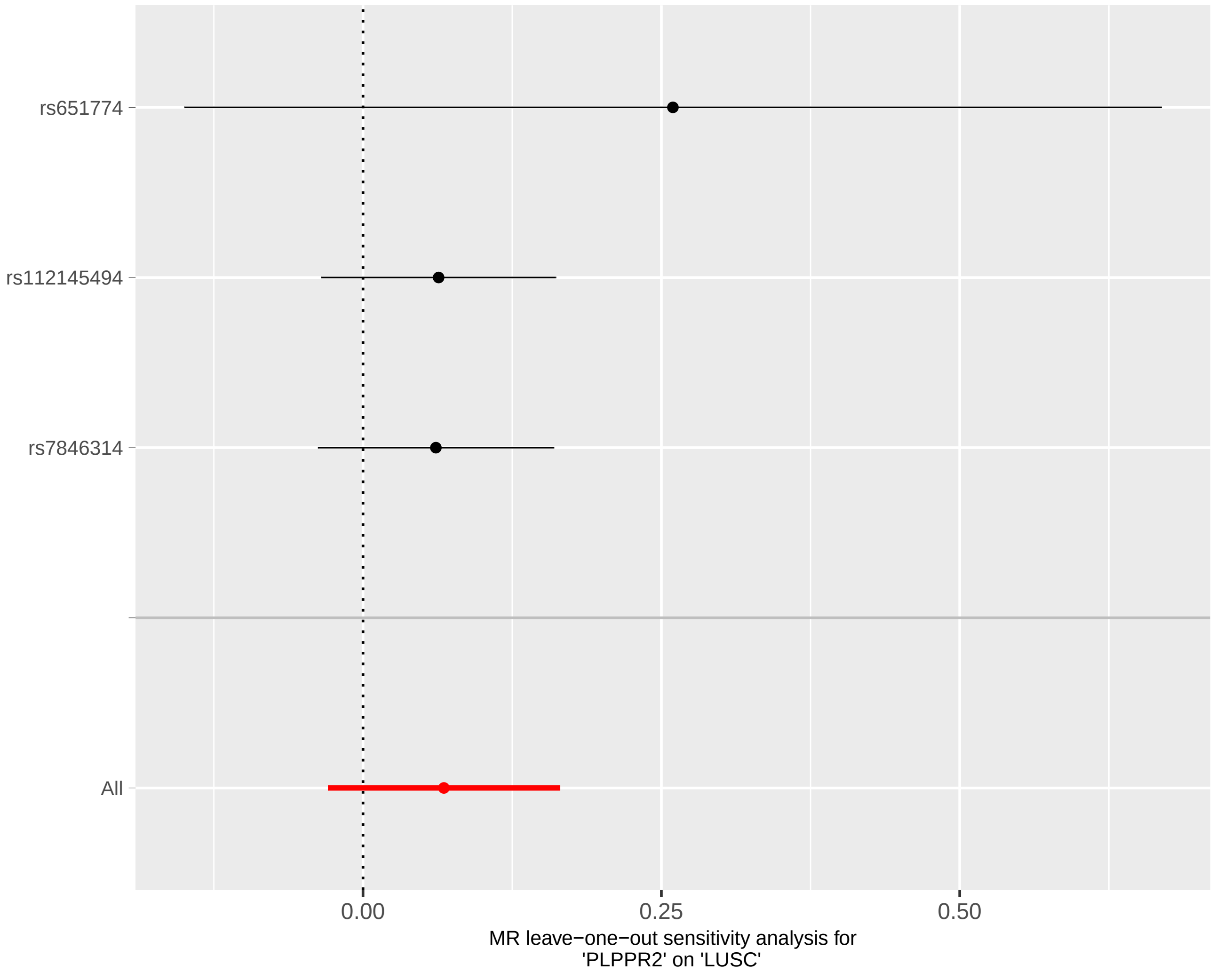
rs651774
rs112145494
rs7846314
All
0.00
0.25
0.50
MR leave−one−out sensitivity analysis for
'PLPPR2' on 'LUSC'

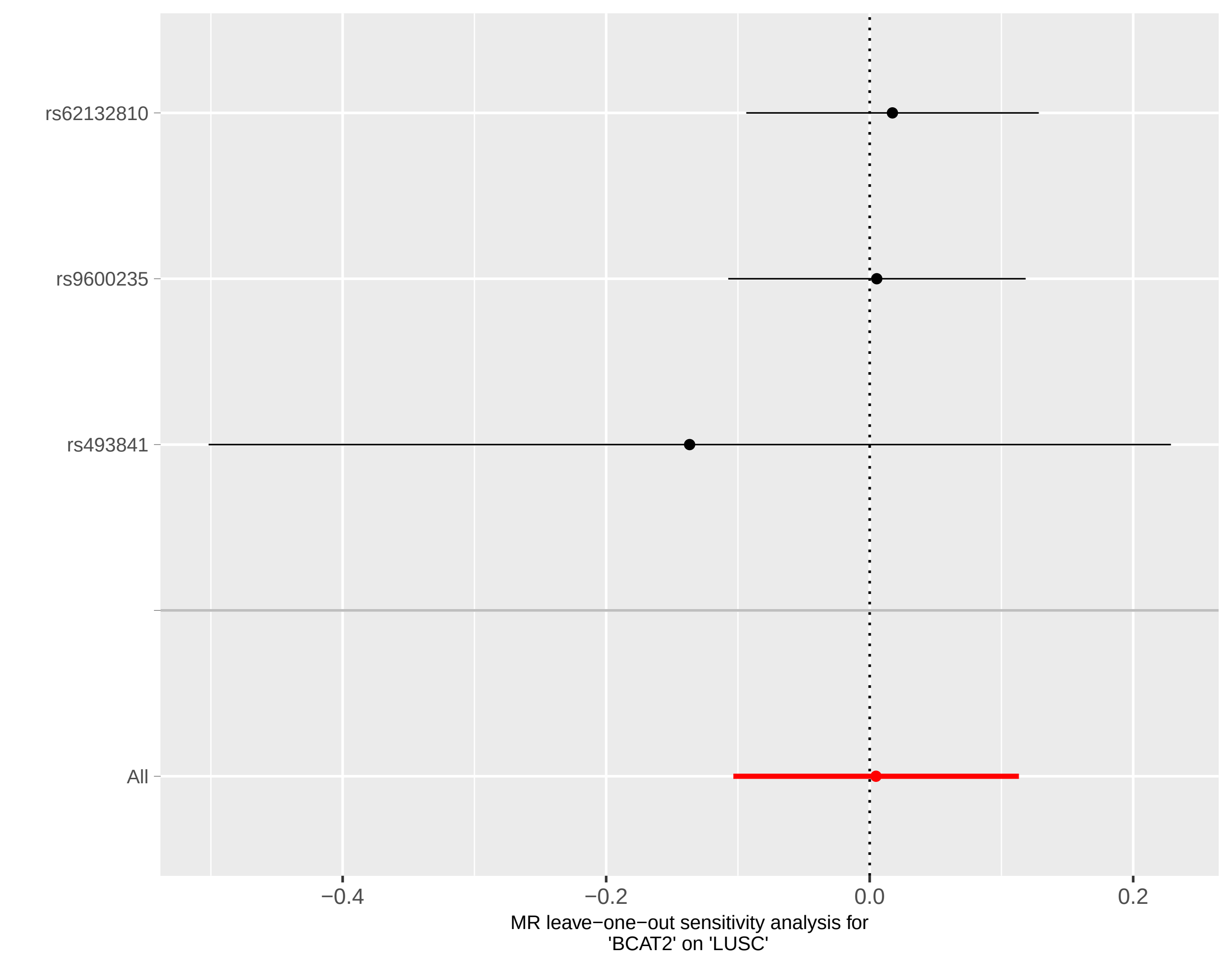

rs62132810
rs9600235
rs493841
All
−0.4
−0.2
0.0
0.2
MR leave−one−out sensitivity analysis for
'BCAT2' on 'LUSC'

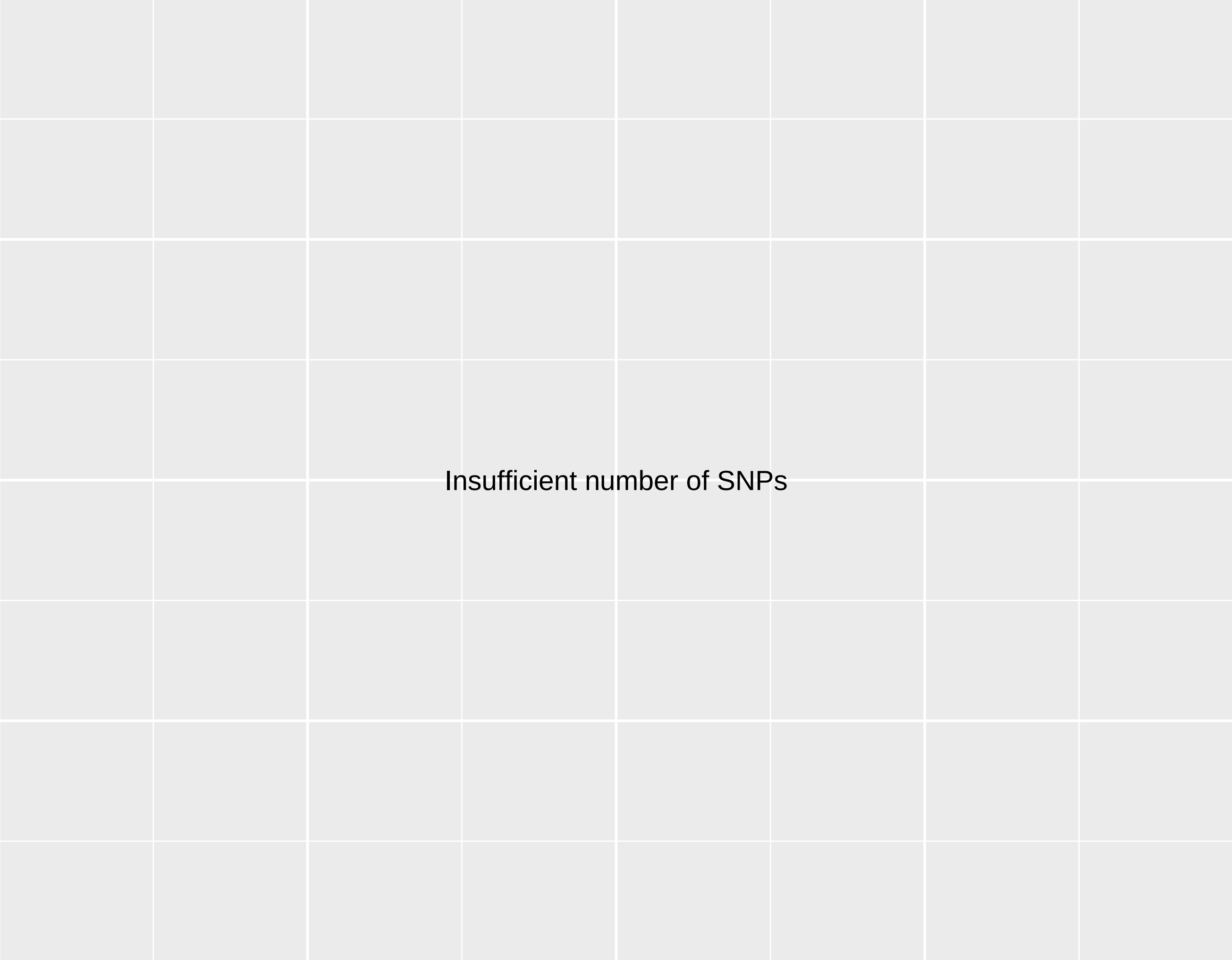
Insufficient number of SNPs

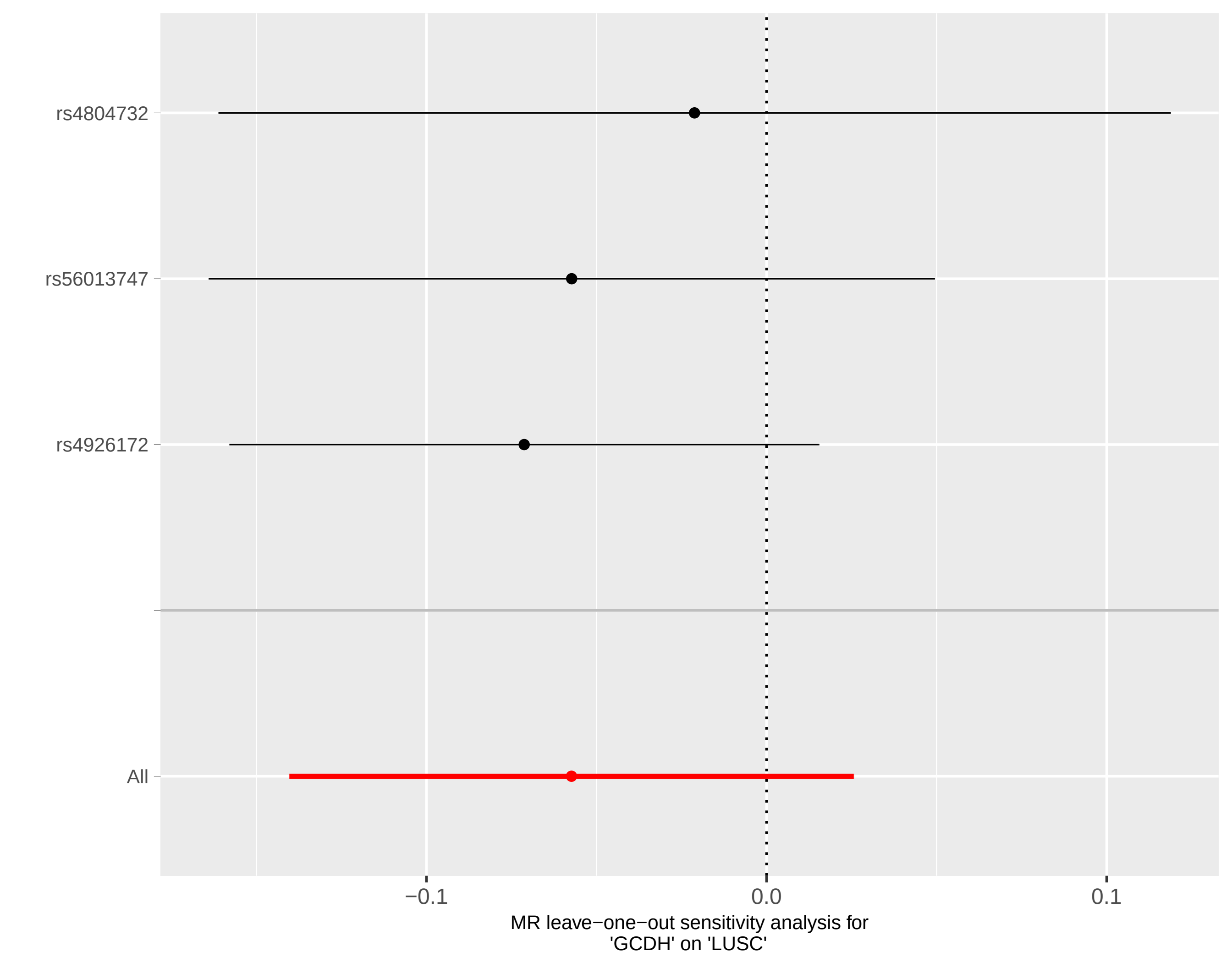

rs4804732
rs56013747
rs4926172
All
−0.1
0.0
0.1
MR leave−one−out sensitivity analysis for
'GCDH' on 'LUSC'

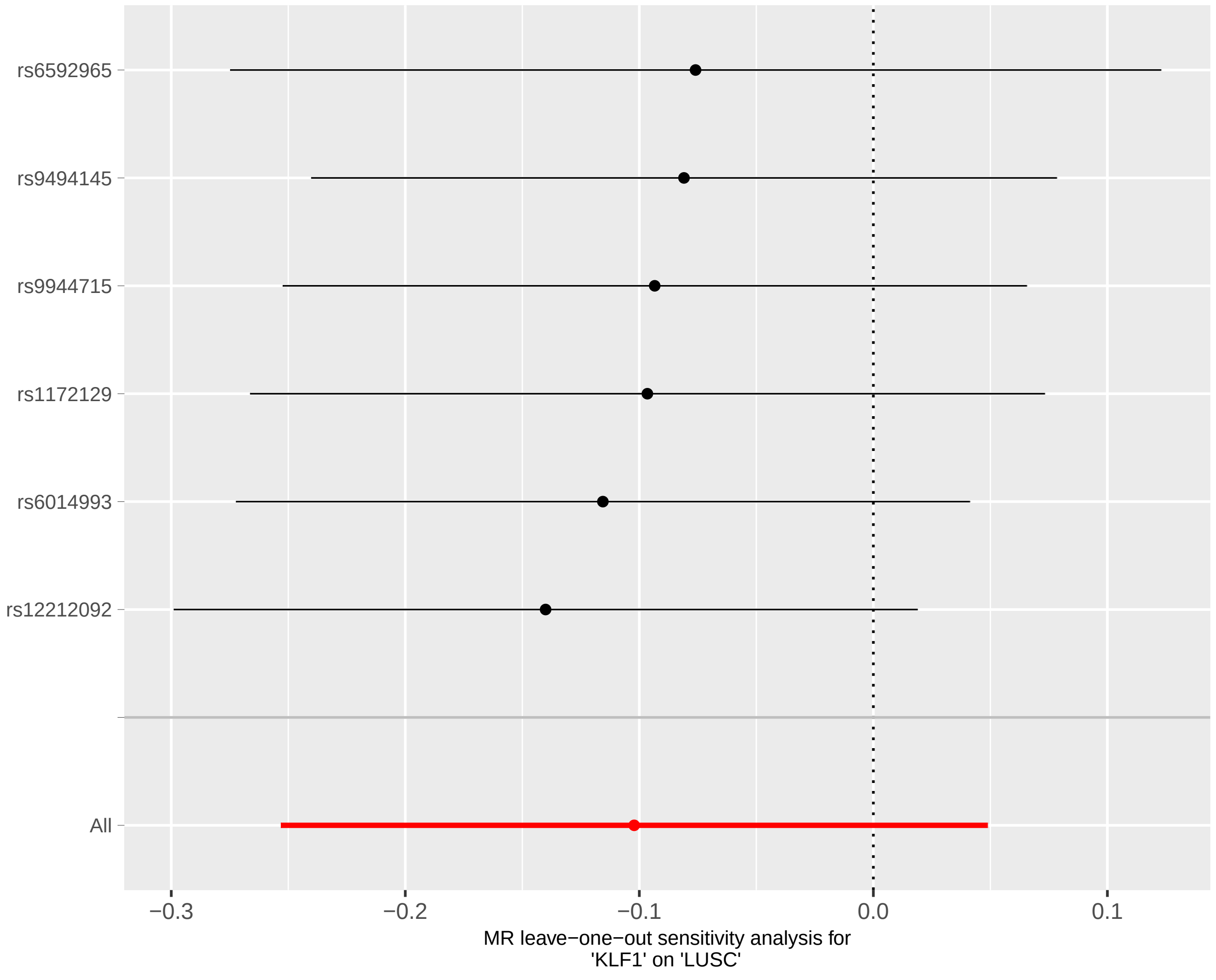
rs6592965
rs9494145
rs9944715
rs1172129
rs6014993
rs12212092
All
−0.3
−0.2
−0.1
0.0
0.1
MR leave−one−out sensitivity analysis for
'KLF1' on 'LUSC'

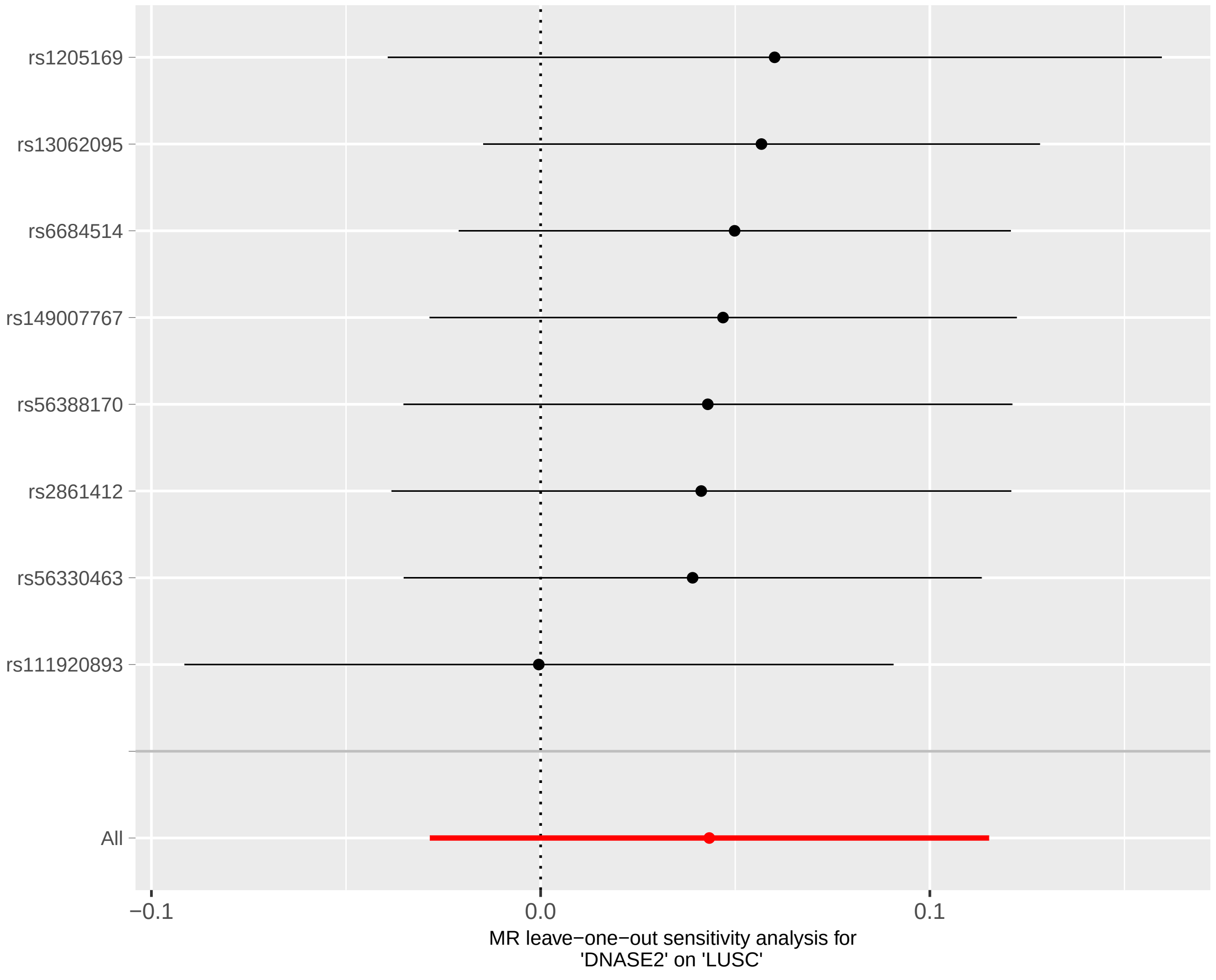
rs1205169
rs13062095
rs6684514
rs149007767
rs56388170
rs2861412
rs56330463
rs111920893
All
−0.1
0.0
0.1
MR leave−one−out sensitivity analysis for
'DNASE2' on 'LUSC'

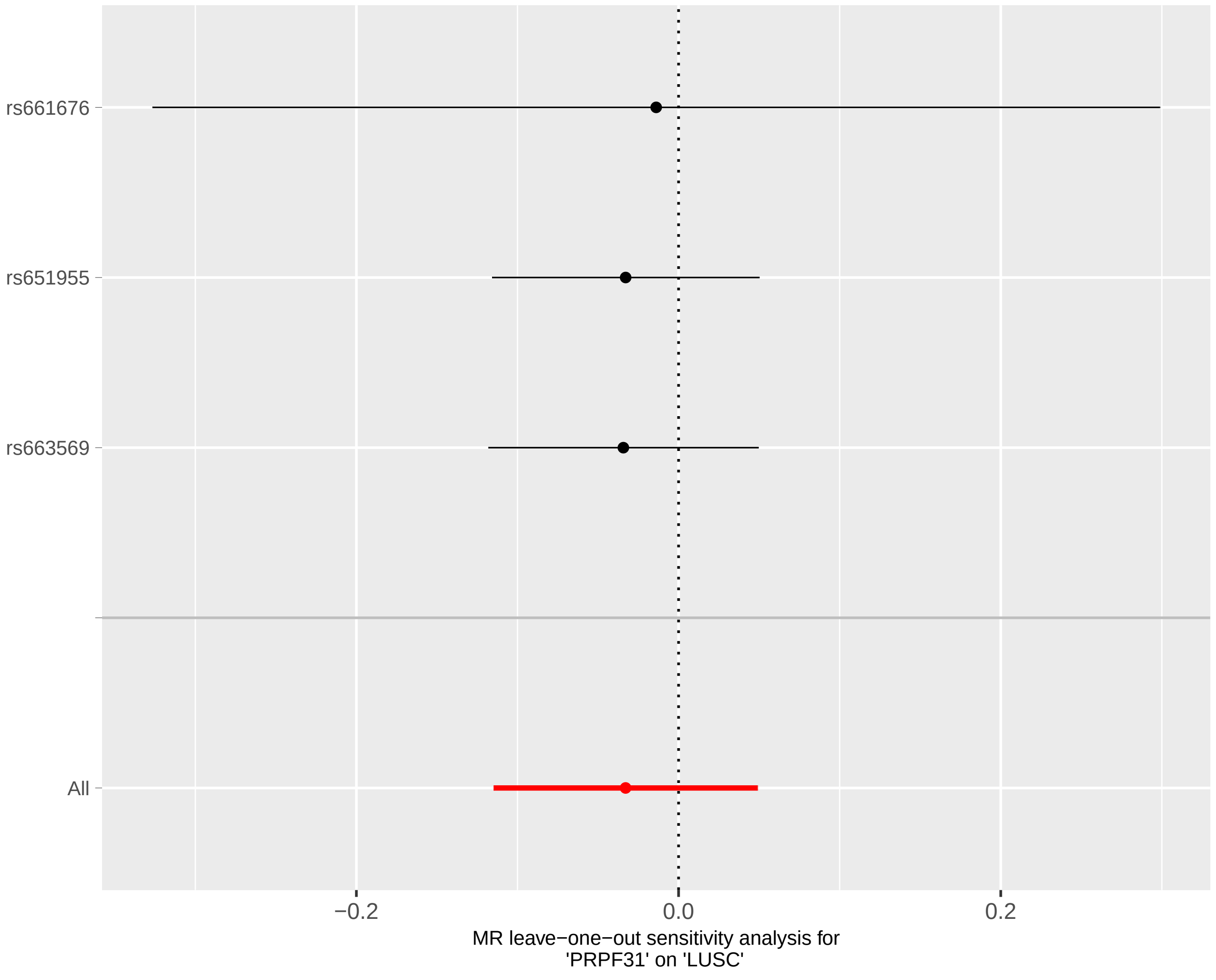
rs661676
rs651955
rs663569
All
−0.2
0.0
0.2
MR leave−one−out sensitivity analysis for
'PRPF31' on 'LUSC'

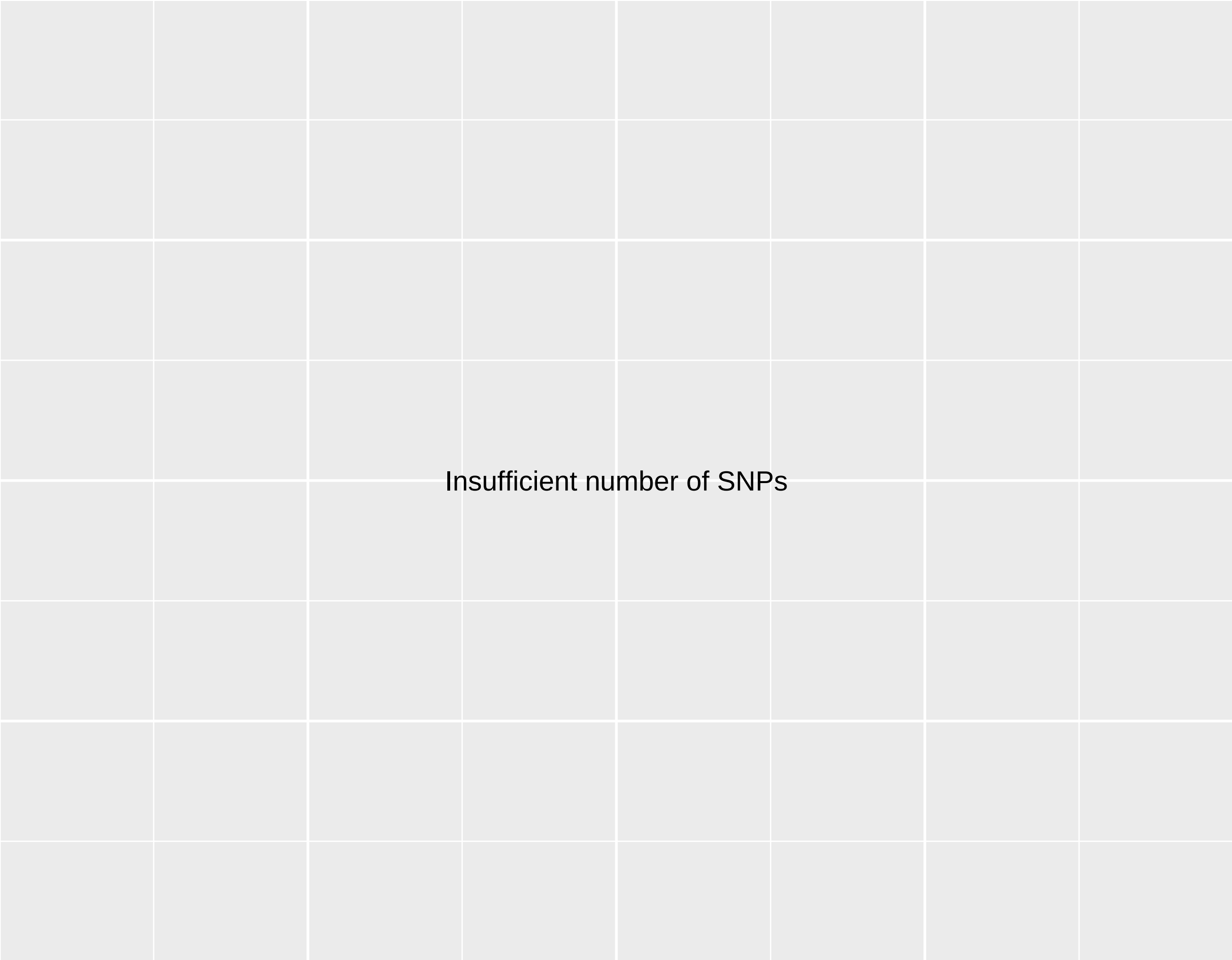
Insufficient number of SNPs

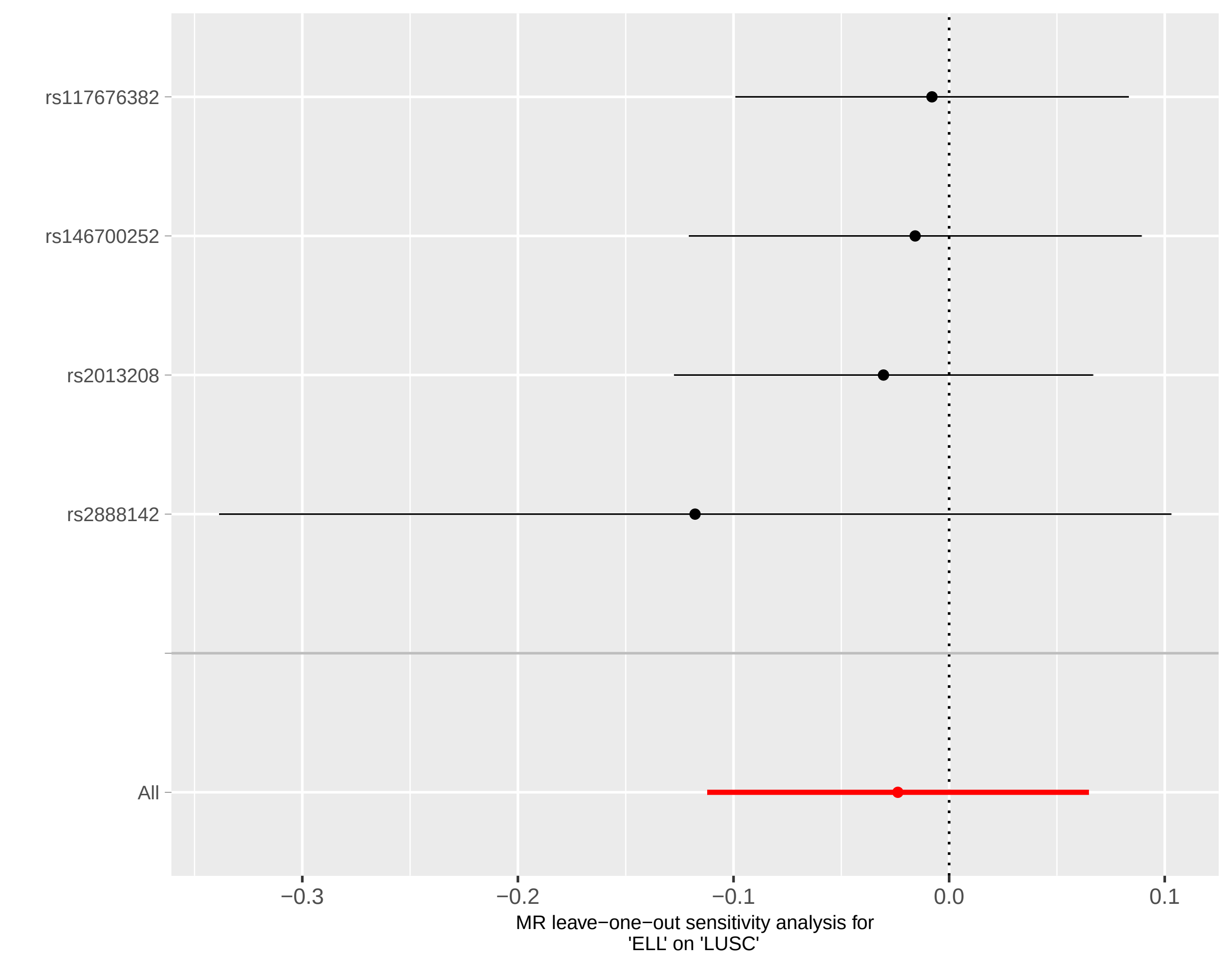
rs117676382
rs146700252
rs2013208
rs2888142
All
−0.3
−0.2
−0.1
0.0
0.1
MR leave−one−out sensitivity analysis for
'ELL' on 'LUSC'

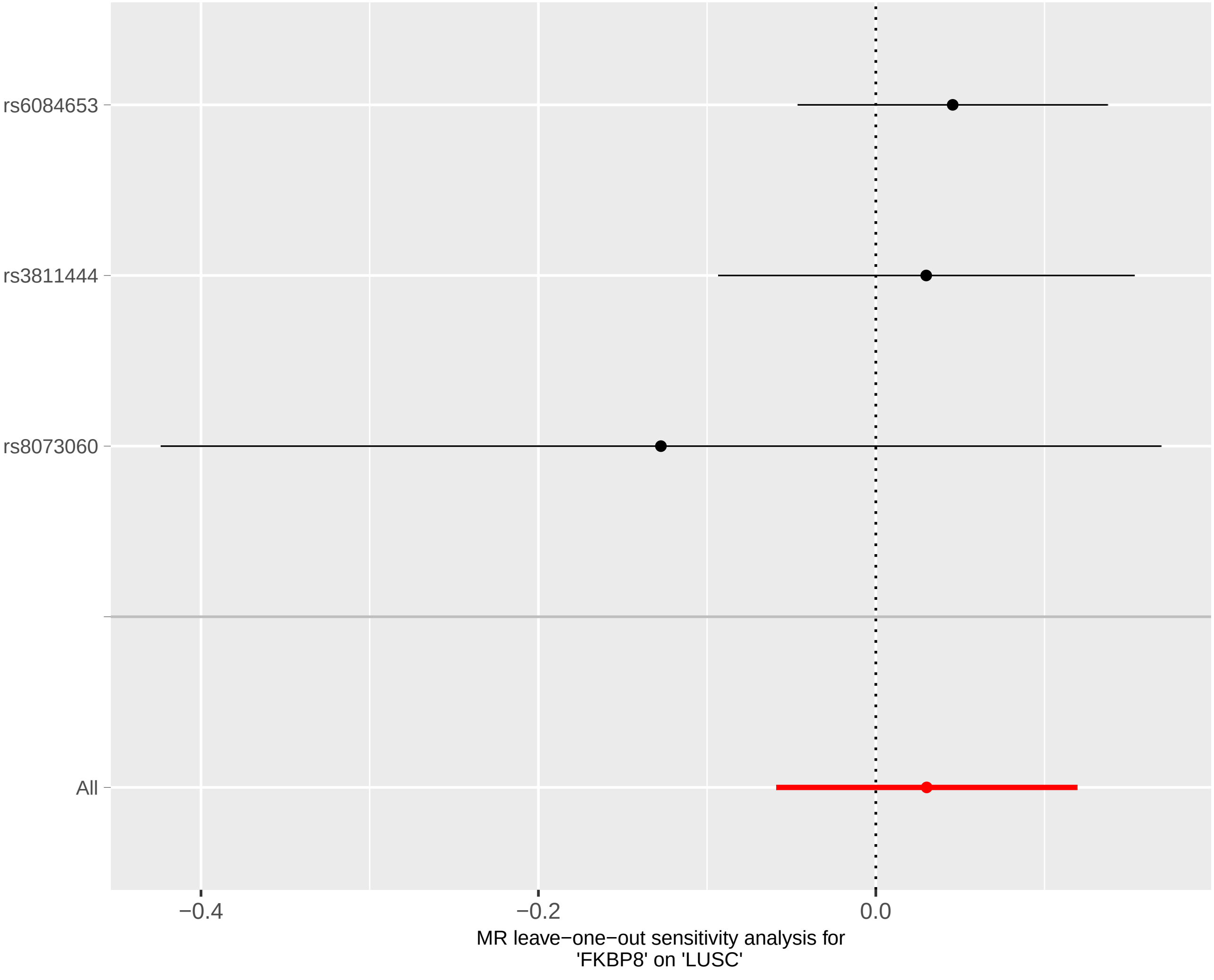
rs6084653
rs3811444
rs8073060
All
−0.4
−0.2
0.0
MR leave−one−out sensitivity analysis for
'FKBP8' on 'LUSC'

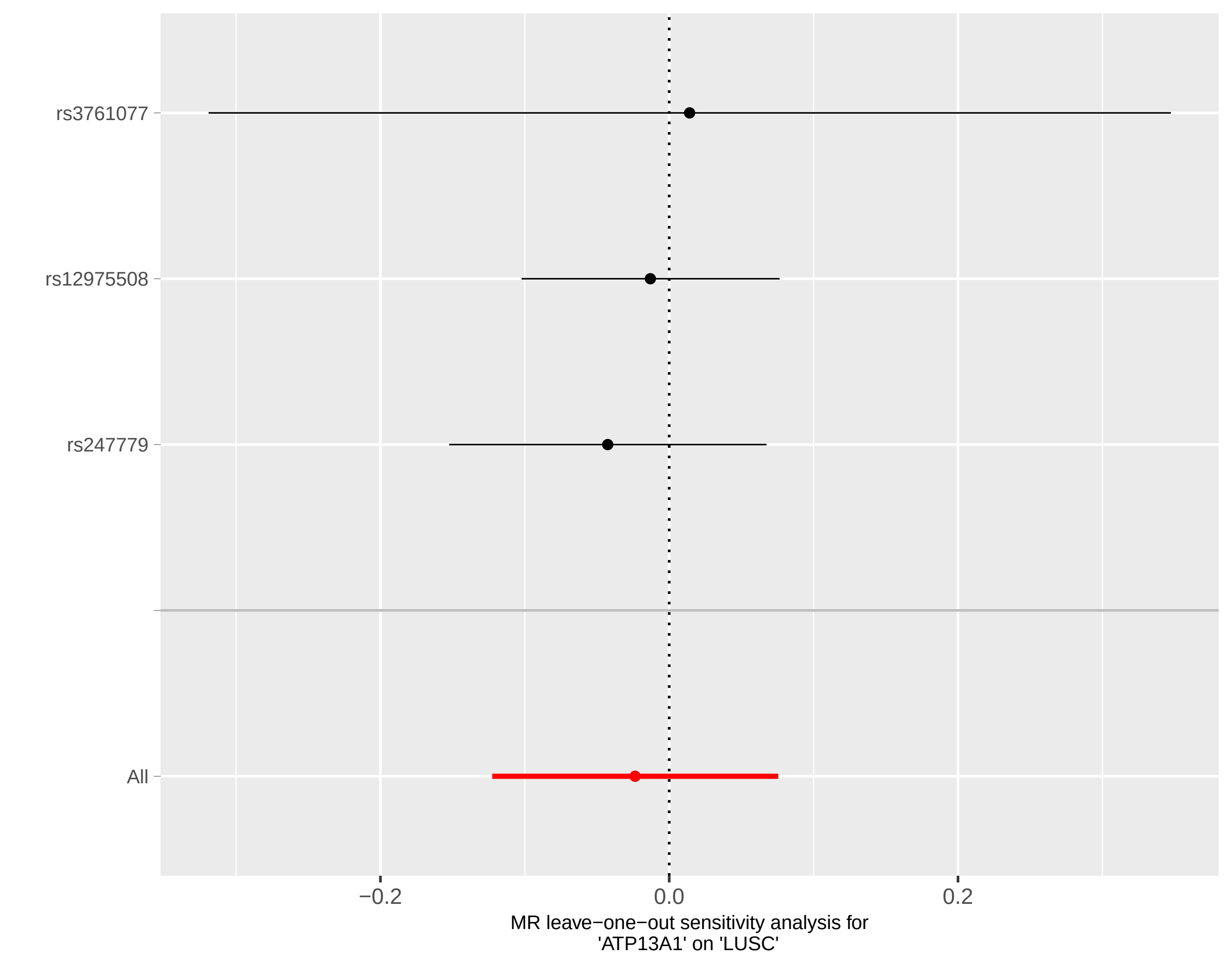

rs3761077
rs12975508
rs247779
All
−0.2
0.0
0.2
MR leave−one−out sensitivity analysis for
'ATP13A1' on 'LUSC'

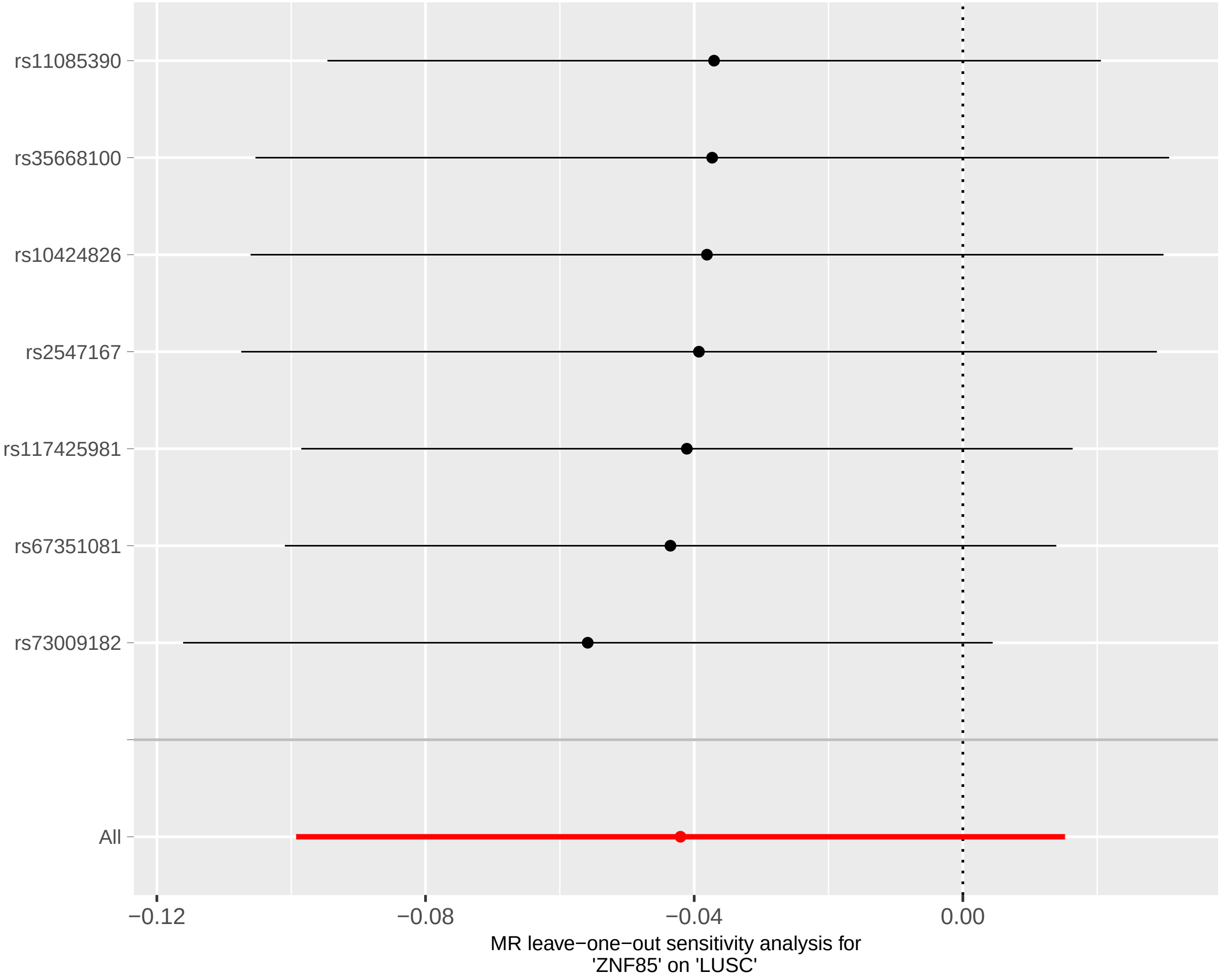

rs11085390
rs35668100
rs10424826
rs2547167
rs117425981
rs67351081
rs73009182
All
−0.12
−0.08
−0.04
0.00
MR leave−one−out sensitivity analysis for
'ZNF85' on 'LUSC'

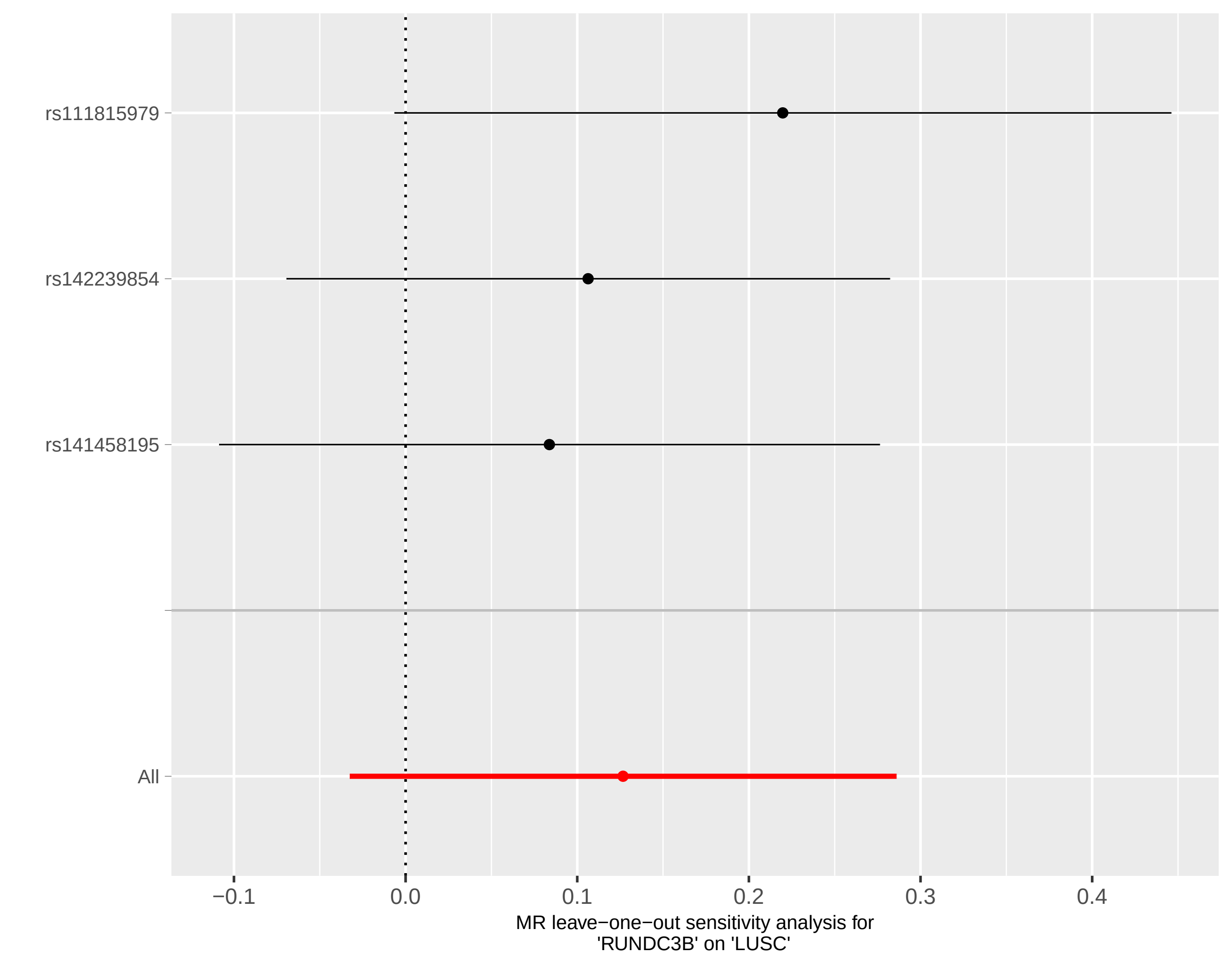
rs111815979
rs142239854
rs141458195
All
−0.1
0.0
0.1
0.2
0.3
0.4
MR leave−one−out sensitivity analysis for
'RUNDC3B' on 'LUSC'

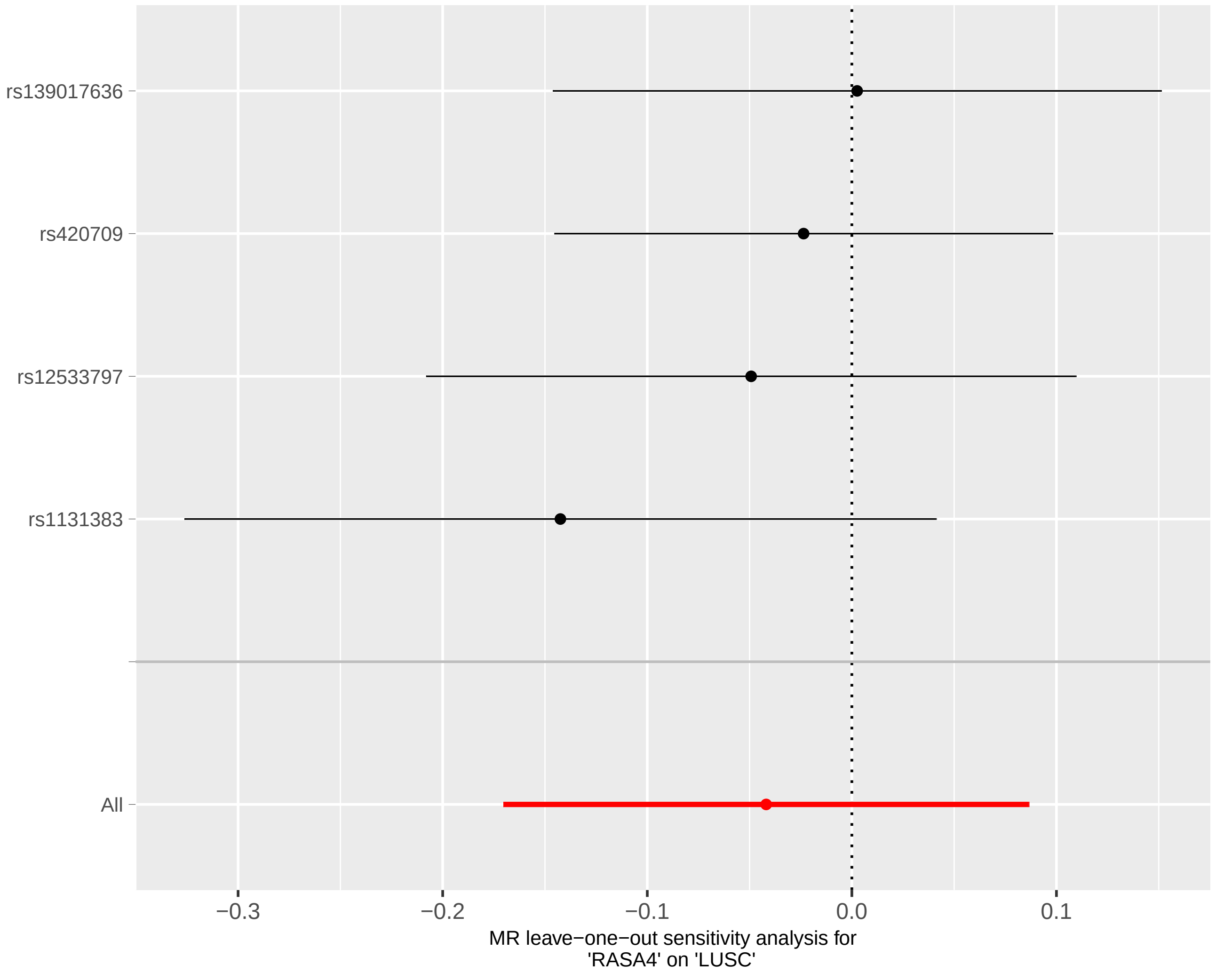
rs139017636
rs420709
rs12533797
rs1131383
All
−0.3
−0.2
−0.1
0.0
0.1
MR leave−one−out sensitivity analysis for
'RASA4' on 'LUSC'

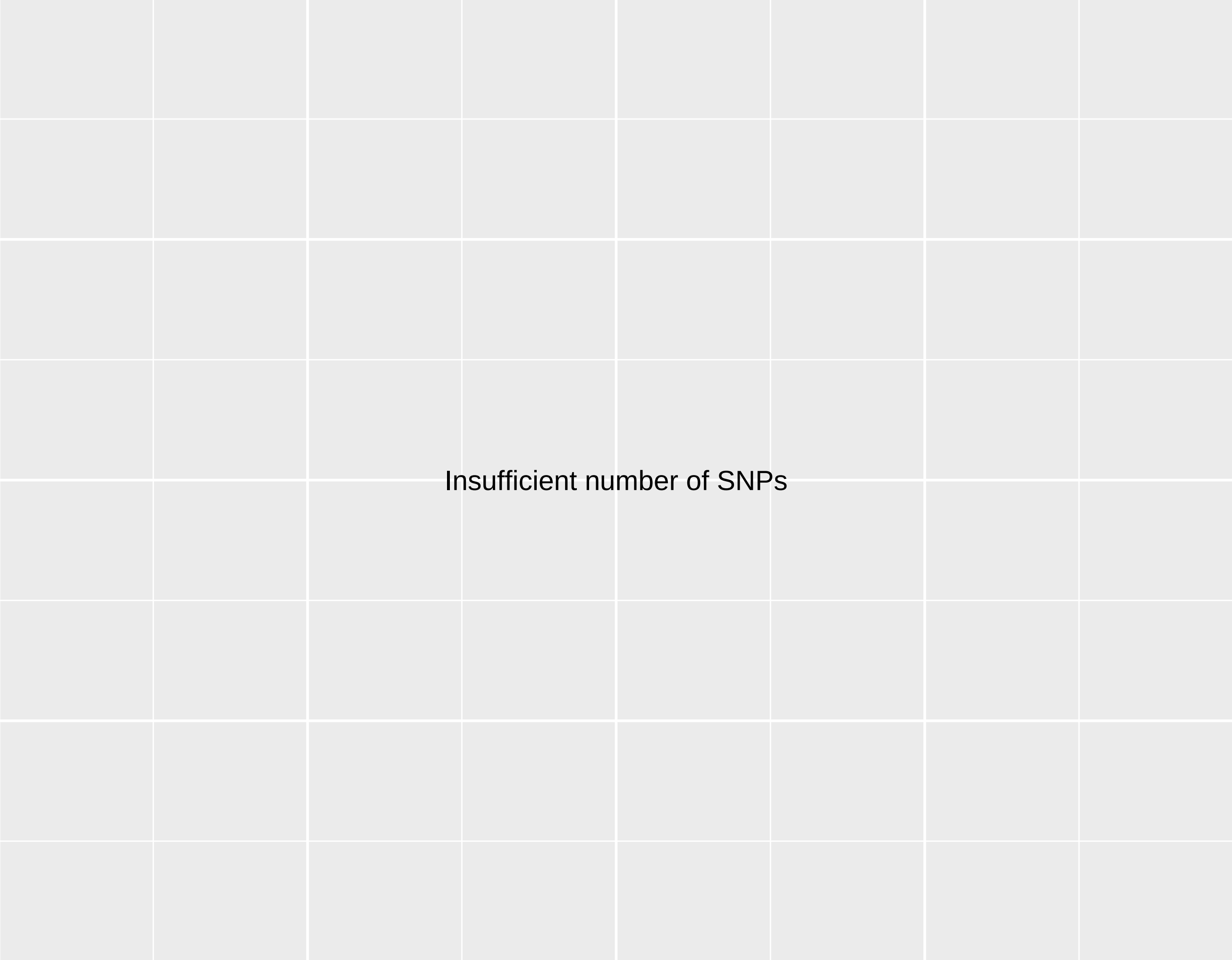
Insufficient number of SNPs

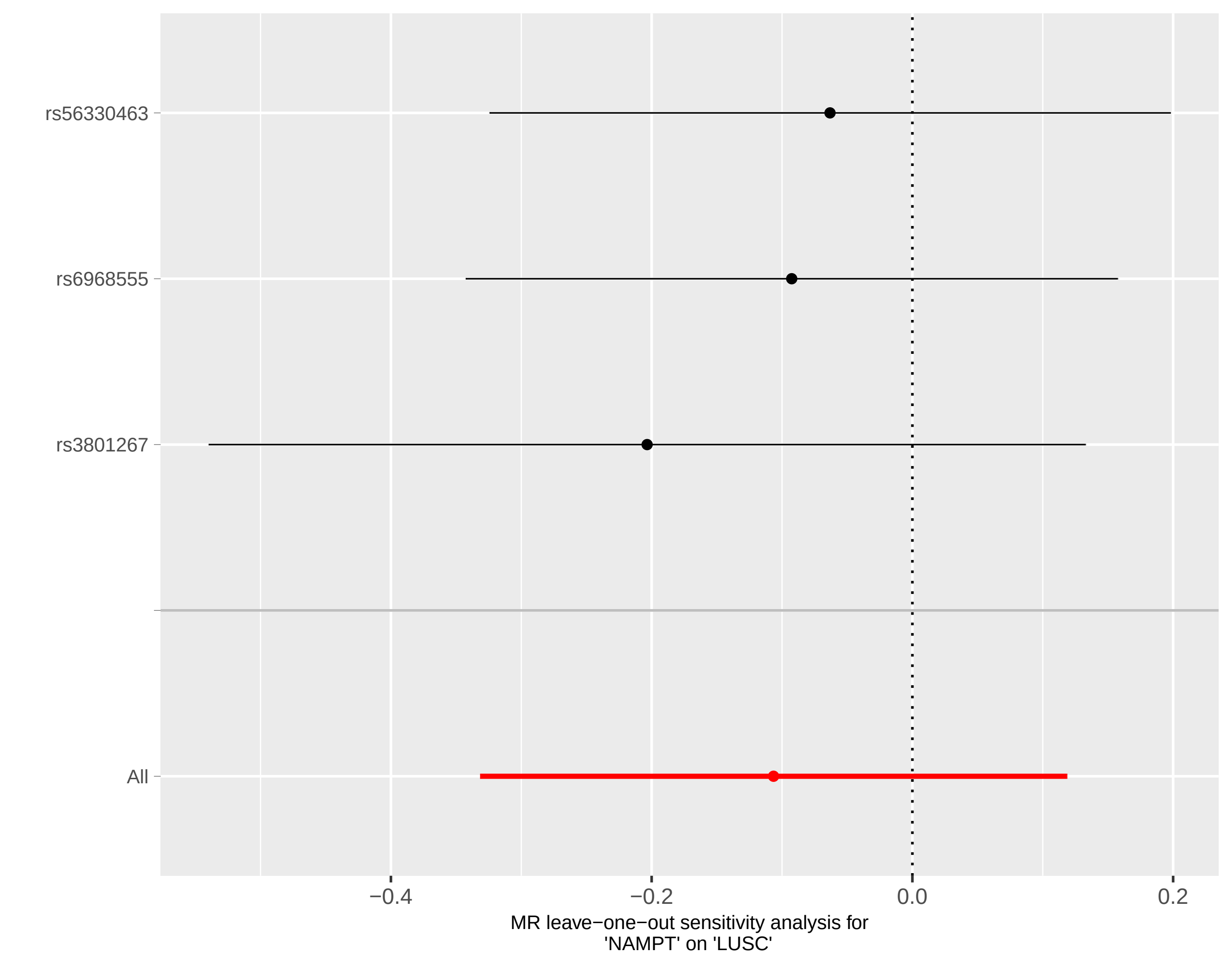
rs56330463
rs6968555
rs3801267
All
−0.4
−0.2
0.0
0.2
MR leave−one−out sensitivity analysis for
'NAMPT' on 'LUSC'

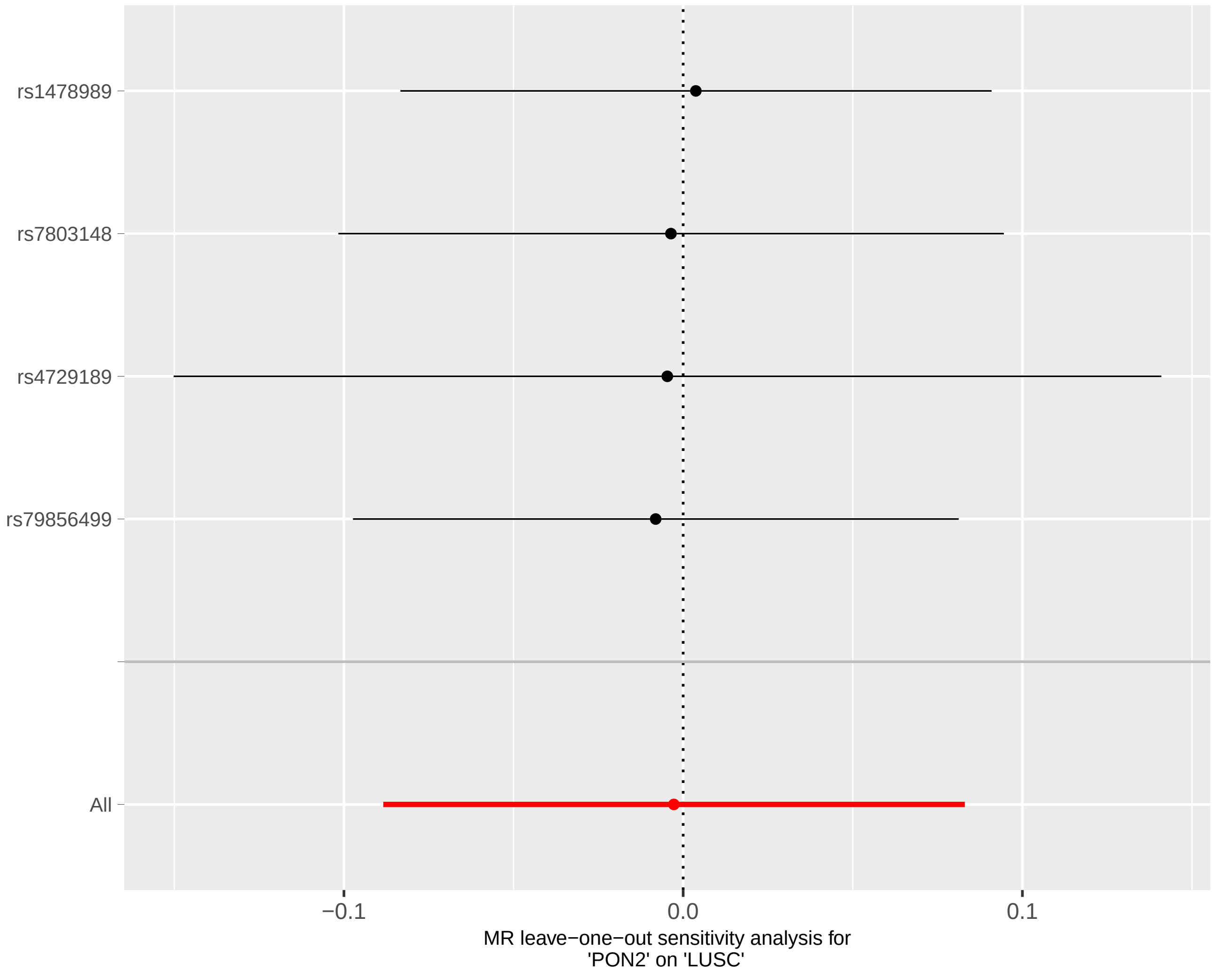
rs1478989
rs7803148
rs4729189
rs79856499
All
−0.1
0.0
0.1
MR leave−one−out sensitivity analysis for
'PON2' on 'LUSC'

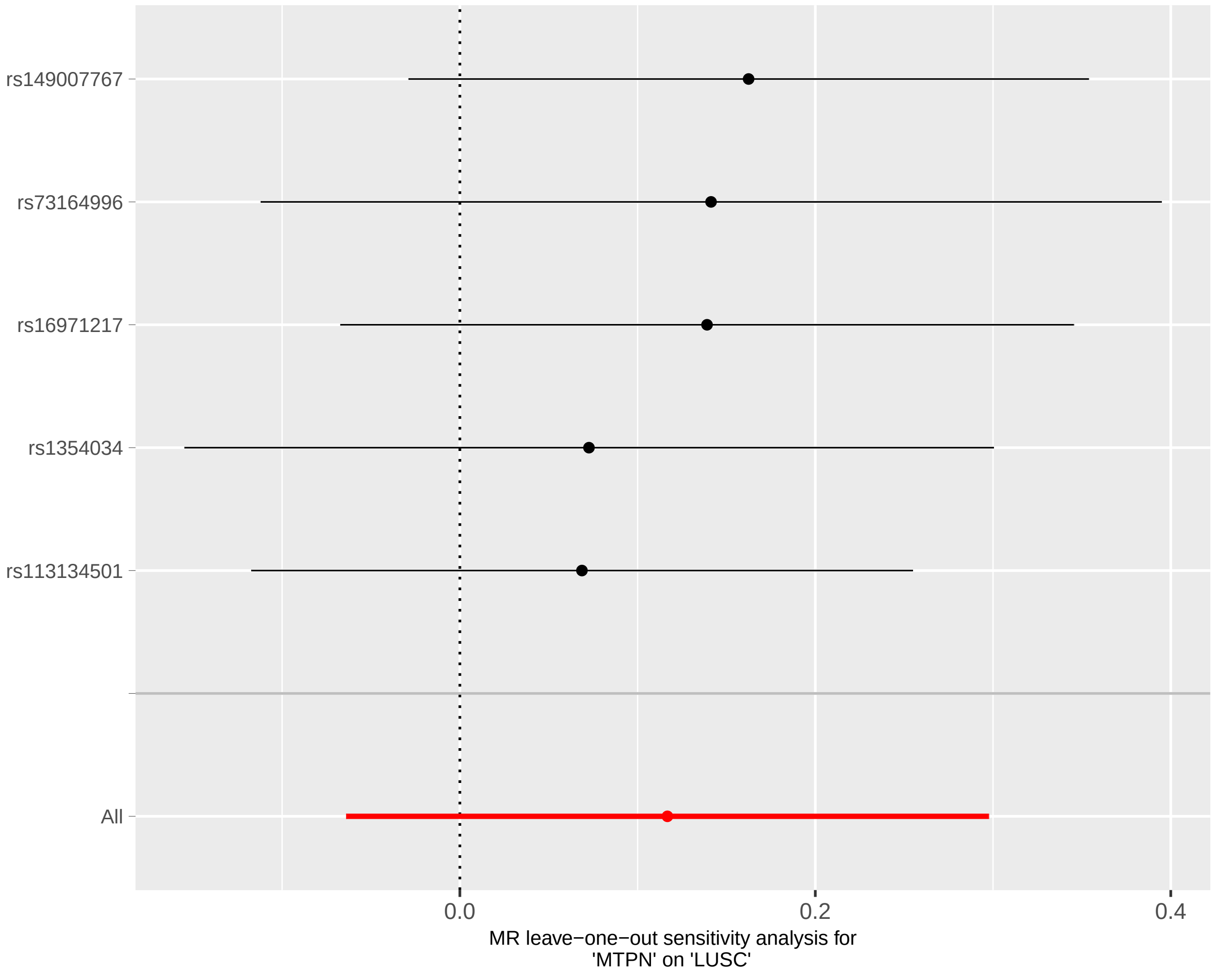
rs149007767
rs73164996
rs16971217
rs1354034
rs113134501
All
0.0
0.2
0.4
MR leave−one−out sensitivity analysis for
'MTPN' on 'LUSC'

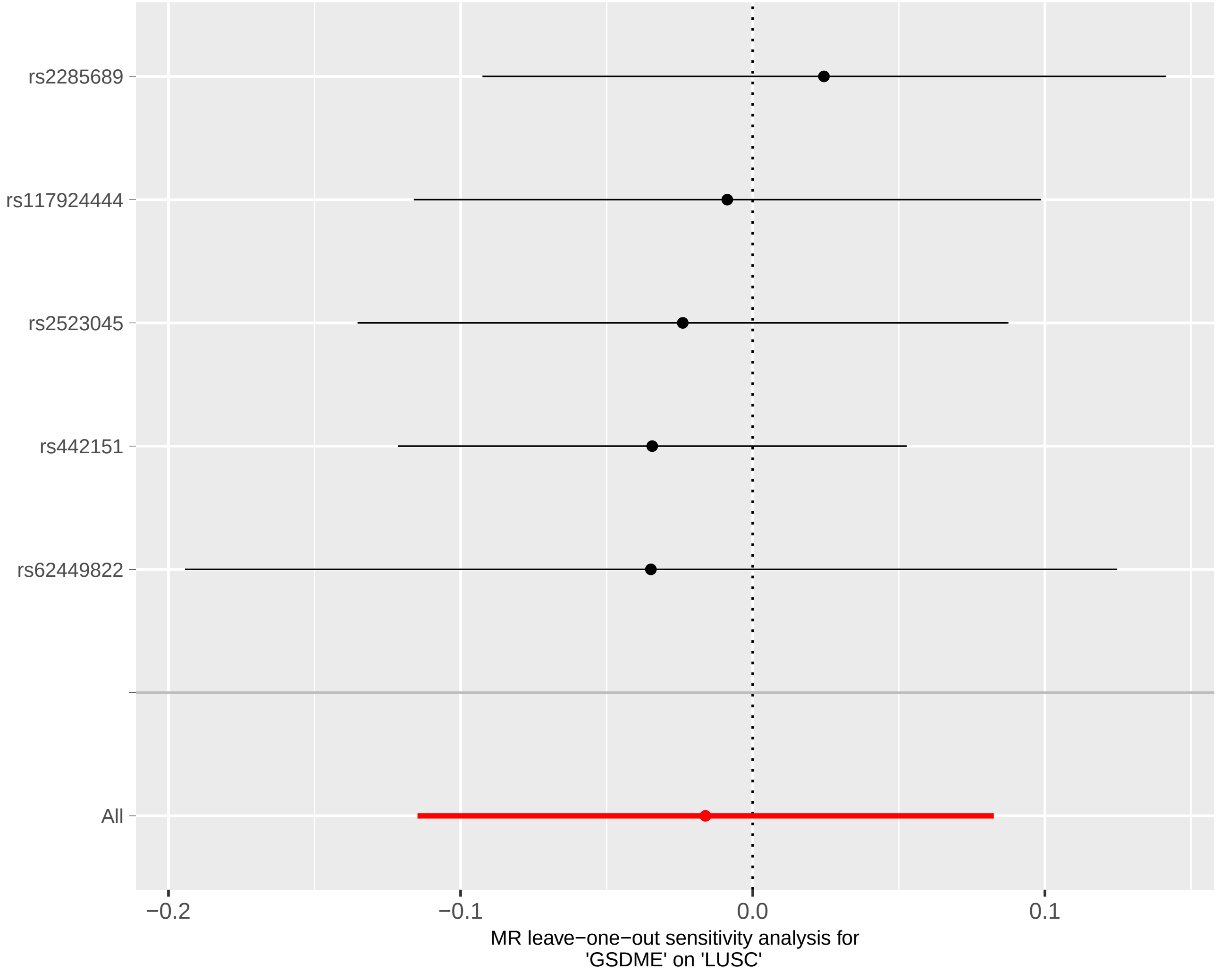
rs2285689
rs117924444
rs2523045
rs442151
rs62449822
All
−0.2
−0.1
0.0
0.1
MR leave−one−out sensitivity analysis for
'GSDME' on 'LUSC'

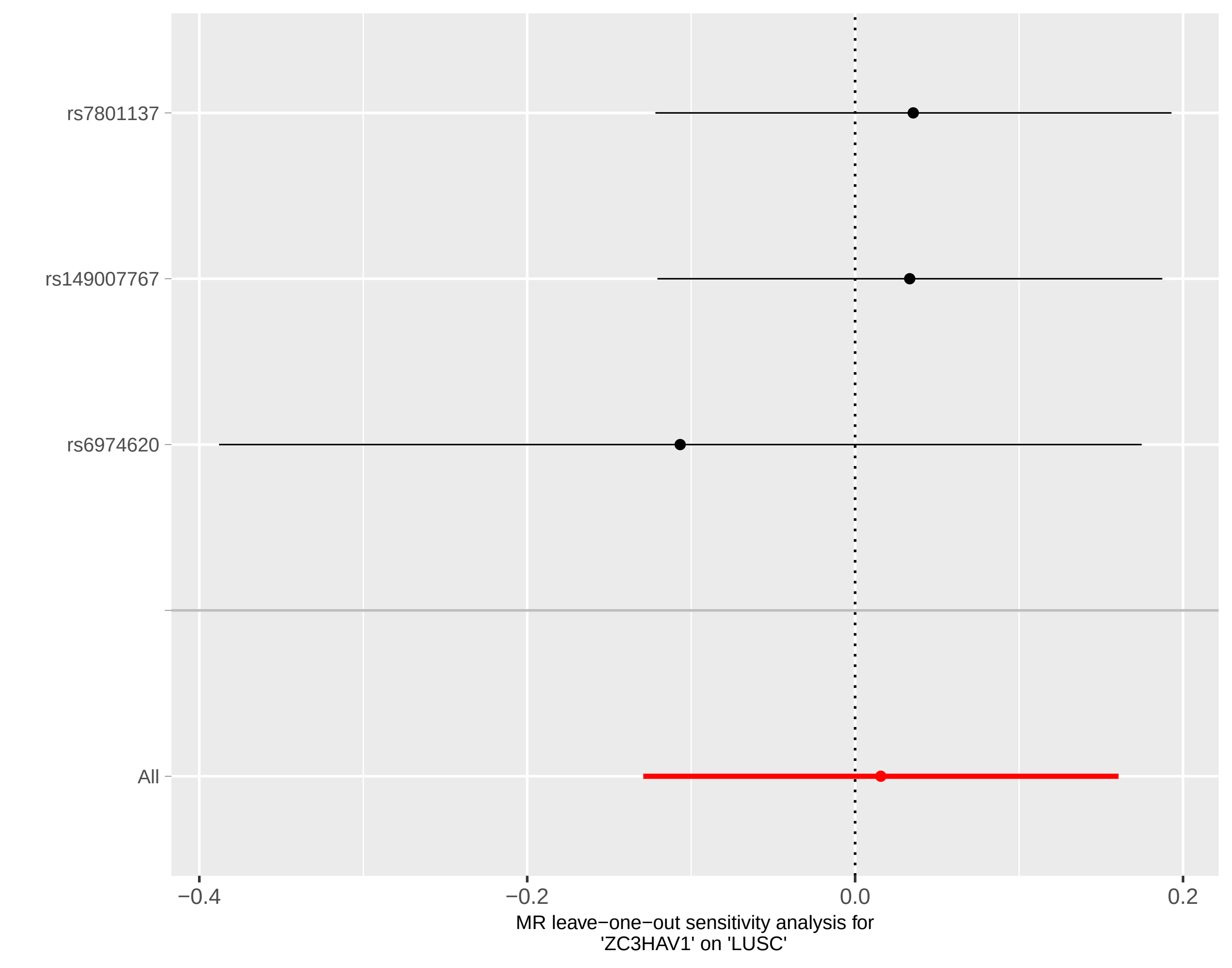
rs7801137
rs149007767
rs6974620
All
−0.4
−0.2
0.0
0.2
MR leave−one−out sensitivity analysis for
'ZC3HAV1' on 'LUSC'

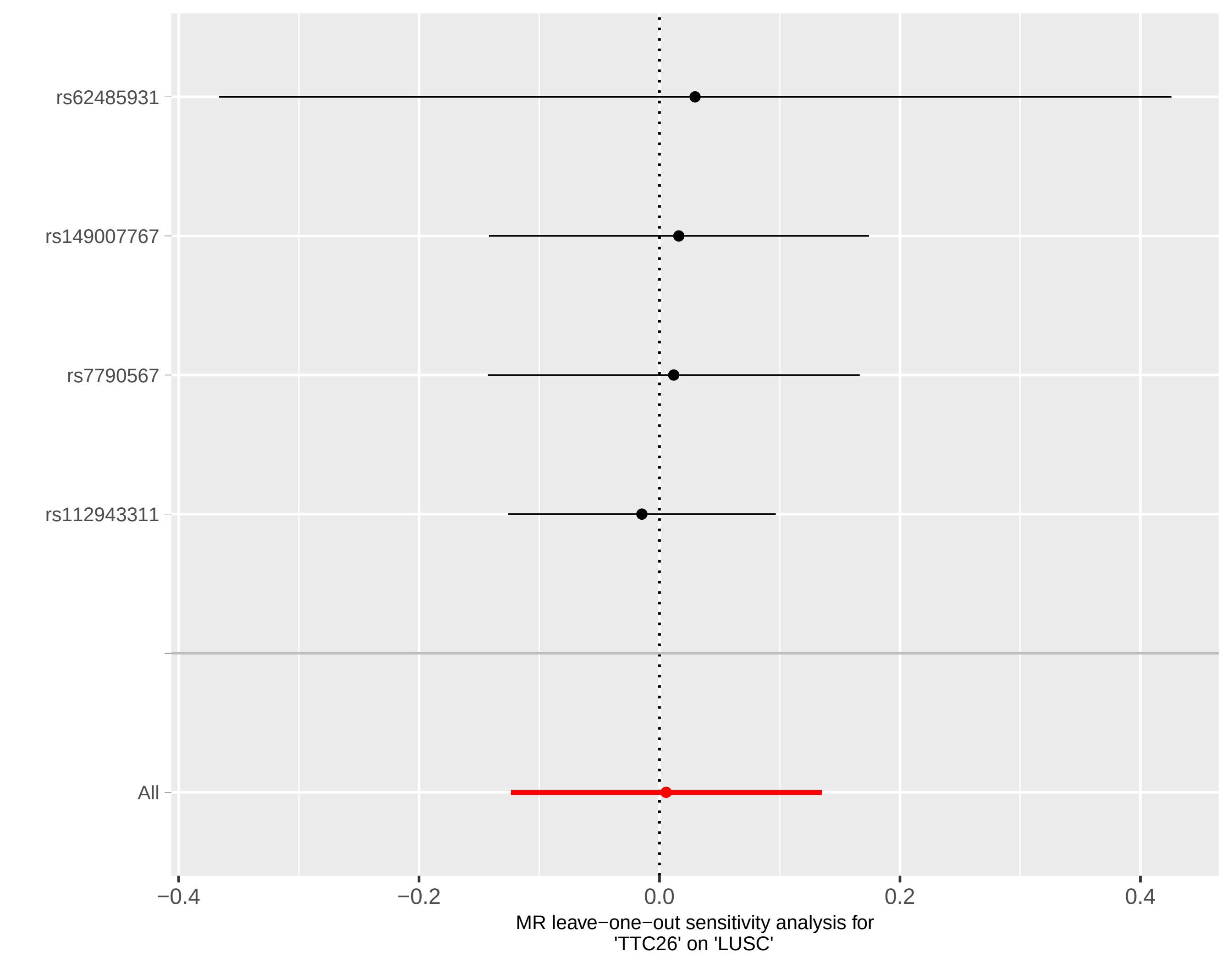
rs62485931
rs149007767
rs7790567
rs112943311
All
−0.4
−0.2
0.0
0.2
0.4
MR leave−one−out sensitivity analysis for
'TTC26' on 'LUSC'

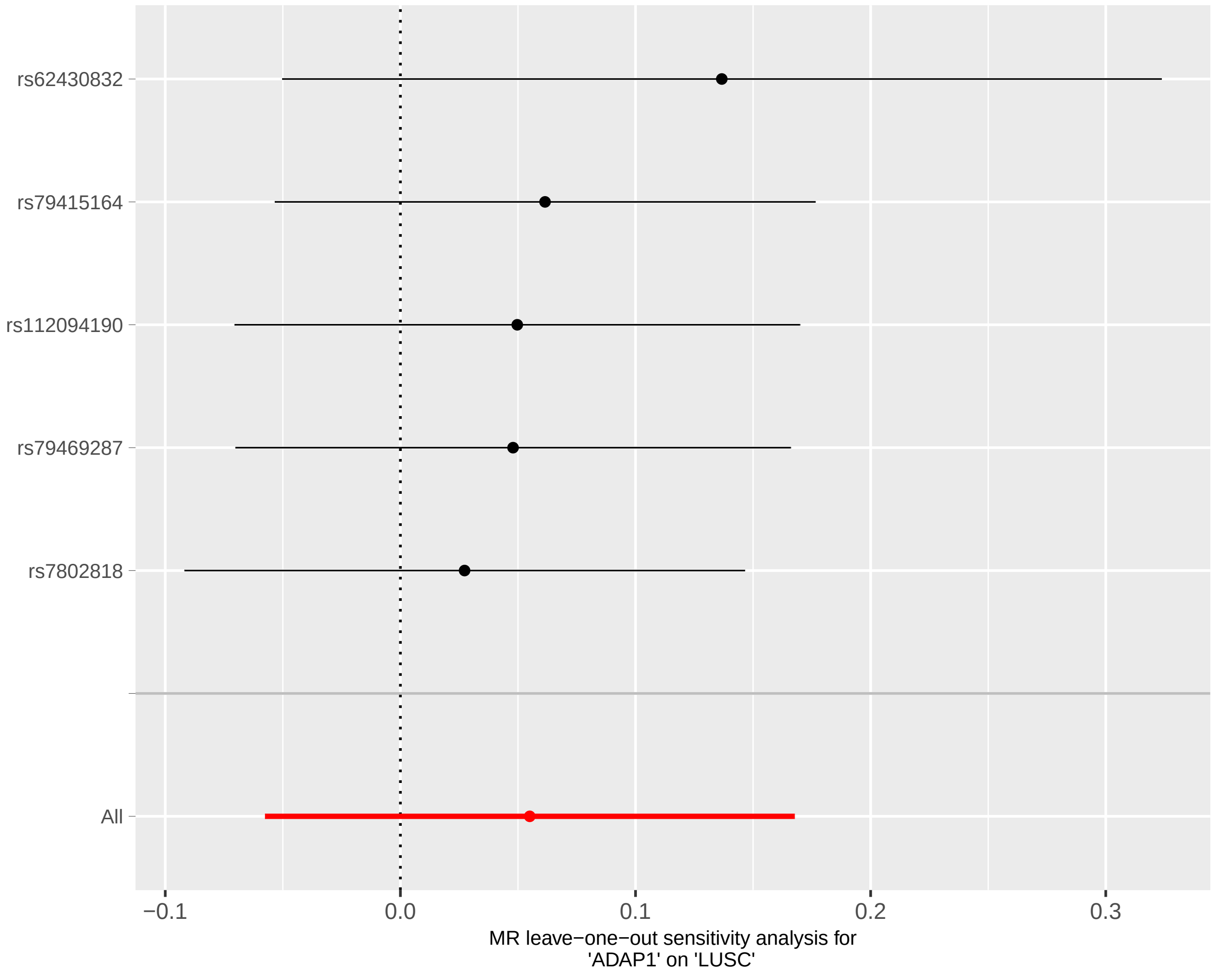

rs62430832
rs79415164
rs112094190
rs79469287
rs7802818
All
−0.1
0.0
0.1
0.2
0.3
MR leave−one−out sensitivity analysis for
'ADAP1' on 'LUSC'

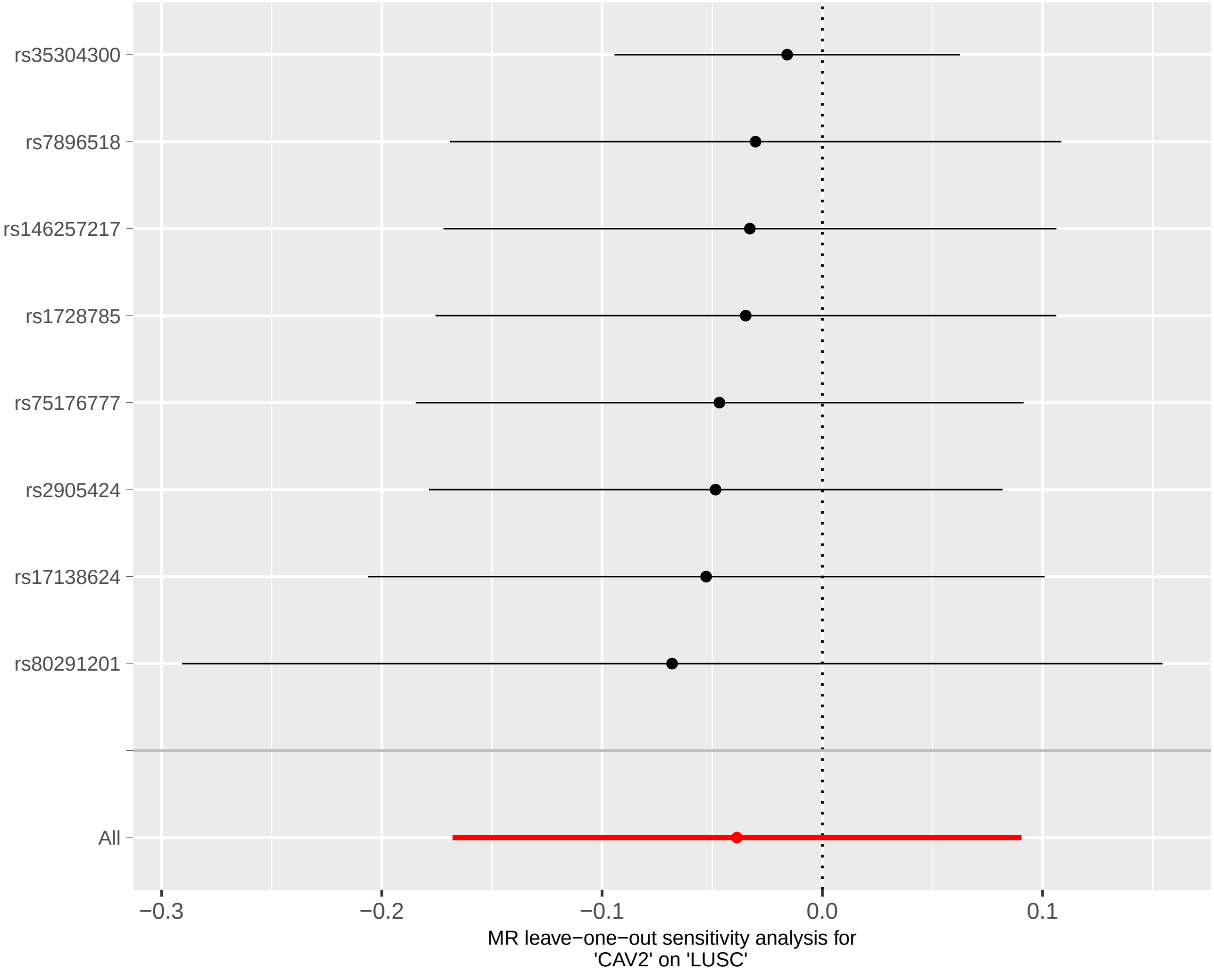
rs35304300
rs7896518
rs146257217
rs1728785
rs75176777
rs2905424
rs17138624
rs80291201
All
−0.3
−0.2
−0.1
0.0
0.1
MR leave−one−out sensitivity analysis for
'CAV2' on 'LUSC'

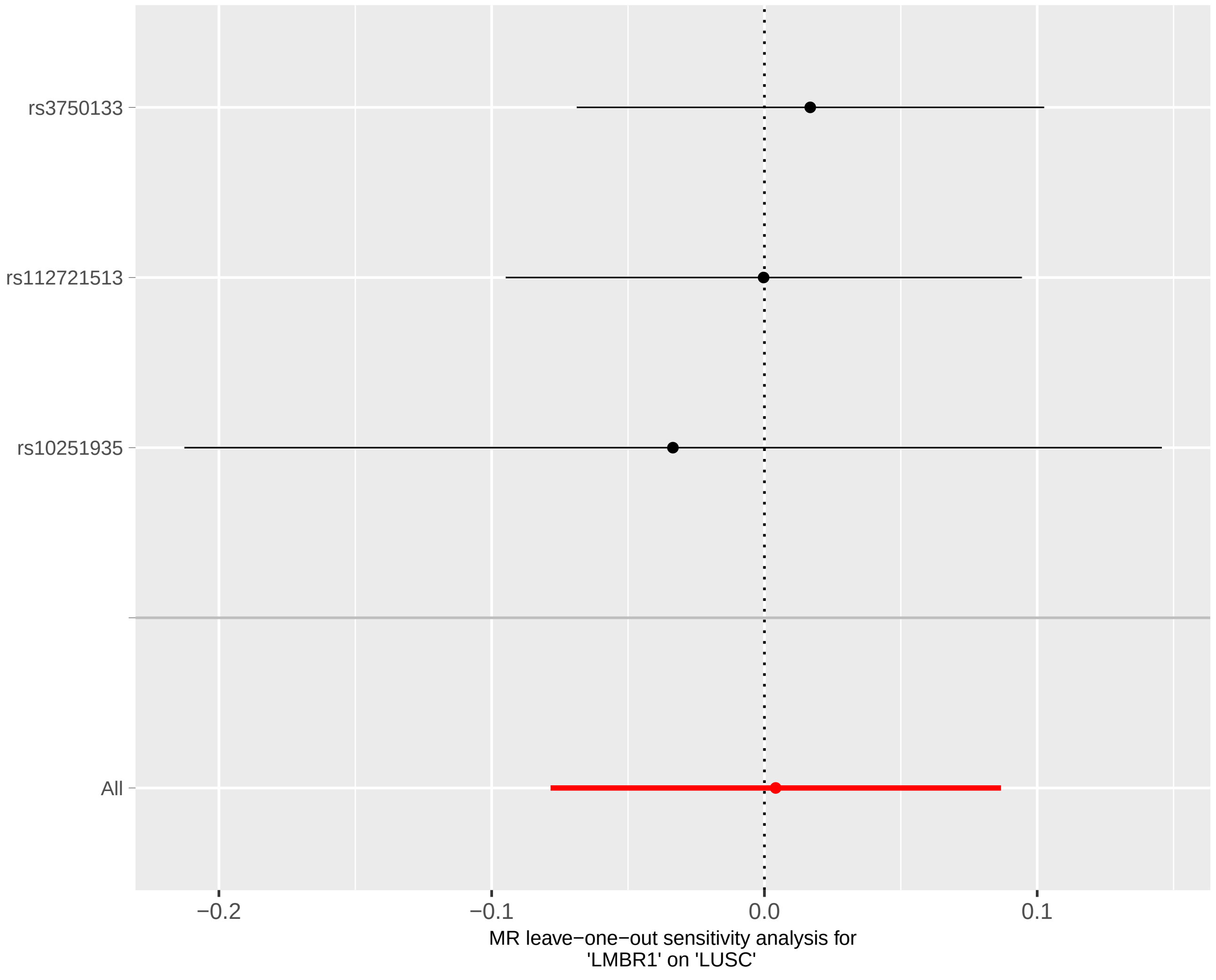
rs3750133
rs112721513
rs10251935
All
−0.2
−0.1
0.0
0.1
MR leave−one−out sensitivity analysis for
'LMBR1' on 'LUSC'

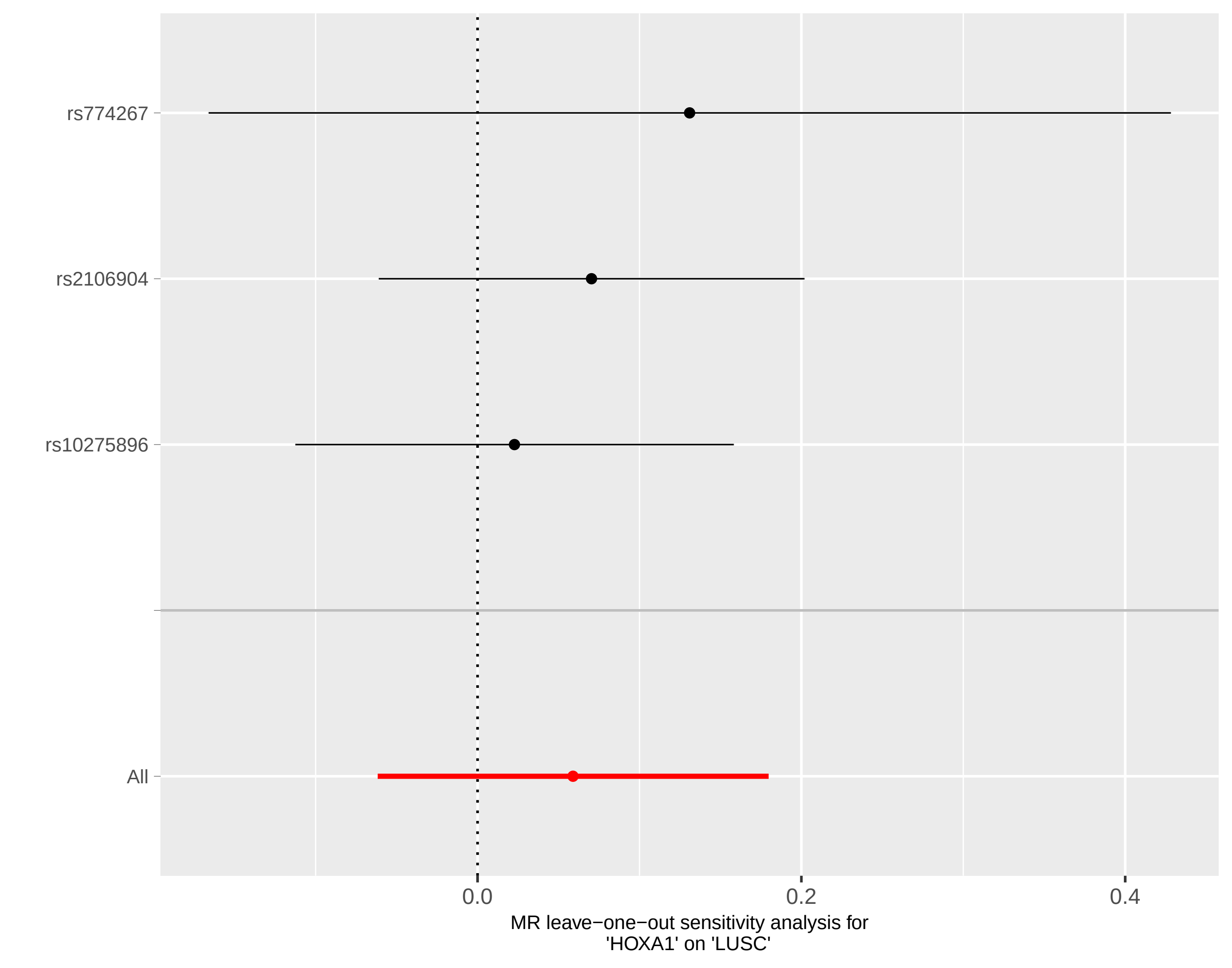

rs774267
rs2106904
rs10275896
All
0.0
0.2
0.4
MR leave−one−out sensitivity analysis for
'HOXA1' on 'LUSC'

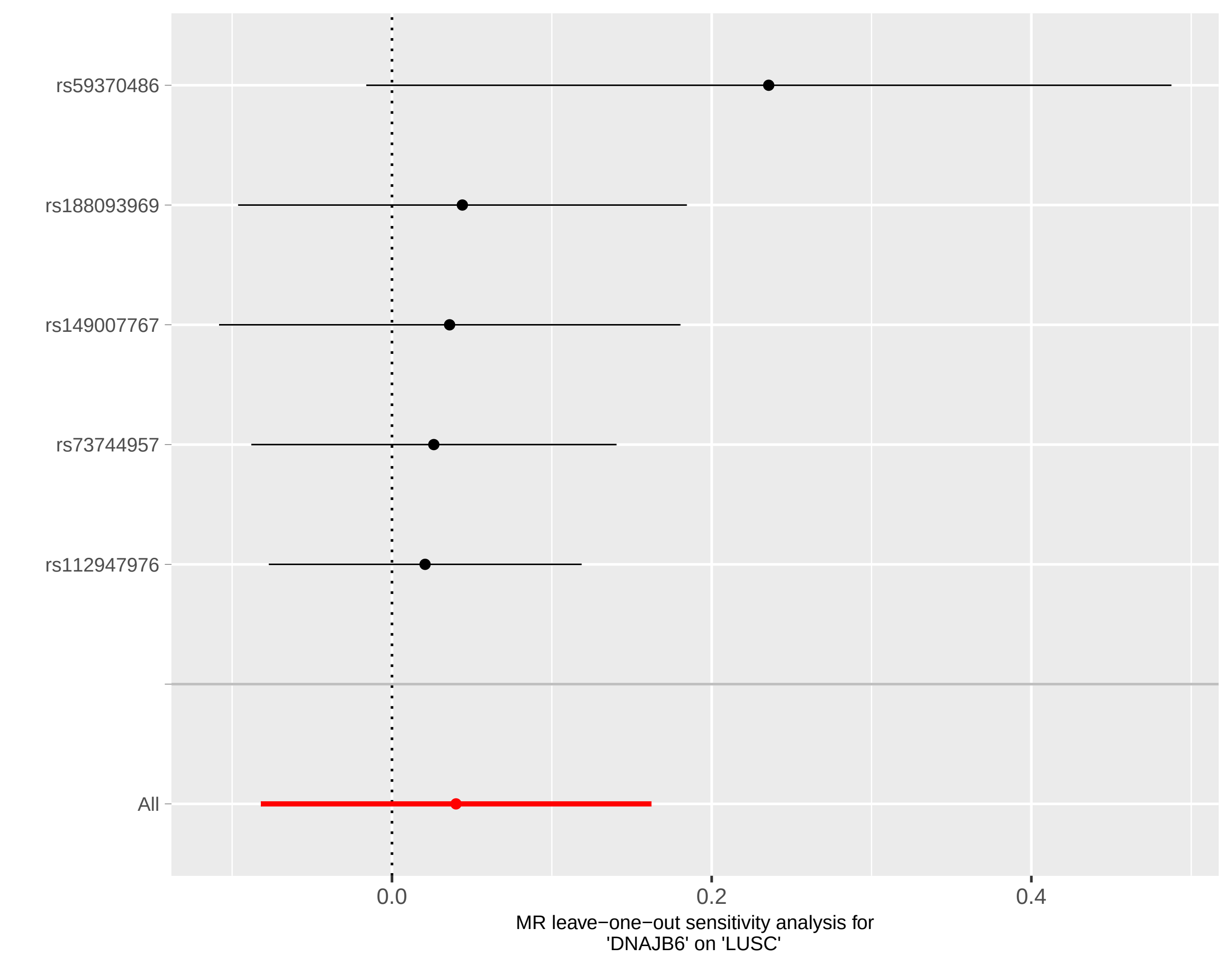

rs59370486
rs188093969
rs149007767
rs73744957
rs112947976
All
0.0
0.2
0.4
MR leave−one−out sensitivity analysis for
'DNAJB6' on 'LUSC'

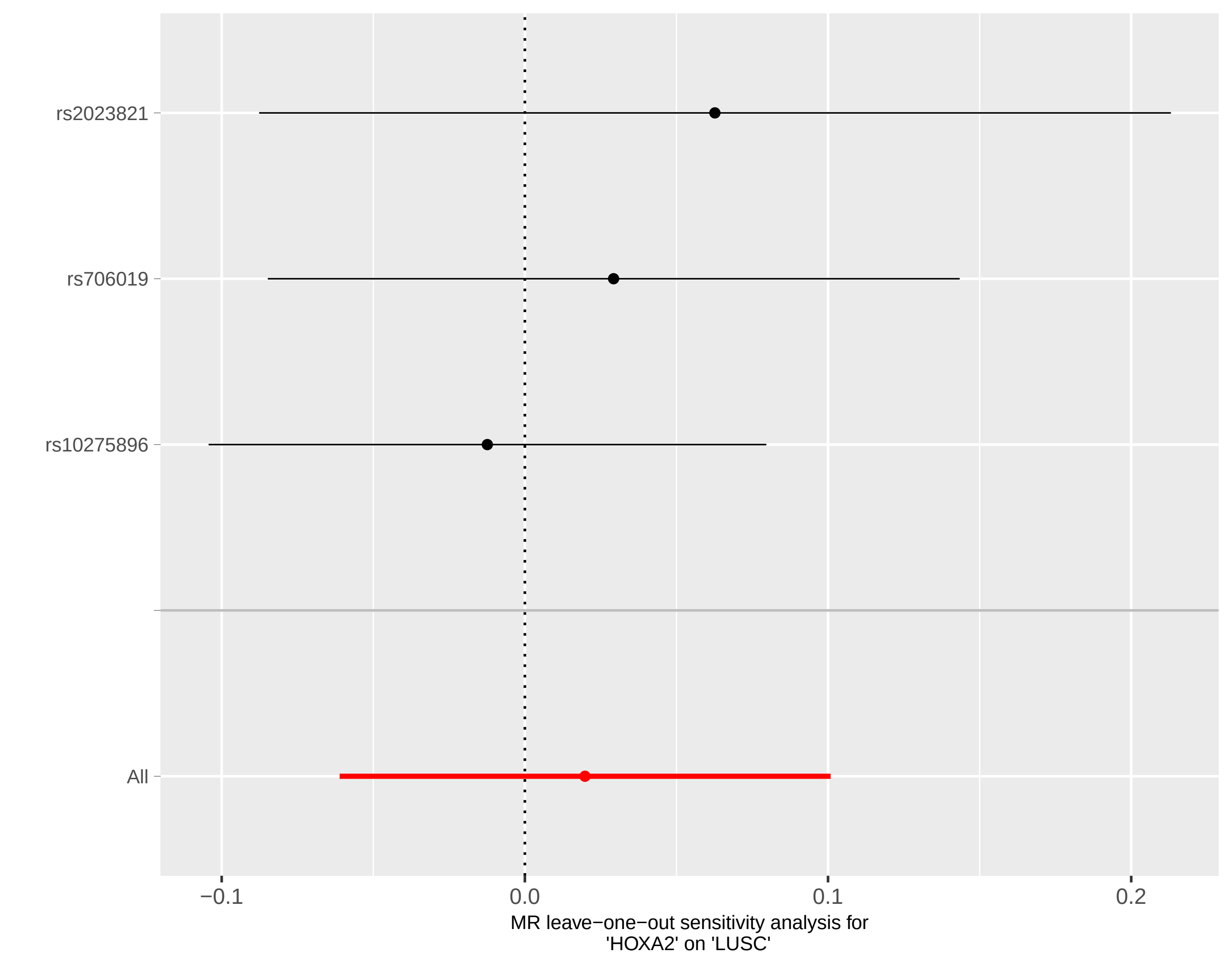

rs2023821
rs706019
rs10275896
All
−0.1
0.0
0.1
0.2
MR leave−one−out sensitivity analysis for
'HOXA2' on 'LUSC'

Insufficient number of SNPs

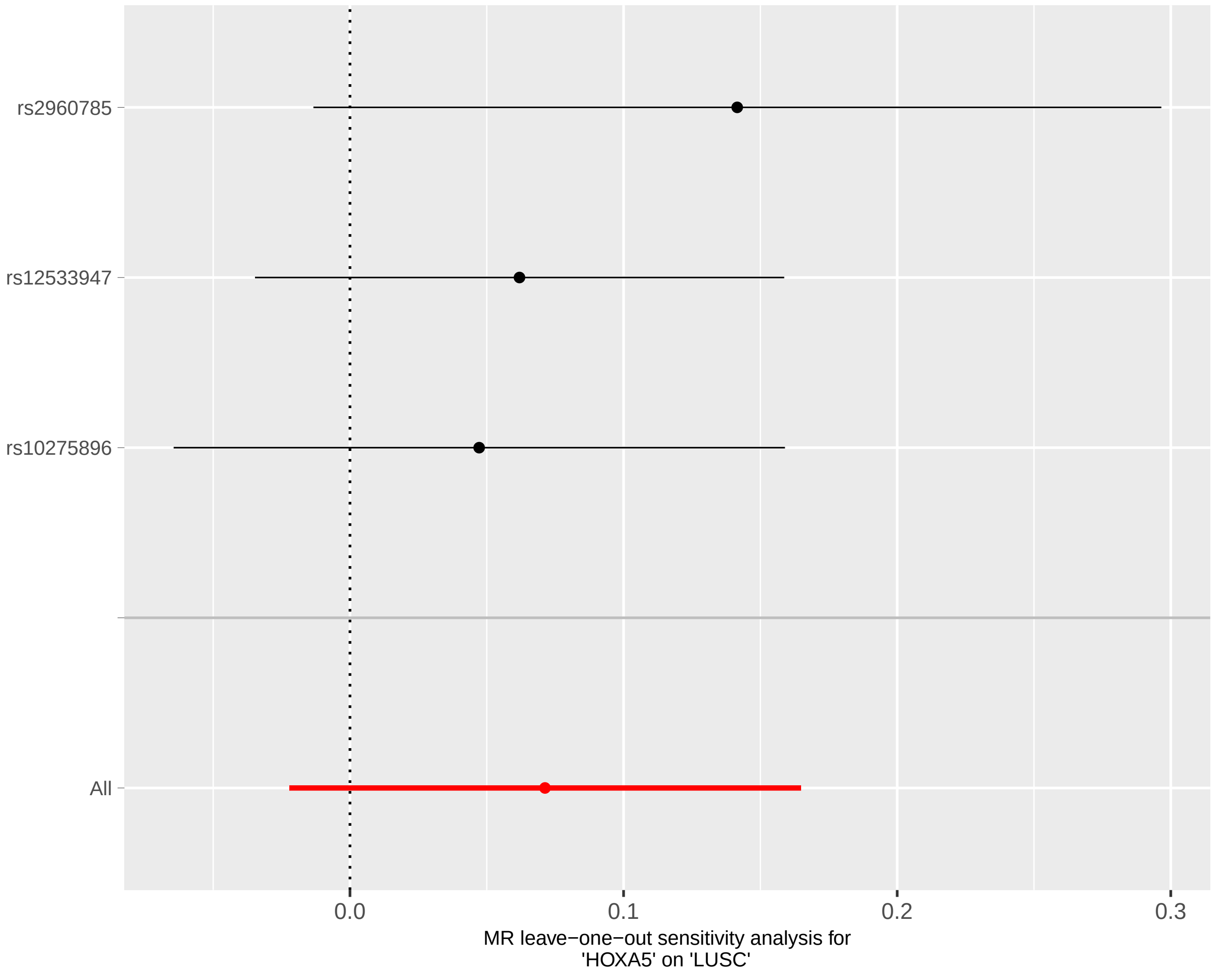
rs2960785
rs12533947
rs10275896
All
0.0
0.1
0.2
0.3
MR leave−one−out sensitivity analysis for
'HOXA5' on 'LUSC'

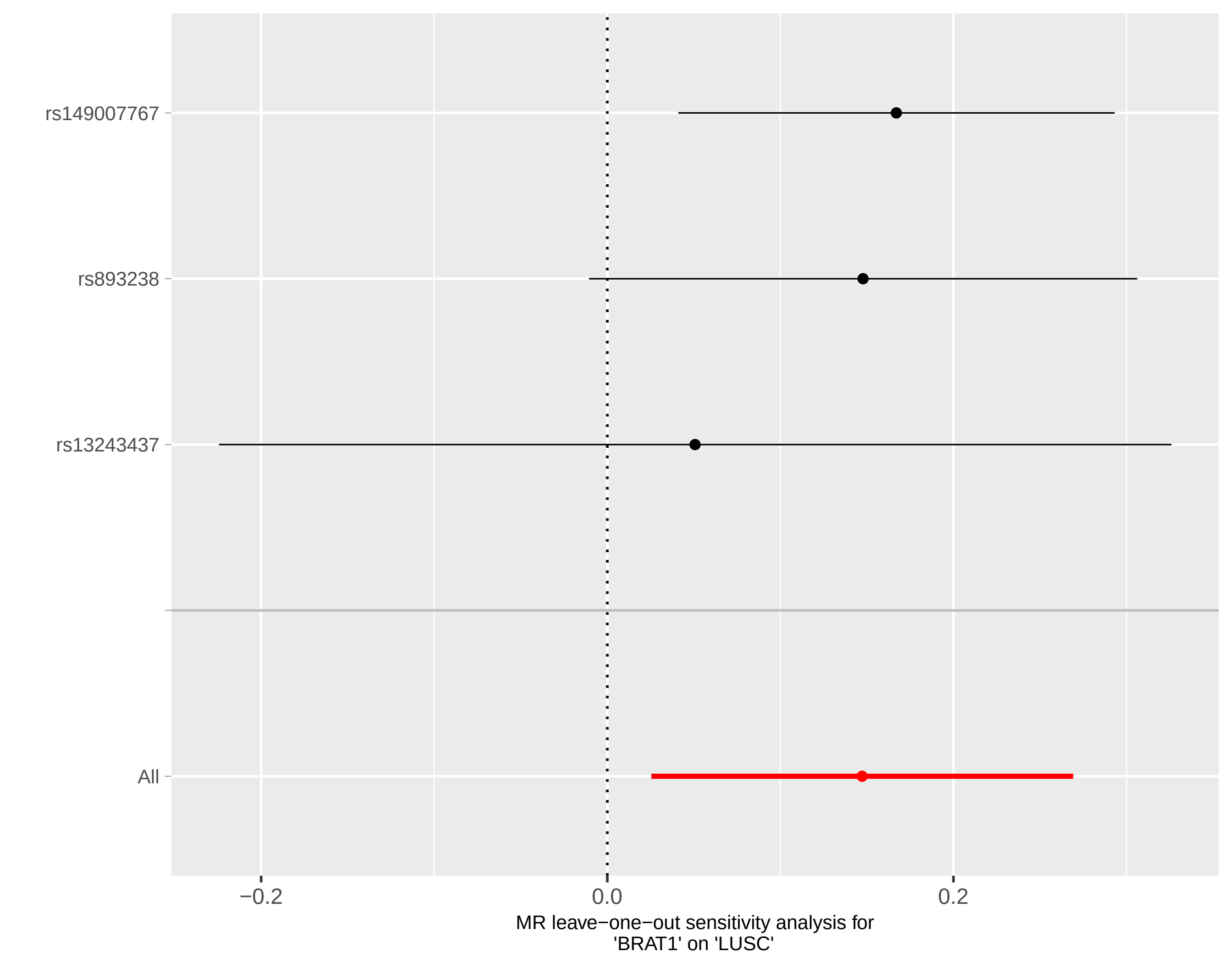

rs149007767
rs893238
rs13243437
All
−0.2
0.0
0.2
MR leave−one−out sensitivity analysis for
'BRAT1' on 'LUSC'

Insufficient number of SNPs

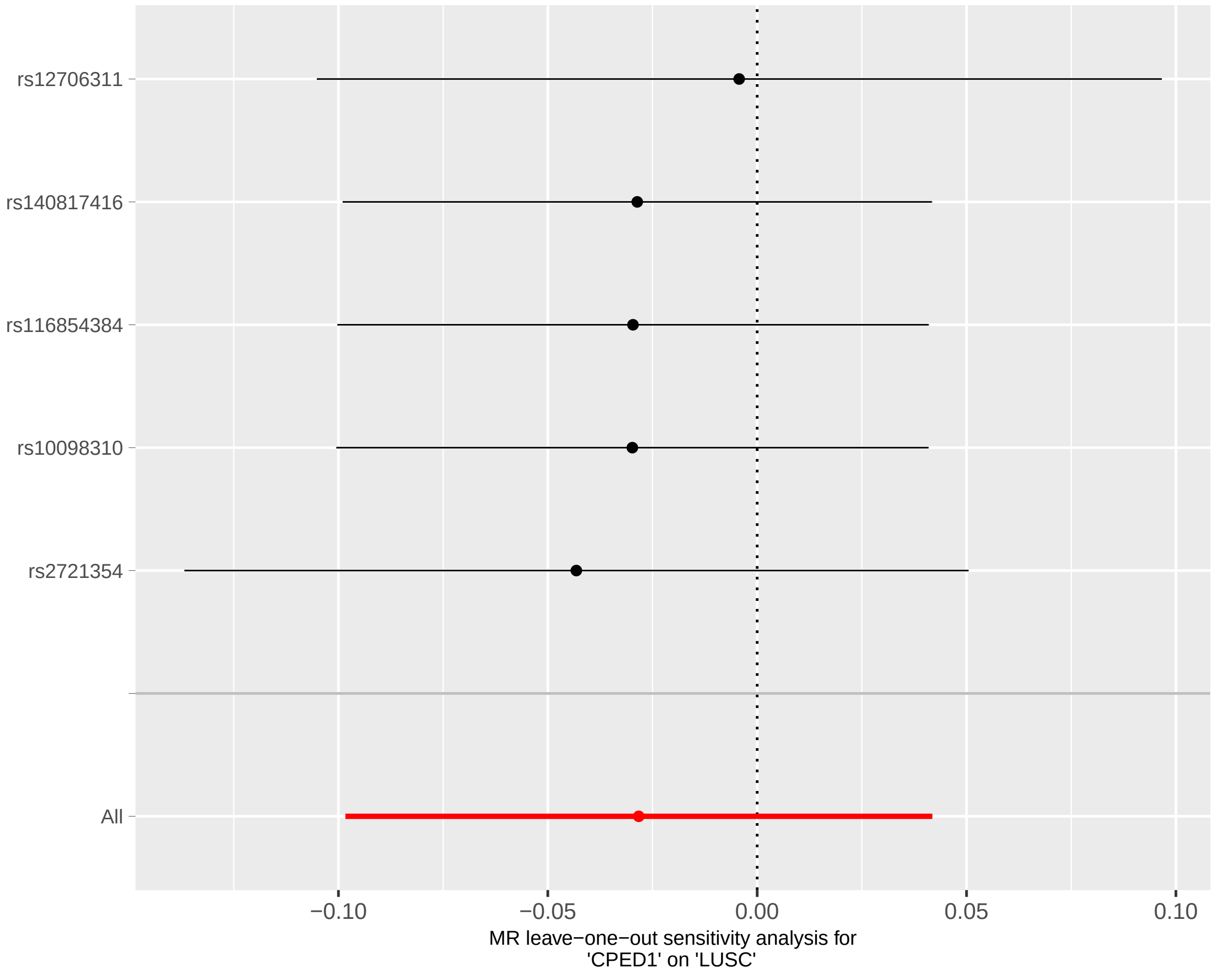
rs12706311
rs140817416
rs116854384
rs10098310
rs2721354
All
−0.10
−0.05
0.00
0.05
0.10
MR leave−one−out sensitivity analysis for
'CPED1' on 'LUSC'

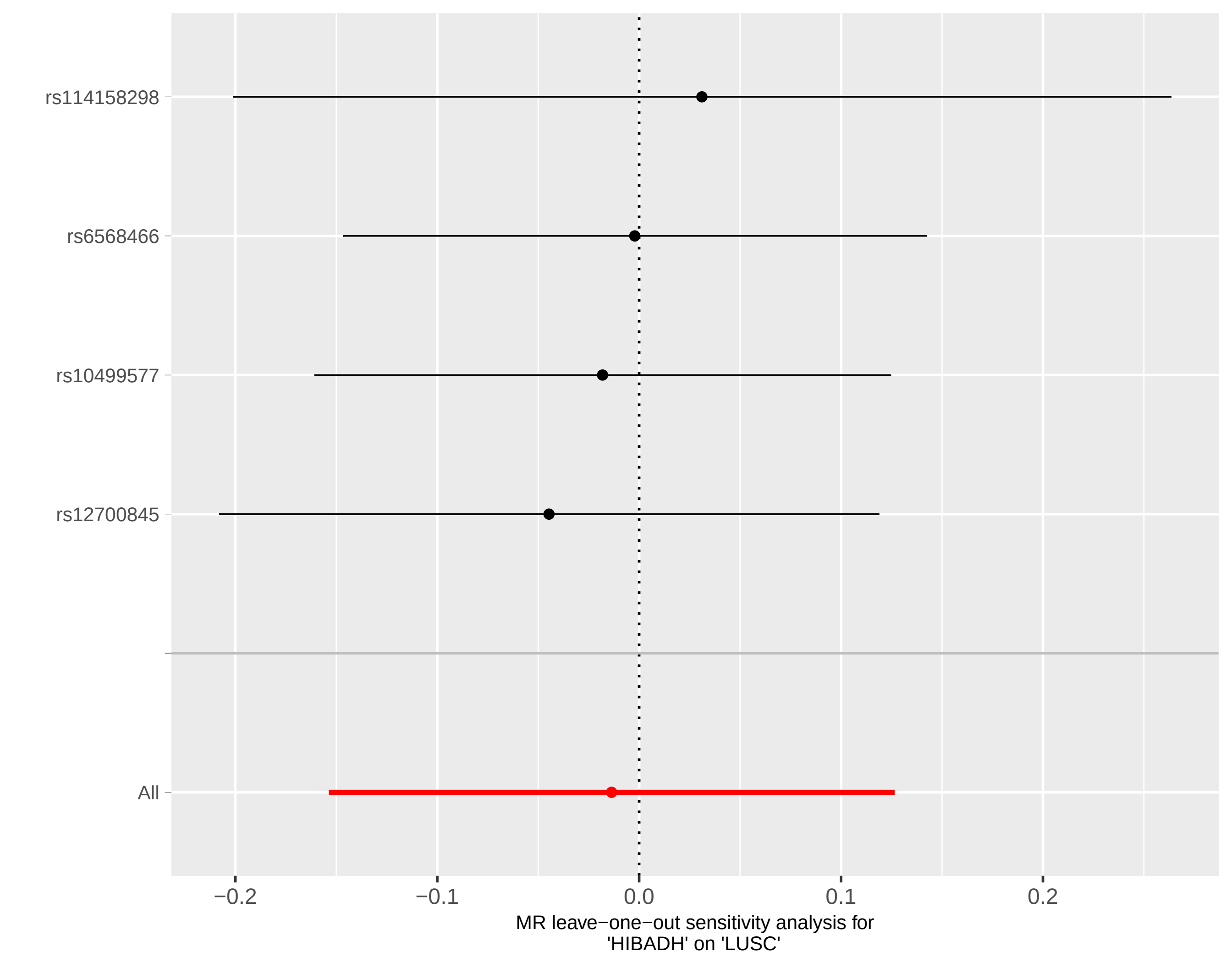
rs114158298
rs6568466
rs10499577
rs12700845
All
−0.2
−0.1
0.0
0.1
0.2
MR leave−one−out sensitivity analysis for
'HIBADH' on 'LUSC'

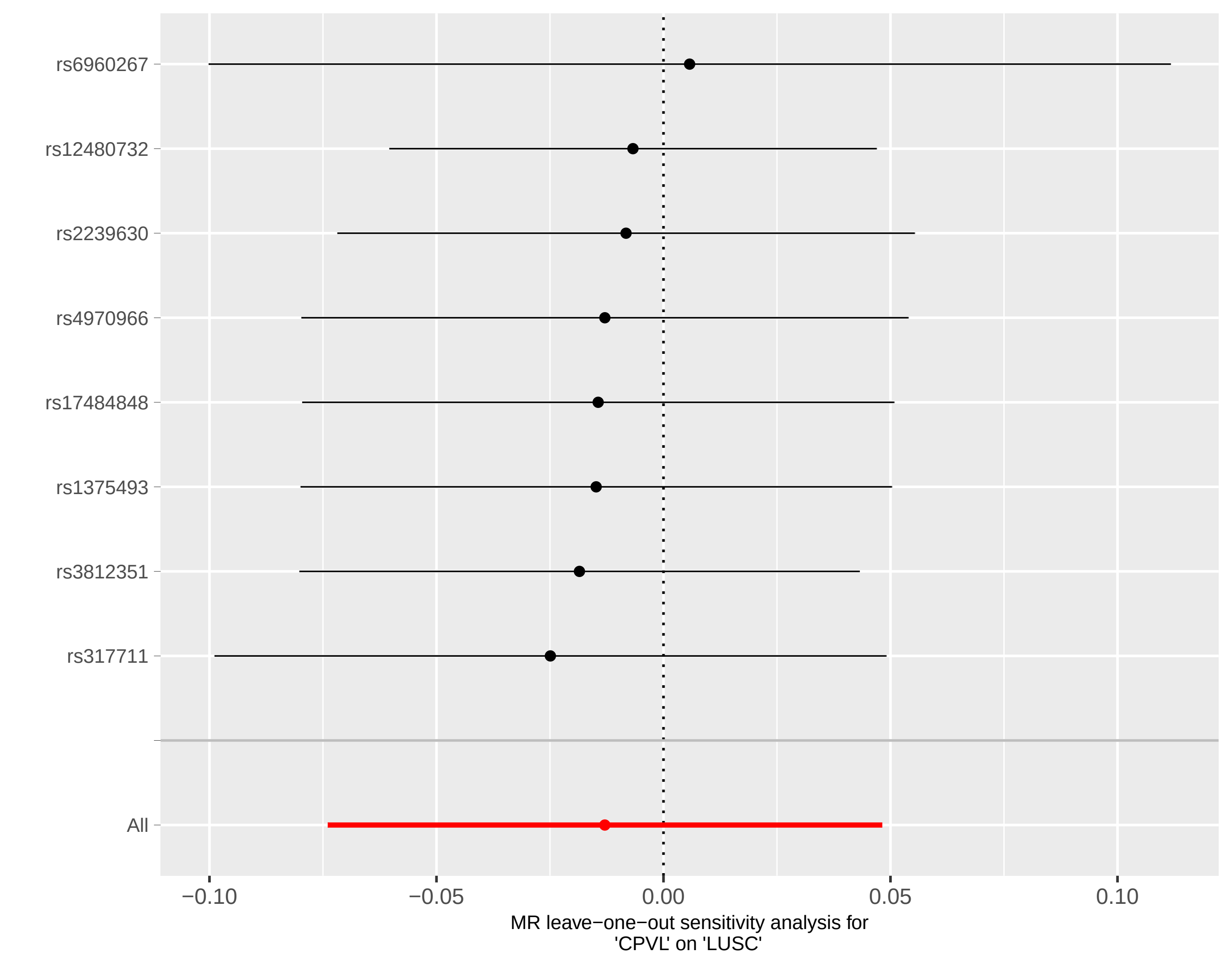
rs6960267
rs12480732
rs2239630
rs4970966
rs17484848
rs1375493
rs3812351
rs317711
All
−0.10
−0.05
0.00
0.05
0.10
MR leave−one−out sensitivity analysis for
'CPVL' on 'LUSC'

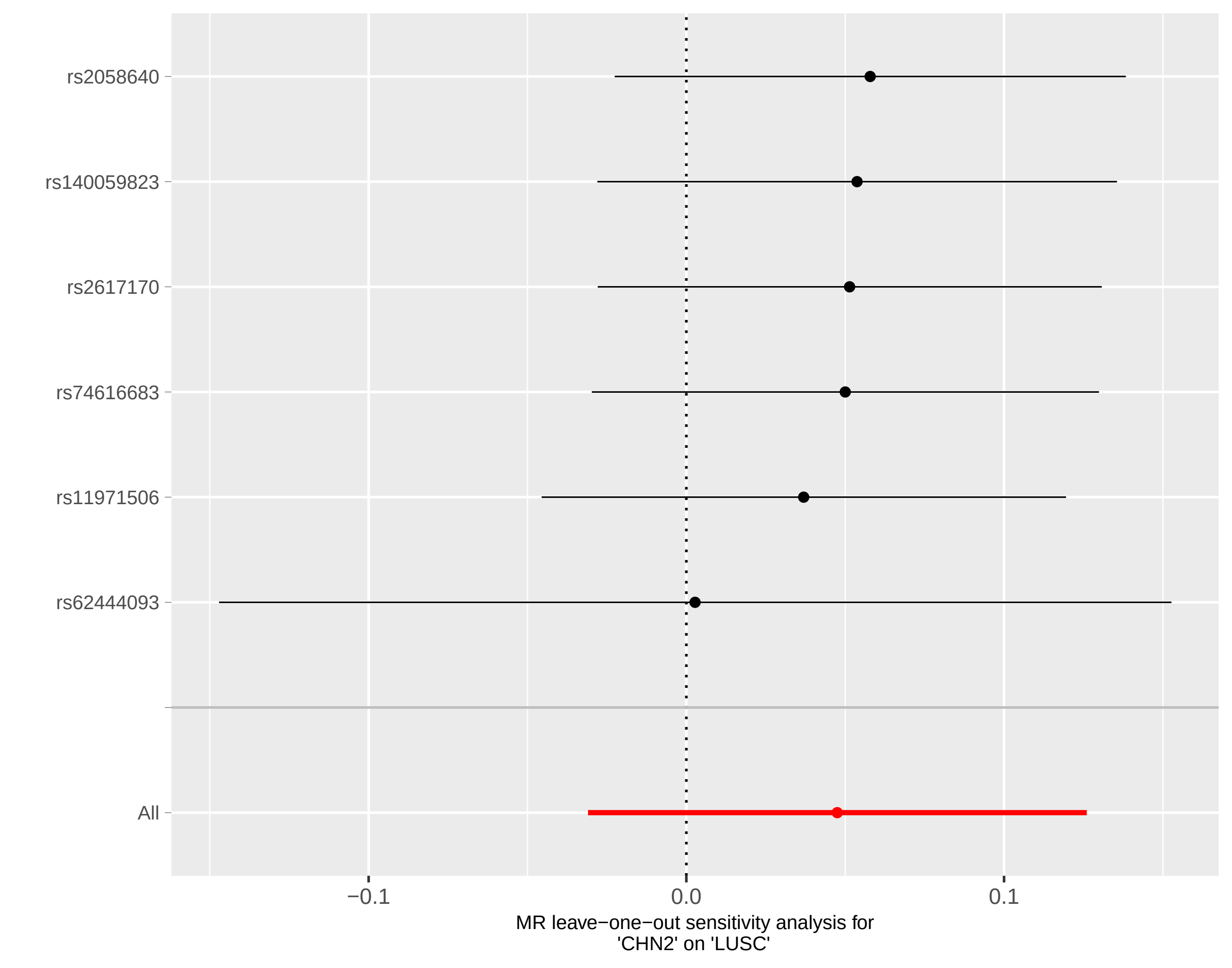

rs2058640
rs140059823
rs2617170
rs74616683
rs11971506
rs62444093
All
−0.1
0.0
0.1
MR leave−one−out sensitivity analysis for
'CHN2' on 'LUSC'

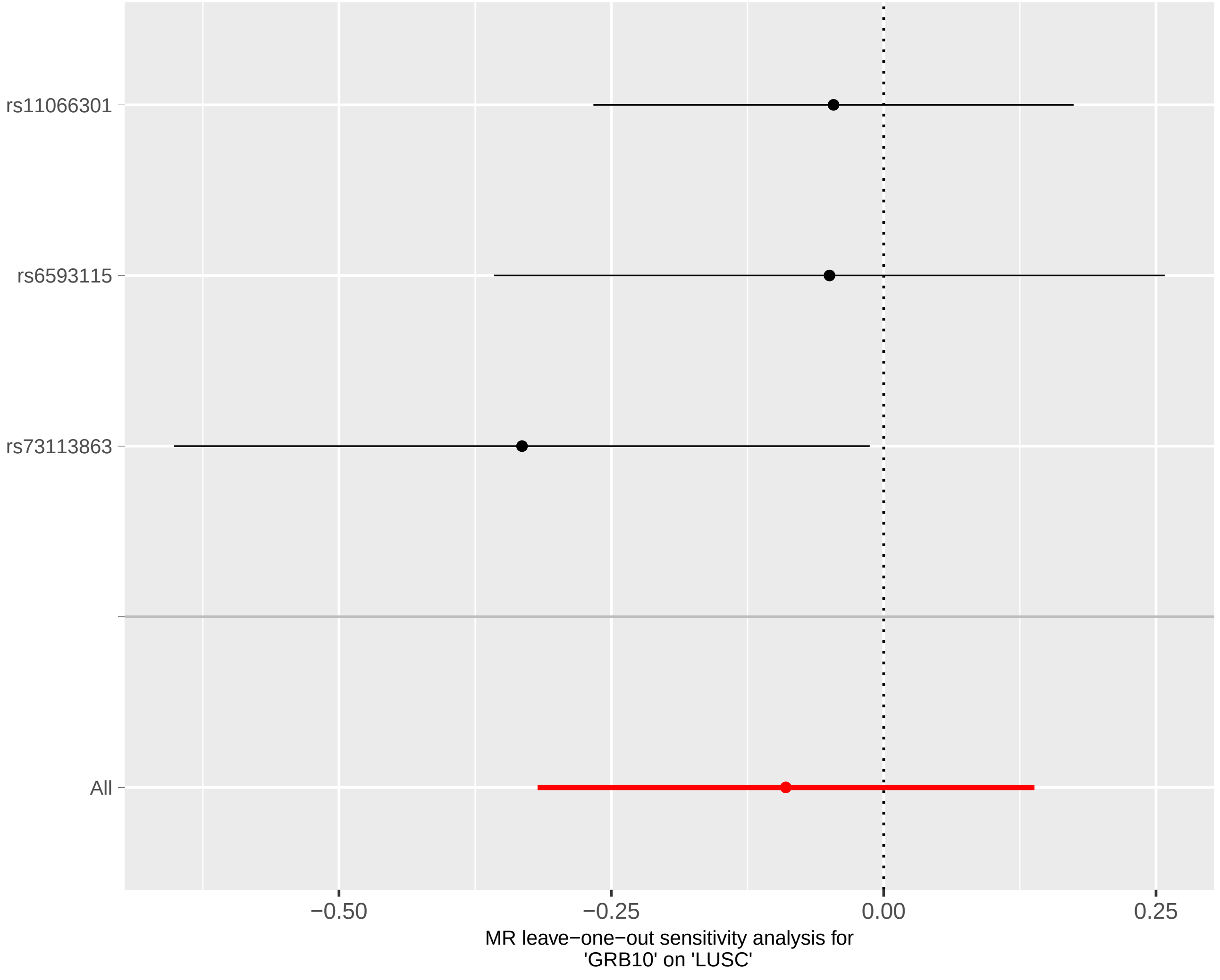
rs11066301
rs6593115
rs73113863
All
−0.50
−0.25
0.00
0.25
MR leave−one−out sensitivity analysis for
'GRB10' on 'LUSC'

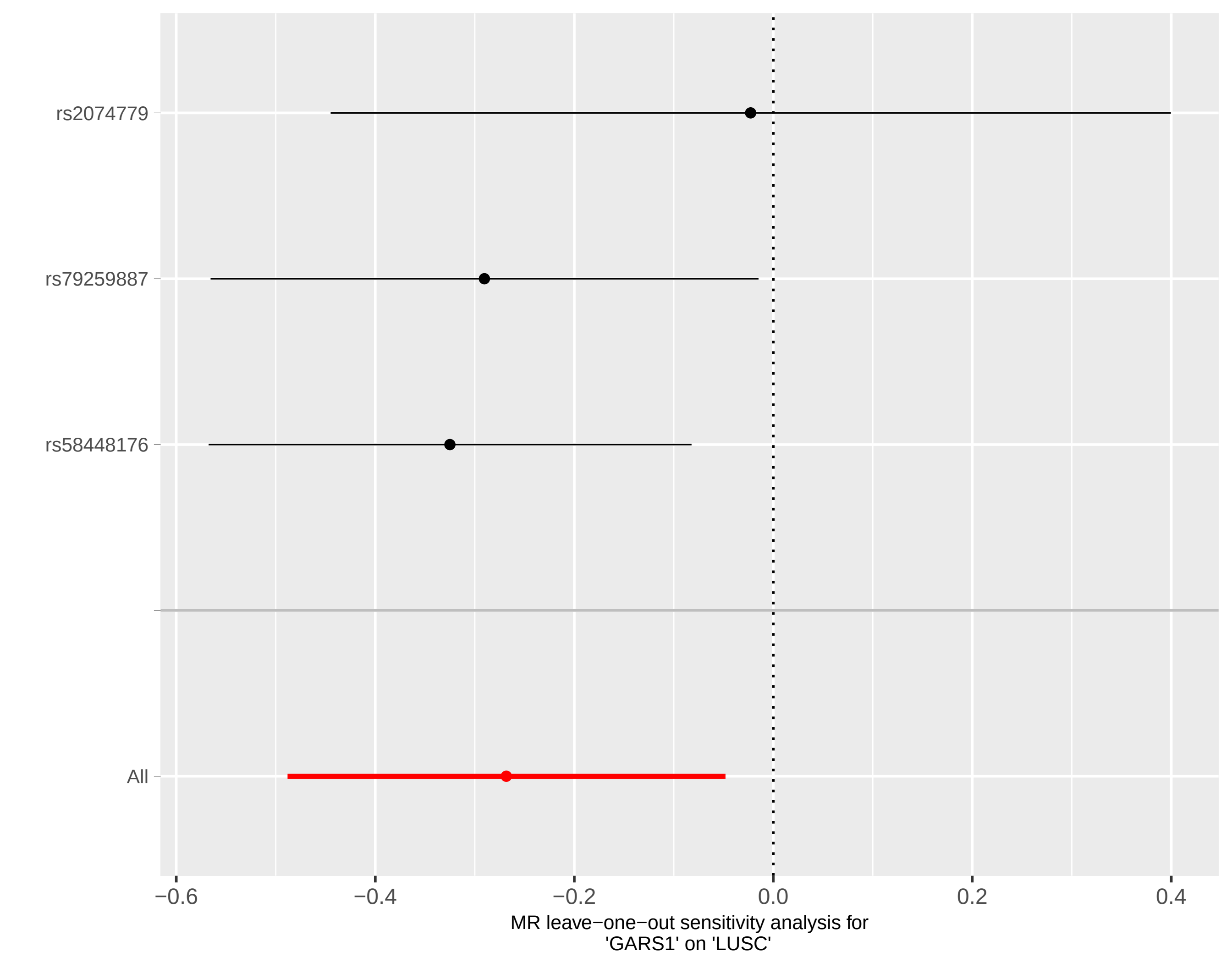
rs2074779
rs79259887
rs58448176
All
−0.6
−0.4
−0.2
0.0
0.2
0.4
MR leave−one−out sensitivity analysis for
'GARS1' on 'LUSC'

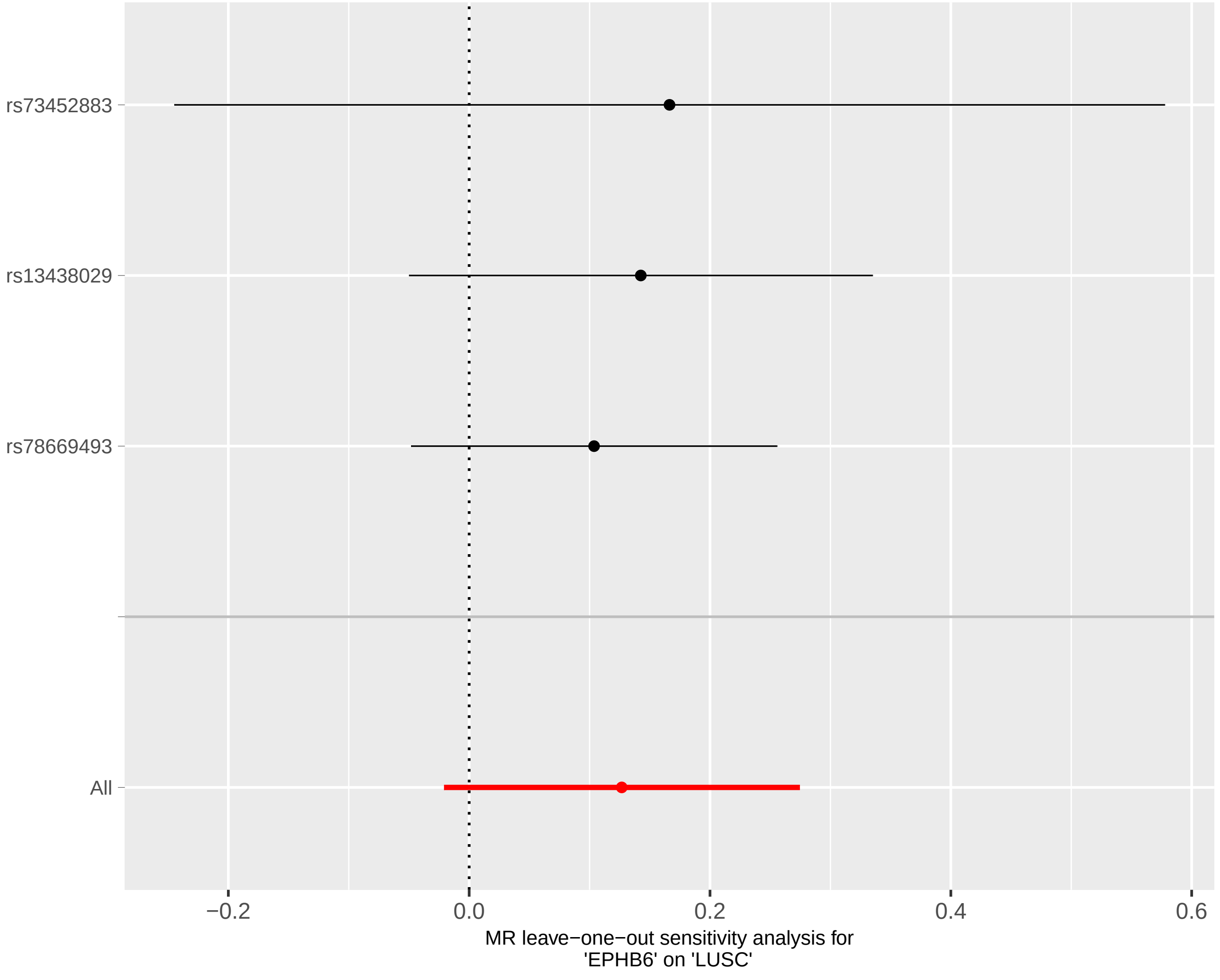

rs73452883
rs13438029
rs78669493
All
−0.2
0.0
0.2
0.4
0.6
MR leave−one−out sensitivity analysis for
'EPHB6' on 'LUSC'

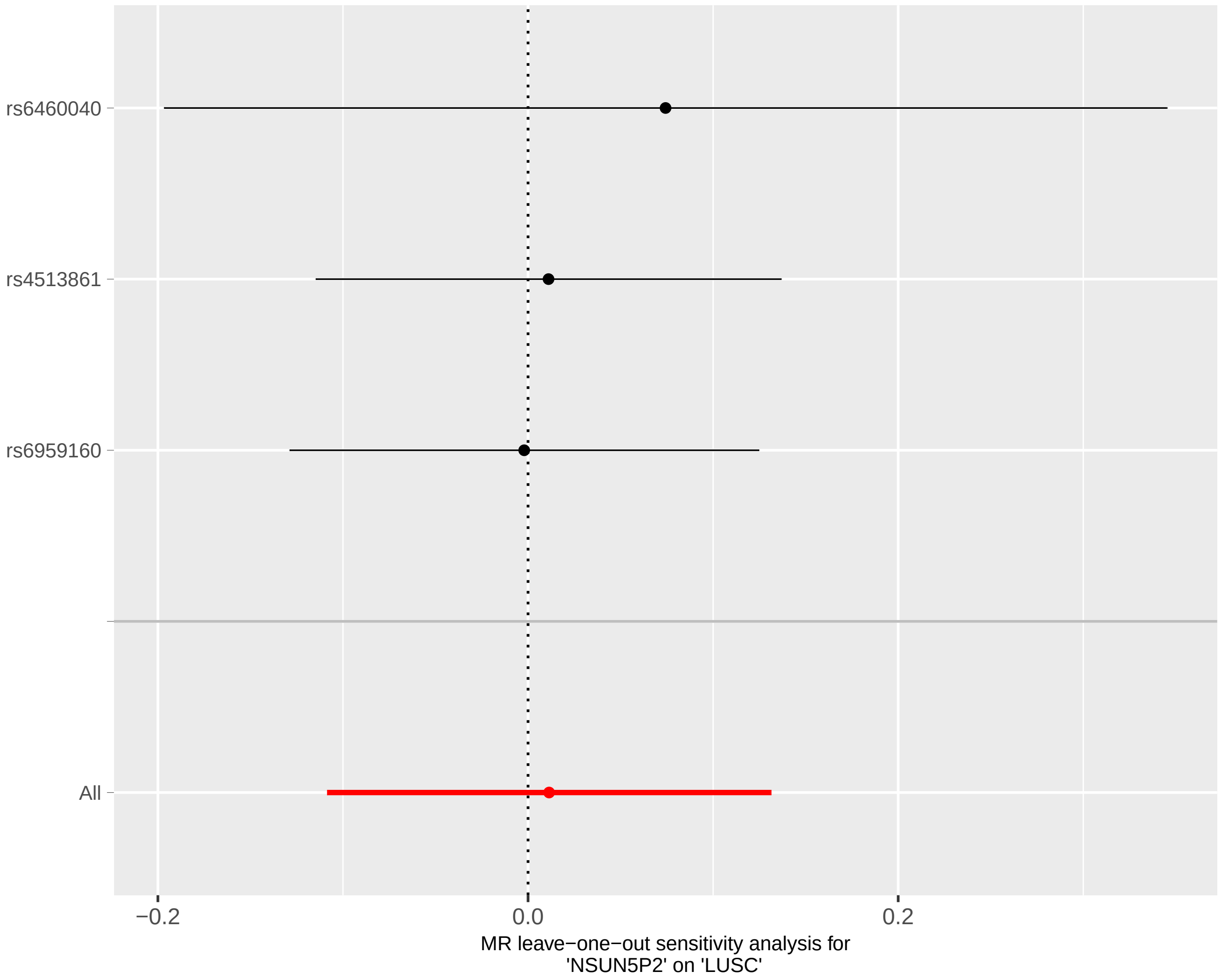
rs6460040
rs4513861
rs6959160
All
−0.2
0.0
0.2
MR leave−one−out sensitivity analysis for
'NSUN5P2' on 'LUSC'

Insufficient number of SNPs

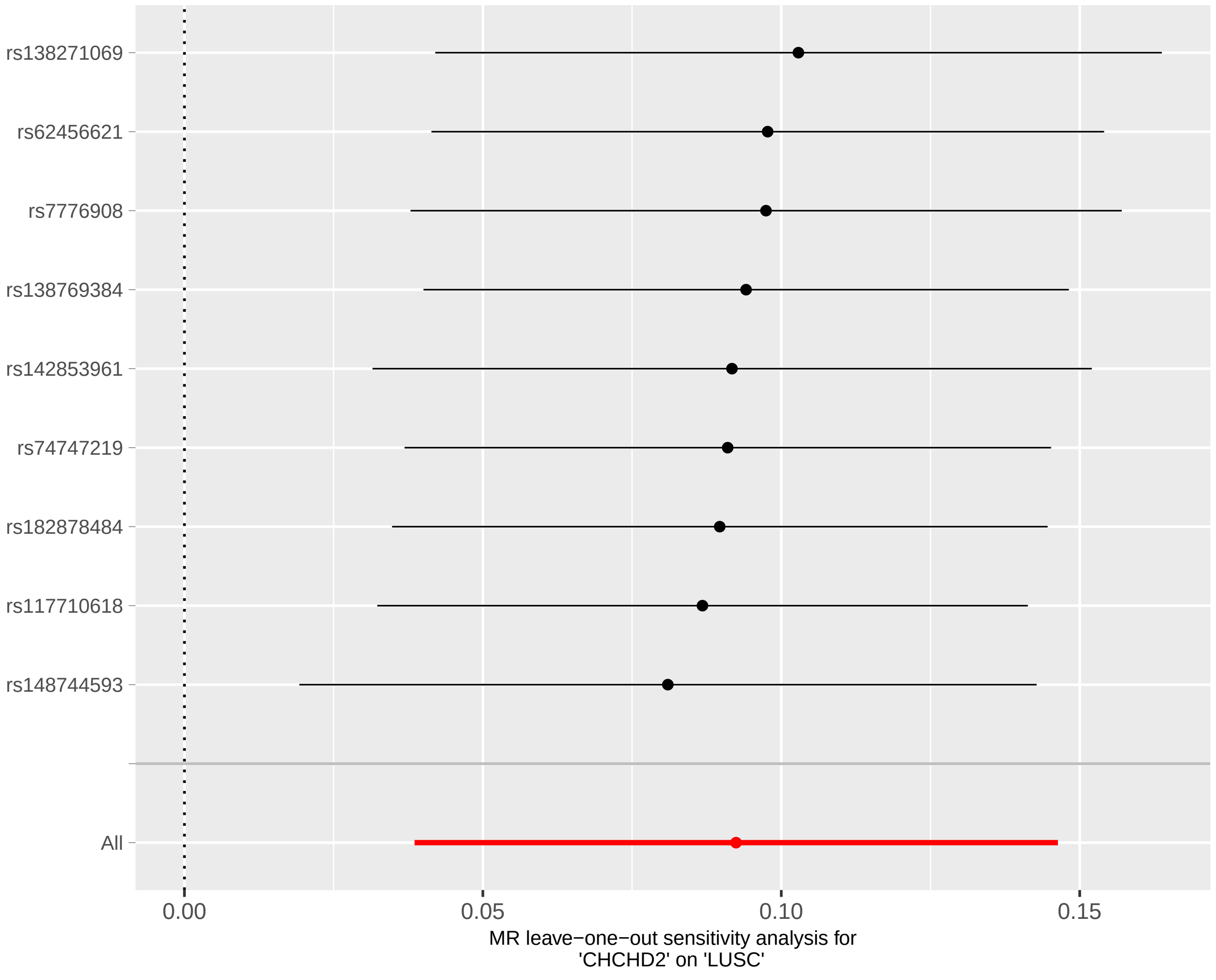
rs138271069
rs62456621
rs7776908
rs138769384
rs142853961
rs74747219
rs182878484
rs117710618
rs148744593
All
0.00
0.05
0.10
0.15
MR leave−one−out sensitivity analysis for
'CHCHD2' on 'LUSC'

Insufficient number of SNPs

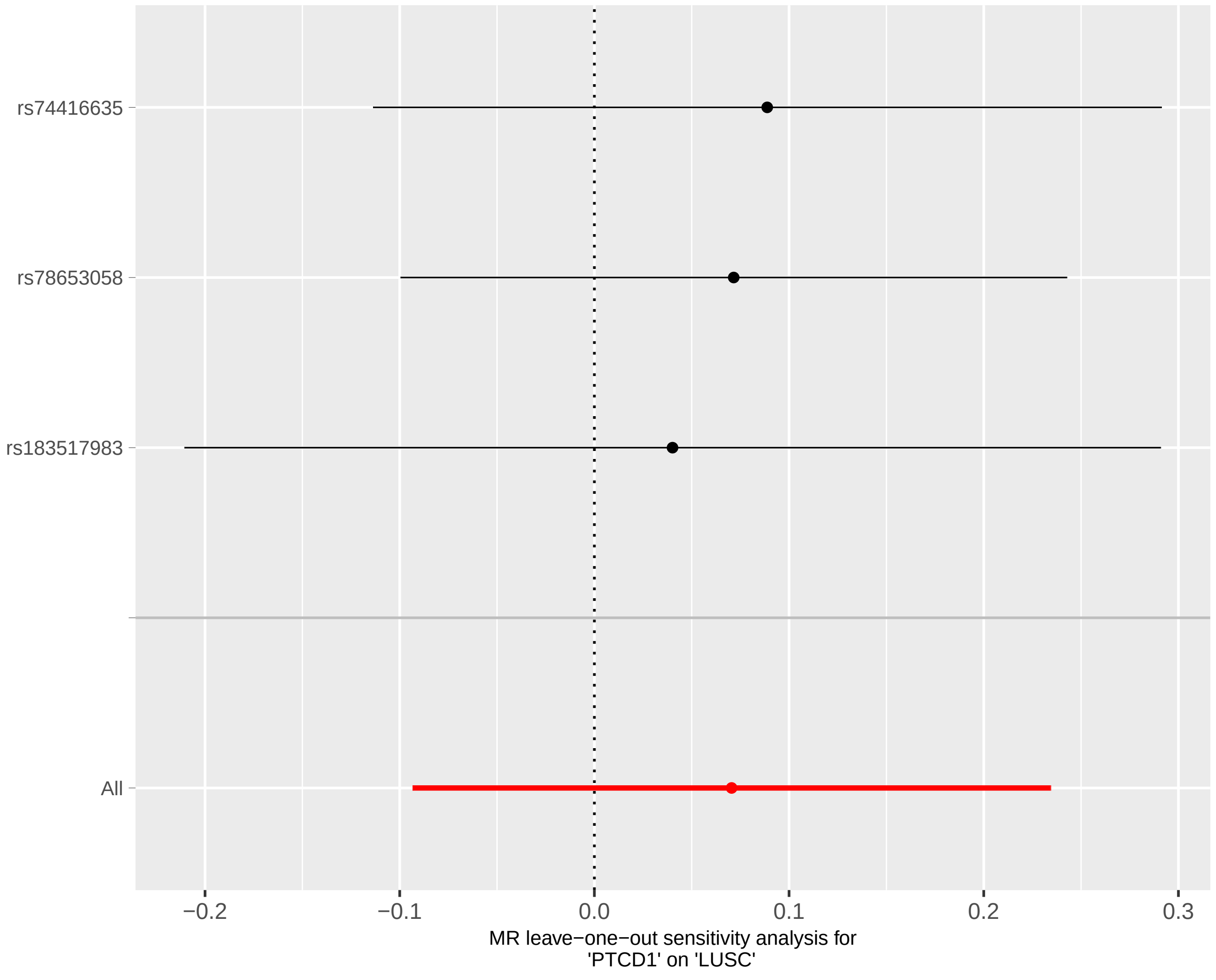

rs74416635
rs78653058
rs183517983
All
−0.2
−0.1
0.0
0.1
0.2
0.3
MR leave−one−out sensitivity analysis for
'PTCD1' on 'LUSC'

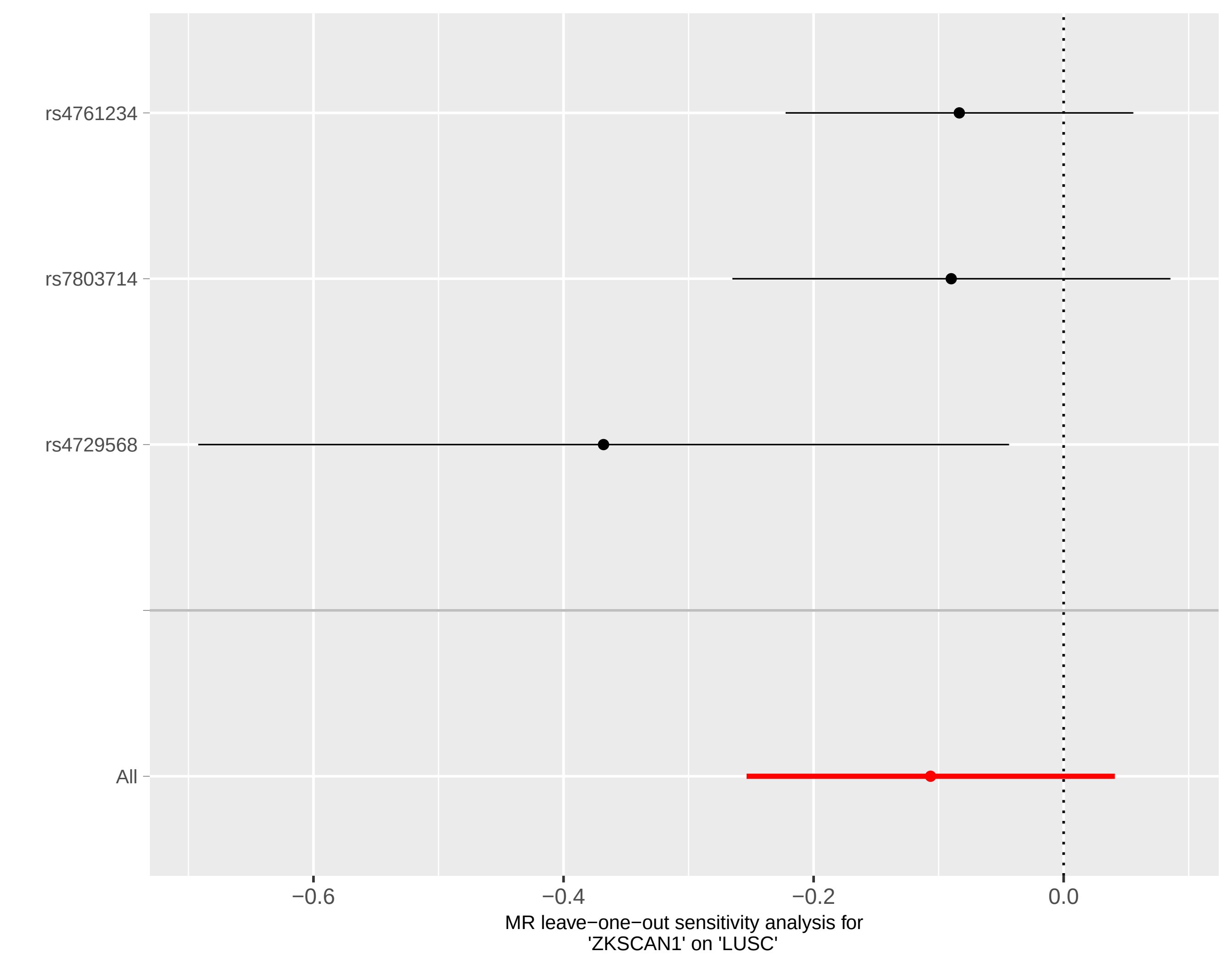

rs4761234
rs7803714
rs4729568
All
−0.6
−0.4
−0.2
0.0
MR leave−one−out sensitivity analysis for
'ZKSCAN1' on 'LUSC'

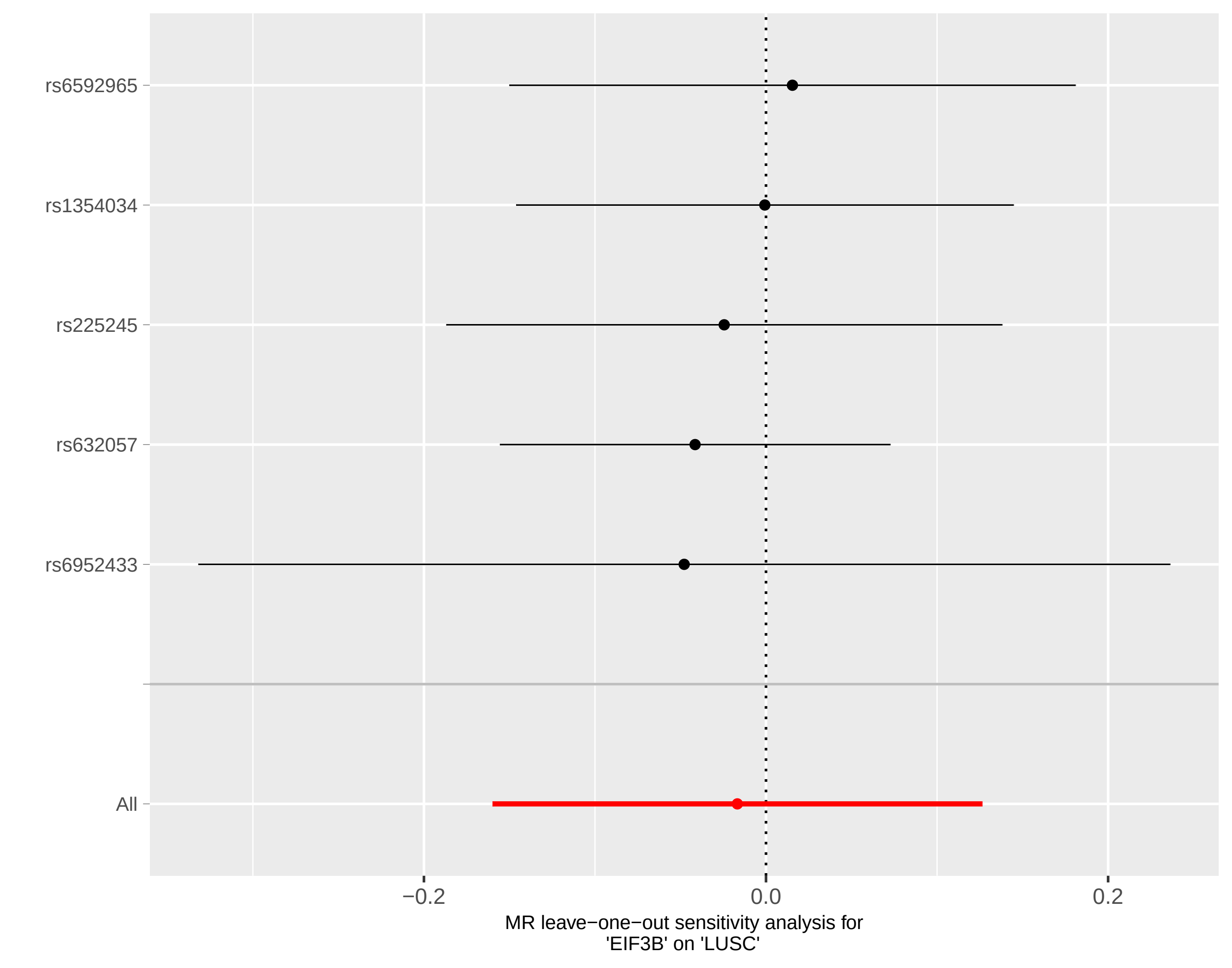
rs6592965
rs1354034
rs225245
rs632057
rs6952433
All
−0.2
0.0
0.2
MR leave−one−out sensitivity analysis for
'EIF3B' on 'LUSC'

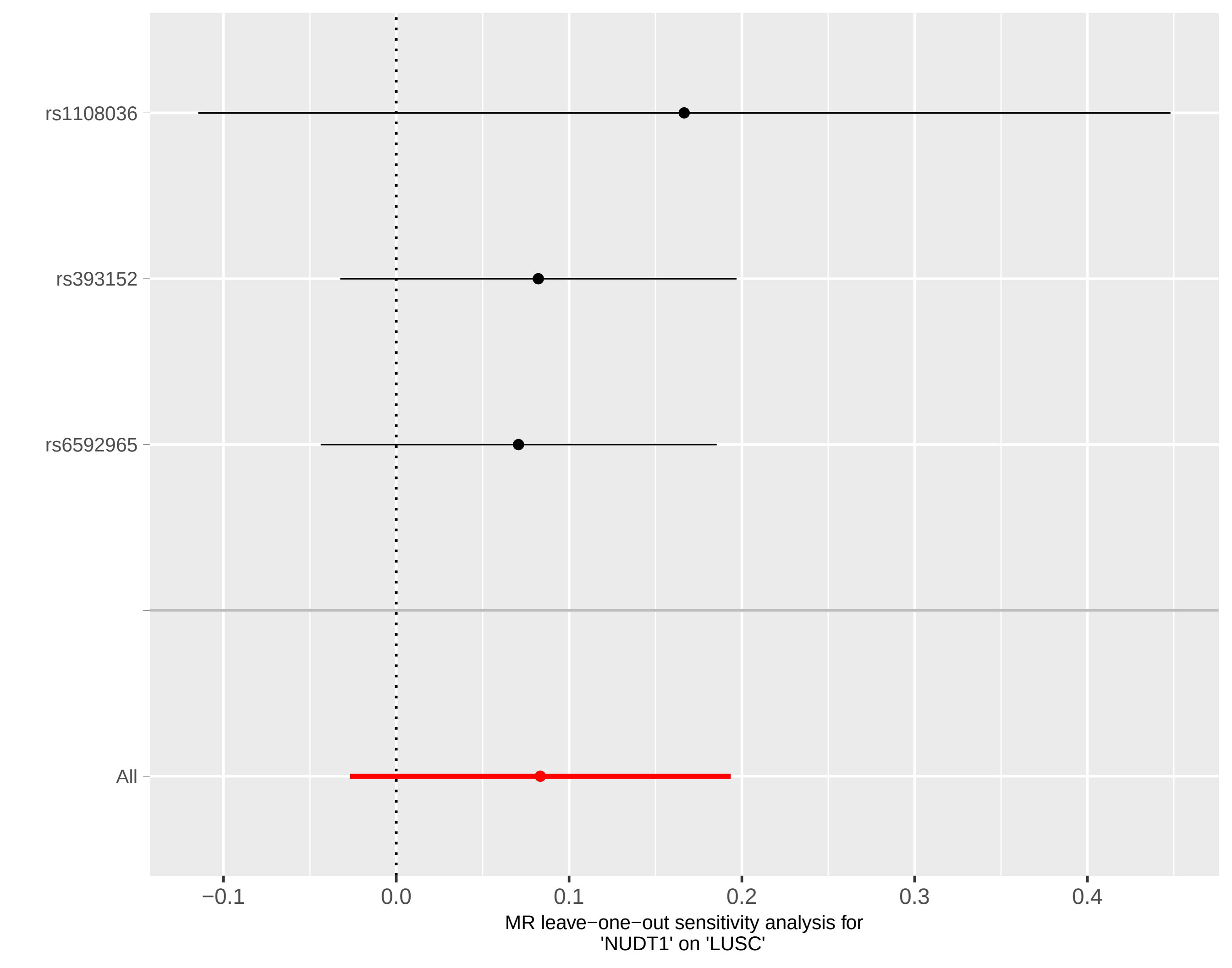
rs1108036
rs393152
rs6592965
All
−0.1
0.0
0.1
0.2
0.3
0.4
MR leave−one−out sensitivity analysis for
'NUDT1' on 'LUSC'

Insufficient number of SNPs

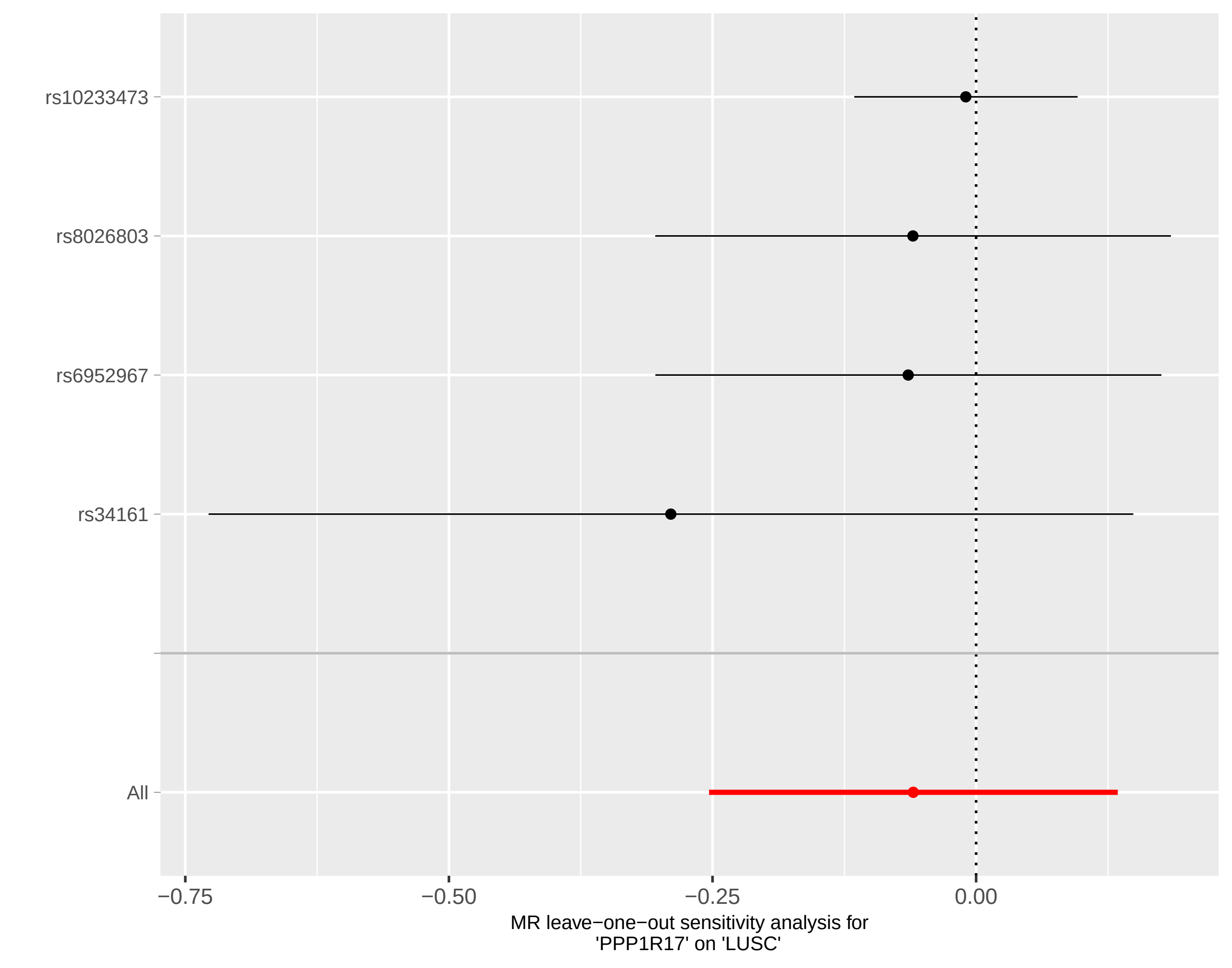
rs10233473
rs8026803
rs6952967
rs34161
All
−0.75
−0.50
−0.25
0.00
MR leave−one−out sensitivity analysis for
'PPP1R17' on 'LUSC'

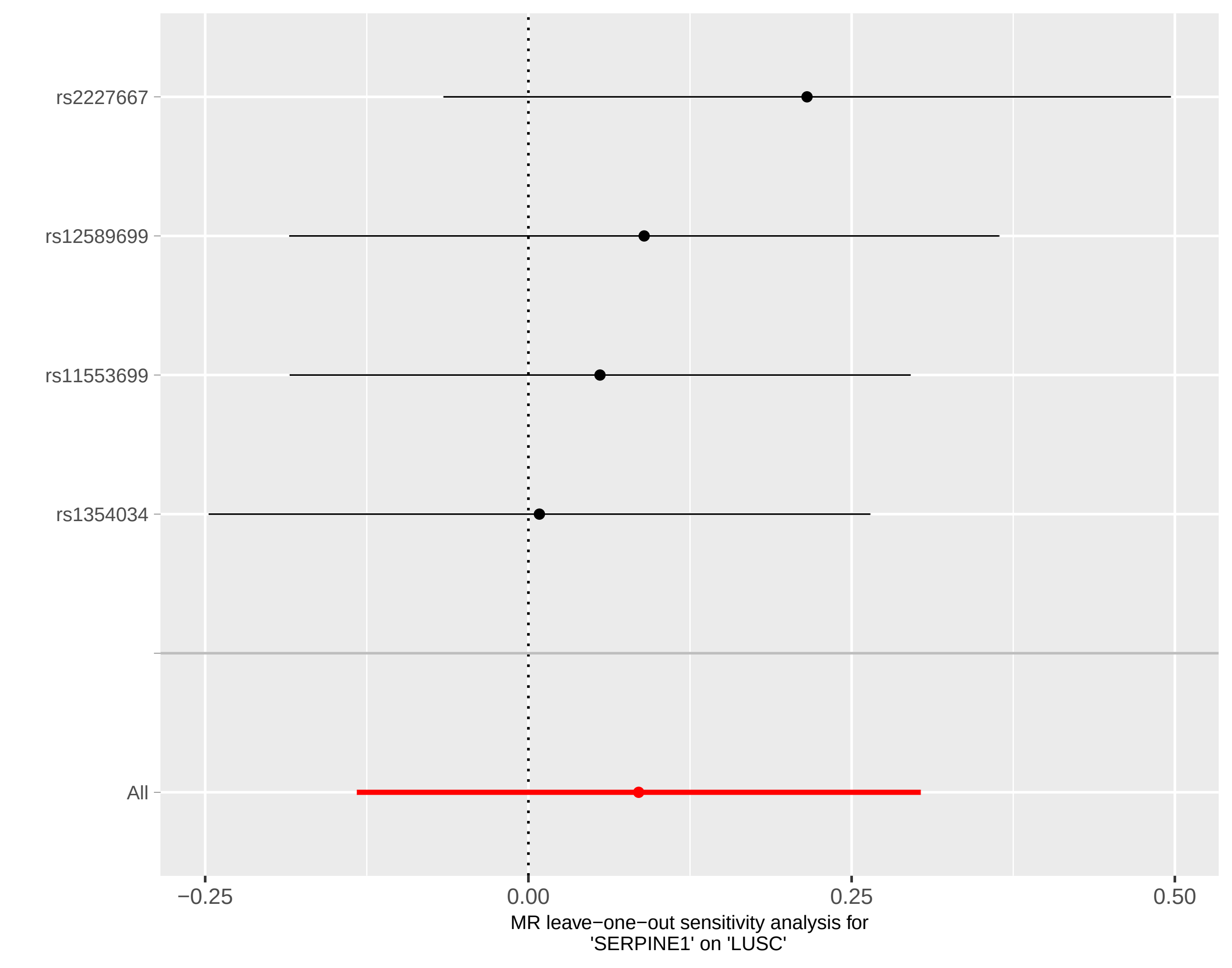
rs2227667
rs12589699
rs11553699
rs1354034
All
−0.25
0.00
0.25
0.50
MR leave−one−out sensitivity analysis for
'SERPINE1' on 'LUSC'

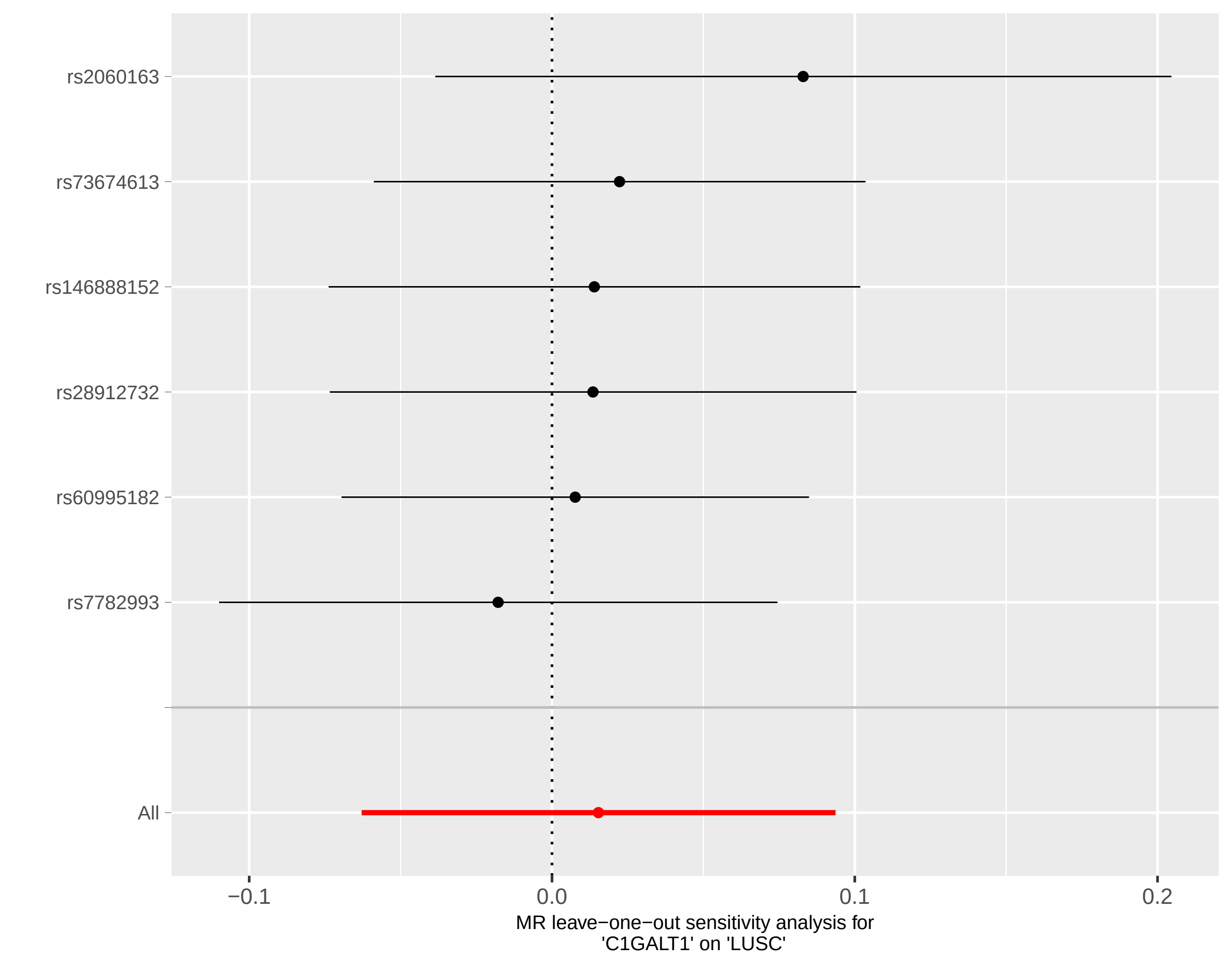

rs2060163
rs73674613
rs146888152
rs28912732
rs60995182
rs7782993
All
−0.1
0.0
0.1
0.2
MR leave−one−out sensitivity analysis for
'C1GALT1' on 'LUSC'

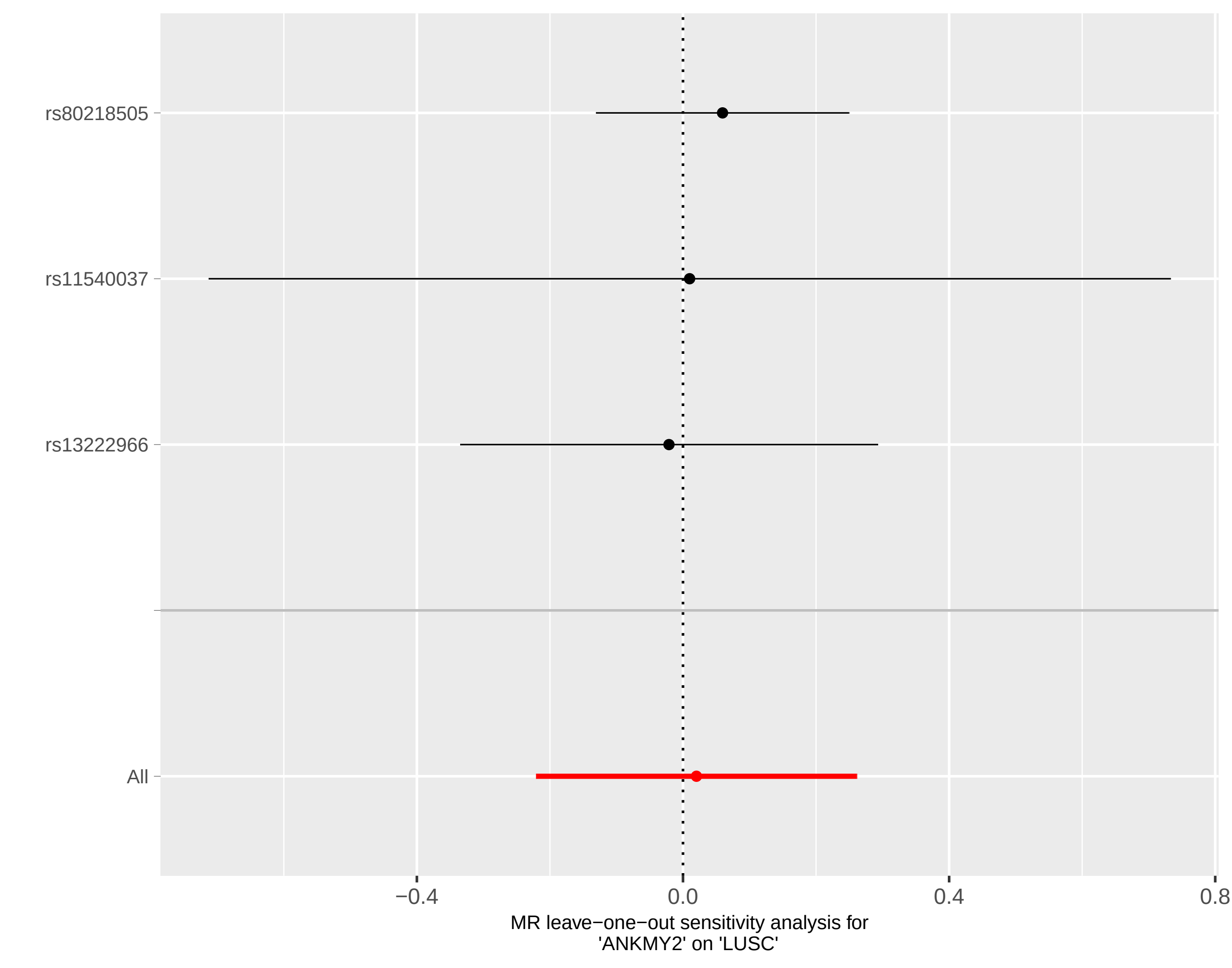

rs80218505
rs11540037
rs13222966
All
−0.4
0.0
0.4
0.8
MR leave−one−out sensitivity analysis for
'ANKMY2' on 'LUSC'

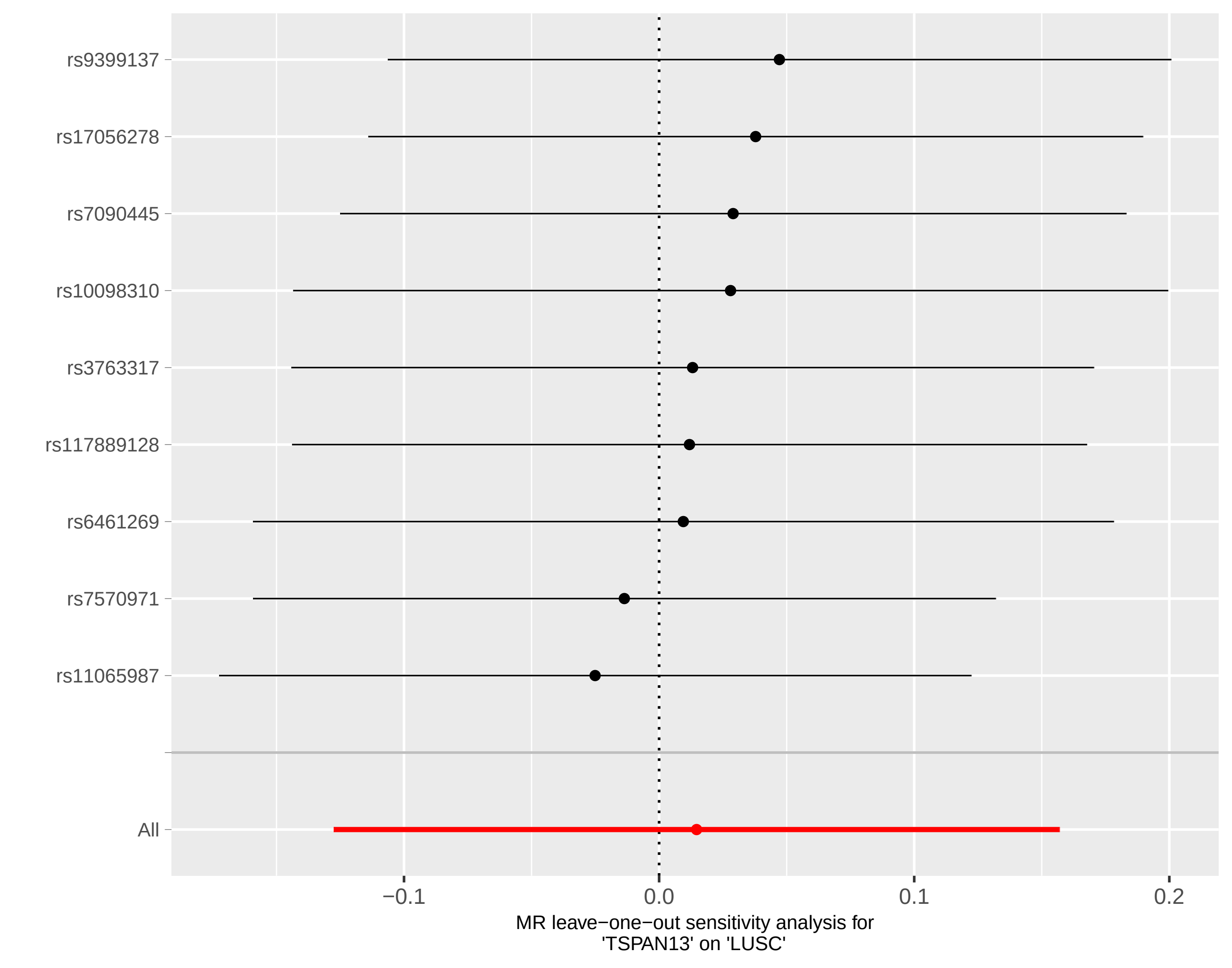
rs9399137
rs17056278
rs7090445
rs10098310
rs3763317
rs117889128
rs6461269
rs7570971
rs11065987
All
−0.1
0.0
0.1
0.2
MR leave−one−out sensitivity analysis for
'TSPAN13' on 'LUSC'

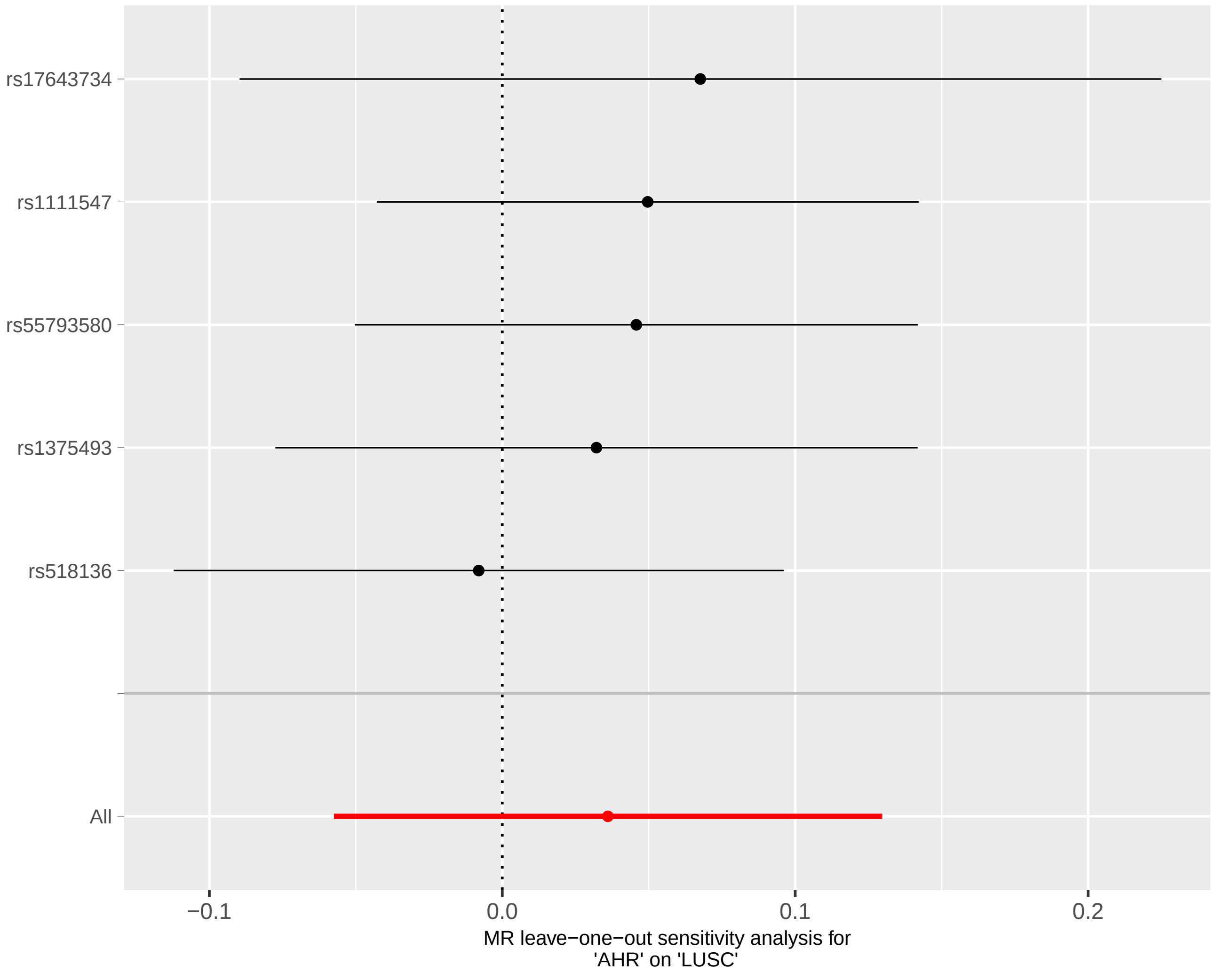
rs17643734
rs1111547
rs55793580
rs1375493
rs518136
All
−0.1
0.0
0.1
0.2
MR leave−one−out sensitivity analysis for
'AHR' on 'LUSC'

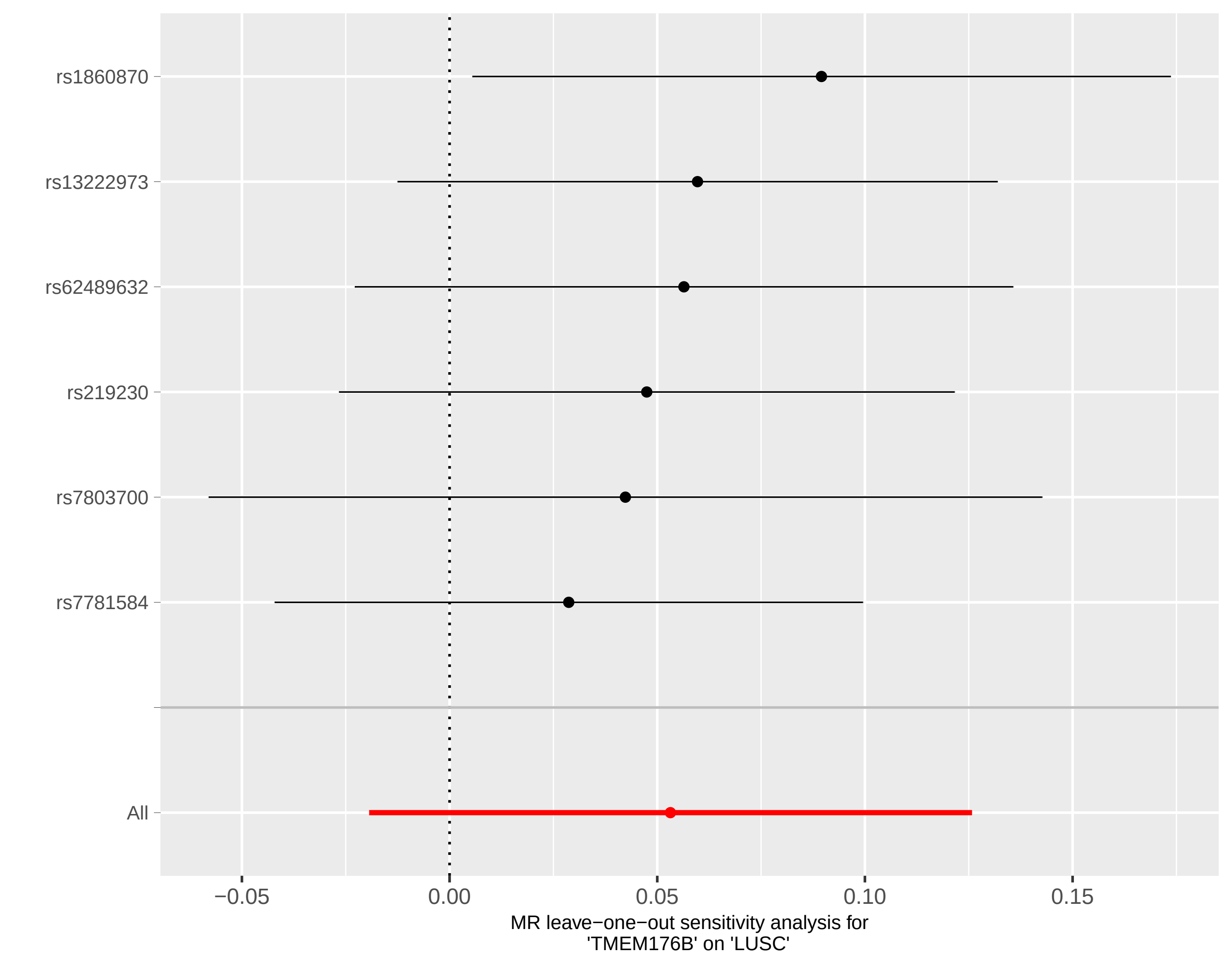
rs1860870
rs13222973
rs62489632
rs219230
rs7803700
rs7781584
All
−0.05
0.00
0.05
0.10
0.15
MR leave−one−out sensitivity analysis for
'TMEM176B' on 'LUSC'

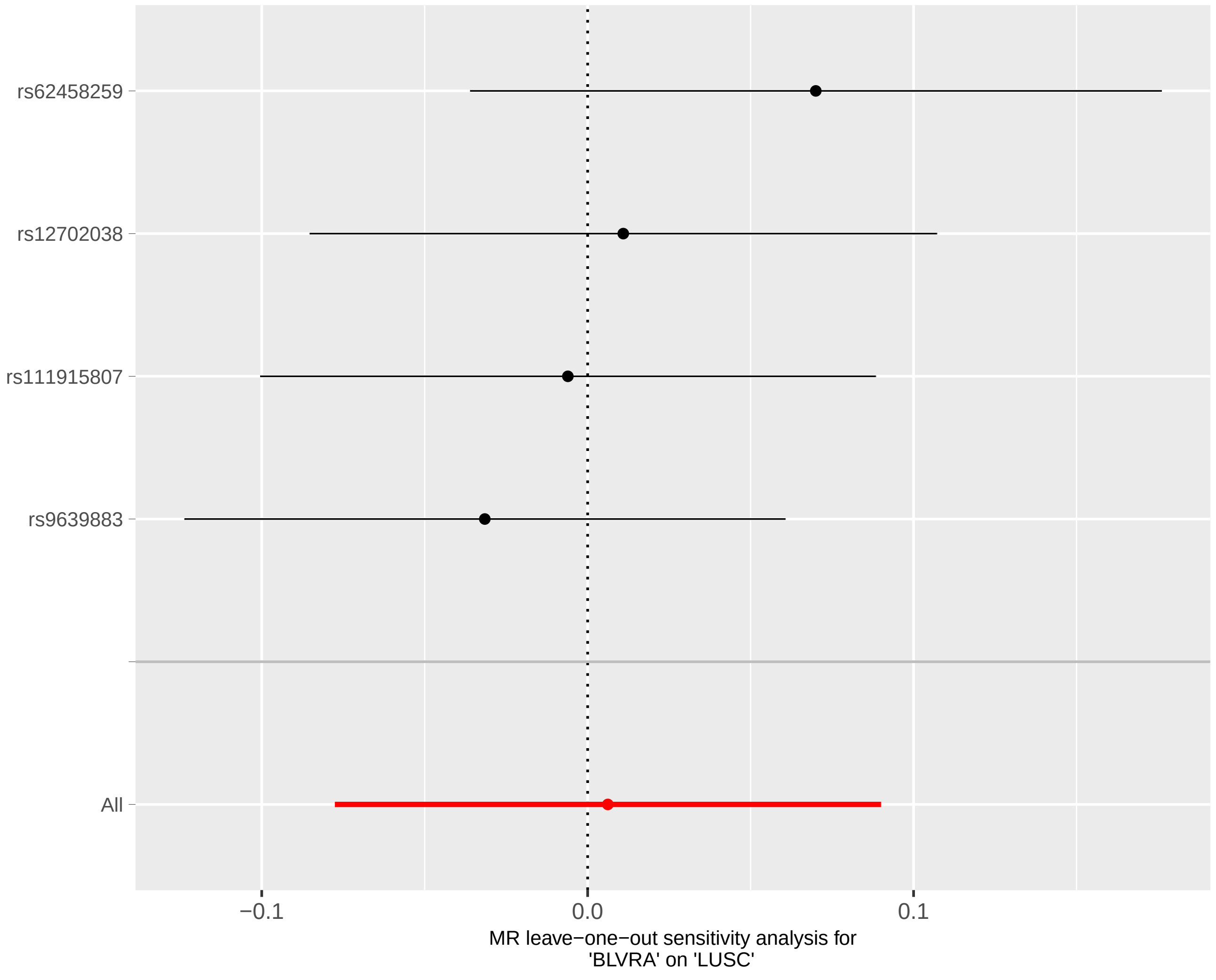

rs62458259
rs12702038
rs111915807
rs9639883
All
−0.1
0.0
0.1
MR leave−one−out sensitivity analysis for
'BLVRA' on 'LUSC'

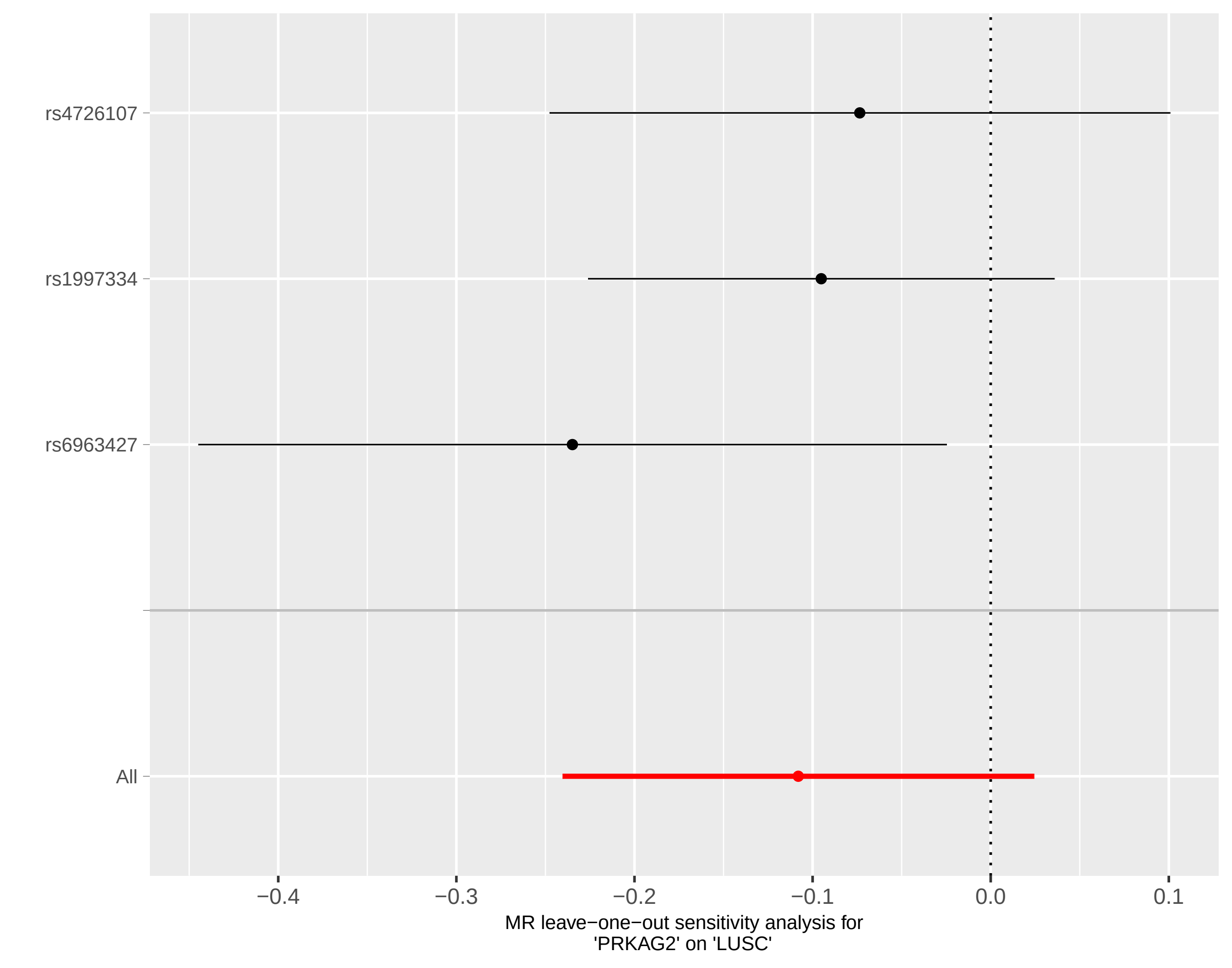
rs4726107
rs1997334
rs6963427
All
−0.4
−0.3
−0.2
−0.1
0.0
0.1
MR leave−one−out sensitivity analysis for
'PRKAG2' on 'LUSC'

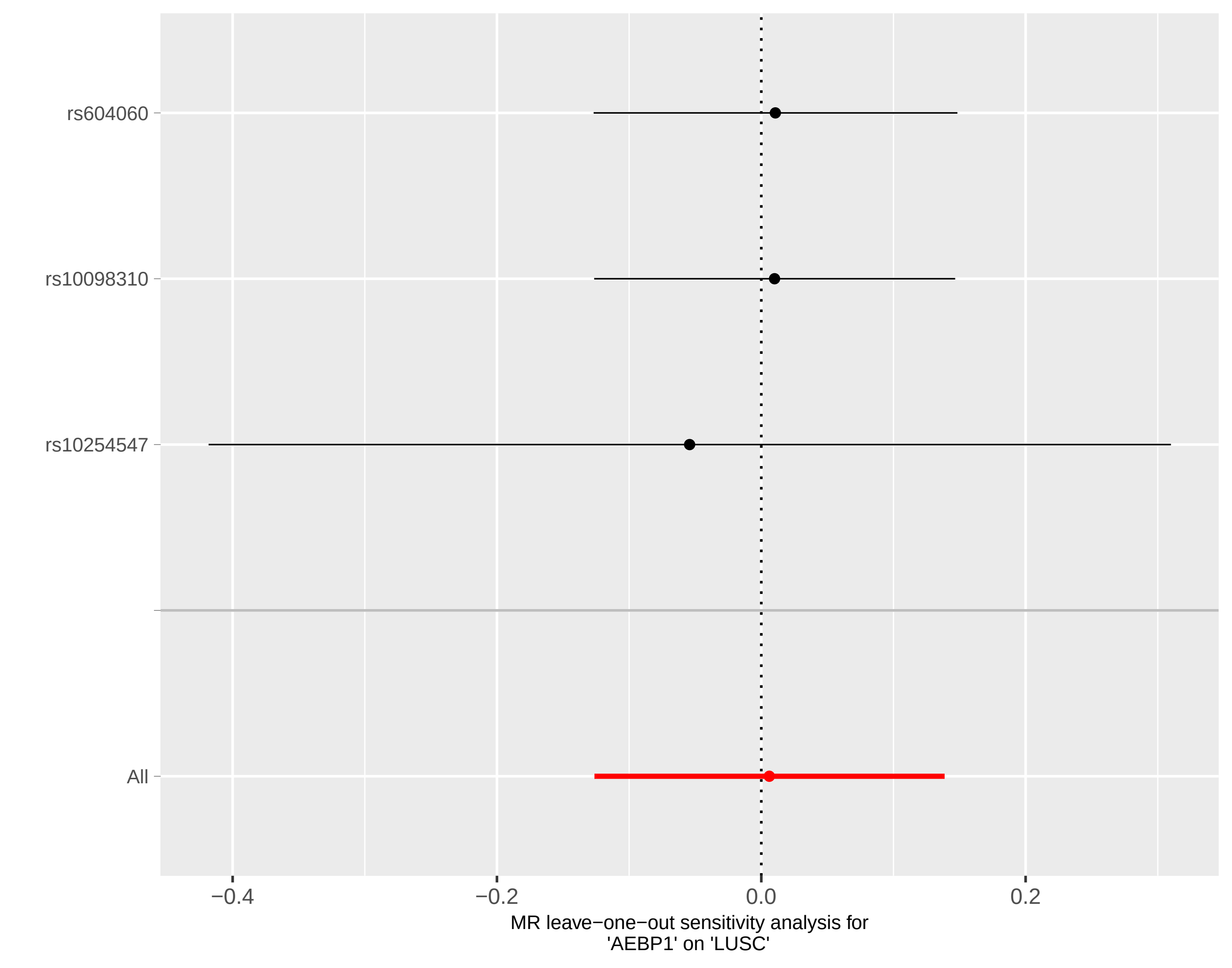

rs604060
rs10098310
rs10254547
All
−0.4
−0.2
0.0
0.2
MR leave−one−out sensitivity analysis for
'AEBP1' on 'LUSC'

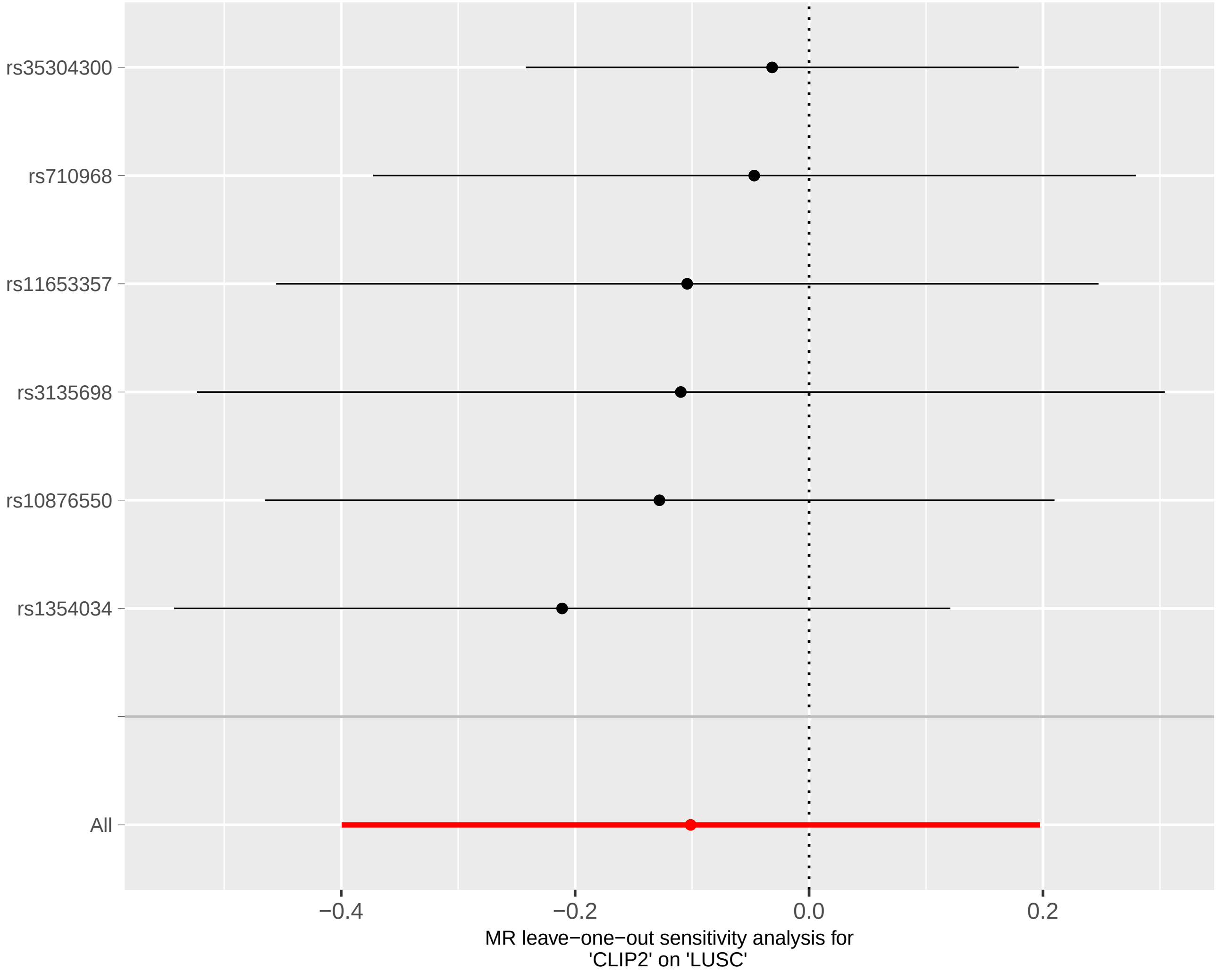
rs35304300
rs710968
rs11653357
rs3135698
rs10876550
rs1354034
All
−0.4
−0.2
0.0
0.2
MR leave−one−out sensitivity analysis for
'CLIP2' on 'LUSC'

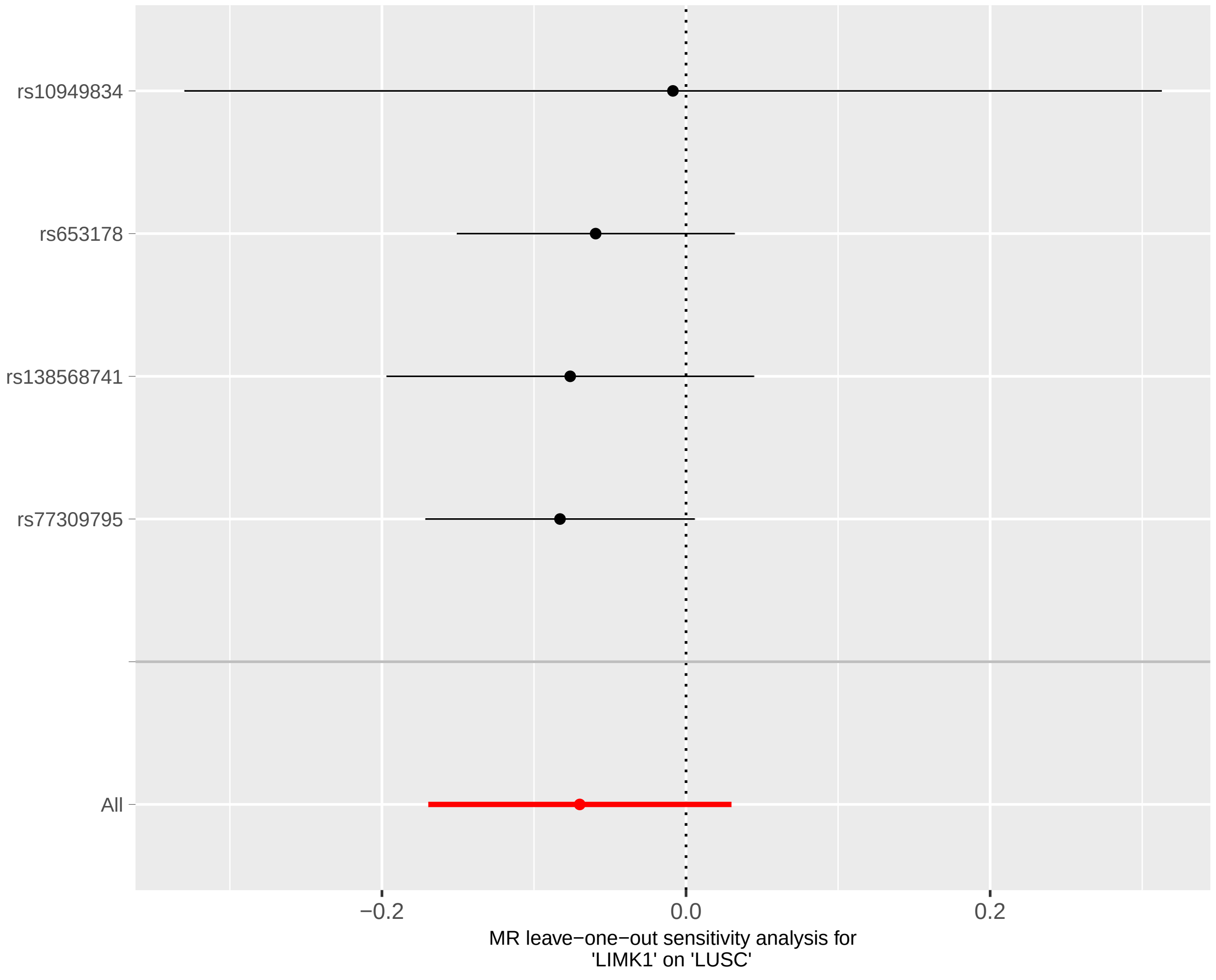
rs10949834
rs653178
rs138568741
rs77309795
All
−0.2
0.0
0.2
MR leave−one−out sensitivity analysis for
'LIMK1' on 'LUSC'

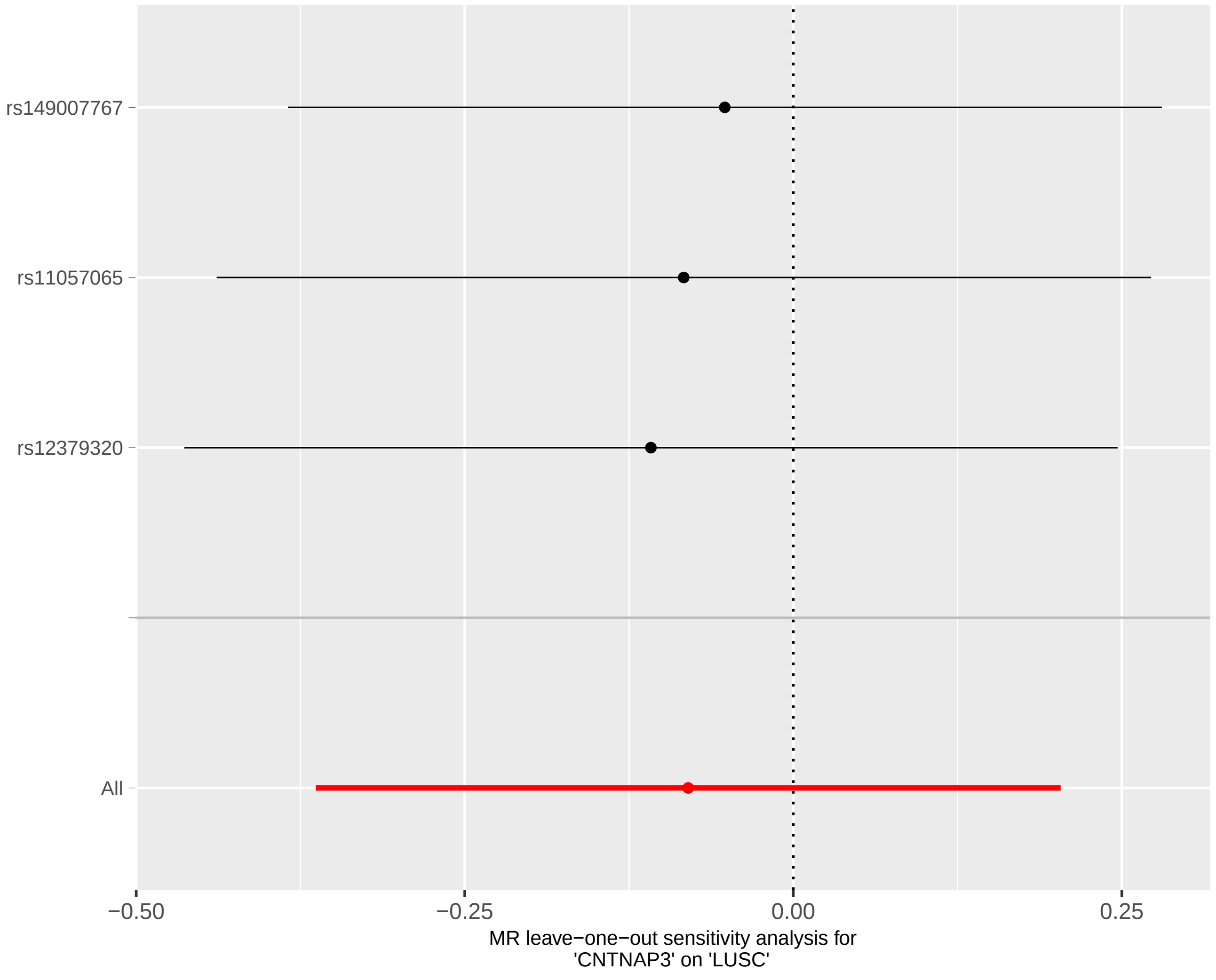
rs149007767
rs11057065
rs12379320
All
−0.50
−0.25
0.00
0.25
MR leave−one−out sensitivity analysis for
'CNTNAP3' on 'LUSC'
